# Supplementary material for: InsectOR—Webserver for sensitive identification of insect olfactory receptor genes from non-model genomes
Source: PLoS One. 2021 Jan 19;16(1):e0245324. doi: 10.1371/journal.pone.0245324 (PMC7815150; doi:10.1371/journal.pone.0245324)
Supplement: S1 File — (PDF) [file pone.0245324.s001.pdf]

>EDS41961.1 olfactory receptor [Culex quinquefasciatus]

MSPNIPPDLQVLKFPLRMLRFVGLWGDRRELVRYASVVLCMSVVLIIPKAALGSGKAGFDSFARNTAELI
FFTEVCVSIGIFASRRGSFERLVEVLRETVLMYEDVELLGEIAAFNRKMERFSKSYAAWIGFWVVLYLGI
PMIFTCVKVVFPGEGDRGDFMLIAELQFYWLDIRHNLLDYAIYLVFCSMAIFCSSYQSTLKGAVILVSIQ
YGTKLFELVAMSIDRLGNVKEGIARKNQLREIVNLHKLAFQYTKHLEDTVCFMMINQILNCILIWCLMMF
YVSTNFGPNAVCVIILFAVLMGEMIAYCVNGSKLAETAAAVGHAVYRYPWYNEPTAMQKDMQLIIERAQK
PTGITAAKFYFVNIERLGLVVQASYSYYLILKKRF

>EDS41960.1 olfactory receptor [Culex quinquefasciatus]

MATTKTTAVTKVDSAVFRRRLPEELQIMPYTLRVLEFVGLWGSWNQFPFFVLFMTTGTMVILFPKAFLGI
GHSEFAIMAKGIAEFIFEANIYLSALIFSTKRASFVKVVNGLAEFFHEVSNADNDCYDLIRTTNVKIRYF
WLFMVLYCATGPIIFCIPSATVTYLRYWDAIGKNLSEPLVYEIPMEQEFYGLDIRKNFIHNQIFLGFSFL
AYCASKFFLLVMITTPFIMIKYNSLTYQLVCERIRKLPHLPGGPKPAGSSTSNRTEVNVPDLAEVVRLHR
QAYEITQQIEDIVHLPVALEYFTCVMFYCMAMFYISTYIDFSLFNIMVLFLASLMETFGYSYLGSELSEE
AAAVGTAIYDLPWYDHSVELQRYYRLIIQRTQRTTGIFGLRFFLVQLTTFANVMQMSYSYFLVLKDVLQQ
L

>EDS41954.1 olfactory receptor [Culex quinquefasciatus]

MFTTQNSHRLPSELEVMPFILRLMEFIGLWGPPGQIVRFLAAFGWGTMIILFPKAVLGIGSDRFDAIAKG
IAEFIFEGSLFVATAIFASKRIAFERLIDGLKEIFQRATNGPHSNDCYDLLLRQNLSINKFVKGYTIYCC
FGPFVFCVPSLAASHFRYWTHSGNQSEPLIFELPMEQEFYGLQIRTNFLHYHIFVLLSLFAYFVCAYFLL
VKVSVHFIMIQYSSSTYRLVALRIRKMTESKEVTQSELIDIVELHRLAYRCSTLVEQICHLPLALEFLTC
ILFWCLTMFYVSNNIDFNLVNVMVLFWLSLLETFGYSYLGSKLTEQADEVGAAIYDLPWFEHSVELQRYY
RLMLQRVQRPTGITGAKFFVVQLATFGSVVQMSYSYYLVLKEALTMI

>EDS45840.1 olfactory receptor [Culex quinquefasciatus]

MELEHYLPEPGSSEQNDKSIFWLRTLSTSLGIWPEKMVGENKSWWKRLYYFMIINAKTITTSMHWYNTYL
QVEFFCANMGELKTITEGLCSFCSITVTGIKIMRLHSFSDEIYIMLQQMKGHEFLKSINFLKKGNNKDIF
TKIDKIMKAKWREVNLNLRLYSLSVGVVAGTYSIVPAVINLVNLFQGNDIPKRFVYKTYYRYMEEAKYHS
PLHELLFCSESLSGFTTWAGVISFDGLYVLLTMHLVTMFSSLNLIIKETSNANFNDAEKQFFLHEFLVAQ
VFTSTSIICVIAFHTSANASERDSQTLVMILYLIAAFYQLLQFCWNGQRVQNESVELPKSVYECDWYKCS
KKFKTTLHILLLDVQKTVDISAYNMFVMSLETYLAIVKTAVSYFTALQTLTEE

>EDS40325.1 olfactory receptor [Culex quinquefasciatus]

MQIEDCPIIGVNVRVWLFWAYLRERKWLSYLLGCIPVTVLNVFQFMNLFHIILSGGTMNKIIIDGYFTVL
YFNLVLRTTFLLTNRSKFQQFFEGIAAEYAKLEKRNDIRLLLEQLTRRARILSKSNLWLGAFISACFVTY
PLFSPDSGLPYGVYVPGVNMQSSPTYEIVFVLQVYLTFPACCMYIPFTSFYCTCTLFGLVRIAALKESLE
RLHQFSSEPKTLLAKVKECLQYHKEIIKYIRDLNELVTFIFLLELLSFGMMLCALLFLLSTSNQLAQMVM
IGSYIFMILSQMYALYWHSNEVLEQSLKIADSLYYNGNWLKFSTPVKKLMILMIARAQRPLVIKVGNVYP
MTLEMFQKLINASYSYFTLLRRVYN

>EDS40310.1 olfactory receptor [Culex quinquefasciatus]

MDLSFMLQHTDSFNLVFEGISVLVAGLDAWFGLVEIIVNRKAWVALMKDVSSRRSMYKSAKISALFDEYY
ERNIMFCKFLYGVYMSTFSYFLLPAILPDPGKYNLPIPATIPHLVPDTNKLYWITFLIQLLMVGIAQHVL
IAQCSSLIIGIMSACCQIRALKIKLEDLNEQIKDPAVKPGTVHESLGEIIYLHASTKDYIRLLQRKAAIV
YLSVFVTCGGIVCSCLNVIAEDLFNSANALMLAGTFSVLVHCFFGNTLLIENDSLPDAIYAIDWYKLPLA
DQKAFKFLLANAQPDAALHGILMPLNMAPGVATPGVASALGKVLPGDINRTIRLGQYGYQNSWFLKLIMK
MAILTGLATPGVASGLGKVLPWGH

>EDS37537.1 olfactory receptor [Culex quinquefasciatus]

MPKTSSVHPFRQSRYEPLQSQRRAFRIFGYYPGDSGFLHWSLVGVFLFHYWSQVQLCYWEFRHGWAKIRE
GEVFVALEVMTPTLSRVGALLKCSFLIAERKSLKKFLDKLVELHDQADENEKPIYKWVTYWSRQFTNFEQ
NFFLVTCLFFSLFPLGVMLFNSIMNPNNPRIFLLPTQVTLPYEYKYSPMFELTFLLMSYITFTPCFMLGG
SDGLFIGVSLLVSSQFRLVQQQLENLEVEESLSEDVPAENKRILKQLKQIVQRHNQAIEMSQEMSSLFVP
NVFTCYTIAAVKLGMACLIMSKIIRTAGSYMTIMQSFVEN

>EDS37168.1 olfactory receptor [Culex quinquefasciatus]

MHFLKRSLPPNQQFLTFGLRALRLIGLWGPDRRQIFRYYLLVLAELVLLVGPKIIFGSGQDGFESFVRNS
AELIFLIEVMISIGIFASRRRSFERLVEVLDQTLQRKWPDSLREEIGQFNRKVELFARVYAAYLFGMLVF
FCVFPVISTFYKVVFVAEEERSDFLLIIYVKFFGLDIERNVHHYMIYILSMICPISASAYQSSIKMIVIV
VVIQYGAKLFDLVAKRVTVLELIKSDQERHDELREIIKLHNLALEYVEHLEETVCFIMINQILSCMLISC
LMMFYITTNFGPNVATVVQLFMVLVGEMIVYCFNGTMLIEKATSVVDALFYYPWYKEPIWVQKVFLRMIQ
RGQRPTGITAAKFYYVDVNRLGVVFQASYSYYLILKNSF

>EDS35591.1 olfactory receptor [Culex quinquefasciatus]

MDDEENLLRVQLFSMALIGVQERETVPSRIYSYFCFYSLLIMDLSMVLFAVQHFGDMVLVCDCLGPGFTA
YLGMVKQHYLSEQRKQLWEIIYALKKLKQIAKPDEIRSIERNNQIDRYLATAYLTSAVITGSHFIVTAIV
KAVYSKVVHGKFVWQLPLLQSTLKFAFFSSFYSYPFDISHPLMFAVLFVWTSATIYMVVFGSVSSDAAFG
GLASNLVVHFKFIQAGFRDRSFEDNDQSLKDLIEYHRHVLDLSRKLISAYRPIMLNNFIVASFLLCVLGF
QLVLFMGSTMMFLYIVFVTAIVIQITFFSYYGSQLSHESALVGDAIYCSNWYETSPKTRRLLLQCLMRAQ
VPVNIRVGFIEASMPTFRAILNSAGSYVALLLSFTDT

>EDS35590.1 olfactory receptor [Culex quinquefasciatus]

MATSTSKIAWTANDGNHRRVRSPDKEKDNFLRVQLFTMALNGIRKHETVSSRIYFYGCFLTMLIMDLAGV
HFAYQNAGEILLVCDCLGPTFTCFLGVVKQYYLDVHREQLWYIIHELRKLKQNATVSDIEMIEKNNKIDQ
FLATAYFASAMGTGTIFIVEAILKGTYNYFIRNCIEWNLPIAISFPFDISHPAIFAFFFIWCSAATYMVV
FSSVSSDAGFGGLASNLVVHFKILQNRFKDRRFEDNDQSLEDLIEYHTLVLKLSRRLMSSFRIIILNNLL
VASVLLCVLGFEMVVYLGTSLMLLYITYITAIVIQIFFFSYYGSQLLYESAAVGDAIYCSNWYEATPKTR
KLLLFCLKRAQVPVNTKVGIMVASLPTFRAVLI

>EDS31360.1 olfactory receptor [Culex quinquefasciatus]

MLFRKQHHFDAMMAFNIRVLTALGFWGESGIELRATLQYLAYTTWFIPPGVLFCVRQQPSSKMMLKSVYE
LVAAGSCIVRIGNLYVFNGTLQKAFYEVQFALGELSRDSSDAVRKVLNHLTISADYICKGYGGMLIIQCL
LFGPAQGLVSILKYFVWGEDPKYSLLEADYLIYNQYSNFNVWLLTMLASTCALYVLVFALLSHETFYWNI
LHHVSCLFKIIRLKILELDDCSTPKQFHEQLSIVVEMHERAFKSTRLLEQAISAMMSFLYLSFITIMCSM
LLVFTVIQPDLGFLLMMSVALQYNVFLIFTFSMLGTELTEASLSVSDAVYSIRWYEKSPAERRLLLFVQM
RAQKQAAITAAKFFYLTRASFATCGCLSVSASSWLGMSAQREALEKKEREIEESGKDRWLRS

>EDS28558.1 olfactory receptor [Culex quinquefasciatus]

MARTSKVVPFGESQYGPLQLQRKTFRIIGYYPGDSGFLHWAMVGVFVFHYWFQIQLSYWEIRYGWIKMRE
GDVLAALEGICPTPSRIDAILKCLILIGERKKLKVLLDKLVELHDQGKAGEKSIYQWANYWGHQFTKFEL
TFFIMTCIFFCLLPMATMIYHAVVAPDEPRIYLLPALVALPYNYAYSPVFEFTFLLLAFITFTPCFMLAG
GDGLFIGVCLLVTSQFRIVQQQLESLGREEPPGTAVNPANPTPAENGQILAQLKLIAQRHNQAIEISREM
SSLFMPNVFAVYTIAAVKIGLACLILMQSEGFKKLIYMFGSLGILTEIYVYSYGGTLLLEESFPGFARLG

>ABW80750.1 odorant receptor, partial [Rhagoletis suavis]

QVFWGPNALFRAVGYDFQRLPRPYWRQILMKAVLIFMILSAICIRIYMFMSLRELIIRDDILNSFRLGAF
IAYGVDSNVKFAYFIFKAHRLRKIYDFLAAEYPQTSSEQKLYKIDIYGFQRAPVMICAYMAVVASIMLSP
LLQSIVTYIIDIYRFGYDAAEYPYLHPIPMPYNFDYYTPRYYIPVYMVESLNGHFSSTTNLGTDLFISIF
SGQLCMQLEYLGYSLETYEPSMEKSEDDCEFLRKWIRKHQLMLGLCADLDEVFGTTLLCKLITNCTYFCI
IVAQLMLEGYGYGFLNFGSFFFLTVAQFFMVCQYGQNLITISEHLSFSAYKNRWYNGSKAYKKMILTIIT
RAQTPANLTAKGFQPISLLTFQIVMSVTYRVFAVLQQVFD

>AHF20367.1 odorant receptor [Aedes aegypti]
MTQSIEFEQTFGFIAKVLQMIGYPSCLAPYPTTFASRLKSSAGFVVCFLMLTYCVFGQIINIGLLMMGYR
QTDQVVEEVAIQVSSTGFCIIGLAKMYSLSYNHTILSWLIADFRVKWNAGELTEKDRSIRDGTLRPTVAI
TTVAALGNIIMVSAFNFQPVVEMIYGRVVTGEWVKLFPYVIWFPFNPTPGAIYYLVYLFEVYSGVIVAVG
NVGFNCIFCLLTSHLSMQLKLLCSWIEDMVEVEDEKGVRSKRKLYRIVRYHQDLIRGRDALQSMFSTTLF
LNFSASSVLMCMQLYLITTAGITLMIKFTLFMLCILMEIFILCYYGEEIFANSSSIAVGAFNSKWYQLEA
SQQNSRFGKNLIPIIQRAQTPMVLTAWKFWPITIRTFSAILQTSWSYFTLLRTVMR

>AID61247.1 odorant receptor, partial [Calliphora stygia]
NSLHLVCQTLALNMTSHWKVFFGNIFPSNPEIGEIGSIELNIWLANITGVPIIGLKKESNGLKFLILMYG
ICTSLLVTFVYTGFEIYDLILNLDDLDKITQNICLSLTHVAGAVKITNLFYRYKDLKIVIDQLKYVTKTY
IKSEKQLETFHNGELENKIGLSIYAGIVGTTGILGMILLFMHPESVAGEIFPYRVILPSWLPLFWQLIYM
GLSVTVFAIQIVAVDYLNVTIINQIRFQLNILNLSFDELTIAAETTLNKKTEHAKTLEILKLEPLARLNS
IIEHHCLLREIRQLTEDIFSQPVLLQFFTSLMIFAMTGFQATVRTGGSNGAVLIYFYCGCIFCQLFVYCW
FGNEVFEQSKTLATSGFNSSWYLFDRRYGKSLLVYLTNAQRPFLFTAGGFMGLSLPSFAGILSKSYSYIA
LLRQIYGR

>AID61245.1 odorant receptor [Calliphora stygia]
MAGKYSERFFNIFYLTRRFSELCGADVIKNDYKICWKTGAIFLLINFAIAFTFYTNYVEVIVNGNYYNLL
KSASILGTGLQGYTKLINILLQQKSLRFLYQEITEIYEIYELKSTAYKDCLRYSITLVKKLLSTLLTLIV
ITTLIIIGIPVFMLIFLDTRIEIMPFKIPYIDIETDIGYYVTFVVHTISVFFGGFGNFVIDSWLFIFAAH
VPLIKNILKCKFDELDKILEANPKDVEKSRAPLKDIFEWHQKYMLFCKIIKEAFFWVIFVQVGTEFFGII
STIVCIFLGIWPPAPAYLLYLFAMFYSYCSLGNIVEVSNDDVTLIIYDSCWYNLTASEQKMVLIMLRESQ
QATGISIGGVSPLSMSTALQLTKTVYTLSMMLKEFLN

>AID61244.1 odorant receptor [Calliphora stygia]

MSANEAELPQPRIKRYFHVQRVCFAILGINPTSLERTVFNHYRVWLPMIVQLLHYIPMVFYAIENINDVV
KVTTALAPIWQAINATLKIIYFVWNRKKIVALVRKLWFWNLEAKDEELVILTIENRKDILFCTSYSMVLN
VTGVAALLAPLLIAGFYAWKGEIFWEYLEPPVKASYGIDKQSVFGYIIVFILNGYGAFFVVYGTISADSL
FSWFMCNIVAQFHILKYRLRQAGGENNGDCSMKTISDCIAYHCRIIELASDFNDAFSVVVFIKFAISCVQ
ICCLAFKLSRGEGELFDQVYHGLFLICLSMQLMLYCYGGQRIMDESESIANEIYDSFHWESLSVANRKML
IFAMMRSQMPCNVCGVFFVANLALYLWVYRTAASMITLLKTIEED

>AID61243.1 odorant receptor [Calliphora stygia]

MVVKRYFYIQQLTFSLCGIDLKATNSKHVVTRPLLCYVPLCFAICHVVAIVHYAFVNRHDYVEVTDSLAL
FCQSLLAIWKMLIFLYKRQGFIEMIHELQLGNSKAGRLELPMIRSENTRDVTFSTIYFTVVLTSVFFAFG
SPIIEAVYFYVSTEELVLRMPHKASYFWNHTQLPGYSVVYMWDMLSIYNLVGISLAIDTLFTWLVSNISA
QFHILCFRFKDTAKAFDDGTNDSLKFMKSIKSCISYHNQTLKLADKLNEVYGEIIFIKFIISCSQICCLV
FRLSRPIESLTAAAYQGFFLASVAIQLILYCYNGQRIRDESLQVATEIYYAFDWSHLPKSCKKLLLIPMM
RSQKASELKGVFFVVDLTLYLWVFKTAGSLIAALKTLEENRI

>AID61242.1 odorant receptor, partial [Calliphora stygia]

MVKNSTTANNQELVEAKRPTNTKPAVSTKQALTYLYRVFRILGLYRTEKYKYAYILYSIFMHCSVTIFLP
ASFIASYSLVSNKNVDYDTLFTSIQVAINVFGCSLKICMLLYLIPHLLTAEPVMAKLDERCVAGDEVELL
YKIKKLGRKLVVSFCITFWSYATSTFIVSMLAGHPPYSLYLPGINWRITNWEYFVSSFIEFLLMLGACSQ
QVANDSYAAVYVCILRAHVNILRVRIRKLGSFNQKTLLEHEEDLKMCIKDHQNLIELYKIISPVISATIF
LQFTITAGILGITLINMLIFARGYSSIIASCFYVLAVVVEIFPLCYYANCLVCDSNLLSVEIFHSSWIQQ
NSRYRKMLIFFMQRSQQTIELKAAKMFPINLNSFISIAKFSFSLYTLIKKMGIKERLGLE

>AID61240.1 odorant receptor, partial [Calliphora stygia]

YTNNPSTSKEMPLTKAKQNLEFLTTQHLNLLKMGIDINTLKRRELFNNYWKFLWLISCTIYLEYALINFV
AHSLSDIDEATGALSIFNQGCLILIKVSMFLAKGDRFLKLIWDMNLLAEKANPEEHKKWLEENRRSQLIG
KMYFYACCVAVACASVVPLLFMAYDYQQNSGFNKKLPFGGKFPFDKGGVTAFAINYMLSLIYIYSLLNMT
VGIDTLYGWYIYSISAHFRILRCKLESTARKLQNNDCSDFTRDIGLIVNYHNATLQFVEGLNTNFGEILW
AEVMLSCLQMCFVAYTLNNDTDVSNMPFNVMVLVAVMMQMIIYCFGGEKIKNESLMLSSEFYLNFPWHKM
SAQQKKLMLLPLLRSQKLSVLRGLFFEVDRNLLVYILKTSFSFNALLSAMKE

>AID61239.1 odorant receptor [Calliphora stygia]
MSAVKNSKTKKNVTIRDIFYLPFKFYTTVGIKLFHWDEYDIMTKWQKFIMAVTLINLAISFTFKVSYIIV
DEFENTVHMTEWSLYMSFATNGFFKTLSVVWGRKTLDRVLKTLERLFPRNDKERRDFKMVEGYEYIQYHS
RIMVYSHWTIALMFMSFPIVQSSVEYIQRRVFVQRLPYILSYPFDTSSDTAYVCAYATQFMGGFTLSCYF
LGSDTLLLHTVYMVVLNFKYLCFRIVNFEPKDYDQDMNELKDVLEKHYLLNDLAKSVNNVFSLSILLNYM
ISIMVIVLIGVQIITGSELFDFIKFVGFFASATIQVYYVCLMSTLLMERSAEVGESLMGQKWYMADVRYQ
RMLTLAIARSQRPAHLTAFKFFMISMESFSNLMTTAYQFFTLLQSRMEEEGF

>AID61237.1 odorant receptor [Calliphora stygia]
MVLDKTIPHIGKFFAIPLNLFFILGFCIIRWKPNEKPKELQYLFILFLVFNSVYNVAGMLSYTIYEPLET
SLEKTAYIIYTTFAGNSVMKFVCCLCNLKKLHQCFKSLEKYYPRTAKEREDYRLDEHLKKMERFNLLLTI
YHFLVTSIFSWFPLIQSTVLYYKNEERSFPYMLPFPMHYIFNERTNLGYAFAYTTQCTGSYAASCMCQGA
DILLLTCVHLINMNLTHLAKTIRDFKPTGTLSDLKQLKQFVTYHNDILSTVNLIDDTFSLSILLNYLCTV
TIMCLIGFQMVIGTNIFHLLKFLLFFMSVLTPVYFISKFGTDMMELSSDINEAFMHHSWYDGHILYQRSL
IMSIRISQKPVHLNAFKFFIISMETFKSLISISYQFFTMIKTSYVEE

>AID61236.1 odorant receptor, partial [Calliphora stygia]
MISKKYFFVQKLTFAGVGIDSNAKNPNLIVKRPMLLYGLLLFSMFHFIIITHFAYIHITDFEDVTDSFPM
LCQLILCICKVLIFLRKRHEILPLIHEVHEINLKAKGEELAIVRRENAKDYFLCSVYLKVVIFSGCFAVL
HPLVNAAYVFITSGELVLNEPNKATYFWNYSNLTGYSAVYVLNCISVYFVCDVSLAIDTLFAWFVSNVMA
QFHIMYYRFEKASIKCSKHRKTNYQPKKFNKTIISCIQYHHQILQLAEKLKQVYGEIIFIKFAIVCTEIC
SLVFRVSRPNDSLPETAYKCLFLSAVAMQLALYCYNGQRIRDESGLVGTAIYCIFDWSNLSKSNKKLLLI
SMRRSQKHCNINGVFFEVDLSLYLWVFKTAASLLT

>AID61235.1 odorant receptor, partial [Calliphora stygia]
MTTKYSHDFAKLFNFTRNFSRMCGADLIREDYKMTALTWFIIAIVNGAIAFTFYTMYVGVAVNHDWTELL
KCLCMFGTGVQGYAKLVNGLLRPEMVRFIYRTITGMYSVYELKTANYHKLLKQSVSLVRKLIIIILLSSVI
LVCLAIIGTPVIYKIIFNERIFIMPFFFPYIDYNTDFGYYLTSVFHVICVIFGIFGNFVSDSWCFAFAAH
IPLMKNILQAKFNELDELLQENDESKDLKQVAEKLFEIFKWHQKYQEFCNTVKELFFWVIFVQVATEFVS
IVCTIVCIFLSIWPAAPVYLVYSFVLFYLHCALGNLVEISNDDVILMIYDCCWYNLSVPEQKMLLIMLRE
SQQADGMSIGGIAPLSMSTALQLTKTFYTFSMMLRQFLN

>AID61234.1 odorant receptor, partial [Calliphora stygia]
KDFENTATICSGWQKQPSFILRSCKQMDPSTITNSKYSKQFEKVFNFTRFFSEICGADVVSEDYRMTWVT
WSLIGLVNGAIVFTFYTMYVGVAINNDWSEILKCLCMFGTGIQAYAKLINGLTRKDKFCFLTKEINGIYS
TYELKSKNYHKLLEQSISLVKKLITILLSIVVLVSLAIIGTPIFYSIVFKERIFIMPFLFPYIDYETDFG
YYLTSAFHVVCVFFGAFGNFVSDSWCFVFATHIPLIKNVLQAKFEELDEMLEEEPQDASKVMDLLMDIFK
WHQKYIVFSNTVKELFFWVIFVQVSMEFVSIVCTIVCIFLGIWPAAPVFLIYSFVLFYFHCALGNLVETS
NDDVTLMIYDSCWYNLNVSQQKMILIMLRESQQAEGMSIGGVAPLSMSTALQLTKTIYTFSMMFREFLN

>AID61233.1 odorant receptor, partial [Calliphora stygia]
PRPRTVNEEVLKLCKMSEDLNKLLYKKSFENKRIITPLVMICMSIGCNIKYAAGFKDPVKLLSLTLVLLS
IVGLGSQFLYIYENLSESMLVYTDAICTSFQTLISISKLFHFAFTQHKFYKLVQIAQNSEILQNFEILEL
NILNKKKIIQEIQEIIQDSWLDIKRQLNFYLCCVFGIAAWYYGSCFAVNIYNICTHSSFAEFELIFPFQA
SFPIWRDNSKVFPYYFIKFIIISSETHISGMSAICFASLYIVISLHTLALLKILRRLVAHSTTENVLPQE
RDKFIMVWAKLHQQIYEYFCQMNSLYYIQSAPLFFVSMLVICLLIFQASVGLGTNADVVIKMALYFSAAG
FEVSMFCFNGQRLTSENERLPVALYNTNWYEENDKYKFITLMMLMRTNRPIAVQVGCFTTMSLVTLLGIM
RSSFSYCLLLREFNE

>AID61232.1 odorant receptor [Calliphora stygia]
MMLNTEAEVETWQAFKNHWILWKFFGLQPPKRDSKWFKPYIAYAIFLNVTVTLLFPTTLIVNLILSKNLT
ELCENLYMTTTDVICNIKFLNVFVMRHKLLKVRNILQRLDARAKTHKEVAILEEGLKLARKCFMTFARMF
CCAIISSQMMVYLSSERILMYPAWYPWDWKASKRNFLYAHSYQLYGLVLQAIQNLGNDTYPPTYLIILTA
HIKALASRIKDLGTNETTTDEDLYKELTDCINDHNTINELFLNIQEIISPTCIAQFMATGLAQCTIGVYL
IYVGLHPSKTLNIIIYFSAVTMEIFILCYFGDLYCQANAHLTESIYDCNWMDRDKKFKQAFLVLLQRSQK
NSSIMAGSLIPVRMPTFVSVMKTAYSVFTVLNKVN

>AID61230.1 odorant receptor [Calliphora stygia]
MEDLENQPPNAITAVQEINPLAKTEEEEQTPDVKQEVENLKKGTGFENVSTVMGTRYLFNGFRFLGVYMP
ENNRVLYVIWSAFINLLVTIFLPVGFAMSFVKMSGEELEIGNLLTSVQVSINVLGCSIKIILMGFLLPQL
LSCEPIINKLDKRCHTKEEMDSIKRFIKEGNRFVVLFSISYWSYSSSTCLSAVIFHRLPYNIYNPFIDSK
VSNLHFYAAVFVEMALIDIACFQQVVDDSYAVIYVNILRTHLDILLKRIKNMNENASTSLEANFVELKMC
IIDHKNIIQLYNTIAPIISITIFVQFTITATILGATLINILLFATNFASIVASCFYVLAVVVEIFPLCYY
AQCLMDESNRLSDVIFHSNWVDQDVRYRKMLIFFIHRSQRTIEFTAGKLFPITLNSFLSIAKFSFSLYTF

IKEMGIKEKFTNK

>AID61227.1 odorant receptor, partial [Calliphora stygia]
KEEVSKRQALLASVIFYSGVINMNYVLLSEILYVIMALVKGENFLEATMTMSYIGFVLVGNFKMFFVYRR
KDDLTKFVHGLQRIFPDTPELQVEYNMPHYLKQCSRITMSFSWLYMILIWTYNLFSIIQYVVYELWLNIR
QVGQTLPYFMYISWNWQNHWSYYLLYAIQDFAGYTSAAGQIAGDLLLTACATQLIMHYDFISYKLASYQV
QRSLEGVDKELAYCQDMQFLKNIIQYHTNLLHLTDQLNDVFGKPLLLNFAASSFVICFVGFQMTIGATPE
TILKLLLFLFSSITQVYLICHYGQHLIDSSTNISNAVYNQNWTAADVRYKKMLILIAKRAQKPAILKATS
FVLISRGTMTEIMQISYKFFALLR

>AID61226.1 odorant receptor, partial [Calliphora stygia]
IMYCAYMSSFHLERHLPDILYDLGILLELILEVFLYFVAIHIVILYMCTFYLNYHKGDLEILVNCLMQII
IYLWSIMVKFYFRRLQPKKLKDLMDFINLKSKTHSAIGFTYVTMDDSWLMSNNWTKRYVCCCFLGTTFWL
ILPIAYGDRSLPLACWYPFDYKQPVIYETMYFLQSVGQIQTAATFSASSGFYMTLCIVVSGQYDVLFCSL
KNILATVAINRRHKIELRELYELQELAAPEINEFYCSKELTCNIDTLFRIIKNRKPHDFHYHFRNALKKC
VDHQRYIVKCLNKIEDFYNPIWFFKTGQVVTLMCLVAFAAVKTTNANSSFMKIISLGQYLMLVAWELLII
CYFGEVIFINSQRCGEAILRSP

>AID61225.1 odorant receptor [Calliphora stygia]
MSERQDLFKYIRRTMYCADMHPIHMERQLPHFSRNLGIWLEFSFEIFLYFVTTHIAILYICTMYLNYQQG
DLELLVNSLMQTIIYLWTIVMKLYFRRWQPKSLKDLIDFINFKYKTRSAIGFTYVTMDDTLAMSNKWIKW
YLYSCYLCAIFWLILPITYGDRSLPLTCWYPFDYKQPVIYEILYFLQSVGQIQVAASFSASSGFHMVLSI
LISGQYDILFCSLKNILATVAINRKNMDLSMLYELQESEAEEINEYYCSKEIICDVNTLIRHKTTYEERH
DFRYHFRNAFKNCADHHRYIVECLKKMDDFYSPIWFFKTGEVIFLMCLVAFVSVKSTTANSSFMKILSLG
QYLLLVAVEMLVICYFGEIIYMNSQRCGDALLRSPWYIHITEMQRDFMIFLQNTKKPVQLSAGKMYTLNI
ELFKSIMTTAFSFLTFLQNMDESNE

>AID61224.1 odorant receptor [Calliphora stygia]
MIDLFGRQRQCLRIMGHHFVRDKSLLLRKWRNIVYFGVLMLVMSAQWPMINYAIYYIDNLELATASLSIC
FTNVLTVIKISTFLSYKWRFVALMTKLETMYQESKDPAAKKILKTANRSALMMVKLYWMSVCSTGMYFMM
SPVLKILWSQIHKTEMVLDLPMPMRFAFDIESFPGYEIAYIYTGLVTLAVVMYAVATDGLFVSFAINLVS
HLKILQKSIEENTFLKSDEELHGDLKSYIEYHNLILSLYNELREIYSPIVFGQFLMTSLQVCVIVYQMVT

HMDTILVLIINITFLTSILLQLFIYCYGGEILKLESLMVGISVQISNWYNLKPSHRRMLVLLMLRSQREA
IIKAGFYEASLANFMAILKAALSYITLIQSIE

>AID61223.1 odorant receptor [Calliphora stygia]
MFNILYRPRLPNGKHVPLNWSLKLYRWTNIICWPLEDNAPYWCHLFDRFLWFLGFVTFVVHNDAELRYLS
VYFNNLDEMLTGVPTYLVLIELHLRAFSLGWRKDDFKRLLKKFYAEIYIEESMNSQLFKKIQRQIWPILT
FSLLYFLALNSYIVTGVYVLSTNKRELLYKMIPPVDYKNNFYIFFPLLMSNIWVGFIVTTMMFGEGNTLG
ILIFNLNGRYIMMREAFNNKVETLLRSNLNSNIVEKYERVLTETLKENIRLNKFAREIQDEFSFRIFVMF
SFSAISLCALGFKVYTSPVNSIGYAFWAIGKIQEILAFGQLGSTIISTTDQLSSMYYESKWETIIERSSH
SPDNVNLMKLVTLSIVTNRNPFHFTGLNFFNVSLVSVVAILQGAGSYFTFLISLR

>AID61222.1 odorant receptor [Calliphora stygia]
MAETYQLYDFMHYPNMAFKMSAIQPFTWSGALTKEQRHSHHHQHQHPQCCTAKWFLTKVWFIFGATNLIY
QTFGMFIYLLAPQSAHMYTAANMEPEYQIALVAQISETGGIMGLTLVAACKMFIMFWHGKRIAKLLEELQ
EIFPREHVQHEPNALYRVQYFAKTSGNLMKRTTIFFIFAFCFYNSLPILEIMYEHFSADQHIMYRYQSNT
WYPWQTKENSKTWWNFTFAYLCQLQSSLTGVSFIMAGEFMLCFFITQMQMHFDYLGNALMSLNAASAQAN
EELKILIVYHIKLLRFSKEINIIFNISFLVNFITSSVAICLMAFSMVMISMSHTFKYSVGLLSFLVFNFF
ICYNGSEFTTSSDELMPSAFYNNWYDGDICYRRMILFFMMRSCESNVLRAYKFATVSMPTFMAILKLSYQ
LFTFVRAVG

>AID61221.1 odorant receptor, partial [Calliphora stygia]
VLHSPIQLKVMAKTCLDRFTKIIKTTRTMASLCGADVLHPNYKLNPLTWFVIFAIIFFFGCTIYTIYVGM
VIERDWKVILQALCLSGSAVQGCTKLITYIYRRFVLFAMNQNLLNIYKEYQNDGDYLKILHYRTDLITKI
LKIVMWFYLATIGGIVVYPLIYGLLYGEKIFVMLFLLFGIDPSTYGGYALHIIVQVIVVGLGAFGNFAGD
MYIFILFLNIPMLKDILKVKCEKLNRVALKTRDPKQTMPLLKDILEWHQDYNKFVQQVEEVYYIVIFVQI
FTSVVSICCTIFSIVIHSWPAAGMYLTYSAVLLYSYCGLGHLVEISNDEVIDIIYCDCLWYELSVPEQKL
ILLMLRKAQSPTTLTVGQIMPLSMSTALQLTKAIYSYMMVLLNFLETDNM

>AID61219.1 odorant receptor, partial [Calliphora stygia]
KEYDIICAKFKSFYTMSQMLKDILKSQEIVNDKILSTFYKISFMTGVNVKYKTGFKDPVKLVNALLVSVS
LVGLCAQYCLVWHNRKESFVESADAICTANQAWISVFKLIYFIFVQHEFYDLLHTATDGSLLYELGIFDL
AINCKQKLLQEIKDILEDSWLHIKHQLNFFTFSCMMACGFYMFSCIFANYYYTHIQPQNFTLQLPMPALF

PMWHDYGMTLPYYPIQYIIAGIENYICGMCAVCFDGIFIIIVVHCSALFEVLHKLLEYATDEDIPQSERV
KYLICCVRLHEQIYRYYLKINSMYRNPSLAQCLLSMLVLCVVMFMANVGLEEDITLFFKMLCFLGAAGFQ
IVIYCYNGQKIITQSEKTPSLWYISSWYNESKQFKYIINMMILRTNRTLYLQVSGFTTMSHMTLLSIVQT
SGSYFLLLKNLSGMD

>AID61218.1 odorant receptor [Calliphora stygia]
MSTANTEILSKPSSINCREFFIINWKSWKLLGIIMPQRDARNRLLHIFWNIFINITVTCMFPIHLMLGIF
LVKATKSELFENVSIFITSAGAALKLIIFASNVKRIIKMEQILQTLDERIQHIDDQLYYTQHIKRHLIYV
QRMYIVVYLAVGFFASLAFIASGEQKLFYPGWMPFNFHQSWWHYSAALGYQLHGIFFQIMHNFANDSFSP
KALCALSGHIQLLYKRVARIGYDPSLTSQENERELNRCVTHQKDLYELFDAIQEIISWPIFCQLFVSLAN
LCVAMVAVLFFVTDIFYRIYYVMYFFGMIMQLFPVCYYGSDFVILFEKLHYAVFSCNWTGQSKRFKRHMI
LFTERTLKMSMAMAGGMFPIHLTTFFATCKGAYSMFAVVITMK

>AID61217.1 odorant receptor [Calliphora stygia]
MTTTKVNVVQQHAEPNSCAFFKPHWLCWKILGITLHIDNSHRHRDIYMLYSIILNILVTICYPLHLALQL
FRSDSMADNIKNLAVCVTCVACSTKFIIYSTKLSTIWQFEQILERLDARITDDVETNYFRRMRNRLRNVG
IVFLSVYLPVGITAELSFMFREERSLLYPAWFPFNWIESTGWFYVANVYQIVGIFFLLLQNYADDTFPPM
ALCMLSGHIKILSIRVASIGYDQNSLHQNEEELNRCVEDQLNLYELYTTIENIISWPMFIQFCVTATNIC
VAMAALLFYVSAPLDILYYFVYFLAMPLQIFPACYYGSDFQCLFDQLHLAIFASNWTTQMLKYKKHMLLF
TERSLKQNVALAGGMVRIHLDTFFTTCQGAYSLFAIIMRMK

>AID61216.1 odorant receptor, partial [Calliphora stygia]
GAIFTASFAVSCCLIFIKISRGFASLADGATSCATAFLYLSTSIATANAFFQRTRVVRMVTFLHEDITEL
LRITDVKEELMLAETVKYLRIVTILMWTPSLTAGFIAYIDCFYRSAFMPETVFNIPQVLNGTAQPILLFQ
LFPFGEVYDNFIVGYLGACYALFLGITTIPCWHTFVTCLMKYIVLKYGIVHKRLKEYDFAKFSLELNPEK
VRNLSERDLLYWHTKMCEFCVTHQLKLRWFTGELQALIRIPVFSDFIIFSVLICFLFYAIAAGNPSNMDY
FFIAIYLFVMSFILWLYHWHATLIAESNDELCFGLYSSPWHRFPLSIQKNIRLMMMESNTPLIMKAIFVE
LNLKTFIDVVRGAYSYFSILRSANMETDDNSI

>AID61215.1 odorant receptor [Calliphora stygia]
MFEDIQLIYMNVKILKFWSLLYDNNWRRYVCLIPTTFLVFTQFYYMFMTHEGIDAIIRNSYMLVLWFNTI
LRAYILIYDSEKYQNLIKDLEKHFYDLMNSNDFYIQSLLDEVNSMGKVMSKGNLFLGLLTCIGFALYPLF

AEEKVLPFGSMIPGVNEYKSPFYEVWYAFQMIITPMGCCMYIPYTTLIVSFIMFGIVMCKTLQHRLRTLK
LFNQNPLLIYQQVINCIKFQKEIISYIEIVNRLTTFIFMLEFIAFGTLLCALLFLLIIVDSSAQALIVCA
YITMIFAQILALYWYANELMEQNLQIAAAAYDTEWFTFEIPVQKNIQLLILRAQKPCSIKVGNMYLMTLE
LFQALLNASYSYFTLLKRVYG

>AID61214.1 odorant receptor, partial [Calliphora stygia]
LKSVNKMSPKSANPLVAQKDFQDFCNLPNVLLSRIGYDFQNKPRPIWLVVLGKCYFLFASISHLYIFMYI
AKATYSMIISADFELSLLLRLISGFNYAVFSTAKFITFHWHRKELKFIYETLKEIFPKTRKEKLIYRVRD
NFWPKWILFIVYFYLGAVAFIATSPLMEGIVLYLGNVSKVGWRRAEFGYFKLYEIEYSFDHRSAFSYLIT
YSMELMHAHIMVTCNICADVWLLCLALQLCMHFDYVARTLEAYEPDEKEFVKDQEFIAELIKRHQILLNI
GDGLHSVFGVLLLLVLMATAATLCCAGIYAITQDLGREFIEYAAFLPITIGQYYLICFYGQQLIIRSNSV
ADAAYNHSWYNGSKSYKKSIFIIIMRSQKEVELNALGLQPICLEAFKMLMGATYRVFALMKETML

>AID61213.1 odorant receptor, partial [Calliphora stygia]
LKPFKLNNMTKVAIQKFTNYQQDEEDEEYGIKNASVDTILAAYKDLRKVLFQRQAPDKISLVYMRNYMRL
LGILPRTWRGETLSYRIINKFIMIMLASFAVSITFDLYDAAQDVLQFGEDLVVLIGIYLIFLKLVLCAYH
AQDIEQIIREFSKMHQYFGQLKHSSNIGKIRKLQRLFYMAELISFFLYISLGILFTAAICLPPILTPNGT
PYRAKYPFEWQTYSDHPLRFTSIYLFQCIMTQFVLLAIVVIDNMGCHIFTQTTLNLQIFCMRIREMGSQP
LGDRDLLEELHKAIQFHQYIIDLIAKINDVYYYNYAAQMAASTFMICLTAFEAMLAQDQPMLAIKFQIYM
FSAFAQLFYWCCTGNMVYYNSLDVADAAYEIDRWYDQSKEFKYCLRFLIQRAQSPLVFRPKPMFCFNFES
FSSILSTSYSYFALLRTMND

>AID61212.1 odorant receptor, partial [Calliphora stygia]
FEFGTLFTTMKTRDHHKALETYYKYQSWIFRLLALWKLPDTVTPRFRIAHRIYYYYILFFWVLSFDTCCF
IQIITNITDLNEVIKVFFIFATALAVLSKFSTIKLKNHLYYELVETIHRPMFRPTNKNEMKLFKQSQNLS
RTVRNSYCSISLCALHVVLMTQYFVDNTELPLSIYNPVNMDQKWRYLLMYLYQYLAVSVCCYMNIAFDSI
SASFLIHIKGQLDILCDRLEHLGFEENNLVDDNKITYKLKNCIKFYEEIVRITRIVENLVSFPITIQIAC
SVLVLVANFYAMSFLSDPGDYANFIKFLIYQLCMLSQIYILCYFSSEVTLKSQEISYYLYCSQWFEWNKL
NRKLTLLMMTRFDLPIRIRSLNPTFTFDLEAFTSIVNSSYSYFALLKRINS

>AID61211.1 odorant receptor [Calliphora stygia]
MYYDLALFHCNVKIWKYIGFIEFKNKYRTTPIIAIVFLTIFCQITNFLFIWHDLSALVMNSFMTAILANS

LVRILIVMKNQNAFIEFMRGIESWYKEAEITNDIVAWSILKEVPKRTVAISKFSLIFGGSGGVLTAFVPM
LMGQRTHPYSVYIFGVDALKSPLYEIIYFIQMFIIIPFIITAYIPFTNLFISWLIFGINILKILRKKFEQ
MPVVNDREQLKCLKALIKYHKRIIRFGQTLEGLVSFVCLVEFVLFTLMLCVLLVCILLVDTTMLRVTTVI
YILCILYALFLSYWHANEFSSESVLIADAVYSIDWINSSVEVRKCVLILLVRCQTSLKISAGGMYPMTLE
AFQALLNAAYTYFNMLRGFMAK

>AID61210.1 odorant receptor [Calliphora stygia]
MLEDNPMLAINVKLWKYFAVIFPGHGNHWRVYAFVLPICVMNAMQFVYLFRMWGDLAPFILNTFFAAAIF
DALLRTCLVIINRDKFEAFILELLSMYKEIELSNDAYGNAILKDTISSARKVSIFNLTASFFDIVGALIY
PLLCDGRVHPFGVALPGVDMTASPVYEIFYIIQFPTPLILTAMYMPFVSLFASFAMFGRTALMILQHKLQ
GICEDGRTEEQQYMALKASIKYYNRLVSYIKDFNSLVTYIVFVEFMLFGAIICSLLFCMNIIETYTQIIS
IIMYILTMMYVLFTYYWHANEMLIESIKVSEAAYSIPWYCCSKRFRKTLLLFIIRTQQPLQIMVGNIYPM
TLATFQSLLNTSYTYFTMLRGLYSQ

>AID61209.1 odorant receptor [Calliphora stygia]
MKLIESLLQSLKKLSKKNIFEMATLKLDGPQPKTKDATVYLFRGLMIVGYLPPKTNLVPFYIWTIFINAF
ITIYMPIGFLLSFLTRSSSFTPSEFLTSLQISVNCFGCSLKMFVFLFLYRRLLSTIKYMDQLDLRVSEPE
DKLQIRKIVAFCNRSNVMFTGLFLSYASSTFLTSVINGKPPYQVFNPFMEWSDRTLFFCIQAAVEYFMID
IHCYQQALLDSYPVIYITIIRTHLHILTRRIRKLGQDSKLSSDERYEALVQCVLDHKNIMAIYNYFSPVI
SGTMFVQFLIIGLILGITTIHIVLFADILAIFASMLYVASILAETFPCSFLANSLMDDSDNISLAIFHSD
WPSEEPRFKQMIAFFLHHTQKTLILTAMKIFPITMNSNINVVKFAFSVYTLMKQMDFGQNLKDSVTGEKL
P

>AID61208.1 odorant receptor, partial [Calliphora stygia]
MSLKTIEDVPLFNTSLRIMKFWSFLLQHNWRRYSCLIPYIMINTTQFLDIYFSAEPIDAVVRNAYIAVLF
FNTILRAVLLCINRFEYEKFMENIRLLYIELMESEDKSVRKLLNETTQASRFISKVNLFMGTCSCIGFIT
YPIFATSRVLPFGMYVPGIEKYESPFYQIFFICQVIITPMGCCMYIPFTNLVVAFILFAILMCKVLQHKL
SNLKDVSNEKAREVIVWCIKYQLELIRYVDTMNNLTTHTFLVEFLAYGAMLCAMLFLLIIVETLAQMIIV
SIYIFMILSQSVIMYYFANELYDQSLL

>AID61207.1 odorant receptor [Calliphora stygia]
MDIKSIEEVPMFISSLRIMKFWAFLLEHNWRRYASLIPYTLLNITQFMEIYFSTEPVDAIIRNAYIAVLF

FNSTLRGVVLCLNRFGFEEFMETIRILYIELRASDEKIIRKMLHETTKTSILVSKVNLVMGACSVMGFLM
YPIFATTKALPYGIYVPGIDKYQSPYYEIFFLIQIVMAPMGCCMFIPFTNLIVAFILFGILMCKVLKHKL
SNLKEVSNEKARAVIVWCVKYQLELIKFVNTMNSLTTHTYMIEFLAFGAMLCAMLFSLVIAETPAQMIII
SIYMFMIFSQSVVLYYFANELYDQSLLVAIAAYECNWFDFDVETQKILKLMILRAQKPCAIMVGKVYPMN
LELLQSLLNATYSYFTLLKRVYG

>AID61206.1 odorant receptor [Calliphora stygia]
MFNPKPKEDPKYRIPHQCIWLKMNGSWPFDYDAERDFYNSHTLYGMLYSIWSWYIICSVGVTIGFQTAFL
VSNLGDIMMTTENCCTTFMGALNFVRLLHMRLNQRSFREIIQQFVDDIWIPKKYNPYIAAECSKKMRTFR
IMTVLLSCLIAMYCVLPLVILFFDVGLDAEEKPFPYKMLFPYNAHRGWRYVVTYIFTSYAGICVVTTLFA
EDSIFGFFITYTCGKFKILHERIDNIVIDSYQLARNRQNENEIQWLYIRLLNKIAYDHNKLIEFSNRLEM
FFNPILLMNFTISSILICMVGFQLVTGKDMFIGDYVKFIVYISSCISQLYILCWNGDSLIQHKLLNIYMI
ATGKVKIYI

>AID61205.1 odorant receptor, partial [Calliphora stygia]
KYILKLCAKPTRKMPLQFLSSNYPLHDYYFYVPNFCLRVMGFWPQAPNTQTKRLWASSNFLMLLIGVLTE
MHAGLTALTYNLEKGLDTLCPAGTSAVTLLKMILISYYRQDLQYVLKKMQTMLYGESTNRQILQQHKKII
RQFSVLAARFNFAPFLTGFITNTAYILKPLIMAWIFWSKGKEIQWTTPFNMTMPSILLRAPLFPLAYIFT
AYTGFLTIFMFAGCDAFYFEFCSHIAALLKMLQADIVSLFAVFENKLILTEEENKYVENRLKIIISRHNE
IIDLTHFFRKRYAVITLAHFVSAALVIGASIFDLMTYTGFGRILYVAYTMAALCQLMVYCYGGSMVAENS
VQISTVIFGCNWFICNPQVRRMILLIMIRSQRTLTMSVPFFSPSLATFASILQTSGSIIALASSFQ

>AID61204.1 odorant receptor, partial [Calliphora stygia]
LLFNANIMFPKFWRDNNNSQATREATDYLFQLYLYLGFRHPLQRHQLHKVYSVLIQLSVCVFLPISFALS
YSYEYSKMTIVQLLTSLQAYINILGIPAKILTITVSMKHLRQALLVTDILDARCQCPKEHEKIRHCAMTG
NRLAIFYCSLYCFYVTATMIASLCNGKPAYALFLPVINWHNSLMELLVHSCIEYMLLNLAVLYQAGIDGY
PVIYIYVIRTHMQILVERVRSLGTNVSSSNDKHYEQLVNCVKDHQALLSLVEIIIPVISITMFIQFTITA
IILGTTLINMLVFADLSGQIGSAVYLLAVLVQTSPCCYQATCLLEDSDQLSLAIFHCNWFNKDRRFRKLL
VYFMMRSQMPITLTALKLFPIHVNTSVGIAKFSFSLYMLIKEMDFGNNLKP

>AID61203.1 odorant receptor [Calliphora stygia]
MFDRFWGRGPNVKATREATAYLYKNFLYLGFQQPQKWKPLYYVYSVLLNFCVCVLYPISFSLSYYYGYRQ

MTPVQLLTSLQVALNVCSLPAKFITIVLNLKSLDRAMDVMDILDARCKRHDELEKIRHCAMLGNRLTVFF
IGFYMFYTALTMVSSVAFGQPPYSVFIPYMDWRNSTLEFIGQSFLEYLLMNFICMHEGGDDVYAVIYIYT
IRTHMQILVDRVKRLGRDDSISKEEHYKQLVLCVKDHQDLLRLLDTISPVISITIFIQFMVTAIILGTTL
INIMIFADFSAQIASVSYFLAVLVQTSPCCYQATCLMEDNEQLSLAIFHCEWFDKDIRFRKMMIFFMMRS
QTPMTLTAMKLFPITLNTSLGIAKFSFSLYTLIKEMDFGQNLKT

>AID61202.1 odorant receptor [Calliphora stygia]
MVLTSNNPDTNLAFYYHWKVWHWVGIKAPQESNLQLYRVYAVLINSLVTFLFPLTLIVNVFFAQNTQQLC
ENLTITITDTIANLKFVNVYLVRGELERIKAILGKLDKRAKNTEEQKILKSAIRISQLSFLIFVRLYTVG
TCLSILKVIFAAERCLLYPAWFGLNWFDNTFIYILVMTYQLFGLIVQALQNCANDSYPPAYLIILTAQMK
ALEVRVRAIGRAENGEERMCLTKEEYLRNLNEFNECIKDYKNILKLFTIIENIISKACLAQFVCSALVQC
TVGLHFLYVVDAADYGAQILSIIFFVAVTLEVFIICYFGHCMSTQSWNLTYAFYSCGWLAQTPCFKKNLL
ITLMRTQRHSIIYAGSYIPVDLPTFVQLMKYAYSTFTLLIRFK

>AID61201.1 odorant receptor, partial [Calliphora stygia]
MQSNLQPTKYVGLVADLMPNIKLMKYSGLFMHAFTGGSPLLKKVYSSIHLVLILAQFIFILVNMALNADE
VNELSGNTITALFFTHCVTKFIYLAVNQKNFYRTLNIWNQVNSHPLFAESDARYHSIALAKMRKLFFLVM
LTTVASAVAWITITFFGESVKFAFDKETNSSITVEIPRLPIKSFYPWDASQGMFYTISFALQGYYLLFSM
VHSNLCDVLFCSWLIFACEQLQHLKGIMKPLMELSASLDTYRPNSAALFRSLSANSKSELIINEEKEPPS
DLDMTGIYSTKADWGAQFRAPTTLQTFNGVNGGNPNGLTKKQEMMVRSAIKYWVERHKHVVRLVAAIGDT
YGAALLLHMLTSTIKLTLLAYQATKITGVNVYAFTVIGYLGYALAQVFHFCIFGNRLIEESSSVMEAAYS
CHWYDGSEEAKTFVQIVCQQCQKAMSISGAKFFTVSLDLFASVLGAVVTYFMVLVQLK

>EDS40324.1 odorant receptor [Culex quinquefasciatus]
MPQTALEQQLLNCPIISVNVRVWHFWSFLVKHDAMRYISIIPVGAMTLLMFADLYRAWGNIEEVIINAYF
AVLYFNAVLRCSILVVNHDKYEKFLMDIARIYKDLQQIEDHEIKSLLQRYTKRARMLSIANLGLGAFIST
CFVVYPLFAGGRELPYGMYIPGVNLFGTPQYEILFFLQVILTFPGCCMYIPFTSFFASCTLFGLVQVKNI
QHQLRKLRIEGLKPSKLIEIIKDHQRVIDYVHDLNSLVTYICLVEFLSFGMMLCALLFLLNIIQHQAQFV
IVAAYIFMIISQIYAFYWHGNELREQSMAIAKVAYGAAPWLEMKPALKKMVLLIILRAQRPLEVTVGNIY
PMTLEVFTSLLNASYSYFTLLRRVYVK

>EDS40323.1 odorant receptor [Culex quinquefasciatus]

MNARTHFSGQSKAKHIRWVQTKVNLYIHAPLFTAANGGSSSGENQQFMSKQPSGMTAAPILDCPIVCVNV
RVWHFWSFVLKHDAMRYVSIIPVGVMNVFMFADLYRAWGNIDENYEEITSIEIVMDYKLLSQMIDDYVVQ
KLANLVLRAVISICYVVYPLFTGTRSLPYGMFIPGVNNFKAPLYQVFFIGQAVLTFPGCCMYIPFTSFFA
TTTLFGLVQIQTLQRQLRTFKDEVVKENRTLVESKLEKCIEDHKRISRYVSDVNSLVTYICLIEFMSFGL
MLCALLFLLNIIFTFYWHANELREESMGIAEAAYDAPWVELDDSMKKKLLLFIARAQQPLEAIGNVYAMT
LAMFQSRLNASYSYFTLL

>EDS40279.1 odorant receptor [Culex quinquefasciatus]
MANRVANSEGQHPKSEPTPPLRFESFTKVIYVIEKISGIVPYDELQQPKHFCSSVFTWLYFWATYIHLVL
SILMQFAFFKKVPMTYLMVMVTCMGFLLVSIVKMSVYKLYENQLNEILDQLKSLYPDHVDAKSAEECRKR
LWFLKLFSVGYFVVVVVFQAILYVTSIWKYFSTGYWEKLLPYNLWYWYDWRQPVVFELTFLHQLWGSTTS
VSCVILLDSLYCIIILLISMQFELLGKRLLANEGNAQELNSCIEQHLQLVELCERFERIYSPSLLVTFLG
SSAIICFSLFVTLIVDDAAESIRFSILLSVYVMNIYLLCYYGSMLMEKSSNVAQHVFNGHWYKMSKKARK
MCLMILIRESF

>EDS40277.1 odorant receptor [Culex quinquefasciatus]
MNDLVRFESFIRVPEIFYGMVGITRYGEPRNTTKARLKQLFFWSSYANTIFCLIIEHIYFFRAAGNFTNF
LELTALAPCIGFTALSIVKIMTIKLNEAKLNGILDRLKELFPVTHLEQTRYRTHQYNLESQMVMKSFSIL
YMILIWIFNLLPLVSMLVNYLMSGVLVRELPYFMWYWYDWHREGLYEITFFHQNWGAFDSAVFNLCTDLM
FCAVILLMCLQFDIIAVRLRAAKDDPQELISCVQLHQTVLELGDQLESIFSPSILVNFLGSSVIICLVGF
QATSQISAFDLFKFVLFLISSLVQVFLLCYYGNKLIEASSQIPYAAFQGEWYLADVRYRKSLLFLMARAG
KWQKLTAMKFSVVSLASFTGFPPRWTTMARIKDTSSQHLPPGSIFERAQS

>EDS30207.1 odorant receptor [Culex quinquefasciatus]
MDPIQEFEQICGWQCRVLKQFGICAYEQSFKPSARTVLLLLLISCYFFIAFYDLHHFLGDLFSFAFALVT
LAYGLIGISRVGHLLANPARFSELMHEAKKTYERSALNQREAKILKRYTSWLKHCVIFYSMAFVAATVAT
GLLPTVIYLWTGQRNLPLGIELPFLDPDSLKGYLLNYLYQISCMLWTPPALIAVQNMCFILVFNVFIQYD
ILLLGLEDLNLLIKENTDGKLDSDVHQKLLQVLQYHQRLTSFSGAIERSFSVQFFVEISSNALQVIVTLF
VANTANWMPGYLIIFLATFQSLMLCFLGALNELKSDQLVAKIYDVAWSEMRLPEQKSIHILLTKSQQASL
LSCGGLLPMNMNLFLKVGFFSEAIKVSVPTRVEIYPK

>EDS29141.1 odorant receptor [Culex quinquefasciatus]

MANRRKLAAAAEFDRILAVQSWILRRLGCDVFDLNYRFSPATWVIVFLASFYMVISAYDLYRFRNDVFNF

AFSLVTLSYGVIGCTRIVLFLRNSRTYAQIVVEARRTYEQVSNEREQEVQERYTRMLKRCVTFYSVSFIG

GCIMGGFFPLAVYWWTGLKVLPFGVILPFTDPDTIEGYQLNYLYQVSCIVWTPPGLTATQNVYFALVFNL

CIQYDVLKLKLEDLDKLIRDGAEYDTIHEKLVEIINWQRHLVDFIAEIDRNFTVQTFVEISSVAMQMVIV

LFVLHIDVWLPGYMVIFVASFQLFVLCILGAMIEFKSDIFTEQIYDIAWHRMRTPEQKMVQFMLAKAQYT

MQLTYGGMLPLNMNLFVTIYKKTYSVFMMLQNM

>EDS29140.1 odorant receptor [Culex quinquefasciatus]

MTYRRPLQVFERITSANRWVLKLLGIDVFNPNFRYSTATWVIWSLASFFILVTGYDLYRFRNDVFNFAFA

LVTLGYAVIGVSRLGFFLGSPAAYSQIFAESKETYRQESSERSREVQQKYTIMLKQCVMLYSGCFLGGCI

IAGMLPFAVYWWTEQKVLPFGVILPFTDPDTMEGYQLNYLYQVSCIAWTPPGLTATQNMYFALVFNICIQ

YDVLQLKLEDLDKLIEDTAEYSVIQQKLVEIIHCQQHLSAFVTEIERIFAVQMFIEISSMAMQIVVILFV

EHIVSAISLQKCQNTLSPFRLQDLWIPGYLAIIVATFQLLIFCALGTFISIKADLFAESVYNVSWHQIRI

PEQQSIKFMMAKSQQSLLLTFGGMLPLDMNLFLSVG

>EDS27308.1 odorant receptor [Culex quinquefasciatus]

MEKNIRLETQKEFEKITGWQLWVLRLMGCGAYEPNFRISFATLVILFLAGAFVVITIFDLFLFQNDVFNF

TFALVTLSYAVIGCTRLGFIIFHPNPYSAAFQEAKQTYLLASSDERDQQVLQHYTKLLKQCISMYSIAFL

GGSVGTAFLPLIVYLWNGEKILPFGVVIPFTDLGTSLGYQLNYMYQVSCILWTPPGLTASQNIYITLVFN

ICIQYDLIFIKLDELDQLAVRNIDGTLDKEVEQKFIEIIKYQQRLNQFVAEVENRFTVQIFVEISCNALQ

IVVTLFVMEIDTWIPGYLVITVATFQSFLYCILGTLIDMKTELFTEKIYNVAWHRMRTSEQNNIKYMLSK

SQQSVLLTYGGMLPMNMNLFLAVYKKIYSIFMMLKNV

>sp|Q7QCC7.3|ORCO_ANOGA RecName: Full=Odorant receptor coreceptor; AltName: Full=AgOr7; AltName: Full=Gustatory and odorant receptor 7

MQVQPTKYVGLVADLMPNIRLMQASGHFLFRYVTGPILIRKVYSWWTLAMVLIQFFAILGNLATNADDVN

ELTANTITTLFFTHSVTKFIYFAVNSENFYRTLAIWNQTNTHPLFAESDARYHSIALAKMRKLLVLVMAT

TVLSVVAWVTITFFGESVKTVLDKATNETYTVDIPRLPIKSWYPWNAMSGPAYIFSFIYQIYFLLFSMVQ

SNLADVMFCSWLLLACEQLQHLKGIMRSLMELSASLDTYRPNSSQLFRAISAGSKSELIINEEKDPDVKD

FDLSGIYSSKADWGAQFRAPSTLQTFDENGRNGNPNGLTRKQEMMVRSAIKYWVERHKHVVRLVSAIGDT

YGPALLLHMLTSTIKLTLLAYQATKIDGVNVYGLTVIGYLCYALAQVFLFCIFGNRLIEESSSVMEAAYS

CHWYDGSEEAKTFVQIVCQQCQKAMTISGAKFFTVSLDLFASVLGAVVTYFMVLVQLK

>ETN58372.1 putative olfactory receptor [Anopheles darlingi]
MAESSEESTEVRFKSFIRVPEGFFDVIGVARYAPTKDRKRLRFVLFWSSFGNTAVCVVLEMIYFVLAARS
GLANFLQLTALASCTGFSALSVAKIMTIKLHETKLKEMLRELESLFPGTAMLQDHYGVHRYYREGQLVMK
SFSVLYMILIWIFNLMPLVSMMIGYRTERVWHKELPYFMWYWYDWHRAGYFEITFVQQNWGGFVSAVYNL
STDLMYCAFILLFCIQFDIVAQRLRHARPDDRAGLIETVRIHQKVIELCNQLERIFSPSLLVNFMLSSVI
ICLVGFQATAGVQPIDLFKFILFLISSLVQVFLLCYYGNKLIVAVGRASQEPLEADIYIFFLVFVSLFTH
RHQSDQISYSAFEGHWINASGSYQKSLLLVMVRSLKPQKLTALKFSVISLASFSKVSRPSGGV

>ACO83222.1 putative odorant receptor [Stomoxys calcitrans]
MSQMLKALLLEKQLENNKMLNIFYRISFMTGVNVKYQATFKDPVKLWNGFLIIVSLIGLSAQYCLVWNNR
SEPFAESADAICTANQAWISILKLIYLLFVQHEFYDLLHTAINGSLLHDLGIFDLAINSKQYLLKEIKTI
LNDSWLHIKHQLNFFTFSCMMACGFYMFSCIFANYYFTHIQPQNFTLQLPMPALFPMWHDYGMTWPYYPI
QYFITGVENSICGMCAVCFDGIFIVIVVHCSALLEILHKLLEHVCDEEIPQSERVKYLLCCARLHERTFR
YYEKINGMYRTPSLAQCVLSMLVLCVVMFMANVGLEEDITLFVKMLCFLCAVGLQIGIYCYNGQKIITQS
EISPVAWYSSTWYNESEQFKYIVNMMVLRTNRTLYMQVSGFTTMSLMTLASIVQTSGSYFLLLKNLSGMD

>NP_001298159.1 odorant receptor 67d-like [Stomoxys calcitrans]
MARPTIIKSPSQRFKKFLDVIKLFAKTCGANIFAEDYRINALTCLITILVNCFMLFNFYTIYVSVAKDNY
HIVLQNLCVVGTAIQGFSKLINAIVYQDLTRFTCSEIEFMYRTFETEEQHYVDVLNTSLKLLKRIIFTIL
KIYGILTIAILISPMIIQMITNERLFILNVFIPGVDVDTTVGFIIIQTFNAACTTFSGFGNFAADTACFM
LGAHTPLMKDIIKCKLIDLDEVLRKHPKDRSRTEPLVKDIIQWHQRYIIFTEKNTSNFFWMIFIQVASSV
MGIISNMVCMFLGGWPVAPLYLLSSFVILFCYCSLGNLVELSNEDMCDNIYECKWYELTVPEQKMILIML
RESQKPNNLSVGGVASLSMNTGLQLTKSIYSVAMLLNNSLN

>DAA80450.1 TPA_exp: odorant receptor 125 [Aedes aegypti]
MLNVLLLCAGIRTQLAVDWTWERPVRYLVHLISAYHSMVMILQAAHVVAEQNDVMDTAFCLIKIVGMGSA
YIKILLLTYHANSVDEVEHFIRSKPMSSGEDQYDSTVRGKFLRSTLVMIRWVLGVLIVDEILFAFPNSQR
NKLFKLPPAMSSLGSGITGWLANFLFVNWMPLIWLSKYLCCTTKLGVLLMGLRVEFKILTHKLEQITRQA
KSIQSVEDHCKFLKKELEVFLYKQAELRRNIELIRPLLGMAFFMIYYYALFFIGTMLYVTHHQGFEFYSL
TFASSVVVTLLECYWWCQLVDSIQDDAESMGNELYDICAGIPYARQSHRTYVEMRTSLMIIWINARHSLA
IDCVGIFSISTAIFVQMLNTSYSVLMFLINMG

>DAA80446.1 TPA_exp: odorant receptor 121 [Aedes aegypti]

MFGKLRLSWCRMNLWFSSRVVKIVSCCFKGQPAGDCFWLLDVLLLLAGIRSESTLNWANERKIRSIINCL
YAYHSFVLIFQLYTSLSTKFDDTSLVVFDMVKVAVTLLSGMRILSIALLREPIASLRNFVTSNRLNSGDA
VFDELERRRFNKFSRAALLVIYGATVLDTILLSVPNSSKDSVLELPPQLVSTGKYASNTLYFLFVGLVGL
SIIPKMFSALSCSQVLLVGMRWKFKMLVHRYESIVNLRFLDVDDYYERIECKVLEAVEQQLEFWSYLQIL
KDLVAKQFFLVHYFSVGAIGSMLYVSRDIGLNILSVAIFASTLVMMLEYFLWCHLVDSLEDVADSVGSRI
FELCAKIPYSPKYRSRYRKLQASLMITWIVARNGISMNCLGLFKISTIAFVGFVNTAYSVLMFLINMH

>DAA80444.1 TPA_exp: odorant receptor 117 [Aedes aegypti]

MFAKIRYAWNRGCAWWSTKINQIRQYFSDEGAAGDCFWWTDVLLLLGGVLNVHTSNWIHERKIRIIFQSL
SVYTVYVFYLRLYEALHEKKDASSLIMEITKVTSISISCLRMVAAAFLIKVLNTIRSFVLSNSISSGDVE
YDEREQNNFNRLAKRAIQVIFSWITADTIFFLIPSSTKDDLFQFPVPSFLIGEKASKILNMICVTSIPAS
ILPKTISCTTCIGVMLIGMRTKLRVLAHRFELISHQSISNEDQLYERIDRDLREFLTQHRMYLSYLKISK
DLVGKAFLLVHYFSIFAIGALLYACHEIERSFMTVVFGAAVSFFLSEYYLLCYLIDSLQDEADSIEQHIF
ELGINLPFRPERRSEYVQLRTALMIIWMNTRNGITMNCMGIFEINTPKFLSLINVAYSVLTFLIKME

>DAA80443.1 TPA_exp: odorant receptor 115 [Aedes aegypti]

MNCVRKWWSSLVAKKRYFWSNKGPGSDCFLWQDVLLLIGGIQSELTLKWNCERYIRFLVSCLFFLQSFFI
FVVLCSTLQTNRDEMFKVVVEVLKFSAFLVAGCKLLLIKLHRKSITNIRTYINDGQTTTGDNSYDRLELT
NFKQRSMTMIRFIYGLIFIDMALLSIPNDITDMAFDIRSDIQPFMSHARNIYRLLFITLLPVGFLPKFFS
SMATVGTLLLGMQANFKVLANRFHSILSQPFVINGTDWEMINHELKDTVKHHLEFWRHFKALKSLVGETF
FLVHYFSIMSIGALCYICQDIGVNFLSFVVLATLAMFLVEYYMFCHFVDSFQDIANCIGEHIFQIAILMP
HNRKNHSHYIGFRTALMIIWLNTRRGVSMDCMGLFNISTVAFLHVLNIAYTVLTFLIQMSQL

>DAA80442.1 TPA_exp: odorant receptor 114 [Aedes aegypti]

MICVRKWWSSVVAKKRYFWSDNGPGSNCFFWQDVLLLTGGIQSELTLKWSCERYIRFLVACLFSLQTFFI
FVVLYSTLQNNRAEMFRVVVEVVKFTAFVVAGCKLLLLKLHRKSITSIRSYINDARMITIDDSYDLLELS
KFKHRSMTMIRGIYGLIVIDTVLLFMPNDTTVMAFDRRSDTLPFMSLERNIYRLLFLTLLPVGFLPKFFS
SMATIGSLLLGMEANFKILANRFRSILSQPFVINGTDWEKMNSELRDTLKQHLEFWCHFKALKKLVGETF
FLVHYFSIMSIGALCYICQAIGVNFLSFVVMATLAMFLMEYYMFCHFVDSFQDVANCIGEHIFQIAILMP
HNRKNHSHYIGFRTALMIIWMNTRREVSMDCMGLFNISTAAFLHVLNIAYTVLTFLIQMS

>DAA80440.1 TPA_exp: odorant receptor 111 [Aedes aegypti]

MFTKIRSGSKRLHFWLSAKGSEVNKLLWINGSFGDCLYLLDVFLLISEIQSEKTAKWTNGRKVRIFANSL
FVFQIVVTILQMVHARDNNNNVEFQIVLSITKLVGMIAICVKWSLLIFQTRQICTVLNFITSNQLDSGDE
AYDELEYKEFNRSACTMMRIIYAMTIANAVLLLVPSAATKQALALPPPLSNYGKLVSCIVYLFSTQLLFL
GTVPKFLSNMACIGMLIMGMRFKLKILAHRYFRMLNQPVVSSEKHFARMERDVKEVLNYQTEYRKHFETL
KQFVEKAFFIAHFYALYSLGTCFYLSHKTGFNVLSLTLISLSVAYILKYYLWCHLVESLQDVANSIGDLI
YEHCVQMPYSRKHHTQYMGMKTSLIIIWMNTKNGYAISCMGMLDISTKTFVSFLNAVYSVMMFLINVV

>DAA80437.1 TPA_exp: odorant receptor 107 [Aedes aegypti]

MFEIYCSTRNRVRATWSSLQVTWHEFWSDGGPGRDCFWWLDVMMLIGGIRSGVPRLKNEDYIRYFTNVLF
FCEGIVFLLQLEHSLNTENNDLPVKMWEIVKFGTWCNTTVKLFLSLLLHERITVVRNFITSDRVNSGDHA
FDDYEYQKFNRYVKIMIGLLSSLIVVDIILLTLPVSSIEEAFASPPQLQKTGKVISGIIHCITVRFVSLL
VHPRCFSNLTSAATLLLGKRAKLRMLSHRFTKLITLSDLSLDIYFEHMSRELREALLQQTEYWRFLGVLK
GLIAEIFVLVHYFAILTIGAFFYITTGTGISFMSFAITTAAIYLLLEYYFLCRLVDSLQDEAEAMAGVIL
ELCTRMPYSREHHSKYIELRTSFMIILLNIRRGMLMNCFELFEISTLAFVELLNTAYSVLAFLISFG

>DAA80434.1 TPA_exp: odorant receptor 104 [Aedes aegypti]

MLHRLRLGWNRVRTETSHFWSDDGPAGNCFWWIDTMLLLAGIRNEWSSRWKGARLVQNMSNALFAFEVAV
LLSQVKEGLDDTWDDVANKLMGILKCSSSILASAKMLVMIHLREPLGILRNYVLDDRVNSGDKVYDEFEQ
RKLNQTARFMFRAMLGLLIAEMTLLSIPNQTMASMFRMPPWGASNYLYHFFVTTLPLGLLPRLLSHMSYV
GILIMGMRMKLGMMAHRYQRMTSASDLDLDQYFEGVNRELRETFKQQVEFLGHFNTLAEAIGKTFLIIHY
YSIFSIGTIIFMGKHMGVNVFSAAFVLMSLALLLEYYVWCYLVDSIHETAASIGDHIYEICAIMPYSREY
HRKYIQLRTSFIIIWINNCNGYSVDCFGLLQLSIIAFVNLLDVAYTVLMFLINMA

>DAA80418.1 TPA_exp: odorant receptor 85 [Aedes aegypti]

MKAIVSFWKTIRYYETDSDYFVLLDMLADLCGFYPPKWKSLAVVAWIALKVGQVVQYAFYTYHCYQSLVV
WRNMLYFSLNINLFIILSVGLFRALTLAYYHRHLITLKNFVNSRQCGKTDNKANYMRKVRFWTNNRLILG
GSAMLVLNAVNWCLTAAFTEDLYQIPFSLQFLPTTMANVIAYYYSFQMLIQNFTYWQSFFQFGSLLSLLN
NELAIISDYFESIFDRAFQVCSEEELELRNGDSLTASKMWETIDKDFRQATKYHSDFIDQVGLLKKVTYF
NFLALLSATAILVTLNSFLCVIDFSSDALGLLVFGSICCMECFFTCRLLDELDDVNEEIAMHAYAMDWMT
SITVPRGNLNSYRSIKRTALIVQAQAQQGFGFRAGGMFDMNSEMFMQIMEMCYSWITFLMQTQESQ

>DAA80415.1 TPA_exp: odorant receptor 80 [Aedes aegypti]
MKLEKFSRKWNGLLHFDNQTDYFSLVDKICLLFEQRPPITKRTAIKVIWTALSVLHGLQYFGFTIQMIRR
MNKSSRNVSELTTITNMLVMLGVSVVRGLCLAYNRNDLIKLKNFVNSKTCQRDNPIAFEQRKNTYQKINK
FLIAYYSISVTNASLYSLTTGFQEDVFKIPYLLERLPEKVALSINVCTSTMHIPWCCTVWYSATQLLSIM
YVLHTELRIIIDQFKHIYEKVAVNHVLQSSDIILLTQEQKTLFLAELNSRFSNALAYHSEFIKYLQLFQK
TSSKNFCILFIVGAIIITVNTGIFILEPTIEQLPLLIVSIQFTTEIYCCCTMFQSLENENNRITQYIYAL
DWINDLGSLTTLDDRKYSKSIHRNALMLLAQVKNGIKFRVGGMFSLDLKTFTDLIRSVYSLLTLMMRTMS
DV

>DAA80414.1 TPA_exp: odorant receptor 79 [Aedes aegypti]
MSNPTRYSFPSNIALPRWLAFINQIQNPLALQKTVDRCLGFIVWDFDTKLSYAKLIFVVIMYIYLVVCCV
CALFLVNPADVHPDYYLKMWFFIGAGASCSLPWIALLPARRHFATIHEFLTDQYRLDPYHPLRYRSRPVI
FLCSVFFFSINTSISVFWCILLQGSCPITFAFRYPGVEPASSLVYLVECFHLGMTANGTMVTTLTTLLSF
IVEFDVLGVELRESFSTMDVDVIRRSVERHQRLLELVNLFRAKIKPYFLIAMGLYLFLVTFSCFLLVVQL
REGDFQALQFNVFNAAISIVTIIMFGVICDMVEDRVRRIGDQVYESEWPLKLVYDQGRGDVYRLQKSSLM
MIIARSQKKVGFTCGDIYQMSTITSMQVLKLCYTAFTMLWNATNE

>DAA80413.1 TPA_exp: odorant receptor 78 [Aedes aegypti]
MSNSIRAAFPSNIVLPRWIGFIHKIQDPLALQKALDQLMGFISWDLTNKIIYIKVFLITYLFVYYVICSV
FIIFLVNPEDVHPDYYLRMWFFIGAGASCIIRWIAMIPARHGFAIVIQHLTELTRQKPYQPLRVRSRPVI
FLCSAYFFAMNTSVSAFWTVLLLGSCPTDYVIHSPILSYASLLLYPMETLINGMVATATTVTILTTMLVF
IAEFDILGYDFREAFSTANSEEIRHCVERHQRLLEMVTLYREKSKLYFLIAMSLYFFLVTFSCLLLVVQL
RQGDFHSVRFNGINAGLSIVSILLYGRICDMLEDRVQDIGNQVYGSDWPLKLFGTRERREVHQTQKSGIL
MVITRSQRKVGFTCGGIIEMSTVTSMQALKLFYTALTILWNATSESEGTQN

>DAA80412.1 TPA_exp: odorant receptor 76 [Aedes aegypti]
MPAARVDCLPNCRSFSFLGHFRDPFAFQKLLDRCIGFIHWDTESAFTRVKILLLVFTGTYYTLSCLCLTR
IDPAEVPLDHFFGMWFLVGGGCSCFSQWYVLAIERRHLAKVIGFLTNLQQNGIDHPTRVRQRSRIVLYSI
VHWTTNVSQTAVWAVTLLFTSSIAHATSNSYLQMLAYIFFPLEIMLIGMTANVSQINTFTTLLVFAVEFE
ILGEDFRQALDKWNVVDMKTCVRRHQKLLEMVMLFRDKLKLYLLLSLQIYFFSITFCCVMLVIQLKTGDN
QMFYTLINLTTALLCLLLFGLFCDYLDLKVAEISDQVFGSKWSERISRDRSMKRNLLMILMRSQKKIKFT

CGDIYAMSIVTCMNVINMCYSAFTLLMNMVQD

>DAA80411.1 TPA_exp: odorant receptor 75 [Aedes aegypti]
MSNLTQWISLKLSRIDDPLLFHRTIDRYVGFLHWNIRTLSTRIKLLAIFLTFIYLVIAVLSIPMIDPATV
PIDFYFGMWFLFGAGGSCFTQWLVLAHERHKLREVIRFLTDLQRRDRHHPMRVRSRPQILVFVVLYWAQN
VGQAVYWAFTLKHTSPIQQASQFAVVKVLASWLYPPIIVMLGQMSDITQVMTFNTLLVFTVEFKILADDF
RQAIDCWNVGELRECVRRHRRLLAMALVFRDKLRMYLMLTLEIYFFLITYACFVMVIQLTGNGGSRSLFT
ILTGFVSVVCLILFGWVCDRLEVKASDVGRQVYNSDWPSNFIYRENRALDYRLTRKNLLIVMMRAQNRIR
FSCGSVLDMSLETSYQLLKLCYSAFTIMLSMIRNWSV

>DAA80410.1 TPA_exp: odorant receptor 74 [Aedes aegypti]
MFKFFRSIKPFLIEKYRKFNASDNSFVVMMFCNHFVGIFDNPDPDPVRIRLLKRFLLTFAIVYVFSEVVS
VFLFEAFSVEQDFGILFMTFGRLLCIVMWSSFAMHHQDLKRTWLFLLNTQQDTPDDRRRKFIRTVNWITL
MFLMQDVLPMVVWAMSGHSESSLQLYNNHLIDQVNALANPIVVVVLTMMFCYSVVVVGSVLPALTLEFHI
LGLDFELLFEDVGSLSDGDNWDAVEAAFKRCVDRHQVLLDTAKALRKQLKIYFLVQLGINFVAIVFSTLI
YIYTQRSNDSSYVFNAFGAMSIMCNLLLYGYLCDRLEEQVAAINRHLYCSNWTGIKFDSTKFGKRYKNLR
RMMLIVMERTQKKVGFTCGNFFGMSLVTCRKVLWFAYTVLAMLMHFLE

>DAA80409.1 TPA_exp: odorant receptor 72 [Aedes aegypti]
MESIRLFVNQVKSKLASLDDESDYFRQMEWAMVVAGIRLPSQNSTYQRLFNCYRILMLLQFSIWVDRVYV
AYTEWNSPGELIGVVAFCLALVMILSRVVLMRVYLKDLLKVRDGLENRLNNHHSEGRVRSYRLIRRFFMV
LEWIYLFDQIILYAFGINEERQYSVPDNLRRLSKRSKLGFDIFICSNHFVFSSVYASILTIMNTVFMGFS
TELENIVSECNGIFERARQQVTVIPLYEGSSEHTEMRENLVFWSTLKNEISLIAERHSELIEHVATVRNL
LKVSFLLIFYTEIVFIGCALFYVKLIGITMNTVIVVSYVASILLECYWFCRLTDNINGTNRAIGFALYNL
DWPIQLCDLPNSRKQYLEIRASLLVIMTRSQQNLGITCGGMFEMSAQAFDELMKMIYSCLMFLLSVTT

>DAA80408.1 TPA_exp: odorant receptor 71 [Aedes aegypti]
MELSYHRSLPPELQIMPFQLRCMELIGLIGPKGRFYRFVLAFGWGTFVILLPKSVLGIGSSELDAIIKGF
AELLFEGNLFIAVASLVPKLPLVKRLLHVLSEIFRQATHDKRDVKDHCYALICEQNSKIDKFCKFYFIYC
CFGPFVFCIPAMVTSYVRYFGTTNNGTNANGSEHLRFELPMEQEFYWLPIRTNFACYHLFLTLSLSAYCV
CSYMSVIKVSTLLIMIKYCSLVYRLVAIRIRELGKLPPGRKQDDTDEDERTKMVKVKEVVEMHEKALEAT
DLVEKVINIPIAMQFMACILFWCMTMVYVSTNINFNLFNVMVLFWLSLIETYGYSYLGTELSEDAKAVGH

AVYDLPWYEDSAQLQRYYRLMIQRSQQNIGVTAAKFFIVGIEKFGKVVNLSYSYYLVLKDVLDRL

>DAA80407.1 TPA_exp: odorant receptor 70 [Aedes aegypti]
MSAIELYRQCIDILRLLSHPVGVSLWNPDQFLTFGSYSIVTQMVIYFWCNFWTVFKYRHDIIHVMEVLNC
AGIAFQLSVKFFIAMNNKFLIRGLLQTIENNLYERYPDRTSSEGEIVFIFARKYNILLKMLAVLYCSTLL
VFALYPLYIYYSEGKLIPLFMFEVSYVDWHTVWGYLLTNFVQVVTYLMGLFGMILADGLLVLLVVHGLVY
IEVFMIHLRDLAKMLQSENVGENEEKIMELWRECLVEHQTIIEYFTDIETVNGGMCLILVFTGVFAICDN
LVLCALTDWYASYLFLLICFVQLTIYFAVGNAVELKSDALDISVVNFPWHLLKIDNQKEYLFLICQMQRP
IILTVYGFSNLNLEAYMTILKALYQFAMMILNFLA

>DAA80406.1 TPA_exp: odorant receptor 69 [Aedes aegypti]
MASLEGYYRIRAPLITISKIMGAEIWTSDKFFQPASYMLMAHMVIYNVCNGYTVLTQISDPVKLMQVTII
FGIASQLIFKFFYAISRKYDLRKMFDIAEETIYQRYSEGNKEEVLILHKTVRYLGIIWKFLALIYSSTLF
VFGMWPIYVYYSTGQMVPLFSYEIPLIDPASSFGYILNMFLHVDIYILGILGSILADYTFIFIVFHAVAN
VDLFILHSKELSDLLIENDPTKNVRAIKEKWNCCMCDHQIATEYLNGTEDIVGLLCLVQVFSCIFTICDA
MLLVALTDWYAAVCFLIVVFGEITIYFLIGNFVELKVDELYASVVSVPWNLLNNIQQKEYGYLLARSQRP
LILTLFGFAPLNFESYMTVLRALYQFFVMIMQSVE

>DAA80405.1 TPA_exp: odorant receptor 67 [Aedes aegypti]
MYQARISNSGAIMAQGSAEELQKSFDIAKKTTYLVGVNPFQADREITARFIFSALLMLAIYVFCCYTLWV
LGSEWQTSLEVFQVLMLNFLGTNKIWLGFMRVHDYYLLFVSTEEIYKKFDEDIRNRPILRKMVRILVAML
KSMVVIYTLSGSLIFLLMTTVVLITKEKVLLLRVYIPFVDHTTPVGYVITTAFHMLMIAFCVNGYLASDS
VFVSTILPIVGYTNSLRREIDNFNASLDEIERNEEEITEQLMRIVKLHQMIVEYECQSVKFFKDSNLVQV
SLQAGLLLVLVFMGLILHYLPAMCATLAVLFELTLYCSLGTVITTKNNQMMVDIYAINWHLLPKSQRMLV
VFMLHRAQNGKNLYVGNFAPMNMTTYVQILKTVYTFLAMLITMMH

>DAA80404.1 TPA_exp: odorant receptor 66 [Aedes aegypti]
MEALDKFMLYTKYVRGLCSVIGLDILDPDYKKGFKTYFTFFLMILYVVLTVNSLLTAKGSTEVLMALSFG
GFFGQCLLKLIFTLANRKQYYVNHTNLKESIYFKYLHGSEKEKSVIYKNVSQLLMLVKVTSLLYLSSIFL
FSLYPAYMYFFENIKVTIFPLLVPGIDIYSAYGYGFTNMIHMFFGVYGLFGALSSDTAFMMFVFHIVSYT
NLLQIHFLSFAEKLTSIEVKYKTKDYAAFCSSEMRELYVSHKEVIDFLSSLKMCYESICVVQVATCVVTI
SLNLFLALMSDWYATYGFLLASLFQLFIYAVLGTLIQLMNDKISTLVYDAPWHLLPNSDKRSFQFLLYKT

QRPIEMFVRGLGPLNVETFTEIMRMIYSSFTMLYSFIVE

>DAA80402.1 TPA_exp: odorant receptor 63 [Aedes aegypti]
MDRFLTTHLRMFDEKRLSLLSSQDFFDEMINFLRVFLTFCGSDVLLMEKFRWNARTWLCFWTLVSFAITL
FYTIVFRSDDIYAILDTLSYSGIAIQGAFKMHGALSRVKLFQQKYLSLKALHARFSKEPDNNMALNKCVL
TITYIFRFFLLIYAAGGLAFFVIPLYVLFVYQKVVLILHVEIPFVDPDVFSGYVITTAYQVMMIALAIAG
ILAADMAIMILVLHIVGIVDIFANKLKELDRMLEDVRYDKQQIHEKVTEICVMHREIIKYEEDLDECYHT
TVFVQVLTSVACLSLALFVVYMTNDWTRAMFLAATFFQLLEFCILGTALTLKNDQARVAIYHTKWYLLTT
SDQRRMQFVLHRSQNAVEMTIGGVALLNMETFVAIIKTIYSYFTMLVTFISKE

>DAA80401.1 TPA_exp: odorant receptor 62 [Aedes aegypti]
MAPKIDLSRLKIRQACNVFKAKYNNDKDFFILHDFYAMVGGVHFNTENATMKRWWNLSRVVMAVTYVLLT
WNICLQFINEKRLEILMNTIQIGAGLIIVLFRTIVIGWNYDFVKSLRRYLNSRRFNREDESSFKIRRQAY
EFIHRIVLLFVSNSCLMTAPIFVIQPTAPLQLPFTLNNHVLQTVAQKIYLLMIIQIVINLATNFFVIVMI
LTGLAAECRILSNAVEKIFEDSISELKNTGPENSSITSDEQFWKAFNRRFNECLTEHRIILQHLTDIRPL
LEGTFIITYYTATLNIAAGAFFLISNLDNINLYIYQICHYTIVLTLECFVFTYFTTRLVSAYQYIGLSAY
KMDWPDQLKYSQLFEHQYRAVRAKLLLMITVGSQDVRFSAGGYFEFTLEKFTDLMNISYSMIMFLWEMR

>DAA80400.1 TPA_exp: odorant receptor 61 [Aedes aegypti]
MFRSTRRRFTNWISNKTKLKPDTDVFFLLDYFLVLSGVQLLTKNGYLRTAWNVYRTLLGVHVLLMSRKIW
SVFQTEDNFQLIANNMLMCVGCLIIFARCIFIVRSSEQIKIVRRFVNERKFKADDSFAMTVRQNTYNNTI
IVTVVMILNCVVQSGMILFTELGEAESLLLPFYLEGLSSSENRIIQKLYSGMFSVYASYASTNFLAVYLP
MSTLKVELKVVVDAFEKIVHRVDERTVGKNMQPLEDLNEAVFWEILRDELTQCIRAHGAVLARVKALKKL
TDPTFLLLYYMTMLFIAIGVIAVLFTPKFDSFNTISLEYTFRYTMEWYVLCYLVSNFNEEHNDIAKKLSQ
FHWGVDLRYSKRFARDYKQIRSMVLMVIMQSQKSLNFSCGGLFELTMGSFTAVINKTYTLTTYFWNIKQR
G

>DAA80399.1 TPA_exp: odorant receptor 60 [Aedes aegypti]
MFDLSHNRFKRYLKHFFKLKPETNHFFIVAFFHAIAGIALPTKRPLWRWIWNVYRSCLVLHYLLCVVRCF
LTIKSGESFNAVLINMHVIFSLTINYTRSLIIGANFKYFVHVKGFINDRKYRNDTKSVEIRQKAYEHSLV
VTMIFVANIVFQSISIPSTGMTNTDPFQIPIDLTGLPHFARKIVEMWYNLLFITATYISASNFLSMYLAM
VGLRAELRVALDSISSIGDYLDYVDGKDGEFEQGEKFWKELHGELKRSIGHHVEVLVHLDVLKNVTNLSF

LLLYYMTMLIVAAGVLILTIYPVVDVFYVFAIDYTIRYLIECFVFCNMVSSLNEVQHAIGETLIHQAWIS
KLKFTKQFSEHYRAVRACILIELMESQRSLRINCGGMFELTLAKFTRIINTSYSLSLFMANFRN

>DAA80398.1 TPA_exp: odorant receptor 59 [Aedes aegypti]
MLNTLRQALRYDSSSNPLKHMIVLHRIIGFNLSSRPAIIVSRLSMVLAGIHALCFAYRVHLVSKRDLGFQ
YYIAALNVVGGYIFAVIRMTGFAWNYDSGFRGIHRFLQEHAFQRNDLRAQRLREQCFMNNQKFTIGMLVA
SVNAIMFFILTDYRMTDQYDIPFELSFLHPTIKSVFNALFGLYLYVIATYFWIPFITLRVVIHTLCVELA
IANEAFGKLFTTSSNRADLLMLHHSSNSSRSSQLTEQIRTDLFWLSLSSELRGLVDHHREILINVDRLCQ
LAAMPFLSETMSCILISSISVFFLLNGESLSLVAISCVLMFESFYVSSLVEALQDVHGEMGAVVYALEWP
AELRYDRRNHHHYKYVSKILQIVLMRSQRRLRFHCGGLFEMSRGTFTFTVKTCYTMLTFLLRMQDV

>DAA80397.1 TPA_exp: odorant receptor 58 [Aedes aegypti]
MLFVIRSYLVGRFRSFHNSDNCFVIMMFCNRLIGVWDYPESDTDRFHLVKRILINFSFIYTVLEVMSLVP
VVKASGVSENISSLFLVSSRVFCLAIWCSLAVYRKDLKRIFMYLLKVQQEAADDSRRKSIRYVNWFTFSF
LVLNLTPLTVWIINGQAGSPISVFGHPWIEGLNIVAYPVAVFLITVMLCYAVILVLSIMSALTLEFYLLG
LDFQQIFEAKPQDGNTINWTTIEQAFNRYIDRHQTLLNKADLFRELLKINFLIQLLVNFFLIVLNSFMYI
LMQRNESSGFAFSVFAVLTLTMNMMLNGFLCDQLEEQVSSINHRLYSSGWTDKLMHTKAFNQRYKNLRQM
VLIVMQRTQKPIGFTCGSLFEMSLITCRKVLWFIYTVLAVLMSLLE

>DAA80396.1 TPA_exp: odorant receptor 56 [Aedes aegypti]
MTTKRMPPFRLMSASLKLCKWLGLWHEVNLSSPCWQTVFIMCSILFWFILPGCLYITRGEKTLRDLLKSI
LEVFAMSVIVSRLMVHMFNRKKLQACFVDLREAISTFENYPHENVQRILRHLLKSADYLVKIYVSIVFIQ
ASVYGVVPAVLTTYQYCTSDEIIRLPSAVMDADYILFDHTTSYWIWMLVTIVSLIVEYLMLGSVSAQECL
FWNLLHHTSCLFKMVCLEIARLDQYTDPNQFRERLACIVPIHEVCFKCARCLENVLNPLLALWYCTCIVQ
TCYLLFAISMIDDIVVIASMMFVLQYTVFLIFSFSMLGAELMEESARVSEAVYNTHWYMRKATESRLLLF
IMMRTNRPVGIRAAKFFFVNRSTFADAMKTAFSYFTIMQRFYGEK

>DAA80395.1 TPA_exp: odorant receptor 55 [Aedes aegypti]
MQRKPPFRLMSASLKLCRWLGLWHDVNLDKPCWQTVFISFCLLFWYILPGCLYITRGGRMLQYLLKSILE
VFSMCVIVLRCVVHMINRKTVQNSFVELEDAISTFENSPYEDVRQMLRHLLKSADYLVKIYVSIVFIQAS
IYGLVPAILTTYRYCNSNETVQLPSAVMEADYVLFDHSTNYWIWLLVTIISLLVEYLLLGTFSSQECLFW
NLLHHVSSLFKVIRLEIARLDQYTDPKQYTERLASIVSTHEVCYRCARSLEIVLSPLLAVLYCTCIIQTC

YLLFVISMIDDLVVIASMIFVLQYIVFLIFSFSMLGAELTEESALVSEAIYNSNWYMRMPAERRLLLFMK
MRADRPVGITAAKFFYVNRSTFAEAMKTAFSFFTIMQQFYGE

>DAA80394.1 TPA_exp: odorant receptor 53 [Aedes aegypti]
MNTNSQPPFRLMSASLKLCKWLGLWHDANLDKPCWQTVFLILCLLFWYILPGYMYIVRGEKMLQDLLKPI
LEVFSMAVIVLRCLIHMINRQSVQECFADLQNAISKFKNSPYEDVQRILRHLLKSADYIVKFYVSIVFVQ
ASLYGFLSAALTTFKYCTSDEIIQLPSAVMDADYVLFDHTVNYWIWLPVTIVSLIIEYLMMISISAQECL
FWNLLHHISSLFKIVHLEIARLDQYKDPKVLSPLLALWYCACIAQTCYLLFFISMVNDVVVVASMIFVLQ
YVVFLIFSFSMLGAELMEESARVSDAIYNTQWYNRMAAERRLLLFMKMRADRPVGITAAKFFYVNRSTFA
EAMKSAFSFFTIMQQFYGDK

>DAA80393.1 TPA_exp: odorant receptor 52 [Aedes aegypti]
MERIRKLVAQLQISVRDDESDYFRTMDYFLILGGIQLLSKNPVHQVLFLCYRMLMTVQFSVWFDRVYVAY
SEWNSTSELIGVISFLLGLVMIVVRAIIIRLYLEDIYAARKYLGAQLNGLRMSESRIQSYRLLRRIGLVL
EYTFFADQILFYAFGIYQDKQYTVPDNLSRLGWQMKLLFNIIISTNHFVFSSVYATILTVQNTLLMGIGA
ELDVILAQCDGIMARVDEKLKNAVDRDGDQRMLYDVRGSRAEYFFWKLMKRELNVTVARHSRLLEQIDIL
GGFFKMNFLVMFYMAIVIIGCASFYVNMLGLTVNTVIILSYVGAILLESYWFCKIADNMNDTNSQIGESL
YNLGWSERMPEMPEARKEYFEIRETLLIVMTKVQQDLGISCGGMFGLSMQAFHELLKMIYSFLTFLLNTT
S

>DAA80392.1 TPA_exp: odorant receptor 50 [Aedes aegypti]
MQFIIRLLQILGFWTQPFQKRSTAKPLGYMVLFFVWLLLPEIIFIVRQEPTFAIVARNAVECLLIANVIL
LIGSTIVHQSKLEESYGNMRFALDTIASNVDSKLLSTVTHLGSSTDRYFKVYVGFEGIITLVYALANPVL
TLTQYIQSGELPPLHAIIESDFYMFDFTSNVWLWLLVVIVGGILLCFLSVDIVSINSLHWSLIHHATALF
KIVGQRLSYLNTFSDEKSQSRELTDIIKMHEIILRSVRLLEEVINIYMLVQFGTCIIMLCIALIVLILSI
DDRDLLVKMVLMLSYVLSHIMLYSLLGTELISASDSVADAVYDVPWYQWTVSEQRKVLFVLGRSQRMTAL
TAGKFFYINRDSFGKTLQTTYSYFTVMKQMYG

>DAA80389.1 TPA_exp: odorant receptor 47 [Aedes aegypti]
MESIIKLLKFCGFWGRPYQKFSLWQPLCHIAVLVVCLLAPGVIFIVRNSSNFASAISAAIESMGFINTIL
LGTTMLYHRSALENAYGDIRIALRIGKSSSIGDVQRNIEFLEKSTNFLFKGYTVFQSVVGTGYALTIPSL
TVVYYVQTGQWPPLHGIFEADFFVFDFTTNVWLWVLVIAVGMFAMLCLISVLVIVSSFNWSFLHYIIGLF

KLVHIRISRLNAFANPQSRQMELIEIVKLQELVYRCARTAEDTLNLFLLTQFGTCVVAICLTMMTLTLAS
NDQDLLIKMILMLAYILFNIFVYSMLGEELIATSTSLAEAAYGTQWYEWSIPEQRNILFIVRRSQKTAAL
TTGKFFAVNRSTFAATLQAAYSNFTVLRQMVHSH

>DAA80388.1 TPA_exp: odorant receptor 46 [Aedes aegypti]
MKTIIKLLHTFGLWSQPYQNHKPWKPLWIIILPTLFLVGPEVAFFIRNHSSFTKATRAAIESVELTNVAI
MAMNHLIHRSALEQSYKELQLALKIMSYDSHIDVQAALDQLKKIIRISSKVYITFQMIISIGYAMSIPML
TVYHFAKTGKLPPLYGIFEADFIFFDVTHNFWAWLLILIVTIVTFLSICLVLAVINSLHWGLLLHVTGFF
KIVYMKILRIDEISDKQSRHKEMIDIVQLQELAYRNARILEQSLNQIMLLQFGMCVVTVCLTMLTLTLAN
DDKDLLIKMGIILVFIFAHLLVYSLLGTELITASSSIADAMYGTQWYEWTISEQRNVLFILLRSQRMTAL
TIGKFFYINRSTLGKILQTTYSHFTVLRQMIDSH

>DAA80387.1 TPA_exp: odorant receptor 45 [Aedes aegypti]
MVYNTSLEAYQDIIRLARRMASFCGVDLLADNYKPNWRTALTFAGCISYLFTSCYSAWYYYPDVFKMLQA
LAPNGIAWQGCLKLFIAVQQRKFFQGRAKYLEEFHRIHVNRPEDNAVLLGLMVKMHLACRVLLSAYITAV
LGFGLYPVYFFVMYGERTFAINVLVPGIDPDSQWGYFLTVSYQIFLLSIAMAGISAFDTTFLIFVCNLAG
LVDVFMNKLKELDRLLEKEKADLAEIRDHVREILIDHYGIVSYESDLDNRYIAINLMQVGSSVACLSISL
FLCYMTNYLPGYAFILGAIFQLLTYCLLGTIFSVKNDDAILAINGTKWYLLEKSQLKMVGFMLHRCQNPS
NLTVGGFAPLNIETFVEIMKTIYQFFAMMINFVN

>DAA80386.1 TPA_exp: odorant receptor 44 [Aedes aegypti]
MQKFKYSYEMYDHNLIYVRWLADVCGVDMMVENYKINYRTVATLFLISFTVVNLGYSCWFHYPNWHVIME
LMMVFSFCLQGANKFYNAYIHRHFFILMYGRLRNLHYKYQHHRENNAQLLLLMQRIHLISKGIFVLFGLG
ACSYFIYPVFNYWRYRRLELLISVRLPGIDADSHYGYIITMAYQIFMMVAAVFGMAAADSAILLFVCSLA
GFVDVFKNELRELDMMLVQNPRDESKIRRKVREICVQHFTVIEYESDLDERYFTTCFVQVVSTTIGLSGA
LFLAYIVRYIPGFALMLVLTAQLLEFCLLGTVLYVKNEEITEAIYGTSWHLMEKPQQRCFALMLHKSQNF
VEMTVGGLAPLNMETFVAIMKSIYSYFTMLISFIK

>DAA80385.1 TPA_exp: odorant receptor 43 [Aedes aegypti]
MNQFQKSYELYDYNLIFIRRLADVCGLDIMAKNYTFNFRTLLILILTIVSTASVLHSYVFYYQNWFRILE
TTVLISLIMQAIVKLYNAYIHRHFYETMYDRLRDFHYKYRNHKNHEQLLLVMEKIHMVTKAFFASYIVTG
CCFFVFPVYVYLTERRRELLISIRIPYIDADSVSGYVVTMCFQSSLLVTFVIGFTAADSVILLFVCSLIA

IVDVFTYDLRELTAMLNETYPNRFAIRKRMRQLFMQQLEIIEYESDLDERYFVSYFIQILSGVMGITITL
FLIYKANYVQGCALLLSLFGQLLEMCLLGTIFSIKNEEITEAFYGMDWYLMDILEQKCIALMLHKSQHFV
QMTVGGLRPLNMETFIVIIKSIYTYLMMLITVFK

>DAA80384.1 TPA_exp: odorant receptor 42 [Aedes aegypti]
MAVKENPYESFERILYWQHLVLKVMGVDGFGPNFRRSALTYFIVFLANLFFVISLIDLVLFRQDVFNFTF
VAVTIFYALIGLGRLAVLVRHLVAPTVLVQSMKTVYQDAVRDPMETVVLQKYTNQLRQCVIFYTVIFMGG
VVLTALLPLPIYWWSGDKILPFGVVLPFVDPESSDGYQLNYMYQVSCMLWTPPGLIASQNFYFALSFAIG
IQYDVLVLKLKALDKLIVDNVDGSLNSEIRIKLIKVVRYQQRLVQFISNLEDLYSYQTFLEVACNAMQIV
MTLFVLHIEFWFPGILIMLVSTFQLFLSCMLGTMNDVKSDLFIQEVYDISWHAMPKQEQKMLKFMLTKSQ
RMQKLSCGGMMAINMNLFLAVYKKIYSIFMMLQNL

>DAA80383.1 TPA_exp: odorant receptor 41 [Aedes aegypti]
MKFPKSANVPRGTSSFESFQRILYWQHITLKMIGCDIFEINFRVSFLTVFIIFLAVLFMVISLINLYFFR
DNIFNFTFVLVTFFYGVIGCGRLGFLLTHSKVSSKLVFEAKKTYELTSSGASETKVLVKYTKMLQQCVVF
FSIAFMLGVVLTAIMPLILFLWNGEKILPFGVILPFTDPNSVDGYQLNYLYQISCMLWTPPGLTASQNMY
FALVFNICIQYDLLILKLQALNELICQNKGEIHDIKIRSKIIDIIESQQRLDNFVTTLEELYATQVFIEV
GSNALQIVMTLFVQHIDMWMPGYLILLIATFQLFVSCFLGTLIDIKSELFTKAVYDISWHKMGKENQKTL
KFMLAKSQCSLQLSCGGMMTLNMNLFLTVYKKIYSIFMMLQNI

>DAA80382.1 TPA_exp: odorant receptor 40 [Aedes aegypti]
MDELSKDLSIIFFLMRFVALWPYDYNRHLPVWLQRYKTSLVLNVLYYLFWQFICLHIAIFHVITVLNSLD
NFDDLFLVMVSTIIYCLMLLINVNFRLYYHKNLVLIEFLKYRFKQRSAAGLNFVKFESSRRYFIRLFKIW
IVTCVLGTMHWAIFPILQREMVLPLQCWYPFEVHRSPYYELAYLGQVLGQLQVGLVYGMTGALLMMYIFI
VCGQFDILCCSLSNVYYTALINRNGNRFKLSASQAVLEKALRNPNDLYIKEVFLEELSKAPRTTTKKLAP
VAEKHQYLHYLQDELATALDECVDHHVLLIHFCRLLEEGFHPFVLLKLGQMLSLLCLLSYMATVTDLSVM
KLMNIGEYFVLTMTELFLFCYLGETLKNQSLKVSGALLKSNWYKCGAQSRQRVIFLLMASQKPLKLTALK
LYSLDFDTYRSVLTAAFSYYTILKKLQNGSRHTS

>DAA80381.1 TPA_exp: odorant receptor 37 [Aedes aegypti]
MASQGELISSVRVILWIYRILGLSRENNQSVRYRIYRWVLNIPFLFAYLFAMIISALHEENSEILWKDTI
FIILTEASMFVKVVTTYCRFQDTFQLLQTSVSEEFSPRCPCEQERHRRVLRHLNGALMGYLTVSVITACS

TAIHIFEGMHKLPTFSWFFGVPYGPDHGLNYLLIASYQVSGMVMHCALNVSGDIQITYLLAIAGIQLDFL
KRRFEDLKEDYLVHLQRRNLIHHNRHMQLVEQFVQDIERVYSPATFTQFCVSAITICATAFRISSIDIRE
NAGVAIGMMMYLLSMTVEIYLPCYYGNEITRKSQRLTNALYSCEWYRFDSETRRTVKMLMIRTNKPMMLK
AGRFFQYSLDTFGTTLNSAYSLFAVLQNTLVDSGKQGQP

>DAA80379.1 TPA_exp: odorant receptor 34 [Aedes aegypti]
MGEFDDFIPSQRVAFWIWKILGIWATDDESPFYRAYRRIYHFFFTGIYLFSMFTSSFFTENSEELWVEIL
FILPTEIAMLTKTIITVYKFETIHRLLQTTISKEFQPTCPKHGKEYDRFFDRFSKVMLMYYFCSVCAAWT
HLGFLFDDRLKLPFFNWFFWVPLDRDHLNNYYILFAYQMIGMMGHCSLNVSGDMNIAYLLSIAGQQLDLL
SCKFASLLVPGTGTTIEKDYYKRTFVEQIQHFAREIERTVSWCVFAQICASGITICAIVFRLSAISIIDH
LGTSIPMFFYMVSMLTQIFLPCYFGNDVTLKSQKLTNALYTSKWYQLAMNDRKDLKMMTLRTSESIRLKA
GGFFNFNLEAFTSTLNTAYSVYAVLNSKNNK

>DAA80378.1 TPA_exp: odorant receptor 33 [Aedes aegypti]
MEQHLKKIKRIFNGQEYPQTICISYSLNMLTYLGQWNHKDSTRAYKIYFYFISLLFLFHFYALVRDLFET
YNDLILFGDNMCVTAGVALVLYKKVYHNYYRENFEEIFGKLQSLSKENDKRITPIRNLLRTYFIQEYILT
ISTIFLGLSLIVAICGHSFLDLTLPIRAKYPTEVDSVEKLVFFSLFQASVSFFLIEGIVFIDGIGGQVMS
QMSLQFHILSLEFRTIGHTLNNDSTCGSGVPNHISMVRQDLHELIQRHQQLIEFGMNVNSLYQPMLMAQL
GCSVSMICLTAFEATLTMHDLFLFMRFAVYTLSVLIQILYWCYYGNRVSYMSTMINDAIIECNWLGSDTS
FKKDLMLTMMRAQKPFKFKVYGYFPISYDTFIAVLSRSYSFFTLFRTVSK

>DAA80377.1 TPA_exp: odorant receptor 31 [Aedes aegypti]
MAPTQNGRDREKFLRVQLLCLALIGIKRHETVSSRTIFHVCFISMVIMDLATILFALEHANDIALVCDCL
GPTFTAYLGIVKQYCLSAHRVELWNIIETLRRLKDYAGTSEIESIERNNKIDRFLATAYLMSASATGSLF
IIAALAKGCYKLIFQNIIEWGFPLSLSFPFKTSHPIVFGVFFVWSSAAIYIVVFCSVSSDASFGGLASNV
VVHFKLLQKRLQDATFADNDENLKQLIEYHSLLLNLSRKIMSSFRVIIINNLLVASVLLCVLGFQLVMFL
GSTLMLIYLMYVTAIVIQITFFAYYGSLLSHESEEVSSSIYCSNWYEASPKTRRILLQCLMRAQVPVNTK
AGFMVASLPTLRAILNSAGSYVALLLSFTDN

>DAA80376.1 TPA_exp: odorant receptor 30 [Aedes aegypti]
MNLTIAYLRMVGYWSHPDRPFRPTQAICLIGVVLFWIVIPELVYIMRQEPNFVTFVRNLAEILIIGIAVP
QASIALVHRPLIEETYSEIQSSLETVSTDPYRDIQRVIRKLKTFSEWIFKGYIGGEVALAGPYFLSIPVT

IILKYFITGALPPLRGVFEADYLLFDYQANVWLWFITVVINLTSMSYVILFLVSSHCISWSLLHKVSGLL
KIISLKIGRLNEFVEDVKRREELTEIIELHEVAYRSARALEKSLNVFMLMLYGMCILNLCVTMVSLSLPN
NDRDLLLKMLVILVYILFHIFVYSMLGTELMYSSTLVAEAFYGTHWYMRSVSEQRIILFALTRSQKMVML
TTGKYFPVNRTTFGMALRTALSYFAVLRQVYGAQ

>DAA80375.1 TPA_exp: odorant receptor 28 [Aedes aegypti]
MVWIKADRSSSLEYDSFFRLPKIFGLLNGVVYNDEKPSSKWSKAKNVYFWISLMHSILVAVLELVYLAKS
VEQNADFVFIMSLVPLVGHGILAIVKLSVQKYYHKEINSILISLKDIYPSTLDDNITKDYSKKILYMKLF
VIFYLVTLIFFNIVPFAPVLHTYFTTGVFEKTLPFFIYYWYDWRRPILYELTFIEQIWVSTASVVANMNI
DLMLCSLILQISMHFDVLSDRLSVLQHNDHKELTKCVERHSVLLDLCLRVENIFSRSMLASFLLSSVIIC
LTGFQVFAQDSINKAIPYATFLFLHMVDVYLLCYYGNLMMEKSLDVSNYAYESLWYLGNRPFQKSILIIL
ERGQRAQTLTAMKFIVINLTCFKTILSTSFSYFTLLKVLNEPMD

>DAA80374.1 TPA_exp: odorant receptor 27 [Aedes aegypti]
MPPSSFSIMSANVRLCRAVGLWYDLTHWRFTWQPIFVILSQVFWFMIPTVAFMIQREKIFAVQLKPILEI
VEIGMIVFRTTAHWYGRRSLTNCFDDLHKAFEQFSVSAHEDIRRTLRHLQRSASYLVKIYVFVVLFQALS
YGPLATFITIVRYCRSDETLVLTSPVLEADYVFFDHLSSFSAWLPSSLISVSVQFMMVISITASECLLWN
LLHHVSCLFRIVRYEISRLDGSSDRKTFRKQFVEIISAHNTAYRCARRLESILSPVVGMLYCSCIFQTCY
VLFVTSVVDDPMLMASMIFILQYTTFLIFSFSMLGTELMGESALVSEAIYTSTRWYEWSVDKRRLVLFVQ
MRADRITGITASKFFYLTRPTFGTAMKTAFSFFTIIRSLFEN

>DAA80372.1 TPA_exp: odorant receptor 25 [Aedes aegypti]
MEDAKFGLIFRFIRRALSVAGCDIFEENWRPSAWTFIVLLFASIFPYFAVVFLLNHHDDMSYERLAESVA
IFITSLDGIYGLLEFIVNRNKWNEVMKNIHSRRFQYKSKTISELFDLYYYRNYQFCKVLYSAYISSAVSI
LLAPFVFPIPEQYDLPAACTISLIEPAEPYFYPVNYIFQAIVIFSVQHVLIAQCLSLVTGIMSACCQIRA
LKIKIDELNEQIADPNIKAGTVRESLGEIIYLHQCTKEFIVIIQRKYGVVYLSMYMVCGGIVCMCLNVIA
QNIFTSATLLTMAGVFSVFVHCFFGNLLLIENDSLPDKIYALDWHELDIAQQKSLKLLLENAQPDSLLHG
ILMPLNMSTFVSIMKAAFSYYSILSKKQNS

>DAA80371.1 TPA_exp: odorant receptor 24 [Aedes aegypti]
MGFLKRLNRFKIFQHSFKEPADFYAHQIKTPNMISKISGLNVFSEDFTVPNKFLFGILLLLGFYFYINAA
SAYEMRNDTEDLINSLTTFGIATQAASKLVIFIIFRKDLNWLHKYTEQLYREECNPRTRELLTDNVFLLS

VILKTMMVGYGFTSFSLDVAPMLVLAFTGSKLLPFGFYIPHIDRFSWFGYIINYMVQIILTVFVTSEDMG
PDCIYMIISMNAFTQIDLIIDSLKEVNRHIEAGDLEVDDHIIKIIQRHQEHLKYLRTVEIIFRMIFFASF
VSLSSVLILSLFAVVTLGWYQGIVFILFVSYQLFFGCFLGTFLEFKNEQLQREIYTISWYKLSIKNQKSL
RFLLQSAQEPVNWTLIFARLNIPTYLQVYKTIYSIFTMLLTVREE

>DAA80370.1 TPA_exp: odorant receptor 23 [Aedes aegypti]
MERFQRYYNRRKNELQSEFKSSRAMYESACDTLIGYFHVSGADRLRGGDYTRLNPRLIFLVCDLTLYIVV
NCWCLTVFWGQLTDVVFCLVTMGIAVQGFAKIANYTDDRLYELHVYNVARFDRVRDYPEARESLQTTAVL
CKVFIKIFSYLFMMLTTFIPVYTIVYSITSRSLQLPFGFFFPWIDHTQLFGYIINLSYHFLQIYEASYGL
LATDTCFLFFIIHAMGQLDVIIIYLKKLDELALEYDKFKNDEELYQLLNDITEKHQEHVEYMSKMDSLLK
PGFFVNFSCMIAETVASLYVQSETDGIWYPGLIVVLLCIVQLFIACALGTIYSTKNDQLIDEIYNISWYA
MPIPAQKSLALILNSSQHPVVLSDGFDAIDLFAFVQIYKKIYTYFTMLQSFN

>DAA80369.1 TPA_exp: odorant receptor 21 [Aedes aegypti]
MDLIETLSRYYVFRYKINSAVQFYHEMVEKIDKYNAIIGANMFKSKSIVLTFPFALAVCMHCIYVYLFTS
SMYYYRDDIEKILINVTTIGFSIQMLAKLYTFLYGRQNMVELHKLNLIYFENNHFGSETVKEALFKNAKF
TYVVLQLVVIYLFTLWILITAPFLLYSVISSKRLLPFVFEWSHSENWIAYSVNFVIQAICFFYVVVGTYS
TDATFIVYLLTGCGQIDAIGAMLQDLNTMIEDGASEQQITEQITRIVKLHQHLMLYMSDLESKFSGYFLT
TLGVLSFIMIVSMCALILINWATGLLLVLIATCQLLFVCSLGTYWQIKCDKLLVDVWSLKWYRLSVRNQK
SFLLLLNGAQAPLNLTAIFTPLDMSAYLSIHRTLYSICMLLIQFTE

>DAA80368.1 TPA_exp: odorant receptor 20 [Aedes aegypti]
MDLLNKLQKYHIFRHDYKGADVAYINTIDRIEYFSGFMGINLFNRVFKFCNFTFLWGVSTLFIYIYLVLT
STYYYRNDIEKALSCVTTFGFSTQGASKIYSFILRRKKVIVIHEMNLAFFKLDIMQNETVKKAFQPSVQL
NHILLTLTIYGYIGLVAIIALTPELYGLIISKCILPFGFEIIHSDAGFAYAINSWFQVNCTYYVAFLTTV
TDGTFILYLLNATGQIDAIVELLHELDDMMMKEHEEQKIDEQLQKIITIHKHHQLYMRKVEHLFNLYFLI
SIASLCFNMSISLAAFVLIDWYLGVVVFCFASSQIFYMCFLGSYYETKSEFLITEIGSFDWYKLSVKNQK
MVNFILATSQSPILVTAILENLNVAAYLKIHKTVYSGIMLLLRVKD

>DAA80367.1 TPA_exp: odorant receptor 19 [Aedes aegypti]
MDVLKRLKLFQKIRAEIGDPQMVYDGITMQLNTAGRKYLGMDVLFVEFSFVNPRYILLLIIMSSFLYADV
EAAALAEDIGGFSYNIAVLGFGLQGFAKFDAYVYHKKSMSELLWSASRFLGENKGNDRLNPLLVDNVSVI

VMIKRFYFKLYGLVFVTVSTFGMITSLINRERSLSFGFQFSFIDTSQLVAFTFTYCYQVICSMMVVISSC
CNDILIGAVYVNAMTMYDCIMSDLRELSKMSEMEQTPVNKRMAEDRMKSIIQQHQQLMQFLDRANEVYSS
YFLMSLASMTGTIAVLLTALVFVRWYPAIVICFAASFQIFSLSLLGTLLLIKGEELVEQVYDINWYNLDL
KVQKSVKLLLLMSQHYKEISFRFGVMNMETYVKSNKMIYSFFTMLVTTKE

>DAA80364.1 TPA_exp: odorant receptor 15 [Aedes aegypti]
MKYFELTEPEAAMPLALRLLETYGLRGGKRKFLQFQVTILWELLMIVIPKIVFGYRSQDLVIRGLSELLF
QLHIMIRISIFAWHRFKYESLIDIIRKVYRKTFSTGGDPTSKSIILKFNQMINKQSKGYFLYIMGCVSLF
SVAPVVQSVIIFMANQSRNGTEKAEYVTMMEQEFYGLDIRGNFGHYAIYVALAGLAHYYSASFFAVTGVI
IICGVRCTILTFKLINVRLSKLHELPKQDIRDELREIIDLHVDALRCIQLLEQIANLAMVIQIIDCVLIW
ISMILYMRNNLGVDAISLMVLFVALTGETYALCDLLTQLTSESLAVTRAIIDCQWYSLPLDVQKSLSFVL
FRAQRKEGITAAKFFFMDIERFGSVAQTSYSIYVVLKDQL

>DAA80363.1 TPA_exp: odorant receptor 14 [Aedes aegypti]
MNYFELVEPEAVMPLALRLLETYGLRGEKRKFLLFQVMIFWEMLMIVIPKIFLGYRSQDLVIRGLSELLF
QLHIMIRICIFAWHRFKFEGLVAIIRRVYKKICSSEGDSTMKADLLNFNVMINKQCKGYFLYIMGCVSLF
TVAPLFQSLGKFITNRRGNATENVEYITMMEQEFYGLDIRGNFSHYLIYVALGSIAFFTSASFFAVTGVI
MNCGVRYTMPMFRLIVVRLNKLHELPEQHIREELRETINLHVDALRCVKKLEKIANVAMVIQIVDCVLIW
ISMILYMRNNLSVDAISLMVLFVVLTGETYALCDLLTQLTNESLAVTRAIIDCQWYSLPLDIQKSLSFVL
FRAQRKEGITAAKFFFMDVERFGRVAQTSYSIYVVLKDYL

>DAA80362.1 TPA_exp: odorant receptor 13 [Aedes aegypti]
MWQPLRKFLAPGPELLSFGLQMLRFIGLWGDRRQVVRYLLVLFSEFIFLIGPKALLGSDKEGFDSTARNI
GELIFLVEVCISIGIFASRRASFERLIVVLENILRRKWPRNLQDEIYRFHRRMEFFARAYALYIGFLLFL
YNCVPIGSTIVKLIRFDESERSDFMLVVELQFFWFDIRRNVVHYAIYMAFCFVAVSCSAYQSTLKGSVIV
VVTQYGSKLFELISKRIDAMRSIERAADRDRELREIVKLHGMALEYVQHLESTISFVMINQIMNCIFIWC
LMMFYVSTNFGPNAANVMLLFLVLMGEMVVYCLNGTALSEQAAGVGHAIYNYPWYKESVAMQKNMQLMIQ
RAQRPTGITAAKFYFVNIERLGLVTQASYSYYLILKNRF

>DAA80361.1 TPA_exp: odorant receptor 11 [Aedes aegypti]
MQLKDEWIQEDDVYDNPLLRLTINGLKYYGILLYKSQPFKELNCFRGVCFTASMLAFNVTQYVDLYQVWG
NIAEMTANAATTLLFTTTIVRILHFYWNRARFNNAIKVADEGVQHLLRFGNAPEKEIFWDNVKYMNRLTA

AFWICALVTANTMCVYALVQYQTLKSMDLFNSTEPFDPPLILRSWYPTDNIVDSFATIYLIQLYIMYVGQ
LIVPCWHVFMVSLMLYARTALMALNYKLAHLEQYAVSGMRSGRKIKCVVDPEEERCNVRKELIVECIQQL
HKIFEYTRELEALTRGAMFMDFVVFSVLLCALLFEASSTNSFVQIFIDICYIMTMTAILFLYYWHANEIH
YQANLLSSSAFMNDWYNYPRSVNRHLITFICYSNKPLDMKAYIVSMSLDTFLAILRASYSYFTILKQAAG

>DAA80360.1 TPA_exp: odorant receptor 10 [Aedes aegypti]
MASILDCPIVSVNARVWRFWSFVLKHDAMRYISIIPVTVMTFFMFTDLCRSWGNIQELIIKAYFAVLYFN
AVLRTLILVKDRKLYENFMQGISNVYFEISHIDDHKIQSLLKSYTVRARMLSISNLALGAIISTCFVVYP
IFTGERGLPYGMFIPGLDSFRSPHYEIIYIVQVVLTFPGCCMYIPFTSFFASTTLFGLVQIKTLQRQLQT
FKDNINSQDKEKVKAKVVKLIEDHKRIITYVSELNSLVTYICFVEFLSFGMMLCALLFLLNVIENHAQIV
IVAAYIFMIISQIFAFYWHANEVREESMNLAEAAYSGPWVELDNSIKKKLLLIILRAQQPLEITVGNVYP
MTLEMFQSLLNASYSYFTLLRRVYN

>DAA80359.1 TPA_exp: odorant receptor 9 [Aedes aegypti]
MSIEKILACPIISINARVWRFWSFILKHDYMRYISIIPVTAMTVLMFTDLYRAWGNIGEVIINAYFAVLY
FNAVLRVLILVHYHEEYESFLEKIADVYREIISMPDEKTKEMVQLFTKRARVMSVSNLGLGAFISACFVV
YPLFTGERRLPYGMHIPGVNKFESPLYEILYVMQAVLTFPGCCMYIPFTSFFASTSLFGLIQIKSLQYRL
ENFKQNGTGKSNKEQRSQLEAIISDHQRVIAYVGELNGLVTYICLVELLSFGMMLCALLFLLVIIEHYAQ
LIIVVSYIFMIISQIFAFYWHANEVREESMAIGEAAYSGPWIELDQASKKKLLLVILRSQVPLEISVGNV
YPMTLEMFQSLLNASYSYFTLLKRVYS

>DAA80354.1 TPA_exp: odorant receptor 3 [Aedes aegypti]
MGFFDLQDCTAVMPFALWLQEKFDLRKSRWKYLRYRFAISCMCIVVIVPKIFLGYRDDIRLVLRGISELI
FQANIALKVVIFVWNQDHFEQMLALLRKWFEKTFSNGYDSEPNKALLRCNRVNDLFAKIYFTYLAVVVNI
FNIGPLIHSTFIYLTFDRENSTEPVELTTHMEQEFYGFDIRTNFLHYMGFTACSILAYFSAAYIMAVEAG
FIYCSCKSCSQCFKSSSKWELEDIIAMHDDAYRCLELLDKNTTFASMVQVINCVLMWCLMGVYLTYNVNY
SAVNVLVLFGLVTFETYARCLLGTEVSQKSFDVYQAVYNFTWHETPVPIQKNLLQVLQRAQKKVGLTMVG
FCYSAYVVMKNCL

>AIO10899.1 odorant receptor 136 [Culex quinquefasciatus]
MTQFTSRAVQTVKKWLKVKCSEMKQNCKNLYSKAFVLDPDGDHFSVVKKMEQFCGFHAKIETTKGAICWA
LLRLIIILARFSHMIGKFILTFIEEDDFEYKFLVHTVTYMMFYNYTQVILLRFSYQDLSSIRKFFNARKYL

KTDPEAHRIRSAAYRKTNWAILGPLPMWTFLWISMVLTGVYKWRVLNIEPKTLASYPILQAIIMYGYPVQ
NLIIAGWYQLPAVLINSVLLGFITELKILTDSCDKIIDNAEIAVEKERLESESTDADALFWKHFKIELDA
RVKAHITVLANLINLRQILKPCLLLYYYTLLMVNAFIICSVKNGYFKDFASSTIIMAVYFNVDFFIICYN
MSQVDDLCSKVGKRIYNLPWPYKLTKTERFVCEYRSIRSTMMVMMMRAQAGMAFSCGGFFEMSMGKFAEL
MDLTYTMVMFVLHLQE

>AIO10898.1 odorant receptor 55 [Culex quinquefasciatus]
MTLLKKLNSYKIFRHSHTEPNELYDSLIVVPNRIAKIIGLNVFSENYKVLTWNWFSLIMMISVYFYCTAI
TAYEVRFDTGDLIYCLVEGGIGIQGLAKIYTYLVYRKELVWIHQYTKQLYHEACNDQTKSMLMDNIFLLK
VAIIVMLMCYAFTSISLITAPMLFSIMTGEKILPFGFYIPNMDRTEWFGYLINYAVHLYDTIYVSAEDMA
ADTIYMITMLSAFTQIDLLMMSLKETSRMLDKDEKDDENLQAHLALVIKRHEEHLKYLRTVETVYRFYFF
ITFVSLASVLIMALFAVVTLSWYQGYIFVGFISYELFFGCFLGTLLDIKVLSL

>AIO10897.1 odorant receptor 132 [Culex quinquefasciatus]
MATYLDRLKAGYHRFVDIRKTGDLFGHYIWTHGNTAGIGLHDPAQRVKRIAFRCLTCVFALQQYILVRDV
VRAIRADDDDLAIRIGMFVIYSALSLLQMISLDRNYGLFLQVRKYFNSRLRRHCGGSQAEAIRGDLFRQS
RRNILVAEIPVVAMSLAWAIWAREEYFHLVLDVGEDYQWIVDFVDQFYGLPLVFWNNSMWIISLTMLSLL
QNAVHELTVIARSFAATFDQAMVESNEGQLPDHFWRAFDTSFKASLAEYEQFLAMIATLRALMSTFFLLK
VLTVESLLAVTCFMTFKVTFQLITAFSYAIVFTVECYIFCNLVEQLNDQREAIVECCQHWRWPFPAEKPA
RLRHVRTMVLITEAHCQQPVRFRCGEMLDVSMEMFRMVLDTCYTLITFLQATKPAQ

>AIO10895.1 odorant receptor 93 [Culex quinquefasciatus]
MVNYLTRLRSAYARFVDIRNADDIFSHYVWVHGNTSGIGLHSPAQRWKQTLFRSISFVFALQQLVLVRDV
IDAMSRTDDLTIRIGMCLIYSTLSMLQLVCMDLRYDLFLRVKDYFNGRRDRCCGGRDADQTRAEFFRQSR
RNLLVAEIPVTIMSLIWAFFAREEYFHLVWNVSEEHQWIVDAIDQFYGLPLVYWNNSSWIISLSMMSTLR
SAVHELSLIAESFATVFARASQKVQHIADPTAKETQFWTTFDQLFRESLKDYEEFLAMIVTLRKLMSLFF
LLKVLTVESLLAVTCFTTFNITFQVFTALAYAIVFTIECFLLCKLVEQLNDQKESIGENLYHWQWPDWLR
HTPERAARMRQMKITTMITAEHCQKTFRFRCGDMLDVSMEMFQMVLNTCYTLLTFLQATN

>AIO10894.1 odorant receptor 64 [Culex quinquefasciatus]
MEYLRKISQLKVFRHSYKDPSDFYNHVLVMPNHVASITGLDVLRTDYKRSSFNFVGLILVILLFFYVTFF
TVYEMRHHVEDLIYCLVTIGICLQVVMKIYTHLYYRDDLLWIQEYTKELFQEETAAHKSILMGYIEVLNV

IAKAVLLAYGSCSCTMLTVPIVFTLLSGHKTLTFGFWLPFIDRFSWHGWILNMVMQIVMSIVIVSEDIGL
DIIYFMTVMCSVMQIDLLKVKLQNVNAKMEKGETDITEELNDIFKRHYEHMELVRIVEKVYRGYFFVLFS
TLGASLVLVLYAIVTLSWIAGYGSGVFITYQLFVFCLLPTLLGTKKEELQREIYDISWYTWTIENQKVLR
FMLEAAQQPNRLSMIFHPLSMPTFTEVIRTIYSILTLLLTFRSD

>AIO10893.1 odorant receptor 125 [Culex quinquefasciatus]
MELKDEWIHEDDVYSNPLLRLTLNGLKYYGILLYKSQPLKKLNCFRGVCFTASMLAFNVTQYVDLVQVWG
DIGEMTANAATTLLSTTIILRIFHFYWNRARFNDAIKFADEGVRHILDYGTPAEKAIFWDNVGYMKRLTA
VFWICALVTANTMCVYALIEYCSVPEAERVEPPMILRSWYPGGHEEENFGAIYAVQLYIMYVGQLIVPCW
RVFIVSLMVYVRAALTILNHKLRHLDGYVASGMRSGRLIRELKDPEEERLNARRELIIECVQRQRKLHEY
TGELESLIQGPVFVDFIVFSVLLCALLFEASVTDSAVQVFIDVCYIMTMTAILFLYYWHANEIQHQSDQL
SKSAFANDWYNYPPKVNRNLLVLICYSIKPRIMKAFIVSMSLDTFIAILRASYSYFTILKQAAD

>ADF42902.1 odorant receptor 10 [Culex quinquefasciatus]
MTAAPILDCPIISVNVRVWHFWSFVLKHDAMRYISIIPVGVMNVFMFADLYRAWGNIDEVIINAYFAMIF
FNAVLRTIFILCNRQDYEDFLQRIAEVYSEIAMIDDHVVQKLVRKFTKRARLLSKANLVLGAVISTCYVV
YPLFTGTRSLPYGMFIPGVNNFKTPLYQVFFIGQAVLTFPGCCMYIPFTSFFATTTLFGLVQIQTLQRQL
RTFKDEVVKENRALVESKLEKCIEDHKRIIRYVSDVNSLVTYICLIEFMSFGLMLCALLFLLNIIENPAQ
IIIVVAYIFMIISQIFTFYWHANELREESMGIAEAAYDAPWVELDDSMKKKLLLIIARAQQPLEITVGNV
YAMTLEMFQSLLNASYSYFTLLRRVYN

>AAL47188.1 putative odorant receptor Or5 [Anopheles gambiae]
MVLPKLSEPYAVMPLLLRLQRFVGLWGERRYRYKFRLAFLSFCLLVVIPKVAFGYPDLETMVRGTAELIF
EWNVLFGMLLFSLKLDDYDDLVYRYKDISKIAFRKDVPSQMGDYLVRINHRIDRFSKIYCCSHLCLAIFY
WVAPSSSTYLAYLGARNRSVPVEHVLHLEEELYWFHTRVSLVDYSIFTAIMLPTIFMLAYFGGLKLLTIF
SNVKYCSAMLRLVAMRIQFMDRLDEREAEKELIEIIVMHQKALKCVELLEIIFRWVFLGQFIQCVMIWCS
LVLYVAVTGLSTKAANVGVLFILLTVETYGFCYFGSDLTSEASCYSLTRAAYGSLWYRRSVSIQRKLRMV
LQRAQKPVGISAGKFCFVDIEQFGNMAKTSYSFYIVLKDQF

>ABB29301.1 putative odorant receptor Or7 [Culex quinquefasciatus]
MNVQPTKYQGLVADLMPNIRLMQGVGHFLFRYVTGPIFIRKLYSWWNLTMILLQFFSIAANLVMNTGDVN
ELTANTITTLFFVHSVTKFVFFAVNAEGFYRTLGIWNNPNAHPLFAESDARYHSIALAKMRKLLVMVMTT

TVLSVVAWITITFFGDSVKGVLDKETNETYIVEIPRLPIKAWYPWDAMSGAGYVFSFIYQAYFLLFSMCQ
ANLADVLFCSWLLFACEQLQHLKGIMRPLMELSASLDTYRPNSAALFRAISAGSKSKLILNEEKDPDSKD
FDLSGIYSSKADWGAQFRAPSTLQTFENGMNGEKGNPNGLTRKQEMMVRSAIKYWVERHKHVVRLVSAIG
DTYGAALLLHMLTSTIKLTLLAYQATKIDGLNVYGLTVIGYLVYALAQVFLFCIFGNRLIEESSSVMEAA
YSCHWYDGSEEAKTFVQIVCQQCQKAMTISGAKFFTVSLDLFASVLGAVVTYFMVLVQLK

>ACH95386.1 odorant receptor 2 [Anopheles gambiae]
MLIEECPIIGVNVRVWLFWSYLRRPRLSRFLVGCIPVAVLNVFQFLKLYSSWGDMSELIINGYFTVLYFN
LVLRTSFLVINRRKFETFFEGVAAEYALLEKNDDIRPVLERYTRRGRMLSISNLWLGAFISACFVTYPLF
VPGRGLPYGVTIPGVDVLATPTYQVVFVLQVYLTFPACCMYIPFTSFYATCTLFALVQIAALKQRLGRLG
RHSGTMASTGHSAGTLFAELKECLKYHKQIIQYVHDLNSLVTHLCLLEFLSFGMMLCALLFLLSISNQLA
QMIMIGSYIFMILSQMFAFYWHANEVLEQSLGIGDAIYNGAWPDFEEPIRKRLILIIARAQRPMVIKVGN
VYPMTLEMFQKLLNVSYSYFTLLRRVYN

>ACH95385.1 odorant receptor 10 [Anopheles gambiae]
MEVLNCPLLSVNVRVWRFWSFVLVHNWRRYISIIPVTALNVFMFADLYRAWGNIEEVIINAYFAVLYFNA
VLRTLILVYNRDKYESFLAGAASVYEEIRAINDDVITKLVSTYTKRARFLSISNLALGAFISGCFVVYPL
FTGQRGLPYGMFIPGVNNFDSPQYEIFYITQLVLTFPGCCMYIPYTSFFASSTLFGLVQIKTLQHQLKTF
RSSEMLNESTVVLNRKLQKLIEDHKRIIRYVQDLNDLVTYICLIEFLSFGLMLCALLFLLNIISVMAQIV
IVGAYIFMILTQIFAFYWHSNEVREESMAIAQASYSGPWLNVDDTIKKKLLMMTIRAQRPLEITVGNVYP
MTLEMFQSLLNASYSYFTLLRRVYN

>ANW10212.1 odorant receptor 91.2 [Culex quinquefasciatus]
MDKLPTRSPELKIVTRLMQSVALWPYDYDRHLPGWLRSRYHLSTALNVLYYLVWLFINLHILVLHVKTIL
DRLDSFQDLFLILVTTFIYCVMVPVSLYCQLYYDKNVELIQMSMLLFRKRSAAGIIYIKFEPTKQFVFKM
FKMWMIGCMFGTMHWAIYPILQREKILPFQIWYPFDVHRSPLYELAFLGQVLGQYQVGMVFGLAGSLLMM
HIFMVCGQFDILFCSLANVYNTAMINSGGYKLKLATARIMYKSSERRPNLYYFNEVHEENLSDAPKTKTK
EIAALPQKHNYLPKLSNELATALDDCIDHHSVLIKFCHKMEECYHPFIMMKLGQIVVQLCLLVYMTTSQD
DLTLMKLMNICEYLMLTMTELFLLCYLGQTMKVQSMKVGDALMMSPWYECGAAFRKRAQLIQINSIHPVR
LTAMKLNDLDFETYYMVLKASFSYYTLLKKLQQ

>XP_021711562.1 odorant receptor 94b [Aedes aegypti]

MVFSQAYKSQIGKPDAKLAMGLLFRMSNFVRARTPNYRGTFAVFRVFMLICGVNFFDEDFMVGPVNMFRF

VGPMLTGYYSLVACLIHLIRYLGDTDVTILSLAAFFAAFEVLIKVGGMALKRKLGARLMNTILEDRSYED

GEVERFTFLKYHTLARKLMYITIISYPFTALMLLSYPVLVGKLDEYVLPVGYSIPFINYKQHPWYTINYL

ITIVQMAWCTLAFIGCDGPFYLYVCYSSCKLEILKSYTEKIGETNDIEEQRTLMRKIIEIHTQVLEFLRD

CSNFYNEIYLTQVLFSIAHICVSLFHVQLKWKNSSYGMLATNVAKMWIFCYCGELVVTKSNELSEAMYTN

RWYQLWSKKDLKVIQFMLANAHRNVGFSFGGFGFLSYDAFAEIMKTAYSCNAFLHNMMN

>XP_021709040.1 LOW QUALITY PROTEIN: odorant receptor 94a [Aedes aegypti]

MFTSSFFTENSEELWVEILFILPTEIAMLTKTIITVYKFETIHRLLQTTISKEFQPTCPKHGKEYDRFFD

RFSKVMLMYYFCSVCAAWTHLGFLFDDRLKLPFFNWFFWVPLDRDHLNNYYILFAYQMIGMMGHCSLNVS

GDMNIAYLLSVAGQQLDLLSCKFASLPVPGTGTTIEKDYYKRTFVEQIQQYNRIYEXNSLNLKIGRFLIP

LLLHSFAREIERTVSWCVFAQICASGITICAIVFRLSAVILATIGYNEYKKNPPTFFSQISIIDHLGTSI

PMFFYMVSMLTQIFLPCYFGNDVTLKSQKLTNALYTSKWYRLAMSDRKDLKMMTLRTSESIRLKAGGFFN

FNLEAFTSTLNTAYSVYAVLNSKNNK

>XP_001662355.3 odorant receptor 85c [Aedes aegypti]

MKKEEIFKEIHQELSIDLKNLQRFTIFLLLTISRNALMGGKFSINLRIVQPTRLILDPSLLLAFKRRQMN

DLVKFESFIRVPEIFFDMIGITRYGEARDTWKARLKQAFFWSSYANTIFCLIIEHIYFIKAAGNFTNFLE

LTALAPCIGFTALSIVKIMTIKLNEAKLNGILDRLSDLFPRSHLDQDRYRTYNYNLESQMVMKSFSILYM

ILIWIFNLLPLVSMLVNYISTGILEKELPYFMWYWYDWHKAGYYEITFFHQNWGAFDSAVFNLSTDLLFC

AIILLICLQFDILAYRLRHAKGDYKELEQCVKLHQSVVELSNQLEGIFSPSILVNFVGSSVIICLVGFQA

TSNISAFDLFKFILFLISSLVQVFLLCYYGNKLIEASSQIGYCAFEGTWYMADLRYQKSLLFVMTRAGQW

QKLTAMKFSVVSLASYSAILSTSFSYFTLLKTIYEPSQK

>XP_021703700.1 odorant receptor 67c isoform X2 [Aedes aegypti]

MFLKKTTEPEPLQVAFRMMSFAGLHEPNSIWRYFFAISWLVGNNILPKALFGSGMEGFDSVARNLAELIF

FGDVCIAVGIFVTRRRPFVRMVQLLKEIFERYKSRECVDEIHRFNRRMDRFAKGYIAYISLLVVLFLIPP

ISWTLYLAIFVSSDQRRHYVLLVEVQYYYLDIRQNIVHYLIYFAFCSTATMCSAYQTCMKAAIFLTSLQY

GAKLFELLHMRINSLDMVKAGKERHEELRQIIELHKMTLRYTELLEETITFILINQILNCMAIWCLMMVY

LSSALKVSDAIYNYKWYQEPAAMQRDLRFMIQRAQKPCGVTAAKFYFVNIERLGIVVQASYSYYLILKNR

F

>XP_021703699.1 odorant receptor Or2 isoform X1 [Aedes aegypti]
MFLKKTTEPEPLQVAFRMMSFAGLHEPNSIWRYFFAISWLVGNNILPKALFGSGMEGFDSVARNLAELIF
FGDVCIAVGIFVTRRRPFVRMVQLLKEIFERYKSRECVDEIHRFNRRMDRFAKGYIAYISLLVVLFLIPP
ISWTLYLAIFVSSDQRRHYVLLVEVQYYYLDIRQNIVHYLIYFAFCSTATMCSAYQTCMKAAIFLTSLQY
GAKLFELLHMRINSLDMVKAGKERHEELRQIIELHKMTLRYTELLEETITFILINQILNCMAIWCLMMVY
LSSNYGPNALNVMVLFLVLIVEMVVYCVNGTRLSENALKVSDAIYNYKWYQEPAAMQRDLRFMIQRAQKP
CGVTAAKFYFVNIERLGIVVQASYSYYLILKNRF

>XP_011493056.2 odorant receptor 4-like isoform X1 [Aedes aegypti]
MVSFEQSFTAVDLILITSGIPSCSSFYPVSIKSALKRNAVFLMAFTLLFYTAFGELVYLVEMLQRDFSFL
EITFQAPCLGYCTIGLMKMLILAVKRNTIAELVQSLQEEWNKSVRSLEHQSICDDVMKPAIRFTTIVAVV
NIVMGLAFTLLPIPEMIYYYSTHGKWVRQLPFLIWWSFDAYSGFVYYFIYPLYVVIGFSGIIIHMGFDCL
FCILSAHLCVHLRILKFDLENLTYGLDSSENMSIKLNPKLFYIVEKHQNILECHDRMNKIFNFALFYNFF
VSSFIICIQGFMVTAASGYTLIKFALFLASFLVELFLLCFYGHHIVESSVLVAEAAYNCLWYNTNHQFRT
IILQMVNKGQIPLSLMAWKIWPVNMNTFANILSASWSYFTLIRTVYAD

>XP_021698208.1 odorant receptor 63a isoform X1 [Aedes aegypti]
MESSLTTYTMEIEYYRPNSNSAEDNDASIYWLRVLSASLGIWPQKLVGDKKQWWKRLYYFMIIMHWYNTY
LQVEFFFHNLGALKTMTEGLCSFCSISLTGIKIMRLNSFSNEINLLLERMRTHHFMQEIKLLKKGKNKPI
FERIDQIMKDKWKEVKINLLLYTVSVGIVASSYSIFPAVINLINLFQGSSNPRRFVYKTYYRGMENLKFY
SPLHEILFSSESLSGYTTFAGVIAFDGLYVLLTMHVITIFKSLKLVIKESTRSIFTDEEKQFYLHECIDH
YTRAIMFMDDINKIFSPIFILQLFTSTSIICVIAFHASANASEGDSQILVMVLYLIAAFYQLFQFCWYGQ
RVQNESIQLPNSVYDCDWYKCSKKFKIKLHLFLLNVQKTVEISAYNLIVMSMETYLSIVRTAASYFTALQ
TLTEE

>XP_021696812.1 odorant receptor 67d-like [Aedes aegypti]
MQQYKTSLESYDAFMRLFRRFSRLCGADIMDDNFRVDYRTITLCSIIASYMVCVFYSVFHYYPNWLDVLE
VFSLMGVGLQGAAKLYNCIDHSEQYRRRNRFLQKFLRIHSDHPKDNAVLMNVMTKIHVFKTILLLSYFFG
MIMIGGFPLGYYLFYGQLKLAMNFLIPGVDPSSSLGFGITQAYHLVINVLTVVGIGAMDLIILVVVASVA
GLVDVYRNKLEELDELLDLEDETGSQVRGMVAEIVELHQEIVRYETNLRDRYEMVNFVQLTSSVSFIALN
LFLFYVYDYYHVIGFLLAGVFQLMEFCLLGTIFSVKNDEMTLAIYNTKWYQLARTEQRSLQLILARSQNS

VELTALNLLPLNVETFVNIMKNIYKCFAMLITYMEPL

>XP_001651373.2 odorant receptor 49b [Aedes aegypti]
MNTNSQPPFRLMSASLKLCKWLGLWHDANLDKPCWQTVFLILCLLFWYILPGYMYIVRGEKMLQDLLKPI
LEVFSMSVIVLRCLIHMINRQSVQECFADLQNAISKFKNSPYEDVQKILRHLLKSADYIVKFYVSIVFVQ
AGLYGFLSAALTTFKYCTSNEIIQLPSAVMDADYVLFGHTVNYWIWLPVTIVSLIIEYLMLGSISAQECL
FWNLLHHISSLFKIVHLEIARLDQYKDPKQFKERLAFIVSIHEVCFRSARCMEKVLSPLLALWYCACIAQ
TCYLLFFISMVNDVVVVASMIFVLQYVVFLIFSFSMLGAELMEESARVSVAIYKTQWYNRMAAERRLLLF
MKMRADRPVGITAVKFFYVNRSTFAEAMKSAFSFFTIMQQFYGDK

>XP_021693467.1 odorant receptor 94b-like [Aedes aegypti]
MKSIVKVLQWLGFWTQPYQNQKRTMMPLVYIVVYILCLIIPAIVYIVRGNHNFASFLTIATETISISNIL
CLFTNTFFNRLNLERTYDGVRFTMNAFLTDGHDEIRTQVNQLDRSSDRFFKIYIRFEVVVGVAYLFYSPI
VSIVQYASSEESPPLHGIFEADFYFFDTTSNFYQWLLIIVISWVVLISLMIMLISITSMNWSLLHHINGL
FKVICLKISYLNEYSDERARTQELFATIELQERIYRCVRSMENALNVYMLIQFGTCIIMMCMTMMTLILA
KDDRDLLIKMVTMLSYIFFHILVYSMLSTELITASTSIADAIYDVHWYQWSVPEQRVILFVLSRSQRMAK
LTTGKFFDVNRETFGKTLQTAVSYFAVLLQLYGSQ

>XP_001654552.3 odorant receptor 43a [Aedes aegypti]
MVLFSRQKSRMTSSPSKDTFPLMATNVKLCQLCGLWGDIAYRSHVWRAVLFSFCLIFWYLVPSFMFMISE
EPSFIVLMKPILELFAITMFVLRISNHVLCRETLYECYRDLQQAYDQFAGNLHEDVGSIVRHVRRSAELL
TKIYFSMVYFQAATYGVVPAITTIVRYSFGNEAVELPSTVLEADFVVFDHKANFWTWLPTMMTSIAVQYG
MLTFMSSNECLFWNMLHHVSCLYKIVYQEIGRLNEYKNPDEFKRQLAIIVEVHEVCFRTSRRLESVLSPA
MALLYFSCIFQTCYVMLVVSVIDDLFLLASMAFILQYTVFLIFSFSMLGTELMDASSLISEAIYNTKWYE
WAAPERRLLLFMLMRSDRVVAISAAKFFHLNRATFGVAMKTAFSYFTLMQRFIGEDRDQGH

>XP_001649199.1 putative odorant receptor 83c [Aedes aegypti]
MHGALTRVKYYQQKYIGVRAMYIQFCKEPDNNLALSKCVLTITYIFRFFLLIYAAGGLAYFIIPVYMLVV
HQKVTLILHLELPFVDPRQLTGYIVTTIYQGVMIILAIAGILAADMAIMILVLHIFGIVDIFSNKIKELD
RMVDCANVNHEAVRRKVTEICIIHREIIKYEEDLDECYHTTVFIQVMSSVSCLSMALFVVYMTRDWTRVM
FIGATFFQLLEFCILGTALTLKNDQARIALYHSKWYLLSISDQQRLKFVLHRSQNAVEMTIGGLALLNME
TFVAIMKTIYSYFTMMITFIE

>XP_020717741.1 odorant receptor 2a-like [Ceratitis capitata]
MTKQEQATPTRLDSGDATRYVWLFWSIIGIHPFKKHRTLYWLYSVLLNFCCSAFFIAFYAVTFFVVSDLL
EILANLSVMVPLIYNTAKQLVIFYHIRRTLPQAALHLHALDRRAEQEPAAREQLKRLVQLSHRIFLTALT
GIGICLTLYAMGGILRHRLPFDGWLPLDWEHSVGAYVAACAYQLFCLIVQCIAALCNDTYTVIYLLLLAT
HLRILNARIASLGHGECTEVENYRQLAACVRDHWACMNFYNSIRPAIAATLFIQFFSTAITLCTSAVAFV
NAEDSVAQLFKFLPHLLVVVCEILPCCWLMDKAALEMQDLTKSLFACRWYEQNQKFRRSLLIFMQRSQKV
EKILAGDLVPVSLETFVNIIKFAFSLFTLLNQIKSK

>XP_012162260.2 odorant receptor 85b-like [Ceratitis capitata]
MNAIKRNTNFLRFTAGPVKYFKVIGICLQPPETLSIKFARFLTVLTTLLLFVHQIAFLVTPGRTFVELSA
AVGLLNYTTVGAGKILFLILNRHLLLKSYTQLQAIYPSEAVERHYKLDRYLLIYKRVETLLYNFFKYILI
VYLVSPIVQSFYDLWSNGAYSYRMPTIIWYPVPLEESLLDSVSQLMLHLDLLAQRILELQPAEQGSMNDL
KAIIEYHQRILTLAQDVNSIFAPSIVFSLASSSFILCFSAYQLLEDVSFIFALKVLLLLGYEMKQVVITC
YYGDKLMDSSANLFNVVYAHDWTDGTPAYKRLVLIMLIRTYRPIALNVAGIADVSLITLKQVLSTSYQIF
AVLKTA

>XP_020717560.1 odorant receptor 7a-like [Ceratitis capitata]
MHRVMEYIFGRRRLVVKSGTNDSFELLFLIWKIIGVEHSRSYGFFQLFHVFCCWALLLYSPAAYNMGFLR
ALKTLPMASALNILQTDINVSILLFKVVIIKFHLKRLRSLRDIFKRLDERYHNPEERAQIDESVAICRRI
IYIYIFVYFTFAFLSWITAIMAGELIYSLWLPFVELIPHQGWQYWARFSVEAFYLYFLILVCLICDVYPA
VYIRAIRTHVHLLAGRISRLGSNPDLSAEENHQELVDCILSHQELMRVVEVVSAVTSLTLFLQFTVAAMI
LCVCMLNALIFADRAGQIMTVGYYMGVLLQTGGACFQASMLEAECVKLPLAIFHCQWLNLDRHSRSLLTF
FMQRAQVNVCFTAIKLFQINLRTNLSLAKFSFTLYTFMNEMGFGGDTNEKIS

>XP_020717141.1 odorant receptor 88a [Ceratitis capitata]
MALQAGRGGEPKLRSIDDLCAILHPIQRYLSINFLDFTRINGRFAIPSSMLLNVGIVLSVLDCMGNITKV
CMAINDRDLTKAQETFAVLGMAFVMTMRGMMLARSRVRLSELYNSIDRIFPNSSELQTHMEVAKTHDYIK
RRFFLLHQGLSFALVLFCTMPAVKLVFFYDFEAQEPVADEFHVNPSWVPFQVKETISSYGYIYVYEVILA
LVAVNMIITWDEVFVVLISQLSVLFFLHFVSSLADELNDLFNLSILVSDMGTAMSICFNLFLVTGAKDYL
QIPSYLTPCFVETWLIYDVSKWGTMLETVTARINEVLYEQKWYDSSVH

>XP_004535567.2 odorant receptor 67c-like [Ceratitis capitata]

MPFAFQQLCFELQLSLKYSVPAMPLKLANNEPAATIQDFVGIPLFLLTFMGVKLFKWTPEEASSKRQLIM
LGVFCVFATYNFATMILYIMYEPLNSSLDITEIILFWGFSLNGMMKLAIMILYRNELKSILRGLGARHPQ
TAEERSIYRLVPYYNKILIYNKYLAAWHLSITTLFSFHPLVASILGYIFRRDSSDGYDFTLPFMMWYYYD
TTKPILYIFSYVVQTFGAFWMSLLFLSGDLLLISLVHLVNMHFDYLIRHIESFQPNGTDEDMKVLGPLLA
YHQEILDYAERIDSTFSLGTLLNYAGSCLVLCLIGLQIVLGSEFLKVVKFIAFLVSTIVQVFFVSYFGNN
LMDLSIGMSDAFYNHPWYDGNYRYSRMLVLPIARAQRYAHLTAFKFFEISMDSFKSLCTTSYQFYTLLRT
SLEEEAG

>XP_020716967.1 odorant receptor 85b-like [Ceratitis capitata]

MAPYFHTREPAATIPDFVGIPFFLISLNGMQLFKWTPNEEASRRKLLLITAFSVIVTYDCVSMLSVFAFV
KLERLDYTTFALYWGYALNSLMKGGTLWFGRRQLEFILKSMVEKHPKTIAERQEYHLVAYFTKIKSFNKY
LTIFHLCTTSLFNIQPMVSSIVEYMGRQDKEEEFKYKLPFIMYYYYNERQPVLYLFSYFLQCMGGFYMSY
LFLGGDLLLMTLVHLVNMHFEYLIRRIESLQPTEDSEKDLNLLGPLVTYHLEILDYVKKIDATFSLSILL
NYIASCLCLCLLGLQIVMGSDLVTVVKFFAFLVSTMVHVYYISHFGNNLIDLSTGISDAFYNHPWTNAKY
KYSRMLVLPIARAQRYAHLTAFQFFEISMHSFKSLCTTSYQFFTLIRTSLEEDFH

>XP_004535438.2 odorant receptor 7a-like [Ceratitis capitata]

MRKFSELFYGEGKENFETNESFELLYWNWTFLGTRLVKPYRVRNLMSSAFAWTCLLTSPLFFFVGIAKMV
NTSSMTELLTLIQAALNVITLPLKTIVIAYYMKRLSSVKPMFKRLDERYNTPREREQIKESVKYSTRIFA
IYFIAYFIYGTMTALLGLTLHSQPLNSWLPFTDWIPMQTLRFWLHFCYEQFTVYLLLLNQVSNDAYACVY
IHALRTHINLLAERVSRLGTNSEFDDEQNFKELIDCIAAHQELLEIVKTVANVFSLTVFMQFTVAAAILC
VCMLNIFIFADTFHQVVTVIYYMCVSLQTLPTCYEASMLEAESAQLALAIFHCNWVDMDKRSRKLLIYFI
QRAQEEISFTALKIFQINLRTNLSIAKFSFTLYAFMNEMGFGENLKDKK

>XP_004534467.3 odorant receptor 45a-like [Ceratitis capitata]

MLVHSHYKSHSICSLCVRRSTGSQKMFHSDLVINGYFQLQKHTFQRLGIDMTTRNASITHIYFLALQIVA
LVTITMPMVIYSCQHLQEIAEVTNAMAPFMQATITLWKIWRVIYRRKMMAELVEDIYSVSTKATKQELTH
LRRENNRERIMNTFYYYSVFNTGVLALIAPVLVSFIQYLRLGEFSYIVVLKATYPIELARPLNYVLIWLW
SAIAIYGVIYGSVSVDSLYSWYIHNLVGNFKILQTKFVTAELIAEVSERRASIYYCLGYHQRLITMSEQL
NIIYQPIVLVQFSLNALQICFLAYQIGSGDVAVVDLPFLFLFMTSVGIQLMIYCYGGQYLQNESENVAKF
ISQTINSTIWPIDLRKVLLFSMARAQRPCKLTGIFFDVDLRLFLWVWRTAGSYVTLLRSVDQQSS

>XP_020716474.1 odorant receptor 49b-like [Ceratitis capitata]

MATERKSMPNVERLFNWNLEAWTRLGYLDKRKRLLACTIISAPIVVSMFVMPTFFMGVNTFEQHIYNFYM
IIVTSSSVARAILIIIKQRKILDLLNDMENWFVEVQEQNDNNALETLNKLTQKVRRYSKYTLWWVIIFGA
FLSFQPICTGYGKFVYDTQIPGIDLHQSPLYEIMYGFQSLWVIPMACVSSISYADTLLIFISFGIFATKQ
LQRKLKDISQMDEQQGLENIKKCVQYHWKIIKFGEDLENAYSLMCLLDFSLYCVTLCMLLFYSVMDFTWA
LMFQAVVVELILTLLIFLTTFLADIFTQESLNVAQTAYDMNWLQRDKAFRVAVLLIILRSQRPLILTAGG
IQPLNLETFLAIMRSSYSFFSVLRGVM

>XP_020716058.1 odorant receptor 94a-like [Ceratitis capitata]

MDLQEYDNNSGGRRIIQVMKLLGLWYYEGSAKMPYLLYSCLLHFTISIPFTIFMAMDVVHATDLEKFTNI
MYLTLTELGMVAKLFNVWFYAKLLVDFFETLSGDKYFELREKDERLKWQHAQRTYARIVLFYVFIGLGAM
FTGFVGVLFSAKYELPFPYAPPFNWHTPHGYWCAYLYELLAMLITFFANYGFDMIQCYMLLQLSLCFKLI
CGRLECMGELRSGTAVSRGFSEQQLYRQFVDIVKLHARIKNLSRLCQTYISFPFLIQIMCSSFVLCFSAY
RLQKLSILSDPMQFLTFVQVNLIMILEIFLPCYYGNEVIAQSSALNNATYNSEWFRCSPGLRKYLVIYMA
MLQRPLRVRAADFFDISLEIFTNTMKNTYSLMALLLNMNN

>XP_020716035.1 odorant receptor 74a [Ceratitis capitata]

MYSIKYLAEDADEKEAWRSLVKMLYRPRLPNGKLIPLSWPLAAYRLLNNICWPLRDNANRLERLFDRFCW
ALGFFIFIQHNDAELRYILSNNNNLDQMLICGPTYLILVEAHLRAFQLGLKKNNFKNFLKRYYAEIYIDK
PTHPKLYANIQKRLRPIWFYSFLYYSTLFSYVITPLTNYLKNVKAPLFKMYYPFDITPNPIYVAVVLSNI
WVGFTVISLVAGEDNILSEVMLHLNGRFLLLQQKLRQNADRLLHNTDGRYIADALQDQIIEAIEENVRLY
EFAKGFEREFSFRIFVNLSFSAGLLCVLCFKVYTNPMASYGFMFWICAKIMEMILVGQLGSTMIYTTNEV
SSTFYECNWELVLMKSTDTKANVRLLKTLALAISTSGKPFVLTGFNYFSVSLTAVLKILQGAGSYFTFLT
SMR

>XP_004531172.2 odorant receptor 7a-like [Ceratitis capitata]

MPLRTFQFYKFTFSLFRYKYLVALILNLLLSKKKITDLFATMADTAINSQRLQTDTLNNGLKKKGDQLAV
RTEHATNYLFNGFRVLGIYMPARRKWLYSLYSLIPNSLVTLWLPLSFVFSYFTMSAEDLVPSSLLTSIQV
AINVIGCSVKIVVMAFLLPKLRKANAYMDRLDARCKDEDEIAELRKIVKQGNRFVVLFAMSYWSYASSTF
IGSVTFGRPPYDLYNPFIDWRKSKLEFVAASLIEFALMDVACFQQVVDDSYAVIYVCILRTHMNILLKRL
GKLATCTEMSLEQNLEELKLCIRDHKNLLGLYNIVAPIISITIFIQFMITASILSATLINIFIFANQFST

QVASCFYILAVVVEVFPLCYYAQCLMDDSDRLSQQIFHANWIEQDVRFRKMLIFFMQRTQRVMELNAGKI
FPITLGSFLSIAKFSFSLYTLIEKMGIRERLGLE

>XP_020715918.1 odorant receptor 59b-like [Ceratitis capitata]
MSNLLQRLLNQLLPSRTTQKSIDVVKQVSPSLSNAELIRIQFERATRTPKERSAPVGRPYQAVHDIHSRD
GLIYLYRSFSALGVLMPDKHKILYCLYALLPLGLITFYLPISFALSYFYLDYSTVKIGNLLTSVQVFIAS
IVGGVKLIVMAFKLPKLRASEAIMHQLDARCKDEDEIEVLRKVVRQGNRVFVLVLICNLIYSTSTFLAAA
SKGRPPYNLYNPVVDWRKSKGAFLWAALWEFILMDGLCTEEAITDSYAPIFVCIMRAHMKTLLMRIQKLG
SNPERTLDENYEDLKMCIKDHKLLLELFDVVHPIISTTYFLQFMTTSLMVGCTLLNIMIFAVDNLARVGH
LAYVMALLMEVYPLCYYGQSLLDDSNRLANTIFHANWIKQNEKFRKMLVVFTQHTQKPMELLAGKT

>XP_020715917.1 odorant receptor 42a-like [Ceratitis capitata]
MNRFRKQSARAAETENNKLALALSKEKLSGLKNGEEQRIARKPAKPASAVREKQGEFGNVFFSLTFLVAS
PASSKEATNYFFKAAFGMGIMLPTRHRILYILYSFAVNSMATLYFPIGFTLIFFTLPEDDLDVSNLLTSL
QVTFDVYAGSIKLIIMAFLLGKLRTSEIVFQQLDNRCRTPDEMNELRKMQQFGRKVIIFYMTIFLIYSSS
TFLGSVTFGYPPYSLYFPFLKWRRSRIEFIIASLLEFLIMDLACLQQTVNDGCPVVYVNILRTHMKILRS
RVEKLCTNAALTKEQNLLELKLCIKDHQLLLELYEIIASIVSITLFLQFTVSAICVGTTLINFVIFANGF
STRVACFCFILAVLIEIYPICYYSQCLITESEGLSDVIFHSNWIEQNKEYRQLLIFFIQNAQRPMSLTAG
KLYPVTLSNFISIAKFSFSLYTFIEKMNLKERLGIE

>XP_020715765.1 putative odorant receptor 69a [Ceratitis capitata]
MSGVFSMNHFLKYPNFTLDLAWSNPFAWSGARQYGYHMLWIRRLLFTFGAANLVYQNFGMMIYLCMPHEV
SNESTIGQITETGGIMGLTMVGASNMFVMYWHADRIALLLEKFQRLFPTKQLQWRAKHANGRLRGVKFPH
SVEYFALKSNKLMKIATTAYLFAFSYYNSLPIVEYLYESLTPGVELKYHYQSNTWYPWQNAHNRKSFIAF
LAAYICQVQSSLTGVAFIMAAEFMLCFFITQLQMHFDYLANALETIDAAGENANEELKFLIHHHGRLLSY
SKEINAIFNISFLVNIFTSSIAICLMGFSMVMISVIHACKYCIGLLSFIVFTFFICYTGKELTDASDKLL
YAAFYGNWYEGDLAYRKMILFLIMRCRIPTVLRAYKFTTVSMPTFTAILRSSYSLFTFFQAMGK

>XP_020715078.1 odorant receptor Or2 [Ceratitis capitata]
MLYSEEIYNWNLVFMRSIGYLGNRRRAYFLLSLPVLICFGAIYGTYKMWSDFDKVIINLFKTSGLLTVTL
RSFVIIQKEKKLYDFFDYISQLYRELQAEGDETTLKRMHEFARKTKRYTKGLFILMIACTFYISLIQVMS
TFGIGLKKFLIEMELPFISVNENPYWDIFSTLQAVWLAPSILLSYISYLCIIFTTISFGILLMKDLQFKL

GNMNEKNDLEAYEYIKNCVKQHVMIIKYHRQMEVLFSLGSCAEVCNFCIIPCVIIVYSTMDYDLAFLMTD
IQLAVIAVSSTFFNFWLANNFCVESLNIAYAAYNSNWIDRNKEFRKYIVLIMTMSQKPLQLTAAGLKPIN
MEFFLAILRAAYSLFTVLQ

>XP_004527093.2 odorant receptor 63a [Ceratitis capitata]
MILEEVEDVYKRNYNSIKVLIRVSFGLGVNLTAPTRFKDSLRIFNVILVTTSILSLYAHWCYLIRHFDNI
PLLAETVCTALQTLISAVKMVYFLFTQRTFYRLIDQALTHEVIRKIEIFKYAFPALYPFWEAKGMTFPYY
HLQMYMTGSAVYIAGICAVSFDGVFIVLCQHAVGLVKVHNLLVLRATSPLIPPERRVDYLRYVIFTYQRV
RVYVQQVQTIYKHVSLSQFVLSLIVFGFVLFEMSFGLESSITIFIRMIMYISAGGTQIIIYCYNGQQLTS
VSEEMPLAFYSCGWYEESKKFKQLLRMMIMRTNRHFYLEVSWFTLMNLATLIALFRMSGSYFLLLRNLQE
S

>XP_012157426.2 odorant receptor 85b-like [Ceratitis capitata]
MHRLLCKFEQVAMRVFPSDVRRGQIGSIEFNIWLSQMTGVPLPPSFVPLRRRFRQIFDRAEVENKLFLVI
YATVIGFTGFTAMILVFINPDMAGKIFPYRVALPAWLPLPVRVAYIGTTDFMFAVQIVTVDYLNIGMMNL
LRCHLNVMKSSFDELNFNVKCMKSDIKRIRDPNERLADIVRHHCVLKSVRDDVEQIFRLPVLLQFFTSLV
ISAVTGFQATIYSSNFKSELIIYFYCFCIFTQLFGYCWFGNEVNEQNKTLAARGYSSSWYYFDNRFRKSL
AIFLVNAQQPFDFTGGGFVALSLPSFTGIMSKAYSFIAVLRQMYDR

>XP_020713417.1 odorant receptor 47b [Ceratitis capitata]
MLHECSDKSTISNTIFNHQSYRRRSRKYSSSKLETILAPIRTLKEMLLSGDAAHPSHTCLYYVRAYIRLL
GLWPSQRGVEQPMYYAYNVLIMTVFSFFVATIIADLYVASSDFVLLGEDLVVALGLYLILFKMILFRMST
ADVDVIVDEFDALHMKFARDTSDSPHIRRIRQLQRSFFLGEASFFCGFFFLSLFLFAAMSLQPLLTHQAL
PFRCVFPFGLHDPDKHHITFVCVYAFQCFCTLYMLVSIVVMDSLGGNSFNQTTLNLQILCESIRHIGYAG
GRSTTITEAVLWRELRENVEFHVKIIELVDGINHTFYWNYVSQMGASTFMICLTAFEALLAKDQPMVAMK
FQTYMFSAFMQLFYWCFMGNRTYYDSMEVATAAYEVYAWYEHSPRLQRNLLFMIKRAQKPLEFRSKPFFG
FTFASFNSILSTSYSYFALLRTMND

>XP_020713013.1 odorant receptor 63a-like [Ceratitis capitata]
MYSQREINDLKVRNHWLIRELKRSSYLIGVNLSTQTFLKGWLKIINVLLIIASCIALYPHWLMIEHAEGN
LSLIAETSTTALQTTTALVKMAFILFKQHRLHELLYKAEYHELLQGIQIFMTDMPIRISLKNEAIRIMDT
TWQEARGQLLFSLISCICIQANYFFYAFFKNLYHHLQGTPNYVYILPFTGYPMFHHKGMSSFYYIMDMFF

GACSLHCAAMCAICVQCTFMVLCKHCCGLVQVQCLMLLRSTSPLVPKVRRVEYLRYCVIQHQQILRFMEG
INQLFRHICLSHFLHSLAIYGFVLFEMSFGLESNKVIFVRMMMYLCAALTCDCMFYVNGQFLSTELEKIP
LACYSCEWFHESREFKMILKMIIMRSNKPFYFQISWFTVMSLATLMGIFKASGSYFVLLRDIDEA

>XP_020712941.1 odorant receptor 7a [Ceratitis capitata]
MSDLLFGRGQIVYKSRHALTYLFNIFTFVGTNPLGGQSYAYYSLYYLYSLLVNFVCCIFCPISFHIGYIK
LLNVLNTNELLSAIQNAIQVSGIPIKIIVIAWYMKRLQSVCEILDKLDENYKRAEDLNSIRRCVRSCCKI
IAAFCVPYYGFELSTIAFGVYQNRAPLTIWLPYFDATRTTWEYWTHVGWDVFIMLFLLAHQLGSDTYPPV
FISVIRLHMQLLVERVKRLGTNKALCREKRYAELLVCINTYGQILSIANIVAPVISITLFTQFATTATTV
LNWFGNMKFPDNIIPMAFFSCQIMQILPCCYYASQLIADWCFLMTHHSLRPIFEQHYRPAVGSVIRATID
QLYTVLTLLDNARPPSAVIATVEQNIKENPNEYIHYREAQLEPCSSTFCKILLKNLGLRTYKTQLVQESK
QNDQLERCTSAK

>XP_020712742.1 putative odorant receptor 71a [Ceratitis capitata]
MTFDNIANSRFLTRALVLLGLWPVITVHGSWLQRNYCFYQLFLQITFTFSFTFLMLLEVICSESLDHATE
VLKFLLTEMALVFKILNTWYYARKAAAFLHEWETGEMFVLRSNAEKNMWAKKQSTFRKIMLGYIYWSISS
AVCALLSCLFINDQALPFPYWTPNHWLEDYYWLMYFYELLTMPFTCLCNIEIDVFQCYLLLHLALCLRVV
GMRLERLANAGDENAITREFLKTIKMHRRINEWIMLSALIICFIIYRMQSVHFSDNPTEYLAMFQYVVAM
SMQIFLPCYYANELTVQSQNLSKSLYNADWTGMSAYNRRLMLLYMQYLKLPLTMNNAYSLLALLLNVSDD
KDKK

>XP_020712741.1 odorant receptor 59a [Ceratitis capitata]
MSSPLISSSSSSPSPSPSPSPSPSPSPLPFPLPQRLAATAVDTRSFFKLHWACFKVLGVVAPTADVFYLVYS
VLLHLLVNLCYPLHLALMLFRSPNSSANIQNLAVCVTCMACSVKFVIYTMKMWRIRELESIVAALDARAC
SPRERGYFLKLRKDMRRITIGFLSIYAFVGVTAELMFIFCNEHNLLYPAWFPFDWRASKLKFYAAHFYQI
VGISYLLLQNFVNDCFPTMALALLSAHIKLLGIRVSQIGHEAKSLDANELELLRCIKDQEHLFNMLNTIQ
NIISLPMFLQFTVTAINICLAMAALFFFVDAPFDRLYYLAYFLSMPLEIFPTCYYGTDFQLLFETLHIEM
YASNWVEQTQKFRKHMILFNERSLKKEVAMAGGMIRIHLDTFVSTCKGAYSLLAVIMKMNE

>XP_004520354.2 odorant receptor 74a-like [Ceratitis capitata]
MRYLPSSYHKPLLPNGRHPPIDWQLYGFVCSNCWPLARDITKTRRIIDMVITAMQFMSESMVLSGETIAM
RNNLDDISFVCMVLAPYLILIELMLRAYNIIYKRNSFRTHIEEFYKKIYVQRTWNPELFEQIRRQHLPTK

YSTFTYIITLVTYVYVPISGLVKNERLVPFPIQFSFDFTVPWPRYVVFLIMSIWTGFAVVGPLVSEANML
AMQILHLNGRYSLLLRDLRKIAREAIEEHEKCKGRDKISVTQRFRYSLFDIIRRNVELNEFAKSLQDQYS
FRVFVMMALSATLLCVLGFLTATLGLTAENIRFVSWIIGKVVELLIFGRLGTTLSTTTNDLSTSYYCCDW
EEIIFHSADAEENRKTMKLIALAIHLNSNPFQLTGLNFFVVNYETVVSILRGAGSYFTVIYAYR

>XP_012161119.1 odorant receptor 7a-like [Ceratitis capitata]
MFDLLKGRGYRELNSRDALIYLFNMLSFVGLNPTAHCRLLYYFYGSIITLFVVVLSPLIFNIGWIRDRNV
LSIMEILNCVQAALNVIGVPIKSIALMLCLDRIHSVEPLLLKLDAHYSKWDDMLRIRQCAIMGNRLVFSY
IVPYMMYETLTVVSAVLGGHAPLTLWLPYVDWHRSSREYWLQVCFDAITLFYLLCHQIINDSYPAVYIYI
VRTHVQLLERRVSRLGYVPQKSEHENCQELQECIVTHQEILRLVHTIQPIISITMFVQFIIAAAIMSITM
INIFIFADLATRLASFVYLICVVLQTAPSCHQASYLQGDCEKLSSSIFHCNWIAQDKQFKKLLIYFLQRS
QADMPLIALKMLPINLATNVSIAKFSFSLYTFIQKMGLGAHLND

>XP_012160723.1 odorant receptor 10a [Ceratitis capitata]
MKFKFLSRTFPLRDYYFYVPQLCLGSMGFWPMDTCRQQAANVGAWMNLIILAIGVFTEIHAGCTVLRTDL
ELALDTLCPAGTSAVTLLKMTLIYYYRQDLAWVLERMRSLVYERDVSINPIKKRIIRAHAVMAARLNFIP
FVMGFITCTSYNLKPLLITLILYMQGQQPMWKLPFNMTMPAFLLRAPYFPFTYIFTAYTGYITIFMYGGC
DAFYFEFCSNAAALLKLLQEDLKSIVSFEEQLVFTAQESTLLEWRLVRFIMRHNDIIELTRFFCKRYTII
TLAHFVSAGLVIGASIFDLMTFTGFGIVIYIAYTIAVLGQLFIYCYGGSLVAESSVQLATVAFGCDWYAC
NPKLRRYVLMIIIRSQRAISMSVPFFSPSLITFTSILQTSGSIIALASSFK

>XP_004533437.2 odorant receptor 67d-like [Ceratitis capitata]
MATKTIRPTQKFAKMIKIVRFISSLVGADVADENYRINIVTVLVILCIIIYFIFTGTTVASVFAENWKYL
LEASCMVGSVLQGITKLISGVGCTKMISSICKELENLYQHYETKGEAYCKVLNEGCERVWYSIKMVGHIY
AAAIYGILLLTGFLILTTNEKVYVMHFFIPGVDVETTYGYLFTLAVHTVVFLAGAFGLFAGDLFFLIFLG
QTQLFRDILVLKVKALNEAAAENAKNTESLLIDIIEWHQYYTDYNKRCNDVFYYIITMQIVTSGISIICT
MYILLMGDWPGAYLYIFVAFCGLYLYCIIGTSTQTCNAEFFDEIYNINWYELDVKCQKMMVFIIKKSQSP
AEIKIGGVLPLSVQTALQITKSIYGLFTMIIGVIEENN

>XP_012159806.1 odorant receptor 22c [Ceratitis capitata]
MLQPLLGSQVPIEQSFFRIPRISARIAGFWPQPAVRPRTWLTVLRFCVNTFAVAVGGFGEVTYGFFYLYD
LFSALEAFCPGVTKVISLLKMTIFFGRHERWQRVIHGLHTLLLLDTSAGKRRIMEPLASFASVLSFVLLA

SGSLTNTFFNVLPLLKMAYFKWRALDMQLLLPFNVILPEVLVNLPYYPATYLVLTLSGAMTVFTFSAVDG
FFLCACVYATALFRILQHDIRNAFAELQEQESSSFEQNMRIQHRLSVLVERHNKIIDLCSDFAAEFSLII
LMHFLSAALVLCFSILDLMLNSASIGVLIYIFYSIAALTQLVLYCIGGTYVSESSLSIAEVIYDVDWYKC
DVRTRRMLLLMMCRAQRAKTIAVPFFTPSLPAFRSIVSTAGSYITLLKTFI

>XP_012157159.1 odorant receptor 2a-like [Ceratitis capitata]
MDKLEALSSRIFPSDPSIGKIGSIEYNVWLAQLFGVPVLGLKKETPRMRIALAVYGVVATLVVTFLYTGF
EIYDMIFCWPNLDKLTQNICLSLTHVAGALKVINIIYRLKEVAGVVRKIEYAARYYVISKNQLKAFYRGE
FENKIPLTIYASLVGFTGILGIAYLLHNPTGVAGEIFPYRVKLPHWMPFGLQLAYMGFSVLVFALQIVAI
DYLNVTMINQIRFQLKILNLAFEELKFVSGQAAHELSLDRRLRTIVDHHNLLRNLRNEVEEIFRLPVLVQ
FFTSLIIFAMTGFQAIVKSENSNGASLIYCYCGCIFCELFVYCWFGNEVSEQSKTLTTSGYNCHWYQFGP
RYKKSLLIFMFNSQKPIVFTAGGFMALSLPSFTGILSKSYTVIALLRQFYGR

>XP_004520908.2 odorant receptor 7a-like [Ceratitis capitata]
MFDLLKGRGRSVFASRDAVIYLFNIFRFLGLNPPPQCRFLYFFYGSIITLFAVLLSPFIFNVGWIRDRHI
LSIMEILNCLQAALNVIGVPIKSITLALSLGRLRSAEPLLMKLDARYTDPEDVARIRGCAITGNRIVFGY
IISYMMYETLTVVSALMGGHAPLTLWIPYVDWHRSAREYWLQVSFDAAMLFFLLFHQILNDSYPAVYIYI
IRTQVQLLTNRVRRLGTGGSSRDDAYHELQDCIITHQEILNLVGVVEPIISATMFVQFFIAAAILGTTMI
NIFIFADFATRIASVTYLFCVLLQTSPTCYYATHLQSDCERLSMSIFHSNWLAQGKRFNQMLIYFLHRSQ
ADIPFFALKLVPINLATSVSIAKFSFTLYTFIQKMGVGKNLKQ

>XP_012158499.1 odorant receptor 85c-like [Ceratitis capitata]
MSIIIRFEEFLRLPSFFSRNIGIILWGQRGKLFDRFMFYFSSINLFLTLLAELWYIISTISTDFITAIMG
LSYVSFVVLAEVKFYYLIKYDMKVSTVLKRLNALFPHTKEEQENIQLIKYLKMSKFYTLFYTVTFMLVIW
TYNLYTVSQRFIYTKILQVREIERELPYPAIYFWNWQDNWSYFMLYISQSLAGWHATCAQILTDLLICIL
ISHLIMHYDHIARSLLNYQSKFAELYGKESTMKCMPKLARVMMEERAVRADMKFLADIIAYHTELLSLTE
SLNDVFGVPLFMKFMSSSAIICFLGFQMTVNRGFDLLTKLALFFILSVLQVYLICHFGQLLIDASTNVST
ALYSQDWTNADVRYQKMLVLIIKRAQRSATLKATNFIIISRATMTEIMQMSYKFFALIRTMYND

>XP_012158498.1 odorant receptor 85c [Ceratitis capitata]
MCENIESFEAFLRIPSFFYRSVGVDLWNTNGGSIQRFIFYFGFLNVNLWLLSELIFAIITVSENFIQATM
TLSYAGFVLVGSIKMYFMWRKKTEMTQFLKLMDEIFPRTAEQQKMMNLRRHLRQSTIVMSGFALIFMILI

WTYNLYPFMQRQIYDCWLDTRSINKTLPYESYIPWNWHNHWSFYLYYVLQSIAGYHSAAGQIASDLVLCA
MATQMIMHYEYVSHKIRSRYRGERKCVDSKSVSCLNALNHWTEEQVATHKDMRWLCETIAYHSNLLSLSD
VMNDVLGVPLLVNFMTSSFVICFVGFQMTMDAEPDYMVKLFLFLFSSLAQIYLICHYGQLLIDASINVAA
AVYDQDWFDLNVRYQHMLVLVVARAQKPAMLKATNFVRISRGTLTDIMQISYKFFTLIRTMYSD

>XP_004518315.2 odorant receptor 83a-like [Ceratitis capitata]
MALADRKTPPTDTEHIGCPVYSTQRRDMFRFMRWNLWFTAMCRLPLEYYFPTCLRCLANTLDWTYEVFLY
FTLLHIDILFMCTIYLNKDKGDLALIVSCMIQTVIYTWALIIKVFFKRVKPKRVAKLMQYLNNECRTHSA
AGFTYVTVKDSVELSKNWISIFLICCYAGVTFWLFVPIFNADRSLPLACWYPIDYKVPVIYESIYLLQTI
GQLQIAGAFGCTSAFYLLACVLFSGQFDVLNCSLKNILATAYINLGKSKSELCKLRDKQYIADKELNQYY
CSKEYKSDLDCLPHLMNVATPKPKTLYAAFKQAFIPCITHHRYILYGLQMLEDIYSLLWLLKTMEVTVLV
CLVAFAWVKSTTAKSFLSILSLSQYLLLALWEMFMICYSGEIIFLNSQRCDEALQRSPWYLHANEIKQDT
LFFILNAQRPFRLTGGKMFDLNVEKFRSILTTSFSILTILQKMDVRPTQPK

>XP_004537973.1 odorant receptor 33b-like [Ceratitis capitata]
MAFEENPESVGSLFRTHWIVWKCLGQVPDPRYPKLFKVYAVLLNVGFGLGYPLHLLLGQLGLQTLEEVLL
NLTISVPVAVCALKFFNIWRNLRKVRHLEKMFNTLNTRINQRDEWIYYRKVTIPNALKVLHLFYFICVGT
ALASELTLLIMGFAYEWRLMYPAYFPFDPYATTGGYVVAHTFQIIGLLVQLAENLVSDTYGGMCLALLAG
HAHLLGKRVAIIGYDNQKTEMDTGRELANCIVDHNMLFDCHSILGEIIGIGMFAQIISASLIMGIVVIYM
VFYVGNAFEYVYYSIYLFGCAMEVFPTCYYATNFEFEFDKLTFMLFSCNWMDQNQSFKKSLMISIEQSLK
TRSFRVGGMFRINLQIFFATCKGAYSVLALALKFK

>XP_004536508.1 odorant receptor 7a-like [Ceratitis capitata]
MFDLIKGRGRTVFASRDAVIYLFNSFRYLGINPPDKYRIPYFLYSAIITFFAVLFSPVIFNVGWLRDRNK
LSVMEILTCVQASLNVMAVPLKCITLAMAQNRLRSIEPMANELDDYYRQPADKVKIKKCAVTGNRLVFGF
AVSYLMYETLTVVSALVGGHAPLSLWIPHVDWHRSTWEYWLQVSFDAAVLFFLLYHQVLNDSYPAVYIYI
IRTQVQLLAHRVENLGYDETKSDDENYKDLLECIVLHQKILKIVSIVEPVVSVTVFTQFLVAAAILGVTM
INIFIFADLTTKIASVTYFFCVLLQTSPTCYHASYLLADCDELRLSIFNCNWIAQNKRFNNLLIYFLHRS
QDSIPFFALKLVPINLATNLSIAKFSFTLFTFIQEMGLGENLKG

>XP_004535437.1 odorant receptor 43b-like [Ceratitis capitata]
MRKIGDLCYGRGKNNVYIKESFRLLFFSWSLTGIAPTKTPRLFNTIFMIICWCGILMCPYCFIAGAVNSM

KTSVITVTLVNLQAALNGIALPLKAITIAVNVKRLRSIDNIFKELDNSYTDPVHHELIKKSVMRCTRLFV
VFLTVYWLYGITSCTAALFSHKYPHSMQIPFIDWLPDSDVKYWLHYILEASYFFFLLLVNLTNDVFPAIY
IKAIRTHLYLLTERVSTIGKKSETTAEQNYDTLVECIISHQKLLRISDTVGDVISKSIFFQLAVYSTILC
ICMLNMLIFADTTYILVTLVYLIPVLSQTIPSCYQASMLEAESTKLSVAIFHTNWWNLDKRCHKLLIYFI
QRSQQEMVFTAVKLFQISLKTNLTIAKFSFTLYTFINKMGIGETWKN

>XP_004534466.1 odorant receptor 45a-like [Ceratitis capitata]
MRIIVLKDITPVVDAFACIGFNIDLNKAKGSFSQPVRYFVLLIGVIAWTAALAAYTMQYLTDVDKMVAAM
TINVQLFLTTSKNLIFLARRKRFLHLNEALERLALNGNESERILWNTTNRFVLPITRAYRISSELTVSFC
VLLPILKLLYYYIFHSEVVLTLPLPGIFPYNYTLPFYFILTTILTILLVYLCAYTIVAIDGLFGWFIYNI
SAHLQIMSLRLEQILQLPIEDPRFHRHFVDLVNYHKEIIRLSLELDAVYAPIIFLEVTSSSLPICFLAYQ
LSYLSDPANVPFMCLLMSSIVIQLMIYCFGGEKVQSECDQLCENIYLLIPWQNLPQKHCRLLLNPLIRSQ
RVLVLTGYFFTANRSLLVWIFRTAGSFTAMLFALKEKDV

>XP_004534465.1 odorant receptor 45a-like [Ceratitis capitata]
MDGLGIGGDGNSGIRAGEIGEKTDANSDSDVGANVTSSTMDNALMQKPDQAITVLTVFLQGVLSVFKSGM
FVLKSGRFIELIRNLDMLAEKEHSAWRLENDWQQRIVSVYYFCCTSTGILYCTVPALVLLYTRYFREQTV
FILPFEASFPYDTNQPLFYILSYIWCISFIIYAIHAIVAMDSLFCWFIFNISAHFRILQQKLTDVSVSPN
ASTDHAIFQQDITQTLFYHQRIIELSAEFDELYAPIVFVEISVSYLKLCFSAYNLINLDDISSLPVIAVG
LVTITFQLCIYCFSGEKIKNASEQFANHIYLSFPWERVPPSLRRLLFVPIMRAQRPMHLTGFLFIVDHSL
LVWIFKTTGSIIGFLSATKKENTNI

>XP_004534194.1 odorant receptor 30a-like [Ceratitis capitata]
MIPPIFCNLYLGHDDLGEAIYDFFTAMIDITGLIRSIVILRKQRKFLNLFGVVESWYEDLKQPNEHKALE
TLNQIVAKVQLYSKCCLYSLLMVDVTYAFEPIITHYGKLVVELQLPSIDLHQSPVYEMVYLIQALWLVPL
TSVNYVSYSNSLLIFTIFGVFATRHLQQKLMEISQMEDDEALANLKQCVVYHSKIIKFGENLEELYSLMS
LLDISLYCISVCLMLVYLTMDFTWPLLFKGVIVILFLTTLIFLTYHVADVLTHESMNIAELAYNTNWMDR
DKEFRSCIQVIIVRSQRPLMLTAGGFQPMNMKTFLAIMRASYSFFSVLRSTV

>XP_004533817.1 odorant receptor 43a [Ceratitis capitata]
MPITRIEDNPLLAINVRLWKFLSVLFARNWLRCAAFVAPVCLMNAMQFVYLYQQWGDLATFILNTFFATS
IFNALLRTCLVIKNRDKFEALIQELVTMYDDIEATGNDYAKRELAAATKAARKISIFNLSASFCDIIAAT

LFPLFQENRIHPFGVALPGIDVTRSPLYEIVYISQLPCPFTLTSMYMPYVSLFASFAMFGKVALKVLQDN
LRNLCDNMQHKSEMELFNLLRSNISYHARISKYVNDSNELVTYMVLIEFMLFSCVICSLLFCINITNSTA
EKISIVMYIGTMLYVLFTYYWQANGILEQSLLVSDAAYEMQWYKCSQRFKRTLLIFIGRTQKPLQIRVGQ
MSPMTMEVFQSLLNSSYSYFTLLHNLYND

>XP_004533438.1 odorant receptor 67d-like [Ceratitis capitata]
MAAEKVSPSESFAKIVKIFRLICSLVGADVCDVNYRINIVTAIVIFCIIIYFIFTATTVASVFAENWEYM
LEASCMVGSVLQGITKLTSGIAFAKEICAMRFELEDLYRLYETRGEEYTAVLHLSCKRVWQVIKMVGQIY
LAAGVGILFMTAIFIVATDEKVYIMHFFIPGLDVHTQMGYLLTMAVHTVVFLAGAFGLFAGDLFFLLFLG
QPMLFLDLLTLKVQALNVAADRCSNEAERLLIDIIEWHQYYTDYNRRCNHLFYYIITMQIVTSGISIICT
LYILLLGDWPGAYLYIFVAFSGLYLYCIMGTKIQICNDAFCEELYNIDWYKLNVKSQKMLVFILKKSQKP
AEIKVGGFLPLSVQTALSITKTIYGIFTMMLRFLDEEN

>XP_004530391.1 odorant receptor 33b-like [Ceratitis capitata]
MLKNVFIFKPAGNAAVDSVACFDIFWMCWKLNGIAVNSNKWYITLYDIAVNIFITIFYPIHLTVGLFMVP
TLADVFKNLAINITDVACSTKHYLFRYKLPKIRELQRLLKQLDERVLAPNEREYFDKKIRLGVRNIMLLF
CASYAADALASAIDVLSKNERELMYPAWFPFDWSANRFTYYGAVFYQIFGVSLQIVQNLAHDTFAPVGLC
VISGQVRLLAMRVSKVGYDESKSLAQNEQELNECIEDHKKLLRIFDLMQDVFWYTQLVQFSSVGLNICLT
VVFLLLFVDNLFGYVYYTVYFISMAIELLPACYYGSNMQEEFQNLPYAIFKCNWIPQRRGFQQNLRIFTE
LSHKQLTPTAGGIINIHLTSFVATCKMAYSLYTMLMNI

>XP_004530390.1 odorant receptor 33b-like [Ceratitis capitata]
MFQRQIETRAIFRRLFMTWRVLGIILWPFNKYLRIIYDILMNIFITFAFPVHLTLGVIFSSNQEQFFTNL
IIGIASVSCTFKHLLWRSRLAEMQQINEILAQLDDRVRVREDYEYYKRSIERLCNFMINFFTRCYFSVGV
TALFIALITGELLYPAFMPLQWRTSFWNYVAAILFQFVGVMLQIVQNIANDVYGPVVLCMISGHVHLLAN
RVSRVGHDTEENTQSNYEELSKCIEDHKLLMSTSKTVERIASLSYLVQFVAVGINLCIGLVYLLFFADNY
FAYVYYTIHITAIMIELFPCCYFGSMLECEFHDLSYAIFSSNWPTQPRPFRRNVVSFTEMTLREVTMYAG
GMIRINLDSFFATCKMGYSFFTVIQTMK

>XP_004530147.1 odorant receptor 7a-like [Ceratitis capitata]
MLDLLRGRGRRVYKSRHALTYLFNVFTFVGTNPGKTRTHKYYTLYYTYSLTVNFICCLFCPLSFHIGYIK
SWHLLNTTELLAAIQNAVQVTGIPIKIFFITWNMRRLQSVIPILDELDENYKSAKDLLKIRKCVRGCCKM

IGFFCLPYYSYEITTIALGVWQNRAPLAAWVPYLDGQRAAWEYWTIVVWDIFVMFFLLSHQLGSDTYPPI
YINIIRTHVQLLVERVELLGSDKTKSAEEHYAELLGCIRTHGQIKRIVNLIAPVISVTLFTQFATSATTL
LNWFGDVEFPENIISFAYFTCLTLQILPCCSSASYLISDCELLPNAIFHCNWIERDRRFRKTLLFFLQRT
QTPLRFSCLKLFVVKLETSVAIGKFAFSLYTLIQGTEVGGKTEN

>XP_004529819.1 odorant receptor 13a [Ceratitis capitata]
MLFNPKPLKDPVNFKFPLQCIWLKLNGSWPVNSKTSSSFKNYCRFLYSLWAWYVVAMVGITIGFQSAFLA
KSFGDIMVTSENGCTTFMGVLNFVRLLHLRLHQRDFHQLIAQFVKDIWITKSTHPAVEQSCARTMRVFQV
ISVLQSCLITMYCILPLVELYMLNANLDQESLAHIDKPFPYKMLFPYDANHGWRYALTYLFTAWAGVCVV
TTLFAEDSLFGFFVTYTCGQFRILHTQIDNIIPAAYAATRAGRGTEADYQRECVRRLDKIAGKHSILFNF
VSRMEEFFSPIFLVNFLISTVLICMVGFQLVTGNNMFIGDYVKFLVYILSSLSQLFVLCWNGDKIIQNSL
EMANHLYACNWESDIVLTNTHTNNNQQMKNPTRIIYYSTGAVFRKNLQFMIMRSQRQTCITAMKFSILSL
SSFSGLMSSSMSYFALLQSFYEDEEN

>XP_004526558.1 odorant receptor 49b [Ceratitis capitata]
MFDDLQLIQMSVRILRFWSLIYEHTWRRYACLSMTTFLVFTQFYYMFRTSEGIDSIIRNSYMLVLWFNTI
LRAYLLLYDREKYEELLRDLENFYYDLKKSKDFYIQDLLNEVNSTGKYMARGNLFLGLLTCFGFAFYPLF
ATERVLPFGSMIPGVDEYKSPFYEFWYIYQMIVTPMGCCMYIPYTSLIVAFIMFGIVMCKALQFRLKTLH
RCRHVDGLIHKNVKECIRYQLSIIDYIARVNGFTTYIFLLEFLAFGTLLCALLFLLIIVDSSAQAIIVCA
YIAMIFAQILSLYWYANELREQNLAIAAAAYDTEWFTFPIPVQKYILLMILRAQKPPAIMVGNTHPITLE
LFQSLLNASYTYFTLLKRVYI

>XP_004526336.1 odorant receptor 46a [Ceratitis capitata]
MITPRKASSIPIIKYTRITMEEPKKIVNCFYERQFKFFKFLGLFGLPPNYSRFCQILFKLYFWHVTVVWM
LLFDISMWVKVIGNITDLNEIVNVFYICSMAIAVMAKFVHIRKKNSRYVAFFARMHNDDLLPANPSELKK
FIKSVHLSCAVRNCYMGLSLTSLALVFVPKLISDPGELPLSIYIPLNVEHTLCFLVAYIFQFVGLSLCCF
LNIAFDSLSASFFIYLKGQLDILSNRLENIGKFQNITQDVITLQLKECIRYYAKLRYITDIMEDLLCIPM
SVQVISSVLVLVANFYAMTFLTDPSDYGTFMKFLVYQLCMLSQIFMLCYFANEVSLRSAQLSYALYSSEW
THCNQINRRLMMLMMAQFDVPIRIKTINRCYSFNLPAFTSIINSSYSYYALLKNMKD

>XP_004521318.1 odorant receptor 7a-like [Ceratitis capitata]
MFELLSGRGIGNCPSSYAFIYLFNTFTILGTNPPSDAGPLYYIWSAFLNTFCIIFSPFLCTVGFIMKYMQ

STITTMQFLSGIQAGTNVLGIPPKCLTLAFSLKRMRSIEPLLDVMDARYTDPEDVALIRQAAIMGNRLVF
GFGMTYLTYMLLTITPPLISGNVPLSIWIPFLDENQSTLHHLMQVVMDLFLMFFLLFHQVVNDSYGTVYI
YVIRTHLRLLIRRVERLCVNGEKSVEDNMAELVDCVTTHQQILSLLTIIEPIISVTMFTQFLIIAIILCV
TMVNMFIFADLSTQIASTFYFMCVLMQTSPCCYFATELKADSERLPLAIFHCRWMDQDQRFRKVIIYFMH
RAQSPIELMAMKLFPINVATNISLAKFSFTLFTFIKEMGVGQDARE

>XP_004521076.1 odorant receptor 67c-like [Ceratitis capitata]
MLPAARTFGEFIRIPIRFYQTIGEDLYEHRSPHRIRRLILKALLYIGFLNFNVLVLGEIIYFVKALNSFA
TVLEATGVAPCIGFSFVADFKQIALTVHRQTLREHLDQMEELFPKTVRQQAQYKLPQRERVMRRVMGVFT
LLCLAYTTTFSVYPALKATVQYWLLGAPTFERNFGFAIWYPYNATGKTWVYWLTYMGQVHGAYLAGVAFL
SADLILVASVTQLCMHFDYISRCLEDFAGASKKCAEEDIKYLQALVVRHAKCLELSEHVNSIFSFSLLLN
FLTASLTICFIGFQVTASSTEDIVKYIIFLTASLVQVFVVCYYGDELMTASLRVGDAAYNQNWFECDTRY
KRLLIILILRSQKPASIRAPTFPPISFNTYMKVISMSYQFFALLRTTYSDKGN

>XP_004520877.1 odorant receptor 49a [Ceratitis capitata]
MDFEKIFWLPNTLYLVVGYDFRQVSKSYLKKILMTAFLILTNITGICIRIYMLIQLRELVLSGDMLNSFR
LGVYISYAFDSIVKFFGFLHNAHRLRKIYESLATEFPQTFSEQQFYQVHKYSFNRSRILIFAYLSVTNSI
LLGPIVQSIIMYIIDAFLYGLSGAKFQCLHPTPITYNFNFCSPRYYIPIYIVEYLNGHFLTTTSLGTDLY
VCTFAAQVCMHLKYLGNSLEGYEPSADNSKADCAYLKEWIKKHQLMLRLCADINDVFGTTLLFKLISNCT
VFCIIVVQLKLEGFGWGFLNFLCFFFVTVAQFFMVCHFGQKLINTSEDVSLCAYKNRWYNGSKAYKTLLF
TIIARSQKSCKLTAKGFQPISLQTFQIVMTMTYRAFAVLQRALD

>XP_004519848.1 LOW QUALITY PROTEIN: putative odorant receptor 85e [Ceratitis capitata]
MQNTAGACSIILYSEKDKPRVCDLFMAQVLAFKATGQIPFNWRWRLGYIYCFIVIAQTFLLAVLFLKSSY
IMLLSGKLEEITDALTMTIIFWFSVYAACYWLLRWRRLMAFLELINQQYWHHSLPGLSFVSWQRTYLLAK
RMTIVWTVACVLGTVLYGLAPLVMGVRALPLKAWYPFDPLQPYVYELVYVMQLSAQIIMGATFGNGSALY
VSLVILMCGQFDVLYCSLKNLSHSARLRCCSGVEILRKEQAALPKSPDDELNQYMYCREHLTNLSILQHL
YTQQPALTLPEALHLGVVQCVQLHRFILDACKELEELFNPYCLVKSIQVTLQLCLLVFVGVAGERSMVRI
VNLAQYVTLTLVELLMFTYFGELLRGHSVRCGEAFWRXQWWTHTIPIRQDILILLANSKRAVRLTAGKFY
AMDIERLRSVVTQAFSFLTLLQKLAAKNQK

>XP_004518582.1 odorant receptor 24a [Ceratitis capitata]

MSIKFLTQSYPTEKSLFLIPKFVLRIVGFYPEQEKSTIRRNAWTMFNLIMLIYGSYAEFMYGVHYLSIDA

VRALDALCPVASSIMSVVKLSFLWWHRVELERLIRRVSVLTAEQDSRLKNNYKRRYFTIATRFSAALLCL

GTCTSTLYTIRAALANYFSYVRGENVPYETPFKMIFPQTLLSKWIFPVTFTFSHWHGYITVAGFTGTDGL

FLCFCMYFGTLLKALQIDLKDLLKDMDCGQHEGLSERDIEECMKKTVMRHNEIIDLIGDFSAVMSSITLT

QFVLSSVIIGTSVVDMLLFSDYGILLYFVHALAVTTELFLYGIGGTTVIECSSQLATAVYDSNWYSHNVE

VKKMVLFMILRTQRSLVIKVPFFAPSLPALTSILRFTGSLIALVKSVV

>XP_004518314.1 odorant receptor 83a-like [Ceratitis capitata]

MILTDAPTAHAAAQLCASPNGCLSNVKRRDLFRYVRWLMWGAAIRPIPFENHLPRRLGNYSAIINVILEI

FLLLTVIHIFVLFILTLYLNYGSGDLEFFIGCSIQSMLYFWAIIIKIIFRRVRPELVRDIMDYVNEKYIV

HSAVGFTYVTMNECLEQAERGIKYFVLSSLVAVIFWLFQPVVYEERTLPLPCWYPFDYKAPFIYPLAYFL

QVIAQLQLALTFVTNSIYFTVLCFLLCGQFDVLNCSLKNILATTYILMGASRKDLIELREHQCNADDEIN

QYFVAEELHINLDCIPHVLTPATTAGTMNFRDAFHCALGQCVDHHIFILNALRKTEKLFSMVWFFKTLEV

TFAICTIAFDVVKSTDDKSFLQVLSLGQYMILVLWEMFMICYGGEIVYINSQRCDLALLRSPWYLHSREM

RAEILFFLLHAQRAFALTGGKFYPLKLEKFQAILTTSFSFYTLLQNMDQRN

>XP_004518300.1 odorant receptor 7a [Ceratitis capitata]

MDKLRAVIFDRVGLSTKDSFDLLYLNWWLNGNTSWKPHRLGHILHMTICWCLKFFAPVTYFKGFLIALST

STITTALYNLQATLDVMVAPFKAVVIAKHMHRLRTLTEVFNRLDDRYHNPRERAQIDEGVIICRQIICFY

CAVYSGYAVMTWLGALIAGKMPHYLWFPYFDSIPNETLRYWLQFTFEALFIHFMLNVSYTNDVFPVIYMR

ALRTHVKLLAERVSRVGSNPELSAEEHHRELVDCIVAHREILYIVDVVGAITSLTIFLQFAMAAATLCAC

MLNVLIFAERIGQIITIIYYMGVLLQTGGSCYQASMLEAESSSLATAIFHCNWLNLDKRSRTLLVYFMQR

AQEDIAFTALKLFQINLKTNLSLAKFSFTLYTFMNEMGLGNDLAKSQS

>XP_019933504.1 PREDICTED: odorant receptor 47a-like [Aedes albopictus]

MLFAHCFRDNVDVFPVGMQTLKFLGLWGEPRRVVRFGCIASWMAFMFIAPKTCLEYGGEGFDSFARGTAE

LIYFTDFISSMVLFAFRRKSYVRMISTLQETFRTLASPGQPISCVNAITNFNRRIFRYSRIYACFIGSCL

IFYVPLPMTATFVNYFAAGNETDSVEFVLPLENKFYGLDTRRNILHYIIYMMMLTPAVCGSASLSIVKGT

VLFTIIRYGATLFELVSLKIADLGDPSNFRDQDDLETQRKRRQDRLQEIITLHQTALEFADLLENTMQHI

LLSQFVNCLLISCLMMFYISSTYGPNVVNMVILFAVLMVEVFAYCYNGDELSEKAAAVANSIYCYPWYLE

PVPVQKAIQLMILRSQRKIGITAAKFYFVDIGRFGVVVQASYSYYLILKERF

>XP_019561179.2 PREDICTED: odorant receptor 63a-like [Aedes albopictus]

MFLKKTAQPEPLQVGLKLLKWIGLHSTTSRKRIWRYYFVVGWLLGNIVLPRALLGSGNEGFDSLVRSLAE

MVFFSDVCIAVGIFATRLRYFERMVQILGGIFERYETKECVEEIRRFNRRMDSFAKVYIAYIVMLVILFN

IPPIIWNLYMAIFVSVEHRSSYVLLVEVQYFYLDIRRNIVHYLVYYVLCSTATVCSAYQSCIKGTIFLTA

LQYGAKLFELLHLRIDRLGKVKAGERRRDELRRIIELHKMTLKYTELLEETITFIMINQILNCMAIWCMF

TVYLSTNYGPNALNVVVLFVVFIVEMVVYCVSGTRLSENALKVSSAIYHYQWYLEPADMQRDLRFIIQRA

QKPCGITAAKFYFVNIERLGIVVQASYSYYLLLKNRF

>XP_019932610.1 PREDICTED: odorant receptor 63a-like [Aedes albopictus]

MFLKKTAQSEALQVGLQLLTWIGLQGTNRKRIWRYYFIVGWLLGNVILPKALLGSGDEGFDSLVRSLAEM

AFFSDVCIAVGIFVTRLRHFERMVQILKEIFDRYETKECVEEIRRFNRRMDIFAKSYIAYIGMLVFLFFI

PSIVWNLYMTIFVSPEHRSSYVLLMEVQYFYLDIRHNFVHYLLYFVLCGAATLCSAYQSCIKGTIFLTAL

QYGAKLFELLHLRISRLDEVEAGDRRRDELRRIIELHNMTLKYTELLEETITFIMINQVLNCMAIWCMFM

VYLSSNYGPNGFNVAVLFVVFIVEMVVYCVSGTHLSENAFKVGSAIYHYPWYLEPADMQRDLRFMIQRAQ

KPCGITAAKFYFVNIERLGILVQASYSYYLLLKNGF

>XP_019932566.1 PREDICTED: odorant receptor 49b-like [Aedes albopictus]

MDILKRLKLFQKTRAEIKDPQAECDGIVLYLNTSARKILGMDVLSLDFSFVNPRYIFLLTIMTSFLYADL

EAAVLADDIGGFAYNIAVLGFGLQGFAKFDAYVYRKKLMYDLVWQCSAFLGKNKGHDRFSTFLVDNVAVI

MIVNKFYYRLYGLVFFTCAFFGILQSVMSGERLLSFGFQFSFLDTTNWVGFILTYCYQVISSLMVVISSC

CNDILIAVVYVNAMSMYDCIMSDLRELSKMSEMEKTQDNKRLAEKQMKSIIQKHQHLMQFLDLSNDVFSS

YFLMSLASMTGTIAVLLTALVMVRWYPAIVICFAASFQIFTLSLLGTLLLIKGEELIQQVYDINWYNLDL

PVQKSLKLLLLISQNNKEISYRFGVMNMETFVQSHKLVYSFFTMLVTTQE

>XP_019891912.1 PREDICTED: odorant receptor 59a-like [Musca domestica]

MTEEPNTKALFKTHFIAWRILGMSPPDNYRPLYWIYSILLNIFVTIGYPLHLIFGLFTSTTMYEIIQNVA

INFTCSVCAMKTIAIWWRFNKVDVMFEIIQRQDQRFTSHEEIAYLRKEVYPPVRRIILLFSILCTFIGIS

GESAVLVTGLLGTWNLMYKAYFPFDVFASTKNYMAAHLYQFIGISYLILQNVVNDTFGASHLCLLRSQVR

MLNIRVTKIGHDPKKSREENNQELLECIKVHKDLLEYRRQLEEIISIYMFFQILIAALNMCVVLVFIILF
VRDIFTLAYYVSYLTSMIFEILPSCYYGTLLEDEFEDLAYALFSCNWPKQTLEFKKNLRIVAEQAKRRIY
VTAWLFRINNNAFLIACKNAYTLFALVMNMK

>XP_011291083.2 PREDICTED: odorant receptor 49a-like [Musca domestica]
MYSVFQQPLTVMATTERYFEDFVNMPCALLRTLGIDFLNISRSLLAKCLMQLYFVLSLLSCFYCTYFVME
MAVREIHCGSGNLPLILRLVDDIFHSLNGLLKSYYFFRIWKSNKSLFNRFCEIFPISMEDRREYRVNDYY
WPRWITCMVYVQCGAIAVIIFSPFAATLKDYFLAILKFGFSDAKFSYHILYEEHTYIVDHQRPTGYIFIY
SVLAMGTQYAVIFNICPDIWLVAYAIQLCMHFDYISRNLENYEPKEERSHKDLEVVAKLVKKHQILLDLA
NDLRKTFSILVLIMLFSTVVTLFGAAVYVLTQGINSNVLGYLAFLPTTLGQYFMVCYYGQLIINKSLRIG
DAAYSQTWYNGCQSYKKSILAILGRSQSQCEINAGGFQTTNLKAFEGVIRMTFQLFAVWRTLMEPK

>XP_019891102.1 PREDICTED: odorant receptor 49a-like [Musca domestica]
MKVTAFSSSALKTAEKELYFDDFVKLPCVLVRTIGYDFIDKPRPLWLRALMLLYLVLCLIFCAWFTYFAW
DFMMAEIAAGANDLALVLRLSVDVIYNVAAIVKSLFFFRNLKSLKSLLQRFRDIFPISREDRLAYRVNDY
YWPKWITTILYMQLFALSIILFLPFVEAVYEYFGALLTVGYANAKFGYYRMYPETTYGINHYNPLGYIIV
YTMDIMNGHYCTVWMMGPDVWLVAFSIQLCMHFDYVSRTLENYKPSKERAAQDLRVLAELVRKHQTVLEL
ADDVQENFSVLILVMLFSTASILFGAAELVITQGITAHVLGYLAFVPTGVGQFYMICYYGQLIINKSLQV
SEAAYNQTWYNGCQSYKKSILTIMRRAQCHSEINAGGFQTTNLMAFESVMRMTYQLFAIWSTMTSSK

>XP_005180067.3 PREDICTED: odorant receptor 49a-like [Musca domestica]
MSIVRVKKARVNFQRDFRDFCHLPNYLMRIYGRDFSERKRTKWQTLLLRLYAVVTVSSHIYCFYFISQQV
FLMFLSGVPNLELFLRLLSGFNYGLFAIMKYLAFKNRITDAAAINRVLREIYPKAGRERILYRVNAFFWP
KWMLTVIYFYFGAVAFIVLSPLLESVIVFVIGVGRLGWNEAQFGYIKLYDIPYSFDHRSPFAYVLTYSIE
LFHAQFVIICNVCGDIWLLCYAMQLCMHLDYLIKILEHYEPRVEHHLRDTQFIAGFSQKHQILLNIADDV
NTVFGVQLLLILISTAATICCAGIYTLTQGVGKELLEYVAFLPCVVGQYYLICFYGQRLVSSSENVGAAA
YNHAWYNGSPSYKKSVLVIMTRSQRSMKLKAYGLSSVSLGSFRMVMSESYRFFAVLKHAVFDKKN

>XP_011296067.2 PREDICTED: odorant receptor 45a-like [Musca domestica]
MTRILKRYFRLQRFIFSGLGLDIAATPEKMVKRPWLMMTPLVMSILLCIANGHYVLDNASDYLEATDSLT
LLCQSLISVWKVIMVIWKRKEFANMIARIERLNVKAEGEELKIVRRENTRDIIFSTTYFVLVLLTGAWSL
LVPIYFAVHVYVTTGEVDLPVPHKATYFWNHEHVKGYSLVYIWDVFIIYFIACSAVSTESMFSWLVCNII

AQFRILMHRLEVASRQVMSTRPMTASHHVDDDDDNPLMGELDPQAGMVDAIIACVKFHRRTLRLTQELNS
LYGAIIFVKFIVSGTQICCLAFHLVRGNNSLFNVAYLCMFLSAAALQLILYCYNGQRLKDESLLVTTKIY
SIFPWSKMPVSTQRMLLIPMIRAQQFSELRGVFFTVDLSLYLWVFRTAGSLIAALKTLEEKE

>XP_019895389.1 PREDICTED: odorant receptor 45a-like [Musca domestica]
MKILKRYFGMQKFAFAALGVEVESMSPAGSERIFRHPIRYAVLFILTVLQYISIGHYSYVYTSDIVSAAY
SIALSCQGVICITKLVIFFFKRQGIVELVRMLQTDAFNAQSEELAIIKEENRKDIRICTLYCIVIYGTTF
FGMTLPFARTILGYLRNGYLVYVTPVASPSLWNYDTVHGYTLVYIITLLRLGTLCFTTIGIDTLYSWLMS
NIVAQFRILTHRFQQAAWATTALDGSEISISEEQHRLINDCIRFHNRTLDLVKELNRVYGAITFVKFVVS
SIQICCSVFFVSSSDSKESAFNLFYQSIFLGAVSMQLATYCYNAQRITDEVSSELVATKVYLIFPWSKLP
IPTQRMLLLPMIRAQRSCEMRGVFFRIDLSLFVWVFKTAGSLIAVLQTIDEAQ

>XP_019895117.1 PREDICTED: odorant receptor 67d-like [Musca domestica]
MAKTLVQRYETIVRLIRIFSGICGANIFNPAFKKNIITWIVIIFIYQYFVFTGYTLYVKIYIDKDRPSVL
QVLCYLGSAVQGYCKLLNFLWNKDDIRYLIYELRDIYEKYDLKHADYRCCLEKNTNRVNRFIKFMATMHL
VITITLIAVVPFYRVVFNERILIMQFLFPGVDPNTAYGYTIITTIHCICILFGSFGNFAADVCFFNIVSH
VPLFRDLLRCKCQDLNEILEEERASEEEGFAEIELLLKDIFQWHQKYMRYITTVKENYFWVVLVEMGTVA
LSIASTLFCLILGKWPGGLTYLTYCFIMLYMYCDLGTIVEITNDGFIDSCYTEIIWYRLSIHQRKMLQMM
LMMTQNTEGLTIGSVIPLTVNTGLQLTKSLYTMTMMLINFLE

>XP_019895282.1 PREDICTED: odorant receptor 67d-like [Musca domestica]
MAGNIQLSPSERFAKFIKVIKLFAGFCGVNSLERDYRVTWVTWLVICVVTSFFVCTFYTIYVGMAIQNNY
SILLQSLCITGTGVQVRSNQFFQGYTKLLNAIFCGKHLRFAFEELTAIYEEYECKRLEYRDNLKENLEMV
KRLIYGLLLINFILIAALFAVPLFYYYVRKEKIDVIPLMIPGINPSNNRIENYIYQFYHICCVIFSTFGN
FASDTFMILIVVHVPMIKNIFKLKFDDMAETMKLHLRNRKKTEPLLRDIFQWHQKTILIIETMQKGFFWV
IFVQIFTSMLNIIFTIVCIFLGVWPVAPVYLLYSFVILYIYCGIGNLVEISTDDITSIIYDFIWYDLTVS
EQKMILIMLRESQSPPTMTIGGVMPLSMNTALQLTKSIYTIAMLLNEFVN

>XP_019895195.1 PREDICTED: odorant receptor 45a-like [Musca domestica]
MFKIPRAPDALPRQPSLRKFLYIQKICFAGIGFDPTSVKRTIFSPWLTFIPLFSILGLLAPMGVYAFKYI
KIDLAKTTAALSPFWQSLLSSVKFFVFMLNRKKIVESVRKVWLWTLEANEEEVEIIAEENRYDARISKFY
FASVYVTGVLAVLAPLAIASVYAWQGYANEEEVEIIAEENKYDARISKFYFASVYVTGVLAVLAPLAIAS

VYAWQGYGFLESLDAPLKAEYFFNIRGSYQAYIFCYVWNCIGIYYVLHGALSIDTLYSWFVHNISAQFRI
LNLRYRQLSERTMMLRAIGEHNEEKFITAIIECVKYHRRIIQMAERFNDVYKGLVFIKFLISCLQLACLS
FQIPSGGEIADLLFSLSFLISVTTQLMLYCHGGQKIQDMSTSVSLAIYEHFQWHDLSVKSKKLLLLTMLR
AQKPCYVRGIFFTTDLSLFVYTELPDHL

>XP_019895194.1 PREDICTED: odorant receptor 45a-like [Musca domestica]
MTSDDLPPLEGVKYYFVVQKFCFTAIGVDALSARRTIVNGFLFWIPNIVQFILSQPLTLYSLQHLEDMSL
VTDAMAPVWQVLMANMKMALFLWHKKEMKKLVRDLWLWNLEATPDELKILEVENRKDTMTSFSFYMTVLT
TGILALTSPFFKAFYRYLKGDNYWDALETPLKGRFHHIFRSYFIDPKETYMGYFIAYMWAFIAIYAVLNT
TLAADSLFSWIVHNISAHFWILRERLKSIAATNREGSHGYGKFRKSIGDCVRYHQRIIDTIDEFNKVFMT
IVFVKFLISCIQIAFLAFQFVRGGDFAGQVFHMLFLMSISIQMMLYCYGGQRIKDESASISVAIYEYFHW
DLLCPKSRKLLLLPLARSQKPCKLTGVFFIADLSLFLWVYKTAGSFVTLMMSVSDTSN

>XP_019894952.1 PREDICTED: odorant receptor 42b-like [Musca domestica]
MEIPNITTVLPQQVQEDEQEPSTSSNKTLKSSHANKSDTNDDSSVQTRHGLRFLFIGFRLLGVYFPKRGR
FLYFLWSLFVNLYATIYLPTGLVVGIITHRDVAIGDMLTSLQVAIDVVGCAIKIVLMYFLLPQLLQCDPV
LERLDKRCTSPEEKDLVRRFISHGNRFVILFGMAYWSYASSTCISAVLFHRLPYNLYNPLLDATASKGSF
VLGVFVEMMPMYLACSQQVVDDSYAVIYTQILRTHLKALVFRLQHLNDDHRNENGVISPEAEERNIENLK
LCIIDHKNIIELYTRVAPVISITLFVQFTITASLLGVTLINILIFATNTASIVASCFYVLAVVVEIFPLC
YYAQCLMNENDHLTEAIFHSNWIHQSKRYRQMLIFFMQRSQKSIEFTAGKLFPITLSSFLSIAKFSFSLY
TLIKEMDIKTHYGLD

>XP_019894764.1 PREDICTED: odorant receptor 67d-like [Musca domestica]
MTVSVVDEYEGIVRLIKLCSGVCGANVFVANYKVNVLTRIVVTFINLYFIFTGYTLYINIFIEKDWTHML
QVICFFGSALQGYCKLLNAIWNKDHLRYLVDDLREVYAEYAPKHDEYRDCLQKSINTAVKCIKLMAFFHV
AITVGLIGVVPFFRFVFNERIFVMQFQLPGVDGDTEYGYLIMNCMHSICIIFGAFGNFAADLCFFTFVSH
FPLFKGILSCKFHDLNDVLEGSDDAKKAECKEMLKDIFRWHQKYMRYITTVKDNYFWVLLVEMATIALSI
SSTLFCLLLGTWPGGQTYLSYCFIMLYIYCGLGTVVEVTNDSFTDLCYTQVIWYKLPAAERKMLSMMLMM
AQKTGGLTIGNDSFTDLCYTQVIWYKLPAAERKMLLMMLMMAQKTGGLTIGAVIPLTVNTGLQLTKLIYT
LTMMLINFLD

>XP_019894621.1 PREDICTED: odorant receptor 63a-like [Musca domestica]

MDTILIDISDKGGRILNPLKWIGMFSGCNIKYKSKFLHPLKILNLFLFVTSILACYGQLYYVWERRHYTF
EIYIEAILIFFQSLISIWKLWMFTFSQDCLFDMMKSVENSETLQNLEIFQLELIDSANIINDITQILNES
WIDIKRQLLLLRFTVFGICSWYTGHSLVSNIYYLYISDENDKEKLEFPFPASFPVWYSNVNSLWHFYLEY
FVVTMQIYLATVASITCSGLFSVISVHCLTMLRVLRTLITYSTSEHVPSQHRTKYLEACVRLHQNLLSFC
SRLNRVYQKPSLGLFISCCLLICLLTFKASVDLGKDISGSIKVCLYLLAAFYELLIFCLNGQRITSESER
LPQAIYSSLWFDENRNFKFMIQIMIMRTNQNIRMDVGGFSRMSLETLLTITRSSVSYFLFLRNCM

>XP_019894515.1 PREDICTED: odorant receptor 13a-like, partial [Musca domestica]

ISKLFRATVPKTFRIYKRNKIPSKLFIVYLCLQLNVSLDFILCSRIKALTCMYVYENSADLGKVVGNMCL
IMIALMVSLRLLYFRGDISRMKRLLTMFAEKIWIDSEAHPKAYERAVRRTKPTFYISLSLWICLVLYLLF
PIIFNLTQGKSPDSNDKPLPFPTVFPYDTQTHWAYIFTYIFLSYAGYIAVSLFYAMDAILAYFISFVAGQ
FEILHADIARLIPECHAEWLRRYGAGAAENGVKLNYLQEMYAKRLHGIAKRHKDIIAFCKELEKFMSFPL
FANYGTSTFLICFVGFQFMIAGLKSFGDFMRFFMFFMAVTGQLFIVCKLGNLLITQSTDTAHYLFACNWE
GGYLSKNSPLLLYPDIMELQELNRNLPLWKDLSYIPANRNFKLKLMFMIMRSNRPVQLSVMQFTVLSLQS
FNKVVSNSLSYFALLKSFLDK

>XP_019894514.1 PREDICTED: odorant receptor 13a-like, partial [Musca domestica]

MAILYKPRCGEDVNFVLPLKVRTFLMINGCWPMEDNANNTNGLWNRVTHPLIFKRCIKRTVPTFYLSITL
WMVLFIYCALPIFVLITTDQTIHSNDKTFPYPMIFPYDPQKPINYILTYMTSIYTGAITVTLFYATDAIL
AIFISFLCGQFEILHGNIARLIPECHAEFLANYRGESTGSKKNDFIFLHNLYVKRLHELATAHDELIRFS
MDLEKLFSFQLMVNVVTSTFQICTNLFQFIVAGRNSLSDFLRFFLFFFSVTGQLYVMCELGTILITRSTD
TANYLFSCNWEGGILSQHSPLLRQVDYITLDSLNTKLPAWRTLEYYPTNRDFRMKLKLMIMRSQRPVHLT
AMKFTVSSLESFTR

>XP_005189142.3 PREDICTED: odorant receptor 83a-like [Musca domestica]

MSIAIRPQLLNRMHKRHHVRDNIIRIESRDKRHDLFQFIRRTMYWAAMYPMSLEHLLPQRIRYLSSFIEV
FYELFLHLVCIHIVILYLCTFYLNNNSGDLELLVNCMMQTIIYVWVIGMKLYFRRMNPRPLEELMKTMNL
QYRTHSIKGFTYVTMEECLIMANKWIKTYVYSCFAGAVFWLIIPITYDDRSLPLSCWYPVDYKKPIIYEI
IYFLQAVAQIQVAAAFSASSGLHMTLSILLSGQYDVLFCSLKNILANVALRMQSTEQQLRKLYKLHEITS
HDTNEFYCSKEKTLDVERLFDAQQLFVETSQDFRHNFRNVFKECIVHHWFILDCLKSMERFYNPIWFLKT

GQAILLLCLVAFVSVKSTTTNSSFLKNLSLGQYLFLVAWEFLVICYFGEMIFYNSQRCGEAILKSPWYLC
MREIKSDLLLFLLRSYRPFKLTAGRMFALNIDWYRWVITTAFSFLTLLQNMDQRDVNVST

>XP_005187966.2 PREDICTED: odorant receptor 42a-like [Musca domestica]
MDIDAPLPKTRDATVYIFRGLNIIGYVPTETNKLAFYMWSGFVNFFVTVYLPVGFLMSFLLRLNTFSPSD
FFTSLQIWVNCIGCSLKMFVFFFLHRRLIESRKFMDRLDVRIDNDEDRLVIRKIVAFSNRSLTLYSSLYL
SYASSTFLVAVINSKPPYQVFNPFFLWKENVWKFTMQAGFEYMMIAFHCFQQALLDSYPVIFITIIRTHL
HILTRRISRLGSISTMTSDERYEALVQCVLDHKNIMGLYSIFCPVISGTMFVQFLIIGLILGITTLHIFL
FADRLAIIASLFYVASILAETFPCSFLANCLMDDSDRISLAIFHSAWHEEEPRYKQMICFFLQHTQKTLI
LTAMKIFPITLNSNINVVKFAFSVYTMMKQMGLGQNLQNVVGKEL

>XP_019893370.1 PREDICTED: odorant receptor 85c-like [Musca domestica]
MKKAATFDDFFKLASFFYRTIGIEPYDEPGVEVKKSKSFAENFIFYSGVINLNYVLIMEIVYVAVAFIRG
ENILEAIMCLSYIGFVIVGESKMFFVFRKKPILSKFVKRLVEIFPQEFELQKTYNLSSYLRQSSRVTIGF
ALLYMILIWTYNLYAMTQYLLYEKWLGSRVVGQQLPYYTYAWWDWHDHWTYYLLYFIHAFAGYTSATGQI
ASDIMLCGFATQIIMHFHYISHVLTNYKVKVDEAKDKQAGRSQDITFLKDIIEYHNCLLELSEQLNSVFS
LPLLLNFSASSFVICFVGFQMTIGVEPDALIKFLSEQLASVFSLPLLLNFSASSFVICFVGFQMTIGVEP
DTLVKLFLFLFSSTAQVYLICHYSQMLMDAN

>XP_019892829.1 PREDICTED: odorant receptor 67d-like, partial [Musca domestica]
MTHRQSDRFKAIVRITKICADICGANVLEHDYRINVRTVLVFVIIILTFVFMSYTIYDGFFVQGDWKIIL
QVLSIGAGTLVQGFVKLLNCIQQQENFRFLIGELYDIYEEYELKHTGYQRHLNKGIHLLSYIMKLCAFIA
VLLVIGMAAVTVVRSLVFDVNQVIVQCLIPGVDHTTPRGFFLTCIVQISFIAVGGFGFYAGDMAFFTPIT
QIVTFQGILRCKMFDLNEVLEKDGEENVKKSTEMLKEVIKFHQRYMVFLTVTQDTYFLVILVQIATYSTG
IICTIFCVLLGAWPGGYVYMIYCFVMMYVYCGVGTLVEVT

>XP_019892807.1 PREDICTED: odorant receptor 94b-like [Musca domestica]
MNVRLHNDGGYDRTYAVRGILRVMKILGLWKWQTEADKETPRHILWLQYVQRLVCHGPFTFVFITLMWIE
ALRANGLDEMGDVLYMSLTEAALIVKILNIWQHSTKASTFLHALRHNAHFALHSGDEVTFWRNAQKKFRY
IIYMYSAGSVFTVISAFAGVLFVTEPQMAFAYWVPFEWQSNRRNYWLAYLYDFVSMVCTAGSNVCLDMMG
CYMMFHVSLLYKVLSFRLQKLRAVKGEDVNEKFKKLILMHKSIRRMTRECEILSSKYVLSQIILSALILC

FCCYRIIKLDIVANFGQFLSMLQFLAVMIFEIFLPCYFGNEITLNSSEIMLDVYRTDWLEYSVANRKLII
LFREFLKRPDKVTIGGYFEVGLPIFTKVVNNAYSFFALLMNVEK

>XP_011292769.2 PREDICTED: odorant receptor 67d-like [Musca domestica]
MNLEDSRNANKLHRPSNRLRKIVRITRICSYICGADVFDPNYCVNIRTYFVLAVINFSILLLSYTMYSGW
VEEGDWAIVLQVLTIGGGTLLQGYCKLINSIRQKDKFRFLLTEVYSIFEEYELKSCDYARHLKKGCHLLS
YFMKLCAVINVMMICGLILVAAAINVIFQKRDLIVYGDVIGIDPSTTSGFYVTFMVQACFLLVGGFGLYA
GDMAFFTPISQVPTLKEILRCKFKDINAAMEGDELQDSRHVSELLKDAVQFHQKYLRFLNTTQDTYYWVI
LTQISTYSVGIVCSMFCIFLGTWPGGYIYLLYCFVMMFVYCGVGTMVDIANEGFIDACYNDILWYKLTAS
DRKSLLNMLILCQNTDGITIGSVLPLSMNTGLRVTKTIYSIAMMLINFFMD

>XP_011292665.2 PREDICTED: putative odorant receptor 85d [Musca domestica]
MRQHQQRKSSKRNQNIKTMAPKATSNGIGLNKFLLQADILAKSIGLIPYDEENDKRSVRYEKLMKFIFIL
NMVNMNFVLFSEIMYVLLAMKNGNNFVEATMNLSYIGFVFVGDIKIISVLRKKPVLTILMKEIEDIYPKD
GRAQKAYQVREYVWRFNLISLGFVIVHEILIWFYNLYIAVSYLIYEWWLQWRVVPRTLPYYFWVPWQWQG
HWSYYVLYVSQNFAGHTCMSGQLANDLLLCVAATQIIMHFEFLAKRLREYRPTGRHVDDLKFLREHIKYH
QAVIHLSALMNEVFGVSLLVNFISSSFVMCFLGFQMTIGVEADTLVMLFMFLFCSLVQILMICNYGQQLI
IKSEEIGHAVYSQEWLNSDLRYRKMLIGIIARSQKPVILRATTFLNVSRSTMTEQLVYENSLDHQLLYED
SLDHQAVHKDSLDPQFIYEDSLGHQLMYEDFLDHQLVYEDFLDVQLTYEGYLPRRLPAARVRLLKRPESP
DRL

>XP_005182622.2 PREDICTED: putative odorant receptor 85e [Musca domestica]
MDPGGRSVHMLANFNRQRMTPSVYFYGGDSDVLYSEHDSGREDDVFKLQLLFMKFMGQVPMQLERRLPLG
WKNVAGMFAKSYCIFCVISNLHLAILYVKTTLDMLHNGELEEITDALTMAIIYSFSTFATCYWLFNAEAL
NSFIGDINANYRHHSMAGLTFVSAEHSIRLAYKVTLYWLIACCVGVVCWALAPLLLRSHTLPLRCWYPFD
ALKPVVYEVVYATQLWCQILMGCIFGNGSALFVSVVLIMLGQFDVLYCSLKNVDYNAQLLAGGDLITLRN
LQRDLPRPADDELNQYALLEEHLTDLTALRVSKPNSRPSLKEALHSSLVECVLLHQFILKSCNTLEGLFN
PYCLIKSLQITLQLCLLAFVGVAGERSTMRTINLVQYLALTLSELLMFTYCGELLSSHSIRVGEAFWRSG
WWLNGNLIKRDIFIFLANSKRVVVVTAGKFYRMDVQRLRSVITQAFSFLTLLQKLAEKNQ

>XP_011296015.1 PREDICTED: odorant receptor 67d-like [Musca domestica]
MATKLKLTPSQRFSNFVRVVKIFAIVCGANIFRPDYRLNALTWFVIGVIATFFIFTSYTMYVGVVIDNDY
TKILQLLCVTGSAIQGATKLVNGLYHASLIRSLIAEILTMYEEYECKDQRYIKYLEHTLSLIKRAVFSLL
NIYSIQTIGVLAVPLFYHLLLGQQIDIIALLVPGIDKHTDFGFYTYQFYHFCVVGFASFGNFANDTLMVL
LIVHVPLMKNILKLKFDALDELLKEFPRDVDRTEPLLREIFQWHQKSTMFAQNCTDTFFWVIFVQIFAST
LAIICIMVCQFLGVWPAAPVYMMYCFAIMYMFCGLGNLIEISNDDLTRIIYDCNWYELTVTEQKMILLML
RKSQQAPTMTVGGFMPLSMNTALQLTKTIYTAAMILNEFVN

>XP_011295835.1 PREDICTED: odorant receptor 1a-like [Musca domestica]
MYNNVDGKTRQDLEFLDVQYRALIRVGLDIGAIRGKDFLNDRGKFLIYGIITTYLQYGLILFAVHIFGVQ
IDKASAALSMFNQGSLLMLKVSILIFKSNRLLKLIWDMNLLATMANEPERETWLSENRFSKVIGNIYSTA
CIASVILSISIPIIFMSYEHFKGLEVSLKLPFDGEFPYEHLGIPIFILNYILSVIYVYTLLCWTIGIDTL
FGWLIHAVSGHFRILRLKVEMAAKKIDEHGNHLDFVQDIGAIVRYHIKTLGFVDALNEIFGQIFWAEVAF
SCLQMCFLIFTLNNGSDKRMIPFNAMVFTAISIQMMIYCFGGEKIKSENEMFCFDIYSKFPWEKMYPSEK
RMMLLPLQRSQQDAALRGLFFELDRNLLVYIYRTAFSYNTLLGAMKE

>XP_011295797.1 PREDICTED: odorant receptor 13a [Musca domestica]
MFNPKPNNDLNYRIPGQCIWLKLNGSWPYNHQEANKDFYSSRYVWGWLYTVWSWYVVWSVGITIGFQTAF
LINNLGDIMMTTENCCTTFMGALNFVRLLHMRLNQRQFKVVIQQFVEDIWINKKQHPHVAAVCSRNMRTF
RIMTVLLSCLISMYCVLPLVVLFFDVGLDADEKPFPYKMLFPFDAHHGWRYIVTYIFTSYAGMCVVTTLF
AEDSIFGFFVTYTCGKFQILHERIDNLVFDAYESVANRQNELEIQECYVKLLNRIAYDHNKLIEFAGKLE
NFFNPILLVNFTISSILICMVGFQLVTGKDMFIGDYVKFIVYISSSLSQLYVLCWNGDSLIQHSLETANH
LYTCNWEGGQIRSTAQHQNSRENNPNSQGKVLQKLFKSGQEIGQIKVWQENYYMPASKKFRQNLEIMIMC
SQRPVKITALKFSTLSLQSFTAILSTSMSYFTLLKTVYDENQEDGPAN

>XP_011295595.1 PREDICTED: odorant receptor 42a-like [Musca domestica]
MARSPGARSSTWSLNNYGQPGLISPSAAGGGNGQQLPYPVKISKKVATKQALTNLYICFRVVGIHVTKSN
PHLYIVYAIVIHSLTTVFTPISFTTSYFRKTDQDFNVGVFLTSIQAVINVYGCAIKILLLIYYKTKLEAA
EKLMDKMDQHCRAEDEIQELFNIRDLGRKIVLGYITAYWTYTTMTYISALVSGVPSYSINLFFLDWKRSK
REFYLASFLEYVLVTWTCLQQVANDSYGTIYVCILRGHVRVLLLRIRKMGRKVDQTADQNLEELKSCIKD
HKDLLELYNIISPVISRTIFLQFSITAVILGITLIXDILTYCIFSSFFLQIAKFSFSLYTLIKQMGIKER
LGL

>XP_011295319.1 PREDICTED: odorant receptor 67d-like [Musca domestica]

MQGYCKLLNAIGNKDNIRYLTDELREIYRKYDLKHTDYRCCLQKSINTVNRFIKCMAIIHFSITMSLIAV

VPFHRVVFNERIFVMQFLLPGIDPNTAYGYLMMNCMHCICILFGSFGNFAADLCFFTIVSHVPLFKDLLR

CKCQDLNDILEEGKDVEQEGIGDCQILLKDIFQWHQKYMIYITTVKDNYFWVLIIEMGTVALSLASTLFC

LILGTWPGGLTYLAYCLMMLYIYCGLGTLVEVTNDGFIDSGYTDVIWYKLPMVERKMIQMMVMMAQNTGG

LTIGSVVPLTMNTGLQLTKAIYTMTMMLINFLE

>XP_005189776.2 PREDICTED: odorant receptor 82a [Musca domestica]

MLDLFAKQRQCLLLMGHNFVRDKSELLKKWHNIKYVSVLLLVVSAQWPIMNYTIYYIDDLQLATASMSIS

YTNVLTVVKITTFLFYKWRFAALMEKLESMYHELQEEESKAILKTSNRYAIILVNIYGNSVGLTGLYFMV

APILKIVWSKIRNTELQLELPMPMRFPFDFESSPGYEVCYIYTGLVTLSVMTYAIAIDGLFISFTINLVG

HLKTLQHFIQSKSFEQNDEDVHKQISFYIRYHNLILHLYQEVRQIYSPIVFGQFLITSLQVCVIVYQMVT

HINTFLVFVINCTFLLSILLQLFIYSYGGEILKNESLMVGVSVQLSNWYNLKPRHRRMLWLLMLRSQRGA

IIRGGFYEASLANFMTILKAALSYITLIQSIE

>XP_011294557.1 PREDICTED: odorant receptor 74a-like [Musca domestica]

MLYRPRLPDGRKVPLSWPIALFRLTNNICWPLEENASWLAVVFDRFCWYLAFILFVITNDAEFRYLRVNI

NNLDEMLTGVPTYLVLIEIHLRAFTLGWRKQDFRRLLEKFYRQIYIESSLHPTIFKNIRSQLMPIFVLSS

LYLSALISYVILPIYFLSIGSRELMYKMIPAFDYSPLWIYLLCCLSNLWIGVIVATMMLGEATVLSTLVF

HLNGRYLMMREKLMAKVDVVLEKKKRDNGNQHIAAEYNKILVETLQENVALNTFAQEIQREYSFRLFVIV

AFMAASLCGLGFKVYTSPMTSIGYIFWAIGKIQEILAIGTMGSTIVTITNQISSMYYESNWELVVFQSED

SKSNARLMKLVQLAIATNSKPFCLTGLNFFTISTTTALAILQGASSYFTFLISLR

>XP_011293740.1 PREDICTED: LOW QUALITY PROTEIN: odorant receptor 85c-like [Musca domestica]

MSNAAKFDDFFKLSRFFYTTIGVEPYNEPGVEVKKSKSFAANLIFYSGVINLNYLLSMEMVYVAVAFVRG

ENILEAIMCLSYIGFVIVGESKMFFVFLKKPILSEFVKRLVSIFPQEVKLQKSCNLASYLRQYSRVTIFF

ALLYMILIWTYNLYAITQYVLYEKLLKSRVVGQQLPYYTYNWWDWQGHWSYYLLYFMHAFAGYTSAAGQI

ASDILLCGFVTQIIMHFHNISHVLTNYKVKIDQAKNRQVGLSKDMAFLKDIIVYHKCLLDLSEQLASVFS

LPLLLNFSASSFVICFVGFQMTIGVEPDTLVKLFLFLFSSTAQVYLICHYSQMXMDASLNVADAVYNQNW

SIADVRYQKMLILMAERAQKPVQLRATTLVLISRGTMTELMQLSYKFFALLRTMYVKK

>XP_011293739.1 PREDICTED: odorant receptor 85b-like [Musca domestica]

MSKLISFEDFLSYANALNATIGLVAYEKPNTKPLKKLIFDVIFWLNFINLNLVLLGELVFVIESVNGRHE

FLEMIMALSYIGFVALGSFKTCIIMQKKSHLTTYARDMNQIFPNASIAVQRELNVRKYLKYSKFFSIMFS

TMCLAMLVFFNFEAITEWLIATELRGDQNAAQHLPYFMYAPWDWTGNHWSYYLLYGIQCWAGHTSVVAQF

SSDLLLYAFIGQLIMHFEAITKDVSNYRLRSCTADMDFLRNIVFKHSILLELSERINDLFGLSLFVNFAT

SAVVMCFLGFQMSIGASFVNLLKLVLFLILMLTQGFLICHFGQLLTDASLSIAYAAFNQNWISSDVCCQK

MLILITERAQKPVILKATTLVPVSRATMTQLLQISYKFFALLRTMYVQ

>XP_011291737.1 PREDICTED: putative odorant receptor 92a [Musca domestica]

MVLETDNSLILFDFIRLPLKFYSAVGIKIFQWDADDIMTTKEKCIFLLLGINFIGCFLAKSLFCVFGEFV

DTMQATQWILYFMFAMNGCCKTISVAIGRKKLYTVLKDIEGIFPATLKERQEFRLAHNYGYIMRHAKIMS

IQHCSIAIMFIAFPLVQSTIEYLTSADSEFVTRTPYIMVYPFDATAGIGYVVGYFSQFLGGFTVSCYFVG

SDMLLMCTIYLVIMQYDYICYRIENFKSRNYEEDMKELKIVLERHNLLNIVAETVNEVFSISILLNYMIS

ILIIVMISIQITKGSEFGLDMIKFVGFFTSASTQVYYICMFGNLLMDYSSRVSESLIGQEWYWTDVRYQR

MLVLAIARSQRPSHLTAFKFFTISMESYGNLMTTAYQFFTLLKAQMEQ

>XP_011291062.1 PREDICTED: odorant receptor 46a-like [Musca domestica]

MNSLEHREAMKTFYKKQSFIFRIFAQLKLSDTVSDRFRLLHRIYFYYILIGWVLSFDISCLIQFISNITD

LNEVIKVFYIFATAMGVLAKFLAIKIKNNLYAELIEAMHEAKFRPTNSRELQLFRESQRLARTVRNFYTT

ISLCALNALLFTQYIIDTTQLPMSIYNPINTDTKLRFVLVYIYQYLAVSVCCYTNIAFDSISASFMIHAK

GQLDILCDRLKHLGMDSETSDEEITAQLKNCVKYYGDIIHIVKIAEDLISFPISVQIACSVLVLVANFYA

MSFLSDFANFIKFLIYQLCMLSQIYILLYFPSEVTSKSEEVPYHLYCSKWANWSASNRKLTLLMMTRFDI

PIRIKSINPTYTFNLAAFTSIVNCSYSYYALLKRINS

>XP_011293472.1 PREDICTED: odorant receptor 43a-like [Musca domestica]

MMEENRMVSINIKIWKFFAIIYPTSDKLWRLYSIQFVTILLNFMQFMFLIEMWGNLAPFILNVFYVSATF

DCLLRTGVIVYNRSKFEAFLAEFDSMYSEIEENGDDYAKGKLKEATEFCRKFSLFNVLASFLDLIGTMSH

PILTGTRTHPFGVALPGIDSAVSPYYEIYFILQLHCPITLSVLYMPFVSIFVTFSSFGKTALQILQHRLK

DIFEIYDDDETRLEALKECAHYYNRLTRFIKVFDEMVTYVILGEFLLFGAIICSLLFCINIIDTMAQFVS

IIMYVGTMLYVLFACYYSANEMLEESLKVSEAAYSIPWYEGTPQFRKTLLLFIQRTQKPLCLTVGNVYPM

TLLIFQSLLNMSYSYFTMLRGLKIQ

>XP_005191885.1 PREDICTED: odorant receptor 45a-like [Musca domestica]
MFNVPKAPDALQPQTSIKKFFLIQKISFAAVGLDPTSIRRTIFRPWLTFIPLVSIIAVLGPMGIYAFNYL
KIDLGKAVSALSPFWQALLSIVKFFVFMLNRKKIVGLVRKVWLWTLEANEEELKIIAEENRGDAKVCTFY
YSMVNITGVLATLAPVAVAAIYAWQGHDFWESLDAPFKAEYFIDIKASIVIYAACFTWNFIGIYYIVNGS
LSIDTLYSWIVSNISAQFRILNLHYHQLSQNIIAHKAMGNHNEEKFLKSIIDCVKYHRRIIQMSERFSEV
YKVLVFFKFLVSCLQLACLSFIIPLGGEIADQLFNLSFLMAVTTQLMLYCHGGQKIQDMSISVNWAIYES
FHWHDLSIKSQKLLLLTMIRAQKPCEIRGIFFKTDLSLFVWVYRTAGSFMTMLMSMEDK

>XP_005191599.1 PREDICTED: odorant receptor 45a-like [Musca domestica]
MTAYAFNNLKMDLGKAISALSPFWQAILSIVKFFFFMVNRKKILQLLRDVWLWTLEATAEELEIIAEENK
NDAKICGFYFAMVNISGVLAHLAPLAVASVYAWQGNGFLNSLDAPLKAEYFFNIRQSYITYIVCYLWNVI
SIYFIIYGSLFIDTLYSWLVHNISAQFRILSLRYRKLSLMMVTHKSSEIQNDEIFMKSIVECIQYHLRIL
EISKRFSEAYQHLVLIKFLISCLQLACLSFIIPLGGEMADQLFNLSFLVAATTQLILYCHGGQKIKDMST
SVNWTIYESFHWHNLSVKSQKLLLFVMMRTRKPCEINCIFFRANLNLFVWVYRTAASFVAMLMSLQNKI

>XP_005190907.1 PREDICTED: odorant receptor 7a-like [Musca domestica]
MSKQTVKVIKKVATKQALTYLYGCFRVMGIHFTKSHTHLYLIYVIVIHSLTTVFTPISFTTSYFRKTDEN
FNMGVFLTSVQAVINVYGCIVKIFFLVYYKKKLEAAEKLMDQMDQHCQADDEIQEIYNIRNLGRRIIIGY
GIAYWIYTTMTYISALASGVPSYSLNLFLIDWRRSKLEFYVASFIEYFLTSWTCFQQVANDSYGTIYVCI
LRGHVRILLLRIRKMGRKVNRTADQNLEELKTCIKDHKELIELYNAISPVISRTIFLQFSITAAILGITL
VNIAIFASSITAMAASAFYIVAVSVEIFPLCYYANCLLYDSDTLATEIFHSAWIGQDRRYRKMLIFFIQR
TQKSMELWAGKMFAINLNTFISIAKFSFSLYTLIKQMGIKERLGL

>XP_005190384.1 PREDICTED: odorant receptor 67d-like [Musca domestica]
MAKTVAQSYDKTILFIKISSAVCGANVLSPAYRMNILTWIVIVCINLYYVFTGYTLYVNIYVEKDWPNVL
QVLCYLGSAVQGYCKLLNAIHNKESLRFLDQELREIYLEYDQKHADYRYCLKTTIDRANKFIKFMIIFQI
LISGSLIGVAPFYRLVFNQRIFVMQFLLPGVDPSTEYGYFVMNCMHCICIIFGSFGNFAADLFFFVVVSH
VPMFKDILTCKFHDLNDLLEEEVADNENNNNNNRIKDVREDFRSLLIDIFKWHQRYLRFIAIVKENYFWV
LLVEMGTVALSLASTLFCLILGTWPGGQSYLAYCFIMLYIYCGLGTVVEVTNDGFIDSCYTEIIWYKLPV
SQRKMLRMMLMMAQNTDGLTIGSVIPLSMNTGLQLTKTIYTMTMMLINFLE

>XP_005190271.1 PREDICTED: odorant receptor 85b-like, partial [Musca domestica]

MSKLISFEDFLSYANALNATIGLVAYEKPNTKPLKKLIFDVIFWLNFINLNLVLLGELVFVIESVNGRHE

FLEMIMALSYIGFVALGSFKTCIIMQKKSHLTTYARDMNQIFPNASIAVQRELNVRKYLKYSKFFSIMFS

TMCLAMLVFFNFEAIAEWLIATELRGDVNAAQHLPYFMYAPWDWSGNHWSYYLLYGIQCWAGHTSVVAQF

SSDLLLYAFIGQLIMHFEAITKDVSNYRLRSCTADMDFLRNIVFKHSILLELSERINDLFGLSLFSLSIA

DAAFNQNWIDSDVCCQKMLILITERAQKPVILKATTLVPVSRATMTQ

>XP_005190201.1 PREDICTED: odorant receptor 46a-like [Musca domestica]

MNSREHRELLEIFYKKQSYVFRLLALWKLPDTVTERFRLLHRFYFYYILFFWVLSFDASCMIQFIANITD

LNEVIKVFFIFATSLAVFAKFATIKLKNHLYAELIETIHEPAYRPVNSREVKIFRQTHRLCGTVRNFYLV

ISLCALNVVMLTQYIFDNSELPLSLYNPINIDTKLRYRLMYLYQYVAVSICCYMNIAFDSISASFMIHIK

GQLDILCDRLEHLGMDQESRDEDITRQLKNCVKYYGDIIHIVRIAENLISFPISIQIACSVLVLVANFYA

MSFLSDPGDYANFIKFLIYQLCMLSQIYILCYFPSEVTAKSEEVPYYLYCSNWVYWNRMNRKLTLLMMTR

FDIPIRIRSINPTYTFNLAAFTSIVNSSYSYFALLKRINS

>XP_005190189.1 PREDICTED: odorant receptor 30a-like [Musca domestica]

MKFLTERKTNKITKYSAKIKRLEDVPMLWFNVRILKFWSVLIDNNWRQYFSYIPFFFLNIFQILDLYYTE

KEINDKIHDTYMTMIIFNTFLRAIVMVTNRRKFSESLEYMKDLYAELIMEYDFEIRQIIRKYSDMVLKVS

KINLTMGILTGLGFSMFPIMAEEREFIFGMYVPYLNEYQTPWYEILLAVQSVLNLSGMCTFIPFAGMFVS

FLVFAMAISKVLQYKLSKLSTEISSKLAERQIIECIKLHLKLISFIDKVNELCSIISLVDCILFVVILCI

MLLSFILVKTVIQKCVIVVYMIMVFTQTFLLYYFSNETYHESLEISTAAYNIDWFNYDVETQKVLQLLLL

RSQKPCAILIAKAYPINLVRLQAMLRVTYSVFTLLDKFYG

>XP_005189494.1 PREDICTED: odorant receptor 7a [Musca domestica]

MEHPDIGEQPALLPQQIQEEQPQPETKSNEIPKLNHENKWDLKAEPPLETRQGLRYLYNGFRFLGIYFPK

RRKGLYLLWSIIVNLYVTIFLPTGFIMGIISVTDENVEIGNLLTSFQVAINVVGCSIKIILMYFLLPQLL

KCEPIFERLDGRCTSREEKDLIRQFVHDGNRLVVLFTVAYWSYSSSTCISAVLFGRLPYNIYNPFIDANA

SRGYFILAVFMEMVPMDIACFQQVVDDSYAVIYTQILRTHLQALLIRLQHLNDDDAADLDDEAQERNVEK

LKLCIIDHKSIIELYNRVAPVISITIFVQFTITASLLGSTLINILIFATNTASIVASCFYVLAVVVEVFP

LCYYAQCLMDENNRLTEAIFHSNWIYQNKRYRQMLIFFMQRSQKVIEFTAGKLFPITLSSFLSIAKFSFS

LYTLIKEMDLKERYGLN

>XP_005189143.1 PREDICTED: odorant receptor 83a [Musca domestica]
MEPIEARRDLFQFVRRTMYWAAMYPLHLDRRLPHYICGLGLFVECFFEMFLYLVSIQIAILYVCTIYLNY
DSGDLELLVNCMIQTIIYVWTIVMKVYFRRVRPHHLEGMVDTINAEYRTRSAIGFTYVTMDQCLDMSNRW
IKTYVYCCFIGTVFWLLLPIAYGDRSLPLACWYPLDYKEPVIYETIYFLQSVGQIQVAAAFSASSGFHMV
LAILISGQYDSLFCSLKNILATVAIRMHSTKEELRKLYELQESTDSELNEFYCSEEITCDINMLVHINAS
PKQALMSSQEFRYHFRHAFAECVHHHWYILDSLKSMEKFYSPIWFFKTGEVILLMCLVAFVSVKSTTANS
SFMKVVSLGQYLMLVAWELLIICYFGEIIFINSQRCGDAILRSPWYLQMREMKNDFLLFLLNSYRPFKLT
AGKMYPLNVERFRGVITTAFSFLTLLQKMDERV

>XP_005188267.1 PREDICTED: odorant receptor 2a-like [Musca domestica]
MTNALTDNNKNIYSKLDTNVAFEYHWKVWRWTGIKPPQDMNPQLYRLYAIVLNFLATVLFPLSLIANVFF
TQNLQQLCENLTITISDCQSNLKFINVFLVRHQLDRIKSILRRLDRRVQDDKEFAVLKSAIATARSSFLI
FFRLYSFGTTLSVVKVALAESRSLLFPAWFGVNWDGNLSTYVVVIVYQFFGLAVQALQNVANDSYPPAYL
VILSAHMRALEIRVKAVGQFRQEGMQQPLTLSAEEQAKCLKEFNECIKDYLNILKLHSIIQRIISKACLA
QFACSALVQCTVGLHFMYVVDAANYEAQLMSIIFFVAVTLEAFVICYFGHMMSLQSSNLTYAFYSCGWLA
QSPEFKRNLIITLMRTQRTSTIRAGSYIPVDLPTFVVLMKYAYSVFTLLIRFK

>XP_005187726.1 PREDICTED: odorant receptor 67d-like [Musca domestica]
MAKTHTERLLKIVRITKFCSDICGVNIYEDDYRINYRTFFVIAVIGTSFSFLSYTMYDGYGKEGDWTILV
QVISLAGGTLLQGFFVLILFLTKQEKYRFLLKECIILYEKYEKMDSDYRVYLNKGIHLLANFMKVCAFIN
FMLVLGMTFVTIFYNLIFGTNETLVYGYCPWVSLETTGGLWTTNMVQALLIAVGGFGLYSGDMSVLTPIS
QIPTFKGIIQCKFRELNDLLDDDHESEMAKKIKTLAALKDILQFHQTYLRFLDVSREAVYWSVFVKVGTC
FIGIAFALFCILLGSWPAGYIYMLYCFVMMQVFCGMGTLVDITNEEFIHSCYNDVRWYDLTISEKKMLNI
MLMMAQNTEGLTIASIMPLSMNTGLQVTKTIYSLTMLLLTFVN

>XP_005187292.1 PREDICTED: odorant receptor 67d-like [Musca domestica]
MNNHAIDYLELWMANNWVSMGFCVFLTFIKEQENLRFLLTECYDIYEKYERMDSDYRVYLDRGVRLLAKL
MKLSAFINAMLVFGMSSFTFLYNFIYGTKATIVYAFAPGLDVATPVGFWATNFIQAGFIAVGGFGLYSGD
MSVLTPISQIPTFQGILQCKFREINQLLDDDYESAEERGIKTMAALKDILEFHQKYLIFLKVSREASYWS
VFAKVGTCIIGIVGALFCIMLGSWPAGYIYMLYCFVMMQVFCVMGTLVQKTNDDFIHACYNDVRWYDLTI

REKKMLNIMLIMTQNTKGLSVGSVIPLSMNTGLQVTKTIYSLTMLLMNFVIENEA

>XP_005187291.1 PREDICTED: odorant receptor 2a-like [Musca domestica]
MALQPMASSSSSASNKIHTWQAFRNHWILWKFCGLHPPKRNSRWFNPYLIYAIVLNVTTTLMFPITLIVD
LILSQNLTELCENLYVTITDVICSLKFINIFTVRHKLLEVRWILERLDVRATTPEQRQELRHGIQTSHKW
FMAFFRFYTCAVITSQLVVYLSKERVLMYPSWFPWDWKASKRNFLFAHCYQVYTVSVQTVQNLGSDTYPQ
AYIVVLIAHIRALGLRIKALGEALSATAAGDVSSPSSSSKKLSDDELYRELVNCVKDHQIVHELYLTIQE
CISKTCLAQFVATGLAQCTIGVYIIYVGSDFSRLLNSFMFFGAITIEILILCYFGDLYCRANDFLIDAIY
DCNWIDKDERFKKALLLLLQRSQQADCLKAGNLIPVRLPTFVKIMKTAYSAFTVLNEVN

>XP_005187290.1 PREDICTED: odorant receptor 2a-like [Musca domestica]
MSVLFSPHPNTWEAFKYHWLLWKWCGLQPPSRDSKWFRPYLAYAIIFNLTTILFPLSLVLDLTLSQNLTE
IFQNLYVTVTVVFSSLKFVNVFLIRRKLLEVRFLLERLDVRANTEEQQQELKNGIAMAHKCFMIFLRLYV
CAITTSQLVVYFSSERVLMYPSWLPWDWRESKRYFLFAICFQIYAVSAQLSQNLGNDTYPQAYIVILIAH
IRALALRIKHLGVVSTSVPAPEGKLSQEDFYRELRQCVKDHEHVHELYLTIQECLSTTCLAQFIATGLAQ
CIIGVYILYVGDDFSRLLNSLVFFGAVTIEILVLCYFGDLYCQANEFLIDAIYATNWMDRDGRFKKALLL
VLQRAQVTNCLKAGNLTPVMLPTFVTIMKTAYSVFTVLNKVN

>XP_005187208.1 PREDICTED: odorant receptor 30a-like [Musca domestica]
MLIETIEDVPLYNNSLRIMKFWSFLLRHDWRRYLSLIPYIILTSSQFVDLFFSTEPMDAIIRNAYLAVLF
FNTTLRGIAVCIHQSRYEDFLERIRVLYIDMMESEDQWVREELQAITLAANNISRVNLVMGTCSVISFLI
YPIFATTKVLPFGIYVPGVDKNISPYYEICFIVQTVMAPIGCCMFIPFTNMIVAIMLFAILMCRRMQRKL
RHLCHVTSEEARATIIWCIKYQTELIRYVNTINDLITYTNLLEFLAFGAMLCAMMFTLVTVETVSQMCLI
CVYILMIFAQSTILYYYANKVFDESLNVGTAAYESEWFDVDVDTQRTLRLLILRAQKPCAILVGRVYPMN
LELLQSLLNTTYTYFTLLRNVYD

>XP_005186825.1 PREDICTED: odorant receptor Or2 [Musca domestica]
MYYNHPLFSFNVKMWKYLGFIEFKRINQALLILIIPCLINMCQVMNIAYNWNDMSVIAIGLFMTAILFNA
LVRITTVMRNQSKFIEFFEMIEQWYREIEMGPDDGAWDLLKHIPRRTRLISILSFSFAAGAAVASATIPL
FLEQRSLPYDMYIPFYDHLKSPMYEILYFMQGFISMPFCVLTYVPFTNLFIAWLTFGISLLQILRYKLES
LPHENDEEMLKQLIELIRFHHRIMNFGQTLESLVSFVCLVELVLFTLMLCVLLASFLVMDNVMSKIATCI
YIFCILYALFIPYWHANEFSWESTKIADAAYNIKWTRSNIKIRKCIAMLILRSQTPLKIKAGGIFPMTLE

AFQALLNTTYTYFTMFKGMMGKEPNVHDRGQ

>XP_005186318.1 PREDICTED: odorant receptor 59a-like [Musca domestica]
MADELNTKALFKTHFVAWRILGMLPPTKYRPLYWMYSVFLNLAVTIGYPLHLIVGLFTTTTAYEVVQNIA
INLTCAFCAMKTIAIWWRFNKLDIMFEIIQRQDERVISEEGVAYVRNVVHPPVRRIILAFTILCSVIAAS
GESSVLFNGLLGNWTLMHKGYFPFDISNNTRNYAIAHLYQIIGLSYMILQNVVNDTFAASHMCLLRGQVQ
MLNVRIAKIGHDPKKSREQNNQEFLECIKIHKDLLEYRRQLEEIISVYMFFQILVAAFNMCIILVFIILF
VKDVFTLIYYILYFSAIVFEILPSCYYGTLLEDEFQDFAYALFSCNWPDQDVGFKKNLRIVAEFASRRIY
VTAWLFRVNNNAFIIAVKNAYALFALVMKVK

>XP_005185739.1 PREDICTED: odorant receptor 33b-like [Musca domestica]
MAFENFYQTNSVENFKMFWFLWRLLGFRGFQNKYANIVHNLVLHVAISFWYPMHLTLGLLSLPNQGEIFK
NLSITITCIVCSMKQLFLRWKIRQMHDIEMLFLELDASVESRQEYHFFTNGPRKHAQWITKLYCTCYMGA
NVAAITMVMLDSQRRLMYPAWFPFDWSSSSQVYWAVLMYQFMGVTTQIVQNLVNDAPAGVLLCLISGHVR
LLGMRVSRIGHDSKKTENENLADLGKLFKLVEDTQSYVQLILYISGGLNICVAVVYLIFFVESLTAYLYY
SAFILAITIEIYPSYYYGSSCQQEFNDLSYAIFCSNWLEQPKRFHKNMRIFVESTLPKVTMTAGGIVRMQ
IENFFAICKMAYSLFTLIRSIK

>XP_005185292.1 PREDICTED: odorant receptor 74a-like [Musca domestica]
MEFHRPLLPNGEIAPLSWEIRLFFVNVSWPMKANAKLFTRIYDKATLVLGFLFFCYQNEAEMHYVVNNIN
DIGLALEGMATYLILVETHLRIYNKGLYKSSFREFLNEFYAKIYMEKSYNIETYLDIQRKLLPTKMCSYA
YMLTLVTYFLVPVLGFFSNAHLVPFKTIFHYDLDIWYFYLPTLCLTLWIGVAVVSQLAAESNLLATIILH
LNARYLHLQSDLKELQTRLASDMKLSTDKVLGEYRREFIEIVKRNVEYNDFAQKFQNQYSFCIFVMMAFS
AVLLCVLAFKAATLGMTTKNITFITWIIGKIVELLVFGTLGSQLIETTDKMSSCYYMANWEDIILKSPKT
TDNIELMKLIILSIELNQKPFSLTGYNYFSVSLATVVTILQGAGSYFTFLYAFR

>XP_005184787.1 PREDICTED: odorant receptor 59a-like [Musca domestica]
MAPSMEINSNEFFKINRTCWKLLGLGMLMVEGHKTNGQRKMSTNLYMVWAIVINLMATCCFPIHLFLGIF
ESENKTSFFDSISITITSIGASTKLLIIAIKMKKILEMQSLLRTLDARITHHEEVRHFRQDIRSRIMNIQ
RLYFVVYCGVGISVLGAFLFSKEQRLFYSGWFPFDWRSSLGNYAAAISYQCIPIFFQMMQTFCNDSFSPI
ALCVLSAHIELLYMRVVRIGQDKNGKMRETTTLQEDEEELNRCVLDQMNLYELYNTMQNIISWAMFIQFF
VSVVNNCVAIVALLFFVTDVFERIYYVIYILAMGIQLFPTCYYGSDFVLLFEKLHYAVFSCNWIGQSKSF

KRHMMIFTERSLRETVALAGGIFPIHLDTFFGTCKATYSLFAVVMTMK

>XP_005184443.1 PREDICTED: odorant receptor 45a-like [Musca domestica]
MFSVPNPPDALPPQNSLKNFFLIQRICFSVIGLDPTSLKRTMYRPWLTFIPLLSLMGLLGPMGVYAFNYL
KIDLGKAITALSPFWQSMLSTIKFFVFMLNRKKIVGLVRKVWSWTLEATEEELKIIDEEIKGDARISLFY
YSMVNITGVLAALAPLAISAIYTFHGRGFMETLDAPFKAEYFYDIRASYMGYILCYTWNVLGIHYILNGA
LSIDTLYSWIVHNIAAQFRILNLRYRQLSEKIIAHQAAGNHNEKEFLKSVVECVNYHRRIIQMSERFSEV
YQGLVFIKFLVSCMQLACLSFIIPLGGEFADQSFNLSFLIAVTTQLMLYCHGGQKIQDMSTSVNLAIYEY
FHWHDLSIKSQKLLMITMIRAQKPCDIRGIFFTADLSLFVWVYRTAASFMTMLMSMQDK

>XP_005182424.1 PREDICTED: odorant receptor 63a [Musca domestica]
MSQLLLDLLKEKQLENNKILNTFYRISFMTGVKIKYKTQFKDPVKLINLFLISVSLVGLCAQYCLVWNKR
KEPFVESADAICTANQAWISIFKLIYLVFVQHKFYELLHTAINGSLLYDLGIFDLAIDCKQYLLQEINTI
LDSSWRHIKYQVNFFTFSCMMACGFYMFSCIAANYYYTNIQPQNFTLQLPMPALFPMWHDYGMTWPYYPI
QYFIAGIENYICGMCAVCFDGIFIVIVVHCASLFEILHMLLEHVDDIPQSERVDYLLCCARLHVRIYNYY
AKINGMYKNPSLAQCVLSMLVLCVVMFMASIGLEEDITLFVKMLCFLCAAGLQIAIYCYNGQKIITQSEK
SPDAWYNCCWYNESKQFKYIIDMMIMRTNRTLYLQVSGFTTMSHMTLLSIVQTSGSYFLLLKNLNGID

>XP_005181900.1 PREDICTED: odorant receptor 30a [Musca domestica]
MQIRSIEDVPLLSTNLSIMKFWSFLLEHNWRRYFALIPYLFINTTQFLDVYFSTEPIDAIVRNAYIAVLF
FNTILRAVLLCVNRFEYEGFMEKIRLLYIELMNSEDPALRKMLQECTVASRFISKVNLLMGFTSCVGFNM
YPLFATSKVLPFGMYVPGVDKYESPYYQICFLFQIIITPAGCCMYIPFTNLIVSFILFGILMCKVLQHKL
RNLKDVSSEKARTVIVWCIKYQLQLINFVDTINDLTTFTFLFEFMAFGAMLCAMLFLLIIVETVAQMCII
CIYIFMIFAQSVIMYYFANELYDQSLKVAIAAYESNWFDFDVSTQKTIKLFILRAQKPCAILVGKVYPMN
LEMLQSLLNATYSYFTLLKRVYG

>XP_005181476.1 PREDICTED: odorant receptor 24a [Musca domestica]
MVPNFLKNSYPLNKQYLLIPRFALRILGFYPESEWNVWLKSWAFFNISILAYGCYAELYYGIYYLPIDIV
MSLDALCPVASSIMSFIKIFFIWWYREQYKQLIEEVRRLTEDQNTLRKEKMKRWYFTIATRLTALVLFFG
LCCSTSYSIRAILTNTLLYLNGKDIVYETPFKMMFPEPLLAMPIYPITFLLVHWHGYITVLSFVAGDGLF
LGFCFYFSTLLKALQQDLTEVLGVIDETKKYRKLTESEKVMSLSKIIRRHNEIADLTMKLSSIMVEITLC
HFITSSVIIGTSVIDLLLFAGGYGSIVYIVYTCAVLSEIFLYCLGGTAVIESSQELAVKAYTSNWYGQSV

RIQKMVLLIIVRSQRHFVVKVPFFTPSLPALTAILRFTGSVIALVKSMI

>XP_005180861.1 PREDICTED: odorant receptor 2a-like [Musca domestica]

MQLRQPKDVGQQLNSVYGLKYLWWNFSIIGIHPPAGVRTHPVWRFLYLVYAVVINFLAGFCLPATMLANL

MLLKSLEEIIGNLSLSMTIAISMTKELAILYCRGGLLKANHYLRLLDERCSAHPRDRMTVMEAVRMCHWY

YTVYISFYGFCAIGFAYIGWSNHTLVYSAWFPNIFANDQTNYLAAYIFQNLAQTFTVFQNGNNDMYPLCY

ITLMIYHVRALADRIQRVGGDAETSAEENVQELRNCIQDHKNVQSYFECIQPAISSTMFQQLWVAAFTLC

LTAINLMAFERTFAEKIFSVVYLGVIVIQIFPACLCVNFMMSETSNLTTAMYKCNWIEQNRNFRRMLIIF

MQRSQKVNVIYAGGLAPVTLQTFVAIIKFSFSMYTILSQMKIQ

>XP_005180133.1 PREDICTED: putative odorant receptor 69a [Musca domestica]

MNTHYRLQDFMVYPNIAFNLAMVQPFRLSGTLEEHQTANRCRGFMKSMLIKLWFVFGAVNLIYQNVGMLA

YLLLPQLSEIFDDVEMVAKISETGGILGLTMVAVCKMFVLFWHGRRISILLQELEEIFPDEKEQFAHPTL

YRVRHFAQTSERLMGRTTKFFIFAFCFYNSLPIAELLYELLLPDQEIKYRYQSNTWYPWQTKDNARTWLN

FIASYVCQVQSSLTGVGFIMAGEFMLCFFITQMQMHFDYLTNALRHLDAASVRANEKLKYLIIYHTKLLR

YSKEINEIFNISFLVNFITSSIAICMMACSMVMLSMAHTFKYSVGLLSFLVFTFFICYNGGEFTDASDAI

MPSAFYNNWYEGDASYRRMILFFILRSCEPNVLTAYKFTTVSMPTFMAILKVSYQLFTFLQAMD

>XP_005179498.1 PREDICTED: odorant receptor 49b [Musca domestica]

MFEDIPLIYMNVKILKFWSLLYDHNWRRYVTLIPPTFLVFTQFYYMFMTEEGIDAIIRNSYMLVLWFNTI

LRAYILIKDRVEYQSLLQDLEAYFYDLDKSNDVYVRNLLSHVNSNGKVMARGNLFLGLLTCIGFGLYPLL

AAERVLPFGSIIPGIDEYQSPFYECWYVFQMLITPVGCCMYIPYTSLIVSFIMFGIVMCKYLQRRLATLS

RFKGQPEWIYDEVIECIKYQKKIIEYCETVNRLTTFMFLLEFVAFGTLLCALLFLLIFVDSAAQAIIVCA

YITMIFCQILALYWYANELKEQNLSIAAAAYETEWFTYEIPVQKLILLMIMRAQKPCTIKVGNIYPMTLE

LFQALLNASYSYFTLLKRVYG

>XP_005179301.1 PREDICTED: putative odorant receptor 92a [Musca domestica]

MTIEPRKFSKYLKITITLNQILIIVLQIIYNLTTQDEGVDVLTNIIYINYNVVALGKLLSMYYRRQTLAK

VLEILDGIYPTQRIEEKYNLNSYFRYYSRIETFIWSFYRLVGPVYVTLPLVQSLKSIWTLGKFTLILPLS

LWKMGDPLDNDWWLTYLFYYLIGAFSSISSGMTITGCDLCLYSLITQLCMHYDLLSQRIMELQPAAGEEN

ATKRLGILTRQHLIVTNVANEINIFSVMSSSFTLCLVAYQMLDDVSIFTIVKAFILLLYESKQVIITCYI
GQKLKECSSLVNASLYAHSWYDGSTRYRRRVLYMLLCTMQPFVLNFMGIADITVITLKEVYGNAYRLFTV
FKSA

>XP_005179127.1 PREDICTED: odorant receptor 88a-like [Musca domestica]
MEKHRLYTLDEFLLKLQPSQRYTRIIYLDFRRENQNKPFRFESLRLLYAALTLLIVDCACNVLKIIFEIR
AQRLSEAKQIGAVWSIAFLCLIRGIFVMFKHKSMLDLTNDLDKIFPRTRLLQNRMNCHKLARYLLIRHRF
LFAYAVVGLSAFIGIPLLKYIVFYDPNSGEPLLDEYHQHASWFPFHLKENPTTYPYMYVSETILTLFGIN
CLFTWDHIYTVTVAQFIMHFEYVNTELARLNAKDTMDVEKSKKFYDDLVEIIKYHQHVLRLGNKLRNTFN
LPLFLTDLISGASICFHIYLIANTDDVIAITLFIFPCFVQVAFAFDNCYQGSRIENVTTNMSQVIFEQNW
YDATLEYRKFVVHFLLFASRPFTLCGYNLFSIDMVHFRGTMMIAYRMFTFLQARGSKVE

>XP_005178484.1 PREDICTED: odorant receptor 10a [Musca domestica]
MFLRFLSRSNPLKEYYFYVPRICLQLMGFWPGSPRSRRILCWAVFNFIILLVGVVTELHAGFSYLNYDLE
KGLDTLCPAGTSAVTVLKMILISYYRQDLEAVLKKMHQMLYGCNEKDMEHKAVYNRIIRQSSVMAARVNF
APFLAGFITCTAYNLKPLILVWIFWSKGKDLMWLTPFNMTMPKFLLEGPLYPLAYIFTAYTGYVTIFTFG
GSDALYFEYCTHIATLLKMLQTDVKLLFRKFEGKLTLTPTEAAYVEEQLILIIKRHNVIIEMTDFFRKRY
SIITLAHFVSASMVIGASIFEMLTYTGFGRFIYLGYTVAALSQLAVYCYGGTLVAENSIYLATVVFKCNW
YICDPKLRRIILMIICRSQKSLNMSVPFFSPSMSTFASILQTSGSIIALASSFQ

>XP_005178182.1 PREDICTED: odorant receptor 63a [Musca domestica]
MMSEKEVQMLKKSNYNKIKELIRISFTLGVNLTSPSTLKDSLKIINIILVVSSVISFYGHWCYTIESIKD
IPKIAESVCTGFQTLISVIKMVYYLFIQRRLYYLLYKAQTHEYIRKIDIFHKNFPMSERLQAKIDEILDA
SWKNINGQLIFYICCCAAIISNYFFMALFQNIYHTWKETPNYEFVLPFPSVYPSWKDKGMSFPYYHIQMF
LGTCSCYISGMCAVSFDGVFIVLSVHGVGLVKVLNMLIENSTSADVPKERRVEYLRYCIYQYQRISDYTD
ELRKIYKHISLTQFLLSLLVWGIVLFQMSVGLESDLMTLVRMIMYISAAGYEIVLYCYNGQRLTSECEKI
PYAFFSCDWFNESKEFQELTRMMILRSNRSFFMEISWFTTMTLPTLMAMIKTSGSYFLLLRNVAE

>XP_005176400.1 PREDICTED: odorant receptor 45a-like [Musca domestica]
MKIVKRYFGIQRRTLTAIGIDVNAFLPNGPERIAKHPLLLLVITVMPVLQYISIGHYAYKNSNNMVTATY
SFSLSCQGVICLTKILIFLFKRRDIVKLVKMLQEDVFNAKSDELVITKEENSRDVLHCTVYGSAVYLWDY
SHLPGYSLVYIWNMMRMYTLAFASVAIDSLFSWLVCNIVAHFRILMLRFQRAAWLTPGLDRPEVSVSREQ

ERLIFDCVRFHNRTLNLVQELNLVYGGIIFVKFVVSSVQICCSAFFLNSFGASQSMAKLMYQFLLLSAVA

LQLMLYCYNGQRITDVSFQVATKVYSTFPWSKMPASTKRMLLPPMIRAQRFSELRGVFFTVDLSLYLWVF

KTAGSLIAALKTLEEDK

>XP_011212981.2 PREDICTED: odorant receptor 43b-like [Bactrocera dorsalis]

MGYLHTLETEPITIQLGILQAIFNILGLPMKAIVITILLTHLRSAELIFSRLDARYQSIASREQIKNCVI

ISTRLLSSVIFVFHFYGSATYLQALLTNGYPLNTWLPFTDYIPQPTIRYWAHFIFEVFHLIFLLTVQATM

DAFPAVYIRNLRTHLNLLTERVSHLGENAELTEEENFEELVDCIVTHQEFLEAKNIVESVCSITLFIQFV

IVAVALCVSMLNFFVFADRQQQVVTVTYYLGVMLQIMPTCYQASMIEADSAKLPDAIFHCNWLAMDKRCR

KLIIYFIHRAQEDITFVALKLFNINLTTNLSIVKFAFSLYTWMSNMGFGQNLKDLLE

>XP_011212447.2 PREDICTED: odorant receptor 45a-like [Bactrocera dorsalis]

MTTRSARVIKIYFLTLQIIALATILIPIAVYSWQHIQEIVEVTNAMAPFMQATISLWKIWRVIYRRKEMA

QMAENIYLISTRASAKELTHLIQENNRERLMNTAYYYSVLNTGMLALAAPVLVSFIQYLRLGEFSYIVVL

KATYPIEYARPLNYFLIWLWTAVAIYGVIYGSVSVDSLYSWYIHNLVGNFKILQSKLVTAESASELSERR

ELIYYCIAYHQRIIAMTEQLNIIYQPIVFVQFSLNALQICFLAYQIGSGVVDTVDLPFLFLFMISVGIQL

MIYCYGGQHLQNESVNVSKSIYQTINSSSWPNELRKVLLISMMRAQKPSKLTGIFFDVDLPLFLWVWRTA

GSYVTLLRSVDQKTM

>XP_019847990.1 PREDICTED: LOW QUALITY PROTEIN: odorant receptor 45a [Bactrocera dorsalis]

MENFADVDKIVAAMTINVQLFTTSGKNFIFLARRKRFLRLNEALERLALKGNKYERELWNATNRPVLPIT

TAYSISCQLTVNICLLLPIFKLLFYYIWYNEVVLTLPLPGIFPYDYTLPFYYILTTILTVLLVQLCANTI

TVVDGLFGWFVYNISAHLQIMRLKLEQLLQLHVDDPNFHRDFVALINYHREIINLALELDAVYAPIIFLE

SRLXSLPICFLAYQLSYLSDPANVPFICLLLSSIVLQLMIYCFGGEKVQNECDELSQNIYLLIPWHKLPP

KHCRLLLIPFIRSQRVLVLTGYFFTANRSLLVWIFRTAGSFTAMLFALKEKEV

>XP_019847876.1 PREDICTED: odorant receptor 94b-like [Bactrocera dorsalis]

MAVKKWSPRNTSSMSRTASANIIIAVLKSLGYWQWTRDPRQPYIEKVERAYRIVLHTTLPFTFIALMLTG

VLLSRDLDEIGSILHVLLTEFSLIVKTLHIWRKGGVAWRFMHEVANDPIYDLRQQSEWTKWQQAQRSFAI

VSNTYFVAATTVVVFACIGAMMTPADVYVLPMNIYVPFDWHHPRRYWYAWTYNTIASLMTATANAMLDLV

NCYFMFHLSLLYKLIGWRLSALRRSANEPPVIEQMSEIFQMHMKVRRLTTECETLVSIPVFSQIILSSFI

LCFCGYRLQQMEIMENLSMLFSTVEFATVMAVQIFLPCYFGNKVTESSDALTDEIFNSDWTTFDVPTRRF

MILYMELLKKPANLMSVNYFIIGVDIFAKTMKNAYSIFALVLNMNN

>XP_019847679.1 PREDICTED: odorant receptor 49b-like [Bactrocera dorsalis]

MTIGRAFIVMCKSKKFLNFFESVDEWYQELHVRFAGGSSLFLFIYYLLTFLHFQREGDDVTLKKAHEYTK

KIKKTSKTVLILTGITIFYIMFVQLLSTAGVGYKKLILDVAFPGVDFYESPLWEMMSILQGLWTAPIVIV

SYVSYLCLTLIAIAFGIFLMKNLQSKLEGMNEMTDEEALKCIKKCVKDHVMIIKYHRDLEVLFSVNSFAD

VCIFAVIPCVIIIISTMDHDMSMLIGDIQLSIMVMISTFLVFWVGNNFCYENENIAKAAYNCNWENRNKE

FRKYIPLIIITSQRPLQLTAGGLKPINMEFFLTIVRCTYSFFTVLFTMTTEGDS

>XP_019847671.1 PREDICTED: odorant receptor 30a-like [Bactrocera dorsalis]

MRRIGYFDQHRLAWLYLISPIFLCLTAYYRTYVIRNDFDEVIVNLFKISGATTTTVRAFIVMYKAKKFLS

FFESIDEWYQGLQVRFAGVSSLFLFICYSLTFLHFQREDDDVTLKKAHEFTKKIKKTSKTVLILAGITLF

YVIFIQLLATAGVGYKKLLVDVAFPGVDLYESPLWEMMSILQSLWIAPIVLFSYVSYLCLTLIAIAFGIF

LMKNLQSKLEGMNEMTDEEALKCIKKCVKDHVMIIKYHRDLEVLFSVGSFADVCIFAIIPCVIIVISTMD

HDMSLLIADIQLSLMVMISTFIIFWVANNFCYESANIAKAAYNCNWENRNKEFRKYIPLIIITSQRPLQL

TAGGLKPINMEFFLTMVRCTYSLFTVLFTMKTEGDS

>XP_019847608.1 PREDICTED: odorant receptor Or2-like [Bactrocera dorsalis]

MTSYENLPLYAVNVKVFVKVGLIDSIGWTKRFLFCLIPIITYVGQIIHIFKSWNEDMGETSMNLHILLLK

THCLIRLWLMVKKPKDFERFFQCVEQWYRDIERNGDPQMVGTLKEITKRTQLLSKMTIYVAAGGTIAAFF

YPLSFDRRKHMITVQYPFVDALQTPFFEFLFLLQVLCLAPIILVLTLPFTNIYLISLMFGELVLKDLCVK

LRNIRSENEETMLQEFKKCIAYHQKIIALCDDLQDLLSMDGFFHVALFGMMLCMLLFFLSMIHDLRLILT

ILTFVSYTTYMLFTTYYYANNLATESLEVANAAYDTPWYRGNLEMRKCVITMIARCQKPLQMKAGGLYPM

TMETFQAILRVSYSYFSLLQGLSQQ

>XP_019847607.1 PREDICTED: odorant receptor Or2-like [Bactrocera dorsalis]

MTSYENLPLYLMNVKVFVKMGLIDSSGWIKRFLYGLILITSFVGQMINVSKTWSEDIGDTSMNFYCLLLV

THCLIRFSIVVKKAEKFERFFQCIKQWYTDIEREGDPQMVGTIQEITRKTQKLSKVTIYVAALATLAAFC

YPLSFDERKHMIEVQYLFFDILQTPYYELFYLMEVVLVTPTILVLYLPFTNILLISLMFGELALKDLCVK

LRNIRSENEETMLQEFKECIEYHGKVVDMCDDLEDLISIDGFFHLALFGMILCMLLFVLSVVHNLRLILM

VFVFVSFNIYMIGITYYYANNLATESLEVANAIYDTPWYRGNLEMRKSVITMIARCQKPLQMKAGGLYPM

NMETFQAILRVSYSYFSLLQGLNQ

>XP_019847598.1 PREDICTED: odorant receptor Or2-like [Bactrocera dorsalis]

MTNYENLPLYAVNVKVFVKVGLIDSTGWTKGLLFCLILIITYVGAIINMCKTWYEDIGETTMNFHRLLLI

THCLIRFCIIVKKKNKFERFFQCIEQWHREIERNDDPQMVGILQEITKRTQLLSKMTIYVAAGGTLAAIV

YPLSFDERRHMITMQFPHFDVLQTPFYEIFFLMQVTWFTPAVLVLSLPFTNIFLISLMFGELVLKDLCVK

LRNIRSENEETMLQEFKKCIAYHRKIVDWCADLQDLLSMDGFFHLALFGMMLCILLFFLSLIHDLRLFLA

ALSFVSFTTYIISITYYYANKLATEVLHAT

>XP_019847427.1 PREDICTED: odorant receptor 47b [Bactrocera dorsalis]

MISLSSKATISNTIASHNSYLTNHSYTHLKHTASKLQTILAPYRVLKEMLRCGEAVQPPHTCLFYFRSYI

RLLGLWPAKRAAENQLYYFYNLLIMVLFSFFMLYLIFFKMILFRMGNVDTDIIINEFDALHIKHARGLSG

GPRNRRILQWQRSFFFGEMCFFSGFYILSLLLFAAMSLQPLLSQQTLPFRCKFPFGLNDPDEHPIAFVCV

YFFQCFCTLYMLVAIVVMDSLGGNSFNQTTLNLRILCENIRHLGIVAAGASSSTSEAVAWRELREAVEFH

QKIIGLMNRINQTFYWNYVSQMGASTFMICLTAFEALLAQDKPMVAMKFQTYMFSAFMQLLYWCWMGNRT

YYDSMEVATAAYEIRAWYEHSPLLQRQLMFIIKRAQKPLEFRAKPLFGFTFASFTSILSTSYSYFALLRT

MSD

>XP_019847361.1 PREDICTED: odorant receptor 7a-like [Bactrocera dorsalis]

MTTTCRLMHIKALGLALVNKTSEIRCNMRQSPRQATAEMPKIGLALAIHNEIATQRDIKIKASSSAKALS

ENIEEEAAEQHVSSQDTTKYLFKSAFGMGLVMPSRYRALYIIYGFLVNFFTTFYFPIGFTLILFTLPDDV

NVSNLLTSLQVTFDVYGGSAKIIIMKFVLEKLRATQILTQRLDKRCRASDEVEELRQMVRFGKKVVIFYL

TIFLCYSASTFLASVSSGYPPYSLYFPFLKWRRSRTEFIIASLLEFIIMDFACLQQTVNDGYPVIYINML

RCHMKILQFRVEKLGTNPMLTQVEHLSELKLCIKDHQLLIELYDTIAPIISITLFIQFALSAVCIGTALI

NIVIFANEFQTQVACSFFILAVLIEIYPACYFSQCLINESDKLADVIFHSNWIEQSPEYRKLIIFFLQRS

QRPMFLTAGKLFPVTLSSFIAKFSFSLYTFIEKMNLKERFGIE

>XP_011209577.2 PREDICTED: putative odorant receptor 85d [Bactrocera dorsalis]

MSDQIIHFESFNTLANIFYTSIGLDAYQKAGQRTNNIRRQLLSIFFIITIANMNITLLSELLYIFMAFAK

NNNFVEATMLSSFVGFVIVGDFKIYSIWRQRARITAMMQALHALYPRTLAEQIKYEVQRALQRYQRFAYA

FVLLHELLVWSYNLFPLLNYFIYEVWLAARVVGKTLPYNCWTPFDWHVNDWRYYPMYLTQIAAGQACLSG

QLANDLLLSAVAVQLIMHYRQLARRIELHVAGGGGGSGSKWRTAATNVCREQDLRFLRSVIAYHQQILNL

SQALNDVFGISLFISFASTALIICFVLFQITIGANIDAIIMLAFFLFCSLVQIFLICYYAQQILEASEYI

SYAVYNHNWFDSDLRYRKMLIYIMARAQKPSKLQATALVIVSMPTMTDLLQLSYKGFAVIRTMYAREPKN

FTK

>XP_019847175.1 PREDICTED: odorant receptor 7a-like [Bactrocera dorsalis]

MFELITGRGIRNAASKDAFIYFFKGCTIVGISPPKYAGPLYYMWSFLVNTICIVIGPITATVGFVIKYMQ

NIITTVQFLSGLQASLNLIGLPVKCLTVTSALNRLRGMEPTLAALDARYTRPEDMALIRKAAVMGNRLVF

FFGTSYLMYMLFTVIPPLINGKAPLSVWIPFYDEHQSTMHFFGQIVYDLFLMGFVLFHQVLYDSYGSVYI

YVISTHLQLLVRRVGRLGTDATKSKDDNLNELVDCVVTHQQILELLATIEPIISKTIFTQFLIISSILCV

TMVNMFFFADRSTQIASTLYFLCVLLQTSPCCYFATELKADSEKLPLAIFHCNWPEQDRRFRKVILYFMH

HAQLSIELMAMQLFPINVATNISLAKFSFTLFTFIKEMGIGQEA

>XP_019846037.1 PREDICTED: odorant receptor 7a-like [Bactrocera dorsalis]

MGYLHTLETEPITVQLNILQAICNIIGLPLKAIAITILLTHLRSAEPNFARLDARYQSVASREQIKNCVV

VSTRLLASVGFMFHFYGSTAYLQALLTRGYPMGEWLPFIDYIPRLTIRYWAHFIFEVFHIVFILTVQTAM

DLFPAVYIRTLRTHFNLLTERVSHLGENPEFTDEDNFDELVDCIVTHQELLEAKNIVSSVCSITLFVQFV

IAAIALCITLLNFFVFADTVQRVVTLLYYFGVIMQITPTCYQASMMEVDSAKLPDAIFHCNWLAMDKRSR

KLIIYFIHRAQEDITFVALKLFNINLTTNLSIVKFGFSLYTFMNNMGFGQNLKELLE

>XP_011203704.2 PREDICTED: odorant receptor 67d-like [Bactrocera dorsalis]

MTTTKVRPTESFGKIIKFFHLISSLVGADVADENYRVNIITITLIICIVAYFIFTGTTVASVFSENWTYL

LEASCMVGSVLQGITKLISAFAFAKEILGIRIELENLYREYEVKGDDYAEALNKSCERVWQVIKMVGQVY

FVAGGGIILITIVLIFASNEKVFLMHFMIPGIDVDTQVGYLMTLTLHTMCFLFGAFGLFAGDLFFLLFLG

QPMLFLDLLVLKVKSLNEAAAENSSNAERLLIEIIEWHQYYTDYNLRCNRIFYYINSMQIVTSGISIICT

LYIILLGDWPGAYLYILVAFGGLYLYCIMGTKIQTCNTAFCEELWNINFYDLEVKNQKMIIPILMKAQNP

SEIKVGGFLPLSVQTALQITKTIYGIFTMMLRFLEESQ

>XP_019845111.1 PREDICTED: odorant receptor 7a-like [Bactrocera dorsalis]

MRKIADLFYGRGKHDFETTESFVLLSRSFAAIGFLPKIPKRIVDVIHQLICWSCIFSCPYLFVSGVVKTM

HSLPITIVLAHLGVAINSIVFPLKAVYIKANIDRVDDIGKIFNALDKRYQRPQDQMQIRDSVKTCTRIFV

VFCIVYWLFGISSWLVALCIHEYPHGNNLPFIDWLPESNLRFWLHFIFEVVFLHELLQMSLTMDSFPALY

IRALRTHMNLLSDRVSRLGLNPDFSDQENFEELVDCIVSHQEILQISDTVGKILSLTTFFQFTVYAAILC

VCMLNMFVFGDASTKLVTLVYLLPVFWQTTPTCYQASMLEADSAKLPLAIFHCNWLALDKRCHKLIIYFM

QRAQQEISFTAIQLFVINLRTNLSIAKFSFTLYTFINGMGFGETLKDRLE

>XP_011199152.2 PREDICTED: LOW QUALITY PROTEIN: odorant receptor 67c-like [Bactrocera dorsalis]

MPEARTFSEFIRIPIRFYQTIGEDLYEHRSPHRIRRLLLKSLLYLGFLNFNILVVGEIIYFVKALNSFAT

VLEATGVAPCIGFSLXSGFQADRLDSAPSNLREHLDQMEESFPKTAIQQVEYKLPQRERVMRRVMAIFAL

LCLAYTSTFSLYPALKAAVQYWLLGAPVFERNFGFAIWYPYNATEKTWVYWLTYMGQVHGAYLAGVAFLS

ADLVLVASVTQLCMHFDYISRCLEEFAGRSAKSSAQEDLQYLQALVVKHAKCLELSEHVNSIFSFSLLLN

FLTASLTICFIGFQVTASSTEDIVKYIIFLTTMLVQVFVICYYGDELMTSSQRIGDAAYNQNWFDCDRHY

KKLLAIIIMRSQKPASIRAPTFPPISFRTYMKVSLNFNQA

>XP_019844437.1 PREDICTED: LOW QUALITY PROTEIN: odorant receptor 35a-like [Bactrocera dorsalis]

MDYFVPLQFDNRPIKLPIQVAGYKFNFLWPLKEDAGILSRLVNNICLSVSVLCYIGTIVGEFTFIGENIA

DIAAVAECLCTSFMGVQYIIRIFVLLSRQRALRKLLRNFYRDIYFTPADDAALYKEINSIMRFMNIFTQF

YYVPMMLIFVLYVYDVASVGLASPDKPFIYRMSFRWYDAQVPLQFIITAIYSGWLTISCVTIWTAEDYTL

CLVLCHASFRYKKLRLDLQQLLEMARADLKCGETPCTNQNLHIAFRRRLREIFRRQQRLNGFVAEAKAHF

THQIFYIMSFGVLLLCVVSFQFQSGPITVASSKYISWLISQTAQFLLIGYFGQMLMDETTELRNSFYCCR

WEDLLVLGDPHSNKLLLGDVQFAIMNSQEPIVFDGMKFFPLTYSTVSAALRSAVSYFMFLNTMNGEN

>XP_019847162.1 PREDICTED: LOW QUALITY PROTEIN: odorant receptor 63a-like [Bactrocera dorsalis]

MYNAAEFAELKNNNRFKIRELRNVSYILGINYGSETSLKKFLRVLNLFLIIICAISLYPRWLMLERADGN

VPLIAETITTMLQTTTSMVKMTFCLFMQGQCRALLKKAENYELLQGIKIFLTDMDIKAELKVEINAIMAT

IWKESRRQLLSCLITCSCILSNYFLYAFFTNLYHQIKKTPNYVHILPFTGYPMFLEKGMASPYYAVEMFI

GGCSLLTCGMCSVSFHCIFMILCKHACGLVKVLCVLLMRSTSLQVPAHRRDEYLRYCVIQHQQTLRFIND

INDLFKHITLSHFLHSLAIYGLVLFEMNFGLETDKTTFVRMLMYIGAALTVDSMYYVNGQFLATELENIP

FVCYSCDWFNESEDFKRTLKMIIMRSNKDFCFQISWFGIMSLTTLMGILKASFSYFLILRDMTDETN

>XP_011201924.2 PREDICTED: odorant receptor 74a-like [Bactrocera dorsalis]

MRYLPISYHKPLLPNGLHPPIDWQLYGFFCANGWPLAAHITKTRYIADIMVTIMQFMSEGMVLIGEAVVM

HDNLDNISFVCTVLAPNLILFEMMLRAYNIIYRRNSFRTHIEEFYKKIYIQRTWNPELFEKIRRQQLPTK

YSTFTYIITLVTYVYVPVSGLIKNERLVPFPINFGFDYTVPWPRYLVFLTMSMWTGFAVVGPLVAEANIL

AMQILHLNGRYSLLLEDLRNISRKSIAEHEKCKRKDNMLVTQRFRYRLYDIIRRNVELNDFAKSMQEQYS

FRVFVMLALSATLLCVLGFLTATLGITAQNIRFVSWIIGKVVELLIFGRLGTTLSTTTDKLSTSYYCCDW

EDIILHSTNAEENKKLMKLIALAIHLNSNPFRLTGLNFSVVNYETVVAILRGAGSYFTVIYAYR

>XP_011200603.2 PREDICTED: odorant receptor 63a-like, partial [Bactrocera dorsalis]

INILIIIASCIALYPHWLMIKQAQDDIPLIAETSTTALQTTTGLIKMAYMLFTQHRFHKLLRKAETHELL

QRIEIFQTGMPIKATLKKDINAIMEINWKQTRGQLLFSLGSCICIMSNYCAISFQGSFMVLCKHSCGLVQ

VLCLLLERSTSNLVPKPQRMEYLRYCIVQHQRTLEFISEVNQLFRHICLSQFLHGLAIYGFVLFEMNFGL

KSNKITFIRMLMYLCAATSCDCMHYVNGQFLANELQKVPLACYSCEWYHETDAFKKTLKMIIMRSNKEFY

FQISWFTVMSLATLMGIFKASGSYFILLRDIDEP

>XP_011212445.1 PREDICTED: odorant receptor 47a-like [Bactrocera dorsalis]

MYEYLRIQQFSFRVIGINLWAQRDQRIASAPCRYYSWTLATAIITLFMGFYIYTSEQDKAIQVLTVFLQG

VLSVFKSGMFVAKGRRFIKLIRSLDMLAAEANVKEGKEWKHENDWQQRIARVYYSCCMSTGTLYCAVPAI

ILLYSQCFNGHATFILPFDAAFPFDTAHPFFYPISYIWCISFIIYAVHAIAAMDSLFCWFIFNISAHFRA

LQRAVETVGAAMTGAEDYASLHGRITRTLHYHRRIIELSAEFDELYAPIVFIEISVSYLKLCFSAYNLIN

LAAAAANTFQTSPSVSQATDYLKSLNLLNISCVAASECIGAAKRNLQQVAEPIKLNANARHLAITGQSAV

LACE

>XP_011212431.1 PREDICTED: odorant receptor 49a-like [Bactrocera dorsalis]

MDFVQFFWFPNALYRIVGYDFQQLPRAHWRKALMKAFLIFTTISGICTRIYMLFQLRELILSGDILNSFR

LGVYISYAIDSNVKFFVFLLNAKRLRVIYQSLSNEYPMTSMEQKLYQVDKYSFKRARIMIVSYLSVTNSI

LIGPMLQSIFMYIIDLFRYGYAAAAFSYLHPTPMSYNFNYCTPHYYILIYISEYLNGHFCTTTNLGTDLY

VCTFAGQFCMQLEYLGSSLEAYEPSMDNSKADCKFLMEWIRKHQLMLDLCSELNEVFGTTLLFKLISNCA

VFCIIVVQLKLEGFGFGFLNFLSFFFVTVAQFFMVCQYGQKLITISENLALCAYKNRWYNGSQTYKTLLF

NIIARAQKPARLTAKGFQPISLATFQIVMTMTYRVFAVLQRALD

>XP_011211752.1 PREDICTED: odorant receptor 94a [Bactrocera dorsalis]

MWAQHIIKLELKMTPSAAEQQERIGVARVLMHFLQILGAWPILPEHHHQNASSTQCRTWLARNYRYLLHL

PLTFTYNTLMWVEALTRWERADHILYISITEVGMMALTLNFWRLDQRAYHFMHELCYSDHLALRNQAERQ

WWRAKQRSFTRIAVCYIGGGAGVLCTAFGATLLVNGYSLPYDYWLPFEWHNAQNYWYAYGYELVAMSLTC

IANVTMDMMLCYYLFHVALLYKLIGMRLMALQHLSERLAVQQLINIIELHKRVKRLTAQCEVLVSLPILV

QIVLSVFILCLSAYRLQSMQINENPGQFFAMLQFASVLTLQIFLPCYFANEITINSDALTTCVYNSNWEE

FSPPTRKLMNLYMELMKRPEQIKAGNFFLVGLPVFTKTMNNAYSLLALLLNMSK

>XP_011210512.1 PREDICTED: odorant receptor 7a-like [Bactrocera dorsalis]

MSKILLVRSATVYKSRDALTYLFNVFTFMGTNPLENRSQRYYRLYHFYSFTVNFICCLFCPLSFHIGYIK

LRHVLTNSQLLAAIQNAVQVSGIPIKILVITWYMKRLRHAFEILDELDVNYTRREDLAKIRECVRRCKKI

VLIFCFPYYSFELTTIALGVAQNRAPLAAWVPFLDGQRAAWEYWTIVLWDAFVMFFLLCHQLGSDTYPPI

FINIIRTHVQLLIARVNRLGRTGALTADEHYEELLGCIRTHVQIVSIAKIVAPVISVTLFTQFATTATTL

LNWLGNVEYPENIISLAFFSCQLLQILPCCSSASQLIADCERLPDAIFHCNWVDQDRRFRRAMLFFLQRA

QNPIRFSCLKLFNVKLETSVAIGKFAFSLYTLIEETKVGTDTEN

>XP_011210110.1 PREDICTED: odorant receptor 22c [Bactrocera dorsalis]

MRRLLGPQVPIERSFFRIPRFSARVAGFWPQSTNRHRSWLTALRFYVNTFAVAVGGFGEVSYGFVYLHDL

FSALEAFCPGITKVISLLKMTIFFGRHKRWQHVINSMHQLLLLDTSAEKRRIVESLASFGSALSFVLLLS

GSLTNTFFNILPLLKMGYYKWQSLEVELLLPFNVILPEMFVNWPYYPATYLVLTLSGAMTVFTFSAVDGF

FLCACVYTSALFRMLQHDIRNAFAELQELEHSTLAQNMRIQHRLAVLVERHNKIIDLCSDFASEFSLIIL

MHFLSASLVLCFSILDLLLNSSSVGVLTYIFYSIAALTQLILYCIGGTYVSESSLKVAEVIYDTDWYKCD

VRTRRMLLLMICRAQKAKTIQVPFFTPSLPAFRSIVSTAGSYITLLKTFI

>XP_011209578.1 PREDICTED: putative odorant receptor 85d [Bactrocera dorsalis]

MIEFGAFMSTANFWYSFNGIVAYDDIYRQPGDAPKQKSFAARFTTPLRQIYSLIGLVNLIWVLIIEASFV

VVNFIENSDFLQAARNFTFMGFVIVSILKILSNLRQRSRISILMRKLYEIYPKQSTDQPPYELQSHLSHY

RRIGFMHAFTHAFTVGTYNFLPMINYLFLAPLLQHTDVVRELPYYCWVPFEWRDNWLYYPLYVSQVCASL

TGLGGYLASDLLFCAATVQLIIHFRKLARDIEAYQAGCSCATADVCTQQAQRDLDFLSAAVYYHSHTLAL

CQLINEIFGLPVLINFISTSFVICFLAFQFSIGVPLDSMVALVSYMICCLVQFYMICSYGQELITTSENI

GHAVYNHNWLVADIRYKKMLIMIIRRAQKPAILKATTFVNISMGTLTDLLQLSYKFFALIRTMYAR

>XP_011209576.1 PREDICTED: odorant receptor 85c-like [Bactrocera dorsalis]

MSTIIKFEKFLELASFFCYNIGIKLWGPNDGFWLNFWLYLTSINLFLTVFAECIYIIMTIRSDFIVAIMT

LSYVSFIVVAYVKWYYLYNYQTERNAFFQRLDALFPHTKSEQESIKLSEYFRLNKLATRGYTITFMVVIW

IYNLYTISQRFIYTQLLHVHIERVLPYQAMYPWDWRDNWTYYVIYVTQGFAGFHATCAQIAYDLLLCILS

IQLIMHYDHISRSLEEYQTKFAEVHGIDINNGLPPLMCAAVELRAVKEDIKFISNIVSYHNELLSLSMSL

NKLFGMPLFVNFFTSSAIICFLSFQMSVTREVDLLMKLAVFLFFSVMQVYLICHFGQLLSDASTNVASAA

YFQDWSYADVRFQKMTILVAQRAQEAAALKATNFITISLDTMTVIMQISYKFFTVLRTMYAD

>XP_011209575.1 PREDICTED: odorant receptor 85c-like [Bactrocera dorsalis]

MSNIIRFEAFLRIPSFFYRSVGVDLWNTNGGPLQNAVFYISLFNVNVWLLSELIFAVLMLTKNFIQATMT

LSYAGFVLVGSIKMYFMWRKKAEMTRFLQLMNTIFPRTETQQKKMNLRSHLRQCTIVMTVFAMIFMILIW

TYNLYPYMQRQIYDCWLHMRSINKTLPYESYIPWNWHDHWTFYLYYTLQSIAGYHSASGQIASDLVLCAM

ATQIIMHYEYVAQRITEYQPQALRAPRHQVKESESYRKDMEFLCDIIAYHANILSLSDIMNEVLGVPLLV

NFMTSSFVICFVGFQMTMDAEPDYMVKLFFFLFSSLIQIYLICHYGQQLIDASSNVSRAVYNHDWIHSHV

HYQRMLVLVTARAQKPAMLKATSFVRISRGTLTDIMQISYKFFTLVRTMYSN

>XP_011209441.1 PREDICTED: odorant receptor 7a-like [Bactrocera dorsalis]

MMANVPSSTVQLEASIAALSRAESTDKPAVRTVQATNYLFKGFRVLGIYMPERRKWLYSLYSLIPNTLVT
LWLPLSFVFSYATMSTEDLVPSSLLTSIQVAINVIGCSVKIVVMAFLLPKLRTANVYMDRLDVRCRVEEE
IAELRKIVQQGNRFVVLFAMSYWSYASSTFLGSVVFGRPPYALYNPIIDWRKSKLEFITASLMEFALMDV
ACFQQVVDDSYAVIYVCILRTHMRILLMRLKRLATSAETNLEENLEELKLCIIDHKNLLGLYDVVAPIIS
VTIFIQFMITASILSATLINIFIFADQLSAQIACCFYILAVVVEIFPLCYFAQCLMDDSERLSQQIFHSN
WIAQDVRFRKMLVFFMQRTQRVMELNAGKIFPITLGSFLNIAKFSFSLYMLIKKMGIRERLGLE

>XP_011208900.1 PREDICTED: odorant receptor 33b-like [Bactrocera dorsalis]

MTNKHKPLHATTTLDTTEAFKYIWSCWRLFGMHRDLYERRLNWIYLILLNLYCGVIYPMLYICSFFTPMD
LSQKLANISVAVPIIYTFGKHVVIVYYIREDLPKALAQLKALDRLAESRPEDRAYMQKMVKNCHLVFFVS
FVSFWFALLSYGVLEIFRHKLPFEGWVPFDWTRSEAAYVGACAIQLIGLGIETTTAICCDTYAVTYLILL
VAHLRVLNGRIERVGSAGATSDAESYRELVACVEYHKECMSYYNSLRPTLSGIYFIQFLSTGLGLSMPAI
AFVGGNFSFSHVIKFLIIFGAIIIEVAPCCWFMDEVLVEMRRLTNAMFSCRWYDQNLKFRKALIIFMQRS
QIAQPILAGNLIPVSLETFTNIIKFAFSLFTLLNQLNS

>XP_011208819.1 PREDICTED: putative odorant receptor 92a [Bactrocera dorsalis]

MNAIERNTNFTRFTAGPVRYFKFLGILLQQPEMPHSKYQRLLTVVTIALMFLHQIGYILEPGRTFAEQSA
AAGLLNYTTVSGGKILFLVYNRRLLLSNHCQLAALYPSAAVERHYKLEHYLRIYAHVQTLLYNFFKYILI
VYITYPIVQSFYDLWSSGVYSYIMPTLFWYPVPLEQSLFVYIVYLLFACFCSFCAGLIILSADLCLFSSV
SQLMLHLDLLAQRIKELQPAEEGSLSALKAIIEYHQKILTIAKDVNSIFAPSILFSLASSSFILCFSAYQ
LLDDVSFIFALKVFLLLGYEMKQVVITCYYGDKLMDSSANLFTAVYAHNWTDGSPVYKRLVLFMLVRTYR
PIALKVAGISDVSLITLKQVLSTAYQIFTVLKTT

>XP_011208732.1 PREDICTED: odorant receptor 82a [Bactrocera dorsalis]

MPEDLFRIQRNCLRVMGHQDIFDNNEASSSDEQKSKSKRQRRCFRHWQALKYVLLLLFMVSAQLPMMNYI
IYHIDDLALATACLSIVFTNVLTVIKTSTFLTYKREFKSLMAEFESMYDELQEAGAKQCLVTVNVGAKRF
VKLYFGACTSTGLYFTINPLVSMIWAKFQAKPIPLELPMPMRFPFDFESTPGYEFAYIYTVFITIVVVMH
ATSVDGLFVSFTTNLRGHFQALQYFIETNTFDKSEALLQRELGIYVQYHVRLLGLAQSVQRVFKPIIFGQ
FLMTSLQVCVIIYQLVMNMGVIMEMVVYCTFLSSILLQLLIYCYGAEFLKTESSAVSTAIQMSQWYNLPP
RHRHVLRLMMLRSQREIIISAGFYEASLANFMSILKAAMSYITFIQSIE

>XP_011207940.1 PREDICTED: odorant receptor 33b-like [Bactrocera dorsalis]

MGKRFFIFKSDVDARADSVACFDIFWMCWKLMGIAVNSKKWYITLYDISVNIFVNIYYPIHLTIGLFLVP

TVADVLKNLTINITDVACSTKHFLFRCKLPKIREIQRLLKELDERVVAPDERNYFNTGIRNVVRRIMLIF

CASYAADVVASAIEVLTKKERELRYPAWFPFDWSANRYTYYAAVLYQTVGVSLQITQNLAHDTFAPVSLC

VMAGQVRLLGTRVSKVGYDMSKTLIEHERDLNECIEDHKKLLKIFDLLQDVFWYTQLVQFSSVGLNICLT

AVLMLLFVDNLFTYIYYTAYFCSMAVELLPACYYGSKMQEEFQNLPYAIFKCNWIGQRKSFQQNLRIFTE

LSKKQFTPTAGGIINIHLTSFMATCKMAYSLYTVLMNMK

>XP_011205499.1 PREDICTED: odorant receptor 94a-like [Bactrocera dorsalis]

MAINKLANFRTLEPVLTFLGLWEGGDSSWFKRQYRYYQLFMHTTITFTFACLMILEFIYSESLDYAIDVL

KYMLVEMAIISKVLNAWYYEQQTAELVNELANSAIFELRTSAEEQMWQKSQKNFRKLTMIYMGTGLNSAF

CALLAAALMGAKELPYALWLPYDWRDTYFWGIYCYECIAMPFTCLCNITIDLFQAYLLLHLTLCFRVISM

RLERLEDAGKEDAITTELLNNIKMHQRVKELALKCEQVISIALLSQIMLTFLILCFIIYNMQNVKTENDI

AQFSENPAHFLAMLQYALIISMEMFLPCYYGNELTVESEKLGFHLYSCDWTAMSAVNRRLIYVYMESLKK

PVVLCAGRFFEIGIPIFSKAMNNAYSVLALLLNVNDDEQH

>XP_011203872.1 PREDICTED: odorant receptor 83a [Bactrocera dorsalis]

MSSNEEKQKPDISATVHDGCSTCRMQRRDMFRCIRWHLWFSAMYRLPLERYFPARLRFLAVTLDWTYELF

LYSTLLHIDILFICTIYLNQDKGDLELIVNCMIQTVIYTWAIVAKVFFKRIQPKRVKELMRYLNEECRTR

SAAGFTYVTFKESVDLSNMWTTVFLICCYAGVTFWLFVPIFNQDRSLPLACWYPIDYKVPVVYEFIYFLQ

TVGQLQIAAAFGCTSAFYVLIAVIFSGQFDILNCSLKNILATTYIILRKPKSELILLREEQSIADYELNQ

YYIAKEYRTDFDCIPHFFEKETPKPENFYEGFKIALRPCIAHHRYVLYGLKMLEDLYSNLSFLKYLEVTL

LVCLVAFVWVKSTAANSFLRLLSLSQYLLLALWEMFMICYMGEIIFLCSKRCDEALQRSPWHLHSGEIKQ

DTLFFILNAQRPFRLTGGKMYNLNLKMFRTILTTSFSILTILQNMDLRQPQPK

>XP_011203703.1 PREDICTED: odorant receptor 67d-like [Bactrocera dorsalis]

MTIKHIRPTASFAKLVKTVRFISSLVGADVSTVNYQVNIITIIVIICIIMYFIFTATTVASVFSENWTYL

LEASCMLGSVLQGITKLISGISRTNEVSGMRLELEELYRVYETKGESYCKVMNACCERVWQLIKMVGLIY

GAAIVGNLLLTSFMLFFTNQKIYIMHFFIPGVDVETSFGYLLTTALHSLCFLAGCFGLFGGDLFFLIYLG

QPELFRDILILKVHELNEAAAQKDNKTESLLISIIEWHQYYTDYNERCNEIFYYIITMQILTSGVSIVFT

MYIILMGDWPGAYLYILIALSSLYLYCIIGTNIQTCNETFFEELYNINWYELDVKERKLMILVLMKSQNP

SEIKIGGVLPLSVQTALQITKTIYGIFTMMLGFLDEEQ

>XP_011201816.1 PREDICTED: odorant receptor 63a-like [Bactrocera dorsalis]

MMSESVEEIYKRNYNSIKVLIGVSFSLGVNLTAPSKIKDALKLFNVIWVVASLLSLYAHWSYFIRHIDNI

PLLAETVCTALQTLISAVKMVYYLFTQRTFYRLLEQTLTHEIIRKIEIFERDFPINRQLKQEVDDIMNGV

WRSARRQLLFYFCCCVGIVCNYFFGALFVNLYHQLKQTPDYEHILPFPALYPIWEDQGMTFPYYPLQMYL

SGSAVYIAGMCAVSFEGVFIVLCQHAVGLVKVHNLLVLRSTSRLIPAERRIEYLRYTIITYQRINIFAQQ

IQTIFKHVSLSQFVLSLIVFGFVLFEMSFGLESSIVIVIRMIMYFAAGGTQIILYCYNGQQLTSVSEEIP

LAFYNCNWYEESGKFKQLLRMMIMRTNRPFNLEVSSFTLMNLATLIALFRMSGSYFLLLRNLQEK

>XP_011201756.1 PREDICTED: odorant receptor 94a-like [Bactrocera dorsalis]

MELHEHDNLSGGRRVIKILKLLGLWHYGGVMRTPYLLYSGLLHSVFTIPYTIMMCMDVVQASDLEKFTNT

MYMTLTELGLVAKLVNVWSYSKLLVDFFTAFTHDKLYQLQDAEERQSWQRTQKNYSRVAFLYFTMSLSTL

ATAFVGVLYSEDYELPFPYAPPFDWRTPRGYWYAYCYELLAMPITCLSNCAFDMIQCYMLLQLSLCFKVI

SGRLERMGTLQECSSTRGFSEVMFHRDFVDIVRLHARTKLLSQQCQTYISFPFLIQIISSSFVLCFSAYR

LQKVPILENPSQFLTLVQANLIMVLQIFIPCYCGNNIIEYSSGLNNATYNAEWFRCSPEMRKYLVIYMEM

LQRPVRVRAGDFFDISLTIFTKTMNNTYSLIALLLNMNK

>XP_011200401.1 PREDICTED: odorant receptor 67c-like [Bactrocera dorsalis]

MTPIFKSSEFVPTVPDFVHIPFFLIKFLGVKLFKWTPDEPITKQQITILGLFTVFSIFNFTSMLLYVVYE

DLETLLDITEFVLFWGFTLNALMKGISMVCFRREIESILKGLIAKHPKTAEERAAYQLVPYFRTINISNK

YLSIWHLSITSIFVVHPLIASIHGYISREDKNESFDFTLPFMMTYFYDINQPLAYAVSYFLQCCGAFHVS

LLFLSGDLLLISMVHLVNMHFGYLIYKIESFQPTGTDADMKVLGPLMVYHNEMLNYAERIDNTFGLATLL

NYVGSCLVLCLIGLQIAMGSEAVIVIKFIGFLVSTIVQVFFVSYFGNNLKDLSTGISDAFYNHPWYDANY

KYMRMLVLPIARAQRYARLTAFKFFEISMDSFKSLCTTSYQFYTLLRTSIEEDGV

>XP_011200400.1 PREDICTED: odorant receptor 67c-like [Bactrocera dorsalis]

MMPSFKSSEPAPTVPDFVDIPLFQIKFMGAKLFKWTPDEPRGKLQITLLGTFCVFATFNFTSMLLFVIND
ELATSLDITEFILFWGFALNAMMKGGTMVCFRRDIEFVLKGLVARHPKTEEEREAFQLVPYFRTINASNK
YLSIWHLSITSIFALHPMVSSLLRYIWRDDTNESYDFTFPFMMAYYYDTNQPLTYAVSYFIQCCGAFYMS
LLFLSGDLLLISMVQLVNMHFGYLIYKIESFQPTGTDADMRTLGPLLEYHNEILDYAERIDSTFSLATFL
NYVGSCLVLCLIGLQIVLGSEALSVIKFIGFLVSTIVQVFFVSYFGNNLKDLSTGISDAFYNHPWYDANY
KYMRMLVLPIARSQRYAHLTAFKFFEISMDSFKSLCTTSYQFFTLLRTSMEEEDS

>XP_011199522.1 PREDICTED: odorant receptor 24a [Bactrocera dorsalis]

MFLKFLSQSYPTEENVFLIPRFALRIAGFYPGDGNSRRIQAWLIFNFVVLVYGSYAEFMFGIHYLSIDVV
RALDALCPVASSIMSVVKLAFLWWHREELERLIKRVTELIATQNSRLKLADKRRYFTIATRLSASVLFFG
TTTSTLYTIRAGIVNYLSHLRGEEIPYETPFKMIFPKPLISMPIFPLTFIFSHWHGYITVAGFAGTDGLF
LCFCMYIGTLLKALQYDTKDLLSDVGCGERKHSSEAEIMESLKMIIARHNEIIDLVKRFSAVMSGITLGH
FVTSSAIIGTCVVDMLLFSDYGVLVYLVHTMAVSTELFLYCLGGTVVIECSSQLATAVYDSNWYTHTVDV
QRMVLLIIIRAQRSLVLKVPFFAPSLPALTSILRFTGSLIALAKSVI

>XP_011199471.1 PREDICTED: odorant receptor 59a-like [Bactrocera dorsalis]

MAFEDVKEIFCTHWTIWKWMGQVNHPKYTKLYKAYSILVNVVFSLGYPVHLVIGLSQEKTIQGSLLNLTI
SLPSVICVLKFYNTWRNFDKVRHLEQMYNTLYARLDHPEDLAYYRKVTAPNAIRVVSAFKVICVGMAVTA
ELTQLYVGFVYGWRLMYPGYFPFDPHGSTAGYVTAHIFQFIGLLTQISQNLMSDTYGAVCLALLAGHTHL
LGQRLARIGYDKDKTREQHNQDFVDFIVDHNMLLNCQRTLVDIIGMGLFALIISTSLLLAIVIIYPMFFV
DNALEYAYYVFFMFGALMEVFPTCYYATHFEYEFEGLTYKIFSCNWVDQNRSFKKNLIVCLEQSLKARYV
FVGGMFRINMQIFIAICKGAYSVFTLALNYK

>XP_011199218.1 PREDICTED: odorant receptor 7a-like [Bactrocera dorsalis]

MFDLVKGRGRSVFASRDAVIYLFNTFRFVGLNPPPHCRLLYYFYGSIITLFVVLLSPLIFNVGWIRDRNI
LSVMEILNCVQAALNVIGVPIKSITLALSLGRLRSVEPLLSKLDARYTEPEDLAKIRACAITGNRIVFGY
IISYMMYETLTVVTALLGGHAPLTLYIPFVDWHRSAWEYWLQSSFDGAMLFFLLFHQILNDSYPAVYIYI
IRTQVQLLANRVRRLGTGNKSQEQTYHELQDCIITHQEILRLVSVVEPIISLTLFVQFFIAAAILGTTMI
NIFIFADFATRIASGAYMFCVLLQTFPTCFYATHLQSDCEQLSMSIFHCNWLSQGKRFNTMLLYFLHRSQ
ADIPLFALKLVPINLSTNVSIAKFSFTLYTFIQKMGVGKNLK

>XP_011198390.1 PREDICTED: odorant receptor 2a-like [Bactrocera dorsalis]

MTLKINSWDAFKYHWRVWDLSGFRGPQRQSVWYIPYKLYTIAITLLFPIYYPICFTVESFLADNLNDFCE
VIYIAMADMTLNIKFLTLFIVRRQLLELRPILKRLDARAKTEEEMNVLQEGIDSAKKCFLIILRLFYSAF
VTSQFMVIFSAEARLMYPAWYPFDYQASRTKFWIAYGYQTIGFLVQCTQACSVDTYPQAYMRVLTAHIRA
LSLRIERIGRQNFSGVSSELMCSKENEMKRNYEELVSCIKDHKTIIELFSTIQKPISGTSMAQFVCTGVA
QCTIGVYMLYVGFNISIMLNMAVFFVSVTMETLILCYYGDLFCQECEELSKAIYNCNWTVQSSEFKKVLC
FFLFRSQRVNVLMAGNWIPVRLPTFVMVVKSSYSIFTLLSSFK

>XP_011209369.1 PREDICTED: putative odorant receptor 69a [Bactrocera dorsalis]

MSEIYSMKHCLKYPYFTLDLAATEPFTWSGARTYSYRRIWLRRALFTFGAINLVYQNIGMLIYLFMPHES
SAQSTIVQVTETGGIMGLTMVGTSNMLVMFWYGDRIAMLLEKFQQLFPTARLQRKAKFTKQSLRGVEFPH
RIEHFVLKSNKLMKLATTLYMFAFAYYNSLPIVEFLYEWTTPGIVWKYRYQSNTWYPWQNERNAKSFASF
TLAYVCQVQSSLTGVAFIMAAEFMLCFFTTQLQIHFDYLANALETIDAAGANANEDLKYLINYHSQLLSY
SKETNAIFNVSFMVNLCTSAIAICLMGFSMVMISLAHAFKYSIGLTSFIVFTFFICYTGKELTETSDKLL
NAAFYGNWYDGNLAYRKMILFFIMRCRIPTELRAYKFTTVSMPTFTAILRSSYSLFTFFQAMGQ

>EDS41965.1 Odorant receptor 13a [Culex quinquefasciatus]

MGNFTHQGTRDLIRTNSVAAVVNELHQAHCNCELELESNSIEIDKLCAAVIAESKQSEISKSDLIEVVQL
HRQAYKVTNLIENICQIPMALQFLTCILFWCLTMFYASTNVNFNLFNVALAFFLSLLETAGYAYLGSELI
EAADSVGAAIYDLPWYEDSVEMQRFYRLMIQRTQQRTCVTGVRFFVVELTSFSKLIDFNLCAPLPLGTKI
EDVSISVAKCDPTCLVPRMGSLELHFNAGFTPLNISSSGLSVTSAVGASVQIFTMPGAISAGTFGDKFSV
TPLGNDKYRVVIRTELAVPKMFDRRGKIVWILKKGNDFSFCIETPVLFD

>EDS41964.1 Odorant receptor 92a [Culex quinquefasciatus]

MPASVFIHHHLPVELQVMPVALRILERIGFWGSWKPFYRHVVLLCYNFAVILAPKVIFGIGTDSYPLIAK
GISEFLFVIIGVTVTPIFAFQRSSFEKVVCGLSDIFYKVTAGNRCDESYAMITGLNKKLDLFTKFVIFYC
IYGTIAFCMKSVVISPWRYWTAIANNSTEPMVFELPTEQEFYGLHNRSNFVHYHIFVTFSFFAYLIVSLF
SVVRITTTVRMIKYSSLTYQLVIVKVQKLAQKKITIADLKELVDFHRQAYIISLLVEKICNIPIALEFLT
CVLFWCSSMFYVTQTIDFNLFNLMIIFLLSVTEIFTYSYLGSELSEQAAAVGKAFYDLPWYDHPAELQRY

YRLIIQRTQRTTVITGIKFFVVELASFSSVVNLSYSYCLVLKNMLE

>EDS41963.1 Odorant receptor 13a [Culex quinquefasciatus]
MTATVFVHHHLPEKLQVMPIPLRILKRIGFWGSPKLFYRYVVLLCYNSAVILVPMAFFGFGSDHFHLIAK
GISEFLYVFIGITVTSIFASHRRTFEQVVYGLNNIFYTVTVGNRCAESAELIAKVNKKLTLFIKLIIILYN
KYGALSFCMKSVAISHWRYWTTKVANNTDSVIFELPTEQEFYGLQNRSNFVHYHIFVTFSIFAYFIVALF
SFIKITTTVCMIRYSSLTFQLVILRVQKLAQKHVTITDLKEVVDLHGQACNIALLIEKICNIPIALEFLN
CVLFWCAALFYLTQTIDSGLFNMLIIFLLSVVEIFTYSYLGSELSEQASAVGKAFFDLPWHEHPAELQRY
YRLIIQRTQRPTVITGVRFFVVELASFTRVVNLSYSFCLVLKNILHQ

>EDS41962.1 Odorant receptor 13a [Culex quinquefasciatus]
MFLQRFFRSNLDVLPVGLQTMELIGLWGEDRRQVAKFGAIASWMTLMILIPKSTLEYGGEGFDSFARGTA
ELIYFGDFISSMVIFALQRGSYVRMINILQDSFRKCAGEDQPESCRKAIVDFNWKIFRYSRIYACFIGSC
LIFYVPLPMTATFFNYFSAAGNGTEGVEFVLPLENKFYWLDTRRNIWHYVIYMVLLVPAVTGSACLSTVK
GTVLFTIIRYGAMIFELVSLKIADLGRGENFEEGKEEYRRKQLVEIIELHKTALEYAKLLENIMSFILLS
QFVNCMLISCLMMFYISSTYGPNVVNMVILFAVLMVEVFAYCFSGDELSSKAAEVANSVAMYPWYREPVT
VQKELKLMIQRAQSTIGITAGNFYYVDIKRFGVVVQTSYSYYLILKERFNA

>EDS41959.1 Odorant receptor 13a [Culex quinquefasciatus]
MSALFKNRPLPKEVDGNKSSKNPLPEELLVMPANLRILEFIGLWGSWDNFYRFIVLFVYAFSIIFLPKLV
DGIGSSDSAAIIKGIAEFVFEASIYVPIAIFAVKRQTFEKLMGEFEDFFRKVTIDENFGACYDIIVDQNN
KIKTFFKFYLVYCLSAPFAYSIPSLFGNHIHYWTRGNTTEPLILELPAEQEFYGMQIRTSFSQYHYFLAI
SVPAHCVSTLFSLLKITTAFFMIKYSSLTYRVVAARVQKLTQLEDIKIADLAEVIELHEQAFQITELVEE
MGHIPIAMEFLTCIVYWCLTMLYASTRIDFNLFNVMVLFFASLMETFGYSYLGSDLTDSADAVGEAVYGL
PWYEHSVELQRYYKLIIRRTQKSTCISGIKFFVVELTTFSNVMNMSYSYYLVLKDVLNTM

>EDS41950.1 Odorant receptor 13a [Culex quinquefasciatus]
MAALPKQLQVMPSTLRILALTGVWGSGRKIYRFGLMFGYGFMVMMVPRFVFGVDSDNLSAILKSIGEVIF
LSSIFVPALVFAGKRGTFEKVVTGLGEIFQKATIGEHFKECFELIEQQNVKIKKFADFLTIYMMFAAFGY
CMPSLFISYLRYFTNDGTNPVVFVLPMEQEFYGLHIRTNLAHYNIFMVFSLLAYMVCSYFTLVKLTLPLF
TIRYSSMTYRLVAIRIRNLTRYKSSAADIKDIIELHQQAFNVTALVEEMCHIPVALEFLTCILLWSLVMF
YISTTMGWDLFSILIVVFSSLVEAFGYSYLGSELSEQAVTVGAACYELPWYESSSAELTLACRRIIQRSL

KATRLTGLKMFSIDLKTYGNIVNISYSYYLILKDVLDVL

>EDS41928.1 Odorant receptor 13a [Culex quinquefasciatus]
MDTIQWGSLPEELQVMPFNLRLMARVGLWGPRFRVYRFWALFTFGTMVILFPKTVLGIGSDDVTAICKGL
AELNFEASIYIPMAIFALKRDAFERVVQGLDEVFREVSFGEESKDCYNMIREQNVKIKKFFKFLVVYCVY
GPFAYCLPAVLISHWRFWNSFEPMVFELPMEQNFYGLQIRTNFTHYHIFVGLSMVAYSFCGFMSLIKIAT
MCFMFKYNSLCYRLIAEKIRKLPQPTDDGSTKVNIGDLEKLVEVHRKAYNVTELIEDICQVPLALEFLTC
VIFWCLSMIYISKKIDFNLINVMIIFCLSLIETFGCSFLGSELAEEAEAVGKAIYDLPWYEHSVELQRFY
RLIIQRTQRPTGITGVKLFVVQRTTFASVIQMSYSYYLVLQDVLNNF

>EDS28738.1 Odorant receptor 94a [Culex quinquefasciatus]
MARFGEYIPSERLTFWIWKILGIWATQDESRLYRAFRWIYHFTFTIVYLFCIFISSFFKETMAELWSDVM
FILLTELAMFTKTVITVRKFETVYRLHRETISDEFRPNSEWETRVHGAFFSRFSTAMLGYYLCSFLTLWA
HLGFLFEYKFPFFSWFFWLPLGRANLTNYYIIFAYQMFGMLGHLSLNVSGDIQHAYLLATAGIQLDFLYE
RLTKLPIPTSNSSIEKDHYRETLIQHIEHYERIYRFVKEIEDTFSMAIFVQICASGVTVNLATDLGTDGA
PMVFYLVAMLTQIFLPCYFGNEVTLKSVKLTNALYTADWFRLAGVSDRKEMAALMLRTNKPIALKAGHFF
NYNLEAFTSTLNTAYSIYAVVSKKNREQA

>EDS28737.1 Odorant receptor 94a [Culex quinquefasciatus]
MEHYFSSISFSFKVLKIFGLWRTNKERYSYKVYRMFFVCLFFLFYLGSIFVSALTVSTVDEFFSKILYIA
LTEIVMAFKTFAGFFKFYTIKQLHHQTHSTNFKPLNAKERKIFNKSIARINRYFWLLLCSTCTVWFNLLA
LFSGQFKLPMFPWMLGIPYGRHLPYNFYFLAVYQTTGMFLHAFINIIHDIQVCYLLEAGSIQLMLLEERF
STTQSKQTGRHNHRKLYIKYMEHFVKITNFVKQVESVWSKAIFSQFCASGITICAISFRLSSLNFTQDFP
NALTSLLYLILMMNQIFMPCYFGNERKEIQMMMKPIVLKAGGFFYYNLGMFTSTLNTAYSLFCVLQRRAS
STRGEM

>EDS28736.1 Odorant receptor 94a [Culex quinquefasciatus]
MESTKPLITSVRVTLWIYRCFGIWRENNQRSWYRTYRWIFTVPFLYLYLLSMVIGAFKKNSEQLWKDDLF
ILLTEFAMLVKTVSTYHNFEEIVKLFNISVSKYFSPMHERKQRSILQNLNQILMSYFIISLLTAFTTSIH
IFQGNYKLPTFSWFFGIPYGPDHPLNYYLIAYYQVFGMIVHCMLNVAGDIQIAYMLATVGIQLEQLERSF
TGLSRIVFRDNRQREFCRCITHYQRVRRFAKQVERIYSAPVFVQFCVSGITMCATAFRLSTISITPDNQP
LIIATAMYLCSMMVEIYLPCYYGNEIILKGQRLTNSLYSCEWYRLDRKFRRYILLLMTLTNGKSITVRAG

CFFRCNLQLFTTTLNSAYSLFAVLQHTLNYKDNS

>EDS28735.1 odorant receptor 71a [Culex quinquefasciatus]

MYHNHATAGWFLKIFGLWHDGNESRSYKVYRIVFLVIFFLLYLGSIVISAVLTSSVEEFFSQILYAALTE
MAMAVKTFYGYYHFVTIYRLHKQAISKDFQPVNAEEDKRSKNLLNKINLYFFGYLLWSTAGVWTNIAFLF
NGQFKLPFFPWLLGIPYGKHLPYNYNFLLFYQLSGIWIHAVLNVIVDSQVGYLLAVAGIQMDFLAARLEA
TQAGRIGQKKFRNTYKQHIKHFNVVNNFVKNVERVYSKSVFSQFCASGITICAIAYRLSSVNFAKEFSTV
LPIGIYLLSMLNQIFLHCYFGNEVTLKSSRLTNALYTSEWYKMAPKDRKNVVMMMTRSIRPITVKAGGFF
HYNLHSFSWVRIVV

>EDS28680.1 Odorant receptor 7a [Culex quinquefasciatus]

MTYTQSKRSWWSQGKSRVFAFRRSLSRDFDYSTDFFFGLDFLMVVSGGRLNSRNVQFRVCWNIYRMLVIL
AAIVTLRNCFLNMTNGSDFEIVLNSVQVSLGILSTVLHMALVLWHYNSLMAVRRYVNRRRFGRSLEPSLV
GIGYAITTVHLVPYMILTGLGAEVKVIAKSFSVIFENTDQRVQWRMEGEPCSVWQTELKMKYYFWKFVQE
EIGECIDLHVEFLAMLNRVRPMLNKMFYLFYYTTALSLACAAIYLATKKSISMIFIHTLIHALLIAFEFM
ILTRMVSWLNEAHQSIGNEVYGLDWAMRLKCDDLFIVEHRSVRKIMTVVMAVSQQPLRLNGFGSEEFTQD
RFWALLNLTYNFYNVLNDYNDCCVVKTAP

>ALV83719.1 odorant receptor OR114b-2 [Culex quinquefasciatus]

MATKKVAFDDPQPAEPPPTSVLKFDSFIQLLRIIGTVCGAVNYDEQHLAKPSRWIKFKRMFFWFTYVHDL
LCIALEIAYFVEAVQRDATLAHLMVLVVCIGFLTYTIIKLTMHKLHEVELNEILIQLKNLYPETLDTKPA
EEYRRHIWFLKLFSVLYIVVLVVFNVIFYAPSITVLIKTGHWEKILPFYLKYWYDWRKPVVFELTFLHQI
WVSSSAVTGVLLADTLYCTIILLISMQFELLGKRLEESELDEPELVKCVEKHLLLIDVCARFERIYSPSL
LVTFVGSSGVICCCLFLTLAGENMGEAVRFSVLLFVYIMNIFLLCYYGSVLMEKSSNIAHHVYQSQWYNN
CKKDQKTFLMILIRGQQEEKMTALKFTTVSLPCFSGILSTAFSYFTLLKAVYEGS

>XP_014101401.1 PREDICTED: odorant receptor 88a-like, partial [Bactrocera oleae]

MAVQKKRTFAAKLCAIDDLCAIQHPYQRYLGLKFLDFKRVNGRFAIPKSMLLNVAVFLAIIDCICNVINI
AKAINERDVTKAQEAFAIFGMGLVLTMRGFMLAQNRDKVLKMYNAIDRIFPRSEHLQQHMEVEKMHKNIK
RRLLILHRSITALGVIFFSVPSVRFVLIYDFESDDLVAEEFHVNASWLPFGIKDKVSTYPYIYMYEIILA
LAAIQMLIKWDQIFVILISHLCMYYEYLGKLLAEMNVQDAMDPTKADAVYKQLHDYIYIQQHLNNLAVEL

NDLFNLSILSSDAGIALSICFNLVLITEATNYLQMTLHTTPLFVEIWLIYDAAKWGTMLETVTARINEII

YEQKWYDCSVRFGKYTLMLLQSTNEPLRLTAFNMLYVNMKHFQDMMMLAY

>XP_014101001.1 PREDICTED: LOW QUALITY PROTEIN: odorant receptor 67c-like [Bactrocera oleae]

MTPLFKSSEFAPTVPDFVHIPLFQIKFLGVKLFKWTPDEPRSKLQITLLGLFIVFSIFSLISMLLYVVYE

DLETLLEVTEFILFWGFTLNALMKGCSMLCFRREIESILKGLIVKHPKTEEERVAFQLVPYFRTINTSNK

YLSIWHLSITSMFVLHPMMASIYGYISRVDKNKGFDFTLPFMMTYFYDVNQPLAYAVSYFLQCCGAFHMS

LLFLSGDLLLISMVHLVNMHFGYLIYKIESFQPSGTDADMKVLGPLMEYHDEMLFYAERIDNTFGLATLL

NYVSSCLVLCFIGLQIVMGSEAVSVIKFIGFLVSTIVQVYFVSYFGNNLKDLSTGISDAFYNHPWYDANY

KYMRMLVLPIARAQRCAHLTAFKFFEISMDSFKSLCTTSYQFFTLMRTSIEEEGA

>XP_014100884.1 PREDICTED: odorant receptor 7a-like [Bactrocera oleae]

MTHIIGRHHFTSDAYVLTALRRRKTTEIRCNMRKLPRRSTADMAKVGLALALHNENATPRDTNSKRKLKA

RSSAKVLSELSEAEAAEHHVSSQDTTKYLFKTACAMGIVMPSRYRPLYVIYGFLVHFFSTFYFPIGFTMI

LFTLPDDVNVGNLLTSLQVTFDVYGGSAKIIVMIFVLEKLRATHVLTQRLDKRCRASDEIAELQQMVRFG

KKVVIFYLTIFFCYSASTFLSSFSSGYPPYSLYFPFLKWRRSHTEFIIASFFEFLIMDFACLQQTVNDGY

PIIYINMLRCHMKILQFRVEKLGTNPTLTKAEQLTELKLCIKDHQLLIELYDTIAPIISVTLFIQFAISA

VCIGTGLINIVIFANKFQTQVACFFFILAVLIEIYPACYFSQCLINESDKLANVIFHSNWIEQSSEYRKL

LIFFLQRSQRPMFLTAGKLFPVTLSSFIAKFSFSLYTFIEKMNLKERFGIE

>XP_014100035.1 PREDICTED: LOW QUALITY PROTEIN: odorant receptor 30a-like [Bactrocera oleae]

MHYCGELFNWSLAFMRRVGYFDQHRLAGLFLISPIFLCLTAYYRTYVIRNDFDEVIVNLFKISGATVTTA

RTFIVMYKAQKFLNFFESIDKWYQELQVLFANRKSNFMFIYYLLTFLFNLYFQCEGNEITLNKAHEYTRK

IKKATKTVLILTSITLFYVIFIQLLATVGVGYKKLLVDVAFPGIDLYKSPFWEIMSIMQSLCIAPITLFS

YVSYFCLFLIAIAFGIFLMKDLQSKLESMNDMTDVEALECINKCVKDHIMIIKYHNDLEVLFSVGSSADV

CIFGIIPCVIIVFSTMDHDMSLLIADIQLSLMVMISTFIIFWVANNFCCESEKIANAAYNCNWEDRNKEF

RKYIPLIIIISQRPLQLTAGGLKPINMEFFLTMVRCTYSLFTVLITMKTEGDS

>XP_014099351.1 PREDICTED: odorant receptor 88a-like [Bactrocera oleae]

MAQQQEWNGVWKLCTIDDLCATQHPYQRYLGLKFLEFKRVNGRFVIPKSNILNVVLTLAVVDCAGNIIKI

VKAINDRDVTQAQEVFAVCGMCLVMMMRGFLLALSRGKLLKMFNAIDSIFPRSQRLQQHMEVEKMHKNIK

RRFFLLHTSLTVSVSAFSALPLAKFMVFYDFKSDNRVIEEFHVNSSWLPFGIKDKVSTYPYIYVYEIMLA

TAGVHIIATWDQVFVILISHLCMYYEYLGKLLAEMNEQEAMDPTKADAFYKQLHDYINIHQYLNNLAVEL

NDLFNLSILASDVGIAISICFNLVLITEATNYLQVINYATPLFIEVWLIYDAAKWGTMLETVTARINEII

YEQKWYDCSVRFGKYTLMLLQSTNEPLRLTAFNMLYVNMKHFQDMMMLAYQLLTFLKSKG

>XP_014099350.1 PREDICTED: odorant receptor 88a-like [Bactrocera oleae]

MALQQKRNGVSNLCTFDDLCAIQHPYQRYLGLKFLEFKRVNGRFVIPKSNILNVILLLALMDCAGNIIKI

GKAINDRDVTQAQEIFAVFGMGLVMTMRGSMIGLNRGNLSKMYNAIDRIFPRSQRLQQHMEVEKMHKNIK

RRFFLLHTSLTVTVSAFIALPLAKFMVFYDFKSDNRVTEEFHVNASWLPFGIKDKVSTYPYIYVYEMMLA

TAGAHMLVTWDHVFVILISQLCMYYEYLGKLLAEMNVQDAMDPTKADAFYKQLHDYINIHQYLNNLAVEF

NDLFNFSILSSDAGIAISICFNLVLISEATNYLQILNYTTPLFVEIWLIYDAAKWGTMLETVTSRVNEII

YELKWYDCSVRFGKYTLMLLQSTNEPFRLTVFNIFYFNMKHFQDMMMLAYQLLX

>XP_014098250.1 PREDICTED: odorant receptor 63a-like [Bactrocera oleae]

MYSITEIKTLRVRNHLLIRELKRISFIIGINLNAQTNLKEWWRIINILFIITSCIALYPHWLMIKQAKGD

IPLIAETSTTALQTTTGLIKMAYLLFSQHILYKLLQKAETHELLQRIEIFQTGMPIKTTLKKEINAIMDT

NWKQTRRQLLFTLGTCICILSNYFFYALFKNLYNYLQGTPNYVYILPFTGYPMFLDKVMGSPYYALDMFF

GACSLLVAGMSAISFQGCFMVLCKHCCGLVQVLCLMLLRSTSTLVPKSQHVEYLRYCIVQHQRTLEFIND

VNRFFRHICLSQFLHSLAIYGFVLFEMNFGLESNKVTFIRMIMYLCAALTGDCMHYVNGQFLANELEKIP

LACFNCEWYHETDDFKKKLKMIIMRSNKKFCFQISWFTVMSLATLMGIFKASGSYFVLLRDIDEP

>XP_014098071.1 PREDICTED: odorant receptor 45a-like [Bactrocera oleae]

MENLTDVDKILAAVTINSQLFTSSSKHFIFLARRKRLLRLNEALERLALTGNKFERDLWNTTNRTVLPIV

TAYSISCQLTVNICVLLPILKLLYYYIWYNEVVLTLPLPGSFPYDYTSPFYFILTLILTVLLVQFCSNSI

IIVDGLFGWFVYNISAHLQIMRLKLEQLLQLHVEDPNFNRDFVAFVNYHREIIDLTLELDAVYAPIIFLE

VTSSSLPICFFAYQLSYLSDPANVPFICLLLASIVLQLMIYCFGGEKVKNECEQLSENIYLLIPWQNLPP

KYCRLLLTPFIHSQRVLVLTGYFFTANRSLLVWIFKTAGSITAMLFAWKEKKVV

>XP_014098069.1 PREDICTED: odorant receptor 1a-like [Bactrocera oleae]

MYEYLQIQHFSFRVIGINLWAQRDQRITSAPCRYLTWTLATAIITLLMGCYIYTSEQDKAIQVLTVFLQG

VLSVIKSGMFVAKGRRFIKLIRSLDMLADEANVKEGREWRHENDWQQRIVRVYYICCMSTGTLYCTVPAI

ILLYSRCFNDHTMFILPFNAAFPYDTGHPFFYTVSYIWCISFIVYAIHAIAAMDSLFCWFIFNISAHFRA

LQRELETVVAASAGAEDDSSLHSRITQTLHYHRRIMELSAEFDELYAPIVFIEISVSYLKLCFSAYNLIN

LSDISGLPVLAVGLVTITFQLCIYCFSGEKIKNVSEEVSDRIYLAFPWERVPPSVRRLLLLPLMRAQRAT

NLTGFLFIVDHSLLVWIFKTTGSIIGFLSATKSENTTS

>XP_014097486.1 PREDICTED: odorant receptor Or2-like [Bactrocera oleae]

MTNYENLPLYAMNVKVFVKLGLIDSSDWTKRFLFSLILVITLVGLVVNMFKTWDEDIGETSMNIHKLLAL

THCSIRCCIMVKKAKKFERLFQSIEQWYRDIERNGDPEILSTLQEINRKTDKLSKMTIYLAAMAALAGLL

YPLSYNTRTHMVTVQYPFIDALQTPFFEFFFLIQVLWLTPTVLVISLPFMNIFLITFTFGVLALKDLCGK

LRNIRSENEETMLQEFKECIVYHRKVIKLCDDLEDLISIDGFFNLALFGMMLCILLFFLSMIDDLRLILV

GLVFVSLNTYMIGITYYYANNLATESVEVANAAYDTPWYMGNLEMRKCVTIMIARCQKPIQITAGGLYPM

NMENFQAILRISYSYFSLLQGLSQQ

>XP_014097484.1 PREDICTED: odorant receptor Or2-like [Bactrocera oleae]

MQNYKNLPLYSINVKIFLQLGLIGPSTRAKQILLAFVPVTTYLGQIINLYKTWSDDIGETGLNFYMLALV

THCLLNSDPEVVRMLQDVTTHTQKLTRIGFYTILTGGLCSYIFPFLFEERKFILDIHYIFFDAKQTPFYE

FFLVLQIVVLVPVFIVIYLPFTNILLTSLKFGELILMDMCTKLKNINNQDETTQLREFKECISYHEKIIT

FRDDLEYLVSIDGFFHVTLFGLMLCMLLFFLSLMFFIFVSAYTLYILLLMQSLKVAYAAYDTPWYEGNPE

LRKCVQIMIARSHKPLEIKAGGLLPMTLENFQAILRISYSYFSMLQGFSQK

>XP_014097127.1 PREDICTED: odorant receptor 33b-like [Bactrocera oleae]

MASSNETFQPVDSVVLYRAFWLCWQVVGISTTSCKYFCGFYDLLINVLVTIFYPIHLFVGLFLNPTPADL

FQNLSITITCFVCSVKHYLLRRKLPQIRAVQALLTDLDKRVEGVEEHAYFEEELVVGAKNVWKLFSIAYG

GANMAAISATLLSKERRLMYPAWLPFQWEANTFSYCAAVIYQIAGVTIQIIQNLANDIFPPMSLCIIAGH

VHLLALRAAKVGADAEKSMKQHTHALIECIEDHKKLVRIFQLTQDTLSQAQLVQFISSGLNMCIVLFYLI

FYVDNVFSYIYYAVYFVSMAIELLPSCFYGSMLTYEFQQLSSAIFKCSWLGQSREFYQNQRIFVQLSLKE

IVPLAGGVIAIQLNSFLGTCKMAYSLYMVCNRMK

>XP_014096877.1 PREDICTED: odorant receptor 63a-like [Bactrocera oleae]

MYNTEEFVELKKYNRFKIRELKAVSYILGINYGSETSLKKFLRVLNLLLIIICGISLYPRWLMLERADGD

VPLIAETITTILQTTTSMVKMTFCLFMQGQCCALLKKAENYELLQGMKIFSTDMCIKTELKNEINSIMET

IWMESRRQLLSCLITCSCILSNYFLYAFFTNLYHQIKKTPNYVHILPFTGFPMFLDKGMTSPYYAVEMFI

GGSSLLTCGMCSVSFHCIFMILCKHACGLVKVLCAILLQSTSPHVPAHRRDEYLRYCVIQHQETLRFIND

INELFKHITLSHFLHSLAIYGLVLFEMNFGLETNKTTFVRMIMYIGAALTVDSMYYVNGQFLVTELEKIP

LVCYSCDWFNESEGFKKTLNMIIMRSNKDFCFQISWFGVMSLTTLMGILKASFSYFLILRDITDEIN

>XP_014095654.1 PREDICTED: odorant receptor 46a, isoform A [Bactrocera oleae]

MDDAKGIVYSFYKVQYLFFKLLGLFDLPAHYSSFWHYMYKLYFWHVAIFWMLLFDISMWIKIVGNISNLN

EIIKVFYLCSMAIAVMAKFVRIRLKNSSYVALFARIHDDDLLPVNVSELEKYTQSSHLSCRVRNSYTYLS

LTSLSLIFITQIISEPSELPLSIYIPISVENFWCYLIAYLFQFIGLSLCCLLNISFDSLSSSFFIYLKGQ

LDILANRLENIGTNLYVDDNMINLQLRDCIQQYVKLRNITEIMEELLSIPMSVQMISSVLVLVANFYAMT

FLTDPSDYVTFIKFLVYQLCMLAQIFMLCYFANEVSLRSAELSYSLYSSEWTRCSQTNRRMMLLMMAQFD

VPIRIKTINRCYSFNLPAFTSIVNSSYSYYALLKKMKD

>XP_014095326.1 PREDICTED: odorant receptor 94a-like [Bactrocera oleae]

MTFTFTLLLWMEIILSGDVDYVFQWMRLAITETCLIVKVLNIWYHAQTANELLQEWDKSDMFALKTLEEQ

KMWQGAQRYFRKVVAVYSLLSICSVFMALFSVFFMDTLALPIPYWMPASWRGSNAWPLFLYEDIVVPFSC

LCNTQIEFFLCYLMFHLTLCLRMIGIRMERLGDQVNDVEITTELENIIKIHQRIEGMAKSCQKIIMWPVL

AQIIFSSLIICCSIYSIQRISFSENPSNFLGFIQYVVAMALEIFLPCYYANEITVESENLSNHLYNCDWT

VMSLYNRRQIFLYMEHLKQPITLYAGNYFQIGLPVFSKTMNNAYSLLALLANVNEEE

>XP_014094968.1 PREDICTED: odorant receptor 67c [Bactrocera oleae]

MSPEARTFNEFLRIPISFYQTIGEDLYEHRSPYRIRRLLHKSLLYIGFINFNMLVLGEIIYFVKALNSFA

TVLEATGVAPCIGFSFVADFKQIAMTVHRETLREHLDQMEELFPKTAMQQVEYKLPQRERVMRRVMAIFT

LLCLAYTSTFSLYPALKASVQYWLLGAPLFERNFGFAIWYPYNATEKTWVYWVTYMGQVHGAYLAGVAFL

SADLILVASVTQLCMHFDYISRCLEEFGGASQKGSAQEDLQYLQALVVKHAKCLELSEHVNCIFSFSLLL

NFLTASLTICFIGFQMTASSTEDIVKYIIFLTASLVQVFVVCYYGDELMTASQRVGDAAYNQNWFDCDRR

YKQLLTIIIMRSQKPASIRAPTFPPISFRTYMKVISMSYQFFALLRTTYSGNGN

>XP_014094554.1 PREDICTED: odorant receptor 94a-like [Bactrocera oleae]

MAVKKWSSRSPSSMSRIAATLFIVHVLKAIGLWQWTRDSHLPYFEKLEHAYRIILHIPFTFIFITLMFTA
VLLSQDLEEISSVLHILLTELALVVKILYFWRQGNTAWRYMDELANDPMYALRQQSEWTKWQSAQRSFAI
VAYTYILCSVAAVVFACIGGIMTPTDVYVLPVNIYVPFEWHHPHKYWYAWTYITLGILLTCVSNIMLDMI
YCYFMFHLSLLYKLIGWRLSDLRRLKNTVGVKVDEPEVIDQLSEIFQMHMNVRRLTTQCETLVSVPILGE
IVFSAFILCFSGYRLQQMNNLENIGMLFSTIQFASVMTVQIFLPCYFGDAVTEHSNALTNDIFNSDWTTF
DMPARKFMILYMELLKRPAYLKSGNFFKIGLPIFAKTMNNAYSIFALLLNMNN

>XP_014094548.1 PREDICTED: odorant receptor 94a-like [Bactrocera oleae]

MTPTATAQRERIGVARVLMRLLQILGLWPIWKESHQKNGSSTQCRNWLTHYYRYLLHVPLTFTYNTLMWI
EALTRWERADHILYISITEVGMMALTLNFWRLEQRAYHFMHELSYSDNLALRNQAERQWWRGEQRFFARI
AVCYIGGGVCVLFTAFGATLLVNGYSLPYDYWLPFEWHNAQNYWYAYGYELIAMSLTCISNVTMDMLLCY
YLFHVALLYKLIGMRLMALQHLSERLAVQQLINIIELHKRVKRLTTQCEVLVSVPILVQIVLSAFILCLC
AYRLQTMQISENPGQFLAMLQFASVLTLQIFLPCYFGNEITINSDALTTCVYNSNWEEFSPPTRKLMNLY
MELLKRPAQIKAGNFFLVGLPVFTKTMNNAYSLLALLLNMSK

>XP_014094455.1 PREDICTED: putative odorant receptor 85e [Bactrocera oleae]

MDTRELGDMSLLYSHDDKQRIADLFVAQVIAFKATGQIPFDVHYGLGYTYCCFVITQSLYLGVLFLKTSY
DMLLNGKLEQITDALTMTIIFWFSVYAACYWLLRWQRLLAFLQHINHHYWHHSLPGLSFVSSHRTFILAK
RITVVWALTCMAGTALYGLAPLVMGVHALPLKCWYPFDPLQPYVYKFVYALQLSAQIIMGATFGNGSALF
VSLVILMCGQFDVLYCSLKNLSYYGRLRACCDVEKLRNEQAALPKTSDDELNQYMYCREHLTNLSTLQRL
YSQQPAATLPEALHLAVVQCVQLHRFILGACKELEELFNPYCLVKSIQVTLQLCLLVFVGVAGERSMVRI
LNLVQYVTLTFIELLMFTYFGELLRGHSVRSGEAFWRSQWWTHTAAIRQDVFILLANSKRAVQLTAGKFY
AMDVERLRSVVTQAFSFLTLLQKLAAKNPK

>XP_014094420.1 PREDICTED: odorant receptor 13a [Bactrocera oleae]

MLFNPKPLKDPINFRFPLQCIWLKLNGSWPLKLNVTGEFEKYFRLLYSTWAWYVVAMVGITIGFQSAFLL
KSFGNITVTTENGCTTFMGVLNFVRLLHLRLHQREFQQLLAQFVKDIWITSSSHPTVERACARNMRVFQV
ISVLEASLITMYCILPLVELYMLTVNTEPNALESAPKPFPYKMLFPYNANHGWRYALTYLFTAWAGVCVV

TTLFAEDSLFGFFVSYTCGQFRILHTQMDNIIPDSYAATRAGHGTEAVYQRECIRRLDGIASKHCVLFNF
VSRMEEFFSPILLVNFMISSVLICMVGFQLVAGNNMFIGDYVKFLVYILSSLSQLFLLCWNGDQIIQNSL
EMANHLYACNWECGVKVAATNANTNAVNNMHPDEDEKTTHSAPIVYYSTSVAFRKKLQFMIMRSQRQTCI
TAMKFSILSLNSFSGLISSSMSYFALLQSFYENEEN

>XP_014094224.1 PREDICTED: odorant receptor 63a-like [Bactrocera oleae]

MLESVEEIYKRNYNSIKVLIGVSFGLGVNLTAPSKIKDALKLFNVILVVTSLLSLYAHWCYFLRYIDNIP
LLAETVCTALQTLISAVKMVYYLFTQRTFYRLLDQTLKHEIIRKIEIFQHDFPINRQLKQEIDDIMNGVW
LSAKRQILFYFCCCVAIVCNYFFGSFFVNLYHQLKQTPDYKHILPYPALYPFWEEKGMTFPYYPLQMYMT
GSAVYISGICAVSFEGVFIVLCQHAVGLVRVHNLLVLRSTSPLIPAERRVEYLRYTIITYQRINFYVQQI
QNSFKHVSLSQFVLSLIVFGFVLFEMSFGLESSIVIVIRMIMYFAAGGTQIILYCYNGQQLTTVSEEIPS
AYYSCNWYEESEKFKQFLRMMIMRTNRYFYMEVSSFTLMNLATLIALFRMSGSYFLLLRNLQEK

>XP_014094223.1 PREDICTED: odorant receptor 63a-like [Bactrocera oleae]

MTSENKEELHKRNYNSIKVLFRVSYSLGVNLTAPNKIKDALKTVFTALQIGMAAIKMIYFFFTHRTFYRL
LDQALTHEIIRKIEILTRFPINRQLKQEIDDIMKRVWPFFVNLYHQLRQTPDYEYFLPVPALYPFWEKKG
MAFPYYHIQMYMTFAALYVSGLGAVSFEGVFMVLCQHAVALVKVHNLLVLHATSSQIPAERRLEYLRYLI
ITYRRISKYLQEIKTIFRHISLVQFLLSLIVMGFVLFEISYGLESSIVILIRMIMYISASISQITIYCYH
GQALTSACEKIPMAYYNCDWYGENKAFKNLILMMIMRTNKQFYMEVSWFTLMNLTTLISLLRASGSYFLL
LQNLQED

>XP_014093775.1 PREDICTED: LOW QUALITY PROTEIN: odorant receptor 85c-like [Bactrocera oleae]

MPNVISFEAFLRIPSFFYRSVGVDLWNTNGGPLQNAVFYFGLINLNIWLLSELIFSVLMVSKNFIQATMT
LSYAGFVLVGTIKMFYMRRKKAEMTRFLQLMHTIFPHTEPQQKMMNLRRHLCQCTIVMSGFATIFMLLIW
TYNLYPYMQRQIYDCWLQVRSVNKTLPYESYIPWNWHDHWSFYLTILYKLSYYTLQSIAGYHSASGQIAS
DLVLCAMATQIIMHYEYVAQTITEYRPQLVDCKISRAPRLDXESERSLCKDMKFLCDIIAYHANILRXLS
DIMNEVLGVPLLVNFMTSSFVICFVGFQMTMDAEPDYMVXLFLFLFSSLFQIYLICHYGQQLIDASSNVA
RAIYNHDWIHSHVHYQRMLVLVAARAQKPAMLKATSFVHISRGTLTDIMQISYKFFTLIRTMYSN

>XP_014093772.1 PREDICTED: putative odorant receptor 85d [Bactrocera oleae]

MSTKIVEFDAFIKKANFWYGFNGIVAYDNFHRQAGDEPKQKNIAARLATVLRQIYCFIGLVNLFWVLIIE

ACFVVVNFIENSDFLQAARNLTFMGFVTVSIIKILSNLKKRSQLSILMRKLYEIYPKQSTDQPPYDLQSH

LLHYRRIGFMYGFTHGFTVCAYNCLPMVNYLFLAPLLQLTDVERELPYFCWVPFEWRDNWLYYPLYVSQV

FASLTGLGGYLANDLLLCAATVQLIMHFRKLARDIEAYQAGSSCATEEVIAQQAQRDLSFLSAAVYYHSR

TLALCELINDIFGLPVLINFISTSFVICFLAFQFTVGVPLSAMVALACYMICSFVQIYMICSYGQQLITT

SENIGHVVYNHNWLVADIRYKKMLIMIIRRAQKPAMLKATSFVNISMGTLADLLQLSYKFFALIRTMYAR

>XP_014092478.1 PREDICTED: odorant receptor 7a [Bactrocera oleae]

MLKLITGRGIGNTASKDAFIYFFNGCTIVGINPPKNAGPLYYMWSFLVNVTCILIAPITGTLGFVIQYIQ

NILTTVQFLSGLQAGINLIGLPVKCLTVTSALKRLCGMEPTLAMMDARYTRPEDLALIRKAAVMGNRLVF

FFGTSYLMYMLFTVIPPLINGKVPLSIWIPFFDKHQSALHFFGQIVYDLFLMFFILFHQSLYDSYGSVYI

YVINTHLQLLVRRVDRLGTDATKSKDDNLNELVDCVVTHQQILELLATIEPIISKTMFTQFLIISSILCV

IMVNMFFFADRSTQIASTFYFMCVLLQTSPCCYFATELRAESEKLPLAIFHCNWVEQDQRFRKVILYFMH

HAQLSIELMAMKLFPINVATNISLAKFSFTLFTFIKEMGIGQHAKN

>XP_014092452.1 PREDICTED: odorant receptor 83a [Bactrocera oleae]

MSSNKEQQEPDLTTTVHEGCLTHRIQRRDLFRFIRWNLWFTAMYRLPLERYFPARLRFLAITLDWTYELF

LYSTLLHIDILFIYTIYLNKDKGDLELIVSSMIQTVIYTWAIGIKVFFKRIQPKRVKGLMRYLNEKCRTR

SAAGFTYVTFNESANFSTISTTIFMICCYAGSTFWLFVPIFNQDRSLPLACWYPIDYKVPIVYELIYFLQ

TVGQLQIAGAFGGTSAFYLLVSVLFSGQFDILNCSLKNILATTYLILRKPKSELLLLREKQNIANYELNQ

YYSAKEYNTDFDCITHLLDVKTPKPEKFYKAFKIALRPSIAHHRYILYGLKMLEDLYSYLWFLKTIEVTL

LVCLVAFAWVKSTAANSFLRLLSLSQYLLLALWEMFMICYAGEIIFLNSKRCDEALQRSPWYLHSNEIKQ

DVLFFILNAQRPFRLTGGKMYNLNVEKFRSILTTSFSILTILQKMDLRQPQPN

>XP_014092042.1 PREDICTED: odorant receptor 7a-like [Bactrocera oleae]

MFDLIKGRGRTVFASRDAVIYLFNSFRYLGFNPPAKYRLLYFMYSAIITFFVVLFSPVIFNVGWLRDRNK

LSVMEILTCVQASLNVMAVPLKCVTLAMAQNRLRGIEPMVTELDERFTTPEDKAKIKQCAVTGNRLVFGF

AVSYFMYETLTVVSALVGGHAPLSLWIPNVDWHKSTWEYWLQVSFDTAVLFFLLYHQVLNDSYPAVYIYI

IRTQIQLLTSRVEKLGYDEQKSADDNYQELLECIVIHQKILKIVKIVESVVSITVFTQFLVAAAILGVTM

INIFIFADLTTKIASVTYFFCVLLQTSPTCYHASYLLDDCDQLRIAIFHCNWIAQNKRFNNLLIYFLHRS

QDSIPFFALKLVPINLATNLSIAKFSFTLFTFIQEMGLGENLKG

>XP_014091805.1 PREDICTED: odorant receptor 67d-like [Bactrocera oleae]

MTVKHIKPTASFAKLMKMARFISSLVGADVSTENYRVNIITIIVIICIIIYFIFTATTVASVFSENWTYL

LEASCMVGSVLQGITKLISGIIRTKTVSDMRLELEELYRTYESKGKTYCKVMNKCCDRVWQLIKLVGHIY

AASIFGILLLTTVMVLATNQKIYVMQFFIPGVDVETSFGYLFTTAVHTVVFLAGAFGLFAGDLFFLIYLG

QPELFRDILILKVKELNEAAAEKDNKTESLLISIIEWQQYYTDYNERCNEIFYYIITMQILTSGVSIICT

MYIILMGDWPGAYLYILIAICGLYLYCIIGTNIQTCNTAFFEELYNINWYELDLKGQKMMILVLMKSQNP

SEIKIGGVLPLSVQTALQITKTIYGIFTMMLGFLEEEQ

>XP_014091648.1 PREDICTED: odorant receptor 74a [Bactrocera oleae]

MLYRPRLENGKLIPLSWPVAAYRLLNHVCWPLQDDASWLQRLFDRFFWAFGFFIFMQHNDAELRYIIRNN

NNLDEMLICGPTYLILVEIHLRAFQLGLKKEAFKRFLQNFYAEIYIDQSSHSKLYANIQKRLRPIWFNSL

LYFSTLSSYVIMPLINYLNNVKVPLYKMYYPFDITPNPIYVAIVLSNIWVGFTVITMVSGEDNILSEVLL

HLNGRFLLLQQKLRQDADRLLHIVDERNIADELRRQIIEAVEENVRLYKFAEDFEREFSFRLFVSLSFSA

GLLCVLGFKVYTNPMASFGFIFWICAKIMEMILVGQLGSTVIYTTNEMSSTFYECNWELVLLKSGDTKSN

VRLMKTLLLAISTSQKPFVLTGFKYFSISLAAVLKILQGAGSYFTFLTSMRK

>XP_014088795.1 PREDICTED: LOW QUALITY PROTEIN: odorant receptor 94b-like [Bactrocera oleae]

MRKLEVLSSRIFPSDPSKGKIGSIQYNVWLAQLFGVPVVGLKTESPRLRIALGVYGVLITLVVTFIYTAF

EIYDMILCXPNLDSLTQNICLSLTHVAGVLKVINIIYRLDEVANVVRRIEYSAKTYVVSKSQLVAFYRGE

FENKIPLTIYAALVGFTGVLGLIYLLYNPIGVAGQIFPYRVKLPDWMPFGTQLAYMGISVLVFALQIVAI

DYLNVTMINQIRFQLKILNLAFEELKLDCVNKGEQREFNKRLQTIIEHHCLLRDLRNDVEDIFRLPVLLQ

FFTSLIIFAMTGFQAIVKAENSNGAALIYCYCGCIFCELFVYCWFGNEVSEQSKTLTASGYASHWYEFDQ

RFKKSLIIFMCNSQTPFVFTAGGFMSLSLPSFTGILSKSYTVIALLRQVYSR

>XP_014086206.1 PREDICTED: odorant receptor 43b-like [Bactrocera oleae]

MGYLQTLKTEPITLQLSLLQTAFNAIGVPVKTIAIVIMRTHFRKVEQIFVRLDERYQSVSSRQQIIKCVV

LSTRIFTTVGFVFHFYGSITYLQAFFTNSYPLHTWLPFIDSISQPTIRYWTHFIFEVFHIVFILTVQITL

DSFPAIYIRNLRTHLILLTERVSHLGENPEFTEKKNYDELVDCIVTHQQLLEAKNIIGSVCSITIFIQFI

VVAIALCISLLNFFVFADSVQQAVTLIYYLGVILQIMPTCYQASMIEDDSAKLPDAIFHCNWLAMDKHSR

KLIIYFLHRAQVDITFVALKLFKINLTTNLSIVKFGFTLYTFMSNMGIGQNLKELLE

>XP_014103551.1 PREDICTED: odorant receptor 2a-like [Bactrocera oleae]

MANQPHSATRLDSSDALRYIWLFWRITGIHPVKKHRYIYYIYSLLLNFTSTVLFISFYVVTLFISNGLLE

ILTNLSVMVPLIYSSTKHFVVFYHIRGELPKAALHLQALDRRVELEPVACTHLKRLVQRCHKIYLAALAG

IVVCLALYALVGILRHKLPFEGWLPFDWEHSLNAYILACAFQLFCLSVQSIYALCNDIYSIVYLLLLVAH

LRILNARIARIGGVCAEQHSELANYQQLAACVRDHWECVKCISPAIAATIFIQFISTALALCTAAVAFVN

ADSIGEQLIKFLPYILVVLCEIAPCCWLMDEAALEMFKLTNALFSCCWYEQNLRFRRSLIIFMQRSQKVE

QILAGKIFPVSLVTFIKILKLAFSLFTLLNQLKS

>XP_014097365.1 PREDICTED: odorant receptor 59a-like [Bactrocera oleae]

MSQPVNSNAFFKIHWLGFRICGGDLSVNKYRLVYLAYALMVTALVTFCYPLHLALALFRNGSVAGNIKNL

AVCVTCIACSLKFLIYTRKLGIMREIEQTFSELDSRVSSEVERKYFAWMRISVRNVVSVFLCAYAAVGVT

AELAFLLSKERSLLYPAWFPLDWRASTRNFYVANVYQFVGISYQIFQNFINDTFPPITCCLLSGHIKLLG

IRVSRIGYDCVRLQDNERELVRCIKDQKNLYKLFDLLQEVMSWPMFIQFTVTAFNICVAMVVMLFYVDTP

FERLYYLIYFISMPLQIFPICYYGSSLQLLFGQLQYEVFRCNWTDQTRRFKKQMMLFTERALKTTTGLAG

GMIKIHLDTFFATVKGAYSLFAVIMKVK

>XP_014096236.1 PREDICTED: odorant receptor 59a-like [Bactrocera oleae]

MDKPSVVDSRQFFHTHWRLWLLLGCVREPVRYQLLYSLYRPIVNALIIILFYPGTILLALYNSGNLNDFLQ

TLPICAAALACSAKYISYYRKLKLVRQAEQVFNALDGEVLLEEDRVFFAGIHRATNLILNTLCGLCAFFF

VITVMASIENRDLAIAIELPFDWHASTASYVGAVALDLFLLACDLLQSLVNDSFPAVALCILSNHTRLLG

ARLSRIGHTSKDVQANIRELQHCIIDHQRLYRLLAIIEEIISMPVFVQYAVTAFQDCFTLITFIFYTNTI

SDKLLYLTYLLALQLQIFPICYYGTACAQSMEDLQQKIFASNWVEQDQVYRRLVTIISQCSLKRTTAYAA

GLIPIHLGTFVKTLKGAYSFYTFVNGVRKV

>XP_013117634.1 PREDICTED: odorant receptor 63a-like [Stomoxys calcitrans]

MHDMTQSVQNSQILEDLEIFNLDLKDKHKIINEICQILKQSWMDIKMQLLVLRMTVFAICSWYSGCSLVI

NIRNFWFNSLEGNFYSQFPFPASFPIWYGHGHRLLAFIVEYFIITMEIYLATVASITYSAIFSVGSVHCL

TLLRVLRKLISYSTTDHVPPEVRVKYMEVCIKLHQEILKFCNELNSLYKMSTLGLFAECCLVICMLTFKA

SLDVGDEKFFAVKVALYLSAALYELIVFCINGQRISSESDLLPFAIYDCLWYNEDSQFQFLTQIMILRSN

HSIAMDAGGLARMSNVTLSTIIRSSVSYFLFLRNCM

>XP_013117585.1 PREDICTED: odorant receptor 67d-like isoform X1 [Stomoxys calcitrans]

MTKEPSQRFREIVRVTRFCANLCGSDVFDPNYRINIRTVLVLGVILFSFSCCGYCIHYSITVKGDWTLVI

QALCMGGGTLVQGFSHMMGFMFRPAELRVLLEETHTLYERHEKIDSDYRLYLNRGVDLLTRLMKICAFFN

TILAVGMSLVTVIYNAIYETRVLIVGIYLDAIDPETERGFLITMILQSCFIAVGGFGLYAGDMGTLTTIT

QIMTFKGLFCCKLRDLNEILLDEPDNKTKSLEALRDIIQFHQQYLSFIERNRSVSFWTIFVKFLTNFFGL

VCTVFCILLGVWPAGYIYMAYCFTMMYVYCGVGTMIDIANDGFIEACYNGVLWYELSALERKMLHTMMII

AQNTKGLSVGPFVPLSVNTGLQMTKMIYSMTMMLFNFVSQ

>XP_013117584.1 PREDICTED: odorant receptor 67d-like [Stomoxys calcitrans]

MIKNPSQRFRAIVRVTRICADICGTDVFQPNYHINIRTVLVSIVIIISFFSCGYCIYDSISVKDDWTLVI

QAMCMGGGTLVQGLSHIVCFIRRSDDVLCLLEETYILYEFYEEKDAGYRLYLNRGVYLLTRLMKICAFVN

IILAVGMSMVTLIFNSIVGQRVLIVGIFLPGLDVDTQNGFLTTMFLQSCFITVGAFGLYAGDMATLTSIS

QIMTFKGILRCKFRDLNEILMDEPDNKCKSMEAIRDRNRVRNTSVYRLKNQNWQIVFLSAQLSISICSFV

NRSRSITYWIIFTKIVTNFFGLVCTVFCILLGVWPAGYIYMAYCLTMMYVYCGVGTKIDIANEGFIEACY

NDVLWYEMAAHERKMLHTMMIMTHNTKGLTIGPVLPLSVNTGLQVTKMIYSMTMLLFKFVE

>XP_013117463.1 PREDICTED: odorant receptor 30a [Stomoxys calcitrans]

MQLRTIEDVPLLSTNLRIMKFWSFLLQHNWRRYSCLIPYIMINTTQFLDICFSTEPIDAIVRNAYIAVLF

FNTILRAVLLCVNRFEYEQFMENIRQLYIELMNSEDKALRKMLQECTMTSRFISKINLLMGFTSCIGFVI

YPLFATSKVLPFGMYVPGIDKYESPFYQIFFISQIIITPMGCCMYIPFTNMIVSFILFAILMCKVLQHKL

KNLKNVSNDKARQVIVWCIKYQLKLIEYVDKINDLTTHTFLVEFMAFGAMLCAMLFLLIIVETVGQMIII

SIYIFMIFAQSVIMYYFANELYDQSLNVAIAAYDSNWFDFDVSTQKSLMLLILRSQKPCAILIGKVYPMN

LQMLQSLLNATYSYFTLLKRVYG

>XP_013117462.1 PREDICTED: odorant receptor 30a-like [Stomoxys calcitrans]

MALQACFIAATIEDVPLYNNSLRIMKFWSLLLRHNWRRYANLIPYILITMSQFVDIYFSKEPMDAMIRNA

YLAVLFFNTTFRAVVLCANRFEFEEFLERVRLLYNDLMKFEDIWVRKKLQEITLAANSISKVNLVMGTCS
VISFLMYPLFATTKVLPFGIYVPGVNKYESPFYEIFFLVQIILAPIGCCMFIPFTNLMVALLLFAILMCQ
VLQRKLRHLKDLNSQEARETIVWCIKYQCQLMRYVNTINDLTSYTFLVEFLAFGAMLCAMMFTLVTVETI
SQMLLICIYILMIFAQSSILYYYANELYDESMNVANAAFESDWFNFDISNQKMLKLLILKAQQPSAILVG
HIYPMNLKLLQSLLNTTYTYFNLLRNVYK

>XP_013117431.1 PREDICTED: odorant receptor 63a-like [Stomoxys calcitrans]

MYAHWCYTVRSITDIPKIAESVCTAFQTLISVFKMIYYLFIQRRLYVLLNKAQTHEFVRQIEIFHTGFPM
CERIKGRVDKILESSWKNINAQLIFYICCCVAIISNYFLMAVIVNIYNSWNGAPPNYEPMLPFPSYYPFW
QDKGLSFPYYHIEMFLGTCACYISGICAVSFDGVFIVLCVHAVGLVKVLIMLIENSTAPDVPKERRVEYL
RYCIYQYQRVSEYMDELRRIYKHISLSQFMLSMLNWGIVLFQMGVGLVNLMTLIRMLMYISAAGYEIVLY
CYNGQRLTTECEKIPYAFFSCEWYNESEEFKQLLGMMILRTSRNFYFQISWFTTMSLPTLMSMIRTSGSY
FLLLRNVAE

>XP_013116841.1 PREDICTED: odorant receptor 94a-like [Stomoxys calcitrans]

MDTTEPQHERIYAAEILLKIMKPIGLWQWLPPNASVEPYMATTVQLLQYGQRLLLQVPFTFIFFTLMWYE
VMQATDIDQVGDVIYMSLAGAALILKVLNIWKYSIEALNFIATLKDSPQFALRTVQEVEFWRRSQRRFRY
IIYLYGTGSCITVVSAFLGVLVMEEPQLAFPYWVPFEWKTVRRNYWLAYFLNMMGMACGATTSVCLDMLG
CYFLFHVSLLYRILRFRLQKFNDVPGENVESKLQSIFKFHKLIRRMTKECELISANFVMAQIILSALILC
FCCYRIQKMEIAENFPQFLSMLQFLAVMFLQIFLPCYFGNEITVNSAKLPTDLYNINWLEFSVSNRKLLV
LFQEFLKRPDKVTIFGYFDVGLPVFTKVMNNAYSVFALLMNVEK

>XP_013116840.1 PREDICTED: odorant receptor 94a-like [Stomoxys calcitrans]

MSRIPIEKFRIQQYKKIDRVSSGRLLILILKVLGLWHWQEEEEQSVLSVILHHLHAWLLHVPMSFAFCAL
MWIEVFRAPDFEEAGKVLYMSLTLSVVIPKTLSFWLLSTRICRFIKDLQVNPMYEFQSQEEILMWHSQHK
LFKNVVRLYLGGSVLAALGAFIGVLFEEDYQLGFPYWVPFEWHNPRGYWLAYLYNVVAMSVACFSNVSFD
MFGCYMIFHIGLLYKLLSLRFHQLQHVDEVKAKDKLMAFVLMHMKIKRATKECEHLVSHYVLNQIIFSAL
IICFSGYRLQKMNIMDNVGQFFSMLQFLSIMILEIFLPCYFANEITVNSSLLLFDIYKSNWLNYSPSTRK
FIILFMDFLKQPVIIKAGGYFEIGLPIFTKVMNNAYTFFALLLNVDEK

>XP_013116457.1 PREDICTED: odorant receptor 4 [Stomoxys calcitrans]

MANLKQSVREMFASNIQEGEIGSVKLNLWLAQITGVPIIGLKEESFWVKTFLLMYGIFTTAVVTFIYTGF

EIYDLVLNWSDLDSLTQNTCLSLTHIAGALKAINLIARLPDLHKVLHQLKYVTKTYIKSEKQLKAFLDGE

LENKLVLSIYMGLVGFTGCLGMVMLYVNPEAVAGKIFPYRVVLPAWMPQQLQLLYMGLSVIVFAIQIIAV

DYLNVTIINQIRFQLNVLNLAFDDLMVEIQPNSAGQDAGSLVLYKYDPMERLKSIIEHHGLLRELRQDTE

DIFSQPILWQFMTSVIIFAMTGFQATVRTGGSSAAVLIYAYCGCIFCELFVYCWFGNEVFEQSKTLATSG

FNSSWYLFDRRYRTSLLIFLTNAQRPFIFTAGGFMGLSLPSFAGILSKSYSYIALLRQIYGD

>XP_013115904.1 PREDICTED: odorant receptor 85c-like [Stomoxys calcitrans]

MKVITTRSLTFLDFIRLPLSVYSAAGVKMFLWDDQDFMSWWEKFLLVFQFVNLSTNFFAKALFFVFGQFE

GTVHLTKWALYFIFANNGFCKVFSVALGRHQLFSVLKDLEKIYPKTHQERQEFRLVPCYQYIMKHSKIMS

IQHFTIALIFVVFPIVQSTIEYLTSDDENANFVPYTPYIMVYPFDVSRGIGYAYAYISQTLGGFTVSCYI

VGSDMLLMCSIYQVIMHFDHLCLRIENFQSKGYEEDMQEISMVLERHNLLNKLAESVNNIFSISILLNYM

ISIFIIVMISIQISTGSDFGLDFIKFVGFFTSATTQVYYICMFGTLLMEHSGQVCEALIGQQWYMADVRY

QRMLVLAIARSQRPSHLTAFKFFTISMETFSNLMTTAYQFFTLLKTQMEEQ

>XP_013115213.1 PREDICTED: odorant receptor 22c [Stomoxys calcitrans]

MLDFLKTTTPITKSFMLIPRISGRMCGIWPQRHYTCIKSSLFVFSTFVVGLGAVGENLYGVVYLDDLVSA

LEAFCPGVTKVISLLKILVFFTYNRQWYDIVQRMRKMLMAENHCKEKMEIVERFASIGSIYSFILITSGM

TTNIFFNIRPLAANLMRYLQNEPLQHVLPFNIIVPEIFVKYPLFPFTYAMLTSSGAMTVFTFSFCDGFFV

CASMYMCGVFSMLQHDIRSIFAELSECETSTVAQNQRFRQQLSAIVERHNAIIDLCTDFTANFTLIVLLH

FLSAALVLCSSLLDLMLNSASLGLLVYAFYSVAALTQLFLYCIGGSYVRESSISVADTLYDIKWYKCDVE

TRKMILMMLHRSQKATTIAVPFFTPSLSAFSSIISTTGSYIALLKTFL

>XP_013114455.1 PREDICTED: odorant receptor 85c-like [Stomoxys calcitrans]

MRIKPQIHSKYLQIAVTLNLFIVVILQIIHNVISEDTEERLPNIIFNNYIIVGIGKILAIYYHRLAMAKA

VDYLRVIYPSKAIERKYQLGDYFKFYSRLEMVIWTFYRMVGPSFLLLPLFQSFINIWLRGKFSFILPLHM

WGFDEANIGESSPWVLYILYYLLGGWCCLSVGMSITACDLLLYGMIIQLCQHFDLNSKQILELSPGNEME

ALEQLKKIGIQHQKIMELAKEVNRIFGPSIIFSVMSSSFILCLVTYQMLDDVPYFTILKSFILLLYESKQ

VIITCYMGQKIMECSSLVNESLYMHNWVDGSVKYRRHVLIMLLCTAQPFVLYIGGIADITLVTLKVVYGN

AYRLFTVFKST

>XP_013113430.1 PREDICTED: odorant receptor 45a-like [Stomoxys calcitrans]

MVTLQYGFINYAFHSITNIDSITSSLSMFNQGVLLLFKGAMLICKGSSMLKLIWNLNSLATAANSDENEK

WVSENRRGELIAKAYLYACWISVACVALVPWLFVVYEYVRGLETHLELPFALKFPFDDGSWPSSLTVYIL

TMLHLRGLSNTSIGIDTLFGWYIFAISGHFRILRDKIAKTAVKIDVYGDHMHFQSDIKIFVRYHDKVAKY

VDDLNDLYGAMSWAEIAMSTLQMCFLLYSLGNDPNPASIPFHFVASASIVLQLMIYCFGGEKLTSENDML

CNDIYMAMPWDKMYPSEIKAILILLVRAQRETILKGLFFNLNQSLLVFIFKTAFSFITLLGAIKE

>XP_013113429.1 PREDICTED: odorant receptor 45a-like [Stomoxys calcitrans]

MSSLAEEKQNLEFLKIQRYAMGILGLDLGVTSRKQLIKSPGTLAFFTFSIVLFQYGFFNYAKDSITDIDT

ITSSLSMFNQGGLLLFKGSMLIHYGGKMLNLIWNLNSLAKEANAEEYEKWLSENRRAELLAKSYFCACLT

SVVCCALLPWLFVIYEYFKGVEPHLELPFAVKFPYDDGGWIASLVIYILTLSLLRALSNTSIGIDTLFGW

YIFAISGHFHILRDKIQKAAMKIDLYDNHLDFQQDIAAFVTYHNHVAIFVEDLNELYGAISWAEITLSCL

QMCFLLYSLGNDPNLASIPFHFLATAAIIIQLMIYCFGGEKLKYENDLLCKEIYMVMPWEKMHLSEKKLI

LIALVRTQKETILKGLFYHLNQSLLVFIFKTAFSFITLLGAIKE

>XP_013113427.1 PREDICTED: odorant receptor 45a-like [Stomoxys calcitrans]

MSAERQNLNWLSVQYRSFMILGLDIGATRREDFIKSLRKLLFYTICMVTMQYGFINFVIHSITNIDSITS

SLSMFNQGVLLLFKGTMIIYKRASMLDLIWNLNYLAKKASEEEYGLWVSENRRGELIAKAYLYACWISMT

CVAIIPWIFVVYEWFQGLEMHLNLPFELMFPYDDGGWLASMANYVLTMLHIRGLANTSIGIDTLFGWYIH

AIVGHFRILRYKIKKAALRIDLHEDHSQFVQDIGDFVEYHKKVLQYVEDLNRLYGAMSWAEITMSCLQLC

FLLYSLANDPNFASIPFHFVASASITLQLTIYCIGGEKIKNENDMLCDEIYMAMPWEKMNPSEKKLILIP

LIRAQKQTLLKGLFFYLNQSLLVFIFKNAFSFITLLGAMKE

>XP_013113426.1 PREDICTED: odorant receptor 1a-like [Stomoxys calcitrans]

MLQANGESRQNLEFLGIQYKSLHAIGLDLGVIRGRDIVKNRQKFTILAVITFYLQCGLFVYAAHMFTIQI

DKASTSLSLFNQGSLLCFKMFILLLRSDRLLKFIWDLNMLATMANDAEEELWLSENRYSKLIAKVYGIAC

LGATIASGLLPIVFILYEHFRGLEVNPFLPFDGEFPYEHLGLPIFILNYILSTIYVWTLLGMTIGMDTLF

GWFVYAVSGHFRILCSKVAVTAAKIAQDDNHADFIRDVGSIVYYHNKILGFVNELNGIFGPIFWAEVAFS

CLQMCFLIFNLNSGTDMRQKPFNLLVFLAISIQLMIYCFGGQKVKSENEALCNDIYYQFPWDKMKPSEKK

MMLLPLMRSQEDTALRGLFFELDRNLLVYIYRTAFSYNTLLAAMKDN

>XP_013112864.1 PREDICTED: odorant receptor 13a [Stomoxys calcitrans]

MFNPKPPDDPKYRIPGQCIWLKLNGSWPFSQEARKDFYSARYLWGWLYTSWSWYVVTSVGITIGFQTAFL

INNFGDIIMTTENCCTTFMGALNFVRLLHMRLNQKQFREIIEQFVNDIWIAKNHHPQIAAQCSRNMRTFR

IMTVLLSCLISMYCVLPLVVLFVDVGLDADEKPFPYKMLFPYDAHHGWRYVATYIFTTFAGVCVVTTLFA

EDSIFGFFVTYTCGKFAILHERIDNLVWDANRIVRHKESELNVQEIYVKLLNRIAYDHNKLIEFSAKLEN

FFNPILLVNFTISSILICMVGFQLVTGKDMFIGDYVKFIVYISSSLSQLYVLCWNGDSLIQHSTETASHL

YSCNWEGASHTTKTVNANDGFMTQAVAVLPLDIAGRVENCIPANKSFRQNLEIMIMCSQRPVKITALKFS

TLSLQSFTAILSTSMSYFTLLQTVYNENQEDTKI

>XP_013110983.1 PREDICTED: odorant receptor 67d-like [Stomoxys calcitrans]

MANSRNIISPYQRFLKLFDIVKLFATSVGANVFRHDYRIGPMTVIVTLAVNSFFALNFYTVYVGVVKEKH

YTVVMQSLCIVGTGIQGFSKLICAISNQGMMRYIASEIEFMYSEYENKSQRYVEQLNKAVKTLKSILLVI

FKIYAILTIALISVPVYYYLILNQKMFIFELQLPGIDKNTILGFFLLQAFNALCVVLSGFGNFAADTAFF

LMVAHTTLMKDILKCKFNDLDDILRQYSRDRTKTETTLRDIFQWHQRYLKFTETNTKIFFWIIFAQVTSS

VMGIIGNTVSMFLGEWPVAPVYLVSSFIIMYCYCAMGNLVEISNDDVTTAIYDCLWYELTVPEQKMILIM

LRESQKTESLSVGGIRPLTMNTGLQLTKTIYTVAMLLNESLN

>XP_013109246.1 PREDICTED: odorant receptor 94a-like [Stomoxys calcitrans]

MSFDTLQNSRTIHKILTFVGLWIEGDIKRVPALVISLLIHVPLTFTYTLLMWMEVIASTDITQATDVLYV

ALTETALIVKILSILRRRHLAKSMFRQLRYDDKFQLKNVQEHMMWRRSFKTFGIVSSLYITTSLSVVWIA

FTVPLFLKEYTLPFPFWTPFDWRQPIYYWYAYIYEVLAMPLTCLSNCTLDMWQCYIMQHLAACLRLSAMR

LQKLGHANENDVEATETVICIIQLQQEVKRMSLICERIVSSPIFAQILLSALVLCFSLYRLQHFNILDDL

GSCLGLILYAICMTLQIYLPCHYAHQLTFESSLLLDSIFSCNWTTMSPYNRRLILLYMNYLKRPIILRAG

KVFNIGLPTFTKTINNAYSFFALLLNMDLM

>XP_013109245.1 PREDICTED: odorant receptor 94a-like [Stomoxys calcitrans]

MSFDTLQNSRSIHRILTFVGLWFDDEIKHVPALVISLLIHVPLTFTYTLLMWMEVIVSTDLYQATDVLYI
ALTETALIMKILSILRHRHLAKSMFRQLRYDDKFQLKNVQEHMMWRRSFKKFNLVSRLYIGCCLFVLCAS
LAVPLFVEEYTLPFPFWTPFDWRQPIYYWYAYIYGALAMPLTCLSNCTLDMWQCYIMQHLAACFRLNAMR
LQKVGNANETDVQVTEAVIYIIRLEQEVKRMALICERIVSGPIFVQIFISALVLCFTLYRLQNFNLLADW
GYCLCLIMYANCMTLQIFMPCYYAHQLTVESSLLLESAYRSNWINMSPYNRRLILLYMNYLKRPIVLGAG
KVFRIGLPTFSKTINNAYSFFALLLNMDVK

>XP_013107983.1 PREDICTED: odorant receptor 45a-like [Stomoxys calcitrans]

MWFWPKALDALPPQDRVQRYFFVQRTCFAAIGIDPTSIRSTIFNRFLAWIPMFCMLIIIGPMGLYALKYI
QTDLDQMASALAPMWQAILSIVKFFLFMWNRKKIVRLVREVWMWSLEVNEKELVLLADEIRYDVYLSMLY
ASSCCCSAVLATLSPFIVALVYGWKGYGFWDSLEEPFKGVYFVNDKKTIWSYTFWYIWTLFAIHYVVNGT
VAIDSLFSWFMRNISAQFQILNLRFQLASDNHNLKEGDNNQPHEDLNKSIIECIKYHQRIIELAEKFNDV
YKHLVFVKFLVSCIQLACLTFQFQQGNSIVTQMFNFSFFITATIQLMIYCHGGQRIKDMSLSVSTSLWEH
FNWHNLHPKSQKLLLLPLQRSQMPCNLIGIFFVADFNLFVWVFRTAASFMTMMLTVDKK

>XP_013107982.1 PREDICTED: odorant receptor 45a-like [Stomoxys calcitrans]

MKTDLSQMVIALSPMWQMILAVNKFFLFMWNREKIVRLVREVYFWTLKANDAELVILSDEIRIDIFISLL
YCSSVCLSALLAVVSPFLTASIFAFKGYGFWDSLELPFKARYFVDPKMSIWSYALNYIWIFFAVYYAVHG
TVAIDSLFSWFMRNISAQFRILNLRFKLAANIVDAEGKGNDRFHEEFKKSIIECIEYHRRVIDLVEKFND
AQDLIFVKILISCVQMACLTFLFQQGGYLHTQIFNISFLITITIQLMLYCHGGQRIKEMAIPNIGIGCRS
PDNFTDVNEVNTTFTNIPVFPKDPSFYDFDFALEVRHRDDVEFSGIEQSDLVSQR

>XP_013107980.1 PREDICTED: odorant receptor 45a-like [Stomoxys calcitrans]

MFRLPSPAVDALPAQKTYEQFFFVQKYAFATVGIDPTSLKRTICRPLFLALPLVAIMTVLGPAFLYAGTC
AVDLNEVAKILTPILQCILAIVKITLFVAQRKEIVQLVRKVWYWNLEANIEELKILSEENRYDQRITGFY
YCSVIVSGTMATLLPFIIAGFFAWKGQSFWLSLHPPFKGVYLMDIHETYIGFIFAFVWDVLAIYCAVNAS
LAIDSLFSWFMRNIVALYRILELRFRMTAKSNALNEYTEEQFKKAIGECVKYHVRVINLTESFNEVYKNI
VFFKFLISCVQIAFIVFQFPNTKEMATHMMNVSFMISLSTQLMLYCHGGQKIKDMSTSINSTIYECFQWS
DISVNSKKLLLLPMIRSQKPCYFTGIFFVADLNLFVWVFKSAGSFLTMMLSMYQEDD

>XP_013107336.1 PREDICTED: odorant receptor 85b-like [Stomoxys calcitrans]

MAKSKRKLAKFDDFLKLSNFFYTSVGVRPYEKPGFIQGTSLLSSFIFYFGVINMNCMLVCEIIYVLRAFA

TGENILQAIMTMSYIGFVLVGDFKMLYVWSQKSALTIFVQRLMKMFPLDLELQKEYRMQYYLSQCTTVTV

GFSMLYMILIWTYNLFAITQYFIYDKWLQTRVVGQELPYTMFYVWDWRDNWSYYLMYFLQDVAGYTSAAG

QISSDLMLCAFATQLVMHYDYVSKTMTNYVVKLGSEDVENMDKKINRNLNAIAIVQAKAQAEDMQFLQEM

IRYHENLLNLSEELNNIFGVPLLLNFATSSFVICFVGFQMTIGAAPDTLIKLFLFLISSIAQVYLICHYG

QLLIDASINVADAVYNQNWSNAEIRYQKMLVLMAERAQKPAQLKATTFVLISRGTMTDLMQLSYKFFALL

RTMYVTK

>XP_013107332.1 PREDICTED: odorant receptor 59a-like [Stomoxys calcitrans]

MSSELNTKTLFSAHFLCWRILGMLPPKNSYRPLYLLYSLVINLGVTVGYPLHLIWGLFTSTTMYEVIQNM

AITLTCTVCAMKTLAIWWRFNKVFVMFEIIRRQDERLTLPHDIRYMRREVYPHVRRIILLFSILCSFIGA

SGELSVLVAGLLGDWKLMYQAYFPFDVYAKTRNYIVAHIYQFVGISYMILQNVVNDTFGAAHLALLGGHV

RMLCMRVSNIGHDVTKTKKENNRELLECIQDHKDLMEYRRQLEEVVSVYMFFQILVAGANMCVSLVFIIL

FVDDIFTLAYYMSYFVSMVFEILPSCYYGTLLEDEFQNLAYALFSCNWPDQSTEFRKNLRIVAEQASRRM

FVTAWLFRINNNAFIIACKNSYTLFALVMNLK

>XP_013107331.1 PREDICTED: odorant receptor 67d-like [Stomoxys calcitrans]

MARNKNHVNITSPLLRFHKIFDILKLFARTCGINVFAEDFRFGRITFAIFNMVQLIIALNAYTLWDSISK

GKFAFALQNVCIMNTGIQSYAKLVTSVYYQKMIRYVCAEIDFMYRTYENEGRHYVKCLNESLNLLIRITI

FMFKLYAVLTVAEIAVPLYYQLYRKERLPIFDVIIPLVDPATDWGFMIHQICTGIATTLTGFGNFAADTS

AIMMLVHTRLMSRIIKCKFDDLDEVIRETPREQRNTEHMVKDILEWHQRYLKFTQANSTLMYWGIFIEIT

AAYISIIFNMVSIFIGGWTVAPIYLFASYCLLLGFCSMGNLIEVSNEELTTNIYDCMWYELSVPEQKLLL

IMLNQSQKPNGISLGGLAPLSLNTAVQLTKSIYTVAMLIHSYLSRQNS

>XP_013106741.1 PREDICTED: odorant receptor 67d-like [Stomoxys calcitrans]

MVVKLSVHRYESITRVIRIFSSMCGADVLDPKYRVNLLTWIVLTFINTFFCCTTYTMYINVYVDRDLTKV

LQTLCLVGSALQGYVKLVNAIWNQHNLRFLTEELHRIYAEYDEKQAHYRRKLSKSVNLVVKVIKFVFFSY

FINVSLFLMAVPVYGIIYKEKIFIMQMFIPGLDHTTEFGYYLLISIQLILMLFGGFGTFAADTFLINFIT

HVPMLKDILRCKFEDLNEALDDENKERTKKYKAILRDILQWHQKYMLFITIIKDTYFWVILVQMTTVGLN

IASTLFVVISAKWPSGPPYMIFCFCTLYIYCGLGTVVENANEDVIYSSYTDVNWYHLPPSEKKMLGMMLM

MAQNTSGLTVGVVVPLSVSTGLQLTKAIYTWSMMLINFID

>XP_013105688.1 PREDICTED: odorant receptor 83a [Stomoxys calcitrans]

MMEPIEERRDLFQHIRLTMYSAAMYPMHLERLLPQFICGMGLIVEWLLELFLYLVCIHIAVLYVCTLYLN

FHSGDLELLVNCMIQTIIYIWTIAMKVYFRRMRPSLLEELVDSINLKYRTRSATGFTYVTMDQSLAMSNR

WIKTYVYCCFVGTVFWLILPIVYGDRSLPLACWYPLDYKAPTIYETIYFLQSVGQIQVAAAFSASSGFHM

VLAILMSGQYDILFCSLKNILATVAVRMGKSKEDLRKLRDLQELREPEINEYYCAEEKTCDVEMLFHSNE

IQEQPDLMTTSNNFHRSFRKTFKNCIEHHWYILDCLKKMETFYNPIWFFKTGEVMLLMCLVAFVSVKSTT

ANSSFMKAVSLGQYLVLVAWEFLIICYFGEIIYINSLRCGDAILRSPWYLQMREMKNDFLMFILRSYRPF

KLTAGKMFSLNVDRFRGVMTTAFSFLTLLQKMDERV

>XP_013104921.1 PREDICTED: odorant receptor 67d-like [Stomoxys calcitrans]

MPMSKRLSNRFRAIVRITRICADICGADVFDPKFRINIRTILVLVIIQLSIVFMAYTVYVGFNVDGDWKV

ILQVLSVGGCTLVQGYCKLVNCLREKNNFRFLINELNSLYEKYEKNDADCRLFLYKGIQQLTFIMKLSAF

VVILMVVGMGLVTLVLSVVFNTHVLIVQCLIPGFDPTTGKGFLWINIIQIWFIAVGGFGFYAGDMAFFTP

LSQISTFRGILKCKFCDLNEILADEPGNKEKSFNGVKDILIFHQRYMVFLEVTRDTYYWVILVQIGTYSI

GIICTMFSIHLGTWPGGYVYMIYCFVMMLVYCHMGSLVEVTNEGVVSSCYNDVNWYELSVSERRMLHTML

RMAQNTAGLTIGSVLPLSMNTGVKITKTIYSIAMMLLNFAD

>XP_013104815.1 PREDICTED: odorant receptor 43a [Stomoxys calcitrans]

MLRDNPMLSINVKLWQYLAVVFPGRGNIWRLYAFVLPVCVMNAMQFVYLFRMWGDLAPFILNTFFAAAIF

DALLRTCLVILNRDKFEALMLELASLYNEIEQSNDVYAKRKLKEATAAARKVSIFNLTASFCDIVGALIY

PLLCEGRVHPFGVALPGVDMTASPIYEIFYVLQFPTPLALTIMYMPFVSLFASFAMFGKTALMILQHRLQ

NIWLEEDDENKFAALRRCIKYYDRLTRYVYNFNSMVTYIVCVEFLLFGAIICSLLFCMNIIETFTQIISI

IMYILTMMYVLFTYYWHANEMLMESVKVSEAAYAIPWYYGNHEFRKTLLLFIIRTQKPLQIMVGNVYPMT

LATFQSLLNTSYTYFTMLRGLYNQ

>XP_013104790.1 PREDICTED: odorant receptor 2a-like [Stomoxys calcitrans]

MSSESIHTWQAFKNHWIVWKCCGLRPPGRDSIWFKPYIVYAVLLNVTVTLCFPTSLIVDLILSKSLEELC
ENLYVTITDVICNIKFINVFTVRQKLLQVRTIVERLDMRAKTPEQRHELKCGIRTAKKCFMIFSRFYACA
VITSQLVVYLSSERVLMYPAWFPWDWRATQRNFLFAHCYQVYAVSVQTLQNLGSDTYPQAYIVVLIAHIR
ALALRIKDLGSHSSTREDASKEDALYRELVACVKDHETIHELYLTIQECISGTCLAQFVATGLAQCTIGV
YVLYVGADFSRLLNSCMFFGAITIEILILCYFGDLYCRANDFLIDAIYECNWMDRDEEFKKAILLLLQRS
QQSNCLRAGNIIPVRLPTFVKIMKTAYSAFTVLNEVN

>XP_013102404.1 PREDICTED: odorant receptor 49b [Stomoxys calcitrans]
MLDDIELIYMSVKILTFWSFLYDRNWRRYATLIPTTFLVFTQFYYMFMTHEGIDAIIRNSYMLVLWFNTI
LRAYILIKDNSKYQRLIKDLENYYYDLQRSNDPYVNGLLTEANASGKLMSRGNIFLGLLTCIGFGLYPLF
ATERVLPFGSIIPGVNEYKSPFYECWYAFQMIITPIGCCMYIPYTSLIVSFIMFGIVMCKTLQYRLKALK
RFSDNSKMIHDEVIACIKYQKKIIEYIKTVNSLTTFIFLLEFIAFGTLLCALLFLLMFVDSAAQAIIVYA
YITMIFSQILALYWYANELTEQNLKIAAAAYETEWFTFEIPTQKSILLLILRAQKPSSIKVGNIYPITLE
LFQSLLNASYSYFTLLKRVYG

>XP_013102265.1 PREDICTED: odorant receptor 82a [Stomoxys calcitrans]
MIDLFARQRQCLVIMGHHFVRDKSRLLQQWRNIKYVGVLLLVMSAQGPMINYTIYHIDDLQLATASLSIS
FTNVLTVIKITTFLLYKWRFVALTRKLETMYQELQEDKAKEELEASNRYATTLVKIYYMSVSSTGMYFMV
APILKMLWSKLRGTQLDIELPMPMRFPFDFESSPGYEVCYIYTGLVTLAVVMYAVAIDGLFLSFTINLVA
HLKILQHYIKTNTFKKSDQELQSDVSFYVRYHNIILDLYKEIRDVYAPIVFGQFLMTSLQVCVIVYQMVT
HINTYLVFVINSTFLCSILLQLFIYCYGGEILKVESLMVGISVQLSNWYNLKPTHRRMLCLLMQRSQREA
IIKAGFYEASLANFMAILRAALSYITLIQSIE

>XP_013101684.1 PREDICTED: odorant receptor 42a-like [Stomoxys calcitrans]
MMESNCKESDIKIHQPDVHRVFDMANVIENAPQPSTKDCTLYMFRGIKIIGYLSTDKYKILYYLWSIIVN
FFVTLYMPVGFLTSFILRLDTYTASGFFTALQIWVNCIGCSLKMLAFFFLYKRLLASTEYMDKLDVRVTA
TSDKWQIRKIVALSNRALTLYATLYLSYASSTFWSAVIKGKPPYQVYNPLFEWSGNTRNFAFHAAIEYAL
ICFHCLQQALLDSYPIVYITILRTHLNILSRRISSLGNDSTMNNCQRYEALVQCVLDHKNIMGLYNIFSP
VISGTMFVQFLIIGIILGITTLHIFLYADALAVVASLFYVASILAETFPCSFLANSLVDDSAALSHAIFH
SAWHNEEPRYKQMLCFFLQHSQKTMQLTAMKIFPITLNSNINVVKFAFSVYTMMKQMGFGANLTSGLGIE
S

>XP_013101482.1 PREDICTED: odorant receptor 74a-like [Stomoxys calcitrans]

MEFHRPLLANGKIAPLSWEIRLFFLNVNWPIKEHAKLFMRVYHQAMLVLGFLFFCYTNEAEMHYLVNHID

DIGLALEGLATYLILVEAHLRIYNKGFYQASFKNFLDEFYQNIYMEEYLDKETYRDIQRKLLPTKMCSYS

YILTVVTYFLVPVMGFFQHSNLVPFKTIFHYDLGIWYFYVPTLLVTLWIGVAVVSHLSAESNLVATVILH

LNARYLHLQQELKELQCKLSSDMKISTDRVLGEYRLAFIDIIKRNVQYNEFAQKFQKQYSFCIFVMMAFS

ATLLCVLGFKAATLGMTTKNITFITWILGKIVELLVFGTLGSTLIETTNNMSSCYYMANWEDIILKSSHT

LDNIKLMKLITLAIELNQKPFYLTGYNYFNVSLATVIAILQGAGSYFTFLYAFR

>XP_013099004.1 PREDICTED: odorant receptor 74a-like [Stomoxys calcitrans]

MLYRPCLPNGKLAPLPWPIAIYRLTNIICWPLEDNASWMAKIFDRFWYILGFAIFLVTNDAEFRYLRLNL

NNLDELLTGIPTYFVLIEIHVRAFSLGWNKNKLKKLLQKFYAKIYIDEPDNPKIYKNIQRQLIPILGFSC

LYLCALFVYSVTPIYLLSTGSRELMYKMIPPMDHTPLYIYLPWLISNMWVGVTVATMMFGEANTLSMMVF

HLNGRYQLMRENFVAKVDKLLKGKDDTDIAMRFRQILVEILNENERLNTFANEVQDEFTFHLFIIFGFMA

ASICALGFKVYTSPMTSIGYMFWAMGKVQETIAFGTMGTSIVTVTDEISSMYYESKWELIMQRSMNPKVN

VRIMKLVNMAITINSKPFSLTGLNFFSVSLTSAVAILQGAGSYFTCLISFRQ

>XP_013098553.1 PREDICTED: odorant receptor 33b-like [Stomoxys calcitrans]

MDNAKEHPNMAGRLQYQTNSVELFEIFWLTWRVLGVNVIGNKFFSFCYDALLNIAVTFWYPVHLTLGLLM

LPTHGDIFKNLSMTITCIVCTLKHISLRLKLKKLHEIETLLDHLDKRVESQEEWDYFTEGPQKTVKNVTK

MYFGIYMGANVAGILTVILDSERRLMYPAWFPFDWKSSFSIYCLTLLYQIFGVTMQIIQNMVNDAFTPVV

LCLLGGHVRLLAMRVRKIGYDPGRDNNHNLDDLKRCIEDHIKLRHLFKTLEDALSIVQLSLFISSGLNIC

VALVYLLFYADTFVATLYYSMFLLAICIELFPIYFYGSVMQMEFEDLTYAIFSSNWAEQPKLYRKNMQIF

LQNTLPRVKMVGCGIVSIQLETFFLICRMAYTSFTLIRTINKDVQ

>XP_013098161.1 PREDICTED: odorant receptor 45a-like [Stomoxys calcitrans]

MPIVKRSFLVQYLSFASVGFDPKALYTQENVRLVKYPWLLAFQISLSALLYMAIFHYVYINSEDYTKTIN

SLSLTCQGIICLTKMGIFICKGKEFVALVTRLKEDISKAKPSDLPIFRVENEKDVLSCSIYSMAVVSTAI

WVMVEPLISMFYTHQEEGSFEYIVPHRATYFWDYHNVQGYSLVYIWDFITTYILALGSLAIDTLFSWLVC

NIVAQFRILVQQFQRAAAMTLPLANGMVAVANAAQERAIVDCIKVHIRTLQLTYDLNRLYGGIIYVKFII
SGLQIGSLAFCLSRGGQSMGKVAYQFLFLTAVAIQLMMYCYNGQRIATESLQVASEIYAAFEWSHLAKST
KKLLLMPMMRSQKFCEIRGVFFTVDLGLYLWVFKTAGSLITALKTLEDE

>XP_013098160.1 PREDICTED: odorant receptor 45a [Stomoxys calcitrans]

MAILKRYFRLQRFIFAGLGIDIKARPENMVKRPVLMMTPLVMSILLCIANGHYVLDNASDYLEATDSLTL
LCQSLISVWKVIMVIWKRKDFARIIARIENLNVRASGEELKIVRTENTKDVIFSTTYFVLVLLTGAWSLL
VPIYFAVYIYMTTGVVDLPVPHKATYFWNHEHVKGYSLVYIWDMFIIYFIACSAVSTESMFSWLVCNIIA
QFRILMHRLEVASKLPLQTNYVAANHHVDDDDDNPDLCEIDANGTMMEAIINCIKFHRRTLRLTQELNSL
YGAIIFVKFIVSGTQICCLAFHLVRGNNSLFNVAYLLMFLSAAALQLILYCYNGQRLKDESLLICTSIYS
TFEWSKLPKSTQKMLLVPMMRAQQFSELRGVFFTVDLSLYLWVFRTAGSLIAALKTLEEKEE

>XP_013097687.1 PREDICTED: odorant receptor 59a-like [Stomoxys calcitrans]

MSSTLLSKDKVNSFEFFHINWKVWKIHGLKIVKRDIHTQQRQPVSRSYFIWTIAANFLATILFPIHLSMG
IFQSQNKSELFDGISISITSIGTTIKFFLMASKMKQIAQMEALIRVLDARVTHQEELNHYRMRTRLNILN
VQKMFLILYCGIGAAVAASFLFSGERRLFFPGWLPFDWRQSIGFYALGILYQLFCIVIQISQNFSNDSFT
PKALCLLSAHIELLYMRVSRIGFDDSHTGGSCKSHLQEQDIELKRCVLDQINPYELYKTIQDIISWAMVI
QLLVSVLNNCVAMVALLFFATELFDRIYYSIYIVGLAMQLFPSCYYGSDFVLLFEKLHYAVFSCNWIGQS
KSFKRHMMIFTERSLRQTMALAGGMFPIHLDTFFATAKATYSLFALIITIK

>XP_013097676.1 PREDICTED: odorant receptor 59a-like [Stomoxys calcitrans]

MSSSILPTAKINSLEFFHINWKVWKIIGLRLFKRDVHTQQRLPVSRLYLSWSIAANFVVTILFPIHLSMG
IFDNKSKLFDGLNVTITSIGATVKFFLMASKMKQIAQMEALIQVLDARVTHLEELEHYRTRIRLNILNIQ
RMYLSLYSGIGVALGASILFSKERRLLYPGWLPFDWRTSTGYYALAMLYQLFGFAIQISQTFSNDSFAPK
TLCILSAHIELLYKRVSPIGFDGPYAGDSGSKAALLQQEEELKQCVLDQINLYELYKTIQDIISWAMVIQ
LLVSVLNNCVAMVALLFFATELFDRIYYSIYIVGLAMQLFPSCYYGSDFVLLFEKLHYAVFSCNWIGQSK
SFKRHMMIFTERSLRQTMALAGGMFPIHLDTFFATAKATYSLFALIITIK

>XP_013097563.1 PREDICTED: odorant receptor 85f-like [Stomoxys calcitrans]

MPEIERYFEDFISLPKLLLTTLGIDFVPQRRMWFMRLLMRIYFFLTLICCVYCTYFVADEILQEIVEGAH

NLPLLLRLFNDLNYNAIGILKSYYFIHHLKAQRNLCSRFEKIFPKTIVERFAYRVNENFWPRWITTILYM

YFGAVALILFSPLAESLIEYVIAFFKVGYADAQFTYHKLYEEQSYVIDHHSPLTYILIYSMEVMNSHYAI

VFNICPDVWLVAYAIQLCMHFDYVSRSLESYEPVAAKSSTDSKMIASLVKKHKILLEMADDLKEIFSLLV

LVMLFSTVATLFCAAVYLLTEGLGKDVIGYLAFLPTSLGQYFMVCYYGQQIINKSLEIGEAAYSQTWYNG

CQSYKKSILAILGRSQRQCEINAGGFQTTNLKGFESVMRMTFQLFTLWRTMMEPN

>XP_013097554.1 PREDICTED: odorant receptor 49a-like [Stomoxys calcitrans]

MLNSVFCLLSQLYCLTFIAKEVFLLIALGVPDLPLLLRLISGLQYAVFALSKFVCFKWRLKDAADVYMKL

MEIYPKTSLEHRLYRVIDFFWPKKILIVIYLYLGSIFFIGISPLLEGVVMYLVDCLRVGLGNAEFAYIKL

YDIPYGFNHRRPFSYMVTYMMEVFHVQFVLIANVCPDIWLLCFTMQLCMHFNYLARIMEEYEPDESAPKK

DQQFIAEFVKKHQIVLNIGQNVKDLFSVLLLIVFMSIATTLCCAGVYTLTQGLGRELLEYCAFLPCAIGN

FFLICYYGQQLATYSEKVADAAYNHPWYNGSQSYKKTILIIMIRASRAVELNAYGLKPICLDAFKMLMGE

SYRVFAVLKQTMLD

>XP_013118805.1 PREDICTED: odorant receptor 88a-like [Stomoxys calcitrans]

MDFRYKSLVTLYVAVAVTLIDFVCNVIKISLYIREQRLQEAKQIAAVWSIESLCLVRGISLVLKQRNMLE

LTNDLDKIFPRDSEQQLRMNCAKFARYLDVRFRIVGLYTLVGVTVFIGTPLLKYLLYYDASSGQVIPDEY

HQHASWYPYHLKDNPRTYPYVYVYEGFNTICSINLVFAWDHIYTVSVAEFLMHFDFVNSELANLDARESL

HQGKRRRKFFSRLLGIIQYHQHVLKLGDKFCNAFNLSLFFTNLVSAASICFHVYLIANSDDYLSIILFSF

PCLVQVAFAFDNCYQGTRIAVATSETANVIFGHNWYEGSVEYRKITYQMLQFASRPFTLSGYNLFRIDMV

HFRMSMMIAYRMFTFLQARGERN

>XP_013113323.1 PREDICTED: odorant receptor 46a, isoform A [Stomoxys calcitrans]

MNSREHRELLETFYKKQSWVFRILALWKLPDTCSKRFTMLHKIYFYFILIFWVLSFDASCLIQFIANITD

LNEVIKVFFIFATSLAVFAKFSTIKLKNHLYAELVETIHGHKYRPVNTREVKIFLQTHRLCRRVRNFYLV

ISLCALNVVMLTQYIFDNSELPLSLYNPINIDTKLRYRLMYLYQYIAVSICCYMNIAFDSLSASFMIHIK

GQLDILCDRLENLGLDYGSDDHEITWQLKDCVRYYADILHITRIAENLISFPISIQIACSVLVLVANFYA

MSFLSDPGDYANFMKFLIYQLCMLSQIYILCYFPSEVTTKSQEVPYYLYCSNWVDWNRFNRKLTLLIMTR

FDIPIRIRSINPTYTFNLAAFTSIVNSSYSYFALLKRINS

>XP_013113178.1 PREDICTED: odorant receptor 46a, isoform A-like [Stomoxys calcitrans]

MKSDYQELVKLFYIGQYNLLKCFALWDLGEEISPFMRRLYRFYFWTILTSWTLPFSCGMLIQLILNIGDV

FEVIKVSIFFATSTAALIKYISIKMGTRDYENIFQMLHQQEFLPQNLREWKEYRKAIDLSRQVWKVYATL

SISSISSFFNTEREFNVSLYNPLNMESDVQYFMMGVYQLLSLTAVCFVNLCFDSLAASFFINIKGQLDVL

GCRLENIGQGVRCSQEEVLRQLKDCIIYYQRLLDLTHTMEELLRVPMSTQIACSVMVLIANFYSMSLVSF

EDLVVFIKLILYQMCMLTQIFILCYFANEISMKSSDISFNLYKSNWYDWNKENRKLVLLMMTRFEQPIRI

KSINRCYSFNLPAFTSIVNSSYSYFALLKRINS

>XP_013108393.1 PREDICTED: putative odorant receptor 85e [Stomoxys calcitrans]

MKFMGQVPIKLENYFPSPLKALCRRLAMCYCVFCVFSNLHLSLLYMKTTLDMLESGELEEITDALTMAII

FSFSTFATCYWLIRSQTLLKFISKVNREYRHHSLAGLTFVSAKQSIRLAHKVSLYWLISCTVGVVSWALA

PLLLGSHTLPLKCWYPFDPLKPGLYTFIYATQVWCQFLMGCIFGNGSALFVSVVVIMLGQFDVLYCSLKN

VTHHAQLLTGEDLNILRKIQQELPKSQDDELNQYALLIEHPTNLKELSVSKMTHNANLGQALHDSLVECV

HIHQFILQSCDTLEMLYNPYCLVKSLQITLQLCLLAFVGVAGERSAMRAINLVQYLMLTLSELLMFTYCG

EMLSSHSIRAGEAFWRSQWWINGHLYKNDILMFLVNTRRMVKLTAGKFYLMDVQRLRSVITQAFSFLTLL

QKLAEKNQ

>XP_013107799.1 PREDICTED: odorant receptor 2a-like [Stomoxys calcitrans]

MPKEVLCLTLDTNSAFQYQWKVWHYMGIKAPENINRKLYQLYAVFINSFVTLLFPLTLIVNVFLAQNLQQ

LCENLTITLSDSLANVKFINVYLVRHELDSIQLILRRLDSRIQTRGEYNILKSAIRKAQTTFLIFLRLFT

VGTVLSVVKIILAEKRSLLYPAWFGVNWEESAFKYAIVISYQLFGLIVQALQNVANDSYPPSYLIIILTGH

MEALEIRVKALGNHADGTLKVSLTAKEQNDCLKELNECIKDYINILKLHSTIQRVISKACLAQFVCSALI

QCIVGLHFLYVVDTADYEATILSVIFFVAITMEVFVICYFGQMMSLQSWNLTYAFYSCGWLAQSPIFKRN

LIITLIRTQRTSIILAGSYIPLNLPTFVQLMKFAYSTFTLLIRFK

>KNC34941.1 Odorant receptor 59a [Lucilia cuprina]

MSTINSREFFIMNWKCWKWLGIITPNRSDPTRVKQLLWNCFINITVSSMFPIHLMLGIFIANASKSEIFE

NIAIFITSVGCILKLIMFAVNIKRIRKLENILQTLDERIQHIEDQKYWLHYVKPQLIYLQRMYIVIYTGK

AFCASLAFVVRREQKLFYHGWLPFNWHQTWWHYTLALCYQFYGIFFQFLQNFSNDSFAPKALCALSGHIK

LLYKRVARIGYDTSIVSEEHEMELNSCVRHQKDLYEFFDSIQQIISWPIFFQIFVSLANMCVAMVALLFF

VTDIFYGIFYITYLLGMIMQIFPVCYYGSDFVMLFEKLHYAVFSCNWTGQTKSFKRHMMLFTERSLKNTM
ALAGGIFPIHLTTFFATCKGAYSMFAVVITMK

>KNC34940.1 Odorant receptor 59a [Lucilia cuprina]
MTIRKVNAAAATHNAEPNSCGFFKLHWLCWKILGITLDIDKLNIHRNTYLLYSIVLNIVVTICYPLHLAL
QLFRSELMADNIKNFAICVTCVACSIKFSIYSTKLPVIRQFEQILKRLDERIKTDVEMNYFRQLRNRLRN
VGLVFLSVYLPVGITAELSVMFREGRSLLYPAWFPFNWKESTVLFYVANIYQVVGIFILLLENYLDDTFP
PMALCMLSGHIEILSIRVSNIGYDKKSLKENEEELDRCVEDQQTLYELYTTIENIISWPMFIQFCVTATN
ICVAMAALLFYVSTPLDILYYFVYFLAMPLQIFPTCYYGSDFQYLFDQLHRAIFASNWTDQTKKYKKHML
LFTERSLKQNAAMAGGMVRIHLDTFFSTCRGAYSLFAIIMRMK

>KNC34511.1 putative odorant receptor 13a [Lucilia cuprina]
MSFLYKPRKKNGDIINFPQVFEKVLIGNGCWLTEGDPKDKWFKFKNILYNIYGWIVYVNLSFIIFCEMAY
FLVNIKDVRKAVESFCPSMIGFFIVVRTLHFRLVTEDLKRLLKSFAEKIWIDGDKYPRIVKRCHRIMRPV
FVYFVLMFNVLILYSIIPIFVFFTTNQSLESKDKRMPYSMIFPYDAQTGYIYFVTYIAAILAGYVVISHF
YALDALLLMFVSYLSGQFEILHGEIVRLIPECHAEWLKRYGVEIGVSIGGNNDVGYDAQPDARMLKLLQD
MYTKRLHELSARHNDLISFSVRLNESISFPLLVNVVNSTFLICFCGFQFLLSTNTAFYLYLCNWEGGQLS
KDSPLLLKPEDMDAASLSAKLPLWKHIKYYPAGKDFCNKLRFMIMRSQRPVQMDAMKFTILSLESFSKIL
SSSMSYFALLKTFLDKQK

>KNC33831.1 putative odorant receptor 49a [Lucilia cuprina]
MSPNSANPLLTQKNFQDFCNLPNKILSSIGCDLLNKPHSIWLSVLKKCYSIIAFLSHAYICIYITKTMFN
MIVSDDFELGLLLRLISGFNYALFSILKAITFFWNIKEFMEIYQVLREIFPETRKEKLMLRVRDYFWPKW
ILFTVYFYIGAVTFIASSPLLEGVILYIVSVFRVGWSRAEFGYFKLYEIQYGFDHHSVFAYIITYGMELM
HAHFMVTFIICSDIWVLCFTLQLCMHFDYIARELQNYEPNEKAYVKDQEFIADLVKRHQILLNIGDGLNS
VFGGLLLLMLMSIAATLCCAGVYTLTQDLGREFIEYVAFLPIVIGQYFLICYYGQQLIIKSHSVADAAYN
HSWYNGSKPYKKSIFIIIMRSQKEVELNALGFQPICLEAFKMVMGATYRVFALLKETMF

>KNC33783.1 putative odorant receptor 74a [Lucilia cuprina]
MFNILYRPRLPNGKHVPLNWSLKLYRWTNIICWPLEDNAPHWLYFFDRFLWFLGFLTFAVHNDAELRYLR
VYFNNLDEMLTGVPTYLVLIELHLRAFSLGWRKNDFKNLLKKFYAEIYIEESVNLKIFKKIKRQMWPILT
FSLLYFLALNSYIFNAVYVLATNKRELLYKMIPAMEYKNNFYVYFPLLMSNIWVGFIVTTMMFGEGNTLG

LLIFNLNGRYLMMRETFKQKVDTILKSNLNGNIVEKYERILRETLKENLRLNKFAREIQDEFSFRIFVMF
SFSAISLCALGFKVYTSPVNSIGYAFWAIGKIQEILAFGQLGSTIISTTDQLSTMYYESKWEIVIERSSN
TPDNVKLLKFVTLSIVTNRKPFHFTGLNFFNVSLVSVVAILQGAGSYFTFLISLR

>KNC33632.1 putative odorant receptor 10a [Lucilia cuprina]
MSLKFLSSNYPLHDYYFYVPKFCLRLMGFWPQTTNTNLHRFWAASNFLILLIGVLTEMHAGFSSLTYDLE
KGLDTLCPAGTSAVTLLKMVLIFYYRKDINYVLKKMHGMLYDEGANDQILRKHHKIIRKFSVLAARFNFA
PFVTGFITNTTYILKPLIMAWIFWSKGKEIQWTTPFNMTMPRILLHAPLFPLAYIFTAYTGFITIFIFSG
CDAFYFEFCSHIAGLLQILQSDIESLFNMFENRLKLSDKENQYVENRLKIIIRRHNEIIDLTYFFRKRYT
IITLAHFVSAALVIGASIFDLMTYTGFGRLIYVAYTVAALCQLMVYCYGGSMVAENSVEISNVIFGCNWF
ICSPKVRRMILLIMIRSQRTLTMSVPFFSPSLATFASILQTSGSIIALASSFQ

>KNC33151.1 putative odorant receptor 94b [Lucilia cuprina]
MTKISPKVCDKPQCDKLSGGRVALTILKCVALWPWRYEYKNENQKLLYSRVQLIHRWILHIPLSLTFCLL
MWVEVFISTDLNEAANVVFMSLALAMYLMKFINIRVYSEKATWFIYEMEHNRMFDLLNIEEVDMWLKYHK
SFRMVSIIYIFGSAFSSFFAFVGALFEEDYHLGYPYYVPFEWRNPERYWYAYVFNWTGILVSCFSNVSLD
MLGCYMIFHVGLLYKLLGMRLSNLNSAKENDAVREFTKLFLMHSCIKRMTKECETLISHYVLTQIIFSGL
IVCFSGYRMQKMNILAEFSQFFSMLQFLSVMIMEMYLPCHYANQITTNSAELLNNIYDCEWLQFSIVGRK
FIRLYMEFFKEPEQLRAGKYFELGLPIFTKVMNTAYSYFSLLLNMDK

>KNC32434.1 putative odorant receptor 82a [Lucilia cuprina]
MIELFGRQRQCLRIMGHQFVRDKSVLLGKWRNIVYFGVLMLVMSAQWPMINYAIYYIDNLQLATASLMKD
SAAKIILESANSTAIMVVKLYWISVCSTGTYFMMSPVLKIIWSKIRKTNAAWELPMPMRFAFDFETFPGY
EFAYIYTGLVTLSVVMYAVATDGLFVSFAINLVSHLKILQKSIENNTFLKSDEELHKDLKSYIEYHNLIL
SLYNELRDIYSPIVFGQFLMTSLQVCVIVYQMVTHMDTILVLIINCTFLTSILLQLFIYCYGGEILKLES
LMVGISVQLSNWYNLKPAHRRMLVLLMLRSQREAIIKAGFYEASLANFMAILKAAVSYITLIQSIE

>KNC32056.1 Odorant receptor 85e [Lucilia cuprina]
MSSNDFHGGSMNVLYSKKDHERRRDLFHLQLFFMKFVGQVPTNLEKYASVQWIGAAKKLARFYCAFSAIS
TLHLALLYIKTTFDMLQIGELEEITDALTMAIIYSFASFATCYWLWRTKSLRNFLENINNQHRHHSMAGL
TFVSVDLSYNLAYKITLYWLRSCMVGVVSWALNPLLLGSYSLPLKCWYPFNPLQPVVYELTYMTQVWCQF
IMGCIFGNGSALFVSVIIIMLGQFDILYCSLKNLDYHAQLMSGEDFKYLQKLQSQLLQGEDDELNQYVYS

KEYLTDLSVFGSKEVRQKNSLQKVLHESLVECVLLHKFILKSCDALEDLFNPYCLIKSLQITLQLCLLVF
VGVAGESSTMRTINLVQYLALTLSELLMFTYFGELLRNHSVRAGEAFFRSQWWPHAHYIKRDIFVFLVNT
KRAVKITAGKFYLMDIQRLRSVITQAFSFLTLLQKLAEKNK

>KNC31304.1 Odorant receptor 33a [Lucilia cuprina]
MAQSNTYLPDSVGVFKPFWLCWRLLGVLSWQRKDITMIYDIFMNLFINIWYPVHLTVGLILLPMHDEIYK
NMSITITCIVCTLKHYCIRWKLREILKIMDLFGKLDKRIHSLEERKYFTKYNIRIAKLLAKLYFSVYMGA
NVAALVALLWDSERRLMYPAWFPFDWSSSSGLYYSAILYQFVGVTILIVLNFTNDAFGPVTLCLFSGQVH
LLSMRVAKLGYDKKKSAKQNENELNLCIEDHIKLINLFNITEDSISYVQLILFISSGLNICVVLVYLFFY
VDNLFAYIYYTTFLAAIAVELFPIYYYGSILQEEFNNLPYAIFSSNWPSQTRSYRQNAVVFGEVALRKIT
MLAGGVVGIRLDSFFAICKMAYSLFAVAMTIK

>KNC30306.1 putative odorant receptor 22c [Lucilia cuprina]
MFDFLQPSVPISKSFMRIPRISGIICGIWPQRKHSCIKLLFFAFNVFVVALGAVGENLYGFMYLNDLVNA
LEAFCPGVTKAICLLKMLVFFVFNHRWYLILERIRTMLMAEQHCKEKMQIVEKLASIASIFSFILLTSGS
FTNMSFNLRPLLANMIRHFQGQDIVNVLPFNIVIPEMFVNYPYYPVTYFVLTLSGAMTVFTFSFVDGFFV
CACMYMCGIFRMIQYDIRTIFDELKGGETSSLAQNQRFRLQLTAVVKRHNAIIDLCSDFAKNFTLIILMH
FLSAALVLCSSILDLMLVSE

>KNC30035.1 Odorant receptor 2a [Lucilia cuprina]
MLNVKRSSNMVSGDQQPDTNMAFYYHWKVWHWMGIKAPMQSNLKVYRIYAVLINFVVTFLFPLTLIVNVF
FAQNTQQLCENLTITITDTIANLKFLNVYLVREDLERIKAILGKLDRRAENLKEKRILQDAIRISQLSFL
IFVRLYTVGTGLSILKVILAQEHCLLYPAWFGVNWFDNTFIYVIVMIYQLFGLIVQALQNCANDSYPPAY
LIILTAQMKALEVRVRAIGHRESDEEHLWLTKEERLRNLHEFNACIKDYNNILKLFTIIENIISKACLAQ
FVCSALVQCTVGLHFLYVVDAADYSAQILSIIFFVAVTLEVFIICYFGHCMSTQSWNLTYAFYSCGWLAQ
KPAFKKNLLITLMRTQRHSIVYAGSYIPVDLPTFVQLMKYAYSTFTLLMRFK

>KNC29287.1 Odorant receptor 43a [Lucilia cuprina]
MYYNLPLFHCNVKIWRYIGFIEYENVYRIIPIMIIVLLTIICEFLHCFVVRQDLGFLILSLFMVAILLNS
FIRIVIVMKNHKKFIKFMKVIESWYKEAELTNDIAAWSILNKLPKRTEAISKFGLIFGSFGGVVTAIIPL
LLSHPHPYSVYIIGLDALKSPLYEIIYFVQMSVLMPFIICTYIPFTNLFITWLIFGIHILQVLRKKFEQL
PGSNDPDQLRSLKALIKYHKRIIRYGESLEDLVSHVCFVELLLFTTMLSILLICLLLVDDIMFQVATVVY

IICILYVLFLSYWHANEYSSESVKIADAVYSIDWTSSSIEVRKCVLILLMRCQTSLKISAGGMYPMTLEA
FQVLLNAAYTYFNMARGWRELLSAVDAETHVCVDKISTIECVKAVVRHNFLKMYLPYVRKNHTQFIVSKV
LMGEAPH

>KNC27745.1 putative odorant receptor 63a [Lucilia cuprina]
MTLVISSILGLGGQYYFIWENRSKSMLTYTDAICTSFQTWISISKLFHFAFTQHKFEKLVKMAQNTEILQ
NFEIFELNIFNKKHITEEIQGILDDSWLDIKRQLNFYIISVFGIVGWYYFSCLAVNIYNTYTYSSPTEFE
LLYRAFPMWRDKGIQFPYYFITYIISGSETHISGMSAVSFAGLYIVTSLHTLALLKILRRLVAYSTTEDV
LPQERVKYILAWAKLHQRIYECVKNIYLLEMYLDQFILFYRYFYEINSLYYIQSAPLFLCSMLVICLLIF
QGSVGLVGINIVIPYTTIVERVLCIYADLCPSITVRLAVYPCKPWAHATDLNFQDNLMKYGRNLLLKMVE
IGPLFRLAPLQQYPTNRGFRLIITLNVLLYQQRLGSDVDVVIKMVLYFSAAGFEVSMFCFNGQRLTSESE
RLPVALYNCKWYEECTEFKFIIRMMLMRTNRTLAIQVGGFTTMSLVTLLGIMRSSFSYCLLLREFNSE

>KNC26548.1 putative odorant receptor 63a [Lucilia cuprina]
MFSDYEIKTLRKRNYTKIKELIRISFSLGVNLRCPSLFKDSLKIINILLVICSGISMYGHWCYITRYIED
LTKIAESICTALQTCVSIIKMIYFLFVQRNLYVLLDKAQSHEIIRQSEIFRNGKVTNVLNSPVSRRLMKI
IRDIMENNWKNIRAQLLFYICSCAVIISNYFFSALFLNMYHQIKGTPNYEHVLPYPSVYPVWESKGAVSF
DGVFIVLCVHGVGLIEVLNVMIEQSTSSDVPKERRVEYLRNCINLYQNTYEYLLNISKMYKHIILSQFLL
SLVIWGIVLFQMNIGLESNKITLVRMVLYLSAAGYEIIIYCYNSQRLTNEYEKIPMAFYNCDWYNESKEF
KQLIRMMILRTNRIFNIKISWFTTMSLPTLMIGKDFHALIN

>KNC26278.1 putative odorant receptor 85d [Lucilia cuprina]
METKGKFQKLLAVKSPKKKLNIRDFYITPFRFYTFVGVKLFHWDEFDIMTKWQKFVLTVTIANLIFSFTW
KIFFLMVDEFESTVQMTEWFLYISFAANGFIKTLSVVWGRKILDRVLKTLGRLFPRNAKECEDFKLTEGY
NFIHYHSRIMVYSHWTIAFMFMLFPLVQSGVEFIHTRVYVQRLPYILAYPFDTSPKPVYIFCYVTQFMAG
FVLSCYFLGSDTLLLHTVYMVVLNFEYLCFRIVHFEPKNFEQDMAEIKDVLEKHYLLNDLAQAVNNVFSL
SILMNYMISIMVIVLIGVQIITGSELFDFIKFGGFFASATIQVYYVCLMSTLLMERSASVGDSLMGQKWY
MADVRYQRMLTLAIARSQRPAHLTAFKFFMISMESFSNLMTTAYQFFTLLQSRMEEGGL

>KNC25816.1 putative odorant receptor 13a [Lucilia cuprina]
MFNPKPKGDPKYRIPRQCIWLKMNGSWPFDCETERDFYNAHTLYVNNLGDIMMTTENCCTTFMGALNFIR
LLHMRLNQRSFRQIIQQFVDDIWIIKKHNPYIAAECSRKMRTFRIMTVLLSCLIAMYCVLPLVILFFDVG

LDAEEKPFPYKMLFPYNAHRGWRYVATYIFTSFAGICVVTTLFAEDSIFGFFITYTCGKFKILHERIDNI
VIDAYFMARNRQDDKEIQWLYLRLLNRIAYDHNKLIEFANRLENFFNPILLVNFTISSILICMVGFQLVT
GKDMFIGDYVKFLVYISSSLSQLYVLCWNGDSLIQHSTETAQHLYNCNWEGENLYLTSAISKYQLAKENA
KNSNNTLSKSLKQQNLCNDLHNIHFIPANKQFRQNLEIMIMCSQRPVKITALKFSTLSLQSFTAILSTSM
SYFTLLQTVYNENKEELEHMT

>KNC25487.1 putative odorant receptor 24a [Lucilia cuprina]
MLPRFLTKNYPLDKHLFLIPRFALSLIGYYPESERTTKVQLWSFFNIVILGYGCYAEFYYGIHYLSIDIP
SALDALCPVASSIMSFLKIFFIWWYREEYKFLIEKVRYLTAQQNSSGKVQMKKRYFTLATRLNALVLFFG
FCTSTSYTLRPIVTNTFLYLKGQPIIYETPFKMMQAIQQDLQSILTDNGTKNNCRYDSEEKVCAALEDII
ERHNEVAELTDKFSLIMVEITLCHFITSSIIIATSVVDLLLFSGYGIIVYVVYTCAVLTEIFLYCLGGNT
VMDSSEDLATKAYSSEWYTHSVKIQKIVLLIMVRSQRAIIIKVPFFAPSLPALTAILRFTGSVIALAKSV
I

>KNC24908.1 putative odorant receptor 42a, partial [Lucilia cuprina]
MASLRMDGPQPQTKDAAVYLFRGLIIVGFMAPKIYVVPFFIWTVFINLFVTVYMPIGFLLSFLTRSSSFT
PSEFLTSLQISVNCIGCSLKMFVFLFLYKRLLKGTKYMDKLDLRVTDSEDMVQIRKIVAFCNRSHIMFAT
LFLTYASSTFLTSVINGKPPYQVYNPFMEWTDAKRYFVIQAGLEYFMIDFHCYQQALLDSYPVIYITIIR
THLHILTRRISKLGQDTKLSSNERYEALIQCILDHKNIMDLYNVFSPVISGTMFVQFLIIGLILGITTVH
IVLFADVLAIIASMFYVASILAETFPCSFLANCLMDDSDSIALAIFHSDWPSEEPRYKQVIAFFLQHTQK
TMILNAMKIFPITMNSNINVVKFAFSVYTLMKQMDFGQNLKESVTGEK

>KNC23897.1 putative odorant receptor 67d [Lucilia cuprina]
MAKNCSDRYNKITRITRMMAAVCGADIFDPNFRMSLLTWTVIFAINAFFCCTIYTIYIGLVVEGDWKLML
QTLSLVGSAAQGYSKLLVALFRRWDMVSMNGKLLNIYLDYENDKDFCTILSTRIDMIIKIFKFVLLIYII
VVSIIVLYPLIYGMLYGEKLFVMQFLLPGIDPLTHFGYVVHNVVHVFLLCLGGFGNFAGDMYIFIFILHI
PMLKDILRIKFEKLNRITLQKQNSSKTLPLLKEIVEWHQNYNKFVKQVEGTYYSVIFVQISTSVVGICCT
IFSIVIHSWPAAFVYLAYSAIMLYAYCGLGHLVEISNDEVIDVIYGDCLWYEMSVPEQKLVLLMLRKSQR
PTTLTIGQIMPLSMSTALQLTKAIYSYMMMLLNFLESDDM

>KNC23896.1 putative odorant receptor 67d [Lucilia cuprina]
MAKNCSDRYTNLIKVLTRIAAICGANIFLPNFRIYWLTWIVIIAINAFIACTFYTMYVGLVVDNDWKVIL

QTLCLMGGAVQGYSKLLGALFHRPAMVSMNQTLFNIYKQYEQENEFLKVLSYRINLASKILKLVLCIYLG
TLPIIVLYPLIYGLLYGEKLFVMQFLLPGIDSSTHIGYIIHNVVHVFLMCLGGFGNFAGDMYIFTLIIHI
PLLKDILKIKFEKLNQVALTNSKKSMSLLKDIVEWHQNYNTFVKQVEQSYYGVIFVQIFTSVMSICCTIF
CIITSSWPAAFSYLLYSAIFLYVYCGLGHLVEISNEDVMDVIYGDCLWYELTIPEQKLVLLMLRKSQRPT
TLTIGQIMPLSMSTALQLTKAIYSYMMMLLNFLESDDL

>KNC23846.1 putative odorant receptor 94b, partial [Lucilia cuprina]
AYNTLEKSCIIIKILTTIGILTNKKLHLKLAKVPPIYYKLMIHIPLTFTYTTLMWLEVIFSSDIYEATDV
LYMVLTETALVVKILSIWYHDALIKSLFNEWQQNAMLKLYTTQEYFMWQRTIKLFGIVAFLYITCSVSVL
CFSFTAVLFLNSYELPFLYWTPFNWKHPSNYWYAYFYELFAMSLTCLSNCTSDMLLCYMMQHLALYFKLI
AMRLENLGNRKDEINSVVTKKLLTIIKCHLKLKSMSKSCECIVSYPILAQILLSAFVLCFSLYRLTNFNF
IEDPVTFLSLIQYAMVMNLQIFLPCYYANKLTIESSRLTNSLYNCNWPEMSPFNRKLILMYMQSLQKPVV
IKAGNFFEVGLPIYAKTMNNAYSFFALLLNMDI

>KNC22741.1 putative odorant receptor 45a [Lucilia cuprina]
MGIDINNLKRRKLLKDFGKFVLLISCTIYLEYGLIRFVAQSVSNIDKATGALSMFNQGCLILIKISVFLT
KGDKFMKLIWDMNLLAMRANPEEYKKWLSENQRSQLIGKMYFYACWIAVGCAGVVPIIFMIYDYKQNGVF
NGKLPFGGKFPFDQFGQIIFALNYILSLIYIYALLNMTVGIDTLYGWYIYNISAHFRILRCKVESVALKL
KNNDNENFIRDIGSIVNYHNKTINFTQDLNNIFGEILWAEVMLSCLQMCFAIYTLNNDDDVSNMPFNFMV
LVAVVMQLMIYCFGGEKIKNESLMLCLDFYLHFPWHKMPAQQKKLMLLPLMRAQTLSVLRGLFFEVDRNL
LVYVSL

>KNC22655.1 Odorant receptor 2a [Lucilia cuprina]
MSFNAKAKVETWQAFKNHWILWKCFGLQPPKRDSKWFVPYIAYAIFLNVTVTLLFPTTLIVNLILSKNLT
ELCENLYMTTTDVICNIKFLNIFVVRHKLLKVRKILQRLDVRAKTHKEVAILEEGIKLARKCFMTFARLF
CCAVISSQMMVYLSSERILMYPAWYPWDWRASKKNYIYAFSYQLYGLIVQATQNLGNDTYPPAYLIILTA
QIKALASRIKDLGTNKNTSEEELYKELTDCINDHNTINELFFTIQEVISTTCIAQFVATGLAQCTIGVYM
IYVGLHPSKTLNIVIYFSAVTMEIFILCYFGDLYCQANIHLTEAIYACNWMDRDKKFKQAFLVLLQRSQK
TNCIMAGNLIPVRMPTFVKVKDFFSSIILMNRSGSVLM

>KNC22499.1 putative odorant receptor 45a [Lucilia cuprina]
MIVQNYFHVQKITFAGVGINPMVADPNDVVKRPLLCYGLLISTFFHMIIIAHYILSHIKEYDEVTDSFPL

MCQAILSIWKMSIFLSKRKDILQLINDLHQLNLKAEQDELTIVRRENTNDAFVSNIYFKIVLATGTFAFI
HPLIYGLFVYITSGKLVLVEANKATYFWNYTHIGGYSIVFLLNFLTSYYVCDVSLAIDTLFTWFVRNILA
QFQILMHRFQLIAKECNKDASNGTGPGSSQYRNPKKYFWPIIKCIQYHRKTLALAERLNQVYVCSEICCL
VFSVSRPNYSVFDAVYKVLFLTAVTLQLSLYCYNGQRIKDESMLVSTEIYTSFDWSHLGKPYKKLLLLPL
MRAQKPSHLKGVFFEVDLSLYLWGISQLGILGFIVNSTNSALFGITPLFVVKLSSNNKALDMISRLKTSL
MLITQHRPRKQKLLSVQFNKNRVNDEKQK

>KNC22427.1 putative odorant receptor 67d [Lucilia cuprina]
MSRKYSQKFKNLFNFSRIFARLCGCDVINVNYKMDWKTLAVFILMNMALGSLFYSNYVEVIVKGDVYNLL
KTTSVVGTGVQGYAKFINAIKQKNNFRFVYNEILSMYETCELKTHAYTKWLAYNLALVKKLLLITFLIVL
MASVGVTIVPLYMLIFMNKRVDVMPFAWPFIDGSTDVGFYVTLVGHISCVIFGGYGNFANDSWLFIFASH
VTLMKNILKCKFDDLDETLNEFPRDPTKSREPLCDIFKWHQKYLIFCNTVKETFFWVIFVQIGFEFLGII
CTIVCMFLDIWPPAPAYLIYLYSLLISYCSLGNLVELSNDDVIEMIYNSCWYNLTVSEQKMILIMLRESQ
KSEGISIGGVAPLSMNTALQITKTVYTMTMMLKESLN

>KNC22027.1 putative odorant receptor 85d [Lucilia cuprina]
MSPKNYQHHTENSLFPMKKSVQLSHFLIQSDILAKSIGLIPYDDETKPQRYQTLMKWIFVIFIINMNFVL
FSEITFVILSMVHGENFIEATMNLSYIGFVFVGDIKIFSVLQKKPLLTILMREVREMYPKERKLQEAFDT
RRYVRRFNLISFGFVIIHEILIWSYNLYTAMSYLIYEFWLGTKIVPRNLPYYAWVPWQWQGHWSYYFLYA
SQDLAGHTCMSGQLANDLMLCVVATQIIMHYRYLAKRIETYEPAKDYESNKEFLSECIQYHQKILSLSVY
MNEVFGVSLLVNFISSSFIMCFLGFQMTIGVQADTLIMLFMFLFCSLVQILMICNYGEELIVKSESIGHA
IYNHNWLNTDTRYRKMLILIIERSQKPALLKATTFLNVSMGTMTDLMQLSYKFFALLRTMYSK

>KNC21971.1 putative odorant receptor 45a [Lucilia cuprina]
MNHNESLPSQPGIKRYFHVQRMGFAALGLNIISMERTLHNYFHVWFPAFVLFAFYFPVLSYAVENAQDLN
KLTTTLSPVWQSIIAHFKILFIMWNRKKVVSLIRKLWYLNLEAKNEEHAIVVAENQKDIIFSTFYNWVAH
ITGLSNLLAPLFVATYYALQGESFWEHLDIPSKGNYIIHKKSMAGYIFVYIWNIIGTYYVVFVCIAAETL
FSWFMFNIVSQFHILKHRFHQAGIENDGNCSSKTISNCIAFHCRVIELADEFNNAYGAVIFVKFIVSCLQ
ICCLAFQLSRGDGELFEKVYHFLFLSTVSVQLMMYCYGGQMIQDESYSIADKIYESFHWETLSLTNRKML
IFSIMRSQKPCNVSGIFFTANLNLYLWVCKYFFKLSI

>KNC21960.1 putative odorant receptor 67c [Lucilia cuprina]

MTQKKMSKHLRTYEDFMKIPVFFYKTIGEEIFDYRSTNRCLSLLLKCLLYAGFANFNIFVLGEIIFFYKA
VQSKDTVLGAIAVAPCIGFSLVADFKQLALVRNKKIVQKHFDQMENIFPNTIEKQERNRLQHYERIMHRV
MIVFSILCLAYTSTFSLYPVCKSFVEYYFLGAEKFERRFGFLIWYPYDPTAKTWVYWLTYLLEVHGAYLA
GVAFLSADLLLVSSVTQLNMHFDYLSMELAQYEPDAKHEEKDLEFLNRIIKQHVNCMELSEHVDNIFSFS
LLLNFLMASLTICFIGFQVTTSSLEVIIMYCIFLLASMLQVFIVCFYGDELMTASLKVGDAAYNQNWFNA
SIKYKKMLLLIIRRSQRPSCITPPTFSAVSFESYMKVISMSYRFFALLRTTYYDD

>KNC21930.1 putative odorant receptor 45a [Lucilia cuprina]
MLSKQYFRVQKIVFTGLGIDATVKDSKYLIRRPVLLYGLIVLSFFHFIIVTHYIWINSGDFVEVTDSMPM
LCQLILSIWKMTIFLYKRREILQLINEIHAINLNAKPDEVFLVHRENSKDNFLCSLYLRLVTITGSFAVT
HPVMYAIYMYASSGVVILNEANKASYFWDFSHLPGYSLVFMLNGFTVYFVCVVSLALDSLFSWFVSNICA
QFHILCHRFEKLALNYSKTTITQDQQQEFFKSLVRGVQYHRQTLQLAETLNQVYGEIIFIKCTIVCIEIC
SLVFRASRPHDSLAEAVYKSLFLCAVALQLILYCYNGQRIKDEVTDAVYCAFDWSSLSKSSKGMLFVTMI
RSQKSSNVRGVFFEMSCEVLKQILNGFALIIIITDSL

>KNC21848.1 Odorant receptor 47b [Lucilia cuprina]
MKKVAIKKFTKHQEITEEEEEEYGLEEASLKSILVHLKDLRKVLRHKQEPGKISLVYMRNYMRLMFIFPR
TWKGESLLYRVINKFLMIMLIIFTVSITFDLYEASQDVLQFGEDLVVLIGIYLIFFKLILTAYYAQDIEY
IICEFAKMHKYFSQLKRSPKISHKIPAICLPPILTPNTTPYRAKYPFEWQSSDEHPIRFALVYIFQSLMT
LFVLLSILVIDNIGCHIFTQTTLNLKIFCILIRDMITQPADIALEELHKVIQFHQYIISLISKINVVYYY
NYTAQMAASTFMICLTAFEAMLAQDQPMLAIKFQIYMFSAFSQLFYWCATGNMVYYDSLDVADAAYEIDG
WYNQSKEFKYYLRFLIQRAQTPLVFQPKPLFGFNFETFSSILSTSYSYFALLRTMND

>AKI29048.1 odorant receptor 88a [Bactrocera dorsalis]
MAPQQEVFGAKSKLCAIEDLCAIEHPYQRYLGLKYVEFKRVNGRLVIPKSNILNFLLFLAVVDCTGNVIK
TAIAINDRDVTKAQEVFAVFGMGLVMTMRGFMLGLNRGKLLKMYNAIDRIFPRSEHLQQHMEVEKVHNYI
KKRFFYLHWFLTVSVCGFIFMPFVKFMAFHGFKSDAPVSEEFHVYASWLPFGVKDKVSTYPYIYVYELFL
ATAASHMLVVWDQIFVILISQLCMYYEYLGKLLAEMNVPDAMDPTKSDAVFKQLHDYIYMHQYLNNLAVQ
LNDLFNFSILSSDAGIAISICFNVVLITEAKNNLQIINYTIPLFVEVWLIYDASKWGQMLETVTARINER
IYEQQWYDSSIRFGKYTLMWIQSTNVPFRLTVFNLFYVNMKHFQDMMILAYQLLTFLKAKGYTK

>AKI29042.1 odorant receptor 63a-1 [Bactrocera dorsalis]

MYSISEIKELRTRNHWRIRELKRISYIIGINLNAQTKCKRWWRIVNILFIIASCIALYPHWLMIKQAEGD
IPLIAETSTTALQTTTGLIKMAYMLFTQHRFHRLLRKAETHELLQGIEIFQTDMPIKTSLKKEINAVMEI
NWKQARGQLLFTLGTCICIMSNYFFYAFFKNLYNHLQGTPNYVYILPFTGYPMFLHKGMASPYYAMDMFF
GACSLLVAGMSAISFQGCFLVLCKHSCGLVQVLCLLLKRSTSSLVPKPQRVEYLRYCIVQHQRTLEFINE
VNQPFRHICLSQFLHSLAIYGFVLFEMNFGLESNKITFIRMLMYLCAATTGDCTHYVNGQFLANELEKVP
LACYNCEWYHETDAFKKTLRMIIMRSNKKFCFQISWFTVMSLATLMGIFKASGSYFVLLRDIDET

>AKI29041.1 odorant receptor 59a [Bactrocera dorsalis]
MSPSPLSLPQQALAAVDTRSFFKLHWTCFKVLGINASTSSAYYLGYSLLLQVLVTLCYPLHLALVLFDSA
DASKNIQNLAICVICVVCSVKFAIYAARMSRIRVLESIIATLDARAQSPCERRYFVEIRKEIRRITLGFL
SIYAAVAVTAELMFLLRNEHNLIYPGWFPFDWRATDLKFYAANFYQIVGVTYQLLQNFINNCLPTIALAL
LSAHIKLLGIRVSQIGYAGESPEANEEELLCCIKDQEQLYNMLSVIQNIISLPIFLQFTVTAVNICLPLA
ALLCYVDAPFDRLFFVVYLFAVPLEIFPICYYGTTFQLLFDKLHVEMFFSNWVEQTHKYRKHMILICERS
LKNQTATAGVIIRIHLDTFVSTCKTAYSLLAVIMKMNE

>AKI29036.1 odorant receptor 43a-2 [Bactrocera dorsalis]
MTSYENLPLYAVNVKVFVKVGLINSTDWTKGLLFFLILIVAYVGQIINLCKSWNEDIGETTMNFHCFLFV
THCLIRLWIVVKKKNKFERFFQCVEQWHREIERNDDPQMVGILQEITKRTQLLSKMTIYVAAGGTLAAIF
YPLSFDGRKHMITMQFPHFDVLQTPFYEIFFLIGVTWLTPAFLVISLPFTNIFLISLMFGELVLKDLCVK
LRNIRSENEETMLQEFKECIAYHQKIIDLCDDLQDLLSIDGFFHLALFGMMLCMLLFFLSMIHDLRLILA
ALSFVSFTTYMLFITYYYANKLVTESLEVANAAYDTPWYRGNLEMRKCVITMIARCQKPLQMTAGGIYPM
TMETFQAILRVSYSYFSLLQGLNQQ

>AKI29035.1 odorant receptor 43a-1 [Bactrocera dorsalis]
MVTAVVDNPMLSVNVKLWQFLSVLFARDWRRCVALVAPVCLMNAMQFVYLYQQWGDLSTFILNTFFAVSV
FNALLRTCLIIKNRDKCEALMEELVTLYDDIQDSDDDYAKSVLAAATKSARNISIFNLSASFSDLIVAMA
YPLFQQQRVHPFGVALPGIDVTRSPLYELIYIGQLSFPFTLSSMYMPYVSSFATFSMFGKAALQILQNNL
RNLCDNMKSKTEEELFEILRKNIAYHARIARYVSDFNELVTYMVLIEFLLFSCVICSLLFCINITTSTAE
KISIVMYIGTMLYVLFTYYWQANGVLEMSHLVSDAAYEMQWYDCSPRFKRTLLIFIARTQNPLQIRVGQM
HPMTMEVFQSLLNNAYSYFTLLHNLYND

>AKI29030.1 odorant receptor 7a-3 [Bactrocera dorsalis]

MRKIADLFYGRGKDDFETTESFVLLFRGWAAVGFLPKIPKRIVDIIHQIICWCSILTCPVWYFAGLIDMM
DDLPITLLLSNLGVAINCIALPLKAIYIKVNMNHLHDINLLFKRLDERYQTPEENIQIRESVKISTRIFA
ACCTLYWFFGISSGLVPLFAHEYPHGNVFPFIDWLPEGNFQYWLHSIVEMVNLQYLLHLQSINDSFPAVY
IRNIRTHIRLLTNRVSRLGLDPDLSDQQNFEELVDCIVSHQEILVISDTVGPIPSLTTFFQFTVYAALIC
VCMLNMFIFGDLKVKVSTLIYLIPVVWQTVPTCYQASMLETDCSKLPEAIFHCNWLALDKRCHKLIIYFM
QCTQEEICFTAIKLFQINLGTNLSIAKFSFTLYTFIKEMGLDAHYNQK

>KFB47530.1 putative odorant receptor Or5 [Anopheles sinensis]
MVLPELQDPFAVMPLLLKLQRFVGLWGERQYRYKFRLAFTSFCILVVIPKLAFGYPDLETTVRGTAELIF
EWNVLFGMLLFSLKLDDYDQLVYRYMDIAKMVFRKTLPPELGDYLVRINRRIDKFSKIYCCSHLCLAIFY
WVAPSTSTYMAYLTVHNKSAPVEHVLHLEEELYWLKIRVSLVDYSIFTVIMLPTIFMLAYFGGLKLLTIF
SNVRYCSAMLRLVAMRVQLIDRLDEEQAEKELIDIIVMHQKALKCVELLEIIFRWVFLGQFVQCVMIWCS
LVLYVAVTGISTKAANVGVLFILLTVETFGLCFFGTELTTESFSVARAVYDCYWYQRSVSIQKKLKMILQ
RAQKPVAISAGKFCAVDVERFGNMAKMSYSFYIVLKDQF

>KFB42114.1 G-protein coupled odorant receptor 66 [Anopheles sinensis]
MEASNKFEQYMTFIRRLCRVLGFDVFNVEWKMNYRSYFSLFLCVQYLWWMIHSAIIAEGISEPLKTLAFL
GFFFQSTLKIYYTLSNHEKFQANYNKLAQSIYEGHIEGTAEQKKVILRVITVVLALVKVTVVIYGFSLVM
FSLYPAYMYFWEDTKVTIFPLHVPGINIYSLYGYSVTNMLHMLVAVYGLFGALASDTAFMMFVLHIVTYV
ELFRVECEKFESELLTSECRDRWHSKEYKALCRLSMLAIYQFHQDIISYMDSLKVCYYQTCMVQVATSSF
SIMFNLFLALTTDWYATYSFLAVSLFQLLIFCVLGNVIQVMNDRLNQVILNLPWYLLPNGEQKRFLFMLY
RSQLPADIDIRGFGPLNMETFTAIMQKIYSAFMMMYSFIEE

>KFB39839.1 odorant receptor 2 [Anopheles sinensis]
MLIEECPIIGVNVKVWLFWSYLRRPRLIRFLVGYIPVALLNVFQFAKLYSSAGDMSELIINGYFTVLYFN
LVLRTSFLVINRRKFEKFFEGVAIEYERLERNNEIRPVLERYTRRGRMLSMSNLWLGAFISACFVTYPLF
APGRTLPYAVSIPGVDVLASPVYEVLFVLQVYLTFPACCMYIPFTSFYATCTLFALVQIAALKQRLGRIR
GGAPSAGESRTLFVELKECLMYHKQIIQYVHDLNSLVTYICLLEFLSFGMMLCALLFLLSISNQLAQMIM
IGSYIFMILSQMFAFYWHAHEVLEQSLGISDSIYNGAWPDFDESIRKKLILIIARAQRPMAIKVGNVFPM
TLEMFQKLLNVSYSYFTLLRRVYN

>EDS45185.1 Odorant receptor 7a [Culex quinquefasciatus]

MEASARSRLWSRCKSRATAYYRALTRDFNYSTDFFFGQDFLMAIAGARLNSTNVRIRRWWNAYRAGGFLE
HFCDGAKSREAGNCTELRAGNGWNGVTMLRMMIILWHYDSLMEVRRYVNRRKFGKNLQLSLGIRSEAFYH
IRKIVFISTTVILTVMTSFLAVDFSDHHYFKIPFIEQFGTVVQKLCQKALNLAFYGVGVMTVFVYLFPYT
ILNGMMSELKVLAQAFSVVFDNTEMRVQRKLELEPGNSRQQDLMKKRFFWKFVQEEFALLNRIRPFLNAT
FLIVYYSTVMSLSSGAIYVSQMENVTIFSLNTLYYCVWVAFECGTLTRTVSLLTESQESIGWEVYNLDWP
EKLEWDDQFQEEYRSVRATMLNVMIVAQQPLGLNCYGFFEFTQDRFYELLNMAYSVYTFFRDFV

>EDS45068.1 Odorant receptor 83c [Culex quinquefasciatus]
MESPGVKPPKASTSLQEFRNSFETVRKVTYMVGINVFTLEPDLNVRFFGSAMFMALIYVLCFYTGWVLRE
AEGYRILEMVMILVLNAQGTNKMWIGFVHKPRYCRLFQSSEQIYESFDSDERNRPVLRDLVAKMNHLLKW
IVIVYASSGLLIVVLVALYAIVAREKFLALTIIVPFVDHTSLTGYLIVYLTHMAMAAFCANGYLASDTAF
IVTVVPIIAYANSLQNEIRNFNLLLQAPERDEDLIAEKLLRICQLHQMIDEFEQEAIAHFKGGCLNDQMI
HDIYEIGWHRLDKPQQQMVAFMLHRAQNAKELSVGGVAPLNLVTYVQGTKIRNF

>EDS45067.1 Odorant receptor 83c [Culex quinquefasciatus]
MTRETSKPTTNEGSLCEFRKSFETVRKASYMIGIDTSTPERDTNIRIIGSNLFLMALYVVCLYSGWFLRA
DWYSLLELSVCLLLTLQGSSKMWTAIVHKVRYFELFQTTERIYERFDSDERNRPILIDVIAKMNLLIKGI
VIVYVSSGLLIVVVVVLYASITRQKFLALTAFIPFVDYTTSAGYILHSMLHLSMIVICVDGYLAADVAFI
ITVVPIIAYGNCLQNEIRHLNLLLQTPQRNEKLITENLVRICQLHQMIVEFEQDAVEHFKFGCAVQILFQ
SGTLLVTIFLTYICGYLQAACIFMALFFQLTQYCALGTIVTAKKNNQMIIDIYDIEWYLLDKPQQRIVSF
MLFRAQTAKDLSVGGVAPLNVVTYVKVSSSLLFCLAYFYQVC

>EDS45066.1 Odorant receptor 83c [Culex quinquefasciatus]
MHNVVNERAAVNAGSLPEFRKSFETVRKASYLVGVALFTRESDLNVRFFGTSTIIALVYGCCLYSGWFLR
SDWNRLLELSVCLLLNLQGTSKLWTGFVQKLRYCELFQTTERIYECFDVDERNRPILREVVSKMNLFIKW
IVIVYRNEKLITEKLVRICKLHQMIVEFEQDAVEHFKYGCAVQILFQAGTLMITIFLSYICGYVQAASIF
MALLFQLTQYCALGTIVTAKNDQMTIDIYGIEWYRLGNTQQQIVVFMLHRAQTAKDLSVGGIAPLNVETY
VKGVDCQSERNSTLVAYATKWWLEEPGNK

>EDS44804.1 Odorant receptor 94b [Culex quinquefasciatus]
MDFVQRLRYNRFFKTSYADPSEFYDSLIVAPSRIAKVTGINVLSKDYKVFTFRLFTVFVGMVIYFYTSFV
TAYQVRRSTEELIFCLVTIGIGFQVPTKVFTCVIYRKEMVWIHQYARDLYDQECSPRTKTKLMSDVFLVS

VIVKVMLLCYAFTSISLILAPLLYTMQTGEKTLPFGFYIPQLDRDTWFGYFCNYAMQIYLTAYVSSLDMG
TDCIYMMTLMSSFTQIDLLKMSLVEMNKMIDNEAEDIDNFFIRVIKRHQEHLKYLKTVELVYRVNFFLTF
ACLSSVLVMAMFAALKLSWYQGYVFIAFLSYQLFFGCFLGTLLAIKNEQLQQAIYKVTWYKLSIPNQKML
QFVLKSSQESVCMSLIFAPLDMSTYLQVYKSIYSLFTMLLTVEDE

>EDS44719.1 Odorant receptor 7a [Culex quinquefasciatus]
MFNRIRQYLSNSWSSHVRMKPKTDFFYILNFYLSIAGVYFPVKNRMFRLTWKVFCVLVILHYVTMQRRLL
QILQTEQSFELLVNGIHVAGGATIILVRSLLIHVNFRHFNQARKYLNERSFREEDVDVARIRQQSYELSV
KVTIIFSINVLSQFVILIGSGITEMDPFLLPFSVKQFSLFEQKLYSNVYSGLFTVYSFIGASNFLTMYLG
LVGLRAELRIAVDSFGKVMDRVNNRLENDPGEQNFWKFLQDELNSCIEHHSTVLNQLKVFKNLTSMSLLV
SYYMTMAHTAIGVIHVLSKPSLDFLSVLAIDFTLRYLLEFYVFCHMVTSLNEEHSKIGLLLSHQPWISQL
RIESKYRSQYRQIRATILNVMIQSQRSLNISCGGIFELSMDKFTTLIKTSYTLMAVVWNMQQGNGLSW

>EDS44718.1 Odorant receptor 7a [Culex quinquefasciatus]
MFNRIRQHFTKFWRTHVQLTPQTDLFFILNFFLATAGVYLPLKNGFIKLCWKVYRVFMILHFAMILRRLL
LTLTTERTFDPLVNGIHVALGVTILLSRSMLIQANFKHFKTVRKYLNERSFRADDPEAVRIRQKSYEMSV
KVTIIFIANILFQSISITSSGMTDMDPFQIPFGMEDFSDFERSLFTGLYSLLWAVYSYVAASNFLTIYLG
LVGLRAEMRIAVDSFGKVMDRVEVRLEQKPGEQNFWKFLQDELNTCIEHHSEVLNQLKVFKNLTNLSFLL
LYYMTMLFIALGVIIVFFRATLDIFTVTAIDYTLRYLIECYVFCHMVSSFNEEHSKIGPTLSHQPWISQL
RCRKEFASQFRQVRATILIVTMQSQRSLNISCGGLFELTMAKFTGLVNRSYSVMMFVWNAKQGHGVAW

>EDS44717.1 Odorant receptor 7a [Culex quinquefasciatus]
MIQSRIARIVQHHASILEELKSLQNLTKLSFLAIYYSAIAFISMSILIVFKMHTFNVAMVNNKISDAVTN
LEWPERLRFSKDFTGEYKSVRTSLLVVMARAQHSLGLSCGGMFELDRTRFKSLVKATYSMVMFLWNVSPN
YAPIAFPISLTQLSPAMKRTVRKIYESLLLVYSHLGSVNFLAIFIPIIELLCELKIIRNSFAGIFQNKPR
YPSSKSAQTTFWIMTQSRINRLVQRHSDIIGELKSFEKLTRTSFLIIYYTAMLYVSMTIIAILNVQSFSM
ATIFLLEHILRMIVECYVFCHLATKLNEAHSQIADKIMNLDWPEMLHYSEDYPKEYKSVRTSLLIVTIRA
QHSLGISCGGIFEMSQDKFALLVRMTYSVLMFLWKFKAL

>EDS44577.1 Odorant receptor 7a [Culex quinquefasciatus]
MTFVNWLNSVRFWLVLRWRIFTHWWLNLYSKTFKFDHGADFFAEVKYLEIFCGFYAKLRSTGDKLWWRSL
RLVIAFRMSHITGKFIFTFLEEDDYRYKVLIHTATYLMFCNCIQMFLLRVAYKEITSIRTFINGRTFLPE

DSEAHRIRWAAYRKNLWTILVPIPSNIFIWIFLFASGAYKWRVVNIGPDTLASIPWLRTSVQLFYWLQYS
VGVEWFQLQTVLVNSMLYGLAGELGVVSYACENLVQSVDFAVQVDLLNTATSSDSSRGEAIFWKHFKIEL
DKCARAHSKVLDQLINLKQILKPCLLMYYYSLLIVNAIIICAVKNGYFGAFTVPATVMVIYLNVDFFFIC
YNMSRLDDLFAREYRDIKSTLKMMLTRAQAGMEFSCGGFFEMSLMKFAELMELTYNMVMFVLQFHH

>EDS44513.1 Odorant receptor 7a [Culex quinquefasciatus]
MANWRTYWYRFRRWVQSRRESYYEFNCDSEFFVGMNICALVGKIQMDSDDRGPKRLWKLFRVAMVLIFGL
IGWRFYLDVVEGELEFVKLINNVFICSTLLVTSVRSALIGWCYGDIGKIRAYCNGRQFLREDPEVFEIRR
KAYFVVKNVTVVILSNLVVFLSAPSLMIDVPAFRLPFTIPNMEWATYVCQKVHILMIMTVGVNFMMNVLI
IFMILFGLITEGKVADCAFSRVFHQALNRQTESGFWNELNDRFDECIEQHSLLLKKIALMRNLFESTFLV
TYYSTTLHIAGGAFFLISSFKSFDMYMLQMINVIVMQIIECFILSRWTTLLNDVHESIGDTVYGLDWPQQ
LQHSERFSAQYEAVRAKIVLTIERCNQPLRDQSDYHGSNTGGNLERIENLVNQHCETD

>EDS44239.1 Odorant receptor 7a [Culex quinquefasciatus]
MDTCAHQLKNRLNRFVLRTAGWYQDQKPFWLMRYLFAINGISLEFKCGGFWRFIWTVHRMLLCLHVMIFL
TDQFVTACAQERSFGRRLFNIHLILLTVVVMLRHLCMTLKITSMGTLKKYCLENEFLRRDEEAMAMREKM
WNRLKRMELGMVCQGSVQFLIFVLTDVQEAISMQFPFDFSDTSVVLQFMCKNLYTMYYACYCLAISMNCY
VLFAIMLCLITELMLIVDAFGSVFKDSLQPRQEYRFDMTKQDILNFWNALEEHIRKCCRQHVEFIAMIRV
LNTFTKETFLLVYGAALGFTAIEIYQILSTKQIGFYEFMILQDTIVFYVEFFCFCWLATKLNDLNNQIAN
RIYEQEWHSEMQYSDDLPEQYRSVKQSLLIVMINAQRSLGVSVGGVYELSLELMVELLHNSYSTLMFLLE
VTK

>EDS44180.1 Odorant receptor 83c [Culex quinquefasciatus]
MHRLPTVPKFTKSWEMFEYNLLFVRRLVDFVGLDFMIENYKFNWRTGSAVFFCVTVFGLSLYNTAFYYPD
MYKICEVSIPLSICLQSVTKLYYGYRHRHFYLETYDRIRQLHLKHQNHEENNAKLLLLIERIHVLSKLMT
LLYTCGGLSYLAYPVLMHFMYQERVLALALRIPFVDADSTTGYIITNLYHVILIIVGCAGISAADIVITL
MVGSLVGFVDVFTSDMNDLDRMLDSGDRNEEFIRDEVKSICGQHQHIIEYESDLDERYIVICFVQVFTSI
SGVVVALFLVYMIQFIPGYMLVLASFVQLLQLCLLGTVLTVKNEQITETTYNLRWYLLRKSEQKCILQML
HKSQSFVEMTVGGFAPLNLETFVSIMNRIYTYFMMLIQFLEQEE

>EDS44179.1 Odorant receptor 83c [Culex quinquefasciatus]
MHRLPTIPKFSKSWEMFEYNLLFVRRLVDFVGLDFMIENYKFNWRTGSAVFFCVTVTSLSFYNMAFFYPD

MYRICEVSVPLTLTLQNVVKLYYGYNHRHFYLETYDRIRQLHLKHQNHERNHEKLLLLIERIHVLTKLMT
IVYFCGGLSYLFYPIFMHFYYHEWVLALALRIPFVDPDSTVGYSITSLFHLTLIIVGCTGISAADIVITL
MVGSLIGFVDVFTNEMHELDQMLDSSERNEKCVREKVRSICLQHQHLIEYESDLDERYIVICFVQIFTSI
AAIVIALFLVYTIQFIPGYVLVLAGFIQLFQLCVLGTILTVKNEQITEATYNLRWYLLEKSEQKCILQML
HKSQHFVEMSVGGFAPLNLETFVAVILSTT

>EDS43416.1 Odorant receptor 83c [Culex quinquefasciatus]
MSVKTSTPEQSLVRNFEISYRLCEKIGLNALDRNYRPNWRTVYPGTVNLFALVQLLYLLGTERADLLQFL
LILPHFLFTAQGFHKFILCTRRSSKIFALRCKLTEIQALLLETKSSGPVICRTLNISRLLQTLIFVFYLA
ADVDLRVLSGFGGSHHPGSDHAMARFLVNTILHAVILLAAFHAYTGYECLFLGLIMPVGAYVDAFGNEVQ
ELNSVLSLKERNDPAIRARLNRIVKLHQLMNEYKAMLEELYNVLILSTIGLNYLGIISTIVVILKSNDRM
AYIFFALMFEILFRICLMGTIITVKVG

>EDS42729.1 Odorant receptor 83c [Culex quinquefasciatus]
MRQFLKRKLVLFNEQGLRSLRAVDIFDELMRFLHHFIAFVGADIFVEHFRWTFQTYVSFFSLVLFAVNLI
YTVTVHWGEWYAVMDTLSISGIGFQGMIKMWSGLSNVEFFRKKRADLRSMHARNGRHPENNYVLLKNVLV
ILYIFRFFSVSYSVAGLFLFVIPGYMLMVRNEEVLLFALEIPTVDPLTHTGYVVTMGYQVFMVFLAIAGI
LAADMGIMIIVLHIVGIVDVFRNTMNELDQLLEDPDCEDDEIHEKVTEICMMHKEIIGYEEELNEHYFFT
VFVQVITSVTCLSMTLFVIFMKGDWTRVMFLFATFFQLLEFCALGTSLTLKVTITSLTLSTHQVYLLCPS
DQRVAVMLHRSQNAVEMTIGGLALLNMETFVEVCIVERIL

>EDS42728.1 Odorant receptor 83c [Culex quinquefasciatus]
MDIRAKLRRQFRLLDEEALRRERAIDAYNEIIVYLRRFLYPVGMDVLDANFHWNWRTSLSLGCLVTYFSF
LVYTVYVYWGELMLTMEAFSITGIGVQGIFKMRSGFKYFDFFNQRYRLLKALHARNSCHQENNRALVFCC
LLIGVIFKVFFIAYLISGTGFLLIPMYMYVMHNEKLMVLKVNVPGFDPTTQLGFLVTTGYHTLLIFMAIA
GILASDLGIMLIVLHIVGIADVFKNGLKELDELLTDEDRTDEEVHEKVLEICIMHRELIVYEEELDSCYA
TIVFVQVITSVACLVLSLFIFYVTQNIGSGLFIIAAFFQLLEFCMLGTALTIKNEEILLALYDVRWNALS
NQQRKLWQFLLHRSQNAVEMTIGGVALLNMETFVEISSPFSTNLNLRLHL

>EDS37921.1 Odorant receptor 94b [Culex quinquefasciatus]
MEFVKRYFVRHRNLWSSEFKNPKALYESASESAMRLADICGAEIFRQNYTRKNGRLALLYADFTLYLVLS
FWCITVLWGQLLDVMFCVVMIGGAVQAFAKIHSYTNPTIHELQMCNLENFQNVRKYDEFEEAMWNAATFC

KFAVIFYAIFAKIMIGLIVAYSIVSSLVGEHYVLPFGYFFPWIDRDTLAGYLINFAYQSTLLVYGYCGLQ
ASDLVFIFFIIHAIARLEIIIVYLKKLDLLTQSPELERNGSVINELLDDIIEKHIQHTGNLSDLDDVLQN
GIYVNFGSLVAQTVFSCYILVTADEIWYTGSAVAFGSALQLFSACLMGTLLSSKNDQLIREIYDISWNNL
PIEAQKSLQLLLHSAQQPMVLSDGFNAVDLFYFVTIYKQIYSFVAMLLNFN

>EDS37919.1 Odorant receptor 94b [Culex quinquefasciatus]
MEFAKRYFVRRRNLWSSEFKNPKALYESGCESVMSLAYVCGSEVLRPNYTRKNRRLALLFADFILYLVLS
FWCTTVFWGRLNDVIFCFVTIGGAIQAFAKISSYTGKGLYDLHLRNLENFKNDRNYDEVQEIMMNVATIC
KLSVTVFKIIFSGMVGLVMFYSIGGSIMGEHYVLPFGYYFPFIDPDTLVGFVVNFSYQLTFIIYAYCGLQ
ASDLVFLYLIMHAIGATETIIIYLRKLNHLISSNGASSCLDLLHDIIEKHIKLTEYSKDMNDLLKMGIFI
NFGSLVAQTVFSCFVLATAEEIWYTGLAIVVLSTVQLFTACLLGTLLSSKHDDIIWMLFP

>EDS37547.1 Odorant receptor 7a [Culex quinquefasciatus]
MADFRRLQELRKWNGAEVPVTKSGPKPILSSILALGMVGNLREFHCDKEYFVHHDLLSAIGGLHLMGDDG
ETPVWWSIYRGFTALIARLRHRCSAATIQVSNHTLLNTVIQKIYIVLIFSATVNAVNNFVTIFMVLKGLL
TEAEVVANSFNRIFDQASVIPLTQEASSSNSDESTFWDQLNQQFAEHINQHCILLGHIQTIRPLLEGTFL
VIYYVTAFNIACGCFFVMAQDKPFNIYTIQVVNLIILQTFECFVYTYLTTKLKDVHSSIGQAVYCLSWPQ
NLKFSERFGPQYRAVRAKLTLIQERCNQDVRFSTGGHFEFTQERFTELMNMTYTLVTFLWEMKKGG

>EDS36623.1 Odorant receptor 83c [Culex quinquefasciatus]
MVQVKAKDTLPMRQFSKSYEMYDYNLLFIRWLADFCGVDMMVEHYRFNYRTALCILALSLSVILSLYTFI
LYYPDIYKISEVLVLTGFILQGSVKFYYGYSHRKFYQVQYGRLRNLHYKYRNHEKINASLLTLMERIHML
SKLIWVVFLLGAMGYTIYPLYMHFANGERVLMIAMRIPGIDTDLLSGYVATSVMQMAMLIVACTGISAAD
TVILLFVANLIAYVDVFTNELDELNAMLNAEVRDEAKIRQQVRVICSQHQDIIEYESDLDERYIMICFAH
VIGSIVAMSTALFLVYMIQFVPGYPILLAVFCQLLEFCLLGTVLTVKNEEIIRAIYSAQWYLLREPERRS
FALMLHKSQNFIEMTIGGFAPLNLETFVAVG

>EDS36622.1 Odorant receptor 83c [Culex quinquefasciatus]
MVQVKAKDTLPMRNFAKSYEMYDYNLLFIRWLADFCGVDMMVERYRFNYRTANCVTVLAFGGSVKFYYGF
IHREFYKIQYGRLRRLHYKYRHNQKLNAKLLLLMERIHILSKFLTIVFHLTGLGYSIFPLYMYFAHGERV
LMIAMRVPWIDADSNAGYIVTSFLQMVMIVIGCTGLSAADTVILLFVTNLIAYVDVFTDALDALNAMLNA
DVRDEWKIRRQVRKICTQHQDIIEYESDLDERYIMICFAQVIGSVIGMSGCLFLVYMVRFVPGYFILLTV

FCQLLEFCLLGTVLTVKNEQIVEVIYSTNWYLLREPERRCFALMLHKSQNFSEMTIGGFAPLNLETFVSI
MNRIYSFFMMLINFIEMDSYAA

>EDS36621.1 Odorant receptor 83c [Culex quinquefasciatus]
MRSFTKSYEMYDYNLLFIRWMADFSGVDIMVENYRFNYRTGLCVAVIWMCAINATYSLVYYYPDYYKMCE
VVLLFGILIQGIPKLYFGYIHREFYKLQYGRLRRLHYKYRDHEKLNANLLLLMERIHVLSKLLAIVFIFG
GLGYSIYPMYVYWANNERVLMIAMRLLWIDADSYTGYIVTSAVQMVMIVITCTGLSAADTVILLFVANLI
AYVDVFTNELDELNAMLNEEIRDETKIRQQVRTICTQHQDIIEYESDLDKRYIVISFAQIIGSVTTLSGY
LFLVYMVNFIPGYGLILATICQLLEFCLLGTVLTVKNEEIIAAIYNAHWYRLQRPELACFALMLHKSQNF
IEMTVGGFAPLNLETFVAIMNRIYSYFMMLISFIE

>EDS34874.1 Odorant receptor 83c [Culex quinquefasciatus]
MAQVSVVKPAFSDPARELFDRIFSNVSRLYGLIGMNVLDPRYRTRGRTLVPWVVTLFATIQVAYFLWRER
SDLLKVMVILPYATFPVQGLHVMLQSVTNPSNFKDLQAKSTEVFELLNENLTNRPRMARVLKVATFLQKM
IIGSYGGSNGFILVAPFIVWLISGTKSFIFLYRIPFVDEWSTVGFLMNLALHAFIIPIAYCKYVGIDCLF
IAMIVPIAAYVDGLVNEIDEINDLMEQAKPDQKVIRTKLIRIAKLHQLLLEYEQMLEDRYNFMALVRIGS
IVLGIISTIVAIYETGDHMAAFFSVFLLMQLTQNCLLGTVITIKNEKLTEALYDIKWYQLPVEEARQLTL
MLQSAQNAPTLSIGKIGPLNVESYESIMRSIYNFVMALFAFVG

>EDS34845.1 Odorant receptor 94b [Culex quinquefasciatus]
MHFYVSLKLKRAVTTDGCKVDRVIYVRMYVQMQYYGARGEFPKIFRTQQTTSAAAVDSFEILLTSEGADI
PARVWAPPGLLVGLRIQPQKLDACRRKLNPFGLVKAYIYSHDTIYDLLQYNMKRFENPKKIPEIHTSLMK
TASWCTISVKLIAPAYFTLAAFTVIFSVVISAFNRRFELPFGFFIPGVDRTTWVGYLLNLAFHTLQAFEA
GAGLLATDLCFFNLMINAIGQLDVLIIGLKKLGEAATSDELAETELLNDIIEKHVEHVKYLSAMESHMKA
GYFINNVCLITELVTCLHVVLTNDEIWFTGISLAICYSFQLVIPCFMGTFLSSKNDELIREIYNIPWNEL
PIPAQHSLQLLLHAAQTPVTLSDGFAPIDLDSFLEIYKKIYSYLAMIQNIN

>EDS34844.1 Odorant receptor 94b [Culex quinquefasciatus]
MNEKSKSLESGFDPPSFGLMQSIRRSLSLKYRTNNPIHFPMTLPLVVAGASIDQHDWSLMWFLSGLSTNV
MELLRRRIRQWRNGLESEFSNPKLLFEKTCDSVSKWSTICGWERFEPNYTRANPRFIFLMGLVKAYMFTH
DTIYALLQYNMKRFENPNKIPEIHNSLMKTASWCTISVKLIAPSYATMAALTVIISLLISAFERRFELPF
GFFIPGVNRATWIGYLLNLAFHTLQAFVAGGGLLATDMCFFNLIINAIGQLDVLIIRLRKLGEAATSGKH

VEAELYQFLREIIQEHMEHVKYLDGVASKSDYLKNKIVYECPHLPLQNDELIREIYITSWNELPISAQQA
LKLLLQASQKRSPYQTDSPRLIWIAF

>EDS34843.1 Odorant receptor 94b [Culex quinquefasciatus]
MEFLRRRFLCWRNGLESEFSSAKKLFEKACHSVTQYIWVCGCERFEPNFTRWNARFVFLMVNICVFMAGF
VKAYMYSHDTIYELLQYNLKRFENTKRMPEIHHSLVNTASLCTASVKLIGPCYASMGAFTIFISIVISLY
YGRFELPFGFYLPGLDRATWIGYLLNLAFHILQVFEAVTGLLAADMCFFNLMINAVGQLDVMVIYLRKLG
GAANTDKYEDALAEPELNELLSELIENHCEHRKYLTKMESLMQAGFFINNGCLIAETVTSLFVILTSDEI
WIPGILIAVVCTFQLIIPCFMGSLLSSKNDELIREVYNTPWNELPFPAQKALGTLLQFAQNPVTLSDGFA
PIDLDSFLEIYKKIYSYLAMIQNIN

>EDS31434.1 Odorant receptor 7a [Culex quinquefasciatus]
MFSALNHFRQRVHGQLAKIRRNLREFHCDKEYFVHHDLLAAIGGLHLMGDDALMYVLMTWRAYQTVTTCH
ELVQLVYNMLVYTETVVVLVRLVLIGSNYTHLGKVRGYINARNFDRQGPCALETRKRSYFVVRNVSLVSL
INLMMVAVPALVFDIDALQLPFQVSSHTLLNTVIQKIYIVLIFSATVNAVNNFVTIFMVLKGLLTEAEVV
ANSFSRIFDQASVIPLTQEASSSNYDESTFWDQLNQQFAEHINQHCILLGHIQTIRPLLEGTFLVIYYVT
AFNMACGCFFVMAQDKPFNIYTIQVVNLIILQTFECFVYTYLTTKLKDVHSSIGQAVYCLSWPQDLKFSE
RFGPQYRAVRAKLTLIQERCNQDVRFSTGGHFEFTQERFTELMNMTYTLVTFLWEMKKGG

>EDS31418.1 Odorant receptor 92a [Culex quinquefasciatus]
MAPVKRYASKQCLDGTPTDAEMYGTRTAFVLARKGGLLIPKLNGPPPPRTISTPPPARMATLPNLLQVMP
STLRLMELTGLWGSPKLIHRFVLMYCYGLLVMLLPRFVFGVGSEDVSAIIKSLAEVIFLVSIFVPTLIFA
AKLRGLERVVRGLGDIFREASNDDQSSECYDLIVKQNVKIEKFFNFLTIYMKFAAFGYCLPSIMITYWRY
FTNDGTSPVIFILPMEQEFYGLQIRTNLVHYHIFMVFSLLAYMVCSYFTLVKLTLPLFMLRYSSMTYRLV
AVRIRNLSVPEMTADDLKKVIELHRKAFEVTELIEEMCHIPVALEFLTCVLLWCLVMFYTSTTMGYDLFS
ILIVVLSSLVEAFGYAYLGSELSEQAEKVGVACYDLPWYVNCSKELKGAFQLMIQRSQKINNSITIPSVK

>EDS31359.1 Odorant receptor 9a [Culex quinquefasciatus]
MLFTKKRSFNGSMSFNAKVLRFLGYGWNDPAKNILERRLFIQIAFYFLWLDPPALAYGCRHQQDQLKLVL
KGFFEVMAVTGITVRMCLFARGSSILRQVFDDVQRALAIVSEDCSGEVRNILDHLEMSADKFAKGYTVGF
TLQCLLYGPVQIVLATGKYFRNGEMPTIAVVQAESCRRLETALFALLGYLYTSCIVQICYLMFIFSIIRD
DLSVLSMMGVAVQYNIFLIFAFSMLGTELIEAVFVYKSLSVADEVYNVRWYERSPEERRLLLSIQMRSQI

EASMTAGKFFCVNRATFAMAFMRGAIERSRFRMKPIPGRTYQSAKLATVISAGPASLHSVTAIGIIRHRG
KITVATISERKPYEFEIHASITPYKL

>EDS31358.1 Odorant receptor 9a [Culex quinquefasciatus]
MLFKKDKPFPMMETTVRLCKFIGLWHDTDLGKVCWQAKFTLASLTFWYIVPEICFVTRQDRELTIRLKGM
IEILAMSIFVVRIGAHVLHRGTLQRCFWDVQETLGQFVDSGHVEVQKMLEHLVSSASFITRSYTTALLCN
ASLYGDIPALIAIGQYLMGADIETFPPTILEADYVLFEHQSNILVWIPVLLVSIATQYSMLTSLAANECL
NWNLLHHVSCLFKMVKFEISHLGECSTSDEFKKRLVKIVTVHDAAYRNALCLETVLSPVMFILYCTFVTK
VCAMMLLVTIVDDFFIIAMMLFVTSYQMFLVFSFSMLGTELTEASSAISEAIYQCGWTERTPSEQKLLMF
VQMRSQRRVAITVWKFFALGRASFSVAIKTTFQYFTIMRQLCEIEV

>EDS29849.1 Odorant receptor 83c [Culex quinquefasciatus]
MRSFTKSYEMYDYNLLFIRWMADFSGVDMLVDDYRFNYRTAICVATVLMGAINCTYSLAYFYPNYYKMCE
VVVLLGILIQGTPKLYFGYIHREFYKLQYGRLRLLHYRYRDHEKLNAKLLLLMERIHVLSKLLSIVFIFG
GLGYSIYPLYMYWAHHELVLMVTMQVPWIDADSYTGYTVTTAMQMVMIVIACTGLSASDTAILLFVANLS
AYVEVFTDELNKLNAMLSAEDRDEEVIRCQVRKICSQHQDIIEYESDLDERYIVICFGLAIGAITTMTVS
IFLAYMNSYIPGYALVVVGFFQLLEFCLLGTVLTVKNEEIIEAIYSTDWYLLAEPERRCFALMLHKSQNF
TEMTIGGFAPLNLETFVAIMNRIYSYFMMLISFIE

>EDS29230.1 Odorant receptor 83c [Culex quinquefasciatus]
MSNKVREISCQAKFNQIFANVSRLLGVIGFDVLDPDFGPNWKMIYPCAIIFCAMLQILYLLNRSDMVQFL
VIVPYAMYEIKLVDIMVQCVAKSGMFQSLKARSDEIFELLCENPTNKQVMDKDLKVAIFLQKFIFGWNVQ
TSVLMIPGPVLVWLVSGKMILFFLYRLPIIDDQSAAGFLLNLIYHAAILLIPFCNSGGIDSLLVALIVPI
SAFINAIANEVFEINELLETDRRDELAIKTKLNRIARLHQLLLEYVSLLEDTFNGMALVRIGTIVLGTIS
AIITFFETGDPMTFVFIPVLFVQLSQCCLLGTIIPVKNEELIEHLYDIKWYLLPEGHAKHVSLMLHQAQN
APTLTIGKYAPLNVESYEQILNSIYSFVTFLITFTG

>EDS27901.1 Odorant receptor 7a [Culex quinquefasciatus]
MEASTRSRLWSRCKSRATAYYRALTRDFNYSTDFFFGQDFLLAIAGARLNSTNVRIRRWWNAYRLVSCLP
MAVVFWNTFDAVRHCAKLEILLSCVQAVIGIGISALRMTLILRHYDSLMAVLRYVNRRKNRVQRKLELEP
GNSRQHDLMKKRFFWKFVQEEFGDCVKLHIEFLALLNRIRPFLNATFLIVYYSTVLSLASGAIYVSQMKS
ITLFTVATLYFCFWVSFECASLTRAVSILTESHESIGWEVYNLDWHEKLEWDDQYRDEYRSVRATMLNVM

TVAQQPLRLNCFGFFEFTQDRFYELLNMAYSMFTFFRKFV

>EDS27531.1 Odorant receptor 83c [Culex quinquefasciatus]
MSSSEWLNGNFQLSYQLLDHIGGNIFDHRWRPNRRTFYTVAVMLLAAVEVLYLLWTDRSDVVKFLEAFQN
AMVVSQAVHMRFILMIYTVTSVVIVLGPILLWIILDEKIFIMVYFLPGIDGNSTEGFLVNFALHAVMLPV
AIGVYVSLECTFMALIIPVGAYVDAFGNEVSKLNSALNLTERNEATICDQLNRIVRLHQLLIEYATTLQE
LYSMAILVKISLVYLGIISAIVVIFKSQDDRIAYIYLMLQFEHLTRYCMMGTVITDKNDQLLEYIADINW
YQLDQGQAKRLILMLLQAQNATSVTIGNFRPLNAETFVEILKSIYSYVVVLNALFD

>EDS27354.1 Odorant receptor 83c [Culex quinquefasciatus]
MFKRVGRVKSASGEELFGRLFANVSRLFALIGVDALDPKFRYKLLTMYPVLAALIAATEILYFLGSTRSD
MIQFMVTLPYASFVTQGIHALALALMNPASFSSLHAKSYEIFKLLNENHGNRSILARPLRIGTVIQSMMT
TLYVVILMFILFLPLAAWWIAGQKILILLFRLPGVDESCTEGFLETMALHVALLPSAFCGFLGIDCLFLA
LIVPIVAFVDAMANEMDELNQLLQSPESSQGHEIRSKLSRLVQLHQLLIKYELTLEEHYNVVVFVKIGAI
VFGMISTILVMFNVRDIMAFIFTIVLFEQLTQYCLLGAVISSKNNQLINHLYDIKWYLLPADQAKHLMLM
LERAQNAPSLTVGKIATLNMELYDNIMKKVYSYVMLMATFLEQ

>EDS27353.1 Odorant receptor 83c [Culex quinquefasciatus]
MYRGAAKERPATAEELFHKLFSNVSRLFDLIGVNPLDPDFGPNRRMICTWIAFLVAVANVTFFLWKQRSD
MVEFLMVVPYVSFVIQALHMMIKAMVNYSNYRLLYAKLKQILDMLNENSTSRKLMAESLQTGTVLQGLLA
VLYTVSVIFILAVPFFIWCISGDKIFLIMYRLPAIDENTTLGFLETMTMHVAILLASFCGFVGIDCFFLA
LIIPIIAFVDGIENEIHELNQLLDLEEASPIQSKLNRIVQLHQLLIEYELMLEDIYNFVMLVKVGTIVIG
IISAILTIFTNQDKMAFVFAILLLVQLTQYCMLGTVISSKNNQLTEHLYDIKWYLLPAGQAKHLVLMLER
AQNAPTLTVGKFATFNMQFYDRIMRKIYSFVMLLATFLESA

>EDS26719.1 Odorant receptor 83c [Culex quinquefasciatus]
MVFILKATTVIYVTAGGAIIFSVVLYSAFTRQHFIAISVFIPYVDHTSSIGYMLTFVVHALMIVFCCNGY
LASDTAFMATVVLIIGYANSLQNEIVNFNHLLTMPKRDEKLISEKMARIGHLHQMVVEFELDAVKHFQGG
CLVQILTQAGAVLICIFLGYICGYVQAFSVLVGLCFQITQYCALGTIVTAKNEQITRDIYDIGWNLLQKP
QQKMIVFMLHRAQVARDLTVGQMAPLNMVTYVKNEQITADIYDIGWNKLQKPQQQMVAFMLHRAQIARDL
TVGQVAPLNMVTYVKVGRRT

>EDS26718.1 Odorant receptor 83c [Culex quinquefasciatus]

MENSNRKVSANAKPTKQNKNHSSRSSLDEFIKAFDTVHKRTSFVGLDFKTTEWDINIRSVGLVLFMLTTY

GLCFYTGWILREDWQSMLETLLILVLNMQGTSKLWVGFVHKVRYSKLFETTEQIYASFDADERNRSILRD

MVVKLTFFLKGMTIMYEMAGGVVVIFVALFVVITLFIPYVDHTSWIGYMITFIVHSLMVVCCCNGYLASD

TAFVYIVVPIIGYANSLQNEIVHFNKLLTMPKRNEKLIAEKLDRIGKLHRMVIDFELHIIEHFQVGCLAE

ISLQAGSVLLCIFLSYICGYVQAYSILVVVFSQIMEYCVIGTIITAKNEQITADIYNVGWHLLEKPQQKT

IMFMLHRAQTARDLTIGKVASLNMITFVKIMKTTYSFFAMLVTVLE

>EDS26623.1 odorant receptor Or1 [Culex quinquefasciatus]

MAFLNTLLFRLFFWKNLWDPRNRLESFKRQLTLLKWIGLWPPLDGSDQRRLLYRIYGWTLRIVFLYAFTL

TQVMFFLNVETVQDLANALYALMTQLTLIVKLRIFCSKIDPIQVLVLRVRCSMFHPQCDAETKEVLRAMN

TTWFFGTLFHVVTYATVTFWAISPALKGEFALLLPSWFPFDYRQSAWVYGAVWFYQTLSLYICATFNVAT

DTMIIGLISHTGGQVGRLGVLFSKMGHVAPGAKRQIDLSCKICNSLSPLLNNIEKLAKIHGEPYAYLIEL

ILFHKEILRFAYEVVDIFNVSIFSQIMASVIIICMTALKVISDRNVVSMIGNLIYLLTMICQILQFCYVG

NDISYSTGKFNEMAIFSNYPEFNKTTARAFLIYLTKVIQPADIKVGKVFKFSLTLSTFLWILKTSYSYFA

VLNSVRS

>XP_011194821.1 PREDICTED: odorant receptor Or2-like [Zeugodacus cucurbitae]

MASYQNLPLYSVNIKAFVKLGLIESNNSTRRFLLGIIIIVTYIGQLTNMFRTWDVDIGETGMNFHVLALV

THYLLRFIIIVRKEKKFERLFQGIEPWYTDIERHGDPHIVSILQKITQKTQRLTRLSFYASVVATLATFI

YSLSFDERRLLVTVQYPFFDVLQTPFFEFFFLIQMVWLVPTSLLVYLSFTNIFLTSLMFGELILKDLCLK

LRNIRSENEMTMLKEFKDCIAYHNKIIDLCGDIEDLISMDAFFHVTSFGMMLCMLLFFLSMIDDLELIPA

VLVMMGFDMYLIGFSYYYANNLATESLEVANAAYDTPWYRGNLEMRKCVLIMIARSQNPLQITAGGLYPL

TMENFQAILRISYSYFSLLQGVSQQ

>XP_011192525.1 PREDICTED: putative odorant receptor 85d [Zeugodacus cucurbitae]

MSNQPIQFERFTILANAYYTSIGLDAYEKTGQRTSNIRRRLLSIFFIFTIANMNITLLSELLYILMAFVS

NNNFVEATMLLSFVGFVIVGDVKIYSIWRQRERISAMIQALHALYPQTLAEQAKYAVERELQRYKRFAYA

FVLLHELLVWSYNLFPLLNYFIYEVWLTWRVVGKTLPYNCWTPFDWHNSDWRYYTMYLTQIAAGQACLSG

QLANDLLLSAVAVQLIMHYRQLARRIEAHVADSRDDSASKRRGESANECAAHDLHFLRTIIAYHQQILNI

SQVLNDVFGISLFINFTSTALIICFVLFQITIGANIDSIIMLAFFLFCSLVQIFLICYYAQQLLEASEYI
SFAVYNHNWFVADLRYRKMLIFIMARAQKPSKLQATSLVTVSMSTMTDLLQLSYKGFAVIRTMYAREPKS
VSN

>XP_011192524.1 PREDICTED: odorant receptor 85c [Zeugodacus cucurbitae]

MAPLRVCPSIMSKIIRFEAFLRIPNFFYRSVGVDLWNTDGGPLQDAVFYFGLLNVNVWLLSELVFAVLMV
SKNFIQATMTLSYAGFVLVGSIKMYFMWRKKAEMKRFLQLMNAIFPRTEQQQKIMHLRRHLRQSTIVMSC
FAMMFMVLIWTYNLYPYMQRQIYDRLLDVRSINKTLPYESYIPWNWHEHWTFYLYYTLQSIAGYHAASAQ
IASDLVLCAMATQMIMHYEYVAQKITEYQPQVGADSGSKNEAYCRDMKFLCDIIAYHANVLSLSDIMNEV
LGVPLLVNFMTSSFVLCFVGFQMTMDAEPDYMVKLFLFLFSSLVQICLICQYGQLLIDSSSNIAHAVYNH
DWVHSHVHYQRMLVLVAVRAQKPAMLQATSFVRISRGTITDIMQISYKFFTLIRTMYSN

>XP_011191673.1 PREDICTED: odorant receptor 94a-like [Zeugodacus cucurbitae]

MAIDTMVNFRTIVRILTLSVTFGFILLLSLEIIYSDGLDHAIDVLKYLLVVMALGIKVLNAWYYTRQITE
VMYEWENSELFVLRNDDEKQMWAKTQKTFRKLGMTSFGLGFNSAMCALLGVLLMGATEQPYALWMPTNWR
DKYYWQMYIYQCLSMPFICFSNVTNDVFQAYLLLHLTLCFRVISMRLERLANAGADGAITAELMNDIKMH
QRVKEMAISCEHIISLSMLTQITLTFLIICFIIYNLANANFREDPVHCLAMLQYALIVSLQMFLPCYYGN
ELTLESEKLSINLYSSDWTGMSAYNRRFIFHYMESLKKPLVLHAGSFFEIGIPIFAKAMNNAYSLLALLL
NVNDDEQ

>XP_011190505.1 PREDICTED: odorant receptor 24a [Zeugodacus cucurbitae]

MLLKFLSQNYPTEKNVFLIPKFALRIVGFYPGDSKSRLMHAWLIFNMFVLVYGSYAEFMFGVHYLSIDVV
RALDALCPVASSIMAIVKLSFLWWHRAELNCIIKRVAELTAEQKSPLKSYYKHRYFTTATRLSAAVLCFG
TTTSTLYTIRAAMVNYSSYLREEKIPYETPFKMIFPQPLLSMPIFPLTFILSHWHGYITVAGFAGTDGLF
LCFCKYIGTLLKALQFDMKDLLSDVDSVTRKSTSEYEFRESLKLIIARHNEIIDLVKRFSAVMSGVTLAH
FVTSSVIIGTCVVDVLLFSDLSGIFVYSVHTMAVTSELFLYCLGGTVVIECSSQLATAAYDSQWYTHSVE
VQRMVLLIIIRAQRSLVVKVPFFAPSLPALASILRFTGSLIALVKSVV

>XP_011189274.1 PREDICTED: odorant receptor 7a-like [Zeugodacus cucurbitae]

MFDLVKGRGRNVFASRDAVIYLFNTFRFVGLNPPPQCRFLYYFYSGIITLFVVLLSPIIFNVGWIRDRNV

LSVMEILNCVQAALNVVGVPIKSITLAMSLERLRSVEPLLSKLDARYTEPEDVAKIRSCAIIGNRIVFGY

IISYMMYETLTVVSALLGGHAPLTLWIPYVDWHRSMWEYWLQVTFDGAMLFFLLFHQILNDSYPAVYIYI

IRTHVQLLSNRVKRLGTANKSQDETYHELQDCIVTHQEILRLVRVVEPIISLTLFVQFFIAAAILGTTMI

NIFIFADFATRIASLTYLFCVLLQTSPTCYYATHLQSDCQDLTMSIFHSNWLAQGKRFNTLLLYFLHRSQ

ADIPLFALKLIPINLSTSVSIAKFSFTLYTFIQKMGVGKNLK

>XP_011188666.1 PREDICTED: odorant receptor 59a-like [Zeugodacus cucurbitae]

MSPSPLSLPPPPPPLAAVDTRSFFKLHWTCFRVLGINAPSSNTYYSVLLQVLVTLCYPFHLALALFSSPD

ASINIQNLTVCVTCVACSMKFVFYATRLSRIRELESIIAALDARARSLCERRYFVQLRKELRRITICFLC

IYTVVGVTAELMFIFHNERNLLYPAWFPFDWRASNLKFYAAHSYQIVGISYQLLQNFVNDCFPTMALALL

SAHIKLLGIRVSQIGHETASLGANEAELLHCIKDQEQLYNVLNIIQNIISLPMFLQFTVTAINLCLGMAA

LLYFVDAPFDRLYYLAYLLALPLQIFPICYYGTTFQLLFDKLHVEMFASNWVEQTHKFRKHMILFCERSL

MSQTAMAGGIVRIHLDTFISTCKAAYSLLAVIMKMNE

>XP_011188299.1 PREDICTED: putative odorant receptor 85e [Zeugodacus cucurbitae]

MDTRALGDTTLLYSHQDKPRIPDLFVAQIIFLKATGQIPMNFGYGLGYGFCFIIIIQSLHMAVLFLKTAY

DKLLDGELEEITDALTMSIIFSFSVYAGCYWLLRWQRLLAFLQCISHHYWHHSLPGLSFVCWHRTFVLAK

RVTIAWTLACVAGTVLYGLGPLVMGAHVLPLKCWYPFDPLQPYVYELVYALQLSAQIIMGATLGNCSALF

VSLIIILMCGQFDVLYCSLKNLNYYGRLRACCEVEKLRNEQAALPKISDDELNQYMYCREHLTNLSTLQHL

YTQEPAVTLPKALHMAVVQCVQLHRFILDVCKEFEELFNPFCLVKSMQVTLQLCLLVFIGVAGERSTVRI

LNLAQYAILTFIEVLMFTYFGELLRGHSVRCDEAFGRSQWWTHSVAIRKDILILLANSKRAVRLTAGKFY

AMDVERLRSVVAQAFSYLTLLQNLAAKNPK

>XP_011188298.1 PREDICTED: putative odorant receptor 85e [Zeugodacus cucurbitae]

MKERAERNTRELESNKLLYSNEDKPRIPDLFVAQVICLKATGQIPMNLGYGLGYIYCFIMIIQSLHMAVL

FLKTAHDKLLHGELAEISDALTMTIIFWFSVYAATYCFLRSQRILAFLQRINHHYWQHSLPGLSFVCWHR

TFVLAKRITVTWTLSCVAATILYGSTPLVMGVRALPLKCWYPFDPLQPYVYELVYALQLSTQVIMGVTLG

NCSALFVSLVILMCGQFDVLYCSLKNLSYYGHLRAFCEVEKLRKEQSALPNTADDEVNQYMYCQEHLANL

STVQHLYTQEPAVSLPEALHLGVVQCVQLHRFILDACKELEELFNPYCLVKSIQVTFQLCLLIFIGVVGE

RQIVRILNLAQYAFLTFVEMLMLTYFGELLRGHSVRCGEAFWRSQWWTHSIVIRKDILILLANSKRAVRL

TAGKFFAMDVERLRAVVTQAFSFLTLLLKLAAKNPK

>XP_011188297.1 PREDICTED: putative odorant receptor 85e [Zeugodacus cucurbitae]

MKDKTARDVRELESSTLLYSDEDKPRIADLFVAQVICFKATGQIPFNLGYGLGYVYCFFVITQTLHMAVL

FLKTSYEMLLNGKLEEITDALTMTIIFWFSVYAACYWLLRWQRLLVFLRRINHHYWHHSLPGLSFVSSHR

TFVLANRMSIVWTVACVAGTLLYGFAPLVMGVHVLPLKCWYPFDPLQPYVYELLYVLQLSAQMIMGATFG

NGSALFVSLVILMCGQFDVLYCSLKNLSYYARLRSSFEVEKLRNEQAALPITSDDELNQYMYCQEHLTNL

STLQLLYTQQSAVTLPEALHMAVVQCVQLHRFILDACKELEELFNPYCLVKSIQVTFQLCLLVFVGVAGE

RSMVRILNLVQYVTLTFIELLMFTYFGELLRGHSVRCGEAFWRSQWWTHSVAIRKDIIILLANSKRAVRL

TAGKFYAMDVERLRSVVTQAFSFLTLLQKLAAKNQK

>XP_011185366.1 PREDICTED: odorant receptor 13a-like [Zeugodacus cucurbitae]

MDYFVPLQFDNRPIKLPIQITGYKFNCLWPLKEDASVLWRLFNNVCLSVSVLCYIGTILGEFTFIAENIS

DIPAVAECLCTSFMGVQYIIRIFVLLSRQRPLRQLLRNFYRDIYFTDADDAALCKEINSIIRFINIFTQF

YYVPMVLILGLYVYEVASVGMASPDKPFIYRMSFRWYDAQAPLQFIITAIYSGWLTISCVTIWTAEDYTL

CMVLCHASFRYKKLRLDLEQLLETARADLRSGETRCTNLNLHIAFRRRLCDIFQRQQRLNGFVAEAKAHF

THQIFYVMSFGVLLLCVVSFQFQSSPISVEWSKYISWIISQTSQFLLIGYFGQMLMDETTELRNSFYCCR

WEDLLALGDLRSNKLLLGDVQFAIMNSQEPIVFDGMKFFPLTYSTVSSALRSAVSYFMFLNTMSEN

>XP_011184786.1 PREDICTED: odorant receptor 42b-like [Zeugodacus cucurbitae]

MPNLIRVGGACIYKSRDSLTYLFKIFTFVGINPSEQQSRKYYWLYYSYSLTVNFICCLFSPLSFHIGYIK

LWHVLNNNQLLAAIQNAVQVTGIPIKILVITWYMKRLRQAFKILDQLDVNYTQHEDLAKIRECVRRCRKI

VLIFCLPYYSFELSTIALGLLQKRAPLAAWVPFLDGQRAAWEYWTIVLWDTFVMFILLSYQLGGDTYPLI

FLNIIRTHVQLLVTRVSRLGRDGALSADEHYAELLACIRTHVQIVSIANIVAPVISVTLFTQFATTATTL

LTWFGNVEYPENIISFAFFSCQLLQIFPCCSSASQLIADCERLPDAIFHCNWVDQDRRFRRAILFFLQRT

QKPMRFWCLKLFVVKLETSVAIGKFAFSLYTFIQESDVGRKSNN

>XP_011184667.1 PREDICTED: odorant receptor 10a [Zeugodacus cucurbitae]

MNFRFLSRTFPLRDYYFYVPKLCLGALGFWPLDTCEPGAFNVWAWVNLIILTIGVVTEMHAGCLALRTDL
ELALDTLCPAGTSAVTLLKMALIYYYRQDLAWVLKRMRDLVYERDGELLANLEKHIVRAHAVMAARLNFI
PFVMGFITCTSYNLKPLLMTLILYVQGREPMWKLPFNMTMPPFLLRAPYFPLTYIFTAYTGYITIFMYGG
CDAFYYEFCSNTAALLELLQNDLKSILSFGGDKFTLTAEESTVLEWRLVQFIKRHNDIIELTRFFCKRYT
VITLAHFVSAGLVIGASIFDLMTFTGFGIVIYIGYTIAVLGQLFIYCYGGSMVAESSVQLATVAFGCDWH
ACNPRLRRYVLMIIIRSQRAISMSVPFFSPSLVTFTSILQTSGSIIALASSFK

>XP_011184142.1 PREDICTED: odorant receptor 83a-like [Zeugodacus cucurbitae]

MSPPSKRNIGLTGSCTNPNGCISNEKRSDLFLYVRWLLFFSAIRPIPFDKHLPRRMHGHSVLVNVIWEIF
LYLVVLHILVLFIITIYLNYDNGDLEFLISCGIQVLIYLWAILIKVTFRRIYPELVNGIVDFVNEEYVQH
SALGFTYVTMKECVDRVNGGIRIFVPCCFSAVIYRFILPIIYNDRSLPLPCWYPVNYKAPFIFQILYFFQ
ILAQLQMSAAFTVSSVYFISLCFLLSGQFDVLNCSLKNIVATTYIYMGASKHELIELRDNERIPGEEINE
FFVAKELPFDLDCLPHILNPADTARTRSFREAFNYALGSCVKQHNFILNALLKLERLYNLLWLFKTLDVT
LSICMGTFDVVKSSDEKSFLQLLSLGQYLFLGLWEIFMICYAGEIIYVNSQRCDEALLRSPWHLHLREVR
ADFLLFLMNAQRAFKLTGGKFYPLALEKFRGIVSTSFSFYTLLQNLDERN

>XP_011184140.1 PREDICTED: odorant receptor 83a-like [Zeugodacus cucurbitae]

MADEETQKTGIPTTVNKGYPPYMERRDMFRFMRCNLWFTAMYRLPLERYFPACLQCLAITLDWAYEVFLY
LTLLHIDILFMCTIYLNKDKGDLELIVSCMIQTVIYTWAIVIKVFFKRIKPKRIRELMRYLNEDCRTRSA
AGFTYVTIKESTDLANVWTTVFLICCYAGVTFWLFVPIFNQDRSLPLACWYPIDYKVPVVYEVVYFLQTV
GQLQVAGAFGCTSAFYLLVSVLFSGQFDVLNCSLKNILATTYLSLKNPKSELITAEIIKKNPKDEHFYEA
FKIAFRSCIAHHRYILRGLKMLEELYTYLWFLKTIEVTVLVCLVAFVWVKSTAANSFLRLLSLSQYLLLA
LWEMFMICYAGEIIFLNSKRCDEGLQRSPWYLHSSEIKQDALFFILNAQRPFRLTGGKMYNLNVKKFRSI
LTTSFSILTILQKMDLRQPQPK

>XP_011183574.1 PREDICTED: odorant receptor 74a [Zeugodacus cucurbitae]

MLYRPRLNNGKLIPLSWPIVAYRLLNNICWPLRDHASLLERLFDRFFWSLGFFIFVQHNDAELRYILVNN
NNLDEMLICGPTYLILVEIHLRAFQLGLNKEAFKRFLQKFYAEIYIDEPAHPKLYASIQKRLRPIWFYSL
LYFSTLSSYVIMPFVNYLNNVKAPLYKMYYPFDITPNPIYVAVVLSNIWVGFTVITMVSGEDNILSEVML
HLNGRFLLLQQKLGQDAERLLHAGDDRNIADDLRQRIVEAIEENVRLYKFAEDFEREFSFRLFVSLSFSA

GLICVLGFKVYTNPMASFGFMFWICAKVMEMLLIGQLGSSLIYTTNEISSTFYKSHWELVIQKSSDTNAN

VRLMKTLLLAITTSQKPFVLTGFNYFSVSLTAVLKILQGAGSYFTFLTSMRK

>XP_011183261.1 PREDICTED: odorant receptor 42a-like [Zeugodacus cucurbitae]

MLCKMSKLQPQLTAVMAKDVKTAQRNTNVQRKPLSVLEAKNLVKTLSEETVEEDVVQPVSTQDTTKYLFK

AAFLMGIVMPSRYRALYVLHSFWVNFLTTFYFPIGFTLIFFTLSDEINISNLLTSLQVIFDVYGGSAKFI

VMMCMLEKLRATQAITQQLDRRCRAADEIAELQKMVRFGQKVVIFYLTIFLCYSGSTFMASFFSGYPPYS

LYFPFLKWRRSHSEFIIASFLEFVIMDLACLQQTVNDGYPVIYINMMLCHMKILQLRVQKLGNNTALTLE

EHLSELKLCIKDHQLLIELYDIISPIVSVTLFIQFTLSAVCIGTTLINIVIFANEFQTQVACCFFILAVL

IEIFPACYFSQCLIDESDNLSDVIFHSKWVEQSKEYRKLMIFFLQRSQRPMFLTAGKLFPVTLSSFVSIA

KFSFSLYTFIEKMNLKERLGIE

>XP_011183038.1 PREDICTED: odorant receptor 88a [Zeugodacus cucurbitae]

MALRQEKYGTAKLDDLCDILHPVQRYLRLNYLDFRRVNGRFAIPNSKLLNICLILAVFDCIGNCIKCVKA

INAGEITKAQEIFAVFGMGFVMTMRGLMLALNRVQLSNFYNKIDCIFPRSAHLQQHMAVEKVHSYIKRRF

YIMHTLMTVTVAAFLTTPGVKFMVFHDFDSDDSVADEYHVNPSWLPFGLKDKVSTYPYVYIYESVLAAAA

VNMIITWDELFVVLISQLCMYYEYLGRLLEEMNVQDALDPTKLDAFYEQLHEYIYMHQYLNKLAVEFNDL

FNFSILFSDAGIATSICFNIVLITDATDYLQIVTYTSPLFVEVWLIYDAAKWGTMLETVTGRINEILYEQ

KWYESSVRFGKYTMMWMQSTNEPFRLTAFNMFYVNMKHFQDMMMLAYQMLTFLKSKS

>XP_011181975.1 PREDICTED: odorant receptor 82a [Zeugodacus cucurbitae]

MPEDLFRIQRNCLRLMGHQDIYDDDNETAGDEQKSKSWQQRCFRHSQTLKYALLLLLMMSAQLPMMDYII

YHIDDLALATACLSIVFTNVLTVIKTSTFLAYKREFKSLMAEFERMYDELHEAGAKRCLVTVNVGAKRFV

KLYFYSVSCTGLYFTIKPLVSMIWAKFQTKPLVLELPMPMRFPFDFESPPGYQFAYIYTILITIVVVMHA

TSVDGLFVSFTTNLRGHFQALQYFIETNTYDKSDALIQRELRIYVHYHVRLLELSQSVQRIFKPIIFGQF

LMTSLQVCVIIYQLVTNMGVIMEMVVYCTFLSSILLQLLIYCYGAEFLKIESSAVGTAVQMSQWYNLPPR

HRHVLRLMMLRSQREIIISAGFYEASLANFMSILKAAMSYITFIQSIE

>XP_011181972.1 PREDICTED: odorant receptor 7a-like [Zeugodacus cucurbitae]

MGISPPKNAGPLYYMWSLIVNLVCIITSPITGIVGFANKYLQDIITTAQFLSGLQAGLNLIGLPVKCATV

TFALKRLRGMESTLAIMDARYTRPEDVALIRKAAVMGNRLTLYDSYGPVYIYVISTHFQLLVRRVGNLGT

DATKSKDDNMKELVDCVVTHQQILELLATIEPVISTTMFTQFLIISSILCVTMVNMFFFADRSTQFASTL

YFLCVLLQTSPCCYFATELKADSEKLPLAIFHCNWVEQDQRFRKVIIYFMHHAQISVELMAMKLFPINVG

TNISLAKFSFTLFTFLKEMGIGQETTN

>XP_011181266.1 PREDICTED: odorant receptor 49a-like [Zeugodacus cucurbitae]

MDFVHFFWFPNALYRVVGYDFQQLARAHWRQVIMKAFLIFTTISGICTRIYMLFQLRELILNGDILNSIR

LGVYISYAIDSNVKFFVFLLNAQRLRVIYQTLYNEYPVTPIERKLYQVDKYSFKRAHLMMVVYLSVTNSI

LLGPMLQSIFMYFVNLFHYGYAVAEFPYLHPTPVLYNFNYCTPHYYILIYISEYLNGHFCTTTNLGADLY

VCTFAGQFCMQLEYLGNSLETYEPRVENSKTDCEFLMEWIRKHQLMLDLCCELNEVFGTTLLFKLISNCT

VFCAIVVQLKLEGFGIGFFNFLSFFFVTVAQFFMVCQYGQKLITISEDLSLCAYKNRWYNGSQTYKILLF

NIIARAQKPVKLTARGFQPISLATFQIVMTMTYRVFAVLQRALD

>XP_011181254.1 PREDICTED: odorant receptor 45a-like [Zeugodacus cucurbitae]

MRITILKDIKPITDAFVRIGLSIELGGSKGIFSHPERYATVMIGVIVWAVALFTYTMEYLADVDKIVAAM

TINVQLCLTTSKNFIFLARRERFLRLNEALERLALTGNNIERELWNTSNRRVLPITMAYSISCQMTVSIC

VLLPILKLLYYYIWHNEVVLTLPLPGIFPYDYTVPFYFILTTILSVLLVYFCVYTICAVDGLFGWFVYNI

SAHLQIMRLKLEQLLQLHVDDPNFQRDLVALVNYHRQIIDLSLELDALYAPIIFLEVTSSSLPICFLAYQ

LSYLSDPASVPFMCLLMSSIVIQLMIYCFGGEKVQNECDQLCENIYLLIPWHKLPPKHCRLLLNPFIRSQ

RVLVLTGYFFTANRSLLVWIFRTAGSFTALLFALKEKEV

>XP_011181253.1 PREDICTED: odorant receptor 45a-like [Zeugodacus cucurbitae]

MTTREARVTQICFLIIQIFALASILIPIAVYSWQHIDDIAEVSNAMAPFMQATISLWKVWRVIYRRKEMA

ELCENIYLISAKASKLELAHLIQENNRERIMNTAYYYSVLNTGVMALTAPVVVSFIQYLRLGEFSYITAL

KATYPIDYARPLNYFLIWLWTAVAIYGVIYVSVPVDSLYSWYIHNLVGNFKILQSKLVSAESIAESTADL

GKRRELIYYCVAYHQRLITMSEQLNIIYQPIVFVQFSLNGLQICFLAYQIGSGVVAMVDLPFLLLFLISV

GIQLMIYCYGGQHLQNESVNVSKSIYQTINSSPWPNELRKVLLISLMRAQKPCKLTGIFFDVDLPLFLAV

WRTAGSYVTLLRSVDQKTM

>XP_011180402.1 PREDICTED: odorant receptor 94a-like [Zeugodacus cucurbitae]

MKLEQFDNISGGRRVIKILKLLGLWHYGGVFRMPYLLYSGLLHSSFTIPYTIMMCLDVVQASNLEKFTNT

MYMTLTELGLVAKLVNVWIYSRLLVDFFAAFTNDKLYKLQDAEEQLSWRRTQGNYARIAFLYFAMSLGAL

ASAFVGVLYSEDYELPFPYAPPFDWRTPRGYWYAYFYELLAMPVTCLSNCAFDMIQCYMLLQLSLCFKLI

SGRLERMGALREDSASRGFSEFRLHREFVDIVRLHARTKKLSQQCQTYISFPFLIQIICSSFVLCFSAYR

LQKVPIQENPTQFLTLVQANLIMVLQIFIPCYCGNEIIQHSSGLNNATYNAEWFRCSPKMRKYLVIYMEM

LQRPVRVRAGDFFDISLAIFTKTMNNTYSLMALLLNMNK

>XP_011180216.1 PREDICTED: odorant receptor 74a-like [Zeugodacus cucurbitae]

MVTIAQFMSESMVLIGEGIVMHDNLDNISFVCTVLAPNLILIEMMLRAYNIIYRRSSFRKHIEEFYKKIY

VQRTWNPDLFEQIRRQQLPTKYSTCTYIITLVTYVYVPISGLIKNERLVPFPIRFSFDYTVPWPRYLVFL

AMSIWTGFAVVGPLVAEPNLLAMQILHLNGRYSLLLQDLRKISKESIVEHERLKGKDTLLVTQRFRYRLF

EIIRRNVELNEFAKSLQEQYSFRVFVMMAMSATLLCVLGFLTATLGLTAQNIRFVSWIIGKVVELLIFGR

LGTTLSTTTDELSTSYYCCDWEDVILHSTDAEENKKLMKLIALAVHLNSNPFRLTGLNFSVVNYETVVSI

LRGAGSYFTVIYAYR

>XP_011179733.1 PREDICTED: odorant receptor 94a-like [Zeugodacus cucurbitae]

MDKLAVLSSRIFPSDPSKGKIGSIEYNVWLAQLFGVPVVGLKAESPLMRIALGIYGVLLTLLVTFIYTGF

EIYDMILCWPNLDDLSQNICLSLTHIAGVLKVINILYRLDEVAHVVRRIEYSAKTYVISKRQLVAFYRGE

FENKIPLTIYASLVGFTGVLGIIYLFYNPVGVAGQIFPYRVKLPDWMPFGLQLAYMGISVLVFALQIVSI

DYLNVTMINQIRFQLKILNLAFEELKLDCGSKKKAHFNHDKRLQTIVEHHCLLRDLRIDVEEIFRMPVLL

QFFTSLIIFAMTGFQAIVKTENSNGAALIYCYCGCIFCELFVYCWFGHEVSEQSKTLTTSGYSSHWFEFD

QRFKKSLLIFMCNSQTPFVFTAGGFMSLSLPSFTGILSKSYTVIALLRQVYSR

>XP_011178893.1 PREDICTED: odorant receptor 43a [Zeugodacus cucurbitae]

MSTAVEDNPLLSINVRLWKSLSVLFARDWRRCVAFVAPICLLNAMQCVYLYQQWGDLATFILNTFFAVSV

FNALLRTCLIIKNRDKFEALMEELVTLYDNIEESGDDYAKSVLAAATKSARNISIFNLSASFSDLIVAMA

YPLFQEQRIHPFGVALPGIDVTRSPLYEIIYIGQLSFPFTLSSMYMPYVSLFASFAMFAKATLQILQNNL

KNLCDNMKTKTEEELFELLRTNIAYHARIARYVSDFNELVTYIVLIEFLLFSCVICSLLFCINIVSTTTS

TAEKISIVMYIGTMLYVLFTYYWQANGILEMSLLVSDAAYEMQWYNCSPRFKRTLLIFIGRTQKPLQIRV

GQMYPMTMEVFQSLLNTSYSYFTLLHNLYND

>XP_011178699.1 PREDICTED: odorant receptor 33b-like [Zeugodacus cucurbitae]

MSSSNPSLQSVNSVVLYREFWLCWHAVGISTAYQKHLCALYDLLINVLVTIFYPIHLIVGLFLNPTPADL

FQNLSITITCFVCSVKHYLLRRKLPQIRVVQALLADLDKRVEDAEERAYFEKQLVVGAKNVVKLFSIAYG

GANMAAISATLLSKERRLMYPAWLPFQWQASTFSYCAAVIYQIAGVTIQIVQNLANDIYPPMSLCIIAGH

VHLLALRVAKVGRDGKKSLKQHNQSLIECIEDHKKLVRIFELTQETLSQAQLAQFISSGLNMCIVLFYLI

FYVDNVFSYIYYAVYFVSMAIELLPSCFYGSMLIYEFQQLPSAIFKCGWLGQSREFYQNQRIFVQLTLKE

IVPLAGGVIGIQLDSFLGTCKVAYSLYTVCNRMK

>XP_011178698.1 PREDICTED: odorant receptor 33b-like [Zeugodacus cucurbitae]

MDSSIDTVNTFKRLFFFWRILGFTTNHNKYLIRLYDIFVTIFATFAFPLHLALGVIFADDKEVVFTNLAI

GISTFACTAKHLMLRPQLSKVIAVNRILQKLDERVQSDEDTHYYIKQMREKCIFMMHFFTVVYFSVAVMA

VLSALWSGKVLYPAYVVVDWHGSTWKYLAVMLFQIYGLNMQIVQNLTNDAYGPMILCLLSGHVHLLSRRI

LRIGHEHETEVERNYAELVHCIDDYKVLMSTTRVVERVISSSYMVQFTAVGINVVVGLIYLLFFADNLFA

YCYYVFHILAIMIEIFPCCYYGSMVQAEFHALSYAIFRSNWLSQSRTFRRAAVTFTELSLKDVTVTAGGM

MKIHLDSFFKTCKMGYSIFTVLQSLK

>XP_011178697.1 PREDICTED: odorant receptor 33b-like [Zeugodacus cucurbitae]

MVRQLDTRAIFTRLFLTWRVLGIIDWPFHRHLRLVYDILMNTVVTFGFTAHLVLGIILSTNQDQFFTNLV

IGIASVSCVFKHLLYRFRMPEMQRINEILGQLDDRVRTKEDYDYYKRLMERPCNFMVNFFTRCYFAVSIT

ALIMALLTGELLYPAFIPLQWRTSVFKYAVGLLFQFVAVSLQIVQNIANDAYGPVLLCMLSGHVHLLSNR

VSRIGHDKPESVKDNYKELSLCIEDHKLLMRYLRTTKAVEHMVSASYLVQFGGVGINLCIGLVYLLFFAD

NYFAYVYYTIHITAIMIELFPCCYYGSMLECEFHDLSYAIFSCNWPMQPRPFRRNIVNFTELTLREVALY

AGGMVRINLDSFFATCKTGYSFFTVIQSMK

>XP_011178078.1 PREDICTED: odorant receptor 2a-like, partial [Zeugodacus cucurbitae]

NLQAIVNNFTIFQLTILHPGENTSKMSHKPLLTATTLDTKEAFSYIWYCWGFFGMYPDLYERRLNWIYLI

LLNLYCGVIYPLLYIGSLLTPMDLNQKLANISVAVPILYTLGKHVIIVYYLRKDLPKALRQLQALDRLAE
RRPKDREYMKRMVKNCHWIFLASSVSFWFALLSYGVLEMFRHKLPFEGWVPFDWQRTEFAYVCACALQLF
GLGIETTNAICCDTYAVTYLVLLVAHLRILNRRIARAGNTGDGSDAENYRELVACVEYHKECISYYNSIR
PTLSGTCFIQFLSTGLGLSMPAIAFVGGSFSFSHVIKFLIIFGAIIIEVAPCCWFMDEVMAEMHKLTNSM
FSCRWYDQNLKYRKALIIFMQRSQIAHPVLAGNIIPVSLETFTNIIKFAFSLFTLLNQLSHN

>XP_011178077.1 PREDICTED: odorant receptor 2a-like [Zeugodacus cucurbitae]

MNNKQDAVGRLDSSDALRYVWLFWRITGIHPTAKYRGIYWLYSLLLNISSSVLFIAFYVVTFFISTDLLE
TLTNLSVMVPLIYTSTKHLVVFYHIRGKLPQAAFHLQALDRRVELEPAACEHLRRLVQRCHRIFLAALAG
IGVCLALYALVGIARHKLPFEGWLPFDWEHSLNAYILACAYQLFCLSVQSIYALCSDTYSIIYLLLLVAH
LRILNARIARIGGACAAQCGEVANYQQLADCVRDHWECMKCISPTIAATIFVQFLSTAFALCTAAVAFVN
ADSSVEQLMKFLPYLLVVLCEIAPCCWLMDEAALEMLKLTNALFSCCCSDGN

>XP_011178076.1 PREDICTED: odorant receptor 7a-like [Zeugodacus cucurbitae]

MSAVQPQQQQQQQHAAASLHSSHAFRYLWLNWRLIGMHPTRRHRLPYYIYSGCINISLGVFLPATMIAKL
FFIENLSQLIGLLYLGVTLTMATAKQWSLWLHRSKLLAVNQYLDKLDARCMPHAVDRQHIRTAIRICHLY
YAGYMFVYELCSSGFAYIGFTLRQLVYDGWFPQFYADEATNLTVTLIYQNFAVMSFFVLQNVNNDMYPQC
YLAMMIGHLRALTARISRIGKDGVLSEDENIAELTNCIEDHKNLLGYFACIRPVISRTIFMQFGITAFVL
CLTAVNYVAFERDAAQMLIAATYIFAVLIEALPCCWYVNSLMEECGELTTALYNCQWYDQNRKFRKMLII
FMQRSQRTMLLMAGDLVPITLQTFLNIIKFSFSMYTILKG

>XP_011177369.1 PREDICTED: odorant receptor 13a [Zeugodacus cucurbitae]

MLFNPKPLKDPISFRFPLQCIWLKLNGSWPLRPQRSGEFERYFRWLYSVWAWYVVAMVGITIGFQSAFLV
KTFGDIMVTTENGCTTFMGVLNFVRLLHLRLHQREFQQLLARFVKDIWITSSSQPIVERACARNMRVFQV
ISALQSCLITMYCLLPLVELYMLTVNTAPDVLESVEKPFPYKMLFPYDANYGWRYALTYLFTAWAGVCVV
TTLFAEDSLFGFFTTYTCGQFRILHIQIDNIIPDSYAATRAGRGTEADFQRESVRRLDRIAGKHCVLFNF
VRSMEEFFSPILLVNFLISTILICMVGFQLVTGKNMFIGDYVKFLVYILSSLSQLFVLCWNGDNIIQNSL
EMANHLYACNWECGVEVAATNADNKRADEQVKMAAPTVYYTTNNAFRKKLQFMIMRSQRQTCITALKFSV
LSLSSFSGLISSSMSYFALLQSFNEDEEN

>XP_011196821.1 PREDICTED: odorant receptor 59a-like [Zeugodacus cucurbitae]

MNNSPPVDSRQFFRTHWRLWLLLGCVREPVHYQLLYRLYSTVVNALIMLFYPGTILIALYNSANLTDFLQ

TLPICAAALACSAKYISYYRRLGLVRQAEQIFNALDEQVLLPEDREFYAGIHRGTNLILNTLRGLCIFFM

AITVMAFASSIEERGLAFAIELPFDWRKSSVAYVGAVGLELLLLSCDLLQSLANDSFPAIALCVLSNHTR

LLGARLARIGHTSKDVQANIREMQRCIIDHQRLYRLQAIIEEIISMPVFIQYAVTAFQDCFTLITFIFYT

NTVSDKVLYLTYLLALQLQIFPTCYYGTACAQSMDDLQQEIYASNWVEQNQVYRRLVTIFSQRTLKSTTT

YAAGLIPIHLSAFVKTLQGAYSFYTFVDGVRKV

>XP_011196690.1 PREDICTED: odorant receptor 47b isoform X1 [Zeugodacus cucurbitae]

MISLSTQATINNTISTHNSYLSNDSNTHLKHTASKLRTILAPYRVLKEMLRSGEAVHPPHTCLFYFRAYI

RLLGLWPAERAVENPLYYAFNVLIMLLFGFFTLCLIFFKMILFRLGNADTDIIINEFDALHVKHFNESHD

SPRNRRTRQWQRSFFFGEMCFFSGFYILSLFLFAAMSLQPLLSQQILPFRCKFPFGLDDPDEHPMGFVCV

YFFQCFCTLYMLVAIVVMDSLGGNSFNQTTLNLRILCENMRNLGNGSTSELVVWRKLKETVEFHQQIIKL

MNRINQTFYWNYVSQMGASTFMICLTAFEALLAQDKPMVALKFQTYMFSAFMQLLYWCWMGNRTYYDSME

VATAAYEVRTWYRHSPLLQRQLIFIIKRAQKPLEFRAKPLFGFTFASFTSILSTSYSYFTLLRTMSD

>XP_011195821.1 PREDICTED: odorant receptor 63a-like [Zeugodacus cucurbitae]

MVFEDVEEIYRRNYNSIKVLFQVSFGLGVNLTAPSKIKDALKLFNVILVVASLLSMYAHWCYLIRHFENI

PLLAETVCTALQTLISAFKMIYFLFRQHNFYRLLDQALKHVIIREIEIFKHDFPINQQLKREIDEIMNGV

WQNARRQILFYFCCCVGIVCNYFFGAFLVNLYHQLKKTPDYKYVLPFPELYPFWEDKGMTFPYYPIQMFI

SSSAVYIAGMCAVSFDGVFIVLCQHAVGLVRVHNLLVLRSTSPLIPVERRVEYLRYTIFTYQRIYTYVQQ

IQTSFKHISLSQFVLSLIVFGFVLFEMSFGLVSNCESSIIIFIRMIMYISAGGTQIIIYCYNGQALTSVS

EEIPMAFYNCNWYEECEKFKQLLRMMIMRTHRYFYLEVSWFTLMNLATLIALFRMSGSYFLLLRNLQES

>XP_011191113.1 PREDICTED: putative odorant receptor 69a, isoform A [Zeugodacus cucurbitae]

MEELKALFPSIAKQKRLAEINESKEGIIGSGGIYRLEYYEEKSRTIMQFITRYFMFAYVAYNSIPVMQLC

FAVITQQEHITYRAQANAWYPWHNHNDHSSFMGFMLSYLTQAIVEYTSIAFVMSGEFIFCFFTTQMLMHY

NYLCSALSALDASAPDAVRQLKALISYHTHLLRLSKLINSIFNLTFALDLIITTFAISLMGLAIVLVNFA

DALMFSAGFSFFLLLGYLFCNNGDEILRETMQINSAIFYSNWYEGSPEYRRLIIFFIMRTKTPCQYQAYG

YTPLSMETYMRILKLSYQMFTSVRAIE

>XP_011191101.1 PREDICTED: putative odorant receptor 69a, isoform B [Zeugodacus cucurbitae]

MSETYTLNDCLKYPYFTLDLASTEPFTWSGAPTHSYRKLWLRRALFIFGALNLVYQNVGMLIYLFMPHES

SAETTIAQITETGGIMGLTMVGTSNMLVMFWYADRIAILLEKFQQLFPTERVQRQVKLEFPHRIEYFAVK

SNKLMKLATTLYMYAFAYYNSLPIVEYLYEWSTPGIVWKYRYQSNTWYPWQNERNSKSFLSFAVAYICQV

QSSLTGVAFIMAAEFMLCFFTTQLQIHFDYLANALETIDAAAPNANEDLKYLINYHSQLLSYSKETNAIF

NVSFMVNLCTSSIAICLMGFSMVMISLAHAFKYSIGLTAFIVFTFFICYTGKELTETSDKLLHAAFYGNW

YDGNLAYRKMILFFIMRCRIPTELRAYKFTVSMPTFTAILRSSYSLFTFFQAMGK

>XP_011191024.1 PREDICTED: odorant receptor 67c-like [Zeugodacus cucurbitae]

MLPPFKSREHAPTVQDFVYVPLFQIRFMGVKLFKWTPEERTSKLQITLMGTFCVFATFNFVSMMLFVIYD

ELPTSLDITEFILFWGFALNAMMKGGTMVFFRHEIESVLKGLIARHPKTEAERVAFQLVPYYKTINASNK

YLSIWHLSITSIFALHPMVASILGYIWREDKNDAYVYTLPFMMGYYYDTNHPFPYAISYFIQCCGAFYMS

LLFLSGDLLLISMVQLVNMHLEYLIYRIESFQPTGMDADMKVLGPLLEYHNEILDYAERIDGTFSLATLL

NYGGSCLVLCLIGLQIVLGSEALSVIKFIGFLVSTIVQVFFVSYFGNNLKELSTGISDAFYNHPWYDGNY

KYIRMLVLPIARAQRYAHLTAFKFFEISMDSFKSLCTTSYQFFTLLRTSIEEEGFQ

>XP_011187028.1 PREDICTED: odorant receptor 30a-like [Zeugodacus cucurbitae]

MYKSQAFLDFFVFVEKWYEEQQRDGEEVTLRKTHEFTQKIRKAAKTLLIVTMIILSYMVLIQLLATVGIG

YRKLIVDVAFPGIDFYISPNWEMMSFLQCLYVAPFTFVSYVSYLCLTLIAISFGIFLMKDLQFKLENMND

MTDLEALNCIKKCVKAHVMIIKYHNHLEALFSVGSFADVCIFGIIPCVIIVLSTMDHDISMLIADIQMAA

MVMTSTFIFFWVANNFVIESENIANAAYNCNWVDRDKEFRKYIPLIIGNSQKPLQLTAGGIKPINMEFFL

TIVRCTYSLFTVLFTMKTGGDS

>XP_011186909.1 PREDICTED: odorant receptor 67d-like [Zeugodacus cucurbitae]

MAVKAIRPTETFTKILNFFHLICSLVGADLSNDNYRVNIITVIVILSIIIYFIFTATTVASVFSEDWTYM

LEASCMVGSVLQGCTKLISAFIFKNKICGMRAELERLYAEYEVKGDEYVKTLNKSCERMWQITKVVGQMY

LYAAVGIVVTIIYFVIATTQRVYVMHFFIPGIDVNTQTGYLITLGVHAVVFMSGAFGLFAGDLFILLFLT

QPMLFVDLLVLKVKALNEAAAQKTNAVQRLLIDIIEWHQYYTDYNKRCNHLFYYIISVQIITSGISIICT
LYIILMGDWPGAYMYILIAFSGLYLYCILGTKIQDCNSAFCNELCNINFYDLEVKSQEMIVLIIMKAQNP
VEIKVGGFLPLSVQTALKITKTIYGIFTMMIRFLEEEQ

>XP_011186895.1 PREDICTED: odorant receptor 67d-like [Zeugodacus cucurbitae]

MVRLISSLVGADVSTVNYRVNIITVILILCIVIYFIFTATTVASVFSEDWTFLLEASCMMGSGLQGCTKL
ISGIFRTKDVSSMRLELEEIYRTYESKGQSYCKVLNESCDRVRKVIKMVGYIYASNIAGIILLTTVLMLT
SDRKIYIMQFFIPGVDADTSFGYLLTTAVHMVVFLAGSFGLFGGDLFFLIYLGQPELFRDILILKVEELN
EAVAQKDDNIESLLINIIEWHQYYMDFNNRCNDIFYYIITMQILTSGISIICTMYIILMGDWPGAYLYIL
VALCGLYLYCIIGTRIQTSSETFFEELYNINWYELDIKKQKMMILILMKSQNPSEIKIAGVLPLSVETAL
QITKSMYGIFTMMLQVMAEEL

>XP_011186833.1 PREDICTED: odorant receptor 59a-like [Zeugodacus cucurbitae]

MAYEAKPPGVKQLFRTHWTVWKWLGQVIHPQYPKLHIAYTVLLNVGFSIGYPLHLLLGLLNLKSLQEVLL
NLTISVPVAVCTLKYFNIWRNLDKVRHLEQTYNTLYARIDHPEEWLYYRKIIIPYALKVLHLFYFICVGT
AITSELTLLIVGFAYEWRLMYPAYFPFDPYASTGGYVAAHLFQIIGLLVQLAENLVSDTYGGMCLSLLAG
HAHLLGQRVARIGYDEQKTQEENNRELVDCIVDHNMLFDCHRTLTDIIGLGLFMQIISASLIMGVVIIYL
IFFVGNSFEYVYYGLFLFACIMEVFPTCYYATYFEIEFEKLTYMMFSCNWMDQNRQFKQNLIVCVEQSLN
TRYFHVGGMFRINLQIFFATCKGAYSVLAVALRLK

>ETN67579.1 putative odorant receptor [Anopheles darlingi]

MSSLWPRDLPDELYITYQFDNLRFIGIYPGFSKAPFWQRLGLYVLDLFCVLQHIAIACDLLEFREDIERF
GDDICLLTAFTLVLGKRWFCRFYIDDLLDFVEQLSLGFDHYRHREQQYVQQLLSHHRLESLIAYMGRMLS
IVLFLAMVAHGLLSNGFILRAKYPFSTDSFLTCGAVFLSQMLFDGYSILTIMLVDLFTILVLSQLSLHFQ
LLSMDFANIGRVLPATVHGPLCRDEAVLAHIRELVLRHQNLLDFGTQVMKMYDSNLMGQFVASIIVICMS
AFELLLAQGNVTLVIRFGMFMVCTFFQILVWCFFGDLIAQKSLSICDGIANCNWIVLDDRQKKDLSFIVM
RAQKPFIIDVYRLFPLTYETFLAILSRSYSMFTVMMEMIE

>ETN65485.1 putative odorant receptor [Anopheles darlingi]

MEVLECPLLSVNVRVWRFWSFVLVHNWRRYVSIIPVTVLNVFMFADLYRAWGNIEEVIINAYFAVLYFNA

VVSDAAKPPIFDYAQCLRTLILVYQRQKYEVFLANAANVYQEIRQIKDDDVIGKLVEDYTRRARILSISN
LALGAFISGCFVVYPLFTGQRSLPYGMFIPGVSNFESPQYEIFYIMQLVLTFPGCCMYIPYTSFFATSTL
FGLVQIKTLQHQLRMFRSTGSNLGRRTPSLDRQLTKFIEDHKRIIRYVQDLNGLVTYICLIEFLSFGLML
CALLFLLNIISVMAQIVIVGAYIFMILTQIFAFYWHSNEVREESMAIATAAYSGPWLEVDNSIKKKLLLI
TMRAQRPLELTVGNVYPMTLEMFQSLLNASYSYFTLLRRVYN

>ETN65297.1 putative odorant receptor [Anopheles darlingi]
MIRRTVTMKVETSPTFVPPQSSIFRLQRKILRAFGIWPLDRPTRRWFVSLQIGVNLVALALCMIGEFLHG
LYAYQQGDLGEAIESICPTVARVSGLLRMVFYLVNEQHLEKVLKSIEVAQQHEQPHERNNSRQIALFGQR
LTFYLFLMMFSAALLYGVTPFLIMAYNWHQGQWPLVKILPFKLALPYDWQRWFPFTLTTLFLNYASIPTI
TSQSGSDALLTGVCLYVRGQFAALGQDIAALSEQQPRIGKTVSVVASEQETRRINRQLQRINARHQRIIE
MVAEVRTAFAPNILLVYMCTALIMCIVCIAMLTVEGVYKFTYLPYALSELAILFLYSYSGTIICESSEAI
QTAAYSFPWYRYDRDTRHLIQMLMIRAQRGSNLNVPFFETSMATFSVIVRTASSYITLMKSFL

>ETN65050.1 putative odorant receptor [Anopheles darlingi]
MADTTPLERFRRTLAWQNRMLAWFGLYVYMGQHRYTYRLVVTLLLCVSFFIFTVYSAIISWGNAAEVMFS
VVTVFYDFVGLTRLAILLSYPDDCYRVVQLAEQMYENDDPGTLLLSSGAASHQQHHHRQAKEQVLIRYTD
LFQHSVTIFTVCFLSSIALVVMLPFVMYIFTGDMILPLGVWLPYMDPREPVGYWVTLGVQLMYIIAGPLA
LTPSQNVYFAFIFNICMQYELLTVQLDELDATIERNSSQVPVNAAAKAAGRVLVRDQLVRIIEYQQKCRN
YIRIIEKCYANQTFVEFFCSSGQVALMFYEFRRTFWLPGLFVMPAAILQMLIQCGLGTLIEMRCDQFTTK
LCAISWLRMERTEQKMYQFVVASAQQPARLTCGGIGVINMNLFLAIYKKVYSFFMMLRNF

>ETN63321.1 putative odorant receptor [Anopheles darlingi]
MSHLVLHEVRYVLVAMGYTLDCFTPKIRYSLRDSAIYWFVTMIPLVFLCEPQFAFLIFDAKDLYKFVSVL
VPCTEIVLTNLKMVICNAKRAKILNLINDVQAEVRELLVVTRKKARVFVIVYTSAFVFICLEYAFMPIWK
WCYYYGFSTQHSNYTIQLPYLQRMFYSIEGNGSFSVTYIFIMVAVYVLALALAGFDSVFTTLVMHITALF
KLLNMEIDQMGMQLRGGAKGQELKATFKAIILKHKTYLAFIDQLEDAFSLVLMVQFLTSSIVICVVLYQL
TLAFGWNEETIKTITYLPGAILQLYVFCWYAQKITEEAELVANHIYSIPWYLGDPAYEKMCITLMAKAQK
PAGVTASKFYMITLQSFQRIISTSYSYFTLLQTMNQQ

>ETN62294.1 putative odorant receptor, partial [Anopheles darlingi]
MDLKEEWILAEAILDNPLLKSTLRGLRYYGLLIYDTQSWKKLHCFRGAVFSLSMVLFNFTQYVDLYQVWG

NVSDMTANAATTLLFTTIIFRVIFFYVHRTRFNNIIRVAHDGVERIRKSEWVEEREILDHNVAYLKRLTA
VFWSCALLTANTMCVYALVEHFIYEQPPGNEALSTIDSITNSTIPVKSTHPATILRSWYPTYNGKANHFV
EIYLIQLYIMYVGQLIVPTWHMFMVTLMIYGRAECSVLNHRLRNLSHYHAPVLASENRAPDRVNNPERRS
LIIDCIQRQSALVAFTKELEQLTRAAVFLDFVVFSVLLCALLFEASMTTSGVQIFIDICYITTMTAILFL
YYWHANEINHCADQLSMSAYKSDWYRYDRSTNKLLQIFIMYSNEPLKMRAFFISMSLDTFLN

>ETN60992.1 putative odorant receptor [Anopheles darlingi]
MLIEECPIISVNVKVWLFWSYLRQPRPFRFLVGCIPVAILNVGQFAKLYYSTGDMSELIINGYFTVLYFN
LIVSPAVVAVVMLRTAFLVINRRKFETFFEGVAREYESLEKNDEIRATLERHTGRGRMLSISNLWLGAFI
SGCFVTYPLFAPGRVLPYGVSIPGVDVLASPTYEILFVLQVYLTVPACCMYIPFTSFYATCTLFALVQIE
ALKQRLGRLHAFQADPRALFRELKECLKYHNQIIQYVHDQNSLVTYICLLEFLSFGMMLCALLFLLSIVR
RRPRATATPARLELDSISDGDTPLFACCPFQSNQLAQMIMIGSYIFMILSQMFAFYWHANEVLEQSLGIC
DAVYNGSWPNFDEKIRRNIILIIARAQRPMTVWHAMTKTRFF

>ETN60957.1 putative odorant receptor [Anopheles darlingi]
MALYLCALVLNIFVSQTFEQLVFYIMYIVLTETAMCLKTWTNYRLFPVIWSLHETTIGTSFQPLDSYEKD
AQDRTLRTFNRLYYIYIFVSHMAAFGTGSHLFSSEYRMPFFPWFFGVPYGPDASLAYYTIFAYQSFGMYF
HMLLNTAGDTQLCYWLQMIGLQLDMLANRFRKLNTNEEFEKSFVPLVLHYEKVYRMLLRVERLFSLAYFV
QFSVSGLVICASAYQVASMLNLNDFSRLMNVFYMMSMTMQIGLPCYYGNEVTLKSYALTKAIYSCNWYNM
KRSNRKKIQTFLIRTNKPFAATAFGYFNFNLPAFTTILNMAYSVYCLLQRKASSNI

>AGS08022.1 odorant receptor 73 [Culex quinquefasciatus]
MSSINLPSFQMMVGTVRWCKLIGLWDGTGSSRYHPRTLTLLLELIFWYILPTCMFVLRRQDRPLTLVMKD
VHDDKKMTHLCCGSLAHGTEAVQRWKSAFYEVQGALDEFARNGQLSEDMRKHLERADVVLARYYIPGNLF
GIFLYGGLPGVLAIGRFVFTGYAEELPATVLEADYVLFDHQSNFWIWLPTLVVSIAVQYGMIIGITANET
FCWNLIHHVSCLFKIVSARVARLDEFEDEEAYQQELEAILETHLVCYRSVCYLEKALYLQMAVLYSACVA
VTCFVLFVVSIVDDLFLLAMMAFVLNYHVFLIFSFSVLGNELMDASTSVAEAIYNIKWYNRSISEQRCLQ
FMIYRSQNAIFITAGKFFKLTRATFMVAMKTAFSYFTIMQQLYAEE

>AGS08021.1 odorant receptor 1 [Culex quinquefasciatus]
MKFAPLQDRMAVMPFTLQYLRLFGLRGDRRNRVHFVLALLYRVLLLNFPKLAFGFRDRIDLVIRSISELL
FQIHIDLRAVLFATKLREFEELAGLLRKVYNKVKTLDADSPERKIIEASNLGINRRSKSYALYVAIAVTV

FFWVPVVQTTAIWLLNRGSNSTDRPEFVTMMELEFYGLDIRGNIWHYLVYASLSSVAHYYSAVYFALSGM
VIFSCIKSIAALFELVSTRLATLHELSGKELREELADLVELHVNGLRCIELLENINNLAMMVQMVNCVLI
WISMFLSISTNFTPEVVSLLVLLIVMTGETYVLCQLATELSHVNLTVAESIHRSEWIQMPVDVQKGLAMM
LQRAQKREGLTAAKFFYMDVERFGRVAQTSYSIFIVLKERI

>AAL35509.1 putative odorant receptor Or3 [Anopheles gambiae]
MPSERLRLITSFGTPQDKRTMVLPKLKDETAVMPFLLQIQTIAGLWGDRSQRYRFYLIFSYFCAMVVLPK
VLFGYPDLEVAVRGTAELMFESNAFFGMLMFSFQRDNYERLVHQLQDLAALVLQDLPTELGEYLISVNRR
VDRFSKIYCCCHFSMATFFWFMPVWTTYSAYFAVRNSTEPVEHVLHLEEELYFLNIRTSMAHYTFYVAIM
WPTIYTLGFTGGTKLLTIFSNVKYCSAMLKLVALRIHCLARVAQDRAEKELNEIISMHQRVLNCVFLLET
TFRWVFFVQFIQCTMIWCSLILYIAVTGFSSTVANVCVQIILVTVETYGYGYFGTDLTTEVLWSYGVALA
IYDSEWYKFSISMRRKLRLLLQRSQKPLGVTAGKFRFVNVAQFGKMLKMSYSFYVVLKEQF

>AAL35508.1 putative odorant receptor Or4 [Anopheles gambiae]
MKFELFQKYSSPDTVLSFVLRLLHIVGMNGAGFRSRIRVGGIFLFYLIFLVIPPLTGGYTDGHQRVRTSV
EFLFNCNIYGGSMFFAYDVATFQAFIQELKSLSVLVCSHSYRLKYKLTRFNRRADIIAKVQTTCMGAVTL
FYWIAPIPSICAHYYRSTNSTEPVRFVQHLEVKFYWLENRTSVEDYITFVLIMLPVVVMCGYVCNLKVMT
ICCSIGHCTLYTRMTIEMVEQLESMASAERTASAIRNVGQMHSGLLKCIRLLNTSIRSMLMLQWLTCVLN
WSISLIYLTNVGISLQSVTVVVMFFLATAETFLYCLLGTRLATQQQLLEHALYATRWYNYPIAFRSSIRM
MLRQSQRHAHITVGKFFRVNLEEFSRIVNLSYSAYVVLKDVIKMDVQ

>AAL35506.1 putative odorant receptor Or1 [Anopheles gambiae]
MKLNKLNPRWDAYDRRDSFWLQLLCLKYLGLWPPEDTDQATRNRYIAYGWALRIMFLHLYALTQALYFKD
VKDINDIANALFVLMTQVTLIYKLEKFNYNIARIQACLRKLNCTLYHPKQREEFSPVLQSMSGVFWLMIF
LMFVAIFTIIMWVMSPAFDNERRLPVPAWFPVDYHHSDIVYGVLFLYQTIGIVMSATYNFSTDTMFSGLM
LHINGQIVRLGSMVKKLGHDVPPERQLVATDAEWKEMRKRIDHHSKVYGTMYAKVTECVLFHKDILSFGD
EVQDIFQGSIFAQVCASVIIICMTLLQATGDDVTMADLLGCGFYLLVMTSQVFIFCYVGNEISYTTDKFT
EFVGFSNYFKFDKRTSQAMIFFLQMTLKDVHIKVGSVLKVTLNLHTFLQIMKLSYSYLAVLQSMESE

>ACS83758.1 G-protein coupled odorant receptor 66 [Anopheles stephensi]
MEAAEKFHQYERYLRTLCNVLGFDVLRKGWKKTFRTYVTIFLCGQYFLWMVWSIIIASDTFELLKSLSFI

GFFFQCSSKMYYTIANAAHYSTNFAGLQETIYTAHMDGTEEQKTVIDRVITVVLLATKATTVLFTSSLFI

FSLYPAYMYFVMNVKVTIFPLYIPGINIYSSYGYGITNSLHMLIAVYGLLGALTSDTAFMLFVLHFISYV

ELFRIECEQFARDLDAFGQQWEYHTVEYKTFCRDWMRALYQYHQQVIVYLSSLQECYHSICVYQVASCSF

SIMFNLFLALTTDWYATYSFMVISWFQLFVYSLLGTVMQVMNDRLNSYISNLPWYLLPTDEQLRYNFMLG

RSQLPAEMVIRSVGPMNMETFTDIMQKIYSAFTMMYSFLVDLG

>XP_021711435.1 uncharacterized protein LOC5568767 isoform X1 [Aedes aegypti]

METVKKFKLIRDIRAEITDPQKTYDDMMHQLNSVAQKYLGMDTLHLEFSFVNRRFIFLLAIMSTFLYADV

ESAVLAGDVGEVAYNIAVLGFGLQGFAKFDAYVYRKESMHTLVWQISAFLNKKKMFDGLNEIVTANVAIM

VLLKRFYIGLYGFVFVSMSSLGLISSLSSGERQLSFGFQFSFLDTSNWVGYLATYIYQVAGILMVVISSC

CNDILIVVLYITAMGMYDCMMFDLRELSKLSQMEKSSANKRIAEERIKSVIQQHQEVLEFLELSNETFSL

YFLMSLVCMTTAIAILLVALVWNRWYAGLVICFAASSQIFALSLLGTLLLVKSEELIDEVYSITWYDMDL

PVQRSLKLFLLMSQHVKEISYRFGVMNMETYVQILPFRLQPPRSEIASSRPYEPVL

>XP_021708610.1 uncharacterized protein LOC23687563 [Aedes aegypti]

MKMLLMIHLKIPLASLRQFIDSNRIYSGDKTYDERVFSKFHNSARKIPQVVFALIGLETILLSIPSSARK

AVFKLPHQLIGAGKHVSFLVNLLYFGRLPLGMCPRFFTNLGSLGALLMGMRAKLKILAHRYQQTFAHLGL

DEKQYFAYMKLEMREIMDQQLEFWRNLNILKDMVGKAFCLVHYFSIYAIGTMLYVSKIMGLNATSVMLVA

STAWLLLEYYVWCRLVESLKEEAELVALDIFEICCLMPYNREHAVQYTQQRTSLMISWISMNNGLTMDCL

GLFQISTIGFVELLNVVYTVVTFLINVN

>XP_011493425.2 uncharacterized protein LOC23687957 [Aedes aegypti]

MDQLCQFHRNLFETNCFSMLEMVLMIAGIPIESPTTNSWKRTIPRISNVISALQLALTIGQFDGNADGLG

SCCVKFLVLNHFAEPIREVQNFIVNRPCDSGDNQHDASIREKFGKSVKRILVAIMTMVAMDMVVISIPSS

QRTKLLGIPKYFLKFGSFCYKSVQFLYGFFIPLIWIPMYLSYPLVLGILLTGLRTEMQILKHSFEHLGHQ

TKSGLFDKRHWLQLKHDIGGLLNQQLLLHRHLRTVRHLVGFGFFIAYYFAVFFIGAILFITKMHDIAFFV

VFLISLFMVVLIECYCWCHLVDSLDDVADGIGQSIYELCAKLPDSREYHSDFVAMRTSLMIIWMNTCNSP

QVKCLGIFRICSEKFVSLCNASYTVFTFFIGMPH

>XP_011493310.2 uncharacterized protein LOC23687429 [Aedes aegypti]

MFTKIGYGWRWVCVRWTAKIMQQISYFWSDKGPAGDCFWWLDVLLLLGGIHSESTATWAHERKIRIICRC

LFAYHAVAFVLQLNDALYEEKKAALVVWELMKVIFIFVAYLKVVLVVQLKGSIATLRQFIRSNHICSGDI
EYDELEQNKFNKIVRITIQVVFVLIIIDTLILSVPNFSNNDLLKLPHLLALTGMFPSYILKILLTSCLGI
SVIPKYFACTACVGAVLIGMRTRLRILAHRFEHISQQDFTSEEKAFECVNRDIQEALAQHLEYWSHLKAM
KIMVSKTFLKVHYFSIVAIGSLIYVCCAMGVNGVTFVIGAGTVSFLMEYYLLCHLVDLLQDEADSIGYHI
FRICAQIPYNSELRSEYVQLRTTLMIVWINTRNGISLNCLGLFEITTFTFVTLIDAAYSVLMFLIKMGKI
VDTDG

>XP_011493113.2 uncharacterized protein LOC23687725 [Aedes aegypti]
MEIKNTLFAIILNYSRESFPSNSNASKVITSFSCRYSMEKVKRLWTRQEFDIDSDYFKLIDSICLLFEQR
PPLTDRCGIKVVWTALSVFHGFQYICYSIQLVRCAYKSPINMEELTSIGNLLVVLTVAVIRGLSLAYHRD
NMLELKRYVNKKSCRRSDPDAFALRKRKYLKINRYAAWFYIVTTMNSFTWAFTTGLQEDIFKIPYLIDGL
SGSTRKSINVFFSLLFVPWCYTVWYSPTQFITLLSILHTELTIIVGQFEGLFHKVIEKYAFDAYELNHMT
KMQINRFWFNLDNEFKNALSHHIAFVRKLSLLRKVSNVNFFIFLCSSTLITTFNIFLFLVEPSLGRVPLL
LMAIQYISETYLCCTMFNNLENENDRINYLVYARDWLNEVIKHRTYDRPYCASIFRNAIFLHQQVGSGLT
IRAGGIFPLNLRTFTSMMKSVYSLLTLLLQSIEMKPNY

>XP_021703812.1 uncharacterized protein LOC110677197 [Aedes aegypti]
MLLLGGLRSAIPRWKNQNYIRYFTNVLFVSQGIVFLLQLEHSLNTYRVDLPVKMWEIMKFATWFSAAMKM
FLSALLHERITVVRQFIISDRVNSGDKVFDDYEYHKFNRYVRLMIALLSGFIVVDSVLLAVPNSSMQNAF
LLPPQLKRTGKIISGIIHCFAVNFLSFGVHPRFFSNLTCAATLLLGVRAKLRMLTHRFTKLIALSDLSSD
NYFDCMSRELREVLLQQTEYWRFLGILKRLIAEVFVLVHYFSILSIGAFFFITTDTGISFMSAAITSAAA
YLLLEYYLLCRLVDSLQEEADAMVSVIFELCARMPYSSEHHSKYIEFRTSFMIIWINARRGMLMNCFGLF
EISTLAFVGLLNTAYTVLAFLISVG

>XP_021698209.1 uncharacterized protein LOC5573676 isoform X2 [Aedes aegypti]
MESSLTTYTMEIEYYRPNSNSAEDNDASIYWLRVLSASLGIWPQKLVGDKKQWWKRLYYFMIIMHWYNTY
LQVEFFFHNLGALKTMTEGLCSFCSISLTGIKIMRLNSFSNEINLLLERMRTHHFMQEIKLLKKGKNKPI
FERIDQIMKDKWKEVKINLLLYTVSVGIVASSYSIFPAVINLINLFQGSSNPRRFVYKTYYRGMENLKFY
SPLHEILFSSESLSGYTTFAGVIAFDGLYVLLTMHVITIFKSLKLVIKESTRSIFTDEEKQFYLHECIDH
YTRAIMFMDDINKIFSPIFILQLFTSTSIICVIAFHASANALEKFLFQKVISIYLHSAEKSSQTVFSFPR
IWCNKSTYALLRIAAPSQL

>XP_021697065.1 uncharacterized protein LOC5578227 isoform X2 [Aedes aegypti]

MFEKLRLSYLWFSSRVVGIVSFCFKGQPVGDCFWLLDVLLLLAVFQLYASLSTKFDDTSLVVFDMVKVAV

TLLSGMRILSIVLLREPIASLRNFVTSNRLNSGDAVFDELERRRFNKFSRAALLVIYGATVLDTILLSVP

NSSKDSVLELPPQLVSTGKYASNTLYFLFVGLLALSIVPKMFSALSCSQVLLVGMRWKFKMLVHRYETIA

NLRLLDVDDYYERIECKVLEAVEQQLEFWSYLQILKDLVAKQFFLVHYFSVGAIGSMLYVSRDIGLNILS

VAVFASTLVLMLEYFLWCHLVDSFEDVADSVGSRIFELCAKIPYSPKYRTRYRKLQTSLMITWIVARNGV

SMNCMGLFKISTIAFVGFVNTAYSVLMFLINMH

>XP_020717798.1 uncharacterized protein LOC110119025 [Ceratitis capitata]

MDFFVPLQHDNSPIILPIQVAGYKFNFLWPLKEDANVFMRLISSLCLGVSILCYIGTILGEVTFVAENFS

DIPAVADCLSTSFMGIQYIIRIFVLLSRQRPMRKLLTNFYRDIYFTSADDGALYKEINTIIRFVNIFTRF

YYTPMVLILGLYTYDVASVGLAYPDKPFIYRMSFRWYDAQAPLPFIITAIYSGWLTISTVTIWTAEDYTL

CMVLCHASFRYKKLRLDLQELFEASRADLRCGEARRKNEDLHTAFRRRLCDIFQRQQDLNGFVAEAKAHF

THQIFYTLSFGVLLLCVVSFQFQSTQMTVEWSKYIAWLISQTSQFLLIGYFGQMLMDETTDLQNSFYFCH

WEDLLSLGDIRSNKLLLRDLEFAIMNAQKPIVFDGMKIFPLTYSTVSSALRSAVSYFMFLNTMNNTN

>XP_019931134.1 PREDICTED: uncharacterized protein LOC109621528 [Aedes albopictus]

MSVGMRSALGRARNWWSAMKVKVQHFWSDGGPGSNCFWWQDVLLLLVGIQTELTSKWSAERIVRYFVNCL

STYHVTMILIQTIYALKHQREDVYAVVIGITKAAAFLVSTLKLLLIKFYQKPIENLRHYITNGNITSGDE

YHDKLELSKFNEKFRVLIFCILGLTVIDIVFLSIPNPATEMVFRLPSHLQPTNPYISFAFRLFFISLLPL

DFVPKFFCCMATIGTLLMGMRTNFKMLSHRYGSILNQPFVIDGTDWRRMNRELKETLAQHVEFWRHLKVL

KNLVGRSFFLVHFFSVLSIGALCYVCHGIGVNFLAFVIIATMAMFMLEYYLFCHFVDSLQDVVNCIGDHI

FEICALMPYSKTNHSQYQGFKTDLMIAWINTRHGLSMNCVGLFDISTFAFLRILNIAYTVLTFLIQLSQL

DTSA

>XP_019932611.1 PREDICTED: uncharacterized protein LOC109622646 [Aedes albopictus]

MELSHYRSLPSELQIMPFQLRCMELIGLAGPKGRFYRFVLAFGWGTFVILLPKSVLGIGSPRLDAIIKGF

AELLFEGNLFIAVASLIPKLPLLKRLLHVLSEIFTQVTHDARSKDQCYELICEQNSKIDKFCKFYFVYCC

FGPFVFCIPAMVTSYVRYFGGAGNGTDNADTNETEQQLLFELPMEQEFYGLQIRTNFAHYHLFLAASLSA
YCVCSYMSVIKVSTLLIMIKYSSLAYRLVAIRVRKLAELPASGQEQNFEEVTDGGDVLKMSMVKEVVDLH
KKALEVTELVEEIINIPIAMQFIACILFWCMTMFYVSTNINFNLFNVMVLFWLSLIETYGYSYLGTELTE
EAKTVGQAIYELPWYEDSAQLQRYYRLMIQRTQQNTGVTAAKFFIVGIEKFGKVVNLSYSYYLVLKDVLD
SL

>XP_019932563.1 PREDICTED: uncharacterized protein LOC109622604 [Aedes albopictus]

MFAFLRVVRESLRYRWLTNIIHCWIQRASNCFWLLDSLLLLAGIRSELTSHWTMERPIRYFVNCLFGCQA
VAAGLRLFHALVHDQENSFLIVCGFLVVISLSFTFMKVFALNCLRKPIDTLRSFIISDRISSDDVHFDEV
QQNNFYRSARGMMLVVYVLIILDTVLLSIPNHSSEGIFGIPPQFSWIEGPVASIVSFLLVSLLAVGFFPK
YLSNVTCVGILLMGMHAKLKILAYRYDRLLNEPVLSYGQYFTRIDREVRGIFVQQLEYWKHLRILKNVVG
KTFFFVHYYAIFSIGTSGYAIQKVGINTLSIVSLASTLIFLLEYYLWCHWIDSLQDVADNIGRTISELCI
RMPYSREYHTQYTGLRISLMITWINTQHAFTMDCLGLFKISTFAFTDLVDTAYTMLMFLVNVC

>EDS41940.1 conserved hypothetical protein [Culex quinquefasciatus]

MQLFPRALRFGELDQVFPIVLRLLRFFRAWGSRRRLLGLYGFFAVHFSVRLLPLIVYLDTSDMQTLIRHL
GEIVFVGMLYPVFVVYVWKIPKLIQLVEILDRAFAKYDQPKEFREEIVKTNKFVRNICRFYFVYTGANIP
VYIVAPILLTFWKYLRWNNPNEPFYFDFPNELPLLNHLNMWHYVICEVLIAPVFFCSAIFLALKSMLYYS
LLQYVSLMFKLVLKRIQLLDKSLSHRRRRRQLNRQVDAVVKHHYLALKCAAILEELISPILLAQFLGCVI
VWCMLIFYMTMSIGDFGALTTLILCEILAFEMLAFSFFGSELTHVSSSVATEIYNFRWYDAPLAIQRKVL
LISVRSQRIVGVTAFKFYYVTIEQFGKAVQTTYSFYLVMKKLFEGQ

>EDS39720.1 conserved hypothetical protein [Culex quinquefasciatus]

MKFYELREPMAAVPFILRVLRFSGLLGCPRGLLRFGLSFLGPWLVIGLPKLICGFGSDLGLNVRGYAEVL
FMCNIDVRMLVFFWHRRKLAEFVEIVQRAFDKVSILSSDSSMYKMILKSNQMMDKSAKSYVLYTLGTSGV
FLVLPALQSCGIYFMNHGNDTVVPKFVTATAHEESGWDVDENIVYYFIHVMLITPMHLLLGLRFATIDTM
IFCGVRSTILLFRLVSAKLEKLHKFSGSTLREQFLDVVNLHVDALRCVQILEGIFSFVVMVQLVSTVIIW
IAMVLCVSNNPNANAINLFVLLILITAQSYILCRLGTELTAESFAVATSSYDCQWIQLPADIRSGVGRIL
QRAQKWEGITAAHFFQLDVERFGAMVQTSYSIFVILRERLMHS

>EDS39719.1 conserved hypothetical protein [Culex quinquefasciatus]

MKSHSPLNCMAVMPFTLRCVKLFGFRGGLHNRAHFLAALAYRLVVINLPKVAFGLSDQIDVAIRSVSELL
FQWHIDIRVVLFAAKVPEFEQLVRILRRVYNKVKTLDSNSSERKLIEDSNRSLDRRSKSYALYVAVAVTI
FYWVPVVQTSTIWLRSLGDNSTTRPKYVMMMEMEFYGLDTRGNIWHYLVYASLSSIAHYYSAVYFALAGM
VIFSCIKSIAALFELVSAKLATLHELSGKALREELAELVELHVDGLRCIKLLEDIHSLVMMAQMVNCVLI
WISMILSISTRFTSETTSLLVLLIVMTGETYVLCQLATELSEVSLTITDSIWNSHWIKLPVDVRKGLAMM
LQRAQKKEGLTAAKFFFMDVERFGRVLQTSYSIYVVLKERL

>EDS39717.1 conserved hypothetical protein [Culex quinquefasciatus]
MRFAPLQDRMAVMPFTLRCLRLFGLRGDRRNRVHFVLALLYRVVVIYVPKLVFGFRDRVDLVIRSISELF
FECHIDLNAVLFAVKLDEFEELLCLLRKLYNKVKTLDANSPERMIIEASNLAIDKRSKSYVVYVAIACTI
FFWVPVAQTTGIWILSHGSNITDRPEFVTMMELNFYGMDNRKDIVHYVIYAAISGVAHYYAAVYFALSGI
VIFGCVKSIAALFDMVSARLAALHELSGRELREELVDLIELHVDGLRCIELLENINNLAMMVQMINCVLI
WISMFLSISSHFTPEVVSLMVLLLVTTGETYVLCQLATELSQSLTITDSIWNSNWIGLPVDVQKGLAMML
QRAQKKAGLTAAKFCFMDIERFARVAQSSYSVFVILKDSI

>EDS39367.1 conserved hypothetical protein [Culex quinquefasciatus]
MEALDKFMQYTNYVRGLCKVLGMDVLGPNYKRNYRTNISFLMIAIYAILSINSFMLADSSIEMLKAISFG
GFFCQCVLKIYFTLTKSEQYHENLTTIKKAIYLDHLDGNERQKRDILKTVDLLHVVVKGTTLLYFSSVIL
FSIYPAYMYFIVDVKVTIFPLYVPGVDIYSAYGYGFTNSIHLLLSIYGLFGALASDTAFIMFVFHIYSYT
DLLMIEFDEFADKLGQIEESKDTKQYEAYCRFKMREILLNHKDIIAYLVSLNNCYQNISSVQVATCSVSI
CLNLFLALVTDWYATYGFLVASVFQLLVFSVLGTIIQLMNDRIIKLIYNLPWHLLPNNEKKSFCFLLFKS
QRPIEILIRGLGPMNVETFTEIMKVIYQAFTMMYSFLIDQ

>EDS32845.1 conserved hypothetical protein [Culex quinquefasciatus]
MLMTIRKVMKLIRNAWRDFASKDCFQLLDILWILGGSLNLPSQGWKRGVFFLWHGLTAFHFGISLFNVVG
KVTTGDSFSMTMWYLSFTAATSVCLIKMLFMWHHDQAIQQVIAYLDSKCNLSGDVQHDRSVREKYFTRAR
ITIRIIFGLMAVYQVLFSIPHPQQDKLYHFPLKYFWCTGMIVVILMNFQAELKILIHKFRAVLERMESSM
STEPFEPENDPDRIQAETVLWNQLSADVGLAIEQHVEFIGFFQSVQPILGVSFLLYYYFALATYASVLFL
ILNEPISFVTIFQATSVLGSVLECYLICLLVDNFQEMNEEIQTIARDICIGMPHSDEHHSSYDGMRATMM
IIILSNDSLISCAGVFDISVETFAGLIDTSYSYLTFLLSFR

>EDS32844.1 conserved hypothetical protein [Culex quinquefasciatus]

MLRFEEVTCKVRHFWTDFGSKDCFLLLDILRILGGSLNLPSRGWKRGVFFLWHGLTAFHFGITLFNVVEK
VTAGESFSMTMWYLSFTAATSICLLKMQFMWTHNKQVKRMIDYVGSKCNFSSDAKHDSSVREKYSTRARN
TIRFVLGLMAVNQVLFSIPHPQQDKLYHFPLLAFLETSWIRQLVQIVYISTIPIIWYSKYFWCTGMIVVI
LMNLQGELKILVHKFSDVLEHMEQFESDNNLDSTQARTLFWNQLNEDVKSALEQHVQFIKYFQSVQPILG
VSFLLYYYFALATYASVLFFILNEPISFGTIFQATSVLGSVLECYLICLLVDNFQEMNEEIQAIARDICI
RMPHSQEYHSDYVGLRTTMMIILLSNNSLISCAGVFDISVETFAGLINASYSYLTFLLSFL

>EDS32841.1 conserved hypothetical protein [Culex quinquefasciatus]
MSLLTMTLCYVKQFLLWYYRESIHKLRTFIVERQFCSGDSSYDSTVRSGFYRNAQNLVTGIFGLILLDQI
MIAAPSPLRKRYFGIPSWFYSYGEATALVVELAFYPTFIIVWVSKLFCSSATVAIVLNGLRTELQILAQY
YGHIMKGLQLEVCSDPSKFHRHFRKSPQTRSWFWSQLRLRTGVGVQHHVHILEYLHLLQPVFEKIFFLIY
YQALIVAGAVFYVTMRDEFTVCSVAVLMCMSISVLECYWWCHLVDSFQDVNNTLSHHLTNQCSQLPHSAD
HHSEYVQMRTTCMIIAERAHRGVEFSCVGMFTISTAAFANLLNVCYSVLMFLINVCDKVDPVKQFQLWMG
SKM

>EDS27262.1 conserved hypothetical protein [Culex quinquefasciatus]
MEYIRKLSQLKVFQHSFKEPSEFYASVLVVPNRVAGVSVGVSLQASMKIFTHLYYRQDLLWIQEYTKKLY
QEECINHKKVLMDFVTMLHVLMKVVVVCYAITASSMIVGPFMLTLLSGQKLLTFGFWIPYIDRNSWFGYG
CNFTLQLILSLFITCEYMGLDIIYFMTMLSSVNQIDLLIIKIKNIGTQIEQTDAKLQESLMEIVKRHEEH
LKFVRTVENMYRGYFFVLYATLGFTIVLVLYAIVTLSWIAGYGSGIFITYQLFIFCLVPTVLEIKKEELQ
REIYNISWYEWSRQNRKSLQLMLQTAQQPNCLSLIFCPLDMPTFVEAMRIIYTILTLLLTFRSGGNSREN
VSLQVSGGMGKFGKRKHD

>EDS25984.1 conserved hypothetical protein [Culex quinquefasciatus]
MTIMEYIQRLERFKFFQHSYKNPSEFYTSLIEIPNKVARVAGLNIFSKDYKVFSWNLACLLTVFVVYGIV
TPFTIYEVRHNVDDLIYCLVTVGIGIQGVMKMVTFLVYRKELIWTQEYTQKIYQEESHNHKQVLMEQVSL
LNIMTRIIITSYIYLGFLLAMGPFLYSLMAGKKALSFGFWIPYLDRFSWPGYLCNVVMQVIMTLFICSEN
LGADVLHFMTMMSSRTQVDMLMIKLRGIKSSDLDIRDKLKEIIRRHQEHLEFVGTVEMIYRSFFFVIFST
LGATLCLSLYAVATLGWVAGSIPCMFIIYEMFVYCFLATILETKEDELQQEIYKVPWYQLSISDQKSLRI
VLEATQQPVCLTLIFYPLNMPTFVDLPATTTPPLATATMRLVTLEWLD

>KXJ84422.1 hypothetical protein RP20_CCG011520 [Aedes albopictus]

MFLKKTAQPEPLQVGLKLLKWIGLHSTTSRKRIWRYYFVVGWLLGNIVLPRALLGSGNEGFDSLVRSLAE
MVFFSDVCIAVGIFATRLRYFERMVQILGEIFERYETKECVEEIRGFNRRMDSFAKVYIAYIVMLVILFN
IPPIIWNLYMAIFVSAEYRSSYVLLVEVQYFYLDIRRNIVHYLIYYVLCSTATVCSAYQSCIKGTIFLTA
LQYGAKLFELLHLRIDRLGKVKAGERRRDELRRIIELHKMTLKYTELLEETITFIMINQILNCMAIWCMF
TVYLSMNYGPNALNVVVLFVVFIVEMVVYCVSGTRLSENCAALDDVACPAIYHQTTCPTVVVDVVETDAS
LISTSRLMNGFKFHSVLLSGDVWALHLLAAAAAAQLALFNSTPFIIAFVHRTMIDRHKINQSPRHDRGAA
AGGAPWISTRPSPG

>KXJ84122.1 hypothetical protein RP20_CCG019769 [Aedes albopictus]
MSRFAFAEGPAQYRPFQLQKRLFRVLGYYPGDDRLAHWGMLLVLFFHYWSQVMLIYWEGKHAWIKIGEGD
LQPALEGICPTPSRFGGILKCCILIWKRKELKQLLDKLKGWFDREEPREKKVNLWATYWGYQFTYWELMF
THLTCVFYCLLPVAAMLFHLMKQPDEPRIYILPFKLALPFDYCKSPIFEITYLIMCYIAYPPIFMMAGGD
GLFIGVCLLISSQYRIVQQELEALGNSVESDHHHRGTGEVTAEENDHVFEQLKLIAQRHNRTIDTTEEMS
RLFLQNVFASFTIAAIKIGIACITVMKAEGPNKLIFVWYSLGILTEIYLYSYGGTQLMEESEELNRTAYD
FPWYRYRKNVRQIIQMMMLRAQKPSRVDVPFFEASVVTFSTVIDWTWFLATVCAVLLVEMLYCDDKSRTW
LPEKRRSYAVTRLCCFDVIQVAYYGYTFILERVHSIT

>KXJ83446.1 hypothetical protein RP20_CCG004843 [Aedes albopictus]
MGGVRRLTNWLKSKLGTMDDESDYFRQMEWALMIAGVQLPSENPSHHKWLFCYRVAILFQCCIWCDRFFV
ACTEWNSPAELIGVFSFVLALVMIISRVILMRVYLKDILKVRSYLETILNKDLTEGRVRSYRLIRRIFLV
LEWLFLCDQIILYTFGVYEERQYSVPDNISRLGPRVKLVFDILICSNHIMLSSIYAAILTILNTLIMGFS
TELENVVFQCNGIFKRVDDQMSSTVSLHAKMATSKFWNIFKTELNMVVTRHAEVIEQVATVRTLLKISFL
FIFYTEIVFIGCALFYVKMLGLTMNTVVVVSYVTAILMECYWFCRLTDIVNDTNHEIGCVLYNLNWPEKL
CDIPNLRREYLEIRATILVIMTRAQKNLGITCGGMFEMSAAAFHELMKMIYSCLMFLLSITTH

>KXJ82818.1 hypothetical protein RP20_CCG010978 [Aedes albopictus]
MQPRGRVVCPHAKMKYFELVEPEAVMPLALRLMETYGLRGGKRKFLQFQVTILWEFLMIVLPKIIFGYRS
QDLVIRGLSELLFQLHIMIRISIFAWHRFKFEDLVAIIRRVYRKTFSPGADSKLKEIILDFNKMINKQSK
GYFLYIMGCVSLFTVAPVVQSVVIFITHQGNNGTDKAEYVTMMEQEFYGLNIRGNFAHYVVYVGLAGLAH
YYSASFFAVTGVVMICAVRCTILTFRLIIVRLSKLHELPKEDIRDELREIIDLHVDALRCIQLMEQIANL
AMVIQIVDCVLIWISMILYMRNNLGVDAISLMVLFVALTGETYALCDLLTQLTSESLAVTRAIIDCQWYN
LPLDVQKALSFILFRAQRNEGITAAKFFYMDIERFGNVAQTSYSIYVVLKDQL

>KXJ82754.1 hypothetical protein RP20_CCG011372 [Aedes albopictus]
MESILSCPIVSVNARVWRFWSFVLKHDAMRYISIIPVTVMTFFMFLDLGHSWGDFQDVIIKGYFAVLYFN
AVRIDDHQIQSLLKSYTARARMLSISNLALGAIISTCFTVYPMFTGVRGLPYGIYIPGVDGYQSPQYEII
YLVQVVLTFPGCCMYIPFTSFFVSTTLFGLVQIKTLQRQLQTFKDGIGSHENKNANLQVIKLIQDHKRII
AYVSELNSLVTYICFVEFLSFGLMLCALLFLLNVIENHAQIVIVAAYIFMIISQIFAFYWHANEVREESM
NIAEAAYSGPWVELDDSIKKKLLLIILRAQQPLEITVGNVYPMTLEMFQSLLNASYSYFTLLRRVYN

>KXJ82313.1 hypothetical protein RP20_CCG014266 [Aedes albopictus]
MRRFQKSYELYDYNLMLIRRLADICGLDIMAQNYKFNLRFLVVSILVLITAASLLYSCVFYFQNWYKMLE
IAILLALVMQALVKMYIAFIHRHFYETMYRRLRDFHYKFRNHKNHEQLYKTMEKIHLTTKLFLALYIFTG
FSFSVHPLYLYLSERRRELLVCIRLPWVDVDSLGGYVVTMCFQSSILVLFVIGFSASDSVILLFVCSLIA
FVDVFKYNLMELTNMLNEFNPDKPTIRKKVRQIVMQQLDIIHYSKCVCLERYSQLRYAFLSTTSNESASI
EASLQNEEITEAIYETSWYLMETPEQKTIALMLHKSQHYVEMTIGGLQPMNVETFVAIMKSIYTYLMMLL
NFIE

>KXJ80035.1 hypothetical protein RP20_CCG027084 [Aedes albopictus]
MNKITRIWRPCFHEFHLSSDYFWLADALCIISEQRPPLTKRASIRFLWTTLSILHAFQYGCFVVQLIHCL
KQSPVNKHQFAAIVNLMIPLAVAVVRGMCLANQRESVLKLKRYINSKICQRDDIRSFELRKQQYRKINRN
LAAFHCVTTANSVVWALTAGLHGDEVFKVPFQLTKEFEGLQNVIDVGYAVLLIPWCFTLWYSPTQFVPIL
SFFNTELRIIVAQFDGLFEKVKVNYTLELNDRAEMSMAQRTRFWAELDVAFKDAISHHSSYISAAIITFN
IFPIIVNPSLEHLPLLLLAVQYACESYLCCSMFSSLESENRRIAKYVYGIDWLCKERYDAANELDRIYRK
SIKQNALILHQRVNKALAIKAGDMFPLTLETFSHMMKSVYTLLTLLLQSMD

>KXJ79577.1 hypothetical protein RP20_CCG000278 [Aedes albopictus]
MINESRLEILMNTIQIGAGLIIVLCRTLVIGWNYEKLMNLRRYLNSRKFYREDEVAFKIRRKVYLFIHRI
VLLFVINSCLMTAPIFIIQPTAPLRIPFKFGSYVLDIVAQKIYLLMIIQIVIDLATNFFVIVMLLTGLTA
ECEILSKAVAELFKTTILKLETTGPSRSSSRTLDEQFWTTLNHQFDKCIVEHRTILRHLIDIRPLLEGSF
LITYYTATLNIAAGAFFLISNLDHINLYIYQICHYTIVLTLECFVFTFFTTKLSDAYKSIGQSAHEMDWP
DHLQHSHQFERQYRAVRAKILIMMTVGSQEVRFSAGGYFEFTQEKFTDLMNKSYSMIMFLWEMRK

>KXJ79464.1 hypothetical protein RP20_CCG000712 [Aedes albopictus]

MIDLFRSIWSRVRKWSSTVSYFWSHNDPADCFLLMDTMLLLAGVQSGLNQKRTSTLIVRYLSNGLFAFIS
IILILQVWSDTQEGWSDVTLKINSLLKITTIVMSCLKVLMINHFREPAQNLKNFILSQTVTSGFKSTDDL
DRRKFNSIALRMLRAMFALLIADLILLSFPNQTMDNAFRMPPLLLQHMGKFSLRILCSFFVCALPLVFVP
RYFTHLSCMGILIMGMRMKLQMLAHRFRRITHLPVVNAKYNLEQINDELREILNQHMEYMGCFKMLANMV
GTAFLLTHYYSLLNIGALVYMWHTMGFNGFSMVYLLTFIALLFEYYIWCRLVDSFQEVADSIGQLVYEIC
VYMPHNADHHSEYHQLRTSLIIIWINNAKAYTVSCYGLLKVSTLAFVDMVDITYTVLMFLINMIDH

>KXJ79342.1 hypothetical protein RP20_CCG001075 [Aedes albopictus]
MANLWLNHLRHHIKNIFKLTPSKNHFCIVSFFHAIACVNLTSKQPLLRWIWNAYRACMVLHYVLCGIRFS
MTVRTSENFNSVMNNMHVIITFTINNMRSFIIFANFEHFARVKNFINNRKYRKDHAGSGEVRRKTYEHAV
KVTMFFVGNVIFQALSFLISGMANTEPFLIPMELGFLPYFAKEAVEFCYSMLFLTAPFFAASNFLSLYLA
IIGVRAELRIALDSIGSIPDRVNQHDYANDANEKFDQGDEFWNVLHSEMKYSIEHHVQVLDHLNIFKNVT
NYSFLLLYYMTMLQIAAGVLIVIFNPVVDLFYILSIDYSFRYTLECFVFCYMVSSLNEIQLTIGDIISHQ
PWISRMRFNKQFGKQYRSVRANLLIELTESQRQLGINCGGMFEFTLEKFTHIIKTSYSITMFLWTFRK

>KXJ78652.1 hypothetical protein RP20_CCG003920 [Aedes albopictus]
MLRCATNTNHRIGTVIDDRHEARALTRADGTAATSSERVSAVRSNRESSSERRRRAVAIIIIIDTRRPAV
NVMRKFRADDPAAFAIRQRTYANTINITIAMILNCFVQTVIVLFTDLANSESLQLPFYLTGVSSLENNLL
EKIHSGMFSVYVYFASTNFLAVFLPLSTLKAEMEVVGDAFDKIMDHVDVRMAALELNEIDEIREEQFWKI
LQEELTQCFIAHTAVLDKVKDLKKLTDPTFLMLYYMTMLFIAVGVMAVLFSPQFDSFNTLSLEYSFRYTL
ECYSFCYVVSRFNEEHNGIVNKLSHFHWGVDLRYSKRFAREYKKVRSMVLMIIMQSQKSLNFSCGGLFEL
TMGSFTSIINKTYTLTTYFWNIKQRT

>KXJ78651.1 hypothetical protein RP20_CCG003919 [Aedes albopictus]
MFCSIKTRFTEWWKHLTKLKPGRDSFFLLDYFLVLAGIHLLTRNGYLRTVWNFYRALLFIHVVLMTRKVM
EIFQTEDNFQLIANNMIMCVGCFIIFARCVFIVTSFKQVNSIRNLVNRREFQANDPTAMAIRQSAFNNAI
FVTLFMAMNSLTQTFMVLFTDLGKTESLLLPFDLVGLSSSENFILQRIYSGMFSIYVFLASTNFLAVYLP
LTALKAEMRIVVYSYSKITNQVEDRMSGLGHDAVDDSNEAHFWEILQDELTQCVRAHAAVLDKVGEFKKL
SDPTFLLLYYMTMLYAAIGVIAVLFTPKLNTFNTISLEYSMRYMLECFVFCHVVSNFNEQHNNIVNKLSQ
FHWGVDLRYSKRFSREYKQIRSMVLMIIMQSQKSLNFSCGGLFELTMGSFTTIVNKTYTLTMYFWNIKKR
E

>KXJ78605.1 hypothetical protein RP20_CCG004018 [Aedes albopictus]
MDGHRNLRWIIVLVLLFTPLILIIIRMTRETTVLMLGYCIISFMAYVLVAFKMTVVRLTFPKAQILIDYF
NAHVFHRDDPVSYRLRRKTYFITWKIFFSVGAFNVCYVVAFMALTNPTSSYVGIGESEVSWYHMALIQAA
AVFSLLHHGIYATSILVVSFLMHWFQTELEILAKAFAKIFHDKPPIALRKSEKRRTVYVNKEEILWTGIE
RRLIYCISRHGEVMELVGVLRQIVEPIFLGLGFYIVTTISTLIFVMIKDHAFNYLGLIHVVVTIIEFYYY
AHLVDDLDEKNNLIAIALYQQDWHGQMKYKLTLEKHYRSVKTMMMIVIMHSQKPFRFTCGGLYKMSVPVF
TTMIETLYFAVTFMIRTIKIQR

>KXJ77288.1 hypothetical protein RP20_CCG007898 [Aedes albopictus]
MESILSCPIVSVNARVWRFWSFVLKHDAMRYISIIPVTVMTFFMFLDLGHSWGDFQDVIIKGYFAVLYFN
AVLRTLILVKDRKLYENFMEGISKFYFEISRIDDHQIQSLLRSYTTRARMLSISNLALGAIISTCFTVYP
MFTGVRGLPYGMFIPGVDGYQSPQYEIIYLVQVVLTFPGCCMYIPFTSFFVSTTLFGLVQIKTLQRQLQT
FKDGIGSHENKNADLQVIKLIQDHKRIIAYVSELNSLVTYICFVEFLSFGLMLCALLFLLNVIENHAQIV
IVAAYIFMIISQIFAFYWHANEVREESMNIAVAAYSGPWVELDDSIKKKLLLIILRAQQPLEITVGNVYP
MTLEMFQSLLNASYSYFTLLRRVYN

>KXJ77275.1 hypothetical protein RP20_CCG007975 [Aedes albopictus]
MESIIKLLQFCGFWSQPYQRHRLRKPLCHIGVIVGCLLGPGIIFIVRNSFDFASAISAAIESMGFVNTVL
LGTNLLYHRSALESAYGDIRLALQTAKYAANADVLQNIEFLEKSTSFLFKAYTAFQSVVGTGYALTIPSL
TLIHYTKTGQLPPLHGIFEADFFMFDFTTKFWLWVVVIVIGSFGMLCLISVLVIVSSFNWSLVHYLIGLF
KIVHSRISCLDDLPDQQSRQKELTEIVQLQELVYRCARTAESALNIFLLTQFGTCVVAIGLTMMTLTLAS
NDRDLLIKMVLMLAYILFNIFVYSMLGQELMSTSTSVADAAYGTRWYDWSLSEQRNVLFVVSRSQRMAAL
TTGKFFVVNRATFASTLQAAYSNFTILRQMLESR

>KXJ77274.1 hypothetical protein RP20_CCG007974 [Aedes albopictus]
MRSIIKLLQWLGFWTQPYQTRSSSKPLAFTALYIGWLLFPGIIYIFRQQPSFAVITRTAVESMSIANITM
LIVSTIVHQPVLEKAYGDVRFGLAAVSDSLDRELQRTIDHLGTSTDRFFKVYIGFQMTVGLIYTLSNPIL
TIVKYVRSGELPPLHGILEADFYFFDYTSNFWLWVLVVGVGGLSLFSIIVVIVSINSLNWSLIHHVAGLF
KIIRQRLSNLNAFSNEMSRKQELVDIIELHEVVYRSARLLEQALNVYMLLQFGTCIIMLCLTLMVLVLAN
DDRDLLIKMILMISFILFHILVYSMLGSELMSASASVADAVHEVSWYQWPVVEQRKLLFVLNRSQRITAL
TTGKFFYLNRETFGMVFVFTVFTKQPV

>KXJ77255.1 hypothetical protein RP20_CCG008015 [Aedes albopictus]

MLNVLLLCAGIRTQLAVGWTWERPVRYLVHLISAYHSVVIILQAVHVIAEKNDVMEAAFCLIKIVGVVSA
YIKILLLTYHANSIDEVELFIRSKPMDSGDEEYDSRVRGKFLRLTLVVVRYVFGVLIVDEILFAFPNSQR
NKLFKLPPVMSSLGSGITAWLANFMFVNWMPLIWLSKYLCCTIKLGVLLMGLRVEFKILTHKLEQITKKA
KTFECVEDHCKFLKDELRVFLEKQAEFRRKFELIRPILGMAFFMIYYYSLFFIGTMLYVTHHQGFEFYSL
TFASSVLVTLLECYWWCQLVDSFQDDAESMGNELFDLSAGIPYARENHRKYVGMRTSLMIIWINARHNPA
IDCVGIFTISTAIFVQMLNTSYSVLMFLINMG

>KXJ76837.1 hypothetical protein RP20_CCG008906 [Aedes albopictus]

MELSHYRSLPSELQIMPFQLRCMELIGLAGPKGRFYRFVLAFGWGTFVILLPKSVLGIGSPRLDAIIKGF
AELLFEGNLFIAVASLIPKLPLLKRLLHVLSEIFTQVTHDARSKDQCYELICEQNSKIDKFCKFYFVYCC
FGPFVFCIPAMVTSYVRYFGGAGNGTDNADSNETEQQLLFELPMEQDSLAYRLVAIRIRKLAELPASGQE
QNFEEVTDGGDVLKMSMVKEVVDLHKKALEVTDLVEEIINIPIAMQFIACILFWCMTMFYVSTNINFNLF
NVMVLFWLSLIETYGYSYLGTELTEEAKTVGQVIYELPWYEDSAQLQRYYRLMIQRTQQNTGVTAAKFFI
VGIEKFGKVVNLSYSYYLVLKDVLDSL

>KXJ76122.1 hypothetical protein RP20_CCG010302 [Aedes albopictus]

MEVTKITGISISFLRMVAAAMLIEPFTTLRRFVSANSVNSGDAQYDECERNKFNGFARTAMTLIVMWITT
DTILFAIPSSTKDELFKFPLMLSGIGETASRILNIVLVSCIPACVFPKTAGCTAYIAVLLKGMRMKLRML
AHRFERITEVPAIYEDEYFDHVNWEVQHALLQHLEYWKYLDILKDLVGKVFLLVHYFSIFAIGAMFYVCR
EVRISFMTVIYIAGLAFSLSEYFLLCYLIDSLQDEADSLEQHIFEICALVPFRPERRSEYVQWRTTLMTI
WINTRNGMSMWCSGLFEINTSQFVAVLNIAYSMLTFLLQMG

>KXJ75420.1 hypothetical protein RP20_CCG011758 [Aedes albopictus]

MLIENCPIINVNVKVWLFWAYLRKPKWYSYLLGCVPVTVLNVFQFMNLFNVIASGSGDMNKIIIDGYFTV
LYFNLVLRTSFLMGNRGKFETFLEGIADEYAVLEKQNDIRPLMDQLTRRARILSKSNLWLGAFISACFVT
YPLFSPDNGLPYGVYIPGVDVHASPIYEIVFVLQIYLTFPACCMYIPFSSFYCTCALFGLIRIAALKRSL
EKIHEFNISPRSLFARIKECLQYHEDIIKYVSDLNELVTYIFLLELLSFGMMLCALLFLLSISNQLAQMV
MIGSYIFMILSQMYALYWHSNEVREQSLEIGDSLYYNSAWLDFDQSVKKKIILILARAQRPLAVICIIRY
GRLLIDRFLLLQIKIGNVYPMTLEMFQSLLNASYSYFTSLRRVYN

>KXJ74893.1 hypothetical protein RP20_CCG012785 [Aedes albopictus]

MDNFLRTHVRMYDEERLARLDSLDIFDEMITFLRVFLDFCGADVLLMEKFRWNFRTWLCFWVLVSFSICL
FYTIAYSSEDIFAIMDTLSLSGIALQGAFKMHGALTRVKYFQQKYMDIRAMYIRFSREPNTNLSLSKCCL
TITYIFRFFLLVYAAGGLAYFIIPVYMLVVHQKVTLILHLELPFVDPNELTGYVITTIYQAVMIILAIAG
ILAADMAIMILVLHIFGIVDIFSNKLEALDRILENPIVDPELVKSKVTEICVMHREIIKYEEDLDECYHT
TVFIQVMSSVSCLSMALFVVYMTRDWTRVMFLGATFFQLLEFCTLGTALTLKNDQARIALYNTKWYLLSI
SDQQRLKFVLHRSQNAVEMTIGGLALLNMETFVAIMKTIYSYFTMLITFIE

>KXJ74892.1 hypothetical protein RP20_CCG012784 [Aedes albopictus]
MDRFLTTHLRMFDENRLTLLSSQDLFDEMINFLRVFLTFCGSDVLLMDKFRWNARTWLCFWTLVSFAITL
FYTIVFRSDDIYAIMDTLSYSGIAIQGAFKMHGALSRVTRLAFFVIPLYVLFVYQKVVLILHVEIPFVDP
DVFSGYVITTAYQVMMIALAIAGILAADMGIMILVLHIVGIVDIFRNKLKELDRMLEDAQYSKQQIHEKV
SEICVMHREIIKYEEDLDECYHTTVFVQVLTSVACLSLALFVVYMTNDWTRAMFLAATFFQLLEFCLLGT
ALTLKNDQAIVAIYHTKWYLLTPSDQKRLMFVLHRSQNAVEMTIGGVALLNMETFVAIMKTIYSYFTMLL
TFISNE

>KXJ74632.1 hypothetical protein RP20_CCG012996 [Aedes albopictus]
MLFSVRSFLAAKYHAFNNSDNSFVLMTFCNLFTISYQIFEIISLSMAIISTGFDGQLGSVYLVTTRVLSF
VMWGSLAVHRHDLKLMWIFLMGLQRRCADHRRRKHIRTINWIMLAFLVQELIPMTVWIIKGHTGSVVSFY
ENDTIEKLNSVFYPVTLFTLTFVFSYALIVGTSIIAALGLEFRMVGSDFEGMFDNLGSLEPSNEDSEHNW
TTMENTFKRCALRHQILLGVTITLRDLLKTNSLIQLLTYFTMVAVGAFVYVFTQTSFGIGVVFFAFCVFA
TSVNLLLYGFVCDRLDDQMISIGHRVYSSGWPDKLIYSRAYARRFKDFRKMMLIVMERAQKSVEFTCGNF
FVMSLITCRQVLWFAYSVFTVMISFLE

>KXJ74631.1 hypothetical protein RP20_CCG012995 [Aedes albopictus]
MIFIIKTYFIGKYRSFHHSNNCFVVLMFCNRLIGFWDNPGADPDRFHPVKRILINFSFMYATLEVISVTM
AIRTTGFNENVGSLFLVIARQFCLAIWCSLAFNRQGLKEIWVYLLSVQQDAVDDSRHKYIRFVNWFTLMF
LILNLLPLMVWVINGQIGSPLSIFGSPWLDRLNTVFYPAAVFLITLMLCYAIIMTLSIMSAFTLEFRLLG
MDFQQIFEEVEPHGEIIVQRWINLEKSFGRCVERHQTLLSLTDSFRQLLKTNFLIQLVVNFCLIVLHFSV
YVLMQRHEVSGFAFSAFAVIALMMNMLLYGFLCDQLEEQVLSINHQLYCSGWTDKMIYSRAFDRRYKNLR
QTMLIVMERTQKKVGFTCGNFFEMSLVTCRKVLSFTYTVVAVLTSVLE

>KXJ74630.1 hypothetical protein RP20_CCG012994 [Aedes albopictus]

MFKFLLSFKTFLVRKYRAFNASDNSFVIMMFCNHFVGIFDNPDPDSARLRLFKRSLLTFALLYVGAEFVS
MCRFRAFSIHQDYGILFMTCGRLLCVFMWSSLALYHQDLKRSWIFLLDSQRRTPDDRRRKFIRTANRITL
LFLMQDVLPMVVWAMSGHTESPLSLYQDDLIDKVNTVVNPIAVLILTLMFCYSIVVVGSILPALTLEFHL
LGLDFERLFEGVGSLDDVESWDTVELAFKLCVERHQVLLDTANGLRQQLKIYFLVQLGINFIAIVFSLLI
YIYTQRSNDSSYVFNAFGSMSILCNLLLYGYLCDRLEEQVAAVSRHLYCSAWTGRKFDTATFGKRWKNLR
RMMLTVMERTQRKVGFTCGNFFGMSLVTCRKVLWFAYSVLAMLMNFLEQAF

>KXJ74448.1 hypothetical protein RP20_CCG013594 [Aedes albopictus]
METIIKLLHSFGLWTQPYQKHKLWKPLGILMSQTVFLVGPGIVFVFRNHSNFTSAIRAAIESIELFNVVC
LAMNLLIHRPALERSYGELQFARQMVACDAQEDVQKALKKLGKITRISFKAYITFQTIISGSYTISFPIS
TVVHYIKTGYLPPLHGIFEADFIFFDVTSNFWAWVSVIIVTGLSFLSLSVVLVAINSLNCGLLLQVTGFF
KIVCMKISRIDEIASRQSRHKELAEIVELQELAYRNARVLEGSLNQIMLLQFAMCIVIVCLTMITLTLAR
DNKELLINMVIILVYIFGHLLVYSVLATDLITTSTSVSDAMYGTQWYDWTIPEQRNVLTVLCRSQRMAAL
TIGKFFNINHNTVGKTLQTTYSHFTVLRQLIDSH

>KXJ73965.1 hypothetical protein RP20_CCG014651 [Aedes albopictus]
MTRGSAEELEKSFEVAKKTTYLVGLNPFIGDRTVTGRFVFSALLMLGIYFLCFYTLWVVGSEWQTSLEVI
QILMLNVLGTNKLWLGFMRSYDYYLLFVSSADIYKIFDADIRNRPILRQMVRILAVTLKSMALIYTLSGS
LILVLMTAVILITKERLLLLRIYIPFVDHTTPVGYAITTALHMLMIAYCVNGYLASDSVFVSTILPIVGY
TNALRNEIDNFNATLHEAEQNEDEIAEQLMHIVKLHQRIVQYEGQAVAFFKETNLIQINLQAGLLLALVF
MGLILHYLPAMCTTLAILFELTLYCSLGTIITTKNNKMILDIYDIDWHLLPKSKQMLVLFMLHRAQNGRS
LCVGNFAPMDMQTYVKILKTVYTFLAMLITVMD

>KXJ73651.1 hypothetical protein RP20_CCG015303 [Aedes albopictus]
MTVIPGLVHFGSLEEVYPLVITLLKRLGAWGSRRQLYGLYGFFLCHFFLRLLPLILYLDAGDIHALVRHL
GEIVFISMLYPVFIVYVWKLPKLMLLIKILRRSFYEYCGPHQPPVYREAIVKTNRFIRNICRFYFAYTGL
NIPVYIVMPPALTFYKYFSWNSNSSEPFYFNFPNELPYMNHFNMGHYITVTSLIAPIFFCSALFLAMKSM
VYYSLIKYVSLMFKLVVKRIRLLDDSISIGGGSRLEQAVDDVIRAHYLALRCAQLLEELISPILLAQFLG
CVIVWCLLLFYITLNVSGVGAMTTLILCEIVAFEMLAFSYFGSELTEISESLAHEIYSFRWYDAPMTVRK
KVLLMSVRAQQIVGITALRFYYVSIEQFGQAVQTTYSFYLVMKKLFE

>KXJ73228.1 hypothetical protein RP20_CCG016235 [Aedes albopictus]

MDGIRRFNNWLKSKLGTLDDESDYFRQMEWSLMIAGVQFPSANPNNRKWLFCYRVVTLFQFSIWCDRFLV
ACTEWNSPAELIGVFSFFLALVMIISRAILMRVYLKDILKVRNYLETILNRDYTEGRVRSYRLIRRIFLV
LEWLYFWDQLILYLFGINEERQYSVPDNISRLGPRVKLTFDILISSNHFMFSSIYAAILTIMNTLFMAFS
TELENIVLQCNGIFERVDEQMADSVGMHENMVGSKFCTILKRELNMIATRHAGLIEQVATMKSLLKISFL
LIFYMEIAFIGCALFYAKMLGLTMNTVIVVCYVTAILMECYWFCRLTDIINYTNHEIGFALYNLNWPEKF
CDMPNSRKEYLEIRATLLVIMTRAQQNLGITCGGMFEMSAAAFHELMKMIYSCLMFLLSVTT

>KXJ72891.1 hypothetical protein RP20_CCG017037 [Aedes albopictus]
MWPRLRKFLAPEPELLSFGLQMLRFVGLWGDRRRIVRYLLVLLSELMFLIGPKALLGSGKEGIDSTVRNI
AELIFLVEVCISIGIFASRRTSFERLMAVLEEILRRKWPQDLQDEIDRFHRRMEFFARAYALYIGFLVVL
FCCVPVGSTLVKLVRFDESERSDFMLVVELQFFWFDIRRNAFHYAIYMMFCLVAVSCSAYQSVLKGSIIV
VVTQYGSKLFELISKRIEAMAKIPKQTDRDRELREIVKLHSLAMEYVHHLESTISFVMINQIMNCILIWC
LMMFYVSTNFGPNAANVMLLFLVLMGEMVVYCLNGTALSERAAGVSHAIYHYPWYTESVQMQKHMQLIIQ
RAQGPTGVTAAKFYYVNIERLGMVIQASYSYYLILKKRF

>KXJ72046.1 hypothetical protein RP20_CCG019061 [Aedes albopictus]
MSAIDLYQQCIRIFKFNSIAVGVSLWNADQFLTIGSYSIMTQMVIYFWCNFWTVHKYRHDMLHVMEVLNS
AGIAFQLSVKFFIAMYNKPIIRGLLQTVEDKLYARYPDRSSREGEIVYKFAKKCNILIKILAPLYCSSLF
VFALYPLYIYYSEGRLIPLFMYEVSYVDWHTVWGYLLTNFVQIFIYVMGLNGLIMADGLVVLLALHGIVY
MEVFMIHLDELAKLLQSENVIENKEKITKMWRECLSEHQTLLEYLLRLFIVNPSDALDISVVNFPWHLLD
GDLQKEYLFLICRMRQPIILTVYGFSDLSLETYMSVGIIS

>KXJ72015.1 hypothetical protein RP20_CCG019151 [Aedes albopictus]
MSHPVRASFPANITLPRCLAFVNRVQNPLALLKTVDQCVGFISWDFTTKLSYAKLIAVVIMYGYLLVSFI
CALFLVNPAEVHPDYYLKMWFFIGAGVACSLPWIAMFPARHHFRTILEFFTDQYRQDPYHPLRVRSRPIV
FLCSAFFFTINTSISVFWCILLQGSCPMVFAFRYSIVERVSPTLYLVQCFHLGMTANGTMVTALTILLAF
IVEFDVLGDDLRECFDTMTVDIIRRGVGRHQRLLDMVNLFREKIKPYFLIAMGLYLFLVTFSCFLLVVQL
REGDYQALRFNVFNAAISIVSIVMLGAICDVLEDRVKQVGKQVYESGWPLKLVHNREQQDIYRWQKSSLL
MVLARSQKKVGFTAGNIFQMSSVTSMQALKLCYTAFTMLWNATND

>KXJ72013.1 hypothetical protein RP20_CCG019149 [Aedes albopictus]
MSARTDFLQNPPAFPILGNLRDPFAFQKLLDRCIGFIHWDTKSAFTRAKLLLLAITGAYYTMSCLCLARI

NPTEVPLDHFFGMWFLVGGGCSCFSQWYVLAIERRHLAKVIEFLSDLQRRGVDHPTRARYRPRIVLYSIV
HWTTNVSQTVVWSATLIFTSSIAHAASSWFVKVLAYVFFPVEIVLIGLTANVSQINTFTTLLVFAVEFEI
LGEDFRRAFDERKMDELRSCVQRHQRLLEMVMLFRDKLKMYLLLSLQIYFFSITFCCVMLVIQLKTGDNQ
MVYTMINLTTALFCLLLFGLFCDFLELKVTEISDQVFSSGWSRSISLNRSMKKNLLMILMRSQKRIKFTC
GDIYAMSIVTCMNVINMCYSAFTLLMNMVQD

>KXJ71975.1 hypothetical protein RP20_CCG019254 [Aedes albopictus]
MKTIAGLWKTVRYYKHDSDFFLLVDFLVDICGYYPPKWNPFALFAWRILKVGQLIQYTFYGYHCYLSAAK
WRNILYFSLNINLFVGLSVGLFRGLALAYYHDDLVRLKHFMNSRNCGKDDAKANYSRKLHFWANNRLVLI
GSTILVLNAIHWCLTTAFTEDLFQIPFSLDFLPSSVANVIIYYYSFQMLIQNLGYWQSFFQFGLMLSLLK
NELLILSDYFESVYDRAFQVCCDNDLELKYEDSSTASKMWEAIRTDFIRAAAYHSDYIDHINLLKKVTYI
SFLVLLSATAIFVTLNSFLCVIDFSSDALGLLVFGSICGMECFFICRFLDELDEINQDLGMKAYAMDWMT
SIKVPPRNMGDYRIVRRTALIVQAQAQQGFGFRAGGMFDMNLEMFMQIMKMCYSLITFLMQTQESE

>KXJ71730.1 hypothetical protein RP20_CCG019855 [Aedes albopictus]
MRSVIKLLQLLGFWTQPYQKRSSAKPLAFTTLYIGWLLFPGIIYIFRQQPSFAVITRTAVESMSIANIIM
LIVSTIFHQPVLGKAYGDVRFALAAVSDSLDRELQRTIDHLELSTDRFFKGYIGFQMAVGLIYCSSNPIQ
TIVKYVRSEELPPLHGILEAESARSLEQALNVYMLMQFGTCIVMLCLTLMVLVLANDDRDLLIKMILMLS
YILSHILVYSMLGSELMSASASVADAVHEVSWYQWPVGEQRKLLFVLSRSQRITALTTGKFFYLNRETFG
MVYLLSQLLLNNQYTVHCTFITINVGSIFTDPPHHDVLFHRADANVWGVIMRESALQPADRVSVMERNHF
RTSTSLTESISARCK

>KXJ71728.1 hypothetical protein RP20_CCG019852 [Aedes albopictus]
MDKMPPSSSKKPAFSMMSTNVRLCRAVGLWYDPTHWRFTWQTVFIISTQVLWFMLPTVAFMIRREKTFAV
QLKPILEIVEIGVIVFRTSAHWNGRRLLTNCFDDLRKTFGRFSGSAHEDIRRTLRHLDQSADYLVKIYVF
VVLFQALTYGPLTTFITIVRYLRKDESLVLASPVLEADYVFFDHLSTFSIWLPSSLISVTVQFMMVISTT
ASECLLWNLLHHTSSLFRIVRYELSRLDGFAEWEPFRNQFAAIANAHDVAFRCTQRLESVLSPVLAMLYC
SCVFQTCYVVFVASVVEDPIVIASMIFILQYTTFLIFSFSMLGTELMEQV

>KXJ71505.1 hypothetical protein RP20_CCG020411 [Aedes albopictus]
MDLIIRALQLIGYWSHPDRSFRAIQAVCLIAVVLIWVIIPELAFIMRHEPNFGIIVRNLVEMLIIGMVVP
QGSIALCFRPLLEKTYSEVGSILDKVSTDPHRDVQRVIKQLKMFSEWIFKGYIGAEVLVALPYFLSIPVT

TIVKYCSTGVLPTLRGVFETDYLLFDPQANIWLWMMTVIANIAVMAYVILFFVSSHCLFWSLLRNVSGLF
KIISMKIGRLDEIVEDVQRHEELIEIVGLHEAAYRGARALEKSLNIFMLILYGMCILNICLTMVALSLPN
SDRDLLHKMTVVMVYILFHILVYSLLGTELMCASIATSEAFYKSQWYMRAVQEQRTILFALARSQRMAAL
TTGKFFYVSRTTFGMALRSAMSYFAVLRQVYGAQ

>KXJ69515.1 hypothetical protein RP20_CCG026720 [Aedes albopictus]
MTTHGVDVYHGLIVRLRWQLRAVGYDVLKIPFKYGKMTAFSSLCLLLFIAFTGYTVVLHWGDWFQVIDTL
SLLGMGSSSVAKALTGLLNYEYYQKNYRILTALYRSNDKHKDNNRRLVYWGMLITYYYRFFLVISVSGGF
GFVLTPVFTYFWYNERMLIINLHIPGVDTVTRTGFMITTLFHLVLIFMAVAGILAADLGCIIVVGHVAGI
ADVFKNALNELDQLTKQKCDERVDDSIHRKVLQICVMHQEIISYEEELDENYGVTVFVQVLSSIACVALS
LFNFYMTYNIGALTFLVYAFFQLFQYCVLGTVLTIKNDEIMIALYDTYWYKLTNTEQKMIGYMLHRSQNA
VEMTIGGISLLNMETFVEVSSVI

>KXJ69110.1 hypothetical protein RP20_CCG000088 [Aedes albopictus]
MSNAVKGSFPANIVLPQCLGFLNRIQDPLALQKVVDRWLGFMTWDFTTKLSYLKLFLMVYIYTYYVICSI
CIMFLINPEDVHPELYIRMWFFIGAGASCLSRWIAVIPSRHHFAGIIKHLTDLTRQKPYQPLRVRSRPVI
FLCSAYFFSINTSVSAFWTVLLMGSCPTDYVFRHPIVDFLSLILYPVETFLNGMVANATTLTILTTLLVF
IVEFDILGWDFREAFNSSNQDEIRLCVERHQRLLEMVTLFREKTKSYFMIAMSLYFFLVTFSCLLLVVQL
RQGDFQALRFNVINASMSIVSILLYGKICDMLEDRVRDVGDQVYGSDWPSKFDSTRGRRKAFLRQKSSIL
MITVRAQRKVGFTCAGIIEMSTVTSMQALKMFYTAFTILWNATSESDGTQ

>KXJ69004.1 hypothetical protein RP20_CCG000550 [Aedes albopictus]
MGQFDNFIPSQRAAFWIWKIYGIWATEDESPFYRTYRRVYHFLFTGVYLFSMFISSFFTENSEELWGEIL
FILPTELAMLTKNVITVNKFETIYALHRTSISKEFQPTCPKHGKEYYLYFDRFSKLMLMYFFVSICAATS
HFGFLFDDRLKLPFFNWFFWVPLDRDHLNNYYLLFAYQMIGMMGHCCLNVSGDMNIAYLLSIAGQQLDLL
SCKFACLPVPRAGTNAEKDHYRRKFVAQIQQYNRIYEFARDIEKAVSWGVFAQICASGITICAIVFRLSS
ISIIDHLGTSIPMFFYMVSMLTQIFMPCYFGNDITLKSQTLTNALYTSEWYQLAMEDRKDLKMMMLRTGE
SIRLKAGGFFNFNLDAFTSTLNTAYSVYAVLNSQEKNK

>KXJ68938.1 hypothetical protein RP20_CCG000962 [Aedes albopictus]
MLETFKQALRYDPTSNPLKHLMILHRIIGFNLSSPLANKVSRLSMILAGMHALCFVYRVYMVSKKDLGFE
YYIAALNVVGGYIFAVIRMTGFAWNYDGFRGICRFLQDHAFQRNDLRAQRLREQCYRNNQKFTIGMLVAS

VHAIMFFILTDYRTTDQYEIPFELSFLHPAIKSTFNALFGLYLYVIASYFWIPFITIRVVIHTLCVELEI
ANKAFGKLFTAASDRADLLMTHHSASSSRSVQLADQLKADLFWLSLRSELRELVDHHRELLINVDRLCQL
AAMPFLSETMSCILISSISVFFLLNGESLSLVAISCVLMFESFYASSLVEALQDVHGEMGTVIYALAWPS
EMRYDRRNHRHYKYVSRVLQVVLMRSQRRLRFHCGGLFEMSRATFTFTVKTCYTMLTFLLRMQDV

>KXJ68766.1 hypothetical protein RP20_CCG001781 [Aedes albopictus]
MGGPLSWLSSFLQATTPNYQGTFAVFRVFMLICGVNFFDEDFMVGPVNQFRFLGPYLSGFYTVIACVLHL
IRYLGDVDSTILSLAALFSAIEVMIKIGGMALKRKLGAKLINIILEDRSYEDGDLEWSVFLKYHTLARKL
MYITIISYPFTALMLLSYPALAGKLDEHMLPVGYSIPFFDHKQQPWYVINYLIIVVQMSWCALAFIGTDG
PFYLCVCYSTCKLEILQSYTAKIGETEDVEEQRRLMRKIIEIHTHVLEYSMKKLKNSSYGMLATNVAKMW
IFCYCGELVVTKSNELSQAVYTNRWYELWSKKDLKAIQFTLANAQRNVGFSIGGFGFLSYVTFTQIMKTA
YSCNAFLHNMMN

>KXJ68741.1 hypothetical protein RP20_CCG001943 [Aedes albopictus]
MGGPLSWLSSFLQATTPNYQGTFAVFRVFMLICGVNFFDEDFMVGPVNQFRFLGPYLSGFYTVIACVLHL
IRYLGDVDSTILSLAALFSVIEVMIKIGGMALKRKLGAKLINIILEDRSYEDGDLEWSVFLKYHTLARKL
MYITIISYPFTALMLLSYPALAGKLDEHMLPVGYSIPFFDHKQQPWYVINYLIIVVQMSWCALAFIGTDG
PFYLCVCYSTCKLEILQSYTAKIGETEDVEEQRRLMRKIIEIHTHVLEYSMKLLRYAGNERGQNVDLLLL
WHLFNLPLGKKLKNSSYGMLATNVAKMWIFCYCGELVVTKSNELSQAVYTNRWYELWSKKDLKAIQFTLA
NAQRNVGFSIGGFGFLSYVTFTQIMKTAYSCNAFLHNMMN

>KNC23892.1 hypothetical protein FF38_05605 [Lucilia cuprina]
MISNWKVLFVNIFPSNPEIGEIGSIKLNIWLANITGVPIIGLQKESNGLKTLILFYGICTSFLVTFAYTG
FEIYDLILNLDDLDKITQNIYPESVAGEIFPYRVILPTWLPPFWQLIYMGLSVLIFAIQIVAVDYLNVTI
INQIRFQLNILNLSFDELTIAKKQDTKALEVFKGEPLKRLNSIIEHHSLLREIRQRTEDIFSQPVLLQFF
TSLMIFAMTGFQATVRTGGSNGAVLIYFYCGCIFCQLFVYCWFGNEVFEQSKTLATSGFNSSWYLFDRRY
GKSLVIYLTNAQRPFMFTAGGFMGLSLPSFAGILSKSYSYIALLRQIYGR

>EAT46338.1 AAEL002479-PA [Aedes aegypti]
MVFSQAYKSQVGQPDAKLAMGLLLWISNFVRARTPNYRGTFAVFRVFMLICGVNFFDEDFMVGPVNMFRF
LGPMLTGYYSLVACFIHLIRYLGDTDITILSLDALFSAFEVLIKVGGMALKRKLGARLMKTILEDRSYED
GEIERFTFLKYHTLARKLMYITIISYPFTALMLLSYPVLAGKLDEYVLPVGYSIPFINYKQHPWYTINYL

ITIAQMAWCALAFIGCDGPFYLYVCYSSCKLEILKSYTEKIGETNDIEEQRTLMRKIIKIHTQVLDSHLR
FAVPCSAALTVCSQPTWPRCGSFVTVVVTKSNELSEAMYTNRWYQLWSKKDLKAVQFMLANAYRNVGFSI
GGFGFLSYDAFAEIMKTAYSCNAFLHNMMN

>EAT42233.1 AAEL006192-PA [Aedes aegypti]
MFLAVQHFVITKYRAFNSSDNCFVLLMFCNRLVGFLINPDDAQDYLRPIKHLLLSFTVIYELFEVFTLII
AIQSTGIDGHIGSLYLLMSLQQNSPDIRRRKHIQTINWFMLMFLLQELIPMGVWTIKGHTGTPLAFYASD
TIDKLNAVFYPITLFSLTLIFCYSLIVVSSIMSALTLEFRLLGTDFEHLFEQVGPLETSSNNDSEHNWKA
VEYNFKLCVVRHQILLGSTLALRSLLKMYSLIQLLIYFLMVAIGAFLYVFTQVSFGFGVIFFAVCVVSVS
VNLLLYGFLCDRLEDQVESVGYRLYSSGWTDKLICSIEHAQRYKNFRKMMLIVMERTQKSVEFTCGNVYV
MSLLTCRHVLWFAYSVFTVLINFLE

>EAT34320.1 AAEL013422-PA, partial [Aedes aegypti]
MATSLRASFPTNVILPRCIPFIDRIQNPLALQKSMDRYFGVIYWGSRPLEFFMKIFSLCFMVGFLLLCFS
CMFWVNPLEVPPDMYLNMWFYLAASTFVILQWITLLPNRHHYAKIVQYLSDLQRTDPFHPFRIRSRAMIF
LSTAFLLAMNTSMAIFWGFLLFGSCPMAFVFDDPFVKKLFSVIYPIQSAYLATLSNGGMVTCFAVLLVFT
VEFSIIGEDLREAFNSGNRDQIRSCIKRHQRLLEMVNLFRDKIRFFLLVVVGLYFLLVTFSSFLVVLQLH
SGDVQALRYNMINAGFSIITIVLYGVVCDLLEETIQEVGNQVYSSAWPSTLILDRKHRNVYNSGKRSLMM
VIARSQQKVGFTCGGIYQMSTVTSIAPAVLLLSVYCTVECNELIMFFGYSVPFDGSANWHLKYICILSCK
SLGSPL

>KFB53971.1 AGAP002639-PA-like protein [Anopheles sinensis]
MAVRKSVPVQVTSFANPEKLSDSEALYNFGLQRKIMLVCGIWPVEDSLERRWYLKSLIVINLVVLALCIV
GEFLHGLNAYRDGNLNEVIESICPTVARISAFFRIVFYVANEKKIRRVLSDIDHAIRNQHPRERVITERV
AYVGHCFTYCLILMMFFAALLYGVTPFLIMAYSWHQGQTPLVKLLPFKLALPYDSQDTLFFVLTTLFLNY
ASVPTITSQGSDALFSGVCLYVQGQLKGIKLDMEALATSEFGRSFGEDLLRADSLETRHVNRELRRISVR
HQQIIELIDDVRTAFSPNVLLVYSGTALIICIVCIAMLIVEGLYKLTYLPYAVAELVLLFLCSYSGTIIR
DSSEAIHTVAYNFPWYRYNRDTRHLIQMMMIRAQHGSNLDVPFFETSMATFSTIVRSASSYITLMKSFL

>KFB53785.1 AGAP009706-PA-like protein [Anopheles sinensis]
MATVESYRNLVKLLCISSKIVGVEVWTAPGRFRPASYYLSFHVIVYFVSTIYTLAKYSNDPLHMMKILIT
LGTAVVLYMKFFIVILRSFEYKSFADLIEEELLKPFENGNAEEVAVLERTGRILWVIGRLMFMCFACSGV

FFGIYTVYEYYVNGALVPLFLYELPYYDWSTTGGYVTNMLFQVNLYAIGVIGASFIEFLFIMFALYTLAY
VDIFLVHLRELRSLLDDVEFVKKNNGSEIRQKWMECILNHQQALRFLDSIEDLFKLMFLGQVWNGVISLC
IGMLLILMTDWYAAYFFVLVIFLDFSFYFLIGHYVELKVDEMYDTINSVPWYKFPIINQKEFIFLLGRQQ
RPMIMTVYGFAPLNFATYTNLGNN

>KFB53784.1 AGAP009706-PA-like protein [Anopheles sinensis]
MATVESYHNLMQMLSISSKIVGVEIWTAPGRYRPASYYLSFHVIVYFLSSIYTLAKYSNDHQHQMKILIT
LGSAIQLYVKFFVAIKRGFEFKEFTELVEQVLLAPFQCGSPREVAVLQRTGRVMWIIFRLIATSFLSTAF
VFGLYPLFAYYANGTVMPLFLYELPYYDWSTTGGYVANMLFQVNLYVLGIVGAIYSDVIFILCALYTMAH
ADIFMLHLHELQEILNDSAFMENDNSAMNHKWKQCMYDHQQSVKFFNSVDDLFNIIILVHVGMGLFSICD
GMLLVALTDWYAAYFFLLVLFFQLTLYFLIGHYVELKVDEMYKTINSVPWYKLPVSNQKEFVFLLCRQQH
PLILMVYGFAPLNFATYMSVG

>KFB52367.1 AGAP004356-PA-like protein [Anopheles sinensis]
MDTERQHGQFPLMELCIRMLRKLGLWNQAPGRTFPIMGHLHVGSYMLVWVIPSLVFIMTSQDNVTLFLKA
VTEQIVFFTIFYKFCSFVYNFRQWESLFYDLQRTFGTAQTDPNAEVQAVLTHVRKVAYYLTRYYCTAGTV
NCFVYGPFPMIYVIIKYAITGTYSVPLSTPIEGSCASNLEEILTGQMLFMYMSTIFSLCLLMTVISVAFN
NTFLLISMLFVISYCLFQTFCFSMLGTEVIEESASVANAIFHSSWYSRSHLEQRDLAFILLRAQRPVKLT
AAKLFVVTRMSFTQVMSSSILRS

>KFB52366.1 AGAP004356-PA-like protein [Anopheles sinensis]
MEHDEHQHGQFPLMELCIRMLRKLSLWNQAPGGSLPIMGYLSVGSYMLVWVLPSFVFIVTSEDNVTLLLK
AVTEQIVFFTIFYKFCSFVYNFRQWESLFYDLQRTFATVQTDPNAEVQAVLTHVRKVAYYLTRYYCTIVT
FNMLAYGTFPMIYVIIKYAITGTYSVPLSTPIEGCCASTLEEILSGQMLFLYTSTIFALCLLMTVISVAF
NNTYLLISMFFVLGYCMFQTFCFSMLGTELIEESASVANAIFHSSWYSRAHLQQRDLAFILLRAQRPVKL
TAAKLFVVTRMSFTQVMSSSILRN

>KFB52312.1 AGAP011631-PA-like protein [Anopheles sinensis]
MELKEEWILADEVFENPLLRSNLLGLKYYGLLLHDTQCFKKLHCFRGAVFTLSMVLFNLTQYIDLGQVWG
SVSDMTANAATTLLFTTTIFRIIFFYAHRARFNNIIRVANEGIKRIVKDAWMDEQNILISNVRYLKRLSV
LFWSCALITANTMCVFSLVQYFIYERPPSGSSEGEKGGRVGASMNSSTTQLYPAKILRSWYPTSPGNDHF
VEIYLIQLYIMYVGQLIVPSWHMFMVTLMIYGRIECNVLNHRVRSLARYHKVHAAERIQIKSNSFDPVDN

PERRTLIIDCVKRQSNLVAFTRELEQLTRAAVFLDFVVFSVLLCALLLEASITTSVVQIFIDICYITTMT
TILFLYYWHANEINVCVGTKWGKKYEKMDNRDVHFQANQLSMSAYNSDWYRYDRTTNQMLQLFIMYSNRP
LKMHAYFITMSLDTFLAILRASYSYFTILKQLTD

>KFB50905.1 AGAP006167-PA-like protein [Anopheles sinensis]
MAAGPGAFEDTLKNTNIMLRMMGIPPCDEPYPDTLLALLKRNIGFIGSFLLLVYTTLGEVIYLVQMFRGS
EVSFLEVTFQTPCIGYCLIGVLKMILLAVRRNTIAELVQLFRTKWKVVIISNENWTVCEETMRPAIRVTS
VTALANVVMGIAFTMLPMAEMLYHWLHSGSWVRQLAFNIWWPFDVFAGTRYFWFSYPLYVIIGFNGIIIH
MAFDCLFCILAAHLCMHFRILKHNIEKVVDVSEEGRAGNEGRLQDAIVEHQDLIGCSAFLQDVFGDVLFL
NFLGSSIIICIQAFMITTVSGYTLIKFVLFMLCFLIELLMLCAYGEDIVQSSGAVADAAYSCKWYEEERL
FKSSILQILHTAQKPIVLTAWKIWPIQMVTFRGPNVAQAQVAHSPWGRADDQSENYFLPASGTVITRTGE
SAFVSWLTSRSRASRCAI

>KFB50423.1 AGAP005760-PB-like protein [Anopheles sinensis]
MQSVRRRIDRLVRGDLVSRVVFVKESFALLRVLGVYRGVRESPFFHRMVYYVPDVFFVLQIATIVWDLAG
VLDDIGLFGDDMCILTGLVLTLVKKWHCIVNIEPLDECVEQFQLYFEHYMNHGEQFVDRIRRQKLNEVAL
LFLSRVLGVVLGGTLILHALLSNGESLILRARYPFSTATPLGYGFVFLCQAFLVSYVLFNVVHIDTVGAQ
MLSQMSLLFHLHRMEFEAIGEGLQLPPDGPLYGDDAIRSRIHGFGDRLKRLYEPNIMAQFVCSMLIICLT
AFELMFSKGDLMQMFRFGAYMVTGFFQIFVWSFFGNRITATSTSIHEATSTCNWIVLDDRLKKDLRFTMM
RAQKPFVIDVYWLFPLTYETFIAILSRSYSMFTLLRTMIE

>KFB50274.1 AGAP011813-PA-like protein [Anopheles sinensis]
MLIFVDRLRIHLGRRLEKRAASVREQYFYIVRILNVMGGLVGGDIFTPNFTAKNWYFKFVLFNIALVFSI
NLFSLYKVYGSLVDFMYCLETILYVGICSVKLYTFVWHKDLILKLHQFIITFMDNFGGSKEEDEHISQTV
HNIYALLVLFACCSCGAAGLIFVYSLLWSALVEYVVPFGFVIPSVSIDNLRGFSVNYALQLFESILTVSG
IVGSECAFFMFLLNACLQVDMMCLELDRLSALCVLNGKGQYTVEIRNRIRTIIEHHIEHLDFMKTMCNLF
MLHFLVVFGCIFFQLISIVVVVVAIPNWYPGYFLFTMLTFQLFFSCALGQLLDLKCDELTVAIYTVPWYN
MEVQDQKALRLLLMASQRPVLISYGFGTVNIRTFFEIYRKTYSIGMMMIGVNEEN

>KFB49096.1 AGAP009640-PA-like protein [Anopheles sinensis]
MLSLIKLSPRWDVHDRRDSFRLQLVCLKYLGLWPPENADERTRSRYIVYGWVLRVVFLHLYVVTQVLYFK
DVKDINDIANALFVLMTQVTLAFKLEKFNYNIGRIQACLRKLNCTLYHPKSQVEYRPVLHSMSEVFWLMI

FLMFLAIFTIVGWTVSPAFDNERRLPVPAWFPFDYQRSTTTYALLFLYQTIGIVMSATYNFSTDTMFSGL
MLHLKGQIVRLGSMVQKVQKLRNKAVKCDDEWKSIRARIEDHSKTQGKTYSEVIECVLFHKNILSFIDEV
QDIFHPSIFAQVCASVIILCMTLLQATGDDTTMAELLSCALYLLVMTSQIFIFCYVGNEISYATNQFTEF
VGYSDYFKFDKATGKAIVFFMQMTMKDVHIKVGTVLKVTLNLHTFLQIMKLSYSYLAVLQSMESK

>KFB48460.1 AGAP004951-PA-like protein [Anopheles sinensis]
MAFSEHHLPESHFRYLRYCGVYPRPTVGSRLRVWYTCVLLCIFVPLHLAYIVHNSTSDLMETCEEIMLMQ
VCTTAMLKFNIFFSNREKMYALLEAFKNIHVRFGEVEELYRFVRCHDMHAKLRRIYIISTTVVASLYVLN
AVTASVTLSLQSGTLRFVTPMNFPYNYQHPVVFGLSFIYNIETMIMTMCISVTVDTCFSELSNDLALHFD
IVGKRFAKLDFSAPVSTTAERELIRLIGYHGELLELAGKMMQQFQQVIFFLLLMVSTILCVLGYEFVIVT
SVSKRLQVVAMAAVFITQAVIYTYNGSLVTEKSAAVSEGIYACNWYEASPAIKKTIYFCLMRAQKPIVMK
SGFIEATLPTLKKILSSSGSYITMLLSLESDV

>KFB48435.1 AGAP004974-PA-like protein [Anopheles sinensis]
MLPDVSVDFFRVQSICLAAIGISRNETRPGRALFGFSFFTVLFMKLGTVLFAVSHIDEIMLLCDCLGPTF
TAYLGLVRQYNLRRHRSELWAIVDEFSMLKGSLAPAEVRIVQRYNRFDRFLAWAYLISAMSTGVLFVCAA
LVRVAISERSDWKLPLLMAFPFDATHPVTFTVLFVWCSIAIFWVVLDCVACDATFGTFSSCLVAHFVIIQ
RRFERIRFDQAPHAPLGALIEYHKHILNIADRVIDAYRNVILNQLLISSVLLCMLGFQLVLSAGSSLVVV
YVAYGTAIVIQVTYYCYYGSQLYHESTQVHDAVFQSNWYEADVRTQKLLINCMMRARKAVNAKSGFAQAS
LPALRAILNSAGSYVALLMSLME

>KFB47528.1 AGAP011469-PA-like protein [Anopheles sinensis]
MVQILPKLKDPLAVMPFLLRIQTIAGLWGDRSQRYRFYLIFSYFCFMVVLPKILFGYPDLEIAVRGTAEL
MFESNAFFGMLMFSFQRDNYERLVNQLQDLATLVFKDLPAELGTYLIEVNKRVDRSSKIYCCCHLSLATF
FWFMPIYTTYSAYRAAANSTEPIEHVLHLEEELYWLKSRTSIVHYTIYAAIMWPTIYTLGFTGGSKLLTI
FSNVKYCSAMLKLVALRVRCLANVKEEKIEKELNEIIAMHQKTLDCVLLLETTFRWVFFVQFIQCTMIWC
SLILYIAVTGFSSTVANVCVQIILITVETYGYCYFGTDLTTESFGVALAVYDCEWYKFSISMRRNLRLLL
QRSQKPLGITAGKFRFVNVAQFGKTRFSEDAGDLHR

>KFB47258.1 AGAP010507-PA-like protein [Anopheles sinensis]
MTKLIYCLATFGFGCQGVMKIYSYIITRRRVIELYMINVKYNERMMNQSDRVKRVLCENATITHILIKGT
MLVYIILVCVTVTIPGLSSIFLSSRILPFGFVLPFLDPETWTGYIWNYAFQVIMSYFYLVLTLGGDITTI

FNLLTAYGQLDALMMLIEECNEQLARNEPAEAIQKKIVDIVQLHQHHRLYLQQLVDFLNPYHFVTVGSTV
PAMVISVLGVMLLKWYPGAVIMFLGSVQIFYICFLGTGLELKTDALTDMVGAIRWDKLSVRDMKHMQLIL
ALTQQPKVLLVATTPLNVTAFLQIHKFIYSLIMMVENTKE

>KFB47253.1 AGAP010505-PA-like protein [Anopheles sinensis]
MNCTVSHDLYIHNLNYIRWCANAFGMDVMAPNFKPNWKTFIAVGIVSIALFGQFYSVWYFWNDVVKLMES
TALYALILQGCTKFYTGLRYYDFFIAMYGRLDRLHQEHRTHPKNNAALLLLMQRIYLLTRLIGVQYAFSA
SVFGLIPPLAYLVTGERMLCVSLVIPFTDPTIPSHYFLNLAWQYYMLMLAIAGFSAAESIILLFVGSLAG
YADILKNEIDELNTILQDAERTDDRSMVKKKIHDIACLHQRILEYENDLEERYYLNNFVQVFSIVLTLGG
ALFLCLVTNSISMYTLAISGVIQLFELCLLGTILSVKNEEIEATFYNSLWYLMDRSEKSTFLILFHRSQH
AVEMTVANMGSLNIVLFVTILRKIYGFSMMLMNFYE

>KFB47252.1 AGAP010505-PA-like protein [Anopheles sinensis]
MNYTVSYDLYIHNLNYIRWCANAVGMDVMAPNYKPIWKTFVALVAVCVGLLGEFYSIWYFWTDVVKLMES
AAIYALMIQGCTKFYTALRYHDFFIAMYGRLDRFHQEHRTHAKNNAALLLLMQRVYLLTRLIGVQYAISV
TVFGCIPPLAYLVKGERMLCFSLVIPFTDTTILSHYFLNLAWQYYILLLALAGFSAAESVILLFVGSLAG
YADVLKNEIDELNSILQDADRTGDRSMVKQKILKIICLHQRILEYEKDLEERYSLNNFVQVFSVMLTLGG
ALFLCIITNSITMYTVVLCVAIQLFELCLLGTILSVKNEEIEATVYNSLWYLMDRSEKSTFLILFHRSQH
AVEMTVANMGSLNIVLFVTILRKIYGFTMMLMNFYG

>KFB46816.1 AGAP011978-PA-like protein [Anopheles sinensis]
MASLFHLGFTLRQKFLERFFIFSDSVDYFNLFNTFGTLFALHYDAKGPRKLFWMAYRALYVLSYLSYCYK
AGWMFNHWEYNTASANVLGALGLCSGAFLRMLLIEHHYPAIEQLQRFLNDRTYQGSDPSWARQRRSRMYR
QNNRFLVILVSAIVLESLCFLARLLLTRREFMLQFNGEVVGGSMVQIVYGTITAGWGIVYVLSFILFYML
MEGFRLEMEILVRSFQHLEDSLFPKHGEFIRTGLPMCEQNELAFWSDLKLRLNERIGRHVELLQNLRKLR
SIVAPFAFLQYYSTFGLIADSFFIVSFEGFSGYSMAYILFASFLILESFLLSRGVEQLTELNHQIGMALY
EFDWPKLMRYSTRFRREYNGIRRTMLLTIVQTQRSLKFTYGAQGEISMNSFAELMQKSYSMLTFMLQLGH

>KFB41716.1 AGAP001012-PA-like protein [Anopheles sinensis]
MSLKKLYRTVYTEQDFFGPFELLLILPGFHLARGWQSLRVRLVFRVMRAVQLFTYLLWIDRFVLALWNAS
VEPEKALHYSNTFGVLSMMLARMLVFKWYMADVERLQKYIRRQRAAAPPTTGSYRKIVTIAIIFQLIGLF
DRVVFGFSSTYRRELYEMPAIVSSFGWLAELLVHVLSFDFAWRWAAAYNTSLTGMNSIMLGLSDELAEIA

QEYQYLMVVEPGIDFWTQLERNIRCTVRRHEQFLQQLDHLKPFLRTTFLLMFYSAAIFLAIGIFIISANG
TSFTYYMMLSGFLVTLLLECYWCCQLVDRLNDEHYRIGELLYGLEWPERLCYSAHDAHHYRQARSSLLIM
MSLSQKSLGITCGGMFEMSSEAFANLVKLTYTMLMFLRDTQHTN

>KFB40824.1 hypothetical protein ZHAS_00008347 [Anopheles sinensis]
MTSLNSEAKFQRLKSLISRHMEYNGVNIIREDWRVSFRTFVCATFVLMQPFVNISSFLYYSHSLDMLTEN
LSLGCSGIQLVVRTYFYLCQRDLCRNVIREIGEQRLLLGLPDNERMEQLFCKSFDWMARYYWIMHASYLS
AITFIAMALVIPDPKKHGLPLAYRLPLLPPDEQDLYWYITFAYHVGLILMAIHYLVPIDGMMMVSLFSAR
TRVHALKVLLEELDEKIGLSEWQRTEHLEANLNRIIELHVSIRRFTRLINNSYQLHYFTVFGTICFVLCL
SVNLIVVTPKNSIYNYLLASICQLFVGCYFGNLLLIENDSLSSCVYCIRWYRMTIAQQKKILILITNTQP
DLQVSALFLPVNMASFVTIIRAAYSFFTVIH

>KFB40732.1 AGAP001912-PA-like protein [Anopheles sinensis]
MEQTAELVGFESFIRVPEIFFTLIGVARYGEPQGTVRAHLKRVLFWSSYANTFFCLIIEHIYFVKAAGNF
TNFLQLTALAPCMGFTALSFVKIMTIKLHEAKLVDILRRLEEMFPKTAALQTRYGVFQYNRESQVVMKSF
SVLYMILIWIFNLLPLVSMVVGYYTDGSWQKQLPYFMWYWYDWHQPGYFELTFLHQNWGGFDSAVFNLST
DLMFCAIILLLCLQFDIVAYRLKHALPDDHEELNGCVRIHQSVIELSNELESIFSPSLLVNFLGSSVIIC
LVGFQATAGISAADLFKFVLFLVSSLVQVFLLCYYGNKLIVASDQIPYSAFEGHWIGASQSYQRSLLFVM
MRSTKLQKLTALKFSIVSLASYSKILSSSFSYFTLLKAMYEPNENIKDM

>KFB40595.1 AGAP002995-PA-like protein [Anopheles sinensis]
MLCASSSHYENFRKLEDFFNDRSFARNHPLTRRIRERCYRWGNCFTLIPQAGVLFVMLQCIYLKQYEKKS
MMLVIRGDTIGTPLTHSLYISFLYFPSLCFFMGCSMVNVCLMGFLAEMEILATQLGELDDTVRRRLADEG
NETCRKSFWIAYHDELRRCAKRHCEIFEMIDHMKQFSSLLFLLQHVFSLSFLVASAYVVLRANALRENII
FVEYPIPLVFLYFIFCLLVEKVQDMNNLIGQRLYGTEWMLQLRYSREFHHEYRSAVCTIAILLGRSQQRI
RFTCGSINEVSMAKFTEFLNLIYTILMFFININ

>KFB40587.1 AGAP002722-PA-like protein [Anopheles sinensis]
MNLNRNFDHPMRFAYLILDTRNLFEFVMVLVPFTEILLTNVKMIICNVKRVKIINLINQLQLEWDEFKKS
EIAEVQQLVASTTKKTRIFVIIYTASFFLIVVEYATLPLFKLLYHRVIMNDNSNYTTKLPYDVRLFYSIE
GNASFSVTYFFILIAAFVLAFALTGFDSMFSTLCMHITTMFKFLKLEIDQLARDLEAAAPQKELHTKLRR
IILKHKSNLSLIDQLEEEFSFFLMIQFLTSSIVVCVVLYELTTVFGWNEDTVKTITYLPGAILQLFLLCW

YAQNITDEASEVADHIYSVPWYLGDVPLQKTILTLMVKAQKPSGVTASKFYMVTLQSFQRIISTSYSYFT
LLQTVNEQ

>KFB38970.1 AGAP009394-PA-like protein [Anopheles sinensis]
MFNFNIDKPPGVPHLVLKILKLGGISGTPNERYRHVPIFLTFTLMIAVPKIFFGYADFESTIIGLAELFF
QINSFVGLIVFLLQNESVNEFIRCSQSLVDDVYKEHHPRIVGHLRVKHDLIHKATKFYTIALICAVNFYI
FSPMISTLWSYYNLQYNNTNMVYTIHMEENFYNVPIRTSLLEYLLFSVCMIWTCCIAAYVGGTKLMTLLN
FISYCTVYFHLVVMKIEESTRLNSIRENEKTIVMMHYKALRCAELMISITAPVLLQQLIFCVLIWSSMLL
YFTVSCAQVARAIYDCKWEQQPPDSMKHLQLILLRAQKPVGITAGGFCFMDMEQFGKVCTNR

>KFB38890.1 AGAP000226-PA-like protein [Anopheles sinensis]
MGKEPHPIATFDKLIQRLRSLLKLLGLDSYEPNYRPNFRTAIVLCLAALYVGITMYDLVHFKDDLFNFVY
VLITFFFATIGIGRVAIFLTCGDALQFLLAETYRTYREARADEREQRVLWWYTNIYRWAVRTYARTFVVT
GTIMPLIPLLRFFVTGELVLPFGVVLPFVDVNSGLGYVLNYIYQVSCIVWTAPGLVSSYCLTLAQVVNIC
TQYDLLAVKLTVLEEHILAHDDDLVARKLRHLIRDQQRVERFVAKVESTYRLLSGVEVLSLGLQVVITLF
VEQFSLWIPGLAVIPLFSGQLFIFCALGAAIQHKSESFAAGVYRLSWHELSRREKQTFRIMLLRSQESQK
LTCVHIHDINLNLFVAVRVTH

>KFB38883.1 AGAP000230-PA-like protein [Anopheles sinensis]
MNFAAIVFRRLYSKLFKLRVDGDFFELLDQWLIFSAIQLKSRRILLRIAFRLYQLLLPVQCIFWFSRVWV
VAVIDRNVSLTVSLICAALAVTSIVIRYALILRCCGRFSAVRAYINSRQYLRGLPEAHEIRERAFRTNNT
ILVVLQAYCLMNITVFMATDMQQHEIFRIPDYIRRTTEPLFWVMHYLMQPMVLTGLATYMGSFIIPNALL
IGLRAELQLVQLGFQRVFQLVDTRAHQVDHQLNPNAREDLAWRALHQELAVCVHEHCKVMQHVREVHQIL
NVSVMVQYYCALLALAVDGFFISYHGFDFVAFTVVVFSLLLVFEWFYRCKLVEDLQTIHNHIGWTLYNAN
WPAWLQCSRRHRTSLRQLRDTLNTVQAISQRSLSCQGSEIVEVSWRAFSNLFKTSYSVLMFLIEMRRLNR
TNLQ

>KFB38050.1 AGAP002126-PA-like protein [Anopheles sinensis]
MAVYLFTLTLNMFVMQTFEQLVLYTMYIVFTEIVMVMKAFIAYYNFDRIYNLYHGTIADAFQPKDELERK
LYRAGIGEIKYYSYLYVLAGNLAVGSSLLYLLHKDYRMPYFPWMFGIEYGPEARMNYGIIFGYQVLGMYF
HMLVTVAVDVQLCYLLGMIAIHLDLLDKRFRMLSTSQEFQHSFIELVQQYEMVHRMVREVQQLYSPAFFV
QFSASGLVICATAFKASSMINLYELTAIQNLLYMLAMMFQMFLPCRFGNEVTRKSNALRTAVYSSQWYGM

RLRERKFLHMLLQRMNKPFTLRGYHFFNYNLPAYTSTLNMAYSVYALLQRMALKRS

>KFB36940.1 AGAP002044-PA-like protein [Anopheles sinensis]
MVLNITQLFSRKERIRYIPPMEAPGQPSKICFLRWVEWMDMTNGINLFERSRYGYVSTGVFWVLQIMQLL
IAYNFFVVCTVTSSLEEFSIQFNQFGGILLTFSRVLCIQYNRKNLQKTAAFINASKFHHLNESANKIYTK
TLHVAKRLLSSLLAIQLLSMVFWFLQNEYQARQMDVLLPMVSYLPFDPTRWSSEAKFAFRLSFYVANTQL
MMAFFGSYIITSCYLLTMTIELRILNGSYETAPTNPDQLVTFLRERVVYKQELLEHIGIIKRQMNLSTLV
ELVFIVCLLTINALRICMTSSNPSEVFMSTSMILIYVLELFQYCWQVDEIELLQERHAFAVYSTPWYHSA
QKTKALLLITIRMSQMPLNFVCGGMYRLSTVLFASVIKLIYSLVMMLLQFK

>KFB36939.1 AGAP002046-PA-like protein [Anopheles sinensis]
MFILQRALKFAQVHFNVGEPSKHFCLLRCLDIVSPFMILERKRSELEIGFKTLCLSILFIHIVGLAYDIL
RQHDIRGALDIYSMLSVFVCIYVRPLCLRSFRESISAMDQLDANPRFRKGTPYGDAIRQQVVHKDNVYLG
SAMAMHSFTVVLYIWHNLTTQNSFLTLVTYWPTDLSDVSPVLDKLAQTAYCLVAFLWGWSHAAGQVTIIV
LLRIAVAEFRVFLESLVRLDEQIEETGKAYPELSHERIVCDLLHEHARRHQELIVVMMRLRKMLRIYTLV
HFSLYMSIVATFMTRMLVITGASSLGTAVSLLATMIFFFETLGMCLLVEQLIQLNRKVNFALYSFDWPKC
LPFGHAIKRTIMLMIMQSSNTNDFSAGGLTKISAELFAKSCRLVYTMMMCMANLANVNAE

>KFB36329.1 AGAP008333-PA-like protein [Anopheles sinensis]
MDTFSTSKPNVEKLFTNRKRQDHEWKRFPAVRMVLFVFRVFYVWPQKRMDPSEQFRYRLKGVAFRCTVIY
LSVAAQAAYIFTIEKRQDLIDGLFVLSTQLVLILKIEFLYKNISKIQDLFYQLDAELYVSRNAEEDLPLA
KARKKTSIFGMLYFMFSDGLITYWLFYALTINGLIVPSWYPFDYTSSYAVYLVILIFQTVCMLWNAAFNI
SWDSLVYILLSLANAQLQRLQIQLVKVRFQAKLKMITVKPVQSQPGRKDLLSSVQVDQKSAEDVYNDLLQ
CIIFHQKLIKFVHQVLELFGGIIVVQLFSSVFIICIAEFHLLTEVNTLGELLRGLPYLICMLLQVYQYCF
HGNEISYTAQKVHQATAFVNYPDMNIKTRKLLILFQQRTAVGIPCYAKNIFKIELSNATFVAIIRSSYSF
LAVLRTMIN

>EDS44900.1 conserved hypothetical protein [Culex quinquefasciatus]
MTFVNWLNSVRFWLVLRWRIFTHWWLNLYAKTFKFDCGADFFAEVKYLEIFCGFYAKLRSTGDKLWWRSL
RLVIAFRMSHITGKFIFTFLEEDDFTYKVLIHTATYLMFCNCIQMFLLRVAYKEITSIRTFINGRTFLPE
DSEAHRIRWAAYRKNLWTILVPIPSNIFIWIFLFASGAYKWRVVNIGPDTLASIPWLRTSVQLFYWLQYS
VGVEWFQLQTVLVNSMLYGLAGELGVVSYACENLVQSVDSAVQVDLLNTATSSDSSRGEAIFWKHFKIEL

DKCARAHSKVLDQLMNLKRILKPCLLMYYYSLLIVNAIIICAVKNGYFGAFTVPATVMVIYLNVDFFFIC
YNMSRLDDLSCNIGDKIYNQPWPYNLTLSDEFAREYRDIKSTLKMMLTRAQAGMEFSCGGFFEMSLMKFA
ELMELTYNMVMFVLQFHH

>EDS44805.1 conserved hypothetical protein [Culex quinquefasciatus]
MIGKLTNLNVLNKDHQVFTWNLAPVVTGMIVYFYTSFDTAYAVRESTEELIFCLVTIGIGIQGAGRIFTF
LIFREKLVWIHQYTTDLYREECNPRTKSFLMDSVFLLSVVLKVMLTCYAFTSISMDVAPLLFTIVSGEKI
LPFGFYLPYLDRFTWFGYFSNYVMHIFMTVYVSSMDMGTDSIYMMTLMSAFTQIDLLKLSLEEMSEMVDQ
KHEDLENFFKKLINRHQEHLQYLRTVEIVYCFNFFLTFLCLASVLIMSLFAVVKLSWYQGYVFIAFISYQ
LFFGCFLGTLLAMKVIALFRRVNPLLIVGFGPFQNEQLQHEIYKISWYKLSIPHQKLLQFILKSAQEPVC
LTVIFAPLDMGTFLQVYKSIYSMFTMLLTVQEEE

>EDS43587.1 conserved hypothetical protein [Culex quinquefasciatus]
MGVYSAISSFFTTTAPNYHGTYAVYRVFMFISGVNFFDDDFMVGPLNIARFLGPAIAAILGTISCMIHLH
RYLDEVDQVILSLAAFFSGTELLIKMCGMAVKRNKGAELLAVILNDRSYEDGPVERAIFVNYHGLARTLM
FITIFSYPFTALMLISYPVIAGKLGEFLLPMGFSIPFISHKQHPWYEINYLIEVVQMVWCAIAFIGLDGP
FYIYVCYATCKMEVMKTYIEQIGDSEDVEQQRGLLRKIIGIHTHVLQFLRDCSDFYQEIYLAQVFFSIAH
ICVSLFHVQLKLKNSSYGMLATNVAKMWIFCYCGELVVTKANEVCEAMYANRWYRLWRKDDLRTVRFVLA
NTQQKVGFSIGGFRFLSYDAFTEIMKTAYSCNAFLHNMLN

>EDS42087.1 conserved hypothetical protein [Culex quinquefasciatus]
MEPTRKGRSTAIEKWIRKLVECSQDPFWLLDKLLVLAGAPFTPRNRSQRIVWILYQTTTYFQLSISLVCS
ISFVIFEEDSVTVIRTFLMFLTMTLSFVKLHLLLQNSSGISKLRGLLEAPGFCSGDPDFDESVRKRFKRT
SRVLIITIAWIVMVQQILSWIPSDTQGIIFKIPAWVAWCFGDRVSLVIKNWYMSLCFTIWCNKLYACTIT
VVVVMLGLEAEQTILAHKFGRIQDSLQNLRQSYWDCDIARKQYWERLRILIRVSFDQQQLLLRHLKELQS
LVQNLFFYVYYSALILFGSDIFIAMSNPSVFMYVAVAGTVLVSSLECFSLCYLVDCLKDAFESISEHMFY
LCARLPYSEDHHNDYLDTRATLQIIARCSRNAVTFGCAGVSEISIAVFTDMLNICYSVFTFLRETI

>EDS38976.1 conserved hypothetical protein [Culex quinquefasciatus]
MAKEPGRSSPAGKILVKEAPEMLAIAHWTGQIPRRPPLPDGVSVSQLIALGSRRKRSKVYWMIAKSEEEV
STVSTVGKMFRLLGVLLVNAFYRFCRHEHCFVVVLNFNRLIGFTGYTWDQPISIVKAGLLIYAILYEILA
IVAIAFFDPGPVSIDQQFGMIFVMNTGLMCVIIWVLLALQRRDLDLVLNRLMELQRQAADDRRRQYIWRI

NCLSLVFLVQNLVQIIIWNNAVDYSCPLQVFRSEIVDRLNVVCYPLAMTLLSLMFVYTIILISAILPALS
IEFLVLGEDFQRIFDNIGPLFETARNANSNWTKLENKMKSCIRRHQELLTLAELLQLRLKLFFLFNLMVD
FFLITFSCFQFGRSRRSDSPRYMYSAFAAVTATINLLMFGKMCDQLETHVSSIKRHLYSSNWVDRLVYSR
VFSPRYKNFRKLMLIVMERTERKVGFTCGNFYAMSLTTCWSVLQFCYSVFTLLLSVFE

>EDS32027.1 conserved hypothetical protein [Culex quinquefasciatus]
MDLIESLKRYRIFQFDYSNAGKVYLEAVDWIDRLNAFMGICYFDPKVSRLQPRFIWGITTLFIYIYLAFE
STYWYRNDVEKLLLCITTHGFSVQMASKVYTFIINRSQVVEVNTMNRDYYEMETTKSVTVQNSHKSSATI
AYILLKMTAACYIILVGLIVLGPAIGGAIVSEKILPFGFELSHSNAWPAYIMNLAFHVNCGFYVAFLTTS
SDSTFILYLLTAVGQIDAISGLLNELNEMLEKNASEEVISLQLRRIFQLHQHHFAYMRMFKNMFQYYFMM
AITMLYFCMSICLAAFVLINWYMGVLVFCFCSAQIFYMCFLGTALQSKTEFLMKEIDSFSWYRLSVPNQK
FAILFLASAQNPILLSAAMVKLNVATYLQVHKSVYSFLMLLLRIKQ

>EDS31931.1 conserved hypothetical protein [Culex quinquefasciatus]
MEFLAAQDPFEAMPFLKKVLTVTGLGWPRRNLRKFALTLLFPWLVVLVPMLGYRFGSQVDLMIRGYSELL
VLFNIEIRVLIFAWKQTEFEDMLAVLQGVFDQVRSYGAKSQEFKIVTVANQAMDKAAKAYMTFPLILLIV
FLMVPLLQTTAIYVMNRRNGTIEQDFITMTEIHFYDLDIRRNIFHYLIYYTTRQNKTCVKGLTVSISGIV
ASGSIRSINLIFDLVIHKLETIHEFSGKELHAHFAEIVNMHADALSCIRIFERLSNVAVLVQMVDTALIW
ICMILCVTNNPTKNSLNLVVLLVMISSETYAFCKLGNELTEKSITVGRSAFEAQWSELPLDIQKGLSLII
TRSQKWEGLTAAHFYPLSIEQFGAMVQTSYSVFVVLKERLQGVI

>EDS31930.1 conserved hypothetical protein [Culex quinquefasciatus]
MEFLAAQDPFEAMPFLKKVLSATGLGWPQRNWWKFALTLLWPWLIIILVPMLGYRFGNQLDLMIRGYSELL
MLFNIEIRVLIFAWKQAEFEDFLTIVQGVFDKVRSLGAKSQEFKMVTVANQAIEKAAKAYMIFPLILSIV
FLVLPLLQTTAIYVMNRRNGTIEQDFITMTEIHFYELDIRRNIFHYLIYYVCAFPSHCIIGFRVSISGIV
ASGSIKSINLIFDLVSHKLEKIHEFSGKELYDQFSEIVNIHADALSCIRIFERLSNVAVLVQMVDTALIW
ICMILCVTNNPTKNSLNLVVLLFIISSETYAFCKLGNELTEKSVTVGRSAFEAQWSELPVDIQKGLSLMI
RRSQKWEGLTAARFCPLGIEQFGAMVQTSYSVFVVLKERLQGVN

>EDS30726.1 conserved hypothetical protein [Culex quinquefasciatus]
MEFVKRLNRFRFFQHSYRNPSEFYDSLIVFPNMVAKITGMDIFSEGYRVISWSLFSVVVLLTVYVYTSVV
TASEVWGSTEDLIFCFVTAGIGLQGLGKLYTFVAWMHQYNKELFRKECNPTVRKMLMDSVFLLSVIVKTM

LVCYVSTSILLDVAPLLFSIQTGEKILPFGFYIPFLDRNEWFGYFCNYAMHVCNTVYVSSEDMGPDCIYM
IMMMCAFTQIDLLKESLTEFNQKIEENEDDLDAFFGEIIRHHRDHLEYLQTIEKVFQLNFLITFVSLSTV
LVMSLFAVVTLSWYQGYVFVAFISYELFFGSFLGTMLEIKKEQLQQAIYAISWYKLSSENQKSLRFMLHA
SQESVSLTLIFAPLNMPTFLQVYKTIYSIFTMLLTVREE

>EDS29875.1 conserved hypothetical protein [Culex quinquefasciatus]
MDGQRSLRWILVLAILTVPMAFFAYQIAVGDALISISYYVISLVFNVITLLKMTLIRLSFDKTRTLMDYF
NEHVFHRDDPWCHRLRRRTYFTTWKIFGAMLTYVLLTALFYLSTTRPSSHYIGLDRTEVTWIHVLLVQIV
AGCTLLHLVIYSVGILLISFLMHWFQTELEIMANAFEKVFHGECPRKVLGRNVLRRARLQREEVRWTGIE
RRLVYCISRHGEFVELNKLLRGIAQPIFFSLGFYIVTTISTLIFVTIIDGIFNLLAIVHVAAVIAEFYYY
AHLVDELEDKRHMIARAIYEQNWPEQMVYSNRVAKHYKSVRTMIVTVIMFAQRPFRISCGGMYKMTVPVF
TTMIETIYFAVTFLIRTVKIQR

>EDS28962.1 conserved hypothetical protein [Culex quinquefasciatus]
MDSFKKLYRYKYKFFQHSQTEPSDFYQSVVITPNNAARFVGLDVFTEGYKFSWRTFCMIVSILVYIYCCV
FTAYEVRCSVKELIFCLVTSGLAPHKTVHVWTFLMFRKELYWFHQYTKDLYREECKDRTKSMLMKNVSLL
SVTTKLMLVAYTCISSAMDIVPIISSAASGEKTLPFGFYIPFLDHTSSPGFTLYVLCSGYGADCIYLIAM
ASSFTQIDVLMSALRELTTEIEAKKSDVGECLNQIIVRHQEHLKYLGTLESVFRIYFLVNFLSWYQGYGF
ILFISYGLFFGCFLGTLLAMKSEQLERAIYDVPWYKMSLANQKSMKFLLNSAQQPVSMTFIFYKLDVPTF
LQMYKMIYSTFTMLLTVRDE

>EDS26557.1 conserved hypothetical protein [Culex quinquefasciatus]
MKFLQSLQRFRMFRVGVQNPGQFYRATIADIEWFNAKAGLNMFNRSVNLLNFNFCWMMLMLVSNIYCAPE
TVVFYRDDIDKVLMGVVTLGFLFQMTSKMYTFVIVRKKVVEIHEMNVEYFDSMLFNDMDVQDSLVENAML
TKFIIQMTSRIFPSMSMTLMFVPLFYSLITSNLLLPYGFEITHSDTWLVYAINWAYQASCSFYVVVITVT
TESTFVLFLLTACGQIDTLITLTNQLNSKINSEESEEEIALQLKKIVKLHQHHKLYIKKVKELFKFYFLI
AIASLCSVMTMSMAAVVLVNWYLGVIMFWFSSCQILFGCVLGTHWQIKNEQLLVKIASFDWYKLSTKNKK
MILMFLKASQTYTDLSAILLPLNVGSYVQIHKKVYSMFMLLVNVKE

>XP_316227.4 AGAP006167-PA [Anopheles gambiae str. PEST]
MVRLSFEETLRNTNLMLLMMGIPPCEEPYPPGVLPSLKRNAGFIASFLLLAYTTIGELIYLKQMFERDVT
FLEVTFQAPCIGYCTIGVLKMVILARGRNTIAELVGLFRAKWTSAIVTGAHWAVCEDTMRPAIRVTSVTA

LANVVMGIAFTILPIAEMIYTHHYTGRWNRQLAFNIWWPFDVLGGVKYYWFVYPLYVVIGFTGIIIHMAF
DCLFCILAAHLCMQFRILAHNFGHVVEVANGAREGDSGSTSRLRDAIRIHQELIGYEGTPLDAGVNQWRN
GYMLVKFVLFMLCFLIELLMLCAYGEDIVESVRHQAVMSESRVIEAFAFKTHQSLGVIDAAYGCEWYREG
SVAFHRSVLQIIHRSQQSVILTAWKIWPIQMSTFSQILQASWSYFTLLKTVYGNK

>XP_001688726.1 AGAP005760-PA [Anopheles gambiae str. PEST]
MEYIYRWILRDDTFAKEVYIKYNFTKLRTLGIYKDASRTRLARRAGFYALELFFLLQLGTIVWDLATVLG
NIGLFGDDMCILAGLLLTLAKKWHCVLYIDELWECVEQMQTYHEHYLLQGEWFVRRMRRQNLQERMLQDA
SKLLATLLASCLMVNVRIYGILVQEFTIFEDGLRLIFYSTQCSLGTLFQRRNAYPACHLSLQHGHHAWLR
VCVRLSGLSDRLRTVQHGADRLYRRASAQPDGATVLHAAQTVRADRGRSSAAPRGPPPRRPVDTGPGVQC
DSFTPAVAVVSIGKTRMSLALFSTPERPLLFTGSFCNRLKRLYEPNIMAQFVCSMLIICLTAFELMFAKG
DPMQMVRFGAYMLAGFYQVFVWSFFGNRVTNMSTGISDATISCNWIVLADGLKKDLRFTTMRSQKPFVID
VYWLFPLTYETFIAILSRSYSIFTLLRTMIE

>XP_315072.4 AGAP004974-PA [Anopheles gambiae str. PEST]
MLAAETVDFFRVQSICLRAIGIARTDSFRGRVLFAVSFFTVLVMMLGTVMFAFKHIDQIMLLCDCLGPTF
TAYLGLVRQYNLLLHRSELWSIVDEFAALKHSLQSSEIRIVQKYNRIDRFLAWAYLITAMSTGVLFVGVA
LVLVFLSEKSDWKLPLLMDFPFDVKHPVTFTIFFVWCSVAIFWVVLDCVACDSTFGTFSSCLVAHFVIIQ
ERFEGLRFDDGNRELKKLIEHHKYILRISDRVINAYKNVILNQLLISSVLLCMLGFQLVISVGTNIMVVY
VAYGMAITIQVTYYCYYGSQLYYESTQVHDAVFKSKWYDASVATQKMLINCMMRAKKPVNAKSGFTQASL
PTLNAILNSAGSYVALLMSLME

>XP_315068.4 AGAP004971-PA [Anopheles gambiae str. PEST]
MSCVEDFLDAQMWYLRYCGVFREKSALGGIRLSVCLGVLVVFMVLQSIYIFQHVHHFAMICDAIPTLVVC
MVAISKFYIFVFHPAALFALIDSFKALQRRASREDLLLFRRSGAFHAKLTKIYMTSALIVGWFYILSAIV
SGISRSLAEGRVHFVAPMAFPHNYQHPLMFALTFLFNCDSIHMSIFISGSVDTCFSELATSVTIHFQLIQ
RQFQAVDFAARTAEDELEVVVAYHKDVLQLCLAMTNLFQYTVFYLLLLDSVLLCVIGYQFVIFMNTPRVL
MLASMAFVMVLQAVIYCYHGSMMYDESLKVADAIYQSNWYEAPPAVQKRLRLCIMRAQKPIVTKGGFIKA
TLPTLKKILNSTGSYITMLLSLETEQ

>XP_320910.3 AGAP002125-PA [Anopheles gambiae str. PEST]
MYDPGRFIFPMRFSMWAWKVCGFFNAPVPKSVAYRVYCYAFYSCIMAVYLFVLLLNVFVPQPFEQRVFFI

MYIFLTETAMILKTLTIYRHFNIVWSLYETTLGVSFQPRDEQERELQQRRLAVFNRWYYAYIFVSHMAAF
GTGSHLLSAEYRMPFFPWFFGVPYWEDAHVAYYTIFAYQSFGMYFHMLLNTAGDTQLCYMMHMIGIQLEL
LGKRFRDLNNCEEFDRSFVPLVQHYNKIHRMLCRVQNLFSPAYFVQFSVSGLVICASAYQVASMLNLNDF
SKLMNVFYMMSMTMQIGLPCYYGNEVTLKSYALTNAIYSSRWYDMPQSNRKSVQMFLVRTNKPFAVAAFG
YFNFNLPAFTTILNMAYSVYCVLQRKAKNV

>XP_320874.3 AGAP011631-PA, partial [Anopheles gambiae str. PEST]
MELKEEWILPDVVYANPLLKRTLLGLRYYGILLGQSQSYKKVHCFRGMVFTLSMVLFNCTQYIDLWQVWG
SVSDMTANAATTLLFSTTIFRIIFFYFHRARFNSIIKAAHDGIERILKDGWTDEQSIVGSNVRYLKRLAV
VFWSCALVTANMMCVYSLVQYLTYEEGPPDGTGTSAANITRKGQPIPPTPILRSWYPTADGKDKHFLEIY
LIQLYIMYVGQLIVPSWHMFMVTLMIYGRTECSVLNHRLCFLERYHTAGKDDPTAPVNNAERRSLMIDCI
KRQASLVSFTRELEQLTRAAVFLDFVVFSVLLCALLFEASMTPSGVQVFIDVCYITTMTTILFLYYWHAN
EIHACADRLSMSAYKSDWYRYDRGTNRMLQIFILYSNRPLKMHAFFISMSLDTFLAILRAS

>XP_311815.3 AGAP003054-PA [Anopheles gambiae str. PEST]
MEQEINQIISFLRRPLKWLGFDVIDPNWKVTPLTMFTIVMFALQHYTSYLYLSTHLDMFDMFTECFSTSA
VALEIAIRMGLLVHQRELRNETIEIIRQQQKVTARFHKLISVTLHALAACYMSTMMFELIPIVSPNPRKS
NLPLPMYLPYMPHDVTPYWHLNYTFITVMNLMCILFLVGIDGLLVLSILAAVHQIKLLKITIQELDIGAE
QAELHRELVRIIQIHQRIQQFIHQLEQTYYIDLLVDFGLVCLILCMGLNVIADEVINAIWFFLIAVVFQL
SLLCFSGNLLLIESDSLSSCVYSIDWHAMPVPEQKLLMVMIAHAQKPQVLRGIFMPLIMSSFLSVIKASY
SYFTLLH

>XP_001230709.1 AGAP009412-PA [Anopheles gambiae str. PEST]
MEKLRPEDPKAVMPYAKRLLQLSGFRQDTEQLGTRIYLNLFIFVAAILIPKVCFPYPDTEAMVRGLSELI
FFTNIFVGLFCFIAQHRPYRELLNAIESFVNIVYPTSQQPESLSERTLIKLNVKINILSVLYCRYIVVAA
FIYWMVPCVVTYSSIHKAEVSMGNESIQSIQYYPNLEESFYWLDNRSSVSGYAAFSAVALIVFAFASYNH
VTKLLTILSTIKYCSTLFHLVSIGIDELNLVSPAHIDRELKKLIQMHQLAIRCDVLLNQTLSYVMALQLA
LGTLTWCFTLLYILIIGLDVTAMMGLLIMMNMTSEMFGYCLFCTELTNTATTISQQIYVFQWEKHSPAVQ
KMVAMIIARGQAPLQIKACGFIPINLELFAKVVKTSYSVLIVLRDFV

>XP_310072.3 AGAP009397-PA [Anopheles gambiae str. PEST]
MIRFFTIHKPPGVPHLALKLLHAVGIANGETFSHRFTAVYLLFIFIIAIPKLFCGYTSFEASVVGLAELF

FQLNNFTGLLLVLVSSKQLQCLVRVGQTIADEVFQCAQTDMRQLLTPDITVYIFATAPICATFWSYVRAA
HRNTTAQYVLHMEEDFYGLQIRSSLRSCLMFAIPMVPTSYVCAYVGCAKIMIVFNFTSYCTVYFRLVALQ
LQYQTRTVPSDRDSIRTIVAMHQQALCCADLLETIVSPIMLMQIVLCVLMSSSMVLYFTFPTMSGHMINV
LLLFLVVSTETFGYCYLGTQLSMESARIAYAVYNGKWEQQPREIAKHLQLILLRAQKPIGITAGKFCFIN
MEQFAKLLKTTYSVFILLRDLL

>XP_310070.2 AGAP009396-PA [Anopheles gambiae str. PEST]
MNFLRQEQPAGMPHISIKLLQLFGVTGSPEERFRILPVMLTYFFFIVVPKCFFGYPDLEITIIGTAELIF
QSNTFCGMFWLFLNRHKLAQFITQVRTFSLTVFRESPPAVVQHLTTQHDFIHKITRIYCIVVMFAAHFYV
LTPLLSTFYAFYGTVRNDNVTVHYTLQMEENFYGLQTRTTTSHYLIFGMFMTPTIYLCAFTGTVKILSIC
NITMYCTLYFQLVQLKLRTATEDNTFNRQEVKSIVVMHQDALNCASLVESITSLVLLQQLVLCVMIWSSM
LLYFTVSGFDLNFINLLVLFAFDTTETIAYCYFGEQLSNESARVAHTVYESGWETQTPDIQKDLQLIIAR
AQRPVGITAGKFCYMNMEQLGIIVKTTYSIYIILRDQF

>XP_310066.3 AGAP009394-PA [Anopheles gambiae str. PEST]
MRFFLIEKPPGVPHIALKLLRLVGVSQTQSERYRYVPMFVLFLLTIAIPKIAFGYPNFETSIIGLAELFF
QTNRFVGVLLLVLYSDSIFELVRQSEFFAKKVLSETSPVAEYLTKMDTQVTKITKLYLTALLVPANFYSY
SPIVATLWKYYNTYENDTVPEFTLHMEESFYRLNVREKLDHYLIFGAIMVPISFLCALVGTAKLVSILSL
IKYCTIYFQLVKVKLEEVGRSQNYASDIRSAIQMHQSALSCADVLHDITAPIMLLQLVLCIMVWSSMLLY
FTVAGFNTQFISLFILFMFDTTETFGYCYLGNQLSDESARVASVVYDCRWEGMPPALRKDLQLIMLRAQR
SVGITAGKFCFMNMEQFGEVVKTTYSFFVVLRDQF

>XP_310061.3 AGAP009391-PA [Anopheles gambiae str. PEST]
MKFFQIDDTREILPIGCRLLMLCGLPRSGRVNRRFWLLCVFFFVFGQIPRFLIKIDEPIALVRVGAEIVY
STYMFLQLIALYARRSDLYRLIDTLQKCVENPYPDDVRAFILITRDTINKSSVMYSKCFFAVCISYIIMP
FMATSAVVVRNRRNQTGEREEYVMPTEMKFYYLDIRFNLLHYSLYFGAVSGLSVIGSLALCTKDVMDFSL
IRTASMLFQATAQQIRNLPPGASQAKLEAIIHSHRSTLKCAAQLQNALNPALLIQITFCTAIWCLMLFYI
LLLGFTSKVMNVCLLLLVLTCETYSYCQLGTQFTSNAEEVLDELQQLARYDQSIPIQKQIYFMIHRSQTR
IELTAGKLFPVNIAQFSEIVKKSYSYYLVLKDIF

>XP_319861.2 AGAP009111-PA [Anopheles gambiae str. PEST]
MKLFQIDDPREVVPIGCRLLKLFGLGRDEKLKLLYWVQCVFYLVFSIIPRVLVQIDDTIMLLRLGSELAF

VSYLYCQILGLYFRRSYLYRLVDLLQTCINKQYSESIDQFVIKSNTKINKLSVTCCKYFTIAYVLYCAMP
PIASTVVYVRNQRNKTAEPEEFIISSEMKCVLYYLNIRFNLLHFSFYTIVICLLTITSAGSLCIKDIMDV
AVIKTTCLLFQTTAMQIRELKDNISQAQLSVVIKSHRDTLLCAQYLQEALNLSLLFQLTFCSLIWCLMMF
YILLMGFDSRILNVLILLLIVTVETYTYCTLGTQLTDKGEEVLMALQQLAWYDQSIPIQKQLLFMIRRSQ
KPIILSAGKIFYANVLQFSEMVQKSYSFYLVLKNVF

>XP_310087.2 AGAP009409-PA [Anopheles gambiae str. PEST]
MLFQALPNDRAVLPLLLYIQERLGLWGESYRARFLFVVVAFSITVCIPKFTTTYTNLETFICSMAELVFI
GNVFGGAMLLWTEYDAFRQFIEQVNSLTKHFYREDPLKEHVLQFNYLIHRYTNRYCFSVMILIMFYLAAP
VITSFVVYFQSLWKSYHLANGTVRTWDISHRQFSLHMEQSFYGLQHRTNILHYTLYIAVIVPMMFVTACT
VHMKVLTIASSVRYAEMLLHIVMLKVDSLPKQKSIREELHDIIKVHQRTLDCIALLVKALRPVLLLQLVF
CVLIWCLMMLYFTITDGLSVKFFNIGIMFLVITIETFSPCYFGTRLSTQAVELSESVYACGWPAMDRDIQ
QRLRMVLHRTQSPVGIQAGKFCFVDMELFQKMVNKSYSCFIVLKDAL

>XP_556129.1 AGAP005495-PA [Anopheles gambiae str. PEST]
MDTEHYKPEDDYGELVQRSTYWVRALATTMGIWPGQYVSMRSHWYRRVYYFMLLMHWLNTFLQTEFFFRN
LGNLGLVVQGLCSFVSITTTGIKVMRIHAYEAEIVQLWQTLQDATFIKKIRFLRKTDRGTIFQRIHQLLA
RQCKEVQLNLRFYTLVVGLVASNYSIIPACSNLYNQFQGNAFNRSFVYNTYYPLLEPIKRRSPLFELLFC
SESLSGYTTWAGVVAFDGLYVAMVLYAASLMRLLRDLLHETANPGLTDAERAFFQRECILHHIRTIQLIE
KINEIFSPVLLVQLFTSTSIICVIAFAASMHADEGDSQTMLMVLYLIAAIYQLFQFCWYGQRLQNEGTEL
PRSVYDAQWELCAQRFKSTQHVLLLYSQRQIEMRAWSFSAMSLETFSTIIRSAVSYFTVLQTLAEE

>XP_321007.1 AGAP002044-PA [Anopheles gambiae str. PEST]
MGTANQPSSICFLRWIGWMDTINGIHLHDDSRLARAFEYTFYALQLVQLLNGYNFAVLCSNDTISLETFA
EQFNQFGGILLTLSRVIAIVGSFPRLKDTAEFINAYKFHHLNERAEEIRAGSIHNAGRFLSTLLAVQIVT
LIFWFVLTELQAHRQDVLLPIFIYLPFDASHWPLVAKVAFRLYAYLAYAQLMLTFFGSYIITSSYLLTLT
IELRILNDSYAAAPTDPQQLVAFLKERVVYKAALLQHIRTIKRHMNGSVLLELLFIVCLLALNGLRLCTT
TTTSDLSELALSSSMILIYLLELFQYCWQVDEMELLHEGQAFAVYSTPWTGAIMQSKPFLLITIRMAQVP
LRFMCGGMYQLSTELFASVVQFIYSLIMMLLQFK

>XP_321153.1 AGAP001912-PA [Anopheles gambiae str. PEST]
MDPPTDELVRFESFIRVPEIFFAMIGVARYGEPKRTLRAYLKHLLFWSSCINTGFCLVIEHIYFVKAAGN

FTNFLQLTALAPCMGFTALSFVKIMTIKLNETKLTDMLHRLDALFPSTVALQQRYGVYQYNRESQVVMKS
FSILYMILIWMFNLLPLVSMVAGYVSDGTWHKQLPYFMWYWYDWHRPGYFAVTFLHQNWGGFVSAVFYLS
TDLMFCAIVLLLCLQFDIVAYRLSHALPDDHQELVGCVRIHQAVIELCNELEHMFSPSLLVNFLSSSVII
CLVGFQATAGITPADLFKFVLFLVSSLVQVFLLCYYGNKLIVASSQIPYSAFEGNWIGASVSYQRSLLFV
MLRSTTVQKLTALKFSIVSLASYSKILSTSFSYFTLLKAMYEPNEKKMK

>XP_321006.1 AGAP002045-PA [Anopheles gambiae str. PEST]
MTVVHRIVSFGYNLLQRHFNVGHPTEQFFLLRCLDVVSPAMLLQRPRSNLEVALKTLCLSVMVAHTIALA
YDFSQQTDVRLAMDMLCMLSLFVSIILRGTCMRQYLAHINALDRLERRPTFRIGTPYADESRRNVALQNS
RYLGVALVFHSLTVTVYVIQNMVRENSFVKIITSFPIDLSERAPVLERVADLCYSLVGYVWGWYHGATQL
TIIVLLRYAITEFRVFLHSLDSLDDQLQQRREQSQGAPDEERILRELLYEHARHHSQLIVVVTHLRTLLR
NYSLVHFSFYMIIVATFMTRVLIIPGRSSFGLAIPLLVTTIYFLETFGMCMLVEMLVQLNRKVSTSLYGF
SWPQYLRYGRTIKRPMMLMIMQANMTKDFSAGGLTTVSAELFAKTCRMIYTMMMFMANMAT

>XP_320909.1 AGAP002126-PA [Anopheles gambiae str. PEST]
MEAEKQDTPTSDDPYRWGDYIRPVRITVWTWKICGLYNGKPQTTRYRVYRAVFNIFLMVVYLFTLSLNVF
VMQTFEQLVLYIMYIVFTEIVMALKAIVTYYKFDQLCDLYRQTLGSDFKPLDAEEEQLHRKGVASINRYL
YPYLVTTNLAVASSCLYLLQDDYRLPYFPWILGIEYGPTKRLNYGLLFAYQVIGMYLHMLINVAIDVQLS
YLLGMISIQLDLIGKRFRSLHTSEQFRESFVGLMNHHGKVQRMTAEIEQLFSAAYFAQFGSSGLVICASA
FKTSSMFNLYELTAIQNLLYMLSMIFQMFLPCRFGNEVTRKSHLLRTSIYSSRWYEMGLQERKTLRMLLQ
RMNKPLTLKAFYFFNYNLQAFTTTLNMAYSLYALLQRNALKKV

>XP_320702.1 AGAP011813-PA [Anopheles gambiae str. PEST]
MYYLEQLRALVRRYLERRSPDPRIQHTFIVQSINRVGGMLGIDIFTPGYSSTNLLLRMVLLNTFTFFWIN
LYSLTTTYGNLVDFMYSFETLLYVGIACIKMYVFIKNKTLILQMHQYMIDFFDHFHGDREQDELLVRTLN
NTTLLSSLFAVCSSSAPGLLFVGSLLWSIAVEYVLPFGFFIPTVGMDTLQGYTLNYGFQMLETTLMVIGI
ISSESAFFMFQQNACLQVDMLRYSKSMCSLFELHFFIVFGCIFFQLVSNVVVIVSVPDWYPGYFLFIMLT
VQLFFSCALGETFNIKSDELTVAIYNVPWYNMEVRDQKAMRLLLMASQNPGRLSYGFGTVNMRAFFEIFR
KTYSIAMMMISVNEEE

>XP_320553.1 AGAP011978-PA [Anopheles gambiae str. PEST]
MVRSQPFRLSLVRKYWDKYFTFTDTVDYFNLLNTFGTLFALHYHTRQPRWTVKKLLWTVYRTLYLLSYLS

YCYKVYWLFSHWQYSTAAANLLGALGLCSGALLRLLLIEQNYPTVHRLQQFLNDRTYLREDPWARTERSK
LYRHNNRFLVVLISAISVESLCFLARLLLTRPEFMLQYGGAVLGGPSVQLAYGMVTACWGIIYVLSFIVF
YTLLTGFRLEMQLLARSFQQLEETLVPTGVGMDDQNEWEYWDNLQQRLTHRIKRHLELLENLRIFRSIVA
PFAFLQYYSTFGLIADSFFVVSFEGFTGYSMAYVLFASFLILESLLLCRGVEDLNDLNRQIGTILYNFDW
PRLLRFSIHYRRQYFSVRRTILLVILQSQQSLRFSYGAHGEISMHSFAELMQKSYSMLTFMLQFQN

>XP_320552.1 AGAP011979-PA [Anopheles gambiae str. PEST]
MIDLVTRIRRKLLALERKLANDLDQFVLLHHLTFLFAIRNDAPRGVGQFMRWCFHLMAPVFVLSSYCYKA
YWYMSHSEYSFALFNVVGTIWIMAGAFVRRLLFDRGLLTRLEQFLNDRSFREDEPLVRAARQHVKVQNNR
YLFAVCLTLVLEASIFSGTNLMLQPEFMLLYQGHAVGGFLIQLLYGWATCLWGSLYVLIFAFIYVLLNVF
RGEMSLLVESFERIDECFHKYRPELNTASAGEREEEFWRELQALIKLNVQRHVELLENLVDFGSILKPFS
FIQYYGSFTLIGYYCFILMYKGVTPLTVVYIAFIVFLVAESFLFCRILSQINDLHARIGTVMCELEWYDK
LRFSTRFASAYRQMRASFLIIIIRSQKPLSFSISAAGTISMARFADLLNSSYSLMTVMFQLKERIIAKLT
SDGNN

>XP_320543.1 AGAP011989-PA [Anopheles gambiae str. PEST]
MYLTQLFASMRTQFLHRITHYTKHSDFFIMQRYFEKIYAIHYTPHGWHDRILWYLYRALYGLIYLSYIHK
THWVLHHPQDSFSTANILGVMWFFSVVILRVAILEWYYPLMLRMQTFLNNHTSYQRTDPWAVGRRARFYR
RTNRVILTVMGINLAETVCFTATNVMKLDEFMLQFRGAIVGGWPVQIVYGVLTMGFGGMYCMGFMMCYLL
LSIFKLEVDILIRSLEEVERSDRLESDFGDSADIFWNNIVDQLRPHMQRLDELFIQLQHLKAVIGPIAFV
QYYSTYLIIADCCLILVSHGLSSFSIVYFISMLVFLTESFLLCHGVENLRDLKPRIASTVYDFDWMLQMR
CPNPRHRAQYRHVRRTLLLLTAQSDQTIQFSFAGIGEISMNSFAQLLEKSYSMLTFLLQFAK

>XP_320542.1 AGAP011990-PA [Anopheles gambiae str. PEST]
MFHPSRWMAKCRVWYAATFKFSADADYFFLVQPLCRSLQLFGNPVRCLQEFGTVRGLFVQLCRFLFLLPY
ASFALKSYWQLNHPLDTNNSILTYGSFVIYMLWAILIWVLNHYEHEMCELRLFFKNPTYQEQSDWAHRIR
AGNYRRWNWMILMFYLFNVINVSIFTLTNAHNRQFHFQTRGEVVGPLYFQIIVEIFTGYLSLGYFVPSCI
TLMTLQLFRTEMHILTTTLKQAANEEQQQQHQHAVDIRCAYRSFCKQFYANIRRHTELLQSIATFCKVF
SPISVVLYYGALITITCTCFYVMKHDVSTTTVCYAGFAVYMLFNTLLFCKSIDSVNDLHTEVGYIMYSEY
WPATLQYADQGLSTETLRPLRRSILIVLQQTLRPLRFGYGVSGSLSMQRFGEFMQQIYSLIMFLAQLN

>XP_320541.1 AGAP011991-PA [Anopheles gambiae str. PEST]

MWHHQVQLWFRFLLGRFTTFTDSSDYFGLYKTLATISAIHYDASCWFDRAIWIVYRSLPILVNISYFYKA
YRLILFPEDNTSAASVIASVWGFTEGTLRICLIELRYGTLASIMSFLNERSYRQQDSLVRQQRATLFGEN
NRIQLILVATMLMEAIWFMTTQLFSRDAFMLQVNGHVVDSIAVQILYGLLSNVWGLIYVLSFAIFYIIMN
TLHLEMSILLDGITSVQFTVMRRLKQRMETLAASGHSSTQVFWSILQPELNSHISRHVDLLDNLKEFSSI
VGPFSFVQYYGTLALIADCGFILSIEGLSANGMIYLLFVTVLVFQSFILCRGIEKLNDLNEAIGQALYSG
FDWPDMLQYDQRFRRQYVTVRHTLMLVIGRSQKGFQCSYGGLGSISMERFAQLMQKSYSLLTILLQFAK

>XP_319640.1 AGAP008894-PA [Anopheles gambiae str. PEST]
MVKFFRCCKARVSPELLSKKNEKPEAFLPSLRMILFIFRMFYAWPDEQSLAPNALWWYRAKGVLFRTFFI
YLSAAAQLAYNFTVTSREELFAGMFVLLTQLVLILKMEFFYKNVSKIQQLIRRLNGKRYQQSGNAEEDMP
LVSARKKSTIFWTLYFMFSVGLATEWLVISLSLTTLIVPVWPVVDHTTPYWVYLMVIFHQYVAIALNASF
NISWDSLVAALFALTNAHLHRLQIQLMKDAKRRMIAVSSFDDNARSVPTKSDADEVYNELLQCIIFHQEV
TGFLREVLDLFSGPLLAQLYCSVFILCITEFRLLTDVNTMADTVQAVIYLLCLVIQVAQYCYFGNEINYM
AQKVHRATAFVNYPDMNIKCRKLLIAFQQITAVGIKCSAKYVFTIQLSMETFVTILKTSYSYLAVLRSMT
D

>XP_319538.1 AGAP003310-PA [Anopheles gambiae str. PEST]
MEASEKFSQFERYIRTLCNIIGFDVMTETWKKSYRTYMSIFLCSQYLILMVYSLIIASDTFELFKSLSFL
GFFFQCSLKMYYTIQQAPQYAVNFCCLKEAIYERHSKGTLAQKTTVARIIDLLVLVTKAISVLYTSSLFI
FSLYPAYMYFVVHVKVPIFPLYIPGINIYSAYGYGITNSFHMLIAVYGLFGALTSDIVFIMFVVHFVTYG
GLFKIECEQFDQDLSGVFQHCEWRTAVYKTFCRQRMRAIYQYHQSVIFYLESMQECYRNICVVQVASCSL
STVFNLFLALTTDWYATYGFIVISVFQLFVYCLMGTVMQIMNERMIDYISNLPWYMLPTEEQKQFKFMLA
RSQLSAEIMIRSVGPMNMETFTDIMQKMYSAFAMMYSFLVDLG

>XP_318795.1 AGAP009720-PA [Anopheles gambiae str. PEST]
MANVEGFRKLMKSLIVYSKVAGVEIWTAPGKFTPASYYVSFHITVYFASTVWTLRKYIDDPIHTMKVLIT
LGTAVQLYIKFFVGHSKAREINLFTAKLEQEVLQRYQNGSEEETDVLRHTGRILWLVYRMISASYIFLAV
AFGMYPAFYCFATGKVMPLFLYELPFCDWSSSLGYAVTICFQINLLAIGVLGAILSDFVFFMYAMYAMAR
ADISIVHLGELKNMLNDPTKNEERTAEIRRKWIQCMHDHQQSTSFFTMIEHIFGLICLTQVATATFSICD
AMLLVILTDWYPTYSYLYVMFVQLSGFFLIGHLVELKNDALYNKVISMPRYKLPVKEQKDFRFLMSRQQN
PMMLTAYGFHPMNFEVYMSVLKRLYQFFVMIMQYVG

>XP_318789.1 AGAP009719-PA [Anopheles gambiae str. PEST]

MANVEEFRKLMKSLIVCSKVAGVEMWTAPGKLKPASYYVSFHITVYFVSTVWTLRKYIDDPIHMMKVLIT
MGTAVQLYIKFFVAHSKAQEVKLFTVELEQEVLKRYQNGSEEETAVLRRTGRILWIVHRLMSASYIFVAV
AFGLYPAFYYFATGKVMPLFLYELPFFNVSSSLGYAVTMGFQINLLAIGVLGAILSDFMFFMYAMYAMAR
ADISIVHLSELENILNDSTKNEEHSANVRHKWIQCMHDHQQSTSFFSTVENIFGLMCLAQVSTATLSICD
AMLLVLLTDWYPTYSYLYVVFVQLSGFFVIGHLVELKIDALYNKIISMPWYRLPVKEQKEFRFLLSRQQC
PMILTAYGFHPMNFEAYMSVLKGLYQFFVMILQYIGRN

>XP_318786.1 AGAP009718-PA [Anopheles gambiae str. PEST]

MANVEGFRKLMKSLIVYSKVAGVEIWTAPGKFVPASYYVSFHITVYFVSTVWTLRKYIDDPIHMMKVLIT
MGTAVQLYIKFFVGHSKAQEIMLVTAELEQQVLKRYQNGSEQEIAVLRRTGRILWIVYRMMSASYIFAAV
AFGLYPAFYYFATGVVVPLFLYELPFFDLSSSLGYAVTMCFQINLLAIGVLGAILSDFVFFMYAMYAMAR
ADISIVHLRELENILNNPSKNEEHSAELRHKWVQCMHDHQQSTSFFSTIENIFGLMCLAQVATATLSICD
AMLLVLLTDWYPTYSYLYVMFVQLSGFFVIGHLVELKIDAMYNKIISMPWYKLPLKEQKEFRFLMSRQQC
PMILTAYGFHPMNFEAYMSVLKVLYQFFVMIMQYIDRN

>XP_318764.1 AGAP009707-PA [Anopheles gambiae str. PEST]

MANVKDFRKLMKSLIVYSKVAGVELWTAPGKFTPASYYVSFHITVYFASTVWTLRKYIDDPIRMMKVLIT
LGTAVQLYIKFFVWHSKPQEVKLFTAELEQEVLKRYQDGSEEETAVLRHTGRILWIVYRIMSASYIFAAV
AFGVYPAFYYFVTGVVMPLFVYELPFCDLSSSLGYAVTMCFQINLLVIGVLGAILSDFVFFMYAIYAMAR
TEISILHLGELENMLIDPNKNEEHTEDIRRKWVQCMHDHQQSTSFFSTIENLFGLMCLAQVATEFLSICD
AMLLVLLTDWYPTYSYLYVMFVQLSGYFVIGHLVEIKIDALYNKIISLPWYKLPVKEQKEFRFLMSRQQY
PMMLTAYGFHPMNFEAYMSVLKRLYQFFVMLMQYVG

>XP_318763.1 AGAP009706-PA [Anopheles gambiae str. PEST]

MATIESFRKLLKYLIVYSKVPGVEMWTAPGKFKPASYYVSFHIIVYFVSTVWTLKKYSDDPIHMMKVLIT
LGTAVQLYIKFFVGHSKSKEVIVFSNKIEQEVFEQNKNRTAGETVLLKQTGYILWFFFRFVTTLATIVVL
AFGLYPLFAYNVNGVVMPLFLYELPYYDWSTTIGYVVNMMFQVNLLVIGTIGAMLFDFLYFMYAMYTMVK
ADIFIIHLGELEKMLNDPLTMKKNQSSVREKWVQCMFEHQQTTNFLNSIEDIFGLMCLAQVSMGVFTICD
AMLLVALTDWYPTYSFLLVMFIELSLYFLIGHLIEQKIDAMYNKIISMPWFKLPVKEQKEFRFLMSRQQC
PMMLTAYGFHPMNFEAYMSVLKRLYQFFVMVMQYVG

>XP_318761.1 AGAP009705-PA [Anopheles gambiae str. PEST]

MATVESFRQLLQPLIFYSKGVGVEIWTAPGKFVPASYYLSLHVAIYFSSTVFTLIKYSDDTLHMMKALIT
LGTCVQLYVKFIIGHKKASELKLLSDNIEQAILQRYENGKAEEIAVLQRTGRILWFIFRFMRTSVSSAAF
GFFLYPVIAYYTTGELMPLFMIELPYYGWTTTIGLAMNMFCQANILVIGTMGAIMSDFLFFMYAMYAMTC
IDIFIVHLCELETLLKEIHIHEKECAKQTSIMRQKWIQCMQDHQQATSFLNTTEDIFGITCLAQVLMGIF
TVCDGMLLVALTFWFPTYCFLLVMFVELSIYFVIGHFVELKIDEMYNSIISMPWYKLPVEEQKEFAFLMC
RQQRPMMLTAYGFLTMNFESYMSVLKGLYQFFVMIMQYVE

>XP_318760.1 AGAP009704-PA [Anopheles gambiae str. PEST]

MYTAIEFYNASFNRLKLSSRLIGAGLWEKQEGILWGRVASVMQIVLFLTLHAWTGYKYRYDALQMLETQS
LICTGAALMIKYFTMIRNPAPVRELSGNIETQMYTKYQEMSPEYPVVLKYGRVLYIAGHIMIGGYFGSLF
IIWINPLIMYFKEGRVMLLFFCEIPYVDWTVMKGYWTTVILQLFFYITGTCGLILVDYLCAYFTINGSLY
VDILRFHLDELSELLASPGYQTRNSPEIMAKVSRKWRMCLVEHQRIVEYYDNFSDLWSMINLAQVGCSVF
GICINMLIIFLTDWYAAYAILFALFIDLSVHFVLGAIIERKVDDLHISLMHFPWYLMDDRRQKEYKLLLL
RAQQPSGMSIAGLTPVNYETYTQIMKMLYQLFALAMNFLK

>XP_317710.1 AGAP007797-PA [Anopheles gambiae str. PEST]

MRWRKDETGLHTPQCTVDLPGDGSHSVSVRKPLLDRDRLGIADGCCVLLCYAGNRYTGELMKFYFEHSIT
HHGPSAQGLAKIEAFTCPELNDLHLYNVARFKAPPRFPEVDEALFHTATMCMVFIRIFAVAFSIVAIAIY
SYAILMPLIEHELSLAFGFYLPFVDYRTPVGFAINWVYQFVQVLEGCIGLMACDSCLLVLIMNATGQMDV
IIVYLKQLTLLIDNNHTGQHDEEIADIIKEIVLKHLEHTKYMTDMDKLLKKQFFINFGCMIFELVASLAI
VVRVPWYPGMAICLICTNQLFINCALGTFLSSKNEKLVEEIYNVNWYGLTTKHQKTLQQILLTSQHPVVL
SDGFSPIDLFNFVEIYKKIYSYLMVLQKVS

>XP_317343.1 AGAP008114-PA [Anopheles gambiae str. PEST]

MFSLGKSHQYLELSYNRIYEVFHWFLKISLLRIFDDDFLVAPVPTTLFQFHETELVVSLGVILLHAYCYR
NDLDTIILSVSAFVSAFELMLKINGMVYRRKQIAAMLRTVLSDRSSLNGPIEAVICGKYQRLARKLLLVT
ILSYLTTGAMLLIYPVVSGGLADRTLPLGYSIPFADYRTHPWYLINYLLQIVQVQWVALVFVGLDGPFYL
FVCYSASQLEILIVYLRQIGESPDNVQEQRRLMRKVFEIHTGLSQFVARCSSIYREVYLMQVLCSIVHIC
VSLFHIQIKFKNGSYGMLLTNVNKIWLFCYCGELVVSKAAEFSTGVYANQWYRLWNRRDLQDILFMLRNA
QRNYGFSIGGFGFLSFATFTAVMKTAYSCNAFLHRVMN

>XP_317124.1 AGAP008333-PA [Anopheles gambiae str. PEST]
MVRLFFSKTRVTKLFTRKDERPEMFLPSLRMILFIFRMFYAWPDEHLEKSALWWYRAKGVLFRVFFIYLS
AATQLAYNFTVTSREELFAGMFVLLTQLVMILKMEFFYKNVFKIQQLIRRLNGKLYQSRNAEEDIPLASA
RKKSTIFWVLYFMFSDGLVTEWLVISLLLTTLIVPVWPRVDHTTPYWVVLMVILYQYMAIVLNASFNISW
DSLVAALLALTNAHLHRLQIQLLKVDANKRMITVSPIDDDARSVPPKSDADVVYNELLQCIIFHQEVTGF
LREVLTLFSGPMLSQLYCSVFILCITEFRLLTDVNTMADTMQAVMYLVCLVIQVVQYCYFGNEINYMAQK
VHQATAFVNYPDMNIKTRKLLIAFQQITARGIKCSAKYIFTIELSMQTFVTIIKTSYSYLAVLRSMTD

>XP_316698.1 AGAP006667-PA [Anopheles gambiae str. PEST]
MFRRLTHPTSPKQQHHRDRACDFTVMPHGLWLLEHSGLWGDPRRKRSFALMLLGTVLLLIVPKIVLGTGS
DSFDSIARSTAEFIFCYNNYLMMAIFAIRRQPFEQLIGTVQLLFDKQRKFQTTDSSRYVVYVNRQIMRYS
RLYIIVQGVYFLIFNLLPAIVTYSAYFASSGDGEPVEFLLPVESRFFFLDIRHSIVHYTVFSLLACPAFL
FTAYLTVVKGLVFIGIIVYNTLQYQLVSRGVRELKSLDPTTTQFRHRLTEIIDQHGAATRCTKLLDSVLN
LMLLVQFTNTVLMCCLFLFYISKNFNSGAVNVLLLFLALTVENLCFSYFGNRLSTENTSVAIAVYSTDWY
NYPPCLQKQFQQMIRHAYIPRGITVGKFHFVDMASFGQLLKAIFSYYLILKELF

>XP_316697.1 AGAP006666-PA [Anopheles gambiae str. PEST]
MVNLFARTPADNFRVMPYNLRLFAMLGLWGDRRKLYRLYALLLLAYVAIIFPKPVRITDRHPFESIVRSV
SELIFAALCYLTIIILAIKSEPFRAVIRKMEQALELFRDKQDQCSQLIIEVNANIHRFSLGYAKLNLLYV
LLFNVIPPIYNYPRYFLQWRMPEDNRTVEFMLPLMQDLYGLDVHHNIVHYTISWVAITPFCVFCALILWF
KGALFMLIRYNTLLYQLVNRLLQQYDRESAGGLLAQKHRRLQQIVQLHYRAIECTKLLDSILSLILLIQC
VGCLLMLCLMLFYITRNHSLNVINIAVLLMSIFIEMMCFSYLGNQLTEENANISHSAFNCRWYDEPIVIR
KYFLRIILQAHRKATITAGKFYNVNIVTFAQLIKTSYTYYMIMKEMF

>XP_315048.1 AGAP004951-PA [Anopheles gambiae str. PEST]
MSQLNEFPAYQFRLIRFCGVVEQPSLRNNVRCLFAFILLCSFGPLHVWYLAKTPVLDLVVTCEEIMLIQL
CFVMLLKFNLFITYRSGMYNLVEAFKRILKCIDKAEFARFVKCSELHAKLLRAYVIGTSIVLLLYELNAI
VASITLSLQQNKVCFVTPFSFPFDYQHPIVFALTFLHNFDAMLVTVCTSVTVDSCFSEMASNLTIHFDIV
RERFEKLDLSAAQPYAEHQLRNVITYHREVLSLAQKMVQLYQQSAFYLLLLVSTILCLLGYEFVMVSNIY
KRMQVAILASIMIGQAAIYTYHGSAISAKSVSVADAIYGTNWYDAPLAVKKLVYICLMRAQKPVIMKSGF
IEASLPTLKKILSSSASYITMLMSLEADLNEKKT

>XP_314480.1 AGAP010507-PA [Anopheles gambiae str. PEST]

MDLTAGIESFNKSMGVSFFKRKHSFVNFFFIFGISNLVLYVVSVLISSYKSRHDIIKLIYCIATFGFCCQ
AIMKIYSFIITRQRVVDLYEINHRYYQHMMGQSIAVKRVLCDNASLIYIVIKVTVLVYLLLVITAMSIPG
LSSIFLADRILPFGFVMPFIDANTLAGYYANYGIQLIMAIYYWIITVGSDITTIYNLLTAYGQLDVLMTI
TEELNEQLERNESLDTIQDKIVEIIRQYQHHRTYLKQLVDFLNPYHFVTLISTVPTMVISVLGLVLLDWY
PGAAIVFLGSVQIFYICFLGTSLELKVGVTNALTLKIGAIHWDKLSVRDMKFMKMVLMMSQKPKMLMAAT
LPLNITAFLQIHKFIYSLIMMLENTKG

>XP_314478.1 AGAP010505-PA [Anopheles gambiae str. PEST]

MTFTLSHDLYVYNLHLIRWFASFVGLDIMAPNYKPNILTFLAFFGLSISLIGEVYTVWYYWPNLVKLMES
AAVYGVFIQGLAKFYTALRYRKFFEAMYNRLDLFHYEYRNHEKNNATLLLLMNRICLLRKFITLQIIGCI
LVLSLNPVIQYIIKRERVLVYAILLPFSDPEITSHYLLNLIVQYYLMIAGIGGFAAAESVLILFVTSVAG
YADVLKNKIDEMNIILLDAQNSRDRTPVKLKLREIVLLHQRVLEYEDDLEKRYYLNNWVQVASSIFNLTG
ALFGCYVSNSFTMYALAIAVVVQLFELCLLGTILSIKNEEIEHAFYDSLWYLMDHSEKKDFLIMFHKSQH
AMEMTVASMAPLNIVLFIAIMQKIYAYAMMLMNFFE

>XP_314477.1 AGAP010504-PA [Anopheles gambiae str. PEST]

MTFTLSHDLYVYNLHLIRWFASFVGLDIMAPNYKPNILTFLAFFGLSISLIGEVYTVWYFWPILDKLMES
AAIFGVFIQGLAKFYIALRYRKFFEAMYNRLDLFHYEYRNHEKNNATLLLLMNRICLLRKLITSQFSISI
LTLMVTPVAQYILKGERFLVYTIILPFTDPEITSHYLLNLVVQYYLLIVGLAGFSAAESVLILFVTSVAG
YADVLKNKINEMNTLLLDAQNSRDRTSVKLKLREIVLLHQRVLEYEDDLDKRYYLNNWVQVASSIFNLTG
ALFGCYVSNSFTMYALAITIVIQLFELCLLGTILSIKNEEIEHAFYDSLWYLMDHSEKKDFLIMFHKCQH
AKEMTVASMAPLNIVLFIAIMQKIYALAMMMMRFSE

>XP_313640.1 AGAP004357-PA [Anopheles gambiae str. PEST]

MDIVSKQGEFPLVKLSTKLLRIIRFWNDSPGQRIPSFGLLVTAIYPLIWLIPSWLFLVSSQDNITRFMKA
ANELIVFSLIFCKLCFYAIHFRRWEKLFYDLQRSFSSVLNNPSLEIQTILGHVTKSTHNLTKYYCSTVSF
NCAAYGLFPMLFIVVKYAVTGSYDVPLSTPIEANYFIPGLRTNFWVWLPVNITLSAMLELHGFALFFVET
FTWNLVYATSCLFRILQIQANELSHQCRNEKEWNGKLKTFIALHDSVLRSAETLEEILSLQMLLLYLSTI
LALCLGMVVLSLAVNEVYVLLTTMAVFGYCIFQTFSFSYLGTELIEQSEAVADAIFHSKWYTQKLNRQKD
MCFLMMRAKRPVKLTAAKLFVVTRDSFTQVIKQAYTIFALMSQVLDDTMG

>XP_313637.1 AGAP004355-PA [Anopheles gambiae str. PEST]

MKPSCKEGVFPLINLSIKLLKIVGFWSKTTGQRISVIGLLAVILYLLAWLIPNWLFIVSSQDNITRLIKA
TNEQILYFIAFFKLYFYVIHYRRWEKLFYDLQIAFSSVMTNPSPEIQGILGHVTKSTHNLTKYYSYVVNF
NCAWYGVFRMLFIVVKYAVTGSYDVPLSTPIEAKYFIPGLHTNFWVWLPVTLGLNVLLEWHSLALVFVET
FIWNLVYATSCMFRILQILANELSDRKRNEGIWTVEFEKFSTLHDSVLRSAGTLEEILSGHMLLLYVSTI
FSLCLSMVVLSLPIDDFILLLTTFVAFGYCIFQTFSFSYLGTELIEQSEAVADAIFHSKWYTQKLNRQKD
MCFLMMRANKPVKLTAAKLFVVTRDSFTQVIKQAYTIFTLMSQFLDNPVN

>XP_313636.1 AGAP004354-PA [Anopheles gambiae str. PEST]

MDSNSKKDTFPLIQISTKMLQIIRLWNESSGQRMSTFGVLLIAVFPIMWLIPSWLFIVSSQDNIKRLMKA
INEQIVFCIVFLKFSFYAIHFRRWEQLFYDLQRSFSTVVNNPSLEIQTILGHVTKSTHKLTKYYCSIVSF
NCAAYGLFPMLFIVVKYAVTGSYDVPLSTPIEGNYFIPGFRTNFWVWLPFNLAQNVVLQCHSFAVITIEC
FTWNLVYATSCMFRILQIQATELLDRSRDKKEWNIKFKTFIALHDSVLRSARTLEAILSGQILLLYVSTI
LAVCLGMVVLTLAIEDVYLLLTTFVAFGYCMFQMFSFSYLGTELIEQSEAVADGIFNSKWYEEDVKVQKD
LSFVLMRAKKPVRLTAAKLFVVTRDSFTQVMKQAYTIFALMSQFLDDIAN

>XP_313200.1 AGAP004278-PA [Anopheles gambiae str. PEST]

METHIGKQHSLERFQAMVAWQNRVLALFGCYMYLGKERVSYRIVPICFMACSFVLLSLYSAIQARGNMGL
AVLSIVVLFYGIIGVTRLAVAISNPAGCYRSIQIAEEMYQRANGSNPAECNVLAKYTDLFCKSVQLYTVC
FVLSVVVLTLMPFALYLLRGERYLPLGIVLPFTAETDDFGFWCTLVVQLAYMYTGPFGLIPSQNIYFAFV
FNICLQYELLIERLKQLDERIRSSNNLKLTVRDQLVKIIQLQQESTNYITHIERFYQLQSFVEFLCNSLQ
VALTLNELHRNFWLPGLFILPIAVMQMLILCSLGTLIELKSDQFKDQLYDIAWPEMDLPEQGMFKYVLKS
AQQPKQLTCGRFAVINMNLFLAIHKKIYSFFMMLQNM

>XP_312381.1 AGAP002558-PA [Anopheles gambiae str. PEST]

MLRPTSEDDQTLLINFRLLERVLRLMAVWPTDFNPYLPQYLRGRFFLSELFDFGYQLFWYFICVHIAAFH
IMSVLTGDLSYDELFLTLITTSIYSILVPLSLYLRLYESNIRQLYEFSQRHFRKRSAAGVHYISISSSYR
FTNKYQFWWIASCLLGTMHWAVYPILSQERTLPFPCWYPVDVHESPMYELAYIFQVLGQLQVSLVFGLAS
ALFMVFVFVVCTQFDMLCCSLSNVRQSAMILNGGYGPELRDYQDNYELDTRDYVLQEVFREDLDTVQGTK
TASRLDQLSPAQSYLMELAPELTCVMEDCIQHHLILLRFCQLLENCYHPYVLLKLLQILLLLCFLSFMAT
VESLSTMKLINVLEYFMLSITELYLYCFLGQTLKNQGLKVGDALWKSPWHLCGASYRRRLLIILMNAQRP
VRLTGLKLYELNLETYYTVLKAAFSYYTIIKKFR

>XP_312289.1 AGAP002639-PA [Anopheles gambiae str. PEST]
MVSFGAAASNPRDAETTPHWDIFKLQRKILLIFGVWPADRLVRPWYVKVLIAINLATLAICMVGEFLHGL
YAYQEGDLSESIESICPTVARISGFLRMVFYLANEEKIHRVLNNISKTLQDEHPREHTITKQLTTLGQQF
TFYLFLMMFFAACLYGVTPFFIMIYNWVQGQRPLVKLLPFKLALPFDSQNLFPFVVTTLFLNYASAPTIT
SQSGSDALFSGVCLYIYGQFQAIRLEVEALAETVDRNTLKSSAAETHRINLELRRISKRHQAIIDLVSEV
RSAFTPNVLLVYTATAIIMCIVCIAMLVVEGIYKLTYLPYAFAELTLLFLYSYSGTIIRDSSEAVQTVAY
DFPWYRYDRNTRHLIQMMMIRAQYGSNVDVPFFETSMASFSAIVRTASSYITLMKSFL

>XP_312288.1 AGAP002640-PA [Anopheles gambiae str. PEST]
MIITVAPVTDARPQTPEDCGMFKFQRKILLIFGCWPPDRLTRRWYVKVLIAVNLITLAICIVGEFRYCLY
AYRNGSLIETIESICPTVARFSGLLRMCWFLRNEHKIKSALNSVVHLIKNEHPRETGYTNRITARDQTIT
KVLFHSSFITAILYGIIPYFMMAYNWFQGQYPLVKLLPFKVVLPFDSQDPTLFVLTTIFLNYASVPTITA
MTSTDALFSGVCLYMDGQFQAIRLELEALAETVDKYTLKSSAAETLRINLELRRINKRHQTIIDVVSEVR
HAFTPTILIVHICAAFMICVISMAMFLAEGINKLTYMPYTFTVLMLLFMYSYGGTVVRESSEAIQTVAYG
FPWYQFDRNTRHLVQMMMVRAKYGCNVDVPFFQTSMATFSVIVRSAMSYITLMKSFL

>XP_312203.1 AGAP002722-PA [Anopheles gambiae str. PEST]
MARLVLHEVRYVLMAMLYISRGMATKIQNSPIDLYVYWFLTFIPIASLCVPQIIFRVKMLKQTKQIIHFY
LSLQFTYLVVDTKSLIDFISVLVPITEILLTNGKMIICNVKRGKIINLINQVQVAWDECAKSEHLEIQTL
ITTTAKKSKIFVIIYTTSFLLICVEYSSMPLFKLIYHSAVYGKQSNYTIALPYLSRFAYSTESTTSFAWT
YFFILIGVYLLALTLSGFDSLFSTLVMHIKMMFKVLKFEIEQLGLDLSAGKSHVELQAKLKQIILKHKTN
LSLIEQLEDGFSFFLMAQFLTSSILVCVVLYELTMVFGWNEDTFKTVTYLPGAILQLFLFCWYAQQITEE
ARLVSDHIYNIPWYLADPKLQKDILTFMVKAQKPTGVTASKFYMVTLQTFQRISSTSYSYFTLLQTINQQ

>XP_311894.1 AGAP002995-PA [Anopheles gambiae str. PEST]
MFSASAEMEWFRQQRHAICHLREGSDYLRYLRPFQLIAGYPINPRPALSKLATVVRLLAYLTYLGTLLHK
ISYVLYRPEDINYVSFVSGGITVLVAVLLLMLIFTVHYDAFQQLGTFLNDRAFARDHPLATGIRDRWYRW
SNGLILGPQCCIVLILLQTWISRQHLKKHTMLVVRGEPVGTEFDHFLYMSCLYFPTVGFFMGCSIVNAML
VGFLGEMELLATCLGELFETVERQVKEEGPTGRPDSRALYWSTLHGELRRCAQRHCAIFTMLPKLQRMAS
FVFLQHHIFSLGLVVAGSYVTLRGPALRENIVLSEYPISVVLEYFIFCQLVEKLQDMNRSIGDRLYGTEW
MLKLQYSRDFQREYRSAALTIRLLIMRSQHRVRFTCGSINPVSMEKFTEFLNLSYSIVMFLLSIN

>XP_311816.1 AGAP003053-PA [Anopheles gambiae str. PEST]

MPRATSNSAANLERLVIRISSHMAVLKLNIIDPAWRPTLRFGIVLFLTALVPVYIWQGIKVYRTRFETLL
EVLSVAGCGWQMFFRMYFYLFQQDRCRQIVQEVRDQRTVYGADRNPRMEKLFRAGTKRMLLAYRVIHLMY
GTSYFFQLGPLIMPDPHKCNLPLALQLPFLPPDRNMVYYCINYAHHLLLNTIGVFILLPMDGVLIVALLN
ICTRIAALQLLLEELDAKLGTVQWQQTAHLDAELNRIIELHIDTKRFARVIYETYQMHFFSMFSVLCFVI
CMCMNVVARDPRSTLIPFGLASTGQLFVICMLGNVLYIVSDRLKDSVYGIRWYRCTVSQQKRLMFLLANA
QPEIVMGAVFIPVTMTSFVTIIRAAYSYFTILY

>XP_310907.1 AGAP000226-PA [Anopheles gambiae str. PEST]

MSEHPIHTFDRLIKRQRLLLKLIGIDSYDPAFRIHSLTFMLVCLALTFFVISLYDLYLFRDDLFNFVYVL
ITIFFATIGIGHISVFLLCSKLLAELLEQSYRTYRLVVDDQREQRILQWYTRLFQRAVDGYTLVFIGTSV
AAGLLPLAIYLLSGDRVLPYGVVLPFVDPDSLVGYELNYIYQVSCIIWTPPGLVASVCMLFGLVLNICIQ
YDILAVKLQDLDELIRSPHPHRDAMIGCKLRSILRNQQRLISFIANIEAAKNVHSAVEVLSLGLQIVITL
FVLQFSLWIPGLVLIPVFTMQLFLFCLLGTIIEDKGVKFSAGVYSLTWNELSKQDKQIFRLLLLSSQQPQ
TLTCAGMTCISLNLFVNMSQKFYSIFMMLRNM

>XP_310903.1 AGAP000230-PA, partial [Anopheles gambiae str. PEST]

SLTPTDAGVQLQTERLWLRALFLLYQLLLPTQAIIWLWRTWAVAYIEHNKPLAISLLCGQFALTSIVARY
MLLLRSYAQLQPIQRHLNEQRFLRGHPRAHALRQQAFRTNNVLMLALMVYGVLNFVVYEASGLHWQEIFR
MPDYLLATNRPLTWTLHLIMRPMTFNGLGAFIATFVSIHTMLTVLHAELLLVEFAFDGLLERVERHVQAA
GAQESPLLWQQFNRELGRCVREHCVVLKQVREVNRVHSFSITVQYYTALLSLAIDAFFISYYGLNFVSVC
VSIFSVLLVFEWYYCCKLVEDLQATNKRIGWTLYNDKWSDWLQYGREQPAALREFRTTLSIILLATQRSL
SLRGSDIVEVSWQTFASMLKTSYSVMMFLIELRRLNR

>XP_310092.1 AGAP009413-PA [Anopheles gambiae str. PEST]

MLRLSPEDPKAVMPFAKRLLRLSGFRQETEQLEKQIFFNLFVYVAALLIPKVCSPYPDSEAIIRGLSELI
FFTNVYVGYYCFVVQHRHYRDLLDEIQSFVNVVYPTSQQPESPSERTLIKLNVKINKISVLYCWYLAAAG
LIYWSTPCLMTYHSVLKAKAEYGPNHPIRFYPNLEGSFYGLDNRTSVYGYAAFSIVALLVFAFASYNNAT
KLLTILSTIKYCSTLLQLVGVEVDNLNHTSSEAIGRELKKVIQLHQLALRCVALLNQTLSFVMALQLALC
ILTWCFTLLYILIVGFNAIATNGLLIMINMTLEMFGYCFFCTELDTTGKIVSRQMYEFRWEQHRPTVQKM
VAMIIARSQTPLQITACGFIPINLELFTKVVKHSYTVLAVLKDLI

>XP_310088.1 AGAP009410-PA [Anopheles gambiae str. PEST]
MTSFKPTDDTDVMPLVLRLLRVIGVWDVPQYRYRYAAVFLSYCFGILIPKLCFGYPTLEASIRGYTELIL
ETNVFAGMLQLYLCYNHFVPLVDELRAFAAIVFQDNQPMLLRTNLTDLNLRIHKYTLCYCLYMCCVCTVY
CVAPLGSNIAGYTAAMADTASNSSTTFAFTLYLEQGFYWLDNRTSLLGYCVCTVFMLPLMYLCAYTATVK
VVAVFNMIKYCQTVLRIVVLKLRLLKTLPDVRQRSDGMSEVWVLHQRALRCAELLELVLQPLLLMQFVLC
ILIWCMMLLYFTVSGLNVKFINMFLLFLFVSIETFGYCYLGTQLSQESINVGQALYASGWYEYDVQMRKH
ISFMIMRSQRRVGLTAAKFCFVDMEQFGAMLNMSYSFFVVLKDAF

>XP_310085.1 AGAP009408-PA [Anopheles gambiae str. PEST]
MLFQELPNDRAVLPLLLYIQERLGFWGDRTTRVRYPVAFVAFWITVAIPKLATDYSDVELFICSMAELAF
VGNVFCGGMALWSVYNSFRQFIEQVIRLTKYLYRDHHPLQTVREQLLQFNHRIHRYTNRYCFSMMILIVF
YLVAPVITSFGVYFQSVWQSHHDTNDTIRIWEISQVAPHRQFSLHMEQDFYGLQHRTNILHYTLYIAVIV
PMMFVTACTVHMKVLTIASSVRYAEMLLHVVMLKVDNLHRIPNQKSIREELHDIIHVHQRTLNCITLLVQ
ALRPILMVQLVFCVFIWCLMMLFFTIADKFSVAFFNLAILFVVITIETFSACYFGTRLSTQAVELSKSVY
GCGWPAMDRDIQQGLRMVLHRTQSPVGIQAGKFCFVDVELFQNMVNKSYSFFIVLKDAF

>XP_310073.1 AGAP009398-PA [Anopheles gambiae str. PEST]
MNFLRQEQPAGMPHISIKLLRVFGVTNHSEERFRIVPVMLAYFFFIVVPKCFFGYPDLEIMIIGTAELIF
QTNSFCGMFLLFLNRRKLAEFIQHARSFSQTVICASPPAVVRHLTTQHDMIHKITRIFCIVVMFAAHFYA
LAPFLSTLYTFYGTVRNKNATMHYTLQMEENFYGLQTRTSGTHYLIFGVVMTPTAYLCAFTGTVKTLTIC
NITTYCTLYFQLVQLKLRTVTQDNTFRQELKSVIKMHQDALNCASLVESITSLALLQQLLLCVLIWSSML
LYFTVSGFNVNFMNLFVLFVFDTTETFAYCYLGEKLSYESARVAHTIYESGWETQTTDIQKDLQLILVRA
QSPVGITAGKFYYMNMEQFGIIVKTTYSFFVILRDQI

>XP_310068.1 AGAP009395-PA [Anopheles gambiae str. PEST]
MNFLRQEQPAGMPHISIKLLRVFGVTGGPDVRFRILPVMLTYVFCIVVPKCFYGYPNQEIAIIGIAELFF
LTNSFCGMFLLFLNRHKLAEFIRHVRTFSLTVLQESPPAVVQHLTTQHDFIHKITRMYCIVVMFAAHFYV
LTPFLSTLYAFYGTVRNENATMHFALQIEENFYGLQTRTTTSHYLLFGMIMTPTVYLCAFTGTVKTVTIC
DTTIHCILYFQLAQLKLRIVTQDNTFRQELKTVIKMHQDALNCASLLESITSLVLLLQLILCVLIWSSML
LYFTVSGFDLNFISLIIILFVFDTTETFAYCYLGELLSYESARIAHIVYKNGWERQHAYVQKDLQVIIARA
QRPVGITAGKFCYMNMAQLGIIVKTTYSFFVILRDQI

>XP_310064.1 AGAP009393-PA [Anopheles gambiae str. PEST]
MVVFDPLDDPLKVLPLPLKLLALLGVNKNPSERFRLYAIYAYLCIALFIPKLCLGYETILQCFRSIAEGM
LSFNTTITFIMLPLKMDNLEDLLKNLKRFTEIVIFNEDYEQILIRLNTAIHKFTKYYFIFTNGIVFAMTS
STIAGMFYTYYAKDSEYSAAFPLIMENRLYLIDSHYNLGHCFVHQALMFFALYILLVMFTAKAGTLFGLI
RFCSTVLGIIVLKIERLSQIGPVDKYTAELNEIIELHQLAIKCSRQLQNILMEILLAQFTGCVFIWCFML
YYVMISGITAEGIAVVAMLIALSTETFIFCLLGNELTLKGLEISTAMYFTNWYDQPVKLQKMVVPIIQQS
QQRIGITAAKFYYIDYNRYGQSLKTAYSFYLLLKDIF

>XP_310060.1 AGAP009390-PA [Anopheles gambiae str. PEST]
MKLLELDNPREAIAIGCRLLKLFGLGRDERFKLVYWLQCVAYLAFSIVPRLLVEIEDMVALMRLIAELVF
VVYLCLQIMALYCRRRQLYRLVDMLQQCIDIPYSEQIESFLIRSNVKINQSSAAYARFFMCVYVLYCTMS
PLASGFVYIRNQRNATGVQEDLYDLDIRYNPLHYSIYAGLIFVLSAISSLSLCTKDVIDIAAIKTVTLVF
GIVTMQIRDLHEQITQERLNRVIKSHSNALSCATQLEQALNLSVLFQFASCSAIWCLMLFYILLMGLDSR
VLSVVLLLVIVSIETYAYCMLGSQLTTQGEDLLMALQQLSWYDQPVPIQRQILLMIRRSQTPLILRAGKL
FSANVVQFGDIVQKSYSFFLVLKNVF

>XP_309621.1 AGAP004067-PA [Anopheles gambiae str. PEST]
MSALVHLAKDHIQRIADGGQFVIVNGMDRFIGFFAWDVKLNATWLKMTLIVFAVGYEITAIAAMALASVK
GVFTERSFTMSFVTMTGAMCIVIWLSLAAFRRDLTGTVKFLQHRQCTIHRQSAARKALMDRVNRYLWLFY
LQNIAQVFFWINLLRNCSPLAVFELSLLDSVNVLLYPLAMTLMSLMFIHTIMIVSTLLSALSLEFYWLGQ
EFEQVFAQCNTFAATARKRYWDGLKRRIDTCVIEHQRLLGQISTLRNNLKLYLLLNLVADFSLITFAGCQ
MVISSEGDQHLYSILAALTACLNMLNFGGLCDLLKIQVHAIKFRLYSSQWTDYLRPVSGPLYPRCRRIRS
SILIVMTRAEHELRISCGSVYDMSLTTCWAVLQFSYSVFTLLLSFFENEPRDQ

>XP_309205.1 AGAP001012-PA [Anopheles gambiae str. PEST]
MQTGGVLSRMRRALGRFYVERDFFRPYEILLALPGFHLVEEFRTSSWKRALFLLSRTVQLLQYALWADRF
YLALVDPSSPPGKALHYGNTFGVLTMMLARMLVVRWYLPHVRQLMEYLRRQRGRIEPAPDTHRLSYRRIV
NIAITFQLIGLADRLVAGFSQTYRQELYEVPANLAELQWPMVVAVHVLSFDFASRWGAAYNVSLTGMNSI
MMGLYDELTSIAQEYGRLLTKEKNGQADIWTSFERNTARAVRRHETFLWQLGQLKPFLQTTFLVMFYSAA
LFLAIGTFMISANGTTTYGVILSGFLFALLLECYWCCQLVDRLNEVNTQIGILLYSLDWPVELQYTKATA
SRYRQARSSLLIMMSKTQKSLGIRCGGMFEMSSEAFASLVKLTYTMLMFLRDTQKPN
