# Supplementary material for: InsectOR—Webserver for sensitive identification of insect olfactory receptor genes from non-model genomes
Source: PLoS One. 2021 Jan 19;16(1):e0245324. doi: 10.1371/journal.pone.0245324 (PMC7815150; doi:10.1371/journal.pone.0245324)
Supplement: S3 File — (PDF) [file pone.0245324.s003.pdf]

>AmOr2

MMKFKQQGLIADLMPNINLMKATGHFMFNYYTDSSTKHIHKIYCIVHLVLILMQFGFCGINLMMESEDVD
DLTANTITMLFFTHSVVKLVYFAVRSKLFYRTLGIWNNPNSHPLFAESNARYHQIAVKKMRILLLAVIGT
TVLSAISWTTITFIGDSVKKVIDPVTNETTYVEIPRLMVRSWYPYDPSHGMAHILTLIFQFYWLIFCMAD
ANLLDVLFCSWLLFACEQIQHLKNIMKPLMEFSATLDTVVPNSGELFKAGSAEQPKEQEPLPPVTPPQGE
NMLDMDLRGIYSNRTDFTTTFRPTAGMTFNGGVGPNGLTKKQEMLVRSAIKYWVERHKHIVRLVTAIGDA
YGVALLLHMLTTTITLTLLAYQATKIHAVDTYAASVVGYLLYSLGQVFMLCIFGNRLIEESSSVMEAAYS
CHWYDGSEEAKTFVQIVCQQCQKAMSISGAKFFTVSLDLFASVLGAMVTYFMVLVQLK

>AmOr4

METKHTEKDLKQAFYVQTFLKIIGAWPIAIESSLGSKIQKWFIISFYLFLQICIVAPCILDVFLKEKNGS
RRINLFMLLISTLNQVFKYVITLNRANELRIAIHEIKKDWLTATPEDRFIFVMNSRIGQRIMLIMAFIMY
ISGLGYRMVLPLLKGKIVLPNNVTIRLLPCPTYFTFFNELVSPYYEMIFMLQLLARFFIYTVLNSTVGIS
LMLSLHMCSLLKILTRKMADLTDGSIISEKIMQQRIVDIIEYQTRIKRFLSNTELITQYFCFYDIGCSTC
LICFIGYSIIVEWENHNIASTVIYFSGLVTCTLMIYIICYIGQLLLDESNNLAQTCITLNWYRFPKKKAR
YLILMIIMSNYPIKLTAAKVVDVSLTTFTDVMKAAVGYLNMLREVI

>AmOr5

METKHTEKDLKQAFYAQSFLKIVGVWPIPIGSPLSSKIRNWFITFFSLFLQICIVGPCILVMFLKEKNGK
RKINLFKLLTNTLNQLFKYIITLNRANELAIAMNEIKNDWLTATSEDRWIFTANSKMGQKVMLIVAVTVY
SSGLGYRMLLPILKGKIVLPNNVTIRLLPCPTYFTFFNELVSPYYEMIFMLQLLAGFFSYTVLNGTVGIS
LMLSLHMCSLLKILTRKMANLTDRSITSENIIQEKIVEIVEYQTKIKRFLGNAELITEYFCFYDIGCNMC
LMCFIGYSAILEWENHNIAAIVVHFMLLGTCIFIIYIVCYIGQLLLDESNNLAQQCITLSWYHFPTRKAR
CLILMIIMSNYPVKLTAAKVVDVSLTTFTDVMKAAMGYLNMLREVI

>AmOr8

MVQIRNAREGINHTFWFAYPLSRMLGYWPLNVPSSAFSKILNSFTIFFSYLLPLIVLIPGLLYVFLKERN
GRRKVKMLMPHINSIAQMTKYTIILRRTKELGKLLDEIKKDWSTATQENRRIFSERASIEHKLTMIVAIT
IYGGGFLYRAILPLSKGRIVLPNNVTIRLLPCPGYFGSLDEQVTPNYEIIFTLQVLGGFVTHTAVCGIKS
ACLMVCMHMCGLLRILTNKLTDLTNDNDERVVQEKIVHIVEYQTRIKEFLNHVDQFVPYVYLIEIFVGVL
ITCILGYCIIVEWEDSDAMAIIAYVALQTTCVFGTFSICYVGQLLVDESESVRQACKTLKWYRLPTKKAR
SLILLIIMSNYPIKVTAGRLVDVSLVTFTSIIKSAVGYMNILQQVT

>AmOr11

MVQIRNAKEGLKHTFWFAYPFSRTLGYWPLVSPSAFTKFFNSFTIFTLYFLELIVLIPGLLYVLQVKNPR
TKIKLLMPHLNSIAQMAKYTIILQRAKEFSKLLDEIKKDWLLATEENRQIFSERASIEHKLTTVIVVTMY
GGGFFYRTILPLSKGKILLPNNMTVRLLPCPSYFGSLNEQATPNYEIIFTLQVLGGFIIYTVLCGTKSAC
LMLCLHMCGLLKILTNKVMDLTNDSDEQVVQEKIVHIVEYQTRIKEFLNQLDQFVPAIYLIEVVIQVLII
CIIGYCIIMEWEDSNAMAMVIYVVFQVTCVIGTFSVCYVGQLLLDESENIRQAYNTLNWYRLPVKKARSL
ILLILMSHYPIKVTAGRIMDLSLVTFTSIIKSAVGYMNMLRTVT

>AmOr12

MARIRNAKDGIRHTFWFAYPFSRMLGYWPLSVSSSAFAKISNYFIIFLSYLLTLIFMVPGLLYIFLKVKN
GRSRIKLLMSHINGIVQMAKYTILLRKTKEIAKLLDEIKKDWMTASEENRQIFSTRASIEHKLTMVVVVT
MYGGGFFYRAILPLSKGKIVLSNNVTIRLLPCPGYFGFLDEQVSPNYEIIFTLQVLGGFVIYTAVCGTKS
ICLMLCLHMCGLLKILTNKVMELTNDKDEKVVQEKIAHIVDYQTRIIEFLNDLNQFVPSVYFFEIILEVL
IICIIGYCLITEWEDNNTMATVIFVIFQITCFIGTFAVCYAGQLLVDESENVRQACSTLNWYRLPVKKAR
SLILLILMSNYPIKVTAGRIVDVSLVTFTSIIKNSVGYMNILQQVT

>AmOr13

MGQPYSLKLVYPLLKILGAWPKSSPSSVLSTILKCCLISICYLIQLMVLIPGILYIFLKEANLGGKIKMF
VPHMNGITQVSKYTILLRQIKEFNIILKEVKRDYSLATDKNMWIFTTRAYIGHKMMIAIAIAMYSSGVGY
RMILPFLKGRILLPDNTTVRLLPCPGYYMFLNEQVTPNYEIIFTIQVLGGFLNYTTLCGTTGITTMLCLH
MCSLLEILINKMNDLTCQSDECEIIVRKKLADIVEYQMKIIDFLNHVEQLTSYLYFCEILEYVCGACVIG
YCLITEWENSNAAALIVYFILEFLCIFCTLTICYIGQLLIDESDKVRQISVTLDWYRLPVNEARGLILVI
IMSNYPIKVTAGKIVDISLITFTDIVKTSVGYLNILRTVA

>AmOr14

MSRVGKAENGMRHTVWFAYPLLRILGAWPNRVSSSTLSKIFNWYLIFTCYTLQLIVLVPGFLHVFLKEKN
GRKKMKMMIPQVNGYLQLCKYSLVLRWTNKLRVLLNEMKEDWLNTTEEDQLIFRAKASFGHRVMSMIAIV
TYSAGLGYRTILPLSKGRILLPNNTTKRLLPCPGYFVFFNEQVSPIYEIIFIIQVLGGLLTYTIMCGTIG
MCVMFCLHSSSLLRILLNKIYQLTKQLDVNEVVVHEKIVDIVKYQTKVKGFLKNVEQLTTYLFLLEIMVE
TSIGCVIGYNVVTEWEDSNAAAMIIHLMMQVSTISCTFIMCYVGQTLIDEGNNVRRMSITLDWYRFPVKE
ARNLILVIIMSSYPVKLTAGKVVDISLATFTDIIKTTVGYLNMLQKVT

>AmOr15

MSRIGNAEDGMRHTIWFAYMLLGKLGAWPNRATSSTSFSRTRNCILIFMCYSVQLIIILIPGLLHFFLKEK
DSRKKVKILIPLINGYLQLCRYSLVLRSANKLCHLLNEMKKDWMNISEEDRLIFRRKASIGHRLMSVVAI
IMYSAGLGYRTFIPLSKGRILLPDNTTIRLLPCPGYYIFFNEQITPNYEIVFTLQVIGGLLSYTIMCGTT
SMCAMLCLHATSLLRILVKKINELTKQPDINESAVHMKITDIVRYQTKIKQFLNDVEHITTYLFLLEIID
ETGIGCVIGYCAITEWEDSDATAAIIYLLLEASVFGVTFTMCYVGQILIDEGNNVRRMSITIDWYRFPAK
EARNLILVIIMSSYPVKLTAGKVVDISLSTYTDIIKATVGYLNMLRKVT

>AmOr18

MNAEKLMIEGKPPNANYKNDLSFNVRLNVWTLRTIGTWPRSPDHSWLETLEHVCLNLFCYELLAFILIPC
SIYIIILEIKDFYNQLKLGSALSFFLMAVMKYCVFIIREDDIRKCVELIENDWKNVRYQEDRKIMLENASF
SRRLIVICGTFMYGGVIFYYIALPLTRAKIVEEGGNLTYRRLVYPFPKVLLDARHSPINEICYTIQLLSG
FVAHNITVAACGLAALLAIHACGQLQILMSWLEKLVDGRKNDNENLDQRLANIVKQHVRIINFIALTEDL
LHEISLIEVVGCTLNICFLGYYSMMEWDSKQPVSGVTYIILLISVTFNIFIFCYIGQLLAEQTVKVGEKS
YMIDWHRMPWKKSLAIPLMISMSHSTTKITAGNIIELSISSFGDVIKTSVAYLNMLRTFTT

>AmOr21

MSSVKIDQDYKSNVNLSIKYSRRISKMIGLWPIFDKISTIHKFLRMLYNTICYCLLMFMIVLGWMYIAFE
VKNIYDGLKFVSLMSFCMLSITKYHLINIHKDDVRECVKRIEWDWKNISYSEDREIMLMNANFGKRLIIV
TTTVTYSGFVFFYIAIPMKIGKIPAPDANISFIPTMFPFPKYIADVRYSPINEIVFFFQFMCGFLVHGVT
SSACSLAAIFTVHACGQIQVMMIWLEHLIEGRLDMCYSVDQRIAKIVSQHVRILKFLSLIEKILQQVSYM
EFLECTVNVCLLGYCAIIEWESNHLTEVVTYVIILITIIFNIFVFCYIGELLADQSRKIGEVTYMIEWYR
LSGKKKLCCVLIIAMSNSSMKLTAGNLIELSMSTFSDVVKTSFAFLNVLRTLT

>AmOr22

MEKSKINSISCIQTNHDYKRNVNLSIQWSRWILKPIGLWPNSSTISTTGKYLYRLINVICYSLISFLSIP
CSLYVILEVEDIYNRIKLFGPLSFCVMAFLKYHLLILHKDNISECIKRIEWDWKNITYSKDIEIMITNAN
FGRRLVIICTFFMYSGFAFYYIAVPISVGKILAEDDNITFIPLVFPFSRFIIDTRYSFINEIVFSIQLIA
GALMHTITTAACSLAAIFAVHACGQMQVLSNWLKHLINGRSDMYNNVDSRIASIVSQHVRILKFLALTEK
ALQQVSFVEFLGCMLNICLLGYYVITEWSSSHLTSAITFFILLISLTFNIFIFCYIGELVAEQCKKIGEI
SYMVDWYRLEGNKKLCFVLIIAMSNSSIKLTAGNMVELCLTTFSDIVKTAVAFLNVLRTLTI

>AmOr26

MMNQLNEQSVLMPVSYARDYEYSIQVNRWLLKPIGAWPNLTKATRTEKLLVKLLNFICHSLIIFTVMPCI
MYIFYEDESLKTRMKAIGPTSHWLMGELNYCCLLMRAKEIVYCIEHIKYDWKTVRRARDRELMIKNAKLG

RFIACIAALCMHSGIMSYTVITGFKKITFQIGNDSYSMYRLPCPFYTNLLDVRFSPMNEIVFALQLLSGF
ISTSVTVGACGLAAVLAMHACGQFNVVMIRSDKLVKDNNEKKQDEQTLHKKLGFIVEHHLRTLSLVWYME
KVMNMICLVELVGCTMNMCILKYYFLTEKSKTILGIYAIVYASMVFNIFIFCYIAEIVTEQGKKVGEKFY
MTEWYQLPHKTALGLVLIISRSSMVIKITAGKLIQISIATFAAVFKASFAYLNMIRTIAM

>AmOr27

MMNQTAITEEIKTNSDYSLQLNRWFLKPIGAWPLFSTTTKFEKTVSLILNIICYAIVILCATPSLMQIIL
AEESFYLKLKTLGPVSHWFVSTVNYTALLMKSKDIRYCFEHMEADWQTIKRMEDQQTMLKNAKFGRYVAA
SCAIFMQGGILCFCFVTILTTETIQVGNETRVLHVLPCAVYKKLVNVEENSINIFMLCFQFVAAAIANSS
TVGIFSLAAVLAAHAYGQLSVVMVWITEFVNQSRNQKKTDDFKEIGIIVERHLRVLNFITYLENIMNRIY
FLELFRCTMIICIVGYYILTEWAEKNVQNLTTYFMMLLSICFNIFIICYIGEILTEQCMKIGEVVYMTDW
YYLPDKTILNLILIIILRSTVVVQITAGKLFNMSIYTFGDVLKTAFAYLNLLRQMT

>AmOr28PSE

MSNRSVAIKTDPDTNSDYCLQLNRWFLKPIGAWPSFPSTTKHERIISFLLNVSCYSSLLFTLIPCLLHML
LEDESFYLKMKVLGSLAHWFVGTMNYTTLLLRGKEIRLCVEHIRTDWQTVTREEDQQVMLKNAKFGRYVA
AVSAAILQSGVNCXCCMTISRTELIQIGNETRIVHVLPCAVYRKLIDVTHSPNSELIIASQFLSGFIVNS
STAGIFSLAAILGAHACGQLSVVMTWITEFVNKSKKREKMIFREIGLIVEHHLRTLNFISCIEETINRII
FLEVFRCCLHICCLGYYILMEWSDYDKRSMIIYFMLFVSVCFNIFIICYIGEILAEESMKVGEVVYMTDW
YYLPDKTILDLTLIIARSSVVVQITAGKLIHMSIQTFTDVIKTGFAYLNLLRQVT

>AmOr29

MKNQQVVITQDDYKRKTNLSIQWNRWLLTPIGAWPNLRKSRIGKCYSLLISIICYSLIGFMLVSCSIFLM
VEINNIYNKLKMVGPLSFFVMTIMKYYFLLFHENDIREGIERIEWDWKNVKHQEDRNIMITYANYGRKLA
FICFFFMLCAFIFYFLIQPFGGGKIVDGNLTFIQLPFPISILIADVRDSPYNEIMLSIQILTGIVMNAIR
SAICSVAAVFAIHACGQMQVLMNWLNHLVEGRSDMSKKIDDRIANIVIQHDRILKFLALTERALQQISFV
EFLGCTANMCLLGYYLIVEWNPKELIVSFTYIAIIASITFNIFIFCYIGELVAEQTEKVGEVAYMIEWYR
IRGKKKLCCVLIIAMSNSSIKFTAGNMVELSIYTFSDVVKTSVAFLNMLRALT

>AmOr30

MEKNRSIIGHDDYERNVNLSIRWNRFLLKSLGTWPNLRESRIGKCYSVLIGIVCYGLISFMLTSSNMFLV
VEVKDTYNRIKMIGPLSFFAMTLIKYYFLTFHEENIRKGIEHIEWDWKNVKHEEDKRIMIEYANYGKKLA
LISIFFVYSAFVFYYFVVPISVGKIRDENLTFIPLPFPSSKLIADMRQSPANEILFSVQVLSGVIIHAIT
ATAVSIAAVFAVHACGQMQMLMNWLECLVDGRSDMNKIVDKRIAKIVVQHDRILKFLALTERALQQISFV
EFLGCTMNMCLLGYYLIVEWNPKEISLSLTYISLLISFTFNIFIFCYIGDLVAEQCQKVGEMTYMIEWYR
LTGKKKLCCVLIIAMSNSSIKFTAGNMVELSIYTFSDVVKTSVAFLNMLRALT

>AmOr31

MTSKSVISEESFDSLCDYSLQLNRWLLKPIGAWPSSSSSSKLERIVSFFLIVLCYGFILFTVIPSLFHIV
LEDENLHMKLKVFGPLSHWFIGGINYTTLLLQNKEIQYCVEHMQTDWKIVNRAKDQQVMMKYAKIGRYIA
ALCAIFMQTGVLTYCVVTAFSTRIIEIGNETRIVHMLPCPVYKELISIDTSPTNEIVLISQFVSGFIVNS
IAVGAISIGAVFTAHACGQLTIIKRWIREYINRSKDNNKNVVINEIGEIVEYHLRILNFIEGIEDVLNRF
CFMELFKSTLDISMLGYYILTEWADHDIRNLTTYFMILTSMSFNIFIICYIGDILMEQCRKVGEVLYMTN
WYYLPYKDILDLILIISRSNAVIKITAGKLTNMSIYTFGNVMKTTFTYFNLLRHVT

>AmOr32

MIDKFASIQQTNNNLSNYSIQLNRWFLKPIGAWPPSPSTTKLEKIISIVLIICCYSSICFTVIPCLLHVM
LEDESFRDKLKVLGPLSHWFIGAINYTTLLLRSKEIRYCIEHMQRDWRIVTRTEDQQIMMKHAKIGRYIA
VFSAAFMQGGVLSNCAVTAFSTQTIEIGNVTKTIHMIPCTAYKKLIAVDTSPTNEIVIASQFLSGFIVNS
SAVGAVSIAAVFAAHACGQLSLLMVWIREFVDHSKKIHDKNIGLNKIGKIVRHHLRTLSFVTGIENVMSG

ICFMELFKCTVNICMLGYYILTAWSVHDIQNMVVFLVILLSMIFNIFIICYIGDILTEQCKMIGEAVYMT
NWYYLPGKDILNLVQIILRSSMVIKITAGKLVHMSIYTFGNVMKTAFAYLNLLRQMT

>AmOr35

MLVLKDSSSVSYSKDWIYSVQINRWLLKAIGIWPLSLCVTTTEKIHSVILTLISIFLIGFLLVPCTLCTL
LDKTGDLDTKIKMIGPFSFCIMAAIKYYVLLSRGSHIGKCIEDIRVDWFRVSSHNCLEDRKIMMENARIG
RSLAIFCAGFMYSGGFFYTTVMPLCTKRTEIIDNEIVRSQAFPIYRGLLDPRTSPSFEIVQLMQCLAGFV
IYSVTVGSCSLAAVFVMHACGQFQILVTKLRRLIDGLKEDKDMENIVHEQRLGNIVEHHLHILGFISQIE
ELLNEICFVEFIGCTLNICFLGYFLLKEWEQSETIGILTYCILLISFIFNIFILCYIGEILSEECKSIGL
SAYMIDWHRLPGKKALSLILISAASNSSTKLTAGKLVELSLSSFCSVLKSSLAYLSLLRTLTT

>AmOr38

MMADDIATVQKEFNNLNEYSIQFNKWFSKTIGVWPLPSSTSKFEKIVTRILIIVCSIITLFVIIPSMLHF
ILVKEDIISKLKSLGPISYCFGGGLNYAVLLLRKNDIRYCIDHIETDWKVITRMTDRQVMLKNAKIGRII
SCCIVGFLQIGTFCFCTILGVFKRTIKIGNNSMEIYVLPSPAYKIPVDTNPGHDIVLCFQYLAAYITSAT
VVSAFSFAIVFACHASGQLTIMIIWIEEFINRPQEENKNVHIDKISVIIKHHMRILSFLERAEHLLSPIC
FMEMFKNILSICLFSYCILAEWSEHNIRILGTYIITVINITLNTFLICYIGEVLTERCKEIGDMVYMTNW
YRLPKKDILNLIMIITRSSVEYKMTAGKIIDMSVITFGNIIKTVFGYLNILRQTTML

>AmOr41

MADDIVAIQKKFGSLNEYSIQVNRWLSKTIGVWPFTSTTSKFEKIMTKILIIVCSIIALFVTVPSMLHFI
LVKEDIITKLKMTGPIIYCIGGGLNYAILLFLRDDIRYCIEHIEADWKTITRTGDRQVMFKNAKIGRIIS
GCIGSFLQFSTISYCTVFGVFKQTIKIGNESMEIHVLPFPTYKIPVDTNLEHGIVLGFQYLTACIMTATI
IIAFSLATVFACHAVGQLTIMVTWIEEFVNRPQEEKKNMRINEISVIIEHHLRILSFLERTEHLLNPIYF
MEMFKNILTTCMLSYCILVEWSGHDIKVLSAYSFTITNIILSLFLICYISEVLNEKCKEIGNIVYMTNWY
RLSDKDILNLIMIIIRSSVEYKMTAGKIIDMSVITFSNIIKTIFAYLNILRQVTIL

>AmOr43

MMADDIAAIQKKFGSLNEYSIQLNRWFSKTIGVWPLPSSTSKLEKIMTKILIFLCWIIALFVIISSLLYF
ALVKEDIISKLKTLGPISYCFGGGLNYAVLLLRKNDIRYCIDHIETDWKAITRTGDRQVMFKNAKIGRII
SGCVAGFLQLSTISFCTVFGVFKRRIKIGNESMEIYVLPFPTYKIPVDTNPGHNIVLGFQFLAAYIMSAT
VVIAFSLATVFACHAIGQLTIMITWIEEFVNRPQEENKNMRVNEISVIIEHHLRILSFLGRTEHLLSPIC
FMEMFKNILSICMLSYCILAEWYGRDVRVLGAYAFSVTCITLNTFLICYIGEVLSEKCKKISNMIYMTNW
YRLSEKDILNLIMIMIRSGMEYKMTAGKIINMSVVTFGNIIKTILAYLNILRQMTIL

>AmOr48

MADDIVAVQKKFGSLNEYSIQLNRWLSKMIGVWPLPSFTSKFEKIMTKILIFFYWIILLFIILASSLHFL
FVKEDIVSKLKTLGPISYCFGGGFNYAVLLLRKNDIRYCIEHIETDWKIIKRMEDQQVMLKSAKIGRIIS
GCIAGFMHIGTFCFCIVLGVLKRTIKIGNDSMEMYVLPFPTYKIPVDTNPGHGIILSLQYLTSYTSSATV
VIAFSLATVFAYHAIGQLTIMISWIQEFVNQPQKQNKNIRIDEISIIIEHHLRILSFLERTEHLLSPICF
MEMFKNILSICMFSYCILAEWSERDIRVLGIYTFAVMNVILSTFLICYIGEVLTERCKEIGNMVYMTNWY
HLPDKDIFNLIMIIVRSGVEYKMTAGKIIDISVITFGNIVKTVFVYLNILRQMTIL

>AmOr49

MADDLAKIEKKFGDLSEYSIQFNRWILKPIGAWPASFYKSRIEKIVSKILIVICWISSLFTLIPGVLHFF
LEKEDIYVKLKILGPLTHWLVGGFNYAVLLLRKDDIYYCIKQICADWNIITKKQDQQVMLKNAKIGRYVA
VFCTVFLQGGVFCTCLALGAFKKTIKVDNETVNIYNLPCPAYNMPVDTNPTHDIILGTQLLSAFICSSST
AGAFSFVAICASHALGQLNLMVIWINEYVNRPKKLNNNAYINKIGIIVEHHLRILSFIARVEHVMSPICF
MEMIKCMVGICMPIYYILMEWSEHNIQNLTVYVMIIISMTYNIFLVCYIGEIITEECKKIGDIVYMTNWY
ELSDKNILNLMMIITRSSMNINMTAGKMTNMSVLTFGKIVKSIFAYLNVLRQITMI

>AmOr50

MTNDINVAKQRSDNLSEYSIKLSRWYLKPLGAWPASSSTTKMERIISQILIVICWCIILFTVIPGILYIL
FVKQDIYVKLKIFGPLSHWCIDGFNYAILLLRKNDILHCIEHLRADWKLITRTQDQQVMLRNAKMGRYIA
AFCAIFMQVIIFFTCFILGIFKRSIHIDNKTVELYNLPCPAYKIPFDTDPTIHDIMLGTQFLSAFVVSSS
ASASFTLATIFTCHVLGQLNIMMIWINEFVDRLQRKENKDNHINKIGVIVEHHLRILSLIARIERITCPI
YFMELFKCMMGMCMPSYYFLAEWSERNIQNLTIYVMVALSMSFNILLVCCIGEILREQCKKVGDMVYMTN
WYQLPDKDILNLIMIISRSSVEVKITAGKIITMSIYTFGNIVKTVFAYLNMLRQITMM

>AmOr51

MRSTNNIDNLPLNDRYESDIQYTFQFCHWILKPLGIYYFIYNQANKFEKILSIILILICFFIIQFVIVPF
GYYILFYEKDMNTKIKFLGPLTFCLSALFKYSYLGIKSSELGHCIKHVEKDWKMLQNEDHRVIMSRYVIM
GRNLITLCAAFMYTGGLSYHTIMPLLSKRKVENFTIRPLTYPGYEAFLNIQKSPTYEIIYCMHCIYVIVV
GNITMAAYSLTTIFITHACGQIKIQMLRLENLKNEKKVLETGIESHLAVVVKNHVEILRFAKNVETTLRE
LFLVEVIVSTLLMCLLEYYCMVEWETSDSAAILTYVILLFSFTFNILIFCYVGELLLGQGSEIATALYEI
EWYNLPGRKARDIILLLVISKYPPKLTAGKIFILSMNTFSVVLKSSVVYLNMLRTITEL

>AmOr53

MHDRSHDNINGQLKNSHYKSDIHYTLQMCQWLLKPIGVWPLIYNQTSRFEQLISIILMGTCFSSLLFIIL
PSGHHILFVEKNLHMKVKAFGPAGFCLSSTIKYCYLGLKGSSFERCIEHMRKDWMMVQDPNHRTIMLKYA
TISRKLITMCAVFLYTGGMSYHTIMQFLSKGKNKDNYTIRPLPYIGYDPFFDTQSSPTYEIVYCIHCFTA
MIMYSISTVAYSLTTIFVTHICGQIQVQIARLQDLVESKEKRKYKDCDPFALIVHDHVEVLRFSNNIEEA
LREICFTEIIECTIDMCMLEYYCIMEWSVGDTITLLTFFTLLISFTFNIFIFCYIGEILTEQCSQIGTVS
YEIDWYKLSPKEAYDLILLISISQHPPKLTAGKIIELSLNTFSTVAKTSVVYLNLLRTVTDW

>AmOr58

MHLFVRDQTNQPRNLNYEKDIVYVTKHNKWILNSIGIWPTVLKGIDEYLPKIAIALSNLVLSFTVIQCVL
HILLEQKDPILRLKILGLTFFSFISLMKYWVLTMRKPKIKLCIEQIQHDWKQVEFERDRKLMLKYGIIGR
NLSMYSIVFMYSGGIIYHTVMHYKLGSYVDEYNRTIKLLIYPTYSRLYDVQKSPVYELVYILQCICGYMF
DAVTVGACGLAALFATHICGQIDIVMAKLEDLVDGKFSKENSNPNIRLIEIIEHHIKILRFSAMVETVLQ
EVCFLEFIGSTFVICLLEYYCITDWQQNNTIGLTTYSLLLISLVFNIFLLCYIGNLLIEKSSNIGIVCCM
IDWYQLPIKTIQGLILMIAMSNSPAKISAAGIADLSLSTFGSVLKTSFAYLNFIRTTIM

>AmOr59

MHPITLNESDCKARNLKYKEDIAYVTKHNKWILKSIGIWPSIFKDVSKFLPKIMFGLCNFVLFFAIIPCI
LYIVIEENDTMIRFKLFGLLSFCLVALIKYWTLLYRKSRIKNCVEQIWIDWEQVELYEDREMMLKYGQMG
RNLMIICAMFTYTGGTIFHTILQYKVGTFIDEYNRTIKPVIYPTYNGLFNVQRSPIYEFVYILHCMCGYV
MHSVTAGACGLTALFATHACGQIDIVIARLNDLIHGKYSKEKINLNARFTKIIEHHLRILRFSATVQEVL
QELCFLECIGSTFLICLLEYYCITDWELNNTISLTTYIILLISLIFNIFILCYIGELLMEKSSNIGLSCF
MIDWYYLPSKTIRGLILMIAISSNPTKISAGGIVDLSLSTFGNVLKTSFAYLNFFRTTIM

>AmOr60

MHLTILNESDYRARNLKYKEDIAYVTKHSKWILKSIGIWPIILKDVAKFLPKIVIGISNFVLLFAIIPCI
LYIIILEEKNNLIKLKLFGLLMFCSIALMKHWALAYRKPKIKNCIEQIQNDWEQVKLYEDREMMLKYGQVG
RNLTIICAVFMYTGGIIYHTILQYEIGTFIDEYNHTIKPVIYPTYSGLFNVQKSPIYELIYVLHCTCGYV
MYSITAGACGLAALFVTHACGQIDIVIARLNDLVHAKYGKGKFNLNARLIKIVEHHLQILRFSATVQVIL
QEVCFLEFIGSIFLICLLEYYCITDWELKNTISLTTYIILLISLTFNIFILCYIGELLMEKSSSIGLSCF
MIDWYHLPVKTIQGLILIIAISNSPTKISAGGIVDLSLFTFANILKTSFVYLNFIRAAIM

>AmOr61

MHLTTLNKNNCKVRNLKYKEDIAYITKHNKWILKSIGIWPSVLKSVSRFLPKIMFGFNNFVLLFSVIPCI
LYIVYEEKNIMIKFKLVGLLSFSLIALIKYWTLTYRKPRIKDCIEQIWIDWEQVELHEDRKVMLKYGQIG
RNLTIICAVFIYTGGSIFHTILQYKIGTFIDEHNRTIKPVVYPTYNALFDVQKSPIYELVYLLHSICGYI
MYSVTAGSCGLTALFATHACGQIDIVIARLNDLIHGKYTKNTFNLNTRLVKIVKHHLRILRFSESIEMAL
QELCFLECIGSTFLICLLEYYCITDWELSNTISLTTYTMLLISLTFNIFILCYIGERLMEKSSSIGLSCF
MIDWFQLPTKTIHDLILIIAMSNNPIKISAGSIVDLSLYTFGGVLKTSLVYLSFLRTTIM

>AmOr62

MGKRKESIDERIRNFMVQKMVLKIIGIWPTNGERSFFGRWIFAVTTQIGIIYILSLEIYRHCLDIDDTMD
AFVMDLSAVISLAKLFILRLNSKHAWVLINSVVEDWSAVHDSRHEYIMTEYLKKGRIVSLMILYLGYASG
FSFIVKALPFGDILPFQMFQNSRNSSMNPDIPLKLNYFLASYCVFGSLPLLHHVCVLLLQGIFIFVNAVA
HCGNDGLFFSLTMHLCGQFEILKTRIAKIEFVDRRKIGPLVKRHCQLAVLVNDLEQTFNMIIFVQLLMSA
LLICVEGFVFLVCLSTKDNIGALKSMVLMVTLLIQLYLYAYAGDALESRTEEIAQAAFHSFWYQSRGRTA
RDLILIICRGNSSYHVTAGKFVFMNIFTFKEILKSSASYLSVLKVMMDT

>AmOr63

MLKKMKTTSNKDFAYAMTPLKFLAWPVGTWPLQVFNTFSIIRATFSTFLLLLMLTILQVELYLDSSNPEY
NLDALILINAGILAVTKVICFHVRSLGLVSNFTSAVKDYKELNSEENRVIVRRHAYMGRAACISLIFCSY
VGCTLFMIVPIVAGDKEEVINVTEESAMKYPVPFENTLILINMPENMYFLIFIVEYLMLLLTTTGNLGSD
SLFFSIVFHLCGQVEILRLEYNKLSNENERTTKHITLLIKRHIYLLKLGDMLNKTISSILIVQLSSSCML
ICTTGFEFILALSIGNIVMIVKTFAVICVLLIQLFAYSYVGEYLKTQTEGLGNSIYFCTWYDMPKNVSHN
ITFIIMRAQHPVLLTAGKFFVINMETYMSILRISMSYLSVLRVMVNS

>AmOr68

MTILQPIFNILTICGCRMPSSCRTSYKRMLYILYATFVLLLLYSFCISQFLNVIINVRTADELCNSFYMF
IASLLSCCKIVALLMNHKAIKIFRRKLEEEPCKPTNTKEVTIQKSFDKNIGSITIYYTVMVEFTVFCMIV
SSLVTDFRNQRLAYEAWLPFNCSAPNYYYYYIAYVHQIIALIGTSLLNVACDVTICGLFVHMYSQQEILK
HRLKESVNVENRLNIGKIVYFHNYLYGYAFMVQEKFKKIIGIQLLSSTLVVCFILYKLANTSLISTKFLE
FVLYLACMMTQIFVYCWYGNQLKLKSVEVVDTIFELDWISLDNRSKKDLINIMRRAMNPIELTCAYIFTI
DLRTFVTILKMSYSTYNFLQRTKVN

>AmOr71

MRILRWTFLLFALCGCFPPSSWTTRLKRYLYKIYAVFSFVALNSFLLSQILDMVYNVKGTDDFSDNFSVT
VVVFVTCFKLITILTRRENILLLCNTLKQEPLSPINTEEFEIFLKFEKLTDWNTLGYFILLMSSSLCILM
GSLLANFKIRKLAFRTWLPYDYSTASAFLLAFAYQVVVATVCTFACVASDTLYSGLLIHISCQFEILEHR
LKNIGSDKNYTMKQCVRHHNHIYKYGEMVNDAFQSIMFFQFCTSLSMICFNFYRIMQIEMDSRYVGTILY
MVCSLMQIFYYCWFSNEVKLKSLELSDMIFRSNWTSLNNNVQRAILLVMRRSMKPIEFTSIYIVSVNLDS
FMTLLKSSYSAFSVLQQSRES

>AmOr72

MHLLRWTFKLFVATGYFLSPKIKSPRKRFLYNVYTVVVTLFLLSFLLTLIMQIVFNVRTADELSENFGIT
ITVFTTICKFINLLFRRGIIISLLDLLQKEPFLPMDIEEIKIHTKYNKLIEKVSIFYTLQNVSCLVALIG
ATLITDFKKKKLTFEAWIPFNYTASWFLFSLTFIHQCGCAVVTSFGISIFDTLFAGLLLQVCCQLDTLVY
RLQNIKEDAIQSLKYCARQHELIYRFTELMNKLFSSILCLQFLISAVAICFSVYRVIYTKTDSQFAGAII
FVFSALIQIFYFCWHGDIAKYKSLEIPDMIFNSNWPNLSNEAKKILLIIMARSLTPVEVVSAHIIPLNLE
SFKRLIKATYSAYNMLQQTK

>AmOr73

MHKLSLSFALLTYGGYWRPTKWPASSYKYHLYNIYSAFMIFLLYFITFCTCVDSLISKNLKTMSEKFSLC
ISVFGVSLKVANLFLQRGKIINIMNSLTKENSIPRDEQEEIIQRRNDNYARKVTIYCEILNESAVFFATV

GQYKRFINTRTLPVSDWIPYDLSSTELYIISLLYQTVGLLICANASVGNETLIAGLMIQAGVQFEIFCHR
AQNLPSLVLTVTRNSNVFAETVNTVFQYMIFLQFTISSVVLCLSIYKFSTVDPLSMNFVWSGFYLCCMLM
QVYLYCWFGNEVTLKSNKVSDAIYEMDWTILPSNVMKDLLLVIARSKKPVKITSGQIFILSTESFMKIMK
ISYSSFNILKNSTMK

>AmOr74FIX

MQGEGYTDVSLKVSQFLLKSAGIWVIGNDAEERQRKFAVFYTLAALIYGIYVNAVDIYHNLDNLAHCVFL
TCNMMCILLGLFKCFVISFFRIEFSRIVSYAQKHFWRLDYDYDEKILFGECQKFCRLWIIVVSMISQSSL
AFYIITPIYENIGKNKSERILPFKMWVDLPLSVTPYYEIMFVIQLLAVEQIGIAYVCSDFFLCILNLHAL
YQFRMMQQELSKIWSAIEQQTTSVAAYTRGCHVALKKCIRRHQSLIEFCNKLEQVFTFPILSHVVVFSLL
MCFDTYEILLADIPTLKRLIFLCHMVASFIHIIFFTYICHGLMEESGNVGLATYSGWWTTLPMNETGRML
RKDIRIIMMKSMRPCYLSRSGFFPMSLETSTALVSSTMSYFTLMRESSMKTN

>AmOr76

MKSKEVRDLSITVTAFYMKIAGFWTSTNYVEERRRNVTMSYTLFAILFAATTEARDLYFSWGNFSDSIYV
ACNIITVSLVLIKLLTSFIYNEELLGIIRYAKTNFWHSNYDTCEKSIMNKCQRTCNYLVFVFTFFAQGTV
LGFILRPILVNRGKNESDRILPFNMWLELPLSITPYFEVMFFVQVVFVYHVCVCYHCFDSLLCILNLHTA
SQFRILQHRFANTCNEKRGKRDEDEESALSFYEYSKLKAYIRQHQALIEYCKKLEQVFNSIVFGQVLLFS
LLMCLDGYLILMEETPFGRRVTFTFHITGCMCQLLMFTYSCDCLIRDSMDIADAAYNCSWSFLPMDKYGK
MIRRDLMFVITRSRTPCCLTACGFFAVSLETYTKVLSTAISIFTILKRYEKEFKSDSS

>AmOr78

MQSENQLDVSITLSTFFLRNIGLWMSDDPGEQRRMRILLVYTVWILLLGMIINGRDLYFTFLYNGDILYA
LTNNVTMVMGLIKIYIILLYKGKFLNLIVHMQQNFWNVNYDYHEKEILDDCRKTCIFFVSSLTTIGICAM
LSYLMTPFAIRSGNNESERMLPFNMWLDMPLSKTPYYEITFLIQAMCVYYIGISNFCFDTVFCIMAVHLA
GQFRILQYRFTKLCDTDNQICKKNLILEEQMQKFHEKFKKYVRRHQALIDYHQKLENVYTTIMLSQVLLF
SVLICLFGYQVLLATASLARRSIFIFLLMGAMFLLFMFTFSCNGVMEQSDNVAVGTYSALWTVMPMEKFG
RMLRKDLIMVIMRSRRVCCLTANRFFPISLETYTKILSTAVSYFTLLSKHVDNS

>AmOr80

MQTESQLDISINLSTFFLKNVGIWMSDNPNEQRRIKMLFLYTIWNLLFGTVVNSRDLYFTLLYDGDILYV
TTNNITMIMGMVKICIILIYKKKFLNLIVYMQQNFWNVNYDHREKQILDDCRKTCIFFVSCVTIMAICAM
ICYIMIPFIAQSGSNESERMLPFNMWINLPISRTPYYQITFLIQATCVYYVGISYFCFDNIFCIMAVHLA
GQFRILRYRFTKLCDMEYGIKENSQSILSKQMHKFYEKFRKCVQHHQALIDFYQNLENVYTMITFGQVLV
FSVLICLFGYQVLVATISFARRFIFVFMLNGSMFLLFMVTYSCNGVIEHSDNVAVGAYSALWTIMPMDKF
GKILRKDLIIVIRRSRRVCCLTANGFFPVSLETYTKILSTALSYFTLLSNRIENSS

>AmOr82

MQTESQVDISMNLSTFFLKNVGVWMSDNPNEQRQIKMLLINTTWILLSGIVINGRDLYFTLLYHGDILYS
ITNNITMIMALIKISIIIIYKGKFLNLIACMQQNFWKVNYDYREKEILNDCRKTCIFFVSCLTTMVICAM
ISYLIIPFIAKGNNESERMLPFNMWINLPLSKTPYYEIMFLIQAMCVYYIGVASFCFDNIFCIMAVHLAG
QFRILQYRLTKLYDVECIEMHKKDSILANRVPKFYEKFRKCVQHHQALIDFYQNLENVYTRIAFGEMLVY
SILICLFGYQVLVATASFARRSIFVFLLNGSTFLLFMVTYSCNGVIEHSDNVAIGAYSALWTIVPMDKFG
RMLRKDLIMVITRSRRVCCLTANGFFPVSLESYTKILSTALSYFTLLSNRVETANDT

>AmOr85

MSSNKVGGDLSITVMTFYMKIVGFWIASNYVEERRRNLTISYTFFAIFFAMATEARDLYFSWGNFGDSIL
IICNLVTVILVLFKISISLMYRNKLHKIIQYAKTNFWNLKYDLHDEQIIINTCKRYSTFFVCIFTFFSQG
TVFSFVIRSLKENIGKNETERIHPFNLWLDESWYMTPYFEMVFIIEILSLYHVGVCYLYFDNFMCIINLH
IAGQFRILQHRFSNVCNEMCEKCCYQLSRKSPYLSICKYAKLKIYIRQHQTLIEYCRKLEMVSNFIIFGQ

VLLFSLLICLDGYLILMEDTSNMSRLIFTFHLISCMCQLLMFTYSCDCLIRDSTNIANATYNSLWSFMPM
DKYGKMLRKDLILVIMRSKSPCYLTALGFFPVSLETYISILSTAISYFTLLRNRAEQTIMDA

>AmOr86

MHATPYSDVSIVVSQFLLKLTGVWMTVNDGEKRRRRIAMAYTFVIQVYGLYLNIGDIYHSWDDLSHCIFL
TCNTLCIVLTMFKFSILFIRRTEFKNLILFARKNFWHLDYDRHETILFTKCRKFCTLWTLTVFSFTQASL
TFYIITPICANIGKNKSERILPFKMWVDFPLSETPYYEIMFVIQLLTVQQIGIAYTCNDNFLCVLNMHVV
CQFRILQHRLTKLWSIIDERADKFNYASKCYEALKECIRQHQSLIEFCDKLEHVYTLPIFGHVVVFSLLM
CFDTYEIFLANVPVSMRLIFFFHMVGSFIHIIFFTYICGGLIEESSNIGLATYSGWWTVLPMDEAGRMLR
EDVKVMIMKSMRPCHLSAGGFFPVSLETSTALMSSTLSYFTLMRESSKDK

>AmOr88

MNGRNVRNLSITVTAFYMKVAGFWVANNYAEKRRRNVAMFVTIFFAFMGISIEGRDLYFAWGDFEDSIFA
GCNVITIVLVLLKIFVLYINNEELLNVVNYAKTNFWRESNYEPHEKKIIDDYRRLCSFLVCSFTFFAQGT
VVCFVITPVFVNNGKNESDRIHPFNMWFDRSLSLSPYYEIIYTIQVLSAYEIGICYHCFDNLLFVINLYT
AGQFRILRYRFENICGKNDDKNYYKVSKSSKYCINEYKSFKTCVQQHQALIEYCKKLEDVFSIIVLAQVL
LFSLLICLDGYLVLMEDTSRAKRVIFTFHLMGCMCQLLMFTYSCDCLMHDSMSVANAAYNSLWPCLPMDK
YGKSLRKDLTFVIMRSRSPCCLTACGFFPVSLETYTGILSVAVSWFTSLKKYEKKLLQYACIANQSLLKE
EESKIIFYHQNI

>AmOr89

MQREVELDVSVNLAAFFLKNVGLWASNDPGHERRRKVILVYTMWCVTLSSVVIIRDVYFTWFYNGDILYV
VTNALSMMMITVKVCVIVVHKEEFINLIVYMQENFWNDNYHDLREREILENCKRTCAFFVSLVTAIGICA
ILSYLATPLIVQTASNNSERMLPFNMWLKLPLSESPYYELMFVYQIMTFYFIGISYFCFDNIFCIMTVHL
AGQFQILRHRFDRLCNAEDRIAEKGAHAREFYDRFKARVPYHQALIDYCEKIENVSPTIILEPVMVFSVI
ICLFGYRILWANAPSTRRSIFIFLLIGAMSLLFMFTFSCNCVTEHSENIAIGAYSALWTAMPMDKFGKML
RNDLIMVIKRSRRVCCLTANGFFPVSLETYTTILSTAVSYFTLLRNNVEKANEA

>AmOr92PSE

MNFLENDVAVSLTSIFMKFVGIWMXQYQQRMRNIMVAYNVIAIFFALWIQTMDMYHSWGNIRACLFSTSN
TLSLILPLLKIFILLCHKQDFFRLVLYMKRNFLXNYDDHERKIVIGCNQKCTFFICFFTFLTIATTASYM
VIPLIVNIGKNESDRVLPFNMWVNLPLSMTPYFEISFVLZVLSLYQIAVSYFCFDNFLCIMNFHVAGQFK
VLQHRISTIADLTIKTEEKKEKLIIDSLHFSNKCYTTFKKYIRQHQTLIAYCRKIEVVFNWIVLEQVLMF
SLLICLDGYQILMADEDIKTRSIFSFHILSCLCQLLMFSYSCDCILRESVSVATAAYEGPWTLPPMTISG
RMMRKDLIVVIMRASIPCCLSGKGYFIVSLETYTSVLSTAASYFTLLRNNIESEIMKHD

>AmOr93PSE

MNFLESDVSXIFMKLVGIWMAGNGZEXITLFTAIIFSGYNLZMYFILGDFSACLFFISIILSSIMLLLKI
IILFSHREDFFHLILYMKRNFLXNYDDHERKMIGCNZKCTSSSVSSRFSRWRPLLLTSSVRLLVKNIGKN
ESDRILPFNMWVNLLLNITSYFEITYTLQXFSLSLYHIGVSYFCFDNFLCIMNLHVAGQFQVLQYRISNI
IYXRFNEKKEKLIVDSXATKCYAIFKKXQHQALIYLIYCRKLEEVFNLIVLEQVLMFSLLICLDGYQILM
ADGDVKTRLIFSFHILGFLCQLLMFSYSCDCIIRWTLXPLLSMTSSRRMIRKDLILVIMRSNVPCYLTGR
GFFIVSLEMYXVLSTAAXFTLLKQRTEATS

>AmOr98

MDMFQKTGKEYSNIFDIPYYKVLKKYLQFLGQDPYQECKYRNIITIIMLISMIAIFIPTTFEIYVSIHDK
NTDAVMECLPNLCASLSSVVKILNVHFNRENFNKLLEFVVKEWDELKLNELHILEEITIQGSKIAHLYRN
TLLSFLILFLLVPMYFPILDMIDALNQTRSRQQLLRVNYMVFNADDYFFYVYLQLAWGAIVIVMIVITVD
SLYIIIIHHVCGLFAVCSYEIQKTVKDLTVFTDIEKCSYKELKNCVIKHKKAIKFYNILNNSSQLSYLLQ

IGINIMGISTTAFQLAVNLDTRPQEAIRNAVFCGANQFHLFVLSLPGQILLDHCAELSNTIYCSMWYKLP
VKIQKMFNIMLMRSKKSCALTVYGLYELNMENFGTTFKACISYFTMMLSLK

>AmOr99

MDRLSVFQKIEKQHNEIYDIPYYKMMEKYIRFLGQDPRQKDEFRNIIVFILIISIASIVIPTTLELYISL
RNKDVDGVIECIPHFIASSISAVKLLNLHFNRQNYNILFHFVTKKWQQLKSTYELNALDETIMQGKKMAQ
LYRNTLFSFLILFLVVPLVSPILDIVHPLNQTRSRQQLLRVNYIVFDIDDYFFYVYLQLAWGSIIVVLTI
IAADWFYILIIHFNSGLFAVCGVQVLEATMNSNLISKDAFSENSSYEKFRTCVIMHNEVIEFYNILNENC
QYSYLIQVGLNMLGMSTTAVQTVINLDRPDVAIRSAVFFGADQFHLFLLSLPGQILLDHCADFANAIYDS
TWYGTSLEIQKMLYMMQIRSKKLCALTAGGLYDMNIENFGITFKTCMSYFTMIMSFK

>AmOr102

MNIFQKTRRQCPDIFDIPYYKMVEKYFQLLGQDPRLKNEFRNFIVTVVVISISGNIVPTSIELYTSLCDK
NMDAVIEGLPHFIAATISAVKILNVYFYRENFDKLFQFVTNEWNKLKLNNELHILDKTIIRGNRTAHLYR
SALLIALVLFLLIPLISPMLDVFLPLNETRPRQQLLKVNYLVFNDDDYFFYVYLQLAWGSIIVVVTSVAV
DSLLILIIHHCSGLFTVCGYQVQKVISNAKSFNGTVLNNYTYEQIKNCVIMHDEAIQFYNILNESNRNSY
LIQVGLNMLAISATAVQAVVNLDRPEEAIRSAVFCGANQFHLFVLSLPGQVLLDHCSEFSNNIYSCIWYR
APVRIQKVLYIMQIRSKKLCTLSAGGLYEMNIENFGITFKTCMSYFTMIMSLK

>AmOr104

MDVFQKTRNKCINIFDIPYYKLLEKYMKFLGQDPRQRDGFRNIIVIVMVASISGILIPTSLELYTSLRDK
NMDAVIECLPHLIAAATSVVKLLNIHFNRENFKKLFEFITKEWEKFELNNQFHVLEEITIKGSKMAQLYR
NTLLSFMVLFLLVPLIFPFLDIVHPLNETRPRQQLFRVNYLIFNHNDYFFYIYLQLAWGSIIVVMIIVTV
DSLYMIIIHHSSGMFAMCGYKVQEATKYQNLFNDRIISENYTYEQLKNCITIHNKALQFYNILNESSRNS
YLIQVGLNMMGISVTAVQTVVNLDRPEEAIRTAVFLGAEQFHLFVISLPGQVLLDHCTELANNIYSSTWY
RIPVKIQKVLHMMQIRSKKPCSLTAGGLYEMNMENFGITFKTCMSYFTMLMSLKK

>AmOr107

MDQQAMEELYLKDNKFFGQLVGVWPDQGKFMKFLMRFIILIVMIIAFIAQISRVAVFYSVDVLSDQIPYI
DLGFALMLKQYNYILNEKKLRELLHNIISDRLVKRSKEEEEIFEIYFKRAMFFCSFYEVSIYSCGFMFLS
MPSIPLIMNVIMPLNESRSRELVYPSYYFVDEQKYYYLITGHMLAVCLGHVFVYIACDINLIHVVHHGCA
LLTISGYHFKHAMDNVDLCNEKYSDELMEKTYAKVSQSIDAHKKAVEYVNKIDACHIHYFFILLGMIIVT
FTGTFIKLTSMEIGGRFFTFCTFTIGQLTHLLFLMVMGQFLIDSNEEVFKTIYDARWYYGSSKTQSLYLL
VLRKCLNPPKLTGGGLIALNLDSFVKVLKTSFSYYTVFRSS

>AmOr108

MNKQAIEDQYLRINKFFGQLVGVWPYQERFTKFCIRLTIFAIIILTLTTQIYQVIVFCTLDALSNQLPYL
NALFILLFKQYNYILNEDKLRDLLNDIIFDRLMVRSKKELEILNMYSRRATTLCIFYEVIVIFSAIMFIM
IPTIPPILNIIMPLNESRDREFIYPTYFFIDEEKYYYPILTYMATVILIVSSVYLACDTNLVQIVHHGCA
LLAISGYHFKHAVDDMKFSNGNYIDLLMDETYKKVKQSIKAHKTAVEYVDKIDACHIYYFLLIIGMIVLA
FTGTFLKLSTMEIEIRFFTFCGYTVAQLTHLFFLTIMGQFLINANDEIFNTIYEAHWYNGSSRTQSLYVL
VLRKCLNPPTLTGGGLIVLNLDSFVQILKLSFSYYTVFRS

>AmOr109

MDERAIEDQYLKINKFFGQLVGVWPYQKKFFKTCIRFITFTIMIFSLATQISRVIVFYSLDVLSDQLPYI
NAGIVTLFKQYNYILNEDKLRELLHDIVSDRLIERSKEELEILEMYSRRTTALCALYKVMVYSCAFMFLV
IPTIPPILNIVAPLNVSRSREFIYPTYYFVDEQKYYYPILTHMIAVILVLSSVYLACDTNLVQIVHHGCA
LLAISGYHFKHAVDDVKFCDGYYIDASMDETYVKIRQSIKAHKTAVQYVDKIDACHIHYFLLVIGMIVLA
FTGTFLKLSTMEVGIRFFTFCAYTIAQLIHLFFLTIMGQFLINANEETFKTIYEADWYNGSSKMQSLYVL
VLRKCLSPPKLTGGGFVALNLDSFVQILKASFSYYTVFRS

>AmOr113

MDSDILEKRFLKITKRFAKLSGIWPDQNKYLKYISWIIIYVISIPSIVVQIARIVHISTANVIVEQSGIA
TAIFLSLLKEANYILNATKVKSLFNDMYMDWRMDRPKKEFEIMSTYAQRGSFLAMFYFINAYCCSLLFLQ
VPWTARLLYMIKSQNTSPPMLYVIPGYYFVDDDRDYYYFIQLHMSLSIIMVANVYVAYDTCYMVFVQHVC
GLLAVAGYRFKHAIDDSASKNSEEKIKETCKKIRSSIQGHEGAIRYLKKIEDTHVNLLFISLGLIIMCFS
ITLLKVVTMDYCLDFYKYSSFLIVQLMHLCYVMIQGQFVIDSCNEIYYSIYEASWYNINPKIQALYILAL
RRSLTPPRLTAGGLIELNMQSFSEVIKLSISYYTVLRST

>AmOr114

MVHLREKTSMNIQKDLKYLYGWNYYTMKFIGIWPEERKWNQASNYLVLIPFLMILCFICAPQTINLTIIS
NDFNLVIENLSMGNITITLSLLKTIAFWINGKPLKSLLNCMANDWIKVTSKTEQETMARIASITRNTIIK
STVMCHTVVAFYVFLRYISMKYNENKLLFRAYFPYDTTVSPNYELTILGQFVAALYAATSYTAVDTFVAM
LILHVCGQLSGIKNELSRLPTYDKKDLKRRLKEIVQKHEYVNRFAETIENCFNVMLLIQILGCTVQLCFQ
CFQAIMSFGGGEAQEYLFFQLMFLLVYVFYVMLQLYLYCYVGERLSVESMEIVNAAYNTEWYTLPTNITK
MLIIVMCRAKSPLTVTAGRFCSFTLQLFSEVLKTSMRYLSVLYAVKDKIKR

>AmOr115

MDFAMGWNRFNLTLLGVYPEPRKMSRNSRLMSSLIFWFTTLVTFTFICAPQTANLILKSTSLDEVIENLS
INIPIVFALIKQIVLRYYKKALTELLGEMLADWSGPIGDQDRETMLRNARLSRAISIVCSTLTYFMLLAF
VSLQVWSNAENASETDLGGLLHPATFPYETSKSPNYEITWLGQLMGTVLTAICYSCFDTFLAVLVLHLCG
QLTVLGTALEDLVNATRRNDYKTFEQRLSSIVNRHNHLSRFAVIVEDCFNITLLVQTLICTAMFCLTGYR
MITSVDREDEADVPIVGIIFFIIHVIYTMLHLFIYCYVGETLLGQSTGIGLSTYHCNWYDLPSRRAVLLM
IVIRRANVSFQITAGKFSPFSLEFFNAVLKTSAGYLSVLLAMKDRLVEGK

>AmOr116

MTNHLEKQIKLKKINSNKHLQNNLSIIYYIGLWPDRVKYKYLYNLYTICSLIFLVGIIIVSEIIYIIINW
GKIEIMMTGLTILMTNSTYAAKVIYIICRYERIKNLVDITNSEIFNRDNDKYKHIISYYNWQGIFHHIAY
QGFASICIFSYSCIPLQSAFSGKSKQLPIAGWYPYNVTSTPIFEIACLHQVLVILINCINNIAIDTLITG
FIIITCCQLTILSYNISSIHYTVESVESSILIEKCIARNNNINIEKSPSKIYNKFYENLKHCVKHSIIIF
DFTKQIQDIFGIIIFFQLFVNCIIVCLAAFNLSQIKNYITPEFFGSLLYICCMIYQIFIYCWHGNELYLH
SMKICLSAYKNNWWNNNKNFNYALLIIMIRTQIPLIIIVGKVMELSLQNFLLILRTSYSIFTLLKTFTT

>AmOr118

MINRPLEYSLRIFGIWPDSPYPKLKIITWIIILPTFLVFQYWYCITHIKLGLIDLLDGLSLTLSNTLVFI
KLIVIWFHKRTFYEILMSMKEDLNNNKHSATENKRIIMDKSMLSSRISNFLISYFAITFFLYSGVALVIF
DEDQGKFLVRMEFPFIATISPRYEIILITQFIFESFIVYGAATSIALIAALILYVGSQIDLFCQNLTFHS
YKKRESQDTIKDIIVRHQKIIQLSKNIETIFTYISLCQFVSNMLVICFISFVLTVSLHTEQTIVLIMKCL
PYYIAVNCEAFILCYTGEYITSKSENINKAVYNFLWYNLKPRDVRIMLMIILRSQKQLTLTAGKFICLSL
EAFANMLKASASYVSVLYARY

>AmOr119

MHTQRDTSEITYSHDTATSRKLFYLLVVVGGQMAHASGHEWMKSIRTLTSIKINYLKYSGLGEIDSSCSR
ILKYAYFVYKVWMLVSMCILAITVFADIYTNMDNLSITTDDGCIFAGIFVVIFKAMNLQIQLESVKKIID
KYHTRNKVMFFGFCVIGACLGFALLCFTPMENGLPIRAKYPLNTTVSPWHEISFFVETCAVSGGLLGIIV
MDSMTTFKCSLITMLLDALSVNFENCNGETKRTICNRHGKEERNDNNNRFLDRYKKCVQFHQRLVVISRD
YNKIYSLSMLVQMISSTSIICLTGFQAVVVGGQSSNIMKYGIYLSAAMSQLFYICWLGNELGYASSTLDK
NQWFSGWCNERLTGIGQVFTLSTVFTRKSIILRASVFYVLSLETFIAIIKRSYSFFTLLNNMDLTDH

>AmOr120

MSNQTNTMNIRNYIFINQLVLKFVGFYPINILRYVICISCIMFIVIPQIIMIYINWNDLNIVMETGSTLL
TILLAALKSIVWIFNRKKLEFFIEFMLTDYWKIIETNVFEYLQEYAIYAKNITKGYFFSMCNALLFFFSL
PIIETLTKNENLNNFTIKNFPFAASYPITFYKFPFYEIAYISQILATSICCLMMLAIDSLIATALLHTCG
HFTVLKENLKNLDTYIYDLTKTNLKTNSKYINKNLYEIKTQIIYIIKHHQLVLWFCDNMEKNFHLILFLQ
AITSSLIICFVGFQISIALTERSKFLESFSHLIVSLFQLLLFCFPGDILIRQSFNISIAAYSMQWYQLPT
FIKDEICMIILRSQRPSFITAGKLYIMHLENFTAILSTAFSYFMMLQSFNTEA

>AmOr122

MNMDVFDKQYRIYRIILKIVGLWPYDKSIYVWIQRICLSMYFLIGVIFQIILLVKSEITLRNYIVTLSAI
FPLLLFFIRYIYYITMFPYVEILFDNIRTEENLLQDTTEIQIQTKYLDISSHIIYIFCCMTFAFIVAAII
FLVNPVILDLRNPLNESRIFYFDLLFLLDDQSAYIKIFLILNFMLNILFGLLSITSTESFTNIFSYYICR
QFNIVNYRIRKIIEDLSTRNLSKIDLKIKDIHRVVDIHCHAIEHVHIATNNAATQYLIAIILCILSFSVN
LYRLYKALITMDNRIEIFGSTLIVIYHLMIAFYNNHCGQLIIDSNLGIFNEFASTWYRIPLKAQKLLLFM
ILRSSMGCEICLSGLFTPSYAGLTSMMSSSFSYCTVIYSIQ

>AmOr126

MNVFDNQYRTYRIILKIVGLWPYDNSIYVRIQRICVLIYFLIGVLVQIFSFVKSEISLRNCIVTFSMTFP
TVLFCLRYIYCLTLFSYAKLLFDDICTEEHLLQDTTEIQIQTKYLDISSHIIYIFCWLSFICAAASCILI
LNPVILDVIMPLNKFRLHYSLIFLSNDRRKCIDIFLVLNSIIIFTFGLLSLICSELLTNIFSYYICRQFH
IVCYRIRKIITDLSTPNLPKTDLKLRDIHRVVDIHCHAIEHIHMAFHTNNAATQYLLAIILFVFSFSINL
YRLYKALITMDNRMEILGSILIVIYHLMMALYNNHYGQLIINSNHGIFNELCASTWYRIPLKAQKLLLFM
ILRSSMGCEICLSGLFTPSYAGLTSMMSSSFSYCAVIYSIQ

>AmOr130

MNVFDNQYRIYRIILKIIGLWPYDNSIYVWIQRLCLLSYFFANIIFQIVSLLRSEITLQNSILILSITCP
LVLFLLRYIGSIACFPTIKIVFKHIRTEENIVQDSIESQIRMKLIDDSHHIINIFFWMTYTTIVIFIIYV
SYPIILDFMIPLNESRTHFIYYITTFSHNQSIYLDILDFNFMFTGIFGLLSVACSESITGIYSYYICILL
KIVSYRIQKIIMYLAMFKLSPKQIDSKLIELYRVVDIHNQTIELIDFMLSTAGIHYIIASLLVVISLAIN
LHRLVNATLIKKNQLEMLFCFTLVAIHLVIIFLNNYNGQIVMNSSQELFDELYNSMWYFMPLKAQKILLL
IMLQSTTKHAFNILGLFTPCYAGFSTMLSSSFSYFTLMYSIQ

>AmOr131

MDVFDKYYHTYRIVLKIIGLWPYNNSVYVWIQRLCISALFLGNIIFQILSLIRSEITLRNCILILSTTCP
LIIILLRYISFIIFFPMVKLLFHHICVEENAVQDLIEIQIRMKYIGNSRHMIEILLRVTFLTITLFSIFL
LYFVTMDFIMPLNEFHRHILLYVTLFSVNRTIYFYILYLNFLFVITFGLLSLICTESIVGLYSYHTGMLF
KIISYRIRKIITYLTMFNVSSKQIDSKLAELHRVVDIHNQAIGFISIIINNSGKQFMMPTLLIVMSMAIN
LHRVVNAITIKKDQLEILITLIIFANHLMIMFLCNYNGQILINSNEEFFHELYIPVWYFVPLKVQKILLL
IMIRSSMACIFHIFGVFIPCYVGFTTMLSTSFSYFTLIYSIQ

>AmOr132

MDVFDKYYHSYRTVLKIIGLWPYNNSIYVWIQRLLLLTLFLGNIIFQIMSLLRSEITLRNCILILSTTCP
LIIISLRYICFILFFPMIKYLFHHMRMEENIVQDSIETRIRTKCINDSCHMIDIFLWMIYAIFAFCIILL
LCPIILDFIMPLNESRIYIAHYITIFSDKRIIYVDILCLNYMFLMIFVVLSIMSTESILGLYSYHTSMLF
KIISYRIQKIITYLTIVNLSSKQIDSKLAELYHVVDIHNQAIQLINIVINNSGKQYMISTLLFVISMAIT
LYRLENAIIVTKDHLEILICLMLFVKQLMIMFLCNYNGQILIDNSEELFDELYFSIWYFVPLKVQKILLL
IMTRSSTTCMFHILGVFVPCYTGFTTMLSTSFSYFTLMYSIQ

>AmOr133FIX

MDVFDKHYYTYRFILKIIGLWPYNNSVYVWIQRLLLLTFYVGNIIFQIVLLLRSKITVRNCILILSTTCP
LIIISLRYIRFILFFPKIKYLFHHMCMEENIIQDSIEAQIRTKYISDSRHMIEILLWMAYATITLYSILG

LCPIIFIILLNESPIRMLHYVTLLSVNGTIYFYILCLDFLFIIIFGLLSMICTETIVGIYIYHTSILFKI
ISHRIQKIIAYLNMFNLLSNQIESKLAELYCVVDIHNQAIQLFNIIIKNSGKQFMISAFLSMISMAISLH
RLVNAITIKKDQLEILISLIIFVNHLVIMFLCNHTAQILINNNEEFFHELYISVWYSVPLKVQKILLLIM
IRSSMACIFHICGVFVPCHAGFTTMLSTSFSYFTLMYSIQ

>AmOr135PSE

MDVFDKQYHSYRTVMKIVGLWPYNNSIYIWIQRLLLLTFFLGNVIFQIVSLLKSEITLRNCILILSITCP
FIIVSLRYVCFIVFFPTIKLLFHHMRVEENIVQDLIEIQIRTKYINDSCHIIDIFFWVACTNITLSSISL
LYFITLNFIMPLNEFRIIHYITLFSVNRTMYFNILCLDFIFVVIFALLSVICTESIIGLYSYHISVLFKI
INHRIQKIITYLTIVNLSSKQIETKLAELYRVVDMHNQAIELIDVMINNSGKZFMISALLLVISTAINLH
RLVNAIIIKKDQLEISISFIFFVNQLIIMFLCNHSGQILIDNSQKLFNELYISIWYFVPLKVQKILLLIM
IRSSTRCMFHILDIFTPCYAGFSKMLSTSFSYFTLIYSMQ

>AmOr136

MNVFDKHYHTYRTLMKIVGLWPYNNSIYVWIQRLWFLMFFFGNIIFQIMSLLTSAITLQNCVLIFSTTCP
LIIVLFRYIGLILFFPTIKLLFHHMCMEEAMIQDSIEAQIRRKYIDDSCYMIDIFFWMTYVGIALCSILL
LCPITLDFIMPLNESRTRIVHYVTIFSDKSIIYMDILCLNYMLLAILVILSATCTESILGLYSYHTSIMF
KIIGHRIQKIVKYLTMFNLSSKQIDSKLAELYRIVDIHNQAIELVDIMINNSGKQFMISTLLSVISMAIS
LHRLLNAIVIKKDELEILISFIFFTTQLVITFLNNNCNQILIDNSQELFIELYISMWYFVPLKVQKILLL
IMIRSSTACMINILGVFTPCYIGFSKMLSTSFSYFTLMHSIQ

>AmOr137CTE

MDIFDKRYCTYRTMLKIVGLWPYNNSIYVWIQRLWLLIFFLGNIIFQVVSLSSEITLRNCILILSLIFPL
TIILVRYVSCVIFFSMIKLLFHHMRMGGNIIQDSTEIKIRKKYINDSCHMMNIFFWIIYGIAALSIIFIL
YPMTLDFIMPLNRTRIRIIHYITIFPYNRTMYLDILSLNFMFVGIFGSLSLACTESIFGLYCFHASILFK
IIIYRIQKIVTYLTMFNLSSKQIDTKLTELYRAVDIHNQTIGFINVMINNSGMQFLLSSLLCVISIGISL
YRLVNAIIARKEKDQL

>AmOr138NP

VVSLSSEITLRNCILILSLIFPLTIILVRYISCIIFFSMIKLLFHHMRMERNMIQDSTEIKIRKKYINDS
CHMINIFFZIIYGIAVLSIIFILYPMTLDFIMPLNKTRIHIIHYITIFPYNRTIYLDILSLNFMFVGIFG
SLSLACTESIFGLYCFHANILFKIISYRIQKIVTYLTMFNLSSKQIDMKLTELYRAVDIHNQAIGFINIM
INNSGMQFLLSSLLSVISMGISLYRLVNAIIVKKDQLEMLISFMILMAQLIITFLCNYNNQILIDNSQEL
LDELYISAWYFVPLKVQKILLLIMIRSSTSCTFHILGVFIPCYTGFSKILSTSFSYFTMIYSIQ

>AmOr139PSE

MDIFDKHQSYHSYRTIMKIIGLWPYNNSIYVYIQKLZLLIFFLGQIIFQXDAVIAFFSISLLYPIILDFI
NSLNESRTRIIHYFTIFFHSRIIYIDILCLNYIFLAIISLLSIICIESMIGLYTIVTTSLFFKIIGYRIQ
KIITXLTIFNLSSKQINSKLVELYHVVDFHNQVIELINAINNSGIQFMMPSILIVISVAXLVNMILIRKD
QLEIFMFFIFLVSQMMIMFICNYSSQILIDNSQELLYDLYISMWYFVPLKVZKILLLIMIQSSITYMISI
LGVFILCHIGFSTMLNTSFSYFTLIXSTQ

>AmOr140TRA

MSQRNERSATTVDMDFFEQPAYLLNKRLLSIIGIWPFQKSLPKLLRQNIVLIFCFTLLIAELRGLYSVWG
VNYDAVIECMPPIISIFQSASMYFNGIFNTKKIKNILLFIKNDHKYYINRPENIILQKYDLQGKKITFYY
ILYVYTTLFVYLLLPTIPLIIDFITSSNHSQKRNFLFELDYGMDKQQYFYYISIHSYIGTAIVANLIASC
DTMYMLYAQHAYALFAIVSYELKTIHILNTNNLINVTDHHLLEKYKNITLLSKDEKKVYRKLFICIKNHQ
NAIKYSNLLESLFTKSILVQLFFNVLCLSITGVETVIKLGNLSEMMRFGSFTFAQAVHIFFLCLPGQRLL
NHSEELHVSACEVTWYIFPKKYQNLYKFLLARSLIFSKLTAFKVTTLSMQTFLAIIQTAMSYFTVLLSTT

>AmOr142

MKNRLTPEKAILFTKLSVALTCSWPPSPLATKAQHLFFNALWCIAFLTSVMLFLPLLAAIYVYRKHPVIL
GKTVSLTAAVAQVTIKMIICRLQQKRFQMLYSEMENFCKQATNEEKIILQRYVDRYKYFHSFYILWSFLT
TIFVICGPLYTVQTFPTHAIYPFSVRRHLYKGLIFFHQSLVGFQVSSGMAIDTQIALLLRYATARFEILG
IQFNNAKSDGEFDACIKKHDELLRYSREIRQSIKFLILATNGTTVIAVIFGSLNLIANQPLILKALYAIV
VFSASVELFMYAWPADSLMHMTMKMATKVYNMDWYGKDIRTQRKILFIILRSQKYESFGINGIVPALSLS
YYGKYLYTSLSYFNALRIMVEDTVN

>AmOr143

MNIRQILYILELIGTFTCTWPINPNISKRRIIFRNIFWIFSILNVILLMTSLMLAVVYFRNDILMSLKTA
SEMAALLEVVLDLILCKWNNSEFQVLIEEVKSFVEMANEYEIKILQGYVNRYKKFFSTVSMGYISTAISF
SLMPLFSAQKLPADGWLPFSTEPFGIYCIIYFNHVYCILQTAFCIFVDFTIVILFSFPAAKLDVLRSKLR
HVNNYDTLVSCIKEHQKIIGFVEDTKATVETLLFKTNVTMGSTVMCGAFPLLNNQSLAAISQFLPLVLSG
ILHLYVIAWPADDLRESSVQFSNSISDIQWLGQSNKMKSCVIFMMMRSQKAFLIRMSNLLPPLSLEYCSN
FITTVSSYFMAMRTMIES

>AmOr145

MGMLNMNIRQVFYILELTGTFTCAWPINPNDSKTYIIIRNILWIFTILNVIFLAISMIFAIFHFRSDIPK
SMKTASEMAALLEVALDLALFKWNNSELQILIEEVKSFLEIADEYEIKILQGYINRYKKFFSTVSMGYIL
PASSFILTPLLSDKELPTEGWLPFSIEPLGIYCAVYVNHVYCILQTLSFCIFVDFTIVILFSFPAAKLDV
LGSKLQNVNNYDMLVSCIKEHQKILGFVENSNATVETLIFKTNITMGSIVICGAFPLLNNQSLDVVTQFL
PLILTGMLHLFVIAWPADDLRESSIQFAESINDIQWLGQLKKMKSCVIFMMIRSQKLFLIRMSSLLPPLS
LEYCSNFVTTISSYFMAMRTMIES

>AmOr146

MFRNATPEKAIAFTQFIVSLSCCWPLPSTATKLQTRCFKIIRSLLFLNSLLLFFPLLYFVYVNRNDNTTF
CKAMSLSLAVVQVPLLSSFCITQYDRFQRLIKEMKFCCENANSYERQVFQGYAKSYATFYGVSAIWFYWC
ALIVVVGTLFISDPFPTNAEYPFPVHFEPVRSIVFVQQALVGFQCSAHLCVNIFCALLLLFAAARFEILM
NELRAVENIESLIKCIEKYYAIRRYAEEVVNSARYTTLITLCICGVESVFGGIIFIGRQPFTVKLQFLTL
SATTLLAVFMCAWPADYLMDVSENTMRAVYESEWYKRSLKLQKFVLFATIPQTPVILKVRCIIPAFSLNY
YCSFITNVLSMFTALRVLMYKDEN

>AmOr147FIX

MLKQVSPEKGIYIIWLSVALSLCWPLPINSTRKQIVCMKILQIGAIISAFMILLPLIYTIYLNLDNLNIF
FKSICLLMGVFQHIVQTITCFIKYDSLQRVVEEMMICIKEMQLNEIMCAYVAKCNIFYGGTIVLIYTTAT
VFILGPTFLPITFPWETEYPFQVNYTSRNFIIYMHQFFFTYQCAAHICVSMFVALLLWFTSARFECLVKE
LQKTTNIEMLIVCLKKQLLLRRYAEDVVNCIRFIIFYTMAVSTIVLTLSGIILITTSSLLVKIQFLTICI
SILLEIYMYAWPADHMYDMSITVLQSVYDSMWYGQTLNMQKLVLITLIYQKPVTISINVVLPQLTLHYHC
LYVSNAFSIFTAIRAIIYDR

>AmOr148

MLKYVTPEKGIYIVWLSVALSLCWPLPASSTRKQIVCIKILQIGAIISAFMVLLPLIYAIHLNIHNLINL
FKCICLLICVFQNIIQTIICFIKYDVLQRVVEEMMTCVKEEQLYKVLCIYVKKCNIFYGGTIVLTYGAAT
VFVLGPTFLPISFPWETEYPFQINDTSRNIIYIHQFFFTYQCAAHICLSLFGALLLWFAAARFECLVEEL
QKITNIDMLIVCFKKLLLLRRYAEEVVSCIRFLVFYAIAVGTFMLTLSGIIMIINSPILVKIQFIIICMS
SLMEIYMYAWPADHMQDASINILRSAYNSIWYEQSLDMQKDLLIIILMYQRPVILSINVLLPELTLRYYCS
YVANAFSVFTALRAVVEDK

>AmOr152

MLKQIISEKTIQIIWFSVAITFCWPISLNSSKTQVFIFKILQIISIINVFMLLLPLLYSVYLHFNDIIIV
SKSIALSVGLIQVIVQTIICFIKYDSLQHVVEEMIIYVKEAQQYEKKIFHKYIEKCHIFYGCSIACIYLT
ATVFVIGPVFSSASFPADAEYPFQVNSTSMKIIIYLQQSLIAFQCAGHACLSIFGALLLWFVSARFECLA
VELQKTTDIGMLIVCVKKQLHIRRYARRVVISFRFIILCAMGVSIFSLTLGGIIMITKSPFIVKVQFITL
ILTLLTEIYMYAWPADHMKDMSINVSKSVYNTIWYEQTLRMQKNLLNILMYQQPIILSINCILPELSLRY
YCSYLSNAFSIFTAIRVIIENNPS

>AmOr153PSE

MVKEMIPEKTIHITWLSVALCWPLSVNSGKTQVFIFKMLQIISIVSACMLLLLSSYSIYFXHGQCRIFKN
YHRFIDVAQNIIQTVICFYIIKEMKICIKETQEYEIEIFQKYIAKFKTVWGCNITCMYLTALAFTIGSVF
ISTSLSCDAEYPFQLNYTLVFAIRYQSFLSYQCAYACADHXLLWFTAPRFECLCVELQNVTNINMLIVCX
YAKKMINWFRFIIFNAIGLSILVFTLASIILIMISICMYIVVCSCMYNFINKNYYMYIWPADYMTDKSIN
VSRKIYDSMXYKQMLKMQKNLLKXLIFQRPVXIYRLZLLSKLILRYYCLYLSNVFSIFTALHVLEDNI

>AmOr154

MLKKVTPENVIYIIRLSVAICCCWPRPFNSTKNQIFAFKVLQISTIISAFMVFLPLLYSIYLHNDNIIHV
FKCICLSIGITQLIVQTLICFIKHNSLQRVVEEMVNCVKQAQQSEIEIFYKYIEKCKIFYGSSIAFSYLA
ATAFMLGPAILPISFPLEAEYPFHVNESLITIIIYMHQSLVSYQCSANVCVSIFGALLLWFTVARFECLI
EEFQKCSNIDMMIACIKKQLQLKRYAEEIINCFRYIVLYGIAVTTFALILCGIILLMNIPLIVKIQFVII
CITIMTEVYMYAWPADYVKNMSINISRSVYELSWYEQTIEMQKNFLNVLVYQKPVIFSISCIVPELSLRY
YCSYLSNVFSIFTTLRVLLEDTSA

>AmOr155JOI

MLKKATPEKIIDIIRFSVAICFCWPYPLNSSRNQIFGFKVLQISTMVSACIMLLPLLYSIYLHNDDVIHI
SKCICISIGVTQLIVQTLVCFIKHNSLQRVVGEMMKCVKEAQQNEIEIFSKYIEKCKIFYGSSIIFSYLT
STAFMLGPIILPISFPFDAEYPFHVNHSLVTIIIYIHQSLVGYQCSANVCASVFGALLLWFTVARFECLI
VEFQKCTDIDMVIACVKKQVQLRSYAKEVIKCFRYIVLYYITITTFALIISCIILLMNVPLIVKMQFIII
CVTIMTEIYIYAWPADYVKNMSINISKSVYELSWYEQTLEMRKYLLNVLIYQKPITFSISCIVPELTLRY
YCSYLSNAFSIFTTLRILLEDNST

>AmOr156

MIEQVMLKRVIYITWLSVALCFCWPVSANTSRNQIIVFRFFQIFTIISSCLGSLPMFHSIYLHQDDIVIV
AKSISIMVVLIQLIVQTTICAIKHDTLQHIIEEMITYMKEAKQYEKKIIQKYVSKCYILYGSAIIISYLT
TTIFILGPIFLPISLPFYTEFPLSLNNTAVYIIIYFHQCFFAYQCSATVCLSIFGALLLWFVVIKFECLI
MKIQNISNKDMMVICIKKQLQIRRYAKEIANCFRHIIFYTIIATSFNMILAGIILIMNPLLVIKIQFMIT
CFTALIEVYLYAWPAQYMDDMSKNVSISAYNLKWYEQTSEMQQNILIMLIFQKPISLSINFLMPKLSLRS
YCAYLSNAFSIFTALRVILKDNSI

>AmOr158FIX

MLKQITPEKSIYIIWLSVALSFCWPLHINSTRKQIMYIKILQISAVVNAFMVLLPLIYTIHLNMHNLINL
FQCICLLICIFKHIIQTVICFIKYNALQRVVEEMMICVKEEQLYDILCMYVKKCNIFYGGTIVLIYGTAT
VFVLGPIFLPISFPWGTEYPFQVNYTTINVIIYAHQFFLVYQCAAHTCLSLFGALLLWFATARFECLIKE
LQKITSIDMLIVCLKKLLFLRRYAEEVVSSIRFLVFYAITISTFTLTLSGIIMIINCPLFVKMEFITISI
SLLVQIYIYAWPADYMQDMSINVLRSAYNSIWYEQTLDMQKTLLIMMAYQKPVTFSINVLLPELTLRYCC
SYVSNALSIFTALRAVVEVT

>AmOr159NP

LTMLMILYPMVYNVILPLNESRKFIILCDIYYFVDPQEYSSIFLLYTLVLIFILYTIIGTQFTIIIFVQH
CCGFFQNCXYRLEHAMDTYKGQNEIEIYYIICTKLIKAIEVYKLAVKFFKWANGIYKIPYTITIFLYVLD

VSVQLYYVVYMLQQLENIYKLCINLILLIRKFCFLFLITYLGQNIENHSNEVFEKCYDSLWYTAPVATRK
LLLIIMINIMKPCQCKMFGGLFKGNIEGFAQIIRICISYFMSLYSTQ

>AmOr160

MRRPISSYVELFYDKNVISWSKRLLGLSGLWPDNRNDVRFFLYITYVVIFTWLEIVTLVQNIHDLEKTLK
NITLSFPTILIVLKAVMFRMNMHLVLPLLTVVKRDVNEGLYRSAEERRTVVWYNVAATLFSTSSALSLFF
VPTLFYAKPIIGCLLSKYNNCTLPFELPMKVNNVYEITKLQTYALFCVYLIPTSTLLTIGATGADSLLVT
LTFHLCSQLSIVAYRMRNVNIEPKIYFPKMKALVERHTELLRLANILANTFSSLMFVQTLGLIFSLCIVV
YQLLMTSESGEDMNTIHFIIYSCAVILLAFCYCFLGECLINESSEVQMACYFTNWYDLPEQYTRSLIFCI
ARAQKPLYLTAGKFYVFSLETFAVIVKASMAYLSVLKSII

>AmOr162FIX

MDGIQHEYLRVNKYLLFVVSAWPYQAILQRSLIGIIVIPIVTAQLILQFGGMMTAIIAGDIESFLESFAP
LAISLMCFVKYINFLYNFNQMKRLMDIMQEDWKFHARLRNEYEILCEHYAIARKITTSFVAFLLGLTTPF
GAMPLLLNIGDALGLCNISDDRPLAFRVEYFVDVDKYYYLLLVHSSIGTLGYTVIVLAINSIIIVYVLHE
CGLCEILRVKLENFVETDAMDIELRPPNKKDKWYQNARDCVLLHKRIIEFAKILEDTNTTSYLLQLGFNM
ICISFTQFQAIINIEDTPKVLRYVSITIALLCDLLFVSWTGQQLSNSTERIFEYTTNGKWYQSSISCRKL
LAIMLSKSIAPLRLTACKLYTLNLESFTTIAKTSVSYTMVLCSLQ

>AmOr164FIX

MAIKSIINRPVEISLRLIGAWPNSSCQILKYIMWTIVMSIFLIFQYSYCIIHIKTATLIDILDCLSITCS
NTLLLLKFIIIWFHKRVLFESLIIIAEDWDNCKFEWNMEIMMQKAILSYRIAKLMLIIFICSIFMYAVST
FFGPDIGASHSDQKKFLLKMEFPFEATVSPLYEIIITIQLVMQFMFATMAGMFMTIIATFVLHIASQLDI
ICDRLSEILDEHKEQELRIRIIKKLIAKHQRTLNLSENIENIFTFISLSQFFFNILVICFVNFILVTSIG
TEQAPTVISKCFPYYIALNFEALILCYTGEYLSSKSENISWIAYNSNWYELSIYEIRVLLLLIMRSQKPL
TLTIGKYMKLSLETFANMLKISASYASVLYALE

>AmOr166JOI

MTSINTISRSVKYGLYFAASWPGASFSILHKFFWTIIFCTLHISQYSYLIMHYKYDALTEIIDNISICLP
HSLVCIKLFTAWTQNTLIRNILLSMEEECQKYAIMDTDNLISKTAYLSYRLTSTIICTCVASTVCYAIGI
FSHQEVNVTSSRELLLKMNLPFDTNKSPIYEFVVIIQYFYQVSAAFVFGVFAAFLLMIVLHVGCQIDIMC
QTLMKTTHRDQKKLKFFIKRHQEIILLAEKIEKFFTYIALSQLISNTLITCCLGYLIVITLHLGNNIILI
KYIMFYVAVCSEAFIYCFAGEYLSIKSKLIADTAYEFLWYNMNPNESRLLIPIILRAQRGFTFTFGKFAT
LSMESFTAIMKASGSYMSVLLAMT

>AmOr167JOI

MIPIRSISHPIVIGLRLIGIWPKSSYEIIVRFMWVIIMMCAQIFQYQYIINHIGFDNLADLIDSVSTTLP
YSLLCFKLISFWTKREIFENILIGMYHDWTNAFATDFIVEDMIKKTELAYYCSNLILSIYAIAVFLYVGV
FLELSHDHDQENRSNLSPELLIKMDLPFTYDESPIYEYVFIVQFIQLFFIASSIAVLDALIITLIFHIGG
QIEILHKTLKNISINDEKPESSRIIIKSLIDRHYRIIIGSEYIESLFSYIALMQLICNTLIICCIGFLIV
VALNSNLKLLIRISFFYIAITLEAFIFSIAGEYLSNKSLSVSISAYESPWYLLSPKNRGVMILLMVRSQR
RLTITAGKFMDLSMQGFANVLKASVSYVSILYAMY

>AmOr169

MKMNFQNLNRLNTFVNAVSGNILPITDMKKRLSIVLKIYSILVWTIELSYLAACILGLFNVSRERALKDS
TVNIVISLEVFVLIVYLHNRENLLRELIGKLNCLLIVDDETLRDVTIGTVKPLEKPLRVYIIASVGSLMI
WASLPLAKIFRKSEFYYTDYQVPAVISNEPFPIGVFIGGVALQIFGSAYTLLRKVSLDLYTMHLILLITA
QYKYLRIKFAAILEQETPKDFFYGGIIWQNVPCEYDKMVKQEMKLLTRHFEIVVEMTVMLKKLLSPNIGI
LYINYVFRFCFLSFMLATSSGMHFEKCLLVSYTIGALIQFYILCYCIQQLLEASTTVADDVVHEKWYLHD
VKFQHIILMITLANKLKCKLSSFRNIDLTLPSFMSILNQAYSVCLLFLKARQS

>AmOr172JOI

MDFEGSQYYNINRILMISVGLWPYERTIYSKMLNILIFFFLSTAIFIQVMSFIMLKMDINLTLNSLSYTS
CTCICIMKYFNCLFHIKDIKNFLDEIKNDWNSLRNIEELRIIHEYSKTIKKITICFVIIIVPLQLVFFLN
VFGNNILDILIPLNHTRPRTVPIEIYYFIDQQKFFFFFGMHLNIITSFGGLVYIAIETISMGMIQHLCGL
LKITSFRISHTFVANIPKISSTERSIIIRKKVMSIVYLHVKIKKYINWIQEKIIITYDLLILLGLIAFSI
FIFELAKSITSRTNTNEIFSSLIFVICITIYGFIPNHFAQEIINHSSNIFIDTYNTEWYKLPIIEQKLLL
FIMQNNLKSLNFVLLGTMIASYNVYAMILRTAFSYFMVIYSMD

>AmOr173JIP

MDFEGSQYYNINRILMISVGLWPYERTIYSKMLNILIFFFLXTAIFIQIMSFIMLKMDINLTLNSLSYIS
CTCICIMKYFNCLFHIKDIKNFLDEIKNDWNSLRNIEELRIIHEYSKTIKKITICFVIIIVPLQLVFFLN
VFGNNILDILIPLNHTRPRTVPIEIYYFIDQQKFFFFFGMHLNIITSFGGLVYIAIETIYMGMIQHLCGL
LKYINWIQEKIIITYDLLILLGLIAFSIFIFELAKSITSRTNTNEIFSSLIFVICITIYGFIPNHFAQEI
INHSSNILIDTYNTEWYKLPIIEQKLLLFIMQNNLKNLNFVLLGTMIASYNVYVMILRTAFSYFMVIYSM
D

>AmOr175JOI

MVELLSYYDFTKFLLTTLGLWPYYNAKYRILYNSIISFTSFSFIVIQCIVFKNSFKNKKMLKYLSMLIMS
LGSFVKYNINWYQLATVKKFMDKIKYDWKIIEMNNETFEIMKKRTCLGKHYVIRFASIFYSSIIIIVFCH
ISTCILDKILPLNETRLKKFPILTEYSILDEKYFSLTVIYLYISLLLIITIFVATESLMILWLQHAASLC
EITRFFVQSATLKSSKDHFDNNIKKYIIKAVITHRRVIQFIQDTRDYNAITYCILLIFAIISLGINLFCL
SQSILVLTKIEETIVSFMCIICELSYMYYMNYMSQQVLDCSNNIIITIFHTNWYKMPVPIQKLILNIMLN
CNKPFLFTFFSIYYPTVEGFASLLKMSLSYFTMLLSMQ

>AmOr176

MLDNYYYRYVRFYSKLVGLWPFDNSKQQTTRKWFIQAMFVLIIVYQVRIMLLCRNNINDLLGCVIIITIN
IIVSIKYYTCIYNYDVMKILFINIERDWAELKCIDDMNILKKYSRNSYLYERYLIWTSWIVYSSVIIVNI
TMDYSAYFINSNVLNEIDYYIFTIFVDIVIIFSLIILMSTDSIYITIMYHVCAVFDITRFKHLFDDIRKM
NVAYRKYLLQNQIVDIVRHHWKCIEYINLLDTYYSISIVLQYVIITIIFTLKFYKVYRCIKTNHIRKLIT
IFIDIVQNYILVFLYSYPATKLTKHSELLLNSAYNACWYETPVYIQKMFKFVLQNCLKSYHLGIPQIFFI
TFESISKMVQFSFSCLILLISLEE

>AmOr177

MDFYEANVVKSYRLYLLLVGVWPYEYSKANEMQRIVYVTCVISFLLVQIFTLFTDKIDHALLYQIITNVL
IVSLILIKYVNIWIKIEEFKEFFDYIRIDLSKLTETEKILIKRTYKKCSYYCQIIFLFCYFVAFVLMIIQ
VFQIVDYETTSRTNRTNYFDRIILTEYFIDREKYSYPIYIHLNVAILLVCSMEIGTDVLHAIIITHIIGI
FDKIGYLTIHLFDEPEITCQKIDKHLIYCRRLVYIVNLYKRNLKFCKKYNSTMVICGAIQLIWGTLIISA
LLLKLRNELLPHNLRIMFIYCFFVSLYLFIIYMLILPYQKVYEISETLFFKAYCGHWYSTSAKTQKLLLF
LMHRFMTPYKFRFKNVVIVVDQTYLSMIRIGFSYFMFVHSIQE

>AfOr1

MGEEIGNTTNYRNIHYKSDAEYTVHVAKTLLTLIGIWPRRNTFVDNVKFYVQIGIVFFLMCFLLLPHVIY
TYFDCENLTKYMKVIAAQIFSLLAIIKFWTIIINREEIRFCLMEMEIQYRDVECEEDRSVMMNTAKIGRF
FTIVYLSLSYTGALPYHIILPLISERIVKEDNTTRIPLPYLSDYVFFVIEDSPIYEMTFVLQIFISSIIL
STNCGTYSLIASITMHCCGLFEVTNRKIETLCKWDNRNLHDRVIDIVQSHLKAIEYSARVGESLSIVFLS
EMLGCTVIICFLEFGVIMELEDHKTLSTVTYFVLMTSIFVNVFIISFIGDRLKQESERIRETSYFIPWYD
FPTEVAKNIKIIILRASRPSSLSGAKILDLSLQAFCDVCKTSAAYFNFLRAMTV

>AfOr6/7

METKYVEKDLKQAFYAQPFLKIIGAWPNAIKSPLSSKIQKWFIISFFLCLQICIVTPCIFEALLKEKNAK
RKIHLFMLATNTVNQVFKYVITLNRSSELKIAIREMKNDWLNATPENRYIFITNSKIGQRIMLIIVVTMY
ASGLGYRMVLPLLKGKIILPNNVTIRLLPCPTYFTFFNELVSPYYEMIFILQLLAGCFIYTVLSGTIGIS
LMLSLHMCSLLKILSKKLVDLADGSITNENIMQEKFVDIVEYQTKIKRFLNNTELITQYFCFYDISFNTC
LICFVGYCIILELENHNIVAIVVHFMLLGTCIFVTYIVCYIGQLLLDESNNLAKTCITLNWYHFPARKAR
FLILMIIMSNYPVKLTAAKVVDVSLTTFTDIMKAAMGYLNMLREVT

>AfOr10

MVQIRNAKEGLKHTFWLAYPFSRMLGHWPLSVSSSAFSKILNSFIIFISYLLQLIVLIPSLLYVFLKEKN
PRKKIKLLMPHVNIIAQMIKYTILLRRMKQIGKLLDEIRKDWSIATEENRQIFSATASIEHKMTSIIAVT
VYGGGFLYRTILPFSKGKLVLSNNVTIRLLPCPGYFGSLNEQVTPNYEIIFTLQVLAGFIIYTAVCSTKS
ICLMLCLHMCGLLRILTNKVMELTNDNDERVVQEKIVHIVEYQMRIKEFLNQLDQFIPAICLIEVFIQVL
IMCIIGYCIIMEWQENNGMGIITYVLVQMTCLIGAFSVCYVGQLLFDESENVRQAYNTLKWYELPIRKSR
SLILLIIMSNYPIKITAGKIIDLSLVTFTNIIKTAASYMNMLQQIT

>AfOr17

MKNISGIAKAEEDLKYATRFVKPIMGMIGAWPISPSTSFFSKVLQRSKHIFTYFLFFLIMIPTLMYVFLK
EKNNKMRLKLMPPIINCSIQFFKYTIILWRRKEIQEGLYAIKHDWIKATEEERLIFRSKTKIGRRVVLIV
AFTMYGGGLCYRMILPLLKGTIVTANNTTIRALPCPSYFLILNEQQSPIYEILFVLQIIAGIAIYAVICG
FCGIFALLVLHAWSMLRILVNKIKKLVDKSDMSEVVLQRKIMDIVEYQMKIRGEFLKNIETITEYICLIE
MIGSTCMICLVGYCILMEWENTNTMAIVIYITIQISIIFCVFILCYIGQMLVDENYIVSQASSTVNWYRL
SIKNMRCLILIIAMSNHPMKLKAAKMMEMSLITFTDIMKVSMGYLNILREII

>AfOr20

MQKSKDKANTANQKFYSTDYEYQKNVNLSIEYNRWLLKPMGVWPNSYTLRSKDYLYWLINIVCYSLISFL
FIPCTLYLFLEIEDFYGKLKQFGPLIFCMMAFVKYYYLIAHKTDIRECVERIKWDWKNITYAKDKEIMIT
YANFGRKLVVVCTFFMYSGFAFYYIAIPISVGRVKTDNLTFIPLVFPFSRFIVDTRYSPTNEIVFSIQLM
AGALMHGITSAACSLVATFAVHACGQMQVLMNWLQHLIDGRLDMDERLDGRIADVICQHVRVLKFLALTE
KTLQQISFTEFLGCTLDICLVGYYVIMESKSNDVTSVITYVILLISLTFNIFIFCYIGEIVAEECRKIGE
ISYMIEWYRLMGNKKLFCILIIAMSNSSIKLTAGNIVNLSISTFTDVVKTAVTYLNVLQKTT

>AfOr46

MSDDLVEIEKKFGSLNEYSIQFNRWILKPIGAWPTSLCTTRNEKIISKILIIILCWSFSLFTLIPGLLHFL
LEKEDIYLKLKTVGPLSHYCVGGFNYAILLLRENDILHCIEHIRVDWSIITKKQDQQVMLKYAKIGRYIA
AFCTAFLQGGVLCTCLALGAFKTTIKNGNETIEIYSLLCPAYKFPVQTNPTHDIILGTQLLSAFINSSSA
AGAFSLAAVFASHALGQLNIMVAWINEFVNRPMESNNDAYVNKISIIVEHHLRVLSFITHIEHLMNPICF
MEMFKCMVGMCMPSYYILAEWSEHNIQNLAVYVMIIISMTCNIFLINYIGEILTEQCKKIGEIVYMTNWY
ELSNKDIFNLMMIISRSSVSVNMSAGKLINMSVLTFGNIVKSVFVYLNMLRQMTMI

>AfOr56

MHFSIRNPINEPRNPNYEKDIAYVTKYNKWVLSCIGIWPIVLKNTNKILPKIVIGINNLLCSFILIQSAL
HIIYEEKDVLLRLKILGLIFFSLISLMKYWALTIHKPEIKYCIEQVQLDWKQVEMENDRELMLKYGMLGR
NLTIYSILFMYIGSITYMSITQYAMGLQINEHQSNQTIRVLIYPTYGYNIQKSPIYEIIYGIQFMCGYVV
DTITSGACGLAALFVTHACGQIDIITSRLDDIVAGQFYNKNLSPNIRLMAIIKHHIRILKFSAVVETVLQ
EVFFLEFAGSTFVICLLEYYCIADWEQKNIISLTSYILLLISLTFNMFLLCYIGDLLIQKSGNIGVVVFM
IDWFHLPTKTIQNLILIMAMSNNPAKLTVGRIVDLSLSTFGNVLKTTFVYLNFLQTAVMQ

>AfOr64_2

MKTTSNKDFAYAMTPLKFLSWPLGTWPLQVFDTFSIIRAMFTTFLVLLMLAILQVELYLDRSNAENNLDA
LVLINGGILAVAKVMCFHVRPVGLISNFTSAVKDYNELNSEENRVIMRRHAYMGRVACASLIFCSYVGST

LFMTVPMLAGDEEEVINVTEESAIKYPMPSENTLTLINIPENMYFVIFIVEYLMLLLTSTGNLGSDSLFF
GIAFHLCGQVEILRLEYNKLSNENERTTKHITLLTKRHIYLLLKLSDMLNETISSILVVQLFSSCVLICTT
GFEFILALSIGNIVMTIKTFIVMCVLLIQLYLYAYAGDALESRTEEIAQAAFHSFWYQSRGRTARDLILI
ICRGNSSYHVTAGKFVFMNIFTFKEILKSSTSYLSVLRVMMDT

>AfOr65

MKTTSNKDFAYAMTPLKFLSWPVGTWPFQVHDTFSLTRTIFSVSLLLLMIAIVQVELYLDRSNAENNLDA
LLLINCGILAVAKVMSFRVRSIGLVSNFISAVKDYNESNDEENRVIMRRHAYMGRVACASLICCSYICST
LFITVPMFAGDEEEVINVTEESAIKYPMPSENTLRLINMPENMYFVIFIVEYSMLLLTSIGNLGSDSVFF
GIVFHLCGQVEILKLEYSKLFNKNEKITEHFVLLIKRHIYLLLNLSKMLNETIGTILLIQLFSSCVLICTT
GEYSFQFILALAIGNIVLTTKILLIMIVLLIQLFAYSYVGEYLKTQTESVGNSVYFCTWYYMPKNISKDI
IFIIMKAQRPALLTAGRIFVVNMETYLSILKTSMSYLSVLRVMVNS

>AfOr69

MQLLRTIYHLLTSCACWRPPFLSPLKNLAYTVYYCYVILLIYGATFCTFVDLLLIVETEDEFCDNFYLTL
AIFISCHKMYSMLVNRENIILITSMLESKPFQPETEEEMNMRNKCDKQARLNAICYAILVELSVMSLSFG
GLLKAESHKLPYRMWLPYNYTSLSAYTFIYTQQVVSLIVSAMIHVACDSFIWALLMHICNQIEIFNCRLK
KIKDHEKNEVTKLCIHYHNLIYRLATTINEQFKMVIFVQFTVSTLTICVNLYILMGTQITFERIMQLAIY
SSCMLTQIYIFCWYGNEVKLKSLDISDMIFKLDWPDLDNTTKHDLLMIMMRASYPIEMTSVHVITMNLDS
FVILLKTSYSAYNLLQSNRE

>AfOr70

MVVQTLQWTRFLLSVCGCVPPTSWKSSFKKSLYNIYTLVIWLLILSLVSTQILDIIINVKNKNEFIENFY
ITLVVFVTSCKMTIILRYRKNILSLIDDLQHEPFSPITHEENEIRTKFNKMNERTSICYTILVLVSATWI
FVRSFFTDFKKRKLTFRAWLPYDYSELLPFTLSYVHQATTSMFCSCQNISCDTLFAGFLVQIYCQFEILE
ERLKNVQQDESNYSAKQCVRHYHQIYKFSRTLNEKFKVILFLQFCAISFILCFNLYRMTTITMIPKLLEA
SLYLVRVLVQILYYCWFSNEVKLKSLEVPGMIFKSDWISWDDKTKKIFLIIMTRATQPFEFTSGYLVTLN
LEFFVALIKASYSVFNLLQRTK

>AfOr74/86like_1

MLYTVASHIYALYINITDIYHSWGDFNYCMFIISNLLIVILSLSKFIILSFRRREFVKIILYAQKNFWHY
KYENYEKLLFMKCQTFCKFWTIIAYFFVQSCIFFYVITPFYVNYGKNKSERTLPVRMWIDIPLSTTPYYE
LLFASQLIALQQIGLTYLSNDNYLCVLSMHLNYQFRILQHRMLTIWSNIKEQKDIVSCTEKCYKALKKCI
KQHQSLLKYCKKLDYVYTLTILNHVIVFSLIMCIAFYEILIADVPLTTRFIFVFSGIGILVHILFFTYIC
GELIEESGNVGLATYSGWWTTLPMNKTGKMLRNDMKIIMMKCMRPCYLSAGGFFPVSLETSTSLISSTVS
YFTLLRDNVEESNEMRPLISNLYIQRINAHKRHALTYQPLSGR

>AfOr75P

MRRPQDKDISIIWTSFLMKIVGLWLAADRNEQRCRDFALIYTVGTLFISVCIAFRDIYHSWGNFSDSVFI
CCNILYVAIVLLKISVLYTHKVEFFNLITFTQRNFWRFYNDPQEVLIIAGCKRICNFSIVLIVFCAQGTC
AGYMVTPLIENIGKNESDRALPFNLWIDFPVGLSPYFELLFIIQILCVYHVATCYICFDNLLCIVNLHVA
GQFRILQHRLKNLVADEGRGDTPRYENCCYEKLRDCVEQHQTLIEYCKRLENIFTVMVLGQVMFXAVVIC
LVGFQLFLAETPASKRASLVVNLGGTFFQLLIFTYSCDNLIRQSVNVGNAVFSGPWVNLPMNKAGALVRK
NLIIVIMRSQKVCCLTAGKFFPVSLETSTAVLSTAMSYFTLLKQSSLDADNI

>AfOr77

MGFLNTDDISMIMTATFMKLVGLWTAKDRHEQRARKFALIYTLATILFALWIEFTDFYYSFGDFSTCLFN
VCNIMYITMPLLKIFIIVSNKKDFFYLIFYTQNNFYKDNYNNKREQQIFTNCRRQCTIFVCFLTFSTKGT
LVCYIISPLVENIGKNESERVLPFNMWVNLPLSTSPYYEIVFTIQALSLYHVGIGYFCFDNLLCVFNLQL
AGQFQILQYKMANIADLIEEKNEKGIVSSPRFAKKCYAAFKNYVREHQALIAFCEKLEKVFSLIIILWQVL

MFSLIICLDGYQILLPESPVRRRLIFAFHLAACMCQLLMFTYSCDCIIQESSSIASAVYDGPWPFLFETT
SGRTMRKDLTLVILRSAVPCCLTARGFFVVSLETYTRVLSTAVSYFTLLRQSTLE

>AfOr80

MEAETQLDVSMSLSIFFLKNVGVWISNDPAEKRRMKILVLITLWNTMLGAVVIFRDIYFTFMYNGDILYV
ITNFLTVMISFVKIIIIMIYKKEFIDMILYMEKNFWNVKYDFQEEEILNNCRKTCIFFVTSVSTIGICAM
ISYAITPIIEYYTENLSERILPFHVWLNLPIISVTPYYEILFAAEVLSIYCVALCYFCFDNVFCILAVHL
AGQFNILKHKFAKLCDTDSKISKKDEESRLAIEHVQVLYERFKEYVRRHQSLINYCERLENVYTVIILGQ
VVISSVLICLFGYQILVNDVSIGRRSIFVFLLLGSMCLLFMFTYSCNSVIVHSENIATGAYSALWTTMPM
NKFGRMLRNDLILVIERSRRVCCLTANGFFPVSLETYTTILSTAVSYFTLLRNNLEE

>AfOr83

MHTKSELDISISLSTFFLKNVGVWMSDNSGEQRRMKMLFVYTIWMLFCGTIISTRDLYFTLLYNGDILYA
MTNTITTIMALIKICIILMYKGKFLNLIVYMQQNFWNVTYDYREKEILDDCRKICIFFITSVTTIGMCTV
ISYLTTPVITQSGSNESERMFPFNIWINLPLVTRTPYYEIIFFIQGVSLYYIGISYFCFDNIFCIMAVHL
AGQFRILRYRLTKLCDTENEIRKKDSQSTLAKQMYKFYEQFKKCVRYHQALIDYYQNLENVYTMITLGQV
LVFGVLICLFGYQVFVATSSTARRFIFVFLLSGSMFLLFMFTYSCNGVIEQSDNVAVGAYSALWTILPMD
KFGRMLRNDLIMVMKRSRRVCCLTANGFFPVSLETYTKILSTAVSYFTLLNNRVENASGS

>AfOr84

MRPTGSRDISIIWTSFLMKIVGLWLAADRCEQRRRDFALIYTVGALFIIVCIGFRDIYFTWGNFSDSVYI
SCNNLYLMIVVLKVSVLYAHKMEFFNLVTFTRKNFWRPYRDPEEKLILAECKRICTIFVVVISFCAQGTC
TGYMVTPIIANIGKNESNRELPFNLWVDFPVGLSPYFEILFTIQILCVYHVGVCYICFDNLLCIVNLHVA
GQFRILQHRLRSLSSAEPGDRRDARKLYAKLKSCVLHHQALTKYCKQLENIFTIIVLGQVLFLAVVICLV
GFQLFLMDTPASRKVSLVLNFAGTLCQLLMFTYSCDDLIQESVKVGNAIFSGPWASLPMDEVGRVLRKNL
VIVIMRSHRVCCLTAGKFFPVSLETSTAILSTAMSYFTLLKHSSLEKMENT

>AfOr87

MGEVSKEThtRVVGVARDSSSRGKDFALLMTAFLMKIVGLWLAESKEEQRRRHLTLMYTVVAILFGVWVQ
FRDFYYSWPNFGNCAYTACNILCLIMVLLKLFVLFVHRKEFIDLLVYTHEHFWHTNYTYNELLLVEKCKR
ICMLCITLINVCAQGTIVSYVLTPIVENIGRNHSDRVLPFNMWVDLPKLSVSPYYEILFVLQVLSLYHVG
VCYICFDNLLCLMNLHAATQFRILQHRLSSLGSSGWDTGGRAFDNRDTSRWSSCIENCYATFKLCVKQHQ
DRITYCQRLNDIFTIIVLGHILVFSLLMCLVGFQVLMANSPPTRRLIFVFHITGSLCQLLLFTYSCDSLI
QESTNVGSAVYSGPWICLPMNRTGRTLRRDLKMVIIRSRKPCCLTASRFFPVSLETCTTVLSTAMSYFTL
MRQSFAN

>AfOr90

MEKELDISVNLSSFFLKSIGLWIADDSTDERRRKGMLAYTVWCTFFSTVITSRDLYFTWIYNGDILYALT
NYTSVMMILLKICVIVVHKGEFINLIVHMQQYFWNVNYDSREKEILNSCKKTCAFFVSSVTFIGICAILS
YLTTPFTARIGNNESERILPFNMWLNLPLSQTPYYELSFIIQIVTLYCIGICYFCFDNVFCIMAIHLTGQ
FRILGYRFTKLCDTEICEKGAESGLAKRVHTCYEKFKEHVRYHQALINFYTKLENVYTMIILGQVILFSV
LICLFCYQVLLANAPSARRSIFIFLLIGAMSLLFMFTYSCDGVIEQSDNVAVEAYSALWTILPMDKFGRM
IRKDLIMVMERSRRVCCLTANGFFPVSLETYTTILSTAVSYFTLLRNNVENDKNDQ

>AfOr91_1

MGFLESDISVRLTSIFMKLVGLWMAANQYEQRMRNAMITYNVVAILFALWIQTMDIYYSWGNLSACIFST
SNTLSLILPLLKIFILLSHKEEFFRLIVYMQRNFLRGNYDDYEREVVFGCKRKCTFFICFFTFFTMATIV
SYIASPIVENIGKNESDRVLPFNMWVNLPLSMTPYFEITFTLQTLSLYQIGVSYFCFDNFLCIMNLHVAG
QFKVLQYRISNIADLIIKTEEKREKLIVDSSYFSSKCYTTFKKCIRQHQTLITYCRKLEAVFNLIVLEQV

LMFSLLICLDGYQILMADGDIKTRLIFSLHVLACLCQLLMFSYSCDCIIRESVNVATAAYGGPWTLLPMT
TNGKMMRKDLIVVIMRSSIPCCLTGKFFIVSLETYTSVLSTAASYFTLLRNNIGSADET

>AfOr91_2

MSFLESDISVNLTSIFMKIIGLWMATNRHEKRIRNITIIYSLIAILFALWIQSMNIYYSWGDFSACLFST
SNILSLTMPLLKIFILLTHKKDFFHLISYMQRNFLHGNYDDYEREVVFGCKRKCTFFICFFTFLTMATVA
SYIFSPLIENIGKNESSRMLPFNMWVNLPLNVTPYFEITYTLQVLSLALSLYHIGVSYFCFDNFLCIMNL
HVAGQFQVLQHRISNIIDSMNKEKEEKREKLTVDSSYLASKCYAIFKKCIRQHQALIAYCRKLEQVFNLI
VLEQVLMFSLLICLDGYQILLANGDVKTRLIFSFHILGCLCQLLMFSYSCDCIIRKSVNVATAAYGGPWP
LLSMTTSGRMIRKDLILVIMRSSVPCCLTGKGFFMVSLETYTSVLSTAASYFTLLKQRAEVIS

>AfOr94

MSLKHRRDVSFSLATFFLRVVGFWLTSSPLEEWLGNVTVMYSIITIVFSMWVQTRGLYFSWGDFGVCTFI
ACNSLGLIMDLLKVFVVFIHKKKFLGLIAYMQKNFWHFDYDQYENSLIADAKQMCVYFVCVFSFFSQSTV
FSYMFMPMISNIGKNESDRMLIFNMWLDLPLSMSPYFEITYVIQALCLYQVGICYLCVDNMFCIMCLHLA
SQFRILQYRLVNLSSVENKEEVDNEENMDSSGRCYAILKNCIRQHQALIQFSITLEEIFTIITLGQVLIF
STLICFVGYQVLLVNLTFSWRISFLCFLITNMCQLWMFTYSCDCMTRESVNVASAVYSIPWTRIPMDKFG
KMIRKDLQFVVVRSRRACSLTGCGFFAISLETYTKIMSTAMSYFTILKQRTIEIENT

>AfOr95

MEKDDFSINLSSIFIKLMGIWMANGRSEKCVRNVTIVYSIIALLFGLWLQITDMYYSWGDFSECIFSMCN
MLSIAAPLLKLITLIVHRKDFFYLILYLQRKFLHGDYDDYERNIVLNCKRKCTFFTCSLTFTTLATVVSY
IINPLVANIGRNESDRALPFNIWIDLPLTITPYYEITFVLEVISLYHIGVSYFCFDNFLCIMNLHVAGQF
QVLQHRISNITDLIIEKEEKREKLIMDSSYFASKCYAIFKKCIRQHQALIAYCRKLEEVFNLIVLEQVLM
FSLLICLDGYLVLMADTSTTTRLIFGLHITVCLCQLLMFTYSCDCIIRESSSIATAANRGPWPMIPMTTS
GRMMKKDLILVIMRSGTPCCLTGRGFFVVSLETYTSVLSTAASYFTLLKQHSEAHS

>AfOr96

MNLKYRKDLAFTVASFYLRVVGFWLTTSRLEECFRIGVVGYTILAITFSAWVQIRGLYFNWGDFSACTYI
ACDGLGLVMDFFKIFSLFIHQKKFLGLMAYMQKNFWHYDYDEEENLIVKDTKKTTAYFVCILTFSSLSSI
FMYMFRPLLTNIGRNETDRILIFNMYLDLPLSISPYYEIAYTIQIAALNQAGSCYFCFDNIFCILCLNVA
CQFRILQYRIANAPILKKKENLDSSKYPSDECYNAFKNYIQQHQALLHFCETLEQIFTIIVVGQILMFSI
LFCFLGYQVILADLTTSYRVSFISYLFAGMCQLWMFTYSCDCITQESAEIASAAYTSPWIDLPMDKFGKM
LRKDLQIVVMRSRRACCLTACGFFPISLETYTKIMSTTMSYFTLLKQRTVDAVDT

>AfOr97P

MTYDGKQKYGKNGKYKSTVFDISYYKTFKKYLKFLGQYPNQSRWNKEFNTNVMICSLISFLIPGLLQVYT
SIVEKNLNALMEIIPIVFATISCAIKLLNHRINKKNFDKLFDFMSEEWEMENDRNQTCILDEFTKQGNKF
AEIYKNVLLSALLLFLLLPLFPSFLDVVFPLNETRQQLQIFKMKYFIKEDQYFYPIYFHSVWSSFVIIMI
TVTIDSLYMLIIHHASGLFAICGYQIAKATECNDININENELFRQCVITHNKAYKFFEIMNKSSRNNYXL
SILLSIIGISITAVQIVMYLHKPEEAFRISLFLMAIQFHLFIITLTGQVIADQSSKLASNIYCTTWYRMP
PNIQKIFHIIQIKSSKPCKLTAGGILELNIENFGIALKTCMSYFTIFLSLQD

>AfOr100

MCIHLVFQIYGGQCLDIFEIPYYKSLKKWLTLSGLYPLRNTIIILVVISIISVTLPLIFAIYTSLCAKNI
DAMLECLPPLGVCVVAMFKLQNIYNNSENFKKLFTFVAKQWHQLKLNNEIRVLEEIVMQGNKMAQIYRNT
LLVAMVIFFSVPLIFPILDIVYPLNETRPRQQLYKVNYLIFNHEDYYFYVYFQLVWSSFVCVIIIIIFDW
LYILIIHHNSGMFTVCGYQIQKIFTEEAFSNIRIYEQFKNCLIVHNEAIQFFDILDESSRNTYFFLVGSN
IIGTSMSAVQIVLNLDKLEVAIKSTVFLIAAQFHLFILSIPGQILLNHYSNLTNNIFMSAWYNMPIEVQK
MFYVMQIRCNKPCSLTACGLYEMNMENFGTALKTCMSYITMILSLK

>AfOr105

MEQISIIPIFYVLQKNNEQEYNAFDIAYYKTLKLYLTICGINPYQNNNISIIIIIMIISVCMSFLCPTSI
QLWEAISNKDFDNIIQNIPQVITVIISMIKILNIYSNKMQFKKLFYSLAQDWKSLESKEELIMLDKFTQY
GSKLALLYRKSLLTFLVIFLSLPLCNPILDIILPLNETRPRQNIFNVNYIILDNYEYFYIVYVHLSCSAA
IIVIIIISVDSLYISIIYHACGLFAACGYQIQKLTKIHTIEKNGPNISSIEYEEFKQCVIMHYKCLQLYD
VLEKCCRNLYLIQMGLNIMIISVTCVEIVVFLDRPEEAIRAIIYVMAQQFHLYAISLPGEMLLNQSSKLA
DKIYDSEWYKIPMKVQKVLHIMQIRSNKPCILTAAGLYEMKIESFGITIKTCMSYFMMFLSLRE

>AfOr106

MDVQRLEERYLKINKIYSIIIGMWPNQNRKMIRCIFVEFIGIAAHLTQGSNIILFFSLSVAMDQVPFLVV
ATLLMIKYNFFIINEQKFKELFTSILNDWQKKKTHEEEMIMEKYADKSVFFILIYVVNAYFCSILFLLLP
FTPILLDIFIPLNESRPRVQMYPAYYYIENEADYYYPILIFSIVSLLTAMCVFIATDTMLIYIVQHACGL
LALAGHRFRNSLNDLYSSRKDSKVDGKIYRRLCYAIKTHKRALAYLAEIEDFYSVNIFMQVGASILCLTV
TMMKIATITWSMETNQYYGYVIAQVVHIFFLTAQGQFVIDSHENVYWDMYEPYWYNLQSKMQAMFVLILR
RNLNPPLLTAGGLVQLNLNTFAKVVKTSVSYFTVLKSV

>AfOr111

MINYRLLNMDKRIIEDQYLKINKFFGQLVGVWPYQQRFTKFCIRFITSVIVVLTLAAQISRVIMFYSIDV
LSDQLPYLDVGFVLLFKQYNYILNEDKLRELLNEIISDRLIKRSKEELEILEMYLKRAKVLSTVYEVSIF
FCGFMFLLIPSIPPILNIISPLNVSRGRELIYPSYYFVDEEKYYYPILMHMIAVALILTSVYVACDTYLV
YIVHHGCALLAISGYRFKHAVDDIKLCGEDYIDSLMDETYTKVRQSIKAHKTAVEYVDKIDACHIHYFLL
IIGMIVLAFTGTFVKLSSMEVNVRFFTFCAFTVGQLTHLFFLTIMGQFLINANDEIFKTVYEAYWYNGSS
RTQSLYILVLRKCLSPPKLTGGGLVALNLDSFLQILKASFSYYTVFRS

>AfOr112_1

MDARTVEKNFLKVNKIFGLITGVWPYQNYRMKMAERFLSVTVMMSGFVTQFAYLVLNPTIDKAATNLPYS
IASFGTFVKMGNYFLDETKLITILNHIFEDWAAIKTKEEYEIMYKYSRRGLFITISYFLHIGITETFMLM
WPMVPPILDIIMPLNVSRKRILLYPAYFWLDDEKYYVLLLGHMIITILMICFIFCACDMNYVYAVQHACG
LLAIAKYRFKNVCKDLNDKEDHAMPLEKIKYKSICESIKAHQHALKYLRLIENSYHTYLFVSMGLLIMAI
SVSLLEVANGKNGSRKLVQATFLFAQLFHTFILTVQGQFVINELQDVYESIYESPWYTFSPRIRSLYVLS
LRSCLNFPILTAGGLIVLNLQSFAEIIKAAVSYYTVMQTT

>AfOr116P

MFRKMINYLKKETKLKKINPNKHLQNSLSIIYYVGLWPDRVKYKYLYNLYAICSLIFLVGIIIVSEIIYI
IINWGKIELMMIGLTILMTNSTYAAKVIYIICQYKRIKNLVDITNSEIFNRDNDKYKHIISYYNWQGIFH
YIAYQGFASISIFFYSCIPLQSVFSEKSKQLPLAGWYPYNVTSTPIFEITWLHQVLVILINCINNIAIDT
LITGFIIITCCQLTILKKCIARNNNVNIEKSLSKIYNKFYESLKYCVEHSIIIFDFTKQIQDIFGIIIFF
QLFVNCIIVCLAAFNVSQIKNYITPEFFGSLLYMCCMIYQIFIYCWHGNELYLHSMKICLSAYKNNWWNN
NKNFNYALLIIMIRTQIPLIIIVGKVXMELSLQNFLLILRTSYSIFTLLKTFT

>AfOr117

MKKPFNKSIDYYILPNKIFCSIAGMWPIDEKSSMFSKIFAYVRLIFGLIVVSSFFIPEIIIIAMNWKNIK
IIAGIGCVLTTITQVLFKMLYLIVRREKTYSLYYKIRSLWNSSNDSKERPYEEFAYWARIFSIIFYSSCM
CNVFTFSIAAAIDYFKFEYNANNTENNRHLPFIVCIITRYGTDISASPNFEIAFFYQIISASICAAVISG
LDTSLMTIILHISGQFKLINIWINNIGIEINCNPNYIRKLKIDLIKCIRHHQQIIHVVNNVNNLFTPIIF
IQLLTSGIEICLSGYAVLDSDSANADLLKFISYFISMGIQLLLWCWPGEILIQESQEIGHVIYLNIPWYN
LPPIYQKYFCLMIVRTQQYCRITALTFQTLSICTLTSVFNTSISYFTLLRQIQQ

>AfOr121

MVTSESKKYSKDYEWAVRLNRFSLNLICLWPVEEQNIRKQSWTKLHIIACFMLITFVCTIPCLCALKQCN
TLMEVTDNLAYSIPLIITTIKFIVISSKKKVLSPIVNMIAKDWLKLKTDYEKDVMIQRARIARIINIFGY
ILMCILIWLLMILPRFGITIRYVMNGTDAKKLFPLPTYYIFDVSETPYFEITYTLQSISLLIAAFCYAGV
DNFFGILILHICGQLTNLRFQLANMKESETSNFVLIAIVKDHIRLIRAANVIENTSTLLFLILLLNFGIC
TCIYGFLIITIYIEEEQFSLLRIIYLICNFTNTFLQTFLYFMAGQMLVTQSEEIHNAAYECEWVSLKYTK
AKSLIIIMARSKKPLYLTAGKLFPVTMLTFCNILKISLSYISFLLTIL

>AfOr122like_1

MDVFDKQYRIYRMVLKIIGLWPYDNSIYVWIQRICLLLYFLSGVIFQIIVLLKSEITLRNYILTLSETFP
LLLFFLRYIYYITIIPYVEFLFNNIRTEEHSLQDMTEIQIQTKYLDISNHIIYFFCCVTCAFIAASIVFL
LNPIILDFLMPLNESRAHFNVSFLFDDQSMYIKIFLILNCTLNVSFGLLCIMATELCTNIFSYYICRQFH
IASYRIRKIIEDLSMPLSKQIDLKLIDIHRVVDIHNDVIKYIKLATNNAATQYLLAIIVCILSFSINIYR
LYNAITTMDDRIEIFGSAFIVMYHLMIAFYNNHYGQLIIDSSLDIFNELYASTWYRIPLKAQKLLLFMIL
RSSMGCELGLSGLFTPSYAGFTSMMSSSFSYCAVIYSIQ

>AfOr122like_3

MNVFDNHYHTYRTVLKIVGLWPYDNSIYVWIQRICLLIFFLIIILFQIIVLVKSEISLKNFIVTLSMTCP
VLLYFLRYLHFITIFSFVELLFDKLLTEEHLLQDSIEIQIQTKYLDISSHIIDIFCWMSFFGIAAVIIFL
LNPVTLDIIMPLNESRTHFNVLFLFDDQSPCIKIFLILNFMLIMLFGLLSLIGTESLTNIFSYYICRKFY
IASYRIRKIIEDLGIITMPKQIDLKLTDIHRVVDIHNQAIELINMGTNATATQYLTAVIICILSFSVNLY
RLYNAITTMDDRIEIFGSAFIVVYHLMVAFYNNHYGQLIIDSSLGIFNELHSSTWYRIPLKAQKLLLFMI
VRSSMGCELCLSGLFTPSYAGFMSMISSSFSYCAVIYSLQ

>AfOr122/130like_1

MDVFHKQYRTYRILSKIVGLWPYDDSIYRWIQRLLLLMLFLCGIIFQIMAFVKSKILTLRSCILIFSTIC
PCVIFFLRYICVLTMFPFLKNFFGHLRMEQNIIQQDLREAQIQIKCINDSSYMINIFILLNLMISLNESH
INYVIYITMFCDDRIVCIYMVYLTLILIIFFGLLSLTCTESLFNILSHHLCGLFNIMSYRIRKTIQELNI
YTSSSKQIELKLKDFHQIVDVHNQAIEFAYASASKMTMQYLLVIVVTTISFAINLQRLFKAIKSMDDPLE
TFTCIILVLYHLVFIFINNYNGQLVIDSSLNMFNEIYISTWYSIPLKAQKLLLFIMLRSSIECAFRLSNL
FTPCYVGFSTMINTSFQYFTMMYSIQ

>AfOr130_1P

MDVFDKQYRIFRIVLKIVGLWPYDNSFYVWIQRLWLINLLSSKYNISVMSLLRSEITLENSILILSTICP
FTLFLLRYVGFIVFFPTIKIVFDHIRTEESIVRDSIESQIRMKLIDDSCHIINIFLWMTYTTITFYSACL
FYPAILDLIIPLNESXYSSYFTTFSHDQTIYLDILCFDVTFITTFGLLSITCSESITGICTYYICMLLKI
LSYRIQKIVTYLATFKLSSEQIDSKLVELYRVVDIHNQAIDFINFTVSTAGMHYIIANLLVVISLAINLH
RLVNTILIKKNQLEMLFCFTLVAIHLVIIFVNNYTGQILMNSSQELFDELYNSMWYSMPLKAQKILLMII
LQSSTTYGFNILDLFIPCHSGFSTMLSSSFSYFTLIYSIQ

>AfOr130_2

MDVFDKQYRTYRILLKIVGLWPYDNSICIWIQRLCLLTYFLGSIIFQVLSLLKSKITLQNFILTSSMTCP
FVLFLLRYVSFMILFPTIKIVFNYIRVEECIVQDLIEAQIRIKFINDSCHLINIFFWMSYATIAFYSLYL
LYPVTLDLIIPLNESRIRLIYYFTTFSHDRIMYLDILCFNITFLIILGVLSIACTETLIGVCSYYICILL
KIISYRIQKIVTYLAMFKLSSKQIDSKLVELYRVVDIHNQAIELTNVAINASAIHYIIASLLAVISLAIN
LHRLVSVSIIKKDQLEMSFCFMLVTIHLMVMFLNNYNGQILIDNSQQLFDELYVSLWYSIPLKAQKILLL
IMLRSSTVCAFNLFGLFTPCYTGFSTMLSSSFSYFTLIYSIQ

>AfOr131like_2

MDVFDKHYRTYHIVLKIIGLWPYNNSVYAWIQRLLLLTLYLGNVIFQIVSLLRSEITLRNCILILSTTCP
LIIILLRYINFIMFFPMVKHLFHHIGMEENIVQDSIETEIRTKYINESCQKIDFYFRVIFSTITLFNISL

LYFITMNFIMPVNQYGILILRYITLFSVNRNIYFYILCLDVMFVVTFGLLSIICTESIIIVYSYHTGMLF
KIISHRIREIITYLTIFNLSSKQIDTKLTELYRVVDIHNQAIELINIMINNSGKQYMMSALLLVISMAVS
LHRLVNAITIKKDQLEILISLIIFANHLMIMFLCNHSGQILIDNSEEFFHELYISVWYFVPLKVQKILLL
VMIRSSTACMFHIFGVFIPCYAGFTAMLNTSFSYFTLIYSIQ

>AfOr131like_3

MDVFDKHYHTYRTVLKIVGLWPYNNSVYVWIQRLLLLTLYLGNVIFQIVSLLRSEITLRNCILILSTTCP
FIIVLLRYVSFIIFFPMLKYLFHHMSMEESIIRDSIERRIQTKYIADSCHMIEILLRVTYGTVISYGMFL
LYLITSDFIMPLNEFHTRVLHYFTLFSVNRTVYFYILCLDFLFVVTFGLLSIICTESIIGLCSYHVGILF
KIISHRIQKIIKYLTMFNLSSKQIDSKLIELYRVVDIHNQAIEFISIVINNLGKQFMIPSLLIVISMAVN
LHRLVSAIIANKDQLEILISLIFFTNQLVVVFLNNHNSQIFINNNEEFFHELYISVWYFVPLKVQKILLL
IMTRSSTTCMFHIFGVFFPCYAGFTSMLSTSFSYFTLMYSTQ

>AfOr132like_1

MNVFDSKHYRTYRTIMKIVGLWPYNNSIYVWIQRLLLLAFYLGNVVFQIVSLLTSEITLQNCILILSTTC
PLVIVSLRYVSLILFFPTIKLLFRHMRMEEAMVQDSIEAQILGKYIDDCSCMIDIFFWMIYEIIAFCAIL
LFYPITLNFIIPFESLRYVILFSINQTIFIDILRLNYTFVMIFAILSITCTESTIGLYSYHISMLFKIIS
HRIQKIVTYLATFDLSSKQIDSKLTELYRVVDIHNQAIQFIDIMTNNSGKQFTMSALLVVISIAINLHRL
INAITIRNNQLESLISLIFFVNQLTIIFFCNHICQIVINNSEEFFKELYISVWYFVPLRVQKILLLIMTR
SSTTCMFHILGIFFPCYAGFSKMLSTSFSYFTVMYSMQ

>AfOr134like_1

MDVFDTHYHSYRTVLKIIGLWPYNNSIYVWIQRLLLFTFFLGNVIFQIVSLLRSKITLRNCTLILSTTCP
LIIISLRYVSFIIFFPMIKHLFNHIRMEESIVQDSIETEIRAKYVHDFCHMIEIFLRVTCATFIFYSVLL
LYVMILDFVMPFNESRIYILDYFTLFSINRTVYFYILCLNFFFVLIFGLLSVISTESILGLFSYHAGVLF
KIISYRIQRNITYLTTFNLSSKQIDTKLAELYRVVDIHNQAIQLINILINNSGKQFLMSTLLTVISIAVN
LHRLVNAIIAKKDQLEIIVSFIFFVNQLVFMFFCNRSAQILINNSEEFFHELYISVWYFVPLNVQKILLL
IMTRSSTTCMFHMFGVFFPCHAGFTTMLSTSFSYFTLMYSIQ

>AfOr135like_3PF

FKNTIEILYNHYKIQYFKPVLTNFXIVSLLRSEITLRNCILILSTTCPLIVILLRYISFIIFFPMIKHLF
HHISMVESIMQDSIETQIRTKYINDSCQRIHFLFRKKYVNFFYNI

>AfOr136like_1

MDVFDKHYHTYRIVLKIIGLWPYNNSIYVWIQRLWLLIFILGNIIFQIVSLLRSEITLQNCTLILFTTCP
LTVVLLRYVGFVIFFPMIKLLFRHIRMEESMVQDSIETQIRTKYIDDSCHMIDIFFWIIYTTTAFCSISL
LYPITLDFINPLNESRTRIIHYFTMFSHHRIIYIDILCLNYVFVAIISSLSVTCTESIFGLYSYHISVLF
KIIGYRIQNIVTCLTIFNLSSKQIDSKLTELYRVVDIHNQAIELIDAINNSGIHMMPSFLIVISMAMNLH
QLVSTIFRKDQLGILIFLISFIVQLIIIFICNYNSQILINNSEELFHELYISTWYFVPLKVQKILLLIMI
RSSTTCMIHILGVFTPCHAGFSTMLSTSFSYFTLMYSIQ

>AfOr137/138P

MDVFDKNYYTYRTVMKIVGLWPYNNSIYVWIQRLWLFTFFLGNIIFQIVSLLNSEITLQKCILILSTIFP
LTIVLLRYVSFIIFFPMIKLLFHHMRMENNIMQDSTEIEIRTKFXINGSCHMMDIFFWIIYATVALSSLF
ILYPITLDFIMPLNESRIRIIHYITIFSYNRTIYVDILSLNFMFVGIFGSLSITCTESIFGLYSFHTSML
FKIIGYRIQKIVTYLAMFDLSSKQIDTKLKELHRVVDIHNEAIGLIDVMINNSGKQFISSNLLCVISVAI
NLYQLVNAIITKKDKLEIFIFSVFLMMQLMIIFLYNYNNQILIDKSQELFDELYISMWYFVPLKVQKILL
LIMIRSSTACMFHIFGVFTPCYTGFSKILSTSFSYFTLIYSIQ

>AfOr138P

MDVFDKHYRTYRTVLKILGLWPYNNSVYVWIQRLLLLTLYLGNVIFQIVSLLRSEITLRNCILILSTTCP
LIITLLRYISFIIFFPMIKHLFQHIDMEESIIQDSVERRIRTKYIDDSCHMIEIFLWITYTTIAFYGMLL
LYPIILDLIMPFNESRMYNYHYFTLFSVNRTIXFYILCLDYIFVITFGLLSILCTESIIGLCSYHAGVLF
KIISHRIQKIIAYLTTFNLSSKQIDTKLGELYRVVDIHNQAIQLINILINNSGKQFLISVLLAVISMAIS
LHRLVNAIIIKKDQFEIFLSLIIFTNQLVFIFLCNHSAQILANSSEEFFHELYISVWYFVPLKVQKILLL
IMTRSSTTCAFHILGVFVPCYAGFITMLSTSFSYFTVIYSIQ

>AfOr141P

MIDEKTKREFNKTIDLNLFXLKLCGIVSCGDGFANRNILAWLAFSCLTIYSISYVHEFITNTTNLTTALE
SIAMIISIVGGHARYMILLWFRDICQTMLNVCETFWSNLKPHEKKIVQSYTRKTTRLTRWYLASCVLTIA
FYAFLVLFGSLFDHSKDFEHITRNDSGLVPSEAGNVLESTNKRHLPYAFFLDVQKTPWYEIVYALQLIGM
FNVGFTCVGVDTVGALFILITCGYFDTIRSRIENLHSFDTSLPSSSLSLLNILSRKITTAKMSDTKTKTS
NSAQMKNLRMCVIHHQLLLNRFCEDIEHLTSGMFFIQVIASTYNISLVGFKLLEDTPDKFKYITQLIILI
IQLFLCNWPADLLLSKSVDISRATYSMPWYRYSYNLQKITNILMVRSQKAVRLTAGKFIGLSLETFASMI
STAASFFTMVRSMN

>AfOr149

MLKQVTFEKVIHIIWFSVALTFCWPLPANSTKNQVFVFRILQIISIINAFMLILPLLYSIYLHFNDIAIV
FQSLSILVGLSQMIIQTVILCFVKYNSLQRVIEEMITCVKEAQQYERKIFCKYIDKCNIFYGSSLIFTYM
VVIVYIMGPIVLPAPFPIDTEYPFHVNYTITRIIIYLQQSLLIVQCAGHVCLSVFGALLLWFTAARFECL
IVELQKITNIGMLIICIEKQLRLRRYARNVVNSFRFMIVYAIGVSTFALTLYGIIMIVKAPLIMKIEAIT
LSFVLLLQIYIYAWPADYMKDMSINVSKSVYNIIWYKQTLRMQKNLLNVLVYQQPIIFSISCILPELSLR
YFCSYVSNIFSIFTAVRVIIEDDQLDPLSNC

>AfOr150

MLKQLTPEKAIHITWLSVAITFCWPLPANSSKIQVFMFKALQIISIINAFVLLLPLLYSIYLHFDDIVVV
FKSIALCVGLSQMIIQTAICFVKYDSLQRVIEEMITYVKEAQQYERKIFHKYIEKCHIFYGCSIICMYLT
GVAFIIGPAFSPASFPADAEYPFQINYKPVKVIIYLQQTLVGFQCTAHICLSVFGALLLWFTAARFECLA
VELKKITNIGMLIVCVKKQLHVRRYAKRVVISFRFIILCAIGVSTFALTLGGVIMIKKAPFIVKVQFITL
ILTLLTEIYMYTWPADHMKDMSMNVSQSVYNITWYKQTLRMQKDLLNVLMYQQPIILSINCILPELTLHY
YCSYLSNAFSIFTAIRVIIEDDPS

>AfOr151_1

MTKQVTPEKAIQIIWLSVASTFCWPLSINSSKTQVFVFRVLQITSIINACMLLLPLLYSAYLHFDDIITV
SKCAGLAIALTQIIVQTSICFVKYDVLQHVIEEMITYVKEAQQYERKIICKYIKKCHIFYGCSIVCVYLT
AIAFIIGPVFLSTSFPADAEYPFQINYTPVKVIIYLQQSLVAFQCTGHVCISIFGALLLWITAARFECLA
VELQKITNISMLIACVKKQLHIRRYAKRVVNSLRFMIVYTIGISIVILVLDGIIMIMKASLIVKIQFIGI
SLTALVEIYIYTWPADHMKDMSIDVSQSAYNVTWYKQTLKMQKNLLNVLVYQQPIILSVNCILSELSLRY
YCSYLSNVFSIFTTIRVMVEDDP

>AfOr151_2PN

MLKQLTPEKXLSTLLELASTLCWPLSANSSKTQVFIFKALQIISIINAFILLLPLLYSVYLHFDDVIIVS
KCVALSIGLTQLITQTAICIAKYDSLQHVIEEMIICIKAAQQYERKIFHKYIEKCYTFYACSITCTYLTT
TVFIIGPAFSPSSFPIDAEYPFQINYTPVKTIIYLQQTLVSFQCAGHICISIFGALLLWFTAARFECLAV
ELQRITNIGMLXACVKKQLRIRKYTQKKVIISFRFIIVYAIGVSTFVLILDDIIIIMKASLIVKIQFITL
SLTVLTEXIYIYAWPADYMKDMVTNVSKSVYNITWYKQALSMQKNLLNVLMYQQPIILSVNCILSELSLH
YYCSYPSNAFSIFTAIRVMIEDNP

>AfOr153

MVKGITPEKAIHITWLSVALTLCWPLSVNSGKTQVFVFRVLQIISIVSACMLLLPLSYSIYLHTDDVIVF
LRTIIVFVGVAQNIIQTVICFVKYYPLQHVIEEMKICVKEAQKYEIEIFQKYIAKLKTVWGCSITCMYLT
ALAFTIGPVFMSTPLPCDAEYPFQLNYTPVFAIVYFHQSFLSYQCLAHMCISMFGSLLLWFTAARFECLC
VELQNATDIDMLIVCIKKQLHLRRYAKKVINGFRFIIFNAIGLSTLVLTLAGIILITNVPKILKYQFVIV
CMTLLAEIYMYTWPADYMTDMSVNVSRKVYDSMWYKQTLKMQKNLLKVLIFQRPVSLSISCILPELTLRY
YCSYLSNVFSIFAALRVVIEDNI

>AfOr154/155P

MSKRMTPETVIRIAWLSVVITVCWPLPMGSGRGRILGFKILQIVSIISDFMLLFPMIYSIYLHFDDIIVS
SQCVCMSIALTQLIIQTFICFVNHDSLQRVIEEMMTCVKEANQQETEIFYKYIEKCGRFCGGSIAWMYAT
AICFSLGPVVLPITFPSKAEYPFRVNYVPVNVIVYMHQAFLSFQGAAHICLSMMGALLLWFAAARFECXA
VELQNCANMPTLIVCVKKQLRLRRYAEQVIDNFRFIVLYAIAVSTFALTLCGIILIINVPILVKMQFITL
CFTVLTEIYLYAWPADYINDMSMNISQSAYNSVWYEQTIELQRNLLSVITYQKPLILSIKCILSELSLRY
YCSYLSNTFSIFTALRVLIVKNSS

>AfOr157

MSRQVTPEKSVYLVWLSVAITFCWPLPANTARKKIVGMKVLLIASIINGCVVILAMFYWIRLHLDDIISL
FKCVCVVLCLLQYVVQTIVCFVKYDTLQRVVDEMMGCINEAKTDEILRAYASRCNTLYGMSLLSIYICGT
SFVFTPLFLPNPFPFETEYPFDVNTTSRSFIIYASHVLAIFQSTGHMCLCTFGAVLLWFTAARFECLIGE
LRSVTSIDALVVCLKKHSRLKRYAEEVVSCIRFLVFHAILLGTFVLTLCGIVLIINSPLIVKAQFIIICV
CILLEIYLYALPADYMYDMSMNISRSVYDSMWYEQKLDLQKALLTVLAFQKPIPVSINVLLPELTIRYYC
SYVSNALSIFAALRTVVDDS

>AfOr159like_1PF1

KYQEDHKIILKNASFNYRLIVICFXMHGGMIFYYIILPFTRTKIVEKGSNLIXEGNKRVCPFPKILLHAC
HSTINEIRYMIQLLCNIIVPACSLAMLXFVIHTCRQLQILMSWFEKILNXRKNNNENL

>AfOr163_1

MMLKTIITCPIEVCLRLIGVWPYSSYRIVQRVFWTIIMGNSTVFQLWYCISYFKTADLLDLLDGITLTLS
NTVLFFKLIILWFNYRIFHNILTIVFEDWNNRALTDQKKQLMVENTHLSSRISNFLFGIYSVTSILYSAS
IALISDDIDNTNNELILNKKKLLLKMKLPFDFTIFPLYEFVIVAQFVFECFVALMAGMLMAFSAALVLHI
GSQIDIICQELIEIPRHYKGKTSYMLKNIIIKHQRILRLSENIKHLFLYTSLIQFLSNILVICFLGFILV
NALGTEQGSTIFIKCFPYYIAANCEAFILCYTGEYLMFKNESIVHAAYNTLWYNLNPRDSRIVLLILIQA
QRKLILSAGNFVTLSVQTFASMQKVSASYISILMTIY

>AfOr165

MLVLNTLSPSVKFGLHFAGIWPGTAFPYLHKLGWLAAMVALQSYQYRYIVMHYKSDNLMSIIDNLSIAMP
FSLVFIKLIVTWVNYGVFCDILSTMKKDCQKYAVIDINNLISKTGQISFYMTTIVMSSYLVSAVFYITGT
LAFQRTNDSISRELLFKMDLPFETSESPNYEFVVTSQFLIHVSAALTFGTFSALLLMVVLHIGCQIDILC
QNLLHISPHICASHLKFFIIRYQEIIIFAERVEKLFTYIALSQLVSNTLITCCVGFLIVIAVHEDNSLPL
LLKSVLFYMVICLEAFIYCFAGEYLRIKSKLIGDTAYEFLWYDLRPNESQLLIPVILRSQKGFTFTFGKF
SSLSLESFTGIMKISASYISVLLAVY

>AfOr168

MNFQNLNRLNAFANMISGNFLPMTNINEKSSIVSKIYFVIVWIIQLIYLAACTLGLFNVPWERSLKDGTV
NMVLLFEVIILNIYLHSRRNLLRELIGKLNHVLINEDEMFRDVTISTIKTLEKPLRIYILVNVTSIMVWI
SSPLIKLFQKNEFYYEDFTMPVVLSEQPFSTGVFICGVFLQLFGGEYLLFRKISLDLYTMHLNLLITAQY
KYLRIKFATVLKEKGETFGKDTWQNIPYDDDTTIRQEMKLLTRHFETVIEMTGMLKKLLSPNIGVLYLNY
VFRFCFLSFMFATTSLLEKLTYTIIVSYTTGALIQFYILCYCIQDLFEASSSIADDVVYEKWYSYDVRFQ
RVILMISLANELKCKISNFQNIDLTLPSFMSILNQAYSICLLFLKTKQD

>AfOr170P

MNFQNLNRLNVLVNMISGNFLPMNNISEKLSIVLKIYFVIAWIIEVIYVTACFLGLFNVSGEKALKDGTV
NIAISFEVIVFNIYLHSRKKLLHKLIGKLNHLLITEDEIFRSVIIDTVKPLEMPLKIYIIASVASLMIWI
LSPLIKLFXKNEFYYEDFIMPAVFSKQPFSNDIFICGVLLQLLGGEDMIMRKISLDIYTIYLCLLITAQY
KYLKIKFAIILKEKREITKDLYKDIIWQNENVETIRQEMKLLTRHFETVIETTIILKKLISPNIGFLYLS
YVFRFCFLSFMFAMTTAKYFEKCLLVSYTLGALIQFYILCYCIQRLFEASSSIADDVVYEKWYFHDVRFQ
RVILMISLANELKCKISNFQNIDLTLPSFMSILNQAYSVCLLFLKAGQD

>AfOr171

MSKELRLYKKYASFVKLFLLIGGMCPITRELNIIYRYIPIWTIFSCFIELCAVGNSSLQNVENIPLLTAS
LILFGTILNVITKASCFFIHRKKLQQVNDILSSILEEILGEIYIKSVILFYLQKFYRLIYVQAILMFITS
IIYSMKPMIIKMFHDANNITNVQYPLPLLGTFPWRINSILIWQLHYFFDVNILWFIFFVSVSVDAFFGFC
MFRISVILRFLSFEFKRSSIDDKNKRNKEYEKSHQQIFRECVEKHVLLLKCRNIIQEIYGPIILLVTITN
AMSMCSIIFQLFQVNGINIYKIGTFMAYLILKLIQTFLYSWPGDVIFTESEFLRRNVYCSCWYDKNTSFA
KYFLLVLAQKPIVLKACSLVQVTMDLLAKIMNTTISYYFLLETMDNK

>AfOr174

MDFEGSQYYNINRILMICVGLWPYERTIYSKLLNIFIFLLLTTAIFIQIMSFITLKMDMSLILTLLSFTS
CTCIFITKYFIFLFYIKDVKNIEELRIIHEYSKTTKGITICFIIIIVPLQLIFFLSIFGNNILDILIPLN
HTRPRTVPIVIDYFVDQQKFFNFFGMHLNLITSFGGLGIIATETISMAMMQHLCGLLKISSFRISHTFVA
NIPRVSFTERNIVIRRKVISIVNLHVKIKNYINWIQEKIIISYDFLILFGLVVFSISMFEFARSVTSKTD
ANELISSLFYIICITIYGFVPNHFAQEIINHSSNIFMDTYTNTEWYELPVIEQKLILFIMQNNLKNFNFV
LLGSMIASYHVYVTILHTAFSYFMVIYSILLKTDE

>AfOr176P

MLDNYYYRYVRFYLKLVGSWPFDNSKQQTIRKWFIQAMFVLIIVYQIRILXIXYNIELLECIIILTINVI
LSIKYYTCIFNYDVMKILFINIERDWIXELNCINDMNILKKYSRNSYLYEKYLIWTTWIIYGSIIVINII
MNYAVYFINVLNDLNKIDYYILMIFLNIFIIFSLIILISTDSICTTITYHVCAVLDITRFKHLFDDLRKM
NVAHRKYLLRNRIVGIVRHHWKCIEYINLLDSYYSIPIVLQYLIATIIFTLKLYKIYECVKINHFRKIII
ILIDIIQNYVFMFLYFCPATKLTKHSEILLNSAYNACWYETFVHIQKMFKFVIQNTLKSYHLGILEIFCI
TFESMGKLVQFSFSCFILLISLKE

>AfOr179P

MDFEGNQYYNINRILMICIGLWPYERTIYSKLLNIFIFLLLSTGIFIQAMSFIMLKMDMNLILTSLSYIS
CTCICIMKYFSFLFYIKDIKVLFDEIKKDWNSLKIIEELRIIHEYSKTIKGINRIFHXXLLPLQLLFFLS
IFGNNIDILILLNHIRPQTVPIIIDYFVNQQRFFYFFGIHLNLITSFGGLGIIATETISMATMQHLCGLL
KITNYRISHTFVANILXVSSVERNIIIRKKVFIYTNCFFFCXSYINSFQENIIMSYNFLMLFGLIAFNIS
MFEIAKSITFKTDTNELISSLFFVICFTIYGFIPNHFAQEIINHSFNIFIDAYNTEWYELPMIEQKLILF
ITQNNLKNLNFVLLGSMVASYPVPQQILRTAFSYFMVIYSMI

>gi|815927561|ref|XP_012248310.1| PREDICTED: odorant receptor 4-like isoform X2 [Bombus impatiens]

MQTELDLDMSMVLSKFFLRSIGLWISENSAEERRMKIMIIYTVWHSIFATVEITRDFYFTIFYKGDILYV
TTNILTVTMGLIKICIIMAHKEEFINLIVYVRQHFWNVKYNFREKEILDDCRKTCTFFVCSVTIMGACAI
IAYLITPVIANAGKNNSERVLPFNIWLNLPVSMSPYYEMIFTLQMINMYQIAVTYFCFDNIFCILAIHLA
GQFRILRYRFSKLCDIEHQINEKDMELTITNYAHTFYKKLKMYVRHHQELINFCDRLENVYTMLILGQVL
VFSVLICLFAYQGLLAAAPLARRSIFIFHLIGSMALLFMFTYSCDGVIEHSEKVAIGAYSALWTIMPMNK
PGKMLRNDLIMVIERSRRVCCLTANRFFPVSLETYNKILSTAASYFTLLRNHLENEAEN

>gi|350427979|ref|XP_003494946.1| PREDICTED: odorant receptor 67c-like [Bombus impatiens]

MNSKLLMLAILQMEMYLDSGDAGKNVDALILITSGVVALSKIIWFRIRPAGLISNFTSAVKDYEELEGQD
KRLIVRKHAYIGRVASATVIFSSYIGSTLYMTIPMLAGNEEKDIVNVTEESTTDYPIPSEYVMDVIQLPD
NLYFMVFIIEYLMLLFTSTGNLGSDSLLFGIIFHLCGQVEILKLEFNKLGNENERIMERFIVLIKRHVYL
LNLAKMLNETISSVLVVQLFTSCILICTTGFQFILDLSIGNIVMAMKTLIVMSCLLVQLFAYSYVGEYLK
RQMESVGDSVYFCSWYHIPKNVAKGIVFVIMKSQDPVSLKAGKFFIVNMETYMNILKTSMSYLSVLRVMV
NP

>gi|350427266|ref|XP_003494705.1| PREDICTED: odorant receptor 49a-like [Bombus impatiens]

MDVFQKTGEHYPNIYDIPYYNTIKKYLRFLGLDPHQKYGLIIVIIMVISMTSGLIPMSIVLYGSLYTKNL
DMVLECLPHLGALLTSVVKILNVHLNRENFRKLFDSITKEWQQLKLSQDLYILEEVTIRGSKMAKLYRNT
LLICMVLFLLVPLLPPMLDIVLPLNETRPRQQILNVNYVIFDSNNYFFYVYLQLSWTSVVVSIIIVTVDS
LLMLIVHHNSGLFVVCGHQIQKNTRHLNSFTNEVMSERYTYKQIRNCVIMHNKAIDFYDILDENNRISYM
IQIGLNMIGITTTAVQTVINFDRPGESIRSAVLCGANQFHLFMLSLPGQILIDHCTELTKQLYNSTWYGA
PVKVQRMLYMMQIRTRKPCTLTACGLYQMNIENFGTTFKTCMSYITMIMSLKG

>gi|350425964|ref|XP_003494289.1| PREDICTED: odorant receptor 13a-like [Bombus impatiens]

MSFIKTDDISVRLTSVFMKLVGLWMATERSEQRIRNITVGYTLVAIMFAMWLQTTDMYYSWGDFSACLFD
ACNILSLTMPFIKILVLLAHKEDFFRLIFYLQRKFLHGNYNDYERKIVISCKRKCTFFICFFTLFTLATV
ASYIINPLVANIGRNESDRVLPFNMWIDLPLTVTPYYEIIFVLQVLSLYHIGISYFCFDNFLCIMNLHVA
GQFRVLQYRIANMPDLMDKVKQSGDKILNTGSSCLANECYSIFKKYIRQHQALIAYCGKLEEVFSLIILE
QVLMFSLLICLDGYQVLMANASTSIRVIFICHILACLCQLLMFTYSCDCIIRESASIATAAYKGPWLILP
MSTSGRMMRKDLTLIILRSHIPCCLTGKGFFVVSLETYTSVLSTAASYFTLLRQRETP

>gi|350425958|ref|XP_003494287.1| PREDICTED: odorant receptor 13a-like [Bombus impatiens]

MSSKKGRDLSITVVSFYMKIVGFWLAKNHVEERWRNFAMIYTTFAILVAIVVETRDLYFTWGDFSGTVYI
VCNLVTIILVLFKILVCFVYKKELLSLIQYAKTNFWHSNYNSHERMIVDRCKRTCTVLVCVFTFFAQGTV
ISYVIGPIQANIGRNETNRILPFNIWLNVPICYMTPYFEIEFIIQVLCLYHVGVCYLCFDNILCIMNLHT
AGQFRILQYRLENMCGENNNEKLSYSICKYMKLKTYIQQHQMLIEYCKKLEQVFNLIVLGQVSLFSLLMC
LDGYLVLTEDAPLMRRLIFLFHITGCMCQLLMFTYSCDCLIRDSTNVANAAYKSLWSFLPMDKYGKILRR
DLMLIIMRSNIPCCLTASGFFIVSLETYTGILSSAASYFTLLRNHASDTS

>gi|350425900|ref|XP_003494268.1| PREDICTED: odorant receptor 13a-like [Bombus impatiens]

MQTEQELDISMRLSAFFLKHIGLWIANDPADERRMKIIFTYTIWNLIFGMIVLSRDLYFTWFYNGDTLYA
LANTMSLILSSVKVCVIVMHKKEFINLVVYMEQHFMAVKYDIHEKEILDNCRKICALFISSVTTIGICAI
LSYITTPFIAQIGNNESTRELPFNMWIGILSQSPYYELIFFAQIMSLSYIGICYFCFDNVFCVMAIHLGG
QFRILRYRFSELCNIKYQISEQDKMSILTKHVHRTYETFRKYVQQHQALINYYNSLENVYTVIILIQVLV
FSVLICLFGYQVLLANTNSARRSIFVFLLIGALSLLFMFTYSCDDVIEHSDNVAIGAYSALWTIMPMNKH
GKMLRNDLIMTIERSRRVCCLTANGFFPVSLETYTKILSTAMSYFTLLSNNVTKNET

>gi|350425896|ref|XP_003494267.1| PREDICTED: odorant receptor 30a-like [Bombus impatiens]

MHTTRYTDISITLSQFLLKLAGVWMTVNNAEERRRRLTMAFTAVIHVYGLYLNLGDAYYTWNDLSHCTFL
LSNTLCIILAMFKLLILNFRRTEFKDLVLFAQQNFWHFKYDHDEKILFMKCRKLCKLWTITACSFTQASL

AFYIITPICANIGKNKSDRVLPFKMWVDLPLSVTPYYEIMFVIQLATVQQIGVTYLCNDNFLCILNMHVI
CQFRMLHNRLLNLWKIIDQKTDKIDYADKCYIALKKCIRQHQSLIKFCEKLEYVNTFPIFGHVVVFSLLM
CFDTYEILLANVSTGTRLIFVFHMVGSFIHIIFFTYTCHGLIEESSNISLATYSGWWTILPMTETGRMLR
EDVKVMMMKSMRPCYLSAGGFFPISLETSTALMSTTMSYFTLMRESSVKNADK

>gi|350425706|ref|XP_003494207.1| PREDICTED: odorant receptor 13a-like, partial [Bombus impatiens]

LGVWPEPRKVSRGSRLLSSIIFWFTTIVTFTFICAPQTANLVLKSTNLDEIIENLSINIPIAFALVKQIV
LRYYKKALTLLLSQMFDDWNEPIANQDRQTMLKNAKISRMISIVCSTLTYLMLFAFISLQIWSNMQSASE
ADLGGLLHPATFPYDISKSPNFEITWLGQFMGTVLTAICYSCFDTFLAVLVLHLCGQLTVLRMALEDLAN
TTKKDNNYARFHERLGFIVNRHNLLSRFAVIVEDCFNLTLLIQTVICTAMFCLTGYRMITSVDQEQADVP
IVGMMFFIIHVIYTMLHLFIYCYIGEMLLGESTGVGQAAYECDWYDLPPKNAISLIIVICRAKVSFQITA
GKFSPFSLELFNAVLKTSAGYLSVLLAMKD

>gi|350425429|ref|XP_003494119.1| PREDICTED: odorant receptor 4-like [Bombus impatiens]

MQTEQDLDISVIFSMFFLRNIGLWISDNRAENRRMKIKLMCTLFNSIDASIVIMRDFYFTWLYKGDILYV
TTNILTVIMGLIKICIILMHKREFISLITYTQRNFWNVNYDTREKEILKNCRKTCTFFVCSVSAIGICAM
LAYLSTPLITNAGKNKSERILPFNVWLNLPVSISPYYEIGFLIQTISLYYIAVSYFCFDNVFCILAVHLT
GQFRILRYRFAKLCDMEHQITEEDTGLMITKHVHTFYEQLKLYIRQHQTLIDFCNRLEDVYTMLIFGQVL
VFSVLICLFAYQGLVVAAPFARRSIFVFLLVGSMALLFMFTYSCDGVIEHSEKVAIGAYSALWTIMPMNK
PGRMLRNDLIMVIERSRRVCCLTANNFFPVSLETYTTILSTAVSYFTLLRNNVEDKNVD

>gi|350424935|ref|XP_003493960.1| PREDICTED: odorant receptor 46a, isoform A-like [Bombus impatiens]

MHTLRWTFALLTLTGLIRPSTWKYLWKRVLYDVYTIVVLLLLFSFETSLILDLVINVDNQDDFSENLYVT
LVLFSSCCKALVLLIYRGNIEILMGVLLEKPFVPVNDEEIEIRTKFEERIEWNSKAYSFVLHFFVAWLWI
GAFLTDFRHGRLKFRAWIPYDYNSPLLFLFSFIHQILATIFSTNLNIVCDCLFSGILIHIYCQFEILEHR
MKNITTDKDYSAKFCAHHHHRIYKFASMVNDNFKMIMSMQFLISTGAVCFNLYRLSVMEFGPKFMETATY
TLCLLMQVFYYCWYGNEVKLKSLEIPNAVLESNLPFLNDSSKKILLLIMRRSLEPIEFTSCHVISMNLES
FAILLKTSYSAYNLLQQSKLND

>gi|350423776|ref|XP_003493588.1| PREDICTED: odorant receptor 67c-like [Bombus impatiens]

MKELSTTFDYYILPNKIFCSIVGIWPIGERSSTCSKIFAYFRLIVSLIAISNFFVPEIMAVAFYWGDVET
VIGIGSNLMSATQLFFKMIYLVARRERVYRLYNEIRILWDSTDDPNERKSYEQIAYQARIVTITFSSCFL
CNLTTFSIATIADYFRFAYNGNNTNDNRHLPFLVWYGTDISASPKFEIAFVGQVMTAMIGLSAITAIDCT
FMTMILHVSGQFILIKTWINKIGFEMSHKSIDMDKFEEDLFKCIRHHQRMIHVVNDVNNLLTPIIFMQLL
TSGLEICLSGYAMLDNGTKITDILKFTSYFISVTVQLLLWCWPGEILVQESQEVGQVVYFSVPWYNLPPI
YRNHVCLMIIRAQQYCSITALTFKVLSIQTLTAVFNTAISYFTVLQQMQQN

>gi|350422520|ref|XP_003493189.1| PREDICTED: odorant receptor 4-like [Bombus impatiens]

MVDVSSEPPISNAFYAHDYEYSIQVNRWLMQPIGAWPKLTKTNRTQRLFAKLLNFICHSLIIFTIVPCIL
HIVYEAESSRMKMKIIGPVSHWLMGELNYCCLLSKTDDIIRCIKHVERDWQVVENASSREMMLKYAKVGR
FIAFIAAFCMHSGVLAFSITKGFKKMMFLVGNDSYFMYPLPCPVYTNLLDARFSPANEIVFVLQILSGFI
VTSVTVGACGLAAVLTMHASGQLNMVVARLDNLVDTKVEEKQEAQTVAQRKLGIIVEHHLRTLSLIASIE
KVMNMICLVELVGCTINMCIIKYSFLTEKSKDMRIVYAIVYASMVFNIFIFCYIGEIVIEQGERVGKKVY
MTEWYRLPHKTALGLVLVISRSSMVVKITAGKFIQISITTFGVVFKTSFAYLNMIRTML

>gi|350422517|ref|XP_003493188.1| PREDICTED: odorant receptor 85c-like [Bombus impatiens]

MMTEAVIVESDSNSNSDYSLQLNRWFLQSIGAWPSSASTTKLEKIVSFILNIVCLSIVILAVIPSLLLMI
LGDESFNFKLKMFGYVSHWIFSGINYTALLTHGKNIRQCIEHIEADWRTVTREEDKNVMLKNAKLGRYIA
GFSVIFVQGSVLCFFFAAALSTVEMQVGNERILHLLPCAVYKKLVNVDDSLTNEIMLLLQLWSTVIANSS
TSGIFGLSAVLAAHSCGQLNVIMVWITEFVNKARERTKTGNFIEIGMIVERHLRTLNFISCIEDVMFKIY
FVEMFRSTMDICVIGYYILSEWADQDIQNLITYFMMLISICFDIFVICYISEILTEQCQKVGEVVYMTNW
YYLPNKVILDLILIIARSSTVVHLTAGKFIQMSVYTFGNVLKTGFAYLNMLQQMTR

>gi|350422508|ref|XP_003493185.1| PREDICTED: odorant receptor 4-like [Bombus impatiens]

MTNEPVITEGDSNSNSDYSLQLNRWFLKPIGAWPSSSSTTRLEKIVSFILNIICFTSVIVTAIPSLLLMI
LEDESIYMKLKTLGPVSHWFVSSANYTALLIRGRDIRHCVEHIEADWRTVTREEDQHVMMKNAKFGRYVA
ASCAVFMQGGVLCFCFVTALTTTEIQIGNETRVLHVLPCAVYKKLVNVDESPANEIMLFLQIWAALIANS
STVGIFSLAAVLAAHACGQLDVIMMWITKFVKEAREQKETSSFRKIGVIVERHLRTLNFISCIEDVMNRI
YFLEMFRCTMDICVIGYYILSEWADRDIQNLFTYFMMLISICFNIFVICYIGEILTEQCQKVGEVVYMTN
WYYLPDKIILDLILIIARSSMVVHITAGKLVHMSVYTFGDVVKTGFAYLNLLRQMT

>gi|350422503|ref|XP_003493183.1| PREDICTED: odorant receptor 4-like [Bombus impatiens]

MTNKTVAIKIDFDGRSDYSLQLNRWFLKPIGAWPSFPSTSRLERIMSFVLIVICYIILICTIIPCLLHII
LENESFRIKLRLIGPVSYLCVGSINYTTLLLRGKDIRYCVEHMQTDWRTVKREKDQQVMLKSAKFGRYVT
ASTAAFMQGGVFCYCFMTALSTEVIQVGNETRIVHQLPYVIYKELIDINESPTNEIILFMQFLTGFIVSS
STLGILSITVVLIAHACGQLNVVMTWITEFVNESRKEKIAPFENIGIIVERHLRTLSFVSSIEETVNRIF
FLEVLRSTLHMCMLSYYIVTEWSDSDIQILTTYSMLLASICFNIFVICYIGETLTEQSRKVGDVVYMANW
YYLTEKRILELILIIMRSSVVVEITAGKIIHMSIQTFSTVIKTAFTYLNLLRQVT

>gi|350422500|ref|XP_003493182.1| PREDICTED: odorant receptor 4-like [Bombus impatiens]

MTNKSAVVEKTFDSLGDYSLQFNRWLLISIGAWPASTSTSRRERIISFILIALCYGFILFTVIPCIFHFI
LEDESIYMKLKVFGPLSHWFIGGINYTNLLLRSNEIRDCIHHVQTDWKIVTRPEDSQVMMKYARIGRFIA
AFCATFMQGGVLTYCVVTAFATQTIEIANETRIIRMLPCAAYKNLIPVDSSPMNEIVLATQFISGFIVNS
SAVAIISIGAVFTAHAYGQLTVLMRWINEFVNRSEDQKKSVEFNEIGEIVEHHLRILSLIAGIENVLTQF
CFMELLKSKLDISMLGYYILTEWAEHDIRNLTTYFMIMASMSFNIFTVCYIGDILTEQSKKFGDVVYMTK
WYYLPNKDIFDLILIITRSNSAIKITAGKITHMSINTFGDVMKTAFAYLNLLRQVT

>gi|350417424|ref|XP_003491416.1| PREDICTED: odorant receptor 13a-like [Bombus impatiens]

MSDQQFSGKEYDELIKPIMITAKIISIWPLEEDSGKGTILFRRFHLFCMFFLAVVMSIAVTADVVHNIDD
LNEATECALICTAFYLCVVRLLVYSLHQKDMFYVVKTMKEDWILSSHEDRTILAKKTMFAFRLAKYFIST
VAMTIVLFMCIPFLEIYAFGSNERVLPFRGYFFVNHTISPVFECLYFFNVTAGGFGGSMIAGATSFNLVV
IMHGSGKFAVLRKRLEALSGEDPNSTAIMSNYVIRHQKAIEYADALERIINVLALGQFIISTGLICFAGF
QITSMMKDKGRLMKYSTFLNSAILELFMFSFSGNGLIDESGAVGDSAYGSGWIGSRFSQSLQIMMMRARI
PSKITAAKFYAMSLESFSAVLSTSFSYFTVLTATEGD

>gi|350415237|ref|XP_003490576.1| PREDICTED: odorant receptor 22c-like [Bombus impatiens]

MREKVAIEEYVRNFLVQETLLKVVGIWPTRNGSSFGRWIFAMMTQISTIYSLSLEVYRHCLDIDDTMDAF
VMDLSSIVSLAKLFIMRRNSKHAYVLINSVLKDWSAVDDSRHEDIMTKYYKRGRIVSLTILYLGYASGLS

FIVKALPFGEILPFKMFQNSANSSANLKGSLEMNYFLASYCVFGSLPLLQRICVLILQAMHIFVNAVAHC
ANDGLFFSLTMHLCGQFEVLKMNFNKFELQQFGCHKRLRFLVKRHCQLLMLANDLEQTFNMIILVQLLMS
ALLICVEGFVFLVCLATRDNVGALKSVILMVTLLIQLYLYAYAGDVLESRSNEIAHGVYDSPWYQPRGHV
ARDLMMIINRGHRSYHVTAGKFLSMNIFTFKEILRSSASYLSVLKVMMDT

>gi|350415235|ref|XP_003490575.1| PREDICTED: odorant receptor Or2-like [Bombus impatiens]

MKAALNKDFAYAMTPMKILSWPVGTWPLQDYNIFSALRVIITSCLLLLMLTIVQSEMYLDSNDAEKNLDA
LVILSCGILAVSKVIRFRIRPAALILNFTSAVEDYNELRDDEKRVIVRKHAYMARVTSVSMIFFAYFSSI
LFITVPMLAEKEEKNVVNVTEESTSEYPIPSENVMALVKIPKNLYFFVFIMEYLMLLFTSTGNLGSDSLF
FGITFHLCGQVEILKLDFKRLKIGSERTREHFNVLTRRHIYLINLAKMLIDTISSILAMQLFTSCILICT
SGLQFILALSIGNIVMAVKTFMVLTALMVQLFAYSYVGEYLRRQMEGIGDSMYFCNWYDIPKSVAKDIIY
VIMRAQEPVFLRAGQFLVVNMETYTSIIKTSMSYLSVLRVMVNG

>gi|350415230|ref|XP_003490573.1| PREDICTED: odorant receptor 24a-like [Bombus impatiens]

MKATLNKDFAYAMTPMKILSWPVGTWPLQDYNIFSAMRVIITSCLLLLMLTIVQSEMYLDSNDAEKNLDA
LVILSCGILAVSKVIRFRIQPAGLISNFTSAVEDYNKLYDQEKGVILRRHAYMGRVAGIGVVLFAYFSAT
LFMSVPMLAGEEVKDVVNVTKDNIPEYPIPSEKVMALIKMPDNLYFIVFIVEYLMLLLTSNGNLGSDSLF
FGIIFHLCGQVEILRLDFTRLSNDNERTIEHFIVLSKRHVYLLKLAKMLNETISSILAVQLFTSCIVICT
SGLQFIIALSVGNIVMTIKSFIVLSTLLVQLFAYSYVGEYLKRQMEGIGDSVYFSSWYDIPTSVAKDIIY
VIMRTQDPVFLKAGKFFIVNMETYMSIIKTSMSYLSVLRVMVTA

>gi|350415227|ref|XP_003490572.1| PREDICTED: odorant receptor 7a-like [Bombus impatiens]

MKTSTSKDFAYAMTPLKILSWPVGTWPLQDYNIFSAIRATIATFFLLLMVTVVQSEMYLDNSDAEKNLDG
LVLITCGSLAASKIIQFRIRPAALISNFTSAVEDYMELRDEEKRLIMRKHAYMARVASASVICFAYFSSI
LFITVPMLAEEEEKDIVNVTEESITEYPLPSENVMAVIKMPDNLHFIVFIVEYLMLLFLSTGNIGSDSLF
FGIIFHLCGQVEILRLEFNRLNNENEKAMEHFISLTKRHIYLLKLAKMLSETISSILIVQLFTSCILICT
SGLQFIIALSVGNIVMTIKSFIVSSTLLLQLFAYSYVGEYLKRQMEAVGNSVYFCSWYDIPKCVAKDIIY
VIMRTQDPVFLKAGKFFVVNMETYMSIIKTSMSYLSVLRVMVTT

>gi|350415157|ref|XP_003490551.1| PREDICTED: odorant receptor Or2-like [Bombus impatiens]

MKESSDEDFAYAMAPLKIMSWPVGTWPPQVYDIFSAIRAIIAILLLLLMLIIVQMELYLDSSDAKKNLDA
LLLITCGILALSKIIRFRIQPDGLVSNFVSAVKDYNELKDQEKRMIMRRHAYMGRLVSASVIFSSCIGST
LYLAIPMLSGDEEKDIVNVTKGSIMDYPIPSECVMALIQLPDNLFFMVFIIEYLMLLFTSTGNLGSDTLF
FGIIFHLCGQVEILRLEFNRLGNKNERTMERFIVLIKRHIYLLNLAHMLNETISSILVMQLFSSCVLICT
TGFQLILDLSIGNIVMTMKGFIVLNAMLVQLFAYSYVGDYLTRQMKGISDSIYFCNWYDISKSMAKDIIY
VIMRAQYPVFLKAGNFFIVNMETYMSILKTSMSYLSVLRVMVNA

>gi|350415148|ref|XP_003490548.1| PREDICTED: odorant receptor 47a-like [Bombus impatiens]

MKASSRKDFAYAMTPLKIVSWPVGTWPLQDYDIFSAMRGTIVIFLMLLMLLIVQTEMYFDRSDAEKNLDA
LLLIACGILAVSKIMWFRIRPAGLISNFTSAVKDYNDLEDQEKRAIMRMHAYMGRVVSGSVIIFAYVATV
LFMIVPVLAGVEEEDIFNVTEEGVPDYPMPSEYVMELIKMPDNLYFIVFIIECLMRLLVSTGNLGSDSLF
FGITFHLCGQVEILKLDFNRLGNENERAMEHFIVLIKRHVYLLNLAKMLNETISSILIVQLFASCILICT
TGFQFILDLSVGNIVMAIKTFIVLSTLLVQLFAYSYVGEYLKLQMEGVGDSVYFCSWYDIPTSIAKNIIY
VIMRSQDPVSLNAGKFFTVNMETYMSILKTSMSYLSVLRVMVNA

>gi|350415138|ref|XP_003490544.1| PREDICTED: odorant receptor Or2 [Bombus impatiens]

MKATSSKDFAYAMTPFKILAWPVGTWPLQLYDTFSAIRAVITIFLLLLMIAIVQSEMYLDTSDAEKNLDA
VIILTCGYLAVSKILQFRIRPAGLISNFTSAVKDYNELNDQEKRAIVRRHAYMGRVAGISVVLFSHFSAT
LFTALPMLAGEQVKDIVNVTEESIPEYPMPSEKVIALVKIPDNFYLIVFIVEYLMLLSTSNGNIGNDSLF
FGIIFHLCGQVEILKLDFKRLRNETEKTREHFSVLTKRHVYLLNLAKMLDDTISSILAVQLFTSCTLICT
SGLQFIIALSVGNIVMTIKTFIVLGTLLGQLFAYSYVGDYLNRQMEAIGDSIYSCSWYDIPKSVTKDIIY
VIMRAQDPVYLKAGKFFILNMETYMSIIKTSMSYLSVLRVMISA

>gi|350412835|ref|XP_003489780.1| PREDICTED: odorant receptor 82a-like [Bombus impatiens]

MTDDPVTVERKFGSLSEYSIQVNRWILKPIGAWPSSNSTTNREKFMSWILNVICWCFSLFTIIPGVLNIL
LEKEDLYLKLKMFGPLSHWCVGGFNYAVLLLRQGDIHYCIKHIRTDWKTITRLEDQRIMLKNAKLGRYIA
CFCAAFMHGSVFCTCLVLGAFKRTIEVRNETILVYTLPCPAYKLPVQTNPGHDIILGTQFLSAFIATSSA
AGAFSLATVFASHALGQLNIMVTWINEFVNQHSKDQNSNARANKIGVIVEHHLRALSLIARIERIMSPIC
FMEMFKCMLGMGMPSYYILAEWSEHNIQNIAAYVMIIISMTCNIFLVCYIGEIIMEKCKKIGDMVYMADW
HHLPDQDIINLIMIISRSSMEVKITAGKIIDMSVLTFANIVKTVFAYLNMLCQTTMT

>gi|350412806|ref|XP_003489770.1| PREDICTED: odorant receptor 82a-like [Bombus impatiens]

MADELVAAKKKYGNLGEYSVQLSRWYLRPMGAWPNSPSTTRREKILAQISIVICWCIVLFTIVPGFLHII
LVKEDIYLKLKTLGPLSHWCVDGFNYAVLLLRQEDINYCIERVRSDWKMITRVQDRYEMWKNAKLGRYVA
GFCAGFMQGTMIYTCVVLGAFRRTIKVGNETMDIYTLPCPAYKFAVQTNPTHDIILGTQFLSALVVSSGA
AGSFSLATVFASHALGQLNIMVTWVDEFTNQTKQQNKQAQINKIGVIVEHHLRVLSLIARIERIMSPICF
MEMFKCMLGMCMPCYYILAEWSEHNVQAMIIYVMVFLSMTFNIFLICYIGEVLKEQCQKVGDMVYMTNWY
QLPDKNILSIIMIISRSHMEVKITAGKIITMSVYTFGNIIKTVFTYFNMLRQTTMI

>gi|350412763|ref|XP_003489754.1| PREDICTED: odorant receptor Or2-like [Bombus impatiens]

MHDRPYTTVDDQLRNIHYENDIHYTLQMCQWLLKPIGVWPLVSNHTSKLEQLVSVILMIMCFSSLLFIIL
PSFYHIFFVEKSVHVKVKLLGPVGFCLSSTIKYCYLGVKGAFFERCIQHVEKDWKMVQDPNHRTIMLKYA
TVSRKLITLCAIFLYTGGMSYHTVMQFLSKERNKKNYTFRPLTYPGYDSFLDTQSSPTYEVVFFLQCFAA
MIMYSVTTVAYSLAAIFVTHICGQIQVQIARLHDLVENEKRKNNGRDSMSVIVHDHVEVLRFSKNVEEAL
REICLTEIVESTIIMCLLEYYCMMEWQNSDAIAILTYVTLLISFTFNIFIFCYIGEILTEQCSQIGITSY
EIEWYQLPAKKAYDLILLISISHYPPKLTAGKIIELSLNTFSSVAKSSVIYLNLLRTVTDW

>gi|350406010|ref|XP_003487626.1| PREDICTED: odorant receptor 46a, isoform A-like [Bombus impatiens]

MDIFYNVETQDDLSENFSVTVTVLITSCKFVSLVLGRKTIINILGLLKKKPFVPENNEEAEIYAKYDNLI
EKIAMFYTIQNAFCVLSLIVATLMTDFKLKKLAFRAWLPFDFTSSWFTFSMTFIYQFVGSMVISAGISIF
DTLFAGLLLQICCQFEILVNRLHNIGGDEILSLKHCVRHHNAIYRFAEMVNNLFSKMMCVQFMVSAAAIC
FNVYRVTESNAGSQLIGSVLFIFSALLQTFYFCWFGDVAKLKSLDIPNMIYHSDWTNLSNDAKKMLMVIM
ARSLTPVEITSAYILPMNLESFKGLIKTTYSTYNMLLQSKSSQ

>gi|350405970|ref|XP_003487614.1| PREDICTED: odorant receptor 24a-like [Bombus impatiens]

MLRQVTPEKVIHIVWLSAALSFCWPLPISSSRTRVLGFRILQISAIISACMLLLPMLYSIYLHPDDLVVV
FKCMCMSTALCQLIVQTTMCWIKQDSLQRTVREMMTYVKETQQYEKEIFYKYITRCDMLCICTIVCMYAT
ATGFSLGPAILPISFPADAEYPFRVNYTPMNIIIYAHQSILSYQCAAHSCVSIFGALLLWFTAARFECLT

VELEKSTSINTLIVCIKKQLSLRRYAEEVLNNFRIMVLFSIIISTLVLTFGGIILIVNSPLLLKLQFVIV
SLTVLTEVFMYAWPADHLREMSANVSRSAYNLIWYKQTLEIQKNLLNVLVFQEPVTLSVSCVLPELSLRY
YCSYLSNAMSIFTALRAAIGDNST

>gi|350405074|ref|XP_003487316.1| PREDICTED: odorant receptor 4-like [Bombus impatiens]

MLKQITAEKVIHITWLSVVISLCWPLPINSNRNKIFCFKILQICSGISGCLLFMPILYSIYLHLNDIVIT
SSCMCLLLGVSQNIFQTLICFIKHDSLQRTVEEMMTYVKEAQQYEKKIFYKYIKKCYVFCGGSMACTYVT
ATCFSLRPIILPVSLPFDAEYPFPINYTSMYIIIYMHHACLCIQTAAHVCVSSFGALLLWFTVARFECLA
AELERSTDFDALVVCVKKQLHLKRYAEEVFNSFRFLVLYAIGVATFGITLCFLMVLVNVPLFVKLQFIGV
CVTVLMEIYLYAWPADYLKDMSMDAPQSIYNSTWYEQKLRMQKSLLNVLIYRQPLTLSIACIVPELSLRY
YCSYLSNAFSMFTTLRVMIHN

>gi|350405070|ref|XP_003487314.1| PREDICTED: odorant receptor 85b-like [Bombus impatiens]

MTKQITPEKVIHITWLSVVITLCWPLPINSDRNKIFCFKILQICSIISACLLFLPMLYFCYVHFDDLIAT
SSCVCLTIGVSQNIFQTIVCCVKHVSLQRIVEEMIVCIKEAEQYEREIFYKYIKKCYVFCGCSIACTYVT
AVGFSLRPAILPVPFPFDVEYPFPVNYTSVYIIIYMQHVCVCFQSAAQICISSFGGLLLWFTAARFECLA
VNLERSTDTDTLIDCVKKQLHLRRYGEEVLNNFRFMVLYAVGVATFALTLCCIMMIVDVPLFVKIQYIGA
CVTVLTEIYLYTWPADYLIDMSMGIPQSAYNSTWYDRRLEMQKNLLNMLTYQKPLVLSIRCIVPELSLRY
YCSYLSNAFSIFTTLRVMIQN

>gi|350405014|ref|XP_003487292.1| PREDICTED: odorant receptor 49b-like [Bombus impatiens]

MFRNATPETAIAFTEFILGLSCCWPLPSTATKSQILCFKILRSVLFLNSLLLFCPLLYAIYVHREDTAMF
CKSVSLTLAVVHVPLHSTYCFSQHDRYQRLIEEMKSCCEKGNSYERQIFQRYVDKYAIYYAASAVWFYWS
PSIILIGTFFISDPFPTNAEYPFPVDFEPVRSIIFLQQSLVGMQCASLLCTNILCALLLLFAAARFEILM
TEICAVNGVKSLIKCVKKYYTLKRYAEEVANTARYTTLITLCICGIESVFAGIIFIGRQPFTVKLQFVTV
SITVLLAVFMCAWPADNLIDVSESTMRTVYQSKWYEQPLRIQKFILFMMIPQSPVILRIRCIIPAFSLSY
YCSFITNVLSMFTALRVVMFQDEDGIPDDI

>gi|350397298|ref|XP_003484832.1| PREDICTED: odorant receptor 13a-like [Bombus impatiens]

MINCYLHTQLLAVQQIGASYICPDNFLCVLNLHVVYQFRMLQNTLVNLWSNIDERTDIVEYSNECYVMLE
KCIPKHQSLIEFSAKLDDIYTLPILSHMVIFSVLMYFDTYEVILADVSPGTRLIFFFHMIGSFTHIIFFT
YICNGLVEESTNISTASYSGWWMILPMTETGRKIRKDTRIMIMKSMRPCYLSAGGFFPVTLETSTALISS
TMSYFTLMRESSIKAAAE

>gi|350397295|ref|XP_003484831.1| PREDICTED: odorant receptor 13a-like [Bombus impatiens]

MSFLKSHDISISLTSTFMKLIGIWTSKNQMERRVRLITLIYAFSIILFALWIEITDTYYSFDDLSTRIYN
VCNILAVLMPLLKMTVLLAHKQEFFHLIVYTQRRFWHENYDEDEKKIYMDCKRKCTVFVCFVIFTTKATL
ICYALSPILENIGRNESDRELPFNMWIDLPLTATPYYEITLLLQLMALYYVGVGYFCFDNLLCVMSLHLA
TQFQILQYKMSRMTDLTNKEKGETNPQLFSGSSANKCYTAFKKYIQQHQALIAYCRKLESVFNLPVLVQV
LAFSLVMCLDGYQILMPGAPTRTRFIFSFQLIACLCQLLMFTYSCDCIMQESTSVALAVYKGPWSFLPAT
ESGMMMRKDLILVTIRSGVPCCITGYGFFVVSLETYTRVLSTAVSYFTLLRQTTQETLNS

>gi|350397292|ref|XP_003484830.1| PREDICTED: odorant receptor 30a-like [Bombus impatiens]

MDKDSEKTKVSPTPACLKRENEASYTKDFALLMTGFLMKIVGLWLTKNKEEERTRQLTLMYTVVAILFGV
WVQFRDFYYSWPNFGDCAYTACNILCLIMVLLKLSVVFIHKKEFIELLVYTHKNFWHTNYSYNELLLLQN
CRKISIVCISLINVCAQGTVFGYVLTPIVENIGRNYSDRVLPFRMWLDLPLTVTPYYEILFVLQVLSLYH
VGICYICFDNLLCLINLHAATQFRILQYRLLYLGETIEKQPNEYAIKETLPSNYLKNYHTVFKCCVREHQ
DRINYCQRLNNIFTYIVLGHIVVFSLLLCLVGFQVLMANSPPTRRLIFVFHIVGSSSQLLLFTYSCDTLI
RESTNIGTAVYSGPWTHLTMDKIGKLLRKDLTMIILRSSKPCCLTASGFFPVSLETCTKVLSTAMSYFTL
MRQSFTN

>gi|350397203|ref|XP_003484804.1| PREDICTED: odorant receptor 13a-like [Bombus impatiens]

MSSRQVKDLSIIITSFYMKFVGFWLANNYVEKRRRNVALSYTLFAVLLSLSTEARDLYFSWGDLGDSIYV
ICNVITIVLVVVKIFILLIYNEELLDIIDYAKINFWHLNYDSHEQMIVDNCRRTCTIFVCVFTFFAQGTV
IGFIARPLLINYGKNESERILPFNMWLPECHLSMTPYFELMFMLQVVCSYHVGVCYHCFDNILCILNLHA
AAQFRILQHRLTNMCNTNDCAEFYEVSEKSSYSIHRYGKLRTYIQQHQALTDFCKKLEDVFNLIVLGQVS
LFSLLICLDGYLILMDDAPATRRFTFAFHITGCMCQLLMFTYSCDCLIRDSANVANAAYKSLWSRLPMDQ
FGKILRKDLILVIMRSATPSCLTACGFFTVSLETYTGILSSAVSYFTLLRNQSNDN

>gi|350396511|ref|XP_003484578.1| PREDICTED: odorant receptor 82a-like [Bombus impatiens]

MTNQPVTIGASAKANSDYGLQLNRWFLKPIGVWPSFPSTTRFEKIVSITLNVICYSSIILTTIPCLVRMF
LEDESIYLKLKSLGPVSHWIVSGANYTTLLLRSKDIRQCMEHMEADWQTVTREKDQWVMLKNAKFGRYVA
ASCAIFMQSGIMCFIFVAAMDTVEIQIGNETRILHVLPCAVYKKLINVDESPTNEIMLFLQAWCTMIANS
STVGMFSLAAVLAAHACGQLNVVMVWITEFVNEPKKTDGPNSIGVIVERHLRTLNFIGYIENLMNRIYFL
EIFRCTMDICILGYYILSEWADQNVQSLITYIMIYISIGFNVFLICYIGEILTEQSKKVGEVVYMTNWYY
LPDKTILDLILIIARSGVVVQITAGKMVHMSVYTFGDVVKTGFAYLNLLRQMT

>gi|350396509|ref|XP_003484577.1| PREDICTED: odorant receptor 85c-like [Bombus impatiens]

MNESTIIKTDAKSHSDYSLQLNRWFLKPIGAWPYFSTTSTLEKVISVSLIILCYVVILFSIIPCVAHLIF
EDDSFYRKVKVFGPLGHWFIGGINYTNLLFRSRNISDCVEHIETDWQIVTKEKQQQVMLKHAKFGRYVSA
ICAIFVHSGIMSYCIVSASSTQIIKVGNETRMMRSLPLGVYNRMIPVDTSPANEIVLVMQFLSAFITDSS
GIGFYTLASVLAAHACGQLSVLTIWISDYVNEAGNRKEDASFRKIGTIVEHHLRTLDFIARIEEVMSWVC
MTELFRCVLAICMVGYYIVTEWSDHDVRSLTSYFIIFASVTFNTFLLCYIGELLTEQCMKVGEIVYMTNW
YILPRKRILELILIIARSSVVIEITAGKLIHMSIHTFGDVMRLAFAYLNILCQMT

>gi|350396456|ref|XP_003484556.1| PREDICTED: odorant receptor 4-like [Bombus impatiens]

MSELGDKEENLKHFVKFIYPPMKLIGAWPKPTATSTFSKAIKWGLIVSAYFLQLLVFVPGVLYLFLKEKN
GKKRIHIMIPHINGFSQLCKYTILLRRTNEFSKILDQLSDDWVDATEDSRHIFRMRANIGHRMVLTVAIT
MYTTGLFYRIIIPLSRGRIVLPNNTTMRVLPCPVYFFFFNEQSTPYYEIIFVLQIMGGFLNYTILCSTMG
VCLMLCLHLSSLLRILMNKMIELTSQLDTSETAVQKKISDIVAYQTKVKGFANSVEEITPYLYFFEIFNY
AIEACIVGYCIIVEWEESNAASIIVYLMFQGICIFCNYAMCYIGQLLINESENVRLMSITLNWYRFPMKK
ARNLILIIIMSNYPIKLTAGKIVDISLATFTDIIKASVGYLNVLRKVT

>gi|815928873|ref|XP_012248903.1| PREDICTED: odorant receptor 9a-like [Bombus impatiens]

MTGSLLTFYFPADIGCNIIRDFTQQFAQIFTSLYENDLGGMLEGMPVVASFEKMFDVIKKDWKLLNDKSQ
THILEEITKQGNKIGEIYRTFVLSCMSGFIVIPLYPAFLDIIIPLNETRQRHQMFRLTYFLDENRYFYPI
YFHSLWCSFVTVMIAVTIDSLYIQIVHHDCAIFAICGQNIITATGSTDVRINETYTERFRQCLTMHKNAL

QLFEMLDDSSRRSFFFQILLTMVGMTITAVQAVINLHRPEEALRIGLFLVGQQFHLLIITLPGQVITDHS
FELTNDIYRSMWYNMPINVQRVLHMMQMRSSKPCKLTAGGIYQMNIENFGIVRT

>gi|815928688|ref|XP_012248811.1| PREDICTED: odorant receptor Or1-like, partial [Bombus impatiens]

MLDLLKKKPFVPENNGEGEIHARYDNLIEKVAVGYTIQITFCVLSLLGMALICDFKLKKLMFPGWFPFDI
TSSWLAFSMTFLYQFLGLVIICIGVCIFDTLFVGLLLHICCQLEMLVYRLHNIEGHEIQSLKHCVWHHNK
IFRFADMVNNFFNKMMFVQFMASAVAVCFTLYLLTDVQDTAQLIGWSTYMFAGICQTFFFCWFGNAAKVK
SLDISNMVYNSDWSTMFLI

>gi|815927557|ref|XP_003494290.2| PREDICTED: odorant receptor 13a-like [Bombus impatiens]

MKSNMSLKYHKDVSFSVAVFYLKIVGLWLSTSLAEKWFRNALVTYTVLAIIFNMWMQLRGLYFSWGDFSV
STYIACNSLGLFMDLFKLLIIFIHKKKFLYLIVYMQKNFWHFNYNQYKKSVLADAKRMCIYFVCVFSFLS
QSTIFSYIFMPLISNIGKNKSDRVPIFHMHLDLPLNVSPYYEMTYLIQALTLYQVGVCYLCVDNIFCIMC
LHVASQFRILQYRIANVLSLRDKVKFDQDTNLDSSDEFYAIFRKCIQQHQALIRFCTTLEEIFTVIILGQ
VLTFSILICFVGYQALLVKLSLSWRISLVSFLTTNICQLWIFTYSCNALIEESMNTANAAYAAPWIYLSM
DKFGEMTRKDLQLVLMRSRRACYLTACGFFPISLETFTKIMSSAMSYFTILKQRTVDTIE

>gi|815927462|ref|XP_012248265.1| PREDICTED: odorant receptor 49a-like, partial [Bombus impatiens]

MMTSITTVLIPMSIALYESLYTKNLDVMLECLPHLAAIVTSFVKIMNVYLNRENFRKLFDSITKEWQQLK
LSQDLYILEEVTIRGSKMAKLYRNTLMVFMVLFLLVPLISPMMDILLPLNETRPRQQLLNVNYVLFDSHN
YFFCVYLQLSWAAIVVSISIVTVDSLLMLIVHHNSGLFIVCGHQIQKKTRHLNSFTNEAMTEHYTYKEIR
NCVIMHTKAIDFYDILDENNRLSYMIQIGLTMIGITTTAVQTVINLDRPGISIKSAVLCGANQFHLFMLS
LPGQILVDHCTELTKQLYGSTWYGVPVKTQKMLYMMQIRTRSPCTLTACGLYQMNIENFGTFNKLFQLVA
KQWEQLKLNGELHVLEEVVKQGNRMAHFYRMFTIIGADWLYMLIIHHSSGLFAVCGHRVQKATVNPNYFT
GDAISENYTYEKIRNCAIMHNEAIRFSDILKESSRGSYLIQVGLNMLSISATAVQTVINLDRPEEAIRSA
VFCGACLFHLLLLSLPGQVLLDHCSDLADN

>gi|815925234|ref|XP_012247254.1| PREDICTED: odorant receptor 33b-like [Bombus impatiens]

MVLVQHACGLLIVTGYRYKNAINDLSFNTKDSEKKAKETYERVRFSIQAHQRAIMFLEKIESTHVTYLFM
CMGIVVLCVSITMAQIATMEICVDFYKFISFLVIQFLHLFCLTMQGQFIINSSDEIYDAIYEASWYNTNP
KTQALYLLALRRSLTPCYLTAGGLIRLNMESFSEVIKLCVSYYTVLRST

>gi|815924108|ref|XP_003493307.2| PREDICTED: odorant receptor 24a-like [Bombus impatiens]

MSKELKIYRKYASFVKRFLLLSGMCPITKKRDVFYRCISIWSIFSSFISLCVVGNFCSQNVQNIALLTAS
FSLFCAILNTTMKACCFFIYQNKLQQANDILSSMLEQALSETDIGSIAFSWVRTFYRLIYLQFTLMAINS
TIHAFKPLITRILYNANNTTNLQYPLPFPASYPWTIDSMLVWQLHYLFDLNIVWNIISVSTGVDGFFSFC
LFRISVMLRLLGFEFEMRFSTDEKNKANKEYEENRKRIFQECVNKHALLLKCRDIVQEAYGPVILLTTIT
SATSLCTLIFQVLQVKGQIMGKIIMCTVFIFMKLLQTFLYAWPADVILIESDRFRHKVYFSDWYKHKDTS
FAKGFTLILAQRSIVLKACDLMQVSLDLFVKVLNTAVSYYFLLETIDKDK

>gi|815923208|ref|XP_003493069.2| PREDICTED: putative odorant receptor 85d, partial [Bombus impatiens]

MKELSNTSIDYYILPNRIFCSMVGMWPIEEKSSTCSKIFAYIRLILALIAINSIFVPEIMMIVSSWGDIT
ILAGVGCVLTTIGQLLFKMIYLIVRRERSYRLYYEIRSLWDTANDSKEMQPYIRVAYWARICTIAFYLSC
MCNVITFSIAGVVDYFRFEYNASSTDNSRHLPFVVWYGTDISASPKFEIAFICQILSSMVCATTICGLDA

SFMTTILHVSAQFKLINTWISNIGTEINCNPNYTRKIKIELMRCIRHHQRMIHVVNDVNNLFTPIIFMQI
LTSGIEICLSGYAMLDNEAEITDILKCTSYFISVTVQLLLWCWPGEILIQENQEVGQVAYFNIPWYNLPS
MYQRHLCLMIVRAQQYCSISALTFQTLSIHTLTTVFNTAASYFTLLRQI

>gi|815920800|ref|XP_003492337.2| PREDICTED: odorant receptor 9a-like [Bombus impatiens]

MKSFYDDWRSAKTEEEKTAMLKMAKPANFISVWCSILTLTMVTAYLSLRSISVYLSDRLHENRDHLSLYP
GYFPYNIRPVPILLMTNFAQVIAGYSATICYTTVDTFIAMLVLHICGQFEILRKKLTRLLDGEEGNRSID
EFRKELVWIITKHEHLNWHAQTIEKCFSTLLLLQMLLCTIEICFQGFLFFNVLIKNENGIFNFQLVFFVL
FVCFILVHVYLYCYIGEMLLIQSREMSNSAYESNWYNVSPSQTKCLLFIMNRSTRPLCLTAGKFGIFSME
LFSTILKTAMGYLSVLLTVANND

>gi|815920798|ref|XP_012245316.1| PREDICTED: odorant receptor 67c-like [Bombus impatiens]

MLCFACAPQTINLPMIAGDSDLVIENLSTNITVMVSLMKTLTVWINGIPLKSLLRYMANDWDAVTTNIER
ETMVNTARITRKITIGSTLMVNIVILAFVPARLSSMKNNDITLFLRGYFPYNTSISPNFELTMIGQYVAA
IYAANTYTTVDTLVVLLIFHVCGQLSILRQDLGRIDSYDKKNIEMKMQKIVEKHEYINRFVLRKFVILLK
ILGNIYSEENKLEFAGRIENSFNMMLLFQMLSCTIQICSQFYQVMMSLGENTVEDMIFQISFLLIYVFYV
MLQLFLYCYMGEKLAAESTEIANIAYSTKWYNLSPKNARWLVIIMCRATSSPLQITAGRFCSFTFALYCQ
TAACLFMSYEAICGDDGLK

>gi|815920796|ref|XP_012245315.1| PREDICTED: odorant receptor 4-like [Bombus impatiens]

MGKNASMDIQRDVRYMYGWNYYMLRFMGIWPEERKWYRAYSYLVLLPCIMMLCFVCAPQTINLTMIASDS
DLVIENLSTNITITLSLMKTIVIWFRGKSLRPLIKCMVNDWDTVMNETERETMVNIAIITRKTTMRSTLM
VNIVVLAFLPARLSNMRYNDSALFFRGYFPYNTSISPNFELTMIGQFVATVYAANTYTAIDTFVVLLIFH
VCGQLSNLRDDLQKIHSYDKKDVETKLRKIIQKHEYINRFATTIENSFNMMLLLQMLGCTIQICSQSYQI
IMSFEEEAMEYMIFQITFLLLYVVYVMLQLFLYCYMGEKLAAESTEIANTAYYAEWYNLPPKSARWLVII
MCRARSSPLQITAGRFYWFTLALYTQVSVNES

>gi|815920792|ref|XP_012245313.1| PREDICTED: odorant receptor 22c-like [Bombus impatiens]

MLKFMGIWPEERKWNRPSSYFVLLPCIVMVCFICAPQTFNLSIIAGDSDLVIENLSTNITITISLMKTMA
VWMKGKPITYTTVDTFVVLLIFHVCGQLSILRDDLRKIHSCDDKNVEIKLQQIVQKHVYINRFAETIEKS
FNMMLLFQMLGCTTQLCSQTYQVLMSLGEEAIEHMILQITFLLIYVIYVMLQLLLYCYMGEKLTVESTEI
ANTAYNAEWYNLPPKNARWLVIIMCRARSSPLQITAGRFCCFTLVLYSQVLKTSMGYVSVLLAMKNK

>gi|815916412|ref|XP_012243371.1| PREDICTED: odorant receptor 9a-like [Bombus impatiens]

MIGSLLTLHYPGFVQIYTSLYAKDVAKFLEAIPVITTLTAVLIKLLNHVIYKENFEKMFHIIKKDWELLN
DKSEAQILKEITKEGKKLGEIYRTFMLSCMSGFIIIPLSPVILDIISPLNETRHREQMFRVTYFLDEDQY
FYPIYFHSLWCAFIITMIAVTIDSLYIQIVHHDSALFAICGQALITARKSTDVDTNKTYTEWLRQCLTMH
NDALQLFEMLDDSSRRSYFFQILLTMIGMTVTAVQAVMNLHQPEEALRIGLFLVAQQFHLLIITLPGQVI
TDHSFELTNDIYRSMWYNMPINVQRVLHMMQMRSSKPCKLTAGGIYEMNIENFGTTFKTCVSYFTVLLSL
GD

>gi|815914351|ref|XP_012242451.1| PREDICTED: odorant receptor 22c-like [Bombus impatiens]

MYLYQFPVMFVALSHISAISLLVNLVLHICGKFSILSYRIQNISTNSNVNLDSVIKEFVTAHVKLITTAN
SINSALQVFLLVELLQTTIRMATIIYMMLLNPSGSFVNTLTYVLYISIIISMLYLYSFMGEQLSNESMKV
SEAFYATDWNNLSLHNQKLLLLVMTLGQQNLHVTAGKFYTFSLYGFIAVMKTSFGYVSLLRTLI

>gi|815912130|ref|XP_012241492.1| PREDICTED: odorant receptor 13a-like [Bombus impatiens]

MSMYLNAIFNTKKMRDVLLFIKNNHNYYANRPEKLILRYYDVQGRKITLYYALYVYTTVVAYITIPAISL
LIDFIIPSNHSEEKSFPIELDYGVDTQHYFYYLFIHSYMTIAMIANLIASCDTTYMLYAQHGCALFAIVS
YELRTVHILDASSLINLKDHRLFENYKNIELLPEDEKKICTKLFLCIKEYQNAIRYCNLVESLFAKSIFV
QLFFNVVCLSIAGVETVMKLGNAADTIRFGSFTFAQVAHIFVLCFPGQRLLNHSEEVYNAACDAMWYIFP
KKCHNLYKFLLARTLIFSKITAFKVATMSMETFLAIIQTAMSYFTVLLSTT

>gi|815911912|ref|XP_012241397.1| PREDICTED: odorant receptor 4-like [Bombus impatiens]

MYPSIRSQTDQSRNPNYKEDIIYVTKHNKWILNCIGIWPTVLKGMSKFLPKLVIGFSNLMPSFIIVQCVL
YIVLEEKNPLLRLRFCSLAWYSLINLMKYWALIARKPDIEYCIKWMQTDWKQVEFQRNRMLMLKYGKIGR
DLTIYSAVFMYSAGMCYTTIMQYGMRMSLKENNRTLRILVYPTYSGLFDIQKSPVYEIVYVFQCIYAFTC
ISVTVGCCGLAALFATHACGQIDVVISQLDDLVDGTFSKKSSNPDTRLMEIVKHHIRILKFSAMIETVLQ
EVCFFDFIGTTLLICSLQYLCITDLQHNNKIGLATYSMLLIGFTVNMMLLCYIGNLLMEKSTSVGISCYM
IEWYRLPGKTMQGLILISAMSNSPAKISAGRIFLLSLPAFGNILKTSFAYLNFVRNTIVL

>gi|815911908|ref|XP_012241395.1| PREDICTED: odorant receptor 13a-like [Bombus impatiens]

MQLSMQHKCDYTPRNLYYKKDIAYVTKHSKWILQSIGIWPAAVGDVKKLLPKIAIALSNFVLLFAIIPCI
LHIIFEEKDTIMRLKLSGLLSFCCTSLMKYWALTLRKPRIKGCIEQVCIDWEQVELHKDREIMLKYGRMG
RNLTIICAVFMYTGGTIYHSILQYAIGTFVDEHNRTIKPLVYPTYSALYDVQSSPIYDLVYVIHCMCGYV
MYSITAGACGLAALFATHTCGQIDIIISRLNDLVRGEYMKETLNLNTRLIEIVERHLRILRFSAAVEMVL
QEVCFLEFIGSTFMICLLEYYCITDWEQSNTISLTTYTMLLISLTFNIFILCYIGELLIEKSSNVGLSCF
MIDWFHLPTKTIHGLILIIAMSNNPVKITAGRIADLSLSTFGSVLKSSLAYLSFLRTIVMQ

>gi|815911906|ref|XP_012241394.1| PREDICTED: odorant receptor 4-like [Bombus impatiens]

MYDRSYTIGDDQLKNNHYQNDIQYTLQMCQWLLKLIGIWPLLNNHTSRLEQLLSIVLMITCYSSIFFIIL
PSGHHFFFVEKNLYMKMKMLGPVSFCVFATVKYSYLARKGAFLQSCIRQLRNDWKRVQDPSHRAIMLKYA
GVSRKLITVCAVFIYTGGMSYHTVAQFLSKERTRENYTVRPLAYIGYDPFFDTQSSPTYEIVFLLHCFAA
MIMYSITTVAYGLAAVFVTHVCGQIQIQIARLQNLVESKDRDLFSVIVHDHAETLKILRFSKNIEDALYQ
ICLTEIVECTINMCMLEYYCLVEWANSDLIATLTYMTLLISFTFNIFIFCYIGELLSEQCSEIGTVSYEI
DWYNLPAKEAYDLILLISISQYPPKLTAGKIIELSLNTFSSVAKTSLVYLNLLQTVADW

>gi|815911898|ref|XP_012241390.1| PREDICTED: odorant receptor 13a-like [Bombus impatiens]

MFTSTRLSTRSRTDQSRNPNYEEDIIYVTKHNKWVLNSIGIWPAMVEGIGKFLPRIAIGLSNLILFFTLV
QCVLHIVLEQKDPLLRLKILGLTCFSFISLMKYWALTIRKPKIEYCIEQLYADWKQIEYQRDRKLMLKYG
KIGRRLTVYSAVFMYSGGIIYHTVMQYAIGSYVDEFNHTIKLLVYPTYSGLYDVQKSPVYELVYVLQCIC
GYVFDTVTVGACGLAALFATHTCGQIDVIMSRLNDLIDGKFSKENSNTSVRLMEIVEHHIRTLKFSAMVE
TVLQEVCFLEFIGTTFVMCLLEYYCITDWQQNNKIGLTTYSLLLISLTFNMFLLCYIGDLLIEKSTNVGI
SCCMIDWYHLPAKSVQDLVLIIAMSNNPAKISAGRIVNLSLSTFASVLKTSFAYLNFLRTALV

>gi|815911896|ref|XP_012241389.1| PREDICTED: odorant receptor 22c-like [Bombus impatiens]

MHLSVRRQTDPPQNTKYEEDIDYVTKHNKWVLNSIGMWPVVAEGIDKFLPKIIIGFSNLVSLFTVVQCIL
HIILEEKDALLRLRLLGLACFASINLLKYWAVIVRKPNIEYCIKQVQTDWKQVEFQKNRMLMLKYGKIGR
DLTIYSAVFMYSAGICYVTIMQYAMGMSLKANNRTIRVLVYPTYSGFFDAQKSPIYEIVYVLQCMCTFVF
NSVTVGCCGLAALFVTHACGQIDVVISQLNDLVEGKFAKKNSDPNTRLMEIVKHHIRILKFSAVIETVLQ
EVCFFEFVGSTFVICLLEYYCITDWQQNNKIGLTTYSMLLVSLTFNMFLLCYIGNLLIEKSTSVGISCYM
IDWYRLPVKTVQDLILIIAMSNSPAKISAGRIFLLSLTTFGNVLKTSFAYLNFVRNTIMY

>gi|815911888|ref|XP_012241385.1| PREDICTED: odorant receptor 4-like [Bombus impatiens]

MRSSSDVNDHLQNSHYKSDIYHTFQFCHWILKPLGIYFFLHDHVNKFEKIVSVLLIIICCSILQFVIVPF
GYYILFYEKDMNMKIKFLGPLAFCVTALFKYGYLGVKSSELGRCVKHVKKNWKMLQDKDHRAIMVRYVTM
GRNLITLCAAFMYTGGLSYHTIMPLLSKKKINENITIRPLTYPGYEAFFDIQESPTYEIVYCMHCVYVLI
TGNITMAAYSLTAIFTTHACGQIKIQTLRLENLRKDGKALKNGIEDHLAVIVSEHVEILRFTKNIETALQ
GLFLIEVMLSTLLICLLEYYCMMEWETSDSVAISTYIILLTSFTFNILIYCYVGELLLGQGSEIATALYD
IEWYNLPGRKARDIVLVLAISKYPLKLTAGKILVLSMNTFGVVRVEILASLPEHAANGY

>gi|815909105|ref|XP_012240198.1| PREDICTED: odorant receptor 4-like isoform X1 [Bombus impatiens]

MILNTISPSVKFGLHFCGVWPGTPFHFLHKLCWVIAIITLQTYQYKYIVIHYNTDTLMNIAENLSIAVPF
SLVLIKLFVTWTNYGLFCDILSTMEEDCQKYAIMDINNLITKTGLFSFYMTSMIMSSYLVSAVCYLAGAI
AFQGTNDSMSRELLLKMDLPFETSESPNYELVVTTQFIIHFSAALAFGTFTALLFMMILHLGCQIDIMCQ
NLTDVFSKTENKLKFFISRYQEIIAFAEKIEKLFTYMALSQLVSNTVNTCCEGFLIVLAVNDENGLAIDI
LIKSIVFYIVICLEVFIYCFAGEYLRTKSQLIGDTAYNMLWYDLRPNKSRLLIPVILRSQKGFTLTFGKF
SNLSLESFTGIMKVSASYMSVLLALD

>gi|815909022|ref|XP_012240158.1| PREDICTED: LOW QUALITY PROTEIN: odorant receptor Or2-like [Bombus impatiens]

MHTGQYTDMSIKMSGFLLKITAALVYGVYVNVVDIYHNLHNLDHCIFLASNTLNIILAMFKLSVLHFYKT
EFSDIIVYAQKHFWHIDYNDDEKILFAECRNFCKLWTVFILLVTESSLSFYAIVPISANIGNNGSERLLP
FKMWVNLPLTVTPYYEIMFAIELLAVQQIGASYLCPEQFLCVLNLHVVYQFRMLQKTLLNLWSNIDEQTD
IADYSNKYYIILKKCIRKHQWLIQLNAQLEXVFTLPILSHMVIFSVLMCFDTYEIVLANISSEKRLIFFF
HMIGSFAHIIFFTYICHGLVEESTNISIASYSGWWVCLPMSKTGKKIRKDMKMMMMKAMRPCQLTAGGFF
PVNLETSTAIFSLQLISSTMSYFALMGESSMRVAEQ

>gi|815909018|ref|XP_012240156.1| PREDICTED: odorant receptor 4-like [Bombus impatiens]

MPAQVFQYRYIMKNVSSGNLADVIESASTSLPYTVLLFKLISFWIKRGIFKNMLVGMYYDWINSSADKAN
VDVMTNKAELAYRCSYSIFGLLMKMDLPFAYYESPVYQYVFLVQFLQLLAVGIGMAILNALIITLIFHVG
GQIEMLHEALENISIKDEKHGSLRNVIKSLINRHYRIIFNSEYIESLFSYIALVQLLCNTVVMCGIGFLI
IVAINSHGDVGIVVKIVLFYIAIMLEAFVFSYAGEYLSSKSLSISASAYGSSWYLLEPRNRRVVILLMIR
SQRRLTITAGKFMDLSMFGFASILKASASYVSVLYARY

>gi|815906995|ref|XP_012239264.1| PREDICTED: odorant receptor 2a-like [Bombus impatiens]

MFNRDNDKYEHITAYYTWQAIFHQITFQTFGFIAVISWGSTPILYQISGTSKQLPMVGWYPYNVTSTPVF
EFTSLHQFMVVCTSCINNIAIDTLATGFIVTACCQLTILNYNISSMHCIIEKKYTLLIDNVSIENSTLKV
YSKMYEDLKHCIEHTIMIFDFSKQIQDVFGTLIFLQLLVDCIVICAILFNLSQMKDYVTSEFLGALLYTC
CVIYQIFIYCWHGNELHFHSMRICSSAYANNWWDNSKDFKHALLIIMARAQWPLILIVGNVMELSLQNFV
LILRTSYSIFTVLRTSTAT

>gi|815904383|ref|XP_012238122.1| PREDICTED: odorant receptor Or1-like [Bombus impatiens]

MLLRLKAFFYFLFSSSWNTLGYMTILMTSNFYLFMESLLTYKKRQLTYRTWVPYDYSSASAFLLTLLYQS
LFTTICSFGCVATDSLYSGLLIHITCQFEILEHRLKNIESNQNYSVKLCVRHHNQIYKSLPTTLYNFFTV
TFSYLRAYRFSEMVNEEFRTIMFFQFYTSLCMICFNLYQITQMEMDSNIIGRILFMNFSLMQIFYYCWFG
NEVKLKVQHYDEECNKTLRLDFLYASHVYSLELSDVIFRSDWTSLNNNVKRAFLMLMRRAMRPIEFTSIY
VISVNLESFMTLLKTSYSVFSVFQQSQES

>gi|815903135|ref|XP_012237574.1| PREDICTED: odorant receptor 67c-like [Bombus impatiens]

MDVFYRQYNTYRILLSISGLWPYHKSIYSTIHRISISVIMLAYIVFEVLSLFKSGITFRNCILTLSTTCP
IMVFFMRYITSVAMSPVVRDALKNVYIFDNLRKTDTTLKDELEVQILMKYVDYATYIISIFLCLCCSWTI
LGALYVFTPITLDLLLPLNESRGRYFSYLTMFSHDRIEYVDMVCVNILVVYTIGLFCLAGTELMLAVFAH
YMCGLFEITSYRLRKTIADLSSSRQMDLNFRDIRHVVDCHRNTLQLVSKTITDAKDEKEIFICIFFLIGH
LAFLYICCYSGQLIIDRSLNVFKDSYNSTWYYMPLEAQKLLLFIMLRSSTESVINIFGFFVASHAGYSKL
LSTSFSYFTMIYSNQ

>gi|815902073|ref|XP_003486905.2| PREDICTED: odorant receptor 49b-like [Bombus impatiens]

MSDDKTKREFEKTIDLNLALLRLAGVVSSERASRDTLASLAFVCMTINTVSYVYEFATSSYTLATVLESF
AMIISLVAGQTRLVILLWLRDSCQTMLNICESFWSTLNLREKKIVQSYTNKAKLLSRCYLFSCVSTIFFY
VLLIQFGSLIFSEPKHFANVTLYGNNSSFVPSEAAKSLQATDDKRRRLPYAFFIDVQETPWYEIVYVVQL
GSMISVGLTCVGVDTTGPLLILIACGYFDTVRSRIENTYSFESPSSLPILASLSIATKTKIATESTSVTR
TESSSRNVGMKNLRTCLNHHQLLLKLCEDIENLTNIMFLIQLITSTYNISLIGFKIVEDNPGKVKFVTQL
GIFIIQLFLCNWPPDLLRSKSEAIGHAGYSMPWYRYPCNLRNPVNMLLIRGQKPVRLTAGKFIELSLGTF
ASMISAAASFFTMIRSMN

>gi|815900272|ref|XP_012236302.1| PREDICTED: odorant receptor 4-like [Bombus impatiens]

MKRLLDIMREDWEIYKKLRNEYDLLCEQYAIGKKITVSFVAFLFGLITPFAAMPLLLNAADALGLCNISD
DRPLAFRVEHFVDVDKYYFPLLVHSYIGTLAYTTIVLAINSIIAVYVLHESGLCEVLRFKLENFVESDVM
DVKLHANKRDDKWYENARDCVILHKHIIEFANILEDANTTSYLLQIGFNMICVSFTQFQAVINIQENAAL
AFRYISVTISLLCDLLFVSWPGQQLSDSTERIFEFTTNGKWYLSSINCRKLLMMMLSKSITPLKLTAYKF
YTLNLESFSAVRFIFYIDRSLNRHQVILKLNDHSGPYRIMIILRECRYIGLATK

>gi|815897750|ref|XP_003485761.2| PREDICTED: odorant receptor Or1-like [Bombus impatiens]

MAQEPGHEWMKPFRKLINLEISYIKYSGLGEIDSSSAFLNCAYFVYKIWMLIAMYIFAITLFLDIYVNRD
NLPISTDDGCIFAGIFVVIYKAMNLQFRQKGITKLLDETLKCTDDLCEFSEVEYIKEIIEKHQVQSKMIF
YGFTALGSILGTALLFFSPMEDGLLIRAKYPFNTTISPWHEISLVIETCAVFGGLLGIIGIDSFVVMICT
LLTVLFDMLNVNFENCGIETKEHTVESYDERIHANIRFNRKKLNNRFLYRYKTCFQFYQRLVCMTNDYNK
LFNLSMFIQMLSSTSIICLSGFQAVVVGGQSSDVMKFGIYLSAAISQLLYICWIGNELNYASWTLDRSQW
LSGWNNERLTNIVKMFTLSTMFTRRSITLKASVFYVLSLETFITIIRRSYSIYTLLNNMQVTDH

>gi|815897156|ref|XP_012249339.1| PREDICTED: odorant receptor 9a-like isoform X1 [Bombus impatiens]

MCGFISFFGPTSMQLYDAIRNKDFDNVILNLPHILTVFISVAKILNIYSNKTLFRKLFNSLEEDWKLLES
KNELQMLDKFTKHGYKLAYLYRRTLLIALVIFLSLPLYNPVLDIIIPLNETRQRNNVFQVHYGVLDNEEH
FYIVYVHLSLCAIIIVVTIISVDSLYITIIYHACGLFAVCGSQIQKTAENNFVEKNGTNIRNIGYDAFKE

CVMMHYKCLQLYDVLEKCCRNLYLITMVLNAIILSVTAVEVIVFLDRPAEAIRAIVYLIAEQFHLYMFSL
PGQTLLDQSVELSNKIYDSDWYKIPTKAQKVFYLMQVRSNKPCILTAAGMYEMNIESFGIVRNNGN

>gi|815895919|ref|XP_003485159.2| PREDICTED: odorant receptor 4-like [Bombus impatiens]

MWPIEQKSSTCSKIFSYIRLTFTLISLISVFVPEIMLIAVNWGNIEILAGAGSIVTTLGQTLFKMFYLIA
RRERSYMLYYEIKSLWDTANDSNEIQSYIQFAYWARICTIVFYSSCVCNVITFSSAAAVDYFRFDYNANS
TSNSRHLPFIVWYGTDISASPKFEIAFICQFLSATLSITSIAGLDCSFMTTMLHVSAQFKLINTWISNIG
TEINCNPNYTQKIKVDLTRCIRHHQRIIHVVNEVNNLLTPIVFLQILTSGIEVCLSGYAILDTGKAEADL
VKFISYFISMAVHLLLWCWPGEILVQESQEIGHAVYFNVPWYDLPPIYQRHLCLMIVRAQQYSSISALTF
QTLSIHTLTAVFNTAASYFTLLQQIQQT

>gi|815895212|ref|XP_012241927.1| PREDICTED: odorant receptor 13a-like isoform X3 [Bombus impatiens]

MLATDGLIATALLHTCGHFAVLRKNVKQLDSYIYRVCIVFYYIYVYNCNVIYFKIIQLLQITRLKTNSKH
INANLYEIKIQIIHVIKHHQVVLWFCDNMEKNFHLILFLQAMISSLLICFVGFQVSATLMEQSKMIKFAS
HLIVAFFQLLLFCFPGDMLIQEVDDII

>gi|815895210|ref|XP_012241922.1| PREDICTED: odorant receptor 13a-like isoform X2 [Bombus impatiens]

MLATDGLIATALLHTCGHFAVLRKNVKQLDSYIYRITRLKTNSKHINANLYEIKIQIIHVIKHHQVVLWF
CDNMEKNFHLILFLQAMISSLLICFVGFQVSAVRIFTDLKIVINHKQLIICYLIHIFALQTLMEQSKMIK
FASHLIVAFFQLLLFCFPGDMLIQEVDDII

>gi|815893781|ref|XP_012250080.1| PREDICTED: odorant receptor 67c-like isoform X1 [Bombus impatiens]

MSEKIGNSTNYRNVHYEADAEYTIHIAKTLLKLIGIWPRRDTFLDNVKMYIQTTIVFSLMCFLLVPHVIY
TYFDCENLTKYMKVIAAQIFSLLAVIKFWTVIFNRKEIRFCLDQIEIQYRDVKCEEDRLVMTNCAKIGRF
FTMMYLSLSYTGALPYHIILPLISERIVKADNTTQIPLPYLSDYIFFTVENSPIYEITFVLQIFISSIIL
STNCGIYSLIASITMHCCGLFEVTSRRIETLHKWNKCDLHDRVVDIVQYHLEAIGYSALIGKSLSIVFLS
EMVGCTIIICFLEFGVIMEWEDHKTLSTLTYFVLMTSIFVNVFIISFIGDRLKQESERIGETSYFLPWYD
FPVDVAKNINTIMLRTRLPSCLSGANILEISLQAFCDVSAHTHYFKYIDRKLFEQRKDYCRFVKLQQLIS
IFCEQ

>gi|815893761|ref|XP_012249992.1| PREDICTED: odorant receptor 4-like [Bombus impatiens]

MENISALTKAEEDMKYATRFVKPILGVVGAWPVSSSSSSFSKILTRIEHILTYFLFLLLMVPTLMHVFLK
ETNNKIRLKLMGPIINCSMQFCAYTILLCRANEIQNGLNVIKQDWITATEENRLIFRSKAKIARRVVLTV
AITMYGGGLCYRTVLPLLKGTIVTPGNVTIRPLPCPSYFVILDEQKSPNYEILFVLQVMAGFVIYAVISG
SCGLSALFVLHACSMLSILVDKMKALVDTRDMSDTMVQRRIMDIVEHQTKIKGFLKNIETITQYVCLSEM
ICGTSLEWENNNATAVLIYSTIQISCTFSVFILCYIGQLLVDENRIVGQASYMINWYYLSRKHMRSLILI
IAMSNYPMKLMAGKMVEMSLATFTDVMKMSMGYLNILRNVI

>gi|815893755|ref|XP_012249963.1| PREDICTED: odorant receptor Or2-like [Bombus impatiens]

MALGAWPLPPESSLCMKISQRVIRFIIISLALFFVSPGFYYVFFKEKSNKRKLQIITTYINSFVQLIKYI
IILYRMKDIRILSEEIRNDWLHSTEENRRLFRENAKIGERVVFIAAFTLYSGGFCYRTILPLTRSSIVLP
NNITIRILPSPTYLPFINEQITPYYEIIFVLQVLSGFCIYTVFSGAIGIMMMICLHTCGLIRILMDKLVD
LTDKSNTSEEIIQEKMANIVEYHGKIKKFLSNGQQLSEYISFLELFNGAGIVCLIGYAVIMEWENLNPVT

IVVCFTLLTTFTFTCYTICSIGQLLLDESNNLAQTCVTLNWYRLPMKQARYVVLMMIMSNDPMKLTAVKV
MDVSLSTFSDIMKASMGYLNMLRNVT

>gi|815893753|ref|XP_003484570.2| PREDICTED: odorant receptor 13a-like [Bombus impatiens]

MSEKIKNIIYRNDHYKSDSEYVMHIAKTLLTLIGIWPRKDTFLDNVKMYIQTVTIFILMCFLLIPHVIYT
YFDCEDLTKYMKVIAAQVFSLLAIIKFWTLIFNRKEIRFCLNEIEIQYKDVKCEEDRLIMLNCAKIGRYF
TMVYLALGYSGALPYHIILPLISERIVKADNTTQIPLPYLSDYVFFVIEDSPIYEITFVVQMCISCIIMT
TNYGIYSLIASITMHSCCLFEVTSRRIEELRKWDKRDLHDRIADIVQCHLKAIEYSAVIGKSLSFVFLSE
MVGCTIVICFLEFGVIMEWEDHRTFSTITYFVLMTSMFVNVFIISFIGDRLKQESERIGETSYSLPWYDF
PLNEAKNVRTIILRTRLPSSLSGAKILDLSLQAFCDVVKTSAAYFNVLRAMAA

>gi|815893711|ref|XP_012249779.1| PREDICTED: odorant receptor 43a [Bombus impatiens]

MTTLSVTLVKVASQANISGQLIKDIVFLLAQLVHIFFFLLQGQFVLNANDEFAESIYNTFWYNTNTRTKL
LLVLVLRSCSSAPNLSAGGLLVFNLKNFSEILKTSFSYFTVLKTT

>gi|815893599|ref|XP_012249301.1| PREDICTED: odorant receptor 33b-like [Bombus impatiens]

MSMASFTVSLVKVASQSEISGVLIKDSVFLLSQLIHILLLTVQGQFVLNSNDEIIESIYDASWYNANRKT
QLLFVLSIRSCLSPPILSAGGLLDLNLKNFAEIIKASVSYFTVLKST

>gi|815893327|ref|XP_012238845.1| PREDICTED: odorant receptor 4-like [Bombus impatiens]

MVCFACAPQTINLLLIAGNSNLVIENLSTNITTTISLMKAMAVWIKGKHFNCQTKWTLKTYSICHFLLLQ
ALKFLVKCMANDWNTTTDKAERETMVNIRRITRKTTIRSTLMANIVLLAFVPARLFSMRYSDNMLFYRGY
FPYNITISPNYELTMIGQFMATFYAATTYTAVDTFVVLLIFHVCGQLSNLRDDLRKIHSYDKKDLEKKLQ
KIIQKHEYISRFANKIENSFNMMLLLQMLSCTIQICSQSYQVIMSFGEQEMEYMILQLSFLLIYVVYVML
HLFLYCYMGEKLTSESTEIADTVYNAEWYNLPPKNARWLIIIMCRARASPLKITAGKFCSFTLVLFSQVS
VDES

>gi|815921554|ref|XP_012245653.1| PREDICTED: tyramine receptor 1-like [Bombus impatiens]

MANQTANYYGDVYQWNHTVSTVDRDTQSEYYLPNWTDLVLAGLFIMLIIVTIVGNTLVIAAVITTRRLRS
VTNCFVSSLAAADLLVGLAVMPPAVLLQLTGGTWQLGEVLCDSWVSLDILLCTASILSLCAISIDRYLAV
TQPLIYSRRRRSKRLAGLMIVAVWVLAGAITSPPLLGCFPRATNRDTKKCSYNMDSSYVIFSAMGSFFLP
MLVMLYVYGRISCVIASRHRNLEATESENIRPRRNVLIERAKSIRVRRTECVTSSVTCDRASDEAEPPST
SKRSGIVRSHQQSCINRVARETKTAATLAVVVGGFVACWLPFFILYLATPFVPMEPPDILMPALTWLGWI
NSAINPFIYAFYSADFRLAFWRLTCRKCFKTRTNLDRSNRKLPAPTNWKKDNMEDVKIGE

>gi|815916723|ref|XP_012243495.1| PREDICTED: allatostatin-A receptor [Bombus impatiens]

MFQEMETLDRIARNFSNSSYNETHKPFDDFDGYNFDRHQVERIVVVVVPIFFGMIGILGLVGNCLVVIVV
AANPGMRSTTNILIINLAVADLLFVIFCIPFTATDFVLPYWPFGNFWCKTVQYLIIVTAYASVYTLVLMS
LDRYLAVVHPISSMSWRTENHAIVAICIAWAMIFAISTPAFIVHGEDMDDLSSENLTACRILPQYSWPLF
QLSFFLMSYLLPLTLICFFYICMLIRLWRTDRVSAESRRGRKRVTRLVFVVVGVFAFCWCPIQVILVTKA
LDVYRLTSATIMVQIASHILAYTNSCVNPILYAFLSDSFRKAFRKIIYCRPRPDQTRQLGPLTKTTRAAS
TGDIL

>gi|815915602|ref|XP_012243011.1| PREDICTED: FMRFamide receptor [Bombus impatiens]

METSLKIFNSTSYYDAGQLLENLSIKETTREPLECKQEINSNGLFDFIIYGVLMNLVGLFGIFGNAISMI
ILSRPQMKSSINYLLIGLARCDTVLIIISILIYGLPVIYTYTGQLFDYKFIVHPKIVRFLYPLSCIAQIA
TVYLTLTVTLERYIAVCHPLQARSFCTYGRARLAVLVIVLFSFLYNLPKFWEVDYYAEVHWKYNVTIYCI
YPADLRSNNLYVTVYVHWMYFFICYLFPFLALVIFNAAIYQRVRKANRDLQQLSRHQRREIGLATMLLCV
VIVFLICNILPLASNIHETFFSDPPHWLVQTGNLLVTINSSINFIIYVIFGRKFKRIFLKLFCNSKLFRP
GRDSPEFQTYDESIVTNMTNIELRNSIRHYHVNRSSTINRNNNVSNGSMRQSVKISARPASPGPCVYYPA
RSPVRSPSQMSATSSTQNGWSKKDVNSSL

>gi|815914631|ref|XP_012242568.1| PREDICTED: dopamine receptor 2 isoform X2 [Bombus impatiens]

MSARSWLRVPRKMNESAVYLLGSEEEANVSSNQLNRSFYNASFPPQNRTYEDLWNLATDRAGLAILLFLF
SVATVFGNTLVILAVVRERYLHTATNYFVTSLAFADCLVGLVVMPFSAIYEVLENRWLFTTDWCDVWRSL
DVLFSTASILNLCVISLDRYWAITDPFTYPSKMSRKRAAILIAIVWVCSSAISFPAIAWWRAVRTEEVPE
DKCPFTEHLGYLIFSSTISFYLPLFVMVFTYYKIYRAAVFQTKSLKLGTKQVIMASGELELTLRIHRGGG
TNTDARHLFRTASSTPEDLQDLEEPLTTMHNNCLTRVPSTRINKQHRGNNFSLSRKLAKFAKEKKAAKTL
GIVMGVFIICWLPFFVVNLWSGFCSQCIWQEKIVFAAVTWLGWINSGMNPVIYACWSRDFRRAFVRILCA
CCPGRVRRRYQPAFRCKPSQRFASGRYYSAYSLHHVRSSRESSCEQTYI

>gi|815913369|ref|XP_012242030.1| PREDICTED: neuropeptide Y receptor type 2 [Bombus impatiens]

MNISWYDENEVIENIWQPTFNNTSTISPSYANGSIYDDFDGKYEVPTAILVVLSISYGTISFLAVVGNSL
VMWIIATSRRMQNVTNFFIANLALADIVIGLFAIPFQFQAALLQRWLLPHFMCAFCPFVQILSVNVSVFT
LTAIAIDRHRAVLKPLSAGPSKLTAKIIIAGIWFLACSLATPMAIARRVTMVRENSINGGDRYKPFCIHV
NLSTKSMFIYSGLLVFLQYVTPLSIISCVYARIALKLWGNKAPGNAEDSRDANLMKNKIKVIKMLIIVVA
LFAICWLPLQTYNTFQYFYSEINNYKYIHYIWFFFDWLAMSNSCYNPFIYGIYNEKFRREFQQRCPFKSR
KWSTNPAIDTTDTDKTQSTRTSIRYEWRRTISNTYPTVSSFHKGMSARDSTQSFIDDHQKNVRNKKGINY
YFRGENHCSHKNEELYVFSSGRHKHTQDLDTEELCL

>gi|815912710|ref|XP_012241742.1| PREDICTED: neuromedin-U receptor 2-like [Bombus impatiens]

MDPTTTLAPKVISSALQTYLQNFTAFTNTTNTVLPLRDSLYIVIPVTIIYLSIFITGTIGNISTCIVIAR
NKSMHTATNYYLFSLAVSDLLLLVSGLPAEIYMVWFKYPYIFGEVFCVLRGLAAETSTNASVLTITAFTV
ERYLAICHPFLSQTMSKLTRAVKLILVIWLMSLLFALPLALQVGVVHHYSNPKMVMCTVKRVLVNYSFEL
STFFFFVVPMILITVLYALIGLKLRKSNMMKRSRGSGESGSCRHHPGKSSRRVLKMLVAVVIAFFICWAP
FHIQRLVATHGTNSEDHMSSNSKLDEFLYTLMTYISGVLYYVSTSINPILYNIMSNKFRVAFMETLSRSC
RIPGLAIRTEQRSYSSLPRSQQRAIGSRNMGTGGTGIIHDSTDCSGNSGHHSLKQNAQPLAEHLNAESTR
NNVDKKDNEDIGRLEDSNEIKNKDVGCCKCSRKSRPARYATVKLSNASQSEKKWWRLLKWFPGLKSLKLA
GRSTCTVPENSPLRRSELRREEISMTLWNETNEQNTV

>gi|815910948|ref|XP_012240985.1| PREDICTED: adenosine receptor A2b-like [Bombus impatiens]

MTIAFRVSAFIVMEFPSNASELASNSTVRAVNASTHSELNLPYTVCEILVAVCAVFGNGLVITVFSKERK
LRRRTNYYIISLATADLLVGLFAIPFAILASIGLPTNLHACLFTVSVLIVLCTISIFCLVAVSIDRYWAI
LHPMGYSRTVRTKTAIGIICVCWIAGTLVGFLPLLGWNAGKKSNEKCIFTEVMDYDYLVFLYFATIIFPA
LLIAAFYTHIYRVVVKQLQQIVTMNPGRRTGNQTTGTMLRLLGAARKREVKATQNLSRIVIFFIICWFPL
YTINCVMAFCPRCRVHDVLMNFCIILSHLNSAGNPLLYAYHLKDFRAALKNFMWRLLFPHSDVKSSVVSV
IHDRGSLVGSQRQFLRQTGGPPSTRVIRSDLLRQVTDVRSRGSFSSPRNVTIRENDAANTSSSSSDRQEN

DNASQNSNDLQRNESDNFENDRITPRSPRITSLELQTYKPLMPLLPAEVDHVEETPPASIFVIEVDVNHV
ESVHEKLDEELMENKDKG

>gi|815908114|ref|XP_012239759.1| PREDICTED: orexin receptor type 1-like [Bombus impatiens]

MSTNVYNDSRISSPEFNSSYNGTMVSSNESFGMDITGDILWYFYDKFHSETNYLLIILYIPVMALAITAN
VLVIAVVFKYHYMRSVTNYFVVNLSVADLLVTTICMPVAVSQAISIVWIHGEIMCKLSSYLQGVAVAASV
FTISAMSIDRYLAIRSPMAFRRVFNRRSTVLVIVALWLVALIIFAPVLRAMTLHSPSMELSNISFHGSWT
MTRNFSQNTSRVSHLPPVFYICSEDFKPLGIQAPLFGAVCFVLVYAIPGFVVILSYSMMGRTLCARKPPF
DCDSVEGSASSQQSFRLVRERRRIAWILLLLAVFFALCWLPYNVLMLLIDLGAVGGKTTRDALSYCLFLG
HANSALNPVVYCVMTRNFRRSISEILCCGTHGFTRRKLRHRSAQGTAVIDDMCAGCSASSTIRRGLLRKR
RMLPGCRCGLPINGHHAVLALRRTATTSSGYDSYLSRHSPHRRCYMLRSLRGRPQMGMEQVQVNKTQETC
YKKNEANQPQINIFITTDEQR

>gi|815907251|ref|XP_012239379.1| PREDICTED: G-protein coupled receptor moody isoform X1 [Bombus impatiens]

MNSRKPDEIPFKMDNLATWLLRASENNTESAIIDGEVSRFPKPLQTFAAVVAILIMITGLAGNLLTIIAL
CKYPKVRNVAAAFIISLCVADFVFCLLVLPFDSIRFIDASWVNIRFLCVLVPFLRYGNVGVSLLSVAAIT
INRYIMIAHHGLYSKVYKKYWIAFMIVFCWLFSYGMQVPTLLGIWGRIDYDPNLETCSIVKDSKGYSSKT
FLFVVGFVIPCVIIVGCYTKIFWVVHRSETRMRKHASPTIKSPHTPGRDTREIKQRRSEWRITKMVLAIF
LSFVACYLPITIVKVSDAEVRYPEFHILGYLLLYFASCVNPIIYVIMNKQYRQAYAGVIGCSRIRASLSP
YGSSAPGHHQQQDYGQDYSKDFSKTMVSTVSIAMNPVRNSRFEE

>gi|815907249|ref|XP_012239378.1| PREDICTED: G-protein coupled receptor moody [Bombus impatiens]

MEYWMASNETGHNGTEETIQEVLRDPGSAVLFVGYPPWLLHFAAGCCILFMVIGIPGNLFTIVALFRTKK
LRNATAIFIMNLSLSDLMFCCFNLPLATSTFWHSSWRHGPLLCQLFPLLRYGLVAVSLFSILAITINRYV
MIGHPRFYPRIYKSRYITPMILSTWTIAFGALVVTWFGNWGRFGLDVAIGSCSILPDVNGRSPKEFLFVV
AFLIPCIAIVVCYARIFYIVRKTACKSRNRNVNASTDAVESNHERRSISPRIQEEELSVVGSSCAASVLC
ANFSSKCTVASPNRLNCSSTPSRSAIEESLSEQLSWEQNSAGSEEEDDEDDDDDDEEGENEEERTRQRRK
RKQRCKRRQKSTSQKLVDDRDRAKPSFEIKIDDIPYVDDLDAAETVLANDRIKIKESAEKASANARNKLE
RMTSRASFVIESALWVQRFNRRASMDGDRLDSSRSNTPDRSANSRRRPTIARRESRFTGVQKFRNSDGPP
RMSNKDKKLLKMILVIFSSFLVCYLPITVTKTFKDAIDWRGLNIASYILIYLTTCINPVVYVVMSSEYRS
AYKNLLLCRNDSGVKNGARRSPG

>gi|815902842|ref|XP_012237452.1| PREDICTED: tachykinin-like peptides receptor 86C [Bombus impatiens]

MDNLFSYGLYNCSTIILRRNITILLMLNRSELLNILENALDSSTEQEVLRDAFLDCFLNYQERPFDLSWW
QKLFWSLIFAAMLLVATGGNVIVIWIVLAHQRMRTVTNYFLVNLSVADLMMSLLNCVFNFIFLLNSDWPF
GVVYCTINNFVAHVTVSSSVLTLVTISFDRYMAIMRPLKHHMSRKKTIMIVFLIWVISSTLAVPCLLYST
TESRRYSNGKTRISCYLVWPDGTYLFSRMEYSYNIVFLSVTYLVPMALMAICYTLMGRKLWGSKSIGELT
YYQKKSIKSKRKVVKMFIIVVVIFAICWLPYQGFFFFLYHHKHLAENSNIPHVYLSFYWLAMSNAMVNPI
IYYWMNNRFRLYFQLIICKRTNCDDSPMHDFVKRTDVITCNSSRPKSTSIRWKHNMAKTQMQNIRMRSIC
INDHKQDTTTV

>gi|815902096|ref|XP_003487004.2| PREDICTED: dopamine receptor 1-like isoform X1 [Bombus impatiens]

MILPQNNLTDGEEDLPEDSFSLLSVLLVGFLFLVLIFLSVAGNILVCVAIYTDRGLRRIGNLFLASLAIA
DLFVGCLVMTFAGVNDLLGYWVFGPRFCDTWIAFDVMCSTASILNLCAISLDRYIHIKDPLRYGRWVTRR
VAVAGIAIVWLLAGLISFVPISLGLHRADEPVMYDDGKEEHPTCALDLTLTYAIVSSSISFYVPCIVMLG

IYCRLYCYAQKHVKSIRAVTKLPDTSMAKSFRAKSTRNKPPKPQTKTKPTSPYHVSDHKAAITVGVIMGV
FLICWVPFFCVNIVASYCKTCISVRAFQVLTWLGYSNSAFNPIIYSIFNTEFREAFKRILTKGARARGNQ
PSTSECGEFRSVVVQKRNGSMIECNISPRSSADSCQVGMMAQRHRDTIVSAI

>gi|815901244|ref|XP_012236722.1| PREDICTED: orexin receptor type 2-like isoform X1 [Bombus impatiens]

MHPLELVIVGWLALVISTLVDAIDYLDDYSAMDYTDESDIDYNTTNCTNSYCISNEEYVDRMINYIFPKF
WDWVLIASHSVVFVVGLVGNALVCIAVYRNHSMRTVTNYFIVNLAVADFLVLLLCLPFTVLWDITETWFL
GLTLCKAVPYLQTVSVTVSILTLTFISIDRWYAICFPLRFKSTTGRAKSAIIGIWAIALLFDIPDLVVLH
TVPPTHIKIKTVLFTQCDISWSQRSQVAFTIVKLIFLYTGPLIFMSVAYWQIVKVLWRSNIPGHNLPSRA
SQMSQIPSTGGGNPEVQLRSRRKAAKMLVTVVITFAICYFPVHLLSVLRYTTTLPSNKWINAISLIAHGL
CYFNSAVNPLIYNFMSGKFRKAFRRTFRCARENGSRIQRGYLASTSNFPRIKSRTTTIRTTFKNNNNLQR
NTEIIPLSAITTIQQNEKHD

>gi|350408093|ref|XP_003488300.1| PREDICTED: octopamine receptor beta-2R-like isoform X1 [Bombus impatiens]

MTTIVTSSESTEVVSSMDVTTILNAISTEDNQFANSSYSNYQKWSVPITIAKGCVLGSIIVTAVFGNLLV
MVSVMRHRKLRIITNYFVVSLALADMLVAMFAMTFNASVQITGKWMFSYFMCDVWNSLDVYFSTSSILHL
MCISVDRYWAIVKPLKYPIIMTKRLAAYMLLACWIMPAFISFMPIFMGWYTTEENKMHRQKHPELCEFKV
NKVYVIFSSSISFWIPCTIMTLTYFAVFKEASRQEKQMHSRMGNVMLLSHRPSKDLNNLNGELNSAGSSK
TLTLNEISTDHLHTPTKDKNIMKMKREHKAARTLGIIMGTFIICWLPFFLWYVITTLCGKSCPCPDIVIA
ILFWIGYTNSALNPLIYAYFNRDFREAFKNTLQCAFCSLCRREPSDLEALDFRRPSLRYDDRTKSIYSET
YIKHIDRRRSSEYGSSL

>gi|350397193|ref|XP_003484801.1| PREDICTED: neuropeptide FF receptor 2 [Bombus impatiens]

MVETTSTQRFTIKLPMLQPPSPLLEMASLRLPDSEEFDIDTRRRSNNGNIPPTTVSAIPQTTGTLLQMRN
DLGDYLNNLIAEAITTNPRTSMEEQGGLLNASKTANLSTAEQIPDRLYRHSMAMSAVYCVAYVLVFVVGL
IGNSFVIAVVYRSPRMRTVTNFFIVNLAVADVLVIVFCLPATLMSNIFVPWFLGWFMCKAVAYIQGVSVA
ASVYSLVAVSLDRFLAIWWPLKCQITKRRARMMIVVIWFIALTTTSPWLLFFDLVSIYDDDPDLRLCLEV
WPHPEDGTLFFLIGNLTLCYVLPTILISLCYILIWIKVWRRHIPSDTKDAQMERIQQKSKVKVVKMLVVV
VILFVLSWLPLYVIFTVIKLGGDVAEREDEILPIATPIAQWLGASNSCINPILYAFFNKKYRRGFIAILK
SGRCCGKIRYYETVAMMSSSTSMRKSSYYVNNNNNNSSTRRTFHGPPVHQESNVSYIFNHTGV

>gi|815914995|ref|XP_012242729.1| PREDICTED: neuropeptides capa receptor-like isoform X1 [Bombus impatiens]

MDVSDKYSFYENISDELEYLEKIRGPKYLSLTLVVPVTLTYVIIFVTGFVGNVITCIVIWRNPTMQTPTN
YYLFNLAVSDLLFLILGLPFELSVFWQQYPWQWGLGICKLRAYVSETSSYVSVLTIMAFSVERYLAIYHP
LRHYGSGLKRSIRSIFGAWLIALIFAIPFAAYIDIDYVEYPQNSKRNSEESAICAMLKENMPNFPLYQLS
CIFFFLIPMVFIAVLYVRIGLRIQSDTLAQNVEGYVHGETKQAQSRKTITRMLSAVVITFFICWAPFHIQ
RLLYVYEDSTYDDINQWVYPLTGCLYYFSTTINPILYNVMSAKYRNAFKETCRCSPSNPSISRVGLSSMR
DSSTICGVRPSQASQVFRERSVNSQRHGMYSASDPVKNKSHEAAQLQADDGEDEKRTNNVPANYFLTADK
NTNEIGASRYLQQTKRSSILIHAKNRRLKYQVSDEDETPSNETHI

>gi|815914993|ref|XP_012242728.1| PREDICTED: neuropeptides capa receptor-like [Bombus impatiens]

METSKNDTNDIHDFWKDLDLKNLTEDEYLATVLGPKYLPLKMVIPLTIAYVTIFVTGIFGNVTTCIVIIR
NPSMHTATNYYLFSLAISDLILLVLGLPNELSLFWQQYPWVLGVSLCKIRAYVSEVSSYVSVLTIVAFSM
ERYLAICHPLRVYAISGLKRPIRFILAAWSIALVSAIPFAIYTKVNLVEYPRDSGNYSANSAVCGMLLPY
MPNFPLYELSSIIFFLIPMLVILVVYTRMGLKIRNSTNHTLNSVMQGAIHGDSRQTQSRKSVIRMLTAVV

ILFFICWAPFHAQRLLYIYAQESDYYPDLNECLYILSGCLYYFSTTVNPILYNLMSMRYRKAFKETICCK
TRMTGRRSWTTRESQICNSNSSGKARSSSFKCSVRYTISQAKEMFRFTTSREGVNRDGNINDNIARKSHV
LTKEDFTSKPLLNRMHSGQQEERNERDPARNTEENKSGSTTSSVL

>gi|815902024|ref|XP_012237087.1| PREDICTED: rhodopsin, long-wavelength [Bombus impatiens]

MISALGPSYEAYAYGAQRFANQTVVDKVPPDMLHLIDAHWYQFPPLNPMWHGILGFVIGLLGFISVSGNG
MVVYIFLSTKSLRTPSNLFVINLAISDFLMMFCMSPPMVINCYYETWVLGPLFCQIYAMLGSLFGCGSIW
TMTMIAFDRYNVIVKGLSGKPLTINGALLRILGIWLFSLIWTIAPMFGWNRYVPEGNMTACGTDYFSRDI
VSVSYILLYSIWVYFFPLFLIIWSYWYIIQAVAAHEKNMREQAKKMNVASLRSSENQNTSAECKLAKVAL
MTISLWFMAWTPYLVINWSGIFSLVKISPLYTIWGSLFAKANAVYNPIVYGISHPKYRAALFARFPSLAC
AAEPAPTDATSTVSGTTTVADNEKSNA

>gi|815898064|ref|XP_012249743.1| PREDICTED: prolactin-releasing peptide receptor-like [Bombus impatiens]

MNDSPILSNGTAVEGWSTTNRPTIENDVTSNSLVQLIFYIFYANIFAFGVFGNVLVCFVVARNRQMQTVT
NLFITNLALSDVLLCALAVPFTPLYTFLGGWIFGRTLCHLVPYAQGVSVYISTFTLTSIAIDRFMVIIYP
FHPRMKIEVCLAVIVGIWIIALLFTLPYGLYMHLEEPYTYCEEHWPNERFRKIFSSITSILQFVLPFFII
AFCYICVSIKLNDRARTKPGTKTSQREEVDRERKKRTNRMLIAMVAVFGISWLPLNIINVIDDFYSPAND
WAYYRICFFMTHCLAMSSTCYNPFLYAWLNDNFRKEFKQVLPCFPKAINGYTPRTTEEFQNNKECSGNNT
IQESLLLNETHIRTSNPEARELIVHETTTNNMSRNLEEMETF

>gi|350417471|ref|XP_003491438.1| PREDICTED: uncharacterized protein LOC100746388 [Bombus impatiens]

MPYPTHYENTWYRMNLNATLQRLRFLLHYINTMSRSNNYTNILLSMNVTKEDINYAKNFLGNLTNYKTSN
DQIGCYCNGTVRDLALEYKNYHGYAALIVCLFGTLANMLNIVVLTRKDMVTAPINRILTGLATADMLVML
EYIPFAIYMYIVLPKRQIFPYGWAVFVLFHMHFTQLLHTISIAITLTLAVWRYIAIRFPQYNSWCTAARC
KTALWSSFLAPFVACAPSYLVFGIRRQAVNENGTLEIAYTVDADYSQHKDFFYQLNFWVLGVVVKLLPCV
ILTVISCWLIKALYRAKGRKQALKSYNQCKNAGTMGNGSVPRRPSKSEKRADRTTKMLVAVLLLFLVTEI
PQGILGLLSGVLGECFFRNCYHNFGEVMDILALLNGAINFILYCSMSRQFRTTFGHLFKPRIMKKWQPAT
QQTDVQSTYV

>gi|350412452|ref|XP_003489650.1| PREDICTED: neuropeptide Y receptor-like [Bombus impatiens]

MNTDLQVNRSLERLQDFEAWNVTNPEDCNYFSESQSIFSTKWFRSIICFLYSTVFVVALTGNGLVCYVVH
SSPRMKTVTNFFIVNLAFGDILMALFCVPTSSISTLILQYWPFGPEVCLTVIYLQAVSVLVSAYTLVAIS
IDRYIAIMWPLKPRLSKRQAQFLILTVWMLAMIISLPIAIVSKLSQPSKKYERCNQYICAEVWPSMENKY
YYSIALLVLQYVIPITVLVFTYTSIAVMVWGKRPPGEAENVRDQRMARSKRKMIKMMVTVVIVFTICWLP
LNILNLLMDNNETLESWAGLPFVWMVLHWLAMSHSCYNPVIYCWMNARFRTGFVTAIGHLPGAHRMLRRE
RQRDNYNASTMGIPLTGINDSGHSVLRRMNTCTTYISVRRKTNGNHTAPVRSASIRNDSFRPVNQPLQRQ
LVYLESHTEEQL

>gi|350406447|ref|XP_003487773.1| PREDICTED: neuropeptides capa receptor-like isoform X1 [Bombus impatiens]

MVHVQRDLVELDVTENILRSVQLYYTPILVYFGSLGNCLSVIVFFGTKLCQYSSSIYLGALAISDTGFLV
SVFVVWLNMVEVGLFNKPGFCQFFIYLTTLCSFMSIWLVVAFTIERFIAVEYPLYRQSMCTVARAKLAVA
ILTILGLILCSPVLWFSAPRLESNEKGNVTECHLAEGLESWAIVYNVIDTVLTFAIPFTVIIILNVLIAR
AIYRHIKIRKSLTNEPRIVKERQPYGQSFRNNLAQTKITKMLLVVSSVFLCFNLPAYVFRIHAFLHFQEE
SNAPRSVELAQQVCNLLFNTNFGINFILFCATGQNFRRAIRCMFLKRFRRRNVTMTQVSNQGKQNGSNFV
RSTTGATRQHAIVFTEPWEEFHELNKAEMNEQL

>gi|350396325|ref|XP_003484515.1| PREDICTED: FMRFamide receptor-like [Bombus impatiens]

MDFNASTSYYVCDLSSFHSYYSRLHGWISLFVCIFGSIANILNILVLTRREMRSPTNIILTGLAVADLLV
MIDYIPYAFHLYLYRRSRRDTFTYGWTIFVLFHSNFAQVCHTISICLTLILAVWRYVAVARPQQNREWCS
YKRTIFAISIAYIFCPVLCIPVYITTEVRQQVEVLDSNGMSIKSRNESLIGNTTNTTLYFVRLTEMAENH
DILKELNFWIYSVVIKLLPCLVLTVVSLKLLQVLLEAKRRRRKLTNIQEQQLEKKKSCRRGDKERQTDRT
TMMLLAVLLLFLLTELPQGILGLFSVLLGPGFFSTCYLMLGDVIDMLTLVNSAINFILYCTMSRQFRKTF
NELFCKNCKIPRSTAKQICIENNGNTGTNHTVTQVTQV

>gi|815909599|ref|XP_012240415.1| PREDICTED: protein odr-4 homolog [Bombus impatiens]

MGRTVYAEQRLHNYLTSLAKPDEYTIGLIVGQSAGQKDYIVHLAKTPPPLGKNVVEETLLSSTTKSEQNT
ATENHIKAVKDIPESWVADHAKHVTRMLPGGMRVLGTFIVGPEDISHNNNNVQKLKSVLTTMQKNLSCNK
YLCGDNNEEHLILYLNSVTQKYTCKSIEIRNEILKPVDWKFQAKATNWHQLEALIDFDRLFLIAANKDPE
TLKKQLQDILKSVSDIIESSLIVIEGEVRSPDDRLELISKNKKDERNCRNNEKNNNDRSIQINLYIPCHE
KNLNSDVRITPCNASIRLIGQLVSRTFVHQKANVEEANAAIKQDIIRSLASRLEMHWDSLIEEENGSPEE
NITLHEPPRRVLIALPESKVTLSDYLFPGEGPQEALLSLQELLDLEVQESNVQKDIEFEADPTEFYSQNK
IDVKTVDLNIDSSGNYQTKIYVVSFSIAVLIVIFAIIIQKLY

>gi|350422524|ref|XP_003493190.1| PREDICTED: uncharacterized protein LOC100748706 [Bombus impatiens]

MPNEAVVIEADSNSNSDYSLQLNRWFLKPIGAWPSSPSTTRLEKIISFLLNTICYSTVIITAIPSLLQMI
LENESINSKLKNLDFLSHLIVSIFTYSVLLLHNKDIRRCVEHMKADWRAVSRKEDQHVMMKNAKFGRYVA
AFCAIFVQGSVLCFCFVTALNTLEIQIGNETRILHVLPCAVYKKLVNVDESPTNEFMIFLQIWSTFIANC
STVGIFSLAAVLAAHACGQLNVVMLWIAEFVNEATVKSRTGGFTKIGIIVERHLRTLNFISYIEGVMNKI
CFLEMLRCTMEICVIGYFIVSEWAEHDVRNLASYVMMFIAICYNIFILCYIGELLTEQCKKIGEVVYMTN
WYYLPGKTILDLIMVIARSNVVVHITAGKLVHMSVYTFGSVVKTGFAYLNLLQQMM

>gi|350422514|ref|XP_003493187.1| PREDICTED: uncharacterized protein LOC100748230 [Bombus impatiens]

MTESVRIEENLYSLSDYSLQLNRWFLKPIGVWPLSASTSRLERIICFVLIFLCYFFVLFTIIPCLLHLIL
EDENIRMKLKILGPFSHWLIGGINYSTLLLRSREMRVCIEHIQNDWKIVTRQKDQQVMMRYARIGRYITV
FCTAFMQVGVLSHCTMAAFATKIIEIGNETRIIRLLPCGFYKSLISIDVSPTYEIILASQFVSGFIANSS
AVGAVGMAAVFAAHACGQLTVLMIWIKEYVNRSKDHSRNVGLNEIGEIVEHHLRVLSFIAGIEDVMNEIC
FMELFKCTMNMCMIGYYILTEWKEHDIQYMPAYLIIILFSMTFNIFVVCYIGETLKEQCEKVGEVVYMTNW
YYLPYKDILNLIQIILRSSMMIKITAGKLVHMSIFTFGNVIKTACAYFNLLRQAT

>gi|350413753|ref|XP_003490100.1| PREDICTED: uncharacterized protein LOC100744254 [Bombus impatiens]

MSKRITPEKVIDIIWFSVALTFCWPLPINSSGKRILVHKILQISSVISACMLLLPLLYSIYLHLDDIIIV
SECICLFLGVSQTIVQSIICLINHDSLQHVVEEMIICVKQARKYEREIFSKHIARCSVFYASSIMCIYLA
ATAFSIGPAILPLSFPSEAEYPFRVNYTPVYVIIYMQQSILSYQCVAHICLSMFGSLLFWFTAARFQCLA
LELKRTADVRTLIVCVEKQLHLRRYAKEVVNNFRFIVLYAIGVSTSALTLCGIILLVDVPPMVKIQFVTV
CLTVLTEIYVYAWSADYMKDMSINVSRSAYDIIWYKQKLEMQKNLLTMLECQEPITLSVSCIIPELSLRY
FCSYLSNTFSIFTTLRIVIEDNAE

>gi|350399566|ref|XP_003485570.1| PREDICTED: uncharacterized protein LOC100741772 [Bombus impatiens]

MIVIIVVTSIASITIPTTIELFASLHNKDMDGVIECLPHFIASSISAVKILNMHFNRQNFHKLFQLVARH
WQELKLNGELHVLEEIVMQGNRMALFYRSTLLSFMGLFLLVPLISPILDIVHPINGTRTRQQLLRVNYIF
FDDDDYFFYIYLQLFCGAAVVVYTIIGADWLYMLIIHHSSGLFAVCGHRVQKATLNPNYFTDEAISENYT
YEKIRNCAIMHNEAIRFSDILKESSRGSYLIQVGLNMLGISATAVQTVVNLDRPEEAMRSAVFCGANQFH
LFLLSLPGQVLLDHCSDLADNIYSSTWYGTPVQIQKVIYVMQIRCKRFCSLTAGGLYEMNIENFGITFKT
CMSYITMLMSLKN

>gi|350396218|ref|XP_003484482.1| PREDICTED: uncharacterized protein LOC100741444 [Bombus impatiens]

MDWQTIEDQCLKANKFFGQLVGVWPNQEKFTKIIIRFVIFIIIITTITAQISRVVVFYSLEVLSDQMPYL
DIGFVILIKQYNYVLNEKKLKGLLNDIVADRLIERPKEELEILDIYSKKATFLSCLYQVSISFCAFMFVL
LPTIPPILNVVAPLNESRSREFIYPSYYFVDEQQYYYPILVHMISVAVILTSVYIACDINLVHVVHHGCA
LLAISGYRFKHAMDNVSFSNEKYLDVLLDETHAKISRSIDAHKKAVEYVDKVDACHVHYFFIILGLIIVT
FTSTFVRLSTMEIGVRYFTFCAFTLSQMTHLLFLTIMGQFLINSNDETFQAICEAKWYNGSSKTQSLYLL
VLRKCLSPPKLTGGGVISLNLESFVQVLKASFSYYTVFRSA

>gi|805825627|ref|XP_012152698.1| PREDICTED: odorant receptor 49b-like [Megachile rotundata]

MVDNFLNAKYDDFELQILMDCKRKSKFFVCIFILFTEVTVLSYACTPLIENLSRNESDKAFPFRMWIKSI
PLQETPYYEICYTVQAVSVYMVGRTYFSLDNILCIINLHLAGQFRMLQYRLSEKYTKNQKKNDESRNLLD
LANNATDIFKSCIRQHQALVEYCEEVDAVFSPCVLIQVLAFSIFICLDGYQMLLVDIFERQIIFVFHFLG
TVCQLVMFSYSCDCIIRESELLATAAFSGPWLQLPSSKRIENLKKDFIMLIMRSYKSCYISGLGFFIVSL
ETSTRVLTTAGSYFTLLQETQTKVD

>gi|805825625|ref|XP_012152697.1| PREDICTED: odorant receptor Or2-like [Megachile rotundata]

MGMEYTSDIAVRLTIFYLKVVGFWFAANRLQQLFRCLTVIYTIVMSVFALWIQSMGIYYCWGDYTMCTYI
FVNILGIAISLLKLCLLLVQKEKFLRLIEFMQRNFWHSNYSQKEIKIFAVNIGKNESERVLLFHMWLDLP
LSTTPYFEIMYVLQVLSLYQCDVGYICFDNIFCVMCLHSAGQFRILQYRLKNVHQLASKHEDNSNNSASY
FSYKCFVVFKDCVRQHQIIIAFCKLFEEVFKVIVLCQVIMFSMLVCLVGYQIFLVDLNLPMRVSLTSFII
SNLCQLWVFTYSCDTMTRESLSVGTAMYEAPWPQLPTDKFGKMIRKDMQIVIMRSKRGCHITACGFFPIS
LETYTKIMSTAMSYFTLLKGSTIDVDT

>gi|805825623|ref|XP_012152696.1| PREDICTED: odorant receptor Or2-like [Megachile rotundata]

METKYSTDIAVRLTFFYLKIIGFWFARSRLQLWFRHLMVIYTFIMNMFTMWLQCMGIYHFWGDFTMCTYI
LVNVSVNIGKNESERVLLFHMWLDLPVSMSPYFEIMYVLQVLSLCQCNIGFTCFDNIFCIMCLHAAGQFR
ILQYRLKNMREMAGIQEDNLKSASYFSSKYFMVFKNCVRQHQMVIAFCTLFEEVFSAIVLCQVIMFSMLV
CLLGYQLFLVDLNLPMLVSLISFISANLCQLWVFTYSCDTMTRESLNVGTAMYTAPWLQLPTDKFGKMIR
KDLQIVIMRSRRGCHITACGFFPISLETYTSIMSTAMSYFTLLKGTTVDVET

>gi|805819496|ref|XP_003707222.2| PREDICTED: odorant receptor 13a-like [Megachile rotundata]

MDLPVATKETPVYELVLTAQFLHQTSTAFSFGTFSALLLMVVLHVGCQVDIMCRTITEDRVKNKEQLKFF
INRHQDIILLTERIEKLFTYAALSQLLSNTLNTCCLGFLIVIAFNIDNGLPILIKSILFYVVVWLDAYLY
CFAGEYLSTKSKLICETAYKCLWYDLHPNESQLLVLPILRSQKGLKLTFGKFSSLSLESFMSMMKASASY
MSVLLAMS

>gi|805819494|ref|XP_012149881.1| PREDICTED: odorant receptor 4-like [Megachile rotundata]

MASTLLAFGQEHHYQGCIKFVGLLDMIDNLSIAMPFSLVCIKLIVVWTNHGVLRDILSTVEEDCKKYAVI
DTNNLIPKTASLSVYFTIMVMSSYVVSAVFYLTGTLTQDTNNNSTRRLLLQMDLPFDTSESPTYELVLSA
QFLHLVTSALTFGVFSALLVMVIMHVGCHVDVMCQAITDDPLRTKEQLNFFINRHKEIILFVDKIEKLFT
YMALSQLVSNTLITCCVGYLIVMSIHTANGPALLIKSVLFYIAICLEAFIYCFAGEYLSIKSKLIGDTAY
ESAWYDLRPSENRLLILLILRSQKGFTFTFGKFSSPSLESFTSIMKVSASYISVLLAMS

>gi|805819492|ref|XP_012149880.1| PREDICTED: odorant receptor 67c [Megachile rotundata]

MAEDWTNCKSEWNLEAMIDKATLSYRITRIMLVSFLSSSILYTLGVFFGSDNDDGTSNPNERKFVLRMEF
PFEATISPIYEVIVIVQIIAQATFAVMAGMLMTLNATFVLHLVSQVDIICERLTQILNDNNEEKSRVGII
KKIILKHQRILDLCNNVDYVLTFISLIQFFLNTVVICFLSFILVTSLNTDEAAIIISKCFPYFVVIHLEA
LILCYTGEYLTTKSKSISWAAYNSNWYQLSIRECRALLLLILRSQRPMTLTIGKYINLSLETFANMLKTS
ASYVSVLYAME

>gi|805818116|ref|XP_003707057.2| PREDICTED: odorant receptor Or1-like isoform X1 [Megachile rotundata]

MDFMENRTKLKKIKPNKHLENSLSIIYYMGMWPSESKYKRLYMLYTVFSFMFLLGIFLASQIAYIIVNRK
SVDKIIAGATLLMTNATHAYKAILIICHHKRIKDLTDITRSETFIQDNGKYEKIVRHYTWQGVFHHIAYQ
SFGLMAVISWGVTPVLNLLTQRSKELSMEGWYPYNTSSTPAFEITSSYQAVAIFLCCINNVAIDTLITGL
ITIACCQLAILSSNIASLNCAENTEPIGINNNTDLEISTSKNYNKLYEDLKSCVEHSNMIFDFSKQIQDT
FGTVIFLQFLVNCIIICLIAFNIAQMKDYIPYVLCGMLMYMCCMTYQIFIYCWHGNELYLHSMNVTFSAY
ANNWWYNSKDFKQAICIIMVRVQQPLILTAGNVMQLSLQTFVRILRMSYSIFTVLQSSANS

>gi|805809810|ref|XP_003705978.2| PREDICTED: putative odorant receptor 92a [Megachile rotundata]

MLPALYGLRKRQTGRATSRAQMKRETASFDRSIGFNLFLLRLAGVISRGERSVERRALACLAFFCMSVYS
VSYVYEFLRRDGRSNANLETFTLITSFVGVQTRFAMLLLHRGTCQTMLTECELLWSRLNSREKKVVGNYA
RRSNRLAITYVVNCQLTIFAYVAVALVTSWLPDDSNVARDAVNASNVSLDLENARHLPYAFFLEVRGSPW
YEIACVLQFFSVTSVGLTSVGVDTTAAMFALIACGHLDVIRSRVESLRSVDSNSVQEDLRNSLIHHQVIL
EFCERIERCTNVILLTQMMLSTYNVSLVGFKLVGDDPNKFKYVTQLLIAVIQLLLCNWPADVLLAKSQAI
AVAAYNSPWYRWSASSRTPIAVLLVRAQKPARLTAGKFVVLSLDTFGSMISTSVSFFTVVRSMN

>gi|805809121|ref|XP_012146759.1| PREDICTED: odorant receptor 4-like [Megachile rotundata]

MGDLELMVENILDNTLGTTTYLMLFLFRFSKMIKHVIATMKRELAEDDFRNNEEKQLYLTYNIISDNFGR
YAVRTTAVIVVMWYSTPMLKLLKGTGNGTGMYRLPFRIHSFFDYEDNFKYYMLLYAYELPIMCIGIFHIT
SISLLLSLVLHVCGKFSILSYRIQNIVEDSENGYQSKVKELVERHVELIMTANALNSALQLILALELLQT
SVRMAVLMYTILLLPSDDLVDIFTYGLYVMIVTLMLYLYSYIGEQLQYESTKVNDAYYDANWLKLSTRDQ
KLLLYCMCNGRRTLSLTAGKFYSFSLFGFIKYMGHRSSSKSPTIPTSSQEIYQPLPLNYLNHYSTKLRTI
ERSLLIITTRKVVDSNDSSNKVCLYTVDHGKKKRQKKAFGH

>gi|805798914|ref|XP_012143986.1| PREDICTED: odorant receptor 9a-like [Megachile rotundata]

MKNKDLAYAMTPLKILSWPIGTWPLQEYNAFSFARFGFTTSVLFLSLLVTNAEFVCTRGSDPDKDMYAVM
LLVCNILALSKITYFRVHSAKLIFNFVSAVKDYNELDNEKKRAIMRQHARMSRLTFVGLMSSATVCSAFL
TFLPVLKRNENSINMTFSELLKYPIPSEQVITSLELPEYLYLLIFTMEFMTLPLISAGNLGSDILFFGIV
FHVCGQAEILKMEYNQVTDGSTEKFHALIKRHGYLLKLTEMLNDAVSSVLAIQLLSSCILICACDMQFLL
IQQDGNIASVARTAITMIILMIQLFAYSYVGNYLKCQMAGVGYSLYSCTWYNFSTNVAKEIIFVILRAQK
PAHLVAGKFTVVNLETFTSILKTSMSCLSVARVMMSR

>gi|805795911|ref|XP_012143140.1| PREDICTED: odorant receptor 49a-like [Megachile rotundata]

MVMYSLPIILAVSVILIQLLNHNINRKKFDHMFDRMSQLWEIAEMNGEVEVLKEVTDEGSQMGSMFRSSI
WIFTIFFLSMPLCPIFLDFILPLNETRAREPLFKLNYMVDMRDHFFIEYFHSAFSSIVVVLITATFDSLY
MVIIHYGCGLFALCGYQVKKAAEIAGTNATICTKNDLFRRCVITHHEAIRFYECMDESTRTGYLIQVVLT
MIAFTVNAVQAVLHMDRPNEFIAILMFMCGQQFHQFALCLPGQVLIDRSLDLINDIYSSKWYQTPVKYQR
TILTMQIRCSKACKLTAGGFYEMNMENFGAIIKTSVSYFTVLLSLRS

>gi|805795881|ref|XP_012143130.1| PREDICTED: odorant receptor 46a, isoform A-like [Megachile rotundata]

MFLCIIVSAITFLQLMDLVLIVENQAEFTDNIYISLSMIIACYKMYSILLSQKNIGVLTNILEDEPFQPK
NEEEIEIRRKFDNKAKQQAVLYTLLVESSVTLYVCVDILKFDDKKLLYRIWLPSELLPSVHIFIYLQQSL
SLIVGSIIHVACDSLIWGLLIHSCCQLEILEARLKAIKRDDDQSAKSSARYHNRVYRYAKMINQEFKMII
FVQFTASTLVVCFSLYIIATAKDMNDRLLVIAVYESCMLGQIFFYCWYGNEVKLKSVQLSDVIFGTDWMQ
FSRDTKKILSVIMRRATMSIEFTSVYIVTMNLESFVSLLKTSYSAYNMLQSSHG

>gi|805795879|ref|XP_012143129.1| PREDICTED: odorant receptor 45a [Megachile rotundata]

MQTLKWSYKILDILGFSLTSHWTSLWRRALYNSYGMILVISLHFMSATQMLDLFNVTNQEDFVDNLYVTL
VFLCDCCKTIMLLRRRGNIAKLIDELKEEPFATLNAEETEIQRKFMQQIERNTITYALIIDVYVVATIII
SFFTDYRHGGLKFRAWLPYDYSSPLLFTVTYVHQMVVMVFATNFIVACDSLFSGLLVNIYCQFELLEYRL
KNVEKFATAVNEVFTAIISIQFIVNTFALCFNHYRLSQLEFGAKFGEAAAFMFCVLAQIFYYCWYGNEVK
LKSLTIVDVALDSTLMSLDNSTKKMFLTITMRAMEPIQFTSIHIVSMNLESFITLVKTSYSAYTMLQQMH

>gi|805795873|ref|XP_012143127.1| PREDICTED: odorant receptor 22b-like [Megachile rotundata]

MMYSLFVAQSLDIAFHVTNLDEFSDNFATTSLLLVDCIKHGVLSMRRDSLIELTDMLSKNLFASVTKREI
KIRERYDKLVELNTNLYAICFFGGAVFAFVTTTIIDFKKRRLIVSVWLPYNYSSATLYLLTSAYEWVAGE
YAIMTSAACDCLYIGLLLHICGQIKVLEHRFQTLTEEGYYSLNQCAAHHYNIYQFVFDQSGSIVESSTIK
WFAKMVNKKFTEVMFFQFAARMLIETLIYSFGTFAQIAYYCWFSNEVKLKSIEFADEIFYSDWVSWGNEA
KKVFLMVLTRSAQPIEFSCISILPINLESFMKLLKTSYSAFNIMQQSR

>gi|805795867|ref|XP_012143125.1| PREDICTED: odorant receptor 22b-like [Megachile rotundata]

METLRWTRVMLSLSGCCLEFYIYTPLQRFLYQIYTIVAKMLLYSLLILQTLDIVLIVENRDEFTENIAIT
SMAFNALMKNTMMSTHRANILTLIKRLERNYFLPITKQEMEIRSRFDRIIESTSKAYTALLGFFAFLVLF
MTIVIGFNKRKLVFRMWLPYNYSSARLYILTSVYEVMVTSYGVSVSIACECLYTGLILHVCCQFEILEHR
FKMLNTNPVYTVNQCASHHHLIYKYAEAVNEEFKGIVSFQFFSSMSMICLTIYQLPYAENSATFLATAMF
LMCVFLQIFYYCLCGNVVKAKSAEFPDEIFSSGWPSWNDSSKKVLLMVIRRSKTPIEFTSMHIVTLSLES
FMSLLKTSYSAFNLMSTTR

>gi|805795339|ref|XP_012142988.1| PREDICTED: odorant receptor 49b-like [Megachile rotundata]

MVQEKFQIIMGIQLASSTLVVCFILYKLTNTPMNSTYLQFVLYMACMMSQIYFYCWYGNELKLKSVEVVD
AISEMDWMSLDNKSQKDLIHIMRRALNPIELSCAYIYTMDLSTFMSILKMSYSVYNLLQRT

>gi|805793982|ref|XP_012142616.1| PREDICTED: odorant receptor 4-like [Megachile rotundata]

MTMIKMFILQARRVQIAKILVYAQQHFWNNDYNPQERHIFKMCVKYSTWFVIFVFSVPSTSLTGYVITPI
FDINSVCVYIIGMGYVCPDILMCLLNVHAVGQFRILQYRMLNFWNIVCKDSDPEKYTEECYMALKKCIRQ
HQLIIVFCNMMESVYSTTIFIHVTIVSVLMCLDWYEILLANPGNAMRLIFIFHMLGSLGHLCILTCSCHY
MMEESSNIVRAAYGGSWSSLPMNQSGRSLRTAMMFIMIRAMKPCCITAAGFFPVSLETFTTILSSTFSYF
TLMRNSFTKDEDE

>gi|805793979|ref|XP_012142615.1| PREDICTED: odorant receptor 13a-like [Megachile rotundata]

MDGSEERQRLAALLWVFYTALYCVYVNVVNINHYWGHDMSCCVFGMSNLLIITMAISKVIVLRIRRLELA
DVVVYAERHFWHYNYSFEEQLIFAKCQRFCKLWIIFVFCILPASISGYVTTPIVYNIGRNKSDREFPLEI
RSDLPLTETPYFELIFTFQYSVYASCNVLCTIMAASKILVLRIRRLELENILADAERHFWHWNYNSEEQL
LFSECRLICKYFIMFVLSIPPASLFGYVITPIVSNIGKNKSERILPLDVWFNFPSTETPYYEFMFTFQII
SVFLVGTAYVSPDTLLCILNLHVIYQFRMLHYRMLRLWKRNNGNTDATKYAIQCSEDLRKCVNEYQSLIE
FCENMEHVFSRTLLIHVAIFSVLMGFDGYEILLSNAPLARRCTFIFHVAGSFIHLGIFTFCCSGLTEESS
KISTIIYFGSWSTLPMDQIGKSIRSKIIIVMMRTMRPCCLTAGGFFPVSLKTFTTLLSSTFSYFTLMRNS
ISGSESE

>gi|805793965|ref|XP_012142612.1| PREDICTED: odorant receptor 22c-like [Megachile rotundata]

MVRNFFHANYDDYETLVLINCKRKCSFFACTFVCFTELTIISYIFTPLIGNLGKNETERLLPFKMWVNLP
MQKTPYFEIGYIAQTNITVNLMPRFVLNYRFQTISFMKDVFLQTLSLCTIGFAYLCIDNMLCIINLHLAG
QFRILQYRLSDKYTAQDQNNVDQESKNFLYLANNASDVFKSYIRQHQSLMEYCDEVEAVFSIVLLIQVLT
FSMLICLDGYLILVAEMAQRKVMFICHFAATVCQLVMFSYGCDCIIRESTKLATAAYSGPWLQLPPTKSV
REVKKDLIMLIMRSNIPCYLTGSGFFIASLETCTKVLTTAASYFTLLQETQNNISA

>gi|805793961|ref|XP_012142611.1| PREDICTED: LOW QUALITY PROTEIN: odorant receptor 22c-like [Megachile rotundata]

MWSLLFGIYLQTTIMYHSLDDVENLIFGLLNLLSVLVPLIKLLALLPRKKKLFGLIAYMVRNFLKADYDD
FETSILTTCKRKCSFFVCSSVCFTELTIVSYVCAPLLVNLFMNESERVLPFKMYLNVPIQATPYFEIAYI
TQVLALCPVGFSYFSLDNVLCIINLHIAGQFRILQYRLSDKYSGQVQNGLDQKSDLYLKDNAPDVFKSYI
RQHQALITYCNELQEVFGIIVLALYSQVMTFSMLICLDGFQVLLLDLIQRKVIFFFHLLTTVCQLIMFSY
SCDCIIRESVNLATAAFSGPWLLLPSSKGIDNLKKDFIMLIMRSNKPCYISGLGFFIVSLETSTRVITTA
GSYFTLLQQTQSNIDS

>gi|805793823|ref|XP_012142572.1| PREDICTED: odorant receptor 4-like [Megachile rotundata]

MHLATQFRILRYRMEILCETTTETEKSSEKLIPCNQVTESYNKFKNCVQQHLALINYCEALEKVFTSITL
VQVMVFSVLICLFGYQILLAQSSIGRRSIYISLLVGSMGILFMYTYSCNGLMVESDKIGEAIYSTMWTQM
PMNEHGRKLRKDLIMVMLRSRRICCMTAYGFFPVSVETYSTILSTAASYFTLLRSRIE

>gi|805784782|ref|XP_012140182.1| PREDICTED: odorant receptor Or2-like [Megachile rotundata]

MEEISKEPRLKSIAFPLVGAFYRTILVEAVLMAGITLLYATRSLLNRLLRDPTNTTEENYPLPFPERFPW
SLESPPIWYLHYAFEFNVCYYIIFVSSGVDAFYGFTMFRMSSVLRRLTLQFEILSARKDDEIRRKEFGEC
IEMHVALFKCRDIIQEVYGPVVLIVATTNALCMCSLIFQIVQVNTEISNAQMAIYTTYFVMKFTQTLMYA
WPGSVIISESELFRDKIYCSYWYKNGDANLIKQFSLILTQRTIVVKACRILLVSLDLFVKVLNTAISYYF
LLKTIDGGK

>gi|805782670|ref|XP_012139624.1| PREDICTED: odorant receptor 13a-like isoform X1 [Megachile rotundata]

MLTMTLAVLKSGVWMFNRKDLECFINFMLTGYWKIVNADVFVYLEEYAIYAKNVSRGYFISMCNALLFYS
SLPIIEILISKSQVSNNNSTIKKFPFTGTSPAAFHKFPFYEMMYIFQMLATNICSLIMLATDGIIAIALL
HTCGHFAVLKQKLKNLDSCIYCITNSEKNSTNIKTDLYDIKIQLMHTIQHHQIILWFCDNMEKNFHLMLF
LQSMTSSILICFVGFQVSTALSEISKLFKSFAHLLVALFQLLLFCFPGDILIRQSCDISTAVFSIKWYKL
PSLIKDEVYMIILRSQKPCSITAGKLYVMHLENFAAVSCIIYLVKKITFLLLQSYFSS

>gi|805766386|ref|XP_003700842.2| PREDICTED: odorant receptor 2a-like [Megachile rotundata]

MSNEFMVQKFISQKELDGLSDYSLQLNRWLLKPIGVWPKSFYTSNIEKIVSLISNMICYCSIVITVVPCI
LHLTFEDIDFRTKLKVFGPMTHWFAGGINYTTLLVRGEEIRHCMKQMQNDWRTVSSPEVQQVMLKDAKFG
RNMATFCTVFMQGGVLCYCAAMALTKEIVQVGNETRTIHTLPIAVYKKLIPVDTSPTNEIVLAVQVLAAI
IVNCTVVGAFSLAIVFAAHAHGQLDVLNMWITEFVDQSRNSSKKVRWNGIGVFVNNHTNVLSFIEHIEHV
MNVICFTELCQCTMSICFLCYYILTEWHEHDIHNLTTYVIILVSVTFNVFIMCYVGELLTKQCNRIADVI
YSTNWYYLPCKNVLDLTVIITRSSMVTKITAGKIIYMSMYTFGAVLKTSFTYLNILRQMT

>gi|805766381|ref|XP_012135246.1| PREDICTED: odorant receptor 4-like [Megachile rotundata]

MASSKPIVVHTELEILGDYSLQLNRWMLKAIGAWPKSHSTKFEKIVSILLIITCYFFVLITVLPCILHLI
FEDDDLRTKVIILGPLSHWFLGGVGYTTLLVHNKEIKWCVDQVKSDWRNATRPEVQQLMLKDAKFGRYVV
AFCATLTEFSTACFTLVRALTTEVIVVGNETRIMRMLPCVPYRELVPVHTSPTYELMLIAQFVSGFVISC
AFVGAFSLATVFTIHAHSQLNVLMMSITELVDESREKSFERDIGAVVRKHLSIFSFISHIEAIMNEICFM
ELFKCTFSICLLGYYILMEWAQQDFRTMTGYISVFSSMTFNIFMLCFIGETIKEQCNKVGEAVYMSNWYY
LREKDVLDLMLIISRSCVETNITAGKIVHMSFYTFTGVRFPNFSAQRNRVNLQTNKKRQA

>gi|805766378|ref|XP_012135245.1| PREDICTED: odorant receptor 4-like [Megachile rotundata]

MEKIASLILIFVCQCCILITVLPCILYIVLDDDDIAEKLMVVGPLSHWFVGGCNYTTLLLHSKEIRFCAQ
QMKSDWMTVTRLKDLQVMLKYSKLGRYVVVFCAAFMQGGVLCFCALRAFTVETVLIGNDTKNVHLVPCTP
SKKVISFDTSPTNEIFIALQFLSGFIVNSTAAGAFSFAAVSAAHAYGQLNVLTDWIKELLNRTGKFDRKI
DSNKIGIIVNNHLRILTFIANIEDIMNKICLMELFKCTVCICTLEYHILMEWANHDYQSVTSFFLVLCSM
SFNMFIVCYIGEKLSEQCKSIGETVYMTNWYYLPNKDILDLIMIISRSHMMTKITAGKVVPMSFDTFASV
IKTGFAYLNVLRQTI

>gi|805766356|ref|XP_012135238.1| PREDICTED: odorant receptor 63a-like [Megachile rotundata]

MQKATAKAEQEYLKNVDLSVRLNRWILKPIAAWPESSTVSPIKKYLGWFVHIICYSLLSFLFVPCLTFLI
LEVNDTYNRIKLIGPLSFFLMSYLKYYLMLLHKNDILKCVQQVQRDWANARHLNDRNIMGMNANYGRKLV
VVSAFFMYSSYIFFYIAVPITVGRIPAQDGNFTFIFLPFPSSMRITDYRQSPVNEIFYFLQCLAGVVLHI
ITVGACSLAATFAVHACGQMQILMNWLGYLVNGRADMSKSLDGRIASIVEQHVRILKFLDLTEKTLRYIA
LVEFLGCTTVLCLLSYYVLVEWSTNDRISSATYAVLFVSFTFNIFIYCYIGELVAEGCRQIAEISYMIDW
YRLLGKRKLSIILIMQMSHSTIKLTAGNIVILSYSKFGDVVKSAFAFLNVLRTVT

>gi|805766339|ref|XP_003700911.2| PREDICTED: odorant receptor 4-like [Megachile rotundata]

MHRVKKPRRRVRPFEKYSLRRIRDACVRRSVNLSSDICNQYRANYRAPRMQESIEQSENVNRSIQLSRWI
LKPMGLWPNSSETSCLEKYLYRLINVACYLLIFYLIIPCGLYMLLDMENLYKEIKLFGPMIFCVAAFVKY
HWLILHGDDIRECMEHIKWDWKNVEYFKDKDIMSVNANFGKRIVTVCALFMFTACTFYFLIIPLNLRRIA
TDNVSFIPVMYPFSKHIVDIRYSPVNEIVFSIQFVAGVLINAITVGSCSLAAVLAVHACGQMEVLMRWLE
HLVDGRSDMSESVDHRIAKVISQHVRILKFLALTEKTFKQISFAEFLACVLNLCLVGYYIITEWNSKNIT

ITVGYVILYTSLAFNIFIFCYIGELVAEQCRNVGQMSYMIDWYRLRGNKKLFCILIMAMSNSSIKLTAGN
MVELSIGTFTDVVKTSVAFLNVLRKFS

>gi|805765827|ref|XP_012135096.1| PREDICTED: LOW QUALITY PROTEIN: odorant receptor 22c-like [Megachile rotundata]

MQKTSVHKKTIDDYEKNVNLSTQWCRWILKPIGVWPTSDVTGTQKCMYRLINVMCYSFLSFLCVPGILYV
ILEVEDVYNQIKFFGPSSFCMMAIMKYYLMILHENDIRDCIQRIELDWKNISHDDDKKIMVEKAKFGRQL
IALCTFFMYAVSRSGFAFYYVVLPTSADKIQAADANLTFIPLACPFSSLIIDTRYSPTNEIVFLLQVVAG
ALIHSIASAACGLAAMLAVHACGQMEVLMNWLKHLVDGRSDMGDTVDARIASVVSQHVRILKYLTRTEGA
LQLISFVEFLGCTLDICLLGYYVIMESKSNDITSTVTYIILLISLTFNIFIFCYIGELVAEQCRKIGEMT
YMIEWYRLPGNKKLCCVLIIAMSNSSITLTAGNMVELSIETFTNVVKTAFAFLNVLRTLT

>gi|805765819|ref|XP_003700910.2| PREDICTED: odorant receptor Or2-like [Megachile rotundata]

MFLKTKSGQVRLKVFGPILNCCMQFIKYTILLLRSKQIRDCLDVIRQDWIEATEEDRSILHSRGMLGRKL
ILTVMLTLYGGGLCYRTIIPLMKGPITLPNNVTIRPMACAGYFVFINEQQTPTYEIVFTLQFFSGIITYA
TSSGSYGIFTVFVLHVCSLLRMLNKKMKSLDEGADLSERTVNHKIADIVEHERKIKGFFENVEAITQYIC
FVDIMGNTCLICLMGYRIITEPQKSDPMFITICIILQTSFIFCSFILCYIGQLLVDENNVVGMTSVMLDW
YRLPIKKARCLVLIIAMSNYPMQLTAGKIVEISLVTFTDIMKMSMAYLNILREVV

>gi|805765798|ref|XP_003700909.2| PREDICTED: odorant receptor 49b-like [Megachile rotundata]

MIHYSLQLFKESSPHQKHYIKHYRSIISAMVYRNASYKLDTEYTIRFPKALLTPIGIWPLYQDDTALRKT
RRQVQIALIFCSMCFLLIPHAIYTYHDCEDLKRYMKVIAAQVFSLLGIVKFWTIIINKNEISFCLTELEL
QYRDVECEEDRKLIRESAKIGRFFAILYLGLSYGGALPYHLILPLLSEKVVKSDNTTQIPLPYLSNYVFF
VIEDSPFYEMTFAFQMFISIIILSTNCGIYILIAGITMHCSGLFEVINRKIDLFMKETNGKLRDRLRFII
QRHVQATEYAAMIEKTFNVVFLSEMLGNTVIICFLEYGVLVEWEDHKTLSTMTYFILMTSILSNVFIISF
IGDRLKQVSTRVGRTAYFLPWFELPMDVVKDVSMVILRTSRPSSLSAGKLFDLSLQGFCDVFKTSAAYLN
FLRTMTA

>gi|805759628|ref|XP_003700090.2| PREDICTED: odorant receptor Or2-like [Megachile rotundata]

MGCLLEVILDLVMYKTYGAQLQILVRTVKTYLKTADHHQITILQSYFDRYKKFFSTIAFPYVSNGFSFTI
MALCTKRGLPVDGWVPFSIERSEIYSCVFFVQSYGIFQISFSIFADFMIVGLLCYIAARLDILSEKMKQV
TDYDLLTSCIKEHQEIIGFFEDTKAAVQTLLFKTNITMGGTVISAVLALLYVRNQSMVVASQFLFIVLAG
CGHMYVITWPANDLKESSLRFAISLNDIRWIGKSREMKSAVLIMMLRSQKPFLIPMAGLLPPLSLEYFSQ
FMSSVFSYFMTMRSMLEL

>gi|805759615|ref|XP_012152339.1| PREDICTED: odorant receptor 46a, isoform A-like [Megachile rotundata]

MFQNTSPEKAITFTRFCLALSLFLPLSSRATKLQTICYKSLKFIVVLSSFFLFLPIINSLCMYQDPTNVS
QSVCFLMGEFQLISNTIIGTVQYDRLQEVIEVMEACCKNATLHEKVVFQRYINTYSPFYGSSAIWFYVTG
MILILGTLVSSEPFPTNAEYPFPVDYQPLETIVFLHQSLVLIQCCAHACINMFCAFLLLFAAARFEILKM
ELREVMNIEQFVMCVEKYYTVKRYAKQVVSVVRLEVLCMLVLSAIAVVFCGIILIQPQPLATKGQYLPVV
GTALLEVFVIAWPADHLLDMSENVMHEIYKSKWYDQDVRMQRDVRVMMLPQKAVAIKVPCLISTLSLNYF
CSFISNVFSLFSVLRIAILKDG

>gi|805759602|ref|XP_012152298.1| PREDICTED: odorant receptor Or2-like [Megachile rotundata]

MKLWIKQHQDTLRYANEINSIIAILVVKSTIAVALNMIICGLVIVRSTYFPNISPFQSKNFIELIKFFTL
TIFSVLRFVICSWVADTMSENANGIAWKIYDSPWTYSSPRIRRMALLIIARCQKPVAIHATGFLSSWNLK
FCAQVLYTNATYLMTLRAVVRE

>gi|805759258|ref|XP_012151297.1| PREDICTED: odorant receptor 83a-like isoform X1 [Megachile rotundata]

MARTPGHKWMVAFRKLINIQINYLKYSGLWVIDPGHPWLSKSAYLVCKGWTLSAMYIFAITLFADICDNI
DNLSITSDDGCVFAGIAVVIFKTMNYQIRQKKIALFVDKVLKCSDDLCEFSNEESIKPIIDKYRLRITVT
FWGFSALGCALGFLLLFASPRQNDLPIRAKYPFNTTTPGWHELSFFIETCAVSGGLVAIVGMDSITIYMS
SIITMLLDILSCNFESCSGRSANNVRSFAIIYICSKSLIKVWFYQENEICLVRNCEERQRDKWDSNKFLH
RYKNCIRFHQRLVTLSKEYNRLFSSSMFVQMLSSTSMICLTGFQAVVVGGQSSDIMKFGMYLSAAVSQLL
YICWIGNELSYSSSVLDKSQWLSDWHHEHLPSIVQVFTLSTMSTRRTLTLKAGIFFVLSLETFIAIVKGS
YSVFTLLNNMQTTDP

>gi|805759031|ref|XP_012150706.1| PREDICTED: odorant receptor 67c-like [Megachile rotundata]

MFHANLFYMVDLKYVYGWNQYTMKFLGIWPEERKWNRPSSYLVFVSVLMMSCFITIPQTINFPLIWGDMD
LVVENLSMANVTFTICILKAIAFWNNGKSLKSLLECMAIDWSTTKSPEQRETMKIIAKTTRTMVMRSTIM
CSTVVFVYVFLRCLTMKYNDSKLFFRAYFPYDVNVSPNFQLTMFAQFVAAMYGAITYTAVDTFVAMLILH
VCGQLENLHGDIKRLLSSQEKHFEVEVGKIVRKHEYLNRFAVTIKDCFNMMLLFQMIGCTTQLCFQCFQV
IMSFDVQEKEYLIIQISFLMIYVIYVMLHLYLYCYVGEKLRAESLDIANAVYNSNWYNLPAKNAKLLILI
ICRSKNPLQITAGGFCSFTLELYSQVRN

>gi|805754639|ref|XP_012138789.1| PREDICTED: odorant receptor 4-like isoform X2 [Megachile rotundata]

MALAENRVQYHQFKLCVITHHKALEFFGYLQKCTQNMFLLVVAINMILISTTAVQILMHMDRPLDSTRFI
LFFLCSHFHLYVISLFGQIVLNHSTVLAKRIYNCNWYEVPIKFQKLLCFMIVRSSKPCILSAGGLYDMNM
QNYAIAVKTCMSYFTMFLSMRE

>gi|805754636|ref|XP_012138785.1| PREDICTED: odorant receptor 4-like isoform X1 [Megachile rotundata]

MRDNPSQSSGVFRLPTKMYSEHVPASGGNKHDTHQHDGRSSKDIRVLKLKPYQLLSILQILMHMDRPLDS
TRFILFFLCSHFHLYVISLFGQIVLNHSTVLAKRIYNCNWYEVPIKFQKLLCFMIVRSSKPCILSAGGLY
DMNMQNYAIAVKTCMSYFTMFLSMRE

>gi|805754623|ref|XP_012138758.1| PREDICTED: odorant receptor 9a-like isoform X1 [Megachile rotundata]

MEGTVKSTRNELRCHNTECIKETLNTVLQLYVAILAKDIDEIIDVLPHIFLVIGSLTKFGNIFLNKERVR
TDYFKASTVYYYTYFLLQIKILLDRIVRDWKTMGSELHVLDEITATGYRLAHLYRVTLLTFTMIFNYIPLI
PPTLDIILPRNESRPRHQLFQVNYVFFDADDHFIFTYLHMFWAGSLTVFVVVTVDSLYMLIIHHASGLFD
VCGYQVEMACREEKVKDAMFKQCLITHHKALEFFNLLQACSQSMNLLLVGMNMLLISTTGVQVKISLAIK
HIEAHTLFSQIILYMDRPLDSVRFVLFFLGEHFHLYIISLLGQIVLNHSLLLPEKIYGSNWDDIPIKFQK
LLFKMIIRCSRPCILSAGGLYDMNMENYGKAVKACMSYFTMFLSMRE

>gi|805754615|ref|XP_012138734.1| PREDICTED: odorant receptor 24a-like [Megachile rotundata]

MFRNSLRLLGVDPYQDNMLSNFIIFLVTCTALGMVIPTFLGFHKALRDKDMDALFESLPYFIASSIGIVK
LANLQLNKANFRKLLNMVADEWENLKLNNELKPLEDLTKLASTVARIYRTCILSSMTIFLSVTLLPSVLD
VILPLNETRPRQQLFNVEYLFFDKDEYYYSVYLQLSWSTFVIVMVIGFVDSLYIIIIHHSSGLFAVCGEQ
IKKVTEMSDAVGGRISDQIYQCKRCMVMHEKALQFYEILKETSTKNYFFQVGLNMMGISVQAVLLVSSLD

KPDQAMKAGVLLISKQFHLFVVSLPGQVLLDHCAQLAKNIYCSKWYRTPLQIQKMIKIMQIKANRPCALT
AGGLYEMSIENFGSTLKSCMSYVTMLLSLKE

>gi|805754598|ref|XP_012138695.1| PREDICTED: odorant receptor Or2-like isoform X1 [Megachile rotundata]

MESVPYMATGSVSAIKLLNHNINRKKFENLFDRMCRQWEISEINGEIHVLNEVTEQGTKMGALYRTSLWV
FMLLFLSLPLSAPILDFVSQSNKTRTKIPLFKLNYIVNTDDYFYFVYLHSVCCSLTIVIVIIAMDSLYMV
IFHYICGVFALCGYQIKKAAETMEKNRTLRSEHQYHLFRRCVNTHHEAIKFYEYMNESTRTSYLFQVGLN
MLGITSTAVQAVMNFDQPQEAFRIIMFLLGQQFHLYTLSLPGQVLLDRSSELSENMKDIFRYFSKWHEVP
VKFQKVLHIMLVRASRPCMLSAGGIYEMNMENFGSIVKTCVSYFTILLSLRD

>gi|805754586|ref|XP_012138654.1| PREDICTED: odorant receptor 49a-like isoform X1 [Megachile rotundata]

MQKGVRRKRHAFDLPCYKVFKEYLIVLGQYPKQSERSNKINVTVVVCCSLSILIPSIFQFVKSLREKDMD
TMMECVPIIATISVSIIKIVNHNINRKKFENMFEHMCEQWEMSELNNEIDVLNEVTKEGSRMGTLYRISI
WGVLVIFLSIPLSSPVLDFILKSNQTRTKIPLFKLNYLINTEDHFYYVYVHSAICSLAVSLVIIVMDSLY
MVIFHYISGVFALCGYQIKRATEIMETDSTLKYEHKYRLLKRCLNTHYEAIKFYEYMDDSTRLSYLFQVG
LNMLGITSTAVQAVMNFDRPQEAFRIAMFLFGQQFHLYTLSLPGQVLLDRSSELSDNIYCSKWHEIPVKY
QVILHIMLTRSIRPCKLTAGGLYDMNIESFGTMFKSCMSYCTILLSLRE

>gi|805754569|ref|XP_012138607.1| PREDICTED: odorant receptor 4-like [Megachile rotundata]

MYRYQIEAASQFRELRYDQFKQCVMTHHKALKFFDNLQECSKNMFLALVAINITIISMTAVQILMHMGRP
LDSTRFILFFLCSHFHLYVISLFGQIILNHSTILAERIYNCNWYKIPIKFQKLLCTMIGRCNRPCILSAG
GLYDMNMENYGKAVKACMSYFTMFLSMRD

>gi|805752323|ref|XP_012144879.1| PREDICTED: odorant receptor 2a-like [Megachile rotundata]

MYSEYVPTVGGNEHDTHQHNGSSSKDMRVLKLTLYQILFILQILMHIDHPLDSSRFVLFFCCCQFHLYVV
SLFGQIILNHSAQLAEKIYGSNWCEVPIKFQKSLCFMIARSSKPCILSAGGLYDMNMENYGKAVKACMSY
FTMFLSMRE

>gi|383861644|ref|XP_003706295.1| PREDICTED: odorant receptor Or2-like [Megachile rotundata]

MGKPSENTIDYYIQPNKILCSIYGLWPTDEERTTSKQVLAVVHLVISVIIVCSVFVPEVIILTTKWRDLS
ILAGAGTLTLTMTQFLFKTIYLTTKRDTAHRLLVELRSLWVTTDDPVERQSYEVFAYWGRISTIGFFMSA
VGSTSLFLVTATLDCFEDVENSNRTNRHLPYDVWQELDFLKSPEFEVIYACQILATLNACCAVCGMDGMC
WTTILYLSGQFRLITTWLNNIGVEMNYKPHPKDCSVKVAADLVKCIRHHQRLSKVVKDVNSLLIPIIFIQ
LLTSGVQICLSGFAVLSNNGGDNLLKFSCFLSSVLVQLVMYCWPGEILIQESQEVGYAAYLGVPWYQLPP
AYRRQLMLIILKSQDGCSISALTFKSLGCHTLTSVFNTGSSYFALLRKMQDTSS

>gi|383861642|ref|XP_003706294.1| PREDICTED: odorant receptor Or2-like [Megachile rotundata]

MRQPSKDTIDYYIFPNKILCSMFGMWPSDKKRSTVRKMLAYLHFVFSVLTVCVIVVPEIILLAVRWRDLK
IFAGAGCIIMSLMQVLFKTVYLTMKREEAHKLYTEFRSLWYLTKDPVERQSYELFAYWARTCTITFGVSC
VGTMIIFFISATYDYITDVNMNAGSRHLPYDVCCMINVQLEVSQTYQMEMRGIDFIDSPEFEIIFVSQIL
ASLFAACAVIGLDSTCVTIILHLSGQFRLITTWLSNIGIEMQYNRIDLHNCPPSVSADLIKCIRHHQRLI
KVLQDVNSLLTPIIFMQVFTSSIVFCLGGYAIISNNAADDLFKFVSYILSMTIQLLLWCWPGEILIEESL
HIGYAAYVNVPWYNLPPFYRRQLCLIVLRSQKTCSLSAFIFHSLSIYTLTNVLNTAFSYFALLRQIQDKN
M

>gi|383861640|ref|XP_003706293.1| PREDICTED: odorant receptor Or2-like [Megachile rotundata]

MRQPSKDTIDYYILPNKILCSLYGIWPSDEKPSTIRTVMSVLHILVSIVVVCSIYVPEIIMLLASWDDLS
ILAGAGSITLTITQFLFKTVYILAKRESAYKLLEELKSLWYSTDNPMERRSYEEFAYWGRTSTISFFVSG
ILTTFTFMLSATLDSMKQGYFDNGTSNRHLPYDVWHGMTYLKSPEFEIIYVGQIMASFACCCAVCGMDGM
LLTTILHLSGQFRLITTWLTNIGIEMNYTTIDLSKRPVKIVAGLMKCIRHHQRLIKTVKDVNIFMTPIVF
VHLLTSGMQICLTGFAVLSNNVGANVFKFISYLGAVMIQLVMLCWPGEILIQESQKVAYAAYLNVPWYQL
PLCYRRHLLFVIMKAQESCCIKALTFESLSSHTLTSVFNTAASYFTLLRQMQETPM

>gi|383861634|ref|XP_003706290.1| PREDICTED: odorant receptor 47a-like [Megachile rotundata]

MGKVSKNSIDYYILPNKILCSMIGMWPFSEERSTCRNAFSCFRLVFSVTAVCTIFVPGIMIIVVKWGDLK
ILTGVGCILTTLAQCLFKTIYLVARKERSHRLYKELRSLWDSTDDPTEMQCYQDLAHVARTCTITFYSCG
ALTSIVFIISAALECMNVDVSNGTDSIRPLPFEVWYGIDVSESPRFEVVFVCQALASTICAVAICSLDTA
CVTAILHVCGQFKLISVWISHIGFETDCNLTNYLVDSRRNLRTDLVKCIRHQQRLINVVKDVNTLMTPII
FIQILTSGIEICLSGFAVFSYDAGGDLFKFISYLASMMVQLLLWCWPGEILAQESQKIGHSVYLNIPWYQ
LPLIYRKKILLMILRSQKYCSISALTFQSLSIYTLTSVFNTASSYFALLRQIQDQST

>gi|383861632|ref|XP_003706289.1| PREDICTED: putative odorant receptor 85e [Megachile rotundata]

MGKVAKDSIDYYVLPNKIMCSAIGIWPPDEEQSFGGRLFVGFRVVFSIAAVCTIFVPEIMMIAVNWGDLR
ILTGVGCVLTTVAQLIFKMIYLIARKERSYKLYKELRSLWDSSHDSKERQCYQGLAYIARNCTIIYHTSG
LLTVAVFTVSAVFDYVKFGQDNNAANRHLPYDVWYGTDVTDSPGFEIAFACQVLAASICTIGVTGLDTTC
ATSILHICGQFRLMCMWISNIGIKINCDSPRTVTTDLIRCIRHQQRLISAVKDVNNLLTPIIFVQVLTSG
IVICLCGFAVLRGTGDDLFKFIVYLTAVMIQLMFWCWPGEILIQESLEVGYAVYLNIPWYNMEPACRRQL
LLVILRSQNVCSISALTFRTVCIHSLTTVFNAAASYFTLLRQMEEKAMSK

>gi|383857773|ref|XP_003704378.1| PREDICTED: odorant receptor 46a, isoform A-like [Megachile rotundata]

MCGCWRPRSWASTYKKLLYTVYTVFVFTLLNTVMVLQIMDLVLNVENQDELSEGFYMMLTMFVACHKMYS
ILTSKKNIVALTRILENEPFLPENEEEVDIRKKFDDIAQKQAYVYTTAIEVAVLFQSCVGALNMGGQKLP
FRMWLPQKYLSLTVLVIANIQQAMSLITGALVHIGCDNLIWGLLLHTCSQIEILEYRLRAIKPGESQSAK
SSARYHDLVYRFAKMINEEFKMVIVVQFTVTTMIVCFNLYLLLTTENMNTKFLIVVLYTCSMLTQIFIYC
WYGTKVKMKSLEVSDMVFRTDWMFFSKDVKRILYVIMRRANVSIEFRSAHVVSMNLEAFVNLLKTSYSAY
NVLNGKT

>gi|383857757|ref|XP_003704370.1| PREDICTED: odorant receptor 22b-like [Megachile rotundata]

MVQCPREERHDVTASSEYINLNQKAGRKLFSACHQTRDGNSIEIRQANRNLIDDTYDKNDMAIFVLSLFL
RSSSKAYTAILMSCLSCIIITVFVIGFTKRMLICRIWVPYNYSSTSLYIVTSMYEGMGACYGISASSACE
CLYTGLLLHICCQFEILEHRFRSLNSNPIYTANHCASHHHLIYKYAEAVNEEFRGIMSFQFFSSMSILCL
TIYHLAYAENSAAFMATGLYMTCVLCQMFYYCFYGDMVKTKSIEFIDKLFQSGWASWNGSSKMVLLMVMR
RSRTPIEFTSMHIVTLNLESFMSLLRTSYSAFNLMSSTR

>gi|383857335|ref|XP_003704160.1| PREDICTED: odorant receptor Or2-like [Megachile rotundata]

MRLLRSKDRSIIWTCFLMKVIGIWIATNRAEQIRRKLALVYTLMALSIALGIALRDMHFSWGNFNDCVYI
GCNILYLMIGFFKIAVLSAHKKNFYDLVVSIERSFIRPSDDITEQKILMECKSVCDMFIVFITFCVQGTC
SGYAVTPLLENIGKPEMERMLPFNLWIDFPTGKSPYFEVLFAIQMLCVYHVAISYFCFDNFLCLVCLNIT

GQFRILQHRLASLNGMKKTDDEDSDAYVSDDAIKYRTKLKSCIRHHQTLINYCRKLENVFTLIVLGEVLF
LAMLLCCLGFQIFLMSSPPARRISLVIGMMGICSQLFMFSYCWDGLVRESVNVGQAAFAGPWSIFSIKEG
GKSLRSDVLIIIMRSNKACCLTAAGFFPVSLETCTAVLSTAMSYFTLLRHSAPDLQTA

>gi|383857333|ref|XP_003704159.1| PREDICTED: odorant receptor 13a-like [Megachile rotundata]

MDQRSEEDFSITTTFFYLKFVGFWLVTNKFEERMKTLSIIYTVTMLSIAVGIEGTDLYYSLDNFGEAIYT
GNNVMTISMVLLKIFISYIHRKKLLRLVIYAKTNFWHLNYEPEEMEIMDNCRRTCTFLICAFSFLTQGTV
AGFIMSCVVENIGRNESDRVFPFNVWLDVPEITLTPYFEIISAIEVLALCHVGVCYLCFDNFLCLMNLHA
ACQFRILQHRLSKVYVIHEKEKDHLTVKYYDTLRGYFKQHQTLIAYCDKFEEAFSVMVLAQVLLFSLLIC
LDGFLVLMDDAPNMRRVIFAFHLMCCICQLLMFTYSCDCLIRESLDVATAVYNSPWTVVPHDNMGKMVRK
DLVLMILRSRVPCCITASGFFVVSLETYTRVLSTAVSYFTLLNQY

>gi|383857331|ref|XP_003704158.1| PREDICTED: odorant receptor 13a-like [Megachile rotundata]

MQGERYTDLSITLSTFCMKYAGLWTAKNYAEERRRKLGITYSIYILCFAMFFEIRDLYFSWGDLSAALYV
ACNLSIVALTSFKVFILVLYRSELADLILYMQEHFWHCEYDEREKAILASCRKTCIFFTISVTFIGECAV
FSYLVTPIVVNLGGNGTVRLHPFNMWLDAVLITPYFEIAFFGQILILFHIGSCYFCFDNMFSIFCLHLAT
QFRILQYRFETMCEISEETVHAYLKFSPTNYAVDVYQKFKVYVQQHQALIDYSTLLERVFTLITFGQVMF
FSVLICLFGYQIVMADSTPERRTIFICLTVGSTSLLFMFTYSCHGLMEQSDLLADAMYSTMWTRIPMNKY
GKMLRNDLMMVILRARRTCCITANGFFPVSLETFTTILSTAASYFTLLRNSVEQQ

>gi|383857305|ref|XP_003704145.1| PREDICTED: odorant receptor Or2-like [Megachile rotundata]

MRLLHSKDRSIIWTLFLIKVVGIWIANNRAEQLRRNLALVYTLTALSIAMGIALRDLHFSWGNFNDCVYI
FCNILYLMNGFFKIAVLSAHKRKFFDIVLCTEQTFMPPTDDQTERKILMDCKRLCDWFIVFIMFCVQGGC
SGYAITPFVENIGKNESERILPFNLWIDFPTGMSPYFEVFFTIQILCVYHVAISYFCFDNFLCILCLNIT
GQFRILQHRLRSLASMQTCEQNLNRSRTKSAIERLIKLKSCIRHHQALINYCRKLENVFTLIILGEILFV
ALLLCCLGFQVFLMFTPPARRISLVLSMMAVSSQLLMFSYCWDGLMRESGNIGQAAFAGPWFVAKMNEGG
RALREDVLIIIMRANKACCLTAGGFFPVSLETFTAVLSTAVSYFTLLRNSAPDLIKT

>gi|383850742|ref|XP_003700935.1| PREDICTED: odorant receptor 13a-like [Megachile rotundata]

MNQSLQPNLNYENDIIYVTKYSRWILVSIGIWPASLKGSAKFLSKIAIGFGNLVLFFAIIPFSLHIVLEQ
KDTVIMLKMLGFLSFCLISVLKYWALAMRKPKIERCIELMQTDWKQVEFRRNRHMMLKYGKVGRDLTILC
IVFMYSGGTIYHTIMQYAIGSFIDEHNRTIKPLVYPTYSALYDVQKIHVYVLVYTVHCMCGYVIYSVTAG
ACGLAALFATHTCGQIDIVLSRLNGLVDDNLMRHDDLNERLIEIVEHHLRTLRFSAMMETVLQEVCFLEF
VGSTFMICLLEYYTIVDWKQSNTIGLTTYTLLLISLTFNIFILCYIGDLLIEKSTSIGTSSFEIDWYRLP
TKTIRGLILIIAMSNNPVKISAGGIVTLSLSTFVNVLKSSLGYLSFLRTVVV

>gi|383850740|ref|XP_003700934.1| PREDICTED: odorant receptor 4-like [Megachile rotundata]

MHARVSNGVSPNKYYDTDIKYTFELCQWILKPLGIYPFVYSNVSKLERGVSVLLLITCCSIIQFVIVPFG
HYVLFYEKDINARIKFLGPLTFCSTALVKYVYLCLKAPAFKRCIEHVERDWKKLQDQVYRDVMINYISMG
RNLITVCAMFFYTGGLSYHTVMPLLSKVKTENVTIRPLTYPGYEAFFDVQRSPTYEIVYFMHCIYVMVAG
NITMVAYSLATIFTSHACGQMKIQILRLENLTDERVTEKRGEDRLTIIVKDHVEILRFTKLVESAFREIC
LIEVIVSTLLICLVEYYCLMEWETSDSVAILTYAMLLISFIFNILMFCYLGELLLEQGNRVATASYETLW
YNLPAKKARDMVLLLAISKTPPKLTAGKIFDLSLYTFGVVLKSSMVYLNVLQTMIEL

>gi|383850736|ref|XP_003700932.1| PREDICTED: odorant receptor Or2-like [Megachile rotundata]

MNALSDLSLENDWMYSIQMNRWFLKPIGVWPLSLSVTTFGKLNSIILALISSFLIGFLLVPCALCTLLDK
TGDLDTKVKMIGPLSFCMMSTFKYYILASRGGKISECIESMRSDWAKTRSAGYEEDREIIIDCARVGRSI
AIICAGFMYSGGCFYTAVMPLYSKRTEIIDNETVRSQTFPIYRRLLDPRTTPSVEIVQFMQCLAAFVMYS
VTVGSCSLAAVFVMHACGQFRILVMKLQRLVDGATGKNSVRTYEHRLGDIVEHHLQILRFISRVEDLLNE
ICLVEVVGCTMNICFLGYYLLTEWEQSEAIGTLTYCLFFISLTFNIFILCYIGEILSEQCQNVGLSACMI
DWYRFRQKKALGLILIVAISNSSTKLTAGKLVELSLSNFCSVFKSSLAYLSLLRTLTT

>gi|383850718|ref|XP_003700923.1| PREDICTED: odorant receptor 2a-like [Megachile rotundata]

MEPSIIRTDLDSLFDYSLQLNKWLLIPIGAWPSTTSKIERVVSLILIVVSYCSILTTVVPCILYVILDDD
LLTQKLMMVGPLSHWFIGGINYTTLLLRKKEIQCCVKHIHMQTDWRSVTKSKDQEIMLKDAEFGRYVVIF
CTAFMQGGVLCYCVITGFTTEVIQVGNETRTIRMLPCAAYKKLIPVDTSPTNEIILFVQFLAGFIVNSST
VGAFSLAIVFAAHAYGQLNVLITWIRELVNESRGRDRNVYLKEIGVVVDNHLRALSFISYIEDMMTEICF
LELFKCTFNICMLGYYVLMHWANRDYQNMTICVVILCSMAFNIFIVCYIGEKLSEQCKNVGDSVYMTNWY
YLPDKHILDLLLIISRSSTVIKMTAGKFVTMSIYTFASVVKTAFAYLNVLRQTT

>gi|383850716|ref|XP_003700922.1| PREDICTED: odorant receptor 13a-like [Megachile rotundata]

MTTPASVVPTDLDSLSDYSLQLNKWLLITIGAWPSTPSTTKIEKIVSFILIVICYCTILTTVLPSILYVI
LADEDLQQKLMVMGPLSHWFIGGINYTTLLLRGKEIQCCIKRVQTDWRTVTRLNDQKIMMRNAEIGRYVV
VCCTAFMQGGVLCYCVITGFTTEVVQVGNETRTVRLLPCAAYKKLIPVDTSPTNEIILFVQFLAGFVVNS
TTVGAFSLAIVFAAHAYGQLDVLSTWITEFVNQSREHDKNAFLNSIGPAVEKHLNVLTFISHIEDMLTEM
CFLELFQCTFGICLLGYYVLLYWALHDLQNMVTCSIILFSMSCNIFVVCFIGEKLSEQCTKVGEAVYMTE
WYHLPGKDILDLVLIISRSSVVTKITAGKIVHMSLYTFTTVMKTAFAYINVLRQTT

>gi|383850686|ref|XP_003700914.1| PREDICTED: odorant receptor 47a-like [Megachile rotundata]

MKKTVTQTTALDTQDYTKNVNLSIQFSRWLLRPVGVWPKSEVTRIDKYFTWLVNSLCSTLLSFLFVPCLV
FLILDVSDSYNRIKLIGPLSFFLMSYMKYYLMLLHKNDILKCVQQVQRDWANARHLNDRNIMGINANYGR
KLVVVSAFFMSSSYIFFYIAMPISVGRIPAVDGNFTFIVLPFPSSTRIFDYRHSPFNEIFFVVQCLAGTI
LHMITVGACSLAATFAVHACGQMEILMNWLEHLISGRVDMKDTVDGRIASIVEQHVRILRFLELMEKLLQ
QISCVECLGCMMNLCLLGYYCIMEWNPKELTFSLTYILLISYFGFNIFIFCYIGELVAEQCKKVGEVSYM
IEWHRLSGKKKLCCILIIAMSNSSIKLTAGNMVELSISTFSDIVKTAVAFLNMLRALT

>gi|383850681|ref|XP_003700913.1| PREDICTED: odorant receptor 22c-like [Megachile rotundata]

MKRPEAYRKPSVIDGYERNVKLSIQLCHWILKPLGVWPRSTNLTVAQIWLYQLINVLCYAFISFLLVPCS
LYVILEVEDVYNQIKFFGPSSFCMMAIMKYYLMILHENDIRDCIQRIELDWKNISHDDDKKIMVEKAKFG
RQLIALCTFFMYSGFAFYYIVLPISVGKIQAADANLTFMPLVFPFSKLIIDTRYSPTNEIVFFLQLIGGA
VMHGVTSAACGLAAMLAVHACGQMEVLMNWLKHLVDGRSDMGDTVDARIASVVSQHVRILKFLELTEKIL
RHISLVEFLGCTLNLCLLSYYVLMEWGTNDIMSSATYAVLLMSLTFNIFIFCYIGELVAEECRKIGEMSY
MIDWYRLPGKRKLSVILMIEMSNSSIKLTAGNIVILSYSKFGDVVKTAVAFLNVLRTVT

>gi|383850526|ref|XP_003700846.1| PREDICTED: odorant receptor 4-like [Megachile rotundata]

MHDRSHDNQVLKNSYYSSDIQYALQMTQWTLKPIGIWPVIYHRVTRREQLMSVVLMIFGFSALCFVLIPS
GHYIFFTGRISTKISLFGPVGFCLSTTVKYVYLGLRASIFGRCIEHVERDWKAVTDQRHRSIMLECTAFS

RKLITMCAAFLYTGGLSHHTLFPFLSKDKQQDNLTIRPLIYPGYDVYVDSQASPAYEIIFCLHLVTALVI
YTISTGAYSLLVIFIMHISGQIQIQITRLQNLKKAQMEKGHRNPLAIIVYNHVEILRFSENVQAALEEIC
FTEVVECTINMCMLQYYCLMSWRSSDGVKIITYFTLLCSFTSNIFIFCYIGEILTEQCSQIGKASYEIDW
YNLPAKNAHDLILLNLISQNPPKLLAGRIIELTLNTFSAVVKTSVAYMNLLRTVTD

>gi|383850524|ref|XP_003700845.1| PREDICTED: odorant receptor 4-like [Megachile rotundata]

MRDRSFDNVVLKNSYYSADIQYSLQMCQWTLKPIGIWPLIYHRVTRREKLISVVLLILGFSLLFFILIPS
CRFIFFEGHISTKVRLLGPVGYRMSTTVKYIYLALRGSIFGRCIEHVERDWRTVQDQRHRSIMLECAGLS
RKLITMCAAFLYTGGLSHHTVHAFFSKDRRHSNVTIRPLIYPGYDAFFDTQASPTYEIIYCMQLLSSLIV
YTIGTATYSLLVIFVMHICGQIQIQMARLENLKNARLENLKNAGLEKGQRNPLSKIVYNHVEILKFSEKV
QKALEEICFTAVVECTINMCLLQYYCLKSWKASDTVQIVTYITLLMSFTYNIFVFCYIGEVLTEQCSQIG
VASYDIDWYNLPAKNAHDLVLLNIVSQNPPKLLAGRIIELSLNTFTAVVKTSVAYMNLLRTVTD

>gi|383850520|ref|XP_003700843.1| PREDICTED: odorant receptor 82a-like [Megachile rotundata]

MIDESTIQQNPIGLSDYSLQLNRWFLKPIGVWPVLTSRNEKIVSLTLNVICYCSILVTVVPCLLRIFLED
DDLLAKLKMTGAMSHCFAGGLNYTTLLLRRKEIHYCIKQINSDWRTVKRPDAQQVMLKDAKFGRSVAAFC
AIFMQGGVFCFSVATVFTMEIVQVGNETRAVHTFPCPAYRKLIPVDNNFLCEIFLAVQFLCVFIVNSTIV
GAFSLAIVFASHVYGQLDILMVWVTEYVNKSEEMHKNRWNQIGVFVENHVKILRFIAHIEQVMSPICLSE
LCQCTLSMCTLCYYIIMEWEEHDVQNLTSYAMILVSVTFNVFIMCYIGEVLMEQCNRVADVVYMTNWYCM
PYRSILDLILIISRSNVVTKITAGKVIYMSIYTFGDVLKTAFTYLNILRQTT

>gi|383850508|ref|XP_003700837.1| PREDICTED: odorant receptor 4-like [Megachile rotundata]

MQKSGKDSEYERHVNLSIQWNRWLLKPIGIWPHSDMSRTKKLYDWFTYVVCYGLISFLFVPCSMYVFLEV
EDLYNKIKLFGPLIFFLMAFAKYYLLIYHGNDIRECIERIKWDWKNITYHDDKNIMLENANFGRRLVTVC
TFFMYSGFASYYIIIPITIGKVKSEDGNLTFIPLAFPFSSLIVDTRYSPTNEIIFSVQLIAGALMHGITS
AACGLGAMLAVHACGQMQVLMNWLKHLNDGRSDMGESVDDRIACIVSQHVRILKYLTHTERTLQLISFAE
FLGCTVDICLLGYYVIMESKSNDITNTVTYAILLTSLTFNIFIFCYIGELVAEQCKKIGEMTYMTEWYRL
PGNKKLYCIMIIAMSNSSMKLTAGSMVELSIETFTNVVKTAFAFLNVLRTMT

>gi|383849003|ref|XP_003700136.1| PREDICTED: odorant receptor 85c-like isoform X3 [Megachile rotundata]

MARTPGHKWMVAFRKLINIQINYLKYSGLWVIDPGHPWLSKSAYLVCKGWTLSAMYIFAITLFADICDNI
DNLSITSDDGCVFAGIAVVIFKTMNYQIRQKKIALFVDKVLKCSDDLCEFSNEESIKPIIDKYRLRITVT
FWGFSALGCALGFLLLFASPRQNDLPIRAKYPFNTTTPGWHELSFFIETCAVSGGLVAIVGMDSITIYMS
SIITMLLDILSCNFESCSGRSANNENEICLVRNCEERQRDKWDSNKFLHRYKNCIRFHQRLVTLSKEYNR
LFSSSMFVQMLSSTSMICLTGFQAVVVGGQSSDIMKFGMYLSAAVSQLLYICWIGNELSYSSSVLDKSQW
LSDWHHEHLPSIVQVFTLSTMSTRRTLTLKAGIFFVLSLETFIAIVKGSYSVFTLLNNMQTTDP

>gi|805808284|ref|XP_012146523.1| PREDICTED: LOW QUALITY PROTEIN: odorant receptor coreceptor-like [Megachile rotundata]

MSDPVNSLTLCKEEPLYDTVNSRTYSERIYINLCTSSNPVTNVKSKEIFKRDWVRVPIECVNRTMMKFKQ
QGLVADLMPNIKLMKASGHFLFNYADGSGKSMQKIYSSVHLVLILMQFAFCGINLVQEREDVDDLTANTI
TMLHFTHTIVKIIYFAVRSKLFYRTLGIWNNPNSHPLFAESNARYHQIAIKKMRILLLAVMGSTVLSTLS
WTILTFIEDPVKKVTDPVTNETMFVEIPRLMVRSWYPFDASHGMAHVMVLIYQFYWLLFSMXERESFRRV
ILFVAPLRLRTNSTFEEHHEAFDGVQRDVGYRCAKQW

>gi|805756685|ref|XP_003699729.2| PREDICTED: G-protein coupled receptor moody [Megachile rotundata]

MEYWMASNATVHNGREEAIQEVLEDPGSAVLFVGYPRWLLHFAASCCILFMLVGIPGNLFTIIALFRTKK
LRNATAIFIINLSISDLMFCCFNLPLATSTFWHSSWNHGVLLCRLFPLLRYGLVAVSLFTILSITINRYV
MIGHPTLYRTIYKPKYLIPMVLSTWIVAFGVLIVTWFGNWGRFGLDTAIGSCSILPDKNGRSPKEFLFVV
AFLIPCIAIVVCYARIFYIVRKTASKSRTPHIAASTDAIDISHEQRSASPRSQEEDPSASRSNYAAGVLD
TNLSPNHLSTNQLNLQVHQESKHSPRANEETRDPKNKKEKYRQENLKEQPKNPPNIFEVYEAGLEIKAND
IPYADSDSPETESFSNKIKESVGNTRSKLERMASRASFILESTLWVQRLNSKVSTGSDQFENSRSNSPER
STAKKAMIVRSESRFTNARRMKNLEPPRMSNKDKKLLKMILVIFSSFLVCYLPITITKTFKDAIDWRGWN
IAGYILIYLTTCINPVVYVVMSSEYRSAYKNVLFCRNDGRKETRKMFD

>gi|805825925|ref|XP_012152840.1| PREDICTED: orexin receptor type 2-like isoform X3 [Megachile rotundata]

MYSIEKTLIVWSTMVSILVDATDYQDDYTSVEYIDESDMDFNSTNCSNIYCISNEEYVDRMMNYIFPKLW
DWVLIASHGIIFVVGLVGNVLVCTAVYRNHTMRTVTNYFIVNLAVADFLVLLLCLPFTVLWDITETWFLG
LTLCKAVPYLQTVSVTVSILTLTFISIDRWYAICFPLRFKSTTGRAKTAIIGIWVIALLFDIPDLIALHT
FPPTHIRVKTILFTQCATSWSQRSQITFTIVKLIFLYTGPLIFMSVAYWQIVRVLWRSDIPGHNLLPRAS
QMSRTPSSGGGNPEIQLRSRRKAAKIYTIMLPSDKWMNNICLVAHGLCYFNSAVNPLIYNFMSGKFRKEF
RRTFRCAQESSSRIQRGYLASTSNLARIKSRTTTIRTTFKNNNNVQRTTEIVPLSAITSVQQNEKHD

>gi|805825923|ref|XP_012152839.1| PREDICTED: orexin receptor type 2-like isoform X2 [Megachile rotundata]

MYSIEKTLIVWSTMVSILVDATDYQDDYTSVEYIDESDMDFNSTNCSNIYCISNEEYVDRMMNYIFPKLW
DWVLIASHGIIFVVGLVGNVLVCTAVYRNHTMRTVTNYFIVNLAVADFLVLLLCLPFTVLWDITETWFLG
LTLCKAVPYLQTVSVTVSILTLTFISIDRWYAICFPLRFKSTTGRAKTAIIGIWVIALLFDIPDLIALHT
FPPTHIRVKTILFTQCATSWSQRSQITFTIVKLIFLYTGPLIFMSVAYWQIVRVLWRSDIPGHNLLPRAS
QMSRTPSSGGGNPEIQLRSRRKAAKMLVTVVITFAICYFPVHLLSILRYTIMLPSDKWMNNICLVAHGLC
YFNSAVNPLIYNFMSGKFRQEFRRTFQECAPSNHSNGHRPNQQLSYAYMGNFERNLDVLSVALKKVVRAF
NVDI

>gi|805824910|ref|XP_012152377.1| PREDICTED: probable G-protein coupled receptor AH9.1 isoform X1 [Megachile rotundata]

MLTGILKGTLLCCGGENSTWNVIIDTNKNVLHESTSLLRIIHRIMIPTIVTTGIFGNLLILLVLSTPQFR
GIAYLYLKGLALAHIGVLTSWIPIFVRLGYGMKNNYPSVFYHAYLELVAVNTFAISSILIMTCLIVDRYI
FIFFPARIRSRNARKSVRSFVLCSFIVGFAASAPLTGMRTVYELQENTGTLFTLRENISVTQHTLWIAYI
WIMEITLRLGPTIILFLLNSFVVKRFLHLNAKKKEFQSVSEKFRTANVPETSLLSRNRGYREEQHLATLM
SVMAVCFILTMIPSILLPFLYHNYDIADVGYLLFRTFAAVTELCNFAVHIVIYFLCSKEFREEFLKLLQN
RCKKRVVEEEFERPVGEIEDYSRHGTTVRLTPEQTKLNSPGSASKLNAMEEDKESEVLNASDII

>gi|805814874|ref|XP_012148323.1| PREDICTED: growth hormone secretagogue receptor type 1 isoform X3 [Megachile rotundata]

MLSTVSSVPLELNSSYYATVSGNNGLDGLSVIESENSTEGLYMLPAYIRTTSMVVCIIVMVLGIVGNLMV
PLVVFRGKDMRNSTNIFLVNLSVADLCVLLICTPTVLVEVNSGPEVWPLGEHMCKAVPFVELTVAHASVL
TILAISFERYYAICEPLRAGYVCTKARATFLCLLAWVAAALCTSPIIWVSQYQEMRVNDLDQRNFVPVCL
TSVDTVGTAVFFLFLVVFFYLAPLLILLILYAFIIGHLVPDSSNTSDTYHARAKRHVITLLLSVVLCFFL
CLTPYRVLILYIIVAPAEQIAAIDRDTFFAVLNFSRIMFYLHSAVDPILYNLMSSKFRRGFLQLCRLTAC
ERSNRSIGTRKTDSTEQENFV

>gi|805814650|ref|XP_012148264.1| PREDICTED: probable G-protein coupled receptor B0563.6 isoform X2 [Megachile rotundata]

MMMDAANGTKTESCFRLEDLEDPRTPFLRRISYGIILPAICCLGIVGNILNLVVLTRRNMQGTVYIYMRG
YSAAALLAIFFCIPFALRVLIHQETGQWSNWPQAFYHAHLELFLGNGCLGVGVMMLLALTVERYVSVCRP
GQHTRPLCGPPHLTVAVISLATFIVYLPSAFRAQITTCLLEPGGPVVYQKRENQLYLDSMFHQVYKVVLE
IIFKVVPAILLAGFNLRIMVAYRRSCERRRRTTLARTASNGVDSRTFAEERRLVLLLGSTNILFVVCVSP
MVILNITLRESNLNNYAYQQTFWR

>gi|805806639|ref|XP_012146104.1| PREDICTED: gonadotropin-releasing hormone II receptor isoform X2 [Megachile rotundata]

MGGTIKVATSTELPRFQINNSDHLELPIDMRFNEGHIVSIFIYSVLMIISAVGNTTVLVLIIRRRRTSKS
RIHNMLMHLAIADLLVTFLMMPLEIGWAITVSWKAGDAMCRIMAFFRVFGLYLSSFILVCISIDRYYAVI
HPLQLWDIDKRGKLMLCLAWGGSIACSMPQMIVFHLETHPNITWYSQCVTFNTFPTYTHEITYSLFGMIM
MYWFPLIVIIYTYTSILLEICRRSKKSEDKIRRSSMGFLTRAKVRTLKMTVIIVTVFFICWTPYYVMSLW
YWIDRHSAYKVDQRIQKGLFLFACTNSCMNPIVYGAFNIRDRNKTSVRPATIETRVTPLTLSLKLLD

>gi|805803772|ref|XP_012145303.1| PREDICTED: tachykinin-like peptides receptor 86C [Megachile rotundata]

MNNLVLYSLYNCSATVLDRNVTFLLKLNRSELLSVLGDALDRSTEHEALRDAFFDCLFNYQERPFDLPWW
QKLCWSLLYAAMLLVATGGNIIVIWIVLAHRRMRTVTNYFLVNLSIADLMMSLLNCAFNFIFLLNSDWPF
GVVYCTINNFVAHVTVASSVLTLVVISFDRYMAIMRPLKHRMSRKRTVIIVFLIWTISSVLALPCLLYST
TESRRYSNGRSRISCYLLWPDGSYLNSKIEYFYNLVFLSVTYLIPMTVMAICYSFMGRKLWGSKSIGELT
YNQKKSIKSKRKVVKMFIIVVTIFAICWLPYQAIFIFLYHHRHFTETSYIQHVYLSFYWFAMSNAMVNPI
IYYWMNNRFRVYFQLVICKWELKNSGNRQLQEFMDQRSDIIPYNSVRFKSSSSRWKHEMTESQTQNFRTR
SSYRRSHKQNATVV

>gi|805793928|ref|XP_012142602.1| PREDICTED: tachykinin-like peptides receptor 99D isoform X1 [Megachile rotundata]

MQAVDVYNSTPDPWNITLWDNYTYYNNTNTTDLQLRNQFILPWWRQMIWTLLFAGMIIVATGGNLIVIWI
VLAHKRMRTVTNYFLVNLSIADAMVSTLNVTFNYIYMLNSHWPFGTLYCKICQFIAVLTICASVFTLMAI
SIDRYMAIMNPLRPHMGRRATLCVAIVIWIVGAILSLPMLLFYTTYTQNFASGEIRVICYGDFPNRDNNG
LSYDEYLYNVIFTILTYFLPIGSMTFTYARIGLELWGSQSIGENTAGQLESIRSKRRVVKMMIVVVVIFA
VCWLPFHVYFIIVSYFPEITNKPYIQEVFLGIYWLAMSNSMYNPIIYCWMNSRFRRGFARFFSWCPGVTV
PAEPSLSRSEALTSRYSCTGSPQTNTRISRNGTSSCTSLERPCTSSTTPTSESRFEHDDSYTNTRNSYIG
LHELGCVEQPRESPDLG

>gi|805772771|ref|XP_012137028.1| PREDICTED: 5-hydroxytryptamine receptor 2A-like [Megachile rotundata]

MIGSSTPRIDRTDPYEAVVDTCYEELLLFAAVNDTSAGCDRVRFTLSGILNDTDYEGKAVNETRLDIENN
GLNNWWAMLALVLVVGTAAGNILVCLAIARERRLQNVTNYFLMSLAITDLMVAVLVMPLGILTLVRGYFP
LPSVYCLVWICLDVLFCTASIMHLCTISVDRYLSLRYPMKFGRNKTRRRVTLKIIFVWILSIAMSLPLSL
MYSKEEDSVLIDGACQIPDPLYKLIGSIICFYIPLGVMLLTYALTVRLLAEQRQNIGGTAGWSSGWLGGP
QGPPPGG

>gi|805770109|ref|XP_012136296.1| PREDICTED: probable G-protein coupled receptor No9 [Megachile rotundata]

MRELNATACAALYERVEWSGPGILVTLIILAIVNVMVVLGNVLVILAVHYTSKLKNVTNMFIVSLALADL
MVGVAVLPFSAMWEVFKVWIFGDLWCSIWLAVDVWMCTASILNLCAISLDRYLAVTRPVSYPQIMSPRRA
RLLVATVWILSFVICFPPLVGWKDKRSQATYNATFAQNGPFNTTTILVPVKPCPWICELTNDAGYVVYSA
LGSFYVPMLVMLFFYWRIYNAAVSTTKAINQGFRTTKGSKVLGSRFDEQRLTLRIHRGRGSVHNGSNNGS
PRSPESNSRSSMKREKVKISVSYPSTETLNTKCNTLERTPSRCSQTSVHYSNGQTHSQLCPTPRSTHLKV
SDINRVGSTRRPSRRSSCESQMTGDDVSLRELAPGPEEKPRVMKMGKRNIKAQVKRFRMETKAAKTLGII

VGGFILCWLPFFTMYLVRAFCPNCINSTVFSVLFWLGYCNSAINPCIYALFSKDFRFAFKRIICKCFCKR
RANTLRRGSDGSQLTTRNDRSPSYSIRVPQQGVSIDDSDPDPNSEPTVHSQSESR

>gi|805767181|ref|XP_012135511.1| PREDICTED: muscarinic acetylcholine receptor DM1 [Megachile rotundata]

MNYTSEPDIFGNESYNASNGLDCGGDTYSYGIWERVMIVIIAVVLSLTTVVGNIMVMISFKIDKQLQTIS
NYFLFSLAVADFAIGLISMPLFTLYTVLGYWPLGPHICDTWLALDYLASNASVLNLLLISFDRYFSVTRP
LTYRAMRTTCKAGIMIALAWGVSMVLWPPWIYAWPYIEGQRTVPVNACYIQFIETNHYITFITAIAAFYV
PVTVMIILYWRIWKETKKRQKDLPYLQAGKQDASKRSNSSDEALDMEDCKRPRSESSTGAEDMNSTHIAV
SYLEKHCPQYKTKYRPLSWMWLKMWCIAWWHSGREDDDDDDDIEGAESSRTGHGYDEATTPLSAETPLTG
TVSRSASLSGIHPTALTVEKTVCIADIHDYKKSGKGGELSTKSISSDSVYTILIRLPTRDTSGMGKYTEG
PSIKMYHDEAVTYQLHSMIEDVEEENGKIFRLGSTISSPSPVIRRPSRMPDIRIPLNTKNIPKALASKVP
ANKSANKKKKKLQEKKADRKAAKTLSAILLAFIITWTPYNILVLIKSITACSWYIPQQLWDFFYYLCYIN
STVNPMCYALCNAAFRRTYVRILKCKWHNRNRAAVDRG

>gi|805765510|ref|XP_012135020.1| PREDICTED: neuropeptide FF receptor 1-like [Megachile rotundata]

MISTTSSSSFNDLYNDTTMSNKSFQVDITEEILWYTYSRFNETNYLLIILYVPVIALAVTANVLVIAVVI
KYHYMRSVTNYFVVNLSVADLLVTTICMPVAVSQAISMVWTYGEVMCKLSSYLQGVAVAASVFTITAMSI
DRYLAIRSPMAFRRVFNRKSTVFVIVALWLVALIIFAPVLKAMTLRDPSQELSNITLHGSWIMAGNFSEN
ISPMSQRPPPFYVCWEDYKLLGVREHLFGTVCFVLVYAIPGFVVILSYSMMGRTLCSRKPPFDCDSVKGS
ASSQQSFRLVRERRRIAWILLLLAVLFALCWLPYNVLMLLIDLSVIGEETVTTDALSYCLFLGHANSALN
PVVYCFMTRNFRRSVAEILRRGPRALARRRPRRKSVQGTAVIDDMCAGCNAGGTMRRGLLRKRRMLPGCG
CGLPIGGHHAVLTVKRTATSSSGYDSFYSRHSPHRRCYMLQSIRKKPHVPVDQAQNANKRQETSYQKNVT
TSQPRVNTLVTTDEQR

>gi|805764641|ref|XP_012134775.1| PREDICTED: cholecystokinin receptor type A-like isoform X2 [Megachile rotundata]

MSFSDHNISDKALKHEYGVQSDMDQLFVSNIPQLSTMTSVTVNITLSNITQSGQADLLETLIIPLYGTIF
LLSIIGNSLVLITLARNKRMRTVTNVYLLNLAVSDLLLGVFCIPFTLLGQVLKNFVFGVTMCKLIPYFQA
VSVSVGVWTLVAISLERYFAICRPLKSRRWQTQFHAYKMIAVVWTASLTWNAPILVFSQLQDIRGVDRTE
STGTVRNSTYDKDDSGTTVVVNSCGASSRLRRGFSSSHGYFHGASQGISSKGRVPRTRIARDPGGNCQGS
EFSHESGQQVRSNGSNCLSRNTEDNSVREKQNSTCPFSRQHVIRSNYMGKSIEAKKKVIRMLFVIVLEFF
ICWAPLHVINTWYLFAPQLVYSIVGSTGISLVQLLAYVSSCCNPITYCFMNRKFRQAFLSLFDCHRCWRV
GCRNSDVATASTKNAAQGGNNSELSGNETTVYLGKASLVARSEVVRLLEEEDRV

>gi|805764638|ref|XP_003700734.2| PREDICTED: cholecystokinin receptor type A-like isoform X1 [Megachile rotundata]

MSFSDHNISDKALKHEYGVQSDMDQLFVSNIPQLSTMTSVTVNITLSNITQSGQADLLETLIIPLYGTIF
LLSIIGNSLVLITLARNKRMRTVTNVYLLNLAVSDLLLGVFCIPFTLLGQVLKNFVFGVTMCKLIPYFQA
VSVSVGVWTLVAISLERYFAICRPLKSRRWQTQFHAYKMIAVVWTASLTWNAPILVFSQLQDIRGGRRKC
REDWPSVGTERAYNLFLDGTLLLVPLIVMSLAYSLIAVKLWRGLRQEIRQSSSCPQRLDRTESTGTVRNS
TYDKDDSGTTVVVNSCGASSRLRRGFSSSHGYFHGASQGISSKGRVPRTRIARDPGGNCQGSEFSHESGQ
QVRSNGSNCLSRNTEDNSVREKQNSTCPFSRQHVIRSNYMGKSIEAKKKVIRMLFVIVLEFFICWAPLHV
INTWYLFAPQLVYSIVGSTGISLVQLLAYVSSCCNPITYCFMNRKFRQAFLSLFDCHRCWRVGCRNSDVA
TASTKNAAQGGNNSELSGNETTVYLGKASLVARSEVVRLLEEEDRV

>gi|383862251|ref|XP_003706597.1| PREDICTED: growth hormone secretagogue receptor type 1 isoform X1 [Megachile rotundata]

MLSTVSSVPLELNSSYYATVSGNNGLDGLSVIESENSTEGLYMLPAYIRTTSMVVCIIVMVLGIVGNLMV
PLVVFRGKDMRNSTNIFLVNLSVADLCVLLICTPTVLVEVNSGPEVWPLGEHMCKAVPFVELTVAHASVL
TILAISFERYYAICEPLRAGYVCTKARATFLCLLAWVAAALCTSPILLMVSYDIEENADGTYTSTCNTVA
DTYWTMGFILTTILVFFVIPLLILVILYTVIARHLMTNPTISRGSSNNLLKYRKQVMLMLGTVVLCFFLC
LLPFRALTLWILLTPKSMIIDLGIEGYFTLLYFCRVMLYLNSAINPILYNLMSTKFREGFLRLCGLGPNR
RKKKKTSADKTGTYTTGSTNYSSNHSDFWRRQSSNKSSSMKVTGSNSSAEKQIKLPLQTAVVSGNIVRKK
QESYV

>gi|383862143|ref|XP_003706543.1| PREDICTED: octopamine receptor beta-1R-like [Megachile rotundata]

MKLNETMEPDASNATDLPGDLPQILVDTGQSNRSNDVYDQQRSHDLEYVLLLTLKATVMAFIIICALFGN
LLVIVSVMRHRKLRVITNYFVVSLALADMLVAIFAMTFNASVELSGRWLFGYFMCDVWNSLDVFFSTVSI
LHLCCISVDRYYAIVQPLDYPLIMTNLRLSTMLSVVWCSPTVMSFLPIFAGWYTTEKHLEYRRNYPDVCV
FQVNKLYAVISSSVSFWVPGIIMIAMYYKIYREADRQERMLYRSKVAAALLNKHLQINGISAGLTTLPTV
DQSSPDPQPEDPPITSSSKMRRERKAARTLGIIMSAFLACWLPFFLWYIITSLCDTCESSDAVVAVVFWV
GYFNSALNPLIYAYFNRDFRAAFRKTLESCCVAIGPVRDLRQVHRKQDLVHSNASSELHVNNQLRTSEMT
NVHIEACI

>gi|383862095|ref|XP_003706519.1| PREDICTED: somatostatin receptor type 2-like [Megachile rotundata]

MNSTVMSTTSVIDYAWKVSQEDSTIQNCEADLPIVSLVNQILYSIVCIVGLLGNTLVIYVVLRFSNMQTV
TNMYIVNLAIADECFLIGIPFLVTTMSLRGWIFGKIMCKAYMTTTSINQFTSSIFLFIMSADRYIAVCHP
ISSPKIRTPFISKVVSLTAWVTSVLFMIPIFLYANAMESERGINCNIYWPTDRGGHTTFTLYTFILGFAI
PLILILIFYFLVIRKLQTVGPKNKSKEKKRSQRKVTKLVLTVITVYVLCWLPYWLMQVALIYTPPKQCQS
KITITSFLLAGFLSYSNSAMNPILYAFLSDNFKKSFLKACTCAVGKDVNATLHIENSVFPRRNKANAERL
QANRLAASGQSRLELEDEDAERGLLISKTSTTTVTMTSRSNITIGNDSKDQPQREKDSMKNGAQLTLLTQ
V

>gi|383860442|ref|XP_003705698.1| PREDICTED: 5-hydroxytryptamine receptor 1 [Megachile rotundata]

MEGKNAITTEGSLLLNLTTIATQDDEDFLRTFPGKNSPYTAAQAILIALVLGSIIVGTVIGNILVCVAVF
LVRKLRRPCNYLLVSLAVSDLCVALLVMPMALLYEISGNWSFGAFMCDFWVSFDVLSCTASILNLCMISV
DRFCAITKPLKYGVKRTPRRMIIYVSLVWLGAACISLPPLLIMGNEHTYSETGAFHCVVCQNFFYQIYAT
LGSFYIPLLVMIQVYYRIFCAARKIVLEERRAQSHLEAHCYLDIEPTVQHHQPSVVNRQLNSDVQTVHGS
PPVKQHRSSSASTTCSGHAVRCFAGGPRKSNESQCPMLQKLEKPILSSSTTTTPPMTSTKSTIVRNHLNS
TCSVTNSPHQKKLRFHLAKERKASTTLGIIMSAFIVCWLPFFVLALVRPFLRDPDQIPAFLSSLFLWLGY
CNSLLNPIIYATLNRDFRKPFREILYFRCSNLNHMMREEFYQSQYGDPINNYEIKAGELEGAERLDNQGI
EAIDVAASAPNESFL

>gi|383852942|ref|XP_003701984.1| PREDICTED: octopamine receptor beta-3R-like [Megachile rotundata]

MEVSEPSAEDVNRSIGRISTTIGIDRLSESLNSTVQSTFEVVTVSQEPSRVIWTVIKGMVMISIIVTALV
GNALVIASVRRHRKLRVPTNRYVVSLAAADFLVAVCAMTFNASVELIGHWVFGRIMCDMWNSLDVYFSTA
SILHLCCISVDRYYAIVRPLEYPAIMRRLTVTCMLCSAWMLPALISFVPIFMGWYTTDEHLKLLREHPEV
CVFVTNPSYSVISSSVSFWIPGFVMIVMYCKIYKEAVRQREALSRASSNTVLNSVHLHRASTSRHHSRAS
HQLLLHPSDASDFGRPISYRTAAELNAENGTSIRQPTKSWRAEHKAARTLGIIMGAFLLCWLPFFLWYVT
TSLCGEACYCPDTVVSVLFWIGYFNSALNPLIYAYFNRDFREAFKDTLKSALPCCASCWKTPSQFV

>gi|805819498|ref|XP_012149882.1| PREDICTED: uncharacterized protein LOC100884046 [Megachile rotundata]

MQVLHVGYQIDIMCQTVTDMPIIDKEQLRFFIKRHQDIIIFTEKIEKYFTYIALSQLFTNTLVTCCLGVM
IVIVFTTGGGAILVMKYLMFFVNIWVEAILCDMLSTIEEECKKYAFIDKTGLILKTIHVSYRLTTSIIVL
YVMVAIIHIIGNATSAHSNETYSLILKMDLPFEINKSPNYELVLTAQIFHQTYTAFTFSVYSALILMVVL
HVGCQIDILCQTIIDVPLRDKKQLEFFISRHQDIIVFTEKIEQFFTYMALSQLLSNILITCCLGFLIIMS
ISGDAELSVFFKSVSFYIALWIEAYVYCFAGEYLNTKILKASASYMSVLLAMS

>gi|805812281|ref|XP_012147625.1| PREDICTED: uncharacterized protein LOC100879426 [Megachile rotundata]

MFTVQTVSAIYNSAAVWGIETSCMTVILHVSGQFKLIKTWINNIGVKIKNEPKDHYYKCPPDIEDGLVRC
IRHHQRLVNVVNELNDLLIPIIFIQLLTSGIKICLSGFAVMNNNTNAELIKAVLYLFGMTTQLLLYCYPG
AGSLVLTIGQTFFKTAYLLTKKERACRLLDELRSLWNSTDDPVEKKSYEVFAYWGRTCTIAFFVSGMSTT
SMFMISGVVDSLNQQDLDNISNRHLPYDVWHEMEYLESPEFELMYAGQIMASFICCFGVLRTLRSGCVVE
DVNTLLAPIIFLQLLTGGMQICLSGFAVLSNNAGDNLIKFIAYLASVTIQIVMYCWPGEILIQESQKVGH
AAYLNVPWYQLPLFHRRQLLLIIIKSQKYCCISALTFKSLSSHTLTNISWQLMHHYCKVCKDVSNTVKLP
IRVLAVLSVKNEMEEITLLP

>gi|805759611|ref|XP_003699993.2| PREDICTED: uncharacterized protein LOC100882905 [Megachile rotundata]

MEGMQTYCKNAKVRERAILQWYVNQFSTFYGVSATWFYTTAIVVILGTFFSSHPFPTNAVYPIAVDYQPL
MSIVFLHQSVIGLQFSASVCVSVLCALLLLFASARFEILKMELREVKKPSELIKCMEKYYTVRRYACDVV
NTIKLLPLCTVILCGVILVFCGIKLIQPQPFTSRCQYLSIVWTALVEVFVCAWPADHLLDIKSGMKLISL
LTEPECYGSSSHAGADLPEIIAKEENLAVIKTAFVMINVILLTVSLVFGIYYYRSDIVILTKSISELTAL
LEVILDLLFCKMNHRRLQGLIGRIRMYLQVADEQENKIIQSYVDRYKKLFSVIAIAYISTGISFSLAPLF
SGQKLPADGWIPFSVEFVGIYWVVYLVQVYCILQTALCIGVDFMITTLFCFTAARLDILGSKMKRVNRYD
LLVSCVKEHQEILGFVDDTKAAVQALLFKTNITMGSALICGAFPLIYNQSLAVTSQFLCMVVSGCGHLYV
ISWPADDLKESSLRFATSVNDIQWIRQPRKMTNLILIMMQRNRKPCLITMGGLLPPLSLEYYAHFLTSIS
SYFMAMRAVIES

>gi|805752310|ref|XP_012144545.1| PREDICTED: uncharacterized protein LOC100878875 isoform X1 [Megachile rotundata]

MNPTLDLIIPLNETRPKEFLFKASYAFIEVDDHFISVFLHLSWIAIVTVYCIAIVDSLYILIIHHVCGLF
DVCGYQIETATQDPELRYDQFKQCVMTHHKALMLFDNLQECSKNMFLVLVAINMTLISMTGVQILMAVGK
PHEMLRFSLFFLCEHIHLYIISLFGQIILNHSAILAERIYSSDWYEIPIKFQKLLCIMILRCSKPVNLNA
GGLYDMNMENYGKIKILLNFILNDWKILRDELPVLDRITEQGSRLAHMYRVTLMWATIGFIYIPLINPTL
DLIIPLNETRPKEQLFGVCYVFINPDDYFTAVFLHMGWTTWVTVYNIITVDSLYILIIHHVCGLFDVCGY
QIETIFKRSNLQHYQFKQCVIHHHKALEFFNYLQECSQNMNLMLVAITMILISTTAVQILMHMDQPLDSA
RFILFFVCSHIHLYIISLFGQIILNHSKTLAERIYSCNWYGIPIKFQKLLCIMILRCSKPAILSAGGLYD
MNMENYGKAVKACMSYFTMFLSMRE

>gi|805809243|ref|XP_012146799.1| PREDICTED: rhodopsin-like [Megachile rotundata]

MQTLVSINTFNTTSHGEVNPSQFSILTTTMGPTFARQYMRFNNQTVVSKVPEEMLHLIDTYWYRFPPMHP
LWHKILGLVMIVLGIIGWFGNGVVVYVFLLTPSLRTPSNLLVVNLAFSDFIMMGFMCPPMVICCFYETWV
LGTLMCDIYAMVGSLCGCASIWTMTAIALDRYNVIVKGMSGTPLTINRALCQILGIWLFGLLWTILPLVG
WNRYVPEGNMTACGTDYLTEDWGSKSYILVYSLFVYYTPLFTIIYSYYFIVSTVAAHEKAMKDQAKKMNV
ASLRSGENQGASAEAKLAKVALTTISLWFMAWTPYLVINYIGIFNRSLITPLFTIWGSLFAKANAIYNPI
VYGISHPKYRAALKEKLPFLVCGSTEEQAAGGEKAAETEGKS

>gi|805791840|ref|XP_003703932.2| PREDICTED: neuropeptides capa receptor-like isoform X1 [Megachile rotundata]

MQYQMPSNNVPHTSTQHRASNVCPTAAADMFENYTSPIVAFLQREDSPNRRDPLYIVLPITVIYAVIFFT
GLVGNVSTCVVIARNKSMHTATNYYLFSLAVSDLLLLISGLPPEMYYIWSHFPYVFGEPFCIIQSFAAET
SANATVLTITAFTVERYVAICHPFISHTMSKLSRAVKFVIAIWLTALFLAVPQAIQFGVTYDHSKNGSAI
MDSARCSIKWVLIAHAFEISTILFFAVPMTIITVLYILIAIKLRRSRLLTASTVKRNHLPAGLNHCESGR
AKGSAQRNVIRMLIAVVVAFFICWAPFHAQRLLAVYAQNTNGEPEDVVVIVYTILTYVSGVFYYLSTTVN
PLLYNIMSNKFREAFKSMLSNHCGRKWSSRKSIPRQPTYSSLSKYPRSTTRQADDRQNSPSISVSDENQK
LTANVQRPTNSNDLNGLERRSQDNRSTSKINRINNHREYGRSASRGSNSSQLTLMTSISRSFNEGNNNMA
AACVNECFLKMQSPPRAVTIGIIAERLRLGTKGLFSHQQKVPTIPLSKPEVTTEQQPRFQSHPSIESANT
ISNSSLQDFDETEFTGTELARYMGELNCDLVT

>gi|805788311|ref|XP_012141145.1| PREDICTED: neuropeptides capa receptor-like isoform X2 [Megachile rotundata]

MEEPIVSTNLSNSTSIQEYVQDITNFYNGTVDNYFQDIVHPPRRDSLFIVVPVTIIYASIFVTGTIGNIS
TCIVIARNKSMHTATNYYLFSLAVSDLLLLVCGLPAEIFLVWYKYPYIFGEGFCVLRGLAAETSTNASVL
TITAFTVERYVAICHPFLSQTMSKLTRAVKWILVIWLVALSFALLPALQFGVIQHKNNPSLVMCTVKRIL
LQHSFELSTFLFFVVPMSLITVLYVLIGLKLRKSNMMKRSQEREGSCRHHTGRSSRRVLKMLVAVVIAFF
ICWAPFHVQRLIAIYGTNSEDHITSNSKWIEFLYLLMTYISGVLYYMSTTINPILYNIMSNKFRVAFMET
LSRSCRIPGLVVRNDQRSYSSLSRSQQRALGAYGSRTVGTGGTGIAHESTDCSGNSAREDSPKQTTALVE
QLKTDSRNVDRKYADKDSEEIRKLAKEVRVVDEHRSTGKYATVKVSNVDEARKKWWGLLKWFPGLKSFKL
AGRNTYTVSENRILKQTELHREEFSMSMWNVREANDQLPV

>gi|805778926|ref|XP_012138649.1| PREDICTED: neuropeptide Y receptor-like [Megachile rotundata]

MTRRDRNPGSMFAERQLVTNPEEKEYAAGGSYRFITRSVYSAETCHNEEQPRELQLGEPAGQRMERNESF
GRDGLRLFHGAIFVVALAGNGLVCYVVYSSPRMKTVTNFFIVNLAFGDILIALFCVPPSFISILILQYWP
FGQELCPTVSYLQAVSVLASAYTLVAISVDRYIAIMWPLKPRLSKRQAQFLILGVWMLAMVISFPIAVVS
KLSQPSMQHQRCNQYICKEVWPTIENRYYYSIALLVLQYVIPIMVLMYTYTSIAVMVWGKRPPGEADNVR
DQRMARSKKKMVKMIVTVVIVFTICWLPYNILMLIIDNNEALSNWSGLPLIWMALHWLAMSHSCYNPVIY
CWMNARFRTGFITAIGRLPGMHRILRTDRAYNNNYNASTIDVPLTDPHGSGHSLLRRMNTCMTYISVRRK
TNGSHTAPARSASFRDDSFRPSTQPLQRQFTCLESHSEEQL

>gi|383864302|ref|XP_003707618.1| PREDICTED: prolactin-releasing peptide receptor-like [Megachile rotundata]

MNNASGAYNDSSRPIWSGVNRWKDPSSEKDATSNGTVQLIFYAFYGNIFVVGVFGNALVCFVVARNRRMQ
TVTNLFITNLALSDVLLCALAVPFTPLYTFLGGWIFGRTLCHLVPYAQGVSVYVSTFTLTSIAIDRFLVI
VYPFHPRTRIEVCLSVICGIWIIALTVTLPYGLYMRLEEPGTEIEMGSESDSDSGTSAKTRTEIYCEEHW
PNERFRKAFSSVTAILQFALPFFVIVFCYARVSIKLNDRARSKPGTQTGERERADRERKKRTNRMLIAMV
TVFGVSWLPLNVVNVIDDFYSWANDWSYYRLCFFMAHCLAMSSTCYNPFLYAWLNDNFRKEFKQVLPCFP
KTSDNRGPTAIEEEFQRDKARNGNNTLQESMLPSQTRIRVSAPEGYELILLEPTTTNASRNIEDQVL

>gi|383854790|ref|XP_003702903.1| PREDICTED: opsin, blue-sensitive isoform X1 [Megachile rotundata]

MLSRNDTLISGPLPFVGEETGHVPSMRERFLGWNVPPEHSDLVHPHWRAFLAPGKYWHIGLALIYIMLLV
MSVVGNCCVIWIFSTSKSLRTASNMFIVSLAIFDIIMAFEMPMLVISSFAERMIGWELGCDIYAVFGSIS
GMGQAITNAAIAFDRYKTISCPIDGRLNSKQAAIIIAFTWFWVTPFTVLPLLKVWGRFTTEGFLTTCSFD
FLTEDQDTKVFVMSIFIWSYCIPLSFIVYFYSQLLKSIRSHEKMLREQAKKMNVKSLVSNQDKERSVEMR
IAKVAFTIFFLFLLAWTPYATVALIGAFGNRELLTPISTMLPALFCKTVSCIDPWIYAINHPRYRQELQK
RCQWMGIREPEIPHDTASSQTEKVKSDDSA

>gi|383853616|ref|XP_003702318.1| PREDICTED: neuropeptides capa receptor-like [Megachile rotundata]

MDDTDDVHDFWKDWDLKNLTEAEYLTKVLGPKYLPLKMVIPLTITYVVIFVTGIFGNVATCTVIVRNPSM
QTATNYYLFSLAISDLTLLVLGLPNELSLFWQQYPWALGVGLCKIRAYVSEMSSYVSVLTIVAFSMERYL
AICHPLQVYTISGLKRPIRFILAAWSIALVSAIPFAIYTKVNLVEYPPGSGNYSADSAICAMLLPYMPDF
PLYELSTIVFFLVPMLIILVVYTRMGLMIRNSAKETLNSVTHGAIHGDAKQVQSRKSVIRMLSAVVILFF
VCWAPFHAQRLLYVYAQESDYYPDLNEWLYILSGCLYYFSTTVNPIVYNLMSMKYRHAFKQTICCKTKRK
SRTANSENCRNNSNGKIRNSSFNKIRYTISQAKEMFRFTASRENMDKDGNMNDNVSRKSCALRKEDPTSK
PLLDRIHSVVQEQNSNSSTAFHTETEKSMSTTTSAL

>gi|805825633|ref|XP_012152700.1| PREDICTED: uncharacterized protein LOC100879986 [Megachile rotundata]

MVTSLVQEMVTPGSSRDQEVTHEGFRDLEKTRSSPTIPQVLELYVAIQAKDIDEVIDILPHIFVVIASLI
KFGNIYLKKDRFKILLDLIVKDWETLTNELHVLDKVTATGDKLAYLYRMTLLSFLVAFNYIPLIPPTLDI
ILPLNETRPRQQLFQVNYIFFDVDDHFFAIYLHMSWAGSLTVFVIVTIDSLYMLIIHHASGLFDVCGYQV
QMACKQKKMEDEMFKQCVITHHKALEFLTS

>gi|805825070|ref|XP_012152457.1| PREDICTED: uncharacterized protein LOC100876377 isoform X1 [Megachile rotundata]

MDAMTFFLYLIAEKFHLFILSLNGQILLNHAVALTDKIFSSNWYDIPVKYQKSLYMMTIRCSKACMLSAG
GLYDMNIENFGKVRVDTSTDKPVTFENTLMTNLLYLKDS

>gi|805823875|ref|XP_012151894.1| PREDICTED: uncharacterized protein LOC105663983 [Megachile rotundata]

MRESGKESMRESGKESMRETFLNSFLSLYPVINALQTSTDPMDVSESAVFLIGLLLAISHIAISIARSKN
IRNNNPNFLPKTHIIMVLELTEDVCKNANDSARRLFQRYMEQFSTFYGVLIIWTYATGMTFILKALIMSD
PYPLNAIYPFRVDYQPLKTIIFLHQSLYICNVSGNSSVNVFCGFLLFFAAARFEILKMKLRNVKNTEDLI
IRMEEYYVVKEYANFAVNIVKFVAPCAVILAVVALLMCGMNLLQPQPLTVKMQYLSVAVTVGVTIFMFAW
PADNLLYQTAYQCRPCKSRYLQRNLPHKKHIKSSKWNNTYT

>gi|805821062|ref|XP_012150587.1| PREDICTED: uncharacterized protein LOC100882786 [Megachile rotundata]

MYFSVKLLCKYCWHCQWTLKEQHLKRLSQSVFHSANAGDLLLFLMLVIGEVQYMFLINLAMQFAADGAAN
ISISIYNTSWYETSIATQKLLQMVILKSNQGFTFTFYNIFDASLRGFSVLFQTSVSYFTVLISLK

>gi|805819185|ref|XP_012149737.1| PREDICTED: uncharacterized protein LOC105663686 [Megachile rotundata]

MRDHFWRRWAAEYLNTLQTRSKWSQPQPNVKPGDLCLITSEVTPPTKWPVGRIIEVYPGSDGKIRAIVTM
EMTLHRVLRNVTFLTVTMSSLIKYHIHWYQANRVSYNIKHYYFQTAVSAEVTDTTTPRKHINNKNMSYLI
KSVIMHREAILFCNEYDNYIKLSYTPLIILAIISLSINLFLSQSMLHSANIGDLLLFLMLVVGEVQYMFL
TNFAVQFAIDGATNISIYIYNTSWYETSIATQKLLQMVILKSNQGYSFTFYNMFYASLRGFSVLFQTSIS
YFTVLISLK

>gi|805815197|ref|XP_012148421.1| PREDICTED: uncharacterized protein LOC100884043 isoform X1 [Megachile rotundata]

MFGVYKTNVYFVTAFLFGVITPFAATTCILNIADAVGFYNLSGERPLTFRIEHFVDVDKYYYPLMVHSYF
GTVAFTAVVVAVDSIIIIFVIHECGLCEILRYRLENCGENHSMDIKIHPDKKRDIWYQDARSCVLLHRHI
IEFADIIENANTMSYFFQLGLNMICVSFTQFQAIVHISTLNQFLRYVSLTISLLCDLLFVSWPGQRLIDY
TERIFTYATNNKWYQSSIKARTLLGIMLAKSMKPLQLTACRIYTLNMENFSTVRTPTHDKSHCFSEFDTT

>gi|805814959|ref|XP_012148352.1| PREDICTED: uncharacterized protein LOC105663513 [Megachile rotundata]

MIINMFCAFLLFFAAARFEILKQRLRDVKSTDEMIIHMKEYCVVKGYANVVVNIAKFITPCSVITNAVVI
VFCGINLIQPQPLAVKCQYMAVVATVTLAVFVTAWPADHLLDKSNNAMKSVFESTWYEQSTKVQRDVQIM
MVPQPPVAIKIPCVISTLSLSYFCSFISNVFSLFSVLRVAISKDE

>gi|805808993|ref|XP_012146726.1| PREDICTED: uncharacterized protein LOC105663312 [Megachile rotundata]

MRDQETQIKDVLILNERVFWICDAWPLNNSYKKFVMYMSYLSVHMVIMYMDLYDVMGDLELMVENIIDNT
IATTTYLMLFLFRFSKLIKNVVAMVKRELAENDFRNNDEKQLYLSYNIISDNFGKYAVRTTAVIAVMWYI
TPMLELLQGSGNDNSTMYRLPFRVHSFLDYEDNLKNYMLMYAYQMPLMFIALLHISSISLLLSLVLHVCG
KFSILSYRIQNIAEDSKNSFQRRVKELVERHVELIMTANTLNSALQYILAMELIQTSIRMAVLMYTILLS
KKVNDAYYDANWLKLSIYDQKLLLYCMCNGRKTLNLKAGKFYSFSLFGFIQVSKSCSTDEKVISSIIVCN
RL

>gi|805805305|ref|XP_012145742.1| PREDICTED: uncharacterized protein LOC105663127 [Megachile rotundata]

MEMTLYRVLRNLTLMTISMGSFVKYYTIWYQANTVKGFIDRIQFDWQTNNDVILQIMQKHNYLGKQLSTF
FTSCVFSTVVIMTTYHISPIILDKIAPLNESRPIQLPVDAETFFDKEKHAITTTITYYFWIAILAIIYIG
TESLTIMICLHAASLFEVTSYYFQRAVSIEVTDTCIPRKCTNNKNMSHLIKSMIMHQETIQFCSVCSSHF
KITYSPMIILGVTSLSINVFFLSQSAIHSANFGEAIFYLLLVIGVIQYMFLINLAIQYVMDSAANISTTI
YNTSWYETSVATQKLLQMVILKSNKDLTFTFYNIFNASLRGFSVYPGSGDVFSRPDNPFSQSFQHIHVVG
GVNYLFFKKKFFMDCLTDYFAGWWPPRALIVVNLFPPFLKGL

>gi|805803754|ref|XP_003705289.2| PREDICTED: uncharacterized protein LOC100883629 [Megachile rotundata]

MDFLNLNRLNAFMNVMSGNMLPVTDKRMKLSVSTRIYWLIVWLVQLSYLAVCVLGARYVPTEISLKDSTV
AMAVSLEGIILSLYMNSRQNLLRRLIEQLNDTLNNGDKLLRSVTTQTVQPLEKVLKLYTIGSIGPVTVWT
LLQFIRVTQTDEFYYVDYRVPAVFSAEPFSLKVFVAGGFFVCLGSTYAIARKVSLDLYMIHMIRLMTAQY
RYLRIKFAEILRGKSDVWTKGPWKGEMEVKEEMRVLTSHYGTIIKMTSTLKKVLAPNIGVLYINNVFRFC
FLSIMLVMTSTEDMEKYLITLYSIGALIQLYMLCFSIQELLEASIAVADDAFYEKWCVYDTSLLRATAIM
SFGSKLECKLTSVRSVDLTLSSFMSILNQAYSVCLLFLKARYSHLRATIKEPYIKDTIAPAGNYTER

>gi|805803739|ref|XP_012145291.1| PREDICTED: uncharacterized protein LOC105663048 [Megachile rotundata]

MDFLNLNPLNAFMNIMSGNMLPVSNKNMKLSVITRIYWPLVWVLQVFYLSVCFLGIRYAPVEKSLRDSTV
STAVTLEGIILSLYMNSRRNLLRRFIEQLNDTLDKGNKMLETITIESVKPLELVLRLYTYGSIASVAVWA
LLQFTIVTQTDEFYYVDYRVPAVFSPEPFSLKVFVPSGLVVCLGNMYAIIRKASLDIYVIHMIRLVTAQY
KYLRRKLAEILRGTSEEAVDGEDKVKQGMRLLAFHYETIIQMSSILKDVLAPNIGVVYINSVFRFCFLSI
MVVMISADDMEKYLVLMYTFGALVQLYMVCFSIQELFEASKAIADDAFYEKWYVYDSSLQRAMVIMTFGS
KLECKVSSIRSIDLTLPSFMSILNQAYSVCLLFLKAR

>gi|805798911|ref|XP_012143985.1| PREDICTED: uncharacterized protein LOC100876205 [Megachile rotundata]

MNTSKNKDLIYAMKPFKILSWPLGTWPLQTYNLFALTRFVFAISVMLVVQVILNSEMCLNPTDVEANLDA
LMFSVVGTLALSKVLSFRFYSTKLIFNFVSAVKDYNELDEENKRMIMRRHAYMSRVMCVALIGIGNVCAW
ILTALPILLKGDQEINVTVEDTITFPLPSKTVILLLNLPENLYLLIFFVQYLMMVIMVTGNLGNDLNSRV
AKEITVKKVILRLTSSDLVFFGIVFHLCGQAEILKMQFTRLINDSGETTENFPSLIKRHDYLLQLSKMLN
DVISFVLVIQLLASCVLISTCGFQFILALNTRNIIIVLKTLFILLTVLIQLYSYSYMGDYLKSQMDGIGY
SLYSCSWYSLPANVAKNIVLVILRAQIPVHLTAGKFFVVNLETFMSILKTSMSYLSILRVMITT

>gi|805795905|ref|XP_012143137.1| PREDICTED: uncharacterized protein LOC100875831 [Megachile rotundata]

MEVDILDIDMSCQKKNDINEKYFLNHLVLQSNGTRKENSSQIPCLKVFKKYLIVLGQYSYQTELSHKVII
TVIVGCVMSLLIPAILQFFSSLRENDIDTAMDCIPVSVSVINSMIKILNHNIYRKKFENIFERMTRLWEI
AEVNGELQILKEVIDEGSQMGTLYRSSLWIFVSLFLCIPLSSFALDIIVPLNESRPRVSLFKLNYIMDME
DHFYIVYLHSACCALNVVLIVTTMDSLYMVIIHYSCGLFALCGYQIQKVAEDMDLNSDNTRISSVKYERL
KRCVKTHYEAIKFYEYMEESTRIGYLFQVGFNMIGITATAVQAVANFDRPAEAFRIVMFLCGQQFHLYAL
SLPGQELMDRSAGLIDAIYSSKWYQTPIKFQRVLEIMQIRCSRPCKLTAGGLYEMNIENFGSMIKTCVSY
FTVLLSLRG

>gi|805795896|ref|XP_012143135.1| PREDICTED: uncharacterized protein LOC100875381 [Megachile rotundata]

MSQALYYFTPSVNSEPFNTNSSKSKGTHKKASELPYFKVFKKYLIPLGQYPYQTERSHKINVTIIVFSTT
SLLIPSMLQFFLSMRERDMDTMMECVPIIVTLLVIMIKILNHNINRQKFLNIFNRMAELWEISEINGELH
VLSEITEEGSRMGSLYRKSLWGFLVLFVSIPLSSPALDFIAPLNETRTKLPLFKLNYIVDMRDHFYIVYL
HSAWCSLIIVLIVTTMDSLYMVIIHYACGLFALCGYQVKKATEMVGPKAIPTINTKAQFNRCVITLYEAI
RFYEYMDESTRTSYLFQVGLNMIGITVTAVQAVMYIDRPEEAFRIVMFLLGQQFHLYALSLPGQVLIDRG
LELINDIYFSKWYQIPVKLEKVLHIMQIRCSRPCTLSAGGLYEMNMTNFGSVCITILPWQYDYYTTTVNI
E

>gi|805795870|ref|XP_012143126.1| PREDICTED: uncharacterized protein LOC100881791 [Megachile rotundata]

MGKIIIYSFFILQVLDIFFNVQNQDEFTENFTLTSMALNVLLKRHMLSTRRTNILSLIKRLDKSHFLPVT
KEEMKIRSKFENIIECATKMYATGLAFFAFSVPFISIVIDFKSRKLYARMWVPYNYSSASLYLLTSSYEV
VASIYGVSISIACECLYTGLILHVCCQFEILEHRFKTLNGKDTRVVNQCASHHNLIYKFADVINNEFKTV
MSFQFFNSTAMICLSIYQLTYAKNSTAFMEIMMYLVCVFLQILFYCLCGNMVKMKVCYWISNATIVSVMQ
FLFAERSRTPIEFTSMHVVSLSLESFMSLLKTSYSAYNLMKTTR

>gi|805793799|ref|XP_012142565.1| PREDICTED: uncharacterized protein LOC105662692 [Megachile rotundata]

MDAVEIQERYLKITKRYLVLGGIWPTQKKSAQYIYRVIIYIVMVPTVLAQLARVIWFFDMTTLLQQLSFF
LAMVLLLIKQAAYIINDAKFKALLDGMYNDWKKDRTKEEIAIMISYMDRGAFYSSIYIASAYFAMMLFLQ
MPWWPRVADILMPLNASRPRGYIFPAYYYVDDDKYYYWIIAYSSIATIITVCGYIACDIYFIYAVQHACG
LFTVAGYAKAIDDFYYGILFIVVGMTIGAFSSTLFWVSSLNFILTDDSNLSDDSSLLYIFSKQLSKMKPC
AQFYEYIVFLTIQLLHIFFLAIQGQFMINRTDIVYNQIYEGQWYNSVPKTQELFILALRTAMNPPQITAG
GVIAMNLQTFAEVVKVSFSYYTVLQTV

>gi|805773782|ref|XP_012137305.1| PREDICTED: uncharacterized protein LOC105662050 isoform X1 [Megachile rotundata]

MCLIMLAFIVLSYYFQKAILEESSALHHRRDWSNKKTMPYIANGVIVHQMARQFVHDVNNRCKVPYVLTI
MCGVIALSLHFFCLSETVLRTADIRETILSVLLIISTLGYMFWLNLAVEFIIDSACSISLRTYSTNWYET
SVATQKLLQLIIILNSHKSGFVFNFLCVYVPSMEGFAVVRTISYYDPLKTIFCIEFSFFSF

>gi|805766770|ref|XP_012135343.1| PREDICTED: FMRFamide receptor-like [Megachile rotundata]

MNFNTSSSHYVCNLSSFHSHYSQLHGWISLFVCIFGSIANILNILVLTRREMRSPTNIILTGLAVADLLV
MIDYIPYAFHLYLYRRSRRDTFTYGWTIFVLFHSNFAQVCHTISICLTLILAVWRYVAVARPQQNREWCS
YRRTIFAIFIAYVFCPVLCVPLYITTEVKEQVFDVSIIPQVCHTISICLTLILAVWRYVAVARPQQNREW
CSYRRTIFAIFIAYVFCPVLCVPLYITTEVKEQVEILDSNGMSVSNRNQSLIDSPLENTTNVTLYFVRLS

ETAKNHDILKELNFWIYSVVIKLFPCVVLTIVSLKLLQVLLEAKRRRRKLTNIQEENSERKKSCRRVEKE
RQTDRTTMMLLAVLLLFLLTELPQGILGLFSVLLGPGFFWTCYLMLGDVIDMLTLVNSAINFILYCTMSR
QFRKTFNELFCKSWKLPRSTRKQIFIENNGNTGTNHTVTQVTQV

>gi|805766376|ref|XP_012135244.1| PREDICTED: uncharacterized protein LOC100876531 [Megachile rotundata]

MHDKPITEQTDPEILSDYSLQLNRWLLKSIGAWPKSSSTTKTELTVSLILTVIGYTTILITVIPAILQLI
LGDIDLHTKVVLVGPMSHWCLGGIGYTMLLMHDEEIKWCVERVKNDWRSIMNPEMQQTMIKNAKLSRYVV
SLCAILMQSAVLGFGVEKALSTQVITVGNETKVVHMLPCATYRKLLPVDTSPTFEIVLVAQLVSGFFVNG
TFAGAFSLASAFTLHAYSQLNILATWITEAVDQSSLNDVGAIVRKHLAILSFISRIEAIMNEICFMELFK
CTFSICLLGFNIILCNKIGEAIYMSNWYYLADKDIHNLMMIISRSCVETKITAGKIVHMSFYTLASVIKS
SFAYTNILLQTT

>gi|805766364|ref|XP_012135240.1| PREDICTED: uncharacterized protein LOC100875960 [Megachile rotundata]

MNSSDKLITNTRYLQDYEYSIQLNRWFLKPIGVWPRASTASIAEKILAIVLNVACHVLIIFTFVPCLLFI
LFEEASFQTRIKAIGPMSHWIMGELNYCFLTMRGQDILHCIEHIGSDWQTVKRASDREVMLKNVKFGRLI
ACLAAVGMNVGVFSYIVIGFRKIVFHVGNESFLMYRLPCPVYTKLLNVKLSPANEIVFALQVLSGFIVNS
VTVGACGLAAILAMHACGQLNVMMSRMDELVGAGVEEQRVVKKKLADIVEHHLRTLSFVWYIEKIMNLIC
LVELVGCTVNICVLEYYIITEKSKDTLASYAIIYASMSFNIFIFCYIGERLTEQCKKVGEKAYSVEWYRL
SHRTALSLVMIISRSSMVIKITAGKIIRISLATFGDVSIKTLIA

>gi|805766358|ref|XP_012135239.1| PREDICTED: uncharacterized protein LOC100875508 [Megachile rotundata]

MQKAPAKSRAGLPEQDYLKNVDLSVRLNRWILKPIAAWPESSTVSPMKKYLSWFVHVVCYFLMSFLFVPC
LIFLILEVNGTYNRIKLFGPLSSPLMSYSKYYLLLLHKNDILKCVKQIEWDWRNMKHLEEKRIMVANANY
ARRIVIVCTFFMCSSYMFFYIAVPITVGRIPAQDGNFTFILLPFPSPMRITDYRQSPVNEIFYFLQCLAG
ILLRVITAGACSLAATFAVHACGQMEILMNWLGYLVNGRADMSKNLDGRIARIVEQHVRILRFLELTEKT
LRYISLVEFLGCTVELCLLGYCVLMCRKIAEISYMIDWHRLLGKRKLSMILIMQMSHSTIKLTAGNIAVL
SYRKFGDVVKSAFAFLNVLRTVT

>gi|805766347|ref|XP_012135234.1| PREDICTED: uncharacterized protein LOC100876418 [Megachile rotundata]

MVPSCTDSVPLSTLSFNRAFIMKKTRVSKEIDEHERNVNLSIKWCRRILTIIAVWPQPPDFTLSQKCFYR
FRNVMIFSFLSYLSVPCIVYMIVEVQDVYNKIKVFGPTSFCVMALIKYYVLIVHESDIRRCITLIELSWK
DITHPEDREIMIKNARYGSQLIALCTFIMYSGLTFYYVSIPIGSGRIEDPEENVTYIPLMFPFPKVIVDT
RFSPTNEIIFSFQMMSDATLHGVSSASCSLASMLAAHICGQMEIMVNWMKHLVDGRSDLPKSVLRAYREN
TSIHIPGGVPGVYNGVVFA

>gi|805766344|ref|XP_012135233.1| PREDICTED: uncharacterized protein LOC105661823 [Megachile rotundata]

MKKTRVSKKIDEHERNVNLSIKWCRRILTIIAVWPQSPDFTLLQKCFYRFRNVMIFSFLSFLCVPCTIYM
IVEVEDVYNKIKVFGPTSFCVMAVIKYYLLILHESDIRRCIRLIELSWKEITHPDDREIMIKNARYGRQL
IALCTLIMYSGLTFYYVSIPIGSGRIEDPEENVTFIPLMFPFPKLIVDTRFSPTNEIIFSFQMMSDATLH
GVSSASCSLAAMLAVHICGQMEVMVNWLKHLVDGRFLELTEKTLRYISLVEFLGCTLNLCLLSYYVLMVR
TNEAVNRFSIDLKMDFVQIFRNGVRTIG

>gi|805759618|ref|XP_012152350.1| PREDICTED: uncharacterized protein LOC105664057 [Megachile rotundata]

MKHVTPEAAIHFTKLSVLLTCSWPPSPLITKTRLLFFNILWCTSLVSALGLFVPLFNAVYEYRKNPIILG
KTVSLCAAVAQVIIKMTMCRLQQKQFQVLYFDMEDFCKRATSSERIVLQRYVDRYKYFHGVYTLWCFLTT
MFVISGPLYSPQTFPTHAIYPFPVEHQPLKCLIFFHQSLVGFQASSGMAIDAQVALLLRYTAARFELLAM
QLHAATSDRELNACIKVHSELLRYTRQVCKCARFLVLATVTTTIIAVIFGSLNLVTKQPLLLKGLYAVIV
FSASVELFMYAWPADNLMRMSEKIATNAYSTKWFEKNIAIQRKIFFMMLRSQKLEFIYVRGIVPKLSLFY
YAQYLYKSTSYFAALRIMVEK

>gi|805759608|ref|XP_012152319.1| PREDICTED: uncharacterized protein LOC100884055 [Megachile rotundata]

MFRNITPEKAIAFTRMSVAPAVFLPLSSQTTKLLTTGYKVFKILVLLNSFLFLLPLINALYTYNDPANVS
ESICFLLAEVQFIVNTIISITHYDRFQGVVEGMQTYCKNANVRERVILQWYVDQFSTFYGVSATWFYLTA
ILVILGTFFSSHPFPTNAVYPIAVDYQPLMSIVFLHQSVIGLQFSATVCVSVLCALLLLFASARFEILKM
ELREVKKPSELIKCMEKYYTVRRYACDVVNTIKYLPMCTVIMCGVILVFCGIKIIQPQPFTSRCQYLSIV
WTALVDVFVCAWPADHLLSISQNVMEGIYESTWFDRGSSMQRDVRIMLLPQPPVAIKVDCIIPALSLNYF
CSV

>gi|805759605|ref|XP_012152307.1| PREDICTED: uncharacterized protein LOC105664056 [Megachile rotundata]

MFRNISPERAIEFTRYSVALTCCWPAQARSRVTRFKICRCLVLLNAFLLFLPLVYSLYTNHNDYVNFSKS
ICLFIAVVQILWNSSICIVHQNNFLELIAEMKECCKKAKPYERSTYQRYVDKYSMFYGVSTIWFYVTALI
ISTGTLFIPQPFPTNAEYPFPVHYQPLKSIIFLHQSFLLLQCSAHVCINTLCALLMLFAAARFEILKIKL
RKVRTSRDLIDCVRMYYIVRRYAKRVSTAVRFIALVTVTMCGVVLVFCGVNFIEKQPFTVQVQFLSLAGT
GLIEVFMFAWPADHLLHLSQNTMQVIYESLWYLRSASMQRDIAIMMLPQAPVAIKIVYIIPIMSLNYYCS
FVSNVLSLFTVLRATIKTEHN

>gi|805754729|ref|XP_012138996.1| PREDICTED: uncharacterized protein LOC100875216 [Megachile rotundata]

MTDTAFDASDQEQVELSTANEQVQEVFDLHHYKPLKKYLTTLGLNPFNETWGSKIGANVMFYTLILGITP
LFIQLCVVIQDTDVDAIIDELPHLLLAVFKILLNFILNDWVMLTRELPVLEKATAQGSRLARFYRVTLIL
AALVFIYIPLINPTLDLIMPINGTRPKQQLFGACYVFVEADDHFAAVFLHLGWTTFVTVYNVSIVDSLYI
LIIHHVCGLFDVCGYQIETATQDPEMRYDQFKQCVMTHHKALLFFDKLQECSQNMFLLLVALNMTLISMT
AVQILMHINQPLDSARFILFFLCAQFHLYIVSLFGQIILNHSTILAERM

>gi|805754618|ref|XP_012138745.1| PREDICTED: uncharacterized protein LOC100875437 [Megachile rotundata]

MLQENGPCSNVYNIPYCKMLRKYLLLFGQDPYQGNGTRNVITVVMVLGLLGVIVPTSIEVYVSLLEKDMD
AVIECMPHLIAAITTIVKILNGHLNKENFRRLYQLMIEEWELLEMNNEVDVLDKITEQGSKIAQLYRSTL
LGALGIFLMIPLIAPLLDVVLPLNETRPRQQLFKVNYVVFDERDYFFPVYFQLAIGAFIIVTGIITIDSL
YMVIIHYNSGMFAVCGYQVQKATKCYDMNSDRTTVAEDDCQRFKRCVLTHHKALQFYEILKENSTNSYLL
QIGFNMIGISVTAVQTVLNLDSDKTEEAVRTAVFLGAEQFHLFVISLPGQVLIDHCSELVVHLYCSTWHQ
APIKIQRMIYLMQIRASKLCILTAGGLYQINMENFASAFRMCMSYITMLLSVRE

>gi|805752326|ref|XP_012144983.1| PREDICTED: uncharacterized protein LOC100878543 isoform X1 [Megachile rotundata]

MPINGTRPKEPLFGVCYVFMKADDHFAAVFLHLGWTTFVTVYNVSIVDSLYILIIHHVCGMFDVCGYQIE
TATQEPELRYDQFKQCVMTHHKALLFFDKLQECSQNMFLLLVALNMILISMTAVQVNMSLYLKLTLHFTN
FADTYAHGSTSGQYQIHSVLSVRTISFVHRQPIRANHTEPQHNISRKNVSMIQLQLVRGTDQVPEIALHD
DRKVQQTLHFERGRTVRYEHGELWKSMYFWKFVTSFNELLFAKGSESVHVVLHHVSVDEGLSVQLSSSTN
HKDNETVVTKFASKSEVT

>gi|805752313|ref|XP_012144604.1| PREDICTED: uncharacterized protein LOC100878875 isoform X2 [Megachile rotundata]

MSTWIPQFLQLCVVIREMDLDAILDEIPHLLLACLSYIKLKNIFLNKKRIKILLNFILNDWKILRDELPV
LDRITEQGSRLAHMYRVTLMWATIGFIYIPLINPTLDLIIPLNETRPKEQLFGVCYVFINPDDYFTAVFL
HMGWTTWVTVYNIITVDSLYILIIHHVCGLFDVCGYQIETIFKRSNLQHYQFKQCVIHHHKALEFFNYLQ
ECSQNMNLMLVAITMILISTTAVQILMHMDQPLDSARFILFFVCSHIHLYIISLFGQIILNHSKTLAERI
YSCNWYGIPIKFQKLLCIMILRCSKPAILSAGGLYDMNMENYGKVCIGYVRKFVTHLHALMDFFLTKGSE
SVHVIFYDVSVNEGVSVNLTISTNDDETVVTDKTIKNGNEKRSAYSVYKGDCISSKKILKHTMKH

>gi|805752301|ref|XP_012144307.1| PREDICTED: uncharacterized protein LOC100878986 isoform X5 [Megachile rotundata]

MVYSGMFIGMLPLVIQFGISISEKDVDGIIDEFPHILTAVGTMIKFCNISLNHQHIKTLLDLILKDWEVL
TNELHMLNKITTQGNRIANLYRTTLLSFLMVFIAIPLIPPALDIIAPLNVTRPRQPMFRANYVIIKVDDH
FFVTYVHMASVASLGVFIIVTVDSLYMLIIHHACGLFDVCGYQVQMATKNSILSYEEFRQCVITHHKALH
FFNTLQECSQNMNLMLVGINMLLISMTAVQIILYIDQPADAMRFILFLIAEKFHLLVISLFGQILLNYAS
SLPEKIFSSNWYEMPVKYQKILYMMIIRCNKTCNLSAGGLYDMNIENFGKVCTDSCIGRAIMVGCMIMLN
LFCLKDS

>gi|805752280|ref|XP_012143908.1| PREDICTED: uncharacterized protein LOC100878986 isoform X1 [Megachile rotundata]

MVYSGMFIGMLPLVIQFGISISEKDVDGIIDEFPHILTAVGTMIKFCNISLNHQHIKTLLDLILKDWEVL
TNELHMLNKITTQGNRIANLYRTTLLSFLMVFIAIPLIPPALDIIAPLNVTRPRQPMFRANYVIIKVDDH
FFVTYVHMASVASLGVFIIVTVDSLYMLIIHHACGLFDVCGYQVQMATKNSILSYEEFRQCVITHHKALH
FFNTLQECSQNMNLMLVGINMLLISMTAVQVTMSLCLNFPNINYITFILQIILYIDQPADAMRFILFLIA
EKFHLLVISLFGQILLNYASSLPEKIFSSNWYEMPVKYQKILYMMIIRCNKTCNLSAGGLYDMNIENFGK
VCTDSCIGRAIMVGCMIMLNLFCLKDS

>gi|805752277|ref|XP_012143815.1| PREDICTED: uncharacterized protein LOC100879096 [Megachile rotundata]

MNLMLVGFNMTAISLTAIQVKISLRLSLTYVNCNIFELQIIMHFDRPADAMRFVLFLIAQNFHLFVISAN
GQILTNHAFLLPDKIFSSNWYEIPVKFQKLLYTMIIRCNRPCILSAGGLYDMNIENFGKVCTNYVGNAYT
CMLMSNLF

>gi|383862949|ref|XP_003706945.1| PREDICTED: protein odr-4 homolog [Megachile rotundata]

MGRTVYAEERLHTYLISLAKPDEYTIGLILGQSAGQKDYIVHLAKTPPPLGKNVVEETLISSTTNTQQDS
TESYIKSVKDIPENWVADHAKHVTRMLPGGMQVLGTFIVGPEDALNNNTNIQKFRSILAAIQKNLSYNKY
LCGNSNEEHLILSLNSITQKYTCKSVETNKTGMLKPTEWKFQAKPTKWHQLEALVDFDRLFLIAADKDPE
TLKKQLQDILKNVSNVIESSLIVIEGEIRSSEDTLETINKNKNGDKTCKNNEKNANDKLIQVDLYIPCQE
KDINLDIRTKPCSASIRLIGQLVSRTFVHQKANIEEANTAIKQDIIRSLASRLEMHWDSLIEEENGSPEE
NITLHEPPRRVLIALPESKITLSDYLFPGEGPQEALLSLQELLDLEVQESNVQKDIELQADPTEFYCQSE
IDLKPTDLSKDTSGSYKARIYFTGFSIAILIVILSIIIHNFINMKSDV

>gi|383857245|ref|XP_003704115.1| PREDICTED: uncharacterized protein LOC100880718 [Megachile rotundata]

MDAVTLQQRYLKLTKAYLLLTGMWPNQNNQILPNNCGISYIWHLQQVARVVRFFSLTVLIQQLGFLLGGI
LLLAKQSAYVGHKAKFETLLHGMFNDWEAVRPKEEVAIMTSYMDRGAFIVLIYAVNVFGCTLLFINMPWL
PRIADILMPLNTSRDRLFIIPACYFVDENKYYYWIIAHMTIIIITAGSVYIACDTCFIYIVQHACGLLGV
AGYRFKEAVDEMSKERKIADLSNETYMKVRRSVQGHVRALRYLRDIDDVHASYLLACLGIVTAAFSATLA

LLSELDICLEFYQGLGFLVVQLMHLFFLTLHGEFVIESNDKAYNQIYEGLWYNAAPKAQALYVLALRASA
AAPQITAGGQIPMNLETFAVIIKASVSYFTMLKST

>gi|383848906|ref|XP_003700088.1| PREDICTED: uncharacterized protein LOC100874989 [Megachile rotundata]

MSAYLHKVWDLLESMGMFTCTWPVDRNASKWKIILRNVHWLVAVVNVLIVITSLILTMYHYKSDIIMTE
AISEMGCLIELTLDMILCKIHSSQIQILVRKVKTYIDTSNEYKNSVIEGYVHRYKIHFLLIVSQFVSTGT
LFNLMALYTKQLPLNGWYPFSTEPDLVFYYIFCVEAYSIFQVSFSIFSDFVIIGLISFIAARLDILSSQM
KQVSDYDLLVKCVKEHHEIIGFFEDTTAAVRSLLFKTNITMGGTVISAVLSLLYVRNQSLYTACQYFGMV
MAGYGHMWIITWPADDLTESSLKFAKSLHDIPWPGKSRRMKSTVLIMMLRCQKPFLITMGGLLPVLSLQY
FSQFLTNVFSYFMAMRSMLE

>Lalb_02620 [mRNA] [translate_table: standard]

MTLNAKVIVQEIAIITTATEAVEGAIIIEEVNIMKNRIQTNDIMLAICWPRTIAGCARCLFEADLASVSR
QGHVFDYIEDDWKMFTNEGELETIMKYANRGKLLNLYMLALVALFIAYPFVAMAWTHQAQSLQIPIYIDY
IVDHDRYVYWILLYIDVQTIVGTSTLLVADFASVIFMFHLCGVFRIVSDRLEKTVASSVLNARNGNKYSS
SVRKQLIAALELHQRLLESRAWPALNMATLVPTIAPSILHQHPSLSWRKDALRPLKAPTA

>Lalb_02845 [mRNA] [translate_table: standard]

MFADVTPKKAITFTKYSVILCCGWPLPSSAPKSHFYFYVIAKVLIALSNISVILPAFWSLLFYIHDPVRT
GKTFGLLTAMCQTIAYIIVCSFQHDHYQRLIEEMTDYLEKAKLYEMVIFQRYLDTYSTFLGITTISFYIA
AGVIVVTTLFTDQPFPVMAKYPFRVDYEPVRSTIFLNDAFVALQNVASVSLNTLTALLILFAAARYDIVM
LELQSAVTVADLKESMKKYHEVKRCALDTIGGVQYVTLITLVSSSVILVNAGLNLIGRRDFLIKCQYVFV
ATTALLEVFSCVLPADRLIEVSTGTMRSVYDVKWYERDLSVQRTVYHMLVPQKPVIISFLFVVPQLSLNY
YCSV

>Lalb_09696 [mRNA] [translate_table: standard]

MRLLGIWPLNSDDILRSSLGRWCFAMLTQVSAICSLTTAVYRHCLDIDDTMDAFLMDLSAMVSLSKVFIL
RFNWKHTYALVNSIVEDWSNVKDLGHRNIMAKYEEKGQLVSMLLLYMGYASGLSFAVKTLPFHLLPFQVF
QFPSNSTSTVDPTLKNKYFLSTYCAFGPLPWSLRSCVLAVQGVTVFVNVVGHCGNDVFFFSLTMHLCGQF
EVLKTKLSEIEIEKPDYRKKIGSLVQRHCRLVLLADDLERSFNVVILVQLLMSILLLCIEGFLMLVFLKA
NDRAGELKCLLIATTLLIQLYAYTYAGDALESQSAGISFAAYDSTWYPYDSTWYRSRGHRARDLALIIHR
GNSPYCITAGKFMPMNLLTFKEILKASASYMSVLKVMMDA

>Lalb_14616 [mRNA] [translate_table: standard]

MRGRSSNQVKSFNDDYENDLNYTLGMCRLLLKPLGVWTFIYDRGSRLERVLSIPLMLICFSSLFFIILPS
VYNMLFMEEDLQNIVKLLGPISFCMFSTIKYCLLGMKGNTLGQCILHLERDWKMVQDPDHRAIMLKQAST
SRFLITICVGFLYSGGMSYHTVMQFLSKDRTNTNVTLRPITYPCFNFLDTQSSPTYELVFFTHCVTAMVM
QTITTAGYSLAATFVTHMCGQIQIQISRLDNLIGEHREKNTFQERIAVIVRDHAEVLRFSKDVEEGLREI
CLTEIVESTLIMCLLEYYCLMEWANNDTVAILTYIMLLVSFTFIIFIFCYIGELLSEQCSQMGPAAYKIH
WYNLSSKNSYDLILLCAVALYPPKLSAGKIMDLSINTFGTVCDFV

>Lalb_14617 [mRNA] [translate_table: standard]

MIERELDSSSDYSLQVNRWYLKSIGAWPSSSFAPRFERIVSVVLIIICYCSMLFTGIPCILHILLEDEDL
RKKLRATGPLINMFMGSIKYSTLLFRGNEIRDCVEHIQVDWQIVSRVRHRLVMTSYAKFGRYVSVFCAAF
TQLGVASYCFVNALSTRSVQIGNETRIVHVLPLEFYKKLLNIDESPTYEIVFVSQFISTSIVNFSSVGAF
SLAVTLAAHACGQLNILMSRIIETVNGERDRDAKILLNETGVVVENHLRILSFISQIEEVMNKICFTEMF
HTSLCTCMLGYYILTEWDDRDYQNLSCYFMILFSMTFNVFLLCYIGQTLTEQCKKVGEVVYMTNWYFLPR
RCILDLRMIIVRSSVMIKITASKMFHMSVYTFGQVSMSYHWNKPNYHTGMARLLELYENFVSF

>Lalb_14618 [mRNA] [translate_table: standard]

ILGIMRDRSADRADGHFTNPSYKSDVKYTLEMCRWVTKPIGVWPFVYSRTSKVEKLVAIVLLTVCFSDLL
FATIPPGHYLIFVEKNVYYVIVKFLGPLGFCISSAIKYLYLVFKGNLFKRCLVHVERDWKTVVDQRYRRIM
VRQSSVSRQLIVLCAIFLYSGGMSYHTIMPFVSKPKMRGNQTLRPLIYPGYDAFFDSQASPTYEIIFSMH
CLSGFIKYSITTGAYSLAATFVTHICGQVQIQIARLENLVESIQKNSDRNPVAVIVREHVEILRFSKNVE
EALREICLTEIVESTFILCVLEYYCLVVISSLNTFIYYLFIFETCRKFPSHLRLCSKIGPAAYEIDWYNL
PYKKAHDLILLSVVSNYPPKLTAGKIFTLSLNTFSSVGDAVIGYLFKSATHSYGLRTKRVPPVKNADVSG
IITYQWLMPFQFTKKLCSHHSRRRIAQIDKDLNPYENERNKT

>Lalb_14619 [mRNA] [translate_table: standard]

NPYYEKDIVYIFKIIRWILNSLGIWPSLFENSNQFQQNVSIGLCNVVLLFAIVPFTLHLTVEEKSWKARV
KLIGLLTFCWISLFKYWSLVSCKPNLKYCIEFIQHDWREVEHSEDRELMLKYGNVGRNLTILCLVFMYSG
GFMYHTIMQYAIGTFVDEHNRTIKPLVYPTYSGLFDSQASPFYELVYAVHSVTGYVIYSITVGACGLAAM
FATHICGQIDIMILRLQNLAQLEENTDLHPRLVRIVQHHIRMLRFSTVVGTLLQEVCFLEFIGSTFMICL
LEYYTITDWEVNNTLSLITYTMLLLSLMFNIFILCYIGDLLVEKTGNVGSLCFMIDWYKFPVETMRSLIL
IIAMSNNPAKITAGHIADLNLSTFGGVSD

>Lalb_14620 [mRNA] [translate_table: standard]

MHDRSRDTVVRTLKKYHEEDDIDYALEMCRWVLKPLGMWSLVYNRTSRWERTVSIILLVTCFLSVILLIV
PLYWEIILGDIGGKARIKRLGATCNCTLSAVKYMYLVLRRATFASCIEYLENDWRTVKNQHHRAIMMKQA
TFSRSLVMLCIIFIFCGGISFHVMLPLSRRRVVGNYTLKALAYPGYDRFFDIQSSPTYEILYCIQCFGGF
VRHNVTTATFALAVFFVTHICGQVQIQILKLEELNIDSEEKNCDMVGEIIQSHTEILRYAKIIAESFSEI
IFMEIMASTFLICLVEYCLLTEWIDSNAIAITTYAVYMISLTFNAMIFCYIGQILTDQCSQIGFASYQVN
WYNLPPTQARNFVLLGTISLYPPKLSGGKVTELSLNTFTSVNPEVVNGLLELAADSYIHLA

>Lalb_14621 [mRNA] [translate_table: standard]

MLGIMRDRSADRADDCFANSNYKSDVNYTLEMCRWVTKPIGVWPFVYSRTSKLEKLMSIVLLTVCFSDLL
FSIIPPGHYVIFVEKNVYYVIVKLLCPIAFGSSSAIKYLYLVFKGNVFKRCLVHVERDWKTVVDQRYRRIM
VRQSSVSRQLIVLCVIFLYSAGMSYQTIMPFVSKPKMRGNQTLRPLIYPGYDAFFDSQASPTYEIIFSML
CFSGFIRYSITTSAYSLAATFVTHICGQVQIQIARLENLAASSQRNTGRNPVAIIVREHVEILRYDREAW
KKKFFLGGVVAIRLETVALQAFKVGQRLGVSWDMIGYKSPTCRAYNENCSKIGPAVYEIDWYNLPYKKAH
DLILLSVVSNHPPKLTAGKMFTLSLNTFSSVGDAVIGCLFKSATQSYRLRTKRVPPVKRNTDVCEAITYQ
WLTEFVRGIALIDIDSNPYENEGSKS

>Lalb_14622 [mRNA] [translate_table: standard]

MTPVTVQEQDIGKPRNPYYEKDIVYIFKHSRWILNSIGIWPSFLKNNNQFQQNFSVGLCNIVLLFAILPF
TLYLAVEEKNLMIRVKLFGFLSFCWICLLKYWVLIACKPNLKNCIEYVQHDWREVEQREDREVMLKYGNV
GRNLTLLCVVFMSSGGFMYHTIAQYAIGSHVNEQNRTIKPLVYPTYSRLFDPQPRPFYELVYVIHSLNGY
ILSAITMGACGLAALFATHICGQIDIMILRLQNLTQLGANTNLHPKLDWEVNNTISLITYTTLLLSLMFN
IFILCYIGDLLVEKTGNVGSLCFMIDWYKFPVETMRSLILIIAISNNPAKITAGHIVDLNLATFGGILKT
SLAYLSFLRTSVM

>Lalb_14624 [mRNA] [translate_table: standard]

NPNYENDINHVFGLSRCILKLIGVWSLFEKQSNTVEHVVSFLVRITLLCIQAFFVIPICLHIFFVEQNPI
MKIKLFGPPSNCVFTVIKFFCMVFNGTTIGRCIEHIKNDWKKVRNPQHRDIMLKQISISKNLTILCFIFI
YTAGISYYTIIPYMNKHRLKGNYTMRKLAHPGYDRFFDVKSSPTFELIYSAHLLSGFFRYNVTAASFSLT
VIFVTHVCGQIQIQLLRLKELYQENMEKNDGPDPLAIIIHDHGSTLKLASDFRESLNELLLTEIFGCTFS
MCLAEYCCLMEWNDMNPVAITTYLIYCTSFSFNAMIFCYIGELLTEECSQVGFASYDVSWYNLPPSKASG
FVLLNLTSLFPPRLVAGKVIKLSLNTFSVVSDLV

>Lalb_14625 [mRNA] [translate_table: standard]

DVNYTLEMCRWLLKILGVWTLVYHRISKSDRALCTATLATCIGILSFTILPSLYSVIFTKNSTDTIIKLL
GPVGLCSFCTVKYMYLILKGKSLGRCVQHLERDWEMVTHPGHRSIMLKYSSISRKLIVLCVVFLYSGGLS
YHTVKQLITKDESTLNSTERPFVYPCFEFINPQASPNYEIIFFVHCLSGLIQYTITSAMYSLAAIFVTHI
CGRIEIQISRLDELIEGSEEKPIFYDRLGIIIQEHAEVLRFSKDVEAALRELNLTEVAESAVIMCVLEFY
CMIEWRRSDAIALLTYFTLLVSFTFNIMIFCYVGELLSEQCSRMGPASYDIQWYNLPPRRAYNLILLSAI
SLEPPKLTAGNIIDLSINTFGAVSDFIMTNVFRI

>Lalb_14627 [mRNA] [translate_table: standard]

NYSVQVNRWFLKPIGIWPIGPDATRSERIFSSLLNIACHSLVVFTFAPCVMFILFEETSLDARIQAIGPM
SHWLMGELNYCCLSFRTNDIFRCIRHMKTDWKTIEKSSDRELMLKDAKVGRSIATIAAVCMNLGVFSYNF
VTGFQKLEFHVGNDTYSMLRLPCPFYSNLMDVRHSPANEIVYFLQLLSGLIVNSVTVGACGMAGVFAMHA
CGQLNVITSQLNNLVTPNEEQTFAKKKLTSIVERHLRTLNFVWYIEKIMHMICLVELMGCTLNICMLEYY
MLTEDSTERMATYAILYVSMIFNIFIFCYIGEKLTTQCLQVGEKAYMTQWYRLPHKTATGLILLISRSRM
VTRITAGKLLPISISTFGDVSI

>Lalb_14628 [mRNA] [translate_table: standard]

MTKQEIISSNSLTRDNDYKKDVDLSIEWNRWILKPMGVWPRSRHVSQLEVCFNWLINAVCYSLISFVFAP
CYLYVSLEVEEVYEKLKLSAPLIFCVMAYIKYYSLMAHTNDIRECIKCIEWDWRNVSHSEDREIMVANAI
FGRRLVKICTFFMFSGFVFFYIAVPMSVGKIAIEGANRTFIPLMYPVSKLIVDTQRNPSNQIFFSIQFVG
GILMNANAAGACCLAVAFAVHACGQMQVLMCWMSHLVDGRVDMSNTTNGRIAKIVSQHESRAKDMTAVAT
YAIILVSLIFNIFIFCYIGELVAEQYRKVGQMSYMIDWYRLPENTKLSVVLIMAMSSSPTNLTAGRLVKL
SLVSFSDVMKTSVAFLNMLRTLTDTRE

>Lalb_14629 [mRNA] [translate_table: standard]

NYKKDVDLSIKWNRWILKPMGVWPRSRHVSQREVCLNWLINAVCYSLISFLFAPGYLYVSLEVEEVYDKL
KLYGPLMFYVMAYIKYYSLMAHTNDIRECIKCIDCDWRNVIHSEDREIMVANAIFGRRLVKICTFFMFSG
FVFFYIAVPMSAGKIAIEGTNRTFIPLAFPVSKLIVDTQRNPSNQIFFSIQLFGGMLIHSIAAGVCCLAV
AFAVHACGQMQVLMCWMSHLVDGRVDMSNTTNGRIAKIVNQHVRIQKFLTVMEKALTQASLVELLGCTVG
ICLLGYYIIVESRAMNMTAIATYAMILGSLIFNIFIFCYIGELVDDQYRKVGQMSYMIDWYRLPKNTKLS
VVLIMAMSSLPTKLTAGRLVKLSLASFSNVSIVILLYV

>Lalb_14632 [mRNA] [translate_table: standard]

HRTNVNLSIQWNRWILRPMGVWPSSGTDSGFEKCSNRLMNLVCYSLISFLFVPCWLFMMLEVENLYDKLK
LFGPLSFCVMAYMKYYSLMTHANDIRECIKYIEWDWRNVKYSKDREIMVANAKFGRRLVKICMFFMYSGF
VFYYIALPIRVGRITVEEQNVTFIPMVFPMTKFMPDTRHRPLNEIFFSIQFFGGIVIHGVAAATCSLVAA
LAVHACGQVKILLRWLEHLIEGRPDMSKSVDKRIAGIVSQHVRILKFLSITEMAMRQISFVEFTGCTLNL
CLLGYYAIMEWDPNDLTATVTYITILVSLGFNIFIFCYIGDLVAEHCNRVGEVAYMIDWYQLWGNRKRCL
ILIIAMSNCSTKLTAGKMVELSLSTFGDV

>Lalb_14633 [mRNA] [translate_table: standard]

MLVEETKISKFAQSNHAKNMDLSIGWNRRILETLVIFIPFGLFGIFEVEGAYNKLKLFGPTSYFLMVYMK
YFNLINKISHFRTCVEQIESDWKNMSYSEDRDIMVESAISGRRLVKICCFLSYIGFVFYYVAIPITTGRV
TDPIHNLSFVPTSFPVAQIVADIRYSPVNEIFLAGQVLGGFVVHGIAVGACTYAAVFAVHACGQVKILLS
WLEHLTGGRTDMSESVDDRIASIVSQHVRILKFLSAAEEALQQVSFVEFIGCTLNLCFLGYYILMEWDIH
DITTVVSYGVILISIGFNLFIFCYIGELIAEQCSRVGEVAYMIDWYNLQGKKKQCLIMIIAMSNSSSKFT
AGGMVELSLSTFGDVVKTAIAYMNMLLALT

>Lalb_14634 [mRNA] [translate_table: standard]

QNIHHKSDFVFTIRVPRILLMLPGIWPLHRGDSILGDVKSVIQVGVMFLLMCYLLVPHVIYTFHDSEDLT
RYMKVIAAQVFSLLGIIKFWVMIFKKKRFRACITEMDLQYANVESEEDRLVMKNSAKVARLFTTIYLGLC
FGGAFPYHMIMPFLSEKVFKTDNTTQIPLPYLSNYIFFVIEDSPMYEITFAVQIAISNMILFINCGTNSL
IASMTMH

>Lalb_14635 [mRNA] [translate_table: standard]

HYESDIAFTVRVAKTLLTPIGIWPLRKSDTFLDRLKLYAHIGVTFTLMCYLLVPHVIYTFHDAEDLTRYM
KVIAAQVFSLLAIMKFWTIIINRKDIRYCLQQMEIQYRDIQCEEDRIVMKNSAHVGRFFTTLYLGLSYGG
ALPYHIILPLMSERVVKRDNTTQIPLPYLSNYVFYVIQDSPFYEITFVSQILISSIILSTNCGIYSMIAT
IATHCCGLFEVVSRQIETMLEYKEAEPYERLKEIIRYHLKAIEFAEMIENALNTVFLSEMLGCTVIICFL
EYGVITEWSDHKILSTMTYVVLMTSIFVNVFIISFIGDRLKQQVSSV

>Lalb_14638 [mRNA] [translate_table: standard]

MTIRSTVSKSVRYGLHFIGIWPDTPFPGLHKFYWISTTIIWQFYQCKYMITHFKTDDLTKTIDCLSMTLP
YTLLIAKLSVVWINHNVLSEMLSTMEEDCVKYGMIDANNVISKTAEFSYRITSLVTVLYLSSASGVAAGN
LAFPRYNETVDRQLLSNMDLPFNTNESPTYELVVTAQVLHQMASAYVFGVFSALLVMLILHVGCIIDILC
DTIVRISSKEIEKLRLVALRHQEIIVFADRIEQLFTYIALCQLLLNTLVTCCLGFLAITAIKTEHGLPLL
LKSSSFYAVLCLEIFIYCFAGEYLNVKNQMIIDAAYQMTWYDLQPDISRQIVFIMLKSQKGLPLTFGKFS
TLSLESFTGILHVGCLIDILCDILAQISAQEEDKLRFVAIRHQEIVDFAERIEKLFTHISLCQLMSNTLV
TCCLGYLVITAVKSDNGLPLLLRSFSAYTVICLEIFIYCCAGEYLNNKVNKFYEFHQVYGK

>Lalb_14640 [mRNA] [translate_table: standard]

FRSTVSQPVKYGLYFVGIWPGTPFPGLHKFFWVLSTTLCQIYQYKYVIAHFNTDGLADMVNCLCFALPYT
MTMVKLIIVWTYHRVLCDILSTMEEDCVKYAVVDANNLISKTADLSYRLTSTLGFFYLAAVSCHSATILA
IPRSNNTMDEKLVMAMDLPFDISESPTYEIVIAAQVIHLTSTAYTFGLFSALLLMMILHVGCVVDILCDV
LAHVSAEGTGQLRFVAAEHQQVILFTEKIEQLFTYISFCQLLSNTLVTCCLGFLAISALETENALPLLFK
FFSAYAAICVEIFIYCFAGEYLNIKSQMIVDAAYQIAWYELQPNISRQLVLLILKCQRGLPLTFGKFSTL
SLDSFTSILHMGCHIDILCHVITKITSSKDKKYFRFVAVRHQELILFSERIEQLFTFISLAQLMSNTIVS
CCLGYLVIIGLQQGSDLPELLKFGVCYVAICIEIFICCFAGEYLNVKAS

>Lalb_14641 [mRNA] [translate_table: standard]

MSRSVLRDIITSMEEDCVKYSVDDSKNLISNAADLSQSVSRTVIIIYVASAGFYAVTTLAAPHSNDTIPR
RLLLNMDLPFNTVESPNYELVVMLQVISILMTSYAYSVFSALLLMMTIQEGGKFSQQIKFIVCYLAICIE
IFTCCFAGEYLNMKNDMIVEAAYEISWYNMRPSMSRQVILLLSRSQKGLCLTFGKFSALSLETFTWVSVL
RQILSTMEDDCARYAALDKNNVISKTADLSFRITSTIPALFMISVCCNAVGSLAIPQSNDSSVRQLLINM
DLPFDTNQSPTYELVVAAQIIYQVMSAYTFSIFNALLLMMYKYMITHFETDGFIETIYTLSITVAYTLLI
GKLVVAWMNHGVLREIISTMEEDCARYAALDKNNVISKTADLSFRLTSTIPALFMVSVSCNAVGALAISQ
SNDSSARQFLMNMDLPFDTNQSPTYELVVAAQIIFQVMSGFTFSMISSCLVMTILHAGCVIDILCYIITH
ISCLDERQFRFVAMRHQEIIQFTQRIERMFTYIALGQLLTNTLIICCLGFLMITVINTDLVISRHEVVPC
FINTIGRLSMKP

>Lalb_14643 [mRNA] [translate_table: standard]

QLFLSIAADSQWDEIAIPIPGALVQLLIMFLNNYGGQSVIDTSSQISTESYNSVWYLIPPESQKMLLLVL
VKSLNGTKINIAGLFVPCYEGFSTVKSFMRSMQNDCDMLDNPIETQILIKHIEKGRRIIFIFLGKNIRQR
VLRKLSKSMADYVNMVGFFYSQKDAHIKLIYVHISVTLFMGLLTLACTEGSSGGGKTQDHIYRTNMAAVN
HPSLLAWTPLSPKTLLYAQSFRSVMPEPWVLGFFPSAASVGSAPHSVATTVHFNAQQTFLSLVKMEVTHP
EEIFVCMLVTSVHVLIIFLNNYSGQAVVNTSSQIFYDTYNSAWYRIPPSSQKMLLLVLLNSLNGVQISFA
GLFIPSYEGLSTVI

>Lalb_14645 [mRNA] [translate_table: standard]

QASLLMLEMEQLDEAVMSFLVVMTHLVVMFLNNYSGQQLANISVNVFNETYNSKWYCIPPKSQKLLLLVL
MKTANELKFNLAGLFSPCYEGFTMVKNFVQYLQNDCTTIQSPVERKILAKYMEKTRHIFLCFLGVSIVGI
TFVSVRIIIPTILRSKYQLYSLQSFGFFYSKQTRESDWASIHLTVTSAIGLLTIACTEGSLAVFSLYLCG
LFEIVGACTIIANEISPPTQYTDLGSVYRCEPLLTRRGESQSSAQFKRPNGGNSVQSLALRQASLLILEG
QQLDETLFAILVVLTHLVVMFLNNYSGQQLANISVNVFNETYNSMWYTMPPKSQKLLLLVLMKSATEVQF
NLAGLFTPCYQGFTTVI

>Lalb_14647 [mRNA] [translate_table: standard]

MFVQLSNAVKVTQATRDLTEAAYFLILQFTYLFLCNYVSQLLINHSSNVFGIIYDTEWYSIPVPVQKFII
LIMKRSLQPCILTLGKMYPPSCEGFAMVKLNNVLHGQYRLFLEINFSFFLDMQHVDVVLHVLVLDTTI

>Lalb_14649 [mRNA] [translate_table: standard]

MDEVRSKWNTTHKDTLEILEKRAILGKQQGALYATLLLVVFRTSSYVLLVTASPDSNVTDPFPVVTDYMI
DQEKYFFFIIMHQNVTFFFVATIYVASETLFIMWLQYGIGLFELMSYHIEKGLCDRPSHVSKKSMDAYRK
QSFATAVTYHTEAKTFLRSLKNTFSFSYSVLLVFAVGSLAINLFRLSTAICETHDIEEMILSFLFSVSEL
VYMFYLNYVVQQSLDYADVLPTIM

>Lalb_14650 [mRNA] [translate_table: standard]

IVRHHAYMGRLACGSLIFFAYFSSTLYSAVALLTREDEDAEQILNVTQEDSFKYPLPSECTLDLLNLPAS
LYHVVYIGQYVLMLLTSTGNVGSDSLFFGIIFHLCGQAEVLKLDFIKFVELGEKKARFDVLVARHQQLLK
LSDHLKDIISSIMIVQLFSSCLLICTSGSDCQKVSDTGISMVGAGRHRDELGRVLGSSGLRMCPVAAATI
LIDFVWKTMVLGFQFIRSLSTNNVVMMVKTLVLVGTMLAQLFAYSYIGEYLKNQFEDVGYYAYCSTWYNL
PQKLSFDLVFVVMRSEYPVHLKAGSFFVINMETYMSIVKTSISYLSVLRVMVT

>Lalb_14651 [mRNA] [translate_table: standard]

NKDFSYAMRPMKTLSWPVGTWPLQKYNFSSGLRSFVSVILVLLMLLILHVEMYLDSSDAEKNLDAVLLIA
CGSLAVWKVVSFRIHSKGLVSNFLSAQKDYAELEEQEKRLIVRHHAYMGRLACGSVIFFAYFSSTLYTTV
ALLAREDEDAEQILNVTQEDSFKYPLPSECTLDLLNLPASLYHVVYIGQYVLMLLTSTGNVGKSLFFFLR
IGRRIAYQLLSRVYLLGSDSLFFGIIFHLCGQAEVLKLDFSKFVEAREKKARFDVLVARHQQLLKLSDHL
KDIISSIMILQLFSSCLLICTSGSDCHQVSDTGISTSRWSRREVIDTRFQFIRSLSTNNVVMMVKTLVLV
GTMLAQLFAYSYIGEYLKNQFEDVGYYAYCSTWYNLPQKLSFDLVFVVMRSEYPVHLKAGSFFVINMETY
MSIVKTSISYLSVLRVMIT

>Lalb_14654 [mRNA] [translate_table: standard]

MNLPKDKDFAYEMAPMKIVSWPVGTWPLQQYNYLTGAKSVTALFLLMIMMLIVHTEVYLDFGNAEKNLDA
LVIITCGVLAISKVACFRFRPAGLIANFFSAVSDYQELRGEDRRAIVKRHAQMGRTASANMLFFSCFSAT
LFSMVPMFAGDDDTTEGFNQTRKDQLNYPIPSECTLELLQVPGNLYALIYIGEYILLLVTATGNLGTDSF
CQLSSVHGLIDDESFERDEEQRMENGRGVAEWVERSTADSVVAGSDSLFLGIVFHLCGQVEVLKLDFSRF
LEDEEDSKKRLTTLVNRHRHLLALAEHLNDTIEYILILQLFSSCLLICTTGFQFILSLHVHNFVMVIKTF
IVMSTLLIQTFAYSYVGEYSKNQFGGVGYFAYSSDWYNAPCCISKNILFVLVKTQCPVYLRAGRFFVVNL
ETYMSILRTSMSYLSVLRVMIT

>Lalb_14655 [mRNA] [translate_table: standard]

MNLPKDKDFAYEMAPMKIVSWPVGTWPLQQYNYLTGAKSVTALFLLMIMMLIVHTEVYLDFGNAEKNLDA
VVIITCGMLAISKIACFRFRPAGLIANFFSAVSDYHELRGKDRRAIVKQHAHMGRMASANMLFFSYFSAT
LFSMVPMFAGDDVTTEGFNQTRKAQLNYPIPSECTLELLQVPGNLYALIYMGEYILLLVTAAGNLGTDSF
SRSRLYYTLLQQRLLAEAVRWLGSDSLFFGIVFHLCGQVEVLKLDFSRFLEDEEDSAKRLTTLVNRHRHL
LALAEHLNDTIGYILILQLFSSCLLICTTGDTILVCINYTNDTLSLPGFQFIMSLHVHNVVMVIKTLIVV
GTLLTQTFAYSYVGEYSKNQFGGVGYFAYSSNWYNAPCCISKNILFVLMKSQCPVHLRAGRFFVVNLETY
MSILKTSMSYLSVLRVMIT

>Lalb_14656 [mRNA] [translate_table: standard]

MKLPEDKDFAYEMAPLKIVSWPVGTWPLQDYNFWTGAKSVIALILLVLTVLILQTELCLGEGDPEKNLDS
VVIGSCGLLAISKVAYFRFRPTGLIANFVSAVKDYQDLQDVERRAIVKRHAYMGRMASVNMLFFIYFGAT
MCVVTPMFLRRGGVMESVNRTEEAKRSYPMPSTFTLELLHVPESMYIWIFLGEYILLLTIAAGNLGNDSF
YHFIAVLVAIKILRRDVRWIGSDTLFFGIVFHLCGQVEVLKLDFSRFLDERKNSVRRFNELVRRHRHLLL
LADHLNDTIGYVLILQLLTSCILICVTGDQSLVKSWGFVIDVPLFPGLQFILSVQVHNVVMVIKTFIAMG
TLLTQTFAYSYVGEYSKNQFGGVGYLAYSSDWYNAPCILTRNIIFVIMETRTPVQLKAGHFFVVNLETYM
SILKTSISYLSVLRLMVTG

>Lalb_14657 [mRNA] [translate_table: standard]

LCVLVAYKKDFLDLLVHLRQHFLESQYDTNEKTILDACKRTCAIFIGSFTFFSHMTLLGYVLVPLIVSIG
KNESERLFPFAVPGSPLYVTPYYEIAFFVQVLVVYEVGICHFCVDNILCIMNLHVATQFRILQYRLSNLK
CENIGDDPLMRVPSATENSYMTFKRCVQHHKTLIAYCNKLEEVFNLIVFAQIFIFSMLICLNGLQILVVS

>Lalb_14660 [mRNA] [translate_table: standard]

DVYNIYFVDYQQAYLFISVNILTVFGGLFKIFVLLPHRRDFFSLVHHLQHNFLNSDYDHHEKDIVNRCSS
LCTIFIGLLTLSAHLTAFCYAASPLIANIGRNESDRVLPFNLWVDLPVSMTPYYEIIFIIEVLSLYQICV
AYFCFDNFLCIMNLHVASQFRVLQYRLTNLRYKQDELNRLGDVKICMLHSTKDVYITFKSYVRQHQVLIA
YCKKLEAIFNLIVLGDILTFSVVICLNGCQALVVDSARRFIFLFFLLTSFVHLLMLTYSCDGVMRESVKV
AAGIYNSPWCYLRFNKYGRMLRKDAILIIMRARVPCYITARGFFPITLETYTKV

>Lalb_14662 [mRNA] [translate_table: standard]

FVFSLCNIVTATLVFSKLITVFLRKPQFFKLIAYMEKRFWHTNYDSYEQALLDKCKQLSVVFICSFNFFA
QGTAFSYSLESSVEYFSATNVTTRSLVFNLYTDVVMKPFIFEMTLLTQYFVLVACGIGYICVDNFICISN
FHVATQFRILQYRILNVHKRTQTEIGIGTPSKLSADRADYCYTVFKDCIRQHQALIRYYEDMEEVFTIAA
LAQILLFSVLICLDGYLMLLGESSPYRRLSFTFHLIGTMCQLWMFTYSCDCVLQESMRIADTVYETIWKN
LPMNTSGKMFRTDLQVVMIRSRVPCCLTASGFFIVSLETYTKVLSTAASYFTLLRQ

>Lalb_14664 [mRNA] [translate_table: standard]

PDSWTSPSKRILYSTYSAFIWFVMYWWTVSQILDLIIQVKTQDEFTDIFYATMANCCICAKFTNVLLTRT
YIANMIDRIEKEPFASLNVEEEEIRTRFDKLARRNAVTYTIIISCYILPTCAMGLFVGFRDHKLLSRAWL
PYDYSSNVSFSTTLIHQVGAQVYAGCITTGSDCLFNGFLMHTYCHFEILNHRLKMIEADKKYTLKRCTYL
HDYIYTYATTVNRRFKIIIMIQSLVSIVTVCCTLYQLTQVHLGSRFIEVALYLTNTLIQIFYYCWYGNEI
KLKVQSCRRQS

>Lalb_14666 [mRNA] [translate_table: standard]

MPTLQCASTILTVIGCHRPPSWTSEPMKFLYKVYRIIILVLVQVLTLITILDIVFNVKNQDEISDNLVLT
IPMLITCCKFCSFLARGDNIMVLINTLQQKPYSPENILEKDVETKFDKHNERVTALYTFSTEACIVMILL
TTLITNGKNKMLAYRLWIPFDYSAPVVYAIIFALQAVSVIISSLMNVTYDALFSGLMFCIYSQLEILGLR
LQNVVKNGKDTARECARHHNFLY

>Lalb_14669 [mRNA] [translate_table: standard]

MFGTVTPEKAITFVRLSIALICGWPQPSVSTKAQVLCSRISKVLAIISALALFLPVLSATILHSGDANVF
SKAAFMAIATGQVFIYALLTAIQHDRFQRLVENITVSIKDAKSYERNVYQRYVDTYYKFYGLTVMWYYMS
AMIVIVGTIFLPQPFPTISEYPFDVDYEPVRIMIFIHQAIVGFQCSASVSLNMFAALLILNAAARYEMLM
IDLKEANSVDALIVCVKKYHAVTRFAKDVINGAQYIAVGTVLLSSMKIVLCGLNIIGRTSIVIKLQFMAL
SWTALMEVFMCALPADALINVSTNAVRSVYESTWYDQVLSVQKTVLHILVPQEPMAISVKVFIPALSLEY
YCSVSI

>Lalb_14671 [mRNA] [translate_table: standard]

MFENVMPEKAMVFIRHSVVLICGWPLPSMATKAQVLCYRILKVLSAISALALFLPVLNAAILHFGDPTNF
SKAVIMAIACAHVLTQIVVSSIQHDRWQRLIEHMTASLKNAKSYERNVYQRYVDSYCRFYALTVMWFYMS
PIVFVVGTIFLPQPFPAISEYPFRVNYEPVKTIIILHQALVGFQCSASVSMNMFAALLLLYVAARYEILM
IDLRESTSIDALTMCVKEYYSVTRFAKDVTDATQYVVWFTILYSSIDLVLCGLNIIGRQPVVVKLQFITL
SWTAMMEVFMCALPADTLINMSTNAVRSVYESTWYNQVLGVQKTVLRILVPQAPIIISLKCVMPRLSLEY
YCSVRVHFIFTLNLYVSTVIIIFKY

>Lalb_14672 [mRNA] [translate_table: standard]

DVLQRLIEEMKASLKNAQSYERNIYQRYVDTYYKFYGLTVMWSYVSPVVVVVGSIFLPQPFPTVSEYPFR
VDYEPVRIIIFVHQAIAGFQCSAAVSLNMFAALLLLYTAAKYEILMIDMRRATSVDALIACVEKYQAVNR
FAKDVVTVTRYIVCTTITCSSTNIILCGFNIIGRQPFIVKLQFVVLSWTGLMEVFMCALPADRLINMRLI
EHMTASLKNAKSYERNVYQRYVNTYYRYYGLTVMWNYMCTMVVIVASIFLPQPFPTISEYPFDVYYEPVR
TIIFLHQAFVGFQCSASVSLNMFAALLLLFAAARYEILMIDLRESTSIDALTMCVKKYYAVTRLAMDVID
ATQYIVWFTILYSSIDLVLCGLNIIGRQPFAIKLQFIFMAGTILMQVLMCALPADVLINMSTNAVRSAYE
STWYDQVLGVQKTVLRILVPQAPIIISIKCFMPRLSLEYYCSVCVQCTFTLNLYVSSAIIIFK

>Lalb_14673 [mRNA] [translate_table: standard]

MFENVTPEKAMVFVRLSIALICGWPLPSTATKAQVLCYRILKVLCGISAIGLFLPVLNAAIFYYGDATHF
SLASIMIIASAHLLTQVLINSFQHDQWQRLIEHMTASLKNAKSYERNVYQRYVNTYCRFYGLTVIWYYVC
ALVIIVASIFLPQPFPTISEYPFDVYYEPVRTIIFLHQAFVGFQCSASVSLNMFAALLLLFAAARYEILM
IDLRESTSIDALTMCVKKYYAVTRFAKDIIDATQYIVWFTILYSSLDLVLCGLNIIGRQPFAIKLQFIFM
AGTILMQVLMCALPADVLMNMSTDAVRSAYESTWYDQVLGVQKTVLRILVPQAPIIITIQCFMPRLSLEY
YCSVCVHFIFTLNFFVLEDVGTAAPGTANSLRHEPASPEPAPVSRRKRKRVEVEEPRTAAAAVMDYLLIA
NREKEYDDIEQFARLIATKLRKLNPINLAMTMKEIFSMVFDVQCWQLAETGGNRSSSDDHLENSPT

>Lalb_14674 [mRNA] [translate_table: standard]

MSFVGILAVLVLFLIPTLLHLKYQVRIIQLFGFFYTERDTRTDLICLHLALTSVLGLLSVSCTEASLAVY
SFYLSGLFEIVGYVQNNMHRFLKFIREFIMMNISNNCIRSCSLLPSEKMLYHTCWQSRLWARSLSIYTAP
EEIVVPILVTLIHLLIMFLNNYSGQSVIDSSSQIFIESYNSVWYLIPPESQKMLLLVLVNSAKAVKINLA
GLFIPSYEGFSMVILLICLCSVFNTLQSDSGNFC

>Lalb_14677 [mRNA] [translate_table: standard]

MYVWLCEWERDKDGFRQLLLAMSELSKPGNLVITLMIVLVHVIIMLLNNYSGQKLMTISVEVFHNTYNSL
WYCLPPKSQKILLFILMKTATEVQFNLAGLFVPCYQGFTTIRCLYENIVKDYTTLSNPIESEMLTKQIVE
TRRVVLLLLALSLGVVLFVVATLLVPTLLHSKFQTHYLRIFGFFCNETGLQTDLICYQLVLVSTIGVIAL
AAAKLRMQRRASEHIWARGGRVRGLARLADGNPAPVPFYNDISLSFCPVFVSSFLDRGHAGLTKAGSGSH
EEHDGVVFSGDRDAVMQLDELDNVLISFQFVLVHVIIMFSNNYSGQKLMSTSGQVFHGTYNSMWYRIPPK
SQKLLLFILMKSAPEVQFNLSGLFVPCYQGFTAVID

>Lalb_14678 [mRNA] [translate_table: standard]

LFDNYDSDCASMNNPIEAKIITNQIIEAKRVIMALLGLSCAGICLAILVLLVPVMLQSSFQIRFLNTLGF
FYLERSQETNLVCCHIFIVTSFGLLTVTCTESSLAVFSSYLCGLFQITSYRIETTVNAMAQSGNQNGLID
IRSAVKLHQRAVQSFYLAVLELEKVDNFIVSLQIFLVHVLIMFLNNYSGQRLISTSVEVFHKTYNSPWYR
VSPKSQKMLLFVLMKSITEVQFNMAGLFTPSFEGFTMVINYICQ

>Lalb_14680 [mRNA] [translate_table: standard]

LLYALATMLCTTNDLFKVCVLVAYKKDFLDLLVHLRQHFLESEYDTNEKTILDACKRTCAIFVGSFTLFT
NVTLIGYILVPLFLSIGKHGSDRFFPFALPESPLYISPYYEIAFFVQALIIYAIDLCHHSVDNILCIMNF
HVATQFRILQYRLSNLKSENVCDGPLKRVPSAAENSYATFKRCVQQHKMLIAYCNKLEEVFGLVGFSQII

IFSMLICLNGRYTALFFLMTSTMQLLMFTYSCDGVIQESLKVAPAVYSSPWAYLPMTISGKMLRKDLVLV
ILRSRIPCCLTAKGFFPISLETYTKVWTTAMSYFTLLRQTMDDLK

>Lalb_14683 [mRNA] [translate_table: standard]

MSKETLNYRRFTNNVERLLTVCGLFPHQQSIVKYLSILVLVSYCLLFYVIFNFFRIHIANISAIIACFSL
LTTTITVAIKIVQFLRRREKLARIGDTLATLFEETSLKQPSSSPVFQYLQPCYRLVCYEFACMFLTVILY
TSKPLIDIAIRNASPSTPFPSVYPWPIVSTASFAGHYLYETSMSLSFPIITTGVDAFFILCSFRVCSVFR
AMAVELKESPKRHTEDWEQLLRKCIERHTTLIECRDGIQEIYGPVVLSVTLTNGLGMCALIFEVLRVKDI
PLEKATALSLYLFGKIIQTLSYAWPGDMITSEDEVFRREVYCNNWYEHSNVRITKLSITILSQRLMVLKA
YNLLVISLDLFAKIMNRTLSYYFLLVTLDDAE

>Lalb_14684 [mRNA] [translate_table: standard]

MPKESPLYQEYADFVKRCLLMCGLYPYQQSIASKYLPIFWILGYCLDFCALINFFRLHITEISAITACLS
VLSGIVNMTIRASRFVICKEKLVQVTDTLEALFEETSMQQPSSSPAFQFMLPFYRLGYYHYVCMVSTAFL
YSTKPLIGVTIRHANRSTPFPAIYPLPIDSTAFFVAQYLLEVSICYSFCIITSGVDAFFTLCAFRMSSVF
RAMAVELEEFPKGRNNVESNEQVLRRCIDRHVTLIKCRDIIQEIYGPIIFSVMMTNCLGMCALIFEVSKA
NAAIAVEKAVAITLYLTGKILQTFMYTWPGHVVTSEEVYCNNWYEDLNTKTGKLTLTILMQRSVILRACK
LMAVTVDLFAKIMNRTISYYFLLVTLDDAE

>Lalb_14685 [mRNA] [translate_table: standard]

MSSNSKTVITSPIVFSLRMIGAEPRSSRRILQRITCSILMMSSLSMQFWYVASYSKNEELTDLLDGLSVI
LSNGGTFVKMVIIWWNHRMFFNALTIIFEDWKNLDSIGRNKRFMVDKALMSLRISNLLIVAYSMSVICYT
ISSLFISGDSGEEELLPSKRKLLLKMRFPFEVMFSPVYEIVIVAQFLLEYFIAFVAGMFMALLAALVLHV
GSQIDIMCQELIDLPNHQQEERLSMFKSLVVQHQKIIHLGECIRDLFINIALVQFLSNILVICCLGFLLV
NSLGTEGGPSIILKFLPFYVAANSEAFVLCYTGEYLISKSDDVRRAVCDMDWYKLNNRDMQLLLLILLRS
QKELTLSAGKFVVLSVEAFATMLKASASYISILLAM

>Lalb_14687 [mRNA] [translate_table: standard]

PQRPNPVLFAGTLCYQSLVIVLCAWAAYDVRNNFQMLLLDMMEWIIILVSSTLALILVWLSDSSKVVIDET
KREIRDGMFLRNDEERRLYHRYNSISWKFGKYATVFQSALVLLMFLRPAIPLLLHAGESNATLSYKLPFQ
AHIFFDYQNHTMRYFLLYVYQIPMIYSGMFHLAEVSYVVTLVLHVCGKFSILCVRIRNVPTKPQSLFRHH
IKSVVNQHLELKE

>Lalb_14689 [mRNA] [translate_table: standard]

LYYMINAVTIFNSLFKLIAIHPYKRKFFNLILHLQHEFIDSEYTEHERRMLLVCQRTAKIFIWSFTILVH
VTVLGYSAIPFFGRSNESDRVLIGNMWMSVIAFSPYYEIVYIIQIFCVIKIGVVYHSYDNFQCLMNLHVA
TQFRILHHRLESLESTYCSGRRLVQMSSNSLSNDSDYGADCYAMFKTYIQQHKALLAYCKKLNEVFNIYA
LGQVILFTLILCLDAYQLLMVNALPSRKMTFAFHMLGCFAQLLMFTYSCDLLIAESLKVAEWAYGIPWTY
FPMNKHGKMMRNDLIFLLVRSSEPCCITAFKFFPVSLETYTTV

>Lalb_14694 [mRNA] [translate_table: standard]

WAAYDARNNSELLLLDISEWAIILTTAVALVIVWLSDSWKPIIDEIRRDIREGVFLRNDEERRLYHRYNN
ISCKFGKYATAFQCALLLLMFLRPALDLLLHAGENSATRSYKLPFQGHIFFDYENHTIRYFLLYVYQIPM
LYTGMFHIAEVSYVITMVLHVCGKFSVLRSRIRNMPTKPSSLFRDHIKSVVNQHIELRQ

>Lalb_14695 [mRNA] [translate_table: standard]

QMRKTMNRIQYDWRNAILNNEDHILKANAEKGWRFLTVCMYMSGILSCQIIPEILNMSISNNITRFIQHV
RGEFFLDQDKYYALILSYLIMSLICTAVTSTAMALQWIMIINHACGMFAIFGYRLEHAFHVSSRKRDRKD
SVKKLLAHTVEYHKEIIKYIAAVNSNYSVYYVLLLIMGIIVLSTFLIQVMIVLSLFSVTVCMYMSGILSC
QIIPEILNMSISNNITRFIQHVRGEFFLDQDKYYALILSYLIMSLICTAVTSTAMALQWIMIINHACGMF

AIFGYRLEHAFHVSSRKRDRKDSVKKLLAHTVEYHKEIIKYIAAVNSNYSVYYVLLLIMGIIVLSTFLIQ
ISFPFSSSTKNIYMFRRLLRSTTPNVEVFLLAMLFAVCLWYIYVDSVSAQKLIDASSKVFRQTYYGEWYN
ASISDQKTLLFIMQNCMQPLTITTAKILTISMEKFGMVR

>Lalb_14696 [mRNA] [translate_table: standard]

LRFVVQTDSARRFIFLFFLLTSFVHLLMFTYSCDGVMRESVKVAAGIYNSPWCYLRFNKYGRMLRKDAIL
IIMRAKVPCYITARGFFPITLETYTKVSKEDIKTVLCPHSTFPFYNAIPHSSAAARSRVTMSPGARSQAQ
WPSRRPRVSRTSNRNEFLCEHS

>Lalb_14697 [mRNA] [translate_table: standard]

EGASPYRRVVFTFHLIGTITQLLMFTYSCDCLLHESLEIAESVYTAQWPLLPMNNSGKMLRSDMQLVIMR
SREPCSMSAGGFFTVSLETYTKVLSTAASYFTLLRNY

>Lalb_14698 [mRNA] [translate_table: standard]

MFDNMERDSNAIKDPAETELVMKHVIETRRVIVLLLGISCAGLVMLVMFFIVIPALFLPNSQTEYLHMLG
FFYPETSMESTVVAIQIVFVTTIALFGVSCTELSLAVFSSYFCGLFEVASYRFETAVNMLANSENQNVLI
DIRSAVKLHQRALESLYLAFLMLEDVENFLISLQIFMIHVLIMFVNNYSGQKLMNSSIEIFTKTYNSLWY
SIPPQSQKLMLFVLMKSITAVQFNLAGLFNPCFEGFNMVINYIYQ

>Lalb_14699 [mRNA] [translate_table: standard]

MNVENYISINLFVLKFVGLYPINIIRYIISVCCIILIVIPLAAHIYKNLDSLNILLETSSVLLTISLAIL
KSLIWFSKRKKLELFIDFMLTDYWRLGETTVFEHLQEYAIHAKKVTRSYLFLIVNALLFFFSVPIIEIFI
NAAYVSNNNSTVLRDFPFVASYPLAFYSFPFYETVYVSQMVATSVCGLMILATDTLIASALLHTCGHFQI
LKENLKQVDSEIYCVRIKSLSFCDNMEKNFHLMLLMQTVASSLILCFVALQVTTTLMDHSKVMKYASHLM
MALFQLLLFCFPGDLLISQSSGISQAIYSIQWYRVSAFLRCEASMVMLRSQKPSYITAGKLYIMHLENFS
ATLSTALSYFMMFRSFNAEA

>Lalb_14700 [mRNA] [translate_table: standard]

LISIQCFFLFVFVSKVMLKILNVMADDWEKPKTDEEKNIMYRYAKMARIVAVLGFIFAGIAILMVIVLPK
FGIYIRHTTDGIDVFPFPTYYVYDVTRSPYYEIIYFGQIFMLVITLLAYNGINILFGTIFLHICGQAENL
RSRIASQRKFGDFGQALASIVSDHVRLIRTVKMTESTFSVIMLTMLLMFAAITCTVMTSIFSIFAEKDGF
SLTRVLYLVLLITTNFIQMFFYFITGQTLLNESEGIYDATYECGWLNLKSNEAKSLILVMARSKKPLFVS
AGKLFPMSYLTFGNVIKISFSYMSFLVT

>Lalb_14701 [mRNA] [translate_table: standard]

RYCGLWVLNPNTESVLRCGYFAWKVFIIATMYIFTITLLADIYTSIDDISVATDSGCICAGMIVVVFKTM
NYQLNQKNIERLISKILKCADDIVEFSVDSDSVTDIIKRHYTFNKIIFHGFRAIGCFLVIALLLFSPVKD
GLPIRAKYPFNTTIFPYREIALAVESVAVSSGVLAILSMDGLTLLMCNYTTMQFDILNVNFENCNRRTAN
NTGSVYRRPNDTFMTRYKTCLRFHQQLMSTTNDYNNIFSPSVFVQMLSSTSIICLTGFQAVVVRHD

>Lalb_14702 [mRNA] [translate_table: standard]

RMAVQLKRIFSPNVTVLYLSNVFRISFISIMIIMSTDVVETCFLVPYAGGAVIQLYMLCSCIQHLLDEST
TMMDSVFHQKWYDHDISLQRSVMLMIMATKLKCEISRIQNVNLTLPSFMSILNKAYSVSLLFLKSRRD

>Lalb_14703 [mRNA] [translate_table: standard]

PQRPNPVVFVWTVCFESFVAVLMAWAAYDLRNNIRILLQNLMEWVVTVSFIITLILVWLSDSSKLIIEEI
KREIRDGLFLRNDEERRLYHGYNNISCKFGKYATVFQFALLLLMFLRPAIALLLHAGESNSTLSYHLPFQ
THIFFDYQNHTMVYFLLYVCQIPVAYSGMFHIAEVSYVVTMVLHVCGKFSILCVRIRNVPTKPPSLFRHH
IKSMVDHHLELKQ

>Lalb_14706 [mRNA] [translate_table: standard]

MALACTWNGIEIELEWNWSEFEMDLPRKFNEFGMELKWVWHGIASARFRCKGRMAAVLKQIFSPNIAALY
INNVFRFCFLSIMVITSADAVLRGFILLYTCGALMQLYMMCFCIQHLLEASTTMMDSVFHKKWYDHDISL
QRSVMSMTLATKLKCQLSRIRSIDLTLPSFMSILNQAYSVCLLFLKSRRD

>Lalb_14707 [mRNA] [translate_table: standard]

QLHFLNLLGFFYTERSLLSAISCWHIVLTTTYGLLILTVTEGGITVFATYISGLLKIASYVSISFSEILS
DGVCFVPCDTSYRLVRHKYISGESLIHSGLHYFQNSKILFRISQFFISITEFSDKDAVLLSIHFVVTYTT
FICGNNYSGQILLDTSAALFHDTYNSLWYRIPPRMQKMVLFTMMKTQAAVELNCAGLFTPCYEGFATVTD
TIFL

>Lalb_14712 [mRNA] [translate_table: standard]

PQRPNPVLFAGTLCYQSLIVVLCAWAAYDVRNNIQLLFQGLMEWIIDVALVFTLILVWLSDSSKLIIEEI
KREIRDGLFLRNDEERRLYHGYNNISCKFGKYGTVFQSAIIALMYLRPFIPLLLHAGESNSTFSYKLPSQ
IHIFFDYQNNTMLYFLLYVCQIPAAYSGMFHVAQVSYVVTMGLHVCGKFSILCVRIRNMPTKPASLFRDH
IKSLVNQHLELKQ

>Lalb_14713 [mRNA] [translate_table: standard]

MFDSMERDRNAIEDPVEMELVLNHVTQTRNVITALKALVYAAVVILITFFILVPAIFLPQSQIRYLRYLL
GFFYREISTETTMVAIYLVFFSTAILVVIICTELLLAVMSSYFCGLFEIVSYRLETTVNKMAESRYQNGL
IDIRSPVDLHQHVLESLYLSFMALEDVVNFLIGLIIFSAHVLIIFLNNHSGQKLMNSSIEIFIKTYNALW
YRIPPKSQKMLLFVLMKTVTEVQFNLAGLFNPCFTGFATVINDIYQ

>Lalb_14714 [mRNA] [translate_table: standard]

LLDNLQHDFVTLNDPLEVQMLMKDSIVQKRIVTVDIALSTTGTLLVGGLLLVPTLMQSDAQLRYLRLIGY
FYNERSMHTNLTCLHMTMTTLYGLLTFVCTEGTLTIVSTYVSGLLHIASYRIKNAVADAVSSDRMKPLDI
RSAVDMHRQAIKLLFVSITVVKETDNTMFNIQVVLAHLAIIFGSNYSGQIAMDTSVKIVHATYDSLWYRL
PPKMQKLILFMMIKAQTKLEISFAGLFTPCFEGFSTV

>Lalb_14716 [mRNA] [translate_table: standard]

MEACSQELGLSYMVTIGTAIVSFALNLCRIFMAITQGKLDETVAPSLIILSHIIMLFLGNHVGQRVTSTS
HGIFNAVYSSEWYNIPLHERKLVNFIMLKSSKEIALCLISLYTANYEGFSMLMVKQEVVKTYSMGTNYIA
TEETGVIPRAVHNIFQIIESKEDWNFNVSVSFMELYQEQLYDLLADKPRNQSIVDIRDDGKNVKVSGMVE
NVVKSASEALQCLTQGSLGRVTGATAMNAQSSRSHAIFTICIHQQKQGDPSTATTAKFHLVDLAGSERSK
KTQATGERFKEGVNINKGLLALGNVISQLGEGGSNAYIGYRDSKLTRLLQDSLGGNSITLMIACVSPADY
NLDETLSTLRYADRARKIKNKPVVNQDPQLLEINRLNKIVKELKLALVDQEIRISCPLEHQELNEKNQSL
LKKIRDLTEKLNENLVEIVIMHERAELAEQSREQIQADMMKILEECKEILNDFDQNPESPAGHRAKVVVY
RYVAAV

>Lalb_14717 [mRNA] [translate_table: standard]

MFSRDNVKYERVVTHYTWQGIFHHITYQFFGTMAVVSWGAKPVIDLLSKRSKELPLLGWYPYNTTESPAF
EITSLYQAVAIILCCFNNIAIDTLVTGLITIACCQLTILNRNIASLNSETRQSINVEKDNDIKASNKQYE
NLKVCVEHSNMIFK

>Lalb_14718 [mRNA] [translate_table: standard]

MIFTWLEIVALLQSVHDLEKTLKNITLSFPTILIVLKTVMFRLNMHHVLPLLAVVKRDVEHGLYQSQEEQ
RTVVWYNVAATMFSTSSALSLFFVPTLFYAKPIVSCFLSNYDNCTLPYELPMKVNPVYEVTEMYTYAFFC
VCLVPVSMMLTIGATGADSLLVTLNFYLCSQLSILSQRVRNIDSDPQKHVSNMRILVERHIELLRLASTL

AETFSSLMFVQTVGLIFSLCIVIYQLLMTSESSGGDVNTIHFIIYSCAVVLLAFCYCFLGECLIAESSDM
QLACYFTNWYELPNEYAKALMFCIARSQKPSHLTAGKFYVFSLETFGVIMKASMAYLSVLRNINM

>Lalb_14719 [mRNA] [translate_table: standard]

DYKWAVTLNRYPLKLLGLWPPENNREESLLDKLRALVVFILMNVILVLPLACMMLLKSNDFMMYLEYTGY
FIPCVISVMKFVIMYSNKTNLVPLLNMMANDWENLKTDIERDVMIRCISISRGFMKFCYATFGPIIFTMT
VLPKLGISYLMATNESAMFPLPTYYVVDVSHSPYFEIVYATQLIIILATSFCYLGVDAFFGTVVLHITAQ
LEILHNRLTSIQSSDQFDHALMDVVVDHIRLSSAIEVIENTYMLLLLVLLLYFGIFNCVYIFEIVYALTG
KSKSDSAGIYYYVGSYTNTLAQMTIYCLAGQLLTTQSEGIYNAAYECNWTDLKSNEARNLILIMVRAKKP
FYLTAGKLFPMNMLTLCNILKISLSYISFLLTKS

>Lalb_14720 [mRNA] [translate_table: standard]

MNFQIYGGEQYDRLIKPIMTTGRIISIWPLPADSSRSTILFRRFHLFCMFFLVVVMSIALTADVIYNLSN
LDEATECALICTAFYLCVVRLLVYTAHQKDMLYVVNTMREDWTVSSYEDRMILSEKTMFAYRLAKFFIST
VAVTIVIFMCVPLLEIYVAGNDKILPFRGYFYVNHTVSPIFECFYVFNVTAGGFGGSMIAGATSFNLVVI
MHGSAKFAVLRKRLEAISGDDPDSSSEMTEFADALERIINVLALGQFVISTGLICFAGFQITSMMEDMGR
LMKYSTFLNSAILELFMFSFSGDGLIEESENVGLSAYSCEWIKSHYCPNLMIMMMRSMVPSQITAAKFYS
MSLQSFSTVLSTSFSYFTVLTATKED

>Lalb_14722 [mRNA] [translate_table: standard]

GSLCILGTLGISTFLKSDIQIRFLYLVGYFYNEKNFRTTLICWYIIITTIHGLLVLVCSEGTVAVLGTHL
SGLLEITRYSKKYMDDMMLPSMLMILVGVSSFGVNIYRVSNTKIKYLELENRSRQIFIILSKTKYNQIVF
RMLQLYISITEVKEIDTVIVSVQVFLAYLAFIAIDNYSGQIVMDNSIQIIEEIYNSLWYRIPTKMQKMVL
FMFMNCHNLVTFNLAGLFILCLQGLAMVKSIYILLFIFS

>Lalb_14724 [mRNA] [translate_table: standard]

MFDKATPEKAVNFARLAVCLNMYWPLEPTTNKFWRVLYNVLKALSLICTIAVFLPLLYSVYLNLKRNDYT
HFSKAACMTLASIHVMGQAAVSFFQHDQLQYLVEEMVDYFRNMKPYEREVYQRYMRKYSPFYGVSTGWNA
LSAFFVMIGPFVTGQPFPMAAEYPFPVDHEIVRSTIYLQHSYVCVQCMCTLNSNMMAAILILFAAGRFEI
LMIDLRAASSIDDLRNCIEQCYVIRKFAQDVVHGVEYITIITLIVSNHCDTGRTMLLFVQRQPIMVKVQF
IFLAGSALIEVYMCAWPVDALMELSETAVRDVYEAKWYDQNVRVQKTVLQMLIPQKPIVISLRFVMAALS
LNYYCSYASNAFSLFTALRVVMENGHDTVPSITSEDNTDCPGNIPPGSLSYDGLILNYTGTSSNYCGAKG
VNLKERNQSEPEQKALATRVNRSQ

>Lalb_14725 [mRNA] [translate_table: standard]

MSIVYLHKKQFVEIISYAENNFWHTNYDPCEKAFLDKCKHMCIVFICSFNFFAQGTAFSYVIEPCVEYFS
NNVTIRRLPFNFYNDFFLKPYIFECTFIFQTSCMIMYGIGYLCVDNFLCITNLQAATQFRILQYRIANIR
KDNDTKEHSGISRSLPAYHADHCYAVFKDCIKQHQALISYCRKVEELFTLIVLGQVLIFSILICLDGYLM
LLVSVRK

>Lalb_14728 [mRNA] [translate_table: standard]

MMKFKQQGLVADLSPNIMLMKISGHFLFNYYKDGSSKFIHKVFCIVHLSLVLLQFGLCGINLMLESDDVD
VLTANTITMLFFTHSVVKLIYFAVRTKLFYRTFAIWNNPNSHPLFAESNARYHQIAIKKMRILLMAVMGA
TIVSTISWTTLTFIGDSVKKVVDPVTNETSFVEVPRLMVYSFYPFDARHGVAHIMMLIFQFYFLLFTMLD
ANLFDTLFCSWLLFACEQIQHLKNIMKPLMEFSATLDTVVPNSGELFKASSAKPVAEIDAPPPPPITPTG
GDSMLDMDLRRIYNNRQDFTANFRPNAGGQYNGTIGPNGLTKNQEMLVRSAIKYWVERHKHIVRLVTAVG
DAYGVALLLHMLATTITLTLLAYQATKINGVNVYSASTIGYLLYSLGQVFMLCIFGNRLIEESSSVMEAA
YSCHWYGGLVIRTNCVLGAMVTYFMVLVQLK

>Lalb_14729 [mRNA] [translate_table: standard]

MGYRVTPRIAIAFTRASVALTRAWPPPLQASRTELFFFNVSWCASFISSIGLLLPLFNAICEYYDEPIIL
GKNVSLFAAVAQVAIKMIVCRLMQQQFQRLFFEMEEFCKHATEKENAVLQRHVDRYKYVHGVYTLWCFLT
TIFVICGPLYSPQAFPTHAKYPFPVEEQPLRSIIFFHQSLVGFQASAGMAIDAQVALLLRYATARFEILA
MKLERVQSESQLNACILEHARLLRYTKSIYRAVRFICFATAVTTNLAVIFGSLNLVTKQPLPLKILYALV
VVSASGELFMYAWPADSLIHQSTGTAMSVYNSTWFEQNVKQQKKVYFMISRSQKREAIQITGFLPQISLF
YYAKFLYTAFSYFTALRVIVEKSNVG

>CbirOr1

MMKMKQQGLVADLLPNIRVMKTFGHFVFNYYNDNSSKYLHKVYCCVNLFMLLLQFGLCAVNLIVESADVD
DLTANTITLLFFTHSIVKICYFAIRSKYFYRTWAIWNNPNSHPLFAESNARYHAIALKKMRLLLFLVGGT
TMLAAVAWTVLTFFEHPIRKIVDPVTNETEIIELPQLLIRSFYPFDAGKGITHVLVLVYQFYWVLFMLID
ANSLDVLFCSWLLFACEQLQHLKQIMKPLMELSATLDTVVPNSSELFKAGSADHLRDGDNPPPPPPPQSD
NMLDLDLRNIYSNRQDFTATFRPTAGMTFNGGVGPNGLTKKQEALVRSAIKYWVERHKHIVRLVTAVGDA
YGFALLLHMLTTTITLTLLAYQATKVNGINVYAASTIGYILYTFGQVFLFCIFGNRLIEESTSVMEAAYS
CHWYDGSEEAKTFVQIVCQQCQKAMSISGAKFFTVSLDLFASVLGAVVTYFMVLVQLKTFDSVRFRRSPX

>CbirOr2

MSANDEGRVFRNLDYKSDTDYVIKVAKTLLTPVGIWPLYRGNSTSDKIKNFLQTGVIFCLMCFLLVPHVI
YTFFDAEDLSRYMKVIGAQVFSLLAIIKFWTMIINREGIRYCLQQMEIQYRDVECEEDRLVMTNSAKIGR
LFTVAYLGLSYGGALPYHIIMPLLADRVVKSDNTTQIPLPYLSNYIFFVVEDSPLYEIVFVSQIFISSII
LSTNCGVYSLIATCVMHGCCLFEVVRRKMETILSNGTDDLHRRLGQVIEHHIQAIRFAEMIEKSLNIVFL
CEMVGCTVIICFLEFGVLKEWEDGKVLPMGTYFVLMTSMYINVYIISAIGDRLKEESEKVGESSYFIEWY
NLPTNIVGYLILMIIRSGRPSTLTAAKIFDLSLQGFCEVCKTSAAYFNFIRAMTT

>CbirOr3

MKIADASPVDERTLFQNFNYKSDTEYVMRIAKILLSPVGVWPLYRNDTILDKIKYFFHTSFVFSLMGFLL
VPHVIYTFFDAEDLTRYMKVIAAQVFSLLGIIKFWTMIINRDEISCCLRQIEIQYRDVECEEDRLVMVRN
AKLGRQFTIMYLGLLYGGALPYHIIMPLVAERIIKEDNTTQLPLPYLSDYIFFVVENSPVYEIFFVTQIL
FSTLILSTNCGVYSLIATCVMHACCLFEVARRHIETVMVDGTDGLHERFGHIIAQHTQALRFSKMIEKSL
NIVFLSEMVGNTIIICFLEYGVLREWEDNQIFGTIIYLILAISILVNVFILSSIGDRLKEESEKIGEASY
FINWYALPAKNMSNLIMMMVRSNRSSTLTAGKIFDISLQGFSDVCKTSAAYFNFIRMITA

>CbirOr4

MDELSSQNVQNEEHFLDFHYAAQVSLWLLKPMGAWPLQQQPTILEIIVYIFSIAITTFLQLFMIIPWFLS
IITANWSFYDIIRTACPLIFVNTIFLRYLLLLFHRDAIRSCINCVAEDWRNVVIAEHREIMLTNAKSGRF
FGMVSVAFMFSSGLPYCVMPLLLPSPVNEDNVTIKVFPNPCELIFVDVQVSPFYEVAYTLDVFACCVFYT
IFCGICSLTAKFVMHACGQCEILMDFFEKLVDGDDKNESTINQRISNVVTHHLRILKFVSDVDKVLNEIC
LAEFLNSSCNICLLGYYVIVDLRNHEPMIQIFVYFVACVSITFNIYIFCYIGEQLVDRAQKIGTTCYMIE
WYQLPNDKARSMIFPIIMSSYPIELTAGKMLKMTMSSFSNILKISMTYFNLLREVSSRDV

>CbirOr5

MVDRKRRSASATDVTKENEQHVSDFRYAVQISVYLLKPIGAWPLDDDEATSWLKIAVHKVSTLIATFLML
FTIVPWIVQIIKEKWSVIQMLRTICPLLFTLTVFARYILLMLHQNQLKSCMDRVADDWRYTIIAEDRETM
LANARMGRTFGIVSVVFMFSSGALFFSLPLVLPSLINEDNVTVRLHPSPCELLVFDSKITPVYEIVYLLQ
TLSGCTIYSAFCGICSLMANFVVHVCGQCDVLTLIFDETVDGGEHNRGPIEGRIATAVTRHLRMLRLVSD
VSGLFTEICLVEFIQASCNICLIGYYIITDLNNNESFVQISMYVLALASIVFNVFMFCYIGDLLAERCQK
LGTTCYTVEWYRMPPRKAVEMIMPITISRYPATLTAGKMMTMTLTTFSDILKTSMAYFNLLREFSIRDAT
RT

>CbirOr6

MIPNEHHEADVKYVVEQSHVVLRVLGIWPSDRQPNTIERIANIFLVIVCYFFLHCDMVPGVLYYMFVDDD
VREKAKMMPPIMYSVMAIAKYGNLIIHERDIRSCLRHIKEDWAVMIVDDAREVMMIKATTGRRLFILCCT
FMYCAGISYNTIVPLSRGSVITDENVTIRPLSCPGYYVFFDPQNSPAYEIVFLLQCLCGVVMYTITVTIC
GLAALFVMHAGAQMEILMRLIESLVDDCKFEQKNIDTKLAIIVEHQIRIRNFLRLVENTLCYSSFVEIVG
CTTIICLVGYCIIVEWEDRNVTALCSYVTSLISVIINIFILCFIGEYITIQADEVSLTLCTLNWYRLPIS
TVRNMVLITAASSVPPQITAGKFIDLSFRTFGDVIKSAVIYLNMLRQFTE

>CbirOr7

MQDIIPSLNVYYVQDLKYARMFVPNILNALGIWPSLGKRKSICKRLVDYLLIFLSYSLIFASLIPSSLFW
VFKGTIRTRVRMFLPLAYTIAAVSKYGILILGQDRIRHCVKYIEEDWKNVANVDARNMMRESARVGRRLV
AISISFYCSSIMSVRLIPLLYLGKIVDQYNVTIRLLPTPAYLFSLDVQATPVYEIMYSLQGAYGIVTASI
STGGIGLITIFVMHACGQLKILMNLMSEFSEDLQQKEVDKKLANIVEHQIRIRNFLQLVDHALCQISFLE
ISIASLIMSIQSYCILTEFENSNSMVIIFFTISYCTATISVFIFCYVGNQLILQAEKVALMSYELEWYRL
PDRKARQLLLVMIMSNKPMTITAGNFLDLSLKTFCDIMKTSFAYLNMLRNTIG

>CbirOr8

MQKVQPPNEYYLQDLKYASTFLPNILNALGIWLSLGKRKSICKRLVDFFFIFLSFGLIFAVLIPGSLFWV
FKGNARTRLRMSTFTMYVIGTIGKYSILILGQDRIRRCWKYIEEDWRNVAKADARNMILECAGVGRRLAA
ICGWFYFCSGLAGRLLPLLYWGKIVDQNNVTVKLLPNPAYLFSLNMQATPVYEIMYSVQGAYGLVTVSIT
ASAPGLIIVFVMHACGQLKILMNLMREFIEEQRQKEMDKKLANIVEHQIKIRSFLELVEHAMHQISFIEI
SNELLIQLLRCNIFRNEWNNSNSMVMIPYTIVCSSAIISMFIVCYTGNQLILQAKTALMTCELEWYRLSD
LKARQMVLLMIMSNKPIKITAGKVLDLSLKTFGNIIKASLAYLNMLRNVIGVFLX

>CbirOr10

MFANRYYEHDIKYTFEMNRFLLRMLGMWPVASTKFPLFGTVTTIVLVTICFALLLFELVPSMLYIFMVLK
DTRRRINVIGNPLFTTVAIIKYGFVVLYKNQVKDCLMAVDKDWRNVVESNDRTSMINNVKISRRLLAMCA
IFVYLTGVGWRIIIPLSKGKIVTAENVTIRVLPCLAYFVVFDVQRSPAYEIIFVVQFFSGFMKYTITVAI
FGFVTLCGMHICAQLNILATLMNNFVNEHHLGSTHKKLVVIVKHQVKTLQFLELVQSTTQYPSLIEILSS
TLLICIVGYYILLYWGTQNTAILFTYSIAMLTFCFNIFIFCYMGEQVIEQGEKIALTLCTLEWYRLPNAQ
ARALILIMAMSNSPVKLQGGKFIDLSLRTFGTVIKMALTYLNLLRTSQF

>CbirOr12

MHEGHLTTPEMICKTLLIVICCIMICFNIVLYTFYCIFNTENDPRAKLKLTAPIIYCMTAFAKYVTFIIH
EGDIKTCLKHVTNDWKFLANVPNARDAMIVNAKTARKLFTVCCTILYFTSLLYHLTPLIEGNIDIDENAT
IKLLINSSQLSILDQRRNHAFEIVFLLQFFGGFIICTLTIALYGLNALLVMHTCAQMTILMTLIKMLSGE
EACKGRNVDERLAIVVEHQTRTRSFLSLVEKTLQHGSLFDVLGCTMLICLVGYCILIEWQDGNIANTCTF
LFALISNLVNIFVLCYIGEHITAVADQVAWTTNMLEWYRLPTNKTRNIVLIIITSHIPLKITAGKLVVLS
FRTFGNVVKSAVAYLNILRTVVE

>CbirOr13

MYERADLCNSNYENDIRYTVQVHRLILSLIGVWPILKKSRYRQTLVKGVIKIVCCFLLSFNLIPWALYMF
LIVDTLTGRLKMLGALCFYSMVPAMYYALMLREDRIKECVRHVEEDWRNVRDVNDRRIMLDNARAGRFIL
IYATLFLFTSGFIYRLIQPIARGKTVIGNITIRPLVQGNYYIFFDPQQSPAYEIVFSLHLLTGIIIYAVM
AAVCGIAALFTMHACGQLEMLAAWLENLSNEDQRPRDCTSAQKLAMIIMHHVRIRKFVDRIQDIIGGMCF
IEIIDSTLILCLLGYYVITGWERNDALSSLTYFIMLISFTFNIFILCYIGDALSTQGSKVHTTSCTIDWY
CLSNKEARYLILIIIATASHPTKLTPGKVMDLSFSSFGTIVRTAMTYLNLLRTVTL

>CbirOr14

MANRTRYLGIRDEPRIYNRDYKDDLQVSVQLNVWTLKPIGTWPKSPDRSWIEAVCCRTLHASCYGLLALI
LIPCGMYIVLEMKDFYNQLKLASALSFFMMSVMKYCALIVHQADIRKCVEYIEGDWRNVRYTEDRNIMLK

NANFGRRLVVICGFFMYGGILFYYVALPLTRAKITDEDGNMTYRRLVYPVPKVIVDTRRSPVNEIFYFIQ
LLSGFVAHNITVAACGLAALLAMHACGQLQVLISWMNHLVDGREEVNDTVDERLASMIQLHVRILNFISL
TEDLLHEISLVEVVGCTMNICFLGYYCMMEWDFKQPVSGLTYLILLVSVTFNIFIFCYIGELLAEQTMKV
CESSYMIDWYRLPKKKGLAIILIICMSNATTRLTAGNIIELSISRFGDVIKSSVVYLNMLRTFTI

>CbirOr15

MQGDPIQKTNSVELVHDHEEDMRLSIQLNRWLLQPLGAWPRSAGISSMERYTYTLVNVICTGLIGFILVP
TVVYMTLEIEDTYNTLKLSGPVSFFVMAIIKYSSLIFRENDIRKGIKYIENDWMNTRYQNDRIIMIKSAK
FGRRLVVICAFFMYGGAVFYYLALPFSKGTITEDDDNLTYRPLVYPVARVIVDARHSPISEIFFWVQCLS
GFVAHSITTGACSLAAVFAMHAYGRMEILIQWIEHLVDGREDLCGSVDNRLAMIVHQHVAILRFIALIDN
ILREISVVEIAGCTLNMCFLGYYTITEWGSQEPATYVTYIILLISLTFNIFIFCYIGELVTEQCKKIGEV
SHMIDWYRLPEKKGLALILIIAMSNSSFKLTAANLFELSLSTFGDVVRTSVAYLNMLRTLTS

>CbirOr16

MSQPLFGAVVTRNVMHQQDSEYSIQLNRWFLKPIGAWPRAGASISIMNKLLSRGIQFTCHSLIASTVIPC
ILYILFEPDVYLKLKAFGPMIHWLMGGANYCSLLSSSHKIRKCVDHMRTDWQMVERMSDREVMLRNARFG
RFVSTFCAVFMQGGVCSYSVITVLTPVIMQIGNITITMRQLPCPFYTDLVDVRYSPANEIVLGLQLMSTV
IVNSVTVGACSLAAVFAMHACGQLNILMIKLDELVDGTEGEFKTNHRKLAVIVEHHLRVLSFVSQIEAIM
HHICLIELLGCTIDICMLGYYTIMEWELHDTKNLLTYFTIFISMSCNIFIFCYIAEILTDQCQKVGEMVY
MTEWYRLHHKTAHDLILIISRSNTVIKITAGKMIQLSIATFGDVMKTAFAYLNILRTVAT

>CbirOr17PSE

MKCATHESAIDTYRRDNDYSLQVNRWLLKSIGAWPESSMKYSVTRNILKTVLRLTCHLLIAFTVVPSMLY
IIFEEKDFRLRLKAVGPFSHCLMGGINYCSLLHHGSHIRRSIEHMENDWRMAKREHDREVMLRNARIGRT
IAGVCALIMQGGILSYNLARGMSPIIEVIGNETVAFGRLPCPSFNMIVDTRFSPVYEVVLVFQFLSIIVV
NNVTIGGCGLTAVFAMHACGQLNMVMLRLHELVNEKREFLSRMETVIRQICFVELVGCTFNLCMLGYYTI
TELHEESANTIISYIMVLISMMFNIFIFCFFGELVMDQCKKVGEAAYMTNWYYLPRKTVFALILIIILRSR
ITVKITAAKVFHMSIATFGVVMQKDSLAYIAPYINSL

>CbirOr21

MCQSVIKDLHDYSVQLNRWFLKPLGAWPRSATTSKGERALSYILIFICFFLIALTAVPCALNVFFEERDI
VLKLRAIGPLSHWIMGAMNYCSLLLRSADIRGCVQHMQRDWQIIKHTRDREIMARNAQLGRLVAGFCAVF
MHGGVFSYNIVCGMTTVVVPIGDNRSVEMIQLPVPSYSKFIDARFSPANEIVLMTQVLSTFVVNSTTVSA
CSLAAVFAMHACGQLDILMARLNRLVEGEDSRTSESLQRRLANIVDHHLRVLSFIARIEDVMHQICLVEL
LGCTFNLCMLGYYTITSWNNIDIKGIVPYIIVYISMTFNIFTFCYIGEILTQQCKNIGETAYMTDWYYLP
HKTARNLILIISRSNVVIKITAGKLVQLSIATFSDVMKTSLVYLNILRTTILTT

>CbirOr23

MTKRNKSNSCRSNNDYSLQLTRWFLIPIAAWPRAAPSTVEKISLQAHVLACYLLIVVVMVPCFLYMSLEE
NDIQMKLNAMGPLSHWIMGTINYWLLLTRSNDIRECVRHMENDWRLVQRADDQWVMLRYARIGRFVAAFC
AVFMQSGTLLFQVVKAMTSMPVVVGNETVLVHPMTCPIYSKFVDTRFSPANEIMLVVQILSCFVVNSITV
GACSLAAVFAMHAYGQLSMLFSWLNELVADEDKGNEFADQKLAVIVEHHLRVLSFISRMESLMQHICLVE
LVGCTLNMCLLAYYTITNWSNFDAGRMTSYFFVYVSMAFNIFIFCYIGEILTEQCKTVGEKAYMTDWYKL
PHKTARGLLLIIARSSNVIKITAGKLFQLSIATFGDVIKTSVIYLNLIRTMTST

>CbirOr24PSE

MTQVKCKPVDIAYENVNDYSLQINRWCLKPIGAWPLKDSAIEKTIAFIQVAACSTTIAIVTVPCILYVLL
EDNSFKMKLSALGPLLHRIIGSISYXTLLKRSKDIRDCIQHMQADWRLVRRVQDREVMLQYAKFGRFIAG
IGAIIMXGGQLLFGTTRAIKTVKITVGNQTFTTHPMTCPAYSRIIDTRFSPMNEIMLVIQFVSAFAVSSA
IVSVCSLAAVFAMHACGQLNVLYAWLNDLIEEYEMGDRSVDQKLAIIVEHHLRVLSLVTCMESIIHKVCF

VMLAGSTLNMCLLGYYVIMCPILHYIYLVSFNIFIFCYIGEILTDQSKNIENMVYMTDWYXLPHKTALCF
ILIIMRSNYTIKMTAGNLVYLLIATFGDMIKTSMAYLNLLRTMTT

>CbirOr25

MTNPKHVASGKLDNDYSLQLNRWFLKPIGAWPCIVSRTTWTIFLPLQIVISWIIVAIITVPCLLHVLFEV
KDIQTRLHVIGPLLHRLIGWVNYWTLLNCSDDIRNCILHMETDWKTMRRTEDREVMLQYAKFGRFLAIVC
GLIMHGGTLLFSIVKSIKTITVTIGNETFLTHPMTCPSYSKIIDTRLSPVNEIAIVMQFTSTLIVSSATV
GACSLTAVFAMHACGQLNLLYSQLNKLVEDQEKENYIAERRLASIVSHHLRVLSFISRVESIIHKIALVE
LVGSTIAMCLVGYYIILDLNALNKAKLISDFIVYVSLAFNSFIFCYIGETLTEQCKRVGEIAYMTNWYKL
HHKTALNLVLIIAQSNNVIKLTAGKLFQLSMTTFGDIIKSSVIYLNML

>CbirOr26

MKSPPQDHLITPYFSYADDTEYSIQPIRWLLRPISVWPVSSIKERILSAVLLIVCVFLIAGTLVPCALAI
FLDQNKDVETKVRDFGPLSNWVLASLKYCSLLSHVGDIRRCLEHVDADWRAVTKHEDREMMLRNAKIGRF
IALFAALFMHSGVFSYSIFQAMTMNESVLGDNASMHSLPFAFYDKILDTTTSPTYEIVFIMQFLSTFVVN
SVAVSTVSLIAVFVMHACGQIKILISLLENFVDETSEKWENSTQGKYAVIVEHHLRVLSFVSYIDKITNI
ICLVEVMGCTLHMCLLGYYCIMDWTQGDKESIVSYVIILISVTFNIFIFCYIGEILSEQCGQIGDTAYMT
NWYLLRRNNAVGFVLIIILKSNVVVKITAGKMIELSLSTFGDVIKSAVAYLNIFRTLVT

>CbirOr27

MRHQKQTTFITSAEDGCKYTIQMTRFFLRTIGVWPLALCETRREKLGCAIVAAISSFLLLFLFIPCILCT
FLMKTDLITKLRFGLGASTYMLMAVAKQYILLTRSKSITECIIQIRKDWDRIMLARETDLEIMLDNVKFG
RRLSIVCTVSMYTAHVLRNVLPFVKPRTQIIGNETVRLLLYPVYRGLFDVRNSPYFEITQIVQMMGGYVI
YTLTISACSIAAVFVMHTRGQIRILMLKMDDLADGKKRQSACNTTSAQRLGDIVRSHVSILSFVARTDDL
LNKMCFADVFGYTFLICFLSLYTMTVWEQRKTIETITFFLIVTCFTFNTFIFCYIGDVLAEECLEIGIKS
YMIDWYRLPGKEGLGIMLMMQMSNATIKLTAGKFMDLSLASFCSIMKTALAYLNLLRTFT

>CbirOr28

MGDNTVDNPVNVSPTYLADNEYSIQIIRWILKTINLWPQPPGASIIKKIRSEFLILTCYCLMFGTMVPSG
LSIFIESQETLDVKLRSFGPLTFWFMAMINYSCLLLHIDDIRSCVEHMKADWRIVRRSEERQVMLKNARI
GRFIVGFCAVFMHSGVLTFNTIRGLSKDVIYTENSSVLVRGLPYPFYSRILDAHYSPAYEFVFLLQCVST
FLFNSVTVAVCGLAAVFVMHACGQLRILMSWLDNLIDGNERNISVRRKFAIIVEHHLRVLNFTSRIENIT
NMICLVELVGCTMHICLLGYYCIMDWVHDNKQNIVSYCMLLTSITFNIFIFCYIGEILSEQGGQIGRSAY
MTNWYLLPGNTALGLTMIIILRSNVALKITAGKFVQLSFVTFGGVIKSALAYLNLFRTFIM

>CbirOr29

MYDQCQLANKIQVYNVHHQRDIRYATQLSRWILKPIGIWHLIYARSSRVEKLFSVTMIFVCFAVLFFVLV
PSSPYVLFSKNNISTKAKLLGPVLFCLTSAVKYYFLGTRGEMIGRCVNQMEDDWRIVRYQDHRKMMLDNA
LVGRRLTTLSVIFLYTGGLSYHIILPFSTKKGLNNTFTNKPLIYQGYDVYFDSQASPAYDIVFSVHCVSA
VIQYNITTAACSLAAIFVMHVCGQVQILMTMLGDLVEGERDKGTSVEERLGVIARYHVRVLRFAVDVEEV
LREMCLMELVAATFIICLLEYYIMTEWENSDVLGIATYFILFISLSFNFLIFCYIGELLVEEYSKIASAV
YGINWYDLPGHKALHLVMIITMSQYPPKLTAGKFVDFSLSTFSTVLRTSIVYFNLLRTVT

>CbirOr30

MRDRSQLPKRVSEVHNAHYEDDVRYTMQLCGWVLKPIGMWYLIQDSCSRNEKIVSIALIVVCFSALCFVL
IPSGLYLFLVKQDVYALIKLLGPVIFCLTSTIKYCYLGTRGAAFGRCLRHVTNDWRLVRDQDHREIMLRG
ALIGRRLTMLCAIFLYTGGLSYHTILPLSSKQNISENFTSRRVPHTYPGYDLWFDPEASPAYEIVFCVHC
VFALITYNITTGGCGLAAIFVTHACGQVQILMIQMSDLVEGKRSKGTVQNRLSVIAKHHVRVLRFSASVD
EVLREICLMELLASCLTICLLEYYCIMEWENSDAVAILTYFILLISFMFNILIFCYIGELLMQQYSTVAS
AVYGINWYDLSGKKALDLKLIIAMSRYPVKLTAGKFVDLSLNTFGAVLRTSVVYLNLLRTVTEYSX

>CbirOr32

MAKIVNIPWSNVNCEGDIVNTLIWNRWLLRVLGIWPLVYPNTTKIEKIIAMFSFVLCWIVLSLFLVLTSI
YTFSERSIMREKMKMLGPLGYVFFSMLKYFFLVARHKSIRQCVQVLSADWRRVEEDDHREIMMRGAEKGH
LLSKFCIAFMYCGGLSYNTIMPFLSQTSEQNVTVRPMAYLGFDILFNLQLMPVYVFAFCLQCVTGIVMFN
ITTVVCCLAAMFVAHACGQVDIVLARVKSLVKGEKRNRVQFEQCMAIIVQHHVRVLRFSANIEDTLREIC
LVEFVGTTLIMCLIEYSLITEWNNSDSIAILTYFFLFVSFAFNIFMFCHIGELLTEQCIKVGYTSYKIEW
YELPGKAALDLMFMITMSRHPVQITAGKLISLSFTNFGNVLKTSVAYMNLIRTAL

>CbirOr33

MSNARERCATSQNSNYQRDIRYAFKLNSWILGSLGIWPLTIRNIGQYVSNITIVFYNFSFVFAIVPFTLY
IMYDAKDINAKLRSCGLLAFCLTAMIKYLILAIRRPKIQLCIEYIKYDWWQVTFVSDREMMLKYAVTGRN
LTIIGALFMYGAGIIYYAILIPINTIKIITNQTLRPVVYPIYSQFGDVQNSPKYEIVYAVHCVCGYTMYT
VTTGTCGLAALFVTHACGQIQMIVSRLQNLLDDKNLEQTQNIHQRIVAIVKRHVRVQRFAAVVEEMLQEV
CLVEFASSVCTICLLEYYCILDWQKNDKVGVVSYSLLFVSFCFNVYILCHIGELLMEKSSQIGSVCYMIN
WYQISPKLVRSIIMIIAMSSHPTKLTAGGMVDLSLSTFGNVMKTSIAYLSFLRTLVMX

>CbirOr35

MGSSRDVHAISPNVSYQEDMRYIFKPSSWILGSIGIWPLTICGAGHHVSKIAIVICNFALSFAIVPCILH
IIYDEKNLNIRLKLSGLLGFCITAMMKYCVLTIRRPDIQRCIEHVKSDWWQVKFSSDRELMLKYAGTSRR
LSTISVSSMYIAGFIYHLILPFCNVHKIDNQTIRPLVYPTYSEFHQSQISPIYELVYLAHCMCGYTMYTV
TAGSCGLAAIFVTHACGQIEVLASRLEDLSHGQNFGPSLDVNQRIAAIVESHVRILRFSAAVRELLQELC
LLEFASSVFTMCLPEYYCIVDWQDSDTVGLTTYFLLFVSFCFNMYILCYIGELIVEKSSQIGLMCFMIDW
YQLPAKSVRSLVLMIAMSSQPIKISAGRMIDISLATFGNVLKTSLAYLSFLRTLIM

>CbirOr36

MFSRGDIVEKLTLSVHRLMLSCVGIWPVKNKDLFMDLRWIIAVFLEVTPMCVYFIEIYLHCNGAKKSFNR
VTPCAAAALALTRLITPRIYREKLLEIVTSMMDDWSMQQDKKVRWVMKKYARMSTCVTMLTFLLVGTIVG
IYIFLAISAIIMKIGNDIDGLNASQEYEVESCVFHSAASNEAFLVAQAMQMFVTGILTFGSTSFFFGLAM
YLCAQFDALGIKLSEFQIGKAQQMITEVIQRHCHLIRLCDCMEESFNANILMYLFVTTTLMCIDGFMLIV
SLRLGDLSMIIHSVTVLLLMMIQLSFYTFAGDCLEMRSTALSYAIYDYNWYQLPTGIAKNFQIMLMRASI
PHQLTAGKFVSLNMLTFKDILKSTVSYLSVLRVMMNE

>CbirOr37

MKEVKDWSDEIYRTLSVYEKVLSVIGIWPLNAGDVKSIVRCLLAILIQISTISSLSLEAYRQCLGTEDMM
EAFLMDLSSVVSLSKLLVVRLTWRHTYFLVTSLVDDWSISRNTRQREVMMKYTNVGRIVSLTMLYLGYAS
GVSFLFMAVPFDKLIPWLNASAPNENSTVIPTYFLATYCVFGSLPTIAHSCVLLLQVAQIFVNATSHCGN
DGFFFGLAMHLCGQFEVLQMDFADIEVEKQACKRKLRMLIGRHCHLIKLADSLEYAFNMAIFAQLLMSVL
LLCVEGMQLIISLKLNDNIAALKHVVLILTMLVQLYLYCYAGDRLESITGTLAHGVYETPWYDFDTKIMK
NLPMVMFRGKVPHQITAGKFIPMNLFSYKEILKATGSYLSVLRVMIDT

>CbirOr38PNC

DAFLTESSSIINMANCFCMYELETQATAEICHLQLDYYKEPLLARYHTEVAHVDRLGSIIFFSLSCVSLV
LFASTLMLANINFPXTSEKQIYNQTYEKKLILTAYCFFGKYISSSVTYCFIEVLQLLQVSVNFTSQCGHV
GFFFDLTMHMCGQFKILQMELAEMGRDCRNKFDILLKRHRFLIHLAYHLERAFTLVILAQLFISMFLLCV
E

>CbirOr39PSE

PNCIFGSYTFFAYRFIKVLQTLRILVTCISRCRNDGFFFGLTMYFEVFRMDFAKMDCDKFFYRNEFNILL
KHRLIYLAHHLQKAFSLVIFLTLIRFTILYILGFQLILYLLMHDTAMKHVLYNVVLLVQLFLSSSTDIGV
PISRTGLCDLXISKRDEGFIIDNFPNINHHHRLTGGKFLTINFMSFKEILKTSASYLYLG

>CbirOr40

MLDKRWNDDIAYAFSIHKLYLEFYGMWPLQTQTVFTKIRWSFYLIIHFTMLPFVMMGLIGNSGTNANLES
MVYLTSTLSGIIKCICIFLGQKKLGPNINAAIDDWLSLKDDEIARKIMRKCAHRTRILTIVLLYSAYTCL
SIYILSVVVINVKQIFFMDSNLVDADTNVTGWKFLIPSGLLSSSITGSQYVMILIVQSIQTFLICATQCL
VDSFFFNVATHLAGQLEILKHKFKIFANKRDTETNYRKKFVDLINRHNELMEFNQNLEDTFHFLILCQLV
MVTIMIALLGLRINLCLNENNQFEATKSSLIMNYLLMQSLVYTYSGDFLQRESEDIFCALYATSWFTLPV
ALMKDLHFAMMRSSIPFRLTGGKFFYVNRETIMCIIKTAASYVSVLRIALKN

>CbirOr41

MLDDHWNDDVAYVFSMHRTLMQVLGIWPLQKKTVFQMMQCSLTNLLQWASFIFLFMEFSGNYRGAGASIE
TILFFTCLMSTAFKCFCVVLNREKLARNINAAIGDWFLAKNDEETYKIMKKHAFTSKLCTSVIVYLTYIC
TASYISVVILMNVKQMFLQDVNVSNGSDSNTSNWIFLIPSGMLSSKLTTLQYTILYILQNLQIIVVCVTQ
CITDSLYISIVLHLTGQLKILKMKFKAFASKPDTEINYRKQLISLINRHCKLTELNQNIEDTFHLVILLQ
LTIATLLLALIGLRTIFCLKNNDYVELTKTIITLNYVFMEALVYSYGGDFVQRGSEDIFHAIFTASWYTL
PATLMKDIHFVMMRSSYPFHLTGGKFFNVNRETMVYVLKTAASYVSVLRIALKD

>CbirOr42PSE

MRTNWNTGIDYGFSMIRKMMWILGVWPLQQNSLVCTFRWIMIFIVESLTFINVLIESFKSCGDVKDALEV
FLIIETLLHAWTNTIFMRIYMKKIVININSAINDWSCSSMRKESYLIMIAYARAGRIVXXXXXXXXXXX
XXXXXXXLLXFLSAVLYFTSIIVGNKQQVIIIDNSTVVLWNFVFPSTCLFKGISYSTYKILFVMQIVQAF
IIFIAECANDSFFFAITMHLCGQLELLKARFVELGRKIEKKNHCRNVLGPWIRRHYQLIIILARNIEDAFN
INILIRLSITTIVIAISGMHIIMSVRHNNYTDVIKSIIFIQYFILQSFLFTHAGDTLQNQSESIISAIYS
ITWHKLSSTTMKDLLFIMMRTKIPLQLSAGKFFYITRSTITNILRTALRIQCAISYTILLSLNRIIIFK

>CbirOr43PSE

MKTNWNDGIDYSFSAIKSLMWLLGSWPLQNDATHTIRWFIIFTAGSLTMINVLIEPFKSCGVPRDGLESL
RMIESCLHSWXNIIFPRIYVKKITINMNAAIDDWSSVSLKKESRLTMMAYARIERLVSLTNLTLGGVGFV
LFFASVFLSNKPKVATNDTTWNFVLPSTCLYKGVSYSMFKALFAIQVIQGAIIIISECAYDSFFFNIIMH
LCGQLELLRIQFLAFNQKHNDKTHNGNILRLLVKRHCHLIALTKNIEDAFNINILLRLLIISVVIAASGI
GVILSLKNHDYKEVMKMLISIQFYMIQTLLCTYAGDSLQNQSEAIIHAIYNSTWHEMPPVMVKDLILVMI
RLKTPLRISAGKFFYLTRSTMTDILKTTVTYISFLQVTVDV

>CbirOr44

MARWKDGKRWKDDIAYAMTPFKLITWPIGVWPLQVYDVYSLIQCVWGTFCSIRTFQSLLVILPCIELCMG
CTDTDRNIDCLMLICCGILGMQKTIWFRIYGNNLANNYNSAIKDYVTIENAKQRSIMRRHAFMARLLCCF
LVSFSYCSCIIYALIPLLGDNKNNQINKTSDDSVLEYPMPSRCALEFFEVPTSMYRIFCLIEAIALVMTS
TCNHGNDTMFLNITLHICGQVEILKTHFINLNLSNPNFNDHLNTLIQRHHHLIKMAKKLAETISSILLTQ
LFISSVLICIMGFQFILALKVHNVVMMEKSVIVQSTFLTQLSLYSFIGDYLKSQMEEVGPSIYQSSWYRF
PAEMMKNLVFIIMRTETPVMFQAGNFIVINLSTYMGILKTSVSYLSVLRVMVEI

>CbirOr45

MANEQWNHDIAYAMTPFKLITWPIGVWPLQVYDVYSLIRCVFGTCCSSLMVILPSIELYMGCTNAEQNVD
CLMLICCGMLGILKIILFRIYANNLIDNYESALNDYLNIENVKQRTIMRRHAFMGRILCFFMMSFSYISC
VIYGLLPFLGDDDSLSINITNENSVREYTIPSRCALEYFNASNSIYRIVCFIETIALILATSANIGNDAM
FLNITLHMCGQVKILRTNFMDIDVTSSRAYDRFNILIQRHIYLIHIVKQLAETISFILLTQLFISSILLC

IMGFQLILSLKVNDIVMTTKSFSVLSAFLSQLTLYSFIGDYLKSQMEEIGLSIYRSSWYDYPRKLTRNMI
FVLMRTESPIALQAGNFIMINLSTYMSILKTSASYLSVLRVMIEI

>CbirOr46

MAGERWKSDIAYAMTPFKFLTWPIGIWPLQVYNIYSLIRCGLATCCMSIIVILPSMEFYLGCTDAEQNID
SLMLICCGILGVLKTIWFRIYADNLTINYGSAVNDYVTIVNVKHRSIMRRHAFMGRILSCFMVCFSYVAV
LIYSFIPLLGDEQDNVVNETNEEAVLEYPIPSRCALQYLNAPMSMYQIYCFIEFIVLLITCTCNHGNDSL
FLNITLHVCGQVKILKANFMDFTVTNLQVYDHFNALIQRHNYLIQLAKKLADTISFILITQLFISSILLC
IMGFQFILALKLNDAVVMGKSLTVLCTFLTQLSVYSYVGDYLKSQMEEVGLFSYQNTWYNLPEKIMKNLI
FIIMRAHYPVKLQAGSFIVVNLATYMSILRTSISYLSVLRIMVET

>CbirOr47

MAWENDVAYSMTPIKLLTIPIGGWPLQEYNNFALARHVLSTCGLSVIVIMAYLELYYNCTNAFAQIDALM
ILVCGILALLKITWFRIYADNLISNYASAMRDYYAIDTEDKRTIMRDHASMGRIISIIALAITYVDSVIF
IVGHMLASGPDNQVNVSVWGHQLGYALPSTCALAYFHISTGSYWIICILQYVYLMITCISNHGSDSLFLH
IALHVCGQLKILKVHFTNFNVTNPKVYERFNVLILRHKHLIKMARKLAETISFVLIVQLFISSVLLCILG
FQFIIAMKTSDYSMMSKSFLVLSAFLAQLAAYSFVGDYLKSQMEEVALSIYYCAWYNLPAKVAKNLVFIM
MWTQLPIKLQAGNFIIVDLVTFMSIIKTSISYLSVLRVMLNT

>CbirOr48INT

MLWNDDIAYAMTPFKLLSWPLGTWPLQEYNKCSLVRSIVSISSLALMIITCGTLAVMKISCFRIYANNLT
RNFTSAVNDYLAIDTEKKRTIMRQHAFIGRVICCSLIFFAYIGSVTFGLVALFAGDKNQVRLNVSKDAAY
PMPSTCTLANFHISTSVYLLIFVVQYILLVLTSTGNLGSDSLFLAITMHVCGQVKLLKDEFMNFGMENKN
INEEFVKLTMRHRHLLDYAELLADTISFVLLAQLLISCILICITGFQFILALKVGDLVMITKTVMVLGCF
LSQLFAYSFVGDYLKYQMEEVAHSIYCCNWQCLSVKFMKNVLFVIMRSQQPVQLAAGKFIVVNIETYMTI
LKTSLSYLSVLRVMLD

>CbirOr49

MDSCKTWNKEMTREFFPYKRIMWPVGSWPVDSSKNFAKLRALFVTVTLALMVAYLSMEIGYHDGANLNLI
IDHFTLITIGTLTIIKISIIRLHRDDLSKYMCNAASDWIYVAQRDHREVMLRYANLGRFVFFSQMCSSYI
VIIPLIAESLLSFVTMSSLQNITLSMSVEEMRVIKLPHDMICPFNAQVACFGICIVQAVQLISLATGNCG
SDVFLFGICMHLCGQLEILGLQLSRFHEGKGNDSWGKTKLVALIERHCLLLNLAKDIVDTLDIILSAQLI
FQASLICIIGLQFIVSLAISDFFLVVRSILSFGILMIQLFLYTYVGEILSLRTQAISTAIYLSKWYDLPI
NITRDICFIMFRATYPVYIRVGKFYNMDLNTFKTILKASASYFSVLRIMFTQY

>CbirOr51

MACWNKNAAYEWSSYKTLAWPVGAWPIEDDTLYSRMRWSFAIISEILLVAELLIEVYLACENSAENPIDT
YVVTASAILVFVKLILLRIRRSTLSLNISSAIQDWCSIKDTKSHEIMIQYARMARIISLSLFYSGFFAFI
LYMLRLLPLVSGTNERTFYLPTSCLFESISTLQYIFLTLYQIIQLFITYAGNCCTEGMFIGVTLHLCGQL
QLLMIDIRRIDWRNKRGNIVEKLVVRHRQLIRLTETIEDSYNIIILAQILVSAILICVTGFGFIESLHIH
DTVMTVKSAVIMFVMLLQSFLYSYAGDNLRDQSDALSFAIYDCNWCNFPPNDVRDLAFIMIKTNIPIRLT
AGKFFYVTLTTFTDILKTAVSYLSALRVMTEKQAVNK

>CbirOr52

MNAKKDLNYAFALSRRWLWLLGVWPDPNASVSNFHRLNMRFIIVMCILSFYVIMPQLRNIICAGGNIGRI
VECIASVNFSFMALSKLYVTWYHNETLRTLMTSVLTDWMTSRNSRERNTMLKFAKSGRNLSFQCYIYGTI
LFMFYILLNLQRMYQNIMLQQQQRKLVYHFNYPYNVQKRPNYEITYLIQISGGTYLAVINCSVDSFILIL
LLHMCAQLINLRVALNDLVNDLANHSISSSKFKECLAAIAMRHEHIIRNVKMINDCYSTVLLIYVLTATF
QICFETFQVFTIITDHLEISALKMFFLLSYATFVIMQLYLYCHSAEKLLTESVGMAYGAYECKWYELSPN
DAKNLMFIVYRSKIPLQLTAGKFGTFSMEMFIMTMKTAMGYLSAMLAMRN

>CbirOr53PN(F)

MPMEFAMGWNKFNLTLLGVWPEPTKTSRTMRCISSFIFWSSSFITFVFICVFIRLPQTAHLILESTSLNK
IVENLLINIPIIFAWTRQLVLWYHGKALTTLVGQMLNDWTQPVPEPNRRLMLRNARLSRHVSLVCSVLTY
IMVSSFIVLQIWANAQNAAETDLGGLIHPATSPYDTQKSPNYQITWLGQFIGTVLTAICYSCFDTFLAVL
VLHLCGQLTMLKTRLRNLVDATRGDNFFAYQDRLSFIVQKHEELYKFAILVEDCFNLTLPGQTMISTATF
CLTGYRIIISMSQNKTELPVVAMVFFVVHVTYTMLHIFVYCYVGEQLLIESTGLAQSAYDCEWYELPSKK
AICLIIIIIRANVSFKITAGKFSSFSLELFGTILKTSAGYLSVLLAMSDRLNEEQ

>CbirOr54NTE

XLNFAIGWNRFNLGLVGIWPGPLQNIKWISGLSRCRFLIASFFMLGFMCIPQSTNLIFIWGNADMMTENL
ATANIPVANAFMKAFVIWRHRKALRLLIDFFYEDWHAPKTLQERATMLRSAKLVRKISIWCSVLTQTMVT
IYIVLRLCLIAKWKSDLERPLVYTAYFPFNINRSPIFEVICVCQIISAYSATFSYTGSDSFISMLVLHTC
GQFENLHAKLKNLADDPHDAKTVEEFRRELTWIVKRHEHLNWFAKTIEDTFNVVFLIQMLSCTIQLCFQG
FQVFRILISEEQDESLTFQLIFLTSFIGFVLAHLFVYCYVGEILLVQSTGMGFSAYESNWFNIPGKEARN
LLFIMHRSTIPLCLTAGKFGTFSLQMFSTIVRTSLGYLSVMLTVTDRTQ

>CbirOr55

MSTEADLEYGYGWNRYTMTFMGLWPENRNLGRISSYRAMVPILMMLCFVCAPQSANLPFIWGDFDLVIEN
LSMGNITITISMLKTVVFWSNSRHLKALVTCMTKDWNLTVDKRDRKTMLDVARITRSLSIYSTLLCQTVL
VTYVAMRCYMIRQIGRQMFFRGYFPYNATVSPLYEFTFVGQILGATYAAITYTTVDTFIATLVLHTCGQL
SNLRRELTDLRAGTSEEFQARLGKIVIRHEYLNRFAETIEDNFNMMLLMQMLGCSVQLCCQCLQAFMSVV
SGEINEFFIFQFIFLVPYVIYVLLQLYLYCYIGERLLVESTQIAYAAYDCSWYDLSAYEVRSLIIIIYRA
RSPLTITAGRFCSFNRELFSEVLKRSVAYMSCLYAMKGTQRNEN

>CbirOr57NTE

LEWAIGINRFTLKILGLWPDERLNRQQRFLANLRAFIIFTTMILVSVIPAYISLMRVWGDMMAIIDNLQV
TLPCSIAALKIIIMWSRKEDLTPIVNMVVADWIRTKTEEERDTMIRQARIARWIVVFGCIIMILEIIIIV
IPPCFGYSMRYLTNITDKPGKPLLLQTYYLRDTTESPYFEIVFVAQAIAVVMCAFSYTATDNFMGIVIFH
ICAQMEILKARLLNLQGFKDFNVGLSTNVQDHLRLIRSVDVIENTFNLMLLALLVYFTILFCLQGFLIIS
IIDEGGNVSFPRICWLVSVLINTFVHMCLYCVVGETLIAKCEGIYYAVYDYAWYLLKPKEARSLMLIMIR
ADKPLYITAGRIFPMTLSMFCSIIKTSAGYISVLLANR

>CbirOr58NTE

LEWAIGINRISLKIIGLWPDDKLNRWQRFLADLRAFTIFITMILASILPGIFALVRVWGDMMAMTDNLQI
GLPFSVTAMKFVIMWIRKKELMPLINMIVEDWIKVKTEQEREIMIKQAKTARMIVMFGGIMMTLASIILI
IPPCFGYSMRYLTNLTDSGKPLLLQTYYLRDMTESPYFEIAFIAQATSIIVAAISYTGIDNFLSVVIFHI
CAQLDILKGRLLNLNTFKDFNSGLSFNIQDHLRLIRSIDAIDNTFNLMLLTLLVYFAILFCLQGFLIIVD
GVGGDVSFMRICWLVSILINTFAHMCLYCVVGEILIAKAEGVYYATYEYAWYSLKPNQAKDLMLIMIRAE
KPLYITAGRIFPMTLSMFCSLIKTSAGYISVLLANR

>CbirOr59PN

ASIIKMIVEDWLKSKTIYEKNAMMKWALRARIIITCTYLVIVLAFILLFGMPIIGKSMALTPNITDSGKS
MLMPTYYIYDVTKKPPPQYELTIISQFISLSTIAILYIGVDNFFGLCVFHICGQLNIMRSRLKHSHGNFR
TVLKNSVIYHIRLLRAINTIEDTYSVILLILFVYFGIMFAFCGFLLITLFEVEENNVSFTRLLFLFLIII
SMLVHMGIYCAVGEALISECNGIYYAVYDFEWYYLDPKEAKDLIPFIIKVSEPVYFTAGKVFPVTMAMLC
NLIKTSAGYISVLLATKRN

>CbirOr61NTE

XFEWAVQLNRFSLKLLGLWPEEAASLQDRLNSNLRLFGVFILINGVCTVPSLHLLLKIWGDINAMMDNFV
FTLPLLTVSVKLLILWWKREALSSLMTMIAHDWIKSKSKDERNVMIRCAQNARLIITFSYIVIFCSLIIL
IISTAFGYTVTHVTNITDTKKPLPLQGYYIYNTSVSPQFELTFFGQCISLTMVALSYTSTDNFLGLLVFH
ICGQLENLTSRLYQMRESKDFAMALRVNVTDHTRLIRSVAIIEDAFTLMLLFLFLYFGSMVCTYGFLVVT
VIIEEKNVSIKKTIFLIMVICTAFTHMCVYCAVGELLIIKYEEVYNAVYEFEWYTLDTKKAKNFIFLLVR
MNKPLYITAGKMLPMTMATFCSLLKTIYQWHILIKVEIN

>CbirOr62PN

AIELNRLSLTLFGVWPKNNETKQNKLMSDIRVIILLTVILWICIIPSLHSLLKSCDDIMLTIDNLQITLP
LLMAVMKLHNIWQKRYDVLPLLNMIKDDWLRPKTSEERNVMIKQAQIARILTLLGCCIVEISVIIASILP
ICGISMIYRTNRTDPGXLFPLQTYYLYDVNNSPLYELTFVLQSFSMVLAGIMYTSTDTFMSLLIFHVCGH
LENLKARIRNLGQFNDFPDALSANVKDHIRLLRSIIIIDDMFNLMLLGLLVYFGMLFTLYGFLFVSITTQ
GRNLSMARLFLFLLGFINTFGHMSLYCILGEILVIQCDGIYEAVYQYEWYKLKPWQAKDFLNIMIQSRRP
LHLTAGRLFPMTIATLCNLLKTSGGYTSVLLAQHH

>CbirOr63NTE

XALSFFIKIEIRLKTLLGIWPENHETKQKKLMSDIRVIITLNLITWICLIPSLHSLLKIYDDIILTIDNL
QYTLPLLMAIIKLSIMWHKKNDILPLLNMIKDDWLRPKTSEERNVMIKQARIARILTIFCCCIMLISAIV
ILVIPLFGISMRYRTNRTDSDKSLPLQTYYVYDKEKSPFFEITYVLQCISATLSGFMYSGTDSFLTFLVF
HVCGQLENLKKRVIDLDKFHDFRNTLSYNIQDHIRLIRSINIIENVFTIMLLGALLYFGILFAFYGFLFG
TMFSQGRNVSVTRLIFIGIVSFTTFTHMCLYCVVGEILLAHCEAVYDAAYEYNWYTLEPKKARNILMIMI
RANKPLYLTAGKLFPLTMAMFCNLLKTSGGYISVLLAHRE

>CbirOr64NTE

XYEWAIEFNRFNLTLLGIWPENNEDKQKKLKSDIRVVLILNIIMWTSIIPTLHSLLKIYDDIMLTIDNLQ
YTLSMLMALIKLIIILWHKKYDILPVLNMIKDDWLRPQTSKEKNIMIKQARIARTLTIFSFFVTLMSGIIV
SFLPLFGISLRYRTNRTDPGRLLPLQTYYLYNVNSSPLYEVTFLLQGFSVMAFATMYSSMDTFMSVLVFH
ICGQLENLKTRIRNFDKFNNFANTLATSVKDHIRLNRAIIIIDDTFNLMLFGLLVYFGILFALFGFLFIS
IITQGRNLSIARLFLTLIAFTNSFGHMCLYCALGEYLVMRCDGIYDAVCQYEWYRLKPKQMKNFLNIMMQ
SRRPLHLTAGKLFPMTVATLSNLLKTSGGYISVLLAHRT

>CbirOr66NTE

XFEWATKLHRISLELIGLWPNSELNFRKKLICNLRVLLAFLMITIGILVPSIHSLIIIRSNIELAIDNLQ
CSLPVLTVVIRIVLFWWKKEALVPIINMIMEDWLKRKSVYERNTMITWAFRARIIITCAYVSLGVAYIIL
IGVPIFGKSMRLTSNITDPGRPMPLQTYYIYDITKRPQFELTFLSQIISCIFALIPYTAIDNFLGLLTFH
ICGQLIILKNRLIHINEQKNFHEELKDCIICHIRLLRAIDTVEDVYNIILLVLFLYFGTLFAFYGFLIIS
LFQDKEHEVSFARMSFLAVIITSLFAHMCLYCAVGEMLMAQCNEIHFAVYNHEWYFLDAKEARKLIPFMM
RAGQPIYYTAGKVFPITMATFCNLIKTSAGYISVLFTGN

>CbirOr67NC

FEWAVKLNRISLEMIGLWPKPEKIRRDELKRNLYVFVVLLMLVIVGLFPCTLSLLRIQQNFTLIIEQLQF
LLPLVTCVIRLVIFWWKKEAVISIMNMIVEDWLNSKNIQEKNIMITWAQKPRKIIMIAYSIMGISYACVV
VLPALGISVTYTRNATDINKILPLQGFFIYDATKTPLYILTYIAQVITFFFAVMAYTGIDNFLGLLVFHI
CGQMEILMARFSCLSKLTKFRNGLESCVRNHIRLLX

>CbirOr69NI

XLEWAIKLNRISLEVLGLWPKVEEVPREKLKRNLHVLAILLLFVFGVLFPCIHSLIKTHEDIMIVIEHLQ
GILPIITCVVRIIVFWWKKEDVIPIISMITEDWLKSRDVQEKKVMITWAQKSRIIVISSYIVMGVTSVCG
VLLPIFGISPTHGVNINDTEKMFPLRVYYFYDVTKSPQYELTYISLSIGILFAAITYTGIDNFLGLLVFH
ICGQIDILSTRLIYLNKFVKFHNSLKNCIWNHTRLLRLVEERNYLSVTRILILICLVITILVHMALYCAV

GEVLIIQCDRMYHAICSQKWYNLDSKKAKNLILLLIRTKVSCYITAGKVFPMTMSTFCNLVKTSAGYISV
LLTTKNT

>CbirOr70NTE

LEWAIKLNRISLEVLGLWPKVEEVPREKLKHNLHVLSILLIFVLSILFPCIHSLVKTHEDIMIIIEHLQC
ILPMITCIVRIIVFWWKKEDIIPIISMITEDWLKSRNVQERNMMITWAQRSRIIVISSYIVMGISCFCGV
LLPIFGLSLTDGVNINDTEKIFPLRVYYFYDVTKSPQYELTYIILTIGIFFTATTYTGIDNFLGLLIFHI
CGQLDILSTRLMYLNTFMKFHNDLKNCVMNHTRLLRAIAIIEDTYNKMLLALFLYFGILFAFYGFLIINL
VQERNYLSITRIISLMCVVITILVHMGLYCAVGEVLIIQCDRMYYAICSQKWYTLDSKKIKNLILLLIRT
KIPCYITAGKVFPMTMATFCNLVETSVGYISVLCTARNNDLKA

>CbirOr71PN

LEWAIKLNRISLEVLGLWPKVEEVPREKLKRNLHVLAILLLFVFGVLFPCIHSLIKTHEDIMIVIEHLQG
ILPMITCVIRIIIFWWKKEDIIPLISMITEDWLKSRNVRERNVMITWTQRLRMIVLASYILTGVAFVSVI
LLPMFGVSLTYGVNINNTEKRFPLRVYYFFDVTSRPQYELTYISLSIGLFFAAVTYTGIDNFLGLLVFHT
CSQLDILSTRFMYLNKFVKFHNGLKNCVMNHTRLLRGIAIIEDTYNKMLLALFLLVEERNSVSITHVTSL
ICVVFVIFVHMGLYCAVGEILVIQCDRMYYAICSQEWYTLESKKARNLIFLLIQTKIPCYITAGKIFPMT
MATFCSLIKTSTGYVSVLFTTRNN

>CbirOr74CTE

MTLVMDTSERSGYRDFEWAVKLNRFSLKLLGLWPMTEDNVPENIMYKSRPLIIILILTFGFLVPSIHSLI
RIRSNIMLLIDNLQYTLPTITCAIRVVVFWWKKEAVTSLLNMVAEDWLKIKSAQERSKMIGKAQTARIII
TCAYGAMMIAFIFTGILPIFGISARYLTNTSDPDKLLPFQTYYLYDVTKRPQYEFTFIVQILCMVFGVMS
YTGIDNLLGLLVFHISGQFDILRNRIMHMNDYIDFRDSVRSSVIDHTRLLRAIAIIEDIFNIILLALFIY
FGILFACYGFLIINLIEKENDISVTRFIYQVCILINTFGHMCVYCAVGEILVAQCEHIHYTVYNYKWYTL
DPRNVKVLLFLMVRSSKPTYLTAGKIFPMTMSTFCS

>CbirOr75PN

FEWAVKLNRAGLTLIGIWPDPEESPREKVMSNIRVFVTFLLVISLLVPSIHSLIKTHSDIMLTTDNLQYT
LPALNTIIKFPIFWWKKKALASIVKMIVEDWLKSKTIYEKNAMIKWALRARVIITCTYFIIAMAFIIFIG
MPMFGKSMTLALNITDSGRSMAAPTYYIYDVTKSPQYELLVNSSSRTVTIVYTGIDNFFGLCVFHICRQL
HIMRNRFKHNHKYFRTVLRSSVKCHIRLLRAINTIEDTYNVILLILCLYFVIMFAFCGFLLITQVKENIS
FTRLLFIIVTIISMLVHMGIYCTVGEALISECNGIYYAVYDFEWYYLDPKEAKDLIPFMIKVSEPIYFTV
ANVFPVTMAMFCNLIKTSAGYISILLATKKN

>CbirOr79NTE

XFEWAVKLNRAGLNLIGLWPTPQESTRQRLINNIRIVFTFLVVTSLLVPSIHSLIKTHSDIMLTIDNLQI
TLPVLSSIIRLPIFWRRKKALTLIINMIVQDWLTTKNIRERKTMIKWASRARIFIICTYIIIVTSIIIMF
VIPIFGKSMTLALNITDSGKSLPLSTFYIYDVTKRPQYELTIINQCISMITAAILYTGIDNFFGLLVFHI
CGQLNIVRNRLKHSHKNFHAVLRSSVRYHIRLLRAINIIEDTYNVILLILFLYYLIMFAFCGFLLVTLFE
DEGNDISVTRLSFLLLIIISMIVHMSIYCAVGEALMTEINGIHYAVYDCEWYILDPKNAKDLIPFIIKVS
EPIYFTAGKVFPITMAMLGNLIKTSAGYISVLLTSRKK

>CbirOr80PN

FEWAVKLNRAGLNLLGLWPNPKENPREKLVNNIRVFLTFLVLINLLVPSIHLLIKIHSDIMLTIDNLQIT
IPILTTVIRLPIFWWKKEAFTSIVNMIMEDWLKTKNIHERKTMIKWALRARIIITITYFVIAIALMLFFV
IGPIFGKSMTSNITDSHRSLTLPTYYIYDVTKRPQYELTIISQCISLTTGSILYTGIDNFFXLLVFHICG
QVNIMRYHFKHSHDNFRVVLRTSVMYHIRLLRAINTIEDTYNVILLILFLYCGILFACCGFLLVTLVGDE
ENDVLFTRLSFPFMIIISLLIHMSIYCAVGEMLMAECNGIYHAVYECEWYSLDPKEAKDLIPFMIKVSEP
VCFTAGKIFPITMTTLCNLIKTSAGYISVLLTARKN

>CbirOr82

MIRSTRNKKEDFEWAVKLNRAGLNLLGLWPNPQESAREEVISNIRVLLTLLVMLTLLVPCVHSLIKTNSD
IMLTIDNLQIIIPILSTIIKLPIFWWKKKAFTSIVNMIVEDWLKSTNIHERKTMIKWALRARIFITCSYI
LITIGLIIFFVIGPIFGKSMTLTSNITDSSRSLTLPTYYIYDVTKRPQYELTIISQCISITTTGILYIGI
DNFFGLLVFHICGQLNIMRNRLKCNHENFRVVLRASVMYHIRLLRAINTIEDTYNVILLILFLFFGILFA
FCGFLLVALFEDEENDISFTRLSFLFLTIISITTHMGIYCAVDETLMTQCNGIYYAVYDYEWYSLDPKEA
KDLILFMIKIRDPIYFTAGKIFPVTMAMFCNLLKTSASYISVLLTSRKN

>CbirOr83NTE

XFEWAVQLNRASLHLLGIWPNPEENPKKKLMSNIRVFVTFVLVISLLVPSIHSLIKIRSDIMMTIDNLQY
TLPVLSAIIRLAIFWWRKEVLMPIMNMVMEDWLKSKTIYERNTMIKWALRERIIITITYLIIMMVIILLF
GMSIFGKSMRLTSNITDSYRSLPVQTYYIYDVTKRPQYELTIISQYISLIIAAMLYTGIDNFFGLLVFHI
CGQLDIMRYRLKHSHQKFRAILRCSVMYHLRLLRTIDTIENIYNVILLILFLYFAILFAFCGFLLVTVFE
DEKNDISFTRLSLSFLIILALLFHMGIYCAVGEALISKIDGIHYAAYDCKWYSLAPKEAKDLIPFIIKFS
EPVYFTAGKVFPITMAMFCNLIKTSVSYVSVLLTTRKN

>CbirOr84NTE

XFKWAVKLNRISLEFVGLWPESKQNFREKLLCNLRTFFAVIIVTIGALVPSIHSLIRIHSNIVLTIDNLQ
FTLPILSVVIKLIIFWWKKETLIPIVDMIAKDWLKPKSVRERNTMVIWALRARIIIICSYVSMAVAYIIF
ITMPIFGKSMRLITNITDPGRPMLLQSYYIYDVTKRPQFELTFISQAFSTFVAVLPYTGIDNFLGLLTFH
ICGQLVILKNRFIHLHDYKDFHEILKDCVTYHIRLLRAIDVIENVYNMILLVLFLYFGILFAFYGFLLIS
LFQDKEDDVSMTRLLYLIVIIMVLFAHMCLYCAVGEMLMAQCNEIHFATYNHEWYFLDSKEAKNLIPFMI
RASQPIHFTAGKVFPMTMATFCNLIKTSAGYISVLFTGN

>CbirOr85PNC

YEWFIALNRACLKLVGTWPETNETAWKTLMTNIRVIIILNLIIWSCIIPALHSLVRIWGDITSMIDNLQY
SLPITVSVIKFILMWRKKMVLIPILKMVKTDWLKLKTEEERVVMIKQGQTARAIMIWDAFVMVFSFILVV
ILPCFGISIRYLTNITDPRRVMPLQTYYVLYDVSDSPFYEVTFLLQSFGVIAAATIYTATDTFMSLLVFH
ICGQLENLKTFILDLDKFSNFTVALSSSVQDHIRLIRFIKIVDNTFTLMLLGLLIYFGILFILYGFLSIT
IITQGHNLSIARLTFLLTSFINTFAHMCLYCAVGEFLVIQYDGIYEAVCQYEWYKLEPRQAKDLLNIMML
TRRPLHLTAGKLFPMTIATLCN

>CbirOr86NTE

XYEWATELNRISLTFFGVWPENHETKQKNLISEIRVIIACNLLIWMWLIPSLHSLLKIYDDITSAIDNLQ
YTLPLLIALMKLFIIRQKKYDILLVLNMIKNDWLKPMTSEERNVMIKQARIARILTLLSCLVVEMFAIIT
SILPIFGISMRYRTNRTDPGKILPLQTYYLYDVNNSPLYELTFVLQSLSMIVVATIYTGPDTFMSLLVFH
ICGQLKNLKARIRNLDQLNNFPDALSASVKDHIRLLRAIIIINDTFNLMLLGLLVYFGILFTLYGFLFVS
ITTQGRNLSMGRLLLFLFGFTITFGHMSLYCILGEILVIQSEGIYEAVCQYEWYKLKPWQAKDFLNIMIQ
ARRPLYLTAGKLFPMTIATLCNLLKTSGGYISVLLAHRSX

>CbirOr88NC

XFEWAIKLNRLTLSMVGIWPNVHGDNCDDYFSDLRTMFSLFLFVFIGMIPAIHSLMRTWGDMIATIDNLQ
FSLPLLTTIIKLVILRWKKLDLTSVLNMIADDWLKDKTDRELHVMKNHARNARAITIFGCVFMTVGFSLL
VFLPCFGMSLRYVTNITDPQKILPLQTYYFYDKDQSPYFELTYVAQGLLLVTAGASYTGIDNLLGLLIFH
LCGQMENLKDKLTNMRQFKTFHSNLALIVRDHIRLIKYFDVVENTFTLLLLGLLLYFGTLFCLYGFLIIA
VFTEGKEMSMMRLIYLISVALNICGHMCLYCVVGEILVTQCDGIYHAAYEYEWYMLRPEEAKTLIIIMIR
ASKPLYITAGRMFPMTMSMFCN

>CbirOr89NTE

KNFEWATHLNYLILNAIGIWPMTHKRVYDKVLSDLRAILTFIIITFIGVIPAIHSLLRTWGDMMLMIDNL
QYTLPLITTVMKLVVIWWKKPDLVLVINMIAEDWIKAKTDKELYIMIKQAQNTRIVTMIGYVFMVMGITL
LVILPCFGKSVRYTTNITDPNKLLPLQTYYLYDKDQSPFFELTFAAQTVVIFMSGATYGGIDTLLGLLIF
HLCGQMENLREKFITMSKFKTFHSGLVFIVRDHIRLIKYFDTIESIFTVLLLGLLIYFSTLFCLYGFLII
AILTERSQMSTMRFMYLVSVMINFCGYMCLFCAAGERLVTQCEAMYYAAYEYEWYKLEPKKAKVLVLLMI
RTSKPLYITAGKIFPMTMSTFCNIIKTSAGYVSVLLAMQS

>CbirOr91NTE

FEWATELNRYSLEFIGLWPKMEETTREKLTANARVFLLIIMVTFVCVIPCIHSLIRVWGDLMSMTDNLQF
TLPLVSMVMKLVIMWSKKAALAPILYMIAKDWLRSKSEEERHNMIRCARIPRMIIICGFVIMFASFILLF
ILPCFGITMRYITNVTDPGKPLPLQTYYFYDTDESPYFELTFIAQGFTLMVSAMGYTAIDSLFGLLVFHV
CGQLENVKGRLTGPEKDPNFEHVLMNTITDHVRLIRCIKVIESTFTLMLLGLFLYFGTLFSLYGFLLVTI
VTDGRHLSLVRLAFLVTVVANIFAHMCLYCAVGEFLIAQCEGVYQAACEYHWYDLEPKQARNLILLMMRA
NKPLYVTVGKIFPLTMNAFCSLLKTSGGYISVLLARRD

>CbirOr92NTE

FVWAVELNRYGLEFVGLWPTSDKAAKHNYISNLRAYIIFIIITFVCVIPLICSLIRVLNNIILVIDNLQF
TLSMMVVLFQFIIMRWKQTALLSILKMMAKDWIVFKIDTQRDVMMKWARTARVIAICGYIVIGFGIIVVI
IPPYFGIPFRKLSNLTDRDKPLVLQAYYFYDTDPSPQFELTFIFQAITIFLAATSYTSVDALLGLVILHT
CGQLENFKYQLANIAASENFDGALRNSVSTYARLIRFTNKIEDIFALMMLGLLFYFGIVFCLFGFLLLSV
MTDEDISDVPLSRICYILCGIIIIFAHTFLYCGAGEIMIEHCNRVYQAIYNLEWYKLEARKARNLVLLMI
RASEPFRITAGKVIPLTMATFCSLLKTTAGYISVLLAKCS

>CbirOr94PNC

FVWAIEVNRFGLDLIGLWPTIDKAAKNSYISDLRVSIIFIIIAFASGIPFIWSLIRVRSDIMLVMDNLQV
TLTIMAGLLKLIIIRWKQTALLSIIKMMAEDWIAFKLDTQRDVMMKWARTARLIVICGYIVTGFGIIVVI
IPPYFGIPFRRLSNLTDSDKPLPLQTYYFYDTDPSPQFELTFLIQAITMFLAAISYTSVDALLGLVILHT
CGQLENFKYQLANMIASENFDSALRNSMITHVRLIRFTNKIEDIFALLMLGLVLHFGIVFCLHGFLVVSV
MTDDQRSDVSFSRICSVIAGVTVLLAHTFLYCGAGEIMTQHCDAVYHAIYDLEWYKLESRKSRNLILLMI
RAGEPFRITAGKVIPLTLTTFCS

>CbirOr95NTE

FVWVVELHRFGLELVGLWPTIDKAAKTSNISDLRGCIIFIIIVFVCAIPFICSLIRVRSDIILVMDNLQV
TLPLMVVLLKLIIMRWKQTALLSILNMMANDWRVFKVDTQRDVMLKWARTARFIVTCGYIMTGCAVIIAI
VFPYFGVPFRRLSNLTDSDKPLPFQAYYFYDTDPSPQFELTFVFQSITMFLAAVIYTSVDALLGLVILHT
CGQLENFKDQLANMAASKNFDSALRNSVLTHVRLIRFTNKIEDIFALMMLGLVIYFGIVFCLYGFLLLNM
MTNGHISDLSLSRICFIICVISILLAHTFLYCCAGEIIIEHCDAVYQAIYDFDWYKLEARKARGLVLLMI
RASEPFRITAGKVIPLTLTTFCSLLKTSAGYISFLLAKCS

>CbirOr96NC

FEWAVELNRFGLKLTGLWPKIDETVKTNFVSDLRVATVFIIIFLSIISFLCALVRIWGDASLMIDNLQLT
LPVLIVLLKLGIMRWKQTAVSLLIKMIEEDWLRVKKEAEREVMIRRARTVRLIIICGYFLMLFAFFVIII
LPSFGLHFRRLTNLTDRNRLLPLQTYYFYDTDKSPYFELTLVAQALTISLGGITYTSVDAFLGLTIFHIS
GQLENFRYQLPNLISCDDFYSALRTNVETHLRLIRFINNVEDTFSILMLGLILFFGIVFSLFGFLMVTVI
TGGEMINISVARIGFMLFGIVTLLTHTLLYCAAGEMIAEQVSYX

>CbirOr97NTE

FVWAIQLNRFGLELIGLWPGNDEVAEVKLASDLRVGIIFVIVTLISGIPLVCSLVRVWGDMILMIDNLQI
TLPLLIVSLKLVVMRRKRMALLSLVKMMAEDWMELKTAKEKDVMIGRAHLARAIVISGYVLMVLAFIVVI
VLPYYGFSLTRHLTNLTDPGKPLPLQTYYFYDINRSPQFELTFLIQAITIFLAAVVYTSVDAFLGLTILH

LCGQLENFKGRIAVLSSCQNFIRVLSNNVMNHLRLIRFASIIEDTFTLMMLGLVFYFGIVFCLHGFLLFT
YVMQVITGDFSFAQLCFPIIGITILFTHTFLYCGAGEIVTNQCEGIYRAMCDLEWYKLESRKAKSLIPLM
IRAKQPFRITAGKIFPLTMTTFCSLLKTSAGYISFLLAQRE

>CbirOr98NTE

FVWAVELNRFGLELIGLWPKTNELAKERFWSDFRVGFIFIMVTFVSGIPLVCALIRVWGDMILMIDNLQI
TLPLLVVSFKLIIMRWKRTVLLSMVKMMAEDWMTSKLDAERNVMIKHARVARLIVICGYVLMVLAFSILI
ILPCFGLHIRHLTNLTDRGKPLPLQTYYFYDTDKSPQFQVTYFLQAIAIFLTAVTYTSVDAFLGVTIFHI
CGQLENFRHRLTNLISYKDFNSALAKNIVAHLRLIRFADSIENMFALMLFGLVVYFGIVFCLYGFLLLTS
ITNKEVNDASFSRTLFVSVGVTTLLTHTFLYCGAGELVTEQCEAVYRAMCDLEWYKLESKRARNLILLMI
RVNEPFRITAGKVVPLTMTTFCSLLKTSAGYISFLLARRD

>CbirOr99NTE

FVWAIELNRFGLKLIGLWPEIDKAVKTSYISDLRAYMIFIIITFVTTIPLMRSLIRVRDDILLVIENLQF
ILPIMMVLLKFLIMRWKQTALLSILNMMANDWIAFKLDTQRNVMMKWARIARLIVTCGYMLTGFGIIVVT
IFPYCGLPFRHLSNLTDRDKQLPIEAYYFYNTDPSPQFELTFLIQAMTMFLIAIIYISVDAFLGLVILHT
CGQLENFKYQLVSMVASDNFDGALRNSVITHVRLIRFTNTIEDIFALMMLGLVFYFGIVFCLYGFLLLSV
ITDKRSDVSFSQICYVIVSVTMLLAHTFLYCGAGEIMVEHCDALYEAICDLDWYKLDSRQSRNLILLMIR
ASEPFRITAGKVIPLTMTTFCSLLKTSAGYISFLLVKCG

>CbirOr101NC

XFVWAVELNRYGLELVGLWPPINKAAKTSYISNLRAYIIFIIITFVCVIPLICSLIRVLNNIILVIDNLQ
VTLPMMVVLLKIIIMRWKQTALLSILNMMANDWIAFKVDAQRDVMMKWTRTGRLIIICGYMMIGCPAIVA
IVFPYFGLPFRRLSNLTDSDKPLPIQGYYFYDTDLSPQFELTFISQAIMIILAAVIYTSVDALLGLVILH
TCGQLENFKYQLASMATSETFDGSLRNNVITHIRLIX

>CbirOr102PNI

YEWAIELNRLSLTLLGIWPENHETKQKKLISDIRVIITLNLITWICFIPSLHSLLKIYDDIMSTIDNLQY
TLPLLMAMIKLFIIWQKKYDILPLLNMIKDDWLRPKTSEERNVMIKHARIARILTIVGYFVMLLTLIFGL
VFPLFGFSIRYRTNRTDPDKSLPIQTYYIYDKDKSPFFEITYILQSISAVLLAFMYSSPDSFLSLLVFHV
CGQLENLKKRVIDLDKFQDFRNVLSYNVRNHIRLIRSINIIDNVFTIMLLGALIYFGILFAFYGFLFGTC
EAIYDAAYEYNWYTLEPKETKNLLMIMIRANKPLYLTAGKLFPMTMATFCNLLKTSGGYISVLLAHRE

>CbirOr103NTE

YEWAVVLNRICLQVFGIWPDNNDIKQRSLMLDIRVIFILIIIICSSVIPTIHSLIRIWGDFMSTIDNLQY
TLPLVMSILKLFIMWQKKEEIAPVLKMVKEDWLKFKTEDERNIMIRRARIARIITICGYVVMFGSFIFAV
VLPFFGISLRHMTNITDPGKLMPLQSYYIYDINNSPIYEITYFIQGISLMLAATVYSSTDNFLGLLVLHV
CGQLENLRKRFLLLDQDDFKNSLSYNVHDHRRLIKSIKIIDDTFTIMLLGLMIYFGLLFALYGFLFVTII
VQRYGLSFPRLAFIVLAFTSTFAHTCLYCIIGEILVIQCNEFYKAACEYKWYELQPKQVQSLLIIMVHAN
RPLYLTAGKLFPMTMSTFCNMLKTSGGYISFLLAHRE

>CbirOr104NTE

YEWAIELNRLSLTLFGVWPQNNEDKQKTLMSDIRVIIIILNIMTWTCLIPTLHSLLKIYDDIMSTIDNLQY
TLPLLMALLKLSILWQKKYDILPLLNIIKDDWLKLKTSKERNAMIKQARIARMLTIYGYFMMLMSVTLAF
LLPLFGISMRYRTNRTDPNNKYLPLQTYYVYDKDKSPFFEITYVLQCISLSLAGGIYTSTDSFLGVLVFH
VCGQLQNLKERVINLDKFYDFRLALSYNVRDHMRLIRCINIIDNVFTIMLLGALLYFGILFAFYGFIFGT
MFSQGREFSVTRLTFIVIISLNTFTHMCLYCIVGEILLAQCEAVYEAAYEYNWYTLESKKARNLLMIMIR
ANKPLYLTAGKLFPMTMAMFCNLLKTSGGYISVLLAHRE

>CbirOr105NTE

YEWAIEANRLSLTLFGVWPKNNETKHNKLMSNIRVVILLNVMIWICLVPNLHYLLKVYDDIMSAIDNLQC
TLPLLIAIIKLFVIWQKKYDVLPLLNMIKDDWLRPKPLTERNVMIKQARIARTLTIFGYFVTQLTAITVL
FLPLFGISMTYRNNKTDTDKLLPVQSYYLYNVSKSPLYEVTFVLQSISVMTAGTTYSGMDTYMSLLVFHV
CGQLENLRGRIRNLDKFNNFADTLSASVKDHIRLIRAIIIIDDTFNLMLLALLIYFSILFALYGFLFVSI
MTQGRNLSVARLLFTMMTFINTFGYMCLYCLLGEFLVSQCDGIYEAVCQYEWYELKPKQAKDLLNIMMQT
RRPFNLTAGKLFPMTIAMLCNLLKTSGGYISVLLAHRN

>CbirOr106NTE

XYEWATELNRINLTLLGLWPENNETKQKKLMSDIRVILIFNIIFWSCIIPTLHSLLKIYDDIMSVIDNLQ
YTLPFLIAVIKLFIIWQKKYEVLPLLNMIKDDWLRPKTSKERNVMLKQARIARIFTIFGFLLIQISAIIV
LFLPLFGISMRYTTNRTDPGRMLPLQTYYLYNVNSSPLYEITFFLQSFSVMAGSTMYSGTDTFMSVIVFH
VCGQLENLKTRIRTLDKFQNFAGALSASVKDHIRLIRAIIIIDDTFNLMLLGLLVYFGILFALHGFLFVS
IVTQGRNLSLARLFLFLLGFIITFGHMCLYCLLGELLVIQFDGIYEAVCQYEWYKLKPKQAKDLMNIMMQ
TKRPLNLTAGKLFPMTIAMLCNLLKTSGGYISVLLAHRN

>CbirOr107NTE

XYEWAIEFNRVSLTLFGVWPINNETKQKKLMSDIRVIITLNVVIWICVIPTLHSLLKIYDDIMSTIDNLQ
YTLPLLIAVIKLFIIWQKKYDVLPLLNMIKDDWLRSKTSEERNVMIKQARIARILTIFGCFVTQTAAIII
LLLSVCGISMRYRTNRTDSDKLLPLPSYYIYDVSNSPLYEVIFVLQSFSFMIAGMMYTGTDTFMSLLIFH
VCGQLENLKARISNFDKLNNFADTLSISVKDHIRLIRSIIVIDDTFNLMLLGLLVYFGILFALFGFLFVS
IITQGSNLSIARLIFTVMTFVLTFGHISLYCVLGEILVIQYDGIYEAVCQYEWYNLKPKQAKNLLNIMME
TKRPLHLTAGKLFPMTIATLCNLLQTSGGYISVLLAHRN

>CbirOr108NI

XYEWAIGLNQACLKPFGIWPENDQRKFILNVRVIIAAAILFSSLIPCLHSAIRVENDIILIIDNLQYSLS
IMIGMLKLFTMYWKKKGECCHLILILRMIKEDWLQPKTTKQRHIMVKHARSARFIMIFGYLTIISSFILL
VFLPCFGVSIRYLKNMTNSARLMPMQSYYLYDSDQSPLYEISFVLQAACVVSTAALFSGIDNFWGLTVSH
ICGQLENLQTRITSLDMFKDFDSTLSYSVIDHIRLIRFIHTLDNAFNSMLLAVLIYFSTLFAFYGFLFSI
ILTRNHNMSIMRILFFTTAFLNIFVHMCLYCTIGEMLGIQVLKTSTGYISVLLTKSATK

>CbirOr109PNI

YEWAIELNRLSLTFLGIWPENHKTKQKKLMSDIRVIIILNLITWICLIPSLHSLLKIYDDIMSTIDNLQY
TLPLLMAIIKLFIIWQKQYDILPLLNMIKDDWLRPKTSEERSVMIKQARIARILTIFGCFIMLISVIFAL
VFPLFGISMRYRTNRTDPDKSLPFQTCYVYDKDKSPFFEITYILQSISSMLLAFMYSSTDSFLSLLVFHV
CGQLENLKKRVIXXXXXXXXXXLSYNVRDHIRLLRSINIIDNVFTIMLLGALLYFGILFAFYGFLFGTMF
SQGRNLSVARLSFIVIVSLNIFMHMCLYCVLGEILLAQLLKTSGGYISVLLAHQK

>CbirOr114

VGTISLTKEDSTPLTNGMDRSNDYNDLEWAIGLNRRMLKFVGLWPQNSEKAREKLSSKFQLLFNAITLVF
VLTIPAIMSLIRVWGDMMLMIDNLQFTLCLLITILKVAIMWSNRAALSPLIDMIARDWMRAKMKEERDVM
LKRAKTTRILAICGGVLILLTLVFGLGFSSLGMSLRLVTNLTDPGRPLPIQTYYLHDVTRSPQFELTFLA
QGIALTISGISYTGVDNFLGLLMLHICGQMENLHLRVRNLRRDPDFKATLKYNVRDHIRLIRSIEIIDDT
FNLMLLGLTFLFGILFCLHGFLIINVLKEGGQLSVMQFVNFVAASVTVLTHMCLYCAVGELLVSQCEKVH
HATYEYMWYTLEPKKAKNLTLMMLRANKPLHVTAGKTFPMTMATFCNILKTSAGYVSVLLANRN

>CbirOr117NTE

MNETRFSAEEKTKIYNFDYAVAMNRWSMRMIGLWPVDTGFSNFLCNISFTIMLICLLPPAAQLFATAKDL
NSITNQIIRTVPFIPLMMRFILIKMQTKNLRVILNSMIIDWTNYRHLPEQCRQIMNYYARRGRLFTLTSI
VFTTLSIIGYTLTPVMNTWLNNLPWNRTREYLPNEGFFPFPVQGSPTFELLYFLQASSLFLSGMVMTAVD
CFLCIIVFHACGQFDVLATALQRYDCSFKHGCTQTRHTRSIVCACLPCIVKRHVHIINYMDIVERSFNVF

IIIQIFGNCFDLAIEGYLFYMSFKSHDINGIITCIIYLITIAYNIVVYCWIGDCIIEKSDNIRTVAYNLE
WYSFPRKHALSIILIMARSRRPCKITSGRFHTMSFSYYKTVLTTLFSYIYLLLTLNNFR

>CbirOr118PSE

MAPTSTIGGPMEFTLRLIWPDPWCRFLQRLWATTIAISLLFQFWYLFSHVMLFLGAGDMLAQKSVGTKXF
VEEKQLIIKMNLLFEYMRRFTKLSWFYNFSCSTPLAMLAGMLNVLIVALVLHIAGQIDIMCYELLKILVA
ENKCNSRIITLRSIVIIREQTSKAFRSIPIKMLVKLVPYYFFVNVEMFIPCFTGEYLSSKVRKGRRA

>CbirOr119PC

MEFATTISPQIKFGLQAIGIWPGSSYSLLYRAFWTITLGVTQTFQFKYIISCIKNNNFPDLVDSVSTTLP
YSLLCLKLIILWLNQRLFNDILTSISRDWHNCDSVSFNMHTMTNKANLSRRYSMLIIGVYWMAVVVYVSV
IMEFNYTNESNKAEDHLFLKMEFPFTYDVSPIREIVMVSQFVQLLSNASVIGMLDALIVTLILHISGQID
IVCRELFKLFSEKYGCKLYKAATREVIRRHQDVIALSDNIENLFSYIALMQFFTNTLVICCIAFVIVTSI
EGNQNYAMLLKSLFFYIAITLEAFIFCFAGEYLSNKSKSVANAAYEVFWYNVKPSESRMLLILILRSQKR
LTLTIGKFNDLSLQVFAS

>CbirOr120

MIPTTTVSRPVEIGLRLTGIWPNSSMLFRLLWTMVMGTGLIFQCLYLLTHFSIAELPNLIDGLSTTLPYS
LLFFKLITLWVKNQIFKDILTAMSKDWAEYSNMYTMVDKAVLSHRCSKLIIGIYATAVLLYSTATIDFRK
QTIDNECRQQLIKMELPFVFCDSPIYEIMVFVQFIHLMAVASTIGMLDALIVTLMLHIGGQIDIIQEQLE
KICSEDSKPHLSKDIVKSLITKHHKIIAFSDKIESLFTQIALMQFLSNTMIICCIGFLIVTSLGTDTGIR
MMIKTTFFYIAITMESFIFCFSGEYLSSKSKMIGDAAYESLWYVLKPEDCRILLFVIMRSQRRLTITAGQ
FMDLSLEGFANSLKASASYISVLYAMY

>CbirOr121PSE

MTPTSTVSHSMQIGLRIVGIWPNASYSLLFRCAWILTRGMIQTGQFXWIIIHFGNEDLSRLMDILSITTE
YTVMILKLMILLLNSRIFYDTLAAMATDWKDASAIHAYHGEEGESMTALFQHDHRASLGGSTFLRHRSSR
IFYHDADDAIEIFIRDFTLKIQLPCDCNESPLFYYISGVSLSCILRHNWLTQLSDHHVSKSFTQAVRYSV
RCIERNISQKXQSTFWRFRREGTDQKIIIFSNERIFCIELIQLKYVDDLLLGIHDRDSEYLIRRXMRXKF
AYXFSINKSYTVLHGCYGYDI

>CbirOr122

MSSTLKLDRNSIGRMSTISESVEVGLRFIGMWPNCTYANINWWTYILSVAIMQYFQYSYILQHFDISDLS
VTIDGLSITLGYSLSLLKLINLWFNRRKLYVILDAMDKDWTDGIAVDSDISMMIRHANLSRQLSNAMIMT
NALAVFFYTIGGPILRSMIYKNEETKRELPIKMEFPFNVDNSPVFELVLVAQLFHDLSVACIIAMLNSLL
VTLVLHVSGQIDIMLQGFLEISAKKHARSSLATIKILINRHQRIIDLAENIEDLFSNVALLQFIWNTLVI
CCIGFLLVISIGTEEGATVITKSLIFYVAIALEAFVFCYAGEYLSAKSKSISDAAYECFWYDLTPSESRI
LMFLMLRAQKRLTITAGKITDLSLEGFTTIMKSSASYISVLCAFY

>CbirOr123

MKRTSTISGLVEFGLRFIGMWPGSTYANLWWLIYMTTIVIVQYYQYMYVCTHFDLSDLSLLMDCLGLTLA
YSLAFVKLFALWWNRRIFHHILAAMEHDWRECAVNDSYVSTMLNVAELSHRCSNVMISINALAAFFLSIG
EHLLQSMNVDDSRANNYSRELPIKMEFPFDVSKSPVFECFLMGQFLYELVLACLVGMVNAILVSLILHVS
GQIDIMRQDLIKISNNYNPSTSLNVIKRFIDRHQKTIILSEHIENLFTYIALMQLLWNTLVICCTGFVII
ITIGTNEGATASIKSVSFYIAITLEVFILCFAGEFLSAKSKSISDAVYESLWYDMPPTDSRILLFIIIRS
QKRLTITAGKVVDLTLEGFTSIMKASASYVSVLNAMY

>CbirOr124

MKRANTISRSVEICLRFIGLWPNSAYATLYWITYMATLVIMQYCQYAYIVARFELNNIWLLMDCLSLTLA
YTLVLFKLIILWWNRRSDIPCFLQNISLRREKECTMNDSYASTMMSMADVARRLSNIVFTFNTFVAFSLS

IGEHYFQSMSNANQIDNSSQVSPRELPIKMELPFDVSKSPIFECFLIGQFFYDTVVACTVGMVNVLLVTL
VLHVSGQIDIMQQNLIEISNKKYDRSIFLAVIKTFIYKHQRIIILSENIENLFTHIALVQILWNTVVMCC
TGFVIIIMIDNGEGVIGLIKPVSYYFAITLEAFVFCFAGEFLSAKSKSIGEAIYGSLWYNLSPSDTRILK
FIMLRCQKRLTITAGGVIDLTLEGFTSIMKASVSYVSVLNAMY

>CbirOr125

MLTSPISPLLRVGLSLIGMWPDTSYGTLSWLFYMMTVVVMQYFQYSFVYAHLDFNNLTKLMDGLGLTLDY
TLTIFKLLSLRFNHRIFAKILAAIDKDVKDCTSEMHECIMTSKANLAQRCSNAIISVNAIATVLYFIDSL
VRRRMVSEDGQFREFPVQVKFPFEAHESPIYEFAVVGLFFHVLETATVIAMLNALILTLVLHISGQIDIM
CQELREISSTSKPYSLSTKSLITRHQKIILLSNNVENFFSFVGLLQFVWNTFVICCIGFMAVISLDMNTE
GKSGVIIQFIIPSVAVTIEAFVFCFAGEYLSTKSKSIGDAAYEAVWYDLSTSECRVLLFLILRSQKRLTI
TAGKVTDLTLEAFTTVMKASASYISVLHAMY

>CbirOr126

MLTSTISPSLRIGLGLIGMWPGSSHATFFWLFYMVTLVVMQYLQYSYVYAHLDVRDPKLIDGLSVTLDYT
LTFLKLLSLWRNRRIFSDILDAMEDDWNDCVTDSYICVMKSKAGLAHRCSNAMMTLNTLSAIFYFIGNYL
SHRAVDGAPREFPMQVQFPFNATNSPIFELIVLGLFLHVWETATVIAMLNSLILTLVLHVSGQIDVMCQG
LREISSTRKSDPLATRSLVERHQRIISLSNSIENFFSFVALIQFVWNTIVICSIGLMIVISLGTDMEGKS
GILIQSIIPYIAVTLEAFVFCFAGEYLSSKSKSIGDAAYETLWYDLSTKECRILLLIIIRSQKRLTITAG
KVMDLTLEGFTSVMKASASYISVLHVIY

>CbirOr127

MAVTSTVSPSLKIGLRLLGVWPGVPLSTFYWLTFMSSILILQYFQYQYIFEHFKISELPNLVDGLPATLD
YTLTFLKLASLWIHRRVIHQILNAMDNDWCECIGVDQYLQVMRVKANISHFCSNAMLSFNAVAGVLYLLG
EYAIRFLYLAENYNDTLRQLPIKVQLPFEINRSPTFELLVIVLFIHVMLNVCTVAVINALILTLVFHVSG
QIDIICQEFRIICTKIPIHGSSTSTLGILIERHNKVILFSDNVEKLFSFIALMQIVWNTLVICCLGFIIM
ISVHNDTSVIVLVKTIFAYTAIMIEAFIICFAGEYLCLKSKSIADAAYESLWYDIPPDHVKIILFMIMRS
QRGLAITAGRITNLSLEAFASIMKASVSYISVLHAMY

>CbirOr128PSE

CHKSTTQNGSYIARRVARVAAFDRSFAHIRVEHIDHTVLSVYNHAFWGSCPISMDDLTMTLNYSLTFFKT
FYKILMVMEDDWHECDNIDQHLCDDHQRQYFTRLLQRYTSINVSSAVLYLFERYAIHLLSLMERRNNTWQ
QLLLKLEIPFEVKQSATFELLAIDFLRIRLHAATVVILKALIFTLIPNIRGQIDMMCREFIKVPVKTLAY
GFSTFAVNTEKMQQNHSLLEGYRRTFLLYRINAAHLKYRSNAG

>CbirOr129PSE(F)

MRATSISTSVEIGLRFVGIWPDLSHGTLIWLTYMTSVAIVLYFQYVYIFNHFDVSNISNLIDALSITLAY
SLGFLKLISLWLNRKIFYNIMLTIDKDWNNVSRDQSLMCIMSSNANLSRYCSNVLISINSTAAICYAATS
LVRHSTDLEGNLNASKRVLPIKMELPSNFWRSVSLFMKYRSPALVGMINSLIITLVLHVTGQIDILRQEL
LMTCNDESSESSSIIAGIRLLITRHQKIITLSDNIEELYSDIALMQFLSNTLVICCIGFTIIASLGKAGV
TIILLKSAIFYVAVTLEAFIFCFAGEYLSAKSKSIGDAAYESLWYNMTPDKCRIILFVILRSQKRLTITA
GNVMDLSLQGFTTVMKASASYISMLHAMY

>CbirOr130

MRSKTDPIIVGTVSRSIEMGLRMIGVWPDSSYTILHRAFWMITLTMAQTFQYWYFIIHVRTDDLSHLMDG
LSTTMSYSLLLLKLTIFWVNRRIFHNILNTMARDRSECATDWAGYLMSRTVYVSHRSSNLIIGLYSMSVI
LYGTGVLIAHSDEPEDELNEQLAVPTRELFLKMELPFESNASPVYEVVMVTQFFHQLAAATIVGVLNALI
VSLILHVGGQIDIMCRGLVEISSGDGMFDLPATSIKTLIHRHQRIIALSADIETLFSYIALMQFMWNTSV
ICCLGFLIVTSIGDSEGSTMLVKSLFFYVVITLEAFIFCYAGEYLSAKSRMIGDAAYEAKWYNSSPKQSR
VLLLLILRSQRKLTITIGKFMDLSLERFTTIIKASASYVSVLHAMS

>CbirOr131

MSSVCVSVEYGLRLVGIWPGTSYAILRKFFYISLMAIFQILQYRYLFLNFGEDLSLLMDVLSANLAYSLL
LIKLISFSLNTRLFDETITCMVEDWKERDISDKHTMTRMACISRRYSNLIIVLYAMSVWFYATGTLLKHK
SDNQSDVRELLLKMELPFAIESSSVYTAVLITQFVHQTSAASMVGVIASMLIILVLHACGQIDIVRQKLS
EITRNDIKPSEAESIMKTLIVRHQRIISFSKNIETLFSNITLIQFVSDTLLICCLGFLIVISIGVSGGST
MLIKSVLFYLVILMEAFISCFLGEYLSTKSKTIGDAAYESLWYNRTPAQNRDLLLMIMRSQKHLSLTVGK
VVDLSLQQFASVVKVSASYVSVLHAMY

>CbirOr132

MRTTTLSRLIRIGLHIFGVWPYVSSTVLFRLYWIVMLSTAQVFQYRYVVVNIHMDDFSQFMDGVSSAMAS
SLLFIKLIILWINQRIFFELLQMMAEDWQNYISNHTNLRLMTNIADLARRMSRWIVGMQMIAVVTYISGV
LAANVNSSEEVKPYARELILKMDFPFNISTNFIYMVISVVQSYHLTLVAYGITIINSLLVTLVLHVGGQI
DILRGWLLTAFSKNSAQSMSEITLRSLITKHQQIILFSENIEDLYTYVALMILLSDTFIICCLGYVVATS
LDTPNAAAILMKSLVFYITINLEVFIYCLSGEYLSAKSKMIGNAAYNALWYDIPTKQSRIVSFIILRSQK
LLTITSGKIMDLSLERFTSVVKASASYLSMLLAMY

>CbirOr133

MEISTVSRVVKIGLRACGIWPYLPTTTLYRFFWIVMLGIAQIFQYQYVVIHYNTDNFSHFMDGVSSAMTY
SLLFVKLTILWANQRTFSDILQMMAVDWENCVLADRSLRITTDKVKLSHRFSKWIIGLQLTVINPIQLRG
TCCNIQRINVSAREYILKMKLPFTVSTSPIYVLITILQFFYLAIYGCGISMVNSLIVTLILHIGGQIDIL
RECLLTAFSKNMMSTVDSITLRTLITKHQQIIIFSENIENLYTYIALMLFVSDTPIICCLGFIIVTSIGT
TDGPAILVRSVLFYLVMNLEAFIYCFAGEYLSAKSKMIGDAAYNSLWYDVATKKNLIILFIILRSQKRLT
ITIGKVMDLSLERFTSVVKASASYISVLLAMY

>CbirOr134PSE

MEVSTVSQVVKIGLRACGIWPYLPSTPLYRFLWIVMLSIALLIQYXYMVIHYNTDNFSYFMDGVSSAMES
LIPHTGGPNLREWLLKAFSKNMISIVNDNIESVDHTKHLSKLLYSRKTLRIFTLILHXCCSYQISSSYAV
YYLGFIISIGTANGLAILVKSVLFYLVMNLEAFIYCFAGEYLRAKSKLIGNAAYNSLWYGVASKIESNYS
IRNPKITETVNNH

>CbirOr135

MKLEDTISQATRVWLEVFGIWPNSSCIFLRRMFWTAALIIEQVGQYQYIIMHLYSTEVSEIMNLLSAAMS
FTLFCIKLGTFWYKQRTFKKILAMMAIDWDKCFRAEFDTFTTINHAKLSQRFSNTIVALFSIAAILYSTN
IVRMGTDKTTNASIRQPLILEMDLPFGDGRFVYELVIIAQFLHLVICSCCIASVNALLVNLILHASGQIE
ILREWLMKIFPKEKERDANLFMVKKAIRKHQKIIIFSEHIEELYSNIAMALFVSDTLVICFLGFIIVTSI
GTPDATRIIIRTVVFYFVINMEAFIFCFAGEYLSSKSQSIADAAYDSYWYESYTTDNRIIPFLIMRSQSQ
LSITIGKITNLSLERFTSIIRVSASYVSVLHAMY

>CbirOr136

MKLENTISQVTRVWLEIFGMWPNSSCIFLRRIFWVVALIFEQVGQYQYIIMHLYSTEISELMNHLSAAMS
FTLFCIKLVVFWYKQRTFKRLLVMMAIDWEKCFNEEVHIFATTNNVKLSQRFANMTVVLFSIAVILYSSN
ILHTGTDKTSNASITQPLILEMELPFHYNRRFVYELVIIAQFFHLWLCSCAIGLLNALLINLILHVSGQI
DILREWVMKIFSKEKGRGASLFMIKKAIRKHQKIITFSEHIEDLYSNIAMALFVSDTLIICCLGFIMVTS
IGTSDAARIIIRTVLFYFVINMEAFIFCFAGEYLSAKSQSIGDAAYDTCWYDSYSTANRIIPFLIMRSQN
QLTITIGKVTNLSLERFTNIIRVSASYVSVLLAMY

>CbirOr137

MTNTSTICFSIKFVLLVFGIWPGVSCVILHRLFWSSTLIITQIFQYAYFILHFHTDNLSDLMDNLSCALA
YSLLFFKLIIFWTNQRKFRDILKTIAADWKDCASDPFSLHVTTSAANLSHRISNMIIGLHMAAVVTYSFG

VLSSNGDDNFNASTVLDRALILRMDFPFDSNSSPIYEIVMVIQFFQLVTHACAIDVLNALIITLILHTGG
QIDILREWLTNVFSKESMHGMTGITMKNLIGKHQKIILFTENIENLYCYIALMQFVSNTLIICSIGFVIV
MSMHSPDMSTMLVKTLLFYIVMNLEAFIFCFAGDYLSTKSQHIADAAYRSLWYNMTVNESRTISFLIIRS
QKRLTITIGKIMDLSLERFTSVIKASASYISVLMAIS

>CbirOr138

METIGAINRSVEILLRICGLWTGSPYAPFCKLYWTFSFSITIILSFQYIIKYLQDTDLPELMETFSVFVV
NILVFCKLVIFWLNQRTLDELLAIMAMDWKKNIDTDLNAHIMAKKANLSRYFSNTLVVIYSGSVLAYSIV
ILATNIFSNESSERSLLIKMNLPFDSNRQFVHEFVMLNQLFQLLMLASADAILNALLITMVLHASGQIKI
LCGWLIKIFPKKEKYNLQITMVEKIITRHQDIIAFSKKIEKLYSYIALVQFLSDTLLICGLGFILATSIG
KPNTWVILIKTCLFYFSMNLEAFTFCFAGEYLSTESKIIGDAAYNSLWYESTSKDTQIILFLIMRSQNQL
TITIGKITDMSLKRFSDIVKASASYISVLLAMY

>CbirOr139

MKVTSTIGRSVEIPLRMFGIWPGSPYVSLCRLFWTIALVTAQYFQYQYIITHIYIADLSDLMDGLSATLA
FTLLFIKLVTFWINQRTFNKILAMMAIDWKKNSGIDFNMIISNATLSHNFSNFVFSFHSIAVIVYSISVL
TVSTSDYEGTDISMRPLILKMNFPFHSDTQLIYAIMLIAQFLYLLLCSIGVSTLNALLIVLILHLAGQID
ILRRWLTEILFSEENGYKPSAVMIKKIIEKHQNIINFSEDIENLYSRIALILFVSDTLIICFVGFVLVTS
IGASNSFFTILKILAFYFVMNFEAFVYCFAGEYLSSKSKMIGEAAYDSSWYQSTPQDSRIILFLIMRSQN
QLTITIGKFMDLSLERFANIIKASASYVSVLLAMY

>CbirOr140

MASKRTINRTIELLLTMFGVWPGISCVIIYRVFWIITLAINEFFHYRYFVTHLSIDNLFDLMDCLSSFLA
HVKLTVKIIIFSLKQRTFIEIIKLMSEDWSDSSNSDVALNKTSSKAKLSSRISNGLIILHTIAALAYVVG
ILLADVDVTDQTVDLPLMMKMEYPFVIDTQREYRLVLVTQFIYVMVCSWGAGLFNALLITLTLHIGGQIN
ILLCWLMEVTPKEIETKRESIVAIIRKHQKIITLSEVIENLYSYIALVQFTSNTVMICSLGFIIVTAIGN
PDASEKIMRSLLFYAVTNIEAFIFCFAGEYLSNKSKAIGNAAYNSAWYDMKITDRRTFLFVILRSQKQLS
FTAGKMMTLSLEGFTNIMKASGSYLSVLLAMR

>CbirOr141

MTRESTINYTLKIMLILCGVWPGRPYITICRAYWVIALGIDEICHFRYLLMHFQYNDLFDLMDCFSSFLT
QIKFITKLIIFWWNEQKFMKILTMMAEDWNDCTGDDVSARETACKVRSANRIINAMFTLHTVTIVAYSIG
IFLADVDVTDQQSELPLLLKVELPVNIHTKHTYKTLLTMQFVHLIMSGCGTGLLNSLLLALILHAGGQIN
ILHSWLIELVPRKEKERDDSIVVMTNKIIQKHQKIISFLEYIEDLYTYIALVQFISNTMLMCSLGFLIAI
GNPDATEQIVRSLLFYAVTNLEVFIFCYAGEYLKNKTTAIGMAAYNSAWYELKPKHSRPLIFIILRAQKQ
LTITVGKIMDLSLECFASIMKASGSYLSILLAMQ

>CbirOr142CTE

MLRKSTLNRTIKFLLILFGIWPGTSYILVCRGFWIITIGFIEFCHYRYFVTHIYSAELLDLIDCTCSFLA
HAKVLIKFIVFWLNQGTFIEMLTMITDDWSDCANSEISMREMVCKAKISDRISNALVTLHTVTIVAYFIG
IIMVDVDIINTTELPLANKLEIPFININTQRTYRLILLIEFLHMIMCGWAIGILNGLLLTFTLHAAGQID
ILRCWLTQRVDENKHGSVVITISKIIEKHQKVIEFSENIESLYTYIALSQFVSNTIMICSLGFLIVMAIG
SPNVVEQILRSVLFYTITSLEAFIFCFAGEYLNNKSKAIGAAAYNSLWYNLKPKDSRVLLFMILRSQKQL
TLTAGKMMDLSLQSFTT

>CbirOr143

MMRKSTINRITLFLLPLFGICPSKSCTLISRVFWIVTSGFIEYCHYCYFLLHLNLENFFDLIDCLCSFLA
HMKLLIDLIAFWINQQKFIETLALITDDWSDCANSDIDVRMATCKAKISDRVTNIILILHIIAIIAYCAG
IILADIDITETNELIFFNKLELPFRINTQHMYKGVLIAEFMHLFLSNLGIGLINTILLTLILHVGGQIDI
LQYRLEQLAFSEIRNEQESIITVNKIIQKHQKIIYFSENIESLYTYIALLLFVSNIALICSIGFLIVTAI

GSPDATQQIIKCILFFTITNLEAYIYCYAGEYLNNKSKEIGLATYNCAWYNLKPKDSRVLLFIILRSQKQ
LTLTAGKMMDLSLQSFASIMNISGSYLSVLLAMQ

>CbirOr144

MIRKSTINRTTEIMFTLFGIWPGTSCIQIRRIFWFITIIIVQFLHYRYLLTHFYSDDLFNLMDCLSSFLA
YAKVMTKFIVFWFNQRKFIETLAMMAEDWNDCPNNEISMQETICKAKLSDRIANAIIILHTLSVVTYGTR
IILADVDITDSTSIPPYIHKVELPFDVNTQRAYKMVLITQFTYLIMCSWAAGAVNALLLTLILHIGGQVD
ILRCLLTDLVSKEDGRKRTIVTTGEIIRKHQKIINFSQNVENLYTYIALLQFASNTIIICSLGFLIVCAI
GTPDATQQIIRSVLFYIITNLEAFIFCFAGEYLNNKSKAIGVAAYNSAWYNLKPEDGRILSFIILRSQKQ
LTLTVGKMMDLSLEYFASIMSASGSYLSVMLAMQ

>CbirOr145

MFANTITRSIEIGLRVAGIWPNVSYTIASRLLWTMTMIAAQTFQYRHMVLHLHSEDLSPLMDGLSATLSY
SLLCVKLIVFWTKQRIFHDVLRVIATDWKECEDVGEALCTMTSVANLCHRFSNSIIGLHSMAVLFYCIGV
VALSNHDGIAERELFLKMELPFESGTSPVYELVMTTQFLHQMTSATVIGVLSALLVTLVLHAGGQIDILR
ERLLEILSKDKKPTMSTITIGSLIQRHQNIIIFTEKIESLYSYIALAQFISNTVVICCLGFIIVNSIGTE
GSSMIIRSLLFYVVINLEAFIFCFAGEYLSMKSKMIGDAAYESLWYDLTPSETRILLFLIMRSQKQLTIT
VGRFTNLSLQQFANIIKLSASYVSVLLAL

>CbirOr146

MKHQVQVDEEYDKLIRPILVISKLIAVWPLKEDRPFSATVFRICHVVFLLFMLLSLSIALTIDIFYNTND
VDEVNERILFGCVCYLSVIRVIVFSFHQKDILYVIEAMRNDWIGSSYEERAILRNKCLLAYRLAKYFITV
IVGNGFVYVLVPIFEMNFLSDVERTLPFHGYFFANQTVSPIYEYLYVVDAIGGLSAGWTVASATSFTLIV
AIHGSVKFMMLQRRLETIKRDDPDVDRVIANCIRRHQDAITFADAFERISNLLVLGQFVTSIGLVCFAGF
QLAIMLHDNIQLYQTLIFLILTILELYMFSLSGNKLIIESDAVGESAYRSDWIGGTFGRSLQIVMIRSRI
PSRITVGKFCDVSLRSFTQVLSTSFSYMMVLFTIMTEE

>CbirOr147PSE

LLSMSIAMTIHASYKMDDVDEMTELILSCCVCYLSLIRVVVFSIHQKEMLYVIETMRNDWIGSSYEERAI
LRNKCLFAYRLAKCFITMVMGIATLFALEPIFKMNFLSDVERTFPFHYIYFNQTASPIFECLYFLDTIAG
LSTVCTISSATSFNLVAVIHGSAKFAILQRRLETVNRNDPDIDRVIVNCIRRHQDAIAFADALESVLNLL
ALGQFVLSIGLVCFAGFQITSMLQNKVQCLKYSGFLCATILELFMFSSSGNELIIESDAVSESAYRSDWI
GGTFSRSLHIMMIRSRIPSRVTVGKFCDVSLRSFTQVLSTSFSYLMVLLTAMSEE

>CbirOr148

MQTAKLARASNIVTWNKWFMTCMGLWPLNVNNFLYVFFTTYMIIYCSMAVRHLLKHFGQAESIIANLTDN
IFLSMIIGKMFICRMSCKDMTKFLKAIKDDFTTHNYTSVQEEMAYLRYNELALIFTKYSMSISAFTATMY
YLRTFIINWRQFLTGNFSYDELPYPVQPFFEIKDTTTYACVSAYLFITVPIIICGYAGADAYVLSMVLHI
CGQYAALAYKVDNLLRHQENYQRNIGNIVIKHRLLITLAEILEKNFTIIFLQQTLGTILLLCLTLFHMIT
NSEHGDNASVVAFILYTFCVSMTIFGYCYIGDCFINESAALRDAFYNTEWYNNPPSCTKLISICMVRAEK
PMVLTAAKFFPLSLSTFTNIVKTSMAYLSILRNFIVKF

>CbirOr149NTE

RASNIITWNSWPLKFLGLWPSKVNDFLYIFFTVYMLIYYTMAVRHLIKQFGHLGNIVDNLTDNILLSMIL
GKMIILRRSCGIMAKFVKAIKDDFSINTYSSVQETMAYLRYNEIALVFTKVSMGTTIITAISYYLRMFIA
NWRQFMSGNYSYELPYPAYFFEIKDTTSYTCVCIYLTLMVPIIICGYAGADAYLLSMILHVCGQFAALTC
KVNNLLKNHKNYRRNFTNIVLRHQHLITLAEILENNFNYIFLQQIVGTVFILCLTSLNMLANSEFGDNTN
FIVFLLYTSCVFMTIFGYCYIGDCLITESSGLQDAFYNIKWYKNPPQHTRLISICMTRAEKPLTLTAGKF
CILSLSTFTNIVKTSMAYMSLLRKIL

>CbirOr150

MRLRIESFYDKQVISWSKLLLSIIGLWPENHNDFRFFFYITYIISCSLLLIVNLVQNMHDMKKVMRYITF
VFPSILIVLKNVMFRWKKDQLLPILAVIKGNVKEGLYQTYDEKYKVIWYNIASTLFTTSSVTSLFFVPAL
YYLAPIFACILSNEYGSGNCTLPYELPVRVNLVYEISGMRSYVLFCVILIPSSTFLTIGATAADSAMISL
TFYLCGQLSILDHRMKNIDLKSSKCHYEMKVLVKRHMELIQLANILADTFSWLMFVQTVGIIFSLCIILY
QLLMTAEGEKEIDEIIHFMMYSIAVVLLAFCYCFLGECLISESLALEWACYSINWYESSSEFVHLLMICI
ARSRKPLCLTAGKFYVFSLETFGTIIKASMAYLSVLKTIT

>CbirOr151

MNIDDYVFINKRVLKFVGLYPTNIARYIVCCVCMFTIVIPQAVQIYQNWRDLAVVLETSSVLLTILLAIL
KSLVWISNRRKMDSFIDYMFTDYWDVMTAHISKHENVYAIYVRKGHLFTKGYLFLICNSLLFFFSLPIFE
SVVTTIKGTSDNSTNNFPFVALYPESYYNFPMYEIVYFSQMLATSLCGLIIILGTDTLIATALFHTCGHFR
VLQGKIEHINGEIGHMLQAHNELNIVRKIKLQIISTIKHHYTILWFCDYMETIFSPMLFLQTLASSLIIC
LVGFQITDAHITANAISKLIKYFSYLLMALFQLLLFCIPGDALIDESSTISRTLYTIDWYELPVLFKIEV
CLLMLRSQKSSKITAGKFYVMHLENFNAVLSTAVSYFMLLRSFNSD

>CbirOr152PSE

VNTWMESFTMILSLLSGQAPLTILLLFRGRLQRLLSTCEELWATLSATEKKCVHDYAKTTQRLTYFYLFG
CAFTIFFYAVASLFVAQRDESSNVTTRVLPYARLVEVSRTPCYEIRCSMINVGVTCVAADTIGPVLILTV
SGHFKVLNSRMLNLSNRACWNKCPRNRAKPDLQLCVQYHQIVFDEAISKAVYCTSWYHFSYALKRSLNIV
ALRSQKAAQLTAGYFVPLSLQTFASVRFITSDSSLFTTFLMLFAN

>CbirOr153

MAVEKRDRFLSIRISRFLMKTVGFWHAKSKAEEQLLNGILSYTICMIGSSLWIEATEMYGTKGDFYAITY
TACSAMPVAVVLMKICFFLKNRKEILKMLRYTEDHFWYAQYDEYGNKLLEDINKKGIILICTFIFFVQGA
VVSYMLAPIIGILILKLHQNVGKNVSDRKLPFNVWIGVPTNVSPNFEIIFFIEVLSLIHSGLLFCCFDVL
LGLLNIYTAGQFKLLQHRLETALERVVCTDIVKSLDQKRKQELYEEIKRCVIFHHELIWYSEKMESIFMY
TTLCQLMVSGILLCVAGFQVFLGRGTLIRRTIYMSHANACVCQLFVVTFTSNDLIEESRAIGHAAYNANW
QVLSHKHDKRIRNAIQMIMVRSIRACSISAGGFFPVSLETFMTVMGTAASYFTLLRNFIQ

>CbirOr154

MTQSKEYRSVRITRLFMKMVGLWYVETPRERLLLRAAFSYAIWAIVFAILVVGVDLYYCIGDFYAVTSNL
CTTLLLIMILVKLGSFMFYRDIVFDLIRFAEKNFWSTTYDEFGAQVLETYDKLGMTMVYIFTFIVYIATF
NYIFAPFFEIQETNVTKKILPFKLWFDFPYHSPYYEVTYTIQSFSTIHSGICTCCFDNFVSTFNIHVAAQ
LKILAHKVEVIVEKCIENTVNGECPPNIMEMALKKLQNCIQQHLTLICYVRNMQRAFAVILLGQLLLSSI
VICFGGFQFLETNVAVRKFLFACHFVGGLIQLFIYTWTCNDIIIQSTAISDAAYNSKWYLLSNNDPGKTL
RKGLIMIMIRARRPCTLTAGYVTVISLDTFMGILSKAMSYFTLLRQMSEEEIKF

>CbirOr155PSE

MIRRTNVTEKILPFKLXLDLARHSPYYEIAYTLPIMAQLENVICIFCFDNFVRXVHVAAQLKILNRKVDI
ITEKCIEDSANEKCQPEIDSIEWVLKKLQNCVQQHLTLIITYVRNMQRVFAIILLGQXPLSSVIICFSGF
SLLVGSRCCQKCIFAVQFVGSLTQLLIYTWTPNDIIVRSAAISDAAYNSKWHLLPGNGSGKSLREGLIMI
TIRARRLCALAAGHFAVTSLDTFTR

>CbirOr156NTE

LQTKIGHIEDLFVHLERIFSIGGIWPFKQTYIRFTIYISYFILYLIMTYANFCNVFGNLELMVMNLVETI
AYTMTFTIIWIVRCSKLLKCLINVVKKDMMERKFEDSEEEKIYYNYNYISKIFTYGSIVGMFITVMLLYF
RPLMLFLMNDQVLHNNTESFMLPYRIHTFFDITDTYTYVLIYLYLFPMLYASMLHMAAICLLVVLVFHIC
GELSILSYRIKNIKVQSMMVKRFRTFVRMHLKIIWMAKSVDDIFNFILLDELLSNSIVLAISMYYVIMNL

AISELATCFTFIFFAAIALVIIYGCCLISDQLTQQCINVLDAYYECDWYEMPLDCKRCLLICMIRGQVML
HLTAGRFYIFSLNSFTNVIKTSLAYLSMLRTVL

>CbirOr158PN

DQNDLDRAAKVLSWNKRGLMSTLGLWPFRSNDLIFSINLGYFSFLMILEYLDLFLFIDDLEHVIMNLTEN
MAFSQIFVRMSMLRLYNNQIGEIIAETMKDFLIRQVIKLLKRXKHLSRITVGQRSSIVKLLMIFVALTAS
SYYLTPIIIILGSGLPNIIINKNVTQILYILPYRFHMFYAIENMLSYVITYGVQLPFVFISGFGQSAADC
LMVTLVFHICGQMAVLALRINNISTDSYNCGHEIRHVVRMHIRLLRMGKTIERAFSAILLTHFLGATSLV
CILGYQPLMNFAKGEKGVLITFIIFQFLVLLILYALCTVGESLLTESIKICEAFYDCRWYDM

>CbirOr159

MARRTISNRLEQFRKLNTLYKTYLKYIGLWMLDSDSPILLRCLYYIYNKLVIIIIFLFEISVLLDIYLNF
DDFLVVTDNVCVFVGVFMIFFKMLIFQAHREQIARLLHATIKSCDQLCKLTTGGEYEILNKYLLICRVIS
VGFGFILCFFLAFALLFFVSVEDGALLIRARYLLNISLYPCHEIEFFMEVYTIYVVLIAITGIDSMQLTI
GMLSLAQFEILNKHFESCGRDNVGRRREVSTEVNCDEDNYESYEARHRGGFAEWFGRSVRDHQRLRVIID
DFNKIYNMCMFVQMLLSITMICLTSFQLLLVTGQSSTFKFSLYLIAAAMQIFFVCWLGNEVMYQSALLTQ
SQWLSGWEDELSNTKTGRLLILSMIFSTRTLNIKAGVFYDLNMETFTTVLKGAYSVFVLLNMYSEDQ

>CbirOr160

MLPLDNSPGKLFFYPHGHPKGERPSNKPVDDALVLRYRVYTRRIRRMLYLGGVLQDRTRSVFWSYVMGLL
VIVMFSSQCFFVMNFCRDHTDNLLLLSKCFGLTCSLLAPVVMSACFLVKREKLMELHEMLDDLFERELAQ
DQETALATLYAFDRPFYLLVLVLASTMLFLYSSPLISMFHQIIHDVQPRKYRLLLPARFLWPLPSSGFFF
FLHVLYQLSSCWMIFTISGVDSLFGFYAFQISSTLRAMSARLRNPRADDVFTGVLKTCVETHNRLLRCGH
MLEDIWGFIIMRILLTNAILMCVLIFEATPFTELTISQASVFVSYMTLKLLQAFIYAWYGGLVTSASEHF
REGIYFGEWTDSSCDRHVRATVIVTMMQKPMIIKAMKVSSINVNMFTTIVNTAMSYFFFLQSLDEGR

>CbirOr161

MDFQNVNPLNIRLNKLSGNLLPMTDRDSSFTLLWKLYGVFVTVTELLLTVVLIPGCIYVSRKKLMQDGLI
CLATTGEMAFMIVRIHKCRDLVRQLIRRLNGIMYVADKNMRNIVTKTLKPTQVPLMFYTAAGMMSTITWC
CLPFVLIFEKSVFWNEDYKMPAFFPKQPFSLKIFILNNLFIMLGCIYMILKKIAADVYMIHLVLLMTAQY
QYIAMRLTMIFHEQNEHGDTKKKRLSEINQKQEREMMALCRHHNAVIHIMFLLKKLLASNFSLIYMINIL
RFCFIGVMLSGIPSTTFWEAAAIFLYASGSVVQLYVMCSCVQKLLDASMEITDKAFHEGWYFCKPSIKWK
FMLIIMANNLECKIAKIENFNLSLPSFMKILNQSYSIALVFLRFK

>CbirOr162

MDFRNVNSLNIRLNMLSGNLLPMTDRGSSFSLLWKLYGVLVWLIELILAICLTLGSIYVSTQKLLEDSLI
CFAVGGEVIFTLLRLYSCQDLVCQLIRQMNDILCTADESMKNIVRETLEPVKVPLMFYIVASLGAIIGWW
GLPFIAILNKNVYCYEDYRVPVAFSKQPFSFGTFVLGDLFVLISTTYMFLKKVGADVYMIHLVLMMTAQY
RYIAVKMAIFHEEEEHSDISTQNKYSSKTNRKKKKEIIALCRHYNALIDATCLLKRILSTNFSIIYVYSV
FRFCFIGILISTIPSSTFLQGISVTFYAVGAAVQLYVLCSCVQQLLNASTEITDKAFHEEWYLVEISLKR
TFMTIIMANNLECKLAKIENFNLSLPSYMTILNQSYSIALLFLRMK

>CbirOr163

MDFQNVNPLNVWINFISGNLFPMTADDTSFSVLWKIYSILLWLLELVQVCMLIPGCILVPKEKALKDGMV
GLVVTFEVTFMVTRIHTCKRLMRQLIQSLNDILHAKDELMRNIVVATLKPMETPLKFYLMAGVLSIIIWS
STPFVLVYQKDVFFYVDFRMPVAYTREPFSTTVFVIGSLVVLMSSIYIFIKKVSVDSYMMHLILMITAQY
RYVALKLSMIFQDKVPRDDGNSYNEKKHRLTMDYRAEKDIKALCRHHNTIVQMTLMLRNLLSLNFSLIYV
NSIFRFCGIAIMVFAIPSTTLLETTVIIMYASGGIVQLYLLCSCVQQLLDATIEITDKAFHEEWYRYGGL
IKRTFIMIIMANNLECKLSTFGKFNLSLPSFMTILNSSYSIALLLLKTN

>CbirOr164

MDFQSVNPLNVRLNLISGNLLPMTADGTVFSTFWKIHSVFVWLLTAVHTSVLIYGCTLVPREKALKDGSV
ALVITVEVIFMVMQIHARKRLVLQLIKKMNEILCLKNEMIKDIVIALLKSMKIPLKFYWMAGVVSIIVWC
SAPFVVILERNYFFYVDYRMPVFYAEEPFSISIFVLGTLILMVSSMYTFTKKVSVDSYMIHMILLVTAQY
KYIASKLSMIFQDGIHNNGPKERYSNVDCYTEKEIKSLCQHHNLTLQIILLLKTLLSLNLSLIYVNSVFR
FCFIGVMILSISSTTLLETSMIIIYASGAIVQLYILCSCVQQLLDAGIEITDKAFHEEWYRYGKSIKHMF
VILIMANNLDCKLSTFDNLNLSLSSFMQILNSSYSIAILLSKTT

>CbirOr165

MDFENVNSMNRQLNLISGNLFPMTANGFTIGWRIYSIAVWLLMTVECIAFFSGCAMVPKEKNINNGMTVM
LFNVEVFSLITRIHVKRGLIMQFIRGMNDSLRTRDKIMKRIVMKVVRQMQTPIKFYLLIGQLTMTIWILL
PLGEIGKKSFFSYVDYKIPNAISKQPFSAMTFVLGIMLTLFADVYLLTKKLSLDSYVVHMVQLMTAQYQY
IALKLAEMFQNGMSENDYKAENKHQHEIDQRMKREIRALCLRHNGVTQMSFILKELLSFHVSVTYICNIL
RYCCIGVMALDTISRPLYEYVMVLLYLFSSIVEFCMVCFCVQELLNASKELTDKAFHEDWFRCRLSIKRT
FLLMITANKLECKLAKVGNFNLALPSFLMIFKQAYAFAVLFLKIRST

>CbirOr166CTE

MDFQNVNPLNVWINFISGNLFPMTADDTSFPVLWKIYSILLWLLELVQMCVLIRGCILVPKERALKDGLI
GLAITIEVVFIVVRIQTRRGLIQQLIQNLNDILRTKDEIMRNIVIGIFKPIKAPFKFYLIGGTASVFMWT
VVPFSLVFHRNVFFYIDYRMPVIFSKEPFSITTFVAGSLLVMTSSVYIFIKKVSVDSYMTHLILLITAQY
KYTALKLAMIFQGAQNNCDDASEAKSYDMNQYAKKKIKIICQQHNAVLHMTFLLRELLSSNLSIMYLNNV
FRFCCIVIMVIS

>CbirOr167

MDFQNVNSLNVRLNMFSGNLLPMTSDDSLFPIPWRIYSVIIWLIEMIYICGMTLALILIPKEVLQDGGTM
GFLVSIEVFVLMMRMNSRRDLISQLIKDLNGMLRSEDETMKNIVLSILKPINTPLEFYWIAGTSSLMIWS
YIPLVVLSKKTYFSYSDFRVPIVLTKQPFTVEIFVLGTILVFIGNLYLFTKKVALDVYMINFVLLMTAQY
HYIATKLAMIFRDAQDTDDKVREGCYFKISSYAERRMRTLCRHHNSVMYITSMLKELLSSNMSLMYLSNV
FRFCFLGIMLISAILSSNLFDTFMISMFISGGLVQLYILCLCVQQLLNASMEITNRAFHEKWYQYESSLK
RIFMLMIMSNNLVCKLSKFEKFNLSLSSFTTILNQSYSIALLFLKMK

>CbirOr168

MADVRIEKLIAFLKTHLFFACCWPLRSTATRYEKTCDMIFRFFSMVHGTVMIVSILYTIYINPSNLLLIM
RLCCQLCTTTEVPLQIMCYTMQYDRLQYVIYELEDYCKRAKPEEVIVFHRYIDNCRSFYIGSVSAFTVTA
LLLCISPAVESTTFPIDVEYPFSMDYLPVKILIYLHQLLLIYQSYTQVCSNVFVGLLLWFVSARCDILSK
KFRTVTEFTELRAHLREHQQLLQYGNEVALSVRYVMLASLTVSTVVIIFTGCTFLSRQPLSVKSTFLIFF
VSSLAKVYLCAWPADRLLSASTDIAHAAYNSMWYNSKAEFQKNFLHTLLRSQQPIIINVPCILPTVSLNY
YASYISTAFSYLTTFRILLEDDDED

>CbirOr169

MKGNKVTVDRVIAFLRVYLALACCWPLPPDATKFQRLCRRILRYFCLANSITIIFSGVWTISKHGDDAFM
VMRVGCQMSAAAQIPLQMILFAMQDERMQVKMFMLVMLSYVMLCCVWGTCSILCCLAITGVSFIFAPLLL
PQPFPLELEYPFDIYQPLQTIIYVHHIIAIFQSVTQVGANTFPALLLWFVAARFHILSTRFCTVTDMKQL
LKCVYEHNILLRYAEEVTHAVRYVALLCITCSTGAVIFGYLTFMSRQPVSVKFIFLMIGLCGFVELYMYA
WPADNVMTMSSDIALAVYNSSWYKDDVAMRKVLMHIIRRSQRPVVVSVPCALPQLSMTYYASYISTVFSY
MAFIRIMIGQE

>CbirOr171

MCVRKRVTLKQVISIVKLSVLFIWFWPLPQSSSKWKVLSMKLYQYSSILFAITVMAPMLYSVMNNLDDSN
HIIKSLFALFPCCHVIWNILCHIVIYQQLQFVTFEMERFCALIKSHEEACIQREYVDKCAHFYGFCIVVF
YMSLFALILGPVVLDQPFPVTAEFPFDASRQPLRIITYLHQVVVGLQIAAHLCVNAYMALLLWVTSARFR
LLTEEIRAATNIYDFIKCIKKHQQLLQYTGKVVFTVRPFALGTIMSSTISVIIFGLLLITRGPVVLKIQC
LFLASCALLEVFMYAWPAEHLIQISSDIAQTTFEMKWYESEHFRKNLQMIIIRSQKPIRIVLPCGFSSLS
FRYYASYLSTIFSYFTTMRIMFEKQKDGV

>CbirOr172

MGIFIEDRLIRMLRVCGTYYTIWPLSSEDGKYKTLFCNFLWWFYTVNILTEVFLIIKSMFHQEGSVNILK
SLVETTYVLEISFGIICCRIRRKQIQKLLFEIEQPHKIWTPREQAIRSKFIRRLYKICIPVWAISVADIL
LYISSPLITNRMFPLNNVYPFPISNIWVHCLTYVHSAIALQQAAIVVFIDLMITVIMWHAAFKFYLLGMQ
INLVDTAEKLRASIIEYQNIISYVREIEHTFNILILKSAVVTFIDVIASSLLVLNNAPIVEILEFFISTF
ISVGRLFICCWAADEITDKAYDVAWQIYTSPCMYGSSIMRQNIYMLIQRCLKPVVISTSGFIPVVSIKFC
GKLLYLTFSFFMALRAIL

>CbirOr173PI

MTLLMQEWVIRHLRVYGTYYTIWPLSSEDGKFKTLFCNFLWWFYTVNMISMSFLIPNTILNQESLVDTLK
TLPVSAYCLEIIFNLIYCRIRRKEIQKLLFEIEQPHKTWTPREQIIKVKFIRRFYKFCILLWIITVIDLA
IFVFSSLISNEMYPINVVYPFPISNNWVYYVTYVDIVIAMQETCVVMLVDLMVTLIMWHAAFQFYLLGMQ
IRFVDTAEKLRASITEXNTEKNIVERVKFFMGSFVSITRLFICCYAAEEITDQAYDVTWQIYTSPCMYGS
RIVRQNVYMLIQRCLKPVVINAGRFIPVVSIQFCGNLLYFTFSFFMGLRTLF

>CbirOr175CTE

MVLIRIRIHYVLRVLQICGAFTSTWPPDPTATKREIFFRDLSWFLSIMNVLSSIPPLILGAWYSRNDIIQ
MMKSLSELTALMEVLFNLILCKIERLRLQVSKISDFFKYSELHEKHTIQKYIDRYSNFSAVVGISYILAA
ITFSCGPFFLPINLPMEAWYPFPTDSLYVRSILYISQVLAILQTGFCITVDFMIAMFFWYSAARLDMLGL
ELQHITHENQIKTCIQKHQEIIRYAFNYLKYKYCDHIIFTMX

>CbirOr176

MTRERDIVPNFHLRVSLSIIRFLGMWLPLSSNYWALFLVYTVCSFIFILGILLAVEIANVFVYWPDLSRI
IAGVPILMTNCVHASKTIIVLHRQKRIQALLDTANSLSRDELHQDLLREYTWKGLFHHAVYQSFGAVAVF
CWGFTPISDLIAGRSRQLPMEGWYPYNTTTSPAFEITAAHQGIAISIACFHNVAMDTLITGLITVACCQL
AILERNILSIDNEKIVCVVQNDIQDDQGNSDVPEGTLSPNQQLKRCAMHNNLIYNFTREIQDIFGTIIFF
QFLSNCLIICLIAFNVSQMKVYIPEVLIGMLTYMCCMTYQIFIFCWHGNELHLHSLRLALAAYSSNWFAG
TEGFKRSLQIVMVRSQRPLTMSAGNIMLLSLDTFVQILRMSYSIFTVLQSSTE

>CbirOr177

MHVLPLSFALLTYTGYWRPVDWPSTSVKYWAYCIYSTAMIFLLHTFALCGLVDCFMIKDVEIFIEKFSLF
LSVLGVCLKVMNLVLRRDEVIGLTDMLVKDVCTPRDNYETEIQQRFDRTAMTITIYCEILNESAVFFATV
AQLKHYIDTHTLPLSDWVPYDISSTGVYWATVLHQTIGLMVCANASVAHETLISGFMIQTCAQLDIFCHR
ARTLPNLLQEAQKYSTSKADQKMRERQLIREFVHHHRYIYRFADRVTAVFTLMILLQFTISSTVLCLSVY
EMSKKNLLSFEFAWSSLYLGCMLMQIFLYCWFGNEVTLKSAEIGSAIYEMDWSMFPTDLMKTLLIIISRS
KRPITITSGYIVTLSNESFMKIIRISYSAYNVLKKYERGX

>CbirOr178PSE

MHILPVSFALFNYTVYXQPVYFPVNLLKYXMYNLYFVFMITLLNTFVFYGLVNIFFMSANLVREQFVNKC
YLYFFRSTAFLARSCISLXLREDKLNKMLLRENCIPKEGHKVSIQAKFDRNARYTITVLRDIPLYVYPLR
NFTCWXWTCSFPVYDXAPFDVWSIYIFMPTLLFQCVTLMLCANSSVTHETLIVRMMIQICSQFETLCHRT
NLLPMLLMNVEKKYKSDQDLIMXERMMIRNLMYHHLYIYKFANMVNVLTLVMFVQFSVSSLVLCMSIYKI

STMTSLLTLDFDVFSYLYTPILTQIFLYCXYGNEVTLKSIDVSNDIYKMNWTILKIWIIKDLIVMTMCVA
KPIKMSSGYVVILT

>CbirOr179

MQILSLNFLLYTIGGVWRPIEWSSTRSKLLYNLFTFYVICSLMFLLVTELLNILFVDNIDDCIMILMILL
SIVSCICKMFIVIIRRDKIINIIGTLREKPCKACTEDEMDIQLKFDRLIRSQSISYLLLALLSLLGGMIG
AILETLEGTLPYKIRVPYDCSSLLSLWLTSLQENIGMIMGTFINVATETSIMGFCLQMCAQFEILKHRLQ
RMVNPTEEIPEHFQAHMPKKTSRLSKHICHHLVIIRNVRMVNDIFSVVIFIQFFASILILCTSLYFVYSH
TIADVAPFIIYAFCMFVQIFVYCWAGNEVMLQSTGLSEGVYDMDWTLMTINERRDLLMIMKRSMKSIKLT
SSFLVTLSLESYSNLLKTSYSVFSLLQQT

>CbirOr180

MQILSLNFLMYTVGGIWRPIKWCSSGLKVLYNIFTILVLLSLYFLVLTQFLDIVLIVDNIDDFTTNSLML
LTIVAVCCKATLVVVRRNAIINLVRMLLKDPYKLRDADEMAIQRKFDTFIRSYSIKYSLLCTTSLTGVTI
GSVLNIIQGYLPYRVWLPYDSNIATMFWITSMQQIISLIFATVINVGTETLISGFILQTCAQIEIFQNRL
HKLMINQTTAYMKQSLASSCKGTSIFSEYIRHHLKIYEYAKTVNIIFNPIFFVQFFSSIIVLCTSVYYLS
QHITESGSATFIVYTIGMFVQIYIYCWSGNEAILKSTSVGDVIYQLDWHLLSVSEKKDLLMIMIRTTIPI
KFTSSFLITLSHESYSNILKTSYSAFNLLQRS

>CbirOr181

MQLLSLNFLMQTIAGIWRPTEWRSSGAKLLYNVFTIFILFLIYFLMLSQFMDSVLVVDNIDDFATNSLMF
VSMIAVCCKATVIVVRRNAIINLVEVLLKEPYKPRDADEVAIQTKFDEFIRSWSRKYLILATSSVTSVTI
GSLLNVMHGYLPYKVWLPYDSSTSPTFWITSIQQIITVIFATIINVGTETLVFGFILQTCAQIEIFQNRL
EKLVIYKTTKYAEHSFASSNKKKATISEYVCHHLSIYNYAKTLNSIFNPIFFVQFCGSIIVLCTSVYYIA
GHITDSESATLLVFTICMFVQIYVYCWSGNEVMLKSVKVADIIYHLDWPLLSVSEKKDLLIIMIRSRIPI
QFTSSFLITLSHQSYSNLLKTSYSAFNLLQQS

>CbirOr182

MQILSLNFFIYTVGGVWRPIEWSSTGAKLLYNVFTFITIVPLYFLMLTQFMDIVLIVDNLDDFATNSLMF
MTIVGVCCKATVAVVRRSEIIDLVQILLKDPCKPRNEAELAIQAKFDEFIRSSSIKYSLLATGSITSFTI
RSLLNVLQGHLPYRVWLPYDSDESLMFWITSIHQIITLIFATIINVGTETLIFGLFLQTCVQLEIFESRL
HKLIINKTAGYREYFSRASSDGKETIIAGYIRHHLSIFHYAKTLNSIFNQVLFFQFFGSILVLCTSVYYM
STHISGSEAATLPAYTVCMFVQIFVYCWSGNEVILKSTSIGNAIYEMNWTLLSISEKRDLLMIMKRSAIP
IKFTSSFLITFSLEAYSSILKTSYSAFNVLQQS

>CbirOr183

MQILSLNFFMHTLGGVWRPIEWSSTGAKLLYNVYTSITIVHLYFLMLTQFMDLLFIVDNLDDFATNSLMF
MTVVAVCSKATVAVLRRNTIIDLVQILLKDPCKPRNETELVIQTKCNEFIRSISIKYSLLATCSVTGVTI
RSVLNVMEGQLPYRVWLPYDSDEPSMFWITSILQIISIIYCTIINVGTETLMFGLFLQTCIQLEIFENRL
RRLVINKTSGYREYLPHKSLDTEEEMNISGYINHHLCIFNYAKTLNRIFSQVLFCQFFASILILCTSVYY
MSMIKGSEAATLIVYTFCMSVQIFIYCWSGNEVITKSTSIGNAVYEMNWTLLSISEKRDLLMIMKRSSMP
IKFTSSFLITFSLESYSNILKTSYSAFNILQRS

>CbirOr184

MQILSLNFLLYTIGGIWRPIEWSSNCSKLLYSLFTFFSIYSLLILLVTQFLDIVFIINDIEDYITNIMIL
LSVISCVTKMFVVVIRRDKIINIIEILQEKPCKACTEDEMNIQLKFDRIIRSHSISYLLLAMVSATGTVA
GGLLDTLKGELPYNTWVPYDYTSSLSLWLTSLQESIAVILGTFINVATETSVLGFCLQTCAQLEILKHRL
QRMVNSTEGIPEHFPLHTPQRMGRVSEYVCHHLSIIRIAKMINDVFSEVIFVQFFASILVLCSSLYHLSS
HAIVDVAPLIVYAFCMFVQIFVYCWAGNEVILKSTELSEGVYQVDWTLITVSERRDLLMIMKRSTRPIKL
TSSFLVTLSLTSYSNLLKASYSAFNVLQQS

>CbirOr186PC

MRILGFTFKILMSCGCWIPNSWRSPHRRLMYHVYTIFILMLINTFTLSQFLDIILTVDNADDFTDNFYMM
LAMIVSCFKMFSLLINRGNIAMLTDILMKGPCKPLEQEEIEIRQKFDKLIETNTLHYMILVELTCASTAV
ASLFTDYRKTQLTFRAWLPFNYSSTMLYHFTYFHQLISLTVGSVLHVACDGLICGLLLHICCQMEILSYR
LKKIVCSPERIRDCVIQHNLIFKFAFMLNKKFRFTITFQFLVSTLVVCFTLYQLTKTSGKFVEIGLYMSC
MLTQIFLYCWYGNEVKLK

>CbirOr187

MHILEKTFIILTICGCWRPNSWTSLYKRILYHMYTVFIFVLVNSFTLSQLLDIILIVDNSDDFSDNFYTL
LAMIVSSCKMFSLLINRNNIAMLINILMEKPCKPFRLNEVEIYHRFDKSIQTNTIHYAILVETTCISVAI
TSLFTNFNKRILTFRAWLPYDYSLPILFYITYIHQLIGFIMGSILHLACDGLIFGLLVHIYGQIEILESR
LRRITHEPDILRECILQHNRILKFAVLINEKFRFILSIQFVVSTLVVCFNLFQITKTTTSSAKYIQLALY
MSSMLTQIFFYCWYGNEVKLKSRQLSNNIFEMEWSTLDSRVKKTLLLIMRRSTVPIEFTSSYIIFMNLDS
FVGLLKTSYSAYNILQQV

>CbirOr188CTE

MHVLDSTFKFLTICGCWRPNSWTSSCKSMIYRMHTIFILALVNTFTLSQLLDIIFIVDNADEFTDNFYML
LAMFVSCCKMLSMLINRKNITMLINIVMEKPCKPLEPDEMEIYHKFDKSAQATTIHYAILVESTCVCITL
TSLLTDYRKQALTFRAWLPYDYSSPILFHITYVHQLISLIVGSVLHVACDGLICGFLVHICCQIEILESR
LRRIVCEPDILRDCILQHNRIFNFAFLVNEKFRFTITIQFIVSTLVVCFNLYQLTRVTTSNEKYMQLVLY
MCCMLTQIFFYCWYGNEVKLX

>CbirOr189

MRILQLTFKILTFIGCWRPESWSSLYLRMVYGAYTIFMIILLYTFLMSQFLDIIWNVDNAEDFTENLYVM
LASVVSCSKMLSLLVNRRNIYALTNILIEKPHKPLQIDEIKIQYKFNKLIQNKTFWYTILVETTCACITM
TSLFTEFRKGNLTYRAWLPYNYSSTIVFCFTYAHQLLSLTAGSLVNVACDSLICGLLVHICCQIEILEYR
LSKLSRDHDVLRDCVLHHDSIFAYAHKLNNEFRMTIAMQFIVSTMVVCSNLYQMTKSTTANASYLPLLLY
MSCMMTQIFIYCWYGNEVKLKSIQLVDNIFAMDWLTLDRNVTRGLLIIMNRATIPIEFTSGYVFSMNLDS
FVGILKTSYSAYNILKKM

>CbirOr191

MEKAKIMEFTFKVLSVVGVWPPDSWTSSFKRAMYNVYSAFITLMIFTLLIPQLLDIILNVKTTEDFADIF
YMLLAVAMSCFKMTNLLINRKNIKMLKNILSERPFIPVEADEMEIRRKYDKSIHQKTFWYTVLAEVTLMW
TALASFLTDFRSGNLTFREWTPYNYSETMFCVIYVRQLISTSMGAMVNIACDSMICGFILHVCCQFEILE
CRLKKVALDQKNLRMGDCVHQHDCIFKFAFMINEHFKPIITIQFIVSMLVVCSNLYQLAKVTLNAESLTL
MLYTTSMLTQILIYCWFGNEVKIKSVQLAANIFEIEWLKLDRSSKKDLLIIMNRSLVPIEFSSAYIITMN
VDSFVSLLKTSYSAYTLLKQMEME

>CbirOr193

MRAMQFPLKILMVAGCWPPVSWSSLCKRTVYNGYTIFVTLMLVTFMFPQIMDIILNVDNADDFTETFYVM
LATVIASCKMFSLLLNRKNIKILTDALAEKPFRPLEPDEIEIRQKVEDTIRTNSIWYIFLVEITCACINL
TSLLTKFRRGDLTYREWTPYEYSSVMYYITYFRQLISLSTAAIVNVACDCIICGLLVHICCEIDILKYRL
RKSLRNRTDFRECVRQHDSIYKFAYTINENFRIILAIQFAVSTLVVCSNMYQIARTSLSAEYIPLMLYTS
CMFTQILIYCWYGNEVKLKSVQLANEIFEMDWITVDKEVKEGLMMIMNRSLVPIEFSSAHIFTVNLDSFV
KILKASYSAYSILQQMK

>CbirOr194

MRVMQFPLKVLTVAGCWPPISWSPLCKRTVYNAYTIFVTLLLFTFMVPQLLDIILNVDNPEDFTATFYVM
LAMVIACCKMLSLLHNRKNIAKLTDTLAEEPFRPLEPDEIEIRQQFDNMIQTNAKVYTVMIETTCACMNL

TTLFINFRRGDLAYREWLPYEYSSGIIYYFTYFRQLVSLTAASIVNVACDCMICGLFLHICCQIDILKCR
LRKCLRNRNDLSECVCQHESIYKFAYMINEKFRVIIAVQFAVSTLVLCSSMFQLARMSMNAEVVPLLLYT
ICMCLQILIYCWYGNEVKLKSIQLADDIFEMDWIASDKGTKENLIMIMNRSLVPIDISTAHILTVNLDSF
VKLLKASYSAYSILQQMEGNKK

>CbirOr195PIC

MPLKLTYTIVMIAGCFRPLSWTSPFKCTIYIIYRLYIISMLCTFTMLQIMDILLDIDNADDFTNNLNMLL
TTSAASYKMFIMWINYENFATLINYLTXNNTLRYTILIETTCSFIALTSLFTDFTHRRLTYREWVPYDYS
SFMAFCFTYAQQMVSTFHCATVNVACDTLLCGFLMHLCCQIEVLEYRLKKLSSNKNSVGYCIRHHNRIFE
FARLVNIRFAKIIGYQFIASMLVICANLYQLTKSSLSANHVQLIMYTSCMLTQIFIYCWFGNKVKI

>CbirOr196

MHILKLTFMIVMSVGCFRPPSWTSLFRRVVYNIYRIFIITILYIFAFLQFMDIVLNVDNPDDFTNNSYMM
LNVSVSGYKLLIMWMNYKNIAALISKFTEEPFKPLDLSEMEIRRKFDKLIWMNTLRYTILIETSWSCSGL
TSLLADFRHRRLTYREWVPYDYSSYMVFCVTYAHQFLSTFYCATVNVACDTLICGLLMHVCCQIEILEYR
LKKLSRNADTLDYCIRHHNSIFEFARLVNTRFTQIIGFQFITSTLIICSNLYQLSKSTMSADNISLIVYT
FCMLTQIFIYCWFGSKVKSKSLELTDRIFQTDWPMLNSSIKNSLLIIMKRAIIPIEFTTAHIISLNLESF
VALLKISYSAYNLIVRMQQE

>CbirOr197

MKFTLLLCTLSGCWLPPSWMSLPKHRMYSIYRILLIVILFTFTISQIMNVLLNIDKFDEISDSIYMMVSI
SVAAYKIIAVWINHKNITEIITIFAEKPFATVESSEEMIQQKYNKAIKKYALWYVILVETTVGIMIVGSA
FTDGVERKLPYRVWLPYDYSPPTIFYPTYIHQLIGMTIAANINIACDSLISGLLQQICCQIEILQYRLTK
VSHDGHVLNNCVRHHDRIYKYAQTVNMNFTKIIALQFVVSMLVVCANLYKLVMATILADFIILMLYTACM
LAQIFLYCWFGNELKLKSKSLSNSIYNITWYTLDDSKKKSLLLIMKRSMIPIEFNSVVIITLNLDSFVSL
LKASYSVFNVLKRAQ

>CbirOr198

MSVMKSTLLICTIAGCWQPPSWKLSFKYIIIYRTYAMFLTSLLYLFTISEFMYIIILNADSSDDYTDSLYM
LLSIFVTAYKQVSMWINYKRIMAIINVLSEKLFASCDSCEVTIQEKFEKMSQTNTSRYLNIVMLSVITIF
SMSIFTNLMKRNLTYKAWIPFDYSSPAVFYIVYAHQLLAMVTSGIVNIACDSLLCGFLLYICCQLEILEY
RLTKMACGQYILRDCIIYHNYIFEYTYAVNVFARIIAIQFVASAIVVCSNLYRIFVAVVENDYITTLIIS
TYTFAILMQIFLYCWFGNKVKLKSLHLANSIYDLEWRTLTVSTKKALLLIMNRAKIPIEFKSISILTMNR
DSYLNLLKLSYSAFNVLYHTR

>CbirOr199

MRVLRFTLLICAFAGCWQPSSWTSLLKHIIYKTYAMFLGSALCIFSISQFMNIMLNVENSDEFIDSLYMM
LTVFVASYKQVYMWRDRKNIMMIINVFTFKLFAPCESYEVTIQRKFEKITKNNTLRYLLLIVIAIASMIM
MSVLTELTKRNLTYKAWIPFNYSSPAVYFLVYIHQLIGMATSGIVNVACESLIFGLLLQICGQLEILEHR
LTKLVHDEGIIGDCILHHNLIFEYAYKVNNMFAKIIALQFAVSMLVVCSNLYRLAMVTDYVTFISLTTYT
CAILTQIFIYCWFGNEVKIKSLHLANKIYNMEWLTLSNSCKKGLLMIMKRATVPIEFSSAYVITMNLDSF
AALLKMSYSAFNLLHQTQE

>CbirOr200CTE

MNVLKFTLIICGFAGCWQPLSWTSLSKNIMYKAYATFLISSLYIFLISQFIHVALNVGNSDEFTDALYMM
LTILVAGYKQVYMWIDRKNFMIIINVLTEEPFAPCESHELMIQEKFEKIAQTNTLRYLILVLMSISSIIL
TSVFTVLTNHNLTYNTWIPFNYSPPAVFFLVYTHQLIAMSTSGVVNVACDSLLCGFLLYICCQLEILRYR
LSKIIHGQDILHDCVYHHNRIFEYAYIVNKKFAVIITIQFAVSMLVVCSNLYRIAMAANYMSYIPLVVYT
SAILMQIFIYCWFGNEVKLKSLHLMNNIYNMEWTTLNSSSKKALLFIMKRAMVPIEFHSSYIITMNLDSF
VAVLKMSYSVFNX

>CbirOr202

MDILPLNFRVLWFCGAWNERSSSNTFIRFLCSCYRYAVFILIYQFTISEIIELIRMRDHIEELTEGLFLA
LTYVALCFKYGNFLARQNQVSMLLDCFRGETCQPKNSEEKMILSRYNRKAEWCARAFMIMCQASCVAHIL
APIVGPQDTDRPLPFKTYLPYSISGLFPYVATYLQHVGAIFYGVLLNVAFDTLVYGFTLHVCGQIELLRH
RLSEIFRNYPDTERTDSGKDAMIDECVKHHLHVHEIVRRIQSLFVWTVTVLFIFSMVTLCTSIFQMSKKE
LLSAKFFSLLLYLGCMLFQVFLYCWYGNELELKSKSIGDAIYSSNWTIATSRERRSLLFMMTISQRGLNL
SYYGIFSLALDTFTWILKTSYSAFNVLQRASM

>CbirOr203

MEHPEERYYKLNRFLLSVNGLSPYQNVWNARLIRLFITVILVSALFTEASTTLTSEVNQELLVAIIPEIL
PTLVVTINLYLRHGNIDKYKKLFDYMWRDWALQKTKDEIKIMHDYAETTRLVMLLFTLQMWIIVVVYIVW
IFTPEILDVISPLNESRPRIESFKLEFFIDKDRYIYFTRFYICFVAIIIITIFLSNFSIVLTFTQHVCGM
CKLLGYRAEHLFCVIENTAGCNLIRGTKIGCRDTAVFVRLHCNVIQFISLIEACHRVPFLCELIGILFNV
SMTLIQILTIAGNIERAITSISSTTMILTYSFISNYMGQRITDMSSNMCEKVYNCAWYNVAVSEQKSVLF
IIRRRFPPLVLTANKFYTMSLQNFGMILQTVISYCMFFRQI

>CbirOr205

MTNEKHAWPSQYYTIPRLYLSLAGIWPYGKLRNRYMCFVPMFTCCFSVVIPQSLYIINNSLNIDEIFESL
PSVIISIIFSFKVANMMINNEKIKMCFETIKADWLSLNTDIEKAILQRQTMYGRHMTIFYTVLMYMTALF
YLIKPVIITLLEDEMANTTKSRISGISKLPFLVDYGEKLNYYFYPIMIHCYLGVFAHIFCTIAVDTLYYI
LVQHVCGIFAIIGHALENINEYTDATDNLHSQLNTTQDDYNKALNCLRRHLHVIEFADLIESMFTKILLI
SVSLNMICGSICGIRVIMNLSNARDIVAPLAIYVAQLSHMFLQFWQAQFLLDYSIIPYESICRAKWYKTS
IKCQKLLLLIMNRTVYPCKITAGKILILSIESFGTVLKTSMSYLTVLRSFQ

>CbirOr206

MTDAKDMWQSRHYIVLKLYLIITGIWPYHKPRVRYVRFAFWFAFGFTILIPQSLFLLILPTTLDDIFECM
PSICVSILCSFKIAIVMLNSDKVKMCFNIMEKDWLSLKTDTERAILQRHAKYGYYTSLFFTVMMHTTVGL
FILKPIMYTLINDATLNITTKSTIPSASKLPFHVEYGQHFNQYLYLIALHCYGGIGGHCVASIVADSLYY
TLIQHACGMFSVIGHMLENIGKNNDINFDLNPDKIDDDNYNKTLNCLRRHLHIIQFAEFIEIIYTKIYLV
SANINMIAGSLVGIQVLMNINKTAKDIVAPLTVYIAQLLHLFLHFWQAQFLLDYSVLPYESICRANWYYT
SKRCQKLLLLIMNRTMKPCKITAGKIMILSIESFAAVVKASVSYLTIFRSLQQ

>CbirOr208

MIERKDVWQSRYYMVPRFFMTLTGLWPYHPILNRCLRFVPIFILCSSILLPQFLYISTMTDTDDLFECMP
PIAFTIIFSLKLAMIMVNTKKIKACLKTIEDDWLSLTDSEKAILQRHAIYGQYLTIFYAVFMQLTGFLYM
LKSIVLILIEDTSNSTRLAVTKLPFRVEYGHTIDQHFYPILVHCYMTVFSHVTATVAVDTMYYTLIQHAC
GMFSIVGQSLERISQNSNADFNLKPDKTTDSDYRKALGCLRRHLHVIELAELIESTFTNIFLVSISLNMI
GGSICGMQVLIHLNDTKDKTAPFTIYVAISRYIAQLVHLFLQFWQAQYLLDCSTIPYESICRVNWYYTSE
RCRKILLLIMIRTILPCRITAGKLVTLSIESFGVVLKTSISYFMVLRSFQ

>CbirOr209

MDFFDTRCFRINKLFLSFVGLWPFQTAFMKFLTLSFSIFGVTIMCVPQIVYLLKHTDDLDNVFELMPTTS
GTVICVMKIISLTRNSGKFKMLLQQTHEDWRNLLSSQETQILTRYSENGRKFTLAYSICIIGFVFCYALL
PLAGPVLDIISPLNGTRPRKMPHPAEYFINQEKYYYVLLLNTYVGYVACVSIAVAADTMYVLLVEHICGM
YGVLCHRLENLTHDEIQWVDDDYIHGHDEIGRRMRCCIQLHERIRLFIEMMESTFALFLLFDVGLGFILH
TSSCVLIVVRMGRSSEILRYVSLVALQSCRLFFNSWAGQEVADHSTGISIAAYNGTWYNAPTEVQKLLIL
LIARSQKASRITIAKLYVINLEGFSMVMRTSISYCTVMISLRETSESM

>CbirOr210

MSVYNSRYYYLNKVLLSILGLWPYQSRLEGNLTLTISLLFIVSFSSLELWSLITGITDLSIVIENLSSTL
INVFLVGGKMANHLYKYKMKDLLDRIEETWKMIPTGPENKILQKYAEESRSFTVYYSLIMYTIWIFYSTM
PVVLRGIYSFLPNQTYEIKFLFRVEHVIDVEKYFNLLMIYAIISVFYIVSVVVAIDCIFVACIQHNTALF
ECIRFNMERIRSVDCAQAETQWMLKPNIADDEVYHIIIGCIKSYKNALNFSSVLSSANSELFLVLLGSVI
VCLSFSAAQLMLLDNQLDAIIRLISLNVAELMHIYYLSLMSQQLINYSTGIQEVIYNCDWYTISLRSRQL
LKFTLLRASKPCQIMAGNLYVMSMENFGSIIKACISYVTMLLSLQ

>CbirOr213

MSVYNSRYYYLNKVLLSIVGLWPYQSRLEGNVMAIITLLFAGGYSGLELWSLIAGITDLSIIIENLSSTL
INIMVIGKMVNNLYNNYKMKDLLDRIEETWKMIPTGPENEILRNFAEKYAEESRSYTVHYALVLYAVWIS
FSMMPIVISRLYTFLPTNETFETKYLYRVEHVIDVEKYFTLLMLLAIIGIFYIVSIAIAVDSVFVLCMQH
NTALFEEVRFNMERIRSLDCVQVEAQWMLKPNVANDEAYHIIIGCIKSYKDALNFFDVLASAYSTFFLLL
LGMVVVCLSFSAAQLLLLDNQLDAIIRIVALNVAEVIHIYYLSLMSQQLIDYSSGIQEVIYNCDWYAISL
RSRQLLKFTLLRVTKPCQITAGKMYVMSMENFGSIIKACLSYFTMLLSLR

>CbirOr215

MSVYNSRYYYLNKVILSIVGLWPYQSRLKGNVMTTITLLFVGSYSSLELWSLISGIKDLSIVIENLSTTL
INFASVGKMANHLYNKYKMKNLLDRIEETWKMIPTGPENEILRNHAEESRIFTIYYTLAMYSAWIFYSTM
PIVVSVIYKVLPANETYEAGFLFRLEHVIDVEKYFTLLMLYAFISVFYILSIVIAVDCIFVLCIQHTCAL
FEGIRFNMERIRSLECAQAEAQWMLKPNIADDEAYHIIIGCIKSYKNALNFSEVLASAYSTFFLVLLGTV
IVCLSFSAAELILLDNQLDTIIRIVVINVAQVLHVYYLSLMSQQLIDYSSGVQGIIYNCNWYAISLRSRQ
LLKFTLLRATKPCQITAGKMYIMSMENFSAIIQACISYFTMLISLQ

>CbirOr216CTE

MSVYNSRYYYLNKVILSIVGLWPYQSRLAGNVMATITLFFVGSYAVLELWCLIAGITDLSIVIENLACTV
SHFLAIGKMVNNLYNNYKMKDLLVRIEETWKMIPTGPENEILRNYAEESRSFTVHYALAMYTIWIFYSTM
PYVMSGLYMFMPTNETYEIKFVFRVEHVVDMEKYFNLLMLFAIISVFYILSIVIAVDCIFVLCIQHICAL
FESIRYNMEHLRSLDYAQAEAQWLLKPNVADDEAYHIIIGCIKX

>CbirOr217PSE

MSISSDIFQSGQYKLNRVLLSLLGQWPFQKARDKRAIFSALSFIGLTQAITQVLALVTLRGDLSATFECI
PPLLIDGVCIIKLLNLVYNMKKIKILIIHIQKDWQSCAIKSEFEILHKFAESGRSITIGYAGGIYAFGSL
FPLLAIIPKILGNDVTSNYSTRPVGFPYHVEYYIDLEKYYYPVLIHNYLATTIRLTIVIASDTCVAILVQ
HCCALFSVVRYRLEHIQKSIEQDKELASLRKDDKVYTNFVYCIRKHKAALAFFLEIGFVILVMSVSAVQA
TNDSLTPQLAIRHGGYITAQLLHLFIACWLGQQVIDHSDRVYTSTYRGEWYESSCKSKKLLNMVMLRSIS
PCTLTVGKIMILSLPSFSAVVRASASYFTVLRSVQ

>CbirOr218

MDKPDQFFFQNTNYRILRILLSVSGLWPFHTLRRRYLTYLVMVLVLGSGFVFQVLGIVEVLNDTFAVIDT
LPLLVFVTTSMAKMFCVVYALPQLKILLIKINEYCLSAKSDEEAKIHNKHAIYARNLGYAYTGFILIHSV
LFIMVTLMLRFVHTSSDKNITSPDNSQVIFVYRVDYMVDLNTYYIPIFIHTATCSFSTAVLIIVFDVLYL
TMVGHCCGLFAAVKYRLENAVDREEGSNCLPLTLTKDISYANVAYCVRRHAETIEFVAAVESMYSLPLFV
HIGESILGLSVLGYQVVTNMGGINNLLRPASYLNGLLINVFFENWQGQKIIDFSEKIFESAYNTKWYNMS
IATRKLLIIIMMKSRTPLVITAGKIMIMSYVTFNAVLRASSSYFMLLRSL

>CbirOr219

MDDVDEFFRQSHYNIIRILLSVGGLWPYHTTSQRCATYVGFLLVLGTGLIFEVLGMIEVWPDSLEVVDCL
PLLVLGAVPLTKLVCAIYTLPEIKILLRKMQEYWYSPKSDEETRILHSYAIYGRNLGYVYMGMLLIHTVV
FILATLLTKFISVESTGNNSSSNSHSTQRGLPYRVNYMVDLETYYVPIFIHTAVCDVIYTILMIMFDVLY
LTLVQHCCGLFAALRYRLKSVSEHGNDDDDSTPISRRNKIFSNIVYSIRRHAEVIQFATVTESIYRVPLF

VHVGANISVLSILGFQVITNTEDINRVLKHVSYLNALLVNTFFENWQGQKMMDSSEEVHESAYNTKWYNM
PTNQRKLLVMIMMRSRKPSRITAGKLVNLSYVNFSAVIRASSSYFMLLRSMQ

>CbirOr220PSE(F)

MDNIDKFFQQSHYNIIRTLLSVAGIWPYQTMSQRCVIYLAILLVLGTGLIFEVLGMIEVWPDSLEVVDCL
PLLVLAIVALFKLVCAMYTLPKIKILLRKMQEYWYSPKSDEETTILHSYALYGRNVGYVYFGMVLTHCTI
FMLITFLSKFISVESTGNNSSSNTQSTQRGLPYHVNYMVDVETYYVPIFIHSLVCDVYYSVLHGVFDVFY
LTLVQHCCGLFAAIRCRLESASEYGNDVSTPISRRDKMFSNIVYSIRRHAEAIQFASATESLHRAPLFVH
LGTNTVVLSILGFQAIVNTADINHVLKHITFLDVLLFNMFFENWQGQNMMDSSEEVHQSAYNMKWYDMPT
NQRKLLVIIMIASIITAGKIIIMSYVSFSAVVRAASSYFMLLRSMQ

>CbirOr221

MDSVQAQYMRINQFLMCLIGEWPYQDNWEKILIQIIFVPAVFSQAVLQGGGMITAWFAGDIDAFMESSSP
FVISLMCICKHINYTYNHGQMRQLTVTMADDWNIYSKFSHEFDILCRSYAIGKRVTIAYAVSLYGSMTPF
LVVPVILNTASYMGLYNISEGRPLMFRTEYFLDSEKYYYPLLVHSYIGTLGFVTIVVAIDSMLVFHIQHE
CGMCEILGYRLARIVDETTLDVDLHLPKEEDVSYRHIKKCVIMHNHIIEYARRIEIANTTSYFFQLGFNM
MGMTFTIFQAVVKLSDPKEALRFASFTICLFSVLFLESWPGQQLSDYTDKIFAYITGGRWYAFSLNVRKV
ISIMLLRSYVPIRITAGKLYTLNLENFSSVVRTSFSYFTVLCSMQ

>CbirOr222

MDFFDSRNYRINKILLSIIGQWPFQSPRTSHTIVIIFVLIVCSQLFVKLCGMFSYIYDLDTVIECLIPIL
IDISGLTKIMNSILYANEIRGLFDQIRDDFLSLRNSSDIEILRKYADNGRRLSTIYVCLLYMVIVIFLLL
PLKPLIFHGANTTTRPLLLHKVKYYYIDIDKYYFLIWIHGYLASLICGTWIIAADATFMIFVQHACGLFI
VTTVRIERVIQQKGCLTRDANSSITEDKEYQNMMRCICDHKAAVRFTDLLEVAYSKHFLFHVGMVMMTIS
VTGVGAIAQLQDKSELLRLTVLSWALLFHLCCECLNAQRIIDYSEYVYNNLINLNWYDTSLRTKKLILFM
MMKIQAPCILTAGGMFVLSMETYATLVKTTMSYFAFLRSAQ

>CbirOr223

MQFFDGRNYRINKVLLLCIGQWPYQTSKSSSAIIVIIVSLAGTQFVAKICGLFSIDDIDVFIDSLSPLVV
DIGCGVKLITCILKAPEIRALFDQIQSDWQSLMTSTKIKILDTYAQNGRKFTIIYASTLYSALILFMLVP
LQSLLLESSLNSTNRPLLHRVEYYIDMDKYYFPILIHGYLTAIVCISIAIATDTMYVIMVQHVCGLFTII
GQEMENIIKKDNKDSLQINLYPTVQDDKPYENIIKSIYAHKRVLRFANLIETAFNQMFLVLAGFNMLIMS
MTGVTAVTNVDKPEEFLRQITFACALLVHLFFESFQAQRLIDHSAHIYIRLMNIAWYQTSIRTRKILLFM
IMRTREPCVLTAGKMFVISMDTFSTIVRTSVSYFTMLRSMQ

>CbirOr224PSE(S)

MPDKTYSRYYVFVYITSSIAGIWPYLKPRTKLFRISLLLLTTLTVIIAQAGFQYMCKLRLQCTAQNFATC
TVPILVIIMMYTIQSNARTIKTLTEHLFIDWEELKSTEEYDIMKSYAKNSRRLFLFFFAHCFLTMCIIML
MSLMSFILDIVLPLNESRPVLLPYPSYYFVDAEEYVFQIFSHTIVAWFFLGIGIMAHDGMFITYIEHICS
SFAVIGYRFEHLFSNEAKGIVKLVQMMNAARELRALLAQLVEDVFNIPNILQLFLITVGLSLTLLQIAQH
EDNILEIVRYMSFISVQMTRLFIFSFEGQKLIDHSLQIRDRIYNSSWYNVSAKSRELVMMVMMKSLRPSF
ISAGKIYIFSLKSFITVVQASISYFAMLSSFH

>CbirOr225

MEVTHNHYYDIVYKISSLTGMWPYLKPRTRIFRVTLLTLVSLTIIIPEVNIRSYQCMCKNMQCTFQAMTA
YLLSAVSLLKMYTFQINIRTIRSLTEHLFTDWKELRTPEEYEIMKSYAENSRRFSLIYLVYCFLAVVIFM
SMSLVPYILNIVRPLNQSRPILPPYRGYYFIDSQEHFFQILWHSIVGWEVVVAAVVAHDSLFVTYVEHIC
SMFALIGFHFEHLFHNNNGTVKILNADSRDVDHKKIAFFVHRHREILECAQHLEDIFTFPFAIQILIVTV
GMSITLLQQNDDYLEATRYVFYVIAEMIHLFFLSLEGQKLIDHSFQIRDRIYHSSWYNVSTKSQKMAMIV
MARSRRPAFFSAGKVYIFSLESFTTILQTSMSYLTVLASFQ

>CbirOr226

MEVTYSHYYDITFKLSSLTGMWPYLKPRTKLFRITLLTIIVLSVLIPQIAQQYICKTDLQCTSENLSSSI
IPITCLVMLYTFLSNNRTFKVLTHHLFVDWKELRNPEEFEIMKLYAENSRRFALLYSVYCTLSLFLFLSL
SLIPFALDIILPLNESRPIVSPYPANYFVDVREHFFKIYWHSILAWEIIIIGIITYMCMYVTYIEHVCSI
FATIGFRIEYLFYGDNDKGESLNFTQYDTFRERIALMVNTHQKAIELAQLLEDAFNIPYGIYTLIVTGGM
SVTLLQITKQNSTTLETTKYVLYVISQLFTLLIPNFEGQKLIDHSLQTHDKIYNSYWYRESTKSQKLIML
MMMKSLQPCCLSVGKIYILSMQSFSTILKASVSYFTMLASFQ

>CbirOr227

MDLPYSRYYSTAYKLSSIAGIWPYLKPKTKLFRLSLVLVATVTVLIPQVAYQYMCKLGLKCTAQNLALFI
VSILVVIMMYTFQSNARIIKTLTEHLFIDWEELKDPEEYNIMKSYAENSNRFSLLYTIYCFLTTYLFILM
SLEPFILDIILPLNESRPILPPYPSYYFVDTEEYFFQIFLHTIMVWTFIVIGIQAHDCMFITYVEHICSS
FAVVGYRFEHLVSNETIGIKNICSDDECCKRVAWFVDKHQKTLEMAQLVEDIFSIPYIMQLLMATIGMSI
TLLQITQYNDNGLETLKYVAYVVIQLIHLFILNFKGQQLMDHSLQMRDRIYNSSWYNVSTKSRELVMMVM
MKSLRPSFISAGKIYIFSLKSFITVVQASMSYFAMLSSFQ

>CbirOr228

MSSTYSQYYDVIKKVSSLAGQWPYQTPRTRLFCVSLITLNTLSVIVLQMVNFVKCDGNLHCIFETMTSYT
LTFVTLVKLFTCYFNRCKIKALIDHFFNDWDKLETPEEYEIMETYAKNGRRYSMGYSLYCFACLALFMCA
SLIPQLLNVVLPLNESRPVLPPYPGHYFVDEEEYFLHIFCHSIVAWEIAMTGIVAHDCMFLSCVEHGCSI
FAVAGFRFERLIYEDTPMKVLRTHWSDSYCKRIVFSVHTHQEAVKFAQFLEDIFSLSLAIQVGLNTVVIS
ITLLQITHQNSDTLESSRYIVYIAGQLIHLFYLSFEGQKLIDHSLQMCNKIYNGSWYELPIKSQKLMLLV
MMKSFRPCCVSAGKIYIFSLQGFCTIVQASVSYFTMLASFQ

>CbirOr229

MEFTRSHFCGITKKVLVLTGQWPYQNRRNRLLGVTVTSIATFSLIIPQVSKIFQCGKNVQCILQVLPTIC
LFTIIIVKLYACHFNASRIKYLTDHLLDDWKKLESPEEYKIMKAYAANGRLFALIFMLWCYIISPVFVSI
SLIPQILDLIAPLNESRPILLPYEAYYFVDEEEYFFYIFFHAVVALEIAITGLLAHDCMLLTYVQHVCSI
FAIAGLRFENIAYKNVNVTNLNNLDNYYNKKIVISVNAHWRALDFADLLENTFTITFLIQIVIVIIGMSI
TLLQISMQSDVLQATRYSAFVVGQLIHLFFFNVQGQKLVNHSLQIRDKIYNSSWFKIPVKSQMLLLYVMR
KSLQPNFISAGKIYIFCLKSFTAVVQTSVSYFTVFASFQ

>CbirOr230

MSSTCNQYYGVIKKVSSLAGQWPYQTPKTRIFCVILVTLNTLSMIIPQTAKFVRCDGDLQCIFESMTSYT
LTGVSLLKLYTCYFNQYKLKILVDHFFINWDRLESPEEYEIVKKYTENSQRFSLGYSSYCFISYALFMCI
SIIPQMLNVILPLNESRPVVVPYPGYYFVDEEEYFLYIFCHAVVSGEIMMTGIVAHDCMLLTCIEHICSI
FAVAGFRFEQLTYDYTTKVLHDYVSDDYRKRIAFSVHMHQKALQFAQFVTDIFSLSLAIQVAINALVISI
TLLQITQRDADTLDALKYVVVYFAAQLIHLFYLSFEGQKLIDHSVQMREKIYDSLWYKTPVKSKNVLLFVI
RRTYQPIFLTAGKILVFSLESFTTILQTSMSYFTVLASLE

>CbirOr233PSE

MRVFAGERCYRLHRIMFLATEFWPYQKPFIRXLQAVFYFSAYCCIYFQRFAAFFTSTCNMDCILKRFSYI
CVTFVFVLCYCSFYFNSEFLKQALKHMQLDWKMFENSDTIKIFEEYLFLSYIFALFVIMMVPTCLSVLMA
LKCKPVILDAIMPMNVSRPRKLEVEAEYFLDKQEYFYFYLMEELVGLGIGFYSALVIATFCITLVRHCCA
TLKIASCIIQNTAIVHTLQIPVTQEIQFMHRSICFSVYLHRRTLKLMKDFMHIMNVWYSPLILISVLGLS
CVLLRLYNAILETNDLYETFVCCVLLYICFMYMFITSFLAXSYTDHSIEILKFTYDSFWYVAPVSIQKLF
LIMQRSIKLHIILMGGLFVFSMEGFSTLLTTAVSYFTVLHTMHS

>CbirOr234PSE(S)

MYVFAGERCYKIHRIMFLFMGFWPYQKPFICRLQAVFFFSAYFCTTFFQLTAFLTTTCNMDCILKRFPYI
CLSLVYVLCYCSFYFNSEILKQALEHMQLDWKMFENSDTRKVFEEYLFLSYVFALFACLICPPFLFSWAA
LECSLVILDAIMPINVSRPRKIEVDYELFLDKEEYFYLYVTQELLGVGIGFFTILVPGTFCLTLVRHSSA
TYKIASCLIQNTVIVHTLQIPVTQQIQFIHRSISFSVYIHRRTFKFMKNFLDTVYVWYFPLILICILCLS
VLLLRLYNALLHINDIDFYDIFVCCVLLNTCFIYMFFTNFIAQSYTEHSIEIVKSTYDTLWYLAPLQIQK
LLLIIQRSIKAHIIVLGGLFAFSIEGFATIFTTAVSYFTVLHTLHLTT

>CbirOr235PC

NVFLTKLLRSSRRMVFVGERCYKLHRIMLIALGLWPYQKPFIWRMQAFTAFLTTTCNTTDCILKRFSYIC
LSCVYVLSYYSFYFNSESIKQMLEHIQLDWKMFKNSDTIKTFEEYLFVSYVFTSCVYIIVFIGTFVFTAI
ECRAVILDVIIPMNNSRPRKLEVDLEMFVDKKQYFFLYIIQEVLTWVIGVCSIVTTGTVLTTVAEHCCAA
YKIA

>CbirOr236PNI

LWPYQKGFICRMQAVFVFSACWQYIFSAFLTTTCNMDCILKRFSYICLSFVFILCYCSLYFNSEVIKQLL
KHVQLDWKMSENSDTIKVFEEYLSLSFVFTLFVIMIVPMSLFVVMSVKCKPVILDAIIPLNVSRPRKIET
DYEFFLDKQEYFFLYIIQEVLAMSIGFFSALIPGTFSVTLIRHFCATYKIASCLIQNTAIVHTLQILVTQ
EMQFMHRRICLSIYIHRRTFTCVKSYMHSVDLWYSPLLLICVLSLSCLLFRLLTTAVSYFTVLHTMHL

>CbirOr237PSE

MVFAGTRCLKLHRIMLMTVGLWPYQKSFILRIQSIFFLSVYCCHVMTFLTTTCNMDCILKRFSYLCITFV
FIINYYSVYFNSDAVKQVFEHMQFDWKMFENSDAIKIFEEYLFESYIFILSLCIIFLLFASFLITMECKA
IILDVIIPMNESRPRKVEIDLELFINEEQYFFFYIVEEALGVGFGIWSMIMTGTFLTTIVKHLCATYKIV
SYLIRDTVTTHTLQLPVVQRIQFMYRNICLSMYIHRRTFEFCKGLLLLFQMWYFPLLLVSVLSLSCILFR
LYNAIMQFNDFYDISLSSSLVLCYLMYMFIQYDTLWYVAPLSIQKLFLIMQKSIKSHKVVVGGLFVASIE
GFSTLVTSSVSYFTVMHAMRS

>CbirOr240PI

MVFAGTRCLKLHRTMLISVGLWPYQKPFIWRIQSIFFFSMYCSHTFFQLTPFLTTTCNMDCILKRCCYIC
ITIVFIMNYYSLYFNSEAVKRMVEHMQFDWKMFENTDAMKIFEEYLFESYIFTLSLCISFVILGSFLIPT
ECRSIILDVIIPMNESRPRKAERDLELFVDEQQYFFFYLLEEILGLGVGCWSILVWYNTLWYVAPLPIQK
LFLIMQKSIKSHKVVVGGLFVASIEGFSTLVTSAISYFTVMHAMRS

>CbirOr241PSE(F)

MVFAGTRCLKAFHRTMLISVGLWPYQKPFIWRIQSIFFFSMYCSYTFFQVTPFLTTTCNMDCILKRCFYI
CFTFIFIMNYYSLYFNSEAVKRMVEHMQFYWKMFENTDAMKIFEEYLSESYILTLSLCIILLLFTSFLIS
TQCRFIILDVIIPMNESRPRKAEVDLELFVDEQQYFFFYLLEEILGVGVGCWSVFAVATLLTTVAKHSCA
TYKIVSCLIQDTVTVHTLQLPVVQRIQFMHRNIRLSVHFHRRTYEFCNDMILLFEMWFFPLLLIGVLALS
CLLFRLYNAIIQFDDVYDISLSCAYLFFFFIYMLVANFLAQSYTEHSVGLLESTYNTLWYVAPLPIQKLF
LIMQKSIKSHKVVVGGLFVASIEGFSTLVTSAVSYFTVMHAMHL

>CbirOr242NI

FTAFLTTTCNTDCILKRFSYISIDCVYAMNYYSFYFNSESIKQMLEHIQLIGKCLKIVTQLKYLKNISLY
RMFSQYLGVVTFVFICVFVFMVIECRPVILDIIIPMNESRPRKVEVDMEIFVDKEQYFFLYIMEEVLLLG
IGLCTVITTGTFLATLGEHCCATYRIASCLIENTVTIHTLAIPVDQKIQFMHRSICLSVYIHRRTMELYL
AIIKANDWYDILVCCVVLYGYLLYMFVANFLGQSYTEHSAELLESTYNSLWYVAPLPIQKLFLIMQTAIK
GHKIVVGGLFTLSIEGFSTLITSAVSYFTVIHAMYL

>CbirOr243PSE(S)

MVFVGERCYKIHRIMFMIMGFWPYQKPFIWRMQAVFFFSAYCCIIFSKFTAFLTTTCNTDCILKRFSYIS
IDCVYAMNYYSFYFNSESIKQMLEHIQLDWKMFENSDTIKIFEEYLFLSYIFALFAIILIPTMISAYMAL
ECKSLILDAIIPMNVSRPRKTEADYELFLDEQEYFFLYIMQEGLGIMIGFYAILVPAAFCFTLTRHSCAA
YKIASCLIQNTAIVRTLQIPVTQQIQFMHRSISFSVYLHRRTLKLMKDFMHAVNLWYFPLVVICILCLSC
LLLRLYNAITDTNDLHNTLVCCVLLYTCFMYMFITSFLVQSYSEHSILILKSTYDTLWYVAPVPIQKLFL
FMQTSIKAHMLVLGGLFVFSMEGFSTILTTAISYFTVLNTMHS

>CbirOr244

MVFMGERCYKIHRIMFMAMGLWPYQKLFIWRIQTVIFFMIYSGCFLGQFMAFLTTTCDMDCFVKRFSYIC
INFIYIGSYYSFYFNSETIKQVLEHIQLDWKMFENSDAIKLFEEYLFGAYILSLFTYLIVPSSLLLFAAL
EYRPVILDAIIPMNVSRPRKTEVDFELLFFDKQEYFFLFIMAELFAITIGFFSLLVPGTFFVTLGRHCCA
TYKIASCLIENTAIVHMLQIPFGQKIQFMHQSICLSLYIHRRTFKFAKDLILSFDLWYFPLILICALSLS
CFLFRLYNSITRFNDFHEMFVSCVILFGYLLYMFLANLLAQSYTEHSVEILKSTYDTLWYLAPLSIQKLF
LIMQESIKAHSIAIGGLFALSMEGFSGLLTTAVSYFTVLHTIHSGS

>CbirOr245INT

MVFTSERCYKIHRIMFMAMGFWPYQKPFICRLQALFFCSTCCFNILFQLTPFLTTTCNMDCILKRFSYIC
LSSVYVLCYCSFYFNSEVIKQSLKHVQLDWKMFENSDTIKIFEEYLFLAYIFEIFACILIPTMISAYMAL
ECKSLILDAIIPMNVSRPRKTEADYELFLDEQEYFFLYIMQEGLGIMIGFYAILVPAAFCFTLTRHSCAA
YKIASCLIQNTAIVRTLQIPVTQQIQFMHRSISFSVYLHRRTLKLMKNFLHAVDLWYFPLLLICVLCLSC
VLLRYDTLWYVAPVPIQKLFLFMQISIKAHIIMMGGLFALSMEGFSTLLTTAVSYFTVLHTMHS

>CbirOr246NTE

XIVFMCVFVFTVIECSPVILDVIIPMNESRPRKFEIDLEIFVDKEQYFFLYIMEEVLLLGIGLCTVITTG
TFLATLGEHCCATYRIASCLIENTVTIHTLAIPVDRKIQFMHRSICISVYIHRRTMEFVKRMLLAFDLWY
FPLFLIGVLSLSCLLFRLYNAIIDTNDWYDILVCCVLVYAYLIYMFVGNFLAQSYTEHSAELLESTYNSL
WYVAPLPIQKLFLIMQTAIKSHKIVVGRLFTLSIEGFSTLITSAVSYFTVIHAMYL

>CbirOr247

MVFIGERCYKLHRMMFIFMGLWPYQKPFIWRIQAVIFFMIFCGCFLFQFTPFLTTTCDIDCILKRYCYIS
ICLVYLMSYYCFYFNSEMIKHALEHVQLDWKMFENSDAIKILEDYLHDSYILALCACAFLFTGMFTFLTV
ECGPVIFDVIIPINETRPRTVEIDLETFIDKDKYFFVYIVEELLGLGTGLCTVVTTGTFLNMIGKHCCAT
YKIASCLIQNTVIVHTLQIPIDQKIQFMHRSICLSVYIHRRTMELINHLVVSLDLWYLPLLALAVLSLSC
FLFRLYNAIMRFNDYYDIFVSSSLLNGYLLFTFLANFYGQAYTEHSIELIESTYNSLWYLAPLPIQKLFL
IMQRSVKSHKIVMGGLFTLSIEGFSTLITSAVSYFTVIHAMHS

>CbirOr248CTE

MVLLTKLLHSSRKMVFVGERCYELHRIMFMAMGLWPYQKPFIWRIQAFTAFLTATCNVDCILKRFSYICI
CFVYVMNYYCFYFHSESVKQTLEHMQLDWKMFKNSDAIKVFEEYLFESYIFTLFIYIISLVGLFIFVIIE
CRPIILDVIIPMNESRPRKIEIDFESFVDKQQYFFFYIMQEVLGVGIGVCSIITTGTFLAMVVKHSCATY
KIASCLIQNTVTVHTLQMSAAQRMQFMHRSICLSVQIHGRTVQFMKGLLQMVDLWYFPLLLVAVLSLSCL
FFR

>CbirOr249

MVVAGARCLKLHRTLFIIAGLWPYQKPFIWQIQAVFFFGAYCSMFFCQFTPFLTTTCNMECILKRFTYIC
ITVVFILNYYSFYFNSEIVKQMFEHMQFDWKTFKNCDAMKIFEEYLSESYIFTLSLCILFLIGASLTTIM
EFKPIILDIIAPMNESRPRKLEIDIELFVFNEEQYFFFLLVLEGVAAGIGLWSLLTILTFITTVTKHSCA
TYKIVSSLIQNTVTVHTLQLPVAQRIQYMHRNICLSVYIHRRTLEFCKGLILSFDMWYFPLVLICVLSLS
CVLFRLYNAIMQFNDLYDIFIPCGMLVCYLLYMFMANFLTQSYTEHSVEVLESAYDTLWYVAPLRIQKLF
LIMQKSIRSHKIVVGGLFVASIEGFSTLVTSAVSYFTVMHAMRL

>CbirOr250PSE(F)

MVFAGERCVKLHRIMLMIVGLWPYQKPVIWRIQSVFFLSVYCCHVFSQFASFLTTTCNMECILKRFSYIS
ITFVFIMNYYSFYFNSEAVKQMLEHIQLDWKMYGNSDAMKIFEEYLFESYIFTLLTYFCFLLAASVMATI
ECRSIILDIIIPMNESRPRKIEMDLEFFVNEEQYLFLYLMQEGLGVGIGAWSVITTGTILTTVAKHSCAT
YKIVSYLIQNTVTIHTLQLPIVQRIQYMHRNICLSVYIHRRTLEFCKSLLLSFDIWYFPLLLVGVVSLSC
ILFRLYNAIIHFNDFYDIVLSCALLFCYLIYMLMANFLGQSYSEHSIELLEFTYDTLWYVAPLPIQKLFL
MMQTSIRSHKVVMGGVFVMSIEGFSTMMTSAVSYFTVMHAMHV

>CbirOr251PSE

MVFVGKRCVKLHRTMLMIVGLWPYQKPVIWRLQSVFFLSIYCCDLLFQFVFITNYYSFYFNSEAVKQIFE
HMQLDWKMFENSDAMKIFEEYLFESYIFTLFTCILLLIAASFTTTIECRSIILDIIIPMNESRQRKIEVD
FELFVNEEQYFFWYLVQEVVGVGIGFWSVLTTGTLLTTVTKHSCATYKIVSYLIQNTVTIHTLQLPVVQR
IQYMRRNICLSVYIHRRTLEFCKSLLLSFDMWYFPLLLVGVWSLSCILFRLYNAIIHFNDFYDIVLSCGL
LLCYLIYMLMANFLGQSYSEHSVELLDSTYDTLWYVAPLPIQKLFLMMQTSIRSHKVVMGGVFVMSIEGF
STMMTSAVSYFTVMHAMRV

>CbirOr252

MIFAGERCFKLHRLMFLAMGLWPYQKSSIWKIQSAFFFSVYCSNLFSQLLPFVTMVCNMDCSLKRFSYVC
ILIACFVSYYSFYFNSEIVMRALQHMQHDWKMFESSDAINIFEEYLFEAYSFVFSALIFLVVAAIIFVIF
ECSPIILDVIAPMNKSRLRKVEVDFELFVDQEQYFVLYLVHEILGATISIWSIITTATFLITVFKHSCAT
YKIASYLIRNTITDYTLQLPVAQKLQFMHQNICLSVYIHRRTFEFCKDFLLSLDLWYFSLLLICVLSLSC
LLLRLYYAIMQFNNFYDLFTCCMFLFCYLVYMFVVNFLGQSYTEHSVKVLESTYDTFWYVAPLPIQNLFL
IMQKSIRSHKMVFGGLFVLSIEGFSTLITAAVSYCTVMRTVHT

>CbirOr253

MVFAGERCFKLHRLMLLAIGLWPYQKSFIWRIQSVFFFSAYCSTLFSQLLIFVTMACNMDCTLKRFCYIS
ITIGCIVSYYSFYFNSEIIKRALQHMQHDWKMFESSDAINIFEEYLFEAYIFVFTALIFVILVTTIFVLF
ECSPFILDVIAPLNKSRSRKLELDIELFIDEDQYFVLYLAQEILGSNVCIWSIITTGTFLATILKHLCAT
YKIASSLIQNTVTVYTLQLPVVQRIRFMHRNICLSVYIHNRTLEFCKDFLLSIDFWYFPLLLICVLSLSC
LLLRLYNAVLQFNDFYDILMSCIFLLCYLSYMLLANFLGQSYTEHSVKILESTYNTLWYVAPLPIQRLFL
IMQKSIKSHKMVLGGLFVASIEGFSTLITSALSYCTVMHAVHS

>CbirOr254

MVFAGERCFMLHRILLIAMGLWPYQKPLIWKIQAVFFFSAYCSILFSQLLMFATIACNIDCTLKRFSYVC
ITIACIMSYYSFYFNSEIVKKALQHMQLDWKMFENSDAMKIFEEYLFESYIFMISANILGAVSGVFFMTI
ECRSIILDVIVPINKSRPRKVEMDLELFVDEEQYFFLYLAQELLGIGISAWSLVTTGTFLITIVKHLCAT
YKIVSYLIQNTVTIHTLQLPIVQRIQYMQRNICLSVYIHRRTLEFCKCLLLSFEMWYFPLLVICILSLSC
LLFRLYNAIMQFNDLHDILMSCVFLLCYLIYMLMSNFLGQSYTEHSVKILESTYDTLWYIAPLPIQKLFL
IMHKSIKSHKVVLGSLFVASIEGFSTLITTAVSYFTFMRAVRT

>CbirOr255PSE

MVFAGKRCFKLHRIMLLALGLWPYQESFIWRIQSVFFFGTYCCFIFFQFTPFLTTTCNMECIMKRFSYIC
ITFAYVMNYYALYFNSKVVKQVLEHMQFDWKMFEKSDAMKIFEEYLSESYILALSLCSVIVLGAFGFIII
ECRSIILDVIAPMNESRPRKLELDLELFVNEEQYFLLYFLQEIIGMGIGIWSIITTGTFLITIAKHSCAS
YKIVSYLMRNTVIYTLQLPVVQRIQFMHRNICLSVYIHRRTMEFCKGLLLSFDMWYFPLLLTCALSLSCQ
LFRIYFVYMIAGNFLGQTYTEHSFTILVAMYDSLWYVAPVPIQKLFLIMQKSIKSHKLVLGGLFTVSIEG
FSTLVTTAVSYFTVIHAVRS

>CbirOr256INT

MVFAGERCFKLHRIMFMAMGLWPYQNLFIRRIQSVFYFGIYCGNTFFQIAPFLTTTCDMDCTLKRVSYIS
ICLVYLLHYSCFYFNSEAIKQILEHMQLDWKMFKSCDAAMKILEEYLYESYLFAIFVHFLIIVVTFVLGT
FESRTFILDIIFPRNVSRQRKLEVDLEFFIDKEQYFILYLIHEVAAVSIGSWAVMTLGTLLGTVGKHCCA
AYKIASCLIQTTVTAHTLQFPVAQKMHFMHRNICLSVYIHRRTMEYNTLWYVAPLPIKKLFLIMQKSIKS
HKLVIGGLFVPSMEGFTTLVTTAISYFSVMYAMHSRKCSYIX

>CbirOr257NI

VFVGERCYNLYRIMFIAMGYWPHNHKSIIWRLQAGFIFGVFCCTFFSGISCTAFLTPCNMACILKRFSYI
CITLIFIFCYYCFYFNSEDVKELLEHIKLDWKMFENSDAIKVFEEYLFDSYILSLFAYLMLPTCVFILVM
IECKPLILDIVIPMNVFRPRKTEMDFEFFVDKEQYFFLYIMQEILTLVTGFYSILIPGTFFVTIGRHCCA
IYKIASCLIANTAIVHTQIPVAQKIQFMHRSICLSVHIHRRAMKFVKDLVQMIDLWYFPLFVICIASLSC
LLFRYDTLWYVAPLPIQKLFLIMQKSIQAHTLKIGGLFTVSVEGFSTILTSAISYFTVYILCIYDHEVAR
KWRKTFNR

>CbirOr258

MLFAGERYLKLHRIIFIALGLWPYQKSFIWRIQAVFFFSAYCSTFFSQSLTFVTMACNMDCTLKRLSFFF
VYFIQILSYYSFYFNSEIIKKALKHMQFDWKLFENSEAMNIFEEYLFEAYIYIFSAIIFVGLASIVFLTF
ECRAVILDIIAPMNECRPRKTEIDLELFVNEEQYFHLYVIQETLGSFIANLSIIAIGMFFAIVAKHLCAT
YKIASCLIRNAVTIRTLQLPVAQRMLFMHRSICLSVHIHRRSMQFTNNLIISFNVWYFPLLLFCVLSLSC
IMFRLYNAIILFNDFFEIFLSCLMFFSCIAYMFVANFLGQSYIEHSAEVLESIYDTLWYVAPLPVQKLFL
IMQKSVKSHKLIMGSLFVLSIEGFSTLITSAVSYFTVMHAMRSRV

>CbirOr259

MVFVGKRCFKLHRIMLMIIGLWPYQKPFIWRIQSVFFFSTYCCTLFFQLSTFVTMTCNMDCILKRFSYIC
ICCVYILSYYAFYFNSEIVRKTLQHIQFDWKMFENSDIMNVFEEYLFEAYIFVFSALIFVILSAIIFTIF
ECRSIIILDVIAPINESRPRKIEMDFELFVDQEQYFVLYLVQEILGGFIGGWAILTAATFLTTVMKHFCAT
YKIASYLIQNTVTVHTLHLPVAQRMQFMHRSICLSVYIHKRTLKFCKGLFFSFGLWYFPLLLICVLSLSC
LLFRLYSALMQFNDLRDIIMSCAILFSYFVYMLMANFLAQSYTEHSVGLLKSTYDTLWYVAPLSIQKSFL
IMQKSIKSHKIVLGGLFVASIEGFSTLITSAVSYFTLLHVMCS

>CbirOr260CTE

MVFVGERCFIFHRILLVAMGLWPYQKPFIWRIQAVFFFSTYCCTLFFQLLTFVTTACNTDCTLKRFSYFS
IYFIHILSYCCFYFNSEIIKKTLEHMQFDWKLFKNSEAINVFEEYLFEAYIVLFSAFIFVLLTAISFVTL
ECRSMILDVIIPVNESRPRKIEMDFELFVDQEQYFVLYLVQEIVGMGIGAWSLLTTGTFLTTVMKHFCAT
YKIASYLIRNAVTVRTLHLPIAQRIQFMFRCICLSVQIHRRTLMLCNSMIISFNLWLSPICGLCVLVLSC
VLFR

>CbirOr261PSE(S)

MVVAGARCLKLHRTLFIIAGLWPYQKPFIWQIQAVFFFGAYCSMFFCQFTPFLTTTCNMECILKRFTYIC
ITVVFILNYYSFYFNSEIVKQMFEHMQFDWKLFENSDAMKIFEEYLFDSYILAYSLCFFFVLATPCATII
ECRSVILDVIIPMNESRSNKLEVDIEFFINKEQYFFLYLVSEVVGVAIGCWSVLSTLTFLTTVVKHSCAT
YKIVSYLMQNTVTVYMLQRPVVQRIQYMHRNICLSIYIHRRTLEFCKSLLLSFNIWHFPTVLIGILSLSC
LLFRLYNAVMQFNGFIDILMSSGFLFGCFLYILMANFLAQSYTDHSVGVLQSTITCIIAVAPLPIQKLFL
IMQKCIRSHKVILGGLFVMSIEGFSTLVTSAISYFTVINAMHL

>CbirOr263

MVFVGERCYKLHRILFITMGLWPYQKLSILRIQAVFFFSTFFCFLFFQLTTIFTSKCDVDCAIKKISYIA
ITLAYIITYYSFYFNYDVVKQALEHMQLDWKTFENSNEMEILNKYIFEAYMIELITCIFIILAIFVLAIF
ECRSVILDIIVPMNQSRPLKVEIPFEFFVDKERYFFLHLVQEIVGAAIGISSISTTFLFLATIGKHCCAT
YKIASLLIENTVTVHTLRMPSPQRMRFMHENICRSVHIHRRTMKFINDLLISFNLWYLPLLLIGVVSLSC

ILFRLHNAMTELDDFYDIAISLLLFSGLLILMFVANFLTQRITDHSAEIFKATYNTFWYVAPLPIQKLLL
IMQKSIKDHKLIVGGLFTPSIEGFSTLMTSAVSYFTVMHAMR

>CbirOr264PI(S)

MVFVERYYKIHRIFLMILGLWPSYKSKHLKRIHIIFIFFLYCSSVVVQLATFLTAECNMECIVIKLSYIL
PSIVYILNYNCFFFNIECIRQSFEHIQLDWETLEDNNEMEILKKYTYVAYVFTIILSISFIAGICAFALF
ECTPIILDVIAPKEKSLPRRISIPIEFFVDQEEHFFLYLISEVLGVFFGIWSTIATGAFLAAIGQHCCGT
YKIASNLIRDTVTNHTLQIPVSQRIRFMYQNIRRAVRIHIRTMQFLNGLLFSFHLWYFPLIVIGVISLSC
TLFRLFNATLRLNDSNEVFISYTMFIGHLIYMFLTNYFGQLIIDHSTEILRETLATSSISYFTVLYTMRT

>CbirOr265

MIFAGARCLKFHRTLLMIVGLWPYQKPFIWQIQTVFFFGAYCCMLFLQFTPFLTTTCNMECILKRFSYIC
ITFIFMLNYYSFYFNSEVVKQMFGHMQFDWKLFENSDAMKIFEEYLSESYIFALFLCFLFVITVPLITIM
EHRSIILDVIAPMNASRPRKREIDLEFFVNEEQYFCFLLVLEGIGVGVGFWTLLTTGTFLITVAKHSCAA
YKIVSNVMQNTVTIHTLQLPVVQRIQYMHRNICFSVYIHRRTMEFCKSLVLSFDMWYFPIVLIGVLSLSC
ILFRLYNAVMQFNGFIDILMSSGFLFGCFLYILMANFLAQSYTDHSVGVLQSTYDTLWYIAPLPTQKLFL
IMQKCIRSHKVILGGLFIMSIEGFSTLVTTAVSYFTVMNAMRL

>CbirOr266

MAVAGAHCFNLHRILFMTVGLWPYQKTFIWQIQAVFFFGAYFSMLFFQFTPFLTTTCNTECMVKRISYIS
ITVVSIMNYYLVYFNSEAVKQVFQHMQFDWKMFKNTAAMKIFEEFLFESYIFALFLCFTFVITSFVLTLM
ESTPMILDVIAPMNDSRPRNLELDFEFFIFNEEQYFFFLLLLEGAAAGFGVWSLLAIFTFFTTVMKHSCA
TYKIVSYLIQDTVPVYMLQLPVVQRIQYMHRNICLSVYLHRRTLEFCKGLLLSFDIWLFPLLLVYVLSLS
CVLFRVYNGIIQFNGFHDVVLSCGLLVCYLGYMFTANWFAQSYTDHSVEVLESTYDALWYVAPLPIQKLF
LMMQTSIKSHKLVLGGLFVGSMEGFSTLVTSAISYFTVLHAMQLVSDEETAX

>CbirOr268

MVFAGERCFKLHRIMLIAMGLWPYQKPFIWRMQAVFFFSVYCFSLFSQFTPFFTRPCNMECILKRFSYIC
NTFVYIMNYYSFYFNSDAVKQMFEHMQLDWKIFKNNDAMNIFEEYLFESYIFAVSTNILVLIGGFGFIII
ECRSIILDIIIPMLTHKSRPRQLEMDLELFVDKEHYFFLYLGQELLVIGIGTWSMLTTGTFLTTVTKHSC
ATYKIVSYLIQNTVTVHTLQLPVVQRIQYMRRNICLSVYLHRRTLEFCKGLLLSFDMWYFPLLLVAVLSL
SCILFRLYNAIIQFDELYGILVSCITLGCYFVYMFMGNFLGQTYTEHSVEVLDSTYDTLWYVAPLPIQKL
FLIMQKSIRSHKIILCGLFVASIEGFSALVTSAVSYFTVMHAMRS

>CbirOr269

MVFVGARCLKLHRTMLMSVGLWPYQKPFIWRIQSVFFFSMYCCNLLFQFTPFLTTTCDMDCILKRFSYIC
ITLVYIMNYYSFYFNSEAVKQMLEHIQLDWKMYGNSGTMKIFEEYLFESYIFAVTLCISLFLGVSGITTF
ECRSIILDVIIPMNESRPRKIEMDLELFINEEQYFFVYLVQQVVGVALGIWSMIMTGTVIITVAKHSCAT
YKIVSYLIQNTVTVYTLQLPVVQRIQHMQRNICFSVYIHRRTMEFSQNLLLSFDMWYFPLLLVGVVSLSC
ILFRLYNAIMQFNDVFDILLSSGILFCYLLYMLMANFLAQSYTEHSLGILESAYDTLWYVAPLPIQKLFL
IMQKSIRAHKVVLGGLFVLSIEGFSTLMTSAISYFTVMHAMR

>CbirOr270

MVFAGERCVKLHRIMLSALGLWPYHKSFIWRIQSVFFFGAHCSTLFSQLLLFITSACNMDCTLKRFCYIC
ATIIPIMSYYSFYFNSENLKRALQHMLLDWKMFENSDAIKIFEEYLFVSYIFTLFSCISVLIAGVIFSAV
ECRYIILDVIAPMNESRSRKIEMEFELFVNEEQYFFLYLVLEIVGVEIGVWLVLSTGTFLTTVAQHSCAV
YKIVSYLIRNTVTIHTLQLPPSQRMQFMRRNIYLSIYMHRRTLELCKGLLLSFDMWYFPLILICVLSLSC
VLFRLYNGIIHSDDFYDILVPAVMLCCYIVYMLLANFLGQSYSEHSVELLESTYDIYWYVAPLPIQKLFL
IMQKSIKSHKIMLGGLFVLSIEGFSTLVTSAISYFTVMHAMRS

>CbirOr272PI

MVFLGERYYNIYRIMLTIIGLWPYQSSIIVQVQTAFFFGAYSTFLFSQLTIFLTTTCSLEVIIKELSYIF
ITMLYIICYNSFYFNAKEVLFSFSIFFLLIVELIPVILDTVVPINESRQRKLQIDFEFFIDEEKYFYVYL
IHEITVVLIGVFTMLATGTLPIAFFRHCCAICKISSNLIENTVTKQTLQVSSHRKIYIMYQKISRAVHLH
RKSIQLTKFFMKTMNKWYFVLLMICAASLSCNLLRLLNAATVLNEFSELIICFGTVMGHFLTMFIPNFIG
QSFIDHHGEMFYAAYNTMWYLAPLPIQKLLLFVMQNSMKAHALLLGGIYVSCLEGFSTLVTTSISYFTVM
YSMTTKQ

>CbirOr273CTE

MVFGERYFNIHRIMFTILGLWPYQKQKILRIQAVFFSSVFFSLVCFQLTPLVTTACDVECIIKKVSYICI
TSVYILNYYSFYFNARTIKQSLEHMQLDWKMLENKNAMKILEEYIYEAHLFTLIVLILVISGVSVFIIFE
CIPVFLDVFLPLNESRLRKTEISFEYFLDDQQYFFLYVIHEFIGLLIGLSSMSITGSYLLVIATHTCAVY
KVASNLMQGTVTEHVLQIPTPQRMYFMYNNIRLSVHIHRRNMKFIDGILLSLQFWYFPLVIIGVVSLSCV
FFR

>CbirOr274

MVLFVRERCFKLHRLMFTILGLWPYQNSCIWRLQAVFFFSVYSSFLIFQLTTFLTSTCNIDCIFKKFSYI
CITIAYITNYYSFYFNSETVKQSLEHIQLDWKMLETSETIKIFEEYLFEAYILALSAYITLIVGSFVLIS
FESTSVILDAFIPINESRPRKLEVDFELFVDQEEYFFLYLIQEMLGVGIGMCSVIATGTFLATIGKHCCA
TYKIASYLIQNTVTIHTLQLPAPQRMQIMHRSICLSVHIHRRTMQFCKRILISFHFWYFPLLIIGVLSLS
CVLFRLYNAIMQFNDFYEILISCLLLYCYLIYMFVGNFIAHSYSEHSGRVLEAMYDTLWYVAPLPIQKLF
LIMQKSIQNHKLVMGGLFVLSIEGFSTLITSAVSYFTVMHAMNDHDTEGRLVKKI

>CbirOr275PI(F)

MAFAGARCLKLHRTLFMIVGLWPYQKPFIWQIQTVFFFGAYSCMLFSQFTPFLTTTCNMECILKRFSYIC
ITFVCILNYYSFYFNSEAVKQMFEHIQFDWKTFKNCDAMKIFEEYLSESYIFTLSLCILFLIGASFITIM
ESKPIILDVIAPMNESRPRKLEVDIELFVFNEEQYFFFLLVLEGVGAGIGLWSLLTILTFITTVTKHSCA
TKIVSSLIQNTVTVHTLQLPVAQRIQYMHRNICLSIYIHRRTLEFCKGLILSFDMWYFPLVLICVLSLSC
VLFRYDTLWYVAPLPIQKLFLIMQKSIRSHEIMLGGLFVLSVEGFSTLVSSAVSYFTVMHAMRL

>CbirOr277PIC

MVFVGERCYKIHRIMFMIMGFWPYQKPFIWRMQAVFFFSAYCCIIFSKFAAFFTTTCNMDCILKKFTYIC
LSFIFVLCYCSFYFNSEFIKQALKHMQLDWKMFENSDTIKIFEEYLFLSYIFALFAIMIVPACLSTLMAL
ECRPVILDAIMPMNVSRPRKLEVESEYFLDKQEYFYFYLMEELVGXCLIQSTEIVHTLQIPVTQEIQFMH
RSICFSVYLHRRTLKLMKDFMHSIDVWYSPIILMCVLSLSCVLLRLYNAILETNDLYETFVCCVLLYICF
MYMFITSFLAXSYTDHSIEILKF

>CbirOr278

MSSIVDRYYKINRILLKALGIWPYQNSKWTHVQALIITSIYLSVLFVQLAVFVTHECTLKIIVDVLSFVF
PTVLMLFLYDLFYFNTDFLKEVLDHLSYDWEFIRSEEEFIIMDDHASTAKIFTHIFLFLSSIGISVMIMS
HIPIPIPTIIDVLLPLNLTRTQGLHLIVEYFVDPSKYFTLILIHLTISICLGVITITSTSLMLLGIGFHC
CALFKLANYRMEIIMDQNILRIPSPEKEQIIYKRMIRAIDIHCRAFELCEYLVMNMQKWFLVIIVVGVTS
LSINLLRFLEAITNLKIVELKVIDLFLPGALVFTHFCYLFFSNHIGQRVADNNDSIFNTIYNLPWYIAPL
NVQKLLIFLLQKGTKTFYLSLSGLFDASYQGFTSLSMTSISYFTVLYSIQ

>CbirOr279

MSSSLKIEDRFYKINRICLKFLGIWPYQNSTKWTRVQTTIIRCMYLSGIFIQLGVFITHECNLDILVDVL
SFTFPAIFILLMYSIFYYNIDTFKELFESIYHDWKIFNTKAELAIINDYANTVKLFTQFFSTFTCIAMIV
TTMIHVIPITLDMILPLNESREQGTHLMVEYFIDPSKYHILIVSHLTLYICLGAIGVMAIALTFLSFGFH

SCALLKLTNYHVKIMMDQTVLQTPSPRKEQVIYERIVFAIAVHRRALQFCKQFVLSLETWFFVITIIGVL
SLSINIFRMLKAITVYNSAIDVFIPCVLVFVHFCFMFFGNQIGQNITDHTTDIFNTIYQLPWYTANLNIQ
KLLLFLLLKGSKTFYVTLDGLIKVSYECFTTLTTSSISYFTVIYSFQ

>CbirOr282

MYGIEEYYYKVNRVFMKTLGLWPYQQPYIAQLQKVLFSSILFSFILVQCLACFTMKFSTDLFLKILSFLF
PTLFATTKYCVFVIRADSVKLLLEEIRNDLTLLKNEVEIDIIKKYADNMRFITMVSIVSSYAGTVCYVMS
QYLLPLLLNVLLPLNASRSLELIGITEYFVNREKFFWALLFHEVVTVYVGVTAVCSTGGTTIMYFLHSCA
LFKVASYRIENAIDKKILAIPDPTRKYLLQQKIVHAILIHRRAIKYIELWSSNFMVPFTILILIGVSSLS
FNLFHLLQLVIVLDDPSEMYFAFLLITLHVGYMFALNYGGQEIQNHGVQLFEAIYNGLWYAAPLHTQKLL
LFLMQKAAVKVTLVCGSIFVASLEGFVTLVSSAVSYFTVMYSTRQ

>CbirOr283

MQDIEERYYKVSDTFLKMIGLWPYQKTKFVRIQNVLIIGILCGYIIVQLLSFLTMQFSVNLLLKVLSFVF
PTLFMVIKYCTFIIQADNVKQLVEQIRHDWKLVKSKLEIDIIKKYAENGRFVTIIVIVCCHCGILCYITL
QLLPLILNVIAPLNESRSVKLIATTEYFLNREKYIWIMLLHEIVTAYLRLITLCSISMTLLMQILHACAL
FNIASYLMENAIPENVLAIHDSLKEHILHQRIAHVVLIHRRAISFIQLWISSFAVPFFIMILVGVCSLSV
NLFHLLELITVTDDVNEMYAVFMSVILHFNFMFILNYAGEIVQNHGMQVFGATYNGLWYVAPLRTQKLIL
FVMQRGTVKVNLTCGHMFVASLEGFLTLVSTAISYFTVIYSTR

>CbirOr286

MHEIEEHYYKINRNFLKMLGLWPYQQSLFARLRKVLFIGILFGYIVAQFLSFVTMQFSANLLLKILSFVS
PTLYVTMKYCLFVVQTDNVKRLLEQIRNDWELVKDKLEIDIIKKYAGSVRFISMCALVFCHCDTLCYITL
QFLPLILDVILPLNESRSLKLIVITEYFVNREKYLWLMLLHEIVAIYLRVITICCTTTTIMMYILHACAL
FKVASYRMENAIQEDMLAMCNPVQEYLLYQKIASVVIIHRRAIAYVELWISSFTMVFTILIVIGISSISF
SLFHLLQVITNTSNDYTDMYAAFISVVAQFTYMFIINYCGEIVQNHGMQVFEATYNGLWYAAPLRAQKLI
LFVMQRAIVKVNLTFGNIFILSLEGFVTLASKAVSYFTVIYSTRQ

>CbirOr288PSE

MHVIAERYYKINRIFLKAIGLWPYQQSYLVEIQKVLCTGILLSFIIVQLLVLVFTTQYNLALVLNVLSLV
FPVLFVTIKYCLFAIQKDSVKELLEQIQDHWKLMVTELEIEIIEKYARNTRSLTLFAMAFCHCAMFPFAI
LHFLPLMLDVILPLNESRPLQFIVTMEYLINQEEYFYIIFLHVGLTFVVSMIAIGITTATIMLYILHAFL
HVGLTGVVAMIAICITTATIMLYILHACALFKVASHRIENAIDQNITYPNAVKQYLFYRRILHAVIIHRK
AIEFIKTFTSNFAIPYDILIIVGVTSLSINLFQFSQLVALTNNVDEAFMVAILIFLHLIYMFLLNYGGQE
VMDHGKKLFNAIYNGLWYTAPLHTQKLLLFMMQKSKVDVILTMGTIFVATLEGLASLISMTLSYFTVIYS
TR

>CbirOr289

MHEFNKRYFKLNRILLKMVGLWPYQQSYFIRIQKGVFTGIFLTFIIVQLLVFLTAIQPNLMFVLKILSFV
FPTMFVTMKYCLFIIHADSMKRLLELIRSDWNFLKDKVEIDILEKYAWYARLLTIIAFAFSYCCLLCAGI
LQFLPVILDLILPLNESRPLHLIVFAEYFINQEEYLYAMLLHEIFAVHLAAIALCCTSTTIMSYILHGCA
LLKVASYRVENAIGEDILAIPSPKKEYLLYRRIVNAVIIHRRAIEFIDVFTSSFAVSYDILIIIGVSSLS
INLFQFLQIITTKNTNEIFVVSGLIITHLSYMFGGNYAGQQITDHGTKLHQATYNAPWYVAPLHTQKLML
FIMQRANVDVTLTFGSIFVASLEGFCSLTSTAISYFTMLYSTR

>CbirOr290PSE(F)

MHAIEERYYQINRIFLKTLGIWPYQHSFLALVQRVLFSTILFTFIIVQLLAFFTMQFSTGLLIRILTFVF
PNLFVTVKYCSFAMKADKVKRLIEQIGNDLDLLKGKFEFDIIKKYADNIRIITILAMEFYKFGISCYLTF
QLLPLILDVIVPLNVSRSLKLIAITEYFLNREKYVCVMLLHEMLTVYIGVITACSTGLIIMTYILHACAL
FKIASYRMKHAIQEDVLAMPNPLMEYFLYQRIVDIIFIHRRILQFLTFSISSFMVTFAFLIIIGVTSLSL

NLFQLLQLMTVTYDIGEISVIFIFIVFQVNYMFVLNYVGEELQNHGMELFQATYDGLWYAAPLRTQKLLL
FIMQKTTVKVTLICGGIFVASLEGFVTLVNAAVSYLTVMYSTR

>CbirOr291INT

MHRIEEHYYQINRIFLKMLGLWPYQQSYLXFCHFGISCYLTLQLLPLILDVIVPLNATRSLKLITITEYF
VNREKYACAILLHEMLTVYVGAITTCSTGLIFMAYILHGCALLKIASRRMKHAIQKNILAMPNPVKEYLL
YQRIVDIVFVHRRVIQFHELSVSSFMISFAFLIIIGVTSLSLNLYQLLQLMTVTYDFGEISVIFILVVFH
VNYMFVLNYGGEEVQNHGMELFQATYNGLWYAAPLRTQKLILFIMQKTTVKITLVCGGIFVASLEGFVTL
VNTAVSYFTVIYSTR

>CbirOr292PI

MHRMEEHYHQIIRIFLKTLGLWPYQHSYLALVQKVLFGGILFTFIIVQLLAFFTTQFSTNLLLQILSIVF
PTLFATVKYCIFVIKADKVFCHFGTSCYLILQLLPLILDVIVPLNASRSLNLLAITEYFVNREKYACVIL
LHELLTIYVGTITLCSTGLIFMTHLLHGCALLKVACRRMKHAIQKNFLAMPNPVKEYLLYERIVDIVFIH
RRAIQFHETTVSGFMVSIAFLIIIGVTSLSLNLFHLLQLMTVTYDIGEISIIFLLVVFHVNYMFVLNYGG
EELQNHGMELFQVTVKMTFVCGGIFVASLEGFVTLVNTAVSYFTVIYSTR

>CbirOr293

MHGIEEHYYKIIRIFLKTLGIWPYQQSYLTLVQRVLFTSILSTFIIVQLLAFFTMRFSTGLLLRILSLVF
PTLLVIIKYCTFVIQADKVKQLIGQVGDDLNLLQDKSEIDIIKKYADSINFITIYGIVFCHFGILCYLTL
QFLPLILDVIVPLNTSRSLKLMAITEYFVNREKYASAMLLHEILTVYTGVMTLCSTGLTIMTYILHACAL
LKIASHRMKHAIQEDVLAMSNPVKEYLLYQKIVDIVFIHRRVMQFISFSVSSFVVSFALLLIFGVISLSL
NLFRLLQLMTVTYDIGEISVIFLLVTLHINYMFAVNYGGEILQNHGMELFEATYNGLWYAAPLRTQKLLL
FIMQKTTVKVTLICGGIFVASLEGFVTLVNAAVSYFTVIYSTR

>CbirOr294

MHEIEERYYQMNQNLLKLLGLWPYQQSYFTLIRKVLFLGILSGYVIAQLLSFLTMQFSVSLLLKILTYVG
PTFFVIIKYCIFIIKADNVKLLLERIRDDWNLLKGKLEIDIIKKYASSVRLISMIAIIVCHFDILCYIIL
EHLPLLLDVILPLNKSRTLKSIAITEYFVTREKYIWVTLLHEIIAIYIRVITLCCTGITMMMYILHACAL
FKIASCRIENAIQENVLAIPNPVKEYLLQQRIAHAVLIHRRAIAYVELWISSFTVTLTILILIGICSLSV
NLFHLFQVLTVSTDFVELYAVFVSVVAHFSYMFVINYGGEIMQNHGMQVFEATYNGLWYAAPPRTQKLIL
FVLQRAIVKVNLTCGHMFIASLEGFLTLTSSALSYFAVIYSTR

>CbirOr295PC(F)

MHEIEEHYYQINQNFLKILGLWPYQQSKIFARLRKVLFLGILSGYIIVQLLSFLTMQFSVNLLLKVFTFV
FPTLFVTIKYCIFITQAENVKLLLEQIRDDWNLLNGKLEIDIIKQYASSVRLISMIAIVFCHFDTLCYVT
VELLPLILDVISPLNKSRSLKLIANTEYFVNREKYIWVMLLHEITAIYIRVITVCCTSVTIMMYILHACA
LFKVASYRMKNALGEDVLVIPNSLKEYLLYQRIAHVVIIHRRAI

>CbirOr296

MHLIEEHYYTINKSFLKMLGIWPHNKSRFILLQRILITFVTVTYISMQWSIFITAECTMPLFVKTMIRSY
PFAIIVMKYFIFMFKSEAIKNMHYQIQQDWKLLRNKLESKIIQQYAHEGQLCTLFIFSFISFILVLVTVI
QFLPNILDVILPLDEPRPRRLLVTAEYFIVKTNDKYYYTRVFHELVFLTMCASMIFATATQLLTFAFHSF
GMFKIASHRIKYSIEESILHVQKFGKEYTMCKTIMHAVTAHRRAMEFSNIFISCFNAPYCVMAILGIISL
SISLYGLIEAATISKSMENLVICFISVIGHLVYIFVASYVGQKIIDYNNDLFKLAYNTSWYLMPVSSQKL
ILFLMQKTSRDFYFMIGLIFVAKMESSTTLMNAALSYVAVMYSCVQK

>CbirOr299PN(F)

MATFSTSGFHMKILLKILSQTLPCLMYILKYLSFVVNNKEIKECYEQICHEWTLLKVRSEINILKYANFS
RLFGVFITVGMFITLAVFGFYQFLPDLLDMIAPLNDSRKHFLTFDAEYFVDQKEYFYPIVTHGLTAVYIG

GTVGIVATGGMLMGYILHICAMLKIASYRLEHITDDVPLVSSVEKDHIIRERIISAVDIHRRXKIEFAEF
VVSSFMIFYFILIGIGILSLTLNMFQRLQFMLLTDDVNGMLACVLLIIGHLNYLFIANMAGQIVTDHNID
IFYTTYTTRWYTVSAQLQKLLLFIMQKTTKNYYFIVGGIFAASLEGFATMMSTSISYFMVIYSTS

>CbirOr300PSE(S/F)

MIFVKSPFYNINRIILLLLGIWPYQKSKFIYVQRLFSVGIFISFVACQLLLFVTEKHTVNLFFTTLSQSL
PILICLLKYNAFFINSENVKILLEQIQHDWNTLNGKELEIMKQYLNNSRLFMIIFTSFATMSVCAFTFIQ
LLPVTLDVIIPLNVSRPRRFYFRLELFINKEKYFYVIAFYTILSLCVSAATIVSTGASLLTFGQHSCAML
KIACYRMEHAMDKYKVLHIAKRKEIQNKIVSAVDIHRKAAEYADILISPFMISYIALIVIGVISLSFNLF
CFFQAFFYTHDMQEVLVSVSLIGGHFLYMFCANYGSQVITDHFEDIFIAAYGIPWYVAPVRIQKLVMLLL
QRSTKTFVLQIGGLFIGSLEGFTTLISTSLSYFTMIYSTR

>CbirOr301

MLFVKSPFYNINRIVLLLLGIWPYQKNKFVYIQRLFSMGILISFIVCQLLLLFTEKYSVDLFLNILSQAF
PPMIYLVKYNAFCINSKAVKLLLEGIEHNWTTLNDKELQIVKQYANTSRLYMITFTSFGTLLLCTFTLMQ
FLPVISDIIIPLNISRPRRFYVRLEFFINEEKYFYVIAFYTILSLTVGAATVVSTGASLLTFGHHSCAMF
KIACHRMEHAMDKYKLLYIAKRKDIQNEIVNAVDIHRKAVEYADILVSPFMMSYLGLIIIGVISLSLNLF
RLFQAFLYTHNMEEALISIFLIGAHFLYMFWANYGSQVMTDHFDDIFTAAYDIPWYAAPIRIQKLVMFLL
QRGTKAFVLQIGGIFIGSLEGFATLLSTSLSYFTVIYATR

>CbirOr302PN

MDVVIMILSYALPSCICMIQYHSFCSNSYIVKRIWEDIYSTWNLMRNETEHKIMRQYKAKAEYHTKLFLT
IIISSCIIYGIFEAMPVILDIILPLNKTRPREIHALLEYFIDKDAYFFPILCHWLIGLIIGCYTIICVST
SLMICVQHISGLFKVANYRIEHSADDYVFCNSIQEKNRIEKFIQNISAAVDIHRTAIERCEYLIDKFNKH
FFFILLKAIITLDEVEIVSSILFTSGHFVIMFGINYYGQQITDYHSEIFTAAYNVRWYVTPVKIQKLLLF
IMQSTTKAYYLNIGNLIVASIEGFSKLVSMAISYFTLIYSLL

>CbirOr303INT

MSLNRRRIESAENRYKINQILLSSXLTSFVTYEVTLDVIIMVLSYAIPSFIYVIQYYSFSFKPYTVKRIW
KDIHNSWDLMKNGAERKIMRQYSIAGEQITILLSLSIITSCVFYAIFEAMPIILDMIFPLNETRPREIHA
LTEYFIDERTYFCPILCHWLIGLMLGAYVVAVTCTLHLVYMEHICGLLKVANYRIEHSLDECALCDSTHG
KNRAIQNITAAVDIHRKAVKRCVFVLDNFNPYFFVILIIGVSSLSLNLFRLLKTILALDKMVQLVSSILF
VTMHFVIMYLANYYGQKITDHHNELFNTTYNVQWYTAPIKIQKLLLFVMQSTTKSYFLNIGKLVIISIEG
FAQLISLSISYFTVIYSLL

>CbirOr304CTE

MDFIEQYYKLNRILLLCLGLWPYQNSAFKKIQIIFYQTLFVSFLVCQFNTFLVKKCNTDVLLKIFTYIVF
DCIYIVKYSAWLLLSNNIKYIFDRMRHDWNILKDQTECEILREYADNARLHTIFFLLLATLCFLGLLTLG
CVPRILDIIMPLNESRPRQLPIIVEYFIDEETYFYAILIHIATAIYAGSVTLAACATMPISYILHVCAMF
KIASYRIKHICGKDVLNMPKDIKQYILYERLIHAIYVHRRAMDLCRILTNTFTIFYFVLLGFGX

>CbirOr305

MDFTEEHYKLNRILLLWLGLWPYDTSFFKKIQIVFFEALFISFVLCQFNVLLVKNCSIVLVMRICMFLFI
ISCVIINYNAGLLLTHTFKYVFNRIRYDWKILKNQAEFEIIQKYADKTKFYTISFALLGTSGCLGVLSLS
FVSPILDVIIPMNVSRSLRLPIVAEYFVDQTRYFYPIMIHIFIVSYVCLMTLVAIIALLIAYVMHNCAIF
EIVSYRLEHMFDEKILEMSKDIREHILYERLVHAVYLHRRATDMANIMTNSYANFYMIFLIIGIGSTTFS
VFHFFYVLEALNDVIEIITSSAIIVFQFYYIFIANYMGQNIIDISVNLFQTTYNTEWYAAPLWLQKLILF
IMQRSSIKSTFTTAGVFDASLEGFAKLLSMSMSYVMFLRST

>CbirOr306

MNFVEHYYKLNRILLLWLGLWPYDTSFLKKIQIICFEATFISFLLCQLNVFLLKNCSAELVMKVLMFLFI
TIFFIVQYNVGVLLTDSFKYIFSRLRYDWNMLQNQKEFDIIQKYADRTRFHTILFTLLALSISLGIIVLC
SVPSLLDIIIPLNESRPSWLPIVAEYFVDQQRYFYAILIHIFIVVYAGSMAIASVAGMMLGYVLHNCAIF
KIASYRIEHIFDEKILQMSKTIGQYVLYERLIHAVHLHRRAVDLTNDLNNSFSRICFIMIIVGMCCIPFS
VFHFFYVLAPLNDVLELLLSCGVLIFQLYYMFIINYVGQDIINISTNVFHTAYNTEWYAAPLWLQKLILV
ILQRSSLKSTLIAGGIVDASLEGFAKLMSMSMSYVMFLRSTRTQX

>CbirOr307

MDFTEEHYYKLNRIIMVCLGLWPYNTSAFRKIQIIFYEALLISFSLCQLNVFLMKDCSIAATMRICMFLF
CNLFFIIKYTGGLLLTDSFKYIFSRLRYDWNILKNQTEFEIIQKHADKTRFYTIIFMLIATSVVLGVISL
AFASTILDIIIPLNESRPLRLPIEVEYFVDQQRYFYAISLHMGMTFYAGMMIMTAITTIFIAYVSHNCAI
FEIVSYRLEHMFDEKILEMSKDIREHILHERLIDAVHLHCRAVDMTNVLTDNFATLFFILIVTGVACTTF
SVFHIFYLMTLLNEIAIIDLISGVGCVLFQFYALFTGNYIGQDLIDISANVFQASYNMEWYAAPLWLQKL
ILFIMQKSIIHATLKAGGLFEGSLEGFARLISMTTSYVMFIHST

>CbirOr308

MDFLGERYYKLNRILLVCVGLWPYDTSPLKKIQIIFFEALFLSILLCQLNVFLVENCDIAKIMKILMFFL
INCIVIIEYNSGLLLTNTIKYIFTRVHYDWTILKNQAELDIIQKYACNARFHTIAFMLLGTFMCAGLFIL
SSIPFILNAIIPLNESRPLWLPIIVEYFVDQERYFFAILTHIFMTTFAGCITLAGIATMHIAYALHNCAI
FEIVSYRMEHIFEENILQMSKDIREHILHERLIHVVYLHRRAIDLTNILTNGFATLYVILLLFGIASTSF
SVFHFFNVLTSLNNVLEVIETSTLILIHLYYLYVGNYVGQSIIDTSTNIFRTTYNTKWYAAPLWLQKVIL
FIMQRSSIRSTLTAASLFDASLEGFATLMSMSMSYVMFMLSTRSQQENMQKNYNYDLVEFDL

>CbirOr311PI

MDFIGEQYYNLNRILLVCVGLWPYGTSSLKKIQIIFFEAIFISFLLCQLNVFLVKNCSIAKVMKILMFVI
INCIFIIKYNAGLLLTDNVFIGIVIGMGIIILSSISFILDAIIPLNESRPLWLPIIVEYFVDQERYFFAI
LIHTFMFVYVGCITIAAIATMLIAYVLHNCAIFEVASYRIEHIFDKTILQMSKDIRQHILYERLIHAVYL
HRRAVDLANILTNSFATLYFILLTFGVASTSFSVFHFFHVLTPLNDVLELIMTSIFIILHLYYIYIGNYV
GQSIIDTSTNIFRTTFNTEWYTAPLWLQKVTLFIMQRSSIKSTLTAGGIFDASLEGFAKLMSMSISYVMF
LRSTRSQQENTQEEL

>CbirOr312

MNFLGHEYYKLNRRLLLLVGLWPYEHSVFKYCQMILCNIIVIMTTVCQIAKLIELRQDVNLVMKLLSPIV
LYVVYIIKYQTFCIVAKKIRHLMKHIEEDWNMLKDKKELEIIGRYTYIGSVCTLCLTILGSLGTLTCFFL
PFAPSILDIFMPLNVSRPRQLLFPGEYFVDQEKYFYAIWLHLDITLSLVVTTLIGTESLYIIYIQHVCGM
FQVASYRLYQAFDKKVLQAYAPEKRAIIVYRKIIEAIYIHNRTIEFFEFLWSTLMVSYSILLTIGITSLV
MNLFCLFYEALFIKQINEIMRLLIFIVGQVFYFFLGNLIGQIVIDHSTGIFQKTYITRWHSAPVRAQKLL
PFIMQRSMKSCKMDMGGIFVSSFEGFASLMSTTLSYFTVLWSVHK

>CbirOr313PI

MNFSGDHYKFNRIFLSVIGLWPHRHITLRQIQGIISSVVLISVTSPQLIKLFTIEYDVTLVLRILSSVLP
FLLFIVKYVTFYFITENALIYMCLFIIICIQYVPCILDVVMPLNNSRRVALLFQVEYLVNHEEYYHVIQL
HLHIGLLVAATTVLATESFCLTLSIHAFAMFKIASYRMQRIIDKNSLNLLIEKHCALYKNVVAAVDGHRR
AIEFSECIKRTFTIPYLALILLGVTSTSVNLFLLFQVITSMTSMDDLMRSIVFVVCHLIYMFLTNYAGQK
FIDHAVDVYQKICNVRWYNAPLRIQKHLLFIMQKAIKSYCMDVGGLYNPSLQGFTTLASASLSYFTVLCS
I

>CbirOr314

MEYFFEHYYRPTRIMLTALGLWPYNDSIFTCIQKIFFALLLGFSICLQIGKIITSVNDLDSLFGILWFAV
PCLVFSLKYATFCIQSKEIRKLIEVIKHDLNTIKRKEEIEILKEYANIGYILTMSILILFYIAIFGFIMM

EVLPKILDIVVPLNESRPVHLLGVATLFFDEEKYFFPILGHMTVALLSETTTIIATETVGLILIQHLCGL
FKIASFRIQHIFDCDLHISVSRKNRIYNANIAEAVIIHNKAIEFSDVFISSLEASYFILYWLGVCSLSIN
MFRLFQAGVKNETHQVYASGFFVFCHFFYMFINHLVPQIMTNHSNDLYYKACNLPWYSVPVQFQKMLPLL
IQKTAKPCKFAIYMLDVSLERFASLVSLSLSYFTVFWSMRY

>CbirOr316

MTSAANSYYRINRILLECVGLWPYQNSNYRRAIMAIVSVLLVSGVVLQLTTFVTAEFSVDLLLKILAHCT
PWLSYMLKYNVLSSNIKKMRDLMEHVRSDWNELNTMQEVEIIKKYSAFGKLITVVATSFIYLCILCVIVE
QISMNLYLDFTTAANESRIREFPALIECFIDHQKYFYPILSYMFLVIVCGQTTMIATETLYMTYTQHACG
LFEIANCRIEQALLITVMQDTASSLEKQSIVWQGIIGAVNMHRRAIEFIEISKANFTFAYFLVLPLGVLS
LSVNLYRLARLLPIKEYEEANVSFFLVFGHLWYFFFVNYLGQKVIDHSGNIFHKTYNAQWYVAPVKVQKL
LWLVMQRSLRHCTNVVGGLFVPSLEGFASITGASISYFMVIYSVH

>CbirOr317NTE

XFIHLSAFGFVLIYLVSTFLSDTAVDVNESNIRNLPGLTEYFIDQQKYFFLILFHISFFVWCGLAVVAAT
ESFYMSLIQHACGLFQIASYRIEQALHKDLVRNVTSIVERNTIINRKLISGINMHKRAIEFVEMSKASYK
WTYVMLGPLGVVSLSINLYRLSLLITIKDYRELISSFVFVLGHSWYMFFVNYVGQEVVDHSGDVFHRIYN
VQWYTAPLKAQKLLLYLMQRTMRHCTIVIGGLFVTSLQGFASLTNMSVSYFMVISSVH

>CbirOr318

MDFDGSQYYKFTRVLLTHLGVWPYCTKRANCVRSIFLSPFLFAPFIFQLSSFVTKEYSVDLLLNILSYAC
TCVYYVLKYFGMVIRSNEIKHFMEQIQSDWNSLQNEEELRIIHRHTRNARDFITITITCFYLIILFVTVV
FLWPNILNIVMPLNESRRSRQFPDAMEYFIDQEKYIYLLTFYSVVIAFVTISVVLSIEAFSVLHIHHICA
MFQIVSYRIECIVKKNEMKSLNLVPKQSNGFYKSIVEVVDSHQSVIEIVSNMKSSFDFVFLISIPLGVIS
LSINLYRLCLYLKTHDIGLISLSGLFVISHFIYIFFGNNNCQKIIDYSDGVFKTLCSTRWYATPVYVQKY
LLLIMCRSMKTSSILVGHIFVPSLQGYATLISSSLSYFMVIYSSYGRSTDETCSYNQFL

>CbirOr320

MDFAGKRYYNIHRILLSSIGLWPYQDPRKKTIQRIISSVILISSVIVQLMKFLTTEYSLDLLLKVLSFTI
PCIVFVLKYISFSIGTETVKDLMENIINDWSLLKTEDEFEIIKKHSDFGRFYTMLFTLAVYSGLSVCVVI
QLAPNFLDFVAPQNESRLHHIPITAEYFVDQQKCYLPILLHIDVIAVVGFTTVMSTESLFAAFVQHAIGM
FAIASYRIEHAFDEVLGINMPRKNCPYCRKIINAIYIHKRAIKFVEFLRSSFAISYIFLICLGIASLTAN
LFRLFLTLRCMDDLEEFIITTLFVFGHVYYIFLGNYTGQRVIDYSTDIFYKTYMSQWYNGPLHAQKLLLF
IMQRSMKSTVMCLGNLFVPSLEGFATITSMSFSYFTVIHSVQ

>CbirOr321PSE

MDFDGSRYYTTNRLMLSSIGLWPYQNGRWSIRIXRFCCLVCFVTCVLSQLLTFVTFEYNLDLLLEVLSFT
IPYLIVILKYVAFCVKSESMKKIMDMIKYDWNTLTNSMEYKIIHRYTYIGATYAQLFALIAYIVPLFITF
GHFVPDFLDIVEPLNQSRSHQMIILAEYFVDNEKYFYFIALHVNVCFFIFQITLMSTTSTYISYIQHVCG
MFQIASYRIEHALDSYEKGNLISERRCVTCARLISAIDIHRRAIMFFEFMADTFIILYFCLLFLGVASLT
INVYCAVITEGIIENFISILSVLIHLFYFFLVNYAGQKVLDHSNIFLSKTYNSKWHMMPLHVQKLILFIM
QRSTKDCMLLVGGIYVASLEGLATAMSASLSYFMVIYSTR

>CbirOr322

MDIFDNKYYGLTRQLMASIGLWPYQKREHQIVQSLCVSFILILSILLQLATFITHEFSTSLFIEVFSFDI
LCIIYALKYIIVYFHSNHVKSLFDQIQRDWKLIKNADELKIMQQYAYNAKFYTIFSGSIVYPGTLAFILT
MFVPDVLDIITPLDEPRPRQLPVQIEFFFDQDRYFYLFSFIFIIVAFLGMTVLMATENMYMLFTQHACGL
FKLLSHRLTRTFDMCSSKITPSKTKCRVCTNLLSVINIHQHCLKFLYDMQYKFSSSYFILSGFGVASLSV
NMFRLFLALNADNVFESVSSGLFVYAHFCYIFWMNYFGQDLIDHGEYLLQQICNAQWYTAPLHSQKLLLM
VLRRSMKSTAIIVGGLFIPSLEGFATLISMSLSYCMVIYSVRT

>CbirOr323PC

MDVYDDQFYGYTRRLLASLGLWPSQSSRYRKVIWIFALSLYVHGIVFQYTTFITRQYNIQLFIELMSLNP
LLFLYIIKHNAVYLNFANVKLLLEQINYDCIAITDSRELEIIQKYASKGKFYTIYSALFMYITTSMFIFS
LCIPDILNFVAPLEEPRLRELPTPVDCFLDQQQFFYFIVSDFALIALIGMTTFIATETMFMMFILHACGL
FAIARFMDFINSSFALSYLLLAIAGVISMTINMVRLFQAIELHISNDIFVSAMYVFTHFLYVFWVNYFGQ
ILLDHSETVYEATYSARWYMAPLHSQKLLLFILQRSSKACNFSVFSGVVIASMDGFAS

>CbirOr324PSE

MICIKKQYFNWNRVLLLPLGLWPDNETKFTRFQARLLFSLLMSSVVFHFSRLFIGKCSIDYVITLISSAT
FFVTLLIVFISFWINMKTMKNLLNYLQHIYDGLKDRNEIAIYDKYGYIGKRVTIILTIPTVCGLCFNSIM
LYSPYILDIVMPKNESYAIHIMEMITKYFRVSEKYYFLVLVHLNATCTAGLIIILIGTGTMMISYFKHACG
MFEIASYRIEQAMAIELLHHINTKTEIVIYKKIICAVDIHRKAMQFAQCFMNKLERPLSFLIIATVLCLS
FNLFRIFQIESPTEKVEEFLLHFLAMIAIFVLMFIANYTGQEITDHSNSVYVTTYNVSXYLAPIRIQKLV
LFLLQRGSKIFTLNVGGLFSASLECFSTLVSASVSYFTFMYSMQR

>CbirOr325PI(F)

MICLEAQYFNLNRILLQGTAQWPFEQSKLVRLQFIINWTILVSAIICQITTFFTSHFTSKFAIKVFSCVL
FLSILTIYYTSFRFNIKTVKDLMEQLHCACTKLREQKEIAIIQEYGRTANRVTAALLFGIIKPAFWAIQI
AIPYLDNVTPINGCRPHRLYIAVEYFIDQEKYYYVILIHMNAALFIGILSLMASATLMLMFFQHACGMFK
IACYRIENAIEINALKNISFNSELSISKKIVYAVDIHREAMQYNIRWYLCPHHVQRLIVLLLQRKAKQFH
ITCGGMFIASYECFATVVKATMSYFTLMHSTRR

>CbirOr326

MICLEVPYFSLNEILLRATGLWPFQQQKLARIQFSLFFSILTTAVIFQITTFATSKCTSKFVVKVLSSAL
SCTMVVIHYSSFYINIETVRNLLTELHHICEQLKDENEIAIIEKYGYKGKRFTTVLTALSASFIFAVLVN
QFCPGIFDVILSINKSRSHHLYLKMEYFLDQEKYLYFILLHISLAISIAVAVLIAIGTMFIAYLQHTCGM
FRIASYRIEYAMGVDMLQNICLTNEHLVYKGIVCAINIHRQATKLSKCLISNFDTTYFFLVIMSVISLSL
NLFQVATFQNDISEIVLPMIIIFFISMYLFFGNYMGQHITDHNNHVFATVYNVQWYMYPLHVQKLMLFLL
QRGGREFHLTCGGLFVASFECFATLVKTAVSYFTVIYSMQ

>CbirOr327

MICLEAQYFNLNRIILQYTALWPLQQSKLVTFQSTLILIIILITAIMCQITTLIMSKVSPKFVVVNVFARAF
FLMLLVVHYSAFRINIKSIKNLLQQLHHACNKLTDKNEIAIIQQYGWEAKRYTAVLVVTFFGEGSAFFLS
QIAVAFYEVNLSINRTRPPRLQIATENILGEHNFFQLILLHIDTAIILGEISWIAMGTFMIAYLQHTCGM
FKIACYRIENAIGINISKDINQKRGPSISEKIICAVNIHREAMRLSEHLISMFDPMYFCLTVFAVITVSL
NFSQISDIVTSGDIRKNISEAIIPIASILIIILYAFFANLIGQNVIDHNNEVYDSAYSISWYMCPLHIQK
LILFLLERKTKEFHLTCGGLFIASFECLATLAKATMSYFAFIYSTR

>CbirOr329INT

MVRLEDQYFNINRILLIAIGLWPYEQTKFARFQFIFFCANLMALIIFQVIAVCSLFILIISQTWMDICDV
VLRINTSRSHNLMIMTEYFVDQEKYYYFILLHINVTVSIAIFSVMAMGTMFLTFFQYSYGMFRIASYRIE
HSLDMDTLKSNMKNVNLVSKKIICAIVIQRQAMNLPKFLVNKFELMMFCQVIIGVTTLSLNMFQAFQIAS
FGNDIIEVVPHLIYVFAITLYMFVSCYIGQELTDHSKEVFLAAYNIQWYLNPVRIQKLILFLLQRGITDF
NVTIGRLFIPSFECFAMLIKTSMTYFTVIHSTR

>CbirOr333CTE

MVCFEDQYFNLNRSLLLVIGLWPYEQTNLARLRFIFFCVNLMSLIIFQLTVFVTSRCTPDVIITVLSVVL
LVVIVKMQYILFSVNIKIIKQLLVQFRNIYGKLKDENEIAIAEKYNYKARRYTATFIMIAVCSIFIFIIA

QTWIDIFDVVLQINKSRSQSLLIMTEYFVDQEKYYYFMLLHLNITVSIAVFIFVAIGTMFITFFQYIYGL
FKIAX

>CbirOr334

MTRLENHHFSLNRILLRAIGLWPYEQSKSFRLHFILFSGILISAIIFQLTAFLTTRCTPDLILKVFSIVF
IFCIFLLQYVSFGINVESVKELLIYLQDIRDELKDKNEIAITDEYGRDAKLCTAVLTMISIVGTFLIAIT
QVWSDVLNVILQINVSRSHNVPIITEYFISQEKYYYLIMLHQTTAVYIGVFTTVAIGTMLIAFYHNICGV
FRIASYRIKHSIIFNMIQNSIKNEALISARIICAVDFHRRAMELTRSMVSTLQVMYCCLILFGVAALSLN
LFRICQIATLKIDFMEMIIPLLMILIVIMYMYIANYLGQEVMDHCKDVFLNAYNSQWYIAPLRVQGLILF
LLERGSTEFKLNVGGLYNASLEGFAMLVKASVSYFTVIYSTR

>CbirOr335

MICLEAQYFNVNRILLQGTALWPFQQSKFAQFQFNIMFIILVTATITTFVTSEITLKFVFKVLPCILFFI
MLVIHYSSFRINMKTLKDLMRQLHRACTKLEDKNEIAIINKYGWNAHRYTIVLIISFLSGAFIFIASQIA
IAFFDISSPTNGSYKHRILYIHIATEYFIDEQKYFYLIVFHMYTALFIGTFSAIAIETLMIAYLQHTSGM
FRIACYRIEHAMKINILENISLSTASSISKKIVYAINIHREAMKLSELLISSFDKTYFCLTVLSVITLSL
NLFQIFQIVTSEDDAMGVSIPVLIVVILIVYMFSANFFGQDIMDQNNEIYIAAYNIQWYKCPQRIQKLIL
LLLQRKAKEFHLTCGGLFIASFECLATLVKATMSYFTLIYSAR

>CbirOr336

MSISFHERYFSYNRIILLAVGLWPYQQSKLVGFQTALFLSILTSFVVFLFSRLSFADYSFEFNIQLLCMS
TYYTFLIIKHLSFWINIEPIRYLLEQFQYIYKTLKNSKEIAIYDKYAIIAKRITIGLTIVAIYNLACFIV
MLCWPYILDIIMPKNETYVQRFIVHMTKDFAIQEKYFYLFLLHLYVASAVGGVSFLATGTMMLSCLKHIC
GILRIASYRFDRAIPATLQSITLKNETIIYKELIYAVDIHRKATECAIFFMNNIGRSLFVITMITVFCMS
LNLYGIFQVKSSLQEIEKTMGHLLFVIFLFIYMFAGNYAGQEITDCNNHIFLTVYNAPWYLAPLQIQKLT
LFLLQRSNKAFTLSIGGLFTLSIESCASLLSASISYFTVMLSF

>CbirOr337

MSTSVQERYFSLNRIILLAVGLWPYQQSKIARFQTALLLSILISIITLMLSRLCFVEYSFEFTINLLCAS
TYFTFFLIKYLSFWINIETIRYLLDNFQYIYKRLKDSKEIAIYNKYGNIAKRITVGFIIVAICNQACVTA
VQCAPYIFDVIMHKNETYARRFIVALTTYFAIQEKYFYLFLLHLNTVMAVGSLALLAIGTTVLSCFKHIC
GLFRIASYRFEQAITATTLQSITLKNKTMIYKEFIYAVDIHRKAIQFAKFFVGGMERSLFIVTMITVLCM
SLNLYGIFQIESPMQEIEKTVGHFQLIIFLLIYMFVANYAGQEMTDYNNHIFLTVYNAPWYLAPLQIQKL
TLFLLQRSNKAFTLSIGGLFTLSIECFASLVSASVSYFTLMLSL

>CbirOr338

MICLETEYFVISKPLLKAIGLWPYQQSALIWFQVILFSGILATVIIFQLTTFVTSKCTTNFVIKILSNAF
FFLTLLTKYTSFRLHINVIKDLLTQLQDTCNQITEPNEIAIIRKYGYNARRYTSIFRTFFLSTVPITIIG
QVLPNIFHSVLSNNESRPHNLQITMEYFIDQQKYFYFILFHLNTAICIGIITTAAVGSTFIAYFQYACGM
FMIASFRIENAMENNKLQDINNKHFILIGLSRAVEMHRQAVKLCDLLICRFETMYFCLIVFGVICLSLNL
LQIFQTKESGEIQEFVLSIVLVAVCGFYMFVANLVGQDITDHNDEIYVTVYNVQWYKAPLHIQKIILFLL
QRGVKSFTLTIGGLFDASFECFATLVKASVSYFTVLYSTR

>CbirOr339

MISLITQYFNLNKILLQAIGLWPFQQTILVQLQFTLLSGILTAAIISQLTTFITSKCTPQCIIKHLSIAL
ALIVALTKYVSFYVHIKPMKELLIQLQYISNELKEKKEIAIMEKYGSNARCYTVILTSFGVLSLSALLLK
QCWSSILDNIVYNNISRPYHLQIKMEYFVNQKKYFYFILFHLNIAICIGIIITAATGTMFIAYFQYTCGM
FMIASFRIERAMENNKLQDINNKHFILIGLSRAIEMHRQAMKLCDLLICRFDTMYFCLIVFGVTCLSLNL
LQIFQTIASGDIQEFVLSIILVVICTLYMFVANLVGQEIIDQNNEVYVTVYNVQWYKAPLHIQKIILFLL
QRGVKSFTLTIGGLFDASFECFATLVKASVSYFTVLYSTR

>CbirOr340

MIRIVEHFRIHRILLLAIGLWPYNPSKFVKLRLLLFILITNSFIIFQFTSFITAECTADHILKVLSVMLF
FLFCDVHYTSYWINAHILKGFVERLQHVYNDLKDENEIAIFNKYGNIADHITVIITSFTACSVLILSLLP
LLPCILGTFLFANESHPLYNIIETEYFVDEEKTFYLTLLHTYASLYIGVTATVGGGLVVLTYIIHICGLF
SIARYRMNQLLILNIHDKTNLKNNMDIDKKIARIVDFHCIAIELSEFYVSNFNGTYFCIILLVVICLSLN
LYQVFQSVLHRTSVEESVFHLIFAVAIIIYSFVSNYAGQEVIDHYNDMFYVAYNIQWYTTPIRIQRLILF
LLQRSCKIYCLRIGGLFNPSLECFASLFAASMSYFTMMYSTQK

>CbirOr342CTE

MICIRSRFFRINRILLFVLGLWPYEQSKFTRLQSILFYIILISFIVAQFAVFTTSKCTLQIVIEILSTIF
FFIICVLKYNSYHSNMDAVKYLLELLQHTYNELKDENEVAIIEKYWTIIKRNVEMLTLMLFCGIFICGCN
TFLPCILHVILPANKSGPHPSLHIMTEYFIDQEKYFYLLVMHKEVASCIGTTAIIATGTMNMIFLQHACG
MFMIASYRMEQATMSLNLKKDNKGNKHLVYMGIIYAIDMHRKAMX

>CbirOr343PSE

MTISVVEQHFSINKILLRAGAIWPYHRTKLVKFHLILVFSILSSCVLFQLTTFLTTECTPEMVIKILCYT
SYASMYVLTYSSFWLNVDTIRCLLEKFQYICDELTDEGEIAIIQLYGNGVKRFTIMVLLCTMSNISIACL
LPFFPRILRTFTSINVSQEHFTVHIMREYFIDQEKYYYCILLHFDVSIFIGTLVTTATGTLLFGCIKYIC
GIFRIASYRIDQAMQTATLHHAVLSNNFILYKKIVRAIDIHRKAVKSVIVLSLCDIITSNFVGTFFIMMI
LHRAIMLRSATEEYVAYVSLTGAPLVYIFFANYAGQNIADHHNYIFTSVYNCKWYITPLYIQRIILFLLQ
KGTNTYYLIFGGILVLCMENAAMMVSTSISYFTVLYSTQQSSRYN

>CbirOr345

MISIVEHFRLHRTFLLVIGIWPYNQSKFVELQFSLFFAVPHSFIIFQLTSFITSECTVDLIIKILSTIFF
FLICEVHHTSFWINAYTVKCFVERLQHVCNDIKDENEIAIIEKYGNSAEYITIILTLFAVCCVIIITVLP
FLPWILGTFLLANESRPFHNMLIVTEYFVDKEKHFYLIILHTYASIYIGVTAMVGGGMMLASYLKHICGL
FSIASYRMEQTIMLNSHEKANGRNEMEMNKKLRHAVDVHRTAIELSEFFMSNFNGTYICLIVIAVICLCL
NLYEVFQTTLLRGNVEEILLHLIFASAIVLYTFVANYTGEEIMHHYNDMFSMAYSVQWYTAPIRIQRLIL
FLLQRSCKVYRLKIAGIFITSLEGFASLSTASLSYFTVIYSTRE

>CbirOr346PI

MISIAQHFRLHRNVLFVIGLWPYNQSKFVKLQLILFLFVQTSFIVFQLTSLITAECTIDLVIKILSTIFY
FLFCVTHYISFWINAYTVFAVCCVFMLSLLPILPWILGTFLFANESQPLYDIQIVTEYFVDKEKTFYLTL
LHTYASLYIGVTAMVGGGMMLIAYLIHICGLFSIASYRMEQSLILNINEKPNLRNKKEIDKKIGHAVDVH
RTAIELSEFFISNFHGTFFCIIAMMVLCLSLNLYQISQSALHQNIDECLFHLIFAIDMLIYSFIANYSGQ
EVIEHYNDIFSVAYSVQWYTAPVRIQKLILFLLERSCKNYGVRVGGLFITSLEGFATLFTTSISYFTVMY
STQ

>CbirOr349PN

MVFCLLTITLMPLFPRILSTFLLVNISRPLYNMQFITEYFVDKEKNFYLILLHTHTSFYIGVIALVGGGL
LGCAFLKHICGLFSIASYRIQQSMRVKLHEKPDLRNEMEIEKKIICAVDIHRTAIEIFQTALIRDKVEEF
LLHSEFAFGLLLYSFLANSCGEEITEHYNDMFSYAYNVRWYTAPIHIQKLILFLLQRRCKPYGLKLGGIF
TASLESFASLLTASVSYFTVIYSTQK

>CbirOr351INT

MAVDIVLQYFNLNRILLLTVGLWPYQRTKLVEFQLYLVFGILVSIIPALLTPLLTLECTVSLLIKILPPV
LITIICTINYFSFIIKAHAVKQIMDQLQNICTNLTDETEVDIMKNYGSKTRSYTASIILFQTVSSGHNVE
EFVVHFSCLSILLLYLFLANYAAQEIIDHYNHIFITVYSIQWYAASICIQKMILFLLQRGAKAFNLNLGG
LFIGSLETAGMLLSTSVSYFTVLYSTR

>CbirOr352PSE

MIYIQAHFNLARILMLAIGLWPYQQTKLVQLQLILISNILTSFVVFQFTTFLTTECSPNLVIKIITCSLF
VVSPMIMHNSFWLNIHTVKYSFEQLQDICNELKDEGEIAIIKRYGNDTKRYTLILFLCATCGIVFYSFLP
LWPQILSTVLPINVSQTQRIMLIMTEYFVDQERYFYLLLLHINTATYIGLIVAIATGTVLLGCIIHACGL
FSIASYRIKQAMTIIMMKNANQENQILMYRKIVCAVNMHCKAMNFLEVLMSAFQGMFFLMIVITVIIGSL
SLYGITSCRDNISEFCLYLTCISTTVVYIFAGNYLAQDIMDHSNDIFVTVGSLQSAATLISASISYFTVL
YSVQKVKARFQREI

>CbirOr353

MTIYIIERYFSFNRIILLAIGLWPYEQSTFAQLQMVVILGILISFDAFQLSRLLFVECTFDFTLKMLSIS
LLFTFYVIKYMSCWVNIETIRYLLEQLQHIYNGLKDKNEIAIYEKYGNTAKRFTVNIIIVEISIMVFGFV
MEIWPYIFDVIMPKNETYARHLIIILISKYYSIEEKYYYLIFLHLNAAHAVGSLILIAVGAIMLSYIKYIC
GMFRIASYRLERAMTTNNTLQNSLKNEFVIYKEIIYAIDIHRKAIECAEYIVSNFEKSYVFIIATNVLCV
SLNLYRVSQIESPMKEKEETLSHFFITLFILLYMFIANYAGQEIIDDNNHVFFTAYNIPWYLAPLHIQKL
ILFLLQRNSKAFTLNFHGLLIASLECFASLASASVSYFTIMYSVQG

>CbirOr354

MICITKQYFNWNRVLLLPMGLWPDEETKFTRFRASLFYCLLMSNIAFQLSRLFFAEFSFDFVIKILSFSA
AFCTLAIFFMSFWINMETMKYLLHQLQHIYDGLKNQNEIAIYEKYGYIGKCISARLTIFTICGLFFNFII
IYSPFILDVVIPKNESYAIFIMKMVTKYFVVSEKYYFLIIVHVNAAFSVGMIVLTGTGTMMISYFKYACG
MFEITSYRIEQAMAIELLRNTDIKKEIVIYRGIICAVDIHRTAMEFAKCFLTKTEVLFFLLVIAAVLCLS
CNLFRILKIESPMEQVVEVLLHIIVVILIVAVTFLPNYVGQEIMDYNNNVYVTTYNVLWYLAPADIQKLI
LFLIQRSSRIFCLTVGGLFTASLECFATIISLLYVSLLDFSRAN

>CbirOr356

MICIESLQIRLNRILLLLVGLWPYQQSRLVRVQLVVLFTILTTFILFQFATFATSKCTSDLSINILSSTL
FCAIFVIKYSSFRINIDIVKSLLEQLQHMYNELMDKNEIIIIEKYGRYAKRYTAIFTLLAIFIAFGLILY
PFSPRILDILWPVNESRQRPSLLFMTEYFVDQERYFYLIFLHANVAICIGGFVMLATGTILITYFQHACG
MFRIASYRIEQAMTIGTLDKGCLGNDNLIYKGLIYAVDMHRKAMKFSDSSVSKFKVMISLLLIFGVICWS
LNLFRIFQVMSFKQNFMEISLPIIFVIVHFTYMILGNYLAQEITDHNNDVFTTVYNVQWYLAPLHVQKMI
LFLLQRSTRTFTLNVGGLFIGSLEGAATLLSTSISYFTVLYSTQCKIHLIVYX

>CbirOr358

MICLEAQYFNLNRILLQGTAQWPFEQSKLVRLQFIINWTILVSAIICQIPTCFTSQFTSKFAIKVFSCVL
FLSFVTIHYTSFRFNIKTLKDLMEQLHCACTKLRDKNEIAIIQEYGRTANRVTAACVVFVGITKLAFWAV
QIVIPYLDNVAPINGCRPRRLYIDVEYFIDQEKYYYVILIHMNAALFIGILTLLATGTLMVMFFQHACGM
FKIACYRIEHAIEINALKNINFNSVPSISKKIVYAVDIHRQAMQITDHLMNRFDTMYFCLVVLSVITLSL
NLFQISQIMAFEDNIREAVIPSVSVCVLSLYVFLGCVLGQNITDHNNEVYAAAYNIRWYLCPQHVQRLIV
LLLQRKAKQFHLTCGGMFIASYECLAMITKATMSYFTLMHSTQR

>CbirOr359

MICLEAQYFNLNRILLQSTALWPLQQSKLVQLQFIINWTLLVSTIICQITTFFTSKFTPTFAVKAFSCIL
PIIVATIHYTSFRFNIKTDLLEQLHYACSKLKDKKEIAIIQEYGRTAKRYTAGFVVLAFSAESALWTIQI
AIPYLDDVTPINGCRLHRLYIAVEYFIDQEKYYYVILIHMNVALFIGTLTVIATGTLMLMFFQHACGMFK
IACYRIEHAIEINALTNISFNSEPSISKKIVYAVDIHREAMKITDHLMNSFDPTYFCLTVLAVITMSLNL
FQISEAIILEGNIGEAIMPTISVVGLFIYTFLGCAFGQHITDYNNEVYAAAYNIRWYLCPQHVQRLIVLM
LQRKAKQFHLTCGGLFIASYECFAMVAKATMSYFTLMHSTRS

>CbirOr360

MTRLENHFSLNRILLLAIGLWPYEQSIFSRLQFILFSVILMSAIVFQLTVFLTARCTPDLILKVSPTVLL
FCIFLIQYISLDFNIESVKELLNCLQDIRDELKDKNEIAITDEYGRNAKRCTAVLIMISMVGTCLFVVIH
FWSDVLDVIFQINISRSHNLPIITEYFINQEKYYYLILMHLAIAVYIGIIVTLAVGTMLIAFYHNTCGVF
RIASYRIKHSINSNMIQNSIKNEALMSERIICAVGIHRQAMKLTRSMVSKLQVMYCCLIPFGVATLSLNL
FRLCLITSLKIDLMEMMPPIVSIFVIIVVMFIGNYLGQDLTDHCKDVFLNAYNIQWYMAPLPVQSLILFL
LQRGATDFKVNIGGLYYPSLESFAMLVKISMSYFTVIYSTR

>CbirOr363

MICLEAPYFNLNRILLQSTALWPFQQSKLVQLHFIINWTVLMVAIICQITTFSTSQFTPKFFLKVFSGVL
FLIVLVVHYSSFRFNITILKDLMGQLHCACTKLRDKNEIAIIQKYGRNAKRYTAALLVVFFILESIFWAS
QIIIPYLDDVTPINGCRPHRLQIAVEYFIDQQKYYYVILLHMNAAIFIGVLTFLAMGTLMIAYLQHTCGM
FTIACYRIEHAMEINILKNISLNNEFSISKKIVNAVNIHREAMKLSEDLITRFDTMYFCLTVLVVITMSL
NLFQIFQILTSEENIIEATVPTVTVLVLILYVFFANFFGQNVIDHNNEVYAAAYNIRWYMCPLRIQRLIL
LLLQRKAREFQLTCGGLFVASFECFATLAKATMSYFTVMHSAR

>CbirOr364PNC

MITFFTREVTSKFAVTISSRLLFYSNMMIIYSAYRFNIKTLKDVLGQLHSACTKLRDKNEIAIIQKXGRN
GSYYTAAILVFYIIATIIFQANQLYYATKFDTYDIIAPINGCRSHRLCIAADYFTDQQYYYYTIVLCMTA
AVSIGVLTIIATGTLMIVYLQHTCGMFRIACIVWNTQWRLMYLKNINNFKNSNSELAICKKIVDAVNIHR
EAIKLSDDLITRFDTMYFCTTVLEVITMSLNLFHISQIITSKDNIQEAVISAISLSVLSEYTVFANLCGQ
YVTDHNNAVYAA

>CbirOr366PI

MICIKKRFFNLNRLLLLPFGLWLEETKFNYFQATLFCSLIMSSIVFQITSLFNQLQYIYDRLKDRNEIAI
YNKYGYIAKYFTTIVITILVCGLFANSVIVYWPYILDAVMPKNESYAIHTMKLVTKYFEVSEKYYFLVLV
HLNAACTAGLIILTGVGTLLISYMKHICGMFEIASYRIEQAMAIELLHNFDIKKFIVMQKIICAVNIQRY
AMNFIDCFMTTLEGTVLALIIVTVICMSVTLFRIFQIVSLEEEVEEVILHLFSVLILLVVMFITNYIGQQ
VTDYSNHIYIITYNASWYLAPIRIQKLILFLLQRSNKIFTLSVGGLFTASLECFTTLVSASVSYFTFMYS
IQQ

>CbirOr367

MTICIEKRFFNLNRLLLLPFGLWPGKKTKFTLLQGTLLCSFLMSSIAFLLSRLFIAECSFDFVVRIFSST
TFYVMLAIPSISFWINRRNIKYLLDQVQYIYDRLKDRNEIAIYDKYGYIAKHITIIVTVLLACGLCFSFV
ILYWPYVLDAVMPKNESYALRTMEIVTKYFKVSEKYYVLVLVHLNMACSVGLITLIATGTMMMSYFKHAC
GMFEIASYRIEQAMAIELLHIFDMKNEIIIYRKIICAVDIHRKAMEFAKCFVTSIEGSFFVLVITTVLCM
SFNLFRVFQIELPMEKMEEVLLHLLSVMLILLIMFLANNTGQEIIDHSNNVYVITYNISWYLAPIRIQKL
ILFLLQRSNKIFTLSIGGLFTISLECFATLVSASMSYFTFMYSMQ

>CbirOr368

MKINYKNQFFLNWLLLLPFGLWPDKETKFTYLQATLFCCLMMSSIIFQLSRLFTAECSFDLIVRILSSTT
FFATLTVPTILFWIHSKTMKYLLYKVEHMYDELHDRNEITICEKYRYIARRFTIIVVILLMCAMTFATVL
LYWPFAFDILIPKNESYTIRTMEIVSKFFVVSKKYYFLVMVHLCSAASAGVIVLSAIGTMLISHFMHACG
MFEIASYRIEQAMAVELLHIFNIRNEIIIYRKIICAIDIQRQAMEFTTFIVTNIETSIFFSTITTVLCIS
FNLFRIFQIESFRDQIEEALAHLSVVTVVLGLMFLTNTIGQEVTDHSNNVYVITYSVLWYLAPLRIQKLI
LFVLQRSNKIFILNVGGLFSASLECFATLISASISYFTFMYSMQ

>CbirOr370

MICLEAKYFNLNRILLQSIALWPFQQSKLVRIQYIISLTILLGALISHIITVLTSTFTLALTIKPFSCVL
FYTILVIHYSSFGVNLKTLKDLLTRLHCACTKLRDKNEIAIIQKYGRNAKCYTAALIAIYISVSPLFWTI
EMLMPYCDIVIPINGCRPQRLSIAFEYCIHQQKYYYVLLLHMNAARAIGILTFIATGTLMIAYLQHTCGM

FRIACYRIEHAMEINIQKGNSGNSESSISKKIVYAVHIHREAMKLSEHLISRLDTTYFCLTVLVVITMSL
TMFQVFQTILSVDNTLDAMVPFINLVILSLYVFFANFFGQNVIDHNNEVYAAAYNIQWYMCPIHVQKLIL
LLLQRKAREFHLICGGLFIASFDCLATMATATMSYFTLIHSTRR

>CbirOr371PSE(F)

MICLEAEYFNLNRILLQSIAVWPFQQSKLVRIQFIISMTILVGAIIYQITTFLTSNFTLKFAIKAFSCAL
FFTFVVIHYSAFQLNIKTLKDMMKRLHCACINLRDKNEIAIIQKYGRDAKYYTAALIVFFVIAKSAFWAS
QIIIPYLDGITPINGCRPHQLYIVAEYFIDQQKYYYVILLHMNAALFIGILTLIAMGTLLIAYLQHTCGM
FKIACYRIEHAMEINIEKDISGNSESSISKKIVYAVHIHREAMELSEHLISRFDTMYFCLTVLVVITMSL
NLFQILLSSDSIMEATVPSLNLLILSLYVFFANFFGQNVIDHNNEVYAAAYNIRWYMCPIHVQRLILLLL
QRKAREFHLTCGGLFIASFDCLATMAKATLSYFTLMHSARQ

>CbirOr373PI

MMMCIKNRFFNQNRLLLLAFGLWFDEETIFTHFQATLLCSLMISSIIFQFSRLFIAECSFDLIMRIFSSM
MFYVLLTIPCISMWINIKAVVLAFSLCGNSVIIYWPYILDIIVPKNVSYAIRAMYFMTKYFKVSEKYYFL
VLVHLNAACTTGLIVMIAMGTMLLSYTQHICGMFEIASYRIEQAMAPELLHNFDIKNRTVICKQMICAID
IQRQAMQLSDCLLSTMHGSLAVVIITTVLCASCNLFQVFQIESPAEELEEVLLHLLAASFVVWIMFICNY
AGQEITDHSNHVYVITYSISWYLAPLHIQKLVLFLLQRNHKIFTMSVAGLFTASLECFATLVSASVSYFT
FMYSMQK

>CbirOr374PSE(S)

MVCIKKRFFNSNRLLLVAFGLWPDEETIFARFQATLCCSLLISSIVFQFSRFFIAECNFDFIVRIFSSTS
FFVVLTVPAILFWIHLKTIKRVLNQLQYVYDRLKDRNEIAIYDKYGYIGKQITTLVIIGLVCTLCGNSVI
VYWSYVFDIVVPKNESYTIRTMQFVTKYFKISEKYYFLVLVHLNAACSTGLIILVGTGTMLFSYFKHVCG
MFEIASYRIEQAMAIELLHNFDIKNRIVICRKMICAIDIQRQAMEFTKYFISSIEGSCFIMILTTVVCLS
CNLFQIFQIESPMEEVEQVLLHLSAVSFVLTILFLSNYVGQEVTDHSNHVYVITYNVSWYLAPLHIQKLI
LFLLERSNKVFSLNVGGLFTASLECFATIYCMYLSYFTFMYSMQ

>CbirOr375

MVCIKKRFFNLNRLLLIAFGLWPDEETIFTRFQATLLCSLLISSIVFQLSRLFIAECSFDFIVRIVSSTT
IYIMITGPPVSYWIHMKTMKCILNQLQYIYDRLKDRNEIAIYDKYGYIGKHITTIVIILLVCCLFGNSVI
VYWPYILDIIIPRNESYAIHMMQFVTTYFNVSEKYYFLIVVHLNAASTIGLIVCVGTGTMLFSYLQHICG
MFEIASYRIEQAMAPELLHNFDIKNKIIIWREMICAIDIQRQAMQCAKCFETSLEGTTFILVITTVLCVS
CNLSQVFQIEALMEEMNEVLIHFSVVSFILTIMFVSNYAGQEVIDHSNHVYVITYNVAWYLAPLHIQKLI
LLLLQRSNKIFSLNIYGLFTASLECFATLVSASISYFTFMYSMQ

>CbirOr377

MVCIRKRFFNSNRLLLAPFGLWPGEETIFTRFQATLLCSFLIGSIVFQLSRLFIAECNFDFITRIFSYMS
LFVMFTSLSVVLWIHMETMKYLFDQLQYIYDRLKDRNEIAIYDKYGYIGNHITTTIITLVVCGLCGTSVI
LYYWPFIFDIIVSKNESYAIHTMQFVSEYFNVSEKYCVLLFMHWTASGTIGLIAFTGAGTMVLSYFQHIC
GMFEIASYRIEQAMAIELLHNFHIKNGIVICRKMICAIDIQRQAMELATHFITSMEGPVFVMVITTVLSM
TCNLFRLFQIESPMERMIEILFHLWAVFFILVILFLANYVGQEVIDHSNHVYIITYNVSWYLAPLHIQKL
ILFLLERSHKIFTLSAGGLFTASIECFATLVKVSMSYFTFMYSIQ

>CbirOr378

MSISVQERYFSWNRILLLAVGLWPYHQSRFARFQAALCLSSLTSFIVFVLSRLYFTEYSFEFTIHLLSIS
MYYTFFLINHIAFWINIETARNLLEQFQYIYKRLKDAKEIAIYNKYGNFANRITLSCIILAVCSAFIITA
IEYWPIIFDMIMPKNETHARRFIVTVTKYFALQEKYFYVFILFMNAVHNIGTVGILATGTMLLSCLKHIS
GILRIASYRFEHAITATLQSITIKNETMNYKEFIYAVDIHRKAMECAKFGVDGMERSFFVITMITVLCMS

LNLYGIFQVESPMQEIEKTAGHFFNIMCLFIYMFLGNYAGQEITDCNNHIFLTIYNAPWYLAPLQIQKVI
LFLLQRSNKAFTLNIGGIFTLSIESFTSLVNASLSYLTLMLSVQKYN

>CbirOr379PSE(F)

MWISVQERYFNFNRIELLLVGLWPYQQSGLARFLRTLCLSILMSVVALMLSRLCFAKYSFEFSINLLCFS
TYYTFYAILYIAFWLNREAIRYLLEQLQYIYNRLNDENEIAIYDKYGNIGKRITIACIIIAVCNQFVVVA
MQCWPYIFYVILPENGTYAGHLLVVVSKYFAVQEKYSYLVLLHINIAISVGAFIFLAVGTMMLSCFKHIC
GMFRIASYRFEQAITTTTLQSITLKNKTMIYKELICAVDIHRKATEFIKFLVSSVDRSLFVVVIVTVLCV
SFNLYGIFHIEPDKQEIEVTLSHLIILCFVFAYMFLANYAGQEIMDYNNHVFLTVYNAPWYLAPLEIQKL
ILILLQRSNKAFTLSIGGLFTLSLECFASLASASISYFTLMLSL

>CbirOr380INT

MICIKKQYFNWNRVLLLPMGLWPDEETKFTRFRAQLFYYLLMSNIAFQLSRILFAEYSFDLVIKILSSSS
FFFSLAIFFMCFWINMKTMKYLLHQLQHIYDRLKDKKEIAIYEKYGYIGKCISARVTIFTICGLFFNFII
IYSPFILDVVIPKNESYAIFMMEIVTKYFVVSEKYYFLIIVHMNATFSVGLIVLTGTGTMMISYFKYACG
MFEITRYNVSWYLAPVDIQKMILFLLQRGSRTFTLNVGGLFTASFECFATMTSASVSYFTFMYSIQQ

>CbirOr381CTE

MCCIKLPSLRVNRILLLVVGLWPYKQDKFVRLQLILFYVIMMSFVIFQFTTLLTLKRTSQIIIEVLSTTF
FFIACMIKYSSFCFNINAIKHLLDLLQHTYDELRDEGEVAIIEKYWNLAKRYTKVLLITAICALSLGIFI
SLLPLIFNIALYTNTSQLYISLFIVKEYLVDQENYIFLIILHTNVAGFMLVIAMLATGTMTIMYLLHACG
MFRVASYRVQQAMTSINRETSTKKNEKCVYVGIICAIDMHRKAIX

>CbirOr384

MVCIKKRFFNSNRLLLLPFGLWLGEKTIVTHLQATLLYSILISSVVFQLSRLFIAECSFDFIVRIFSSTS
LYAMLTSLPIIFWIHMKTITYLFDQLQYIYDRLKDRNEIAIYDKYGYFANHVTTIIIILLVCGLCGNSVI
IYLPFVLDIVVPKNESYAIRTMQFVTEYFNVSEKYYFLVLVHLTAASVTGAIVFTGTGTMLFSYLQHVCG
MFEIASYRIEQAMASELLHKCHIKNRNVICRKMICAIDIQRQAMEFAKHLITSMEGTLFVMIITTVLCMT
CNLFRIFQIESPMERMDEVLIHLLHLWAVLFILIIILFLSNYVGQEVTDYSNHVYEITYNVSWYLAPLHIQ
KLILFLLERSHKIFTLSAGGLFTASIECFATLVKVSMSYFTFMYSIQ

>CbirOr385PSE(F)

MICIKKRFFNSNRLLLVAFGLWPSEETIFTRFQATLLCSFLISSIVFQLSRLFIAECNFDFIVRIFSSTS
IYVMITGPSILFWMHMKTIKYILNQLQYILTTGLKDKKRKFAIYDKYGYIGKHITTIIIILNACCLCGSS
IMVYWPFILDIVVPKNESHAIHTMQFVTKYFKVSEKYYXLVLVHLNAACTTGLIAFTGAGTMLLSYLQHI
CGMFEIASYRIEQAMAIELLHTFHIENRNVICRKMICAIDIQRQAMEVAKHLITNTEGTIFVLVVTTVLA
MSCNLFRIFQIKSLMEQMGESLLHLFAVSFILILLFLCNYVGQEVIDRSNHIYVITYNVAWYLAPLHIQK
LILFLLQRSNTIFSLSVGGLFTASLECFATLVSASISYFTFMYSMQ

>CbirOr387INT

MTSINTQYLYMHRISLITLGLWPYHRTILVKLQSFLFSLLTISIIIFQLTTFLTTECTINFIIKVFSRTL
FLLLCVIQYNSFWINTHIVKHLLENLQYVCSKLNDKNEIAIIKKCGQIAKYVAIGFILVAMGSVSISISA
LPLLRISFLTNKSKLHLKMLIMTEYFVDQEKYMYFILLHLYSAICIAVATLVGTAILMTGYFIHFCGLFD
IASYRLKQAMRINSYEVTNRGNKNKIYKKISHAVDIHRTAIEFTEFFLYNFKIAFSFIMAIIVICLSLNL
FQVSTVSMSCFTVMYSIQN

>CbirOr389PSE

MTICVVDQHFSLNKMLLRAVALWPYRRTKLIDFHVFLILVIFISFILVQLTTFLTTECTPEMIIKSLCYT
SDASMYVLTYTSFWLNVDTIRCLLEKFQYICDELTDEGEIAIIKQYGNGAKRFTIMFSLCIVSNISITCL
LPVFPRILRTFTSINVSEEHFTVHVMREYFIDQEKYYYCILLHFDVTIFIGGIGCMATGTLLFGCIKYIC

GIFRIASYRIDQAMQTATLHHAVLSNNFILYKKIVRAIDIHRKAVKLCDIMTSNFVGTFFVMMIVCIISL
SLNLYGSTSTQCAVAYVSLTATPLVYIFSVNYAGQKITDHHNYIFINVYNCKWYRTPLYIQRIILFLLRR
GTNNNYLVFGGLAVLSLERAATMISTSISYFTVLYSTQQSP

>CbirOr390PI(F)

MICIKTEYFNLNRILLLAVGLWSYQQSIVQFQLFVFSSILNSYIIFQLLSFLTAECTPDFIISVLSHVSL
LTVTTIHYNAFWINISTIRYLLDQLQDIYNKVKDANEIAIIKTYGKNAKRVTANIICYRIHQAITLEKIF
LQNDLMIYKEMIYAVDMHRKAMXXILSSIIFVCCLSLNLLGIVWTTSSNFNIKKVFIHFGTLLPIVISMF
LSNSIGQEITDHNNHVFNTVCNTKWYIAPLQTQKLILFLLHINNKPFTLNIRGILASMECFSTLMKTSIS
YFTVIYSMQ

>CbirOr391

MSISVQERYFSLNRIILLATGLWPYQQSKFARFQAALYLSMMISFIIFLLSRLCFTEQSFEFTLHLLSMS
TYFIFLLIKYISFWINMKSIRYLLEQFQYIYKRLKDSKEIAIYNKYGNNGKRITIGLIIVGICTQTYLIA
IQCLPHILDMIMPKNETYMHRFIVIVTEGFAVQEKYFYLLFLYLYAVLTAGAVTLIAVGAMLLSCLKHIC
GMFRIASYRFEQAVTATLQNITLKNKTMFYKEFIYAVDIHRKATEYAKCVLGDMERSLFVVTMVTVLCLS
LNLYEVFQIKSPMQEIEKTMGHLLLIIFLFIYMFAANYAGQEITDSNNHVFLAVYNAPWYLAPLQMQKLT
LFLLQRNSKAFTLSIGGLFTLSIECFTSLVSASVSYFTLMLSLY

>CbirOr395PI(FS)

MDTHFLYIHRLSMVAIGLWPYHRTMLVQLQCSVFSLTLISFIIFQLTTFLTTEWTIDFIVEILSLSIVIL
ICAILYNSVWINTRGVKRILNNLQYICSDLKDEKEIAIIKRYGYIAKCATIGMTLFVMCFFFIHNSFANS
AANFRHLFPRNKSEPYRNIYIRTEYFVDEEKYFYFILLHLYAVQYIAGGTLLVAGTLVAGYFTYCCGLFN
IASILEFYLYNFEDTWFLLIVLVVICLSLHLFGIFQAISIVFKMENFVLHFGFTLGILLSSFASNYVGEA
ITEHYNYIFSTAYNVRWYDAPIRVQRLIFFLLQRGTMSYAMKFGGVYTLSLENFATLSTASISYFTVIYS
IQK

>CbirOr396CTE

MAICVVQQYFSINKILFRVVALWPYHRSKLVEFHLSFFLVILISFIAAQLATFLTTECTSHMAIKIFSLV
LYFTLYLIKYLSFRVNAHTIRCLLEKFQYTCDELTDEGEIAIIKNMGTRRNILPLKFPKYYYCILLHMDV
NFFMGTTVLTRILRTFTSTNVSEEHFMIHIPTEYFINQEKYYYYIVLHMDASVFIGSIVLPATGTLLFGC
MKYMCGLFRIASYRIDQTMKTPMFQIAGLSKDYVIYKTIVHAIDIHRKAIEFCNMIKSNFVGMFFIVIVI
SVTSLSLNLYE

>CbirOr397PN

LTTFLTTEWTIDFIVDILSTSLFLLLCVIHYNSFWINTHVVKHVLENLQYICSDLKDENEIAIIKRYGHI
AKCMAIGSILLMMCSLFLLSLLPILPRISGTSFLVNKSQLYRTYIRTEYFVDQEKYFYFILLHLYAAQYI
AGGTLIGTGIVVVGYLIYFCGLFDIASYRVGQAMRKNSNEVTNRENKREIDKKIRHAVDIHRTTLEFTDF
FLYNFEGTYILIITIIVLCLSLHLFGIFHNVSFVFRMEKFVLHCGFTFAILGISLGGNYISQAITDRYNN
IFSSAYNIQWYIAPVRVQRLILFLLERGAKPYEIKYGGLYTASLENFATLSTTSISYFTVICSI

>CbirOr398PSE

RNRLVAIGLWPYHRTMLVQLQSCVFSLTTISFIIFQLTTFLTTEWTIDFIVEVLSVSLFTLLCAIQYNSL
WINTHVVKRLMEHLQYVCSELKDENEIAIIKRYGHIAKWITIGLTLSAMCVLIIFTLLPILPRIFRIFFV
VNKSEPFRNVYITEYFVDEEKYFYFILLHMYATQYIAGGTLLAAGIVVMGYLTCVCGLFNIASYRIEQAM
LLNSDEVTNRKNKREIDKKLSHAVVMHRTALEFTDFFLYKFEGTYLLIIALIVICLSLHLFGIFYSVNFV
FRMEKFVLHCSYALGLLGCSLGGNYISQAVSDHYNYIFSSAYKVRWYIAPVRVQRLILFLLQRGTKPYNV
KFGGLYTTSLENFATLSTRSKDFEFDIYYKGHLPE

>CbirOr400

MRVDAQTNVNGINAQFLYIHRISLIAIGLWPYHRTMLVQVQSSLFSFSTRLLTTFLTTELTVDFVIKVLS
TTLFLLLCVIQYNSFWINTHVVKRVLENLLYVCSELNDENEIAIIKEYGHFAKRVAIGFILLAMYGVFVC
SFLPILPRIFGIFFLVNKSESHHRNTYIMTEYFVDQEKHFYFILLHLYSALFIAVATLLGTAILMTGYFI
HFCGLFNIASYRIEQAINSHEVTNRKYKSEMRKKITNAVDIHRKTIEFIDFFLYNFEGTFILIIMIIIC
LSLNLFRIFQALSENSIENFVLHSILMGAIFVYSFVGHYTGQEVTDHYNYIFSTAYNVVWYTAPVCVQKL
ILFLLQRSSKPYGLKFGGLYITSLESFASISTASMSYFTVMYSIQK

>CbirOr402PSE(S)

MDTHFLYLHRISLVAVGLWPYHRTINDYKIRVHICIIIILFLLLLHILLTTFLTTEWTIDLIIEILSISL
FLLLCIIQYNCFWHNTHGVKHILENLQYVCSELKDENEIAIIKKYGHIAKCFAIVFTLLTICGLFILTLL
PFLPRIFSIFSLVNESKSYCNIYIRTEYFVDQEKYFYFILLHLYVAQYITGGILLGAAILLAGYITYFCG
LFNIASYRIEQAMQIINSDEICNWKNKMEIDKKLSHAVDIHRTALEFTEFYLYNFERTYFLIIAIIVICL
SLHLFGIFQAVRFVFRIEDFVIHCGFTLGILGCSLGGNYLSQAITDHYSYIFSTAYNVRWYIAPVRVQRL
ILFLLQTGAKAYDIKLGGFCTLSLENFASLSTASISYFTVIYSIHK

>CbirOr404

MDTQFLYIHRLSLVAIGLWPYHRTMLVQLQSSVFSFMLISYIIVQLTAFITTEWTLDFIVEILSTLFFLL
LCAVQYNCFWINTHAVKRILDNLQYICSELRDDNEIAIIKRYGHIAKCIANGLTLLGMCCLIIFTLVPIL
PRIFGILFLVNKSEPYRYIYMKTEYFIDQEKYFYFILLHLYAVQYIGGGTLLGSGIVLTGYFTYFCGLFN
IASYRIKQAMQIINSDKICNWKNKRDIDKKISHAVDIHRTIIEFIEVSLYTFEGTYFLLIGLIVICLSLH
LFGIFQAVCVVYNMADIVIHCSFAVGILVCSLGGNYVGQAVTDHYNDIFSTAYNIRWYITPVRVQRLILF
LLQRGTKPYGIKLGGLYTLSLENFASLSTASISYFTIIYSLQK

>CbirOr405PN

FTTFLTTEWTIDLIVEILSTSFCVLLSVIHYNFLWINSRLVKHVLENLQYVCSELKDENEIAIIKRYGHI
AKYLAIGLILLGMCCLIIFTLVPILPRIFDIFFLVNKSEPYRYIYMKTEYFIDQEKYFYFILLHLYAVLY
IGGATLVVAGIVLTGYFTYFCGLFNIASYRIKQAMQIINSDKICNWKNKRDIDKKISHAVDIHRTGLEII
EFYLYNCEGAYFLIVTIIVISLSLNLFGLFQAACFVHRMEDIVLHCIFAVGILGCSLAANYIGQTVTDHY
NYIFSTAYNVRWYIAPVRVQRLILFLLQRGTKSYTIKLGGLYTLSLENFATLSTASLSYFTVIYSTQK

>CbirOr406

MSCIDTQFFYLHRISLVTLGLWPYDRTMLVQLRSCVFSLITISFIIFQLTTFLTTEWTIDFIVEILSISL
FFLLCIIPYHFIWLDKHLVKHMLENLQRICSELKDENEIAIIKRYGHIAKCVAIGLTLLAMWGLFVFTLL
PILPRIFGIFSLLVNKSESYRKIYIRTEYFVDEEKYFYFILLHLYAAQYIAVGTLIGAGILVTGYSTYFC
GLFNIASYRIKQAMRINCDEVTNQKNKREINKKISQAVDIHRTALKLIEFLLCNFEGTYFVLIAIIILCL
SLQLVGIFYAISFAFRMENFLIHCGFTVGILTCSLGINCVGQAITDHYNYIFSSAYNVRWYVASVRVQRL
ILFLLQKGTKPYNMKFGGIYTISLESFASLSTVSISYFTVIYSMQK

>CbirOr407PSE

MSCIDTQFLYIHRISLVTLGLWPYENIILTTFLTTEWTIDFIAEILSTSLFLLLSAIFYNSFWINTHIVK
HMLENFQYMCSELKDKNEIAIIKRYGYIVKCIAIGLILLTMWGLFFFTLLPILPRIFGIFFLVNKSETYC
TMYITTEYFVDEEKYFYFILLHLYAAHYIALGTLLGIGIMSTGYFTYFCGLFEIASYRIEQAMRINSDKV
TNRKDKRKIDKKISHAVDIHRAVVELTEFFIHNLEGTYILTMAIILICLSLNLFGIFHAVSFVFSMENFL
LHCGCTLVILTASFGGNYAGQKITDHYNYIFSSAYNVRWYVAPVCVQRLILFLLQKGTKPYRMKFGGLYT
TSLENFASLSTVSISYFTVIYSIQK

>CbirOr408NTE

XYGMFNILTTCLLPVFPRILRTFTSTNVSEKHFMIHIPREYFIDQEKYYYCILLHMDVSLFMGAIVLVAT
GTLLFGCMKYMCGLFRIASYRIDQTMKTPMFQIAGLSKDYVIYKTIVHAIDIHRKAIEVCDIIKSNFVGM

SFILMLISVTSLSLNLYGLHQAVMFGNATEEYLVYAKGLFVTLLYIFICNYIGQELTDHYNHMFISIYSV
QWYVTPLHIQKIILFLLQRGTKNFYFVFGGILVMSIENATTLVSASISYFTVLHSMQQPDIIT

>CbirOr409

MTICVVDQHFSLNKILLRALALWPYHRTKLVELHLVLLFFIFISFILVQLTTFLTTECTPNMRIRIFSFV
LYFTIYLIKYSCFCINSDTIRCLLDKFQYICDELKDEGEIAIIKEYGNEAKRFTLIMSVYSISNALILCL
LPIVPRILGIFTSINVSEEYFMIHVPREYFIDQEKYYYYILLHMEANFFIGAIVLLATGTMLFGCIKYIC
GLFRIASCRIDQTMETSMFQSAGLSKDCVIYKKIVHAIDIYRKAVELCDIIKSNLVGSCFSLMLVGVISL
SLNLYGLHQALMLVSVTEECLMYVTLTVVILIYMFIASYIGQEVSDHHNHVFISVYNIKWYVTPLYIQRI
ILFLLQKGTKNFYITFGGQFVMSMESATTMLSTSISYFTIIYSTQQDT

>CbirOr410PI

MDTRTRVTCIDTQFLYIHRISLIAVGLWPYDRTMLVQLQSSLFFLITTSIIIFQLTTFLTTECTIDFIIK
ILSTTLFLLVCVIQYNCFWINTRAVKHVLENLLYICSKLHDKNEIAIIKKYGYIAKCVAIGFIFLAICGI
ITFSLLPILPQIFGIFYLVNKSEPYRNIYITTEYFVDQEKNFYFIFLHLYLSHYIAVSTLVGTGILITGY
FIHFCGLFNVASYRMQQVMRRNSHELIYRKHKSEIEKKISHAVDIHRATINIIEFFLYNFEGTFILVTAI
IVICLSLHLFGYNVKWYTASVRVQRLILILLQISAKPYGLNVSGLFITSLENFASLSSVSMSYFTILYSI
QK

>CbirOr412PC

MTCIRTRYCNWNXVLLPFGLWPDKETKFTRFQARLFCCFLMSSIAFQFSRFFKAECRIDNALRLLSSVTF
YSTHAIMFISFWINTNNTKYFLDQLQHIYDELKDKNEIAIYEKYGYFGKRLTITLIGFRTCTAFGNSIIL
CLPYILDIVMPKNESYGIYIMEIMTKYFIVSEKYYYLIFTYLNISCSAGIIAYVATTTMFISFFKHICGM
FEIASYRIEQVMAIELLLDINTKNEILIYKKLIYAVDIHRKAMEFAKCFMKKLEGSLFFLIIATVLCLSF
NLFGIFHIESPTEKVEEVLLHFTAVAFIMVALFVPNNIGQEITDHSNSVYVTSYNVSXYLAPIHIQKHVL
FLLQRSNRIFTLDVG

>CbirOr413PIC

MVICIVTEHLRLMRFYLLAVGLWPYYRTNLVRLQFFLCFSILLSFVIFQLTPFLVDDCTVNIIIKNLSIT
SYFSIYMIAHVAGWASDDKIKGLLEHLQLICNQLKDENEFAIIKKYGNHAKRYAAVLTSFSMCSMFGIML
LPFWPRILTIVSLINESQTYAKVQITTEYFINQEKYLYLILLHLDTVLWIGSSTLMGIGLLLLGYCQHMC
GIFSIARIFKNMLIEGDIETCMLHIIIIGILFILLYLLNSLGQDITDHSNYVLSTAYNVKWYTAPLRIQK
LILVLLQRGNKTFTLTIAKMLLLSRESFAT

>CbirOr415

MDTQFLYIHRLSMVAIGLWPYHRTMLVQLQCSVFSLTVISFIIFQLTTFLTTEWTIEFIVEILSTSFFIL
LSATVYNSFWRNTHVVKRVLKNLQYICSDLKDENEIAILKRYGYIAKCATIGMTLFVMCISFIGILLPIL
PRIFGIFFLVNESEPYRITYIKTEYFVDEATYFYFILLHLYAVYYIAGGTTLVAGIIVAGYFTYCCGLFN
IASYRIEQAMRIKSDDLTNPTNKRQVDKKISHAVDIHRTTLKILEFYLYNFEETSFLIIVLIVICLSLHL
FGILHTVCFAFRIEEFLLHFGCAIGFLGFSFAGNYVGEAITDHYNYIFSTAYNGQWYVAPVRVQRLILFL
LQRGTKSYGLTFGCLYTLSLESFASLSTTSISYVTVIYSIQK

>CbirOr416PC

MDTQFLYIHRIILVAVGLWPYHRTMLVQLQCFVFSLTLSSSIIFQLTTFLTAEWTIEFIVEILSTSLFVL
LCATAYNSFWSNTHVVKRVLQNLQYICSNLKDENEIAIIKRYGYIAKCVIIGMTLFVMCFFFIVTLLPIL
PRIFGIFFLVNESEPYRNIYIRTEYFVDEEKYFYFILLHLYAVQYIAGGTTLVAGTFLTGYFTYCCGLFN
IASYRIEQTKWINSDELPTRMNKRQVNKKKKLIHAVDIHRTSLKTIEFCLYNFEKTCFLIIVLIIICLSL
HLFGIFQAISFVFRMEDFVLHCGFMLGILGCSFGGNYMGQAITDHYNYIFSSAYNVRWYVEPVRVQRLIL
FLLQRGTKPYCMKVGGLYIPSLETFAS

>CbirOr417INT

MDTQFLYIHRLSMVAIGLWPYHRTMLVQLQCSVFSLTLISFIIFQLTTFLTTEWTYDIIVEILSIVLLSL
VCAILYNSFWINTHVVKRVLKTLQYICSDLKDENEIAIINRYGYIAKCATIGMTLFMMCFFFIVTLLPIL
PRIFGIFFLANKSEPYRNIYIRTEYFVDEEKYFYFILLHLYAVLYIAAGTILATQTAMLGYFIYCCGLFN
IASYRIEQAMRIIDEITNRTNKRQVDKKISHAVDIHRTTLXXFYLYNFEDTWFLLIVLVVICLSLHLFGI
FQAISIVFKMENFVLHFSFTLGILLCTFAGNYIGEAITEHYNYIFSTAYNVRWYDAPIRVQRLIFFLLQR
GTMSYAMKFGGVYTLSLENFATLSTTSISYFTIIYSIQK

>CbirOr419

MITIETQYFNWHRILLLAVGLWPYERSNFVKFQCYFFLAIPTSFIIVMLTPFLTTECTFTFIIKVSAIVF
VFMVCMVTHNSFWVNACTVRCLLEKLQHMCNELKDQNEIAIIKKYGNTAKRYTTVLILLVMCSLFLLPFL
PLSTRILGTFLHLNESQQYRKLYIVTEYFVDQQKNFYFILLHMYAATYIEMTALIGPGALIIGYLKHICG
MFKIASYRIEQAMSSNIFDKIYLRDKVEIHKEIISAVDIHCKAMEFANFLIFNFKDPFVMSQMFNVLCLS
LNLYCIFQAVSFKDNIEEFLIHFTLATGIFLYSFVANYAGQEIIDHYNYIFSAAYNFQWYLTPISIQKLI
LFLLQRRSKPFNLKMLIFIPSMECFASLSITSISYFTVMCSLQQ

>CbirOr420PC

MDTQFLYIHRISLVASGLWPCNRTMLVQFQSFVFSLTMISFIIFQLTTFLTTECTLDLIIEILSISFFQL
LCVIAYNSFWSNAHVLERLLKNLQYICSDLKDKNEIAIIKRYGHIAKCVAIAFTLLTICGIFILTLLPIL
PRIFDIFFLVNVSEPYRNIYIRTEYFVDEEEYFYFILLHLYAVTYIEGVTLLGGAVTMTGYGIYSCGLFN
IASYRIEQAIRINSDEVTNRKNKREIDKKISHAVDIHRTSVEYTECYLYNFNGTYCMLTVILVICLSLHL
FG

>CbirOr423NTE

LTTLLTTEWTIDFIVEILSTSLFVLLCAIIYNAFWIKTHVMKRILTKFQYICSDLKDENEIAIIKRYGYS
AKCVAIGFTLLTIGAFFILSLLPLLPRLFDIFYLVNKSEPYRNIYMRTEYFVDQEKYFYFTLLHLYAVLY
IECGVLLGGAIVMTGYGTYSCGLFNIASYRIEQAMRISSDEVTNRKNKKKIDRNISHAVNIHHTALEFIT
FYIHNFEGPCFVIIAIIVISLSLNLFGISRAVCFLHRMEDFVIHCIFAVGIVICSFGANYIGQAIIDHYN
YIFTTAYEVPWYVASVRVQRLILFLLQIGAKPYRINFGGLCTMSLEDFASLSTVSLSYFTIICAIQK

>CbirOr425PC

MTICVKTQHFHLNRILLLLVDCGNQTYEQSKFVYLQLILFFGILDSFLTVFLTIQFTLDLVIQVLSVVSF
NVIFIITYNSFWANAYTVRALVEQLQHMCNELKDKNEIAIMQKYGNNAKRFTIILIFILVCYLISFNMLP
VVPYFLDIILRLNKSNLPMQFILTEYFVDQEKYLYLILLHTNVAFSIRGTVFAAIGLMLLLYVEHGCGIF
QLASYCLEKALGRNVLENVNVKKEMMLYREVVYAVNIHREAMXXYIYIFLSNYCGQQVTDHNNHVYLTAY
NVYWYTAPIRVQKLILFLIERSMKSFNLKVVGLLVASIXCFVS

>CbirOr426PI

MAICVVEQYFSINKILFRVVALWPYHRSKLVEFHLSFFLVILISFIAAQLATFLTTECTSHMAIKIFSLV
LYFTLYLIKYLSFRVNAHTIRCLLEKFQYICDELTDEGEIAIIKEYGNEAKHFYLKISLYGMFNIITTCL
LPVFPRILRTFTSTNVSEEHFMIHIPREYFIDQGKYYYCILLHMDVSLFMGATVLVATGTLLFGCMKYMC
GLFRIASYRIDQTMKTPMFQIAGLSKDCVIYKNIGLKASPGYDVWKRHGGIFSLXLKVYLLYIFICNYIG
QELTDHYNHMFISIYSVQWYVTPLHIQKIILFLLQRGTKNFYFVFGGILVMSIENATTLVSASISYFTVL
HSMQQPDIIT

>CbirOr428

MTICIKERYFSFNRISLLAIGLWPYQQSTFAQIQMVFIFGILISFITFQLSRLLFVEWTLNFTLKMLSIA
SLFTIYAIKYITTWINIETIRYLLERLQHIYNELKDKNEIAIYEKYGNTAKRLTINLLIVDISILSVGFV
MEIWPYIFDVIMPKNETYARHLIILMCKYYSIEEKYYYYIILHASAATAVGVFALAAIGTIMISCIKYIC

GMFKIASYRLERAMTITNTLQNISLKNEFVICKEIIHAVDIHRKAMEFTEYLVSNFEKSYAFMIGIAVFC
VSLNLYRVSRIEPMKDKEETVVHVAITFFTLFYIFIANNVGQEIIDNNNHVFFTAYNIPWYLAPLQIQKL
ILFLLQRSSKAFTLNFQGLFIASLECFASLASASVSYFTIMYSVA

>CbirOr429PSE

MICIRKRYFNWNRALLLPLGLWPDRQTKFTRFQTRLFCCFLMSSIAFQFSRLFKAECSTDHAIIIISCAT
FYVMLTIMFICFWINMKNTKYFLDRLQCTYNGLKDRNEMAIYDKYGYIGKRLTIILTVLIMGAVFGNFII
LYLPYILDIVMSKNESYAIHIMEMVTKYFIVSEKYYFLILVHLNATCSAELIVFTATATTLMSIFKHICG
MFEIASYRIEQAMTIELLRDLNTKNEILIYKNLICAVDIHRKAMVFAKCFMKKLEGSFFFLIIATVLCLS
FNLFGLFHIESPTEEMEEVLLHNGAVIIILVVLFLANYTGQEITDHSNSVYVTTYNVSXYLAPIHVQKHV
LFLLQRSTRIFTLDVGGLFSASVECFTTLVSASVSYFTFMHSMQ

>CbirOr430PSE

MICIKKRYFNWNRLLLLSLGLWPDKETKFTRIQARLLFSLLMSSVPFHFSRLFIEECNIDYVIKIIASVT
FFIMLIVIFICFWTNMKSMKYLLNQLQHIYHELKDKNEIAIYDKYGYSGKRFTIRLMIFAVCSLFFAGTM
LFSPYILDIVMPKNESYAIHIMKLITKYFRVSEKYYFLSFIYLNAACSAGVIVVIGTGTMILSYFKHACG
LFEIASYRMEQAMAIELLHDLNTKNEIIIYKKIICAVDIHRKALQFAKCFMDKLQGASFFLIIATVLCLS
CNLFELFQTQSNTEEVEEVILHFFAVIGIVLFLFVTNYSGQEIIDHSNNVYVTTYTVSXYLAPIRIQKLV
LFLLLRSSRILTLNVGGLYVASLEGFATLVSASVSYFTFMHSVQ

>CbirOr431INT

MTCIRKRYFNWNRILLLPLGLWPDKETKFTRFQARLFCCFVMSNIAFQFSRLFKTECGIEHAIRIICCAT
FFVMLMIMCICLWINMKTIKYFLDQLQYIYDELKDKNEIAIYEKYGYFGKRLTITLIVLLMCGVFTNCVM
IYSPYILDVVMPKNESYTIHMMEMVTKYFIVSEKYYFLFLVHLNVTCSAELIVLIATATMLMSVFKHICG
MFEIASYRIEQAMTIELLRDHNTKNEIIIYKKIIYAVDIHHKAMQFAKCFMNKIEGSFFFLIIATVLCLS
FNLFGIFHIQSPLEEKEEVLLHLLAISSMLVGLFLANYTGQEITDHSNSVYVTTCVHQYLTSPSCTQCSX

>CbirOr432PSE

MTCIRKRYFNWNRALLLPLGLWPDEETKFTRFQARLFCCFIMSNIAFQFSRLFMAECSIDHAIIIISCAT
FYVMLAIIFICFWINMKIIKYFLDQLQHIYDELKDRNEIAIYEKYGYFGKRLTITLIVVTVCGTFSTAII
VYAPYILDVVMPKNESYAIPIMEIVTKYFIVSEKYYFLILVHMNITCSAELIVMIGTGTMIVSYFKHACG
IFEIASYRIEQAMTIELSHDLNTKPEIVIYKKIICAVDIHRKAMQFAKCFMKKIEGSIFFLIIVTVLCLS
FNLFGVFHIESPMEEVVEVVLHLFAIFTMLAGLFLANYTGQEITDHSNSVYVTTYHVSXHLAPIRVQKLV
LFLLQRGSKIFTLDVGGLFSASLECFATLLSASVSYFTFMYSMQQ

>CbirOr433

MAVDIVLQYFNLNRILLLTVGLWPYQRTKLVEFQFCLFFGILVSFIAVQLMSLLILECTISLVIKILSPA
LFFTMFAIKYSSFIINASSVKYLMEQLQHICNNLKDENEVAIVKNYANKTRTYTASIILYTVCNLLIFIL
LPVLPTAAGIILFINESQLHHTVYIMTEYFVDREKYFYLILLHMDVAVCIGAIAVISTATMLIGYCKYIC
AMFKISSYRIKHAVLDAESFHWRNADMIHKKIKCAVDIHRKGMEFASFLISRFDWLFFLLVAVGVLSLSL
NLFCLFQTISLEYGVEEFIIHFVYVSIILVYMFLANYIGQEVTDRYNYIFVTAYTVQWYKTPLYVQRLIL
FILQRGNKTFGLKVGGLFTGSIECFATLSTTSISYFTVMYSVQQ

>CbirOr434IC

MVICIVTEHFSLTRIHLLSIGLWPYYRPKFVRLQFFLCSSILISFIIFQIKGLLEHLQLICNQLKDENEF
AIIKKYGNHAKRYAAVLTSFTMCSMFTFMLLPFWPRLLKIVLLINESQTLGKLQITTEYFINQEQYLYLI
LLHIDTAVWIGSATLVGTGLVIIEYCKHMCGIFSIASYRIEHAMSMLENSSLDTELKMYKEMTYAIDIHC
TAIKYSELLTLSFENICLPLALLIVISTSLNLFGLTKASVSYFTILYSMQQ

>CbirOr435PNI

MTTFMLAKISLKYVLKVLSRIFFLMFSLIHYSSFRFNIKYIKKLMQQLHYACTKVTDKNKISIIQXYGWE
ARRYTAALIVALSSEGCVFLVSQIVVAFSSVVSPINGSQPPRLQIATENIMGEQNFFHLILLHIDTAMIF
GAVLLLTMGILMIAYLQHTCGMFKIACYRIENAMEINIQKSINIKREPSISEEIICAVNIHREAMKLSEL
LISSFDAMYFCLTVLAVMALSLNIFH

>CbirOr436

MTVYIVLQYFNLNRILLLIVGLWPYQRTKLVKFQLCLVFGIFVSFIPAQLTPLLTLECTVDLVIKILPPI
LIVIMFAIKYMSFIINARFVKQMMEQLQHICTNLTDENEIAIMKNYGNKTRTYTASLILYAFCNLIIFIL
LPFLPKIAGIILFINESQLHHTVYIMTEYFVDREKYFYLILLHMDAAVCIGAIAVIGIATMFVGYMKHAC
AMFKICSYRIKHAIMLDAESVQLRDYMIHKKIKYAVDIHRKGIEFSTFVISSFEWLFFLLIAIGVLCLSL
NLFCLLQTVSSGHNVEEFIVHFSCVSIILIYMFLGNYAAQEIIDHYNHIFITAYNIQWYAASIRIQKMIL
FLLQRGAKAFNLNLGGLFIGSLESAGMLISTSISYFTVLYSTRQN

>CbirOr437PSE

MSISIQERYFSLNRILLLTVGLWPYQQSKFVSCQAALCLGILTSFVILLLSRLCFLEYSFDFTIHLLSAS
TYFAFILMNYISFWINIETIQYLLEQFQYIYNRLKDSKEIAIYNKYGNIGKRITISITVGICNHAYLAVI
QCGPYILDMIMPKNETYSRRFILVMSNYFAVQEKYFYLFLLHLNAVFAVGSLALIAVATMLLSCLKHICG
MMKIASYRFEQAILTMLQSITLKNKTMTYISIAKIFVDDMERSLFIVTMSTVLCMSLNLYALSQGESPVQ
EIERTIGHLLLIIFLFTYMFIANYAGQEITDSNNHIFLTVYNASWYLAPLQIQKLILFLLQRSNKAFTLN
LGGLFTLSIECFASLINASVSYFTLMLSL

>CbirOr438

MTICIVDQYFSINKIFLRAVALWPYHRTKFNEFQWFFFLVILITFILAQLATFLTTECTPNMTIKIMSLA
LSFTLYLIKYVSFRVNADTIRYILERCQYMCDELTDEGEIAIIKECGHEAKRFTMLMLLYGASNIMITCF
LPVFPRILRTFTSINMSGEQFMIDIMREYFVDQDKYYYYILLHMDVSYIIGIIVIPATGSLLFGGMKYIC
GLFKIASYRIDQTMETPMFQSAGFSKDYVIYKKIVHAVDIHRKATELSDKMISDFVGTCCLIMLVSVTSL
SLNLYGVHQAMMLGSAMKEYLVHVKCISVSVMYMFIGNYIGQEITDYHNHIFSSIYNMKWYGTSLYIQRI
ILFMVQRGTENFYIVFAGMFVLSMENAAMLFSTSISYFTVLHSIQ

>CbirOr439

MDTQFLYIHRLSMVAIGLWPYHRTMLVQLQCSVFSLTLISFIIFQLTTFLTTEWTLDFFVEILSTSLYLL
LWVIQYNSFWINMHVVKRLLENLQYICSELKDENEIAIIKRYEHIAKHIAIGLILLLMCVLFIFTLLPFV
PRIFGIFFLVNKSEPHRNMYMRTEYFVDQEKYFYSILLHLYAALYIVGGILIGTGILMTGYSSYFCGLFN
IASYRIEQAMRINSDDIINRMNKREIDKKISHAVDIHRIAYECNEAFLHNFEGTYFLIIMILVICLSLNL
YGIFRTVCFVYRMEDFVKYFGFAGGILGCSLAGNYMGQAITDHYYYIFSSAYNVRWYVAPVRVQRLILFL
LQKGTKPYNMKYGLYAASLETFATLLSASLSYFTLLCSIQK

>CbirOr440

MICIKTKYFNLNRILLLAVGLWPYQQSRLVQLFVFNSLLNSIIIFQLLSFLTAECTPDFIITVLSSVSVY
FVSIIQYNSFWSNISTIRYLLDQLQDIYNKLKDENEIAIMETYGRNAKRLTANISLLTMGSIVIFLSLPI
WVRIIDIFLILNNSQHGMIQLTTEYFIDQEKYFYLILLHIDAATCIAGSGLTAIGLIYCAYFKHICGMFR
IASYRIKQAITLEKIFLENDLMIYKEMIYAIDMHRKAMKFSEDLLNSIEGSVFLSIISSVCCLSLNLFGI
VWTTSTDFNIQKVVIHFGVVLMIVICMFLANSTGQEITDHNNHVFTTVYNTEWYIAPLQTQKLILFLLQR
NNKPFILNIRGIVISSMECFTKLMKASISYFTVIYSIH

>CbirOr445

MIRIVDHLRIQRILLLALGLWPYNQSKFVQFQLLLFFIVSNSFIIFQLTSFLTSECTVDFIVKLLSIVSF
FLACTAHYIAFWINMYTVKHLMDRLQHACNELKDENEIAIMKKYANSAERIAIVITSLTVCALLSVTLMP
LLPRILSTFLLVNISGPLYTKQFVTEYFVDKEKNFYLILLHTCASFYIGVIAFSGGGLVGCAFLKHICGL
FSIASYRIEQVMKLNIHEKTDVRRKEMEIEKKIICAVDIHRTAIELSELFISSFEGPYFCGLVVVITSLC

LNLYEVFQTALIRDKIEEFILHFGCAGSLLIYSFLGNSCGEEITEHYNDMFSYAYNVRWYTAPIHIQKLI
LFLLQRSCKPYGLKLGGIFIASLKGFASLSTTSVSYFTVIYSTQK

>CbirOr446

MIRIVEHFRIPRILLLTVGIWPYNQSTFVQFQFLLFSIVSNSFIIFQLTAFLTSDCTVDLVIKILSTTSL
LLMGIVHHMSYWINTYTVKYFVERLQHICNELRDENEIAIIKKYANSAEYIAIFITLYTACCGFIITFTQ
IFPRILSTFLLVNISRPLYNVQFVTEYFIDKEKNFYLIFIHICASLYIGTVAFAGTSLISFTYFKHICGL
FCIASYHMEQSMMLNIHKKTNLWNNMEIDKKIIHAVDIHRTAIELSEFFISNFNGTYFCVMLISVLCLSL
NLYKLFLTVLFQDEIEEFLLHFIFATAILIHLFLANSCGEEITENYNNIFSAAYSVHWYAAPIRIQKLIL
FLLQRSCKTHGIKLGGIFIASLEGFAALSATSLSYFTVLYSLQK

>CbirOr447PSE

MRVEVRTRVTCMDTQFFYIHRISLVAIGLWPYHRTMLVQLQSCVFSFIMISCIIFQLTTFLTTEWTIDFI
IEVLSTSLVVFLCAIQYNFIWINTHVVKHILENLQYVCSELKDENEIAIIKRYGHVAKCISIGLTLLAMC
GLFILTLSPILPRIFGFFFLVNESESYRNIYIRTEYFVEEEKYFYFILLHLYAAQYIAIGTLIGGGILVI
GYSTYFCGLFNVARYRIEHAMRINSDEVTKXKNKREIDKKISHAVDIHRTVLEFIEIFLYNFEGTYLLII
AIIVICLSLNLFGIFHAIGFVFRLEKFLLHCGFTLSTLTCSLGINCMGQAITDHYNYIFLTAYNVRWYIA
PVRVQRLILFLLQKGTKSYSMKFGGVYTMSLESFASLSTTSISYFTVIYSIQK

>CbirOr448PSE

MRVEARRRVTCMDTQFLYLHRISLVALGVWPYHRTMFVQLHACLTPFLTTECTIECIIEILSTSLYLLLC
LIHYISFWINTHVVKHVLEYLQCACSELKDENEIAIIKRYGHIAKCVAIGIILLAMCGLFIMTLLPFLPR
IYGTFFLVNKSEPYPNTFIITEYFIDEEKYFYFILLHQGAAYYIAVGTMMAGIVVTGYCTYSCGFCAIAS
YRIDQAIRITSDEVTNRKNKSKINKKISHAVNIHHTTLEFIEFFLHNFEGTYTLLLAIVVICLSLHLFGL
CQAILFADRMQEFVLHYTFTLSILICTLGGNYVGQAVTDHYNFIFLSAYNVRWYIAPLRVQRLIVFLLQK
GTKPYSMKVGGLYTISLENFASLSTAAISYFTVLYSLQN

>CbirOr449PN

VKYILEKLQYVCSELKNENEIAIIKRYGHIAKYVAIGLTSLAKWSLFIFTLLPILPRIFGIFFPVNKSET
YRNIYITTEYFVDEEKYFYFIMLHLYVAHYIAGGTLIGAGVLLTGYFTYFCGLFNIASYRIEQAMRINSD
ELTNRKNKRKIDKKISHAVDIHRTALEFTEFFLYNFEGICFLIIAIIVICLSLNLFGIFQAVRYVYNMQE
FLVHCGFTVGILTCSLGVNCVGQAITDHYNYMFSSAYNVPWYVASVRVQRLILFLLRRGTKPYGLKFGGL
YTISLESFASLSTASLSYFTLLCSIQK

>CbirOr450

MCVETRTRVTCIDTQFLYIHRISLVAIGLWPYQRTMLVQLQSCVFSLIMLSFVMFQLTTFLTTEWTIDFI
VEILSTSLFILLCAIQYNSLWINTHVVKHILENLQYVCNDLKDANEIAIIKRYGHFAKCVAIGLTLLAMW
ALFIFTLLPILPRIFGIFFLVNKSEPYQYIRTEYFVDKEKNFYFILLHLYTAQYIAGGTLIGSGILATGY
STYFCGLCNIASYRIEQAMRINSDEVTNRKNKREIDKKISHAVDIHRTAFEFTEFFLHNFQGTYVLLIAI
IVICLSLHLFGIFQAVRFVFRIEDFVIHCGFTLGILGCSLGGNYLSQAITDHYSYIFSTAYNVRWYIASV
RVQKLILFLLQRSAKPYDIKFGGLYTASLENFATLSTASISYFTVIYSIQK

>CbirOr451PN

LTTILTTEWTIDFTVEILSTSLFHVMCLIMYHSFWINTQVIKRLLKNLQYICSELKDENEIIIIKRYGYI
AKWLAIVFTLSMMGGLFVVTLLPILPRIFGIFFLINDSMPYRNIYIRHEYFVDEEQYFYFILLHLYVSQF
AAGGTLLGGAIVMTGYVTYFCGLFNIASYRIEKAMRINSDEVSNGKNKREIDKKISHAVDIHRTALECTE
FCLYNFEGALCLIIAIIVICLSLHLFGIFYAVGFVFRIETFVLHCGFLVGILTCSLGLNCVGQAITDHYN
YIFSSAYNLRWYIASVRVQRLILILLQTGTKPYSVKFSGFYTISLESFASLSTASISYFTVLYSIQK

>CbirOr452INT

MDTEFLYIHQISLVAIGLWPYHRTILVQLQSSVFSLTMISSIIFQVYRIEQAMRINNDELTNRKNKRKID
KKISHAVNIHRTALELTEFYLYNFEGTYCLIIVVIVICLSLHLFGIFHAISFVFRMENFLLHCGFTVGIL
TSSLGVNCIGQAIINHYNYMFSSAYNVRWYVAPIHVQRLILFLLQAGTKPYSVKFGGLYTISLESFASLS
TASISYFTVIYSIQK

>CbirOr453PI

MICIVEHFRIQRSLLFAIGVWPYNQSKYVRFQYSLFLVVTNSFIIFQLTPFITSVCTVNLIIKLLSTIVF
SLVCVIHQISFWINTDVVIAVCCVFIVTLMPIYPRILSTFLLVNISRPLYNMQFVTEYFIDKEKNFYLIL
LHTYAFFYIAIITLVGGGMMVYAYLKHVCGLFSIASYRIEQSMILNIYENTDLRNKMIEKKIRLAVDVHR
TAIELSDLFTSSFNGTYFCLMVVAVLCLCFNLYEVLETALLRDKVEEFLLHLIFASAVLLYSFLANTSGE
EIIEHYNNMFSTAYNVQWYMAPIRVQKLILFLLQRSCKMYGLKTGGLFIASLEGFASLCTASVSYFTVIY
SVQK

>CbirOr454INT

MICIVEHFRIQRILLLTIGIWPYNQSKFVRFQFSLLSVVTNSYIIFQLTQFITSELTVERIIKILSSTLF
CLVCAIHQLSYWINADIVKCFLERLQHACNELKDENEMAIIKKYATSAEYISILVLLFAACWAFIVILMP
RIFSTFLLVNISRPFYNFQFITEYFIDKEKNFYLILLHAYASAYISLTALIGGGLIGCAYLKHICGLFSI
ASYRIGQSMIVNIHEKTNQWNKMEKKIRLAVDAHRTAIEVFQTALLRDKVEEFLVHLIFATAVLLYWFLA
NVSGEEIMEHYNYMFSTAYNVQWYMAPIRVQKLILFLLQRSCKMFGIKTGGLFMASLECFASISTASVSY
FTVLYSVQK

>CbirOr455

MICIVEHFRIQRILLLTIGIWPYNQSKFVRFQFSLLSVVTNSYIIFQLTQFITSELTVERIIKILSTIFF
SFLCLIHQVSYWINANVVKCFLERLQHVCNDLKDENEIAIIKKYATSAEYISILVLLFAACWAFIVILMP
RIFSTFLLVNISRPFYNFQFITEYFIDKEKNFYLILLHAYASAYISLTALIGGGLIGCAYLKHICGLFSI
ASYRIGQSMIVNIHEKTNQWNKMEKKIRLAVDAHRTAIELSEFFMSSFNGTYFCLTVLSILIVCLNLCEV
FQTALLRDKVEEFLVHLIFATAVLLYWFLANFSGEEIMEHYNYMFSTAYNVQWYMAPIRVQKLILFLLQR
SCKMYGLKTGGLFIASLEGFASLSTASISYFTVIYSVQK

>CbirOr456

MICIVEHFRIQRILLFAIGVWPYNQSKCVQFQYLLLSVVLNSFVIFQLTPFINSELTVDLIIKLLSTIFF
SLLCLIHQVSFWINIDVVKGFVERLQDVCNDLKDEKEIAIIKKYANSAEYISILVILLAVCCAFIITLMP
IYPRILSTVLLVNISRPLYNMQFVTEYFIDKEKNFYLILLHTYAFLYIAIIVLVGGGLMGCAYLKHVCGL
FSIASYRIEQSMIANIHEKTDQWNKMKKKIRLAVDAHRTAIELSEFFMSSFNETYFCLMVVAVLCLCLNL
YKVCQTVLRDKVEEFLLHLILAISMLVYSFLANSCGEEITEHYNNMFSSAYSIEWYRAPIRVQKLILFLL
QRSCKMHGIKIGGLFTASLEGFASLSTASVSYFTVIYSMQN

>CbirOr457PSE

MDTQFLYIHRLSLAAIGLWPYHRTMLVQLQSSVFSLTMISYLIFQLTTLLTTEWTIDFIVEILSTSLFVL
LCTILYNSYWINTHVMKRLLNNLQYICSDIKDENEIAIIKRYGYSAKCVAIGFTCGFFSLSLLPILPRIF
GIFSLVNKSELHHNIYMRTEYFVDQEKYFYFILLHLYAVQYIGGGILLGESIVMVGYGTYSCGLFNIASY
RIEQAMRISSDEVTNRKNKKEIDRKISHAVNIHRTALEFITFFLHSFEGTYFVLIAIIVISLSLNLFGIS
RAVFVRKMEDFVIHCIFVVGILTCSFASSYISQAIIDHYNYIFATAYEVPWYVASVRVQRLILFLLQIGA
KPLSSVSLSYFTIICSIQK

>CbirOr458

MDTQFLYIHRISLVAIGLWPYHRTMLVQLQSCVFSLTMTSFIIFQLTTFLTTECNIELILEILSTSLFVL
LCAILYNFLWINTHVVKHVLENLQYVCSELKDENEIAIIKRYGHIAKCVAIGFTSLTMCGFFIVTVLPIL
PWIFGIFSLVNESKPHRNIYIRTEYFVDQEKYFYFILLHLYANQYIGGGILLGGAIVVTGYFTYFCGLFN
IASYRIKQAMQIINSDEICNWRTRGRFDKKISHAVDIHRTALEIIEFYLYNCEGTYFLLIVITVICLSLN

LFGIFQAVHFVFRMEDFVLHCGFTVCILTCSLGGNYMAQACIDHYNYIFLTAYDVRWYIAPVRVQRLILF
LLQKGAKPYNIKLGGLYTLSLESFATLSTASMSYFTVMYSMQK

>CbirOr459

MVCIRKRFFNLNRLLLHAFGLWSDEQTIFARFRATMLCSLLISSIVFQLSRLFILEYSFDLIVRIVSSTT
FFAMFTVAPFSLEINMKTIKYLLDQLQYIYDQLKDRNEIAIYDKYGYSGKHFTTIIIILLVCALCGDFVI
VYWPYILDIVVPKNESHTSPMIQFLSEYFHVSEKYQFLVLVHFNTAMTAGSIVFVGTTTMLFSYFLHICG
MFEIASYRIEQAMAIELLHNCHIKNRIMIYRKMICAIDMQRQAMEILKYIVTVIDETAFILVITSVVCMS
CYLFRVFRIESPMERLEEVLVYLLDVSALLTIMFLGSYVGQEIIDHSNHVYVITYNVSWYLAPLHIQKLI
LFLLQRRTKSCTLSVGGLITASFECFATLVKVSMSYFTFMYSVQ

>CbirOr460

MVCIKKRFFSLNRLLLLAFGLWPDEETIFTRFQATLLCSLLISSIVFQLSRLFIAEYSFDFIVRILSSVT
FFATLTSLPLSFWINIKTIKYLLDQLQYIYDQLKDRNEIAIYDKYGYIGKHFTTIVVIFLVCGLCSNSGI
VYWPYILDIIVPNNESYAIHTMQFVTKYFNVSEKYYFLVLVHLNAAITTGLIVSVGTETMIFSYLKHICG
MFEIASYRIEQAMAPELLHTVDIKSRIVICREMICAIDMQRQAMQCTKCFVTSMEGTVFILIIINVLCMS
CNLLRIFQIESPMERMDEVLLHLLAVSTVLTITFACNYVGQEVTDHSNHVYVIIYNVAWYLAPLHIQKLI
LFLLQRSNKIFSLSVGGLFTASLECFATLVKASLSYFTFMYSMQ

>CbirOr461

MICIKKRFFNWNRFLLLLFGLWPYKETIFTNLLGTLVCNYLMSIIVVLLLRFYFVECSFDFVVKMLCGVT
FYAILAIPPITMWMKTKDVRYLLDQLQHIYNQLKDKDEIAIYDKYGYIAKRMTIRIIILIMSGLYFIGVM
VYWPYTFDIIMPKNESYATRIMEFVTEYFVVQEQFHFFLILHLSTVSFTGSVLTTAVGTMIISYFKHICG
MFEIASYRIEKAMAIELLHNFDMKNGIVICRKIICAVDIQRQAMEFATASVNSLEGSVLALVIIIVLCMS
LNLLRIFQIESPAERIEEVLLHLSVVIIIMCSTLLANNSGQEVTDYSNHVYVTIYNVPWYLAPVNIQKLI
LFLLQRSSKIFTLNISGIFVASLKCFATLINASMSYFTFMYSVQ

>CbirOr465

MYVEARTRVTCIDSQFLYLLRISLVAVGLWPYHRTMFVQFQSCVFSLTTISFIIFQLTPFLTTECTIEFI
IEILSTSLYLLLCVINYISFWINTHVVTHILENLQYVCSELKDENEGAIIKRYGHIAKCVAIGLTLLAIC
ILIIFTVLPILPRIFDIFFLVNTSESYRNIYITTEYFVDKEKYFYFILLHMYAAHYIALGTLIGAGILWT
GYCIYFCGLFAIASYRIEQAMRINNSDEITNRKDKRKIDKKISHAVDIHRAAVELTEFFIYNLEETCILL
MAIILICLSLNLFGIVHAINFVFSMENFLLHCGFTLTTLTASLGGHYAGQEITDHYNYIFSSAYNIRWYV
APVRVQRLILFLLQKGTKPYRMKFGGLYITSLENFASVLTASISYFTVIYSIQK

>CbirOr466PSE

MICIRKRYFNWNRILLLPFGLWPDKETKFTRFQARFFCCLLMSSIAFQFSRLFNAKCSIDRAIRLLSSAT
RLCSSVKYILDQLQHIYDGLKDRNEIEIYEKYGYFGKRLTTKVIVLTMCGTFCNSIIIYSPYILDIVMPK
NESYAIHMMEMVTNYFFVSEKYYFLILVHLNAACTTELIVLGATTTMMMAIFKHVCGMFAIASYRIEQAM
TIELLRDLNTKNEIVIYKKIICAVDLHRKAMQFAKCFMNEMEGSFFFLIIATVLCLSVNLFRIFQTESLT
KEMEVVLHFLAVLGMILGMYLANYTGQEITDHSNYIYIITYNVSXYLAPIQVQKQVLFLLQRSTRIFFLD
VGGVFTASLECFSTLVSASISYFTFMYSMKK

>CbirOr467IC

MILVETQYLSLNRILLLTLGLWPYERSKLIRLQLILLYSTVISFIIFQGKHLLEQLQDMYDELTDEIEIA
IIQKYWSMARRNTEVLTLILLCGIFVGVFNAFLPNILDAALSTNKSQSPSTLHIMTEYFIDQKKYFYLIM
MHKEAASCIGVTAIVATGTMTMLYFQHACGMFMIASYRIEQATSSINLSDSNKRNKDLTCMGIIYAIDMH
RKAMRFVNHILSAFHIYYFFMIAAAVISVSFISFR

>CbirOr468

MIYIQAHFNLTRILMLAIGLWPYQQTKLVQLQLILISNILTSFVVFQFTTFITTECSPNLVIKILTCSLF
AASPMIIHNSFWLNIHTVKCSFERLQDICNELKDESEIAIIKRYGNDTKRYTLILFLCAMCCVVFVGFLS
IWPQILSTILPINVSQTQRIMYVMTEYFVDQERYFYLILLHINTATYIGMIVAVATGTVLIGCIIHACGL
FSIVSYRIKQAMTINMMENANQENQILIYRKIVCAVNMHCKAMNFLEVLMSAFEGLLFLLIVIYVTVMSL
SLYGIASYRDNTSELFLYLTCMTTAVLYIFIANYVAQDVTDHNNDIFVTVYDFQWYTASLRVQKMMLFML
QRGTKAFCVNIGGLFVGSLQNAATLISASISYFTVLYSMQN

>CbirOr470

MDFAGERYYRFNKILLNCFGLWPSQTRRVKILFVFFSAIFFSFIFVQFSALVTSKRDLDLVLKVLSHVLP
VLIYTMNYNAYYFNAKKVKLMMTEVCNDWNALDDKREIEIIEKYTYFMNIYTISIMVFLCICMVLFVFLE
IWPIMLDFVVPLNESRPRKMHVVAEYFIDREKYFPFMLLHEIVACLVGGVTLLSTGTICMIYASHVCGML
KIVSFRIEHALGKNVLQHSTSQVERIICGRIVRAVELHRKILKFFDFMISTFVVPFGILIVIGVTSATVN
LYRLLQPSTMKNISEFTSSLMLLIVHFLYMFVANFIGQIITDHSTDIYNTTCNVLWYVAPQSSQKILLFL
MHRTMKSVKPTIGNLFVASLEGFATLSTTSLSYFTMLYSLQ

>CbirOr471NTE

LSRLFFAECNFDLVSKILSSSSFFVTLAILFMCFWINMETMNYLLHQLQHIYDGLKDQNEIAIYEKYGYI
GKCITARLTIFALCGILSTCIMIYSPHILDIVMPKNESYAIHTMEMVTKYFVVSEKYYFLILVHTNAACS
VGLIVLVGTGTMMISYIEHVCGMFEIASYRIEQAMAVELLHNTDIKNEIVICRGIIYAVDIHRKAMEVAK
CFITKTEVLFFLLVIAGVLCLSCNLFRIFKIESPMEQVEEILLHFFAIIVMLVGSYLANYVGQEIIDHNN
HVYVTIYNVSWYLAPVQIQKLILFLIQRSCRIFTLNVGGLFTSSLECFATLVSASVSYFTFMYSMQQ

>CbirOr473NTE

DFVLNILSSSCLFSCGLIEYSAFWINNDIIKHAIKKLQHMCNELQNENEIAIIKKYGDRAKSLTKIFTMF
AMCCIFSAIFLQIWTHLYYTDMSINTSRSLRTMYIMTEYFIDREKYCYFIMLYEYAAYCIAGFAAVGTGT
MLLAILQHMCGMFSIVSFRIEQAVTTNMLSDLNQQNQTIICKKIVDAIDIHRKTMEFCNFLTSNFEGSFF
FLILVGMICLASSLVRVVSSISDMKQIVIPVISISICYVYLFISNYAAQEVMDHNNRIFDTAYNVQWYAA
SVRIQKMILFLLQRGSKAFNLNLGGLFIGSVESAAMLMSTSISYFTFLYSTRQD

>CbirOr474

MVCIKKRFFNPSRLLMLPFGLWLDEETIFTRFQATVLCSLLISSILFQLSRLFIADCNFDFVVRIFSSAT
FYAMLTIPPILFWTRMKTMKYFFDQLQYIYDRLKDRNEIAIYDKYGYIAKQFTIILIIILLVCCLFGNSVI
VYWPYIFDIIIPKNESYAIHTMQFVTKYFKVSEKYYFLVVVHLNAACTTGLIVLAGTGTICFSYTQHICG
MFEIASYRIEQAMTTELLYNFDIKNRIVISRKMIFAIDIQRQAMQFAKHFVTSIEGALFIMVLTTVLCVS
CNLTQVFLIQSLAEKMEETILHLSAVGFILTTMFLTSYAGQEITDHSNHLYVITYNVSWYLAPLHIQKMI
LLLLQRSNKIFTLSVGGLFTGSLECFATLVSASVSYFTFMFSMQ

>CbirOr475

MVCIKKRFFNPSRLLLLAFGLWPDKETIFTRFQATFLCSFLISSIVFQLSRVFIAECNFDLIVRIVSSTT
FFTMLTILPFSFWVNIKTITYLLDQLQYIYDRLKDRNEIAIYDKYGNTGKQFATIVIILLVCGLSGNSVI
IYWPYILDIVVPKNESYAIHMMQFVTEYFNVSEKYYFLVVVHLNAACSTGLIVLAGTGTIFFSYLQQTCG
MFEIASYRIEQAMATELLHNFDIKNTIGICREIICAIDIQRQAMQCAKGFITSMEGTIFVLVITLVLCVS
CNLFRIFQIESPMERMDEVLLHFSAVFIVLMIMFLSSYVGQDVTDHSNYVYVITYNVAWYLAPLHIQKLI
LFLLQRSNKSCTLSVGGLFTASLECFATLFQASMSYFTFMYSMQ

>CbirOr476PI(F)

MDTHFLYIHRISLIAIGLWPYHRTILVQLHSCVFSFTIISFIIFQYRIEQAMRINSDEVTNRKREISKKI
NHAVGIHRTAFEFSEFFLHNFQGTYILLIAIIVICLSLHMFGICQAVRLHRMQTFLLHCAFTVSILICSL
GGNYMGKAITDHYNYIFSTAYNVRWYVAPVRVQRLMLFLLQKGTKPYLKYSLYSTSLENFAS

>CbirOr477

MDFVGEHYYNYTEHYYKLNRILLVCVGLWPYDTSFLKKIQIIFFEALFMSFLLCQLNVFLLKNCSAELVM
KVLMFLFITLFFTVQYNAGVLLTDTFKYIFSRLRYDWKMLKNQTEFDIIQKYADRTRFHTTLFTLLATSV
CLGIVVLCSVPSILDIIIPLNESRPSWLPIVAEYFVDQERYFYVIVIHIFIVLYAGCMVLVASGGMILGY
VMHNCAIFKIAIYRIEHIFDEKILQMSKHVREYVLYERLIHAVHLHRRAVDLANILTNSLATVYFILLIC
GVASTSFSVFHLFHVLTPLNDILELLACCGVILSQLYYVFLGNYIGQDIIDLSTNVFRTTYNTEWYTAPL
WLQKLILFIMQRSSIKSTFTAGGIFDASLEGFAKLMSMSMSYVMFLRST

>CbirOr478

MYKTSISQSVQEYFSWNRITLLAVGLWPYQQSRFARFQAALCLSILTSFVVFVLSRICFVEYDFEFTIDM
LSLSTYYSFFLMNYISFWINIETIRYLLEQFQYIYNRLKDSNEIAIYNKYGNIAKRITLACILIGICNHA
YLTVIQCGPYILDVIMPKNVSYARHLIVTLTTYFAVQERYFYLLLLYLNAVCTVGTVVILAVGTMLVSCF
KYICGMLRVASYRFEQAITAMVQSITIKNETMIYKEFICAVDIHRKAMEIAKIVVGDMERSLFIVTMSTV
LCMSLNLYGILQVESPMQEMEKLIGHCVNIICLFLYMFISNYSGQEIMDSNDRIFLTVYNAPWYLVPLKI
QKLILFLLQRSNKAFTLDIGGLFTLSIESFASLVSTSVSYFTVLLSLR

>CbirOr479PSE

MSISVQERYFNFNRITLLAIGLWPYQRSKCAQFQVALCLSILTSYVVFVFSRLYFIEYSLEFIIQLLSSS
TYFIFLLLNYISFWVNIETIRYLLEQFQYIYKRLEDSKEIAIYNKYGNIAKRITVSCIIVGTCNQAYLIA
IQGCPYIFYVIIPKIVTYARHFIEFVTKYFAIQEKYFYFFSSYLDVVLTLGGMSILAVGTMLVSCFKHIC
GMLRIVSYRFEQAMTATLQCVIIKNETMIYKEFIYAVDIHRKVMEFTKIFVSDMERSLFVVLIIAVLSMS
LNLYGVSFLHTILLMSDLRSILQYVIRKKLYMFAVNYNGQEIMDCNNHIFLTVYNTSWYLAPLQIQKLIL
FLLQRSNKTFTLNIGGLFTLSIESFASLVNASISYLALMLSLY

>CbirOr481

MIFSVIQYFNLNRILLLMIGLWPQKQTKLVRLQIILCYFILTSFIVVQFMTFATSKISLQLIIDVFPAAF
FFLSYMIKYSSFYFNRDVVKYLLDIVQHTYNELKDESEIAIIKKHWNIARRYTKVLTIFSIFGVFGLISV
PILPHIIDVSWPANKSRRCSSSQILTEYFIDQEKYFYLILVHMYAALYVGAAAMVSTGAMSVVYFQHVCG
MFKVASYRIKQSMTNNVLQSDCLQKENLICKGIICAVDMHRKALKILELFLSKFNIFYFFLIPSAQISLS
FSLFQVFNGVLSGYNIVRLISPCIYATVHYANVCVANSFAQKITDYNSHIFVTVYNVKWYVAPLRTQKLI
LFLLQKGTKVFNLVVGGLFTASLEGLAVLTNATLSYFIVIYSAG

>CbirOr482NTE

MICIQTQDFNINRILLLLIGLWPYQRSKMVHFQVILFFGIMACSVILQAITLCGLLIFISLTIWPRIIDI
DIVLLINDSQPCHARHAIITEYFLDEERYFYLVLLQMYVTFFIGGFTMTATGTMLLGYIKYICGMFKIAS
YRIEQAMQINIFDRIYLRDKAEIHKEIIRAVDIHCKAMEFANFLISNFQDTFVMSQLFNVLCLSLNLYCV
FQADSFKDNVDEFLLHFMLANGIFVYSFVANYAGQEIIDHYNYIFSAACNIQWYVAPLRTQKLILFLLQR
GNTIFNLRIAGVFTGSFECFATMTKQAISYFTVIYSTQQ

>CbirOr483INT

MIYIQTHFNLNRILMLAVGLWPYQRSKFIEFQMILFFGILTSFVIFQVSAIIQRYGNNAKRFTFIIILFA
ICGVFTISFLSIWPCILGVILPLKASRSRSAMQVVTEYFIDQEKYFYLILLHMNVTIYIGATAVIATGTM
LMGYLKHICGMFSIARYRIEQAMKFDTLQNTGLEDENVIYKKLIYAVDIHRKAIEFSEFLISSFEGSLFL
LIGVGVSCVTLSLYGVISHKSDVAELLFHFTGTTCTLVCIFVVNYAAQNVTDHNNDVFTTVYNVKWYMAP
LNIQKMMLFLLQRGTKVFNLNIGGVFVASLESAATLTSLSISYFTVLYSTRK

>CbirOr484PSE(F/S)

MVCIKKRYFNWNRLFLLPLGLWPDKETKFTRFQAKLLFSLLMSSAAFQLSRLFSVECHFDSAVTILSSSS
FYIALTIIFMCFWINMKTMRYLFEQLQHIYDGLKDKAEIAIYDKYGYIGKCLTIRLMIPMVCGMFSNFII

VYSPYILDVVMPKNESYAIHVMEMVTKYFIVSEKYYFLVLVHLNVACTAGLIVTVGTGTMMLSYFKHACG
MFEIASYRIEQAMAIELLHDLNTKNEIVIYKKIICAVDIHRKAMELFAKCFITNTEGTLFFLILTGVLCL
SFNLFRIFQIKSLVDEVEEVLLHFFVVILVLVGSFLANYVGQEMMDYTNHVYETTYNVSWYLAPLDIQKM
ILFLLQRGSRTFTLNVGGLFTASFECFATLVSASVSYFTFMYSMQQ

>CbirOr485PI

MTICIVDHYFSINKILFRAVALWPYHRTRFNEFQWFFFLVILITFILAELTPFLTTECTPYMAIKIFSFA
LSFTIFLIKFISFRINADTIRYLLEKFQYICDEITDEDEIAIIKEYGYEAKRFTLIVSVYGVSNAITSSV
LPVVPRILRTFTSINVSDEHFMIHITREYFVDQEKYFYYILLHLGASFLIGTIVIVGTGSLLFGCMKYMC
GLFRIASYRIDQTMETPMFQSAGLSKDCVTYKNIERAIDIHRKAIELHQVIMLKSVIKEYIPLVSNIIGT
LVYVFINSYVGQEITDHHNHMFISVYNAKWYRTPLYIQRIILLLLQKGTGNYYVVFGGIFVLSMENAATL
LSTSISYFTVLSSVQNP

>CbirOr486

MKVSLRERYFNFNRIMLLSIGLWPYQQSTLTQLQLVVLLSILISFIIFQFSRLIFVDYTYGFVLRMLSIS
SLFVLYVTKYISFWINIDAIKCTLERLQDVYNELTDKNEIAIYEEYGKLAKQYTIKFILVQASILSFAFV
MESWPFIFDIVIPKNDTYTRHLINSLTSYYLIQEKYFFFVLLHINAAHAVGSFVLVGVGALFVSYGKCIS
GMFKIACFRLERAMAADTLQNIYLKNHFVTNKYIIGAVQIHRKAMEFADYLIYCFEKSLLFIAGTTVFCV
SLNLYRMSQIEKPLEEIEETLTHFFIMFFVLVYLFIGNYIGQEVIDNNDELFITVYNIPWYQAPLHIQKL
ILILLQRNCKPFALNFHGIFVASLECFAMLVSASVSYFTIMISLQ

>CbirOr488

MIRIVETFRIHRILLLAIGLWPFNQSKFVQLQLPLFVVVPNSFIIFQLTSFITAECTVDLIIKILSTIFF
FLLGEVHQISFWVNAYTVKCFVNRLQYFCNELKDENEIAIIKKYGNSAEHITIAVTLFSGCCLLILSLLP
ILPWILGTFLLVNESRPLYNMQIVTEYFVDKDKTFYLILLHTYVSVCIGIIAMCAAGMMLVAYLKHVCGV
FSIASYNMEQTMMLNVHEKPNLWRRSRNKMEIIKKLRRAVHVHRIAIELSENFTTNFNATYFFIVGIVVI
CLSLNLYQIFQIVLHRSYVEELLLHLVFAAAMLVYSLVANYTGQEIIEHYNDMFTVAYNIRWYTAPVRIQ
RLILFLLQRSCKIYGLKIGGLFIASLEGFASLFTASISYFTVLYSTQKR

>CbirOr490PSE

MICIVEHFRIQRILLFAIGIWPYNQSKFVRFQYSVFLIVTNSFIIFQLTPFITSKCTSDFIIKILSNIFF
CLSCVIHQISFXINIDVVKSLMECLQHACNDLKDENEIAIIKKYANSAEYITILVTLMAGCCAFIMTLMP
IYPRILSTFLLVNISRPLYNMQFVTEYFIDKEKNFYLLLLHTYAFVYVAIIALVGGGMIVCAYLKHISGL
FSIASYRIEQSMILNIYEKTDLRNKMIEKKLCLAVDVHRTAIELSDLFTSTFNGTYFSLIVISVLCLCLS
LYELLETALLREKVEEFLLHLIFASAILVYYYLSNSCGEEIIQHYSNMFSTAYSIQWYTAPIRIQKFILF
LLQRSCKMHFIKIGGLFITSLEGFASLCTASVSYFTVIYSVQK

>CbirOr491

MSISVQERYFSYNKIILLAVGLWPYQQSQVVRFQTTFFLSILISFVILMLSRLRFTDYSFEFNIHLLCTS
TYFTFLLIKYISFWLNIETIRYALEQFQDIYKRLKDSKEIAIYNKYGNIGKRITIALIIVAICNHVYLTA
IQCGPYILDMIIPKNETYAHHFIEVMTNSFAVQEKDFYLFLLHLNAVLAIGSIAFVAVGTMLLSCFKLIC
GMFRIASYRFDRAILATLQSIGLKNETRIYKELIYAVDIHRKAMQFAKVFVGDMERSLFFVTMITVLCMS
LNFYAIFQIESPVQEIEKIMGHLLLIIFIFIYMFVANYAGQEITDYNNHMFLTVYNTPWYLAPLQIQKLT
LFLLQRSNKAFALSLGGLFTLSIECFASLVSASVSYCTLMLSF

>CbirOr494PNC

IKCLLEQLQYIYNDLKDKNEIAIYDKYGNIAKRITITFIIGEISCLCTLSGMQWWPWFLDMIIPKNETYT
HHLIVLLSKYYDVQEKYYYFILLHLSATITVGSFALVAVATMMLSYAKHICGMFRIASYRLEQAMTINTL
QSISLKTEIVIYKEIVYAVDIHRKAMKFAKFLINSMERSFLSVIMITVLCVSFNLYRISQTESFTENVEE

TLIHLLIITFTLLYMFLANYTGQEITDYNNHVSFTAYNIPWYQAPLHIQKLILVLIQRSNKTFTLNIGGL
FTLSLECFAS

>CbirOr495

MLANILREYNINKILLSCLGLWPFQHKFARRVLSIVCFTIAISYLPFEILTLYIHRHNGQMIAECLYQLV
VTTAFLVKLLNQIWNRDKFRRLYMIMENHWNIFTNDLEARILKNYSNIAHKFTILYSVTLYSMVTMFIII
PSVGPMFLDVVLPLNESRSRNFAVYAEYGIDQNRYFVSILLYTSVMIVVGVSIMVAVDTMHVACTAHACS
LFQLIGRQIENIISNVRTDEEINKTKCRTRYELFDEELIYREYVVCLKKHQLALEYVDMLNATHRIVGVS
FLLLIVAAFSLTGVRIVYVLDQLEEMIRFTFIIVGALLQLMIVCYSGQKLMDESQNIFHQAYATEWYNFS
PRLKSLLSIILYRSIVPCRLTAGNMFPLSMAVFAGVIRTAMSYFTAFLSLKE

>CbirOr496PSE

MLANFQREYNINRIFLSSIGLWPFQNKIMRNVLRTFCILLEISYYPFELILLYDHWDDSKLVFDGLYQIT
MSVCFFARIINQLWNHEKLQKLYLTMDEHWDIFTNETDVRVLKDYSTLSRKFTIYFSILVYIMTSAFIIV
PLTPIFLDFMLPLNESRPRFFAIEIEFRLDKNEYFLPLYFYTTTAIVVGITIVVAVDAMHITCTTHACSL
FASVSQQVENMILKVNNNNKISDHEHCMNTEFESLEEEIMYREYITCLKKHQLAIEFVDILESTYQGFSI
CLLLLIIAILSLIGVRIVDVLDQVGEVTRFTFLIIGDLVVLLIVCYPGQRLMDESQDVFHRAGRNLDILI
YISIATYAAVVRISISYFTAFLSLKD

>CbirOr497IC

MLTNFLREYNANRVFLSSVGLWPFQNKIMRNFLRTFCILLEISYCPFEVILLYDHWDDSKLVFDGLYQLT
VSVGFLVRIMNDLWNYDKLQKLYQAMDEHWDIFTNKIDVRVLKEYSTFSRKFTIYFSILLYIMSSAFIIV
PLKPIFLDIILPLNESRPRFFAIELECRLDKNEHFLLLYFYTTTIIVVGITIIVAIDTVYVISTTHACSL
FASVRIVYVLDQVEEVIRFAFLIIGASMILLLVCYPCQRLMDESQNVFHRAYAAEWYNYSPRLKYLLIIT
LYRSNIPCGLKAGNMVPLSIATYAA

>CbirOr498

MVYIETEYFSFNKILLLAIGLWPYRQSKIYQFQIIFFSSILMSAILFQLYSTVTTQIASPNKKSFRVLVT
IILKIHKNNIKNSLNKCVKILLMQLQHIHNKLNDANEIAIIKVYAYNTKRYTVILIIILLIGSIFLFILSQ
VCPNIFDIILPINKSRHYRMQFIVEYFIDQKKYFFFLLLHMDTAFLLAGIVTLAIGLLIMGYIQYICGIF
NISSYHIKHAIKSNMLQNINSKNENLIFCNKERIIHAVDIHRQARNLCILLMSKFELMFLFTTPLVVLSL
SMNLFRISQVALSEDDIMDLVLPFIAVCFCTMYMFLAHYIGQDVTDHNNHIFGTAYDIQWYVAPLHIQKL
ILFLLQEGTKDFTLNIAGLFIGSLECFATLAKASLSYFTVIYSMRK

>CbirOr500PSE

MSISVQERYFSWNRILLLAVGLWPYHQSRFARFQAALCLSSLTSFIVFVLSRLYFVEYSFEFTINLLCMS
MYFTFLLIKYISFWINIETQFQYIYKRLKNSKEIAIYNKYGNIGKRITVGFIIVAICDLACLIAMQCWPF
IFDMIMPKNVTYARHLIVTVTKYFAIQEKHFYLVFLHLNAVAVVGGFSFLAIGTMLLSCFKHICGMFRIA
SYRFEHAITITLQSITLKNKTMVYKEVICAVDIHRKATEFAIFLEKSMDRSFFIIIMVIVLCMSFNLYGI
FQIESPMQEIEKTTEHLLFIIFIFIYMFVGNYIGQEITDYNHHVFLIVYNVPWYLAPLQIQKLILFLLQR
NNKVFTLTIGGLFTLSLECFASLVSASVSYFTLMLSLQ

>CbirOr501PI

MRISVQERYFSFNRITLLIVGLWPYQQLALARFLTTLCLSILISFVGLIFSRLYFVDYSFDFTINLLCVA
TFYTFFVIKYISFWINRKAIRYLLEQFQYTYNGLKDENEIAIYDKYGNFGKRITVGFIICNQSVIIAMQC
WPYIFDVIMPKNGTYVGRLVAVVSKYFAVQEKYSYLLLLHVNITSTVGSFVLLAIGTMILSYVKHICGMF
RIARFAKFLISSMNRSLFFVIMIYVLCVSFNLYGIFHNEPAVQEIEQTLVHLIIIGFVFAYMFIANYVGQ
EIMDYNNHVFLTVYNAPWYLAPLQIQKLILILLQRSNKAFTLSIVGLFTISLECFASLASTSISYFTLML
SL

>CbirOr503

MICIEKRFFNSNRLLLLPFGLWPCKETKFTRFRPTLLCSILISAIAFQFSKLFIAESNFEIFIRLLCSTT
FYIILTIMYISFWFNIKSIKYLLDQLQHTYDRLKNKNEIAIYDKYGYIAKRMTTTIIILLMSGLCIITLI
VHCPYIFDAVMPKNESSHFAIRTTEIVAEFFIISEKYYFLILMYMTAACYGGLIAMIAIATLFMAYLKHT
CGMFEIASYRIEQAMAIELLHNFDMKNEIVIYREMKRAIDIQRQAMEFAKCLMISIGTPFFFLIITTVFC
VTVNLFRIFHIESPAEKMQEVALHIFVIFTIVLTMFMGNNTGQEITDYSNHVYLITYNTSWYLAPIRVQK
LILFVLQRSHKIFALNVGGLFSGSLECFATLISASMSYFTFMYSMQH

>CbirOr504

MTFAKSSFYNINRILLLVLGIWPDQKNKCMYVQRLFVWGIFTSFIICQLLLFFTEKYTVSLFFNTLSQSL
PIMVCLLKYNAFCSNKKSVKLLLEQIEHDWITLDDRELEIMKQYASTSKLYVVTFISFAVATICIYAFLQ
LLPAILDFIIPLNISRPRYLYVRFEFSIDEEKHFYAITFYTVVSLFVASATVISIGTSILTFGNHSCAML
KIACYRMEHVMDKYKSLHFVKRNEIQNEMVRAVDIHRKAMEYTNILISTLMLLFFGLLVTGVISLTLNIF
HLFYAFFDINSMEEILISTFLICAHFLYMFWANYAFQQIADHFHEIFIAAYAIPWYVAPVRIQKFIIFLL
QRGTKNYTMQIGGVFTGSLEGFATLLSTSLSYFTVIYTTR

>CbirOr505PSE(F)

MISIVEHFRLHRTLLLTIGLWPYNQSKFVELQFLLFFAVPNSFIIFQLISFITSEYTVDLLIKILSTIFF
FLFCEVHHTSFWFNAYALKCFVERLQHVSGELKNENEIATIKKYGKSGEHITIILTLFTVCCLIIITILP
FLPWIFGTFLLANESRPLHNMLIVTEYFVDKEKIVYLILLHTYASIYIGVTALVGGGMMLMAYLKHICGL
FSIASYRMEQTIMLNTHEEANRRNEMEMNKKLRHAVDVHRTAIELSEFLTSSFNGTYCCLIVVAIICFVL
NLYQIFRSALLLFNIEEILLHLVFTFSPLLYGALANYIGEEIIGHYNDMFSIAYNIEWYTAPICIQRLIL
FLLQRSCKAYGLKIAGLFIASLEGFNSIITVSISYFTVIYSTQK

>CbirOr506PSE

MCVKARTRVTCIDTQFLYICRLSLVAIGLWPYLLLFSNTTFLTTECTIDFIVEILSTSLFFLLCAIQYTS
FWINTHVKRLFENLLYIGSELKDENKIAIIKRYGHIAKCAAIVFTLLTICILIIFILLPFLPRIFGIFFL
VNKSELYHNVYITKYFIDEEKYFYFIFLHLCSTHYITGGTLLVAGIIVAGGPIYVCGFFNISSYRIEQAI
RRNSNKVTNWKTKREIDKKIGHAVDIHRKALEFSEFFQQNFEGTYFLLIMLIVICLSLHLFGICQAVRLY
RMQAFLLHCAFTVSILICSLGANYIGQVVTDHYNYIFSTAYNIRWYIAPVRVQRLILFLLQRGVKPYSVK
FGGLYILCLETFATLSTASISYFTVIYSI

>CbirOr507

MICLEAEYFTLNRILLQSIALWPFQQSKLVQFQSIIILTILISAIVCQIPAFMTSKLSFKDVLKALSRIF
FLMILVVHYGSFRINIKSIKKLMQRLHCACTKLTDKTEIAIIQQYGWEAKRYTAILVVGFSGEGCAFFLS
QIAVAFSDVVLPINESRLPRLQKTTEYIIGEQNFFPLILPHIDTAMIIGVISWIAMGTLMIAYLQHTCGM
FKIACYRIENAMEINIPKNSNLKIGRPISEKIIYAVNIHREAMKLSELLISRFDTMYFCLTVLAVITLSL
NFFEISETVTSKEKITEAIFPTASVLVIILYVFFANFIGQNVTDHNNEIYNAAYNMRWYKCPLHEQRLIL
VLLQRKAKEFHLTCGGLFIASFECLATLVKATMSYFTLIYSAR

>CbirOr508PSE(FS)

MYKTSILVQEYFSLNRIALLAVGLWPYQQSKFAPCQAALCFILFTSSVIFLLSRLCFVEYSFEFTIHLLS
ISTYYTFVLMNYISFWINIKTIRYILEQLQYIYNRLKDWKEIAIYNKYGNIAKRLTLACIIIGICNQAHL
IVMQCWPYICDMIMPKNVTYARHLIITVTTYFAVQERYFYLLLLYLNAVCTVGTVAILAVGTMLVSCFKH
ICGMLRIASYRFEQAITATLQSITKKNETMIYKEFIYAVDTHRKAMEIAKSFVGDMERSLFIVTMITVIC
MSLNLYAILQVKSPMQETEKIEGXSIFTIICLFIFMFATNYAGQEITDCNNHIFLTVYNAPWYLVPLKIT
EVDLVFITKKNKAFTLNIGGLFTLSVESFASLVSASVSYFTLMLSLQD

>CbirOr509PSE

MRYLLEQFQYIYNRLKDSNEIAIYNKYGNIAKRITHLVGICNHVYFMVIQCSPYVFNMIMPKNVTYVRHL
IVSVTTHFGVQERYFYLVLLYLNATVTVGTAAILAVGTMLISCYKHICGMLRIASYRFDQAILATLQSIT
LKNKTTTYKEFIYAMDIHHKATELAKIIVDDMERLFFILIMITVICMSLNLYGHILLLSFVFSCICLSNH
TGQEITDCNNHIFLTVYNAPWYLVPLKIQKLILFLLQRNNKAFTLNIGGLFTLSIERFASLMSTSVSYFT
VTLSLQ

>CbirOr510PI

MAICVVQQYFSINKILFRAVALWPYHRTKLVEFHLTFFLVILISFIAAQLATFLTTECTPHMAIKIFSLV
LYFTLYLINYLSFRVNAHTIGCLLEKFQYICDELTDEGEIAIIKEYGNEAKRFTVIISLHQAMMLGSATE
EYLVYVKCMLVILIYMFVGNYIGQEIADHHNHMFISIYNIKWYVTPLYIQRIILFLLQRGTRNFQIVVGG
LFVVSMESATMLLSTSISYFTVLHSIQQNDVTK

>CbirOr511

MTICVVDQHFSLNKILLRAVALWPYRRTKLIDFHLFLVLVILISFILVQLTTFLTTEWTSDVAIKILSSA
LYSTIYVVKYNSFWFNANTIRCLFEKFQYIYDELTDDSEIAIIKEYGHEAKRFTILMSLSGVFSSITMCL
LPIFPRILGIFMSINVSEEHFMIHIPREYFIDQEKYYYCILLHMDVSFFMGAMILVATGTLIFSCIKYIC
GLFRIASCRIDQTVECPMFQSAGLSKDFILYKKIVHAIDIHRKAIELCDMLKSNFVGTCFLLVLVGVISL
SLNLYGAMMLGSATEEYLVYVKCMLVILIYMFVGNYIGQEIADHHNHMFISIYNIKWYVTPLYIQRIILF
LLQRGTRNFQIVVGGLFVVSMESATMLLSTSISYFTVLHSIQQNDVTK

>CbirOr513PSE(F)

MDTEFLYIHQISLVAIGLWPYHRTILVQLQSSVFSLTMISSIIFQLTTFLTTEWTIDFIVEILSISLFLL
LCVILYTSFWINMHVVKRLLKTLQHICSDLKDETEIAILKRYEHIAKCVAIGFTLLTICGLFILTLLPLL
PRIFDIFFFVNESEPYRNIYIRTEYFVDEEKYFYFILLHLYAAQYIAGATLLGRAIVITGYGIYSCGLFN
IASYRIEQAMRINNDKLTNRKNKRKIDKKISHAVNIHRTALEFIEFYLYNFEGAYFLIILIIVICLSLHL
FGIFHAISFIFRMETFLLHCGFTVVILTSSLGVNCIGQAIINHYNYMFSSAYNVRWYVAPIHVQRLILFL
LQAGTKPYSVKFGGLYTISLESFASLSIASISYFTVIYSIQK

>CbirOr514

MSISVQERHFSLNRIILLAVGLWPYQRSKFVRFQAALCLSILISYVVFVLSRLCFTEYSFVLTIHVLSIS
TYYTFFLIKYISFWLNIETIKYLLEQFQYIYERLKDSKEIAIYNKYGNSAKRITLACIILAVCIMLSIIA
IECWPFIFDMITPKNETYARRFIVTMTKYFVLQEKHFYLFLLLINVVTAVGSVGILAIGTMLLSCLKHIC
GMLRIASYRFEQAVTATVQSITIKNETIYKEFIYAMDIHCKAMEFIKFFMGGMERSLFIVTMITVLCMSC
NLYGIFQIESPVQEIEKTAGHLFNITCLFIYMFIANYAGQEITDCNNHIFFTLWNAPWYLAPLQIQKVIL
FLLQRSNKAFTLNIGGLFTLSIQSFASLVSSSISYLTLMLSVQ

>CbirOr515

MTICLKTQHFNLNRILLLLVGLWPYEQSKLVHLQLILFFGILSSSVIFQLTVFLTAQFTIDLVVQVLSVV
SLNVTFMITYNAFWANTHTVRLLGEQLQHMCNELRDGNEIAIIQKYGNDAKRITIIFIFLLVCYVISFNM
LSFMPYFLSAILHINESNHPMQLFLTEYFVDEEKYFYLILLHTNVAFSIGGTAFAATGLMLLSYVEHSCG
MFQIASYRLEKALGNVLENISAEKEMMQYRQIVYAITIHRDAMEFSTLFISNFGNSFFGVAVVGVLCMSL
NFFGISRTLLHKEHFMQLAKHFIIAFSIVMYIFLSNYFGQQVTDHDNHVYLTAYNVYWYKAPIRIQKLIL
FLIERSTKSFNLRVVGVLVASIERFASLMKTSISYFTVIHSLQ

>NvOr1

MMKMKQQGLVADLLPNIRVMQGVGHFMFNYYSEGKKFPHRIYCIVTLLMLLMQYGMMAVNLMMESDDVDD
LTANTITMLFFLHPIVKMIYFPVRSKIFYKTLAIWNNPNSHPLFAESNARFHALAITKMRRLLFCVAGAT
IFSVISWTGITFVDESVKRIVDPETNETTIIPIPRLMIRTFYPFNAMSGAGHVFALIYQFYYLIISMAIS
NSLDVLFCSWLLFACEQLQHLKAIMKPLMELSATLDTVVPNSGELFKAGSADHLRDSQGVQPSGNGDNVL
DVDLRGIYSNRQDFTATFRPTAGTTFNGGVGPNGLTKKQEMLVRSAIKYWVERHKHVVRLVTSVGDAYGV

ALLLHMLTTTITLTLLAYQATKVNGVNVYAATVIGYLLYTLGQVFLFCIFGNRLIEESSSVMEAAYSCHW
YDGSEEAKTFVQIVCQQCQKAMSISGAKFFTVSLDLFASVLGAVVTYFMVLVQLK

>NvOr2

MTSKVSPKLEKAPLAYVNEQYLADTEYVVRVAKTLLMPIGIWPRYGDNSTLSNAIIYIRVCLIFCLMLFL
LTPHFIWTWFKAEDLRKLMKIIAAQVFSSLAVLKFWTLILNKQDIRYCLEIMENDYRVVESEEDRQIMLK
NAKIGRFFTTAYLGLSYGGALPYHIIMPLLQPRVLRSDNTTMIPLPYPSEYVFFIVEDSPLYEIVFVTQI
LISSIILSTNTGVYSLIACVVMHCCCLFEVTSNRAEKLLRGMKYDKSKISPELGKKLSELIDFHVKAIQY
AETMENALNIVMLSEMGGCTIIICFLEYGILQDLEDREYLGMVTYIMLMTSIFVNVFILSYVGDKVKEQS
EAIGFSAYSMQWVDLPNEFIMKDLKFVMARANQPTRLTAGKLFDLSLQGFCDVAKTSMAYLNFLRTLEIT

>NvOr3

MAEMKRMEDVFAYYDERMKKPGPSCSNEKFEEDVKYATALNRRIANAIGIWPIFTSTGARLGFDICVKTL
KNAAVYILLSFLLVPGILHIVVEEGKLKAKILKTGPMILNTMALLKYSVMLFRKSQIQECLKQLESDWRK
AGNDELRALMRRNTAVGHRLSRVCVATFYVGGIFYRLIKTLLTPIRYTKDGLMIKPLPSPLYKGLFRFNT
SASPVYETIFATQMMSGFVVHSTTVTTCSYAVLLATHACGQLDIVVYLLKRLIEDDGDNGRLTRVGNEAV
DRKLRVIVQLHLKVLRFISSVEDLMNQICLVEILGGSTILCLTSFYFIVDLQSNDALGLFTYMVMITSLI
ALLFTYCYVGEIVSDKAKKVGAKTYMINWYDLPPKKGLCIGLIISVAHSPVQLTAGKMLELSMYNFGCIM
KSTAGYLNLLRTITD

>NvOr4PSE

MSRYFLRSIGLWSSSSSRSQAYLHKFLNLLLIVITYLLIMFILVPCALHTFIEEPNMAIKMKLIGPMSFA
LMAVTKYASLTQRTAEIAQCFRHVEEDWENGCAMEKKRVVAVIYRHAKIGRSLSIFSGIFMYGGGIFYHG
IMPMAAVQTFSASIDNSLSLNTSSVLEVAKKPRILTFPTYNAWLNIEISPIYEIVYLLQCFSGFVLDTVT
VGTCSLAAVFVTHTCAQLELVMMLIRRYIDGDVGPDDVPYPKDATTREARLAVIVTRHIRALKFAARVEK
YLNGICFVEFIGCTMNICFIGYYCLTEWERKEPISTTTYFILLAPFTTNILXEQCTRVGSIYHTIDWYNL
PGKSASDLFFIIAVSRYPAKLTAGKFVDLRLVTFSNVMKSAFTYFNLIRIVII

>NvOr5

MLGEKSHYAVQLNRLFLTPIGVWPIGRDAPLVQRLLKRLAIIGCYLLMSYLLVPTALHTFLEEPDPAIKL
KLIGPMSFHLMAIGKYVSLVGRTEEISACFEHVEEDWKMYSDKNAKPELEMMKRNAKIGRFLIYLCAAFM
YGGGFFYHMIMPLSVGRLVTKQVRAERYLQLVAENASTDNIDVEPVRVLSYPIYGLLAKLDYVTLLLVQF
VAGFVLYTITIASCSLAAVFANHVCGQLEIVMSLLRDFVHDNEDNPRIYALADDAATVERSRSDKFAEIV
QRHLRALNFASRVEKNLNAICFVEFIGCTLNICFLEYYFITEWENQNTVSTMTYCILLISFIFNIFIFCY
IGELLTEQSKKIGEVTYAINWYTLSGKRAVDLIMIIMIASCYPARITAGKMVNLSLGSFCNVIKTSATYL
NLLRTMML

>NvOr6

METQSTKRIENDRGFNYAVKLTRLLMISCGIWPAKFSTSFQKCLRPILIIICFFIMFFQLIPFCLFMFLI
IKDMRIRLKLLGPLGFSLTSLFKYVVVVIKNREIAKCVQIMVDDWHQLNSTEDRKAMLINAKTGRVLTMV
CMFLMYGGGMPYVTIVPLTKGVTMVGNVSYRHLAYPSYYIFFNPHVRPIYDVIFATHCICGFTRYTITCA
VYSIVIICVMHICSRIAITSSMLQRLADDSDGRLLGTAVKHHLDILKFATKLENIFKEIFLAEVLGSTYQ
ICLLGYYFITEYEQRAGIATATYLFLFMSFVFNIFILCYIGQILTEQCESIATTAYTSKWYQLSGREARS
IILIVHWNRRRVVLTAGKMLTLSLESFSSIVKAAGGYLNILRTAVANSN

>NvOr7

MIESVYKLKMNEDNKQNLERNQGFDYAVQLTRLLLMPCGIWPAKFSSRVQRFLRPFLIVACCFVMLFLLV
PVCLFMFLIVRDVRIRIKLLGPLGFSLMSLFKYAVVIIRSREIEKCIQNMLDDWQQVASDEDRDTMFENA
RTGRVLTMVCMFLMYGGGMPYVTIVPLAKGATMVGNVSYRALAYPSYFIFFNPYIRPVYDVVFLTQCLCG
FTRYTITCGVYSVVIICVMHICSRITVTSSMLQRLADNYDNKLMGTVVKHHLKFLNFAAKLDNIFREIFL

VEVMGSTGVICLLGYYFITEYEQRESIATITYFFLLMSFVFNIFILCYIGQVLTEKCESIAKAAYTTKWY
QLTGKEARSIVFIVSCNHRPVELTAGKLLKLSLNSFSSIIKAAAGYLNILRTAIVNSS

>NvOr8

MYSAMDNHDIKDLINAEDFEYAIQILRWLFQPMGIWPLKSAAYPSFLRPISIVISFWSAAFLIIPGILSV
IRVQNDFALRLRLIGPVSFCLVTSFKYFSFLVKNRQFYAYLINVALDWREMKKNNHNRIIMLRKTQISRF
FMTSCSICMYLSGMSYNILLPLTKAPTQVGNVTFKNLPYIGYYIFFDQYADPYYYVVFVMQCMSSFFCYS
TCCGVCCISIQSVLHISGRCDITSIMIKNLNGNCNEKALKAVVEFQLQSLKFAREIEKLLNQMFLVEFVG
STFNICLLVYYFMGDFKENDTVGTMTYVLLFISFTFNIFIFCYLGEHLTEQCASAGAAVYTMDWFRFSAK
KSRDLFLIVLFCQRPVVITAGKMVNFSLLSFASLMKASAAYLNMLYKMG

>NvOr9

MVKICPDMNSEDVSAIKNAQGYAYAVQLTRWLLLPLGLWPTKSIIYQKILRPVAILLCLFIMLFVIIPLC
LFIFLVVKDLGIRLKLIGPLGFGLMSLFKYVVVIVKQRDVASCFLGMAVDWQELSSLSDRKVMLRNAKTG
RLLTIICVIFMIFGGMPYITVLPLTKGPIMRGNVSLRPLAYPSYFVFFNPQIRPIWDYVFVTHCMCGLVR
YSVTCGVYSIAILCIMHICSQITITSSMLDRLVENFDNMLLGKIVTQHLRFLKFASKLEDLFNQICLVEV
LGSTCIICFLGYYLITEYEQREPIATVTYFLLLCSFVFNIFILCYIGEILTEQCESIGTTAYMIRWYHLS
GKEARNVVLIIASTQRPVVMTAGKMVNLSLQSFTNVIKASASYLNMLRTVTANAN

>NvOr10

MSKQNLISNLIELKSIRNAQDFEYAVQITRWLLQPLGIWPMKSSTFFSSILRSLSIATCTFLLGFLLVPC
CLHMFLIEKDLGVRLKMIGPLSFCLMNIFKYYAVLIKDGQISSCIVDMAGDWHRLEGSEQRGYMLENAKT
ARVFTTICALFMYGGGLPYSTILPLTRDAIIVGNDSYRHLAYPSYFIFFNPHIRPIYDLVFFAHCLCGFV
MYSVTCGVCSIAILCIMHICSQCSITSATLRSLTSDVDEKTFGKIVTQHLRSLKFASKLEKILNDMCLVE
LIGCTFNICMLGYYFITEFEQSETVGTITYSLLLISLTFNIFIFCYIGDLLTEQCENIGEVAYMINWYQF
SGKDARNIILIVASTQRPVVLTAGKMVTLSIRSFCNVIKASVTYLNMLRTLTASES

>NvOr11FIX

MTSSVSWQHLVVVGRFEGIDMVTDSKTESGYDIAVGPCRFFLRIFGIWPDPFVESSALEHLRALIVSSTM
VFFATITQTAKNVQVWGDLNVVTEISINSEVPTSTAAVKVAGIWYYRRALRKLMIQITNDWRSPHGPIEL
EIMEENARFSRLVSATCIFLADATFAAQFIIAVLRIDAQNRLQANGTDIDRPLYMRASFPYDSQISPNYE
LTLAGQLFSNFFAATTYTSIDSLFIVLMLHLCGQLSVLRLKFPGLKGCSGNEFAEKIAYIHRRHDQLTVC
ANTIEEVFNKTFLLQIITSSFVLCMLGYQIMTITNGNNIPFMELVFMIYYVICFLFITFTYCYMAEKLRE
KSVDISDAAYQCDWYDLTPQQSKQLIIIMLRARRPLQVTAGKFVAFSLSLYCSILKSSGGYMSMLLAIQQ
RLGTS

>NvOr12

MADKKGYEEAIEATRAVLRAFGVWPNRHKISENWLSRSHFLAPAFLIICFINIPQTLKIIKVWRNLNEVL
DILVTANIPSFVALIKLLCVRYNKKVIGLLLVSMENDWKSLKTLVETRIMWKNGKLGSLITLVIYTLTCG
SYVAYVIMITYINVGGSKQEDVITLNESKKLRPLYMRSYFIYDVQKTPVYEIIWIFQFVSMGVATFTFMA
VDSLFAVLMMHLCGQLINLQERLKNFTNMLGQTKTRNFSYQLSTIVSRHEQLNRFAKAIENAFNTMFLVQ
MLLSGMVLCLQGYQIVIILTGRDTVQIIELLFMVYYTLCFAFSLFVYCYIAEILRIESMEIGNAAYHCDW
YDLSAFERRLFILTIIRSKTPFEITAGKFAAFSLEFYCSILKTSGGYLSVLLAVQDRLAA

>NvOr13

MTVEIAEDSMERIVALSDDQNVDGYNHAIGPCRFFLRLLGTWPDPYGNVDSWTTSARCLVITATMFLFAT
ISQTVKMALSYKDLNLVTEILTNCNIPTTIATIKIASIWYYRWVLRDLVRQIIEDWEMSHDRHESAIMWR
SAKISRIFSIGCMFMTEGTLLTQCVVGLFRPISYAFKTDLNQSIEWPLYMKGSFPYDVQSSPNYELSILG
QLLSNVFASTSFSSADSFFIVLMFHLIGQLSILKLTILDLPSKIENSDDRSKFIDRFAFVHMRHNRLWRF
SMAIEESFNTMFLIQMIPCIFALCTQGYQLIMIMDADNVSLMELIFMIYFLVLFLFTIFTYCYVTEILRC

KSLELSYAVYDCDWTILPAKEARILLLILVRTQHPFEITAGKFASFSLPFYCRILKTSAGYLSMLLAVKK
RSEQVASKVVL

>NvOr14

MSKKSGFDVAVGPSRAFLCFVGVWPNPEGSETTFETIQCIIVTLTMIIFANIAQTVKVFMVWGNLNSVIE
ILTTADMPIFVALMKFLVAWYNRKVLKGLVILMMEDWSRSYSSSNLDSMWRTARFSRKLSAVCIGLAQGT
ITAQFIMVVVFDVNNKGEAERTLYMISYFPYDTQVSPNYEITWLGQCFSNIFAAGAFSAVDAFFAVLVLH
LCCQLSILRKELVMLADHHKKQGDNSEEFSRKLARIVEKHEYFNSFAKTIEDSFNTMFLSQMIASSLALC
LQGYQLVMIITNTEGKLPVFQLIHMIYFTCCFSFSLFVYCYVAEELRFESTELDYAAYDSDWYNLPPKDT
KLLLLLMHRSRKPLEITAGKFCAFSLRLYCSILKTSGGYLSMLLAVKDRLVVEAD

>NvOr15

MAEKEQGFQTAFSVTRFVMRFQGIWPGVDKPRTGFSRFQFIPAALMMVFFINAVQTMELTRVGGDLNMII
DILTFADIPIFIALVKHVGIAYNNKVLYKLLYLISEDWKEVTKESEKKVMWQKARLSRIFTMIEVSLGLG
RLFIHTIRMTYAMLHPTSFDPTGKLIRPSYMRGYFIYDSQSTPIYEITWGCQFVATAFGGCAFASADALF
VALVFHLCGQLTNLQTEFREVGKNTSGKKLEFVRSLARIIKKHRRICHMADTVEYCFNKIYLVQVSSSSV
IFCLHGYSLVTILFDQDDVVVELIVMTFFTLGFIYSMFVYCYVAECLSTESLALSSAIFDNTWYDLPPKH
AKLLLLPLQRTGKPLIVTAGKFVVFSLNLFSNIIKTSAGYLSMLLALREKL

>NvOr16

MDDKEGFEVAVKASRTILRVLGIWPNHHERTESWLSRSYFIMPTFILVYFTSFPQTMEIIKVWGDLNSVL
ELLTTFDIPNLISLIKILSVWYNKKVLGLLIMAMENDWKSLKTVFELRVMWKNVKLGRLITLAIYLLTYS
TVATYVVMAVYITANAYKQEFILTPDNSTKLRPQYMRAHFAYDVQKSPVYEIVWIFQCIAMHLAGLSFMA
IDSLFSILVLHLCGQLINLQERLKNVTENLTKRHNLSYQLSRIVMRHEQLDRFAKAIENAFNTMFLAQIL
LSGVVLCLQGYQIVIILTSRDTVQVTELLFMIYFILCIAFSLFIYCYIAEILRTESTEIGNAAYECNWYD
LPACETRLFILTMIRSKTPFEITAGKFTAFSLQLYCSILKTSGGYLSMLLAVKERLAL

>NvOr17

MSDKEGFDVAIQTSRTILRFLGVWPDPKRKESWIYSGHFLIPAIVMFYFVNIPQTMMVTKVWGDLNAVLE
VLTTSDIPIGIALFKMLGIWYNRDVLGQLVVSMSEDWKSVKSPEERDVMWRNARLSRLLSVTIIGLAEGT
IVAQFAMVIYFNVLEARQYSLTKDNVTARFRPLYMSAQFFYDAQKSPNYEIHWLFQCSSTIFAASAFSSV
DAFFAVLMLHLCGQLNNLREKLKKLPKQISDKGGGSFVEKLSEIVTRHDHLDRFGNAIEDAFNVMFLVQM
VASSMVLCLQGYQLVMITTAGDGIPLFELIFMIYFTCCFTFSLFVYCYVAEVLRTESMEVGNAAYESNWY
DLPSCETKLLMLVIIRAKKPFKITAGKFAAFSLGLYCSILRSSGGYLSMLLAMKDRLAS

>NvOr18NTE

GFDVAIGATRAVLCFLGVWPDIKRKKLISRTRFLLPTLIMLYFAIIPQTTMAIKVWGDLNAVLEVLTISD
ISIAIALFKMLGLWHNKDVLGQLVVSMSEDWRSSKTEKEFQVMWQNARMSRLMSITIIILAEGTIMAHFT
MALYFTMLESKQYSLTKLNATTRFRPLYMSAEFFYDIQSSPNYEIIWLFQFLSTMFAASAFSSVDAFFAV
LVLHLCGQLNNLKEKLKNFPKRHEEGEIQSFLHMFSDIVVRHEALNKFANTIEESFNIVFLVQMVASSLM
LILQGYQIVILATSEGSIPFFELIFMAYFTCCFTFSLFVYCYVAEVLRTESMEIGNAAYQCNWYTLPPSE
AKCLTLIMIRAKQPFEITAGKFAAFSLELYCRILKSSGGYLSMLLAVKDRLAV

>NvOr19

MTTKEEGFDVAIGITRFVMRTHGIWPGFSVSKAGIMRYAYLPAALMLLLFVIIPQTVQVIFVSRDLNAVL
NVLTLGNVPVGIALAKLLGVSYKQNVLHQLILSVCEDWKHTTKESELVVMRLNARKSRMFSIICIVLSEG
TAMAYSARMFYAAFSTHTKAQATGIDDCEKPLFFIGKFPFDPQSYPNYQITWTLQIIATFLAAGAFSSVD
ALFVTLVLHLCGQLTNLQAAFSEIGEENAEKGTMFVSKLSKLIERHRKINVFADIIEYSFNMMFLVQVLS
STLLLCLQGYLFMIILSGQDGLLVEMIFISYFTICFTFSIFVYCYVAELLQEKSLQLGYAIFYSKWYNLP
AKKARLLIISIVRCKRPLEISAGKFCIFSLNLFCNIVRTSAGYMSVLLAVKDKIT

>NvOr20

MARKEEGFDVAVGFSRFFMRLHGIWPGDTSSKFTWARFAFVPPAVIILMFINIPQTVQIFFVGGDLNAIL
DILTLANVPLGIALAKILGVSYNHNILRQLIVSVSGDWKHTTKKSELQVMWRNARISRTFSILFIGLAEV
TVLANTARMFYILYSTRSEAESSGIKNYKKPLYYTGKFPYDAQSSPNFEITWVMQILATILAAGSFMAVD
ALFVTLVLHLCAQLTNLQTAFRKIGEDKHEKEVDFMSKLSKLMKRHRKINEFADIIEYSFNMMFLFQVMS
STFLLCLQGYLFVILISSQKVILVELIFMVYFIICSSCSIFVYCYVAEILREESLQLGNAIFYSKWYNLP
ANKARLLIIAILRVQKPLELSAGKFCIFSLNLFCNIVKTSAGYISVLLAVRDKIVQP

>NvOr21

MKIRKSGYDECVGFTRLIMTIIGTWPGAEYSQHWYARYMFSIPLFFSMFFMIIPQTRMLLHVKDDLNYII
EILTTADVMIIVACLKLIGVWYNKKDLRYLLNEIEKDWTITEKEEQHVGNAMWENVKLGKFIMNGYAVLT
YGTVVLYAAGMLLLMNSQKIEDFDNENITQSRLMFVRSKFPFETQGSPTFEIIWFLQFLAAVMSIAAFTT
FDGFFIFSILHVCAQLVNLQCNFRNLISRCRLTKRTFVQHMRDLVERHIHLQRFTQIIENNFNKVFLMQM
IGYSVTLCLQGYQLVISLTENSEQNFITIAFILVYTTANILSLFVYCYVAEKLRKESTEIFYAVCAMPWH
EVKPEESKMIVNIMYAAKHPFEITAGKFAVLSFSYFVKVLKTAMGYLSMLLAMKSSHKM

>NvOr22

MMANNNKLGFDESVGVTRWTMNVIGLWTLDERRDLQTRFRSLLPAFLILFFIVIPQTRKATLAHDDLNLM
LEILTTADIIEGICLLKIFGLWYNKADLKKLVIQISEDWTHTNNDEQGIMWSNARLSKFVCLFCISSSSG
SVLTHAIVFLVTNVGANETRSLFLISQFPFNTQHSPVYEIVCFCQFAGALLSTFIFSSFDGFFVFSILHF
SSQLSNLNIRIRSLTEKTSGDKCQFVESLKSVVKHHQHLISYTDIIEYNFNKIFLVQIFATSIVLCLQGY
QFVMIISESGTKLLTSLIFILVFTTGNVLSLFMYCYIAEIIRNESQRLLRAVYEMKWYTLPAKDSCLLLI
VMCRLKMPVEITVGKFAPFSLEYFASVVKTSVGYLSVLLAVRNKIND

>NvOr23

MEVKTLVKSDTQISISNNLNGLSGFDHSVKVTRVISRMCGVWPGFEEKKSFTERFFFIVPGMVTFFSITL
PQLRRVMIHRKDLSTVLELMTTGIVMELISILKLLAIRYNQSGLRWLLRRMVDDWKIYDKGQYYKIMWVY
ARHTNTIVTICIALTTGNIAAQIIRQYAIYIIERHYSSANETVIKPTILKSDFYFNEQIEGIYELVVAAQ
ILGGFSVAFSFTAFDGFFVCSIMHVSGQIHKLQMQIEDLVQCYERREGAFSEVLGPIVHRHRDLRGYAAV
IEENFNKIFLVQMLVTSVFLCLQGFEFAMVVAEGGTEMVPHLIFIVCFVASNLVSIFTYCFVAEQLRTQS
NQLFRSIFQIRWYDLTPKDSRLLIIIMVQTKKPIEITVGKFVPFSLDYFCSVLKTSAGYLSVLLSMKDRL

>NvOr24

MEESPGFLHAFGICRTCLTMSGLWSDTHFKKSKKFVISVLYAANVFVILTFMNVAQTVKLFLIWGDFDEM
SQIISTSDFSVGMLVVKMFVFRSYRKALALLIEFVEKDWLDLKTISEEETMEQNAHTANKIYLTCFFLGN
SAVNSYTLLRLGQEMSFLPGPPDKRQPLFDAYFPYDDKRSPAYEITWLMQYAGIALANLAFTGMYCLFVG
LMLHLCGQFANLRIKLIEAVSRKEGESEKKSDGAKTFRERLAFIVERHNSLNKYAQVIEKIYHWIFFVEI
LSSTIQMCSQWFMLVTVISNTQGGLPYLQIGFLLIFTAHSGFHLFACCYAAERLQNESLSIFEAAYSCEW
YNLSPQDAKMLLFIMQRTKTPLRVTAGKLCVFGLELFAKILKTAGGYLSILLAMRDRLVIDEEPI

>NvOr25

MDGKRGFDHAFSLCRINLGTVGLWPNSKNGKGHQEVASLIFFIISLFTIIVFVNLAQTVKLIMIWGDLNH
MIDNISTANLPIAVVVFKMLTFRRYKKTLTRLLGIAMDDWCTKKTSREAENMSKNARTARKMSLVCVVLG
FGSVNGQLAVRISQELDILPGQTEKRLPMLSSYIPYEYQTSPAYEITWFMQYLGAVLATLVYSGVYCVFV
GLVLHLRGQVANLRFMFESVDDPEEDKGKNFRRRLRSLVERHESLNRFAEDIENIFTLMFLAEILSCTIQ
ICLQVFLLVTLMSNDNGGVPILQILFMMVYAMHVGTHVFICCYVADKLRDESLSICDLAYNYEWYRLPAR
DARLLLFIMLRAERPLEVTAGKFCAFSLRLYAQILKTSGGYLSMLLAVKDRSTNF

>NvOr26

MDKKRGFDHTFGMCSINLGIVGLWPNSKNTKFQEFRSNVSFVFAIFSVSVFISMSQTAKLIMIWGDLYQM
IENISTANLPITVTVFKMLIFRSHKKVLGELLALAIGDWCTKKTEEETANMCANARLAHRISMICVFLAG
GTVSIHAVLRTCQELDIMPGPPEKRLPLFSSSYVPYDYKSSPIYQVTWLMQLTGTSCATLVFSGVYCAFV
GMVLHLRGQVANLRLKLENICEIREKGEGLVEARRDFRKKLGFIVERHLVLNRFAADLETVFTLMFLAEL
LSCTIQICLQVFLLVTLLSNIKHGFPILELFFLMVYIMHVGTHVFICCFVADKLREESLLICNSVYNYQW
YKLSAQDAKMLIFVMHRGDRPLAMTAGKFCAFSLQLYAQILKTSGGYLSMLLALKDQS

>NvOr27

METKAVAMTDSRAQVSNYFPDSSGFHKSINITRTISRVCGIWPELEEKKSIAARYYFIVPTIVIFFTMTV
PQVRRAVLHRKDLSAVLELMTTGIVMELIALLKLLGIRLNESGLRWLLRRMIDDWKTSNSKERNIMQEYS
NLTRFIMTLCITLTIGNVVAQTTKQFAIYFMERYQSMANETVIKPTFLKSDFYFNEQPEGIYEAVVAAQI
LGGFYVAFAFTACDGFFVFSILHVSGQICNLQLQIEGLVQNHEQRRCSFIKVLAPIVVRHRDLRGYAAVI
EENFNKIFLVQMIATSIFLCLQGFEFAMVITKSGSEMVPYLMFILCFVASNLVSIFTYCYVAERLREQSE
NLFRAIFEIRWYDLAPNDSKLLIIIMTQTKTPIEITVGKFVAFSLGYFCSVLKTSAGYLSMLLAVQDRL

>NvOr28

MDGEKGFLYAFGMCKKSLTLIGLWPKSKSSNYAEAVVVLRFTLTTLLIVSFVNIVQTIKLLAVWGDLDAM
TDIISTANLPIAVAVFKMMVFYKHRKAFEPLLSFVEADWKSYKTDSDMTNMWSNAQTTRRISMICVILGA
GTVNGHLFIRLGQEAKILPGKDGATRLSFVDSYFPYDYSPTPIYEITWAIQYIGAALATCAYSGIYCLFV
ALMLHLCGQFSNLRKKLRRVVTNEDDKRKFVEKLAEIVKRHENLNNFARVIEKIFNLMFLAEILGCTIQF
CMQGFFLLTLSSKEGMGLPILHILFMVIYVLHIGTHLFICCYVSEKLQDESVSIVRAAYNCEWYNLSAKD
AMLLVMIMNRAKKPLRITAGKFCAFSLSLYAQIFKTSGGYLSMLLAVRDRIT

>NvOr29

MDDKKGTSFIHAFGLCRINLTVLGIWPTLRSSKRDETAALFRLVLSLTIIILFINTVQTIKLFIMWGDLD
AMTDIISTANLPIGLMVFKTFVFLYHKEALVPLLSFVQTDWSNFKTVSEAANMWSNALAARKISLLCVVI
GWVTVNCHLAIRIGQELRFMSGKNGLTRLPFFDSYFPYDYTPSPVYEITFVIQYIATMLATFGYSGLYSL
FVALMLHLCGQFANLRDRLYTVTQKKAGVTFQQRLGYIVMRHQCLYNFAQVVEKMFNLMFLAEILGCTIQ
FCMQGFFLLTLSSKEGMGLPILHIMFMVVYVAHIGTHLFICCYVAEKLQDESVSIAKAAYECQWYHLSPK
DVMLLIMIINRAKDPIEMTAGKFCTFSLSLYAQIFKNSGGYLSMLLAMRDKIT

>NvOr30PSE

MSKSIEKATSMDNNKGFDYGFGICRMALTIYGLWPRLKNATYYDKSSSRSFRLIVWFILNILFINLVQTI
QLIMWGDLFAMTDIISTANLPIGLVVFKTFVFIYHKKALLPLLLCAQSDWDSPKSASEDMNMWSNTRIAR
RLSLICLTIGISTVNCHLVVRVCQEIKFIPGKTTSKLELFFNSYFSFDYTHSPIYELTVTIQYVATILIT
IGYSGLYGLFVALMLHFCGQFANLRAKLDRIAQEVDGTKFCKNLEEIVIRHQFLYDCAQEIENIFNIIFL
GEIIGCTVQFCLQGFYMFTLNTDDMAALILYIIFMIFFMGHIGSHLFICCYVSEKLKVESTSIAKAAYEC
QWYDLSPKDIMLLVMVINRAKDPIEITAGKFCTYSLSLYAQIFKTSGGYLSMLLAVRDKIT

>NvOr31

MDDKDGFEYAFGVCRKELIIFGMWPKPNDTMDHKVFAIFRLVLCIALNFIFINLVQTIQLFIMWGDLFAM
TDIISKASLPIGLVLFKTLVFIYYREALLPLLAYASSDWKKPKSSLEAANMWSNARTARQLSITCLFIGL
SAVNYHMAVRICQELRIIPGKTKVERELYFNAYFPYNYTESPAYELTFAMQYFATVLATFSYSGLYGLFV
GLMLHLCGQFANLRVKMDKVAKQADSAKFRQNLTAIIIRHQFLFRFSQIIEKIFNVIFLGEILGCTIQFC
LQGFFLCTLSTEDVGLLVMYIFFMVFFIGHIGSHLFICCYVSERLQDESVSIANAAYKCQWYHLPAKDVM
LLVMVINRAKDPIQITAGKFCVFSLSLLAQIFKTSGGYLSMLLAVRDKIT

>NvOr32NTE

EANFAVNLSRLCLCFVGVWPNLQCSRFFEIVSNAVFSVSSILIIYFIVIPQTTKLFYSDRDLDIIVDILC
TADIPIIVALIKMFVLRYDSNVMRYLLDQLFKDWKSPQTESRRATMRDSAKSGRMIAAACMGIAYGTSNV

FFFLRLIMLRQKQARTGHRAFLFESHFPFDVFNSPWFEVTWIIQAFCTFSATTAYSGIDSFFAVLVLHLC
GQLIIILGDELNSLAFQKDTEGIKEDIAKIVKRHYELNKYAETIETTFNKMFLAQIIACIVQFCLQGYQII
SILIDKSLELPYFQLIFMVMYISYMSLDLYIYCYVAEKLRGKSIEIADAAYNCKWYNFQKKEVSDLILIM
KRAQYPLDITAGKFCSLSLRLFAIIGKTSLGYLSMLLAVKERIN

>NvOr33PSE

MEIQDFDFVISWNRRILNIVGIWPEPETKNSLFSKCNFVWAAFLIFYFVSAPQTADLFIICSNMDLVVEN
LSTANIAITTTLLKLFVFWFNREDLVPLIAYMRKDWIKTRKREHLAEMRAVGRISRSMTIHLVILTQIMV
FVYMLLQIYLNVSGFFPTRRLFYRSYYPYDTSYTPVYEITWISQYYGASISVIAYVGVDSFIGTSILHVC
RQXAILKYELEHLLDPADDEKYNLLNSVKKKIKFITRRHEHLNEFAGTVEDIFNMMFLVQMTSLTILLRF
QGFQVINSMSSGKGTGLFQIIFMFLFIISVILLSYLYWYVGEQLNVKTTELGIAAFDCDWFTLPTSEVKS
LGFILLRTKNPLALTAGKFCSFTMVTFCE

>NvOr34PSE

ALTQLYHRIPLVVDNLTTSCAALTSTIKLFLLWNSRQAFQPIIHTAGLDWSRLKTTREQETMIRQARRAR
LLAIIGYAIMFVSAXSSRLPPYASSTTSPTSPRTGSYRCRRADPFETSRSSYFELTYATQLVAGSFVGFS
ISVPDNFFGALVFRASAQCEILGEKMKGLLDGQVDGRMLFRQRLGRLVDTHVHLLRXIDGEKEIVTLILT
QVLCLSLRIVCCLGFGVIRGLVPTQYTLKKIGVYYVMHRLQSIASDDNESPPIVHEILAEHACYSSPASY
ARDLIPIMVRSNVPVQLTAGKFFALSLNAYAXILKTSAGYISMLLAASVVNKHVDRLAESAYCDVLFYRN
R

>NvOr35

MSDKIEDAQKKLETREQRLRDFKWALGLNRLSLRLMGVWPGDDEAEGLGRLAILLRVPFMIAAMFFCLFL
PQMGALALVIHELPLVIDNLMTSCAAFTCCIKLYFVWRSKQVLRPVIQSVSADWLRPKLDWEREAMIREA
SRARIFTVSGYAVLAGCYTGFAFAPLFGFDIRMISNITDYGEKHLLVQSYFPYDYSKSPNYEITQVSQLI
AGFFIGMSVSVPDNYFGALLFHASAQFEILGANLENLVRQDDKALRSRQFNRRFGIFVDRHVHLMTMVTA
VEYSFSFVIMAQIFCMSIMVCSLGFQILGMIEGTTADKPSLLQVLTLLGTLFTLMMHTLVDCFACETLEL
RSAGIFENVYNSRWYTVPKQSVAKDVIPMMVVSKNPRKLTAGKIFTLSLATYCSILKSTAGYISMLIAVN
RR

>NvOr36

MERSQKNQLQDFDWALGLNRFSLRLMGIWPADQDESSKSLLTVSRIPLMILVLLCGLFLPQMWALALVIE
QLPLAIDNLMTSCPAFTSCIKLFFIWRSKTILQPVIDSALQDYLRPKSKSEETAMQREALRGRLVTIADY
SIMASCYVGFIFMPMLGFNVRIINNLTDCDTQRVLLVQSYFPYDYARSPAFELTHLLQLAASFFVGMAIS
IPDDYFCALLFHASAQFEILGLQIESLPIDGSKSGRLLSGFIERHVHLNRMVSAVERSFEFVIAAQIFCM
SIMVCCLGFQVLRMLDSAAEKPTPVQILTLGGTLFTMLLHTFVDCFASENLAARSSELFFKIYSSRWYSL
SWSKMRCLVPMMLVAKTPRQIRAGKILSMSLATYCSIIKSTAGYISMLIAVSGR

>NvOr37

MESLEKYRSQEFDWALGINRVSLRLLGIWPADQDESSKSLLTVSRIPLMVLVIFGGLFLPQMWALALIIE
QLPLAIDNLMTSCPAFTSCIKLFFIWRSKTILQPVIESVLQDYLRPKSEWEELTMRREASKGRLITIADY
SLMTICCVGFIILPTLGFHVRIVNNVTDYASYGNRALLVQSYYPYDYYESPAFELTNLVQLTAAFFVGMT
VAIPDDYFCALMFHVSGQFEILGLQIENLMGKDDAKEGVDWSLLGSFVERHVHLNRMVATLEKSFEFLIA
AQILLVTVMVCCMGVQVLRTLNGAGEKPSPFQILTLSGTVFYLLLHTFVDCFVSESLTSRSSEIFFKIYS
CRWCALPWNKVRCLLPMMLAAKTPRQIRAGRIMPLSLATYCSIVKSTTGYISMLVAVSGR

>NvOr38

MKSNNAHESFFAYLNWAIGLNRLSLRLMGIWPDDSVAETKLFTTILRIPLIISVMMLCIVVPQMYALILV
RNNLLLIIDNFMTSFPTLIGCAKFYFLWRSKEVLRPVVCSVTEDWLRPKSDLECQKMRDAAVVARLFTVG
GYSLITGSLMGFIIAPLCGLNIRVEQNITDYGRQPLLVQSYYPYDYSQSPNFEITHSSQIVAACFVAMSL

AVPDNYFGALVFHISGQFQLLGLNFEHFIKQNEKIVGIMAVRDFNKSLGVYVDRHVHLIRMVAIVEKSFN
FIILIQIFCLCVMACCLGVRILSAIGNPNDKTAVIQIINLGATLISLMIFAFSNCYASETLASRSAEIFQ
QVYSSDWYKIPKRSTCCYLIMIMIMSKNPQMLSAGKILYLSLSTFCIILKSIAGYLSVLIAQSN

>NvOr39PSE

MDRQQRGLRGATIRRRKNNHQDFEWAFGLNRICLNFIGVWPNEVDANTVRVKASSIKYLRIPAMLILILV
GLIVPQMYAITKVYKRLQLVLDNLTLSYCTITSFIKLFFLWKSRGVYGPMLRSALLDWQSSQGSLWQYEI
MRKQARRAQIFTLSGYIVMLFSFIVFIIFPIFGLSIRIINNITDTAGEDRFLPIQTYYPFDANRSPYFEM
IFVSHVIICSGAASCFSIPDYFFGALVFHASAQCEILAVKVQQLFPPQVEAGISNTFLENKFFRTKIKQL
VQRHVHLISFVEMIENSFNLIILAQTICLTLIICCLGFNIIMSLGTESNDSPILPVVSLGGSTLTILMHM
LVYCFASEILAMHSEGISEAAYDCDWYTLPANCAREIIPIMVRASYPLQLSAGKFFYLSLNAFLSIVKTS
FGYVSVLLAVST

>NvOr40PSE

MDRRRSTLRTDSTKGNQDNNQGIVLREYFEWAFSLNRICLKAVGVWPSELDELDTRGVSNNRNHLRIPIM
LFTLWFGLITPQLYALAKVYRQLQLVLDAHXYCTLTSFIKLFFLWRSRRVYGLILRSALIDWSGSRSSDW
KYETMYKQAYRAQIFTLGGYLVMYLLLRLLHVLSIFGXRIRIINNITDPGGEYRFLPIAYYPFRYDRSPY
FEITYATQMLVGCCAASTFTIPDYFFGVLVFHASAQCEILAAGIRELCLEELDTSSTEKPRELGIFRDIX
FVNNIKKSFNMVILAQTFKLVLVICCLGFNIVKATGNNSESLPILPIVTLGGSTITMMCHMLVYCFASEV
LAMHXRTIGTACYGSDWHLLTVDCARTIIPIIARAQHPLQLSAGKFFYLSLNAFLSVVKTSFGZVSVLLA
VGA

>NvOr41

MRTRVKRLKVSRSSKTKGGFSYEFRIYKIITWPAGLWPLERDNIFNVLRFLLAASSQMFIVVAALVEIYR
KCGNVADVLDYYALSIAFWLSFVRLVLVRIHLAKIHKICYNARKTWARIKDPDLVKIMISHAKTGKRFYY
LQMSIAFVIVTLYVFNPLILRRYDAANLPMQTVCTFNNADVLKHTAVYFIETLSFVYLAVGFISIDLLFL
GIAMHLCGQLKILQKEFSEIVGKSTSQADCIRYVISLSRRFQRVVELTDDIRKTFSEILLVNFVVNLFLI
TSQSVTLLLALKINNYFLAVKCSQTFPILLIEMFLYCYVGELLRHAFDDIPRAIYSSRWYLLPPKIRRGY
LLHVMAQASKTFDLTAGKMIRMNMCTFIQLVRSIVSFFSLLLLMFDK

>NvOr42PSE

IFIVTLVSEEIYGTCGTADDLLDYYAFFGCFIMSLIKLVCIRLHFIEMYRNCLYAQKNWTKVADTNVLKI
MQRYANVAKKFYHVQMFAAYCVVSFYVANPFILPRTDASNLPIQTVCSFRNLTDREYVIVYVVQSMSLLY
LAFGLIGVDVFFFGIVMHLCAQLKILQIEFNTIDKLTWRLDNIRLMALYSKKHYQLIMLANDLKTKFRVS
YSCDNLYXSATFFIALKINNKFLAFKSIQSFPILFLEVFLYCYAGQLLQYEFSNITNSIYNSRWYILPPK
LRQYYLLFGMTAGNKVFKLTAGQLLNVDMMLFIYFVKTMFSFFSLLLLMLDKRAEKLILCL

>NvOr43

MKCFDSYDKSNFKTDPQHAINFFKKLGRFWTIWPVSANASRFTKVYHECSLWFIIINLFFASLTLWMSVC
VYHKYPILMAKNLSQLMIISDSFTHLVLYRINRSELQVLVQEVFDFMKNSKQNEKYIMRKHYNRFLGHYT
ILIVLYVIASLAFFCGAFILGKKFPMDASYPFSTDSILVSSIIFTHQTFSIVQNSVLIMIDLLVITLFWY
AGARIKILGYKFKIVDSNEKLKNCIKEHQKIIQYVASIVKAVRFILYKTISIVAIIIISAGLQLLYYDAK
VVISQFSLIIIVACFRIITYSSTIEEMNQLNEDLRWTVYKSSWFCITSEMKQCLQIFIHRCQIPLVTIDG
QLLNIMSLAFFAKLIYSTVSHLTTLRAIIERS

>NvOr44

MAFKISPEKAFTFTKLSVFFTAIWPPNCNDSSFKIKLANIFWIYSIISAMCLLIPMLASVLVYKDNPMIV
SKSICLSCAVIQVIAKAIVCRHHQKKLQFLVKELTHFLKKAKKEERQLIEKYINRRAIFHMTFTLCCFGS
SFFVICGPIFLPFSLPADAVYPFSVNSSPIWEIIYVHQASVGIQASSGMCVDNLVAYILWYTGVRFESLY
YKFKHIKDSKEMLQCIKEHQYLLRYGTTVADTFRYVIFTTVLSVTAGLAFAGIYLFSPQPIFVKGQFVVV

SISVVVNLYVTALPSNNLISMCHKVGDVVYESLWVGDSPSIMKHWIFIIQRCQKPVVIAIPGLIKELSLQ
FYSSVLCSTFSYFSALHVIMTKE

>NvOr45

MKFLIRPKFFFKTLRLIGDMIAIWPKHIGAKKTMIMFHEVKWWLSFTNATGLLIPLVLGVYYFRNDSITM
TKTLSELTALCEVFINLIQCRLQEKKFQVILYEIENFIENSNEQEESLLQDYLNRYKTLQLFVGSSFIST
AILFSCIPIFTSQLLPADAWYPFSVEYFPIRVFLYITQVLAIFQTGFGICVDLTVATMLWYSAVQIELLE
KNVHKAVSKAELRECARRHQEIIEFTDNIKKGIKFIILKTNATMIIVVICGAFQLIHHEPLEVLLRFTLM
VLAGCLRLYVSAKPADDLKENSEQLARTAFQTALMQKSTSNSKIGLMLAFRCQKPIVLSVTAVIRAYTLQ
YYASFLSRTVTYFVNLRAVLDD

>NvOr46

MRKMKCTFTFSIISVYETLKYFGLLTSIWPIFSKNRYLWVFMKAVYYFILVNYLCVFIPLILMTLFNINT
SATVTITAVEQMIIVEAVYNLLYTRFYSAQFKSAVKEIEEFFKNSSPKERYILDRYATTRTFFNIYIAIN
YFVAIMSFNFGQLFLKDRPYPLNLWYPFTIKSQVIVVIIYIHQVIVITHTLILIVFDLIVQIFLWTLAAR
FELLQADFKKTASEMDLKCNIQKHQYLIRTTEAVIDFTKYMILKVFLAVTILVISSTLQILHRGPSTIIV
QFFFIMKIASMRAFAYCWAGHSLAEKTGGLARSIYNSYWINQTQRMKTNVLIVMQRCQKPTVVKISGIIS
SLSFRFCVNYFYMIYSAFMTLRAVLEV

>NvOr47

MSSWRMGSIKDIMMNVFMMKVIGVALAIWPLKSAGKRWYYAFLQEMVYRFFHVNFWLLVIPSLWSIYKKR
HNLASVLTSVTQLTIVFEILAIMVLSRRQAARLKTLLTMAYDYVSVADDKVYPVVYKYVRKAQIIFGIIT
IAYALILLTYLVQAFIENKPPIYAYYPFDIKSPVVWICVYGNQLLCTFYAAVVIIMDAMMMFMIFVTSIR
LELLQNDFKKVKDYPDLVKCIRTHQDIIWYIKEVYCIKKYMVLKMLISIAIYIMCEGLQLFALNLSWGMR
FQVSLLFGIGLFRVYIYAACSQDLISSGLDLGYFVYSSLWYNQSHSVMVAKAFVICRCQKSLGIRVCGIT
DDLNMKFLANFLYRVFSYTMTLRAIIKTLR

>NvOr48

MMINKKVTDKLVTIVSTLLNIFLHIDYLFHSFKILKFFSRLFALYPLNSDCTKLEILYDNFVWLFIHFHF
WVAAAATLVAIYKARSDLSIWLIAFSELIIIIEIIVAMILYRLQRSRLKILMHIFEDFVKDPDDSKIQLI
RRNAKEHIKVFSILALLFIIVVIMYVYRALSTRPYQLLLSGYYPCTSDSLIIWLVVFFHQCILVIYSPST
FASDSIVTVLIFAAIIKLQKIRPRFRNIENYAQLVGCINEHQHIIWFVQEINYVIRLFVFKSIFCLAALQ
LGVGVILFMPNISVFTRIQFLLLFTVTIFRIYIYSYCAEILTKSGLDLGFAVYSSRWYDQRRKMVLAKSI
IICRCQKPLLIAINGIIPALGMRYLARFLYLTFSYIITLQAMTRT

>NvOr49PSE

MIGNKFTNKLVTIVPVTLIDIISYLDYLFYNFKVLKLFARLCALYPLDSGCSKLEIXYDNFVWYYIQFNL
WTALVATFTAMYKARSDLSSWLNSFSESIIFIEIILLMILYRLQRPHLQILLQIFDDLIKDPDDFKLLLI
RQSARKHLKGFSTVAVLFTMLAVIYVYKSFSTKPIQFILSGYYPYTSNSMMLWIIVLYHQLTILLYVPSI
FASDFIVTLLIFGAIIKLRNIRPEFENIRNHNQLICCIDEHQHIIWFVHKINYVIRFFILKTIFCLGALQ
LGVGVILLRPDICAITRIQFLVIFAITISRIYIYAYCAEILTKSGSDLGFAVYSSRWYVQRHNMVLAKSM
IICRCQKPLVISVAGIIPVLGMGYLXRFLYLIFSYIIALQAITKNHYL

>NvOr50

MFKKIKPNFTVQKNFNILCNCMKILGTWPVHRRHNKIFTCLNHSLWWFYVVNHMMLLLPTMQTFYNTTKD
IISASYSLIEITGIVESMVILITFKLQGSRIQLLLQIIKNQIVVKKPKPALNNRNVHASVFAIIAVLYVI
VVYMYIHKPATLINKGFIMTTCYAFPTEDIRTKIAIYCNQLIALMHTSVVLVTDGVAVLFIYTCAIKLKT
LEIRLKKAPDWTKLKYDIAEHQTILLVIEETNSLAGVLVVKTVICFMCYSISAGVQIINQHVVTAQMLHQ
FIIIAIVYLRIYICAETAEMLLTVNGDMLFTVYSIAFSTPNIVKVKSLIIMRCQKVPKIYVNGLMAALNR
AYLRSISYATFSYFMTIRAIVSK

>NvOr51

MRIHLNAKIAYQYLRLSATLMATWPLSDATKKYRNVFYNMLWWLYLTNHLIILYLTLNTIITHNKNHLTV
FYTWLEISFMTENIIVLVSYKLQETKWKQLLYTSKMTINRTEDNIRLENSDLYPKVFATLFIFFIVIIIS
YVNKAETYERGLIMTTRYPFEIKSIGLKLFLNLSQFITLLHASSILITDAIVVLLLYTCTIRLKIVEQKF
RACKYYRHLKLHIYEHQKTLLLIEDTNLLVSKTVLKSIASFMSYSIGGGLVLYNKNTSPLQLVQICLVIC
VIYLRVYVCAEIAEKMISANESIGFTIYFTKWYEESAKDINAKNIIIQRCQKLPRIYINGFMQSLNRNYV
RMITYATFSYFMTIRKIINKTANCVDC

>NvOr52PSE

MQNKYEKSGSKNRSMEMVGYTVISLCLKCLGVYTVSSTNDKLWIRVYSSLWWFYLLNHVVFFLATAYAFF
TSKLDVISLSYSFMEGLFVLECIILLLHFKFQGPRLRNLLVNANVQVSRKKTLTILNNNNTYILIASLIA
LIYVISLSLYANKPKTVYYRKYFTQARYPFEINSTFATILIWCHQSIAVLHMLLIPTSDALVVLLLYACT
VQFKILESNIRKGRSRKKLQAYVREYNDIFLTVEETNRLVRVIVIKTVVYFMTFSISTGIQILNXSFKHS
FRKMQLMVTAIVSLRFYVCAESAEKMVSSVESIGLAMYFTSWYEEKLKFLIAKLMIIRRCQSLPKIYLSG
FMPKLDRQYLGTVAYTTYSYFTMIRSLVYKYAIENDQPTLSATFNKGRIQ

>NvOr53

MQNKFKLRDRTNKFTFMFKSYSGVHLGFLCVNFYMKCLGIYPLPSTVSKFWTRVYNLLWCFYLSNHLLII
FPTFYAFGSTTQDIAVATFSLMEGLCMIECIVLLIHFKYQRSDFKILLSLVHHELNKKKRIITLDNGNVY
IIAFVLIAIMYVLIIFNYIQRPETVRYHKLLTTARYPFSTRAATIKIILSCHQIVVLLHMTIILTSDGLA
VLLTLICTVRLKNLETKISNEKRGKLPKRIREHQQILLQVEETNLIVRIIVIKTVFCFMVFSISTGLQIF
HKFEIIQIFIVMIVFLRFYVSAESADNMATCANNLGIAVYSTAWYEEKTKIRIAKTIIIQRCQKSPRIFI
TGFMSELNRKYFLVVAYATYSYFTMIRTLISKNK

>NvOr54PSE

MNIYIPHNKSFCSLRFIGTCLGIWLTDNKKWTCKNDYLFWFCILNYIVSILPLFYSLYLNRRNVSAALYT
WIELSGVIKMMAVITIYSKFQRHRLNARLIALFVPTEQLLAIDKVKIVKKYANTYTLMFLAVLLLYMITM
SIYLAIEQTSNWIRIRXMTAVYPFRFYCYAVKVLTCANZAFVQMHSVILPTFDGISILLIFMCTYRIKVL
DHDFKNAZNFIELRKCIQEHENILXMVKEINLIIRFMIFKTVITFMSNVIACDLHILNNVPVTQSTFQVS
TIFLVCTEIILCAECADNMTTAGEDMEFTIYSTPWYEEEPKIMIIKSIILSKCQKAPVISINGVMSTLDR
KYLATIMYATVSYITTLRAVIENDKIL

>NvOr55PSE

MRIYISPKKSFRVLRFVGIILRVWPVNDTKWNYRTDIFFWFCIINCALLWVPLCNALYLNHKNAHGNSCY
FMDXVSWFAEIIFVLTYSKYKKRQLYVCKVIKVKYVERCVYLKKTNIIKQYATIYAMIFFSVMLFYLLAF
FVYMSIERPNTGYNTLITTAVYPFDINSYXVAKAILYCNQIFLLTYAAIAPTFDGISVLLLFVCTHRIKI
LDHNFRLAKTLSDLAQCIREHDDILYTIKETNSIVRIVVFKTVCSFMSNVIPAGLQILNSVGLSQSILHI
CTILLVYTRIVLCSESAGNMTNAAKDLVFTIYSSLWYNEEPKIVLTKTFILQKCQNVPTIHINGLMSGLG
RKYLLTIVYSTFSYLTTLRAVTSNERQ

>NvOr56

MSVYISPNRSFRVLRFLGTHLRIWPDDNKKWNFKTDVFFWFCVINYVLLLLPLFNALYLNRKNVVAASNT
WIEVSGYAEVLAAFIYSKYKRVQLYVLLCEAEKYLLFKKVTIIKKYANTYAKIFLLVIVFYLFTVFVYWS
IEKPITGYEHLITTAVYPFNIRSHPIKGLIYCNQTFNLVYSSILPVFDGISVLLIFNCTHRLKILEHKFK
LAKTSSDLSECVREHDDVSRTIKETNSIVRFLVFKTVCSFTSNVIPGGLQILNNVALSQSICQVCIILLV
YSRIVLCAECAGNMTDAGEDLLFTVYSTLWYNEEPKIVSMKIFIIQKCQNIPAIHIKGIMSGLGRKYLLT
IMYSTFSYLTTLRTVTSDEKS

>NvOr57PSE

MSVYISPHRSFRVLRFLGTHLRIWPDDNKKWNFKTDVFFCLINYVLLLLPLCNALYLNRKNVIAASNTWI
ELSGYAEVLAAFIYIKYKRNQLYARKTNIVAKYFLLKKSMIIKEYANTYAKIFLLVILFYLFAVLVYSSV
EKPITVSPIYYFLKTFVXRTFSNYNLFDEPKIVSMKIFIIQKCQNIPAIHITGVMSGLGRKYLLLIMYST
FSYLTTLRTITRNEAQ

>NvOr58

MTIQSVLRRKVDVLLKAIALNKMCVSPKMILLVIKFAAMYLAIWPLDSSGKHWNTAFDCLWWFYVVNNVL
VIIPTLLAFYSSRRDIIAAMFSWLEILALLEALIIILANFRYYRSRMQPILKEAVDYIGSANSRRQLCLEK
RASIITTTFGVIVALYIAGIIIYIYRPAVTEWDGMLTTAYYPASMRSPFADVFIYITQLTALLHNGVLIV
SDAFTVLLLYVCTVRLEVLQKNILRVADYDELKLWIREHERVLRLVTDTNMVVRINISKTVISFVGYSVG
AGLQIISPTVTIVSFQRFALVIAMNAMRLFFSATFADDLVNSSNSLINTIYSTIWYKDNRDMKIGKIIIM
LRCQKLLRISVGGIMPVLGKPYLTKILYTSVSYFMTFRAITGN

>NvOr59

MSRNLYHDAKIKCKMNVITYLLKCLNFLRFMGKIYAVWPLKTDDNIRWRFVYECLWWFYFLNYLVAASFT
LNTCGHASDDITIASFSWLEFVSMVESIIILINYKCYHVTLQLLLTEVEDYLTLADEKKQWVLKEKASIF
AVMMCIITFLYFVLVVLYFTNPAITAWETFLTTSYYPPAIRSPVMDVFLFSNQLIVMCHTSVIVNLDAMV
VLLIYICSVRLKVLAADLESVNDDEELKQRIREHQHILCLAKKTNIAVRLVVSKTVICFISYTVGAGLQL
VNPTATVASLQRFGIVLLINYVRLIMNATSADELLTVSRNVGLSIYSTDWYGESKIVTSSKFIVMLRCQK
LVRIHVDGVMPALTLTFITGIISTSISYYTTLRAVTRQN

>NvOr60

MSRVLQSDSSSREGKHVWSKDAKFALMLNKFIVWPLGLWPLECDDAFSRFRNFYAVVSQVWMIGTQATAA
YLGCGDVADTVDFVMMTACALMALSKIVTIRLHMSKVHTVFVSALDDWLAVDVDKSRDVLIPFAKTGRFV
FYLQMVSAYMSNTLIIIGALPFLIPPAANGTWANVSETLQSRQLPMRTGCMFAGYRDEIYGSLYVYESVM
IMITAHGNVGCDVLFFILAMHLCGQIELLKTDVLKIGEDEKVPGEWKNKIVECVHRHIRLLGMAKALNKV
VSGVLVIQLLLNAGLNLMLGIRMLIEIKRGSIFNAVRPMIGFNVLMLQLYLLSYASDRLSSQAESILDAV
YDSYWYKLPAKLRRDLYFVTMRANKPIYFMAGHFYAMNIENFMNILKASFSYFSILRIMFQA

>NvOr61

MGGKSEVDEAFAVYRAFLWAIGVWPLEEKSFSQILRYIVAAVVQVTFLLHTFTEILLNNGKVSDMVDVFF
FSSAAFLTFAKHTYLHLHKDAIRENLRCYLDDWSNTKDEHFLRIMREHVKIYKYQFHIYNLCGYVGTTLF
MCRSILINILAKRQLGPGESYNYQFICQTSYLSQDTLAKYYPIIMAIQYIQCMYCCTSGACTDCFFFGLV
FHLCAQFEILKIKWERLGTKDFGVTAVHDRVKVNALIARHKELVKLGENLESGFNNTILVQLMISIVLIC
MSGCSILVAIMRNDHVTMLISTNSISFMVTETLIYGYASDYLVTQSESIVQAVYSSSWYDMDSSVKKDIV
FVMMRAKIPLHITAGKFFCVTRNTIVQLLKTSVSYLSVLRLTLEMSHQEGQL

>NvOr62

MCANIFIGHHQFGLRVVGSWPGKSQLPGFYFAIGIMLFFLIFEILNITEVYHDLEELMDNLVSTIGVVLG
LFKFITVRVKRRKLKTVINKIFDDWKTDSQFVSEMMVKNCTRSQLVSKFVIFLYNSMNFTYFLRTVISHI
FDEVQDRKFLAQVTFPIVDGRQTPLYEIIIFFQFITASVCFNSQALVEGLLATLVLHACSKVDVVRREIL
NFSTICKTDKNDKKDILKTLRKLSEEHFKFIEFSEDIQDIFSYVSFFHIFFLTLIQVVSGYMFIDGLERG
TKPVNLIHYAILTTSFLVSAGYYCIAGEYLTSQSEIIFNELYNCYWYEFPSSYKKAICFMLLKARKPVKL
TVGKFSTLSLIYLTSIMKTSFSYLSLVRAVR

>NvOr63PSE

MMKTNIIKKPHRIGLKMIGVWPGVTGWSGFMFIMGWLFFTLIFMIWDVFIIYHDLELLMNNLLLNCFGXS
SKQSPVFEVVCFFQFAIAVISANAHALIEGVLTVLVLHAGTKVHLLEKEIQKFSAICQSKTDKEITSKAI
RGLIDKHLNFIKFVKEVKDIYYFVSFVHVFLSLFXHVIVGYMFIDTLESGDRSIKLFLYGLFTTRALAST

TIYCIVGEYFMNQSMRIYGELYNTAWYDFDVKNIKAVTFMIMKARNATSLKSASFGQLSLFYLTGVIRTS
FSILSLTRATR

>NvOr64

MSDKIVMRHVRVALQVIGLWPGYTSSVGFVIAITWLLTCLTFQLWHAAVVFSKLDALMGNLGATMAVATA
TLKLIAFHVKGRNVKIVIKEILNDWAYENRSSNCEVMVQNTKRAKYLTKWITGAYNATVITYLVNAIIAY
CSGITEQRLYVLPSKFPSFCKQSPVFEIVCFFQFSAALISTNVQVLVEGMLTVLVLHAGTKVFLLQKEIQ
KLSVICQSKTNNKEVISKSTIALINKHLNFIKFVKEVKDIYYFISFVHVFTFTFLHVIVGYMFIDTLERG
DRSIKLFLYGLFTTRALASTTIYCIAGEYLMNQSMRIFDELYNSAWYEFDVPNIKAITFMIMKARNATSL
TPASFGQLSLFYLTSVIRTSFSILSLTRATR

>NvOr65

MSILSRHVKIGLYAIDAWPGVSSSGLFFLVMAYMTFSLIFQILNTTEMITQLDLLMNNLQTTMPVILVVL
KLSVFRVKCRSARLIIADMLSDWKCINETKERKVMMKNAKIAFYLSSTIAICYNGLILSYLLKAILAYET
ENIYDRKYVMQATFPINAKSSPVFEMLCLFQFTVSVFAANGHAILEGLLTTSVLHANTKAFGVCQEITKF
AKSCEANKSRKNIVEAKRRLIKRHLYFINFAEKIQETYAYISFFHLFLMTLINCIVGYMFINLTINKDNI
SALLLCIAYMFTALSAVGSYCIAGEYLMSQGSLIFEKLYDCPWYKFKPVDTKTFIIMLMKSRHSVTITAG
NFGDLSLVYFTNIIKTSVSYLSLVRAATN

>NvOr66

MIPIFNKPLECCLKVAGFWPYDFNMLGPVAITSMLVTTLPFQCWNAFALTENLVVLMDSLSDIFTEVLIY
IKIFILWNHRREIRDLLEEIGKDWSIKSIPTEWENIADYCRIICNIDVIVYASASILYYPDLLMSYFGKP
VNERHMLFQSYYPFDYRRSPIYEVINIVYFFQGILMIIADSVSKTLFISMIFHVSSQIYELRNNLEQYSR
HSNDGYENKNFKRLKLVVQQHLKILSLVRRIDHIYSYVALFQIVFSSIIICVTGFVIITAMESANIMLLV
KFMTFIIAMLAQVSYFCFAGQYLLNKGESIVEMINSSFWYNSQCKDVKVLIFVLTNAQKPLTVSGANIFN
LSAETFTMIVKTSASYLSVLRAMYTQ

>NvOr67

MILLINKPLEYSLKLSGFWPFEFNIIGSLALISTLVTTLPFQCWQAFNFTNDFVLLMDSLSDILAEVLIF
LKLFAMWKSKSCITIILREIFDEWSTEKIPDEWKTLAYYSRMFCNIDTLVYFSAAASYYPDLLMSYFGKP
IENRKMLFQSCYPFNYLGSPTYELINLMQMIQAVAMMAADSLSKTLLVALILHVIANIDLLKNEIRIYST
NIANTCNHTNNKKSTVDLKQVISQHRKILYLVQSIDNAYSYVSLFQIVFSTIIICVTGFVIVTAMESANI
ILLFKFILYIIVMLSQAFTFCIAGQYLRNEGESIIHEIYDCLWYYTEPKEIKSLIFVLKSAQIPLTLGGG
KLFELSTNSFTMIVKTSVSYLSVLRAVCV

>NvOr68

MKIPIIGIPLEYTLKLAGLWPDQSNILGSIVMGSALVTMIPFQVWDTINVSDNLVMVMDNLSNILSEVLL
YTNFIVLLLNKSYLDDLLREIADDYKNNIVTEKWLKLDQNSRRFCNYDYGMYLGACCLFYLQFALMYTQM
PSEDRIMLLKAYYPFDYKSSPVFEIMCFIQVIQGLLMCSIQALSESLLIALVSHVSGHIDLMNKQINVVS
KSYDGQNSLTLKLVIKSHLKVLNLVNKIESVYTYVSLTQVCLSTFIICVTGFVVLTMNSANEIVVMIKYI
MLYFTLLWQSFSFCFAGQHLLNKSDMIPYQVYDALWYKAEATEMKAILFIIKRAQTPLSLSAGKFIALSA
QTFTLIIKTSFSYLSVLKASYA

>NvOr69

MKIPMVYWPLEYTLRINGLWPGENNILGSIVTASGMVLILPFQVWDAIKTIDNPILLMDSLSDIMTEIAL
YAKLIIMWFNRRYVVDVLKEISNDCNQNDVSQNWTLLNYNARRFCKYDYSWYISATLLYYIQLVTMYIEV
PVDGREMLLKSYYPFDYKSSPTYEIMLFLQIIILAMSMAIANAMTESLFIVLILHACSYVDLLLDEIKIFS
DNCNKKVLNITDSNNMRFYVHVILKRHIQLLESVKKIENIYSNVSLVQMFFSVITICVTGFVMITALESK
DIVLLIKFATFIWFLLWQIFSFCFAGQYLLNKGETITGAMYDSDWYNIESNDVKAISFIIKKTQRPLSVT
AGKYIPLSVTSFAAIVKTSFSYLSVLRASYVE

>NvOr70PSE

ALMALINKILDDRQAKKTIDESRVMINNFERSKSLGTFIIWLYNDILLTITLKPVISYIYDSVVNRQLIA
PVAFPKFFGDPKQSPTYEIVLIIIGQIGTTLFAVNIVILTYAILEGLLALSVFHACSIMDSVRQEIVKFS
NVCRLQSGNKVLIMTAMRRLIQVHIYSDEFSDDVDDLFTVIAFFHIFFVTLAQFFCAYMFICNIEDGGDA
VKTIHYGVMTIIMLISCGYYCITGEYLTSQNELFTVEIYNCFZNDFPFSQQKAIKFVLAESQRPVCLTFA
KFDQLNFLFLIKXNFSSFSYLSLVRKVH

>NvOr71

MYDEIFIRPHKISLKLIGAWPGYAKLTGFFLVIGSSSVLLFFALWNTIEVFGNLELLVDNLVNVIGIIVG
FFKLTTLRVKRRNLIFMVDTMFEDWQTSKKTIEELNAMKDHFERSKWLCKSIIMLYNSLILTFLLKPVRS
YMNDSIEGRQYLAPVSFPKFIDAKQSPIYEIVIIGEIGTAFFCINSHALVEGLLASTVLHASAKIAAVRQ
EIIRFSKVCRSQNSNKRLIISATRRLVQVHLSCNEFSETIVDIFAVISFFSILLMTLAQVFSGYMFIFNI
ENGGETVQTLHYGFLTIVFLVSSGYFCIAGEHLANQSELLTMEIYNCFWSEFRIPEQKAIRFILAQSQRP
VRLTLGKFDELNLVYLTKIIKTSFSYLSLVRRVR

>NvOr72

MDDEIFIRPYQISLKLVGAWPGCAKLSGFFFVTGWSSILLFFALWNTTEVYENLDFLVDNLVNVIAVVVG
LLKLTTLRVKRRTLMTILNKMLEDWQTMKMIEEFKAMTDNFERSKWICKSIVMLYNSLILTFLLKPAISY
MNDSVEHREYLAPVSFPKFMDAKQSPMYEIITAGEIVTTFLCLNSHALIEGLLASSVLHACSKVDAVRQE
IVKFSDVCRTQSGDKMLKLTAIRRLVNVHVNCDEFSENVENIFTVISFFHISLLTLMQVLSGYMFILNLE
EGGEILQTLHHGLIIIVILVSCGYYCIAGEYLTNQNELLNVEIYNCFWTEFPVPQQKAIKFILAKSQRPV
RLTFGKFDQLNLLCLTKIIKTSFSYLSLVRQVH

>NvOr73PSE

MGDTYNKNMQSIFITIIILMRLLGMWYVSNKREKIIADVLLGIVLASMIFSCMVQTNEFYHARKSLMLICS
AAPTFFVLFSEQMKLLVFCKNRERVMKLNKYTYEHFWNGKYNRKETKIFNDCNSWCIKALACFLVTMQCI
SVHLMLAPYLEPSDNSTSGEKIFPFPVYVNYSIFETPTYEILYVLEVLGLYGVVLCVMSFPIFLLVTNMF
TAVQFKMLNLRMRSLCQFPRNKNDDVNLQQQTNAYEKLKECIRKHQSLIHYVNEMENLYCYAMLGQILAS
IFQLSSTSITILLSNQGEESLNKAVLRILILVASLMQFYFYAYSSHEILTESEKISEAIYSSDWYQITHP
RYRKNFSLLVQIVMRMSMSFFTVMRQTIG

>NvOr74PSE

MGDTYNKNMQSIFITIIILMRLLGMWYVSNKREKIIADLLLGIVLVSMIFSCIVQTNEFYHARKSVMLLCS
AAPTFSSLSSELMKLFVCFKNRERVMQLNEYMYENFWNAKYNREEXKIFNECNSWCIEALACFLVTMQCI
SVHLMLAPHLEPSDNSTSGEKIFPFPLYVNYPIFNSPVYEILFVLEVLGLYGVVLRVLSFPIFLLVTNMF
TAVQFNMLNLQMQLLNELPKNKNGNVNLQVQEHAYEKIKDCILEHQLLIHYVDEIESLYSYAMLVQIFAS
ILQLSSASITILLSNQGEESLNKAVLRISMLVGSMMQVYFYAYSSHEILTESEKISDGIYSTGSYQITHP
RYQNYFSLLVQIIMRMSMSSDSIYSIIERVSISFVCKLILTSYTEYKFVKRY

>NvOr75PSE

MEKSNVSKKMQSIVITVVLMKVLGMWYVSNRKEKMIADALLSAVTASVLFSFVIQTNEFYNARNSLMMMC
SVAPTFCSLSSELIKLLVFCKNRQRVMKLNKYMYENFWNVEYNKEETVLFNKCNNWCIKALICYLILLQI
IVVHLTIAPYLEPRHNTTSREIELPFPIYIDYPIHETPVYEILFILEVFGLQGVILCLLAFPIFLLVTNM
FMAVQFKMLSLRMRSLCKFPNNDNSDNVDIKLQMHTYEKLKDCIRKHQLLIHHVDEMENLYCYAMLGQIF
ASICQVSSTSITILLSNQGEESMNYAVARIEILAASTLQFYIYAYSSHAILTESEKISEGIYSSDWFRIT
HPSYQKHFSLLVQIVMRMSMSFFTLLRQTIA

>NvOr76

MSTKKIASSIDSFLWPNRYTLEFLGFWPPEPGTSSISKYFAAFRIVFSILAIGFLFVPEIMMVVVFWGDI
TVLTGVGCVSTTLAQLNFKMLYVLARRRRFCRAYRKTRELWSMTDHESELRKGLEKLAGQAKKYSIAFFF
TCFCNNISFTTLSVVVWLNYNAQENKSLLERRLPFDVWFGFDLQRTPNFELVFVGQSISAIFCCFGIVGL
DTAMMALILHVCGHFRVIGARLRAIGQGMHNDVQSKNSVEYLHTSPKLAIWQCIQYHQQMIKFAEEVRSL
LSPIIFVQLLTSGLEICLSGYAVIVNSDAGNYGDLVKCTGYFLSVFIQLIIWCWPGQILIQDSSEIGRIV
LHDLPWWDMATEQQRQFVFVIFRTQKECQITALGFQVMSMSKLTDVFNTAGSYLALLRRVYEKETEE

>NvOr77

MAGRGSSVRIDEYLWPNRYLLELFGTWPTDYDGRTLASQLFVNFRVCFVFVAITGVLVPEILMIIVYWGD
LDVLTGVGCIATPVSLILFKVAYMIIRRNRFHGVYSNLRRLWLAIDDAEEFEPLEELARLAKRVTIGFFL
SCFSNNVSFTTAAVIDWVNYDETRNDSTPRHLPFDVWFSFDVERSPNFEIAFGCQVISSLYCCTGIVGID
ATMMTFILHICGHFRTIAAKWRAIGSKILDNEKYSKSGQVMPVKKDINQILRQHSEMLRIAEEVRRLLAP
IIFMQLLTSGLGICLSVYAVTMNGSKGADLFKFIVFFVSIFVGLIIWCWPGQLLMQDSAALGDVVCYELP
WHLLGVAEQRNLAFFIMRAQKECQITALGFQVLSMNKFTEIFNSAGSYFALLRTIHEKQLEAQ

>NvOr78

MARSFASFDEYTFLNRWGLTFLGIWKSDAEARGGPLRRFLHRLHVTILFTLLMLLLLPQWMDMYVLWGNI
DANAETFVLNVFTITALLKLWCFLSARQIFEQVIDTMKENWRRTMSGDEPGRKTHREILLDMAGKARDYT
KRYGLLMYSTATMYFVSPFVGMQRDNVRIRKYPFFGWYYFDRFSNLYYGICYASQVIIGIVVGTSNYAMD
SIFLVAIYHTCARLQMLQHDLKKIGEDRENRSPEEIVQLIRLHQREIRDAKRLTKIFNGSSLQQLLVSCV
IICIIGFKLIIALNDGGFEFLVYVAFMFVALLQIFLYCRPGDELIVQSTAVGYAAYQSHWTSLEAESIRK
IMFMILRSQTSLKMTAGNFYVLSLPNFTMILRMSMSFLSLLRAMYRKSDGFG

>NvOr79

MRIGARRASRMESTTEASGIMREYDDCIFLNRLGLTMVGIWPLEHNASRLRIVLRRIHLGAIYVLMLSVV
IPQWFDIYCLWGNIDANTETFMSNVFMIAVMIKISNFLNSMRLFEDVLRTMRLNWLDVMRLSSGELEKKE
IMQGLSMKARSRGRVYGLVVVMTGAMYGLMPLIGSNKVASLRDRSYPFFGRYLFDRNSDTVYRLCYLSQL
MSGSVTAVANFATDAIFLFCVYHFCAQLRILQTDLLKLGGPRFDSREALVQLIRRHQKEIRNVRALQSLF
SISSLQQLFLSCLMICLNGFKLIVSLCNREVDILMYIVCLPVTLFQILFYCQPGNELIVQSQSLDEAIQQ
SHWVNLDRLSKRQLFFMIQRSQKPLAITAGKIYVLSLENFMRIVKTAMSALSVLQAMYRKTGS

>NvOr80

MHCSYSFFLVTAFAMWRPRSWDDSKILTALYTLYSILSFTVYYTFLISQILDIVLLAENIQQITENMIQL
INVVNVSQKSLCFFLKRKKIIRFMDYFFEDMTLPQSPREKEIQKSFDDESKGNSQKLFVLYSVSVVMYVY
MPFFISKREDRVLPYRAWRPYSLDNVNYYYLAYLHQSWSVTIAATGNAATETLVSGFMIQICAQFEILEH
RFMQLPKILKEMRENGESESTVLATERSIIIKLIHHHWRIFEMTELFNDIFVFVILSQFVTSITVLCVST
YNLALCKSVNNDFVTIFMYLLCMLLQIFMYTWYGNEITLRSCDLGNRIFLSEWRSLNPPTVKNLLIIAQR
TMKPIILSSGYVITLSNVAFTSIVKTSYSVFNVLNV

>NvOr81

MHILSLTFTFFKIYGFWRPLSWKSPTLGFLYDVYTFVMFMIVFTFALSQLMSIILTVQTVDEFTSSSFIL
LSIVSACFKASNLLLKRKSLVRLLNVLISTTCKYQDDDEKMIQDMFDKKARRNTVWYMALIQSSVFMITL
QSIFINIPQKTLPFPAWLPYNYSNTRLYAISYTHQVIGNAASATLHAANDALISGIMLQICAQLEILKHR
ILKLPTIVLKMNSGKEAPMNTVASKESELLGNIIKHHNCIFQFSKDINDTFSMALFAQFFIAALVICSSV
YELSKIVLLSSDFVALLSYLSCMLVQIFLYCWYGTAVTMKSWSVGDTIFATDWSPLSMGLKKSLLIVMIR
AKKPIELKTGKIFTLSILTFAKIIKASYSAFNFMQQA

>NvOr82

MRVLPITFGILTVCGFWRPISLESSIPKQMYNCYSIFMCFLIYTFTLSHLIDIVISAADFESLTGSCFML
LSMMNVCCKMTNILYFRKNIVELLQILASDHCTAKDVVERDIEKKFHKRARSVTLCYWILTETTCMLITL

RTFFGSSKQILPFKAWIPYEITGLAVYWTTFFHQTIAHVAAANLQIANETLICGLMIQACSQLEILKYRL
KKIPDESKIDKFPLQSTVNNAQNTNKKDTKTLLVNCIDHHRRIIEFSEKLNSTFNVILFVQFAISSLVLC
SSVYLLSKMKLVSVHFMSLSLYLSCMLYQIFLFCWYGNEVILQSLDLGNAVYHMDWTILSTEDKKKLLIV
ILLVRKPIQFTSSFLVSLSIESYCKILKTSYSVFNLLQRTSI

>NvOr83PSE

LSACGTWQPLHWQERRRLRYLYELYSAIAVTCVNVIVLLQGALLFLNEFDVNFVADILFTLLCAVSIATK
SLNFLIKRDRVIDLANMLTRSCCLPRSAVEIQMEKESDEFLSXFAIHFNGLAQVAVALLAAMPLVQEPGE
RDLPYRMWLLYDIHDALNYWITYAVIAGVIIIGVLINVSVDVVISGFVLKACMQLDMLKHRLNRLSGIVR
RAEEKGAASRQALISFEQATLRQAVLHHDYIIKSRDIRRGDSRTVFAGALAFSIICXYILTIGKVAVLEK
ILAIVYLICTMGELFAYCWFGNEITVKSLEFSTDIYNIDWTALSQKTKKMLIIIMMRTNKPIVISYGYLI
VLNIDSFKSILKVTYTAFNVIKESS

>NvOr84INT

MMRKLTFAFKVLSMCGVWLPVDWTSPRRIFYYKIFSCVVLIACYTIVTLQCLLLSVTEFDFSFIADILFT
FLTSITVCTKTMNFIAKRPDVIRLADMLLLGCCLPRDKEEAAIQKQCDEFIRVFTIALNIMAQSTLACWL
LIPLFQSPELRSLPFQIWLPYDTRGSRNFWITYAYEIWPMIMGVLVNATVDVVVSGFILQACAQLDILKY
RLTRLPELVKQAEESNASLVSIRKFERTTLEMASRHHAHIIENVNETFNMIIVEQFFASSLILSVIIYAL
TKSNVNTLQYVMDVGYLLCMLGRVFQVTIYRIDWTLLSAEASRHLLFIMLRTSKPIVMSCGHFVVLNVES
FKSILKVSYSAFNFLKESAD

>NvOr85

MRSLTFTFKVLSLCGIWLPLHWQSHRRLRLFYKIFSISTVVLTNIFILLQGLLLALSEFDWQFLAEILFT
LLTAFSVSFKATNFLMRRDKIICLADMLLKSWCIPRNAVEIEMESRINEFLRVFTIYFNALAQLSLACLL
IMPLVQDPDKRELPFRMWLPYDIRNQWNYWSTYVIEVGPMIVGILLNVTTDVVVSGFVLQACIQLDMLKH
RLNKLPNIVKVAKRKRLASEEVVRSFERKTLHQAARHHDYIIKYAKVVTETFDVVIVEQFFAGALIFSVI
IYVLTIGKVPILQKLMSVGYLICMLGELFAYCWFGNEITLKSLEFSDDIYKIDWMALSDSSNKKLIFIMM
RATQPIIMSYGHLVILNIESFKSILKITYTAFNILKESTSTT

>NvOr86

MLELPYKLLILTGIWMPEDWTHKHQKLGWLIFSIISIGLVFMQFSSLVIFLMISKSCAQFFERVFLIPAG
VSSLQKIYIFITHRKELIDLGKMLLKDYCIPRNFEELSIQHRYEELIRVLTLVCFVLVNITMMNLLVLPL
VTNGENRTLPMNVWLPYPVDSDASYWLTYTHQTLGTLLLGTGAVGSTLMINGFMHQVCCQFEILSSRFQK
LPQIIKRLQLLKKPNHLIYEYEKKSMKQYVQHHLYIFRVADTINDIFKSVIFQQFCISSIVVSASIFQLS
TRPDKDMEFIMVFCYLICVLVEFLIYSWFGNELMLESLHFQTSVYQIDWTALSIGSGKDLVFIMMRASKP
VIMYCGHFIILSLESYLGILKASYSVFNILRRSSN

>NvOr87

MHILYLPFKLLTLTGIWMPEDWTQKQQKLIWVLYSMVSIGLVFMQLSSQIGYLMQSKTWAQVNERLFFIP
TGISSVHKIFIFIVHRKDLISLGNMLLKEYCIPRNAEELSIQERYNEIIRVLTLACAFLVNVTMMNLVTL
PLVTSGDNRTLPMRVWLPYKVDSDMSYWLSYAHQTVGIVFVGTGAVGSTLMINGFMYQVCCQFEILSSRF
QNLPLIIEKFQSLKKPNQLIYRYEKRVMRQNIRHHLYIFRFAEALNKIFKSVIFQQFCLSSIVVSVSIYQ
LSTRPEKDLEFIMVFFYLVCVLVEFLVYSWFGNELMLESLNFQQTIYEIDWTSLSTRSSRDLVLIMMRAS
KPIIMYCGHFIVLSLESYIGILKVSYSVFNILRMSEE

>NvOr88

MHTILQLPFKLMTLTGIWMPKEFTSQYEKQGWTLYSIASITLMAIQSLTLSITLILSENSEQFFETLFIV
PTGLQNLQKIYVVVAHRKKLMDLEKMFSNDYCIPRNVEELLIQRKYDENIRILTLSCIILMNLTVANLIA
SPLFDAYFTTMNTRTLPMRIWLPYKMDLNIIFWLTFIQQSVGVIFVGYCIISTTLMINGFMYHVCCQFRI
LSCRFKKLPQVIDYFRSLKKPYNVIYQYERRAIKQNVQHHLCIFRIAENINDTFKSVIFQQFCISSIVVS

ASIFQLSTRQEIDMEFFMVLFYLICVLVDFYIYSWFGNQLMLESLNFQRSIYEIDWTTLSTNAGKDLVFI
MMRASKPILMYCGHFVVLSLESYVGILKVSYSVLNLFRRSK

>NvOr89

MEIIEQLEKMRILQVPFKVLTWSGVWMPEDWTQNQRKLKYNLFSFVCIGLMTIQSCSLTVYLMMSKTWSQ
FVETLFLIPPGLSNLQKIFVIMLHRKKVIDLVNMFENGHCIPRTADEWSIQQRYDATIRVVTLVCFVLVN
VTMVNMVTTPLFLKADERILPMKVWLPYSIETDFFYWLSYMHQTLGVTLVGSGIIGSTLLINGFVYQVCC
QFEILSSRLEKLPQIIRNLRSLKKSDHLVHQYELKLIKQIVQHHLYLFSIAETVNEIFKSVIFQQFCVSS
IVVSASIFQLSTKPDTKTEFIMVLFYSICLLVELFIYCWFGNKLMFESLNFHQAVYDADWTVLSNESGKD
LMFIMMRASKPIIMYCGHFIVLSLETFLSILKVSYSVFNVLRRSHG

>NvOr90PSE

QALPAEVWTPYNHTSTSSLYWLSYAINSICAVSMGNLSIGTDVAIYGLMLATNAQLELLAHRLVNLSNZA
NTEKGIMLTGPSRVRRENFLLGQSVRYHVFIYDIYQLTKDTSASSPAFYVMLFYLSGLMTQLFMFCWFGN
EVTVRSQELGMTILEASWTGLSTQSLKAILLISLQRLGLFLYLEGTLLRSLWRRLKRILKVSYTAFNVLR
DSTYEE

>NvOr91PSE

MDGFNPAFEILTCFGFWKPTSWPTPWSERFYDGIRVMLVSLLYYLALGQILRLLLDTISIDEMADTLFSM
MSTVNACCKLTNMHLRNKQIDELMDMLRIEWTKPGNKEENVIYNGFNDIIGYVTLVEPATLLDLSIPIIV
GKAEQSLPLEVWTPYNXNVRRIDGQLVHWHRRYYIYGLMLATSAQLELLAHRLVNLSKYANKEEGMMLSR
SSRLRKENYLLAQSVRHHVFIYDVHQLTKDASASSPEFYMMLFYLSSVMTQLFLFCWFGNELMIRSQELE
TTILESDWTTLSLQSTRSILLISLRTSKPILISRGYFVPFSLETFKRILKVSYTAFNELRDSSYYDE

>NvOr92

MQSLKVSFTILTYCGIWQPIYWTSGWHRTSFNFCRVVFRPLPYLLASAQLARIALVDMSFEELTEVIFIL
LSIVNICCKSVSILMRRADLIKLTKMLGIVSASPQDSDEFNIQHQYHQFIRYVTLSSLVLVEITAITFLI
PPFFQPENNRTLPFKIWLPYDYSMDKLFWITYFPESITIILASLISVSSNTLIFGFLIEACGQFELLNHR
FMTMPLYIEDFAKGEKITTYEVCKLEKQLLSRNIRHHTFIFEFVDIFKKTFSSAIIGQYIVSSLVISTSV
YQLSTNTTMDVVFFTNLLYLMCMLLEFFLYCWFGNELTVKSEDFGRKVFRTNWLALSTKSNKDIFVAMLR
SSKPIIVSTGFFAVLSLESFMKIIKLSFSAFNVLRTASDYQ

>NvOr93

MHVLPESFMMFTCAGVWQPVHWSACDSRFLLYKLYTLFSIVLVYTLTISELMGAILLTQSLEDFTDISFL
LISTISVCCKIASIIARRDRVIHLTEMLLEVQCIPKNVRELEITRKFDKIARFTALSCIVLAEATVVVMS
TGPLFQKAENRTLPFKSWLPYDSTTTPCTFWLSYVHQTAAIVLCATVNVANDSLICGFMTHSCSQLELLN
RRLLELPRAVKLKMKKLPRRLMCNVEAMIVSRHVKHHVHIFKFAENINVIFTPVILVQFCMSSIVLSLSV
YQLAVRSANGIQFITMVMYLTCMLVQFFMYCWFGNEVTLKSVEFGQAIYNIEWTSLQVQTSKDLMIMMIR
AKRPIIMSSGALVTLSIKSFTSILKASYSTFNVLQRSSHN

>NvOr94

MHVLPEAFNLATYIGLWEPTHLESSIARCFYKFYTCLSFALIIILTMITQILAMLFFTKTLDEFAETAYML
LSAINASVKGVVILLRRKHVIDLAEMLLKKECVPINATEKRVCSYFNKISRYTVLSCIVLAEGTISALAL
LPVVFEQGELVLPLRAWYPYNAGSGLGYWLSYLHQAMALTLIAAYDVANDTIITGFMVQACAQLELMTCR
FHRFSWRGSNAVMRNGARHQLRLFEKRMVAQSVRHHLLIFRFTEIINSIFAPVILVQFCLSSGVLCITVY
QMSASKSNGLKVIVLSLYLVSMLVEFFLYCWFGNEVTLKSLGFNIAVCEMDWTAMHVQTLKELLIIMVRS
TSPIFLSCGPLIKLSLESFTNILKISYSAFNVLKQFD

>NvOr95PSE

MLSVQFNVMTIAGIWCPPNWPSTWMRILYKLYSFVVNMLMYTLGLFQLARLIFVKQSFKEFNDTFFLLLS
TNFTCIKTACHLLIQKRVINLTNMFKEDCCIVRRDGEIKVQKKFYDISRXPYDLSSPVVYZLSFLHQSIA
AILLAAIGIINDALIAGFMLQVSDQLEISAHRIQEMPNYVTEALKVRKSSDEVRLLEKRLLEENVKHHNH
IFRIADSLCMTFYEIVVCQFLVSVLAICVSVYQISVGSSNKVELITFVLYLVCMLQQLFVYCYFGCEITK
QSKNIGSEILGMDWSSLNNSVVLSIXLTCHTFYNIKQKSLHIDSYIV

>NvOr96

MLSIHFQVLTISGVWCPNHSSSVQRIFYKCYSFIVVVLMYSLALSQLARIIFVKQSFNEFNDTFFISLST
NFACFKAASNLVNQKQIVSLVNMFKHNCCLAHNDSERSIQKQYNDSCRKIIISLLILVETSAFFVVVAPL
CGTMDNQDLPYQVLLPYDLSNKLFFWLTFVHHSFGAVLFTAISITNDAVITGFMMHVCGQLIILQHRFAL
LSRSLANEVSKKGRITDFDMMLERHWLRQIVYHQNHISNIAKKICSTFNEIVICQFFISGLEICVSVYQL
SVRNNNTVELCTYAIYLMVMLGQFFVYCYFGNEITLQSKITHRAIFDIDWTSFSLSLKKDLTLIMLYSSK
PIAMSCGPFAHLTLESFTNILKTSYSIFSVLKTAT

>NvOr97CTE

MHTIRLPFTILKYIGIWKPNSWKSTWKGYQYDCWSLAVMMIMYSFVATELIAVVKLISDHYDEISDTLFL
LLTTAGVSIKSMNFLANRDNMAHLGDMLLKSCCIPKNLNELKIQKKFDDVNRFVTLMCITMVYGNIMTMM
TIPLLQSKEHRNLPFKTWLPYDTRSNSLNYWLSYLHQGLGLACCGTMGVMIVNIITGFMQQACAQFEILD
SRWRNLPKIVEIARSRWSDSAADEHENIMLSQYIRHHIHVYEYMNEFVKTFNIIILVQFCVSSVVITISV
YQMSIKSLGLEWFMVFGYAVSMLTEFFLYCWFGNEVTLKSMDFALKIYDTEWNLLNIKSWKLILFVTHRT
RKPIVVRCYNYIVLSLDTYVN

>NvOr98

MNNQQMNEDLAPIPFRILKFCGWWRPLNMSTWRRAVYSCFTVIMLTLLVTITLTVLIGVTQMSATDDLFA
DNVFLMFALINSVFKATNVLLSRRRFIKMLEIVQDTRWRDLRNDEEIEIQDRYRKTIRKISVYFTTAVFV
AIILRVVAPLLDLSDEIKLPVDAYCPCDIRHSSCYWTLYWHQALGTGVATLTHAAKDCLISALLLQTCAQ
LEILKNRLLSIADTCVVAGNKTGAADRVEKLEQKLIGDCVRDHESIFEFAKILNDSLNVMLFGQIAVTIP
NLCLSIYLLSTQKIASMDFMMTTQFFSAVVIELFFFCWYGNEVTLNSLDVENAISEMDWTLLSTRSKKDL
LMMMVRTSRPILFRVGPIMNMNIDSFLSIMKTSYSAFSVLQSTGD

>NvOr99

MKFQDSIEYQLLPIPFMVLTLCGTWCPENWSKKRKRIYKCVTTVLVSLGIIILLVEMLVFIIVKSGKDNID
LENIFATICIAVGLYKKINILYHRPKLMNFISNYTKNEWNKPKNFEEATIHLNILSETRYISYAYAAFIL
VSIIFRSITPILESGTFIILPLDACYPYNADNFIAFSLTYLHQIISGVTLTCMHIGTDTLFVGLLLQMNY
QLHILKNRLRQLGNSKTYKNNTQTIKDRELFIKSKISQRVREHESIFRFGYDLQKTFKPILMAQMVVVVP
SVIINVYFLSIYTDRLNLKYFMTFFFALVSLMQIYMFCWYGNEILLSSSDVGDALYESNWFALDQSTKKI
MLTMITRSSKIFLISAVAIIPLDIDTFIKIMKTSYSAFNLLQRTTAQ

>NvOr100

MHEKLIAIQKANVEYELLPFQFLLLTIWGIWHPKDWPVRLKNISNIIFIVVFCLDIIICFEMSIYLVLSI
GTNDFKLVNIFFTSATITGIYKAIKTMQIRESFRTILLNYFNYEHLCSLNTKERMIRESNQAQIRKVTVI
YSASMAGIFALNAIAPALSQPDSTMQLPVDAWYPYSIQKSLNYWLTYFHQIILGSSLICVHIGTDTLFVG
LLLKLVCQINILRYRLQSLTSLCSKNFEHFNAMGRKFIYRYIHHQNEIYEFSKVLNNKFQAVLLIQVITS
IPNLCINVYTLSKYSGIINMDYISIFFNTTSSLIQLFITCWYGNEVLLSSLQIKKSIYEMDWTKLDVPTK
KLLIVIMARSLRPIAFSVAHVIPMNIESFIKIIKISNSAFNVLQQT

>NvOr101

MHLKFVGILRSNIEYELLPFQFVVLTVWNIWCPKDWPRRLKNTSIILFIVILILNFIMCTEMLIYFILSI
GTEQFKLTNLFFVSASITGVYKSLKIMKNRKIIRCFVRNYFNHQWIKLLDDEENEIHEKINTRIRHITVT
YFISMISIILMKDLGPIAESGLAIQLPADGWYPYDIENSVLFGITYVHQVILGSFVICAHVGIDTLFVGL

LLKLLGQINILKHRLQILGNSLDHKMISLNKFESFQTVQKHLILECIHHHKRIYRFGEDLNKIFQEMLLI
LVVSSLPNICINIYALSSNLKNINMDYIATFFSTTSAFIQFFIACWFGNEVSLNSVEVRNAVYAMDWNKL
DTPTQKLLIVVMARSLKPIEFSVGYIIPMNVDSFLKIIKASYTAFNLLQQTSSS

>NvOr102

MHSTLAAIVKNNIEYKILPFQFFLLTFLGIWCPSNWSLKSKTAHNVYFTFIFFLDFLICIEMFIHFVSSF
GTDNFKLINFFLVSANITAVYKSIRLMQNREVLRYFIISYFDYEWTKSHDSVEHEINSKIDLRIRRVTVI
YSASMIGIVLLKAMSPIAESNGISLPVDAWYPYSIEKSRWFWITYLHQVILGSSAVGAHIGIDTLFVGLL
LKTSGQIHLLNYRLRNLMLLKECNFAKLKEYSEKNVVLRCIYHHKRIYRFGGDLNDKFQEILFILVVSSL
PNICINVYSLSSYKGNINVQYIATIFSTTSALLQFFIACWYGNETTFDSLQVINAVYEMDWTNFHVSTKR
LLIFIMLRASKPLKFSVAYIIPMNLDSFIKIIKASYTAFNLLQQTTN

>NvOr103

MDLSQCLEYRALPMQFYIFTLSGVWCPSNWTSLLKLSYNMYTTTIAISGILFWASMFVNLIITKNESEYF
YENVFAISTLTYAMYKEFFVLKKRKEIQQMLKLSFDDEWYRPFDNREIQIIDHYAHETRWVTQVYAIGII
AGLATKAIMPMLNSNSAWVLPIEAWYPYNTSNLKNYLFAYTQQLMGGIPLICLHISVDSLFVGLILQMCI
QLKLLQYRLQKTFSTDIDLQEEKNIERNIKISDVIIANYAFKHQCIFRLGNYLNQEFRGILAGQVMITIP
NICINVYLLSQHRGGITLHLVDSFLCFTTCLMQIFLYCWYGNKIILLSIDVANTAYTTNWLSLNISSKKK
LLTIMVRATRSIQFAAGTFIMNIDSFIEIIKTSYSAYRVLQKTS

>NvOr104PSE

MEKYTNPGVTRILKSNIEYKLLPFQFLLLTFWGIWRPQTWSLQIKNVHEMYFALIFFLDSRISIEMLIYF
ILSFGTDNFKLINVFFVSANITGVYKAIKIMKNRENIRNLIKSYFDYEWMNPRDAHEDKIHKKSZHENXZ
VTMTYSTSMIVIVFLKALSPLAEVNIIIPLPVEAWYPYPIGNPYWFWITYLNQVILGSSAVSAHVGIDTL
FVGLLLKSSCQIDVLKYRLQNLTPINIKIKLIKNEHLKIXIFECIHYHERICRFGDDLNSQFQDMLIILK
IPNICINVYALSAYRDYINLQYIATFFCTTSAFLEFFIVCWYGNDVSLNSIQVKNAIYEMDWTICCMFQL
KKFIIIMARSLKPIQFSVGSVLPMNLDSFIM

>NvOr105

MIIRKTLEHQVLPIPFHILTLWGIWCPEHVQPRLRRFYFAFTCIVIISEILLTTEVFINLIIIIRNKRFE
LDVFFILTSLMNGLYKALNILLTRKRIAKLITIGFEDRWRFPRDDSEKKILQNYKFESWRIHLIYAGACL
AGVTIKLVGPMMKQNADIEFPAPAWYPYDTNKPVYFWLAYVQQMFVGGATISMHIGADTMLSGLMLQSCI
QLKLLKHRFKHFFQHYEQVKGRLHLSSTKRKVEIALMKQYICDHQFVYSYANKINRNFSGWLIAVLIVVV
PNICINVYLLSFSKIGLNVDFITSLGLFSISLFQIYLPCWYGNEVMLHSSEIANSIYDMDWVRLSPTARK
TLIIVMIRSSKPIQIRAGYFVSMNLRSFLSIMKTSYSALSVLQQTT

>NvOr106

MTTTTIIERTGSADVYGIENRLFSISFNVIKLSGFWRPTTFRKPFDYLYEMYTLFCLVGILMLIATIIVD
NVVTEKSIRSLIENLYLILTVSNGISKLCNIYHRRDRVISMLQRSSEDRWSVHRDEEEARIVEESIESES
YIIRFCIYLVTINNVSNALNPILNPDPEHDLMVDAYSPCDRSKSALCFWTAYLYQVFGYVSTSLVHVGCD
CLVFNFVDRLCAHLKILEHRILQLPDLVEANACDEIRYLKSCIEDHHSICEGIKELNDTFYETIFIQFVT
SISVLCTNIYLLSMQDLFSAEFIAVFVYLCCAFVQNFFYCWYGYKVSVNTLHISDAIFNMNWCILKRESK
KILSYVMMKTSQKVFLFNSAVVTLTPESFVNILKVSYSAFNILQQTK

>NvOr107

MEVMDTIKSTDIMPLPFFYLKLSGAWKPSSWPSYLRLIYDSYTILMTFFIMKVIIVTEILYVIFAEENQS
KVLKDNVYIICTFINGWFKMFNLICRRKNIANLVKGCIAKQWNPPRDNYESSVLAATKQTSRKITLAHAS
VVGSCVVSTLLNSVLSSPPFLPVDAWYPCNITLPICFWTSFVHQSIGYTVTAIVHVANDNIVVGFMMQIC
AQLNVLNRRLLLVHVEVEKAARQQKDQSQITSLETTLVNDCIVNYRDILKFAEQLSETFIETIFIQFCAG

LSVICTSVYVLTTLNIFSFEFFGMFLYLWCMLGQMFLYCWFGNEVVLNSSKLFHSIYNMDWIKLQSQTQT
KLLFMMLVASSPIQLFRGAIIRVNLDAFINILKFSYSAFNILH

>NvOr108PSE

YITLAYATSVGTCVLIIILLNPTVSADAWATPVYSWIPCNINFSSCFWACYLHQSVGTATIAIVHVACETL
VTGFMLQICAQLNVLNHRVLSIDLKIRNLTLREKDENRILMAETFLTNACVADHNGILKFSKLLSETFIQ
VLVIQFCASLTVFCTSIYMLTKIKVNTLDFLLMTTYLICMLNQILLYCWYGNEVMLNSQKLVQSIYNADW
IALHGKTQKTLLLMMLVASSPIQLFEGAIIKVNLDAFLNILKFSYSAFNILHSEDHN

>NvOr109PSE

YISLSYATLVCGNCLNFIIIPIVSATWTPPLYAWYPCNMSISICYWSCYLHQSTGFLTIGVVHVACETLV
TGFILQICAQLEVLNHRVWSINFKIQNLALREKDKNTIFLYESFLTNACITDHNSILKFAELLSETFLQV
LFIQFCASLSVIGTSIYLLTTIKVYSADFVITTLYLICLLNQMLLYCWYSNQVLLNSRELFRSIYNMDWI
SLHSKTQKTLLLMMLMASSPIQLFKGAIIKVNLDAFLNVLKFSYSAFNILKNPSKDLN

>NvOr110

MKEMNPAKSVDVLSTSFLYFKLIGAWRPLNLPKWLRVIYDLFTISMVILMYEMLIVTEILAIIFAEENRL
KVFQDIVHITITHVSGCFKMLFVINRRQSIMLLVNGCVAKQWYPPRNELEATILTKHNNLSRRITLTYAT
LVGASLLAAVLNPILYSTRVLTIATWYPCNISLPICYWSSYAHQTMGILAMAIAHVATDSLIVGFTIKIC
TQLNVLNQRLLSINFQLENTSARCQKSQEQSLALEAILVNECIVNYKDILRFADLLSRTFIEIVFIQFCV
GLTVICSTVYLLAKLSIFSYDFFGLFLYLGCMLMQMFLFCWYGNEVVLDSTKLFHTIYNINWIELQIQTQ
SKLLLMMLVASSPIQLFRGAIIKVNLDAFINILKFSYSAFNLLQKSS

>NvOr111

MDEIIDPIKRTDVLPISFLYLKLVGAWKPLDLPKCLRLIYDLFTIFMVIFICKLLIISDILCVVFAEENR
FAVFKGIVHVTITHLSGWIKMLHVLSRRRSIMLLVNGCVAKQWNPPRDRHEASILTSFDNSSRRTTIAYT
IQVSAAVSMLVLSPVFSSTWFLPIDNWYPCNISSPICFWPSYVHQSMGIVAIAVAHVATDTLIVGFMIQI
CTQLNILNHRLLSIHIKLEDTARRQKNQEQISAVETLLVNECISHYIDILKFADLLSKTFVEVVFIQFCV
GFSVICSIVYLLAILSILSFDFFGTLFYLGGMLSQMFIYCWYGNEVVLNSTKLFHTIYNMNWVAFQIKTQ
KKLLLMMLVALSPIQLFEGAIIKVNLDAFINVLKFSYSAFNILQKSS

>NvOr112PSE

SNVGDVEKKTFPRTFALLIIGGLWAPTTIKZRALFICYQIYAVFYFISALMMIITIVIDNILSDDKSMEY
LMENWZKLIVFFNGLQRITNLAVRRDKILHLLRSNIMSGRWQIIEKSRISEIFILKIWGSLIFVNGISSW
LNPIVHENPENKLMYECYSPRDRRTPTCFWIAYAYQLFGYLVLSAANVGTDCLIYNFIDRINAHMMIFLN
RLLKLPTRVRDTAAQDDVVAFHYENKYIRECIEDHHDIYESIEELNRIFNELISIQFLSCISLLCMNIYF
LSKQELFSLPFIAVSAFL

>NvOr113

MMSEIFPTTYFFLKISGFWRPYSLKLPLYLCYQIYTAFSFATVLSLIIILLVLYCAFAHDKFLELLLENMY
LVISFSNCISKTSNIILRKKNVEKLLQWIREKRWLAERDLEESCIVAHSKLMEKAIPQFCTLLVCANGIG
NLMNPIIRANPDKKLVIEAYPICDRSRPVCFWLTYLHQCFGFVIVNVIHVACDCLIYNFIDRTCAHLKIL
GHRLQKLPVLVKGIRHQGIDTVEFEKSYVIDCIKNHQGIFMFIRELNDTFCETVFFQFLSSILVLCTNIF
LLSKQELFSPEFIAVFSYFCCVLAQNFFYCWYGYKLSVNSLAFVDAIAKINWVELDMKTKKMLVYMMLIT
SNKVELFNNAVVNLSPASFINIVKISYSAFTVLQRTSHKEMKI

>NvOr114

MLRTEELLANSRANERRNERVGIEDQVFPATFLLLKAAGVWTPTTLKLRSQYMCYRIYSAFCFISVLALV
VTVSIENVVSSNASILESWYMLVIFSHGLLKIKNLQWRRVKVIHLLKECIMNERWSIARNQDERAIINES
KRAEKFITHLWLSLLLVNGLGNALNPLIHENPNNSLIFECYSPCDRSLPSCFWTAYAYQLFGYAISSTVH

VGCDCLIFNFIERINAHTMIFIDRLQKLPSRVVEGKNEGCLDASRHEARLLKECIQDHRRIYESVEELNN
TFYEVVTIQFLTTISIVCTNIYFLSKQELFSADFIGVLVFLVCVLTQNFIFCWYGYKLSESSSYIVNAIF
NMDWLVLNKRSKGLLLFAMMSASNEIKIFHNALVNLSPETFLQFVKMSYSAFNLLQQSN

>NvOr115

MHQAIKIANGRIDADGIPNVEGLEKRVFPRTFLLLIVGGMWAPTTIKSRALFACYQLYTVFCFVSVCMLI
ITILIDNVLSDDKTMESLVEYAYMLIVFSNGLVRIINLVSRRDKILRLLQGNIMLDRWQSLRDDEELAII
AESKVSEKLVLKIWGSLILMNGISNAVNPIIHENPDNTLMFECYSPCDRSVSYCFWMTYSYQLFGYVIMS
VAHLGVDCLIYNIIDQINSHYKIFLNRLLKLPARVREKARDDVAAALRYENNYIKECVADHHSIYKAAGE
LNDIFNELVFIQFISCISLLCTNIYFLSKQELFSPPFIAVFAFLCCALTQNFFFCLFGNKLSQTGSEIAG
AIFGMDWQELQKETRRKLLFIMLLTSKGIALFNNAVVNLSPETFLKLVKVSYSAFNLLNQSTHK

>NvOr116PSE

MRRTSEIEVADIDGVEKKIFRRTFILLIAGGLWTPTTTKSQALSTCNQIYTVFIFVSVLMLIVTILIDDT
MEALIEYGHVLIVNLARSNIVLNKWLNCRDDEELSDNCQIRNLGXLVLKIWGSLILVNDISNTLNPIIRE
NPENTLMFECYSPCDRSISSCFWMTYSYQLFGLYXNAAHVGVDCLIFNFIDRIDAHYKIFLXQVAQIAGS
SQGKARDDVAAALLYENNYIKECVADHHSIYXSSKIADAIFGIDWQDFQKETRRKLLFIMLLTSKEIDLF
NNAIVNLSPETFLRLLKVSYSAFNLTRMTIPPRRTLFYIQL

>NvOr117

MDLLPVHFRTFQFFGLWYNDPCSYRLIKLVHRSLIVLLIVHLSLFQMIALFSAKRNVDEYTNTLFLALTY
FVHIYKTLVFMAKNRSVNEMLDEFRSDICRTRGPEEEHILAKHVQRANWAYSGRMILTLFAGSIRVVLPI
LIGFSTGKLELLPFDTYFFNVKHLVQYALVYVLQTLAIITVIVTDVCLDSTPCACMILACAQLEICRHRI
KHDNMVLYENSEDGPGRGFNEEMALKEYVKHYVLIQEAVHRIQSVFIAIVLPIFSSALLTLCTSIFQLAQ
KNHTTGEYCFIILYLCCLLVQTFSLCWFGNELQSKGEIVTSAVYETDWTVLKPCLKKSYRYLMFMGQNKF
IISFHGQCTLTLQTFIWMIKTSYGAFNLLKQVADT

>NvOr118

MDLLPMHFRTFRFFGLWYDDPRSYGLAKLVHRSLVVILIVHMSLFQMIALFSVKHSVDEYTNTLFIALTY
FVNIYKILAFMAKNRSVNEMLDKFRLDVCRTRDAEEERILAQYLHTANWTYSARMILTLCTGVIQIVVPF
LIGYFTGKVGLLPFDTFFFNVEDLAQYALVYALQAVAIVTVVITDVTLDSTPCACMILACAQLEICRYRI
KHDNNVIANEVTGDGEMNSECKPGKELREKMALKKYVKHYVLMREIVDRIQSVFISIVLPIFCSALLTLC
FSTFQLAQKNQTTGEYCFIITYLCCLLVQIFCLCWFGNELQFKGEIVSNAVYETDWTVMKPRIKKSYWYL
MFMGQNKFIISFHGQCTLTLQTFIWMIKASYGAFNLLNQVADTKY

>NvOr119

MDLLPMHFRTFQFFGLWYSDWRSYRFFKLVHRSLELLLIVHVCLLQIIALFSVKHSVEEYTNSLFIGLTH
FANIYKTVVFMVKNQSINEMLDKFRLDICRARGREEEQILAKYLHKANWTYSARMILTLFCGSISIVVPI
LVGIFTGKLELLPLDTYFFNVNDLKQWTLAYVLQSLTVITVVVTDVCLDSTPCAFMILACAQLEICRHRI
KHDNMASHEIGKDVPRKGYKEEMALKEYVKHYVLIREVVYQIQSVFISIILPIFCSALLTLCTSIFELAQ
VIVFLFNYHTTGEYCFIMSYLCCLLVQIFCLCWFGNELQLKGEIVSNAIYETDWTVMKPCTKRDYWYLMF
MGQNKFIISFHGQCTLTLQTFIWMIKTSYGAFNLLTQVADT

>NvOr120PSE

MDLLPVHFXQLFGLWYDDPRTYRPAKLVHRSLVVLLIIHFSLFQMIALFSVKRSVDEYTNTLFFAMTFFV
HIYKMLVFLAKNRSVKEMLDEFRSDICRTRGPEEENILAKYVRRANWIYGARMILTLLCGSIRCVLPILI
GLSTGEIGELPFGTYFFNISDSLQFTLAYVLQTLAVVAAVVTDVCLDSTTCTCMILTCAQLEICCHRIRC
DNSNKAVDADSDIAEVFIRKDKDENCFKEYARHHITIQKIVQRIQSAFIPIVLPIFCSALLTLCTXNHSV
VEYCFITIYLCCLLFQIYCFCWYGNEVQLKGELVSDAICQTDWTTIKPSTRKIFWYIMFMGRNKLVISFH
GQCALTLQTFIWMIKTSYGAFNLLKQVSDSE

>NvOr121PSE

MDILPLNFQTCRMCGMWFESNSYYLPKMIYRLLVFLIIFQFTLSQIIELLTMHGSVDDFTEVLFLTLTFV
ALCLKVLNFTTZRNEMVDMLDDFRTPICLAKSPEEKDIIQKCSKTTKKIFLSIMSLSQSTGLVLLVIPFL
TSETREIPLPFKSYQPYEISSATNFWITYALQIFAAIYGVLLNVSMDTLVYGFIIMATGQFEINCYRLEN
STCSMRDCIEHHVLIQDIVYKIQHFFIYVVVPLFLFSLVTLCTSIFQMSQKQVASFEFLSLTMYLACMLC
QVFLYCWFGNELELKSNAVANAIYCSAWTDFTRREKRNISFIMLSAKSGSKISYHGQCSLSISTFAWIVK
TSYASLNLLQQASSDK

>NvOr122

MDVLPLNFRTLWLCGIWHEENEKLTVPRIAYRFLVICLMFYFTFTLSAVVFVENSNVSELTEAIFLAVTY
ITLCLKIVNFAFRRAEMIEILHDFRHPYCKAEHSEESEILKGYSKQARKMYIYLMAFVMSDVAYFWSTFA
FKVSKNIMELPYHTYQFYNMSSKAILFSTAALQATSVLYSVSINISFDTMTAGLLILTTGQLELNAHRLS
KLGEHNVDSMNGYIAHNVLINGTVDKIESFIKTVVIPFLFFSLLSICASVFQLSEYSVFSLEFLGLFSFA
ICILLQVLVYCWFGNELMLKSEAVTDAIYRSDWTMLSPQNRKSLQVMMICNKDGRTVSFGGQCSLTLETF
VWILKTSYATISLLNRVSA

>NvOr123PSE

MDIVPLSFRTLYYCGIWHEKDEVMTFPKLLYQYFVTLTIFYFTFIQGAVIFEAQLDVNEFTDALFLTLTF
VTLCFKIINMIYGRSEINSFLRDFRNERCKPLKSDEADILDVYSKKAKKIYKVMMSMAIIDTLFFVVVNI
IKVLNNISNKYVFFITNFIQIGTMCXAEYKHVLRYNDTAGVLMFTAGQFKLNAYRLAQLDQNNIHLLNQY
IAHNRLINGIVTKIQSFFIRAVIPFFLFSLISICSTTFQVSQHSIFSFKFLGLITFATCVLLQVFLYCWF
GNKMKIKSTAILSAAYNCDWITLDPKTRRSLYIIMLCNQNGRTVSYSGQCSLTLDTFVWIVKTSYAMZAS
CNK

>NvOr124

MDILPLNFRILRYCGIWYELPEHLWLVKIVYKIFVVVVIFSFTLSELIELALTYDDLQNLTECLFLTLTF
LALCFKMINFMCRQESLKALLNTFRDEICQPKTLEEKDIIEKNRSMLRLFCISYFSLGILSGSTLVFVPF
ASFKSSKIELPIKTYQPYDVEDFVLFSLTYFHQILSMYLGVLINVSLDMLVCGFIFLTCGQLDLCYYRIV
SSNMYTMNNNIRHHAVTKDIVKKMQSFSIVVVVPLFIFSLITLCTSLFLMPEKEIMSFEFITLFIYLTCM
LTQIFLYCWFGNELQLKSKTISDAVYHSNWTRLTPKLRRNLLFTMFISQNGLMISFHGQCSLSINTYVSI
LKTSYAAFNLLRKTSNTLGV

>NvOr125

MDMLPSFRVLAYLGIWIEEGSSFVFLRRLCGLFLSNTIFYFTLTEVIELYLLRNNIEELVDVMFLTVTFA
MLCLKILNFNFRHKGLLNLLTDFRMDVCKARSPEEENILNKYTTKILNIFQNILVLSQATGIFFCVLPFI
TLEPADYEIPYKTYQFYDDTTAMGFTITCVIQFIALIFGIFINVSMDTMIYGFIILSTGQFELISYRINK
SSKENDRALLKQCIMHHNCMNNLVKKTTNLFMTVIAPLFFFSLLTLCASIFQMSQNDIISLEFLGFAMYL
SCMLCQVFLYCWYGNELKLKSADLVNEVFGSDWTVLEYTEKKTLYLLMLSAQRPCDISWRGQCTLSLETF
VWIMKTSYTAFNLLQRASDK

>NvOr126

MDVLPLNFRSLQYCGIWYEFPEHLWLIKTVYRTFIVVVIFSFTLSELIELALTYDDLQNLTECLFLALTF
LALCFKMINFMCRQESLKALLNTFRDEICQPKTLEEKGILAKYQNILKKVFIFYMSLGLMSGSSLLIVPL
VSMENSRISTLPMKAYQPYDVEDTILLNITYFYQVFSTWIGIIINVSLDLMVCGFIILICGQLDLCCYRI
LCTKTKMFHDNNVRHHAVIAEVVRRVKSFFIVVIVPLFIFSLITLCTSLFQMPEKEVLSLEFFSLFMYLS
CMLFQIFIYCWFGNELQLKSKTIVDAVYQSDWTDLTPKLRRHLLFTMFISQNGLTISFHGQCSLSINTYV
SILKTSYGVFNLLQKTSNI

>NvOr127INT

MDVLPLNFRILQYCGIWYEYPEHLWMVKTVYKTFVVVVLFSLTLSELIELGLISNNVHESTECLFLSLTF
LTICFKIINFMCRQDSLKEILDAYRVDIFRPKTAEEKQIIVNYQNVISTFFVIYLTMALMAGTCMILVPI
ISNEDLMLYSITYFHQILSFLFGILINVCMDMLVCGFVILACCQLDLCGHRIGQNQMDIPAKDHITHHIL
IGDVVKKVQSFFIVVVVLLFSCSLIILCTSLFQMPQQNIMTLEFFTLFMYLMSVLYQIFVYCWFGNQLQL
KSKSISDAIYDSNWADLTPHKRKDYLFSMFMSQNGFTISFHGQCSISIQTYVWIVKTSYGAYNLLQKTSA

>NvOr128

MDVLHLNFRVLQYCGIWYEYPENLWLVKMVYKTLIVVMLFCFTLSELTELVLNRNNVHDLTECLFLSLTF
LTCCFKMINFLCRQEGLNRILNAYRADVFQPKTTEEKQMTTQYQNLISKFFMVYLIMALLSGICLSLIPI
ISSASNETQFPAKSYQPYNTQDSTLYLITYFHQILSIFFGIFINVSMDMLVCGFIILACCQLDLCCYRIS
LNKKDTSTNDHVVHHVLIGHAVNRVQSFFIVIIVLLFIFNLIVLCTSLFQIPQKNIMTLEFFTLFVYLVG
ILFQVYVYCWFGNQLQLKSKTISDAIYESNWPDLTPCKRKDYIFSMFMSQNGFTISFHGQCSLSIKTYVW
IVKTSYAVYNLLQNTST

>NvOr129

MEIYDSRYFVHAKRFQELLGIWPYQSRLKNNCSWVILSFLFIAMIIPQIVGLSVHAGKDSKRTLECTFGT
CYMLAIYMKLLVACADKDKAKFIFEYTARNFKKINDNDERKILIEYSERGRLIGVVYTIFVLAALGVFVV
VPLAPGILDVILPLENGTRSKFFILNGEFLVDKTEYFIEIYAFDSICCIVTVLIICATDPLYAAILEHCL
AIFAIVKLRLRKYRVRKGLKCVSADEAEYEAIIRAVQLHREIIAFIETIQNNCSLYFAFEMGVTLISFTV
NFVLAVLKTPDLFDRLRLAMVLFAQAVHLFYITWPGQKLIDHGEDLFKETYFNDWYKSSVKCQKALRFMS
LRCSKPCKLSGAGVYVLNFAYTLLILKTSASYITVVAQFEYKIVA

>NvOr130

MEIYDSRYFVFNKRFQMALGIWPYQSRVKNSITYAGLVLVMIIMLIPQFIRLNTYLGKDIEKTMENIFIF
FYVFGIFVKLFTAHFAEDKLKILYESTAKNFETYTDAVEAEIMKRYSERGRLLTFVFLLYMISAVAVSVV
LPMCPIVLDSTDPLDQPRPRMFILNGEYIVDKYEYYFQIYTLDIISVFLMICILCATDPMYAAIVEHCLG
LFSICKYRLRNFNKSCGLQMVEHAEAERYGGDYAYAALVRAILLHKDIIKFTEIIQTSYSLYFLLEMGAT
IGILTSSSVVVVMKLKQPLELLRWSLFLFGVILHIFFLTWPGQKLIDFSSDIFQEAYLNDWYKSSLKCQN
LLKFMSLRCSRPCELSGGGLYIMNFINFATILKTSASYITVFSSV

>NvOr131PSE

MEIYDSKYFFFNKRFQQALGIWPYESRAKKNAHVRNPGADRHRCTLVPQIARLSTSIGKDLEKTTENVVI
LLYTYXIYIKLFVAVYSEDQMKTMYESTARNFQVYTDETEKKILKDYSLFLCKLIGHQLYMIQALMIFIS
LPLTPVSLDIIMPRPRMFILNGDYFVDNDIYYYQIYLFDSIACAATVFIMVSTDPCMYAATVEHCLALFS
ICKYRLEIFNRNQNDSKTSITEKPRHDYAYYALKEAIILHKEILKYHEILQASYSLYFLLIMGATIGSVT
MNSAMILVKTHNPFVLIRYTLVLIGIMGQLFYLSXPGQKLIDYSSEIYRHIYLNDWYNSSLKCQNLLIFM
TLRCTRPCTLAGGGIYVMNFINZAAILKTSASYIMTVFASL

>NvOr132

MEIYDSRYFVYNKRFQTALGVWPYQSRVKNAIICGFLLLVMIALVVPQIIRLKMYIGKDKDKSMENVFGL
FYIFAIYVKLFTAVYAEDRLKILYESTARNFQIYTDKMEKKILHENSERGRLITLVFIMYMMTALIVFIL
LPMYPIMTDVIVPLDHPRARMFILNGDYLVDRDEYYFQIYVFESTSAALTVFILCSTDPMYAAIVEHCLG
LFCICKYRLNNFNKPRRTEIIEKANAESQVDEYAYTALVEAIQLHKNILKYTKIIQTSYSLYFLLEMGAT
MGLLTSTSIIIVMKLYRPLDCIRYFLVLIGLLLHIFFLSWPGQKLINVSGDIFQDTYHNDWYESSLRCQR
LLRFMSLNCSKPCQLSGGGLYVMNFVNFARILKTSASYITVFSSF

>NvOr133

MEIYDSRYFIHNKRFQKALGVWPYQSRAKNIVVCGLLLLLMLGMLLPQIVRLKKYAGKDSDKMMENIFIL
FYIFGIYIKLFTAVYAENRLKVLYESTAKNFQIYTGEAERRILYEYSERGRLLTLAFIVYMLPAVTVYVM
LPMCPIIMDAAKPLDHPRYRMFILNGDYLVDEYDYYFYIYAFDSMAAIVTVAIMCATDPMYAAIVEHCLG

LFSICKLRLKNFNKPNGTKAIEKTYYYSETCGDEYAYAALVKVVQLHKDIFKYTEIMQASYSLYFLLEMG
VTMGVVVCNSVIIVMKLSQPLELVRWSLVLIGGLLHIFFLTWPGQKLINFSGDIFQDTYLNDWYESSLRC
QKLLKFMSLRCLKPCELSGGGLYVMNFINFATILKTSASYITVFSSF

>NvOr134

MEIYDSKYFIHNKRFQMALGVWPYQNRVKNLSICGVLLLVMFGMLIPQLLRLRTYLGKDIDKSMENIFIL
LYTFGIYIKLFTAHIAENKMKILYESTAKNFETYTDEAEKKIMKQYSERGRLITLAFLIYMVLALILFVM
LPLYPIIMDATIPLDLPRPRISVLNGDYLVDENDYYFQIYVFDSIACTLTVFIMCSTDPMYAAIVEHCLG
LFSICKYRLKNFNKSCGMRMVERADAERYGGDYAYAALVRAILLHKEILKYTQIIQTSYSLYFLLEMGVT
VGILTATSVIIVMKLERPLDCLRYFLVFIGLLVHIFYLTWPGQKLIDFSGDIFQDTYLNDWYKSSLKCQN
LLRFMSLRCSRPCELSGCGLYVMNLINFAAILKSSASYITVFSSV

>NvOr135

MELFDSRYFIINNTCMKLLGIWPYSSHVKNYLRRCGLGLFLLSCYLPQFIPLYMYFGEDMDQMIQNIGVI
LYVFGTSVKLITGVTAKDRMKIVYEKTARDFQTIVDKEERNILFEYSERGRTLSITFIIYMWIALAIYVG
LPMGPLVLDYFIPLQNGSRERGFVWKGEYLVDPDKYYLTIYAVELFSSVLSVTILSSVGPMYQAIVEHCL
GLFVIVKFRLQICTRGGKKAEEESYRLIVKIIRLHNDIIEFTRIIEASYTSYFFIEMDITISLVTLISVN
LISRLDYLFDSIRHIFILLGVMIHMFYLTWPSQKMINHSTDLFHDTYSNEWYNCSIRCQNLLKFVALRCV
EPSQLTARGLYVMNFENYASLVKTSASYITVLLSFR

>NvOr136PSE

MEIFDNRYVVINKTCMKLLGLWPYSSRVKNYCRRCGLGIFLFSCYLPQFIRLYKYFGEDMDEMIQNIGVI
LYIFGISVKLITGVTAEERMKTVYENTARDFQTIVDKDERNIFLEYAERGRTLSITFIXIKTHTLIAFVC
LPLGPLVLDYFIRLQSGSRERVFIWKGEYXVDPDKHYYKIYAIEMFSSLLTVAILSSVGPMYQAIVEHCL
TSIZCFXYRLQIYTRGGKKADEETYGLIVRTIRLHTEIVEFTRIIEASYTSYFLIEMGITILLVTLVLAI
LISRLDRQFDSLRHIMILLGVMIHMFYLIWPSQKMINHSTDLFHDTYSKEWYKSSIRCQNLLKLMALRCA
EPSQQTARGLYVMNFENYASLVKTSASYITVLLSFR

>NvOr137

MDIFDRRYFVLNKALLRSTGLWPYEDRRKKLYIRTFVNLILGICVIFPQIVRIYNYFGVNMNMVLEHAAV
LMYITSIYLKFLTSVYYEEKLRVVYDNIAKNWQVIKDENEINILIQYSENGRLLTIGYTMYIIAAFCSYV
FLPIVPVLLDVFNPLNQTRSRFYILGGEYFIINNVEDYGKVYAFDCLAVIVTVWLISAVDSMYAASIEHC
LGLFAIVKLRLQTCTRSICDGQKDECYKMIVRLIRMHKDIIKFTDILESSYSSSFLILVGINVIFLSFEC
IIVLTRFGQAMEMMRYSMIMVGIVVHLFYISWPGQKLIDFSLGLFQDAYLNEWYTCPTRAQKLLGLMTLR
CSKPCQLTAGGMYVMNFSNFAKIVKTSMSYMTVLASFR

>NvOr138INT

MEIFERRYFTLNKTLLSSAGLWPYQNRRKKFCIRSFVNLIIGIFVIFPQIVRIYSYLGVNMDLMVEHSAV
LLYIMTIYLKFLTSVFYEEKLRVVMNLFAFQYIIAACLAYILLPMAPILLDVFVPLNQSRPRFYILGGEY
FIIDKVEDYGKVYMFDVLAVIVSVWLICAVDSMYAASIEHCLGLFAIVKLRLRMCTQPSCEGPRGRDASY
KLIVRLIRMHKDIINFTDILESSYSSSFLILVGINVIFLSFECIIVLTRFGQAMEMMRYSMIMFGIVVHL
FYISWPGQKLIDLSLGLFQDTYLNEWYACPTRAQKLLGLMTLRCSKPCHLTAGGMYVMNLSNFAKIVKTS
LSYMTVLASFR

>NvOr139

MEIFDQHYFSVNKALLKSTGLWPYESRRRKFCIRTFINLILGVFVIFPQLVRIYNYFGVNMDMVLEHAAI
LLFILTTYLKFLTSVYYEEKLKVVYDNIAKNWQAIKDENEVNILSQYSESGWFLTISYIMYIVIAASAYS
LLPMAPVLLDMIDPLNETRPRLYILGGEYFIVDNVEDYGKVYAFELVPAAVTVWLICAVDSMYAASIEHC
LGLLAIVKLRLQMCTQPSCDSRKDVSYRLIVQLIRLHKDIINFTDILESSYSSSFLILVGVNVLFLSFEC

IIVLTRFGQTMELIRYSMIMVGIVVHLFYLSWPGQKLTDLSIGLFQDAYLNEWYTCSTRAQKLLNLMILR
CSKPCQLTAGGIYVMNFSNFAKIVKTSMSYMTVFASFR

>NvOr140

MEIYDSRYFIINKTLMTKMGLWPYQHPLKKFLVRTFLVVFIFVSSMPQLYGLKKNFGVHMDKIIEHLALL
MYIYGIKLKLVTSILSEKKLKKVYENIMENWQQIKDVHERAILVEYSERGRTLTIGYIMYMTSALLFFII
LPITPMVLNVIKPLNESRPWDFIMHGEFPVNDMHAHYGEIYLFDSLACIATVLVFCTVDSMYATCIEHCI
GLFAIVKSRLDLSTKFVNRQGALGIKRDDKVYDLIVKTIKLHKKIINFTHILESSYSTSFLILMGMNMLY
CSLVSVLLIIKSDALMERIRYGTILLGLLIHLFYISWPGQKIIDLSTGLFEDAYSNEWYETSIRSQNLLK
FMRLRCLTPCQLTAGGIYVMNFANFASIIKTSTSYITVFASFT

>NvOr141

MDIYDTYVLNINKKLLSFVGIWPYEEKKKNKFTRVFYLITMFCIIVPQMIGFYQHFGVDIDELLENTGTI
FFTLSIYTKLFTSIIFENKLKILYDSVAKNWKNITEKHEREILVKYSERGRMLTLGYITYNFAAVIVYTT
MPLMPFLLDIILPLNESRPSMFILNGQFYVDKHEHYKKLYAFDCLCIFVIVPAALAVDTMYVACTEHCLG
LFAIIKYRLAMSDKFISTRDIYLTEEKDSSYRWMIHTIRMHIDILKFANILDKSYSSSFVILMLINTVYV
SVLCVLVLISLDKPLNLIRYYMLLVAICIHLFYLSWPGQKLIDHSEGLFRDAYNNQWYEGSAKSKTLLKI
LTLRCVEPCLITAGGLVTMNFATYLTIMKKSVSFITVFSSFR

>NvOr142

MTMDFYNSRYFSINRRMMTIMGLWPYQDFKTKLFIRTFLAIVLGIALIPQIISIVKYTNEDSDKVIQGIA
TLLYVTGITLKILTTITSEKKIEIVYRNIVDNWKLLDDENEIRTMTEYSEFGRLLTIGYVTYMFFALGLF
VTMPMLPMMIDVISPINGSRPRIFILDGEYIADKNENYGKVYIFESLTCIMSVFVFSTVDSTYAVCVEQC
VGLMAVVRLRLKLATAKAARMKYKSDSDEHDIPYQLVSSSAKLHIKAISFARILDSSYSVNFLLSMGSNV
MILSVGSVVILINLGRPMEFIRYSMIFIGLMIHMFYLSWPGQKVIDSSQGILYDAYNNEWYECSKKTKTL
LKFMMLRCIEPCQLTAGGLYVMNIANFGSLAKTSMSYITVFASFR

>NvOr143

MDFLDSRYFILNKKMLHILGIWPYQKRLERYAIRSVYFFFMGVSFVPQILCVKKYFKVDSDKFIRGVTTL
LYLSGVSLKLTIAILMNGKIQIVYSKVADNWKMFTDKDEIKTLLEYSEVGRMLTLGYVVYMVLAVIVFIT
MPYLPVVIDIVFPINGTRPRLFVLDGEYIVDKYENYNKIYIFESVCSVVSVPIFCTIDSTYAVCVQQCVA
LLAIVKLRLKVATKYTKNYLRDHKYNDASQQLIIKSADLHNKVIEFAQILETSYSMVFLLLMGMNCLILS
VGTLVILVNLNNPLELSRYIMIFIGLMMHMFYVSYPGQQLIDRSSAIFNDAYNNEWYECSIKSQRLLAFM
MLRCTKPCELTAGGIYTMNLENFGSLVKTSISYIAVFASFT

>NvOr144PSE

FKVMYDDIRRNWDTITEKNERNILIEYSERGRLLTIGYIXQLKAQTLPFRFDVLTCSTSAFVVMAVDTMY
ATCTERCIGLFALVKYRLQLPNKLTVNEYSDGLKKEDESYRWMIDTIRLHSKILKFTNTIETAYSASFLL
IMGINILYYGILSVLVLVNLDNPLNSIRYIGLMIGPMVHLFYLSWPGQKLIDHSTGLYRDAYCNDWYEGL
VQSKKLLSFFTLRCSEPCLLTAGGLVICMNMENFMMIMKKSVSYITVFSSFR

>NvOr145

MDKLKQSTVDIDTINNIFGNTYFKINKELQELVGLWPYQKGFSVRVVQTIMLFVLSFIMIPHLNGIRVWC
GKDLGICSENIAATIYLSGCFLKYLVVLLCKRDISKVYEKIAINWLTINDPNERVILDKFSSLGKLKSIG
YTVYVSAAGIGFSQFALLPFAFDYFSPLQNGSRPKIRIVRAEFFVDPIEYYWHIYATYCIVTFVSAFTII
SIDTSYTAVVHQNLGIFNIVKYRLSLAKKAVGTSKDLAYEQIISAVRLHQDSLGFNNLIEVTYRVCFLLL
ILVCISFLTFGAITILENSDNWIDIVRLGSIEVGAVIHLFYLSWPGQLVVSESEELYYYTYNNEWYNLSA
ESKTLLHFMMLRCINPCCLTAAGLYVMNFENYGAIIKSTVSYITVLSSFRE

>NvOr146

MNGFETSSLNSKIINEVFDNTYFKINKILQELIGIWPYQKRFDALIKQFIVILILSMVAMPHINGIRVWC
GKDLGLCAENLAGVTYASGVCTKYFVVTRSKDQMTKVYEKITSNWLTITDPDERVILNKFALLGKYKSIG
YVGYITVAAICFSQLGLLPILIDVVLPLQNGTREKLRVVKAEFGVDPYDYYWHIYGAYCAISVVSSGVLM
AIDTSYTAVVHQNLAIFNIVKYRLTQAKRAVNTVKDVAYEQIISAIRLHQDSLEFNNLIEHTYDVSFLIL
ILICVTFLTFGAITIMEESDSYLDMFRLSLLECGVCIHLFYLSWPGQLVVTESEDLYYYTYNNEWYNLSE
KSKTLLKFMMLRCMKPCCLTAAGLYVMNFENYGAIIKSTVSYITVVASFRED

>NvOr147

MTNEISQEKLNRVFDSHYFYLNKKLQIVSGLWPYQSRKRKFIHKLTMLCFLGTALVFLLNGLRHWCGVDI
DVCGENLVGLIYVISVLSKLFITSLYEEKFKIIYTRLAINWLELTDPQEHNILISFARQAKIKTVVYFVY
MAAAGVGFCQIPMIPVFLDFINPLNETRPKILFVKAEFIFDPYKYFYQLYAFFIGCAASAVFIVCSIDTT
FTAVVHQIIGVVSIIKYRLNCATVSFNPNKDVSYKLIVHAIQLHKEVLQFSDLIEKSYNIFFLVLTGLTV
VFLSTGAIVMLVRVGAMLDLIRLVLVLIGAILHFLFLTWPGQNLIDHTSDLFAAIYATEWYNVSERSKKL
LSIIMLRSLKPCVFTAGGLYVMNLENFGSIMKTAVSYMAVVSSFR

>NvOr148PSE

MKKEEVAFDDSWLKINKELNVFNRPERGKIFRSIIVLAVFITGFLSHVFGTALQCRRILVLHRHCSGIIA
KFIVLMVSKEKALESILLNELLSKISLDWNGITDPYERSILEECSKSGRPKMWLYFVYCVLVGFALCQMF
ALASLMRIILPLNESRPKILAIKAECPFNYYYELYSIYCDVAVTSVSVTDQTYVMILQQSLGLFQIVKHK
MQKTTMYNNQDEVMVLAITLHKSVLQFLELTESTYQSAFLLFMVATVAFLSFGLVIMIKHSKEIIDLIRM
TMIVXSHIFPQQQARTISYZSZYPTKWYNVL

>NvOr149

MNKEEVDEAFNDSLLKINKELNIFNGLWPHRPDGDKLFRRIIVLTVLISVTLPHVLGMFIQCGRNMALCG
ENICGFCYCSGVIAKFIVPIVSKEKFITLYEKIALNWKEITDPYEQSILEEFSKLGRLKSWLYFVYCAVA
GFAFCQMTALPALMDIILPLNESRPKILVTKAEYPFDPFEYYYELYFLYCTAAVVSVSVLASTDSTYSVI
IHQSLGIFGIVNHRLQKAAKHKNQEESYRVMVSAIELHKSALEFLELIESTYQSAFLIFIFVTVAFLSFG
SLIIVEHSEEIIDLIRMTLIEFGAMIHIFFISWPGQLVIDHSENLFLSTYTTKWYNMSKKGKMLLLFMMM
RCLKPSFLTAGGFYIMNFENYGSIVKTTLSYVTVALSFH

>NvOr150CTE

MDVQKVEEVYDQHLRINKTLQIHSGLWPYRPIKEKLIRRTIVFIMMAIFIFPHINGMRTWCGKNMDLVLC
GENFEGVLYLGVVTLKYYVTILSEPRIMQIHKQIAKNWSTLTDSEEQSILIKHSKQGKIVTILYLVYLYA
AGFAFSQVSMVPVILDYIWPLNESRPKILIVHAEYIIDPLQYYFELYGAYCILTFVNLTVLISIDTAFSS
IVYQNLGVFSIVKNRLNAVTKRPNEKKDNAYELIVGAIRLHNSALQFNDLIEMSYRKTFLIILGYTILFL
SVGGVILIEQTNIMQFIRLAMIEVGVMIHLLYLSYPGQKVLDYSSSICQESYTNEWYLISKKSKILLKFM
MLRCIKPSVLTAGGLYIMNYENYGI

>NvOr151

MDEREIDLLYDNYYFKLNKKLQIITGLWPYKSRKYKLGIRAVVYATLSLVMIPLCNGFRTWCGVNLDICG
ENLVGICYTMLIFLKYWVTTHSEERLKNVYRLVAKNWMEITDPHEHEILVDYAKQGRLKTIGYTAYVVVA
GIGFCQIPMISVILDIIIPLNVSRTKILFKGGEFILDPYQHFYKLYVYFVITSFVIMTIIIAIDTNYTII
IHQILGLLTIVKHRLQRLAIPMNLKKDNSYHAIIKAIHLHNDALQFVDLIESSYSCLFLVFIGFTIIIIS
ISTSIMMAQIGKLLNMIRVAMFVLGASLHFLYINWTGQQMIDHSKELYLNVYSNEWYNLTKEAKTLLKIV
MLRCLKPSKFTAGGLYTLNLESFGTIMESALTYVAIMSSFR

>NvOr152PSE

YMMIAGLGFCQIPMIPIVLDLIMPLNESRPKILFVKGEYIIDPFKHFYKLYVVYFAITSLTTVTVVSAIDT
TYTACVHQILAIFSIIKHRIHTATISFDSKNDISYDIIVKAIHLHTAALQFIDLIDLSYSLSFLILTGFT

IVFISIGTVVVMVHMGAMLDLIRLALFVIGSLVHFLFISWPGQLVIDHSNDLFVSVYANEWYVISARAKT
LLKIVMLRCIKPCILTAGGLYVMSLENFGVILKSALSYIAVVASFR

>NvOr154

MDIYDSRYYKTNIFYLKLLGLWPFDDFLNKRVRRILIIIAVVSLIIPQVIRLFEEWGRDIDIVIEVIGSL
IYFSGCQIKYLSFLRVEAKMKYLYNKIAEHWKSLSSKDEIKTLEEYGEIGRGLTLGYIIPINIILVIYIS
LPLLPLLLDVIDPQNETRPKQFPYFAEYFIDDQKYYFELTIHGWIVCILSVQIYGTFDTTYTQCVQHACG
LFGIVEQRLRKATKLASSNAFSTQEEKDEKVYDKVIDAILLHKEAIQFVNLIEDCYSFSYFFVVTLNTAV
VSLAAVDTMLNLENGNTKQMVRIGALYIGFSFHLLYNMSPGQRVIDSSTNIQNAAFHCDWFNASSKTKTL
IRIIMLRSLTPCQFTAGKLIVLHLESFAFVFKNSISYVTVVGSMR

>NvOr155INT

MDIFNSHFYKTNSFYLKLLGLWPLGDVSNNRIKRVTIISLTISLMIPQVIRLVEEWGNDMDIVIEVIGSL
IYFTGSQIKYITCVSVESQMKFLYEEIARHWKTLTSEEEKKILSQYAQDGYDLALGYLISINIILVGYIT
LPLAPMLLDIIDPLNETRPKAFPYFAEYFIDDQKYYFELTVHGWIVCILSVQIYGTFDATYTQLVQHSCA
LFAIVEYRLNKATKIEPSISSDFVGLIEKCYTFVFFGVITLNTAVVSLAAVDTMLNLDKGNHKQMIRIGL
LYIGFSFHLLYNMYPGQKVIDSSTRIQQAAFHSDWFNTSPKTKQLIRIIMLRSMMPCTLTAKTLVVLDLE
SFAFVFKKSISYVTMIGSMR

>NvOr156

MSESKTLKIFESDYYRTYKNSVKLIGLWPHENIHKKRITRFFITALLTTFMILQGIRLYEELGNDIDIVL
ELIGSIAYFSGCICKYLTTIKAQAALQFLYEQIQGHWDTITNKRERQILEQSASESQFLSKFYMGASYVA
LVVYTASPVLVPIILDIALPLNESRRKTFPYFIEYFIDTEFYYYQLMVHGTICFTISVLVYISIDTMYAA
CCQHLCGLFDIVEHRLKEAVKTNSNRINLEPDRTDILMHKLLNEAITLHQDSIEFAVLIENTYALCYLLV
LGLNLAVIVLAAVDIVINLDDTNQIIRLSILYIAFSFHLFFNSVPGQKIHDKSVNVMNSAYFSEWYNLPL
NARKLIQLIIHRSLNPCQFTAGGLFVLNIENFGSIMKSSMSYITVLASIR

>NvOr157

MDIFNSRFYRTNCFFLKLLGLWPLGDVSNNRIKRVTVVSLVVSLIIPMVIKLVQEWGNDIDIVIEVIGSL
IYLSGSQLKYISCASVQSQIKFLYTEIERHWNTLTNEEEKKILKQYARDGYNLSFGYLMLLNVILVGYLL
VPFTPMLLDLIDPLNETRPKAFPYFAEYFIDNQKYYFELTVHGWIICILSVQIYGTFDATYTQLVQHSCA
LFAIVEYRLGQATKMVASDEDSSHKDTDKVAYNMMVGAINYHKQAIQFVGLIEKCYSLLSFLIIIILNTAV
VSLAAVVTMLHIEKGNQKQAIRIGMLYVAFSFHLLYNSYPGQKVIDSSTRIQEAAFHCEWFNTSSKTKQL
IKIIMLRSMVPCTLTAKTLVVLDLESFAFVFKKSISYITVIGSMR

>NvOr158

MSESKRLEIFESDYYRMYTNALRLIGLWPFECTYRQRIIRFFIIILLITFTILQGIRLYEEFGQNLDIVL
ELIGSITFFIGCILKYIVTIQTQSMFQFLYEQIQSHWEIVTNRERRILEQSANDSQFFTKLYMGAAYGAL
IVYVSTPIIVPNVLDVVIPLNESRAKTFPYFIEYFIDTEVYYYQLMAHGTLCFTISALVYVSIDTMYATC
SQHLCSLFDVVEYRLEKASKTDSKMNVNLDLNGNDKNIYKLLNEAIVLHQDSLEFALFIENTYASCFLPV
LGLYLTSIVIVAVDIVINLGDMNQIIRLSILYFAFSFHVFFNCVPGQKIHDKSVSIMNSAYFSEWYNLPL
EAKKLIQIIIHRSSIPCKFTAGGIVVLNVENFIVIMKSTLSYITLLSSIR

>NvOr159

MSVRKDMHVFESQYYRIYKNSVKIIGLWPYENIQIKRVIRISIILLLISLVILQAIRLYEELGRDLDIVL
ELIASLSYFAGCLSKYITTIRAQSAFRFLYDLIAGHWQIITDIKEREILEESTRQSQTLCLSYMVAAYSA
LVVYSTMPAIAPAVLDIVIPLNESRKKTFPYYAEYFIDDEAYYYQLMGHGTIVFTVSVMVYVSIDTMYAC
CAQHLCGLFSIVEYRLQEALRTDDKLHLEPPERDKLTHKKLHEAIILHKDSIEFAFLIENTYALCFLLVM
GLNLTVIVFTAVVIIINLGDMKQMIRLTLLFGAFSFHLFFNCVPGQKVHDKSISIMNSAYFSEWYNVSLK
SRKLIKFVMHRSLNPCQFTAGGLFVMNMENFGSIMKSSMSYVTVIASIR

>NvOr160

MSVYSSDYWKMPVLAQKFMGVWPFNNRQYDKCMRVFVYVALYSLIVPIGIRLVEELGVNTAIAIENLVGQ
MYLNAAVIKFSMTILFKEKHKQIYELIARDWKMTSDKEELEIMEKHAAIGRTISLAYGICCCSTAGAFLM
IPTLLPLLDYVAPLDNSSRPVVLPYYAEYYIDQRKYYLPLMLKALVAGMISMTVFITYDMAFAMCVQHVC
SLFDIINLRLQRASQLGSQLGSQLGRSARTSASYDSGVFRLIQKAIELHQIVIENVSSLENAYNLNWFFI
LLLNTTAVGGALLVVLLKLGHPEDLVRYGMFFAAIFIHKYFIFLPGQKIINYSLEVFEYSYSCEWYNLSA
ECKVLIKIMMLRSIRPLNLTGGKMFLLCMETYSAMLKAGMSYFTVFASTQSF

>NvOr161

MELFESNYWKLTVFLQKLIGLYYFQSLWKNIVAWIYVYTFTLSFIIAIGVRLYQEIGIDINIVTENLVAE
MYLIIVFAKLTTSVVYMKDLKRLYKSIANDWRVMSDEKELKVMHEYTDIGRKSVQLYSGYMIIGIAIFLS
LPISAPLWDYIVPLENATRPNALPYYAEYGVDQEKYYFPLMGQAVFGGIGTGMLLVTFDLGFILTVQHVV
ALFALVWYRLDQAANLSLSVERGKIDFIKADRSAYEYTVKAINLHQTVLGYVDLVENCYNAGWLVVLFMN
MLLCGGGLAVLLMKTDRPEELLRYFTVLFAGFIHFYYIFLPGQKIINSSLEVFDKCYASRWYNLSEKSKS
LIKIMMIRSLRRCELTGGKMFILCMDTYCNMMKTGLSVFTVLR

>NvOr162

MFDSKINTQDDFNLDIFETTDYKLYKDGMKLIGLWPFESSTKKTLKRAFVLISMISVLISIQIRFVEELN
QNIDIVLQSAGSEILSIGCIAKFVTTLRAEDSFRVLFIQIAKQWASITDETECKILADNVKLCHPLCTFY
RVMAVFALSSYACLPSFGPVIMNILLPLNETRQKRIPAPAEYFVDEEKYFYILFSHGMILYMLVCVLYVT
IDSMYSCIVHHTVGLVGIVTYRLQNIIDLDITSSPKNHTNNLEIRRRLRRAITLHKESIEFAENIEATYS
LCFIIVMFVNLFSMVFTAACGIRTLHYDKVESFRWLMLYGSIIFHLFFNSNPGQNLFDKTSEIINTLYFT
AWYDSGISTSNKRIIQIMMIRCLRPCQLTAGGLLVLNMFNFGAIVKTSFSYITMLLSVG

>NvOr164PSE

VMSVFALAAYASLPSFGPVIMNILFPLNETRRKQLPAPAEYFVDEEKYLYILFLHGMICCIFVPILYVTI
DSMHVLCXYTVGLVGIVTYRLQNIIDLEVTSYQEYHANNLEIRKRLKRSITLHKEFIEFAEKIETTYSLC
FIIVMFVNLFAMVFTAACAIRSMYIDKADSFRWFILYGGIIFHLFFNSNPGQNLYDKSSQIVDMLYLTEW
YXLGVSTGNKRIMQIMMIRCSQPSQLTAGGLLVLNMENFGAILKTSFSYITVLISKG

>NvOr166

MLSFKIQAKVNNGDVLGVGYWKLNKLMLKSVGLWPYQKSSTKMCIRTFIFIAIYSMMIPQIIRTFEEWGK
NSEIVIENITGFLYFQVVITKYVTSCIAESNLQYLYVRITEDWNHFRDEGEQKVLSHFASHGRFLTIGYS
VYLYTAGIAFTTLPCLIPAVLDLIIPLNDSRQKVLCFYGEYFIDQRVYYYELLLHTFVCVMCTIMLFTTI
DAAYACCIEHVIGLFNIVDYRLNQAFNLVKDKYDTKSEVMRSEIHKCVLRSIEVHNHSIEIVELIQTTYT
TCFFFTTGISLICLSLGTVDMMLSVNNYINFARVFFAWCGIVIYFFYISMPGQRIIDASSDIFNSVYFSG
WYDFPLKTQRLLKFMMMRCSVPCQFTAGPLLVLNLENCGVILKTAMSYCTFVFAIS

>NvOr167

MEEHEILNNEYFKVNRFLLKLTGLWPYQKRHVKLIIRILYICAIHSMMIPQVIRTVEEWGKDFEIVLENI
VGFIYLQCVLAKYIITFTAEPQLVFLYKKMAFDWTRYIEAEEQLSLQRAASNGQLMTIIYSVYVNFAGVG
FATLPGTLPTILNIIAPLNESRPTKVLCFYAEYFIDQEEYYYQLLFQTFIGVMSTVFINATVDTLYVICA
HHSDGLFNIVSYRFQKAFNKSQERYQVKSRNLVAAKNLDEEIHEYVLTAINIHNESIEFINLIQSTYTLY
FFIQMSLTIISLSLATVVAMMNLHDIINLIRIFFIWCGIILNLAYISIAGQQIIDTSLQIFDSAYFCGWY
NHPLKTQRLLKFIMLRCSRQCQITAGPMLVINLESCSNILKSSLSYCTFMIAVS

>NvOr168

MLDSNEVFNNKYFILNKRLLMVNFIWPYQTKHMKKFVNTFIIVAIHTMMIPQIIRSVFEWQLEERNVEIL
VENFAGFLYFLGTLSKYYTTIYLEKKLLQLYQQITYDWKGLQDDNEQAVLLRSWDIGRTLTIFYMGYMFT

ACISFVTLPSAVPLMLDLILPLNESRGVNLCYYAEYFIDQQKYFIYLLLHTFICVSFTILIVTAVDSTFV
SIVYHAVGLFDILEYRMRNVLRFVEDDYQNNKFLSKEELHKYVVDSVKMHKKTIEFSEIVQRIYNDCFFF
VTVLVLVCLSVGTIDLVMNLNNPINLIRVGFVWLGIIMYTFFISWPGQKLINCSHELFNTVYACGWYGCP
TKTKYLIKFMMMRCTVPCRLTAGPLLTMDLVTSGNILRTALSYFTVVASFS

>NvOr169INT

MELMVEKKTKSEIKEEYIFSNKYFIFNKMMLKSTGLWPYQDVWLKRGMMILYITGFFSLMIPQMRFIYEE
ITWNWGEINDSGERAVLGRFCNIGRILSIFYTAYIHICMFFFVWSPALGPMILNTFSNATYKKSICIDAE
YFVDQDEYFSYIFSHTVFVSLLSASILTAFDSTFVLITQHGIGLLHVLKYRLNKISKIFEMSEYNFKDNT
IIHTKIVSCIILHKKSLQFLKVIGSTYDLYHFFIYSLLLASLSIFTIDVVVNLQDFATVLRLLITWSALV
MYMFYFNWPGQKIINSSEEIFEVIYLSNWYSYPLKTQKLIMFMLLRTGKRIFLKAGIFEMSFVTVGH

>NvOr170

MELKVERKAKSGLREEDIFNNKYFILNKKLLALVGLWPYQDARLKRVVRILLVLCIYSMMIPQMMKGIEE
CREKNPNPEIILENISGFFYFQGVTAKFLTAILTEDKLKYVYEEVMKDWKRFTDKNEIAILCKFAHVGRV
LTVVWSIYAAMSCLLFVTLPAVIPMILNIILTRNETFKKSLCIYCEYYIDQDKYFFYIFLHHIIAGIATI
FLTIGIDTSYVNCVQHVLALFNVSRYRLKVAFDTIHHSKKNDYNLKTLENNVHSYVVSSIRLHQRSIKFV
DTIQSAYNIVFFIVCALLLFGISIITVDLVWNVHNPINLIRIACLWMGTIMYMFYSNWPGQKLIDSSNEL
FDAIYTCGWFEFPMKTKILIRFMLLRSIDPCRLTAGPLLQMNFESCSLILRSAMSYFTVLVTTG

>NvOr171PSE

MIKGIKECQEKNPSPEICLENLSGFFXSQIISTKFVTSILTEHKLKYVYEXKDWKELSNENEVAILRTFA
IIGRLLTLIWSVYTIISDLAFVTMPAVIPMLLNAILARNKTFDKSLCIYCEYFVDQVKYFFYIFTYHIIT
GIVTIFMTLGIDTSYMNCIQNILALLNITRYRLKVAFDTISHYTAKKELDLKVIENQTHDYVVSSIRLHQ
RSTXFIDTIQSAYNVVFFIVCVILLFGISTLTADLIWNSHIPINLMRITCLWMGAILYIFYS

>NvOr172PSE

MLDSSEIFSNKYFILNKKMLIVIFIWPYZKKYLRIFVNTFIILAIHTMMIPQINGMRTWCGKNMDLVLCG
ENFEGVLYLGVVTLKYYVTILSEPRVYLYAAGFAFSQVSMVPVILDYIWPLNESRPKILIVHAEYIIDPL
QYYFELYGAYCILTFVNLTVLISIDTAFSSIVYQNLGVFSIVKNRLNAVTKRPNEKKDNAYELIVGAIRL
HNSALQFNDLIEMSYRKTFLIILGYTILFLSVGGVILIEQTNIMQFIRLAMIEVGVMIHLLYLSYPGQKV
LDYSSSICQESYTNEWYLISKKSKILLKFMMLRCIKPSVLTAGGLYIMNYENYGI

>NvOr173

MDIFDGRYYKTSKWFLEFLGLWPFQSNRRRYVTCFIFVFMTATVVFPQVLLLIELKTSNFNILIENSLSI
IFGFACLLKYGVTFASRSRLQTLLTQIASDWQRLTDKAEIDILSQYGEEGRYLVLFYTVYVFLAWVTCNF
VPFIPPLLDILLPLENGTHDLVYPFYADYVFFKQTDYHYESCLHVFFVYFGTTSLFAGMDTIYVATVKHS
CGLFAITWLETMARTGKSNRSNYSIKPNSVVHHDMVEAIVMHNETIRFVELLEDSFSLCFLMVQCMIVAG
LATLCFYMMRIYDKTFNMCQFSTFTVGLVIHLLYLHWVGQKIIDSSDKVFYSTYYSDWYLISRNERQLTK
IILARSLYPCQLTAGKISVLSMETFGALMKTSMSYCTVLLSVS

>NvOr174PSE

MEIFDSRYYKSCKVLMEMLGLWPYQALKRKRLTYIIFYSLHGSLLLPQVIRLLQTKDFNIIVENTISILF
LFVCIAKYAVSYTSGPNFKLLFTQIAADWQNITDKSEREILSKYGNDGRFASLFYTFYVFWAWATYNMMP
FLPPILDIVLPLPNGSHPLLLPFYANYIFFEQLDYHYETALHAFFVYFFATSLFAGIDTIYVSTVKHTCG
LFAIIZLETLAKTDENKASIEFRSDKVVHHDLKNTIIMHNDTIRSCRLLEESFSLCFLLIQSLCVVTIAL
GGFYMLCIYNDAYKLLRVVAFYTGTVLHLLYLHYVGQQIIDSSEKIFNSAYSSEWYLISTNARKLTKVIM
IRSLYPCHLTAGKITSLSMETFGSLMKTSMSYFTVLLSLE

>NvOr175

MDIFDGAYYKSCKWFLSTLGLWPYQTDNRKRISAVIFFVVNISLAIPTGALLYKLWLKSFTVTFENTVSV
MFAIGCCAKYVVTYTSSQRLVRLFKQIASDWQRITDTTELSILTKYSEKGKFLITFYQVYVWFGWTVYTL
MPFIPYFLDKVSPLNESRPLLMPFYADYIVFDQADYHYTSCFHIAFVYISSALLFCGVDATFVMSVQHTC
GLFAIICHRLEGEKIKKESEYAQNVMKTLSEEELREVVIFHNNCITCSGLLEDSFNLSFLILNSMSVLGL
ALSGVYFIYIYEDYYKFVRIMAFFVGLIIHLLYLNWVGQKIIDSSEDVFLAAYCSKWYVISTSARAFIKI
IMVRALEPCRLTAGGLSTLCMESFGILIKTSVSYFTVFLSVA

>NvOr176PSE

ALMLIQKWGKDFTITFENSGSLLFACASLSKYLVTYSSAKRLELLFLQISKDWQRITDIKERQILMKFYE
EAKSLVVFYTIYAFAAWLVYTSIPFIPFILDLVHPLENGTRPLFMPFYADYVFFDQNDYHYSACGHVAVI
YFTSFLLYSGVDGIYVLTVKHTCGLFAITCARFICRGSIYSCIIRKYYVSIFRYHFLKEDILYFYENTFL
LLRVGAFLFGVILHLLYLNWIGQKIIESSSQVFDSTYKGNWYTVSIRSRKFINMIMYRSLKLCELTAGTM
TTLSMETFGILMKTSMSYFTVFLSVT

>NvOr177

MDLFDGQYFKLNKIFLTICGLWPYQSKLRRRITFAMLASSTLLFIFTLVAGILSQSKFDFVNTEETFIFI
FYCSAGLLKCTILYNQQNKIKKLYERIATDWKQLTDTSERDILRSFLLEGRKLNFIIMISCSSAFIIYSC
VDLLPRILKEKSEYHRPHSFPYYFRPMVINEKLYDLQVAVHITVIVFYAGFAYMSAIATYISSVKHVCAL
YEIARYRLQNAVSYDKSNNSLLELVEDTSIVPKLVKVIDMHAQALRGIQIIEKVFSADFFVLEASSLTAL
ATDVYELKYCRANVRTFIRAKLLTPIVIIYLFFVNCSGEQVIQACNDMRITAYYIDWYRTSSRARVFVLM
IMRRTLNPKYLTAGTIVMIISIENFAAIITTAWSIGTILLTT

>NvOr178PSE

MDLFDSQYFKINKRVLTICGLWPYQSKLGKRINFASLASSTILFIFTLIAGIISQSPVEFVNTVETSMGI
SFCSTGLLKCAIFYKQQNKVKELYERIATDWMKLTDDSERVILQSFFXVGRSLNFITMVYSFAAFVVFSC
FEFLPRIFYKESZYHKPHSFPYYFRPMVIHKKFYDLQVAVHTTVIVIYSGLTYLSAIATYIFSVKHVCAL
YEIARYRLQNAIVYDISNKPLQELIDDTSTIPKLIKVINMHKQALRATQRIEKVFSTDFFVLDASSLIAL
AIGIFXLNYYKGNFREFVRALLVVSIIIIYLFFVNQSGDRVIQASNNIHITAYNIDWYKSSSRVHLFVLM
IMRRTLKPEKLTAGTIMMLSIENFTA

>NvOr179

MDSFDSQYFEINKRVLTICGLWPYQSKLGKRITFAMLASNTFLFNFTLIAGILTHSPGEFVNTAETLVGL
FFCSTGLLKCAILYNQQNKIKKLYERIATDLKKLTDNSERGILRSFLLEGRRQNFLTMVYGVSAFIVCSC
VEFLPRIFNEESEYHRPHSFPYYYRSMVIHEKFYDLQVAVHGTVIMLYSGLTYMSAIATYISSVKHVCAL
YEIARYRLQNAIIYDKHNYPLQELTDDTSTIPKLIKVINMHKRALRVTKKIEKVFSADFFVMEASCLIAL
ASGIFELNYFRGNVRAIIRPLLVMPIVTIYLFFVNQSGQQVIQACNDIHTTAYNNDWYKASARVRIFVFM
IMQRTLKPENLTAGSILILSIENFATILRAAWSFGTIMLTTLKHSPSRNEDA

>NvOr180

MDLFYNQYFNINKHVSMICGLWPFQSQFGRRISYMIFAMSTFSMIFSLTAGIISQLNPDLLNILETCVAL
FFCVCGFLVCTILYNQKNQIKRLYERIAADWENLTDDLERDVLRTFLLEGRKLIFITLVYSFPAFSLFAC
ITFLPRMFSEESTKLCLHSFPYYLESMVIDKNLCNLQVSLHYSVALGYVGLSFLSVGATYICSVKHVCAL
YEIARLRLENATVRYGNYDPLGELTDETSIIHNLIEAIDMHKNALRGIQIIEQVFSTGFFIIQIFGLSLL
AILICELKYHEGEITEMIRFMLVLSVFVIYLFFMNWSGEQVIQSCDDIQKTAYDIDWHRISSRTRIFVLM
IMQRTLKPVHLTAGNMMILSIQNFGTILKSAWSFGTILLTTQKSV

>NvOr181

MDLFDSQYFKINKLALTVYGLWPYQSEIGRIINHVIFVVTSFSMIFAMAAGIQSQVNAELKNILETVVAL
VFCGAGLVKCTILYNQRNQIKKLYERIAADWEKLTDTSERDILRAFLLEGRHSIVITIVYAVPAFCLFIC
VEFLPRIFSKESAKHRLHSFPYYYKSMVISENTYDLQVCVHLMVVIIYVGFSYLCASATYISSVKHVCAL

YAIACQRLRNAIVYRKNSTPLKELIEDTSVIPNLIKVIEMHKEALRGIQIIEQVFSAGFFVFEISALTTI
AILIFDLNYHQGNPFQMMRVLLILSVFVLYLFFMNWCGEQTIQSCNNVNAQAYNIEWYGISLKARVFVLM
ILRRTLKPIHLTAGTIMILSMENFGTILKTAWSFGMILLTTQTSARNKDPNFFGY

>NvOr182

MDLFDSQYFKINKRVLMICGLWPYQSILGRRIAFAMLANSIFLFVFTLIAGVISQSQLDIINTEDTIIIT
FFCLLGLLKCTMFYNQQNKIKNLYECIATDWNKLTDSSEHNILRSFLLDGRKINFITMVFCSSAFMIYSC
IDFLLRIFNKESEYQRQHSFPYYFKPMVIYEKLYDWQVALHVTVIVIYSGLAYLSAIVTYISSVKHVCAL
YEIARHRLQNAIIACDKINHPLQKCIEDISLIPKLIKVIEMHEQAVRGIRIIKKVFGADFFVLTVFCISA
LTIGTFELNFCRADIHSFIRVLLLMPIIMIYLFYVNYSGEQVIQACDDMYTTAYNIDWYKTSSKTRIFVL
MIMRRTLKSEYLTAGTMIMILSIKNFATIIKTAWSFGTLLLTTQKHRKNEDANFVAENTLI

>NvOr183

MTLINSHYFKLNKLLLTFCGLWPYQTKLKRRINYTTFAIITLSMIFSLAGGIQSELNTGFMNISESIIAL
LFFSTGFLKCTIFYNQRNQLKILYEQTAHDLKKMTHHLERDILQAFLLEARNFNVVSLVYSIPIYIVFAI
ATYLPQVFGFTNESAKYELHFFLYYKPMIIHESVQDLQVLIHATISTIYVGSAYLCVSATYISSVKHVCA
LFEIARYRLKNVIVDHSNNNRRGLMKNASVISNLIKVIDIHEKALRGVQRIDNVFNASLFILEVTALSAV
TILIFHLNYHQGNFRQMTRYSTILSAFVSYLFFCNWFGEQVIQSCNDIRETAYNVNWYNMSLRARMFVLM
IMQRTLKPVHLTAGTVVILSMENFSAFLKTAWSFGTLLLTTQKPSPKENSIFFEY

>NvOr184PSE

LEVFDINCSSSIYYPTKWISLIVNILWPYQSTLRSISNYVIFTVTLVSMVIAMVAGIYSETKLLNILKGS
LPIIFCSGALVAYMLLYSRKKEIKERTELMATDWMELADDSECKIMXNFFLEARSAINITIGYGIPVYGL
FFGVTFMPRIFNKKSLEQCLRSFPYYFKLIVIDENTCDLQVCVHFALAILFSSFAFLTISSTYIISVKHI
CGLYGIACHRLRNAELVLDNKSLDALKKSSEDYDTTVIHSLIKVIDVHKEALRGIQIIEKHYSFGFFFLE
IGAFVVLAILMFEINYHKEHISELLRFLPLLLLFTTYVFFMNWCGEQVIQSCDDPRISVYNMDWYRTSSR
IRIFVLMIMQRTTKPVHLTAGTVMLLSIKNFATILKTASSFGMLLLTTQKPGKNKDADYLGY

>NvOr185PSE

GCGIPAFLLFVCITFLLRIFSKQSLDQCLQGFPYYLRSMVINEKTCNLQICVHCSVAIILASFAFLSISA
TCITSVKHVCALYSIAXHRLRNAEVYFKDNGPLKDSIEDTPIIPSLIKVIDIHKKALRGIQRIEKYFSLG
FFSXIGAVLAFLAVLMFEVKYHGGNVNELLRFSPILLIAVIYVFFINWCGEQVRESCNDLRISTYNIDWY
RISSRVHIFVLMIMQRTAKPKHLTAGTVMMLSIENFATIIKTAWSFGMLLLTTQKQGRNKGANFLEY

>NvOr186PSE

MSSEPKIFDNPLLARNKTLLIHYGLWPYQSKLAKRCKYSIFSITSISLIIPMLQGLRDDTSTDIVIFFET
VVATLMVFGGFAQCLIMYARENKMRNLFNEIDANWHKLTDYKEREIFLHFARRGRALVTLFTVSTISCYF
VFMILNNIPTILGEATKDASKTFPYYSKRWIIDERLRNLQIFLHVIIVTFYSGMVYIVAITTYIYSIKHF
CGIYAIVAYRLKNLTSTRRVMPRNKFSAKYDVLAELFDIVNKHKEAIKGVRAIGGIFRRSFFVIEICFLC
AFAMILFDIHSNLFNLRLLLRICFIASIFFMHTFYINYGGEMVIHFSDKIRMATFFIQW

>NvOr187

MKLNGYDKFYYTLHKYVLTCIGLWPYQSRMSKRFFFITYGISSCSLIIALIAGLSEKWSTDPVIILENML
GIIFLTSSTAESSILYMHESKIIEFYDKIKTDWKKLTNKKEIEILQMHTRKGQFVSTAYIVYGIPAFAIF
GFVTFLPPILDPPSRVEYSHIFPYYFYCMIINEDFRYYQIVLHCMVSFSYASVSYLAVNCTFAKCVNHVC
GIYAIICYRLQNAIEPTVVRGPFNKLKNSKLIRFNLIDVIAKHREVIHGVDMIEQIFSTGFLVIEIAGFS
GIALVIADILYNQKNAYQLFRIMVVTAIFLVYIFYINWMGEQIIQVSDDVRLTAYFIDWFTLSIEAQEII
HMIVWRSCKTNKLTAGSFVALSLENFLSMLKTSWSVATVLLSAHRSQKNAHFTGYGITNSFTNSSST

>NvOr188

MNIFDFPQRHLLTCLGLWPYQSKFTQRIFFTCAILSFFSLFVAMAAGLGEEWSTELVIIYETIVALFVIF
GGLAKCIVLFCRKHQMKSLYDQIRKDWQELTNEKEAAILQSFMLKGKAQIILYVVCAIPGYFIFVALTYV
PIISSEDASKDYSHTFPYYTDLLILSKRFRVYQVFIHAGLGIFCGGITYVAFMAMYITCVRHVCALYAIV
RYRLENMVKSQDKLMDKLNDDEEVIPGLLEIICTHKRAIKRVRLINRIFSRTFFMVEICLLICLALLIFD
VKYNQHNVRLVIRMLMIALMFIVHVFCMNYCGEQVIQFSTDVQYAAYFMEWYMISSRAQKILIMILCRSS
NPDYLTAGNMALSLKNFASIVRTSWSMATVLLTTQKVNRPSYSIS

>NvOr189

MEEPDLDIPYMKLQKLLMNCCGLWPYNSRLINRLIYSFFLLILISTIVPLGLGLIEEANNDIVTYFESLV
SVVTMFGGVAQITMLRTIRNHLVKHLYKKITADWQTLKDAKEIKILSAFSFKGRSLTFLYMLITMSSYVI
YLMLIYIPLANDKATSWDYSKIFPYYSKHWIISERVRHLQVTLHGCFGIFYGGVAYVVGMALYICCCKHV
CGMYAIVGYRLKRLIISCEVTSSGKLRDDVLVYNLYAIMDQHKEAIKGVHLLARLFSHSFFLIQLCLLVC
LSFLIFGVQYNIYSHKGMVRMFPASIIFVAHVFFMNYGGEQIIYYSSKIHTTTHFMQWYLLSVKSRRILL
MLIRRSCKPEQLNAGTMTLSLVNFTSIIKASWSMGTVLVSAHRKH

>NvOr190

MPMEEPDSSIPYVKIQKFLMNCCGIWPYNSRLVNCLIYSFFVVFSFTTMTPLSLGLNEEANNDIVTYFET
LVAVVAIFGGFAQITMLGIRNHLLKCLYRKISTDWRTLKDARETEILSAFSIEGRSLTFLFMLITISSYV
FYLLLTYIPLINDEVASSDYSEIFPYYSNDWIISDRMRHLQVILHGCFGIFYGGAAYIIVMALYICSFKH
ICGMYAIVGYRLKRLVTSCKLTSSGELRDDDAVSKLYAIIDQHEEAIKGVRLLVRLFSRFFFIIELFLLI
CLAFLIFVLQYDIRSSKVIVRIFLASIILVTHVFFMNYGGEQIIHYSSKIHTTTHFMQWYLLSVKCRRIL
LMVIQRSCQSEQLSAGIMTLSLENFTSIIRASWSMGTVLMSAHRKE

>NvOr191

MDIFQSSYYIRCNRYLSFCGHWPYQSLRNRIRNFVLLMFLMSTILIPQIIKFWQLRHNIHVFVAALPSML
YYCAFLFKNSFSMLQSKEIKKVLEKIKSDFQRYKDEDLKILHKYSGQANKINTFYTVYMFMAVGGYSMLP
LTLHVMDIALPKNESRLPTKPRLINYNIEAFDENIFFIIIHGVIVDTAVIVFIIGFETLCFSFSYHVCAL
FVIVTNKIRDSIDERITSKHSEVDQDIFYRNFVKIVIMHKDALDFVDTVETALSVLNLFAIGFAMMPLTI
TGFEFILSKGNVGEMARWSLFAFGEIVHLFYYNWPGQKIRDHSLCVYQSCYAIEWYKEEIPDKCKKLLNL
MMLRGQKPCSLTAGKVYILGLENFAAVMKVSMSYFTVLSSVM

>NvOr192

MDILKSSYYIRCNKYLSFYGHWPYQNVIVKIRNQIVIMLLIMSIFLPQFMKMIEIRHYFHYFILSLPSLL
YYTQFIAKNVFAFVGRKQIKNVLDKIQQDFQVYKGEDLAVLHEYSKKAQKFNKFYTVYMFMVVGAYSMLP
FTLYMLDTFVPLNYSRLPYKPRLVKYCITTFDDNILFIIIHGGIADMMAIVFVIGFDTLFLSFAYHICAL
FVIVTHKIRDAVNDEIDSQNSTINECSNLREDISYRNFVKTITLHKYVLTFIDTIETAFSPLNVISIALA
MVPLTITGFEVVMNKGNPGEMLRYAMYAIAEMIHLFYYNWPGQKIRDHSMLIYEACYATNWYRKDFSVRS
KKIMNLMMIRSQKPSYLTAGKIYVLGLENFAAVMRVSMSYFTVLSSVT

>NvOr193

MEDNVLDGPYYVYCKNYLSSFGTWPLQSYKKKVLLRTLMYLGCSSALIPHVTKAYELRNHLEYFFLCIPS
IIFYVQVLTKISCMILNEDKCKELIKQIKSDFQSYTGDNLRILNEYAEQARKVNHVYIYYFMGTVVVYNT
SAFVPLLLDLLVPLNETRPRPVLRLMKYNIQRIENNFFVTTLHGFVLNILGMMLIMGFDTLLLNCSQHAC
ALFQIVMTELKDTIDKHKIEATSDTAKDTNSRDVFYQEVVKVIIKHKHAIEFVDLVESIYAMANLLVIGI
TLGSITLAEFETVQHKDNHEIAFRYAIFTSGELLHILFHNYPGQRIKDHSLMVYQSCYNCEWYREGITDE
CKKLLSFMMLRSQKPSCLTGGGLYVLGLENYATILKASLSYFTFLSSV

>NvOr194

MDIINGPDFVYSRICLRPFGLWPFQDPKSKLISRVITLMAVSTVLIPHIMKTYEFRNDFHILLMCIPSLL
YYAHYITKFLYIAFREEKVFRNVLERIKDDFVTFRGESLNHLTNYSEEARKFNTFYMMYLCSTVVIYNVT

AFIPHMLDFVFPLENATRPRHAARLVKYNIHQIDNNFYFVLIHGMIFDVVAIAIIIGFDALFINCAQHAC
ALFKIVVVELRKSTKLDEKMSNSASDLVTLQCRQDIFYAKLVRTIIAHKHAIEFTDNLESTYALVNFLMI
GIAVATITLTEFETIVHVNEVDIMCRFAFFSGGELISMLYQNWPGQRIKDHSLRVHASCFECEWYREDVS
YKSKRLLMFMMLKSEVPSALTAGKLFILDLQNYVKIFKASLSYFAFLSSVAKVSSN

>NvOr195

MKMKDNVLDGPYYVYSKNNLSPFGMWPLQSYKKKVLLRTLIYLGCNSVFIPHVCKAYEVRNNFQYFFICI
PSVIFYIHVMLKMACMILNEDKCKELFKQIKNDFEIYTGESLRILNKYAEQARKVNRVYVYYFMGTIVAY
NTLAFMPLFLDFLVPLNETRPRIITKHMKYNIKRIENNYFVTTLHGYVFNILGMMVVVGFDSLLLSYTQH
ACALFQVVRNELKDTIDKHEIEVTSHAAKDANSRDVFYQEVVKVIIKHKHAIEFADLVESTYAVTNLLVI
GITLGFITLVEFETVQHKDNRALGIRFAIITIVELLHILFHNYPGQRIKDHSLRVHQSCYDCEWYREGIT
DECKKLLSFMMLRSQKPSCLTGGGLYVLGMENYATILKASLSYFTFLSSV

>NvOr196

MAYAEEPRENILESSTFLYSKSSLRVFGLWPYQEPKQRLICRTSTAVLIGSLLIPTICIVLEQWRNFDYV
VLGLPSFLYYVEFVTKYMYLAANQKKLEQIFGHIKNDFDTRKDRKLEILKDYATETRLFNHIYAAYLIIV
VVMFNLSFYQPHFLDLIMPLNESRPRPIPRLARYYVTSLDESFNFVVLHGLVIDWYSMIFFLGHDTLLVN
CAQHACALFKIVINDIQDCLVVQKNDKADDEDQFYRRISNTIDLHKWALEYTAMVDKMYMYVNLCIIGVS
LLAITLSQYQTAIHLDNTDLVIRYSFFSIGELVHILYFNWPGQRIRDHSLSIYQACYNCEWYRDDISYRC
KKLLKLMMARSQLPSNLSAGKLYVLGYENFAQVLKASLSFFTVLLSVN

>NvOr197PSE

LDNSKIMLENTVALIFTTVCMSKYCITYSSTKQLDLLLLQIVKDWKTFEDKTERKILEKNTGQGTTLVLI
YTYYLLFAWMIYTFMPFTPYFLDQIFPLRNGTRPIQLPFYADYVIFNQLDYHYWCCGHIAVVYFTSFFLY
SGVDGAYVLTVKHVCGLFAITCNRIETMGNSQKTDEARYEQKNYSTVKGEMAGAVILHNETIRSVDLLEN
SFSKCFLIVEGLCVIGLALESVYMLFIENEIFKFLRSGAFIIGVIIHLLYLNWVGQQVIDSSSKIFYSS

>NvOr198

MDKERNMDRMNFYYIYSRISMLSLGLWPYQSWSSMMTLRSLWIIQHISIMLPELIKIYENRGHFNLLIES
LPPFTYNIVMAIKFTNGVLNQRKLKSILEKIKYDWNKFTDKKEIEMLCYYSHRGKSLNTVYIGLVAVVLL
SYMLLPMLPAVLDLINPLNESRPKSPLYMVEFYIDQDKYFYSVLTHAYITSLAGVLPLFATDLLFSNCAH
HACGIIKILGRRIENILSEEPALKRSYKVDDEKKAIACVIEHQNIIKYCESINSLYTTSFFLILSISIGL
MSVTGFVTLIKMNEEFKDCIRFAMFTFAQIFHQFCYYFLGQSVLNHEEKLKDYVSNFNWYKASPKTKFII
KFMIMRTLKPTKIRAMIFPLTLENFTSLMKTTMSYFTVIKSTR

>NvOr199PSE

GIWPLQSKSSKVIFEFLWITQEFIITGAESIKLVEIYKEIDLXPFIYNRLNDIVFANGIINQHKMKILRR
KIQNHWDSLTDESEIQILAKTVKFGMLINVGFIXIIPLLPLVLDIVZSLNESRPLKSPFTCEFFVDPDKY
YFTILAQNYISALASPLPLVGMDLFFINCCNHICRIIGILGFRIDNITKTEALNKESKSTYKQLCDCMDM
HQIIFDFCDSINDTFSTNFLVMLFINMSLMSFTGVAAVIKMDDFSQINDVMRFGVFTTAQIFHLFCFNYM
GQSILTAIEILEEKIYNVNWYELPLESRVLVQVMILKNYRPTVIQGGIFPLSLPSFTAVMKTSISYFTVI
KSTR

>NvOr200PSE

MSILGFWPSRIVLRCFWVFQHMSIMIPEAIKLYENRNNIDLVIEGTAPFTYNTTMLIKFLNGIFNLEKTK
SVLEKVKNDWNTLLDDRZTEILSYNCSSGKFLNTVYIYMAWLTLVSYMFFPLTPMILDFISPLNETRPKS
PLYLVELYIDQDKXYCDNINEIYNISFFIILIVELLEMSVTGTAXVSRCQSHTAIFSTSHHTQIFHVFCY
FFLGQNVLNYSENLRDTAYNLNWYAASTKTKYLIQFIIMKSLKPCLISVSIFPLTLENFTTLMKTVFSYF
TVMNSTR

>NvOr201

MDKERNMKFYFVYTRLSMLCLGIWPYQSWSSMLTLRSLWVIQHISILLPEGIKIYKNRKNLNSIIDGLPP
FIYNVVIAIKFINGIINQHKIKSILEKIKNDWNQLSEKKEIEMLRDYSDTGKAFNTVYLSLVTVILLSYM
LIPMLPAALDLVNPLNESRPTSPLYLVELYIDQDKYFYSVLTHAYITSLAGILPLFAIDSLFSSCAHHAC
GMIEILGGRLENIINEEASIKEIDNNEEEKNAIACVIEHRGVIKYCESINSLYSTSFFFVLSFAIVMMSV
TGFVAVIKMGEEFKDSIRFAMFTFAQIFHMFCYYFLGEIVLHHEEKLKDYASNLNWYKASPKTKYIIKFM
IMRALKPTTMRAIIFPLTLENFTTLMKTTMSYFTVIKSTR

>NvOr202

MEQHYSIRTYFKLNRVFMVSSGVWPYQPLHVARIIRLLWITQHISIMTPEIIKLIEVRGMADLLLECIPS
VFYNIVIAVLYGTTIHHQRKIKELIEKIQKNWITISKKSEVEILTRYSNMGIRIGWLYIGALYFTLFIFC
LFPLSPIVMDYVNPLNVSRQRLPLYRVQFFVDDKKYYWTILMHAYTTTMIGIIPLLTVDLFLANCTQHIC
GMMLILGKRLEKTMETTKLVVNKLDDNIYKDIRKCTILHTEILDFIEDINYIFSTAFGILLAVLTFLTSF
TGIVVLIKWGDWNEVIRFGMFTMAELFHAFCYSYHSQDVIDHNNQIHKSIMNSGWYKSSMRTRVLVQMMF
LRSNKPCLINCIIFPLSMENFTTILKTMFSYFTVVKSCRF

>NvOr203

MDGKQCLQTYFIVNKVFLFSCGTWPYQHTIFAKTFRYFWITQQIVIMAAKSIKLYEIKNDTDLVIEAVAS
FFYNISITIKFVNQVINEHKVKIILEKIQDDWKSLEDDSEIKILSYYARLGKLFNFMYIGAVYSALISYM
VLPLTPIILDFIVPLNESRPKQPLIMAEFFIDQDKYFYPLMIHAYLSVLYGIIPLLGTDTLYMNCVHHSC
GMLKILGNRIRNILNSSSRELSNKIKYEKMVKCIIQHQNIIEFCNNINETYSTSFLIVLCFSITLMSFSG
VATVIKLGDNFNDVIRFGFFSVAQIFHLLCYNYMGQNVLNYGEELRAQIYNTNWYEASLKTQRLVKFMMA
KNMHPIILRANIVPLCLPNFTRVIKTSMSYFTVLQSTR

>NvOr204

MEKENDCIRAYYKLNKFSMTLLGHWPYQSENSVKIVTFLWIFQHLSILLPELIKFVEIRNNVDYVILAFS
PIIYNIVVGIKFVNGSLNRHKIKITLDTIQSDWKSLRTEEEARILANYSSFGKLCTVGWAWICTTTIICY
LLFPFTPFVLDLIRPLNETRPRQLIYMVEFFIDEDKYFYEIQIHSYATTLIGFIPLISIDTFYAASVQHA
CGMFAILGHRLRRINGAMSKSKKRSDEDAYREIVSCAIQHDKILQYCDNLNDTYTDSFFYIMGCNMISLS
FCGVLLILMWGRIYDMLRNGIFTFAQIFHLFYYSFQGQVLSDRSLMISDCVYDSEWYTASLRTRKIMTMV
SMRSLKPFLLTAKVYVMSLPNFTLVIKTSMSYFTVLKSSR

>NvOr205PSE

MILEQTDIRDGIGPKTYFKYNSMFARGIGVWPFQSKLSKAVYFTLWVAQELILAIAELIKFTQIYTDVDL
LLEAVPPFLYNRGNDVLLANGVINQKKIKALLIKIQKHYNSLTDESELQILDETTKFGHFLNLGYIGLIY
AAVLQWMLLPLAPLLLDYIKPLNESRSLQPLFMVEFYIDPDKYFYTILAQNYVCALVSPISIIAMDLIFM
NCANHICGMIWILGFRIDNISKNVEMKGQSKEREYKQLCDYIIMHQNIFDFCDDINTAFSTSFLLALFIN
MVVMSFTGMAMVIKMDDFSQFNDVMRFGVFTVAQIFHLFCFNYMGQSILTAIEVLDLKXHDVKWYNVSLK
SQVLVLLMILKNYRPTVMQAFLFPLCQETLKSFMKTSMFYFTVLKSTR

>NvOr206PSE

LYIFQEKYLIYFAWQMVSTLKNGADDSPPFLRIVKKMTTRLIYFKNLKINIFMIXYFEHVYCNNIGNIFY
QNIIVSWNHTSKEKEIILTRLYTECCLKTMIVHTISINILFQRRPVLYDIIKYLKESNITCYNKELPFCF
EVFADQEKYFDYICMLLFLMSCTLLIALSHELIFLQSVQDIIGSFEITEXGLSKIVKDMALIZYZKKLKC
KMLACIFRVVELYNAILXNIQLINTAFCTNYYEHLLFNCLIFRAFXXCSHEWYKLPKRLKGLLRLMILRR
AIKCHFTVGSTFVLSSETLCR

>NvOr207

MIFSFKDKFFPYYAWQTVTVLEKAAGWWPFQNRTKNMTLRLMHFGNLLLIIIMCSVRLFQEYKQKKLYIV
VENTVILVMIICTKMKVIMFFINEKQRKIFYENVLIHWKDTNDEEEMMILKQYAKLGLKTIINYAISVNI
LFQGVPVLSDVINYLNDPNITYLKKELPIYIEVYIDQEKYFYQLYVVLFFMTCAALLLAFSHELTFFQSV
QHINAMFKIIEIRIIRLSKIVKRTECGLDTFKEADRKIFVCISRAVDLHNAVLNNIKFINSSFGATYLVV
LLFNCLIFGASLFLIFNNSDQKIHLIRYGLVFLGLSVHFFIIFWPGQKIMDGSESLFNVCCSCDWYKLSK
RSKNLLRIMMLKSVMQCQITASGMFVLSFETYVKLFKTGLSFVAVFS

>NvOr208PSE

MHXILFKGENQYFLYHPWQVVTSLEEANGTWPFQNKRKNIILRLLHMGNITAMFIMFSIRLWQEYKNKNF
HIIMENSLALIICFCVQVKIIMLCINEKQRYSFYKQVLALWKDTSDEEEIMILKQHADFGFKTLKIYIII
SSTIYQVIPILSGVLRYLTDPNITYYQKELPFYIEIYIDQEKYYYHIFIAMFIMILAAAIITFSHELTFL
QSVQHIIASFKIIEFSLIRLSKIAGEVESGLNTLKDTDKIVLTSISKIVDLHNATLNNIKYIDQAFGTTY
FIILLFNSLLFGSSLVLINNNLGDTLYVIRYALIFTGLTTHFFIIFWPSQRITDANESLFQVCCSCEWYK
LSKRPINSLRIIMLKSSMQCEITAIGMFVLSLETFSKLFKTGLSFFAVFARN

>NvOr209PSE

FKDKYFLYYAZQAVTVLZKVSDCWPFQNQTKNVILRSMHFGNIMMIILVAXLMSRTIIMKNNKFKYYLRI
RTSHTMPFENQIKKMIIRLIHFRKRFYEQIFVQWKDTSDEEEIMILRQYATSGLKTIIRYTTSISIYFQT
VTMLLTLMKYLMNPNITNYQKQLPFYAEYFVDQEKYFYQLYLLLYFMIGAAFIVTFSHELTYFQLVQHTM
ASFKIIEXLRLSRLLKIVKSVDCCLGRFNDADQKIFTSIRGAVDLHNVTLKNIKIINRSFGTTYLFVLLL
NSVMVGSGLFVISKNIDDIFDLIRYGLSFLGLTTHFFIIFWPGQKIIDSSESLFNFYYSCKWYKLSKQSK
RTISIMMLRSFGQCQITASGMFVISLETYGKLFKVGLSIFAVFSQN

>NvOr210PSE

MRKIFSLNRDXDDFFPSQYWHLLKRLEKLAGFWPYQNKIKNTLIQSLNMIFLTIMILAFVARIPIEIKLQ
NHAIAIEASVGAMTAFCVVMKSCIMSVNDKSKKLLYAHVVSHWRNVSLKNEKSILSRYAHNGHRIGVTYV
VCLAIVCVFNVPIGITYIEYWLAENRTDLKIQTLFYAEYFVDQDKYFYEILTFNVIVTINGYFANCAHDF
AFIVTIQYASSLFKIIIXXTVAYIENIFNLSWYFVISTNIMILAPGLVLIMRVZSIEEGIPYILALVVYF
THLFTIFWPGQIVSNSSSLLYYACWNCQWYRLSRRSKFLIQMMMRRSSKCCILTGGSIFVLNLETFKKVF
QTALSFFSVFANI

>NvOr211

MGKWRESKSEFYEDRLFVINKKFLQILGRWPYQAKRSRIFLLSLYFVGILTVVVAELIHFVRVIRIKDIP
KIIDCLPALIFTYGAMVMIFNSMIKFAEMRQILGKIEENWLSARDAEEYALLKEFAEEGRFLIIGYTLCI
IGSLATYSLEPLVPQILDKLSPLNESRPIKTFLEVEYLVDHRRYYTAIYLHNMQAACWVILTVLAIDAYF
VMIVQHACSMFAVLGLRIGKIGSGSPERIASGRIIECLKLHKSCIEFAGLIESSFTASLLLQLFLNMVVI
SVTGVQTINRREQPGEMLKFSLSSLSVMTRLFYISWPGQKMIDHSLRVRELVCSVAWYELPAGSRRLLLL
MLLRSSRACHITAGGMFPMNFETYCRLMQTSMSYFTMILSTQ

>NvOr212PSE

FAGIIYYAHQDIWVKALENFVAILFFGAILSEFNLNYLLKERVRLLYKKIAYDWVMLSDKNELDILHTYS
RFSRTLTMIYXNYALLAFILILFVPLAPIIMDNIMSLKSGTRIRVETIHPYYFDMIDVSKYYYHAYVLHF
QLVGSYDZIAYLSINSMHATCIQHVRGLFAIVCYRLEKLERCDNISETSEKTSIGLIDDRKVIQNIKAIN
TLYSSSMXYIHMIANSFGLNFIMFISFTVVGISIIMFDLLFYHDHPFEKIILLGLIIGILMFFFVLSWII
QKLTDRSEKVFIQV

>NvOr213PSE

MRLPITGYSVFDGPYYWINKILLDGFGLWPETSRERKFMSFVFFNISTFSLLIPTFAGMIFYGKRKIWVK
ALENFVAILFFGAILSEYNLNYLLGKRVKLLYKKIADDWKMLSDKAELDILHKYSRFSKTITMIYICYAL
LAFILILFVPVVPVIMDQIIPLKNGIRIRVETIHPYYFDIFDINKYYYHMYVFHFLLVGSYGSIAYLAIN

SMHAACIQHVRGLFAIVCYRLEKLGKSDSMAEAPCGLINDSKVVHNIKAVNILYNSSIXCIHMIGNSFGA
NFIVFISFTVIGIAILMFDLLFYQDHPIQKILVVGIIVGVLMFFFVLVWITQQLTDSNENVLLQVYSCHW
YNLSVKGQKLVYLLMMRTFKTTKIRAGGLIEINIHLFESVLKTSLSYAMVMQKLNIS

>NvOr214PSE

MALLSTDFSVFDGPYYRINKILLKAYGLWPETCNKTTKRTIFGFLNLATFGLIIFIFAGMMHYVNENIWV
KALENFVAILYLIAIVLEYNINYLLENKVKLLYEKIEYDWKMLTDQNELDILHKYAQFSRKLITIYIYYA
LVACILFLILPLAPIAMDYIIPLKSGIRIRIQTIHPYYFNIFDVNKYYYYVHVFHVSAVGFYVSAAYLTL
NSMHVACIQHVRALFAVACYRIEKLGHHDNTSETSAGVIDNKSVNQNIRSVNNIYNNCKSX

>NvOr215PSE

MRKQSVNLAIFNGSYYGIIKTLHVSYGLWPELGKTRKFISLILYFTTTLSLYIPIIAGIIYYLHRSEWVI
LLENVVGILYSLVTLLKYISCHMFEERIKRLYNRIVCDWENLTNKNEVDILHNYSKYSRTFAIFYIIYSA
IGWIIILCLPFIPIIMDQLAPLQNETRIRIELYHPYYFGIIDMNKYYYHXYYAYIFHGQVIYGISTIAIV
TVDTMFAACMQHVCGLFAILCYRLKQIGSNTKGNEMRVSDDKQVYRDIKFANEMYNNCRXFIDGIWNSFG
ITILNFVSSIIFATAVLLFDLLFYRIHLYERIILSILIMGILLLFLLLSWLFQHVSNSSEXVFKAVCCSC
WYNLSVKEQKLMYLLMLRTFNSIKLRAGGLIQINLRQYAWVVKTAISYAMVMQSANT

>NvOr216

MTISETDLAVFDGPHYSLNKKLLIMFGLWPTLSRTRKVICLIFFTMIDLSLYASFADGINYYRKQKKWMY
VIEDTISIIYLSVTWIKYVTSYIFESRIKLIYEQIAADWKSLIDEEEIQILNNYSAFARLLTVLYVFYAI
IATTLFYVLPFLTIVIDRIKPLENGTRFRAQPYHQHYFDLIDNEKYYYHMYIGHGYVVSIIVTVAVMAID
TMYAANIQHACGLFAIVRHRLSKIGILNGEREYEFRVVDDKKVYESIRAVCEMHKNSIKIVELIWDSFSI
SFLIFMGCSLVGMGMLMFNYIFNMIHPIEKVVGTGLLLGIAILLFYMNWIAQQLTNSSDEIFIAVYSNRW
YNLSIKGQKLIYSLLQSNANSVTLRAGGIAEMNLQQFAAILKTAMSYATVMMSMNG

>NvOr217

MGIPDPENPLAIFYTDYYKYNRKLLEICGLWPELSRPRKIIMMILFALLMTSLIIPMGAGAIHYFHKGRI
MYVVEDLIGLLYLTVASSKYFTYSVFEGRILRLYHQVGEDWRTTTDEEERKILQEYSEFARLLSIIYFVY
AAIGAFYFNMSPYLPLGLDRWMPLESNESRVRIRTYHPYYFDLIDAEKYYYECYFFHGNSVVYVSTMVGL
SVDSMTAFNVQHICALFHIVGHRLRKIGSTLEINAKGEKIARVDDITVVRQIKHVCGMHRTSIDSVELLQ
SSFGMNWLVFLIGTVTGIALLMFDLIFSMKHPLEKMTGLVIFIGIQILIFYINWIAQKLTDSTEQIFLAA
CETCWYNLSVKGQKLVYFMMQKNIIPLTLTAGGIAELNFQQFASVSKTSMSYAMVILQMNDE

>NvOr218

MPVETKDQRKSYDFLSISSSHLTFMRLSSFLPLKGKSFFHPLSLLLQLWDHFIVIGYNVMWQGYGIRMIQ
RGDVEVDFICEDIITVGFTIRYLLLCAKREKLCCLVESCEKLWDLLKDGEVIFVRQFARKGYYFRNFILI
NAMLMAALYSVTAPFVRLPPIEANGTERKILPFRFFMDIQKEPAYSIVFAFQSILLQFIDLMIVSTETVS
LYLIMMACGYLRSVRNRLLSFKGNDDNTSEKGEAALKFVIDCAHFHQQIMIFCEDIERMTRTLFFFACFC
PIYNVSITGIVLFNNDEDKYKFLPLLCYNFFQFFLCQWAPEHLAVESEDIALAAYSASLRPQAPSHREKI
NRILYFMMMRAQKPVQLTAGGFVDLSIETFGAMTKSAFSFFMVLRKFRS

>NvOr219

MSTKAEDSHTFQSISASHLNYLRLCSLLPWQGKGFSHPFSLLFQLWNHLAIFGFNAMWHGYGIRMLQRGD
VEIDLICEEITVLDITARYFLLLIKREKLGRHIETCRKLWSYLKAGEDMFVSQFERKGYYLRNFVMINSV
MVTTAFIITATFVRLPPLEANGTERRMLPARFFMDVQEDPAYSIVFASQSILLLSVDVMIGSTQTVSLYP
IMMACGYLRSVRNRLLSLEGSDNGTDAKGEATFKFVVDCAHFHQQIIIFCEDIERMTRMLFFFACFCPIY
NVSIAGIVILNSNEDKIKFVLLLVYNFFLFFLCQWAPEHLTVESRAIAEAAYFASLQPLASSYREKINWI
LYFMVVRAQKPIQLTAGGFAPLSIQTFGAMTKSAFSFFMVLRNFRT

>NvOr221

MRGAMSAEPKDLRESFTFLSISSSHLIFMRMFSYLPLKGKSFSHPLSRLLQLWNHFAVFGFNAMWQGYGI
RMIQRGDVEVDFLCEDIITIGFTIRYIVMRIKREQLCRLVESCEKLWDLLEDGEAVFVRKFERKGYYFRT
FILCNALLMAGSYSIAAPFVRLPPLEANGTERKILPFRFFMDVQEEPAYSIVFVLQSIALQFLDFMMVMT
ETISLYLIMMACGYLRSVRNRLLNLKGSDSDPSEKGEAALKAVVGCAHFHQQIMIYCEDLSKMTETLFLI
SCFCPIYNVSVTCLVILNTEEDNLKFVPLMLYNFFQFFLCQWAPEHLTVESDNIAEAAYFASLQPQAPSH
REKINRILYYMMMRAQKPVQLTAGGFAPLSIKTFGAMTKNAFSFFMVLRNFKN

>NvOr222

MTTKTKDGYTFLSISSSHLIFLRLSSFLPLKDKSFSHPLSILLQLWDHFVVMVYNSMWTGYGIRMILRGE
MEIDFICENVVVMGFTVWYIVIQMKRQQFCSLVKFCEKLWSYLEVGEEVVVRQFERKGHYFRNFMLFNLL
LMCTLFITTAHFIKLPPLEANGTERKILPFRFFMDVQEEPAYSAMYTLQFFVCYFVVFMIASAETVSLYL
IMMACGYLRSVRNRLLSLEGNDDDTGEKGEAAFKLVVGCAYFHQQILIFCKDIERMTRTLFLFACFCPIY
NASITGIVLLNNDEDKFKFILNLFYNFFQFFLCQWAPEYLSEESEVIAEAAYFASLQPLASSHRQKINRI
LFFMMMRAQKPVQLTAGGFVKLSIETFGAMSKNAFSFFMVLQNFRS

>NvOr223PSE

VRGATSIKTKDGHTFLSIFSSHMIFLRLSSFLPLEGKSFSHPLSLLLQLWGHFLVVMFNVMWHGYGVRMM
QRGDMEIDFIWEEIVTIGFTRRYLVLFINRRQLCHLVESCEKLZDLLEDGETVFXGYYFRNLVMFNMMFM
TTVFSVTAVFVRLPPLEANGTERRMLPFRFFMEVHEEPPYSAVFVSQVVFVYFLSVVFVSVDTVSLNLIM
MACGYLRSVRNRLLNLRVNNDDKSEKGEATFKIVAGCARFHQQVLIYCENIGRMTQTLFLISSFCPIYNA
SVAGIKLLDNDKDKYKFMANLVYNFFIFFLCQWTPHYLTEESEDIAAAAYSASLQPZAPSHRQKINGILY
YMMMRAQKPVQLTAGGFVQLSIETFGAMTKNAFSFFMVLQNFRT

>NvOr224

MPGAMSAETKNTFLSISSSHLIFLRLASFLPLRSKSFSHPLSLLLQLWDHWSVLAGNMMWSGYGIRMTLR
GEMEIDFICEDIIMVGFTMRYILLATKRKKLCHLVESCEKLWDYLEIGEDALVRQFERRGYYYRNFMMLN
LLLMCTLYIVTAHFATLPPLEANGTERRMLPFKFFMDVQEEPAYSIAFVSQSVVTYFICFMFVSTETVPL
YLILMACGYLRSVRDRLLSIEGSDDDTSERGEVAFKFVAGCAHFHQQIMIFCEDIKHTMRTIFLFACFCP
IYNLSITGIKLLDNDEDKFKFIVILVYNFFQFFLCQWAPEYLIEESEDIAAAAYSASLQPQALSHREKIN
GILYFMMMRAQKPMQLTAGGFVRLSVETFGAMTKNAFSFFMVLRNFSS

>NvOr225

MSTEMEDPHESYTFLSISSSHLIFMQLSSFLPLKNKRFTHPLSLLLQLWGHFVVFASNVFWTGYGIHMVM
HGEVEVDFICEEIVVLDFTARYILLIVNREQLCCLVKSCGRLCSYLEAGEDIFVRQFERKVYYFRNFVII
NSLLVSTVFDVTAYFTRLPALEANGTERRMLPARFFMDVQEQPAYVVTFVMQVILDYYLDFLIASTGAAP
FYLIMMACGYLRSLRNRLLNFKGGDYDTSEQGEAALDTVIGCAHFHQQMMIFCKNIERMTQTLFLFACFC
PIYNVSITGIAILNSDEDILKFTPLLVYNFIQFFICQWASEYLAEESEAIAEAAYFASLQPQVPSHRERI
NRILYFMMMRAQKPVQLTAGGLVNLSIQTFGAMTKSAFSFFMVLRD

>NvOr226

MSAKKKVQKEGDTFLSLSWSHILFLRVASFLPLKGKSFSHPLSLLLQLWDHINVIAFTSLWQGYGYRMIK
RGEMEIDFICENIITIGFTLRYIILCLNRELLCHLVESCEKLWDLLEDGETVFVRQFERKGYNFRNFFFG
NLMFMATLYTITAAFVKLPPMEPNGTETRMLPFRFFMDVQENPGYAAAFVFQDVVVFYTDVIFASAETVP
LYLVLMACGYLRAVRNRLLKIEGNDNDSSEKGEAALKVVVGCAHFHQQIMIYCEEIGQMTKTLFLVSCFA
PIYNVSIAGIKLLENDEDKFKFIVILVYNYFQFFICQWAPEYLTEESESIAVAAYSASLRPQAPSHRQKI
NGILYFMIMRAQKPVQLTAGGFVNLSIQTFGAMTKSAFSFFTVLRNFSG

>NvOr227

MPTGMDSLTTTFQSISSSHLMFLQLSLFLPLESKSFSQRLISNLLQLWNHLMVIVYNVSYAGYGIGMALR
RDIEIDYICEQIVVETFSARYILLCFKRAQLRRLIESCKRLWGYLEVGEDIVVRQFERKGHFFRHFLILS
SLMAVTSYVVTAHFLRLPPLEANGTERKMLPFRFFMDVQEGPAFNAMYALQIINSYYLVFMFASVETVSL
YLIMMACGYLRSLQDRLLSLITEMNEDDLSKNGEATFNVVMGCAHFHQKIMIFCKDVDQMTRTLFLFACF
CPIYNMSITGIKLLESDEDKFKYASLLFVNLFQFFSCQWAPEFLIIESEAIATAAYFASLQPFAPSHREK
INRILYFMMMRAQKPIQLTAGGFIKLSIETFGAMVKSAFSFFAVLRSFRT

>NvOr228PSE

MTTEMDNAKPEATETYTFLSISLSHLWFLRLASFLQLKDKSFSHPLSLLLQLWDHVSVVIFNSMWTGFGI
RMIIRHEVDVEYICEEVAVNAFCLRYLVVCFKRVQLCRLIEFCERLWEYLEVGEDMVVRQFERKGHFFRQ
LILINVLAVATLYVITAHFIRLPPLEANGTERRMLPYRFFLEIQEEPAFSIVYVMQIVVVYFVNTMFASV
ETVSLYLIMMACGYLRSIQNRLLNLAGSYDDSDAKGEAAFKRVTDCARFHQQIMXFCEEVDRMTRMLFLF
SCFCPIYNLSITGIKLLENDEGKFKFAAILSLNLFQFFACQWAPEFLINESEAVGTAAYFASLQPFASSH
RERINRILYFMMMRAQKPIQLTAGGFIPLSIQTFGAMVRSAFSFFMVLRSFRT

>NvOr229

MLIEKKIESFTFLSISSSHLTFLRMAAFLPLDNRSFHHPYSRLLQLYGHICIFIYNTMWTGYGYRMISRR
EFEIDYICEQMVVEGVCLRYIVLCAKREQLCALVESCKRLWSYLRSGEDVIVRQFERKAYFFRNFMLINS
ILVVMLFIGTACFVRLPPLEVIGTERKVLPFRFYVDVQEDPMFSAVYALQAVVCTTISFVIASIETVSLY
LIMMACGYLRSLRNRLLSLAENEDDAILAGETSFRLVVGCAHFHQQIMIFCEEVDRMTRTLFLFACFCTI
YNMSITGIKLLENDENKFKFGAILSLNLFQFFTCQWAPEFLIIESEAIGKAAYFASLQPMASSHRERINR
ILYFIMMRAQKPIQLTAGGFIKLSIQTFGAMVKSAFSFFAVLRSFSST

>NvOr230

MEREPIKYEDISRLYYRLFRTMGILPSSSSRRTTLLRVYFHVTIVLYYSMSMFDGLRMLGHNDIEIEYVF
EEVVIHGICARFLILSCRREELAELLLSCEKLWRMLKPGEDRVVKSYEKIARYLAHYITWTTLVAIFFYI
VAARIVKLPPAEVNGTERRMLPFRFYVDVQRQPWYDIVTVLEIVVVLNIAMIVSTIETTGPFLITMACGY
LRSIRNRLLAIADEAEGRGEISRLSTIRVVSCVKFHQKIMRFCQDIEKLTSSVLLVQVVCTAYNISLVGF
RILKNDPNAVKFVPLLLLNLLQLFTAQWIPEHLLSESKAIANAAYSASLLHPEYEPRANRALLFVMLRAN
RPVQITAGGYMKLSLETFKRMLTSALSFFTVLRSINDGAGDEGE

>NvOr231PSE

ISAEKSVRPSFDELTSLYVRLFRLVGLLPFEAGSFDFLGTRLLAVNFLLFAGLDCSMFICYTINHLKQGE
RDVEIFCEIVPVVGTFVKYAILLSNRNELREIFVDGREIWECCTDSERQVVEGFEKKIRMLFKCLFSTTM
LNIIFYFMKMLTTRLPPLEPGGPERKVLLFKYYVDEVSTSPAYELVVTLQTAVCYNFAIVLLVQDIFVPL
LIMISCGYLRVVQNRLLNICELDSKDDALDEGIDDCCKFHQSTLDMCLKIKKLSPTLYLVHILCTGYVLS
LFGLKFSXSDPDRFKSLSSMMILVVQLYICQWAPDQLIRESEAVSTAAYFANLSRIGSPGNRKILSIVMQ
RAHKPVILTAAGFVRLSIESFGSMVTSAVSFFAALRSISIRDEEQ

>NvOr232

MRLHEINSFERVPASGTIRKFVEFRETDGSLRIFSPPHRGFTFGEPRLQLSLLQDASHLLRVYWPTQQCS
GVVEVDSIFVMVMAVSTIMRYYILVYHRFDFRDTMDACRVIWNDCTPNEHQIVRWFERKTWMLFKLLAGS
GMFINVFCSIGSIVVRLPPDEPNGTERRLLPYRWFIEDREYHWLGYELIFGLQVLITHHLTVIAATVDTA
GPLLMMISCGFFKALQERFFAAAARNEMILCKDKLEFKQTIVSCSKFHQSVLVLCKKIEVMTRMIFMVQL
ICLGYNISLIGLKLTGTDPERFQYIPNLVLCLCQLFITQWASDYLLEQSEEVATAAYFATLMSLDARIGG
LLLTMVIRAQKPVQMTAGGVIKLSIERFGSLITNAISFFMVLRNFTTQV

>NvOr233

MRVHEINSFERALASDEIRKVAEFQESDGGLRIFPSTHRGVTFGKPRLQLSQLPDASHLLHVHRTPHQRH
GAVELDSVFVTVMATSTIVRYYILVYHRFEFRDAIDACRDIWEDCTPSEHQIVRWFERKSWILFKLLAGS

GLLINIFCSIGSIVVRLPPDEPNGTERRMLPYKWFIEDREYYWMGYELIFGLQVLILHHLTVLTATVDTA
GPLLMLLSCGFLKALQERFFAAAVSNEKFFIEDKLSYQPLLTSCSKFHQNVLNLCRKIEVIMRMIFMVQL
MCLGYNISLIGLKLAGNDPERFQYIPNLVLCLCQLFITQWAADYLLEQSEGVATAAYFTTLMSLDPRIGG
LLLTVITRAQKPVQITAGGVINLSVERFGNLITNAISFFMVLRSFTA

>NvOr234PSE

MSEKSRQDECGKLHVFLLKLIGLLSFDGRSLNFPGTLLLSLYVHCSHLLVTSMYVCNVAKTGLGDGEYDI
ETVAETVGLCGVHCRFLIMFFNRERIAKLLNESKKLWTELDEFEVAIIRFISYZVXSLPYPFYADVQSTL
WYELSVTGQMLGMIGICLSSSGVDTAAPFFIMVACGHHRSLCDRLQNLASAVDDDDQVVKVDAVKKIIEC
IVYHQRMIKFCKEVEKLTNSLFMVQLISTTYNMSLIGLKLVGDDPEKFKYLTVLSLLMCQLFMCQWAPDL
LVSESESLARSAYFVPGSGDESKKLAKLIHILMMRSQRPLQLTAGGWINLSMECFGSMITSAMSFFTVLR
SIH

>NvOr235PSE

MSSSRASKSFNASSRIQLSLFRLMGVLSFEKSRPLTSRLLSGWFYLCYCFFGSMFLNTCVHNCLRGYYNL
EQISETVILLGSVGRFLLLSGSRREMERLLTSAEDLWRVLEQGERTLVARFVDISRKVVYAYLVASCLMC
SFYVGITPILQGNGZCVLPFEFYVEVQSTPWYELVLILEGIAMFSLALVSSLVDITAGPFLILMGCGHLR
TLGHRLRNIDSRLDGRPAASSRQYLAELTSCIRYHQMILSYCDQVQRVLGGVFVTQLISTTYNISLLGLK
VIGSDPDKSKYVMLIGVLMLQLLFLQWAPDMLVEESKRLASDSFLVPPIGRENRQIGQLICIFAMRSQRP
IEMKAAGYLELSMESFGAMLTNILSFFTVLHSIN

>NvOr236

MEETSAFYRRIRRIQTRVLRLAGLVPFENRTLIFAGTILMSIYVNFAFTAVSSVYIWAFFEDCLNKRFNP
DITSELFSFVGFHFRFMYIFSRRRKLGEMLGYAESLWERVRSEEKVHVRLFVRKVSKLSVCYSGIILTTI
TLYVLSSQLPQLTAAATNETVHRVLPYPFYVDVQSSPRYEILLGAQIVCLLTVTQTSVCVDTAIAFLIMI
ACGHFRLIQVRLGVIARHIEENEDKRKSQRSVGKNGEVIEAEAEMDEEDFERTDDRVRERVKELVMHHQE
ILSFCDDIKNLSSEIFMIELISTTYNLSLIGILLAGNMPLAEKFKFAPVLFILTTQLFVCQYPPDLLIQE
SEAVANAAYFVPPFRRDRRRIDRILLSLLTRSQTPYQLRAGGQIPLSIESFGNMIRGAVSFFTVLRSFN

>NvOr237PSE

MSSKELAKYRKYAQNARNLLLPVGLWPYESSGFIYRFLSVFAGMSLVLLSYTALNYCFINVTDITKVTRS
FSWCISIWTMLMKIVLFVVHRDRLLDINETLSKTFERELKSFGLESVMLQRLSVFTGVYYTMVVALAMAF
SLFAVIPLIFMITDRXFGVIYYLIYAYEALLAFLFFCVGCGTDTAFGFWVFQICGQLRILAVKFSKLEPG
KDYAAGLKECLEKHEMLLRCRDHLQRVFGFLVIWLYVTISIVLCEYIYKMSKMTLQLSFWQSVMIFTFAT
KFLQAFTYAYCGSIIDIESEKLLYAIYDCHWPGSGDRRIMSDVLSLLTHKSMALRSYNFFIVSMEMFMKI
VNAAVSYFFLLRTFDT

>NvOr238PSE

MEVELAKYKRYKRDLRFMLICSGLWPNYEKHPRIFRAFLSFCSGFLNGLVSFGILSFCIINATNINLLTR
GLGLFFSFSSAFLKVCTLSLHQNDLQKLNDGVSASFARDLEIPENRPHLLAHFRTFSKFFYTFDYSVAMN
VLLYALIPLSLLRHGKYIRMYPQVFFYIYDYEAGGAIHWTLYAFELLAGFFLWSVTCGVDSFFGLYSLHV
VGEMRLLSHRFQNLKSSKEYAKDLKDCVERHVELIKXSVCWERVFGLSGPFGWAITCAVVLCALIFQATE
TKNLSTLKVCYLICYSFLKLVQAYSYAWFGNIVAVESKSCLEAMYNAQWPGSGDTRFMNDVLIVLTQKPF
AFKARNVMLLEMDMFQKIVNTSVSYFFLLRTLEETLDHDS

>NvOr239PSE

MESGLLKYRTYSRNVRAFLAFSGIWPETNRLLSRILAFAALFSNSVVALTSZXVIIFLLHHKELAHLNGQ
LSKRYTEDLSNPDNRSRLLIRVAPFSSILYTLIITGCLTILMYRLAQTRQIPPDVPAYEPGGVLHWSXYG
LELVAGMFLCSVTTGVDCSFGMYSLQVCGELRLLZLKRFEELGASKEYKKDIKQCVGRHHVIVSAKNKLE

STFGLLTIWIAVSAGTVLRVRLXFQFTEAIKVRSTGLHMGVMSMYIVLKFLQGVFTYAWYGDIIAEESDL
CLHSIYDSYWPDAFDPHFRKGVLFVLLRKPLTLXAKGCMHIQLDMFSKIVNTTVSYFFLLQTLNDESEQ

>NvOr240PSE

MEHEVKKYKKYKSDIKFMLIGSGLWPDFYQHPKHFRTFLSVCSAFASGATSYGIVAFCVDNVTNINVLTR
GLGLMISFSSTWLKVVMLAVHKNDMLSLNKGVSAQFEKDLEKPEYRPHLLAYFSSFSRFFYVFDYSVALN
IGMMVFKPLLSLRQGKYVRTYPVLVPFKYEPGGVMHWSIYAFEALSGYYCWSITVGVDSVFGLYALHMVG
ELRLLSHMMENLKASSNYKRDLKECVERQMMLMRSQQLMQRVFGFLAIWLAVTCAIVMCAITFQATEIRS
MXLPYRAIYLVCYCFLKLVQAYSYAWFGNVIAVESELCLNAIYNSHWTDSAGDLSFMNDVLIIQSQNVMR
FNAIGCMIVRLDMFSKIVQTSVSYFFLLRTLDESLVDTS

>NvOr242

MIMEKEVEKYKKYKSNLKFMIVSNGVWPDYEKHPYCVRKFLNFCSISSISMTNYCMMLFVIATTTDVRSF
TSFFGLLLGGFGNLFKVCALTMNQKELHALNEGISASFERNLRVPENRPHLLANFPMFSKFFNFLSYSTL
GTIGFLTVIPLLHLRHGTYSRMWPILLPFSYEPGGTIHWIIFVFELVVSFFAWITTCGVDCLFGLYSLHI
VGEMRLLSSRFQKLEWSENYRKDIRSCVKSHLLLLKTLSQMQEAFGDLAVWFAFNSAASLCTLVFQFSQL
TVMNPARVLYLLCHTCIKLVQAYSYSWYGNIITVESEVCLNAAYNSHWPNHGDKHFMRDVLIILLQRPMV
FKAKSFIALRLDLFARIANTTLSYFFLLQTLDEKV

>NvOr243

MELELLRYRAYTHNVIWFLKSAGLWPEGHPVSRKIRSMVTLFSTFVVMVTVSNFSFQNVSNVMVLTRGMS
LAVSFSSAFSKVALFLLNYEDLVYLNEHLTGIFERDMKKPEYRPDLLKNVKTFHRFMYTHVASLTFTLIM
YVIGPLLALRKHGKYVRVFPAIYPFAYEPGGLVHWILYILEVLGATCLWSVTSGVDCVFGVYALQVCGEL
RILAKKFKELGAIENYREKLNDCIRRHHVLIKAKNKLDNIFGLISIWLAISGALVLCSLIFQITELIKAK
SSYLRVVHLSVYLLPKFLQIFSYAWYGNLIAEESTGCLEAMYDSHWTDSLDKNFKSDILIVLVQEPLTLI
AMGCMVIQLDMFTKIVKTSVSYFFLLRTLNEK

>NvOr245

MDQKRFKYKAYERNVIWLLKSAGLWPEAHPVPRKILSLVTLFTSFVVMVTATNYSFQNVGNVRMLTKGMS
LAVSFSSVFSKIAFFILHQEDLLYLNKHLTGGFMRDMKRPENGPALLSNVKTFNRFLYMHAVSVAIAMIM
YSITPLLVLRKHGKYIRTFPSIYPFAYELGGLVHWIIYAVEVSAAATLVTVSAGVDNLFGFYALQMCGEL
RMLAHRFRDLRAGNNYKDNLKDCIERHQVLINAKNKLEDIFGLITIWLAISGSLVLCSLIFQVSELIKNH
VSYLRIAHVCAYLLPKFLQIFLYAWCGNLIAEESKICLYAMYDSHWPDSHNTNSKRDILIVMSQEPLSVV
AMGCMVIQLDMFAKIVKTSVSYFFLLRTLSAENE

>NvOr246

MEHDVKKYFKYKRGIVFMLSASGVWPNYTSHPAAVRLFLNICSALASGCMFYCIVNFCLNYATNINAFTS
CLGLMIGFFSTFIKVIILPMQKEDLQSLNEGVSASYERNLRIVKFRHHLLAHFPMFSRFFYLYSYSVGMS
VLLLTIMPLLALRQGKYVRMYPQLVPFSYEPGGSLHWSIYAFEVFCGFYLWSVTSGVDSVFGLYALHMVG
ELRLLNVRFQMLKSSNNYAKDLKSCVDSHIMLMESRHKLQRIFGFLAIWLAITCAIALCALVFQALQAKH
ATIIRIIYLCGHCFLKLLQAYFYAWYGNIIAIESDACQSAIYESQWPGSGDKRFMNDVLVVLSQTPMIFK
AKQWMPLRLDMFSKVVHTSVSYFFLLRTLDES

>NvOr247

MELELAKYKSYARHVITRLIFAGIWPESNKTIKTILYFISFTSTLTVSVTSINFGIQNANNVILLTKGIG
LASAFSSVFSKALLLPLHQEDIIFLKNRLTTKFMSDMETIEYRADLLSSVHVFSAFFNMHEAMVAFAMFM
YCFVPLYVLFKHGTYLRTYPCLYPFSYTPGGLVHWLIYALEVAGAISVWTITVGADCGFLMYALELCGEF
KILARKFTELKAGDGYKRNLKECIERHHLIIEAKNRLEDSYGLIVIWLALSGAFLLCSLIFQITELYDNH
GSYVRIAHLCSHLVAKNLQIFMYAWYGNLIADESKAFLNAMYDSHWPEACDKNFKNDILIVLTQEPLVVV
AKGCMYVQLDMFTKIVKTSMSYFFLIQTLAN

>NvOr248

MMDNEVASYVKYSSYLKRLTAFIGLWPDYQKQMPAISLLLSIQAAFSSFTTFCFIAYSCYLDSADIGAFT
SYIGGLVGYLTTVMKIFVLGIQQKNLKKLNNGISASFEANLKVPENRQYLLAHLPMSLRFFYTYAITTGS
SLALLVLIPLLLLRHGVYVRMLPLTLPFSYKPGGMVHWMFYLYEILCGWNLWTVAVGTDNLFSLYCLHIV
GELKLLSSRFRNLKSSKNYRKDMKDCIQSHMLLMKTFLKLQKVFGFVVMWFAITCALCLCSLVFQAVEMD
KVSVMRVFYLFNHSFVKLLQAYLYTWCGNIITVESEICLNAAYEAHWSDSGDKRFMKDILTVVLQRPMVF
KANKFMELRMELFLKIVNTSVSYFFLLRTLDDDS

>NvOr249

MESKVARYAKYKRDIKCLIVASGIWPHYEKHPHVLRKLLSFCSAFCSGSTFYCIVAFCFKYATNINIFTS
CLGLMIGFFTTFIKIVILSMRQEDLQSLNEGVSKSFENNLKLPENQPHLLYHFPSFSRFFYLYAYVVGIS
FVFLASTPLSIMLRYGKYVRMYPQLMPFAYEPGGSVHWAVFGFEMFTGFYLWSVTIGVDSIFGLYALHMV
GQLRLLGSRFQNLKSSSNYDKELGECVRSHIQLMKSRHKLQRVFGFLAIWLAVTCAIALCSQVFQALHMR
NTTPVRALYLFGHWFIKIVQAYSYSWYGNIIAVESDLCLNSMYYSHWPGSGDKRFMADVLIILSQKPLVF
KAKQLMELRLDMFLKIVHTSLSYFFLLRTLDENPKAGT

>NvOr251

MERDIQTYKVCSENVTLCLIFSGVWSATHPVLKKIAFFVTFFSTFSIMAHTLNFSLHNAQNVRILVRGLA
AASSFLSISSKAFLFLQHQNDLNYLKDYLTEKFMSDMKNPENLPDLLSNMRMFAVFVTMYKTTIAFIMSM
YCIVPLFSFLKYGKYLRVYPCLYPFSFVPGGVVHWLLYGWESTGALSAWAISVGTDCAFGMYAIQICGEQ
RVLARKLKDLRVGSNYTRELRDCMERHHLIITAKNTFESLYGLISIWLAISGAIVLCSLIFQVTEYLENR
GGYVRAIIFFAHFSGKMMQVFMYAWYGNLINEESLAFPRAIYSSHWTDCCDTRFKNDILIVLAQRPLIVT
ALGCMNVQLDMFAKIVKTSISYFFLLQTLKAKTEEK

>NvOr253

MELEMAKYNSYSNTIIWSLICSGLWPKGHYVLKKILSCISFLSITTIMTTAINFSFQNARNVQLMTKGMG
TAVSFSSVFSKIVMVLYHQNDFIYLKKHLTTRFKRDLEQTENRQDLLFNVHIFTKFVNTHEASMAFAMFM
YCIGPILALYRHGKYVRTFPCLYPFHYEPGDVVHWVIYGLEVTGATVIWFITIGVDCGFCMYALELCGEF
KVLGRKFRELRVADDYKEKLRDCIERHHLIINAKNRLEDAFGIMAIWLALSGAFLLCSLIFQITEILENH
GSYLKIAHLCSHLLAKYLQIFMYAWYGNLIADESQSFLYSMYSSHWIDACDKRFKSDILIVLVQEPLMLV
AKGCMNIQLDMFLKIVKTAMSYFFLLQTISSEE

>NvOr254PSE

MEQEVVKYKAYSSNIIWCLSFAGLWPHTHPVPKRIFSFVSFFSTLAILMSTTNFVFQNGRNVILVAKGLC
PAVSFSSVFSKVLLFLLHQDDLVYLNKHLTSKFMIDMEVSEYRKEQLSIMRFFAKFVRAHEASLTAAMSM
TSXVPIIIFLKHGLYVRTYPCLYPFSYEPGGLIHWLLYTLEAVGAVCIWSTSIGVDCGFAMYTLQLCGEL
KILAKKFKELKLGKDHKEKLRDCIERHHVIISAKNRLEEAFGLVSIWLAISGALVLCSLIFQISELSENK
NSFFHMGHLCAQFLAKFLQIFMYAWYGNLVAEESLAFLDAMYSSHWPDSCDTQFKNDILIVLVQKPLVLV
AKGCMNIKLDMFAKIVKSSMSYFFLLQTLNADTDRKH

>NvOr255

MEVELKKYKRYYRDIKLLLVVSGIWPNFYPILDRVVSIVAAISTLLLTMALLNFCAHHVANIMILTKSMG
IAISFFSSFLKICIFLSHHDDLVYLNDYLTSSHTSDLSNPDDRSHLLEKFSSFSKFFYTLTIAVALTFVL
NTIAPFFALKRGKYLHIYPVIFPFDYEPGGSVYWSLISLELTAGFFVWSVTSGVDSVFGLYALQMCGELR
VLAKRFEELRATGDYRMRMRECMDRHHLLMRSRDILEKVFGFLAIWLAVTSALVQCSLVFQAKVEFKTLS
PFKIGFFFFYILMKLVQAFTYAWYGNLIAEESALCLNAMYNAHWPGSGDVRFMNDVLIVLSQKPLIFKAK
SCMSLHMDVFTKIMNTAVSYFFLLQTLDEGSVRHL

>NvOr256

METELRKYERYSRDLKCLLVLSGIWPDFHPIIQPLLGCFAAFVCFVTVIAFLNFSIHHITNVVVLTKSFG
LVISFFSSFLKICVFLWHHDDLVYLKAALTDRFNTDNLNKSFRRFTLAKVNVFANLFYILTIAVGLTTGM
AVVLLIISLRHGKYVMLYPSIFPFSYEPGSRVYWILLMVELFANLFVWAVTSGVDSVFGWYTLQICGEFR
VLAHKFQNLKSSENYRDDLKECVERHYVLMKTRDVLQDVFGFLTILLALTSAIVQCMLVFQAIQVFKNLS
LGMMVFLIAYITLKVVQAFIYAWYGQLIAEESEVCLGAIYNARWAGSGDTRFMSDVVIVLSQKPLIFRAN
GCMSLKMDIFIKILNTSVSYFFLLQTLDEGSEHHQH

>NvOr257

METELRKYERYSRDLKWLLVLSGVWPDFHPVIQPMLGCFAVLVCSLTAIAVLNFSIHHITNFVVMTKSCS
IAIGLCLSTLKLCACLWHHDDLVYLNTSLAASFNADNQNKSFRRFTLAKVNVFANLFYILTIAVGLVIVM
GLVFMILSLLHGKYVLVWPSIFPFSYEPGGWVYWILLTVQLLANFFAWTVPSGVDSVFGWYTLQICGEFR
VLAHKFQNLKISENYQDDLKECLERHYALMKSGEVLQDVFGFLAILVGLSSAIIQCMLIFQAIQVFQQLS
FGMMILIFAFITLKHVQVFIYAWYGQLIADESEDCIEAMYNAQWAGSGNIRFMRDVLVVLSQKPMIFRAK
GCMLLKMDMFIKVLNTSVSYFFLLQTLDEGLQN

>NvOr259PSE

MENELRKYKTYRRDIKCLLCVLSGIWPDFHSIIQPMFGVFAAVVSLITVTAFLNFSIHHITNVNVLCKSF
SLVISFFSTFLKICVFLZHHSDLVYLNTILNARFEEDTRNESYRRFTLARVNIFTKTFYAVSISVGLATG
TAFVLLIISLGHGKYIMLYPSIFPFGYESGGRLYWILLSIELFTNFFVCAVTSDXDSIFGMYTMQICREF
RVFAHKFEILKTSENYRNDLKECVERHHELIRTKEVLHDVFGLLSIWLAIRSAVILCILIFQAIQVFKTM
GLGVIYIVGYIMLKLVQSFIYAWYGKLIADESENCIEAIYNARWAGSGDARFMTDVIIVLSQNPLVFRAK
GCMSLKMDIFIKIFNTSVSYFFLLQTLEEGSQH

>NvOr260

MELEVRKYENYSRDIKRLLIVSGIWPNFYPVLQRFVAVLAICCTAMTFMGAFNFCLEHVSNVVVLTRGMG
LLFTLLSTGMKICVFLHHQKDLIHLNQHLSARFLDDLQNKAYQSHVLARLPAFSELFYSLTYTIGSTAFL
TTILIPLLALRHGKYIQVCPSIFPFEYAPGGLVYWLLQLTEALAAFFVWAVTSGVDSAFGLYTLQMCGEL
RILGSKFESLRVSDKYREELRECIERHHLLMKARDSMEKTFGLLAIWLAVSSAVIQCTLVFQADEVAKSM
NPLRIGFFFLYIVLKLLQAFMYAWYGNLIAEESAMCLNAIYNARWAGCGNSRFMTDVLIILSQKPLVFTA
KGCVSLKMEIFSKIVNTSVSYFFLLRTLDEGSQN

>NvOr261

MEAKLAKYARYRNVVRRLLLLSGIWPHLEDTCRLYRVLTFSATFVIAALGAKVFAYCIDNIAHVSLFAKG
MSNAFSFYTSVLVYLVYRKDLVMLNDCLGRRFEDELKREDRRPLLLQSISVYTRFMCIVAGLTATALVFY
TLVPLVFIFKYKKLTQIYQGRYPFAVEPGGRVYWCVCFVESISVVFVWNVVCSVDNAFGLHSFRMCGLLR
SLADRFAKLQPDDPGYIVELRDCVRTHQLVLRAKEALQRVYGLVVLWTYVTSAIIMCSILYQADQAKKHM
TVTRVIFFTSYITLKLLQSFTYAYYGSLVSQESEKCQNAIYTSNWPGSGDLRLMKDVLIIQSQRPIVLRA
NGFFIVSMEMFEKIVNTTISYFFLLQAVEEK

>NvOr262

MMMELELLRYKAYTQNVIWFLKLAGLWPESHPVPKKILSTITLSSILVIVLTVSNFSFHNLGNIMVFTSG
MCMAASSTSAFSKVALFLLHREDVVYLNKHLSGGFMRDMDEPDNRPDLLSNVKTFERFMVTHVISVAIAM
FTYSVRPLLVLRKHGKYIRSFPAVYPFAYEPGGLVHWILYAVEVSGTASLWTVTIGVDCVFGVYALQVCG
ELRILSRKFRELRADDNYKEKLKDCIRRHHVLINAKNKLENIYGLISILLITSTTLVLCSLVFQVSELMK
TNSYLRTAHLCVYLIPKFLQIFTYAWYGNLIAEESGACLDAMYGSHWTDSCDKNFKNDILIVLAQEPLAL
VAMGCMVLQLDLFAKTVKTAVSYFFLLRTMNEGSE

>NvOr264

MKTKDESLQPNIFLQHYLNINSKMLRYMGLVVRTKGNKTDSKSKILERLPTYATNLISIIDAFFQMRWIM
DLWQRDNDLVMQITTSGISNIVCICKGFRLAYCREDIQTLFEKLATIWDQTCVPEDIRDTIVKKAQSTLV

FCRCYIVMMLGLGICFALPPMKNFLIQYFARKEMNHTYDYSERVFLVRYPFEINSSSIYFSVLFEEQWVL
FCSALYWVCCDTLFAQLTTHTSLHFEILQYDIEAVVNRENDEDRLKQSMIDFVKRHRELLRICHMIEKLF
SPVIFTTMLLTSINICVNVFELREMISEAKLGDALLHGFHLVNIFFQLLVYCIFAERLTQQAGTIANATY
NCKWTEKNNKLRIYLQILIMKSQKPFHCTAYGFFPIDHKTITIIVNRALSFYMMLETTN

>NvOr265

MKTEDKSAPLTPDFEDYTKINSLFLRCMGMGIGTDGNKKDRRSQIIERVPTALINVLCLLDSVFQVQWVS
ELWKTDKKLVLQILTNALSNIVCLCKGFQLAYSREDLQTLFEDLAMIWRKRIPHHEIRDEILRGAQKTLV
FCRCYISMILVLGLCFGLPPLKYFILQFTDRNANRTYDYTERIFLVRYPFDVNNLTAYNFIFMEELWVLY
SAAIHWMCCDTLFVQLTSHTSLQLKLLHYDIEASGNTEDERQFKENVMDIIKRHQELLRICDLIEDVFSP
VLFVIMLLTAMTMCVNLFELREMLLEAQYVGAILHSFHLINVIFQLLIYCVYAETLTEQAGSIAEAIYNC
KWTENSHEVRTNLRMCIMKSQKPFYCTAYGFFPIDHRRITYIFKTAMSYYMMLHQTTS

>NvOr266

MKTEDSPSVTPAFEDYTKLNSLLFRCMGMGIGTDGNKRDKRSQIIERVPTVLINIICLLDFVYQMQWIND
IWKTDKKFVLQILTNALSNFVCLCKGFRLVYNREDLQTLFEDMAVIWRRRMPRHEIRNEISREAQKTLVF
CRFYVIMILFLSLSFCLPPLKYFILQFTDRNANRTYDYTERIFFVRYPFEVNNLKVYNFLFIQELWVLYA
AALHWMCCDTLFVQLTSHTSLQFKILHYDTETSDNTKDERQSRKNIVDIIRRHQELLRICDAIEDVFSPI
IFIIMLLSAITMCVNLFELQEMFLEAQYAGIALQSFHFMSVFFQLLVYCDYAETLTEQAGSIAEAVYNSK
WTENGHVLRMNLQMCIMKSQKPFYCTAYGFFPIDHQRITTILKTAMSYYMMLYQTTS

>NvOr267

MSQPTEDDLEYYFAFNLKLLALVGFKCSMDKKEKGLGFVNKLPSYIMCIQGTILSLFEVYLLRDIYKDED
KTIVMQVLSQGVENTLNVCKGFFLAYSIERMENVLQEIKFLWNTYRPSPDNRKIILAEAQQTYSYCKIYF
CVLASCCTSYFLCYLPALFKLAQQYRDREANNYTYDFSQRLLLLKYPFDIPSIPIYFLVELQEGFYLFYA
AALFFVSGDTLFAQTVTHICLQFKILKFDIDAMFNPENTGEKDHLNLVTFIKRHRDLLRVCALIEEVFSP
IILSMMLLSSIALCVDLVGIRGTMEKNNYEETAVVITLMMLTLLQILFYCTFAEKITEETRSLADTMYGC
NWTMKNNKLGLYIHLMILRAQKPFQCTAYGFFPIGHSQLTTIINTAFSYYMMLQTTS

>NvOr269

MKTSVNQPTEDDLEYYIGFNLKLLSSIGLKCSLEKNLKSLGLINKLPTFIMCIHGLIFFIFENYFIRDIW
SSDKTLAMQILSQEVSNIQCISKGFFLAFAIERMQNVFQEMQYLWKTYRPSQDNRKKILLGAHQTFSFCK
IYFFVLLSCSISYFLCLIPSLFNLAQQYRNREANNYTYDFSQRLGLVKYPFEIPNIPTYLLIVFQEAFYL
FYTAALFWVSGDTLFAQSVTHICLQFKILKYDIDATFNREDMRDHLSLVTIVKRHRDLLRICKLIEEVFS
PIILSIMLLSSLNLCVNVVGIRGTIAKENYQETAINVTIFMLTFLQILFYCTFAEKISEETRSLADTIYN
CDWTVKNYKLRFYIQLIIMRCQKPFYCTAYGFFPIGHLQLTTVLNTAFSYYMMLQTMN

>NvOr270PSE

ALAAQTLGQLVCNTQCITKGLLFAFSIEKLQPVFHEIRILWEKYQPEEVIQKSIVKDADRTLTFCKCYVT
ANFSCLVSFSLPMGVQLFLQCQSRVATNHTYDVSQTMLLVKYPFEISDTSTFAIVIFLEEFLLVMNVVFW
VSSDATFAQTTTHLCLQFKVLKRDIEKMFNYEGPDGKEILLKLVHRHRELLRICMLIEDIFSPIIFFHRV
LXSINMCVNVIGTRETISNRVYSDTGVYATMLLMTIFQIFFFCVFAEKISETTTSLANMVYNLNWTSKDN
KLGFYXFIIIRAQRPFYCTAYGFFPIGHQRLTSIIRASFSYYMMLQTTDNK

>NvOr271

MKMTAINPEDYFGLNIKLMSLCGLRCSMTKTIGSFINKVPTFLANLVGIIYLVFQATFVMEAVRLRDVAL
TSQILSQLVSNIQCITKGFLFAVSIEKMQSILYEIRSLWERYQPDIEIQESILDDADRTLNFCKYYVIAN
FSCVLAYALPLVLNLFMQYQARESTNHTYDLSQMILLVKYPFEVTKVSRFIILVLLEEYLLVVSVIIWVS
SDTLFAQTTTHICLQFKVLKQDIEKTFNYGGPNSKEILLKLVHRHRELLRMCMLLEDVFSPIIFFTVFLS

SVNMCVNVIGTRETISDKTYLNTGIYATILTMTIFQILFFCIFAEKISEETTSLADMVYNLNWTAKDNQL
GFYIYFIIVRAQRPFYCTAYRFFPIGHQRLTSIIRASFSYYMMLQTTDNK

>NvOr273

MQTNTEEKAITAVDAEYYFDLNIKLMSLIGLKCSMTETVTKFIYKIPTFLTNVLGIIYLIFQISYVREAV
RSHDTSLAAQILSQTVCNIQCNSKGFLFVISIAKVQAILHEIRILWETYPPDDEIQKSILLVADKTVTFC
KYYVTANLSCVLAYALQMGLNFFMQYQAREATNHTYDFSHIILLVKYPFVVTEIPTFITLFLSEEFLLIM
GATLWAIIDTLFAQVTTHICLQFKILKRDIQEKFNTEGSNDKEILLKLLRRHRNLLRICMMIEDIFSPII
FFTVILSSVNMCVNVIGARETIASKAYFETCIYASIFLMTIFQIFFFCIFAEKLSDETTSIADTVYDLNW
TTKDYKLRLYLRFIIVRAQKPFYCTAYGFFPIGHQRLTAIIRASYSYYMMLQTTDGK

>NvOr274PSE

MQTKERHKSKVLNIEYYFYLNIQVMSLIGLRCNGPTITGFVHKIPTYVANVLSVIYLMSEIYFVSDPVYS
SDMGFIIQTLSQSVSNIQCFTKXFLFATAIAKLQVIYNEIKFLXQKHPPKDNIRVLIFNLADQTMNFSKN
YMMAICSCILZYYTPIAINLVKHALQDSSSNHTYDFTFPIYSIILFIEAVFLIVQTVFWVDSDTLFTQIT
THLCLQFXVLKHDIQSTFNIEGSKSKEILIGLIKRHRKLIKICMLTEDIFSPMIFXVMLLSSVNMCVNVI
GTSAAVKDESYLDAGIYATMLLITVFQFFFFCILQKELXEETRSLADIVYNLNWAMKDYDLRLYILLIIM
RAQKPFYCTAYVFXPIVHQKLTSXLKLLYDLYMMLRTTADK

>NvOr275

MQSKERDKPKVLDIEYYFDLNIRVMSLIGLRCDGPKITGFVHRIPTYTSNTIAILILIFEICLMSDPVCS
SNMELTIQTASQTVSNIQCVSKGFLFVNAIEKLQVVYNELQVLSQKYPLEDEIQVLVFDIAEKTMNFCKY
YAIAICSCILFYYTPIVVNVIVYILQDPSTNHTFDFTQTLFYLKYPFTIKTFPIYSTIVSIEAVNLIAQG
IFWFLGDTLFAQVTTHICIQFKILKHDIQKTFNDEGSKSKEILIGLIKRHRQLISMCMLTEDIFSPVIFS
VMILSSTNLCVNIIGASTAINDGDYMNAGVYATILLITVFQIFFYCIFAEKTTEETRSLADTVYHLNWAM
KDHHIRLHILLIIMRAQKPFYCTAYGFFPIGHQKLTSILSTAYSYYMMLRTTANV

>NvOr276

MQTKEETIEVNAQYYFSLNLNLMSMIGLKCNMTENVGRFYHRIPTFITNVCALMYQSMTVYYLVEAISAK
NTSLSIQIISQLVSNIQCFTKGFFLAFGINKIQFILQEKQILWKKYPPNNNNHHTILGIAQQTLTFCKFY
VVAIFSCVMSYDVPLAINIFMQYLKRESTNYTYDLSRRVILVKYPFEVTEISTYVILCLQEALFVFIQCI
FWVNSDTLFAQVTTHIGLQFKILKCDIEAAFNRDDAKNKEILIELVNRHRELLRICMLIEDVFSPIIFCT
VFLSSINICVNVIGVRETISEKAYLDTGIYFTMLLITLFQIFFFCIFAEKLTEETRSLADAVYNLNWTIK
DYKLRVYINLIIMRAQKPFYCTAYGFFPIGHQKLTGIISTSYSYYMMLQTTDK

>NvOr277

MKRLAKVQSTKDDIEYYFGFNLKLLSQIGLKFSMNEKTDKFTFLQKLPSYIFLVEGMILFILEVYLIRDT
IQSDTLLSIQIMSQIISNLQSVSKGFLVLNKAKSIKNVLETLGIIWRKYPLNNSDRALLNAPSKIISLSK
IYWGIAVALLVIYDLPPFVIFFMQYQNRDAMNHTYDLSQTILLLKYPFNITRKSTFFFLISQEAFVLYAS
GVYWIGSDALFAQFTTHICLQFKILKCNTKEVFNRGSKEAHSSLIDLIKRHRELLKICEMTEEIYSPIIF
STMLFSAINMCVNVVGVRETITRGFYQETGVYLFLFLVTFAQILLFCIFAERITEETKSLADLAYNLEWT
KEDHKLRVYILFIILRAQKPFSCTAYGFFPIGHKKLSSIINASFSYYMMLQTMS

>NvOr278

MKSQENIFTKDDIEYYFDFIFKSLNTLDLKFSISKKTDEFKFRHKLPTIIGCLIGLIIFFLEIYFIRDAL
HNHTILPIQIFSQVISNFQSISKVILIVYKVNKIQQILEKIGVLWKTYTPDEGNRAVLYNTLQRTLSICK
IYYAVLIATVLIYYVQPIVNFVGQYGARNSINHTYDYSQTLVIIKLPFKVTQKRYFFVISQEAYLLYMSG
VYWGCSDTFFACFTTQICYHFKILKYHTKAFFDEKNNNSRLNLVTLIKRHQELLRLCVLIEDVFSPIIFS
TILFSAMNLCVNVIGVQETILNGSYRQAGIYLFLFIITFSQILFYCAFAEATMEEAWSLADLAYNLEWTS
KDYKLRYYIHVIILRAQKPFHFTAYGFFPIGIQKLTSIINASFSYYMMLQTVS

>NvOr280

MKTQENDLSIEDDIECNYSGYIFKAFHFMGLKLSLKKKTDGFKFVHKLPTTIGILQSIVVFFLQMNFIRD
VVQCDSNPPIQIISQVISNIQAGLKTLLVFKKIEDIQRMLETLGEFWKKYSPDKNYRVVLFRELGKTSSL
CKYYFGTLVGIMIAYDVQPLVYFLTYYFEQNATNHTYDLSRRILLVKYPFEITRKSTYCFLLSQEAYLLY
ITAIYWANGDTLFAQFTTHICLQLKILKYETGKFFNQSNQEGRSDLLILIRRHQELLSMCDMIEDIFSPI
IFSTMLLSAINMCVNVIGVTETIAAGSYEETGIYTFIFIATFLQIIFYCVFAETLTEETRSLSDFVYNLE
WTSKDYRLRFLIQVIILRAQTPVYCTAYGFFPIGHQKLTSIINASFSYYMMLQTVK

>NvOr282PSE

MRINEKRPKQAVPDIEYFFRANLKLLSSVGFKCSVDQKCEKFKFRHRIPTYITNIEGIVLFALEIYFIVD
VFKTDTLLTMQIVSQIISNIQCICKGYLMANTIDEIQQIFENLGTCWRKYNTKEADKVLILGRAYTAMKF
CKLXRMFIQYLNREATNHTYDFSTRILLLKYPFEISNVSIYILTNFLEGNLVFICTIFWASSDSLFATVT
SHICVQFKVLKNDITSAFNDSTLKERFILVNMVNRHRELLRMCYLIEDIYSPIIFSTTLLSAVNMCVNVY
CFRETIAKGYYLETGVSLSLFIGTFLQIFFYCIFAESLTEETRSIADTVYNLKWTTRDHKLRFYIQLIIM
RAQNPFYCTAYGFFPIGHKQLTSILNTAFSYYMMLNTMSN

>NvOr283

MQIRVVENLTLTKHDIKYFFKENLKLLSKIGFKCSLTKKSEKFKFHHKIPTYIANFCGLIVFALQIYFVI
DKIQTNTVLAMQSLSYAVINVQSILKGFMTANSIENIQQIFENLGIFWQKYMSRKPGRELILDRAYKTIS
LCKFFFVMAIVCYFLFVMQFLIKFSIQYLNREATNHTYDFSNTVDLIKYPFEIPNLPVYFLLISVEINYL
FVCIVFWCNTDSLFVTLTSHVYVQFKALKLDTTLAFNNSMLKERSILIDMVNRHRELLRMCYLIEDTYSP
IIFSTTLLSALNMCVTVYAVREYIDKGYYLEMGIPLFLFIGASLQILFYCIFAESLTDETRSVADSVYNL
KWTTKDNKIKFYIQMIIMRCQKPFYCTAYGFFPIGHQQLTSIISAAFSYYMMLQTMSN

>NvOr284PSE

MQSTEVKKMASLNPEYYFDLNIKLMSLCGIKCSMTETIGSFINKIPTILSNLVGLVYLMFQVNLLREIRE
HLRDTVLAVQILSQIVCNVQCNIKGLFFLLSINKVQYMLHEIRILWESYPPDDELQKSIFLIADQTLTFC
KYYITVNLSCILAYALQMGXNFFRQYRTHQHGSNHTYDLSHIIILVKZPFVVTGVPTFITLFFTEEFLLI
MYATFWAIIIDTLFAQVTTHMGLQFKILKRDIEEKFNNKSSNRKEFLLDQVRRDRNLLXVCRMIEDXFSP
IVFFTVFLSSVNMCFNVIGTRETILNKDYFDMGIYATILTMTIFQIFFFCIFAEKLSDEXTILADTVYNL
NWTTKDYKLRFYLQVIILRAQKPFYCTAYGFFPIGDXRSILRASCSYYMMLQTTDSK

>NvOr285

MKIVGEKSSPNKDIEKYLGLNLKMLSCIGLDVSLENDDVIQERRILEKMPIFMTNGLGIFAAILQISLIT
DSMTHNRMFLATQVSSHLFSNMLCISKGYQLATAIAKLGEILREIALIWKQNPLNDEFHRNILSDAAKTL
LFCKVFVVVTLCAVFGFGLPPLQNLFFQYLHARNSANHTYDYSQRVFIIEYPFPIQDVLTYSSVLLEEEY
LLLASGLYWVCCDTLFAQLTTHISLQLEILQYDIETLINRESAEDRLNENFIIIVKRHRKLLSICELIES
VFSPVILTTVVLSGMNICMNVFELSKTISEGNYAEAALHAFLFMNTFLQIVFYCTFAEKLTEQTSFVANS
IYNCKWTEKNCKFRVYLQMLIIRSQNPFYFTAYGFFPIGHKRLTTVINTAFSYYMMLQTTS

>NvOr286

MINHPPQNNDIREYLGLNLKMLSFIGLEFNLNNDRPIKKSKFMQILPIFMTNVVSLTIAALEITFIAFVL
RNHEEHLAVQICSELFSNILCIGKSLRMATAVASIQTALDEVSILWAKHRPNQHCKMEIMKKARNTLNFS
RWYLGFIITGIAGFALPPIHNFVYHYFIRDANNYTLAFSKRIFLLRYPFEIKNVPLFFFVLTEEGYILLI
SAMHWVTCDTLFAQITTHTSIQLKILHYDIGALINHETVEHRLKAKILIIIRRHQCLLRVCRLIEDIFSP
VILTTVLLSALNICVNIFETKAMNAEGNYARAALHANLVLVLFLQILFYCSFAETLTNQTSAIAESVYNC
KWTEKNHKLGFYLRMIMMKSQSPFYCTAYGFFPIGHARMASIISTSFSYLMMLQSMS

>NvOr287PSE

MTRSYETLESPSKDQGIRRYLGLNLKMLLFIGMEFSLNNDRTIKKSRFVQILPILMTNVISLSVRIILKY
SLYSQIYIIQRQRDSYYYSNNCNCRCYLGVVISGITAFGLPPIYNFVYHYFIREVKKQTMAYSKRVFLLR
YPFRVKNEPIFFSVLAKEEYLGXSCDTLFVQLTTHISLQFKILHYDVRTLINREVPYRLKMNIIIFIKRH
QGLLRTCRLIEDIFSPVIYSXVLLSALNICVNIFEARAMNAEGNYAGTVLYVILVLILFFQILFYCSFAE
TLTDKTSAIAESVYNCKWTEKNHKLRFYLQMIIIKSQRPFYCTTZGFFPIGHARLASIISTSFSYLMMLQ
SVTQ

>NvOr288

MKKTILQEYDKENQKAFDEAKTLITWNKYLMSALGLWPSHRYDFIFVSLFCYYIFHFLLDYAAFYFALRS
FNLIKIIGATMENVTMAQIFLRLYTMRRYNRQYGEILEEFTRDFSVKNYKSEEERNTFLSYNSRSKFFIK
IVVIFLGVTAILYFTKPLIRQLSLSKNVNTTKAFTYDLPYRIHLLYKITDIQTYIATYISRIPILYIIGF
TQTAMDCLTLTVIAHLCGQLGVLSIRISNLDVVNKSNELNEIIQRHQKLIKIGLRLRRMYRLCLLGHFLG
ATIAICILVYQVLISIAAGQKTNLVTFFVFGFLNIFRLYTHCWVGEYLIHESINVSHAYYRCKWYKLPLK
DQKSFIICIKRSQQPLSLMAGNFSHYSLVMFTNVMKSAMAYLSFLRNFI

>NvOr289

MMQNHQLQGQAELDDSSQVFRYNYILLTTLGLWPASLSDVRFFLNFGYFCYEMLLEYLDLFLFIDNFENV
LMNLTENMAFSQIFIRMLMLRIYNSELGEIIGDAKKDFDAKNYTEEERKTFVAYHVKSRTFMKLLITNTA
LTASSYYVKPLLGQMGELMEYANSNGENSTFIFMLPYRFYTFYELNDAQTYFWTYGSQLPFVFISGFGQS
AADCLMVTLVYHVSGQMAVLALRIASIDTHPSKCTQEVQKIVKAHIRLLRMGKVIQRTFSATLLGHLVGA
TSLVCILGYQILTSLANGERAILISFFAFIFLVLLVLYAHCTVGESLITESERVSQAYYDCEWYNMSKEN
ARIIILCMARSQKPLQLTAGKFSMFCLQTLTDSIKASMGYLSVLRTVM

>NvOr290PSE

SAELVFVWNKKLFAAFGVNPLQFNSLLYLFCLFYSTMHFILSCAALPQNLHNLNNMIDAITEVGSMITIL
AKFVLYRINRKSLAGLLVDIEKDYSIDKYENELEVRMFIDYVFKAKAFFQLTVPVSIASAVMYFFRPFAM
GGFDSANLTTFVLPYRMIFFYPITELSGYLGALFYLIPVIPITAFGLVADANLMLAIAAHICGQLSVLSR
SLLHFAKDGTSFQQKLAKTIRQHQRLIRMAKTLDSVFNLLMLQQVLSVTFFICFVSYSMLINWSQRESAL
ILTFLVCMVSMSFLLFAYCYAGQCLIDESTFLGNALYTSTWYKLEPSNVKNYIIFMKRTQKPLVITGGRF
YIYSLSSFLAVMKSAMAYLSVLRTLI

>NvOr291

MNISGSEQTILAKYKNDLQKASKILTWNRRLLSLLGLWPESPMDLLFCASAVYYIFYLGLIFVSFVLYLK
KKILNVSIFIALLSYGHISARLLLLRRHNRTFGVLFAEMKQDYELRNYKSDQELRVFLKYNILAKSMIKF
LLFCSTFFAIVFYVKPLLMTYNIHRAIRKSHRNATAPFVLAQNSFYQFYKITTVKKYAINYVSMLPFSVL
TGFINCATDCLVLTIGCHLSGRLAALSHRIRNVEFCNGSQEFKAVIRLHQQVLRIGDMVENSLNTLMTCH
ILTAGVIMCFILYKTLIYLRPGKRIHLIHIVILLSLNIVRLYFHCCVGEFLMQESRVVHEAFFECKWYTM
LLQDRKLIVLNLLRSQRPIRFAARGLGTFSIELFSEVLKSSLGYLSVLRNVI

>NvOr292

MNLYESDVNQKSLTKCKDDLKNASKILTWNKRLLLLLGLWPESPMDFLFCASAVYYIFYLGLDFVSFVLF
LRKKILNVSIFIQLLAYGHISARLLLLRRHNKTFGILFTEIKQDYELRNFESDQELRMFLKYNRPAKTMI
KLLFICSTFFGVVFYVRPFLTTYFVHRAIRKAHRNVTAPFFWNTYFYKFHKITTINVYAMHYVSEFPFSI
LTGIISCATDCLVLTLGCHLSGRLAALSHRIRNVNFRNGSQEFKAVIRLHQQVLRIAEMIEDSLSSLMLC
HILVASILMCIVLYKTLICLRPGKRIHLINTVILLFLNIVRLYSHCCVGEFLIQESRAVQAAFYECKWYT
MPLQDRKLIIILNLLRSQRPIRFTAGSLGTFSIQLFSEVLKSSLGYLSVLRNIV

>NvOr293

MEFVRKAYGDKQRKRQASSKCISRADRVFKRCVFFHKFVGIWLEKDRSQRLLDRLKGYVSAAFTLGICIF
QIVMLSVESSSVVTVLQNNLLILIRKTKEIAAPLHASKIKERKIVDRWLNNQDKILKILLTSYTFTFSSY

SLFPLLKENGLPFTGRLPAICYVNPWYPTIFAAQLVFIIFRFFCVLSNDILCITFLCQLCSELELVKHLI
VELGNGKDRNVKQIIIRHAMVLDYGEIICETYSATLIMQHLNCSIFLCLSGLVTMKTSDMFALLKIGSLS
LIGIITMLIICFVGEMVMSSSLEIASTIESSVYKDYRNDVANLKLLNFMLMRAQKPLCMMVCTQGKLSLR
FFSENINKVASFFIYLKTLVE

>NvOr294

MNIKSAESSLQSTTFEYEAIFHKVVGVWPGDDYFLARYSRIRGYVLAAFAVVVCVFQFTALLEANSDDVP
ENDFINLMRMTKEINEFSTLTDEEETIHIDWQSVQDKLMKIISRYYFLTVIGLYFVAPMFRNALPLRGIV
PEVLRVTPWFQMIYVLQCLLLLSNVITSISSDAFSVTFMCQLCKQLELVQCSIKHLGSHTKVNLAETINR
HAVALDYGQRVCNTLKTMFLLQHIFISMFLCFAGVIVLNTQNSLILMKMIVISVIFVSTLLIICFVGETI
TSSSLKIASATESSNYEIFLGDVSTLRTVSFILCRAQKPLRMAVSLSGSMNLSFFTETMNKLVSAFMILR
TMME

>NvOr295

MGLIEVLESRKIFLWICGLWPKEYQHKPKLSQMKLYFIWFNMLMMCLLVFAGLVAIATPDNIPQASIRLP
RKRMMKVIMETLKLEETSKFENDNDLEIIRSWRRTRDGVLKYHMRIYGFISVAYCFLPIISQVNTYPAQT
IIQASLFVSPWYEFFYGFHCAQLFLYLFIIIATDGLSMILIFKLCEELQRFECLLFERHRADDATLSEKY
KSRGEFLRCIIRKHCTILDYGESICNLLTGALFTQYFLLSGTLCFSVFTILSSNSSAMANQMSIMAGTCI
VQLFMISLAGELVSTRSLALADALLQSDFCCSIFGELKSSELRQTVLMQLRMQKPLKLSIGTLGVINIEF
FSRIMKGVYSFTMLLRTSYV

>NvOr296

MNVGEEYDKLALPMTLSSRVVGSWPSRAELEGQGGRSVLVHRLHRYLAIVSIYLMSMGVAAEVIVFFGED
MNETIECALISSAFFMALTRIITFASHQPEMLYVVETMREDWIRSTDEERAILRDKCLFAFKLAKFFAIS
VTITCSAFILMPMLELKFVENAKRMLPYRGYFFFNHTVPGVYEYVYLVNSMLGVLGCSTIACATSFSLIT
SIHGAAKFAIVQKDFERIDQVTWNNSEIVGRCVRRHQECIRFAETVENIINVLALAQFVISTGLICFAGF
QMTTMLTDRARFTKYASFLNAAVTELFIFSYGGQSLKSESEEVAEGVYSSNWIGSALSSNLRLIVLRSRK
PCTITAGKFYDMSFESFLKVLSSSFSYFTVLLAMEEE

>NvOr297PSE

MTTAVEEYDNLALPMIISGQACGSWPMRAELEGERSLRVLLHRLHRFLAILFLYLISAGVTVEVIVFFGD
DMNETIESALVSSAFYMTFARVLTFARYQPQMLYVVZTMREDWLRSTSEERAILRKKCLLAFKLTKFFAL
SVVTTGISFVIIPLLELRFKEDAKKVLPYRGYFFYNYTQPGVYGYTYLANSMVGALGCMSIAYGTSFSLI
STIHGAAKFAIVKRDFEKIDRTTWTDHKIVGKCVRRHQECIRFAETVEDIINILALAHFVISTGLMCFGG
FQLTTMLEDRARLTKYTSFLNTAVTELFIFSFSGQSLKSESEDVAEFAYTSNWIGSVLSTNLRMIILRSR
KPCTITAGKFYDMSLESFLKVMSSSFSYFTVLLAMREED

>NvOr298

MLGKSSLNSKIPIRERDFNYSMKLSRITLSIIGLWPFRENIRCSNFKFVVILVSILMTLLSSLTFVYQTD
DDDKMFHSLINSLYMLMTLVKLLMMRCKNDKLEVILSEMRIDWRKYERFSDGNKRLVDLYTGKARTSSFV
CIFFMEFSITTYFISRVAYALQQPAKIREWDLPYTAVYPFEVTSSLFVPMYLWQVFSAMCLGSVTISIDC
LLVTTACHATGQLAALCENIKSYGHEQRHRDETLSSEIECSCIRCIIERHVDIVRYCRLVEDAYNLILLT
EFIGTTFQFCLQMYIIVEHSHDKNIVGLLSFCIYLLVFNFRLFMYCNVFDAMVEMGEKVGASAYDISWYD
FHPEAVRQLMFCILRANKPLNVTAGKFFSLNRNSYKNVIMTSSSYASVLLSIK

>NvOr299

MRTYNFRRVYSAVKLRLLKVVSLLKMKTESSNARLKGKNVTFCTEVFECDEILQLDNELGLIQWSLKLMG
IWPFWTRFSNVKFFLCGAILAFNVVGCFSGILNVNSDIEQFIECLLYFNVNLATLLKFLIVKYKRRSIEF
ILRCILDDCSRYSHLSVSCRSRVAGNIKKRKLMTLTLALFILAPVAGKRSFLSMLWSTAYIFSAFAITKY
IEYRDDLAMIRELPIFSALPTFVRHSQIFYLALLSGLFGILMSTLVIVTIDSVFAILMIHATNQFIVLSE

ELKAYREDHLDACYKISKNMRNCKCMRCIIDGHVNILRYTLNFYWYSKFILYKIDSVFSIVHIAVILLSM
NLHLFMYCVTSKSMTDASEQIGIRAFKMKWYSFQKTTVRSIVLMTLRSQIPCYVTVAKFINLSLETYTSV
LKTSISYASVIIIAREHLVNER

>NvOr300

MGKGKVRSFSDYFWLSQGMLKFCGVLPMPERGLFVNYFLIMLSISSLVFLFFPGFYIIAFHGSEINAAAK
VDIIAGEALEIWVTTIKALVLLPCRQTMLSVSRRATRLLVDIEDEKEQQLAEPYARRGYYLLYGFGGTVF
FALLSIVIKPFGQQVQYGANGTILASKDLPYSIGIVHENQQLFNAWWIGQCFAGIIAIIAIIGIDTTLAI
FVLHACGHFRILRSRFQAVAENSSSRRMSVSGRDDRRRLIDLIDKHQEIIQFVSTIIIIRYVITLRKCFL
FSIKLLRRNRIRLQSGGTDSNDTQHLSDLRIRLQFAGGKSLYIYIYTSSQVGFEAYNLRWYDWIEDDKSL
VTFLITRSQKPMLITAGRFTSISLETFSAVLSSAFSFFSILRKTL

>NvOr301FIX

MFVRIIIILNEINFLGNMDSEIYDSEYYHEVKLLLTYFGLWPNLSRFRKVVSFIAMVAMPISLVIPMSFGL
KRAIRLKEPIQIIEDTIGILYFLAITTKYICIFIFEGRMIVVYEQIASDWKKIKDKNELEYLHGRAKEGK
IITILYLGYGAVGCTIFASTPYLPLFLDLVIPLNVSRDKIYPYYADYVIVDSEKYFYTLYTLHGIFIIIL
VTMSAISIDCLFIMMVKHSVGLFQIVCYRLKKIGEEHNEKPHECKRLMDDKIIHTRMKEIFDSHKSSIEC
VDAIQASFDVSFLFIMTMSGVGVSLILFDLLLNLDDLTQILRINSMMFGVYIAVFVICYAAQMTLNSSEI
VFNDTYCGYWYNISPNARKYTQMVMVRSMKPCIITAGGLINMNLQSFFAILKTSVSYATVMLSMQEESNM
QN

>gi|528747958|gb|AGS43074.1| odorant receptor Orco [Cephus cinctus]

MMKFKQQGLVADLMPNIRHMQFSGHFMFNYYNDTGGSTKLFHTIYCSIHLFLILLQFGLCCVNLTLERAD
VDDLTANTITVLFFAHSIIKLAYFAVRSKLFYRTLGIWNNPNSHPLFAESNARYHAIALTKMRRLLAAVG
AATILTVCAWTGITFVGDSVKKVTDPVTNETMTVEIPRLMLRSWYPYDASHGMAHVLTLIYQFYFLLITT
MDANSLDVLFCSWLLFACEQLQHLKQIMKPLMELSATLDTVVPHTNELFKAGSTDHLRDTQGTQPMAPPP
NENMLDMDLRGIYSNRQDFTATFRTAAGMNFNGGVGPNGLTKKQEMLVRSAIKYWVERHKHIVRLVTAIG
DAYGVALLFHMLITTVSLTLLAYQATKVNTVDVYAATVIGYVLYTLGQVFLFCIFGNRLIEESSSVMEAA
YSCHWYDGSEEAKTFVQIVCQQCQKAMSISGAKFFTVSLDLFASVLGAVVTYFMVLVQLK

>gi|528747956|gb|AGS43073.1| odorant receptor Or4b [Cephus cinctus]

MEVLPLCFKLWTLSGVWRPMHYSSPVSKSLYTVYSLAVLITLYSLTFFEFLDIVFNFGSLDDFANTAFLL
LSMIIICCKATNTLKKRAQIIEIHDLLRAEICRGQNTTENLMLEKFAKTCRSNTLNLFTMMMSCAMCLTL
ESLLYHVNDRLLPMKIWLPYSLSSLTLFSLSFLYQVVTMTLANAITVANDTYITGLMIEICAQLEILKLR
LVELHDPKTLQMDENYSFNREKDILKMCIQHHNHIFKVKANVEKAFNSIFLIQIVTSTLVFCVTALTFLK
HEILSVELASVILYFFTMMFQLFLFCWYGNKVILKSTEVRSAVFEMDWIPLPQAIKKDFIFLMMRTNIPI
RFTSGYVVTLSLDSFMAILNYRTPH

>gi|528747954|gb|AGS43072.1| odorant receptor Or4a [Cephus cinctus]

MRTLPVCFVIFKFWGLWRPSDYLSWMKILYNIYSLLIVVIMYSFMLFEFIDVFINIKSIDDFTMNSFMLV
TFTNACWRVAKILAGRGKILKVINLLTSDYCVAVDKREVEIKEGYDGIARWNTLRYVILVQITVALMLIV
PLSNESRKRILPFRSWLPYDLSSLKLFWLSYVHQCIAIMAAAYINVATDSFISGLMIQICSQLDILKYRL
IKLPRLYNINHRDEFLSSLTHCVLHHEHIFKFASAVESVSNPITIVQFCASAVALCSSVYQLSKHSVDSA
KFMPLILYLLCMLFQLFFYCWYGNEVILKSMEVRDAVYEMDWILLDNNNKKALLMMMKRSERSIQIKSGY
FIALSVEPYIKILKASFTAYNALQQISN

>gi|528747952|gb|AGS43071.1| odorant receptor Or3l [Cephus cinctus]

MEQTISVYWWKLAPRTTYFRFSIWMSFFTLNISLLYVDLYEVFGNLEQMLLNLSDSVIKSLILAKLLLFR
FSEPLARLITDAQEDITAGEFGSLEEKKCFLEYYQRGKIFYQITMSCVVFAVHVYFFKGLETYLFSEVAG

TTVILGLTCYNIIANSGDLADISTLCCFALYASSMILLLYGYCFVGECLIHESTKIHEACAQCTWYSMPL
IYQKALIMCMLCAQRPLQLTAAKFYVFSLDSFSNVIKTSIAYVSMLRTVV

>gi|528747946|gb|AGS43068.1| odorant receptor Or3i [Cephus cinctus]

MVGIESCKRILSTEDKVQTEQNKNYKSDIEYALKLNRWLLNPISLWPLPSHVSALKKIQFKFIRTMGWFF
LAFLIIPTSLHTILVQTDPNVKLKMIGPLGFCVMVIVKFFVFVVRAKNIKICVDHVVADWRNVNTIEDRE
IMIDSARIARLFTAASALFMYGGGISYTIILPLTRSKSLSSDNVTIKVLPYPCYFFFFDPQISPIYELIF
LAYILCNIVRYSTTSGVCSLAVVFAMHVYGQCQVLMRLLDDFVDGEREKSSTLHQRLANVVVRHLRILRL
ISTMENIFNEIFLIEVVGCTFILCFLGYYCMTDLTESETVGLIAYFLLLISLTFNVFAFCYIGELITNQC
MQIGQAAYMTEWYRLEGKNASNLIALIIISNRPVFLTAGRMINLSYNSFCQVIKSSLAYLNILRQVTM

>gi|528747944|gb|AGS43067.1| odorant receptor Or3h, partial [Cephus cinctus]

MHGQDDFDRAANIMTWNHWLLAMLGFWPEKPRDIWFWINFGYFAYHMTMEYVDLFLFIGNLEHVIMNLTE
NMAFSQIFIRMLMMRVYNRQLGELIMEMRKDFQAHNYRSVDEQKIFLSYNSKSKTFMKLLMAFVALTASS
YYLKPILGNLGNDPIESESTNSTLTFELPYRFYLLYNVNDTHTYMITYLSHLPFVFVSGFGQSAADCLMV
TLVFHVCGQLSVLTLRISSINSDPSQCSQNIKDVVVTHQRLLRMGQTIDKAFSAILLGHLVGATSLVCVL
GYQILTNFAHGQNADLATFLTFAFLVLLVLYAHCTVGESLVQESTRVYEAWYDCNWYNMPTENARLIILC
MSRSQKPLCLTSGKFGIFCLSTLTDVLKTAMAYLSVLRSFL

>gi|528747940|gb|AGS43065.1| odorant receptor Or3f [Cephus cinctus]

MDHEIEEFKIKTAVQNKNFDSDVEYAIKLNRWLLKPFGIWPLNSSSTKFDRGISVISSFICCLLLFFVMI
PSFVVMFVSEKDFKGRLEIVGPTSFIIMVVLKYFFLITRGDTLKMCIDTILGDWSNVQAKEERKIMFRNA
KIARLFTIICVSFMYCGGIFYSIFLPLITAKSLTNGNNLTIRILPYRCNFIIFDPYRRPIFDIVYVVHCF
CSVIMYSITTGICSLAAKFVMHACGQCEIVMSLLENLIDDDKQCSDIVESKLATIIVQHLHVIRFVTRVE
DLLNEVCLVEFLGCTMNMCLVGYYIITGFESADTVRFITFTLLFISFTFNIFVFCYIGQILTNHCHQIGE
ASYMIDWYRFPGTQARFLILLIGVANRPIRLTAGKMVQFSFPCFCNVIKAAMAYLNIIRTVTI

>gi|528747938|gb|AGS43064.1| odorant receptor Or3e [Cephus cinctus]

MEHKRGWAKIKSDIPNKNYDTDVEYAIKLNRWLLKPIGVWPLELSSSRTERIVTITTAVTCCLLMSFVLT
IPCCIEMFSSQKDFKSRLEMLGPSSFCVMAVIKFFFFVIRGKEIRFCIDSVVTDWRNVDVPEERNIMLRK
AKSARFLTTVCALFMYYGGIFYTTYLPLTTAKALSTENVTIRILPYRCNFILFDPYAPLFFDIIYFLQCL
SAAFMFTLTSGVCSLAANFIIHACGQCQIIGLLLENLVDGRGNTSTTLEKRIAVVIVRHLHLLRFVTRVE
DVLNEVCLVEFLGCTLNICLLGYYFITGLETADTARFVTFALLFISFTFNIFIFCYIGQLLTNHCHQIGE
ISYTIDWYRIPGAQARFLILLIAIANRPVTITAGKIVQLSFPCFRDVLKAALAYLNMLRKVTT

>gi|528747936|gb|AGS43063.1| odorant receptor Or3d [Cephus cinctus]

MTVHSLSPTGNCKGLKEGQASYSWCVRNLRTLMVFTGIWPMEPPTLLLNVSYYYNATTFTLVICGMMAGA
ISVMDNYDLLVDNLSINLIFTEIFIKCILIKIYSKPLSRVLSLMKFDWISLKGRGPIHGDVLQSENIMLL
HANIPRIFFIAYTALALVAWTATVVAAVSRKSSKMQIDASNAFPMPSWYPFEMHSTPNYEMLLTFQVIIG
CSIAVSSAAVDSLLVTAVFHVCGQLEILRKYFENLHSSETALEETEKKVAAAIKRHSKLIDLCDLIEDCY
SQITLSQLLVASLNVCLSGFGLLLAIESGNIKVFLKFLLLLIAMLQQILIYSVTGDYFVI

>gi|528747934|gb|AGS43062.1| odorant receptor Or3c [Cephus cinctus]

MHHTMSNTREKDPDTSFLKYADFHINLLRKSGFYSMKGISNKINKEPTIWEVLLVLTISTCGFFIIILEF
RSVAVSLGSDTGFVIAVLSGTLTATLSMSKGLTILTSHREVRELLLRLSGFWEKSIERPENVDVMVQMAN
RASYLSKCYAATVVIMCSSYCMNPYVSVITQFLFTKTANNSYNFTATTFPTVYPFDLSYFPKYVVWILFE
QAVCLLMTLHWIACDTLFPMCATHLAIQFQILRRDLERTTEVDELREIVKKQIILFQSCDILENIFSPII
FLTIIMTSTIMCACIFQFEKTLSCGVYLEIIKYVTHMMSLFVEILIYCGFSNVLSDQTELLYHAAYNSEW
TDRSKKYKSIIYFLILRSQKPFQCTAYHFFPVGLVQITTILTTAVSYFTLLKTVTSESDDKVICS

>gi|528747932|gb|AGS43061.1| odorant receptor Or3b, partial [Cephus cinctus]

MNMPEAMKVLTWNKWVLEFLGIWPSNESLFTFSFFFFLVSMATCFVYADLIYRISDFKYVVENLTENIVL
TLLCCKIGLYRLNRRLMKEILLDIKMDYAIELYNTEEQKSIFLAYNRLSKSFIKYSVTTTTVATVLYYIQ
PLMDHANSHRKLTENSSITYILPYHMRMFFNITESSLYYYVYAYEGVLVPIIACGYSGTDCLLVTLTLHL
CAQISVLANQVENLNGDFRKFHSHLKQVVTKHSRIISLSVKLRTAFAYFLLVQLVGATLVVCLAIYNLLK
NYATGHTAQLFGFIFYGSSVTVQLLGYSFIGERLMTEGKECILQLQMVRFVTDIRQSC

>gi|528747930|gb|AGS43060.1| odorant receptor Or3a [Cephus cinctus]

MHNPLAPPPLEVTSEHFRFSFESSTFCEASSRFTEWKSELKNLTIAILNEPLNMYLTIDSDILWLSKRVL
SLAGIWPESPNNFRFFIYLLYLSLFNCAEFAALVLNLYWMNFDKSVRNMTESIPTAMVILKTAMFRRNMQ
LLLPLLSEVRADKFSVEEEPGIAWLYNIMGKLYTRLSVVLIFIVTTMLYAVPLSQWIVAKSNNLTSTYEL
PYQMYFGFEINDLRSHVLACLSLLPMSTVLTIGCTGSDTLLVVLIFYLCRQFVLLSVRIRNVETDPLIHP
TKMKQLIERHANLIGMATALNKTYSSLLLVQTMGLSFVICIVAFELLTMAEVGEETNTLSFIIYSLAVVT
LLFSYCFLGECLIHESSSIHNACYFSNWYRLPPDLARPIIIPIMRSRKPLHLTAGQFYVFP

>gi|528747928|gb|AGS43059.1| odorant receptor Or2j [Cephus cinctus]

MYNLCSTMPLVMVLVKISNFLFHWNVMMYLISFAQNNFWCDPDDDFTRETMKRCDKYGKIFVYLFTNLVL
FAVLDYIFAPVVENFHRNETDRILPFTLWVNLPVTVTPYYEITYTIQSLSTLYTGICTCFFDNFISVLNI
YVAGQLEILGHRVEAVADTCIDITMKDYVSEKSKLGLSLTLKNLTRLYKSTSNPDILYRTNGTCFHFDST
WTINTFQFCDLRRWFSAHGEKQRIILSLVRWQHCCPVEFHFLQATMSFLRKFTFICHFMAGLTQLLLYTW
SFCHTMKLENR

>gi|528747926|gb|AGS43058.1| odorant receptor Or2i [Cephus cinctus]

MSNLIEGYTSTKISQILITLIGMKRGKTKREQLLIDGLLVYIFATVLIAIFVENSDLIYSRNDLYALTYN
APCSFAVTYDFVKLLIFTYKRRELYELHKFTEDTFWNKDYNELDKAILDKCDSTSAIGMCVLSVLATIVA
IHYLTGPYWDAVGTNTTERTLPFRVVFDLPLTVTPYYEISYVIEVIGAFSVGLCSVAFASYLFYTCTFVS
GHFKILQRELENVCEVELKILITKSSYSDNDAKLAYEKFKKCIVQHELLIGYLGKLESLFSYIFLMLVLC
IVIILCFSGFQFILGDGTSKLHRQILSAEYIVTTLVETGLFAFSCNEIFEASAAIGEAAYRCKWYKLPCD
ENGRALRQGMTIMVMRSYKPCSLTVGKFCPMNLQVFSSVLSTSLSYFTVLRSMNESE

>gi|528747924|gb|AGS43057.1| odorant receptor Or2h [Cephus cinctus]

MWDIYKYPFNFNEAVYIICNILTPGIVLFKLSMIRLNRKSLYELIDICQTKFWHDDYDEFGIAILQNCET
KCVLLITSYMSFALFTAITYTVRSIIDNIGKTGTDKILPFTMWLNETMARAPYFQLLFIFEGIILCYLGV
GFFCIDNFFCIINIHVAGQFKILQGKLERLCGPNDREDEKKDRGIWIRKNPAAVFQEFRSCVQLHKMLIY
YVEKVEAIFSLIILCQVILSSILMCLAGFQAVSDDNSASQRCIFTAYTIGCFFQLLLYTSTSNE

>gi|528747922|gb|AGS43056.1| odorant receptor Or2g [Cephus cinctus]

MKLVGIWVPKDNNEQWIMNCALSYTILVILLAVAIEGFDIYYCWGNFYATTYTACATMPVIIVLAKIFFL
LLRRKLVMELIEFTEKNFWQGDYDSYGMSVLDDIDRKGVLLMCTFIFFVQGTVIGYVLTPIIENIGKNES
DRVLPVTLWINIPVTTTPYFEICFVMESLTIVHIGICFFCFDIFLCILNIHAAGQFKMLQHRFALVYDGD
NKQEMPITDKLHFVKDSEEIYIKFKDCVKHHKILIDYTEKVQSVFTFIILCQILISSLMMTMAGFQALLT
HGSIIRRLIFIAHTSGCFAQLLLFTSTCHEIIIESGGIADAAYNADWANTPYDDVGRSLRIGLQLVMIRA
SRPCHLSAGGFCHVSLDTFTAVIFCPALILSQNXRLQKRLLETAF

>gi|528747920|gb|AGS43055.1| odorant receptor Or2f [Cephus cinctus]

MTTLLSTMAPLMSITSGTSRYLLAVYNKVLIERILEVNKQVWNCATKQQDYETLKNYGLRARALTAMLFG
SASLTVVFFLATPILAITNEPMDFYSNSTGNFALRRALPLPSPFETFSSPYYELAYVAQGIATCYLGVAV
STVDALSTVLVIHGCAQFKLLNNRLLGIVRQDLQSASNYYGHDQRARFFKTSFRFHRSILEYCYTLEAVV

SISNLISVLTCMCAISFCAVNIVRGGADLLKFLGLFMVFIMQMLFVCWPADYLSSESTNIAFAFYNCSWY
NYEKSIGSSIQIAIARSQNPVALTAGKFVIMSLETFTSILSSAMSFFAVLKSI

>gi|528747918|gb|AGS43054.1| odorant receptor Or2e [Cephus cinctus]

MNRNNFVRDNVMIYKCLGSWPLDCVARRLENTRYILGLTLIAVITVFNGLQYIDLFIEWGDWGFISENIS
VSFIYSIFVSKIYVFYSRRREIMEMVLEIDNYVRKTYKDGHQSNXQILQSCEDIARRIKIVFGWSALLTV
IILHTWPIASMIFKKDPNLRMLGVPAYFPFSLNSTNNYAIAYVVEIVTASVMSLHTVNFDTFFISCILFA
IGRLRILHNSINNIKKIAPSDLQMKFSKNETYQAELQKIHSPIIV

>gi|528747914|gb|AGS43052.1| odorant receptor Or2c [Cephus cinctus]

MSCSYLRDLLLTDNMEVAMQSLAFMITGFGNTLHYILIAKSRAKLCDVLATFEDLWGLLEVQEKRVLMSY
VRDAKKLTYFFMSQCAATVFLYVGAPVIFGNGFVRVNGNITERMLPYSLIFECKDSPCYEILYILQILTV
INIAITYIGVDTIGPVLILTVSGHMKIIQNRIMSLGSSEEFXNFKNDKVKRISDYEISHFQYFGKSFEAC
VKYHQTVLKLCKDIEKVTNKAFLVQLITSTYSISAIGFKMNDSDKSKYVTQIVLSLVQLFLCNWPPDVLQ
NESQAVAYAAYFMPWYRCSRDVKKSTEIIIMRGQRVVRLTAGNFVDLSLETFIRMVSSALSFFTLLRSIE

>gi|528747912|gb|AGS43051.1| odorant receptor Or2b [Cephus cinctus]

MDVPLHGGEEYDQFIKPVMNTMQIISIWPLAADCGSYEYLLRICHQILMFFVTGTMSIVVTADVIHNWGN
MDTATECSLIASAFVLCFLRLMVYTYHQKDMRYVVETMRSDWADASYEEKQVLKEKCDFAFRLAKYFIAT
VAITIAFFMTVPMLETYILHSEEKILPFRGYFFLNHTLSPNYEMIYIFEIIAGSFGGSMIAGVTSFNLVV
IMHGAARFSLLQKKLESLNRNDPDVNKLMVKCIKLHQDAIKFADALEDIINVVALGQFVTSTGLVCFAGF
QLTSMLEDRGRLMKYSTFLNSAILELFIFSFSGNELIVESEAVGDAAYRSDWASSSFTQSLRILMMRATL
PSRITAAKFYSMSLESFSAVLSTSFSYFTVLKAVSEE

>gi|528747910|gb|AGS43050.1| odorant receptor Or2a [Cephus cinctus]

MVNLVKGYMSTKISQLLMTLIGMKRGKTKREQLLMDALFVYILATVLSAIWLENSELFYSRNDLYALTYS
APCCFTVTFDFVKLMIFTYKRHELHELHKFTEDTYWNKDYNELDKAILDKCDTTSAIGMSILALTSAILA
FHYLTGPYLDNLGTNTTERTLPFRVVFDFPITVTPLYQILYFIEVIGTISIGICSVAFASYLFYTCIFVS
GFFKILQRELENVCEVELESVNTKSSYNNNDTMLAYKKLKKCVIQHQLLIWYLGKLEGLFSYILLMLVLC
AVIILCFAGFQIILGDGTTKLHRQILSVEFIMAALAETVLFAFSCNEILTASAAIGEAAYRCKWYKLPCD
EYGRALRQGMTIMVMRSYKPCSLTVGKFCPMTLEVFTSILSTSLSYFTVLRSMNESE

>gi|528747908|gb|AGS43049.1| odorant receptor Or1d, partial [Cephus cinctus]

MYFKVILSELMNFLNTASPFEKAKLQKHVDQCTPFYLFILILFSLTAVAFSCGPIVMDRPFPAEAWYPFS
TNPLPISCSIYVLQIMVIAQAAMCIHMDFMIAFLLWYAVASFEILGEKFRRVENEVDLRNCIVQHQKLIV
FVREINRVFYVMILKTPLSMTISIICSSVQLIHHEPLPVLSQFILLLVVCSIKLYITAWPADSLIHASEN
IASSVYESSWIGKSSTFLKSLNIVIRRSQKPLIISITGILPPLSLRFYAS

>gi|528747906|gb|AGS43048.1| odorant receptor Or1c [Cephus cinctus]

MGIANNIELLNTSVGRRWSSYQTVLEYKMEVECLKLAGLWYLSSDTSDCVKVIYWIYNKIVFAAIAIFSI
GLLTDIIINYKDLVTFTDSGCIFVGISVVSFKAVVFQCKKSRIDRLVHSVRQCESWTSDLVDPAAMSLLK
KYKIRERVTILGFSCLGCVLVIALIFFVPRETGELPIRCWYPFDTTVTPMHQIVFGIQSFAVAVGMIAII
GMDNTVFVLCGRVLFQLEILAANFQSCSLDINASGGRSETNPHKDFTCFRHTIDCGGFFERYRKCVMHHQ
YLILLVDEVNDVFGSSMFSQLLSSSLIICLTGFQSLLVVGHGTNFFEICYLPRSSFQPIALLVLLRK

>gi|528747904|gb|AGS43047.1| odorant receptor Or1b [Cephus cinctus]

MFLLKYPMQYTSFCLKIICCWPISKNASNLRKNFDFLHFLFVISGFILISIQALVYAIIQANNPQEIIET
LSTQVIYLHAAGKVGIMRLHRHKFSRLLHGVERVQTTASGELLRYHKEYTRVGYVAYWIYTISVFIITLD
YIVQPLWKKSQFLPTGAWYPFDYKKSTFFYTLAYFQQIICITFSGCASTTEITFGVFIFFACARLKVLQR

KFQQLSDNSNGNEKILRRRICNYVQQHCDILRYINDVNETYTFIILVLFLSILLTVCCTSFLIINVTEHS
LDSIFINSLLMVASAVQLLFYYLPGHILIEEAKTIAESVYYSGWESLSINCRKLLLQIMVYSASPINLRN
GKMGLLILENYTTFLTTAASYLTSLRSIVGTV

>gi|528747902|gb|AGS43046.1| odorant receptor Or1a [Cephus cinctus]

MDFSSVNRFNGLVNFVCGNLLPLTDDATKFSKAQKLYSAFTWILEFTYFLVTTFGIFFTTNQRIFQDTTV
NQAVVIEILILGFYMNLRRGLIYRLIGQMNSVLINVETLKECVEKTVKPLQRPLKLYTIFATVTVVLFCG
SPIYKVFKKDQFSYNDFRIPAYIPGEPYSTGLFIAGILFETLGGFYTILKKASIDIYLIHVITLLTAQYK
YLSLELINIINGKNENINKSDLESLDNERNEKIEKTVQIELKKWIRHREVVMEIGKILKNLLVLNVVYVY
LNCIFRFCFLGFLLISSSGDYFIQALVCSYTVVCLMQVYVLCFCAQNLLDSSTAMTHDAFYEKWYAYGPS
TKRIFSMILISNKMECRLSMCGVVDLTLPTFMAILKNSYSACLFLLKVK

>gi|452887659|gb|AGG17946.1| olfactory receptor 13 [Microplitis
mediator]

MEEERSRDERDLESSVRAFYWGKWMSKWIGVWPLAPNFYLFNVTFAYFTAVMLLEYVDLFFCLPNFEKVL
DNLTENLSFTIIYVRTLMLRVHNYKLGTAIRECLKDSSVSAFRNSKEIDIFVQYTKEGKFFAKFVIAFAA
MTETSWYLRPITSHTAIRVIADNETLNNVTLKFSLPFHFYVFYEINSIKTYALTYLSHGPFVPINGFGTA
SANCFLIALSFHISGRLAVLAERIKTLKDNPDSYKRELKLIIDEHIRLLRMGEDVKISYGVNLLVYLLNG
TILLCIIGYQILLTLTVGPRTNIMPFIVYIMTMYMVISIFCILSENLIAESNKVCEAFWACGWYDMPPDC
ISDVYYCIARSQKPLALTAGKFLTFGYGTITDVTRTAVGYLSVLRNFLLLEE

>gi|452887657|gb|AGG17945.1| olfactory receptor 12 [Microplitis
mediator]

MIKRPIETHRKYLDKCIHCIRFCGIWKFDSSASTYHKLMNLFGKIFNPTLLIFHFLTLFADIVANYNDLI
IIADDGCFLAGSFTVCFKAYEFHLLNNIYMKIINDVHDSVDVLQKSCDLGVLTIIKQYIFFETLDFFLLI
YVVTVLGVGLIVLLPLTRGGLPVRAIFPFDVTKPLMHKIAFFIQAYNISFGLVTIVALEYLSWGLMRWTI
VQLKVLSSNYRNCNSDKVPIASFNVTKNTYNKIKNFNILKVDDEDIEIHNFIVFEEKELNSINDCFNWRF
RTCIRHHQRLIKIIYDLNDIFTVSLLIQLGVSTFLMCLNGYLAFMFPHDNQRLIRSVLYLVAGFVQLLYW
CGFGNELKFQANDLTTSQWMSGWEDKFDGGIKNLVTTSMIRTMQPLEVRAGGLFILSMETFLSILKTSYS
VFVLLTTVSDEE

>gi|452887655|gb|AGG17944.1| olfactory receptor 11 [Microplitis
mediator]

MQNEKEELPSCEKLFSLELAALKFVGLSSLRNGFRKENMNIPVKFYEIFLFICAASILSGFFISSALTVH
MLLQSDFILACEIATFIFAGAVTVSKVLRIWSYRIELIEILRELNELWEKIVKHRLNLKENILNMLNESR
PIRYGYCFIASSLNLSYALRPYFHMLVYFVKQSENKTIDLTVTTYPLLYPIERGTWLGYLLCVTYEQSIL
YFGGIYWIMCDTLFILLTSHICVHFMIISNDFNNLHINYDKNNNESFQLIEDLSRRHQKMFVLCQRIETL
FSPIILLTVVFNGIDLCLCIFALDKDLSDGNWAKVASSVTHALTLFFQIVIYCEFSHVATEETSKVGEAI
YNSSWIYFDKKMKKMLLIIMMRASKEYKFSVFGILILDREQLTQIVKTTMSYFTMLRSFS

>gi|452887653|gb|AGG17943.1| olfactory receptor 9 [Microplitis
mediator]

MYSSNSTQVIADFFKQPCYRVNVLTLRICGLWPYQSKFEGKLLRFLWAFFVSSQMIPQVCVCITDFGMDT
LTSTIPPFTVAIIAGAKMIISTLKVNQIIELLVTIQKDWSKLKSETECDIMKKHLDQGKKLTLFISAWYY
NSMVVFLLLPMRPKVMTWLGLSKGPADFDFPYPVNYGVDHDKYFYAIETHISFCSTLVITTIIAADTLFI
VFVQHICGVFKIISYRLENLVSSSSLDIDLHPNKINDRAFWVISDCIKKHNKAIQFAELLENSYCWIFFI
SVGFETLLMTFSGVQLVSQMGNIDGLFRYGPFAFGQLVHVFIENAISQQLINYSGEINDAISMIKWYTLS
MRSRKLLTFMIMRSQVICSVSAGKMFIMSMETFGMILKTTMSYFTILSSMQDD

>gi|452887651|gb|AGG17942.1| olfactory receptor 10 [Microplitis mediator]

MSTENTQLDADIEYNLKLTKWILKPLGVWSIVTKDYRFISKLSSVFIIIILNSFIMFITFMPCCVHMIYRE
TDPAVKIILMGPFGFCLTNCCKYYFIIFRSNIITHCLENLKSDWARPKTKQDRLEMIKNVNIGHNITKLC
AIFMFSGGVSYHTIMPIWSGSTVNEANETIRPLVYPGNEIFVNCQETPIYEFIFILHLTCGMVMQTITTA
ACHLAAVFASHVCGQVDILKSQLQNLVDKESLKIDGTIENRIASIIQSHVRILDFSTNIEKMLREICLVE
VGASTLIICLLEYYCMTEWSNSETINILTYLMLLVALSFNIFIFCYIGELLKKQCNSIGESTYMINWYKI
PRNKGKQLMLIIASSNNQRKLTAGGMMELSLRSFGNIIKTSVAYLNMLRTVTE

>gi|452887649|gb|AGG17941.1| olfactory receptor 8 [Microplitis mediator]

MDVLQENFSVLFYLGVWKPLDCTGIKSFLYNLYTLFITSISYTFLLSQILDLIISTKTVSDFTNNIFIVS
AILTGCLKIFRFIRSRNTFINIINNFKRGLFKPANNDEIIIWNKYARITRLVTIGATTSLIIGLIVMSYA
LCSFNIPQRQLLYRAWLPYNYSSLPIIYWLSSMEQLATVHILAGINFSFDLIFFGTMLNICAQINILKLR
YKVALSHIYSINDSINNNDLGELRDVSKLIREYTDSHDSIIKLFNSAHHLFSTIVTIQYCTSSVAICTSA
FNVTKMKFFSFQFFSTALYINNVMIELFILCVSCNEVTLEFADLGNTFYDCQWYAINNANKKSVAIMMTN
TIKPIYFTCGYVIHLSLDSFTSVLKLSYSIYNVLQSAD

>gi|452887647|gb|AGG17940.1| olfactory receptor 7 [Microplitis mediator]

MDIYDKPYYKITKISASLIGRWPYQSSRQSLVIVTVIWSAFILQAIPQIIAIVTHFDDREVLLEALAPFI
IDIMFVAKYMNSIYNAELMVTLFERMKKDWKLLSSAKEKRILEYHANIGRLISTGYAGFAYTTTAIFLSE
PILPRIINYFSKSNESVPLKFALPLEYIIFEKENHYWLMLAITNMFAINMIIVTISCDIMFITFVQHVCG
LFAVVGFRIENSPTGKITDSNHRAVSLRKNSQDFSYKHLVSCIRSHRRALEFVKLLEETFTGTFGVVVAL
NLPMISITGLQLITQSNTVEQTLKYLMFALAQVLHLFFDCFLSQNLTNMSSRIPQCIANMKWYNISKNSQ
KLTLLMTMRSQTPCKLTAGKIMELSIENFGMMMKTSGSYFTMLLSMQ

>gi|452887645|gb|AGG17939.1| olfactory receptor 6 [Microplitis mediator]

MTLIESCWMVFTWLGLFRPIKWKGLKARIYDLYTAIVLFFNYSFFICGVMDIDFTHLNFFADIDLITLML
QYIENTPKILCMILNRNALIEIDFKLQHDHFKIKDEDEKKIQNKFDKFSRYVLLAYSALQATSLVYYTTG
RILAMESPVILPYRSRIPFNYSSSGKIYMLTALDQLYSVSSLICINGAFNLVFTSTMYQICTKIRILKHR
FKVIIQQLEHDGELGNNDNKNLRDVMRKNDATTDKFESQLIANWVESHIALINLYDYAKSVFAKAVFIHY
VINSIVMCTLAYILSHCEIDNIFFGNVCYFSVKCTQQFLQCSSAHQITLEFEDLRDMIFSTNWFATKITI
QKSIIIIMFKSIVPIEFVSGYFVTLSLDSFKRILKLSYTIYNVLEG

>gi|452887643|gb|AGG17938.1| olfactory receptor 5 [Microplitis mediator]

MDFWGNSDWRFLRFQLCTLGVWPFQKSNFKRVVGFFVILSVQSITLPEVIKFTYIWHDMEEFADCFPLIG
IHFVCTIKWMCCVVNMDKIIALLNMIKSDNLSKELTEEEHQILRDTGKINRLFVLVYSIWIYVIAILFLV
FLPLIPVTLDIILPLNESRPKIELYHTDYLFGPVKYSWVISLHQCIISPFPTIIIIATGSLYCNCCQHAC
GMFEVIGYRLKNLDITIEAAMNKKNLGYYADTEIFYKSLVTCEQMHQRMLGYVEKFQDIYSLTLCFSMTT
SIITLCITGLEAIIKKDQFFEVIRYVTCGLAEIADVFILCWYGQKLIDSSDYLYLCACQGNWYKYSAKSQ
KMFISMIVRFSTPCSITLGKLYRVSLECFSTIMKTTMSYFTVINSLREEE

>gi|452887641|gb|AGG17937.1| olfactory receptor 4 [Microplitis mediator]

MDFFDSHYYKGMKILLCLIGRWPYQTLKERVLITIILSILWSYTMFHRMLVYSNYDNYSSKHEVILETIS
PLIVDTITFAKYITTICKMDTIINLLESIKQDWKIYTNKEEKKILEYYANLGKILSLGYVGAVYMVTVLF

MTEPIVEQTFFKLFQNETIPKRFSIPIYWKTPDIEKYYYYLISFQTLNTNFIISITCASDAMFINLLQHV
TGLFSVTGYLLENVPIEENSEENGQKKIKDVAYEHYVRCMRSHKRALEFAENLESIYVWCFGIVITLNMP
VMSVTAMQLTTGTSNVIQSMKYGTFAGVQLLHLFFYCFMSQKLLTSSSVIPECAMNGKWYLCSVKAQRLV
TLVIMRSQISCQLTAGKILVLSMETFTSIVKTSGSYFTMLVQMRNV

>gi|452887639|gb|AGG17936.1| olfactory receptor 3 [Microplitis mediator]

MDIYERPYYKISKNFASFIGQWPYQSRLHSFMCGSVLWTLFIIQVIPQIIAAVVNSDDQELLLESVSPFI
TDGIYIAKYVNTIRKAKMIRRLFEKVREDWKVPKNNDEKLVLESYLKMGRFLSIGYAAFVNMGVIIYIMD
PVLSAIVNIISKSNDSMPLKFSVPMRFIMFDEEKYYWLLLILSNTCVIFIINVIICCDVIFITVVQHVCG
IFAVVGFRLEHSPSDTVSPDLIEGTRFSMNSQDISYKHFVSCIRDHRRALEFSELIESTFAISFGISVGL
NLPLMSITGVQLLTQSESMRATLKYIMFTGGQILHLFFDCYMSQKLTDMSSRIQHSVARANWYENSVKSR
KLLILMTLRSQVPCKLTAGKIMELSIENFGMMMKTAGSYFTVFLSMR

>gi|452887637|gb|AGG17935.1| olfactory receptor 2 [Microplitis mediator]

MHHKLINILSKILRYNGIWPVESTTAISFKLLNLILRFFNFCIFVFLTSIIMADAIANHSDLSLITDNLC
FLIGCFETMSKAFKFYTEYNNIIKLINDIYEPIDKLKTINNVEIMTRVNKLSRFECRQFYILCGVVALLI
SARVFGADRANREFPVRAIFPFDKSKSPNYQLIFILISYGVAFIDVSLFTLDLMIVVIMRYLTLQLEILI
SNYKHCRVGLIRNIARNIPSNGSETVESFYEIATVTDNDDDDNGDDGIKNFVIFEIHRKDINNINTFDWR
LKQCIKHHQKIVQMMVVLNDCFSFCVIVQIMTSTILICLNGFQILLGNDDRHLLVRRIIAINAVLLQLFF
WCWYGNKMSTVADSLTYNQWMCGWESEFKRGVSNSVTTSMILSLRSLELRAIGLVPLSLQTFVSAIKKSY
SVLILLLTVVED

>gi|452887635|gb|AGG17934.1| olfactory receptor 1 [Microplitis mediator]

MVTVYEYEEMMKPIKMVNRLISIWPLEENDNSILSRLRIFHRISMFILILIQTVAVTADIVHHWGSMKEV
TECALIATAFYLCVLRLTVYTIHDKDLQTSVQIMKNDWIKFSGEDELTLKEKCLPIIKLAKFFIMTVFST
IGLFMVAPILEVKILGMEEKKLPFRGYFFENQTITPAYGGLYLLGVTAGGFGGSMIAGATTLNLILVMHG
AAKFMVVRKNIESLKSNSENSITFIDCVRGHQDAILFAERVENTINVLVLGQFVISTGLVCFAGFQITEM
AEDRGQLMKYTSFLNSAIFELFLFSYSGNELLTESDAISQSCYASNWVGTSFAKSMQIVMTRSLSPCKIT
AVKFYDMSLANFSSIFSFSFSYLTVLRTMEAE

>gi|119888030|gb|ABM05966.1| olfactory receptor [Microplitis mediator]

MMKTKHQGLVADLMPNIRLMQISGHFMFNYYGEGKKLMHKIYCSVHLFLILLQFGFVAINLVKEKEDVDD
LTANTITILFFLHTLIKIVYFAARSKLFYRTLAIWNNPNSHPLFAESNARYHSIALTKVRRLLFCVGAAT
VATTISWTTLTFFEDPHVERLNKETNETYIEEIPRLLVRSWYPFDARHGVAHIGMLIYQIYWLFICTVDA
NSIDVLFCSWLLFACEQLQHLKAIMKPLMELSATLDTVVPNSGELFKAGSADHLRDNDGVPAEPAMNGDN
MLDMDLRGIYSNRQDFTATFRPTAGTQYNGGVGPNQLTKKQEMLVRSAIKYWVERHKHIVRLVTAIGDAY
GVALLFHMLITTITLTLLAYQATKVNGVNVYAASTIGYLLYSLGQVFLFCIFGNRLIEESSSVMEAAYSC
HWYDGSEEAKTFVQIVCQQCQKAMSISGAKFFTVSLDLFASVLGAVVTYFMVLVQLK

>gi|861722551|gb|AKO90017.1| odorant receptor 53 [Microplitis mediator]

MVVLKDIFTILFYVGLWKPATWHGRKSILYTLYTSCIVIMASTFLITEVMDLIFVTSNIVEFTNNVFMMS
AVISSFIKTIIIIRHRKIIADIIDVLKIYLSKISGNEEIIIIDRYTRLIKFMNRSFLCTALFGVSLMVYV
ASSQNISQHILFYRAWLPYNYSQPMAYWMTTTGQVLTIYVLTIIYTVFILLFSGIMFNICAHINIFKYHL
QITFSDEYYHSRNDKRRCISKKENDKKIIHDCVETYLSIRRLFNTVKNLFSSIMLYQYSVGSIIFCTSAY
NMLQVEIFSAHFFSITLYMLNMMTELFIICITCNQITLEFQGVPNALYHSLWYVTDNNNRKSIVIMMSYT
LKPVYFTCGYVIDLSLDSFTNVLKLSYSIYNVLQSTF

>gi|861722548|gb|AKO90016.1| odorant receptor 52 [Microplitis mediator]

MEYNQDFKYAVAWNRTSLRFVGLWPEPNDGFFTKLKGWLGAWSIFMTIYLPQSTLAYVNWGDMNAVIESL
SINGPILIAIIKIIIFRHYRDVLKLAIVTMTKDWNELRSKEEYKVMLKTAKISRIISVTSTIITNTLFIA
FVFFKIWIGMQLMKRTDLDPRLSVGLLYPGYLPFDSRIMTYFIPTWIAQCFATCFSMTAYAAFDTFVSCM
VLHICGQLAVIGVSLKNLINDDVKVDSKVFWIKFSEIIKHHEEINKLGLMIENSFNSILLPQMFVCTVTF
CLQGFAMITSFIDPSAGEISIFEMLFSIVYVFYTVMHLFVYCYVGDYLSFESTLIGQSYYKSKWYELPVI
KSRSLMFIGHRARRPLLLTAGKFCAFSRNLFLAVLKTSFGYLSMLLAVKQEKISDT

>gi|861722545|gb|AKO90015.1| odorant receptor 51 [Microplitis mediator]

MKRKEKKNSQTKKKINLTVFDDIEYTQQLLSILAIWPLVTKSSTIKKILSWIHLLVIIGSLIWNILFRCI
FIYIYVKKFDDQIMLIGPTFFRVIILLKYLAIIYHRKTIKKIFNHIQTDRSGVECQEQQNIMMRNVQINR
HVTLVFAIFMYSSGTFYNFVMPHILPLLTHRGSRNTSQRLTIFPGYNVLSDVQKSPIYEITFFFHIFSVF
AGFSVLVLACNIAVVLVTHACGQIQIIIGQLNSLIDDFTNNEKTLYIKFSSIISRHIRVIQFSNNIIKDA
LYETCLVEIGASSVLICIVEFLLLKMIENQNYANMIPYAMLLASLTLSILIICYFSELLESQFIEAGIQA
YSINWYQLPPKARKYLILIIIISQRSYKITAGGIIDLSYIAFVQILKTGFAYLQLLRATK

>gi|861722542|gb|AKO90014.1| odorant receptor 50 [Microplitis mediator]

MKFFNHPDWRIGKLMLCSFGGWPYQSQHSRRILNFISIFTVQSIFIPEIFRLTRIWNSMEMIVECVPMIS
LHIVANIKMFNCIINLNKVKVLLEDIERYYQSDLCKSELRILYKDRHSHKKVISVYVFYIYSIAVVFASI
PVLSKVLDLIAPLNESRPKVYLYPAEYFVDQDKYSTYIYIHGYMALPITMTLCTAYDFLYSACGHHVCSM
FKIAGSRLKNFIDNKIAWSKNESLDRNYRQDEVYKSLVECVKMHKFILNYVDCYQETFSDSLFLVIGVNM
LALCITSLQSLITMNQFHDAVSYIVFAFGQLTHLFLLNYQGQNIINHSENFYNDAYQTNWHEFSRKSRKL
YILIIMRSSEFSVIRAGKIYVMSSDSFSNVLKTAMSYLMVLNSLR

>gi|861722539|gb|AKO90013.1| odorant receptor 49 [Microplitis mediator]

MEVHSRDQRDLDKGVRVFTWARLMSKCIGVWPLEPNYHLFNICFFYFTYIMITEYINLYYCLPNFKKVLG
NLVENLAFTHIYVRTLMLRIHIDKLRDIISESLKDYHASAYKNSDEVNEFLIYVRKGKFFVKAAGIFVIS
TATSWYIRPITSPSLPNNETAAFTYILPYKFHIFYKISNYRTYVLTYLSHAPFAIISGVGAVTSAWLLIM
LSFHVTGRLAILAKRINSLKDKNGGYRSHLDEIISEHSRLLEMGEGIKSSYAIALLIYFVNGTILLCIIG
YQILVTITLGVKHNLMPYFVFILTVYLVISIFCILSENLLAQSNKVSEAFWSCEWYKMPQDCVKDITFCI
LRSQKTLGLTAGAFLTFSNSTLTDVTKTAMGYLSILRNFLIVE

>gi|861722536|gb|AKO90012.1| odorant receptor 48 [Microplitis mediator]

MEVHSRDQRDLDKGARLFTWARFISKGIGVWPLEPNYYLFNICIFYFTYIMITEYIDLYYCLPNLKKVIN
NLTESLAFTQMYVRAVMIRVHIKKLHHLMSEALKDYHVSAYKNSDEVYEFMSYVKRGRFFVKSVTIFILS
TTTSWFLRPITSSTPSTSMSAPDNETAAKFTYILPYKFHVFYEINNYRTYVLTYISHGPFPYVSVLGAIT
SAVFLIILSFHVSGRLAILARRINALNCKNEGFRTDLTDIIAEHTRLLEMGEEIKISYAVALLVYLVVGT
TQLCIIGYQILVLITMGKQHSLMPFFVFILTTYLLISIYCILSEHLLAESKKVSEAFYSCEWYDMPQDCI
KDISFCILRSQKTLGLTAGAFLTFSNSTLTDVTKTSMGYLSILRNFLLNEQ

>gi|861722533|gb|AKO90011.1| odorant receptor 47 [Microplitis mediator]

MSEKATNSIITSKIVNSKYALDANRWILKILGVWHFAIDSSYFHKVIALCHIIICTFLLSFVVIPGILFI
FVIVKDVTTRLRISGVFSFCVMGVIKYYYLIKSNKQIGNCVKQFNSDWAQINDIKDKTIMVKYARFGRNS

SIICAAFMYGSCMFYACILPHVSGVFKNKNDPTERTFAYPCHFIVFNQYESPAYEIVFSIHCCCAFVLAS
ISNAACNLITVLITHACGQLEILMVWLNDLDSCQQNEVYAEKYSKIIKQHVKTIRFIVKIENLFQQICFV
EVVGCTLIICLVGYYVLLDWNQKDTGGMTTYVMLLISFVYNIFLYCYVCELLTAKCKLISESTYLTRWYQ
IPENFARGLVLTIAISQNSNPIKAGKLIPLSINTFGTVMRTSVVYLNFLRKLME

>gi|861722530|gb|AKO90010.1| odorant receptor 46 [Microplitis mediator]

MKVNPSDQNSTVIQNQSSEPENIVNNEQDFEWAIHYHRKVLKFCGIWIYSNSNRWYLKLITDLHSLFIIG
GVLICLTTPEAMALVKIWGNLTLIVDNLLSSAALISTQIKLFVLWTRRKAIARIVEAVKSDWLEPKTEAE
RKIMRRYARIARIMMVCGLSNIAYNLITFHGSVLFGFVYRTVNNITDIEGYLIPTQSVFPFDITIGYRCW
IIRIVQALQCFGAGITYTAIDVFCGMSVLHNCGQLEILADKIKDLVNPDEPRVFQELLKTIVLRHYRIIG
LIEEIRNIFATVLLLLVLCFGILFSVIGFLIASSFESDGTKVPVSQMNFYIGYILFFVGLLFVYSWVGEN
LLSHSEEIHVAVYSCNWTDLEPHQIAQLIIILVRAQRPLEITIGKFAPATLNTFAQILKTSAGYISVLLA
RNG

>gi|861722527|gb|AKO90009.1| odorant receptor 45 [Microplitis mediator]

MDVFDSMHWRTTKKLLSAIGLWPFQPVIQRIVVGAIVYFIIQSIFICVFLKLIVAWGNLAETLYSVPILV
YFSMIQVKITNCHLNLHKAKFLLLRVKRDWESKLEDSEFEILRNDGRIHKIIMDVYFSGLICVATVYIVL
PLMSPLLDIIIPLNETRERILPYPAEYFIDIQKNFFMLYPHGAIVTPIALTVLVGFDSLYAGFVQHACSM
FTIIGRRLENLTVDRNNIDEEKNSLNERHGLQSFITCVKMHKDILQFVKLVEKYYSNYFFVLLGVIVVGL
SAAGFQFVVLLSGVGEKVRCLWYAMGQVTHLFFLSYVGQKLIDHSQVINASLSAAKWYDYPQKMKPLIIL
MLMRGKRVSTVSAGKIYVMSIENFSSVIKASMSYFAVLTSMES

>gi|861722524|gb|AKO90008.1| odorant receptor 44 [Microplitis mediator]

MENDQKELDEGVKAFYWAERMSRCIGLWPVTPNYYLFNICLLYFSVLMVLELIDLYNSVYDIDKLIDNFT
ENLASTHMYARILMLRVHNYRIGEMITQAMKDYRISAFKNSYEIKVFMEFVNKGKFLIKGLFIFIMSTEI
SWFLKPLTTPSSSDNSIVNANKTFPQFILPYNVYIFYEVNSIKRYVLTYLSFMPMVYVSGIGHSAVDCIL
VLLVFYISGKLSVLTMRIDALKNNQYDCRKELKEIIAEHSRLLKMGDEVKDVYSTGLLVYLVNGNLLICI
IGYQILINYMTGPNSDLLQYFVYIGATYFMIANFCIISEHLTAESNKVCEAYWNCEWYNMPQDCVKDIIY
CIVRSQRPLALQAGKFSTFSIVTLTDVTKTALSYLSVLRNFLIAE

>gi|861722521|gb|AKO90007.1| odorant receptor 43 [Microplitis mediator]

MSKSRVSDYKAYRGLTKTLLTVGGLWPYSNSNIFYRTLPYIQIIILNLGMALAILGFVRDHFSNIALVTRG
MGVMTSFLTTILKVTCLVINKKDLMELHGNLDPYLNDLLKNSPSTEVILKDINSFKFLSWGLTLFAFIIL
HCHQHEIELINYPLIYPSVYPWKIISNGWIYKMNYVFETLASLILFFVTASVDSLFTLYVFQMVGLLREI
SYSIRRLDEKNVNRDSVICKCITQYEKLMRCREILEKIYGPVILWIMTTNAVVLCTLLFQISQMKSISVT
RGLLFTTYITLKMIQTFMYAWSGSCLTDESENYKDAVYAAHWYGNKRFMTSVIIMLAQRPLTLTACNFST
VSLKIFVMVLNTTVSYFFLLQTLDYQE

>gi|861722518|gb|AKO90006.1| odorant receptor 42 [Microplitis mediator]

MAILERNIFILTLVGVWKPNHWKGFKAALYYFYICLVTIVNHSFLLSGVLDFELRNIDVVVIIDNLSLLI
CVFTIRYKIITLLYYRKFIEEFVNCFERDPFRAKDNEEEQIYVQFDKRTKTLSILYAGLFTVAVSWYSVG
HVLRMSPPNVMPYQGWFPYNYTVYKYYWPTVIYQLYAVCSGAWVNLAYDSLFCSILYYVCAQTHILKHRF
SVLAENLQKINEENDGNDNKEIERKMIGDWVDYHNNILDLVKFVKSFFSTAIFVQYAASSLLICSIAYTL
SHTETRSLNFAGNFFYLIAMTIQIFFQCIAADQVTVEFADITNALYSTNWYNLSNNAQKSLAILAEPLKP
TLINSGYFVILSLDSFTKVIKLSYTIYNVLE

>gi|861722515|gb|AKO90005.1| odorant receptor 41 [Microplitis mediator]

MKQTATKEGLGRKGFSVTPETAISFTRITVYLTCIWSPSTSSTKLTFILFEIFLWFSIFLSLGLLFPLIV
SIIKFIDDTFVVMKSFILISGIVNFVIKVIVCRIYRQELQELGASLNDFVRNSNENEKFYLQKYVDKCWK
FHGYMTCSYYLTTSAVLMGPLVLPEKFPSDAVYPFPVDHPVVATIVYLHQCIVGYQCAAGMALDCQAALF
LWYLSARFEILISETVNIASHKEIQDFIKTHQDILKFGKQVIRPIRLIVLTTVTMTKVGMIFGAIVLISD
EPITVKVQFAILVVSATVNIYVCTWAADNLLTVSSSTISNEIFHVSAIHPPAMKKLWLTVIHRALKPITI
EVPGFLETLSNEFYSNFLSTAFSYFAAMHAVVNS

>gi|861722506|gb|AKO90004.1| odorant receptor 40 [Microplitis mediator]

MSMQYSQDFKYALAWNRTSLKIVGLWPEDDDGIITKSLGWFCACLIIIIIYLPQSASVYVYWGNMDAVIE
CLSVNGPVFITIVKIIIFRYYRRVLKRVIDTMAEDWSSSRSNEEYAVMLKTAKISRAISVTSTIITNSLF
VAYIFFKIWEGLEMSKRTDLDPRLSVGLLHPAYFPYDTKKIKFFVPTWIAQLVATLFSMTAYAVFDTFVS
CMVLHICGQLAVVGISLQNLINEDTNSDPNYFWSKFSKTVERHEKLNELANVIEDSFSSILLPQMIICTV
TFCFQGFAMITSFIDPLAGKVSILEMLFSIVYVFYTVLHLFVYCYVGDYLSFESSIIGQSYYKSEWYKLS
QDKSRSLMFIGHRARRPLKITAGKFCAFSRNLFIRILKTSFGYLSMLLAVKRDKSA

>gi|861722503|gb|AKO90003.1| odorant receptor 39 [Microplitis mediator]

MTAGMIAARIDIDVFLEAIPTVLADVICGIKMFNFSVNAAKMKKLLLTMEEDWKIYASGPENKILNEYAH
FGRKVTIYYTGALYGTLAPLVLVPITPLILDVIAPMNESYPKHLMFQQIEFLVDADKYFFPLFIHNYMGT
VAFLTIIIAIDTMLMVYIQHGCAKFAILGLCLERIATNADQNIDRHTSEFDDIDYREIVKCIVIHNRAIE
FANLIEEANHLSFLIVIGINIIMMTTSALVAVFKLAMNETEIAGRFAFFTLGEICHIFYSSWQGELILKH
SESIFYFVYQANWNNTSTRSQKLMVPLLLRSAIPCRITAGKMFEMSLKSFSMIVKTSFSYLTVFASMRV

>gi|861722500|gb|AKO90002.1| odorant receptor 38 [Microplitis mediator]

MKTLPYSFMILEYFGGWKPLKWGTSIKGKLYNMYTLTVAVTLVTFCLSCIIDLFYTANIEDIVHNMSMSF
TLIVVCSKLTLVTVKRNEIIRIIKLLDINICRVRCAKEANIQLKYDEKAKNIVKKYGGLVCGAVFAVTGA
SILENIPTKTLPFNGWFPYRHDNSTGFWVAYFHQNIAHFYVAMIGFSFDTVVYGVLLQNCSQLQILKNRL
ENFVEIINEEKLKDKINGLSRTIRECEHKFIKQCTHHHWIILQFSQESNDLFAPIIFLQYLSSSLILCLC
VQLLTKLAFMSPEFIFIVVYLGCMLTQIFLFCWYGNEVIMESSEISSAIYKMNWQVLTNKTKKDLLFMKT
QSILPIKFTSGHLIELSLDSFTKLIKFSYSAYNLLHQK

>gi|861722497|gb|AKO90001.1| odorant receptor 37 [Microplitis mediator]

MEVLTFNFFLLSIMGVWKPRGWRGIKAILYNINRSIVVIVNHIFLLSGILDLEFKNVDLDAFVDNLALIL
AMVVVRQKIVCVIQNRTGVKHILDSLAKGPFKLRTHQEKLIFSRFDDFARNIFTYYPLVFMSSLLTYSSG
HMAVMDPPYVLPYKGWFPYNYTRTTKIYWTTAIYQLYAVFTTATINLILDLLLPCIMCYMCGHIHILKHR
FKEMIEKLLVMSENNVPQEKIISTERKLLGEWIEYHIDILRLVKFTNELFSSVVFVQYTVSALLLCTIAY
LMSHTDTMTMSFAGNLAFFTAMFIQILLPCYCADKLSYEFLDISTGIYDTNWHYLSNNIRKSVVIILRKS
YRPVIITSSFFIVLSLESFTKVVKLAYTIYNVLE

>gi|861722494|gb|AKO90000.1| odorant receptor 36 [Microplitis mediator]

MEILTTNFFVLSLVGLWKPRGWSGIKAILYHFYSSIVIFANVSFLISGIMDLEFTNIDIAAFIDNVSLLL
SLVTIRQKTACAIGNRGDIKEIIDSLGRSPFKPQDEEEENIVKRFDDLTGYILKYYPVLFTVAITWYSIG
HMFIMDPPYVLPYRGWFPYNYTTTGVYWLTAVYQLYAICSAASINLAFDPLLPCIICHMCAQVHILKYRF

GVMLKKLEVISDNEPRYVIASAERKLMGEWVDYHISILNLIKYVNSTYSKVIFVQYTASSLILCTVAYVL
SHMDALSMNFAGNFFYFIAMNFQIFFQCFCANQVTLEFLDITTALYDENWFNLSNNVRRSMTIILCEPFR
PVLFTSSFFIVLSLESFTKVIKCAYTVYNVLQ

>gi|861722490|gb|AKO89999.1| odorant receptor 35 [Microplitis mediator]

MAILKESFCVLTGIGLWRPVEWQGIKGAFYNCYTLLVLLNSITFIISESMELIFFNDGIFDFFNNSSMLI
TVIGMCGKLITVVTNRETIIKMIERFHRSPFSPRDYEEEIIHKNFNQKIRFNTLAYIVFFEASVTVYTVG
KIFEDRPPGVLPCRAWLPYDYSNNIIIYWMTASQQLLTVVMTANVDIAYDTLFPGMMMQVCIQINVLKHR
FRLTLDALENISDDKMDPVMVKTVEKKFFSEMVDTISHVFGPMIFIQYSFSSVVLCSSVYALSQMVPFSP
EFCACSVYILCMFFQILYICLSGNRVTLEFAKLGTAMYDTYWFALSNNAQKHIIMMMMSSVKPIIFASGH
VVTLSLESFKRLLKLSYTIYNVFQQSS

>gi|861722487|gb|AKO89998.1| odorant receptor 34 [Microplitis mediator]

MEDLTQEQKDLNEGAKMFDWGKWISKGIGVWPLAPNDYLFTTTFLYFTAVMTLEWVDLYTCLGDFEKVVD
NLTENLAFVHIYVRTLMLRVHIDKLRDVMTESLKDYRTSAFKNSTEIKLFMTHINKGKVFAKVVITFIAM
TEVTWYLQPLTTPSPPVDNRDNETISILLPYHFYVFYEINDFKTYVLTYLSHGPHVVISGFGHATSDCFL
IILVFHLSGRLAVLAERINALKNKPEMNGIQIKSIIAEHIRLLKMGENIRSAFATALLAYLFNGTILLCM
IGYQILVNFMTGPNSDLMQYFIFILATYFIITVFCIVSERLIFESTKVCEAYWNCGWYNMPREHINDIMY
CIVRSQKPLALQAGKFAYFGNSTLTDVTRTAMGYLSVLRNFLIVN

>gi|861722484|gb|AKO89997.1| odorant receptor 33 [Microplitis mediator]

MQVLTFNFFLLSIMGVWKPRGWHGIKAALYYIYQTITIIILNHLCLLSLLLDLQFKNIELGDLIDNLALVF
TIIIIRQKIVCIIGNRPGITHILDSLKNSPFKLEDSKEEFIFSRFEKLARNIITYYPLIYLSTLSVHSTG
FISVMDPPYTLPYKGWFPYNYTRTTKTYWVTAVYQIYIVLTMGSINAISDILLPCIICYMCGHIHILRYR
FQVMAEKLRIMSKNNEPKDKIISTERKLMGDWVKYHIDILNLVKFTNEIFSSVIFIQYTVSSLLLCTIAY
LLSHMEPTTMRFAGNSAFLTAMFFEILLPCYCADKLTFEFLDISTGIYDTNWYHLSNNIRKSIVIILRKS
YRPVTMTSGFFIVLSLESFTKVIKLAYTIYNVLE

>gi|861722481|gb|AKO89996.1| odorant receptor 32 [Microplitis mediator]

METTATKNDNTNQNKNELQLTKTNKYENVDYKSDADYAVVVARTLLTPLGIYPLHGSDTSLSKFLIAIQI
IIVFGLMLFLLVPHFIWTFFDAEDLKKLMKIIAAQIFNSLALIKFWTMIIHKKELRNCLIQLENNWKNVL
CEEDRVIMIKNAKIGRFFTIAYLSLSYGGALPYHILLPLTAERIVKEDNSTQIPLPYPTDYVFFVPEDSP
GYEMLFVTHIIISTMILSTNCGIYSLIATYIMHACCLFEVVCRHLDEFSKNNTNNFKTELTWIVENHNRA
IQFAETLESSLNIVFLCEMVGCTVIICFLEYGVIVDWEDGKLLGLVTYVILMTSIFVNCFIISFAGERLK
EQSIKIGESAYFAEWYLLPKGLVYDFMLIMIRSSKPASLSTGKVSDLSLAGFAGLVKTSAAYLNFIRAVV

>gi|861722478|gb|AKO89995.1| odorant receptor 31 [Microplitis mediator]

MLFKATPEFAIAFTKLTSILGSSWPHYKNATKCQLIVFNIKWWFFWFMSITAFLPMCYAAYNNTKNILSF
TKSLCDAANCSQAFIKMLLCKIHYRKLQFLFYEMEKYVEQARANERELFISYIKRCGRLHVSIMISAVMA
AVIIIIAPIGMPQPFPNVAEYPFPVDGHPTFEIIYLQQSIATIHCMSIPVFDCQIALLLWYAGARLELLG
DEFRKVTDNQQFVACVKKHQYLLWFIQEIIMSSRHILATTVVMCTIAVITSGVHIVGKEPLADKVTSVIL
STGLSAVLYLCAWPAEHLAQMCENVGAALYCSTWIKNSKESNKNIFIVIQRSQKPETIQVPGILPILSLT
YYATFLSKTFSYFTTLRVVLDKMED

>gi|861722475|gb|AKO89994.1| odorant receptor 30 [Microplitis mediator]

MLFKATPEFALKFTKLIALLGTSWPNYEGTPKWKLVVFQIRWWSTFFLAITACLTMCYAACNQYQNILNL
TKSLFDISNTSQTFVKMFFCKVHYKRMQYLLCDMEKYVTKAKPHERDLFIKYIKRCGKLHLTVMGSGLLI
IHIIILAPIALPQPFPNIAEYPFPIDGHPTYELLYLHQSCATIHCLSIPAFDCQIAMLLWYAGARLELLS
EECKTITDNKQFVECIKQHQYLLWYIQEITTSSRHILATTGCTCILTAISSGVHIVSNEPVAFKVPFMIS
WVIVSSTLYITSWPAENVLQMCEQVGMALYESPWVQNSKELNSSILFVVQRSQKPSTIEVPGILPVLSLR
YFAMFLSRTFSYFTTLRVLLDKINLDMEIPAED

>gi|861722471|gb|AKO89993.1| odorant receptor 29 [Microplitis mediator]

MYHKLLNKLVRILRYNGIWPVESTVRSYKLLNLIFRLFNLSIIVIMMLLTIADAIANFNDISLITDNLCF
FVGCSEALTKGIKYCIEYKNIVKLMNDIYGPIDIINKKNNTEVMKGINEIARFENRQFKIIFGIVSLLIV
ARVLGADFKNKGFPIRALFPFDATATPYYHLIYLLISYGVLLVDYTLLGVDLMVVVIMRYLTIQVDILRA
NCRHCDIESTRRNIVINGYDDKNNENTDIRNFVGFEIEHEDSDGKDSFDDRLKRCIIHHQKVIYMLNGLN
DCFSFCVVVQILGTTVLLCLNGFQIIMGRDIHLFMRRVLASTAALLQLLLWCWYGNKLSAAADSLTINLW
MCGWEDNYKHGLRNFISIPMTLSLQTLELRAIGVVPLSLQTFVSAIKTSYSVLVLLLTVAKDE

>gi|861722468|gb|AKO89992.1| odorant receptor 28 [Microplitis mediator]

MTRSLKLQDQETFDQVAKVLKWNKWLLSTLGLWPQSPNTFIFTVNFSYFVYHMAMEYLDLFLFIDNLEHV
IENLTENMAFTQILVRIAMLKKYNRQLGEVVNEAFKDYDARIYRTDEERQVFIDYMKKAKLFIKLLCAFV
TMTATSYYAKPITSPPPPPEGELDVEMENATMSFILPYRFHLFYQVNDSRTWALTYLSHFPFVFVSGFGQ
TAADCLMVTLVFHVSGKLASLAIRISEINTEPGVCKQELRSIIIEHDRLLKMGQSIEEAFSETLLAHLIG
ATSLVCILGYQLLVNYARGQGADLVTFFVFIFLVFLVLYAHCVVGESLITESFKVCEAYYDCLWYKMPKE
SSKTIVLCMARSQKPLGLTAGKFGAFCLSTLTDVVKTAMAYLSVLRTFLVIE

>gi|861722465|gb|AKO89991.1| odorant receptor 27 [Microplitis mediator]

MKFFEQSYFTLPRNFARSIGRWPYQSSLQSFLIGIVIISAFILQVGPKILADIVHSDDQELILETLAPTI
TNVMAFAKYINTFVNARMLKILFERIKDDWESVTDKKEKIILESYAGFGKLMATGYAGFVYAATVQFITE
PVLPIILNNILRTNLSAPHKFADPMEWIIIDKEKYYWILLSNSSVCIMVILTVLISYDVIFITFVYHACG
LFAITGHRIENLPHDENFKIINRNTNSLKNSRDVHYKHLVSCIRIHRMALKYVDLIESTFAGCFGVVVGL
NLPLMSITGVQGMKFFRLLHNRMTLQQKIKYVMFTGAQMLHLFFECFLSQQLTDMSLRVQQHIANGNWYD
ISTKSQKLLILMTMRSQVPCILTAGKIMELSVESFGMMMKTSGSYFTVLLSMQ

>gi|861722462|gb|AKO89990.1| odorant receptor 26 [Microplitis mediator]

MDIFDAPYYRIMKNSAKLIGQWPYQSREKRIIIIIIVWAFFFMQFVPQIIAIVVHIDDPDILFEACSNMA
VDFVTAIKYINTICKTNLIKKLHDRVIIDWSLMLNDEEKSTLEKHTNLGYLFSSGWAGFAYMSATIFVLE
PVFPRILNVFISVNATDPFKLALPLEYIIIDREKHYWIMLFVSTVFVYNIIIVLVSCDIMYITFVQHVCG
LFAVVGCRLVNTPINENYSERHKAGDYLSNSKDIPYKHLVSCIRSHRRALEFAELLEDAYCISFGLTVGL
NLPVISVTGFQIITQFNTIQQLLKYASFTITEILHLFFECFMSQRLTDMSLEMQKSIAEVQWFDNSIKSR
KLLIIMTMRCQVPCKLTAAKIMDLTIENFGMMVKTSGSYFTMLLSMQ

>gi|861722459|gb|AKO89989.1| odorant receptor 25 [Microplitis mediator]

MIFKATPEFAIAFTKFSTLVGTCWPNYKNAPKWKFVLFQIRWWLTFCLSVSAFLPMCYAAYNHWRNILSF
TKSLFDAANTSQTFIKMILSKIHYKRLQYLLYEMENYVTNAREDERELFIVYIKRCGKLHLFVMIFGFMA

ILIIIVAPIGLPQPFPNIADYPFPVDESPAFELVYAHQSAATLHCLSIPVFDMQIALLLWYSGARLELLA
REFKTVTDNKHFVECVKKHQYLLWYIQEIIISSRYILATTSVTCVIAVITSGVHIAGNEPVGFKIPFAGA
SSIIAIILYISAWPSEHLIHMCEGVGTALYESEWVQNSKALNNSMLIVMHRAQKPSTIEVIGVMPILSLP
YYATFLSKTFSYFTTLRVLLSKVEMD

>gi|861722456|gb|AKO89988.1| odorant receptor 24 [Microplitis mediator]

MELDNIKLRHLEPYYNIKVSLTLTKYIGTWPPVLEPYRSIYLLYTCVSFIFILGIYLTVQTVNLFVIWGN
IELMIATGFLLMTNSIHAYKVFVILGNQKRIQVLLDKLSTTNYYHNDDKYERVFTYYAWQGLYHHIAYQS
FGTVAVLCWGLTPLADAVAGNTRRLPMEAWYPYNTKKNPAFEITSGHQAVAVLIACVHNIGMDTLVTGLI
NAACCQLEIIKQNLKNVDLDFEYQIDKCDYEDFMNKQINKIIKHSNEIYK

>gi|861722453|gb|AKO89987.1| odorant receptor 23 [Microplitis mediator]

MFKINPEFAIAYTKLTVTLVCSWPPGRNSSRLDFLLFRIKWWISWLMGIFLVIPLIYAAYIDRRNVLEFT
KSLCLAVSCGQCAVKMFFCKLQHHRIKFLLDEMEEYVKVAEPFEREIFLGYIKNCGLVHVTLNVCSLVAS
VGVILGPLVLPQSLPTEAKYPFSVENHPNYEIIYIHQAFAGILCSSIGSIDCQIAMLLWFSIARLELLSL
EMKNITNVYQFHNCVRKHQFLLWFVDEVIKAGRNLVATTVIMTTFAVILGGVHIVGNEPMLVKLQFVIIV
GGFSMLLYVTAWPSEILTRMCQNIGWTIYNSEWIRNSKELNKGIEFVIQRSNKPAVIYISGIFPAISLNY
YATFLSKTFSYFTTFRIILAKLE

>gi|861722450|gb|AKO89986.1| odorant receptor 22 [Microplitis mediator]

MDNSLDSFLRINRLFLSSLGQWPQQEQISKIFTLINAVFFLITQAYFQTGGMIAAKCDQPIFMESIAPVL
ISFMCLVKFVNFNYNADKMRKLLEIIQADWNSINDLEELKILNSWAKDSRKNTIMYAGALYGTMAPFMLG
PLVPIFCKLMPAGVLPANSSIVLEKPVLFHVEYFYDLEKYYYPLLIHSYFGTMAYMTVAVAIDSMFMVYV
QHACAIFAVIGNRLEHLADDSSINFYYNPHILNDEPYKRMIECIVQHSKALQYAQMIQSANSLSFFFNWD
SICSLLLSVDFNWPSQRLADESARISETTTRCAWYLTSMRSRKLLQLFIMRSSVPCQLTAGSFYTLNMQN
FSAVVRTSMSYFTVLTSVQ

>gi|861722447|gb|AKO89985.1| odorant receptor 21 [Microplitis mediator]

MDYLSSPYCRLNKILLSCLGEWPYQTSTQRRFIRSTIYFFSASIIIPKIIKLIKVWGNLDMIIECIPMLL
LDAVNFVKVVNGFINFRKMRELFDRIQDDWGLNYSKREFEIMQNYAEDGKKLSQFYASYMYATMLIYFCM
PIIPKVLDIVLPLNTTRPELYLFEAEYFVDQHKFYYPILIHAYITCAVAVSMLVAFDTEYAIQALHGSGI
FSALRYKLENLVIKDDEADYKNDEKIKQSTYNMVVQCAVLHKRALDYADLLESSRVTCFFFVLLVNIAAI
SITGVQTVMKLDQPTEAIRFGVYTLAQITHIFYNSYPAQMLFDNSWKTSDAIFAGNWYRAGSKSKNLLHM
MIMRSRIPCKLTAGKIYLMSLENFTGVVKTSMSYFTVLLSFR

>gi|861722444|gb|AKO89984.1| odorant receptor 20 [Microplitis mediator]

MENTSIESRQSVLSMAIAGLRLCGIWSLDSSSPIFLKLLHNISNIFGIITLIIFVGTLTIDLLLNSNDLL
IATDDGCYLAGISVIVFKVYQFHRHHKRIKNLTDATYQPIYVFWKSTDIGVKTVLRTNKFYEDLGFTFFV
SLGGFLVIALIFFVPTEEGALPIRGAYPFNTTISPMHEVAFCLQIYAVTYGLMVIVLMDGMGLGIMRWLN
VQCIILASNYRNCRTNQNNSFYLESRDDLSKIASIEDDNNNVTDIYDEPDSNITTFCPFDEQDHAGMSDC
FIGRFKKCIKNHQRLLNTIDELNACFSSCMLMQLFASFSMICLTGFQAVLGATTKTSLIKFVLYLGAAFS
QLLYWCWFGNELLYEVFILPHD

>gi|861722441|gb|AKO89983.1| odorant receptor 19 [Microplitis mediator]

MDVFEEPFYKMIKNFSHLIGQWPYQSSRKKFTIVTLIWIAFFMQFIPQIIAIVIHFDDRDVLFEAFSSMV
IDFAFIIKYLNAIYRAGLMKELWESIRRDWTLLLNDVEKRTLQHHANLGNFFSMGYAGLAYMSTTIFVTE
PIFPRIVNIFVETNETIPLKLALPLEYIIIDIDKHYWLILTITNIFVFNIIIVIISCDIVLITFVQHVCG
LFAVVGCRLESTPFDENYLEGQKGEDFLSNSNDIPYKHLVSCIKGHKRALEYAERLERAYTLSNGIVSGL
NAPVMSITGFLMITESSTIEQLLKYATFAISQMSHLFFLCFMSQRLADMSLRIQENIGNATWYNNSLKSQ
KLLVLMLMRSQVPCKLTAAKLMDLFIENFAVVVKTFASYITMLLSM

>gi|861722438|gb|AKO89982.1| odorant receptor 18 [Microplitis mediator]

MYSILPGSFVVLQAIGLWKPPEYNNSPILNYYYRLRTFITFFLIYSFTITGITGLILTTKDIADVTSDCF
ILLSIFAICGKIANIIWSRNEIIWIIDTLNSEPCKPLNNDEIIIQQKVDRLIWHSTLFYGILTEITVFMV
TFGTLLLQLPIGTLPYNTYLPWDYSHGYLYWVAYGYQIISVCLSANSDIGFDTLVPGLMLQITAKLEILK
YRFINLVDTLKLTQWNGVNDKSYHNFRIENKLIADYVKCHLIILKLADTINKTFDKVILLQFFISSIVLC
ISVYNLAFLDVFTTEFTSIILYLCCMLMEIFILCAAGNQVTIVSSTLSDAIYHTDWINLDTSAVKSLMII
MNRGLKPIIFSSGHIIKISYDSFKTPIKLSYSSYNVLQRT

>gi|861722435|gb|AKO89981.1| odorant receptor 17 [Microplitis mediator]

MENIPIDIHRKFLNINITILRYSGVWPLLPTAKIGWKVFNFIYRIFNLTVFIFYLITLGADAVTNYKDLT
IFGSDGCYFFGTCMCVFKACKFWASYHKIIKLIVDVYDPIDVLVRSADPGILMNIKKSYYQESIAFWGFS
TLCSFFHFSVIFLIPREKGILPIRAIYPFDTKISPNYELAIIYQAYCLAYALCVTIALDITTIGFIRWST
LQIAALTSNYKNSNPNVTKRASLVTSSSDARKIIEKLNKIKITDDDVEIETFLPLDYHETKYFINDLFLS
RFTTCIKNHQRLIKIIRDLNAVLSPLMLVQFATSTCIICLNGYQMILAETMTYNQWMSGWECAYGKVKSN
ELRNLVTIAMMPAIKPFAFNAVGLFALSMPTLLAVVKSSYFMLILLTTVTED

>gi|861722432|gb|AKO89980.1| odorant receptor 16 [Microplitis mediator]

MKLPAHTKGYKVTPEGAVNFIRVTVYLTCISFPLTEKTKVRINYEIILWLSIFLSISLFAPLLASIIKYS
DDTFIVMKSFILMSAISNYVIKVIIVRIYHKELQQLGSALDEFIKKANESEKVILQRYVDNTWKFHGFMT
CSYYLTATAVLLGPLILPQKFPTDAVYPFPVDNQIISYIVYLHQCIVGYQCSAGMALDCQAALFLWYLSA
RFEILISEAKNVETFDELRNYIKKHQIILLYAKELIRPTRLIAFVTVVMTKIGMIFGGIVLISDEPVVIK
IQFAILVISTTINIYVCAWAADNLITVSSTAMSNAIFEISWMHAPKLRNFLQTVIHRTQKPVVIKIPGLL
ETLSNEYYAQFLSAAFSCFAAARVVVSS

>gi|861722429|gb|AKO89979.1| odorant receptor 15 [Microplitis mediator]

MEAFRLHLFFLSILGVWKPQGWHGIKAFLYSIYGSTVVIFNHIFILSGILNLTKFKHVSLDVFIDNFSQM
LALIVVRQRIICVIENRNSISQIIESTDKYPFKLRDRQEKLIFSKFSKLAKNILIYYPIVHMCIILVHTV
GHISVMDPPYALPFQGWFPYNYTRKTKIYWATATYQLYAIFSEGSIDLILDLLLPCILCYMCGHIHILRH
RFGVMTEKLQIMSENNEPREKIDSAERKMTAEWVEYHIDILRLVELVKKIFERMIFVQYTVSSLLLCTLA
YLLSHTKCTTMTFAANFSFFMAMFIQILLPCYCADKLTFEFLDISTGIYNSNWYQLSNNIRRSVVVILRN
TYQPVTITSGFFIILSLESFTKIIKLAYTIYNLLE

>gi|861722426|gb|AKO89978.1| odorant receptor 14 [Microplitis mediator]

MEKKTINDHTSIKLMRLCMNGIGMWSIEKRRDEIISNIVICYTIATLTVGLIVETTDIYYCLGDLREMSY
VAPCLLNVIVELFLMGTFVINRSEVIAFSDYTTREFWSIPYIESERKLLDDCNRKSVKIIIAFIVVIQLV
VWQYITIPIIESYGKNASERTLPYNLWFTFIPFKETPYYEICFFLQSAATLTTGVCATAFATFLFTINLY
ATGQFKILQQRLESSCQVYNIEKIKSVEQINLIAEESYANLRKCVELHNVLLKYITRLENLYCQIMLVET

LACVFLICTTGFQIVLGVDSSILRTSRSALYFCCLVTQLLLYSWSCHEIIIESLEVAEAAYRAYWYSLSW
SKYGKSFRQALLIIITRSRRPCVLTVGKFVPMSLETFTAVFNSALSYFTILRQMTEEMENS
