# Supplementary material for: InsectOR—Webserver for sensitive identification of insect olfactory receptor genes from non-model genomes
Source: PLoS One. 2021 Jan 19;16(1):e0245324. doi: 10.1371/journal.pone.0245324 (PMC7815150; doi:10.1371/journal.pone.0245324)
Supplement: S5 File — (PDF) [file pone.0245324.s005.pdf]

>AcepOrco
MMKMKQQGLVADLMPNIRVMKMFGHFVFNYYDDNSSKYLHKIFCCANLFLLLLQFGLCAVNLIIESADVDDLT
ANTITLLFFTHSIVKIVYFAIRSKYFYRTWAIWNNPNTHPLFAESNARYHAIALKKMRLLLFLVGATTVLTAV
AWTTLTFFEHPIRKLVDPITNETTIIQLPQLLVRSFYPFDASKGITHILMLIYQFYWVFFMLVNANSLDVLFC
SWLLFACEQLQHLKQIMKPLMELSATLDTVVPNSSELFKAGSADHLRESNENNQPPPPSAAAQGDNMLDLDLR
NIYSNRQDFTATFRPTAGMTFNGGVGPNGLTKKQEMLVRSAIKYWVERHKHIVRLVTAVGDAYGFALLLHMLT
TTITLTLLAYQATKVNGINVYAASTIGYVLYTFGQVFLFCIFGNRLIEESTSVMEAAYSCHWYDGSEEAKTFV
QIVCQQCQKAMSISGAKFFTVSLDLFASVLGAVVTYFMVLVQLK
>AcepOr1
MLFHRDITVTLSVHRFALSWVGIWPVREKNIFMDLRWIIAVFFEAIPMSVYFTEIYLHCNSKKSLDSITTGAA
GALALTRLITPRIHREELLEIITSMMDDWAMQKDKKIRWIMEKYATMSIRVTILTFILVGIIIGGYAVMAISA
LTAKTGQLDNNRSQSCVFRTASLQQAFMVIQAMQMFITGIVTFGTTSFFFGQVMHLCSQFDALCIKLSEFRVS
EARRAIAEAIQRHCQLIRLAECIEESFNTNVLLYLFVTSTLMCIDGYILIASLSVGNLSMIIHSISVLLLLLI
QLSFYTFAGDYLEMKSTALCYATYDCDWYELPASIAKDFQIILMRASIPHQLTAGKFVVMNMITFKDILKSTA
SYLSVLRVMLD
>AcepOr2
MKEARNWNDESYYALNVYEKVLGIIGIWPLNAGELKSIARCSLAILIQISTIGSLSLETYWQCLDTEDMMEAF
LMDLSSVVSLSKLLVIRFTWKHTYILVTSIIDDWSMSRNKQQREVMMRYTHIGRVVSLTILYLGYASGVSFLF
MAVPFDSLIPWLNVSKANDNDTMVTTYFLATYCVFGSLPTITYSCVLLLQAAQIFVNATSHCGNDGFFFGLAM
HLCGQFEVLQMDFAGIEMEKQNCKRKIRMLIIRHCHLIRLADNFEYAFNMAILTQVSMSVLLLCVEGMQLIIS
LKLNDNIAAIKHVVLILTMLVQLYLYCYAGDQLENVTGRIAYSAYDSPWYDFDVKIMKDLPMVILRGKLPHQT
TAGKFLPMNLFSFKEILKATGSYLSVLRVMIDV
>AcepOr3
VLPFARMTDKYWNDDIAYVFSTHRLFLKGYGLWPLQKQTIFTKIQWGFCLIAQLMILPCLTTEILWSNQDASS
NIENITFFASTSTGLTKNLCLIVSQKRLSMNINAAINDWLSVKDNMETRKIMKKYAVQSKILTFTLLYSLYVC
LGMYIAIVIFINLKQIFFTDLNLVNVNTTNWLLLIPSGPLSHLITGPQYAIILTIQIVQSCVLSFLLFTVDSF
FFNVTIHLTGQLEVLKNNFKTFTNELNTEANYRKKFVNLINRHSLLIELYQNLEDTFHFLILYQIVILTILLA
LTGLRLNICINEKNHVETTKSIFVLNYLIMQSLIYTYGSEFLQKESENIFYTLYTTSWFMLPSTLMKDLHFVM
MRSSITFRLTGGKFFYVNRETMMYVLKIVVSYVSVLRRALKN
>AcepOr4
MGLTVKWNAEISYVLTIYKYVLSILGLWVLDDKIFSRIRWFMSTIIELSATISLSLEIIRHCRGYEDALDAFL
SASSSITSISKLLLHRVNWRHKLILVESIINDWTFVKNIYTRDIMLKYARIGRRGSSVFFYLGYSSCVFLFSS
FIFANFVNFDLPWSFEEQIYNKTSEMRMFLAAYCVFGSYTSIAYGFVEVLQILQIIVNCISHCGHDGFFFNLT
MHVCGQFEVFRMEFSEIENNKEFFDRNKLGMMLKRHHRLIYLAYHLQEAFNLVILIQVLLSVILLCVEGFQFI
FTLSMHSTYAAMKHLLFIAVLLLQLFLYCFAGQTLELQSQGLASAMYDLPWYNFDLNVMKNLPIIILRSTKPH
QLTAGKFLAINFVSFKEILKTSASYLSVLRVMLET
>AcepOr6
IIRVLPLARMTNKRWNDDIAYVFSTHRLFLKVYGLWPLQKQTVFTKIQWGFCLIAQFMILPCMMMELLWSSKD
VISNIESITFFASTSIGIIKNLCFIVSQKRLGININAAIDDWLSVKNNVETKKIMKKYAVKAKILTFTLLYSL
YVCLGMYIAVIIFINLKQIFFTDMNLVNVNTTDWLLLIPSGPLSQLITGPQYAIILTIQIVQLCVLSFLLFTV
DSFFFNVTIHLTGQLEVLKNNFKTFTNERIITEANYQKKFVNLINRHSLLMELYQNLEDTFHFLILCEIVIVT
ILFALIGLRLNICINEKNHVEAIKSVFVLNYLIMQSLVYTYGGEFLQKESEDIFYMLYTTSWFTLPAALMKDM
HFAMMRSSIPFRLTGGKFFYVNRETMMYVLKTAVSYISVLRIALR
>AcepOr7
MKTNWENGMNYGFSVIRMMMWIVGVWPLQWNDIVCTFRWIVIFIVESLTVICTLIESFQNCGDTKDVLEAFIN
IEFYVHSWINVVLARIYMKKIAVNVTSAIDDWSFSSREKQSYLIMTGYARISRMIAVTQLIIGFIAISVYLIA
IVLNKQQVVTIGNETVTLWNFIIPSSCLFRGISYSTYKTLFLMQILQVSIVYIGQCANDNFFFAITMHLCGQL
ELLRIRFVELVEKDTNERTYYRNVLGSWVRRHYKLIILAKNIEDTFNVIILFRLTITTIVIAVSGMRIIMSIK
NYDYTNVLKSMLSAQLFIVQSFLFTHAGETLRKQSESIFWAIYSTTWHNMPPSIIKDFILIMMRTKISLQLTA
GKFFYVTRRTTTDILKTALTYISFLRVTMEE
>AcepOr8
MKTNWNSGMEYGFSLIRMIMWIVGVWPLHHDDIICTFRWIIIFIEQSLTVICLLIELFINCGDIKDALEVFLV
MEANLHSWINIIFARIYMKKIAVNVTSAIDDWSSLSIKKQSYLIMTGYARIGRMITVSQLMIGFIAAVVYFST
IFFGNRQQVVIIGNNTEALWNFVLPSTCLFRGVSYSTYKALFLMQILQAFIIYTAECSSDCFFFAITMHLCGQ
LKLLRIRFIELIDKSINERNHYHSILGSWIRRHYKLIILARNIEDTFNLNVLFRLSITTIFIAVSGTRVIMSI
KNHDYTNVVKSIIFVQFYIVQSFLFTHAGETLRKQSESIVLAIYNTTWHKLPPSVVKDFIFIMMRTKIPLQLT
AGKFFYVTRSTITDILKTALSYISFLQMAVE
>AcepOr9

MVYWNKDAIYALSSYKILAWLVGVWPIDDNIFCSKIRYLFVIVSEILLMTTLLMDVYLACENSSEDPIDTYVV
ASSAMLGIIKLTMLQIQRSTLSINLCSAIQDWCNIKDARSREIMIQYARTAKIISLSLFYCGFVSLTFYLLRL
LSFINATANGRTFVLPMTCLFESVSNLQYVLITFYQVIQLFVTYAGNCCTEGIFVGITMHLCGQLKLLMSDFH
QIDHKYKYKGGNIIEEFVVRHRKLLKLTETIEDSYSIIILTQIFTSAILICITGFGLIVSWHIHDIVMTVKSI
VIMIVMLMQCFLYSYAGDNLRDQSEALSFALYDSNWCDFSPNDIRDLAFIMIKTNIPIRLTAGKFFYVTRATF
TDILKTAVSYLSALRVMIDKQEINSY
>AcepOr10
VIGGSYSYYLNPMASERWKDDVTYAITPFKLVAWSIGIWPLQVRNTYSLISYVFGIFSAVLLIVLPCFEIYLG
CTDAETSIDCLMLIFCGILGVLKIIWFRIYARNLANNYSSAMKDYVTIENAKERAIMRKHAFMGRTLCCLILG
FAYFSSIMYGLIAILDEYKHVNITNENIMPEYPIPSKCFMKFLNAPISMHKIFCLVDVVALILASTTNHGNDA
LFLNATLHVCGQVKILRANFLNFDIKGPHIYNRFNVLIERHKYLIKLARELAEMISFVLLIELFLISILLCIM
GFQLILQLGENNVVLITKNIMVQSTFLTQLTLYGVIGNYLRSEMEEIGLSIYRSTWYNFPAKLTRHMIFILMC
SDSPVALQAGNFIVVNLATYVSILKTSLSYLSVLRVMVK
>AcepOr11
MTNIRWKDDVSYAMIPFKLLTWPIGVWPLQVYNIYSLLRCIFATCCVSLMVILPSIELYTNCNNAEQNVDSLM
LLCCGILGILKTVWFRIHAKNLTNNYNSALNDYLMIKNAKHRAIMRKHASTGRILCCFLMCFSYFSCLLYTII
PFLGNANNKINVTSEAMLQYTVPSRCALEYFNAPTSMHTILCLIEAISLTLTSTTNHGNDSLFFNITLHICGQ
VEVLKAKFIDFDVTRPQVYERFLTLIKRHSYLIRLARELADTISFILLIQLSIISLQLCIMGFQFILALKDND
TAMAAKSIMVQSAFLSQLTLYSFVGDYLKSQMEEVGLCVYQNIWYDFPTKLTRNLIFVIMRSESPVTLRAGHF
IVVNLSTYMSILKASITYLSVLRVMVET
>AcepOr12
MASCKTWNKEMSREFSLYKRVMWPLGSWPLDHNRNFAKLRALLLIVTQTMMIVYIGVDINFHNNITLVMVIDH
LMLASCGTLTIVKIALIRLHRDNLLKNLVNASNDWTYIIKQNHRQVMFRYTNIGRFVFFFQMSSSYFVIMPLI
IESLLSFATIPSRNITLIAKSEKEMQAMQLPQEMICPFDAQIVCFSMCILQSIQLISTCTGNVGSDVFLFGVF
MHLCGQLEVLSLELQQFHERKKNGCWKRSKMVMLIERHCLLLNLAKDIVDVLNIILIAQLILHALFICLIGLQ
FILSLVNSDFVLIMRSISSINIVMIQLFIYSYMGEILSSKTQAISYAIYLSKWYDLPTNILRDVCFIIARANV
PVRIKVGKFYIMDLDSFKNVLKISASYFSVLQIMLVQ
>AcepOr14
MKTNWNSGIDYGFSTIKSMMWILGLWPLQQNNVVYTIQWFIIFITGSLTMINVLIESFKSCDAVRDGLDILRL
IESCMHAWSNIVFPRIYMKKIAINLNSAIDDWSSPFMKKESRIVMMGYARAGRLIALTHLIAGTTAGALWFIS
VFLSNKQKAAITDNDTVATWNFVIPSTCLYKGVSYSTYKILFMMQVIQGSLILISECACDSFFFGITMHLCGQ
LELLGIQFTESNKKHYDEKRHRNVLGPLVKRHCQLIALTRNIEDAFNINILLRLLIISVVIASSGVGILLSIK
QQDYKEMIKMFMSIQFYMVQTFLYTYAGDTLKNRSESIVYAIYSTAWDEMSPIMVKDLIFIMMRMKTPLRISA
GKFFYLTRNTMTDILKTTLTYISFLQVTINE
>AcepOr15
MSSNDDIAYAMTLVKHLTVPIGAWPLQEYNKFALLRHILSSFGLSVVVIVQYLELYYNCTSATANLDALTLFS
CGILALTKIIWFRIYADNLICNYSSAMNDYVTIDTEEKRIIMRNHAFWGRIICIIALLISYVDSVIFIVGHAQ
LSSEETKINITIFGHQAGYAIPSTCTLAHFHISTDLYLVIFILQYIYLVIMCISNHGSDSVFLHIVLHVCGQL
EILKANFINFDITSPKVYERFNTLILRHDHLIRMTRKLAEIIRFVLMVQLFISSMLIIIVGFQFIIALTTNDY
GMMSKSFLVLSAFLIQLTLYSIVGDYLKMQMEEVAQSVYHSFWYDLPIKVTKNIIFIMMWTQFPIKLQAGYFV
VVDLGTYMSILKTSISYLSVLRVMIE
>AcepOr16
MTWNDDVAYAMTPFKLLTLPLGVWPLQKYNTFALIRSIISGSSLTGMMIMLFLEIIFGSSDAYVKLDALMLMF
CNVLSVLKLLSFRIYANNMIRNFTSAINDYLAINNKKKHAIMRQHAFMGRIISCSILFFAYLASTIFCLVPII
ISDEENIQVNVSIKNQASDLPIPLTWSLGSYHLSANLFFVISIVQYFLLVLNSNCNCGSDSLFLAITTHVCGQ
MEILKIEFVNYGVKSKNIFEDFSKLASRHHYLMEHAELLVEVISFVLLVQMLFSCLIICLIGFQFILALKSHD
VVMITKTITVMGALLLQLFFYSFVGDYLKCQMEDIAQSIYSCNWYCFPLKLMRNVLFVIMRSQQPVQLLAGRF
FVVTIETYMTILKSSLSYLSVLRVMVDS
>AcepOr17
MSWNDDVAYAMTPFKVLTLPLGVWPLQKYNTFSLVRSIVCGFSMTLMMIILFLEIIFGSSDAYIKLDALMLMS
CNVLCVLKLLSFRLYADNLIRNYSSAVNDYSTIDSEEKRTIMRQHAFMGRMICYSTIFFTYLASSIFTLAPMI
TDDENVHVNISIKNQAAKLPVPITFLGNLQIPTGLYFVISTVQYFILLLTGTSNCGNDSLFLAITLHVCGQLE
LLKIEFTNYGVKSRNINEYFSILTLKHRYLISLAELLSDVISFVLLVQVLFSCIIISLIGFQFILALKSHDVV
MITKTISVLSGLLLQLFFYSFVGDYMKCQMEQIAHSIYSSNWYYLPTKLMRNVLLVIMRSQQPVQLLAGRFFV
LNIKTYMTLLKSSLSYLSILRVMVE
>AcepOr18
MSWNNDVAYAMTPFKLLTLPLGVWPLQKYNTFSLVRSIVCGFSLTVMMIMLFLEIIFGSSDVYIKFDALMLMF
CNVLCVLKLLSFRLYADNLIRNYSSAVNDYSTIDTEEKRTIMRQHAFMGRMICYSIVFFAYLASSIFTLAPMM

TGDEDIHINVSIKNEAAKLPVPITFLGDLQISTDLYFIISTVQYFILLLTGTSNCGNDSLFLAITLHVCGQLE
LLKIEFSNYGVKSKNINECSILILRHRYLIELAELLSDVISIVLLVQVLFSCLIISLIGFQFILALKSHDAVM
ITKTITVLSTLLLQLFFYSFVGDYMKCQMEQVAHSIYISTWYYLPTKLMRNVLLIIMRSQQPVQLLAGRFFVV
NIKTYMTILKSSLSYLSILRVIME
>AcepOr19
MSWNDDMAYAMTLYKFLTLPLGVWPLQKYNTFSLIRSIVCGFSLSMMLITMLLEINFGNGDAYVKIDTLMVMS
CIILATLKLLSFRLYADNLICNYSSAVDDYSTIDTEEKRTIMRRHAFMGRIICYSTLFFAYLASSIFMLMPMI
AGNKDIQVDDASKTQASELPMPLTFLGDLHIPSSLYFVISTLQYIILMLTSTSNCGNDSLFLGITLHICGQLE
LLKIEFINCDMKNKNINEDFSIMALRHCYLIKHAELLADVISFVLLVQVMSSCLIFSLIGFQFILALKSHDIL
MLTKTTTVMSALLLQMFFYSFVGDYLKCQMEEIADSIYNSNWYYLPTRLMRNVLFVIVRSQQPIQFLAGRFFV
VNIKTYMVILKSSLSYLSVLRVIVES
>AcepOr20
MSLNDDLAYAMTPLKLLTLPLGIWPLQKYDIFSLVRCIVCIFSLTVMMVVLFFEINFGSGDAYVKLDGLMVMF
CAVLSVLKLLSFRIYADNLVRNFSSAINDYFAINTEEKRTIIRRHAFMGRMICYSIIFFAYLASSIFMLAPML
ADNNDVQMNVSIKNQASELPVPLTWALGNFNLSTNLYLLISVVQCILLMLNSTSDCGCDTLFLAITLHVCGQI
ELLKIEFINYGMKNKNINEDFSKLASRHHYLIEHAKLLVNVISFVLLVQMLFSCLIICLIGFQLILALKSHDA
VMITKTITVMSTLLLQLFFYSFVGDYLKCQMGEIAYSIYSCDWHCLPMKLMRNVLFIIMRSQHPVQLLAGKFF
VVNIETFMAILKSSLSYLSVLRVMLDA
>AcepOr21
LNFAIGWNRFNLSLVGIWPDPLQNPKQKSGLSRCRFIIASFFMLGFVCIPQSANLVFIWGNVDMMTENLATAN
IPVANSFMKAFTIWRKRKVLKLLVNFFYQDWYTPKTSGERTIMLKNAKLMRKISIWCTILTQTLLIIYIVLKI
SAIIFSPDDSSRPMIYTAYFPFDVIKSPIFELICACQILSAFSGTVAYTGSDSFISMLVIHVCGQFENLRERL
KNLVNDPNGIKTSQEFKSELRQIVMRHEHLNWFAKTIEDSFNMIMLIQMLSCTIQLCFQGFQVFRIIIKKEEN
ESMTFQLIFLGIFVGFILVHLYIYCYVGEMLLVQSTEIGFSAYESNWFNVPGKEARNLLLIIHRSTAPLSLTA
GKFSTFSLQMFSKIVRTSLGYLSVLLTVTERKD
>AcepOr22
LNFAIGWNRFNLSLVGIWPDPLQNPKQKSRLSRCRFMIASFFMLGFMCIPQSTNLVFIWGNADMMTENLATAN
IPVANSFMKAFTIWRKRKVLKLLVNFFYQDWHTPKTFEERTIMLKNAKLVRKISIWCTILTQTMVTIYIVLRI
STIIRFSSSDPARPMLYTAYFPFDITRSPIFELICVCQMLSAYSATVSYTGSDSFISMLVLHVCGQFENLRER
LKNLINDPNGIKTSQKFKNELRQIVMRHEHLNWFAKTLEDSFNMMMLIQILFCTVQLCFQGFQVFTILIKEEE
NESMTFQLIFLIIFVGFILVHLYIYCYVGEMLLVQSTEIGFSAYESNWFNISGKEARNLLLIIHKSTTPLCLT
AGKFSTFSLQMFSKIVRTSLGYLSVLLTVTERKK
>AcepOr23
LNYAFTLTRQWLWIFGIWPDPHIPLSEFRRPSIRFIIVTCTVSFYVSGPQIMNVLRAWGNLSRILENFVSATF
SLMGVSKLIVTWYHGETLRPLMASIMTDWITSTSDWERNTMLKISRRGRSLSYRCCLSAACLTTFYLSLHLLK
FFKTINQSQRNLVYRIGNLQSSPFYEITYFIQLSGGAYSILANYTIDSFVSMLVLQVCAQLINLRTTLNNLIN
ELVSKSISSSRFREGLAAIAVRHNHLIRNAKIIDGCYSPLLFMHVLAATFQICAITFQVFTIITDNMKVPVIK
MTFLSFYFVVLLTNMYAYCYSAERLMTESTKMTYGVYECKWYDIPPKDAKDLMFIVYRSRIPLRLTIGKFGIF
SLEMFGTVLKTSMGYLSALLAVRD
>AcepOr25
LNYMFTTTRQFLRLFGVWPDPHMPLSDFRWPSIRLIIVICNISLYIFVPQIMNVIRVWGNMTRMVEYFVSTNS
SLMAMCKLIVTWYHGKKLQQLIVSIMTDWMTATNWERNIMLKSARRSRNISFRCCATVTGLIIMSFCLYTIRF
IKIVHLPYRTLIYRMDNIQKSPTYEITYCIQFLGGMYSLLAIYTIDSFVSILVLHTCSQLTNLRMTLNNLVNE
ITNDSISSSRKESFRTGLAAIVIRHEHLIRNAKTIDSCYSSLLFMNILLVILQICFIAFQIFTMITDHLKIPM
IRIIFLSSYITYMMTHLYAYCYSAEKLMAESTNMAYGIYECKWYNLPSKDAKDLMFIVYRSKIPLKLTAGKFG
IFSIEMFGSTIKTAMGYLSALLTIR
>AcepOr26
INYAFAMSRQCLRILGVWPDPFVSDSKRLNIRFMIVACILSFYVIMPQFTNMILAWGNVARMVEYVASANFSL
MALCKLVGTWYHGETLRTLMISIMTDWMTSTNNRERNTMLNIAKRGRTLSVRCFITMTGTVTFYICLNLLKFY
RNVHQPQRNLVYRFSYPYNTQNTPNYEITFFTQLSGGMYSAMINCTIDTFISILVLHICAQLINLRMALNNLV
DELAKGSISSSRFKKGLIAITMRHEHLIRNANTVDECYSAVLFVHMLAATFQLCFESFQVFTIVTSHLGIPII
KITFLSFYFSLVLTHLYFYCYSAEKLSTESTNMANGVYECKWYDIPPRDAKNLLFMIYRSTIPLKLTAGKFGT
FSLEMFGTTVKTSMGYLSVLLTMMD
>AcepOr27
MHVLKFTLTICTIAGFWQPPSWTSLLNHIIYKTYAMFLVLSLNIFAISQFMNIIMYVNNSDDLTDSLYMMLTV
FVAGYKNICLWIDHKNVRMLVNVLRKKPFKPNEFHEITIRQQFDKRIQNVAMGYLILVGSAVVVVIISSIFMD
LSKGNLTYRAWIPFDYSTPIAFTFVYTNQMIGMSCSGLVNVACESLVCGFLLHICCQFEILEYRLKNINQNQN
ILCDCINHHNYIVKYAHTINDKFAKIIAVQFGVSMMVVCSNLYRMAMANEYMKFIPLIMYTGCMLAQIFIYCW

FGNEVKLKSLQLANSVYNMNWLELSNSNKKSLLIIMKRSTFPIEFSSAYIITMNLESFVALLKMSYSSFNLLH
QTQE
>AcepOr28
MRVLKFTLLVCAFAGCWQPISWTSLFKHIIYKTYAMFLVSSLYIFSISQFMNIVLNVENSDEFTDSLYMMLTV
FVAGYKQVYMWIDRKNIMVIINVLNEKPFAACEIQELKIQQKFEKMVQNSTLRYLILIMATIISIVLTSVIME
FSNRNLTYKAWVPFNYSYPALYFLVYTHQLIGMATSGIVNVACESIICGLLLHICCQLEILEYRLTKMTHGED
VLRNCVSHHNRIFEYAYVVNNMFAKIIGLQFAVSMLVVCSNLYRIAMATDYVTFISLMMYTGAILTQIFIYCW
FGNEVKTKSLQLMNNIYNIQWPSLSNNNKKGLLIIMRRAMNPIEFSSAYIITMNLESFVALLKMSYSAFNLLH
QTQD
>AcepOr30
MIVLQFTLVLCTVCGCWQPLTWTSQSKYIMYNSYRILLICIASAFTISQIMNIVLNINNFDEISDNIYMTLAV
FIATYKIVTMWIIKEHVITIVNVLMEEPFKPSESCEVIIRQRYNKTIRKYALCYYGLVQITVICIILNAIFMD
FMKGNLTFKAWVPFDYTSPIIFFFVFTHQIIGMSICATVNVACDSLISGLLQEICCQLEILEYRLKISYDQHS
LCNCIRHHDRIYEYAYMVNRRFAKIIAFQFAVSMLVVCANLYKLASISIVMINGNLVTLIMYTACMLSQIFLY
CWFGNELKLKSIGVANSIYNMKWHIFDNKNKKALLLIMRRSMVPIEFNSAVIITLNLDSFVSLLKSSYSAYNL
LK
>AcepOr31
MIVLEFTLTLCAVSGCWQPLTWTSLFKYMMYNSYRILLICLLCTFTISQVVNIILNINNFDEISDNIYMTLTV
FIATYKITTMWVIKEHVITIINVLKKKPFKPSESCEVIIRQKYDKTIKKYAVWYYGLVQVTVICIILNAIFMD
FIEGNLTYKAWVPFDYTLPIIFLLMFTHQMIGMSICAAVNVACDSLITGLLQEICCQFEILEYRLTKILYEKH
ILRDCVRHHNRIYEYAHIVNRRFAKIIALQFAVSMLVVCANLYKLAAISLLMINGSLVTLILYTACMLSQIFL
YCWFGNELKLRSTKVANSIYNIKWQELDNKNKKSFLLIMRRSMIPIEFKSAIVITLNLDSFVALLKTSYSAYN
ILKRAQ
>AcepOr32
MPVLKFTLAVLAVAGCWRPTSWTSLFRHMIYNAYTTSMLITLYTFAMTQIIELILRADNADTIGDALFNALIS
LLACYKAIAMRINHNSITMLISNLVEKPFKPVDLNENMIREKFDKRITNNTLYYLILSSITVIYMILLSIFTN
FKNGTLMYRAWLPFNSSISALFYLAYAHQIFSLIFNGLIHPICDNLICGLLLHICCQIEILEYRLSNIANDQE
NLRNCVRHHIYIFEYAYILNDKFSKIIPSEFLTITVVMCYNLIHMAFRTSNTVSCIQDVMVIASTLAPIFYYC
WFGNEIKLKSLQLSDSIYNMEWTMFSNNMKKGLLMIMNRATIPIEFISADIIPVNLDSFIKVLKTSYSLFNVM
LQSRK
>AcepOr33
MHILKLTRTIIIVAGCFRPFSWTSLFKQTIYNIYRLYVISMLYFFSMWQFMDIVLNVDNPYDFTNNLNMLLTS
SAACYKIFIVWLNYEKIAALINCLIEEPFKPLNLSETKIQRQYDKIIRNNTLRYTSLIGTTCSFIALMSLFTD
FRHKRLTYREWVPYNYSSYMTYYFTYAQQMISTFHCASVNVACDTLLCGLLMHICCQLEILEYRLKNISNNEF
TLGFCVRHHNRILEYAQLVNVRFTKIVGFQFMTSTMVICCNLYQLTDSALSANHIPLIMYTCCMLTQIFIYCW
FGNKVKFKSLQLTDSIFHIEWPILNNNIKKSLLVIMKRVIIPIEISTVYILTINLDSFVALLKTSYSVYNLLV
RVQ
>AcepOr34
MHILKLTRTICMITGCFRPQSWTSLFKWIIYDIYRLYVISMMYTFTFSQIVDIVINVDNPNDFTNNLNMMLNM
SASCYKFYIASLSYKNIVTLINYLTEEPFKPLDLDEIKIRRQYDKIIRNNTLRYAIMIATAWTFMTLMSLLTN
FRHRRLTYRGWIPYDYSSYIKFCLTYGQQLLSTFHGASINVACDTLLCGFLMHICCQIEILEYRLKKHLCNQF
SLGYCIRHHNRIFEFAQMVNTRFTQIIGFQFMASTMVTCSNLYQLTKSTLNADHFALIMYTCATVIQIFVYCW
FGNKVKLKSLHLTNSIFQMEWSVVENSIKKNILIIMKRAMIPIEITTVHILTINLDSFVALLKTSYSVYNLLV
RIQ
>AcepOr35
MSSLKFIFAILTIAGCWRPFSWTSLIKHALYDAYTLLVISVMYSFTFSQFMDVILNVDNPDDFTNTLYTMLTM
FAANYKILNLFVNHESFAKLIQNLTEGTFKPLLLVEIEIQRKFDKIIQNIAKCYTILIIITYTGHVLISLLTN
FKKRQLTFRGWIPYNYSSFMLFCLTYVHQYIGVIFACLVSVACDSLIVGLLLHVCCQITILQYRLKSLINGQN
TLRDCVRQHHHIIKFAYATNKRFTRTIAFQFVASTFVVCSNLYQLTRTKLIVDFIAFFAYTLCILVQIFIYCW
FGNKLKLTSLQLVDSIFEIDWIALDLKTKKSLLVIMTRAMKPIEFNSAYILNMNLDSFVALLKTSYSAYNLLS
QVQE
>AcepOr36
MNSLKFTLAILTIAGCWRPFSSTLLIKHTLYDAYTLLVISIMYSFTFSQFMDIILNVDNPNDFTNTLYAMLTM
FAADYKILDLFVNHESFAELIQNLTERTFKPLLLFEIEIQRKFNMIIQNMTKCYTILIMITYAGHVLRSLLTN
FKKGQLAFRGWIPYDYSSFVLFCLTYAHQYVGLLFACLVSVACDGLIVGLLLHVCCQITILQYRLKGLINGQN
TLRDCVRQHRHIIEFANATNERFRRIIAFQFVASTFVVCSNLYQLTRTELIVDFIAFFAYTLCVLVQIFIYCW
FGNKLKLMSLQLVDSIFEIEWIALDHKTRKSLLIIMIRAMKPIEFNSAYIFNMNLDSFVTLLKTSYSAYNLLS
QIQE

>AcepOr37
MQVLKFTLKILTVVGCWPPNSWTSLCKRTIYNVYTISIILLLFTFMLPQLMDIVLNVNNANDFTDTFYIMLAM
IISCCKMVGLLINRNNIGILTNILTQKPFIPLEADEIEIRQKFDKTIQTNTLWYTILVETTCACVMLTSLFTD
FRKGNLTYREWTPYNYTSEVIFYIIYARQLISSTIGSMVNVACDSLICGLLLHVCCQLEILECRLKKNFLGQN
NLRECVRQHDCIFKFALMVNEKFKIIIAIQFIVSTLVVCSNLYQLAKMTLIAQCFPLILYTCSMLTQILIYCW
YGNEVKLKSVQLIVNIFGMGWLTMKQNRKQSLLIIMNRSLIPIEFSSAYILTMNLNSFVSLLKTSFSAYNILQ
QI
>AcepOr39
MHILKFTFKLLTIFGCWRPDSWLSLHKRIAYYLYTSIIILLLHTFMLSQLMDLILTVDNTDNFSDNFFVLLAM
FISCCKLLILLTNRKNIIMLIDMLMEKPYRPSKSTEMEILYKFDKDIEINTRRFVYLGAVTMSCIILSSLSMN
FKNRKLTYRAWLPFDYSSMILFYLMYIHQLISLAIAAFINVGCDTLICGLLVHICCQIEILTCRLKKIISYSN
VLRGCVYQHYHIIRLLAYIVNTKFRLIIIIQFITSTLIICFSLYQISKTTTKAKYIEMTLYISSMLTQIFFYC
WYGNEVKIKSHQMIDNIFEMEWLTLNKNKKKSLIIIMRRTIVPIQITCAYIIPMNLDSFMGILKTSYSTYNLL
EKIR
>AcepOr40
MHILKLNFKLLTIFGGWRPDSWSSLHKRVAYDMYTSIIIIILNTFVLSQLMDVILTVNNADDFSENFFILIAM
FISCCKLFILLMNRKNIIMLIGILMKEPCRPSRSTEIEILYKFDKSIQINTRRFICLGTVTCLCVVLTSLSVN
FRIRKLTYRAWLPFDYSSTLLFYLMYAHQLICLIAGGFLNIGCDTLICGLLVHICCQIEILTYRLKNIVSYSN
VLRNCVYQHYYIFRLAFIVNAKFRLTIAIQFITSTLVVCFSLYQLSTATTKAKYIEMILYISCMLTEIFFYCW
YGNELKIKSHRMIDNIFEIEWLALDKNRKKSLMIIMSRAIVPIQITCAYIVPMDLNSFMSLLKTSYSTYNLLQ
QIRE
>AcepOr41
MYILRYTFNLLTIFGCWRPDSWSSLCERITYHVYTSIVILIVNTFMLSVLMDVIWTINNGEDFLDNFFPPIAT
FISSCKVFILLMNRKNIIMLINILRQKLHRPSSSTEMEILNNFDKSIQTNTLRFIYLGIVTMMWIALSSLYIN
FKNRKLMYKAWLPFDYYSSTVLFYLTSAHQVISLTIAMFVNIGCDTLICGLLVHICCQIEILTYRLRNIIFCS
DFLRVCLYQHYHIFRFALIVNEKFGMTIAIQFILSTMMICFNLYQISKTTVKTNYIQLILVTFCLLTQIFLYC
WYGNEVKIKSYQIIDDIFKMEWLILDDNIKKSLMMMMMRALVPIQINCVYVIPMNLESFMSILKMSYSTYNLL
QQV
>AcepOr42
MQVLDSTFKFLTICGCWRPDSWTSSCKRVIYHMHTFFVLVLINTFTLSQLLDIILTVDNADDFTDNFYMLLAM
IVSCCKMLSMLINRKNIALLINILMETPCRPLEPEEMEIHNKFDKGVQANTIHYAILVETTCVCITLTSLLTD
FRRRTLTFRAWLPYDYSSSILFYITYAHQLISLIIGSVLHVACDGLICGLLVHICCQIKILESRLRRIAYEPG
ILSECILQHNRIFDFAITVNEKFRFTIAIQFMVSTLVVCFNLYQLTRSTTTNAKYVQLVLYMCSMLTQIFFYC
WYGNEVKLKSQQLVSNIFDMEWCILDQNVKKILLLIMRRSTVPIEFTTAYIISMNLDSFVGLLKTSYSAYNIL
QQI
>AcepOr43
MFILTFTFKILMICGCGRLDSWPTPYKRLVYHVYTIFVMLLIYTFMLSQLMDLIIIVDNSNDFTDNFYVLLAM
IVSCCKMLALLMNRSNIEMLIKTLMRKPFRPVEPDEMKIRQKFEKLIQSNTLYYTILVETTCLSVAVTSLLTE
FKKGNLTFRAWLPFDYSSSQLFPFVYAHQLISFTMGSVHHVACDSLICGFLVHICCQIEILEHRLRKSARDPN
ILRECVLQHNNIFKFARIVNKKFRITIFIQFVVSTLVMCFNLYQFTKSTALRTKYMQLIMYTCSMLSQIFFYC
WYGNEVKLRSRQLINNIFEMEWFKFNETAQKTLLMVMRRAVVPIEFTSACVISMNLDSFVGLLKTSYSAYNIL
KQTQ
>AcepOr44
MQVLNFTFRILMICGCWIPDSCTTLCKRLVYHFYTILILLLIHTFMLSQLMDLILTVDNPDDFTDNFYMLLAM
IVSCCKMFTLLMNRSNIAILIDILVRKPCKPIQSDEIKIQQKFDKHVQTNTLFYAVWVETTCLCIAVTSLLTE
FRKGRLTFRAWLPFDYSSSSLFHIVYAHQLISLTAGSILHVACDGLICGLLVHVCCQIEIIECRLQKVAHDQD
ILRKSVLQHNHLFKFARLMNEKFRLTIVIQFVVSMLVVCVILYQFTKSTASRAQYMQLMMYMGCMLSQIFFYC
WYGNEVMLKSRQLINSIFEMEWFELNKQTKQSLLMIMRRSSRPIELTSAYVISMNLDSFVGLLKTSYSAYNVL
KQM
>AcepOr45
MSLLEFTFKILSSCGCWIPNSWMSPHRRLIYHVYTIFILLLINTFTLSQFLDIILIVDNSEDFMDNFYMLLAM
IVSCFKMFSLLINRNNITMLTNILINKPCKACDTVEIEIQHKYNKLIETNTLYYMILVELTCASTAVASLLTD
YKKEKLTFRAWLPFDYSSTMLFHFTYFHQLISLTVGSVLHVACDSLICGLLLHICCQLEILSCRLKNIAHNPE
ILRDCVIQHNLLFKFAFLLNKKFRFTITFQFIVSTLVVCFTLYQLTKTSGKFVELGMYMSCMLTQIFLYCWYA
NEVKLKSLQLVNDLFEMEWFTLRQDIKKDLLTIALRSRMPIEFNSAYIISMNLDSFVGLLKTSYSTYNILQQT
QN
>AcepOr46

MDINNYVFINRKVLKFVGLYPTNIMRYIICCMCMITIVIPQGLQIYQNWQDLSTVLETSSVLLTILLAILKSL
VWISNRRKMDPFIKYMLTDYWNILTTYISEKHTDAIYVKKGYLYTKGYLFLICNSLMFFFSLPIIEIVIAVIK
GTNNNTIKHFPFLALYPEYYYNFPMYEITYFSQMIATSLCGLIILGTDTLIATALFHTCGHFKVLKKKLKILA
LCKLISLIQHTYLEENTMQKIKLHIIDIIKHHYTILWFCDYMETVFSPMLFLQTLASSLIICLVGFQIATANI
TASTISKSIKYISYLIMALFQLLLFCIPGDALIYESSKIYKTVYTITWYELPVSFKTEICLLMLRSQKSSKIT
AGKFYVMHLENFNAVLSTAASYFMLLRSFG
>AcepOr47
KRMDFKNANFLNNQVNLISGNLLPIVSDNSRFSVIWRIHGVTVWLIELIHTIALIVGMVLAPKEKSLKDGTIA
VVIILEASFMLTRLYLRKKLMEEIIEKMNDILQNADEIMKDIIKSAIKPIIMPFIIYGVTGVVTVATWTIQPI
LLVFEKSIFYYVDYNLPTAFNSEPFSSRVLIFSTIFMTIGSSYLFLKKFGVDVYMMHLVLMLTAQYRYMAVKL
TILFRDLQNYHDETKKKYSMEDQWTERELRKLCIHQNNVLNMSFILRKLLSVNFSLLYFNNVFRFCFIGILLS
TVPSMSFAEGISVTSYAMASLMQFYLLCSSVQTLLDASTEITDKAFDEGWYQFGPSTKSTFILLIMSNNLECR
IAAIGKFNLSLPSFMTIINQSYSIALLLLRA
>AcepOr48
IRMNFQNVNSLNVRLNLLSGNLLPMINRDSFSSFWKIYSVFVWILQFILIIAMIPGCIYVPMEKALKDGLLTI
VIFIEIFFVVLRICIRKNLAYKLIQKLNEILYIADETMKNVIITTLKPVKIPVNFYLSSGIVAIIGWSCMHFV
LIFEKNLFYYEDYRMPAAFSEQPFSFRVFLLGDLFILICMIYMFVKKVSVEIYMMHLVLMVTAQYRYIAMKIA
MVLQEKNEEDKSRTGHSPKHYWKKEKEIKALCRHHNDVIYLTLLLKKLLSLNFSLMYVMSVFRFCFIGIMFSS
VTSTTFGEAISIAMYTSGATVQLYILCSCVQQLLDASTELTDKAFHENWYLLEPSIKHIFILIIMANNLECKI
ATFKNFNFSLPSFMTILNQSYSIAILFLKMK
>AcepOr49
MDFRNVNPLNVKLNRFSGNLLPMTSDDSLFPVAWRIYSAIIWLIEMIQTSTVVTGILIVPRNKTLQDATVGLV
VTIEVFFLLRQMHVHRDLVTQLIQKLNEILCSEDKTMKIIVKSTLKPVEIPLKFYCIVGTGSIFVWCCTSFTV
IFKKNYFLYEDYRLPIVLSKQPFSMEMFLLGNCIATIASVYMFIKKVALDVYMINLVLLVTAQYRYIAVKLTT
IFRENVPQNQNNESEKKYSIVDSLAILIKMKALCRHHNAVVQITLMLKELFSLNMSLMYLTNVFLFCFLDVML
INTVLSKAPVEGIMIIMYISGGLVQLYILCSCVNQLLNASVEITDKAFHEKWYQFGPSLKHMLRMMIITNNFE
CKLSISEKFSLSLPSFMTILNQSYSFALLFLKMK
>AcepOr50
MDFQSVNPLNVHLNALSGNLLPLRPNDSRFPITWKIYSALIWLIEVIQTIALIPGLILVPREKALKDGTVLCV
VTVEMFFMAVRIHSRRQLVNQLIQKLNDILRFSDETMKNVVTTTLRPMENPLKFYWLSGWLGVFVWACLPFLL
SFKEISFFYEDYRMPAVFSKQPFSLDVFLLGSVFILIGNMYVFVKKVGMDVYMIHLVLLITAQYRYIAIKLAV
IFRDGNSLNKLNESHQKYCSGINHWIKKEMITLCRHHNNIVHLSSMLKKLLSLNFSMIYVNNVLRFCFIGIML
STIPSTTILEALSIVMFACGSIVQFYILCSCVQQLLDASQQMTNKAFHEKWYQFGPSVKRTFMLMILGNNLEC
KLSMCDKFNLSLPSFMIILNQSYSIALLLLRV
>AcepOr51
MTIIYLKFQRILYILRVLGTFTNTWPLYPDVGKNEYILRNIYYYVATFILMTVWIPMIISAYKNRNDVGVLMK
NMSHIAAITEAVLNSILCRIKKKQLQDLMINIEKFAKVSKNYEKAVLQKYMNRYGMFISTIAISFVMAGITVI
CAPMFLPLEFPLDVWYPFSTKPPLLKFILYFMQIFAIIHTVFCIGVDVMIAVILFYSSAKLEILAFEIQQTTN
EIYLISCIKKHQEITKFISKTQYAIQHILFKTTFTMGFTVISGCFPMLYIQSHILIPQFLSMIMAALQRMYIT
AWAANDLKEISIQFTWSVYSASWIYESQKVKSNIFMILQGSQKPILISMSGLLPALTLEYFASFLTSVLSYFM
TMRAAITT
>AcepOr52
KIMNANWSHYYDVIKKVSSLSGQWPYQRPIMRLFCVILITLSTISMIIPQIAKFVRCDGNLQCIFETMTSYML
TTVTLIKLYTCYFNRCKIKILIDQLIIDWNELETPEEYEIMKKYAKNSRRYSLGYSLYCYFAVYVFMSVSLIP
QILDVVLPLNESRPILPTYPGYYFIDERKYFFYIFSHAIMAWEIAMTGIVTHDCMLLTYIEHVCSIFALVGFR
FERLTYKHNDAMRILHFHTSDMYHKQIAFFVYTHRKALKFAQLIEDTFSLTLAIQIALNTIMISLTLLQQQAD
ILEMIRYVMYVIGQLIHLFCLSFEGQKLIDHSLQVRNKIYKSLWYEISPKSQKMLLFVMQKSLRSIFLSAGKV
YTFSMENFTMIVQTSMSYFTVLSSLE
>AcepOr53
NNYYDIVKRISSLAGQWPYQSPKTRLLCVSLMTLSTFSINIPQMAKFITCNKDLPCIFETMTSLMLAIMSLVK
LYTCYLNRYKMKDLTDRLYVNWNALETSEEYNIMRKYAKNGRRYSLGYSLYCFVALYLFLSMSLIPQLLDVVL
PLNKSRSILLTYPGYYFVDEREYFFYIFFHAIVAWEIAMTGIVAHDCIFVTYVEHVCSMFAVVGFRLEHLFYN
RNDQAKTINTDDAYRKRIAFIVHVHRKTLKYAQLLEDTFNVSFAMQILIVTIGMSITLLQMMQQNNSNILESM
RYALYIIGELIHLFFLSFEGQKLIDHSLQIRDKIYNNCWYKVSIKSQKLITLIMIKTLRLSFLSAGKIYIFSL
ESFTMVLQTSMSYCTVLASFQ
>AcepOr54
MDNVDKFFRESHYNIIQVLLSVSGLWPYHTTGKRCAIYFVFLLVLGSGMTFEVLGIIEIWSDSFEVIDCLPIL
VLSTVSISKLICAIYMLPEIRILLTRMRQYWYSPKSDEETRILLSYALYGKKLSYVYTGDHTVVFLFTTLLSK

LLYTESSESDPSSNVHKQVGLPYYVNYILIDPKKYYVPIFVHVITCVISYTILMLTFDVLYMTLIQYCCGLFV
ALRYRLENAFECEGNDLQVPARNKLYSNIVYSIRRHTEAIQFSLAIESIHRIPLFIHVGANISVLSILGFQVI
TSTEDINHLLKHTSYLSALLLNTFFENWQGQKIIDSSEKVYESAYNAEWYKMPIAQRKLFIMIMMKSRKSLKI
TAGNFVNLSYVNFNAVLRASSSYFMLLRSIQ
>AcepOr55
LDWAIGINRISLKILGLWPDDKLSRRQKFLADLRAIIIFTTMLCAAVIPGILALLRVWGDMVAMADNMQIGLP
FSVTVLKFIIMWFHKKDLEPVINMIVKDWLRTKTVQERDTMIKQARIARIMVMFGCTMMTLACIILIIPPCFG
YTTRYLTNLTDPGRPMLVQTYYLRDITETPLYEIVISAQAISILMAAISYTGIDTFLCLLVFHICAQMEILKE
RFLSLNNFKDFDIGLSFNIKDHLRLIRLSVDIIDNTFNLMLLTLLVFFAMLFCLQGFLIVNIIDGGGEGVSIM
RICWLVSILINTFAHMCLYCVVGEILIAKGEGIFYAVYNYTWYLRTPKEAKNLMLIMICAEKPLYITAGKIFP
MTLSMFCSLIKTSAGYISVLLANR
>AcepOr56
MISVVDRYFNLNRTLLLILGLWPYEKSKFVGLQIICIFSILITCSTFQLTAILTSYSTEDIIKVLSFALPFMC
CIIQYNSFYINSETIKKLMDQIQHVYNDLRDSNEIAIINKYGNNAKQYTVVLTMILICSISVCIVISLCLEFI
DTVLSTNVSRSHHVSISTEYFVDQQKYFYLMFLHLSVGFIVGGTATVATGSLLLAYLLYFCGMFKIASYRIKK
TIHIYTLKDLSIQKESLVNKHLIRAVNIHRKCMTFSNYLKSRFEISFVFLMALGVLSISLNIFRLFQIVLLKY
NTAELLLYLSITFACFLYMFLCTLIGQEIIDHNNYVFITAYKVQWYVTPLPIQRLILILLQRGNKSFCLKVGE
LIVASLESFATLLNASLSYFIVIYSMK
>AcepOr57
MNDKEDVCQSRYYFVLRLYLTISGLWPYHKLRDRCIRFVSLFVFSFTILIPQILYLLIGSTTLDDIFECAPSI
FISILFSFKILVIMLNNEKLKICLNIIQKDWLSLNTNVEKIILERHTKFGQYLETSYAVFMHTTACLFMLKPI
VLTLTANDVFNLTKSSITYASRLPFHVEYGEKFNQYIYPIAVHNYVAVVAHSFATIAVDGLYYSLIQHACGMF
SIIGNTLENIGKNNEENFDVKSIKVKDDNYSKTLHCLRRHIIVIEFAEHIESLFTKIFLMTLNLNMIVSSISG
IQVLMNLDKGANDIIGPLTIYVAQLIHLFLHFWQGQFLLDYSVLPYESICRANWYYTSQRCRKLLLLMMYKTV
IPCRITASKLMILSIDNFATVVKTSLSYLTVFRSMQ
>AcepOr60
MIERKDILQSRYYTIPRLYMTLVGLWPYHPIRNRYLHFVPTFTICSIILIPMLLYVHIALRDLDDLFECMPTI
LITIIFSFKLASLMANSKKIEACLKTIEDDWLLLKTDSEKAILQRHAIHGQYLTIFYAVFMISTGLLYMFKSI
VLIMIEDTSNSTRLDATKLPFRVEYGYRIDQYFYPIMIHCYLTVYSHVTATVAADTFYFALIQHACGMFSIVG
HMLEHIGENINENFNLKPHKMNDNDYNKALSCLRKHLHVIKFAELIDSTFANIFLVSVSLNMIGGSICGIQVL
MNLNDAKDIVAPLAIYIAQLTHLFFQFWQAQFLLDYSVIPYESICRANWYYTSERCKKLLLLIMSRTISPCRI
TAGKVATLSIESFGVVLKTSISYFTMLRSFH
>AcepOr63
MDVFDTRYFRVNKLLLSFFGLWPTQSTSDKNKRLCCTLFGILILLLPQIAFLFKRMRRLNDFYDVLPTFLGTC
ICLLKTIGLQWQTEKFRMLIQHVCYDWCLLAKYNDIWILMEYLERSRMFTLVFLIFTLTGAASFVTAPLTIPL
FDSILMSNVTRTKQMPHPTEFFLDVEKYYYILLTLTIVGYSVCCTVIVATDTIYLALLQHTCGTLAILSYRLK
RLATHDKSKKCFDPTSKENRDVQNMVNCIQLQVRIERLIYLIESTFATCLITDIGLGILLQCTACVMIVTRTE
LVRNGPLVLIQSLRFFFNSWLGQKIIDHSSQISVAAYNGMWYQICSEAKKMLLFLLMKCQKPYHITMVKLYVI
CLENYGSLMKTSASYITVMVSLNSDDM
>AcepOr64
MSFFDNRYYYLNKRFLAVIGQWPFQSRLKSNMMFAITSLSTFSFISFQLWGLAAGITDLSIIMENASQILVNS
MIAIKLINCVFTNDKMKVLLEDIKKTWKIEHTDAEKKILQHYAEKSRTFTIGYAIILYATWLFYSTTSIVVSG
IYMILPINETYTAKFLYRMDHVLDIDKYFKLLMLHGFISVFYLSSVPLAVDTTFTLCTHHICALFECLRYNIE
RIRGLDFVLLEPNIKDDEAYHNIIGCIKSYQHALKFSDVLSSNYATSFFFLLGNVIISLSFGAAQLIMIDNQL
EEIVRLFAVTLAQLLHIYFLSLISQRLIDHSSGLQNVIYSCDWYKISRRSKHLLSFTLLRSTKPCQLIAGNMF
VMSMENFSSILKVSLSYFTMLTSLQ
>AcepOr66
MSFFDNRYYYLNKRFMIIIGQWPFQSRLEGNMLFAAAFLFILSLIAFETWGLVAGIMDLNIIMENASPLLVDC
FMMLKLMNCVLTNNKIKELLKDVEETWKIKPTGPEKEILQHHAEKSKIFAIRYAIVFYAVWLFYTLTPVVISG
MYKILPTNKTYNVRFLYRLEHVIDMDKYFNIMMLHRFISVFYIVHVPIALDTMYTLCIQHICAFYNIERIQGS
DFVFLQPNIEDDEAYRHIIFCIKSYKYVLKFSDLFISNYATSFLFQLSIGIISLSFSAAELIMIDLQLDQIIR
IFSSNLAQLIHTYFLCLISQGLIDHSSDFQNVIYSCDWYKISRRSKQLLRFTLLRSTKPCQIIAGKMFVMSME
NFSSILKISLSYCTMLMSLQ
>AcepOr67
MSLYDSHFYHFNKTSLCIIGQWPFQSRLKNNVMFAFTVFFIFSLATLELWGLIAGIADLSIIMENTSPLLVCT
FIITKLINSISNNYKLQIKELLEHVEETWKVIHEGPENKILMYYAEENKIFIKRYAIGLYAMWLFYTTPPIIV
SGIYTLLPTNETYSARFLYRLEHVLDMDKYYNLLMLHGFISVFYIVSVPIAVDSLFTLCIQHVCALFRCMKYN
MEQIRGPEFTLLNPDIADDKAYHSIISCIKLYKRIVKFSDLLSSTYATSFLIMLGNVVICLSFGTAELVMVDN

QFDEIIRILAANVAQLLHIYYLSLTSQRLIDYSNELQNVIYSCYWYTISLRSRHLLRFTLMRATKPCQIKAGN
IFVMSLETFGGLLKMTMSYFTMLTSVQ
>AcepOr68
NDIFQSRLYKINHILLSLLGQWPFQKNRHRSIIFVIISLIGITQAIAQVLALVTLRGDFDAIIECMPPLIIDF
VCITKLVNLTCNIKKIKILLIHIQRDWRSWTIKSEFEILHKFAESGRTITIAYAGGMYAFGSLFPFLAIIPKI
IGKNVTSEYSTRPVGFPYHVEYYVDLEKYYYPILIHNYLATAIRLTTLVATDTLVTILVQHCCALFSVVRYRL
EFIRKSIEQDKELALLKEDDKFYKNFTYCIQKHKDVLRFARGLDAIYTKAFFFEVGLIILAMSMSALQATSRT
INPHLAIRHASYITAQLLHLYIVCWLGQQIIDHSDRVYTSTYRGEWYESSLKSKKLLNMIMLRSTSPCTLTVG
KIMILSFPSFSAVVRASASYFTVLRTV
>AcepOr72
DQFFQDSHYNIIRLLLSISGLWPFHTRNKRYAIYFAMVLILGSGLIFQILGITEIWHDPFELLDALPFLFFTL
VIISKTFCTIYALPEIKILLIKMHEYCLSPKSDEEEKIQNSHALYGRNLGYAYTGFLLSHTILYLFAPLLTKL
SYVESNKTNELANEELKSQTGLPYRVNYMIDLNTYYVPIYIHCSVCNFIYTILLCVFDVLFLIVIEYSCGLFV
ALRSRLEIALVSKLTMTNDKCYSNIVYSIRRHAETIQFVAIVDSAYSLALSIQTGLTVFITSFLGYQVIKDDQ
DVNYLVKLFVYLNASLFITFFINWQGQKIINSSEKVFESAYNSEWYSMPIATRKLLITIMMRSEKPSGLRIGK
IVVLSYLTFNTVLRTSLSYFMLLRSL
>AcepOr73
MVDIDQFFQDSHYNIIRLLLNISGLWPFHMRNKRYAIYFAMVLILGSGFISQILGITEIWHNPFELLDIVPSL
FFSVIAISKTFCIIYALPQIKMLLIKMHEYCLSSKSEEEEKIQNSHALYGRNIGYAYTGIFLSHTILYLFAPH
LTKLFYVESNKTNELANKELKFQTRLPYRVNYMIDLDTYFVPIFIHCSVCVLIYTVLLCVFDVLYLIVIEYSC
GLFAALRSRLEIALVSILVSRLTMTKDKCYSNIVYSIRRHTETIQFVAIMESACNLALFIQTGLTVLIISFLG
YQMIKSVQDINHLVKLFAYLNATLFATFFINWQGQKIIDSSEKVFESAYNSEWYSMPIAARKLLIMIMMRSEK
PSELTMGKIVVLSYFTFNTVLRTSLSYFMLLRSL
>AcepOr74
MGDIDQFFQNSHYNIIRLLLSISGLWPFHTRNKRYAIYFTIVLILGSGLIFQILGITEIWHDPFELLDTVPSL
FFAVVIISKTFCTIYALPQIKMLLIKMHEYCLSPKSAEEEKIQNLHALYGRKLGYTYTGFLLSHTILYLFAPL
LTKLSYVESNKTNELANEELKSQTGLPYRVNYMIDLDTYYVPIFIHCSVCDLIYTILLSVFDVLYLIVIEYSC
GLFVALRSRLEIALVSKLTMTKNKCYSNIVYSIRRHTETIQFVAIVESVYSLALSIQTGLTVFIISFLGYQVI
KNVQDVNRLVKLFAYLNAVLFTVFFVNWQGQKIIDSSEKVFESAYNSEWYSMPIAARKLLIMIMMKSEKSSGL
RIGKIVVLSYITFNAVLRMSSSYFMLLRSL
>AcepOr75
MNSVQGQYMRINQILMCLIGQWPFQEGWEKFLIQFIFVPAVFSQAIVQGGGMITAWFADDIDAFMESSSPFVI
SLMCIVKHINYTYNHEQMKKLTFIMADDWNIYSKLSDEYNILCKNYEMGRKVSIAYAVSLYGSMTPFLVVPVI
INTVSYMGLYNISDKPLMFRTEYFIDSEKYYYPLLVHSYIGTLGFVTIVVAIDSMLVFHVQHECGMCEILGYR
LARIVEADTLDINLYTSKEEAMSYGYIKNCVIMHNHIIEYARRIEIANTTSYFFQLGFNMMGMTFTIFQAVVK
LSDPNEALRYASFTVTLLSVLFLESWPGQQLSDYTNKIFAFTTNGRWYQSSLRIRKVISIMLMRSYVPIKITA
GKLYALNLANFAAVVRTSFSYFTVLCSMQ
>AcepOr76
MDFFDGHNYRVNKILLLAIGQWPYQSSRTSHAIVIVIVTIVCTQIFAKLCGMFSYIHEMDIVIECLVPLMVDV
SGMTKIMNSVLYMNEIRALLDRIRDDFYSLRNSNDIKMLEKYADSGKKSSTIYVCVMYILTVAFLLMPLQPLI
LQVTNVTRPMLHRVEYYVDMEKYYFHILIHGYFTAIICVTSIVATDAIFVIFMRHACGLFIITGSRIEQAVQE
IYLTSDVNPPIIKDTAYRNMIQCVHDHRAAIRFVNLIEIVYSKHFLFHAGLNMIAMSVTGVGAVTKSNDLSEL
LRLIAVSCALLFHLCFECINAQRLIDYSGYLHTNLINLNWYNASPRTKKLVLFMMMKTQPPCVLTAGGMFVLC
METFATIVKTAVSYFTFLRKAH
>AcepOr77
MESSQDEYVTSSNANYQRDIQYIFKPGNLILGSIGIWPIAIRGIERYAPKIAIVVCNSALAFAIVPCILHMIY
DQKDLNIRLKLFGLLGFCTTAMMKYCVLVIRRPKILRCIEHVKSDWWQAKFSSDRELMLKYATTGRRLSMISA
TSMYVAGFIYHTILPFCIEHKVDNQTIRPLVYPTYSKFFKTQASPIYEMVYLAHCVCGYTMYSVTAGSCGLAA
IFATHACGQIEVIISRLEDLSCGKNFEQLSDVNQRISLIVKSHVRILRFSAAVEGILQEVCLLEFASSIFTMC
LPEYYCIVDWQDSDTVGLTTYFLLFVSFCFNMYILCYIGEQLVEKSSQIGTRCFMIRWYQLPAKSVRSLVLVI
AMSSHPIKISAGRMIDLSLATFGNVFKTSLAYLSFLRTLVV
>AcepOr79
NYQQDIQYIFKLNNWILGSIGLWPVSFQGIGRHAPRIAIALGNFALSFAMVPCALHIVYDEKDIIMRLKLCGL
LAFCLTAMTKYCILVIRRPKILRCIEYVKHDWWQVTFRSDRELMLKYATIGRNLTIIGASFMYTAGIIYYGIL
IPFFSETKINNQTIRPLVYPTYNEFYQFQISPIYEIVYVAHCMCGYTIYSITAGACGLAALFVTHACGQIQVI
MSRLKILLNDENSNIHQRIAIIVKDHVRIVRFSAVVEEVLQEVCLVEFTSSVCTICLLEYYCIVDWKDDNKLS
LATYFLLFISFCFNIYILCYIGELLMEKSIEIGSMCYMINWYQLSPRSVRSLVLIIAMSSHPIKLSAGRMADL
SLTTFGNVLKTSVAYLSFLRTLVM

>AcepOr80
MAKLNIPWSNVNSERDIIKALMWNRWILRILGIWPLVYSNTTKIEKILATISFALCWSALSFLLISMIIVTLS
KHSATSDKIKVLGPLCYVFVVTLKYFFLILHHKSIQQCIRILSSDWRTIQQENHRKIMIKNAAKSHVLSKFSI
IFMYCGGLWFHVMMPFLSHTINKQNVTIKPMPFAGFNNIFNFHLMPVYVFVLCAQWFSSIILFNVTTAVCCLA
AMFVSHACGQIEIVMDRVESFRKDTQSNHMKQCMAIIVKHHVQSLRFSVDIDKILREIGLVEIVGATFIICSL
EYYCITEWNNNDSIAILTYFFLLISFVFNVFTLCYIGEQLTSQCSKIGYTSYEIEWYHLPGKIALDLTLIISI
SHHPVKISAGKIVSLSFSSFGSVLKTSIAYLNLLRKFI
>AcepOr81
MAKLNIPWSNVNSERDIINALVWNRWILRILGIWPLVYSNTTTIEKIIATISFALCWSALSFLLIPMAIFTLS
EQTTINDKIKMLGPLSYVLISTLKYFFLVIHCKSIRQCIHVLSTDWRAIQQQNYRKIMIKNVAKSHMLSKICI
MFMYCGGVCFQSVMPLLTHTTIDKQNITIKPIPCPGFDIIFDLHFTPIYVFVFCAQWFSGIVLFNVTTAVCCL
AAMFVAHACGQIEIVMARVENFVKNAQSSRMKQRMAIIVKHHIQSLRFSVNIDNILREICLVEIVGTTLTICL
LEYYCIMEWNNNNGIAIITYFFLLISFVFNVFTLCYIGEQLTNQCSKVGFASYKIEWYHLPGKIALDLILMIS
ISHHPIKITAGKIINLSFSSFGSVLKTSIAYLNLLRTFV
>AcepOr82
MAKLNIPWSNVNSEKDIINALIWNQWILRILGIWPLVYSNTTTIEKILATISFALCWSALSFLLIPMVIFTVS
ERTTVNDKIKMLGPLSYVLISTLKFFFLIIHRKSIRQCINVLSTDWRAVHQQDYRKIMIKNAAKSHVLSKFCI
MFMYCGGLCFHTVMPFLTHTTIDEQNVTVKPIPYPGFDIIFDMHFTPAYVFVFCAQWFSGIVLFNVTSAVCCL
AAMFVAHACGQIEIVMDRVESFIKGTQSSRMKQRMAIIVRHHIQSLRFSVSIDNILREICLVEIVGTTLTICL
LEYYCIMEWNNNDGIAILTYFFLLISFVFNVFTLCYIGEQLTNQFSKVGFASYKIEWYHLPEKLALDLTLIIS
ISQHPIKITAGKLMNLSFSSFGSVLKTSIAYLNMLRTLV
>AcepOr83
MAKLNISWSNVNSERDIIKALMWNRWILRILGIWPLVYSNTTTIEKILATILFALCWSALSFLLIPLIIFTLS
ERTTIYEKIKLLGPLSYVIIATLKYFFLVIHRKNIRQCINMLSTDWRAVHQQNYRKIMNKNVTKSHMLSKFCI
MFMYCGGLSFHTVMPFLTHTTIDEQNVTVKPIPYPGFDIIFDLHFTPAYVFVFCAQWFSGIVLFNVTTAVCCL
AAMFVAHACGQIEIVMARVENLVKNVQNSRMKEHMAIIVKHHMQSLRFSVSIDNILREICLVEIVGATLNICL
LEYYCLMEWNNNDNIAILIYFFLLISFIFNVFTFCYIGEQLTNQCSKIGFASYKIEWYHLPGKTALDLTLINS
ISQHPIKITAGKIINLSFSSFCSVLKTSIAYLNMLRTLV
>AcepOr84
WPNANHEKDVIDILIWNRWLLRILGIWPLIYPDTTMIEKILATFSFASCWIVLGLFLILTSIYTFTDRSIMGE
KMKMLGPLGYVFFSMLKYLFLVIRHKSIRRCVQILSTDWRMVQEKYHREIMMKDAEKGHLLSKFCIVFMYCGG
LSYNTVMPFLSQTSDANKQNDTVRPMAYLGFDIVFDLQLMPIYVFAFSLQCFIGIVMFNITTSVCCLAAMFVA
HACGQIDIVIARVENLFKGERDHIKFEKYMAIIVQHHVRTLRFSAGIEDTLRELCLVEIVGTTLIMCLVEYSL
ITEWNNSDSIAIFTYFFLFVSFVFNIFIFCYIGELLTEQCSKIGNTTYTIEWYNLPGKVALDLMLMINMSGHP
VQITAGRLLSLSFANFGNVLKTSVAYLNLLRTAI
>AcepOr86
MSYRSSLPERIHNIRYEQDIYYAIQLSRWILQSIGIWHIIYGRSSQSEKLLSLMLIFMSFFGLCFVLVPAGSY
FLFYDEDIQSKIQFLGPVSFCLTSVIKYCFLGTRISTIGKCIEHVESDWRIMQYQNHRRMMLRNVLIGRRITM
MCVIFLYGGGLFYHTILPFTSRTKTDGNFTSKSLLYPGYDLYFDSQTSPTYEIIFCMHCLSAVIQYSATAAVC
SLAANFATHARGQVQILVMLLDDLINGKRTEGTNMEKRLSFITKHHVRVLRFTTDVEKILREVCLVELVTATL
MICLLEYLFLMEWENSDAVSIMSYILLLIALTFNVLIFCYIGELLVEEFGKIGLAVYEVNWYDLSGHKAMDLI
MIMMKSYYPPKLTAGKFCDLSLNTFSAVLKTSVVYLNLLRTVTQ
>AcepOr87
MRDRFQLIERTLKMYNIHYKHDIRYTMQLCRWMLKPIGMWHPIYGHSPSQNEKFISIILIVICFSALCFVLIP
AGFYTLFREKDISIKIKLFGPVGFCLTNTIKYCYFGARAAAFGRCIRHVEDDWRIVHHQTREIMLKQVLGRRL
TTLCAIFLYTGGLSYYIIMPLSSRQEINENYTIRINTYPGYDMFFDPTASPAYEIMFCIHILFAMISLQITTA
SCSLAAIFVTHACGQIDILMLLLDDLVEGKRSKNTTKTPGKRSKNVKKRLSAIAKHHVRILRFSASVEEVLRE
ICLLELLTYTLTICLLEYYCLTEWKNSDAIAILIYFILLISFTFNVLIFCYIGELLVEQYSKIGAATYEIDWY
NLSGNKALELVLVIAMSHHPPKLTAGKFIDLSMNTFSAILKSSVVYLNLLRTVTE
>AcepOr88
MRDRSQLPDRIHNIHYKHDIYYAMQLCRWILKPIGIWHMIYGRSSQSEKLLSLILIFVCFFGLCFVLVPAGPY
TLFREKDINIKVKLFGPVGFCLTSAIKYCFLGARASSIGRCVEHVESDWRIVRHYDHRRMMLRNVLVGRKLTT
LCVIFLYTGGMSYHTIMPLSSRTKTNGSFTSRPLVYPGYDLYFDSQASPAYEIIFCMHCLSAMIQYSATTAAC
SLAASFATHACGQVQILMTLLDDLVDGKRTKGTNVEKRLRLITKHHVRVLRFTADVEKILREICLLELVAATL
IICLLEYYCMTEWANSDAVAILTYFMLLISLTFNILIFCYIGELLVEEFGKIGSAAYEVNWYDLPGHKAVDLV
MIITMSYYPPKLTAGKFCDLSLNTFSTVLKTSVVYLNLLRTVT
>AcepOr89

MPDYSKSKSIKSITDGWNYSIQMNRWFLKPIGVWPLTLCETTVEKIGCMILTVISCLLICFLLIPCTLCTILV
DTDLDTKIRMIGPVSFFLMAAMKQYILIIRSENIDECIRHIRVDWSRVALNHEKDREIMVNNAKFGRWLSSIS
AIFMYSAGIFFTTVMPICARRTEIIDNETVRSLSFPIYRGLFDPRTTPSFEIAQFTQALAGYVIYTITISVCS
LTAVFVMHACGQFRILMLKLEDLADGKERKSANSTHEGRLGDIVEHHIRILSFIARTEKILNEICLVDVVGCT
LNICFLGFNMMTEWEHRETLGTMTFCSLLISFTFNIFILCYIGELLAEQCIQIGMKSYMINWYRIPNKGAIGL
TLIMAMSNATIKLTAGKFMDLSLASFCSIVKASVGYLNLLRTFYI
>AcepOr90
MGDDLADNSSISPYLVNKYNEYSIQIIRWILKAISVWPRSVNASLIEKIRSDFAIFICYFLIITTMVPNGLSM
LIDSQMSYETRLQNFGPLTFWFIAMVNYSCLLMHVDDIHNCVEHVKMDWLIIKRFEDQKVMLKSAKLGRFIAG
FCAVFMHCGVFSYNVVQGLSKSILYMENSSIVVRGLPYPFYNKILNVHFSPAYEFVFFLQCLSSFVVNCVTVA
TCGLTAVFVMHACGQMKIMISWLENFIDDKKEERTSLRQRFAIIVNHHLRILSFVSRTEKIMNVICLVELIGC
TMHICFLGYYCIMDFIDGNHQSILSHSMVLSSITFNIFIFCYIGEILSEQGEQIGKSAYMTNWYLLPGKTAQG
LILIILRSNAVLKITAGKIVQLSFSTFGDVIKSALAYLNILRTVLL
>AcepOr91
MSNSKHAPKRKTSEKHIDYCLQFNRWFLKPIGAWPQKNVPSLVYKIIILLQIFICLSIITVPTIPCLLYVLFE
AKNSQQKLNPMLPLINRFMSSMHYWVLLKRTDDIQKLIRHMETDWNLVQRIGERKVMLQHAKFGRFVTIICGI
MMQGSCLLFGLGQSMRTATITIGNETFTTRSMTCPIYSKIIDTRFTPQNEIAVILQQLVILVISTCTVGGCSL
AAVFAIHACGQLNVLYLWLHELIENQTEKNDKSEQKLAAIVEHHLRILSFISQLESIMHKPAFVELTGCTTIM
CLLGYYTATIWETFDKTKLISYLFSYLSMSFNIFIFCYIGEIVTEQCKLVGEMAYMTDWYNLHHTTARGLILI
IARSNNVVKITAGKLFHLSIATFSDVIKTSFVYCNFLRTVAM
>AcepOr93
MSNSKHAPGRKISEKHIDYSLQLNQWILKSIGAWPQISASSSLYKIIILLQIFICLSIIVVPTIPCILYVLFE
AKNIQQKLRPMIPLIHRFMSSMHYWVLLKRSDDIHKLIRHMETDWNLVQKIGEREVMLQHAKFGRFVTIICGI
MMQGSSLLFGLGQSMRTATIIIGNETFITHPTTCPIYSKLIDTSFTPLNEIAIVLQNVIMLLLSFCTAGGCSL
AAAFAIHACGQLNVLYLWLHELVENQTEKNDKVEQKLAAIVEHHLRILIWETLDMIKLISYLFPYVSVGFNIF
IFCYIGEIVTEQCKLVGEMAYMTDWYYLHHTIARDLILIIVRSNNVVKITAGKLFHLSIATFGDVIKTSFVYC
NF
>AcepOr95
MSNSKCALESDKLKTRTDYNLQFNRWLLKPIGAWPQINVSSSLCKIMILLQIFICSSIIAVTTIPCLLYVLFE
AQNIKQKLHAVGPLINRSVSSIHYWVLLKRSDDIQILIRHMETDWNLMQRIDEREVMLQHAKFGRFITIICGI
MMQSGCLFVGFGQSMKTATIIIGNETFTTHLMTCPIYNKIIDTRFTPVNEIALVFQNLVLLVISFCNAGGCSL
AAVFAIHACGQLNVLYAWLHELIKNKAKENGKAERNLAAIVERHLRILSFISQLESIMHKVALVELVGSAITI
CMLGYYIITAWETFDVTKIISYGILYLSLCFNIFIFCYIGEIITEQCKLVGEMTYMTDWYNLHHKTARGLILI
IARSNDVIKFTAGKLFHLSITTFGDIIKTSMVYLNFLRTMT
>AcepOr96
MSNSKCTPKKEGSKKHIDYSLQFNRWLLKPIGAWPKINTSSSVCRIVILLQIFICSSIIAVPTIPCLLYVLFE
AENIQQKLNTMMPLINRFMSSMHYWVLLKRRDDIHKLIRHMEIDWNLVQKIDEREVMLQYAKFGRFITIICGI
MLQGGCLLFALRQSMKTATITIGNETFITHLTTCPIYSKIIDTRFTPLNEIALVLQNLVILVISFCTAGGCSL
AAVFAIHACGQLNVLYAWLHELIENNAKENDKTEQKLAAIIEYHLRILSFISQVESIMHKAALVELTGCTIIM
SLLGYYTITAWETLDTGKVTSSLIVYLSMGFNIFIFCYIGEIITEQCRLVGQMVYMTDWYNLHHKTARSLILI
IARSNDVIKITAGKLFRLSIATFGNVIKTSVVYLNFLRTMT
>AcepOr97
MSNFKRAPESDISKKHIDYSLQINRWFLKLIGAWPQINASSSVYKIMILLQIFVCSSVIAVTTIPCLLYVLFE
ADNIKQKLNAVAPLLHRVMGSVHYWVLLKRSHDIDKLIRHMEADWNLIQRIDEREVMLQHAKIGRFIVIICAV
FMQSGLFLFSLGRSMKTTTIIIGNETFKTHPTSCPIYSKIIDTRFSPVNEIALILQNVTMFVASFSTVGACSL
AAVFAKHACGQLNVLYAWLHELVENQKKSDTNQKLTAIVEHHLRTLSFISRVESIMHKASLAELMGCTIILCL
MGYYTIMAWETFDTAKITSYVVIYLSMGFNIFIFCYIGEIITEQCKRVGEMAYMTDWYNLHHKTARGLILIIA
RSNNVIKITAGKLFHLSIATFGDVIKTSMVYLNFLRTMT
>AcepOr98
MSNSKHVPESDIPKKNIDYSLQINRWFLKPIGAWPQINSPSSVYKIMILLQIFICSSIIAVPTIPCLLHVLFE
ANNIKQKLNVSGPLLHRALGSIHYWVLLKRNRDIHKLIQHMEADWSLIQKIDEREVMLQHAKFGRFVVIICAV
FMQGGLFLYSLGKSMKTITIIVGNETFKTHPTNCPIYSKIIDTRFSPVNEITLVLQNLTMFVASFSTVSICSL
AAVFAIHACGQLNVLNAWLRELVENQKRNDTNQKLAAIVEHHLRTLSFISRVESIMHKASLAELMGCTISLCL
IGYYTIMAWETFDTAKITSYIFMYLSMGFNIFIFCYIGEIITEQCKHVGEVAYMTDWYNLHHKTARGLILIIA
RSNNVIKITAGKLFHLSIVTFGDVIKTSMVYLNFLRTMT
>AcepOr99
MSTSKCASEYNRSKKYNDYSLQFSRWFLKSIGAWPQINASSLVCKIVILLHIFICSSMIASNMVPCLLYVLFE
KVNIKLKLSAVGPLLHRVMGSVHYWVLLKRSDDIHKVIRHMETDWRLIQRLDNREIMLQQAKFGRFVAIICGI

IMYGGTILFSIAKAMKTIIINVGNETFTTRPMTCPVYSKIIDTRFSPVNEIALVLQNFAILVVSSSTVGACSL
AAVFAIHACGQLKVLNAWLHELVQKKKNQKKKNDTAERKLAAIIEHHLRILSFISQVESIMHKAALVELAGCT
VVMCLLGYYTITAWETLDTTKITSSLIVYFSMGFNIFIFCYIGGIITEQCKDVGEMAYMTDWYNLHHKTARGL
ILIIARSNNVIKITAGKLFHLSIATFGDVIKTSMVYLNFLRTM
>AcepOr100
MSNSKCAPEKIIPEKHINYSLQFNQWILKPIGAWPQISASSSVYKIIILLQIFICLSIIAVPTIPCLLYVLFE
AKNIQQKLNPMMPLINRFMSSMHYWVLLKRRDDIQKLIQHMETDWCLMQRLDEREVMLQHAKYGRFITIICGI
MLQGGCLLFSFVQSIRTVIIIIGNETFTTHTISCPIYSKIIDTRFTPMNEIALILQNMIVILVSFCTAGGCSL
AAVFAIHACGQLNVLYMWLHELVQNQAKENDKIERKLAAIIEHHLRILSFISQVESITHKAAFVQLTGCTVIM
SLLGYYTVTGWETLDTGKITSSLTVYLSMSFNIFIFCYIGEIVTEQCKYVGETAYMTDWYNLHHKTARDLVLI
IARSNNVIKITAGKLIHLSVPTFGDVIKTSVVYLNLLRTMTL
>AcepOr101
MSNFKCASENDRSKNHNDYSLQFNRWFLKPIGAWPQTDELSLVWKILVLLQIFICSSIVASNMIPCLLYVLFE
KDNFKLKLNATVPLLHRVMGSIHYWVLLKRSGDISNLIRHMETDWCLVQRFDREIMLQQAKFGRFVTIICGIM
MQGGCLLFGIGHLMKTATISIGNETFTKHVFPKTCPVYNKIIDTRFSPVNEIVLILQNLTMLIVSSSTVGACS
LAAVFAIHACGQMNVLYEWLHELVKNQEKRNDKTKQKLGAVVEHHLRILSFISQMESIMHKISFVELTGCTVV
MCLLGYYIIMAWETLDKAKITSYFILYLSTGFNIFIFCYIGGIVTEQCKLVGEMAYMTDWYNLHHTTARSLIL
IIARSNNIIKITAGKLFHLSIATFGDVIKTSMAYLNFLRTMT
>AcepOr102
MSNFKCAPESDRSKKHNDYSLQFNRWFLKPIGAWPQTDESSLVWKILVLLQILICSSIVASNMIPCLLYVLFE
KDNIKLKLSATGPLLHRIMGSVHYWVLLKRSGDISNLIRHMETDWCLIQKFDDREIMLQQAKFGRFIAIICGI
IMHGGTILFSLARAMKTVTIVVDNETFTTHPMTCPIYNKIIDTRFSPVNEIALILQNLAMLIVSSSTVGACSL
AAVFAIHACGQLNVLYEWLHELVKNQEKRNDKAKRKLATIVEHHLRILSFISQVESIMHKVSLVELTGCTMAM
CLLGYYTIMAWETLDTAKITSYFIVYLSMGFNIFIFCYIGGIVTEQCKLVGEMAYMTDWYNLHYTTARSLILI
IARSNDVIKITAGKLFHLSIATFGDVIKTSMAYLNFLRTMT
>AcepOr103
NLKHDLRNVDFKHINNYSLQINQWCLKPIGAWPITSTSSTLEKIAAIVLIIFCSLTIAIVTVPCILYIILENQ
TIIAKIGAFGPLFHRIMGSISYWTLLKRSHDIHNCIQHMEIDWQLIQRIGDRETMLQYVKFGRFLAGISAAIT
QGGQLLFGTARAIKTTTIMVGNETFKTRPMTCPTYSKILDTRFSPINEIMLTIQFVSAFVVSSAIVSVCSLAG
VFAMHACGQLNVLYAWLNELVIDYEEKGNQSVEQKLAAIVEHHLRALSFIARVESIMHKVCLVILMGCTLNMC
LLGYYSIMNWGAFDAAKILSYITLYTSMGFNIFIFCYIGEILTEQCKNVGNVVYMINWYQLPHRTALCLILII
MQSSNVIKITAGKLVHLSIATFGDVIKTSMAYLNILRTMT
>AcepOr104
SNLKRILINFKNVNDYSIQFNRWFLISLGVWPQRSASKIKQIVVSMQIFIFSIVVAVIMIPCTLYVCFEKKKF
GIRINAIVPLMHRIMGSINYWQLLTHNKDIQHCILHMETDWKLVRKMDDREVMLRYAKIGRFITKFCAIFMHS
SAIFAIAVKAMKTTTVIIGNETFKMYPMTCPPHSKIVDIRFSPTNEILLSVQLLSTLIVSSSTVAICSLAAVF
AMHACGQLNVLHTWLKELAEEKTYHLAEQKLAVIVEHHWRVLSFVVQIENIMHKACLGELMGCTLSMCLLGYY
SILNWANFDAAKIFACISFYISYTFNIFIFCYIGEVLTEQCKNIGEIAYMTNWYKLHHKTALKLTLIFMQSNY
VIKITAGKIFRLSIATFGDIIKTSTAYLNFLRTNMT
>AcepOr105
VNERVMSHLDASDYSIQLNRWFLKPVGAWPRSASTTIKEKVISIVLIIICYSLIAYTVVPCILNILLEETDMH
KKLQAVGPLNHWSMGGVNYFSLLFRSRDIYRCVEHVRTDWLIITDIEDRQIMLKYAKFGRFVAGLCAIFMHSG
VFSHSVMQGIIPTFVYIENVSVSIHILPCPTYSKFIDITKSPAGEIAFTVQLISSIVVNSVTAGACSLAAVFA
MHACGQLNILMRWLDQLDNRKQQDMIQHRLAIIVEHHLRVLSFVARMEALLNQICFVELLGCTFNLCMLGYYI
ITGWNTIETKMTIACMMIIYISMTFNIFIFCYIGETVTEQCKQVGEIAYMTNWYCLPHKTARSLVLIISRSSN
VIKLTAGKLFHLSIATFGDVMRTSMIYPNMLRTMTDS
>AcepOr108
VSELIISHRDASDYSIQLNRWFLKPVGAWPSLASTLVKEKVISTILIIIICYSLIAYTVIPCILNILFEETDVQ
KKLRAVGPLNHWCMGGMNYFSLLFRRRDIHRCVEHMKTDWRTVTEIGDRQVMLKYAKVGRFVAGLCAIFMHGG
VFSHSVLQGTTPTFECIGNVSVSVHVLPCPSYSKFVDTTQSPAGEIALTLQLISSIVVNSVTVGACSLAAVFA
MHACGQLNVLMRWLDQLDDRKQQSTVQHRLAIIVEHHLRVLSFVARMEALLNQICFVELLGCTFNLCMLGYYV
ITGWNVIEKKTSVAYMIIYISMTFNIFIFCYIGEIVTEQCKQVGETIYMTNWYRLPHKTARSLILIISRSSCV
IKLTAGKLFHLSITTFGDVMKTSMIYLNMLRTITAS
>AcepOr109
VSEPVTSNRDASDYSIQLNRWFLIPVGAWPSSASTTIRKKVIATILIIICYALIAYTVIPCILNILLEETNVQ
KKLLAVGPLNHWCISGINYFSLLFRSRDIHRCVEHIKADWRTMTEIADRQVMLKYAKVGRFVAGLCAIFMHGG
VFSHTVLQGATPKFECIGNVSVKVRVLLCPSYSKFVDTTQSPAGEIALALQIISSIIVNSITVGACSLAAVFA
MHACGQLNVLMRWLNQLNDQKQQETVQHQMAIIVEHHLRVLSFVARMEALLNQICFVELLGCTFNLCMAGYYV

ITKWSILETKMSMDILITCIIYLSMTFNIFIFCYIGETVTEQCKQVGETAYMTNWYHLPHKTARDLILIISRS
SNVIKLTAGKLIHLSIETFGNVMRTSMIYLNMLRTVTAS
>AcepOr110
YSERILTSIKFTYRSNNDYSLQLTRWFLMPIAAWPQATISRTVKRISLQAHVLACSSLIAIVMVPCMLYVSLE
EKDVQMKLNAMGPLSHWIMGTINYWLLLTHSDDIRKCVQHMEMDWRLVRRNDDQDVMLRYAKIGRFVAGFCAI
FMQSGTFLFTVAKAMTSMNVMIGNETMSVHPMTCPIYSKFIDTKFSPANEIMLAVELLSCFIVNSVTVGACSL
AAVFAMHAYGQLSMLFAWLNDLVEDEDKGNNFAEKKLATIVEHHLRVLSFISRMENVMQNICLVELVGCTMNM
CLLAYYSITNWSDFDAARITSYIIVYVSMAFNIFIFCYIGEILTEQCKNVGEKAYMINWYELPHKTALGLILV
IARSSNVIKITAGKLFQLSIATFGDVIKTSVVYLNILRTMTPSS
>AcepOr111
MTYSDHVTRGTKFSYKSDNDYSLQLTRWFLIPIGVWPQMSTATKVKRIFSHIHILTCASLIAIIMVPCLLYVL
LEEKDTEIKLNIASPLSHWMMSMINYCLLLTRSDDIRQCVLHMETDWRYIRKIEDRQIMIRQAKISRFITGFC
AVFMQSGTFLFAIAKSLSTTIVIVGNETVLIHPMTCPMYTKFIDTRFSPANEIIVGVEWLSCFIVNSVSVSTC
SLDAVFAMHAYGQLDMLFSWLNDLVDKEGNENAEQRLAVIVEHHLRVLSFISRMETIMQQICLVELLGCTLNM
CLLAYYFITNMDSFDMAKTISYAIIYLSVTFNIFIFCYIGEILTEQCKNVGERVYMINWYELPHKTALGLILV
ITRSSNVIKITAGKLFQLSIATFGDVVKTSMVYLNMLRTMS
>AcepOr112
MCRSMIKDLHDHSVQLNRWFLKPIGAWPQELTTSSSEKAVSRILIFICYFLIAFTVIPCALNIFFEEKDVELK
LRAAGPLSHWFMGGLNYCSLLLHSADIHKCMQHMEMDWRIMKQSRDREIMARNAKLGRFVAGFCAICMHGGVF
FYNIVSGMTTVMVSIGNNQSISMLQLPCAFYNKLIDTRYSPANEIVLITQMLSGFIVNSTTVSACSLAAVFAI
HACGQLDILILRLGKLIEGEDAEKNEPVQRRLANIVDHHLRVLRFIGSIEDVMHQICLVELLGCTFNLCMLGY
YSITWWNKIDRKGIMAYIIVYISMSFNIFIFCYIGEILTQQCKKVGEIAYMTDWYHLPHKTAMSLILIISRSS
VVIKITAGKMVQLSLVTFSDVIKTSLVYLNMLRTVT
>AcepOr113
MTIASDKPPSVLYRHNEHHDYSLQLNRWFLKPIGAWPESRVTTISERLLLKIIQIICYALIAFTVIPSMMYFY
LEEQDLDTKMRLVGPVSHWVMSSLNYSSLLWRGKDIRCCIQHMENDWCIVHRNVDRGAMLRYAKFGRSMAAFC
AVFLHCGVFSYSVVNSLTPIIIVVGNETVSTRRLPCPFYSKLLDISHDPMNEIVLMIQFLSGFIANSVTVSAC
SLAGVFAMHVCGQFAVLYTWLNELTNEEKRTTKNKLANIVEHHLRVLNFISRFEKIMNQICMVQLVGCTMNLC
LLGYCSIKEWDEKNTKTITTYGILFVSMSFNIFIFCYIGEIITEQCRKVGEMAYFINWYYLPHKTTLDIMLII
SRSRVIIKITAGKLIHLSLMTFGDIIKTSVAYLNILRTV
>AcepOr114
MQKSSFTQKSNLVVTGYDHEKYAQISIKQIRWLLKSLGIWPNSLKSSSLIKKYVRVLMNIIYLGIMAFLFIPC
VFYVVLEVQDTYNTLKFAGPLSFGLMVIMKYCSLALHGRDICVCIEHIKIDWRNTWYHDDRAIMTKNAKFSRR
LVMINAFLTYSGAIFYLIAMPIGMGKITDKNSNLTYQPLVYPVARIIVDTRHSPINEIFFWTQFASGIISQTV
AAAACSLTAVLAVHAYSRLEVLMQWIVHLVDGREDFSNNVDDRLAIIMQEHVRILYFIKLTEKVLHQISLVEV
VGCTFNICFLGYYTIMEWEGGFAITYIILLMNFVFNLFVFCYIGELVAEQCKSVSETSYMINWHRLPGRKALA
IVLMIAMSNSSIKLTAGNIIELSMSSFGDVMKTSVAYLNMLRTLTT
>AcepOr115
MMRKSPSTKKVNSTTVYDREKDAQINIQQIRWLLKSIGIWPNSLKSSSSIKKYVRVLMNVIYLGMVAFLFIPC
VFYVVLEVKDIYNILKFTGPLSFGLMVTMKYSSLALHGRDVRICIEHIKTDWRNTWYHGDRVIMTRNAQFGRR
LIIINAFIAYGGITFYHIAMPLSMGKITERNSNLTYQPLVYPVARMIVDTRYSPINEIFFWTQFASGVISQTV
ATAACSLTAVLAVHAYGRLEILMQWILHLVDGREDFSNNVDERLAIIVRQHVRILYFIKLTEKVLRQISLVEV
VGCTLNICFTGYYTIMEWDNDFAIYITYIVLFMNFTFNLFVFCYIGELVAEQSKRVSEISYMIDWHRLPGRKA
LAVILMIAMSNSSVKLTAGNIIELSMISFGDVIKTSVAYLNMLRTLTT
>AcepOr116
MMRKSSCTQNSNPVATVYDHEKYARLSIQQIRWIMKSIGIWPNSLKSSSSIKKYVRVLMNIIYLSIMAFLFIP
GVFYVVLEVEDIYNTLKFIGPLSFCLMTIMKYSSLAFCRRDIRVCIEHIKIDWRNTWYHDDRAIMTKNAEFGR
RLIVINGFFAYSGAIFFHIAMPISMGKITESNLTYQPLPYPVSRIIVDTRHSPINEIFFWTQFVSAVVSQTAV
TATCGLIAVLAVHAYSRLEVLMQWIVHLVDGREDFSNNVDERLAIIVREHVRILCFIELTEKILHKISLVEVV
GNTLNICFLGYYTITEWENGFAITYIILLMSFVFNLFVFCYIGELVAEQCKRVSEVSYMIDWYRLSERKALAI
ILMIAMSNSSVKLTAGNIIELSMISFGDVIKTSIAYLNMLRTLTT
>AcepOr117
MKRVSSEIIVNNYKRDNDYSLQLNRWFLKSIGAWPEIQTNSMIKTVLINILRIICHSLIAFTVISAILYILFE
EKDFRLRLKAIGPTSHILMGGINYCSLLHHNNRIRTSIEHMETDWRMVKKEYDRELMLRNARVGRVIAGICAL
ILQGGVICYNIARGMSRISVKIGNKTIETGRLPCPSFNKIVDTRLSPVYEIVLALQCLSTIVVNNITIGACGL
AAVFAMHASGQLNVVMLHLEELVTERQDLFQLRLANIVEHHLRALRFLSHLEAIMSEICFVELIGCTFNLCML
GYYTITEWHEESINTIITYIMVLTAMMFNIFIFCFIGELVSDQCKKVGEVAYLTDWYKLPHKIILGLILIIILR
SRIVTKITAGKIFHMSIQTFGVVIKTSVAYLNMLRTLTM

>AcepOr118
MSYSFRVSSVDMRNIAYQQDNEYSIQLNRWFLKPIGAWPGINTGIAGKMLSYGIQFVCHSLIAFTVVPCALYI
IFEPDVHLKLKAFGPMIHWLMGGANYCSLLMRSCKIRKCVDHMRDDWRYVGTRRDREVMLQNAKFGRFVSAFC
AVFMQGGVCSYSIITTLTPANIQIGNVTIITHQLPCPFYTELVDTRYSPANEIVIVLQLLSTIIVNSVTVGAC
SLAAVFAMHACGQLSILMIRLDELVDETGSEYKITRRKLAIIVEQHLRVLEWKLHDTKNLLTYFTIFIAMSCN
IFIFCYIAEILTDQCQKIGEMAYMTEWYRLHHKIAIDLILIISRSNAVIKITAGKMVQLSIATFGDVMKTSFA
YLNILRTVAT
>AcepOr120
IKSIMADSSKSKYKNDFNYAVQVTRNVLRMIGVWPISSNASNVERIATRLQNVSCYFLFTFIIVPSLLLIFLK
EHDFKRSVRVLGPLLNCGMGCLKYSLIMYHAKEIQSCLKQVLHDWRDTVDWKNRKIMLSKAKIGRRFAIISAV
FMYVGGLSYRTLVPLSKGRMLTSMNITVRALACPSYFVKFDEQTSPAYEIVFILQFFAGLITYSVTVGAAGLA
AFFVMHICGQLSILIGKFKHLNDIPEPEDRPVAILLADIVEHQIKVKTFLKQVEETMRYILLVEMTGSTILLC
LTMYYVIMEWERSDSTAMLTMFVILTSFTFSIFTNCYVGQLLTDQSIKVGSMTSTTNWHRLPHKRARTLILIM
AVSNIPAKISAGKMIEMSLPTFSNIIKTSLAYFNLLRKFIT
>AcepOr121
IIMEKANPVIVVNDYKKDLRLSIQLNRWILKPLGAWPKSIKISFIERYAYMLVNVICISLIGFLFVPSAIYLV
LEMDDAYNILKLTGPLNFCLMAIIKYSSLIFRENDIRNGIEHIANDWINTRYSDDRIIMIKSAKFGRRLVTIC
AVFMYGGATFYYLALPLSNGKITADGGNLTYRPLMYPVSSMIVDARRSPISEIFFFVQCLSGFIVHSITTGAC
SLAAVFAMHAYGRLEVLMQWIEHLVDGREDLCDNVDERLAMIVQQHVRILHFITLTDKMLREISMVEILGCTL
SMCFLGYFVITEWERKDMTSYVTYVVLYTSLTFNIFILCYIGELVAEKCKKIGEKSYMIDWHRLSGRKGLDLI
LMIAVSNTSIKLTAGNLFELSLSTFGDVVKTSVAYLNMLRTLT
>AcepOr124
LTTSKYDNDFNYAVQVTRIILRLIGAWPNPNSSMERIATRFQNVICHFLFGFIIMPSLLRVFLKEHEFKRRIR
LLAPIVNCSMGWMKYFLILNHAKEIQSCLNRAHQDWSNTVDGSDRKAMLSTARVGRKFAIFSAIFMYTGGVGY
RILIPLSKGRMLTPENITVRALALPSYFIKFDGQVSPAYEIVFTLQAFAGVLTYSTGIGAAGLAAFFIMHVCG
QLSIIMGKLQNLNNISNSNDQAVAMLLADIVQHQIKVKSFLTQVEATMRYVWLVEIMGSSLLLCLSGYYIIME
WQNNDSTAMLTNSVMLTSFIFSIFTYCYVGQRLTDQSIKVGLMTSTTKWYHFQYKRARSLILIMAVSNIPAKI
SAGRMIDISLPTFSNIIRTSMAYFNMLLKF
>AcepOr126
LKLIMKSSSTMSKRDNDFDYAIQVTRIILRSIGAWPSPNYVSNMERIATRFQNVICHFLFGFIIVPSLLQVFL
KEHEFKRRVRLLAPVVNASMSWIKYFMILNHAKKIQSCLNRAHQDWSNTVDGSDRKAMLSAARVGRKFAIFSA
AFMYTGGLCYRILMPILKGRMLTPENITVRPLALPSYFIKFDGRVSPAYEIVYTLQAFAGVITYSTRVSAAGL
AAFFIMHVCGQLSIIMGKLQNLNNISNSNDRAVAILLANIVQHQIEVKSFLAQVEATMRYVWLVEIMGSSLLL
CLSGYYIIMEWQNNDLTVMLIMSVMLISFILSIFTYCYVGQRLTDQSIKVGLMTSTTNWYHLQYKRARSLILI
MAVSNIPAKISAGRMIDISLPTFSNIVKTSMAYFNMLLKF
>AcepOr127
MYPNENYVNDIKYTIQLNRVICRLLGIWPETKSSFLEHLKKISLILGCYFLLGCELIPTILYIVFVEKRTRIR
LKLISSVMFTILAVLKYSSLVLSKNQVRNCLARMKDDWRNVTSTSARTSMIHKAMTARRLLILCGIFMYISGL
YFRTIVPLSKGKTITNQNITIRHLPCPSYFVLFNGQISPAYEIMFFIQFFSGFIKYTITVVICSLAALFIMHT
CGQLEILMTLINSLINETEEKNLDKKLAFTVEHQIKMRNFLRLVQSTLQYTSLLEVMGCTIIVCLLGHDVITE
WEDQNVISMCSYLILLTSIGFNIYIFCFIGEQLSIEGEKLALTVCTLAWYRLPNAKARALILVIATSIVPAKL
KAGKFFDLSIRTFGDVVKTAVTYLNFIRKMME
>AcepOr128
MPQNEHYQNDILYVMQPIRNLLLALGAWPTIKKERSVYQKAYNLLLICISYTLLFCDFVPSILYWFIEATSRI
RLQMIPFLLYDFMSVGQYGIFIFRYNQLKRCLMHVEEDWENVLNVDARNIMLKSARIGKRLITICGIFMYSAV
ITFRTILPLFQGKIITDQNITIRHFSCPGYFFSLNVQVTPVYETIFIIQFVIGFIPVSIVTSACGLTAIFVEH
ACGQLKILISLMTTLVQKQWQKEHEVNTMLAEIVNHQMRVRNFLRLVQHTLQEVYILEIAVNTASLCILVYFM
LVDWENRNIATVCTYCISITNVTIHTFLFCYTGEQLTSQAEKVAIASCELEWFRLPDKKARSIILLIRMSNAP
SKISAGIFIDLSLKTFGNIMKTAGTYFNMIRNVID
>AcepOr129
MSQNKHHQSDILYITQPTRNILLALGAWPSINKGESIHSKVHNLLLIFMSYSLLFCDLIPGILYWLMKATTRV
RLQMIPLLLYDFMSVSQYGIFISRYDQLKRCLKHVEEDWENILSVDARNIMLKSARTGKRLVTICAFFMYSGV
ITFRGILPLFQGKIVIDQNVTIRRFACPGYYFSFDVQVSPVYETVFIIQFLTGFITVSIVTCACGLTAIFVVH
ACGQLKILISLMTGLVQKEWQEECETNKKLAEIVEHQIRVRNFLRLVQHTLQEIYMLEIVINTTTICLLVYFM
LMDWQTRNIGTLCSYLISIINVIIHMFLFCYTGEQLTGQAEKVAITSCMLEWYRLPDRKARSVVLLIIMSNMP
TKISAGQFIDLSLKTFGNVMKTAGTYFNMLRSVID
>AcepOr130

MPQNKHHQNDISYITQPTRNILLALGAWPFVSKGRSVYPKAHNLLPICISYIFLSSDIIPGVLYWLMAETARV
RLQTIPLLLYDFMSVSQYGIFIFRYDKLKRCLQHVEEDWKNVLSADARNIMLKSARTGKRLVMICAIFMYSGA
FTFRTILPLFQGKIVIDQNITIRHLACPGYFFSLDVQVSPVYETVFVIQCLTGFIAVSIVTCACGLTAIFVVH
ACGQLKILIGLMRELVQKQWQEEREMDMKLAEIVEHQTRVRNFLRLVQHTLQEIYVVEYLVNTITICLLVYFM
LVDWQNRNIPTLFTYWLCIVNVTTHMFLFCYTGEQLTGQAEKVAIASCELEWYRLPDKKARSVVLLIIMSNAP
TKISGKFIDLSLKTFGDVMKTAGTFFNMLRNVID
>AcepOr132
MFANKYYERDIENAFGINRFFFRLLGIWPFSRTNSLLFELVETFILVFACFAFLFGELIPTILYVFMVLTDFR
LRLKVMASGIFTIIEIIKYSYAILYKSQVKNCLILVDKDWRNVINPNDRNSMIDKVKTSKRLIVICAIIVYVT
GMAVRMIIPLSVGKIVTSQNITIRPLPHVAYLVIFDVQRSPVYEIIYFVQFLAGLIKYTITVTTFGFMTLCAM
HFCAQSDILVTLMNNFVNENQSENLNKRLATVIEHQIKIRNFLQLVQSTTQYPSLIEILGSTVLLCFAGYYII
MEWEDHNVIRLCAYISVLIMFCFNIFIYCYMGEQIIEQGEKVALTTRTLEWYRLPNTKARALILLIIISENPL
NLKAGNLIDICLRTFGNVAKMSVTYLNFLRSV
>AcepOr133
MFANKYYEHDIENAFGINRFFFRLLGIWPFSRTNSLLFELAETFTLIFACFAFLFCELIPTMLYVFMVLTDFR
LRLKVMASGIFTITEIIKYSYAVLYKSQMKNCLILIDEDWRNVINSSDRNSMIDRVKTCKHLIVICAIIVYVT
GMAVRMIIPLSVGKIVTSQNITIRPLPHVAYLVIFDVQRSPVYEIVYFIQFLAGLIKYTITITTFSFMTLCAM
HFCAQSDILVTLMNDFVNENQPENLNKRLAIVVERQIRIRNFLQLVQSTIQYTSLIQIVGSTVMICLVGYYVI
MEWEKHNVVNICAYISGLIMLWYIIFIYCYMGEQIIEQGEKVSLTTCTLEWYRLEDATARALILLITISENPL
KLKAGNFIDISLRTFGNVAKMSMTYLNLLRSVV
>AcepOr134
MFANKYYERDIENAFAMNRFFFRLLGIWPFTHANSFLLELMETNALILTCFTFLFGELIPTMLYVFMVLTDFR
LRVKVMASGLYTLIEIIKYSCTLLYKNEVRNCLKFVEEDWRNVINPSDRVSMIDRVRTSKRLIMMCAIFMYFS
GMGFRIIIPLSTGKIITSENITIRPLPHVAYLVLFDVQSSPVYEIVYIVQFLSGLIKYTITVTTFGFMTLCAM
HFCAQSDILVTLMNDFVNENQPKDLNKRLAIVVERQIRIRNFLQLVQSTIQYTSLIQIVGSTVMICLVGYYVI
MEWEKHNVVSICAYISGLIMLWYIIFIYCYMGEQIIEQGEKISLTTRTLEWYRLEDATARALILLITISENPL
KLKAGNFIDISLRTFGNVAKMSMTYLNLLRSVV
>AcepOr136
LLGVWPFYRKKSTREKIWKYLLILIYHILLYSVLIPGALFWLIEKRARVRVRTIPLLLYGIITCGKYGSLIFH
EKNIKRCLQHIEEDYKVVNAKARNTMIKSAKIGRRLVTLCAIFMYGSGLFFRLILPFAKGQIITSQNVTIRPL
PCPAYFIFFNVQVSPTYELIFAIQVLSGIVTFSITTGLCGLATLFVMHACGQLKVLINLMTNLVEEKWHEKRE
LNRKLARMVEHQIRFRNFLQLIENTLHQACLIELMGCTMLICLLGYFIIMEWENSNRIAMFSYFSSITSMMIN
VFMFCYTGEQLTIQAEEVARKSCVLEWYRLPNKDARGIVLVIIISNLPAKITAGKIVDLSFKTYGDVIKTAVT
YFNMLRKMT
>AcepOr137
MFHNKYYKDDMIYITHLTRNILSLLGIWPSNKKDSICAKVWRYFLISICHILLYCVLLPGIFFWLIEKRTRVR
IQTIPLLLFVLMACSKYINLLFHEKKIKRCLEHIEEDYKVVTNAEARDTMITSAKIGRHLVSLCAIFMYSSGL
SFRLILPFARGKVITPQNITLRPLPCPSNFFFFDVQVSPTYELIFAIQVLSGIVTYSITTGLCGLATLFVMHA
CGQLKVLINLMTNLVEEKWHKKRELNKKLARMVEHQIRFRNFLQLIENTLYQACLIELMGCTMLICLLGYFII
MEWENSNRIAMCSYFSSITSMMINVFMFCYTGEQLTIQAEEVARKSCVLEWYRLPNKDARGIVLIIIISNLPA
KITAGKIVDLSFKTYGDVVKTAVTYFNMLLKMTD
>AcepOr139
MILNEYQKIDVKYVFEQSHVVLRMLGIWPSIDRQPNIIEKIINIFLIIVCYLLLHCDMVPGILYYAVVDDEPS
EKVKMMPPILYSVMAIAKYSTLLVYEYDIRNCLRHIKEDWGTVVVDDAREVMLSKASNGRRLFTLCCTFMYCG
GVSYNTIVPLSRGSIVIDENTTIRPFSSPGYYVFFDPQNSPAYEIVFLQQVLCGFVMYTITVAICGLAAVFVM
HASAQMEILIRQMENFVDESEFGQRNICAKLAVIVEHQIRIQNFLQLVENVLRYSSLVEIVGCTTLICLTGYC
IIGEWNNGNLPAFCTYVTALTSVIINIFILCYIGEYITAQAEEVGLITCTLDWYRLPTNVARDMILVIISSNI
PPRITAGKFIELSFKTFGDVIKSAVIYLNILRQLTE
>AcepOr140
MVDRKQGKTLNTLAANVTKQNELHVLDFYYAVQISVCLLKPFGAWPLANDETSKFKIVLHKASMMIATFLLIC
TIVPMMLHMMKEKDVFLIIHLMCELIFTLMAFAKYVLLLWHQDQLRFCVDYVANDWRYAIIAEDRNIMLANAR
IGRTFGITSVIFMFSCGTLYYLQPIATSNLVNEDNVTVRLHPSACEFLVFDSKVSPAYEIVYFLQLLCGFTAY
SAFCGICSLMANFVAHICGQCDVLMSLLKELVDGGKRNSGSIDNRIAVVITRHLHLLRFVSHVRNLFTEICLV
EFMNASCNICLLGYIIITDMNNNESFVQIFTYFFGLVSVIFNVFMFCYIGDLLKERCQQIGTICYTIEWYRMP
SRKAIDLLMPIIMSRYPATLTAGKMLTMTLITFSDILKTSMAYFNLLREFSSRD
>AcepOr141
MIASMSPVDNRALFQNFNYRSDTEYMLRVAKILLTPVGIWPLYGNDSTMYKIKYYLQTSIVFCLMCFLLVPHI
IYTFFDAEDLTRYMKVIAAQIFSLLGIIKFWTMIINKNNIKYCLKEMEIQYRDVESEEDRLVMVKNAKVGRQF

TMMYLVLLYGGALPYHIIMPLMADKVIKEDNTTHLPLPYLSDYVFFVVEDSPFYEILFVVQIVFSTMILSTNC
GVYSLIASCVMHACCLFEIARRHMETLLVGGIDDLHERFNWIITNHMRALRYVEMIENSLNIVFLSEMILCTI
IICFLEYGVLKEWEDNKFFGVIIYFTLVTSIFVNVFTLSTIGDRLKEESGKIGEVSYSIDWYTIPTKNVNSLI
MVMIRSNRPSTLTAAKMFDISLQSFCDVCKTSMAYLNFIRMLT
>AcepOr142
MSLKDFTLFRNRNYKADTEYVVKVAKTLLTPVGIWPLYRGNSKSDKIKNYLQTGVIFGLMCYLLVPHIIYTFF
DAEDLTRYMKVVAAQVFSLLAIIKFWTMIINREEIRYCLQQMEMQYRNVECEEDRLVMMKSAKIGRLFTVTYL
GLSYGGALPYHIIMPLLEDRIVKEDNTTQIPLPYLSDYVLFVVEDSPFYEITFVSQILISSIILSTNCGVYSL
IATCVMHSCCLFEVVRRQMETVLDDEINNLHKRFGRIIEHHMQAIKFAEMIEKSLNIVFLCEMVGCTIIICFL
EFGVLKEWEDGKILNMGTYFVLMTSIFVNVFIISAIGDRLKEESEKIGESSYSIKWYDLPSQIVKDLILVMLR
SNRPSTLTAAKIFDLSLQGFCEVCKTSAAYFNFIRAMTT
>AcepOr143
MIPTSTVDNLMEFTLRMIGIWPYSSYRFLMRIIWTMTMTIWQFLQYWHLFSHIGSDTLPNLMDSLSLCLSNSL
LFLKMSLLWLNGRIIYDMVARIAEDWNECSSNHSKMQLMISKAILSHRISKYSIGTYTVVLLLFGAGNMIVQK
TMGSEQLVEEKQLIVKMKLPSEFDASPIYEIVVVTQFFVQFTLALLAGMLNALIVTLILHVAGQIDIICHELL
EIPIAENKYDSRIILLRSIVSRHQRIIAFADSIEDVFCYMALMQFLLNTFVICFLGFVIVTSFGTHDGNILMV
KIIPYYAVVNLEAFILCFSGEYLTSKSKSINQAAYNSFWYKLKTKESKILLLLIMRSQKELTMTAGKFVDLSL
EGFTSILKASASYISVLHAMY
>AcepOr146
MGLKTTISSSVEFGLRAIGIWPGSHNILFRTFWTISLGLAQTFQFKYIVACIETANFLDLVDSVSTTLPYSLL
CFKLIIILWQNQRLFNDILTSMSRDWRNCHTVAFDVRIMTNKATLSHRCSMLIIGVYSIAVVVYVSVIMEFNSI
NSESGRGELFLKMEFPFVYEFSPVYEIVMFIQCIQLLSNAWVIGMLDALIITLIFHISGQIDIVCQGLLELFS
KDYEYKSYKDAKSAVIRKHQNVIAFSNNIENLFSNIALMQFFTNTLVICCIAFVILISIEGNKRFNMLLKSLF
FYIAITLEAFIFCFAGEYLSNKSKSVANIAYEALWYNAKPSESRNLLILILRSQKRLTLTIGKFNDLSLEVFA
SILKASASYVSVLLAMS
>AcepOr147
KMIPTSTVSRPVEIGLRLTGIWPNSSIFFRLLWTLVMGTGLIFQYHYLLVHFSIKELPNLIDGLSTTLPYNLL
FFKLVVLWINNRLFIDILTAMSNDWSEYSNMYAMIDKAILAHRCSKVTIGVYSTAVLLYSTASINFRKQTNGN
CRELLIKMELPFEFCNSPIYEIVECVQFVHLMAVASAIGMLDAFMVTLMLHIGGQIDLMQQEVEEIYLKNNKY
NFPATILRSLINKHHKIIAFSESIESLFSHIALMQFLSNTLIICCIGFLIVTSMGTDEGIRMLVKTSFFYIAI
TLEAFIFCFAGEYLSNKSRTIGDAVYESVWYTLKPQDCRILLFVIMRSQRRLTITAGKFMDLSLEGFTNSLKA
SASYISVLYAMY
>AcepOr148
TSTIALSVQIGLRSIGFWPNTPCGLLFRCLWMLTIGIVQTFQYWWIILHLRTDDMSYLMDGLSVTIEYSVMSL
KLIIILWFNSRIFYDVLAAMAADWREATSNEMHIMMSKANLSRRFSNVIIGLHSVAVMCFGIEVLASHTNDYDA
DHGIETPIRAFTLKLQLPLQCNESPLYELVMSLEFLHQLASATATGILNSLLITLILHTSGQIEILCDTLRNI
SSKMNNQQLIFCMKEAIGKHQKIITFSDKIDKIFSYIALIQFMSSTLLTCCIGFTVITSINAQDSDTIDGTAL
MKAIVFYMAAAVEAFTFCFCGEYLTAKSKMIGDAAYKSVWYDFAPKESKLILLIIILRSQRRFTITAGNMMDLS
LEGFTSILKASVSYVSVLNAMY
>AcepOr149
MLELRKDTIGRASTISESVEVGLRFIGMWPHCVYANINWWTYIVSVAIMQYFQYAYIFAHFDLSHLSVFVDGL
SITFGYSLSFLKLINLWFNRRKLFAILDTMNKDWSDAIHSDMSTMIRYANWSRQCSNVMITTNALAVFFYVIG
GLILRSMIQKNDDSIRELPIKMEFPFKVDYFPIFELILILQFFHDLSVACIIAMLNALLVTLVLHVSGQIDIM
LQGFMEISSKHTSRSSLDAIKDLVNRHQRIIDLSEDIEDLFSNIALLQFIWNTLVICCIGFVIVISIGTEEGA
TTITKSLIFYVAITLEAFVFCYAGEYLSAKSKSISDAAYECLWYDLTPSECRILMFLMLRSQKRLTITAGKIT
DLSLEGFTTIMKSSASYISVLSALY
>AcepOr150
MKRTSTISRPVEIGLRFIGMWPDSAYATLYWLFYMATMVIVQYYQYTYVFTHFDLSDISLLMDCLSLTLAYTL
AFLKLFVLWWNRRTFYYIVKAMNEDWKECVINNSYKSTMMSVSDLSHRCTNVMISINALAAFFLSIGEHLLQS
MGDVSGVDNNSRELPIKMEFPFDVSESPIFECFLIGQFLYELVLASIVGMMNALLVSLILHVSGQIDIIQQDI
NKISNSKYDSSLSLIVIKGLIYKHQKIITLSENVENLYTYIALMQLLWNTLVICCTGFVIIITIGTNANATTS
IKSVSFYIAITLEVFILCFAGEFLSAKSRSISDAVYESLWYNMPPTNSRILLFMILRSQKRLTITAGKVVDLT
LEGFTNIMKASASYVSILNAMY
>AcepOr152
MKRANTISFSIEIGLRFIGMWPDSAYPNFYWFSYMTTVAIVQYYQYTYIFVHFDLNDLWLLMDCLSLTLGYSL
AFLKLLVLWWNRRVFYYILKAIDEDWSEYVINDSYKSTMVSMADLSRRFANVSFSIYAFSAFFLSIGEHLLQS
MDDQFGNSSRELPIKMEFPFDVSKSPIFECFLIGQFLYDLVIASVVNLINALLVALILHVSGQIDIMQQDLFE
ISNKKYDHSTFLPVIKNLICKHQKIITLSESIENLYTYIALMQVLWNTLVMCCTGFVIIIVVSSGEDTTNLIK

SVSYYFAIMLEVFVYCFAGEFLSAKSKSISNSAYEALWYNMPPSDSRIILFMILRCQKRLTITAGRFMDLTLE
GFTSIVKASVSYISVLNAMY
>AcepOr153
MKRTSTISRLVEFGLRLIGMWPDSAYPNLYWSMYMTMLGLYQYFQYSYIVTHFDTSNLSILTDCLGLALANTS
AFLKLFFLRWNRRTFYNIVAAVKRDWNDRVVSDSYSTMMAIANQSSRYSVVLIGLHFLTGFSLSIGAYMFRTI
NTDSEASTREFPVKMQFFFVVSESPVFECILFGQIFFLMLIAPVVGMINALLATLILHVGGQVDIMRQAIMDV
HTNDKLGTSLIVLRDLIHRHRKIITLSNDIETLFSFIALLQLLWNTLIICFCGFMIILAVSSNKGIMVLIKSM
FLYTVKTLEVFVFCYAGEFLSSKSKSISDAIYESLWYNMIPSDSRILLLIMVRSQKRLTITAGKIFDLTLEGF
MSVMKASASYMSVLHAMY
>AcepOr155
MLTSTVSPLLKLGLCLIGMWPDFSYRTFFWFFYMATLVIMQYFQYSYFFAQLGTNDFSKLMDGLSITLDYTLT
FLKLLSLWHNRRIFSDILGAMNDDWNNCSTHTCMMMNKANLAHRCSNVMLILNSLSVIFYFVGNYMNHRTVST
DFREFPIQIQFPFHATDSPIFEFIVLGLFLHVWETAIVIALLNSLILTLVLHVSGQIDIMCQRLREISTTQKS
HSSAMKSLIEMHQRIISLSKNIDNFFSFVALIQFVWNTIVICSIGIMIMISLSTDVEGKSGLLIQSIVPYIAV
TLEAFVFCFAGEYLSTKSKSISDAAYETLWYDLSISECRILLLIIVRSQKRLTITAGKIMDLTLEGFTSVMKA
SASYMSVLHVMY
>AcepOr156
IMSTVSPAVKIALQCLGIWPDVSYSTIYWLSFMSSMLIVQYFQYSYIFDHLSTSELSNLIDGLPMTFNYSLTL
FKLSSLWINRRIFYKILATMDNDWCECINIDQHLYIMTIKANISHFISNALLSFTAIVGVLYLLGEYAVRFVF
LIENYNDTIKQLPLRLQFPFETQQSPIFEFLVVTIFLQAMIHVCTVAILNGLIFSLVLHASGQIDIICQEFKN
IFKNSLLHEFSLSMLIEKHNKIISFSEDIKKLFSFFALMQIVLNTLTMCCLGFFTIICIHNEIAVFLLVKAIL
SYCVIMSEAFVICFAGEHLTLKGKLIANATYETLWYDMPINTNRIIIFIIMRSQKQLTITAGKMMDMSFETFT
NIMKTTASYISVLNAMY
>AcepOr157
MSITSTVSPALRFGLRFFGFWPGVSYSTVYWLSFMSSMLIIQYFQYLYTFDHLKISELSNLADSSLFTLDYSL
TCFKMTGLWIHRRVLHKILADMNNDWRECTNIEQHLHMMTIKANTSHFLFNALLSCNMIITIIYLLGDYVICS
IFLNENYNNTVRQLPIKIQFPFEIQQSPKFEFIAVIIFIHTLLQVWTNTIVNGLVFTLALHVSGQIDIICQGF
KNISENILSYGSSTSSLGMLIERHKRVISFSENMERLFSFIALMQVVWNTLVICCLGCLFIISIHNETGIFAL
MKTIFAYFAITMEPFIICFAGEYLTHKSKLIASATYEMLWYDMPSNYNKIIVFIIMRSQKRLAITAGKMMDMS
FETFTNIMKASASYVSVLIAVY
>AcepOr159
QMITSSISPALKVGLRFLGMWPGDSYSTIHWLTFMLSMLIIQYFQYLYIFGHLKMSELSNLVDGLIVALDYSL
TLFKLTSLWTHRRVFHKILAAMDNDWREYINEDKHLYLMTIKANVARFTSNILLSVNAIVAVLYLLGDYVIRF
VFFTENQNDTLRQLPIKIQFPFETQQSPTFEFLVVTLFLHVAIHACTIAILNGLMLTLVLHISGQIDIMCHKF
RNISKGSLLHGSFESFFGLLIEKHNKIIIFSNNIETLFSFIALMQVIWNTLVICCLGFIIIISIYNETGIFVL
VKTVSGYFVIMIEAFVICFAGEYLSLKGKSIANAIYESMWYDMPSNKNKIIVFIIMRSQKRLAITAGKMMDMS
LETFTRIMKASASYVSVLNAMY
>AcepOr160
MVITSTISPVLKIGLQILGVWPRVSYSTIQWLSFTLSTLIIQYFQYLYIFEHLKISELSNLIDGLTVTLEYSL
TLFKLIGLWIHRQVLHQILVAMDNDWRECINMDKHLYMMTIKANIAHFISNALLSVNATVALLYLLGDYVIRF
VFLNEDQNHTLRQFPIKIQFPFETQQSPMFEVLVVTIALHVMLHVSILCILNGLVLTLVLHVSGQIDIMCYEF
RKISRSTLLHESVVSLFGILIERHNRIISFSENIEKLFSFIALMQVVWNTLVICFLGFFIIICIHSETGVFVL
IKTILSYFAIMAEAFIICFAGEYLSFKGKLIANAIYESLWYDIPSRQSKIIIFIIMRSQKRLAITAGKMMDMS
FDTFTNIMKASVSYISVLNVMY
>AcepOr161
VVTSTVSPVLKIGLQLLGVWPGVSIPYWLIYICSILIVQYFQYRYVYEHFKISELSNLVDSLPASLDYSLTIF
KVVILWIHRRVVYQLLAAMDNDWHECVDIDQHLYVMIIKANTSHFYANIMISIYIIVGVFYLLSGYIVHFVYE
IEDYNNTLREFPIKVHFPFENQQSPIFELLALTLFLHVLLNTSTVSIVNALISTLVLHVSGQIDILCQEFKTI
SAKVLPDKTSTSTLGILIERHNRIFWFSDNIETLFSFIALMQVIWNTLVICSLGFIIIISFYIETSVTTILKS
IFAYLAVILEVFILCFAGEYLNLKSKSIADAAYESFWYDLPLNQRKILMFIIMRSQKQLMITAGKITSLSLTT
FTSIIKASASYVSVLHAMY
>AcepOr162
MTITSTISPVLKIGLQLLGVWPGMSISYWLINILISILILQYYQYRYVLEHFKISQLSDLVDSLSAALSFSLI
IFKITSIWIHRGVLHQLLAAMDNDWRKCVDTDQHLYVMTIKANISHFYSNVMISINILAAVSYFFGGYAIRFV
HQSGDYNNTLGQFPIKVQFPLKVQQWPIFELLAVTQFLLILFNSYMLSVINALISTLVLHVSGQIDILCQEFK
TISVKTLPYKTSTSMLGILIERHNRIFWFSDNIENLFSFIALMQVIWNTLVICGLIFIIIIKSIADAAYESFW
YDLSSNNKKIISFIILRSQKQFVITAGKITNLSLETFTSIIKASASYVSVLYAMY
>AcepOr163

MTITSTISPVLKIGLQLLGVWPGMSISYWLINIFSILILQYYQYRYVLEHFKISQLSDLVDSLSAALSFSLII
FKVTSIWIHRGILHQLLAAMDNDWHECVDTDQHLYVMTIKANISHFYSNVMISINILAAVSYFFGGYAIRFIQ
SGDYNNTLRQFPIKIQFPLKAQQWPIFELLAVTQFLLMLFNSYMLSVINALISTLVLHVSGQIDILCQEFKTI
SVKTLPYKTSTSMLGILIERHNKIFWFSDNIENLFSFIALMQVIWNTLVICGLIFIIIISFHIETGVSVIIKS
VFSYLAVIAEIFILCFAGEYLNLKSKSIADAAYESFWYDLSSNNKKIISFIILRSQKQLVIMAGKITNLSLET
FTSIIKASASYVSVLYAMY
>AcepOr166
MIIRSTVSPMLKIGFQLFGIWPGVSYSTIHSMSIISSILIIQYFQYLYIFKHFKISEISNLNDSLNVTFTYGL
TIFKLIYLWIRRQVFHQILIAMDNDWRECINMDEHLYIMTIKANLAHFISNSILSVSTIIAIPFFLGEYIVHS
VFLTEDQNDTLRPLPMKIQLPFDTQQSPIFEIVYVTLFLHSIIIFVYMVCIVNGLISTLVLHISGQIDILCHE
FKNISKSISLHGSTVSLFRMLIERHNRIISFSKNIEKLISFIALMQVVLDTLIICGLGFFAIIGIHNEMGVFV
VTKTIFAIALILAEVFIICFTGEHLNYKSKSIINAAYELPWYDISLHQSKMLILIIMRSQKQLTISAGKMMDM
SFETYTNIIKTSVSYISVMNALY
>AcepOr167
VAKSTVSPALKFSLRLLGVWPEVSYSIFYWLIFMSSILIIQYFQYLYILDHFKISEFSNLIDGLSLTLDYSLT
FVKLASLWIHRRVFHKILAVMDNDWRECINIDEHLNMMIIKATVSHFYSNAMLSFNGVAAVLYVLGDYAIRFV
YISKDYNDTLRQLPIKVLLPFETEQSPIFELLVAIMFLHIILVSLLVAVLNGLIFTLVLHVSGQIDIICQEFK
NISENAFPYQSSAFTLKVLIKRHNKVYAFSENIEELFSFIALMQVVCKTLVICCLGFVIIISVHNEIDFYLFV
KAAVAYIAVMIEIFIICFAGEYLSLKSISVADAAYKSLWYDMPINQTKIISFVIMKSQKRLSITAGKMMDMSF
EALTNIMRASASYVSVLYAMY
>AcepOr168
MRATSISTSVEIGLRFVGIWPGLQYGTITWFTYMMSLVFALYFQYVYIFDHFDVNNISNLVDALSITLAYSLS
FLKLISLWLNRRIFYDIVLTMEEDWTNVNIYDKSVSCIMASNANLSRYCSNVLISINAAAAICYSVTNFLRLS
TDFKENLNISSRVLPVKMKFPFKVDVSPLFELLAVGQILHVVSIATLVGMMNCLIITLVLHVSGQIDILRREL
LTICGNGISQRDSIVTSVRLVIIRHQKIITLSENIEELYSDIALMQFLSNTVVICCIGFTIIASLVRDGAIVV
ILKSAIFYVAVTLEAFIFCFAGEYLSAKSKSVGDAAYEALWYNMTPAECRILLFVILRSQKRLTITAGNVMDL
SLEGFTSVMKASASYMSVLHAMY
>AcepOr169
MENEIDSTNIVCWSVQYGLRMIGVWPGTSGAILLKVLSISSMIVFQIFQYQHVIVHFEEEDLMILMDALSVTF
AYTLLLIKMLIFAFNSRLLNEIITCMIKDWKECDISDKYTMTRIAYVSRWVSNIIICSHMTSVFLYAIGTIMK
IKNENQTDVRELIIKMEIPFKIESTSVHVAVLVSQFIHQTSAAGMVGVINSLLIILVLHVCGQIDIMRQKLSE
ITQKNIERKNIEQDVNENIVKDLIVRHQQIITFSKNIENLYSNIALIQFVSNTLVICCLGFLIVISIGTPGGS
MMLVKSVFFYFVMSLEAFVYCFVGQHLSTKSEMIGDSVYESFWYELSPSQNRDIYIMIIRSQQNLTLTIGKVM
ELSLRQFANILKASASYVSVLHAMY
>AcepOr170
MNNEIKSANTICRSVEFGLRMIGVWPNISCAIFRRICCISSMAIFQTFQYWYLIMHFGESNIFLLMDVISATL
TYSLLFIKLIIIIFNVSLLDDIIAHVIEDWKKRDISDEYMMNRMAYVSRWFSNLIICSHAVSVFFYAMGTLLA
HRNSNQTDARELILKMELPFKIETTSVYLTVLITQFVHQVSAASMLAVLNCLLLTLVLHACGQIDILRQKLSE
ITRKNTKQHMNDIAKILIIRHQKIISFSKNIETLFSNIALIQFVSITLVLCSLGFVIVTSIGVPGGSPMLVKS
VFFYILVNMEAFTVCFLGEYLSTKSKMIGDAAYEALWYELNPIQNRDILFIIVRSQKFLTLTIGKVAELSLKQ
FANIMKASASYMSVLHAIY
>AcepOr171
MNNGTELENIVCRPIKFGLRLIGVWPGTSYAILRRVFCISSMAMFQIFQYWYVILRFGEEDLLFLMDVLSATM
AYSLLLIKLIIFTFNARLLNKIIAHTVVDWKERDVYDEYTMTRMAYISRRFSNFIIALYALSVFLYATGTLLR
FKSNNQTDTRELLLKMELPFEIKSTSVHIAVFVTQYVHQTSAASMVGVMNSLLITLVLHVCGQIDILRQKLCK
ITQKDIKRGINDRIMKMLIIRHQKIISFSKNIEDFFSNFALIQFVSNTMVICSLGFLIVISVGVSDGMPMLIK
SVFFYIVISMEAFIFCFLGEHLSTKSQEIGDAVYELLWYELNPNQNRDILILMIRSQKHLTLTIGKVMDLSLK
QFASMVKASASYVSVLYAMY
>AcepOr172
VRMNFQNVNSLNVRLNLLSGNLLPMINHDSSFSFFWKMYSVFGWIINFILIIVMISGCIYVPMEKALKDGLHA
IVVFMEILFAILRICTRRNLAYQLIQKLNEILHIADRTMKNVVIETLEPIKIPLNFYLSSGIVAIIGWSCMHF
VLIFEKNQFCYEDYRMPAVFSKQPFSFRIFLLGDLFILIVTIFMFIKKVSVEVYTMHLVLMVTAQYRYIALKI
AMICQEKNGDDKSRTGHSPKHYWKKEKEIKALCRHHNDVIYLTLLLKELLSLNFSLMYVMSVFRFCFIGIMFS
SVTSTMFGEAISIAMFTSGATVQLYILCSCVQQLLDASTEITDKAFHENWYLLEPSTKHIFNLIIMANNLECK
IATFKNFNLSLPSFMTILNQSYSIALLFLKMK
>AcepOr175
MTDKWLKTYRTYKRFFRTLLIINGCWYMPTKSGKLMYYWSICVFLSMIIITMISLHMSYIVRHNMKIMVKSGG
VVLSGFGAIIKVLTFKINRDTLINYHLTLNDLFEKELMQNEKVQTMIFSSLRAISILTYIYFVFLIGIIVAYS

KSSYIYLIQNLLYFHLPTNYTLPLSKGFGWFWNVPDNFLYHIHMFYETVLITLSCMMACGVDSAFGFYVVYQIS
STIRAMMFALTNPLSTEKFSDLLRTCAIKHQKLLQCRNTLEHVYGPIIFFHIGSNAVVLCFLIYDFTSLSVLN
FENISVSVTYGGVKLLQTFIYTWYGTYLTNAGEEFRKGIYFSKWLNSNLDCHVRTNVILMMMQKPMTINAVFS
PVNVTMFTSFVNTTMSYFFLLQSM
>AcepOr176
MINKQLKEYRTYQHFLRSLLTITGCWHIPTKFGKSMYYWSVCVLLLMIIYSMLSLRMIYIARHNMRIMMKYVN
FVISGLSTVLKVVSFIINRESLINYHRTLNDLFEEELMQNEKISTIIFSSLRTIYTMAYTYFAILVVMMFAYY
KSSCICVIHNFLHFHSSTNCTPPISGGFGLFSIDSDNFLYYLRLFYEPGLMTLSSIITCAVDSTFGFYVVYQFS
STLRAIISTLMNPLSTEKFSDLLKMCAVKHQKLLQCRNSLEHVYGPIVFWHVVTNAMLICVSMYDFTSLSVFN
FRNLSSTVIYAAIKLLQTYMYAWYGTFLTNAGEDFRKGIYFSKWPNSNLDRHIRTNIILMMIQKPMTVNAIFS
PIDVIMFINMVNAAISYFFLFKSIN
>AcepOr177
DLTEALNLVIWNKRILSFLGLWPLKPNQFLFIFFIIYMIIYCIMGAGHLIKNVDQPEVVIANLTDNILFGMIL
GKMFICKRSCEIMIEFLKSIEIDFTTRMYDNVQEKKAYLYYNKIALLFVKISTSMAGLAATMYYLRTFFENWS
AIVSGNFSYKLPYPVHPFFEIKDTPTYICMCVYLALGIAIIVCGYAGPDAFVLSMTLHVCGQFAVLSCKVNHL
LKDNENYRRHINNIVLRHYHLIRLAEILESNFNIICLQQTLGTLLLLCFTVYHTISTSAYGDETTLLAFALYV
ACVISTILAYCYIGECLIIESTTLREAFYNSDWYNNPPSTSKLINICMVRSEKPLMLTAGKFCVLSLNTFTSI
VKTSMAYVSVLREFI
>AcepOr178
FQDQKDLDRAAKVLSWNKWLMSMLGLWPFQSNDLIFSISFVYFSFLLLILEYLSFFFYISDLELVIMNLTENVA
FLQIFVRMSTLRLYNDEIGEVITEAMKDFDEANYKTAEEIKTFVTYYAKSRIFFKLMMIFVIITALSYYLTPI
LIILGNGGLPEIVTIENVTQIIYLLPYRFHVFYAIENIRMYTITYVWQMPFAFVSACGQSTADCIMVTLVYHI
CGQMSVLALRINNINTEVCDCGPEMRHMMLMHVRLLRMGKIIGKAFSATLLAHLLGATSLVSILGYQMLSNLS
KGETGVVPLLLKFFLFIFLVLLILYAHCLVGENLLTESSKVGEAFYNCRWYDMSKNNARMLILCMARSQKPLC
LIAGKFTMFCLSTLTDVLKTSMGYLSVLRSLL
>AcepOr179
LQNKVDYIEDLFIYLERIFSVGGIWPSERSYVRFAIYITYYIHYFIMAFTEFYNVFGNLELMVMSLVETVAHA
MTFSIVWLIRCSNLLKIIINEIKKDVAERKFENSEEEIIYYNYNRISKIFTYGSNVGMLITISLLYFRPLMYL
VITNQGNTLPLHNSTVSLTLPYRIHSFFDTTNLRTYTLMYLYLFPMIYIAICHMAAICLVVVLVFHICGELAI
LSYRIKHIGENSQIMLVHRIRNIIRMHLKIIWMAKSVDNTFNMILLDELFGNSVVLAISLYYVMMNLDISEIP
TCFTFIFFALTALVMLFGYCLIGDQLAQQCISVQNAYYQCNWYQMPLNFRKYLLICMIRGQVILYLTAGRFYV
FSLNSFTDV
>AcepOr180
FEDQKDLDRAAKVLSWNKRLMSTLGLWPFQSNDLIFSINFGYFTLLLILEYLDFFLYISDFEHVIMNLTENIA
FVQMFVRMSTLRLYNDEIGEVIMEAMKDFDKANYKTTEEIKTFVTYYAKSRIFFKLMMVFVTITASSYYLTPI
LIILGNGGLPEIVTIENVTQIIYLLPYRFHVFYAIENIRTYTITYAWQMPFAFISAFGQSTADCIMITLVFHI
CGQMSVLALRINNIDTELCNCESEMRHVMLMHIRLLRMGKIIGKAFSATLLAHLLGATCLVSILGYQMLTNLS
KGETGVVPLLVKFFIFIFLVLLILYAHCTVGENLLTESAKVCEAFYNCRWYDMSKNNARMLIPCMARSQKPLC
LIAGKFTMFCLSTLTDVLKTSMGYLSVLRSLL
>AcepOr181
PEEHYYKFIRFLLSVSGLWPYQSEWSAHLTRVVISVVMLSSVFVQISSMFTSEITLDFIVDGIPSLLFVLGSL
SNLYSRIIHIDKFRELFERMWQDWALQKTHYEIKIMHEHAEISRLFTLYYIIMMYINLVGYNIWMFIPEILDI
MLPMNESRPRRQPFHTEFFVDEEQYFYFIRFHICLVVTITPIVYAASTTLFLILTQHTCGMCELLGHRAERLF
CVVKDKARYNLIQVSEITCGNISVFVQQHYNIIQFVEIIEICHTVPFLIDLINLVILMSLTLIQIIITISDMER
AIRSIAVFCSVLYYVFIPSYMGQKITDTSTSLCGKIFNSTWYNAVVLKQKTLLIIMMRCYQPLILTACKFYVM
SLQNFGMILQTTISYSMFIRQV
>AcepOr183
MEHPEECYYKLNRFMLLLTGLWPYQSKWSTRLVRAVITIILLSSVIFQLLSFFMSNITANFIIDMIPAFMPVM
GSLSQMYARVGHVDKLRDLFEHIRNDWKLRKTNYEAKIMQKHAETSKLLTLYYLLMQYVAVIGYNAWLFLPDI
LDIISPINESRPRILPFKAEFFIDQERYFNLIRSHTCIVIFILSLIFLAVSTLYVALTQHACGMCELLGYRAE
RLFYVVEDAVEYDSIRPEINYRNMAVFVRLHCNIIQFVDMIEFYHTIPFLMDIIELVLTMSLALTQMLTITDD
MERVVRSISVTILTLIHLFVSNYMGQKVTDASSNACEKVYNSVWYSASVSEQKLLLLIMKRRFHPLILTACRF
YMMSLQNFGMILQTAISYCMFMRQL
>AcepOr184
MTRNKEYRSVNITRLFMKVVGLWYVETPKEKLLLRVVFGYTVWAIVFAILVEGVDLYHCIGDFYAVTSNLCAT
LLLIMILVKLGSFMFYRDMIMDLIHFAEKNFWGVTPYDEASAQILEGYDKLGMTMIYTFTFMVYIATFNYIFA
PFFEPQEKNETEKVLPFKLWIDFPYHSPYYEITYTIQSLSTMHSGICTFCFDNFVSTFNIHAAAQLKILAHKV
EVIAEHCIGDTMDQKCPLETDVTVLTFKKLRDCVKQHLTLICYVRNMQRVFAIILLGQLLLSSVVICFGGFQF

LATDVIIRKCIFAFHFVGGLIQLLIYTWTCNDIIVQSTAISDAAYNSKWYLLPDNDLGRAVKRGLITIMIRAR
RPCILTAGNFAVMSLDTFMGILSTAMSYFTLLRQMSEDDV
>AcepOr185
MMKNVKKDKFLSIRLVRFLMRVTGFWPAESKTEKRLLNGILSYTICISSISLWLEATEIYLGKGDFYALTYTA
CSSMPVVIIMLKIIFFLRYRKELLNMLKYTQDNFWYAQYDEYGSKILEKINKKGLILMCTFIFFVQGTVCTYI
LTPILENIGKNESDRILPFNVWIGTISTIVSPNFEIMFTVEISALIHCGICFCCFDNLLGLLNLHTAGQFKIL
QHRLRSILMRVERTGSVRLFNEKQKQKVYEKLRECVILHHELIWYSEKMEQIFMYSTLCQLLVSGIMLCVAGF
QVFLARGTLIRRLIFIAHTNGCFVQLFVVTLTATDLMNESRAVGDAAYNANWQVLSHEENRGVRTAVLMIMMR
SARACSISAGGFFPVSLETFMAVLSTAASYFTLLRKFVE
>AcepOr186
MICIETQYLSLNRILLLAIGLWPYRQSKITQFQFVFFSVILMTSIIFQLTTFITTNLTSDVIVTILASASIFT
IFMIKYYTFRVNIELVKKSFMKLQHICNELKDKNEIAIIEKYGYIAKCYTAALTIIGIFLIFISIIIQFWRIF
DIGLSTNISQPSRVFFVTEYFIDQEKYYLLILLHINIALFIGGAAMVATGTMLIAYFKFICGMFKISSYRIER
AVNINIMQNINIKNKILRFEDLICAIDIHRQAIKLSKNLLSYFEIMFFCLAQIFVVSLCFNFVRIFQIILTEK
AINEALLPMLYAAIFILYLFISNSIGQNMTDHNNYVFATAYRVEWYIAPLHIQKIIFFLLIKGAKNFHLNLGG
LFIPSLECFTTLVKASVSYFTVILSTQ
>AcepOr187
MIHLEIQYFTINRILLLVIGLWPYQQSKFTRFQFIFISTILTAGIVFQLTTLMTSKCTSNLIRKVLSSTSFFI
FCIISYNSFCINIKAMKDLLMQLEHICNQIKDKNEVAIINEYNYTARRYTIILTVLVIGLGFFCFIGQYLSNC
LTVLPKNISHLPIMMEYFIDQEKYFYLILLHSYAIICIGIVVILATGTMLLTYLQYVCGMFRIASYRIEHAIN
FNIRQNISQKIKILMSEDIICAVDTHRRTMKLSKHLMSTWEVTIFCLIVLGVACLTLNLFQIFQIASSENTVS
KFLSLLYVISMIFCMFLSNYIGQNITDYNNYVFSTAYNVQWYRAPLRIQRMIMFLLQREAKEFTLNVGGVFNA
SMECFAMLVKTSISYFTVIYSI
>AcepOr188
EIQYFSFNRILLLVIGLWPYQQSKFTRFQFILFSIILITGIIFQLTTLITSKCTLNLVSKVLSSTSFFVVCII
NYNSFSFNIEAVKDLLMQLEHVCNQVKDKNEVAIIEEYNYTARRDTNMLTLFGVIAVFICTIDQHLLNSFIVL
PKNISHLPIMVEYFIDQEKYFYLILLHFYAITCVGGNVILAIGTLLLTYFQHICGIFKIAGYRIEHAININIR
QNITPKNEILMNEGIICAIDIHRQAMKLSKHLMSIFEVMMFCLIIVGVSCLTLNLFQSASSENTINEFCFPML
CVTVSIIYMFVSNHIGQNIIDHNNYVFSTAYNVQWYRAPLRIQRMILFLLQRETKMFTLNVGGLFNASMECFA
TLVKTSVSYFTVIYS
>AcepOr189
MVHLGIQYLSFNRILLLVMGLWPYQQSKFAQFQFIFFSTILISGIIFQFTTLITSKCTSNLINKVLSSTSYFI
FCMICYDSFSVNIKAVKDILMQLEHICNQVKDKNEVAIIEEYNYIARRHTIILTIFCTSGSICIIGQHLSNNF
IVLPKNISRLPIMMEYFIDQEKYFYLILLHTYAIICVGGTAILAIGAVILTCVQYVCGMFRIASYRIEHAINI
NSRQNITLKNEILMSEGIICAVEIHRQAMKLSNLFVSIFEIMMFCLIVFGVACLSLNLFQVSFLASSEKTVSE
FCLPLLYVVTMILCLFLGNYGAQNITDHNNYVFSTAYNIQWYRAPLNIQRMILFLLQRKAKEFTLNIGGLFTG
SMECFATLVKTSISYFTVIYS
>AcepOr190
MIHPEIQYFSFNKILLLVIGLWPYQQSKFTRFQFILMSTILTAGIVFQLTTLITSKCTSNLINKVLSSTSFFI
ICIINYNSFCFNIKDIKDLLMQLEYICNQVKNKNEIAIIKEYNYTARRHTIILTILAVIIGFFCLIGQYLSNK
LTVLSKNISHLPIMMEYFIDQEKYFYLILLHTYAIICIGIVAVLATGTMFFTYLQYICGIFKIASYRIEHAIN
INIQQNIMQKTKILMSEGIICAVDIHRQAMKLTKHLMSIVEVMILCLIVFNVICLTLNTFQIFQISSSENIMN
EFCLPLMYAISIILYLFLTNNIGQNIINHNNYVFSTAYNVQWYRAPLRIQRMILFLLQRETKVFTLNVGGLFN
ASMECFATLIKTSVSYFTVIYS
>AcepOr191
MVHLRIQFFSFNRILLLVIGLWPYQQSKFTRFQFIFFSTILTAGIIFQFTTLMTSKCTLNLVSKVLSSTSFFV
VCIIYYNSFNFNIKAVFAVIIVFICTIGQHLWNSFIILSKNISHLPIMVEYFIDQEKYFYLILLHFYAITYIG
VFAILAIGTLLLTYLQYICGIFKIAGYRIEHAININIRQNITPKNEILMNEGIICAIDIHRQAMKLSKHLMSI
FEIMMFCLIIVDVSCLTLNLFQVSFLSSENIINEFCSPLMYAVFIILHMFLSNNIGQNIIDHNNYVFSTAYNV
QWYRAPLRIQRMILFLLQRETKMFILIVGGLFNASMECFATLVKTSVSYFTVIYSTR
>AcepOr192
MVHLGIQYFSFNRILLLVIGLWPYQQSKFTRFQFILFSNILTAGIIFQLTTLMTSKCTLNLVSKVLSSTFTFI
ICLISYNSFSFNIEAVKDLLMQLEYVCNQVKDKNEVAIIEEYNYFARRDTIMLTLFGVIIVFIYTIGQHLLNN
FIVLPKNISHLPIMMEYFIDQEKYFYLILLHFYAIICVGGIAVLAIGTLFLTYLQYICGIFKIAGYRIEHAIN
INIRQNITPKNKNLMNEGIICAVEIHRQAMKLSNLLVSIFEVLAFCLIVIVVACLSLNLFQVSFLASSKKSVS
EFCLPLLNVIATILCLFIGNYGAQIITDHNNYVFSTVYNVQWYRTPLHIQRIILFLLQRKAKEFTLNIGGLFT
GSMECFAMLVKTSISYFTVIYS
>AcepOr193

EIQYFSFNRILLLVIGLWPHQQSKFTRFQFILISTILTAGIVFQLTTLITSKCTSNLISKVLSSTSFFIVCIT
SYNLFCFNIKAVKDLLMQLEYICNQIKNKNEIAIIKEYNYIARRHTIMLTILAVIVGSFCLTGQYLSNDLTVL
PKNISHLPIMMEYFIDQEKYFYLILLHSYAIICIGIVAILATGTMFLTYLQYICGIFKIASYRIEHAININIQ
QNITQKTKILMSEGIICAVDIHRQAMKLTKHLMSIVEVMMFCLTVFGVICLTLNLFQISSSENIINKFYLPLM
YAVSIILYMFLSNNIGQNIIDHNNYVLSTAYNVQWYRAPLCIQRMILFLLQRETKVFTLNLGGVFNASMESFG
MLVKTSVSYFTVIYS
>AcepOr194
MIRLEIQYFNLNKILLLAIGLWPYQQSILTRFQFVFLSSILTASIIFQLTSFITLRCTFDLIAKVVSSVSFFT
VYLINYSSFYINIKNVKNLLMQLQHMYDELKDEKEIAIIKKYGYNAKYYTTLLTAAGICGIIVSIIFQCWTNM
FDISSSINVSQPHRIQFITEYFVDQEKYFFLILLHLNVALYIGIITTIAAGTMLIGYLQFIFGMFKISCYRIE
RAIKIFTSQNITLKNKISKSENLICAVDIHREAMRLSKYLVTSFEIMFFSLTGVFIISLCFNFLRIFQIASSG
EAVKEAFLPVTFIFVNIMYLFIYNFFGQNIIDHNSQVFITAYSVQWYVAPLHIQRMILFLLLKGAKDFTIIIG
KIFISSIECFATLIKASISYFTVVLSMQ
>AcepOr195
LNRILLLIIGLWPHQQSKFVQVQFIFFFAILSTGIIFQLTPLIILKCTSELVTKVLSSVSFFTLFIIQYIAVH
LNIDVVKNLLVELQHIFNDLRDENEIGIVEKYSCVAKRYTIVLTAIVLCTIFAIILAQFWSNITDINESTNTS
QHHLLIMTKYFVNQEKYFYLILLHMYAVLCIGITIMLGIGTMVITYIEHICGMFRIASYRFDHAVNINILHNS
TIKNMILMTEGIIYAIDIHRQAMKLSKHVVSTFELTILCFTGCVVACFSLNVFQLFQITSSENNINEFLLPCI
YAFISILYMFIANYVGQNIIDHNKHIFVTAYNVQWYRTPLHTQRLILFLLQRGVQEFSLNIGGVLDASIEGFA
TLVKTAISYFTVMHSVQ
>AcepOr197
EYSNLNRILLLAIGLWPYQQSKFTRFQCIFLYTLLWTGIIFQLTPLITFKRTLNAIIKMLSSVSFYIMFIIQY
SAFCLKIKVVKNLLMQLEYIHNKLKDNNEIIIVYKYSYIAKRYTVILTAFGICALFIIIYQIWYVAKVDVSMN
TSQSYHIFCTTVIEYFVDQEKYFYWILLHMYTVICTGSIIMLGIGTMLITYIEHICGILKIASYRIEHAVNID
IPRNYDKQKILMIKGIISAVDIHRQAMKMTKYFMTTFEIMISCFTGCVVICFSFDLHQFFQIVSSENNKTNVS
ESLLSFIFASVCIIYMFIANYIGQNIIDHNNHVFITAYNVQWYKTPLYIQRMILFLLQRKAKEFSLNIGGMFD
ASMEGFATLIKASVSYFTVMHSTQ
>AcepOr198
MIYPEIHNNLNKILLLAIGLWPYQQSKFTQLQFIFFCAILSAGIIFQLTPLIILKCTIDLVIKVLSSVSFFIL
CLIKYNAFHLNIEIVKNIFMELRYILNELKDKNEIAIVYKYSCTAKRYTVTLIVLGICGTFSLIIAQIWSFIA
DVNAMNTSQLHHLSFMTEYLVDQEKYFYWILLYMYTVLCIGQIITLGIGTMLITYIEHICGIFKIASYRIKHA
LNIDIPQNIMIKNKILMMEGIIYVVDIHRYAMKLSKHFMTTFEIMMSFITGCVVVCFSFNMFQIASSENNKNN
VIEFLLPFLLAFTSILYMFISHYFGQKIINHNNHVFVTTYNIQWYKTPLHIQRMILFLLQRRTKKFSLNLGGI
FDASIESFATLIKASVSYFTVLNS
>AcepOr199
MIHPETQYNNLNKILLLAIGLWPYQQSKFTQFQFIFFCTVLSAGIIFQLTPLIILKCTLDLVIKVLSSVSLFI
LCMIQYNAFYLNIEIVKNLLAELRYIFNELKDKNEIAIIYKYSCIAKRYTITLTIFGVCGIFIIIVIQIWSFI
ADVNTMNTSQSHHLLIMSEYFIDQEKYFYWILLHMYGELCVATAIMLATGSMFVSYLLHTCGMFRIASYRINH
AVNINTIQNITLKNKVFMTKSIICAIDIHRQAMKLSKHLVSSFEITLLCFTGGVVVFFTLNLYRIATSISNFN
EFFLLIMFMSVSIIYMFLANYMGQIIIDHNNHVFLTAYNVQWYRTPLHVQKIILFLLQRDTKKFTLNIGGIIE
GSVEHFASLVKASVSYFTVIYS
>AcepOr202
MICLETKHFRLNRFLLLAIGLWPHQRSKLAQIQFIVLFTILTTFIVFQFTTFLTLKCTADLIINVLSSAFFFI
CYAIKYHSFWINTHTMRVSLEQLQHVCNEIKNKNEIAIIEKYGHIGKHQTRIITIFAACSISIYLFLPIWPRL
FDVILAINNSQSRSAAIYIATEYFVDQENFSYLILLHTNAAFCIGATAMVATGTMLIAYLKHICGMFSIASFR
IKKALKINMQQDVNEEKMIIIYKGIICAIDIHRKATEFSQVFIKSFEGSFFCLIAAGMVCLTSTLVQIISYNS
TEQLLLQLVCISILYIYMFLSNYIAQDITDHNEYVFATVYNVEWYVAPLHIQKMMLFLLQKGTKAFHLILGGI
FIASMESAATLMGTSISYFTVLYST
>AcepOr206
MICLETKHFRLNRFLLLAIGLWPQQRSKFAQIQFIVLFTILTTFIVCQFATFLTLKCTADLIMNVLSSAFFFT
CYAIKYYSFWINTNIMRVSLEQLQRVCNEIKNKNEIAIIEKYGRIGKHQTRIITIFAACGVPIYLFVPIWPRI
LDVILTINNSQSRSAALYTTTEYFVDQENFSFLILLHTNAASCIGATAMVATGTMLIAYLKHICGMFSIASFR
IKKAMTINMQQDVNEEEMIITYKGIICAIDIHRKATEFSQVFIKSFEGSFFCLIAAGMICISSTLVQIITYNS
TEQLVLRLRYISILYIYMFLSIYIAQDITDHNEYVFATVYNVKWYMAPLHIQKMTLFLLQKGTKAFHLILGGI
FVASMESAATV
>AcepOr207
MICLETKHFRLNRFLLLAIGLWPHQRSKLAQIQFIVLFTILTTFIVFQFTTFLTSKCTADLIINVLSSAFFFI
CYAIKYYSFWMNANTMRVSLEQLQRVCNEIKNKNEITIIEKYGRIGKHQTRIITIFAACGIFIYLFVPIWPRI

FDIILAINSSRSRSAAIYTATEYFVDQENFSYLIFLHTNAASCIGATAMVATGTMFIAYLKHICGMFSIASFR
IKKAMAINMQHEEKMNEEKMVIIYKGLICAIDIHRKAIEFSQVIIKSFEGSFFCLIVASMICLTSTLVQIITY
NSTEQLVLRLIYISVLYLYMFLSNYTAQDITDHNEYVFATVYNVEWYVAPLHIKKMMLFLLQKGTKTFHLILG
GIFVASMESAATLMGTSISYFTVLYST
>AcepOr210
FIETQYFSLNRILLLAIGLWPYKQSKLIQLQLILFFGILTSCIIVQLMTFLTAEYTLNFVIKIFSYISFLILF
IIKYNSFWINTRTIRYLLEQLQHIYNEIKDENEIAIIEKYGLNAKHYTIRIIQITLCCALAIIFVPMWPCILD
IVMPMNESRPHPLIPIMTEYFVNQEKYFYLILLHIDVATCIGAIAYIATGTMLIGYLKHACGMFKIASYRIEQ
AMMNKILKKNVKNEITIYKAMIHAVDIHRKAMKYAEFLISNFEGSFLFLITVSVCCLSLNLFRIFQSITFGNS
KEEFLLHFTIAGVILLYMFLANYAGQEITDHNNHVFSTAYNIRWYTAPLSIQKLILFLLQRGSKTFSLNVGGL
FQASLKCFASLTSTSISYFTVIYSTQQ
>AcepOr212
MIDKRLQTYRTYQRFTRILLTICGCWYMPTKSDKSMHYYSICVLLTMIMMTMVTLHTSYIHRHNMGNMMKNVG
FAITGLSAILKVVSFTINRGSLINYHRILNDLFEEELMRNDKIRTIIFSSLHTMYILTYGYFALATTLLLLYF
APSYLLIIRGLLHFHLSTNYTLPISRGYGHFWTVPDNFLYHLHLVFETTLTGLSGLMACSVDSFFGFYVYQFT
STMRAMNFRLTNPLPTEKFLDLLRMCVAKHQRLLRCRDTLEHVYGPIVFWHIVTNAILLCGLMYDAMPFSDFK
RISMFLTYAVVKFVQTFTYAWYGTVLTNASEDFRNGIYFGEWFNSSLDHHVRTNVILIMMQKPITINAIYSPV
NITIFTNFVNATMSYFFLLQSIGDKDG
>AcepOr213
MEKLIAFLKVDLLFACCWPLPPTATKCEKIRNKIFRYFSILHGMIMMIAILYTIYSNRSDLFLIMKLCCELCT
TTEVPLQIICFSIQYDRLQYVIYELEDFCKRAKPEERNIFHRYINSCKSIYIGSICAFTVTGLLLIISPIVEP
HPFPIDIEYPFSVDYQPLKIIIYLHHILLIYQSYAQVCSNIFIALLLWFVSARCDILSNRFRAVTKFTELRAC
IEEHQELLWYGRKVTLSIRYVMLASLAVSTIVIIFTGCTFLSRQPMSVKSTFLIFFISSLAKVYLCAWPADHL
LSASTDIAHAVYDSIWYKGKVEFQKNFLHTLLRAQQPIAVNVPCMLPTVSLHYYASYVSTAFSYLATFRIILE
ESDE
>AcepOr214
MEGKVTLDRVIAFLKIYLIFACCWPLSPNATKSQRLLRSAFQCFCVTNSILIAIAAVWTLCNHSDDMLLVMKL
GCQLSAIIQVPLQLILFAMQKKRLQFIILEMETCYQRAQEYEKKIFQQYIDKCKLFYGIILCWLAMTGISVIL
TPLFSSQLFPCETEYPFDVQLQPLKTIIYVHQILIAYQSVIQVSTNTFPALLLWFVAARFEILSVQFRTMTSM
KELVNYTRKHSLLLRYAKEVSHAIRYIALLCVTFSTGAVIFGYLTFMSQQPWTVKWTFLMIAFCGFVELYMYA
WPADNVISTSSGVASAVYDSLWYDNNLAMQKILIHVILRSQRPVTISIPCALPNLSMNYYASYISTIFSYMAF
VRIMIGQE
>AcepOr216
QDCPVNDYYTINRSLLLCIGLWPYQKSTFRYIAITFTTIMLVSFVAFQLMTFITEYNVDILLHVLAYSIPWLG
YMLKYSIVCLNIKRMRDLMKRILCDWNELNNLQEIEIIKKYAEIGRFITLIATLFIYVSFFCIILVQLSNFFF
NTTSDKNESHLRQLPILVECFIDQQKYFFLILFLIYFVAICGLTMVVATETINMSYVHHACGLFEIASCRIEQ
TLFKNTIQNAIHIPFSSEQSSIMCQGVINGFNMYKKAIEFIEMLKRNYIWEYSLLLPLGVLSLSINLYRLSQL
ITSKKYYEMIVSFLFVSGHFWHMFFCNYLGQKVIDHSSDVFHRVYNVQWYVAPLRTQKLLLLMMQRSMRHCTI
VINGLFIPSFEGFATLASMSISYFAVI
>AcepOr217
QDCPVNDYYTINRSLLLCIGLWPYQKSAFRYIAIAFTMIMLVSFVAFQLMTFITEYNVDILLHVLAYSIPWLG
YTAKYSVVCLNINRTRDLMKRILCDWNELNNLQEIEIIKKYAEIGRFITLIATLVVYVSCFCVILIQFLSNFF
FNTTSNKNESRPRQLPILVECFIDQQKYFFLILILICFAGLCSFTMLVATETVNMSYVHHAYGLFEIASYRIE
QTLFKNTIQGIASSSEHSSIICQGEINGFNMYRKATEFIEMLKNNYIWEYSLLLPLGVLSLSINLYRLSQLIT
FKKYYEMIVSFLFVFGHFGNMFFYNYLGQKVIDQSSDVFHRIYNVQWYLAPLRTQKLLLLMMQRSMRHCTIVI
NGLFIPSFEGFATEVSTSISYFVVIFSLF
>AcepOr218
QDCPVNDYYTINRSLLLCVGLWSYQKSTFRYVAIAFTMIMLVSFVIFQLTTFITTEYNVHILLHVLAYSIPWL
GYMSKYSIVCLNIRWMRDLMKRILCDWNELNNLQEIKIIKKYADIGRFITLIATLFVYISIFCIILVQFLSNF
FFNTTSDKNESHLQQLPILVECFIDQQKYFLLILILLCFIVICSLTMVIATETVNMSYVHHACGLFEIASYRI
EQTLLKNMIQDAILASSSEQSSIMCQGIINGFNMYRRAIEFIEMLKRNFIQYSLLLPLGVLSLSINLYRFSQL
ITSKKYYEMIVSFVFISGHFWYMFFCNYVGQKIMDHSSDVFHRIYNVQWYVAPLRTQKLLLLMMQRSMRHCTI
VINGLFIPSFEGFATLVSMSFSYFAVIFSLY
>AcepOr219
QNCPVNSYYTINRSLLLCIGLWPCQKSIFSYITIMFTTIMLVSSVILQLMTFITTEYNVNIFLQVLAYSVPWL
GHTLKYNVLCLNIRQMRDLMKRLLSDWNELNNLQEIKIIKEYADIGRFITLIATLFIYISIFGFILIQFLPNF
FFNTTSDKNVSQPRQLPILIECFIDQQKYFFQILFFICFAIIYGFTMIIATETINMSYTCHAYGLFEIASYRI
EQTLFENTIQGIASSSEKSSITMCQGVINGFNMYRKAIEFIEMLKRNYTWAYSLLLPLGVLSLSINLYRLSWL

IVSKKYYEMIVSFLFVFGHFGHMFFYNYLGQKVIDHSSDVFHRIYNVQWYIAPLKAQKLLLLMMQRSMRHCTI
VVNGLFIPSFEGFATLVSTSISYFAIIFSLF
>AcepOr220
CTEMTCTADSYYIINRNLLLCIGLWPYQRFGFRCILITLMIIILTSSTVFQFATLFTREYNMELLLKILAYSI
PWLSYVLRYNIHWLNMKKMRELIERIRLDWNELNNAHELEIIKKYSAIGRLITFFTILFVYLAVFSFILIQLL
INFVLDITTATNESRIRHLSAEIEVFIDQQKYFTPLLLYLFLIVLCGITTVIAMETLCMSYTQHACGLFEIAN
YRIKQALHKDMIRDVISVTEKNSIICQEIISAVDIHRKAIEFIEMTKVNFEWMYFIAIPFAVLTLSINLYRLS
RLITTEAYGDLITTLLFVLGHFWYLFFCNYIGQEVIDHSSDIFYKTYNIQWYIAPLKVQKLLLFVMRKSMRCC
TLMIGGLFIPSLEGFATLISMSLSYFMIIYSIQ
>AcepOr221
KMKSFGKHYCRIQQILLIIILGLWPYNNTLYRCIQRIFISTLILFSILLQIGKIITSASNLDNLLQILSFAVPC
LVVTTKYATFCIKSEDMKKLIDEINYHWNSIKCKEELKILQQYSYTTKLLTIMAIGACFLYLNLCLFLMVQIL
PKFLDIMIPLNESRPLKLLGLATFFFDQEKYFIPIFIHMTIALLVEATTILGTETMSLVYIQHACGLFKITSL
RLKHAFDCEIQISAEKHKIYYTNIINAVISHNRAIEFFDFFNSCLEISFFFLFLLGTSSLSINMFRLFQAGLK
KEIEEFFLVAIFVIIHFIYMFTINLLMQMLTNCSADIFYKICELPWYSVPIPLQKLLQFMMQRSVKNSKFIMC
SVFSGSLESFASFVSCSLSYFTIFLSI
>AcepOr222
MDLLEERYYGLNRNLLSAIGLWPYDNIKIRRLRYALSLLIIISLPGTQLIKLYISEYSSNLLLKILSYNIIFI
ICCIKYITFYAVYKNIIEFQERIQNNWNALKDNQEIKIICKYGNNGKLLTIFITIFVYTFSLFYIFTQYVSIL
LDIVVPLNVSRPRELLFLAEYFIDHEKHFYIITIYIAIALLVAMTCIIATESFSLTNALHAFGLFKIASYRMK
HILSEINPHMCLTKKHTIYRNRIIGAVDFHRRAIEFSELLKASFGQVYLIIFVMGICSASINLFNFFRILMTE
KEILEIIKSVFLISVHIVYFTLSNYAGQEFINSDTHFYRMICNTKWYNVPLKIQKLILFLIQKTTKCYKVDAG
GMFNPCLEGLATSLNMSVSYFMVLYSV
>AcepOr225
MDFAGERYYKLNKILLICFGLWPYQTRTLKKIQIIFFQTLFISFLICQCNTFLIKKYSMDHILKTLLFIIMTC
IFIVQYNACLLLTDNIKYIFDRVRYDWNILKAQTELEMIRKYASYAKLFTIGYAFLIISSSLGFLVIICLPYI
LNVIIPMNESRPRQTLILVEYFVDEETYFIAILIHIIIAIHAGNITVMSVASLHIAYALHTCAMFRIASYRIE
NIFNENIQQMPKDIKQYILYNKLVQAIYVHRRAIDLANILTNSFATLYFVLLGLTVALMSVSFLNVSFHNAVI
SLEKVDLLIFGTYISIELYYVFLANYIGQDVIDSSISIYQATCNAHWYAAPLCMQKLILFIMQRSCRKSALTA
GGLFDASLEGFAKVL
>AcepOr227
MISFVDQYYNINQKLLTIVGLWPYQKWRFRIFQIILFPSIFISFLIAQLATFITSEFNVNLMLKVLSQAFPCL
IYFLSYISFLLNNKDIKIFFEWIRYEWSTLKTTDEINILRKYADFSKRLIVIMTVALILFLNFFGLFQFLPNF
LDIIVPLNESRERHFSFLAEYFVDQKRYFYPILTHNLLSVYTGATTVFCTGTMLIGFVFHICAMLQIASYRLK
HISDNIPLVSKSDKDYIIRKRIINAVDIHRRALEFAEFILSSFANFYFILIGIGISSLTVNMFQFLQLMMFTN
DTNGILACGLLIIAHFVYMFFGNLAGQIVTDHNINILNTTYTVQWYTISLQAQKLLFFIMQRNTKNYYFVIGG
IFAASLEGFATVM
>AcepOr228
MRLVEEHYYTINKTFLKVLGIWPHDKSRLVFFQRVLFLTLVVTYIVLQLSVFVTTKYNMILFIKTMSLAFPSI
IITMKYCVFIFKSKTIEILHHYIQEDWKIIRNKLEYDIIEKHAHDCRNYAIFVICKNSIWNIGLLMIALIIIQ
FFPVILNFVSPLDKPRPCKPLITAEYFIFHDNHFYIKVFHALIIITLCASIMVATATQLLLFTYHSFGMFKIA
SHRIEHFIDDHVLCMSNCEKERVICKRIVDVVIAHRRATEFSNTFISSLNVPFCVIITFGVFSLSINLFRFVQ
AVTILKNMEDILISFFAILGHLIYMFVANYLGQKAIDCNNEIFRLIYGASWYLMPVTSQKLILFLMTRIGKDF
HYMIGFIFVAKIENFAMLVNTALSYVAVMYTI
>AcepOr229
MSQYSYKTYALQERHYKIYQIILKTLGLWPYEQTYLTWLHKMLFAGILLTFLLVQLLIFITNQYSVNLLLKVF
SFFIPLTFIVIKYIVFIIQADDLKILLEQIHNDWNLLKDKLEIDIIEKYACNMRLFTIVMLAYCYISTLLCAL
FELLPLIFNIVLPLNESRPFHLLVLTEYFVNQERYIHYLLLHEILTAYIAITALWGICVIIVMFIMHACALFK
IASFRIENVIERNILMIPSPKREYLLSQKIIHAVVMHRRAIEYTDFVISTYTTLLAILIVIGISSLSINLFQF
FQLIKFTTDIRIISIAIIFIIYHLIYMFILNYGGQELMNHGLHFFKVSYNGLWYAAPLHTQKLLLFIMQRGTI
NTVLVCGRIFVASLEGFASLTSTAVSYFTVIYST
>AcepOr230
RYYNIYRIMLTAIGLWPYQTPIVMQVQSVFFLGAYCFTLLFQFTTFLTTTCNLEFIFKEFSYIFVTILYIVNY
NSYYFNSKEVKYLLKQVKVDWNSIEDNNEIKILKKYAIENRLVSLIFIFIAAFSLCFFIIVELIPVILDAAMP
MNTSRLRKVKIDFEFFIDEKQYFYIYFIYEIITLLIGMLTVLATGLLSLAFLRHCCATFKIASNVIENTVTKH
TLQIPAYRKTRVMYQKINRAVHIHRKSIEFSTFFMNIMNKWYSAVMLISVISLSCSLFRLLNALTISNEISEI
TMCIGMVGAHFIVLFAPNFVGQILTDHSAEIFNAAYNTLWYLAPLSIQKLLLFLMQNSLKTHTLIIGHFYVTS
LEGFSTLITTAISYFTVIYT

>AcepOr232
YYNIYRIMLTAIGLWPYQTPIITQMLSVFFLSAYCFILLFQFTTFLTTTCNLEFIFKELSYIFITLLYIVNYH
SYYFNSKEVKYLLKQIKLDWNSIENNSEIRILEKYAIENRLMSLILSFIIAFAIFLIITIELIPVVLDIIAPM
NKSRLRKIKMDFEFFIDEEQYFYIYLIHEVILMLIGMFTILATGTISLAFLSHCCATFKIASNIIENTVTKRT
LHIPAYRKTHVMYKKIIRAVHIHRKSIQFSTFFVNIMSKWHFAIVVISVISLSCNLFRLFNALTVSNEISEIT
ICFGMVVAHFLFIFAPNFIGQLIIDHCAEIFNAAYNTLWYIAPLSIQRLLLFVMQNSLKSHNLTIGHVYISSL
QGFSTLITTAISYFTVIYT
>AcepOr233
LFHICVLQFTIFLRTTCNLEFTFKELPFICFTLLSITTYNSCYFNSKEIKYLLEQIKLDWNSFEDESEIRILQ
KYAIEYRLVSLIVGFFNAFCILLVIIIELTPVILDAVVRMNNSQPRQTKINFELFIDQEQYFYIYLIYEIIAI
LIGLLTITGTGTLSLAFFRHCCATFKIASNIIENTVTKHTLQIPAYRRTHVMYQKIIRAVHIHRKSAQFSTFF
LNIMNKWYFALLIISVISLSCSLFRLLNALILLNEFFEIIFCITLVEYHFIMIFLTNFVGQSITDHYAEIFNA
ACNTLWYLAPLSMQKLLLFVMQNSLKAYTVTIGHIYVKSLEGFSARITTAISYFTVIYT
>AcepOr234
YYNIYRIMLTAIGLWPYQTSIIMQVQTMFFLGAYYFILLSQFTTFLRATCNLEFTFKELPCIFITMFCTITYN
SYYFNSKEIKYLLEQIKFDWNSFEGENEIRILEKYATENRLVLILGFFSALGMFSIIIIDLTPVVLDVIVPMN
KSRPRKIRMDFEFFVDQEQYFYIYLIHEIMTLLIEVLTVLAIGTLSLAFFRHCCATFKIASNIIGNTVTKHTL
QIPAYRRTHVMYQKIIRAVHIHRKSAQFSTFFLNIMNKWYFALTIISVISLSCSLFRLLNALILLNEFSDITL
CIAIVECHFVVMFVINFVGQSVIDHYAEVFNAAYNTLWYLAPLSMQKLFLFIMQNSLKTYTLTIGHIYVTSLE
GFSTLITTAMSYFTVIYT
>AcepOr235
LFHIYVLQFMIFLRTAYNLEFIFKELPLICFTLLDIIIYNSYYFNSKEIKYLLEQIKLDWKSFEDENEIRILQ
KYASENRFVSLILCFFVAYCIFFAIIIDLTPIILDAVVPMNKSRPRQIIMNFELFIDKEQYFYIYLIQEIVLF
MIGLFTFLGIGTLSLAFFRHCCATSKIASNIIENTVTKHTLQIPAYQRTYVMYQKIIHTVQIHRKSAQFSMFF
LNIMNKWFFAIMIISVISLSCSFFRLLNALILLNEFSEIILCIGLIECHFMVMFVINFVGQSVIDHYAEIFNA
AYNTMWYLAPLSMQKLLLFVLQNSLKPYTLTIGYIYVTSLEGFSTLITTAISYFSVIYTM
>AcepOr236
YYNIYRIMLTAIGLWPYQTSIIMQVQTMFFLGAYYFILLSQFTTFLKTTCNLEFTFKELPCIFIYMFCAVTYN
SYYFNSKEMKYLLKQIKLDWNSFEDENEIRILEKYAIENRLVLLILGFFSAFSIFLIVIIELTPVVLDIVIPM
NKSRPRKIRMDFEFFIDQEQYFYIYLIHEIMVMLIGVLTVLATGTLSLAFFRHCCATFKIASNIIRNTVTEHT
LQVPIYRRTHVMYQKITRAIHIHRKSAQFSTFFLNIMNKWYFALVIISVISLSCSLFRLLNALILLNELSEIT
LCIGIVGWHFVVMFVTNFVGQSIIDHYAKIFNAAYNTLWYLAPLSTQKLLLFVMQNSLKTYTLTVGHIYVTSL
EGFSTLITTAISYFTVIYT
>AcepOr238
YYNIYRIMLTAIGLWPYQTPIIMQVQTVLFLSAYYFVLLSQFTIFLRTTCNLEFTFKQLPCIFMTICGIIMHN
SYYFNSKEMKYLLEQIKLDWNSFEDESEIRILEKYAIENRLVSLILVCIALIIILFTAIVELTPVVLDAVVPMN
KSRPRQIRIDFEFFIDQEQYFYIYLIYEIIAMLIGLLTVTGTGTLSLAFFRHCCATFKIASNIIENTVTKHTL
QIPAYRRTHVMYQKIIRAVHIHRKSAQFSTFFLNIMNKWCFALLIISVISLSCSLFRLFNALTLLNELSEISF
CIFIITCHIVVMFVPNFVGQSVIDHYAEIFNAANNTMWYLAPLSMQKLLLFVMQNSLKSYTLTIGHIYVTSLE
GFSTLVTTAVSYFTVIYS
>AcepOr239
YYNIYRFMLTAIGLWPYQTSVIMQVQTVFFLGAYYFILLFQFMTFLTSTCNLEFIFRELSYIFMTMFGIITYN
SCYFNSKEMKYLLKQIKLDWNSIEDESEIRILEKYAIENRLVSLILSFIVAFAMFLIVLIELTPIVLDAVIPM
NKSRPRQIKMDFELFIDKEQYFYIYLIQEIVLIMIEMLTVLATGTLSLAFFRHCCATFKIASNIIENTVTKYT
LQIPAYRKTHVIYQKIIRAVHIHRKSAQFSTFFLNIMNKWYFALVIISVMSLSCNLFRLLNALILLNELSEIT
LCIGIVGCHFVVMFVTNFVGQSVIDHYAEIFNAAYNTMWYLAPLSMQKLLLFVMQNSLKAYTLTIGHIYVTSL
EGFSTLITTAISYFTVIYT
>AcepOr240
YYNIYRIMLTAIGLWPYQTPIIMQVQTVLFLGTHYFILLSQFMIFLRTTCNLEFIFKELPCIFMSLLDIIIYN
SCYFNSKEIKYLLEQIKLDWNLFEDENEIKILEKYIIENRLVLLFLGFLDAFYIFLIIIIELTPVILDAVVPM
NKSRPRQTKINFELFIDKEQYFYIYLIQEIVLLMIGSFTFLGIGTLSLAFFRHCCATFKIASNIIKNTVTKHT
LQIRAYQGTHVMYQEIIRAVYIHRKSVQFSTFFLNIMNKYFFALMTISVISLSCSLFQLFNALTVSHEVSEIA
VGIIMVTAHFMLMFLFNFVGQSLIDHSAEIFDAAYSTLWYLAPLSIQKLLLFVMQNSLKTHTLTLGYIYVASL
EGFSTLVTTAISYFTVIYT
>AcepOr241
YYNIYRIMLTAIGLWPYQIPIVMQVQSVFFLSAYYFILLFQFTTFLTTTCNLEFIFKEFSYIFITILYIINYN
CYYFNSKEIKYLLNQIKLDWNSIVDNNEIRILEKYAIENRLVTLILSFCVAFGMFLLITVELIPIILNTVIQC
PMNKSRPRKVIIDFEFFIDENQYFYIYLIHEIVLMLIGMFTVLATGTLSLAFLNHCCATSKIASNIIENTVTK

HTLQIPDHRRTRVMCQRINRAIHIHRKSIQFSTFFMNIMNKWLIATGLIVVMSLSCNLFRLFNALTVSHEISE
IALGICMVTAHFMLMFLSNFVGQSLIDHSAEIFNATYSTMWYLAPLSIQKLLLFVMQNSVKTHIVKIGYIYVL
SLEGFSTLVTTAMSYFTVICT
>AcepOr242
YYNIYRIMLTAIGLWPYQTPIIMQVQSVFFLGTYCFILLFLFTSFLTTTCNLEFIFKQLSYIFIIILSIIFYN
AYYCNSKEVKYLLQQIKLDWNSIEDSHEIRILEKYSNENRLLSLILVAVACAISFIIIIELIPVVLDAVIPMN
ESRPRKVKIDFEFFIDKEQYFYIYLIYEIITILIGIFTIFATGALSFTLVRHCCATFKIASNVIQNIVTKRTL
QIPDHRRTHVMCQRINRAIHIHRKSIQFSTFFVSIMNKWFFAMMIIVVMSLSCNLFRLFNALIESHEISEIAL
GIIMVTGHFVTMFLSNFVGQSLISHSAEIFNSAYNTMWYLAPLSIQKFLLFVMQNSLKTHTLTIGYIYVASLE
GFSTLVTTAVSYFTVIYT
>AcepOr243
YYNIYRIMLTAIGLWPYQTPIVMQVQSVFFLSAYYFILLFQFTTFLTTTCNLEFIFKEFSYIFVTILYIIHYK
CYYFNSKEIKYLLNQIKLDWNSIEDNDEIRILEKYAIGNRLVTLILSFCVAFGIFLLMTVELIPIVFDTVICP
MNKSRSRKILIDFEFFIDEDQYFYIYLIHEIVLMLIGMFTVLATGTLSLAFLSHCCATSKIASNVIENTVTKH
TLQIPAYRKTHVMCQRVNRAIHIHRKSIQFSTFFMNIMNKWFFAIGIIVVMSLSCNLFRLFNALTGSHEVFEI
ALSIAMVTAHFMMMFLFNFVGQSLIDHSAEIFDAAYSTMWYLAPLSIQKLLLFVMQNSLKTHTLKIGYIYVAS
LEGFSTVINI
>AcepOr244
MVFSGERYYNIYRIMLTAIGLWPYQTSIIMQVQSMFFLGTYCFVLLFQFTTFLTTTCNLEFIFKQLSYIFFTI
LCIVYYNAYYYNSKEVKYLLQQIKLDWNSIKDNEIRILEKYANENRLLSLVLVVVAFAIFLVIIVELIPVVLD
AVIPMNKSRPRKVKINFEFFMDEEQYFYIYLTYEIITMLLGIFTILATGTLSFTLVRHCCATFKIASNLIEKT
VTQHTLQIPAYHKTRVMYQKINRAVHIHRKSIQFSTFFINIMNKWFFAILVISVISLSCNLFRLLNSLTVSNE
ISEIVMCIGMVIIHFIMLLVFNFVGQSISDHSAEIFNAAYNTMWYLAPLTIQKLLLFVMQNSLKTHTLIIGHI
YVTSLEGFSTVINI
>AcepOr245
YYNIYRILLTMIGLWPYQTTIIMQLQSVFFIGTYCFILLFQFTTFLTTTCNMEFILKELSYILITILCFITYN
AYYFNFKEVKYLLKQIKLDWNSIEDNEIRILGKYANENRFLSLIFSFVVAFAIFFIIIIELIPVVLDAVTPMN
KSRPRKVKIDFEFFIDEEQYFYIYLIYEIITVIIEGFTVLATGLLSYVFIRHCCATFKIASNLIEKTVKTQHT
LQIPSYQKTHMMCQRINRAVRIHRKSIQFSSFFMNVMNKWYFTLMIICVMSLSCNLFRLLNALTLSNEFSEII
MCIGMVIGHITSLFVFNFVGQSISDHSAEIFNAAYNTTWYFAPLSIQKLLLFVMQNSLKSHTLTIGHIYVSSL
EGFSTLMTTAVSYFTVIYTM
>AcepOr246
RYYNIYRILLTAIGLWPYQTPIIMHVQSVFFLGTYCFILLFQFTTFLTTTCNVEFIFKQLSYIFITILCIVFY
NAYYFNSKEVKYLLQQIKLDWNSIEDNSEIRILQKYANENRLLSLISSIIVAFIIFFIIIIELIPVVLDSVSP
MNKSRSRKVKIDFEFFIDEEQYFYIYLIYEIITIVIAVFTVLATGMLSLAFIRHCCATFKIASNLIEKTVTQH
TLQISSSQKIHVMCQRINRAVHIHRKSIQFSTFFMNIMNKWYFTLVIIGVMSLSFNLFRLLNSLIVSNEFSEI
IMCIWVVTCHTIILFVFNLVGQSVSDHSAEIFKATYNTMWYLAPISIQKLLLFVMQNSLKTHTLTLGHIYVTS
LEGFSTLITTAVSYFTVMYTT
>AcepOr247
MVFSGERYYNIYRILLTAIGLWPYQTPIIMQVQSMFFLGLYCFIFLFQITTFFTTTCNFEFIFKELSYIFITI
LCITMYNSYYFNSKEVKYLLKQIKLNWNSIKDNSEIRILEKYENENHLLSLTISFVIIFVIFFIIIIELIPVI
LDAVTPMNKSRPHKVKIDFELFIDEEQYFYIYLMYEIVTLIIETFTVLGPATLSFALFRHCCANFKIASNLIE
KTVTQHTLQIPAYKKTHVMFQRINRAVHIHRKSIQFSTFFMNIMNKWYFTLSVISVISLSCNLFRLFNSLTDA
SNEFSEIVMCIFMVISHIISLFAFNFVGQTVSDHSAEIFNAAYNTMWYLAPIPIQKLLLFVMQNSLKTHTLTL
GYIYVTSLEGFSTLMTTAVSYFTVMYTTTMK
>AcepOr248
MVFSGERYYNIYRIMLTALGLWPYQTLFMQVQSAFFFGAYYFILLFQFTTFLTTTCNLEFIFKELSYIFITIL
YFITYNSYYFNSKGVKYLLKQIKLDWNSIEDNSEIRILEKYANENRLLSLILGFVVAFAVFFIIIIELIPVIL
DAVTPMNKSRPRKVKIDFEFFIDEEQYFYIYLMYEIITIIIGIFTILATGTLSFALIRHCCATFKIASNLIEK
TVTQHTLQIPACQKTTHVMCQRINRAVHIHRKSIQFSAFFMNIMNKWYFPLVIISVISLSCNLFRLLNALTVS
KEFSEIVMCIGMVTAHFMVLFASNFVGQAVSDHSAEVFNAAYNTMWYLAPLSIQKLLLFVMQNSLKSHTLTTG
YIYVSSLEGFSTLITTAISYFTVIYTT
>AcepOr250
MEFLGYHYYKLNRIFLLLLGLWPYGDSFLKRIHIIFFIFTILFLIIAQLLKLFTIEFNLEYVLTDLSFAIPSI
IYLLKYSTFYIQSQRIKELNEHIRIDWNVLQDEEEVEIIRKYAKTARRYMYTFVGVVYPSVIVYISIPFLPDI
LDIVAPLNESRTRQLPFLVEYFLDEQKYFYPILLHIIIALVMGITTVLATETLALAYFYHVCGMFEIVSYRII
CALDKNVSLSLTLNKENIIRVKLIEAVELHQRTLEYFEYLSSTLSVSYFILSILGVASLSINLFRLCQVTILP

DERKDLVPLHVVFVFAQFSYLFICNYMGQKIIDSSTGIFRKTYDTQWYVAPVQMQKLLLFMMQRSMKSCNFIV
GDLYCVSLELFTTLASLSLSYFTIIYSVQ
>AcepOr252
MDFDGSRYYTLNRMMLSSIGLWPYQNMWHIRIQRISCLFCVISCIITQLSTFVTSEYNLDLLLEVLSYTIPCF
IVILKYVAYCMKIKSIRMLMEIIKHDWNTLKNKVEYEIIKKYTYIGAFYAQLFAIMTYSVPIIFGCIHFIPNL
LDFVAPLNQSRPHHILILAEYFIDSDEYFYLILLHMVVWCFTIQITLMSTTSIYVAFIQHACGMFKIASYYIE
HAFSDDYKKRNPASGKRCIVCNRIISAIDIHRRAIEFFESLKNTFVIMYFFLLVLGIASLTVNLCRAVFAKGI
VENILSVISLFVHLFYFFFVNYSGQKILNHSNDFLSRTYNSKWHTIPLHAQKLILFVMQRSSKNCVLLIGGLY
VLSYEGFATAMSLSMSYFMVFYS
>AcepOr253
MDFFNNEYYFTRHLMESIGLWPYEKPERRIVRGMCVSFVLTQSILVQLATFITHDYNISLFIDVFSFNFLCII
YVLKYNVVYFNSNHVKNLFDQIQCDWNSIKNVDEQKIIKKYALKTRFYAIFSGSIVYPGTFIFILFVYMPDFL
NIISPLDEPRPRQLPAQIELFIDQEKYFYLFSLIFTITAFLGMTVLMATENMYMILVQHACALFELTSFRLTL
AFECNTSLSTPSKTKCRFYIKLLSALNIHQHCLKFIENIQHKFSTSYFILFVFGVASGSVNMFRLLDAVTTHN
LFETILIGLFVYSHFCYGFFMNYFGQDVIDHSENFFRQIYSTRWYTAPLYAQKLLLITLRQSIKNSNIIVGGL
FVMSLEGLATLFSMTLSYCMVMHSV
>AcepOr254
KMNLFESRYFNLNRLLMSFIGLWPYQTLGTRRIKMILISFFLAEGITAQLMSFVTIEYSRDLLTKVFSFSFII
LICTVKYNMLYFKSEQVKYLFEQVLNDWHALTDAEEIKIIRKYANKGRFYTIYAGFVLYFGILFFTVLFFIPD
VLDIVAPLNKPRQHQLPLAIELIYDLEEHFLLFILNFLIISLIICTILLAVETLYMICIQHACGLLKLTSYRI
LNAFDDQLQLMSISKKSKCTICIKLSKAIKIHKRSLKFIECLCSTFSASYLFLCIFGVASLSINLFEFIKTIE
SKDTNQSLYFGLFISAHLGYMFWVNYFGQDLIDNSADVFAQTYNVKWYMVSTHVQKKILFILHRSSKNILFDV
GNMFVFSLDGIATLINSSLSYSMLLYSTR
>AcepOr255
MISLEIQYFNLNKILLLAIGLWPYQQSILTRFQFIFLSSILTASIIFQLTSFITLRCTFELVAKVVSSASFFT
VYLINYNSFCINIKSVKNLLMQLQRIYDELEDEKEIAIIKKYGYNAKYYTTLLTATGICVIIVSILFQCWMNM
FDVTLSINVSQPLRIQFVTEYFVDQEKYFFLILLHINVAFCIGTIATIAAGTMLIGYLQFIFGMFKISCYRIE
RAIKILISQNITLKNKISKSENLICAVHIHREAMRLSKYLVTSFEIMFLSLTGVFIISLCFSFLWIFHIVSSG
EAVREAFLPVTFIFTNIMYLFICNFFGQNIIDHNSQVFITAYSVQWYVAPLHIQRMMLFLLLKGTKDFTIIVG
GILISSIECFATLIKASISYFTVMLSMQ
>AcepOr256
MLVNILREYNVNRIFLSCLGLWPFQRKLARRLLPMFCFILEISYLPFEILTLYINRYNGQVIFESLYQMVVTL
AFLIKFLNQIWNRDKFHHLYEIMESHWNIFTNDLEVRILKSYSHISQKFTVSYSILMYTMMSMFIMIPSLGPM
FLDVVLPLNKSRLRNIAIYSEYGIDQDKYFVPIFLYTSIMITVGITIMVAVDTMHIACTSHACSLFQLTGQQV
ENVTSNVLIDNEDNQIRHCTNTEYKMFNEEMIYREYIICLKKHQLALEYVNILNDTHKIVGISFLLLIAAVFS
LLGVRIVYVLDQLEELIRFTFIIMGALLQLMIVCYSGQKLMDESQNIFHRAYAAEWYKFSPRLKSLLIIIILYR
SIVPCKLTAGNLFSLSMAAFVSVVRTGVSYFTALLSFKN
>AcepOr257
MESAQKYYGISVTKQLLLLIGQWPYQEKRIRLFRMSLLTVAVVSLTAPQVAKLIQCDGNTRCILLTIPTLMLM
IMILVKLYTCHFNRSKIKILTDHLISDWEKLQGAEEYEIMKTYAANARLLSLAYCLYYYIVSSTFISISLISP
IMNVILPLNESRPIFMPFEAYYYVDGEKYFFYIFFHTLVSLEISLTAIVAHDCMLLVYIEHVCSIFAVAGFRF
ETLSRNEHDIMSKSLDDIYGRKIADSVYAHRRALWFAELLKNTFYVTFAIQIMIVTVTMSISLLQIAMQIEEI
TETMRYTAFVISQLVHLFCYSLQGQKLIDHSLQMRNKIYNCSWYNIPVKSQRLLLYVMRRSMQPNFLSAGKIY
VFSLKSFTTVVNSSVSFLQYL
>AcepOr258
MENNREYYYDISKWYLMLVGQWPYQKLKESLFFLIFILFLETSALVTQIAKFVICDNAQCIYETLPPHMLTVM
ILVKIFTYQFNSRKIKDLTDRLFVDWDMLETVEEHNIMRNYARNGRWYALIYGSYVYVSTISFTTTSLAPRIL
DIVFPLNTSRPIMLAYPAYYFVDENQYFYYIFLHMLLTSSVCMTGLIAHDSMFFIYIEHICGLFAVVGFRFEN
VSHKYSSTEKSLIDCLDNGYHRNVVFSIRAHRKVLRFAKLLEDTFSISFAVQLLIVTVGLSITLVQLHDLAEA
MRYFVFIIAQLFHLFCLSFQGQKLISHSLETCDKIFRGSWFIIPMKEQRLLLFVMRKSIEASVLTAGKIYVFS
LENFTAVVQSSMSYFTLLSSFDV
>AcepOr259
MKTRRSYYYDINKKYLMLVGQWPYQKPKEKLIYFTLIIIILAISSIIPQGAKFGVCKTAQCIYETLPPYMLAMM
ILLKIFTYHFNNEKIKDLTDRLFVDWNILETKEEHDIMRKYAEKGRWYALTYGSFIYASNIIFATTSLVPRVL
DIVFPLNISRPIMLPYPAYYFVDENQYFYYIFLHMLLTSSVCMTGLIAHDSTFFVYVEHICGLFAVIGFRFEH
VSHKRSAMEKNMINYPDGMYQKNIMISISAHRKALQFVQFLESTFTISFAVQLLLVTIGLSITLVQLHDLAEA
MRYFLFIFGQLFHLFCFSFQGQKVIDYSFEIRDKIYHSSWYTIPVKEQRLLMFVMRKSIEACVLSAGKIYVFS
LENFMTVVQSSMSYFTLLSSFDV

>AcepOr260
YYSITKWLLLFCGQWPYQKNRARLFQLSTVTLVIFSLIFPQVAQIFYCDGNARCIQLTIPVLMLMVMLLVKLY
TCQFNSSKIKSLTDFVCDDWEKLNSAEEYEIMKKYATNARLISFAYILYMYSCSSASILFSLVPKLLNIVLPL
NESRPIIMPFDVYYFVDEEKYYFYIIFHIYMSIIISFTTATAHDCTFFIYIEHVCSLFAVTEFRLETLSHKDR
NDANNLSYNRKIIVFIHAHWRALRFAKILEDIFCTAFAVQMLIVVVTLSISLVQITLLYGDINAMLRYLMYIF
GQVLHTFCFSLQGQRLINHSIQLHDKIYNSSWYEIPAESRKLLLFVMQMCIKPRFLSAGKIYVFCLESFTMVM
QSSVSYFTVLVSFQ
>AcepOr261
YYSITKRLLLFCGLWPYQEKRTRLFRVHSLTLMIFLLIAPQVAQFFHCNGNARCIKLTIPMFMLMITFLVKLY
TCQINISKIKSLTDHICDDWEKLNSAEEYEIMKKYATNAKLITFAYLIYVFSCCGGYTLFSIIPKLLNIVLPL
NESRPVNLPYQAYYFVDKDKYYFYIIFYVCVFGSVATMMVIAHDCIFFIYIEHVCSLFAVTGFHLETLSFNNH
NDTNNRLDDRKIYNRKIAISIHAHWRALRFADILDDIFCTAFAIQMSIVTVTLSITLLQVALLYGEINETLKY
LAFIFAQVIHTFCFSIQGQRLIDHSSQLSDKIYNSSWYEVPAESRRLLLFMMRRSVQPCFLSAGKIYVFSLKS
FSTVMQSSVSYFTVLASFQ
>AcepOr263
LEWAISLNRCMLKIIGLWPKESKIRCEELLSKIRFLFNVIMLIFILTIPALMSLIKVWGDMILMIDNLQYTLP
LLITMLKVFIMWYNKGALSPLIDMIVKDWIKVKMEEERIVMLKQARITRLLAICGALMILSTLLITFGSFLFG
KTLRHVTNFTDPVGKHLPIQTYYPHDISNSPNFELTYLIQVIGLTTSGLSYTAVDNFLGLLILHICGQMENLY
LRLLNLGKNSNFKELLKHNVKDHIRLIRSIETIDNTFNLMLLGLLFFFGILFCFDGFLIISIIERGGYLSLMQ
LIWYMSATTCVLMHTCLYCAVGELLVTQSEKIHRATYEYVWYTLEPKAARNLTLIMLRTKKPVNITAGKTFPM
TMSTFCNLLKTSMGYVSVLLANANR
>AcepOr264
MIDKQLQAYRTYQRFLQTLLTICGCWYMPTKSGKSTYYWSICTLLAMITYTITNLNMSYNLRHDLRNMMKAIG
LTTAGISTILKVTSFMIHRGSLINYHLTLNDLFEEELMQNGKIRMTMFSPLRTMYTMAYTYFIVMTAVILIYF
APSYIIIIRHFLLHFHLTTNYTLPLTKGLGYFWTVPENFLYHFHLFYETSMVILSCATATSVDSMFGFYVYQF
SSTIRAITFKLINPPLTEKFSNLLRICVAKHQRLLQCRQTLEYVYGPIIFWHIIINAVHLCALIYDTMLFVDF
NLSNALAFLSFAAVKLIQTFTYAWYGTILINAGDDFRKGIYFAEWPNSNLDHHMRTNIILMMMQKPMTINTFF
SPVDVIMFTNV
>AcepOr265
VVMQERVTLKKTIAIMKLSLFVIWFWPLPLGTSKCKILCVKLYQYICILMTAGIIASVIYGLVKNKNTNNLDS
FMRSLFSLVTLSHVIGNILCHLIIYQRLQFVTFEMENFCALIKPHEETIVQREYVNKYSNFYGFCISLFYMSL
FGLFIGPVMLDQPLPTAAEFPFDASHQPLRAITYIHQIIVGLFISAHLCVNAFMALLLWLTSARFKLLTEEFR
TITNIYNLAKCIEKHQKLIKYAEDVTLTVRPFAMVTVLFTTVALITFGLVFIAKVPLSLKIQCVLLSSCALLE
VFMYAWPAEHLIHVSTNVAQAAFETDWYAKSEYLRKNLQMVILRSQKPILVTLPCGLPSLSLRYYASYLSTIF
SYFTTMRMMFEEKEFG
>AcepOr266
KLNFKKVIFGVKLSLWITWSWPMPKDATKFKITCMKLYQNLCIILAICMEASMIYTIVNHINDFAIFVELVVQ
QAPILHAICNFIFHKVNYHHIQNIAFEAEYFCDSMKPREEVVIQRYIDKYAAFYGVSSVIFYFLVIAACAIAS
IMHQPFLMLNEYPFDVYYEPLRTIIYAHQAIVGFIVSGQLCLNTFVATLLWFASARFEIIIEDLRTFTNVYQL
VKCVKEHQKILEYTAEVIIVARCFALTCICCSTVNLIIIGVSFLTNQSLLLRFKYTFMILACLAEVFMFAWPA
ERLIFLSNDISQAAFDTHWYNQSDEIRKLLQIIMLRSQKVITIAVPYITPSISFNYLTSYCSTIMSYFTTLRV
MLNTD
>AcepOr267
LIMWSKLTLKKVIAVVKLSLIPTWCWPLPKDTIKLKVICAGIYQYFNIIVLSSVAAGLINAMRNHFNDPVIIA
KSISVLCPAIQVVCNIICCRINSCRLQLVTFEMENFCELLKPREEAILQRYINKCVYFYGGSMFWIYLSAVFI
ISGPVTLDQPFPTNAEYPFDVYHQPLKSIIFIQQTIACVQGAAQLCMNIFMALLIWFTSARLEIFIEKLQKIT
TISELKKCIQEHQNLLKYAEEVTILIRPIALSTVSFSTIALIIVGLILLTDQPLSMKVQCVGIIFSGLSVVFM
YTWPAEHLIHISDEIGQAVFDTEWYQQPVALRKTLQIIMLRAQKPIIISVPCVMPALSLKYYASYLSTIFSYF
TTLRVTMQN
>AcepOr268
MTETLTLEKIIAFLKVDLTFACCWPLPMKATKFQKIRDKIFRLLCCLNGIIMSISLIYTLSKKYDNMILAMKV
GCELSASWQIPIQITLFTLQSDRLQIIIYEMKDYIQQAKLEERNIFQRYIKKCKLFYGTTMCLMTATTIIMWF
GPLLLAQSFPIEVEYPFDVNKQPLRTIIYLHHIMAVYQSRTQVCSNIFIALLLWFTVARFEILSHKFQKSTSI
SEFIICIQLHQRLLRYAKDVTMTVRYIVLSSVGFSTIAVVFSGLTFLSQQPLTIKTQFFAIAASALIEVFICA
WPADYLLRMSNDIGHAGYESSWYSKDISLQKNLLYTVSRCQHPVTLTVPCLLPTLSLNYYASFLSTTFSYLTT
FRAMLAKKDGLQM
>AcepOr269

MKETLTLEKIIAFLKVDLTFACCWPLPMKATKFQKIRNKIFRLLCCLNSIIMVISLIYTLNRKYNNMIFVMKV
GCELTAFVQIPIQITLFTLQSDRLQIIIYKMEDYIQQAKSEERNTFQRYINKCKLVYGTTMCFITVTAIIVSF
GPLFLAQPFPIEVEYPFNVNKQPLRTIIYLHHMMIIYQSRVQVCSNVFVALLLWFTAARFEILSHKFQKITSI
SEFTICIQLHQRLLRYAKDVTMTIRYVALSTIGFSTIGIIFSGLTFLSQQPLTIKTQFFAIAASALIEVFICA
WPADYLLRMSNDIGHAGYESSWYSKDISLQKNLLYTVSRCQHPVTLTVPCLLPTLSLNYYASFLSTTFSYLTT
FRAMLAKKDGLQ
>AcepOr270
MRKKVMLKEVISAVKLSVFPIWCWPLPQDATKFKMFCMTLYHCLSIIVTTGLKLSLIYGITNHLDDPPVLVNQ
ILILSSIIHTTFNFIFHITHYHHIQIATFEIVNFCNVMKPHEEVVIQRYIDKCIIFHGTSIFIFYWLTFITIA
VIPALQYQPFPTLAEYPFDVSYQPVKTIIFIQQSMAGIMVAGQLCMNVYMAMLLWFVSARFEILTEELKKTAN
VYQLFECIKKHQKLLIYAKEVTNAARPFAFTTIYCSTISLIVVFLLLITHQPIALIFQFSGLGVIGIAEVFIY
TWPTEHLMYTSKNVAQTAFYILKNNHLIEVWKCLQIIIMRSQKPITMSIPCILPELSLNYFTSYLSTILSYFT
TLRVMMDDDN
>AcepOr271
MTETLTLETIIAFLKVDLTFACCWPLPMKATKFQKIRDKIFRSLCCLNSIIMSISLIYTVIKKYDMFVVMKVS
LELTAFLQIPIQITLFTLQSDHLQIIIYEMEDYIQQAKSEERNIFQRYINKCKLFYGTALCLITTTAIIMCFG
PLLMTQPFPIEVEYPFIVNKQPLKIIIYLHHIMAVYQSCVQVCSNVFVALLLWFIAARFEILSHKFQKITSIS
EFIICIQLHQRLLRYAKDVTMTIRYIALSTIGFSSIAVIFSGLTFLTQQTLTVKTQCFAISTSALIEVFICAW
PADYLLRTSNDIGYAGYESSWYNKDISLQKNLLYTVSRCQHPVTLTVPCLLPSFSLNYYASFLSTTFSYFTTF
RAMLAKEDGLQM
>AcepOr272
KTTPKNAITFIKVILALSFSWPLSKSASKFQITCFKILRSLFCMNTVVLSIPIAYTLYRNDYDLPRITKLWCT
LGAMIQVSVEMTQCALQYDRLQYLISEMERTLERAELYEKNIYQRYIDRCAMFYMSSTAAVFLGAGIGVIVPL
TAADQIFPTEAKYPFNVEHEPIKTIIFVHQFIAVWQCFSTVCIGSFISLFIWFAAARFEILSQQFRMVTDIYD
ITVCVRQHIKLLRYAQEIIIAFHSVLLTIIIICTWAIVASGLTIVSQCSLTDKIQFMALCISALMEVYACAWP
ADRLIDMSTNVAQAVYESLWYNQDKIFQKNLNFILLRSQIPTTICVSILPALSLQYYASYVSTAFSYLMTLRM
VFMEEDSGIKN
>AcepOr273
IKITPKTAIAFIKVIVALSFAWPLSKNASKFQVIRFKILRTLLCINAVVLSVPTVYTLYRNDYNLARITKLWC
LLGAFIQIPLEITQCALQYDRLQYVISEMEHNIKCAQSYEKKLYQQYVDRCAIFYVSLTAAVFLGASITATLP
FIAANQIFPTDTKYPFDVEYEPVKTIIFLHQFIVIWQCFSIVCIGSFVGLLIWFAAARFEILSEQFRMVTDIY
DITVCVRQHVKLLRYAQEVIIAFRSVILSLIIICTWAMVASGLTIVSRSTLTDKIQFMALCISALMEVYACTW
PSDYLKDTSTNVAQTIYESSWYNHDKIFQKNLNFILLRSQTPTTVSVSILPALSLQFYASYVSTAFSYLMTLR
MVFVENNGFK
>AcepOr274
MRRKVTLKEVISVVKLSLFLIWSWPEPHNATKLKMLCMKLHHYLGIITVISLQVPLIYGITNNLDNPAVLTKQ
VLMLSSVIHAMFNFIVHIINYHHIQNVTFKMIQFCDLMKLHEEIVIQRYIDKCIIFHGTSIFIFYWLTFTAIA
VIPALEHQPFPTAAEYPFDVSYQPLKTIIFIQQSIAGIIVAGQLCINVYMALLLWFASARFEMLTKELEKTTN
VYQLFKCIKKHQKLLKYAKEVTICAGPFAFITICCSSIGLIVIFLLFISHQPILLIFQFFGMGIIGISEVFMY
TWPAEHLMYMSKYVAETTFHMLENNHLIKMWKCLQIIIMRSQKPVTVSMPCFLPILSLNYFTSYLSTILSYFT
SLRVMMDNDN
>AcepOr276
MIMKKNLTLKKVIAIVKLSLFITSCWPLPKDTVKLKIICVGLYQYLCLILTLGLIVGLTNTVKNHLNDPLIMA
KSIIIICPMVHVVCNIISCKINSRRLQLVTFEMENFCQLIKPHEEATIQQYISKCIYFYGGSMLWIYLSAVFV
ITGPVTLDQPFPTNAEYPFFVYQQPLKSVIFMHQSIILLQISAQLCMNIFMALLLWTTSARFELLVKKLRTIT
NVYDLIQCIRQHQKLLHYSNSIKMIEIKNYLITIISHFRLPIIYIVVIIIIINQPISIKIQCISFIFSGLSEV
FMYTWPAEHLIHISGEIIQQAVFDTQWYKQPVALRKYLQIIMLKAQNPIIIAIPCVMPALSLNYYTSYLSTIF
SYFTALRIIMQN
>AcepOr277
MDLLPVNFYVLRFCGVWKEHKNSNLVIRFISFCYRYTIAAIIYHFTIFEMAELIRMRNDVEDLTESLFLVLTF
MCLCLKYLNVFMRQYELRALLDCFRANLCQPRNSMEESILKQYDLKAKRITCAFMFISQISGFLVLIGPLMTQ
NERQLPMKMYVPYSMMELFPYLLTYLEQVAVVFYGVSLNVAFDSLVYGFIIHTCGQIELLCYRLSETFRFLQE
NNKEKEHDVIEKFAIAECVRHHVSVCNIMHRIQSLFMWIITILFFFSLITLCSSVYQMSNKNFFSVEFFVFLV
YLGAMLFQVFSYCWYGNELDLKNKSIAHAIYASDWTVISAKQRKSLSFMMMISQGGRILSFYGICSLVLSTFT
WILKTSYSSFNLLQQAT
>AcepOr278
MDILPLNFRVLWFCGAWSEKKDNNDLFIRFLSFCYRYAIVILIYEFTISEVIELIRTHDDIEGITEGLFLALT
YVALCFKYGNFLVRQNEVSMLLDCFRRETCQPRNSEERMILIKYDRKAKWCVRTFMSISQATCIALILAPIVG

PQNTDRPLPFKTYLPYSISGLYPYLATYLQHVGAIFYGVLLNVSFDSLVYGFTLHVCGQIELLCYRLSKIYKD
HPKQYQIDMDKGTMISECVRHQLYVHEIVRRIQSLFVWTVMILFIFSMVTLCTSIFQMSKKSLLSVGFLSLTL
YLGCMLFQVFFYCWYGNELQLKSKGIGDAIYSSNWTIATTRDRKSLLFVMTISQKGLKLSYYGIFSLALDTFT
WILKTSYSTFNVLQQTSM
>AcepOr279
MDILPVNFKALRFCGAWKEREDNNMCVGFLRLCYRYAIFLLIYEFTVLDVIEVIRTRDHIQELTEGLFMGLTF
LTLCVKYANFLLRGNELSDLLNCLRVKRCQPRNSIEKLIIEEHSRRAKWSTMSFLMISYVTGLGFIITPALGL
LKEGKRVLPLKTYIPYSVSNLLPYLVTYLQQFLAVFYAIMLNVNFDCLVYGFTIQVCAQIDLMCSRLTDSLKN
IGGISSGRKPETNALIVECVKHHLLVSNLVKKIRELFIWTVMIFFFFSLLIVCTSIFLISKKKLLSFEFLSMF
LYLSGILLQLFYYCWYGNELELKSKSIATTVYSCNWTMATPQERRSLMLIMISSQKGIMFSYHGVFTLSLNTF
AWICRTSYSAYNLLQGASN
>AcepOr281
MQILSLNFLMYTISGLWRPIKWSSKCSKLLYSMFTFSTIYLLSYLMLTHLLYIIFVIDNVEDFASTSPFFFST
ISLMFKAVTAVIYRDRIINTIEVLQNEPCKACNKDETDIQMKFDHTIRSYSIYYLLLCTFSAVGAVTAGMLDI
LEGQLPYNMWVPWNCTSFFSFLFTSLQEIISLIVATIVNIATETIVLGFCLQLCAQFEILEHRLQRMVKRREE
EIFPKSSLNKVSREISKLSKHISHHLCIIRLAGTINGIYSKVIFVQFFVSILVLCSIVYHLSSHLTLTDVATL
IAYTFSMFVQIFVYCWAGNEVMLKSIGLSEIVYHMDWMLMTNSEQKDLLMIMKRCIKPIKFTSSFLVILSLES
YGNLLKMSFSAFNVLQQF
>AcepOr282
MQMFSLNFLMYTIGGVWMPIEWSSNIAKLLYNVFTTVVLVLLYFLMITQFMDIILLVDNIDDFATNTLMFLTI
VAVTCKATIVVVRRKAIINLVQVLLTAPYKPRNEDEMTIQTKFDKFIKSCSIKYSLLATSSVTGVTVRSVLNA
MQGQLPYRVWLPYNFNVSPMFWITSVQQIITVIFVTIINVGTETLVFGLFLQTCAQFEIFESRLQKLVTNKTN
KLMKNKIRYLEHSSPSSNEEKTIISKYIHHHIKIYKYAKTVNVIFNQVLFVQFVGSILVLCTSVYYLSAHKTL
SETATLIVYTICMFVQIYVYCWSGNEVILKSMSIGDAIYHMNWPSLSVNERKELLMIMLRSTLPMKFTSSFLI
TLSLQSYSSILKLSYSAFNVLQK
>AcepOr283
MQILSLNFLIYNLCGMWRPNEWSSNGAKLLYNVFTFMVIFSEYFLVVTQFMDIILIVDNIDDFATNTLMFLTI
VAVCCKATIVVIRRNAINNLMQLLMETPCKPRDEDEIAIKTKFDTFIRSCLIKYMLLSMSSLTGVTIGSVVNI
MQGYLPYRIWLPFNYTIPLAFWTISIHQIVTLIFATMINVSTDILVFGLFLHTCAQFEIFESRLHKLVISKTI
NYLGNAISSLNKDKTEISECIRHHLSIYKYAKMVNVIFNQVLFVQFFASILVLCTSVYYLSIHIRNFSGILPF
LVYTIGMFVQIYIYCWSGNEVILKSMSVGDAVYCMDWSLLSVNEKKQLLMVMVRSTIPIKFTSSFLITVSLQS
YSSILKLSYSAFNILQK
>AcepOr284
MQILSLNFLIHSLCGIWRPNEWSSNGAKLMYNVFTFIVIFSEYFLVLTQFMDIVLIVDNIDDFATNTLMFLTI
VAVCCKATVVVVRRNAITNLVQILMKTPCKPCDEDEVVIQTKYDMFIRSCSIKYSLLATSSVTGTTIGSVLNI
MQGHLPYRIWLPYNYNVSTTFWAISVHQIITVIFSAMINIGTDTLVFGLILHTCAQLKIFESRLHKLIINRTI
RYLENTFSPSNKNKIGISECIRHHLSIYKYAKTVNIVFNQVLFVQFFASILVLCTSVYYLSIHITELSGTATF
LVYTIGMFVQIYIYCWSGNEVILKSMSVGDAIYYMDWPLLSINEKKELLMVMIRSTIPIKFTSSFLITLSLQS
YSNILKTSYSAFNVLQK
>AcepOr285
NFFMYTICGIWRPIEWSSKGVNFLYNVFNYIILLLEYFTMLTQFLDFLFVVNNIDDFIANSIWFVSFVAMVCK
ATVVVIRRNAIISLIQVLLKGLCKPQDEDEIAIQTKFDQFVRLCSIKYLLLAILSLTGVTIGSVLNIMNGQLP
CRIWLPLDYNVSLVFWIISIQQVIAICFGTIINVATETLVFGLILETCAQLEIFENRLHKLINKKTIKYLGNA
LYSLNEDKTGISKCIRHHLSIYKYAKTVNVIFNQMLFVQFFASILVLCSAVYYLSMHITELSRIATFLVYTIG
MFIQIYIYCWSGNEVILKSMNVGNAIYFMDWPLLSVNEKKELLIVMIRSTIPIKFTSSFLITLSLQSYSNILK
TSYSAFNVLKK
>AcepOr286
MQMFSLNFLIYTICGIWRPIEWSSNGANFLYNVFTYIVLFLEYFLMLTQFLDFLFVIDNIDDFVANSIMFVSV
AAVVCKATVVVIRRNAIISLIQVLLKDPCKPQDEDEIAIQTKFDQFIRLCSIKYLLLAMISLTGVIIGSVLNI
MQEQLPCRIWLPLDYNVSLVFWIISIQQVVAIIAGTIINVGTETLVFGLILETCVQLEIFENRLHKLINNKTV
KYLKNAFCSLNEDKTEISKCIRHHLSIYKYAKTVNVIFNQILFVQFFGSILILCTCIYYLSTHITELSGIGTL
LVYIICMFVQIYIYCWSGNEVMLKSISIGEAIYCMDWSLLSIIEKKELLMIMIRSTIPIKFTSSFMITLSLQS
YSNILKTSYSAFNVLQK
>AcepOr287
NFFIYTVGGIWRPVEWSSNGAKLLYNVFTFIIISSEYFLVLTQFMDIVLIVDNIDDFATNTLMFLTIVAVCCK
ATVVVVRRNAIINLVQILLKAPYKPRDEDEVAIQTKFDKFIKSCSIKYSLLATSSVTGTTIGSVLNVMQGHLP
YRIWLPYNYTVSMFGIISIHQIVTLIFATVINVGTETLIFGLILQTCAQFEIFKSRLHKFITKKTVTYLGHAR
SLSNEDKTWISECIRHHLSIYKYAKTVNIIFNQILFVQFFSSILVLCTSVYYLSMHIKELSAAASLLIYTICM

FVQIFVYCWSGNEVMLKSMSIADAIYRMNWPLLSINEKKGLLMIMIRSTIPVKFTSSFLIILSLQSYSNILKT
SYSAFNILQK
>AcepOr288
MQIFTLNFLMYTIGGIWRPVEWSSNGAKLLYNIFTFIIISSEYFFLLTQFMDIIFIVNNVDDLATNILIFLSM
VAVCCKTTVVVVRRNAIINLVEVLLKAPYKPQDEDEIIIQTKFDRFIRSCSIKYSLLATSSITGGTIGSVLNV
MQGYLPYRIWLPCNYNVSTIFWTISIHQMVTTIFATMINVGTETLVFGLILQTCAQFEILENRLHKLIINKTV
KYLEHASCLLNENKADLSECICHHLSIYKYAKAVNVTFNQVLFVQFFSSILVLCTSVYYLSIHIKNLSATASF
LAYTICMFVQIYFYCWSGNEVIFKSTSIADAIYNMNWPLLSINEKKGLLMIMIRSTIPVKFTSSFLITLSLQS
YSNVLKTSYSAFSVLQK
>AcepOr290
MQILSFNFFLYTITGLWRPIEWSSKCSKLLYNVYTFFTLYLFTYFFLTHFLYVIFVVDNLENLASCCSIFLGT
ITLFCKAIIIVIRRDRIINLIKILQEEPCKACNEEEINIHIKFDHLIRLHSMPYIILCTISLIGTIIGGILYM
LEGAIPYGFVWVPWECTSFLLFCVTSLQEIAAVTIGIIVNIATETTVLGFCLHTCARFEILNHRLQRMIKGEK
EGMRKSSINNISRLSKYVSFHLCIIRLAEMINDVFSPVIFIQFCISILVLCSAAYYLSSNITLLNVINFTAYV
FCLFVQIYVYCWAGNEVTLKSVELGDEIYHMDWILMTKNEQLDLLMIMKRSTKPIKFTSSFLVTLSLESYSNL
LKATFSAFNLLQQ
>AcepOr292
MHTLPLSFALLTYTGYWRPIHFTSIKYWTYTIYSIVMNFLLYSFTFCGLVDCFILKDLETFIEKFSLFLTVLG
VSCKVINLAVRRDEIIGLSNMLLQDICIPRNDQETDIQRRFDRNAKIITIYCEILNESAAFFATVAQLRYFIN
TRTLPLYDWVPYDISSTTAFWATMLHQTIALILCANASVAHETLISGFMIQICAQLDILCYRARTLPELLQEA
WQYSNSKEDFKMRERQLIREIVHHHRYVYRFAERINTVFTLMIFVQFSISSTVLCLSIYKMLTKNLLSLDFAY
NLSYLGCMLMQIYLYCWFGNEVTLKSTEIGNAVYEMDWPMLSVDLMKTFLVIIIRSMRPIKITSGYVVTLSNE
SFMKVNEMRY
>AcepOr293
FMWAIELHRLGLEIIGLWSNSEKFKKSLWPKIQIGVILILLIFISIPTICAIIRVWGDMILLIDNLQITIPIL
IVSVKYVILRWKQTVLWSIMNMIAEDWMAFKLDGERNVMIKRAQIARFIMIIGYVLAIIGFLSVIIPPYFGIQ
IMYDTNFSNRSKLLPLETFHFYDTDKSPQYELTFFVHIITTLLAAIIYMSVDMLLILIIILHICGQLENFKYRL
LSLVSCKNFNKVLNNIIATHLRLIRFAEKIENIYSLMMLIMVFYFGIVFCLSGFIFTVFLTDKKMDDIIVIKV
YYSTILVIALLMNTFLYCGAGELIIEHCNAVHRAICDLEWYKLESREARNLIILMIRTHQPFRITAGKIIPLT
MATFCSVLKTSSGYISFLLTK
>AcepOr294
FLWAVNLHRFGFKMIGLWPKPNKCTEKSLWSEIWIGIIFILLIFISNVPMIYAVIEVWGNMVLVIDNLHTTLP
QLIISVRYIIMRRKQTVILSIVNMMAEDWIAFKQDKERNVMIKQAQTARLIMIIGYVFMIVAVLAVIIPPCFG
IPVIYVITNFTGVNKPLPLKTYHFYNTDKSPQFELTFFIHSFTTLLGGIIYMCVDIFLILIIILHICGQLENFR
CRLTDLISCKNFNEVLNNIIETHLRLIRFADNIENTYSVMMLALIFHFGIVFCLSGFLFTIIFTDKNVDKADI
AQGYFSLLLLVILLMNTFLYCGAGEIITEQSNAVYRAVCDLEWYKLKSSKARNLIMLMIRAKYPFYITAGKIF
PLTMATFCNILKSSIGYISFLLTK
>AcepOr297
FMWAINLQRLGFEMVGLWPKPNKCTKKCLWPKIWVGIILILLIFVSYVPMIRAIIQVWGNMVLVIDNLHTTLP
QVIVSVKYIIILRKRTVVLSIINMMAEDWMAFKLNEERDVMIKRAQTARLIMMIGYVIMIIAFFTTVILPYFG
FVIMYVTNSTDRRKSLPLETYHFYDTEKSPQFELIFFIQMVTLLLAGTIYMSVDIFLVVIVLHICGQLENFRR
RLINLISYKNFNKILNHIVATHLRLIRFADNIENTYSLMMLILVLHFGVVFCLTGFLFTIILADGKIDKTVII
QGYFSILLFIIFLMNTFLYCGAGELVTEQCNAVHRAMCDLEWYKLESRKARSLILLMMRTKYPFCITAGKIFP
LTMATFCSILKTSMGYISFLLTQHG
>AcepOr298
FMWAVNLHRLAFEMVGLWPKHYKCIKKNLWAEIWVAIVIISLIFISNIPMICTIIQVWGNMLLVIDNLHTTLP
QIMVSVKYVIIRRKRKVLLSIVNMMAEDWMAFKLNRERNVMIKRAQTGRLIMMIGYIIIITGFFTVNFLSLLD
IHVLYVTNYTDRRRLLPLETYHFYNSYKSPQFELTFFIQILTTLFAAVIYMSVDIFLVVIVLHICGQLENFRC
RLIDLVLYKDFNKILNDIVTFHLRLIRFADNIENTFSLMMLVLIFYFGVVFCLSGFLFTIIIFDGKIDKSVIV
QGYFSILLLIILLMNTFLFCGAGELVTEQCNAVYRAMCDLEWYKLESRKARSLILLMMRAKYPFSITAGKIFP
LTMATFCSILKTSMSYISFLLTK
>AcepOr300
FVWAVELHRLGLKLIGLWPSTDEISKKKLGSNICVGFIFITVIFISGVPLVWALIRVWGDMVLMIDNLRITLP
LIVVLLKFIIMRWKRKVLLSIVNMMAEDWISLKLNTERDVMIKRARSARLLIIFGFVLMTFAFIMLIIFPCFD
IQIRHITNLTDRKKPLPLQTYYFYDTDKSPQFELTFLVQAVTISLAGIIYTSVDAFLGLVIFHICGQLENFRC
RLVKLILCKNFNRALNNSVVYHLRLIRFADNIEKTFSLMMLGLVIYFGIVFCLCGFLLVSVVTNQNINNANVA
QACYMAIAALILLTHTFLYCGAGELIIEQCAALYRAMCDLEWYKLESSKARNLILLIIRANEPFHITAGKIVP
LTMTTFCSLLKTSAGYISFLLAK

>AcepOr301
FVWAIELNRFGLELIGLWPKTNELVKDKYYISDLRVIIIFIIITFISGIPLVCSLVRVHRNMLLVTDNLQITL
PLIIVSLKLVIMRWKRSALLPIITMMAEDWMTLKLDTERNVMMKRARTARLIVICGYVLMILAFTVIIIFPCF
GVPFRRLTNLTDRDKPLPLQTYYFYDTDKSPQFELTLVIQAITIFLAAITYTSVDAFLGLIILHICGQLENYR
SRLINLVSCKDFNNALRSNVIAHLRLIRFAGKIEDTFTLMMLGLVFYFGIVFCLYGFLLLTVVTDDETNGIPF
SQILYAMVGIANLLIHTFLYCGAGELITKQCEAIYRTLNDLEWYKLESKKARCLILLMTRASEPFHFTAGKII
PLTMTTFCSLLKTSASYISFLLAYRS
>AcepOr302
FVWAIELNRLCLEVVGLWPKINGAAKNTFVSDLRMGTIFFIVTFLSIIPLLCSLVRVWGDMILIVDNLQVILP
LLVVSLKLIIIRWKRTAIALLVKMMVEDWMELKMDAERDVMIKRAQTARLIVICGFFLMIFVFIVLIILPAFG
LHFRYITNLTDQERLLPFQAYYFYDIDKSPQFEIALVIQAATMFLGGMTYTSVDAFLGLLILHICGQLENFKH
RLTNLISSKDFDSALCNNVRTHLRIIRFANNIEDVFALLMLGLVFYFGIVFCLFGFLFVTVITGNEIGHMSLT
RVCFLVIGIFTLLAHTFLYCGAGEIIAEQCEAVYSAICNLEWYKLEPRKKKYLILLMIRANEPFRITAGKIFP
LTMTTFCSLLKTSAGYISFLLAKR
>AcepOr303
FTWAIKLNRLGLELLGLWPKSDEISKNNFSSDLRMVIIFVIVTFVSGVPLACSLIRVWGDMILMIDNLQITLP
LLVVSLKLIVLRWKQTALVSIIKMMAEDWMELKTRKEREVMMNRAQIARTIIISGYILMVLAFIVVIILPYFG
LPLRHLTNLTDSDKPLPLQTYYFYNIYKSPQFELTYIIQAITIFLAAVTYTSVDAFLGLTILHLSGQLENFKG
RITNLISCQNFIYNLSHNITNHMRLIRFAGIIENIFTFMTLGLLFYFCISFCLHGFLLLAVITEGSNFSAARL
CFPLMGITILFSHAFIYCGAGEIVTMQCEEVYYALCNLEWYKLEPKKARSFILLMIRASQPFHITAGKIFPLT
MTTFGSLLKTSAGYISFLLANQ
>AcepOr304
FTWAIKLNRLGLELIGLWPKSEVPKNNFSSDLRVVIIFVIVTFISGIPLTCSLIRVWGDMLLMIDNLQVTLPL
LIVSLKLVVMRCKRTALLSIIKMMAEDWMELKTKKERDVMMRRAQIARIIVISGYILMVLAFIMVIVLPYFGL
SLRRLTNLTDPGKPLPLQTYYFYDVDKSPQFELTYIIQAITVFLGAVTYTSVDAFLGLSILHFSGQLENFKSR
ITSLISCQNFIYILSHNIMTHIRLIRFAGIIENTFTFMMLGLVFYFGIVFCLHGFLLLTVITEKTDFSFARLC
FPLIGITILFSHTFLYCGAGEIITMQCEEVYRALCDLKWYKLESQYARSLIVLMIRASQPVRITAGKIFPLTM
TTFCSLLKTSAGYISFLLANQ
>AcepOr305
FEWAVKLNRIALNFIGLWPKTAKNTRQKLMCNIRVLIVFLSVTCGVLIPSIHSFIRIYGDIMLMLDNLQFTLP
AISCSIRIVIFWWKKEAIIPIMNMIAEDWIKSKSVQDRNVMIRTAYTARIIITCAYCIMGLACFFIIILPSFG
ISMRLIPNITDPGKPMPLQTYYIYDITKRPQYEITFISQAVYILLAIMSYTGIDNFLGLLIFHICGQLDILKN
RLTCLDKYINSRNMLKSCIIKHIHLLRAIGVIEDTYNITLLALFVYFAILFAFYGFRIIILFDEGNNLSITHL
IYFICNVFTIFSHMCLYCALGEILMARCNEIYYAAYNNKWYFMDPKIAKDLLFLLIRGTKPVYLTAGKVFPMT
MATFCDLMKTSAGYISVLHTTK
>AcepOr306
FEWAVKLNRITLDFIGLWPKTAQNSRQKLMCNFRVLIVFTAITFGVLIPSIHSFLRIYGDIMLMIDNLQFTLP
AISCSIRIAIFWWKKEAITPIIDMIAEDWIKSKNEQERNIMIRKAYIARMIVACAYCIMAVGCLFVIIPPGFG
IPIRLTTNITDPGRIVPLQTHYIYDVTKQPQYELTYISQTIYIVLAVLSYTGIDHFLGLLVFHISGQLNILKN
RLINLNKYINSHDMLKTCVARHIRLLRAIAIIEDTYNVTLLSLFLYFAILFAFYGFRIINLFDEGNDLSFTHL
IFFMSTFLNLFVHMCLYCALGEFLVAQCNEIYYAAYSNEWYSVNPKIAQDLLFLLIRGAKPIHLTAGKIFPMT
MMTFCSLIKTSIGYISVLHTTRSH
>AcepOr307
FEWAMKLNRFTLDFLGLWPKNIQNPWQKLMCNFRVLIAFLGITFCLFIPSIHSLIIVFGDILLILDNLQITLP
VTSCLIRIIIFWWKKKAVIPVINMITKDWIKIKNDQERNLMIRRAQTARIIIICSYCIMGLQWFFISVLPIFG
VTMRLTPNITDPGRVPMPLQSHYIYDITKRPQHELTFISQAVYIAIGMMAYTGVDNFLSLVVFHICGQLDILE
NRVQHLDKYKNYPKILKRCIEKHIRLLRAIDIIEDTYNGILLSLFIYFAILFAFYGFRLISLFDEGNYMSITH
LIYFISSIINIFTHMCLYCVLGEILVAQCNKVYYAAYSNEWYTIDPKVAEDLLLLMTRGSKQVCLTVGKMSPV
TMATFCNLVKTSVGYISVLHTTR
>AcepOr309
FEWAVKLNRFTLNLIGLWPKTAQNSRQKLICNFRVLITFLGLLGLVIPCIHSLIKIFGDGLLMLDNLQFTLPV
ISCLIRIMIFWWKKEAIIPIMNMIAKDWIMLKSNQERNMMIRRAQNARIIIICSYSIMALACFFVAVLPIFGM
SVRLTPNITDPGKIPMPLQTHYIYDITKRPQYELTLIGQIIYVAIAMMTYCAVDNFLSLVIFHISGQLDILEN
RMQYLDKYINYHKILKFCLTKHIRLLRAIDIIEDTYNGILLSLFIYFAILFAFYGFRVISLFDEGNNISITHS
IYFISTVINIFTHMCLYCILGEILVAQCNKIYYAAYSNKWYTMDPKVAEDLLLLMTRGSKQICLTVGKVSPVT
MATFCSLIKTSVGYISVLHTTR
>AcepOr311

FEWAVELNRFSLELIGLWPKSKQNTWEKRLCNLRVLLIFLLIIILALMIPAIHSLIRVHFDILLVTDNLMCTLP
IITCVLRLVIFWWKRKAVALIIHLIANDWTKTTTFQEKAMMIAKAQIARIIVIFGYGIMGVGMIIMFGLPLFG
FSVRYLSNVTADLEKPFPIQTYYIYDTTRSPQYELTYAVQCIILFFCTVCYTGIDNFLSLSVFHISGQLDILR
NHLLHLHIVANFNNVLKICIMEHIRLLRFINHVEDIFSIILLILFLYFGILFAFYGFWITNMLESRQYLTVLN
LVYIICVVINTFGHMCMYCAVGEILTAQHDRIHYAVYFNEWYTMNARNTRALVLLMLRTSKPLHLNFGKLFPL
TMATFCSLLKTSASYISVLLTTRN
>AcepOr312
FEWAVKLNRFSLELIGLWPRFEQTTWEKRLCNLRVLLTFLFIIFVLIIPAVHSLIRVHFDILLVTDNLMSTLP
ITTCVLRIVIFWWKRKAVASIIYLIANNWMKTTTSQEKKMMIAKAQIARIIITFGYGMMAAAITVMSILPMFG
YSVRHLSNITADLDRPLPIQTYYIYDTTRSPQYELTYAAQCIILFFCTVCYTGIDNFLSLSVFHISGQLDILR
NRLLHLHIVANFNNVLKSYIKEHIRLLRAINHVEDIFNIILLILFLYFGALFAFYGFWIINMLESRQHLTVLN
LVYIICIVINTFGHMCMYCAVGEILTAQHDRIHYAVYFNEWYTMNAKNTQDLVLLMLRTNKPLHLNFGKLFPL
TMATFCSLLKTSAGYISVLLTTRS
>AcepOr313
FEWAVKLNRLSLELIGLWPRFEQTTWEKRMCNLRALLIFLLIIFVLMIPSIHSLIRVHFDILLVTDNLMGTLP
IITCVLRLVIFWWKRKAVAPIIHLIANDWRKTTTSQEKAMMITKAQIARIIVTFGYGMMGAAIIVMLILPIFG
YSVRYLSNVTADLERPLPLQTYYIYDTTKSPQYELTYVVQSIVAFLCTVCYTGIDNFLSLSVFHISGQLDILR
NRLLHLHIVTNFHNVLKNCIMEHIRLLRNKSSIILPICSTIVLIILMSYTYVSLITTYITVIYMLESRQLLTF
SNLIYFVVVLDIFSHMCIYCAVGEVLTTQYDKIHYAVYFNEWYTLNARNTRDLVLLMLRTSKPLYLNFGKLFP
LTMATFCSLLKTSAGYISVLLTTRS
>AcepOr314
FEWAVKLNRFSLELIGLWPRFEQTTWEKCLCNLRVLLTFLFIIFVLMIPALHSLIRIHFDILLVTDNLMSTLP
IITCVLRLIIFWWKKKAVAPIIQLIANDWTKTTTSQEKAMMIAKAQIARIIITFGYGMMAAAITVMAVLPIFG
YSVRHLSNITADLERPLPIQTYYIYDTTRSPQYEMTYAVQSIASLLCIMCYTGIDSFLSLSVFHISGQLDILR
NRLLHLHAVANFNNVLKNCIMEHIRLLRAINHVEDIFSIILLIIFLFFGILFAFYGFWTINMLKSRQLLTFSN
LIYFIVVLDIFGHMCMYCAVGEILTTQCAKIHYAVYFNEWYILNARNTRDLVLLMLRTSKPLYLNFGKLFPLT
MATFCNLLKTSAGYISVLLTTGS
>AcepOr317
YEWAIGLNRINLALLGVWPKNTETKRKKLISNIHTILMLNTVICGCYIPSIHSLFKVWGDIMSMIDNLQYTLP
MSVSIIKLFIMWLRKEDILPLLSMIKDDWLKPKMIKERDIMIKRARIARIFTMFGYFMMLVSFNLCVIFPVFG
ISMRYLTNKTDPGKLVPLQTYYIYDRDKSPFYEITYVMQSLGLSAAAIMYTGVDSFLSLLVFHVCGQLENLKI
RIIHLDKFQNFENALSHSIQDHIRLIRFITIIDEIFTLMLLGTLFYFGILFAFYGFLLGTMITQGRDLSLPRL
TFIITVFVNTFGHTCLYCAVGEILVAQCEGIYEAACKYKWYNLESKKAKSLMMIMIRANKSMYLTAGKLFPMT
MSTFCNLLKTSGGYISVLLAHQE
>AcepOr318
MHTLPLSFALLTCAGYWRPINLTSIKYWAYTVYSIAMNFLLHSFTFCGLVDCFISEDLKTFIEKFSLFLSVLG
VSCKVTNLATRRDEIINLTDMLLDDICVPRNAHESNIQRRFNRNAKIITICCEILNESAAFFSIAQFRHFVNT
RTLPLYDWVPYDISSTMIFWATLLHQTIIIMICANTSVAHETLISGFMIQICAQLDILCHRARILPDLLQEAW
KCSISKEDFKMRERQLIREIVHHHRYIYRFAKRINAVFTLMIFVQFTISSTVLCLCIYQMLTTNLLSVEFAYS
SSYLGSMLIQIYLYCWFGNEVTLKSTEIGNAVYEMDWPMLPVDLMKTLLIIITRSVKPIKITSGYIITLSNDS
FMKIIKISYSAYNVLQGS
>AcepOr319
MHTLPLSFALLTYIGYWRPINLTFIKYWAYIIYSIVMNFLLYSFTFCGLVDCFITEDLETFIEKFSLFLSVLG
VSCKVMNLTLHRHEIIGLTDMLLQDICVPRNDCEMDIQRRFDRNAKIITICCEILNESAVFFATVAQFRYFIS
TRTLPLSDWVPYDISSSTAFWATMLHQTIGLIICANASVAHETLISGFMIQTCAQLDILCYRVRMLPSSLREA
HKCSISKEDFKAREQRLIRELVHHHHFVYRFAERINAVFTLMISVQFTISSTVLCLSIYKMLTKNLLSLEFAW
SSSYLGCMLMQIYLYCWFGNEVTLKSTEIGNVIYEMEWPMLPVDLMKILLLIITRSKRPIKITSGYMITLSND
SFMKIIKISYSAYNVLQGS
>AcepOr320
MEHQIYADKEYNELIKPIMIAAKFVSFWPLKRDHSTSAEVFKTCHVLWFFFVLISMCISVTADIVNNFNDLDE
LTACALTGSALYLATLRLIIYTASQKDMLYVVEIMRKDWTCSSYEDRVVLKEKCLFAFRLAKCFIIMVIVTVT
TFSCIPILEAYVFGREKIFPFRGYFFVNQSVTPVYECIYVFNVMGGFFGGGTIVGATSFNFVAITHGSAKFAV
LRKRLEAMNSNDPNADRAMANCIKDHQNAIIFADALERIINILALGQFVISTGLVCFAGFQITSMMKNKARLM
KYSMFLNSAILELFMFSFSGHALIDEANNSDAVSKSAYCSYWIGGTFGRSLQIVMMRSKVPSRITAAKFYSMS
LESFSQVLSTSFSYIMILKTASEE
>AcepOr325
MTRKNTFNHTLKFMLALCGIWPGASFVLPCRIFWIVSLTVTLFCHYRYFLTHVHSAEILDLMDCLSSFLAYSK
IIIKFLVFWLNEQKFVEILTVMAEDWNDCSNNDISIRETTRKAKISDRFTNAIITLHTMAIVAYCIGIILADI

DVSDTTKELPLVNKIEIPFDINTQFTYRTVLIAEFLLMILCGWATGITNSLLLTLILHTAGQIEIMRHWLMQL
VPRKNEDKHKSVAATTSKIIQKHQKIISFSRNIEILYSYIALLQFVSNTIMICSIGFVIVTAIGSPNAVEQIM
KSILFYITTNLEAFIFCYAGEYLNNKSKEIGVAAYNCEWYDLKFTESRILLFIILRSQKQLTLTIGKMMDLSL
QAFTSIMNASGSYLSMLLAIQ
>AcepOr327
MRSNRTINRTVELMLTMFGVWPGISCVIFYRVFWMITLAINQFFHYSYFVTHFHFDNLFDLMDCLSSFLAHIK
LTIKLIIFSLKQRKFVEILITMAQDWNDCDNDGIALRETTDKTKLSSRICNGLIILHTIAAFSYVIGILLTDA
DVTDRTVELPLMMKMEYPFVIDTQRKYSLVLVTQFVFVMVCSWGAGLFNALFLTLTLHVGSQINILLCWLSKL
GSKDIEDKHDSFVSVTTKIIQKHQKIINLSENIENLYTYIALFQFTSNIVMICSLAFLIVTAIGSPDATDQIV
RSLLFYAVTNLEAFIFCFAGEYLNNKSKAIGNAAYNYSAWYDMKAKDSRVLLFIILRSQRQLKLTAGKMTVLS
FECFTNIMKASGSYLSVLLAMK
>AcepOr329
MRSNRTINRTVELMLIMFGVWPGISCVILYRVFWMITLAINQFFHYSYFVTHFHFDNLFDLMDCLSSFLVTVK
LTVKLIIFSLKQRKFIEILTTMTEDWNDCDNDGVALCETASKTKLSSRISNGLIILHTMATFAYVIGILLADA
DVTDRTAELPLMIKMEYPFVIDTQRKYSLILVTQFVFVMVCSWGAGLFNALFLTLTLHVGSQMNILLCWLTKL
GSKDIENKHNSFVTVTTKIIRKHQKIINFSENVENLYSYIALLHFTLNIVMICSLAFLIVTAIGTPDATEQIV
RSLLFYVVTNLEAFIFCFAGEYLSNKSKAIGNAVYNSTWYDMKAKDSRVLLFIILRSQRQLKLTAGKMTVLSF
ECFTNV
>AcepOr330
MKVKNTVSPATEILFRIFGIWPNTSFILLYRLFWTISIVFEQIGQYRYIVKHFHLMELFEIIIILSLAVTMTR
FFIVLVLFWYKQRTFSKILTMMAIDWEKCSNTKFSMFVTSYNAKLSERFVNMMIILHSIAVILCNTHILVKHV
DDGNASNVSTRLFVLEMELPFDANQRFVYESVIIIQFFHFLLCSNVVSVFNAFLINLILHIGGQIDILRESLL
GIIPKKDKDSSSHFTVKEVIKEHQKIITFSEHIEHLYSYIAMVLFVSDILIICCLGFTIVASIGRPDVLKNII
RVLLYYFAINMEAFIFCFAGEYLSAKSKSIGDAAYASLWYELDSRTNRTMLFLIMRSQNQLTITVGKIMNLSL
ERFTSIIKTSGSYISVLLAMY
>AcepOr331
MKVRNIVSPAIEIWFRIFGIWPNMSCASLCRLLWTILIIIEQVLQYRYIIMHFHLIEFTEIMHILGSTMTYTI
FLIKLVIFWCKQRTFNRILIMMAIDWEKCFNTKFSMFAICNAKLSQRFVNMTVILYSTTVLLMSSKIFLKHAD
DNIASNISTRLFIIDMDLPFDANQRFVYELIIIAQFFFSLLCADANCLLNALLINLILHIGGQIDILRENLKE
IFPKNDKNPSHFEAKEAIREHQKIITFSKYIEDLYSYIAMVLFVSDSLIICCLGFTIVASIDRPDASESIIRN
FLFYVVINMEAFIFCFSGEYLSIKSKSIGDAAYASFWYESDSRDSRIILSLIMRSQNQLTITIGRIMNLSLGQ
FTTIVKASASYISVLLAMY
>AcepOr334
IEMSTISRAVKICLHAYGIWPYLSSTVLFRLFWIVMYGTVQFFQYHYVAIHFYTDSFSDFMDSVSSAMTYSLL
FIRLNILWANQQTFSNILQMMAMDWKNCVLTDCSLRITINKAKLSHRISNWIIGLQLVAVTLYSCGVLAANIG
NVQRMNVSAREHILKLKLPFQIDTFPIYVCVTILEFFHIITCGCGISTINSLIITLILHIGGQVDILYDWLLK
VFSKNTMNTVNRITMKTLIIKHQQIIMFSENIENLYTYIALMLFVSDIIIICCLGFIIITSISTPGGAAILVK
SILFYIMMNLEAFIYCFAGEYLNAKSKLIGDAAYNSLWYDITIEKKRMILFMILRSQKRLTITIGKIMDLSLE
RFASIVKVSASYISVLLA
>AcepOr335
MTTISRLIKFGLHIYGVWPYVSSTIVFRLYWIIMLSMAQVFQYGYVIMNIHMDDFSEYMDGVSSAMTSSLLYI
KLVILWTNQRIFFDVLQMMSEDWQVHTSNCYNSRIMTDMANVARRTSRWIVGMQIGSATFYSVGVLAANANSP
EKLEPYARELILKMEFPFNISTDFIYATVQTVQFYHLFLVACGITIINSLLVTLILHICGQIDILREWLKKIF
SKNSADLMDEITMKSLITKHQRIIVFADNIEMLYTYIAMMMLLSDTIIICCLGFIIATSLDTPNAAAILVKSV
LFYISMNVEAFIYCFCGEHLSAKSKMIGSAAYDSLWYDFSAKESQTVLFLILRSQKRLTITSGKIIDLSLERF
TSVIKASLSYMSVLIAMY
>AcepOr338
LQNKIDRIEDLFVHLERIFSIGGIWPFKQSYIRFAICISHFTLYLIMAYADFYDVFGNLELMVMNLVETMAYS
MTFLLVWLIRCSNLLKLLIHVIKKDMVERKFENFEEERIYYNYNFISKIFSYGSIIGMFVTVVLLYLRPLVYL
LTINQASRNSTESFMLPYRIHLFFDINNTHTYILMYLCLFPMIYFSICHMAAICLMVILVFHICGELSILAYR
IRHIKECSQTMIADRIRNFVRMHLKIIWMAKSVDNTFNLILLDELVGNSVVLAISLYYVIMNLDVSEIATCCT
FTFFALISLVMLFGYCLIGDQLTQQCISVQDAYYECNWYEMPLDCKKCLLICMIRGQVMLYLTAGKFYIFSLN
SFTDV
>AcepOr339
MQFFEGQNYRINKILLSCVGQWPYQTNRSSNAIIIIIVSLAGTQFIAKICGLLSIDNIDLFIDSLSPLVVDIG
CGVKLITCILKATEIRALFDQIRYDWQLLITSSQIKILNNYAQNGRTFTIIYASAFYSALVLFMLIPLQSLLL
GSSSNDTTRLLLHQVEYYIDMEKYYLPILIHGYTTAVVCVSIAIAADTMYVIVVQHVCGLFMIIGQQLENIIK
EDNLEINFNLSIREDKPYENIVSSIHAHKRALRFASLIEAVFSQMFFVVAGFNMLIISMTGVTAVSNMNKPEE

FLRQITFSCALLVHLFFESFQAQRLIDHSTYIHASLMNIAWYQTSARTRKILIFMLMKTREPCVLTAGKMFVI
SMDTFSTVRR
>XP_016905109.1 PREDICTED: odorant receptor 94b-like [Apis cerana]
MIDFHRSKQVGGQSSNIMKYGIYLSAAMSQLFYICWLGNELGYASSTLDKSQWFSGWHNERLTGIGQVFT
LSTVFTRKSIILRASVFYILSLETFIVIIKRSYSFFALLNNMDLTDH
>XP_016914913.1 PREDICTED: putative odorant receptor 71a [Apis cerana]
MDIFQETRNKYINIFDIPYYKLLEKYMKFLGQDPRQRDDFRNIIVIAIVTSISGILIPTLLELYTSLCDK
NMDAVIECLPHLITGVTSIVKLLNIHFNRENFKKLFEFITKEWEKFELNNQFHVLEEITVKGSKMAQLYR
NTLLSFTVLFLLVPLVFPFLDIVHPLNETRPRQQLFRVNYLIFNHNDYFFYIYLQLAWGSIIVVMIIVTI
DSLYMIIIHHSSGMFAMCGYKVQEATKYTNLFNDRIISENYTYEQLKNCITIHDKALQFYNILNESSRNS
YLIQVGLNMMDISVTAVQTVVNLDRPEEAIRTAVFLGAEQFHLFVISLPGQVLLDHCTEMADNIYSSTWY
EIPVKIQKVLHMMQIRSKKPCSLTAGGLYEMNMENFGITFKICMSYFTMLMSLKK
>XP_016915034.1 PREDICTED: odorant receptor 63a-like [Apis cerana]
MDRFSVFQKIEKQRNEIYDIPQYKMIEKYIRFLGQDPRQKSESRTIIVFTLIISIASIVIPTILELYISL
RNKDMDGVIECMPHFIASSISAIKLLNLHFNRQNYNILLHFVIKKLQQLKSTYELNALDETIMQGKRMAQ
LYRNTLFSFLILFLLVPLVSPILDIVHPLNQTRSRQQLLRVNYIIFDIDDYFFYVYLQLAWGSIIVVLTI
IAADWFYILIIHFNSGLFAVCGVQVLEATMNSNLVSKDAFSKNSSYEKFRTCVIMHNEVIEFYNILNENC
QYSYLIQVGLNMLGMSTTAVQTVINLDRLDVAIRSAVFFGADQFHLFLLSLPGQILLDHCADFANAIYGS
TWYGTSLETQKMLYMMQIRSKKLCALTAGGLYDMNIENFGITFKTCMSYFTMIMSFK
>XP_016921242.1 PREDICTED: odorant receptor 30a-like [Apis cerana]
MHTSESKKYSKDYEWAVRLNRFSLNIICLWPVEEQNIRKQSWTKLHIITCFMLITFVCTIPCLCALKQCN
NLMEVTDNLAYSIPLIITTIKFIVISSKKKVLSLIVNMVAKDWAKLKTDYEKDIMIRRARIARIINIFGY
ILICILIWLLMILPRFGITIRYVTNGTDAKKLFPLPSYYIFDVSETPYFEIIYALQSISLLIAAFCYAGV
DNFFGILILHICGQLTNLRFQLANMKESEASNFILIAIVKDHIRLIRAANVIENTSTLLFLILLLNFGMC
ACVYGLLIITIYIEEEQFSLLRIIYLICNFTNTFLQTFLYFMAGQMLVTQSEEVHNAAYECEWVSLKYTK
AKSLIIIMARSKKPLYLTAGKLFPVTMLTFCNILKISLSYISFLLTIL
>XP_016922450.1 PREDICTED: odorant receptor 4-like [Apis cerana]
MDFEGSQYYNINRILMICVGLWPFERTIYSKLINIFIFLLLSTAIFIQTMSFITLKMDMNLILTSLFYTL
CTCICIMKYFSFLFHIKDVKALLDEIKKDWNSLKNTEELRIIHEYSKTIKGITICFIIIIVPLQLVFFLS
IFGNDILDILIPLNHTRPRTVPIEIYYFIDQQKFFYFFGMHLNIITSFGGLVYIAIETISMAMIIHLCGL
LKITSFRITNTFVANIPRVSFTERNIIIRKKVISIVYLHVKIKSYINWIQETIIISYNFLMLFGLIQFSI
SMYELTKSIISNNTNELISSLFFIICIAIYGFIPNHFAQKIINHSSNIFINAYNTEWYELPVIEQKLILF
IMQNNLKNFHFVLLGSMVASYDVYVTVMKMIKIIIVSFIICSRLLKQNHFRYYVRHFRILWSFILLILLI
EFDKTDE
>XP_016922939.1 PREDICTED: odorant receptor 49a-like isoform X1 [Apis
cerana]
MLKQLTPEKVIHIIWVSVALTLCWPLPANSSKTQVFMFKALQIISIINAFILFLPLLYSIYLHFDDIIIV
SKCAAVSISLTQVITQTIICFAKYDSLQHVIEEMIICIKAAQQYEEKIFHKYIEKCYTFYACSITCMYLT
TTAFIIGPAFSSASFPIDAEYPFQINYTLVKIIIYLQQTLVGYQCAAHVCLCIFGALLLWFTAARFECLA
MELQKITNIGMLIACVKKQLHIRRYAKKVVISFRFIILYAIAVSTFVLILDGIIMIMKASLIVKVQFITL
SLSMLTEIYIYAWPADYMKDMSTNVSKSIYNITWYKQTLRMQKDLLNVLVYQRPIIFSVNCILPELSLHY
YCSYLSNAFSIFTAIRVVIEDDP
>XP_016922948.1 PREDICTED: odorant receptor 67a-like [Apis cerana]
MIEQVMLKRVIYITWLSVALCFCWPVSANSSRNQIIVFKFLQIFTIISSCLGSLPMFHSIYLHQDDIIIV
AKSISIVVVFIQLIVQTTICAIKHDTLQHIIEEMITYIKEAKQYEKEIIQKYVAKCYILYGSAIIISYLT
TTIFILGPIFLPVSLPFYTEFPLSLNNTAVYIIIYFHQCFFAYQCSATVCLSIFGALLLWFVVVKFECLI
VKIQNISNKDMMVICIKKQLQIRRYAKEIANCLRHIIFYTIIATSFNMILAGIILIMNPLLVIKIQFMIT
CFTALMEVYLYAWPAQYMDDMSKNVSISAYNLKWYEQTSEMQQNILIMLIFQKPISLSINFLVPKLSLRS
YCAYLSNAFSIFTALRVILKDNSI
>XP_016903989.1 PREDICTED: odorant receptor 4-like [Apis cerana]
MLKQVTPEKGIYIIWLSVALSLCWPLSINSTRKQIVCMKILQIGAIISAFMIFLPLIYTIYLNLDNLNMF
FKCICLLMGIFQHIVQTITCFIKYDTLQRVVEEMMICIKEMQLYEIMCVYVAKCNVFYGGTIILIYTTAT
IFILGPTFLPITFPWETEYPFQVNYTSRNLIIYIHQFFFTYQCAAHICVSMFVALLLWFTSARFECLVKE
LQKTTNIEMLIVCLKKQLLLRRYAEDVVNCIRFIIFYTMVLSTIVLTLSGIILITTSSLLVKIQFLTICV
SILLEIYMYAWPADHMYDMSITVLRSVYDSIWYDQTLNMQKLVLTTLIYQKPITISINVVLPQLTLHYYC
LYVSNAFSIFTTIRAMIYGR
>XP_016904167.1 PREDICTED: odorant receptor Or2-like isoform X1 [Apis
cerana]

MHKPCTNVSSYIFERRYFSRFYTIVGGVEMKNDDFSINLSSIFIKLMGIWMANGQSEKCVRNMTILYSII
ALLFGLWLQITDMYYSWGDFSECIFSMCNMLSIAAPLLKLITLVVHREDFFYLILYLQRKFLHGDYNDYE
RNIVLNCKRKCMFFTCSLTFTTLATVVSYVINPLVANIGRNESDRVLPFNIWIDLPLTISPYYEITFILE
VISLYHIGVSYFCFDNFLCIMNLHVAGQFQVLQYRISNIIDSIDKEKQEKKEKLIVDSCYFASKYYAIFK
KCIRQHQALIAYCRKLEEVFNLIVLEQVLMFSMLICLDGYLVLMANTSTTTRLIFGLHITVCLCQLLMFT
YSCDCIIRESLSVATAANRGPWPMIPMTTSGRMMKKDLILVIMRSGIPCCLTGKGFFVVSLETYTSVLST
AASYFTLLKQHSEAHS
>XP_016904168.1 PREDICTED: odorant receptor 13a-like isoform X2 [Apis cerana]
MHKPCTNVSSYIFERRYFSRFYTIVGGVEMKNDDFSINLSSIFIKLMGIWMANGQSEKCVRNMTILYSII
ALLFGLWLQITDMYYSWGDFSECIFSMCNMLSIAAPLLKLITLVVHREDFFYLILYLQRKFLHGDYNDYE
RNIVLNCKRKCMFFTCSLTFTTLATVVSYVINPLVANIGRNESDRVLPFNIWIDLPLTISPYYEITFILE
VISLYHIGVSYFCFDNFLCIMNLHVAGQFQVLQYRISNIIDSIDKEKQEKKEKLIVDSCYFAKQVLMFSM
LICLDGYLVLMANTSTTTRLIFGLHITVCLCQLLMFTYSCDCIIRESLSVATAANRGPWPMIPMTTSGRM
MKKDLILVIMRSGIPCCLTGKGFFVVSLETYTSVLSTAASYFTLLKQHSEAHS
>XP_016904169.1 PREDICTED: odorant receptor 13a-like [Apis cerana]
MNGRNVRNLSITVTAFYMKIAGFWVASNYAEKRWRNIAMFVTIFFVFMAITIEGRDLYFVWGDFEDSIFA
GCNVITIVLVLFKIFILYINNEELLNVVNYAKTNFWRETNYDAHEKKIIDDYRRLCSFLVCSFTFFAQGT
VVCFLITPVFVNNGKNESDRIHPFNMWFDRSLSLSPYYEIIYTIQILTAYEVGICYHCFDNLLFVINLYT
AGQFRILRYRFENICGKNNGDNYHKFSKSSYCIDEYKSFKNCVQQHQTLIEYCKKLEDIFSIIVLAQVLL
FSLLICLDGYLVLMEDTSSAKRIIFTFHLMGCMCQLLMFTYSCDCLMHDSMSMANAVYSSLWSYLPMDKY
GKLLRKDLMFVIMRSRSPCCLTACGFFPVSLETYTGILSTAVSYFTLLRNQSTDTS
>XP_016904171.1 PREDICTED: odorant receptor 13a-like isoform X1 [Apis cerana]
MRVEKELDISVNLSSFFLKNIGLWIADDSTNERRRKGMLAYTIWCTFFSTIISSRDLYFTWIYNGVRVYY
ICMKLLAVTDILYALANYMSVMMILVKICVIVVHKSEFINLIVYMQQYFWNVNYDSREKEILNSCKKTCA
FFVSSVTFIGICAILSYLMTPFTARFGNNESERVLPFNMWLNLPLSQTPYYELSFLVQIITLYYIGICYF
CFDNVFCIMAIHLTGQFRILGYRFAKLCNIEHEMCEKDAALAKHVHKCYEKFKEYVRYHQALINFHTKLE
NVYTMIILGQVIVFSVLICLFCYQVLLANAPSARRSIFIFLLIGAVSLLFMFTYSCDGVMEQSDNVAVGA
YSALWTIMPMDKFGRKLRRDLIMVIKRSRRVCCLTANGFFPVSLETYTKILSTAVSYFTLLSNRVENANG
S
>XP_016904174.1 PREDICTED: odorant receptor 13a-like isoform X1 [Apis cerana]
MSLKYRRDVSFSLATFFLRVVGFWLASSRLEEWFANATVMYSIITIIFSMWVQMRGLYFSWGDLSVCTFI
ACNSLGLIMDLLKIFVVFIHKKKFLGLIAYMQKNFWRLDYDQYENSVIADAKQMCIYFVCVFSFFSQSTV
FSYMFMPIISNIGKNESDRMLIFNMWLDLPVSMSPYFEIIYVIQALCLYQVGICYLCVDNMFCIMCLHLA
SQFRILQYRLANVSSVEDKEGVDNEENMDSSNKYYVILKNCIRQHQALIQFSVTLEQIFTIITLGQVLIF
STLICFVGYQVLLVNMTFSWRISFLCFLITNMCQLWMFTYSCDSMTRESVNVASAVYSIPWTHIPMDKFG
KMIRKDLQFVVVRSRRACCLTGCGFFDISLETYTKIMSTAMSYFTILKQRTIEVENT
>XP_016904176.1 PREDICTED: odorant receptor 13a-like [Apis cerana]
MQAEYRLDISINLSTFFLKNIGVWISRDPSEQRRMRMLLICTVWMLLLGIVINTRDLYFTLLYNGDILYV
VTNNITLIISLVKICNIIIYKGKFLNLIVNMQENFWNVNYDYHEKEILDDCKKICIFFISSVTTIAICAI
ISYLMTPFIAQSGNNESDRMLPFNIWINLPLTRTPFYEIIFFIQAISLYYIGISSFCFDNIFCIMAVHLA
GQFRILQYRLTKLCDTKHEIREKESTLTKQMHKFYEQFKECVRHHQALIDYHENLENVYTMITLGQVLVF
SVLICLFGYQVLVATASLARRSIFVFMLNGSMFLLFMVTYSCNGVMEHSDNVAIGAYSALWMIMPMDKFG
RMLRRDLIMVITRSRRVCYLTANGFFPVSLETYTKILSTAVSYFTLLNNRIENANGL
>XP_016904178.1 PREDICTED: odorant receptor 13a-like [Apis cerana]
MQSENQLDISITLSTFFLKNIGLWMSDDPCEQRRMRMLLVYTVWILWLGMIINGRDLYFTFLYNGDVLYA
LTNNITMVIGLIKIYVILLYKGKFLNLIVYMQQNFWNVNYDCHEKEILDDCRKTCIFFVSSITTIGTCAM
ISYLMTPFAIRSGNNESERMLPFNMWLDMPLSRTPYYEITFLVQAMCVYYIGISNFCFDAVFCIMATHLA
GQFRILRYRFMKLCDMDNEIHKKNSILEERMHKFHEKFKEYVRRHQALIDYHQKLENVYTTIMLGQVLLF
SIIICLFGYQVLLATASLARRSIFIFLLMGAMFLLFMFTFSCNGVMEQSDNVAVGTYSALWTIMPMEKFG
KMLRKDLIMVIMRSRRVCCLTANGFFPISLETYTKILSTAVSYFTLLSNHVDNA
>XP_016904179.1 PREDICTED: odorant receptor 30a-like [Apis cerana]
MQKEKYTDVSLKVSQFLLKSAGVWVIGNNAEERQRKFAAFYTLVTLIYGIYVNVVDIYHNLDNLAHCVFL
TCNIMCILLGLFKCFVLSFFRVEFSRIVSYAQKHFWRFDYDDDEKILFGKCQKFCRVWIIVVSMISQSSL
AFYIITPVYENIGKNKSDRILPFKMWVDLPLSVTPYYEIMFVIQLLAVEQIGIAYVCSDFFLCILNLHAL
YQFRMMQREISRIWSVIDEQTTNVTAYTRGCHVALKKCIRRHQSLIEFCNKLEQVFTFPILSHVVVFSLL

MCFDTYEILLADIPTLKRLIFLCHMIASFIHIIFFTYICHNLTEESGNVGLATYSGWWTTLPMNETGRML
RKDIRIIMMKSMRPCYLTAGGFFPMSLETSTALLSSTMSYFTLMRESSMKTN
>XP_016904180.1 PREDICTED: LOW QUALITY PROTEIN: odorant receptor 13a-like [Apis cerana]
MQTDNQLDISINLSTFFLKNVGVWMSDNSDEQRRMKMLFIYTIWMLFCGTIISTRDLYFTLLYNGDILYA
MTNTITTIMALIKICIILTYKGKFLNLIAYMQRNFWNVNYDYREKEILDDCRKTCTLFISSVTTIGMCTV
ISYLTTPVITQSGSNESERMFPFNIWINLPVTRTPYYEIIFFVQAVSLYYIGISYFCFDNIFCIMAVHLA
GQFRILRYRLTTVCDTEMREKDSQSTLAKQVYKFYEQFKKCLRYHQALIDYHQNLENVYTMITFGQVLVF
SVLICLFGYQVFVVSYLYFXICXNNWKEYKXYVVKFKKLYLYFVYKTLCKIDDLKXWTIMPMDKFGKILR
KDLIMVIKRSRRVCCLTANGFFPVSLETYTKILSTALSYFTLLSNHIENST
>XP_016904181.1 PREDICTED: odorant receptor 30a-like isoform X1 [Apis cerana]
MHATPYSDVSIKVSQFLLKLTGVWMTVNEGEKRGRRIAMAYTFLIQVYGLYLNIGDICYSLDDLSHCIFL
ICNTLCIVLTMFKCSILFIRRTEFKNLILFARQNFWHLDYDRQERLLFTKCSKFCTLWTITVFSFTQASL
TFYIITPICANIGKNKSERILPFKMWIDFPLSETPYYEIMFLIQLLTVQQIGIAYTCNDNFLCVLNMHVV
CQFRILQHRLSKLWSIIDERADKINYASKCYEALKECIRHHQSLIEFCDKLEYVYTLPIFAHVVVFSLLM
CFDTYEIFLANVPISMRLIFFFHMVGSFIHIIVLTYICGGLIEESSNIGLATYSGWWTVLPMDEAGRMLR
EDVKVMIMKSMRPCYLSAGGFFPVSLETSTALMSSTLSYFTLMRESSKDK
>XP_016904184.1 PREDICTED: odorant receptor 13a-like [Apis cerana]
MRSTRGRDISITWTSFLMKVVGLWLAADRDEQRRRDFALVYTVGALFIIVCIGFRDIYFTWGNFSDSVYI
SCNNLYLMIVVLKVGVLYAHKMEFFDLVTFTRTNFWRSYRDPEEKLILAECRRICTIFVVVISFCAQGTC
TGYMITPIIANIGRNESDRELPFNLWVDFPVGLSPYFEILFTVQILCVYHVGVCYICFDNLLCIVNLHVA
GQFRILQHRLRNFNVTGDRESCRANICYTKLRSCVIHHQALTKYCKQLENIFTIIVLGQVLFLALVICLV
GFQLFLMDTPASRKVSLTLNFAGTLCQLLMFTYSCDDLIRESVNVGNAIFSGPWTGLPMDKVGRVLRKNL
IIVVTRSHRVCCLTAGKFFPVSLETSTAVLSTAMSYFTLLKHSSLKMENTT
>XP_016904185.1 PREDICTED: odorant receptor 4-like [Apis cerana]
MQREVQLDVSMNLAAFFLKNIGLWMSDDPGYERRRKVILVYTMWCIMLSSVVISRDVYFTWLYDGDILYV
VTNALSMMMIAVKICMIVIHKEEFINLIVYMRENFWNDNYDFHERGILENCKRTCAFFVSLVTTIGICAI
LSYLATPLIVQTVSNNSERMLPFNMWLKLPLSESPYYELMFVYQITTFYFIGISYFCFDNIFCIMTVHLA
GQFQILRYRFGKLCSAEDRVSEKDTESRFYHRFKAYVRYHQTLIDYCEKIENVYTMIILGQVLVFSVLIC
LFGYQILLANAPSARRSIFIFLLIGAMSLLFMFTFSCNGVIEHSENIAIGAYSALWTAMPMNKFGKMLRN
DLIIVIERSRRVC
>XP_016905270.1 PREDICTED: odorant receptor 46a-like [Apis cerana]
MYNMLINRENIILVTSMLESKPFQPETEEEVDMRDKCEKQARLNAIYFAILVELSVMSLSFGGLLKAENH
KLPYRMWLPYNYTSSSAYIFIYTQQVISLIIGAMIHIACDSFIWALLMYICSQIEIFNCRLKKIKHEKNK
VTKLCIHYHNLIYRLATTINEQFKMVIFVQFTMSTLTICINFYILTGTQITFERIMQLTIYSICILIQIY
IFCWYGNEVKLKSLDISNMIFELDWPDLDNSTKRDLLMIMMRASYPIEITSIHVITMNLDSFVILLKTSY
SAYNLLQGNRE
>XP_016905287.1 PREDICTED: odorant receptor Or1-like isoform X1 [Apis cerana]
MRILRWTFLLFALCGCFPPSSWTTRLKRYLYKIYAIFSFTALNSFLLSQILDMVYNVKDTDDFSDNFSVT
VVVFVTCFKLITILTRRENILLLCNTLKQEPLSPMNTEEFEIFLKFEKLTDWNTLGYFILLMSSSLCILM
GSLLANFKIRKLAFRTWLPYDYSTASAFLLAFAYQIVVATVCTFACVASDTLYSGLLIHISCQFEILEHR
LKNIGSDKNYSMKQCVRHHNHIYKYGEMVNDAFQSIMFFQFCTSLSMICFNFYRIMQIEMDSRYIGTILY
MVCSLMQIFYYCWFSNEVKLKVRYCQVKDTNMTHDYDFLFFFFFVPQSLELSDMIFRSNWTSLNNNVQRA
ILLVMRRSMKPIEFTSIYIVSVNLDSFMTVSIRKISSCTHLQVKTYCATYLCCSY
>XP_016905289.1 PREDICTED: odorant receptor Or1-like isoform X3 [Apis cerana]
MRILRWTFLLFALCGCFPPSSWTTRLKRYLYKIYAIFSFTALNSFLLSQILDMVYNVKDTDDFSDNFSVT
VVVFVTCFKLITILTRRENILLLCNTLKQEPLSPMNTEEFEIFLKFEKLTDWNTLGYFILLMSSSLCILM
GSLLANFKIRKLAFRTWLPYDYSTASAFLLAFAYQIVVATVCTFACVASDTLYSGLLIHISCQFEILEHR
LKNIGSDKNYSMKQCVRHHNHIYKYGEMVNDAFQSIMFFQFCTSLSMICFNFYRIMQIEMDSRYIGTILY
MVCSLMQIFYYCWFSNEVKLKSLELSDMIFRSNWTSLNNNVQRAILLVMRRSMKPIEFTSIYIVSVNLDS
FMTVSIRKISSCTHLQVKTYCATYLCCSY
>XP_016905291.1 PREDICTED: odorant receptor 46a-like isoform X1 [Apis cerana]
MVVQALQWTRFLLSVCGCMPPTSWKSSFKKSLYNIYTFVIWLLILSLVSTQILDIIINVKNKNEFIENFY
ITLVVFVTSCKMTIILRYRKNILSLMDDLQHEPFSPITHEENEIRTKFNKMNERTSICYTILVLVSATWI

FVRSFFTDFKKRKLTFRAWLPYDYSELLPFTLSYAHQATTSMFCSCQNISCDTLFGGFLVQIYCQFEILE
ERLKNVQQDESNYSAKQCVKHYHQIYKFSRTLNEKFKVILFLQFCAIAFILCFNLYRMTTITMIPKLLEA
SLYLIRVLVQILYYCWFSNEVKLKSLEVPGMIFKSDWTSWDDKTKKIFLIIMTRATQPFEFTSGYLVTLN
LEFFVALIKASYSVFNLLQRTK
>XP_016905297.1 PREDICTED: odorant receptor 46a-like [Apis cerana]
MTSLHQTLDRLITISYTVMVEFTVFCMIVSSLVTDFRNQRLAYEAWLPFNYSAPNYYYYYIAYVHQIIAL
IGTSLLNVACDVTICGLFVHMYSQQEILKHRLKEIVNVGSRSTIGKIVHFHNYLYGYAFMVEEKFKKIIG
IQLLSSTLVVCFILYKLANTSFISSKFLEFVLYLACMMTQIFFYCWYGNQLKLKSVEIVDTIFELDWISL
DDRSKKDLINIMRRAMNPIELTCAYIFTMDLKTFVSILKMSYSTYNFLQRTKES
>XP_016906830.1 PREDICTED: odorant receptor 13a-like [Apis cerana]
MSEFRNKEYDQLIKPIMITGKIISIWPLAENSSRITITFRRFHLFCMFFLVIVMSVAVTADVVHNMDDLD
EATECALICTAFYLCVVRLLVYSFHQKDMLYVVNTMKQDWISSSDQDRLIFAEKTVFAFRLAKYFITTVA
ITIVMFMSVPILEIYVIGSSDKVLPFRGYFFINQTVSPIFEFLYLFNVTAGGFGGSMIAGATSFNLVVII
HGSGKFAVLRRRMEALSGADPNSTTIMGDNVIRHQQAIKFADTLERIINLLALGQFVISTGLICFAGFQI
TSMMEDKGRLMKYSTFLNSAILELFMFSFSGNGLIDESEGIGESAYNSGWIGSRFHKSVQIMMMRSKIAS
KITAAKFYSMSLESFSTVLSTSFSYFTVLTATKNE
>XP_016908549.1 PREDICTED: odorant receptor 13a-like isoform X1 [Apis cerana]
MDSDMLEKRFLKITKRFAKLSGIWPDQNKYLKYISWIIIYVISISSVVVQVARIVHISTVNVIVEQSGIA
TAIFLLLLLKEANYILNATKVKSLFNDLYMDWTMDRPKEELEIMSTYAQRGSFLATFYLINAYCCSLLFLQ
VPWTARLLYMIKSQNTSPPMLHVIPGYYFIDDDRDYYYFIQLHMSLSIIMVVVVYVAYDTCYIVFVQHVC
GLLAVAGYRFKHAIDDSASKNSEEKIKETCKKIRSSIQGHERAIRYLKKIEDTHVNLLFISLGLIIMNFS
ITLLKVVTMDYCLDFYKYSSFLIVQLMHLCYVMIQGQFVIDSCNEIYCSIYEASWYNINPKIQALYVLAL
RRSLTPPRLTAGGLIELNMQSFSEVIKLSVSYYTVLRST
>XP_016908625.1 PREDICTED: odorant receptor 22c-like isoform X1 [Apis cerana]
MINYRLLNMDKRIIEDQYLKINKFFGQLVGVWPYQQRFIKFCIRFITSVIVVLTLSAQISRVIKFYSLDV
LSDQLPYLDVGFVLLFKQYNYILNEDKLRELLNEIISDRLIKRSKEELEILEIYLKRARVLSTVYEVSIF
FCGFMFLLIPSIPPILNIISPLNESRGRELIYPSYYFVDEEKYYYPILMHMIAVALILTSVYVACDTYLV
YIVHHGCALLAISGYRFKHAVDDIKLRGGDYIDPLTDETYTKVRQSIKAHKTAVEYVDKIDACHIHYFLL
IIGMIVLAFTGTFVKLSSMEVNVRFFTFCAFTVGQLTHLFFLTIMGQFLINANDEIFKTIYEAHWYNGSS
RTQSLYILVLRKCLSPPKLTGGGLVALNLDSFLQILKASFSYYTVFRS
>XP_016908626.1 PREDICTED: odorant receptor 22c-like isoform X2 [Apis cerana]
MINYRLLNMDKRIIEDQYLKINKFFGQLVGVWPYQQRFIKFCIRFITSVIVVLTLSAQISRVIKFYSLDV
LSDQLPYLDVGFVLLFKQYNYILNEDKLRELLNEIISDRLIKRSKEELEILEIYLKRARVLSTVYEEKYY
YPILMHMIAVALILTSVYVACDTYLVYIVHHGCALLAISGYRFKHAVDDIKLRGGDYIDPLTDETYTKVR
QSIKAHKTAVEYVDKIDACHIHYFLLIIGMIVLAFTGTFVKLSSMEVNVRFFTFCAFTVGQLTHLFFLTI
MGQFLINANDEIFKTIYEAHWYNGSSRTQSLYILVLRKCLSPPKLTGGGLVALNLDSFLQILKASFSYYT
VFRS
>XP_016908627.1 PREDICTED: odorant receptor 67c-like [Apis cerana]
MDKQAIEELYLKDNKFFGQLVGVWPDQGKFMKFFMRFTILIVMIIAFIAQISRVAVFYSVDVLLDQIPYI
DLGFALILKQYNYILNEKKLKELLHNIISDRLVKRSKEEEEIFEIYFKRAMFFCSFYEVSICSCVFMFIS
MPSILPLIMNVIMPLNESRSRELVYPSYYFVDEQKYYYLITGHMLAVCLGHTFVYIACDINLIHIVHHGC
ALLTISGYHFKHAMDNVGLCKEKYSDELMEKTYVKVSQSIDAHKKAVEYVNKIDACHIHYFFILLGMIII
TFTGTFIKLTSMEIGGRFFTFCIFTIAQLTHLLFLMVMGQFLIDSNEEVFKTIYDARWYYGSSKTQSLYL
LVLRKCLNPPKLTGGGLIALNLDSFIKVLKTSFSYYTVFRS
>XP_016908628.1 PREDICTED: odorant receptor Or1-like isoform X1 [Apis cerana]
MDKQEIEDQYLRINKFFGQLVGVWPYQERFTKFCIRLTIFAIIILTLTTQIYQVIVFCTLDALSNQLPYL
NALFILLFKQYNYILNEDKLRELLNDIVFDRLMVRSKKELEILNMYSKRATTLCIFYEVIVISSAIMFIM
IPTIPPILNIIMPLNESRDREFIYPTYFFIDEEKYYYPILTYMAIVILIVSSVYLACDTNLVQIVHHGCA
LLAISGYHFKHAVDDMKFSNGNYIDLLMDETYKKVKQSIKAHKTAVEYVDKIDACHIYYFLLIIGTIVLA
FTGTFLKLSTMEIEIRFFTFCGYTVAQLTHLLFLTIMGQFLINANDEIFKTIYEAHWYNGSSRTQSLYVL
VLRKCLSPPTLTGGGLIALNLDSFVQILKLSFSYYTVFRS
>XP_016908629.1 PREDICTED: odorant receptor Or1-like isoform X2 [Apis cerana]
MDKQEIEDQYLRINKFFGQLVGVWPYQERFTKFCIRLTIFAIIILTLTTQYNYILNEDKLRELLNDIVFD

RLMVRSKKELEILNMYSKRATTLCIFYEVIVISSAIMFIMIPTIPPILNIIMPLNESRDREFIYPTYFFI
DEEKYYYPILTYMAIVILIVSSVYLACDTNLVQIVHHGCALLAISGYHFKHAVDDMKFSNGNYIDLLMDE
TYKKVKQSIKAHKTAVEYVDKIDACHIYYFLLIIGTIVLAFTGTFLKLSTMEIEIRFFTFCGYTVAQLTH
LLFLTIMGQFLINANDEIFKTIYEAHWYNGSSRTQSLYVLVLRKCLSPPTLTGGGLIALNLDSFVQILKL
SFSYYTVFRS
>XP_016908637.1 PREDICTED: odorant receptor 22c-like isoform X1 [Apis cerana]
MDVQRLEERYLKINKIYSIIVGMWPNQKRKTIPRIFVELIGILAHITQGSNIILFFSLRVAMDQIPFLIV
ATLLIIKYNNFIINEQKFKELFISILNDWQKKKTHEEVMILEKYADKSLFFILIYVVNAYFCAILFLILP
LTPILLDIFIPLNESRPRVQMYPAYYYIENEADYYYPILIFSIVSLLTAMCVFIATDTTLVYIVQHACGL
LALAGYRFRNSLNDLYSSKKDSKIDEKICRRLCYAIETHKRALAYLTEIEDFYSINIFAQVGASILCLTI
TMMKIATITWSMETNQYYGYVIAQVVHIFFLTAQGQFVIDSHDNIYWDMYEPYWYNMQYKIQAMFVLILR
RNLNPPLLTAGGLMQLNLNTFAEVVKTSVSYFTVLKSV
>XP_016909063.1 PREDICTED: odorant receptor 13a-like isoform X2 [Apis cerana]
MLNMPMLYLQFYELKTIHILNTNNLINIRDYHLLEKYKNIKLLPRDEKKVYRKLFICIKNHQNAIKYSNL
LESLFTKSILVQLFFNVLCLSITGVETVIKLGNLNEMMRFGSFTFAQAVHIFFLCLPGQRLLNHSEEMHV
SVCKVTWYIFPKEYQNLYKFLLARSLIFSKLTAFKMATLSMQTFLAIIQTAMSYFTMLLSTT
>XP_016910285.1 PREDICTED: odorant receptor 4-like [Apis cerana]
MATSIVHFEIRKDNDQDFNSSLLDHRAISYCIGGGLNYAILLFLKDDIRYCIEHIEADWETITRTNDRQV
MFKNAKIGRIISGCIASFMQVSTLCFGIVFGVLKQKIKIGNESMEIHVLPFPTYKIPVDTNLEHSIVLGF
QFLTGCIMTATVIIAFSLATVFACHAAGQLTIMVTWIKEFVNRPQEENKNMRVNEISVIIEHHLRILSFL
GRTEHLLSPICFMEMFKNILSICMLSYCILVEWSGRDIKALSAYTFSVMNITLSTFLICYIGEVLTEKCK
EIGNMVYMTNWYRLSDKDILNLIMIITRSSVEYKMTAGKIIDMSVITFGNLVKTIFAYLNILRQMTIL
>XP_016910302.1 PREDICTED: odorant receptor Or2-like isoform X2 [Apis cerana]
MKSRKSSPFPVTMRSGVTFNLSFVRPIIKKMSRIGNAENDMRHSIWFAYMLLGKLGAWPNRATSSIFSRT
RNCILIFMCYSVQLVILIPGILHFFLKEKDSRKKVKILIPLINGYLQLCRYSLVLRSAKKLCYLLNEMKK
DCTNISEEDRLIFRSKASIGHRLMSVVAIIMYSAGLGYRTFIPLSKGRILLPDNTTIRLLPCPGYYIFFN
EQITPNYEIVFILQVIGGLFSYTIMCGTTSMCAMLCLHASSLLRILVKKINELTKQPDINESAVHMKITD
IVRYQTKIKQFLNDVEHITTYLFLLEIIDETGIGCVIGYCAIREWEDSDSTAAIIYLLLEASVFGVTFTM
CYVGQILIDEGNNVRRMSITIDWYRFPAKEARNLILVIIMSSYPVKLTAGKVVDISLSTYTDVRIQKLDF
SIFYIKLILLGSNFIFQIIKATMGYLNMLKKVT
>XP_016910308.1 PREDICTED: odorant receptor 4-like isoform X3 [Apis cerana]
METKYVEKDLKQAFYAQPFLKIIAAWPIVIESSLSSKIRKWFIIFFSIFLQMCIIVPCILVMFLKEKNGR
RKINLFMLLTNTLNQVFKYAITLNRANELRIAIHEIKKDWLTATPEDRFIFVTNSRIGQRIMLIIVVIMY
TSGLGYRMVLPLLKGKIILPNNVTIRLLPCPTYFTFFNELVSPYYEMIFMLQLLAGFFIYTVLSGTIGIS
LMLSLHMCSLLKILRRKMINLADGSIISENIMQEKIVDIVEYQTKIKRFLGNTELITQYFCFYEISCNTC
LICFIGYCIILEWENHNIIAIVVHFMLLGTCIFVTYIVCYIGQLLLDESNNLARTCITLNWYHFPTRKAR
CLILIIIMSNYPIKLTAAKVVDVSLTTFTDVSMKKVTFLSSNFIHSAKYIF
>XP_016910313.1 PREDICTED: odorant receptor 30a-like isoform X1 [Apis cerana]
MSRVGKAENGMRHTVWFAYPLLRILGAWPNRVSSSTLSKIFNWYLIFTCYTLQLIVLVPGFLHVFLKEKN
GRKKMKMMIPQVNGYLQLCKYSLVLRWTNKLRVLLDEMKEDWLNTTEEDQLIFRAKASFGHRVMSMIAIV
TYSAGLGYRTILPLSKGRILLPNNTTKRLLPCPGYFIFFNEQVSPIYEIIFIIQVLGGLLTYTIMCGTIG
MCVMFCLHSSSLLRILLNKICQLTKQLDVNEVVVHEKIVDIVKYQTKVKGFLKNVEQLTTYLFLLEIMVE
TSIGCVIGYNVVTEWEDSNAAAMIIHLMMQASVISCTFIMCYVGQTLIDEGNNVRQMSITLDWYRFPVKE
ARNLILVIIMSSYPVKLTAGKVVDISLATFTDVCIKILNCVSHISYMDLILYFRS
>XP_016910315.1 PREDICTED: odorant receptor 4-like isoform X1 [Apis cerana]
MARIRNAKEGIRHTFWFAYPFSRMLGYWPLSVSSSAFAKISNYVIIFLSYLLTLIFMVPGLLYIFLKVKN
GRMRIKLLMSHINGIVQMAKYTILLRKTKEIAKLLDEIRKDWMTATEENRQIFSTRASIEHKLTMVVVVT
MYGGGFFYRAILPLSKGKIVLPNNVTIRLLPCPGYFGSLDEQVSPNYEIIFTLQVLGGFVIYTAVCGTKS
ICLMLCLHMCGLLKILTNKVMELANDKDEKVVQQKIANIVDYQNRIIEFLNELNQFVPSVYFFEIILEIL
IICIIGYCLITEWEDNNTMAAIIFVVFQITCFIGTFAVCYAGQLLVDESENVRQACSTLNWYRLPVKKAR
SLILLIIMSNYPIKVTAGRILDVSLITFTSVSIKTLDSCYYFFTYFLLLTF
>XP_016910317.1 PREDICTED: odorant receptor 4-like [Apis cerana]

MENISEIAKAEENLKYATRFMKPILVTIGAWPISSSTSFFLKVLQRLGNIFTYFLFFLIMIPTLMYVFLK
EKNSKVRLKLMGPIINCSMQFFKYTIILWRRKEIQEGLHAIRHDWIQATEEERLIFRSKMKIGRRVVLIA
AFTMYGGGLCYRTILPLLKGTIITADNITIRPLPCPSYFIIINEQQSPIYEILFMLQVMAGMASYAVISG
TCGISALLVLHACSMLRILVNKIKKLVNKSDMSEMTLQRKIMDIVEYQMKIKRFLKNIETVTEYICLIEM
IGGTCLICLVGYCILMELENANTTAVVVYITLQISIIFCVFILCYIGQMLVDENYIVSQASSTINWYRLS
IKNMRCLILIIAMSNYPMKLKAARMMEMSLITFTDVMKMSMGYLNILREVI
>XP_016910319.1 PREDICTED: odorant receptor 4-like isoform X1 [Apis
cerana]
MVQIRNAKEGLKHTFWFAYPFSKMLGHWPLSVTSSAFSKILNSFIIFISYLLQMIVVIPGLLYVFLKEKN
PKKKIKLLMPHLNSIVQMIKYTILLRQMKQIGKLLDEIRKDWSIATEENRQIFSTTASIEHKLTTIIAVT
VYSGGFFYRMILPFSKGKIVSNNMTIRLLPCPGYFGSLDEQVSPNYEIIFILQVFGGFVIYTVVCSTKSI
CLMLCMHMCGLLKILTNKVMELTNDNDEQIVQEKIVYIVENQIRIKDLPFRFLNQLDQFIPAIYLIEVFI
QVLIMCIIGYCIIMEWEESNGMGILTYVIVQLTCLIGTFSVCYVGQLLLDESENIRQAYITLKWYQLPIR
KSRSLILLIIISNYPIKVTAGKIIDLSLVTFINIIKTAVSYMNILQQIT
>XP_016910322.1 PREDICTED: odorant receptor 4-like [Apis cerana]
MPRIRNAKEGINHTLCFAYPLSKMVGCWPLNIPSSTFSKIFNSFIIFISYLLSLIVLVPGLLYLFLKEKN
GRRKIKMMMPLMSTIAQMTKYTILLRRMKEFSKLLDEIKKDWSTATQENRQIFSTRASIEHKLTTVIAIT
IYGGGFFYRMILPLSKGRIVLPNNVTIRLLPCPGYFGSLNVQITPNYEIIFTLQILGGFIIYTALCGVKS
SCLMLCMHMCGLLRILTNKVMELTSDKDEKVVQEKIVYIVQYQTRIKEFYNYVDQFVPYVYFIEMIVGVS
ITCVLGYCIIVEWEDSDAMAITAYIVLQVTCVFGTFSICYAGQLLVDESENVRQACNTLNWYRLPTKKAR
SLILLIIMSNYPLKVTAGRIIDVSLVTFTSIIKSAVGYMNILQQIT
>XP_016910325.1 PREDICTED: odorant receptor 85b-like [Apis cerana]
MVQIRNAKEGINHTFWFAYPLSRMLGYWPLNVPSSAFSKILNSFTIFFSYLLPLLVLIPGLLYVFLKERN
GRRKIKMLMPHINSIAQMTKYTIILRRTKELNKLLDEIKKDWSTATQENRRIFSERASIEHKLTMIVAIT
IYGGGFLYRAILPLSKGRIVLPNNVTIRLLPCPGYFGSLDEQVTPNYEIIFTLQILGGFVTHTAVCGIKS
ACLMVCMHMCGLLRILTNKLMDLTNDNDERVVQEKIVHIVEYQTRIKEFLNHVDQFVPYVYLIEIFVGVL
ITCILGYCIIVEWEDSDAMAIIAYVALQTTCVFGTFSICYVGQLLVDESESVRKTCNTLKWYRLPTKKAR
SLILLIIMSNYPIKVTAGRLVDVSLVTFTSIIKSAVGYMNILQQVT
>XP_016910326.1 PREDICTED: odorant receptor 4-like [Apis cerana]
MVQIRNAKEGLKHTFWFAYPFSRTLGYWPLVSPSAVTKFFNSFTIFILYLLQLIVLIPGLLYVLQVKNAR
TKIKLLMPHLNSIAQMAKYTIILQRAKEFSKLLDEIKKDWLIATEENRQIFSERASIEHKLTTVIVVTMY
GGGFFYRTILPLSKGKILLPNNMTVRLLPCPSYFGSLNEQATPNYEIIFTLQVLGGFIIYTVLCGTKSAC
LMLCLHMCGLLKILTNKIVDLTNDSDEQIVQEKIVHIVEYQTRIKEFLNQLDQFVPAIYLIEVVIQVLII
CIIGYCIIMEWEDSNAMAMVIYVVFQVTCVIGTFSVCYVGQLLLDESENIRQAYNTLNWYRLPVNKARSL
ILLILMSHYPIKVTAGRIMDLSLVTFTSIIKSAVGYMNMLRTVT
>XP_016910327.1 PREDICTED: uncharacterized protein LOC107996663 [Apis
cerana]
MGQPYSLKLVYPLLKILGAWPKSSPSSVLSTILKWCLIFICYLIQLMVLIPGILYIFLKEANLGGKIKMF
VPHMNGITQVSKYTILLRQIKEFNIILKEVKRDYSLASDKNMWIFTTRAYIGHKMMIAIAIAMYSSGVGY
RMILPFLKGRILLPDNTTVRLLPCPGYYMFLNEQVTPNYEIIFTIQVLGGFLNYTTLCGTTGITTMLCLH
MCSLLKILINKMNDLTCQSDECETVVRKKLADIVEYQMKIIDFLNHVEQLTSYLYFCEILEYVCGACVIG
YCLITEWENSNAAALIVYFILEFLCIFCTLTICYIGQLLIDESDKVRQISVTLDWYRLPVNEARGLILVI
IMSNYPIKVTAGKIVDISLITFTDIVKTSVGYLNILRTVA
>XP_016910330.1 PREDICTED: odorant receptor 22c-like [Apis cerana]
MQKSKDKAKTANQKFYLKDYEYQKNVNLSIQYNRWLLKPMGLWPNSYTSRSKDYLYWLINIVCYCLISFL
FIPCTLYLFLEIEDFYGKLKQFGPLIFCMMAFVKYYYLIFHKTDIRECVERIKWDWRNITYAKDKEIMIM
YANFGRKLVVVCTFFMYSGFAFYYIAIPISVGRVKTDNLTFIPLVFPFSRFIVDTRYSPTNEIVFSIQLM
AGALMHGITSAACSLVATFAVHACGQMQVLMNWLQHLIDGRLDMDERLDGRIADVIRQHVRVLKFLALTE
KTLQQISFTEFLGCTLDICLVGYYVIMESKSNDVTSVITYTILLISLTFNIFIFCYIGEIVAEECRKIGE
ISYMIEWYRLMGNKKLFCILIIAMSNSSIKLTAGNIVNLSISTFTDVVKTAVTYLNVLQKTT
>XP_016910386.1 PREDICTED: odorant receptor 4-like isoform X1 [Apis
cerana]
MHLITLNESNCKARNLKYKEDIAYVTKHSKWILKSIGIWPSVLKDVSKFLPKIVFGLCNFVLFFAIIPCI
LYIVIEENDTMIRFKLFGLLSFSLVALIKYWTLLYRKSRIKNCVEQIWIDWKQVELYEDREMMLKYGQMG
RNLIIICAMFTYTGGTIFHTILQYKIGTFIDEYNRTIKPVIYPTYNGLFNVQRSPIYEFVYILHCMCGYV
MHSVTAGACGLTALFATHACGQIDIVIARLNDLIHGKYTKNTFNLNTRLVKIVKHHLRILRFSESIEMAL
QELYFLECIGSTFLICLLEYYCIIDWELSNTISLTTYTMLLISLTFNIFILCYIGERLMEKSSSIGLSCF
MIDWFQLPTKTIHDLILIIAMSNSPIKISAGNIMDLSLYTFGGVLKTSLAYLSFLRTTIT

>XP_016910387.1 PREDICTED: odorant receptor 4-like isoform X2 [Apis cerana]
MHLITLNESNCKARNLKYKEDIAYVTKHSKWILKSIGIWPSVLKDVSKFLPKIVFGLCNFVLFFAIIPCI
LYIVIEENDTMIRFKLFGLLSFSLVALIKYWTLLYRKSRIKNCVEQIWIDWKQIGTFIDEYNRTIKPVIY
PTYNGLFNVQRSPIYEFVYILHCMCGYVMHSVTAGACGLTALFATHACGQIDIVIARLNDLIHGKYTKNT
FNLNTRLVKIVKHHLRILRFSESIEMALQELYFLECIGSTFLICLLEYYCIIDWELSNTISLTTYTMLLI
SLTFNIFILCYIGERLMEKSSSIGLSCFMIDWFQLPTKTIHDLILIIAMSNSPIKISAGNIMDLSLYTFG
GVLKTSLAYLSFLRTTIT
>XP_016910390.1 PREDICTED: odorant receptor 4-like isoform X1 [Apis cerana]
MHLFVRDQTNQPRNLNYEKDIVYVTKHNKWILNSIGIWPTVLKGIDEYLPKIAIALSNLVLSFTVIQCVL
HILLEQKDPILRLKILGLTFFSFISLMKYWVLTIRKPKIKLCIEQIQHDWKQVEFERDRKLMLKYGIIGR
NLSMYSIVFMYSGGIIYHTVMHYKLGSYIDEYNRTIKLLIYPTYSRFYDVQKSPVYELVYILQCICGYMF
DAVTVGACGLAALFATHICGQIDIVMAKLEDLVDGKFSKENSNPNVRLIEIIEHHIKILRFSAMVETVLQ
EVCFLEFIGTTFVICLLEYYCITDWQQNNTIGLTTYSLLLISLTFNIFLLCYIGNLLIEKSTNIGIVCCM
IDWYQLPIKTIQGLILMIAMSNSPAKISAAGIVDLSLPTFGSVLKTSFAYLNFIRTTIV
>XP_016910391.1 PREDICTED: odorant receptor 4-like isoform X2 [Apis cerana]
MCSTHIIRTKGSDTKTENPRFNFLFFYLIDEVLGFNDTQTENQTLYRTDTTRLETEFERDRKLMLKYGII
GRNLSMYSIVFMYSGGIIYHTVMHYKLGSYIDEYNRTIKLLIYPTYSRFYDVQKSPVYELVYILQCICGY
MFDAVTVGACGLAALFATHICGQIDIVMAKLEDLVDGKFSKENSNPNVRLIEIIEHHIKILRFSAMVETV
LQEVCFLEFIGTTFVICLLEYYCITDWQQNNTIGLTTYSLLLISLTFNIFLLCYIGNLLIEKSTNIGIVC
CMIDWYQLPIKTIQGLILMIAMSNSPAKISAAGIVDLSLPTFGSVLKTSFAYLNFIRTTIV
>XP_016910417.1 PREDICTED: odorant receptor 4-like isoform X1 [Apis cerana]
MSASSTRLLQTVAFQLSNNMHVSVQDPINEPRNPNYEKDIAYVTKHNKWVLASIGIWPTVLKNIGKILPK
IVIGFNNLLCFFTLIQSALHIILEQKDTLLRLKFLGLIFFSFMSLMKYWALTIRKPEIEHCIEQVQSDWK
QVEIENDRELMLKYGIIGRNLTIYSILFMYLSGIIYISIMQYAMGSQINEYNQTIKMLIYPAYGGYNIQK
SPTYEIIYGVQCICEYVFDTIASGACGLAALFVTHACGQIDVVMSRLDDIVAGQYKKNSNANIRLMEIIK
HHTKILKFSAVVETVLQEVFFLEFVSSTFVICLLEYYCITVRFFFMSFHIFYMIFLHLHQDWEQKNIISL
TSYVLLLISMTFNMFLLCYIGDLLIEKSGNVGVAVFMIDWYHLPTKTIQNLILIMAMSNTPAKLSVGRIL
DLSLSTFGNVLKTTFVYLNFLQTAVM
>XP_016910418.1 PREDICTED: odorant receptor 4-like isoform X2 [Apis cerana]
MSASSTRLLQTVAFQLSNNMHVSVQDPINEPRNPNYEKDIAYVTKHNKWVLASIGIWPTVLKNIGKILPK
IVIGFNNLLCFFTLIQSALHIILEQKDTLLRLKFLGLIFFSFMSLMKYWALTIRKPEIEHCIEQVQSDWK
QVEIENDRELMLKYGIIGRNLTIYSILFMYLSGIIYISIMQYAMGSQINEYNQTIKMLIYPAYGGYNIQK
SPTYEIIYGVQCICEYVFDTIASGACGLAALFVTHACGQIDVVMSRLDDIVAGQYKKNSNANIRLMEIIK
HHTKILKFSAVVETVLQEVFFLEFVSSTFVICLLEYYCITDWEQKNIISLTSYVLLLISMTFNMFLLCYI
GDLLIEKSGNVGVAVFMIDWYHLPTKTIQNLILIMAMSNTPAKLSVGRILDLSLSTFGNVLKTTFVYLNF
LQTAVM
>XP_016910419.1 PREDICTED: odorant receptor 94b-like isoform X1 [Apis cerana]
MTDDISAIQKKFGSLNEYSIQLNKWFSKTIGVWPLPSSTSKFEKIMTRILILFCWIIAILDTISGLLHFV
FVKEDIITKLKTLAPISYILGGGLNYAVLLLRKNDILYCIEHMETNWKTITRMTERQIMLKNAKIGRIIS
CCIGAFMQVGTLCFCTVLGVFKRTIKVGNESIEIYVLPSPTYKIPVDTNPGHDIVLGFQFLAAYITSATV
VSAFSFATIFACHASGQLTIMIIWIKEFINRSQKENKNRIDEISVIIEHHMRILSFLERAEHLLCPIYFM
EMFKNILTICLFSYCILAEWSEHNIRILSTYIFSIINITLSIFLICYIGEILTERCKEIGNMVYMTNWYR
LPKKDILNLIMIIIRSNVEYKMTAGKIFDMSVITFDNKNSFWIFKYITSSNDVINCTCIKYIKKYVSNIY
SK
>XP_016910423.1 PREDICTED: odorant receptor 4-like isoform X2 [Apis cerana]
MTDDISAIQKKFGSLNEYSIQLNKWFSKTIGVWPLPSSTSKFEKIMTKILIFFCWIIALFDTISGLLHFV
FVKEDIITKLKTLAPISYILGGGLNYAVLLLRKDDIRYCIDHIESDWKAITRTDNRQVMFKNAKIGRIIS
GCVAGFLQFSTICFCTVLGVFKRTIKIGNESIEIYVLPSPTYKIPVDTNPGHDIVLGFQFLAAYITSATV
VSAFSFATIFACHASGQLTIMITWIKEFINRSQKENKNRIDEISVIIEHHIRILSFLERAEHLLCPIYFM
EMFKNILTICLFSYCILAEWSEHNIRILSTYIFSIINITLNIFLICYIGEILTERCKEIGNMVYMTNWYR
LPKKDILNLIMIIIRSNVEYKMTAGKIFDMSVITFGNIIKTVFGYLNILRQVTML

>XP_016919362.1 PREDICTED: odorant receptor Or2-like isoform X4 [Apis cerana]
MIAIVQVELYLDRSNAENNLDALLLINCGILAVAKVMSFRVRSTGLVSNFISAVKDYNESNDEENRVIMR
RHAYMGSDSVFFGIIFHLCGQVEILKREYSKMFNKNEKITEHFILLIKRHIYLLNLSKILNETISSILVI
QLFSSCVLICTTGFQFILALSIGNIVLTIKILIIMCVLLIQLFAYSYVGEYLKTQTESVGNSVYFCTWYD
MPKNISKDIIFIIMKAQRPVLLTAGKIFVVNMETYISILKTSMSYLSVLRVMVNS
>XP_016910425.1 PREDICTED: odorant receptor 22c-like isoform X2 [Apis cerana]
MEKQQCVIAQDDGKKANLSIQWNRWLLAPIGVWPNLRKSKIGKCYSLLISIICYSLIGFMLVSCSMFLMV
EIKKLYNRIKMIGPLSFFLMTFMKYYLLLLHENDIREGIECIEWDWKHMKHQEDRNIMIAYANYGRKLVF
ICTFFMYSAFAFYYLVLPFSVGKIEDGNLTFIQLPFPSSSLIADIRYSPYNEIVLSVQILTGVVMHAITS
AACSIAAVFAVHACGQMQVLMNWLDHLVDGRSDMSKAIDDRIANIVIQHDRILKFLALTEKALQQISFVE
FLGCTANMCLLGYYLIVEWNPKEIILSVTYVALIISITFNIFIFCYIGDLVAEQCQKVGEMAYMIEWYRL
TGKKKLCCILIIAMSNSSVKFTAGNMVELSIYTFSDVVKTSVAFLNMFRALT
>XP_016910426.1 PREDICTED: odorant receptor 22c-like [Apis cerana]
MVNKLEIESMSYIRTDHDYKRNVNLSIQWSRWILKPIGLWPNSSTISTTGKYLYRLINVICYSLISFLSI
PCSLYLILEVEDIYNRIKLFGPLSFCVMAFLKYYLLILHEDNIRECIKRIEWDWKNITYFKDREIMIMNA
NFGRRLVVICTFFMYSGFAFYYIAVPISVGKIPAQDDNITFIPLVFPFSKFIIDTRYSFTNEIVFFIQLV
AGVLLHTITTAACSLAALFAVHACGQMQVLSSWLKHLINGRSDMYNNVDSRIASIVNQHVRILKFLALTE
KALQQVSFVEFLGCMLNICLLGYYVIMEWSSSHLTSAITFFILLISLTFNIFIFCYIGELVTEQCKKVGE
ISYMVDWYRLEGNKKLCFVLIIAMSNSSIKLTAGNMVELCLTTFSDIVKTAVAFLNVLRTLT
>XP_016910430.1 PREDICTED: odorant receptor 4-like isoform X2 [Apis cerana]
MHLITLNESNCKARNLKYKEDIAYVTKHSKWILKSIGIWPSVLKDVSKFLPKIVFGLCNFVLFFAIIPCI
LYIVIEENDTMIRFKLFGLLSFSLVALIKYWTLLYRKSRIKNCVEQIWIDWKQVELYEDREMMLKYGQMG
RNLIIICAMFTYTGGTIFHTILQYKIGTFIDEYNRTIKPVIYPTYNGLFNVQRSPIYEFVYILHCMCGYV
MHSVTAGACGLTALFATHACGQIDIVIARLNDLIYGKYTKEKINLNARFTKIIEHHLRILRFSATVQVIL
QELCFLECIGSTFLICLLEYYCITSSSIGLSCFMIDWYYLPSKTIRGLILMIAISSNPTKISAGGIVDLS
LSTFGNVLKTSFAYLNFIRTTIM
>XP_016910431.1 PREDICTED: odorant receptor 4-like isoform X3 [Apis cerana]
MHLITLNESNCKARNLKYKEDIAYVTKHSKWILKSIGIWPSVLKDVSKFLPKIVFGLCNFVLFFAIIPCI
LYIVIEENDTMIRFKLFGLLSFSLVALIKYWTLLYRKSRIKNCVEQIWIDWKQIGTFIDEYNRTIKPVIY
PTYNGLFNVQRSPIYEFVYILHCMCGYVMHSVTAGACGLTALFATHACGQIDIVIARLNDLIYGKYTKEK
INLNARFTKIIEHHLRILRFSATVQVILQELCFLECIGSTFLICLLEYYCITDWELNNTISLTTYIILLI
SLIFNIFILCYIGELLMEKSSSIGLSCFMIDWYYLPSKTIRGLILMIAISSNPTKISAGGIVDLSLSTFG
NVLKTSFAYLNFIRTTIM
>XP_016910433.1 PREDICTED: odorant receptor 4-like isoform X1 [Apis cerana]
MHLTILNESDYRARNLKYKEDIAYITKHSKWILKSIGIWPIVLKDVTKFLPKIVIGISNFVLLFAIIPCI
LYIIFEEKNNVIKLKLCGLLIFCSIALMKHWALVYRKPKIKNCIEQIQNDWEQVELYEDREMMLKYGQMG
RNLTIICAVFMYTSGAIYHTILQYEIGTFIDEYNHTIKPVIYPTYNGLFNVQKSPIYELIYVLHCICGYI
MYSITAGACGLAALFATHACGQIDIIIARLNDLLHVKYGKEKFNLNARFTKIIKHHLQILRFSATVQVIL
QEVCFLEFISSMFLICLLEYYCITDWKLNDIISLTTYAMLLISLTFNIFILCYIGELLMEKSSSIGLSCF
MIDWYHLPVKTIQGLILIIAISHSPTKISAGGIVDLSLFTFANILKTSFVYLNFIRAMIT
>XP_016910436.1 PREDICTED: odorant receptor 4-like [Apis cerana]
MMNQLNEQSVLMSVSYARDYEYSIQMNRWLLKPIGAWPNLTKTTRTEKLLVKLLNFICHSLIIFTVMPCM
MYIFYENESLKTRMKAIGPTSHWIRAELNYCCLLMRAKEIVYCIEHIEYDWKTVRRARDRELMIKNARLG
RFITCIAALCMYCGVISYIVITGFKKITFQIGNDSYSMYQLPCPFYTNLLDVRFSPMNEIVFALQFVSGF
ISTSVTVGACGLAAALAMHACSQFNVVMAKSDKLVKDNNEKKQDEHTLHKKLGFIVEHHLRTLSLVWYME
KVMNMICLVELVGCTMNMCILKYYFLTEKSKTVLAIYAIVYASTVFNIFIFCYIAEIVTEQGKKVGEKFY
MTEWYQLPHKTALGLVLIISRSSMVIKITAGKLIQISIATFAAVFKASFAYFNMIRTIAM
>XP_016910437.1 PREDICTED: odorant receptor 49b-like isoform X1 [Apis cerana]
MRSTSNIDDLPLNDRYESDIHYTFQFCHWILKPLGIHYFIYNQANKFERILSVILILICFFIIQFVIVPF
GYYILFYEKDMNTKIKFLGPLTFCLSALFKYSYLGIKSSELGHCIKHVEKDWKMLQNKDHRVIMSRYAIM
GRNLITLCAAFMYTGGLSYHTIMPLFSKRKVENFTIRPLTYPGYEAFLNIQKSPTYEIIYCMHCIYVIVV
GNITMAAYSLTAIFITHACGQIKIQTLRLENLKNEKKVLETGIESHLAVVVRNHVQILRFAKNVETTLRE

LFLVEVIVSTLLMCLLEYYCMVEWEISDSAAILTYVVLLFSFTFNILIFCYVGELLLGQQGSEIATALYE
IEWYNLPGRKARDIILLLVISKYPPKLTAGKIFILSMNTFGVVLKSSLVYLNMLRTITEL
>XP_016910446.1 PREDICTED: odorant receptor 4-like [Apis cerana]
MVDRSYNNSRSKNIHYENDIHYTLQMCQWLLKPIGVWPFVYDRTSRLEQLISIILMAMCFSSLLFIILPS
GHHIFFVEKDMHLKVKLLGPVGFCLSSTIKYCYLGMKGVFFEQCIKHVEKDWKMVQDPSYRIIMLKYATI
SRKLIIMCAVFLYTGGMSYHTVMQFLSKEKVNNNYTFKPLTYLGYDPFFDTQSSPTYEIIFCMHCFAAMI
MYSVTTVAYSLAAIFVTHICGQIQIQTTRLQNLVENKDKKNNCDPFALIVHDHVEILRFSKNVEEALREI
CLAEIVESTIIMCLLEYYCMTEWQNNDAIAILTYFTLLISFTFNIFIFCYIGEILSEQCSQIGIISYEIN
WYKLPAKKAHDLILLISISQYPPKLTAGKIIDLSFNTFSSVVKTSVIYLNLLRTVTD
>XP_016910447.1 PREDICTED: odorant receptor 22c-like [Apis cerana]
MSDDLAEIEKKFGSLNEYSIQFNRWVLKPIGAWPASLYTTRIEKIISKILIIILCWSFSLFTLIPGVLHFV
LEKEDTYLKLKTVGPLSHWCIGGFNYAVLLLRKNDILHCIEHIRVDWNIITKKQNQQVMLKYAKIGRYIA
IFCTAFLQGGVLCTCLALGVFKTTIKNGNETIQIYNLLCPAYKLPVQTNPTHDIILGTQLLSAFITSSSA
AGAFSLATVFASHALGQLNIMVAWINEFVNRPMELDNDVYVNKISIIVEHHLRILSFITHIEHLMNPICF
MEMFRCMVGMCMPSYYILAEWSEHNVQNLTVYVMIIISMTCNIFLICYIGEILTEQCKKIGEIIYMTNWY
ELSNKDIFNLMMIISRSSITVNMSAGKIINMSILTFGNTVKSVFVYLNMLRQMTMI
>XP_016910448.1 PREDICTED: odorant receptor 22c-like isoform X1 [Apis cerana]
MADDLAKIKKKFGNLSEYSIQFNRWILKPIGAWPTSLYKSRIEKIVSKILIVICWISSLFTLIPGVLHIF
LEKEDIYVKLKILGPLTHWLVGGFNYAVLLLRKDDIHYCIKKICADWNIITKKQDQQVMLKNAKIGRYVA
IFCTVFLQGGVFCACLALGAFKKTIKIDNETVNIYNLPCPAYNMPVDTNPTHDIILGTQLLSAFICSSST
AGAFSFVAVCASHALGQLNLMMIWINEYVDRPMDLNNNAYVNKIGIIVEHHLRILSFIARIEHVMSPICF
MEMIKCIPGICMPIYYILMEWSEHNVQNLTVYVMIIISMTYNIFLVCYIGEIITEECKKIGDIVYMTNWY
ELSDKNILNLMMIITRSSMNINITAGKMTNMSVLTFGKIVKSVFAYLNVLCQITMM
>XP_016910450.1 PREDICTED: odorant receptor 22c-like [Apis cerana]
MSTMKNQQVVITQDDYKRKTNLSIQWNRWLLTPIGAWPNLRNSRIGKCYSLLISIICYSLIGFMLVSCSI
FLIVEINDIYNKLKMVGPLSFFVMTFMKYYFLLLHENDIREGIERIEWDWKNVKHQEDRNIMITYANYGR
KLAFICFFFMLCAFIFYFLIQPFGGGKIVDGNLTFIQLPFPASILIADVRDSPCNEIMLSIQILTGIVMN
AIRSAACSVAAVFAVHACGQMQVLMNWLNHLVEGRSDMSKKIDDRIANIVIQHNRILKFLALTERALQQI
SFVEFLGCTMNMCLLGYYLIVEWNPNELIISFTYIAIIASITFNIFIFCYIGELVAEQSEKIAEVAYMME
WYRIRGKKKLCCVLIIAMSNSSIKFTAGNMIELSIYTFSDVVKTSVAFLNMLRALT
>XP_016910452.1 PREDICTED: odorant receptor 4-like isoform X2 [Apis cerana]
MADDIIAIQKKFGSLNEYSIQVNRWLSKTIGVWPLSSTTSKFEKIMTRILIFFCMIIALFVTIPSLLHFM
LVKEDVISKLKMIGPISYCIGGGLNYAILLFLKDDIRYCIEHIEADWETITRTNDRQVMFKNAKIGRIIS
GCIGSFMQVSTISYCTVFGVFKQTIKIGNESMEIHVLPFPTYKIPIDTNLGHGIVLGFQYLTACIMTATV
IIAFSLATVFACHATGQLTIMVMWIEEFVNRSQKNKNVHVNEISVIIEHHLRILSFLERTEHLLSPICFM
EMFKNVLTICMLSYCILVEWSGRDIRALTAYSFTIINITLSMFLICYISEVLTEKCKEIGNMVYMTNWYR
LPDKDILNLIMIITRSGIEYKMTAGKIIDMSVITFSNEWSGHDIRALSAYASAVMNISLGTFLICYVGEI
LTEKCKEIGNLVYMTNWYRLPKRDILNLIMIITRCSMEYKMTAGKIIDMSVITFGNIVKTIFAYLNILRQ
MTIL
>XP_016910453.1 PREDICTED: odorant receptor 4-like [Apis cerana]
MMNQVAITEEIKINSDYSLQLNRWFLKPIGAWPLFSTTTKFEKIVSLILNIVCYAIVILCATPSLMQIIL
AEESFYLKLKTLGPVSHWFVSTVNYTALLMKSKDIRYCFEHMETDWQTIKRMEDQQTMLKNAKFGRYVAA
SCAIFMQGGILCFCFVTILTTETIQVGNETRVLHVLPCAVYKELVNVEESSINIFMLCFQFVAAAIANSS
TVGIFSLAAVLAAHAYGQLSVVMVWITEFVNQSREQKKTNDFKEIGIIVERHLRVLNFITYLENIMNRIY
FLELFRCTMIICIVGYYILTEWAEKNVQNLTTYFMMLLSICFNIFIICYIGEILTEQCMKIGEVVYMTDW
YYLPDKTILNLILIIILRSTVVVQITAGKLFNMSIYTFGDVLKTAFAYLNLLRQMT
>XP_016910454.1 PREDICTED: odorant receptor 4-like isoform X1 [Apis cerana]
MSNRSVAIKTDPDTNSDYCLQLNRWFLKPIGAWPSFPSTTRHEKIISFLLNVSCYSSLLFTLIPCLLHML
LENESFYLKMKVLGSLAHWFVGTMNYTTLLLRGREIRLCVEHIRTDWQTVTREEDQQVMLKNAQFGRYVA
AISAAILQSGVLCFCCMTISRTELIQIGNETKIVHVLPCAVYRKLIDVTHSPNSELIIASQFLSGFIVNS
STAGIFSLAAILGAHACGQLNIVMTWITEFVNKSKKREKMIFREIGFIVEHHLRTLNFISCIEETINRII
FLEVFRCCLHICCLGYYILMEWSDYDKRSMIIYFMLFVSVCFNIFIICYIGEILAEESMKVGEVVYMTNW
YYLPDKMILDLILIIARSSVVVQITAGKLIHMSIQTFTDVIKTGFAYLNLLRQVT
>XP_016910455.1 PREDICTED: odorant receptor 4-like isoform X2 [Apis cerana]

MSNRSVAIKTDPDTNSDYCLQLNRWFLKPIGAWPSFPSTTRHEKIISFLLNVSCYSSLLFTLIPCLLHML
LENESFYLKMKVLGSLAHWFVGTMNYTTLLLRGREIRLCVEHIRTDWQTVTREEDQQSGVLCFCCMTISR
TELIQIGNETKIVHVLPCAVYRKLIDVTHSPNSELIIASQFLSGFIVNSSTAGIFSLAAILGAHACGQLN
IVMTWITEFVNKSKKREKMIFREIGFIVEHHLRTLNFISCIEETINRIIFLEVFRCCLHICCLGYYILME
WSDYDKRSMIIYFMLFVSVCFNIFIICYIGEILAEESMKVGEVVYMTNWYYLPDKMILDLILIIARSSVV
VQITAGKLIHMSIQTFTDVIKTGFAYLNLLRQVT
>XP_016910457.1 PREDICTED: odorant receptor 85b-like [Apis cerana]
MSSVKTNQDIKNNINYSIKYSRLILKMIGLWPTFEKISTIRKYLQWLYNAICYCLIMFIIISGWIYISLE
VENIYDRLKFVSLMSFCMLSVTKYHLINIHKDDVRECVKRIEWDWKNISYSEDREIMLMNANFGKRLIIV
TTTVTYSGFVFFYIAVPMKIGKIPAQDANISFIPTMFPFPKYIADVRYSPVNEIVFLAQFICGFLLHGVT
SSVCSLAAILTVHACGQIQVMMVWLKHLIDGRLDMCNSVDQRIATIVNQHVRILKFLSLIEKILQQVSYM
EFLECTMNVCLLGYCAIMEWESNHLTEVVTYLILLITIIFNIFIFCYIGELLANQSRNIGEVTYMIEWYQ
LFGKKKLCCVLIIAMSNSSTKLTAGNLIELSMSTFSDVIKTSFAFLNVLRTLT
>XP_016913695.1 PREDICTED: odorant receptor 4-like [Apis cerana]
MADDIVAIQKKFGSLNEYSIQLNRWFSKTIGVWPLPSSSSKFEKKITKILILVYWIIVLFIVIASFLHFI
LVKEDTISKLKTLGPISYCFGGGVNYAVLLLRKNDIRYCIDHIEADWKVITRMGDRQVMLKSAKIGRIIS
GCIAGFMQIGTICFCTILGVFKRTIKIGNDSMEIYVLPFPTYKLPVDTNPGHAIILGLQYLTSYVMNATV
VIAFSLATVFACHAIGQLTIMITWVEEFVNQPQKESKNTRIDEISVIIEHHLRILSFLERTEHLLSPICF
MEMFKNILSICMFSYCILAEWSEHDIRVLGIYAFAVMCITLNTFLICYIGEVLTERCKEIRNMVYMTNWY
RLPDKDILDLIMIITRSDMEFKMTGGKIIDMSVITFGNIIKTVFAYLNILRQMTML
>XP_016914987.1 PREDICTED: uncharacterized protein LOC107999574 [Apis cerana]
MLKKVTPEKVIYIIRLSVAICCCWPRPFNSTKNQNFGFKVLQISTIISAFMVFLPLLYSIYLHNDNIIHV
FKCICLSVGITQLIVQTLICFIKHNSLQRIVEEMVNCVKQAQQSEIEIFYKYIEKCKIFYGSSIAFSYLS
ATAFMLGPAILPISFPLEAEYPFHVNESLITIIIYMHQSLVSYQCSANVCVSIFGALLLWFTVARFECLI
EEFQKCSNIDMMIACIKKQLQLKRYAEEVINCFRYIVLYGIAVTTFALILCGIILLMNVPLIVKMQFVII
CITILTEVYMYAWPADYVKNMSINISRSVYDLSWYEQTIEMQKNFLNVLVYQKPVTFSISYIVPELSLRY
YCSYLSNVFSIFTTLRVLLEDTSA
>XP_016915036.1 PREDICTED: odorant receptor 85b-like isoform X1 [Apis cerana]
MIRLKTIITCPVEVCLRLIGIWPYSSYRIMQRIFWTIIMGNSTIFQLWYCISYFKTADLLDLLDGITLTL
SNTVTFFKLIILWFNYRTIHNILTIVFEDWNNRALTDKKKQLMVDNTRLSSRISNFLFGIYSVTCILYSA
SIALISDDIDNTNNELILNNKKLLLKMKLPFDFTIFPLYEFVIVAQFVFECFVALTAGMLMAFSAALVLH
IGSQIDITCQELIEIPRYYRGETSYMLKNIIVKHQRILRLSENVKHLFLYTSLIQFLSNILVICFLGFIL
VNALGTEQGSTIFIKCFPYYIAANCEAFILCYTGEYLMFKNESIVHAAYDMLWYNLNPRDSRIVLLILIQ
AQRKLILSAGNFVTLSVQTFASALGTEKGSTIFIKCFPYYIAANCEAFILCYTGEYLMFKNESIIEAVYN
MLWYNLNPQDSRIILLILIQAQRQLTLSAGNFVTLSAETFASMQKVSASYISILMAMY
>XP_016915037.1 PREDICTED: odorant receptor 85b-like isoform X2 [Apis cerana]
MSLKTIITYPIEVCLRLIGIWPYSSYKIVQRVFWIIVMSISTVFQLWYCISYFKTADLPDLLDGITVTLS
NTVTFIKLIILWFNYRTLHNILIVVFEDWNNRVLTHKKKQFMIDNTYLCSRISNFLFGIYSMTCILYSIS
TMLISNDTNNTNNQLILNNKKLLLKMKFPFDFTIFPLYEFIIIAQFVFEYSVAFTAGMLMAFSAALVLHI
GSQIDITCQEIIEIPSNKEEISYVLKNIIIKHQRILRLSENVKYLFLYTSLIQFLSNILVICFLGFILVN
ALGTEKGSTIFIKCFPYYIAANCEAFILCYTGEYLMFKNESIIEAVYNMLWYNLNPQDSRIILLILIQAQ
RQLTLSAGNFVTLSAETFASMQKVSASYISILMAMY
>XP_016915038.1 PREDICTED: putative odorant receptor 92a [Apis cerana]
MAIKSIINRPVEISLRLIGAWPNSSCQILKYIMWTTVMSIFLIFQYSYCIIHIKTATLIDILDCLSITCS
NTLLLLKFIIIWFHKRVLFESLIIIAEDWDNCKFEWNMEIMMQKAILSYRIAKLMLVIFICSIFMYAVST
FFGPDIGASRSDQKKFLLKMEFPFEATVSPLYEIIVTIQLIMQFMFATMAGMFMTIIATFVLHVASQLDI
ICDRLSEILDAHKEQELRIRIIKKLIAKHQRTLNLSENIENIFTFISLSQFFFNILVICFVNFILVTSIG
TEQAPTVISKCFPYYVALNFEALILCYTGEYLSSKSENINWIAYNSNWYELSIYEIRVLLLLTMRSQKPL
TLTIGKYMKLSLETFANMLKISASYASVLYALE
>XP_016916412.1 PREDICTED: odorant receptor 67c-like isoform X1 [Apis cerana]
MVHLREKTSMNIQKDLKYLYGWNYYTMKFIGIWPEERKWNQASNYLVLIPFLMMLCFICAPQTINLSLIS
NDFNLVIENLSMGNITITLSLLKTIAFWINGKPLKFLLNCMTNDWIKVTSKTEQKIMARIANITRNTIIR
STVMCHIVVVFYVFLRYISVKYNENKLLFRAYFPYDITISPNYELTLLGQLVGALYAATSYTAVDTFVAM
LILHVCGQLSGIKNELSRLPTYDKKDLETRLKEIVQRHEYINRFAETIENCFNVMLLIQILGCTVQLCFQ

CFQAIMSFGSGEAQEYLFFQLMFLLVYVFYVMLQLYLYCYVGERLSVESMEIVNAAYNTEWYTLPPNITK
MLIIIMCRAKSPLTITAGRFCSFTLQLFSEVLKTSMRYLSVLYAVKDNIKY
>XP_016917690.1 PREDICTED: uncharacterized protein LOC108001261 isoform X1 [Apis cerana]
MLKYVTPEKGIYIVWLSVALSFCWPLPASSTRKQIVCIKILQISAIISAFMVLLPLIYAIHLNVHNLINL
FKCICLLICVFQNIIQTIICFIKYDVLQRVVEEMMTCVKEEQLHKILCIYVKKCNIFYGGTIVLTYGAAT
VFVLGPTFLPISFPWETEYPFQINDTSRNIIYVHQFFFTYQCAAHICLSLFGALLLWFAAARFECLVEEL
QKITNIDMLIVCFKKLLFLRRYAEEVVNCIRFLVFYAIAVGTFMLTLSGIIMIINSPTLVKIQFIIICMS
SLMEIYMYAWPADHMQDTSVNILQSAYNSIWYEQSLDMQKNLLIILMYQRPVILSINVLLPELTLRYYCS
YVTNAFSVFTALRAVVEDK
>XP_016918120.1 PREDICTED: odorant receptor 9a-like [Apis cerana]
MRRPILSYVELFYDKNVISWSKRLLSLSGLWPDNRNDVRFFLYITYIVIFTWLEIVTLVQNIHDLEKTLK
NITLSFPTILIVLKAVMFRMNMHLVLPLLTVVKRDVNEGLYRSAEERRTVVWYNVAATLFSTSSALSLFF
VPTLFYAKPIIGCLLSKYNNCTLPFELPMKVNNVYEITKLQTYALFCVYLIPTSTLLTIGATGADSLLVT
LTFHLCSQLSIVAYRMRNVNIEPKIYFPKMKALVERHTELLRLANILANTFSSLMFVQTLGLIFSLCIVV
YQLLMTSESGEDMNTIHFIIYSCAVILLAFCYCFLGECLINESSEVQMACYFTNWYDLPEQYTRSLIFCI
ARAQKPLYLTAGKFYVFSLETFAVIVKASMAYLSVLKSII
>AechOr3
MIPTSTVSRSIEIGLHLTGIWPNSSIFFKLLWILVMGIGLIFQYYYLLTHFSTKELPNLIDGLSTTLPHSLLF
FKLIVLWANHRIFKNILIAMSNDWHKYSNMYAMIDKAVLAHRCSKLIITVYSIAVLLYSTASINLNKQSDDDC
RELLIKMELPFGFCESPIYEIVMFVQFVRLIAVASAIGMLNALLVTLMLHIGGQIDLMQQEVEEIFVKDNKRD
LSIIIVKSLIDKHHKIIAFSESIESLFTHIALMQFFSNTLIICCIGFLIVTSLGTDEGVRMLVKTLFFYIAIT
LEAFIFCFAGEYLSNKSKTIGDAVYGSVWYNLKPQDSRILLFMIMRSQRRLTITAGKFMDLSLEGFANSLKAS
ASYISILYAMY
>AechOr4
VSRRDKMTAISTVALSVQIGLRFVGIWPNASYALFFRGVWILTTGIVQTFQYWWIIIHLGTDDMSHLMDGLSV
TMEYSVMFLKLIILWLNSRIFYDILATMAADWREAAISEIHTMRNKANLSRHFSNVIIGLHSAAAFSYGIGVL
VSHSNDYNIDGDKTPAREFTLKLQLPFESDKSPRYELVMCLEFCHQLASSAMTGVLNSLIVTLILHASGQIEI
LCDALRDVSSDKNNQRFVISIMKELIGKHQKIIVFSDQIEKIFCYIALIQFLSSSLVICCLGFMIVTSISTQD
SDTIDSSSLMKAIVFYMAATVEAFIFCFCGEYLSAKSKMISDAAYESIWYDFKPNECKLILFIILRSQRRLTI
TAGKIMDLSLEGFTSIIKASVSYISLLHAMY
>AechOr7
TSTVALVVQIGLRGIGFWPNTPCALLFRCLWILTIGIVQTLQYWWIVIHFRTDDMSHLMDGLSVTIEYSVMSL
KLIIILWFNSRIFYDILAAMAIDWREATSSEMHIMMSKANLSRRISNVIVALHAASFVCFGIEVLASYTDDYDD
DEIETPVRVFTLKLQLPSQCNESPLYELVTGLEFFHQLVASTTTGVLNSLLITLILHTSGQIEILCDILKNIS
SEMNNQQLAFSMKEVIGKHQKIITFSDKIEKIFSYIALIQFMSSTLLTCCIGFTVITSIDTQDSDTVDGTALM
KAVVFYIAAAVEAFIFCFCGEYLTAKSKMIGDAAYKSVWYDFAPKESKLVLFIILRSQRRLTITAGNMMDLSL
EGFTNILKASASYVSVLHAMY
>AechOr14
MLRSTVSPLLKMGLSLIGIWPGASYGTLCWFFYMTTLVVMQYFQYSYVYEHFDFNNLTKLMDGLGLTLDYTLT
ILKLISFWFNRRIFADILIAMDDDWKDNRTNLRECVMMNKANLAHRCSNAMISVNALATFLYFIESHVRGYTR
SKNGQFRRFPIQVQFPFEVYKTPVYELVGVGLFFHVLKTAIIIAILNALILTLVLHVSGQIDIMCQELKAIPS
MIKSSSVSTKSLVVRHQKIISLSKNIESFFSFVALLQFVWNTFVICAIGFMVSLGMNMTSKFEILVQFIIPYF
AVTIEAFVFCFAGEYLSTKSKAIGDAAYETVWYELSTSECRILLFVILRSQKQLTITAGKVMNLTLEGFTNVM
KASASYISVLYAMY
>AechOr18
IMSTVSPTVKIGLRLLGIWPYSTTYWFSFMSSMLIMQYFQYLYIFEHLSMSELPNLVDELPMTLDYSLTIFKL
TSLWIQRRDLHKILATMDNDWRECININHHLYIMTIKANISHFISNTLLSFTAIVAALYFLGEYAIRFVFLIE
DYNDTSRQLPLKLQFPFETQQSPMFELLAITIFLHVMLHVCTVAILNGLIFTLVLHASGQIDIICQEFKNISK
NTLLYGSFVPSFGMLIERHNKIISFSKNIEQLFSFIALMQVVWNTLVICSIGFVFIVSIHNEVSVFVLVKTLF
AYCVITIEAFIICFAGEHLSLKSKLIANATYETLWYNMPLNTEKIIIFIIMRSQKRLTITAGKMIDMSFDTFT
NIMKASASYISVLNAIY
>AechOr19
MTITSTISPALKFGLCFFGFWPGVPYSTIYCLSFMSSMLIIQYFQYLYIFDHLKISELSNLIDSSIITLDYSL
TFFKLIGLWIHRRILHKILKDMDNNWHECINIDQHLYTMTTKANIAHFFSNALLSCNAIITVIYLLGDYVIRS
VFLNENYNNTVRQLPIKIQFPFKIQQSPVFEFIAVIIFLHTILQVWTNTILNGLIFSLALHISGQIDIICEEL
KNFSKNSLCYGSFTSSLGMLVEKHKRVISFSDNMERLFSFIALMQVVWNTLVICCLGFAFIMSIHNKTGVFAL

MKAVFAYLAITLEAFIICFAGEYLSYKGKLIASATYGTMWYNMPSSHSKIIVFIIMRSQKRLAITAGKMMDMS
FETFTIIMKASASYISVLIAMY
>AechOr24
MIVTSTVSPVLKIGLQVFGVWPGKSHSIPYWLIYIFSILIVQYFQYRYVFEHFKISEISNLVDSLPAALDYSL
TILKVTSLWIHRRVIHQLLTAMDKDWQECVNIDYHLYVMTVKANTSRFCSNIMFSINTIALVSYLLSGYAIRF
IYLSGDYNNDTLRQLPIKVQFPFETQQSPIFELLAIILFLHVTLNSCTLSILNALISTLVFHVSAQIDILCQE
FKNLSAKILPHKTSTSTLALLIERHNRIFLFSDKIEKLFSFIALMQVIWNTFIICSLGFIIIISLYVETGVIT
IIKTIFTYLAVIVEVFILCFAGEYLNFKSKLIANAAYESLWYDLLSNQKKIITFVIMRSQKQVTITAGRIMSL
TLETFTSILKASASYVSVLHAMY
>AechOr25
MIITSTVSPLLKIGLRLLGVWPGVSYCIPYWLIYVFSILIVQYFQYQYVFKHFKISELSNLVDSLPAALDYSL
TMIKVITLWIHRRIIHQLLTIMDKDWRESVNINQHLYVMTIKANKSQFCSNIMFSINTIATVLYFLSYGYAVR
FVYSSGDYNDTLRELPIRVQIPFKTPQSPIFELLVVIQFVHVMLIGWTLFIINALISALVFHVSGQIDILCQE
FKNISTKVFPHKTCTSALAILIERHNRIFLFCDKIEKLFSFIALMQVVWNTFIICSLGFIIIISFYIETGGIT
IVKIIFTYLAIIMEVFVLCFAGEYLNFKSKSIANAVYESLWYDLLTNQKRTLTFIIMRSQKQVMITAGRITSL
SLETFTSILKASASYVSVLQAMY
>AechOr29
ILDGTVSRSIEIGLRVIGVWPNSSFTVLRRAFWMITFTMAQTFQYRYFVIHVRTDDLSHLMDGLSTTMSYSLL
LLKLTIFWLNRRIFHDILTIMSQDRSECVTEWAIYSMSRTTDVSHRSSNLIIGLYSMSVFIYGTGVLVAHSDE
SDEQLAVHARELFLKMELPFESNASPTYELVMITQFFHQLSAATIVGVINALIVSLILHVGGQIDIMCQGLVE
ISSDDTFDLQTSTIKALIRRHQRIISLSADIETLFSYIALMQFLWNTLVICCLGFLIVTSIGDTQGSTMLIKS
LFFYVVITLEAFIFCYAGEYLSDKGRMIGNAAYETKWYKLNPTQSRILLLLILRSQRKLTITIGKFMELSLER
FTTIIKASASYVSVLHAMS
>AechOr33
MATLSRLVKFGLHVYGIWPYASFTVVFRSYWIIMLSIAQVFQYRYVIMNIHMDDFSQYMDGVSSAMASTLLYT
KLVILWTHQRIFFDVLQMMSTDWQDYTSNSYISHIMTDTANVAHRTSRWIVGTQVVAVFLYIAGVFAANINDP
ERLEPYARELILKMELPFNISTNFIYMTVQFVQIYHLLLVACGITIINSLFVTLILHTCGQIDILREWLSKVF
SKNSDSMNKIIMRSLIMKHQRIIVFAENIETLYTYIALMILLSDTLIICCLGFIIVISLDTPNAAAILVKSVL
FYISINLEAFIYCFCGEYLSAKSKMIGNAMYNSLWYDFPAKEGRTILFLILRSQKRLTITSGKVVDLSLERFT
SVVKASLSYISVLLAMY
>AechOr36
MKIKNTVSQVIEIWLRIFGIWPNMSCVSLCRLFWTVSIIIEQIFQYRYILMRFHLIEFSEIMHILGSTMTYTI
FLIKLVIFWYKQRTFNKILTMMTIDWEKYSGTKFSMFATCNAKLSQRFANMTVILYSTAVIFFSSKIFVKHVD
DSNASNISTRLLILEMDLPFDANRRFVYESVIITQFINLLLCADANCLINALLINLILHIGGQIDILREGLKE
IFPKNGKCNPSHFTIKKIIKKHQKIITFSEHIEDLYTYIVMVLFVSDTLIICCLGFTIVASIGQSNATKSIIR
NFLFYVVINVEAFVYCFAGEYLSAKSKSIGDAAYASLWYKSDSRDSRIILSLIMRSQNQLTITIGKIMNLSLG
QYSTIVKASASYISVLLAMY
>AechOr40
MKAKNTISPAATEILFRIFGIWPNTSFVLLRRLFWIISIVFEQIGQYRYIVKHFHLMELFEIIIILSSAVAMT
RFFIVLVVFWCKQRTFSKILTMMAIDWEKSSTKFSIFATYNAKLSERLVNMTVFLYLIAAILTGTKIFVKHMD
SGNASNVSTRLLIVEMDLPFDTNQRFVYESVLIIQFFHLLLCSNVMGVFNALLINLILHIGGQIDILRESLIG
IIPKKERDSSNHFTIKEIIKKHQKIITFSEHIEHLYSYITMVLFVSDILIICCLGSTIVASIGRPDVLKNIIR
VLLFYFVMNMEAFVFCFAGEYLSAKSKSIGDAAYASLWYESDSRTNQIILFLIMRSQNQLTITVGKIMNLSLE
RFTSIIKTSGSYISVLLAM
>AechOr41
MKVKNTVSPVIEILFRIFGIWPNTSFVLLRRLFWTVSIVFEQVAQYRYIIKNFHLMEFIEIVTILSSVMSYTI
FLIKLIIFWYKQRTFNSLLTMMAIDWEKCSRKKFSMFVTSYNAKLSERFANMTVILYSTAVILYSTHILRKHV
DDGNASNISTRLLVLEMDLPFDTNKRFVYESVIIVQFFYLLLCSDAIGLLNASLINLILHIGGQIDILRQNLM
AIFSKKEKDNLSHFMIKKIIKKHQNIITFSQHIEDLYSYIAMVLFVSDTLIICCLGFTIVASIGGSDVLKNIT
RTLLFYLVMNMEAFIFCFGGEYLSAKSKSIGDAAYASHWYESGPRNSRIILFLIMRSQIQLTITIGKIMNLSL
ERFTSIIKASISYISVLLAI
>AechOr42
MKQTPTSTINRTIEIPLRIFGIWPGSPYISFCRLFWTIALVVTQSLQYRYIILHLHTVDLSDLMDGLSATLSF
SQFSSKLIIFWFNQRTFDKILTMMAMDWKKNSAADFSTRVRTNKAILSQNFAKTVFGIFSIAITLYTASVFTF
DTNNLEETDLSMRPLILKMDFPFNSNTQFVYGLVLVTQFFCTVLCGCTVVMLNVLLVVLVLHLDGQIEILSKW
FTKIISKQNGHRLNLAIIRKIIEKHQSIIIFSKRIENLYSDIALILFVSDSLIICCIGFVLVTSIGRPNAATI
IMKSLGFYLIMNFEVFMFCFAGEYLSSKSRAIGDAAYDSRWYQCRFQDSQIILFLIMRSQNQLTITVGKFMNL
SLERYVSILKASASYVSVMLAVY

>AechOr45
MTRKSTINRTLKLTLTLCGIWPGTTCVIICRAYWIIALVIDDICHCRYLLMHLHSNDLFDLMDCFSSFLTQVK
LTIKLIIFWLNERKFFEILTMMAEDWNDCIDNNINVRETACKAKLSDRIANTMFTLHTLSIIGYSIGVLLADV
DITEQSELPFLLKVELPVNINTKRAYKMFLTMQFVHLILCSCGTGILNALLLTLTLHVGGQVNILRCWLNKLV
LQGSEGCESVAVTTTRIIRKHQRIIIFSEYIEDLYTYIALVQFTTNSILICSLGFLIVTAIRAIGSPDATEHI
VRSLLFYTVTNLEAFIFCYAGEYMKNKSKAVGNAAYNSVWYEMKPKNSRILIFVMLRAQKQLTLTVGKIMDLS
LESFTSIMKASGSYLSVLLAMQ
>AechOr46
MTRKSTINRTLKLMLTLCGIWPGTTCVIICRAYWIIALAIDEICHCRYLLMHLHSKDIFDLMDCFSSFLTQVK
FSIKLIIFWLNERKFFEILTIMAEDWNDCASSNINMRETACKAKLANRITNAMFTLHTLTIVAYSIGVLLADV
DISEQSELPLLLKVELPVNINTKRMYKMLLTMQFVHLIMSGCGTGLLNALLLTLTLHVGGQMDILRCWLSELV
PQKNERYEAVTVTTNRIIRKHQRIINFSEYIEDLYTYIALVQFTSNTVLICSLGFLIVTAKAIGSPDATEHIM
RSLLFYTVTNLEAFIFCYAGEYIKNKSKAIGNAAYNSAWYEMKPKNSRNLIFVILRAQKQLTLTVGKIMDLSL
ESFTSIMKASGSYLSVLLAMQ
>AechOr47
MTRKSTINRIMFRMLPWCGIGSDGIIILISRLFWIITLGFIGLCHCLYFLSHLNAQNFFNLVDCLCSFLAHAK
VITKLIAFWVNQRKFVETLTLITDDWSDCAKNDIGMRVMTDKAKISDRITNIIFILHTVTVAAYCIGTIIADV
DITDKIELPLINKLELPFRVNTQRMYRVVLTIEFIHMILTNWASGVINIIFLAMIIHVGGQMDVLQRWLAEFI
PKEIENEQKSVVITTNKIILKHQKIIQISDSIESLYTYIALLIFASNTILMCSLGFLIVTALGTDDVVEQMMK
CLLFFMSTNLEAFIFCYAGEYLNSKSKEIGFATYNCAWYNLKSKDSRILLFIILRSQKQLTLTAGKIMDLTLE
SFTSIMNASGSYLSVLLAMQ
>AechOr48
MNHQNPLNRTAKFILTLSGVWPGASCVLFSRTLFVVSVIIIQCCHYRYFMIHMHSATLWEFMDILPVVMAHSK
MLFKCLVFWLNQRKFIEVLTIMQEDLSDCVNNDISLRETASKAKTSERITNLILILHTMTVSGHGIGVILANV
DVTDNTTTELRFFTKIEVPFDINTQRTYRCILITEFFFLMMWSWSSGAINSLLIILTFHIAGQIDIMRYWLTH
LVSSKDESKDESISIATTKIIYKHKKIIRLSEYIESLFTYIALVLFVSNSVMICLLAFLVVTAIGSSNASEQL
IKSFLFYIITNLEAYMFCYAGEYLKNKSKEIGFAAYSSAWYDMKSKDSRVLLFVILRSQKPLTLTAGKMMELS
LQSFTSIMNASGSYLSVLLAM
>AechOr50
MIRKRIIDRTLEIICTLYGIWPNSRIPFCKLFWIIITIIVQFYHYRYFLIHFNSDELFNLMDCLSSCMSYMKF
TTKVIVLWINQRKFYKTLEMMTEDWDSCADNEIYMNEMIRKAKLSNHINIVIVLLQLVSAFTYCVNIILADVD
VTDHTSELLYIHRMELPFDVNTKRTYKIILFTQTVICIMVSMGTGVINTLLVALVLHVGGQMNILCYCLMNFA
TKGNEKKHQSITTDEIIQRHQKIINFSKNVEKLFTYIALVMFMVNSILICSLAFIIVSAIGTPDAVEQIVRSV
FFFAGTNLEAFVFCFAGEYLNNKSKAIGNAAYNTAWYNLKSKDSRILSFIILRSQKQLTLTVGKMADLSLEYF
TSIMNASGSYLSVMLAMQ
>AechOr51
MSANTITRSIEIGLRIVGIWPGATYAIISRLFWTTTMIVAQIFQYRHMAMHLNSEDISQLMDGLSATLSYSLL
CVKLIVFWTKQRIFNDVLASIATDWKECGDALYSMNNVANLSHRFSNLIIGLHSTAVLFYGIGVIALRSDDVI
DAADRELFLKMELPFDSGTSPIYEVVMTTQFLHQMTAATVIGVLSALLVTLVLHVGGQVDILREKLVILPKAK
KTSVSMIIGSLIRKHQNIIIFTEKIENLYSYIALAQFISNTLVICCLGFIIVNSISANEGSSMLVRSLLFYVV
INLEAFIFCFAGEYLSIKSKMIGDAAYESLWYDLTPNENRILLFLIMRSQKQLTITVGKFMNLSLQQFANIIK
SSASYVSVLHAL
>AechOr52
MEYPEHYYKINRLILSFYGLWPFQSKWSAHLMRASTIIIILLSTAIFQLLTFSTTNITTDFIIDVMSEFIPATA
CLGHIYARIKRIDKLRDLFERIRDDWKLQKTSFEIKIMHKYAETSRLFSLYYLIFYIIMIACNLQIFLPYILD
VILPMNESRPRTQPFQLEFFTIEEQYPTLITFYVFIMVVISSIIFCANSVLCVILTQHACGMCELLGYRAECL
FYNVEHNKNLTTQYDLIRSKINYGNMAIFIQMHYNIIEFIGTIEIYHTVPFLLDIIELILVLSLALTQVLTIG
SDIGRAFRSISLAIIALIYLFISNYMAQKITDMSLNVCEKVYNSAWYNANISDQKPLLLIMKRRFRPFILTAC
KFYVMSLQNFGMILQTAISYCMFMRQ
>AechOr54
PKEYYYKFNRFILSVSGLWPYQSEWSAHLTRAVITVFMLSSAFVQISSMFTSEVTLDFIVDGMPSLLLTLGSL
SNLYSRIIHIDKFRELFEHMWQDWALQKTHHEIKIMHEHAEISRLFTFYYIIMIYINIVGYNIWMFIPEILDI
ISPMNESRPRRQPFNAEFFVDEEQYFYFIRFHICLVIIIIPIVYVASSTLFLTLTQHTCGMCELLGHRAERLF
CVVKDEARYDLIQVSEITCGNISVFVQQHYNIIQFVEIIETCHTVPFLIDLINLMILMSLTLIQIIITISDMER
AIRSIGVFCGILYYVFISSYMGQRITDMSSNLCGKIFNSMWYNAVVSEQKTLLIIMMRRYQPLILTACKFYVM
SLQNFGMILQTTISYCMFIRQI
>AechOr55

ERGTITPNFHLRVSLTIVYFLGTWPPVTGKFRLPYLLYTICSFTFILGILLSSEIANVLMNWGDMSKLTAVMT
LLMTNCTHASKVVVLLRRQARIQTLLDTADSPTFSRYDKEYEDLLISYTWKGIFHHAIYQSFGGIAVFCWGFT
PIADFVAGRSRQLPMQGWYPYNTTATPAFEITAAHQGIAIIIACFHNVAMDTLMTGLITVACCQLAILERNII
SIDNKASIHGMQNDNDKNKPLERRTLAYQLLKRCAMHSNIIFHFTNEIQNIFGTVIFFQFLSNCMIICLIAFN
VSQMNVYIPAVLIGMLTYMCCMTYQIFIFCWHGNELHLHSMRLVTAAYSNNWFSNTERFKRGLQIMMIRAHRP
LTLSAGRVMLLSLDTFMQIMRMSYSIFTVLQGSAA
>AechOr56
MICIVNRHFNYHRILLLMTGLWPYQRSKLVQFQSILFSGILISFVLSQFAVFLSAECTLDMAIKVLSVALIAI
MCMVNQNSFRNNTQIIKQSLEELQHICDELEDENEIAIIKKYGNNMKRISAIMISFHMLSLSYMIFLSIVPYI
PGIDFINESQARHLFQNMAPVFYVNEKKYFYLLLLHANAAIQIGGTALLATGLMILGYAKHVCGMLRIASYRT
KKAFMLNIIKNDNLKNETAIYKEIILAVDIHRKAIKFCEFAFSNFRGSHFFLVMIAVLCLSLNLYGITEFLSS
KNDTAQLSAHCIRVILLFEYFFMVNYIGQDITDHHNQVFSTVYNVRWYVAPINVQKLILFLLQRGSRTFIVHL
GGIFGLSFEFFATLTKVSISYFTIVSSIQQ
>AechOr58
LEWAISINRCMLKIVGLWPQESKDQHEELLSKIQFLLNIILLIFVLAIPALMSLIRVWGDMILMIDNLQYTLP
LLITMLKVFIMWYKKEALSPLINMIVNDWIKVKVEEERIVMLKQAKIVRLLAVCGILMILSTMLITVLSFPFA
QTLRHVTNLTDPIGKPLPIQSYYLHDVSSSPKYELTYLIQTIALTTSGLSYTAVDSFLGLLILHICGQIENLH
LRLLNLGKDSNFKAVLKYNVKDHIRLNRSIETIDNTFNLMLLGLLFFFGILFCLHGFLIVSIVNRGGHLSILQ
LVWYTSATICVLMHMCLYCAVGELLVSQSEKIHRATYEYVWYTLEPKAARNLTLIMLRTKKPLNITAGKTFPM
TMSTFCNLLKTSAGYVSVLLANRN
>AechOr64
LQNEVDHIEDLFVHLERIFSIGGIWPFKQTYIRFAIYISHFTLYLIMAYTDFYDAFGNLELIVMNLVESMVYS
MTFPLVWLIRCSNLLKLLIYIIKKDMVERKFENFEEERIYYNYNFISKIFSYGSLVGMFITVVLLYLRPLVYL
LTINQASQNSTEYFILPYRIHPFFDISNTHTYILMYLCLFPMIYISVCHMAAICLMVILVFHICGELSILAYR
IRHIKEYSQTMILDRIRSFVRMHLKIIWMAKSVDDTFNLILLYELVGLSVVLAISLYYVIMNLDMAKIATCCT
FTFFALISLVMLFGYCLTGDELTQQCLSVQDAYYECNWYEMPLGCKKSLLICMIRGQVMLCLTAGKFYIFTLN
SFTDIIKTSLAYLSMLRRIM
>AechOr67
VSMDFQNVNSLNVRLNLFSGNLLPMVNHDSSFSFFWKLYSALFWIIQFIMTIAMVPGCIYVPIEKALKDSTVC
FIVFIEMFFLILRIRARKNLIYQLIQKLNKILQTADEIMKDVVTTTLKPVKTPLNFYLSAGVISIIAWGCLPF
VLIFEKNLFCYEDYRIPAAFTKQPFSLKIFVLGSLFVIISAVCIFLKKASVDVYMVHLVLMITAQYRYIAVKM
AVIFQDEGRDDESQKIHFSELDRKKEKEIRTLCRHHNDVIYISLLLKELLSLNFSLIYVNSVFRFCFIGIMLS
SIVSTTIWEAILIIVYSLGAIVQLYILCSCVQQLLDASGKVTDKAFHENWYLLRPAIKRIFILIIMANNLECC
KIATFENFNLSLPSFMMILNQSYSIALLFLRMK
>AechOr68
MDFQNLNPFNVRLNLLSGNLLPMTDNSSFPVLWKIYSAIVWLFELVYTVTLMAAFFVVPKEKSLNDGVLAFIT
VLESGLIVVRIHTTQTTLLQQIIRKMNEILNIDNKNIKNIVITNLKPMETPFKLYFVCGTFAVFVWFCLAFSL
VFEKDTFYYVDFKSPAVYSKEPFSINIFLLGSVIITISNVYIFLKKVSVDVYTIHLISLVTAQYQYIASKLVL
LFQNSNQQDNSGFSENNFGVQNFSLKKEIKNLCQQHNTIIHTMLMLKKLLSLNIFLIYVNNVFRFCFLGIMAV
KISVSFIEGFMVFVYACGTITQFYILCASFQKLSEASSEITDKAFHENWYQFNLSIQRIFLMIIASNNINIKL
SLFERFNLSLPSFMSVLNQSYSIALLILRM
>AechOr69
MQSAMKHYYNINKIFLSKLGGWPTQSKFMKILLPTIITSFVFSIGFLQFVRLSERWQTITEDCECFIVMLITF
GAYCKLFTIVFKNKNLEHLLSLIDYHWRIFTHSLEIQIMHEYAIVGRKMTISYAVMIYSLMSLYMLIPVTPQL
LDLFIPLNKSRSYKYLFDIDYSFDREVYYYPVLLHSYLTTVLTMSLMVITDTSYMSLAQHACSLFAAVGHRLE
NLVPESNSKKNNYFVEDVKDVICDKSNSRDYEDQTYHELVLLLRKHQLSIQYARLLNSLFEMYSFMLLFITIL
IMSLLGIQIISLMNHKEEMVRYVAMVIGAFIHLFVLSYPGQKIMDHSADIFHKAYNMLWYKTSRRTTRLLSIL
LYRGFVPCTLTAGKMYVLSMANYASMMQASMSYFTALSSFR
>AechOr70
MQSAVKYYYDINKILMSKLGTWPTQSIFEKTMLPAIFIIFVASIGFLEFIKLSEVWKNLTEDCESLFIFLLAV
GAHVKLFLLIIKNKNIVHLLSLIDYHWRVFTHFSEVKIMHEYALLGRKMSLSYAVIMFSLMSLYLMVPLIVPI
LNFLKPLNTSRPYIYLFDVDYSFDREIYYYPVLVHSYIVTILGVTSMVINDTSYISLTQHACGLFAAIGYRLE
NLTLTINLKKNSFVKDVKVTYNNCESNNDIIYHEMVLLLWKHQLSLEYVDLIESFFKIYLLIMIIMNFIGMSL
LGFQIIIFMNKKTELLTYIAMLIGGFIHLFVLSYPGQEVMDHSADIFYKAYNMLWYRTSRRMTQLLSILLHRS
FVPCTLTAGNMYVMSLQNYASVLQATLSYFTTLSSFK
>AechOr73
MDINNYVFINRKVLKFVGLYPTNIVRYIICFTCMITIVIPQGLQIYQNWQDLSTVLETSSVLLTILLAILKSL
VWISNRRKMDPFIKYMLTDYWNILTTYISKKHTDVYAIYVKKGYLYTKGYLFLICNSLMFFFSLPIIEIVIAV

IKGTNNNTIKHFPFLALYPEYYYNFPMYEITYFSQMIATSLCGLIILGTDTLIATALFHTCGHFKVLKKKIKN
ISTLQIDFIQHTYLEENTMQKIKLHIIDIIKHHYTILWFCDYMETVFSPMLFLQTITCVLFFSLSDLRYESSI
FFLKIIDKKYYQTKLFVNNLLISTYLLIYSLLINDSSMIYKTVYTIAWYELPVSFKTEICLLMLRSQKPSKIT
AGKFYVMHLENFNAVLSTAASYFMLLRSFGSDET
>AechOr75
MQILSLNFFIYTIGGIWRPIEWSSNGAKLLYNIFTFMIISSEYFLLLTQFMDIIFVVNNVDDFATNTLMFLSI
VAVCCKITVVVVRRKAIINLVQILLKAPYKPQDEDEIAIQAKFDKFIRSCSIKYSLLATSSVTGTTIGSVLNV
MQGHLPYRIWLPYNYTVSTTFWIISIHQIVTLIFATVINVGTETLVFGLILQTCAQFEILESRLHKLIINKTI
KYLGRESSLLNENETKLSECIHHHLSIYKYAKAVNVTFNQVLFVQFFSSILVLCTSVYYLSMHIKELSAAASL
LVYTICMFVQIFVYCWSGNEVMLKSTSIADAIYRMNWPLLSINEKKGLLMIMIRSTIPVKFTSSFLITLSIQS
YSNILKTSYSAFNILQK
>AechOr83
SNYYGVVKKVSSLVGQWPYQNPKTKLICLSFVTLSTFSIVIPQLAKFVICNGDMQCIFQTMAAHMLTIITLVK
LYTCHLNRYKIKVLIDRLFLEWDELETPEEYEIMKRYAKNGRRYSLGYSLYCFISAFLFLCMSLIPPLLDIVL
PLNESRPILSVHPGYYFVDEKEYFFYIFWHAVVVWEIAVTGIVAHDCMLLTYIEHVCSIFALGGFRFERLMYK
CNEYESDTITVLHSRASDTFRKRIAFSVYTHRKALKFAQLIEDTFSITLALQIALNTVMISITLLQHNASVFI
VVRYVVYVLSQLIHLFCLSFEGQKLIDHSLQTRDKIYNSLWYKTSPKSQRMLLFVMQKAFHPVFLTACKIYIF
SRENFTMIIQTSMSYFTVLASLE
>AechOr85
MEPSREHYYDISKRYLTLIGQWPYQKPKESLFFLILILFFDVSILVTQAARFFVCNNMQCIFETLPPHLLAAI
IPVKIFTYQFNNQKIKDLTDRLSIDWNMLETKTERDIMRRYAENGRWYVLIYSSYVYISTISFTTTSLMPRIL
DVIDPLNTSRPVMMPYPAYYFVDENQYFHYIFLHMIICSTICLTGLIAHDCMFFTYIEHICGLFAIVRYRFEH
VSYKRSNMEKNMIDCTDYIYYKNIVISIHVHRKTLQFAKLLEDTFSISFAVQILLVTICLSITLVQLHDSAEA
MRYFVFIMAQLFHLFCFSFQGQKLINHSLETCDNIYHSSWYKIPVKEQRLLLFVMRKSIEACTLTAGKIYVFS
LENFTMIVQSSMSYFTLLSSFNV
>AechOr88
TWRHYYNIVYKISSLTGTWPYLKPRARIFRVGVMTIIMGTVLVPQFAYQFMCKTNVQCICEAMTSYLLTCTSL
LKVYTCQLNIRTIKSLTQHLFIDWKGLKTSEEYEIMKLYAENSRRFCVVYTVYCMLAVTTFMSLSLVPFALDA
IWPLNESRPITPPYPGYYFVELREYFYKIFWHSIIVWEITIVGIVAHDCMFVTFVEHVCSMLALVGFYFENLF
NKGDKTMKIANNSNDTYRERIAIFVNKHQDALKFVEHIENTFTIPFGAQMIIVTIGLSITLLQFTQQNGDILI
SIRYVLYVLAQLFHLFCLCFEGQKLIEHSIQTCDKIYSSTWYEVSMRSQKLIVMVMMKSVRPSFLSAGKIYIF
SLENFTTVLQTSMSYFTVLTSI
>AechOr89
YYSISKRVLLFCGQWPYQEKRARLFQLSTVTLVIFSQTFPQVAQIFNCDGNARCIQLTIPVLMLMVVFLVKLY
TCQFNSNKIKSLTDFLCDDWEKLNSKEEYEIMKKYATNARLITFAYVLYLYVFSSAFILLPLVPKLLNIVLPL
NKSRPITMPYNVYYFVDEEKYYFYIIFHMFVSTIIALAAVTAHDCTFFIYIEHVCSLFAVTGFHFESLSRKNR
NDANNLSHNRKIAVSIHVHWRALRFAKILEDIFCIAFAVQMLIVVVTLSISLVQVALQYGDIKEMFRYLSYIF
GQILHTFCFSLQGQRLINHSIQLRDKIYNSYWYKIPVESQKLLLHVMRRSMEPCFLSAGKIYIFSLKSFTTVM
QSSVSYFTVLASF
>AechOr91
YYSITKRLLLLCGLWPYQEKRTRLFRVHSLTLIIFSLIVPQVAQFFRCNGNGRCIKLTIPMFMLMIIFLVKLY
TCQFNIIKIKNLTDHICDDWKKLNTTEEYEIMKKYAANAKLITFIYLIYVFVCCGGYVLFSIIPKFLNIVLPL
NESRPVNLPYQAYYFVDEEKYYFYIIFYTCVFGSVATMMVIAHDCTFFIYIEHVCSLFAVAGFHLETLSLNDC
NDANNRLDNRKIYNRKIAISIHAHWRALRFAKILEDIFCIAFAIQMLIVTAILSITLLQVALLYGEFNETVKY
LAFIFAQVIHTFCFSVQGQRLIDHSLQLSDKIYNSSWYEIPAESRRLLLFIMRRSMQPCFLSAGKLYVFSLKN
FSTVMQSSVSYFTVLASFQ
>AechOr92
NSFASVIRVNIALLRLSGVVSYENGSIHQSSLQNILITIAYGCLFAYAGLYTYEFVLHTVYLDTWMESFAMIL
SLVGGQARFTILLLFRSRFQRLLATCEELWTMLNATEKKHVRDYVKPTRHLTYYYLFSCAFTIFFYALANLFM
GQYDDALNATIRTLPYICPVEVHRSPYYEVMYIAQLCSMIHVGLTCAGADTVGPVLILTICGHFKVLNTRILH
LNDPNRVRSKGSCSVSMKSLEIMRERNDTKNHVKSNFEACVYYHQTMFELCKEVENLTTSIFLTQLLGSTYNV
SLVGFKLAGDDPDKFKYATQLFIAMIQLFLSNWAANVLVMESQDIARAMYFTPWYRFPYQLKRSVNIIVMRSQ
KPAQLIAGHIIPLSLQTFASMVSSAASFFTMIRSMN
>AechOr93
MDFKNVNFLNYRMNTLSGNFLPIATNNSRFSVFWRTYSIVVWLIQLSHTIALIFGIILAPTENSLKDGILSVV
VTIEAFFMLTRLHTRKKLIKEMIQKMNDILQSTDEIMKDTIKSAIKPIILPFVIYGVTSVISVTIYHAMQPIV
LTFEKFTFFYVDYNLSTAFSTKLFSNRILILSIILKTIGSVYLFLKKFGVDVYMMHLVLMLTAQYRYVAIKLT
MLFRDLHNYNDKSRKEHYPIEDRWTEGELRKLCLHQNTVLNMSFILKKLLSVNFSLLYINNVFRFCFIGILLS

AIPSMTFVEGISVTSFAIGSLTQFFLLCSSVQSLSDASTEITDRAFDEGWYQFGPSMKRIFILLIMSNNLECR
IAEIGKFNLSLPSFMTIMNQSYSVALLFLKA
>AechOr94
TMNTNWSHYYGLVKKLSSMSGQWPYQKPITKLFCMILMTLSTISVIIPQTAKFVICDGNLQCIFQTLSSYMLT
TITLVKLYTCYFNRCKIKVLIDQLFVDWNELETPEEYEIMERYAKNSRRYSLGYSLYYYFGMYTFISLSLVPQ
VLDVVLPLNESRPILPIYPGYYFVDERKYFFYIFSHAVMAWEIAVTVILSHDCMLLTYIEHICSIFTLVGFRF
ERLIYNNTMKMLHSRTSDMYCKQITFSVYTHRKALKFAQLIGETFSLTLTIQLVLNTIMISITLLQVRQAGIL
ELIRYMMYVIGQLIHLFCLSFEGQKLIDHSLQTRDKIYNSFWYETPLKSQKMFLFIMRKSFQPIFLSASKIYI
FSMENFTMIVQTSISYFTVLSSLE
>AechOr95
METICSRYYDIVYKISSLTGMWPYLKPKTRIFRITLLTIILLTILIPQIAYQFMCKKSLHCTLQAMTAYLLSF
VALLKVYTFQFNIHTIKDLTQHLLCDWKELNTYEEYEIMKSYAANSRRFSLIYSVYCFAAIIVFMSMSLIPHT
LDIILPLNESRPILPPYRGYYFVDIQEHFFQIFWHAIVAWEIVIAGIIAHDCLFVTYVEHVCSKFAITGFHYE
HLFHDCKNKMEIVSSINPDDITIYYKRVAFLVRKHREALEYAQLLEDTFTMPFAIQILIVTIGMSITLLQITQ
DKSDILEAARYVFYIGGQLIHLFFLSFEGQRLIDHSLQIRDKMYKSFWYKASMKLQKIIMLVMMKSLYPSFLS
AGKVYIFSLQSFTMVLQTSMSYFTILASFQ
>AechOr96
MNVLTFTFKILTICGCWPPNSWTTRYKRIMYDVYTVLIVLLINTFTLSQLMDLILTVDNADDFSENFYVMLAM
FVSCCKMFSLLRNRNNIAMLIDILMKKPCRPTEHDEIEIRQKFDKIVLTNTLCYATLVELTCAFALGTSVFKD
YRKHNLAFRAWLPFNYSSPMLFRIAYFHQSISLTAGSILHLACDSLICGLLMHICSQLEILECRLKKTINKPH
IFRECVIQHTCIFEFALIVNEKFRLTITVQFLVSMLVVCFNLHQLTQTSVLSAKYVQIVLYMFCMLTQISFYC
WYGNEVKLKSRQMVNNIFEMQWFVLDYHVQKDLSIIMMRSIVPIEFTSAYVISMNLQSFVSLLKTSYSAFNIL
QQMR
>AechOr103
MHVLQLTFKILTIVGCWRPQSCSSLYLRIIYDAYTVFMIILLYTFLISQFLDIIWNVDNAEDFTENLYATMAS
VVSCSKMLSLLVNRKNINMLINVFIEKPYRPLEIDEMRIRYKFDRLIYINTLYYTILVETTCACITMTSLFTM
FRKGNLTYRAWLPYDYYSSNIIFCLTYAHQLISLTAGSLVNVACDSLICGLLMHICCQIEILECRLSKVSNDD
GTLRDCVRHHDSILQYALKLNNKFRMTIAMQFVVSTLVVCSNLYQMTKSTSLNASYLPLILYMSCMLTQIFIY
CWYGNEVKLKSIQLLDNVFAMDWMTMNRNLKRNLLIIMSRAAIPIEFTSAYVLSMNLDSFVGLLKTSYSAYNI
LKQV
>AechOr107
MQVMQFPLKILTIAGCRPPVSWSSLWKRTVYNVYTIFVCLLLLTFMLPQLMDIILNVNNADDFTDTFYIMLAM
VIACCKMLSLLLNRKNIEILTDALVEKPFRPLEPDEIEIRQKYNNIIRTNSIIYTVLIETTCGCMNLTSLFTD
FRQGNLAYREWILFEWSDTVYYFTYFRQLMSLTAASIVNVACDIMICGLLLHIYCQIEILECRVRKSLRSRGD
LGECVRQHDHIYKFAYTMNEKFKIIIAVQFIASMLVVCSNLYRLARTTLSSKYIPLMLYTFCMCLQILIYCWC
GNEVKLKSIQFSDEIFGMDWITAHQKVRENLIMIMNRSLIPIEFSSAHILTVNLDSFVKLLKMSYSVYNIL
>AechOr111
MNILKLTYLTIIISGCFRPQSWTSLFKKIVYNIYRLYVMAMLYTFTILQIMDIVLCVDNADDFTNNINMMLTT
LASCYKIFIFSINYENFVTLINYLTEEPFKPLDSDEMKIQRQYDKIIRNNTLRYTILVTITCAFVILSSLFTD
FRHKKLKYREWIPFNYSSYMVFCFTYAQQMVTACHSAIVNVAIDSLMCGFLIYICCQIEILEYRLKKFLNNQL
ILGYCICHHNRIFEFAQIVNIKFTKIIGFQFMASMMIFCSNLYQLTKSTLNADHFSLILYTGCMLTQIFIYCW
FGNKVKLKSLQLVDNIFQMEWSIMDNSVKKSLLIIMKRAMIPIEISTVYILTMNLDSFVTLLKTSYSVYNLL
>AechOr112
MHTLKLTFLIVMFVGCFRPLSWTSLFMRVIYNLYRLFIVTILYIFAFLQFMDIMINVDNPDDFTNILYMALNV
SVSGFKLLIMWLNYNNVTTLITVLNKEPFKPLDLSELKIRQKFDKIIRSNTLRYSVLIASSWTFMSLMSLLTD
FKHRKLTYREWVPYDYSSYMMFCITYAHQILSTFYCASVNVACDTLICGFLMHVYCQIEILEYRLKKILNDQS
ILDYCVRHHNSIFQFACSVNAKFSQIIGLQFIISTLIICSNLYQLSQSSLNADSIGLIGFTCCMLTEIFLYCW
FGHKIKSKSVQVADSIFQIKWPLLNNNVKTNLLIIMKRATVPIEFTTAHIISLNLDSFVALLKTSYSAYSLLV
QVQEK
>AechOr116
VQMLKFSLMLCAFAGCWQPITWTSLFKHIIYKTYAIFLISSLYLFLISQFINITLNVGNSDEFTDALYMMLTI
FIAGYKQVYMWVDRKNFMIIINILNEKPFAPYETHEIMIQEKFEKLVQNNMRRYLIIVLMSIASIVMMSVSTD
FMKRNLTYKAWVPFDYSSPVIYFVVYIHQLIAMSTSGIVNVACESLLCGFLLHICCQFEILEYRLTKLPHDQN
SLRDCVRHHNRIFEYAYTVNNMFAKIIAVQFAVSMLVVCSNLYRIAMARDYVSFIPLIMYTSAILVQIFIYCW
FGNEVKLKSLQLINSIYNIEWLALSNSNKKDLLLIMKRAMTPIEFTSGYIITMNLESFVVLLKMSYSVFNLLH
QTNE
>AechOr120

TIIMITPVKVINFTKISVALSCAWPPPPNATKFNIILFKILWYTSYLSNILLLLPLLSSIYEYRDDPVILAKS
VCLFCAVLQVTIKMMVGYIQQNCFQMLYHEVEMFCKQANEKANIILQYYVDNYKHIYSFYTLWCYITSIGVIC
GPLFLPQEFPTNAKYPFSTQPPIKYIIYLHQSLVGLQAAAGMCADCNIAILLFYSAARLELLVQKIRNVKNEN
ELDSCIKLHDEILRYINKMISTIKPLIFTTIMTTIMGVVFGSLNIVTEQPIMIKLQYSIVVFTASVELFMCAL
PADNLMHMSSKICVGAYESQWFQGSVNMQKKILQIIFRSQKSEIIHINGILPALSLRYYA
>AechOr121
MKRMNFENVNLLNSRVNMLSGNLLPIASDNLRFSVIWRTYSAAIWLIQLTHTIALIFGIIMASKENGLKGSTH
TILLTIEALFMLTRLYSHKRLMEEMIQKMNTILQNTDEIMKDIIKSAIKPIIVPFIIYGVISMISIIIYHAKQ
PIILVFEKFIFFYVNCNLLATFSSEPFSNNAMISSTVLKIVGTIYLFLKKFGMDVYMMHLVLMLTAQYRYTAT
KLAILFRDLQIYNESRKEHYPMENRRIERELRKLCLHQNIVLNMSFILKKLLSVNFSLLYINNVLRFCFIGII
LSTVPLMNIAEGISVTIFGIGTLTQFFVLCSSVQTLSDASTKITDKAFDEGWYQLGPSLKRIFILLIISNNLE
CKIAAIGKFNLSLPSFMTIINQSYSIALLFLKAR
>AechOr122
MLVHVLREYNVNKILLSHLGLWPLQNKFVRRWLPISYLVIHISSLPFEILTLHDQLKNMRRVFENFYYIVCSI
LFIIKLSNEFMNHDKIRYLYKAMENHWNIFTDEFEMRILKNYSILSQKFTLIYAIALYIITIIYLMVPIKPIL
LDVILPLNESRPRFFMLSIEYKIDKDKYFVPLFCYNIIVVVTGVAILIGIDAIYVTRTIHACSLFSIISQQFE
KIISQTGINMKVSKGGCYMNGMNALFKKSASEQLIYDEYIICLKKYQLALKFASVLNSMYRIVSLTSLLLCGA
IISLAPLLMVYELDKFGQLLQYIVIIIGTLLLLIILCYPGQKLLDESQNVFYQIYAVEWYTFSPRLKSLIIIT
LHRSLEPCSLRAGNLCSMSIGTFAKVLRTSISYFTTFLSFK
>AechOr131
MKIAPTLDRVIAFLKIDLLFACCWPLPRTATKFQIICDRIFRYLSMVNGAFMIIEIIYSISNHLDNVLLLMQL
ACALGIFCEVPLQIFLFTKQHDRLQNVICEMEDYYKQANNEEKDVFQQYINKYILFYGTTLTLTTAITFAGCI
IVPLIRSRKFPLEIEYPFRVDYQPIMAMLYFHQVLGMYQVTCQVSANVFLALLIWFTTARFEILTNKFRTVTK
YSDWKICIQEHQVTLRFAKEMSSSIALVVLSSLGVSTIALIFGGVTFLSRFPLSVKFQYIVVCLTSLVKVFLC
AWPADHLMRISSNVAEAAYDSLWYNQNIESQKIMLHTLLRCQRAVVISVPGLLKALTFQQYTSYVSTAFSYLT
TFRIILSEET
>AechOr135
MRRKVMLKEVISVVKLSLFPVWGWPLPQDTTKFKIFCVKLHHYLCIILGLGLMVPLTYGITNYLDDPITLVNQ
ILVLSSTSHAIANFIFEIVNYHRIQNVTFEMVHFCDLMKPHEEVVIQRYINKCIMFHSICLFFIYFPITIAVP
VLLHQPFPVIIKYPFNISYQPVKTIIFIHQSMMAIMIAGQLCMNILMALLLWFTSARFEILIEELRKITNVYQ
LFKCIKKHQELLKYAREVTIAARPFAFSTICCSTVCLILVFLLVIKRQSTTMIFQFFGISIIGTLEVFTYTWP
AEHLMYMSKDVAEATFNMLENDHLIKIWKCLQIIIMRSQKSVTVSIPCLLPILSLNYFTSYLSTVLSYFTTLR
VIMDD
>AechOr136
LTLKKVIAIIKLSLFITWCWPLPKDTVKLKIICAELYQYLCLILTLGLIVGLTNTVKNHLNDPLIMAKSIIII
CPMVHVVCNIISCKINSRRLQLVTFEMENFCQLIKPHEEATIQQYISKCVYFYGGSMLWLYLSAVFVITGPIT
LDQPFPTNAEYPFVIYQQPLKSIIFMHQSFVCLQASAQLCINIFMALLLWTTSARFELLVKELRTVTNVHGLV
QCIRQHQRLLQYAQEVVAIVRPFALTTISLSTFALIIIIGLIFITGQPISIKIQCVGLTFSGLSEVFMYTWPA
EHLIHITGEIIQQAAFDMQWYKQPVALRKYLQIIMLKAQNPIIITIPCVMPTLSLNYYASYLSTVFSYFTTLR
IIMQN
>AechOr137
MDFQNVNPLNVRLNMISGNLLPMTADNSSFPIFWKIYSILIWLLEILQTCVLIPGCFFVPKEKALKDGIIGIV
VGMEVICTIIRICTCRGRMKQLIQKLNDVLNVDDEMMRSIVIETIKSMECPLKFYCSAGVMSIIAWSSQPFML
VFQRNSFFYIDYKMPVVYTKEPITTSMFVFGSVIVMMSSVYIFTKKVGADSYIINLLLLVTAQYKYIALKLSM
IFQDKFLQNDHSKSDTEKCYLNRDDYAEKEIKVLCRHHNTVIQIMLMLRKLLSLNISLIYVISVFRFCCISLM
INSIPSETLWEICSVIMYGIGGVVQLYIICSCMQQLLDASVNITEYAFHEKWYQYGKNMKRTFMFMIMANNLE
IKLSTFEKYNLSLSSFMTILHIVNKLFII
>AechOr142
MAKLNIPWSNVNSERDIIKALVWNRWILRILGIWPLVYSNTTKIEKILATISFALCWSALSFLLISMIIITLS
KHSATNDKIKVLGPLCYVFVVTLKYFFLILHHKSIQQCIRILSSDWRTIQQEDHRKIMIKNAAKSHVLSKFSI
IFMYCGGLCFHTVMPFLTQTSIDEQNVTVKPIPYPGFDIIFDMHFTPAYVFVFCAQWFSSIILFNVTTAVCCL
AAMFVAHACGQIEIVMDRVESFIKGAQSNHMKQCMAIIVKHHVQSLRFSVDIDKILREIGLVEIVGATLIICS
LEYYCITWYHLPGKIALDLTLIISISHHPVRISAGKIVSLSFSSFGSVLKTSVVYLNLIQKFI
>AechOr143
MAKLNIPWSNVNSERDIIKALVWNRWILRILGIWPLVYSNTTKIEKILATISFALCWSALSFLLISMIIITLS
KHSATNDKIKVLGPLCYVFVVTLKYFFLILHHKSIQQCIRILSSDWRTIQQEDHRKIMIKNAAKSHVLSKFSI
IFMYCGGLWFHVMMPFLSHTINEQNVTIKPMPFAGFDNVFNFHLMPVYVFVLCAQWFSSIILFNVTTAVCCLA

AMFVAHACGQIEIVMDRVESFIKGAQSNHMKQCMAIIVKHHEWNNNDSIAILTYFFLLVSFVFNVFTLCYIGE
QLTSQCSKIGYTSYEIEWYHLPGKIALDLTLIISISHHPVRISAGKIVSLSFSSFGSVLKTSVAYLNLIQKFI
>AechOr145
MSSIRDKSVKSHNYNYEQDIRYIFKLNHWIMGSLGIWPIPIRGIGRHMSKIAIAIFNFALSFAIVPCTLHIIY
DQKDLDVRLRLTGLLGFCLTVMTKYCIFVIHRPKIYRCIEYVKNDWWQVTFKSDRELMLKYATTGRNLTIIGM
CFMYTAGIIYHLILPFCVEHKIDNQTIRPLVYPTYSKFTQSQISPIYEIVYVAHCMCGYTIYSLTAGACGLAA
LFVTHACGQIQVIMSRMENLLNDKNSNIHQQIAIIVKDHVRIVRFSAVIEEVLQEVCLVEFTSSVCIICLLEY
YCIVDWKDDNKISLATYFLLFISFCFNVYMLCYIGELLMEKSIEIGSMCYMINWYQLSPRSVRSLILIIAMSS
HPIKLTAGRMANLSLTTFGNVLKTSVAYLSFLRTLAM
>AechOr146
MSSIRDKSARSRNSNYQQDIRYVFKLNNWIMGSLGIWPIAIRGIGKHMSKIAIAIFNFALSFAIVPCALHIIY
DQKNINVRLRLSGLLAFCLTAMTKYCIFMIHRPKIHACIEYVKNDWWQVTFKSDRELMLKYATTGHNLKIIGE
CFTFTDDVYPTYSKFTQSQISPIYEIVYVAHCMCGYTIYSVTVGACGLAALFVTHACGQIQILISQLEDLLAG
ERFKQSPNVHYQIAAIVRNHVKIIKFAAKVEEVLQEVCLVEFTSSVCTICLLEYYCIADWTADDRLSFATFSL
LLVSFCFNIYILCYIGELLIEKSTQIGSICYTINWYQLSPISARSLILIIAMSSHPIKLTAGRMVDLSLKSFK
SVLQTSLAYLSFLRTLVI
>AechOr148
MLENFLREYNVNRVFLSSMGLWPFQNKSIRNFLRTFFFLIEISYCPFEILLLYDHLDNPQMIFDASYQFMMSI
SFIGRQINEFWNQDKLRRLYEAIDQHWDIFTNDMEVQVMKNYSMLSRKFTKYYSMLMFSSMLILSLIPLTPIL
LDTVMPLNESRPRFFAIEVEFRVNKDDYFLSILCYTTVIILIGANVVMGVDAMHIACTAHACSLFAAISKRIE
NLISKANNNKESSKCRYRMNMELDPLNEKLMYREYIICLKKHQLAIEFVNTLESSYQGISLLLLLLLIGTISL
IATRIIYVLDQAGEVIKFTFIFIACLITLMIVCYSGQRLMDESQSIFHRAYAAEWYKFSPRLKSLLIITLYRS
NVPCGLKAGNMVPLSIATFAAVMRIAMSYFTAFTSFKE
>AechOr149
MLANFLREYNVNRVFLSISGLWLFQSKNVRNSLRTICLLMEISYYPFEILLLYDHWGDPQMVFDACYQFIFTT
AFIARVLHNIWNQDKLQQLCIAIDKHWDIFTNDVEVRIMKDYVMLSRRFTIVFSMLLFFTVLIFVSIPLIPVL
LDTMLPLNESRPRVFAIEVEFRVNKNDYFLIMFCYTTIVVIIGLNISIGVDTMHFACTAHACSLFAAVSKQIE
NIISKAYNNNKVSKCGYHVNMELNPFNERIIYREYITCLKKHQLALEFVNILESSFQGLSLLLLLLILGNISL
IGVRILYVLNQMQQLIKFTFMFTAMLIDLMIICYSGQRLMDESQDIFYRAYAAEWYKFSPRLKSLLMMTLYRS
NIPCGLKAGNMIPLSIATYAAVIRIAMSYFAAFKSFSE
>AechOr151
MICLEKHFRLNRFLLLTIGIWPHQRSKSAQIQFIVFFTILTTSIVFQFTTFLTSQCTADLIINVLSLAFFFIC
YAIKYHSFWINANTIRVSLDQLQHVCNEIKNKNEIAIIEKYGRIGKHQTIIITILAACAISIYFFLPIWHRIF
DVILAINNSRSRSAAIHIATEYFVDQERFSYLILLHTNTALCIGATAMTATGTMLIAYLKHICGMFSIASFRI
KKAMAVNMQQDVNEEKTVIIYKRIICAIDIHRKATEFSQVFIRSFERSFFCLIAASMVCITSSLVQIISYNNT
EQLVFELLKISVLYIYMFLSNYIAQDITDHNEYVFATVYDVEWYVAPLHIQKMMLFLLQKGTKAFRLILGGIF
IASMESAATV
>AechOr152
MICLETKHFKLNRFLLLAIGLWPHQRSKLAQIQFIVLFTILTTFIVFQFTTFLTSKCTVDLIINVLSSAFFFI
WYAIKYHSFWINANTMRISLEQLQHVCNEIKNKNEIAIIEKYGRIGKHQTIILTILAACCISIYLFLPMWPRI
SDVILAINNSRSRSAAIHIATEYFVDQEKFSYLILLHTNAALCIGAITMVATGTMLIAYLKHICGMFSIASFR
IKKAMAINKQQDVNEEKMVIIYKGIICAIDIHRKATEFSQVFIKSFERSFFCLIAAGMICVSSTLVQIISYNT
TGQLVLQLIYLVDLYIYMFLSNYTAQDITDHNEYVFATVYNVEWYMAPLQIQKMMLFLLQKGTKAFRLILGGI
FIASMESAATLMGTSISYFTVLYSS
>AechOr153
QHFSLNRILLLMVGLWPYQQSKFVLFQQLLLFSILTSFIVFLLIKFLIVECTFDFVITIFSFISFYVVSIIKY
NSFWINNQTVRNLLDQLEHVCNELKDKNEINIMKRYESNAERYTAKIILITIVALSIIIFMLMWPCILDIINP
KNESRSYHLKLMMAKYFIGQEKYFYLTLIHLNVALCVGAIAIIATGTMFIGYVKYTCGIFKIASYRIERAMTI
NNLQKKFEKEITIYRDIIYAVNVHHKAMELCKSLISCFEGSFFSLIVIGVLNLSLNLCRIFQTISVVNDKEEI
ILHFIIVIITLSYMFLANYAGQEITDHNDHIFFTAYNVRWYIAPIHIQKMILFLLQIGNKAFGMQIGGLFVAS
INCFASLVSASISYFTVLYSTRQ
>AechOr155
MISIESKYFHLNRILLLALGLWPYYQSNLVRIQTILYFGILISYIIFQLSTFLTAECTPELIIKVLSTVIFCI
MYIINYNAFWVNTDNVKCLMERLQQICNELKDENELNIMKEYGNNVKRCTTIFILFAMSCIFIGILTQILWII
FDIILQTNGSHSRQMLHIMVSESFIDEEKYYYIILLYTNVVICIGAITLTAIGTMLRGYLIHVCGMFKIASYR
IEQAFKYSIKIFDSIENEIMIYKEIICAVDIHCKAIKYSEFLLSSFEGSFLALTVISVICLSLNLFAIFHSVS
IGNKEEFLFHLLIVSGIFVYMFIANYTGQEIMDHNNYIYITAYNVRWYVAPVYTQKLILFILQRSGKAVAVNF
GNIIVVSLECFAMLLKASMSYFTFMYSTQ

>AechOr156
MICLETKHFRLNRFLLLAIGLWPHQRSKLAQIQFIVLFTILTTFIVFQFTTFLTSKCTANLIINVLSSAFFFI
SCAIKYHSFWINANTMRASLEQLQHVCNEIKNKNEIAIIEKYGRIGKHQTRIITIFAACCISIYLFLPMGLHI
SDVILVINNSRSRSAAIYIPTEYFVNQENFSYLILLHTNAASCIGAIAMVATSTMLIVYLKHICGMFSIASFR
IKKAMAINMQQDVTEEKMIIIYKGIICAIDIHRKATEFSQVFIKSFEGSFFCLIAAGMVCLTSTLVQIMSYNN
TEQLVLQFMYISVVYLYIFFSNYFAQDITDHNEYVFATVYNVEWYIAPLHIQKMMLFLLQKGTQAFHLILGGI
FIASIESAATLMGTSISYFTVLYSA
>AechOr157
RRGVAGMTRGTTSNRLEQFEKLNIVHLSYLKYVGLWTLDSNSSAFLRCSYHVYNKSVLAIMLVFMITLFADIC
MSFDDLSIVTDDGCIFAGIIVVFFKVMIFQTRHEKIIRLLRKIIDNCIELCKFPIGVEDEILDKYLLLCRVTF
YGFSVLGFFLVIALLFVVPVENGELPVRAQYPFDTTVYPWHGIGFFVEACTVSIGVIAIIMMDGLLTNLCNLF
LVQLEILNAHFKNCGSNEWYDGTPSDIDKQNRSSTIDENSCYGAFSTTEASWKKDNYDPDAIRCGSFAKPFGR
SIRNHQRLLAIIDDFNEVFSAGMFVQMLSSTSMICLTGFQAALVIGQSSNTLKFAVYLMAALSQLFYFCWLGN
EVKYQSASLTQSQWLSKWNDDISAKPGRLLILSIIFSRKAINLKAGVFYALSMETFTAILRGSYSFFALLNTM
HSEDNQ
>AechOr158
MDFQSVNPLNVRLNALSGNLLPLRSNDSRFPITWKIYSALIWLIEIIQTIALIPGLILVPRKKALKDGTVLCV
VTIEMFFMAVRIYSRRQLLNQLIQKLNDILRFSDETMKNVVMTTLRPMENPLKFYWLSGWLGVFVWACLPFLF
IFKKIFFFYEDYRMPAVFSKQPFSLDVFLLGSVLILIGNMYVFVKKVGMDVYMIHLVLLITAQYRYIAIKLAV
IFRDGNSLSKLDESHQKYCSGINRWVKKEMIALCRHHSNIVHLSSMLKKLLSLNFSMIYVNSVLRFCFIGIML
STVSQQMTNKAFHEKWYQFGPSVKRTFMLMILGNNLECKLSMCDKFNLSLPSFMTILNQSYSIALLLLRV
>AechOr159
FMWAVNLHRIGFEMVGLWPKLNKCTEKNLLSKIWVGIIFILLIFVTIVPMVHAIIEVWGNMTFVISNFQTTLP
FLIASLKYAIIRRKQTAVLSIINMMADDWIAFKQNRERNVMIKRAQTARLITIIGHVCVVIAFLTSIILPYFG
ISVVYVANLTDVRKPLPLKMYHFYDTDKSPQFELIFFIQIVTMLFAATIYMSVDVFLVIMVLHICGQLENFRC
RLIDLILCKNFNEVLNNVIKTHLRLIRFADNIENTYSLMMIVLVFHFIIVLCLSGFSFTIILTDKNINTADIA
QGYFSLLLLFIVLMNTFLYCGAGELVAEQCNAVYRAICDLEWYELESSKARNLILLMVRAKYPFYITAGNIFP
LTMTTFCSILKSSIGYISFLLTK
>AechOr161
FMWAVNLHRLSFEMVGLWPKSNKCTKKNLSKIWVGVIFVLLIFVSNVPMICTVIEVWGNMIFVINNLQTTLPL
LIVSVKYVIIRQKQTVLLSIVNMMAEDWMAFKLAGERDVMIKRAQTARLIMMIEYVLVIIGFLTLIILPCFGI
QVTHITNSTDRRKFLPLATYHFYDTNKSPQFELTFFIHFVTTLLAITIYAFVDVFLILMILHICGQLENFRCR
LINLISCKNFNKVLNNIVGTHLRLIRFADNIENVYSLMMLVLVFHFGIVFCLSGFLFIIIFADIKIDKSIIAQ
GYFSILIIIVLLTNTLLYCGAGELVTYQCNEVYRAMCDLEWYKLESRKARSLILLMMQAKRPFCITAGKIFPL
TMATFCSILKSSIGYISFLMTK
>AechOr163
FEWAVELNRYSLEFIGLWPKMKETTQEKLMSNVRVLLLIIMVTFFGVIPCIHSLIRVWGDLMSMTDNLQFTLP
LVSMIMKLIVMWSKKAALAPLLYMIAKDWLKLKSDEERKIMIRCARIPRMIIICGFIIMFASFILLFILPCFG
ITMRYITNVTDPGKPLPLQTYYFYDTDTSPYFELTFVAQGVTLMVSAMGYTAIDSLFGLLIFHVCGQLKNLKG
RLMIGSEKQSNFDHVLADAIMDHVRLIVVTDGRHLSFIRLSFLVTVVANIFAHMCLYCVVGECLIAQCEGIYQ
AVCDYHWYELEPKQARNLILLMTRANKSLYVTVGKIFPLTMNAFCSLLKTSGGYISVLLARRD
>AechOr166
MDDVDKFFRESHYNIIQVLLSVSGLWPYHTTGKRCAIYFAFLLVFGSGMTFEVLGIIEIWSDSLEVIDCLPML
VLSTVSISKLICAIYTLSEIKILLTRMRQYWYSPKSDEETRILLSYALYGKKLSYVYTGDHTVVFLFTTLLSK
LLYTESSEDDPSSNVHKQVGLPYYVNYILIDPKKYYVPIFVHVIICVISYTILMLTFDVLYMTLIQHCCGLFV
ALRYRLENAFECEGNDLQMSAKKLYSNIVYSIRRHTEAIQLILGFQVITSTEDVNHLLKHTSYLSALLLNTFV
ENWQGQKIIDSSEKVYESAYNAEWYKMPIAQRKLFIMIMMKSRKSLKITAGNFVNLSYVNFNAVLRASSSYFM
LLRSIQ
>AechOr172
MLHNEYYKDDMIYITQLTRNVLSLLGVWPNYNRRKTIGEKIWKYFLISVSYILLYCVLIPGALFWMIEKRTRV
RVQTIPLLFYGFMASGKYSNLVFREKNIRRCLKHIEQDWRIVTSGERRDMMIESAKIGRRLVTLCAVFMYGSG
LSFRSILPFAKGKIVTAQNITIKPLPCPAYFFSFDIQVSPAYELIFAMQFLSGIVTFSITTALCGLAAVFVMH
ACGQLRILVDLMRNLVEGQWQEKEVDKKLAKMVEHQIRIRSFLQLVENTLQQACLIELMGCTMIVCLLGYFII
MEWENSNSIAMCSYVITLTSLMINMFMFCYTGEQLTVQAERVANTSCELEWYRLPDKKVRGIVLVIIMSNMPT
KITAGKIMALSFKTYSDVVKTAVTYFNMLLNV
>AechOr174
MLRNEYYKDDIIYITYLTRNVLSLLGVWSFYRKKSTREKIWKYFLILICHILLYSVLIPGALFWLVEKRPRVR
VRTIPLLLFGFIACGKYSNLVFHEKNIKRCLQHIEEDYKVATNAKARNTMIKSAKIGRRLVTLCAIFMYGSGL

SFRLILPFAKGKIITPQNVTIRPLPCPAYFIFFDVQVSPIYELIFVIQILSGIITYSITTGLCGLAAVFVMHI
CGQLKILMSLMRNFVKEKWQEKREVNRKLAIMVEHQIRIRNFLQLVENTLQQACLIELTGCTMNACILGYFII
IEWENSNPLAMSSYFTALMSMLINMFMFCYTGEQLTVQAEKVARTSCMLEWYRLPNKDARGIVLMIIMSNLPT
KITAGNIMDLSLKTYGDVIKAAVTYFNMLI
>AechOr175
MFANKYYKNDIEYAFAMNRFFFRLLGIWPFTRANFFLLELMETVALILMCFTFLFCELIPTMLYVFLVLTDFR
LRIKVMASGLFTLTEILKYSCTLLYKNQVRNCLKFVDEDWRNVINPSDRISMIDRVRTSKRLIMMCATFVYLT
GMAVRIIVPLSIGKIVTSENITIRPLPHVAYLVILDVQRSPVYEIVYFIQFLAGFIKYTISITTFSFMTLCAM
HFCGQSDILVTLMNDFVNENQSKNVNRRLAIVVERQIRIRNFLQLVQSTIQYTSLIQIMGSTVLICFVEYYTI
MEWENHNVIRMCTYISGLIMLSYNIFIYCYMGEQIIEQGEKVSLTTRTLKWYRLEDATARALILLITISENPL
KLKAGNFIDISLRTFGNVKCILIKIWTVTYLNILRSV
>AechOr178
MFANKYYEHDIENTFVINRFFFRLLGIWPFPRTNCLLPELLETVVLVFVCFAFLFGELIPIILYVFMVLTDVR
LRFKIMANGIFTIIEIIKYGYAVLYKNQVRNCLVLVDEDWRNVINLKDRISMIDRVRTSKRLIVICTVFVYLT
GVCARMIIPLSVGKIVTPQNVTIRPLPCVAYLVIFDVQRSPVYEIVYFIQFVAGFIKYTILVATFSFVTLCGM
HFCAQSDILVTLMNDFVNENQPEYLDKKLANVVEHQIRIRNFLRLLQSITQYPNLIEILGSTVMLCYVGFFII
MEWEDHNVFRLSLYFSGLTMFCFNIFIYCYMGEQIIEQGEKIALTACTLEWHRLPDVKARALILLISISEKPL
RLKAGNFIDLTLRTFGNVVKMSVTYLNLLR
>AechOr183
MNVNILHRDDVRRTVNVTRGVLNLIGLWPLPGNSSTLRIIQTKMLRILSQVLLYFIIVPGVLTMFLKESNARR
RIKMIAPICSCSMAVLKHTLMIYRSNRIKDCIEHIDKDWNKASLIGDRRIMMSNSKIGRSLAIFCVAFVYGSG
FSYRTIVPLSRGIIVTPQNVTIRPMGFDGYYVFVDPQKTPAYEIIFSLQFVSGFVHCSATSGASSLAILLVLH
ACGQLKILITRIEDLTQAKYFSDKYANGKLAAVVRQHIRIKSFLSKVEEILQYICLVEVVGCTFLLCLLGYYI
IMEWENNNAVSMLTYTILLLTFIFNIFILCYIGELLTDQSTKMYITFCTFDWYRIPHKTARGLVLIIAMSSIP
SKITAGKFMDLSLNSFGAIMRTSVAYLNILRTTNI
>AechOr187
ESSSRYLNVHHKSEPYNQNYENDLRNNLQLNVWIMKIIGTWPLTHSSIEMLWYRVLNVICYILLALLLIPSGI
YIVLEIKDFYNQLKLGSALTFFFMAVLKYCALLLRENDIRKCVDYIKSDWRNVRYTDERRIMLQNANFGRRLV
VICSVLMYGGVTFYFVAVPLTRAKIKEEDTNLTYRRLVFPVPSVIVDARRSPVNEIFYFIQLFAGFIAHNITV
AACSLAALLAMHARGQLQVLMSWINHLVDGREGVNDTTNERLAKIIQLHMRILNFISLTEELLHEISLVEVVG
CTLNICFLGYYCMMEWDFKKPVSGLTYLILLTSLTFNIFIFCYIGELVTEQIIKVRESSYMIDWYRLPERKSL
AIILIICMSNATTRLTAGNMIELSISSFGDVIKSSVAYLNMLRTFTT
>AechOr188
IMERTSSIATIHDHEKDVQLSIQLNRWILKPIGVWPKLTEISRMERYAYGLVNVICTSLIGFLFIPSVIFMLL
EMDDVYLILKLSGPLSFCLMAVVKYSSLIFRENDIRSGIRHIESDWNTLHSNDRIIMIRNAKFGRRLVSICAF
FMYGGAVFYYLALPFSKGKITESDGNLTYRPLVYPVASVILDARHSPISEILFWIQCLSGFIAHSITAGACSL
AAVFAMHAYGRLEVLIQWIEHLVDGREDFCNNVDERLAMIVQQHVRILHFISLTDKVLREISIVEIVGCTLNM
CFLGYYSLTEWETKETTSYITYIVLLISLTFNIFIFCYIGELVAEKCKKIGEVSYMIDWHRLSERKSLALVLI
IAMSNSSIKLTAGNLFELSLSTFGDVVKTSVAYLNMLRTLTA
>AechOr190
MTERTNLRNFNYEDDIRYTVQVHRFIMGLIGVWPNMEKSGKWARLLRGLLRTACCFLLSFNLIPWVLYMFLIL
DTFKKRLRMIGGFFFYSMVPAMYFTLMLREDRIKKCIRHLQEDWRNVWDANDRKIMLDQARAGRFVIICTLLF
LFTSGFTHRLIKPILRGTIVIGNVSIRPLVQGNYFIFFDPQQSPAYEIVFSMHLVTGIVIYIVTTSVCGITAL
FSMHACGQLKMLTVWLENTAIENQWSKCAIAQRLAAIVMHHVRIRKFLHQIQDVVGEMCFIEIIGSTLILCLL
GYYVITGWESNDTLSSMTYGIMLVSFTFNIFILCYIGELLSSQGSEVNTTCCTIDWYCLPSKEARYLILVIAM
ARYPTKLTAGKVIDLSFNSFGAVIRTAMAYLNLLRTVTL
>AechOr192
MYGTITEKTNPVATVYDYKKDMKLSIQLIRWMMKIIGTWPKTTVISSIERYAYTLLNVICIGLISFLVVPAVI
YIVLEMDNGYLILKLSGALSFCVLAIVKYFSLIFHEEDIRNGIKYIESDWINTQYHSERTTMIRSAKFGQYLV
AICMFFMYGGAVFFYLALPFSASKITEDDGNLTYRPLVYPVARVIIDVRYSPVREIFFWMQCLSGFLAHSITA
SGCSLAAVFAMHAYGRMEILIQWIEHLIDGRKDLCDNMNERLAIIIEHHVRILRFISLTDRILREISVVEIVQ
CTVSMCLLGYNIIMEWESQKTTSYITYTVLLLSFTFNIFIFCYIGELIAEKYKKIGEVSYTIDWYRMSGKKSL
DIVLMIAMSNTSIKFTAGNIFELSFTTFGNVVKTSVAYFNMLRTLS
>AechOr193
MSYSFHVSSMRNIAYQQDNEYSIQLNRWFLKPIGAWPGTNIGIAGKVLSRGIQFVCHSLIAFTVVPCALYIIF
EPDVHLKLKAFGPMIHWLMGGANYCSLLMRSYKIRKCVDHMRADWQYVGTVRDREVMLQNAKFGRFVSAFCAV
FMQGGVCSYSIITTLTPANIRIGNVTITMHQLPCPFYTELVDTRYSPANEIVIVLQLLSTVIVNSVTVGACSL
AAVFAMHACGQLSILMIRLDELVDETESEHKVTRRKLAIIVEQHLRVLSFVSQIETIMHQICLVELLGCTTDM

CMLGYYTITEWKLHDTKNLLTYFTIFIAMSCNIFIFCYIAEILTDQCQKIGEMAYMTEWYRLHHKIAIDLILI
ISRSNAVIKITAGKMIQLSIATFGDVMKTSFAYLNILRTVAT
>AechOr196
MMRKSSCTQNSNPIATVYDHEKYARLSIQQIRWIMKSIGIWPNSFKFSSPIMKYVRVLLNVICVGIVTFVFIP
CVFYVVLEVEGTYNTLKFIGPLSFCLITIVKYSSLAFRRRDIRVCIEHIKTDWKNTWYHDDRAIMIRNAEFGR
RLIVINGFFAYSGAILFHIAMPFSMGKITERNSNLTYQPLPYPVARMIVDTRHSPINEIFFWIQFVSAVISQT
VVTATCGLIAVLAVHAYGRLEILMQWILHLVDGREDFSNNVDDRLAIIVQEHVRILCFIELTEKLLHKVSFVE
VVGNTLNICFLGYYTIMEWEGGFAITYIILLMNFVFNLFVFCYIGELVAEQCKRVSEISYMIDWHRLSKRKAL
AIVLMIMMSNSSVKLTAGNIIELSMISFGDVIKTSVAYLNMLRTLTT
>AechOr205
INERVMSHLDASDYSIQLNRWFLKPVGAWPSSASTTIKEKVISIVLIIICYSLIGYTVIPCILNILLEETDMH
KKLQAVGPLNHWSMGGMNYFSLLFRSRDIYRCVEHVRTDWRMMTDTEDRQVMLKYAKFGRFVAGLCAIFMHGG
VFSHSVMQGIMPTFVYIGNVSVSIHVLPCPTYSKFIDITKSPAGEIAFTVQLISSIVVNSVTAGACSLAAVFA
MHACGQLNILMRWLDQLDDRKQQDMVQHRLAIIVEHHLRVLSFVAQMEALLNQICFVEVLGCTFNLCMLGYYV
ITGWNVIEKTTSVAYMIIYISMTFNVFIFCYIGEIVTEQCKQVGEAVYMTNWYHLPHKTARNMLRTMTAS
>AechOr206
VNDPVMSQRDASDYSIQLNRWLLKLVGAWPSSASTTVREKVISTILIIICYSLIAYTIIPCILNILFEETDVQ
KQLRAVGPLSHWCMGGMNYFSLLFRRSDIHRCVEHVRTDWRMMTEIGDRQVMLKYAKFGRFVAGLCAIFMHSG
VFSHGVLRGMMPTFVYIGNVSVSVQVLPCPTYSKFVDITKSPAGEIALTVQLLSSIVVNSVTAGACSLAAVFA
MHACGQLNVLMRWLDQLDDRKQQDMVHHRLAIIVEHHLRVLSFVTQMEALLNQICFVELLGCTFNLCMIGYYV
ITGWNTVETAMTIACMMIIYISMTFNIFIFCYIGETVTEQCKQVGQIAYMTNWYHLPYKTARGLILIISRSSS
VIKLTAGKLVHLSIATFGDVIRSSMIYLNMLRTMTAS
>AechOr210
MSNTKSAPENKISKQYNNYSLQFNQWFLKPIGAWPQKNMKSVWKIFTLLQILICSSLIALITAPCVLYVLFEK
VNIKQRLNVIGPLLHRLMGMINYWVLLKRSGDIHKLIRHMETDWNLAQRIDEHELMLQYAKFGRSVSIICGLI
MQGGTFLFSLTRALKTTTIIVGNETFTTYPMTCPVYSKIIDTRFSPVNEIALVLQFMSTFVLSSSTVGACSLA
AVFAIHACGQLNVLSIWLHELVQNQEKKNHITQKKLALIVEHHLRVLSFISCVESIMHKVSLAELMGCTTSMC
LLGYYTIMAWETLDTAKLTSYFIVYLSMGFNIFIFCYIGEIITEQCKYVGEVTYMTDWYNLHHKTARDLILII
ARSSNVIKITAGKLFHLSIATFGDVIKTSIAYLNLLRTMT
>AechOr218
MSNLKRASEDNRSKKYNDYSLQFSRWILKSIGAWPQINAPSLGYKIMILLQIFICSSIIAVPTIPCILYVLFE
ADNIKKKLSTLGPFINRFMSTIHYWVLLNRSNDIHKLIQHMEKDWSLVQKIEEHEVMMQHAKFGRFVTVICGI
MMQGGCLLYGLGQSIKTVTITIGNETFTTHPMTCPIYSKIIDTRFTPLNEIVVVLQQLVILVISTCAVGACSL
AAVFAIHACGQLKVLYLWLHELVENRTKKNDKAEQRLAAIVEHHLRILSFISQLESIMHKPAFAELTGCTTLM
CMLGYYVAMSWETLDTTKLTSYLFPYLSTSFNIFIFCYIGGIITEQCKLVGEMAYMTDWYNLHHTTARGLILI
IARSNNVVKITAGKLFHLSIATFGDVIKTSVAYCNFLRTMT
>AechOr219
MSNSKHAPERETPEKHIDYCLQFNRWFLKPIGAWPQINAPSLGCKIMILLQIFICSSILIVTTIPCILYALLE
AENIQQKLSIITPLVNRFISSMHYWVLLKRSDDIHKLIRHMETDWNLMQSVGEREVMLQHAKFGRFVTIICGI
MMQGSSLLFGLGQSMRTTTIIIGNETFTTHATTCPIYSKIIDTRFTPLNEIAIVLQNVIILILSFCTAGGCCL
AAAFAIHACGQLNVLYAWLHELIENQTEENHKVEQKLAAIVEHHLRILSFISQLESIMHKPAFVELTGCTTIM
CLLGYYVATIWETLDIIKLISYLFIYLSMSFNIFIFCYIGGIVTEQCKLVGEMAYMTDWYNLHHTIARDLILI
IARSNNIVKITAGKLFHLSIATFGDVIKTSFVYCNFLRTMT
>AechOr220
MKKLPQNSPIIFNYIENMEYSIQPIRWLLKPISVWPVMDSSITEKISSVVLLITCIFLIVSILVPCALAIFLD
ETKDVNMKIRDFGPLSNWMLASLKYTSLLVHVGDIRQCIKHIETDWRAITKFEEQEMMLRNARIGRFIAIFSA
VFIHSGVFSYSIFRGMTLSESVVKGDNASERSLPFAFYNKILDTTTSPMYEIVFIIQCLSTFVVNSVTVSACC
LTSVFVLHACGQLKILMSLLDSLINERNEKTDSSQQKFAVIVEHHLRVLSFVSHIEKITNVVCLVEVGGCTLH
MCLLGYYCILDWNQNEKEGIVAYGIILISVTFNIFIFCYIGEILSEQCGQVGEIAYMTNWYLLPGNTALDLVL
IILRSSIVVKITAGKMIELSLSTFGNVIKSALAYLNILRTLI
>AechOr224
MSYRSSLPDRIHNVRYEHDIYYAIQLCRWILQSIGIWHIIYGRSSQSEKLLSLMMILMSFFGLCFVLVPAGSY
FLFYDEDIEIKIKFFGPVSFCLTSMIKYCFLGTRISTIGKCIEHVENDWRVVQYQNHRKMMLKNVLMARRITI
LCVIFLYGGGLFYHTILPLSSRTKINGSFTNRLLMYPGYDLYLDPQASPAYEIIFGMHCLSAMIQYSATAAAC
SLAASFATHARGQVQILVTLLDDLVNGKRTEGTNMEKRLSFITKHHVRVLRFTTDVEKILREVCLVELVTATL
MICLLEYFFLMEWESTSDAVGIMSYILLFVALTFNVLIFCYIGELLAEEFGKIGSAAYEVNWYDLPGHKAVDI
IMIMMKSYYPPKLTAGKFCDLSLNTFSVVLKTSVVYLNLLRTVTQ
>AechOr226

MTDKKDIWKSRYYLVLRTYMIISGIWPYQSLRDRYIHFIPTFTFCFSILIPQLMYVLIASNINEIIESITAAM
ISVIFSYKVASLMFDKNIKSCLKIIEEDWLSLNTDVERSILQHHTEYGQYLSTSYAVFMHMTQVFYLLKPVIL
TLLETDIANSTKTITSKLPFRVEYGVDIERYFYPITIHCYLAVFAHVFSTIAVDSLYCILIQHACGMFSIIGH
VLEEIGKNNDINFHLKRNKIQDDDYKKALDCLRKHLQAIKFAELIESMYTKIFLISININMIGGSMTGIHMVM
NLDKGIREIAAPIAIYIAQLVHIFLQFWQAQFLLDYSIVPCEPICRGNWYYTSKRCQNLFLLIMSRTISPCRI
TAGKIITLSIESFGTVLKTMMSYFTVLLRSFQ
>AechOr227
MICIETQYLSLNRILLLAIGLWPYRQSKITQFQFVFFSAILMTSIIFQLTTFITTNLTSDVIVMILAPASVFI
IFMIKYYTFFVNIEPVKKSFIKLQHICNELKDKNEIAIIEKYGYIAKCYTVALMIIGICALFVGIIIQFWNMI
DVGLSTNISRSSRLYFVTEYFIDQKKYYFLILLHVNIALFIGGAAMVATGTMLIAYFEFICGMFKISSYRIER
AMNIKVMQNINIKNKILRFEDLICAIDIHRQAIKLSKSLLSYFEIMFFCLAQMFVVSLCFNFARIILTEKATK
EALLPVIFAAVFILYLFISNSLGQNMTDHNNYVFATVYRVEWYITPLYIQKTILFLLTKGAKNFYLNVGGLFI
PSLECFTTLTKASVSYFTVILST
>AechOr228
MIHPEIQYFTINRILLLVIGLWPYQQSKFTRFQFIFFSTILISGIIFQLTTLMTSKCTLNLISKVLSSTSYFI
FCIISYDSFCIKIKAVKDLLMQLEHVCNQVKDKNEVAIIEEHNYTARCHTIILTVFAVIAGSFCSIGQYLSNG
LILPKNISRLPIMMEYFIDQEKYFYLILLHVYAIICIGVVAILAIGTMFLTYLQYIYGMFRIASYRIEHAINF
NIRQNITQKTKILMNEGIICAVDIHRRAMKLSKHLMSTIGVTIFCLIAFGVACLTLNLFQIASSENTVSEFYL
PSLYVVVMILCMFLSNFVGQNIIDYNNYIFSTAYNVQWYRAPLRMQRMILFLLQRETKEFTLNVGGVFNASME
CFAMLLKTSVSYFTVIYSIR
>AechOr229
QYFNFNRILLLVIGLWPYEQSKFTRFQFIFFSAILTSGIIFQLTTLMTSKCTSNLVSKVLSSTFFSIMCMIKY
NSFCFNIETVKDLLMQLEHICNQVKDKNEVAIIEEYNYKARRYTIMITIFTIIAGFVCTIGQYLSSSFIVLSK
NISRLPIMMEYFIDQEKYFYLTLLHIYTIICVEAAAVLAIGTMLITYFQYVCGMFRIAGYRIEHSININIQQN
ITLKNKILMNEGIICAVDIHRQAMKLSKHLLSIFEVMMFCLIIFGVACLTLNLFRIASSENTINEFCLPLIYV
VTSILYMFLSNYIGQNIIDHNNYVFLTAYNVQWYKAPSHIQRMILFLLQREAKEFILNVGGLFNASMECFATL
VKTSVSYFTVIYS
>AechOr230
MIHLGIQYFSFNRILLLFIGLWPYQQSTFTRFQFILISTILTAGVVFQLTTLITSKCTSNLISKVLSSTSFFI
SCTIIYNSFCFNIEAVKDLLMQLEHVCNQVKDKNEVAIIGEYNYTARRNTIMLTLFAVITVSICTLGQHLSNS
FIVLPKNISHLPIMMEYFIDQEKYFYLILLHFYAITCVGGIAILAVGTLLLTYLQYICGMFKIAGYRIEHAIN
INVRQNITPKNKILMNEGIICAVDIHRQAMKLSKHLLSTFEVMMFCLIIFGVACLTLNLFQVSFLVSSENTVS
DFCLPMLCVVVSNICMFVSNHIGQNIIDHNNYVFSTAYNVQWYRVPLRIQKMILFLLQRETKAFTLNVGGLFD
ASMECFATLVKTSISYFTVIYSMR
>AechOr232
MVHLSNQYFSLNRILLLVIGLWPYQQSIFTRFQFIFFSIVLISGIIFQLTTLMTSKCTSNLVSKVLSSTSFSI
FCMIPYNSFGVNIKAVKDILMQLEHICNQVKDKNEIAIIEEYNYTARRYTIIFTIFCTTGSICIIGQHLSNSL
IVLPKNISRLIMMEYFIDQEKYFYLILLHFCAFICVAGAAILIPGLMILTCVQYICGMFRIASYRIEHAININ
IRQNITLKNEILMSEGIICAVDIHRQAMKLSNLLVSIFEVTMFCLIVCGVICLTLNLFQVSFLAPFENNVSEF
CLPLLCVVVMILCMFLGNYIAQNITDHNNYVLSTVYNVQWYRAPIHIQRMILFLLQRNAKEFTLNIGGLFTGS
MECFATLVKTSVSYFTVIYS
>AechOr233
MIHLEIQYFSFNRILLLVIGLWPYQQSKFTRFQFILISTILTAGIVFQLTTLITSKCTSNLISKVLSSTSFFI
FCIIIYNSFYFNIKAVKDALMQLEYICNQLKNKNEVAIIEEYNYTARRQTIILTILAVIAGSFCFIGQYLSNG
LILSQNISRLPIMMEYFIDQEKYFYLILLHSYAIICIGGIAILAVGTLFFTYLQYICGMFKIAGYRIEHAINI
NIRQNITPKNKILMNEDIICAVDIHRQAMKLSKHLLSIFEVTMFCLILFGVICLTLNLLQVSFLASSENTISE
FCLPLLYAVVIISCMFIAGCGTQNITDHNNYVFSTVYNVQWYTAPLHIQRMILFLLQRKAKEFTLNVGGLFTG
SMECFATLVKTSTSYFTVIYSTQ
>AechOr235
ENQYFDINRILLLAIGLWPYQQSRFTRFQHIFMSAILTTVIIFQLTTFLTLNYTSDLFMKVLSSASFFVTLVI
KYNSFRFNIKSIKDLLMQLQEIHYKLKDVNEIAIVKKYDYIAKRYITLVTTLGICIVFACIILQFWVNVIHLD
LSMNVSHYIFLKMEYFIDQEKYFYLILLHINVAICIASVVVIAIGTLCITFFQHICGMFKIASYRIEYAININ
IQQSVMLKNKIWMIKNIIYAVDIHRKSIKLVNHFTSTFEKIVFILVVFGVACLSLNLFQLTLFENNVEKVLEP
FTYVLVCIVYMYIANLLGQTITDHNNLVFITAYNVQWYKTPLCIQRMILFLLQVGTKEFVVNISGIFDASLEC
FATLIKTSLSYFIVIYS
>AechOr236
EVQYSNLNRILLLAVGLWPYQQSKFSRFQCIFLYILLWTGIIFQLTPLITFKHTLNAVIKVLSSVSFYIMYII
QYSAFYLKIKVVKNLLMQLEYIHNKLKDNNEIIIVYKYSYIAKRYTDILIAFGICALFISATFQIWYIAKVDV

SMNMSRSYRTVIATEYFVDQEKYLYWILLHMYIVLCTGSIIMLGIGTMLITYIEHICGIFKIASYRIEHAVNI
DIPQNIIIKKNCLMIKGIISAVDIHRQAMKMMKHVMTTFEIMISLITGCVVVCFSFNLYQIVSSKNNKTTVNE
SLLSFLFASVCIIYMFIANYVGQSIIDHNNHVFITAYNVQWYKTPLYIQRMILFLLQRKSKEFSLNIGGIFDA
SIEGFATLINTSVSYFTVMHSTQ
>AechOr237
LNKILLLTVGLWPYQQSKFTQLQFIFFCTILSAGIIFQLTPLIILKCTLNLVIKILSSVSFFILCMIKYNIFH
LNIEIVKNLFMELRYTLSELKDKNEIAIIYKYGCIAKHYTVMLAVLEICGTFSLIIAQIWSFIADVNIMNTSQ
SHHLFIMTEYLVDQEKYFYWILLYMYTMLCIGSIITLGIGTMLITYIENICGIFKIASYRIKHALNIDISQNI
MIKNKMLMEGIICAVDIHHHAMTLSKHFMTTFEIMMSFITGCVVVCFSFNMLQIVSSENNKNNVIEFLLSFFF
ASTSVIYMFIGHYFGQKIIDHNNYVFITAYNIQWYKTPLHIQRMILFLLQRRAKKFSLNLGGVFDASIEGFAM
LIKASVSYFTVINSIR
>AechOr238
MVPPEIEYNNLNKILLLAVGLWPYQQSKFTQFQFIFFCAILSAGIVFQFTPIIILKCTLDLVIKVLSSVSFFI
LCMIKYNAFHLNIEIVKNLFMELRYILNELKDKNEIAIIYQYSCTAKRYTITLTSFGICGMFSLIIIQIWSFI
ADVNTMNTSQSHHLLIMTDNFVDQEKYLYWILLYMYTVLCIASFIMLGIGTMLITYIEHICGIFKIASYRIKH
AVNTNVLQNIMTKNKILMIEDIICAVDIHRQAMKMTKHFMTTFEIMMSCITGCLVVCFSFNLFQIASSENNKN
NISEFLLPFLFASTSILYMFIAHYVGQNIIDHNNHIFVTAYNVQWYKTPLHIQRMILFLLQRRAKKFSLNLGG
IFDASIESFATLIKASVSYFTVMHS
>AechOr240
MIHKQLKAYRKYQQFLQNLLILCGCWYMPTKSGKSIYYWSIYVFVAMILYTMMSLHMCYILRHNLKVMMKFVG
ITITGLGAVLKVVTFMINRDSLINYHRTLNNLFEEELMKNEKIQTIIFSSLRTIYTLAYTYFAILVVVIFAFF
GSTYIFIIHNLLYLHLTTNYTLPLSRGIGWFWPIPHNFLYHLHLFYETSLIILSTMTACGVDSAFGFYVYQFS
STIRAMTFALTNPLSTEKFSDILRTCVVKHQKLLQCRNTLEHIYGPIIFWHIVTNAVLLCSLMYDLISLPDYN
FYNVSASLVYAVIKLLQTFIYAWHGTVLTSAGEDFRKGIYFGKWPNSSLDHHVRTNVILIMMQKPMTINAFFS
AVDVIMFTNSVNATMSYFFLLQSIE
>AechOr241
MDFQNVNPLNVRLNIISGNLLPITSDGSSFHITWRIYTIIIWIIELFQTCATICGFVLVANRETLQNAGTVSI
VLSIEVFILLTRMYASKDLASQLIQKLNSVMHSGDETMKNIVHSTLKPLEIPLEFYCIAGTGSVIVWCSMPLI
LIFKKKSFFYEDFGMMAAFSKQPFSTKVFVLGNLIETVASAYIFLKKVALNVYMINLVLLLTAQYRYIAIKLA
AIFHYNTSQNEGHEFQKENYSVNLLVEKKIRKLCLHHNTIILTTLILKKFLSFNMSLIYLNNIFLFCFVDIVF
INAITSGAFFEGSVLIVYLCGALIQLYILCFCVHQLLEASKELTDKAFHEKWYQFGPSLKHIFRMIMVSNNLK
TKLSISEKFNLSLPSFLAILNQSYSIAILLLKVK
>AechOr242
KMNLFESRYFNLNRLLMSFIGLWPYQTLGTRRIKMILISFFLAEGIAAQLMSFITIEYSRDLLTKVFSFSFII
LICTVKYNMLYFKSEQVKYLFEQVLNDWHALTDVEEIKIIQKYANKGRFYTIYAGCKNKIHIMQIYAFTFSRK
VLYHFTISAIPRQHQLPLVIEPIFDLEEHFLLFILNFLIIALIILTILLTVETLYMICIQHACGLLKLTSYRI
LNAFDDRLQLDISKKSKCTICIKLSKAIKIHKRSLEFIECLCSTFSVSYLFLCIFGIASLSINLFEFLKTIES
KDTNQSLYFGLFIGAHLAYMFWVNCFGQDLIDSSADVFAQTYNVKWYMVSTHVQKKILFILHRSSKNILFDVG
SMFVFSLDGVATLINSSLSYSMLLYSTR
>AechOr243
MDFFNNEYYFIRHLMESVGLWPYEKPERRIVRGMCVSFILIQSILAQLATFITHDYNISLFIDVFSFNLPCII
YVLKYNIVYFNSNHVKNLFDQIQCDWNSIKNVDEQKIIKKYVFKTRFYAIFSGSFVYPGTFIFILFIYMPYFL
NNISLLDEPHPRQLPVQVELFIDQEKYFYLFSLIFIVTAFLGMTVIMATENMYMMLVQHACALFELTSFRLTL
AFECNTSFSTSSKTKCRCYIKLLSALNIHQHCLEFIENIQHNFSTSYFILFVFGVASGSINMFRLLEAVITHS
LYKAILTGLFVYAHFCYGFFMNYFGQDVIDHSENFFRQICNTRWYTAPLYAQKLLLITLRQSMKNSKIIVGGL
FVMSLEGLATLFSMILSYCMVMHSV
>AechOr245
MEFLGYHYYKLYRIFLLSLGLWPYGDSFFKRIYPIFCIFTILFLIIAQLLKLFTTEYDFEFVLTDLSFTIPSI
AYLLKYSTFYIQSQRIKELIEHIQIDWNVLQNEEEVEIIRKYAKSARRYMYAFVGLTYFPTFVYISIPFLPDI
LDIVAPLNESRTRQLPFLVEYFLDEQKYFYPILLHTIVTIIMGITTVVATETLTFAYIYHACGMFEIVSYRII
YALDKSISMLLTSNKENTIRVKLIDAIEIHQRTLEFFEYLTSTLSLSYFILITIGVASLSINLFRLCRIIISP
DQRNDLVALYIIFVFSHFYYMFICNFMGQKIIDSSTEIFRKAYDTQWYMAPLQMQKLLLFMMQRSVKSCNIVM
GGLYFVSLEQFTTLVSMSLSYFTIIYSVQ
>AechOr247
MDLAGNIYRYYNIHRIFLSSVGLWPYQNSTMKKIQWIISSIILTSTIITQFLKFITTEYNLALLLKVLSFAVP
CMVFVLKYVSFCFGSKTVKNLMKTVISDWHLLKTDTEIEIIKKHSGFGRFYTLFFALSIYSSLFSYFFIQFIP
NFLDIVAPQNESRLHNIPLAAEYFVDQQEYYLPILLHIDLIALIGFTIVISTESLFTAYVRHAVGMFEVASYR
IEHVFDEVLNIMTSKRCCPYCTKIISAIHIHRRATQFVEFLRSGFVISYFFLLCLGVTSLTTNLLRLFFATQY

LSNLEECITAVLFVLGHICYIFFGNYTSQKLIDQSTDVFYKIYVSQWYDAPLHAQKLLLFMMQQTIKGTAISV
GGIFIPSLEGFATIFSMSVSYFTVICSI
>AechOr248
RYYKIYIFLLCSLGLWPSQTTKCIHIKPIFVYILLHSFLFVEFCTLLTSQYNFNVMLRFLSHFLPAVIFTMKY
HAYHINSKKVKLLMDEVQNDWNALDDKKEIEILEKYAYIMNVFTIITTLLLCTSLIVFSFTEFLPILLNIVVP
LNESRPCNTHVPMEYFIDPKKYFLLMIIHEIFALTTGGFTMIATGATTLAYAQHVCGMLKIVSFRIERVLDKN
MLRNSSSQIERIICNRIVRAINLHRKTLKFFDRIVSYFTIPFAILITTGVVSMSINLYYLLQPSTRQNIAELL
SSISIVGAHFFYMFGANFGGQIVTDHSDDIFNATCNALWYIAPLSSQKIFLFLMHRTMKSFKPVLCNLFVASL
EGFATLITTSLSYLTILYS
>AechOr252
YYNIYRILLTIIGLWPYQTTIIMQLQSVFFLGAYCFILLFQFTTFLTTTCNMEFIFKKLSYILITMLCFITYN
AYYFNSKEIKYLLKQIKLDWNSIEDNNEIRILGKYANENRLLSLIFSFVVAFAIFFIIIIELIPVVLDAVTPM
NKSRPRKVKIDFEFFIDEEQYFYIYLIYEIITVIIEVFTILATGVLYYAFIRHCCATFKIASNLIEKSVTQYT
LQIPSYQKTRTMCQKINRAIHIHRKSIQFSSFFINIMNKWYFTLVIIYVMSLSCNLFRLLNLLTLSNEFSEII
ICIGMVTGHIISMFLFNFVGQSISDHSAEIFNAAYNTMWYLAPIPIQKLLLFVMQNSLKTHTLTLGHIYVPSL
EGFSTLMTTAVSYFTIMYT
>AechOr253
YYNIYRILLTAIGLWPYQTTIIMQVQSVFFLGAYCCILLFQITTFLTTTCNMEFILKELSYILITILCFTTYN
AYYFNSKEVKYLLKQIKLDWNSIKDDSEIRILQKYENENRFLSLISSFVVVFAIFFIIIIELIPVILDAVTPM
NKSRPRKVKIDFEFFIDQEQYFYIYLVYEIITLIIEIFTVLATTTLSFTLIRHCCATFKIASNLIEGTVTQHT
LQISAYQKTLVMCQRINRAVHIHRKSIQFSTTFFMNIMSKWYFALVIICVMSLSCNFFRLLNALTLSNEFSEI
IMCISMVTGHIISMFVFNFLGQSVSDHSAEIFNAAYNTTWYLAPLSIQKLLLFVMQNSLKSHTLTIGHIYVSS
LEGFSTLVTTAVSYFTVIYT
>AechOr255
YYNIYRIMLTAIGLWPYQTPIIMQVQTMLFLGTYYFILLSQFMIFLRTTCNLEFTFKELPCIFMSLLGIVTYN
ACYFNSKEMKYLLEQIKLDWNSFEDESEIKILEKYAIENRLVALFLGFFDAFCMFFIIIIELTPVILDAVVPM
NKSRPRQTKINFELFIDKEQYFYIYLIQEIVLIMIEMFTVLAIGTLSLAFFRHCCATFKIASNIIENTVTKHT
LQIPTYRRTHVMYQEIIRAVHIYRKSAQFSTFFLNIMNKCFFALMTISVISLSCSLFRLFNALTVSHEISEIV
VGIAMVTAHFMLMFLFNFVGQSLIDHSAEIFDAAYSTLWYLAPLSIQKLLLFVMQNSLKIHVVKLGYIYVPSL
EGFSTLVTTAISYFTVIYTM
>AechOr257
MAFSGERYYNIYRIMLTTIGLWPYQTSIIMQVQTVFFIGAYYFILLSQFMTFLRTTCNLEFTYKELPCIFITM
FCTIIYNSYYFNSKEMKYLLKQIKLDWNSFEDESEIRILEKYAIENRFILLIICFSNAFGMFFIVIIELTPVV
LDAVVPMNKSRPRKITMDFEFFIDQEQYFYIYLIHEIMAILIEILTVLATGTLSLAFFRHYCATFKIASNIIE
STVTKHTLQIPAYRRTHVMYQKIIRAVHIHRKSAQLIFLCYSRFSTFFFNIMEKCVISLSCSLFRLFNALILL
NELSEITFCIGVVGFHFIVMFVTNFVGQSVIDHYAEIFNAAYNTMWYLAPLSIQKLLLFVMQNSLKANTLTVG
HIYVFSLEGFSTVI
>AechOr260
YYNIYRIMLTAIGLWPYQTSIITQVQTVFFLGAYYFSLLSQFMTFLRTTRNLEFAFKELPCIFMVMFSIITYN
SWYFNSKEMKYLLEQIKLDWNSFEDESEIKILEKYAIENRLVVLFLGFFYAFNMFLFIIIELTPVILDAVVPM
NKSRPRQTKMNFELFIDKEQYFYIYLIQEIMTMLIGVLTVFAIGTLSLAFSRHCCATFKIASNIIENTVTYST
KHTLQIPAYRRTHVMYQRIIRAVYIHRKSVQFSTFFLNIMNKWYFALMIIMVISLSCSLFRLLNALLLNELSE
IILCIGLVVCHFVIMFVINFVGQSVIDHYAEIFNAAYNTMWYLAPLSMQKLLLFVMQNSLKSYTLTVGYIYVT
SLEGFSMLVTTAISYFTVICT
>AechOr261
YYNIYRIMLTAIGLWPYQTPIITQMLSVFFLSAYCFILLFQFTTFLTTTCNLEFIFKELSYIFITMLYIVNYH
SYYFNSKECMIFLFLTFSVIIAFAIFLIITIELTPVVLDAITPMNKSRLRKIKMDFEFFIDEEQYFYIYLIHE
VILMIIGMFTILATGTLSLAFLSHCCATFKIASNIIENTVTKRTLHISAYRKTHVMYKKIIRAVHIHRKSIQF
STFFVNIMSKWHFAIVVISVISLSCNLFRLFNALTVSNEISEITICFGMVVAHFLFIFAPNFIGQLVIDHCAE
IFNAAYNTLWYIAPLSIQRLLLFVMQNSLKSHNLTIGRVYVSSLQGFSTLITTAISYFTVIYT
>AechOr262
MVFSGERYYNIYRIMLTAIGLWPYQTSIVMQVQSVFFLGAYCFILLFQFTTFLTTTCNLEFIFKELSYIFITM
LYVVNYNSYYFNSKEIKYLLKQIKVDWNSIKDNNEIRILEKYAIENRLLSLILGFFIALSAFFIIIVELIPVI
LDAVIPMNISRPRKIKMDFELFIDEKQYFYIYLIHEVIIILIGMLTLLATGTLSLAFVRHCCATFKIASNVIE
NTVTKHTLQIPAYQKTRVMYQRINRAVHIHRKSIEFSTFFMNIMNKWYFAVVIISVISLSCNLFRLLIVLTVS
NEVSEIIMCIGIVLAHFLFLLVPNFIGQSLTDHSAEIFNAAYNTMWYLAPLSIQKLLLFVMQNSLKTHTLIIG
RIYVTSLEEFSTLITTAISYFTVIYT
>AechOr264

VSQFPYEMHAIERYYKINRIILKTVGLWPYQQSYLSQIQKILFISILVTFILVQLLAFITNQYNTNLLLQILS
FVFPILIDTTKYCLFIIQANNLKQLLEQIQDDWNSLKDKLEINIIEKYACNARLFTIILIVYCYFGLIFCGIF
QFLPMILDVILPLNDSRPCKLFVVTEYFVNQEKYFYVMLLHETLAYIVGTATLCSTSATIMTCILHTCALFKI
ASCRIENAVKKSTLIIPNPKKEYFLYRRIVHAVVMHQRATKFAELLTSSFAVLFFILIIIGVSSLSFNLFQFL
QLITFTKNINQAFIVATLIFLHLNYMFVANYGGQELLNHGLKLFKATYNGLWYAAPLRTQKLLLFIMQKGTIN
VSLTCGGIFVASLEGFATLVNATVSYFTLIYST
>AechOr265
MSQSSYKTLALQQRNYKIYQIILKTLGLWPYQQTYLTWLHKVFFAGILLTSLFVQLLVFVTKQYSIDLLLKIL
SFFFPITFVTIKYLVFIIQEDDLKILLEQIQNDWNLLQDELEIDIIEKYACNMRLFTIAMMAYCHISMFFYMF
FDLLPLILNVVLPLNESRPFQLLILAEYFVNQERYGRYLILHEILTGYITMNTLCGIGVIIVMYIMHACALFK
IASYRIENAIEKCILMIPSPRRKYLLCEKIIHAIIIHQRAIQYTNFVISTYTTLLTTLIIVGVCSLSISLFQS
FQLIILMNDIRAITVATMITFCHLSYMFVLNYAGQELINHGLYFFKASYNGLWYAAPMHTQKLLLFIMQRGTI
NTVFICGKIYVASLEGFASLTSMAVSYFTVMYST
>AechOr266
VSALSYEMHEIGKRYYKINRLVLKMLGLCPYQQSYFTHIHKVLYASILLTFILSQLLIFITQYNVELLFKILS
LVLPVLFSTIKYFLFIIETNSLKHLLAQIQNDWNSLKDKLEIDIIEKYANNVRIFTISMIAFCYVFIFFFGIL
QNLPLILDIMLPLNESRPRQLLLITEHFVNQDKYIYVIMLHEFLIGYIGLTTIGGTAATIIIYVTHVCALLKI
ASYRIENAIERNILVIPSPKREYLLHQRIVHAIIMHQRAIEFNEFMTSIFLIPFAILIVVGVSSLTFNFFRFF
QLITSSSISKIIVSAALIFTHLIYLFIANYCGQAVINNGVNLFNAICNGLWYAAPLSTQKLLLFIMQRGRKNL
TISCFSIFVASLDGFATLTSMAVSYFTVIYST
>AechOr267
MISFIDQYYDINQKLLTLMGLWPYQKWRFRIFQITLFPSILISFLIAQLTTFITSEFNVNLMLKVLSQAFPCL
IYFLTYISFLLSNKDIKIFFDWVRYEWTTLKTTDEINILRRYAGFSKRFMVTMTVLLILFLNFFGLFQFLPNF
LDIIVPLNESRERHFSFLAEYFVDQKRYFYPILTHNLLAIYIGGITVISTGTMLMGFIFHICAMLQIASYRLK
HISDNIPLSKSDKDYIIRKRIINAVDIHRRALEFAEFILSSFANFYFTLIGIGISSLTVNMFQFLQLMMFTND
TNGKLACGLLTMAHFVYMFFGNLAGQIVTDHNINILNTTYTMQWYTISLQAQKLLFFIMQRNTKNYYFVVKDL
ATLLLDRLMSMSISYFTVIYSTR
>AechOr268
MYIFDDYYKFNQTLLRILGLWPYGKTKFERFRAMFFYMLLISYTIVQFTQLAIIDFNTNVIIRILSDALPTFL
FVIQFNMFFFETKKQMLTDAQEIKIVEHYVHQGKNITITFLLLLILAIFLLLIMEFQTDILDFFQPLNKSRTH
YLSFLQEYHININARIQFYSLFFYSLISMIIGLVSVISVSMMLLLIALYCCAIFKICSYRIVQFMDKKEIIAC
FNKKSIIVERIIRIVKLHRKAQKIFKLFMSSFTISFLSTLIIGTSSVIANLYSFMNAVIYMDKWTETLIRTFY
IFTHQFYLFGTTFMGQQIVNHADELFNAIYMSLWYKAPVTVQKLLLFIMQISSKGLVPNIGGVYVSVMESFTS
VTNKSISFMMVIYS
>AechOr269
MTVLESWYYNVNRITLMICGLWPYQNTKFRYVRAMFILGIFICFVVYQFRLLFIYEYNFKLALKICSDIFPAI
LCILQFIAFLIKPKEVKKLLNQICEEWNALKDSKEIEIAERYGTFMRRLTETLFLCGLLSWLFLVLMHLIPIN
INKSAIANESFLRQDVLMQQLDKERYIYLFLTIVFIGVFVMTTTTSMVLTYMRHVCAMLKITRYRIEHSLDGN
ALHISISQQNRLMYQKLISAVNIQRRAMEFTQRALDSFKESYFFLIGIGVVSLAFNLLHAMKAALIIDNVNDL
AISTFYVIIHFFYIFIGNYGGQTITDHNADVLKALYHVQWYMAPIQVQKLILFLMLKNTKYFGLIIGGIYVAS
LEGFITLTSTSLSYFMVIYSTQ
>AechOr271
MDFAGERYYKLNKIFLICFGLWPYQTRTLKKIQIIFFQTLFISFLICQCNTFLIKKYSMDRILKILLFIILTC
IFIVKYNACLLLTDNIKYIFDRVRYDWNILKAQTELEIIRKYASYAKLFMIGFILLIISSSLGFVVIVCLPHV
LNVIIPMNESRPQQTLILVEYFVDEETYLIAILTHIMVAMYAGNMTIMSIASLHIAYALHTCAMFRIASYRIE
NIFNENIQQMPKNIKQYIFYNKLIQAVYVHRRAIDLANILTNSFATLYFVLLGLDVALMSVSLLNFYNAVISL
EKVDLLIIGACIMIQLYYVFVGNYVGQDVIDNSISIFQATYNAHWYAAPLYMQKLILFIMQRSYRKSALTAGG
LFDASLEGFAKLMSMSISYVMVLQSMGAHKENHE
>AechOr272
MNFYGQRYYRINRFFLEMTGLWPYNNSMYIYIYRAFTIFVLISTSITQFIKLQNTVHDLDDFLYSLTYTIPCF
IYVINFLNFIIKIDRIKDMMNRIQHDWNTLKDERECKVIRYHTIVGMRYTMIFFAFLLPLLFSYILSFYLPSF
LDFVYPLNESRLRKLPLLAEYCILDEQKYFYSILVHQLLSIVLGLTAIIATSSLNMLYLLHACGLFEVTSYRL
KHVFDDGMFSVNTNEKYAIIHARIVKVVNVHTRTVEFLDYLMSTFKVTYFIMFVLGIIAITINMFRLIEAILA
MNDFGELIATGSFSGGQYAFLFFVNYFGQKLTDRSILMFDKVYDIPWYAAPVHIQKLFIFILQRTVKGYVLNI
GGIIMASLESFASMASLTLSYLTMLYSVR
>AechOr273
MNFVGDRYYKLNRHLLLLVGLWPTNENSIYKYCQMILCNIFIIFMMICQITKMLTLKRNIDVMLRLLSFTVLC
IIYIIKYQTFCIVARKIKCLMGRVEEDWNMLKDKKELEIIERYTCIGSMCTLSLTILGFVTMLSCSFLPLIPS

ILDIVTPLNISRPRQLLFPGEYFVDQQKFFYVILLQVDVTIGLIIITLIATETLYVTYIQHVCGMFQIASYRM
DQAFNDKLLQGYTSEKQAIIIYKRIIEAVHIHKKALEFSEFLWSTLGISYSILLLIGITSLIMNLFCVSYTSL
ILFSKKVNEIVLIGFFILGHIIYLFVGNYLGQILIDHSTDIFQNIYITRWQGAPIQAQKLLPIIMQRSLRSCK
MVVGGMFVPSLEGFAALMSTTLSYFTVLWSVHK
>AechOr274
MEFPEEQYFKLNRILLSIIGLWPYDNFQTRYIRFILSLLIMISFTSTQPIKLFVSEYSFDLFLDIFSYNIIFL
ICSIKYITFYAVLKNIKEIRERVQNDRSVLTDNQEIKIICKQESIGKLFTIFIIILVYIVIFFYIFMQYISIL
FDIVMPLNESRPRKLLFPMEYFIDQQKYFYVITIHIAVGLLFLATSGVATETFSLVNALHAFGLFKIANYRMK
NILSGINPQKKITEKYAISHNRIMAAVDFHRRAIEFSDLLKTSFGQVYLILFILGVCSISINFLNLSRITEKE
ILEVIKYAFIIVLHMLYFTFSNYAGQEFINCDTDIYRTICDTKWYNAPLKVQKLIFFLIQKTTKCYKIDAGGM
FSPCLEGFATGLNMSISYFMVLRSI
>AechOr276
MDFNIDHYYKLNRIFLSVIGLWPHRYITLRQIQCVVSSFILISVTFFQLIKLITMKYDVDLILKVLSSALPFM
LFTVKYISFCFIIENIKELMQQMQNDWNALNDINELEIFHRYAKTARLFTTTIATLIYTVLILVFCIQYMPSF
LDIVTPLNTSRQAELLFQVEYFLDQKKYYYMIQFHLDVGLIVAMITILSTESFCLTLSIHAFGIFKITSYRME
HVINKGVSNTFMEKYYIFHDNIVTAVNGHRRAIEFSEIFKSTFAIPYLALILLGVSSSSINLFLLFQIIMSVS
TMDDLIKSVAFVTCHLIYMFSTNYAGQKFIDHDADIYKKICNIQWYNAPLKTQKQILFIMQKTIKNYHIDVGG
LFSPSLEGFTMLVSASLSYFTVLCSVR
>AechOr277
MELFEEKYYRFNHILLSSIGLWPHDNSNIKQIQVILSLLIYISFLTVQFTKLFVPEYSLDLLLEVFAFNFLIL
IWFIKYITFFSVIGNVKQLWQYIRNNWNILIDDREIHIMHKYADIGRQSTIAIGMCVYFGCFGFTLVQYIPDV
LDIVKPLNESRPRILLHQAKYFANQKYFYIVMMHEIIAILISGTTGVAAETFLLVNSLHAFGMFKIASYRMER
MLNVDVSQLSTAKSYIIFYDKVVAAVNIHRKALEFSELLQTSFGLSYLFMFMMAICSATASLFRLFRIMTMQQ
EKIEVIKLFCYIVFLFLFLIIGNFVGQEFTNCDNHVHRMICNTKWYNAPLKIQKFILFLIRKTVKNYKIDAAG
MFSPCLEGLAMAMSLLLSFFTILCSI
>AechOr280
QNCPVNSYYTINRSLLLCIGLWPCQKSVFRYITIVFITIMFVSCVVLQLMTFITTEYNIDIFLHVLAYCIPWL
GYTLKYNVLCLNIRRVKMRDLMKRILCDWNELNNLQEIKIIKEYADIGRVITLITTLFIYVSIFCCILILFLS
NLFLSITFDKNESHPRQLPILIECFIDQQKYFFQILFFICFTAVYGFTMMIATETINMSYTYHACGLFEIASY
RIERTLFENMVQGIASSSERSSIMCQGIINGFNMYRKAIEFIEMLKRNYKWAYSLLLPLGVLSLSINLYRMLF
ELFYCLGHFGHMFFYNYLGQKVIDHSSDIFHRIYNVQWYVAPLRAQKLLLLMMQRSMRHCTIVVNGLFIPSFE
GFATLVSTSISYFAIIFSLF
>AechOr281
MTFALRWQDCPVNSYYTINRSLLLCIGLWPYQKSTFRYIAIAFTTIMLISSVVLQLTTFITSEYNVDILLHVL
AYSILWIAYILKYSIVCLNIRRVKMWDLMKRILCDWNELNNLQEIKIIKEYADIGRFITLIVTLFAYVSIFLF
ILIQFLSNFLLNTTSDKNISHRQFPILIECFIDQQKYFFQILFFICFVAVYGFTMVIATETINMSYIYHACGL
FEIASYRIEQTLFENAIQSIASSSKQSSIMYQGIINGFNMYRKAIEFIEMLKTNYIWQYSLLLPLGILSLSIN
LYRLSQLITSKKYYEMMVSFLFVSNHFCYMFFCNYLGQKVIDHSSDIFHRIYNVQWYVAPLRAQKLLLLMMQR
SMRHCTFVVNGLFIPSFEGFATLVSMSISYFAVIFSLF
>AechOr283
MVFAGSRYYKINRILLTCIGLWPYYTQRAKYAYCIFLFLLLLFNIFCQLSSFITKEYSVNLLLNVLPYNIPTI
YFTFKYLIMLINGNKVKELIEQMRYDWNSLKKKEEFKIIQNRTNIGRFCTIIMLIWTYTILFLFILMFFSPNI
LNVVMPLNKSRKLRQLPDSMEFFIDKEKYIELLTSYFFLILTIFITITVTIETFLQMCIHHTCAMFQITSYRI
ECILNRNQTKSLVSQEKFNNHKNIVEAVNSHRNAIEFIDSVKSTFSVAHVFGSSLGVILLSINLYLLCKYLMA
KDIYSMSISFLYIVTQFCYMFFFNYMGQQVIDYSNNIFKKTYNTRWYATPLNTQKCLIIITYRSMKTYTLTIR
FGLFVPSLEGFASLISRSLSYFMVLYSL
>AechOr284
MISIVDRYFNLNRTLLLIVGLWPYKKSKFIGLQIICIFSILITCIIHLLTIILTSTCSTEDIIKILSVALPII
CYIIKYNSFYINNETAKLMDQIQHVYNDLRDSNEIAIIDKYGNNAKQYTVMLTMFLICSIPVSMGPFLLELID
IVLSTNVSRSYHLQICTEYFIDEKYFYLLFFHVNATVNIGSVALVATGSLLLAYFQHACGMFKIASYRIKKAI
HICTLKDLNTQKEILIYKDLIRVVDIHRKSMTFSNYIVSRFQTSYMFLIVFGVFSISLNIFRIFQIVLFEYNT
GELLQCFAITSFCLVYMFICNLVGQQIIDHNNYIFVTAYKVQWYVTPLFIQRLILILLQRGSKSFHLKVGGLF
VASLECFATLINASVSYFVVMYSMQ
>AechOr285
MISIVDRYFSLNRTLLLIIGLWPYKKSKFVGLQIICIFSILITCIIFQLTTIVSSTCSTEDIIKVLSTAFAFS
NFVIKYNSFYINTLKNTLMKIKNLMEKIQHVYNDLRDSNEIAIAEEYGNNAKQYTVMLTKILICSLSVFMSTR
VWLEFSDIVLSTNVSRSHHLQISTEYFIDQQRYFYLPPGLYNITNKLITSISLCTNIIFTSCYSYQLLNYLHS
YRIKRAIHTYTSKNLNTQKEILIYKDLIRAVDIHRNSITFSYYFISRFQISFMFLIVTGVLTLSLNIFRIFQI

VSFQYNTEEFMIRFLIIFICFLYMFLSNLVGQQIIDHNNYIFVTAYEVQWYVTPLPIQRLILIIILQRGIKSFG
LKVGGLFVASLERFTTLINASISYFTIIYS
>AechOr286
MISVVDRYFSLNRTLLLIIGLWPYKKSKFVGLQIICIFSILITCIIFQLTTIFTSTCSTEVIIKVLSTALAFS
NFVIKYNSFYINIIKNLMEKIQHVYNDLRDSNEIAIAEEYGNNAKQYTVMLTIILIFSLSVFMSTSLWLEFSN
IVLSTNVSRSHHLQISTEYFIDQQRYFYLLFFHINATFIIGCVVIFGTGSLLIAYLQHVCGMFKIASYRIKKA
IYTYTSKNLNTEKEILVYKGLIRAVDIHRNSIKFSNYFISRFQISFMFLIIIGVLTLSLNIFRIFQIVLFEYN
TEELMICFIIISICFLYMFISNLVGQQIIDHNNYIFVTAYEVQWYVTPLPIQRLILIIILQRGIKSFGLKVGGL
FVASLECFATLINASVSYFTIMYSMR
>AechOr291
LNYIFNPIRQWLWIFGIWPDPQIPLNEFRRPSIRFIIVMCSVSLYVFGPQMMNVIRAAGSVTRMVENFSSANF
SMLAVCKLLVTWYHGKTLQSLIISIMTDWMTSTKEWERNTMLKITRSGRNLSFKCCALATSSITCHISLQLLR
FFKTIHQPERNLVYRIETIQKSPNYEITYFIQLFGGMYSAFANSIIDSFISMLILQVCAQLINLRTMLNNLVN
KLANKSISSITFREGLAAIVVRHDHLIRTAKTIDSCYSSVLFVHVLSTTFQMCFITFQVFTIITNNMNVSIMK
MIFLTLYFNLVLFSLYAYCYSAEKLITESTRMAYGVYECKWYDLSSKDAKNLMFIVHRSRIPLKLTAGKFGTF
SLEMFGIVLKTSMGYLSALLTMRD
>AechOr293
LNYMFMTHQWLWVFGIWPDPHMSLNDFRWPNIRFIIIVCNISLYVSAPQMMNVIRAWGNMTRMVENFVSVNFS
LMAICKLLVTWYHGKTLQPLIVSIMIDWMTSTTNWERNTMLKIARRGRNLSLRCCLSALGLMIFSMSFHLLRL
FKNIHQPRRNLVYQLEIIQKSPNYEITYIIQLFGGAYSIFANYMIDSFVSVLVLHVCSQLINLRLTINNLVNE
LANNSISSSRKERFKKDLAAIVVRHEHLIRNAKTIDGCYSSVLFMNLLLTTLQMCFIAFQIFTMITDNLKIPI
VRMLFLLFYITFMLTHFYTYCYSAEKLMAESTNMAYGVYDCKWYGIPSKNAKDLMLIVYRSTIPLKLTAGKFG
IFSLEMFGIAIKTAMGYLSALITLQN
>AechOr296
YNWVITLNRSCLSLLGGWQFNETPRRKLMINVRVIIILNIIIWSCIVPSFHSLIRIWGNIMSMIDNLQYTLPL
LSSIIKLILLWQKKKVLVSVLKIVKEDWIKLKTEEERAVMIRQTRIARLIMIWGYLIMVFSFILVVILPSFNI
SMRYITNITDPGKVLPLQTYYIYNVSKSPFYEVTFILQGFSLMTAAITYTGTDSFMGYLVFHVCGQLENFKMR
ILNLDKFIHYEKALSSSVQDHIRLIRFIKLIDSTFNLMLLGLLLYFGILFALYGFLFIITQGSNLSILRLIYI
LIAVINTFTHMCLYCVVGEFLVIQCEEVFEAIYHYKWYNLKPKQAKNLLIIMMLVNRPLYLTAGKFFPMTMAT
FCNLLKTSGGYISVLLAHRN
>AechOr297
YEWAIGLNRTNLALLGVWPKNNETKQKKLISNICTILMLNTAICGCYIPSIHSLFKVWGDIMSMIDNLQYTLP
MSVAIIKLSIMWLKKKDVLPLLNMIKDDWLKPKMIKEKDVMVKRARMARIFTIFGYFMMLASFNLCVVLPVFG
ISIRYLTNKTDPDSKLLPLQTYYIYDRDKSPFYEITYIMQGLGLLAIAIMYTGVDSFLSLLVFHVCGQLENLK
IRIIHLDKFQNFENALSQSVQDHIRLIRFINIIDEIFTLMLLGTLFYLSILFALHGFLLCTMVTQGRDLSVPR
LTFIMTVFVNTFGHTCLYCAVGEILVSQCEGVYQAAYKYKWYNLEPKKAKNLMMIMIRANKSLYLTAGKLFPM
TMSTFCNLLKTSGGYVSVLLAHRE
>AechOr299
MICVETRYFGLNKSLLLAVGLWPYQQTKFVRFRFIFFLSILTTAILFQFTVFFTSKCSSDLVLKILSVTLSLL
MFVINYISFGFKMKVVKDLLAQLQNVYIKLQDEYENAIMEKYGYTGKCYTAALIIFGICSVFTPITAEFLLTT
SNVVLPINASHAHRMQFTTEYFIDQEKYFSLILLHINAAFCIGLFAIIGIGTMLFVYFQFTCGMFKISSYRIE
RAMKINILKNISAKNEKFIFEGIICAIDIHRQAMKLSKMLMSKFEIMLLCQTAVAVTSLSTNLFRIFQISSSQ
ANAKELLFPFIFVSICILYMFLANYTGQNIIDHTNYVFITAYSVQWYMTPIYIQKMILFLLQRGTKDFTLNLK
GLIVGSFECFAMLVKASISYFVVIHSTQ
>AechOr300
MIRIETEYFSLNKFLLLVVGLWPYKQSKLICLQFVFLLSILISCILFQLTAFLTTKHTFDVVIKILSIAFFCI
IVVVVYISFAVNIKNVKFLLSELQYIYNDLKDEYENVIIEKYNYNAKWYTVVFTILGACSVSTLFVAQFWTSI
IDNTLSENVSQSRYLLITTEYFIDQENHFYLIVLHIYAAISIGCIAMVATGTMLIAYFQHICGMLSISSYRIE
HVMRINMLQNSTLKNENLIFKGMIYAIDIHRQAMKLSKLIVSKFEIMLFCIITVGVICLSLNLFRIFQIDIFE
DNVNEFLFSLTFVVASIMYMFIANYIAQDIMDHTNHIFVTAYNVQWYTAPLHIQGIILFLLQRGTKNFTLNVG
GLFNGSLECFAMMVKASVSYFTVMYSTR
>AechOr301
MTCTKDRYFSLNRILMLAIGLWPYEQSKLIRLQLTLHYGILGSFIVFQFTTFVTSKYDLQLTIEILSVTFFFN
LFLIPYLSFNFNMDVVKHLLELLQDTYDELRDENEIAIIEKYWNIAKRYTEILTLLFIFTNILFIFSSFLPHI
IDTMFSGNESQLQPSLKIVTEYFIDEETYFYLIIILHADVAFFIGSLAMLATGAMLLTYMQHVCGMLKIASYRM
EQAMTISIRRMSDIKNAHLICKGIIYAIDIHRKALKIVQITVSSFNTTFFIMVLNGTVFASLNLYRIFREISS
GYIGEKVIQCFIILNIHYIYTFLSTNIAQQIIDHNNRIFVTVYNGYWYVVPLHVQKLILFLLQRGTKTLKIMI
GGLFIGSLEGFATLVSASFSYFTLIYYTNQ

>AechOr302
MICLETRHFSLNRILLLVVGLWPYQRSLIIQFQLILFFGTLTTFIIFQFTTLLTSKCTSEHILKVFSTSFFFV
CFAIKYNSFWINADTVRSLLEQLQDVCNELKDENEIAIIEKYGSKAKCYTTAIILFGMCGECTLTFLQIWPYI
QHFILLNNSSRLNPSVYIVTEYFIDQEKYFYFIMLHALAANYIGSIAMIATGTTLLAYLQHTCGMFSIASYRI
EQAINILQKDDLKNESKIYKEIIYAVNIHRRAMEFTDFLISNFEGSFFFLIAIGVTCLSLNLFQAASYRDNIE
QLILPLLIIIILYVYLFVSNWTAQEITDHNEYVFATVYNVQWYIAPLHIQKMMLFLLQRGTKAFHMILGGIFV
ASLESAAALMSTSISYFTVLYSTK
>AechOr303
MICLETQHFRLNKFLLLAIGLWPLQRSKFAQIQFTVLFTILTTFIVFQFTTFLTSKCTTDLIINVLSSAFFFI
CYAIKYHSFWINANTIRISLEQLQHVCNEIKNKNEIAIIKKYGRIGKYQTKIITIFAACSVSTYLFLPIWPRL
FDVILAINNSRSRSAAIHIATEYFVDQENCSYFILLHTNAALCIGATAMVATGTMLIVYFKHICGMFSIASFR
IKKAMAINKQQDVNEEKMVIIYKGIICAIDIHRKATEFSQVFLKSFEGSFFCLIAAGMICVSSTLVQIISYNS
TGQLVLQLIYISVLYMYMFLSNYTAQDITDHNEYVFATVYNVKWYVSPLHIQKMMLFLLQKGTKAFHLILGGI
FIASMESAATLMGTSISYFTVLYST
>AechOr308
MIDKRLKAYRTYKRFFRTLLIINGCWYMPTKSNKLMYYWSICVFLSMIIYTMISLHMSYVVRHNIKIMVKFVG
VALSGFSAIIKVLSFKINRDSLINYHRTLNDLFKEELMQNEKVQTVIFSSLRAISTLAYIYFVFLIGIIVAYS
RSSYIYIIQNLLYFHLPTNYTLPLSRGFGWFWTVPDNFLYHIHMFYETGLITLSCMMACGIDSAFGFYVVYQIS
STIRAMMFAFTNPLSTEKFSDLLRTCAVKHQKLLQCRNTLEHVYGPIVFWHIVSNAVLLCFLIYDFISLSVFN
LENVSSSLSYAGVKLLQTFMYTWYGTFLTNAGEEFRKEIYFSKWLNSNLDCHVRTNVILMMMQKPMTINAVFS
PINVTMFTNFVNTTMSYFFLLQSM
>AechOr311
FEWAVKLNRFGLEIIGLWPKSGQTTWEKRVCNLRVLLTFLLIIFVLVIPAIHSLIRIYSDILLVADNLMCTLP
ISGCLLRLVIFWWKRKAVAQIIHLIANDWTKTTTAQEKAIMIAKAQIARIIITFGYGMMGTAIIVMLVLPIFG
YSVRNLSNVTEDLEKPLSLQTYYIYNTRRSPQYELTYAAQCIVLFFCTICYTCIDNFLSLSVFHISGQLDILR
NRFLHLHIVANLNNALKSCIMDHTRLLRAINHVEDIFSLILLILFLCFGTLFAFYGFWIMNILESRQQLTFLN
LAYFVSIVINIFGHMCMYCAVGEILTAKCDRIHYAVYFNEWYTLNARNTRDLVLLMLRTNKPLHLHFGKLFPL
TMATFCSLLKTSAGYISVLLTTRS
>AechOr316
LRMRSNMDWNTDTTNILKFHKHFLGIIGLWVLNEKNVFSRIRWFVSTVVETSTWIIMSLEVIQSCNGNEDAMD
AFLSSSSSITSLVKLLLHRVYWKQKFVLVESVIQDWTYVKNSHFRDIMLRYARIGRLGSSIYFYIACAAVVFA
FSVMLSNINLPWVSEKQIFNETYERKLMLAAYCTFGNYYTSSFAFCAIEALQFVQILVNCISQCGNDGFFFDL
TMHMCGQFVILRMNFNALGCDKFSYHNKLDVLLKRHYRLVRLSYYMERAFTLVILAQVLMSVLVLCVEGCFLL
LLSLEINDAFTAMKHGVYIIALLIQLFLYCFAGQTLEFQSKELAYAIYESPWYSFDVNMMKNVPLIILRATNP
QQLTAGKFVPINFMTFKEILKLSASYLSVLRVMTRT
>AechOr318
LRMKSNMDWNTDTTNILKFYKNLLGIIGLWVLNEKNIFSRIRWFLSTMVEMSTSIIMSLEMIRHCNGHEDAMD
AFLLSSSSIISLVKLLLHRVYWRQKFILVESVIHDWTYVKNSHFRDIMLKYARIGRLGSSIFFYLGCASVISF
VSSVVLANINLPWISEKQIYNETNEKKLMLAAYCTFGKYYTSFFTYCTIEVLQFVQIVVNGISQCGNDGFFFD
LTMHMCGQFAILRMNFNALGCDEFSCCNKLDILLKRHYRLVYLSHYLERTFTLVILAQILMSVIVLCVEGRFL
LLLSLEINDALTAAKHGVFIISLLVQLFLYCFAGQMLEFQSKELAYAIYESPWYTFDMNIMKNLPLIILRAAS
PQQLTAGKFVAINFMTFKEILKASASYLSVLRVMMKT
>AechOr319
IIRVLPLARMMNKRWNDDVAYVFSTHRLFLKVYGLWPLQKQTVFTKIQWGFCLITQFMILPCLTTELLWSSKD
ASSNIESITFFASTSTGLIKNICLIVSQKKLGMNINAAIDDWLSVKNNVETRKIMKKYAVQSKILTFTLLYSL
YVCLGMYMAVVIFINLKQIFSTDMNLVNVNTTDWLLLIPSGSLGQLITGPQYAIILTIQIVQTSLLSFLLFTV
DSFFFNVTIHLTGQLEVLKNNFKTFTKEPNTEANYRKKFVNFVNRHNLLMELYQNLEDIFHFLILYEIVIVTV
LLALIGLRLNICINEKDHVEAIKSVFVLNYLIMQSLVYTYGGEFLQKESEDIFYMLYTTSWFTLPAALMKDVH
FAMMRSSIPFRLTGGKFFYINRETMMYILKTAASYISVLRIALR
>AechOr323
VIDSSYSHYLNPMASERWKDDVAYAITPFKLVAWSIGIWPFEVHNVYSLIRYVVGISGPVFMLIKSSFEVYLG
CTDAETSVDCLMLICCAILGVLKAIWFRIYAKNLATNYKSVMEDYLTIENIKERAIMRKHAFMGRTLCCLVLG
FGCFSSIMYGLNAILNEYKHVNITKDTMLEYPVPSKCFMRYLNAPVRMHKIFCIIDVIALILASIANQGNDAL
FLNATLHICGQMKILRVNFLDFDIKDLHIYDRFNVLIERHKYLIKLARELAEMISFVLLIELFIISILLCIMG
FQLILQLGENNVVLITKNIMVQSTFITQLTLYSVIGDYLKSEMEEIGPSIYQSNWYNFPAKLTRHMIFILMCS
DSPVALQAGNFIVVNLITYVSILKASLSYLSVLRVMVK
>AechOr326

MASERWKNDVAYAMTPFKLLTWPIGVWPLQVYNIYSLIRCVLVTCCMSIIVILPSMEFHMGCTNTEQNIDGLM
LACCGVLGVFKTICFRIYAKNLTDNYSSARNDYLTIKNIEYRAIMRKHAFMGRILSCFMVCFSYISVTIYSLI
PLLGEDLVDDQDVQINRTNEDIVLDYPMPSRCALEYLHVPRSMYEFICIFEFIVLVLTCTCNHGNDSLFLNIT
LHMCGQVKILKANFVNFVVSSPQVYDRFNDLIRRHNYLIELAKELTESISIVLLTQLFISSILLCIMGFQFIL
ALKTHNVVVMGKSAMVLCTFLTQLSIYSFIGDHLKSQMEEVGFFIYQSNWYDLPTKLARNLIFIIMRTRSPAK
LLAGNFIVVNLATYMSILKTSISYLSVLRVMIEI
>AechOr327
MSDDVWNDDIAYVFSTHRTFMQILGIWPLQKRTIVTIIQWNVMIFLQLSPLIFLFMEFTGNNGDTGSNIDAII
YLSCTLNLNLKYIAIAANGGKLTKNIKAAVADWVSAKNDEKSYKIMKKYAFKSRLYTLLMLYSSFVCGSLYLL
SVLVMNLIEIFLQDQMMNVSNDRLFIIPCGEFGNKINGLQYSMVIGFQTVQLLMICIMQPIGDSFYINVTLHL
TGQLKVLKKKFKNFASKPDTQINHRKRFISLINRHCELTELHQNLEDTFNLIILFQLVIVTLLLALLGLRIIF
CLKHHYYVELMKSILVLNYMLMESLLYCYGGDFIQKGSEDIFHAMFKASWFTLPATMMKDLNFAMMRSSYPFR
FTGGKFFYTNCEAIVYILKTAASYISVLRMALKD
>AechOr333
RYYSVVRFYLSISGLWPYHRLRDRCIRFVPLFVFSFTILIPQILYLLIGSITLDDVFECVPPIFISITFSSKL
LFIMLNSEKVKTCLNIIQKDWQSLNSVEKVILERHSKYGQHLATFYAVLMHTTASLFVVKPIMLTLMTDDIIN
ITKSSIPFESRLPFRVEYGRKFNQYIYPIAVHSYVAVVAHSFATVVADALYYTLIQHACGMFSIIGNVLENIG
KNDTDESFDTKSDKIKDDNYYKTLHCLRRHLGVIEFAEHIESVYTKIFLINLNFNMIIGSLYGTKMLINFDKN
VNDIVAPISMYFGQLIHLFLHFWQGQFLLDYSILPYESICRANWYYTSQRCRKLLLLMMYRTVIPCKITAGKL
MILSIDNFAMVVKTSLSYLTVFRSMQ
>AechOr334
KKDVEQNRRYAVLRLYLTISGLWPYYNLRDRCICFVLLFILCSSIAIPQALYLLSSITLDDIFECLPSLIISI
TFSSKLLIIMLNSKKVKICLNIIRKDWLSVNTDIERIILQRHTRYGQYLAAFYAVFMHMTGCLFVLKPIMLTL
MANDIFNVTKSVPRLPFRVEYGEKFNQYIYPIALHSYIAVFAHTFATVAVDGLYYSLIQHACAMFSIIGNTLE
NIGKNNEDIFYTKRAKIQDNNYSKTIHCLRRHPLVIEFAEHIESLFTKIFLINLNLNMIIGSLTGIQVLMNLD
KSARDIAGPITMYIAQFLHLFLHFWQAQFLLDYSILPYKSICKANWYYASHRCRKLLLLIMNRTTSPCKITAG
KIVILSIESFATVVKTSLSYLTMFRSLQ
>AechOr335
MFDKKDVEQNRRYAVLRLYLTISGLWPYYNLRDRCICFVPLFAFCSSIAIPQTLYLLIGSITLDDVFESLPSL
IISIIFSSKLMIIMLNSKKVKICLNTIRKDWLSANTDVEKIILQRHTRYGQYLAVFYAAFMHVTSGLFIFKPI
MLTLMTNDIINATKSSVPFISKLPFRVEYGEKFNQYIYPITVHSYTAVFAHVFATIAVDGLYYCLIQHACGMF
SIIGYTLENIGKNNDDIFDVKQAKIQDNNYSKTLYCLRRHLLVIEFTEHIESLFTKIFLINLNLNMIAGSFTG
IQVLMNLEKSANDIAGPITIYIAQFLHLFLHFWQGQFLLDYSVLPYESICRANWYYASHKCQKLLLLIMNRTT
SPCKITAGKIVILSIESFATVVKTSLSYLTMFRSLQ
>AechOr336
MFDTKDIERNRRYALLKLYLTISGLWPYHNFRDRCIRFVPFFVFSFSIAIPQVLYLLIGSVTLDDVFESMPSL
IISILFSSKLLIIMLNSKKVKICLNTIQKDWQLVNTDVERIILQQHTRYGQYLATFYAVFVHTMTTLFMLKPI
IVSLITDISNTTKSSIPIASRLPFNVEYGEKFNQYIYLIAMHNYVAVFTRSFATIAVDNLYYVLIQHACGMFS
IIGNVLENIGKNNANDFNAKSDKIKDNNYNKTLHCLRRHLDVIEFAENIEALFMKIFLINLNLNMICGSLAGV
QVLMNLERNASDISVPIIIYTAQFIHLFLHFWNAQFLLDYSVLPYRSICRANWYYTSQKCQKLLLLIMNRTTI
PCKITAGKIVILSIESFTTVLKTSISYLTMFRSLQ
>AechOr339
MQSIVLRYYRINKIFMSQIGIWPYQSRTKRILIPSTIVLISISVLIAEIICMFDTWGNVNIAVECMISTIIII
GCFTKLFNLILNIKKTRHLFSLIEYHWQVFTNSTDIEIMQDYVTFGRKVLILYSIYIYTSLALFMIIPMSPQI
MDIVMPLNNTRPRKLLIEVEYRVDREKYYYPILFHSYVAIVLIISIIVCVDTTYISYVEHGCSLFAAIGYRLE
HVISKGHIDKTSYFAKSKERTYQDVKIEDTCFKEKEIFYEFVTCLRKHQLAIKYVCILESSFTLSTGIQLLCN
IVGMSLIGVQVINNLDSIENIIRYTASCLATFFHLLCMSLPGQRLMDHSLDVFNSVCRSHWYTFSLKTKKVLR
ILLYRSIVPCTLTAGKLYVMSMANYSSVVQTAMSYFTTFSSL
>AechOr340
MNPEMEKYYAINKLFLSGIGVWPYQRRVLKVFLPCLLSTIHYASFVTQVLLVYDTWGNMDVIVEAIIVITCIF
GASTKLINIIVNNHKFRRLLQLMCEHWEFFNSEFERHIMRYYASISQKVTKYFGVYFIVFTIFYLLIPLIPRI
LDVVKPLNESRPLIYVYSVEYRVDKEKYYYPILLHCYATSLITIIILFTIDTTYIMCVLHVCSLFIAISHRLE
NITVKAETKSDNNKKYVGRHCHLLMEKHGSTGNDYRELIMCLKRHQLALEHVQILNSTFTQAMFILLSLNMLN
MSVIGIQLIKNLEHINEIIRSIFFICAVFIHLVCMCIPGQLLIDRSTEVFDKAYGSSWYTFSIKTRRLLRILL
YRSLSPCTLTAGKMFILSMTMCSSMMQTAMSYFTTFLSMR
>AechOr341
MDFFDTRYFRINKFFLSFIGLWPYQTSFIKLLTQSFAIFGVTIMCAPQIAYMFKHVDDLDNMFELMPILAGTV
ICIAKIISLTCNSEMFKGLLQHMRDDWNNLLTSEETQILTHYAEKSRTLMLAYSISVIGFVFCYALLPLTGPV

FDIILPLNETRPRKLPHLADFVILDQEKYYYTLLLILYVGYVVCVSIAVAADILYIFLVEHICGMYGVLCHRL
RNLAAHDDLRWIDGDYIHEEIRRYIQHCIQLHERIRLLVFIEMMDSTISLFLFFDVGLGFLLHTSSCIMIIVR
MGSSEIMRYVALMLMQSCRLFFNSWAGQEVTDHSVEVSIAAYDGIWYNASVKVQKLLLFLIARSQKASQITIA
KLYVINLEGFSKVMKTSVSYCTVLISLR
>AechOr342
MNVFDTKYFKINKLFLSIIGLWPYQTTFTRTLKTTLTTVAVIVMCAPMFLFIFKHPNDLDHIIELLPTMVGTI
TCGVKFVSLIYYFEKFRDLFQQFYDDWMNVSKKAKQIFRNYMEISWRFTSIYTVWMATFVIGFALLPLVNPLL
DVLSPLNITRPKKFIYPAEMLVDHEKYYYILLTIMYYSYFVACVVTLAIDTIYFAFIEHACALFDILNYHLKN
MVKGRSKQEAHHDSSSKQNNIVQHLIRCILLHIKIKAFIDLINSTFAIYLFFEVGLGFVMHCTLCTMSILQMN
KTVEIIIYVALVVCQTARLFFYGWQGQQIADHSIDVYISAYNGAWYETSNTAKKMLLMLILRSQMISEIKVAK
SFVMNLENFTVIMKASLTYCMVMLSMRE
>AechOr344
MDVFETRYFRVNKLLLSFLGLWPMQSNKKKFFYCTMFGILILLLPQIAFLFKRMKNLNDFYDVLLTFVGTCLC
LFKTISLHWQTEKFRMLLQHVRHDWCLSVKYNDIWILMEYMERNRIFTLIYSIFASTSIASFVTAPLTMPLLQ
SILISNVTRSKRMPHPTEFFLDMEKYYYILLAITFVGYAVCGMIVIATDTIYFALLQHTCGTLAILSYRLAAY
DKSKKCFNRNPTSKEDTDVENMVHCIQLQIRIERLIYLIETTFAICLSSDVGLGVLLQCSGCVRIVTCTELAR
NGPLVVIQSIRFFFFTSWLGQQIIDHSSQISVAAYNGIWYQMSLEAKKMLLLLIMKCQKPYHITAAKLYAICLE
SYSSFMKTSVYYYVTVMVSFNSD
>AechOr345
MSFFDSRYYYLNKRFLAVIGQWPFQSRLEGNMMFAITSLFIYSLTAFEFWGLAAGITDLSIIMENASPLLVNS
IIIIKLVNCVFTNDKMKELLEDIEKTWKIKHTDSEKKILQHYAEKSRTFTIRYAIVLYTTWLFYSTTSIMISG
IYTILPINETYTVKFLYRMDHVLDLDKYFKLLMLHGFISIFYIVSVPIAIDTTFTLYTQHICALFECLRYNIE
RIRGSDFILLEPNIKDDEVYRDIIGCIKSYQHALKFSDMFSSNYATSFLFQLGNVIISLSFGAAELIMVDNQL
DEIIRILFANLAQLIHIYFLCLISQRLIDHSSGFQNVIYSCDWYKISRRSKQLLRFTLLRTTKPCQIIAGNMF
VMSMENFSSVLKVSLSYFTMLTSLQ
>AechOr346
MSFFDNHYYYLNKRFMIIIGQWPFQSRLEGNMMFATAFLFILSLIAFETWGLVAGITDLNIIMENASPLLVDC
FMMLKLMNCVLTNNKVCILTFCHIKELLKDVEETWKIKHTGQEKEILQHHAKKSKIFTIRYAIIFYAVWLFYT
LTPVVISGMYKILPTNKTYNIRFLYRLEHVLDMDKYFNIMMLHGFISIFYIVSVPIALDTTYTLCIQHICALF
ECLRYNIERIQGSDFVFLQPNIEDDESYHHIIDCIKSYKHVLKLSDVLSSSYSIAFLFLLGNVIISLSFGSAE
LILVDKQPDEIIRVLSGNLVQMLHIYFLSLISQKLIDHSSELQNVIYSCNWYEISTRSKDLLRFTLLRATKPC
QIKAGKLFIMSMENFSSVVQTSLSYFMLLISLR
>AechOr349
MGDIDQFFQNSHYNIIRLLLNISGLWPFHTRSRRYTIYTILMLIFGSGFIFELAGIIEIRHNFFEVIDNLPLL
FFAILIISKILCAVRTLPKIKILLLKMQEYCLSPKSDEETKIQNFHAQHGRKLGYAYTGFLLGHSVLYLFATL
LSRVLHVESEEADETNNVQRGLPFRINSMIDLDTYYVPIFIHCSICEFMYMFLLTVIDVLYLTVVEYCCGLFA
ALRYRLETALVFENEKLTIMFTKDKSYANIVYSIRRHTEALQFIAIVESIYSLPLFIQIALTVLLLSLLGYQI
INNMENIGGGINNYVYLNGLLLNVLFENWQGQKIVDSSEKVFKSAYNTKWYNMPVTARKLLIMIMMRSEKPSA
LKVGKIIVLSYVTFNAVLRLSSSYFMLLRSL
>AechOr354
MSWNDDMAYAMTLYKFLTLPLGVWPLQKYNTFSLVRSIVCGFSLSVMLITMLLEINFGNGDAYVKIDTLMVIS
CIILATLKLLSFRLYADNLIRNYSSAVNDYSTIDTEEKRTIMRRHAFMGRIICYSTLFFAYLASSIIMLTPMI
AGNKDIQVDDVSKTQASKLPMPLTFLEDLHIPSSLYFVISTLQYTILMITCTTNCGNDSLFLGIALHICGQLE
LLKIEFINCDMKNKNINEDFSTMALRHCYLIKHAELLADVISFVLLVQVMGSCLIFSLVGFQFIFALKSHDVV
MITKTISVLSGLLLQLFFYSFVGDYMKCQMEQIAHSIYSSNWYYLPTKLMRNILLVIMRSQQPVQLLAGRFFV
LNIKTYMTILKSSLSYLSILRVMVE
>AechOr356
MDILPINFKALRFCGVWKERKDDNICVRFLRFCYRYTIFFLIYEFTIFDVIEVIRTRDHVQELTEGLFLGLTF
LTLCVKYMNFLLRESELSDLLDSLRVKMCQPRNSIEKLIIEQHSRRAKWSSILFMMISYMTGFGFMITPALGL
LKGGERVLPLKTYIPYSVSNSLLYLATYLQQFLTLFYGIMLNVTFDCLVYGFTIQVCAQIELMCCRLTDSFKN
SGRISSGQNRETSASIVECVKHHLLVSNLVKKIRELFIWTIMIFFFFSLLIVCTSIFLISKKKLLSFEFLSMF
LYLSGMLLQLFYYCWYGNELELKSKDIATAVYSCDWTMVTPRERRSLMLIMISSQKGIMFSYHGIFALSLNTF
TWICRTSYSAYNLLQQASN
>AechOr360
FIWAIRLHHLALEMVDLWPKSNKCTKKSLWAEIWVGIIFILLIFVSNVPMIYSVIQVWGNMVLVIDNLHTTLP
QVIASLKYVIIRRKQKVLLSIVNMMAEDWMAFKLDRERNVMIKHARTARLIMMIGYVIVFLSFLTINILSFFN
IHVVYATNSTYRRKSLPLETYHFYDTDKSPQFEFTFIIQAVTTLLTATIYMSVDIFLIVMILHICGQLENFRC
RLINLISCKNFNKVLNDIVATHLHLIRFANNIENTYSLMMLVLVLHFGIVFCLSGFLFTIILTDRKIDKALII

QGYFSILIFIVLLMNTFLFCGAGELVTKQSNAVYHAMCDLEWYKLESKKARNLILLMMRAKYPFCITAGKIFP
LTMATFCSILKSSMGYISFLMTKHG
>AechOr361
FMWAVNLHRLGFKIVGLWPSNKYTKKSLWPEIWVGIVFILMIFVCNIPMICAVIEVWGNFIFVIDNLHTTLPQ
LMISIKYIIIRRKQTVLLSIVSMMAEDWMTFKLEGERDVMIKRAQTARLLMMIGYILVVMSVLTLIILPCYGI
QVMYVANITDVRKLLPLKTYHFYNTDESPQFELTFFIHTLTALLGGTIYVCVDFFLVLTVLHICGQLENFRCR
LINLISCKNFNKVLNNIVATHLRLIRFADNIENTYSLMMLISVLHFCIAFCLSGFLFTIILSDKKINKAVIAQ
GYYSILFLIFLLMNTFLYCEAGEFVTDQCNAVYRMMCDLKWYKLESRKARNFILLIIRAKHPFRFTAGKIFPL
TMVTFCSILKTSCGYISFLLTKR
>AechOr370
FEWAVKLNRFTLNLIGLWPKTAQNFRQQLMSNFRVLVVFLGVTLGLLIPSIHSLIRIFGDIILMLDNLQFTLP
TISCSIRIVIFWWKKEAIEPIMNMIVEDWVKLKNAEERNIMIRRAQSARIIITFAYCIMGIGCFYLIVLPSFG
ISMRDTTNITDPGRPMPLQTHYIYDVTKSPQYELTFISQSVYIIIAMMSYSGIDNFLGLLVFHICGQLDILKN
RLTNLDKCKNSHKILNSCITRHIRLLRVIDIIEDTYNVILLFLFVYFAILFAFYGFRIITLFDEENDISFSHL
IYFASTVINIFAHMCLYCALGEILVAQCNKIYYAAYSNKWYIMNSKVTQNLLILMIRGSKPVYLTAGKVFPVT
MATFCSLVKTSVGYISVLHTTRN
>AechOr371
FEWAVKLNRFTLDLLGLWPKNVQNSWQKLMCNFRVLVIFLGLTVCLVIPSIHSLIRIFGDVLLMLDNLQFTLP
VISCVIRIMIFWWKKESIVPIINMVTKDWIKSKSDQEKCFMIQRAQSARIIIICAYCLMGIQCFFLVIPPTFG
MSMRLTPNITDPGKPMLVQSYYVYDITKRPQYELTFLSQVIYIVIALMIYTGIDNFLSLLIFHISGQLDIIKS
RLTCLDKYTNYQKVLKCCIDKHLRLLRAIDAIEDMYNNILLSLFIYFAILFAFYAFRVISVFNEGNHLPVIRF
IFFTLTILNLFGHMCLYCILGEILMAQCNNIYYAAYNNKWYTMDPKTTNNLLILMTRGSKPIYLTVGKIFPVT
MTTFCSLVKTSVGYISVLHTTR
>AechOr373
FEWAVKLNRFTLNLIGLWPRTAQNPWQKLICNFRVLVVLLGLTICILIPSIHSLIKIFGDSSLMLDNLQLTLP
LISSLIRIIIFWWKKEANIPIMNMIVKDWITLKSDQERSMMIQRAQNARIISICSYCIMALAWFFITVLPIFG
MSIRLTSNITDPGKLLPVQTRYIYDITRRPYYELTFISQVIYAGIAMIAYSGIDNFLSLLIFHVCGQLDILKN
RLTHLNKYMNYHKMLKSCIANHNRLLRTIDVIEDTYNIILLSLFIYHVILFAFFGFRMISLFDEGNDMSITHL
IYFFSVVINLFAHMCLYCALGEILIAQCNKIYYAAYNNKWYTLNSETIEDLLLLMTRGSKSVYLTAGKVSPVT
MTTFCSFVKTSVGYISVLHTIKS
>AechOr374
FEWAVKINRFSLKLIGLWPKTEYNIQETLAYSIRPLIIVLIIIIGVLIPSIHSLIRIHSNIMLLIDNLQFTLP
AITCAVRIIILWWKKKAILWILNMIAEDWQRLKNAYERSMMIKRAQTARIIIMCAYSAMIIAFSFVVVLPICG
ISIRYLTNVTDPGNLLPLQTYYPYDVSKRPQYELTFFIQSIAIFFAILSYTGIDNFLGLLVFHICGQLEILRY
RITHLDNFINFHDILKKNVIDHIRLLRAIAVIDDTYNIILLVLFLYFGILFACYGFLIINLIEKGNNISITHL
TYEVCILVNTFGHMCVYCAVGEILMAQCNQIHNAVYNNKWYTLEPRSAKCLIVLLIRSNKSNYLTAGKVFPMT
MATFCNLIKTSASYISVLLTTRT
>AechOr375
FEWAVELNRFSLELIGLWPKSEQNTWEKRLCNLRVLLTFLIIILAFVIPAIHSLIRVHFDILLVTDNLMCTLPC
ITSLLRLIIFWWKKKAVAPIIHLIANDWIKTTTSQEKAMMIAKAQIASIIVTFGYGIMGAGMIIMIVLPLFGY
SVRYLSNIIADSERPFLIQTYYIYDTTRSPQYELTYVVQSIAAFLCTACYTGIDNFLSLSIFHISGQLDILRN
RLVHLHIVANFNNVLKSCIMEHIRLLRFINHVEDIFSIILLILFLYFGTLFAFYGFWIINMFENRQHLTVLNL
IYILCVVINTFGHMCMYCAVGEILTAQYDRIHYAVYSNEWYTMNARNTRDLVLLMLRTNKPLHLNFGKLFPLT
MATFCSLLKTSTGYISVLLTTRS
>AgifORco
MMKTKNQGLVSDLMPNIRVMQMSGHFMFNYYSEGKKFLHKIWCCINLFLVVLQFAFCGINLIMESDDVDDLTA
NTITILFFLHSIVKIIYFAARSKLFYRTLAIWNNPNSHPLFAESNARYHSIALTKNRRLLFFIGAVTVLSTLC
WTGITFVGESVKRRIDPDTNETMIIPIPRLMVRSFYPFDASHGFLHIAMLAFQFYWLFICMVDANSLDVLFCS
WLLFACEQLQHLKAIMKPLMELSATLDTVVPNSSELFKAGSADHLRDTTGTAPPATPANGDNMLDLDLRGIYS
NRQDFTATFRQTAGMQYNGGVGPNGLTKKQEMLVRSAIKYWVERHKHVVRLVTAIGDAYGVALLLHMLVTTIT
LTLLAYQATKVNGINVYAATTIGYLLYALGQVFLFCIFGNRLIEESSSVMEAAYSCHWYDGSEEAKTFVQIVC
QQCQKAMSISGAKFFTVSLDLFASVLGAVVTYFMVLVQLK
>AgifOR2
MVFFDNKNWLLTKVLLSVVGAWPFQSQQTRIVLGFSSILIILSLLIPEIFGLIKERQNSSTVIECATIMMLHV
AMLVYLTHVLLNHKKMKQLLMEIEQFWNSTLLKNEICILEKLTLANRKFTHSYIWFVLVSTVLFASLPIIPKI
MDIIVPLNESRPTIFIYQSEYFIDPIKYENLIFIHSYIGTMYPTIVLVGYDSLYSNLAQHSSCMFEIISNHVK
NTQLADKNIYGDDNSLSQYDYDAIFYIDCIQRHRVAQKFTSTIKSVYNIPLFFMLFLNMIAVSLAGCQVLLTS

DQPSQAMRFFMNAACALIHLYFLSWVGQKVIDSSSKLYQSIYQSEWYQANNTTQKFLIIFMMACREPCNLTAG
KLWILSSENFTVVLKTAVSCFTLFRSTQ
>AgifOR3
MQLLQESFFILKCTGLWKPASCYGWRSWLYNLYIFIIRFNLWMFVITQSLALVLTSNSIEDFTNILFLLLSVL
VVCGKINNMLSGRKLMDKLINSFNKYPFKPETDDEINIQNRFNRINRINTILYWAINGITAAVWCIRILLDLK
PTYILPYGGWFPYDHTKPIIYWFTAVSQLMAPVMAANMTGGYDSFFCGLMMQLCAQVEILKHRFNNKLDNLEK
KNLINLNPSVHKNIEKKIFSDIIEHHIAILRMAYDVNSFFSKIVFLQYSLSSIVLVASVYILAGLKPGSLQFT
SGLIYLLCMFYQIALLCTSGHEISLAFEELGDSIYNSNWLNLTSNKSQKNMIIMMSRMLRPFVIKSGHFVTLN
MDSLKYLVKLSYSIYNLL
>AgifOROR4
MDTSTPSYLAINKLLASLSGVWPHQPIVTKNIVFIIMLILTISQSYFQIYGAILARSNVDLFLETIPNILVDF
SVAAKVVNCFFNSKKMKKLLITLEKDWIKFNSEGEIQILNEHGVRARKLTLTYFSVICGTITPFMVIPLVPII
YNNFAPANGTLPKQMLYAQYDYLFNLQVSYYPVLIHSYIATFAFINDIIAIDTMCMVFVQHGCALFSIIGYNL
EQIEINSTIKNDINHSRFNDKAYHQMSECIRRHNHALKYAKLIEDAHNVSYFFQIGIDMICFSFTGFQIVDKL
DTAPDVSIRFAAFTSTQLAGLFLLSYPAQQLFDYSVKIYDDIYNAGWYKTSLRTRKLFIIMSMRSLIPCQITA
GKFYVLDLANFGKIVKTSLSYITVLISMK
>AgifOROR5
MKKATSMETPQYLRFNKNLLACAGLWPHQQKLSKIIFFIILLFLIISQTAFQIIGAILARENVDLFMEAIPAI
LGGIAIAVKIMNFYLNGHKMKKLLVLIQNDWIEYKNGPESLILKKYAKFSNKLTLMYFGIIWSTMTPFLTIPL
VPLIFNIFVSQNNSIQRQHLYAQYEYVFDSDKNYYPVLIHSYIGSFAFINAVVAIDAMITMYIEHACALFSII
GYNLQVMQNNHDVDVNLNPHEINDGAFKNVINCIKKHDRVLQYVKLLDNANSISYFFQLGIDIICLSFTGFQI
ATKIDNSPDAAMRFAAYSTSLLVVLYFLSYPGQQLIDHSLKICHNIYKANWYCTSLRTRKLLIFMAMRSSIPC
QITAGKLYVMDITSFKLVVKTSISYVMVLLSVQE
>AgifOROR6
MDNSNNWIKFEKLLNLEKYILQIFGLRSMKTLFLNKPHAKNIYETIYFSIPLTIMIGLIFLNILYVLSVWKDE
VDSAINMISTIFNQFVVIPKGIIIWFYQEELYNILNNLHEKWNTSFTRTEIRDQILKRAKKITKFRNIYVFSF
AILTITYMYPPIRNSLIYFNQNTNNSLDYSQTMWPTEVYPIKIDTFFKYLCISTIEICQAFFYIIYCHVGDIF
YIQIFTHISIQFQVLANDVITTVETKKNGNSKTLLIELISIVMRHQELYELIDKMNNQLYSPVIFVTFIIGSI
NICLNIFEFQRAIDKGNYYDASRNVILTVSICFGIIIYCVQAQIICESAELIPEAIYNSQWSNIDKNSILTFQ
IIMSRCQQKLTCGAYGLVNIDHEQITQVCRRTASFFVFLDSIQ
>AgifOROR7
MRTSYSNAAVHRRIADASILGMKFSGFWNFSNNPNTIEKLFNLIYKYIMKLIIYTFATTLVADLISNTDDLKI
FIDDGCFFAGIFVIIFKLTTFGIYENNINELIEKIFKPIDYLKNLNDKDVTTLINENIFFERCTAYGFASLAV
CLLITMIFFGNHKDGELPIRAKYPFNSTEGINYTTAFTIQATGVTIGLTGIMAMDTIVLGLCRWTKFQFDVLY
SNFQHCGDDIANSKKQQQKKLINNDKTGHNNSEIFKNFVAFNSNENHHLGFSERFKICIKNHQRLIGIIESVN
NLFGLNMFVQICGSSFIICFAGYQALLGSNGMRDLMKFSCYCGTGFSQLFIWSYFGNNLLCQGDLLSDGQWFS
GWEKQNSRDLKNLLTIPMIRTRKPLELKAMNFFTMSIETFMMILRASYSIFTLLRTMEMK
>AgifOROR8
MVKQFLHRKILNMQIDGLRLCGFWSLNPTLPAYVKILYYVYNKVVLSFVLIFIGTLTADLIINFNDLLIVTDD
GCYLAGMMVILFKIYIFNDKQEKINQLVEKVYQPMDIIAKSSDPGVRSRLRISNFLEQLLFYFFIGLAIFLAF
ALLVLVPRQDGELPIRSAFPFNTKISPKHEIGYFLQSAAICYGLFLIVCMDSMVTEMGRWINFRMKILTSNFR
MCDSKQHDRGAFYSTDATFEFLKNTSIYQITDEHQKIRSFIPIKLDEVNNFEDSYQLRFKTCLQNHQDIIHAV
DDLNAIFGSSMLMQLFASFSMICLTGFQAVLGSTSKTSLMKFAVYLGAAFSQLANWCWIGNEISYESTILFES
QWLSGWEKQISDPSIRTLMTIPLIRTKKPLQLKAGKFYTMSMTTFTGILRNSYQFFALLTTATDAQRQ
>AgifOROR10
MTSHEYKPINAFKINLNIFTAEGIWPAGLKNKNLRYPYAVYRLLSPSIWFGSFFIFQLIQVIIVINDMAKLMD
SMYLMVTYFAILSKFMCFILYRKRIEKLFNTLDESIFIPRQKKHFDYINEAMRIIRRDTIFFLSSGISATIFW
TFYPLFDKHQEEKQLAYVMWCPIDVSTSPIFELVYTYQVIAITYNTMFNTMADTTICGLFRMMSGHLEILAQD
YSELFDDNFISPGDIVDKKNNINKGLSNYKNDNEDIDEDKYLIVKKPNVTHEKLSYRVAACVEYAQAIEKFVN
EVNDVFQGGILTQFIASCLIICATAFALTMVPVASVQFVSLLQYQGCMIIQIFIYCWRGNELTLKFSELSNAI
FKCNWQLCNGHFRRSMDIIMSRSQKPMILVVAGLFSLSVDTFIGIIKSAYSFFMFLKEVQTTAAED
>AgifOROR11
MVEFKEHIKKEDLFKFETYITRLFSILCFMKISPSYSEISFIKKLCSTCFVIISLGCSTSGCILQIMSFRNVR
DMPSFSQRVIELCFMGLGTYKWYHCILKFDALKEIIKLLNNCHQVGYLINNNDKEYKSYHQEMLDAKKASEYF
MIVWFPFTILGVVQWCLTPLTSDFYINKDIDRNNKNISLLRSYPNGATFPIEIDTLTKYLLMFLFQSLGILFS
AIGISGCDVILISLIMYICAHLRYLNNCLIDENNIQLILKSKNPSEKLKEKLKHCVIYHDQILKVHENYLSYS
NGAMFAQCIENIIALCLVSLQASTMHYSPGIDFIVAFTSMIEYSMGISIELFAFCYYGTKLEELGHEVSDSFF
FSNWDKLKIDKKCQDIQDKIQNIIKLGIMRAQKPIIITGGPFYILNLETFKTLAYMSISNAIVLRQMADG

>AgifOROR12
MKNKSQSIPASARKNIRYSTELSRWVLTILGIWSNKNNKNIINQILSIVLIFMCYFIICFSLVPCGLYTFISE
KDIHLKMKAIGPLSFCVMALSKYFLLVTRKKDIYNCFKHIYHDWRQVQLINDENIMLENAGTGRFITIICAAF
MYGGGFFYNTIMPFAVGSFVTADNKTIKPITYPVYDPLFSAQETPNYQIVFTLQWVSGFIMYSITIGACNLAA
VFAFHACGQLKIVISRLDNFVDRNHNYDDVLKNKMGVIIELHLRTIRFILRVEDILNELNLIEFLGCTFNICM
LVYYFMSEFEKAETISTITYCVLLVSFTFNIFIFCYIGEMLTQQGKKLGLAAYNNMNWYELTTKNSRGLILIM
AMSNCPCSLSAGKMVNLSYASFASVIKTAMTYLNIIRTTVL
>AgifOROR13
MFKIVNNAINDWQSVLNHESKNIMKKFAKKARKVFFYQMGFSFIIINMMILDSLPVSHDNLNLTADSQLLNSF
PMKTTYYFGYIELSWYKYTALYFLQIIQLIFCVIGNVGNDCYFFGMAMHLSGQFVTLQLQTKDFETEINKNDN
CTNYIENFVEKHQHLIDIGNCLKDSFNSILIVELVNNACSILMMGIQMILNIQSGDNVMVADAIVIILVVIMQ
LFLYCHAGEELKHYFESLRFALCECSWYNMPNYNVKNILFIMMRTSKTFNLTAGSIYHINMDTLTKILKAMGS
YFSVLQALFIK
>AgifOROR14
MVASTFLLMTNSVHAYKICVIIKNQKRIQLLFDTISSELFTKNFDKYERIFTWYAWSGIKHHIAYQSFGTMAV
FCWGFVPISNAITGNERQLPMDGWYPYDTKLSPAFELTCGHQAVAVIIACCHNIGMDTLINGFINVACCQLEI
IKQNILSIDDGDKNVHQEINQSVQHSVVVMKFIDEVQSIFGTVIMYQLIANCLIICLTAFHVVQMTVYIPAEI
FGMMMYMCCMTYQIFIFCWNGNELTIQSETLAFTAITSNWWKFDKKYKRSLGILITRSHKPMIFKAGPLINLS
LQTFMKVLRMSYSFFTLLQSSTNPH
>AgifOROR16
MAFTQILVRIAMLKKYNRKLGDIINKAIKDYDYKLYKTTEEKKVFIDYIKRAKLFCKLLCIFVAMTASSYYVK
PITSPPPQPPPSSTINDDDFIINNLTKSFILPYRFYLFHQVNDFKMYVITYVSQFPFVFVSGFGQTAADCLMV
SLVFHICGKLAVLSIKISTINPNINFCKKQLNEIIIEHNRLLKMGQDIEEAFSETLLIHLVGATSLVCILGYQ
LLTNYAKGQNADLATFLIFIFLVFLILYAHCVVGESLITESNKVCEAYYACQWYNMPHDTSRGIILCMIRSQK
PLGLSAGKFKTLCLSTLTEVLKTAMGYLSVLRSFLAV
>AgifOROR17
MNISEDDDNKKVEEIQEEFEHLNDLLDWNRKFLIFGGIWPLENTKFRAAIFTAYMTFHLFTEYSGVVEVVGNF
EAMVLGIIESSMQSMVLSKLFVFRHSKNLRVLISAMKEDFQENNYNDLNEKKIYLKYNYYSKLYFKLSVPYVL
TIAALYYARPLLTSLILGTFGVNDSLILPFQTKLFFPIDNTQTYFYVYIWYCPMVYLLACHNAFICILITIIL
HICGQLAVVEYRIKNLKYDHEKDHNQMIFKTLVQRHQRSIWMAKTFDTSFNVVLLIDLIGSTVLIGLLSYSIV
TESETLETSFVYIYICAIIATLFLLFGYCYVGECLVENSTRVHEVYYECEWHKMSLKFQKSLSFCLMETEKPL
TMTAGKIFVFSLSGYAYVIKSAMGYVSMLRNVSM
>AgifOROR18
MYEAKGNKEIKQATVKWNEDTIYALGLYKRVTTLVGIWPTNNRILTGIQIGVYITFHLSSVFIFFQKFLEYGN
CGTTMEIVDTLSVCMASVQICIKSFIAWFKNHEFRHVVDSAVMDWSCVEEEQSHSVMLVYARKGRVLLMCQMA
VGFSTAFPLMLDRFPKIVEVIDEVKNESIFVRNIPLLPRCWISLTMSEYSYLAYWTLVVIFIWTLSIATVCCN
VFVYGFGLYVYTQFEILSTSVNIINGNENISEQRRLIKMFAKRHNHLLNIANDLENGSTFIILSEVTVIIFVG
CIAGVMLLIGLRDHDSQTIIAMVIRLTLVMMQLFIYNFMGECLSNQTEKLQTAIYNCPWYELSSRTAKDMKLI
MIRSNNPFRLTAGKLSDMNLISFKSVVKSMFSFFSVLRLMLQN
>AgifOROR19
MDLQNKNKETSIKWNKDAIYALGLYRRLSTILGIWPSENRISSNIRIGTLIILQLTTVSIFIERIIVSGNCGT
IQEIVDTIVISISSTQISLKCLLICCQHRKFKRIISSALEDWSSIIDQQSRKIMLKFGHVGRVLFTCQVITGV
ATSSSLIINELPKLVELPDENNSSITLLRTIPLAPSCWVSTTMSSYLYYTYWTSVALYIFYVGMATVSCNLFV
YGLGLHVFVQFELLYLSLDTVFENHSHFQQKKIFKKFVQRHDEILEIANDLEDASTGVILSEVAAIIVVGCVS
GVMLLLAQHKGDSETVSAMAVRLVLVFIQLYIYSYMGEKLSTQADKIQDAIYNCPWYTFSPLVARDLKFIMVR
SNYPFYITAGKFLPMNLMSFKAVVKSMFSFFSVLRLMLQN
>AgifOROR20
MSMSLIIILLLIKGNCGAITEFVDALSLIICGLTSVIKVVIPRINYKKMSAIIQSAVNDWQKIDHHDKLHFKTM
QHFSNIGRVVFIIQMSGAYIVGFPLIVMKLPFVVNYFHLNKDESLLLINKTILTTSSNVGQVPIGPTCWIPTN
MSPSIYFLYFISQSIQLFFVCSASIGGDTYFFGIGLHIWGQLRILNNSLDKFNKALLKEKILLNEFIKRHVHL
LTLIKYFEDIYRYIILAQVGTDVLLTCISGIVLLMTVQYGDPIVIGGLVIRIYLVYVQLFMYCFIGEKLTEES
NKLEISIYHDIPWYNMDKKIVNNIKFIMMRINYSYKLTAGKMYTMNYANFIGIIKAMASL
>AgifOROR21
MTWSDKEQKSFEKVSLVISRVEWQLKVAGSWPFEKTLWSNIKFIIIVLFYVTHLILAYGDLIMVFSNIEKAVE
NFSSTGIQTLIFFRIITLKLNEKLHNLFAATIEDISENNYKDYHEKRLFIKYHIFSYKYFMIGTWFASGSAFC
WYIKEIPGYIILRFNNETAILPPPFRIFIPFDVTSINRILIVYICETPVIVLSHLIILTSILYSLIVSNICAQ
LSLLSHRMKINLSDKNEYNLTLKRELFFKRHVDKHCQLLSMVRETNNVYDIQLFFELAVLTILLALLIHSESE

SINKADYTGVFFIGTFICVLLMIIFITCYLGECLHDEVNNLRNTYWYCLSYKLTIQDRKNILICMAIVDKPLH
VTAGGFYIYSLNSFLMIIKSSMAYVSILRQME
>AgifOROR22
MFAALVADIDIFLESVPTVLADVVCYLKYFNFFYNGSKMKELLITMEKDWKKYTSPEECQILDKWARFGRKVT
IYYAGALYGTLVPMGLVPLVPVVLDVVAPINGSYPRHLMFQQIEFLFDYEKYFFPLLIHGYFGTMAYLTIIIA
IDTIFMVYIQHACAKFEILGLFLDRVANDDDAIKNRHCSKFDEIDYNDMVNCIATHNHAIDFSNLIEEANYVS
FLFIIGINMIMMTASALVAAFKIDNPDIAGKFVAFTLGEVFHLFYSSWQGQLLLEHSESIFNQVYQAKWNNAS
VRTQRLIIPLLMRSAQPCKLTAGKMLTMSLTSFTTVVKTSFSYLTVFTSMRA
>AgifOROR23
MEHYYKVNEFLLSSIGQWPYQKKYSKLFFYGSTVFLLVTQGFLQTGGLIAARCEPAIFLEALPPVMISLMCIV
KYINFIYNADKMKDLLDAIKSEWNSLTEEKEIEIFKNWAYDSRKSTLMYAGAVYGSLVPLMISPIIPFFIDLI
PDNSNDTIPRLTMFHVEYFIDVDKYYYPLLIHSYFGTMAYITVVVAIDTMFMVYVQYACSIFVILGHRLENLV
RTDILMANANPNLIDDEPYRKIIKCIIKHSDAIEYAHLIESANSVSFLIQLGINMICISFTGLQSALKVDQPH
EAFKFASFTIGQTFHLFFESWPPQRLADESERINEYTMRAPWYNTSLRCRKLLQLIIMRSRFPCQLTAGNFYI
LNLQNFSAVVRSCMSMFTLLSSTQ
>AgifOROR24
MESSIPSYLKINKALLGMFGLWPHQNIFTKILLSFILIFFTSTQTIFQIWGALLAREEWELFLETIPNILAGF
SIFAKMGNFFFNNHRMKKLLEQLEDDWRELKSSAEKEILNEYGVSARKFTLTYLGIMWGTMTPFLVVPAVPLV
INIFLPNNETLPRQMLFVQYEYLFDLKKYYYPVIIHSWIGTCIFMNIVVALDTTCMVYVEHAAALFKCVGHNL
NEIENTDEIKFDVNPKLSDDKAHKEIGQCMKLHNRVLKYSELIEDAHYISYFFQLGFMMICMSFTGFQIATKI
HTAPDVSIRFAAMSSSQLAIMLFQGFIQGQQLFDSSAQVYHDIYNSKWYNTSMRTRKIQNLMSIRASKPCQLT
AGRFYILSYENYGIVVKTAMSYVMVLLSMEEED
>AgifOROR25
MKSSTIPLYLRINKFLLIISGIWPHQEFVSKILFTFFMLFFTGTQSLFQISGALFARENAAMFLETIPNILVD
FSVGAKIANFFFNKNNMKKLMVLLQDDWILFDNKSEIEILKKYGAYARKFTLTYLAVVYGTMMPFMIIPIAPI
MINIFISDNSSLPKQLLFVQYEYLFDIKKYYYPVMLHSYLGTCAFINVIVALDTTCMVYVQHCCALFRVLGYN
LSKIDNETSHNFAFKPTKYYDKSHEEMIKCMELHQRILDYAKLIEDSHYISYFFQLGINMICISFTGFQIATK
FSIAPDVSIRFAAFTSTQLAFLFLQSFPGQQLSDSSSQVFQDVYSAKWYKTSMRTRKLFLLMSMKSAKPCYLT
ALHFYKLGYENYGMVVKTSISYVMVLLSME
>AgifOROR27
MLFLLAPHLIYTYFVAEDLKRLIKIIAAQFFNSLALIKFWTMIINKKQLRWCLNELEDSWRDIASDEDRKIMI
KNAKIGRIFTVAYLSLSYGGALPYHIIMPLVAPRIIMPDNSTLIPLPYPSEYIFWVPRDSPGYEMLFVGQILI
SSIILSTNCGIYSLIATYIMHACCLFEIVQRHLKYILESSVEGQFNDNLAAVIDKHNQAIYYAGLLEKSLNIV
FLSEMAGCTVIICFLEYGVLLEWGDGDVLGLTTYFMLLTSIFVNVFIISYIGERLKEMSLKIGECTYDLDWYN
LPKKMALDLMVIMVRANQPSTLSAGKLFDLSLVGFADVVKTSAAYLNFIRAMV
>AgifOROR28
MGFKITPENALNFAKGRVWIISSWPPSTSGGNKWKMIWFNVRWWISFVFLLLLFFPLLNGVYVFRNDSLVMIK
AVTLSAANLQAIIKMAISRYQWKNLKSLHTDIDDFLKNSTPKEREFIQFYVNKGSFFHLFFTVTTWLVCLALY
IEPIVFNQPFPTDAFYPFNVDNIILHNVLYLQQALSIFIIASALIVDSQVAILIWFACARFAIIGERFEHIKN
EDELKDCIQSHQNVLRYSDNVRKAVRFIAVGTITTTTIGVICGLLTVISKQPVSVKIRVAAIIGCAATELLIY
SWPADDLIQISQVIGDKIYRSEWLEKSHKLRNMVQFVIHRTQKPVYISVPGIIPNLSLQYYTNFLSSTFSYFT
TLRIVLAKEETSYEQ
>AgifOROR29
MKFKATPEAAITYIKFSVIILFTWPPGLGASKRTQFLFEVGWFISWLISILLVIPLGYAAYDQRKNTLNFTKS
VCLAISCAQVVAKMLIAMCQRNRFQILINEMENFVKNSKNNDRKILVNYVKRVSVPMIVFNFLSISACVGVIC
GPLVLDQPFPTEAKYPFSVDKHPVFDLIFLHQAIAGFQCGSIGAIDCQVALLLWYMVARLDMLTFQLKNINKL
SDLKNCVRTHQYLLWYIKELVQCARYFVLTSIIMTTLSIIFGGIHIIGEQPLTVKLQFIIIDCGFSYLLYHSA
WPAENLIRASERIGSILYEINWTKNSQEFNKIMMIIIQRSQKPTTITIAGFVPRLSLSYYATFLSKTFSFFTT
LRIILTKMEAQDDHQ
>AgifOROR30
MSVFSYNIMIFRLLGMWYPEDNNSAWKKIFYIFYTTLIVSIYYTNAISQIIILFGSLDDAKEFSGASFITITR
ILVCYKMYNILSKRKYIIHIINTLETGSFKARDSVELSIQTKFSRKIKLLIITYGALCATTLTALLYVSILVD
IPQRKLAFEAWLPVNTSIPIFYWIVFIYQHFSGYCGTSISVSFDTVLVGSMILLCAQLNILKYRLRHGHNNNE
IENKSEKKYLIDCIEHHKAIYELAELSNEAFSNAIFLQYFASLIVLCVSSFQLSQEKPFTKKFNDLILYLICQ
LLQVFMFCYWATQVQVESENIITGVYDCEWLSLSITTKKNLILTMVRALKPIQYSSGYVVNLSLTEFTNLLRM
SYSIYSILQQSNSRTR
>AgifOROR32

MLKYTILLLTCVGLWQPTDWAKGWKTYLYTIYTFFVFLVVYSSTFSEFIYFAISNKGPIEVARQSFMLLTMIC
DCIKAATIVMNKCAIFDLIMLLEKDPYQPEINNKNKIMKEYDDKINKRSIAYILMFAITGSSCMTAYSIKNIP
KRAFITNIWVPYNHTTPIGYWFTYTCQITAHLYGASINAASDTLVMSIMIQICCQFSILQHRFKILPDVMSNI
KKYDDEKSIILETMKLSECVEHHLQIYQWSKRCNNIFSGIIFLQYSISLNVLCVSVFTLSKIEYNNLNFVGLV
IYLCCMLTQIFMLCYTGSQVTTESIQVGDAIYDMDWGQFESSTKKHLMMIMKRTMHPVVFTSGHFVTLSIDSF
TVLIKLSYSAFNFLNRSSE
>AgifOROR33
MDFLPGSFIIFRIVGLWRTSHNNIYIYRLRTVLTLFLLYTFVGCSIIGIAMKHDDMERVASDCFLMISVLGCC
GKSVNVLICRNKILNMIDILQNDPCLPRDDIETDIQKKFDTFIWKSTIIYGILTEITAGMLTFGTLLLDLPEG
ELPFNTWLPYDHSHGFIYKFAYGQQIISVMTSANIAIAFGTLVPALILQVCAKLNILKHRFTNITNYIYKQQL
NNNDLSFEEKLNREKELIADFVRCHLTIIKLAETINSTFNLMIFLQCLISATVLCTSVYKLVTVPLFSSEFSS
IVLYLACMLSEIYMLCAAGNEVILVSGSISDAIYCADWTKLNISTIKSLVLIMNRAMHPVTFTSGYIVILSYD
TFKSLLKISYSTYNVLQQKG
>AgifOROR34
MSILSYNVMIFRLLGLWYPENSITTRKLFFYKLYTGLLVVTLYTFTLSQLIKMFTSLDNPDDFSKASFMSITM
TATCFKVYDMLSKKKLLARLINTLDNGSFKSRNSIESSIQSNFFNKIKLLIFLYGTLNESTATTITLASIIRD
APQRALAYKAWLPFDESKPTFYWIAFIHQHFGGYCAASMSVAFDTMVVGLMISVCAQLRVLKYRIHHGYNTVT
YEQKKNYENDYLVDCIQHHNAIFQFAELSNYIFSKAIFFQYFASSIVLCVSTFSLSQEKTFSKEFNNLIMYLA
CMLSQIYVLCNWSTNVHIESTSIATGVYESDWPSLLIGSRKNLVIMMIRTLRPIQYSSGYIVNLSLTAFANLL
KTSYSAYNILQKSKSRTE
>AgifOROR35
MIILVYTNTLSQIIDLFVSFKNLKQFINNAFILLSTIGAGVKAAHLIWQRKTIINLMKIIQSHPCIPQDEDEQ
IVQQKFNNKIKRMNFAYICLFVCTITTLTIASFFRDIPMKQLYYHAWIPFDYSSKFRFWMVYVHQVIAHGFDA
SLHAAYDTLAPAMMIQTCAQFELLNLRFEKLPQSLKKKIHKFEGNNNVMINSQHLKREEAKKIGECVSHHIQI
IEFSKKNNSIFGTSIFLQYSVSSLVLCMSVLRLSQLNTFSPDLASVFLYLISMISQVFIPCFAGNQLTVQSSQ
ICDAIYSMDWTTLTISTQKSLVLIMSRSQKPVQFISGRIIPLSMNSFNNVIRISYSIFNVLHGSSAI
>AgifOROR36
MSVFSYNIMIFRLLGMWYPEDNNSAWKKIFYIFYTTLIVSIYYTNAISQIIILFGSLDDAKEFSGASFITITR
ILVCYKMYNILSKRKYIIHIINTLETGSFKARDSVELSIQTKFSRKIKLLIITYGALCATTLTALLYVSILVD
IPQRKLAFEAWLPVNTSIPIFYWIVFIYQHFSGYCGGSMIVAFDTMLVGLMISICAQLKILKYRICHGHNTFT
EKNYLIDCIEHHKAIYEFAEMSNKIFSFSIFFQYFASSIILCASTFKLSQEEPFTEKFNNLLLYLIIMLLQIF
MLCYWATQVQVESENIITGVYKCDWPSFSIETKKSLLLTMVRARIPIQYSSGYVVNLSLAEFTNLLKMSYSIY
NILQQSNTNSR
>AgifOROR37
MSMLSYNIMIFRLLGLWYPEENNFTWKRLFYKFHTTFLVSMFYTLTISEVISLCGSLDPSEFLDASFLSITRV
LICWKIYNILSKSKYIIHMINTLETGSFKARNLVESSIQIQFSQKIELIIILYGGLIEITTTTIIYGSIVTDI
PQHKLAYKAWLPFDISKPTFYWIAFIHQHFSGYCGGSMIVAFDTMLVGLMISICAQLKILKYRICHGHNTFTE
KNYLIDCIEHHKAIYEFAEMSNKIFSFSIFFQYFASSIILCASTFKLSQEEPFTEKFNNLLLYLIIMLLQIFM
LCYWATQVQVESENIITGVYKCDWPSFSIETKKSLLLTMVRARIPIQYSSGYVVNLSLAEFTNLLKMSYSIYN
ILQQSNTNSR
>AgifOROR38
MSSGSFVVFQIVGLWRSPNIKSSIYNNFYRLRTFFSVFLLYSFVGCSIIGIAIKHDDVKTVTNDCFVMLSVLA
CCGKSINILKCRKTILNIIEIMQNDPCSPRNEHEIDIQKKCDSFIWINTVIYGILTEVTAVMLTFGTLFIDLP
EGELPFNTWLPYNHSHGFTYKFAHGQQIISIMTSANIAIAYDTLVPAMILQVCAKLNILKHRFSNFTKMINNQ
INKSSTSIDKLKKEKQLIADYVKCHLIIFKLAKTINNTFSIVVFLQCLISTLVLCVSIYNLASVELFSSEFTN
IILYLACMLSEIFILCAAGNEVTLVSQSISDSIYETDWTDLNTSTIKSLVLIMSRTMKPIIFKSGYVIELSLD
SFKSLVKISYSTYNVLQQTS
>AgifOROR39
MSRDCFLPFAVLGCFFKSLNIVGSHDTITYIIQTFMNDPCLPRNSKEILIQKKFDKFIWRYTVAYGVLTETTA
IGLTIGTLWLDLGPGVLPFPAWLPYKHEDGFWYWFAYYYQLLAAIISANFAIGYDTFITSLMIQIVSKFKILQ
HRFENFYEINKTDNKYDNIFKNEKAFLFDCVKFHEILFKLSNIINETFDFVIFMQCSLSVCVICVTVLNIDKY
QTFSQEWTTVILYLCVILSEIFVLCAAGNEVSFVSSQLGEAVYSMNWTDLDKSTIQSLILIMNRSSHPIVFTS
KNIVTLSLDSFKTLIKISYSTYNVLHRT
>AgifOROR40
MDDVMNFLPACFFVFYIIGCWKSKNYTTSISICFYWLRTIIVIFLLFTFTVSSLLGVAMSKKNIQEMSNDCFL
PFAVLGSFCKSLNIVGSHDTITYIIQTFMNDPCLPRNSEEILIQKTFDKFIWQCTVAYGIMVEVTSIGLTFGT
LLLDLGPGVLPFPAWLPYKHVDGFSYWFAYSHQLLAGINAATFAIGYDTLIPSLMIQIVSKFKILQHRFENFY
AINKTDNKYDNILKNEKSFISDCVKFHEILFKLSEIINENFDFVIFMQCSLSVCLICVGVLNIDKYQTFSQEW

TAAIFYLGCMLSQIFILCAAGNEVTLVSSQLGEAVYSMNWTYLDTSTIKSLILIMNRSLHPIVFTSKNIVTLS
LDSFTNLIKISYSTYNVLHRT
>AgifOROR41
MLKYTILLLTYCGLWQPSDWIPGSWKTYLYTIYTYLMILLLYTITFTEFLYLVTSTAEIEEIANNSFLLLSMI
GVCVKAATVIINKCVIDDMNILLGKDPFRPQTIAEKRIQNKYNDIINKSTFIYMSMIEVTSFVMVFSISIKNI
PNRVLSCNAWVPYDYTTSPISFWLTYVLQLTVHAYGASINAAFDTLIPSLMYQICCQFSILQHRFKILPDVIR
NMKEFDEKRTIKLNECVEHHLKIYEWSKRCNNIFGGVIFLQYSISLNVLCVSVFTLSKIEYNNLNFVGLVIYL
CCMLTQIFMLCYTGSQVTTESIQVGDAIYDMDWGQFESSTKKHLMMIMKRTMHPVVFTSGHFVTLSIDSFTVL
IKLSYSAFNFLNRSSE
>AgifOROR43
MILLLYTITFTEFLYLVTSTAEIEEIANNSFLLLSMIGVCVKAATVIINKCVIDDMNILLGKDPFRPQTIAEK
RIQNKYNDIINKSTFIYMSMIEVTSFVMVFSISIKNIPNRVLSCNAWVPYDYTTSPISFWLTYVLQLTVHAYG
ASINAAFDTLIPSLMYQICCQFSILQHRFEKLPDVMSNIKKYDDEKSIILETMKLSECVEHHLQIYQWAERCN
QIFSGIIFLQYSISSVVLCVSVYLLTKIEYGNPEFLPVVMYTMCMVIEIFILCYSGNQVTIESINVGHAIYDM
DWTQFELSTKKNLMMIMNRTLHPVIFTGCHFVKLSIDSFTSLIKLSYSAFNLLQSG
>AgifOROR44
MLKYTILLLTYCGLWQPSDWIPGSWKTYLYTIYTYSVFLTVYSIALLELLYLVTSTDEIEEIASNSFVSFSMI
SASAKALTIFMNKSVIFDMIILLEKDSYQSKIDCKNIILKKYNEMINKSSLSYIIMVEVTMIVLVVTFSIINI
PNRVFTTNILVPFGEITPFRFWFIYIFQNIAHAYATSIHGAFDTLLPSMIYQICCQFSILQNRLKIIPDIIHN
IKKFDQTKKIILEKNKLSECVEHHLQIYEWAKKCNNIFGAIIFLQYSISSVVLCVSVYLLTKIEYGNPEFLPV
VMYTMCMVIEIFILCYSGNQVTIESINVGHAIYDMDWTQFELSTKKNLMMIMNRTLHPVIFTGCHFVKLSIDS
FTSLIKLSYSAFNLLQSG
>AgifOROR45
MLIFETGYYRLNKIGLMICGAWPRQSKIVAYTIRVIIMLLFISSFIPQAVYSYNFWGNFNEIVPVSTTQAALL
LIALKFLFLVGQKNMVHRVINQIQDDWKIFSKIPEIKKIMNHHATDAYNYTLFYMYFLIVLGSSYVGFVFIAP
VMDIISPLNYTREMTPLYKTNYGVDSEKYFFSIYAHGFIITTIDVICIWTSDTFIIMMVQHCCTLFSIVGILL
QQLNAKNCNYNENHERKIITQAIIVHKYAIEFAEFIEKSVTKLYGFVFLLTMIFMSITGFYVVLIMEENGNEG
MRMVAFTIGQAIHLFFSTLPSQSLINSSENVFYSVYNCQWNELSIKSQNSLCIMLIRSSKSTGLTASKFYPIN
LENFGNIIKTSISYFTVLKSM
>AgifOROR46
MSILDSPYFSLNKRVLKFYGQWPYQSKLQNRCHRIVVFLAVSSILLPKLIKMIESRHDIDEFILCMPMVFLHI
EGLLNLTAWCLNGDKIENLINRIQNDWEILKTDKERQILVEYWKRGRSSISYYAIPMATVLIIFLISPGLPKV
LDIIAPLNESRPHELPYETEYFVDQDDYYLYILIHAYMTVPVSLGLTVLFNSVISSWIHHAAGMLAIVAYQLE
TIHDIPESDERFSQFEKNQSIQKRLAYSVKLHNNAIEFIETVESSASTVLFIVIIGSTLILTVTGTVAVIKLN
HPNESSRYMSFSLATLGHLYYISYVGNQIIIASESINRACYASEWYTLPIKYQKILIPIMARTNRPCTITAGK
LYVLSMDNFSELIKKAMSLFTFINSTTR
>AgifOROR47
MTVFETGYYKINRVSLCLCGVWPEQNSFQAYIARFLLWGGMATIMIPEYVCLYKAWVYGSFMEDFLPCIPTQL
LITAVAAKYVVFAIYKKTFQDTIDKMRVDFEFFGNTPARKILKEYADKGRTRTIFYGCFLFMILTTFVVPAFL
PVVLDKFRPLNETRIRLKLLDTDYGVDPDEYFVLITIHGYIASTFLVLMLWSVDSFIMIMVQHCCSLFVIVGF
TLKKLDQGNKKHTEKFENDVLINALQVHQQAIEFADFIEASLKNMYGIVIIENMLAISITGLETIKQMDQTDQ
AMRFAAFAFGQMVHLFFNSLPGQELVDHSTGLFDLVYDCQWESLSIKSKKKILFILMRSAKPKSLTAGGFYEL
NLQHCGAVLKTSMSYLTVLSSMRQ
>AgifOROR48
MEFFDNPNWIMTKYLLSVLGGWPLQQATIGRTILRTVIVLLVISSLLPQLFIVFTRLNDIDTVIESSAIAFVH
FISITNAIYCFLNTETEKKLLIQIKNLWESRLSAIERGYLEVYGNAGKKITHIYAISIYTTGMITLSTTIAPK
LLDIILPLNETRPTVFLYPAEYFIDEEKYAMFILIHSWILTPIQSTIIVGFDTLYAHYVQHLCSWYLIVGHRM
KNISTALDKINKNNNLFDENKRIILSFEKCINEHIKALEFTSNIQSIYGTPLFFIIGFNMIQISMTGCHTISK
IDDPPEAFRYGAFTIAQFVHLFFLSWPGQKIIDHSGELFEAIYETRWYDLPGKTKQLMILIMIKCQTPCVIKA
GKIIEVSLESFAIILKTSISYFTVFTSMQ
>AgifOROR49
MSILESPDFLLSKRILKLYGQWPYSSGVINYIKRIYCFVAVISILIPKLIKIYESRHDVDAFILCMPMVAVHI
EAIINLTSWCTSGEEIKYLIETIKRDWDILKSGDEIAILDGHWKRGRSNINNYAYPMVGITLVFMTSPAIPLV
CDIIAPLNESRPRVFLYETEYFVDQDEYYFHILIHEYMTVPLSIALTVYYNTVLGSWIYHATGMLAIVSYQLE
NMHVLSEGDKAFKEHEKNQNIYERLTYCIKLHSSTIEFIEIIESSNSLALFLVILICTLIFVITGVVSIVRMD
HRYVGFSIGVLGTLFFLSFIGNEVIKASEAVHIASYHNEWYNFPISYQVMLMKIMMRSEKPCTISTGKILDLS
MENFSELLKKAMSLFTFINSTR
>AgifOROR50

MSILESPYFSLNKRVLKFYGQWPYQSKLQNRCHRIVVFLAVSSILLPKLIKMIESRNDIDEFILCMPMVVLHI
EAMLNLSSWCFNDDNMYKLMDKIQNDWESLKTDKEREILDEHWKRGRSTINNYAIPMATVLIIFLISPGLPKV
LDIIAPLNESRPHELPYETEYFVDQDDYYLYILIHAYMTVPVSLGLTVLFNSVISSWIHHAAGMLAIVAYQLE
TIHDIPESDEQFSQFEKNQSIQKRLAHYIKLHNDAIEFIETVESSSSTVLFIVVIGSTLVVTVTGTAAVIKMN
HINESSRYMSFSLGTLGHLYYMSYVGNQVIKASESINTACYASEWYTLPIKYQKILIPIMVRTNRPCTITAGK
LYVLSMENFSELLKKAMSLFTFINSTR
>AgifOROR51
MFVIMEVWESVYYRTNKRALQIYGQWPFYSKFRTRCHRIIIYTILLSILTPKMIKFIESFGDIDETIECLPMI
ATHFVSQTKFVAWTFTSSEMKKLIIMMEKQWKEITSIEDRKLLDYYAKRGQVTTRLYAIALFGVMGVYLATAG
LPKLLDKINPLNETRPKIYLYKTEYFVDQDDYYKEILIHAYLTVPLSVGVIIYFDNMLACYISFANGMFAIVS
KHLKNIHVLSETDQMKSTIDKHKSIFNRLKYCIKIHSSTIEFIELAESSTTLALLFVAGLNTVVITVTGIVSV
MKIDKPSEASRYMAFTLGGIFHLYYISFLGNQLIQGSASVHRACYDNEWYTLPINYQKLLIPIMMRSKKPCQM
TAGKLYIMSMDSFSDVLKKAMSLFTVLNSAR
>AgifOROR52
MTIFEAGYYKINRMGLCLCGIWPEQNLFLAYIARFFLWGGIISSMVPEYVYLNKVWTNGSFLEDVVPCIPTQL
VIILVATKFIVFARDKETFQNAIDKMRYDCEFYMDLPAGKILREYGEKGRTRTIFYGLFLLTILTTFLVPAFL
PVVLDKLMPLNETRIRFSLFDTDYGVDPDEYFVLITIHGYIVSVFLIFMLWSVDSFIMIMVQHCCSLFMIVGF
TLKKLDRTNKKTFTKKFENNVILSAIQIHRQAIEFADFIEASLKNMYGIVIIENMLAISITGLETIKQMDQTD
QAMRFAAFAFGQMVHLFFNSLPGQELVDHSTGLFDLVYDCQWESLSIKSKKKILFILMRSAKPKSLTAGGFYE
LNLQHCGAVLKTSMSYLTVLSSMRQ
>AgifOROR53
MDEKKKLQAYVAFSEMTKKLMLVAGLWPVANPTLFYSIKPLIALSIALCSAIVITNFTIVNIKNIAIMTKGLS
LATSFYTTSLKIICFALNRKDAMVLHNTLANYYIVYLNNENLKKLVLTGFSGIRRLSTTFSTLVFLSILTYAV
KPMISILIQLLHNDGNKIVLMLAMPAKYPWNSIDNGQLFYWINYLFECSAAVCLFVVTCSADSLFSFYVFQMS
GHLRVMSCQLNFHEENINHHIIIRECVIKYGDLIKCRDILQKIYGPIILWMIISSALVICALIFQMAELEKVT
VGEVLLFTAYSGSKLLQTFMYAWSGSVLTSESENFLDAVYQSNWVGKNRKRFQTSILILLTQKPIVLIACKYF
AVSIDMFVMVLNTSLSYFFLLQTMAEKQN
>AgifOROR54
MDTVATSYHKINRKFLKSLGIWPYQSSLSKKFFILLTLFLTITHGYLQTAGMFAALVADIDIFLESVPTVLAD
VVCYLKYFNFFYNGSKMKELLITMEKDWKKYTSPEECQILDKWARFGRKVTIYYAGALYGTLVPMGLVPLVPV
VLDVVAPINGSYPRHLMFQQIEFLFDYEKYFFPLLIHGYFGTMAYLTIIIAIDTIFMVYIQHACAKFEILGFS
NLIEEANYVSFLFIIGINMIMMTASALVAAFKIDNPDIAGKFVAFTLGEVFHLFYSSWQGQLLLEHSESIFNQ
VYQAKWNNASVRTQRLIIPLLMRSAQPCKLTAGKMLTMSLTSFTTVVKTSFSYLTVFTSMRA
>AgifOROR55
MKKATSMETPQYLRFNKNLLACAGLWPHQQKLSKIIFFIILLFLIISQTAFQIIGAILARENVDLFMEAIPAI
LGGIAIAVKIMNFYLNGHKMKKLLVLIQNDWIEYKNGPESLILKKYAKFSNKLTLMYFGIIWSTMTPFLTIPL
VPLIFNIFVSQNNSIQRQHLYAQYEYVFDSDKNYYPVLIHSYIGSFAFINAVVAIDAMITMYIEHACALFSII
GYVKLLDNANSISYFFQLGIDIICLSFTGFQIATKIDNSPDAAMRFAAYSTSLLVVLYFLSYPGQQLIDHSLK
ICHNIYKANWYCTSLRTRKLLIFMAMRSSIPCQITAGKLYVMDITSFKLVVKTSISYVMVLLSVQE
>AgifOROR56
MSRNNIANLKSSYRWTKLNFDIVGSWPSSSSNIGQFRAFINSVLITTLILIPRFSALYYIKNYLDGVVFNASG
NLIYIVSIIKMFVIFKNQKVLAKILDDILSEWNNSLENSNYHVVEKYANISKNISILSLLISWTVCVMGCLAQ
RYSNIEIDDSVERHPRLAEDMFWSKLKKIVKRHHELIQLSVTLEKCFNKVLLLQLLTCCLTFASQGYLMIGKL
IRGDMTFFQTEFAVAYTTYTVVHFYFLCYAGESLIQTSMKIGFAAYEAEWYDLSTNEGKLLMFVTLNSMRSLK
ITTGKFAILSYELFITVIRTSLSYLSVLLAAKDSKS
>AgifOROR57
MNSVSFLLAITLSAPMSRNNIANLKSSYRWTKLNFDIVGSWPSSSSNIGQFRAFINSVLITTLILIPRFSALY
YIKNYLDGVVFNASGNLIYIVSIIKMFVIFKNQKVLAKILDDILSEWNNSLENSNYHVVEKYANISKNISILS
LLISWTVCVMGCLAQRYSNIEIDDSVERHPRLAEDMFWVSYFPYDIHATKIIFALHWTLQAYASVVGATIYVA
FDCYCCFLILHLTGQLTILQIDLRNIHLKYKSKNYNTNNLQSKLKKIVKRHHELIQLSVTLEKCFNKVLLLQL
LTCCLTFASQGYLMIGKLIRGDMTFFQTEFAVAYTTYTVVHFYFLCYAGESLIQTSMKIGFAAYEAEWYDLST
NEGKLLMFVTLNSMRSLKITTGKFAILSYELFITVIRTSLSYLSVLLAAKDSKS
>AgifOROR58
MNILYTISVWQDDVDSAITMLSIVFTVMVTITKGIIIWFYQEELYNILDNLHEKWNKSWTRIEIRDQILETVV
RVTKFRNIYVVALIGLFITYTYPPIGNVIIYLYKNTNESMDYSQTMWEAEVYPIEIDTFLKYFYLSTMDIYQS
VVYIICCYVGDIFYVQIFTHISIQFRVLANDITTTVNTNKNGDSKKLSMELKHIVMRHQELYKLFDIMNDGLY
SSVIFTSFVIGSINMCLNIFEFQKAVDEGDYYAASRFLFLTVTIFSGIILYCVQAEIICASAEFIPEAIYNSQ
WSNIDKNSILTLQIIMTRCQRKLTCGAYGLVDIHHEQITQVC

>AgifOROR59
MAQKKQLKNIAKCRKRLKLVTMALGIWPQKNPNTNFYCIQSYIVVVYLVIVYSAVLNFMYQNRHSFTIVLKGA
SLSLSFSIVTLKVVVFIYRRKKLREVEDIMEEILKSQLRKSDELVTTMLGPLDYFAKHISFGIFFCGYAIGTV
LYSFPIISLVKQHINNVHPKKYTTPYPTIYPYEIVGGSTLWWAHFAFESILSLIFCSIGTSTDNLFGYYCSHI
MSQFRALNYEMENIKLDDQLKVNITDIVIKHHKLINCCDLLMETYGEIIIGLLITTALILCCLVFQISQMTQI
TIGQVIWFTVYICYKLSQALIYAWAGEKVIEESEKFRMAIYTCSWETQYRRDLGKYLIIMMSQRPVSFTACGI
VSVDAKLFTSILNAALSYFFLLQSLNE
>AgifOROR60
MKRQAELQSFKSYSSGIRILLLFMGLWPVENSNVFYKLIPYFLGSVLLIVTSASMNFAIHYHYNLMIALKGVS
ISLSYLATTVKVICYVIHRKKLMKLEKTLHELLSEQENNKNNDELIKTALTPVFNFRRLSVSLSFCSFSVVTT
YFLTPVISMIKQYKNNIRPIKYLLPYPTLYPYAIEGGSLLWIIHFIIETYACFTLFTITASVDTSFAFYSSRV
IGQFRVLSNNIRSLKFKGNYEKRIQGYIVHHRKLITCCELLQDIHGPIVLAILLTTALIMCSLTFQISQMETI
SIKQTILFVVYIAVKLAQTWVYAWSGEMIAAESEDFRKAVYECGWETSDNKTVKHCILFMLMQKPLILQACNY
TQISAQLFVAILNTTVSYFFLLQTINDD
>AgifOROR61
MKEQEDKLKEVKKKVRIFRLVMSGVGIWPYENPTIYYRLLACLVAFLLLQTFCGCINFIVANSDNLMIMLNGM
GLSLSISCVMLKGGSLIYFRKDNIRMNKKLEELIDKQMKGPEDVCNVMLEPMVVFARKIFYLIYCLGFGMVLV
QWLRPTVAMTKQYMHGYNITYRRPFPSVYPWKIEPGSDLWKFHFFVDSITTWFYFSIGISTDNNFTHQAAQII
GQFSALNYEISNLKVEDVNNDKIKDFVERHQDLMDLCQTLGDCNGLIVLVLTLSAAIILCTLSFQCSKMETIT
VGELTWLLTYIFYKLLQIFLFAWSGEIIKKKSEELRDNVYGIAWTDKADPKVNHAVRFMMHQRPVVLQALGIK
PISAELFSGVVNTSMSYFFLLRTISE
>AmOr3
MSVKTARNIRDYHNIHYRSDAEYTVRVAKILLTMVGIWPRRNTFSNNVKFYVQTTIVFFLMCFLLLPHVIYTY
FDCENLTKYMKVIAAQVFSLLAIIKIWTILINRNEIRFCLMEMEVQYRDVECEEDRLVMMNTAKIGRIFTIVY
LFLGYGGALPYHVILPLISERIVKADNSTQIPLPYLSDYVFFVIEDSPTYEITFVVQMFTSFLIMSLNYGIYS
LIASITMHCCGLFEVTNRRIETILKNRDLRGRIADIIQSHLKAIEYSALVGKSLSIVFLSEMLGCTIIICFLE
FGVIVEWEDHKTFSMVTYFVLVTSMFVNVFILSFIGDRLKQESERIGQTSYFLPWYEFPTEIAKNIRIIILRA
SRPSSLSGAKMLDLSLRVFCDVFKTSAAYLNFLRTMTV
>AmOr4
METKHTEKDLKQAFYVQTFLKIIGAWPIAIESSLGSKIQKWFIISFYLFLQICIVAPCILDVFLKEKNGSRRI
NLFMLLISTLNQVFKYVITLNRANELRIAIHEIKKDWLTATPEDRFIFVMNSRIGQRIMLIMAFIMYISGLGY
RMVLPLLKGKIVLPNNVTIRLLPCPTYFTFFNELVSPYYEMIFMLQLLARFFIYTVLNSTVGISLMLSLHMCS
LLKILTRKMADLTDGSIISEKIMQQRIVDIIEYQTRIKRFLSNTELITQYFCFYDIGCSTCLICFIGYSIIVE
WENHNIASTVIYFSGLVTCTLMIYIICYIGQLLLDESNNLAQTCITLNWYRFPKKKARYLILMIIMSNYPIKL
TAAKVVDVSLTTFTDVMKAAVGYLNMLREVI
>AmOr5
METKHTEKDLKQAFYAQSFLKIVGVWPIPIGSPLSSKIRNWFITFFSLFLQICIVGPCILVMFLKEKNGKRKI
NLFKLLTNTLNQLFKYIITLNRANELAIAMNEIKNDWLTATSEDRWIFTANSKMGQKVMLIVAVTVYSSGLGY
RMLLPILKGKIVLPNNVTIRLLPCPTYFTFFNELVSPYYEMIFMLQLLAGFFSYTVLNGTVGISLMLSLHMCS
LLKILTRKMANLTDRSITSENIIQEKIVEIVEYQTKIKRFLGNAELITEYFCFYDIGCNMCLMCFIGYSAILE
WENHNIAAIVVHFMLLGTCIFIIYIVCYIGQLLLDESNNLAQQCITLSWYHFPTRKARCLILMIIMSNYPVKL
TAAKVVDVSLTTFTDVMKAAMGYLNMLREVI
>AmOr6
METKEKDLKQAFYAQPFLKIIGAWPILIESSLSSKIQKWFIISFSISLQMCIVVPCILVMFLKEKNGRRKINL
FMLLTNILNQVFKYVITLNRANELRIAIHEIKKDWLTATPEDRFIFVTNSRIGQRIMLIIAVITYSSGLGYRM
VLPLLKGKIVLANNVTIRLLPCPTYFTFFNELVSPYYEMIFMLQILAGVFVYTVLSGTIGISLMLSLHMCSLL
KILRRKMIDLADGSITSENTMQKRIVDIVEYQTKIKRFLGNTELITQYFCFYEISCNTCLICFIGYCIILEWE
NSNVVAIVVHFMLLGTCILVTYIVCYIGQLLIDESNNLARTCITLNWYHFPTRKARCLILIIIMSNYPVKLTA
AKVVDVSLTTFTDVMKAAMGYLNMLREVI
>AmOr10
MVQIRNAKEGLRHTFWFAYPFSRMLGHWPLSVSSSAFSKILNSFIIFISYLLQMIVVIPSLLYVILKEKNPKK
KIKLLMPHLNSIVQMIKYTILLRQMKLIDKLLDEIKKDWSIATEENRRIFSRTASVEHKLTSIIAITIYSGGF
FYRMILPFSKNKIVSNNMTIRLLPCPGYFGSLDEQVSPNYEIIFILQVFGGFVIYTAVCSTKSICLMLCMHMC
GLLRILTNKVMELTNDNDERVVQEKIVHIVEYQMKIKEFLKQIDQFVPTIYLFEVFIQVLIMCIIGYCIIMEW
KESNGMGLITYVIVQMTCLIGSFSVCYVGQLLIDESENIRQAFIALKWYQLPVKKSRSLILLIIISNYPIKVT
AGKIIDLSLVTFITIIKTAVSYMNMLQQIT
>AmOr18

MNAEKLMIEGKPPNANYKNDLSFNVRLNVWTLRTIGTWPRSPDHSWLETLEHVCLNLFCYELLAFILIPCSIY
IILEIKDFYNQLKLGSALSFFLMAVMKYCVFIIREDDIRKCVELIENDWKNVRYQEDRKIMLENASFSRRLIV
ICGTFMYGGVIFYYIALPLTRAKIVEEGGNLTYRRLVYPFPKVLLDARHSPINEICYTIQLLSGFVAHNITVA
ACGLAALLAIHACGQLQILMSWLEKLVDGRKNDNENLDQRLANIVKQHVRIINFIALTEDLLHEISLIEVVGC
TLNICFLGYYSMMEWDSKQPVSGVTYIILLISVTFNIFIFCYIGQLLAEQTVKVGEKSYMIDWHRMPWKKSLA
IPLMISMSHSTTKITAGNIIELSISSFGDVIKTSVAYLNMLRTFTT
>AmOr19
MNMEHFIVEKKSYNASYKNDLFFNVQLNVWTLRTIGTWPKSLDRSWLETIEHVCLCFLNYVLLAFILIPGVMY
FLLEMKDFYDQMKLGSALSFFLMAVMKMCVFIIRENDIRKCIECIEDDWKNVKYQEDRKIMLENASFSRRLIV
ICGAFMYGGVVFYYIALPFTRAKVVEEGGNLTYRRLVYPFPKALLDARRTPANELLYTIQLLSGFVAHNITVA
ACGLAALLAMHACGQLQILMSWLEKLVDGRENDDENLDQRLVNIVEQHVRIINFITLTEDLLREISLVEVVGC
TINICFLGYYSMMEWDTEHLIRGMTYIILLTSVTFNIFIFCYIGELLAEQTVKVGEKFYMIDWYRMPWKKSLA
ISLIISISRSTTKITAGNIIELSISSFGAIIKTSFAYLNILRTLTS
>AmOr21
MSSVKIDQDYKSNVNLSIKYSRRISKMIGLWPIFDKISTIHKFLRMLYNTICYCLLMFMIVLGWMYIAFEVKN
IYDGLKFVSLMSFCMLSITKYHLINIHKDDVRECVKRIEWDWKNISYSEDREIMLMNANFGKRLIIVTTTVTY
SGFVFFYIAIPMKIGKIPAPDANISFIPTMFPFPKYIADVRYSPINEIVFFFQFMCGFLVHGVTSSACSLAAI
FTVHACGQIQVMMIWLEHLIEGRLDMCYSVDQRIAKIVSQHVRILKFLSLIEKILQQVSYMEFLECTVNVCLL
GYCAIIEWESNHLTEVVTYVIILITIIFNIFVFCYIGELLADQSRKIGEVTYMIEWYRLSGKKKLCCVLIIAM
SNSSMKLTAGNLIELSMSTFSDVVKTSFAFLNVLRTLT
>AmOr30
MEKNRSIIGHDDYERNVNLSIRWNRFLLKSLGTWPNLRESRIGKCYSVLIGIVCYGLISFMLTSSNMFLVVEV
KDTYNRIKMIGPLSFFAMTLIKYYFLTFHEENIRKGIEHIEWDWKNVKHEEDKRIMIEYANYGKKLALISIFF
VYSAFVFYYFVVPISVGKIRDENLTFIPLPFPSSKLIADMRQSPANEILFSVQVLSGVIIHAITATAVSIAAV
FAVHACGQMQMLMNWLECLVDGRSDMNKIVDKRIAKIVVQHDRILKFLALTERALQQISFVEFLGCTMNMCLL
GYYLIVEWNPKEISLSLTYISLLISFTFNIFIFCYIGDLVAEQCQKVGEMTYMIEWYRLTGKKKLCCVLIIAM
SNSSIKFTAGNMVELSIYTFSDVVKTSVAFLNMLRALT
>AmOr31
MTSKSVISEESFDSLCDYSLQLNRWLLKPIGAWPSSSSSSKLERIVSFFLIVLCYGFILFTVIPSLFHIVLED
ENLHMKLKVFGPLSHWFIGGINYTTLLLQNKEIQYCVEHMQTDWKIVNRAKDQQVMMKYAKIGRYIAALCAIF
MQTGVLTYCVVTAFSTRIIEIGNETRIVHMLPCPVYKELISIDTSPTNEIVLISQFVSGFIVNSIAVGAISIG
AVFTAHACGQLTIIKRWIREYINRSKDNNKNVVINEIGEIVEYHLRILNFIEGIEDVLNRFCFMELFKSTLDI
SMLGYYILTEWADHDIRNLTTYFMILTSMSFNIFIICYIGDILMEQCRKVGEVLYMTNWYYLPYKDILDLILI
ISRSNAVIKITAGKLTNMSIYTFGNVMKTTFTYFNLLRHVT
>AmOr32
MIDKFASIQQTNNNLSNYSIQLNRWFLKPIGAWPPSPSTTKLEKIISIVLIICCYSSICFTVIPCLLHVMLED
ESFRDKLKVLGPLSHWFIGAINYTTLLLRSKEIRYCIEHMQRDWRIVTRTEDQQIMMKHAKIGRYIAVFSAAF
MQGGVLSNCAVTAFSTQTIEIGNVTKTIHMIPCTAYKKLIAVDTSPTNEIVIASQFLSGFIVNSSAVGAVSIA
AVFAAHACGQLSLLMVWIREFVDHSKKIHDKNIGLNKIGKIVRHHLRTLSFVTGIENVMSGICFMELFKCTVN
ICMLGYYILTAWSVHDIQNMVVFLVILLSMIFNIFIICYIGDILTEQCKMIGEAVYMTNWYYLPGKDILNLVQ
IILRSSMVIKITAGKLVHMSIYTFGNVMKTAFAYLNLLRQMT
>AmOr33
MMTSKSVPIEQDNHSLSNYSVQLNRWFLKSIGTWPLSPSTTKLEKTISFLLIICCYCFICFTVIPCLLHIILG
DDSFREKLKVLGPLSHWFIGGINYTTLLLRKKEIRYCIKHVQRDWRIVTRMEDQQVMIKHAKIGRYISMMCAA
FMQGGVLSYCAVTAFSTQTIEIGNETRIVHMIPCIVYKKLIATDTSPTNEIVIASQFVSGFIVNSSAVGAVSI
AAVFAAHACGQINLLMAWIRQLVNHSNVNNKNVGLDKISNIVRHHLRILSFITGIENVMSGICFMELFKCTMN
ICMLGYYVLTAWIDNDMRNLIVCSVILFSMIFNIFIICYIGDILTEQCKMIGEAVYMTNWYYLPGKDILDLIQ
IILRSSMVIKITAGKLVHMSIYTFGNVMKTAFTYLNLLRQLT
>AmOr34
MMIDKFVPIEQDNHSLSNHSVQLNRWLLKSIGAWPSFSSTTKLEKIISFVLIICCYCFICFTVIPCLLHVILE
DDSFHEKLKVLGPLSHWLVGGINYTTLLLRNKEIRYCIEHMQRDWEIVTKTEDQQVMIKHAKIGRYITMFCAA
FMQGGVLSYCAVTAFSTQTIEIGNETRIVHMIPCVVYKKLIASDTSPTNEIVIASQFVSGFIVNSSAVGAVSI
AAVFTAHACGQVSLLMAWIRQFVDHSNIQDKNIVLNDIGEIIRHHLKILSFITGIENVMSGICFMELFKCTVN
ICMLGYYILTAWTGHDIQSLIVFSVILFSMIFNIFIICYIGDVLTEQCKMIGEAVYMTNWYYLPGKDILNLIQ
IILRSSMVIKITAGKLVHMSIYTFGNVMKTAFTYLNLLRQMT
>AmOr35
MLVLKDSSSVSYSKDWIYSVQINRWLLKAIGIWPLSLCVTTTEKIHSVILTLISIFLIGFLLVPCTLCTLLDK
TGDLDTKIKMIGPFSFCIMAAIKYYVLLSRGSHIGKCIEDIRVDWFRVSSHNCLEDRKIMMENARIGRSLAIF

CAGFMYSGGFFYTTVMPLCTKRTEIIDNEIVRSQAFPIYRGLLDPRTSPSFEIVQLMQCLAGFVIYSVTVGSC
SLAAVFVMHACGQFQILVTKLRRLIDGLKEDKDMENIVHEQRLGNIVEHHLHILGFISQIEELLNEICFVEFI
GCTLNICFLGYFLLKEWEQSETIGILTYCILLISFIFNIFILCYIGEILSEECKSIGLSAYMIDWHRLPGKKA
LSLILISAASNSSTKLTAGKLVELSLSSFCSVLKSSLAYLSLLRTLTT
>AmOr36
MTDDISAIQKKFGSLNEYSIQVNRWLSKTIGVWPLPSSTSKFEKITTRILILFCWTIAVLDTTSGLLHFVLVK
EDIIIKLKSLAPISYILGGGLNYAVLLLRKNDIRYCIDRIEADWKVITRMADRQVMLKNAKIGRIISCCIVGF
MQLGTFCFCTILGVFKRTIKIGNDSMEIYVLPSPTYKIPVDTNPGHDIVLGFQYVAAYITSATVISAFSFATV
FACHASGQLTIMIIWIEEFINRSQKENKNRIDEISVIIEHHMRILSFLERAEHLLSPICFMEMFKNILSICLF
SYCILAEWSEHNIRILSTYILAVINITLNTFLICYIGEVLTERCKEIGNMVYMTNWYRLPKKDILNLIMIITR
SSVEYKMTAGKIIDMSVITFGNIIKTVFGYLNILRQVTML
>AmOr37
MMADDIATVQKEFENLNEYSIQFNKWFSKTIGVWPLPSSTSKFEKIMTRILILFCWIIALFDAISGLLHFVLV
KEDIIIKLKSLAPISYIFGGGLNYAVLLLRKDDILYCIEHMETDWKTITRMTDRQIMLKNAKIGRIISCCILA
FMQVSAVCFCTVLGVFKRTIKIGNESMEIYVLPSPIYKIPVDTNPGHDIVLGFQYLAAYITSATVVSAFSFAT
VFACHASGQLTIMIIWIKEFINRPQKENKNRIDEISVIIEHHMRILSFLERAEHLLSPICFMEMFKNILSICL
FSYCILAEWSEHNIRILGTYIFAVINITLNTFLICYIGEVLTERCKKIGNMVYMTNWYRLPKKDILNLIMIIT
RSSVEYKITAGKIIDMSVITFGNIIKTVFGYLNILRQTTML
>AmOr38
MMADDIATVQKEFNNLNEYSIQFNKWFSKTIGVWPLPSSTSKFEKIVTRILIIVCSIITLFVIIPSMLHFILV
KEDIISKLKSLGPISYCFGGGLNYAVLLLRKNDIRYCIDHIETDWKVITRMTDRQVMLKNAKIGRIISCCIVG
FLQIGTFCFCTILGVFKRTIKIGNNSMEIYVLPSPAYKIPVDTNPGHDIVLCFQYLAAYITSATVVSAFSFAI
VFACHASGQLTIMIIWIEEFINRPQEENKNVHIDKISVIIKHHMRILSFLERAEHLLSPICFMEMFKNILSIC
LFSYCILAEWSEHNIRILGTYIITVINITLNTFLICYIGEVLTERCKEIGDMVYMTNWYRLPKKDILNLIMII
TRSSVEYKMTAGKIIDMSVITFGNIIKTVFGYLNILRQTTML
>AmOr39
MMADDIATVQKEFDNLNEYSIQFNKWFAKTIGVWPLPSSTSKLEKIMTRILILFCWITTLFVTISSLLHFTLV
KEDIIIKLKSLAPISYCFGGGLNYAVLLYRKSDILYCIEHMEVDWKAITKTADRQIMFKNAKIGRIISCCIAA
FVQISAVCFCTVLGVFKRTIKIGNESMEIHVLPSPTYKIPVDTNPGYGIILGLQFLTGYIMSATVVIAFSFAT
VFACHTIGQLTIMVTWIEEFINRPQEENKNVHIDKISVIIKHHMRILSFLERAEHLLSPICFMEMFKNILSIC
MFSYCILAEWSEHDIRILTTYTFAVMNLIFSTFLICYIGEILTERCKEIGNMVYMTNWYQLHDKDILNLIMII
VRSSVEYKMTAGKIMDMSVITFGNIIKTVFGYLNILRQTTML
>AmOr40
MADDITAIQKKFGSLNEYSIQLNRWLSKTIGVWPLPSSTTKFEKIMTKILIFLCWIIALFVITSSLLHFTLVK
EDIISKLKTLGPISYCFGGGLNYAVLLLRKDDIRYCIDHIETDWKAITRTGDRQVMFKNAKIGRIISGCIASF
MQVSTICFGIVFGVFKQKIKIGNESMEIHVLPFPTYKIPVDTNLEHSIVLGFQFLTGCIMSATVVIAFSLATV
FACHAAGQLTIMVTWIKEFVNRPQEENKNMRVNEISVIIEHHLRILSFLGRTEHLLSPICFMEMFKNVLSICM
LSYCILVEWSGRDIRALSAYTFSVMNIALSTFLICYIGEVLTEKCKEIGNMVYMTNWYRLSDKDILNLIMIIT
RSSVEYKMTAGKIIDMSVITFGNIIKTIFAYLNILRQMTIL
>AmOr41
MADDIVAIQKKFGSLNEYSIQVNRWLSKTIGVWPFTSTTSKFEKIMTKILIIVCSIIALFVTVPSMLHFILVK
EDIITKLKMTGPIIYCIGGGLNYAILLFLRDDIRYCIEHIEADWKTITRTGDRQVMFKNAKIGRIISGCIGSF
LQFSTISYCTVFGVFKQTIKIGNESMEIHVLPFPTYKIPVDTNLEHGIVLGFQYLTACIMTATIIIAFSLATV
FACHAVGQLTIMVTWIEEFVNRPQEEKKNMRINEISVIIEHHLRILSFLERTEHLLNPIYFMEMFKNILTTCM
LSYCILVEWSGHDIKVLSAYSFTITNIILSLFLICYISEVLNEKCKEIGNIVYMTNWYRLSDKDILNLIMIII
RSSVEYKMTAGKIIDMSVITFSNIIKTIFAYLNILRQVTIL
>AmOr43
MMADDIAAIQKKFGSLNEYSIQLNRWFSKTIGVWPLPSSTSKLEKIMTKILIFLCWIIALFVIISSLLYFALV
KEDIISKLKTLGPISYCFGGGLNYAVLLLRKNDIRYCIDHIETDWKAITRTGDRQVMFKNAKIGRIISGCVAG
FLQLSTISFCTVFGVFKRRIKIGNESMEIYVLPFPTYKIPVDTNPGHNIVLGFQFLAAYIMSATVVIAFSLAT
VFACHAIGQLTIMITWIEEFVNRPQEENKNMRVNEISVIIEHHLRILSFLGRTEHLLSPICFMEMFKNILSIC
MLSYCILAEWYGRDVRVLGAYAFSVTCITLNTFLICYIGEVLSEKCKKISNMIYMTNWYRLSEKDILNLIMIM
IRSGMEYKMTAGKIINMSVVTFGNIIKTILAYLNILRQMTIL
>AmOr44
MADDIVAIQKKFGSLNEYSIQVNRWLSKTIGLWPLTSTTSKFEKIMTKILILLLCWIIALFVTTLSLLHFILVK
EDIITKLKMIGPISYCVGGGLNYAVLLFLRDDIRYCIDHIETDWNAITRTQDRQVMLKNAKIGRIISGCIAGF
MQLDSICFCTVLGVFKQTIKVGNESIRVYILPYPTYKVPVDTNPGHSILLLLQFLTTCIMSTTVVIAFSLATV
FAYHAVGQLTIMVTWIEEFVNRPQEEKKNMRIDEISVIIEHHLRILSFLGRIEHLLSPICFMEMFKNILSICM

ISYCILAEWSGRDVRALSTYAFCVTCIILNTFLICYIGEILSEKCKKISDMIYMTNWYQLSDKDILNLIMIMI
RSGVEYKMTAGKIVNMSVITFGNIIKTIFTYLNILFQMTML
>AmOr45
MADDIAAIQKKFGSLNEYSIQLNRWLSKTIGVWPLSSSSSKFEKIMTKILIFLCCIIALFVIIPSLLHFTLVK
EDIISKLKTLGPIGYCFGGGLNYAILLLRKNDIRYCIEHMKADWKAITRTDDQQIMLKNAKIGRIISCCFAAF
MQFSTVIFCAVFGVFKRTIKISNESMEIYVLPFPTYKIPVDVNPGHNIVLGFQFLAGYITTGTVIIAFSFATV
FACHAVGQLTIMITWIEEFVNRPQEENKNVRVEEISVIIEHHLRILSFLERTEHLLSPICFMEMFKNILTICM
LSYCILAEWSGHDIRALSAYASAVMNISLGTFLICYVGEILTEKCKEIGNMVYMTNWYRLPKKDILNLIMIIT
RCSMEYKMSAGKMIDMSVITFGNIVKTIFAYLNILRQMTIL
>AmOr48
MADDIVAVQKKFGSLNEYSIQLNRWLSKMIGVWPLPSFTSKFEKIMTKILIFFYWIILLFIILASSLHFLFVK
EDIVSKLKTLGPISYCFGGGFNYAVLLLRKNDIRYCIEHIETDWKIIKRMEDQQVMLKSAKIGRIISGCIAGF
MHIGTFCFCIVLGVLKRTIKIGNDSMEMYVLPFPTYKIPVDTNPGHGIILSLQYLTSYTSSATVVIAFSLATV
FAYHAIGQLTIMISWIQEFVNQPQKQNKNIRIDEISIIIEHHLRILSFLERTEHLLSPICFMEMFKNILSICM
FSYCILAEWSERDIRVLGIYTFAVMNVILSTFLICYIGEVLTERCKEIGNMVYMTNWYHLPDKDIFNLIMIIV
RSGVEYKMTAGKIIDISVITFGNIVKTVFVYLNILRQMTIL
>AmOr50
MTNDINVAKQRSDNLSEYSIKLSRWYLKPLGAWPASSSTTKMERIISQILIVICWCIILFTVIPGILYILFVK
QDIYVKLKIFGPLSHWCIDGFNYAILLLRKNDILHCIEHLRADWKLITRTQDQQVMLRNAKMGRYIAAFCAIF
MQVIIFFTCFILGIFKRSIHIDNKTVELYNLPCPAYKIPFDTDPTIHDIMLGTQFLSAFVVSSSASASFTLAT
IFTCHVLGQLNIMMIWINEFVDRLQRKENKDNHINKIGVIVEHHLRILSLIARIERITCPIYFMELFKCMMGM
CMPSYYFLAEWSERNIQNLTIYVMVALSMSFNILLVCCIGEILREQCKKVGDMVYMTNWYQLPDKDILNLIMI
ISRSSVEVKITAGKIITMSIYTFGNIVKTVFAYLNMLRQITMM
>AmOr53
MHDRSHDNINGQLKNSHYKSDIHYTLQMCQWLLKPIGVWPLIYNQTSRFEQLISIILMGTCFSSLLFIILPSG
HHILFVEKNLHMKVKAFGPAGFCLSSTIKYCYLGLKGSSFERCIEHMRKDWMMVQDPNHRTIMLKYATISRKL
ITMCAVFLYTGGMSYHTIMQFLSKGKNKDNYTIRPLPYIGYDPFFDTQSSPTYEIVYCIHCFTAMIMYSISTV
AYSLTTIFVTHICGQIQVQIARLQDLVESKEKRKYKDCDPFALIVHDHVEVLRFSNNIEEALREICFTEIIEC
TIDMCMLEYYCIMEWSVGDTITLLTFFTLLISFTFNIFIFCYIGEILTEQCSQIGTVSYEIDWYKLSPKEAYD
LILLISISQHPPKLTAGKIIELSLNTFSTVAKTSVVYLNLLRTVTDW
>AmOr55
MHFSVRNLINKPRNPNYEKDITYVMKHNKWVLISIGIWPTVLKNIGKFLPKIVIGINNLMCFFILIQSALHII
LEQKDTLLRLKFFGLIFFSFMSLMKYWALTIRKPEIEHCIQQVQSDWKQVKMENDRELMLKYGIIGRNLTIYS
ILFMYISGIMYISFMQYAMRLQINNDNQTNKVLIFPAYSNSIQKSPIYEITYGIQCICGYVLDSVTSGACGLA
ALFVTHACGQIDVVISRLDDLVAGQFYKKNSNPNIQVIKIIKHHIKILKFSAVVEKVLQEVFFLEFISSTFVI
CLLEYYCITDWEQNNIISLTSYALLLISLTFNMFLLCYIGDLLIHKSGNIGVAVFMIDWYHLPAKTIQNLILI
MAMSNSPAKLSVGRIVDLSLSTFGNVLKTTFVYLNFLQTAVMQ
>AmOr59
MHPITLNESDCKARNLKYKEDIAYVTKHNKWILKSIGIWPSIFKDVSKFLPKIMFGLCNFVLFFAIIPCILYI
VIEENDTMIRFKLFGLLSFCLVALIKYWTLLYRKSRIKNCVEQIWIDWEQVELYEDREMMLKYGQMGRNLMII
CAMFTYTGGTIFHTILQYKVGTFIDEYNRTIKPVIYPTYNGLFNVQRSPIYEFVYILHCMCGYVMHSVTAGAC
GLTALFATHACGQIDIVIARLNDLIHGKYSKEKINLNARFTKIIEHHLRILRFSATVQEVLQELCFLECIGST
FLICLLEYYCITDWELNNTISLTTYIILLISLIFNIFILCYIGELLMEKSSNIGLSCFMIDWYYLPSKTIRGL
ILMIAISSNPTKISAGGIVDLSLSTFGNVLKTSFAYLNFFRTTIM
>AmOr61
MHLTTLNKNNCKVRNLKYKEDIAYITKHNKWILKSIGIWPSVLKSVSRFLPKIMFGFNNFVLLFSVIPCILYI
VYEEKNIMIKFKLVGLLSFSLIALIKYWTLTYRKPRIKDCIEQIWIDWEQVELHEDRKVMLKYGQIGRNLTII
CAVFIYTGGSIFHTILQYKIGTFIDEHNRTIKPVVYPTYNALFDVQKSPIYELVYLLHSICGYIMYSVTAGSC
GLTALFATHACGQIDIVIARLNDLIHGKYTKNTFNLNTRLVKIVKHHLRILRFSESIEMALQELCFLECIGST
FLICLLEYYCITDWELSNTISLTTYTMLLISLTFNIFILCYIGERLMEKSSSIGLSCFMIDWFQLPTKTIHDL
ILIIAMSNNPIKISAGSIVDLSLYTFGGVLKTSLVYLSFLRTTIM
>AmOr62
MGKRKESIDERIRNFMVQKMVLKIIGIWPTNGERSFFGRWIFAVTTQIGIIYILSLEIYRHCLDIDDTMDAFV
MDLSAVISLAKLFILRLNSKHAWVLINSVVEDWSAVHDSRHEYIMTEYLKKGRIVSLMILYLGYASGFSFIVK
ALPFGDILPFQMFQNSRNSSMNPDIPLKLNYFLASYCVFGSLPLLHHVCVLLLQGIFIFVNAVAHCGNDGLFF
SLTMHLCGQFEILKTRIAKIEFVDRRKIGPLVKRHCQLAVLVNDLEQTFNMIIFVQLLMSALLICVEGFVFLV
CLSTKDNIGALKSMVLMVTLLIQLYLYAYAGDALESRTEEIAQAAFHSFWYQSRGRTARDLILIICRGNSSYH
VTAGKFVFMNIFTFKEILKSSASYLSVLKVMMDT

>AmOr63
MLKKMKTTSNKDFAYAMTPLKFLAWPVGTWPLQVFNTFSIIRATFSTFLLLLMLTILQVELYLDSSNPEYNLD
ALILINAGILAVTKVICFHVRSLGLVSNFTSAVKDYKELNSEENRVIVRRHAYMGRAACISLIFCSYVGCTLF
MIVPIVAGDKEEVINVTEESAMKYPVPFENTLILINMPENMYFLIFIVEYLMLLLTTTGNLGSDSLFFSIVFH
LCGQVEILRLEYNKLSNENERTTKHITLLIKRHIYLLKLGDMLNKTISSILIVQLSSSCMLICTTGFEFILAL
SIGNIVMIVKTFAVICVLLIQLFAYSYVGEYLKTQTEGLGNSIYFCTWYDMPKNVSHNITFIIMRAQHPVLLT
AGKFFVINMETYMSILRISMSYLSVLRVMVNS
>AmOr64
MKTTSNKDFAYAMTPLKFLSWPLGTWPLQVFNTFSIIRAMFSTFLVLLMLAILQVELYLDRSNAENNLDALVL
INGGILAVAKVMCFHIRPLGLISNFTSAVKDYNELNSEENRVIVRRHAYMGRVACASLIFCSYVGSTLFMTVP
MLAGDEEEVINVTEESAIKYPMPSENTLTLINMPEKMYFVIFIVEYLMLLLTSTGNLGSDSLFFGIAFHLCGQ
VEILRLEYNKLSNENERATKDIILLTKRHIYLLKLSDMLNETISSILIVQLFSSCVLICTTGFEFILALNIGN
IVMTIKTFIVMCVLLIQLFAYSYVGEYLKTQTEDLSNSVYFCTWYDMPKNVTQNIIFIIMRAQHPVFLTAGKF
FVVNMETYMSILKTSMSYLSVLRVMVNS
>AmOr65
MKTTSNKDFTYAMTLLKFLSWPVGTWPFQVYDTFSLTRTIFSISLLLLMIIIVQVELYLDRTNAENNLDALLL
INCGILAVGKVMSFRVRSTGLVFNFTSAVKDYNESNDEENRMIMRRHAYMGRVACTSLISCSYVCSTLFITVP
MLAGDEIQVINATEENAIKYPIPSKNALEIINMPDNLYFVVFIVEYMMLLFTSIGNLGSDSVFFGIVFHLCGQ
VEVLKREYSKLFNKNEKITEHFILLIKRHIYLLNLSKMLNETISSILIIQLFSSCVLICTTGFQFILALSIGN
IVLTIKILIIMCVLLIQLFAYSYVGEYLKTQTESVGNSVYFCTWYDMPKNVSKDIIFIIMKAQRPVLLRAGKI
FVVNMETYISILKTSMSYLSVLRVMVNS
>AmOr66
MKTTLNKEFAYAMTPLKFLSWPVGTWPFQVYDIFSLTRTIFSISLLLLMIAIVQVELYLDRTDAENNLDALLL
INCGILAVAKVMCFRIRPVGLVSNFSSAIKDYNELNSEENRVIVRRHAYMGRVACASLIFCSYAGSTLFMTVP
MLAGDEEEVINVTVESAMKYPIPSKNILAIINMPENMYFVVFIIEYIMLLLTSTGNLGSDSLFFGIAFHLCGQ
VEILRLKYNKLSNENERTMKHISLLTRRHIYLLKLSDMLNETISSILVIQLFSSCVLICTTGFEFILALSIGN
IVMMIRICIAMCVLLIQLFAYSYVGEYLKTQTESLGNSVYFCTWYEMPKNVSQNITFIIMRAQHPFLLTAGKF
FVVNMETYMSILKTSMSYLSVLRVMVN
>AmOr67
MKTTSNKDFTYAMIPLKFLSWPVGTWPFQVHEIFSISRTIFSISLLLLMVVILQVELYLDRSNAENNLDALLL
INCGILAVAKVMCFRIRPIGLVSNFSSAIKDYNELNSEENRVIMRRHAYMSRVACASLISCSFIASTLFMTVP
MLTGDKKDIINVTEKSIIKYPIPSKNALAIINMPENLSFMVFIVEYMMLLFTSTGNLGSDSLFFGIVFHLCGQ
VEILKLKYNKLSNTNERTMEHIILLTKRHIYLLNLSKMLNETVSSILVIQLFSSCVLICTTGFQLILTLTFGN
VVLTIKILAEISILLIQLFAYSYVGEYLKTQTEGIGNSVYFCTWYDMPKNVSKDIIFIIMKSQRPVLLTAGKF
FVINMETYMSILKTSMSYLSVLRVMVNS
>AmOr68
MTILQPIFNILTICGCRMPSSCRTSYKRMLYILYATFVLLLLYSFCISQFLNVIINVRTADELCNSFYMFIAS
LLSCCKIVALLMNHKAIKIFRRKLEEEPCKPTNTKEVTIQKSFDKNIGSITIYYTVMVEFTVFCMIVSSLVTD
FRNQRLAYEAWLPFNCSAPNYYYYYIAYVHQIIALIGTSLLNVACDVTICGLFVHMYSQQEILKHRLKESVNV
ENRLNIGKIVYFHNYLYGYAFMVQEKFKKIIGIQLLSSTLVVCFILYKLANTSLISTKFLEFVLYLACMMTQI
FVYCWYGNQLKLKSVEVVDTIFELDWISLDNRSKKDLINIMRRAMNPIELTCAYIFTIDLRTFVTILKMSYST
YNFLQRTKVN
>AmOr72
MHLLRWTFKLFVATGYFLSPKIKSPRKRFLYNVYTVVVTLFLLSFLLTLIMQIVFNVRTADELSENFGITITV
FTTICKFINLLFRRGIIISLLDLLQKEPFLPMDIEEIKIHTKYNKLIEKVSIFYTLQNVSCLVALIGATLITD
FKKKKLTFEAWIPFNYTASWFLFSLTFIHQCGCAVVTSFGISIFDTLFAGLLLQVCCQLDTLVYRLQNIKEDA
IQSLKYCARQHELIYRFTELMNKLFSSILCLQFLISAVAICFSVYRVIYTKTDSQFAGAIIFVFSALIQIFYF
CWHGDIAKYKSLEIPDMIFNSNWPNLSNEAKKILLIIMARSLTPVEVVSAHIIPLNLESFKRLIKATYSAYNM
LQQTK
>AmOr73
MHKLSLSFALLTYGGYWRPTKWPASSYKYHLYNIYSAFMIFLLYFITFCTCVDSLISKNLKTMSEKFSLCISV
FGVSLKVANLFLQRGKIINIMNSLTKENSIPRDEQEEIIQRRNDNYARKVTIYCEILNESAVFFATVGQYKRF
INTRTLPVSDWIPYDLSSTELYIISLLYQTVGLLICANASVGNETLIAGLMIQAGVQFEIFCHRAQNLPSLVL
TVTRNSNVFAETVNTVFQYMIFLQFTISSVVLCLSIYKFSTVDPLSMNFVWSGFYLCCMLMQVYLYCWFGNEV
TLKSNKVSDAIYEMDWTILPSNVMKDLLLVIARSKKPVKITSGQIFILSTESFMKIMKISYSSFNILKNSTMK
>AmOr75
MRRRGSKDVSIIWTSFLMKIVGLWLATDRNEQRQRDFALIYTVGTLFISICIAFRDIYYSWGNFSNSVFICCN
ILYVAIVLLKISVLYAHREEFFNLIAFTQKNFWRLYDDPQELLIITGCKKLCNFSIVLIIFCAQGTCAGYMVT

PLIENIGKNESDRALPFNLWIDFPVGLSPYFELLFILQILCVYHVATCYICFDNLLCIVNLHVAGQFRILQHR
LKNLGNAIRDETGLPRYEKCCYERLKDCVVQHQTLIEYCKRLEDIFTVMVLGQVMFLAVVICLVGFQLFLADT
SASKKASLVLNLGGTFFQLLIFTYSCDNLIRQSVNVGNAVFSGPWVNLPMSKAGILVRKNLIIVIMRSQKICC
LTAGKFFPVSLETSTAVLSTAISYFTLLKQSSLENM
>AmOr76
MKSKEVRDLSITVTAFYMKIAGFWTSTNYVEERRRNVTMSYTLFAILFAATTEARDLYFSWGNFSDSIYVACN
IITVSLVLIKLLTSFIYNEELLGIIRYAKTNFWHSNYDTCEKSIMNKCQRTCNYLVFVFTFFAQGTVLGFILR
PILVNRGKNESDRILPFNMWLELPLSITPYFEVMFFVQVVFVYHVCVCYHCFDSLLCILNLHTASQFRILQHR
FANTCNEKRGKRDEDEESALSFYEYSKLKAYIRQHQALIEYCKKLEQVFNSIVFGQVLLFSLLMCLDGYLILM
EETPFGRRVTFTFHITGCMCQLLMFTYSCDCLIRDSMDIADAAYNCSWSFLPMDKYGKMIRRDLMFVITRSRT
PCCLTACGFFAVSLETYTKVLSTAISIFTILKRYEKEFKSDSS
>AmOr77CTE
MNGLLNGGDASMTMTAAFMKLVGLWTAKNRREQRARKFALIYTVAAMLFALWIEFTDFYYSFGDFSTCLFNTC
NIIYITMPLLKIFVIVLNKKDFFHLIFYTEKHFYKDNYDEHEQRIFTNCRRQCIIFVCFLTFSTKGTLVCYIV
SPLVENIGKNQSERALPFNMWVNLPLSTSPYYEIIFTIQVLSLYHIGVGYFCFDNLLCVLNLQLAGQFQILQY
KMANIVDLLKEKNEKRIINTSYFAKKCYEAFKKCIREHQALIAYCEKLEKVFSLIILCQVLTFSLIICLDGYQ
IIL
>AmOr80
MQTESQLDISINLSTFFLKNVGIWMSDNPNEQRRIKMLFLYTIWNLLFGTVVNSRDLYFTLLYDGDILYVTTN
NITMIMGMVKICIILIYKKKFLNLIVYMQQNFWNVNYDHREKQILDDCRKTCIFFVSCVTIMAICAMICYIMI
PFIAQSGSNESERMLPFNMWINLPISRTPYYQITFLIQATCVYYVGISYFCFDNIFCIMAVHLAGQFRILRYR
FTKLCDMEYGIKENSQSILSKQMHKFYEKFRKCVQHHQALIDFYQNLENVYTMITFGQVLVFSVLICLFGYQV
LVATISFARRFIFVFMLNGSMFLLFMVTYSCNGVIEHSDNVAVGAYSALWTIMPMDKFGKILRKDLIIVIRRS
RRVCCLTANGFFPVSLETYTKILSTALSYFTLLSNRIENSS
>AmOr81
MQTESQVDISMNLSTFFLKNVGVWISDNPSEQRWRNMLLGYTTWILLSGIIINGRDLYFTLLYNGDILYATTN
NITMIMGLVKICIILMYKKKFLNLIVYMQQNFWNLNYDHCEKRILDDCRKTCTFFVSSVTSMAICAMICYLMI
PFIVQSGKNESERMLPFNMWINLPVSRTPYYEIIFFIQAMCVYYVGISTFCFDNIFCIMAVHLAGQFRILRYR
FTKLCDVEYENSQSILSKQMQKFYEKFKKCVQHHQTLIDFYQNLENVYTTITLEQVLVFSVLICLFGYQVLVA
TASFARRFIFVFLLNGSIFLLFMVTYSCNGVIEHSDNVAIGAYSALWTIMPMDKFGKIFRKDLMIVIRRSRRV
CCLTANGFFPVSLETYTKILSTALSYFTLLSNRVENA
>AmOr82
MQTESQVDISMNLSTFFLKNVGVWMSDNPNEQRQIKMLLINTTWILLSGIVINGRDLYFTLLYHGDILYSITN
NITMIMALIKISIIIIYKGKFLNLIACMQQNFWKVNYDYREKEILNDCRKTCIFFVSCLTTMVICAMISYLII
PFIAKGNNESERMLPFNMWINLPLSKTPYYEIMFLIQAMCVYYIGVASFCFDNIFCIMAVHLAGQFRILQYRL
TKLYDVECIEMHKKDSILANRVPKFYEKFRKCVQHHQALIDFYQNLENVYTRIAFGEMLVYSILICLFGYQVL
VATASFARRSIFVFLLNGSTFLLFMVTYSCNGVIEHSDNVAIGAYSALWTIVPMDKFGRMLRKDLIMVITRSR
RVCCLTANGFFPVSLESYTKILSTALSYFTLLSNRVETANDT
>AmOr85
MSSNKVGGDLSITVMTFYMKIVGFWIASNYVEERRRNLTISYTFFAIFFAMATEARDLYFSWGNFGDSILIIC
NLVTVILVLFKISISLMYRNKLHKIIQYAKTNFWNLKYDLHDEQIIINTCKRYSTFFVCIFTFFSQGTVFSFV
IRSLKENIGKNETERIHPFNLWLDESWYMTPYFEMVFIIEILSLYHVGVCYLYFDNFMCIINLHIAGQFRILQ
HRFSNVCNEMCEKCCYQLSRKSPYLSICKYAKLKIYIRQHQTLIEYCRKLEMVSNFIIFGQVLLFSLLICLDG
YLILMEDTSNMSRLIFTFHLISCMCQLLMFTYSCDCLIRDSTNIANATYNSLWSFMPMDKYGKMLRKDLILVI
MRSKSPCYLTALGFFPVSLETYISILSTAISYFTLLRNRAEQTIMDA
>AmOr87
MGAKAVAKVVAHSKLVARHSNKDFALSMTAFLMKIVGLWLAKNEQEQRKRRLTLMYTVIAILFGVWVQFRDFY
YSWPNFGNCAYTACNILCLIMVLLKLFVLFVHRKEFIDLLVYTHENFWHTNYTNNELLLLQNCKRISMLCITL
INVCAQGTIVSYVLTPIVENIGRNHSDRVLPFNMWVDLPTLFLSPYYEILFVLQVLSLYHVGVCYICFDNLLC
LMNLHAATQFRILQHRLSDLGSGWDTRRSFNKIDRETSWSSCMENCYATFKLCVKQHQDRITYCHRLNDIFTI
IVLGHILVFSLLMCLVGFQVLMANSPPTRRLIFVFHITGSLCQLLLFTYSCDSLIQESTNVGSAVYSGPWICL
PMNRIGRTLRRDLRMVIIRSRKPCCLTASRFFPVSLETCTTVLSTAMSYFTLMRQSFAN
>AmOr88
MNGRNVRNLSITVTAFYMKVAGFWVANNYAEKRRRNVAMFVTIFFAFMGISIEGRDLYFAWGDFEDSIFAGCN
VITIVLVLLKIFVLYINNEELLNVVNYAKTNFWRESNYEPHEKKIIDDYRRLCSFLVCSFTFFAQGTVVCFVI
TPVFVNNGKNESDRIHPFNMWFDRSLSLSPYYEIIYTIQVLSAYEIGICYHCFDNLLFVINLYTAGQFRILRY
RFENICGKNDDKNYYKVSKSSKYCINEYKSFKTCVQQHQALIEYCKKLEDVFSIIVLAQVLLFSLLICLDGYL

VLMEDTSRAKRVIFTFHLMGCMCQLLMFTYSCDCLMHDSMSVANAAYNSLWPCLPMDKYGKSLRKDLTFVIMR
SRSPCCLTACGFFPVSLETYTGILSVAVSWFTSLKKYEKKLLQYACIANQSLLKEEESKIIFYHQNI
>AmOr89
MQREVELDVSVNLAAFFLKNVGLWASNDPGHERRRKVILVYTMWCVTLSSVVIIRDVYFTWFYNGDILYVVTN
ALSMMMITVKVCVIVVHKEEFINLIVYMQENFWNDNYHDLREREILENCKRTCAFFVSLVTAIGICAILSYLA
TPLIVQTASNNSERMLPFNMWLKLPLSESPYYELMFVYQIMTFYFIGISYFCFDNIFCIMTVHLAGQFQILRH
RFDRLCNAEDRIAEKGAHAREFYDRFKARVPYHQALIDYCEKIENVSPTIILEPVMVFSVIICLFGYRILWAN
APSTRRSIFIFLLIGAMSLLFMFTFSCNCVTEHSENIAIGAYSALWTAMPMDKFGKMLRNDLIMVIKRSRRVC
CLTANGFFPVSLETYTTILSTAVSYFTLLRNNVEKANEA
>AmOr91
MSFLESDVSVSLTSIFMKLVGLWMAADQYEQRLRNISVTYNLVAILFALYLQTTDIYYSWGNFSACLFSVSNT
LSLILPLLKIFILLSNKEDFFRLIVYMQRNFLQGNYDDHERKIVFGCKRKCTFFICFFTFFTMATIVSYIAGP
IIGNIGKNESDRVLPFNMWINLPLSMTPYFEITFTLQVLSLYQIGVSYFCFDNFLCIMNLHLAGQFKVLQYRI
STIADRVIEKEEKKEKLIIDSLYFSNKCYTTFKKYIRQHQALIAYCRKLEVVFNWIVLEQVLMFSLLICLDGY
QILMANGDIKTRLTFSFHILACLCQLLMFSYSCDCIIRESVSVATAAYGGPWTLLPMTISGRMMRKDLIIVIM
RASIPCCLSGKGYFIVSLETYTSVLSTAASYFTLLRNNIESDN
>AmOr92PSE
MNFLENDVAVSLTSIFMKFVGIWMXQYQQRMRNIMVAYNVIAIFFALWIQTMDMYHSWGNIRACLFSTSNTLS
LILPLLKIFILLCHKQDFFRLVLYMKRNFLXNYDDHERKIVIGCNQKCTFFICFFTFLTIATTASYMVIPLIV
NIGKNESDRVLPFNMWVNLPLSMTPYFEISFVLZVLSLYQIAVSYFCFDNFLCIMNFHVAGQFKVLQHRISTI
ADLTIKTEEKKEKLIIDSLHFSNKCYTTFKKYIRQHQTLIAYCRKIEVVFNWIVLEQVLMFSLLICLDGYQIL
MADEDIKTRSIFSFHILSCLCQLLMFSYSCDCILRESVSVATAAYEGPWTLPPMTISGRMMRKDLIVVIMRAS
IPCCLSGKGYFIVSLETYTSVLSTAASYFTLLRNNIESEIMKHD
>AmOr93PSE
MNFLESDVSXIFMKLVGIWMAGNGZEXITLFTAIIFSGYNLZMYFILGDFSACLFFISIILSSIMLLLKIIIL
FSHREDFFHLILYMKRNFLXNYDDHERKMIGCNZKCTSSSVSSRFSRWRPLLLTSSVRLLVKNIGKNESDRIL
PFNMWVNLLLNITSYFEITYTLQXFSLSLYHIGVSYFCFDNFLCIMNLHVAGQFQVLQYRISNIIYXRFNEKK
EKLIVDSXATKCYAIFKKXQHQALIYLIYCRKLEEVFNLIVLEQVLMFSLLICLDGYQILMADGDVKTRLIFS
FHILGFLCQLLMFSYSCDCIIRWTLXPLLSMTSSRRMIRKDLILVIMRSNVPCYLTGRGFFIVSLEMYXVLST
AAXFTLLKQRTEATS
>AmOr98
MDMFQKTGKEYSNIFDIPYYKVLKKYLQFLGQDPYQECKYRNIITIIMLISMIAIFIPTTFEIYVSIHDKNTD
AVMECLPNLCASLSSVVKILNVHFNRENFNKLLEFVVKEWDELKLNELHILEEITIQGSKIAHLYRNTLLSFL
ILFLLVPMYFPILDMIDALNQTRSRQQLLRVNYMVFNADDYFFYVYLQLAWGAIVIVMIVITVDSLYIIIIHH
VCGLFAVCSYEIQKTVKDLTVFTDIEKCSYKELKNCVIKHKKAIKFYNILNNSSQLSYLLQIGINIMGISTTA
FQLAVNLDTRPQEAIRNAVFCGANQFHLFVLSLPGQILLDHCAELSNTIYCSMWYKLPVKIQKMFNIMLMRSK
KSCALTVYGLYELNMENFGTTFKACISYFTMMLSLK
>AmOr100
MCIIYEEQCLDIFEIPYYKSLKKWLILSGLYPPKNIIIILVAISIISVTLPLMFAIYTSLHAKNIDAMFECLP
SLGVCIVAMFKLQNIYNNSENFKKLFTFVAKQWYQLKLNNEIRILEEIIMQGNKMAQIYKNTLLLSMTIFFFV
PLIFPILDIVYPLNETRPRQQLYRVNYFIFNHEDYFFYVYFQLVWSSFVCVIVIIIFDWLYILIIHHNSGMFA
VCGYQIQKIFAEKVFSNIHIYEQFKNCLIVHSEAIQFFSILDESSRNTYLFLVGTNIMATSISAVQVVLNLDK
LEVAIKSAVFLIAAQFHLFILSIPGQILLNHYSNLKNNIFMSSWYNMPIEVQKMFYVMQIRCKKPCSLTACGL
YEMNMENFGTALKTCMSYITMILSLK
>AmOr102
MNIFQKTRRQCPDIFDIPYYKMVEKYFQLLGQDPRLKNEFRNFIVTVVVISISGNIVPTSIELYTSLCDKNMD
AVIEGLPHFIAATISAVKILNVYFYRENFDKLFQFVTNEWNKLKLNNELHILDKTIIRGNRTAHLYRSALLIA
LVLFLLIPLISPMLDVFLPLNETRPRQQLLKVNYLVFNDDDYFFYVYLQLAWGSIIVVVTSVAVDSLLILIIH
HCSGLFTVCGYQVQKVISNAKSFNGTVLNNYTYEQIKNCVIMHDEAIQFYNILNESNRNSYLIQVGLNMLAIS
ATAVQAVVNLDRPEEAIRSAVFCGANQFHLFVLSLPGQVLLDHCSEFSNNIYSCIWYRAPVRIQKVLYIMQIR
SKKLCTLSAGGLYEMNIENFGITFKTCMSYFTMIMSLK
>AmOr109
MDERAIEDQYLKINKFFGQLVGVWPYQKKFFKTCIRFITFTIMIFSLATQISRVIVFYSLDVLSDQLPYINAG
IVTLFKQYNYILNEDKLRELLHDIVSDRLIERSKEELEILEMYSRRTTALCALYKVMVYSCAFMFLVIPTIPP
ILNIVAPLNVSRSREFIYPTYYFVDEQKYYYPILTHMIAVILVLSSVYLACDTNLVQIVHHGCALLAISGYHF
KHAVDDVKFCDGYYIDASMDETYVKIRQSIKAHKTAVQYVDKIDACHIHYFLLVIGMIVLAFTGTFLKLSTME
VGIRFFTFCAYTIAQLIHLFFLTIMGQFLINANEETFKTIYEADWYNGSSKMQSLYVLVLRKCLSPPKLTGGG
FVALNLDSFVQILKASFSYYTVFRS

>AmOr110
MDKQTIENQYLKINKFFGQLVGVWPFQERFIKTCMRFIVSVIMLLDLATQISRVIVFYSFDVFSDQIPYLNAA
IICLFKEYNYVLNENKLRELLNDIISDRLIRRSKKELEILELYSRKATTLCILYKVMVYSCAFMFLVIPTIPP
ILNIVAPLNVSRSREFIYPTYYFVDEQKYYYPILMHMIAAILVLSSIYLACDTNLVQVVHHGCALLAISGYHF
KHAVDDVKICDENYITLMDETYTRVRHSIKAHRTAVEYVDKIDACHIYYFLLTIGMIVLTFTGTFVKLSTMEM
GIRFFTFCAYIAAQLTHLFFLTIMGQFLINANEEIFRTIYEARWYNGSSKTQSLYVLVLRKCLTFPKLTGGGL
IILNLNSFVQILKASFSYYTVFRS
>AmOr115
MDFAMGWNRFNLTLLGVYPEPRKMSRNSRLMSSLIFWFTTLVTFTFICAPQTANLILKSTSLDEVIENLSINI
PIVFALIKQIVLRYYKKALTELLGEMLADWSGPIGDQDRETMLRNARLSRAISIVCSTLTYFMLLAFVSLQVW
SNAENASETDLGGLLHPATFPYETSKSPNYEITWLGQLMGTVLTAICYSCFDTFLAVLVLHLCGQLTVLGTAL
EDLVNATRRNDYKTFEQRLSSIVNRHNHLSRFAVIVEDCFNITLLVQTLICTAMFCLTGYRMITSVDREDEAD
VPIVGIIFFIIHVIYTMLHLFIYCYVGETLLGQSTGIGLSTYHCNWYDLPSRRAVLLMIVIRRANVSFQITAG
KFSPFSLEFFNAVLKTSAGYLSVLLAMKDRLVEGK
>AmOr116
MTNHLEKQIKLKKINSNKHLQNNLSIIYYIGLWPDRVKYKYLYNLYTICSLIFLVGIIIVSEIIYIIINWGKI
EIMMTGLTILMTNSTYAAKVIYIICRYERIKNLVDITNSEIFNRDNDKYKHIISYYNWQGIFHHIAYQGFASI
CIFSYSCIPLQSAFSGKSKQLPIAGWYPYNVTSTPIFEIACLHQVLVILINCINNIAIDTLITGFIIITCCQL
TILSYNISSIHYTVESVESSILIEKCIARNNNINIEKSPSKIYNKFYENLKHCVKHSIIIFDFTKQIQDIFGI
IIFFQLFVNCIIVCLAAFNLSQIKNYITPEFFGSLLYICCMIYQIFIYCWHGNELYLHSMKICLSAYKNNWWN
NNKNFNYALLIIMIRTQIPLIIIVGKVMELSLQNFLLILRTSYSIFTLLKTFTT
>AmOr118
MINRPLEYSLRIFGIWPDSPYPKLKIITWIIILPTFLVFQYWYCITHIKLGLIDLLDGLSLTLSNTLVFIKLI
VIWFHKRTFYEILMSMKEDLNNNKHSATENKRIIMDKSMLSSRISNFLISYFAITFFLYSGVALVIFDEDQGK
FLVRMEFPFIATISPRYEIILITQFIFESFIVYGAATSIALIAALILYVGSQIDLFCQNLTFHSYKKRESQDT
IKDIIVRHQKIIQLSKNIETIFTYISLCQFVSNMLVICFISFVLTVSLHTEQTIVLIMKCLPYYIAVNCEAFI
LCYTGEYITSKSENINKAVYNFLWYNLKPRDVRIMLMIILRSQKQLTLTAGKFICLSLEAFANMLKASASYVS
VLYARY
>AmOr119
MHTQRDTSEITYSHDTATSRKLFYLLVVVGGQMAHASGHEWMKSIRTLTSIKINYLKYSGLGEIDSSCSRILK
YAYFVYKVWMLVSMCILAITVFADIYTNMDNLSITTDDGCIFAGIFVVIFKAMNLQIQLESVKKIIDKYHTRN
KVMFFGFCVIGACLGFALLCFTPMENGLPIRAKYPLNTTVSPWHEISFFVETCAVSGGLLGIIVMDSMTTFKC
SLITMLLDALSVNFENCNGETKRTICNRHGKEERNDNNNRFLDRYKKCVQFHQRLVVISRDYNKIYSLSMLVQ
MISSTSIICLTGFQAVVVGGQSSNIMKYGIYLSAAMSQLFYICWLGNELGYASSTLDKNQWFSGWCNERLTGI
GQVFTLSTVFTRKSIILRASVFYVLSLETFIAIIKRSYSFFTLLNNMDLTDH
>AmOr120
MSNQTNTMNIRNYIFINQLVLKFVGFYPINILRYVICISCIMFIVIPQIIMIYINWNDLNIVMETGSTLLTIL
LAALKSIVWIFNRKKLEFFIEFMLTDYWKIIETNVFEYLQEYAIYAKNITKGYFFSMCNALLFFFSLPIIETL
TKNENLNNFTIKNFPFAASYPITFYKFPFYEIAYISQILATSICCLMMLAIDSLIATALLHTCGHFTVLKENL
KNLDTYIYDLTKTNLKTNSKYINKNLYEIKTQIIYIIKHHQLVLWFCDNMEKNFHLILFLQAITSSLIICFVG
FQISIALTERSKFLESFSHLIVSLFQLLLFCFPGDILIRQSFNISIAAYSMQWYQLPTFIKDEICMIILRSQR
PSFITAGKLYIMHLENFTAILSTAFSYFMMLQSFNTEA
>AmOr122
MNMDVFDKQYRIYRIILKIVGLWPYDKSIYVWIQRICLSMYFLIGVIFQIILLVKSEITLRNYIVTLSAIFPL
LLFFIRYIYYITMFPYVEILFDNIRTEENLLQDTTEIQIQTKYLDISSHIIYIFCCMTFAFIVAAIIFLVNPV
ILDLRNPLNESRIFYFDLLFLLDDQSAYIKIFLILNFMLNILFGLLSITSTESFTNIFSYYICRQFNIVNYRI
RKIIEDLSTRNLSKIDLKIKDIHRVVDIHCHAIEHVHIATNNAATQYLIAIILCILSFSVNLYRLYKALITMD
NRIEIFGSTLIVIYHLMIAFYNNHCGQLIIDSNLGIFNEFASTWYRIPLKAQKLLLFMILRSSMGCEICLSGL
FTPSYAGLTSMMSSSFSYCTVIYSIQ
>AmOr126
MNVFDNQYRTYRIILKIVGLWPYDNSIYVRIQRICVLIYFLIGVLVQIFSFVKSEISLRNCIVTFSMTFPTVL
FCLRYIYCLTLFSYAKLLFDDICTEEHLLQDTTEIQIQTKYLDISSHIIYIFCWLSFICAAASCILILNPVIL
DVIMPLNKFRLHYSLIFLSNDRRKCIDIFLVLNSIIIFTFGLLSLICSELLTNIFSYYICRQFHIVCYRIRKI
ITDLSTPNLPKTDLKLRDIHRVVDIHCHAIEHIHMAFHTNNAATQYLLAIILFVFSFSINLYRLYKALITMDN
RMEILGSILIVIYHLMMALYNNHYGQLIINSNHGIFNELCASTWYRIPLKAQKLLLFMILRSSMGCEICLSGL
FTPSYAGLTSMMSSSFSYCAVIYSIQ
>AmOr128

MNVFDNQYRTYRTVLKIVGLWPYDNSIYVRIQRICVLIYFLIVVLVQIFSLVKSEISLRNCIVTFSTTFPTLL
FCLRYIYCLTLFSYAELLFDNVHTEEHLLEDTTEIQIQTKYLDISSHIIDIFCWMSFICVASTCIFMLNPVIL
DVIMPLNKFRLHFSLIFLSNDRRTYIDIFMVLNLIIIILIFGLLSIVCSESLTNIFSYYIYRQFDIVSYRIQKI
IADLSMPNLPKTDLKFRDIHRVVDIHCHAIEHIHMAANNAATQYLLAIIVCILSFSVNLYRLYKALITMDNRM
EIFGCILVVAYHLMIAFYSNYCGQLIIDSNLGIFNELYASTWYRIPLKAQKLLLLMMLRSTVGCELHLSGLFT
PSYAGFTSMMSSSFSYCAVIYSIQ
>AmOr129FIX
MNVFDNQYRTYRIILKIIGLWPYDNSIYVWIYRLCLLIYFLVVVLVQIFSLAKSEISLRNCIVTLSTTFPTLL
YCLRYIYCLTLFSYTELLFDNIRTEEHILQDMTEIQIQTKYLDISSHIIDIFCWLSFICVAATWIFILNPVTL
DVIMPLNKSRIHFSLIFLSNDRRTYIDIFMVLNLIIIILIFGLLSLICSESLTNIFSYYVCRQFDIVSYRMRKI
IVNLSMPNLPKTDLKLRDIHRVVDIHCRTIENIHIASTNTATQYLLAIILCILSFSVNLYRLYNALIIMNNRM
EIFGSALMVMYHLMIAFYNNHCGQLIIDSNFGIFKELYASTWYRIPLKAQKLLLFMMFKSSVGCELRLCGLFT
ASYAGFTSMMSSSFSYCAVIYSIQ
>AmOr130
MNVFDNQYRIYRIILKIIGLWPYDNSIYVWIQRLCLLSYFFANIIFQIVSLLRSEITLQNSILILSITCPLVL
FLLRYIGSIACFPTIKIVFKHIRTEENIVQDSIESQIRMKLIDDSHHIINIFFWMTYTTIVIFIIYVSYPIIL
DFMIPLNESRTHFIYYITTFSHNQSIYLDILDFNFMFTGIFGLLSVACSESITGIYSYYICILLKIVSYRIQK
IIMYLAMFKLSPKQIDSKLIELYRVVDIHNQTIELIDFMLSTAGIHYIIASLLVVISLAINLHRLVNATLIKK
NQLEMLFCFTLVAIHLVIIFLNNYNGQIVMNSSQELFDELYNSMWYFMPLKAQKILLLIMLQSTTKHAFNILG
LFTPCYAGFSTMLSSSFSYFTLMYSIQ
>AmOr131
MDVFDKYYHTYRIVLKIIGLWPYNNSVYVWIQRLCISALFLGNIIFQILSLIRSEITLRNCILILSTTCPLII
ILLRYISFIIFFPMVKLLFHHICVEENAVQDLIEIQIRMKYIGNSRHMIEILLRVTFLTITLFSIFLLYFVTM
DFIMPLNEFHRHILLYVTLFSVNRTIYFYILYLNFLFVITFGLLSLICTESIVGLYSYHTGMLFKIISYRIRK
IITYLTMFNVSSKQIDSKLAELHRVVDIHNQAIGFISIIINNSGKQFMMPTLLIVMSMAINLHRVVNAITIKK
DQLEILITLIIFANHLMIMFLCNYNGQILINSNEEFFHELYIPVWYFVPLKVQKILLLIMIRSSMACIFHIFG
VFIPCYVGFTTMLSTSFSYFTLIYSIQ
>AmOr132
MDVFDKYYHSYRTVLKIIGLWPYNNSIYVWIQRLLLLTLFLGNIIFQIMSLLRSEITLRNCILILSTTCPLII
ISLRYICFILFFPMIKYLFHHMRMEENIVQDSIETRIRTKCINDSCHMIDIFLWMIYAIFAFCIILLLCPIIL
DFIMPLNESRIYIAHYITIFSDKRIIYVDILCLNYMFLMIFVVLSIMSTESILGLYSYHTSMLFKIISYRIQK
IITYLTIVNLSSKQIDSKLAELYHVVDIHNQAIQLINIVINNSGKQYMISTLLFVISMAITLYRLENAIIVTK
DHLEILICLMLFVKQLMIMFLCNYNGQILIDNSEELFDELYFSIWYFVPLKVQKILLLIMTRSSTTCMFHILG
VFVPCYTGFTTMLSTSFSYFTLMYSIQ
>AmOr133FIX
MDVFDKHYYTYRFILKIIGLWPYNNSVYVWIQRLLLLTFYVGNIIFQIVLLLRSKITVRNCILILSTTCPLII
ISLRYIRFILFFPKIKYLFHHMCMEENIIQDSIEAQIRTKYISDSRHMIEILLWMAYATITLYSILGLCPIIF
IILLNESPIRMLHYVTLLSVNGTIYFYILCLDFLFIIIFGLLSMICTETIVGIYIYHTSILFKIISHRIQKII
AYLNMFNLLSNQIESKLAELYCVVDIHNQAIQLFNIIIKNSGKQFMISAFLSMISMAISLHRLVNAITIKKDQ
LEILISLIIFVNHLVIMFLCNHTAQILINNNEEFFHELYISVWYSVPLKVQKILLLIMIRSSMACIFHICGVF
VPCHAGFTTMLSTSFSYFTLMYSIQ
>AmOr134
MDIFDKHYYSYRTVLKIIGLWPYNNSIYVWIQRLWISALFLGNIIFQIVLLLRSKITVRNCILILSTTCPLII
ISLRYICFILFFPMIKYLFHHMRMEENIIQDSIEAQIRMKYIGDSRHMIEIFLWMAYANITLYSILGLYLIIF
IMPLNESPIRMLHYVTLFSVNGTIYFYILCLDFLFVIIFGFLSIICTETIIGIYIYHTGVLFKIISHRIQKII
TYLTIIDLSSKQIDSKLAELYRVVDIHNQAIQLINIVINNSGKQFMISTLLSVISMAISVHRLVNAITIKKDQ
LEILISLIIFTNQLVFIFLCNHTAQILINNSEEFFYELYISVWYFVPLKIQKILLLIMIRSSTACMFHIFGVF
VSCHAGFTTMLSTSFSYFTLMYSIQ
>AmOr135PSE
MDVFDKQYHSYRTVMKIVGLWPYNNSIYIWIQRLLLLTFFLGNVIFQIVSLLKSEITLRNCILILSITCPFII
VSLRYVCFIVFFPTIKLLFHHMRVEENIVQDLIEIQIRTKYINDSCHIIDIFFWVACTNITLSSISLLYFITL
NFIMPLNEFRIIHYITLFSVNRTMYFNILCLDFIFVVIFALLSVICTESIIGLYSYHISVLFKIINHRIQKII
TYLTIVNLSSKQIETKLAELYRVVDMHNQAIELIDVMINNSGKZFMISALLLVISTAINLHRLVNAIIIKKDQ
LEISISFIFFVNQLIIMFLCNHSGQILIDNSQKLFNELYISIWYFVPLKVQKILLLIMIRSSTRCMFHILDIF
TPCYAGFSKMLSTSFSYFTLIYSMQ
>AmOr136
MNVFDKHYHTYRTLMKIVGLWPYNNSIYVWIQRLWFLMFFFGNIIFQIMSLLTSAITLQNCVLIFSTTCPLII
VLFRYIGLILFFPTIKLLFHHMCMEEAMIQDSIEAQIRRKYIDDSCYMIDIFFWMTYVGIALCSILLLCPITL

DFIMPLNESRTRIVHYVTIFSDKSIIYMDILCLNYMLLAILVILSATCTESILGLYSYHTSIMFKIIGHRIQK
IVKYLTMFNLSSKQIDSKLAELYRIVDIHNQAIELVDIMINNSGKQFMISTLLSVISMAISLHRLLNAIVIKK
DELEILISFIFFTTQLVITFLNNNCNQILIDNSQELFIELYISMWYFVPLKVQKILLLIMIRSSTACMINILG
VFTPCYIGFSKMLSTSFSYFTLMHSIQ
>AmOr137CTE
MDIFDKRYCTYRTMLKIVGLWPYNNSIYVWIQRLWLLIFFLGNIIFQVVSLSSEITLRNCILILSLIFPLTII
LVRYVSCVIFFSMIKLLFHHMRMGGNIIQDSTEIKIRKKYINDSCHMMNIFFWIIYGIAALSIIFILYPMTLD
FIMPLNRTRIRIIHYITIFPYNRTMYLDILSLNFMFVGIFGSLSLACTESIFGLYCFHASILFKIIIYRIQKI
VTYLTMFNLSSKQIDTKLTELYRAVDIHNQTIGFINVMINNSGMQFLLSSLLCVISIGISLYRLVNAIIARKE
KDQL
>AmOr138NP
VVSLSSEITLRNCILILSLIFPLTIILVRYISCIIFFSMIKLLFHHMRMERNMIQDSTEIKIRKKYINDSCHM
INIFFZIIYGIAVLSIIFILYPMTLDFIMPLNKTRIHIIHYITIFPYNRTIYLDILSLNFMFVGIFGSLSLAC
TESIFGLYCFHANILFKIISYRIQKIVTYLTMFNLSSKQIDMKLTELYRAVDIHNQAIGFINIMINNSGMQFL
LSSLLSVISMGISLYRLVNAIIVKKDQLEMLISFMILMAQLIITFLCNYNNQILIDNSQELLDELYISAWYFV
PLKVQKILLLIMIRSSTSCTFHILGVFIPCYTGFSKILSTSFSYFTMIYSIQ
>AmOr139PSE
MDIFDKHQSYHSYRTIMKIIGLWPYNNSIYVYIQKLZLLIFFLGQIIFQXDAVIAFFSISLLYPIILDFINSL
NESRTRIIHYFTIFFHSRIIYIDILCLNYIFLAIISLLSIICIESMIGLYTIVTTSLFFKIIGYRIQKIITXL
TIFNLSSKQINSKLVELYHVVDFHNQVIELINAINNSGIQFMMPSILIVISVAXLVNMILIRKDQLEIFMFFI
FLVSQMMIMFICNYSSQILIDNSQELLYDLYISMWYFVPLKVZKILLLIMIQSSITYMISILGVFILCHIGFS
TMLNTSFSYFTLIXSTQ
>AmOr140TRA
MSQRNERSATTVDMDFFEQPAYLLNKRLLSIIGIWPFQKSLPKLLRQNIVLIFCFTLLIAELRGLYSVWGVNY
DAVIECMPPIISIFQSASMYFNGIFNTKKIKNILLFIKNDHKYYINRPENIILQKYDLQGKKITFYYILYVYT
TLFVYLLLPTIPLIIDFITSSNHSQKRNFLFELDYGMDKQQYFYYISIHSYIGTAIVANLIASCDTMYMLYAQ
HAYALFAIVSYELKTIHILNTNNLINVTDHHLLEKYKNITLLSKDEKKVYRKLFICIKNHQNAIKYSNLLESL
FTKSILVQLFFNVLCLSITGVETVIKLGNLSEMMRFGSFTFAQAVHIFFLCLPGQRLLNHSEELHVSACEVTW
YIFPKKYQNLYKFLLARSLIFSKLTAFKVTTLSMQTFLAIIQTAMSYFTVLLSTT
>AmOr142
MKNRLTPEKAILFTKLSVALTCSWPPSPLATKAQHLFFNALWCIAFLTSVMLFLPLLAAIYVYRKHPVILGKT
VSLTAAVAQVTIKMIICRLQQKRFQMLYSEMENFCKQATNEEKIILQRYVDRYKYFHSFYILWSFLTTIFVIC
GPLYTVQTFPTHAIYPFSVRRHLYKGLIFFHQSLVGFQVSSGMAIDTQIALLLRYATARFEILGIQFNNAKSD
GEFDACIKKHDELLRYSREIRQSIKFLILATNGTTVIAVIFGSLNLIANQPLILKALYAIVVFSASVELFMYA
WPADSLMHMTMKMATKVYNMDWYGKDIRTQRKILFIILRSQKYESFGINGIVPALSLSYYGKYLYTSLSYFNA
LRIMVEDTVN
>AmOr143
MNIRQILYILELIGTFTCTWPINPNISKRRIIFRNIFWIFSILNVILLMTSLMLAVVYFRNDILMSLKTASEM
AALLEVVLDLILCKWNNSEFQVLIEEVKSFVEMANEYEIKILQGYVNRYKKFFSTVSMGYISTAISFSLMPLF
SAQKLPADGWLPFSTEPFGIYCIIYFNHVYCILQTAFCIFVDFTIVILFSFPAAKLDVLRSKLRHVNNYDTLV
SCIKEHQKIIGFVEDTKATVETLLFKTNVTMGSTVMCGAFPLLNNQSLAAISQFLPLVLSGILHLYVIAWPAD
DLRESSVQFSNSISDIQWLGQSNKMKSCVIFMMMRSQKAFLIRMSNLLPPLSLEYCSNFITTVSSYFMAMRTM
IES
>AmOr144
MGMLNMDIRQVLHILELTGTFTCTWPINPKDSKKYIIIRNILWTFTILNVIFLTISLMLAIFHFRSNIPKSMK
TASEMAALLEVVLDLVLCKWNNSELQVLIEEVKSFLEMASEYEIKILQGYINRYKKFFSTVSMGYILPASSFI
LMPLFSAQELPAEGWLPFSIEPLGIYCVVYVNHIYCILQTSFCIFVDFTIVILFSFPAAKLDVLRSKLRHVNN
YDMLVSCIKEHQKILGFVEDTNATVETLLFKTNVTMGSTVICGAFPLLNNQSLDVVTQFLPLVLSGMLHLFVI
SWPADDLRESSIQFAESINDIQWLGQSKKMKSCVIFMMIRSQKLFLIRMSSLLPPLSLEYCSNFVTTVSSYFM
AMRTMIES
>AmOr145
MGMLNMNIRQVFYILELTGTFTCAWPINPNDSKTYIIIRNILWIFTILNVIFLAISMIFAIFHFRSDIPKSMK
TASEMAALLEVALDLALFKWNNSELQILIEEVKSFLEIADEYEIKILQGYINRYKKFFSTVSMGYILPASSFI
LTPLLSDKELPTEGWLPFSIEPLGIYCAVYVNHVYCILQTLSFCIFVDFTIVILFSFPAAKLDVLGSKLQNVN
NYDMLVSCIKEHQKILGFVENSNATVETLIFKTNITMGSIVICGAFPLLNNQSLDVVTQFLPLILTGMLHLFV
IAWPADDLRESSIQFAESINDIQWLGQLKKMKSCVIFMMIRSQKLFLIRMSSLLPPLSLEYCSNFVTTISSYF
MAMRTMIES
>AmOr146

MFRNATPEKAIAFTQFIVSLSCCWPLPSTATKLQTRCFKIIRSLLFLNSLLLFFPLLYFVYVNRNDNTTFCKA
MSLSLAVVQVPLLSSFCITQYDRFQRLIKEMKFCCENANSYERQVFQGYAKSYATFYGVSAIWFYWCALIVVV
GTLFISDPFPTNAEYPFPVHFEPVRSIVFVQQALVGFQCSAHLCVNIFCALLLLFAAARFEILMNELRAVENI
ESLIKCIEKYYAIRRYAEEVVNSARYTTLITLCICGVESVFGGIIFIGRQPFTVKLQFLTLSATTLLAVFMCA
WPADYLMDVSENTMRAVYESEWYKRSLKLQKFVLFATIPQTPVILKVRCIIPAFSLNYYCSFITNVLSMFTAL
RVLMYKDEN
>AmOr149FIX
MLKQMTFKKIINIIWFSVALTFCWPLSANSTKIQIFVFRILQIISIINAFMLIVPLSYSIYLHYDDIAIIFQS
LAILVGLSQMIIQTVILCFIKYNFLQRVVEEMIICIKEAQQYERKIFCKYIENCNIFYGSSLTITYLVVIIYI
MGPIVLPTPFPVDTEYPFHVNSTIIKIIIYLQQSLLIFQCAGHLCISIFCALLLWFTAARFECLIVELQKITN
IGMLIICIKKQLRLRRYARNVVNSFRFMIVYAIGVSTFALILYGIIMIVKAPLIMKIESVTLSFVLLLQIYIY
AWPADHMKDMSINVSKSVYNIIWYKQTLRMQKDLLNVLIYQRPITFSVDCILPELSLRYYCSYVSNIFSIFTA
VRVIIEDN
>AmOr152
MLKQIISEKTIQIIWFSVAITFCWPISLNSSKTQVFIFKILQIISIINVFMLLLPLLYSVYLHFNDIIIVSKS
IALSVGLIQVIVQTIICFIKYDSLQHVVEEMIIYVKEAQQYEKKIFHKYIEKCHIFYGCSIACIYLTATVFVI
GPVFSSASFPADAEYPFQVNSTSMKIIIYLQQSLIAFQCAGHACLSIFGALLLWFVSARFECLAVELQKTTDI
GMLIVCVKKQLHIRRYARRVVISFRFIILCAMGVSIFSLTLGGIIMITKSPFIVKVQFITLILTLLTEIYMYA
WPADHMKDMSINVSKSVYNTIWYEQTLRMQKNLLNILMYQQPIILSINCILPELSLRYYCSYLSNAFSIFTAI
RVIIENNPS
>AmOr153PSE
MVKEMIPEKTIHITWLSVALCWPLSVNSGKTQVFIFKMLQIISIVSACMLLLLSSYSIYFXHGQCRIFKNYHR
FIDVAQNIIQTVICFYIIKEMKICIKETQEYEIEIFQKYIAKFKTVWGCNITCMYLTALAFTIGSVFISTSLS
CDAEYPFQLNYTLVFAIRYQSFLSYQCAYACADHXLLWFTAPRFECLCVELQNVTNINMLIVCXYAKKMINWF
RFIIFNAIGLSILVFTLASIILIMISICMYIVVCSCMYNFINKNYYMYIWPADYMTDKSINVSRKIYDSMXYK
QMLKMQKNLLKXLIFQRPVXIYRLZLLSKLILRYYCLYLSNVFSIFTALHVLEDNI
>AmOr155JOI
MLKKATPEKIIDIIRFSVAICFCWPYPLNSSRNQIFGFKVLQISTMVSACIMLLPLLYSIYLHNDDVIHISKC
ICISIGVTQLIVQTLVCFIKHNSLQRVVGEMMKCVKEAQQNEIEIFSKYIEKCKIFYGSSIIFSYLTSTAFML
GPIILPISFPFDAEYPFHVNHSLVTIIIYIHQSLVGYQCSANVCASVFGALLLWFTVARFECLIVEFQKCTDI
DMVIACVKKQVQLRSYAKEVIKCFRYIVLYYITITTFALIISCIILLMNVPLIVKMQFIIICVTIMTEIYIYA
WPADYVKNMSINISKSVYELSWYEQTLEMRKYLLNVLIYQKPITFSISCIVPELTLRYYCSYLSNAFSIFTTL
RILLEDNST
>AmOr157
MRRARPEKSVYLVWLSVAMTFCWPLPPDTARKRIVGMKVLLIISIVNGCAVILPMLYWIHLHLDDIISLFKCI
CVALCLVQYVAQTIVCLVKYDTLQRVVDEMMGLIEERRMYEILRAYASKCNTLYGASIASIYVCGTSFIFAPL
FLPNPFPFETEYPFHVNTTTRIFIIYASHVLVIFQGTAHMCLCMFGALLLWFTTARFECLIGELRGVTSVDTL
VVCLEKHSRLKRYAEEVVSCIRFLVFHAILLGTFVLTLCGIVLIINSPLIVKAQFIIICVCILLEIYLYALPA
DYMYDMSMNISRSVYDSIWYEQRLDLQKALLTVLAFQKPIAVSINVLLPELTIRYYCSYVSNALSIFAALRTV
VE
>AmOr158FIX
MLKQITPEKSIYIIWLSVALSFCWPLHINSTRKQIMYIKILQISAVVNAFMVLLPLIYTIHLNMHNLINLFQC
ICLLICIFKHIIQTVICFIKYNALQRVVEEMMICVKEEQLYDILCMYVKKCNIFYGGTIVLIYGTATVFVLGP
IFLPISFPWGTEYPFQVNYTTINVIIYAHQFFLVYQCAAHTCLSLFGALLLWFATARFECLIKELQKITSIDM
LIVCLKKLLFLRRYAEEVVSSIRFLVFYAITISTFTLTLSGIIMIINCPLFVKMEFITISISLLVQIYIYAWP
ADYMQDMSINVLRSAYNSIWYEQTLDMQKTLLIMMAYQKPVTFSINVLLPELTLRYCCSYVSNALSIFTALRA
VVEVT
>AmOr159NP
LTMLMILYPMVYNVILPLNESRKFIILCDIYYFVDPQEYSSIFLLYTLVLIFILYTIIGTQFTIIIFVQHCCG
FFQNCXYRLEHAMDTYKGQNEIEIYYIICTKLIKAIEVYKLAVKFFKWANGIYKIPYTITIFLYVLDVSVQLY
YVVYMLQQLENIYKLCINLILLIRKFCFLFLITYLGQNIENHSNEVFEKCYDSLWYTAPVATRKLLLIIMINI
MKPCQCKMFGGLFKGNIEGFAQIIRICISYFMSLYSTQ
>AmOr162FIX
MDGIQHEYLRVNKYLLFVVSAWPYQAILQRSLIGIIVIPIVTAQLILQFGGMMTAIIAGDIESFLESFAPLAI
SLMCFVKYINFLYNFNQMKRLMDIMQEDWKFHARLRNEYEILCEHYAIARKITTSFVAFLLGLTTPFGAMPLL
LNIGDALGLCNISDDRPLAFRVEYFVDVDKYYYLLLVHSSIGTLGYTVIVLAINSIIIVYVLHECGLCEILRV
KLENFVETDAMDIELRPPNKKDKWYQNARDCVLLHKRIIEFAKILEDTNTTSYLLQLGFNMICISFTQFQAII

NIEDTPKVLRYVSITIALLCDLLFVSWTGQQLSNSTERIFEYTTNGKWYQSSISCRKLLAIMLSKSIAPLRLT
ACKLYTLNLESFTTIAKTSVSYTMVLCSLQ
>AmOr165FIX
MLVLNTLSPSVKFGLHFAGIWPGTPFPYLHKLGWLAAIAALQSYQYRYIVMHYKSDNLMSIIDNLSIAMPFSL
VFIKLIVTWINYGVFCDILSTMEKDCQKYAVIDINNLISKTGQISFYTTTIVMSSYLVSAAFYITGTLAFQRT
NSSISRELLFKMDLPFETNESPNYEFVVTSQLLIHVSAAFTFGTFSALLLMMVLHIGCQIDILCQNLLDIPHI
STSHLKFFIIRYQEIITFAERVEKLFTYIALSQLVSNTLITCCVGFLIVIAIHEDNGLPLLLKSVLFYMVICL
EAFIYCFAGEYLRIKNESIIEAAYDMLWYNLNPQDSRIVLLILIQAQRQLTLSAGNFVTLSAETFASMQKISA
SYISILMAMY
>AmOr166JOI
MTSINTISRSVKYGLYFAASWPGASFSILHKFFWTIIFCTLHISQYSYLIMHYKYDALTEIIDNISICLPHSL
VCIKLFTAWTQNTLIRNILLSMEEECQKYAIMDTDNLISKTAYLSYRLTSTIICTCVASTVCYAIGIFSHQEV
NVTSSRELLLKMNLPFDTNKSPIYEFVVIIQYFYQVSAAFVFGVFAAFLLMIVLHVGCQIDIMCQTLMKTTHR
DQKKLKFFIKRHQEIILLAEKIEKFFTYIALSQLISNTLITCCLGYLIVITLHLGNNIILIKYIMFYVAVCSE
AFIYCFAGEYLSIKSKLIADTAYEFLWYNMNPNESRLLIPIILRAQRGFTFTFGKFATLSMESFTAIMKASGS
YMSVLLAMT
>AmOr167JOI
MIPIRSISHPIVIGLRLIGIWPKSSYEIIVRFMWVIIMMCAQIFQYQYIINHIGFDNLADLIDSVSTTLPYSL
LCFKLISFWTKREIFENILIGMYHDWTNAFATDFIVEDMIKKTELAYYCSNLILSIYAIAVFLYVGVFLELSH
DHDQENRSNLSPELLIKMDLPFTYDESPIYEYVFIVQFIQLFFIASSIAVLDALIITLIFHIGGQIEILHKTL
KNISINDEKPESSRIIIKSLIDRHYRIIIGSEYIESLFSYIALMQLICNTLIICCIGFLIVVALNSNLKLLIR
ISFFYIAITLEAFIFSIAGEYLSNKSLSVSISAYESPWYLLSPKNRGVMILLMVRSQRRLTITAGKFMDLSMQ
GFANVLKASVSYVSILYAMY
>AmOr168
MNFQNLNRLNALANVVSGNFLPMTNINEKSSVISKIYFVIVWIIQLMYLASCTLGLFNVSWERALKDGTVNMV
LLLEVIILNVYLHSRKKLLRELIGKLNQILINEDEIFRNVTISTTKMLEKPSRIYIIVNVISIIVWISSPLIK
LFQKDEFYHEDFVMPAVFSNQPFSTGVFISGVFLQLFGGEYLLFRKISLDLYTMHLNLLITSQYKYLRIKFAT
ILKENGESAKDNDKTIRQEMKLLIRHFETVIEMTGILKKLLSPNIGILYLNYVFRFCFLSFMFATTSLSEKLT
YTIIVSYTTGALIQFYILCYCIQDLFEASTSIADDVVYEKWYSYDVRFQRVILMISLANELKCKISNFQNIDL
TLPSFMSILNQAYSICLLFLKTKQD
>AmOr169
MKMNFQNLNRLNTFVNAVSGNILPITDMKKRLSIVLKIYSILVWTIELSYLAACILGLFNVSRERALKDSTVN
IVISLEVFVLIVYLHNRENLLRELIGKLNCLLIVDDETLRDVTIGTVKPLEKPLRVYIIASVGSLMIWASLPL
AKIFRKSEFYYTDYQVPAVISNEPFPIGVFIGGVALQIFGSAYTLLRKVSLDLYTMHLILLITAQYKYLRIKF
AAILEQETPKDFFYGGIIWQNVPCEYDKMVKQEMKLLTRHFEIVVEMTVMLKKLLSPNIGILYINYVFRFCFL
SFMLATSSGMHFEKCLLVSYTIGALIQFYILCYCIQQLLEASTTVADDVVHEKWYLHDVKFQHIILMITLANK
LKCKLSSFRNIDLTLPSFMSILNQAYSVCLLFLKARQS
>AmOr172JOI
MDFEGSQYYNINRILMISVGLWPYERTIYSKMLNILIFFFLSTAIFIQVMSFIMLKMDINLTLNSLSYTSCTC
ICIMKYFNCLFHIKDIKNFLDEIKNDWNSLRNIEELRIIHEYSKTIKKITICFVIIIVPLQLVFFLNVFGNNI
LDILIPLNHTRPRTVPIEIYYFIDQQKFFFFFGMHLNIITSFGGLVYIAIETISMGMIQHLCGLLKITSFRIS
HTFVANIPKISSTERSIIIRKKVMSIVYLHVKIKKYINWIQEKIIITYDLLILLGLIAFSIFIFELAKSITSR
TNTNEIFSSLIFVICITIYGFIPNHFAQEIINHSSNIFIDTYNTEWYKLPIIEQKLLLFIMQNNLKSLNFVLL
GTMIASYNVYAMILRTAFSYFMVIYSMD
>AmOr173JIP
MDFEGSQYYNINRILMISVGLWPYERTIYSKMLNILIFFFLXTAIFIQIMSFIMLKMDINLTLNSLSYISCTC
ICIMKYFNCLFHIKDIKNFLDEIKNDWNSLRNIEELRIIHEYSKTIKKITICFVIIIVPLQLVFFLNVFGNNI
LDILIPLNHTRPRTVPIEIYYFIDQQKFFFFFGMHLNIITSFGGLVYIAIETIYMGMIQHLCGLLKYINWIQE
KIIITYDLLILLGLIAFSIFIFELAKSITSRTNTNEIFSSLIFVICITIYGFIPNHFAQEIINHSSNILIDTY
NTEWYKLPIIEQKLLLFIMQNNLKNLNFVLLGTMIASYNVYVMILRTAFSYFMVIYSMD
>AmOr174JI
MDFEGSQYYNINRILMICVGLWPFERTIYSKLLNIFIFLLMSIAIFVQTMSFMMLKVDMSLMLTSLSYILCTC
IFSMKYFSFLFHIKDVKEYEELQIIHEYSKTTKGITICFVIIVPLQLVSFLSIFGNDILDILIPLNYTRSRTI
PIVIFVDQQKFFYIFGTYLNLITSFGGLGIIATETTSMAVMQHLCGLLKILSFRISHTFVANIPRVSFTERNI
IIRKKVMSIVYLHVKIKKYINSIQEKIIMSYNFLLLFGLIEFSISMYELTKSIISNNTNELISSLFFIICITI
YGFIPNHFAQKIINHSSNIFIDAYNTEWYKLPIIEQKLLLFIMQNNLKSLNFVLLGSMVASYDVYTTILRTAF
SYFMVIYSIDSTN
>AmOr175JOI

MVELLSYYDFTKFLLTTLGLWPYYNAKYRILYNSIISFTSFSFIVIQCIVFKNSFKNKKMLKYLSMLIMSLGS
FVKYNINWYQLATVKKFMDKIKYDWKIIEMNNETFEIMKKRTCLGKHYVIRFASIFYSSIIIIVFCHISTCIL
DKILPLNETRLKKFPILTEYSILDEKYFSLTVIYLYISLLLIITIFVATESLMILWLQHAASLCEITRFFVQS
ATLKSSKDHFDNNIKKYIIKAVITHRRVIQFIQDTRDYNAITYCILLIFAIISLGINLFCLSQSILVLTKIEE
TIVSFMCIICELSYMYYMNYMSQQVLDCSNNIIITIFHTNWYKMPVPIQKLILNIMLNCNKPFLFTFFSIYYP
TVEGFASLLKMSLSYFTMLLSMQ
>AmOr176
MLDNYYYRYVRFYSKLVGLWPFDNSKQQTTRKWFIQAMFVLIIVYQVRIMLLCRNNINDLLGCVIIITINIIV
SIKYYTCIYNYDVMKILFINIERDWAELKCIDDMNILKKYSRNSYLYERYLIWTSWIVYSSVIIVNITMDYSA
YFINSNVLNEIDYYIFTIFVDIVIIFSLIILMSTDSIYITIMYHVCAVFDITRFKHLFDDIRKMNVAYRKYLL
QNQIVDIVRHHWKCIEYINLLDTYYSISIVLQYVIITIIFTLKFYKVYRCIKTNHIRKLITIFIDIVQNYILV
FLYSYPATKLTKHSELLLNSAYNACWYETPVYIQKMFKFVLQNCLKSYHLGIPQIFFITFESISKMVQFSFSC
LILLISLEE
>AmOr177
MDFYEANVVKSYRLYLLLVGVWPYEYSKANEMQRIVYVTCVISFLLVQIFTLFTDKIDHALLYQIITNVLIVS
LILIKYVNIWIKIEEFKEFFDYIRIDLSKLTETEKILIKRTYKKCSYYCQIIFLFCYFVAFVLMIIQVFQIVD
YETTSRTNRTNYFDRIILTEYFIDREKYSYPIYIHLNVAILLVCSMEIGTDVLHAIIITHIIGIFDKIGYLTI
HLFDEPEITCQKIDKHLIYCRRLVYIVNLYKRNLKFCKKYNSTMVICGAIQLIWGTLIISALLLKLRNELLPH
NLRIMFIYCFFVSLYLFIIYMLILPYQKVYEISETLFFKAYCGHWYSTSAKTQKLLLFLMHRFMTPYKFRFKN
VVIVVDQTYLSMIRIGFSYFMFVHSIQE
>BtORCO
MMKFKQQGLVADLMPNIRLMKATGHFMFNYYTDNSTKTIHRIFAVVHLILMLMQFGFCGINLIFEKEDVDDLT
ANTITMLFFTHSVVKVVYFAVRSKLFYRTLGIWNNPNSHPLFAESNSRYHQVAVRKMRILLLAVLVTTMLSAI
SWTSITFIGDSVKKVIDPITNETTYVEIPRLMLRSWYPYNASHGMAHILTLIFQFYWLVFCMADANLLDVLFC
SWLLFACEQIQHLKNIMKPLMEFSATLDTVVPNSGDLFKAGSAEQPRDHDPLPPTTPTAPGENMLDMDLRGIY
SNRTDFTATFRPTAGMTFNGSVGPNGLTKKQEMLVRSAIKYWVERHKHIVRLVTAIGDAYGVALLLHMLITTI
TLTLLAYQATKINAVDTYAASVIGYLLYSLGQVFMLCIFGNRLIEESSSVMEAAYSCHWYDGSEEAKTFVQIV
CQQCQKAMSISGAKFFTVSLDLFASVLGAMVTYFMVLVQLK
>BtOr1
MKKVISVDMSEKIGNSTNYRNVHYESDAEYTIHVAKTLLKLIGIWPRRDTFLDNVKMYIQTTIVFSLMCFLLV
PHVIYTYFDCENLTKYMKVIAAQIFSLLAVIKFWTVILNRKEIRFCLDQIEIQYRDVKCEEDRLVMTNCAKIG
RFFTMMYLSLSYTGALPYHIILPLISERIVKADNTTQIPLPYLSNYIFFTVENSPIYEITFVLQIFISSIILS
TNCGIYSLIASITMHCCGLFEVTSRRIETLHKWNKCDLHDRVVDIVQYHLEAIGYSALIGKSLSIVFLSEMVG
CTIIICFLEFGVIMELEDHKTLSTLTYFVLMTSIFVNVFIISFIGDRLKQESERIGETSYFLPWYDFPVDVAK
NINTIMLRTSLPSCLSGANILELSLQAFCDVCKTSAAYFNFLRAMTV
>BtOr2
MSEKIKNIIYRNEHYKSDSKYVIHIAKTLLTLIGIWPRKDTFLDNVKMYTQTVTIFILMCFLLIPHVIYTYFD
CEDLTKYMKVIAAQVFSLLAIIKFWTLIFNRREIRFCLNEIEIQYKDVECEEDRMIMLNCAKIGRYFTMVYLA
LGYSGALPYHIILPLISERIVKADNTTQIPLPYLSDYVFFVIEDSPIYEITFVVQMCISCIIMTTNYGIYSLI
ASITMHSCCLFEVTNRRIEKLRNWDKRDLHDRIADIVQCHLKAIEYSAVIGKSLSFVFLSEMIGCTIVICFLE
FGVIMEWEDHKTFSTITYFVLMTSMFVNVFIISFIGDRLKQESERIGETSYFLPWYDFPLNEAKNVRTIILRT
RLPSSLSGAKILDLSLQAFCDVVKTSAAYFNVLRAMAA
>BtOr3
MMKIKEAKDKKGIEKDLKQSLRYVEPFVVALGAWPLPPESSLWMKILQRVIRFIIIFLALFFVSPGFYYVLFK
EKSNKRKLQIVTTYINSFVQLIKYIIILCRMKDIRILSEEIRNDWLHSTEENRRLFRENSKIGERVVFIVAFT
LYSGGFCYRTILPLTRSSIVLPNNITIRILPSPTYFPFINEQITPYYEIIFVLQVLSGFCIYTVFSGAIGITM
MICLHACSLIRILIDKLVDLTDKSNTSEEIIQEKIVNIVGYHRKIKKFLSNGQQLSEYISFLELFNGAGIICL
IGYAVIVEWENLNTITVIVCFTLLTTFTFTSYTICSIGQLLLDESNNLAQTCVTLNWYRLPMKQARYVVLMMI
MSNDPIKLTAVKVMDVSLSTFSDIMKASMGYLNMLRNVT
>BtOr4
MIKTKEESNKEEREKDLKQSLRYVEPLLMVLGAWPLPPESSLCMKIFQRVISVISIFLALFVICPGFFYIFFK
AGGTRRKMELLSAFINSLIQLIKYIIILNSMNDIRTLLREIRNDWLYGTEENRRLFRENAKIGDRVVSIVAIT
MYCGGLCYRTILPLSRGRITLPNNTTIRLLPSSTYLPFINEQITPYYEIIFVLQVLSGLFIYTVFSGAIGIMM
MICLHTCGLIRILTDKLMDLIDKSNTSEEIIQEKIVNIVEYHRKIKKFLSNGQQLSEYISFLELFNGTIIIGL
LGYCVILEWESHNTIGLMVYITLLTTFTFTSYTICSIGQLLLDESNNFARTCVTLEWYRLPVRKTRYMIMIII
MSSDPIKLTAAKMMDVSLLTFSDIMKGAMGYLNMLRKVN
>BtOr5

MSRLGNKEENLKHFVKFIYPPMKLIGAWPKPTATSTFSKAIKWGLIVSAYFLQLLVFVPGVLYLFLKEKNGKK
RIHIMIPHINGFSQLCKYTILLRRTSEFSKILDQLSDDWVDATEDSRHIFRMRANIGHRMVLTVAITMYTTGL
FYRIIIPLSTGRIVLPNNTTMRMLPCPVYFVFFNEQSTPYYEIIFVLQIMGGFLNYTILCSTMGVCLMLCLHL
SSLLRILMNKMIELTSQLDTSETAVQKKISDIVAYQTKVKEFANSVEEITPYLYFFEIFNYAIEACIVGYCII
VEWEESNAASIIVYLMFQGICIFCNYAMCYIGQLLINESENVRRMSITLNWYRFPMKKARSLILIIIMSNYPI
KLTAGKIVDISLATFTDIIKASVGYLNVLRKVT
>BtOr6
MSRIRNAEEELKHSLRLLYTLLRIVGAWPHFVSTSVFSTISKWCLISICYFLQLLLLIPSLIYIFGKETNSRK
RLKLLVPHINGLVQLTKYTFLLYRMHDFKELMETMKNDWLNATEENRKIFRENTEIGYKMLLGLAILMYSTSF
CNRIIVPLWKGRIILPDNTTIRLLSYQSHFTFFDVHRTPNHEIIFTLQILGAFVIYTILCGSLGLISLLCVHM
SSLLRILANMMIELSDQPDTSENAVYKMIKDIVEYRTKVKKFSDKVEHIMSYISCLEIFNGTCIACLVGYCII
MEWENSDTVAVIIYIMFQTICMFIVFTMCYIGQLLINESENVRQISVTLNWHRFSSKEVRCLIPVMIISNYRI
KITAANIIEISLATFTDIMKASIGYLNVLRNSVK
>BtOr7
MSKSSAMKNTSQLTKREEDMKYATRYVKPILGAIGAWPVSFSSSFMSKILLRTEHILTYFLFFLIIVPTIMYV
FFKEKNNKIRLKLMGPIINCTMQFCKYTILLWRTNEVQKGLDVMKQDWITATDENRLIFRSKAKIARRVVLTA
AITMYGGGLCYRTILPLLKGPIITPDNITIRPLPCPSYFVILDEQKSPNYEILFLVQILAGFVIYAVISGSCG
LSALFVLHACSMLRILVDKMKALVDMRDMSDTMVQRRIMDIVEYQTKIKRFLKNIETITEYICLTEMMGGTCL
VCLVGYYILMEWENNNIAAVLIYVTLQISCTFCVFILCYIGQLLIDENQIVGQASCMINWYHLSTKHMRSLIL
IIAMSNYPMKLMAGKMIEMSLATFTGVMKLSMGYLNILREVI
>BtOr8
MENISALAKAEEDMKYATRFVKPILGIVGAWPVSSSASSFSKILTRIEHILTYFLFLLLMVPTLMHVFLKETN
NKIRLKLMGPIINCSMQFCAYTILLCRASEIQNGLNVIKQDWVTATEENRLIFRSKAKIARRVVLTVAITMYG
GGLCYRTVLPLLKGTIVTPGNITIRPLPCPSYFVILDEQKSPNYEILFVLQVMAGFVIYAVISGSCGLSALFV
LHVCSMLSILVDKMKALVDMRDMSDTMVQRRIMDIVEHQTKIKGFLKNIETITQYVCLSEMICGTSLVCLVGY
YIVMEWENNNATAVLIYSTIQISCTFSVFILCYIGQLLVDENRIVGQASYMINWYYLSRKHMRSLILIIAMSN
YPMKLMAGKMIEMSLATFTDVMKMSMGYLNILRNVI
>BtOr9
MKRGNIVETSNNASYKSDLRFNVRLNVLTLRTIGTWPRFLDDSWWETIEHVFLNLLCYGLLGFILIPGFMCLA
LEIVGFYNQMRLGSALSFFLMAVMKYCVFIMREDDIRRCVKLIEDDWRNVKHNEDRKIMLKNASFSQRLIIIC
SVFMYGGVVFYYIALPLTRAKIVEEGGNLTYMRLVYPFPRTIVDARRSPINEIFYTIQLLSGFVAHDITVAAC
GLAALLAMHACGQLQVLMSWLEKLVDGRENDDESLDQRLANIVEQHVRIINFIALTEDLLREISLVEVVGCTM
NICFLGYHSMMEWDFNHPVTGFTYIILLISVTFNIFIFCYIGEVLAEQTVKVGQKSYMIDWHRMPWKKSLVVP
LMISMSRSTTKITAGNIIELSISSFGNVIKTSFAYLNMLRTFAI
>BtOr10
MQTSDIKNTKFRITDYEYKRNVNLSIQWNLWLLKSIGLWPYSNSISRIRRYFYWFINIACYSLISFLFIPCVL
YIVLDIEDTYGKLKQFGPLIFCAMAFAKYYSLIVHKADIRECLERIKWDWKNMTNREDREIMTVNASFGRKLV
VVCTLFMYSGFVFYYIAIPISVGKIAAENESLTFIPLVFPFSRFIVDTRYSPTNEIVFSIQLVAGCLMHGITS
AACSLAAAFAVHACGQMQVLMNWLKHLVDGRSDMSERVDGRIADIVCQHVRILKFLTLIENTIQQISFAEFLG
CTLDICLVGYYVIMELKSNDVTSALTYMILLISITFNIFIFCYIGEIVTEECRKIGETSYMIEWYRLQGNKKL
CCVLIIAMSNCTIKLTAGNIVNLTINTFADVVKTAVTFLNVLRKTT
>BtOr11
MSSTKVDQDYKKGVNLSIRSSRWILKLIGVWPNSRNVSSVKKYFHVLLNAIYYALIMFLLLPGSLYVILEVED
VYDRIKLFGPLSFCVMALLKYYLLILHEEDIRECVERIEWDWQNITYPKDRELMMTNANFGRKLVIACTFFMY
SGFIFFYIAVPMSVERIPVEGTNATFIPMVFPFSRFIIDTRYSPTNEIVFSIQFLAGALMHGITSAACSLAAI
FAVHACGQMQVLMTWLNHLIDGRLDMHDCVDQRIAKIVSQHVRILKFLSLIERALQQVSYVEFLGCTLDICLL
GYYIIMEWNSNHLTDVMTYSVLLVSLTFNIFIFCYIGELVADKSRKVGEMTYMIEWYRLYGKKKLCCVLIIAM
SDSSRKLTAGNIVELSMSTFSDVVKTSVTFLNVLRTLT
>BtOr12
MQKSVTNDASYLTTDHDCKRNVDLSIRYSRWILKPIGVWPTSPNILMAEKYFYRFINTVCYSLICFLLVPCSL
YLILEVKDVYNIIKLFGPLSFCVMAFLKYYLLILHEDDIRECIKRIEWDWKNITYSEDREIMITNANFGRRLV
IICAFFMYSGFAFYYIAIPISVGKISAGDVNVTFIPLVFPFSRFIMDTRYSPINEIVFSLQLVSGCLMHSITS
AACSLAAIFAVHACGQMQVLTSWLKHLIDGRSDMYNNVDSRIAIVVSQHVRILNFLALTEKTLQQVSFVEFIG
CMLNICLLGYYVIMEWSSSHLTSAITFFILLISLTFNIFIFCYIGEVVAEQCKKIGETSYMVDWYRISGTRKL
CFVLIIAMANSSIKLTAGNMVVLCLTTFSDIVKTAVAFLNVLRTLT
>BtOr13
MNKKKPVMTEHDYKRNVDLSIQWNRWLLVPIGTWPNLRKSILGKYFSSLINVICFGLIGFMLVSCTLYLVIEV
EEAYHKIEMIGPLSFFLMTFMKYYLLILQENHISEGCERIEWDWKNIEHHEDRNIMAQNANYGRRLVAICTFF

LFSGFAFFYVVVPASVGQVVTEDGNLSFVPLPFPASRLIADVRSSPSNEIIFSIQTLTGVVMHTVTSAACSIA
AVFAVHACGQMQVLMNWLEHLVDGRSDMSNIVDERIASIVTQHNRILRFLALTESALQQISFVEFLGCMMNMC
LLGYYFIVEWSSNDIMHSITHMILMISFSFNIFIFCYIGELVAEQCKKVGEMTYMIDWYRLTGKKKLGCILII
AMSNSSIKFTAGNMIELSISTFSDVVKTAVAFLNMLRALM
>BtOr14
MQKQKSVVTEHDYEKDVGSSIQWNRWLLIPIGAWPNFRKSMIGKYFSSLINILCFGLIAFMFVSCGLYALIEI
EELYHKIELLGPLIFFLMTFIKYYLLILHENDIREGCKRIEWDWKNIEHQEDRNIMTVNANYGRRLAAICTFF
LFSAFAFHYIIVPISMGKVVDESGNLSFASLPFPVPGFIADVRYSPCNEIFYFIQTLTGAVMHTVTSAACSIA
AVFAVHACGQMKVMMNWLEYLVDGRPDMSNTVNGRIAKIVIQHNRILKFLALTENALQQISFVEFLGCMLIMC
LLGYYLIMEWNSKDIMLSITHMALMTSFTFNIFIFCYIGELVAEQCKKVGEMTYMIDWYRLTGKKKLGCILII
AMSNSSIKFTAGNMIELSISTFSDVVKTAMAFLNMLRALT
>BtOr15
MLDLTSEPRISNVIYARDFEYSIQVNRWLMQPIGAWPKLAKTTRTQRLFVRLLNFICHSLIIFMIVPCILYIA
YEAESSKTRMKAIGPVSHWLMGELNYCCLLSRINDIVRCIKHVERDWQMVENESDRELMLKNAKVGRFIALVA
ALCMHSGVLAYTVTNGFKKMVFHLGNDSYSMYPLPYPFYTNLLDVRFSPANEIVLTLQLLSGFVVTSVTVGAC
GLAAVLTMHASGQLNVVMARLDNLVNTKEEEKQEEQKAAQRKLGIIVEHHLRTLSLMASIEKVMNMICLVELV
GCTMNMCMIKYYLLTEKSKDVIIAYAILYASMVFNIFIFCYIGEIITEQGERVGKKVYMTEWYRLPPKTALGL
VLVISRSSMVVKITAGKFVQISIATFGAVFKTSFAYLNMIRTMM
>BtOr16
MNESMIIRTDAKSNSDYSLQLNRWFLKPIGAWPYFSTTSTLEKVISVSLIIFCYIVILFSIIPCVAHLIFEDD
SFYRKVRVFGPLGHWFIGGINYTNLLFRSKNISDCVEHIETDWQIVTKEKQQQVMLKHAKFGRYVSAICAIFV
HSGIMSYCIVSASSTQIIKVGNETRTMRSLPLGVYNRMIPVDTSPANEIVLVMQFLSAFIADSSGIGFYTLAS
VLAAHACGQLSVLTICISDYVNEAGNRKEDTSFRKIGTIVEHHLRTLDFIARIEEVMSWVCMTELFRCVLAIC
MVGYYIVTEWSDHDVRSLTSYFIIFASVTFNTFLLCYIGELLTEQCMKVGEIVYMTNWYILPRKRILELILII
ARSSVVIEITAGKLIHMSIHTFGDVMRLAFAYLNILCQMT
>BtOr17
MTNQSVTIGANAKANSDYGLQLNRWFLKPIGVWPSSPSTTRFEKIISITLNVICYTSIILTTIPCLVRMFLED
ESIYLKVKSLGPVSHWIVSGANYTTLLLRSNDIRQCMEHMEADWQTVTREKDQWVMLKNAKFGRYVAASCAIF
MQGGIMCFIFVTAMDTVEIQIGNETRILHVLPCAVYKKLINVDESPTNEIVLFLQAWSTIIANSSTVGIFSLA
AVLAAHACGQLNVVMVWITEFVNEPKKANGPKGIGVIVERHLRTLNFIGYIENLMNRIYFLEIFRCTMDICIL
GYYILSEWDDQNVQSLVTYVMIYISIGFNVFLICYIGEILTEQSKKVGEVVYMTNWYYLPDKTILDLILIIAR
SSVVVQITAGKMVLMSVYTFGDVVKTGFAYLNLLRQIT
>BtOr19
MSESAIIKANAKSHSDYSLQINRWILKPIGAWPYFSTTSTRERVISLFLIVLCYVVILFNLIPCVAYLIFVGD
SFYRKVKVFGPLTHSFLGGVNYTNLLFRSRNISDCVERIETDWRMVKKEDEQQVMLKHAKFGRYVSTMCAIFV
HSGIMSYCIASASNVQIIKVGNETRTIRTLPFDVYNKMIPVDTSPANEIVLVVQFLSAFIADSSGIAFYTLAS
VLAAHACGQLGVLTIWINDYVNEARNRKQDTSFRKIETIVKHHLRILDFIARIEEIMSWVCMTELFRCVLAIC
MVGYYIVMEWSDHDVRSLTSYFVICASLTFNTFVLCYIGEVLIEQCMKVGEIVYMTNWYFLPRKRILELILII
ARSSVVIEITAGKLIHMSIHTFADVMRLAFAYLNILCQMT
>BtOr20
MTNEPVITEGDSNSNSDYSLQLNRWFLKPIGAWPSSPSTTRLEKIVSFILNIICFTSVIITAIPSLLLMILED
ESIYMKLKTLGPVSHWFVSSANYTALLMRGRDIRHCVEHIEADWRTVTREEDQHVMMKNAKFGRYVAASCAVF
MQGGVLCFCFVTALTTTEIQIGNETRIIHVLPCAVYKKLVNVDESPANEIMLFLQIWAALIANSSTVGIFSLA
AVLAAHACGQLDVIMMWITKFVKEAKQRKKTSSFREIGVIVERHLRTLNFISCIEDVMNRIYFLEMFRCTMDI
CVIGYYILSEWADHDIQNLSTYFMMLISICFNIFVICYIGEILTEQCQKVGEVVYMTNWYYLPDKIILDLILI
IARSSVVVHITAGKLVHMSVYTFGDVVKTGFAYLNLLRQMT
>BtOr21
MSNEAVIIEADSNSGYCLQLNRWFLKPIGAWPSSPSTTRLEKIVSFILNIICFASVIVTAIPSVLLLILEDES
INLKLKTLDFLSHLFVSSFNYSTLLLHSKDIRQCVEHIEADWRSVTRVEDQHAMMKNAKFGRYVAASCAIFMQ
GGILCFCFVTALTTVEIQIGNETRVLRLLPCAVYKKLVNVDESPANEIMLSLQIWSALIANSSTVGIFSLAAV
LAAHACGQLDVIMAWITEFVKETSSFQEIGVIVERHLRTLNFISCIEVVMNKIYLLEMLRCTMNICLIAYYIL
SEWDEHDIRNLTSYFMMFVSICFNIFVICYIGEILTEQSKKIGDAVYLTNWYYLPNKEIHNLILIIVRSSMVV
QLTAGKMIQMTINTFGNVVKTGFAYLNLLQQMM
>BtOr22
MPNEAAVIEADSNSNSDYSLQLNRWFLKPIGAWPSSPSTTKLEKILSFLLNIICFGTVTVTVIPSLLLIILED
ESINFKLKALGFVSHWIVSSLNYTALLLHSKDIRQCIEHMETDWRTLIREEDQHVMLKNAKFGRYVAAFCAIF
MQGSVLCFCFVTALNTFEIQIGNETRVLHVLPCAVYKKLVNVDESSRNQIMLFLQVLSAIIANSSTIGIFSLA
AVLAAHACGQLNVVMLWINEFVNEARGEKTSSIQIGAIVERHLRTLNFISYIENVMNKICLLEMLRCTMDICV

TGYYILSEWTEHDIQNLSSYFMMLVTICYNIFVICYIGEILSEQCKKIGEVVYMTNWYYLPGKTILDLIMVIA
RSNVVVQITAGKLVHMSVYTFGSVLKTGFAYLNLLQQMI
>BtOr23
MSNEAVIIEADRNSDYCLQLNRWFLKPIGAWPSSPSTTRLEKIISFLLNTICYSTVIITTIPSVLQLILEDES
INLKLKSIDFVSHLIVSSFTYSVLLLHNKDIRRCVEHMKTDWRAVTRKEDQQVMMKNAKFGRYVAAFCAIFVQ
GSVLCFCFVTALNTLEVQIGNETRILHVLPCAVYKKLVNVDESPTNEFMIFLQIWSTFIANCSTVGIFSLAAV
LAAHACGQLNVVMLWIVEFVNEAKVERRADGFMEIGIIVERHLRTLNFISYIEGVMNKICFLEMLRCTMDICV
IGYFIVSEWAEHDVRNLTSYSMMFVAICYNIFILCYIGELLTEQCKKIGEVVYMTNWYYLPGKTILDLIMVIA
RSNVVVQITAGKLVHMSVYTFGSVVKTGFAYLNLLQQMT
>BtOr24FIX
MTTEVVIIEGDSKRNSDYSLQLNRWFLKPIGAWPSSPTTTRLEKIVSFTLNIVCFSTLILTAIPSLLVIILED
QTFNFKLKTFGFVSHWFVSSFNYAVLLMHNKDIRKCVSYIEADWQTVTREEDQHVMLKNAKIGRYIAAFTAIF
VQSSVLCFCFVTALNTVEIRIANETRILHVLPCAVYKKLVNVDEIPTNELMLFFQIWSTVIANFSTIGIFSLA
AVLTAHACGQLNVITLWIVEFVNETRVEKKTGGFIQIGVIVERHLRTLNFISYIEDVMNKICLLEMLRCTMDI
CVIGYYILSEWDEHDIRNLASYFMMFVTICFNIFIICYIGELLTEQCKKIGEVVYMTNWYYLPSKTILDLIMV
IARSNVVIHITAGKLVHMSVYTFGSVIKTGFAYLNLLQQMM
>BtOr25
MVDMSSEPPISNAFYAHDYEYSIQVNRWLMQPIGAWPKLTKTNRTQRLLTKLLNFICHSLIIFTIVPCILYIV
YEAESSKTRMKIIGPVSHWLMGELNYCCLLSKTDDIIRCIKHVERDWQVVENASSREMMLKYAKVGRFIAFIA
AFCMHSGVLAFNVTKGFKKMMFLVGNDSYFMYPLPCPIYTNLLDARFSPANEIVFVLQILSGLIVTSVTVGAC
GLAAVLTMHASGQLNMVVARLDNLVDTKIEEKQEAQTVAQKKLGIIVEHHLRTLSLIASIEKVMNMICLVELV
GCTINMCMIKYYFLTEKSKDMRIVYAIVYASMVFNIFIFCYIGEIVIEQGERVGKKVYMTEWYRLPHKTALGL
VLVISRSSMVVKITAGKFVQISITTFGIVFKTSFAYLNMIRTML
>BtOr26
MMNETVIVESDSKSNSDYSLQLNRWFLQSIGAWPSSPSTTRLEKIVSFILNIVCLSIVILTVIPSLLLMILGD
ESFNFKLKMFGYVSHWIFSGINYTALLTQGKNIRQCIEHIEADWRTVTREEDKNVMLKNAKLGRYIAGFSVIF
VQGSVLCFFFVTALNTVEIQIGNETRVLHLLPCAVYKKLVNVDDSLTNEIMLLLQMWSTIIANSSTSGIFGLS
AVLAAHSCGQLNVIMVWITEFVNKARERKKTGSFIEIGMIVERHLRTLNFISCIEDVMFKIYFVEMFRSTMDI
CVIGYYILSEWADHDFQNLITYFMMLISICFDIFVICYISEILTEQCQKVGEVVYMTNWYYLPNKVILDLILI
IARSSIVVQLTAGKFIQMSVYTFGNVLKTGFAYLNMLQQMTR
>BtOr27
MTNKTVAIKIDFDGRSDYSLQLNRWFLKPIGAWPSFPSTSRLERIISFVLIVICYIILICTIIPCLLHIILEN
ESFRIKLRLIGPASYLCVGSINYTTLLLRGKDIRCCVEHMQADWRTVKREKDQQVMLKSAKFGRYVTASTAAF
MQGGVFCYCFMTALSTEVIQVGNETRIVHQLPYVTYKELIDINESPTNEIILFMQFLTGFIVSSSTLGILSIT
VVLIAHACGQLNVVMTWITEFVNESRKEKIAPFENIGIIVERHLRTLSFVSSIEETVNRIFFLEVLRSTLHMC
MLSYYIVTEWSDSDIQILTTYSMLLASICFNIFVICYIGETLTEQSRKVGDVVYMANWYYLTEKRILELILII
MRSSVVVEITAGKIIHMSIQTFSTVIKTAFTYLNLLRQVT
>BtOr28
MTNKSAVVEKTFDSLSDYSLQFNRWLLISIGAWPASTSTSRRERIISFILIALCYGFILFTVIPCIFHFILED
ESIYMKLKVLGPLSHWFVGGINYTNLLLRSNDIRDCIHHVQTDWKIVTRPEDSQVMMKYARIGRFVAAFCATF
MQGGVLTYCVVTAFATQTIEIANETRIIHMLPCAAYKNLIPVDTSPMNEIVLATQFISGFIVNSSAVAAITIG
AVFTAHAYGQFTVLMRWINEFVNRSEDQKKDVEFNEIGEIVEHHLRILSLIAGIENVLTQFCFMELLKSKLDI
SMLGYYIITEWAEHDIRNLTTYFMIMASMSFNIFTVCYIGDILTEQSKKFGDVVYMTKWYYLPNKDMFDLILI
ISRSNSAIKITAGKITHMSINTFGDVMKTAFAYLNLLRQVT
>BtOr29
MTESVRIEKNLYSLSDYSLQLNRWFLTPIGVWPLSASTSRLERIISFVLIFLCYFFVLFTIIPCLLHLILEDE
NTRTKLKIIGPFSHWLIGGINYSTLLLRSREMRVCIEHVQNDWKIVTRQKDQQVMMRYARIGRYTTVFCATFM
QVGVLSHCTMAAFATKIIEIGNETKIIRLLPCGFYKSLISIDVSPTYEIILASQFVSGFIANSSAVGAVGMAA
VFAAHAYGQLTILMIWIKEFVNRSKDYSRNVGLNEIGEIVEHHLRVLSFIAGIEDVMNEICFMELFKCTMNMC
MIGYYILMEWRDHDIQYMPAYLIIILFSMTFNIFVICYIGETLKEQCEKVGEVAYMTNWYYLPYKDILNLIQII
LRSSMMIKITAGKLVHMSIFTFGNVIKTACTYFNLLRQVT
>BtOr30FIX
MVVLLNPSTHSWDKDWMYSIQINRWLLKSIGVWPISLCITPTEKSNSIILTLISSFLISFLLVPCALCTLLVK
TDDPEAKIKMVGVLSFCVMAAIKYYILASRGVMIGKCIENIRSDWDRIHAQLRQEDREIMKENAKIGRSLAIF
CAGFMYSGGFFYTTVMPLCTERTEIIDNETVRTQAFPIYRGLLDPRTSPSFEIVQFMQCLAGFVIYSVTVGAC
SLAAVFVMHVCGQFGILVKKLQRLVGSLEEEKNLNVHEQRLGDIVEHHLHILGFISQIEDLLNEICFVELIGC
TVNICFLAYHLLTEWEQNDTIGTLTYCTLLISFTFNIFILCYIGEILSEQCRNISISAYMIDWYRLPQKKALG
LILIFAVANSSTKLTAGKIVELSLASFCSVLKSAFAYLSLLRTLTI

>BtOr32
MTDDPVTVERKFGSLSEYSIQVNRWILKPIGAWPSSNSTTNREKFISWILNVICWCFSLFTIIPGVLNILLEK
EDLYLKLKMFGPLSHWCVGGFNYAVLLLRQGDIHYCIKHIRTDWKTITRLEDQRVMLKNAKLGRYIACFCAAF
MHGSVFCTCLVLGAFKRTIEVRNETILVYTLPCPAYKLPVQTNPGHDIILGTQFLSAFIATSSAAGAFSLATV
FASHALGQLNIMVTWINEFVNQHSKDQNNNARANKIGVIVEHHLRALSLIARIERIMSPICFMEMFKCMLGMC
MPSYYILVEWSEHNIQNITAYVLIIISMTCNIFLVCYIGEIIMEKCKKIGDMVYMADWYHLPDQDIINLIMII
SRSSMEVKITAGKIIDMSVLTFANIVKTVFGYLNMLCQTTMT
>BtOr33
MADELVAAKKKYGNLGEYSIQLSRWYLQPMGAWPNSPSTTRREKILAQISIVICWCIVLFTIVPGFLHIILVK
EDIYLKLKTLGPLSHWCVDGFNYAVLLLRQEDINYCIERVRSDWKMITRVQDRHEMWKNAKLGRYVAGFCAGF
MQGTMIYTCIVLGAFRRTIKVGNETMDIYTLPCPAYKFAVQTNPTHDIILGTQFLSALVVSSGAAGSFSLATV
FASHALGQLNIMVTWVDEFTNQTKQQNKQAQINKIGVIVEHHLRVLSLIARIERIMSPICFMEMFKCMLGMCM
PCYYILAEWSEHNVQAMIIYVMVFLSMTFNIFLICYIGEVLKEQCQKVGDMVYMTNWYQLPDKNILSIIMIIS
RSHMEVKITAGKIITMSVYTFGNIIKTVFTYFNMLRQTTMI
>BtOr34
MRSSNDVNDHPQNNHYKSDIYHTFQFCHWILKPLGIYFFLHDHVNKFEKIVSVLLIIICCSILQFVIVPFGYY
ILFYEKDMNMKIKFLGPLAFCVTALFKYGYLGVKSSELGRCVKHVKKNWKMLQDKDHRAIMVRYVTMGRNLIT
LCAAFMYTGGLSYHTIMPLLSKKKINENITIRPLTYPGYEAFFDIQESPTYEIVYCMHCVYVLVTGNITMAAY
SLTAIFTTHACGQIKIQTLRLENLKKDGKALENGIEDHLAVIVSEHVEILRFTKNIEIALQGLFLIEVMLSTL
LICLLEYYCMMEWETSDSAAISTYIILLTSFTFNILIYCYVGELLLGQGSEIATALYDIGWYNLPGRKARDIV
LVLAISKYPLKLTAGKILVLSMNTFGVVLKSSLVYLNMLRTVTEF
>BtOr35
MHDRSHATVDDQLRNVHYENDIHYTLQMCQWLLKPIGVWPLVNNHTSRLEQLVSVILMIMCFSSLLFIILPSF
YHIFFVEKSVHVKVKLLGPVGFCLSSTIKYCYLGVKGAFFERCIQHVEKDWKMVEDPNHRTIMLKYATVSRKL
ITLCAIFLYTGGMSYHTVMQFLSKERNKKNYTFRPLTYPGYDSFLDTQSSPTYEVVFFLQCFAAMIMYSVTTV
AYSLAAIFVTHICGQIQVQIARLQDLVENEKRKNNGHDSMSVIVHDHVEVLRFSKNVEEALREICLAEIVEST
IIMCLLEYYCMMEWQNSDAIAILTYVTLLISFTFNIFIFCYIGEILTEQCSQIGTTSYEIEWYQLPAKRAYNL
ILLISISQYPPKLTAGKIIDLSLNTFSSVAKSSLIYLNLLRTVTDW
>BtOr36
MYDRSYTIVDDQLKNNHYKNDIHYTLQMCQWLLKLIGIWPLVNNHTSRLEQLLSIVLMITCYSSIFFIILPSG
HHFFFVEKNLYMKMKMLGPVSFCVFATVKYSYLARKGTFLQRCIRQVKNDWKRVQDPYHRQIMLKYAGVSRKL
ITMCAVFIYTGGMSYHTVAQFLSKDNTKENSTVKPLAYIGYDPFFDTQSSPTYEIVFFLHCFAAMIMYSITTV
AYGLAAVFVTHVCGQIQIQIARLQNLVGSKDRDLFTVIVHDHAETLRFSKNIEDALYQICLTEIVECTICMCI
LEYYCLMEWANSDLIATLTYLTLLTSFTFNIFIFCYIGELLTEQCSQIGTTSYEIEWYQLPAKRAYDLILLIS
ISQYPPKLTAGKIIELSLNTFSSVAKTSLVYLNLLQTVTD
>BtOr37FIX
MYDRSYTIVDDQLKNNHYQNDIHYTLQMCQWLLKLIGMWPLVNNHTSRLEHLLSIIVMIMCFCSIFFIILPCG
HHFFFVEKNIYMKMKMLGPVSFCVFATVKYSYLALKGAFLQRCIRQLKNDWKRVQDPSHRAIMLKYAGISRKL
ITMCAVFIYTGGMSYHTVAQFLSKERTRENYTVRPLAYIGYDPFFDAQISPTYEIVFFLHCFAAMIMYSITTV
AYGLAAVFVTHVCGQIQIQIVRLQNLVESKDRDLFAVIVRDHVKILRFSKNIEDALYQICLTEIVECTINMCM
LEYYCLVEWANSDLIATLTYMTLLTSFTFNIFIFCYIGELLSEQCSEIGTISYEIDWYNLPAKEAYDLILLIS
ISQYPPKLTAGKIIELSLNTFSSVAKTSLVYLNLLQTVADW
>BtOr39
MHLSVRQPQNPNYEEDIVYVTKHNKWILSTIGMWPTVVKGIGKFVPKIIIGLSNFVSSLNVLQFILHIILEEK
NTTLKVRFLGLICFASTNLMKYWALIARKSNIEYCIEQVQIDWKQVDFQRNRMLMLKYGKIGRDLTIYSAVFM
YGSEMLYITIMQYALGSMLKENNRTTRVLVYPTYSGLLDVQKSPIYEIVYVLQCMCTLLFNSVTVSCCGLAAL
FATHACGQIDIIMSQLDDLVDGKFAKKNSNPDTRLTEIVKHHIKILKFSTMIETVLQEVCFFEFVGTTLVVCF
LEYYCLTDWQSNNKIGVATYSMLLVSLTFNMFLLCYIGNLLLEKSSDIGISCYLIDWYRLPPKTVQDLMLIIA
MSNTPVKISAGRIFLLSLPTFGNILKTSFAYLNFVRNATM
>BtOr40
MNMHLSVRYQTDQPLNPKYEEDIIYVTKHNKWVLNSIGMWPAVLEGIGKFVPKIVIGLSNFVSFFSVVQCVLH
IILEEKDPLLRLRLLGLACYSSTNLMKYWALILRKPNIEYCIEQIQTDWKQVEFPRNRLLMLKYGKIGRDLTI
CSAAFMYTGLVCYVTIMQYAMGLNMKANNRTIRVLVYPTYSGFFDAQKSPIYEIVYVLQCMCTFVFNSVTVGC
CALAALFATHACGQLDVVISQLNDLADGKFSGKNSNPSTRLIEIVEHHIKILKFSAMIESVLQEVCFFEFVGS
TFVICLLEYYCITDWQQNNKIGLATYSMLLVSLTFNMFLLCYIGNLLLEKSTNIGISCYMIDWYRLPVKTVQD
LMLIIAMSNSPVKISGGRMFLLSLPTFGNILKTSFAYLNFIRNTLM
>BtOr41

MHLSVRQPRNPNYEEDIVYVTKHNKWVLNSIGMWPAMLKGIGKFVPKIVIGLSNLVSVFNVLQFVLYVILEEK
DTSLKLRFLGLICFASTNLMKYWALIARRPNIEYCIKQVQTDWKQVEFQRNRTLMLKYGKIGRDLTIYSAVFM
YSSEMCYITIMQYAMASVLRENNRTTRVLVYPTFSGFFDAQKSPVYEIVYVLQCMCTLLFNSVTVACCGLAAL
FATHACGQIDVVISQLNDLVDGKFANKNSKPDTRLIEIVENHIRILKFSTMIETVLQEACFFEFVGSTLVICL
LEYYCITEWQDNNKIGVATFSMLLVSLTFNMFLLCYIGNLLIEKSTNIGISCYLIDWYRLPPKTVQDLMLIIA
MSNNPVKISAGRIFLLSLPTFGNILKTSFAYLNFVRNATL
>BtOr42
MHLSVRHQTDPPQNTNYEEDIVYVTKHNKWVLNSIGMWPAVLKGIGKFVPKVVIGLSNIVSFLNVVQCVLYIT
LEENDPLLRLRLLGLACFASINLMKYWALIARKPNIEYCIEQVHTDWKQVEIQRNRILMLKYGKMGRDLTIYS
AVFMYSAEICYVTVMQYAMGLNMKENNRTIRLLVYPTYSGFFDAQKSPVYEIVYVLQCMCTFLFNSVTVGCCG
LAALFATHACGQIDIVISQLDDLVEGKFSEKNSNPNTRLMEIVKHHIRILKFSAMIETVLQEVCFFEFVGSTF
VICLLEYYCITDWQQNDRIGLATYSMLLVSLTFNMFLLCYIGNLLIDKSTSVGISCYMIDWYRLPIKTVQDLI
LIITMSNSPAKISAARIFILSLPTFGNVLKTSFAYLNFIRNTIIY
>BtOr43
MHLPVRRQTVPPQNTNYEEDIIYVTKHNKWVLSSIGMWPAVVEGIDKFLPKIVIGFSNLVSLFTVVQCILHII
LEEKDALLRLRLLGLACFASINLLKYWAVIVRKPNIEYCIKQVQTDWKQVKFQKNRMLMLRYGKIGRDLTIYS
AMFMYSAGMCYITIMQYAMAMSLKANNRTIRVLVYPTYSGFFDAQKSPIYEIVYVLQCMCTFVFNSVTVGCCG
LAALFATHACGQIDVVISQLNDLVEGKFAKKNSDPNTRLMEIVKHHIRILKFSAVIETVLQEVCFFEFVGSTF
VICLLEYYCITDWQQNNKIGLATYSMLLVSLTFNMFLLCYIGNLLIEKSTSVGISCYMIDWYRLPVKTVQDLI
LIIAMSNSPAKISAGRIFLLSLPTFGNVLKTSFAYLNFVRNTIMY
>BtOr44
MHLSVRRQTDQLQNPNYKEDIIYVTKHNKWILNCIGIWPTVLKGIGKFLPKVVIGFSNLVPFFTIVQCVLYIT
LEEKNPLLRLRFCSLAWYSSINLMKYWALIARKSDIEYCIKWVQTDWKQVKFQKNRMLMLKYGKIGRDLTIYS
AVIMYSAGMCYTTIMQYAMRMSLKENNRTIRILVYPTYSGLFDTQRSPVYEIVYVFQCVYAFMCLSVTVGCCG
LAALFATHACGQIDVVISQLDDLVDGTFSKKSSNPNTRLMEIVKHHIRILKFSAMIETVLQEVCFFDFIGTTL
LICSLQYLCITDLQYNNKIGLATYSMLLIGFTVNMALLCYIGNLLMDKSTSVGISCYMIEWYRLPGKTMQDLI
LIIAMSNSPAKISAGRIFLLSLPAFGNILKTSFAYLNFVRNTIVI
>BtOr45
MFTSTHLSTRSQTDQLRNPNYKEDIIYVTKHNKWVLNSIGIWPAMVEGIGKFLPKIAIGLSNLILFFTLVQCV
LHIVLEQKDPLLRLKILGLTCFSFISLMKYWALTIRKPKIEYCIEQLYADWKQIEYQRDRKLMLKYGKIGRRL
TVYSAVFMYSGGIIYHTAMQYAIGSYVDEFNRTIKLLVYPTYSGLYDVQKSPVYELVYILQCMCGYVFDTVTV
GACGLAALFATHTCGQIDVIMSRLNDLIDGKFSKENSNTSVRLMEIVEHHIRTLKFSAMVETVLQEVCFLEFI
GTTFVMCLLEYYCITDWQQNNKIGLTTYSLLLISLTFNMFLLCYIGDLLIEKSTNVGISCCMIDWYRLPAKSV
QDLVLIIAMSSNPAKISAGRIVNLSLSTFASVLKTSFAYLNFLRTALV
>BtOr46
MHLSMKHKCDYTPRNLYYKKDIAYVTKHSKWILQSIGIWPAVVGDVKKFLTKISIALSNFVLLFAIIPCILHI
IFEEKDTIMRLKLSGLLSFCCTSLMKYWALTVRKPRIKGCIEQVWIDWEQVELHKDREIMLKYGRVGRNLTII
CAVFMYTGGTIYHSILQYAIGTFVDEHNRTIKPLVYPTYSALYDVQSSPIYDLVYVIHCMCGYVMYSITAGAC
GLAALFATHTCGQIDIVISRLNDLVRGEYMKETLNLNARLIEIVERHLRILRFSAAVEMVLQEVCFLEFIGST
CMICLLEYYCITDWEQSNTISLTTYTMLLISLTFNIFILCYIGELLIEKSSSVGTSCFMIDWFHLPTKTIQGL
ILVIAMSNNPAKISAGKIADLSLSTFGSVLKSSLAYLSFLRTAVM
>BtOr47
MKVPSDKDFTYAMTPLKMLSWPVGTWPLQEYDIFSGIRAIIAISFLLLMLMVMQMELYLDISDAKKNLDALIL
INCSILSLSKIIRFRIQPDSLILNFVSAVKDYNELKDQEKRVIMRRHAYMGRLVSASMIFSSYIGSTLYMTIP
MMAGDKEKDIVNVTKESTTDYPIPSECVIALIQLPDNLYFMVFIIEYLMLLFTSTGNLGSDTLFLGIIFHLCG
QVEILKLEFSKLGNENERTMERFVVLIKRHVYLLNLAKMLNETISTILAVQLFSSCVLICITGFQLILDLSVG
NIVMTIKEFIILNAMLVQLFAYSYVGEYLKLQMEGVGDSLYFCSWYDIPTSITKDIIYVIMRSQDPVFLKAGR
FFIVNMETYMSIVKTSMSYLSVLRVMVNA
>BtOr48
MKESSDEDFAYAMTPLKIMSWPVGTWPLQVYDIFSAIRAIIAILLLLFMLIIVQMELYLDRSDAKKNLDALLL
ITCGILALSKIIRFRIQPDGLVSNFISVIKDYNELKDQEKRVIMRRHAYMGRLVSASVIFSSCIGSTLYLTIP
MLSGDEEKDIVNVTKESSTDYPMPSEYVIALMQLPDNLFFMVFIIEYLMLLFTSTGNLGSDTLFFGIIFHLCG
QVEILRLEFNRLGNKNERTMERFIVLIKRHIYLLNLAHMLNETISSILVMQLFSSCVLICTTGFQLILALSIG
NIVMMMKGFIVLNAMLVQLFAYSYVGDYLTRQMKGISDSMYFCNWYDISKSMAKDIIYVIMRAQYPVSLKAGN
FFIVNMETYMSILKTSMSYLSVLRVMVNA
>BtOr49
MKESSRKDFAYAMTPMKIVSWPVGTWPLQNYGIFSAMRGIIVISLVLLMLAILQTEMYLDSGDAGKNVDALIL
ITSGVVALSKIIWFRIRPAGLISNFTSAVKDYEELEGQDKRLIVRRHAYIGRVASATVIFSSYIGSTLYMTIP

MLAGDEEKDIANVTEESTTDYPIPSEYVMAVIQLPDNLYFMVFIIEYLMLLFTSTGNLGSDSLLFGIIFHLCG
QVEILKLEFNKLGNENERIMERFIVLIKRHVYLLNLAKMLNETISSVLVVQLFTSCILICTTGFQFILDLSVG
NIVMAMKTFIVMSCLLVQLFAYSYVGEYLKRQMESVGDSVYFCNWYHIPKNVAKGIVFVIMKSQDPVSLKAGK
FFIVNMETYMSILKTSMSYLSVLRVMVNP
>BtOr50
MREKVAIEEYVRNFLVQETVLKVVGIWPTRNESFFGRWIFAMMTQISTIYSLSLEVYRHCLDIDDTMDAFVMD
LSSIVSLAKLFIMRRNSKHAYVLINSVLKDWSAVNDSRHEDIMTEYYKKGRIVSLTILYLGYASGLSFIVKAL
PFGEVLPFKMFQNSANSSANLKGSLEMNYFLASYCLFGPLPLLPQICVLILQAMHIFVNAVAHCANDGLFFSL
TMHLCGQFEVLKMNFTKFELQEFGCHKKLRFLVKRHCQLLMLANDLEQTFNMIILVQLLMSALLICIEGFVFL
VCLATRDNVGALKSVVLMVTLLIQLYLYAYAGDVLESRSNEIAHGVYDSPWYQPRGHVARDLMMIINRGHRSY
HVTAGKFLSMNIFTFKEILRSSASYLSVLKVMMDT
>BtOr51
MKVSTSKDFAYAMTPLKILSWPVGTWPLQDYDVFSGIRAITATFFLLLMIMIVQSEMYLDNSDAEKNLDGLVF
ITCGSLAASKVIQFRIRPAALISNFTSAVKDYNELRDEEKRVIVRKHAYMARVASASMIFFAYFSSILFITVP
MLAEEEEKDIVNVTEESTSEYPIPSENVMALVKIPENLYFIVLIIEYLMLLFTSTGNLGSDSLFFGITFHLCG
QVEILKLDFQRLKIEGERTREHFNVLTKRHIYLIKLANMLNETISSILVMQLFTSCILICTSGLQLILALNIG
NIVMVIKTFIVLSTLMVQLFAYSYVGEYLRRQMEGIADSMYFCNWYDIPKSVAKDIIYVIMRAQEPVFLRAGQ
FLVVNMETYTSIIKTSMSYLSVLRVMVNA
>BtOr52
MKAALNKDFAYAMIPMKIMSWPVGTWPLQDYNIFSAIRVIITSFLLLLMVTIVQSEMYLDSKDAEKNLDALVI
LSCGILALSKIIRFRIRPAALISNFTSAVEDYNELWDEEKRVIVRKHAYMTRVASASMLFFAYFSSIVFITVP
MLADEEEKNVVNATEESTSEYPIPSENVMALIKIPENLYVIIFIIEYLMLLFTSTGNLGSDTLFFGITFHLCG
QVEILKLDFQRLKIEGERTREHFNVLTRRHIYLIKLANMLNETISSILAVQLFTSCILICTSGLQLILALSIG
NIVMVIKTFMVLSALMVQLFAYSYVGEYLRRQMEGIADSMYFCNWYDIPRSVAKDIIYVIMRAQEPVFLRAGQ
FLVVNMETYTSIIKTSMSYLSVLRVMVNG
>BtOr53FIX
MRATLNKDFAYAITPMKIMSWPVGTWPLQDYNILSAMRAIFTIFLVLLMLMIVQLEMYLDSSDAEKNLDGLVL
ITCGILAMSKILQFRIRPAGLISNFTSAVKDYNELNDQEKRVIVRRHAYMGRVAGISVVFFAYFGSTLFMTLP
MLAAEEVEDIVNVTEDNTPEYPIPSEKVMELIKMPDNLYFIVFIVEYLMLLLTSNGNLGSDSLFFGIIFHLCG
QVEILRLDFRRLSNDNERTIEHFIALSKRHVYLLKLAKMLNETISSILAVQLFTSCIVICTSGLQFIIALSVG
NIVMTIKSFIVLSTLLVQLFAYSYVGEYLKRQMEGIGDSAYFSIWYDIPKSVAKDIIYVIMRTQDPVFLKAGK
FFIVNMETYMSIIKTSMSYLSVLRVMVTA
>BtOr54FIX
MKVTLNKDFAYAMTPMKILSWPVGTWPLQDYNIFSAMRVIITSFLLLLMLTIVQSEMYLDSNDAEKNLDALVI
LSCGILAVSKVVRFRIRPAGLISNFTSAVEDYNKLYDQEKGVILRRHAYMGRVAGIGVVLFAYFSATLFMSVP
MLAAEEVKDVVNVTEDNTPEYPIPSEKVMALIKMPDNLYFIVFIVEYLMLLLTSNGNLGSDSLFFGIIFHLCG
QVEVLRLEFSRLSNENEKAKEHFNVLSKRHVYLLNLAKMLDDTISSILAVQLFTSCILICTTGLQFIIALSVG
NIVMVIKTFIVLSTLLVQLFAYSYVGEYLKRQMEGIGDSVYFCSWYDIPKSVAKDIIYVIMRTQDPVYLKAGR
FFIVNMETYMSIMKTSMSYLSVLRVMINA
>BtOr55FIX
MKTTSNKDFAYAMIPFKILSWPVGTWPLQHYDIFSAIRAIITSFLLLLMITIVQSEMYLDSSDAEKNLDAVVI
LTCGYLAVSKVLQFRIHPAGLISNFTSAVKDYNELNDQEKRVIVRRHAYMGRVAGISGVLFAYFSATLFTTLP
MLAAEEMENMANVTEESIPEYPIPSEKVVALVKIPEHLYFIVFIVEYLMLLLTSNGNLGNDSLFFGITFHLCG
QVEILKLDFKRLRNENERTKERFSVLTKRHVYLLNLAKMLDDTISSILAVQLFTSCVLICTSGLQFIIALSVG
NIVMTIKTFLVLSTLLVQLFAYSYVGDYLKRQMEGIGDSIYSCSWYDIPNSVAKDIIYVIMRTQDPVYLKAGR
FFIVNMETYMSIMKTSMSYLSVLRVMIST
>BtOr56
MKASRSKDFAYAMTPLKILSWPVGTWPLQDYDIFSATRAIIAISLLLLMLTILHIEMYLDSSDAEKNLDGLAL
ITCGILAVSKVIRFRIRPGGLISNFTSAVKDYDELKDQEKRVIVRRHAYMARLACGSVISFAYFTSTIMMTLP
MLVEEEDEGIVNVTEESIPSYPIPSEYVMAIIQLPDNLYFIVFIVEYLMLIFLSTGNLGSDSLFFGIIFHLCG
QIEILRLEFDRLNNENEKAMEHFTSLTKRHIYLLKLAKMLSETISSILAMQLFTSCILICTSGLQFIIALKIG
NIVMTIKTFIVSTTLLLQLFAYSYVGEYLKRQMEGVGNSVYFCSWYNIPKCVAKDIIYVIMRGQDPVFLRAGK
FFVVNMETYMSIIKTSMSYLSVLRVMINA
>BtOr57
MKTSMSKDFAYAMTPLKILSWPVGTWPLQDYDVFSAIRATIATFFLLLMVTVVQSEMYLDNSDAEKNLDGLVL
ITCGSLAASKIIQFRIRPAALISNFTSAVEDYNELRDEEKRVIMRKHAYMGRVASASVICFAYFSSILFITVP
MLAEEEEKDIVNVTEESITEYPLPSENVIAVIKMPDNLHFIVFIVEYLMLLFLSTGNIGSDSLFFGIIFHLCG
QVEILRLEFNRLNNENEKAMEHFISLTKRHIYLLKLAKMLSETISSILIVQLFTSCILICTSGLQFIIALSVG

NIVMTIKSFIVSSTLLLQLFAYSYVGEYLKRQMEAVGNSVYFCSWYDIPKCVAKDIIYVIMRTQDPVFLKAGK
FFVVNMETYMSIIKTSMSYLSVLRVMVTT
>BtOr58
MSVLQPAFNILIVCGCWIPPSCRTFYGKLLYAAYTAFVIFLLCSFCISQFLNVILNVRTANELSDSFYMFIAS
ILSCCKIFTLLVNHKAIRILSRKLDEEPCKPVDEQEITIRRRFDKSIGSITIYYTIMVMLTVACMILFSFLTD
FGDRKLAYKAWLPFNYSVSNCYYIAYAHQIIALIGTALLNVACDMLVCGLLVHVCGQQEILKHRVKELKKESR
PDIGKIVRFHDYLYGYVSMIQQKFQEIIGVQLLSSTFVVCFILYELSNAPVNSKYLQFVLYLTCMMTQVFFYC
WYGNQLKLKSVEVANAIFEADWISFDNSSKKSLINVMRRATKPIELTCAYVFTMDLKTFVDILKMSYSTYNLL
QRTKES
>BtOr59
MHLLRLTYRFLTICACWRPPLLSPLKNVAYTVYRYYVILLVYGGSICQFIDLIFIVETLDEFCDNIYLTLTFF
ISCLKMYSILSNRKNIIVITDMLESTPFQPETKEEIEIREKCEKQARSNAFYYALLVESSVVVMSVLTLLGAL
YKGEHHKLAFRMWLPWNHTSTATYSFIYSQQILIHAFNGLLHVACDSLIWTLLMFICNQIEIFGYRLRKIEQG
TNDCKLCIRYHNLIYRFATMINEEFKLMIFVQFAVSTLTICMNLYILTGTNVSLEMIVKIIMFSSCMLTQIYI
LCWYGNEVELKSLEISNMIFEIDWLALKETTKRDLLMIMMRARSPIQMTSVYVVTMNLKSFVILLKTSYSAYN
LLQGM
>BtOr60
MHTLRWTFALFTLTGIIRPSTWKYLWKRVLYDVYTIVVLLLLFSFETSLILDLVINVDNQDDFSENLYVTLVL
FSSCCKALVLLIYRGNIEILMGVLLEKPFAPVNDEEIEIRTKFEERIEWNSKAYSFVLHFFVAWLWIAALLTD
FRHGRLKFRAWIPYDYNSPLLFLFSFIHQILATIFSTNLNIVCDCLFSGILIHIYCQFEILEHRMKNITTDKN
YSAKFCAHHHHRIYKFASMVNDNFKMIMSMQFLISTGAVCFNLYRLSVMEFGPKFMETATYTLCLLMQVFYYC
WYGNEVKLKSLEIPNAVLESNLPFLDDSSKKILLLIMRRALEPIEFTSCHVISMNLESFAILLKTSYSAYNLL
QQSKLND
>BtOr61
MHVLRWTFKLFIASGYFLPPTLKSPTKRFLYNLYTVFVTLYMWSYCLTLIMHICYDVETQDDLSENFGISVTA
VITSCKLINLVIGRKTIIHMLDLLKKKPFVPENNGEVEIHAKYDNLIEKVAMFYTIQVVCCVLALVGTTLVSD
FKLKKLMFPAWFPFDFTSSWSAFSITFIYQLVGLMIIATGISMFDTFFAGLLLQICCQFEMLMNRLHNIEGNE
IQSLKNCVWHHNTIFRFAEIVNKFFNKMMFVQFMVSTVAICFTLYQLTEANDSLQIIGWASFMFSALMQTFYF
CWFGDAAKVKSLDISNTVYNSDWANLSNNARKMLVIIMARSLTPVEITSAYILPLNLESFKGLMKTTYSAYNM
LVQNKSSR
>BtOr62FIX
MTVRTLQVSRVLLSIAGCLPPSSWTSPFTKSLYKFYTLFVWLLILSLVSAQILDIIINVENQDQFSDNFYITL
VVFVSGCKLSIILKHRESILSLIESLEREPFSPMNDEEEKIQMKFNRTNERIAICYTILVEVAAIWIFVRAFL
TDFKKRKLVFRAWLPYDYSELLPYTFSYTYEVATSLLCSCQNVASDTLFAGLLIQINGQFEILEERLRNIEED
SNYSAKQCVKHYQQIYKFSKTVNEKFKIILFLQFCTIAFTLCFNLYRMTNITMLSKFLEASLFLIRIIAQILY
YCWFSNEVKLKSLQVPSMIFKSNWASWDTKTKKILLVIMTRATHPIEFTSGYLVTLNLDSFVALMKTSYSVFN
LLQQTK
>BtOr63
MHVLRWTLKLFIASGYFLPPTLKSPTKRFLYNLYTVFVTLYLWSYCLTLILDICFNARTRDDLRDNFSISVTI
LITGCKFVSLVLGRKTIINMLDLLKKKPFVPENNGEGEIHARYNNLIEKVAVGYTIQVIFCVFSLLGTTLMGD
FQSKKLMFRAWFPFDITASWFAFCMTFLYQFVGLVIVSNGVCIFDTLFAGLLLHICCQLEMLVYRLHNIEGNE
IQSLKHCVWHHNKIFRFADIVNNFFNKLMFVQFVESAISICFTLYLLTDIEDTAQLIGWSSFMFAAIFQTFYF
CWFGDVAKVKSLDISNMVYNSDWPNLSNDARKMLVVIMARSLTPVEITSAYILPMNLESFKGLMKVAYSAYNM
LLQSKSAE
>BtOr64
MHVLRWTFKLFIASGYFLPPTLKSPMKRFLYNLYTVFMTMYLWSYSLTLIMDIFYNVETQDDLSENFSVTVTV
LITSCKFVSLVLGRKTIINILGLLKKKPFVPENNGEVEIYAKYDNLIERIAMFYTIQNAFCVLSLIVATLMTD
FKFKKLAFRAWLPFDFTSSWFAFSMTFIYQFVGSMVISAGISIFDTLFAGLLLQICCQFEILVNRLHNIGGDE
IQSLKHCVRHHNAIYRFAEIVNNLFSKMMCVQFMVSAAAICFNVYRVTESNAGSQLIGSVLFIFSALLQTFYF
CWFGDVAKLKSLDIPNMIYHSDWTNLSNDAKKMLLIIMARSLTPVEITSAYILPMNLESFKGLIKTTYSTYNM
LLQSKSSQ
>BtOr65
MHILRWTFLLFTLCGCYPPLSWKTPLQKFLYEIYTIFSFLLLNSFLLFQILDMICNVENTDDFSDNFSVTVVV
FVTCLKLFTILIHRANFLLLCNTLQKEPFLPMNEEEFEILLKFEKITDWNTLGYMTLLVISDFFILVVSLLAN
FKNRKLAFRAWIPYDYSSLSAYLLTFFYQSLFTTICTFGCVASDSLYSGLLIHINCQFEILEHRLKNIESNQN
YSVKLCVHHHDHIYKFGEMVNEEFKMIMFFQFGTSLTTICFNFYRITQIEMDSRFVGTLLYMACSLMQIFYYC
WFSNEVKLKSMELSDMIFRTDWTSLNNNVKKAFLMLMRRAMRPIEFTSIYVISVNLESFMTLLKTSYSAFNVF
QQSRES

>BtOr66
MHRLPLSFALLTYCGYWRPTKWPHTSLKYQLYNVYSVLMILLLYFFAFCSCVDSFTSKNLNAMTDKFSLCVSV
VGVCLKVANLFIRRGKIINVMNMLLNENCIARDDQEERIQRENDDYARKLAIYCEILNESAVFFATVGQYKKL
MSMRELPVSDWMPYDLSSQKVYTISLVYQTVGLLICANTSVANETLIAGLMIQVGTQFEIFCHRARNLSFSLT
SARRNTMSNEEFKIRCNKIIGNLIRHHHEIYKFAETVNSVFQYMIFLQFCISSIVLCLSVYQFSTVDPFSMNF
LWSGFYLCCMLMQVYLYCWFGNEVTLKSNKVRDAIYDMDWTMLPTDVMKNLLLIIIRTDKPVKMTSGHVVVLS
SQSFVSIMKMTYSSYNLLSSSTSK
>BtOr67
MHILRWTFLLCTLCGCFPPSSWKTPCRKFLYKIYTVFSLLVLNTFFLCLIMDMIYNVENIDDFSDNFHVTVGS
FLTTIKVSVMLMYRESFVRLRDTLQKEPLLPMNQQEFEILLRFDKVTDWNTLGYMTILMTSNFYLFMESLLTY
KKRQLTYRTWVPYDYSSASAFLLTLLYQSLFTTICSFGCVATDSLYSGLLIHITCQFEILEHRLKNIESNQNY
SVKLCVRHHNHIYKFGEMVNEEFRTIMFFQFYTSLCMICFNLYQITQMEMDSNIIGRILFMNFSLMQIFYYCW
FGNEVKLKSLELSDMIFRTDWTSLNHNVKRAFLMLMRRAMKPIEFTSIYVISVNLESFMTLLKTSYSVFSVFQ
QSRES
>BtOr69
MHTGQYTDVSIKMSGFLLKITGILKATNSSAERRRKFMVVYTVAALVYGVYVNVVDIYHNLHNLDHCIFLASN
TLNIILAMFKLSVLHFYKTEFSDIIVFAQKHFWHINYNDDEKILFAECRHFCKLWTVFILLVIESSLSFYAIT
PISANIGNNGSERLLPFKMWVNLPLTVTPYYEIMFAIELLAVQQIGASYLCPEQFLCVLNLHVVYQFRMLQKT
LLNLWSNIDEQTDIADYSKKYYIILKKCIRKHQSLIQFNAKLEQIFTLPILSHMVIFSVLMCFDTYEIVLANI
SSGKRLIFFFHMIGSFAHIIFFTYICHGLVEESTNVSIASYSGWWVCLPMSKTGKKIRKDMKMMMMKAMRPCQ
LTAGGFFPVTLETSTALISSTMSYFALMRESSMRLTEQ
>BtOr70
MPAERYTDVSIKMSEFLLKITGLSRTTNSAEEIRRKLTIVYTIAALVYGVYVNVVDIYHNMDNLDHCIFLASN
TLNIVLAMFKLSVINFYKTEFSDMIVYAQKHFWHFNYDDNDKILFAECGRFCKLWTVFLFFVTQTALSFYAVV
PISANIGNNNSERLLPFKMWVNLPLTVTPYYEIMFAIELLAVQQIGASYICPDNFLCVLNLHVVYQFRMLQNT
LVNLWSNIDERTDIVEYSNKCYVMLKKCIRKHQSLIEFSAKLDDIYTLPILSHMVIFSVLMCFDTYEVILADV
SPGTRLIFFFHMIGSFTHIIFFTYICNGLVEESTNISTASYSGWWTILPMTETGRKIRKDTRIMIMKSMRPCY
LSAGGFFPVTLETSTALISSTMSYFTLMRESSIKAAAE
>BtOr71
MMRNNDISIIWASFLMKIVGLWLATNRNEQRRRDFALIYTVGALFISICIAIRDIYHTWGNFSDSVFICCNIL
YVTIVFLKIGVIYKHKIEFFNLITFTQKNFWRPYHDPQEILVVADCKRICNIFIILMIFCTQGTCAGYMVTPL
IANIGRNESDRILPFNLWVDFPVGMSPYFEILFTIQILCVYHVGVCYICFDNLLCIVNLHVACQFRILQHRLR
SIDNATKDQIEEYESDAKLSCYSNMCYTKLKNCVQQHQMLIEYCKKLENIFTLIVLAQVMFLAMVICLVGFQL
LLVDTPTSKKASLVLNLCGVLCQLLMFTYSCDDLMRQSVNVGNASFSGPWPILPMNEAGATVRKNLLIIIMRS
HKICCITAGKFFPVSLQTFTGVLSTAMSYFTLLRNTSLDATNS
>BtOr72
MDKDSKRTKVSPTSARLKLENEASYTKDFALLMTSFLMKIVGLWLTNNKKEERIRQLTLMYTVVAILFGVWVQ
FRDFYYSWPNFGDCAYTACNILCLIMVLLKLSVVFIHKKEFIELLVYTHENFWHTNYSYNELLLLQNCRKISI
VCISLINICAQGTVFGYVLTPIVENIGRNHSDRVLPFRMWLDLPLSVTPYYEILFVLQVLSLCHVGICYICFD
NLLCLINLHVATQFRILQYRLLYLGETIEKQPYEYAIKEILPSDYLKNYHTLFKCCVREHQDRINYCQRLNNI
FTYIVLGHIVVFSLLLCLVGFQVLMANSPPTRRLIFVFHIVGSSSQLLLFTYSCDTLIRESTNIGTAVYSGPW
THLTMNKIGKLLRKDLTMIILRSSKPCCLTASGFFPVSLETCTKVLSTAMSYFTLMRQSFTN
>BtOr73
MSFLKSHDISISLTSTFMKLVGLWTSKNQLERRVRLITQIYAFAIILFALWVEITDTYYSFGDLSTCIYNVCN
ILAILMPLLKMTVLLAHKHEFFRLIAYTQRRFWHENYDEYEKRIYMNCKRKCTVFVCFVIFTTKATLICYALS
PILENIGRNESDRELPFNMWIDLPLTATPYYEITLLIQLMTLYHIGVGYFCFDNLLCVMNLHLATQFQILQYK
MSRMTDLTNKGKGETNLQLFSASSANKCYTVFKMYVQQHQALIAYCGKLESVFNLPVLAQVLAFSLVMCLDGY
QILMPGAPTRTRFIFSFQLIACLCQLLMFTYSCDCIIQESASIALAVYKGPWSFLPPTKSGMMMRKDLILVTI
RSGVPCCITAYGFFVVSLETYTRVLSTAVSYFTLLRQTTQETLYS
>BtOr74PSE
MHTEQYTDISIKISRLLLKIAGIWKSANKTEDRQRKFAVFYTMITSAYSMYVNVADICRNLNDLDXRCIFLAA
NVLNTILAVFKISIINLYKTEFSYILYTPKNIFWYLNYDLDEKILFAECKKVCKLWSLFAILVIQIIVSFYAV
ASICANIRSNSSERTLPVAMWVDLPIYVTPYYEIMFVIQLASVQQICVAYMSCDNFLCILNMHVICQVRILHN
KLLNLWKIIDQQIDKIDYTDKCYAALKKCIRQHQSLIKFYERLEYVYRFPIFGHIVIFSLLVCFDTYEILLXV
SPGTLIFVFYMFGSFIHIIFFTYTCHGLIEESSDISLATYSGWWTILPITETGRMLQEDVKMMIMKTMRPCYL
TAGGFFPVSLETSTALMSTTMSYFTLIRESSLKMKN
>BtOr75FIX

MSSRQVKDLSIIITSFYMKFVGFWLTNNYVEKRRRNVALSYTLFAVLLSLSTEARDLYFSWGDLGDSIYVICN
VITIVLVVVKIFILLIYNEELLDIIDYAKINFWHLNYDSHEQMIVDNCRRTCTIFVCVFTFFAQGTVIGFIAR
PILINYGKNESERILPFNMWLPECHLSMTPYFELMFMLQVVCSYHVGVCYHCFDNVLCILNLHTAAQFRILQY
RLTNMCNMNDCAEFYEVSKKSSYSIHRYAKLRTYIQQHQALTDFCKKLEDVFNLIVLGQVSLFSLLICLDGYL
ILMDDAPATRRFTFAFHITGCMCQLLMFTYSCDCLIRDSANVANAAYKSLWSHLPMDQFGKILRKDLILVIMR
SATPSCLTACGFFTVSLETYTGILSSAVSYFTLLRNQSNDN
>BtOr77
MQTELDLDISMVLSKFFLRSIGLWISENSAEERRMKIMIIYTVWHSIFATVEITRDFYFTILYKGDILYVTTN
ILTVTMGLIKICIIMTHKEEFINLIVYVQQHFWNVKYDFREKEILDNCKKTCTFFVCSVTIMGACAIVAYLTT
PVIANAGKNNSERVLPFNIWLNLPVSMSPYYEMIFTLQMINMYQIAVTYFCFDNIFCILAIHLAGQFRILRYR
FSKLCDIEHQINEKDMESMITNYAHTFYEKLKMYVRHHQTLINFCDRLENVYTMLILGQVLVFSVLICLFAYQ
GLLAAAPLARRSIFIFHLIGSMALLFMFTYSCDGVIEHSEKVAIGAYSALWTIMPMNKSGKMLRNDLIMVIER
SRRVCCLTANRFFPVSLETYNKILSTAASYFTLLRNHLENEIEK
>BtOr78
MHTTRYTDISITLSQFLLKLAGVWMTVNNAEERRRRLTMAFTAVIHVYGLYLNLGDAYYTWNDLSHCTFLLSN
TLCIVLAMFKLLILNFRRTEFKDLVLFAQQNFWHFKYDHDEKILFMKCRKLCKLWTITACSFTQASLAFYIIT
PICANIGKNKSDRVLPFKMWVDLPLSVTPYYEIMFVIQLATVQQIGVTYLCSDNFLCILNMHVICQFRILHNR
LLNLWKIIDQKTDKIDYADKCYIALKKCIRQHQLLIKFCEKLEYVNTLPIFGHVVVFSLLMCFDTYEILLANV
STGTRLIFVFHMVGSFIHIIFFTYTCHGLIEESSNISLATYSGWWTILPMTETGRMLREDVKVMMMKSMRPCC
LTAGGFFPISLETSTALMSTTMSYFTLMRESSMKNGDK
>BtOr79FIX
MQMQRDLDISMVLSTFFLRNIGLWITDYPTEERRMKIMILYTIWNSVFATIVITRDLYFTVLYKGDILYVITN
ILTVTMGLIKICIIMKHKEEFIKLIMYVQQNFWNVKYDFREKEILDNCKKTCTFFVCSVTIMGTCAILAYLTT
PLIANVGKNKSERAFPFNMWLNLPLSVSPYYEMIFTLQTISMYYVSVSYFCFDNVFCILAIHLAGQFRILRYR
FAKLCNMERRMKENDMESALTKHAHVFYEKLKSCIRHHQALITFCDRLENVYTMLIFGQVLVFSVLICLFAYQ
GLLAGAPPARRSIFVFLLVGSMALLFMFTYSCDSVIEHSEKIAIGAYSALWTIMPMNKSGKMLRNDLIMVIER
SRRVCCLTANKFFPVSLETYTTILSTAVSYFTLLRNRVEDEIVN
>BtOr80
MSSKKGRDLSITLVSFYMKIVGFWLVKNHVEERWRNFAMIYTTFAIFVAIVVEMRDLYFTWGDFSGTVYIVCN
LVTIILVLFKILVCFVYKKELLSLIRYAKTNFWHSNYNSHERMIVDRCKRTCTVLVCVFTFFAQGTVISYVIG
PIQANIGRNETNRILPFNIWLNAPICYMTPYFEVEFIIQVLCLYHVGVCYLCFDNILCIINLHTAGQFRILQY
RLENMCGVNNNGKLSYSVCKYMKLKTYIQQHQMLIEYCKKLEQVFNLIVLGQVSLFSLLMCLDGYLVLTEDAP
LTRRLIFLFHITGCMCQLLMFTYSCDCLIRDSTNVANAAYKSLWSFLPMDKYGKILRRDLVLIIMRSNIPCCL
TASGFFVVSLETYTGILSTAASYFTLLRSHANDMS
>BtOr81
MSSKEVKDLSITVTSFYMKFVGFWLTNNYADKRWRNIAMSNTVFFIFVAITIELRDLYFTWGNFEDTIYTACN
VVTIVLVLLKTFVIFIHNDELLYLINYAKTNFWHSNYDSHEQMIINTSKRICTFLVCSFAFFAQGTVASFILR
PILVNYGKNESDRIHPFNMWLDDSLMLSPYFEIVFVIQIFSACLVGTCYHCFDNLLFVINLHTASQFRILQYR
FSNMCNINDREHYTTLESYTVDKYATFKTYVKQHQMLIEYCNKLENVFSVIALVQVTLFSLLICLDGYLILME
EIARIKRLTFIFHVMGCMCQLLMFTYSCNCLIQDSECVMNATYKSSWSPLPMDKYGKMLRKDLMFVMMRSRAP
CCLTACGFFAVSLETYTGILSSAVSYFTLLRNHAGDT
>BtOr82
MQTEQELDISMRLSAFFLRHIGLWIANDPADERRMKIIFTYTIWNLIFGMIVLSRDLYFTWFYNGDTLYALAN
TMSLILTLVKVCVIVMHKKEFMNLVVYMEQHFLAVKYDFHEKEILNNCRKICAFFISSVTTIGICAILSYIST
PFIAQIGNNESTRELPFNMWIGILSQSPYYELIFFAQIMSLSYIGICYFCFDNVFCVMAIHLGGQFRILRYRF
SELCNIKYQISEQDKMSILTKHVHRTYETFRKYVRQHQALINYYNTLENVYTVIILIQVLVFSVLICLFGYQV
LLANTNSARRSIFVFLLIGALSLLFMFTYSCDDVIEHSDNVAIGAYSALWTIMPMNKHGKMLRNDLIMTIERS
RRVCCLTANGFFPVSLETYTKILSTAMSYFTLLSNNVTKNET
>BtOr83
MSFIKTDDISVRLTSIFMKLVGLWMATERSEQRIRDITVGYTLVAIIFAMWLQTTDMYYSWGDFSACLFAACN
ILSLTMPFIKILVLLAHKEDFFRLIFYLQRKFLHGNYNDYERKIVISCKRKCTFFICFFTLFTLATVASYIIN
PLVANIGRNESDRVLPFNMWIDLPLTVTPYYEIIFVLQVLSLYHIGISYFCFDNFLCIMNLHVAGQFRVLQYR
IANMPDLMDKVKQSGDKILNTGSSCLANECYSIFKKYIRQHQALIAYCGKLEEVFSLIILEQVLMFSLLICLD
GYQVLMADASTSIRVIFICHILACLCQLLMFTYSCDCIIRESASIATAAYKGPWLVLPMSTSGRMMRKDLTLI
ILRSHIPCCLTGKGFFVVSLETYTSVLSTAASYFTLLRQRETP
>BtOr84
MSLKYHKDVSFSVAVFYLKIVGLWLSTNLVEKWFRNALVTYTVLAIIFNMWMQLRGLYFSWGDFSVSTYIACN
SLGLFMDLFKLLIIFIHKKKFLYLVMYMQKNFWHFNYNQYEKSVLADAKRMCIYFVCVFSFLSQSTIFSYIFR

PLISNIGKNESDRVPIFHMYLDLPLNVSPYYEMTYLIQALTLYQVGVCYLCVDNIFCIMCLHVASQFRILQYR
IANVLSLKDKVKFDQDTNLDSSGEFYAIFKKCIQQHQALIGFCTTLEEIFTVIILGQVLTFSILICFVGYQAL
LVKLSLSWRISLVSFLTTNICQLWIFTYSCNALVQESMNTANAAYATPWIFLPMDKFGEMARKDLQLVLMRSR
RACYLTACGFFPISLETFTKIMSSAMSYFTILKQRTVDT
>BtOr85
MQTEQDLDISVIFSTFFLRNIGLWISDNRAENRWMKIKLMCTLFNSIDSSIVIMRDFYFTWLYKGDILYVTTN
TLTVTMGLIKICIILIHKREFISLITYTQQNFWNVNYDTREKEILKNCRKTCTFFVCSVSAIGVCAMLAYLST
PLITNAGKNKSERILPFNVWLNLPVSISPYYEIGFLIQTISLYYIAVSYFCFDNVFCILAIHLTGQFRILRYR
IAKLCDMDHQITEKDVELMITKHVHTFYEQLKLYIRQHQTLIDFCNRLEDIYTMLIFGQVLVFSVLICLFAYQ
GLVVAAPFARRSIFIFLLIGSMALLFMFTYSCDGVIEHSEKVAIGAYSALWTVMPMNKPGRMLRNDLIMVIER
SRRVCCLTANKFFPVSLETYTTILSTAVSYFTLLRNNVEDKNVD
>BtOr86
MIDRDIDLNNIYAWNYNLLKFMGIWPEERKWNRSSSYLVLFPFLTMVCFACGPQTINLSIIAGDSDLVIENLS
NNITFMVSIMKTLTVWINGIPLKSLLGYMANDWDAVTNNIERETMVNTARITRKITIGSTLMVNIVILAFVPA
RLSSMKNNDITLFLRGYYPYNTSISPNFELTMIAQYVAAIYAANTYTTVDTLVVLLIFHVCGQLAILRQDLGK
IHSYDKKNIEMKMQKIVEKHEYINRFAGRIENSFNMMLLFQMLSCTIQICSQFYQVIMSLGENTMEDMILQIS
FLLIYVAYVMLQLFLYCYMGEKLAAESTEIANIAYSTKWYNLPPKNARWLVIIMCRARSSPLQITAGRFCSFT
FALYCQVLKTSMGYVSVLHAMKNQ
>BtOr87
MIDRDIDLNYMYGWNYNMLKFMGIWPEERKWNRPYSYFVLLPCIVMVCFICAPQTFNLSIIAGDSDLVIENLS
TNITITISLMKTMAVWIKGKPLKSLLRCMANDWNTVTNNADREKMVNIARITKKITIRSILLANIVIVAFLPA
RLSSMIYNDKELFYRGYYPYNTTISPNFELTLIGQLMAALYAAITYTTVDTFVVLLIFHVCGQLSILRDDLRK
IHSYDNKNVEMKLQKIVQKHVYINRFAETIEDSFNMMLLFQMLGCTTQLCSQTYQVLMSLGEEAIEHMILQIT
FLLIYVIYVMLQLLLYCYMGEKLTVESTEIANTAYSAEWYNLTPKNARWLVIIMCRARSSPLQITAGRFCCFT
LVLYSQVLKTSMGYVSVLLAMKNK
>BtOr89PSE
MLSFLFPRLQSNTQSVSEADLGALHPATFPYDISKSQNFEIAWPGQFMSSVLTAICYSCFDTFLAVLVLYLCG
QLTVLRMAHENPTYATKKNNYAKFYERLRFTVNQDNQLSRFAAILEDRFNFAILIRVIICTSLYCLTGCRMIT
SVNQVQADLPIVGMIFLIIHVIYTALLLLTYDHVGEMLRGQSAGVGQSVYDCNWHLPPKDIISLIIVVCRAKV
SFQGTAGKFSSFSLEFDT
>BtOr90PSE
MTDSDIDVKYMCGWNYYMLRFMGIWPEERKWYRAYSYLVLLPCIMMLCFVCAPQTINLPMIASDSDLVIENLS
TNITITLSLMKTIVIWFKGKSLRPLIKCMVNDWDTVTNEAERETMVNISIITRKTTMRSTLMVNIVVLAFLPA
RLSNVRYNDSALFFRGYFPYNTSISPNFELTMIGQFVATVYAANTYTAIDTFVVLLIFHVCGQLSNLRDDLRK
IHTYSKKDVETKLQKIIQKHEYINRFAATIENSFNMMLLLQMLGCTIQICSQSYQIIMSFEEEAMEYVIFQIT
FLLLYVVYVMLQLFLYCYMGEKLAAESTEIANTAYYAEWYNLPPKSAKWLVIIMCRARSSPLKITAGRFYWFT
LALYTQVLETSMGCVSVQYAMENKQ
>BtOr92NTE
LKTAIGWNRWNMELIGIWPEPRRTDERLSHFKALFYLSIITIFGTGPQSANLFFIWGNLELVTENLSTANIPG
INAMIKLIFAWYYKDTFKPIMKSFYDDWRSAKTEEEKAAMLKMAKPANFISIWCSILTLTMVTAYLSLRSITV
YLSDRLHENQDRLSLYPGYFPYNIRPVPILLMTNFAQVIAGYSATICYTTVDTFIAMLVLHICGQFEILRKKL
SRLMDGEGGNRSIDEFQKELVWIITKHEHLNWLAETIEKCFSTLLLLQMLLCTIEICFQGFLFFNVLIKNENE
IFNFQLVFFVLFVCFILVHVYLYCYIGEMLLIQSREMSNCAYESNWYNVSPSETKCLLFIMNRSTRPLCLTAG
KFGIFSMELFSTILKTAMGYLSVLLTVANND
>BtOr93
MDFAMGWNRFNLTLLGVWPEPRKVSRGSRLLSSIIFWFTTIVTFTFICAPQTANLVLKSTNLDEVIENLSINI
PIAFALVKQIVLRYYKKALTLLLSQMFDDWTEPIANQDRQMMLKNARISRLISIVCSTLTYLMLFAFISLQIW
SNMQSASEADLGGLLHPATFPYDTSKSPNFEITWLGQFMGTVLTAICYSCFDTFLAVLVLHLCGQLTVVRMAL
KDLANTMKKDNNYERFHERLGFIVNRHNLLSRFAVIVEDCFNLTLLIQTVICTAMFCLTGYRMITSVDQEEAD
VPIVGMIFFIIHVIYTMLHLFIYCYIGEMLLGESNGVGQSAYECEWYDLPPKNAISLIIVICRAKVSFQITAG
KFSPFSLELFNAVLKTSAGYLSVLLAMKD
>BtOr95
MIDRNIDLNYVYGWNYHMLKFMGIWPEERKWNRPSSYHVLLPFIMMVVFACAPQTINLLLIAGNSNLVIENLS
TNITTTISLMKAMAVWIKGKPLKFLVKCMANDWNTTTDKAERETMVNIRRITRKTTIRSTLMANIVLLAFVPA
RLFSMRYSDNMLFYRGYFPYNITISPNYELTMIGQFMATFYAATTYTAVDTFVVLLIFHVCGQLSNLRDDLRK
IQSYDTKDVEKKLQKIIQKHEYINRFANKIENSFNMMLLLQMLSCTIQICSQSYQVIMSFGEEETEYMILQLS
FLLIYVVYVMLHLFLYCYMGEKLTSESAEIANTAYNAEWYNLPPKNARWLIIIMCRARASPLKITAGKFCSFT
LVLFSQVLKTSMGYVSVLHAMKNK

>BtOr96FIX
MDIFGEKQTKFKKIHPNKHLQNSLSIIYYLGFWPEWAKNKYLYNIYTTFNLTFLLGIIIVSNIVYIIINWGNI
EAMMASLSILMTYSTYGTKVIYIICRRRRIKDLIDITNSEMFNRDNDKYEHITAYYTWQAIFHQITFQTFGFI
AVISWGSTPILYQISGTSKQLPMVGWYPYNVTSTPVFEFTSLHQFMVVCTSCVNNIAIDTLATGFIVTACCQL
TILNYNISSIRCIIEKKYTLLNDNVSIENSTLKVYSKMYEDLKHCIEHTIMIFDFSKQIQDVFGTLIFLQLLV
DCIVICAILFNLSQMKDYVTSEFLGTLLYTCCVIYQIFIYCWHGNELHFHSMRICSSAYANNWWDNSKDFKHA
LLIIMARAQWPLILIVGNVMELSLQNFVLILRTSYSIFTVLRTSTAT
>BtOr97
MKELSNTFDYYILPNKILCSIVGMWPIEQESSTCTKIFSYVRLTFTLVSLISVFVPEIMLIAVNWGDIEILAG
AGSIVTTLGQTLFKMFYLIARRERSYMLYYEIKSLWDTANDSKEIQSYTQFAYWARICTIVFYSSCVCNVITF
SSAAAVDYFRFDYNASGTSNNRHLPFIVWYGTDISASPKFEIAFICQLLSAALSITSIAGLDCSFMTTMLHVS
AQFKLINTWISNIGTEINCNPNYKQKIKVDLTRCIRHHQRIIHVVNEVNNLLTPIVFLQILTSGIEVCLSGYA
ILDTGKAEADLVKFICYFISMAVHLLLWCWPGEILVQESQEIGHAVYFNVPWYDLPPIYQRYLCLMIVRAQQY
SSISALTFQTLSIHTLTAVFNTAASYFTLLQQIQQT
>BtOr98
MKELSTTFDYYILPNKIFCSIVGIWPIEKRSSTYSKIFAYFRLIVSLIAISNFFVPEIMAVAFYWGDMETVIG
IGSNLMSATQLFFKMIYLVARRERVYRLYNEIRILWDSTDDPNERKSYEQIAYRARIVTITFSSCFLCNLTTF
SIATIADYFRFAYNGNDTNDNRHLPFLVWYGTDISASPKFEIAFAGQIMTAMIGLSAITAIDCTFMTMILHVS
GQFILIKTWINKIGFEMNHKSIDMDKFEEDLFKCIRHHQRMIHVVNDVNNLLTPIIFMQLLTSGLEICLSGYA
MLDNGTKITDILKFTSYFISVTVQLLLWCWPGEILVQESQEVGQIVYFSVPWYNLPPIYRNHVCLMIIRAQQY
CSITALTFKVLSIQTLTAVFNTAISYFTVLQQMQQN
>BtOr99FIX
MKELPNTSIDYYILPNRIFCSMVGMWPIEEKSSTCSKIFAYIRLILALTAINSVFVPEIMMIVSSWGDITILA
GVGCVLTTVGQLLFKMIYLIVRRERSYRLYYEIRSLWNTANDSKEMQSYVELVYWARICTIVFYSSCMCNVIT
FSIAGVVDYFRFEYNASSADNNRHLPFVVWYGTDISASPKFEIAFICQILSSMVCATTISGLDASFMTTILHV
SAQFRLINTWISNMGIEINCNPNYTRKIKIELMRCIRHHQRMIHVVKEVNNLLTPIIFMQILTSGIEICLSGY
AMLDSGTAKADLVKFISYFISMGIQLLLWCWPGEILVRESQDIGQVVYLNVPWYDLPPIYQQHLCLMIVRAQQ
YCSISALTFQTLSIHTLTAVFNTAASYFTLLRQIQEK
>BtOr100
MKELPNTSIDYYILPNRIICSMVGMWPIEEKSSTCSKIFTYIRLILALIAINSVFVPEIMMIVSSWGDISILA
GVGCVLTTIGQLLFKMIYLIVRRERSYKLYYEIRSLWDTANDSKEMQPYIRLAYWARICTIAFYLSCMCNVIT
FSIAGVVDYFRFEYNASSTDNNRHLPFVVWYGTDISASPKFEIAFICQILSSMVCATTICGLDTSFMTTILHV
SAQFKLINTWISNIGTEINCNPNYTRKIKIELMRCIRHHQRMIHVVNDVNNLFTPIIFMQILTSGIEICLSGY
AMLDNEAAITDILKCTSYFISVTVQLLLWCWPGEILIQENQEVGQVAYFNIPWYNLPSMYQQHLCLMIVRAQQ
YCSISALTFRTLSIHTLTTVFNTAASYFTLLRQIQEK
>BtOr101
MINRPLEYSLRLFGVWPDSSYPILKIIIWTTVMLTFLVFQYWYCITHIKSSLIELLDGLSITLSNSLFFLKFT
IIWLHKRTFYEILTTMFEDWNVDNSTARNKRIMSDKAILSFRISNFLIGYFAITFFVYAGLALTLFDEDQLAS
DSKQRKFLIRMEFPFAATISPRYEVILAIQFIFESVMVYGAATSIALIAALILHVGSQIDLLCCKLTEISHNC
DKEKLQKHIIDDIVGKHQRIIQFSKNIEKIFTYISLCQFVSNMLVICFISFILVVSSHMDQATVIIMKCFPYY
IAVNCEAFILCYTGEYLTSKSEDITKSVYNFLWYELKPQNARVILLMILRSQGKLTLTAGKFLCLSLEAFANM
LKASASYVSVLYAMY
>BtOr102
MAIKGIISYPVEITLRLIGAWPNSSCRIFKYIIWTTVMSTFLIFQYSYCLTHIKAADLTDLLDGLSVTFSNTL
LLLKFIIVWFHKQTFSEILIIMAEDWDNCKSEWNMDVMMQKAILSYRIAKVMLIVFTCSISLYAVTTFFVPDN
IEASHSTEKKFLLRMEFPFEATFSPIYEIIVTIQLVIQPIFTLMAGMFMALIATFVLHLASQIDILCDRLTEI
LNNHSEEQLRITVIKNLIVKHQRIISLSENVESVFTLISLLQFFFNTVVICFVGFLLVTSLSSGQAPAVITKC
FPYYIAVNFEALILCYTGEYLSSKSEDIGWTVYNSNWYRLSICETRALLLLILRSQKPLNLTIGKFMNLSLET
FANMLKASASYVSVLHAME
>BtOr103
MILSRSISRPVVIGLRLIGIWPGSSYEIIVRCIWVANMMSAQVFQYRYIIKHINSGNLADIVDSVSTTLPYSL
LFFKIISFWIKRGIFKNILIGMSRDWIDTSTAKLNVHVMINKAELAYRCSNLIIGVYASAVCIYGGMFLEFSR
QDQDDGFNITSRQLLIKMDLPFAYYESPMYEYVFVVQFLQLLATGISIAMLDALIITLILHIGGQIEMLHEAL
ENISIKDEKHGSLRNVIKSLTDHHYRIILNSEYIEKLFSYIALMQLLCNTIVICCIGFLIIVAVNSYGDIKIL
MKILLFYIAITLEAFIFSYAGEYLSSKSLSVSSSAYGSSWYLLEPRNRRVVILLMIRSQRSLTITAGKFMDLS
MIGFATILKASASYVSVLYAMY
>BtOr105FIX

MILNTISPSVKFGLHFCGVWPGTPFQFIHKLCWVIAIITLQIYQYKYIVIHYNTDTLMNIAENLSIAVPFSLV
FIKLFVTWTNYGLFCDILSTMEEDCQKYANIDINNLVTKTGVFSFYVTSMIMSSYLVSAVSYLGGAIAFQGTN
DSMSRELLLKMDLPFETSESPNYELVVTTQFIIHFSAALAFGTFTALLFMMIVHVGCQIDIMCQNLTYAFPTN
ENKLKFFIGRYQEIIAFAEKIEKLFTYIALSQLVSNTVNTCCEGFLIVLAVNDENGLAIAILIKSVVFYIVIC
LEVFIYCFSGEYLRIKSQLIGDTAYNMLWYDLRPNKSRLLIPVILRSQRGFTLTFGKFSNLSLESFTGIMKVS
ASYMSVLLALY
>BtOr106
MIPSKSISRPVVIGLRLTGIWPGTSYEIIARCIWVAIIMPAQVFQYRYIMKNVSSGNLADVIESASTSLPYTL
LLFKLISFWIKRGIFKNMLVGMYYDWINSSADKANVDVMMNKAELAYRCSYSIFGVYIISVFMYAGVFLQFIR
QDQDDFNITSRQLLMKMDLPFAYYESPVYQYVFLVQFLQLLAVGIGMAILNALIITLIFHIGGQIEILHEALE
NISIKDEKHGSLRNVIKSLINRHYRIILNSEYIESLFSYIALLQLLCNTVVMCGIGFLIIVAINSHGDVGIVV
KIVLFYIAIMLEAFVFSYAGEYLSSKSLSISTSAYGSSWYLLEPRNRRVVILLMIRSQRRLTITAGKFMDLSM
FGFASILKASASYVSVLYARY
>BtOr107
MAPINTISRSVKYGLHYAASWPGAPLSVLCKLFWMIVLGVGQTHQYNYIIKHYKVQTLIEIIDNISICLPFSL
VCIKLVIAWTHQGLLHSILSTMEEECQTYAVMDTNNLISKTAHWCYRLTNIIISTTIASTVFYVIGVFTSEGV
NATAPRELLLKMDLPFDTSKSPTFELVIIVQYFYQASSAFIFAVFTGLLLMIVLHIGCQIDVMCQTSSAISYK
NEKQLKFFISRHQEIILFAEKIEKFFTYIALSQLITNTLIICCLGYLIVLIIHIENGFPMFMKCVVFYISVCS
EAFVYCFAGEYLNIKSKLIADTAYEFLWYDTHPSKSRLLIPVILRSQRGFSFTLGKFANLSMSTFAAIMKASG
SYISVLLAMT
>BtOr108
MRKHFYSLSRGRTSYVKMAQEPGHEWMKPFRKLINLEISYIKYSGLGEIDSSSAFLNRAYFVYKIWMLIAMYI
FAITLFLDIYVNRDNLPISTDNGCIFAGIFVVIYKAMNLQFQVEYIKEIIGKYQVQSKMIFYGFTALGSILGT
ALLFFSPMEDGLLIRAKYPFNTTISPWHEISLAIETCAVFGGLLGIIGIDSFVVMICTLLTVLFDMLNVNFEN
CGIETKEHTVEIYDERIHANIRFNRKKLNNRFLYRYKTCFQFYQRLVCMTNDYNKLFSLSMFIQMLSSTSIIC
LSGFQAVVVGGQSSDVMKFGIYLSAAISQLLYICWIGNELNYATWTLDRSQWLSGWNNERLTNIVKMFTLSTM
FTRRSITLKASVFYVLSLETFITIIRRSYSIYTLLNNMQVTDH
>BtOr109
MNIRNYVFINQLVLKFVGLYPISIVRYVICISCIMLITIPQIIMIYTNWNDLNIVMEIGSTLLTISLAMLKSA
IWMFNRKNLELFIEFMLTDYWKIIEANVFEYLQKYALYAKTITKGYLISMCNALLFFFSLPIIEILITKHEDS
DNFTMNFPFAASYPMAFYKFPLYEIAYASQILATSICCLVMLATDGLIATALLHTCGHFAVLRKNVKQLDSYI
YRITSLKTNSKHINANLYEIKIQIIHVIKHHQVVLWFCDNMEKNFHLILFLQAMISSLLICFVGFQVSATLME
QSKMIKFASHLIVAFFQLLLFCFPGDMLIRESFSISTAVYSIQWSQLATFVKNELCMIIARSQRPSYITAGKL
YIMHLENFTAILSTAFSYFMMLQSFNSES
>BtOr110
MSDDETKREFEKTIDLNLALLRLTGVVSSERASRDTLASLAFVCMTINTISYVYEFATSSYTLATVLESFAMI
IPLVAGQTRLVILLWLRDSCQTMLNICESFWSTLNSREKKIVRSYTNKAKLLSRCYLFSCVSTIFFYVLLIEF
GSLIFSEPKHFANVTIHGNDSSSVPSEAVKSLEVTDDKRRYLPYAFFIDVQETPWYEIAYVVQLGSMVSVGLT
CVGVDTTGPLLILIACGYFDTIQSRIENSYSFESPSSPPILASLSIATKAKTATETTLVTGIESSSRNVGMKN
LRTCLNHHQLLLKLCEDIENLTNIMFLIQLITSTYNISLIGFKVVEDNPGKVKFVTQLGIFIIQLFLCNWPPD
LLRSKSEAIGHAGYSMPWYRYPRNLQNPVNMLMVRGQKPVRLTAGKFIELSLETFASMISTAASFFTMMRSMN
>BtOr111
MEMSDQQFSGKEYDELIKPIMITAKIISIWPLEEDSSKGTILFRRFHLFCMFFLAVVMSFAVTADVVHNIDDL
NEATECALICTAFYLCVVRLLVYSLHQKDMFYVVKTMKEDWILSSHEDRKILAKKTMFAFRLAKYFISTVAMT
IVLFMCIPFLEIYVFGNNERVLPFRGYFFINHTISPVFECLYFFNVTAGGFGGSMIAGATSFNLVVIMHGSGK
FAVLRKRLEALSGEDPNSTAIMNNYVIRHQKAIEYADALERIINVLALGQFVTSTGLICFAGFQITSMMKDKG
RLMKYSTFLNSAILELFMFSFSGNGLIDESGAVGDSAYGSGWIGSRFNQNLQIMMMRARSPSKITAAKFYAMS
LESFSAVLSTSFSYFTVLTATEGD
>BtOr112
MKKPISSYVELFYDENVISWSKRLLNLSGLWPHNRNDVRFFFYVTYVVIFTWLEIVTLLQNIHDLEKTLKNIT
LSFPTILIVLKAMMFRLNMHLVLPLLTVVKRDVKLGLYRSEEERRTVVWYNVAATLFSTSSVLSLFFVPTLFY
AKPIVSCLLSKFNNCTLPYELPMKVNNIYEVTGVQSYALLCVYLIPASMLLTIGATGADSLLVALTFHLCSQL
SILAHRIRNISIEPQIYSPEMRALVERHIELLQLASILAKAFSSLMFIQTLGLIFSLCIVVYQLLMTTDSGED
INTIHFIIYSCAVILLAFCYCFLGECLINESSEMQVACYFINWYDLPEQYTRSLIFCIARSQKPSYLTAGKFY
VFSLETFGVIVKASMAYLSVLKSII
>BtOr113PSE
LARTVNIFSYILVYILIRLLMILPRFSIVIRYVTNGTDAGKLLPLPTYYSYNVSKTLYFEIMYVVQCINLLIA
SFCYIGVDNFFGVLILHIFDQLENLRFHLANMKDSRYGYETSNHVLGATIEDHIRLIXAINVIENISTLLFLV

LLINFNICACIYRLLLITASELIYRIMKDNCETNRKVNDSDIILIFLKYSEGVHNTAYECEWVSLKFTKAKSL
IIIMTRSKRPLHLTTGKFPLTMLKXILKISFSYISFLLIMLH
>BtOr114
MSKELKIYRKYASFVKRFLLLSGMCPITKERDVFYRCISIWSIFSSFISLCVVGNFCSQNVQNIALLTASFSL
FCAILNTTTKACCFFIYQNKLQQANDILSSMLEQALSETDIRSIAFSWVRTFYRLIYLQFTLMTINSTIHAFK
PLITRILYDANNTTNLQYPLPFPASYPWTIDSMLVWQLHYLFDLNIVWNIISVSTGVDGFFSFCLFRISVMLR
LLGFEFEMRFSTDEKDKANKEYEENRKRIFQECVNKHALLLKCRDIVQEAYGPVILLTTITSATSLCTLIFQV
LQVRGQIIDKIIICIVFIFMKLLQTFLYAWPADVILTESDRFRRKVYFSDWYKHKDISFAKGFTLILAQRSIV
LKACDLMQVSLDLFVKVLNTAVSYYFLLETIDKDK
>BtOr115
MDFRDLNPFNIFLSTLSANVLPMSSRETKLPIFFKIYSIIIWLIELTYFIGCVVGILTVSREKALKDGTVNLV
IAIEVLVLMIYMHNRKNLLRGLIGKLNHLIEGNKILRNMIVSALEPMEKPLKIYTVASVGSLALWISLPLIEV
FRKNEFRYSDYRVPFVLSKEPFSLNVFVGGVIFQIVGGTYTLIRKISLDIYTMYIILLTTAQYKYLRMKFATI
FEQKPETLNGSYNDSTRQNVSFRKKRMIQEMRVLTRHYETVVEIAVMLKTLLFLNVGVHYINNVFRFCFLSFM
FSTNMFSEKCLIILYTIGALIQFYILCYCIQELLDASNAVADDVIYEKWYLHDVPLQRALLMIISANKLECKL
SNSRHIDLTLSSFMSILNQAYSVCLLFLKSRPD
>BtOr116
MGKRVAQLERAILFTKLSVALTCSWPPSPLATKNRLLLFNALWCTAFASSVALFLPLLAAIYEYYKSPIILGK
TVSLASAVAQVVIKMIICRLQQRRFQMLYFDMENFCKHATKTERMVLERYVHKYKYFHCIYILWSFITTAFVI
CGPLYSSQTFPTHAIYPFSVKHQPYNSLIFFHQSLVGFQASSGMGIDTQVALLLRYATARFELLGIQLRNAKT
NSEFNVCIQKHIDLLRYTKEIRLSIKYLVLATIATTTTAVIFGSLNLIANQPLILKTLYATVVFSASVELFMY
AWPADGMMRMSERTATSVYGTAWYNRDISVQRKVLRIILRSQKLETIGISGIVPQLSLSHYAKYLYTSLSYFN
ALRIMVGDPSPL
>BtOr117
MSMFNVNIRQVLYIVELAGMFTCTWPIDPNSSKKQIFFRNIRWSFAILNVVFLTISLVFAIFHFRHDIPILMK
TVSEVTALLEVLFDLILCRRNNAHLQVLTGKVKAFLEVASEHETKAIERYMDRYKQFLSVTAMGYITTAISFS
LAPFFSAQELPADGWLPFSTESLGIYCMVYVNQVYCIFQTAFCIGVDFTIAVLFSFSAARLDILRMKLRHVNS
SDILVSCIKEHQEIIGFVEDTKATVETLLFKTNATMGSAVICGAFPLIYNQSLAVMSQYLSFVVSGCTRLYVI
SWPADDLKESSVQFAKSITDVQWLGKPKDMKSSMLIMMQRSQKPLLIRMSGLLPPLTLEYFANFITTVSSYFM
AMRSMIES
>BtOr118
MLKQITPEKVIHITWLSVVISFCWPLPINSNRNKIFCFKILQICSGISGCLLFMPILYSIYLHLNDIVITSSC
MCLLLGVSQNIFQTLICFIKHDSLQRTVEEMMTYVREAQQYEKKIFYKYIKKCYVFCGGSMACTYVTATCFSL
RPIVLPVSLPFDAEYPFPINYTSMYVIIYMHHACLCIQTAAHICVSSFGALLLWFTVARFECLAAELEKSTDI
DALVVCVKKQLHLKRYAEEVFNSFRFLVLYAIGVATFGITLCFLMMLVNVPLFVKLQFIGVCVTVLMEIYLYA
WPADYLKDMSIEAPQSIYNSTWYEQRLGMQKSLLNVLIYRRPLTLSIACIVPELSLRYYCSYLSNAFSIFTTL
RVMIHD
>BtOr119
MTKQITPEKVIHITWLSVVITLCWPLPINSDRNKIFCFKILQICSIISACLLFLPMLYFCYVHFDDLIVTSSC
VCLTIGVSQNIFQTIVCSVKHVSLQRIVEEMIVCIKEAEQYEREIFYKYIKKCYVFCGCSIVCTYVTAVGFSL
RPAILPVPFPFDVEYPFPVNYTSVYIIIYMQHVCVCFQSAAQICISSFGGLLLWFTAARFECLAVNLERSTDT
DTLIDCVKKQLHLRRYGEEVLNNFRFMVLYAVGVATFALTLCCIMMIVDVPLFVKIQYIGACVTVLTEIYLYT
WPADYLMDMSMGIPQSAYNSTWYDRRLEMQKNLLNMLTYQKPLVLSIRCVVPELSLRYYCSYLSNAFSIFTTL
RVMIQN
>BtOr120
MLRNVTPETAIAFTQFILGLSCCWPLPSTATKSQILCFKILRSVLFLNSLLLFCPLLYAIYVHREDTAMFCKS
VSLALAVVHVPLHSTYCFSQHDRYQRLIEEMKSCCEKGNSYERQIFQRYVDKYAIYYAASAVWFYWSPSIILI
GTFFISDPFPTNAEYPFPVDFEPVRSIIFLQQSLVGMQCASLLCTNILCALLLLFAAARFEILMTEIRAVNSV
KSLIKCVKKYYTLKRYAEEVANTARYTTLITLCICGIESVFAGIIFIGRQPFTVKLQFVTVSITVLLAVFMCA
WPADNLMDVSESTMRSVYQSKWYEQPLRIQKFILFMMIPQSPVILRIRCIIPAFSLNYYCSFITNVLSMFTAL
RVVMLQDEDGILEDI
>BtOr121
MFRNVAPKTAIAFTQFILGLSCCWPLSSTATKFQILCFKILRSVLFLNALLLFCPLLYAIYVHRYDTVMFCKA
VSLALAVLQVLLHSFYCFNQYDRYQRLIEEMKSCCKEANSYERQVFQRYVDKYAVYYAASAAWFYWCGAIVPI
GTFFLPDPFPTNAEYPFPVDFEPVRSIIFLQQSLVGMQCSSLLCTNIFCALLLLFAAARFEILMTEIRAANSV
KSLITCVKKYYTRKRYAEEVANTARYTTLITLCICGIESVFAGIIFIGRQPFTLKLQFVTVSVTVLLAVFMCA
WPADNLIDVSENTMRAVYQSKWYEQSLRTQKFILFMMIPQSPVILRIKCFIPAFSLNYYCSFITNVLSMFTAL
RVVMYQDED

>BtOr122
MPKRMTPEKVIDIIWFSVALTFCWPLPINSSGTRILVHKILQISSVISACMLLLPLLYSIYLHLDDIIIVSEC
ICLFLGVSQAVVQSIICLINHDSLQHVVEEMIICVKQAREYERDIFSKHIARCSVFYASSIMCIYLAATAFSI
GPAILPLSFPSEAEYPFRVNYTPVYVIIYMQQSILSYQCVAHICLSMFGSLLFWFTAARFQCLAMELKSTADV
STLIVCVEKQLHLRRYAKEVVNNFRFIVLYAIGVSTSALTLCGIILLVDVPPMVKIQFVTVCFTVLTEIYVYA
WSADYMKDMSINVSRSAYDIIWYKQKLEMQKNLLTVLECQEPITLSVSCIIPELSLRYFCSYLSNTFSIFTTL
RIVIEDNAE
>BtOr123
MPKRTTPEKAIDIVWFSVALSFCWPLPINSSGTRTLVHKILQISSVISACMLLPPLLYSIYLHLDDIIIVSEC
ICLFMIVSQIVVQTVICFINHDSLQHVVEEMIICVKQAREYEMEIFSKHIARCSVFYASSMVCIYLTATAFSI
GPAALPLSFPSEAEYPFRVNYTPVYVIIYTQQSILSYQCAAHICLSMFGSLLLWFTAARFQCLAMELKKTSDV
STLIVCVEKQLHLRRYAKEVVDNLRFIVLYAIAVSTSALTLCGIILLVDVPPMVKIQFVTLCLTVLTEIYVYA
WSADYMKDMSINVSRSAYDTIWYKQTLEVQKNLSIVLVYQEPVTLSVSCIIPELSLRYYCSYLSNTFSIFTAL
RVVIEDNAE
>BtOr124
MSKQITPEDVIEFTRLSVALSFCWPHADDSGRSRGSYKIAQICIVINAFLILLPSLYSIYLYLVDISLHLEAL
FKCINLTIYTMQLIIQTCICWKKRDSLQRIIDEMVKCVNEAQKFERDIFMAHIVKYNIIYVGYVVAIYATLTF
FIFGPLVLPIPTLVEVEYPFQVNYMPVNFIVYLHHSSVCLAVTAHLCIGVFGALLMWFAAVRFECLVVEIQKT
TNIRMLVVCIKKQLHLRRYAEEVVGCFRFIILYVLGMTTFTITLCGILLIMDSSVTTKVELIDASAFCLLLTF
MYAWPADYLQDASINVSRSVYYMEWYKQPLEIRKYILTVLVHQKPVTLSVGCFMPELNLRFYCSFVSNAFSFC
TALRTMVQDH
>BtOr125
MSKQITPEDVIQFTRLSVALSFCWPSADNSDRNRGSYKIAQICIVINAFLILVPSLYSIYLYLGDISFYLDAL
FKCITLTIYSTQLVIQTCICWKKRDSLQRIIGEMVKCVNEAQQFEREIFIAHIVKCNVLYSAYVVAVYASLTF
FMVGPLVLPIPTLVNVEYPFEVNYIPVNIIIYLHHSSVCLTVTAHLCIGVVGALLMWFAAARFECLVVEIGKI
TNIRMLIICIKKELFLRRYAEEVVGCFRFIVLYVLGMTTFTIALCGILMIMDSPITTKMELVNSSVFCLILTF
MYAWPADYLQDASVNVSQSVYDMDWYEQSSEIRKYMLNVLVHQKPVTLSVGCFMPELNLRFFCSFVSNALSFC
TALRAMVQDESR
>BtOr126
MSKQITPEGVIRFTWFSVALSFCWPLSDDSSRSQVLGFRILQISAVVNAFLMLVTSIYSFFLNLDNMTALFRC
IFQVIYTAQLILQTFICWKKSDSLQRIIREMMKCINEAKQYEREIFDAYIAKCNIFYGSYVVILYIATLCFML
GTLFLSSVLPLSIEYPFPINYTAVSVVINLHYIILFITCAAHVCMCVFGALLLWFTAARFECLAVEFEATTNI
SMLIYCIKKQLYLRRYAEEVIGCFRFMVFYFLSLATFFVTGLGIILVIDTSTITRMEFVSHSSFSLMHVYMYA
WPADLVQDISENASRSAYDMKWYEQSLEMRKYILNVLVYQKPVTLSVGCLMPELTLRFFCSFMSNALSFCTGL
RAMVQDEPE
>BtOr127
MLRQVTPEKVIHIVWLSAALSFCWPLPISSSRTRVLGFRILQISAIISACMLLLPMLYSIYLHPDDLVVVFKC
MCMSTALCQLIVQTTMCWINQDSLQRTVREMMTYVKETQQYEKEIFYKYITRCDILCICTIVCMYATATGFSL
GPAILPISFPADAEYPFRVNYTPMNIIIYAHQSILSYQCAAHSCVSIFGALLLWFTAARFECLAMELEKSTTI
NTLIVCIKKQLSLRRYAEEVLNNFRIMVLFSIIISTLVLTFGGIILIVNSPLLLKLQFVIVSLTVLTEVFMYA
WPADHLREMSTNVSRSAYNLIWYKQTLEIQKNLLNVLVFQEPITLSVSCVLPELSLRYYCSYLSNAMSIFTAL
RAAIGDNST
>BtOr128NTE
VFQKNERRTSTVFDISYYKVFKKYLLFLGQFPNQSHWSKKLNLTVMIGSLLTLHYPGFVQIYTSLYAKDVASF
LEAVPVITTLTAALIKLLNHVIYKENFEKMFHIIKKDWELLNDKSQTHILEEITKEGNKIGEIYRTFMLSCML
GFIIIPLTPVILDIISPLNETRHREQMFRVTYFLDENRYFYPIYFHSLWCAFVITIIAITIDSLYIQIVHHDS
ALFAICGQALITARKSTDVDTNGTYTEWLRQCLTMHNDALQFFEMLDDSSRRSYFFQILLTMVGMTVTAVQAV
MNLHQPEEALRIGLFLVAQQFHLLIITLPGQVITDYSFELTNDIYRSMWYNMPINDQRILHMMQMRSSKPCKL
TAGGIYEMNIENFGITFKTCVSYFTVLLSLGD
>BtOr129NTE
VFQKNERRTSTVFDISYYKVFKKYLLFLGQFPKQSRWSSKFNVTVMTGSLLTFYFPAFAQIFTSLYEKDLGGM
LEGMPVVASVSAVLIKLLNHEIYKENFEKMFDVIKKDWKLLNDKSQIHILEEITKQGNKIGEIYRTFVLSCMS
GFIVIPLYPAFLDIIIPLNETRQRHQMFRLTYFLDENRYFYPIYFHSLWCSFVTVMIAVTIDSLYIQIVHHDC
AIFAICGQNIITATGSTGVRINETYTERFRQCLTMHKNALQLFEMLDDSSRRSFFFQILLTMVGMTITAVQAV
INLHRPEEALRIGLFLVGQQFHLLIITLPGQVITDHSFELTNDIYRSMWYDMPINVQRILHMMQMRSSKPCKL
TAGGIYEMNIENFGITFKTCVSYFMVLLSLKD
>BtOr130

MDVFQKTGEQYPNIYDIPYYNTIKKYLRFLGLDPHQKYGLIIVIIMVISMTSGLVPMSIVLYGSLCTKNLDMV
LECLPHLGALLTSVVKILNVHLNRENFKKLFDSITKEWQQLKLSQDLYILEEVTIRGSKMAKLYRNTLLICMV
LFLLVPLLPPMLDIVLPLNETRPRQQILNVNYVLFDSDNYFFYVYLQLSWTSIVVSIIIVTVDSLLMLIVHHN
SGLFIVCGHQIQKSTRHLNSFTNEVMSERYTYKQIRNCVIMHNKAIDFYDILDENNRISYMIQIGLNMIGITT
TAVQTVINLDRPGESIRSAVLCGANQFHLFMLSLPGQILIDHCTELTKQLYNSTWYGVPVKVQRMLYMMQIRT
RRPCTLTACGLYEMNIENFGTTFKTCMSYITMIMSLKG
>BtOr132
MDVFQKTGEQYPNIYDIPYYNMIKKYLRFVGLDPHQKNGLITVIIMMTSITTVLIPMSIALYESLYTKNLDVM
LECLPHLAAIVTSFVKIMNVYLNRENFRKLFDSITKEWQQLKLSQDLYILEEVTIRGSKLAKLYRNTLMTFMV
LFLFVPLISPMMDIFLPLNETRPRQQLLNVNYVFFDSRNYFFCVYLQLSWAAIVVSISIVTVDSLLMLIVHHN
SGLFIVCGHQIQKKTRHLSSFTNEVMSEHYTYKQIRNCVIMHNKAIDFYDILDENNRISYMIQIGLTMIGITT
TAVQTVINLDRPGISIKSAVLCGANQFHLFMLSLPGQILVDHCTELTKQLYGSTWYGVPVKTQKMLYMMQIRT
RSPCTLTACGLYQMNIENFGTTFKTCISYITMMLSLKG
>BtOr133NTE
VFQITGGPEIFQVPYYSKLERYIQLLGQDPRHKSSTRNIIVVIVVTSIASITIPATTELLVSLHKKDMDGVIE
CLPHFVTSSASVVKILNMHFNRRSFNKMFQLVAKQWEQLKLNGELHVLEEVVKQGNRMAHFYRNTLISFMVLF
LLVPLVSPILDIVHPINGTRTRQQLLRVNYIFFNDDDYFIYIYLQLFWSSVVIVFTIISADWLYMLIIHHSSG
LFAVCGYRVHKATVNPNNFTGESMSENYTYEKIRSCAIIHNEAIRFSDILKQSSQGSYLIQVGLNMLGISATA
VQTVINLDRPEEAIRSAVFCGACQFHLLLLSLPGQVLLDHCSDLADNIYSSKWYKAPMQIQKVLYVMQIRCKR
FCSLTAGGLYEMNIENFGITFKTCMSYITMMMSVKD
>BtOr135NTE
VRQKTGNPEILHVTYYNKLERYIQLLGQDPRHKSSTRNMIVTIVVTSIASITIPTTIELFASLHKKDMDGVIE
CLPHFVASSISAVKVLNMHFNKRNFSKLFQLVARQWQQLKFNNELHILEEVVMQGNRMAHFYRSTLVSFMVLF
LLVPLISPILDIVHPINGTRTRQQLLRVNYIFFNDDDYFFYIYLQLFWSSIVVVFTIIGADWLYMLIIHHSSG
LFAVCGYQIQKATANPNYLTRESIFENATYEKFRNCVIMHKEAIQFYNILNESSQGSYLIQVGLNILSISTAA
VQTVVKLNRPEEAIRSAVFCGACQFHLFLLSLPGQVLLDHCSDLANNIYSSKWYKAPVQIQKVLYVMQIRCRR
FCSLTAGGLYEMNIENFGITFKTCISYITMLMSLNG
>BtOr136NTE
VLQINEGSEIFHVPYYNKLERYIQLLGQDPRHKSSTRNIIVMIVVISIASITIPATIELLASLHNKDTDGVIE
CLPHFLASSISAVKVLNMHFNRRNFHKLFQLVAKQWQELKLNDELHILEETVMQGNRMAQFYHNTLVSFLGLF
LLVPLIFPILDIVHPINGTRTRQQLLRVNYIFFNGADYFFYVYVQLFYAAAVVVFTIISADWLYMLIIHHVSG
LFAVCGHRIQKATLNSNNSTGTAVSEKYTYEKIRNCAIMHSEAILFYNILNGSSQGSYLIQVGLNMLGISVTA
VQTVVNLDRPEEAMRSAVFCGASQFHLFLLSLPGQVLLDHCSDLADNIYSSTWYGTPVQIQKVLYVMQIRCKR
FCSLTAGGFYEMNIENFGITFKTCMSYITMLMSVND
>BtOr137NTE
AFQETDRQSPDIFDVPHYKMLEKYLQLLGQDPRQKNGFRNFIVTAVVISIIGNIIPTSLELYTSLCDKDMDAV
IDVFPHFMAATISAIKILNIQINRQNFNKLLHFVAKQWEQLKLNNELYVLNQTVMQGNKMAQLYRRSLLTALI
LFLLVPLVSPILDVVLPLNETRPRQQLLKINYIIFDDADYFFYVYMQLAWGSIMVVVTIISVDSLYILIIHHN
SGLFAVCGNQVQRATTNLDTNQIISESYMYKQIRDCVITHNEAIQFYNILNESSRTSYLIQVGLNMLIISATA
LQAVINLDKPEEAIRSVVFCGANQFHLFVLSLPGQVLLDHCAELGNNIYGAMWYKTPVQIQKILYMMQIRSGK
LCSLTAGGLYEMNIENFGTFKTCMSYFTMLLSLK
>BtOr138NTE
AFQKVKTRSTDMFDIPYYKMLEKYLQVVGQDPRQRDIFRSIIVTIMVTSITGILVPTSLEVYRSLYDKDMDGV
IECMPHLIAAITSIVKILNVHFNRENFRKLFEFVRKQWEELKLTNDLRVLEEVTIQGSKMAQLYRNTLLIFMV
LFLLVPLLFPILDVVLPLNETRPRQQLFRVNYIFFDHEEYFFYIYLQLAWGSVIVVMIIVTVDSLYILIIHHS
SGLFAVCGHQVHKATSSSIIFSTEAMTETYTYEQIKNCVITHDKAIEFYNILNENSRSSYLLQVGLNMMGISM
TAVQTVANLERPAEAVRTGIFLGAEQFHLFVISLPGQVLLDHCSDLANNIYGSTWYKIPVKIQKMLLMMQIRS
KKLCTLTAGGLYEMNMENFAITFKTCMSYFTMLMSLRE
>BtOr139NTE
VFQANSKQDFNVFDISYHKPLKQYLSICGINPYQEDRTSNIIVIAIMCSFMSFFAPTSMQLYDAIRDKDFDNV
ILNLPHILTVLVSVTKILNIYSNKTLFRKLFNSLEEDWKLLESKNELQMLDKFTKHGNKVACLYRRTLLIALV
IFLSLPLCNPILDIIIPLNETRQRNNVFQVHYGILDNEEHFYIVYVHLSLCAIIIVVTIISVDSLYITIIYHA
CGLFAVCGSQVQKTAENNFVEKNGINIRNIGYDAFKECVMMHYKCLQLYDALEKCCRNLYLITMVLNAVILSV
TAVEVIVFLDRPAEAIRAIVYLIAQQFHLYMFSLPGQTLLDQSVELANKIYDSDWYKIPTKAQKVFYLMQVRS
NKPCILTAAGIYKMNIESFGITVKACMSYFTMFLSLRE
>BtOr140
MDKATVVDEYMKVVKICTMIIGIWPDQSKSSKLVMRTIIYIISVLSFVTQIANVVHFFNMNVLLNQICFLTAL
SGVLLKEGLYIIKATEYKMLLTAIWKDWSTDSHSNEFHIMAEYAKKGALLSWFDCGIGVVCIVIFLQLFLTPL

VLDIISPMNETRDLLTIYPAYYYIDDRKYRTIINLHMVYTYILGIIVYVGCDTSYMCIVQHACGQLAVAGHRF
KNAIFDLSIVNETSAIQDEIHERVLRSIRQHQYAIDYLKTIQSIHSTYLCICIGLVMVTFSVTLVKVAAQSEI
SAEFIKDVVFLSSQLTHIFLLTVQGQFVQNANDEVTESIYDALWYNSNNKTKLLFVLALRNCLNPPTLSAAGL
IILNLKSFSEILKTSVSYFTVLKST
>BtOr141FIX
MDKATVVNEYMKVVKICTMMGGIWPDQSKVSKLVMRMIIYIVIVISLVTQMANVVRFYSLHTVVEQVCFFNAM
AGALLKQGNYIVNAAEYKMLLTAIWKDWSTDRLIDEFEIMVEYAKRGAFFSRLYCGLGVFCAICFIQLSLSLY
ILDIISPNNETRDLIYIYPAYYYIDDRKYRTFISFHMTYTVISTFFVFVGCDASYIYMVQHACGQLAVAGHRF
KNALSDLSIDNEKGGMQDKSYERVLHSIREHQYATNYLKSIQTSHSVYLCICIGMSMASFTVSLVKVASQSEI
SGVLIKDCVFLLSQLIHILLLTVQGQFVLNSNDEIIESIYDASWYNANKKTQLLFVLSIRSCLSPPILSAGGL
LDLNLKTFAEIIKASVSYFTVLKST
>BtOr143
MDWQTIEDQCLKANKFFGQLVGVWPNQEKFTKIIIRFVIFIIVITTITAQISRVVVFYSLEVLSDQMPYLDIG
FVILIKQYNYILNEKKLKGLLNDIVADRLIERPKEELEILDIYSKKAIFLSCLYQVSISFCAFMFVLLPTIPP
ILNVVAPLNESRSREFIYPSYYFVDEQQYYYPILVHMISVAVILTSVYIACDINLVHVVHHGCALLAISGYIC
SKCLVTSIYIFRFVSNTYCYYHDNFVALHYSILHICIKIYVDKVDACHVHYFFIILGLIIVTFTSTFVRLSTM
EVGVRYFTFCAFTISQMTHLLFLTIMGQFLINSNDETFQTICEANWYNGSSKAQSLYLLVLRKCLSPPKLTGG
GIISLNLESFVLKASFSYYTVFRSS
>BtOr144
MNRVIIEKRFLKITKIFAILSGIWPGQNKIKFILWALVHITMLSSVIVQVARIIHIGTLEVVLEQSSFIGAII
LMIIKHGNYILNAKKLKSLLNDMSEDWATDRLKEEFAIMTTYAYRGTTLAMFYFVNACICTVLFIQMPWTVRL
VHMLKPHNTSSPILYTIPAYYFVEDDRKYYYYIQMYLALSVYVVLIVFVGCDTCYMVLVQHACALLTVAGYRF
KNAINDLSYNGRNPEKGAKEIYKKLRFSIQGHQRAIMFLRNIESTHVTYLFMCMGIIVLCVSITMVEIATMDP
CWDFYKFIGFLIIQLLHLFCLTMQGQFIINSSDDIYDAIYEAQWYNANPEMQAFYVLALRRSLTPPRLTAGGL
IQLNMQSFSEVMKLCVSYYTVLRTTS
>BtOr145
MDVEAMEKRFLKINRMFGVLTSVWPYQKPFPRLIQRIIVLTILFTSFVTQTAYLILFPSIRGIVTSMPYYILV
LGTFVKMGNYFINETKLRSMLNHIFTDWATIKSKEENDIMITYSQRGLLLTLSYALHALITGILMISWPLVPP
ILDILMPLNESRQRMFIYPAYYFVDHEKYYNILAIHMVTVMCMTGFVYCACDANYVYAVQHACGLLAITRYRF
RNVTEGVLDHYKNDAKLSKFNYRNVCKSIQAHQHALRYLKLIETNHHTYLFISVGMLIMCICVSLLQVANEKN
DSWLVQCIFLFAQLFHTLILTGQGQFVINGLDSVFNSIYESPWYTFPAKIRALYILALRSCLSPPLLTAGGLI
VLNLRSFAEIIKAAVSYYTVMKTK
>BtOr146
MNRATFEKRFLRITKIFGKLSGIWPDQNKVKFSLWAMVHITMASFVFVQVARIVHIGTLKVVIEQSSIIGTGI
VMVVKHGNYILNAKKLKSLLNDMSEDWAIDRLKEEVAIMTTYAYRGTTLAMFYLVNACICAFLFLQLPWTVRL
MHMIKSHNTSLPMVYAIPAYYFVEDDRKYYYYIQMYLGLSIYIVVIVFVGCDTCYMVLVQHACGLLTVAGYRF
KNAINDLPFNARNPEKEAKEIYKKLCFSIQGHQRAIMFLTKIESAHVIYLLLCMGIIVLCLSITMVQITTMEI
CLDFYKFVSFLILQFLHLFCLTMQGQFIINSSDMIYNAIYEASWYNANPKTQALYILALRRSLTPRYLTAGGL
IELNMRSFSEVIKLCVSYYTVLRST
>BtOr147
MNRAALEARFLKITKIFAKLNGVWPDQNKVKFILWAMVYITMGSSIIVQVARVIHIGSLEVVIEQSSLIGAGF
LMIIKHGNYVLNAKKLKSLLNDMSEDWAIDRLKEEFAIMTTYANRGSILAMLYFINACICAFLFLQLPWTARL
VHMMKPHNTSSPMLYTIPAYYFVEDDRKYYYYIQMYLGLSIYVVLIVFIGCDTCYIVLVQHACGLLTVTGYRY
KNAINDLSFNTKDSEEMAKETYERVRFSIQGHQRAIMFLEKIESAHVIYLFMCMGIIVLCVSITMAQIATMEI
CVDFYKFISFLMIQFLHLFCLTMQGQFIINSSDEIYDAIYEASWYNTNPKTQALYLLALRRSLTPCYLTAGGL
IRLNMESFSEVIKLCVSYYTVLRST
>BtOr148
MNRATLETRFLKITKIFAKLGGIWPDQNKIKFILWAMVHITMGSFVVVQVARVVHIGTLEVVIEQSSLIGATI
LMIIKHGNYILNAEKLKSLLNDMSEDWAIDRLKQEFSIMTTYANRGSILAMFYFVNACICAFLFLQLPWTMRL
VHLIKPHNTSPPMLYTVPAYYFVEDDLKYYYYIQIYLGLAIYVVLIVFISCDTCYMVLVQHACGLLTVAGYRF
KNAINEFSFNTKISEKKVKETYDRVRFSIQGHQRAIMFLEKIESAHVTYLFMCMGIIVLCVSITMVQIATMEI
CMDFYKFTSFLIIQFLHLFYLTMQGQFVINSSDEIYDAIYEASWYKMSTKTQALYILALRRSLTPCYLTAGGL
IQLNMQSFSEVIKLCVSYYTVLRST
>BtOr149FIX
MNKTAIEKRYLKVVKTCSMIAGIWPDQSKFSKLFTRIIIYIISVLSLITQMANVICFFSLNTLIEQVCFFNAI
IGALIKQGNYIINAAEYKMLFNAVWKDWSTDRLSDELDIMIEYAKKGAFFSWLYFGVCICCAISFLQLSLTPI
VLDIISPMNETRDLMYIFPAYYYVNDQIYRTSISFHMMCTIISTCLVYIGCDTSYMYIVQHACGQLAVARHRF
ENAILDFSIVSETSVVQDKIYERVVHSIRGHQYATNYLRTIQSSHSAFLFITVALGMTTLSVTLVKVASQANV

SGQFIKDIVFLLAQLIHIFFFLLQGQFVLNANDEFAESIYNTFWYNTNTRTKLLLVLVLRSCSSAPNLSAGGL
LVFNLKNFSEILKTSFSYFTVLKTT
>BtOr150
MDVFYRQYNIYRILLSVLGLWPYHKLIYSKIHRISISVIMLAYIVFQVLSLFKSGITFRGCIVTLSATCPVAI
YFMRYVTSVAMFPVTKYIFDNLRTINTTLKDQLEVQILMKYVDYSTYIISIFLLLCCLWILFATSFVFTPITL
DLLLPLNESRRRYFSYFTMFSHDRIEYLDIACVNILVVHTIGMLSLAGTELMLVLFAHYMCGLFDITSYRMRK
TIAGLSSSRQIDPNFRDFRHVVDSHTNTLQLIDYALSNYMVHYLPPCSVFLVSFSVCLHRLTNAITNANDQTE
IFICSMFVIIHMVILYLCHYSGQILIDRSLDVFKETYNSTWYCMPVEAQKLLLFIMLRSSTESVIDLFGFFAA
SHVGFSKMLSTSFSYFTMIYSLQ
>BtOr151
MDVFYRQYNTYRILLNVLGLWPYHKSIYCTIHRISISVIMLAYIVFEVLSLFKSGITFRGCIVTLSTICPITV
FFIRYVTFIALFPVTKYIFDNLRTINTTLKDQLEVQILMKYVDYSTYILFIFLCSSCSWTLLAASYVFTPITL
DLLMPLNESRRRYFSLLTTFSHDRIEYVDMVCVNILIVHTIGLLCLAGTELMLAAFAHWMCGMFEITSYRLRK
TIADLSLSRQIDSNFWNFRHVVDCHRNTLQLIDYALSNYMVHYLPPCSVFLVSFSVCLHRLSNAVTNVNDQTE
IFISSMFVISHIVLLYLCHYSGQILIDHSLDVFKETYNSTWYCMPVEAQKLLLFIMLRSSTESVIDLFGFFAA
SHVGFSKMLSTSFSYFTMIYSLQ
>BtOr152
MDVFYRQCNTYRILLSVVGLWPYHKTIYSTIHRILISVIILAYIVFQVLSLYKSGITFRGCIITLSATCPITA
ILMRYVSSVAVFPATECLFDYLRTTDTMLKDQFEIHILMKYVDYSTSIIHIFLCLCCSWTLLAASYVFTPITL
DLLMPLNESRKRYFSLLTMFSYDRIEYVDMVYVNILVVYTIGLLCLAGTDLTFVVFAHYICGMFEITSYRMRK
TIAGLSSSRQIDPNFRDFRYVVDHHRNTLHFINYLLSNYTIHYLLPSAVAVVSVSVSLHRLSKMITDAKDEKE
IFMFIFFLISHLAFLYISCYSGQIIIDRSLDVFKESYNSTWYRMPVEAQKLLLFIMLRSSTESIVNIFGFFVA
SHAGFTTLLSTSFSYFTVIYSSQ
>BtOr153
MDVFYRQYNTYRILLSVLGLWPYHKSIYSTIHRISISVIMLAYIVFEVLSLFKSGITFRGCIITLSATCPITA
ILMRYVSSVVVFPATEYLFDYLRTTETMLKDQYEIHILMKYVDYSTSIIPIFLCLCCSWTLLAASYVFTPIIL
DLLMPLNESRKRYFSYLTMFSHDRTEYVDIVYMNILVVYTIGLLCVAGTEQIFAVFAHYMCGMFEITSYRMQK
TIAGLSSSRQIDPNFKDFRYVVDNHRKTLHFINYILSNYPIHYLLPSAVAVVSVSVSLHRLSETITDAKDEKE
IFICFFFLIGHLAFLYICCYSGQIIIDRSVNLFKESYNSTWYCMPVEAQKLLLFIMLRSSTESIVNIFGFFVA
SHAGFTTLLSTSFSYFTVIYSSQ
>BtOr154
MDVFYRQYNTYRILLSVLGLWPYHKSIYSTIHRILISAIMLAYIVFQVLSLFKAGITFLGCVVTLSATCPIMV
FFMRYVSSVATLPVNIYLFDNLRRMDTMLKDELEVQILMKHVDYSTYIISIFLCIWCSWTLFTASYVFAPITL
DLLLPLNESRRRYFSLLTTFSHDRIEYVDIVYVNILVVYTIGLLCLAGTELMLAVFAHWMCGMFEITSYRLRK
TIADLSLSRQIGSNFWNFRHVVDCHRNTLQFIDYVLSNYMLHYIPPCSVIVVSFSVSLHRLSNAVTNANDQTE
IFISGMFVISHIVLLYLCHYSGQILIDRSLDVFKETYNSTWYCMPVEAQKLLLFIMLRSSTESVIDLFGFFAA
SHVGFSKMLSTSFSYFTMIHSLQ
>BtOr155
MDVFYRQYNTYRILLSVLGLWPYHKSIYSTIHRISISLIMLIYIVFQVLSLFKSGITFRGCIVTLSAICPVMV
IFMRYVTSVAMFPVTIYLFDNLRTIDIMLKDQLEVQILMKYVDYSTYIISIFLCLCCSWSLFGALYVFTPITL
DLLMPMNESRRRYFSYLTMFSHDRIEYVDIVYMNILVVYAIGSLCLAGTELMLAVFAHYMCGMFEITSYRMQK
TIAGLSSSTQIDPNFKDFRYVVDNHRKTLHFINYVLSNYPIHYLLPSAVAVVSVSVSLHRVSKTITDSKDEKE
IFICIFFLISHLAFLYICCYSGQIIIDRSLDVFKESYNSTWYCMPVKAQKLLLFIMLRSSTESVINIFGFFVA
SHAGFTTLLSTSFSYFTVIYSSQ
>BtOr156
MDFLGSKYYNINQQLLLLTGLWPYEKSKLTVILNFYMLAVFMVGLIIQLTNIFTSVYNIDDFISYSAYMIITA
MYILKYCTFCCQTKKIKILFELVKHDWNDIQHNENELDMLRSRTSTGKLITIVATTIIVMTSILFAISQLLPV
IIDIMIPAKESRSIHSANMTFIIIRYKNSSYFALFSLIIVNILGAIVMTTTETTYVILCQHLVGMFTVAGNKI
EHALDCDKFELSPTRKNIVIFLRIVNAVNTHKRCLTFIKGLTNTFVLTYTILLFLGMVSLIVHLIYVYELIFI
AFNIKKALIISVVIILHFGILYFGNFTSQNIRDHHAAIFFYAYSAKWYEAPVWIQKLYLFLMIETTIDIKIIL
GFYSPSVEGFFRLVNLSVSYLMAFHSMQRTK
>BtOr157INT
MVTSELNVLEPSQKDDIKKGYYRYTTIHLLITGLWPYKHTKFTRVHRIICASSFFNNCILQLIIYINTSFTSS
LFSHDLLDVVISLSFCLLYSTLWHKQDIIKEIMDIVQQDWRTVRNLEEFNILTKHAKEGIRHSKYFCLFMYST
MMIGTVFYAINFTELLTVPDLHLLPIWMQTIVNQKFLRYISYCIFCTVMFLANATSIAAESISTMTFQHLRSL
FEIASNRIKEAFIYNSLHDSTSSKTFKMTESLKSSIHLHVKCMINYVLLYIIISMIEIVCLFRVNYTTQTMLD
AYNNYNIEWYEAPLHVQKIILFMMLKYSFPLSFNMFGVFSACMEGFSSLAKMTVSYFMVIYSTMR
>BtOr158INT

MVETLAYYSFSKTLLRGLGLWPYHNKRFKVLQNSFISLNLFLFIMAQCFVFKTREFMLKDVSTLIMSFSYFVR
YNINWYQLPTAKKMMDKIKEDLETKNGEVLQIMKDHASIGRRYGIRLSLIIYVSLTFVILYPLLICIQDKSTS
LNQICFTRFYIATEYLMLEEKCFLLTVLHSYLSTVIFVTIFLATESLVIMWLQHAVSLCKITRYYVKRATLRN
AKNHFDRRTKEDIAKVVFAQSRSVQFFQELQGNNVLTYGVILVSVIISLGINLFCLSQSLLSLSEIEEEAITY
CLFIICQLTFMFYINNMCQQLLDYSNFHTNWYETSLTTQKLVLNIMLRSSKPLLFTISGFYDGTLNGFLLFLK
ASISYFMMISSIQ
>BtOr159
MVEPAAFYKRSIFLLSWVGLWPYNNTKFRWLHNNIICFIFLSVLFALKTAEFTLYVLSNRLFPIILCSYVVVR
YNMFWYQINVVMELMEQVKYDWKMNQGEPLKILKKYAALGKRYSVVLIALIYLGAFIPITCHVFSYILCIIVS
TNITCQYKFPMAMEYFVDEKKYSFFITSHQYFLLILDATILAGIETLMVTWFQHASSLFAVVRYHIKKAVFED
CVQSHVSRRYLNRNSKRNIEIVINAHRRAMEYAYNLRDRYMFSYGTFLLFVTICLSINLFLLTQSAFLYRNME
ETIIHLVVLSCMLTCMVFFHYMVQFTSDAADNIVTIIYNTNWYEASVPLQKLLLIIMIRSINSFTCTFFGFYS
ASMEGVSMILRKSISYFMVMMSMK
>BtOr160FIX
MRSDFQKMDNFAGSHYYSLNRILISAIGLWPFNRTIYTKLLNVLVILLFLLCVFGQLVKLIMAKLSTALRWKI
VSYIAYSCIFVVRYIGYLYYIKDVKWIFQRITYDWNSLKNEAELLIIHEYTKTMKSLSIGFGLILGPMILTFF
LKTFSNDILDLLIPMNYTRPRVLPMMIEYFIDQEKFFYYIAIYINVTISLGALSVFVTEVNSMAVVYHICGLL
KIVCYRINHIYVSNIPGVSPAQRSMIIHKQIISIVKLHTTIKHYVDFIQLKVMPLYNVFCVFGLITFILALFQ
FAKAMSIRNDIEEFVASLCTVITSVLYAFTVIHFAQKLTDHSSDIFQQLYNIEWYNLPVVEQKVVLFLMRNSL
KDLTFIIGGVIVASHDMFTMYVRTAFSYFMVVYSFD
>BtOr161
MDSAQYEYLRLNKYLLFTIGIWPYQTTLQRSLVAIVFIPIIVAQVILQGGGMVTAITANDIDSLLEGFAPFMI
SLMCLAKYINFFYNFKQMKRLLDIMREDWEIYKKLRIEYDLLSEQYAIGKKITISFVAFLFGLITPFAAMPLL
LNAADALGLCNISDDRPLAFRVEHFVDVDKYYFPLLVHSYIGTLAYTTIVLAINSIIAVYVLHESGLCEILRF
KLENFVESDVMDVKLHANKRDDKWYQNARDCVLLHEHIIEFANILEDANTTSYLLQIGFNMICVSFTQFQAVI
NVQENAALAFRYVSVTISLLCDLLFVSWPGQQLSDSTERIFEFTTNGKWYLSSINCRKLLMMMLSKSITPLKL
TAYKFYTLNLESFSAVARTSFSYTMVLCSVQ
>BtOr162FT
MIIGMDFFEQPAFRLNKRLLSFVGVWPFQSTLPKLSRQSVVLIPIFVLLTAEIRGLSNAWSVNYSLAIECMPP
IIALFLSMSMYLNAIFNTKKMRDVLLFIKNNHHYYANRPEKLILRYYDVQGRKITLYYALYVYTTVVAYITIP
AISLLIDFIIPSNHSEEKSFPIELDYGVDTQQYFYYLFIHSYMTIAMIANLIASCDTTYMLYAQHGCALFAIV
SYELRTVHILDASSLINLKDHRLFENYKNTELLPEEEKKISTKLFLCIKEYQNAIRYCNLVESLFAKSIFVQL
FFNVVCLSIAGTVMKLGNAADTIRFGSFTFAQAAHIFVLCLPGQRLLNHSEEVYNAACEAMWYIFPKKCHNLY
KFLLARTLVFSKITAFKVATMSMETFLAIIQTAMSYFTVLLSTT
>BtOr163FT
MSRNSERNAITNMDFFEQPAFRLNKRLLSTIGVWPFQRTLTKLSRQGIVLFLNITLFIAEVRGLVNVWSISVD
SIIECIPPMVTIFLQVASYFNLIFNTEKFKDILLFIKNDHNYYMNRPENMILQYYARQGSKIILYYVSYIYIT
ITAFVLIPTVSLINELINHSEERSLPIEIDYGIDIQEYFYYLFIPLYISMFIVPHVIASCDSTYLLYVHHASA
LFAIVSYELKTIHIFDTSSLINLKDYNLLEKYKNVELSPDEQKKIFKKLLFCIRRHQNAIRYSNLVESFFTKS
ILAQMFCNVVSLSIGGVETVLNLGNTRNVMRFGALALAQAIHIFILCFPGQTLLNHSEEVYAAACEVVWYIFP
KRCHNLYMFLLTRTMVFNKITAFKLSVMSMETFLSIIQTAMSYFTVLMSTT
>BtOr164TRA
MNFFEQPGFRLSKRFLSIIGVWPFQSTLSKMLTQGIVYLFCVSLFIAESRALSNEWNVNKNSRIECIPPLMTI
SFCLSSHLNLIFNKEKVKDVLLFIKNDHNYYMNRPENKILQFYTAQGNKVVLCYLTYICSTVLAFLMIPTISL
VTDLIVPSNHSKEISLPVIADYGVDTQQYFYYVFICICITVFIIGVVIFTTYSAYMLYVQHACALFAIVSYQL
RTMHILDTSSLINLKDRHLLEKYKNVELSQEEKEKIFRKLLLCIKEHKNAIRYSNLLESLFTKFILTQIFFHI
ICLSIGGVGIVLNLGNTDEITRFGSLALVQVIHIFILCLPGQRLLNHSEEVYVATCEIVWYMLPKKFHNLYMF
LIARTMIFSKITAFKIAVMSMETFLAIIQTTMSYFTMLLSTI
>AGS43046.1 odorant receptor Or1a [Cephus cinctus]
MDFSSVNRFNGLVNFVCGNLLPLTDDATKFSKAQKLYSAFTWILEFTYFLVTTFGIFFTTNQRIFQDTTV
NQAVVIEILILGFYMNLRRGLIYRLIGQMNSVLINVETLKECVEKTVKPLQRPLKLYTIFATVTVVLFCG
SPIYKVFKKDQFSYNDFRIPAYIPGEPYSTGLFIAGILFETLGGFYTILKKASIDIYLIHVITLLTAQYK
YLSLELINIINGKNENINKSDLESLDNERNEKIEKTVQIELKKWIRHREVVMEIGKILKNLLVLNVVYVY
LNCIFRFCFLGFLLISSSGDYFIQALVCSYTVVCLMQVYVLCFCAQNLLDSSTAMTHDAFYEKWYAYGPS
TKRIFSMILISNKMECRLSMCGVVDLTLPTFMAILKNSYSACLFLLKVK
>AGS43047.1 odorant receptor Or1b [Cephus cinctus]
MFLLKYPMQYTSFCLKIICCWPISKNASNLRKNFDFLHFLFVISGFILISIQALVYAIIQANNPQEIIET
LSTQVIYLHAAGKVGIMRLHRHKFSRLLHGVERVQTTASGELLRYHKEYTRVGYVAYWIYTISVFIITLD

YIVQPLWKKSQFLPTGAWYPFDYKKSTFFYTLAYFQQIICITFSGCASTTEITFGVFIFFACARLKVLQR
KFQQLSDNSNGNEKILRRRICNYVQQHCDILRYINDVNETYTFIILVLFLSILLTVCCTSFLIINVTEHS
LDSIFINSLLMVASAVQLLFYYLPGHILIEEAKTIAESVYYSGWESLSINCRKLLLQIMVYSASPINLRN
GKMGLLILENYTTFLTTAASYLTSLRSIVGTV
>AGS43050.1 odorant receptor Or2a [Cephus cinctus]
MVNLVKGYMSTKISQLLMTLIGMKRGKTKREQLLMDALFVYILATVLSAIWLENSELFYSRNDLYALTYS
APCCFTVTFDFVKLMIFTYKRHELHELHKFTEDTYWNKDYNELDKAILDKCDTTSAIGMSILALTSAILA
FHYLTGPYLDNLGTNTTERTLPFRVVFDFPITVTPLYQILYFIEVIGTISIGICSVAFASYLFYTCIFVS
GFFKILQRELENVCEVELESVNTKSSYNNNDTMLAYKKLKKCVIQHQLLIWYLGKLEGLFSYILLMLVLC
AVIILCFAGFQIILGDGTTKLHRQILSVEFIMAALAETVLFAFSCNEILTASAAIGEAAYRCKWYKLPCD
EYGRALRQGMTIMVMRSYKPCSLTVGKFCPMTLEVFTSILSTSLSYFTVLRSMNESE
>AGS43051.1 odorant receptor Or2b [Cephus cinctus]
MDVPLHGGEEYDQFIKPVMNTMQIISIWPLAADCGSYEYLLRICHQILMFFVTGTMSIVVTADVIHNWGN
MDTATECSLIASAFVLCFLRLMVYTYHQKDMRYVVETMRSDWADASYEEKQVLKEKCDFAFRLAKYFIAT
VAITIAFFMTVPMLETYILHSEEKILPFRGYFFLNHTLSPNYEMIYIFEIIAGSFGGSMIAGVTSFNLVV
IMHGAARFSLLQKKLESLNRNDPDVNKLMVKCIKLHQDAIKFADALEDIINVVALGQFVTSTGLVCFAGF
QLTSMLEDRGRLMKYSTFLNSAILELFIFSFSGNELIVESEAVGDAAYRSDWASSSFTQSLRILMMRATL
PSRITAAKFYSMSLESFSAVLSTSFSYFTVLKAVSEE
>AGS43058.1 odorant receptor Or2i [Cephus cinctus]
MSNLIEGYTSTKISQILITLIGMKRGKTKREQLLIDGLLVYIFATVLIAIFVENSDLIYSRNDLYALTYN
APCSFAVTYDFVKLLIFTYKRRELYELHKFTEDTFWNKDYNELDKAILDKCDSTSAIGMCVLSVLATIVA
IHYLTGPYWDAVGTNTTERTLPFRVVFDLPLTVTPYYEISYVIEVIGAFSVGLCSVAFASYLFYTCTFVS
GHFKILQRELENVCEVELKILITKSSYSDNDAKLAYEKFKKCIVQHELLIGYLGKLESLFSYIFLMLVLC
IVIILCFSGFQFILGDGTSKLHRQILSAEYIVTTLVETGLFAFSCNEIFEASAAIGEAAYRCKWYKLPCD
ENGRALRQGMTIMVMRSYKPCSLTVGKFCPMNLQVFSSVLSTSLSYFTVLRSMNESE
>AGS43059.1 odorant receptor Or2j [Cephus cinctus]
MYNLCSTMPLVMVLVKISNFLFHWNVMMYLISFAQNNFWCDPDDDFTRETMKRCDKYGKIFVYLFTNLVL
FAVLDYIFAPVVENFHRNETDRILPFTLWVNLPVTVTPYYEITYTIQSLSTLYTGICTCFFDNFISVLNI
YVAGQLEILGHRVEAVADTCIDITMKDYVSEKSKLGLSLTLKNLTRLYKSTSNPDILYRTNGTCFHFDST
WTINTFQFCDLRRWFSAHGEKQRIILSLVRWQHCCPVEFHFLQATMSFLRKFTFICHFMAGLTQLLLYTW
SFCHTMKLENR
>AGS43060.1 odorant receptor Or3a [Cephus cinctus]
MHNPLAPPPLEVTSEHFRFSFESSTFCEASSRFTEWKSELKNLTIAILNEPLNMYLTIDSDILWLSKRVL
SLAGIWPESPNNFRFFIYLLYLSLFNCAEFAALVLNLYWMNFDKSVRNMTESIPTAMVILKTAMFRRNMQ
LLLPLLSEVRADKFSVEEEPGIAWLYNIMGKLYTRLSVVLIFIVTTMLYAVPLSQWIVAKSNNLTSTYEL
PYQMYFGFEINDLRSHVLACLSLLPMSTVLTIGCTGSDTLLVVLIFYLCRQFVLLSVRIRNVETDPLIHP
TKMKQLIERHANLIGMATALNKTYSSLLLVQTMGLSFVICIVAFELLTMAEVGEETNTLSFIIYSLAVVT
LLFSYCFLGECLIHESSSIHNACYFSNWYRLPPDLARPIIIPIMRSRKPLHLTAGQFYVFP
>AGS43062.1 odorant receptor Or3c [Cephus cinctus]
MHHTMSNTREKDPDTSFLKYADFHINLLRKSGFYSMKGISNKINKEPTIWEVLLVLTISTCGFFIIILEF
RSVAVSLGSDTGFVIAVLSGTLTATLSMSKGLTILTSHREVRELLLRLSGFWEKSIERPENVDVMVQMAN
RASYLSKCYAATVVIMCSSYCMNPYVSVITQFLFTKTANNSYNFTATTFPTVYPFDLSYFPKYVVWILFE
QAVCLLMTLHWIACDTLFPMCATHLAIQFQILRRDLERTTEVDELREIVKKQIILFQSCDILENIFSPII
FLTIIMTSTIMCACIFQFEKTLSCGVYLEIIKYVTHMMSLFVEILIYCGFSNVLSDQTELLYHAAYNSEW
TDRSKKYKSIIYFLILRSQKPFQCTAYHFFPVGLVQITTILTTAVSYFTLLKTVTSESDDKVICS
>AGS43064.1 odorant receptor Or3e [Cephus cinctus]
MEHKRGWAKIKSDIPNKNYDTDVEYAIKLNRWLLKPIGVWPLELSSSRTERIVTITTAVTCCLLMSFVLT
IPCCIEMFSSQKDFKSRLEMLGPSSFCVMAVIKFFFFVIRGKEIRFCIDSVVTDWRNVDVPEERNIMLRK
AKSARFLTTVCALFMYYGGIFYTTYLPLTTAKALSTENVTIRILPYRCNFILFDPYAPLFFDIIYFLQCL
SAAFMFTLTSGVCSLAANFIIHACGQCQIIGLLLENLVDGRGNTSTTLEKRIAVVIVRHLHLLRFVTRVE
DVLNEVCLVEFLGCTLNICLLGYYFITGLETADTARFVTFALLFISFTFNIFIFCYIGQLLTNHCHQIGE
ISYTIDWYRIPGAQARFLILLIAIANRPVTITAGKIVQLSFPCFRDVLKAALAYLNMLRKVTT
>AGS43065.1 odorant receptor Or3f [Cephus cinctus]
MDHEIEEFKIKTAVQNKNFDSDVEYAIKLNRWLLKPFGIWPLNSSSTKFDRGISVISSFICCLLLFFVMI
PSFVVMFVSEKDFKGRLEIVGPTSFIIMVVLKYFFLITRGDTLKMCIDTILGDWSNVQAKEERKIMFRNA
KIARLFTIICVSFMYCGGIFYSIFLPLITAKSLTNGNNLTIRILPYRCNFIIFDPYRRPIFDIVYVVHCF
CSVIMYSITTGICSLAAKFVMHACGQCEIVMSLLENLIDDDKQCSDIVESKLATIIVQHLHVIRFVTRVE
DLLNEVCLVEFLGCTMNMCLVGYYIITGFESADTVRFITFTLLFISFTFNIFVFCYIGQILTNHCHQIGE

ASYMIDWYRFPGTQARFLILLIGVANRPIRLTAGKMVQFSFPCFCNVIKAAMAYLNIIRTVTI
>AGS43066.1 odorant receptor Or3g [Cephus cinctus]
MVEIELRKRILSTENKVQREQNKNFKNDTEYALKLNRWLLNPISLWPLSSHVSALKKIQFKFIRTSCFIF
LAFVIIPACLHTILVQKDPKVKLKMIGPLGFCVMVIVKYFVFVVRAKNIKICVDHVVADWRNVNTTEDRE
IMIDSAKIARIFTVGAALLMYGGGCSYTMLLPLTASKSVSSSNVTIRVLPYPCYFFFFDPQVSPTYEIVF
SAQILCNIVRYSTTSGVCSLAVVFAMHVYGQCQVLMRLLEDFVNGDREKSSTLHQRLANVVVRHLRILRL
ISTMENIFNEIFLIEVVGCTFILCFLGYYCMTDLTESETVGLIAYFLLLISLTFNVFAFCYIGELITNQC
MQIGQAAYMTEWYRLEGKNASNLIALIIISNRPVFLTAGRMINLSYNSFCQVIKSSLAYLNILRQVTM
>AGS43067.1 odorant receptor Or3h, partial [Cephus cinctus]
MHGQDDFDRAANIMTWNHWLLAMLGFWPEKPRDIWFWINFGYFAYHMTMEYVDLFLFIGNLEHVIMNLTE
NMAFSQIFIRMLMMRVYNRQLGELIMEMRKDFQAHNYRSVDEQKIFLSYNSKSKTFMKLLMAFVALTASS
YYLKPILGNLGNDPIESESTNSTLTFELPYRFYLLYNVNDTHTYMITYLSHLPFVFVSGFGQSAADCLMV
TLVFHVCGQLSVLTLRISSINSDPSQCSQNIKDVVVTHQRLLRMGQTIDKAFSAILLGHLVGATSLVCVL
GYQILTNFAHGQNADLATFLTFAFLVLLVLYAHCTVGESLVQESTRVYEAWYDCNWYNMPTENARLIILC
MSRSQKPLCLTSGKFGIFCLSTLTDVLKTAMAYLSVLRSFL
>AGS43068.1 odorant receptor Or3i [Cephus cinctus]
MVGIESCKRILSTEDKVQTEQNKNYKSDIEYALKLNRWLLNPISLWPLPSHVSALKKIQFKFIRTMGWFF
LAFLIIPTSLHTILVQTDPNVKLKMIGPLGFCVMVIVKFFVFVVRAKNIKICVDHVVADWRNVNTIEDRE
IMIDSARIARLFTAASALFMYGGGISYTIILPLTRSKSLSSDNVTIKVLPYPCYFFFFDPQISPIYELIF
LAYILCNIVRYSTTSGVCSLAVVFAMHVYGQCQVLMRLLDDFVDGEREKSSTLHQRLANVVVRHLRILRL
ISTMENIFNEIFLIEVVGCTFILCFLGYYCMTDLTESETVGLIAYFLLLISLTFNVFAFCYIGELITNQC
MQIGQAAYMTEWYRLEGKNASNLIALIIISNRPVFLTAGRMINLSYNSFCQVIKSSLAYLNILRQVTM
>AGS43071.1 odorant receptor Or3l [Cephus cinctus]
MEQTISVYWWKLAPRTTYFRFSIWMSFFTLNISLLYVDLYEVFGNLEQMLLNLSDSVIKSLILAKLLLFR
FSEPLARLITDAQEDITAGEFGSLEEKKCFLEYYQRGKIFYQITMSCVVFAVHVYFFKGLETYLFSEVAG
TTVILGLTCYNIIANSGDLADISTLCCFALYASSMILLLYGYCFVGECLIHESTKIHEACAQCTWYSMPL
IYQKALIMCMLCAQRPLQLTAAKFYVFSLDSFSNVIKTSIAYVSMLRTVV
>AGS43072.1 odorant receptor Or4a [Cephus cinctus]
MRTLPVCFVIFKFWGLWRPSDYLSWMKILYNIYSLLIVVIMYSFMLFEFIDVFINIKSIDDFTMNSFMLV
TFTNACWRVAKILAGRGKILKVINLLTSDYCVAVDKREVEIKEGYDGIARWNTLRYVILVQITVALMLIV
PLSNESRKRILPFRSWLPYDLSSLKLFWLSYVHQCIAIMAAAYINVATDSFISGLMIQICSQLDILKYRL
IKLPRLYNINHRDEFLSSLTHCVLHHEHIFKFASAVESVSNPITIVQFCASAVALCSSVYQLSKHSVDSA
KFMPLILYLLCMLFQLFFYCWYGNEVILKSMEVRDAVYEMDWILLDNNNKKALLMMMKRSERSIQIKSGY
FIALSVEPYIKILKASFTAYNALQQISN
>AGS43073.1 odorant receptor Or4b [Cephus cinctus]
MEVLPLCFKLWTLSGVWRPMHYSSPVSKSLYTVYSLAVLITLYSLTFFEFLDIVFNFGSLDDFANTAFLL
LSMIIICCKATNTLKKRAQIIEIHDLLRAEICRGQNTTENLMLEKFAKTCRSNTLNLFTMMMSCAMCLTL
ESLLYHVNDRLLPMKIWLPYSLSSLTLFSLSFLYQVVTMTLANAITVANDTYITGLMIEICAQLEILKLR
LVELHDPKTLQMDENYSFNREKDILKMCIQHHNHIFKVKANVEKAFNSIFLIQIVTSTLVFCVTALTFLK
HEILSVELASVILYFFTMMFQLFLFCWYGNKVILKSTEVRSAVFEMDWIPLPQAIKKDFIFLMMRTNIPI
RFTSGYVVTLSLDSFMAILNYRTPH
>AGS43074.1 odorant receptor Orco [Cephus cinctus]
MMKFKQQGLVADLMPNIRHMQFSGHFMFNYYNDTGGSTKLFHTIYCSIHLFLILLQFGLCCVNLTLERAD
VDDLTANTITVLFFAHSIIKLAYFAVRSKLFYRTLGIWNNPNSHPLFAESNARYHAIALTKMRRLLAAVG
AATILTVCAWTGITFVGDSVKKVTDPVTNETMTVEIPRLMLRSWYPYDASHGMAHVLTLIYQFYFLLITT
MDANSLDVLFCSWLLFACEQLQHLKQIMKPLMELSATLDTVVPHTNELFKAGSTDHLRDTQGTQPMAPPP
NENMLDMDLRGIYSNRQDFTATFRTAAGMNFNGGVGPNGLTKKQEMLVRSAIKYWVERHKHIVRLVTAIG
DAYGVALLFHMLITTVSLTLLAYQATKVNTVDVYAATVIGYVLYTLGQVFLFCIFGNRLIEESSSVMEAA
YSCHWYDGSEEAKTFVQIVCQQCQKAMSISGAKFFTVSLDLFASVLGAVVTYFMVLVQLK
>XP_015605172.1 PREDICTED: odorant receptor Or2-like isoform X1 [Cephus cinctus]
MNRNNFVRDNVMIYKCLGSWPLDCVARRLENTRYILGLTLIAVITVFNGLQYIDLFIEWGDWGFISENIS
VSFIYSIFVSKIYVFYSRRREIMEMVLEIDNYVRKTYKDGHQSNVQILQSCEDIARRIKIVFGWSALLTV
IILHTWPIASMIFKKDPNLRMLGVPAYFPFSLNSTNNYAIAYVVEIVTASVMSLHTVNFDTFFISCILFA
IGRLRILHNSINNIKKIAPSDLQMKFSKNETYQAELQKCIHQLLSEAIAEHQKIILFCQVIDSMLSWIMF
IGFFIYSVMLCFVGMRIIMIGPSIKLLQLMEYLAIMMTQIFLYYWHGNELKLESLKIHDAAYNCDWYSFD
RNSQQTIMFIMMRGQKPIYLTAGKFYYVTLETFLSLLGGSYSYFAVMQQLYDS

>XP_015605187.1 PREDICTED: uncharacterized protein LOC107272485 isoform X3 [Cephus cinctus]
MNRNNFVRDNVMIYKCLGSWPLDCVARRLENTRYILGLTLIAVITVFNGLQYIDLFIEWGDWGFISENIS
VSFIYSIFVSKIYVFYSRRREIMEMVLEIDNYVRKTYKDGHQSNVQIGPSIKLLQLMEYLAIMMTQIFLY
YWHGNELKLESLKIHDAAYNCDWYSFDRNSQQTIMFIMMRGQKPIYLTAGKFYYVTLETFLSLLGGSYSY
FAVMQQLYDS
>XP_015609891.1 PREDICTED: odorant receptor 22b-like [Cephus cinctus]
MLQTSARLDTLSFRLAEHVDRCSAKRLGGINGPKEEAQRSLGSCVEHHVLIRNGVGKIETLFGGVIGILF
ATSLITLCTTVYQISKQDMPSVQFFALQLYLACMLFQTFFYCFYGNELQSKSRSIGDTVYKSDWIKFSPK
EKKSLKLIILMSQRELNVSCNGIFILSLSTFMWIIKTSYSAFNLLQQIAN
>XP_015610240.1 PREDICTED: odorant receptor 13a-like isoform X1 [Cephus cinctus]
MENQESFDEMIRVNLFLLKFSGIIRASVGKISLTEILSVLAFGLLAAMSCSYLRDLLLTDNMEVAMQSLA
FMITGFGNTLHYILIAKSRAKLCDVLATFEDLWGLLEVQEKRVLMSYVRDAKKLTYFFMSQCAATVFLYV
GAPVIFGNGFVRVNGNITERMLPYSLIFECKDSPCYEILYILQILTVINIAITYIGVDTIGPVLILTVSG
HMKIIQNRIMSLGSSEEFENFKNDKVKRISDYEISHFQYFGKSFEACVKYHQTVLKLCKDIEKVTNKAFL
VQLITSTYSISAIGFKMVGNDSDKSKYVTQIVLSLVQLFLCNWPPDVLQNEILIHFKSQAVAYAAYFMPW
YRCSRDVKKSTEIIIMRGQRVVRLTAGNFVDLSLETFIRMVSSALSFFTLLRSIE
>XP_015610246.1 PREDICTED: uncharacterized protein LOC107275027 isoform X7 [Cephus cinctus]
MENQESFDEMIRVNLFLLKFSGIIRASVGKISLTEILSVLAFGLLAAMSCSYLRDLLLTDNMEVAMQSLA
FMITGFGNTLHYILIAKSRAKLCDVLATFEDLWGLLEVQEKRVLMSYVRDAKKLTYFFMSQCAATVFLYV
GAPVIFGNGFVRVNGNITERMLPYSLIFECKDSPCYEILYILQYFGKSFEACVKYHQTVLKLCKDIEKVT
NKAFLVQLITSTYSISAIGFKMVGNDSDKSKYVTQIVLSLVQLFLCNWPPDVLQNEILIHFKSQAVAYAA
YFMPWYRCSRDVKKSTEIIIMRGQRVVRLTAGNFVDLSLETFIRMVSSALSFFTLLRSIE
>XP_015610247.1 PREDICTED: odorant receptor Or2-like isoform X1 [Cephus cinctus]
MTIQKISFDDVIMLNRVTLRGVGILKFPGFKKSSSKIIQGFLIALMMANALYLMISMSYVLIFSNHEMTT
LLSTMAPLMSITSGSSRYLLAVYNKVLIERILEVNKQVWNCATKQQDYETLKNYGLRARALTAMLFGSAS
LTVVFFLATPILAITNEPMDFYSNSTGNFALRRALPLPSPFETFSSPYYELAYVAQGIATCYLGVAVSTV
DALSTVLVIHGCAQFKLLNNRLLGIVRQDLQSASNYYGHDQRARFFKTSFRFHRSILEYCYTLEAVVSIS
NLISVLTCMCAISFCAVNIVRGGADLLKFLGLFMVFIMQMLFVCWPADYLSSESTNIAFAFYNCSWYNYE
KSIGSSIQIAIARSQNPVALTAGKFVIMSLETFTSILSSAMSFFAVLKSI
>XP_015584747.1 PREDICTED: gustatory and odorant receptor 24-like isoform X1 [Cephus cinctus]
MIKIIHSFSRTMSAFRTSRVAPKYGMNDFIGGMQPRIMYIDQMNNKEKISHPMRIPIEAFKKHDKGELDV
VYDNIKPVVTVIRIMGALPITRTRSGITRFKLASNAMIYSAVVYFALTAHVLYVAWNRIRIVGAVEGRFE
ESVIAYLFIVYLMPNFLIPLLWYETPKHAECFNHWKEFQIFYKRITTRDLPINLKRRALWTAILVPILSA
AAMIGTHLTMINFSIWQIIPYVYVTAFINISGAYWYIHCAAISRSANVLAQDFKHALRNNVQATTVAEYR
ALWLHLNRITRKIGVMCCYSFTILTIYLFFSLTLSIYGLFSQLQDGLTIKDAGLTLSACSNIILLHFICD
QAHAASQHVRVHFQKKLLLVEISDLNPDAQTEIDMFLRATEMNPSDMSLGGFFDVNRNLFKSFLGTMVTY
LVVLLQFQISLPESKNNDNATMDNI
>XP_015586850.1 PREDICTED: odorant receptor Or2-like [Cephus cinctus]
MIMLTEVVCCLLLCAATVFHAQKFSNNAYLIHAIAVMAKLFVASFVCEMLSHQSEQVGNSIYLANWVEYS
SDIRKDVAIIISRGQKPVTFTYQFDQMGFKIFLLILKASTSYYMLLRQTLGEELVSVS
>XP_015589015.1 PREDICTED: odorant receptor 4-like isoform X1 [Cephus cinctus]
MDHEIEQCKIKTTVQNKNVDSDVEYAIKLNRWLLKPFGIWPLNSSSTQFERLVSVISQFVCCFLLLFMMI
PSIIEMFVSEKNFKARLDILAPTSFSVTVAIKYIVFMTRGRQLKTCIDTIINDWGNVREKQEREMMIRNA
RIARLFAIICVSFMYCGGIFYSIFLPLITAKSLTSGNNLTIRILPYRSNYILFDPYVRPVFDIVYIAHCF
CSFVMCSITTGICSLAAKFVMHACGQCEIVMSLLENLIDDDKQCSDIVESKLATVILQHLHVIRFKCLLR
FATRVEDLLNEVCLVEFLGCTMNMCLIGYTILTGLESTDTTKFITYSVLFLSFTFNIFILCYIGQILTNH
CHQIGEASYMIDWYRIRGIQARFLILLIGIANRPMRLTSGRMIQLSFPCFCNVIKVAMTYLNVLRKVTA
>XP_015589018.1 PREDICTED: uncharacterized protein LOC107264835 isoform X3 [Cephus cinctus]
MDHEIEQCKIKTTVQNKNVDSDVEYAIKLNRWLLKPFGIWPLNSSSTQFERLVSVISQFVCCFLLLFMMI
PSIIEMFVSEKNFKARLDILAPTSFSVTVAIKYIVFMTRGRQLKTCIDTIINDWGNVREKQEREMMIRNA
RIARLFAIICVSFMYCGGIFYSIFLPLITAKSLTSGNNLTIRILPYRSNYILFDPYVRPVFDIVYIAHCF

CSFVMCSITTGICSLAAKFVMHACGQCEIVMSLLENLIDDDKQCSDIVESKLATVILQHLHVIRFKCLLR
FATRVEDLLNEVCLVEFLGCTMNMCLIGYTILTCHQIGEASYMIDWYRIRGIQARFLILLIGIANRPMRL
TSGRMIQLSFPCFCNVIKVAMTYLNVLRKVTA
>XP_015589025.1 PREDICTED: putative odorant receptor 85d isoform X1 [Cephus cinctus]
MERKTLYKIAPNEYNKDYQSNVEYAIKLNRWLLKPIGIWPIRSSSKLSNIISFIYSGLCCFIMIFMVVPT
CIEIFTGEQSLKSKLEIFGPTNFCAMAIIKYCVFAFRSRKLKVCIDFIIADWREVSNKEERVIMFRNVYI
ARSLTGVCVTFMFAGGIFYNILLPLLTSNSLSSGNVTIRMLPYRGNYILFDAYVGPFYHMVYLMHCLCAI
IMYSVTTVVCSLATKFVMHTCGQCQIVISLLKNLIDGNQNCSRTLDDRLAVVVVRHLRVLRFASHVENIL
NELCLLEFVGCTINLCLLGYYFITEFENANTLGLITFFLLLVSFTFNIFIFCYVGELLTDHCHQIGEASY
MIDWYRLSGRQGRFLIMIIAIANRPINLTAGKMIQWSLMCFTNVIKVAVAYLNIIRKITS
>XP_015589027.1 PREDICTED: uncharacterized protein LOC107264841 isoform X1 [Cephus cinctus]
MKCCLDKMTTDCRDIDPQDHMIMKENVNIGRFLTMIYSATTFGNTFFYIFIMPNFTETIVDDRNVTLRSL
PYPSDYYLFDVQFTSAFEIIYFIQSLVAYITCTIRSGTCSLAANFVTHACGQCEMIVTHINDLVDGYREE
LNTLDNRMRDIVQHHSRLIRYFSDTVI
>XP_015591754.1 PREDICTED: odorant receptor coreceptor-like isoform X1 [Cephus cinctus]
MDYEYNPEEAFAINFGFLRVDGAWPTGITNPILRFLYRIYGFCVLSIFTVLYFSTEIILMTVIWGDVQAM
VDCSFLFLTHLSHSVKLTNFLLRQKRLDRMIFQLREPMFVPKRPEHIAIMKAAVLNAERETKMFLTITFG
TIICWSILPLLSQKEVKELPLMGWFPFDTTQSPAYGFTYTYQVISVLLNACVNAMMDTMTSGLLILMGAQ
LDMLKENLQSLTDDASTMSQAEMKYIQAVKMKPRGTMFIDIRQIQYDGKTDGSVEKIVKSKHEYGKLDLN
RNFDTDNQFENVMRYEDILHIRVVTCAEHFKALVKYIYNVRDVFQIGIMAQFLASSIIICLTCFKLSLVS
PASVEFASMLQYMACMLFQIFCYCWHGNEIIEKSSELLSAPYGSQWVSGSKKIKQSIQIMMLQMKTPLKL
VVGGFFTLSIDTFFAIIKSAYSFYLVLKQMNE
>XP_015593532.1 PREDICTED: odorant receptor 13a-like [Cephus cinctus]
MKDTAIVGKPVEIGLRLIDSWPGASNRIGRLTVWSLMMAALIFQYWDAVSVFNDLDNLMDNFSVTITETL
FFVKLIIVYKNRRYVNEVLKHMSEDWNSVKSVEEWTVMTEHAKLSRIFYIWALGLYVGTVVLFLPVVINH
YNAADINDRKFVLPSEYPFQSKVSPVYEVLCCVQFLQAVLTAAGNALTESLLVTLVLHAGSRLVLLRKDI
SRFSDISRTVDDRRKILLAGNVLVANHRRVIEFSDKIEDLFSYISLVQVLSSTLIICTIGFMFITSISST
KNILTLMKFGLFILGELWETLAYCLAGEYLSNQSQSISQAVYECPWYKMQPRDSKMLMMIMIRAQKPLRI
TAGKFIFLSLDNFTDILKTSLSYISVLRAIY
>XP_015593744.1 PREDICTED: gustatory and odorant receptor 22-like isoform X1 [Cephus cinctus]
MLFYILSMCSTGYTLMLRFRFFNNPDLPFDDYLFNLIFICVAAAHWILLSAALLSAKRIAEYLSTWNEFQ
RHYHSVTRRRIQLNFKKRATILLMATIIHGIIHPLIQVLLLGTFTFSEATSYEYIIFLQGYLSVFWESQC
RSIIMTARGLRESMKKELTTNPLTIRVEQYRILWQHISDVINKFSRASHIIFGCYSMCLYTIVMVSGYCL
LSHLLSEREQIFSNKLGGYLVAILFHAMNLFVLCYCSHQMRREVVEFVVEDLTELKVHRLKFNQQKEVFL
FLGSISMNDPRLILLGNHTIGMSTISNVCLLPMLLIQYFISLCIKNVYNFVFAVRIHVGDVLPCFTSV
>XP_015594067.1 PREDICTED: odorant receptor 85b-like isoform X1 [Cephus cinctus]
MRHRVEISSSSRETPWIFSSSGAFEPFVSVTMKTKKDKCRDMKFVTNIHRLVLAPIGLWPDVENIKKDKV
LKFKIISSILLVLVFVYLPQTILLVTRIDDFDLVIQILATGEIVCSCALIKIIILYFNRQALGKLLKYME
EDWMNSVNANEETKGILLKKGKFVRRFCISNILFSYLTFLLRVLVKIQFHITKTSECETHRGLEFFFPSY
FPKYMLKSPNFELIFIGQIVATVIVINIYIGCDNFLVLLIMHVSARSTILRALLRDLPLKVHPKDSIKFM
KELGIIVRRHEHLNRFASMIDDNFNIILLAQMTLLAALLCFLCFQLIMKVQVQNAEVSTMEIVFLIFFIL
ATLIPLYIYCYFGEILQHEVNVIFPNIILQHYFFSHNSITAQNEKIRNAAYECLWYIREPIDARSVILIM
DRTVRPMKLTAGKFRSFTMSTFTDILKTSMSYLSVILATMQEPVDN
>XP_015594069.1 PREDICTED: odorant receptor 85b-like isoform X2 [Cephus cinctus]
MRHRVEISSSSRETPWIFSSSGAFEPFVSVTMKTKKDKCRDMKFVTNIHRLVLAPIGLWPDVENIKKDKV
LKFKIISSILLVLVFVYLPQTILLVTRIDDFDLVIQILATGEIVCSCALIKIIILYFNRQALGKLLKYME
EDWMNSVNANEETKGILLKKGKFVRRFCISNILFSYLTFLLRVLVKIQFHITKTSECETHRGLEFFFPSY
FPKYMLKSPNFELIFIGQIVATVIVINIYIGCDNFLVLLIMHVSARSTILRALLRDLPLKVHPKDSIKFM
KELGIIVRRHEHLNRFASMIDDNFNIILLAQMTLLAALLCFLCFQLIMKVQVQNAEVSTMEIVFLIFFIL
ATLIPLYIYCYFGEILQHENEKIRNAAYECLWYIREPIDARSVILIMDRTVRPMKLTAGKFRSFTMSTFT
DILKTSMSYLSVILATMQEPVDN

>XP_015594070.1 PREDICTED: odorant receptor 13a-like isoform X1 [Cephus cinctus]
MCNTDMKFATDFSRLALEFFGFWPDTEYSQASKHLKLKCIITILFLLGFLYLPQSALLLMNIGDLNLVIQ
ILATGKIIISCAIIKIIILHIKREDLRRLLKSMNEDLVDYANEEIREIMLKNAIFVRKMCISYSVTICIT
MLLYIVVKIFFYFAKISDNDTYPGLDLFVPSYLPNYLFNSPSFELIYAGQIVAIMIAISAYSRSDSLLVM
LIMHVSTQFTILRSSLKNLPVKVGTTSSTFMCELGVIVRRHEHLNRFASKIDNIFNMILLLQIVLCSVLL
CFQSFHFLVKLKDKNAELSIIELVFLCFYICPVLIQLYIYCYFGEMLQSQNEETRRTAYECLWYILDLRD
ARTMIIIMARTVRPVQLTAGKFSPVVMSTFTAILKTSMSYLSVLLATINDPKDN
>XP_015594072.1 PREDICTED: odorant receptor 13a-like isoform X3 [Cephus cinctus]
MCNTDMKFATDFSRLALEFFGFWPDTEYSQASKHLKLKCIITILFLLGFLYLPQSALLLMNIGDLNLVIQ
ILATGKIIISCAIIKIIILHIKREDLRRLLKSMNEDLVDYANEEIREIMLKNAIFVRKMCISYSVTICIT
MLLYIVVKIFFYFAKISDNDTYPGLDLFVPSYLPNYLFNSPSFELIYAGQIVAIMIAISAYSRSDSLLVM
LIMHVSTQFTILRSSLKNLPVKVGTTSSTFMCELGVIVRRHEHLNSFHFLVKLKDKNAELSIIELVFLCF
YICPVLIQLYIYCYFGEMLQSQNEETRRTAYECLWYILDLRDARTMIIIMARTVRPVQLTAGKFSPVVMS
TFTAILKTSMSYLSVLLATINDPKDN
>XP_015594074.1 PREDICTED: odorant receptor 22c-like isoform X1 [Cephus cinctus]
MLKKHIASSDLKYVINWIRISLKVVGLWPNSVDEMNWLSQYGFLIVSFLILVVISTPQIISLIMVWPDLN
LMMKNLVLLNVPVTTSMIKCFIFGFSKQDVRPIVDLVVAAWMRPKSDQERALMMKYATIAKIIYVPFSIA
AYASILVNVAIPFVVYQRAENISDYINEPFFQTVIIYNIQNSPIFELTCIVVVIMITLACTAYTVIDGFL
AMSVLHLCCQFTNLRNELKSVMTEATMNDGNQFLEKIGLIVKRHNELIGFSKRVASSYAWTLLVQMTASM
LECCFFGFGFIWVFQNDQSSIAETVFLICAIFVAFAGLYGYCYVSELLCSENEAIGDTVYDSCWYNLSPK
DARCLLFIISGSRKQLFVTAANFCYYSHSLFAAVRNFPTIQKSICYAETFVLL
>XP_015594572.1 PREDICTED: uncharacterized protein LOC107267405 isoform X1 [Cephus cinctus]
MYLTIDSDILWLSKRVLSLAGIWPESPNNFRFFIYLLYLSLFNCAEFAALVPNLYWMNFDKSVRNMTESI
PTAMVILKTAMFRRNMQLLLPLLSEVRADKFSVEEEPGIAWLYNIMGKLYTRLSVVLIFIVTTMLYAVPL
SQWIVAKSNNLTSTYELPYQMYFGFEINDLRSHVLACLSLLPMSTVLTIGCTGSDILLVVLIFYLCRQFV
LLSVRIRNVETDPLIHPTKMKQLIERHANLIGMATALNKTYSSLLLVQTMGLSFVICIVAFELLTMAEVG
EETNTLSFIIYSLAVVTLLFSYCFLGECLIHELITRFDLPEFLDSQRLLFFKLVPIAARPRQANNHPDHE
ITKTLALDSRTILRIFPRNIYIYHEGLHGVSLGLERN
>XP_015594673.1 PREDICTED: uncharacterized protein LOC107267447 [Cephus cinctus]
MRAYAKSGRYLCIGLTIMMYATAAFFFAEPIYYLIVERRTNIAERYFPYPSSYGTDLMKETPFFEVLSWM
QIIGGNFCLTANYATDSSITVTLLHLCGQLEVLANAWRDLFKITGNDKKLINYQVQELVKRHLHLIK
>XP_015594781.1 PREDICTED: odorant receptor 13a-like [Cephus cinctus]
MCENNTFLENLLFVRTYRLAKLVENTFTMIIFLQLMISSILIALIGFNFVQSLQKSDFLQFSLMISYMIS
QSLFVFLYCYSGQKLRDQSIAISTGIYMSNWDDMLTSHVGNDFIIVMMRSQRQLGITAANLYTMDYSMFM
NILKTAGSYVSILRAMY
>XP_015595241.1 PREDICTED: uncharacterized protein LOC107267730, partial [Cephus cinctus]
FNVVFIFLLGYGFDTVTAPNFEIVFVFKTISYMICSCGVTGLDGTFLTVFVYVCGQFQLIQAWLRKIGAE
INKCIQHHQKIIEVADELEDLLSPITFIQFIMNGLEICLSGYAISVDDNYMDLIKISNYLISIMVQLIVW
CWPGDILIEESSAVANLVFHDVPLYRLPKILHFVVIRGQKYSRIVPYDGGYVQETKEC
>XP_015595442.1 PREDICTED: putative odorant receptor 85d isoform X1 [Cephus cinctus]
MDITSLSNHKLPKISSYYNVKLTLLVNKYFGYWPSLNVNQRFYAIYTFFSFSLTIGAILLAELVYVIVYI
RDFNKLTSISLLLFTNVVHVSKIQNLIRKRKQIRHLLEYVETGQFSNETDKYERIVTYFSWFGLFHYAIN
VLFGMGAITGWSIISLFSVDNDKIDRRLPSDGWFPYDPLKSPAFELTCVYEIIILHVCAFHNLALDHLTV
GFMSVVCCQFVILNCNLQSIGDDVLNAENIKNDLSNSLKNKENDDLIYKRLVKCVEDHMTIIQFAKDVEN
IFNLPVFLQFLESCLVICVTAYQISQDSNKGTSEILGNFSYLMCMVYQLFVYCWYGNSILLLSESVVQAA
FSGNWWKSSERYKRALRLMMVRAGRPLTITTGNLLTLSLSTFMGILKASYSFFTVLQSTNKKI
>XP_015595561.1 PREDICTED: odorant receptor Or1 isoform X1 [Cephus cinctus]
MEIQNMGFYESRKKSVKPIKTNVHLQVSLSIIRYMGTWPPQGRYRNLYFVYSFLVLNIILGSFLFAQFGT
IIMIWGDIEKMVSCGALLMTNIAHAFKVVVMLRHQKSIQILLDSLQDELFTRNNERFKFIAYEYTWKGIF
HHVAYQSFGTVAVLCWVFTSISDLIIKHMKRLPIPLWYPFNVTNTPAFELITLQQTVGVISGCFHNVAMD

TLITGLITVACCQFELLKTNIITIGSNVNHEFCKISHNEINVTNVEDNENKVWQEMRKCIAHNNKIIEFS
KEIQSLFGTAIFLQFLANCVIICLTTFNMSQTTVFVPSEILGTVAYTCCMVYQIFIYCWHGNELWLQSES
VLRMSFTSNWWEHNRRYKKALQMVMLRASRPVILTAGKLLKLSLETFVASNIFFQILRVSYSLFTVLKTS
TDH
>XP_015595604.1 PREDICTED: odorant receptor 94a-like [Cephus cinctus]
MEMYILDRHYMKTNFQLMSLLGIWNPYSQGFSYWIYEFYSILVFLFIMITYTYFEILELIKVNLTLDLIT
ENLCISLTHICGIIKIINLYLRKDNIYTIMDIMERQIRQPCSGIIGRVQKESIMSATWTIETTIKYFAII
ITFAEISSLCAPILDGSMWHGQLPFRAHLNILKGKMEICCMISNDDLQDMKTDTSNLSSIELRKCQMMRS
NIELNTCISIHQNILRCLTILESMFNVMILLQFATSLLIICLTGFQINANTTNIPRFMTMICYLCCVLLE
LLLYCWHGNEVIIQSSNLKLSAFSCNWMEANSKFCKTLHIFLTRVQKPMILTAGGLSNLSLSSFTTIISR
SYSYIAVLNQMNQ
>XP_015596472.1 PREDICTED: odorant receptor Or1-like isoform X1 [Cephus cinctus]
MGIANNIELLNTSVGRRWSSYQTVLEYKMEVECLKLAGLWYLSSDTSDCVKVIYWIYNKIVFAAIAIFSI
GLLTDIIINYKDLVTFTDSGCIFVGISVVSFKAVVFQCKKSRIDRLVHSVRQCESWTSDLVDPAAMSLLK
KYKIRERVTILGFSCLGCVLVIALIFFVPRETGELPIRCRYPFDTTVTPMHQIVFGIQSFAVAVGMIAII
GMDNTVFVLCGRVLFQLEILAANFQSCSLDINASGGRSETNPHKDFTCFRHTIDCGGFFERYRKCVMHHQ
YLILLVDEVNDVFGSSMFSQLLSSSLIICLTGFQSLLVVGHGTNFLKFAIYLGAAFSQLLYWCCLGNELS
HQSSLLLESQWKSGWEMQPAKKVLHPMVFSMMRSNKKLELKASNFFIMSTETFITILSKSYSIFALLNNM
IQ
>XP_015596477.1 PREDICTED: odorant receptor 67a-like isoform X2 [Cephus cinctus]
MGIANNIELLNTSVGRRWSSYQTVLEYKMEVECLKLAGLWYLSSDTSDCVKDLVTFTDSGCIFVGISVVS
FKAVVFQCKKSRIDRLVHSVRQCESWTSDLVDPAAMSLLKKYKIRERVTILGFSCLGCVLVIALIFFVPR
ETGELPIRCRYPFDTTVTPMHQIVFGIQSFAVAVGMIAIIGMDNTVFVLCGRVLFQLEILAANFQSCSLD
INASGGRSETNPHKDFTCFRHTIDCGGFFERYRKCVMHHQYLILLVDEVNDVFGSSMFSQLLSSSLIICL
TGFQSLLVVGHGTNFLKFAIYLGAAFSQLLYWCCLGNELSHQSSLLLESQWKSGWEMQPAKKVLHPMVFS
MMRSNKKLELKASNFFIMSTETFITILSKSYSIFALLNNMIQ
>XP_015596478.1 PREDICTED: odorant receptor 83a-like isoform X3 [Cephus cinctus]
MGIANNIELLNTSVGRRWSSYQTVLEYKMEVECLKLAGLWYLSSDTSDCVKCKKSRIDRLVHSVRQCESW
TSDLVDPAAMSLLKKYKIRERVTILGFSCLGCVLVIALIFFVPRETGELPIRCRYPFDTTVTPMHQIVFG
IQSFAVAVGMIAIIGMDNTVFVLCGRVLFQLEILAANFQSCSLDINASGGRSETNPHKDFTCFRHTIDCG
GFFERYRKCVMHHQYLILLVDEVNDVFGSSMFSQLLSSSLIICLTGFQSLLVVGHGTNFLKFAIYLGAAF
SQLLYWCCLGNELSHQSSLLLESQWKSGWEMQPAKKVLHPMVFSMMRSNKKLELKASNFFIMSTETFITI
LSKSYSIFALLNNMIQ
>XP_015599573.1 PREDICTED: gustatory and odorant receptor 22-like isoform X1 [Cephus cinctus]
MHRNNGDEDDRYRDQVQGNIFKVQQDKANTNGGANKPAVFLGTHGRFEEEAVRVAPKYERKNQFSYNEDR
PPEIDAKLLDEHDHFYDDTKALLVLFQIMGVMPIQRGQGKSSLCQREMINLTCTSKNDLGVTTFEWTSPA
TLYAFCLWIAETVIVILVGRERIENTLEPGKPFDEYIYNIIFLCILAPHFLLPVASWTYADEVAKYKNMW
THYQLRYYKVTGSTLEYPNLKRMCVSLCLASWIISIVVVVSQFYLQPGFTMWHTSAYYHIIAMLDCLCAL
WWINCTAIGSASKGLALNLHKALENTGSAALLVEYRSLWLDLSHMMQQLGKAYANMYGIYCLIMFVTTII
AGYGAFSEILDHGFSLKELGLFVIAAYCMTLLYIICNEADHATRKVGLEFQERLLNVNLAAVDVQTQKEV
EMFLVSIYNNPPIYHLNGYAVINRELLSSSIATMATYWVVLMQFKLSLIRRTVQTSFVDTSSAKNLT
>XP_015599574.1 PREDICTED: gustatory and odorant receptor 22-like isoform X2 [Cephus cinctus]
MHRNNGDEDDRYRDQVQGNIFKVQQDKANTNGGANKPAVFLGTHGRFEEEAVRVAPKYERKNQFSYNEDR
PPEIDAKLLDEHDHFYDDTKALLVLFQIMGVMPIQRGQGVTTFEWTSPATLYAFCLWIAETVIVILVGRE
RIENTLEPGKPFDEYIYNIIFLCILAPHFLLPVASWTYADEVAKYKNMWTHYQLRYYKVTGSTLEYPNLK
RMCVSLCLASWIISIVVVVSQFYLQPGFTMWHTSAYYHIIAMLDCLCALWWINCTAIGSASKGLALNLHK
ALENTGSAALLVEYRSLWLDLSHMMQQLGKAYANMYGIYCLIMFVTTIIAGYGAFSEILDHGFSLKELGL
FVIAAYCMTLLYIICNEADHATRKVGLEFQERLLNVNLAAVDVQTQKEVEMFLVSIYNNPPIYHLNGYAV
INRELLSSSIATMATYWVVLMQFKLSLIRRTVQTSFVDTSSAKNLT
>XP_015599657.1 PREDICTED: odorant receptor 23a-like isoform X1 [Cephus cinctus]
MANINNCFLCTSFFLRTIASWPGTTRSISNFRKLFNALHVFLVISLITSTILQVSMYIIKNPLDAKVITE
ILSAQCVYFHVIIKVIMYNVHRKKLIKLLTSVKTSLESNVKPLVNNVETYMKIGIIIYGSYALSIVLLAL

DYILSPILQSERVLPALMWYPFDYTNSTLFYGLAYAHQITAIQLSGCTLGVEITFGMLVFHCCAQLQYLQ
YLLQTLILGCHAERSRRTIRVRTAKCIRYQNEILSSINDIDDVHTYIVLVLFCTATVTVSCLGFQLLNIE
TLSVNSLAKFMLNAGCVLLQLLFYYLPASILTSEAMKVEMAVYRCNWESLLPDQRKDILFILMRSMHVPR
LTVGRISSLHLENYMKFLTTTASYLTSLQAATGATS
>XP_015602105.1 PREDICTED: uncharacterized protein LOC107271048 [Cephus cinctus]
MEEDYTSIKIIRVYMKFIGMWHTENPCEEYIYIGLIIYISMTCVVAGIVTGLDLYHSLGNLYAISNATPC
TETTALALGAMVILTRNRRKLIQLAKYMNENLWRSGKNPVATDVLDKCNRHSLWIIAIFLSISQLMVVHY
VSGPIFDMMDENVTEKTLPFKLWISQEIIPYSRTPYYEIVYTVQSLATVSVGVTTASVGIFFCTTNIYTA
GQLEILQINLGTLCEIDFEKFAIDVNDEIALMHKIYENLKEYIKRHLILISVIEQMEDMYCYIMLAQALS
STSLICLTGFQLVTVSDMTTHRKLLCCEYFLAINCQFFLFSWVCHQVIMRSLEVADGAYNANWHKIPYTG
KGKDIRDGLMLIILRSHRACSLSVGKFCPLSLETFASVLSTAMSYFTVLRQGDYDSYGMSVLDDIDRKGV
LLMCTFIFFVQGTVIGYVLTPIIDLLLSKTYYRNYSTAVVQCNTATIRIRRDMGLDRDHYFSIRVTRFFL
RLTGFWIVESKRDEMVIDFALIYTRVAVLLAAVILSLDLYHLSEDLYVGYFYSFGILKINNTIPIYYTDD
MVFTICNLMTVLTVVFKLLIMTWHRNSVANLISFSLNEFWHKDYKSTEMDILKNCERQCIVLILAFIMSA
CGTGCSYMMRPIIENIGKNETDRIHPIKVWLDLPLATTPYFEIMYFVEILITTQVAICFFCFDIFLCVLN
IHVSGQLEILQSRLECIYDENRIESPKMYSDEKLKNLEKSSIAYNKLCNCIKYHEMVIWYVQQIERIFTI
IILGQMLTSSLLTCLVGFQLLSGIVEITKRYIFIAYMCGCIVQLITYTFTCNEITVASLQLADVAFHADW
YVTPYDKGGRSIRHGIKFLVQRTQRPLCLTAGGFFPVSLDSFTKVIILENIMIAMWLHYVASPMA
>XP_015602106.1 PREDICTED: uncharacterized protein LOC107271049 [Cephus cinctus]
MDTHRNKYLSIKVTKFFMKIVGIWLPESKHEQFVLDVSLFVTIAGTVLSILFEMWDIYKYPFNFNEAVYI
ICNILTPGIVLFKLSMIRLNRKSLYELIDICQTKFWHDDYDEFGIAILQNCETKCVLLITSYMSFALFTA
ITYTVRSIIDNIGKTGTDKILPFTMWLNETMARAPYFQLLFIFEGIILCYLGVGFFCIDNFFCIINIHVA
GQFKILQGKLERLCGPNDREDEKKDRGIWIRKNPAAVFQEFRSCVQLHKMLIYYVEKVEAIFSLIILCQV
ILSSILMCLAGFQAVSDDNSASQRCIFTAYTIGCFFQLLLYTSTSNEIIDESLGVANAAYRAHWYLLPFD
KTSKVIRGCLVLVILRARRPCSLTAGRFFAISLETFTKVISTTISYFTLLRQPLELNSREEKSVMTVSQV
TEMSHHSNIGVSLAVIKYSMKLIGLWKADNTLDIFLMSSIFIYTISMGIAGIVFTVTDLFYVFDDIYAAV
NIICPTIAIFNNTVKLIIFAINRRQVLNIIERLENSIKNETYEEYDIRAVKDCERQCIILVITFVILTQG
AASNYVIPPLFEIFSGNGSRNLPVTIHFGGFAYGESPYFEIGFCIEAMIGFSSALCLVTLNNFLTTTNLH
LACQFKILHNKLKSTCIADLDMNFNASRDAYSKLKKCIKTHKMLIDYTAQVEDVYTYIILMQMFASSVVI
CAAGFQLFFFSGSMLRFVLSIFFFLTSVGEFFLFSWSCNEIIVASATVGDGGYNTTWYSLSATGYGKAYR
DGLQLLIMRSHRPCYLTAGKFCIISLESFSTVMTTATSYFTLLRNFVDEDEFRNK
>XP_015602107.1 PREDICTED: uncharacterized protein LOC107271050 [Cephus cinctus]
MKFHRQYDTSRVSIMTSKESEYRSVKFMRILMKIVGMYYTENPRERLLLRVALTYAIIAILFALAVEFVD
LYHCLGDFSAVMYNLCSTMPLVMVLVKISNFLFHWNVMMYLISFAQNNFWCDPDDDFTRETMKRCDKYGK
IFVYLFTNLVLFAVLDYIFAPVVENFHRNETDRILPFTLWVNLPVTVTPYYEITYTIQSLSTLYTGICTC
FFDNFISVLNIYVAGQLEILGHRVETVADTCIDITMKDYVSEKSKLGLSLTLKKFKSCINQHQILISYIE
QMERAFTLILLGQLILSSFVICVGGFQLMATMSFLRKFTFICHFMAGLTQLLLYTWSCNQISEKSLYISQ
AAYNTRWYLLPYDETGKSLRNGILFLMLRAQRPCQLTAGKFSPITLQTLTAILSTAMSYFTMLRQMSEDD
FDGRQENNVVTVTHVTEMSRRTDIGVSVKIIKFSMKLIGLWKADNTIDKVVTILIYTCLLTLMTIGVMLS
LIDFFYTLDDIYSAVNIFCAMIAILNLIGKLTIFGINRQKVFDIIDRIEDTIENGKKEEYGINAIIDCER
KCVVLVIILGILTQGATANYVILPFVENFNGNTSVKSLPIQIHVTKLSHSESPYYEIGFVLEAAIGVSAA
ICSVTINIFLMTTNLHLVCQFEILHNKLKWTFSNDLDITDSNISRYAFENLKKCIKIHMMLIDYTAQIEN
ACSYMILVQMLSSGLLMCTSGFQIFFFSGTILKFVFGTSLFLTSIGEFFLVSWSCNEIILASAKVGDSAY
NTTWYGLSATGYGKAYRDGLQLLMMRSHRPCYLTAGKFCIVSLESFNAVITTATSYFALLRKFVDDAELR
N
>XP_015602117.1 PREDICTED: odorant receptor 13a-like isoform X1 [Cephus cinctus]
MASHANKYLSIKLVRFFMKLVGIWVPKDNNEQWIMNCALSYTILVILLAVAIEGFDIYYCWGNFYATTYT
ACATMPVIIVLAKIFFLLLRRKLVMELIEFTETKFWQGDYDSYGMSVLDDIDRKGVLLMCTFIFFVQGTV
IGYVLTPIIENIGKNESDRVLPVTLWINIPVTTTPYFEICFVMESLTIVHIGICFFCFDIFLCILNIHAA
GQFKMLQHRFALVYDGDNKQEMPITDKLHFMKDSEEIYIKFKDCVKHHKILIDYTEKVQSVFTFIILCQI
LISSLMMTMAGFQALLTHGSIIRRLIFIAHTSGCFAQLLLFTSTCHEIIIESGGIADAAYNANWANTPYD
DVGRSLRIGLQLVMIRASRPCHLSAGGFCHVSLDTFTAVLSTAASYFTLLRQIGAETVMDT
>XP_015602121.1 PREDICTED: odorant receptor 49b-like [Cephus cinctus]
MPTIVEGYTSTKISQLLMTLIGMKRGSTKHEQLLINTLLVYIFTTVLIAISIESSDLFYSRNDLYALTYN

APCSFTVTFDFVKLLIFTYKRHELHELHKFTEDTFWNKDYNELDKAILDKCDTTSAIGMSILALVSTILA
FHYLTGPYWDALGTNTTERALPFPVVFNLPLTVTPYYEILYGTEAIGAISIGICSVAFASYLFYTCIFVS
GFFKILQRELENVCEVELESVNTKSSYNNNDTMLAYKKLKKCVIQHQLLIWYLDELEGLFSYILLMLVLC
AVIILCFSGFQIILGDGTTKLHRQILSVEFIMAALAETVLFAFSCNEILTASAAIGEAAYRCKWYKLPCD
EYGRALRQGMTIMVMRSYKPCSLTVGKFCPMTLEVFTSVLSTSLSYFTVLRSMNESE
>XP_015602122.1 PREDICTED: putative odorant receptor 92a [Cephus cinctus]
MSNLIEGYTSTKISQILITLIGMKRGKTKREQLLIDGLLVYVLITVLIAMCIENSDLIYSRNDLYALTYN
GPCSLTVTFDFITLAVFTYKRQVMHEVCKFTEDTFWNKDYNELDKAILDKCDSTSAIGMSILGVLGIGVI
IHYLTGPYWDIVGTNTTERTLPLRVVFNLPTTMTPYYEILYGTEVIAGISIGICSIAFACYLFYTCIFVS
GYFKILQRELENVCEVELKRVNTKLSYNNNDTTLAYEKLKKCVVQHELLIWYLGKLESLFSFVLLMHVLC
IVIILCFSGFQIFLGNQATKLHRQILSAEFVMAALASTALFSFSCNEIFEASAAIGEAAYRCKWYKLPCD
ENGRALRQGMTIMVMRSYKPCSLTVGKVCPMTLQVFKSVLSTALSYLTVLRGMNENE
>XP_015602123.1 PREDICTED: uncharacterized protein LOC107271058 isoform X1 [Cephus cinctus]
MSHHSDIGVSLKITKYSMKLIGLWKADNRTENFFMTLIFFYTSGMVVVGVIYLVTDLFYVLDDIYKAVNI
ICPTIALINITGKLIIFIINRDEVMNILKRIENAIKNEIYGEYEINAVKDCERQCVGLAITFGILTQGAT
ANYVILPFVENFTGNATRKTLPVPMHFGNLPYDESPYFEMGLCIEALISISTGACLVSLNNFLTITNLHL
ACQFRILHHKLKLTCKSDLDTMGFNDSRDAYSKLKKCIKTHKMLIDHTGQVEHVYTYVILMQMLSSDILI
CTSGFQVFFVIFWNDTEIHI
>XP_015602815.1 PREDICTED: odorant receptor 13a-like isoform X1 [Cephus cinctus]
MIQQFQIEKFIYLNKWFLNIVGLYPQKSWRFVTSLFCMLLLVIPQFVQIYLFCTDLSVILETSSVLFTIL
LAMLKGAVWIFNGKSMEDLVGFLFNEYWKIIESFDNSKTLVKYAGYYICVYYSYAIYIFCFSWIFHWKDI
SGFRYANKMTKGYTFLIINALLFFFSLPPIEILILKFNGTYKSSDKHFPFPATYPDFLKEFPYFQIVYFS
QIIATMTCALVILATDTLIATALFHTCGQFVVIQQKLELLSSNDGHETGEMIKRKIILIVKHHQMVIGFS
KKMERVFSPMMFLQVFASSMIICLVGLQVSTTFTNQYKLVKYFSYLLMALFQLLLFCWPGDQLLLQSGKV
CQSAYFTKWYQFGKERRGEIQLMLLRSQKLIGITAGKFYLMSLENFNVILSTSMSYFMVLRSFNSSEGK
>XP_015602817.1 PREDICTED: odorant receptor 13a-like isoform X2 [Cephus cinctus]
MIQQFQIEKFIYLNKWFLNIVGLYPQKSWRFVTSLFCMLLLVIPQFVQIYLFCTDLSVILETSSVLFTIL
LAMLKGAVWIFNGKSMEDLVGFLFNEYWKIIESFDNSKTLVKYAGYANKMTKGYTFLIINALLFFFSLPP
IEILILKFNGTYKSSDKHFPFPATYPDFLKEFPYFQIVYFSQIIATMTCALVILATDTLIATALFHTCGQ
FVVIQQKLELLSSNDGHETGEMIKRKIILIVKHHQMVIGFSKKMERVFSPMMFLQVFASSMIICLVGLQV
STTFTNQYKLVKYFSYLLMALFQLLLFCWPGDQLLLQSGKVCQSAYFTKWYQFGKERRGEIQLMLLRSQK
LIGITAGKFYLMSLENFNVILSTSMSYFMVLRSFNSSEGK
>XP_015604266.1 PREDICTED: odorant receptor 9a-like isoform X1 [Cephus cinctus]
MTGKVILPKFDNYILLNKFGLRLLGLWPIPEDSAKWKFTLKKLHVSLVYVLLLSLLVPQLLDLYILWGDV
DANVENLCTSLTTFTVLAKLTNIVASRGVFQKSLATMKENWDTIMRHDNCPEERREILLKMSKVGFVFTRN
YCIIMYITAGMYFLRPLVVGSGTKSPHVKEYPFCAWYYYDQFSNLTYGIFYFSQVIIGFFCGTGNFTLDS
LCLVMVYHACAQLRILQKQISQLTNDGSDDILIVERVRQLVKLHKKNIENARNLEAVFSGASAQQLLVSC
VIICVIGLKLIVSLNDAFQLIMYVAYMQLVIFQIYLYCSPGDELINQSMEIGRAAYMASWTNFPKYATHS
LLMMTIRAQRPLRITAGRFYVMSIPNFTSILKTSASYLSVLRVLYD
>XP_015607537.1 PREDICTED: odorant receptor 4-like isoform X1 [Cephus cinctus]
MDISGATIVLKWNEWLLDFLGLWPLNLNNAKFSFFFIYIIIQCFLQYAALVDNIFDLSYVVANLTETVVF
CMIVLKLVIYRINMKRLHELIRIIKEDYSHELYKTAKERMIFMKYNSLSRMIVQCFSILCVCAAVLFYIQ
PLICYLLAYRDSTGNSSSAFVLPYHIRLFFNLTEARTYYIIYACEILIIPMSACGYVGPSCLLITLVLHI
CGQLSILATQVECMTYDPKTIQQQLKQIVIKHSHLISLCATLNSTYSIFLLQEVIGITVLLCLGSYNITV
NHVLEETGEFLTFSCYVFTVFVQLLGFCYMGECLVNESINLCDAFYNYEWYNASAVHRKLLLMCLIRSQR
PLVLTAGKFFTFSLENFTSVTRNLLINFSPVLFFNQLALQTFLFAGYENINGLFVGTSKIHIKQS
>XP_015607539.1 PREDICTED: odorant receptor 4-like isoform X2 [Cephus cinctus]
MIIPGATKVLQWNHWLLGFIGLWPFDLNNSKFIFFFAYAMVHTFLQYGDLIEHISDLNHVVANLTETIII
NMLIFKMSIYRINTRQLRELIQNIEKDFSTELYNTADEMTIFLKYNSLSRTIVQCFSIMCLISPILFYIH
PLLSHLLAYNDSMGNSSIAFVFPIHFRLFFNLTEERTYYIIYACEILLVPTCTCGYNGPICLMITLVLHT
CGQISILASQVKSMIHDPKAVHQQLKQIVIKHRRVISLVANLQSAYSAILLPEVSGMTFVICLGSYNVIT
TSAVTDSSKFLKFLFYILTLTFQLFSLCYIGECLITESTNLYNAFCNYEWYNVSPDHAKLLVMCLLRSQR

PLTLTTGKFFTFSLESFRIVSQMTIMNFCQGLLVDKFTI
>XP_015607542.1 PREDICTED: odorant receptor 85c-like isoform X1 [Cephus cinctus]
MNMPEAMKVLTWNKWVLEFLGIWPSNESLFTFSFFFFLVSMATCFVYADLIYRISDFKYVVENLTENIVL
TLLCCKIGLYRLNRRLMKEILLDIKMDYAIELYNTEEQKSIFLAYNRLSKSFIKYSVTTTTVATVLYYIQ
PLMDHANSHRKLTENSSITYILPYHMRMSFNITESSLYYYVYAYEGVLVPIIACGYSGTDCLLVTLTLHL
CAQISVLANQVENFNGDFRKFHSHLKQVVTKHSRIISLSVKLRTAFAYFLLVQLVGATLVVCLAIYNLLK
NYATGHTAQLFGFIFYGSSVTVQLLGYSFIGERLMTESLKLSDAFYNCKWYVLSPIYVNLVLFCILRAQV
PLVLTAAGFCTFSLQSFTDVVKTSMTYLSVLRHFV
>XP_015607546.1 PREDICTED: odorant receptor 4-like [Cephus cinctus]
MDISDATKVLKWNKRLLDVLGLWPLNLNEVKFSFFFIYITVQCFLQYADLAEYIYDFNYVVRNLTETIVL
SMIFLKIFIYRIISNELRELIQYIQEDYSEIVYSTANEIKTFLQYTLLSKRIVQCLLITCGFTTVLFYMQ
PLTTQLLAYNEGNSSTSFILPFHIRLFFDVTQARIYYIIYACEIFVVPLVLCGFIGTDCLLITLVLHICG
QISILTMQVGILLDDPRNLREKLKRIVIRHCRLLRLFANLQSAYSAFLLQELFGITFLMCLGSYNVIATS
AVTDSSNFIRFLFYILTLIFQLFGMCYIGECVTTESSCLCNAFYNCEWYNISPDHAKSFLMCILRSQKPL
TLTAGNFFTFSLVNFTSVVRTSIGYLSVMRKFL
>XP_015607547.1 PREDICTED: odorant receptor 4-like [Cephus cinctus]
MPYHIRLFFNLTEARTYYIVYACEIFMIPLVTCGYAGPSCLLITLVLHICGQVSILTCQVENLIKNPETI
HHQLKQIVLKHRRLINLCMNLNSTYATFLLQELIGITLLLCLGGYNIIATPILDETGHFLAFLLYTVTVI
FQLLGFCYIGECLRNESKSLCDAFYNYEWYDEPRVNVKLFLMCVTRSQRPLVLTAGEFFIFSLENFTSVM
KTSMAYLSVLRKFI
>XP_015607548.1 PREDICTED: uncharacterized protein LOC107273647 [Cephus cinctus]
MCDIFPFAFSYYAAAVILGYADLFRNTDDLEYIFWSLQESICFTQIIVKLATLRYYNHEFGKILSAAIID
FNEYNSPTYYLMPLVIQLKAAESANASTTYKLPYYIYVIFNVQSMQSYIIAYIIELPTAIISAFSNTATD
VLMFTLVFYICGQLSVLAIRLRNLDKNRGYYNIRTLVKHHIRLLR
>XP_015607549.1 PREDICTED: odorant receptor 2a-like [Cephus cinctus]
MYEFLIVFVLFSHCVVGQYLYLESVNIHDAFYNCKWYDMSREDKTIIKFCIARSQQPLCLTAGQFGTFSL
EMLTNVLKTSMAYLSLGQLVFGIYVPN
>XP_015608388.1 PREDICTED: odorant receptor 2a-like isoform X1 [Cephus cinctus]
MRSVIAAHPRVSDSRQLKIVGRRNDISDMEILPLCFKLWTIGGFWRPLDYYSPMSKFLYNCYSFVSFLGL
YSLMLFEFLDIVFNFGNLDDFTSIAFMFMTVLSDCLKAANTLGKRSRIIEIQDMLTDETCIAHDTEEQMI
LERSAKVCRSNTQYLGILIMSFVVFLFVGSLLFNVNERILPVKVWLPYSLSSLTSFSLSYLYQIWVSMLA
AFMATANDTYITGLMIQICAQLDILKYRLLSLPRSGDSERNANSSVSQKEIILKMCIRHHNHIIKVAATV
EETFSSIIFFQCFVSTTVICVATFILLKSDFMSVEFIITLMYFPPLLFELLIYCWYGNEVTLKSTEIRDA
VFEMDWIPLPVSMKKDLILVILRAGRTIKFTSAYVLTLSLDSFMAILKLSYSAFNVMQQSSKD
>XP_015608436.1 PREDICTED: odorant receptor 24a-like isoform X1 [Cephus cinctus]
MDISGAADILTWNKWVFSFLGIWPLDDSNKLFLFHYIYFMIHTCLEYVELFKYLNNLEYVVANLTENLVI
TLTFLKISAYRINKNELRKMSNDINDDFKEESYKDSEEKALFLEYNAIAQKFMKVGIPITLIAAVLYYLR
PLTGHVIVDTSHLGNTSHSYIMPYRITLFFEITNFQMYVFMYAFEILNVPIIAFGFIGTDCLLITLVLHL
CGQLAVLSHQVRNLTMNPQNFQNEFRVIVMKHLRLIRISKSLDTAFNQILIQQLVGMSLLLCLAGYNLIA
MSDSGQNVHLITFIFYACSVSMLLFAYCLIGEYLINEVISSVSYFLQQFLTLLLLVVSSQYFVLEHKDPR
CFLQFYMVQQATLSAGTIFDLYGICSTSAGTNCWKNLFIVFKYFCACK
>XP_015608437.1 PREDICTED: odorant receptor 24a-like isoform X2 [Cephus cinctus]
MDISGAADILTWNKWVFSFLGIWPLDDSNKLFLFHYIYFMIHTCLEYVELFKYLNNLEYVVANLTENLVI
TLTFLKISAYRINKNELRKMSNDINDDFKEESYKDSEEKALFLEYNAIAQKFMKVGIPITLIAAVLYYLR
PLTGHVIVDTSHLGNTSHSYIMPYRITLFFEITNFQMYVFMYAFEILNVPIIAFGFIGTDCLLITLVLHL
CGQLAVLSHQVRNLTMNPQNFQNEFRVIVMKHLRLIRISKSLDTAFNQILIQQLVGMSLLLCLAGYNLIA
MSDSGQNVHLITFIFYACSVSMLLFAYCLIGEYLINESTKIQDAFYNSTWYNRPPYQLELFLICMVFVQR
PLVLTAGKIYSLSLSTFARVAKNSMAYLSVLRKFV
>XP_015608438.1 PREDICTED: odorant receptor 45b-like [Cephus cinctus]
MDMLDAIKVLTWNKWVLEFLGIWPFNESHLIFSFFFFLISSATCLVYADLIHRFSDFKYIIENLTESIVM
TLVCCKIGLFRLNRRILNEIVLDIKDDFVKDLYDTEERKSIFLFYNKLSKTFVKFSIGITTFSTILYYAQ
PLIDHVMSYRNFTGNSSAAYVLPVHIRIFFNITELPLYYTIYAYEGILVPIVACGYAGTDCLLVTLTLHL
CGQMSVLANQVENVVGDLPSFPKQIRYLVIRHSRIISLSVKLRTAFAYFLLVQLVGATLVVCLAIYNILT

NYATGLIAQLLTSIFYGFTVTVQLLGYSFIGERLMTESLKLSDAFYNCKWYVLSPMYVNLISFCILRAQV
PLVLTAADFYTFSLQSFTDVVKTSMTYLSVLRHFV
>XP_015609190.1 PREDICTED: odorant receptor 4-like isoform X1 [Cephus cinctus]
MTVHSLSPTGNCKGLKEGQASYSWCVRNLRTLMVFTGIWPMEPPTLLLNVSYYYNATTFTLVICGMMAGA
ISVMDNYDLLVDNLSINLIFTEIFIKCILIKIYSKPLSRVLSLMKFDWISLKGRGPIHGDVLQSENIMLL
HANIPRIFFIAYTALALVAWTATVVAAVSRKSSKMQIDASNAFPMPSWYPFEMHSTPNYEMLLTFQVIIG
CSIAVSSAAVDSLLVTAVFHVCGQLEILRKYFENLHSSETALEETEKKVAAAIKRHSKLIDLCDLIEDCY
SQITLSQLLVASLNVCLSGFGLLLAIESGNIKVFLKFLLLLIAMLQQILIYSVTGDYLSSKSTAIRSAIY
KMKWYELPPSLSKALMLIAIRAERPLVVTAGKFFPMSLENFTQVSSHCAKLQNRVGLVRKLVVTFR
>XP_015605665.1 PREDICTED: odorant receptor 30a-like [Cephus cinctus]
MAFQVTLPRAMQFLLVCGKISCTLPPNFESRRYKSIIHEIMWLISMSVTMSLIIPLFLAMYHFGEDLAVL
TKSVSESMTATDPLLNMIFCKLERARIQVILSELMNFLNTASPFEKAKLQKHVDQCTPFYLFILILFSLT
AVAFSCGPIVMDRPFPAEAWYPFSTDPLPISCSIYVLQIMVIAQAAMCIHMDFMIAFLLWYAVASFEILG
EKFRRVENEVDLRNCIVQHQKLIVFVREINRVFYVMILKTPLSMTISIICSSVQLIHHEPLPVLSQFILL
LVVCSIKLYITAWPADSLIHASENIASSVYESSWIGKSSTFLKSLNIVIRRSQKPLIISITGILPPLSLR
FYASFITKALSFITTLKSVISE
>XP_015606176.1 PREDICTED: odorant receptor 13a-like [Cephus cinctus]
MRDFLEQIKTDYDTPQDDFEAKIYKRYADRTRKFSIGYAVWCHASFSLYIIGPFLNHTEERPFPGHTKYP
LDEDKYYWFIVSHQSICTFTVSAITAAVDALFVMLVHHICAKFAVLGYNLEKIGEDLSESPSAQDELETY
KKIVNCVKKHKEAIGFADSMESAFSIPTFINLTMNMIVMSTSGFVMLANVHSIPNLVKYIGLSVTLTVHL
FFMSWPAQEMMDHSAEICEFAYFASWYRTSQRSKNLLKFLMMRSQVPCKLTTGKLYVMSLENFCAVLKTS
MSYFTVLKSLRD
>XP_015606884.1 PREDICTED: odorant receptor 46a, isoform B-like [Cephus cinctus]
MVYKMDILPLCFTIFQFCGFWRPINYTSWSKFLYNCYTVFVTFCIYSFTLSQAINSITVLDDVNNLTNNT
FMLVTMISVCCKIANILVKREAVINAINMFLENFEDLVEQKIKKKYDLIAWSVTRNYSILVTITVICYIV
IPLVINDIEKRVLPNRAWYPYDLSSLTLFWLSYVHQSFALASAAFINVANDTFIPGLMIQNCAQLEILEH
RVEKLIILTRSKKTENETKKEYKEILEKCIRYHNRIIKFAKIIEKMFEPVIFVQFFVTVLTLCSSMYQLS
RHSTLIVEIINLIFYLISMLFQLFFYCHRGNELFLKSIEVGDNLYKMDWLSLRPSRRKDLLIIMIRTNKP
IQLTIGGIITLSFSAYISILKASYSVFNLLQQTSEK
>XP_015606887.1 PREDICTED: odorant receptor 67c-like [Cephus cinctus]
MSPFALYFRIYKFCGVWRPTDSSRCFKAFYNIYSALSLFGMCSLTILQFTEIITNTHNTDDITTNSFILL
SMVNSCFKAVCVLRHRNEIIGLIAMLVGSIYYKARDPEEIKIQEKFNNILWLNSRRYLILVTTSVLSNSI
GSLFSHLKHRTLPYKVWLPYSLSSLKLFWLSYIHQCASLSIAAYINVTTDLFIIGMMIQICEQFEILKHR
LISTNCEKTSSKSNGTILEECIRHHNYIIRCAGGVKHEFSAVVIVQLTVSAVVLCSNIYKLSRCTVNTTE
FYNLILYMSCILVQLLIYCWYGNEVMLKSLEIGDAAYNMKWTELSVIERKTLAIIIQSTSRPVIFKFAFT
ISLSLDAYMNILKASYSTYNFLQQFSMD
>XP_015606890.1 PREDICTED: odorant receptor 49b-like [Cephus cinctus]
MCLYLANVLFQLFVYCWYGNEVILKSTEIKDAIFQSNWVCLKSSTKRNFIIVMIRAQRAIEFTSGYVITL
SLESYVAILKASYSVYNVLK
>XP_015607077.1 PREDICTED: odorant receptor 4-like isoform X1 [Cephus cinctus]
MQVTMDPTTEINYALGITRICMQALGLWPRTSNKLRLTIIAIYLLALTEWFAGQYAYYITKKLDWMPFIV
KIKHTVVAHMFIGKYLLAIIAGSQFGECLSALERDWHNVKLTYEIEIMRIYATKGRYYALICFILSFTTV
TLQLIVPLTPLSESRAFPYPSHYWFDYQSSPYYEIVYTLKIFSSTILAACNVTVDGFIIILIMHVIGQLE
LVSEHIRNMEVMKSTIETCVQRHRVVIWMAETLENSFNLVFLCHFMVSAIVMCLAGYSLMKSVERNESLE
SVISMFFIGSIAFHLFLYCYVSDKLTEKSLQVGYSAYCCNWYQHECKQSKPLLILMIRSSRSLKLSAGKF
IVLSLENFTDVRDYLVVNFKAEIFWYIKKYFLQVLKTTGAYLSVLRKF
>XP_015607529.1 PREDICTED: odorant receptor 4-like isoform X1 [Cephus cinctus]
MDISEAEKVLTWNKWLLDFLGIWPTNKNNTKFYFFFTFITVQCCLQYADLIEYIDDFEYVLVNLTESIVF
TMILLKIVQYKINTGRLIELIRDIRTDYSKELYRTEEEMAIFIRYNSFSKIILYCLSISGVITSSLYYIR
PLSSYLLAHNDPTGNSSSSFILPYHVRLFFDLTEVPTYYAIYACEIWVIPMVVCGFMGTDCLLITLVLHV
CGQFSILTIQVENLTHDPRELHDELKRIVIKHRRLIRLFANLHSAYSTFLLQELVGITLLTCLGSYNVIV
TPILKETTYFLIFVFYIISIISQLYGFCYIGECLIIESECLCHAFYNSEWYNAPPHHGKFILMCILRSQR
PLALTAGNIFTFSLESFTHVRQKLFINFDSKHVLLSVIHRNSFALTGFKNINGLPISYTKLLINGSQEIS
GSYNPIYIHN

>XP_015607532.1 PREDICTED: odorant receptor 4-like isoform X4 [Cephus cinctus]
MDISEAEKVLTWNKWLLDFLGIWPTNKNNTKFYFFFTFITVQCCLQYADLIEYIDDFEYVLVNLTESIVF
TMILLKIVQYKINTGRLIELIRDIRTDYSKELYRTEEEMAIFIRYLLAHNDPTGNSSSSFILPYHVRLFF
DLTEVPTYYAIYACEIWVIPMVVCGFMGTDCLLITLVLHVCGQFSILTIQVENLTHDPRELHDELKRIVI
KHRRLIRLFANLHSAYSTFLLQELVGITLLTCLGSYNVIVTPILKETTYFLIFVFYIISIISQLYGFCYI
GECLIIESECLCHAFYNSEWYNAPPHHGKFILMCILRSQRPLALTAGNIFTFSLESFTHVRQKLFINFDS
KHVLLSVIHRNSFALTGFKNINGLPISYTKLLINGSQEISGSYNPIYIHN
>XP_015607535.1 PREDICTED: uncharacterized protein LOC107273642 isoform X6 [Cephus cinctus]
MDISEAEKVLTWNKWLLDFLGIWPTNKNNTKFYFFFTFITVQCCLQYADLIEYIDDFEYVLVNLTESIVF
TMILLKIVQYKINTGRLIELIRDIRTDYSKELYRTEEEMAIFIRYNSFSKIILYCLSISGVITSSLYYIR
PLSSYLLAHNDPTGNSSSSFILPYHVRLFFDLTEVPTYYAIYACEIWVIPMVVCGFMGTDCLLITLVLHV
CGQFSILTIQVENLTHDPRELHDELKRIVIKHRRLIRLFANLHSAYSTFLLQELVGITLLTCLGSYNVIV
TPILKETTYFLIFVFYIISIISQLYGFCYIGECLIIEASRIDCRKHIYILFGKFHACFKNINGLPISYTK
LLINGSQEISGSYNPIYIHN
>XP_015609350.1 PREDICTED: odorant receptor 82a-like [Cephus cinctus]
MGDAFMTLSLIVVCFKEPHILTRCIHVIGIRDMAMMKFASFESVFQLVKINVLSVRFVSMCLYLASVLFQ
LFVYCWYGNEVILKSTEIKDAIFQSNWGCLKSSTKRNFIIVMIQAQRAIEFTSGYVITLSLESYVAVGIN
QV
>ARN17885.1 odorant receptor 10 [Cephus cinctus]
MDHEIEQFKIKTTVQNKNFDFDMEYAIKLNRWLLKPFGIWPLNSSSTKFDRGISVISPSICCLLLFFVMI
PSFVAMFVSEKDFKGKLEIVGPTSFIIMVVLKYFFLITRGDTLKMCIDTLISDWRNVHAKHTFLPVLTAK
SLARGNKLTIRLVLFFSCNFILFDPYVRPIFEIVYIAHCFCSVIVYSITTEISSLAAKFVIHACGQCEIV
IPLQNIIDSDKRCLNVVEKNLLLLFCDIYMRSGKIIILQVKGLLERPPFYGFLTILRMNIQYIFVTNVED
LLNQICFVEFLVGTTVYMGKRKCTEAHYSLFLWTLLNSILHLYMKKCHQIEEPSYMIH
>ARN17894.1 odorant receptor 22 [Cephus cinctus]
MDISGATKVLQWNNRLLRFLGLWPFDLNNVKFMFFFTYVTVQCFLQYAGLLEYISDLNYVVANLTETIII
NMLIFKISIYRINTRQLRELIQNIEKDFSTELYNTADEMTIFLKYNSLSKTIVQCISIVCGATAVLFYIR
NSSSAFLMPYHIRLFFNLTEARTYYIVYACEIFMIPLVTCGYAGPSCLLITLVLHICGQVSILTCQVENL
IKNPETIHHQLKQIVLKHRRLINLCMNLNSTYATFLLQELIGITLLLCLGGYNIIATPILDETGHFLAFL
LYTVTVIFQLLGFCYIGECLRNESKSLCDAFYN
>ARN17900.1 odorant receptor 29 [Cephus cinctus]
MNDNNDIPKIVFKWNKLFLSIGGNWPLEPAYFRFSIWMSFFTLNISLLYVDLYEVFGNLEQMLLNLSDSV
IQSLILAKLLLFRFSEPLARLITDAQEDITAGEFGSLEEKKCFLEYYQRGKIFYQITMSCVVFAVHVYFF
KGLETYLFSVWNNETTTSFVLPYRTKLFFNLTSPNTYIILYILEYPMVYIIACQGATICLLVTLIFHVCG
QLAIVSYKIQHLRGNLPEHENRIIKSLIEKHVKYVRMARSLDDTFQFVLLLEVAGTTVILGLTCYNIIAN
SGDLADISTLCCFALYASSMILLLYGYCFVGECLIHESTKIHEACAQCTWYSMPLIYQKALIMCMLCAQR
PLQLTAAKFYVFSLDSFSNVIKTSIAYVSMLRTVV
>ARN17902.1 odorant receptor 31 [Cephus cinctus]
MVGIESCKRILSTEDKVQTEQNKNYKSDIEYALKLNRWLLNPISIWPLSSHVSTLTKIQFKFIRTCCWVF
LAFLIIPTRLHTIFAKTDRAVKLKMIGPLGFCVMVIVKYFVFVVRAKNIKICVDHVVVDWRNVNTTEDRE
IMIDSARIARLFTAASALFMYGGGVFYTSVLPLTASKSLSNDNVTIRVLHPFYFFTYDPQTTPMYELVFL
VHTLSDVVMYSTTSGVCSLAVVFAMHVDGQCQVLMRLLNDFVDGNREKSSTLHQRLANVVVRHLRILRLI
STMENIFNEIFLVEVVGCTFILCFLGYYCMTDLTESETFGLIAYFLLLISLTFNVFAFCYIGELITSQCM
RIGQAAYLTEWYRLGGKNASNLIILIIISNRPVLLTAGSMMILSYNSFIQVSMKNNVIIFWLFPDTFQSE
I
>ARN17906.1 odorant receptor 35 [Cephus cinctus]
MEILPLCFKFWTICGLWRPVRYYSSVSKFFYDSYSCVTVLVLFSFTLLEFVDIALNFGNVDDFTSNAFLF
LTMIGVCYKVAHTFMKRLQIICIRDMLYDEICRPENMEEQRILDNCANACRSNTVNLGIIIMSSIVFFLV
GPLLHDVNDRVLPFRAWLPYSLSSLPVFYLTYLHQSLAIISAGLVSVASDTFISGLMIQACGQLDILKCR
LANLHESHNFEMAINDTFHREEAILKMCIRHHNHIFKFAALVEKTFNHVVLFQCFISAFVVCTTTLTLLN
HQLLSIEFITIIVYFLSIVFQLLVYCWYGNEVILKSIEVTDAVFEMDWIRLPASMKKDLIMIMMRAERAI
KFTSGYVLTLSLDSYVAILKLSYTTFNVLQRSS
>ARN17909.1 odorant receptor 38, partial [Cephus cinctus]
SYQEIFYRLCRDLVFLTYLQSERKKSVLPTTDYIFFLSSLIFSVWCHASFSLYIIGPFLNHTEERPFPGH
TKYPLDEDKYYWFIVSHQSICTFTVSAITAAVDALFVMLVHHICAKFAVLGYNLEKIGEDLSESPSAQDE
LETYKKIVNCVKKHKEAIGFADSMESAFSIPTFINLTMNMIVMSTSGFMLANVHSIPNLVKYIGLSVTLT

VHLFFMSWPAQEMMDHSAEICEFAYFASWYRTSQRSKNLLKFLMMRSQVPCKLTTGKLYVMSLENFCAVL
KTSMSYFTVLKSLRD
>ARN17910.1 odorant receptor 39 [Cephus cinctus]
MSSLTESVYTKYEDKPEESRSDFENLDITLRWNRFSLRQLGIWPDPELFGGRSLRKYKFLFPAFFMLAFT
IIPQTTLLDMVWGDLDLVVDNLCVANLPMISALMKLLILRFNSKALRPVLACITEDWKNVNNPGKREVLL
KYGKIAKIISLVFTFLIYMTVISYVVLQIYLNYQTKIINGTDTSRRFLFQSHFPYNTQVSPSYELTWLIN
LLTLDNILAILVLNVCSQYKNLQIDLKNVLENMDTYKETFIKKLSTIVRKHNHLNRLAILLRNKVLYKLV
EIVLSFFCFKGVLIQLINHLTVCFSCKCLLLRFSFVFRAFKVFLSCFLFSCYIGDKLHTEVRLFISNFQY
YPNLEEREARESLLVMLRANSPMRITAWKFSSFSMVTFAAVGNNWGTQRKRVKH
>ARN17923.1 odorant receptor 52 [Cephus cinctus]
MDYEYNPEEAFAINFGFLRVDGAWPTGITNPILRFIINEVSFIVVKSVICQNNYTVSVMNCCRFLYRIYG
FCVLSIFTVLYFSTEIILMTVIWGDVQAMVDCSFLFLTHLSHSVKLTNFLLRQKRLDRMIFQLREPMFVP
KRPEHIAIMKAAVLNAERETKMFLTITFGTIICWSILPLLSQKEVKELPLMGWFPFDTTQSPAYGFTYTY
QVISVLLNACVNAMMDTMTSGLLILMGAQLDMLKENLQSLTDDASTMSQAEMKYIYNVRDVFQIGIMAQF
LASSIIICLTCFKYHQHL
>CobsOr2
MDILPLNFRVLWFCGAWNEEKENNIFIRSLNLCYRYAIVTLIYEFTISEIIELVRTHDDIEDLTEGLFLSLTY
IALCLKYGNFLTRKKEMSVLLDCFRKETCRPRNSEERIILLKYDRKAKWCVKFFMSISQATCIALILAPIMGP
QKSDRPLPFKTYLPYSISGLYPYLATYLQHAGAIFYGVLLNVSFDSLVYGFTIHVCGQIELLCYRLSKIFKDH
PEQYQLDSSKGAVISECVRHHLHVHEIVRRIQSLFVWTVTILFIFSMVTLCTSIFQMSKTRILSVAFLSLTLY
LGSMLFQVFFYCWYGNELQLKSKGIVDAIYSSDWTIATIRDRKSLLFVMAISQKGLKLSYYGIFSLALDTFTW
ILKTSYSTFNVLQQTSM
>CobsOr3
MDLLPVNFYVLRFCGVWEERRTDTRLIVRFISFCYRYTIAAIIYHFTISEIIELVRMRNDVEDLTEGLFIVLT
FICLCLKYFNILVRQCELRSLLDCFRTTLCQPKNSTEESILKQYNLKAKKVTYAFMVISQISGLLVIIVPLMS
QDERQLPLKMYVPYSIAELPLYLLTYLQQGVAVFYGILLNVSLDSLVYGLIIQTCGQIDLLCHRLSKAFRFLQ
EKNHEREKHDTIEKLAIAECVRHHISVYDITCRIQSLFMWIIAILFFFSLVTLCSSIYQMSNKELFGVEFFTF
VAYLGSMLFQVFSYCWFGNELDLKTKGIAYAIYASNWTVISTKQRQSLSFLMMISQRGKVISVYGICSLVLST
FTWIIKTSYSAFNLLQHAS
>CobsOr4
MDLLPVNFCVLRLCGAWKEREDDGLIVRFISFCYSRYAVIALIYYFTISELIELVLARNDVEALTEGLFLAIS
YITLCFKYFNFLTREHELRALLDCFRAKLCQPRDSAEMSILKQYDRKAKQTACLYMMMCQITGTLMITMPLLT
KNERSLPCKTYIPYSVAAFLPYVLTYLQQSATVIYGILLNVSFDSLVYGFIIQACGQIELLCYRFTETIRFLK
ENNEEKKHQAVDSFAIADCVKHHISVYNITNRIESLFVWTTSTLFFFSLVTLCTSIFQMSKRDLFSPEFFTLL
SYLGSMMSEVFIYCWYGNELDLKSKSVAQAIYTSDWTIISVEQRRTLLFVMMMSQRGRILSSYGFCSLILDTF
TWIIKTSYSAFNLLQQASH
>CobsOr5
MAKKILLKDVIFVVKLSVFIIQCWPLAKNATRLRVVFIKIYHFMCIILLAIMAASCIYTANIKRDTFIVFSNL
IMYTSAMFHSINNFIWYNINYTFIQKVTFEMIHVSESMTAHEKIVFQRYIKKCLPFYSVSLIYFYSLTVGTIT
VVPLLAHQPFPTVIEYPFDVLYQPLHTIIYLKQSICGFFVSAHLCVNVYMALLVWFTTARFKVLAEQLRMAVN
VYELFECIKTHQKLLKYADNVSIVVRAFAFTTICCSTVGLITLFLLVLKEQSLIVISWALAVTVSGLLEVFMY
TWPAEHLMHTASEIGHVAFEILDNHYFVKIWKCLQIIIMRNQKLIQISIPCLMPALSFNYFSA
>CobsOr6
KIINFRNVNLLNSRVNVLSGNLLPVSGDSQFSVIHRIYSAAVWLNELIYMSALIAGIMLSPKEKGLKDGSLPV
AVWMEATVMLTSLLSRKKLMREIVRKMDDILQNADEPMNDIVKAAIKPIITPFIIYAVTSVISVMIWTGQPVV
LAFKKSVFYYIDYNLPAAFSTEPFSSRVLIPSTIIMTTGGVYLFLRKFSMDVYMMHLVLMLTAQYRYTASKLT
ILCRDLQSYDDKSQRERFMQDRLIEKELKRLCRHQHNVLSISLLLKKLLSVNFSVLYVNNVFRFCFVGVLMST
VPSLSLAEGISIVSFSMGAVMQFFLMCSSVQTLSDASTEITDKAFDESWYQLSQPMKRIFILLIMGNNLECKI
AAIGKFNLSLPSFMTIMNQSYSIALLLLRAK
>CobsOr7
KKMDFNNVNLLNSRINVLSGNLLPIKADNSQFSVFARVHSAAVWLSQVVYIVALYAGMVVTPKEKSIKDGTVA
VVISVEAAFMLTNLYSRKKLMREIIQIMNEILQSADDVMKDITKTALHPIITPFVIYGATSVLSITVWTGQPV
MLALNQSDFYYTDYNLPAAFSAEPFSSRVFVSSTIIMTVGSVYLFLKKFGVDVYMMHLVLMLTAQYRYAAVKL
TMLCNSLQNYDEESQKEHFRKENLRVKKELRKICHHLRTLLNMSFILKKLLSVNFSLLYVNNVFRFCFVGMLL
STVPSLSFSEAISIISYAMGSVMQFFVLCSSVQTLSDASTKITDQAFDEDWYHLEPSMKRTFMLLIMSNNLEC
KIAAIGKFNLSLPSFMTIMNQSYSIGLLFLKA
>CobsOr8

MNFQTVNPLNVQLNKLSGNLLPVTGDDSLFPVSWRIYSAVIWLIEMIQTSAVIPGVLSVPREKTLQDATVSIV
VTIEVFFLLRQMHANRDIVIQLIRKLNDVMQTEDETMKMIVKSTLKPVQLPLKFYWLAGTGSLMTWCCMSFMI
IFKKNCFLYEDYRIPIILSRQPFSMHIFLLGNCVVSIASVYMFIKKVALDVYMINFVLLVTAQYRYIAVKLST
IFQENISLDRNNESEKERSAIDSLAILKMKALCQHHNTVVQITLMLKKLLSLNMSMIYLTYVFIFCFLNVMLI
SAILSMSFIEGIMILMYISGCLVELYILCSCVNQLLDASVEITDKVFHEKWYQFGSPLKHMLRMMITSHNLEC
KLSVSERFNLSLPSFMTILNQAYSFALLFLKLK
>CobsOr9
SMDFENLNHLNARFNLFSGNLFPMTNGKSFSFFWKIHSAFSWLTQIILIIVMIPGYIYLPIEKLFEDEMSGFG
EIFDAIMFLIQCYYISLRIHTHKDMLYQLIQNVNEMLHITDGIMKNIMPSILNPVKTPLNFYWSTNLAATSLW
GSLAFLLLFEKDQFRYEDYGIPVAFTKQPFSRTTFFFGSVFIYISSLYMVLKKIGVDFYVVHLVLMVTAQYRY
IALKYAAICQEESESNEFGKKYSQEVRQRKEKEIKTLCRHHKNVIYTTSLLKRILSFNFTLTYITSIFRFGFI
GILIIGIPSSNFVDALWIVIYATGATVQLYILCSSVQQLLDASIEITDQAFHESWYLLQPSVKRIFLLTIMAN
NLDCKISAIDKFNLSLPSFMTILNQSYSIALLFLKMK
>CobsOr10
ISMDFESVNTLNVRFNLLAGNLLPMTNYSSFSLFAKIYSVVTWLIELIFIITVVVGCIYLPIEKTLEDGMTRF
SEILDGIIFTFQTTFMVLQILSHKKLIHQFIQKLNEMLHIADETMKNTVTATLQPVKYPIKYYWTTGIGSTTF
WNLIAFLLMFEKDLFYLDDYIIPIAISKQPFSSTIFAIGTSFITISAVLMVIKKVSVDLYMMHLILMTTTQYK
YISVKLAKVCQIEKNDNKFKENYSHELNRKKELEIRALCRHHRDVINIASLLKTLLSSNCTLMYVVSVFRLCL
IGVVLASMSETSFWTTFSMMTYGSGVTVQLYILCSCVQQLADASTEVTDKAFNENWYLLPPTAKRIFLLTIIA
DNLECKISTNDKFNLSLPAFMSILNQSYSITLLFLKMK
>CobsOr11
MDFQNVNSLNAWLNLLSGNLMPITNNSSFPISWKLYGTVIWLFQLFYTVTLIASFFFVPIEKTLNDGLTVLIV
IMEENSIILRIHQHSSLIQQLIQKLNDAFNIKDENLKNIVTANLNPMKTPFKFYFVTVFPSLIVWFSLPNILI
LERNTFYYVDYKAPAIYSKEPFSTNIFVLGNTIMTVANLFIFLKKISVEVYIAHLISLVTSQYQYIALRFVLI
FQNYDQPNINRDIRNVCRQHKNILYITQMLRKFLSLTICIIYLNNVFRFCFLGFMMSRISVSLFDGFMIFIYA
SGSVIQFYIICNCFQKLSEAASEITDKAFHENWYKCNSSVKRVFLLMIMASNNLTLKLALFERFNLNLSSFMS
ALNQSYSIALLLLKM
>CobsOr12
SMDFENVNRLNVKLNLISGNLLPMVDRDSSFSLFWKFHCVVVWIMQLINAIAMVPGCMYVPIEKVMKDGMICF
VIFFEMSFLLLRIHARKNVVCQLIRKLNEILRAADDTMKNVVTSTLDPVKIPLNFYWSAGVASIIAWECIPFV
LLFEKNIFLYEDYRIPIAFTKQPFSLKVFVLGSLFIIISSIYLFLKKVSVDTYMVHLILMVTVQYRYFALKIA
MICREDEGKDSQELNRRKEIEIKALCRHYRDMIYITSLLRKLLSLNFSLIYVNSVFRFCFIGIMISSITSTTF
WEAISIMIYASGAVVQLYILCSCVQQLLDASTEMTDKAFHENWYLLQPPVKRIFILTIMANNLECKIATFEKF
NLSLPSFMTILNQSYSVALLFLKMK
>CobsOr13
MDFQSVNPLNVRINLISGNLFPMTTDNTRFPVVWKIYSAFVWFVISVIVIGYFFGLVNVSKRKAISDGMIGTV
FIVEVFFMVARIHMRKNLVLQLIQNMNEILRVQDETMRRTVIKSLKLMHSPFKFYWLSGAVTAIIWIIVPVGT
VFKRNFFFYEDYRLPFAISKQPFSTEIFLIGNLLLVFCSVYVLIKKAAVDIYMLNFVMLMTAQYQYISLKLKE
VLRKEYSPNKLDETKRTNYSEIDFERKMEIKRICRHHSTVIRMSAMLKELLSLSFSLIYVNNILRFSFVGVML
LNTALLKTVIERIAMVCFACGELIQFFILCFNVQKLLDASREITDRGFHENWYQSRPTIKHTFMLLMLSNKLT
CKLASFENFSISLPSFMTVRSRTFVILLTWLRNLLLIFLNFFFLTMFQVMNQAYSIALLCLKIVKK
>CobsOr15
DKSATLQNSSYKRDIKYVFKLNNWILGSLGIWPVAIRGIGRHVSKISIAIWNLALSFAIVPCALHIVYDEKDI
IIRLKLGGLMAFCFTAMTKYCIFVIRRPKIDRCIEYVKNDWWQVTFTSDRELMLKYATTGRTLTIIGASFMYT
AGIIYHLILPFCSEHKFNNQTIRPLVYPTYSKFGQSHISPVYEIMYVAHCMCGYTIYSVTAGACGLAALFATH
ACGQIQILVTRLEDLLTGERFQPNSNVHHRIAVIVKNHVRILRFATVVEEVLQEVCLVEFSSSVCTICLLEYY
CIVDWKADDRIGLTTYFLLFVSFCFNIYILCYIGEVLMEKSTKLGSICYMINWYQLSPKSVRNLILIIAMSSH
PIKLSAGRMVDLSLTTFGNVLKTSVAYLSFLRTLVM
>CobsOr16
SYQQDIEYVFKLNSWILGSIGIWPISLRGAGRYLSNFAIAFGNLAISFALVPCVLHIIYDEKDIIMRLKLCGL
LAFCLTAMTKYCILAIRRPKILHCIESVKNDWWQVTFKSDRELMLKYGTIGRNLTIISAAFMYAAGIIYYMIL
IPFFSAKIIHNLTARPFVYPIYSKFSHVQITPIYEIVYVAHCMCGYTAYSITAGACGLAALFVTHACGQIQLI
MSRVDLLNSKSSNIHQQIAVIVKDHVRVIRFSAVVEEILQEVCLVEFSTSVCTICLLEYYCIVDWQDNNKLSL
TIYFLLLISFCFNVYMLCYIGEFLMEKSSQIGSVCYMINWYQLSPKSARSLILIIAMSSHPIKLSAGGVVDLS
LLTFGNVLKTSVAYLSFLRTLVI
>CobsOr17
NTNYEKDILDILIWNRWLLRVLGIWPLIYPNTTTVEKILAMFSFASCWTVLGLFLILTSIYTFTDRSAMGEKM
KMLGPLGYVFFSMLKYLFLVIRHKNIRRCIKILSTDWRMVQEECHREIMMRDANMGHLLSKFCIVFMYCGGLS

YNTVMPFLSQSFNVNEQNITVRPMAYSGFDIVFNLQLMPVYIFVFSLQCFIGIVMFNITTSVCCLAAMFVAHA
CGQIDIVIARVKDLFKGKSDHMKLENCMAIIVEHHVRALRFSINIESTLRELCLVEVIGTTLIMCLVEYSLIT
EWNNSDSIAIFTYFFLLISFIFNIFIFCYIGELLTEQCSKIGYISYKTEWYNLPGKMPLDLMLIINMSRHPVQ
ITAGRLISLSFANLGNVLKTSVAYLNLLRTA
>CobsOr18
HDRSRLTRKMIEKYNVHHDHDVHYTMQLCRWVLKPIGMWYPIYGHPSRYEKLVSIALFVTCFSALCFVLIPAG
LYTLFREKDINIKVKLFGPVGFCLTSTIKYCYLGARAAEFGKCIRHVENDWRVLRHRDHRGLMLKNALIGRRV
TTLCAIFLYIGGLSYHTIMPLSSRRRVNENYTIRIHTYPGYDLFFNPEVSPAYEIVFCIHCLYALITYNITTA
ACSLAAIFVTHACGQIQILMTMLDDLVDGKWSKGTTVEERLSVIAKHHVRVLRFSTNVEEVLREICLMEIVAS
TLIICLLEYYCLTEWENSDAVAILTYFILLISFTFNISIFCYIGELLVEQYSKIGSAVYEINWYNLSANKATD
LVLIIAMSHYPPKLTAGKIFDLSINTFGAVLKTSVVYLNLLRTVTE
>CobsOr19
MNDRSRLPAKMHNVQYEYDIRYAMQLCRWILKPIGIWHLIYGRSTQNEKLLSLILILACFSGLCFVLVPAGPY
TLLREKDINVKVKLFGPVGFCLTSAIKYCFLGARVSAIGRCVEHVENDWRVVRYQDHRKLMLRNALIGRRLTT
LCVIFLYTGGMSYHTIMPLSAKTKTNDSFTSRPLVYPGYDLYFNPQASPTYEIIFCMHCLSAMIQYSVTTAAC
SLAAIFATHACGQVQILMRLLNDLVDGKRTKGTVEDRLSSITRHHVRVLRFTANVEKVLHEICLMELVAATLI
ICLLEYYCMMEWANSDAVAIFTYFILLISLTFNILIFCYIGELLMEEYGKIALAAYEINWYDLPGHKAIDLVM
IITMSNYPPKLTAGKFCDLSLNTFSTVLKTSVVYLNLLRTVT
>CobsOr20
MLDRSKSSSDERITDGWNYSIQLNRWFLKPIGAWPWTLCETSIEKIGCMILTVASCFLICFLLIPCTLCTILV
DTDLDTKIRMIGPVSFILMAVVKQYILIARNEDIGECFQHVRTDWNNVALGHETDREIMVNNAKFGRWLSSVS
AVFMYSAGIFFTTIMPICARRTEIIDNETVRSLSFPIYRGLFDPRTTPSFEIAQFMQALAGYVIYTITIGVCS
LAAVFVMHACGQFRILMLKMEDLADGKKRKSASSTHEERLGDIVKHHIRILGFITRTEELLNEICLVDVVGCT
LNICFLGFNMMTEWEHRETLGTMTFCSLLISFTFNIFILCYIGELLAEQCMQIGAKSYMIDWYRIPNKGALGL
TLVMAMSNATIKLTAGKFMELSLASFCSIVKAAVAYLNLLRTFYV
>CobsOr21
MENDLASNVNVSTSTSLVEKYHEYSIQIIRWILKAINVWPRPANISITEKICSDFAIFICYFLIIVTMIPNGL
IFIDSQESHEIKLFNVGPFTFWLIAMVNYSCLLIHVNDIHDCVEHVKTDWRIIKKFEDRKVMLKNAKLGRFIA
GFCAVFMHSGVFSYNLIQGVLTKHTLNLGNSSITVRALPYPFHIKTTNVYFSPTYEFVFFLQCLSAFVVNSVT
VATCSLTAVFVMHACGQMKIMISWLEKFMDNRNEENTSINKKFAIIVNHHLRILSFVSRTEKIVNVICLVEVV
GCTMHICLLGYYIMMDFIQGNKGILSHSMVLFSITFNIFIFCYIGEILSDQGEQIGKSAYMTNWYLLPGKTAQ
GLVLIISRSNAGIKITAGKIVQLSFSTFGDVIKSALAYLNILRTVMI
>CobsOr22
MMKLPQNPQIAFNYIENKEYSIQPIRWVLKPISVWPVTDSSFADKILSVVLLIACVFLIAGTLVPSALAIFLD
ETKDIEMKLRDFGPLSNWVLASLKYVSLLMHVGDIYQCIKHIESDWRNVTNLEDQEVMLKNARVGRFIAIFSA
TFLHTGVFSYGLFRGITLSESAAVGDNVTVRPLPFAFYNNMLNATVSPIYEIVFAIQCLSTFVVNSVTIGTCS
LTAVFVMHACGQLKILMTLLDNLINEKNEKNPSQQRFAIIVEHHLKILSFVSHVEKITNIVCLVEIVGCTLHM
CLLGYYCILDWHQDEKEGIVAYAIILISVTFNIFIFCYIGEMLSEQCGQVGETAYMTNWHLLPGNTALDLVLI
ILRSSIVVKITAGKMIELSLSTFGNVIKSALAYLNILRTLM
>CobsOr23
MSNSKCAPESNAAKKHVDFNLQLNRWYLKPIGAWPQVDTLSSIWKTLTLLCIFICAGTITVVTGPCMLYILFE
KDSVKAKLNAIGPLLHRLMGSVNYWVLLRRSEDIRKLVRHMETDWSLIEKPDDREIMLQHAKFGRSVTMICGL
IMQVGTILFSIARAMKTATIVVGNETYTTHPMTCPMYNKLIDTRFSPINEIALALQFLSGIVVNSSTAGACSL
AAVFAIHACGQLNVLHRWLEKLVGSEKEENHIVKRKVSIIVEHHMRILSFISRLESIMHKVSLVELMGCTINM
CLLGYYTIMAWETLDAAKLTSYAIVYLSMGFNIFIFCYIGEIITEQCKHVGEMAYMTDWYNLHHKTALGLVLI
IARSSNVIKITAGKLFHLSIATFGDVIRTSMVYLNLLRTLT
>CobsOr24
MEKSKPISRSASFKKNTEYSTQLCRWFLKPIAGWPQMNDLNVAYGILGLLHVLICVTVVALIMIPCTLFVLLE
DANIKIKLGTIGPLIHRIIGTVNYWMLLQHSNDIRNLIRHMESDWKIINRNQDRDVMMQHAKFGRFVAGMCGV
IMHGGAFLFSIARAIKTVPVTVGNDTFRMHPMTCPLYSQIIDTRFSPVNEIALTVQFMSTFIVSSSTAGACSL
AAVFAMHASGQLNVLYTWLSELEDNEKGGNSTEQKLAVIVEHHLRILSFVARVENVMNKISLIELMGCTINMC
LCGYYLILAWDDFDATKITSYINVYFSMGFNIFIFCYIGEIVTEQCKYVGEMAYMTNWYNLHHKTALGLVLII
VRSSNVIKFTAGKLFHLSIATFGDVIKTSMVYLNLLRTLTM
>CobsOr25
TNPKRDLINVDFRHINDYSLQINRWCLKPIGAWSFMSMSSTLQKIVTVTQIVVCSLTIAIVTVPCILYVLFED
QAITTKLNALGPLLHRIMGSISYWVLLTRSHEIRNCIEHMETDWQLVRRIGDRETMMQYVKFGRFLAGVNAAI
TQGGQLLFGTARAIKTTTIMVGNETFKTHPMTCPAYSGLIDTRFSPINEIILIVQFISAFVVSSAIVSVCSFA
GVFAMHACGQLNVLYTWLNELVADYKKKGSQSVDQKLATIVEHHLRVLSFIARVENIMHKVCLAILMGCTLNM

CLLGYYSITNWGAFDGAKILSYIILYTSMGYNIFIFCYIGEILTEQSKHVGEIVYMTNWYLLPHKTAQCLILI
IIRSSNVIKMTAGKLINLSIATFGDVIKTSMAYLNILRTI
>CobsOr26
SDSKRILIDFENINDYSIQLNRWFLISLGAWPQTRSSSRIKKIVVSLQIFIFTSAVAIIMIPCMLYVWFEKED
IKTKLNAMLPLIHRIMGSVNYWMLLTRTKDIQLCIKHMEMDWKIVRRNDHEVMLQYAKIGRFMAGFCAMFMHG
STFIFTAARAMRTTTFMVGNETFRTHPMTCPVYSKIVDVRFNPANQIMLGVQFLSAFVVGSSIIATCSLAAVF
AMHACGQLNVLQTWLNELAEEKKSHLAEKKLATIVEHHWRVLSFIAQIESIMHKACLAELLGCTLNMCLLGYY
SIMNWASFDGMKIISYIITYISMSFNIFIFCYIGEVLAEQCRNVGQIAYMSNWYKLPHKTALGLVLIIMQSSR
VIKITAGKLFRLSLATFGDIIKTSLAYLNLLRTMT
>CobsOr27
ESVKLHFNASDYSIQLNRWFLKPVGAWPSSTSTTIGEKIISTILIIICYSLIAFTVIPCILNILFEETDVHRQ
LKAVGSLSHWTIGGINYFSLLFRSRDIRRCVQHMRTDWQTVTKIEDRQVMLKYAKIGRFVAGMCAIFMHSGVF
FHSLLQGITPTYKTIGNVSVLIHILPCPSYSKLVDNTKSPESEIALTLQLMSAMVVNSVTVGACSLAAVFATH
ACGQLNVLMRWLDQLDDRRHQDAVQHRLAIIVEHHLRVLSFVAQMEALLNQICFMELLGCTFNLCMLGYYVIT
EWNVTEKRLSIAYIVIYISMSFNIFIFCYIGETVTEQCKQVGETVYMTNWYRLPHKTARGLILIISRSNNVIK
LTAGKLVYLSIATYGDVMKSSMIYLNMLRTMTTS
>CobsOr28
MTYSERVLTDIKFTYKSNNDYSLRLTRWFLMPIAAWPRTNTSMAEKVSLQAQVLACLFLIAVVMIPCMLYVSL
EEKDVQLKLSLMGPLSHWIMGTINYWLLLTRSDDIRECVQHMETDWRLVRKTDDQDVMLRYAKIGRFIAGFCA
IFMQSGTFLFTVAKAMTTMAVIVGNETVLVHPMTCPIYSKFIDTRFSPANEIMLAVELLSCFIVNSITVGACS
LAAVFAMHAYGQLSMLFSWLNDLVDDKDNGKYLAEQKLATIVEHHLRVLSFISRMENIMQNICLVELVGCTLN
MCLLAYYSITNWSDFDAARITSYIIVYMSMAFNIFIFCFIGEILTEQCKNVGEKAYMTNWYNLPHKTAVGLVL
IIARSSNVIKITAGKLFQLSIATFGDVIKTSMVYLNILRTMT
>CobsOr29
MTYSNHVARGIELPSRSDNDYSLQLSRWFLLPIGAWPQINGDATRVKRLISHMHVCVCTFLVAIVMVPCLLYV
LLEEKDFEIKINVMGPLSHWIMGTINYFLLLARSDDIRECVLHMETDWRFVQKIEDREIMMRQAKIGRFVSGF
CALFMQSGTLLFVLVKSISTVVVVVGNETVSMHPMVCPIYTKFIDTRFSPANEIMIVAEWVSCFIVNSVTVGA
CSLDTVFAVHAYGQLNMLYSWLNELVSESKGNEDAEQRLAIIVEHHLRVLSFISRMETVMRYICLVELLGCTM
NMCLVAYYFITNMDSFDKAKIMSYVIIYLSMAFNIFILCYIGEVLTEQCKNVGEKTYMINWYKLPHKTALGLI
LVIARSNNVIKMTAGKLFHLSIATFGDVIKTSMIYLNMLRTM
>CobsOr30
MCRSVAKELHDYSVQLNRWFLKPIGVWPRTSTTSSCERIVAQILIVVCLSLVSFAVIPCTLNICFEEKDIELK
LRAIGPLSHWLMGGLNYCCLLLHSTDIRQCMRHMETDWRILEQSRDREIMARKARLGRFVAGFCAIFMHTGVF
SYSIISSLTTVKVSIGDNQSVSVLQLPFPSYSKWIDTRFSPTNEIVFGLQLVSGFIVNSTIVGACSLAAVFAM
HACGQLDILTLRLNMLVKGDHTKKGESVQQRLAVIVNHHLRVLRFITRIEDVMHQICLIELFGCTFNLCMLGY
YSITWWDKIDKKGIVAYIIVYISMSFNIFIFCYIGEVLTQQCKKVGETAYMIDWYRLPHKTALGLILVIARSS
AVIKITAGKLVQLSIATFSDVIKTSLVYLNILRTVT
>CobsOr31
PSALYRGNENDYSLQLNRWFLKPIGAWPHTRATRITEKLLSKIIQIICYAFIAFTIVPSMLYFYLEEQNLDTK
INWVGPVSHWIMSVINYTSLLWRSKDIRCCIDHMASDWCMVNRNVDREVMLKYAKFGRSVAGFCAVFMHSGVF
SYSVVNSLTPIITAIGNETVSARRLPCPFYSKLLDTSRDPANTIVLLIQFMSGFIANSITVGACSLAAVFAMH
VCGQLAVLYTWLNELLDEKQTAEHKLANIVEHHLRLLNFISRFEKIMNQICMVELVGSTMNLCLLGFCSIKEW
NARNTKTIATYVTVAISLSFNIFIFCYIGEIITEQCKKVGEMAYFTKWYRLPHKTALGMVLIIILRSSGVIKIT
AGKLIQLSLMTFGDVIKTSAAYLNILRTI
>CobsOr32
SDKSVSALYRGIEHDYSLQLNRWFLKPIGAWPDTSGTTIKEKILSRFIQMICYALIVVTIVPCILFLYFEEGN
FDLKMDSFGPMTHWIMSGMYYTSLLCHSKDIRYCIEHIERDWCAVSRNVDREEMFRYAKFGRSVAGFCAVFMH
CGVFSYSAVNSLTPMIVAIGNETIAARRLPCPVYSKLLDTSHDPANGIVLLIQFMSGFIANSITVGACSLAAV
FAMHVCGQFAVLYTWLNELIDEKQTAESKLANIVEHHLRVLNFISRFEKIMNQICMVELVGSTMNLCLLGYNS
IKEWNARNTKTLVTYVIVIISLSFNIFIFCYIGEIITEQCKKVGEMAYLTKWYRLPHKTALGIVLIISRSSVV
TKITAGKLIQLSIKTFGDIIKTSAAYLNILRAI
>CobsOr33
MHESPSDPWSSSIVVIHDHERYVRLSIQQIRWIMKSIGAWPRPRESSSPINKYVNALTNVICMGLVIFLFVPC
VLFVVLEVEDTYSTLKFTGPLSFFTMVFMKYSSLILRRRDIRACIDYIKRDWMNTRRHDDRAIMIKSAEFSRR
LVMICAFFTYGSAVFYHIAMPISTGKITEEGSNMTYRPLVYPVARAIIDTHRALISEIFICVQCVAGFVTQSI
NAAACSLTAVLAMHAYGRLEILMQWIVHLVDGRDDLSSNLDERLATIVEEHVRILRFISLTEKIIHEISLVEI
IGCTLNICFLGYYVIMEWHIAEFANQVTYILLLTSFTFNIFIFCYIGELVADQCKRLSEISYMIDWHRLPGRK
ALAVILMIAMSNSSVKLTAGNIIDLSFSSFGDVIKTSVAYLNMLRTLTT

>CobsOr34
EITVNVYKKDNDYSLQLNRWFLRSIGAWPELKTNSTIKNTLMNILRLMCYSLITFTAVPSILYIFFEAKDFHL
KLKAIGPTSHLLMGGINYSSLLRHNERIRWSIKHMETDWRMAKKEHDREVMLKNARVGRVIAGVCAFIMQGGV
LCFNIATAMSRIAIIIGNETVKTGGLPCPAFNKIMDTRLSPIYEITFALQCLSILVVNNITIGACGLAAVFAM
HASGQLNVVMLRLEKFVSEKQDLQLGLANIVEHHLRALRFLSQLETIMRQICFVELVGCTFNLCMLGYYTITE
WHDKGMNTISYIMVLTSMMFNIFIFCFIGELVTDQCKKVGEVAYMTNWYKLPHKTVRSLILIIVRSRIVIKIT
AGKIFHMSIQTFGAVIRTSVAYLNMLRTLTM
>CobsOr35
TVIGKMNPVASIRDHTEDVQLSIQMNRWLLKPIGVWPKSTDISWIGRLAYFIINVTCTSLISFLFIPSAMYMV
LEMNDTYLILKLSGALSFCVMAVMKYSLLIFREKDIRSGVQHIKNDWMNTQYYGDRNIMIRNAKFGRRLVVIC
TFFMYGSAVFYYFALPFSVGKITDERENLTYRPLIYPIAKVIVDARYSPISEILFWVQCISGFIAHSVTAGAC
SLAAVFAMHAYGRLEVLIQWIKHLVDGREDLYNNVDERLAMIVQQHVQILRFISLTDKVLREISFVEVVGCTF
NMCLLGYFSMTEWETREPARYITFMVLLVSLTFNIFIFCYIGELVTEQCKKVGMVSYMIDWHRMEERRGLALI
LMIAVSNTSIKLTAGNFFELSLSTFGDVVKTSVAYLNMLRTLT
>CobsOr36
IKLIMKDLLKVNRYEKDYNYAVQVTRGILRMIGVWPISRHASVTEIIFTRLQIAFCYFLFAFIIVPGLMLVFL
KEHDFKRRVRLLGPILNCWMGCVKYSLLLYHTREIQSCLKQARQDWKCTVDSADRRAMMGKAKIGRKFAIISA
TFMYVGGLSYRTIVPLSKGRMLTPMNTTVRALSCPSYFLGFDEQTSPAYEIVFTLQFFAGLLTYSVTCGAAGL
AAFFIMHVCGQLSVLMDKLQHLNDIPEPDDRPVAILLADIVEHQIKVKCFLKQVEESMRYVWLVEIVGCTILL
CLTGYYVIMEWESSDSTAMLTMAVILTSFAFSIFTNCYVGQLLTDQSIKLGSMTSTTNWHRLPHKRARTLILI
MAVSNIPAKISAGKMIEMSLPTFSNIIKTSVAYFNLLRKFIT
>CobsOr37
VIMKKANPLLTVHDRKKDLQLSIQLNRWILKPLGAWPKARGTLNIERYAYAMINVACVSLIGFLFVPSAMYLV
LEMDDAYNILKLTGPLNFCIMAVIKYSSMIFREQDIRSGIEHIACDWTNTQHPNDRAIMVRNARFGRRLVSIC
AFFMYGGATFYYVALPFNNGKVTEEDGNLTYRPLMYPVARVLIDTRYTPIGEIFFLIQCLSGFIVHSIATGAC
SLAAAFAMHAYGRLEVLIQWIEHLVDGREDLYDNVDERLAMIVQQHVRILHFVSLTDKILREISVVEIVGCTL
SMCFLGYYIVMEWESKELTSYVTYIVLYISLTFNIFILCYIGELVGEKCKEVGEVTYMIDWHRLSERRGLNLI
LMIAMSNTSVRLTAGNLLELSLSTFGDVVKTSVAYLNMLRTLT
>CobsOr38
VTSETNPATVADDHERDMQLSVQLNRWILQPIGVWPKLTEISYTERYAYGLVNVMCTSLIGFLFVPSAVYMAL
EMDNTYHILKLSGPLSFCLMAVVKYSSLIFRENDIRRGIKHIESDWITTRHNGDRVIMIKSAKFGRRLVAICA
FFMYGGAVFYYLALPFSIGKITESDGNLTYRPLVYPVARVIVDARHSPISEIFFWIQCLSGFIAHSITAGACS
LAAVFAMHAYGRLEVLMQWIEHLVDGREDLCDNVDERLTMIVQQHVRILRFISLTDKVLREISVVEIVGCTLN
MCFLGYYTLTEWESKETTSYITYTVLLISLTFNIFIFCYIGELVAEKCKKIGEVSYMIDWYRLSEKKGLALVL
MIAMSNTSIKLTAGNLFELSLSTFGDVVKTSVAYLNMLRTLT
>CobsOr39
NPFHYLNIHKNPKLYNQNYKNDLRVSMQLSVWVMKIIGTWPSRNSWIETLWQRALNIICYALLALLLIPTLIF
IVLEINDSYIRLKISSAISFFFMSVMKYCALLLHENNIRKCLDWIKQDWRSVRYAEERKIMLENASFGRRLVA
ICNFFMYGGVFFYWVVVPLSRPKIVEENSNLTYRMLVFPVPKVIVDVRRSPVNEIFYFIQLSAASIGSTITIA
SCSLAALFAMHACGQLQVLMSWLNNLVDGREGVNDTTDERLAEIIQLHIRIVNFILVTKTLLQEISLIEIVGC
TLNICFLGYYCMTEWDFKQPVNGLTYLTLLLSLTFNIFIFCYIGELLKEQITKVRESSYMIDWYRLPKKKSLA
IILIMSMSNATSKLTAGNIIELSISSFGDVIKSSLTYLNLLRTFT
>CobsOr40
PCNQNYKNDLRVSMQLNVWVMKIIGTWPLSHSSWIETLSYRALNVICYVLLALLMIPSGMFIFLEIKDFYNQL
KLGSALTFFFMAAMKFGALLLRENDIRRCVDYIEGDWRNVKYSEERKIMLENASFGRRLVIVCGIFMYGGFLF
YWVLVPLTRPKIVEEDGNLTYRRMVFPVPGTIVDTRRSPINEIIYFTQLSAGFISHNVTVAACSLVALLAMHA
CGQLQVLMSWMNHLVDGRDGINDTTDERLAKIIQLHVRILNFISLTEELLHEISLVEVVGCTLNICFLGYYCM
MEWDFKQPVSGLTYLILLISLTFNIFIFCYIGELLTEQTMKVRESSYMIDWYRLPKKKGLAIILIICMSNATT
RLTAGNIIELSISSFGDVIKSSVAYLNLLRTFTT
>CobsOr41
MIERINSWNSNYESDITYTVRVHKLIMGLIGVWPNLEKSRMQTRVLKGFLRTACCFLLSFNLIPWILHMFLIL
DSFKTRLRMIGALCFYSMVPAMYCTLMLREDRIRICMKHLQEDWRNVRDTNDRRIMLDEAKTGRFIIVCATIF
LFTSGFTYRLITPIMRSTIVIGNVTIRPLVQGHYFIFFDPQQSPAYEIVFSMHLVTGIVIYLVTTSVCGTTAL
FTLHACGQLKLLATWLENLTSEDRRSKDHAVTQRLAAIVIHHVRIRKFLHQIQDVVGEMCFIEFIGSTLILCL
LGYYVITGWERNDALSFTTYAIMLTSFTFNIFILCYIGEVLSTQGSKVNTTLCTIDWYCLPSKEARCLVLVIA
MARYSTKLTAGKVIDLSFNSFGDAVIRTAMAYLNLLRTVTL
>CobsOr42

KTSKQNTNYNYAVQVTRLILRLIGVWPIPSNASIVERIMTRTQNVICYLLTAFLIVPGLLRIFLKEHEFKSRI
RLTAPIINCGMACLKYILLMYHAREVQFCLDQVSQDWNDAVNENDRRIMLSNAKSGRKFAIYSALFMYIGGLS
YRILIPLSKGRILTPMNTTVRVLACPSYFVFFDGGVSPAYEIIFTLQIFAGVITYSVRCGIAGLAALFVMHVC
GQLSIVVDKVHSLDYVPKSNNRAVMILLADIVERQNKIKKFLKKVETTLQYVWLAEVTDSAILVCLSLYYIIM
GLENNQFSDVFTMTVMLASFAFALFTNCYVGQRLTDQSIKVRLMIAASKWYRFQYKTSRTFVLLMAVSNIPTK
ISAGKIVEMSLPTFSNIIKSSVAYFNILRNF
>CobsOr43
MHVNIVHDENIKRTVHVTRGVLNLIGVWPLPGDSSYIRIVQTKMLRVLCQALLYFIFIPGILKIFLKESNARR
RLKMIGPMCNCLMAVLKHMMLIWRSDRITDCIKHIEEDWRTTDLIEDRKIMLGNSRIGRSLAILCVAFVYGSG
FSYRTIVPLSRGVIVTPQNVTIRPLGFAGYYVIVDPQKTPAYEIIFSIQFISGFVQYSVTSGACSLAALLVLH
ACGQIKILITKMETLTNIEYSDKSANVKLAAVVRQHIRIKSFLSKVEEILQYVCLVEVVGCTFILCLLGYYII
MEWESNDAVSMLTYAILLFTFIFNIFILCFIGELLTDQSTKMYLTFCTADWYRIPHKTARGLVLIIAMSSVPI
KITAGKFMDLSLNSFGAIVRTSVAYLNILRTTSI
>CobsOr44
YPNKNYKSDIKFTTQLNRVFCRLLGIWPETETSFLKSLERFSLILGCYFLLSCELIPTILYIIFVEKRARVRL
KLISSIMFTVLSVLKYSSLVLSKNRVRDCLARVKNDWRNVAGANTRNSMLEKAKTARRLLILCGIFMYTSGLY
FRTIVPLSKGKTVTSQNVTIRYLPCPSYFVFFNGQISPAYEIMFFIQFCSGFIKYTITVAICSLAALFAMHLC
GQLEILMTLIDNLINETEVKNLDKRLALIVEHQNNMRNFLRLVQSTLGNTSLLEVMGCTIILCLLGHDIITEW
EDQNVISMCSYLILLTSIGFNIYIFCFIGEQLSVEGEKLALAVCTFAWYRLPYTKARALMLIIAMSIVPTKLR
AGKFFDLSIRTFGDVVKTAVTYLNFIRKIME
>CobsOr45
MPRNLSHQNDILYITRPTRNILLALGAWPSIGKERSVYPRVHNLFLIFISYALLSSDIIPGVLYWLIEGTARI
RLQMIPLLLYDVMSASQYGIFIFRYDQLRRCLKHVEEDWQNVLTADTRDIMLKSARMGKRLVTICGVFMYSGS
LTFRIIIPLSQGKIVTDQNATIRQFASPGYYFSLDVQASPVYETVFIIQCLTGLITVSVATSACGLTAIFVVH
ACGQLKILIDLMRDLVQKQWKNECEVNEKLIKVVEHQIRVRNFLRLVQDTLQEVYLMEVLVNTVTICLLVYFM
LVDWQSRNITILCSYVISITNVIIHIFLFCYTGEQLSTQAEKVAITSCELEWYRLPDKKARSIVLLMIMSNAP
TKISAGKFVDLSLKTFGDVMKTAGAYFNMLRNVVE
>CobsOr46
MFANKYYERDVENAFAMNRFFFRLVGLWPLQPVDSFIPNAVESIVMAITCFAFVVGELTPTALYTAIKITDVR
LRLKAAGSVIFAVVGLIKYSYMLFNKSQVRNCLMLIDKDWQNVINSDERILMIDQVRFSKRLIMICVVFVYMT
GVFVRMLLPFYTGKIVTPDNVTIRPLPVVAYLVIVDVQRSPFYEIVFFVQFFGGFFKYTLTVAVFSFMTVCAM
HFCAQSNVLVTLMNDLVNESRPEYVNKKLAIIVEHQIKTRNFLQLVQYVTQYPSLIEVLGSTVMLCFAVYYIT
TEWEDHNFLRMFTFIIALAMFTFDLFIYCYMGEQVISQEEKVSLTACTLEWHHLPSAKARALILLIIISEHPL
KLRAGNFIDLSLRTFSDVVKMAVTYFNLIRS
>CobsOr47
ERYKDDVVYVTHLTRNICKTLGIWPSTNKKESHGDKTWKKFLIIFSYALLYSVLFPGFFFWIIEKRIKIRLLT
FPLLLFVSMASTKYGHLIYRENNIRLCLKHMEEDYKTVPTSKARDTMIESAKIGRRLVTLCAIFMYGSGLSFR
LILPFAKGKIVTPQNITIRPLPCPAYFFSFDVQVTPIYELIFAMQVLSGLVTFSITTGLCGLAAVFVMHACGQ
LKILTTLMRNLVEEQWQDKKEVNKKLAEMVEYQIRIRSFIQMIENTIQQACLIEVMGCTTIVCLLGYFIIMEW
ENSNSIAMCSYFTSVTSMMINMFLFCYTGEQLTVEAEKVARTSCTLEWYRLPDKEARGIVLVVIMSNMPIKIT
AGKIMDLSFKTYGDIVKTAVTYFNMLV
>CobsOr48
MLRNERYEDDMTYITHLTRNVLSLLGVWPAYNRTISTGERIWKYFLIAISYILLYCVLIPGALFWLIEKRTRV
RVQTFPFLLYCFMASSKYSNLVFRERNIRRCLRHIEEDWKIVNGVEARDTMIESAKIGRRLVTLCAVFMYGSG
LSFRSILPFAKGKIVTAQNITIKPLPCPAYFFFFDIQVSPAYEMVFATQLLSGVVTYSITTGLCGLAAVFVMH
ACGQLKILVNLMRNLVEERWEAEHEVDRKLARMVQHQIRIRSFLHLVESTLQQACLIELMGCTAIVCLLGYFI
IMEWENSNSIAMCSYFTSFTSMMINMFMFCYTGEQLTVQAEKVARTSCVLEWYRLPNKEARGIVMIIIMSNMP
TKITAGKIMDLSFKTYGDIVKTAVTYFNMLLKVA
>CobsOr49
MLRNENYKDDMIYITRLTRNVSSLLGVWPTYNKRRSTGEKVWNYFLISMSYILLYSVLIPGGFFWLIEKRPKV
RVQTIPLLFYGFMASGKYSNLIFRERNIRRCLKHIEEDWRIVNSVEARNTMIESAKIGRRLVTLCAVFMYGSG
LSFRSILPFAKGKIVTAQNITIKPLPCPAYFFSFDIQASPAYELIFAMQFLSGIVTYSITIALCGFAAVFVMH
ACGQLRILVDLMRNLVEEQWQEKQEVDRKLAKMVEHQIRIRSFLQLVENTLQQACLIELMGCTAIVCLLGYFI
IMEWENSNSIAMCSYFITVTSLMINMFMFCYTGEQLTVQAERVASTSCELEWYRLPDKKARGIVLVIIMSNMP
TKITAGKIMDLSFKTYGDVVKTAVTYFNMLLKVAN
>CobsOr50
MFANKNYKRDVEYVFELSRFVFRLLGIWPYARTNSRLSETLETAVLIVVSYVILTCELVPAILYVVIVQKKTR
ARLKVIATTLFTIITMFKYSQLILNRNRMKNYLAQVEDDWRNIADKRDHDIMINEARIGRRLVIICAIFMYSS

GVSFRTIIPLSYGKIVTEQNTTIRLLSCPGHFILFDVQLSPAYEIVFVMQLFSGFIKCSITTAIYGFTSLFVM
HICAQMEMLIILMNNLVNKKELKNVNEKLAAVVNYQIKIRNFLQMIQNSIQYASLLEILGCTIIVCLLGYFVI
MEWEDNNAVALCSYLVGLTSISFNIFLFCFIGEQLSKKAEKVALTACTLEWYRLPDIKARSLILIMIISNSSI
KLKGGKFIDLSFKTFGNVVKTAVTYLNLLRA
>CobsOr51
MILNQHYKTDIKYVVGQSHVVLRILGIWPSTDKLPSLIEKTANILLVIICYFFLNCDMVPGALYYAVVNDESR
EKFKMMPPILYSIMAIGKYSNLLIHEEDIRSCFRHIEEDWRTIAVGDAREVMLSKAVVGRRLFVLCCTFMYCG
GLSYNTVVPLSKGSIVVDENTTIRPLSCPGYYIFFNPQNSPAYELVYLQQVLCGFFMYTITVTMCGLAAVFAI
HAYAQMEILIQMMKSLIDASGRQNVGTKLAVAIKHQVRLQNFLQLMENTLSYSNLVEITGCSVIICLCLYCIR
LEWEDMNLVAMSSYAAALTSVVINIFILCYIGEYVTSQADEVSLTIRTLDWHYLPTNLARDMTLVTAVSNISP
RLTAGKVIDLTFTTFGDVIKTSVIYFNMIQQLTE
>CobsOr52
ASMSPADKHALFRNFNYRSDIEYVVRVAKILLVPIGVWPLYRDDTAMNKIAYFLHTSVIFCLMCFLLVPHIIY
TFFDAEDLTRYMKVIAAQVFSLLGIIKFWTMIINKNNIKSCLQEMEIQYRDVECEADRLVMMKNAKIGRQFTI
VYLGLLYGGALPYHIIMPFLADRIVKDDNTTHLPLPYLSDYIFFVVEDSPFYEILFVVQILFSTIILSTNCGV
YSLIASCVMHACCLFEVARSRMESLISDNDRFHERFDWIVTHHLRALRYVEMIESSLNFVFLSEMVGCTIIIC
FLEYGVLKEWEDNKLFGVVIYFILAISIFVNVFTLSSIGDRLKEESVKIGEASYSIDWYTIPTKNVNNLIMVM
VRSNRPSTLTAAKMFDISLQSFCEVCKTSMAYLNFIRMLT
>CobsOr53
MPTEDPQPFKNHNYKADTEYVVKVAKTLLTPVGIWPLYRGNSTSDKIKNYIQTTFIFTLMCFLLIPHVIYTFF
DAEDLTKYMKVIAAQVFSLLAIIKFWTMIINREGIRYCLQQMEMQYKNVECEEDRLVMTKSAKIGRLFTVTYL
GLSYGGALPYHIIMPLLADRIVKEDNTTQIPLPYLSNYIFFVVEDSPLYEITFVTQILISSIILSTNCGVYSL
IATCVMHSCCLFEVVRRQLDTVFNVGTDNIQKRLGQIIENHMQAIKFAEMIEKSLNIVFLCEMVGCTIIICFL
EFGVLKEWEDGQVLPMGTYFVLMTSIFVNVYIISAIGDRLKEESDKVGESSYFISWYNLPTKIVKNLILVMLR
TSRPSTLTAAKIFDLSLQGFCEVCKTSAAYFNFIRAMTT
>CobsOr54
MQFFDGRNYRINKVLLSCVGQWPYQTNRSSNAIIIIIVSLAGTQIIAKICGLLSIHNFDLFIDSLSPLVVDLG
CAIKLVTCVLRATEIRELFDQIQYDWQLLITSPYIKTLSNYAQGGRTFTLIYAGSFYSALTFFMLVPLQQLLL
DSSLNASTRPLLHQVEYYIDMEKYYFPILIHGYLTAIICVSISVAADTMYVIVVQHICGLFMIIGQQLDNLIK
TDNLEIDLNPPIKDDKPYENVVNSIRTHKKALRFASLIEAVFSQMFFLMAAVNLLIISMTGVTAVTNMDKPEE
FFRQITFSCALLVHLFFESFQAQRLIDHSSLVHTSLTSVPWYQTSSRTRKILIFMIMKTREPCVLTAGKMYVI
SMDTFSTIVRTSVSYFTMLRSV
>CobsOr55
ANTITQAIEIGLRVVGIWPDSDYITVSRFFWTTTMIFVQIFQYRHMVLHFNSNDVSELMDGLSETLSYSLLCV
KLIVFWTKQRIFNDVLENIATDWKECGDALRSMSNMASLSSRFSNLIVGLHSIAVMFYGIGVIALHNDHGDTG
NREFFLKMELPFEAGTSPIYEVVMTTQFLHQITAASVIGVLSALLVTLVLHAGSQIDILRDRLLEILSKEKKP
AVSLITMGSLIRRHQNIIVFTKKIESLYSYIALAQFVSNTIVICCLGFIIVNSIGSDHGFSVLLKSILFYVVI
NLEAFIFCFAGEYLSMKSKMIGEAAYESRWYDLTPSENRLLLFLIMRSQKQLTITVGKFTNLSFQQFANIIKS
SASYISVLHAL
>CobsOr56
MIRKTTINRTIEVVFTLFGIWPDTSCISFCRIFWIITIIIVQFYHYRYLQVHLHSDDLFNLMDCLSSFLAYMK
VMTKFVMFWINQRKFIKTLEMIREDWDNCADSEINMHQTMCKANISNRITNAMIILHTLSAIAYSMRIMMTDV
DVSDRTSKPPFIHKMELPFNVNTQRTYKMILIMQTVYVVMCSWAAGAVNALLLTLILHIGGQMDILHAWLINF
APQDSGRKHELITTDKIIQKHQKIINFSKNVENLYSYIALLQFASNTIMICSLAFLIVSAIGTPDAVEQIIRT
LLFYAITNLEAFVFCYAGEYLNNKSKAVGNAAYNSTWYNLKPKDSRILSFIILRSQKQLTLTVGKMADLSLEY
FASIMNASGSYLSVMLAMQ
>CobsOr57
QDPLDRIMRFMLTLSGILPGASCVLFWRMYMIVSITFGLYGIYLYIMNRINTVTLWDLMECLSFVLAHSKVIF
KCLMFWINQQKFLEILAMMKEDWNDCAHDDVSLKETTRKVKTYDRIARVILILHTLSIIGFSSGVFFANDDIT
NNVTEVHFNTKIDFPFEINTQRTYKLILVIESLVLFTFSWSTGTVNCMLLILIMHIAGQINIVRRWLTEIAFS
ENTGKDKSISITMTKIIQKHKKIIRFSKKIQSLYMHIALVQFLLNTIMICAIAFLVVTAVGSPDAVKQIVKCF
FFYVITNLEAYIFCFAGEYLKNKSREIGIAAYSTAWYNMKSKDSRVLLFIILRSQKELTLTAGNMMELSLQSF
TSIMNISGSYLSVLLAM
>CobsOr58
QDPLDRIMRFMLTLSGILPGASCVLFWKMYIVVSLIFRLYNIYLYVTNRINTITLWDLMECLSVLLAHSKVIF
KCLVFWINQQKFLEILTMMKEDWNDCAHDDVSLKETERKVKTYDRIAKVILILHTLSIVGFSSGVILANDDVT
NNATEVHFITKINYPFEISTQRMYRLMLLTEMLLLFTFSWSTGAVNCMLLILIMHIAGQINIVRRWLTKLEFS
EDTGKDKSSPITMTKIIQKHKKIIRFSRNIQSLYMHIALVQFLLNTIMICALAFLVVTAVGSPDAVKQIMKCF

FFFVITNLEAYIFCFAGEYLKNKSIEIAIAAYSTAWYNMKSKDSRVLLFVILRSQKELTLTAGNMMELSLKSF
TNIINISGSYLSVLLAM
>CobsOr59
MVRKSTPNRTLKFMLILCGIWPGAPCVLLCRVFWVVSLTVTIFLHYRYFMTHVHTAEILDLMDCLSSFLAYSK
IIIKFIVFWLNQKKLVEILAVMTEDWNDCTDSISIRETERKAKMSDRIANAIVTLHTMTIVAYCIGIILADVD
IANTTEELPLINKLDIPINITTQSTYRIILIVEFLFMILCGWAAGITNSFLLTLILHTAGQIEIMRYWLGQLV
PRKNETKSIAKTTNKIIRKHQKIIGFSKNIENLYSYIALLQFVSNTIMICSLGFLIVTAIGSPNALEQILKSF
LFYTITNLEAFIFCYAGEYLNNKSKEIGAAAYNCAWYDLKSTESRLLLFIILRSQKQLTLTVGKMMDLSLQSF
TSIMNASGSYLSVLLAMQ
>CobsOr60
MTRKSTINRTLKLMLTLCGIWPGTSCVIICRVYWIIALATDEICHYRYLLMHSHSHDLFDLMDCFSSFLTQVK
FSIKLIIFWANERKFLEILTMMAEDWNDCVNSDVNMRETAYKAKLADRITNALFTLHTLTIVAYSIGIFLTDV
DVVNQSELPLLLKVELPVDINTKRMYKTLLAMQFVHLIMSGCGTGLLNALLLTLTLHVGGQMDILRCWLNDLV
PKENEERSESIVAMTNKIIRKHQKVISFSEYIEDLYTYIALVQFTSNTVLICSLGFLIVTAITCAIGSPDATE
HIVRSLLFYTVTNLEAFIFCFAGEYLKNKSKAIGNAAYNSAWYEMKPENSRNLIFLILRAQKQLTLTVGKIMD
LSLESFTSIMKASGSYLSVLLAMQ
>CobsOr61
MASTRTINRTVKLMLIMFGVWPGAPCMMFYQVFWMITLTISEFLHYQYFVTHFRFHNLFDLMDCLSSFLALLK
VSVKLVIFSIKQRKFVEILTMTAEDWDEFDSTGAALRETARKAKLSSRICNGLIIFHTISATVYVTSILLADA
DVTDRTTELPLMMRMEYPFVIDTQRKYRLVLATQFIAVIICSWAAALFNALFLTLTLHVGSQVNVLLCWLTEV
GAKDVEKSHDFFVTFMTKIIRKHQKIISLSESIENLYSYIALTKFISNTLMICSLGFLIVTALDSPDATEQIM
RSLLFYTITNLEAFIFCFAGEYLSNKSKAVGNAAYNFAWYNMKAKNSHVLLFIILRSQRQLKLTAGKMAILSL
ECFTNIMKASGSYLSVLLAMK
>CobsOr62
MASKRTINRTVELMLIMFGVWPGISCVILYRVFWLITLAINEFLHYQYFVTHFHFDNLFNLMDCLSSFLAHVK
LTVKLIIFSLKQRKFMEILSTMAEDWTDCDNGVALRETEIKAKLSSRICNGLIILHTIGALAYVIGILLADAD
VTDRTAELPLMMKMEYPFTVDTQRKYRLVLATQFVFVVVCAWGAALFNALFLTLTLHVVSQINIMLYGLTEIG
FKDAKETRDSFFTVATRIIRKHQKIITLSENIENLYSYIALLQFISNTVMMCSLGFLMVTAIGSPDATEQIVR
SLLFYTVTILEAFIFCFSGEYLSNKSKAIGNAAYNSAWYNMKSSDSRVLLFIILRSQRQLKLTAGKMMVLSLD
SFTSIMKASGSYLSVLLAMK
>CobsOr63
MKVEKAVSRTLEIYLRIFGIWPNTSGILLLRVFWTVMIAFEQIFQYRYIVMRYHSIDFSKVIDVLSAAMTYTI
FSFKLVIFWYKQRTLSRILTMMAIDWEKCTRTKFSMFATTCNAKLSQRLVNMTVIIYSGTVIFYSIGVIFQQT
NNDSATNGTKQFILDMDLPFDANQRFIYESVIVVQFIHLLLCADGMGLLNIVLINLVLHISGQIDILRNSLME
VFPKKKKRSPSRFTIKEIIKKHQEIIAFSEQVEDLYSYIAMVLFVTDTLIICGLGFTIVASIGEPNASKNIIR
NIMFYFVMNMEAFIYCFAGEYLSVKSKSIGDAAYDSLWYESDCKDNQIVLLLIMRSQNQLVITIGKVMNLSLE
RFSSIVKASASYLSVLLAM
>CobsOr64
METSTVSRLVNIGLRAYGIWPYLPFTVLVRFFWVVILSVVQVFQYRYVVTHFQRGNFSDLMDGVSSTMAYSLL
IVKLSILWVNQRTFFNILQMMALDWKKCVLTDCSLRITISRAKLSHRFSNYIIVLQLIAIILYCCGVLAVNTG
DIQRLNVSAREHILKMKLPFRIDTSPMYVLTMVFEFIHLTMVAGGIALINSLIVILIVHIGGQIDILRDWLLK
TFSKNVAHIVDKVTIKTLIEKHQQIIIFSKNIENLFTYIVLMLFVSDTLIICCLGFIIVTSIGTPNGPAILVR
SVLFYVVINVEAFTYCFAGEYLNAKGKMIGDAAYDSLWYDGTTKKSRIVLFIILRSQKQLTITIGKIMDLSLE
RFASVVKASASYVSVLLA
>CobsOr65
TTFSRLVRFGLHIYGIRPYKTTTVVFRVYWVIALSIAQTFQYRYCVLNIHTDDFSSYMDGLSSAMAYSLLCIK
LIILWTHQRKFFDLMQMISLDWKENDTTNCTSRIMTRSKKLARIASRCIIGVQAVSVCLYSTSIVVSGYTPEK
LEPYNRELILKMVLPFNISTELIYETVQLVQFYNIFFVAFGITSINSILVTLILHVCGQIDILRYRLTNIFSK
NSADSVDDITMQKLIIKHQQIITFAENIETLYTYIALIMLLSDTIIICCLGYIIVTSLNTPNVIAILIKCMLF
YFAVNFEAFIYCFVGEYLSAKSKMIGDAAYDSHWYNFPAKESRMILLLIMRSQKRLTITSGKIVDLSLEQFTS
VVKASLSYISVLLAMY
>CobsOr66
MSKDRETANVVCRSVELGLRLIGVWPGTSYAILRRVCCITSMVVFQTFQYQHLIMHFGEKDLLLLIDVLSITM
AYSLVLIKLIIFAFNTRLLNAIIVRLADDWKERETSDGYTMTRTTYISRRISNFMIALYALSVFLYATGTLLK
YKSNNQTDTRELILKMELPFKIKSTSVHVVVLIIQYVHQTCTASMVGVINSLLITLVLHVCGQIDIVRQKLSE
ITRKNVKLSGNESVVKRLIIRHQKIIDFSKNIEVLFSNIALIQFVSNTLVICCLGFLIVISIGAPGGSSMLIK
SVFFYIVISMEAFIFCFVGEYLSSKSQKIGDAAYESLWYQLNPNQNRDILIMIIRSQKHLTLTVGKVMDLSLK
QFASIVKVSASYVSVLHAMY

>CobsOr67
MSNEKKSVSTICRSVEFGLRVIGVWPGTSCAILRRVCYLLSMAVFQTFQYQYLIIHFSESNIFLLMDVLSATL
VYTLLFIKLIIIVFNVRLLDDIVAHVNEDWKKRDVSDEYVMNRMAYVSRWFSNLIISSHAISVLLYAAGTLLR
HKSSNQTDARELLLKMELPFKIESTSVYFTILVTQFVHQVSAASILSVINCLLLSLVLHVCGQIDVMREKLCK
ITRSNIKQDENKSIAKMLIIRHQKIISFSNNIETLFSNIALVQFVSNTLVLCSLGFVIVTSIGVPGGLPMLVK
SVFFYILVNVEAFVYCFLGEYLSTKSKTIGDAAYEALWYDLNPVQNRDILFIIVRSQKYLSLTIGKVANLSLK
QFTNIVKASASYMSVLHAMY
>CobsOr68
MENEVDSMTTVSRSVEYGLRMIGVWPGTSCAILLKVLSISSMIVFQVFQYQHVIVNFGEEDLTILMDALSVTF
AYTLLLIKMLIFSFNARLLNEIMRCMIKDWKECDISGEYTMTRTAHVSRWVSNVIIFSHMTSVFLYAIGTLMK
IKTENQTDNRELIIKMEIPFEIQSTPVHVAVLVTQFIHQTCAAGMVGVLNSLLIILVLHVCGQIDIVRQKLSE
ITQKTMERKNVGQNVNESIMKMLIVRHQEIIGFSKNIEALYSNIALIQFVSNTLVICCLGFLIVISIGAPGGS
MMLVKSVFFYIVMSLEAFIYCFVGEHLSTQSEMIGDSAYESLWYELNPTEKKDIHIVIVRSQKHLTLTIGKVL
ELSLKQFANIVKASASYVSVLHAMY
>CobsOr69
MICDGTVSRSIEIGLRVIGVWPDSSYMVLRRAFWMITLTLAQTFQYRYFLIHVRTDDLSHLMDGLSTTMSYSL
LLLKLTIFWINRRIFYDILTMMAQDRNDCVTEWAVCSMSRTIDVSHRSSNLIIGLYSMSVFLYGTGVLVAHTD
DPEEQLAVPARELFLKMELPFESNTSPAYELVMITQFFHQLAAATIVGVFNALIVSLILHVGGQIDIMCRGLV
EISSGDDAFDLRNSTIKALIRRHQRIIALSADIETLFSYIALMQFLWNTLVICCLGFLIVTAIGDTQGTTMLI
KSLFFYIVITLEAFIFCYAGEYLSAKGRMIGDAAYEAKWYNSNPTQSRILLLLILRSQRKLTITIGKFMDLSL
ERFTTIIKASASYVSVLHAMS
>CobsOr70
MRATSISTSVEIALRCVGIWPGLSYGTVSYFLYMISLVFALYFQYVYIFDHFDASNISNLIDALSITLAYSFG
FFKLISLWSNRRLFHYIKISIEEDWNNVDDHDKSVSYVMVSNADLSRRCSNTLISIHAAATICYSVANLIQLE
FKDNFNVSSRALPVKMEFPFEIDSSPLFEFLVVGQMFHMASIAVLVATIDCLVITLVLHVSGQIDILRQELLT
LVTHSDKISQCDSIFASIRILINRHQRIILFSSSIEELYSDIALMQFMSNTIVICCIGFTIIDSLAKEGVTAM
LKSAIFYVAVTLEAFIFCFSGEYLSAKSKSIGDAVYQTLWYNLTPAQCRILLFVILRSQKRLTITAGNIMDLS
LVGFTSMMKASASYMSVLHAMY
>CobsOr71
ITTSTVSPALKIGLQLLGIWPGVSHSALYWLTFMSSIIVIQYFQYLYILEHFRISELSNLVDGLSLTLDYSLT
FLKLASLWIHRRVFHKILAAMDNDWRESINIDRHLSTMVIKANVSRFYSNAMLSFNGVAAVLYVLSDYVIRFV
YLTKDYNSTLRQLPIKMLLPFETDRSPIFELLVVILFLHVVLISFTVAALNALVFTLVIHVSGQIEIICQEFR
NMSTDVFLYRSSAFILGTLVERHNKVYAFSENIEKLFSFIALMQVVWKTLVICCLGFIIIISLHNDTDVLVFV
KAILAYIAVMIEAFIICFAGEYLSLKNKSVADAAYESLWYDMPPNQSKIISFMIMRSQRRLSITAGKMMDMSF
EALTTIMKTAASYVSVLNALY
>CobsOr72
MVITNTISPVLKIGLQFLGIWPDVSYSAVHWFGFMSTLIIVQYFQYAYIFQHLKISEFSNLIDALTVTVDYSL
TFFKLIGLWLHRRVFHQILADMDSDWRECNDIDQHLYLMTIKANISYFFSNLMYSITMIFTVLYLLGDYMINF
IFVNKDYNVTSRQLPIKLYLPFETEQSPVFELLVVLIFIHVMLHVSILTILNGLILTLVLHVSGQIDIMCYEF
ENISKNALLHKYTVSLFHMQIKRHNKIILFAENIEKLFSFIALMQVICNTLVICFLGFFFIISLYNESDVFVL
VKTVLAYLGIMCEPFVICLAGEHLSFKSKLIADATYESLWYELPSRQNKIIIFMIMRSQRRLAITAGKMMDVS
FETFTNIMKASISYISVLNAMY
>CobsOr73
MIIAKTVSPVLKIGLRFLGIWPDVSYSAVHLYSFILSMLIVQYFQYVYIMEHLKISELSNLIDGLAVTFDYSL
TLFKIFGLWMHRNVFHKILADMDNDWLVSANIAEHSYMMTIKANISHFISNVIFTFNTIVGVFYFLGDYAIRS
IIQSADYNNTLRQLPIRVQFPFDTQQSPLFEILVATLFIHVMLHVSIISILNGLILTLVAHISGQIDIMCHEF
KNISETVLYRESTSITLETQIKRHKRIISFSENIEKLFSFIALMQVIWNTLVICCIGFIIIISIHNDTGVFVL
VKSVLAYVVIMADSFVMCFAGEHLSLKSKLIANAIYETSWYNMPVRQSKVIIFIIMRSQRRLVITAGKMMDMS
FETFTSIMKASASYVSVLNAMY
>CobsOr74
IIPSSVSPALKAGLQFLGMWPNRHSIVHWLGFMLSMLVSLYFQFLYIFDHLKLSELSNLVDCLIVTLDYSLTM
LKMTSLWVHRRVFYKILIDMDNDWRECINADHHLCMMTIKANISHLVSNALLSLNAIVAILYLLGDYVIRSVF
FTLSYNDTFRQFPIKIQFPFETQESPIFEILVVTLILHVIMHSCAIAILNGLILTLVLHVSGKIDIICYEFKN
VSKSVLLNESAVHLFGALIERHNKIISFSKNIESLFSFVALMQVVWNTLVICCLGLIFIISIHNQTGIFILVR
TICAYFVVMIEAFVICFAGEYLSLKSKLIGNAIYETMWYDMPLGQNKIIIFIIMRSQKQLTITAGKMMDMSFE
TFTSMMKASASYISVLNAIY
>CobsOr75

MTVTSTVSSALTIGLRFFGIWPGVRYSTVYWFSFMSSLLIVQYFQYLYVFNHLKISELSNLVDSLIATFDYSL
SFLKLGSLWMHRRIFNRILTNMDKDWFECANIDQHLHIMRIKANISHLFSNSLLIFNTIVAALYLSGDYIIHF
IFLSGHYNDTLRQFPIKIQLPFEMQCTQSPIFEIIVVIIFVHAMLQVWTIATLNGLIFTLVLHVGGQIDIICH
ELKSFSKDTLLRYFPVSSFRMLVERHNKVISFSENIEQLFSFIGLMQVVWNTLLICCLGFVLIISVYNETGVF
MLVKTVFAYLAITTEAFIICFAGEYLSLKGALIARATYETLWYDMPSDRSKYIIFIIMRSQKRLAITAGKMMD
LSFETFMTIMKASASYVSVLYAMY
>CobsOr76
MKRSTTISRAVEIGLRFIGMWPDSAYPNLYWSMYMTMIAVLQYHQYLYVIVHFDMSNLSILTDCLGLALANTL
AFFKLFCLWWNRRIFYNILAAMDRDWNDRVISESYSILTTVAAQSRRYSFVLIGIHILAGFFLSIGAYAIRTM
NKADNSREFPIKMEFSFEVLESPLFECVLATQIFYTLSLASVVGMINALLATLVIHVGGQVDIMKQAIMKVHG
DVDELGTSLTVLSNLIQRHRKIIALADDIENLFSVISLLQLLWNTLIICCAGFMMILTISSGKASMAILMKSM
FLYIAKTIEVFVFCYAGEFLSSKSKSICDTVYESLWYNMMPSDSRILLFIMVRSQKRLTITAGKVFDLTLEGF
MSVMKASASYMSVLHAMY
>CobsOr77
MKRSTSISRTVEIGLRFIGMWPDSAYPNLYWSMYMTMVVVFQYHQYSYVVAHFDVSNLTSLTDCLGLALANTL
AFFKLICLWWNRRIFYNILTAMKRDWNDRVISDSYSVLAVVAAQSRRYSYVLIGIHILAGFFLSIGAYTIRMM
TKIDNSREFPIKMKFSFEVLESPLFECVLATQILCILSIASVVGMINALLATLVIHVGGQVDIMRQAIMEVHS
TNDLTISLNVFRDLVRRHRKIIALSNDIESLFSTISLLQLLWNTLIICCTGFMIILALSSSKAAMAILMKSMF
LYIAKTLEVFVFCYAGEFLSSKSKSISDVVYESLWYDMVPSDRRILLFIMVRSQKRLTITAGKVFDLTLEGFM
SVMKASASYMSVLHAMY
>CobsOr78
MTRLNTISRSIEIGLRFIGMWPDSAYPNLYWFSYMTTVVIVQYCQYAYIFVHLDLNDLWLLMDSLSLTLAYSL
AFLKLLVLWWNRRIFHYIVKAMNQDWSECVINDPYRTTMTNMADIARRFANVMFSFYAFSAFFLSIGEHLLQS
MDDASQLNRSRELPIKMEFPFDVSRSPIFECFLIGQFLYDLVIAFVCGLLNALLVALVLHVSGQIDIMQQDLV
EISNGKYDNSTFLLVIKNLIYKHQRIITLSENIENLYTHLALMQVLWNTLVMCCTGFVIIIIVNSGEDTANLI
KSVSYYIAIVMEVFVYCFAGEFLTAKSKSIGDAIYESLWYNLPPSDSRIVLFMMLRCQKRLTITAGRFIDLTL
EGFTSIMKASVSYVSVLNAMY
>CobsOr79
SRSHYYSVIKKIASLAGLWPYQNQKMRIFCLSLVTLSTFSIIVPQMAKFVVCKGDLQCIFQTLAGHMLAVITL
IKLYTCCFNQYKIKILIDWLFMEWDELKTPEEREIMQRYAETGRRYSLGYSLYCFITGFLFICLSLIPPILDV
ALPLNESRPVLPAYPGHYFVDEREYFTYTFLHAIVAWEIAVTGIVAHDCMLLTYIEHVCSILTLAGLRFERLM
RKRNDHVNALYSHAGPSDVHRKEITFSVCTHRKALKFAQLIEDTFSLTLAIQIVINTIMISITLLQITQQNAG
ILIMVRYVLFVLSQLIHLFCLSFEGQKLIDHSLQTRDKIYNSPWYKMSAQSQKMLMFVMEKALNPIFLSACKI
YIFSIENFTTVVQTSMSYFTVLATLE
>CobsOr80
NHYNIVKIVSSLAGQWPYQKLKTRLFCVGLITLSALSINVSQMARFVVCDKNLQCIFETMTSLLLTTMSLVKL
YTCYLNRYKMRDLTNHLFIDWNTLETSEEYKIIARYAEIGKRYSLGYSLYCCFAVCVFMSVSLIPQVLDIILP
LNKSRPILLTYPGHYFVDEREYFFYIFLHAVVAWEIVISGIIAHDCIFVTYIEHVCSMFNVVGSRFERLFCNH
NNEATKFINTDNTDNTYCKKIALIVHAHRNALRYAQLLEDTFTVPFAMQILLVTIALSITLLQMVQQDDSSNI
LQTIRYTLYVFGQLIHLFFLSFEGQKLIDHSLQIREKIYNSCWYKVSVKSQKLITLVMIKSLRLSYLSAGKIY
IFSLESFTTVLQTSMSYCTVLASFQ
>CobsOr81
VTITMNANCNHYYSMVKKISSISGHWPYQKSTRNLFSVVLITLSVISIIIPQIAKMVNCNRDLKCVFEATTSY
MLSVTILVKLYACYFSRCKMKVLIDELFIDWNELETPEEYEIMKRYAKNTRRYAIGYVLYCYFALYVFLLMSL
IPQVLDVVLPLNESRPRLSAYPAYYFVDESKYSYYILLHAIIAWKIALTGLVSYDCMVLTYIEYVCSIFALIG
LRFERMICNKTADVFHPHTCEVNRKQIAFFVHTHQKALKFAQLIEDGFSLAYAIQVAINTIVISITLLQITQQ
DANILEVIRYVFYVAGQLIHLFCISFEGQKLIDHSLQTRDKIYNSLWYETSTKWQKMILFVMQRSLQPIFLSA
GKIYIFSMQNYSHVLQTSMSYFTVLSSFD
>CobsOr82
VTITMNANYHYYYSVIKKISSLSGHWPYQKSMKNLINVVLITLGVISIVIPQIAKSVKCNGSMECIFETTTAH
VLSFLILVKLYACYFNRCKMKIIIDQLVNDWNELETPQEYEIMTRYAKNTRRYIIVYFVFWYVTLYVNLSICL
IPQMLDAVLPLNESRSKLQILPAYYFVDENKYYYYILLHAVLAYKFISIGTISHDCLLLTVVEHICSIFALSG
FRFEQIICNNTVNVFHPHTCDVNRKKIALFVHAHLKALKLTQLMEDAFSSALALEVASNTIMISVTLLQITKQ
DANILHKVRNICYVVGQLVHLFCFCFEGQKLINHSIQTRDKIYNSVWYETSTKWQKMILFVMHKSLEPVSLSA
AKIFIFSMENFSTVLQTSMSYFTVLSSID
>CobsOr83
METTCSHYYNIVCKISSLTGMWPYLKPKTKVLRIALLTVILLTILIPQLAYQFMCKRDLNCTFKAMTAYLLSF
VALLKVYTFQFNTRTIKDLTRHLFCAWKELNSSEEYEIMKSYATNSRRFSLIYSVYCFAAIFIFMSMSLIPYA

LDIVLPLNESRPILPPYRGYYFVDEREYFFQILWHAIVAWEIVIAGIIAHDCLFVTYVEHVCSMFAITGFHYE
HLFHECKKKMELMSSINRDDTYSKKVAFLVRKHRKALEYAQLLEDTFTVPFAVQMLIVTVGMSITLLQITQET
SDILEATRYVFYVIGQLIHLFFLSFEGQRLIDHSLEIRDKMYSSFWYKASMKLQKLVMMVMMKSLQPSFLSAG
KIYIFSLQSFTMILQTSMSYFTVLATFQ
>CobsOr84
MDINDYVFINKKVLKFVGLYPTSVVRYIICCMCMIAIVIPQGMQIYENWQDMSIVLETSSVLFTILLALLKSL
VWISNRKKMDPFIEYMLTDYWNVMTYISNKHAHVYEIYVKKGRLFTKGYLILICNSLMFFFSLPIIDIIIAEN
KDIIGLNSTKHFPFLALYPDSYYNFPMYEIVYLSQMVATSLCGLVILGTDTLIATALFHTCGHFKVLQKKIKN
INTNFDIKHAYIAENMLKIKFHIIDIIKHHYKVLWFCDYMETVFSPMLFLQTVASSLIICLVGFQIATADITS
NMLSKSVKYFSYLIMALFQLLLFCIPGDDLIYESSMIYKTMYTIAWYKLPILLKTEIHLLILRSQKPSKITAG
KIYVMHLENFNGVLSTAVSYFMLLRSFGSDETTVTL
>CobsOr85
FVWAVELHRLGLELIGLWPSSDTVSKKIIGVEIHVGFIFVTVVTISGVPLVSALVRVWGDMTLMVDNLRITLP
LIVVSFKFVIIRWKRKVLLSIVNMMAEDWIVLKLNTERNVMLRKARSARLLIIFGYALMILAFIMLIIILPGFG
IEIRHVTNLTDQTKPLPLQTYYFYDIDKSPQFELTFLVQAITITLAGIIYTSIDAFFGLVIFHICGQLENFRH
RLVNLVSCKNFCQTLHDIIMYHLRLIRFADNIENMFSLMLFGLVLYFCIVFCFCGFLLLSVVTDGKTNDANIL
QACYMTVAIFILLTHTFLYCGAGELITIQCEALYRAICDIEWYKLESTKARNLILLTIRANEPFHLTAGKIVP
LTMATFCSLLKTSAGYISFLFAK
>CobsOr86
QTYRKYQRFIQTLLALSGCSYMPTRFGRIAYYWSVLSLLVAITYTILSLHASYVHRHNLAIMMKYIGVATSAL
GTLLKVAKFMTNRDSMIIYHRTINDLFESEYIRNEKIQAIMFSSLPPITIIAYIYSMFILALMVTYVMPAYSA
IFRGLYHWHLTTDYILPVTKGYGYFWTVPDNFWYHFHLLYETVDLLLSCLVACGVDTAFGFYAYQLAAILDAM
YYRLTNPLPTENFSDTLRTCATKHKKLLLCRDILEHMYAPIVLWHIVSNAILLCALIYEVSSVCKIIHANVII
YLCVSASYAMIKFLQTFSYAWHGTILTDAGEKLRKGLYFSEWHKSRLDRHVRTNVILMMMQKPMTIHAYFASV
DMILFTNFVNTTVSYFFLMQSV
>CobsOr87
MTSKELKAYHTYKRFLEVILIICGCSYIPRKSNKYTFYWPFFVFSCVFSYVILNLRVPFVTKYNLTIITRYLT
RATSFFGATIKVGTVLINRKSLISHHQMLDNLFEEELRQDKKIQTIMFTSLRKIYILSYTYSGSAIIVIIFYY
TPVFIFIIREFIRDFRLTTNYTLPSGFKIYPWTIPDNLKFYHLMYETITSIFCGITSCAIESVFGLYVYQSVS
ILNAMSFRLMYPLPTESYSNVLKTCIAKHQKLLQSRNTLERIFGPIVFWHIVTNAIILCGLMYEASTIKIVDS
KEFATFMTYIITKMFQCFMYAWHGTLISNAGESFRTGIYFGKWTDSNLDRHVRTNIILMMMQKPMTIYAFVSS
INVVMFTN
>CobsOr88
MIEKKLKAYCTYKRCIQILINICGCTYMPTKFNKLSYFWSILVIILMMSFVILDLKVCYKLIHDLVMLLHVFT
LGVSLFTSALKVMIFLFNRNYIRQYHQMLDNLFEEELARNDLIRTVMLSSLRKIYSLAYMYYAFEAGIYLSYY
LPPCLIIIRDLLHFRLTTSYSLPCKPLYFWTIPDNFLYHFHLMYEMSTAFISCVVASSIDTMFGFWIYQIAST
MDAMTYMLTNPPSTERYSDIIKMCVIKHQKLMQCCDMLGHIYGKIIFWHIISNAVLLCALMLQASTFTEFTVS
NVSMFTTFVTIKLIQSFIYAWHGTVLTNASENFRIGIYFGKWLNSSLNSHREQHCYDVDAKITDHNAVFSSVD
VVMFTNLVNTALSYFFLVRSV
>CobsOr89
MEFTVDQYYKLNRLVLSIYGLWPYHSRTVNAWIMRTFTILFLIWATTTQVIKICTTELTADFILDCLPIIIPN
IGAIAQFVYRIIINDRLKDLLDQIKTDWNHARSQNEIEIMQTNATLAKITTKWFLLYMFVGISVYMLSTFIPQ
VLDVFLPLNESRSREHPFHAEFFLDDEKDFYTIRFIMYFGILFVLGVILANGTIFVVYMQHVTGMFTILGYRA
ELSFNDKKLLPMNQFVREKHYGSVALFVQDHRSILQFVDIIQSCYGLNLFLEFLSLMILIGLTLVQVIKFSGT
SDRSIRSAAYITGQLTYMFMYSYMGQQLIDKSTQLSMKIYNARWYRIPISKQRMMLYIMLKCVNTITINAYNI
YVLSLESFSAIVQSAVSVCMLLR
>CobsOr90
MNERKDIWQSRFYTVPRTYMSLIGIWPYHAFRDRCLLFVPMFTFSITIIVPQLLYLLIAATNLDDVFSCTPSM
WITIIFSFKIGWLMLNNSKLKTCLRTMEDDWLSLNTDIERSILRRHTAYGRYITLTYGIFMQCVGILLITKSL
VVMILEDTSDATISSLVAESKLPLRIEYGKTLNQYLYPMALHCYLAVFSHISITIAVDSCYIALIRHACGMFA
IVGYTLEHIGKDSDSNFDLKPDKMTDDNYNKALACLRRHLHVIQFAELIESTFTNIFLVSVCLNMIGGSMIGI
QVVLNLNDTKDIVEPFAIYIAQLIHLFLQFWPAQFLSDYSILPYESICRSNWYYTSKRCRKLLFLIMNRSVLP
CKITAGKVVPLTIANFGTVLKTMMSYFTMLRSFN
>CobsOr91
MIEQRDIWQSRYYAIPRLYMTLAGLWPHHPTRDRYLHFVPTFTICSIILIPMLLYVHIAMTDLDDLFECMPTI
LITIIFSFKLASLMGNSDKIKTCLKTIENDWLSLNTDSEKAILRRHATYGQHLTIFYAAFMITTGLLYMFKSI
VLIMIEDTLNSTRLAVTKLPFRVEYGYKIDQYFYPILVHCYLTVYSHVTATVAADTFYFALIQHACGMFSVVG
HMLENIGEETDANFDLKPGKINDHNYNKVLNCLRKHLHVIEFAELIESTFTNIFLVSVSLNMVGGSICGIQVL

MNLNDAKDIVAPLAIYVAQLTHLFFQFWQAQFLLDYSVIPYDSICRSNWYYTSERSRKLLRLIMNRTVLPCRI
TAGKVATLSIESFGVVLKTSMSYFTMLRSF
>CobsOr92
MRALFNTRYYSITKLFASIVGIWPYQSQSKKRIIQSVSIFVMLTFIPPQIKRLYDVWGKDIDAVCMCIPPIVT
ILVSMAKVMTTIRFEAKVKELLQHIEIDWEINPAAKEYEILVNYTLKARTLCIIYSTMLCNGLMCFIMTPLIS
PMLDVILPHNDTRKRSLAYDLDYGIDLQTNWLWLWLHSSTTSTATILNIIGADLLYVTLTVHSCCLFAIVRHK
LEHVPDSIKKHVMLSPENYNYNIKCYMKYEDQRSISACVQLLHCIYTWCFLTVLGLNLIIISISAVQLVSRLF
QWDGMAMCIMFICGQLTHLFFLSLMAQYLLDQSSSVHESTYSCFWYNMPIQIQKDIVLILMRSRKPCKIMAGK
LFVMSLENFCTIVQTAMSYFTVLASFR
>CobsOr93
MDLFDTRYFKINKFFLSFASFWPYHSYFVKLLTRSFITFGLTTMLVPQITYILRQTNNLDSTFELMPVLSGTV
ICIAKFVSTIQNPELFKAVLQHMRDDWNNLLTKEETHILTRYAEKSRKITLAYSTCVIGFTLCYSFLPLTASI
LDIISPLNETRPKKFPQLMDFVIVDQEKHYYALLMLIYLDNFVLLSIVVGTDTLYILLVEHICGMYSILCYRL
ENLKIHDKWIDNDCTYEEANRCIRDCIQLHKEILLLVFTKMVEHKIALFLFFDIGLGFLFHSSSCVMIIVRTG
SSEIIRYVGLVIMQSCRLFFSNWAGQEVNDHSVQVSIAAYNGIWYNTSVKVQKLLLFFIARGQKASQITVAKL
YDVNLKNFTTVMKTSVSYCTVMISLR
>CobsOr94
MTRTNTISFSVEVGLRFIGMWPDSAFPNLYWGAYMTTLITVQYYQYAYIFEHFDINNLWLLMDCLSLTLAYSH
IVLKLLVLMYNRRIIYFMVRAIVEDWNESLANDSYKSTMTNMADISRRFSNTMFCAYICCAFFMSIGGYVLQA
MSEANEPDNNKSRELPIKMAYPFDTSKSPIFECFLIEQFLYDMVLALLVGLVNALLVALILHVSGQIDIMQQD
LLEIYNKKYDPSTFLLLMKDFICKHQKIITYSENIESLFTVIALMQVLWNTLVMCSSGFVIVTIISSGENTST
LVKPLIFYTLITLEVFVYCYAGEFLSAKSKAIGDAIYESLWYNLPPSDCCIILIMMLRCQKRLTLTAGKVVDL
NLEGFTSVVKASASYVSVLNAM
>CobsOr96
MSELGKGAVERASTISESVEAGLRFIGMWPRCVYANVNWWTYIVSVAIVQYFQYSYVLAHFDISDLSVFVDGL
SITLGYSLSFLKLINLWFNRRKLYVILNTMDKDWSDGIAVYSDVSIMIKHANLSRQCSNVMITTNALAVFFYV
IGGPILRSMIQKDNRESSTRELPIKMEFPFEVDKSPIFELILIAQFLHDLSVACVIAMLNALLVTLVLHVSGQ
IDIMRQGFKEISTKNNASRSSLTVIKNLINRHQKIIDLSDNIEDLFSNIALLQFIWNTLVICCIGFVIVISIG
TEEGATMITKSLIFYVAITLEAFVFCYAGEYLSAKSKSISDAAYECFWYDLTPSECRVLMFLMLRSQKRLTIT
AGKITDLSLEGFTTIMKSSASYISVLSALY
>CobsOr97
SSTISLSVQIGLRVLGFWPNTPCLLLFRCFWILSIITVQIYQYWWIIIHFKTDDMSYLMDSLSVTIEYTVMAL
KLTILWVNSRIFYDVLAAMAADWKEVTSNEVHIMTNKAILSRRFSNVIIGLHSVAVCCFGIEVLASHPDDVNG
VETPVRAFTLKLQLPSRCNESPLYEVVLCLEFFYQLISSCVSGVLNSLLITLILHTSGQIEILCDELKDISSE
KKNQQLNLSMKALIGKHQRIITFSNKIERIFSYIALVQFLTSILLTCCIGFTLITSFSTMQDSDTIDSAALIK
AIVFYMAATVEAFIFCFCGEYLSAKSKMIGDAAYKSIWYDFKPDESKFVLLIILRSQRRLTITAGKIMDLSLE
GFTSIIKASASYVSVLHAVY
>CobsOr98
KGKMTIVSTVGLSMQIGLRSIGIWPNAPYAFLFRGAWILTTGIVQTFQYWWVVIHFGNDDMSHLIDGLSVTVE
YTLMSLKLIILWLNSRIFYDVLAAMAADWKEAAINEMQTMTSKANISRYIANVIIGLHSAAVIVYVIDVLVFR
TSNYNVDGIETSVREFTLKLQLPFECNESPLYEVIMLLEFLHQLVASATNGVLNCLIITLILHISGQIEILCN
ALRDISLGKNNQEELILSIKALISKHQKIIVFSGSIEQIFCYIALIQVLSSTLVICCLGFMIVTSINTQDSNT
IDSSGLIKSLIFYMVVTIEAFIFCFCGEYLSAKSKMIGDAAYNSIWYDFKLNECKLVLFIILRSQRRLTITAG
KMMDLSLEGFTSIMKASLSYVSILHAMY
>CobsOr99
MIPSSTVCRPIEIGLRLTGIWPNSSILFKLVWILVMGIGLIFQYHYVLTHFNTKELPHLIDGLSTTSPHSLLF
FKLIVLWLNNRIFKDILTMMSNDWHEYSTMYTMINKAVLAHRCSKLIIGFYSTAVLLYSIASVNFGTIDNNCR
ELLIKMELPFEFCESPIYEVVILVQFLRLTAVATAMGMLNALMVTLMLHVGGQIDLMHEKVKEICPKDSEDIE
YYDLPTTVTRFLINKHNKIITFSENIESLFTHIALMQFFSNTMIICCIGFLIVTALDTDEGIMMLVKSFSFYI
AITLEAFIFCFAGEYLSNKSKTIGDAVYESAWYILKPRDCRILLFVIVRSQKRLTITAGKFMDLSLEGFTNSL
KASASYISVLYAMY
>CobsOr100
MIPTSTIGGPMKFTLRLIGICPDSSCRLLMRVVWTTVMVVSQFLQYWYLFSHVGSDTLPDIMDSMSLCLSNSL
LFLKLSLLWLNGRVIYNILEMMTEDWNQCASIRSKMQPMISKAILSHRFSKCSIVAYSIVLLLFATGNIIAQK
NAANSGQTVEEKSFIIKMKLPSECNTSPFYEIVMIMQFLLQLTSALVAGMLNAFIVTLILHIAGQIDIVCHEL
LEISVADDKHDSHMVALRSVVIRHQRIVAFAENIENVFCYMALMQFLSNTFVICFLGFVIVNSLNSPDADAVM
MKIIPYYAVVNLEAFILCFSGEYLSSKSKCINQAAYNSFWYKLKPTESKIIFLLIMRSQKELTMTAGKFVDLS
LESFTSILKASASYVSVLHAMY

>CobsOr101
MLSNFVREYNINRVFLSFSGLWPFQDRLVKNVLRTFCVLLEISSYAPEILLLCDHWDNSQMIFDAGYQFIFTT
SFVGRGLHNIWNQDRLQQLCIAIDNHWDIFTDDLEVQVMKNYSSLSRKFTKLFAGLLIVMLLTFVAIPLAPVL
LDVIMPINESRPRIFAVEVEFRLNKEDYFIFIFGYTTAIILVGINIVMGVDAMHIMCTAHACSLFAAVSKQIE
NIISKANNNKLNKCGYLILDSVNEEIIYQEYIICLKKHQLAIEFVNILESSFQGLSLLLLLLLLANISLIGVR
ILYVLQIEELIKFGLMFILLLSDLLIVCYSGQRLIDESQNVFYRAYAAEWYNFSPRLKSLLIMTLYRSNIPCG
LKAGNMIPLCIATYAAVVRIAMSYFTAFKSFKD
>CobsOr102
MLENFLREYNANRVFLSITGLWPFQNRYLRNILKTFFFLLEISYCPFELMLLYYHWDNPQMVFEGSYQLAMSM
SFIGRQINMFWNDTKLQRLYQTIDEHWNIFTNDTEVEVMKNYSTLSRKFTKYYSMLMFSTMLIFMIIPLTPIL
LDIVVPLNESRPRFFAVELELKVNKDDYFIPILCYTTIVVLVGATVVMSVDAMHIACTAHACSLFAAISKQLE
NINLKANNELKEPGDYMKFNRLNEEIIYREYIICLKKHQLAIKFVNTLDSTYQGLSLLLLVLLVGTISLISVR
IIYVLNEVRQVIKYTFALTACLVTLMIVCYSSQRLMDESQSIFYRAYAAEWYKFSSRLKSLLIITLYRSNVPS
GLKAGNMIPLSIATYAAVVRIAMSYFTAFTSIKE
>CobsOr103
MLENFLREYNNNRILLLTTGLWPYQNKLVRSLLWTFCFLLELSYYPFEILLLYDHSDDAQLIFEGCYQILILT
IFLVRHLKDCLNRGKMRWIYEAIDRHWSIFTDDIEVRIMEEYSILSRKLVTYYTIIICGTLLVIIILPLTPIF
LDIIVPLNESRPRLFAVEIEFRVNKTDYYFPIYCYISAVIIVGSIITLGTDTMHIICASHACSLFAAVSKQIE
NVTLKVNDDNGDIKENKHGINKNVDLCVSQEEMIYQKYIICLKKHQLAIEFVDMLNSSYKECSLAILIIILLGV
LTLSGLRIIYVLNQFRILIKFLLVLITTLFALIVVCYSGQRIMDESQKIFYEAYTAEWYRFSPRLKSLLRIIL
YRSSVPCKLRAGNIIPLSIATYATVIRMSMSYYTTLLSIQD
>CobsOr104
LRILSLNFLFYSLSGFWRPVQWTSKLSKTLYGVFTFISGYLTFYMFVTHFMYIIFVVETLNDLASCCFHILAI
VNLISKELTVVTRRNRLINLIEMLQEDPCKPCDKEETDIQLKYDYMIRSYSMPYIIICSMSMMNCWIGGVLFM
LEGQIPYGLAWVPWDCSSFFIFFLTSIQVMLGTFLAIFVNIATETSILGFCLQICARFEILKYRLQKMINHNE
WENISSFTNKTSRLAKHVSHHLYIIRLAEMINDIFDHVIFFQFSTSILILCSTLYHLSLNSVLLDVLVLSAFT
VCIFLQIFFYCWGANEVMLKSVEFGKDIYDLNWILMTNSERKDLLIIMKRSAKPIKFSSSFLITLSLESYTNL
MKASFSAFNLMQQ
>CobsOr105
MQLLSFNFLIYTYAGIWRPVEWTSTCAKLLYNIFTSVILFLEYFLGLTQFLDILFVIETVDEFVANSLMFASI
VIVCSKATIIILRRESIINLVQTLLTAPCKPRNRDEVIIQTKFDNFIRSWSLRYFLLAMSSLTSVTIGSVMNV
MHGILPYRVWLPYDLNVPTVFWSISIQQIITLIFATIINVGTETLVFGLFLQTCAQLEIFENRMYKLVTGNTT
TESSLASPNKKKKTISEHIQHHLSIYKYAKTVNVIFNQVLFVQFFGSILILCTNVFYMSAHINESQTATLLMY
TICMFVQIYIYSWSGNEVILKSTNIGDSIYHLDWTSLSVNEKKELWMIMMRSTIPIKFTSSFLITLSLQTYSN
ILKTSYSAFNLLQQ
>CobsOr106
MQLLTLNFSMYTVGGVWRPMEWSSIGAKLLYSVFSIVIVFSQFFLTITEFLDIIFVVDNIDDFATTTLMFFTM
LAVCCKATIVIVRRNEIINMMQLLLKKPNKPQDEIEEAIQTKFEKYIRSRSIRYSFLATGSITGLTVGSVINI
LHGQLPYRIWLPWDYHIPLIFYTLGIHQMITLIFACIINVGTETLVPGLFLQTCAQLEILENRIQKLIINKTV
KYLRHTLATPNKDEMEISDCIHHHLSIYKFAKTVNVIFNEVLFIQFFSSILVLCTSVYYLSTHVNDPFATASL
IMYTICMFAQIFVYCLAGNEVILKSANTGDAIYRMDWPSLSVNDRKELLMVMVRSTNPIKFTSSFLITMSLQS
YSNILKMSYSAFNILQK
>CobsOr107
LNLLTFNLLMFTFCGIWRPMEWSSVRAKLFYRVFSIFVVFSIYFLTTTQFLDIIFVANNIDDFATNSSMFFSI
MAVTCKATVVIVRRKEIINLIQTLMEKPHKPQNKDEVTIQTKFDKYIRSSCLTYMILATGSITGLTVGSILNI
LHGHLPYRIWLPFNYSVRVFYILAGHQMITLTFVATVSVAAEIMVYGFFLQTCAQLEIFEDRLRKLIINKTTN
NKYQGNARSLNEEKTEISDCINYHLSIYKYAKTVNIVFNEILFFQFFGSIIVLCTSVYFLSKHIKDLSAIIFL
LMYTVCMFAQIFVYCWAGNEVILKSANTGEAIYCMDWPSLSVNEKKELMMIMARCEIPIQFTSSFLVTMSLQS
YGSILKTSYSAFNLLQK
>CobsOr108
NFLMYTISGVWRPIEWTSIGARLLYGAFSSFVVFSQYFLTTTEFLDIIFVVNTMDDFATNSLMFFTMMAVTCK
ATIVVARRQEIINLIQTLLEKPHRPENEDEVAIQTKYDKYIRSGSLTYIILASCSITGLTVGSILNILHGHYP
YRIWLPFNYSVRVFYILAGHQVLTLLFGATISVGAEIMCYGLFIQTCAQIEIFEDRIRKLIIHKTAKYQGHTR
SLNKNKMKISDCINYHLEIYKYAKTVNTVFNEILFFQFFGSILVLCTSVYFLSKHIKELAAIIFLLMYTVCMF
AQIFVYCWAGNEVILKSSSTGEAIYCMDWPSLSVNEKKELMIIMARCEIPIQFTSSFLVTMSLLSYSSILKTS
YSAFNLLQ
>CobsOr109

MQLLTLNFLIITVCGIWRPIEWTSFGAKLLYNIFTVVIVLSQYFMTITEFLDILFVVNNIDDFAANTLMFFSM
MAVCCKTTIILTRRNEIINLVQSLLEKPHKPQNQDEVAIQTKFNEFIKSCSLRYSFLATCSVTGLTVGSIINI
LHGHLPYRIWLPWNYSMRMFYILAAHQMITLAFAATIDVGTETIVFGLFIQTCAQLEIFEDRLRKLIINKTSK
YLGRTCSLKKEKTNISDYIHYHLSIYKYAKTVNVIFNEVLFCQFFGSILVLCTSVYFLSMHIKEPSAIIFILM
YTICMFVQVFVYCWSGNEVILKSASTGEAVYRMDWTSLSITEKKELMMIMIRSNTPIKFTSSFLITMSLQSYG
SILKTSYSAFNLLQK
>CobsOr110
MELLTLNFFMYTVCGIWRPMEWTSFGAKLLYNIFTVVIVLSQYFMTITEFLDILFVVNNIDDFATNTLMFFTM
MAVCCKATIVVTRRKEIYNLIQTLLEKPHKPQNKDEVAIQIKFNKFIRSCSLGYTFLATCSVTGLTVGSIINI
LHGHLPYRIWLPFNYSVRVFYILAAHQMITLAFAATINVGTETMVLGLFIQTCAQLEIFEDRLRKLIINKTTN
NKYHGHAHSLNKNKTEISDYINYHLSIYKYAKTVNIVFNEVLFFQFFGSILVLCTSVYYLSMHIKELSAIVFL
LMYTICMFAQIFVYCWSGNEVILKSASTGEAIYRMDWPSLSVTEKKELMMIMARTNIPIKFTSSFLITMSLQS
YSSILKTSYSAFNLLQK
>CobsOr111
MQILSLNILLCTLCGIWRPIKWSSVYVHFLYSLFTCFVLFMEYFYWCTQMLDLIFTVNNVDEFVANSIYFITL
IAVACKCIVLITRRDAIINLIQILLKAPCKPQNEEEIAIQTKFDVLIRTWSLRYIYLVIGSATSIIIGSIFNM
RQGQLPTRVWIPWDLNTPLIFWIMSIQQILSVIFGSIINLGTETLIFGFLLQTCAQLEIFENRIYKLINNKAV
RFLGGSQSKMNKSDLEISNIIQHHITVYNYAKLVNVTFNPVLFVQFFVSTLVICTCVYYISTRVRMTETGSLV
MFTVCMFVEIFIFCWAGNEVMLKSMIVRESIYHTNWPLISVNEKKELMMIMMRSTIPIKFTSSFLITLSLQSY
SNILKMSYSAFNVLKK
>CobsOr112
MQMLALNFLIYKFCGLWRPVEWSSNIAKLLYNAFTFVVLISGYFTVFAQLMDIVLIVDNIDDFATNTLMLLTV
VAVCCKTTVVVLRRNAIINLIQILLKAPCKPCDKDETIIQSKFDKFIRSYSIKYLLLVTTSVTGTTIGAVLNV
MHGHLPYRMWLPFNYDIPAVFWTVSIHQIVTNIFCAAINTGTDTLIFGLFLQTCAQFEIFESRLRKSIFNKTI
RYLNRALSSSEDKPGISECVRQHLNIYKYAKTVNVIFNPVLFVQFSGSILILCTSVYYMSQHITDRSESLTLL
VYTIGMFIQIYMYCWSGNEVILKSTSLGNAIYCMNWPLLSSNERKELLIIMLRSSIPLKFTSSFLITLSLQSY
SNILKTSYSAFNVLQ
>CobsOr113
MQLFQLNFSMYTVGGVWRPIDWSSNIVKLLYNAYTFVVLSLLYFLMITQFMDIIFVVDNMDDFATNTLMLFTM
VAVCCKATIVVVRRKAIKDVIQMLLTPPCKPRDEDEIEIQKKFNKFIRSSSIRYTLLATGSVSGVTVRSMLNA
IQGYLPYRVWLPYDHNASLPTFWITSMQQIITVIFCTIINVGTETLVFGLFLQTCAQFEIFESRLRKMIFDKA
KKSVKNEIKYPNHSSFSLNKNKTIISKYVDHHLKIYKYAKQVNIVFNQALFVQFFSSIIVLCTSVYYLSTHSK
LSETATLIIYTICMFVQIYVYCWSGNEVILKSMSVGDTIYCMNWTALSVDEKKELLMIMLRCTLPIKFTSSFL
VTLSLQSYSSILKISYSAFNVLRK
>CobsOr114
MRILSLNFLLYTITGLWRPMKWTSNCSKLVYSLCTVITIYLTVYMQMTLFLHIVFVSETLEEVIAISPLCIST
ITIFFKIFAVIINRDQLINLIETFESKPCKACNKDEIDVQMKFDRSIRSYTIYYILLCTFSCSGAVAAGIFDI
LERRLPYNAYVPWDCTSLLAFLISSLTLIICLIVVTTINAATETTILGLCLQTCAQFEILKHRLASMAKREVL
SPKSPLNNTFRKTCTLSENVSHHLCIIRLVQEINYLYRHVIFVQFFVSILVLCSTVYHVSSHLTVKTLTTLIV
FLFSMFVQIFIYCIAGDQVTTKSFGLSEVVYNIDWTFMTISERKDLLMIMKRSTKPVKLTSSFLVTLSLESYG
NLLKATFSAFNLLQQF
>CobsOr115
DLVRASKLIIWNKRFLSCLGLWPCKENQFLFTLSTMYMIIYITMAVNHLIKNMRQMEIVVANLTDNVLFTMIL
GKMIMCKRSCKIMNKFLRSVENDFSTEMYNNVQEKTAYLYYNRIAIAFVKFSTFIAGLASVLYYLKKFFSNWS
ATVSGNISYELPYPVDPFFEIKDTSTYACICIYLAIAVVIIVCGYSGPDAFVLSMTLHVCGQFATLSCKVDTL
LRDHENYRRHISNIVLRHHHLITLAEIIEDSFNMICLQQTLGTVILLCFTLYFTLTNSEVGEDANIIAFVLYI
CCVISTIFAYCYVGECLIIESTRLREAFYNSDWYNNSSSRTKLIGICMLRAEKPLMLTAGKFCMLSLNTFTSI
LKTSMAYLSVLRKFI
>CobsOr116
IEDLFVYVERILNFGGIWPLKPTYIRFSIFILYFSMHLVMVFGNLIDVLGNFELMVMNIMEMMAYLVTYIMVC
VVRANALLKDVIINVKNDLKERRFENAEEEELYYRYYYMSKIFLQVSVTSITGSIIILYLSPLISFVLGNSEE
LNNTYVLPYRVHLFVNMDNTRTYALMYFYLCPTIYMSMSHMTSICILVILVFHICAHLSILSYRIRNIPTYSY
GEISGRIRACVQMHYKIIWMSRSLNQIFKLVLLDELIGNILVLAISMFYALVNIDAGQLAVSFVFGFFGSIAL
VMLYGYCFVGDQLMQQCINVQQAYYQCKWHDFPNSCKKSLLICMLRAQVTLRLTGGGFYIFSLYGFTEIMKTC
LAYISMLRTLV
>CobsOr117
DQKDLDRAAEVLSWNKRLMSMLGLWPFKSNDFIFSINFGYFSFLMIMEYLDLFLYIGDLEHVIMNLTENMAFS
QIFVRMSMLRLYNSQIGEVITEAMKDFDRTCYKTAEEVKAFITYNARSKIFVKLLTAFVALTASSYYLTPIII

ILGIGGLPEVMISENVTQIIYLLPYRFHLFYAVENMRAYTITYAWQMPFVFVSGFGQSAADCIMVTLVFHICG
QMSVLALRINNINTELCDCKGEIRHVVLMHIRLLRMGQIIGEAFSATLLAHLLGATSLVCILGYQILTNFAKG
ERGVLVTFLIFQFLVLLILYAHCTVGESLLTESAKVCEAFYDCRWYNMSKENARMIVLCMARSQKPLCLIAGK
FTMFCLSTLTDVLKTSMGYLSVLRSFL
>CobsOr118
LQNKAEHIEDLFVHLERIFSIGGIWPFKKTYVRFAIYILHFTFYLAMAYSDFYDVFGNLELMVMNLVETVAYS
MTFPLMWLIRCSNMLKLVVNVIKKDLVERNFENTEEEKIYYNYNFISKMFMYTSLVGMLITVILLYLRPLVYL
LMTTGQASHNKTESLMLPYRVHPFFDTSTTHAYVLMYLCLFPMIYNSICHMAAICLIVILVFHICGELAILSY
RIRHIGEYSQAMLIDRIRSFIRMHLKIIWMAKSVDNTFNFVLLDELLGNSIVLAISLYYVIMNLEISEIATCC
TFTFFAIIALVMLFGYCLIGDQLTQQCISVQDAYYDCNWYEMSAGCKKCLLICMIRGQVMLYLTAGKFYIFSL
NSFTDV
>CobsOr119
LDWAIGINRISLKIVGLWPDEKLNRRQKFFADFRAIVICIMMLFSSVVPGLVALLRVWGDMMAMADNMQIGLP
FSVTVLKFIIMWFHKKDLEPVISMVIKDWLRTKTTRERDTMIKQARIARITVIFGCIMMVLACIILIIPPCFG
YTTRYLTNLTDPGRPMLVQTYFLRDITETPYYELVITAQALSIIMAAISYTGIDTFLSFLVFHICAQLEILKE
RLLNLNSFKDFNTGLSFNIQDHLRLIRLSIDVIDNTFNLMLLTLLVFFAMLFCLQGFLIVNIIDGTGENVSFM
RVCWLVSILVNTFAHMCLYCVVGEVLIAKARIFYAVYNYAWYLRTPNEAKNLMLIMIRAEKPLYITAGRICPL
TLSMFCSLIKTSAGYVSVLLANR
>CobsOr120
MNSDMETYYDINKFFLLQIGGWPYQKKVLKILIPCLLSMILYSVYVIEALLLYETWGDVDIAIECVITITPVM
VSNIKLINIVINNDKFRRLLQLMNEHWKLFHNKNERHILRYYANIGRKITTYVAVYFVTTMIFYLLIPLIPKI
LDIIIPLNESRPLAYVFPAEYKVDKVKFYYPIVFHSYVTTITTIIILFTIDTTYIVCVLHACSLFTAISQNLK
NITKDTKIIINYDKKKHTGSKFHLLMEEHDAASNDYLEFMMCLKKHQLALEHAQTLDSIFTHATFVILFLNVL
LLSIVGIQLINNLGRTEEVIRYIIYSFAVFIHLVCMCIPGQLLIDRSTEIFNEAYSSEWYTFSLKTKKLLRVL
FYRSFVPCTLTAGKMFVMSMTMCSSVLQTAMSYFTAFLSIK
>CobsOr121
MNDKRLQMYRKYQGFIRHVLIISGCWYMPTKSGKPRYYWSLCVLLVAITYALLNMHASYFHRHRLAIMMKYIG
ITISAFGVILKVGSVLINRSSLIHCYQTLKDLFEEELTQNDQVRAIIFSPLPTIRILAYIYSAILIAMMMAFF
MPAYLSVIRDLCHLHLTTNYSLPISRGYGYFWTVPNNFLYHFHLLFETSGAAVSSITACSMDSAFGFYVYQFA
STMRAMTFRLTNPQPNEKFSNVLRTCVAKHQKLLLCRNTLEHVYGPIVFWHTVTNAVLLCALIYEMSSLSEIN
IFSVSISLTYGMIKCLQTFTYAWYGTILTDAGEDFRKGIYFGEWHNSRLDRHVRTNVILIMMQKPMTINAFFS
SVDMIMFTNFVNTTVSYFFLLQSV
>CobsOr122
IDKRLQAYRIYKRFIRILLTICGCSYSSTKSKSTNYSVYVLLMMILYAILNVHMCYFHRHNLPIMMKCIGITI
STFGAILKVGTFLTNHNYLTSHHEMLDDLFEEELVRNEKIRMIMFLSLPTTCFLAFTYSAIIIACMLAFYMPT
YLLIIHDLCHLNLRTANYTLPVTKGYGYFWTVPNNFLYYFHLLYETNTISFSCLTACGMDSAFGFYVYQFSST
MRAMTFRIMNPPPTEKFSDVLKACVVKHQKLLPYRDMLEHMYGPIVFWHIVTNAVLLCALIYEMSSSFSDFNI
ASSFVSLTYAAIKLLQTFMYAWYGTVLTDAFEDFRNGVYFGEWHNYRLDHHVRTNIVMIMMQKPMTINAFFSS
VDMIMFTN
>CobsOr123
TIAPNFHLKVSLTIIYFLGTWPPVTGIFRLLYLFYTGCSFIFILGILLAAEIANVLANWGDMSKLTAVATLLM
TNSTHASKVLIIIKKMTRIQALLDAANSAAFSRYDEKHKDLLINYTWKGIFHHAVYQSFGGIAVFCWGFTPIA
DLLAGRSRQLPMEGWYPYNITASPAFEITAAHQGIAIIIACFHNVAMDTLMTGLINVACAQFAILERNIISID
NEKGIRGMQSDNESKFEIEGRILSYRLLKRCAMHSNMIFHFTNEIQDIFGIVIFFQFLSNCVIICLIAFNVSQ
MTIYIPAVLIGMLTYMCCMTYQIFIFCWHGNELYLHSMRLVTAAYSNNWFSNSERFKRSLQIVMIRAHRPLTL
SAGKVMSLSLNTFVQIMRMSYSIFTVLQGSAA
>CobsOr124
MIFHKDITVTLSVHRFALSCVGIWPVRERNIFMDLRWIIAQVPLNLIPMCIYFTEVYLHCHGAKRSFDSLTPG
AAAALALTRLITPRIHREELLEIVTSMMDDWAMSKKNEKVRWVIKKYATMSTRVTVLTFILVGIIVAGYAAMA
ISSVIPKISHLSNETDYMNVTHEDGSQSCVFRSASSRQAFMFLQAMQMFTTGILTFGTTSLFFGLAMYLCAEF
DTLSIKLSEFQVSEARRAIAEAVQRHCHLIRMAECMEESFNASVLVYLFITSTLMCIDGYMLIVSLPLGNLPM
IIHSTSVLLLMLIQLSFYTVAGDYLEMRSIALAYATYDCDWYKLPADTAKDFQIILMRASIPLQLTAGKFVVI
NMITFKDILKSTASYLSVLRIMLGE
>CobsOr125
MKKPKGWNDEARYALNVYEKALGIIGIWPLNAREFKSVARCSLAILIQISTIGSLSLEVYRQCLGTEDMMEAF
LMDLSSVVSLSKLFVIRLTWRHTYVLVTSIIDDWSVFRDTRQRDVMTRYRNVGRIVSLTILYLGYASGASFLF
MALPFDSLVPWLNISKSSDNETDVTTYFLATYCVFGSSPATTYSCILLLQVAQIFVNATSHCGNDGFFFGLAM
HLCGQFELLEMDFAGIKTDKRTCKKKLQMLIDRHCRLIRLADSFEYAFNMAIFAQVSMSVLLLCVEGMQLIIS

LKLNDNIAAVKHVVLILTMLVQLYLYCYAGDQLEYVTGKIAYSVYESPWYDFDVKVMKDLPMVMLRGKLKHQT
TAGKFLPMNLFSFKEILKATGSYLSVLRVMID
>CobsOr126
VIRILPFARMTDKRWNDDVAYVFYTHRLFLRVFGLWPLQTKTMFTKLRWGLSTMTQLVSLPLVMIGFFWSDQD
AGSDIEYLTHFTGPSLAITKSLCLFVNQEKLGMNINAAIDDWISVEDNEKAKRIMKKYATRARILTFTLLYSV
FGCFFAYISVILFINVKQIFFTDKNLADGNVTQWMFIFPIGSLSNLINRKQYTMIMTFQAVEVFIQSLILCVS
DCLFFSVTMHLSGQINVLKNKIAVFAHKPDTETNYQKKFVELLNRHSELTKLYHNLEDSFNIYILIELVMNTI
MLAFIGLRINICLNKKYYAEAIKSTCIANYILINSLIFTYGGEFLQQDSEDIFHALYAASWYTLPLALMKDIQ
FTMMKSNIPFRLTGGKFFFVNRETMMNILKTAASYVSVLRIAI
>CobsOr127
EWNAETSYVLTVYKYLLGALGLWVLNEKNLFSRIRWLTSTVIELTTIISLSLEVIHHCRGPEDTLEAFRSASA
SVTSISKLLLHRINWRYKLILVETIINDWTYVKSSYSRDIMLRYARVGRLGSSVFFYLGGASCVFLFSPVMYA
KVHSLWASKEPSYNETSERRLFLAAYCVFGNYTSFGYDFIQILQIFQILVNAFSHCGNDGFFFDVTMHVCGQF
KVLRVDFSQIKDKELLSKERLGIFLKRHHRLIYLAHNLQTAFSLVILSQLLMSVMLLCVEGFQLILTLSMHNN
YAATQHLLFIFVLLVQLFLYCFAGQTLEFQSQELVFDIYNLPWYNFDVSVMKSLPLMILRATHPHRLTAGKFL
PINFVCFKEILKASASYLSVLRVMLKT
>CobsOr128
IKSNWNSGMDYGFSVIRKMMWLVGVWPLQHDDMVCTYRWIIIFIIESLTVITLILEPLQNCGDTKDLLEVFLI
IETGFHSWMNVVFARIYMKKLAVNVNSAIDDWSSSLTQKNSYKLMTRYARLGRIITVSQLMCGLIAAIVYNIT
IVVTNKPQIVIIGNETINLWTFVIPSTCLYRGISYSTHKALLLMQIVQSFILFVAECASDSFFFAITMHLSGQ
LELLRIRFVELIAKSGINNYRRILSTWIRRHYKLTMLAKNIEDTFNVIILIRLLITTIVIAVSGLRMLVSAKH
QNFVDVVKSMIFVQFFIVQSFLFTHAGETLREQSESIVSTIYGTKWHKLPPVIVKDLIFIMMRSKIPLQITAG
KFFYVTRSTTTDILKTALTYISFLQVTMD
>CobsOr129
MVHWNKDAIYALSSYKILAWPVGVWPIENDIFYSKLRYFFVMVSEILLTVTLLMDVYLSCENSSVDPIDTYAV
TSSAVLVIFKLTLLQIQRSTLSNSLYSAIQDWSSIQDTKSREIMIQHARTGRMISLFLFYSGFIALTFYILRL
LPVLNSTFEERAFVLPMSCLFKSVSNLEYVLITSYQVIQLFMTYAGNCCTEGIFVGVTMHLCGQFKILMIDFQ
RIDRYKRKHEKGNIVEELVVRHRKLLKLTNNIEDTYNIIILIQIFSSAILICITGFGLIVSWHIHDIVMTMKS
IVIMIVMLMQCFLYTYAGDNLRDRSEALSFALYNCDWCSLPPNDIRDLAFIMIKTNIPIRLTAGKFFYVTRAT
FMDILKTAVSYLSALRVMIEK
>CobsOr130
VIDNSYSHYLNPMASERWKDDVAYAITPFKLMSWSIGVWPLEARNIYSLVRFVLGICSSIFMFIVPSVELYLG
CTDAETNVDCLMLICCGILGLMKTIWFRLYTRNLANNYSSVIRDYLTVEDTKERAIMRKHAFMGRTFCCFMVC
FSYFSCVLYGLIAMLSGNKNVNMTNEDAIFEYPVPSKCIMKNLNVPASTHKIFCIIDTVAMVLASTANHGNDA
LFLNITLYICCQVKILRANFLDFDTESPQIYDRFNVLVKRHKYLIKLARELAEMISFVLLIQLFIISILLCIM
GFQLIVQLGKSNVILITKNLMVQSTFLMQLTLYGVIGNYLKTEMEEIGVSVYQSTWYKFPSKLRKTLILIIMC
SESPVTLQAGNFIVVNLATYVSILKASLSYLSVLRVMVK
>CobsOr131
MANKQWIDDVSYAMTPFKLLTWPIGVWPLQVYNIYSLLRCIFTTCCVSLVVILPSIELYMGCSDAEQNVDSLM
LICCGILGILKIICFRIYSRNLANNYSSALNDYMMIENAKQRAIMRKHASTGRILCFFMLGFSYFSCVIYSLN
PFIGNGINNEINVTNDNVLEYPIPSRCALKYFNAPTIIYKIFCFIEAISLFLTSTTNHGNDSLFLNITLHVCG
QVEVLKSKFVNFDFTRPEVYERFTELIKRHSYLTRMARQLSDAISFVLIIQLFIISIQLCIMGFQFILALKVN
DAVMAGKSIMVQGTFLLQLSLYSFIGDYLKSQMEEIGLSIYQNVWYKFPVKLTRNIVFVIMRTETPVMLRAGH
FIVINLSTYMSILKASISYLSVLRVMVE
>CobsOr132
MASEQWKNDVAYAMTPFKLLSWPIGVWPLQVYNIYSLIRCILATCCMSIIVILPSMEFRMGCTNAEQNIDGLM
LACCGVLGVLKTICFRIYAKNLTDNYGSALNDYLTIENAEHRVIMRRHAYIGRVLSCSMVCFSYVSVVIYALI
PLLGDDLASNQESQINITDEDTVLDYPMPSRCALEYLHVPTSMHTVICFMEFIVLVLTCTCNHGNDSLFLNIT
LHMCGQVKILKTNFINFNISSPQVYGRFNALIQKHNYLMELAKKLAESINFVLLTQLFISSILLCIMGFQFIL
ALKMNNVVVMGKSLMVLCTFLTQLSVYSFVGDYFKNQMEEIGLFIYQSSWYNLPIKLMKNLIFIIMRTRSPVK
LQAGNYIVVNLATYMSILKTSISYLSVLRVMID
>CobsOr133
MSDDVWNDDVAYAFSIHRTFMQILAMWPLGKKTVFTLIQWTLLLLWMLSPLVFIYMAITGSGNDASANIDVTL
YFACTVALSLKYVFITVNGRKLAKNINTAIVDWSFARNNKKTYQIMKEHAFQSKLCTLIMLYSAYICGGLYIL
VVIGVNLKDIFLQKQMMNVSNDDVDTKNRLFIIPCGEFGNKITSMQYAIILIIQFIQLIIICTAQSIADSFYI
NVSLHLSGQLNVLKTKFKTFANKPESQTNHRKKFINLVNRHCELMELHQNLEGTFHPIILSQLVITTISIALL
GLRIIFCLKSNNYMEFIKSIFVLNYLFMQALLYCYGGDFVQKGSEGIFHAMFTTSWFTLPTTLMKDINFAMMK
SSHSFRFTGGKFFYVNRETMIYILKTAASYVSVLRIALKD

>CobsOr134
MKTNWNSGIDYGFSIIKSMMLLLGQWPLQNNVIHTTQWVIIFIAGSLNVLNVLIESFKSCTVERDGLEILRLI
ESCMHAWLNIFFSRIYKKKIAININAAIEDWSSTTMEKESRMVMITYARAGRLIAPILISVGGVGGVLWFASV
FYSNKQQAAIVDNSTVNTWLFVIPSTCLYEGISYSTYKILFVIQVIQGSLVLISENVYDSFFFSITMHLCGQF
ELLRMQFINVSQQLYGKKYRGNVLGPLIKRHCQLIALANNIEDIFSIIILVRLLIISIVIAASGVGIVLSLKQ
QNYEEMIKMIASIMFYIVQILLYTYAGDTLQNQSESIAYVIYGSAWYKMSPIAMKDSIFIMMKMRTPIRIRAG
KFFYITRNTITDILKTTLTYISFLQVTIDK
>CobsOr135
MSWNDDISYAFTLHKFVALPIGVWPLQKYDIFSRMRSVVCGLSLSAMMIVLILEVTCGDNSMYTKLDAIMLLT
CNLVSVLKLLFYHLYADNLIHNFSSAVDDYLAIDTEKKRAIMRRHAFLGKIICYNVVFPAYVASTIFMIMPLI
TSNESAQVNISIKNPAENLAFPMMWTLRDFHISQNSYIWISIVQYILLMLNSSCNCGNDSLFLAIMLHVCGQI
ELLKIDFSKFGVTSKNLNKDFSMLISRHCYLMNNAELLIEVISVVLLVQILISCLLICFIGFQLILGIKFHDM
VMITKTSTVLITLLLQLFFYSFVGDYLKCQTEEIAFSIYSCNWQNFSMKLKRNILFVIMRSQTPIQLLAGRYI
IINIETYMSILKSSLSYLSVLRVMVD
>CobsOr136
MVWNADLAYAFNLYKIITVPLGIWPLQNYNAFALFRSIVCGSSLTIMMTLVFLEIVYGNSNAYVQLDNLEIVF
CSILAILKLLCFRIYANNLIRNFSSAVKDYLAMDTEEKRTIMRRHAYLGRVICYSVLSLSYLATLLFMLMALI
ADNEEVQINVTISQIDELPVPLTFLGEVYMSTTVYLLVSTLQYIVLTLTVTSNCGNDSLIFGIILHICGQIEI
LKIEFTKYDVNDKNKDFSTLASRHYYLIEHAEFIVDVISVVLLLQMLFSCLFVSLMGFQFILALKTNDAGMIV
KTLSVLSALMLQLLFYSIIGDYLKCQMEDIAHSIYSCNWYDFPVKLMRNVLFVTMRSQQPVQLLAGRFFTVDV
KSYMVIVKSAMSYISVLRVMVD
>CobsOr137
MTWNTDMAYAFNPYKILTLPLGIWPLQNYNTFALIRSIVCCSSLTVLMILVFLEITCGNNNAYVQLDNLEIIF
CSILALLKLLSFRIYANNLIRNFSSAMKDYLAIDTEEKRTIMRRHAYMGRLICYSVLSLAYLASILFMLVALI
ADNEEVRANVSKSQMSQLPVPLTFLGEVHVSTGVYMLISIFQYMVLTLTTTSNCGNDSLILAIILHICGQMEV
LKIEFNNYDVKNKSKSFSALALRHYYLIEHAELLVNVISFVLLLQMLFSCLFVSLMGFQFILALKTNDAVMIF
KTTIVLTALMLQLFFYSIVGDYLKYQMEEVGHSIYNCNWYDFPLILMRNVLFVIMRSQQPVQLLAGRFFVVDI
KSYMAIVKSAISYISVLRVMVD
>CobsOr138
MKLSSPKFGQVLQFLQICNSVVCTWPHDSDVSKLIFVLRKMYYSFTILNTCITKMTLIYAIYKYWHDITTIMK
ILAELTCTTELLINVFICKLKRVHIQHTLMEIKNFLRTSNDHERIILQKYIDRYAFFTTFVILCYFIAIITFC
CTPIFTAKKFPTDGLYPFPTESPLVIFIIYAMQVYAVVQCGFCVSVDFMFAVFFLYSAARLEMLCLEIQTARN
EEHIDTCIKKHQEIIKFVDGVKYIVYYSLLITNFMMAIGAICGTFPIIHKQMGTIEFIFLVIGGCQRLYITAW
PTDDLMENGKQIATGIYNISWIGSSQSIIKKICFIMHRSQKSIVINMGYLLPTLSLKYCAKYLITLLSYFMTM
RAIIYD
>CobsOr139
MEHPGEQYFKLNRHILSITGLWPYQKKWIAYLLRIIIIVILVSSIIFQISSFLIFDISVNLVMDIMTEVLPTM
GSLNHMIARIKNMDKLRDLFDHVWNDWRLQEKHYKIKILRKRAKITKLVTFYYILIQYILVIVYNLWLFTPDI
LDIISPMNESRPRQFTLKVYFFIDEEQNINLIRVHASIVIFIFPLIFIVSSTLFTLFAQHVSGMCELLGYHAE
RLFCVVKDMTHCNLTRSNINRENTIIFIRIHHNIIQFTDKIEFYHTVPFLLDLIGIVLLTSVALTQIVLLTSD
DDFERIFKSVSVAVMSLIYVFILNYMGQVIMDTTANVYEKAYNSAWYLANISEQKSLLLIMKRRVHPLVLTAC
KFCKISLQNFGKVLQTMLSYSMFMRQL
>CobsOr140
EEHYYKLNRFLLSVSGLSPYQSRWSAYLIRIVFTVLMVSSLFVQISYMITSDVTIDFIVDAFPSFLIIMGNLS
NLYTRIIHVDKFKELFERMSKDWALQKTQDEIKIMHDYAETSRLSTLYSLIAIYINIVGYNVWLFIPEILDIV
SPMNESRPRKRPFNVEFFIDEDRYFYLIRSHICLVIFTMPIIYVTCSTLFLTLTQHVCGMCKLLGYRAERLFC
VVKDKMTRDIIQESQVNYKSIAVFVQQHYNIIQFVDIIETCHTLPFLLDFLGLIILMSLTLIQVLTISGIERA
IRSIGVFIASLSYVFLFCYMGQRITDMNSSIFDKIFNSIWYNAAISEQKSLLIIIMRRYHPLVLTACRFYVMS
LQNYGMILQTAISYCMFIRQ
>CobsOr141
EERYYKLNQLLLSISGLSPYQSKPSAFLIRFSFTIVMLSSLFVQISSMCTSDITLDYMVDGMQEFIIVLGPMV
NIYTRSSYVDKFGELFERMSKNWALQKTQYEYEILHQYAKISRLLTLISSLALFVCHIPYGCLPISPEMLDIV
LPRNESRPRKQPFKNNNYYFVDEDEYLYSIRFHACLVMFMLPPIYVTCCSLFVTFTLHICGMCELLGYRAERL
FCVIEDKTVFNLTQRSQITLKNIAGFVQQHHNIIQFVDIVGTSHTISFLTDLIGIVILMSLTLIQLLTISSFE
KALRSISVAVIALSYVFISCFMGQQITDLSSNISEKLFNSTWYNAVISQQKALLIIMMRRYHPLVLTACRLYV
MSLQNYGMILQTAISYCMFIRQ
>CobsOr142

MHAAVKYYYNINKIFMSNLGIWPEQSVFMKIVLPTIITSFFFCIAFLEFVRLCEIWEKLTEDCESFIMVLLAF
GASIKLLLIVFKNKNVEHLLSLIDYHWHVFTHALEMKIMHEYALVGRKMTISYAVIIYSLMSLYMLIPVTPKL
MDIFLPLNQSRPYKYLFDVDYSFDREVYYYPVLIYSYLTTVMAVSVMVVTDTSYLSLAQHACGLFAAIGCRLE
SLTSEVNFNRTSYHIKYTKVPNNERNSANEDKIYRELILLLWKHQLTIEYVNLLESLYEIYSFSMIFIHIIVM
SLLGVQIMSLIDRKEEMIRYVSIGIGGFFHLLVLSYPGQEIMDHSADIFHKAYNMIWYRMSRKTTKLLSVLLY
RSFMPCILTAGKMYVLSFQNYASVMQGTFSYFTALSSFK
>CobsOr143
MQSAMTHYYNINKMLMTKMGIWPKQHVFVKVALPTILTALIFSIAILEFIKLSEIWKRLAEDCESCIICLLGL
GAYIKLFIVVIKNKDLEYLMSLIDYHWRIFTHTLEVEIMHEYALVGRKMTITYSIACYSLAIVFMMMALTPQI
MDLIIPLNESRPYIYLFDIDYSFDRDTYFYYVLLHAYVTIILAITTMLITDTSYMMFAHHASSLFAAIGCRLE
NLTTVMNLKKKSNYHITDIIASSKCEPPNYDETVYRELVLILRKHQMSIEYVGMLESLYSMYSFSMILLHFIV
MSLVGVQVINFMSEKTILVRYLAMVIGGFIHLLVLSFPGQEIIDHSSEVFHKAYNMMWYKTSRKTTKLLSILL
YRSLEPCVLTAGKIYVLSFQNYASVMQATFSYFTTLTSFQ
>CobsOr144
MQSAVKRYYSINKSLLSKLGGWPTQSRFMKILLPTIITSFIFSIGFLEFVRLSETWRSMTEDCECFILIIITI
GGYIKLYIIVLKNKNIEQMLSLIDYHWRVFTHSMEVQIMHEYAVIGRKMAISYAVMIYSLMSLYMLIPVTPQL
LDLLMPLNKSRPYKYLFDVDYGFDREVYYYPVLLHSYLTTVLTMSVMIITDTSYMSLAQHACSLFAAIGYRLE
NLISEKNSKGSGRFKDADAMCRWKSRDYENEIYREFVSLLRKHQLSLQYVRLLDSSFELYSFMLLFITIIIMS
LLGIQIISLMNRKEEMIRYVAIVIGAFIHLFVLSYPGQRIMDHSTDVFHKAYSMLWYKTSRRTTQLLSILLYR
GLTPCTLTAGKIYVLSMANYASMIQAAVSYFTALSSFR
>CobsOr145
RKKAVGMTRGANDSRLEQFRKLSLLHLTYLKYVGLWALDSNSSVFFKYSYYVYNKSILTVILVFMVTLFADIC
SSFDDLSIVTDDGCIFAGIVVVFFKVMIFQTRHEQIVRLLQKTIDACDRLCKFPIGGEDEILDKYLLICRVTF
YGFSTLGFFLVIALLFLVPVENGELPVRARYPFDTSVYPWHGIGFFVEACTVSIGVTAIIGIDSLITNLCNLF
LVQLEILNAHFKSCGENRRADDNDKRDDSGATGRNACHGVSSTVAACYGKDNYNLDAIRRGFAKRFGLSIRNH
QRLLAIIDDFNKIFSAGMFVQMLSSTTMICLTGFQATSVIGQNSNIYKFAIYLMAAVSQLFYFCWIGNEVIHQ
SASLTQSQWLSKWNDVLSVKETSRLLILSIIFSRKSVNLKAGVFYVLSMDTFTSILKGSYSFFAVLSTMHVED
>CobsOr146
YYNIPKRMLSIIGQWPYQKERRMKRFRLSLVTAFIFSMAIPQWIYNFRCPSMRCFLQNLPSCMFPAMAILKLY
TCKLVISKIKRLTDYLQDDWKEIKNPEECEIVRKYALTARLHNLTYCIFFVTTTNIFAMTSLIPQMLNLISPL
NESRPLIMATEVYFFVNSEDYYFYILFHTILSANVMLTIIFAHDCTFMAYTEHVCGLFAVVGFRFENLSRNDS
NDATSPEDCKAYNQMIGVSVHIHCRALQFAELLEETFCVTFAAQMLVIVLTLSITLLQMALQLDDIIAIMKCI
LYVIAQILHVFIYSLQGQKLIDHSLHIRDKIYKSRWYDIPVRSQKLLLHVMQKSLQPSYLSAGKIYVFSLKSF
TTVLQSSVSYFTVLASF
>CobsOr147
MENLRHYYDVVYKITMLTGAWPYLKPTARILRVGLVTLTIITIFIPQIAYQFTCKSNLQCTFESMTSYLLTIV
ALIKVYTIQLNNYKMKDLTQHLVVDWKTEKTPEQLKIMKLYANTSRQLSYVLSGVIIFFSLPLVPFILDVMWP
LNQSRPVISPYPGYYFVDTREYFFKIFWHSIISWEIIFTAVVAHDCLLMTYVEHICSMFTMVGLDYESLFSNC
DKAMEITNNYKEICRKKVVLLVHAHKEALRLVELLENTFSIPFAIQMLLAIIGMSVTLLQITQQNDIIKSCRY
TLYVVGQLIHLFWFSFEGQKLIDHSLQMSDKIYNSSWYEVSASSQKLIILVMMRSTRPSVLSAGKIFIFSLES
FTTALQTSMSYFTVLAT
>CobsOr148
YYYDVCKWYLTIVGQWPYQKLKERQIFLTLMTLLAITSLIPQGARFGICESAQCIYETLPPYMLGLILLVKIY
TYQFNSRKIKDLTDRLFVDWDMLETKEEHDIMRKYAEKGRWHALMYGLIIYMSTIIFAITSLVPRILDVIFPL
NTSRPLILPYPAYYFVDENEYFYYIFFHMLIACSICMTGMIAHDTMFFVYVEHICGLFAIVGFRFEHVSHKCK
IIKKNAINYSSAVHKNIVISVSAHHKALQFAQFLENTFTISFAIQLLFVTIGLSITLVQLHDLAEASRYVLFI
IGQLFHLFCFSFQGQRVIDHSIETRDKIYHGLWYTVPAKEQRLLMFVIRRSIEASFLTAGKIYIFSLENFTTV
VQSSVSYFTLLSSFDV
>CobsOr149
MEINQNHYYDVVKRFLRLVGQWPYQKRKESLFFLIFALFFDANVLFTQAARFFVCDNLQCIFETLSPHVLAAI
IPVKIFTYQFNRRKIKYLTDHLYVDWNFLESEEERDIMRKYAENCRWHVLIYSSYVYISTVSFTTTSLVPRIL
DIIFPLNDSRPIMLAYPAYYFVDENEYFYYIIFHMIICAIMCLTALIAHDIMFFTYVEHICGLFAVVRYKFEH
AVNERAKSRTEKNTIDPDYLYYKNIAVSIRAHQKTLRLAKILEDTFSLSFAVQLLLITLCLSITLVQLHKSAE
AIRYFVFFMAQLFHLFCFSFQGQKLINHSLETRDNIYHSLWYNIPVKKQRLLMFVMRKSIEANTITAGKIYVF
SLEHFTTIVQSSMSYFTLLSSF
>CobsOr150
MICVDCLQIRLNRFILLAIGLWPYQQSKLTKLYLILCVGIMATFIVLQLLMILKCSVDALINLFSSILMYILF
SIKLVAFIVNNKPVKYMLEKVQHLYNELTDKNEISIIKRYGSYAKLYTITLTSISIIGILVIFFYSFWPNIFD

IVFIGNGTRSRLTIPFSIVYFVDQEKYDYLILFHMNACIFIGYCALVGLGTFLIMYLQCVCGMFKIASYRFTR
VTMLSRISGQQKENLRNVDLICKGLSYALDMQCKAVEYFNLMASGFGVMYLFLVTAGILFISLNLFRASSIIF
FNSNVVLEIILTVLFTLTVIIYTLVCNWISEEVVNHNNDVYVTTYDIQWYMAPLQIQKMIVFIMLRSNKTFSV
SFCGGLITGSIENAAMVLSTAISYCVVLRS
>CobsOr151
MVSIISRYFKFNRYIMLAIGIWPYQNSKFSQVQVITIFSIILSYIIFQFTTLLTRDCTIDFIIKIVSDACLFI
AVLSIYCVTWLQTDNIRSLMNTLQHTYDQLKNPDEIAIIEKYSNTANTYINIFTTCAVCGLFFVWIFPIWPQV
VNIFLPINSSRRHLVIETEYFINPDKYFYIILLHSNVSMGVADIIVIAISAMIIKILKYTCGMFNIASYRIEN
AISRNIQQNISKTKIIKIHKEIICAVNIHCTAIENILNILKSIEIPTLVLIMFGMISLITSLTKIVISDNIEE
QCTHIILVFGIYAAILLGNSLAQEITDENNRIFITVYTVPWYLAPLRIQKTVLFLLQRGTKIFSLTLYGLIEG
SFENAATLISTSISYFTFLRATR
>CobsOr152
MNCLDTHFRLNRFLLLAIGLWPLKRSKFAQVQFIILFSILITFIIFQFTSLLTSRCTVDLIVNILSSTFFFIC
LAIKYNSFWINADTMRLSLEKLQHTCNELKNEDEIAIIEKYSRIGKIQTIAITMFGAMSLCSFLLLTLGPYAF
NIVFSVNNSQSRIVMVLKTEYFIDQEKYFYLIVLHGNAALFIGATAMVATGTMMIAYLKYICGMLTIASYRIK
EAMTINVQQDIDHEKMIICYRGIVCAVDIHRKAIEFSQVFIKSFQGSFFFLIAAGMICLSNNLVQIALYVNTL
KQFILSLTFVMVLYVYMFLSNYTAQEITDHNEYVFATVYNVLWYVAPLQIQKMMLFILQKGTKAFHLVLGGIF
IASMESAASV
>CobsOr153
MICLETRHFKVNRTLLLIIGLWPYKRSLIIRLQLILLFGTLISFITFQFTTFITLKCTSDHILKVFSTTFFYM
CLAIKYNSFWVNNNIVRALLEQLQDVCNGLKDENEVAIIKKYGKKGNQYTNIIILFAIMGEGVITFIELWPYI
KYKVLHNNESGLNPSTYIVKEYFIDQEKYFFLILVHTLIASYIGSIAMVATGTMLIAYLQHTCGMFSIASYRI
EQAINMLQNDILKSNKQIYKDISYAMITYAVDIHRRAMEITDFLISNFEGSFFFLIVIGVTCLSLNLFRASSN
MDNIEQLILPILFIIILYVYLFLSNHTAQDITDHNEYVFATVYNVQWYVAPIHIQKMMLFLLQRGTKAFHMIL
GGIFVASMESAATLMSTSISYFTVLYST
>CobsOr154
MIDLKSRFSLNRILLLAIGMWPYTKSKLVQFQSTVYYGILGSFIMFQLTAFTTSEYDLHLIIEILSSTSFFSN
FIITLTTFLLNIDIVKYLLELIQCTHDELRDEGEIAIIEKYWKIGKFYTQGLTCIGIFCVTSFILSPYLPYVI
DIVFPINTVNESQLRPSLEIVTEYFFFDQKKYFYLIVLHANVSFCIGILVSIGTGTLLIVYMQHVCGMLKIAS
YRIDQIMAIGIKQMSTAKGASLIHKSIFYAVDMHRKALKTIKVFISAFNITFLILILDGLFYASLHLYLIFQE
LLTGHIGEKMIQSFIMLNYYYMYTFLANDMVQKIMDHNNDVFVAVYNVKWYLAPLHIQKLILFLLQRGTKAVH
IYIGGLFMGSLEGFATLASASMSYFTFIYYANQ
>CobsOr155
MISIEVQYFSFNKILLFAVGLWPYQRTKLVQFRLIFFLTILITFVLFQLTAFLTLKCTSDLVVKILSTMFSYN
IIITKYVSFALNMENVKKLLIQLQHVHDRLQDRYEKAIMDKYGYTGKRYTVALIIFGVFSTFIPIAVDFFMMP
SGILPINNSQTRRIQYTMEYFVDQEKYITLILIHINVAFCLGLFALIASGTMFFVYFQLTCGMFKISSYRIKR
AINISILENVKIKNEKVMFKGIIYAVDMHRQAMRLSNMFMSKFEITMFFLILFGVASVSLNLFQISQIGPSMS
ASNIKELFILFIFVVICVLYMFLANYTGQNVIDHTTNIFVTAYNVQWYRTPIYIQKVILFLLQRRTKNFTLNL
KGIFIGSFECFAMLVKVSVSYFAVIYS
>CobsOr156
QCFNFNKILLLTVGLWPYQRTKLIQFQFIVFLSILTTAILFQLTAFITSDPSSSLFMNVLSTASFFTTFVIKY
TFFGFNFEAMKYLLTQLQQTYNNLKDKYEITIVEKYTYIAKRTTVILALFGLCNIITLISKFWLSYIFDVSLS
RNVSMSRYLIITMEYFIDQEKYFYLIILYPYVAIIIGISTLIATGSMMVAYLQHTCGMFEISSYRIKRVIKLN
MPQSVTLRHKNFTEVKEIIYAIDMHRQALKLSKLLVAKFEIMLFFLIIFGVISLSLNLFQISKLESSNIDKYF
ISFLFTATSIIYMFVSNYTGQKVMDHNNHVFLTAYNLQWYLTPLHIQKVILFLLQRGNKNFCLSVGGLFDGSI
ECFATLVKASVSYFTVMNS
>CobsOr157
MMKKDVQYLNIIKFLLLFVGLWPYQQSSFTKFQHIFISVLLSAFIIFQLTPFITIKCTLDFVTKNLSSTTFFS
MFIIVYNSFCFNIKNVKDLLAQLQHSRNKLMDKNEVAIFKKYDSISGRYTIGLTILGIFGILATVIAQIWLNL
SNVDLSANKSRTNYFLFTMEYFIDQQKHYYLIMLHSHVAVYFGTILMVATGTMMIMYIHHTCGMFRVAKYRIE
HAVNFNIRRNMIPNSAIFMAQGIICAVDMHRQAIKLNKNFMSVLEKMMFCLIACAVACLSLNMFQVTFEKDVK
KLVMPFTRTLVCVIYMYLANLMGQIVIDHNNQVFDTAYNVDWFKTPVHVQKMILFLLQRRSKEFALNVGGVYD
ASIQNFATLVKASVSYFTVICST
>CobsOr158
QYFRLNKTLLLIVGLWPYQQSLFTTFQYIFISTILISFVACQLTTFLTLKCTLELFLKVFCPVSFFVVILIKY
NLFYFRLKSVKNLLMLAFYVQNELKDKNEIAIIKKYDCISRRFTVGLTIVATCSICGVNIMQFWLYIFHINLL
PANVSRFDHLPFEMEYFIDKRKYIYLITLHVCAAVCVGPFVLMAIGAMMIAYIHFTCGMFTIASYRIKNAINF
NIQKRITSKSKIWMTEGIICGINIHRQAIKLNKLFMSISEVIMFCLIACGVICLSVNLFQLVSSKNDIGKFYL

SFMISSACTVYMFLANLMGQIVIDHNNNVYTTAYNAQWFKTPLHMQKMILFILQRRTKDFSLNIGGLFDASME
GFSTLVKASVTYFTVMHS
>CobsOr159
YFRLNRVLLLTVGLWPYQQSSVTKLQYILICAILVAFIIFQMTTFVTLSCTSDLLIKILSSTFFFAMFIIKYI
LFRFNIEGVKDLLMQLQGVYNKIQDKNEIAIINRYDYIAKCYTVVLTLFGVCTIPLGILFQYSMNVTNINSPI
NGSRKYYFMFTMEYFIDQEKYYYLYILHINAALCIGMIIMIATGTMFITYIQHTCGMFKIASYRIERAINRNI
NKTFTLERKTLMTEGTICAVDIHRQAIKLNKNFMSVLEKMMFCLIACAVACLSLNMFQLAFEKDVKKLVMPFT
RTLVCVIYMYLANLMGQIVIDHNNQVFDTAYNVDWFKTPVHVQKMILFLLQRRSKEFALNVGGVYDASIQNFA
TVL
>CobsOr160
MIKNKQECLNLIRILLIIIGLWPYQQSNFTTFQYIFVYGVMIAFITFQLTMFLTSKCTLNLLLKILSSVSLFI
LCVIKYNVFHFNRKNIKNLLMHLQDIYNKLEDKNEIDIMRKYDYTAKRYTIALIMYGICTIIVHMLFQYWINM
ADVALSINESRSYYFSCNMEYFIDEQNYFYLITLHINTAYCLAAFIIIGSGTTIIMVLLYICGLFKIASYRVE
HAINININIERNISLRNIIFMTKGIAYAVDIQRQGVRLSKYLMLVLKKMNYWLIICLVICLSLNLFQIALSKTNI
KDVLMPFGYACVCVMYMFLANLMGQFVSDHNNHVFITTYNVQWYRTPLHIQKMILFMLQIGTKKYYLDIGGVF
SASIEGFATLVKASISYFTVIHSTQ
>CobsOr161
MIRLEVQYFNFNKHLLLAIGLWPNKRSKLTFFQSILISGILSTSIIFQLTSFITLKCTFDLVIKVVSSALFFT
VYLIQYNSFCVKIKNVKNLLTQLQHVYDELKDEEELAIIRKYGYNAKRATVVLTGITLCLVSFFLIIQCWTKI
FDVVLSINITRTRRMEFVITEYFVNQENYLFFILLHINVAFCIGTAVILATGTLLIGCLQLILGMFKISSYRI
ERAIKIYIPQDVTVKNRSLKSESLIHAVVIHREAMRLSKYLIFSFETMFISLAGIFIISLCFNFFRIFQIATS
KKTINEIFFPVIFAIFNIVYLFVCNLCGQNIIDYNNHVFATAYNVQWYLAPLHIQKMILFLLLKGAKDFTVVI
GGIFVSSIECFASLIKASVSYFTVMLSV
>CobsOr162
RYYRLNKLLLLPIGLWPDQQSKFSRFQRILFSIILSTSVILQATPVIKLKFSMDFITKSLSSVLFFAMPLIKY
NAFFLKFKVVKIILVELQYKLDKLKDENEIAIISKYSCIANRYTIALSLVTLFIGFIIQVWFNLINVDPSINV
SQIRLPIKMEYFVDQEKYFSLYMLHTYVFLCIGGFTLVATGTILITYLQYICGMFSVASYRIDRAMNINVLKN
ITSKNKILMTKSIICAVKIHRQAIKMSKQLMTIFEIMMFGLIICGVVSVSINLFQIASKTNIKELFFPFVFVN
ITVLYMFISNYIGQNIIDHNKHVFSTVYNVQWYRAPLHVQKMILFLLQKETKEVTLNVGGLFNASIECFATLF
KASISYFTVIYS
>CobsOr163
QSFSFNRILLLVIGLWPYQRTKFTRFQCICCFTILTSSIIFQLTRLITSKCTANLICEVFSSVSFFIVSLAKY
NSFCINTGAIKDILIQLEHVRNQLKDKNEVAIIDEYTYNARQYSIGLTILCAFFTFVFITLQYFPSILIFPVL
SKNVSQSRHVPIKMEYFIDQEKYFNLILLHVNVTFCIALVSILSIGTMLITYFIFTCGMFKITCYRIKRALNC
NIQRNVTTKRKNLIVEDIKYAVEIHLQAMKLTTHIMSIFETMIFLLIVFGVICLALNLFEMMSSEINMEKFIL
PVVNVAISIIYMFISNYIGQNIIDHNNDVFHTAYTAQWYESPLQIQKMILFLLQKGIKEFTLNVGGLFNASME
CFAMLVKTSVSYFTVIYS
>CobsOr164
MICIESQYFNINRILLLTIGLWPHQQSRLTQFQFFSLFFIILTGIIFQLTTFLTMTKYTSYLIIKVLSSTSFL
IICLLKYYTFYINVEVVKQMLLDLQHMCDELRDKNEIAIIEKYGYTAKCYTVAFIGIAVSAASFLFYTQMYIM
YDNALLKNVTSLHHLLFQTEYFINENNYMFFILLHMDMALSISTITMVATAAIFNACFKIFCGMFQISSYRIE
RAVKINILQNVALKNKICKSEELICAVNIHRQVLNLTATFLDKFEMMFFFLAGLFVFSLSFNFLQISQTILAH
KTFKEAVLSFLIACVNIIYLFVANLLGQNMTDYNNDVFNTVYNVEWYKTPLQMQRTMLFLLIKGAKNFTLNIG
GLFISSLECFALLVKASVSYFTVVFAIQ
>CobsOr165
MICIETQFFSLNRILLLCIGLWPHQQSRITRFQFIFLFVILLTGIIFQLTTLMTTAKYTSYLITKVLASSSFF
IICLIKYYTFYVNVEVVKKLLLDLQCICDELKDKNEIAIMEKYGYIAKNYTVALIVIGIFATCVIFTAQFYIT
YENILLKNVTTRHRLPFETEYFVNLNSYFFIILLHMDVAICIGVAAMISTGTILITCFTIFCGMFKISCYRIE
RAVKINILQNVALKNKISKSEELILAINIHRQVITLSKNFLAKFEIMFFLLAQIFVVSLCFNFVQIFQIFVDQ
MSFKESIMSFVFIVGNILYLFISNFLGQNITDHNNYVFNTVYRVNWYETPLQIQRMMLFLLLKGAKNFTMNVG
GLFVPSLECFATLVKASVSYFTVILSTQ
>CobsOr166
MHTLPLSFALLTYTGYWRPVELTSVKYWAYVVYSIVMIFQLYLLTLCGLADCCIIKNIDTFIEKLSVAVSVLG
VTCKVTNLTLRRNEIIGLTEMLLKDICIPRNDCEVDIQQRFDRSAKRITIWCEILNESAVFFATVAQFSYFIR
TRTLPLFDWTPYDVSSTIAFWVTMFQQTIALILCANASVAHETLISGFLIQTCAQLDILCHRARTLPDSLREA
WKCSNSKDDFKARERRLIRELIYHQLYVYRFADRVNAVFTLMICLQFTVSSIVLCLSIYKMLAKSLLSMEFAW
CLSYLGSMLMQIFLYCWYGNEVTLKSTEIGSAIYEMDWSVLPADLMKTLLMIITRSKKPIKITSGNIITLSNE
SFMKIIKISYSAYNVLQRS

>CobsOr167
MSFFDNRYYYFNKRLLCILGQWPFQSRLKSYILFGVASIFILSLTAFEMWGLVAGITNMNIIMINLSPLLINS
FVIVKFFNCALTKHYMKEFLEDIEETWKVKHIGPEKEILENFAEEYMSITKPYMIVIYTLWFLYCGPPVVINK
IHQLLPTNETYTPEFLYRIEHVLNLDKYFNLLMLHAFIAVFYIITTVIAVDITFTMCILHICALFECLRYNIE
RIRGSDFVHLEPKIEDDKAYHDIINCIESYKHALKLSDILSANYSTAFLFILGNIVISLSFGAAELVMLDTQL
EELIRIFSATMGQVLVIFYLSWISQKLIDYSSSFQNVIYSCDWYNISLRSKHLLKLTLLRAAHPCQVKGGKIY
VMSLMTFSWILKGSLSYFTVLTSLQ
>CobsOr168
MDFAGGHYYRINRILLTCLGLWPYYTQCTKYVYCIFLFLFLLSSTVFQLSSLVTKEYSIDLLLNVLSIVGPSI
YCALKYFATFINRNKVKELIEQIRYDWNSLKEEEELKIMHKRATNGRFYTIVMLLWLYSTLFLLILMLFSPSI
LNIVIPLNESRQSWQLPTAMEFFIDREKYANLLTLYLFLIAFISTSIMIATETLMIMYVQHICSMFQITGYRI
ERTLNKSHIKWLISSEKFNSKSIVKAVDNHRSAIKFVDFLKRTFSISHFFAIPLGVTSLSINMYLFCECIMAK
NIGGASMSFLYIATHFLYIFFCNYIGQKVIDYSDDIFKKICSTRWYEAPLKIQKCLVMITYRSMKPSTLTVCL
GLYLPSLQGFATLVSSSLSYFMVIYS
>CobsOr169
TFISKWQNCHYCPINRYYAVNRILLLCVGLWPNQKSSSRHIAVTFTTMTLVSGIVFQLTPFITHEYNVNILLQ
ILAYTIPWIAYTLKYHFSCLKMRQFRDILERVRCDWNQLSNTHEIEIIKEYAAIGRFVTLIVTLFIYVSIFGF
IIIMFLSNVLLDIMSSKNESCPRQFPILIECFIDQQKHYFLIFFQLTVIVVSGFTTVAASETLNMSFVQHACG
LFEIAGHRIDQALHENKVQGVASTAKRNSMIYQGIISGFDMYKKAIEFVEMLKVICKWTYSALLPLGVISLSI
NLYRFSQLIASKEYFETVISFIFILGHFWYMFFTNYMGQQVINHSSDMFYRTYNVQWYVAPLKAQKLLLLIMQ
RGVRHCTLVIDGLFVSSFEGFATLASTSISYFAIICSVF
>CobsOr170
CTEMTHTSDRYYDLNRKLLLCIGLWPYQRFGFRCVIIMVMTTILAGGVIFQCTTFVTKKFSVELLLKILAYSI
PWFSYMLKYNVLCFNVKKMQKLVERVHFDWDQLHNARELEIIKRYAAIGKFITLITTLFIYLSTFGFIFIHLL
ANFVLDFTTAANESRVRRLPAEVECFIDEQKYFILLLFFAFLVVVCGLSTVVAIETLYMCYTQHACGLFEIAN
WRIEETLHRGMARNIISVAEKNLIMRQGITHAVDIHRKAMEFIEMSKDTFKWMYLITIPVTVLSLSINLYRLS
RLISSNEYQDTVTTFLFVLGHFWYMFFCNYVGQEVIDHSGDIFYKTYNAQWYMVPLKVQKLLLLVMQRSLRHC
TITLGGLFIPSLEGFATMISMSLSYFMVIYSVQ
>CobsOr171
MEHSEEKYYKFNYILLSSIGLWPYDNNATIKAIQMILSVLIFMSFFIIQFLKLLTPEYSLDLLLEVLALNFFI
LIFFVKYITFSFITGNVKQLRKNMQSNWNVLVNDQEIDILHGYAIIGKHFTVALMICAYFASGCFIVIQHYPN
LLDIIDPLNESRPRILLHRAEFFENQQNYFYLTMLHEVLGILISGTTGIATETFSLSYSLHAFAMFKITSYRM
KHMLSMDVSQIPIAKSYIIFRNKISAAVDIHRRAIYFSDVLKASLGLSYLFMVIASIGSATVSLFRLFRILTI
HQEKLESIKLICYTFFLLLFLFIGNFVGQEFINRDEHVHRMICNTKWYNAPLKIQKFILFLMRKTTKSYRVDA
AGLFSPCLESLATAMSLMLSFLTILCSI
>CobsOr172
MNFGADHYYKLNRIFLSTIGLWPHSYITLRQIQCFVSVFILLSTTFPQLLKLITTKHDVELILKVLSSALPFM
LFTVKYVTFYFVTEKIKGIMQQIQNDWNALKDKGELDIIRQYAKIARSFTTTIATVIYVSIGGVIFIQYVPII
LDIVVPLNETRQVELLFQVEYFLDQEKYFHTIQFHLDVGLILAAITIMSTESFCLTLAIHAFGMFKITSYRME
RVIDKNAPNTLMEKHCIFHNNIVTAVNIHRRAIEFSEFVKSTFAIPYLALILLGVTSSSVNLFLFFQVIMSSS
TMEDLLKCIIFVICHFIYMFTTNYAGQQFIDHDADIYIKICNVQWYNAPLKTQKLILFLMQKTIKSYQINIGG
LYCPSLEGFTALASASLSYFTVLCSLR
>CobsOr173
MELPEEQYYGLNRIFLSLIGLWPYDDIKTRFIRIMLWLLICSLMIVTQWMKIYISTYSSDLILHVLSYNLILI
ACSVKYITFFAVCENIKEFRARIQSNWNSLKDNQENEIICKHGLYGKLFTIFIAVSFGSVITAYILIQYSPIL
FDIIMPLNTSRPRKLLFDGEYFIDSEKYFFVISIHMCIGLLAELMCATATESFTLANAVHAFGLFKIASYRMK
HVLDEVNSTMCATKKYFVTRNKLIAAVDFHRRALEFSELLRTSFGPAYIVLIVLGICSTSINLFHIIREIMAD
NKEVIEIIKSAFLIVVNVIYFVLGNYAGQEFINSDTLYYNTICKTNWYNAPLKTQKLILFILQKTTKCYKVDA
AGMFIACLEGLATSFSMSFSYFMVLYSV
>CobsOr174
MNFVGERYYKLNRILLLCLGLWPDQVSLLKSTQVIFLEITFASSIICLCNTFFVKQFNLNLVTQVLMYILFNC
IFITKYNVCLLRVDNIKYIFHRIQCDWNMLTSQTELKVIQRYAENGKLLTLGYLIMAIITAIAFIILQFLPSI
FDVIVPINESRSRPIIITVEYFVDEETYFFPILTHVIISQFAGTMMIIAIGTILTSCVLHASAMFKIASYRIE
HIFDANGQLIPKDLKQYIFYNNLTHAIYAHRRAVDLANFMTKSFATLYVLLLGFGVAAISLCVFNCINAVTSL
NVTESLMFGGVLTLSLYYVSIANYAGQTIIDSSTAIRLATYNSEWYTASLRLQKLILFVIQRSNKQSALVCGS
VFCASCEGFATIMSMAISYVMVLQSMGAHKEN
>CobsOr175

KYYNVNRLLLLICGLWPYQESKFKNIQAAFYVTILLSYIILQLTTFIVAECTINLVTKVLSIVLPMFVCILKH
GAFFYNNQKMRHLTNLMWYHWTIIQDEQEVAILEKYTKFSKRFTIYMLHFFVLGMFILIIGHMLPIILDVIEP
LNSSRPRHFYILMECFIDEEKYFFWLLLHTIVTISTALMMIISVGTMLMSYTFHACAMFKIASYRIGDAMTET
SVETSSLQKDCAICQKIINGVRIHRKAIEFANLIVSDVRKPLFVMLSVGMISLALNLFQVGVFAALSANNINE
LFTTFIFVTTQICYLYAGNYAGQIITDHYSKVFNTTYDSRWYTAPLRAQRLLLFIMQRTSKNFSFVLGGIFVI
SLKGFSTVASMSISYFTVIYS
>CobsOr176
MTVLESQYYNINRITLLICGLWPYQSTKFRYVRAIFILGVFVTFVIYQSVLLFISEYNFKLALQICSDIFPAI
LCILQFIAFLIKPIEVRKLLNQICDEWSGLKDSKEVEIAKTYGTFTRRLTQALLLCALFCWLFLIAMHIGPII
GSNNAAANESFLRQNYDSMQEFQKSYILKERYTTLFFIVVLVGVFVMTTTTSMILAYMRHVCAMFRITCYRIE
HSLDSNTLHMSICQKNRLMCRKLISAVNIHRRAMKFTQRALDSFKESYFFLIGIGVISLAINMLHALQAALTA
NNANELAISTFYVIIHFVYIFIGNYGGQTITDHSADVLKTLYHVQWYVAPIKVQKLILFLLLKNTKQFGLVIG
GIYVASLEGFITLTSMSVSYFMVIYSTQ
>CobsOr177
MNVFDEYSKFNINLLRILGLWPYQKTKFERFRAQFFNILLITYMIVQFTQLVIVDFSVNVVFKILSEAFPTCL
CVIKFNMFFFDPTKVEIINNHQMLTDPKEIKIVEHYIYQGKIITILLTLGLVILSFLILILVLTPDILDFFRP
LNKSRTHYILMLENYNITEGIQFYYFYTYAFASTFIGVFSIMAVSMMLILCSLYNCAMFKICSYRIEHSIDKK
ILACSNVRIVIVGRIIKMVKLHQRALKLFKLLITNFTVPFLALLVLGLFSFVSVMYRFLIAVTITHKMYDIIF
SLIFIMAHYLYLFGSTFIGQHLVNHADELFAAVNLSLWYETPVTMQKLYLFLIRVTSKSIVPNIGGVYVSVME
SFTSITSTAISFLMVIYS
>CobsOr178
MFSNHEETNFPENRYYKLNQMLLSAVSLWPYRRSKFTNISHIFHFVIMTSFVFFQLTSFITIQFTLDLFIMVL
SYAIPSCLYIVQYVSFCTNRNGVKRIWKDIHDSWSLLKNETERKIMQQHSSSGELMTMLLALSVVTSIILYMF
TELLSSILDFIVPINGTRPHELHALTEYFVDQTTYYYPILCHWILSLCFGCFVFLATGTLELVYVENICGLMK
VASYRIECSLNKYALNNSEQKNYAAYRYTITAAIDIHRRALKCSQFFEDNFQAYFFVLVIIGVISTSLNLFRL
LRAIMMQNMYQLIASTLFIIFHFLFLFLGNYYGQKITDCNNEVFYTVYNVQWYDAPVKIQKLFLFIMQNTTTP
YILNIGNLIFASVEGFFKLVSMSMSYFTVIYTLM
>CobsOr179
LVVENYYTVNKTFSKILGIWPHDQSRIVLLQRVLIIILTVTYIMLQLSVFFTSKYNLDLFVKTMSFVFPFILI
MLKYCVFIFKSNTIKILHHLIQEDWKIMQSKLEYDIMEKHTYDCQNNIISLFRIGGGLFTGMVVVQFSPIIFD
FILPLDEPRSRKTLITVEYFIPLDNIFYTAVIHEVIVAGLLILILITTGTQLLLCAHHSFGMFKIARHRMEHS
IDERVLRMPNCHRVICEKIFHVVIAHRRAMEFSNIIMSSFNVPYCIILILGVLSLSVNFFRFVQAISTVSKNL
EDTLLSCSTTIGHLLYMFVANYVGQKSIDYNNDVFRSVYGSSWYLMPVTSQKLILFLLRKTNKDFYYSIGFIF
VAKMENYAMLLNTALSYVAVMYSI
>CobsOr180
EMYALWEGYYKINKIVLKLVGLWPYQPTYLTQIYRVLMTTMFFTAILVQLLVFVTTQYNKELLFRILSFVLPI
VFVVMEYMLMIIKAESLKKLIEDIENDWNSIKDKLEIGILEEYANYIKTLTIYAIAICYISIFIFTLFQRLPL
ILDVIFPLNESRPYQHFILTEYFVDQDQYIQYIMLHQCLVYCIGFITVWSIGLSLIIYLTHMCALLKLTSYRI
ENVFEKNILAISNPKRKQYLLHQRIVRAVVMHRRAIEFNETVQSTFLFPFGFLVVIGITSLTFNCFVLSQQVV
SDDMNNTFITVIIILTHIIYLFVGNFGGQIIINNGADLYQATYNALWYRAPVSTQKLLLFIMKKASTYISLSY
FDILVASLQDFATVMSMALSYFMVIYS
>CobsOr181
MRLIEKHYYTINKSFLKALGIWPHNNSQFISLLQQLLVLTLTLTYIGMQLLVFTTTKYNMVLFIKVSSFIFSF
IVITLKYCTFMLKSKSIQNLHYFIQEDWKLIQNKLEFNIILKYAHNGQFCAHFLFCKAALILFLYLSIINYKI
MFIVFVVIALSFMQFLPYILDIVLPLDEPLPRKIIITAEYFDPENKYFVTEALHEILLIALVASIMFATASQL
LVFCFHTLGMFQIVSHRIQYSIEDSVLHASNSEKEYAIYKRVVQAVIAHRRTMEFFNILISSFNVPYCIIAII
GLISLSVNLYGLVEAIMISKNMNYILLYFIMTLNHLIYMFVTMTFGQKVNDSNNELFTLLYNTSWYMMPLSSQ
KLILFLLQKTGKELYAVVGMIIVAKMETFASLLNASMSYVAVMYS
>CobsOr182
VSQFSCEMHAIERYYKINRIILKLVGLWPYHQSYFSQLLKVLFVSILVTFIFAQLLPFTTQQYNTSLLFQVLS
FVFPILIDTTKYCLFIIQANNLKRLLDQIQDDWNSLKDKFEIKIIKKYACNARLFSIVVMIYCYFGLLSCGIV
QFLPIILDVLLPLNDSRPHYLLVATEYFVSQEKYFHLMLLHEALAYIIGTTALCGTSAIIMTCILHACALFKI
ASCRIENAIKKSTLMIPSPKREYFIYRKVVHAVIMHQKASKFVELLTSSFGTLFSILIIILGVSSLTFNLFQFL
QLITLTKNTGQASMVAILILLLHLNYMFVANYGGQELLNHGLKMFKTTYNGLWYAVPLRTQKLLLFIMQKETLN
IGLTCGGIFVISLEGFATLANAAVSYFTLIYS
>CobsOr183
MVFSGERYYNIYRIMLTAIGLWPYQTSIGMHIFCFGTYCFTLLFQFTTFLTAACNLEFIFKEFSYILVTMLYI
VSYNSYYFNFKEVKYMFEQIELDWNAIEDNNEIKILEKYAFESRLLALFLVLIALSVFLIIIIELIPIIFDAI

VPMNESRPRKIKLGFEFFIDDQRYFYIYFIFEITVVLTGMFTILATGSLSLAFFRHCCATCKIASNLIESTVT
NHTLQVPACRKVHVMYKKINRAVRIHRKSIQFSTFIMNSMNKWYFAIVLISVISLSCNLFRLLYALTTLNEIS
EIIMSIGMVTANFMLIFVPNVIGQTLIDHCAEMFNAAYSTMWYLAPLSIQKLLLFVMQNSLKTHTLTIAGIYV
SSLEGFSTV
>CobsOr184
MDLASNMHRYYNIHRILLSSIGLWPYQDPKMKKIQWVISLIILTSSVIIQFMKFLTAEYSLDLLLKILSFATP
CMVFILKYLTFCIGSETVKHLLERVVSDWTLLKTDAEVEIMKRHCDFGRLFTWVFALGVYFGLVCYLLIQFAP
NFLDIVSPRNESRLHYIPLTAEYFADQQEYYLPILLHIDVLAFIGFTTVISTESLITVYTRHAIAMFEVSSYR
IEHAFDQVLDMMTSRECCSHCGKIKNAVHVHRRALQFVDFLESGFVISYFFLLCLGVTSLTASLLRLFLAAQY
LDDIEECLITMLLVFGHIYYIFNGNYTSQKLIDQSTEVFCKIYSSQWYNAPLHSQKLLLLMMKQAIKGASLTV
GTVFVASLEGFASITSLSFSYFTVICSI
>CobsOr185
MEFHGYRYYKVYRIFLLPLGLWPYGDSHFKRIQTIFCTLTILFLIIAQLLKLFTMECTFEFVLTDLSYAIPSI
VYLLKYVTFCIQSRKIREFMECIRVDWNELQDEEELEIIRKYARTARTYLYAFVGVSYPGTVAYISLPFLPDI
LDIVAPLNESRTRILPFPVEYFLDEQKYFYLLLSHSIVAIIIGIVTIVTTETLTFAYFCHICGMFEIVSYRIM
SAFGKSLSVLLTLNKENTIRIKLIAAVKIHQKAFELFEILTSTLSLSYFTLIILGVASLSINLFRLFQVTTLS
EQKKEIVLYIVFVIGHFYYMFMCNYMGQIVIDSSTDIFKKACDTQWYATSSRTQKLMLFIMQRSMKSCKIVMG
KLYYVSLEQFTTLASMSLSYFTVIYSVQ
>CobsOr186
MDFFNNEYYFTRNLMESIGLWPYEKPERRIVRMVCVSFVLTQSLVLQLTPFITHEYSTSLVIEVFSFSILCLI
YVLKYNTVYFNLKHVKNLFDQIQGDWKLIQDMDEFEIIKKYAHKTRFYAIISGLIVYSGTLAFLSMTFIPTFL
DFILPLDNPRPRRLPILIECFLDQDKYFYYILLGIIMASLLGMTVIMATENMYMIFVQHSCALFEVISYRLTY
AFDTYSKTTSPNIKRKLCTKLLSAFTAHQHCLEFVETMQYKFSISYFALFVMGVGSASINLFRLFDAVTRQNI
FEAVAAGLFVYAHVCYAFFMNYFGQDIIDRSEYFFQQVYSTQWYMAPVCTQKLLLVTLRQSTKNSKICVGGLF
VMSLEGLATLISMSLSYCMVMYSV
>CobsOr187
KMNLFESRYFNINRLLMSLIGLWPYQKPRSRRIKMILVSILLAEGITAQLMSFITIEYSRNLLTKVFSFSFII
LIGTVKYNVVYFKSEQVKHLFEQVLCDWRGLTDAKEIEIIQKYANKGRLYTIVSGLSLYFGMLLFIGMFFIPD
ILDVMAPLNEPRPHQLPIVIETFFDQEKHFLFIILNFFVIAGVILTILFTVETLYMICVQHACGLLQLTSYRI
LTAFDNRLQQMNMSKPKCIVCVKSTNAIRIHRRSLEFVDCLCSTFSISYFFLCIFGVTSLSINLFELLKAIES
KQKSQMFYSSIFVFGHLCYMLWVNYFGQKLIDNSADVFTQTYNVRWYKASPHVQKNILFILHRSSKNVLFEIG
NIFVFSLDGVATLINSSLSYSMLLYSTR
>CobsOr188
MDFFDGHNYRVNKILLSAVGQWPYQSSRTSQVIRIVIVTVVCSQFLAKLCGMYAYIHDMDIVIECLVPIMVDV
SGMTKIMNSILCINEIRELLEQIRNDFCSLRNSNDIKILQKYADSGKRSSTVYASVLYTMTVVFMLVPFQPLI
LRVANATTRPMLHRVEYFVDMDKYYFPILFHGYFTAVICVTSIVASDAMFVIFVQHACGLFIITSSRIEQAIR
EVCLTADANLPIMKDNAYQNMIQCVRDHRAAIRFVPFTNLMEMVYSKHFLFHSGLNMIAMSVTSVGAVSKADD
LYEFLRLVAVTCALSFHLGFECINAQRLMDYSGYLHTNLVNLNWYDASLRTKKLVLFMMMKTQLPCVLTAGGM
FVLCMETFATILKTAVSYFTFLRSAH
>CobsOr189
MDSVQSRYMRINQFLMYLVGEWPYLETWEKFLIQSVFVPAVFAQAVIQGGGMFTAWFAGDIDAFMEGVSPFVI
SLMCICKHVNYTYNHDQMKKLAFTMVEDWNIYSKFTHEYNILCKNYAMGRKVTIAYAVSLYGSMTPFMVVPLI
LNTASSLGLYNISEGRPLMFRSEYFIDREKYYYPVLVHSYIGTLGFISVVVAIDTMLVFHVQHECGMCEILGY
RLERIIEGDSLDINLYPNKQEAIAYRYVKNCVVLHNHIIEYAKRIENANTTSYFFQLGFNMVGLTFTIFQAVV
KLGDLNVALRYASFTICLLSVLYLESWPGQQLSDYTNKIFSYITGGRWYQSSLRVRRIINIMLLRSYVPIKIT
AGKLYTLNLANFSAVARTSFSYFTVLCSMQ
>CobsOr190
MRRVDQFFRKSCYSILRILLIPCGIWPFQALSKRYAMYIGFTFLFGSGISFEILCLIHVWPDFLDVFDCLSML
FYALSSTIKLIYAVYKLSKIKLLLLNMQEHWCSPKSDQEARILRSNVLFARKFGFVYTTIILAHAVIYGITPL
VGKFTYDSIDVSNETQDAQTESVHHLSYILDLQMKYIPLLIQGFLYEFFFTLILISFNVLYLMCVQHCCGLFE
ALKYHLENAFNFGNNDNDVISIQDTCSSKIAYGVRRHAEAIQFAATVESLYSVPFFVQLAMNVSLLSIVGFQV
VTNAENINRLMPYGTYLNGLVLNMFFENWQGQKIMDCNQKIYESAYKLKWYNMPITSQKLLIIIIMRSRKPLI
ITAGKIFVLSYVTFSTVMRTCFSYLMILRSM
>CobsOr191
DIDQFFHDSHYNIIRVLLSVSGLWPFHARARRYAIYLAMMLILGSGFIFQTLGIIEIKHDSFEIIDATPLFFF
AIVTISKTFCAIYSLPKIKVLLVKMQEYCLSPKSYEESKIHNSRAQYGRKLGYAYMGFLLGHSIVYLFSTLLI
RFIYAQSEETNETSNVQIGLPHRVNYMVDLDPYYVPIFIHSAVCDFSFTFLLVVFDVMYLMVVEHCCGLFAAL
RYRLENALVFDNGSTVTTAGYKCYRNIAYSIRRHTETMQFIAIIESIYSLPLFIHVGLTVLIISVLGFQVINN

TENIKGLLRPVAYLNGILINVLFENWQGQKIIDSSERVFESVYNSEWYNMPIPARKLLIMTMMKSEKPSTLKM
GNFVVLSYVTFNTVLRTSSSYFMLLRSL
>CobsOr192
DIFQSRLYRLNRKLLSLLGQWPFQKDRDRRVILATISFIGLTQAVAQILALVTLSGDFDATIECIPPLLVDFV
CVIKLTNLVCNIEQIKLILIHIQRDWQTWAIQSEFEILCRFAESGRSITIGYASGMYAFGSLFPFLAIIPKLT
GKNITSEYSTRPVGFPYHVEYYVDLEKYYYPVLIHNYLSTVIRLTIIVACDTYVAVLVLHCCALFSVVRYRLK
YVRKSIEQDRKLASLEEDDKFYKNFVYCIQKHKDALRFARCLDNIYKKPFFLEIGSVILAMSLSALQATSGII
NTQIAMRHAAYIIAQLLHLYIACWLGQQVIDHSDRVYTSTYRGEWYESSPKSRKLLNMIMLRSTSPCTLTVGK
LMILSLPSFSAVVRASASYFTVLRSV
>CobsOr193
MSFYDNRYYHFNKSTLCIIGQWPFQSRLKKTIMFSILVSFLSTLIGLQLWGLIMAIPDFGIVMDVVPSMLINN
FIVIKLINCLCTKYKLQMKQLLEYVEEAWKIMHTRPECKILELYAEESRSLIVRYMVAFYSIWIFYCATPIAI
SKIYTILPTNKTYTPRFLYRVEHVLDTDKYYNLLMLHGFISVFYIVTVVIAVDCFFVLCTQHVCALFKCIKYN
VERIQGSDFVLLKPNIADDEAYHALIDCIKLYKHALKFADLLSSAFATCFFITLGNVVISLSFDAAKLTSINS
MDEVIRIFAANMGQLLHIYSLSSTSQRLLDHSTEMQEVIYNCNWYKISLRSRHLLQFTLMRTTKPCQIEAGKM
FVMSMENFSSILKVSVSYFTMITSLQ
>CobsOr194
MIDVVNRYFGLNRILLLMIGLWPYEKSKCAVIQIICVYSILISCIIFQLTTFFTSKCTAEFIIKILSITFAFC
SIIVKYNAFYVNSRTIKYLIRQLHHIYNDLRDKNEIAIIERYGSKANCYTASLTLLMFVGASFFTGIQFWRNL
KNVILSSNVSDSHQLPFDTEYFVDKEKYFYLIFLHLRNVKNCQVKLYNLFIYFNVKRFVIISNLIIHSYRIER
AIQIYNSKTRKARNEILAHDDIARAVDMHRKTMTFATDLISSFEISLMFLILFGVLVLSLNIFLIFQIALSKY
RIEKLLINFILACNSFLYMFAANFFGQQVIDHYNHVFATAYKVQWYKISLYIQRLIIILLQRGNKSFGLRLGG
LFVASIECFAMLFNASMSYFTVMYS
>CobsOr195
MIDVVDKYFSLNRTLLLIIGLWPYEKSKCVVIQMICIFCIVITCIIFQLTTLLTSKCTPDFIVKVLSIVIAFS
SFIVKYISFYVNSETIKCLARQLHHIYNDLNDNGEIAIVERYGNNAKCYTIVFTIIFTTGLFSFVGLQLWADF
KHTILSSNLSHSHELLIDTEYIIDEEKYFYLMFLHINMTIIIGCIIILATGAMLVAYLQHACGMFKIASYRIE
KAINIYYSVSKNIKVQNEMLAHDDIVRAVDMHRKAMTFSTHLISSFEMSFMFLIVIGVLSLSLNIFRIFQVAL
IEHKIRKLFLNFLAASYCLLYMFMANFIGQQIIDCYNDVFATAYKAQWYKTSLYVQRLIIILLQRGNKNFGLR
VGGLFIASIECFATLFNASMSYFTLMYSVR
>CobsOr196
MINVANRYFSLNQTLLIIIGLWPYKKSKCDVIQVICIFFILITGIIFQLTTLFTSVCTPEFIITILSAVTIFI
SFLIKYNAFYVNSKTIKDLMKELHHIYNNLRDNDEIAIVERYGSNGIYYTTGLTIFITSAIFVFVGIQFWASF
KHTVTSNLSYSHQLLIDSEYFVDKEKYFYLIFLHINMIIVIGFTTILATGSMLIVYLQHACGMFKIASYRIKK
AIQICNSIPKNVKVKNTILIHNGLVCAVDIHRKAMIFSNYLISSFEMSFMFLIVVGVLALSLNIFRIFQIVLL
EFNLYKFFINLVFASSCFVYMFMANFVGQQIIDHNNHVFATAYKAQWYKTSVYIQRLIIILLQRGNKNFGLRV
GGLFIASIECFATLFNASMSYFTLMYSVR
>CobsOr197
IMDKNFNFNQILLLIIGLWPYQKSKFAQIRYICIFIICTTGILFQFTAFLTSNCTADFIIKVLSSVFPMISVE
IKYISFFVNNKAVKYLINYLQCIYANLKDHNEVIIIEKYGSKAKRYTIALTTLMIAQALIHLIVQMWPSFCNI
IVSINGSQKRSLFMVTEYFINQEKYFYFLFLHMYIFYYVGIFIIIATGTSLYAYLQYACGMFKIASYRIESAM
EIYTSQKIPKNENLLHKSIIYAIDIQRRAISFCECIQTTFEVTFIFLIAFGVLALSLNIFQVYQILSSGRDIE
ELLMPYFSSVSCILYMFFANVIGQEVTDHYNYMYDTTYKTQWYLAPLHIQKLVIFLLQRGNKSFGLSVCGLFV
SSLECFATLVNTSISYFIVMYSV
>CobsOr198
VFRYRVYAYRIRKMLYLGGVLQDRTRSVAWSYFTGLLVIFMCFSQCVFIINFCRDHTDNLILLIRGFGLTCSF
IAPVLMAACFLVKREKLIELHDTLNNLFERELVQDRENKATVLATLYAFDKPSYIFCFTLALTVMLFLYQSFL
SIARHIVDHAKPKKLPLPAKLLWFIPTGDGLPFYLHLLYQIASLWWMIFTIGSVDSLFGYYAFQISSILRAMS
ARLANQHQNREMFAVVLNMSVHTHHRLLRCGHMMSDIWGVIILRMLFANAVLMCALIFEASPFTHLTIGQFFL
FISYMALKLLQTFIYAWYGSLITSASEHFREGIYFSEWPDSSLDRVVRANIMVTMMQKPMIIKVLHLSSVNVN
MFTNIVNTAMSYFFLLQSLDE
>CobsOr199
MIYIKTEHFKINRILLLSIGLWPYERTRLVKLQMIFIFGILLSNVTYQFATLVFTKCTPSLVINVLSVALFFS
TFVITYYHFWINSHDIMIGLDILQNICNNMRDKNEIAIMKKYGDIAKRFTTIFILFHMSNVATGLLVTCLLHF
LDAFLHDDTSQSNRVLQKLLPKFFIGQESHMYLILLNFVVSISIGGTALIGVGSVILSYIEYTCGIWKVASYR
IEKAMMIDMLKNEISMYKELILAVDIHRKGVKFITLVFSNFKQLLFILITIKVLCLSFNLYRISETISNEGDI
EQCYFHVLVTCGIFVFLFVANYVGQEITDYNNHVFYSAYNIRWYLAPLHVQKLILFLLQNGSRTFCLSIGGIY
SLSNELFAMLTKASISYFTVVYSMQ

>CobsOr200
MISIKRQLKYNRILLLSIGLWPYQQSKFVQLQLILFFGVLISCIVSQFTVFVFTKCTVDTAIKVLSAALYVAM
CTIQYNSFRINIHIVKQLLDELDHIFKELTDENEITILKEYGKTAERFTIIIMMINCVNLTSLILMPIVAYIP
GNIFINEARLQHVVQNMMPAYFVNEKQYWHIILLHLILVGIIGGSAVVATGMMLVGYFEHACGMFKIASYRIK
KALMTNVKSVKLKDEIIIHKEIILAIDIHRKAIKFSQYMFSNFQGSHFWLLIVGVVCLSLNLYGISETMLTND
VEQFITHFVFISATFVYFFIANYIGQKVTNHNEHVFFTVYNVRWYVASLHVQKLILFLLQRGTKTVYLYLGGI
FMLSFELLATLMKASISYFTVVYSTQK
>CobsOr201
MICIVTQHFNLNRICLMLIGLWPYECSKLVRFQTFFCFTILISSVVYQLAVFISEDCTINLILKVFSIALLFF
MYVIEYNSFRINRQIIKWSLDQLQHICDELKDEKEIDIMKKCGDDTRRYTILLILLDVINLIVIPLLPFLLYA
YDVLLRINKYHISNVIHMLIPKHFLGRDNYIYLIILHSGSSIAIGGLILIATMTMCVAYIKHACGMFKIASYR
IEKAIAINMLKNSSLENEFMMYREIIHAVDIHRKAMKSTILFFSGFQRSRFILLIIGVLTLSLNFYEISEIIS
YGRDIYDCLFHFLIIIDIFAYVFLFNYAGQEFTDHNEHIFTTVYNVQWYVTPIHVQKLILFLLQRGNKTVSLN
FGIVFVLSMELFAALAKASISYFTVVCSMQ
>CobsOr202
LQWAIGLTRYLLKTLGLWPEKLKNRREDLLSKLSLLFNMFIVIFVLTIPALTALKKVWGDMELMIDNLQYTLP
LLISILKVFTIWYNKEALSPLIDMIIEDWMKTKMKEERNVMLKQAKIVRLVTVCGVFMILGLVTIVFSAFFYG
QMINYTTNLTDLIGKPLPIPTYYLYDISSSPKYEITYLIQLIGLIACGLYYTAIDNLLVFLVLHICGQIENLH
LRLLNLRAESNFKVILKHNVKDHIRLIRSTEIVDDTFNLMILGLLFLFGILFCLHGFLIINVIKIQSDSLSTV
NCIWYISSSVCVLMHTGIYCAGGEFLVTQSEKIFYAAYEYSWYNLEPKVAKELILIMLRAKKPLNITAGKTFP
MTMSTFCNLLKTSAGYVSVLFANNN
>CobsOr203
LSYAFTLSRRCLWILGIWPDPFVPLSDFQRLSVRFIIVTCILCLYVIVPQLTNMILAWGNVARMVEHVASANF
SLMALCKLVGTWYHGQTLRVLMTSVMTDWMTSKDHERNTMLNIARRGRVLALRCYVASSCTVVFYVSLNILKF
HRNKYQLHRPLVYKFAYPYNTEKSPSYEITFFTQLSGGVYSALINCTIDCFVSILVLHICAQLINLRTVLNKL
VDDLAKKVITSGEFKKSLATITMRHEHLIRNAKTVDDCYSAVLFIHMLAATFQLCFESFQVFTIVSNHLNISV
IRVAFLSFYVLLVLTHLYIYCYSAERLLIESTGMAYGVYECKWYDISSKDAKSLMFMVYRSTIPLKLTAGKFG
TFSLEMFGTTVKTSMGYLSALLTMMD
>CobsOr205
LNYAFALSRQSLRLLGIWPDPFVPMSDSHRLSIRFVIVSCFLCLYVFMPQITNVMLAWGNITRMVENITAANY
SLLALCKLISTWYHGKTLRGVMAMVMTDWLTSKNDGERNTMLNIARRGRTLSLRCYGVAGFGCMFFLFMNFLK
FRCSMHQPQRILVYHFSYLYNINKSPNYEITFFIQILCGLYTALVNSSIDSFVSIILLHICAQLINLRTALRN
VVDELAKGSISSSEFKKELGAITMRHINLIRNAKAVDSCYSLVLFIHMFAATFQLCFESFQIYTIVSNHFNVS
IIKMAFLLLYLGTVLTHLYIYCYSAERLLIESTSLAYGVYECKWYDISPKDAKSLMFMAYRSTIPLRLTAGKF
GTFSLEMFGTTVKTSMGYLSALLTMMD
>CobsOr206
INYAFSLSRKGLRILGIWPDPFAPMSESHQYSLRLLIITCILCLYVFMPQVTNMILAWGNVTRMVENIASANY
SMLALCKLVCTWYHGKTLRMLMTSVVTDWMISKNDEDRSTMLNIARHGRTLSFRCYITSSGALLFYLYINIMK
IYRNIHQPHWILVYHFAYFYNIRKSPNYEITYVIQLLGGIYTAFINSTVDSFISILLLHICAQLINLRSALNN
LVDELAKKSISSAEFKKRLATITVRHEHLISNAKTIDDCYSTVLFINMFAVTFQLCFQAFQIFTMITSNLNIS
VMRVSFLSFYVLLVISHLYIYCYSAERLLIESTSMAYGMYECKWYDISPKDAKNLMFMAYRSTIPLKLTAGKF
GTFSLEMFATTVKTSMGYLSALLTMMD
>CobsOr207
LNYAFSLSRRWLWMLGVWPDPFVSLSEFHRIRFVIVTCIVFLYVFVPQITNMILAWGNVSRMVENIASANYSL
LALCKLVCTWYYGETLRSLMTSVMTDWMTSRSNHERNIMLNIARRGRILSLRCYLASLGTVMFYLYINFIKFY
RNMQQRHWILVYHFAYFYNVRKSPNYEITFIIQLLGGIYTAFINSTIDSFISILVLHVCAQLINLRNVLNKLV
NELAEGSITSSEFKKGLAMITTRHEHLIGNAKTIDGCYSTVLLVNMLAITFQLCFESFQIYTIVSNHFNVPLF
KTAFLSFYLFVILTHLYMYCYSAERLLIESTNMTYGVYECKWYDISSKDAKSLMFMVYRSTIPLKLTAGKFGN
FSLELFGTMVKTSMGYLSALLTIMD
>CobsOr208
LNYVFALSRQWLWIFGVWPDPDISLSEFRRANIRFLIVSCTVFLYVSAPQMMNVIRAWGDVSRMVENFASANF
SFMGACKLVVTWYHGETLRPLMASIMADWMTSTSNWERSTMMKIAKRGRSISFKCCMAAVGTIIFYLSFHLLR
FFKTLHQSRRYLVYRLPFETIHKSPTYEIIYFIQLSGGTYSVLANYSIDSFVSILVLHVCAQLINLRMTLNNL
VNELATKSISSSKFREGLAAIIVRHEHLIRNAKTIDGCYSSMLFMQVLATTFQMCVITFQVFTIVTDNLKVPV
VRIIFLSFYIALVLTTMYAYCYSAEKLVSESTKMAYGVFECKWYDLPSKNARDLLFIIYRSTLPLRLTAGKFG
TFSIELF
>CobsOr209

MNREMDKYYAINKLFLSRIGCWPYQRKVIKILIPSLLTIIHCSTFTTQVLLLYKTWGDIDIAVECMISMAFVF
VGSTKLLNIAINNNKFRQLLQLMNKHWEIFNGEDERNILSYYACISLKIAKYYGGYILISLILYLFIPLVPRI
LDIVVPLNESRPLVYVFQGEYGVDKEKYYFLIVLHSYIASLNTITAVFTVDITYIASVLHACSLFAAISHRLN
SLNGQEEIKTGDEKKTHIVAHNHSLRDEDISISNDHYKLIICLKKHQFALEHVQILNSIFTKATFILLSLNVL
MLSVVGIQVLNNATHTDEIIRYAFLAYGTFLHLIFMCIPGQLLIDRSTEVFEKAYAAAWYTFSVKTKRLLSIL
LYRSFVPCTLTAGKMFVMSMTMCSSVTQTAMSYFTAFLSIR
>CobsOr210
DSFASVVRVNIALLKLSGVVSCGSNKACQFCSQNILMVIAYGCLFAYAGLYTYEFALYTVYLDTWMESFAMIL
SLVGGQARLTIVLLFRDRFQRLLTICEELWPTLNAIEKTYVRGYVKTTRHLTYYYLFGCAFTIFFYAVASLFL
GQHDGSSNATTTRTLPYACPVEVHRSPYYEIMYVVQLCSMINVGLTCAGVDTVGPVLVLTVCGHFKVLNARLS
RINERDSGRKGSRSTSRSAATIVPSRERNDHSPENRAKFNLDACVYYHQMMLELCKEVESLTNTIFLTQLLGS
TYNVSLVGFKLVGDDPDKFKYTTQLLIAVIQLFLCNWAANVLLTESQDVARAVYFVPWYHFPYRLRRSVNIIA
MRSQKPAQLTAGYIIPLSLQTFASMVSSAASFFTMIRSMN
>CobsOr211
MRVLKFTLLICAVTGCWQPDSWTSLFKHIAYKTYAMFLCSALYIFSISQFMNIVLYVQTSDEFTDSLYMMLTV
FVAGYKQVYMWTDRKNIKVVIDIFNEKPFAACDAREVMIQEKFERMIQSYTLRYLSLILATIMSIILMSLFTA
FPTRNLTYKTWVPYNYSHPAIYFLTYCHQLVGMAASGIVNVGCESVICGLLLHICCQFEILEYRLTKLQDNEK
ILRDCVNHHNRIYKYAYTINHLFVRIIGLQFAVSMFVLCSNLYRIAMATDIAVFVSLMMYTGAILAQIFIYCW
FGNEVKVKSLQLASNIFRINWKNLSNSTKKGLLIIMKRATTPIEFSSAHIVTMNLDSFVSLLKMSYSVFNLLH
QTHE
>CobsOr212
SMLKFTLMICAFAGCWQPLTWTSLFKQIIYKAYAIFLITSLYIFLLSQFINIVINVGNSDEFTDALYMMLTIL
VAGYKQFYMWIDRKNVLVIINVLTEKPFAPYEKHEVMIQEKFEKLILNNTRRYLTIVVMSISSIVLMSVSTVF
MSKNLTYKAWIPFNYSSPTVYLAVYIHQLIAMSTSGIVNVACESLLCGFLLHICCQFEILGHRLGKLMHDQSS
LRDCVCHHNRIFEYAYTVNNMFAKIIATQFAVSMMVVCSNLYRIAMATDYASFIPLIMYTSAILVQIFNYCWF
GNEVKLKSLQLVNSIYDIEWPALSNSNKKDLLLIMKRAMIPIEFTSAYIITMNLESFVALLKMSYSVFNLLHQ
TQE
>CobsOr213
QNMLKYTLTVCSVAGFWQPLTWTSMFKHIIYKTFAAYLISTAYICVFSQFINIIINVDNSDEFTDTLYMMLTI
LVAGYKQVYIWIYRKNFLDLIKILTEKPFAPCDSSEKIIQEKFEYFIKRNTRRYLTVVMMSVAAMILMSVSTD
LKERNLTFKAWIPFDYSSPTVYFFVYTHQLIAMYDSAIVNVSSESLLCGFLQHICCQFEILGYRLTKVKDDES
FLRDCISHHNRIFEYAYTVNNLFAKVIAAQFPVSMLVVCSNLYRLAMTTDFVSFVALVSYTSVMLVQIFNYCW
FGNKVKLKSLELINSIYNTEWPELNNSSKRDLLLIMKRAMTPIEFTSAYIITMNLESFVALLKMSYSVFNLLH
QTQE
>CobsOr214
MLKYTLTVCSVAGFWQPLTWTSMFKHIFYKTFAAYLISTAYICVISQFINIIINVDNSDEFTDTLYMMLTILV
AGYKQVYIWIYRKDFLDLINMLTEKPFAPCDAIEIEIQEKFEYFIKRNTRRYLIVVMIAVVAMILMSISSDLK
ERNLTFKAWIPFDYSSPTVYFFVYTHQLIAMYDSAIVNVSSESLLCGFLQHICCQFEILGHRLTKVKDDQNLL
GACISHHNRIFEYAYSVNDLFAKIIVAQFPVSMLVVCSNLFRLAMVTDLVSAFALVMYTNVMLVQIFNYCWFG
NKVKLQSAELINSIFNIDWPDLNNSNKKDLLLMMKRTMSPIEFTAAYIISMNLESFVALLKMSYSVFNLLHQT
QE
>CobsOr215
MIVLQFTLLLCTISGCWQPLSWTSPRKYILYSSYRIFLICLITTFTLSQIMNIAFNIDNFAEISENIYMTLTV
FCGTYKLIAMWITKKHVVTIINYLTKESFKPSAEHEEVIRRKYNKMIKKYAFWYYGLVQLTVILIIILNAIFMD
FLKGHLTYKAWIPFDYTQSVIFYFVFAHQMIGMSITAAVNVACDSLVAGLLQEICCQLEILEYRLTKILHDRH
SLRDCIRHHNRIYEYAHMVNRRFAKIIALQFAVSMLVVCANLYKLASISITKINGSFLALIMYTACMLSQIFL
YCWFGNELKLKSTGVVNSIYNMEWQKLDNKSKKILVLIMRRSMIPVEFNSAVIITLNLDSFVSLLKASYSAYN
LLKRSQ
>CobsOr216
MPVMKLTLTVLAVAGCWRPPSWTSLLRYMYNIYSSSIILILYTFAMTQIMEMILNADDIDASGDALFNTVISL
LACYKAIIILRINHGNIILLIDNLVQEPFRPMDLSEIMIREKFDKRITNNTLGFLILVFITAFYMIVLSIFTDL
KNGDLLHKAWLPFDRSISALFYLAYVHQLLTLICIGLVHPTCDNLICGLLLHICCQIEILEYRLSNIANHRGN
LRDCVRHHIRIFEYAYMLNDNFAKIVPSEFAMITVIMCFNLVNMALKSSDSISYVQNVMAVACTLAPIFYYCW
FGNEVKLKSSIIRSIYNLEWPIFSNNIKKGLLMIMHRATIPIEFTSRDIMPANLDSFVLKTSYSLFNVLIHSQ
>CobsOr217
PVLKLTLAVLAVAGCWRPSSWTSPFKHAIYNAYSAAIILILFTFATSQIMELVLNAANTDAVSDLLFNAVTSL
LACYKAIVIRINHDGITMLTNDLVKEPFKPTNLNEIIIREKFDKRITNNTLDYLILILITVFYMISLSLFTNF
KNGSLMYKAWVPFDYSTSSLFYLAYAHQVLTLICIGLVHPTCDNLICGLLLYISCQIEILEYRLSNIATGRQN

LRDCVCHHLHIFKYAFMLNDKFSKIVPGEFAMITVVMCYNLVNMAFKSSDAVSYIQDIMIVASTLAPIFYYCW
FGNEIKLKSIQLSDSIYNLEWTSLSSNVKKGLLMMMNRTTIPIEFTSADIIPVNLDSFVMVLKTSYSIFNVLI
HSQ
>CobsOr218
MQALKFTFLIVTFVGCFRPLSWTSVFRRTVYNLYRIFIITILYIFAFLQFMDLLLNVDNPDDFTNILYMALNV
SVSGFKLLIMWLNYSSITTLIITLDEEPFKPLDPGELRIRQKIDKIIRSNTLRYSILIATSWTFMSFMSLLTD
FRQRKLTYREWVPYDYSSYMLFCITYIHQFISTFYCASVNVACDTLICGFLMHVYCQIEILEHRKKILDNQSI
LRSCIRHHNNIFEFAYLVNAKFSQIIGLQFIISTLIICSNLYQLSQSSLNTDSIGLIGFTCCMLTEIFIYCWF
GHKIKSKSVQLADSVFQIKWPIMSNNVKRSLLIIMKRTITPIEFTTAHIISLNLDSFVTLLKTSYSAYSLLVR
IKEK
>CobsOr219
MHILKLTYTIVMISGCFRPQSWTSLFKRTVYNIYRLYVITMLYTFTILQIMDIILYVDNPDDFTNNINMMLTI
LASCYKIFIMCLNYENIVALINYLTEEPFKPLDTNEMKIRRQYDKIIRNNTFRYSALVATTCTFVISSSIFTD
FRQKRLKYREWIPYDYSSYVIFCLTYAQQMLSACHSAVVNVATDSLMCGFLMHICCQIEILEYRLKKILSNQL
TMGYCVRHHNRIFEFAQIVNIKFTKIIGFQFMASMMIICSNLYQLTKSTLNTDHFSLIMYTCCMLTQIFIYCW
FGNKVKLKSLQLTDSIFQMEWPLVDNNIKKSLLIIMKRSMEPIEISTVYILTMNLDSFVTLLKTSYSVYNLL
>CobsOr220
VNSLKFTFALLAVGGCWRPSTWTSPIKHKLYNAYALLIISLMYSFTITQFMEAVLNISSPDDLTDVFYTMVAL
FSACWKVLNMWVNHESFADLIQGLTKGTFKPLLSIEIEIQRKFDKTIQNNTKRYFLLIIITFFGHVLVSLMTS
FRKRQLPFRGWVPYNYSSFAIFCFTYGHQYVGIIVSCFIHVACDSLIVGLLMNLCCQITILRHRLRAIVNGQT
TLGDCVLQHCHIIKYAYMTNARCTTIISFQFVASTFLACSNLFHLSRITLNATFIILFAYTFCILIQIFIYCW
FGNKIKLMSYHLVDDIFESDWVMLNNKTKKGLLIIMMRAMKPIEFISAHVINMNLNSFVALIRTSYSIYNLLV
QMQD
>CobsOr221
ILKHTLMLMMVAGCWQPLSWISSFKNILYNAYVLLLMLLLYAFAISQIMAIIVNTANPEEFTGVLYMMMAVFV
GIFKISSMWISRKNIANIVNTLTERPFLPMIDDEVKIHRTFEKLIRNNTLFCFVLVESTCVVIALTSFLTNFR
TGDLTYKVWLPFNYSSSTLFPLVFTHQMISMAICALINLACDCFICGLLMHICCQIEILECRFNKSRTWTDLR
DCVRHHNLIFDFASVVNERFARIIALQFVSSMLVVCSNLYQLAQTTLNAKYLPLVLYTVCMMIEIFIYCWFGN
EVKLKSLQMIDRIFEVDWSESNNRFRKAYLMIMNRATIPIEFTSAYLFSMNLESFVSVLRLSYSAYTLLQRF
>CobsOr222
MQTMQFPLKILTVAGCQPPKSWSSLCKRTLYNMYTILMCLLLITFMVPQILDIILNVDNPDDFTDTFYIMLAM
FIACCKMISLLLNRKNIKMLINALVQKPFRPLEPNEIEIRQKYDNIVRIHSISYTVLIELTCGFMNLTSLFTD
FQKGKLAYREWIPYESDAVFYFTYFRQLISLTVASIVNVACDIMIWGLLVHIYCQIEILECRVKKSLRGQGDL
GECIRQHNRIYKFAYTMNEKFRFIIAVQFIASMLVMCSSLYRLAKTTLSMKYIPLIMYTFCMCMQIFIYSWYG
NEVKLKSIQFSDEIFGMDWIAADKKAKESLILIMHRSLTPIEFSSAHAITVTLDSFVKLIKMSYSIYNIL
>CobsOr223
MQVLDFTLKILTIVGCWPPNSWTSMYKRVIYNIYTVFVILLLFTFMLPQLMDVVLNVDNTDDFTDTFYILLAM
IISCCKMVGLLINRKNIEILTNILTQKPFIPLEADEIKIRQKFDKTIQINTLRYTVLVEATCACVALTSLFTD
FRKGNLTYREWTPYNYTSEAIFCVIYVRQLISTTIGSMVNVACDSLICGLLLHVCCQIEILECRLKKISHGQN
SLRDCVRQHDCIFKFAFMINEKFKIIIAIQFVVSTLVVCSNLYQLAKVTLSAQCFPLILYTCSMLTQILIYCW
YGNEVKLKSVELVTNIFEMGWLTMKESKRQNLLIIMNRSLIPIEFSSAYILTMNLDSFVSLLKTSYSAFNLLQ
QM
>CobsOr224
MRILHLTFKILTFIGCWRPQSCSSFYFRIIYDAYTIFMIILLYTFLISQFLDIIWNVENAEDFTENFYATLAS
VVSCSKMLSLLVNRKNINTLINVLVEEPYKSLNVDEMRIQYKFDRIIYINTLCYTILVETTCACITLTSLFTV
FRKGHLTYRAWLPYDYYSSTFVFYITYAHQLVSLTAGSLVNVACDSLICGLLVHICCQFEILGYRLKKISNDH
NILRNCVRHHNSILEYALILNSKFKMTIAMQFVVSTLVVCSNLYQMTKSTSLNASYLPLLLYMSCMLTQIFIY
CWYGNEVKLKSTQLLDNIFAMNWVTMNKNLKESLLIIMNRATIPIEFTSAYILSMNLDSFVGLLKTSYSAYNI
LKQV
>CobsOr225
MPILQYTFKLLTICGCWRPDSWSSLNKRIAYYIYSVIIVLSMYTFMLTQLMDMILIVDNADDFSDNFFILIAM
FLTCCKVLVVLVNRNNIVKLIDILMEKPCRPIRSSEIKILYKFDKNIQINTQRFVYLGLVTFLCILLSSLSTN
FRNKKLTYRAWLPFDYSSTLLFFLTYTHQMVGLLTAAFLSIGCDTLICGLLVHICCQIEILLYRLRKIISYSD
IFRDCVRQHHRICRLALIVNAKFRLTITMQFIISTLVVCFSLYQINKTTRKDKYVEMILYMACMLTQIFFYCW
YGNELKIKSHQMIDNIFGMEWLSLDKDKKQSLMIIMRRTLVPIQITCAYIFPMNLSSFMSILKTSYSTYN
>CobsOr226
MRILDSTFKFLTFCGCWRPDSWSSPCKRIIYHMHTFVVVMLINTFTLSQLLDIILTVDNADDFTDNFYVLLAM
VVSCCKLCSMLVNQKNIATLTNILTERPCKPLEQKEMDIYLKYDKSAQTNTIHYAILVESTCVCITLTSLLTD

FRRKTLTFRAWLPYDYSPPMLFYITYVHQLISLIIASILNVACDGLICGFLLHICCQIKILESRLKRIAHKPD
ILNVCIQQHNRIFDFAVTINDKFRFTIASQFIMSTLVVCFTLYQLTRSTSTNAKYIQLALYMCSMLTQIFFFC
WYGNEVKLKSRELVNYIFEMDWCTLDVNVKKALLLIMRRSTMPIEFTSAYIISMNLDSFVGLLKTSYSAYNIL
QQV
>CobsOr227
MFILAFTFKLMMVCGCGFLGTCATPYKRLVYQMYSIFVMLLIHTFMLSQLVDLVMVVDNSDDFTDNFYVLLAM
IVSCCKMFALLINRSNIEMLIGILANKPFRPVELDEMKIWEKFEKLIQSKTLYYTILVEATCLSVTITSLLTE
FKKGNLTFRGWLPFDYSSPRLFPFAYAHQLMSFTMGSVHHVACDSLICGFLVHICCQIEILTHRLKKSAYNPE
ILRECVFQHNHIFKFAHLVNDKFKITIFIQFIVSTLVICFNLYQFTKSSALKTKYMQLILYSCSMLSQIFFYC
WYGNEVKLKSRQLMNNVFEMEWYNFNKSSQKTLLMVVRRAAIPIEFTSASVISMNLDSFVGLLKTSYSAYNIL
QQTQ
>CobsOr228
MRILALTFKILMVCGCWVPDSWRTPYKRLVYHVYTVFIILLIHTFMLSQLMDLILIVDNADDFTDNFYMLLAM
IVSCCKMFTLLMNRSNIAMLIDILEKKPCRPIQSDEIEIQQKFDKHVQTNMLCYAICVETTCLCIAVTSLLTE
FRKGRLTFRAWLPFDYSSSSLFRIVYVHQLISLTAGSILQVACDGLICGLLVHVCCQIEIIECRLRKLADKRP
DILRESVLQHNHVFKFARLVNEKFKLTIVIQFVVSTLVVCFNLYQFTKSTALRAQYIQLIMYMGCMLSQIFFY
CWYGNEVKLKSRQLVDKIFDIEWFELSKHNKQSLLMIMKRSSRPIELTSAYVISMNLDSFVNV
>CobsOr229
MRVLDFTFKILTGCGCWTPNSWTSPCKRLLYRAYTIFIFVLISTFTLSQFIDLILIVDNADDFTDNFYMLLAM
IVSCSKMSCLLINRNNIILLTDILQEMPCKPVEPDEVKIRKKFDKIIELNTLHYAILVLFSTLYIVVQSCFTI
YWEKKLTFRAWLPYNYSTTILFHFTYFHQLIGLIAGALLHVACDSVICGLLLHVCCQLEILKSRLKRITFNPE
ILRDCIVQHNVIIKFAVMVNKKFRLTITLQFVVSTLVVCVTLYQLTKANAKIIQSGLYMSCMLTQIFLYCWYG
NEVRLKSVGLITDLFEIEWLKLGSERQKDFLTITKCGGIPIEFTSGYIIPMNLNSFVALLKTSYSTYNILQQM
RDTS
>CobsOr230
MLTNISREYNVSVVLLSFLGLWPVQSKLTKRFLPPFCFILSISFLPLEILTLYKHGHDGQLMFECLYQIVITL
IFLVKLINQFLNYNKIQRLYESMENHWNIFTDDIEVRILKKYSHVAYQFTVSYAVIMLVMASMFITIPSLGPM
LLDVILPLNESRTKNIAVYSDYGVDQDEYFVPIFVYTTVMIMVGINILVATDTMHVSCTVHACSLFRIIGYEV
ENVISIARMGEQVNNIQRTKTGYERFTEKQIYQEYILCLKKHQLALEYVNILNDTYKYVGISFTLFMGSLFTL
IGVRIVYVLDQIGELIKFAFIIMGAMLHLIIVCYTGQKLMDESENIFHRAYAAEWYNFSPRLKSLLVIICHKS
FVPAKVTAGDLFPLSMEVFATVLRTSGSYFTTLLSLK
>CobsOr231
MTNKKDIWKSRYYLILRTYMTISGTWPYRRLCDRYIHFIPTFIFSSSILIPQIMYVISGGDINNMIEGTTAAM
ISVIFSYKVATVMFSKNVKSCLKMIENDWLSLNTDEEKSILQYHTLHGQYLTTSYAVFMHMTQVFYLLKPVIL
TILETDVANSTKTSASSLPFFVDYGVDIDRFFYPITIHCYLAVFAHVFSTIAVDGLYCTLIQHACGMFSIIGH
MLEDIGKNNNIHFDLTLNKTKDDDYKKALNCLRRHLQAIEFAELIEATFSHILLISVNLNVIGGSMTGIQMVM
NLNKGARDAAAPMAIYIAQLVHIFLQFWQAQFLLDYSVIPYDSICRGNWYYTSKRCQRLFLLIMTRTVSPCKI
TAGKIITLSIEGFGTVLKTMMSYFTVLRSFQ
>CobsOr232
YADKEYDDLIRPIKVAAKFVSYWPLKRNHSTSAKLFRTFHTLLMFFMIISMCAAQTADIIHNLDDLDEMTACA
LMASAFYLSLTRLIVFSLHQKDMLYVVETMRTDWTCLSYEDRIILKEKCLFAFRLAKTFAYMVTFTVGFFAFV
PILETFILGKERILPYRGFFFYNQTVSPFYESIYVFNVMGGFIGGGSIIGATTFNLIVIIHGSAKFAILRKKL
EMINSNDPDADKDVIACIKDHQNAISFADALERIINVLALAQFVISTGLVCFAGFQATSMMENKARLMQYSMF
LNSAILELFMFSFSGNALIDEASIPDAVGESAYCSHWIGGTFSRSLQILMLRSKVPSKITAAKFYSMSLQSFS
QVLSTSFSYIMFLMTVNEE
>CfOr2
NINYQRDMRYIFKPCSWILGSIGIWPITFRGIGQHVSKIALVLCNFALGFAIVPCILHIIYDEKDLNIRLKLS
GLLGFCLTAMMKYCVLAIRRPKILRCIEHVKNDWWQVVKFNSDRELMMKYAATGRRLSIISTTSMYIAGFIYH
TILPFCTVHKAGNETIRPLVYPTYSEFYQTQISPIYEIVYLAHCICGYTMYSVTAGSCGLAAIFVTHACGQID
VITSRLEDLSRGKNFQQSSDVNQRIAAIVSGHVRILRFCAAVDEILQEVCLLEFASSIFTMCLPEYYCIVDWQ
DSDTVGLTTYFLLFVSFCFNMFILCYIGELLMTKSSQIGSVCFMIDWYQLPTKAVRSLVLVIAISNHPIKISV
GRMIDLSLATFGNV
>CfOr4
AMPQNLSYHRDIQYVLKLNNWILCTIGILPFTTRRIGRHVFKILIAFCNFIISFAMVPCALHIIYDQKDITIR
LKLFGLLAYCITAMTKYCILTIRRPKILYCIEFVKKDWWQVTFRSDREQMLKYAAAGRKLTIILTSFMYSSSV
IYYLILPFFSEHIINNETVRPLVFPIYSKFRQFQISPVYEIVYVAHCMCEYTLCSVTVGTCGLAALFVTHACG
QIEVILSRLEDLVNGKNFMQNPNIHRRIAAIVKSHVRVVRFAAVVEEVLQEVCLVELSSSLCTICLLEYYCIV

DWQDDDRISLATYFILFVSFCFNVYMLCYIGELLMEKSSQIGHICYMINWYQISPKFARSLILIIAMASHPIK
ISAGRIADLSLLTFVNILKTTLAYLSFLRTLVM
>CfOr5
SAFRKESAISQNFNYREDIQYVFKLNNWILGSLGIWPIATRGIRQHASKIAITLCNLALAFAIVPCALHIIYD
EKDIIMRLKLFGLLAFCLTAMTKYCILAIRRPKILRCIEYVKSDWWKVTFKTDHTIMLKYATTGRNLTIIGAS
FMYTAGIIYHIILPFCSEHKINNQTIRPLVYPTYSKFRQSQISPIYEIVYVAHCMCGYTIYSVTAGACGLAAL
FATHACGQIQIIISRLENLLEGENFKQSPNVHQRIAAIVKNHVRVVRFAAVVEEVLQEVCLVEFSSSVCTICL
LEYYCILDWQEDDRIGLATYSLLLVSFCFNVYILCYIGELLMEKSSQIGYICYMINWYQLSPKTARSFILMIA
MASHPIKISAGRMADLSLSTFGNV
>CfOr6
MPSAKDEHDSSYRRNIWNVFKLNNWILGLIGIWPVTIRGIGRHAYKIAIAVCNFTFSFALVPCALHIIYDQKD
IIIRLKIGGLLIFGFAAMIKYCILAIRRPKIHRCIEYMKSDWWQVTFKSDRKVMLKFAAISRNLTMIGASFMY
TAGIIYYLILIPFFFEHKVNNQTVRPLVFPIYSKFHQFQISPIYEIVYAAHCMCGYIIYSVTSGTYGLAALFA
THACGQIEIIVSRLEDLLSGESWKQSSKIHQRIAAIVKDHVRVVRFTIVVEEVLQEVCLVEFISSICTICLLE
YACIVDWQQDNKFGLATYSLFLVSFCFNLYILCYIGELLMEKSSHIGYICYMINWYQLSPKSTRSLILIIAIG
SHPIKISAGGMVDLSLLTFGSVLKASMAYLSFLRTLVM
>CfOr7
MPSAKDEHDFSYRRDIRSVFKLNNWILGSIGIWPVTIRGIRRHVYKIAIAVCNLTFSFALVPCALHIIYDEKD
IIIRLKIGGLLLFCLISMIKYCILAIRRPKILRCIEYMKSDWWQVTFKSDREVMLKYAAIGRNLTIIGASFMY
TAGIIYYLIVVPFFFEHKVNNQTIRPLVFPIYSKFHQFQISPIYEIVYVAHCMCGYMIYSVTAGACGLAALFA
THACGQIEMIVSRLEDLVNGARCKQSPKIHQRIAAIVKDHVRIVKFAIVVEEVLREVCLVEFISSVCTICLLE
YSCIVDWQQDNKLSLANYFMFFVSLCFNIYILCYIGELLMEKSSQIGYTCYMINWYQLSPKSSRSLILIIAIA
SHPIKLSAGGMVDLSLLTFGNVLKTSVAYLSFLRTLVM
>CfOr8
MPNVQDEHDSSYREDIRNVFKLNNWILGSIGIWPVAIRGIGRHTSKIAIALCNFALSFALIPCALYIIYDEKD
IIMRLKLCGLLAFCLTAMTKYCILTIRRPKILRCIEYVKSDWWQVTFRSDREVMLKYAAIGRNLTIIGASFMY
TAGIIYYMILIPFFSDNKVNNQTVRPLVYPIYSKFRQSQITPVYEIVYVAHCMCGYTIYSITAGACGLAALFA
THACGQIEMIVSRLEDLLDGTRFKENSEIHQRIAAIVKDHVRVIRFAIVVEEVLQEVCLVEFTSSVCTICLLE
YYCIVDWQEDNKLSLANYFLLFVSFCFNIYILCYIGELLMEKSSQIGYICYMINWYQLSPKSARSLILIIAMA
SHPIKISAGRMVDLSLSTFGNV
>CfOr9
MRDRSQLPEPIIEHNVHYEHDIRYTMQLCRWVLKPIGIWHLIYGHASRNEKLISLALIIMCFSALCFVLIPSG
LHTLFYEKDINIKVKLFGPVGFCLTSTIKYCYLGARGAAFGKCIRHVENDWWAVQYQDHRKMMLKNALVGRRL
TMLCVIFLYTGGLSYHTILPLSSRQVSGNVTHRPLTYPGYNLFFDPEASPVYEIVFCIHCLFALITYNITTAA
CSLAAIFVTHVCGQLQILITLLDDLVEGKRNNNTTVEQRLGNLTRHHMRILKFSDNIEKVLREICLMELVTST
LIICLLEYYCLTEWENSDATAILTYFILLISFTFNILIFCYIGELLVQQYSKIGSAVYNINWYDLSGNKALSL
VLIIMMSHYPPKLTAGKFFDLSIYTFGVVLKTSVVYLNLLRTVTQ
>CfOr10
MHDRSRLPDKIYNVHYEDDIRFTMQLCCWILKPIGIWHFVYGRSSQYKKVLSIMLIFACFSVLCLVLVPSAPH
TLLREKDINNKLKFIGPVAFCLTSAIKYCFLGMRGTAIGRCIEHIEHDWQVIQYQNHRKIMLRNALVGRRLTM
LCVIFLYTGGMSFHTIMPLSSRTKTNGSYTDRPLVYPGYDIYFDSQASPAYEFVFLLHCLSAVIQYSATTAAC
SLAAVFATHACGQLQILMTLLDDLVDGKENKNTTVGKRLILITRHHMRVLRFTTDVEKVLHEICLIELVAATL
IICLVEYYFMTEWENNNAVAILTYFILLISVPFNFLIFCYIGELVVEEYSKIASAAYEVNWYDLSGHKALDLI
LIITMSHYPPKFTAGKFCDLSLNTFSTV
>CfOr11
NHSKSLESIMDGWNYSIQLNRWFLKPVGAWPLTLCETTMEKISCVILSMISCFLICFLLVPCTLGTILVDSDL
DMKIRMIGPISFFLMAVVKQYILIARSERITECIRHIRADWNRITLNREKDRQIMLDNAKFGRWLSFVSAVFM
YSGGFFYTTLMPLCAKRTEIIDNETVRLLSFPIYRGLLDPRTSPSFEIAQFTQTLAGYAIYTLTIGVCSLAAV
FVMHACGQFRILMLKLENLADGKERKSGKTPEERLSDIIQYHIRILSFITRTEELLNEIFFVDVVGCTLNICF
LGFNMMTEWEHRETLGTMTFCSLLISFTFNIFILCYIGELLAEQCTQIGIKSYMINWYYLPNKGALGLILVMS
MSNTTLKLTAGKFMELSLASFCSIMKAAMAYLNLLRTFYV
>CfOr12
MGHDSMNNPDIVASTYLVDNEYSIQIIRWILKAINLWPRSTDISIVEKALSKFLIFVCYFLMITTMIPSGLSI
FMESQETFEDKLRSFGPLTFWFMAMINYSCLLMHVDDISSCIKHVKTDWRLIKKIEDRQLMLRNAKIGRFIAG
FCAVFMHSGVFSYNVARGFSKDVLYMENSSVIVRALPYPFYSKILNAHFSPAYEFVFFLQCFSTFVVNSITVA
ACGLAAVFVMHACGQLKILMSWLDNLVDGQNEERHSMRQRFAIIVKHHLRVLSFVSRTERITNIICLVELVGC
TMHICLLGYYCIMDWVQDNKQNIISYCIILTSVTFNIFIFCYIGEILSEQGEQVGKSAYMTNWYLLPGKTALG
LVLIIILRSNTALKITAGNIVQLSFSTFGDV

>CfOr13
SAQLCSCACEANMKSSLQHVPIIFNYIEDKEYSIQPIRWLLRPISIWPVSNSIKERILSMVLLLLCIFLIIST
LIPCALAIFLDETKDVEAKVHDFGPLSNWALASLKYCSLLMHVGDIRRCIEHIESDWRAVTKIEEREIMLKSA
RIGRFIAIFSAIFVHSGVFSYSIFQAMTLNKTIADNVSVHSLPFAFYDKILDTTRSPAYEIMFMIQCLSTFVV
NSIVIASCCVTSVFVMHACGQLKILMSLLDNFIDENEKRDFSQQKFAVVVEHHLKVLSFVSHIEKITNVVCLV
EIGGCTMHMCLLGYYCILEWNQDSKEGIVAYVIILISVTFNIFIFCYIGEILSEQCDQIGETAYMTNWYLLPG
NSALGFVLIILRSSIVVKITAGKMIELSLSTFGIVIKSALAYLNILRTLI
>CfOr14
MTCSERVPMSIKLTHKNNNDYSLQLTRWFLIPIAAWPQKCTSTTEKISLLAHVLACLFLIVIIMVPCLLYVSL
EERDIQIKLSAMGPLSHWIMGIINYWFLLTRSDDIRECVRHMEMDWKLVRRIDDQDMMLRYAKIGRFIAGFCA
VFMQSGTLLFVVAKAMTSITILVGNVTTSMHPMTCPIYTKFIDTRFSPANEIMLVVELLSCFIVNSITVGACS
LAAVFAMHAYGQLNMLFSWLNNLVMDENKGNEYAEQKLAAIVEHHLRVLSFISRMENIMQNICLVELVGCTMN
MCLLAYYSITNWSDFDAAKIMSYIVVYVSMAFNIFIFCYIGEILTEQCKNVGEKAYMTNWYDLPHKTALGLVL
IIARSSNVIKITAGKLFQLSIATFGDVIKTSVVYLNILRTMTP
>CfOr15
MIYSERIPTSIKFTYKNNNDYSLQLTRWFLTPIAAWPRICTSTIDRVSLQAHILSCLSLITIIMVPCLLYVSL
EEKDIQIKLSVMGPLSHWIMGTINYWLLLMRSEDIRECVRHMETDWKLVRRIDDQEVMLRYAKIGRFIAGFCA
VFMQSGTLLFVVAKAMTSITILVGNVTTSMHPMTCPIYTKFIDTRFSPANEIMLAVELLSCFIVNSITVGACS
LAAVFAMHAYGQLNMLFSWLNKLVADEENEYADQKLAAIVEHHLRVLSFISRMENIMQNICLVELVGCTINMC
LLAYYFIT
>CfOr16
MTYSEHEATNIQLTYKNNNDYSLQLARWFLMPLGIWSRISMTTKAEKFISYVHILVCSFLMTIVTVPFFLYVW
LEEKDIEIKLSMIGPLSHWIMGMINYCLLLAYTNDIRKCVQHMEMDWRLIKNNEDQQVMLQQAKVGRFVSGIC
AIFMQSGTFLFAIVKSLTTTIVIVGNETISMRLMACPIYSKFIDTRFSPANEIMQVIEILSTFIVNSVTVSIC
SLDAIFAMHAYAQLTVLFSWLNKLVDKNNENNFAGRRLAIIVEHHLRVLSFISRMESIMQNICLVELLGCTMN
MCLLTYYFITNFNTLDVAKLMSFVIIYLSMAFNIFVFCYIGETLTEQCNNVGEKVYMINWYELPHETALGLVL
IIARSNNVIRLTAGKFFQLSVATFGDVSITYTSHL
>CfOr17
MCRSVTKELHDHSVQLNRWFLKPIGAWPRSTTTSSSEKAVSRALIFVCYFLIAFTVIPCALNIVLEEKDVELK
LRAIGPLSHWLMGGMNYCSLLLRSADIHRCMRHMEMDWRIIRRSQHREIMVRNAKLGRFVAGFCAIFMHGGVF
SYSIVSGMTTVMVPIDDNRSVPMLQLPCPSYSKFVDARFSPANEIVLIMQLFSCFIVNSTTVGACSLAAVFAM
HACGQLDILTLCLDKLVEGEGVKKSDTVQRRLADIVDHHLRVLRFIARIEDVMHQICLVELVGCTFNLCMLGY
YSITWWNKIDAKSIAAYIIVYISMSFNIFIFCYIGEILTQQCKKVGETAYMTDWYRLPHKTALGLILIISRSS
SVIKITAGKLIQLSVATFSDVIKTSLVYLNILRTVTT
>CfOr18
KVMSASDRSDEYHNYSIQLNRWFLKPIGAWPESRATTIADRILSRIIQITCYILIAFTVVPCMLYFYFDEQEL
DIKMNSVGPVSHWIMNGINYSSLLWRGKDIRRCIEHMESDWCTVSRIEDRVVMLKYARFGRSVAGFCAVFMHC
GVFSYSVVNSLSPMIAIIDNQTVIMRRLPCPFYSKLMDTSRDPINEIVLAMQFLSGFIANSITVGACSLAAVF
ATHACGQFAVLYSWLSELVDEEEEKRSVECKLANIVEQHLRVLNFLSSFEKIMNQICLVELVGCTLNLCLLGY
CSIKEWNARNTKTIATYSILFISMSFNIFIFCYIGELITEQCKKVGEMAYFTDWYRLPHKTALGMILIISRSS
AVIKITAGKLIQLSLITFGDVIKTSAAYLNILRTVTM
>CfOr19
MKHTSSKSIVEVYRQDNDYSLQLNRWFLKSIGAWPESTNSMIKNILIKILQLTCHSLIAFTVIPSILYILFEE
KDIRLKLKAIGPTSHCLMGGINYCSLLHHNDRIRKSVEHMESDWRMMKKKQDREVMLKNARVGRVIAGICALI
MQGGVLCYNIARGMSRIIVIVGNKTITTGRLPCPSFNKIVDTRISPIYEVVLVLQCLSTFVVNNVTISACGLA
AVFAMHASGQLDIVMLRLEELVDKKQELTLANVVEHHLRALKFLSRMEAILRQICFVELVGCTFNLCMLGYYT
ITEWYEESMNTIITYIMVLTSMMFNIFIFCFIGELVANQCKKVGEAAYMINWYYLPHKTVLGLILIIILRSRIV
IK
>CfOr20
QKSNVVASTYGYEKDIQLSIQLNRWILKPIGVWPKSAKTSWIEKYGYMLINVMCTSLIGFLFIPCAVYITLEV
EDTYNTLKLSGPLSFCLMAVIKYSSLIFRENDIRRGIEYIKNDWMNTRYYEDRIIMIRNAKFGRRLVVLCAFF
MYGGAVFYYLAMPFSNGKVTESDSNLTYQPLVYPVARVIVDARYSPVSEIFFWVQCLSGFIAHSITAGACSVA
AVFAMHAYGRMEVLMQWIEHLVDGREDLRGNLDDRLSMIVQQHVRILHFISLTEKILREISVVEITGCTLNMC
FLGYYVITEWNNKEPARYITYIVLLISLTFNIFIFCYIGELIAEQCKKISEISYMIDWYRLPKRKGLALVLII
AMSNSSVKFTAGNFFELSLSSFGDVVKTSVAYLNMLRTLTS
>CfOr21
QNSKALTSTYDYKRDVQLSIQLNRWILKPIGVWPKSEKTSWIEKYIYMLVNVICTSLIGFLFIPCATFAALEV
KDTYDTLKLSGPLSFCIMAVIKYSSLIFRENDIRRGVEYIENDWMNTRHFEDRIIMIRNAKFGRRLVKICALF

MYGGAVFYYLAMPFSNSKVMDSDRNLTYRPLVFPVAKVIVDVRYSPISEIFFWVQCLSGFIAHSITAGACSVA
AVFAMHACGRLEVLMQWIEHLVDGREDFHDLDERLSMIVQQHIRILRFISLTDKVLREISIVEIAGCTLNMCF
LGYYTIMEWDSNDPARYITYIVLLLSFIFNIFIFCYIGELIAEQCKKIGEVSYMIEWYRLPGRKSLALVLIIA
MSRSSIKFTAGNYFELSLCTFSDVVKTSVGYLNMLRTLT
>CfOr22
MFESADLHNAKYEDDIRYTVQVHRLILGLIGVWPIFKKSRLQKRFLKGFVRAMCCLLLSFNLIPWALYMFLIL
DTFKSRLKMTGALCFYIMVPTMYCTLILREDSIRKCMKHMEKDWQNVKDENDRKIMLDRAKAGRFILICATLF
LFASGFTYRLIQPIFRGKIIVNGNVTIRPLVQGHYYIFFDPQRSPAYEIVFSIHLLIGIFIYIIMASVCGVTA
LFTMHACGQLEMLSTWLENLLNEPQWSQSHVIARRLAAIILHHIRIRRFLQHIQHLIGEMCFIEIIGSTLVLC
LLGYYVITGWEQNDALSFLTYAIMLVSFTFNIFILCYIGEILNIQANKVYITCCTLDWYWLPSEQARYLILII
AMANYPTKLTAGKVIDLSFSSFGGV
>CfOr23
MKLITERSSKTIQESENHFNYAVQVTRVILRMIGAWPIPKSNAKKIAIRLQNVICYFLFAFILVPGLLLIFLK
ERDVKRKVRLIGPLLNCWMGCMKYSLFIYHAKKIQSCLEQIQQDWQSXRMLTPMNTTVRVLSCPSYFVKFDEQ
ASPAYEIVFTLPGMLTYSVTCGGAGLAAFFIMHVCGQLSVLIRKLQHFNNITELKDRTVATLLANIVEHQIKV
KNFLKQVEEAMQFIWLVEIVGSTILLCLVGYYVIMDWESSDSTAMLTMFVVFISFTISIFTNCYVGQLLTDQS
IKVGLITSTINWYCLSYRRARSLILIMAISNIPIKISAGGMMEMSLPTFSNIIKTSMAYFNLLRKFVT
>CfOr24
MKLITERSSKTIQESENHFNYAVQVTRVILRMIGAWPIPKSNAKKIAIRLQNVICYFLFAFILVPGLLLVFLK
ERDVKRRVRLMGPLLNCWMGCIKYSLFIYHAKEIQSCLEQAQQDWQSTVNWNDRKAMLSKARIGRQFAIFSAA
FMYIGGLSYRTIVPLSKGRMLTPMNTTVRALSCPSYFVKFDEQTSPTYEIVFTLQFFAGMLTYSVTCGAAGLA
AFFIMHVCGQLSVLIGKLQQLDDMTEPEDRTVATFLANIVEHHIKVKKEIFINNIFKNFFMTIFQCIIIEWES
SDSTAVLTMFVMFISFTISIFTNCYIGQLLTDQSINVGLITSTINWHRLSYKRARSLILIMAISNIPAKISAG
RMMEMSLPTFSNIIRTSMTYFNLLRKFVT
>CfOr25
MKLITGQSSTTIRANENHFNYAVQVTRVILRMIGAWPIPKFASNAKKIAIRLQNAFCYLLFAFILVPGLLLVF
LKERDYKRRVKLIGPLLNCWMGCMKYSLFIYHAKEIQPCLEQVQQDWQSTVNWNDQKAMLSKARIGRKFAIFS
AVFVYIGGLSIRTIVPLSKGRMLTPMNTTVRALSCPSYFVKFDEQASPAYEIVFTLQFFAGLLTYSVTCGAAG
LAAFFIMHVCGQLSVLIAKLQHFTDMTEPEDRTVATFLANIVEHHIKVKNFLKQVEDAMQFIWLVELVGSTAI
LCFVEYYVIMEWGNSDSTAMLTMFTHFVMLISFIISIFTNCYVGQLLTDQSIKFGLKTSTINWYHLSYQRARS
LILIIAISNIPAKISAGGMIEISLCTFSNIITTSMTYFNLLRKFIT
>CfOr26
SIYDNEKDMHISLQLNRWILKSIGAWPKSAKTLLIERCVYLLLNVICSSLIGFLSIPCAVYMLLVDDAYHIVK
IFGHLNFCLIAIVKYFLLILREDDICRGIEHIKNDMNTRYYDERMIMIRSAKFSHRLVVICSFFMYGGXVFYY
LAVPFINGKITEDXNLTYRPLVYPVAKVIVDVRYNPVSEIFFMQCLSGFVTHSIIAGACSVAPVFVMHIYGRL
EILVQXKHLVNGREDFYGIVDERLSIIVQQHIGILNFVSLMNKTLREISIVEITELNMCFLGYYTITEWDSSR
SARYITYIVLLLSLTFNTNIFLFFCKKIGEVSYMIEWYRLPGKESLALVLIIAVXRSSIKFTAGNYFELSLSI
SADVVRTSVSLNMLRTLT
>CfOr27
LIILGLISVCCLLPLICTIPWVLYMFLILDTFKSRLKMTGTHCFYIMVPTMYCTLILREDSIRKCVKHMEKNWX
CQSDVNDRKIMLERAGCFILICTXLYFSNGFTYRLIQPIFRGKIIVNXDVPIRPLVHGHYYIFFDLQXGPAYE
IMFSIHLLIDIFVYVIIANVSGVIALFTMHACNXEMLATWLNLLNEAQWSQSHVTARRLAAIILYHIRIHRFL
QHIQHLIGEMYFIEIIGSTFVLCLLGYYVITGWKRNDAHSFLTYAIMFISFTFNIFILSYIGEILNNQRNKVY
ITCCTLDWYLINKLVILXSNCNYPLKLTASKVIDLSFDSFG
>CfOr28
MKLITERSSKTIQENENHFNYAVQVTRIILQMIGAWPIPKFASNAKKIAIRLQNVFCYFLFAFILVPGLLLIF
LKERDFKRRVKILGPLLNCWMGCMKYSLFIYRAKEIQSCLEQVQQDWQSTVNWNDRKVMLSKARIGRKFVIFS
AVFMYIGGLSYRTIVPLSKGRMLTPMNTTIRALSCPSYFGKFDEQASPAYEIVFTLQFFAGMLTYSVTCGAAG
LAAFFIMHVCGQLSVLIGKLQQLDDMTEPEDRTVATLLANIVEHQIKVKKIIVIFFETXXXLCFVEYYVIMEW
GNSDSTAMLTMLTHFVMLISFTISIFTNCYVGQLLTDQSIKVGLITSTINWYCLSYRRARSLILIMAISNIPI
KISAGGMIEMSLPTFSNIITTSITYFNLLRKFI
>CfOr29
AHTIHRNDIRRTVQVTRYVLNLIGVWPSQDNSSGFRLAWIKMFRILCQILLYFIFVPGVLKMFLKERNTRRRL
KMIGPMCNCLMAVLKHAVLICRGDRIKDCIRHIEEDWRKLNLAEDRRIMMGNSRIGRSLAILCVAFVYGSGFS
YRTIMPLSRGVIITLQNVTIRPMGFDGYYVFFDPQKTPAYEIVFIIQFLSGFVQYSVTSGTCSLAALLVLHAC
GQLKILIARMKNLTQIKQFCDKNANQKLAAVVKQHIRIKSFLNKVEEILQYTCLIEVIGCTFILCLLGYYIIM
EWETKNAVSMTTYTILLLTFIFNIFILCFIGELLTDQSMKMYVTSCTLDWYRIPHKTARGLTLMIAVSSVPIK
ITAGKFMDLSLNSFGAIVRTSVAYLNILRTTSI

>CfOr30
HINTFHHDDIKRTVQVTRGVLNLIGVWPSHSTPSAFKIIKINLLRILCQALLYVVFVSGGLKIFFKELNMYRR
LKVIGPMCNILMTILKHIALIYQGKRLKDCIRHIEEDWKKVNSIKDDRRIMVDNSKIGRSLAILCVTFMYGSG
FSYRTILPLSRGVIVTPQNVTIRPLPFDGYYLFIDPQKTPAYEIIFIIQLLSGFVQYSVTSGACSLAALLVLH
ACGQLKILITRMEDLTQIEQFPDKNTNRKLAAIVRQHIRIKSFLKEIEKILQYTCLVEVIGSTFLLCILGYYI
ITEWGDNGMMPIVIYTTVLLTFTFNIFILCFIGQVLTDQSIKVYITSSTLDWYRIPHKTAHGLILIIAVSSIP
IKITAGKFMDLSLNSFGAIMRTSVAYLNLLRTTS
>CfOr31
MFVNKNYKNDIKYSMQLNRFVFHLLGIWPCMETEPSFFENLCRILLILSCYLLLGCELIPTILYVILIEKRTR
VRLKLISSVMFTILAVLKYSSLVFGQNQMRNCLMYVRDDWQNVANSSARDSMIYRTRTARRLLLLCGIFMYSS
GLYFRTVVPLSKGKSINHQNITIRHLPCPSYFIFFDGQISPAYEIMFFIQFFSGFIKYTITVAICSLAALFAM
HLCAQLKILMMLMNNLINEPEEKNLNNRLAAIVEHQTRMRNFLQLVQNTLQYTSLLEVMGCTMIACLLGHDIL
TEWEDQNVIAMFSYLVLLTSIGFNIFIFCFIGEQLSVEGEKLALTACTLAWYRLPDAKARSLILVIAMSNIPT
KLKAGKFIDLSIKTFGDVVKTAVTYLNLIRKVME
>CfOr32
MSGKDDCYYNIIYITQPTRNIMRALGVWPSIGREKSICKKFYKLLLISFSYIFLSCDLIPAIFYWIMEETTRA
RLQIIPVLLYNFMAAGQYGIFIFRDDQIRQCLKHVEDDWRNIINADARSMMLTSARTGRRLVAFCSVLMYGGA
LTFRTILPLSQGTFVTEENITIRLLACPGYYFSIDVQVSPTYEILFVIQCLSGLISASIATGTCGFTAIFVVH
ACGQLKILINLMKKLVDKQWRAGYEVDDKLAELVEHQIRVHNFLRLVQHTLHHVCLMEIMANTITICVLVYLM
LMEWQSNNTAGVCTYLLCLTNVTIHIFVFCYTGEQLTVQAEQVATTSCELEWYRLPSRKARSVVLLMIMSNTP
TKISAGKLIDLSLKTFGQIMKTAGAYFNMLRSITE
>CfOr33
NIIYITQPTRNIMRALGVWPSNDRERSMRLQACNFLLICISYTFLSCDLIPGILFWLMEKSTRIRLQTIPLLL
YDIMSVSQYGIFIFRRDQLKRCLKHIEEDWENIINVDMRNIMLKSAKMGKRLATICGIFMYTGAFTFRTILPL
SQGKTVTDQNITIKPLACPGYFFSFDVYVSPVYEIIFTIQCLTGFIMVSVVISACGLTAIFVVHACGQLKILI
SLMRSLVQESWQEEHEVDKKLAQIVKHQIRMRSFLKLVQHTLQEIYLIEIMVNTITICLLLYFMILDWQSKNI
AILCTYLLCIMNVVVHMFIFCYTGEQLTGQAEKVSIESCELEWYRLPNRTARSVILLMIMSNTPTKLSAGKFA
DLSLKTFGDVSK
>CfOr34
FIYHATRNVLFALGAWPSIDHEESMRPQACNFLLICVTYTLLFCELLPGILYWLMEKSTRIRLQTIPLLLYDI
LSVSQYGIFIFRRDQLGQCLKHVEEDWNNIISADMRNIMWKSTKTGKYLAKICGIFMYTGAVTFRTILPLSQG
KTVTDQNITIRPFACPGYFFSLDIYVSPVYEILFTIQCLTGIVMVSIVISACGLTAIFVVHACGQLKILIGLM
RNLVQESWLEEHEVDKKLAEIVKHQIRVRSFLKLVQHTLQEIYLIEIMVNTIATCVLLYFMILDWQNKNITVL
CIYLLSITNVTTHIFIFCYTGEQLTGQAEKVAIESCELEWYRLPNKKARNVILLMIMSNTPTKLSAGKFVDLS
LKTFGDVSK
>CfOr35
MSWNKYYKDDILYITQVTRNVLSVLGVWPSSNRARSTGKKFFKYLLICASYTLLYGVLIPGIFFWLIEKRTRI
RIQTFPMLLYCFMATSKYGNLIFRENQVRQCLKHIEEDWKIVTSTEARDTMIESAKTGRRLVALCGAFMYGSG
LSFRSILPFAKGKIVTAQNITIKPLPCPGYFFSFNSQVSPTYEMIFAMQFLSGLVTYSITTGACGLAAVFVMH
ACGQLRILIDLMKRLVEEQWQEKEEVDRRLAEVVEHQIRIRNFLRLVEHTLQQICLIELMGCTIIVCNLGYCI
IVEWEKSNTIATCSYFMSLTSMLINMFMFCYTGEQLTTQAEKVANTSCELEWYRLPDRKARGIVLVIIMSNLP
TKITAGKIMDLSLKTFGDVVKTAVTYFNMLRNVTD
>CfOr36
MFANKYYESDIKYTFEINRFFFRLIGMWPFPRMNSFIPEIMETVLLNIACFTLLLSEIIPTIIYIFIVLKDIH
LRLKVMGIMLFTIATTIKYGYVLLYKKEIKNCLKLIDEDWRNVVNSSARILMIDRARIGRRLIIMCAIFVYLN
GVAMRVIVPLSSGKIITPQNITIRPLPSVAHFIIFDVQRTPAYEMVFFLQSCTGIVRYTVTVATFGFITLSVM
HFCSQLDILVTLISNFVNEHKKEYLNKRLAVIVEHQIKTRNFLRLVQNATQYPSLVEIMGSSVLMCFVGYYII
LDWENHNAIRICSYIFAIVMFLFNVFIYCYMGEQVVEQEKKIALTVCTLEWYRLPNEKAKALILIMGISDTSL
NLKAGKFIDLSFKTFGNVVKMTVTYLNLIRSI
>CfOr37
FANKYYESDIKYTFEINRFFFRLIGMWPFPRMNSFIPEIMETVLLVIACFTLLLSELVPTMIYVFIVLKDIRL
RLKVIGSVLFTIITTVKYAYVLFYKNQIKNCLKLVDEDWQNVINSSARNLMIDRVKIGRRLIIMCAVFVYING
VAMRIIMPLSSGKIITPQNVTIRPLPSVAYFVIFDVQRSPAYEIVFFLQSFTGVIKYTVTVATFGFITLCVMH
FCAQLDILVTLMNNFVNEHQEEHLRKRLAVIVEHQIKTRNFLRLVQNVTQYPSLIEITGSSILICFGGYYIIM
EWENHNAIRLCTYIIAVVMILFNVFIYCYMGEQVVEEEKKIALRLCTLEWYRLPDEKAKTLILIMAISNTSLS
LKAGKFIDLSLKTFGNVVKMAVTYLNLLRSFE
>CfOr38

MFANKYYESDIKYTFEINRFFFRLLGIWPFSRRNSFIPEIMETVMLTIACFTLLLSELASTLIYIFVVLKDIR
LRLKVIGSMLFTIATTIKYSYVLLYKNEIKNCLKLVDEDWRNVNSYARNSMIDRVRIGRRLIIMCAIFVYLNG
VGIRIIMPLSSGKIVTSQNITIRPLPSPAHFIIFDVQHSPAYEIVFLLQSFTGVIRYTVTVATFGFITLCVMH
FCSQLDTLVTLINNFGNERKEKHLDKRLAIIVEYHIKTRNFLRLVQNAAQYPSLVEIVSSSILICFGGYYTIM
EWENHNTIRLCSYIIGLVMLLFNVFIYCYMGEQVVEQERKITSTLYTLEWYRLPDKKVKALLLIMAISDTSLN
LKAGKFIDLSFKTLGNVVKMAVTYLNLLRSL
>CfOr39
MFANKYYESDIKYTFEINRFFFRLLGIWPFPRTNCFLPGIVETVLLTIACFTLLLSELASTLIYIFIVLKDIR
LKIKLVGSMLFTIASTIKYGYVLLYKNEIKNCLKLVDEDWQNVVNSSDRISMIDRVRIGRRLVIMCAVFVYLS
GIAVRIVMPLSTGKIITPQNITIRPLPSAAHFIIFDVQHSPAYEIVFLLQSVTGVIRYTIIVAAFGFITLCAM
HFCSQLDILVTLINNFGNERQKEHLNKRLAIIVEYHIKTRNFLRLVQNAAQYPSLVEIVSSSILICFGGYYTI
MDWKDHNIIRLCSYTIGLVMLFFNVFIYCYMGEQVIEQEKKIVSTLCTLEWYRLPNKKAKALILIMAISDTSL
NLKAGKFIELSFKTLSNVFKMAVTYLNLLRSL
>CfOr41
NEYYQDDITFITQLTRNVLSTLGVWPPLNRKRTIMERIHKFLLICITYALLYSVLIPGFLFWFFEKRTHVKIQ
MIPLLLFGFMTVTKYGNLIFRERQIKRCLKHIEEDWRNVINMNARNMMIESAKTAKRLVALCGVFMYSSGLSF
RLILPFAKGKIINAQNITIRPLPCPGYFFSFNSQITPNYEMIFAIQFLSGLVTYSITTGACGLAAVFVMHACG
QLRILIELMRHLVKDQWQKREDANKKLAAVVKHQIRIRYFLQLVEHAMQQICLIELIGGTTIICTLGYCIIVE
WEKSNTIATCSYWLXCMMINMFLFCYTGEQLTTQAEKVAIASCELEWYRLPDKSARGIVLLMIISNLPTKVTA
GKVMDLSFKTYGDVIKTSVTYFNMLRNVVD
>CfOr42
MPCNEHYQDDIIFITQLTRNILSTLGIWPSLSRKKTTRERIYKFLLICISYTLLYSVLIPGFLFWFFEKRTHV
KIQVFPLLLFGFMAISKYANLILREGQIKRCLKHIEEDWRNVTNMNARNTMIESAKTGRRLVALCGAFMYSSG
LSFRLVLPFAKGKIINAQNITIRPLPCPGYFSSFNSQASPSYEIIFAIQFLSGLVTFSITTGVCGLAAIFVMH
ACGQLKILIELMRHLVEDQWQEREDANKKLAAVVEHQIRIRYFLQLVEHTMQQICLIELMGCTTIVCILGYCI
IVEWEKSNTIATCSYFMSIISMMINMFLFCYTGEQLTAQAEKVASASCELEWYRLPDKNARGIVLVMIISNLP
TKITAGKIMDLSFKTYGDVVKTAVSYFNMLRNVID
>CfOr43
MLHNQHYQDDIMYITQLTRQVLSLLGVWPSLNKRKSISERAWKFLLISSSYILLYCVLIPGLLFWLIEKRPRV
RVQTIPLIFYGFMATGKYSILVCSEGRIRRCLKHIEEDWKFLISMHARDSMIESAKIGRRLVTLCAAFMYGSG
LSFRSILPFAKGKIVSAQNVTIKPLPCPAYLFSFDIQVSPIYETVFAIQFLSGIVTYSITIGICGLSAVFVMH
ACGQLKILMDLMRNLVEIQWEEDQELNRKLAAMVEHQIRIRNFLRLVEHTMQQACLIELMGCTTIVCLLGYFI
IMEWENSNSIAMCSYFITLTSLMINMFMFCYTGEQLTVQAERVANTSCELEWYRLPDKKARGIVLVIIMSNLP
TKVTAGKIMDLSFKTYGDVVKTAVTYFNMLLNVTD
>CfOr44
MIDRKQLTNTDRVTKRNICESYISDFYYAIQISLCLLKPIGAWPLDDDKTSRLKVALHKLSMMIATFLLIFTI
VPWIMQIIKEKWSVFLILRTICPLLFMLTVFTSYILLLWHQDEFKFCIDHVADDWRCGIIVEDRNIMLANAKV
GRRFGIVSVAFMFSGGMLYGMLPMVLPNMVNANNVTVRLHPSPCEFLVFDSKTSPAYEIVYFLQLLSGCTAYS
AFCGICSLMAHFVTHVCGQCDVLMAIFEETVDGGKHNDGSIENRIATAVTRHLHLLRLVSNISNLFTEICLVE
FVNASCSICLICYYIVTDLNNNESFIQIFMYLFALASIVFNIFIFCYIGDLLKERCQKVGTACYAIEWYRMSS
KKAIDLIIPIMISRYPATLTAGKMMTMTLMTFSDILKTSMAYFNLLREFSSRD
>CfOr45
LHRKQKVAFVNTHIANKMKQNKNHISDFYYAVQISFRLLKPIGAWPLRQQATKIEVIIHGLSIAVATFLQFFS
VASWITCIITTKWSLYEILRTACPLIFTFTVFLRYLLLLLNQNKIKSCIDQIAEDWRNVTIIEDREIMLANAK
SGRYFGIISIAFMFGSGIPYNCMPLVLPPIVTEDNITIRLLPNPFYEIAYVLVALSCITAYTTFCGICSLTAK
FVTHVCGQCDILMHVFEELIDGGNRNPGTVDQRISTAIIHHLRILKFVSDVDKVLNGICLAEFINASCNICLL
GYYVIMDWNNQESMLQIFVYFVAFISITFNIYIFCYIGERLVDRCQQVGIKCYMIEWYRLPQNKARNLMFPII
MSNYPVELTAGKMVKLTMNSFSNILRTSMAYLNLLREVSSRDIM
>CfOr46
QNDKWILDFYYAAQVSFWLLKPIGAWPLEQRATKIEIIIYSLSIVLAMFFQLFMIIPWIICIVTAKWSMYEIL
RTACPLIFSITVFLRYLLLLFRRDEIRSCIDHVVEDWRNATIIEDRKIMLANAKSGRSFGIISAAFMFGSGIP
YTCMPLVLPSVVTEDNVTIRSLPNPSELLFLDNQVSPIYEIVYVLETLSCCTLYTVFCGTCSLTAKFVTHACG
QCEILMYIFEDVIDGGNRNQGTIDQRISFAITHHLRIFRFVSEVEKILNEICLAEFLNASCNICLLGYYVIMD
WNNHESMLQIFVYFFAFVSITFNIYIFCYIGEQLVDRYQKIGVKCYIIEWYRLPENKARNLIFPMIMSNYPIE
LTAGKMTTMTISSFSNILKMSMTYLNLLREVVSRE
>CfOr47
MSSTNASRTLFRNFNYRSDAEYVVKVSKILLTPVGVWPLYKTDSTFDKMKYVLQTSFMFSLMCFLLVPHIIYT
FFDAKDLTRYMKVIAAQVFSLLGIVKFWTMIINRDDIKHCLQQMEIQYRDVESEEDRSVMVKHAKIGRQFTIM

YLGLLYGGALPYHIIMPLVADKIVNEDNITQLPLPYLSDYVFFVVENSPFYEVLFVTQIVFSTIILSTNCGVY
SLIATCVMHACCLFEISRRHMETFLIDETNDLHKRFGQIIMHHMRALRFTEMIEKSFNFVFLSEMVGCTIIIC
FLEYGVLKEWEDNQMLGTIIYFILVISILVNVFTLSSIGDRLKEESIKIGETSYFINWYALPAKNVNGLVMVM
IRSNRPSTLTAGKIFDVSLQGFCDVCKTSAAYLNFIRMIAA
>CfOr49
MASKRWKDDIAYVTTSYKLVSWPIGVWPLQVYNFYSLLRSILSTCFAAIVVIIPPIEMYMGCTSAGQNVDCIM
LSFCGSLAVLKIILFRIYASNLINNYKSALNDYLTIGNIKERIIMRKHAFIAKIVSFPLLCFSYFCCIVYTLT
PFMNHDENNQNVTAEDIVLEYPIPSKCTMKYFHAPINMYKIFVIIQAISLIVVTNANLGSDALFINVTLHVCG
QMKILRSHFANLDVTCPQIYDYFNKLIQRHIYLIGMIRELAEVISLILLAELFIISICICIMGFQFIIALKDK
DSVMIGQSLMAQSVFLVKLSVYSFIGNYLKSLMEDIGYSIYQIAWYEFPIKLMRNLVFIFMQTEAPYMFQAGN
FILINLTTLVSILKTSFSYLSVLRMMLET
>CfOr50
KRWKNDIAYAMTPFKLIAWPIGVWPLQNYNFYSLLRNIFLTLCGLMVILPSIELCMGCTDAEQNVDCIMLICC
GMLGVLKMIWFRIYANSLIDNYSSAMNDYLTIENVDERAIMRKHAFIGRIVSCPMLCLSYFSCMIYGLIPFLG
YDEGNQINITRSMNEDAILKYAIPSGCTMEYFHAPASMYKAFCLIQVVAMILSTNAHIGNDALFLNITLHICG
QVKILRDHFVDFNVTSPRVHDRFNALIQRHCYLIMLTRKLADMISFILLIELFIISILLCVMGLQFMIALKNN
DTVMMTKSLMVLCTFLSQLTVYSLIGNYLKSQMEDIRFSIYQSAWCDFPAKLMKNLIFIFMQTEYPVALQAGN
FIMINLSTYMSILKSSFSYLSVLRVMLET
>CfOr51
MASCNIWNKEMAHEFSLYRRTMWPLGSWPLDHDRNFAKYRALLVIIIQSIMVIYISIGYNKDGILSIIVDQLV
LASCSMLSIIKITLIRLHRDDLMKNLCNAADNWTCIARQEHRQVMLRYTNLGRFVFFFQMGSAYVVVVSLAFG
PLLSFAMSSSLQNVTRFEEQMELPHEMTCPSDVPIVCYGMYLLQTIQLMFTAMGNVGSDVFLFGICMHLCGQL
EILSLELLQFHKGMKNRYSTRMKMMALTERHCLLLDLADSIVSTLDTILIAQLILHASLICLLGLQLIVSLAV
HDFAVVGTSIMSFNVLMIQLFLYSYMGETLSSKTEAISQAAYLNDWYDLPRNIVRDLCFIIARANVPVHIRAG
KFYNIDFNSFKNVLKASVSYFSVLQIMFTQ
>CfOr52
MASCKIWNKEMAYEFSLYRRIMWPVGSWPFDRNNNFAKFRALFIVITQTIMVIYISIGYNKNAVLNIIVDQFV
LASCGILTIIKITLIRLHRDDLMKNLCNAADNWTCIARQEHRQVMLRYTNLGRFVFFFQMGSAYIVIAPLIVG
SLLSLATSFSLQNVTTSEDQMQLPQEMVCPSNVPVVCYGMYLLQSVQLISTSTGNVGSDVFLFAVCMHLCGQL
EILGLELLRFHKEKENRYWKRMKMITLIDRHCLLLNLAKDIVYLLDVILIAQLILHALLICLIGLQLIVSLAI
HDFFLVWRSIMSFNILMIQLFLYSYMGETLSSKTQAISQAAYLSEWYDLPTNIMRDLYFIIARANVPVRIRAG
KFYNIDLNSFKNVLKASVSYFSVLQIMLI
>CfOr53
MVYWNEDAVYALSSYKALAWFVGTWPIEDNTLYSKLRWLFAIVSEILLVITLLMEVYLACENSSGDPIDTYVV
TASAMLVIVKLTLLRLQRSTLSTNLFSAIQDWCSVEDAKSRDIMIQHARMARIISLSLFYSGFFAFMLYMLRL
LPVVTANERTFYLPTSCLFESVTSLQYVLITFYQVVQLFIAYAGNCCTEGIFVGITLHLCGQLELLMIDFQQI
SRRRHKQKKGSLVEEFVVRHRRLLRLTETIEDTYNIIILTQIFTSAILICITGFGLIESLHVHDTIMTTKSIV
IMIVMLLQSFIYSFAGDNLRDQSEALSFAVYDSNWCDFSTDDIRDLTFIMIKTNIPIRLTAGKFFYVTRATFT
DILKTAVSYLSALRVMIGKEGANN
>CfOr54
MKTHWNNGMDYGFREIRVLMCMFGMWPLQQNNLVCTFRWILTFIIESFTVTSVMIDYFKNCDDIKDSLELFLI
IEASSHALINIIILARIYKKRIAINVSSAIDDWSLSMKKQSYVTMMEYARLGRIIIILSQLMIGIICSFLYFPIA
FIRSKQQVATIGNKTLSLWIFVFPTSCLFKDISYSTYKTIFVMQILQGFIMYVSECIGDSFFFAITMHLCGQL
ELLRISFVEVGRKITGRIHYRNFLGQWIRRHYELIIILARNIEDAFNLNLLIRLSIITVFIAISGMRIIVSVKH
QDYTDVMKSLLFVQYYIIQSFLFTHTSDVLRNKSESIISTIYDSTWYEFSSTAMKDLILIMMRTNIPLQLSAG
KFFYITRSTVTDILKTALTYISFLQATMEE
>CfOr55
MKTHWNNGMEYGFRQIRTMMCMLGTWPLQQNDLVCTFRWILIFIVESFTVTSMLIDYFKNCGDIKDSLELFLI
IEASSHALINIIILARIYKKRIAINVSSAIDDWSLSMKKQSYVTMMEYARLGRIIIILSQLMIGIICASLYFSIA
FIRTEQQVLIGNKTLSLWNFXLPTSCLFKDISYSTYKTIFVMQILQAFIIYVSECAGDSFFFAITMHLCGQLE
LLRISFVEVGRKITGKIHYQNFLAQWIRRHYKLIIILARNIEDTFNLNLLIRLSIITVFIAISGMRIIVSIKHQ
DYTDVMKSLLFVQYYIIQSFLFTHTGDVLQNKSQSIVSAIYDSTWYEFSSTAVKDLMLIMMRTNIPLQLSAGK
FFYVMRSTTTDILKTALTYISFLQATMEE
>CfOr56
MKTHWNNGMDYGFREIRAIMCMLGIWPLQQNDLVCTFRWILIFIVESFTVTSVLIDSFKNCGDINDNLELFLI
IEACFHAWLNVFLARLYKKKIATNVSSAIDDWSLSMKKQSYVTMMEYARLGRIIIILFQLMIGIICAFLYFPIA
FIRSKQQVAISEIKLYLYGIFALPTSCLFKDISYSIYKAIFVMQILQGFIIYMSECVGDGFFFAITMHLCGQL
ELLRVNFVEVGRKITGKIQYRNFLGQWIRRHYELIIILARNIEDAFNLNLLIRLSIITVFIAISGMRVIVSVKH

QEYIDVMKSLLFIQYFIIQSFLFTHTGDVLRNKSESIVSAIYDSTWHEFSSTTMKDLILIMMRTNVPLQLSAG
KFFYITRSTTTDILKTALTYISFLQATMEK
>CfOr57
KTHWNNGMDYGFRTIRVMMCMLGIWPLQQNDLVCTFRWILIFIVETSMLIDSFKNYGDKEQSEFFLLIEACFY
AWLNIILARIYKKRIAINVNSAIDDWSLSIKKQSYVIMMKYALGQIITLSQLMIGIIYAFLYSTAVFIGNKQQ
IVVTIGNDTVFIIHXISSYLFKEISYSTYKAIFVIHILQRLMXISERDSFFFAITMHLCGQLELLRINFVEVE
RKMAGRNHYQNFLRPWIRRHHELIILARNIEDXFNLNLLIRLSIITVFIAISGMRIIVSVKYQDYTDAMKSLL
FVQYFIIQSLFTHTGDVLRNKSESIVSAIYDSTWHEFSXMKNLILIMMRMNIPLQFNAGKFFYITRSTTTDIL
KTALTYISFLQATMEK
>CfOr58
MASPAQWNAETAYVLTIYKYLLGIIGLWVLDEENVFSRIRWFISTMVEMTATISLSLEVIRHCHGYEDAFEAF
LSASSSVISILKLLLHRVNWRDKLILVQAIVHDWTYVKNPHSRDIMLKYARTGRLGFSVMFYVGCASCVFLFS
LFIFANLDLPWISLEQQNYNKTSERRLLLSTYCVFETYTSLAYGFVEVLQTLQIFVNCISQCGNDGFFFDLTM
HVCGQFEVFRVDFVEISSKQSLSRNKLGLLLKRHHRLIDLAHHLQKAYSLVILSQLLMSVMLLCIEGFQLILT
LSIHNRFATMKHFLYIVVLLIQLFLYCFAGQTLECQSQGLAYAIYETPWYNFNVSVIKDFPLMILRAAHPHQL
TAGKFLAVNFDSFKEILKASASYLSVLRVMIDT
>CfOr59
PDGKMSAKRWSDDFAYAFSIHRIFLKIYGLWPLQEQTLFTKIRYVFCVTAQFMILPFVTLDLMWNNENAGTGG
IESILYFVSTVLGMIKHVCIAIGQKKLSINLDAAIDDWLSTKENEETRKIMKKYAARARILTLMLLYSGGGCF
SIYMSAIVFINLKQIFFTDPLSADANTTYWMLLVPSGPLATSITGSQYVILLIFQIVQTSLVCSTQCVIDSFF
FNITLHLAGQVEVLKNKFKIFANDSNTEANYRKKFVSLVDRHGELMKFYQNLEDTFHLLILVQLVMVTIMLAL
IGLRINLCLNEKDHVEAAKSIVVLNYLLMESLVLTYGGDFLQRESEGIFYALYATSWFTLPVKLMKDLHFAMM
RSSIPFRLTGGKFFYVNRETMMYILKTAASYVSVLRIALR
>CfOr60
MKKVRNWNDETHRAINVYEKVLGIIGVWPLNAGEPKSIVRCSIAILIQISTIGSLSLEAYRQCLGTEDMMEAF
LMDLSSVVSLSKLLVIRLTWQHTYILVTSLIDDWSISWDARRREVMTRYTNVGRVVSLTILYLGYASGMSFLF
MAIPFDDLIPWLNASKANNNATVMPTYFLATYCVFGSLSGIVHSCVLLLQAAQIFVNATSHCGNDGFFFGLTM
HLCGQFEVLEMDFADIEVDERVCKRRMRMLIGRHCRLIKLADSLEYAFSMAIFAQTLMSILLLCVEGMQLLIS
LRLNDNIAAIKHIVLILTMLVQLYLYCYAGNQLESISERLAYSVYDSPWYDFDVKIMKNLPMVMLRGVIPHQI
TAGKFLPMNLFSFKEILKATGSYLSVLRMVID
>CfOr61
MIIHRGTTIITTLLVPKLALSCVGIWPVKKKDFFMDLRWIIAVFLEASTVLPMCIYFTEIYLHCNGTKKSFDS
LTTGAAAMLALTRLITPRIHREELLEIVTSMTDDWATQKDKRVRWIMKKYATMSTRVTTLTFILVGIIVSVYT
SMAISAITGKKHGNEIENANTSREDRIRESCVFRSESSRQMFMIVQAMQMLITGISTFGTTSFFFGLAMHLCA
QFDALCVQLSEFRVNQAHRAIAEAVQRHCQLIRLANCMEESFNANILMYLFVTTTLMCIDGFMLIVSLHLGNL
SMIIHNSSVLLLMLIQLSFYTFAGDCLEMRSTALSYATYDCDWYELPTNVARDFQIILMRASIPHQLTAGKFL
PMNMIMFKDILKSTASYLSVLRVMLNE
>CfOr62
MASERWTNDFAYAMTPFKLITWPIGVWPLQVYDIYSLLRCILGTFCASLVVILPSMEIYMGCTDVEQNIDCLM
IICCGFLGALKITWFRIYANSLIINYNSALNDYLTIDNTKDRDIMRKHAFVGRILCSSLLILTYCCCLIYGII
PILNYDISNRINITNEDMILEYALPSRCALKYFNFPSSMYKIFCLIETVVMILASTTNLGNDALFLNITLHIC
GQVNILRIRFINFDVISPRIYDRFNVLVERHRYLITLARELANLISFVLLIELFIISILLCIMGFQFIYALKI
NNTVMMGKSLVVQMLFLTQLTLYSFIGNYLKSQMEDIGLSIYQSAWYSFPKKLARNVIFILLQTKYPVALQAG
NFIIVNLSTYVSILKSSFSYLSVLRIIV
>CfOr63
KDDVACAMTPFKLITLPIGVWPLQVYDIYSLLRCILGTFCASLVVILPSMEIYMGCTDVEQNIDCLMIMCCGF
LGVLKTTWFRIYANSLIINYNSAINDYLTVDNTKDRDIMRKHAFIGRTLCSSSLTIAYISCLTYGIIAILNYD
MLIRNRINITNEDTTLEYVIPSRCTLEYLNFPTYMYHIFCLVETVLILLATTTNLGNDAMFLNIILHVCGQVN
ILRSHFLNFDVTSPGIYDRFGVLVKRHCYLITLARELANLISFVLLIELFTISILLSIMGFQFILALKVNNTV
MVGKSFMVLSAFLTQLTLYSFIGNYLNSEMEEIGLSIYQSIWYNFPRKMAKNVIFILMQTKSSVALQAGNFIE
VNLSTYVSILKTSFSYLSVLRIMV
>CfOr64
MARERWKDDITYAITPLKLITWPIGVWPLQVYDIYSLLRCALGICCASLVVILPFMEIYMGCTDVEQNIDCLM
IICCGFLGALKTTWFRIYANSLIINYNSALNDYHTIVNTKDRDIMKRHAFIGRTICSSLLTIAYICCLTYGLI
PILDYDISNRINKTNEDTTLEYVIPSRCTLEYFNFPTYMYTIFCLIETVVMLLAATTNLGNDALFLNIILHVC
GQANILRIRFINFDVTSPRIHDRFNELIQRHHYVIMLARKLADLISFVLLIELFIISILLCIVGFQLIFAFKV
NDTVMIGKNLIILSGVLTQLTLYSFIGNYMKSEMEEIGLSIYQSAWYNFPRKLVKSVIFILMQTKSPVALQAG
NFIVVNLSTYVSILKTSFSYLFVLRIMLE

>CfOr65
MARERGKDDIAYAIIPFKLITWPIGVWPLQVYDIYSLLRCVLSTCCVSLVVILPSMELYMGCTDVGQKIDCLM
LICCGILGVLKMTWFRVYPNSLIINYNSALNDYLTIENTKERDIMRRHAFIGRILCFSLLAGAYIGGLAYAII
PFVNYAKGNQINITNEDVILEYALPSRCALEYFNFPISMYKISCLIQMIVLILAPTTNFDNSNDALFLNIILH
VCGQVNILRNRFIKFDVTSSRIDNRFNELIQRHRHVIMLARELADLISFVLLIELFIISILLCIMGFQLIFAL
KVHDTVMISRSSFILSVFLIQLTLYSFIGNYLKSEMEEIGHSIYQSVWYNFPRKLIKSVIFVLMQTQSPVALQ
AGNFIVINLSTYVTILKSSFSYLSVLRIML
>CfOr66
MTREQWKDDVAYAITPFKLITWPIGVWPLQVYDIYSLLRCALGICCASLIVILPSMEIYMGCTNVDRNIDCLL
LICCGILGVLKTTWFRIYANSLIINYDSALNDYLTIDNIKERLIMRKHAFVGRILCSSLLTLVYFSCLIYGIV
PILNHDINNQINATNEDMTLEYAIPSRCALEYFHFPTSMYKIFSLVETVVMLLAATANLGNDALFLNIILHVC
GQANILKIRFINFDVTSPQIYDRFNKLIQRHRYVIMLARKLADLMSFVLLIELFIISILLCIVGFQLIFALKV
NDTVMIGKSSIVLSGVLTQLTLYSFIGNYLKSEMEEIGLSIYQSAWYSFPRKLIKSVIFILMQTKSPVALQAG
NFIVVNLSTYVSILKTSFSYLSVLRIMLGV
>CfOr67
RCIDITTKPSASQLLTSVTSTMTREQWKDDVAYAITPFKFIAWPIGVWPLQVYNIFSLLRCVLGICCASLLVI
LPSMEMYMGCNNVERNIDCLLLICCGILGVLKTTWFRINANNLIINFNSAINDYQTINNIKERDIMKKHAFIG
RILCFFFLTIAYCSGLTYTLIPSLNCDKGNQINITNEDMQYNRPSRDLEYLIGISNLPTYIYIIYSVIENIVL
ILATTANLGNDALFLNITLHVCGQVNILRIRFIKFDVTSPRICDRFNELIQRHHYVIMLARELADLINFILLI
ELFIISILLCIMGFQLILALKFNNTAIVGKSCLLLSGLITQLTLYSFIGNYLKSEMEDIGLSLYQSTWYNFPK
KITRNVVFILMRAKAPVALQAGSFIVINLSTYVSILKTSFSYLSVLRIML
>CfOr68
WNDDIAYVFSTHRALMKIVGIWPLQKKTRFTIMQRSLAIFMKLMIFSFLFMELTGNHRDASTSIETILYFACT
MILTLKNFCIIANQRKLARNIDGAISDWLSAKNDEESYKIMKEYAFKSKMFTSVILYSGFICTALYIFAVIFI
NVKQRFFQDTYISGTMEWVFLIPSGDLSKIITGSQYLILIIFQNFQLLILCLMQCVSDSFYINITLHITGQLK
ILKAKFKTFASKPDNVENNRKHLSKLVNRHCKLAELNKNIEDTFHLIILFQLVIVTLLLALLGLRIIFSLQNN
DYIELAKSVLVLNFMFMEALVYCSGGDLIQRESEDIFRAMFMTSWFTLPATLMKDLRFAMMRSSYPFRLTGGK
FFYVNRETIIYVLKTAASYVSVLRIAL
>CfOr69
MASERWKDDIAYAMTPFKLLTWPIGVWPLQVYNIYSLIRCVLATCCMSIIVTLPTMEIHMGCTDAGQNIDSIM
LIFCGILGVLKTVCFRIYAENLTNNYGSARNDYLTIRNTEHRAIMRRHAFMGRLLSCFMVCFSYFSVTVYSLI
PLLGDDEDNQMNVTDEDIVLEYPMPSRCALEYFSVPESLYKIICLFEFIVLILTCTCNLGNDSLFLNITLHVC
GQVKILKASFIDFDVSSSQVYDRFNALIKRMSYLIDMAKELANAISFVLLMQLFLSSILLCIMGFQFILAFKM
NDIVMMGKSLTVLCTFLTQLTVYSFVGDYLKSQMEEVGLFIYQSVWYDLPGKLSKNLIFIIMRAQSPVKLQAG
NFIVVNLATYMSILKTSMSYLSVLRVMVET
>CfOr70
MTNNRWKDDIAYAMTPLKLITWPIGVWPLQVYDIYSLLRCGFGTCCAILVVILSSMEIYMGCTDVEQNIDCLM
IICCGLLGVLKTTWFRIYPNSLITNYDSALNDYLTIEDTKERDIMKKHAFVGRFLCCSLLGISYFNCLIYGIV
PILDYDMNNQINITNEDMTLEYAIPSRCALEYFNFPMSMHKISCLVETVIIILSTTTNLGNDILFLNIILHVC
GQVNILRVHFINFDVTSPCIYNRFNALIRRHRYLIALVKELADLISFILLIELFIISVLLCIMGFQFIFALKV
KNIAMVGKSLIALSLFLSQLSLYSFIGNYLKTEMEEIGFSIYQSAWYSFPKKLARNVIFILMQTKSPVALQAG
NFIAVNLSTYVSILKTSFSYLSVLRIMLE
>CfOr71
MAIEQWKEDIAYAMTPVKLFTWPIGVWPLQVYNIYSLLRCIVSTCFASLVVILPAMEICMGCTDVTQSIDCLM
VICCGLLGVLKLIWFRHYANSLIVNYNSALNDYLTIDDTNERKIMRKHAFMGRILCSCLMSLAYLPNLVAGIA
PILVYDNQINITNESIALDYAMPSKCALEFFHFPASMFKISCLLEIVVMLTGATANIGNDVLFLTITLHVCGQ
VNILRSHFINFDVTSPRICDRFNALIQRHQELITLARELSDLMSFVLLIELFIISILLCIMGFQIIIMLKVND
MVMVGKSLMALSAFLIQLSLYSFIGNYLKTEMEEIGLSIYQSAWYSFPTKLARNIIFILMQTKSPVALQAGNF
VVVNLSTYVSILKTSFSYLSVLRIMLE
>CfOr72
MTSEQYKNDIAYAMTPCKLMSWPVGVWPLQVYDIYSLLRCVLGTCWTSLIVILPAMEICMGCTDVMQSIECLM
TICCGSLGVLKATWFRIYANSLIMNYSSALNDYLTIDNIKERKIMRKHAFIGRILCTSLMTIAYSSNLLAGIS
KILAYDSNRINITNESIILDYVIPSRCALEYLNFPTSMFKIVSLVETAALMLGSTTNLGNDLLFVNITLHVCG
QVNILRLNFINFDVTSPRICDLFNALIQRHQELTTLARELSDLMNFVLLIELFFISILLCIMGFQIIIMLKNN
DIVMIGQSLIIISAFMIQLSLHSFIGNYLKSEMEDVGLSIYQSAWYSFPTNLQRNVNFILMQTKSPVALQAGN
FIVVNLSTYVSILKTSFSYLSVLRIMLE
>CfOr73

MTSEQYKNDTAYAMTPFKLITWPIGVWPLQVYDLYSLLRCALGTCCASLVVILPSMEICMGCTDVTQSIECLM
IICCGLLGVLKTTWFRIYANSLIINYSSALNDYLTIDNIKERKIMRKHAFIGRILCSSLMSIAYFSNLISGIV
PILDYDSNQINIINESMTLEYAMPSRCALEYFNFPTNMFKIFCLLETVAMIFASTTNLGNDALFLNITLHVCG
QVNILRLNFINFDVTSPRICDRFNALIQRHQELITLARELSDLMSFVLLIELFIISILLCIMGFQIIIMLKDN
DIIMIGKSLMTLSAFLIQLTLYSFIGNYLKSEMEEIGLSIYQSAWYSFPTKLQRNVNFIVMQTKSPVALQAGN
FIVVNLSTYVSILKTSFSYLSVLRIMLE
>CfOr74
MAIEQWKDDITYAMTPIKMITWPIGVWPLQVYDIYSLLRCIVSTCCASLIVILPSMELYMGCTDVEQNIDCLT
IICCGLLGLLKTTWFRIYANSLIINYDSALNDYLTIDNAKDRDIMRKHAFIGRILCCSMMGFSYLSCLTYAII
PFFDYVHSNRINMTNEDKMLKYALPSRCALEYFNFPTSMYIISCLIETVVVIFSTTTNLGNDALFLNITLHVC
GQVNILRLHFMNFDVTSPRIYDRFNVLIQRHCYQELITLARELSDLMNFVLLIELFFISILLCIMGFQFIFAL
KVNDIVMIGKSLMTLSAFLIQLTLYSFIGNYLKSEMEEIGLSIYQSAWHSFPRKLARNIIFILMQTKSPVTLQ
AGNFIVVNLSTYVSILKTSFSYLSVLRIMLE
>CfOr75
MAIEQWKHDMVHAMTPFKLITWPIGVWPLQVYNIYSLLRCALATFCASLIVILPSMELYMGCTDIEENIDCLT
LICCGLLGVLKTTWFRIYANSLIINYNSALNDYLTIDNIKERDIMRKHSFVGRILCLSLLVFTYFSCLIYGVT
PFLNYNQDNFINLTNEDTVSKYAIPSRCALEYFNFPTSLYKISCLIEAVILIIAATTNLGNDALFLNITLHVC
GQVNILRLHFMNFDVTSPRIYDRFNALIQRHCYLIMLVRELADLISFVLLIELFIISILICIMGFQVILALTV
NDIVMIGKSLMTLSAFLIQLTLYSFIGNYLKSEMEEIGISIYQSAWYSFPRKLARNVIFILMQTKSPVALQAG
NFIVVNLSTYVSILKTSFSYLSVLRIMLE
>CfOr76
TMARERWKDDIAYAMTPFKIITWPIGIWPLQIHNAYSLLRSILSTCCASLAVILPSMELYMGCTDVDQNIASL
TIISCGLLGVLKMAWFRIYAKNLIDNYNSALNDYLTIENTKERDIMRKHAFIGRFLCCSMCGFCYFGCVMYGI
IPLLDYDKNNQIKNENMILEYGLPSRCALEYFNFPTSMYEISCLFETVIMILAATANVGNDGLFLNITLHICG
QINILRIQFINFDVTSPRIYDRFNALIVRHQNLITLARELADLISFILLIELFIISILLCITGFQLIFALEVS
NTVMIGKSLMALSVFLIQLTLYSFIGNYLKSEMEEIGLSIYQSAWYNFPTKLARNVIFILMQTKSPVMLQAGN
YIVINLSTYVNILKTSFSYLSVLRIIV
>CfOr77
MARERWKDDIAYAMTPFKIITWPIGIWPFQIYNIYSLLRSALGTCCASLIVILPSMELHMGCTDVEQNITCLT
IICCGLLGMLKMGWFRIYAKNLIDNYNSALNDYLTIKNTKERDIMRTHAFIGRFLCCSMLGFSYFGCVIYGVT
PFFYNPDNRINITNRDTILKYPIPSRCVLEYFNVPTGMYKISCLVQAVILTIAATANFGNDGLFLNITLHVCG
QINILRIHFINFDVSSPQIYDRFNALILRHQNLITLARELADLISFVLLIELFIISILLCITGFQFIFALKVN
NTVMIGKSLMALSLFLIQLTLYSFIGNYLKSEMEEIGLSIYQSAWYSFPKKLTRNVIFILMQTKSPVMLQAGN
YIVINLSTYVNILKTSFSYLSVLRIIV
>CfOr78
MTSEQYKNDIAYAMTPFKLITWLIGVWPLQVYDICSLLRCVFGICCAILFVILPSMELYMGCTDMEENIDCLA
LICCGLLGVLKTIWFRINANSLIINYNSALNDYLTINNIKERDIMRKHAFIGRILCFSFVVFTYFSCLIYGVT
PFLNSNQDNFINITNRDTVLKYAMPSRCALEYFNFPTSMYRIYCLIEVVILVIAATTNLGNDLLFLNITLHIC
GQVKILRLRFMNFDVTSPQIYDRFNALIQRHCYLIMLARQLTDLISFVLLIELFIISVLICIMGFQVILALKI
NDIVMIGKSSITLSAFLVQLTLFSSIGNYLKSEMEEIGLSIYQSAWHSFPRKLARNVIFILMQVKFPVVLQAG
NFIVINLPTYVSILKTSFSYISILRIMVGV
>CfOr79
MISEQYKNDIAYAMTPFKLITWLIGVWPLQVYNIYSLLRCVFGICCAILFVVLPSMELYFGWTDIEKNIDCLT
LICCGLLGVLKMTWFRINTNSLIINYNSALNDYQTIDNIKERDIMRKHAFIGKIFCSSLLAFTYFSCLMYGVN
SFMNYNQDNRINITNRDTILKYPIPSKCVLEYFNFPTSMFKIYCLIEIVILVTAATANLGNDVLFLNITLHIC
GQVNILRLRFMNFDVTSPRIYDRFNALIQRHCYLIMLARKLTDLISFVLLIELFLIGILICIIGFQLILALKV
NDIVMIGKSVITLSGFLIQLTLFSSIGNYLKSEMEDIGLSIYQSAWYSFPRKLTRNVIFILMQVKSPVMLQAG
SFIIINLPTYVSILKTSFSYISVLRIMVGV
>CfOr81
MANERWKNDIAYAMSPFKLITWPIGVWPLQVYNIYSLFRCSLGTCCAILFVIFPSMEVYLGCTDVEQNITCLT
TICCGLLGVLKSTWFRLYANNLINNYDSALNDYLTIDNTHDRDIMRKHAFVGRIICCSMLGFSYFNCVIYAIT
PFLNYDQGKGINVTNEDKILEYALPSRCTLEYFNVPRSMYKISCLIESIVMVLGTTTNLGNDALFLHITLHVC
GQVNILRLHFINFDVISPRICDRFKALIQRHQHLITLVRELADLISFILLIKLFMISILLCVMGFQFIFALKI
NNIVMIGKSVVALSLFLTQLTLYSFIGNYLKSEMEEIVLSIYQSAWYSFPRKLARNVIFIIMQAKSPVALQAG
IFIAINLSTYVSILKTSFSYLSILRLMLE
>CfOr82
MASERWKNDIAYAMVPFKLITWPIGLWPLQVYNIYSLLRCVMGTCCASLIAILPSMELYLGCTDVEQNIVCLT
SICSGLLGMLKTIWFRIYAKSLINNYNSAVNDYLTNDNTKEREIMRRHAFIGRILCYSMLGFSYISCVIYGII

PFFNHYRDTWINITNEGVILEYAMPSRCALEYLNFPTSMHTISCLLETIITILATSANLGNDVLFLNIILHIC
GQVNILRIHFINFDVISPRICDRFNVLIQRHQHLITLARELADLISFILLIELFMISLLLCTTGFQFIFALKV
NNTVMIGKSLMVQIIFLTQLTLYSFIGNYLKSEMEEIGLSVYQSAWYSFPRKLTKNVIFILMQTKHPVVLQAG
NYIVVNLITYVSILKTSFSYLSVLRIMLGV
>CfOr83
MVSERWKNDIAYAMTPFKLITWPIGVWPLQVYDIYSLLRCGFGTCCASLIVILPSMELYMGCTDIEQNIVCLT
SICCGLLGTLKITWFRIYANNLTNNYNSALNDYLTIDNTKDRDVMRKHAFIGRILCYSMLSFSFSSCLIYGIN
PLLHYNQGNRINVTNENTILEYAIPSRCALEYLNFPRSMYKILCLVEIIIMILAAIANTGNDALFLNITLHVC
GQINILRIHFFNFDATSPRIYDRYNALIQRHQDLITLAKELAELISFILLIELFIISILLCMMGFQFIFALKV
NNTVMVGKSLVVVSVFLSQLSLYSFIGNYLKSEMEEIGLSIYQSVWYSFPRKLAKNVIFVLMQTKCPVALQAG
NFVVVNLSTYVSILKTSFSYLSILRIILGV
>CfOr84
MASERWKDNIAYAMVPFKLITWPMGVWSLQVYNIYSLLRCIVSTCCASLIAILPSMELYMGCTDIEQNIICVT
TISCGLLGVLKTIWFRIYAKSLINNYNSAVNDYLRIDNTKEREIMRRHAFIGRILCYSMLGFSYISCLIHGII
PYLNYFQGNWINITNEDMMLEYAVPSRCALEYFNFPTSMYTISCLLETIILVLAASANLGNDALFLNITLHVC
GEINILRIHFFNFDVTSPRIHDRFNALIQRHQELITLARELADLISFVLLVELFIISIILCMTGFQFIFALKA
NNTAMIGNSLMLQIIILTLTLYSFIGNYLKSEMEEIGLSIYQSAWYSFPRKLVKNVIFILLQTKYPIALQAGN
YIVVNLSTYVSILRTSFSYLSVLRIMLE
>CfOr85
MVSERWKEDIAYAMVPFKLITWPIGVPLQVYNIYSLLRCIVGTCCASLIAILPSMELYMGCTDVEQNVVCLTS
ICSGLLGVLKTVWFRIYANSLINNYNSAVNDYLTNDNTKEREIMRRHAFIGRILCYSMMGFSYISCVMYAINP
FLHFDQVNQINITNQDIILEYAVPSRCALQYFNFPTSMYKISILVETVIVILATTANIGNDVLFLNITLHVCG
QVNILRIHFFNFDVKSPRIYNRFNALIQRHQDLIRLARELVKLISFVLLIELSIITMSLCMIGFQFIFALKIN
NTVMIGKSLAVLSVFLTQLSLYSFIGNYLKSEMEEIGLSIYQSACYNFPRKLTKNVIFVLMQTKYPVALQAGN
FIAVNLSTYLSILKTSFSYLSVLRIILEV
>CfOr86
MASERWKDDIAYAMIPFKLITWPIGSWPLQVYDMYSLLRCGLGTCCASLIVILSSMELYMGCTDVEQNIVCLT
TICCGLLGVLKITWFRIYANSLINNYNSALNDYQTIDNTKERDIMRKHAFIGRILCYSMLGFSYSSCVIYAIN
PFLHYDQDNRINITNEDIMLEYAIPSRCALEYFNFPTNMYKISILVETVLVILATTANIGNDVLFLNITLHVC
GQVNILRMHFFNFDVKSPRIYNRFNALIQRHQDLIMLARELVDLISFVLLIELFIITILLCMIGFQFIFALKI
NNTIMIGKSLMVLSVFLTQLSLYSFIGNYLKSEMEEIGLSIYQSAWYNFPRKLTKNVIFVLMQTKYPVALQAG
NYIVVNLSTYLSILKTSFSYLSVLRIILEV
>CfOr87
IFYLHKFCYTCCCYSLVVILSFMEVYLGCTDVEQNIDYCLTIICCGLLGVLKTTWFRIYANSLINNYDSALND
YLTIDNTKDRDIMRKHAFIGRILCCSMMGFSYFSCLIYAITPFLNHDQADQINITNEDKILEYGLPSRCTLEY
LNFPTSMYKTVCLVEIFIMILATTTNLGNDALFLNITLHVCGQVNILRIRFVNFDVKSPRIYDRFNVLIKRHR
YLITLARELADLISFILLMELFIISILLCIMGFQLILALKNNNTVVVGKSLMILSAFLTQLTLYSFIGNYLKS
EMEEIGLSIYQSAWYNFPRKFTKNIIFILMQTKYPVALQAGNFIVVNLSTYVSILKTSFSYLSVLRIML
>CfOr88
SERWKNDIAYAMTPFKLISWPIGVWPLQVYDIYSLLRCALGTFCASLVAILPFMELYMGCTDIEQNLDCLTLI
CCGFLGVLKTTWFRIYANSLINNYDSALNDYLTIDNIKERAIMRKHAFIGRILCYFLLGFSYFSCLMYAIIPF
LDYEQDNRINITNRDVVLKYTVPSRCALEYLNFSPSMHKISCLVEAFIMIMGTSANPGNDALFFNIALHVCGQ
INILKIHFANFDVKSPQIYDRFNVLIQRHRYLIMLARELADLISLVLLMELFIISLLLCIIGFQLILALKINN
TVIVGKSLMILSTFLTQLMLYSFIGNYLKSEMEEIGLSIYQSAWYNFPGKLARNIIFILMQTKYPVALQAGNF
IIVNLSTYVSILKTSFSYLSVLRIIL
>CfOr89
MASERWKDDIAYAMTPFKLISWPIGVWPLQVYNFHSLLRSILSICFAVFVVTLPFIEMYMGCTNAGQNVDCIM
LSCSGTLAVLKILWFRIYANNLINNYKSALNDYLTIEDIEERTIMRKHAFAARIVSFPLLCFSYFCCIVYTLT
PFMNHDENNQNVTAEDIVLEYPIPSKCTMKYLHAPINMYKIFVIIQAISLLTVTNANIGNDALFINITLHICG
QVKILKTHFANLDVACPQICDDFNKLIQRHIYLIGMIRKLADVISFILLVELFIISVCLCIMGFQFIIALKNR
DTVMMGQSLMAQTVFLITLSVYSYIGRYLKSQMEDIGHSIYQNAWYEFPVKLMRNLVFIFMQTEGPAMFQAGN
FIAINLSTLVNILKTSFSYLSVLRIMVDT
>CfOr90
MASKRWEDDIAYAMTPIKLVTWPIGVWPLQVYNFYSLLRSIVSVFLSATVMFLPPIEIYMGCANAERTVDCLM
LSFSGLLAVLKITFYRIYANNLINNYKSAVNDYMTIDNAKERDIMRKHVFLSRIASFPLLFCSYYSCIAYTLV
PFMNHGEINQNVTDENIMLEYPVPSKCAMKYFHVPISMHKIFVIIQGIALVAVTNANKGNDALFINITLHVCG
QMKILRSHFANLDVTSPQIYDNFKKLIQRHIYLIGLIRKIIDVINYTLLLELLITSICLCTMGFQFLIAMKEK

DTAKMGQSLMIQIVYLVSLTGYSFIGRYIKSQMEDIGYSIYQIAWYEFPMKLMRNLVFIFMQAEGPAMLQAGN
FVVVNLSTLVNILKTSFSYLSVLRIMLEI
>CfOr91
MASERWKDDIAYAMTPIKLITWPIGVWPLQVYNFYSILRSIFGICLSGIVMILPPIEVYMGCTNAEKSVDSIM
FTFAGLLGVLKTIWFRIYANNLINNYKSAVKDYIAIKNVEERIIMRKHVFMSRIASFPLLFCSYYAAVVFTLT
ALMMNHDERNVTDENMMLEYPLPSKCTLKYFHAPTSMYKIFFIIQSMSLIIVSNCNKGSDALFINIILHICGQ
MKILRSHFANVDATNSQIYDRFKKLIQRHIYLIEMTRKLVDVMSFILLLELFITSICLCVMGFQFIVALKEKD
VVKMAQSLMVQIVFLVTLSVYSFIGRYLKSQMEDIGYSIYQIAWYEFPMKLTRNLVFIFMQTEGPAMLQAGNF
IVVNLSTLVNVLKTSFSYLSVLRIMLDT
>CfOr92
MGWKDEVAYAMTPLKILTVPIGGWPLQEYNKFALARHILSICGLSVVVIVQALELYYNCTGANANLDALTLLA
CGILALLKIIWFRIYADNLICNYSSAMNDYLAIDTEQKRAVIQKHASWGRMISIIALLITYVDSLIFIVGHAS
VSSEEGKINITILGHQAGYAVPSTCTLAHFHISKSTYLVIFMLEYIYLMIMCTSNHGSDSLFLHITLHICGQL
KILKTSFINFDVTSPDVYKRFKQLILRHDHLIRMARKLAEIVSFVLVVQLFISSMLICIVGFQFIVALTTSDF
SMMSKSFMVLSAFLAQLTVYSIVGDYLKTQMEEVAQSVYQCAWYNLPVKLTRNIAFIMMWNQFPIKLQAGNFI
VVDLESYMSILKTSASYLSVLHVMMNT
>CfOr93
SLTMMNVLIEPLKNCDVPRDGLEILRLIESCIHGWINIVFSRIYMRKLAININSAIDDWLISFTKKEFRVIMM
AYARTGRLVSLTHVSVASIGSILWFASVFLSNKEKVSTIDNNTAWNFVLPSTCMYKGVSYSTYKALYVMQIVQ
GFLILISECACDNFFFSITMHLCGQLELLRIQFIEICGKRDGEKHRWNILGPLVKRHCQLIELTRNIEDAFNI
NILIRLSIISVVIAASGIGIILSFNSHDYKEMIKMVMSVQFYLVQTFLYTYAGDILQDRSESIIYAIYSSTWH
EMSPIMVKDLIFIMMRMKIPLRISAGKFFYLARTTMTDLLKTTLSYISFLQVTLNK
>CfOr95
KRWKDDVAYAMTPFKLITLLIRVWPLQIYDIYSLLCCILGTVCASLIVILPSMELYMSCTDVEQNTDCLMLIC
CGLLSVLKTTWFRIYANSLIINYNSINYNSALRDYLMIDNTKERDIMQKHAFIGKILCSSLLVFAYFSCLXYG
IIPFLNYNQSNRIITNRNTILKYTVPSRCALEYFNFPTSMYRFSCLVETDIFIEIILCNSKSMHFPKGNDAMF
LNFTLHVCGQVNILKTHFLNFDDTSPRIHDRFNVLIQRHRYLIMLIRKLVDLISFVLVIELFLISILLCIMGF
QLILALKVNNTVMVGKSFMVLSGILTQITLYXFIGNYLKTEMEEIGLSIYQSAWYNFPRKVARNVIFILMQTK
APIALQAGNFIVINLSTHVNILKTSFSYLSVLRIMLE
>CfOr96
MTNERWKDDIAYAMTPFKLITWPIGVWPLQVYDIYSLLRCALGTFCASLIVILPSMEMYLGCTDVNQNVDCLM
MICAGMLCVLKITWFRIYPNSLIINYNSALNDYLTIDNTKDRDIMRRHAFISRALFSSLFAMDYFCCLIYGII
PILDYDINNRSNITNEDATLEYVIPSRCALEYFNFPTSMYKMCCLVETAVMILATTANIGNDALFLNITLHVC
GQVNILRIHFINFDVTSPQICDRFNALIQRHQDLISLTRELADLISFVLLIELFIISILLCIMGFELILALKA
NNTIMVGNKLFALSGFLTQLALYSFIGNYLKSEMEEIGLSIYQSTWYNFPKKLARNVIFILMQTKSPVALQAG
NFIVINLSTYVSILKTSFSYLSVLRIILEV
>CfOr97
MARERWKDDIAYAMTPFKLITWPIGYIVTLAKTTMNFMLIMQRPVENLDLTEXEEIKILLLLLLIFDNTIFGE
MFLSTFCYINYNSALNDYLTINNTKDRDIMRKHAFIGRILFSSLFAMAYLCCLIYGIIPILDINNQINVTNED
TTLEYVIPSRCALEYFNFPPSMYKIWCLVETVVMILATTANIGNDVLFLNIILHVCGQVNILRIHFINFDVTS
PRIYDRFNALIQRHQDLISLTKELADLISFVLLIELFIISILLCIMGFELILALKANNTIMVGNNLIALSGFL
TQLTLYSFIGNYLKSEMEEIGLSIYQSTWYSFPKKLARNVIFILMQTKSPVELQAGNFIAINLSTYVSILKTS
FSYLSVLRIILE
>CfOr98
MARERWKDDIAYAMTPFKLITWLIGVWPLQVYDIYSLLRSVLGTFCASLIVILPSMEIYMGCTDVEQNIDCLM
IICCGILGALKITWFRIYANSLIINYNSALNDYLTIDNTKDRNIMRKHAFIGRILCSSLLAIAYFCCLTYGIV
PILDYDINNRINITNEDATLEYVIPSRCALEYFNFPTSIYKICCLVETVIMILATTANLGNDVLFLNITLHVC
GQVNILKIHFINFDVTSPRIYTRFNALIQRHQDLITLARELSDLISFVLLIELFIISILLCIMGFQLILALKV
NNTVMIGKSLMALSGVLTQLTLYSFIGNYLKSEMEEIGISIYQSAWYSFPRKLTKNVVFILTRIKSPVTLQAG
NFMIVNLSTYVSILKTSFSYLSVLRIML
>CfOr99
MASERWKDDIAYAMTPFKLITWPLGVWPLQVYNIYSLLRCVLCIFCACLVAILPSMEIYMGCTDVGQNIDCLM
LICCGFLGVLKMTWFRIYANSLIINYNSALRDYQMNDDTKERDIMRKHAFIGRTIFSSLLIFAYFGCLMFGIV
PILNYNISNRINMTNEDITLEYIIPSRCTLKYFPTSMYKIFCLVETVLVMLASTINFGNDALFLNITLHVCGQ
IKILRIRFINFDDTNLRICDRFNALIERHRYLITLTRELANLTSFVLLIELFIISILLCIMGFLFIFALKVKN
ITMVGKSLVALSLFLLQLSLYSFIGNYLKSEMEEIGISIYQSTWYRFPRTLTKNVIFILMQIKSPVTLQAGNF
IIINLSTYVSILKTSFSYLSVLRIMLGV
>CfOr100

MASERWKDDIAYAMTPLKLITWPIGVWPLQVYNIYSLLRCVLCTFCACLVAILPSLEIYMGCTDVGQNIDCLM
LICCGFLGVLKTTWFRIYANSLIINYNSALRDYQTIDDTKERDIMRKHAFIGRTIFSSLLIFAYFGCLMFGIV
PILNYNMSNQINMTNEDMTLEYVIPSRCALKYFPTSMYTIFCLIETVLITLASTTNFGNDALFLNITLHVCGQ
IKILRIRFINFDDTSPRICDRFNALIERHRYLITLTRELANLISFVLQIQLFIISILLCIMGFQFIYALKVND
TAMIGKSLVVQILFLTQLTLYSFIGNYLKSEMEEIGLSIYQSTWYNFPKKLARNVNFILLQTKSPIALQAGNF
IVINLSTYVSILKSSFSYLSVLRITLGV
>CfOr101
MTNERWKDDIAYAMTPFKLITWPIGVWPLQVYDIYSLLRCALSTFCASLVVILPSMEIYMGCIDLKQNIDCLM
LICCGFLGVLKMTWFRIYPNSLIISYNSALHDYQTIDDIKERDIMRKHAFIGRIISSFLLSMAYFCCLTYGII
SILDYNINNRINVTNEDTTLEYVIPSRCVLEYFNFPTSMYKICCLVETAIIILVSTANIGNDALFFNITLHIC
GQVNILRIHFINFDVTNPRIYDRFNALIQRHQDLISLTRDLADLMSFVFLIELFIISILLCIMGFELILALKA
NNIIRAGNKLFGLSGFLTQLTLYSFIGNYLKSEMEEIALSIYQSTWYNFPKKLANDVIFILMQTKSAVELQAG
NFIAINLSTYVSILKTSFSYLSVLRIILEV
>CfOr102
SEYAHGWNRYTMMFMGIWPENKNFDRASSYKAVVPILTMFCFICAPQSANLLFIWDDFDLVIENLSMANITIT
ISMLKTAIFWSKGRSMKILISSMKKDWNMTVDKRERKIMSDIAKITRNLSIRSTIMAEIVVIAYVTYRYIVIR
YTGRQLLFRAYFPYNVSNSPSYELTFFAQIIACMYAAVTYAAVDTFIATLVLHTCGQLANLRQELINLHNCTK
TRFQTKLRKIVRKHEYLNSFAETIEDCFNMMLLIQMVGCSLQLCFQCLQAFMAIIGEINEFLAFQIIFLMIYV
VYILLQLYLYCYIGERLLVESTKIAYAAYDCSWYNLSAYEAKSLIIIMCRAQSPLQITAGRFCSFNRELFSEV
LKKSVTYMSCLYAINSL
>CfOr103
LHYAFTLSRQCMRIVGIWPDLDVPLNVCRRPKIAFIFATCIMTLYVLIPQVLNLLRTSGKSVSQMVELFVAPN
ITFMAICKLIITKYHGDKLRILIASSMTDLMTSKNNWERNTMLNLVRTGRKISITYFVIAIAIIIFACYIRLE
NVFQNIHQPRRYLVYRFDYIQKSPNYEITCFIQICGAIYAIFGNYSVDSFISILLLHICAQLINLRTALNNLI
NKLNNKPISSSKFKKGLAAIIVRHEYLIRNVKTINDCYSSVLCIHVLCGSFQLCLVAFQTSTMIADNSNVHLI
KIIFLAIYISFILTQLYVYCYAAERLLMESTNMAFGMYECKWYNIPAKDAKDLMLIVYQSAISLKLTAGIFGN
FSMELFGIAIKTTMGYLSALLTI
>CfOr104
LNYALQLSRQCLRLIGVWPDPHISLSDFRRIRIRFIIAICAVSIYIFTPQAINLIRAWGNVNRMMECFVAANF
SMMAISKLVVTKYHGKKLRTLIASIMTDWMTSNNNSERNKILKLGKNSKNLSFGYFIATVGTTMLAVYVRLEG
VFRNIHKPRRHLPYRFDYIQKTPNYEITCFIQICGGIYTIFGNYSVDNFISILILHICAQLINLQTTLNNLVD
KLKNNPASSSKFRKGLTAIIIRHEHLIRSAKTIDDCYSTVLVIHMMGASFQLCLVTFQIFTMITDNSDFSPIR
TIYLIFFVSLVLMQLYIYCYASERLLTENINMAHTAYDCNWYNILAKDARDLMFIVYRSMIPLKLSAGIFGNF
SLELFGIAIKTSMGYLSALLTIRE
>CfOr105
LSYAFTMSRQCLWLLGVWPDPQVSLNIFRPNIRFMIVTCILSLYVIVPQLTNMIRAWGDVGRMIEYVASANFG
LMALCKLVATWYHGETLRTLMTSIVTDWRISRNNWERDAMLNIARRGRSLSFKCCLAATCTVTFYVSFNLIKF
YRNMYLPQRSLVYRFAYPYNIQKSPNYEITFFIQLSGGVYSAIINCSVDCFISILLLHVCAQLINLRTTLNNL
VSELANRSISSSKFKEGLTAIAIRHQHLIRYAXTSDEIKRYKYLFSNSSLIMKYLNIKFNIFMITDKLDISVI
KITFLTFYITLVLTHLYIYCYSAERLLTESTNIAYGVYECKWYNIPAKDAKILMFIVHGSTIPLKLTAGKFGV
FSIEMFGTTVKTSMGYLSALLTMKD
>CfOr106
LNYAFTTSRQCMRLLGIWPDPNVPLNVFHRSKVGFMLAMCIMSLYVFTPQVINVIRAWGNVSRMVEFFVAANF
SLMALCKLIITRYHGEKLRMIIASIMTDWMTSKSQLEQKTMLKLARSGRSLSFGYFVIVIGTLIAAYYAHIGS
IFRNIHQSRRYLIYRFDYIQKSPNYEITYFIQLCGGTYAIFSNYSVDSFISILLLHMCAQLINLRTTLNNLID
ELNNKPISSWTFRKGLAAIIIRHEYLIRRTKTIDDCYSPVLFVHMLSATFQLCLVTFQIFTMITDNLDVSFIK
IMFFAFYILLVLIQLYIYCYAAERLLTESTNMAYGVYGCKWYNISAKNAKDLMFIVYRSAISLKLTAGKFGNF
SLELFGIAVKTSMGYLSALLTIRE
>CfOr107
LNYAFTTSRQCMRLLGIWPDPNVPLNVFRRPKIEFMLATCIMSVYVFTPQIINTIRAWGNISRVVELFVTANF
SMMSIGKMIITRYHGEKLRLLISSMMTDWMTSTSNWERNIMLKLAKTGRRLNFGYFIAAIGTITFAFYVRLEN
VLQTMHQPRRYLPYRFDYIQKSPNYEITTFIQICGGAYAVLGNYSVDSFISILLLHICAQLINLQITLNNLID
KLDNKSISSLTFRKGLTAIIIRHEHLIRILGYWYSCVSVIIYHIMYYFTILLLIKKYYFQMITDNFNISIIKV
IFLTIYIFLVLTQLYVYCYAAETLSTESINMAFGVYNCKWYNIPAKDAKDLMFIVYRSVISLKLTAGIFGNFS
VELFGIAVKTSMGYLSALLTIRE
>CfOr108
LSYAFALSRQCLRMLGVWPDPCIPLSNFRRPSIRFITVTCILSLYVIMPQLTNMIRAWGNVIHMVEYIASANF
SLMALSKLIATWYHSETLRTLMTSVMIDWVNSWNNPERNTMLRLARRGRSLSSRYYAFATITVSFYMCFNLLK

FYRNIHQPQRRLVYHFVYPYNSQKSPNYEITFIIQLCGGLCTALINCTVDSFISTLLLHICAQLINLRMALNN
LVDELANKSISSSKFKEGLTAIAIRHIHLIRDARTIDNCYSAVLFAHMLAATFQLCFETFQVYTIITDKLDVS
TFKMAFLLFYVILVLTQLYIYCYSAERLLTESSGMAHCVYECKWYNIPAKDAKNLIFIVHGSSIALKLTAGKF
GNFSMEMFGTTVKTAMGYLSMLVTIKD
>CfOr111
FEWATHLIRLMLNPIGIWPKTYKSVYDKFLADLRAISAFIFLIFVGAIPSIHSLVKVWSDMILIIDNLQFTLP
LLTATIKLVIIWWKQKNINLIFYLVTIINMIFEDWMKANTDKELKIMIKRARSIRILTMIAYICVFLGVGLIV
TLPCFGKSVRYIKNVTDSVKILPLQTDYFYDKNRSPYFECTFVAQIFMALICAVCYSGVDNLLGLFIFHLCGQ
MENLREKLINIGQFNSFNDDLVFIVKDHXIRIIKCFDIIERTFTLLLLGLLFYFSTLLYLYGFLIIKIFTEGN
EMSTFRIILLVDICAYFAVGEILVTQCKNIHQAAYEFECYNLIPKEVKTCILIMIRANKPLYITGGKMFPITM
SMFCSLIKTSAGYMLVLLA
>CfOr112
FEWATKLNRFALNIVGLWPKVDENACDRFLSDLRMIFSVLLILFVGIIPAIYSLMRTWDDMMAIIDNLQFTLP
LLIAIIKLVVVWWKKIDLTLALNMIADDWLKAKSKKEQDVMIKCSRIARTILIFGYIFMVLGLNLIVFLPCFG
TSLRYVTNITDPVKILPLPTYYFYDKDQSPFYELTFMAQVLLLIVASVSYISVDNLLGLLVFHLCGQMENLKE
RLINMRQYKTFYDGLTFIVQDHIRLIKYFVIIEDTFTLLLFGLLLYLGTIFCLYGFLLVTVLTEKKEMSMIRL
IYLIAAMLSICGHMCLHCIVGEILITQCEGIYDAAYDYEWYTLEPKKARTLILIMIRTNKPLYITAGKIFPMT
LSMFCNV
>CfOr113
YEWSIKLNRTCLRMLGIWPKNKESKRQKLITNVRVIVMLNIGIWFCIIPSVYSLFKVWGDLMLMIDNLQCTLQ
FMMAMIKFFIMWQKKTDIIPLLNMIKHDWLKPKAVQERNVMMKRVKFASKLTIFGYFTTMMSCLLFTVLPVFG
VSMRYLTNKTDPGRLMPLQTYYFYDRDESPFYEITYALQTISICMFAAAYTGTDCFLSLLVFHVCSQLENLKI
RVIDLDRFNNFENVLPNIIQDHLRLIRLSCLDNVKDFFLFYFAYYIKLFYRISFLQMITQRGDVSVLRLTFIM
TVFINMFAHTCLYCAVGEILVAQCEGVYEAACELKWYMMEPKKARNLLIIILICTNKPMYLTAGKMFPMTMATL
CNLLKTSGGYISVLLAHR
>CfOr114
YKWCIKLNRMCLKMLGIWPKNKETKWEKLMTNMRVIILLNIGIWVCIVPGVYSLVKIWGDIMSVIDNLQYTLP
CVIGTIKFFILWQKKKDILPLLNMIKHDWLEPKTMQERSVMIKRARLACMFTIFGYFIMFMSFFLFTILPVFG
LSLRYLTNKTDPGRLVPLQTYYFYERDKSPLYEITYALQTIGISIVAAAYTGTDCFLSLLVFHVCGQLENLKM
HIINLDKCNNFESVLSRSIQNHIRLIRSINIIDNIFTLILLGALFYFSILFAFYGFLFCTIITQHDNISVLRS
SFIMTTFVNSFAHTCLYCAVGEILLAQCEEVYKAACELKWYMMEPKKARNLLTIMICTNRPMYLTAGKLFPMT
MATFCNLLKTSGGYISVLLAHR
>CfOr115
YEWFITLNRACLRLLGMWPETEKSGRRRWMTDARVIVILIIIIWSSAIPTFHSLIRIWGDITSMIDNLQYSLP
LLISIMKFVLMWQKKNALIPILKMIEKDWTRLKTEKEQAIMVRRAQTARLIMIWGYFVMFISFILVVIFPGFG
MSLRYLTNITDPGRLMPLQTYYLYNVNNSPFYEMTFVLQGFSLMAAAPIYTGTDTFMGFLIFHVCGQLENLRA
RILDLEFNRFDSLLFNVREHIRLIRSIKIIDDTFTLMLLGLLVYFGILFALYGYLFENIMTQGRNLSIARLIY
ILISFMNTFTHMCLYCAVGEVLVIQCNGIYEAVCQYKWYNLKPKQAKNLLFMMMQAKKPLYLTAGKLFPMTMT
TFCGLLKTSGGYISVLLAHR
>CfOr116
YEWFITLNRACLGLLGIWPEIDERNGQKKWMIDARVIVILIIMLWNSIIPTFHSCIRIWGDITSMIDHLQYCL
PLLIAIIKFVLMWQKKNALIPILKMVKEDWTKIKTKKERAIMIRRAQTARLIMIWGYFVMFISFIALVIFPSF
GMSLRYMTNVTNSDRSMFLPTYYMYNINNSPFYEITLILQGFSMMAGAIIYTGTDTFMGFLIFHVCGQLENLR
ARILDMEFNHFELSVNAQEHIRLIRSINTIDDTFTLMLLGLLIYFGIVFALYGFLFIMTQGRNLSIARLIFIL
ISSMNTFTHMCLYCAVGEVLVIQCNGIYDAICQYKWYNLKPRQARNLLFMMIQARRPLYLTAGKIFPMTMTTF
CGLLKTSGGYISVLLAYRN
>CfOr117
FEWAVKLNRCTLKIIGLWPETEQTTWEKCMCNLRVFIVFIIVIFWWKKEAVSSLMDMIADDWVKPKASWEKII
MITRAQTARTITIFMYSMMGICLSISIFMPACGFSMRYLTNITDPGKPLPLQTYYYICDITKSPQYELIYISQ
AVSMFLGVLPYTGIDNFLSLLIFHICGQLDILKNRITHLDKFTNYAKALKNCVMDHTRLIRSIAIIEDTFNLM
LLALFLYFGILFAFYGFLILNVSKAFEXLLIYKILHYAFYTRKNNFIFANIINIILYIYIYCNRIHYAAYFNK
WYTLNPKDARDIIFLMIRTNEPLYLTAGKVFPMTMATFCNLIKTSAGYISVLLTTK
>CfOr118
FEWAVRLNRFSLELIGLWPDCIQTTWKRYMCNLRTLLVFLVVLSVIVIPALHSLIRNHSDIMLVIDNLQFSLP
MTTCLLRIGIFWWKKEAVAPVIDMIANDWMKARKPQEKMTMIAKAQTARMIITFGYTFMISGWIVTVILPMLG
YSVRYLTNITDPGRPLPVQTYYIYDVTKSPQYEITFIFQAIAMFLCIMPYTGIDNFLSLLIFHISGQLDILSN
RLMRLNDIANYNDILKSCVMDHTRLLRTIAVVEDTFNIILLILFLYFGTLFGCYGFVLMSMLADGHHLSISYL

AYFLSVITNTFGHMCLYCAVGEILAAQCDRIHYAVYCNKWYTLDSKDARSVILLMAKSNKSFYLNIGKIFPLT
MATFCNV
>CfOr119
FEWAVKLNRFSLRMIGLWPKTDNNVRKMSVYNFRPLIIILMLTINALIPCIHSLIKIRTNIMLLIENLQFTLP
TLTCIIRLIIFWWKKEAVIWILNMVAEDWLKMKSAQERKVMIKKAQTARIIITCGYCMMMTVFILITVLPISG
ISMRYSYLSNITAPSTLLPLPTYHAYDVTRSPQYELTFVLQTVSMVFATMAYTGIDNFLGLLVFHICGQLEIL
RNRIEHLNKFADIHYTLKNNVEDHTRLLKAIAVIENIFNIMLLVLFLYFGILFACYGFLVINLIEKGNNISIS
HLMYEVCIVINTFSHMCLYCAAGEVLVIQYDQLHYAVYNYNWYTLDPRNARNLIFLMIRSSKPIYLTAGKVFP
MTMATFCNLIKTSASYISVLLTTKT
>CfOr120
FEWAVKLNRISLELIGLWPQLEQNFREKLLCNLRIFVAFLAFWLLIPAIHSLIRIHSNIMLTIDNLQYTLPLI
SSIVRLIIFWWKKRALVIIVNMIAEDWLKSKSLYERNVMIKWAQRARIIVICAFFVMGIACVSLVGTTIFGKS
MRLTPNITDPGRPLLLQSYYLYDITKRPQYEITLMFQIISLFIVIIPYTGIDTFLGLLVFHICGQLDILKNRL
VHLHEQKNFSDILKDSVMCHIRLLRYPVGIVEDAYNIILLVLLLYFAILFAFQGFLLVNLFDEEDIPTIRLSI
LLVGITNIFIHMCLYCAIGEILMTRYDAIHYALYNNEWYFLDPREAKDLILFMIKTGVPVYFTAGKIFPMTIA
MFCSLLKTSAGYISFLLTTRS
>CfOr121
FERAVKLNRISLGLMGLWPTTRQNSREKLMCNLRVLVIFLVIITSLLIPSVYSLIITHSDLMQVVDNLQFIVP
TVNSVIKIMIFWWKKKAIATIINMMIEDWLRSKSTKERNAMVRNAVIARAIIAFAYCLMGLAYVFIIVLPIFG
ISIRCLTNITDAGRSMPIQTYYIYDVTKTPQYELTYIMISISVFCAMTCYAGIDNFLGLVVFHICGQLDFLRH
RFLRMNKFMNFHTILKSCVRDHMRLLRAIAVVEDIYNIIFLALFLCFGLLFSFYGFLIINLLAKENDVSITRL
VYLISNVINIFGHMCLHCAIGEILMAQCDKIYYAVYSQEWYILESSEARDLIPVIIKSRKPVYLTAGKVFPIT
MATFCSLIKTSAGYISVLLRMSV
>CfOr122
FEWAVKLNRIALDLIGLWPKTPQSPRQKLLCDFRVLVVFLGITLGVLIPAIHSLIRIQGDIMLMMDNLQFTLP
AVSCSIRIVIFWWKKEAIVPIMDMIAEDWLRSKTARERRIMIRRARSARIIITCAYGIMLVACFFIIILPGFG
LSMRLTPNITDPGRPMPLQTHYVYDVTRRPQYELTFISQAIYILLAMLAYTGIDNFLGLLIFHICGQLDILKD
RLTYLDKYINSGDMLRSCVAKHTSLLRAIAVIEDTYNITLLALFVYFAILFAFYGFRIISLFDEGNDLSLTHL
MYLISNVFNIFAHMCLYCALGEILVAQCNKIYYAAYSNKWYSVDSRNARDLLPLLIRGAKPIYLTAGKVFPMT
MATFCGLLKTSAGYISVLHTTKS
>CfOr124
FVWAVKLNRLGLELIGLWPRSDETTNNKTKIASDLRIAIIFVIVTFVSGIPLICSLIRIWGDMILMIDNIQVT
LPLLVVSLKLIVMRWKRTALLAIVNMMAEDWTELKTVKERDVMIGRAQITRTIVISGYVLMMLAFVVVIVLPY
FGLLLTRHLTNLTDPGKPLPLQTYYFYDTDPSPQFELTYVIQALTIFLAAITYTSVDAFLGLTILHFCGQLEN
FRGRITILTSYQNFTYILSNIVMKHLRLIRFANMIEDTFTLMMLGLVIYFAILFCLHGFLLLTVIAEETFSFS
QLCFPLMGITILLTHTFLYCGAGEIVTKQCESLYRAMCDLEWYKLKPREARNLILLMRRANQSFRITAGKIFP
LTMTTFCSLLKTSAGYISFLLAKRG
>CfOr125
FVWAVELHRVSLELIGLWPETDKTTKDSFLSDLRVSIIFVIVTFLSVVPLICSLVHIWGDMILMIDNLQTTLP
LLMVSLKLIIIRWKRTAISLLVRMMAEDWVDLKIDRERNVMIRRARAARLVGICGYVLMIFAFTVLIIILPSFG
LHFRHITNHTNHGRLLPLQAYYFYDTEKSPQFELTLGIQAVTMFLGAITYTSVDAFLALTIFHICGQLENFRY
RLANLVSCKDFDSALRDNVQTHIRLIRFAANIEDTFTLLMLGLVFYFGIVFCLFGFLLVTVFTGDNISNMSPQ
RVCFVVIGIVTLLAHTFLYCGAGEIITKQCKAVYRVICDLEWYKLEPKKGRALILLMTRASESFHITAGKVFP
LTMTTFCSLFKTSAGYISFLLANR
>CfOr126
FVWAVELNRFGLELVGLWPRINKVAKDNYTSDLRVGIIFVIIMFISGIPLICSLIRVRNDMILVIDNLQITLP
LIVVSLKLVIIRWKRTAFLSIVKMMAEDWMAPKIDAERNVMIKRAQTARLIVICGYVLMIFAFTSIIVFPCFG
LPFRRLTNLTDRDRTLPLQTYYFYDTDKSPQFELTLLVQAMTIFLAAITYTSVDAFLGLTILHICGQLENFRR
RLISLSSRKDFDCALRNSVIAHLRLIRFANKIEDIFALMILGLVVYFGIVFCLYGFLLLTVVTNAENVSPSRI
IYVIVGVVTLLAHTFLYCHAGEIITKQCDAIYCALYNLDWYKLKSTEARNLILVMIRASEPFRITAGKVIPLT
MTTFCSLLKSSAGYISFLLAKQ
>CfOr127
FIWAIELNRWSLELIGLWPNTDGIVKRKLGPDIRAGFIFIMILFISGIPLVCALTRVWGNMALMIDNLRITLP
ILTVLLKLVIMRWKQSGIILLCIIDMIAEDWLALKLSVERNVMLKYARRARLIMICGFIILISLFILVVILPY
FGLPFRHLTNLTDRSKPLPLQSYYFYDTDKSPQFELTFFAQAVTIFLVIIIYIAVNAFVGCVILHICGQLENF
KGRLNNLISCKNFNRILSNSIVIHLRLIRFANNTENAFTLMLFVLIFQFGIGFCLCGFILLSVMTDENLNVAN
FTLISYMIVAVVSLFMQTFLYCFGGDLITEQCDAVYRTICDLEWYTLESRKARNLILLMLLAKEPFRITAGKI
LPLTMTTFCSLLRTSAGYISLLLAKRN

>CfOr128
FVWTIELNRRSLDLIGLWPNMNEIIKRRVGSDIRAGFTIISLTIILAIPLIYALIRVWGDVGLMVENLRITIP
LLTLLLKFIMMRYKQSVLSSIINMMKEDWMTLKLEVERNIMIKRMRTFRLIVICAYILMICTITAISIFPYFG
LSFRSLTNLTDGDRPFFLQTYYFYDTDKSPQFELTYLYQIITAYLAVIVYISVDSFLGLVILHVCGQLENFRG
RFVNLMPSKEFDKVLSDNIEIHLRIIKYVNKIEDIFNLMMLGSITYFGIIFCLSAFLIIILITNEKINTKNLL
QIYYMVATIIIFFLQTFFYCYLGELMAEQCEKLYRAVYDLEWYQWKSTQARNLILLMIQTQEPFRITAGKIVP
LTMTTFCNLLNTSVSYISVLLAIQN
>CfOr129
FVWAVELNHLGLGLIGLWPTNATKRRIELDIRVGSIFILIMLISGIPSISALMRVWGDLELMIDNLRITLPLI
IVSLKLVIMRWKQTVFSSVLNMMVDDWMVSKLNAERNIMIKYARTARLFAIYTYVVTVSTFSLIIVPPCIGQP
FRYLTNLTDRNKPLPLQTYYFYDTDKSPQFELTFFIQALTILLTLLVYLSVDGSLGLIVLHTCGQLENLRHRL
VNLVSCKDFDRALNSNIMTHTRIIRFVYNIEDIFTFIMLGLILYFAIIFCLYGFQSLIVITEGKINDAGILRV
GYIVFVVVILFVQMFLYCYVGELITEKVTIYYTLCELEWYKLKSSQAKNLIILMIRIQKPLRITAGRIVPLTI
TTFCSFVKTSIGYISFLLTQ
>CfOr130
FVWAIELNRPSLELIGLWPNMDGIVKRRFGSDIRASFAFIMIAFILGIPLVHALMRVWGDMTLMIDNLRVTIP
MITILLKLVIMRWKQSVLLSVINMMKEDWIALKLDVERNVMMKRMRTSRLIVICAYILMVSALIMMNILPCFG
LSFRHLTNLTDRDRPLLLQTYYLYDTDKSPQFELTYLTQIILMILVIIIYTSVDAFLGLVIFHICGQLENFRG
RLDNLIANKDFNKNLSNNVMIHLRLIRFLNFLMLHVFFLMIAKICYLCNMYYVLYVLYVIQMIKNEKINENGT
SFLRIYYILAVIIIFFMQMFFYCYTAELLTKQYEAIYRTVYDLKWYKWEPKQARNLILLMIQTQKPFCITAGK
IFPLTMTTFCNLLKTSAGYMSFLMAIQD
>CfOr131
FVWAIELNRPSLELIGLWPKINGISKRTLGSNIRANFTFIMITFVLGIPLVHALTRVWGDMVLMIENLRITLP
MLSVLLKLVVMRRKQSVLLSVIKMMKEDWITLKRNTERDIMIKHVQISRLIVICAYILMTFTLIMITIFPCFG
LSFRRVTNLTDRDRPLLLQSYYFYDTDKSPQFELTYLTQVIGMFLAVVIYTSVDSFLGLVIFHICGQLENFRS
RLISLDAGNEFNKTLSNNVVTHLRLIRYVDKLEDMFNLMMLGTIIYFGIIFCLSGFVLIVMINNEKINAANFL
RIYYMTAIITIYFMQMFFYCYAGDLITEQCEAVYHAVYDLEWYNWESKQARNLILLMIRVQQPFRITAGKIVP
LTMATFCSLLKTSAGYISFLLAIQN
>CfOr132
FVWAIELNRPSLELIGLWPKIDGISKRSIGPNIRVSFAFITITFFLIIPLVHALTRVWGNMVLMIDNLRFTLP
FLTVLLKLVIMRWKQSVLLSVINMMKEDWIALKLDVERNVMMKRMRTSRLIVICAYFVMVCTSIVITIFSCFG
LSFRRLTNLTDRNRPLLLQTYYFYDTDKSPQFELTYLTQIITLFLGLIIYASVDTFLGLVIFHICGQLENFRG
RLINLIAGKEFNKALSNNIVNHLRLIRYVDKLEDIYNLMMLGSVIYFGIIFCLCGFVFIVMIKSEEINVANLL
QIYYMIAAIIVYFMQMFFYCYAGEIMIEQCEAVYHAVYDIEWYNWESKQARNLILLMIRVQQSFRITAGKMVP
LTMTTFCSLLKTSASYISFLLAIQN
>CfOr133
FASAIELNRLSLELIGLWPTTDRISKKSLGSNIRAAFAFIMITFFLIIPVVHALTRVWGDMTLMIDNLRITLP
MLTCLLKLAVMRWKQSVLLSIINMMKEDWMMLKQDAERDVMMKHMQTSRLIVICAYFMMVCGAFVVIIFSCFG
LSFRRLTNLTDRSRPLLLQTYYLYDTDKSPQFELTYLTQIITLVLGLVIYVSIDTFLGLVVFHVCGQLENFRG
RLINLIAGKDFNKVLSNNVVIHLRLIRYGLHILTFFYAIYNKFLFFIFKFFILINGSYLNSLFMIKNEKVTEN
VTSFLRIYYTIVVIIIFFGQMFFYCYIAELVSEQYEAVYRTIYNLEWYKWKPKQAKNLILLIGRVQVPFHITA
GKIVPLTMTTFCSLLKTSVGYISFLLALQN
>CfOr134
FLWAIDLNRQSLELIGLWPNMDGVVKRRFGPDIRASFAFIMIAFILGIPLVHAFMRVWGDMILMIDNLRLTLP
IFSILLKFVIMRQKQSVLLSVINMMKEDWMTLTQVAERDVMMKRVRISRLIIICSYVLMVFTLIMITIFPYFG
LSFRHLTNLTDRNKPLILQTYYCYDTDKSPQFELTYLTQVIGMFLAIIIYISIDSFLGLVIFHICGQLENFRR
RLVNLDANHEFKEALSYNIETHVRLIRYIDIVEDIFNLMMLASMTYFSFLFCISGFVLIVMINNEKINAASFS
RIYYVIVIIIILFAQMFLYCYGAELITEQ
>CfOr135
FVWAVELHRLGLELIGLWPNCKTDETAKKGHGSDIRMGLAFITVILASGVPLIWALLRVWGDMVLMIDNLRIT
LPLIVVSIKFVIMRWKRTVLLSIVNMMAEDWTTLKLATERDVMIKRARTARLLVIFGYVIMMLAFVMLIILPC
FGIQIRHLTNLTDRNKPLPLQTYYFYDTDQSPQFELTFFAQVITISLAGIIYTSVDAFLGLVILHICGQLENF
RRRVINLVSCKDFNNALSNSVVTHLRLIRFADNIEDTFTLMMLGLLSYFGIVFCLCGFLLLTVVTDKKVSNAG
LPQACYMAVAALILLSHTFLYCGAGELIIQHCNAVYRAVCDLEWYKLESRKARNLIILMIRIHEPFRITAGKI
VPLTMTTFCSLLKTSAGYISFLLAKR
>CfOr136
FKWAVELNRYSLELIGLWPKMEETTREKLMANVRVFLLIIMVTFVCVIPCIHSLIRVWGDLMSMTDNLQFTLP
LVSMIMKLVIMWSKRAALAPILSMIAKDWLRLKTDEERKIMIRCARIPRMIIICGFVIMFASFILLFILPCLG

ITMRYITNVTDPGKPLPLQTYYLYDTDKSPYFELTFLAQGVTLMISAMGYSAIDSLFGLLVFHVCGQLENLKG
RLTDEKDPNFDRVLADAVTDHVRLIRCVKVIESTFTLMLLGLFLYFGTLFSLYGFLLVTVITDGRHLSLVRLL
FLIMVVVNIFGHMCLYCIVGEFLITQCEGIYRAACDYRWYKLEPKQARNLILLMIRANKPLYVTVGKIFPLTM
NAFCSLLKTSGGYISVLLARRD
>CfOr137
LDWAIGINRISLKILGLWPDDKLSHWQKFFANLRAFIIFITMFITSVIPGMLALLRVWGDIVAMADNLQIALP
FSVTVLKFVIMWFRKGDLVPIINMIVNDWIKTKTVQERDTMIKQAKIAKAIVMFGCIMMSFASVALIIPPCFG
YSIRYLTNLTDGAKPLLLQTYYLRDMTESPYYEIAFVAQATSIIMAAISYTGIDNFLGLLVFHICAQLDILKV
RLLNLNDFKSFSIGLSFNIEDHLRLIRLSVDVIDNTFNLMLLALLVYFAMLFCVQGFLLVTIIDGVNGDISFM
RISWLISILINTFVHMCLYCVVGEILIAK
>CfOr138
IEWAIGLNRCMLKIVGLWPQNSKDTREALLSKLRLLFNVITLIFVLTIPALVSLIRVWGDMMLMIDNLQYTLP
LLITVLKISIMWCKKKALSSLIDMIMSDWIRVKVKEERRIMLRRAENTRVLALCGGIMILFAVLITIPTFFGL
TVRHVTNFTDPGRPLLIQAYYLHDVSKSPQFELTFLIQAIALTLSGLSYTGIDNFLGLLILHICGQMENLHLR
LLNLGNNSEFTTTLKFNVKDHIRLIRSVQVIDNTFDLMLLGLLFLFGILFCLHGFLIINIVNQTGHLSFIQLT
SYILASVCVLMHMCLYCAVGEFLVTQSEKIHRATYEYVWYNLEPKTARNLMLIMLRTKKPLYITAGKIFPMTL
ATFCNVSYT
>CfOr139
MTRKSTINRIVMLLLPLYGIWPGRSMILIIRVFWVITIAFIEFCHYLYFSTHLNIQNFFNLVDCLCSFVAYAK
VLIKLVAFWVNQRKLVETLTLIMDDWSDCAKSDIGMRVTMHKAKLSDRITNAILILHTMSIFVYCLGVIIADA
DVTDQTIELPFVNKLTLPFNINIQNTYRLILIAEFVHMIFSNWLLGIVNAILLTLVLHMGGQVEILQSWLSQL
VPKKIGNKEESIVTSTNKIIRKHNKIIQFSENIEILYTYIALLLFASNTILMCSIAFLIVTAIGTPDATEQIL
KSILFFWNTNLEAFIFCYAGEYLSNKSRAIEFATYNCPWYNLKSKDIRILLFIILRSQKELTLTAGKIMDLSL
KSFTSIMNASGSYLSVMLAMQ
>CfOr140
MIRNSSINRTIEVLFTLFGIWPGTSCILLCRIFWIISIIIVQFYHYQYLLTHFYSDDLFNLMDCLSSFLAYTK
VMTKFIAFWLNQREFIKILAMMTKDWHDYVNSEIGMQETICKAKLCERITNAMLLLHTLSVVAYGTRVILANV
DITDRTSQPPYIHKMEFPFDVNTQRVYKMIVIAQFVYVIMCSWAAGAVNALLLTLILHIGGQMNILRSWLMDF
TSNESVKKRTIFTTNQIIEKHQRIINFSEKVENLYTYIALLQFASNTIMICSLAFLIVSAIGTPDATEKIIRS
LLFYAITNLEAFIFCFAGEYLNNKSKAIGIAAYNSAWYNLKPQQTRILLFIILRSQKQLTLTVGKLTNLSLEY
FASIMNASGSYLSVMLAMQ
>CfOr141
MDESNEIHKIIMRKSTINRTTKFVLTLFGIWPNINISYVMFCRIFWSVTILIVLFCHYLYFLTYYHSDDVFDL
LECFSNFLGFFKFMTKITFFWFNQRIFYEILMMMTKDWNDCSKSDIEIHEAVNKAKTSNLIANAVIILHIVTV
LLYGDIILAKIDVTNRTIKLPHIYKIEVPFNINTQRTYKIVLIVELIHVVMCSCGTGILNALLLILVLHVGGQ
INILHCWLTKLISKENKRKSIAIMMKKIIRKHQKIIYFAKNIDSLYTFIAFMQFVSNTIMICIIGFVIITALG
HPNATKKILKAISYYSVTNIEAFIFCYAGEYLINKSKAIGLAAYNIAWYELEPEYNRLLLFVIIRAQKQLTLT
VGKMTDLSLQCFASV
>CfOr142
IMMRKSTINRMTKFVLILFGIWPDVSYVIFSRIFWTVTIAIVLFCHYPYLLAHLHSDDVFDLMDCFSNFLGFL
KLMIKIIFFWLNQRIFHEILTMMAEDWNDCTKNDIEMHEAINKAKTSNRITNVIMIIHTVAVLLYGDLILAKV
DVTDRTINLPHIYKIEVPFSINTQRTYKVVLIVELIHLLVCSWGMGTLNALLLTLILHVGGQINILRYWLTEL
ISKENKSIAIMMKRIIRKHQKIIYFTKNIESLYTFIAFIQFVSNTMMICLIGFLIITALGDPNATKKIVKAIS
YYSVTNMEAFIFCYAGEYLINKSKAIGLAAYNISWYELEPEYNRILLFIILRAQKQLTLTVGKMTDLSLQCFA
SV
>CfOr143
KSTINRTTKFALILFGIWPNISCLMFCRIFWTVTILMVLFCHYLYFLTHYHSDDVFDLMDCFTSFLGFLKLMI
KFAFFWLNQRLFNEILTMIAEDWNDCTKSDIEMHEAINKAKVSSHLANAIITLHTVVVLFYGIGVILGSVDVT
NRTIELPHIYKAIVPFSINTQRTYKIVLIVELIHLITCSWGLGILNALLLILILHVGGQINILCYWLTESISK
ENKHKSIAIIMKKIIQKHQKIIYFAKNVESLYTFIALMQFISNIMMICIIGFLIITALGSSNATEKIMRAISY
YSVTNVEAFIFCYAGEYLINKSKAIGLAAYNLAWYELEPEYNRIILFIILRAQKQLTLTVGKMTVLSLQCFAS
IMNSAGSYLSVLLAMQ
>CfOr144
NESDERYEVMTHKSNINRAAKFVLTFLGIWPDISCVMFYRIFWIVTIFMFLFCHYLYFLAHFHSNDVFDLMDC
FSSFLGFLKIIIYFIFFWLNQRIFKEILTMMAEDWNDCAKNDSEMHEALNKARISNRITNVIMILHILSPILY
GVSMLASVDITDRTVELPHIYKIEIPFNINTQYTYKIMLIVELMHLVMCSLSLGIINVLLLILILHIGGQLNI
LHCWLANLIFKENNCKSIAIIMKKIIQKHQKIIYFAKNVENLYTFIACMQFVSNTVTICIVGFLIITALGSSN

ATEKIMRAISYYSVTNVEAFIFCYAGEYLINKSKAIGLAVYNIVWYELEPEYNRILLFIILRAQKQLTLTVGK
MTDLSLQCFASV
>CfOr145
TESKEVMKHKSTINRAAKFMLTLLGIWPDISYVMFYRMFWTVTMLTFLFYHYLYFLAHYHSNDVFDLMDCFSS
FLGYSKIIMYFVFFWFNQRIFDEILTMMAEDWNDCTESDIEMHETVNKAKMSNHITNVIMTLHVISPVLYGMN
IILANVDITDHTVELPHIFKMEIPFNINTQCTYKVVLIVELIHLVMCSLSLGVINVLLLILTLHIGGQLNILH
RWLAKMIFKENKHKSIAIIMKKIIRKHQKIIYFAKNVENLYTFIAFMQFISNTIMICIIGFLIVTALGNSNAT
EKIVRMIPYYSITNLEAFIFCYAGEYLINKSKTIGLVAYNIAWYELEPKYSRSLLFIMLRAQKHLTLTVGKMK
NLSLQCFASVRFLHIISSYLSVLLAMQ
>CfOr146
EEKYEVMANKSTINRAAKFVLTFLGIWPDISCVMFYRIFWTATLLTLLFYHYLYFLTHYHSNDVFDLMDCFSS
FLGYLKIIIYFIFFWVNQRIFDEIVTMMAEDWNDCTKGDIEMHETVNKAKMSNRITNAIITLHTVGPIIYSLS
IILRVASVDITDHTVELPHIYKMEIPFNINTQSTYKIVLIVELIHLVMCSWALGILNALLLILILHAGGQLNI
LHYWLAKLILKENKRKSIAIIIKKIIRKHQKIIYFAKNVENLYTFIAFMQFISNTIMICIIGFLIVIALGNSN
ATEKIVRMIPYYSVTNLEAFMFCYAGEYLINKSKAIGLVAYNIAWYELEPKYSRSLLFVMLRAQKHLTLTVGK
ITNLSLQCFAGIMNSAGSYLSVLLAMQ
>CfOr147
EKYEVMTHRSTINHMTKFALILFGIWPDTPYVIFFRIFWTVTVAIVLLCHYLYFLTHYHSDDVFDLMDCFSSF
LGFFKLMLKFIFFWLNQRIFDEILTMMAEDWNDCAKSDIEMNEAVIKAKISNRIANAVMTLHTVVPLLYGVGV
ILANVDATDRTIELPHIYKVEVPFIINTQCTYKIVLIVELIHLVMCSWALGILNALLLILILHAGGQLNILHY
WLTKLILKEKHKSIAIIMEKIIQKHQKIIYFAKNVESLYTFIAFMQFVSNTVMICLIGFLVITALGTANATEK
IVRTIPYYSVTNIEAFIFCYAGEYLINKSRAVALAAYNIAWYELEPKYSRILLFIILRAQKHLKLTVGKMMDL
SLQCFASV
>CfOr148
LMMSANTITRSIEIGLRIVGIWPGATYVIASRFFWTTTMLLAQIFQYRHVVLHCNSEDLEQLMDGLSATLSYS
LLFVKLIIFWTKQRIFNNVLARIATDWKECGNAEDCGNAWCNMSNVAGLSHRFSNLIIGLHSVAVMVYGIGVV
VLRSDNDVNDRELFLKMELPFESDLSPVYELVMTTQFLHQITTATVIGVLSALLVTLVLHAGGQIDILRERLL
AILPKSQKEKAAISMVTIGSLIRRHQNIIVFTEQIEDLYSYIALAQFISNTLVICCLGFIIVNSIGEHEGSSM
LVRSLLFYVVINLEAFIFCFAGEYLSIKSKMIGDAAYESLWYNLSPNENRILLFLIMRSQKQLTITVGKFMNL
SLQQFANIIKSSASYVSVLHAV
>CfOr149
MIPQNILNRKVKFIFTLCGIWPGISCVLFYRMFWIITMTIAISYLLSYLLAHLYTAELMDLIDCLCLMLAHIK
VISKCFIFWLNQKSLIEILTLMAEDWDDCTDNDISMRETSKKAKLSDRLVNAIFILHTTTVSAYCIGLFLSDM
DITDQMIELPFIGKLKVPFHINTQRVYKLTIVAQSLHMILCGWVAGITNVLLLTLTLHTASQIDILRYWITQL
TSCESKLIVITIKKIIQKHQKIVTFSENIECLYTYIALVQFVSNTVMICSLGFLIVTAIGNPNAMEQIMKSLL
FYTITNLEAFIFCFAGEYMSNKSKEIGVAAYNSTWYDLKSKDSRVLLFVMLRSQKQLTLTAGKMMDLSLESFK
NIMSASGSYLSMLLAMQ
>CfOr150
MAPKNTFNRTLKFMLIFCGVWPGVSCIVLYRTCWIITVTFILFCHYRYFLTHVYSAELLDLMDCMSSFFAYSK
VIIKFAVFWFNQQKFVEILEMIREDWNDCIDGDVISMRETTRKTKISDRITNAIVTLHTMTIVAYCIGIILAD
ADVTDHTTELPLLNKLEIPFDINTQCMYRFVLIIEFLLMLFYGWAAGITNSILLTLILHTAGQIDIIRCWLMQ
LVPRKEEYKHVSYHIITSKIIQKHQKIISFSKNIESLYTYIALLQFISNTIMICSIAFVIVTIIVYKIIILSI
INSLNNYSKEIGIAAYNSAWYDLKSKDSRVLLFIMLRSQKQLTLTVGKMMDLSLESFTSIMNASGSYLSVLLT
MQ
>CfOr151
MTHKSTINRTLKLMLTLCGIWPGTSCVIICRAYWIIALATDNICHYRYLLMHLHSSDLFDLMDCFSSFLTQVK
FMTKLIIFWLNERKFAEILTIMKEDWNDCCSSDINMRETMCKAKLAGRITNAMFTLHTLTIVGYSIGIFLADV
DVTDHQSELPLLLKVTLPIDIKTKRRYKILLSAQFIHLILSGCGTGLLNALLLTLILHIGGQMDILRCWLNEI
VIKKNKERGESIVTMNKIIRKHQKIINFAEYIENMYTYIALLQFTLNTVLICSLGFLIVTAIGSPDATEQIVR
TLLFYTVTNLEAFIFCFAGEYLKNKSKAIGNAAYYSAWYEMKPENSRNLIFVILRAQKQLTLTVGKIMDLSLE
SFTDIMKASGSYLSVLLAMQ
>CfOr152
MTSGRTINRTIELLLTAFGVWPGISCVLLYRVFWMITLVINQFFHYRYFITHFHVNNLFNLMDCLSSFLAHVK
LTFKIIIFSLKQREFIGILTTMSEDWSCCGDNGIVLHETKRKAKLSSRICNGLIIILHTIAAFAYVIGILLADA
DITDRTTELPLIMKMEYPFVIDTLRKYRLVLTTQFMFVLACSLGAGLFNALFLTLTLHIGGQINILLRWLTEV
RSIEIGRMTKIIQKHQKIISFSEKIENLYSYITLLQFTSNTVMICSLAFLIVTAIGSPDATEQIMRSLLFYAV
TNLEAFIFCFAGEYLSNKSTAIGNAAYNSGWYDMKTADSRILLLIIILRSQRKLKFTAGKMTDLSLECFTNV
>CfOr154

MKVENILSQTIEIWLRIFGIWPDSSCISLRRLFWIIALALEQIFQYQYIIINFYSIEFFEVMGMLGEAMTFSI
LIIKLVIFWCKQRTFSKILTMMAIDWKKSLNTEFSMFVTSNAKLSRRFANVTVALYSMAVIFHSSNIVKHTDE
NKVFNTSMRPLVMNMNLPFDLNQTYVYVLIIIIQFVHLLLCSCATGLLNALLINLILHIGGQVDILCEWLIDI
FPIKKKHSPNLFVIKKIIKKHQQIIKFSEHIEDLYSNIALALFISDTLIICCLGFVMVTSVGTPDAIKIIMRT
LLFYFVMNMEAFAFCFAGEYLSTKSNSIGNAAYNSFWYESNSKNNQITLFLIMRSQKQLVITIGKVMNLSLER
FSSIIKASASYISVLLAMN
>CfOr155
MKVENVLSRTIEIWLRIFGIWPDSSCISLRRLFWIIALAIEQIFHYQYIIIHFYSIEFFEVMGILGETMTFSI
IIIKIVTFWCKQRTFCNMLMMMAIDSEKCSSREFSMSVMTRNAKLSRRFANLTLGLYSMAVILHSSHIIVKHT
GDDKISNTSTRALVMDMNLPFDLNQTYVYVLIIIIQFAHVLLCSCANGLLNALLINLTLHIGGQIDILCEWLM
DIFPIKEKHKPNVIIIKKIIKKHQQIIKFSEYIEDMYSNIALALFVSDTLIICCLGFVIVTSVGTPDAVKIIM
RTLLFYFVMNMEAFAFCFAGEYLSTKSNSIGDAAYNSFWYESNSKNNQITLFLIMRSQKQLTITIGKVTNLSL
EQFTSIIKASASYISVLLAMN
>CfOr156
MKVKNVLSQTIEIWLRIFGIWPESSCVSLRKLFWIIALATEQILHYQYIITYFHSIEFFEVMSVLGETIAFSI
LLIKLVIFWCKQRTFGKVLTMMAIDWEKCLNAEFSMFVTTWNARLSRYFANVTVALHTINIIIYRSVIVKQTD
DKASNTSTRPLLMNMDLPFDFAQTYIYELIIIIQFVYLLLCACANGLLNALLINLTLHVGGQIDILCGWLTEV
FPMKERCENPNLFMIKKIIKKHQQIITFSRHIEDLFSNIAIALFVSDTLIIGCLGFVIVTSIETPDAVKIIMK
TLLFYLVVNIDAFAFCFAGEYLSTKSNSIGDAAYNSFWYESNSKNNRIISFLIMRSQTQLTITIGKVTNLSLE
RFSSIIKASASYISVLLAMN
>CfOr157
ILLRIFGIWPDASHIPLRRVFWTIAIIMEQVLEYKWIVVHFYTNEPFEVMKFLSEAMTYTIMFLKIIIFWVKK
RTFVRILTMMSIDWENRIIDEVSMITTTHNANLCRRFNNGTIILYTIAVLFHISNVYTNFMDEQTSNTSTRPL
VMNMDLPFDLSRTWVYVSVLIIQFIHLILCAYINGLLNILLINLILHVGGQIDILRKWLMEMFPTERRLSEES
SLSPLIVEKVIKKHKRIITFSEYIEEMYTNIAMVLFVSDTLIICCLGYILVASIGTPDGLTIIMRTILFYVVS
SMEAFIYCFAGEYLTNKTSSIGDAAYNSCWYEGNSRESKFIVFLIMRSQKQLTITIGKIMELSMERFTSIMKA
SASYISILIA
>CfOr158
MEMSTVSRVVKIGLRTYGIWPYLPSTALCRLLCIVLLSAAQIFQYRYVLINYHTDSFSNFMDGMSSAMTYSLL
FMKLAILWINERTFSDMLQMMAMDWKNCVLTECSLRITSNKARLSYRFSNWIIGLQMIAITLYSCGVLAVNAG
DVQRMNVSAREHILKMKLPFKVNTFPVYTLVTIFEFFHLMMCGLAISVINSLIITLILHIGGQIDILRDWLLK
AFSKNMITVDRITMKMLITKHQRIIMFSENIENLYTYIALILFVSDTLIICCLGFIIVTSINTPGGLAILVRS
VLYYLVMNLEAFIYCFAGEYLTAKSKMIGDAAYDSLWYDVTSKQSQIIHLIILRSQKRLTITIGKIMDLSLER
FTSVLKISASYVSVLLAIY
>CfOr159
STISRLVKFSLHIYGIWPYVPSTVLFRLYWIMMLSTAQVFQYRYVIVNIYMDDFSELMDGISSAMASSLLYIK
LVLLWSNQRIFFDLLQMMSADWQDRQDTVYNLRIMAKTANAAQRASRWIIGLQIFSVFNYAGGVLANNMDKEE
PYKRELILKMELPFNISTNSIYAAVQSVQFYHLILVAYGITTVNSLLVTLILHVSGQIDILRERLMKVFSKST
VDSEEITIQSLLSKHRQIIIFSECIENLFTYIAFIILLSDTIIICCLGYVIVTSLDMPNVAAILVKSVVFYIT
INIEAFIYCLSGEYLSAKSTMIANAAYDSLWYNFPSKQSRIILFVILRSQKRLTITSGKIMDLSLERFTSVIK
ASASYLSLLLAMY
>CfOr160
QTTISRLVRFNLHIYGIWPYVPSTVLFRLYWIIMLSTAQVFQYRYVIVNIHMDDFSELMDGIGSAMASSLLYI
KLVLLWSNQRIFFDLLQMMSADWQDRQDTINSRVMTETQNAAQRASRWIIGLQIFAVINYTAGVLANNLDKEE
PYKRELILKMELPFNISTNSIYTAVQSVQFYHLFFVACGITTINSLLVTLILHISGQIDIFRERLMKAFSKSV
IDSEEITMQSLIVKHQKIIVFSESIENLFTYIAFMILLSDTIIICCLGYVIATSLDMPNVAAILVKSVVFYIT
INIEAFIYCLSGEYLSAKSKMIGNAAYDSLWYNLPSKQSRIILFVILRSQKRLTITSGKIMDLSLERFTSVIK
ASASYLSLLLAMY
>CfOr161
NEKIDSTNTVCHSVEFGLRAIGVWPDTSYAILRRILYISSMIVFQIFQYRYLIMRFGKEDLFILMDVLSITLA
YSLVLIKLIIFACNAHLLNEIITRIVEDWKRHDVSEEYTMTRIAYLSRRFSNLIITMYMISVFLYATGTLLRY
NSNNQTDARELILKMELPFEMKSTSVYIIVLITQFIHQISAASTTGVLNSLLIILVLHACGQIDIMRHKLSAI
TQKNIERGITETIMKTLIIRHQRIISFSKDIEVLYSSIALIQFVSNTLVICCLGFLIVISIGVPGGSAVLVKS
ILFYVVICLDAFIFCFIGEYLSTKSRMIGDAAYESLWYESNPNLNRNILLMIVRSQKHLTLTAGKFMDLSLQE
FANIVKASASYVSVLHAMY
>CfOr163
VNNEIDSMSTVCQSVEFGLRAIGVWPDTSYAILRRILCISSIIVFQSFQYRYLIMHFNENDLFILMDVLSGTL
IYSLLFIKLIIFMFNAHLLNEIITHVVEDWKRHDTFEESIMIRIAYVSRRISNLIITLYAMTVFCYATSTVLR

YKIGNQTDARELIFKMELPFEIKSTSVYIVVLVILFVYQTSAASTTGILNSLLITLVLHVCGQIDIVQRKLHE
ITRKNIKQNITESIMKKLIIRHQKIILLSKNIESLFSGIAFIELFSNTLIICCLGFIIVVSIGVPGGTTVLLK
SLVFYIMICLDAFVFCFTGEYLSTKSRMIGNAAYESLWYESNPNLNRNVLIMIVRSQKHLQLTAGKFMDLSLQ
QFTNIVKASASYVSVLHAMY
>CfOr165
INNEIGSMNSICQSTEFGLRAIGVWPDTSYAILRRILCISSMAVFQTFQYQYLFMHFEEEDLYILMDVLSGTV
AYSLLLIKLIIFAFNAHLLSEIIAHIVEDWKERDVSEEYTMTRIAYISRRLSNLIIIMYAMTVFLYATSTVLR
YKSTNQTDTRELILKMELPFEMKSTSVYIAVLVIQFVHQTSAASTEGVINSLLITLVLHTCGQINIVQQKLSE
ITQTNIERGITENIMKTLIIRHQKIISFFKNIERIFSNIALVQFVSNTLVICCLGFLIVVSISVPNGTTVLVK
SVLFYIAINLDAFIFCFVGEYLSTKSRLIGDAAYNSLWYDSNLNQHRNVLLMIMRSQKHLQLTAGKFVDLSLQ
QFANIVKASASYVSVLHAMY
>CfOr166
TMNTVCHSVEFGLRVIGVWPNTSYAILRRILYISSMAMIQIFQYRYLIMHFGDEDLFTLMDVLSATLAYSLLF
IKMIIFTFNVHILNKIIACIIEDWKIRDISEEYTMTKVAYISRQFSNLIITMYAMSVFVYATGTLFRYKSSNQ
TDTRELILKMELPFEIKNTLMYIAVLITQFIHQVSGASMEGVFISLLITLVLHVCGQIDIVRQKLNKITRKNI
EQGVTVSNMKTLIVRHQKIISFSKNIESLYSGIALMQFVSNILVICCLGFLIVISVGVPGGSTMLVKSLFFYT
VVCIDAFILCFLGEYLSTKSRMIGDAAYESLWYESNPNQKRDVLLMIMRSQKYLTLTVGKFVNLSLQQFSNIV
KASASYVSVLLAMY
>CfOr167
MSNDMGTMNTVCHSVEFGLRAIGVWPDTSFAALRRILCISSVAVFQIFQYRYLILHFGDEDLFILMDVLSATL
AYSLLFIKLIIFTFNAHMLDKIIACIVEDWKIRDISEEYIMTRIAYISRRFSYLLITMYAMSVILYAAGTLLK
YKSNNQTDTRELILKMELPFEIKSTSVYIIVLFTQFVHQTSAASTIGVLNSLLITLVLHVCGQIDIVRQRLNE
ITRKNIKQGVTVSIMKTLIVRHQRIISFSKNIESLFSSIALVQFVSNTLVICCLGFLIVISIGAPGGSTMLVK
SVLFYVVICLDAFIFCFVGEYLSTKSRMIGDAAYESLWYESNPNQNRDVLLMIIRSQKHLTLTVGKFVDLSFQ
QFANIVKASASYVSVLHAMY
>CfOr168
MNTVCHSVEFGLRIIGVWPDTSYAILRRILYISSLAMFQIFQYKYLIMHFGEEDIFTLMDVLSVTLAYSLLFI
KLIIFTFNAHLLNDIIARIVEDWKKDDVSEKHTMTRVAYISRRFSNLIITMCAIAVIVYAIGSLIRYKSGNQT
NARELLVKMELPFEIKSTSVYIAVLVIQLVHQTSAASIEGMLNSLLVTLVLHTCGQINIVQQKLNKITQTNIK
RGITKNVMKTLIIRHQKIISFFKNIERIFSNIALVQFVSNTLVICCLGFLIVVSISVPNGTTVLVKSVLFYIA
INLDAFIFCFVGEYLSTKSGMIGDAVYESLWYQSNPSQNRDVLLMIIRSQKHLTLTVGKFMDLSLQQFANIVK
ASASYVSVLHAMY
>CfOr169
MNTVCHSIEFGLRVIGIWPDTSYAILRRILCISSLAMFQIFQYRYLIMHFGQEDIFIFMDVLSVTLAYSLLFI
KLIIFTFNAHLLKEIIARIVEDWKRHDVSEKHTMTRVAYISRRFSNLIITMCAIVEFIYATSTLIKYKSSNQT
NARELLVKMELPFEIKSTSVYIAVLVIQLVHQTSVASTEGMLNSLLITLVLHVCGQIDIMQQKLNKVTRKNKE
QDAAKNIMKRLIVRHQKIISFAKDIENLFSTITLIHFISNTLVICCLGFLIVISIGVPGGTTILVKSVLFYAI
ICLGPFIFCFVGEYLSTKSGMIGDAAYESLWYQSNPSQNRDILLMIIRSQKHLTLTVGKFVDLSLQQFANIIK
ASASYVSVLHAMY
>CfOr170
MCIEMSTMNTVCQSVEFGLRVIGVWPHTSCAILRRILCLSSLAIFQIFQYRYLIMHFGEEDIFILMDVLSITL
AYSLMFIKLIIFTFNAHLLHEIIACVVEDWKRHDVSEKYTMARVAYFCYRLSNLIITIYAMSVFVYASGALIR
YKSSNQTDARELLVKMELPFEIKSTSIYIAILITQFIHQMSTASTEGVLNSLLISLVLHVCGQIDIVQQKLNE
ITRQSIEQGAAKGIMKKVIVRHQKIISFCNNIEGLFSNIALIHFVSNILVICCLGFLIVISIGVPGGSIVLVK
SVLFYVAICLGAFIFCFVGEYLSTKSRMIGDAAYKSLWYESNSNQHRDVLIMILRSQKQLTLTVGKFVDLSLQ
QFTDIVKASASYVSVLHAMY
>CfOr171
MNTVCHSVEFGLRAIGIWPHTSYAILRQILCISSMAIFQTFQYRYLFMHFDEEDLFILMDVLSGTISYSLVFL
KLIIFAFNTHLLNEIIVHVIEDWKKRDVSEEYTMTRIAYISRRFSNLIIIIYAMSVLLYAIGTLFRYKNSNQN
DARELILKMELPFEIKNTSVYIAVLITQFVHQTSAASTEGVLNSLLITLVLHACGQIDIVRQKLREITRKNIE
QDATESIMKMLIIRHQKIISFSKNIENLFSIIALIHFVSNTLIICCLGFLIVVSIGVPGGTMVLAKSVFFYLA
ICLDAFIFCFVGEYLSIKSRMIGDAAYESLWYESNSNQNRDVLLMMIRSQKHLTLTVGKFADLSLQQFANIVK
ASASYVSVLHAMY
>CfOr172
MKNGKDSMRAVCRSVEFGLRAVGVWPGTSYAILRRFFCIFSMGVFQTFQYQHLIMHISEKNLPLLMDVLSATI
TYSLLLVKLIIFAFNASLLNKIITVMVDDWKEREISDNYTMTRIAYISRRISNFIIVSHALSVFLYATGTLLR
HRNDNQTDTRELILKMELPFAIDSTSIYVAVLVIQFVHQTSAASMAGVMNSLLITLVLHVGGQIDIVREKLSK
ISRKSIERSTSDSIVKTLIVRHQRIISLSKNIEALFSNIALIQFVSNTLVICCLGFIIVISIGVPGESAVLVK

SVLFYILISLEAFVLCFVGEHLSMKSEMIGDAAYESLWYELNPNQNRDIFFIILRSQKHLTLTVGKVMDLSLK
QFASIVKASASYMSVLHAMY
>CfOr173
MKNEMQSMTTVSRSVEFGLRAIGVWPGTSCAILFEVLSISSMVIFQIFQYRYAIVNFGQEDFTLLMDALSVTF
AYTLLLIKMIIFALNARLLNKIIEHVVKDWEECNILDKYTMTRMAYISRRFSNMIIFSHTISVFLYATGALLK
QKDDNQTDARELIVKMELPFEIEMLSTPIYVTILITQFLHQSSAAAMVGVLNSFLITLVLHACGQIDIVRQKL
SEITRKNDERDATQSIMKTLIVQHQRIIAFSKNIEALFSNIALIQFVSNTLVICCLGFLIVISIGVPDGSMML
IKSVFFYIVMSLEAFIYCFVGEYLSTKSEMIGDAVYKSNWYELSPSQNRDILLMIIRSQKHLTLTIGKVADLS
LKQFADIVKASASYVSVLHAMY
>CfOr174
IILDGTVSRSIEIGLRMIGVWPDSSYAFLHRAFWMITLMMAQTFQYRYFVVHIRTDDLSHLMDGLSTTMSYSL
LLLKLTIFWINRRIFHDILMMMARDRSECATDWAVCSMSRTIYVSHRSSNLIIGLYSMSVFLYGTGVLVAHAD
ETDDDEVDDVQLTVPARELFLKMELPFESNVSPVYEVVMVTQFFHQLAAATIVGVLNALIVSLILHVGGQIDI
MCRGLEEISSDDDVFDLRTTTIKTLIRRHQRIIALSADIETLFSYIALMQFLWNTLVICCLGFLIVTSIGDTQ
GSTMLIKSLFFYVVITLEAFIFCYAGEYLSAKGRMIGDAAYEAKWYNLSPTQSRILLLLILRSQRKLTITIGK
FMDLSLERFTTIIKASGSYVSVLHAMS
>CfOr175
MRATSISTSVEFGFRFVGIWPGLPYGTFTWYTFMTSIVIAMYFEYVYIFDHFDIDDISNLIDALSIALACSLS
FLKLISLWSHRRIFYDILLAMDEDWSDVLNHDRSMLHTMSSNANLSRRCSNVLISINATAAVCYAATSFTRRS
ISLEENFNDSLRVLPLKMQFPFEVNASPFFELLAVAQFFHVVSVAALTATINCMIITLVLHVSGQIDILRRDL
LAICCDEDSQRDSIITSIKHLITRHQRIITFSDNIEELYSDIALMQFLSNTVVICCIGFTIISSLAKDGGTEV
LLKSAIFYVAVTLEAFIFCFVGEYLSAKSKSIGDAAYESLWYYMTPADCRIILFVILRSQKRLTITAGNVMDL
SLEGFTTVMKASASYMSVLHAMY
>CfOr176
IATSTISPALKIGLQCLGVWPNVPYSTVYWFLVMLSTLIVQYFQYLYVFSHFKLSELSNLVDGLSSTLESSLM
FIKVASLWKHRRILHQLLAAMDNDWRECNDVHQHLNIMTIKAGISHFCSNAMFSFNTFASVLYLLGDYVIRFV
YLTKDYNNSLRQFPVKAQFPFETEQSPIFELLVLGLFLHVMSDSFTIAIVNGLIFSLVFHMSGQIDIICQSFR
TISKSIHDRSSISTLGTLIERHNRVVLFSEQIGKLFSFITLMQIAANTLVICCIGFLITISIHNESGFFVLLK
AILAYVAIMIEAFIICFAGEYLSVKSKCISDAAYKSLWYDLPPREGKAISFMILRSQKRLVITAGKITNLSLE
TFAKIIKASASYVSVLHAMY
>CfOr177
IATTTISPSLKIGLQLLGMWPDVPYSAVYWLMFMSSMLIIQYFQYLYVMGHLKMNELSDLVDSLPATLDYTLT
LFKMISLWIHRRVLHKILIAVDNDWHECVNVEQHFHVMTIKASISHFCSNAMLSFNAIAGVLYVLSDYMIHFV
SLVDDYNDTLRQLPIKIELPFEYEQSPIFELLVVILFLHTMLHVCAVAILNGLIFTLVLHASGQIDIICQEFR
NISDKTSLYDSSAPILRMLIDXKVISFSNNIEKLFSFIALMQVVWNTLVICCLGFIIVISVNGGTSVMILIKT
IFAYFAIMIEAFIICFAGEYLSLKSKSIADAAYETFWYNMPSSQSKVMTFIIMRSNKRLAITAGKMTDMSFEA
FSSMIRASASYISVLHAMY
>CfOr178
MSTSTISLSLKIGLGFIGMWPGSSYGTHLWLFYMATLLAMQYFQYSYVFVHLNKNDFSKLMDGLSVTLDYTLT
FLKLLSLWHNRRIFSDILDAMEDDWNNQVTDSHVYVMTSKANLAHRCSKAMMILNSLATIFYFVGSYLSHRII
SAGEEPREFPIQMQFPFDQASDSPIFELIYLATFFHVWETATVIAMLNSLILALVLHVSGQIDIMCQELREIS
STKKSHPSATRSLIERHQKIISLSNNIDNFFSFVALIQFVWNTIVICSIGFMIVISLGTDIEGKSGILIQSII
PYVAVTLEAFVFCFAGEYLSSKSRSIGDAVYEIHWYELSTNECQILLLIIVRAQKQLTITAGKVMDLTLEGFT
TVMKASASYISVLHAMY
>CfOr179
MPTSTISPLLRMGLRVIGMWPDSSYGMFCWLFYMTTLLIVQYFQYSYVYAHLEFDNLIKLMDGLGITLDSTLT
FMKMISLWFNRRIFVDILVAMDNDWKDATDLHKCVMINKANLAYRCSNAMIFVNIVATVLYFIDSYARIRIFS
KDGQYPKFPIQIQLPLKAHETPVFDFIVLGLFFHVLETAIAIAVLNSLILTLVLHVSGQIDIMCQELKEIPSI
FKSKKFSTKSLIERHQKIISLSNNIENYFSLIALLQFVWNSFVSCCLGFMISFDMNMENKSGVFTQFIIPYLA
VSAEAFIFCFAGEYLSTKSRSISDAAYETVWYDLSISECRILLFLILRSQKRLPITAGKVMDLTLESYTTVMK
ASASYISVLHAMY
>CfOr180
MSMSTVSPLLRIGLRAIGMWPGSSYGTFWWLFYMTTLIIMQYFQYSYVYAHLDFGNLTKLMDGLGLTLDYTLT
ILKLISLWFNRRIFADILTAIDDDWKDRPTDLRQCVMMDKANLAHRCSNAIVSVNAVATVLYFIDSHVRRHTI
SKDGQHREFPIQVQFPFETHETPIFEFVVLGLFFHVLETATVIAILNSLILTLVLHVSGQIDIMCQELKEISS
TLKSKVFSSKSLIERHQKIISLSNNIENYFSFIALLQFIWNSFVICCLGFMVIIVSMNIESKSGIFIQFMMPY
LAVSIEAFVFCFAGEYLSTKSKSIGDAAYEAVWYDLSTSECRILLFLILRSQKRLTITAGKVMDLTLESFTTI
MKASASYISVLHAMY

>CfOr181
MKRANTISRSVEIGLRFIGLWPDSTYATFYWFSYMTSIVIVQYYQYAYVLGHLEINDVWLLMDCLSLTLAYTL
AFFKLLVLWWNRRIFYRIVKDMDEDWKNCIVNSSYMSTMMTMADLSRRFSNIVFTVNGIGAFFLSIGEHLLQS
MDDANRINNSSRELPLKMEFPFDVSESPIFECFLIGQFIYDLVIAFVVGLMNALLVTLILHVSGQIDIMRQDL
AEISKHNYDHNTFVIIIKDLICKHQRIIALSENIENLYTEIALMQILWNTLVICCTGFFIIITINTTKDISTL
IKSVNYYIAITLEAFIFCYAGEFLSAKSMSISDAIYESLWYNVSPNDSRILLFMMLRCQKRLTITAGKIIDLT
LNGFASIMKASASYISVLNAMY
>CfOr182
GAMKRTSTISRPVEIGLRFIGMWPDSAYATLCWLMYMVTMVIVQYYQYAYVFAHFDLNNIPLLMDCLGLTLAY
TLAFFKLLALWWNRRRFYFILAAMDRDGRECDINDSYASMMINVADVSRRCSSVMISINALAAFFLSIGEHLL
HSLNDVNRVDNNSRELPIKMEFPFDVSESPIFECFLIGQFLYELLLASIVGMVNALLVSLILHVSGQIDIMRQ
DISEISNKYDPNTSLIIIKTLICKHQKIITLSENIENLFSYIALMQLLWNTLVICCTGFVIIISIGTDESATT
SIKSVSFYIAITLEVFILCFAGEFLSAKSKSISDAVYNSLWYNMPPSDSRILLFVILRSQKRLTITAGKVVDL
TLEGFTSIMKASASYVSVLNAMY
>CfOr183
LDRGTMERASTISESVEAGLRFIGMWPHCIYANINWWTYIASVAVVQYFQYSYILEHFDISDLSIIIDGLSIT
LGYSLSFLKLINLWFNRRKLHVILDTMDKDWNDGIAIHSDVSTMIRHANLSRQCSNVMITTNALSVFFYTIGG
PILRSVIHKNDQETITRELPIKMEFPFNVDNSPIFELVLVVQLFHDLSVACIIAMLNALLVTLVLHISGQIDI
MRQGLLEIPSKNHSSLAAIKVLISRHQRIINLSDNIEDLFSNIALLQFIWNTLVICCIGFLIIISIGTEEGAT
MITKSLIFYVAITLEAFVFCYAGEYLSAKSKSISDAAYECLWYDLTPSECRILMFLMLRSQKRLTITAGKMTD
LSLEGFTTIMKSSVSYISVLRALY
>CfOr184
KMILANIVALSVQIGLRVVGIWPNAPYTLLFRCGWIFTTGIIQTCQYWWIIIHFGTEDLSHLLDGLSVTMEYT
VMLLKLIILWLNSRIFYDALAAMVSDWKEATINDMHTMTSKANLSRRFSNVIIGLHSVGAFTYGIGVLVSHSD
DHDADAIEPVRMQREFTLKMQLPFECNESPLYELVMSLEFLHQLASSAVTGILNSLIITLILHASGQIDILCD
SLKKISPEKNNRKLAVSITKKLIGKHQKIIIFSNKIEKIFCYIALIQFMSSTLVTCCLGYMIVTSISTIQVSD
TIDGSALMKAIVFYMAVTVEAFIFCFCGEYLSAKSKMIGDAAYKSIWYNLKPNDSKLILLIMLRSQRRITITV
GKVMDLSLEGFTSIVKASVSYVSVLHAMY
>CfOr185
MIPTSTVSRPVEIGLRLTGIWPNSSIFFRLLWSIVMGTGLIFQYRYLLTHFSIEELPNLIDGLSTTLPYTLLF
FKLIILWINNRIFNDILRTMSNDWYEYSSIYTMIDKANLSHRYSKLVIGVYSTAVLLYSIATIDFRKPINDDC
RQLLIKMELPFVFCESPIYEFVACVQFVHLMVVASTIGMLDALIVTLMMHIGGQIDIMQQQIREICPKDNEYD
LSITIVRSLIKKHHKIIAFSDNIESLFSSIALMQFVSNTMIICCIGFLVATSLGTNEGVRMLIKTAFFYIAIV
LEAFIFCFSGEYLSNKSRTIGDATYESLWYALKPRDSRILLFVIMRSQTRLTITAGKFMDLSLEGFTSSLKAS
ASYISVLYAMY
>CfOr186
MSFTTTMSPTVEFGLRAIGIWPGSPRSMLYRTCWTISLGLAQTFQFRYIVACIKTNNFLDLVDSISTTLPYSL
LCLKLIILWLNQRLFNNILTSISRDWRNCDFIACNMRIMMNKAYLSHRCSMLIIGVYSMAVIVYSSVIIELNN
IDTDVEPAEKELLLKMQFPFVYEFSPLREIVMFVQFIQLLSHASIIGMLDALIITLILHVSGQVDIVCRGLFE
LFSGKHEYKSYKDATSAIIRRHQDIIAFSTDIENLFSYIALLQFLTNTIVICCIAFAIVTCIQSDQGYALLLK
SLFFYIAITLEAFIFCFAGEYLSNKSKSIANTAYEVLWYDAKPNESRILLILMLRSQKRLTLTIGKFNDLSLE
IFMSILKASASYVSVLLAMS
>CfOr187
LQTKVGDIEDLFIHLERIFSIGGIWPFKQTYIRFAIYISYYMLYLIMAYIDLYDVFGNLELMVMNLVETIAYT
MTFMVVWLIRCSNLLKHVINAVRKSIMEQKFENSEEERIYYHYNYTSKIFTYGSIAGMLITITLLYFRPLLRF
STSNQVLHNDTQFFMLPYRVHIFFDDTTNNYTYILMYLYLFPMFYVSICHMAAICLLVILVFHICGELSILSYR
IKNVRTYSQDTIVDRIRSFVRMHLKIIWMAKSVDNTFNLILLDELLGNSVVLAISMYYVTINLQISEMATCFT
FILFAIIALVMLFGYCLIGDQLTQQ
>CfOr188
RRNRVGMSQKTARNRLDQFRELSALHITYLKYIGLWAVDSDSSALLKCLYFAYNKFILTIMLVFMVTLLADIC
SSFDDLSIVTDDGCIFAGIVVVFFKVMIFQTRRAQIVRLLRETIDGCDQLCKFPVGDEGEILGKYLLLSRVTF
YGFSTLAVFLVIALLFLVPVEDGELPVRALYPFDTTKYPWHAVGFFVEACTVSIGMTAIIGMDSLHTNLCNLF
LVHLGILNEHFKSCSSNGIERDDLGFRFKISSSTVEERNREKDNYDSDEIRRGSSNVFVERFRRSIRNHQRLL
AIIDDFNKVFSAGMFVQMLSSTSMICLTGFQAALVVGQRSNICKFSIYLAAAVSQLFYICWIGNEVIYQSALL
TQSQWLSGWSDELSSKTGRLINLSMIFTKKTLNLKAGVFYVLSMETFIAILKGSYSFFTLLTTMEND
>CfOr189
NKYLSIRMARILMKTIGFWPAETKKGKLLLKGTLVYTLFAFALALWIEATELYLTTGDFYALTYTACTATPVV
IILLKLSFFLYHREELLRMLKYTEDNFWYVQYDAYGSKLLEKIDRKGKILLFTFTFFTQGAVFTYMLAPLVEN

RGKNESERILICNIWVGIPTNVSPNFEIIFFFEVVAFIHSGLCFCCFDNLLGLINMHTAGQFNILQHRIETIL
QKVERGGTVELFNKKGKVYEVYEEIIECITIHHKLIWYSEAMERLFMYTTLFQLLVSSVLLCVSGLQIFLGQG
TIVRRMIFIAHAIACFFQLFVVTATSNDLIEESRAIGDAAYNANWQVLCHNDNKGVRNAILMIMKRSMRPCSI
SAGGFFPVSLETFMTVLSTAVSYFTLLRNFV
>CfOr190
MTQGKEYRSVSITRVFMKMVGLWHVKTPREQLLLRIAFGYAVWQIIFAILVEGVDLYHCIGDFYAVTSNLCTT
LLLVMILVKLGSFMFYHDMIMDLIRFAEKNFWNVTYNEVDTRILEGYDKLGMIMVYTFTLIVYIATFNYICAP
FFDHQGLNKTEKILPFKLWIDFPYHSPYYEITYVIQSLSTIHSGICTCCFDNFISTFNIHTAAQLKILAHKVE
TITEDCINNVIDQKHLSETDIATLTFRNMQECVQQHHTLINYVYNMQRVFTVILLGQLLLSSIVICFGGFQLL
AAEVAIRKCIFAFHFVGGLIQLLIYTWTCNDIIVQSTAISDAAYNSKWYLLPNNGPGNAVRKGLIIIMIRARR
PCTLTAGSFAVMSLDTFTGILSTAMSYFTLLRQMSEE
>CfOr191
KKQATTDALVIRYRTYSRRVRTMLYLGGVLQDRTHSVIRTYIIGFLVILMCLSHSIFLLNFSRDYADNLTLMV
KCFGQMSSFIAPALMSACFLIKREKLLELHETLNDLFERELEQDQETTLAILHAFDRQSYIMFFITTSLILSH
VCPPLIFIIYQSVRHIEPKGYRLPFLAKFPWIVPINGGFLFYLHFLYHFFIGWWVIFTINSVDSLFGFYAFQI
SSILHAMSIKLRSNLRSREVLKICIQIHGRLLQCSYILEDIWQLIILRMLVTNACVICALIFEASQFTDITMN
EVFSFIFFIALKLLQTFIYAWNGSSITSASEYFREGIYFSDWPNSSLDHHIRANIIVSMMQKPMIIKTLKISS
VNVNMFINIMNTAMSYFFLLQSLDEGG
>CfOr192
VNFQNVNFLNNGANIISGNLLPMTDDNSFSIFCRIYRAFVWSIEVIHAISLIIGMTVVPKEKALKDGIISIVV
IMETSFLLASIYTQQKLTIQVVRKMNEILRNADMIMVDLVKTALKPIILPFIIYGVASVISVTIWTIHPIILI
FEKRDTFYFEDYNSPAFFSPEPFSVHILILSTVIMTTGAVYLFLRKYSLDVYMMHLVLMLTAQYRYIAIKLTL
LFQKPQDDNKNSQKKCHPMTDQWAEKELRALCQHQNNVLFISIMLRKLLSVNFSLLYLNSVFRFCFLGILMTT
IPSLTFMEGISIVLFAAGSIMQFFLLCSSVQTLSDA
>CfOr193
MDFQNVNPLNILLNAVSGNLLPMTTDNSSFSIVWKMYSALVWLLEITQMCVLIPGCFILPKEKTLKDGLIGIA
VTMEVVYTVMRIHTRKALVQRLIQKLSNIMRVRDELMRSTVRETLKPIEIPLSFYWREGLVSIIMWTTAPFTL
VFQKNSFVYMDYRMPVIFSKEPFSTSIFVFGSLVVMLSSMYIFTKKVSVDSYIINMILLITAQYKYIALKLSM
IFQDVRLQSNCNSSNEKKDYPETNFCAKEKIKFICQHYNAVFHVTLMLRELLAVSISIIYLNSVFRFCCIAIM
VISIPSSDSIERAVFIMYASGGVVQLYILCSCVQQLLDASNEITDQAFHEPWYRFEGSIKRTFMFLIMANNLE
LKLSMFKRYNISLASFMTILNQSYSIALILL
>CfOr194
KFKSIKHVDDKIMRTTIMNTIKPIEIPLKFYWTAGTMSIIIWSSIPFMLIFQKDTFSYVDYRMPVVFSKEPFS
INIFVLGSFVIMISSMYIFTKKVSVDSYVINLMLLITAQYKYIALKLSMIFHDKTPQINHNNFNQEKHYSETN
YYAEKQIQSLCHHHNAVIHVSLMLRKLISLNFSLTYIISVFRFCCIAIMMISIPSTTLLEAFLMIMYASGGVV
QLYIFCSCVQQLLDASIEITDQAFHEGWYQFKSSIKRMFMFMIMSNNLELKLSTFEKYNLSLPSFMVILHTIN
VYLIIPNK
>CfOr195
MDIQKVNPLNVRLNMISGNLLPMTAKNLSFPVLWRIYSFLVWSLAIVQTCITIPGCMYVPKEKALKDSLIAIV
VTIEVVFLVVQIHARRELMQRLIQKLNNLLRIEDKMMESVVMETLKPMMTPLRFYWMAGIVSIIIWSGVPFTL
IFRRDTFFYVDFRMPIAYTKEPFSTSIFVIGSFSIMISSMYTFTRKVSVDSYMINLILLITAQYKYIALKLSM
IFDDDISQTNHDSSNKRKHYSKNYYTEKQMKSLCQHHNAVIRVTLILRKLLSLNFSLIYVISILRFCGIAIMM
ISIPSTTLLEGSLIVMYASGGIVQLYIICSCVQQLLDASIEITDQAFHEQWYRFGASIKRTFMFMVMANNLEL
KLSTFEKYNLSLSSFMTILNQSYSVAIILFKTS
>CfOr196
MDFQTMNSLNVWLNLLSGNLLPMTVNSSFPIFWKIYSVFVWLLELVYMGTAIFGCFYVSKEKALNDGLLSLIV
TVEGIFMVTRIHAQRRLVQQLIRKLNDLLRVEDRTMKRIVMTNLKPMEIPFRLYLVGGSFSVFLFYCICFQLV
FEKDTFFYEDYKTPAIYSREPFSTDIFLMGSLIALISNMYVFFKKVSVDIYMTYLLALITAQYQYIALKLVSI
FRDGGHLQYNNGGAQENQPTIDFFMEKEIRTLCRHHNSVTRITLMLKKLLSLNFSLIYINNVFRFCFVGIMLT
KISTSFLEGFMVLLYGCGAILQFYILCSYVQKLMEASMEVTDKAFHENWYQFNISIKRTFMLVIIASNLELKL
STFEKFNLSLPSFMAVLNQSYSIALLLLKMN
>CfOr197
MDFQSINPLNVRINIISGNLLPLSKNDSRFPVMWKMYSIFVWLLEIIQAIVLIPGIIFVPREKALKDGTVTCV
VTIEVFFMVMRIQAHRKLMNQLIRRLNDILCVADETMKNIVTTTLKPMGDPLKFYLLVGWLSVFIWCCLPFLL
ISEKVAFLYEDYRTPAIFSKQPFSIDVFLVGSVLLLLSNVYIFLKKVGVDIYMIHLVLLITAQYRYISTKLAI
IFRDGNPQSEFDDSCQEYQLKDQWVRKEMEALCRHHSSVIHLSSMLKKLLSLNFSMIYVNSVLRFCFIGIMLS
TISSTNFLEGSSIVMFASGSIVQFYMLCFCVQQLMDASRKVTDEAFHEKWYQFGPSVKRTFMLIILGNNLGCK
LSMCDKFNLSLPSFMTILNQSYSIALLFLRVK

>CfOr198
MDFQNVNLLNVRMNIISGNLFPMTTDSRFSIGCKIYSIIVWLITISVITLFFLGFTMVSKEKVIADGMISIVF
IIEVFFMFIRIHTCKDLAMQLIQKINDILRAQDETMKCIMMTTLKIAHSSSKFYWINMMINITMWISLPLIVI
KKDSFYYEDYRLPFIISKEPFSFKIFVLGTLFLVITSLYIIMKKAAAHFYMMHLVLLITVQYRYIAVKLQKVF
QKENSQKNKINSRTKFCSEKIDLWTEREMKSICRHYNIVINKRNFFKLMLMILSNIIMKLFQITVYIISFFVP
IYRIYWQSLITLFERITIIMFAFGEMLQFYILCSAIQQLLDASTEITDMAFHEKWYQYGSSIKRTFLLMILSN
NLKCKVATMEKFSLSLPSFMTSINQAYSITLLCLK
>CfOr199
MNFQSVNPLNVRLNLFSGNLLPMTNYNSPFPLLWKMYSMFVWIIELIIGATLIPGCMYVSTEKVLKDGMICFA
VFIEMTFLIARIHIYKNVAHQLIRRLNDILHVADETMMSVVTATLKPVEAPLNFYWSIGVISIIAWTCIPLVL
VFKKNLFYYEDYRIPAAFSKQPFSLEIFLLGSVFLMVSAVYMFLQKVGVDVYMIHLVLMTTAQYRYIAMKIAM
IFHANDEDNKEYSSELNQRQEREFKVLCRHHNSVIHITSLLKELLSLNFSLIYMNSVFRFCFIGIMLSMIPST
TFWEGISIIMYASGAVVQLFILCSCVQQLLDAASFI
>CfOr200
MIMQKKITLRKTIAIVKLSLFVIWFWPLPQDASKRKVLCMKLYQYITLLLMIAVLASMVYALVRNLDDLELVV
KSSLGIFPCSHVIWNILCRIVIYQRLQCVTFQMENFCASINAYEEAIIQRDYIDKCTILISVNFYGFCITSFY
TSLFALFVGPILQDELLPAPADFPFDASRQPLRAITYIHQIVGGLYIAAHLCVNASMALLLWLALARFKLLIE
DLRKITNIYDFMKCIEKHQQLLEYAKEITFVVRPFALGTVFFSTVSLIVFGLIFITGVSLGLKIQCVFLAVSA
LLEVFMYAWPAEHLIHISNNIAQIAFETNWYDESENFRRNLQMIILRSQKPILVTLPCGLPSLSLHYYAS
>CfOr201
MARFHLTPQQLLWILEILGVLSSTSPKPISGRFYNILRNIFWYLICGNFIFQLIGEILYIYHNQDDIIMILKT
ILIAACVTDGLLNLILCHIQREQLQHLLEEIKNYLQTANENDINILQKHMNRYMLISAANILLLSCAGLIICF
RPLVTNDRFPVDVWYPSFMDIPLTIRSFLIYVSQIFSGIECVLAFNTDITIATFICYFTARLEVLQHNLKNTK
TKDFIHACIKEHQDIIKLVEVTQATIQYLILKFNLTMGITVVCSFFPLIINQPLAVKGQFMFTLLSACQRFYI
GAWSANDLIERSEQIAYNFSFEDYMTPETINDILFIIIRSQKPLTISMASFLPVLSLEYFGNFMTKAFSYFTA
LKSLL
>CfOr202
MVLIRIKIHQVLRILEICGTFISTWPPKSTAGKREIFLRDLGWTLAILNVLGSLPPLILGAWYSENDIIRMMK
ALSELTALMEVLLNLILCRIGRLRLQKLLAHLRNFLKHSKLHERHIIQKYINRYSGFYAFVGISYILAAITFS
CGPLFLSINLPMEAWYPFSTETPYTRTILYILQVFAILQTGFCITVDFMIAIFFCYSAARLEMLGQELQQITH
ESHVKTCIQKHQEIIKKKLQCFSSLYIATFLTKIVTLVVKSNIDSILYISQQIAWSAYSIPWFEKSHETITNI
FIFIQRSKKPLLISMGGMFPVLSVQYYAH
>CfOr203
MTIIQFKFQQVFYFLRVLGTIAXTWSPQPDSRKIKLFLRNFYYCIAIFIYVTTWVSIWVINAYKTRNNDVDEF
MMNLSHTLLDAXSLLDATLNSILCTIKRKQLQNLVIHIKEFMNISKDREIVILQKYINRHTTFISIVAISFTM
AGVTIICAPLFTPKEFPLDTWYPFSVEPLWLKFICYVTHIXTHTVFCLNVDIIATFFLYSAARLEMLAFEIEQ
ATDEKHAIFCINRFISKTQETLQHVLFKANFTMAFAVISGGFPILYLESYVQLPQFISTAIAALQRXYITASA
ADDLREVSTQLPWSVYGASWIGKTQKMKNDIFIMLQKSQQPCLISLGSLLPNLTLKYYLFIIIILFVTTILSY
FTTMKAVI
>CfOr204
MVLIHLKFQQIFHLLRVFGTITNTWPPHPNIGKNKLLLRNFYYCISIFIFATVWIAMLMNAYKTRNNDVGELM
KNISHMTSIMEAILNSILCTVKRKQLQNLVIHIKEFTEILKDCDKIILQKYINRYIAFISTVAISFSMAGITV
ICAPLFMPLEFPLDVWYPFSVKSLLLKFILYIMQIFTIAHTVFCLNVDIMIAVFFLYSSARLEMLAFKIKRAT
DNDHVISCIKQHQEIIEFISKTQQTLQHILFKTNFTMAFTVISGSFPMLFLQSGVLIPQFISMALGALQRMFI
TAWAADDLREISTRLSWSIYSASWIGKTRKMKNDIFIMLQKSRRPCLIFMNGLLPALTLEYYANFVTTVLSYF
MTMRVVIAS
>CfOr205
MAMKITPVKAILFMKMSVALSCTWPPSPKITKSNTILFKACWYICYFCSILLLLPLLSSVYEYRDDPVILAKS
VCLSCAVLQVTIKMIVCRIQYTSFQMLYHDMETFCKQADDKTNITLQRYIDNYKCTYGSYILWCYLTAIGVIC
GPLFLPQQFPTDAKYPFSVEHHPVKSIIYLHQSLVGLQASAGMCIDCSVAILLFYSAARLEMLVQEIRNAKSE
CELDACIKLHGEILKYISKMISVVRPLVLTTITMTTMGVVFGSLNIITEQPTIVKIQYSIVVFSAGVELFMCA
FPADNLMHMNSRICLGAYESKWFQRSVSMQRKIVQIIFRSQKPEVIRINGILPALSLRYYARFLYTSYSYFTA
VRIMVNEKII
>CfOr206
MRGKMMLQKVIFYVKLSLSPICWPLSKDASRLEMTCVKLYHYLSIILIMMLQPSLIYTIRNHFDDFLILIECI
LELSSTIHSMANFIFYRINKHRIQHVTFEMIHFSEVIKPHEDIIIQRYVDKCVIFHGASILIFYVAGIIFNIT
PIVTNQPFPMLAEYPFSVYYQPLKSIIYLHHSVVGVQIAAQLCTNIFMALLLWFASARFEILIDELRKTTDVY
NLAKCIRKHQYATKITLAARPFALITTCCSTVCTVIICLFLLTNQSKESLTITAKQFGFVLSCFSEVFMYTWP

AEHLIYKSQDVAQAAFDTPWYNQLKYXRCLQIIIRRSQKSLTIAIACIMPILSLSYFASYCSSVVSFFTTFRV
FLNNEN
>CfOr207
MRGKMMLQKVIFYVKLSLSPTWCWPLSKDATRLEMICVKLYHYLSIILAIISQPPLIYTIRNHLDDFLILVEC
ILQLSSTIHSIANFIFYRINKHRIQYVTFEMIHFSEVIKPHEDIIIQRYVDKCVIFHGASILTFYVAALIVII
GIPILTNQPFPMLAEYPFNTHYHPLKSIIYLHHSVVGMHITAQLCTNVFMALLLWFTSARFEMLTDELRETSN
VYHLAKCIRKHQFLLKYATEIILAVRPFALIAICCSTVCMVILCLLFLSNQSTGFLIIAKIIGLILSGLSEVF
MYTWPAEHLIYKSQDVAQAAFDTPWYDRSIEVRRCLQIIIRRSQKSLTIAIACIMPILSLSYFASYCSSVVSF
FTTFRVFLNNEN
>CfOr208
MSRKLTLENVITFTKLSLVISLSWPLPATATKRQVVRFRLLRFLTYMNIWCLFVPLSMTLQDWSDHSDICIKS
IPLIAGSAQAFIEMWICHGQHKHLQLLIAEMESYCKHAIGYEKDILQQYVDRYAMFYATAAMWFYLTAFAVVC
SPPFSSDTFPTYAKYPFNVNYQPLKTVIYVQQSTVGIQMASVLCINILIALLLWFASARFDMLCNELRMISNV
YELTQCIRKHQQVYANDVTHSVRFLVLTIVGCSAAAILFVGLTLVSLQQPLIIKMQFCVLIFTCLSQVFMCCW
PANNLLVASSNIALAAYESLWYSRNVYMQKNLLLILLRCQKPVAVTVTCIIPVLSLRYFGSYISTAFSYFTTL
RVMFEEN
>CfOr209
LIMWKNATLKNVIATVKLSLFITCCWPLPANTIKFKVICVKLYQYLCMILTLGITVGLCNTIRNHLDDPLIMA
QSIMIMCSTIHVIFNIVSCKINSYRLQLVTYEMENFCELIKSHEEAIIQQYIDRCIYFYGGSIVWVYLTTIVI
ITGPAALDQTFPTNAEYPFDIYHQPIKFIIFIYQAFICMQCGSQICMNIFIALLLWFTSMRFKLLSEELRAIV
DIHDLIQCIQKHQKLLKYAEEVVSVVRPFAFLAISISTFALIIIGIILITQGQPLSMKIQCVGLIFGGLAEVF
MYTWPAEHLIHISNEVGQTAFNTQWYKLSITLQKNLQIMIQKSQKPIIVAIPCVMPTLSLNYYASYLSTIFSF
FTTLRMVIQNTQD
>CfOr210
EILTLEKVLAFLKVDLTFAFCWPLPVRATKSQKIRHKIFRWCCCMNGMLMSISLIYTLYLEHDNMLLLMKGGC
ALTAFLQVPIQITLFTLQSDRLQVIIYEMENYIQQAKSEEKNVFQKYIDKCKLFYGLTISWIAITAIAIIFGP
FLLPQPFPIEVKYPFYVSQQPLKTIIYLHHAVNVYQSYVQVCSNVFVAVLLWFIAARFEILSHQFRKVRSFSE
FKICIQLHQQLLRYAKEVTKAVRYITLSTIGFSTVAVVFSGLTFLSRQPLTIKAQFLTVGASSLIEVFVCAWP
ADYVLSTSNNVGYAAYESLWYKKEVSLQKDYMLNRXQQPVSVTVTCMLPTISLNYYASYLSTAFSYLTTFRVI
F
>CfOr211
IFMKYDKLLHVLHICGLFTTVWPLNSKAGKFKILSYNVLWFIYTLNMIIQYYLVTKSILPAYRKNFINIMKTL
VELIYNTEVIFNFLYCKMQRKRLQKLLYEIEQPDKIWMPYEKIVWLKFMHHTRIKYFSLAIIYIISACILILS
PMISDRFLPLNVVYSFPISKPWIYCAAYLHHIFSIKQASVTIILDLMIISTMWHATFKFNLLGIQMRSMPFTI
NKLRASIIMYQNIFNYIREIDCIASYILFKLTIVASMYVITCGLLILNNASIVDISLFLMVTSTAIWRLFVCC
WSAQAITDMAYNISWQIYDSSWISASSNVRRTIFMIIQRCQKPVAISGTNFIPVVSIKFCGSVLYATFSYFMA
LQAILH
>CfOr212
MACSVTFEKLIVFLRMHLAFACCWPLPPTATKMQITYDKFFRLFNGLHSMLMIYAVAYRIISQYNNTLVIMQL
GCVLGTLCEVPLQIYLFTQQHDSLQTIILKMENYSKHANSTERNVIQEYINKHIVIYGITLTLMTVALVAMIL
VPLILARPVLELEYPFSIDYQPMRGIIYLHQSFALYQVYVQVCATVFLALLLWFSTARFEILANKFRIATEYS
DWKACIQEHQELLNCGYSALFIMILCQFKMNTFPIVDKIIIFFIRFPMFVKMQYSIICCSSLAKVFLCTWPAD
HLMSMSCYAGDAAYNSLWYEHEIASQKIMLYTLLRTQRPVIVSVPGLVTALSLQHYASYVSMAFSYLTSFRAM
LSND
>CfOr213
MKKITLEKVIAFLKVDLLFACCWPVSRDATKFQIVCDRIFRVISSLHAILLMIELIYTIIYRTESIQMLMQST
CAVGILSEVPLQILLFTLQHDRLQVVIFQIENYYHQAKTEERNVFQKYIDRYIYLYATTLGLITVGLFISFLD
PLLRGFDTFPLVIKYPFPIDRQLLRAIVYCHHMFGIYQIYCQVSSNVFLAFLLWFTSARFEISNKFRTTTKYS
DWKRCISEHQELLRFAQEISLSISYIILLSLGISTYSLVFGGVSILSRIPLSVKAKFFIVCVSSLLKVLLCAW
PADYLMTISSDIGDAAYDSLWYKHGIDSQKMMLYILLRCQRPIIITVPGLLAALTFQHYTSYISTAFSFLTTF
RIILSDN
>CfOr214
DTLTIEKLLAFLKADLLFACCWPLPPTATKWEKIRNKIFRCFSVLHGLIMIIAIIYTIYANRSDLFLIMKLCC
ELCTTLEIPLQIICFSMQYDYLQYVIYELEDYCKHAKPKERNVFHQYIDSCKSIYIYSISAFTVTGLLLLLSP
IIESHPFPIDTEYPFSVDYQPLKTIIYLHHMLIIYQSYTQVCANIFVALLLWFVSARCDILSSKFRAVTEFAE
LRACIEEHQELLWYGKKVTLSIRFVMLASLTISTIVIIFTGCTFLSRQPMSVKSTFLIFFISSLAKVYLTAWP
ADHLLSASTNIAHAAYDSIWYNGKVNFQKNFVHTLLRSQQPIAVNIPCMLPTVSLNYYASYVSTAFSYLATFR
VILEEDD

>CfOr215
MEDKVTLGKIIVFLKIYLTFACCWPLPSNATKLQRFLRIAFQYFCCANSVILCVAAAWTLYKHSDDALLVMKL
GCQFSAVSQVPLQIIFFAFQDKRLQFIVLEMENYYKQAQKYEKEIFQQYVDKCMPFYGIILGWLAMTGVSVIT
APLFSSQSFPSEAEYPFDVQHQPLKTLIYAHHILTAYQSVIQVSANTFPALLLWFVAARFQILSTRFRTMTNM
KELINYTREHYILLRYAKEVSLAVRYIALLCVTFSTGAVIFGYLTFMSRQPWSVKWTFLMIAFCGFVELYMYA
WPADHVISTSSDIASAVYDSLWYNDDLAIRKILVHVIRRSQHPVTISVPCALPDLSMNYYASYISTVFSYMAS
VRLIMGQE
>CfOr216
MKGKVTLDRVIAFLRIYLMFACCWPLSPNATKLQRLFYGALRYFCCTNTTIFIITVIWTFCQHNEYMFMMKLG
CELSAALQIILQLILFIIQDKRLQVRFSFVLIFYVKKSCYYIYQEIFFKFNIKCXIGCVFCLVIMTALSMITT
PLFSPQPFPCATKYPFDVHNQPLKTIIFVHQILTVYQSMTQVSANSFPALLLWFVAARFHILSIRFRTMTSME
ELIKNTQEHYSLLRYAKEVTHAIRYVALLCVTFSIGAVIFGYLTVISRQPWSVKLTFLMISFGAFVELYMYAW
PADHVISTSSDIASAVYDSLWYNDDLVMRKLLINVILRGQHPVTVSVPCALPSLSMSYYASYVSTVFSYMASV
RIIMGQE
>CfOr217
MIKKRGTIEPNFHLRVSLSIIYFLGTWPPHASKYRILYLFYTICSFIFLLGILLASEIANVIANWGDMSKIVA
FATILMTNSIHASKVFIILHRQGRIQALLDIANSPAFKRHDKKYQDLLTRYTWKGIFHHAAYQSFGTIAVFCW
GITPIADLIAGRSRRLPVEGWYPYNVTRTPAFEITAGHQGIAIIIACFHNVALDTLVTGLITVACCQLAILER
NIISIDNQKNRQGDKNKSFLEVLSYQQLKKCIMHSNMIFFFTREIQDIFNIIIFFQFLSNCIIICLIAFNVSQ
MKVYIPAVLIGLVTYMCCMTYQIFIFCWHGNELHLHSTRIVMAAYSSNWFSNNVSFKRSLQIMMLRAHRPFTL
SAGNIMLLSLDTFVEILRMSYSIFTVLQGS
>CfOr218
MHTLPLSFALLTYTGYWQPLNLTPIKYWAYTVYSVVMIFLLLSFTFCGLVDCFMIKDLETFIEKFSLFLSVLG
VSCKVMNLVLCRDKIIGLSNMLLTNFCVPRDNHEMDIQRKFDRNAKTITIYCEILNESAVFFATIAQFRYLAN
TRTLPLSDWVPYDISSTAVFWATVLHQTIGLIVCANASVAHETLISGFMIQTCAQLDILCHRARMLPNLLRKA
QKSGISKEDLKAREQWLVRELIHHHRYVYRFAERINTVFTLMILVQFSISSTVLCLSIYKMSTKSLLSLEFAW
SLSYLGCMLTQIYLYCWFGNEVTLKSTEVGNAIYEMDWPMLPVDLIKTLLLIIIRSKKPIKITSGYIVTLSNE
SFMKIIRISYSAYNVLQS
>CfOr219
MPVLKFSLTVLAVAGCWRPTSWTSLSRNIMYNAYTLSVILILYTFAITQIMELILNADDADTFGDALFNIIIS
LLACYKTIVIRKSHGSIITLINNLTETPFKPLDLNENMIQEKFNKRITNNTLYYLVLVLVTSFYLILLSLFTD
FKNGTLLYKAWIPFDYTISALFYPVYIHQILTLIFIGLVHPTCDSFICGLLLHICCQIEILEYRLSNIVNAQE
SLRDCIHHHIRIFEYSYMVNENFARIVPSEFIMITVVMCYNLIHMALTSSTIISYIQNLMIISCTLAPIFYYC
WFGNEVKLKSLQLSDNIYNIEWTILNNNMRKGLLMIMNRATIPIEFSSANIMSMNLESFVMVLKTSYSLFNVL
IKSQKQ
>CfOr220
MSVLKFSLTVLAVAGCWRPTSWTSLFKTIMYNTYSLSVILTLYTFAITQIMELILNADDADAFGDALFNVITS
LLACYKAIVIQKSHESIITLINNLAETPFKPLDLNENMIQEKFNKRITNNTLCYLVLILITNLYMILLSLFTD
FKNGTLMYKAWIPFNYSMSALFYPVYIHQLITLMFSGLVHSTCDSIICGLLLHICCQIEILEYRLSNIMNARE
NLRDCVHHHIHIFESYIYTGSTKSKLLIQKFLTFSRIIEKIRVTLIRHNSAKKIVQISFLICRDKSIFYCNYN
EICDKNSLQLSDNIYNTEWTILNNNVKKGLLMIMNRATIPIEFTSANIISMNLESFVMVLKTSYSLFNVLIKS
QKQ
>CfOr221
MRPMQYTLTVLAVAGCWQSATSLFKYMMYNAYAMLIIILMLYTFSVSQFMDLVLNVSNSGEFTETLYLLLIVLM
ACYKAIIYNMNXHKNVAMIIEYLTENPFAPLDKNEKIIREKFDKMVENNTFXILVLLTCVYIIILLSLFTEFRK
ENLSYRAWLPFDYSISAIIYYLTYTHQMMSMTLNGIVNLACDSFTCGLLLHVSCQIEILEYRLSKIANNKKNL
RNCVYHHNCIFEYAYVVNNKFAKIIAIQFTVSALVVCSHLYHLAVTSSSTRYIQFIMCIAYMLAAVFYYWFGN
VKLKSLQLSDSIYNMESRLSNSTKKDLLIIMNRSLIPIEFNINLDSFVMMLKISYSIFNLLTQTQ
>CfOr222
MHILNLTFFIVTFVGCFRPPSWTSLFKRVIYTLYRLFIIIILYTFAFLQFMDIVLNVNNPDDFTNNLYMMLNV
SVSGYKLLIMWINYTNIATLINKFSEEPFKPLDSGELEIYRKFDKLIRMNTLRYTLLIETSWSCSGLTSLLAD
FRHKKLTYREWVPYDYSSYMVFCITYAHQFLSTFYCATVNVACDTLICGLLMHVCCQIEILEYRLKKLVKNQD
TLSYCVHHHNSIFEFAYLVNARFSQIIGFQFITSTLIICSNLFQLSKSSLSVDNIALIIYTCCMLTQVFIYCW
FGNKVKSKSLQLADNVFQTEWPVLSNNVKKDLLIIMKRAIVPIEFTTAHIISLNLDSFVALLKTSYSAYNLLV
RVQEE
>CfOr223
MQVLKLTCAIVMIAGCFRPLSWTSLFKRTVYNIYRLYVISMLSFFVICQFMDIVFNVDNTDDFIDTLNMMLTS
SAACYKIFIVWFNYERISTLINYLTKDPFKPLDPCEMEIRQRYDRMIRNNTLRYTLLIETTCFFIALTSLFTD

FRHRRLTYREWVPYDYSSFLTFCFTYAQQMSSTIHTATVNVACDTLICGFLMHVCCQIAILEHRLKKLTKSEI
TLGYCIRHHDWIYKFAQLVNTRFTHIIGFQFTMSMMVICSNLYQLTKSPLDADHIPLVMYTSCMLTQIFIYCW
FGNKLKIKSIQIADKIFQINWLILDNNVKKSLLIMMQRSTIPIEICTAYIITLNLESFVALLKTSYSACNLLI
Q
>CfOr224
MSVMKFTLTILAIAGCWRPFSWTSLIKHALYNAYTLLIISFLYSFTFTQFMDLILNVDNPDDFTSTLFTMLTM
CVSCYKIFSMWMNHENIATLIQALTMGLFKPVVPVEIEIQRKFDKMIQTYAMCYTIMIIISCAGNILISLLTN
FKERKLTFREWVPYDYSSYVIFCLTYTHQYLGIIASCFVNISCDSLIIGLLLHLCCQLTILKYRLKNITNDQS
ILRECVRHHHHIIEYAHATNARFSKIIAFQFLVSTFVVCSSLYQITKMVLSAYQISLITYVLCILAQIFIYCW
FGNKLKLTSIQLVNSIFEIEWITLDNKIKKSLLIIMNRAIIPIEFISAYLLNVNLDSFVGLLKTSYSAYNLLI
QIQE
>CfOr225
LLKLTLAFLTIAGCWRPASWISLYKYRLYNIYTTILILLLYTFAISQFMDIILNVDNPEEFTSVLYIMMTVCV
ASFKISSMMMNRKNVADIINTLTDKPFKPMIIDEIKIRQNFDKMIRNNTLYCFVLVGSTCVCIALTSLFTNFR
RGNLTYKAWLPFNYSSPVLFYLTYAHQLTSMATCALVNLACDCFICGLLLHVCCQIEILEHRLNNKSHTRETL
RDCVRHHDLIFDFASTVNERFAKIIAIQFITSTLVVCSNLYQLAQTTLSAEYLPLVLYTVCMLIEIFIYCWFG
NEVKLKSLQLTDRIFEMNWPKLNNSFKKTFLMIMNRATIPIEFTSAYLFSMNLESFVGLLRTSYSAYTLLQRL
>CfOr226
MRVMQISLKILTVAGCRPPVSWTSLGKQTIYNTYTIFVSLLLFTFMLPQLMDIILNADNPNDFTNTLYVMMAL
TNACCKMVSLVMNRKNIEILIEKLIEKPFRPLESDEIEIRQKFDNIIQANTIIYAVMIEVTCGYMTVTSLFTD
FRRGELAYREWIPYEYSSGIMYYIIYFRQLISLTAASVVNVACDCLICGLLLHIYCQIEILECRLKKCLRDGG
NLGECVYQHNRIYEFAYIVNEKFRTIIAVQFSISMLVMCSNLYQLAKNTLSAEYIPMIAYTICMTIQIFIYCW
YGNEIKLKSNQLTDEIFGMDWINVDKKMKENLIMIMNRSLKPIEFSSAYIFTVNLDSFVKLLKTSYSAYSILQ
QI
>CfOr227
MQVLELTLKILMAGGCWPPNSLASLYKRTVYNAYTVFITSLLFTFMLPQLMDIILNVDNTDDFADTFYIMLAM
IMSLCKMTGLLMNRQNIGTLTNILIQKPFIPLEADEIEIRHKFDKVIRTKTLWYTILVETTCAYTALTSLFTD
FRKGNLTFREWTPYNYSEVVFYVIYARQLISTMFGSVVNVACDTLICGLLLHVCCQIEILECRLKKISLGRNN
LRECVYQHDSIFKFAFMINEKFQIIIGIQFIVSTLVVCSNLYQLAKITFSAESFPMILYTCSMLAQILIYCWY
GNEVKLKSSELSANIFKMEWLTLDQNEKAGLLVIMNRSLVPIEFTCAYILTMNLDSFVSLLKTSYSAYNILQQ
MGTT
>CfOr229
MHILPMPFALFTYSGYWRPVHFPVNSLKYRMYNIYSAFMFLLLQLFVFYGIVDTFILSASLPEFVNKCYLFLT
ILGVSCKVIHVFICRGKIIELDKMLLEDNCVPRNIEEILIREKFDRYISRLTIACEIFNESSAMFGTLAQFYI
LLKTRSLPVYNWAPFDLSSIYVFLPMLIFQCVALMLWANTSVAHETLTSGMMIQICAQFEILCHRARILPTLL
MEAEKNSKSDEDLITREKTIIRDLIYHHLYVYKFAHMVNATFTTMMFIQFSIISLVLCMSVYKLSTITSLFTL
NFAHKFSYLCSMLVQIFLYCWFGNEVILKSIDVSTAIYEMDFTKLRVRVMKDLMIIMMRASKPVKISTGYIVT
LSTESFMSILKISYSTYNFLKDS
>CfOr230
MHILPVSFALLSYTGYWRPVHFPVNSLKYWMYNTYSVFMFILLQSFVFYGIVDTFVLSASLEEFIDKCYIFLT
IFGIFCKVMHLFIRRKKIIDLDKMLLKDNCIPRDIEETLIKKKFDRHVRQLTIGCEILNEFTAIFATFAQFYT
FLKMRNLPVFNWAPFDLSSIYVFLPTLIFQCVALMLCANSSVAHETLIAGMMIQACAQFEILCHRAHILPALL
MNAEKKSKSDEDLAAREKTLIRDLIYHHLYIYKFAHTVNAAFTMMIFVQFSLISLVLCMSVYKLSTITSLFTL
DFAHTFSYLCSMLMQIFLYCWYGNEVTLKSIDVSTAIYEMDWTTLRVRVMKDLMVIMMRAGTPVKMSSGYIVI
LSTESFMSILKISYSTYHFLKDS
>CfOr231
MHILPVSFALLSYTGYWRPVHFPVNSLKYWMYNTYSVFMFILLQSFVFYGIVDTFVLSASLDEFIDKCYLFLS
VFGVSCKIVHLFIRREKIINLDMMLLKDNCIPRDIEEILIKKKFDRHARQLTILCEILNESCAIFATFAQFHT
FLKTRSLPVYNWAPFDLSSIYVFLPMLIFQCVALMLCANSSVAHETLISGMMIQICAQFEILCHRAHMLPTLL
MEAEKNSKSDEDLVVREKTIIRDLIYHHLYVYKFAYMVNATFTMMIFVQFSFISLVLCMSIYKLSAMKSLFTL
DFAYTFSYLCSMLTQIFLYCWYGNEVSLKSIDVSTAIYEMDWTTLRIRVMKDLMIIMMRASKPVKISSGYIVT
LSTESFMSILKLSYSAYNFLKDS
>CfOr232
MHILPVSFALFTYTGYWRPGHFPVNSLKYWMYNIYSTFMFILLQLFVFYGIFDTFISASLEEFVDKCYLFLSI
FGVSCKIVHLFIRRGKIIDLDKMLLKDNCIPRDVEEKLIKKKFDRHARQLTIGCEILNEFTAIFATFAQFYKS
LKTRSLPVYNWAPFELSSIYIFLPILIFQCVGLTLCANSSVAHETLISGMMIQICAQFEILCHRAHKLPALLM
EAEKNSESDEDLAAREKTIIHDLIYHHLYIYKFAHTVNAAFTMMIFVQFSLISLVLCMSIYKLSTMTSLFTLD

FAHTFSYLCSMLMQLFLYCWYGNEVTLKSIDVSTAIYEMDWTTLRIRVMKNLMIIMMRAGTPIKMSSGYIVIL
STDSFMSILKISYSTYNFLKDS
>CfOr233
MQTLSLNLFLYTISGMWRPIEWSSKCSKMLYSMLTCFTMCLLVILMLTQLLNIILVIDNVDDFAKNSLMFLSI
VCVLFKAIAVITRRDELINLIETLQKKPCKAYNEEENDIQLKFDCMIRSYSIKYTSLASFSVTGGIIGGLLNT
LEGQLPFRMWVPYDYTSPFLFWLTSIQELVATLFATIVNVATETTVLGFCLQICAQIEILKHRLQKMMKSSKK
EKTLISLNDASNGTDRFSEHILHHLCIIRLAKIINKVFSQVIFVQFFASILVLCTSLYHLSSHVTIIDIISLI
IYVLCMFVQIFVYCWAGNEVILKSTGLSEAVYEMDWILMPINKQKDLLMIMKRSTRPIKFTSSFLVTLSLESY
GSLLKASYSAFNLLQQ
>CfOr234
MKILSFNFFLYTISGMWRPIEWSSKCSKMLYSMLTCFTMYLLIIFTLTQLLDIILVIENVDDFATTSLLLLST
VSVLFKATAVITHRDEIANLIDTLQKKPCKVYTKEESNIQMKFDCTIRSYSIKYTSLTLLSVTGGIIRGLLQI
LEGQLPFRMWVPYDYTSPFLLWFTSIQTFVAVIFATFVNLATETTVLGFCLQICAQIEILKHRLQRMMKSSEK
KTSRISLNDESNEIGKLSEYILHHLCIIRLAKMINKVFSQVIFFQFFASILVLCTSLYHLSSHEAIIEIISFV
IYLLCMFVQIFVYCWAGNEVILKSTGLSEAVYEMDWILMPINKQKDLLMIMKRSTRPIKFTSSFLVTLSLESY
GNLLKASYSAFNLLRQ
>CfOr236
MSILTFTFQILIICGCWPPNSWTSRYKRIMYAVYTVCIVLLISTFMLSQLMDIILSVNNTDDFAENFYMTLTS
IVCCYKMFSLLRNRSNIAMLIDILMKKPCLPIELDEIEIRQKFDRLIQKNTLYYATLVELTCAFALVTSFFRD
YRKDKLPFRAWLPFNYSSPMLFEIAYVHQSISLTAGSVLQIACDSLICGLLMHICSQIEIFECHLRKIVNNSH
FLRECIMQHTCISKFAFMVNKKFRSIITVQFVVSMLVVCFNLYQMTQMTINAKSIQIMLYMCCMLTQISIYCW
YGNEVKLKSQQLIYNVFEMEWLTLDHNVQKSLLIIMTRSMIPIEFTSAYVISMNLESFVTLLKTSYSAYNILQ
QMQN
>CfOr237
MRVLEFTFTILTICGCWTPDSWTSSYKRLLYHIYTIFIFLLISTFTLSQFLDLILIVDNPDDFTDNFYMLLAM
IISCFKMSSLLVNRDNIAMLTDVLMKKPCIPIEPDEIEIRRKFDKLIEVNTLHYAILIEFSSSSSVIQSLLTD
YWKGKLTFRAWLPFDYSSTILFHFMYFHQLIGLLVGALLHVACDSLICGLILHICCQIEILNYRMQRVIRNPK
ILRDYVIQHNLMIKFAFMMNKKFRLTITFQFIVSTMVVCVTLYQLTKTNAXLIQLGLYMSSMLTQIFLYCWYG
NEVKLKSMQLTNNLFEIEWFMLREDVKKDLLTITRCGTVPIEFTSAYIFPMNLDSFVGLLKMSYSTYNILQQM
RNENVE
>CfOr238
MRVLDFTFTILTLCGCWTPDSWISSYRRLLYYVYAIFIFLLINTSTLSQFLDLILIVDNPDDFTDNFYMLITM
IVCCFKMSNLLVNRDSIAILTDILTKKPCKPVEPDEIEIRQKFDRLIEVNTLHYAILVELSSLYTVIQSLFTD
YWKGKLLFRAWLPFDYTSTVLFHFMYFHQLIGLLVGALLHVACDSLICGLILHICCQIEILNSRMKRVIHNPK
ILRDYVAQHNLMIKFAFMVNEKFRLIITVQFIVSTLVVCVTLYQLTKTNAKVLELVLYMSCMLTQIFLYCWYG
NEVKLKSMQLINNLFEIEWLALEKDVKKDLLIITRCGILPIEFTSAYIFPMNLDSFVGLLKTSYSTYNILQQM
RDEAVE
>CfOr239
MSILVFTFKILTSCGCWIPNSWTNSFYRRLMYHVYTIFILLLINTFTLSQFLDIILTVDNPDDFMDNFYMLLA
MIVSCFKMFSLLINRSNIAMLTDILMSGPCRPLEPAEIEIRQRFDKLIETNTLHYMILVELTCASTAVASLLT
DYKKEKLTFRAWLPFDYYSSATIFHFTYFHQLISLTVGSVLHVACDGLICGLLLHICCQIEILSYRLNKIAHN
PRMLRDCVIQHNHIIKCAFLLNKKFRFTISFQFLVSTLVVCFTLYQLTKTSGKFVELGMYMSCMLTQIFLYCW
YGNEVKLKSLQLINNLYQIEWFTLEQNIQKDLLTIIICSKVPIEFSSAYVIPMNLDSFVGV
>CfOr240
MGLLDFTFKILMICGCWIPDSWTTPYKRLVYHVYTIFIMLLIHTFMLSQLMDLILTVDNAEDFTDNFYMLLAM
IVSCCKMFTLLMNRSNITMLIDILTRKPCRPDYSDEIEIQQKFDRLIQTNTLYYAILVETTCICIAVTSLLTE
FSKGRLTFRAWLPFNYTSPLLFRIVYIHQLIGLTAGSVLHVGCDGLICGLLVHVCCQIEIMECRLRKIGSNRD
NLRKSVLQHNTVFKLARLINEKFRLTIVIQFIVSTLVMCFNLYQFTKSTASRAKYMQLIMYMGCMLSQIFLYC
WYGNEVKLKSRQLVNNIFEMEWFELDNYTKQSLLMIMTRGTIPIELTSAYVISMNLDSFVHV
>CfOr241
MRILDFTFKILTICGCGRPDSWTTPYKRLVYYVYTIFILLLIHTFMLSQLMDLIMTVDNSDDFTDNFYVLLAM
IVSCCKMFALLINRNNIDMLIDTLMRKPLQPIESDEMEIRQRYERLIQSNTFYYVILVETTCLCVTITSILTE
FKKGKLTFRAWLPFDYSSPLLFLFVYAHQLISFTVGSVHHVACDSLICGLLVHICSQLEILECRIRKGTRNPK
ILQDCALQHNHIFKFAHMVNDKFRITIFIQFIVSTLVVCFNLYQFTKSTALRAKYMQLILYMCSMLSQIFFYC
WCGNEVKLRSRQLVNNVFEMEWFELNENAKKILLLIMRRGIVPIEFTSASVISMNLDSFVGLLKTSYSAYNIL
QQTQD
>CfOr242

MHILDSTFKFLIICGCWRPDSWTSSCKRVIYHMHTVFILVLINTFTLSQLLDIILTVDNPDDFTDNFYMLLAM
IVSCCKMLSMLMNRKNIAILTNILTEKPCKPLESNEMEIYHKFDKGVQANTIHYAILVETTCVCITLTSLLTD
FRKRMLTFRAWLPYDYSLPFLYYITYAHQLISLIMGSVLHVACDGLICGLLVHICCQIKILESRLRRIAREPD
ILHDCILQHNRIFDLAFAVNEKFRFTITIQFVVSTLVVCFNLYQLTRTTTTKAKYIQLALYMCSMLTQIFFYC
WYGNEVKLK
>CfOr243
ERKKFKMHILELTFKVLTICGCWQPESWTSVHKRIIYRVYTFLVILLINSFVLSQFMDIILIVDNTDDFCDNF
CVLLPMIIVCYKLFSLLGSHKNIIKLTDILMKKPCKPLKPNEIKIYYKFDKGMQINTFHYTAMCMVTCVIITI
TSLLTNFGQKKLTYRAWVPFEYSSMTLFCILYIHQLIGLTMGALVNVACDSLICGLLVHVCCQYEILTYRLKR
IMLHSDGLRNCVQQHCKIFRLAFLVNTNFRMVITIQFLMSMLVVCFNLYVISLSKLDARCIRLALFMGCMLTQ
IFVYCWFGNEVRLKSRQFIDNIFEIEWLTLDKNLKKSLIIMMKRTVMPIEITSAYTISLNLDSFMNVLKTSYS
IYNLLQQMKE
>CfOr244
EQKKLKMNVLEFPFKVLTICGCWQPQSWTSIYKRIMYRMYTILIIILIVNSFTLSQFMDIILIVDNTDDFCDNF
CILLPMIITCFKLFSLLANRKNIIKLIDILTKEPCKPLKLNEIEIYYKFDNSIQLNTFHYTAMCLITWVSITF
TSLMTNFGERKLTFRAWVPFEYSTMMLFCLLYIHQLIGLIVGALLNAACDGLICGMMVHICCQFEILTYRLSR
IMLYSDGLRNCVQQHCSILRFAFLVNAKFRIIITIQFLMSMFEICFNLYQITLSKLDARCIRLALFMSCMLVE
VFVYCWYGNEIKLKCLQFVDKIFEMEWLTLDNKLKRSLIIMMARTIIPIEITSAYTISMDLDSFVGVLKTSYS
AYNLLQQMNE
>CfOr245
MRILEFTFKLLMIFGCWRPNSWTSIYKRIVYYVYSSIIILLLNTFMLSQLMDIILIVDNTDNLSDNLFLFAPI
VTTCCKLFILLLNRKNIIMLINILIEKPCRPLTSNEMKILSKFDKSIQSNTRRYTYLVTITSIFIVLTSLSAN
FKKRQLTYRAWLPFNYSSMTSFFLIYIHQLICLIVSGYLNVACDTLICGLLVHICCQIEILTYRLKKIMFYSD
ILRDCIRQHYYIFRLAFVINVKFRLTLTIQFIISMLVICFSLYQLSNRTAKAKYIEMVLYMICMLTQIFFYCW
YGNEVKLKSHQMIDDIFDIEWLTLDKNITKSLIIIMKRAVLPIQITTAYIIPMNLDSFMGLLKMSYSTYN
>CfOr246
LRVLQFTFKILSIVGCWRPESCSSLCMRIVYDTYTVFMVILLYTFLISQFLDIIWNVHNTEDFTENFYATLAS
VVSCSKMLSLLINRNNINTLTNILIEKPYRPSDIDEMKIRHKFDRLIYTNTFFYVILVETTCACITMTSLLTE
FRKRNLTYRAWLPYNYNSSTIVFCLTYAHQLISLTAGSLVNVACDSLICGLLVHICCQIEILEYRLSKISNGY
NNLHDCICHHDGIFNYALKLNKKFRMTIAMQFIVSTMVVCSNLYQMTKSSTVDASYLPLLLYMSCMLTQIFIY
CWYGNEVKLKSIQVVENIFGMNWLTLDKNFKQSLLIIMNRASIPIEFTSAYILSMNLDSFVGLLKTSYSVYNL
LKQV
>CfOr247
MRVLQFTFKILTIIGCWQPESWSSCMRTVYDVYTVSIVILMHTFLITQLLDIIWNVDDAEDFTENFYATLATF
VSCSKMFSLLMNRNNIDTLTNILVEKPYRPLDIEEMIIWHKFDRLVYINTLCYTILIVTTSICFTLTSLLTEF
RKRNLTYRAWLPYNYNSSTIVFCLTYAHQLISLIAGSLVNVACDSLICGLLVHICCQIEILEYRLSKISNGYN
NLHDCICHHDGIFKYALRLNKKFKMTIAMQFIVSTMVVCSNLYQMTKSSTVDASYLPLLLYMSCMLTQIFIYC
WYGNEVKLKSIQVVDNIFGINWLTLDKNFKQSLLIIINRASIPIEFTSAYILSMNLDSFVGLLKTSYSVYNLL
KQV
>CfOr249
MQILSLNFLIYTVVGIWRPIDWSSNVAKLLYNAFTFIVLVLEYFLMLTQFMDIVFVVDNIDDFITNTVMFLSI
VGVCFKATIVIIRRNSIISLVHILLKPPYKPRNEDEMAIQMKYDKFIKSCSIMYVLLVISSVTGVTAGSVLNI
MQGHLPYRVWLLYDIKVYLIFWITSIQQILSVILDAIITVGTETLVFGLFLQTCGQFEIFENRLHKLIINKIA
SYLNHLPASPNNKKVTISKYIHHHLCIYKFAKTINIIFNQIFFVQFFGSILLICTSVYYVSMHMMESRSATMI
IYTCSMFVQIYFFCWSGNEVILKSMNVGDAIYHTEWPLLSISDKKELLMIMMRSTIPIKFTSSFLITLSLQSF
SNILKTSYSAFNILQK
>CfOr250
MKILSFNFFLYTISGMWRPIEWSSKCSKMLYSMLTCFTMYFLIIFTLTQLLDIILVIENVDDFAMNTSMLLSF
MNVLFKAITVITHRDKIVNLIEILQKEPCKVYSEKEINIQMKFDCMIRSYSIKYASMCLLSVIGGIIKGLHQI
LKGHLPIRMWVPYDYTSPFLLWFTSIQTFVALIFATIVTTATETTILGFCLQICAQIEILKHRLQRMMKSSEK
KTSRISLNDESNEIGKLSEYILHHLCIIRLAKMINKVFSQVIFFQFFASILVLCTSLYHLSSHMTITYIISMI
MYVLCMFVQIFVYCWAGNEVILKSTGLSDAVYEMDWISMPINKQKDLLIIMKRSTRPIIFTSSFLVTLSLESY
VNLLKASYAAFNFLRQF
>CfOr251
MQILSFNFFLYTISGMWRPIEWSSKCSKMLYSMLTCFTMYLLIIFTLTQLLDIILVIDNMDDFAKNSLMFLSI
ISVLFKATAVITRRDEIVNLIETLQKKPCTVYNEEESDIQIKFDCLIRSYSIRYTSLASFSATGAVLGGMFNI
LEGELPYRMWVPYDYTSPLLFWFTSIQEVVAVIFGTIVNVATETTVLGFCLQICAQIEILKHRLQTMMKSNKK
KEIPISLNNTSNGRSILSEHILHHLCIIRLAKIINKVFSQVIFVQFFASILVLCTSLYHLSSHMTITDIISLI

IYVLCMFVQIFVYCWAGNEVILKSTGLSEAVYEMDWVLMPISEQKDLLMIMRCSTRPIKFTSSFLVTLSLESY
GNLLKTSYSAFNLLQQ
>CfOr252
MQLFSLNFLMYTVGGVWRPIKWSSNVAKLLYSIFTSIILVLLYFLMLTQFMDILLVVDNIDDFATNTLMFLTI
VAVTCKATIVVVRRNAIIKLVQTLLEPPCKPRNEDEMAIQKKFNKFIRSCSIKYSLLATSSVTGVTVRSVLNV
TQGYLPYRIWVPYDTDTSPMFLITSIQQIITVVFVTIINVGTETLVFGLFLQTCAQFEIFENRLHKLISNKIA
KYLNPASSNKGRAIISEYIYHHLSIYKYAKTVNVIFNQVLFVQFFGSILVLCTSVYYLSAHITESESATLIVY
TICMFVQIFVYCWSGNEVILKSISVGDTIYHMDWPLLSISEKKELLMIMMRSTLPIKFTSSFLITLSLQSYSG
ILKISYSAFNVLQK
>CfOr253
MQILSLNFLIYTVVGIWRPIDWSSNVAKLLYNTFTFIVLVLEYFLMLTQFMDIVLVVDNIDDFATNTLMFLSI
VGVCCKATIVVVRRNSIINLVQILLKAPYKPRNEDEVAIQMKYDKFIKSCSIVYLLLVISSASGVTAGSVLDI
MQGHLPYRVWVPYDTNVSPIFWIMSIQQILSVFFAAIISVGTESLIFGLFLQTCAQFEIFECRLHKLMNNKIA
NYLNHFPVSPNNEKVTISKYIHHHLCIYKFAKTVNIIFNQIFFIQFFGSILLICTSVYYVSTHIMESRSATMX
VYTCSMFVQIYFFCWSGNEVILKSINVGDAIYHTEWPLLPISDKKELLMIMMRSTIPVKFTSSFLITLSLQSF
SNILRTSYSAFNILQK
>CfOr254
MQILSLNFLIYTIGGVWQPIEWSSNSAKLLYNAFTFIVLVLEYFLTLTQFMDIVLVIDNIDDFVANSVTFVSM
IGVCCKATTIVIRRSAIVNLIQVLCSKPCKPQNKDEETIQTKFNEFIRSWSIKYSLLVVSSFTGVIIGSVLNI
MQGHLPCRIWLPYDINASPLFWIIFVQQILSVFFASVINVGTETLIFGLLLQICAQFEIFESRLRKLVARKTT
KCLKYSSIPSDNERLIISQYIHHHLSIYKYAKMVNIIFNQVLFIQFCGSILVLCTCVYYLSAHITEFESATLI
IYTICMFIQIYIYCWSGNEVILKSKSVGDAIYHMDWPLLSVNEKKDLLIIMIRSTIPIKFTSSFLITLSLQSY
SNILKTSYSVFNL
>CfOr255
MQILSLNFFMNTIAGIWRPIDWSSNTAKLLYNAFTFIVLILEYFLMLTQFMDIVFVVDNIDDFATNTLMFLTI
VAICCKATVIIIRRNSIIRLVQKLLKAPYKPQDEDEMAVQTKCDKFIKSWSIKYIVLVIGSVTGITIGSVLPY
RVWLPYDTNASLIFWITSIQQIVSTIFGAIINVGTETLIFGLSLQTCAQFEIYEIRLRKLVTSKITNYLNHLP
ASSKIRKVAISEYIHHHLCIYRFAKTINIIFNQILFIQFCGSILILCTSVYYVSTHMMESESATLIVYTFGMF
VQIYVICWSGNEVMLKSMSIGDAIYHMDWPLLSTSEKKELLIIMMRSTIPIKFTSSFLITLSLQSFSSLLRTS
YSAFNILQK
>CfOr256
MEILSLNFFMYTIAGIWRPIDWSSNVAKLLYNTFTFIVLILEYFQMFTQFMDIILVVDNIDDFATNTLMFLTI
VAVCCKATVIVVRRNSIISLVQILLSTPHKPRNEDEMAIQTKYDKFIKSCSIKYSILVASSLTGLTIGSVLNI
MQGXLPYRVWLPYDTNASSIFWIISIQQIVSVYFGAIISVASDSLIFGFILQICAQFDIFENRLHKLMTNKIS
NYLNQLPTSNIEKIAISEYVHHHLCIYKFAKTTNIIFNQIFFIQFFGSILLICTNIYYVSTHMMEYGSATVII
YTFGMFVQIYFFCWSGNEVILKSMNVGDAIYHMDWLLLSPREKKELLIIMMRSTIPIKFTSSFLITLSLQSFS
SILKTSYSAFNVLQK
>CfOr257
MQRLSLNFLMYTISGIWRPIEWSSNCAKLLYNVFTTFIIVAVYFLMLTQFMDIVFIVDNIDDFATNSLMFMTI
VSVCCKATIAVIRRNAIIDLVDMLLKDPCKPQNEAEGAIQMKFEEFIRSCSIKYSYLSTSSVTFFTIRSIINI
TQGRLPFRVWLPYDCNKPLMFWITFVHQFITSIFATIISVGTDTLICGLFLQTCVQFEIFECRLQLAINTKAQ
CPEYFSQISSDKGKTMSKYIKHHLSIYNYAKTLNSIFNQVLFCQFFGSILLLCTSVYYISTHITGSEVATMSM
YTVCMFAQIFVYCWSGNEVILKSNSIGNAVYNMNWFLLSINERKELLMIMKRSTIPIKFTSSFLITFSLQSYS
NVLKTSYSAFNVLQQ
>CfOr258
MDILPVNFKALQFCGAWKEREDDNMCVAFLRFCYRYAVVFLIYEFTILDAVEVIRTRDHIHELTEGLFLGLTF
LTLSVKYANFLLRENKVSELLDYLRIKMCQPKNSTEQLIMEEHNRKAKWSTVSFMMISFATGLGFMITPALGL
LTKNEHVLPLKSYIPYSVSNLFIYLATYLQQFITLFYGIMLNVSFDSLIYGFIIHTCAQIELMCHRLTENLNC
ISSERKSQTNVSIEECVRHHLLVKILVKKMQELFIWSVMVFFFFSLVIVCTSIFLISKTRLLSFEFLSMLLYL
SGMLLQLFYYCWYGNELELKSKGIATAIYSSDWTKVTPQDRKSLIFIMINSQKGIMFSYHGIFALSLNTFTWI
CRTSYSAYNLLQQASN
>CfOr259
MDILPVNFKVLWFCGAWKEQEDDNIILSCLHFCYKYAIFFLIYEFTIFEVIELIRMRDRINELTEGLFLASTY
ITLCLKYANFLLRKNDVSEVLDCLRVKLCQPRNSTEKIIIETHNRKAKWSTLSFLIMSQATAVGLVMAPILGL
GKGEWFLPTKSYVPYSISEIFPYVATYLQQAAALFYAIMLNVSFDSLVYGFTIHACGQIELICSRLTDNNKAS
INFQKKSDATISIEECIRHHILVHTLVKKIGALFIWTVMILFFFSLIILCTSIFLISKTKLFSIQFLSLTLYF
SSMMLQIFFYCWYGNELELKSKSIANSIYLSNWTLTTLHERKSLILIMINSQKGLTFSNNKIFALSLDTFTWI
FKTSYSAFNILQQVSN

>CfOr260
MDLLPINFFVFRFCGIWKEHENSNLIIRFAIFCHRYMIAVLIYHLTIFEIIELIRIRNSMEAVMEXFFXXXFT
FVSLCLKYLNFSMRQCELHALLSCFRVKICQPRDFAEKLILKQYDRKAKGIVCFYMLMCQTTGLMFMIMPLLI
PDEKSLPFKTYIPYSITTLLPYVLTYLQQSATLIYGILLNVSLDSLAYGFIIHTCGQIELLCYRLTEIFQFLQ
ENNKKNAYAIESFAIAECAKHHILVYDIIYRIESLFMWNVAALFFFSLINLCTNIYQMSKKELFGPEFFSFIL
YLGSMMFQIFTYCWYGNELDLKNKNISSAIYTSNWMTISTKQRKNLLLMMMMSQKGRILSFYGICALILSSFT
WIIKTSYSAFNLLQQSSN
>CfOr261
MDILPVNFKVLWFCGAWREQKNDNIILGSLHFCYKYAIFFLIYEFTIFEVIELIRMRDRINELTEGLFLASTY
ITLCLKYANFLLRKNDVSELLDYLRVKMCQPRNLTEKMIIETHSRKAKWSNLSFLIMCQATTVGFVIAPILGF
GKDEWILPTKSYVPYSVSKILPYAATYLQQTAALFYAVMLNVSFDSLVYGFTIHACGQIELICCRLTNNIRAS
VNFGKNSDVTASIEECVRHHILVHTLVKKIGELFIWTVMVLFFFSLIILCTSIFLISKTKLFSIEFLSLILYF
SSIMLQIFFYCWYGNELELKSKNIGNSIYFSNWTLTTSHERRSLILIMINSQRGLTFSNNRMFALSLDTFTWI
FKTSYSAFNLLQQASN
>CfOr262
MDILPVNFKVLWFCGAWKERKNDNFVLGCLHFCYKYAIFFLIYVFTIFEIIEVIRTRDQIDELTEGLFLASTH
ITLCLKYTNFLLRKKDVSELLDCLRVKVCQPKNSTEKMIIKMHIRKAKWSTLSFLIMSYTTAMGFVIAPIILG
LSKNEWILPTKSYVPYSTSETLPYVATYLQQIASLFYAIMLNVSFDSLVYGFTIHACGQIELICCRLTNNIRG
SVNFQKDSDSTASIEECVRHHILVHTLVKKVGALFIWTVMVLFFFSLIILCTSIFLISKTKLFSIEFLSLTLY
FSSMMLQIFFYCWYGNELELKSKSIANSIYFSNWTLTTSYERRSLILIMINSQKGLTFSNNKIFALSLDTFTW
IFKTSYSAFNLLQQASN
>CfOr263
MDILPLNFRVLWFCGAWREESNNGLFVRFISFCYRYSIVILIYEFTISEVIELIRTHDHIEDLTEGLFLALTY
VALCIKYGNFLARQDEVYTLLDCFRGETCQPKNFEEKMILIKYDRKAKWCVRAFMSISQATCIALVLAPIVGP
QDTDRPLPFKTYLPYSIVGLYPYLATYLQHIGAIFYGVLLNVSFDSLVYGFTLHVCGQIELLCYRLSEIFKDY
PDMAQYRLNSNKGGVISQCVRHHLCVHEIVRRIQSLFVWTVMLLFMFSMVTLCTSIFQMSKKKILSVGFLSLI
LYLGSMLFQVFFYCWYGNELQLKSKSIGDAIYSSNWTTATIQDRRSLLFMMSISQKGLKLSYYGIFSLALGTF
TWILKTSYSAFNVLQQTSI
>CfOr264
MDLLPVNFFVFRFCGIWKEHKDSNLIIRFAIFCYRYIIAILIYHLTIVEIIELIRIRNDVESVTESLFVVLTF
MSLCLKYLNFSVRQCELRALLDCFRTKICQPKDFAEKSILKQYNRKAKEISCVYMFMCQITGLLFLIMPLLIQ
DKRSLPFKTYIPYSTTTLLPYVLTYLHQSIGLIYRILLNVSLDSIVYGFIIHTCGQIELLCHRLRQIFQFLQN
NNEKNAIAIESFAIAECVRHHILVYNIMYKIQSLFMWNVAALFFFSLINLCTNIYQMSKKKVVSLEFFYFNMY
LGSLMFQIFTFCWYGNELDLKNKNISSAIYTSNWMTISTKQRKNLLLMMMMSQKGRILSFYGICALILSSFTW
IIKTSYSAFNLLQQAS
>CfOr265
MEGDMLKYYSRNQYFLSQMGIWPYQPRMIKILLPCFLVGAEMSVLATQILLLYNTWGDLSMTIEGIITSILLV
GATTKLVNVVTNNKKLQYLLQVMNEHWRLFHSECELHILRYYATIGQKVTKYYSVYINIFVVLFMLIPLMPKV
LDVIIPLNESRPVIYVLEGDWGVDKDKYYFLILLHCYLAAVISTRCMVNVDTMYMMCVLHGCSLFNAIGMSLE
NILCKTKLVQDEEIMNIEQAVIKEYNSQDYCEMIACLRKHQLAIKYTQMLDSTFKSATFLILVLNIMILSLIG
LQLINKFGQTQEVIRFGCIAMGSVTHLLSMCLPGQLLLDKSIEVFDKAQILRVYNAQWYMFSLKTTKLLSVLL
YRSVVPCTLSAANMYIMSMTTFSSVMQTAMSYFTTFLSV
>CfOr266
MHAEMEEYYFINKFFLSRIGAWPYQHKVLKVLLPCFLTIVQYSVIATEIILLHDTWGDVDIAVESVIIIIPIV
GASTKLINIVVNNDKFRHLLRIMNEHWSIFNSESERYILKYYAKIGRKVTKYYAVYCCTILVLYLFIPLSPRI
LDMVIPLNESRPLKYIYQAEYRVDKDKYYYPILFHAYMSSVITVGIILSIDTMYVICVLHACSLFTAISHRLE
NIVGQTDVKTDDNEQIRTRTHYHLSIERYDSINEDYRELMICLRKHQLALEHVRILDSTFTHAMFILLSLNVL
IMSVIGLQLINKLGHTEEVIRYICVTVGAFTHLVCMCFPGQLLIDRSAEIFEKAYCSQWYTFSIKSRRLLKIL
LYRSLVPCTLTAGKMFVMSMTMCSSVMQTAMSYFTTLGSLK
>CfOr267
MQSDILRYYHVNKIFMSQIGVWPYQNRAIRILIPTVLTFIDISYVAAEMIRMFDTWGDVDIAVECIISTIIVF
ACFTKLFNLSFRINEMRYLFSLIEYHWQVFNNSTDVEILQNYVVFGRKVVIFYSIYVYVSMILYLLMPMSPQI
LDIMMPLNESRPRKFLFEVEYRIDREKYYYLILFHSYVAVIGVMSIVVCADTTYIAYVQHGCSLFAAIGYRLE
HIVSREYELSQMNYFTDNEERKCRDMEVDDIYVNQQTVYRELVICLRKHQLAIQYARLLESSFMLSTGIQLSC
NMLALSLIGIQAKYAVISNLDSTEDLIRYLSLCAGAFFHLLWMSLPGQRLMDHSMKIFDKACRSHWYTFSAES
KRLFRILLYRSNVACTLTAGKIYVMSMENYSMVVQTAMSYFMTFSSL
>CfOr268

MEFSLDRYYKLNRLLLSGIGLWPYQSATNAWIQRVFAISFMIWGVIVQIAKMCMSELTMDFTLDCLPILIPNI
GILLQFIDRIIINDKLKSLLDHIKIDWERTRSRNDIKIMRKYAASARLITILFSFYTTMGLSVYIFITILPQI
LDVFLPLNETRSREHPFRMEFFIDEEKFFYLIRIQMYLIIILMMVTILANGTVFVVYTRHASGMFTILGNRAE
HLFSERSSKFGRSNRRTEKEYRNIVLFVEDHRNVIQFVDVIRSSYSLSLFGEYLFFMIVIGLTLVQIIKFSGL
SDRPIRSIAFMVGQLFYLLMFSYMGQHIIDTSIELSTKIYCGKWHNMSVWKQKIMLFIMIRCMRAVSINSYSI
YTLSLETFAMIVQSGISICMLL
>CfOr269
MIEERDIWQSRYYAIPRFYMTLAGLWPYHSIRNRYLHFVPIFTICFFLLVXKLICVLYIFLYIFIVRKHLKCF
LISNFYNIHIYNVFFYFEIIKSCLKTIENDWLSLNTDNEKAILQRQTAYGRYLTIFYAIFMQLTGFLYILKSV
VLIMIDDTSNSTKLAVTKLPFRVEYGHKIDQYFYLILTHNYLTVFSHVTATVATDTFYFILIQHACGMFSVVG
HSLERIGKDSNNSFDSKPDKINDVNYYKVLDCLRKHLHVIEFAELIESTFADILLISISLNMIGGSICGIQVL
INLNDAKDIIAPLAIYVAQLTHMFLQFWQAQFLLDYSVLPYESICKANWYYTSERCRKLLLLIMNRTILPCRI
TAGRVVILSIESFGVVLKTSMSYFTMLRSFH
>CfOr270
MTERRDVWQNRCYIIPRVYMSLIGIWPYHAFRDRCLLFVPMFTFSLTILMPQLLYLLITATNLXRDVFSCTPS
MWITIIFSFKLGSLMINNKKLKTCLETIEDDWSSLSSDMERAVLRRHSAYGQYITMTYGVFMQFVGVLLILKS
LLVILIEDTSDATITSLAAESKLPFRVEYGEKFGRYLYPMTIHCYLAVFAHISITIAVDTFYIALVWHACGMF
AIVGNTLEYIGKDSDNNFDLKPDKIVDGNYSKALECLRKHLHVIQFAELIESTFSNIFLVSVCLNMIGGGMIG
TVKHIFQVILNLNDAKDIVGPLAIYIGQLIHLFLQFWSAQLLLDYSIVPYQSICRSNWYYTSKRCRKLLFLIM
NRSVLPCRITAGKIVALSIESFGTVLKTSMSYFTMMRSFN
>CfOr271
TAQRSIWQSRYYMVMRMYMTLVGIWPYLTVNTRYLRFVTTFMFSMGIAVPQILYLLNDSTDIDDVFECVPSIT
IALMFSLKLVNVMIHSEKVKVCFKTMEEDWLSLKTDVEKAILRQYTEYGQYMSTSYALIIILVPALSHLLKPII
TTLKENDIENITKSSISRSSKLPFRVEYGEKLDQYFYVIMVHCCLAVFAHLVATVAVDSFYYTVIQHACGMFS
IIGHMLENIGKNDDANLEENNIKDNNYSIALECLRKHLHVLEFAGLIESIFTNMFLISVSCNVMMGSLSGIQV
IMNLNSAGNIATPLSVISTHQINSFLELTKIFLSQDNFFLSNNMDLVLCYSCRARWYRTSRKCKKILILIMSR
TMLPCKITAGKVVTLSIESFSAVLRTSMSYLTVLRSFQ
>CfOr272
ITAQRSICQSRYYMVPRIHMTLVGIWPYNTIGIRYLRFVTMLTFSIGIVVPQILYLLNDSTDIDDVFECVPSI
TIALMFSLKLVNVMIHSEKIKVCFKTIEEDWLSLKTDVEKAILRQYTEYGHRMSLSYALIIILVPALFHLLKPV
ITTLMENDIENITKSSISRASKFPFRVEYGEKLDQYFYVIMVHCCLAVFAHLVATVAVDSFYYTVIQHACGMF
SIIGHMLENIGKNDDANLEENNIKDNNYGIALECLRRHLHVLEFAGLIESIFTNMFLFTISCNMVLGSFSGIQ
VIMNLNGAGNIATPLSVYFAQLTILFLQFWQGQFLLNSNDVPYESICRARWYCTSRKCKKILILIMSRTMLPC
KITAGKVVTLSIESFGAVLKTSMSYLMVLRSFQ
>CfOr273
PNDIFQSRQYKYNRILLSLLGQWPFQKKRDRWIVFLAVSFIGITQFISQILALVTLRGDLDATLECVAPLLVN
CLCVVKLTNLVYNMKGIKILLMHIQGDWQSWNIDSEFKILYRFAESGRSITIRLCHLGGMYAFGSLFPFLAII
PKIIGKNVTSNYSTRPVGFPYHVEYFVDLDKYYYPVLIHNYLATAIRLTIIVASDTCVIILVQHCCALFSIIR
YRMEHIRKAIKEDKQFAFLEEDDKVYKNFIHCIRKHEDAIQFANCLEAIYKQALFVEVGLIVSMMSLSALQAT
NDTLTPQVAVRHGGYIMAQLIHLFXLYCWLGQQIIDHSDHVYTAIYRGEWYESSSKSKKLLNMIMLRSISPCT
LTVGKIMVLSLPSFSAVVRASASYFTVLQSVQ
>CfOr274
MSFYDNRYYYINKTLLSLIGQWPFQSRLESNIILVIMLFFSCSLTVLELWGLIAGINNLSIIMENASPLLINS
IIFMKLINCLCNKNKMKNLLEHIEETWKRTQVGPQNKILQNYAEQNRTLIIRYACEKNIVVFKKKICNLQLTI
TYLFLKQLCVLFTPIDSSHYFVHKSIKKLLYQTFFLKVPLSILKLKFIISSQLHSRPILRLTEALLEKTFDTK
VSYFFTHSVYICIYICIYNFMLNTYKFSELLSSTYATYFLFLLGNVVIASSFSAAELIIADLQLDEIIRIVSC
NIGLLLHIFYLSLMSQRMTDHSSRFREVIYSCNWYKISQRSKKLLRFTLLRASKPCQIKAGKMFVMSMENFSS
IIRACMSYVAVLTSLQ
>CfOr275
MSFYDNHYYYINKMLLSLIGQWPFQSRMESNVMLVITLFLSCSFTSLQIWGLIAGIRDLNIIMETASPLFISI
LVLLKLINCLCNKDKMKHLLKRIEETWKRIQIGPENKILQNHAEQNRTLIIKYASTLYILSTLYSTMPVVVKT
LHSLLPTNETYATRFLFRLDHVLDVDKYFNLLMLNGILGIFFLMSVWVAADGMFILCTQHVCALFETVQYNVK
RIQGSDFVIDEPNIMDDEAYHVIISCIKSYEYALKFSELLSSTYATHFLFLLGNVVVAVSFSVAELIMMDIPL
DETVRLTACNIGLLLHIFYLSLMAQQIIDYSDKFQEVIYSCNWYKISQRSKKLLRFTLLRASKPCQIKAGKMF
VMSMENFSSIIRACMSYVAVLTSLQ
>CfOr276
MSFYDNHYYYINKMLLSLIGQWPFQSRLEGNIMFVITLFFICSLTILELWGLIAGITDLSVIIENVSPLLVNN
SVFIKLINSLYNNYKMKDLLEHIEETWKMTQTKPERKILESYAERTRTLTIQYAITIYAMWISYSTMPFVIKG

IHLLLPSNETYPVKFLYRLEHVLDVDKYFNLLMLHGIISIFYIVSIPIAVDTMFILYVQHTCALFKNIWYVHN
MKNIRSSDFEMLKPDIANDETYHIIIKYVKSYKYALRFSDLLSSTYTTCNFFLLGNVIISLSFSAAELIMVAN
ELDEIVRIIASNMAQLIHIFYLNLISQRLIDHSSRLQEAIYNCDWYKLSLRSRHLLRFMLMRAIKPCYIRAGK
MYVMSLENFSSILQVSMSYFTMLTSMQ
>CfOr277
MICIKIRHLSLQRILLLAVGLWPCQRSRLVQFQVILFSSIVISIIVIQFASFVTEEFTLDVVINVLSVGLVFI
MFAIIYNSFCYNFAEIIEHPLEELQDICNELRDEKEIVIIEKYADNMKRFTFIFVLWHVCNLLIVSSIILLPI
TSHIFGFAQHINEAKSREILYKHIPKYLVDRKNYLYLILLYIDASLCIGGTALIATGLTILTYFKYVCGMIRI
ARNRKMQFNILIFKIDLWKKNYSVKLIVACSLSNKILFLVNYLILXIKIYFFYAIERYGLFVDTYKFAVKKII
YXSINLVLFACNKSLYNVQWYLAPLRIQKLILFLLQRNSKTISLNFGGVFILSLEFFASLTKISVSYFTVLYS
LQ
>CfOr278
MISIKNQHFNVHRILLLAIGLWPCQQSKLVKFHLILFFGILISFTVFQLIIFVTEEFTLDLVINVFSIGLVFA
MYTIMYNAFRYNFTEIVERPLEELQHICNELKDKKEIAIMKKYGENMKRFTFFITVWYICNLIIVLLIISLPI
MPDIFGVVLHISESQSRDILYKHIPKYFVDRKNYLYVILLYIDVTVCIGATAMVGTGLMLFAYLKHACGMLKI
ASYRIEKVMNMRNNVCPKIETMMYKGIIYAIDIHRTAIKFSTFLFSYLHFSLFFLIITAVVCLALNFYTVSFF
HIMVLHRDEVDQFFIHLIMVATIFAYLFFLNYVGQEFTDHNDNVFFTIQYSVVTPLRIQKLILFLLQRGNKAV
NLNFGGVFILSLQFFATLTKVSLSYFTVMCSVQE
>CfOr279
MEHNRNYYYDISKRFLWMVGQWPYQKPKTRLSFMALVVIVLANCLFTQIAQIFVCEDTQCIFQTLPPHLLVWN
SLVKVLAYRFNSQKIKDLTDHLFVDWDTLKTQEEREIMKKYADNGRWYSLIYASYCYVSTVSFITTSLVPRIM
DIVFPLNTSRPIMLAYPAHYFVNEEQYFYYISFHMLITALICVTGLIAHDCMFFIYIEHVCGLFAVVGFRFKH
ALDKQDITRSNLIDCSDNLYHKNVTFSIHAHREALQFAKLIENAFSVPFAIQLMISTMSISVSLLQFSMQLND
LMEAMRYFVYILAQLFHLFCFSFQGQKLIDHSLEICDKIYNSAWYEIPLKGQKLLLITMRKSIEASTLTACKI
YVFSLQNFTMVLQTAMSYFTVLASF
>CfOr280
MKYNNNYYYDINKTFLSIIGQWPYQKPKAKLFFLVFALIFLSNSFVTQMAYMFIHRDNMDCILQILPFFFLMI
NIIVKVLTYRLKSEKIKALTDHLFADWNMLETRKEREILRKYAENGRRYTLIYSCKMYMYVTAISYAITSLMP
RIMDIVFPLNTSRPIMLLWPAYYFVDEKKYYYYIYCDMLIILMVWLAVLIAHDSMFFIYIQHVCGLFAAIGFR
FKYLICKRDKKSLIDCPDDIYHKRIVFSIHAHRKALQFAILIENVFSKSFAIQLAVNTVSISISLLQFSIHLH
DMAEAMRYCLYIIAQLFHLFCFSFQGQKLINHSLETHNKIYNGVWYEMPVKEQKLLLLVMRKSIEASTVTACK
IYIFSLQNFTTVLQSAMSYFMMLASFN
>CfOr281
RSMESNMEYYYNINKRLLSLIGQWPYQKPKEKWAFLILILIIVTNLLITQVAQFFICEDAQCIYQTLPPHMLG
IMLLVKIFTFYFNKQKIKVLTDRLFIDMDMFENQDERKIMKRYAESGRWYTLTYASYVYIATLSFATTALIPR
ILDIVSPLNTSRPIVLAYPAYYFVNEEKYFYYIFCHMFVTAGLGLTGLIAHDCMLFAYIEHVCGLFAVIGFRF
EHMSYNHDSAKKNIINYSHDICYKNIVFSIHAHRKALQFAVLLESTFTVSFAIQILIVTVGMSITLVQFTMQL
YNLAEAMRYMVFIVAQMFHLFCLSFQGQKLINHSLETCDKIFHSSWYEIPVKAQRLLLMVMRKSIVASTLTAG
KIYIFSLESFTMVLQTSMSYFTVLSSF
>CfOr282
LKVMDTQNHYYNIVSKISSLTGVWPFLKPRARLFRVTLMTITIFTIFIPQIAYQFTCNRDLHCIFEGCSSYLL
SSIAALKVYTFHLNNNTIKDLTRHLFVDWKKVGNPEEYEIMKSYARNSRRFSLIYSIYCSMAVFMFMSMSLIP
FVLDIVSPLNQSRPILSPYPGHYFVDIREYFLQIFWHSLVAWQILTTGIIAHDCMYVTFVEHICSMFAVIGFR
FERLFHNHDEPTEIVDLNDMYRKKVAFIVRTHRESLKYAELLEDTFNMPFAAQILMVTIGMSVTLLQISRQDG
DLLDLIRYVLYVVGQLIHLFLLSFEGQKLIDHSLQTRDRIYNSAWYKASIKSQKLLMLVMIKCLRPCVLSAGK
IYIFSLESFTMILQTSMSYFTVLASFQ
>CfOr283
MKSVWNYYYSITKRMLLLAGQWPYQRKKERLLRMSLMTLTSLSMIVPQIGKFIQCDKDVQCILTVIPTHLFQL
VVIVKLYTCQFNNSKIKDLTDKVYSDWKSVEIPEECEIMKTYAAKARLFTLIYTSYYFIAAPIFVLITLTPQI
LDIVLPLNESRPILMPYEAHYFVRDDTEYFFYIFVHTLVGIIILCIAILAHDCMILTCIEHVCGIFAVAGFRF
ENLAYNENIDIMNNNLDNIYNQKIALSVHAHWRALQFAELLEDTFSITFIIQILINTAVMSVTLLKIAVQLDD
AMEAVRYIAFVIGQLIHLFCFSLQGQRLIDHSLQMRDKIYNGSWYKIPVKSQKMLLHVMRQCLQPNFLSAGKI
YIFSLKSFTTVLQSSMSYFTVLTSFE
>CfOr284
LKTMDATWNYYYNFVYKISSLVGLWPFLKPRTRIFRVTIFTIALFNISIPQIAYQFTCKKDLHCIFEGSTSYL
CTIVIVLKMYTFQLNINTIKDLTRNLFVDWKKIRNPEEYEIMKSYARNTRRFSIVYTVYCSMAVFTFMSMSLI
PFVLDIVSPLNQSRPVLPPYPGYYFVDIRENFLQIFLHSLIVWQILMSGIMAHDSMYVMFVEHICGMFSVIGF
RFERLSYNHDELTEIVDPNDIYRKKVAFIVRMHRECLKYSKLLEDTFNMPFAAQMLIATIVMSVTLLQISRQD

GELLLDLIRYMLYVIGQLIHLFILNFEGQKLIDHSVQTRDRIYNSAWYKASIKSQKLLMLVMIKCLRPSVLSAG
KIYIFSLENFTAVLQTSMSYFTVLASF
>CfOr285
HYYNTIHKISSLSGLWPFLKPRVRIFRVTLFTITVFTIIVPQIAFQFTCKEDLQCTFKSISSYLLTISIMLKV
YTFQLNINAIKDLTRHLFVNWEKAESPEEYEIMKSYAQSSRRFSLIYSVYCIIAVFIFVSISLIPFVLDIVSP
LNQSRPVLLPYPGYYFVDIHEYFWQIFWHSLVAWQILTIGMIAHDCMYVTFVEHICSMFSVIGFRFEHLIYNY
DKTKEIVDLNDMYRKKVAFIVRMHRESLKYTKLLEDTFNMPLAAQMLMVTAGISLTLLQLSRQDSDLLDLIRY
MLYVIGQLIHLFILNFEGQKLIDHSVQTRDRIYNSAWYKASMKSQKLLMLVMMKCLRLSVLSAGRIYIFSLQN
FTMVLQTSMSYFTVLASFQ
>CfOr286
MESNIVYYYDINKRFLSLVGQWPYQTPKEQRSFLILIMIIIINTMVTLVAKFFICKDAQCIYASLPPNMLAVA
VLVKIFTFYFNKQKIKDLTDRLFMNWDMLENQEEREIMRKYTKTGKWYALIYACYIYIGLMLFASIALIPRIL
DIVFPLNTSRPIELVYPAYYFVNEEQYFYYIFCHMIISAELSLTAVVATDCMLFVYMEHLCGLFAVIGFRFEH
MLYKYNNVKKLMINRSDDMYCKNVMFAVHVHREALQFAILLENTFSLSFGIQMLIAVVGMSISLVQLHDFGEA
MRYMLFIGGQLIHLFCYSFQGQKLINHSTGICDKIYNGSWYEIPITAQRLLLMVMRKGIEASTFTAGKIYVFS
LANFTAVLQTSMSYFTVLSSFN
>CfOr287
LKTMDATWNYYYNFVYKISSLVGLWPFLKPRTRIFRVTVFTITSFTAFIPQIAFQFTCKEDLRCTFKASTSYL
YTILIMLKMYTFQLNINTMKDLTRHLFVDWKKAENPEEYKIMKLYAQNSRRFSLLYLIYCSMGIFIFVSISLI
PFVLDIVSPLNQSRPVLLPYPGYYFVDIREYFLQIFWHSLVAWQILTTGIIAHDCMYVTFVEHICSMFSVIGF
RFEHLIYNYDKTKEIVDLNGMYRKKVAFIVRMHRESLKYTKLLEDTFNMPFAAQMLIVTVGMSLTLLQLSRQD
SNLLDLIRYMLYVIGQLIHLFILNFEGQKLIDHSVQTRDRIYNSAWYKASMKSQKLLMLVMMKCLRLSVLSAG
RIYIFSLQNFTMVLQTSMSYFTVLASFQ
>CfOr288
MKYIWNYYYSITKKMLLFAGQWPYQKKREKLLRTILLITTELSVMITQVGRVIQCDKNVQCILIGLPTYLVHI
VIMVKLIACQFNSNKIKYLTDQLYFDWKTLENPEEYEIMKTYAAKARLISLTYALYYLICCPLFIFTSLIPKI
LDIVVPLNESRPIIMPHECHYFVSDDTEYFYYIFLHAFISMIILVIPLLAHDCMILTYIEHVCGIFAVAGFRF
KNLAHNTGIENNNIENVYDKKIVSSVHTHWRALEYNCSWYKIPVKSQRLLLNVMRRSLQPNILSAGRIYIFSL
KSFMTVLQSAVSYFTVLASFQ
>CfOr289
MQCIWNYYYNITKKMLSLTGQWPYQTRGEKLPRMAFVTIVEISMLIPQVGKFIQCNQNLQCILISLPTTLVYS
VIMIKLLTCQFNSKKIKHLTDQLYIDWKHLKSPEEREIMKMYAARARLISLIYSSYYFICCPMFIFISLTPKI
LDIVMPLNESRPILMPHEAHYFVNDDREYFYYIFFHASISMIIIVMALLAHDCVILTYIEHVCGIFAVAGFRF
ENLAHNIDIENENIDNIYTQRIALSVHTHWRALQFAEFLENTFSVTLITQMFIIIVAMSVTLLQMVMQVGNSI
EMVRYAASVIGQLIHIFCFSLQGQKLIDHSLQMQDKIYNCSWYKIPVKSQKLILNVMGRSVQPNILSAGRIYI
FSLKSFMTVLQSAVSYFTVLASFQ
>CfOr290
MQTIWNYYYNVTKKMLSLTGQWPYQTRGEKLPRMAFVTIVEISMLIPQIGKFIQCDQNLQCILISLPTTLMYT
VILIKLLTCQFNSKKIKHLTDQLYIDWKHLKSPEEHEIMKMYAARARLISLIYSSYYFICCPMFIFTSLIPKI
LDIVLPLNESRPMLMPNEAHYFVSDDREYFYYIFFHVFISTFIILTGLLAHDCVILTYTEHVCGIFAVAGFRF
ENLARNTDIKNDKIDIQSKDVQCQIYTQKIALSVHAHWRALQYAEFLKNTFSVTLVIQMFIVIVAMSVTLLQM
AIQLEIIETTRYMAFVTGQLIHIFCFSLQGQRLIDHSLQMHDKIYKCSWYKMPVKSQRLLLNSMRRSLHPNIL
SAGRIYVFSLKNFMTVLQSSVSYFTVLASF
>CfOr291
MEFVWNYYYSITKRMLSLVGQWPYQKRKERLPRMTIVFLTELSVLVTQVGRFIQCGKNLQCILIGIPTYLLHT
VITVKLLTCQFYSSKIKYLTEQLYNDWKNLKSQEEYEIMKTYGARARLISLIYSSYYYISCPLFVLFSLTPKI
LDKLLPLNESRPILLPHECHYFVDHREYFYYIFFHVLISTFIVLTGLLAHDCVILTYIEHVCGIFAVAGFRFE
NLTHNTDIENDKINVQSKYIQCKTYNQEIALSVHVHWRALQYAEFLKNTFSVTLLIQMFIVIVAMSVTLLQMV
VQLENIIETTRYMAFFTGQLIHIFCFSLQGQRLIDHSLQIHDKIYNCSWYKIPVKSQKLLLNIMRRSSQPNIL
SAGRIYVFSLKSFMTVLQASMSYFTIV
>CfOr292
MESVWNYYYSITKRMLSLAGQWPYQNRRERLLRVILVTMTEFSVIVPQMGKFIQCDRDVHCIFMTIPTYLMHT
VIIVKLYTCQFNSGKIKKLTDQLYSHWKHLESPEECEIMKMYAAKARLFSFIYSSYYVICCPMFVLISLTPQI
LDIVLPLNESRPILLPYEAHYFVHDDREYFYYIFFHALIAIMIVITGVLAHDCMVLTYVEHVCSIFAVAGFRF
ENLTCNNAADLKINNSLIGMYNQKIAISVHAHWQALQFAELLEDTFSVTFAIQIAIVTVAMSISLLQMAVQLD
DILETIRCTAFVVGQLIHLFCFSLQGQKLIDHSLQMHDKIYNCSWYKIPVKSQRLLLNVMRRSLQPNILTAGG
LYIFSLKSFTTVLQSSVSYFTVLASFQ
>CfOr293

MISIKSRHFSLHRIFLLAIGLWPCQRSKLVQFQLILFFSILVNFVIIVFSSFVTEEFSLDLFINIFSIGLFFI
MFAILYNSFWYNFTTIVERPLEELQNICNELRDEEEIAIIKKYGDNLKRFTFTIMLFYICNLIVVLPIVSLPI
MRRLFEFILHINISESHYVIYTFAPKYFLARENYLYLILLFTDASICIGGTAMFATGMMLIAYLKHACGMLRI
ASYRIEKAINMRNNVWPKNEIIMYKGIIYAIDIHRTAIKFSTFLFSYLHLSHFFLIVVGVICLSLNFYAISKI
VSHGDKVDKFLFHFIIVTVIFVYLFLANYAGQELTDHNNDVFFTIYNVQWYGTPLRIQRLILFLLQRGSKTMN
LSLGGISTLSLEFFATLTKASISYFTVMYSAQQ
>CfOr294
MISIKNRHFNVHRILLLAIGLWPCQRSRLVQFQMILCFGILVSSIGFQLLTLITTEFTFDHVINAFSVGLVFT
MYVIMYNAFWYNFTEIVERPLEELQHICNELKDKEEIVIMKKYGDNIKRFTFYALVLYICDLIILLSAISLSI
MPNTFTVALHISESELRHILYKYIPKYFVDRKNYLCVVLLYFDAIVCIGATTFLATGLMLFAYLKHACGMLRI
ASYRIKKAINIRNNICPKNEAIMYKGIIYAIHIHRTAIKFSTIIFFSLHLSLFAFVIVAVICLTLNLYAVAKI
ALHENSIDQFFHIIIIIGIFVYLFFFNYMGQELTDHNNDVFLTVYNVQWYVAPLRIQKLILFLLQRGNKNVNL
NFGGVFILSLEFFASLMKVSTSYFTVIYSMQ
>CfOr295
MISIKNRHFNVHRILLLAVGLWPCQRSRLVQFQLLLCFGILVSSIGFQLLTLITVEFTFDHVINAFSVGLVFT
MYVIMYNAFWYNFTEIVERPLEELQHICNELKDKEEIVIMKKYGDNMKRFTFFSFIFKIKFLNNKSNTLISIL
KIHAINKIYNNESELRHILYKYIPKYFVDRKNYLCVVLLYFDAIVCIGATTFLATGLMLFGYLKHACAMLRIA
SYRFEKAINIRNNICPKNEATIYKEIIYAIHIHRTAIKFSTFLFSYLHFSLFFFIIIAAICLSLNLYAVAKIA
LHEKSIDQYLHIMIIIGIFVYLFFFNYIGQEFTDHNSDVFLTVYNVQWYVASLRIQKLILFLLQRGNKNVNLN
LGKMFILSLEFFASLMKASTSYFTVMYSVQ
>CfOr296
MDAFDTRYFRINKLLLSHVGLWPHKSSSNKNIILYCTIAGILXLALPQIAYLFKHATSVNDFYDVLPPLSGAC
ICLMKLISLFHNFNKFRMLIQHVQYDWRLLENHENFRILMEYLERNRAIPAFISTTATSYITAPLTVPMLDII
LALNVTRPKELPHSGEFFVDLXYYYILFAITCLGYYVSCTSVIAIDTIYFALLQHSXGMLAVLSHRLENCIIN
NKSNRVDCNGISKWDKDVENIIQCIQLQIRIERLIQLIESTFAMCLILDIGLGVIFQCTARVMVINISHNLCI
IVQIVTHTNTMEIMRYGQLLLLQNSRFFLNNWIGQEIINHSSQIPIAXYNGMWYQTSLKVKKMFLYLLMKSQK
PYRIIMLKLYXISLEDYTMLMKTSVSYITLMVSLNSDD
>CfOr297
MSFYDNRYYYLNKTFLSIIGQWPFQSRLEGNVMLAIAILFICSLTALELLGLIAGITNLNIVMENSSPLFINS
LIIVKLINSLCNKHKMKDLLENIEEIWKMTPIGPESKILRNYAEQNRIFTIQYAVALLSSGIFYSTMPIVVSG
IYSFLPTNENYTARFLYRLEHVLDVDKYFNLLMLHGIISIFYIAYVPIATDCMFILCIQHVCALFEVIFSRNR
YKINRIRSSDFVILKPNIEDDEAYHVIIDCIKSYKHALKLVLLVSFYCFFSFYFKFSDLLSSVYGLIMVDNQL
DETIRIVSAIMGLLTHIFYLSLTSQRLIDHSSELQEAIYSCEWYKISLRSRQLLKFTLMRATKPCQIKAKMYV
MSLENFSSILQVSMSYFTMLTSIQ
>CfOr299
MDFFDTRYFRINKLFLSFIGLWPYQTSFMKILTLSFAISGVTIMSWPQIAYLLKHLDNLDLDGVFEVMPTTSG
ALICAVKIISLTRNSEKFKVLLRQIYEDWRDLLTNQETQILTRYANNARKFTLVYSISIIGFVFCYAMLPLTA
PVLDIISPLNESRPKKMPHAAEFFVNQDKYYYVLLLNTYMGYIACVSIAVAADTIYVTLVEHICGMYDILCYR
LKNLITHDDLEWIHDNHVCGHDKIGQRIRCCIQLHEKIRLLVFIEMMESTFALFLLFDVGLGFILHTSSCVMF
VVRMGRSFEILRYVALVLLQSCRLFFNSWAGQEVTDHSAGISIAAYNGMWYNAPIEVQKCLILLIARSQKPSQ
ITIAKLYVVNLESFSKVMKTSVSYCTVIISLREMS
>CfOr300
MQGDMLRYYSRNQIFLSQMGIWPYQSMVIKILLPSFLVATEVSTFITQVFLLHNTWGDMNMTIEGIINTILLV
GATAKLINIVINNKKLQHLLELMDEHWRLFHSECELHILKLYANYGQKLTKYYSVYINIFIVMFMTIPLMPKI
LDIIIPLNESRPVIYVIEGDWKVDKEKYYYPILLHCYLTVVVTIRIMVHVDTMYMVCVLHGCSLFNAIGIRLE
NIFDKTKFMKGETVMNKEFLLREEHEDYCEMIACLKKHQLAIKYTRILDSTFRSVTFLILFLNVLILSLIGLQ
LINKLGQTQEVIRFGCIAVGSITHLLSICFPGQLLLDRSIDVFNKAQVSNAQWYMFSLKTRKLLSILLYRSLV
PCTLSAANMYVDDYISLLSFKVMQTAMSYFTTFLSV
>CfOr301
MINLETQHFHINRILLLIIGIWPYQRSPLIELQLILFLGTLISFIVFQLTTFITSECTVELIYKVLSSALFFI
CLAIKYNSFWINADILRSALKQLEDIFNEIKDKNEIAIIKQYGKRAKHITIGILLVSMCCQLIFVLLYICPYV
YYIMILNNSRSHSSSYIMTEYFIDEEKYSYLITVHRHVACLIGLLAMVATGTMLITYLQHTCAMLSIACYRIK
LALDISRNSDLKNEQKSCKDIIRAVNIHSNAIKLSNFLITNFEGSFFFLIAAGMVCLSCNLLRIASFTDNIGQ
LILSLTFIIILYVYLFLSNYTAQKITDHNEYVFATVYNVHWYIAPIRIQKMILFLLQKGTKSFHLILGGIFVA
SMQSAASLLNTSISYFTVFYSTQHN
>CfOr302
MICLETQHFNINRMLLLIVGLWPYQRSFLIELQLILFFGILITFIVFQLTTFITSECTLGIINKVLSSSFFFI
CLLIEYNSFWINTDIVSIALKQLQEVCNEIKNKNEIAIIKKYGSQAKCITIGLILLVICFQFIFILLYIWPYI

RHIMILNNSQHSPPHIITEYFIDQEKYSYLITVHRHAACLIGSLAMLATGTMLITYVQHICAMFSIACYRIK
LAIDISHENNSKNDQKSYKDIIHAINIHRNAIKLLNILVSNFQGSYIFLIAAGTISASCNLFRVNFFMSFISI
EQLILSLLFLSILYVYSFISNYMAQQITDHHEYIFITVYTVRWYMAPIHIQKMVLFLLQRGIKSLHVTLGGIF
VGSIQSFASLTSTSISYFTVLYSTMHD
>CfOr303
MICIDCLHISLNRKLLLAVGLWPYQQSKFVRFQLILLFAILTSFIIFQFINFVSFMTSKCTPDLIINVLSSAL
FYIMFLIKYSFFHINIEVAKCLLEQLQNIYNELTDKNEIRIIKKYGIYAKRYTAGLILVVIFSAPGLILYPFW
PYFFNILLSINVSRSHLSLKKMYVTEYFVDQKKYFYLILLHANAALYVGVFTILAIGTIFIVYQQHACGMFRI
ASYRIEKAMAVDISQKGNLRNRNLIYKELIRAVDMHREAMRFSDSAVSRFKVMYSLMILVGVISGSLNFFRIF
QALSFGYDAEELLLPLISVSTYLSIIFVGNYIAQEIMDHNNDVFISAYNIQWYAASVNVQKMILFLLQRSTKV
FNINIAGLFVGSLEGAATLLSTIMSYFTVLYSTR
>CfOr304
MKNIKSDYFSLNKILLLVIGLWPYQQSNFARFQFILLSSILTTNVIFQYIILLLQRWTPDFTKDLASIFMFTL
FMIKYNMFSVKAIKDLMEQLLHVCNELKDEKEIAIIDAYGCNGKRYTIALTVLVVFTISTFIVMSFWSDILNV
FLSTNTSRSYNLIITKHLIDQKNYFYLILLHNVISFSIGLTASLATGTMFFTYLQYVCGMFKIASYRIKQAMN
VDMLRNQKNILILKSLIYAVNIHRQAIKLSKHLQYSCETMMFYLIAFGVSSVSLNLFRVSFLCFIIASFEEGL
ILPFLYTVIAILYMFLANYIGQIITNHNHYVFNTAYNVQWYIAPLHIQRIILFLLQRNSKSFTLSVGGLFVAS
IECFATLVKTSVSYFTVIYSTR
>CfOr305
MISLETQHFKINRLLLLIIGIWPLQQSNLTRFQFIFCSSILITSIIFQCTPLISQKCTPDFVAKVLSSASFFA
LFVVKYNMFCINMEAVKDLLEQLQLICNELKDNNEFAIINEYGCSAKRVTAALTICGICSVFAIIAAQFSSKI
FDFILPINVSQIHRIPITVEYFVDQEKYSYWILLHINVAFCVGATAXLNINKRTIIRMQHNYNTCIILIYLFS
YRIEHAMNNNMLRNNNLKNKFLIFEGLIFAVDMHRHAMKLSKHLLSSIEPMMFCLIIFGVASVSLNLYRIFQI
TSLDDTSLNVKELIMPSLCAGITILYMFLSNYIGQHVTDHNHDIFVTVYNIRWYVAPLHIQRMVLFLLQRKSK
EFILNVGLFVVSIEFFATLVKTSVSYFTVIYSTQ
>CfOr306
LEKESRFFKLNRILLLAIGLWPNQQTTFSRIQFFLFSIILLTSLLFQYTPLISQRCTPDFAKILSSATFFSLF
VIKYILFSVNMENMKDLLEQLQLICNELKDENEFDIINEYECSATRLTAALTICSISGIFILAVTQFWSNILD
IILPINASRPYYIPLQVEYFIDQEKYSSWILLHISVAFLVGIIAMVGIGTTIIAYLQYTCGMFKIASYRIELA
MSNNMLQNSNVKNKFSILEGLIWAVDIHRQAMNLSTNIQLNIEPMVCCLIIFGVASMSLNMYQIFQIISADDI
SLYNVKELIIPLLYAIIIFLYMLLSNCIGQYITDHNRDIFVTVYNVQWYIAPLYIQKIILFLLQRKSRDFVLN
YAGLFVISLEFFATLVKVSVSYFTVIYSVQ
>CfOr307
MIRFEIQYLKLNRTLLLAIGLWPYKQSNLTRLQFVFLSAILTSSFIFQCTPFISQKCTPDFVVKILSSASIFA
VFVIKYNMFCINIKAYKCAINYIIKIYHLYVRRKNKNIKYKYIKLHPYHLCSILCSIGGICAIIVGQFWSNIL
NIILPINISISQSTPFIVEYFVDQEKYFFWILLHINVAFCVGATAMVGIGTMLIAYFQYICGMFKIASYRIEH
AMSNNKLRDINPKNKLSIFEGLIWAMDIHRQAMNLSQHLQSSIKPMLFCLIICGVISMSFNLYRIFQITSADD
KLLDNIKDLIVPVICALVTILYMFISNYIGQDITDHNHNVFVTVYNVRWYIAPLHIQRMILFLLQRRSRDFIL
TVGIFVSSIECFATLIKVSVSYFTFIYST
>CfOr308
MICVTTQYFSLKISRMLLLVIGLWPYQQSKFDRLRFIFFLSILISAVLFQFTTFLNSKYTADFVIKTLSSALF
FTLFVIKYIFFAINTETVKDLLIQLQYVYDQLKDENESAIVEKYGYNAKRYTLALTTLSICSISALIVVQFWS
NIIDIILPLNVSRPHRLPIMTEYFVDQEKYFFLILLHLNAAFCTGIAVVVATGTMLIAYLVHICGLFKISSYH
IKHAMRESMLRNYSPKSDILILEEIRYAVDLHRQAMKLSKTLVSRFEKMLFCLIAVGVISLSLNLFQIFRVFS
IAGNVDEILVPSLFAFACILYMFCANYIPQNLTDHNNLVAVTVYNVQWYIAPLYVQKLMLFLLLRNAKDFTLN
IGGIFVASLECFATLVKASVSYFTVIYSTQ
>CfOr311
MISIQTQHFTFHRILLLAVGLWPYQQSRLVQFQLVLFFSILISFIIFEVRLYFHNHNLIYNYIILIFMIIPIY
LLLIILNILDTHISYHNNYLCKNIELLYICASVVVGSVTILPHIFRFVLYINVTQPHVMTQLLPGYFSARDYF
YMLLYMDVASFIGETALIGAGLMLLSLLKHICGIFKIASYRIEKAITVKTKNIDVEKKIDQNEIIIYKKIIDA
VDIHREAVRLIVFLSGSFQKSHLLLILILVICLSLNLYAVSIAMYASNGDFGQLLIHFLIAFLMFIYMFCINY
AGQEITNHNDHVFSTVYNVQWYSTPLHVQKLILFLLQKGSKTFNMNFGGVIILSFELFATLTKASLSYFTVMY
SMQE
>CfOr312
MICIKTRHFRLLRILLFIVGLWPCQRSRLVQFQPILVFSILLSFIVIQFMIFITAEFTFDLLIEILSRGSFCT
MYVIIYNAFWYNFTEIVEYPLEKLQHICNELKDENEIAIIKNYGDNLNRFTFIITLFHMCNLIIVLPIISLPI
MPRTFGLALHINESQSRHMLSKFIPEYLIDRGNYLYAVLYLNAAIFIGGTVMVAIGLMIIAYFKHACGILKIA
SYRIVKAINMRNNVCLKNEAIMYKEIICAINIHRTAIKNFHKFVYINNSLYKCLYKETEEFLVHFVIVCIIFV

YLFLLNYFSQEFMDHNTDIFFTVYNVEWYAVSLRIQKLILFLLQRSNKTININFGGIFILSLEFFATLTKVSI
SYFTVMCSMQE
>CfOr313
MICIKIRHLSLQRILLLAIGLWPCQRTKLVQLQLILFSSILNSSILIQFSFLFIQEFSLDLIINGLSIELIFI
MFAIMYNFCCYNFTEFIEYPLEKLQDICNELKDEKEIVIIKRCGDNIKRFSFIILLWHTCNLIIGSSIILLPI
ITRIGFDVHINESKRDVIYNYILVPKYFIDRENYLYLILLFTDASLCIGGTAMVAIGLITFTYLKHVSGMLRI
ASYRIKKAINMWNNVHQKKETVMYKGIIYAIDFHRTAIKFSTFLFSCLYFPSFFLVIIAVICLSLNLYALSKN
ILYGNDISEVCMHFTIVIVIFIFMFFFNYIGQEFMDYNNDKTISLNFGGVFILSLEFFASLMKASISYFTVMY
SMQ
>CfOr314
MICIETHFTFQRIILLVVGLWPYYRSRLVQFQLNLCFGLLISFIIFQLTVFIYAECTPDFVIKVLSVLLVFIM
YAIEYNSIRINAQAVKYLLEQLQHVYNQLKDENEIAIIKKYGTNAKYFTIMFIMVYICASVTVGSITILPRIL
RIVLHINVTQPRVMTQLMPEYFTDSESYFYIILLYMDVAFFIGATTLVGKGIILISLIKHICGIFKIASYRIE
KAITVKMTINKDHKNEIIIYKKIIHAVDIHREAIKLTVFLSDNFQKSHLLVISIDVICLSLNLYAIAVYASKG
DNFGQLLMHFLIAIVMLVYLFFVNYAGQEITNHNDHVFSTVYNVQWYSTPLHVQKLILFLLQKGSKTFNMNFG
GVITLSFELFATLTKASLSYFTVMYSMQE
>CfOr315
MISIQIPHFTFHRIFLLAVGLWPYQQSRLVQFQLVLFFSILISFIIFQLTVFISIECTSDLVIKVLSIALLLI
MYAIVYNSFRMNTQAVRYLLEQLQHIYNELRDDNEIAIMQRYGNTAKRYVTILLSLYICITVIVILLQFLPRI
LGSVLHINVTHPRMMQQLIPKYFVDQKRYIYLILMYMDAALLIGATALVGIAMMLLSYLEHACGMFRVASYRI
EKALTITMTTNSSLASEITIYKEIIHAVDIHRKAIEYSLFLLSSFRESHILVIVVGVICLTLNLYAIAVNVSH
GGDTEQLLLHFLIAVVLFIYLFTANYFGQEITDHNDHIFCTAYSVRWYAASVKVQKLILFLLQRGSKTVTLNF
AGVFSLSIKLFATLIKASLSYFTVIYSMQ
>CfOr316
MISIQTQHFIFHRIFLLAVGLWPYQQSKLVQFQLVLFFSILISFIIFQLTVFISAEYTSDLAIKVLSIVLLLI
MYTIEYNSFRMNTQAVRYLLEQLQHIYNELRDENEIAIMQRYGNTAKRYVAIFLSLYICISVIVTLLPILPRI
LGFVLHINATHPRIMQQLIPKYFVDQKRYIYLILMYMDAALLIGATALVGIAMMLLSYLEHACGMFRVASYRI
EKAMTTLYIYIYIYIYIYIYIMFLYVSYSFLRFSLFLLSSFRESHILVILVGVICLTLNLYAIAVNVSHGGDT
EQLLLHFLIAVVLFIYLFTVNYFGQIITDHNDHVFYTAYNVRWYTASVQVQKLILFLLQRGSKTVTLNFAGVF
SLSIKLFATLIKASLSYFTVIYSMQ
>CfOr317
MISIQIQHFIFQRIFLVAVGLWPYQQSKLVQFQLVLFFSILISFIIFQLTVFLSTECTSDLVIKVLSIVLLFI
MYTIEYNSFRMNTQVVRYLLEQLQHTCNELRDKNEVAIMQRYENNAKYNATMLFYLYICIAVITTLLILRRVL
GFVLHINATHPRVMQQLIPKYLINQKRYMFLILMGAAILIGGTALVGIGMMLLSYFEHVCGMFRVASYRIEKA
MTITIPMNSSLANQIIICKEIIHAVDIHRKAIEFSLFLLSSFRESHILMILVGVICLTLNLYAIAVNVSHGGD
TEQLLLHFLIAVVLFIYLFTINYFGQKITDHNDHVFYTAYNVRWYTASVQVQKLILFLLQRGSKTVTLNFAGV
FSLSIKLFATLIKVSLSYFTVIYSMQ
>CfOr318
IDSQHWKLNRILLLLLIGVWPLQQSNLTRFQFIFLSIVHITSIICQLMTFFTTKCTPDRVVTVLSSVLMFSICG
IKYNLFFLNMETMKDLLIQLQNVYIGLKDKHEIAIMKKYSCNAKRYTLILTILGVCVAFTSVIILSPRIFHII
LPMNVSLSYRLPIRMEYFIDQEKYSYLISLHISMAMCIGTIALIAIGTICVACLQYICGMFRISSFRIKRAVH
IDMLQNIKATKGNLILEGIISAVDMHRQAMKLSRLLLSTMETIMFCLILLGVITLSLNLFRIFQVISTGDEFK
EFIIPSVYVVAITLYMFLANYFAQDVTDHNNDIFATVCDVQWYAVPLHIQKLILFLLQRSSKDFTLCIGGLFV
ASLECFATLLKTSFSFFSFMYSTQ
>CfOr319
MINLETQHFKLNRILLLIIGLWPLQQSNLTRLQFIFLSTILTTNITFQLMAFVTLRCTPDLIANVLSSTCFSS
TYAIKYNLFHFNIGAMKDLLMQLQNICNGLKDENEITIMKKYSCNAERYTIVLTIHTVCSVFIIIVGFIGSSI
FYMILPMNVTRSRRLPITIESFIDQEKYYYLILLDIIMAILIGAIDMKAIGTMLIAYFQHTCGMFRISSYRIK
RAMYTNTLGNIKWKKENLILKGXISAVDIHRQAMRLSRLLASKIETMLYCLIMLGVISLSLNLFRIFQIMSSE
NDIKEYMFPLFTTITSILYMFLANYIAQDLTDHNNDIFATVYNVQWNVAPLHIQKVILFLLQRGAKDFTISVG
GLFVGSLECFATLVKTSVSYFTVIYSTQ
>CfOr320
RILLLLIGLWPLQQSNLTRVQFIFLSIIHITSIICQLMTFVTTKCTPDRVVTVLSSVLMFSICGIKYNLFPLN
MEATCKHMNINIIYNMSKKSQIEINFQNYNFCYLSLAVCCVFTTSIIILSPGIICIILPINVSLSNRLPIKME
YFIDQEKYSYFISLHMSIAICIGTMTSAAIGTMYVTFLQYVCGMFRISSFRIKRAMHINMLQNIKAKKGNLIL
EGIISAVDIHRQAMKLSRRLLSTMETMMFCLIMLXVMTLSLNLFRIFQVMSTGDEFKEFVLPSVYVVAITLYM
FLANYFAQDVTDHNNDIFVTVCDVQWHAAPLYIQKLILFLLQRGSKDFTLCIGGLFVASLESFSTGKIININISL
KY

>CfOr321
IDSQHWKLNRILLLLIGVWPLQQSNLTRFQFIFLSIIQITSIICQVCSDTCYLCYIIIMKDLLVQLQNVYIGL
KDKHEIAIMNNYICNAKRYTLIFTILAVCLSFTSVIILSPKILYIILPMNVSLLNRQPIRMEYFIDQEMYDYL
ISLHISMSICIGTIAMAAIGTIIVVCLQYICGMFRISSFRIKRAVHINMLQNIKATKGNLILEGIISAVDMHR
QAMKLSRLLLSTMETMMFCLIMLXVMTLSLNLFRIFQVISTGDEFKEFIIPSAYVVAITLYFFLGNYFAQDVT
DHNNDIFATVCDVQWYAAPLYIQKLLLFLLQRNSKDFTLCIGGLFVASMKCFATLLKTSFSYFTVMYSTQ
>CfOr322
IDSQHWKLNRILLLLIGVWPLQQSNLTRFQFIFLSIIQITSIILILVMFRLNFILFFHYFNILSTLQVDSLCM
KDLLVQLQNVYIGLKDKHEIAIMNNYICNAKRYTLILTILAVCVLFTSVIILSPKIVYVILPMNVSLLNRQPI
RMEYFIDQEKYDYLIPLHMSMSICITTIAMVAIGTIYVACLQYICGMFRISSFRIKRAVHINMLQNIEATKGN
LILEGIISAVDMHRQAMKLSRLLLSTMETMMFCLIILGVVTLSINLFRVISTGDEFKEFIIPSVYVVAITLYM
FLGNYFAQDVTDHNNDIFATVCDVQWYAAPLHIQKLLLFLLQRGSKDFTLCIGGLFVASLECFATLLKTSFSY
FTVMYSTQ
>CfOr323
MIDLEIQHFKLNRYLLLIVGLWPLQQSNLTRLQFVILSSTIMTIIILQVRQYSSYTYFNLFFHYILIHYDLII
LFFLYHKKYSCHIKFMKDLLVQLRNICNGLEDKNEIAIMKKYSCNANRYTTVLAILGICGIFIVTISMHWSRI
LYIILNVNISLSHHLPIKIEYFIDQDKYFYLIQLHIIMLLCIGITVTVAIGAMLITFTQYFCGMFRISSYRIK
RAVHIDVLENIKAKKENVTLKGIICAVNIHRQAMKLCRLLETTINPMFFSLITTGVVSLSLSFFQIFQIISIG
GNIKEFIFPIIIMLVIMIYMFLANYLAQDITDHNNDIFVSVYNVQWNVAPLHIQKVILFLLQRGTKDFTVCAG
GLFVGSLECFATLVKASVSYFTVISSAQ
>CfOr324
MIDLETQHFKLNRFLLLIIGLWPLQQSNLTRFQFIVLFNILMTSIIFQVWYFLCVYFTFTLYFNINYIIIFYC
FLSYILININYIKDLLMQLQDIYDRLKDKNEIDIVKEYSYNAKRFTIVLTIFSICGLSFIIIISWSSVFYPIT
YPINVTQTRHLPMRIKYFIDQEKYFLLILLHFNATMCIGMIAVLAIGAMLITYLQHTCGMFRISSYRIKRAMQ
INTLENIKMKKENLILKGIISAVDMHRQAMKLSGLLVSKIQTMIFCLIIIGVIILSLSFFRISQIVSSGDDVM
EFVCLFIFITAGIIYMFLSNYIAQDLTDHNNDIYNTVYNIQWNVTPLHIQKIILFLLQRGAVDFTVSVGGIFV
GSLECFATLVKASVSYFTFLYSMQ
>CfOr325
MMCLETQHFKLNRFLLLTIGLWPLKQSNLTRFQFIILSSVLTTSIIFQLTIFVTLRCTPNLVVKVLSSTCLFS
ISAIKYNLFSLKMEAVKDLLIKLQEIHDGLKDKNEIAIMKEYSCLAKRYTAVLTILGLYSSFSIIIALFWSSI
FYLILPINISISHHLPIMMEYFIDQEIYFYLIPLHISVAMFIAMTVAIAIGAMFVAYDQHICAMFKISSYRIK
RAVHIITLGVNKSKKENLILKGIISAVNIHRQAIKLSEFLVSKIQTMLFCLIIVGVIFLSLNLFRIASSGDDV
EEFIFPFQFVTFSIVYMFLANYIAQDLTDHNNHIFTTVYDVQWHIAPLHIQKMILFLLQRGAKDYTINVGGLF
VGSLECFATLVKASVSYFTFMYSTQ
>CfOr326
MINLETQNFKLSRILLLIIGLWPLQQSNLIRLQFIFMSIILIAGVIFQLTAFITLRCTLDLIARVLSCVFFSM
FFVIKYNLFHFNIKAVKDLLMKLQDICNGLKDENEIIIMKQYSRYGKRYAIAFIIFAVCCSFSVTIAMFWSSI
LQITLGTNVTQSRRLPITMEYFIDQEKYFYWILLHIGVASFIGTIVILGIGSVLIMYVLHICGMFRISSYRIK
RAMHINMLENIKPRKENLMLKGIISAVDIHRQAIKLSNFLVSKIETMMLCLIICGVLTLSLNLLRIFQIASYG
DDVKEFLIPFILIIIDIVYMFIANYLGQNITDHNNDVFATVYDVQWHVAPLHIQKVILFLLQRGTKDFTISIG
RLFVASFGHFTTLVKTSVSYFTVVYSTQ
>CfOr327
MISLETQHFNINRILLLIIGLWPYQRSLLIELQLILLFGILISFIVFQFTTFITSECTLDLIYKVLSSAFFFI
CFAIKYNSFWINADILRSSLKQLQDIYNEIKDKNEIAIIEKYGSKAKRFTFRIILLVIGYQCTFILLYMWPYI
LVLTSKSRLRSSPYIMIEYFIDQEKYSYLITLHRHAACFIGIIAMAATGTMLLAYLQHTCALFSIACYRIEQA
IGTSQKSGFANEQKSCKEIIRAVNIHCHAMKLSNFLISNFVGSYFLLIAAGVICLSCNLLRIASFTGNIGQLL
LSIIHLSIHYIYLFLANYSAQEVTDHNEYVFATVYNVRWYMAPIRIQKMIQFLLQKGTKSFYLILGGIFVASM
QNAASIASTSISYFTVLYSTR
>CfOr328
MICVESRYFNINRILLLTVGLWPYAQSKLVRTQLILLYGILTSFIVFQLMIFVTSRYTLQYAIKILSVTSLFI
FYIIQYSKLSFNMDVVKHLFILLQCTYNELRDESEVAIIEKYWNIGRHYTEMLTLLAASCIIAFILSPFLPQI
FAAVLSGNESRLHISLQIVTDYSIDQERYFYLIMLYTNIAFCIGILALLATGTMCITYVQHICGMLRIASYRM
ERIMKVDTRSISAPKSANLIYMGIVYAVDMHRKAIKFIKIFIFSINKFLFFLIMDGLLTASLNLYQIFQELSF
ECSVAKIIQHSIFLTIYYIYTFVANYCAQQIMDHNNHIFATVYNVQWYIAPLHIQKLILFLLQRGTKTFENLV
IIGGLFIGSLEGFATLVSTSVSYFTFLYSTKQ
>CfOr329
MICVTTQYFSLNRMLLLVMGLWPYQQSKFDRLRFIFFLSILISAVLFQFTTFLNSKFTADFIVKILSSALFFT
TFVIKYIFFFINTETVKDLLAQLQHVYDQLKDEYENAIVEKYSYNAKRYTLALTTLSICSISALIVVQFWSNI

IDIILPLNVSRPHRLPIMTEYFVDQEKYFFLILLHLNAAFCTGIAVVVATGTMLIAYLVHTCGLFEISCYRIK
YAMRKSILQNYCPKNDMLILEEIRYAVDLHRQAMKLSKTLVSRFEKMLFCLIAVGVISLSLNLFQIFRVASIA
YNVDEIFFPSLLAFISILYMFCANYIPQNLTDHNNLVAVTIYDVQWYIAPLYVQKLMLFLLLKDAKDFTLGVG
GLFIGSLECFATLVKTSVSYFTVIYSTQ
>CfOr330
MSFYDNHYYYINKMLLSLIGQWPFQSRLEGNIMFVIMLFFICSLTILELWGLIAGITDLSIIMENVSPLLVNS
SVVIKLINFLYNKYKMKDLLEHIKETWKMMKPETERKILESYAERTRTLTIQYAIGIYSMGMSYATMPFVIKG
IYLLLPSNETNQVRFLYRLEHVIDVNKYFNLLMFHGIISIFYILSVHIANDAMFILYVQHACALFKNICCNVK
HIRRSDFGMLKPDIANDEAYHIIIKYVKSYKYALRFSDLLSSTYATCNFFLIGNVIIALSFSAAELIMVDNQL
DEIIRIIAANVAQLIHIFHLNLTSQRLIDHSSNLQEAIYSCDWYKLSLRSRHLLRFMLMRAIKPCYIRAGKMY
VMSLENFSSILQVSMSYFTMLTSMQ
>CfOr331
MINLETQHFKLNSILLLIIGLWPLQQSNITRLQFIFLSTILTSNIMFQLMAFVTFRCTPDLIANVLSSTCFSS
MYAIKYNLFYFNIRTVTADFFVRDFDIYRSPLRGRNNIYMYVYMIVLETYTLNWILTVCSVFILIVGFIGSSI
FYMILPMNVTQSRHLPVTIEYFIDQEKYFYLILLHIIMAILIGAIAMIAIGTMLIAYFQHTCGMFRISSYRIK
RALHINMLGNIKWKKENLILKGIISAIDIHRQAMQLSRLLASKIETMLFCLIMLGVVSLSLNLFRILQIMSSK
NDVKEYMFPFLSTITVILYMFVANYIAQDLTDHNSDVFATVYNVQWNVAPLHIQKVILFLLQRSIKDFTISVG
GLFVGSLECFASLVKTSVSYFTVIYSTQ
>CfOr332
MINFETEYFKLNKILLLAIGLWPRKQSNLTRLQFIFLSSILTTNLIFQCTIFISQTCTPDLILKVLASVFFFA
LLVIKYNMFAVEAMKDLLEQLLHVYNELKDEKEIAIIHKYGYNGKRFTIVLTVFAAIGIMAFIVASFWSDILN
IILSSNTSRAHHLLIRTEYLIDQEKYFYLILLHIIVTVCIGGTAALAIGTILFTYLQHVCGMFKIASYRIERA
MSITMLQNINLKKQILIFRGLICAVDIHRQAMKLSKRLQYSCEKNDERLIAFGVISVSLNLFRIASSKEELIL
PFLYATISIIYMFLANYIGQLITNHNHHVFVTAYNVQWYIAPLHIQKIILFLLQRNSKNFTLSVGGLFIASIE
CFATLVKTSVSYFTVIYSTR
>CfOr333
MINFETEYFKLNKILLLAIGLWPRKQSNLTRLQFIFLSSILTTNLIFQCMIFISQRCTPDFIIKILASVFVFA
LFVIKYNMFSVKSMKDLLEQLLHVYNELKDENEIAIINEYGYNSKRFTIALTVFAVIGTVAFIVASFWSDILN
IILPTNTSRAHHLVIMTEYFIDQEKYFYLILLHAILSLCIGGIAVLAIGTILFTYLQHVCGMFKIASYRIERA
MSIDMLRNINLRKQILIFKGLICAVEIHRRAMKLAKRLQYSCEKMMSCLILFGVTSVSLNLFRVIFCTNIWHK
TKSIVSIVLPFLYTSLAIIYMFLANYVSQLITNHNHHVFVTAYNVQWYIAPLHIQKIILFLLQRDSKSFTLSV
GGLFVASIECFATLVKTSVSYFTVIYS
>CfOr334
MNNIKAEYFSLNKILLLAIGLWPYQQSNFTRFQFILLSNILTTNIIFQCTIFLSQKCTSDLITKVLASVLFFT
LFIIKYNMFYINIRTVKNLLEQLLHVCNELKDENEIAIINECKYNSKCYTIALTVLAVVSIIAFITASFWSNI
LDIILSTNASRSHHLLIMTEYFIDQEKYFYFILLHNIASVCIGTIAMLATGTILIMYFQYTSGMFKIASYRIK
RAMNVDMLQNIKQKNILIYKGLICAVDIHRQAMRLSQHFISSFEAMMFCLIIVGVVSVSLNLFRIFQIASFTK
NVKELITPIIFAIATIVYMFIANYVGQTITNHNQHVFITAYNVRWYFAPLYIQRMILFLLQRNSRNFTLNIGG
LFDASIECFATLVKTFYFTVIYSTR
>CfOr335
MVNIETEYFNLHKILLLAIGLWPYKQSNLARLQFIFLSTILTTFLIFQCTIFISQTCTPDLILKVLASVFFFA
LLVINYNMFALLKQQMKDLLEQLLHVYNELKDEKEIAIIHKYGYNGKRFTIAITILAAISTMTFILASFWSDI
LNIILPTNTSRAHHLVIMTEYFIDQEKYFYLILLHTIVSFCIGVISALAIGTILFTYLQHVCGMFKIASYRIE
RAMSIDMLRNMSLRKHMLIFKGLICAVDIHRQAMKLSKHVQYSYEKMMSCLVAFLVTSVSLNLFRVSFLFFSQ
NHKQKFILPFLFTILIIMYMFLANYIGQLITNHNHHVFVTAYNVQWYIAPLHIQKIILFLLQRNSKNFTLTIG
KLFVASVECFATLAKTSVSYFTVIYSIQ
>CfOr336
MVNIETEYFNLHKILLLAVGLWPYKQSHLARLQFIFLSSILTTYLIFQCTMFLSQRCTPDFIIKVLSSVFVFA
MCVIKYNMFCVKAMKDLLEQLLHVYNEIKDENEIAIIHKYGYNGKRFTIAITVLAAISTIAFIVASFWSNILN
IILPTNTSRAHHLLIKTEYLIDQEKYFYLILLHAILSLCIGGIAVLAIGTILFTYLQHVCGMFKIASYRIERA
MSIDMLRNINLRKSILIFRGLIYAVDIHRKAMKLSKRLQYSCEKNDERLIAFGVISVSLNLFRIASFDEELIF
PFLFTSLAIMYMFLANYIGQLITNHNHHVFVTAYNVQWYIAPLYIQKIILFLLQRDSKNFTLSVGGLFVASIE
CFATLAKTSVSYFTVIYSTR
>CfOr337
MSSNHCETDFAENHYYRINQIFLSLLDLWPYGRSKFAKIKNVLYLTIMLSFLFVQMTSFLTIQLTMNSIVMIL
SYAVPSITFIVQYYSFYAKSYAVKRMWKNIRDSWNLLKDEAERDIMRQYSSHGELHTILITLSVIGTLLLYAF
FEFMPTILDVIFPLNETRAHELHALTEYFVDEEKYFHIILCHWLIGTYFEGFVCIATYTLQLAYIHHICGLLK
IASYRIEHSLDTYILYNSIQEDHTAIQNICAAVNIHRRALECSEFFMVTFAPTFFIIIVISVASLSFNLFRLL

EALILLENVNELIMSILFVGFHFLLIFLGNYYGQIITDHNNEVFDKTYNIRWYMVSVKIQKLLLFIMQNTTKL
YVLNIGHLIIISTEGFTKLASLSTSYFTLIYALV
>CfOr338
MSSNHCETDFAENRYYRVNQIFLSMLDLWPYRRSKFAKINCIFHYAIMLSFLFVQWTTFLTSQLTINTIIMNL
TYAIPSFSYIILYSSFYAKSKEVKRMWKNMRDSWKLLKNETERDIMRQYASYGELYTKLIALGVIAFLLLYAF
FELMPTIFDVIFTLNETRPRELHALAEYFVDEEKYFYVILCHWLIGTYFTGFVVIATGTLHLVHVQHICGLLK
IASYRIKNSLDKYILWNSIQEDETIIQNIRGAVNIHHXCEFIVITFTSTFFLLIVVIVASLSLNFFRLLNALI
LLDDIRELFTSILFITLLFAFMFVINHCGQQITDHNSEVFEKTYNIQWYMVPIKIQKLLLFIMQNTTKLYIVN
IGRLIIVSIEGFTKLMSMSVSYFTVIYSLV
>CfOr339
MSSNYCDTDFAENHYYRLNQIFLSMLNLGPYRRSKFAKINSVFHYAIMLSFLFVQWTSFLTFQLTMNTVILNL
SCAIPSLSYIILYSSFYAKSKAVKRMWKNMRDSWKLLKNKAERNIMRQYASSGKFLTKLMALSVTMCILLYAL
FEFMPTILDVIFALNETRPREIHALTEYFVDKRKYFYVILCHWLIAINFGGFVVIATCTLHLVYVHHIYGLLK
IASYRIKHSLDKYILCNSIREEDQTAIQNICAAVEVHRRALESCEFVLVTFTSTFFLLVIITVASLSLNLFRL
LKALILLDDVNELIISIMIISLHFAFMFMGNYYGEQITNYNSEVFEETYNIRWYMVPVKIQKLLLFIMQNTTK
LYIVNIGRLIIVSIEGCTKLISMSVSYFTVIYSLL
>CfOr340
MSSNRCDTDFTENRYYRINQIFLSMLNLGPYRQSKFAKINSVFHFAILLSYASVQWTTFLTFQLTINTVIINL
AYAIPSLSYIIEYYSFYAKSNEVKRMWKNIRDSWKLLKNEAERDIMRQYASSGELQTKLAALSVTMCILLYAL
FEFMPTILDVIFPLNETRPRELHALTEYFIDKRKYFYAILCHWLVGTYFSGFVVIATCTLHLVHVQHICGLLK
IASYRIKHSLDKYILCNSIREDQAAIQNICAAVDVHRKALECSEFIVITFTSTFFLLVVLIVASLSLNLFRLL
RALILLDDVSELIIAILFTSLHFAFMFVANYYGQQITDHNSEIFEKTYNIQWYMVPIKIQKLLLFIMQNTTKL
YIVNIGRLIIVSIEGFTKLISLSISYFTVIYSIM
>CfOr341
MDFFDGRNYRVNKILLSAIGQWPYQSSKTNYIITIVITTITCSQLLTKLCSMFSYIHDMEIVIECLTPIMIDI
SGITKIMNSMLCVNEMRTLLDQIQADFRSLRNSGDIEILQKYADSGRKFSIIYIYTLYIVMVIFMLTPLPALI
LHVANETRPMLHRVEYYVDIDKYYFPILIHGYLTVLICVTSIVATDAMLVIFVQHACGMFIITGSRIKRALQG
ELTDANLSVVEDKAYRNMIQCVHDHRAAIRFVLVNLIEAAYSKYILFHTGLNMIAISITGVGAVTKLNDPSEL
FKLIAVSWALLFHLCLECLNAQKLMDYSGYLLHTSLVNLNWYDASPRTKKLVLFMMMKTHSPCVLTAGGMFVL
CMETYATIVKTAVSYFTFLRSAQ
>CfOr342
MEHPEKRYYKWNRFFLLATGLWPYQSVWSARLNRGISIVIILCMVFVQLNSIFTINITREFMVIVVEVLAVTI
GILYHLFAHMRHIDKYKQLFDHMWQDWALQKTNYEIRIMHQYANITKLVTFYYALLMYGVEVTYFTWLFMPEI
LDVVSPLNESRPRKQPFDYNFFIDEEQYFYFIRFLIFIGCTFVPIIFLATSTLFLAFTQHVCAMYKLLGHRAE
HLFYVIGSTAEIDLNRKARIKCEKIAILVRMHYNIIQFVFLINVYYIFNILTDMSSTLCQKLKLFQIYFLICI
SILNVFVILLIKNCLVVMISKXKNKWSYRYNSAWYDAVISEQNSLLLMMRRCLHPLVLTACKFYVMSLQSFGM
VLQMTISYCMFIKR
>CfOr343
MEYPEEYYYKFNRFLLSVTGLWPYQSKWSACFARTIITVIMLSAIFTQISSVFTSELNMDFVIESLPMFVPTV
GNLCQLYSRIFYVDKIKELLEHMWNDWALEKTDIESKIMHQYAKTTRLVMIYHSLLLYIIVTAMAISMFLPEI
FVILPVNKSSQRLEAIHMEFYLDKERYIYLIMSCVCIVLFLVPLVFLASSSLYLVLTQHVCSMCEVLGYRAER
LFYIIEDKVEGELIRKTKISCKNIVVLVQLHYNVIEFVNTIETCHTIPFLMDLVGVVITISFWLIQILTIFEN
VKRACASIGLIIASLCYLAIPNYMGQKVTDMTSSICEKVYNSAWYYASVSEQKSLLLIMGRRFRPLVLTACKF
YAMSLPSFEMILQMGVSYCMFMRKV
>CfOr344
MEHLVERYFKLNRFLLFVTGLWPYQSEWSAYLMRIINFAVLLISAFIQMMTILTSELTLEFLTSVLPMILPTL
GFMMQMYLRIRFIDKLKKLFEYMWDDWALQKTHDEIKIMYEHAETTKLWTLGYFSFYYLAITIYSVWLFTPEI
LDIISPINESRPRIQLSYMRFELFIDEDRYFYFIRFYICIMSFVTPLISMACSTLFVVFTQHVCAMCELLGYR
AERLFCTVGNRGNRKKCNLFCETKINCEKITVFVRLHNNIIEFIGTINSYYTVPCLMDLVGFILLTGITVFQI
LSAVKIEQAIRSIILTSMLLCHMFMHNYMGDKVTDKSSNVCEKVYNSAWYDTLILEQKLLLLIIRRRSNPLVL
SAWFYIFSLSNFGLILQTVFSYCMFIRQI
>CfOr345
MQGNMLRYYSRNQFFLSQIGIWPYQSKMIKILLPCFIISSLLSILVPQVFLLYNSWGDMNMTVEGIVNIILVT
GATTKLVNIVTNNKKLQYLLQVMDEHWRLFHGKCELHILKRYANIGQLITKYYSVYINIFVVLFLSIPLIPKI
LDVVIPLNQSRPFIYVLEGDWGVDKEKYYYPIMLHCCLAIIVGTRCLVNIDTMYVVCVLHGCSLFNAIGIRLE
NILSKAKLMQHEQIMSLEYAVIKEYYSEDYHEMIACLKKHQLAIKYIYMLDSTFKHATFLILFLNVMILSLVG
LQLINKFGQTQEVIRFGCIVMGSITHLLSMCLPGQLLLDRSIEIFDKAQVSKSAQWYMFSLKTTKLLSILLHR
SLVPCTLSAANMYIMSMTTFSSVMQTAMSYFTTFLSV

>CfOr346
MEGDMLRYYYRNQIFLSQMGIWPYQHRMIKILLPCFLVLSEASLFVTQILLVHNTWGDMNMAVEGIVNMILLT
GATTKLLNIVINNKKLQYLLELIHEHWQLFHSESELDILRYYANIAQKVTKYYSVYINIFVAIFMSIPLMPKI
LDVIIPLNESRPSIYVIEGDWGVDKEKYYYPIMLHSYLAVIISTRCMVNVDTMYIACVLHGCSLFNAIGNRLE
NIFDKVKLAKNEIQMNKKHSIIKEYNSEDYHEMIACLKKHQLAIQFVLWKNFIININFIYIXIMIIIYNILNF
IYVICNKLINKFGQIQEVIRFACLVMGSVTHLVSICLPGELLLDSSLEVFDKAQYCNVITMYNAQWYMFSLKT
KRLLSILLHRSLVPCILSAANMYIMSMTTFSSVMQTAMSYFTTFLSV
>CfOr347
VFYQLLVFVTTQYSMNLLIKILSIVFPILFVTVKYCMFMIQADSVKKMLERMQDDWRLLKNKLEVDIIETYAY
NIQFSSIVSIVIVVFCSFLLFILQFLPLILDVILPLNESRPFRTFIITEYFISQEKYIYIILLHEVLACLIAV
IALYGTFITILTYMWHACALFKIASYRIENAIEKNTLMIPIFGRQYLFYQRLVHAILIHRRAVEFTKLISFNF
TVLFAILIIVGVSSLSINLFQVSVLCLTSINNRSFEDIFIVILLIVIHISYMFIVNYGGQKVTDHGMEVFKAT
YSGLWYIAPLHTQKLLLFIMQKGNINVTIIWGRLYIACLEGFASLTNAAVSYFMVMHST
>CfOr348
KMHAIEERYYKINRIILKTLGLWPYQQSYLTQIHKILFAGILWTFILVQLLVFITTQYNINLLLKILSLVFPT
LFGTIKYILFIIRQIVKQLLEQVQNDWLLKNKLEIDIIEKYACNSRLFTMIVMAFCLLGVIFCTIFQLLPMIL
DIIIPLNKSRPCQIIAVTEYFINQEKYIYAILLHEIITVLIIAITIFGTGATIMMYILHACALFEVASYRIEN
AIEKNILAISDPRRERFLYRRIVHAVVIHRRAIEFTEFLTSTFMVSYAILIIVGISSLSFNLFQFLQMVTLTN
NINEAFIFGIFIITHLIYLFFANCAGQEITDHGIKFFKATYNGLWYAAPLHTQKLLLFVMRKGKINVTIGCGS
LFIASLEGFAMLTNTAVSYFTVIYST
>CfOr349
VSQLSYKMHATEEYYNKINQLILKILGLWPYQQSYFAGIQKVFFISILSTFILAQLLLFITTQYSTNLLLKIF
SFVLPILFCTIKYCFFIIRADSVKQMLERNRDNWKLLKDKLEIGIIKKYSYFANLFTISLMCLCCSGLIIFII
IQFLPLIFDFVLPLNESRSYRLIVITEYFVNQEKYIYPILFHHIVAIIVAIIALCSTSSTFFTYLLQTCALFS
IASYRIENAMKWNILAISSPTKEHLLYRKIVHAVIVHRRAIEFIKFFTSEFTILFAILIVVGVSSLSLSLFQF
LQLITLMNNITHTMVLALLIGIHIIYMFDANYAGQIVTDHGMKLFKVTYNGMWYAAPLQIQKMLLFIMQEGMV
DINIRCGGIFAASLEGFATLVNAAVSYFIMIYST
>CfOr350
QFLCKMYATEERYYKVNRVLLKILGLWPYQQSYFTRIHKMLFAGLLCTFILVQLLVFVTTQYSINLLFRILSI
VFPVLFVTVKYCLFIIQADNVKKMLERMRNDWRLLKNKLEFDILEKYFYSIQFFSIILIVIGIFCFLLLSILQ
LLPLFLDVLFPLNESRPFRIFIITEYFINQEKYIYIILLHEILVCFVGLITLYGTFLTIMTYVCHACALFKIA
SCPIVNVIDKNVSMIPTFEKHYLFYQSIVHAVLIHRRAIEFTKLFTFKFTLLFAILIFVGVTSLSINLFQLTL
INNSNFEDGILVIIFIIIHLSYMFIGNYGGQKVTDHGMEFFEATYNGLWYTAPLHIQKLLLFIMQKRNINGTL
VFGRLYVVSLESFASLSNAAVSYFLVMYST
>CfOr351
MSIVEDYYYTILSKFFLKILGIWPHDKSRLVLLQQVLIIISTVIFVLLQVTIDFYNIVIIIYIFYIICSLLPI
LYYIDNFFFVKIKYKYITSEYIIFIIQEXLQIFKKSVDLRIFIKYFKIFVLCVHILVLASFGIIIVSLLLFHP
IILNIILPLDEPRSVKLLIKVEYFVLQDEYIYFKILYGGLMSLLCVSMILATSMQMLIFVFHSIGMFEITSHR
IKYSMEDNILQNISNFKKEYAFCENLIHAVIAHRRAMKFSELCVSTFNITFCILALLGVLSLSINIFRFLNAI
SISKDLEDAVISSGAIIFYLLYMFIANYFGQIITDYNYEIFERVYDTPWYLISIPSRKLILFLMQKTGKEFNL
RIGFIIVGKVESFAMLLNSALSYVAVMFSI
>CfOr352
MSSNRCETDFVENRYYRINQIFLSMLDLWPYRRSKFAKINYVFHFTLMLSFVFFQLTSFLTLQLTMTSIVMVL
SYAVPSFIYIVQYYSFYAKPNEVKRMWKDIHDCWNLIKNKAERDIMRQYASSGELHSILIALSITMSIALYIF
LELMPTLLDIIFPLNETRPRELHALTEYFVDEKRYYYAILCHWLMGLCFGGFVGMATSTFQIVYFQHIYGLLK
IASYRIEHSLDKYVLCNSIQEANATIQNISVAVNMHRRALEYSEFFMVTFAPTFFIIVIISVASLSLNLFRLL
RAVILLQNINELISSILFVNIHFLMMFLANYYGQIITDHNNEVFEKTYIIRWYTVPVEIQKLLLFIMQNTTKL
YVVKIGSLIVASIEGFTKLTSLSISYFTVIYSLL
>CfOr353
MIEVENQYYNINRILLLICGLWPYENTKFRYVRVVFLCGILISFMIYQMIILFIYKYNFKLVLKISADMLPFA
VCMVKFITFLFNSKKVKQLLDQIYEDWNALKNSEELEIAQKYGKFTKRLTKILFLCTLFGLLIVMLVRYIPIF
INSSTTVDKFFLKQDDISDVYLDKKKYIYFFITSFVGVFVITSTITMIIAYMRHICMMFKITCYRIENSLNEN
ILHMSISQKEKMMYQRLISAINIHRRALQFAEFTLYTFKRCYLMMIGLGVLSLAVNLLNMLQAILILDNINDL
ILTSFFIILHFIFFFVGNYGGQIITDHSVDILTTLYNIQWYMAPLRIQKLILFLILKNTKSYSFLIGGIYVAS
LEGFITLTSMSLSYFMVIYSTQQ
>CfOr354
MNFLGNKYYKLNRRLLLLVGLWPYDHSLFRYYQVIFCNITVALMVTFQIAKLIRLRRNVSLMLKLLPLILPCF
ICIIKHQTFCIFAKKIKYLMDHVEQDWNMLKNKKELEIIERYTYIGNMCTLIFTIIALVAISIITFLPFVPIV

LDIFMPLNYSRPRQLIFPVEYFIDQQKYFYVICLHFNITLSIILATLVSTESLYVTHVLHACGMFQIASYRMN
QAFDKKLLQNCIPEKRKIIVCKGIIEAIHIHKRALEFSEFLWSTLAISYSILLIIGIISLLINLFCLLQAVLF
IKKMNDIIILIVFTFGHFFYLFLGNYVGQILIDHSAGVFENIYITRWYSAPLQAQRLLPIIMQRSMRSCKMVV
GGTFVPSFEGFAALMSTTLSYFTVVWSVQ
>CfOr355
MNFLGNKYYKLNRHLLLLVGLWPYVHSLFRYCQIIFCNIIVAFLMVFQIAKLLTLKQDVNLMLKLLPPILPCL
ICIIKHQTFCIVAKKMRYLMDHVEQDWNMLKNKKELEIIERYTYIGSMCTLALTIVGLVAILIIMFLPFVPIV
LDIFMPLNYSRPRQLLFPVEYFIDQQKYFYVICLHFNITASLVLITLVGTESLYITYILHVCGMFQIASYRLH
QAFDNKLLQNCTLEKQSVIVCKGIIEAIHIHKRALEFSEFLWSTLSVSYSILLIIGITSFLINLFCLLQTVLF
IKEIKDIILLIGFNFGHFVYLFLGNYVGQILIDHSSSIFENTISIKIFKFYILISKLLPIIMQRSMKSCKMVI
NGMYVSSFEGFATLMSTTLSYFTVIWSV
>CfOr356
MDFEGSKYYELNRILLLLVGLWPYDKSKFKLIKILILSSFFITCSAAMIAKIITSTKDMEVMLRTMSLAVPAV
IIIVKYITFLLLPASIKKLMELVQNDWNVLKDAKELEIIQKYAHFGRLCTIIFSVFGYACVFGVLITIYIPSI
LDIVAPLNVSRKQKLPIEVEYFINEQKYFYAIFYHIYVTLILSITVILATETLYVSYVHHACGMFKIASYRLQ
HAFDQDALQISSFVRNAMIRTKIIAAIKIHKRALEFYDFLWSKLALSYFILILIGVVSLSINLFRVFQAIVIV
KNTDELLMSSICVAAHLIYIFCGNYAGQVFIDHSMEVHENVYDTQWYRVPLREQKLMLFILHRSMHSCKLITG
GTFVLSLEGFTSLLATSISYFTVLSSVQ
>CfOr357
EQYYILNRVLLSMIGLWPYDDFKVRQLRFILTLLTLILFTSAQLMKLFISKCNLDLLVEILPFNMIFIAISVK
YVTVYVMIKNIKEFQERLQRNWYALTDNREIDIIRKQAIIGRLFTIAIIMFGCGILFSCFILQYISILLDIVI
PLNESRPRELLFPAEYFIDQQKYFPIVTIHAGIGLFVVTISIIATESFSLANALHAFGLFEIASYRMKHILRK
SDSQMCITKQYIRHRIIAAVDFHRRAIEYSELLKASFGRMYLVLFVVMVCSTSINLFNVILSNNINRKNIVKC
IFSIIVYVILITLGNYAGQKFIDYDTHYYRTICNTKWYNAPLKTQKLILFLIQKTTKCYKVDAGGMFSPCFEG
LATSFSMTISYFMVLSS
>CfOr358
MNFNGDYYYKFNRIFLSAIGLWPHRYITLRQIQGIISSFILISVTIPQLIKLITTKYDADIILRVLSSALPFM
LFIVKYVTFYFVTDDVKKDNINNINLYFFIKINNSRQMRMHRESKVRKFYTALIYVCITMVICIQYVPSFLNI
LVPRNKSQRVELLFQVEYFIDQEKYYHTIQFHLDIGLILAAITILSTESFCLSLAIHAFGMFKIASYRMEYII
DENVSNIFIKKHCIFYNNIVAAVNGHRRAIEFSEIIKSTFAIPYLALILFGVTSTSVNLFLFFQVIRSNVISD
LMRSVIFVVCHFVYMFATNYAGQKFIDYDIDVYKKICNIQWYNAPLRAQKQILFIMQKAMKSYHMDVGGLYSP
SLQGFTTVIKKFVNYTIV
>CfOr359
MEFPEEKYYRLNYIFLALIGLWPHKNSSINQIQVVLALLIYLIFIISQLIKLFTTKYNFDLLLEILSYDVLIM
AWTAKYITFYVIIKDIQKIRKCVRDNWSILTDDQEVDIMRKYTDNGKLFTIIIAMSVYFGIFLFILLQYIPIL
FDIIMPLNESRPRKLLIEAEYFVDQQKYFHVLTMHINIVILLLSATTGIATESFALINAVHAFGMFKIASYRI
KHMLSENVLQAKCIAQKYIIFHHRITAAVDVHRRALDLSELLKITFGRSYLLVITIIICSGSINLLHLFRVIT
MKQEILEIIKAVSLIFCHFLFLILGNFAGQEFINYDSYVHQTICNTEWYNAPLKAQKLIILFMLQKTTKTYRVD
AGGMFSPCLEGLVATMSLLFSFFTVLGSI
>CfOr360
MSFYDNRYYYINKMLLSLIGQWPFQSRMEGIVMLVITLFLSCSFMSLQIWGLIAGINDLNIIMENASPLFIDV
FIFMKLINCLYNKDKMKHLLERIEETWKRIQVGPENKILQNHAEQNRTLIIRYASIMYMLGVFYSAMPIIVKV
LYWLLPTNEAYTTRFLFRLNHVLDVDKYFTLLMLNGILGVLFLMSVWVAADGMFILCTQHVCALFETVQNKVK
RIQGSDIVMDEPNIMDDEAYHVIIDCIKSYKYTLKFSELLSSTYGTHFLFLLGNVVVAVSFCIAELIMRDIPL
DETVRLIACNTGLLLHIFYLSLMAQQIIDYSDKFQEVIYSCNWYKISLRSRKLLRFMLLRASKPCQIKAGKMY
VMSMENFSSILQTSMSYVAVLTSLQ
>CfOr362
FVPKWQDCAANSYYRINRFLLICIGLWPYQNSSFRHIVIVFMTAILMSSVIFQFTTFITTEYNTDLLLQILAY
SIPWLAYMLKYNILCLNVKKMQRLMEQVFRDWNALSSVQEMEIIKRYWTIGRFITLITTLFIYLCIFGFVLVQ
FLSNFFLDIVMTGNQSRSRRLPVKLEYFLDQEKYFIPLSFHVFLTVLCGLTTVVATETLFMSYAQHACGLFQI
ASCRIEQALHTKMLQSVTSSAERNLIVCKGIISAVHMYRRAIKFMEMSKANFKWGYLTLLPLGVLSLSVNLYR
FSQLIISEEYYELIISILFVLGHFWYMFFCNYMGQEVIDHSGNIFYRTYNTKWYVAPLKAQKLLLLVMQRSMR
HSSIVIGGLFVLSFEGFATLTSMSVSYFTVIFS
>CfOr363
MTFVPKWQTCAANNYYTINRILLTSVGLWPYQNSNLRYIIIVFMIIILSSSVIAQFTTFITTEYNWDLLLQIL
AYSIPWLGYTLKYSVLCFNIKKTEGLMEQLQRDWNALNTVQEIKIIKKYWAIGRFITLATTLFMYLCIFSFVM
MQFLSNFLLDIIMTRNQSHSRRLPVKLEYFVDQEKYFILLLFHVFLIVFCGLTTVIATETLYMSYTQHACGLF
QIASCRIEQALHKNMLQSVTSSAEKNLIVHEGIISAVDMYRRANKFVEMSKANFYWAYILLLPLGVLSLSVNL

YRFSQLIVSEEYYDLIVSILLIIGHFGYMFFCNYLGQEVINHSSDVFHRTYNVQWYLAPLRAQKLLLLMMQRS
MRHCTIVVGGLFIPSFEGFATLTSMAISYFTVIFS
>CfOr364
MDFGGSYYFRIHRIILIWLGIWPYHTRANYIRCIFVSPFLLSGIIFQLSLITNEFSVEYFLHIFSFITPTLIY
IIKYFLLFFKINASKKLMERVRVDWDSLKEKEELKIMHKHANSGRRYTFITMIIMYTMIFLHTLIFFSTHIFN
TLIPLNKSRTNREHLWHLPNMMEHFIDREKYIYLLIVYLLLIGFIGATVLLVVDSLCIMYIQHITALFQITSY
RIERIVSKSRMKSLGFSKKSDTYENIAKTIDSHQRAVEFLNSMQSMYEIVYMFLIPISVVTLSINLYQVNFYL
EQTIHIISGTEGIVIWLGYIACHFSTMFLHNYFGQQIIDYSNNIFKKLCSIQWYMTPLYTQKCLLFMMRRNMK
SSKIMISSVFTASLEGFATLLNATLSYFMLIYSKR
>CfOr365
MFDKKDIWNSRYYIIPKLYMTISGIWPYDGLRHRCMCFVPMFTFCFSIMIPQLMYVLTATDLNDIFESVPAVL
ISIIFSCKIAGMMLSSKHIKSCLKMIKDDWLSLNTDVERTILARHCEYGRSLTTFYVVFMHMTAFFYLLKPIV
LTLLEDNTTNATNSTISGLSKLPFQVEYGEKLDQYFYPITIHCYLGVFAHVFSTLAVDTLYYTLIQHVCGMFS
IVGYMLEDIGKNSDANFHMELDKMEDVNYHKAFNCLRRHLHAIQFAELIESTFTNIFLVSISLNMICGSICGI
QVVMNLSNARDIAAPLAIYIAQLTHLFLQFWQAQFLLDYSIVPYESICRGKWYYTSGRCRKIFLLIMNRTISP
CKLTAGKIVTLSIESFGTVLKTSMSYFTMLRSFQ
>CfOr367
MIDLETQHFKLNRILLLTIGLWPLQQSNLTRFQFIIFFSVLMTIIIFQLMSFITLRCTSDLITNVLSSTCVFS
IAAVKCITFCFNLENVKDLLVQLQNLYDGLKDKNEIAIIKKYSCNGKRYTTAFTILAICFIFIFVAVFWLRIF
YIIYPINITQSHRLPITVEYFIDQEKYFYLILLHISVALCIAGTAMVAVGSMIIVYFQHTCGMFRICSYRIKR
AVHINLLENIKQKKGNLILKGIISAVDIHRQTLTLCRLLESNFEVMLICLILIAVISLSLNLFRIFQIASSKD
DIKDILFPFAFVTINILYMFIANYLAQDLTDHNNDIFTTVYNVQWNAAPLHIQKMILFLLQRGSKDFTISVGG
LFVGSLECFATMVRASVSYFTVMYSTQ
>CfOr368
MIGLETYNFKLNRILLLIIGLWPLQQSNLTRLQFVFMSTFLMTSIMFQLTTFLTSKCTPDFVAKILSSTSFFS
LIAINYNLFYLNMKPVKMIVHKLSSLIAIFYIIHPINITQSHRLPITVEYFVDQEKYFYLMLIHIIVSLCIGT
IVMIAVGTMLLVCFQYTCGLFRICSYRIKHAVHVNSLENIKLKKGNSMLKGIISAVDIHQQAINLCGLLESNI
EIMLFCLILNTVITLSLNFFRIFQIVLSRYDIMEIIFPFLFVTVSTIYMFLANYIGQDITDHNNDIFVTVYDV
QWHVAPLRIQKMILFLLQRGTKDYIINVGGLFAGSLQNFATLVKSSVSYFTVIYSTQ
>CfOr369
GLVIYNQIKSNNLIQIIEYKIIYLQFTTLLTSKCTSNLLIDVFSSASFYIIFVIKYSWFRINININIVKYLLEQL
QKIYNQLTNENEICIIRKYGNYGKSYTIGLILIAILVIPVPLSYPFWLYFLDTPVTINDSRSRFPLPHNTEYF
VDQKKYFYWILLHANAALLIGIVTILATGSIFIVYLQYACGMFRIASYRIEQSLVDISRKGNLQNNNLIYKKL
IRAVDMHREAMRFSDSSISRFKVMFSFMIAVGVISTSLNLYRISQILSFGYDVKKLLFPLIRVIIFMFYMFIG
NYVAQKIIDHNNVVFATAYNIRWYATSLNVQKMILFLLQRRTKVFSLNIAGLFVGSLEGAAMLLSSEISYFTV
LYSMQT
>CfOr370
LQFTTLLTSKCTPDLLIDVFSSASFYIIFVIKYSWFRINININIVKYLLEQLQMIYNQLTDKNEICIIRKYGKYG
KRYTVGLILIAILVIPVPLSYPFWLYIFDSPLIINDSRPRFPLPHSTEYLVDQKKYFYWILLHANAALLIEIV
AILATGSIFLVCQQYACGMFRIASYRIEQSLVVDILQEGSLQNKNMIYKKLIRAVDMHREAMRFSDSSISRFK
VMFSFMIAVGVISTSLNLYRISQILSFGYDVKKLLFPLIRVIIFMFYMFIGNYVAQKIIDHNNVVFATAYNIQ
WYTTSLNVQKMILFLLQRSTKIFSLNIAGLFEGSLESAAMLLSTEISYFTVLYSTQT
>CfOr371
MIWIDCLYININRILLLLVGLWPYQQSKLFTTLLTLKCTPDLLIDVFSSASFYIIFVIKYSWFRINININIVKYL
LEQLQKTYSQLTDENEICIIRKYGKYGKRYTVGLICKITVFVFSIPLLYPFWLYFLDIPVTINDSRPHFPLPH
STEYFVDQKKYFYWILLHANVALLIGIVTILASGTILIVYQQYACGMFRIASYRIEQSVTFDTSQKGSLRNKN
LIYKELIRAVNMHREAMRFSDSSISRFKVMFSFMIAVGVISTSLNLYRISQMLSFGYDVTKLLFPLSRVITFM
WYMFIGNYVAQEVMDHNNNVFITVYNIRWYTTSLNVQKMILFLLQRSTKIFSLNIAGLFEGSLESAAMVRDTY
IRIYNMF
>CfOr372
IKTQYFNLNRILLLAVGLWPYQQSKFVRLQIFLFFSILTSFIIFQLTALITAECTPEFIIKVFSTAMFFGVYV
IKYYSFWMNAHSVKCLLEQIQNICDELKDENEIAIMEKYGSNAKYFTTISLAICGVFIAILLPIWPQVLGSDL
HINESQPRQTVQIVTEYFVDQEKYFYLILLHKNIVICIGATTLTGTGTILVGYVIYACGMFRMASYRIENAMT
TKILNNINLENQTIIYKQIIDAVEIHCKAIKFSEFLTSAFKGSVSLLVIINVISLSLNIFGVSFFLKILKFIF
FSNSGNLLFKNVTYIXIFYKIRRIQIINHNNHIYFLAYNSRWYIAPLHAQKLILFILLRGSKTVNLNIGGLVV
ASLECFTMLIKLSMSYFTVMYSMQE
>CfOr374

MICINCLNISLNRILLLAVGLWPYQQSKLVRFQLILVYAILTTCIMFQFTALLTSKCTSDFLIDVFSTALLYV
MFLIKYSWFHINIEVIKCSLEQLQNIYNKLTDKNEITIIKKYGSYAKRYTAGLICKTITFSFIKFLYLFYPYI
LDIVLSINESRSYSPVLVTEYFIDQEKYSYLILLHIMAALFVGIITVLATGAIFIVYLQYACGMFQIASYRMK
QAITIGNSRKGNLSRNKNLIYKELIRAVDMHREAMRFSDSFVSRFKVMFSSLIVVGLTSASLNSFRIFQALTL
GYDIEEFLVPLVSMITYISYMFLANYIAQEIMDHNNDVFDTVYNIQWYAVSLNVQKMILFLLQRGTKAFNLNI
AGLFVGSLEGAATLISTGISYFTVIYSTR
>CfOr375
MICIDCLHMNLNRMLLLAVGLWPYQQSKLVQFQLILFFTILITCIIFQFTTFATSKCTSSLLINVFSSALFYI
YFLILYSWFHINMEVVKCSLEQLQNMYNELTDKNEITIIKKYGNYGKRYTAGLILTVVFFAPSLGLYLHWPYL
VNRLLPINDFRSRSSLFVTEYFIDQEKYSYLILFHTLTALFIGIITIIATGTLFIVYAHYGCGMFRITNYRIE
QVMAIDTLQKKSILRNKNLIYKKLIRAVDMHREAMRFSESFVSKFKVMFSCLIAIAVASASLNLFRISQALSF
GNNVDELLFPLITVGIYIAYILIANIMTQKIMDYNNNVFDTAYNIQWYAVSLNVQKMILFLLQRGTKVFSLNI
AGLFVGSLEGAAKLLSTMMSFFTVLYSTR
>CfOr376
IKTQYFNLNRILLLAIGLWPYHQSKFVRFQAFLFFSILTSFIIFQLTAFITTKCTPEFIIKVLSNIMVSTAYI
IQYNSFWMNTHNLKCLLEQLQNVCNELKDKNEVAIFEEYGNISKYLTTISFAMCGAFNALFLPIWPQILGSDL
HINVSQSRQTMQIVTEYFIDQEKYFYFILLHKNVVICIGATTLTGTGTMFIGYFIYICGVFRIASYRIERAMT
TKIRENINIENQTMIYKDIIDAIEIHRKAIKFTDFISSTFSGSVSLLIIIGVVSLSLNIFGIFRNASLGDKES
FLLNFLFVLVILICMFVGNYAGQEITNHNNHVYFAAYNSRWYIAPLHAQKLIFFILLRGGKSVNVNIGGLIVA
SLECFTNLTKTSMSYFTVMSSMQE
>CfOr377
MIHIKTQHFSLNRILLLAIGLWPYQKSKFVRLQVIVIYGILISFVIFQFTALITTKCTLDFIIKIISTAIFFS
FYIIKYNMFWINIHRVKNMLEQLQDICDKLKDENEIAIILKYGNNAKHCTAIFTSLAICSTFIAILMPIWPQI
CDTVSHINGSRQNHTIYIVTEYFIDKKEYFYLIILHINAVMCIGATTIIATGTTLLGYIIHVCGMFKIASYRI
EQAMAPKVLENINVTNRTMIYKKIFYAVEIHRKAMKFANALISNFEGSFLLLIILSVISLSLNIFAVFRNALL
RNKEGFFLHLIAMNIIFSFLLIGNYAGQEVINHSNHIYFTAYNVQWYVAPLRAQKMILFILQRGNKTYSLNIA
GLYVPSLECFAMLTKASISYFTVLQSMQ
>CfOr378
IKTQYFSLNRILLLAIGLWPYHKSKLVRLQEVMFFGILTSFVIFQFTVLITRKCTLDLFIKIFSNAMFFSFYI
IIYYMFWINTHHVTKFQLCRHYEYRLECEKNNLHKFFFETFIGYAVCSTCIAILLPIWPQIFGNVLHINESRL
QSQIIYIATEYFIDKKQNFYLSLLHTSAVFCIGAVTFTATGTMLLGFIIHVCGIFRIASYRIEQAMTTKILEN
INLETMIIIYKKIFYAVEIHRSAMKFVNVLVSSFEGSFLLLIVLIVTTLSVNIFAVSLHYNASHGNKEAFLLH
LIFVFVIVLVMFLANYAGQQIINYCNHIYITAYNVRWYEAPLHAQKMILFILLRGNKSFTVNIAGIFVISLEC
FAMLTKASMSYFTVLHTTQQ
>CfOr379
MISIISQHFSLNRILLLAIGLWPYQKSKLVWLQEILFFGILLSFVIFQVPLITTKCTVDFILKILSTAIIFNF
YVIVYNMFWINIHHVRNLLEQLQYICNDLKDENEIDIIIKYGNFAKRFTAIITWYTVCVICILILLPIWPQIF
GNVLHINESRLQSQTIQIVTEYFIDKKKNYYLSLLHMSAVYHIGAITFTATGTMLLGFIIHVCGIFRIASYRI
EQAMTNKILENINLKTMIIIYKKIFYAVEIHRSAMKFVNVLVSSFEGSFLLLIVLGVTALSVNIFAVSVYYNA
LHGNKEAFLLHLIFVFVIVVYMFAANYAGQQIINYCNQMYITAYNVRWYEAPLHAQKMILFILLRGNKSFTVN
IAGIFVISLECFAMLTKASMSYFTVLHTTQQ
>CfOr380
MICITSQHFSLNRILLLAIGLWPYQKSKLVWLQEILFFCILLSFVIFQLTAFITTKCTVDLIIKVLSTAIVFN
FYVIIYNMFWINIHHVKNLLEQLQYICNNLKDKNEIDTILKYGNNAKHCTAIITLISICSTFFATLLPIWPQI
SNTVLNVNESRLQSHTVYIVTEYFIDKKKYSYLILLHANVTMCIGGTTVIATGTMILACIIYACGMFKIASYR
MEQVMSTKMLENINLTKNQTMIYKKIYYAVEIHRKAMKFTNTMLSNFEGSFILIITFSVISLSLNIFAIFRNV
SFGNKEAALLHLLIVFLIFIYIFIANYAGQEITNHSNHIYSTAYNIRWYKAPSRIQKIILIILQRGSKSFNLN
IAGLFVLSVECFATLTKTSLSYFTVIYSTQQ
>CfOr381
MICIETQHFHLNRILLLAIGLWPYQKSKFVQLQVILNFGILTSFVIFQFTALITTKCTLSFIIKIFSTTIFFS
FYVIFYNMFWINTHNVRNLLDQLQYICNDLKDGNEINIILKYGNNAKRYTAIFTSFATCIAFIATLMPIWPQI
LGTVLYINESQLQINTIQITTEYFIDKKKYFYLILLHTNVVICIGSTTVTGIATIILSWFIHICGIFRIASYR
MEQAITPKMLENINMKDQNMIRKKIFYAVEIHRKAMKFANALISNFEGSFLLLIILSVISLSLNIFAVSSFIV
FFEAFLLHLLIVIIILLVMFLANYTGQEITNYSNHIYSTAYNIRWYKAPSYVQKMILFILQRGNKTFNINITG
LFVLSIECFAMLIKASMSYFTVMYSTQ
>CfOr382
MVCIKTHFNLNRKLLLAIGLWPYQKSKFVLFQGILFFGILLSFVIFQLTALITTKCTSDFIIKVFSTTVIFNF
YVIKYSMFWINAHNLRYMLEQLQYICNELKDENEIAIIINYGNNAKRYTAIFILVNIFCTVIAISMPIWPHIL

GILHINESQLEIRKIEIVTEYFVDKKKYFYLILFHTNAVFCIGGITITATGTLLLGYMIHICGLFQIATYRME
QAISTKMFENINIKSQTLIKEKIFYAVEIHRRTMKSIDFMSSSFKGSYLLLAFFGIVSLSLNIFAIFRNATLE
KKETFILHLIFVFGTLLYMFIFNYCGEEITNYNDHIYSAVYNSQWYMAPLHVQKMILFILQKSSKTFNCTIAG
LLVISMKLFATLTKTSMSYVTVIYSTQQ
>CfOr383
IRTQHFNLNRILLLAVGLWPYQKSKFVRFQGILFFGILSSFIIFQLTALIKKDCTSEFVIKVFSITMIFNIYA
IKYNMFWINTHNLRNLLEQLQYICNELKDENEIAILIKYGNNTKRYTAIFTLLTICLTLSTVILLIWPLIFGT
VLHINESQVITMLTEYVVDREKYFYLILLHVNIVICIGSTTITATGLMILGCIIHICGLFKIASYRMEQAMTT
KMLQNINLENQTTICKKIFYAVEIHRKTMKFTNLLFSSFEGSFFLLIAFLVTSLSLNMFGIFHNASLGDMESV
LLHLIIVLIMLLYMFVANYAGQEVINYSNHIYSTAYNVRWYEAPLNVQKMILFILQRSRKNFHIKIAGLFIIS
LECFATLIKASMSYFTFMYSTQ
>CfOr384
IETQYFRLNRILLLAVGLWPYQRSKLVQFQATLLFGILISFIILQFTVFFTTKCTPDLVIRILSFILFFIANN
LKYFLIKVKCLLEQLQQIYNELKDENEMVIIKKYGYNAKCYTIRIIILITVCSVFVISFLSIWPRLLDIIVPMN
ESRPHYEIEFIAEYFPDQEKYYYVIILHMNAAICIGGIAFIATGTMLIAYLKHACGMFKIASYRIDQAIAINF
LQNIEKNEIITYKKITYAVDIHRKAMKFAEYLISNFEGSFFVLIAVTVCCLSLNLFQIYRAVLLQDNEKEFLL
HFLIVFIILLYMFLANYAGQEIIDHNNHIYSTAYNIHWYVMPLHVQKLILFLLQRSSKSFSLNIGGLFVASLQ
CFASLSSTSISYFTVIYSTQ
>CfOr385
MTCFVNQHFRLNRILLLAIGLWPYQRSLLIRFQQILLFGILASFIVFQLTKLSTSEYKTDLIIKILSTALFFV
MFMIEYNSFWINSQTMKNLLEQLRNICNEFKDENEIIIMKKYGNMAQRYTAIIIVMSTCGTFIIFIIPMWSHI
LDIILPMNESRSRFTIHVLTEYFVDQEKYFYLILLHMNAAICIGMITVVGTGTTLLTILAHICGMFTIVSYRI
EQAMTINNLQNKSKNENVICKGIIYAVDIHCKAIEFSRYLTSSIQGSYFLLIGIGVLSLSLNLFEMYQILSGD
GDKEAFFIHFLFIFIILLYMFVTSYVGQEIIDHNNDIYYTAYNVCWYVAPLHIQKLILFLLQRGSKVVNLNVG
GLIAASLNCFASLASASVSYFTFIYSTHE
>CfOr386
MICIVSQHFRLNRILLLAIGLWPYQQSLLVRFQQFLFLSILGSFIVFQLTIFLTSNCTFDYVLKILSTLLTFT
IFIIKYNSFWINSQTVKNLLEQLQNICNELKDENEIIIMRKYGTNAKYYTLKMIVVSMCSIFTVSFMSMWSHI
LDIVLPLNESRHSKIQILTEYFIDQEKYFYLTILHINAAFCIGATTIIGTGTMLIAYLKHACGIFTIASYRIE
QAMTTNSNLKNKNVIYSRIICAVDVHCKAIEFSKSLISSFEGSFFILIGFGVITLSVNLFEMYQVLSLGSGKE
EFLWHFVLIIVILLYMFLANYAGQEIIDHNNHIYYTAYNVRWYVAPLHIQKLILFLLQRGSKVVNLNVGGLFI
ASLNCFASLASASISYFTFIYSTQ
>CfOr387
IVSQHFRLNRILLLAIGLWPYQQSLLVRFQQFLFLSILGSFIVFQLTIFLTSKCTFDYIIKILSITLFSTIFI
IKYNSFWINSQTVKNLLEQLQNICNELKDENEIIIMRKYGTNAKYYTIKMIVISMCSIFTVSFVSMWSHILDI
VLPMNESRSHSTVQELTEYFIDQEKYFYLTFLHMNATLCIGTTTIIGTGTMLIAYLKHACGIFKIASYRIEQA
MTINSFHSNLKNKNVIYNRIICAVDVHCKAIEFXSLNLFEMYQVLLFGSDKEEFLLHFVMAIVILLYMFLANY
AGQEIIDHNNHIYYTAYNARWYVAPLHTQKLILFLLQRSNKIVSLNVGGLFAASLSCFASLASASISYFTFIY
ST
>CfOr388
MLVNILREYNINRKFLSCLGFWPFQRKLARYSLPIFCFVLELSYLPFEIITLYMHRRSKQMIFECLYQMTVTI
AFLVKLVNQLWNRSKFQRLYETMENHWNIFTNDFEVQIMKNYSGISQKFTISYSILIYVMSTMFITIPSLGPI
FLDIVLPLNESRPRHLALYAEYGIDQNKYFVGIFLYTTIMIMVGLTIMVAVDTMHIACTAHACSLFQVIGHQI
ENVSNMREVDQTGYRANTEYKLFNEKVIYREYILCLKKHQLAIEYVDILNDTHKIVGISFSLIIGMIFSLLGI
RIVYVLDQVEEIIRFFFIITGALIQLMIVCYSGQKLMDESQNIFHRAYAAEWYKFSPRLKSLLIIILYRSVVP
CKLTAGNLFPLSMVIFATVIRTGISYFTAFLSFKD
>CfOr389
DIMLVHVLREYNVNKIFLSRLGLWPFQSKLVRDLLPTFYLMLEISFYPFEILMVYHHRDDTQMVFEGCYQLVI
SSAFLVRLWNEIWNRNKFQCLYEAMNDHWNIFTNDLEVRILKDYSIISQKFTIFYSIMMYLLSSMFIVIPLTP
AFLDIVLPLNESRPRILAVEVDSRIDKDKHFVPLFCYTTAIIVVGISIMVGADTMHFTCTNHACSLFAIIGEQ
IENIKLKLDMEKCECCMNKKFKSSNERAIYQEYITCLKKYQLALKFVDILNSTHQTVAVFFLLLIGATLSLIG
IRIVYVLDQMEEMIRFMFIIMGALLQLMIMCYSGQKLIDESQNIFYRAYAAKWYMYSPRMRSLLIITLYRSNI
PCSLTAGKLIPLSMTTYAAVVRAGMSYFTAFLSIKD
>CfOr390
KGYLQTMNSTWNHYYSVVEKVSSLAGQWPYQKPKTRLLCSSVVTLITFSMIVPQMAKFVKCNGDLQCIFQTLT
AYLLTVVTLVKLYTCYFNRCKMKVLIDQLFIEWDDLQTPEELEIMKKYAEKGRRYSLGYSLYCFISVYLFMSL
SLVPQLLDVILPLNESRPILPAHPGYYFVDEKKYFYYIFSHAIVAWEVAMTGIVAHDCMLLTYVEHVCSIFAI
TGSRFKQLTCKSANSVTILHSTNDTYRKRIEFSVHTHRKALKFAQLIEDTFSLTLAIQIMLNTVMISITLLQV

TQEDADVLVILKYVVYVIAQLIHLFCLSFEGQKLIDHSLQTRDTIYNSPWYKTPAKSQKMLLFVMRKSLQPMF
LTAGKIYIFSLENFTSIVQTSMSYFTVLSSLE
>CfOr391
IRDCLQIMNSTWSQYYDIVKRVSSLSGQWPYQRSKTRLLCVCLVTLSTFSMIIPQIAKFTICDGDLRCIFETM
TSYMLTSVTLVKLYTCYFNRCKIKMLIDRLFVDWDELETPEEYEIMKRYAENSRRYSLGYSLYCFSSVCLFMC
ISLIPQLLDVVLPLNESRPILSTYPGYYFVDEKKYFFYIFSHAIVAWEIAMAGIVTHDCMLLTYIEHVCSIFA
IVGFRFEQLTYRHTDSTSILHPQLTDTCKRIAFSVQTHRKALKFAELIEDTFSLTLAIQLALNTVMISITLLQ
ITQQKGDFLEAIRYIFYVFGQLIHLFCLSFEGQKLIDHSLQIRDKIYNSFWYKTSAKSQKMLLFVIRKSFQPM
FLSAGKIYIFSMESFTTVVQTSMSYFTILASLE
>CfOr392
METTCNHYYNIVYKIASIAGIWPYLKPKTRMFRVTFLTMILLTIFIPQIAYQFTCKEMQCTYQAMTAYLLSII
VMLKMYTFHLNNRTIKDLTHQLFYDWKTLESTEEYKIMKSYAENSKRFSLLWSVYVFMAVIMFMSMSLIPYML
DIVLPLNESRPILPPYRGYYFVDIGEYFFQIYWHSIVAWEIVVTGVIAHDCMFVAYVEHVCSMFAIAGFRYEH
LFYYHKEKEAATVTDGSNTDDTYDKRVAFLVYTHREALKYAQLIEDTFTIPFAIQMFIVTIGMSISLLQITQQ
DDILEAIRYVFYVIGQLIHLFCLSFEGQKLIDHSLQMRDKIYNSSWYKTSIKLQKLIILVMMKGLRPSFLTAG
KIYIFSLESFTTVLQTSVSYLTVLGSV
>CfOr393
LSQLSYKMHAKEEYYNKINRIVLKILGLWPYQQLYFVGIQKLFVVFILSTFILVQLLLFITTQYNTNLLFKIL
SFILPTLFSTIKYCFFIIQANSVKQMLERHRDNWKFFKDKQEIDIIEKYSYIARLISIILMGLCCAALPVLLI
LHTFPLIFDFILPLNESRPWQFLVATEYFFNQEKYVYAILLHHSLAIAIAAITLCSTSSTFIMSVLHICALFN
IASHRIENAMKWNILAISDPKREHLLYRKIVHAVIVHRRAIEFTKFLTTEFAILFAILILVGVCSLSLTLLQF
LQLITLTNNITEAFIFAVLILVHLTYMFGANYAGQIITDHGIKLFKATYNGMWYAAPLHTQKMLLFIMQKGMI
NVNLRLGSVFTASLEGFAMLTNAAVSYFTVIYST
>CfOr394
EMDFIGERYYKINKIFLNCLGLWPSNNKTTGRIKLKPIFCFVLFVSFLFVQLCTFITSECNVHLILNVLSHIL
PVFMYTVEYYAYYFKAQNVKLIMDEIRTDWNSLNDKKEIDIIEKYSYKMNIYTIIFTLFFCISLIIFTFIEFL
PIILDIVVPLNETRPRSMHVEAEYFVDPEKYFPLMVLHELIACLVGFSTLVATGTIMMAYAQHGCGMLKIVSF
RIQHVLRKNMIFKHSSPRTERIIRDRIIRAIDLHRRILKIESLIYLKVTYNCLNIVILRLKEKLFAIICINFT
LFLPSTLQNMKDLFTSTVIVLGHFLFMFLANYMGQIITDHSVDIFYALCNISWYIAPLRSQKLL
>CfOr395
MNFVGYSYYNIHRILLSSVGLWPYQDSRMKKIQIVISFIILMSSIIIQLMKFVTTEYSLDLLLKISSFTVPCM
VFVLKYVSFCMGAGTVRNLMENIISDWNLLKTEVELEIVKKYSDFGRLYTIFFAMTVYSALFVYILIEFTPNF
LDIVAPRNESRLHSIPLTAEYFADQQKYYLPILLHINVIALVGFTTVISTESLFVAYVQHAIGMFEIASYRIE
HAFDEILGLNTSRKNCSYCTKIISAVNIHNRAIKFIEFLVSGFATSYFFLVCLGIASLTANLVRLFLSAQYLD
DLEECIIAVLLVFGHIYYIFLGNYTGQRLIDHSTDVFHRAYVSQWYVAPPHAQKLLLFMMQQSIKGNSISVGG
LFVPSLEGFATITSMSVSYFTVILSI
>CfOr396
MEFLGYRYYKLYRLSLLSLSLWPYGKSLLKQIHAIFCIFLLISATVTQLLKLFTMEYDLELILTILSFAIPSA
IYLVKYIAFYTQSQKIRELMEQVQNDWNALKDEQEVEIIRKYTQSGRKHLYAFAGLVYPGTIGFLLTPFLPDI
LDVIAPLNESRTRHLPYLAEYFLDQQKYFYPLLLHANITLIIGIVTVISTETLFFAYVYHICGMFEIAGYRIE
HALDESMSILSTLNKENAIRTKIINAIEIHQRAIEFFECVSSTFALSYFILIIILGVASLSVNLFRLFQVAVAI
SDEKESFISFMIFVVGHFYYMFICNYMGQKIIDNSTGICRKTYDTQWYTAPVQIQKLLLFMMKKSINSCKFVM
GGIYSVSLENFTTLASMSLSYFTVIYSVQ
>CfOr397
MDFDGNRYYTLNRVMLSSIGLWPYQKTWFIRIQRFSCVTCVISGIIVQLLTFTTFEYNLDLLLQVLSFTIPCL
IAILKYVTYCVKIESMRELMDMIKYDWNTLKNRMEYEIIRKYTYMGAFYSQLFTLCAYISPLFFIFIHFIPNL
LDLMAPLNQSRPHQLIILSEYFVNIDEYFYPVLLHMVVYIFIILITLMSTTSIYVAYIQHACGMFEIASYRIE
HALDDYEKENLLSTKCCIICSRIISAINIHRRAIEFFEFMKNTFVTMYFFLLLLGVASLTVNLYHAVIIQGLI
ENIFPITNVFIHLFYFFCVNYSGQKVLDHSSEFLSRTYNCKWHVMPLHVQKLILFIMQRSSKNCTLLVGGLYV
ASLEGFAMTMSTSISYFMVIYST
>CfOr398
MDVYDDRFYGLTRRLMIFLGLWPYQNPKHRKLLFIFVSIIYVQGLIFQYMTFITHQYNMKLFIEMLSFHTILV
LFIVKYNTVYLNFTNVKLLFEQINYDCITITDTQEIEIIQKYASRGRLYTIYFGLFIYIGTILFMLTLCIPDV
LNFIMPLDKPRLRLLPTPVECFLDQQKYFYVIVSNMSVIAMMGMTTFIATETMFMIFIQHACGLFAIASHRIT
HAFDDCKDNSVISAKLSRAIKIHGRSLQFIEFMNSCFAVSYLVLVTVGIVSISINILRLFQAIESNIFGDILA
SSLYVLSHFCYTFWVNYFGQYLSDHSENMAEGTYNVQWYAAPLRIRKLLLIILQRSMKTCTFNIFSGILIASM
EGFASTISIAVSYFTVLYSL
>CfOr399

MDAYDDRFYGLTRRLMIFLGLWPYQNPKHRKLLLVFVWIIYMKSLLIQYMTFIMHPHDMKLYIEIISFHTLLV
LFIIISNIIYLNSTNIKKLLDYIQRDWNLIKDTNELKIIQKYAYKARFYTVFSALIVYPGTSIFISMIFVPDI
LNILMPLDKPRLRQLPVQVEVFFDIEIYFYVFFLFFITVVFLGMTILMATETMYMTFIQHACGLFELVSCRLT
CALNTNLSGTTLLKTKCKLCTKLLHAFLAHQHCLEFIKIVQYEFSTSYFILCILGVASLSINMFRFFHAIEIH
NIVEVISTGLFVFAHFCYTFYINYFGQDLIDQSECFFQQIYSTQWYTAPICAQKLLFIALQRSAKTSKIVIGS
LFVASFEGFATLISMSLSYCMVIYSV
>CfOr400
MDFFNNEYYGLTRNLMASVGLWPYQKSEHRIVRVFCISCILIISGLVQLTTFTTHEYNTTLLIEVFSFTIICI
VYVMKYCIVHFNSNHVKNLFDQIQRDWNLIKDIDELKIIQKYAYKARFYTVFSGLIVYLGTFIFLSMMFVPDI
LDILMPLDKPRLRQLPFQIEVFFDTERYFYFIAFLLIIINFLGMTVLMATETMFMAFIQHVCGLFEMKFLGFL
DESYSKILFLYINIFYYKERKEIFIENMYCAFSISYFFLCAFGVTSLSINMFRFLHAVTIHNIFEIISTGLFV
FAHLCYTFYVNYLGQDLIDQSECFFQQVYNTQWYTAPLYAQKLLFITLQRSAKSSKLIIGGLFVASLLHLKLI
SMSLSYCMVIYSVRI
>CfOr401
YFQFTTFLNSKYTADFVVKILSSALFFTTFVIKYIFFAINTETVKDLLTQLQHVYDQLKDEYENAIVEKYGYN
AKRVTLMLTTLSICSISALIVVQFWSNIIDIILPLNVSRPHRLPIMTEYFVDQEKYFFLILLHLNAAFCTGIA
VVVAIGTMLIAYLIHICGLFKITSYRIKHAMRESMLQNYSPKNDILILEEIRYAVHLHRQAMKLSKFLVSKFE
KMLFCLIAVGVISLSLNLFQVXSNFKLFKSVKMXFIFYNITEYKIYNVQWYVAPLYVQKLMLFLLLGNAKDFT
LDVSGLFVASLECFATLVKTSVSYFTVIYSTQ
>CfOr402
DINNYVFINRKVLKFIGLYPTNIVRYIMCCVCMFAIVIPQAMQIYQNWNDLAIVLETSSVLLTILLAILKSLV
WISNRRKMDSFIQYMLTNYWEIMTAHVSKGANIYAIYVRKGSLYTKRYLFLICNSLIFFFSLPIIEIFVTMIK
STNDNNTSTKHFPFIALYPKNYYNFPMYEIIYLSQMVATSLCGLIIILGTDTLIATAVFHTCGHFKILHKKIEN
INTEIDLIQHVYIEENIRKIKLQIIDIIKHHHLVLWFCDYMEKIFSPMLFLQTLASSLIICLVGFQIATVNTA
IINSKSIKYVSYLIMALFQLLLFCIPGDALIYESSMISRTMYTIAWYELSTLFKTEVCLLMLRSQKSSKITAG
KFYTMHLENFNAVLNTAISYFMLLRSFSSDETNVKL
>CfOr403
MVSNEPFASVIRANVMLLRFSGVVSYKDGIIRRTNSQNVLSAIAYGCLFAYAFLYTYEFALHTVYLDTWMESF
AMILSLVGGQARFTLVLLSRSRFQRLLAICEELWTTLNATERKCVRDYVKPTRSLTYYYLFGCAFTIFFYAVA
SLFMGQHDDSSNATTRTLPYACPVQVHRTPYYEIMYALQLSSMINVGLTCAAADTLGPVLILTVCGHFKVLNS
RILSLSDRAYSKSSCPTSDSLEIMRGVSRNRVKSDLKNCVHYHQMVLEFCKEVEKLTNGIFLTQLVGSTYNVS
LVGFKLAGEDPDKFKYTTQLSIAMIQLFLCNWPADVLLTESQDVARAMYFTSWYRFSYQLKRSINIITMRAQK
PTQLTAGYIVPLSLQTFASMISSAASFFTMIRSMN
>CfOr404
MISFTAQNFKLNRILLLSVGLWPLQQSNLTRLQFIFMSVIITAGTLFLLTAFITLRCTPDLVTEILSYVCFYS
TFVIQYNLFRFNIKDMKNFMMQLQNVYNGLKDKNEIAIMKKYGYIASCYTTAFTIASVCGIFGYMLIIFPDIM
HIIIPTNVTQSHRLPVPMDYFXDQEKYFYWIVLYLSMGVFIGTVSTIAIGSILIAYIQCTYGMFKISRYKINI
IITVIRCAMHINMLKNIKSKKENSILEGIISAVNIHRQAMKLSKFLESKMETMMFCLIMSGVLSLSLNLFRVF
QIVSSGYDIQELLMPFASVSIAILYMFIGNYATQNVTDHNNDVFATVYDVEWHERPLHIQKIILFLLQKGAKE
FTLGAGGLFVGSLECFTTLVKTSFSYFTVIY
>CfOr405
QLLCLFKVWPDFFEVFDCLSMLFYSITSTFKFIYTIYKLPKIKMLLVKIQEHWCSPKTDQETKILRSYLLFAR
KFSYIYAVFFFSFNXLYIFVFQSIISKIYLLKNLTLCNFALTFYRHSVQFSLNSQYFENRYKTSLMYLNKMNI
FIIIXALIFQHLYTILNFRYRLERALEFEDNGIDNILITAFARNKCYSNIVYSVRRHTEAIQFATAIESLYRF
PFFIHMVTNGSLLSIVGFQLMTNKENINRLLPYASYLNALMLNTFFENWQGQKIIDCNEKVHESAYKLQWYRM
PVVSQKLLIMIMMRSKKPLTITAGKLIVLCYVTFNAVMRTAFSYFTLLRSVQ
>CfOr406
IEDNRNYYYDVSKKFLWMVGQWPYQKPKTRLSFFALLVTTLIICIITQTAHIFLCKDSECIFETLPAHLLMWN
SLVKVCTYQFNSQKIKNLTDHLFVDWDILETREEREIMKKYAESGRWSSLIYAWYYYVSTVSFIMTSLIPPIM
DILFPLNISRPIILAYPAHYFINEEQYFYYIFCHMLMTGVICMTGLIAHDCMFFTYIEHVCGLFTVVGFRFEQ
ILYKRNIAKRKLIDYPDDVYNKNIAFSVHSYRRALQFAELVEKVFSISFAMQLMIATIGLSVTLLQLHNLMDT
TKYFVYIFAQLFHLFCFSFQGQKLIDHSLQICYKIYNSAWYEIPVKGQKLLLITMRKSTKASTVTACKIYVYS
LQSFTTVLQTAMSYFTVLASFS
>CfOr407
MISLETQHFKINRLLLLIIGLWPLQQSNLTRLQFIFCSTVLITSIIFQLMTFVTLRCTPYHIIKILSSTCFSS
TFVIKYILFYFNMKIVXDSLLKMIIFKKQYLLEVDRETGKIFMSIIGVCSTFAIIIALFWSRILYITLSINIS
PLRRLPIKVEYFIDQEKYFYLILLHISVALCIGAITMVAIGTLLIAYLQHTCGMFRISSYRIRHAVHVNTLEN
IKSKEKNLILKRIISAVNIHRKAMKLSGFLISKIQTMLFCLIIIGVISLSLNLFQIFKIVSSGNDVKEIIFPF

IFVTISILYMFLANYMAQDIMDHNNDIFATVYDVKWHVAPLHIQKVILFLLQRGAKDFTLSVGGLFVGSLECF
ATLVKTSVSYFTVIYSTQ
>CsolOr2
MNDFNSAYWKLTKFLQNFIGVWPYSSNSNDKFKRIMIYMAMYSLAVPVVLKLIEEVESHIDIVIENVFGELYI
LIVLMKYTMCIINSNKLKKIYKLIRKHWTMTTDNEELHILKKFANVGRLLSITYAVFCFSVGGSFVIMPLIYP
LLNYISPLENTTRPLQLPIYVQYYIDPQKYYALLMIKICVTSMFSVVVCIAHDTAYIMCIYHTFSLFEIVILR
LQRLADMCYDIETSKHSINFDYKVYHRIIKSIDLHQSIIENIQCIEKAFTINLFITVIANTAATGGGLFVVFM
KLSSPIDLVRYGLFVTVISVHKYFMFLPGQTIINYSLLVFRLCYACRWYNFSLKCKILIQIMMARSQQPCYLT
GGKVFILCLETYSALMKKGISFFTIFVSIQ
>CsolOr3
LKISGLLPSKFSSTFLFILLSHLITTLPFQLWKVCTSRDNTSYFMMNLTSIIGQTLVYIEFSIMWMNRRRIKE
LVQEIIEYWKRNERSEDYENIVKNFRIYCIADIIVYCSAELIYIINTLSLYLNAHVEDRYLMYDLHYSFNYRL
SPFFEIIFLIQILQFTTLIILDLLIKALLVAYTSYVSVRINRLKFYIKQYSNNVIKEREINNNVTSTILKLII
KEHQHILKLVQQIDDIYNLIGLFRFGFSHIAIGVSGFTIIIVNYVYIMKCVITIIILTIQALSLCITGQQLCN
EGDSIIDMLYNCIWYNNSKMKEIKMLSFLILKSQIPLIISAGYIFKFSIKNFVQILKTAFSLLSVLRAVY
>CsolOr4
MNDKLSDNKPLNFHRECCFNIFLLKINGLYPIDGFFGNCFFNVILVILVNISHLFIITMDAVDVVYDTQIGII
DIEVIAGCIGLSGLYVRFVIFCYRRAEVFALFKESEKLWNQLKESEKCIVSKYMRFMKTLTRIYLISYILALL
MYVATNIMLASSISINGIPHKHLMFGKTVYVRKPFFEIISVLEILSAGVMTTSSGYDMAAPFIITMAAGYFKT
LFYRYENAQKQHSFNNDDQMFKDIIACIIYHQKIDRFCTNINKFTKSLFLIQLISSTYNLSILLLVVILNPKE
DLKNGPIIIVQALQFFLCHWTSDVLLSESLDIRKSVYMIPSVNRKDTKLSKLIQILLMKLQNPVQLTAGGFIN
LSVECFGNMMSSVVSFFLVIHSFTN
>CsolOr5
SFESVNKINILLMKIAGLLAFESRSINSIGTTTIIIYAHFSIVFIICMYISTALMNAYRRIMNFEVIAGCIGL
SGLYLRYVLLCFKRSNIATLLVESEKLWGKLQEAEKPIIRGFVRTIGTISICYLGFNFSWSAMCITITQFHGT
TNDTSAAVRRELPYSFALDLQDSPSYEIMFIVQIITSFGYTLVVAGFDMAPPYFIMTAAAHFKMLCSRFEEMQ
KQDRLRRADEAIEDVITCIAYHQMIDRFCKDISKITESFFMVQLLSSTYNLSILLYAIITDNQNNFQLLPVVG
LQTLQLFSCHWISEILRSESKRLIKSTFIVPSMFERNMKLSKIIQIVILKSQNPVQLRAGGYINLSLECFGEM
VTSAISFFFIL
>CsolOr6
MDILPDHFRMLRYLGLWYQADESFWIIKEIYSIVTIIMIFSFICLQVAATSVLANGNLKIFIETLSIVLTYIS
FFFKMINFKIQRKNMNKLLDSFRMEICQPKTPQENNIILEYKNLTTKILTYYRSLAILAGIFLILLPILKQEL
HKRVLPFNIYSPVDLTNPTVYALVYFYEVLMNIISVLLHIVLDSMGVSFICLISGQLELCCYRIVTWSENVEK
FSRKKSIKYIVMHHNLIKDIFQRTENFFMIIIIPFFSICLLVICLVLFQLVQKNVSFSEHGVLALYFFTLINE
VFLFCWFGNQLTVKSEKILNAVYKSNWLELTTKDRKSYYFAMLMGAKGLTMSYHGLCQLSVKTYAWIIKMTYT
VFNLLQ
>CsolOr7
MQVFPITFKILTICGIWRPKNFDTFIKKYIYYCLTVLICFLVFTFTLSHLIDIIVCANNFEDLAESCFMVLSM
LNICCKTINILYLRNDILQLLKLLINSHCQVVDEIERKIEIKFYNKIRFVTLFYTILAEITCILITIRSFFAS
TTNTLPFRAWIPYEIQTQFHFWIVFFHQTIAHVVGANIQIANDTLVYAMLIQTNMQLQILKNRLKNISNNFEG
RDVTKCSTKNINDSKKLLVKCVKYHRCILEYSEKLNNTLRIIIFIQFVVSLLVLCSSVYLLSKMKLVSMQFIS
LMLYLFCMLYQIFLYCWYGNEIILQSMDLGNVVYIIDWTNLKIKDKKNLLILMLLFCKPIKFTSTFLVTLSID
SYCKILKTSYSIFNLLQRISL
>CsolOr8
MLFTQFKVLTIVGIWYPPDWTSNWKKNFYKLYSFSINIIMLTLGLSQLSRLLFVKQTFKEFNDTFFILLSTNF
TYFKATCHILIRKRIINLLKMFQENYCIPDNDDEKDIQKKYDDFSRIMTLALLTLVEVTAFSMIIAPFNNQSD
HELIYKVLVPYDLSTPLNYWLTFIHHTLGAILFAAVAITNDALIAGFMLQVCSQLKLLEHRITNLPSHILKAR
EQNKSENIIRGLERELIKQNVQHHNHIFRLAKIVCMTFIEIVICQFFVSGFAICVSIYQISAGTSNKIELITF
IFYLMCMLQQFFVYCYFGNEVTIQSKNFNDTVFGMDWTSLSIELKKNLTIILLRSSKPIQMLCGPFIHLTLES
FKTIIKTSYSVFNVLQ
>CsolOr9
MLPLHFTVLAMTGVWCPPKWLATRKRIFYKFYSSIILILSEFLILTQVARILFERQKFADFNQIFYIVLSTNL
ATIKAIFNLLIQERILKLINMFTEYYCIAQNSIETDIRKKCDKYCRILELSILLAMNGTTACIVIINLSGFIS
DTKSLIYEVLVPYNISKPVLFWMTFIHHSFQSFILTSISITIDILIASFMLQLCAQLQLLEYRIKALPSWVSK
AQSNYVSNENIRKMQALYMAENIKHHVHILRISEKICSTFKEMVIAQYITSALSISVCIYQISTEKESQMQLL
IYCIYLCCMMTQLFIYCYFGNEVTIQSQSLRTSIFHIDWSLLSLEVKKELLIIFIRTSVPIEMFKGVFMTLTL
ESFINILKKSYSVFTVLK
>CsolOr10

NLKIQRFIGRLFAIWPLDEKAKNWEIYVNECIWYFFHINLWLLILPIFTAAYKERFNLPSFVSSINQLLIISE
TLFLMILFKLGRLRFRVLLQNSNEFFTFMKKRIFLLHKFAKFTKMYLVILHFTGVICLLVVFIFVYRSIAVKP
LRFPTNAYYPFSFNSSIIWNLFYIHQITSVYYVTVNIFIDSIIILLFFTSAMKLKLLQIEFQNVETYSDLVKC
VRRHQEIIWFIQETNSVIRYIILKTIISLCCFFVSEAVKLLNSHVSSILQVQFLLLFMVGISRLYFYSYCAES
ITTLGVDLAFAVYCTMWHKQTQQMKISKCIIISRCQKSLNISVAGIMPSLNMRFLRRLLYLTFSYIVSFRAVT
K
>CsolOr11
QDFDIVINWERYILKFIGVWPEIETRFPLQFIGTFFIIVYFIILPQMADLYIIHTDMVLVSENLATANIAINI
AILKLLTLRYNKKGLWTLINFMKEDWKNTKNKNHLIVMLEMGGLAKYINKMLIILTQVMLSIFIIMNIFVNLT
GYLPSRQLFYRSYFPYNALDTPIYEITCFWQSFGGVLAAMAYAGADSFIGILVLHVCGQLSIIEYELRHLVDP
IVNGNNNALDLIHEKLKFITTRHEYLNKFTQSIEDIFNLMLLAQFVCLSIQLCFQGFTIYNALTNKTGITYAQ
LIFLIVFFVEIFVLFYLYCYVGEQLCIKTIGIRNAAYECKWYHLPTNQIKVLAYIILRTNTPLRITAGKFCTF
SLVTFSEV
>CsolOr12
LLCIGAWPKQSESFIVRVLSKILCFSSILIMIIFANMMQTAQLILYWGDLNMLITILSTADLPIANSMMMLVL
FSYKRKGLQDITLMAKNDWIKLKTNKELEIMNESAKTAKSLSKFCIIVGQCSCLSYLISHIYLQCTIYSGVRL
SYLVSYIPYEHEKSPNYEITLIFQCLSSFIATAAFIGIESFFIIIVMHVCAKLNILSNRIQSLTLEDLDLKKN
KNYVHIKKEISFIVEQHNDLFRISSEIEDIFTTINFMQLVNFTLIFCLLGFRAVTMLFEKTGTYMEIIFVIGF
FTYLSSNLFVCCYVAEQLRSKSIDVGHATYQCSWYQMETSEIKSLMLILYRSQKPFQISAGKFFIFSLNTFTS
LMKTSASYLSVLIAIKDK
>CsolOr13
YNYVFGLCRLNLAIVGLWPASNNSKHQELFSLIVFIVTVTTIISFITIPQTLQLIRVMGDLNAVIDNLLSNLP
IAMVVLKMFEFRRHRKELVITLSLVIDAWNARKTKIETRNMREDAAVTRKILLICMTLGFLSIEGLLLMTIGQ
DIDIHAGHADRKFPMISSYFWYDINSSPVYEITWLLQYIATVLTTIAHTGVTGIFIGLVLHLRCQISNLRLRL
EKLDIYDRNKMIRKKIEFIVMKHENINKFAKGIENIFNFMFLVEVFSCTVLLCMQFYVLVTLTTNDSKKYPII
STLLLYLLNVGAHLFVCCYTTDKLRDEGLSISQSAYNYEWYNLSVSDAKLLIIVMQRAGKPLQISAGKLFPIS
MRLYSQIIITSVRYLSILIALRDK
>CsolOr14
KIGFYFAFGISHMNLSTVGLWPNMNYGKYRNFTSMCLFIISISMVLIFGPITQTIKLAMIWGNFVEMSDNIST
ANLPLAVTVIRMIICISRKKVIQSLLSIVIEDWNGKKTKRELDNMKKNAILSRKFSYVLLALGFSTVNGHCLV
RISQEFDLVPGPSEKRLPLISSYFPYNYKLSPVYEITWFFQYVGASLATITYAGLYCLFVSLVLHLQGQITNL
RYNLENLQASSGDNEEMNGVELRNAIKYVIKRHVRLNRFNIFNIIFLLEIISCTVQICLQIFIMVTLLSKEKN
LPIFQIIFMVIYISDIGTHVFLCCFIADKLREESLSIHESTYNCHWYNFSTQNTKLLILLMHRARTPLEVTAG
KFCTLSLKQFTQILKTSLAYLSMLLAVTDRA
>CsolOr15
GFEMCVSVTRALMIGLGIWPGTKDVHWYLRCRFLISIFIMVFFINIPQTRMMFHVWDNFQVLMEILTSANLLT
ILACVRLFGLWYYKEDLNYLVIKIAEDWRESGKEKQRVMWTNVKFSRGIILGCVILSNGTVIAYAIERILVLD
FSPSVLDYMDNESSTRHMFYLSKFFFETKDSPIFEICWVFQLIGSILGANTFSSFDGFFIFSILHLCGQIENL
QTEFKEVVNQSRKTKQGFAESLGSIVKRHNQLNRFMNCIEDNFNKVLLIQIVGFSISVCLQGYHIVLIITETH
DKNLAAIIYLISYTFGNITRIFLYCFVIEKLRDESTSIFYIIYDLNWYDLKPHESRMLINIMHHAKFPPRITA
GKFSDFSLEYFTTVMKTSLGYLSMILVI
>CsolOr16
KTGIDIAIGVSRKFLKFIGIWPELKEKKNWLSRSYFLIPTFIMIYFCNIPQTIMITKLWGDLNAVIKILTVCN
ISGSIAVCKLISFWYQRNVISQLIMFMIDDWDSQKSKENLTIMWTNAKLSRIISITIIALAEGTILIYVLTII
INQSNDVKENDYNLLLDNNISKTFRPLLVNGYFSYDVQSSPYYEIIFICQFFSILFAATAYSSVDAFFAVLIF
HLCGQLTNLKNHLIRLPKETSTDRNDTFLKTLSIIVLKHEYLERFANTIEDSFNLMFLLQMIMSSFVLCLQGY
QLIMITTDSTISIIDLIFMIYFTLCVSFSLFLYCYLAEILKNKSLEIGDAVYESDWYHLPYFKSKDLLLLMVR
AKQPFKITAGKFVVFSLEHYCAICKSAGAFLSMLLAMKSRL
>CsolOr17
LKSNTGFDVAVGPCRAFLSFLGAWPDPAAPENLFSLCRVITASLTMFLFAIVAQTTMVLKVWGDLNAVIEILT
TADMPICVALMKFLVAWYYREVLKDLVKTMSDDWQSQYTNQDFEEMWQRAQFSRKLSIVCIGLAQGTITVQFL
MVVVFDINNKGQIERPLYMTSYFPYDTQNSPNYEITWIAQCFSNIFAAGAFSAVDAFFVVLVLHLCGQLAILK
RSLIELTDDLQHKSIKLEFTRKLANIVERHEFLNRFAKTIEDSFNMMFLAQMITSSFALCLQGYQLVMIITSN
EDKLPLLQLIHMIYFVCCFSFSLFSYCYVAEQLNYESTEIHYAAYKSDWYNLCPKETKLLLLLMHRARKPLEI
TAGKFCVFSLELYCSILKTSGGYLSMLLAVRNRLV
>CsolOr18
GYNYAIGPCRFLLRLLGAWPDFHYQRSWISFICILITIITMLLFCVISQSVKMFLSWRNFNLIIEILINCNIP
TIIATTKIFSIWYHRYVLKDLLLQIIEDWKMVHVEQELIIMWTNARISRILCIGCIFMTQATLLCQCTVGIFN

ILSYISNMNPNDTMSKPLYMAGSFPYNVQVFPNYELSIFGQILSNIFASTSFSSVDSFFIVLMYHLIGQLSLL
KLALFKLPNKIENAEDKKMFFDKFSLLHRRHNQLCRFSMSIEESFNMMFLIQMVPCFFGLCVLPYKLIQASLY
TIYIISADYISIVELIFVVYFFLLFLFTNFLYCYIAELLHGKSIEVSYAVYNCNWTILPPKEARLFIFILLRT
QIPFEITIGKFTTFSLQFYCHILKTSAGYLSMLLAVRNR
>CsolOr19
NFDDYIFLNRWALTFLGMWTIDNRFDHGVAKILHRLHITFLFVLMLLLIIPQWLDLYIFWGNINANAETVVLN
VFTITAILKLLCCILTANVFNDVLMSLKSDWNETMDTSNPAKEKHREILLKMASSAFNYTKQYAFIMYVTALL
YFISPFVGMQPGDLRIRKYPFFGWYHFDRFSNLNYGICYVSQVMSGTVVGTTNFAMDSIFLVSLYHHSAQLQI
LQHDLAELVSAGCNSANVINNLIKKHQKYIRISQMLQVIFNGSSLQQLLISCVIICVIGLKLIIALEDGGYES
LIYIMFMGLALLQILLYCQSGDVLISESLQVGYATYQSPWTSVNLSSIRSISLMILRSQKPLTISAGKIYVLS
LQNFTMV
>CsolOr20
FGIEDRGFPLSFMILKISGYWKPTIFKKPFNVIYDIYEIYCVANVLLLMITIILHNIHAENSIYFLIENLFLI
ITIFNGLCKVCNIYFRRSNILDILQILMDVPWTVLRDVEETKIIKKSVTAEKYISRFCISTVIVNIISYASKP
IMNPNPDYQLVIDAYALCDRSTPLCFWLSYVHQISSYSSTCLVQVGYDCVLFNFIQRISCLLKILEYRILKLP
NLVEIQAFGYHDEINYIKECIKEHHSICKAVKALNDSFYETVFIQLISSILTMCTNIYLLSMQDIFSSEFNAV
FIFLCCLFTQNFLFCWFGFKISSNSLHVSNSIFNMNWCILQKESKTMLAYVMLKTAQPIYLFSKSIIRLTPES
FINIVKVSYSAFNILQHT
>CsolOr21
TYEEISSPHIMFFKIFGLVPLEKKLFNFIDAKFLSIYWVIISILHNTMTMTYGINMLIKKRFEISYISELLVG
SSTTIRYILIYCKRQFLRIITMCRELWPHILPSETVIMRSFERIIFYIWATISVVIFIIFSSFTLSSVMLKY
EPIFMNETQPRTMPFKFYVDIYDRSWYIVAVLYQIILESHVSLLTVGMEPIEFYLILTACGYLRSLQTRLILM
SEGQNEINTNSKENPYTIELMYHEIVKCAKFHQSIMLYSEEVEDFMRSIYFVCVATNIYNISFVGISLLKETD
EQSVKWFILLIWNIAQFYTYQWSPEYLLTESEAIVYGAYHLRSKLIGQHRKSDKILHFLIMRAQKPIQLTAGG
YIKLSLETFTNMIKSAISLFTLLRTF
>CsolOr22
TYEEISRPHIRLFQLVGFIPFEGKTLEFIGTKLLNVFWSLMVLSYKSMFVGYGIQMLRRKRFEIDYISEQIIV
ENICVRYALFFYKRRKFVELIKMCKQLWTYLTPDETTIIRRYERKAYYIWNIMMINVFSAVTIYILTAHFISI
PSETMNGTQLKTLPFRFFADIQEDPLFTIVYVYQGIVTYSIVVINTAAEATGYYVILLACGYLRSVRTRLRSL
AERQDKLNSNDKENQNKTTELIYKDVLQCAKFHQNIMVYCEEIEQFMRNISFVGVFTTIYNISMIGIKMFQHD
GQFLKYMIIMIMNVFQFFTYQWSPDGLLRESDAIANAAYSTSLKQVGNHKSINKILHFLIMRAQKPMQLTAGG
FVKLSMETFGVMIKSAFSFFALLRN
>CsolOr24
TYEELSRPYIKCFIVLALLPTKKKLFYFITTKFLTFFWHTNATIHNITVLLYGFKMLIQKRFEISYISELLIG
GSATIRYLLLYFKQSEFIKLIDKSKELWSHLLPSESIILRSFERKIMYICLVLVVLVYILFTMFVLSAAFMNK
PKIYDNETILKELPFKTYGDVPVGPLYVILFILQVIVEFNITTITIGMEPIAFFLILTACGYLRSLQTRLILM
SERQNEINMNGKENLYTIQLIYHEIVKCAKFHQSIMVYCEDLERFMRSIYFVCVATNIYNISFVGISLLQETD
EQTGKWIIFLLWNIIQFYSYQWSPEYLLKESEAVVYAAYLIRLKLIGQHPKTDKILHFLIMRAQKPIQLTAGG
YLKLSMETFANMIKSAISVFTLLR
>CsolOr25
MLNKKLANEIKLNYIFMMMLGIWPTNKDTSIVIKIVQRMLLFVFVTSVLLQIVSGILYTLYEEKDMMIKLKLA
GPIIFCSDSLSKILSLIKNINEIGAFFKHVEDDWDSYSSVDTKEEYEIMKNNAKLGRVIASVCAIFMYVSGFL
YHFFMPITGNGIFPVHLVTPINLQSENNSDIVKTRTLIFPIYKGFFTIDRKTTYLICFCTQVIGGFAIYTVNV
AVCAIAAGFVVHVCGQLQIVVLMLHRLIDTKHKDSKNNTIAPKYSSQIKMAKIIEQHLRALNFAERLKKNLNI
PFFLQFVSCTLNICLLEYYIVVGKEIQTLLITFTYFTLIVSLMFNIFIICYIGELLKEQNEKVRYFTYKIEWY
TLPNNRATDLIMIFLITSACPAKITAGFVIDLTFRTFTGVIQKSASCFQLLRTVIV
>CsolOr26
IELDNLRNIKDFRYNLQITRWLLQLLGIWPIKSLFYENIQCLLFIIICIFLFMFLLIPSCLQAYFEKDAMERV
MMIGPIIFFTMNTLNYGIILVKKHDIHACIIQIAIDWHKIKRIENKRIMLKNAKVARFFITLCIYFTFGGGMI
YISVLPIVKNIIDSSSKNNLAFPSYFFFFNPQVRPMYDIVLGLQILSTFLMFAVVTSIFSIIINSIMHASSQC
TIITAALKKFGTSGEEKPIKIIVKHHLRVLKFITKLENTLNYACFIELLGCTFNLIMLGYYLIIHFQNKNIWG
IISITLLLISHGFNLLIFCFAGEHLTQKCENIGKVAYSINWYELCNSDKQNITFIMVISGRSIVLTAGKMTIL
SIYTFCNVMKAFVTYLNVLRT
>CsolOr27
MMLSINKTINICLIITGLRPWRCSFIGPIIMLSFTVLTLPFQLWYFVRMDNIVIAIDNLSTILLCLLILLKYS
VMWINKRIITELLQEIVDEWNKIVIPNKCKTIAYYCDLFCKMDLYMYIVCSIFYHIQSLSRYFYLNVHNRESF
LTSYYPFDYQQSPYFEIIYTMHLIQSVALIATDSLSKTLLIVYISHAIIHVRLLKSYVNEYFKSYVYLNNKNN
SGNYHNLKMVIVEHVKISTLVKKIDNCYTYVSLLQIGINNIIIAISAFTILTNEELVKKIILMKLITFTMIIL

SQTVTFCLIGDFLESEGNSIANALYNGVWYNVKPTEIKVIAFIIEYSQNPLLLTGGKFFKISISSCLRNLKTS
ISYLSVLRVI
>CsolOr28
MHNLTLHVKFGLRAIGAWPGMLSSRLYYIILILFVLSLIFQFWHLVKVIQKLDKLLLNLGTTISVTSVVLKLV
VFRFKYRSIKIIINELIEDWENKNEIKINKNFTMKTSKLATYICYIVAICYTTTVLVISIACIMTYNAEPYDN
REFIVYSYFPMDAKKSPVYEIIYILHLITCLFTCFYHSTIEGLLIISVAHSRSKAFNVCTKFSKLIEMYRTEN
NRKHILKNKQELIECHLNFITFTDCIQDIYSYVAFVHLFLITILNGVIGFLIINFSNIDVDKNFTLFKIIPFV
FTCMWAVGTYCFAGEFLTSQGTLVLKTLCNCPWYIFKPSDVKLLIFMIMRSQQSVKISVGKFGTLSFIYLTQI
IKTSVSYMSVARVAS
>CsolOr29
MDLYETRYWKFTIRLQKIIGFWVFQCNWKDYILWTYTYLSILSFMIPVAWNIYNEIGINEIIVLENSISQMYL
IGASIKLCISIILKSKLKIIYILLANTWNMMADAEELKIICGYLEIGRRKVIIYTVFMFSTSILYLIIPISAP
LLDYISLHENISRTKIFPIHIEYGISKEKYYYPLLLHFIFGGFPTIMVIVIFDLGFILIVENFAALFALVSYR
LSKSLKTAEDIENHVITFSKGDKIAYFHLVNAINLHQQAIGYVNLMEDCYNIPWVIVIFINLLLAGSGFAIFI
FKEDQPDELIRYGPIIIAGLIHFYCLFHSGQRIIDYSSQVFDNCYSCKWYNLSTKSIYLVKMMMMRSFRYSTL
TAGNLLCLRLNTFTKMLKSIVSIFTV
>CsolOr30
EMKIRFTVKMGFRVLCVSMKIFGIWPIPNSPRAHCIFYRFLWWFYLLNHLILILPTFHTFLNNNTGNVTLASI
TWLEMTGMMECMVILINFKIQEKRLKLLRVTKNQLNSTEASISLENANLYVIIVSTIAILYIIVMYMYGNRPD
RSTILTTTRYPFSIESTALKVLIFCNQFIAMSHTAVVMVTDGIFVLFSYVCAVRLKLLEKKLKTAKINKELKQ
YICEHQNILFLIEETNVLVGIMIVKSIICFMSYSIGVGLQIIDPKVSNFEVMNNFLTIVLIYLRLFISAESAE
TMATVTADIERTIYSTSWFAETRKAVVVKIMIIQRCQKLPRIHVNGLMPKLNRHYIGRIFYLSYSYFMAVRTI
INK
>CsolOr31
MDILNSRYFIINKTLMINCGVWPYEKPIKNIIKRAFFFIILLAMFVPQLLGLKKHLGLNTDNAIEHIMIILYI
IIILIKLITSITTQTQLKLVLDSIIYNWSIFNDEHEKSILIKYAEKGRKLTIFYITYMITALIGFITIPITSI
FLNIINPLNVTRPRIHVFNGEFLIDMQKHHNKIYIFDCCSCIITSILNCAADCMYAVSVEYCVALFAIVKFRL
YMSLTSIDQNKLHPSKQNDVSYNLIVKTIKLHKEAINVLNILESSFSFRQLILMGINMVIFSLTSVLILTKTG
QTLQILRYTLVLIGMFIHQFYTIWPGQKVLEHSEHLFHEAYTTEWYQTSSRTKILLKFLILRCCKPCQLTAGG
IYILNFANFLSVFKTSISYITVFYSFT
>CsolOr32
MILFNTLEHRLLPVPFYFLTISGVWCPNDLKLWIKHVYSYFTLTVIVTNILLMIAIVIYIVKKSVNTEFDSNA
YFVLTSVSIGIYKGINIVLNRKIILNLITTCFQKCWLSQDCTEKKIILNVSKRGWKIQVFYAFIVALTIGIKL
LHPMTNDDVKLPTSAWYPYNIDDPLYFWISYIHQIFVGTLSLMLHIGSDTMVSGFMIQLCIQLQLLKYRFENL
NQLLQRINDHSHSHSAHAKMEKMLMIQYIQLHKFIYRFSNRMNSIFSGFLASLLIIVVSNISINAYTLSINEI
RLDLNTLCLCLMIIISLFQICIPCWYGNEIMLNSLEIQTAIYNMDWINISSDTRKLLTIIMIRAAEPIRIVIA
KIFLMNINTFLHVLKISYSASSVL
>CsolOr33
MHFYNSIEYHYLPIPFAILTICGTWCPENFSTISKQIYKLFTIVSVFLGIILFVEILIDVILSNGTIYFKLDN
IFAVLVFSVGLYKTINVLCYRSMILSFITDYVDNQWYRPRNVKEIGIQQNMKSKTRWLFAVYTILIITSMILR
SLTPILESKKFINLPFVAWYPYKIDNPLSFWLTYCHQIISGATLSCMHLSTDTLFVGFLLHMNCQINILIVRL
KDFGNESAIGNDSQSEIHNEVMMKMTMAKLVREHQRIYRYGYTLQKTFEIILMGQLIVVVPNFLINIYSLSIY
IERLDLKYFMTIFFTIISVMQIGMLCWYGNDILLGSKDIGNALYECNWTTVNQQTKKILLMMIVRTSKPLFIS
VINVIPINISTLLRLVKMSYSTFNLIQRTSAQ
>CsolOr34
MNENIAPIPFRILKFCGLWRPVTWSSWKKRAYCSFTALVLFMLISMMMAVFIRVCEMPMTEDVFAENIFLMFA
LINAIFKAINLLISRGRFIKLLNMIQNKRWQNLRNAQEIEIREKYNKTIRKISIYFTSAVSIAIVLRLTSPLF
EPNDEIKLPVESYCPCDISNAGCYWAIYWHQAIGTGIATLTHASKDSLISALLLKTCFQLEILKNRLLSIPKL
CASAKRSDDDEDASARIPSLESRLITECVRDHESIFKFANLLNDTLNVLLFGQLAVTLPNLCLSVYLMSKQSI
GGMEFMMTFQFFMAVVIELYFFCWYGNEVTLNSLEVENAVHEMDWTSLSIKSRKELLFIMKRTSKPILFSVGP
IMNMNIDSFLSIMKTSYSAFSVLQST
>CsolOr35
QAKMTFNTRIIQWNKLLMSPLGLWPENFSNLRFYINFLYMGYFVSLEYLDLWVFIEDFDRVLLNVVENAAFTQ
IMLRMYILYSYNRSIGALFNEINDDLISKEYTNEERKIFITYQVKSVIFIKLLVISTALTATSHYANPFIGQI
SEIVEYAGSSSENSSIVFRMPYCFYQFYEIKDVSTYFWTYGTQLPFVFVSAFGQCSSDCLMVTMVYHISGQMA
VLALRITNIDTDPKRCTIELQKAFRLHIRLLRMGKDVQEIFSVILIGQIISSLLLVCTLGYQILLNLMIGDNF
GLSSLIVFIFLVFLILYTHCTIGENLIIESNRVAEAYYNCNWYQMSNENAKMILLCIHRAQKPMQLSAGQFKT
FCLMTYTDTIKTSLGYLSVLRKTL

>CsolOr36
LKYAIDFTKYSIWILHFWSFPQNISKIQKIVLEISLFLSLISTIHYLISLILAVYVYQDDSEIMTKNVCFCFA
IYQLATKQICIRLRRDKLQVYNLFNEMENFIQYASHSDKLILMKYITKCKTIHIGIALWSYSTAVTFIVRPFL
TKYPFPTDAVYSFPTNNIYSIIFIYAQQSLAAVQTSAAVLLDCLIAVLLWFVCARLEILSLSLRNCDNYKDLL
IHINQHQNLLRYVNDVNETIDLFVISTLVTSVGGVIFSTIQLVSPDMSLAEKAQYGIVAVTASFGLFICSWAA
DSMLDMGYVVGVNAFNCKWYGMEQRSKTCVQRLVMQCQCPLKIEANPLLPNLTLEFFAQFALLTFKIFTSLRV
>CsolOr37
INYQMNSKKFVSYIKLMGKITAIWPLNNDANKTQVYRFEMMWWMQFIINNSVLIPLLLGVYFFRKDSLKMTKS
LCEFASVNEIFVNFLISKIKRKNFQEIFKEIEDFIGNANHREEMLLKKYINKYKNLQIATTIIFQMTASLFII
KSVFNSELLPLDTWYPFSYESQAIRIVLYIIQIIGILQTSLGIIMDLMLSIIFFYTAVQIELIEYKVNEAKSK
TQLIKCVQSYQKLKIFINKMKNSIQLLILKTNVGMVFVIINAALQLLQQESIGSSTAFVSMLIAGCVRVYITA
QPAQDLYESGQNLAERTFQISMLQNSVSNAKIGFMLAIHCRQPIVISVPGLIKAYTLQYYAGFLSTSVSYFVN
LRTALD
>CsolOr38
MDILPFHFRTLQLCGMWYEDNRFVILKIIYRITAMFVVFTVIIGQFIRLITMQDNIDDLSDNLFITLTFISFC
LKILNFIIRRHEMEDLLHDFRLPVYQSRSFEEEKIILEYSNTAKNIFFFFLCITQISGIILLIMPILISTDNV
FYLPYKTYRPYSTSNISFWITYAMQVFTSFYGISLNVSLDTMTYGFIILTVGQFKINSYRLINCTLSIKDFII
HHKLIQNTAIKIESFFICVIVPHFFFTLLVICCSIFQISQKPVASYEFFTICMYIICILWEIFFYCWFGNELG
LQSQSVADSLYSSKWLSCTTQEKRNLWLAMLYAQSGSNISFHGQCTLSLRTFLWIIKTSYALFSLLKQIS
>CsolOr39
MDILTSKYYFRCNKFLRFCGHWPYQKPIIKILNQTLLMITILTIFIPQMIKMFEMRHDFQGFILSIPSFLYYG
NFIFKNIFATILRKEIKKVLEQINYDFKVLYDKDLKILDKYYKKAYDINTYYIVYMLVAVFFYSMLPFTLHTM
DIVFPKNESRLPQSPRLIKYYINIFDDNIYFITFHGFLCDTLSIIFLLGFDTLYFTFVYHACALFVIVTTNLH
DSIIMVNQCANIESKEHHKKKEELFKQKFFKCIILHKYTLEFVDKLQSGFSNLNIFSIVCAMIPLTITGFEAI
LYNKNLSQMVRFIFFAVAEIIHLFYYNWPGEKIQEHSLLVYKACYNCKWYENSISNKYKRLLSFMILRSQKPC
LLTAGTIYILNMENFSAVIKVSVSYFTVLSSLM
>CsolOr40
IMDSSKVFSDPYFKLIKKLLIHIFLWPYQKKIVKAFGQIFIVFLVYSIIIPQMIRAIEELYEINMNMQIIIEN
LSGFFYFHLILTQYFTFIFSENKLKNIYILITKSLAELSNVDEKNILIHYWKIGHLSTRFYMVYVLIVFIIFV
MMPVAMPSLFNHFLDNNETYRKSLCYYAEFFIDEDEYFNYLFVHSFVCIYVTVIINITVDTIFIKSIYHAVGL
FNIIDFRLKCVFDIVNDYLQYKSKNKNRLVHKHVLDSIQLHNKTIEFTNHVQSTFSQCLFINTGIVLLLLSIG
TLDIIMNFNNHMNALRIGFVWIAVVLYLFKISWAGQMIVDINCKILKSIYFCGWYNLPPETQYLMKFMMMRCN
IPFELKAGPLLNLNVESFANVLKMSLSYFTVLLSVS
>CsolOr41
MEYEVKKYEKYKNNIKFLLIGSGLWPDYQKHPKNLKNFLSICSAVSSGCTTYGIIAFCISNISNINILTRGLG
LMISFASACLKVCILALRKNDLLSLNKGISAHFEKDLQIPKFRPYLLAHFSEFSKFFYIFDYSVALNLTMLML
TPMLMLRHGKYVRALPQLIPFNYELGGAVHWSIYVFEILSAYYCWSITVGVDSVFGLYAMHMVGELRLLSARF
ESLISSKNYRKDLKECIDRHILIIDSQHLMQRIFGFLAIWLAVTCAVVMCAIIFQATGIQNMTVYRAIYLICY
CFLKLVQAYTYAWFGNIIAVESEICLNSIYNAHWPETGDLRFMNDILIIQSQNPLRFIASGCVIVRLDMFSKI
LHASISYFFLLRTLD
>CsolOr42
MEYEVKKYENYKNYIELMLIASGVWPTFCKHPYILRRSLSIISVFTSGFIFYCIVAFLVKNVNNINIVTGCLG
LMIGFFTTFIKVCTLVIHEKHIRELNEGISTTFENDIKVPELQPHLLAHFRIFSKLFNIINFLLCISIFLLII
MPLLALRNNKYVRTYPQVLPFSYEPGGFIHWSIYAFEILGGLYLWTVTSGVDSVFGLYALHIVGELRVLGSAF
QSLKFNTNYKKNLKKCIDKHFLLMQSRCKLQQVFSFLALWLAVTCAIAMCGIVFQATQTKNKSIVKLMYWTGH
FVLKLVQGYSYAWYGNIISVESEICLYSIYKASWPGSGETCFMKDVLIILSQKPLIFIAKGCMSVRLDMFLKI
LHATISYFFLLQTLQQ
>CsolOr43
MEEKLKKYKKYQCYIKYLLVCSGLWPNSKKYPNAFRIFLSMCVGFSAIFISFGTLLFCLNNATNINRLTSGLG
PFMSFFTVTVKVIFLSLHQRDLQSLNEGLSKYFARDLEIPEFRLHLLAHLPTFSMFFYIFNYSVLMNVVLYAL
VPLSLISHGKYVRMYPQVMPFSYELGGLVHWLIYVFEVISGFFLWCSTSGGDSVFGMYSLHIVGELKLLSHRF
KNLKFSNNYKCELKYCIDRHIELMKAKQTLERIFGFLAIHLSISCAFVLCALIFQATETKHFTVLKVCYLICY
SFLKLVQAYSYAWFGNIIYVESQICLDSIYNAYWTDSGSVEFMNDVLIILSQKPLRFNAKGVMILELNVFSKI
VNTSVSYFFLLRTLEE
>CsolOr44
HDKLNQKVFDEARILILWNKYLMSTLGLWPNNKSDLIFVSLFCYLCYAFILNYIALYYALKSLNLVNIIGTTV
ESITLLQICIRLYTMRRYNKYYGTLLNQFLLDFSIKNYKNDEERSMFLSYNVRSKFFIKIGVVSIVLTAMLYF
TKPLIRQLHFTKNFNSTVTINYDLPYRIHILYKVTNIKRYIITYISQIPLLFIIIFTQTSMDCLTLSIILHLC

GQLGVLSIRINNLNVMNEMKEFRQIIQRHQELIIIGLKLKAVYRLCLFVHFIGASVGICILIYQVQLNVSYGQ
MTNLLTFFGYGFLNTFRLYTHCWVGEYLIHESVNISNAFYQCQWYDLPIKDQKSIIFCITCSQQPLSLVAGSF
GDFSFTMFTNIMKSAMAYLSFLRNFI
>CsolOr45
DSSSFYTETHINILVFKVMGLVAFKNQDFNFFKTRIASIWFHLSYNFINSVYVYLFVQDAFNYKYNVELISEM
IILLCYQIRFIIFYLRRAELSSLISYSKSLWQYLQPFEQQFVIKFITKIKIMISIYLTLCVMLYGCFLIVNQL
LQSPENDNNTVIKELPFHFYVDIQNTSLFKIMYIIQCLIALGVGFTNYGVDTIGIFLIMMACGYLRVLQYRLQ
NISITYNTKKIGTKDFVKNILACIKFHQLILEYCNKIQILLNPVFVTQLVGSTYIMSIVALNLIGVSKNAKST
KFKCVTLLFLLLIQLLYLESSPQILINECKAVANETFIMPPFGYKDRQANVLLYILLMRTQHPVEFKAAGYFK
LSMESFGLLISDIVSFFAV
>CsolOr46
MGVRSEGDNAFGVYRVFFWFIGVWPLEKKNYFKIFRFSMAIIFQASFLMHSLAELYLNNGKISDIVDILLFFA
SALLSTIKHIFLNIHGDKIAENLKCYTKDWTDVKDETSLKVMRAHVKIYKYQHIIYNSVGYIGTTLFLTRTIL
LNFINQRQLGSNEEIEYQLISHTSYISQEFLTKYYIILIIIQYAQCIYVCTCGACTDCFFFGLVFHLCAQFEI
LKLEWEKLGVDDSNDRNTVESNDLKIKVNILVIKHQKLIKLGKNLEESFNGVVLIQLLISIILICMSGCSILV
AMMVRDYMTILLSTNCISFMISESFIYAYASDYLLSQSESIVQAAYSSGWYNLENRLKRNFIFIIMKAQNPLY
ITAGKFFYITRNTAIQLLKSTFSYISVLRLTFEVSH
>CsolOr47
NKDAVAAFRLTKFITWPLGVWPLRCDDLFSRIRFYLIIFIVFIWLTWERILITIKKCGHLDSYGDDILIIILCA
IMAMSKIIVLRIYKDNMYTMCTSIIDNWINITDKKYIKILKPFTIIGFLLAYQQLIITYFLTMIYTMQPVKGL
IVMYISEIKNKTQWENASDFGTNLLPPYIECIYRIVPEQIYVLIYIFESIAYYSSGQANIGISILFFMLTMFI
SGHFAVLKYDIMRVNTNMKESINLEEEIHSIVDRHSNLLHLAEILNNTISEIILLELLLNCGLNLALGMQILI
AMKLGQNITRFILCFTVLMLQVFLLNYAGETLTTHTEAIRDCLCTINWYEFPPKIQKDIYFMIMRAHKPIYRL
AGRFYVLNYENFTNIVKTCFSIFSIFRLMFE
>CsolOr48
NKDAVAAFRLTKFITWPLGVWPLRCDDLFSRIRFYVCVIFQFWLFWEQILITINKCGHFDSYGDDILIIILCSI
MALSKIVALRIYKDNMHTICTSIIDNWINITDKKYIKILKPFTRTGCWLAYQQLIVNYSVTMIYILQPLTEHV
VMENTSDFEIRRLPAHSECIYGILREQIYVLIYIFESIGFYSSGQAVVGISILFFMLTMFISGHFAVLQYDIM
RVNTNLKESINLEEEIHSIVERHSNLLDLAEILNNTISEIILLELLLNCGLNLALGMQILIGMKLEQNITIVL
LTFIIITFQIFLLNYAGETLTTQTEAVRDCLCTINWYEFPPKIQKDIYFMIMRAHKPIYRLAGRFYVLNYENF
TNIVKTCFSIFSIFRLMFE
>CsolOr49
NKDAVAAFRLTKFITWPLGVWPLRCDDLFSRIRFYVCVIFQFWILWEQILITINKCGNLDSYVDEILIVLCSI
MALSKIVALWIYKDNMYTMCTSIYDNWINITDKRYMKILRPLTKIGCLLAYQQLIITNILIMIILLQPLKGHI
VMKNTSDFEIKRLPAHSECIYGILREQIYVLIYVLESIGLFICGQGNLGISILFFMLTMFISGHFAVLKYDIM
RVNTNLKESINLEEEIHSIVERHSNLLDLAEILNNTISEMILLDLLLNCGLNLAVGMQILIAMKLGQNITRFI
LCFTVLMLQVFLLNYAGETLTTHTEAIRDCLCTINWYEFPPKIQKDIYFMIMRAHKPIYRLAGRFYVLNYENF
TNIVKTCFSIFNIFRLMFE
>CsolOr51
NKDAVAAFRLTKFITWPLGVWPLRCDDLFSRIRFYVCVIFQSLLTLDQMMVMISNCGDFDYFIDEMISEICSI
MGVLKAVTLRIYSDDIYNILTAALNKCFSIKDDESSKVLKPFMKVGCSITYVQLIMCHFTVIIFLIRPLNELL
FNFISQINNNLDRNTTLDHQLLKLPLHSECIYRNLQIETYILVYIFECIGLFMCGQGNVGISIFFFMLTLIIS
GHFAVLKHDIMKINANLKATSNWEEEIYSIVQRHSELLDLADTLNKRISEVLLVEILLNCLVNLILGMRILIA
MKLGQNIIRVILSFTVLMLQVFLLNYAGETLTTQTEAIRDCLCAINWYEFPPKIQKDIYFMIMRAHKPIYRLA
GRFYVLNYENFKNILKTCFSLFNIFRLMFE
>CsolOr52
MKVLKMYFNILTCFGFWIPIFWTYKWCGKLHNFICVFLILLPYWLAFGQITRILFDVKNLDEMTETLFILLTT
INVCCKGANFLLHREEIIDLMKLLTLDWAKPHNIQELDIYNSCNNIIRMVGLIDLVLGESTLFFGLIAPIFDD
KRQYTLPAQVWLPYNYTTSPIIYWLTYIMNSVCAISIANLSVVMDVIIYGFMLITGCLIDLLDNRLVHLEKNM
KHYSTEHPVTSTSVNQEARLLKQIIHNHIFIYEFADKFIRIFSLSICSQFVVSSVALSVSIYQLSADTSTTNP
DFYMKLLYLCTMMTQLYLFCWFGNELTLKSQELGRSIFKCNWTLFHIESVKSLLLISIRSTKPIVISCGCCIT
FTLDTFSKILKVSYTAFNVLR
>CsolOr53
AFLVGTATGLWFPRHCKSTLAKKLYTVYTYISFTQIILVLITQCIFLLYYVKNIYDFTETSFMMITIVCAVIK
AINFLRHRTLIIHLCEILIMNDFKPQSNREKNLCSHYNKLSRNLELFCMVVGSITAVSISAWPLIAAKYELDL
PMKAWVPFDIKISKNYLFAYIHQSMSILMVACYHFTVDLLMKDLIYYTSVQLDFVIYRLQDLSVKTSEAIKAN
IPMNEIRIMEKKFISRCVQHHIMIYAYAEKLNEVLFPIILTQFTTSFSILCVTGYLLIKSSILETLRFISYVI

AMLVEIFIYNWYANEVSTKSLDFNLCISEINWTAMHVQSMKEITIMMIRSTSPIVFSCGSLIQLSLTSFMKIL
KISYSAINLL
>CsolOr54
ITDFMWAVGLNRFGLKVMGVWPINEDNESKSFFTELRVPCMILIMFIFIILPQMFGLVMIINDIELVINNLMT
NFTACLCCVKLFFIWYNKIELQPVVQSMIIDWERPKKKWEVEIMKQKAFWMRNFIIFDYISLAICYLGFATGP
LIGFDMRLISNITDYESHHLAIQSYYPYDYNRSPHFELTHITQIIGGFFMSMSMNIPDHYFGTLVFHTSAQFL
ILNSNITDFIQQNDGILGNTNDIDKKLRIFIERHAYLMRTIAVLEKSFTFIIASQIFLMTVLVCCTGIIIILNL
LEYKCITVQIIVLGGTLINQMLHTFFDFFASESLATSSSDIFFNIYNSCWYILPKRIIRSFILIMIISQSPKQ
IKIGRIFTASFKIYCNIINLILRYISMLFAFTRK
>CsolOr55
INVYYQINKISMLLIGQWPYQSKNLAKFLTYIWIFQYVSILIPQFIKLIEVIDNIDHTIECFGPIIYNLGVGI
KFCNNIFNRKQIKALFDEIQKDWNNITDETEIILLSKYASFAKFLNFGYNYMCYSTMCCYLFLPLSSFIMDII
KPLTDTRPKNLVIFVEFFVDEEKYYFYIMIHSYITTIFGVLAVVSTDMFYTSCVQHACGMLSILGRRLKMINK
DTNIIRKFENDFYKELEDAVTQHNKIIKYCERINKTYGDSFFFILGLNMFAMSFTGALIIRKWGLYYDIIRNG
MFVGSQLFHLLYYSIQGQHLIDQSLMISDCIYESGWCTISLRMKKIMTIMIMRSLKPITVSAKVFIISLSNFT
MVTKASVSYFTVLKSS
>CsolOr56
YFQINQYFMSCCGIWPYQSKKSARLIRIFWSFMHISVLQIEAFSLFRYWDNTDTVIQITILFFYNIILSIIFS
NSIHHQTKIKYLLEKICINWKSLSDKKEQEILFRYSEIGKIISRICIGTTIGSLFFIIVPPMVLFIQDELQYY
KGTRTELNLFIQYPIENNEYFYLFLIHALTSGILGIFPLLSLDSFTVNYVQHVCGMFAILGRRLESTIKNERN
KLYDKTYQEILNCIILHNNILDLVDEFNDIVGTSFAIIILILIFLTCTSSIAIFILWENAKEVMRYLLFTFLE
LLHMFCFAFLSQDLMNHNNKIYKCIFNSGWYKSSAKTGGLIKMMFLRCSRPCLIQCMTYPLTLENFLSVLRHT
FSYFMVVRS
>XP_011307735.1 PREDICTED: uncharacterized protein LOC105269299 [Fopius arisanus]
MKENSELMKYNLLTSSIKFWMLFVGIWPIPNPPIAYRALPIFIVSGNVFLSIALFRFAIAHISNMQLMVK
GVSLGMSFITIAFKVIIFTLCRDKIIDLLGVVKNYHEDSLADENLGQLALKGMSGFRRSSLLLFFLIACG
AVSYCIAPIIFIAIQLGHHLQTIHYILPLPALYPWEIRPGGILYQVTYVFEVCNLMSLLFTSCGVDSLFG
YYILLINGQLRVMGYQLKHLSGNNVCHAFIPRFVDKYIVLQQCCDGLQRIYGPVILWQEALSAPKYLLIM
GYSGSKILQTYLYASAGSNLTAESEALMESIYCSDWQETGRRFRTSILVMLTQKPVRITAAHCIIVSNDM
LIMTLNTAVSQVKGCLYPVGLWPSEKPKLWYRLLPYAQLFLSAITVVAIMNYLVHHIRNWNIVIRVISLL
ASTSLYIFKLSQLMIHRREILDISKILNDYSSRIINEERSLNFAWEWVKVCLTCGRSIIIMVYGSFFVLL
VIIPAVVIFIQEMGHTENIKYTLVYPTMYPWDTSTNGVAYRITYLLESLTALSCCHVTSGVDNLYLFWIF
QTTGQLRAMSYRLHHIRDGDNYDDILKDNIIQYGTLMRIRNQLENIYGPIIIFCNGSAAVLLCTLIFQLS
KASSLTKIQIARFTLYAFGKIFQVYLFTWPGTFLAAESENYLRSVYLSDWINHPSCTSFVMTTLAQKPLT
MKACHVSVVSVEMFAKMIQTTVSYSLLLKTVAPESCCCLFVVCTQGTSIRIIDESSMLFVQVLCLAVNVK
KIMWFYNTVDDLCNQFLSDERFHKFVLNDVTIYRYFFWFHTSLCGFSGTIPLVMSMVSVMNQKIHDVHPI
KYNLIIPGMYPWNASINEIVYGFHFGLESYTLMWTFYVGALVDVLFSFSLLQMTIPLRGMSHAIIHLCDE
SDYEDTLRRCLIQYRTLIECRNIIENTYGLIILGVAITGPIALCSLAWQVTQMETISNFQKFRFAVHGVA
KILQVFSYSWSATMLKGKSEDFLGQVYFSKWQGNRSLMNTVFTILIQRPLTIRAGHMPDVSLSMFVFVMK
TTASYYLLLQTMEQKAS

>XP_011307734.1 PREDICTED: uncharacterized protein LOC105269298 [Fopius arisanus]
MDISVGNPEREKIRLKEFQRFRRNLKYWAYVSGSWPLQDPHFFYRALPFLIFFSNLYICIQEFRFVVAHI
TNIGLMTGGFSMGLSFCSVLVKVGFFRLHRVKLIELHAILEAFLKESLADHHQRYLVLEKFATFQRLMTI
LCICVLFGCGFCIAGPLIFFIVQIKKKVKPLEYILPSPATYPWTTSDGGLIYLLTFIYESYNVVTLGFVT
IAVDGLFEFYIFSIIGQFKVLGYRISNLTVEDNPHDVIRQWVKRCVMLKRCSHMLQTTYGPIILWQVISN
SMIICMVLFQIMSMTSVTLGRYALTFGYTGTKIIQTYIYSWAGSSLTAESEALTQAAYSCNWQKKEYQRV
RTSILIIILTQKPLILVAVSCVYISLDMFLMTLNTALSYFFLLQTFDQKASYELTSQVNSQQSRVMGQNLL
TSYTSCAKNVKHISIISGIWPSEKPNIFYKSLPYFIISVLLTISCATMNFAYVHIHNINSVMKSMSISLS
YLNGIMKVVCYLVYRQELKELNDTITELFKRCQGDDQLLLHTLSRFTLFKVLAILHMCAVFIVLGIYCIM
PIIIIIKEYSNGIEPIRYFLPYPAVYPYDVPGGSGLYVLHYAIQGFGCFFLFSTATSIDSIFAIHSSQII
GQLRALSYEMRCFTFRDGYEKYLQELVEIHRKLIRCCKLLEVIHGPIVLSMVVTTAIILCCLIFQISQMA
TISLKQISFFTLYMSVKLLQTWIYALAGTIITTECDNFRNDVYGTRWENSGKKSAGYHVSIILMQRSIYL
KACNYTPISMNLFTGVVLFKFHSKKLFKVHTILVNFHEKSLNDNNLRPLALEKLSGFRILTQILSTCVLF
GCVFCIVGPLLFCLSQIRQKVKTLTYILPTPAAYPWHTRDGGYLYALTYIYEAYNVIAIVGQLRVLDYQM
RNLKLSDSPMEVVREWVEKFLVLRRCCKVLQRMYGPVSGISIGRYLLIFGYSGTKIMQTYLYSWAGSTLT

VESEAVTKSVYSCNWERSNSQRLRTSILIIILTQKPLVIVAAGCVYISLDMFLMTLNTAVSYFFLLQTFEE
KAS

>XP_011301746.1 PREDICTED: uncharacterized protein LOC105265755 [Fopius arisanus]
MSLFLGAPAVPILMDLWIPLNESRSRIFLYQTEYWIDQDEYYFSILFHAYFTVPVLFACIIFFDNLFSIF
ISHVCGMCAILKAHLEGIHIEGASDNRGSVHERLHLCADMQTNILEFVQYLETTCTVPFLILVGGNIMVI
TLTGMMIVIKDGEISEMIRFASFNIGTIFHLFWSSWQGHTLIVESESIFLSVYQSEWYTLTPKAQRMLLP
IMMRSANPCQITAGKCYVMSMESFGATAGMVAAIVDVDVFLEAIPTVLADVVCYFKYLNFTINAGKMKKL
LLIMEEDWKRYTYGEELDILKEYAQFGRKVTVYYAGALYGSLVPLMMTPLVPIVLDIVMPMNVSYPKHLM
FQQIEFLFDFEPYLFPLILHGYIGTAGYLTIIIAIDTMLMVYIQHACAQFSIIRLMLDRLARVDPDADRH
TPEFDEIDYKNMAACIERFCDLIEEANYMSFTFIVGINMLMMTSSALVAVFKMDNPDVAGKFVAFTIGEM
FHLFYSNWQGQLLQEHSESIFSDVYRARWNYTSVRTQRLIIPLLMRSTKPCTMTAGKMYTMSLRSFKSSD
FYLRVNGAFLSAIGLWPYQQKLQQSVFFILAIFFLATQGFLQTGGLIAAWCDTPIFLESLAPVLISVMCV
IKFINFIHNGRKMKELIDMLRRDWLELKDEFEISILRRWSEDSRKNVLLYAGAVYGSMAPFMLGPLIPPL
LRLIPKGLIDINPNMTMAKPLMFHVEYFIDINKYYLPLVIHSYFGTMTYITVVVAIDSMFMTYVQRACAI
FNIVGYRLEKLVDDYNLDVNLNPKIEDDVSYRRMTECVTRHAQAIQYSQMIESANSTSFLLQLGLNMVTI
SFTGFQAATKLSRPEEAFGYASFTCAQTFHLFFESWPAQRLVDESTRLVEYSIKTSWYKISFRSRKLFHL
LIMKSMEPCQLTAGGVYILNMENFSAVVKTSMSYFTVLCSTT

>XP_011312549.1 PREDICTED: uncharacterized protein LOC105272228 [Fopius arisanus]
MDFWDKPCYSVTKTISSLMGLWPYQSKKELIIHRTIIFFLFIIQVIPEILAVCHNSADFNIIMDSVGTFI
MDAGFLINASTYFWNFKKMRILLDDIQENWKIFPEKRGRHILDKYAKDGGKLAIVYAVGICGSGIFFTTE
PLQWRLLNSVFATNFNVPLFCTPMEYPIIDVEKYYYGLLFIQEISVAVVISMVVVYDLLFFIYSQHACGL
FAALGYAIENLPVGRHSESHNRGFRYVKRCIQIHNRAIRFSNLLEDIVVWNFFLMMGLNMIIMSLTAVQV
VSNMDAIGRLLKNCVFVASQLVHLFITCFMCQRIMDHSSDLQKKIANSRWCFTTVKTQQMIKLMILRSQI
PSQLTAGKIMVMSLETFTVIVKTSASYFTEMEFWNRQYYSCLKFLSCLLGLWPYQSPKEILIHRTIAIIL
FVVQITPQMRIMLDRIREDWNIFLADKGLKILERYARQGQKFSLLYAGSLYISGIFTTLKPLQLRLMNIL
MKSNSSVPKFFMPMDYPLFNVDRYYYALFCLSELCVTIIIVMVLAYDLFFFLCAQHICGLFAALGAAIEK
ASTKPHLNDEIYNYFKSCVLIHRRTIEFAQLLDEILRWNTFILLGLTMIDMSMIELQVKYVEFKYLCKKL
VLHLDDIGEVLKNLVFIASVMTRLFIVCFVAQRVTDHSCDFQKNVMNSRWSDAEIKSQKLIKFVLMRSQI
PCRLTAGKLLVMSLELFTLIVKTSGSYFTMLLAMQ

>XP_011311288.1 PREDICTED: odorant receptor Or1-like [Fopius arisanus]
MVLLHENLQHLHYIGLWRPVDWPLNSWRTRVYRLYTIFVISALYWFAITETMSLFTIVETIEDLSDSSFM
LLTTIGVCFKVNMMVSKKGSIESLISKLEDHPHRPINTDEMKIRENFNGRIRLISRTFGTIGEVSVTIMT
VSVFFQGIPYGDLPYKAWIPYDYSTPLLYWFTFCLQLIVVIYLANIVIAFDTTLIGLFDFLETSPQSSCL
NVMVLLQESFLALQCVGLWPPLVWKSREIKTIIYGGYTTLMIIIIHWFTLSESLSLVTEEVEDVQDFSNN
CFMLLSMISICVKAIVMLSRRSDIVGLLNALEYYPHGPMGDQEQKVQENFNQQIRFYTLLYAGHLAVFAW
LISILTFFQGIPFGVLPYKAWVPYDYSQPLVYWFTFCLQVFSLFAGANMNIGFDTVVPGFFMQICAQFNI
LKCRLRRTIDEFEEKTLELSGAGAVLIYERQIINCVDFHQAILEMSDRVNSIFNPIIFVQYSASSIIICV
SVFMISQVPLFSPQFMSLLMYVSSMLLQIFMLCAFGNQVTIECQSLTVNIYSTKWYLLTNRAQKYLILMM
ARTLKPVIFMSGHIITLSLSSFRNLLKQSYSAYTLLQQFSD

>XP_011309892.1 PREDICTED: uncharacterized protein LOC105270562 [Fopius arisanus]
MAERILYDWRALATGEDIHSCQYLDDISYIAFDNGVPRTFYGFRAMLRTLINATKDQLAEHLYENHEEKK
LYLKYNSLSKLYYKFSVPYVMTAACVYYLRPLVTSLLVGNFGTNDSMILPFRITLPYTILDTRMYWMTYA
YLSPMIYLLACHNGWICVLITVQLHICGQLSIVEHRIRNIVHVTDHDTSHAIFKSLVDRHSKSIWMAKSF
DDSFHFILLLDLIVMTVLLGLTSYVIIIGHGVSESSTAPVFGIAGTATLLLIYGYCIVGESLISESSKVH
AAYYECMWYESSANFKKAVMICMLSSQEPLRMTAGKFFVFSLTASNSNGYFSFTIFHFLILHMSRWQWPF
QEDESQLREAKQLFAWNRMVFIIIGLWPLEPTIYFFHTWLVYFAFHLSMGFVDLVLVFGDLEEVVANVSE
TALESMIIVKMVVMKYSDSLREAVMMARDGLMEDKFLQSKEKKIYLIYNAIAKKFFKWAVTFAFISAILY
HLKPMETRFKAALANETVPMLLPYRSHLTFQLTDMTTYILIYLYQSPMIYIHTFHTAAVCFLITLVLNAC
GHLAILARRIRRIQPDNSTSVDHQLINSVQRYLQIVRFAKLIDKSFWIILLEELVTTTVCLGLASYNVLV
NADLADTTTFMTFVMYVFTMLLLIYGYCFAGEYLITESMNVHEAYYECNWADLSFNCRKSLTLCLIGTEK
PLQLRAGGFYTFSMAGFTGIMKTAMAYLSMLRTLISAPEGSGVETHNPHTHAHPIRNRDDGGG

>XP_011309750.1 PREDICTED: uncharacterized protein LOC105270482 [Fopius arisanus]
MDIFTEGHYKRNRILMISVGIWPESSIFSKIFARTFALVILFSMLLPQYAFLKQPERNISDFVSVIVNEM
AIILVCMELCYVASNMKKAMFEMDEPAKMAGFLIFAVGQITHLLFLSIPGQELYDHSSRVFNTIYSTNWI
KLSTEHQKMILIMLMRSMNPSYITAGNFYRMNVENFKNYAFLNQPDKHLNDLISCLIIQLVILTVVLKIC
YIALNMRKVHTQRLAKRILLAELIESSFTMMFAFVVLINMILISMTAFETVLKVDDPGETVRFVLFIIGQ
IAHLFCTSLPGQELLDHSNRVFTDIYNSNWFDIAGGEQRMLLFMLMRSMKPSYMTAGKFYTLHIENFKNV
LRTSMSYFTVFLSVR

>XP_011308178.1 PREDICTED: odorant receptor Or1-like [Fopius arisanus]
MVLLRESLWFLRYCGMWPPVHDSWWSHVYSLYTIYIIIVMYWFTLSELLNLLMTTDDIEDFSDTCFMLLS
TVAICAKILITLVKKSEIREVLSALESYPHKPMSPEEQNIQNSYDRQVRFITLFYGVATEITVWCMTIFT
FFQGIPFGVLPYKAWIPYNYTGAKMYWFTYYQQLLSVLLAANLDIGFDTIIPGFILQICAQFNILKCRLH
RVVNEFDNMRSKKFGNSPEIYEAEIIECIKFHRAIIEIAKRINSIFSSIIFIQYAASSIIICVSVLLISQ
MPLFSPKFLALFMYLSCMLLQIFMFCAAGHEATIQCQSMTNSIFGTKWYLLSDRVKKYLMLMMLRTLRPV
RFYFGLWPPLHLESSSWKYRIYSIYTIYIVIFISWFSLSELVNLLMTTDDVEDFSDSCFMLLSILTVCAK
ILVILMKRSEIRDVLYALENYLHKPINSEEQSIQNEYDRQIRLLTRFYGGFVELTVWSMTISTFFQGIPF
GVLPYKAWIPYDYSGVNMYWITYYQQLISVLLSANVNIGFDTLIPGFMIQICAQFNILKWRLHRVTSEFG
DLSKNYEIRIVECIKIARRINSIFTVVIFIQYAASLIIICVSVFLISQMPLFSVQFLMLFLYVSCMLLQI
FISCAAGNEATIQCESLIESIFETKWYLLSNRAKKNLMLMMIRTLRPVRFVSGYLIVLSLDSFGKLLKMS
YSMFTVLQRSSK

>XP_011305106.1 PREDICTED: uncharacterized protein LOC105267740 [Fopius arisanus]
MRECMLRCSGIVIFAIFMCHLTILLHYIEPLIENRWRNESERELPFKFYSDLPITLSPWFEILFVIQILT
SYPNCCCYFCFDNFLCQMNLTAVGLFMILHKEIREICDDDPNSLCQSSEDNVRLKFIECVRRHQQLIAWV
EELNEIYRGLTLGVVILLSILICLELLQLMLFAGKNTMFTFHYFIYAGGSAIQLYFYTMTCDNLTGVSLA
VSDAAYDVRWFSIKSERSKNQLVKDLSMIIIRSQKACSLTVGKFSPVTLQTFTSISNTLSIYIKTNTGST
LSVDLDPKWDIKNVKEVISGRIGLAPEDIKIIFAGKELHNSTIIEECDLGQQSILHAVKNPPRRLKSTLN
AQRISESLAESLELNESGSKPLNETLTDLPLNEGETTEVVESPEPRGKRAHFYVYCAAPCKSIEPGKLRV
RCSSCGSGAVTVDRDPQSWPDVLLSNRITVHCENDSCDLSPDSQIPYAHFFFKCGNHPSRGENDEAIPLY
HIRPNLRRVPCLACTDAKDVVLVFPCEAAHVTCLDCFKDYCNVKLGERGFEFDGSNGYYTLPCPAGCENS
FIREVHHFRLLDQHQYEQYQRFGAEEYVLRVGGLLCPQPDCGMGIIPPEPVDPGDAEACRKIQCIGGCGY
VFCRRCLSGYHLGDCGHAEKINSNAQGRGGYSVDPDRAKDAKWDEASKKVIQKSTKPCPKCRTPTERDGG
CMHMKGFLVLFLTLRTAMAPRPGDSWAVILTAFFLKSCGFWPADSEYGKKIMDFLMFYTISGVLCSIGVV
AVDVYKSYSDDFDRFLYCSLIIFTAGFGFFKLIYFYVKRKQLLTVISHAKDYFWNVDYDDYGSAVMKKCM
LRGTGIVSFAVFMCYLTMLLHYIEPLIENRGRNESDRELPFRFYSDLPISLSPWFEILFATQIIVSYPNC
AAYFCFDNLLCQMNITAVGLFIILHKEMREICDDDQNVSSVSNEPNARIRLIRCVQKHQQLISCIENIND
LYRSETLGVVILLSLVICLEMFQIMVSTDNNPMYTFHYCVYAGGSMTQLYFYTMTCDNLTEASVALSDAA
YDARWFLMESGRSKNFLVKDLSMVIMRSQKACSLTVGGFSPVTLQTFTSICNTAFSFLTLMRQSLQKNV

>XP_011302146.1 PREDICTED: odorant receptor 22c-like [Fopius arisanus]
MMFSGWHCCICYFCFDNFICQINITLVGQFIILQKELRELYDHRVALLSEDESYIRRKLAQCVEKHQQLI
DFTKRVNELYRNVILGVVVLLSLLICLELFQLMTVSHSPIKIQILPIFSAVLLQTVGETLTKIHYCVYAC
GSITQLYFFTLTCNDLTEASLAISDAAYDVQWFLMKSKTRRNALMKDLQLVIMRSQKINSLYVGGFSPVT
LMTFTSDVMYCLVAIICDTINNAKFFTFMLKRKEYHNLLRMARDTLWAGFETEYGRKVLKDCENQAMIFV
IVFATFAQCSGILYLLEPILLNIQNNSTDVKERLFPFKIWFDLPIYESPNFEIWFLMEMFVVYHACILYF
CFDNYLILVNIFITGQFSILKNRLEVLYNKKNIQSITSCCRDVKIHEDDSLISISQEFKSCIRQHQLLIG
FVDQVESVYTLMNLISVLLYSGMICLSGYQLIVPGNTLMRRIKFVAFTLGCLTQLLSFSFTCNNVSVSSV
DLSDGPYNSVWYTKNYLSKGRSQTKDFIIVIMRSQRPCYFTGAGFFPVTLDTLKSVLTTAFSYLTLIRQS
AQ

>XP_011298923.1 PREDICTED: uncharacterized protein LOC105264046 [Fopius arisanus]
MTMFIVTVPKIFLPWFHHEQMRNIIQMIIDDWSNLSETKSRKIMGRYAFWGRLAYIIQISAVFIIVLELT
VTRHPSFMVESSENGSLSVRDIVLGPSCWITPSMSTPMYLMYYYIVLSGLCGAALIYAGFDAFMFSTALH
ICGQFEILNSSIERMTDKDDYGILKHRIKEYSQRHAKLLALSNQLNDIVSLIILLELLSNGFLICISGIS

LLTNFKNGNLDTDDFNVGVRVYVCYMALFMYSFVGEKLSIQAEKSQIAIYNRPWYNMSATIAKDMLFIIM
PAIVYPGFDAFMFSIVLHICGQFEILNSSTENITDEDGYWEQRYKIKEYSQRHAKLLALSTQLDEIINVI
ILFTLLSNGFSICIAAISLLTGIKNGNLDNENFNVGIRICVCYVVLFMYSFVGEKLSIQAAKSQIAIYKV
PWYNMPTTIAKDIQFIIMRSNSACNLTACGIMTMNYEAFKYTTRVMFSCFSVLQLALA

>XP_011298920.1 PREDICTED: uncharacterized protein LOC105264043 [Fopius arisanus]
MEENKKSFELIYFFNVHLAATFICLTLEILNNCRGVEIVLDLFVLSVFSLLACVKGLIVHHHIHNMSENV
RSAIADWSLLSQTDNSQNKELMTKYARIGKLVCFSLMIPASGGTLSWIILALPLPMFLPANSSNVIKNFP
LQTACTFDPITTSKFYYLIFGLQMYQLVTTCLGNCGNDVFFFGMSMHTCGQLEILQNDLTDIEIEKEESN
NWRKLKGMVKRHVHLIELVDKLEGTFNMIILAQLIMSAVLICIMGLQVIIALKTGDMFAAVKANIVLSSL
MSQLFLYSYGGDCLTTQNASVALAVDKSPWYKASAVTMKNVAFIMMRANKPIYVTAGKFFDMTLSTFMEI
LKASVSYMSVLRITMLATTGTIKLITQCGSSEEMIDTFLMLVVSLLLLAKCCLLYLHRDKLAFIIRSSTE
DWIMVENAQYKKMMSERRSFCETVAKMFYSGGLFVLAIYLAKILIFDEINVIDEESMNSTSVKLQRRYLF
PDGCVFDGIGNYLYYLIITNQAIQMSITVSINLGGDTLYVALTLHLCEQCEIMKLKFKSFGQYDSLEKNH
EHLKALISRHQKLMIRAEKLEDIYNEIILMQMLMSIMLISVAGFSFLVSLNDGEMLAAAKNICLMQFMLA
QSYIYTFPADDLKDQADGLLHALYSSQWCDMPAHIMKDIGFMMMRINVPPYYTAGKFFYLTKQSYMTVVK
TAASYLSVLRIMIK

>XP_011298919.1 PREDICTED: uncharacterized protein LOC105264042 [Fopius arisanus]
MSFLICSILIPEILRVFTVGGNMNLLVECISTLLLHLLVMVKMINCISNLETEKELLMQIESHRKFYSSA
PEVKVLNSYANRSRTITHVYIYYIYATTVMYMGTPMVPKILDSILPLNESRPTIFLYEAEYFVDRSAYKT
WILLHSYVVTPLPATVVVAFDSLYFHLAEHACSLFLIASKRMERLAQDMEFWKSSDQIISAKRDDVGDAV
VLCIMQHMKALKFADLLRTAYTKALFFILGINMLAMSITGFQTITKLDEPSEAIRFGCYTIAQLIHLYFL
SWPGQRLMDHSQRIHSAAYQGCWYNIPVHLQKLLILTMLRGSKPSVLTAGGFYVMSLQSFSLVLKTSASY
FTIIKLITVRDDLQKIIACVPILALHSLTITKMLNCLFSIEQNKLLLLEIQEDWQRNLSPGDVEILEKNA
NQNRVITHTYIYYIYATTLMYLMGPMVPKVMDVLVPLNESRPALEIYQTEYFVDPEKNEIPILIHAYVIT
PFPSTIIVAFDALYCNYVNHACSMFEIVGKRLEAIINDIDEIEHGLTPISEKSIHNSLKECIKQHRKSLQ
FAHLLRMTYSKCFLSIVIINTVALSITGYQVVANLGETSEILRYGTFSIGQIVHLFFLSRPAQKLMDQSS
RIHWSAYQGCWYRIPIRSKKMLILIMIRSGKPSILTAGKLYVMSSQSFARVVKTSMSYFTVLLSMR

>XP_011298917.1 PREDICTED: uncharacterized protein LOC105264040 [Fopius arisanus]
MDSTTGISRKMLICQLVSAFISDLPLLLCGLGNSEEFNRESNLTEIFRAIPLPTRCFYKDILDFYIEIYA
LQVLQIIALVMANLGCDCYFFGIGMNLVSQIRRFANDLERFEPQSDELKNQQTLRVMIRQHTHLIQMAAH
LEEIFSFVIALQVLASVYQISSAGVQVLLSIRWSDPATAMSYSIYVCIFLMQLAIYNYAGECLSSSISSL
QRSLYQCSWYHLSPKASRDFLFIMTRCGKRFHLTAGRIIPMNLESYKSIIKTLGSYFSVMQAILHPSGND
YESLICHSDVLTGEIFQLTMMISIAGEMHLDCAAVSDRIQNISLFSCALMTIVKVIIIRTNNSKMLDVIL
SAMDDWSNTQTASEENEMLSCAKLGRTICLFQMIASYITTVPIILGGFAVSAAVNTSNSTDPVINYLPLG
TVCLFGEMSSGFYTVLYITQSIQLLTTCTGNIGCNCFFFGLTMHLSGQVQKLTDDMEVFGKNGEDTDRHG
QFVSLVKRQNHLLELAQNLEATFNVIILVELSALTYEICLIDCLLNICISDNDSVVIKQLIVILALQMVV
NLRMGNTVVIINSIIMLQILHLQLFLYSYAGERLSSGFENLGSAIYNCQWCDLPCKITKEFVLVMMRSYK
TFTLTAGKICVMNLESFKNIVKAVGSYFSVLLAMFD

>XP_011313580.1 PREDICTED: putative odorant receptor 85d [Fopius arisanus]
MCGVLKNHLADIGGDDTDNPVDILERIKLSAILQTNVIGFINDLESSYTIALLTLVGVNMMVITMTGMMV
IINVHDPSQIIRYTAFNSGNIFHLFWCSWQGHNLIVQSESVFTSVYQNNWYRIPCKYQKMLLPLMMRSLT
PCQITAGKLYVMSMNSFGGAMRMTLSFLTLLLSVR

>XP_011313358.1 PREDICTED: odorant receptor 30a-like [Fopius arisanus]
MILFCLTISFTMARLKEQTYDEAVSLIRSYLSIFGLWIYPNSSLFRKRCSHIPICFINCCLCICVIIPEM
WALYRIRKDMELVIGNLMVSLTFYLSIIKTFFLLFKREALRIFFKKIEEDWKARMTRKEQNMLVKRWKLI
KLISISAFVIAVTLSLAHNSTVYFNFVVRVLTNLTDTIPGRPLVFSSVYPFDTQPSPRYEVLVFIEQLEG
IVACITAIIPDVLYAAFVFHACGQLEILSSRIEKIGENTSENFNQFLKSSVTHHLRIIKFVENIDNLFNG
FILGLILYFTLVFCSQAFMAMTVTGRSGNKDEMMLKLYRLCSPAGIDMESADLLNAAYNCNWIDLPPNKA
ISLCMICLRSQKPLGITAGKFLPVTLNTYAKGGNYLGFLRDRLITVVVDGPADNAVWFQHDGAPAHTSVA

VVDQLDEWFPDRWIGNRSDRDRGGRR

>XP_011313322.1 PREDICTED: odorant receptor 22c-like isoform X1 [Fopius arisanus]
MQLQWNRDMQYALGFSKVCCQVLGIWPTQCNGVLPFLRRLLLLISQIVACVFLLDRLIRYGNCGLATHLI
EAWCGITGFTGTAIKIVIFSVHKDRIYRIIQLAMLDWANARETSVRQIMSQYAQRGRIAYIAQIGAAAMI
VFEISLTRLPCFTVDSFENNYSSTRNLILGPPCWIPSTMPASLYFVYYFSTFIGLWVIVFFYTGCDAFMI
AAALHICCQLEILNASLENLSDEDSHTHQRFRINEYSKRHNKLLVLGNELNHAVQLIIFADIFSNMSVIC
ISGIAILVNVRNENMNNDILNLVLRMCLGYTGLFMYSYVGEKLSSQADKSRTILYACPWYNMPMDIVKDI
GFMIMRNNSFCYLTIGGVFVMNYESFKRLTKLMFSYFSVLKLMLT

>XP_011313081.1 PREDICTED: putative odorant receptor 85e [Fopius arisanus]
MFEILKFHLETLSMECPQNQEEAVTNSKIIMEKFRRFSDLQTQILKFVKNLEASYNIALLVIVGVNILTI
VLTGITAIIKKSQPSEMIRMVFTSAGGVCHLFWISWLGHALEVQSEKIFIAAYQGRWYYLPHQMQLMLLP
IMMRSLIPCHLTAGKFYIMCMESFGRAVKMMMSSFMVLISLR

>XP_011312993.1 PREDICTED: odorant receptor Or1-like [Fopius arisanus]
MNVLPFSFGLFTFCGVWRPLSWSTGLKKCIYDCYSVFVVSCLYSYCLMEFADTIRSLSEISEFINASFML
VSVINTSCKTANLFIKRNQLIQLLSILDNNLCRSRGTEERAIQEECNRRVKINFFFFCIVEIAVFTSTV
SSIFENIPERTLPFRVWVPYYNNSEFAYWCLYLQQVIVGHGFQSTIAIGNDTLVAGMMLLTCAQLQILKF
RQENLPSFFKELKEEQGSYTEEMQRQLLKEIILHHKTILRFAETANDLFSTIIFIQYAISCYVLCISVYR
VAQMEVNNPEYPFTVFYLLCMTTQIFYFCWYGNEVILEV

>XP_011312816.1 PREDICTED: uncharacterized protein LOC105272397 [Fopius arisanus]
MAKAKIGTYDWAVGLIRSYLSIFGLWIYPNCTLLRRTCSYIPICALNCYLCICVLIPEMWALYKIRKDME
LVIGNLMVSLTFYLTIFKSFFLLFKREAFMAVTVTNRSENKGEILLKLVVFVCYTSYLMFLTFLYCWASE
TLSDKSKYIYKAVYNSNWTVLPANDATLINMICLGSYENLQVTAGKFLPITLNTYAKGQIDPLSVPTLAW
TLTNTPSQPEKKLSSETAKQSHEPDVLS

>XP_011312550.1 PREDICTED: odorant receptor 82a-like [Fopius arisanus]
MEFWNQNHYSCLKFLSCCLGLWPYQSMRESLIFRIIAVFLFLFQTIPQVKKILDRIRNDWEIFKDDNLLE
ILRSYTRQGHKFSALYAVSLYTCSLFLTLAPIELRLINILVKSNMSIPKFFIPMEYPIMNIEKYYYEILF
VTELSVSVIMLMIITYDLFFFLCAQHICGLFAALGVAIEKVPLTKTCEKDYGYDYLRRCIQIHERAIGHT
RLLDEILRWNTFILLGLTMIDMSMIELQLVLHLDDIGRVLKNLIFIGSVMTRFFVVCFVSQRVTDHSIDF
QINVINSQWCDTSLKSQKLIQFVMMRSQKPCQLTAGKLLIMSFELFTVIVKTSGSYFTMLLAMQ

>XP_011312428.1 PREDICTED: uncharacterized protein LOC105272176 [Fopius arisanus]
MWRKKFNEYSNIKNYHWEIQLVRWAFRSVGVWPEVGRANGVKLFINLTIIIAILFIPQSYTLFLADNSNE
LCSAIAGLLLRTYIITAMILFRLKSKVRQSILSEMKINWSTPFTQQDTKIMQYYAGLSRKIGLIMIVAQS
ISLYFIILNPLVVLAVVKEKKLTELSFVSHILSLSNDWIFAGFYICAIVPIAICRIIMVGADLSYVTFCL
HTCGQLHILGNRIREYQGKIRHSSGDDILRSCSCLKCIVDSHAQISQFVHKIDNYFNIVILLHFTALMMM
LSSGAVLILKWFLAGLYNEMGLYITFQLSAFSKIFLYCWSTEKCQQASDALNEISYSVDWSTKTGNVRKN
LILIIRHSRIPFKLTAGKFFVLSLKLFRQFIFTAISYMSVLRAITRDH

>XP_011312061.1 PREDICTED: odorant receptor 49b-like [Fopius arisanus]
MYLLFELCNTTLLLGLCTYSIIANIAANEMEIVLNYVVYVVNVLIFVFINCHMGQCLNDQSMILFTTLSE
FEWFNLPLAFRKFSIICMLQAQNPLQMTAGHFYQFSLYSFTQILKTSMAYASMLRTIV

>XP_011312060.1 PREDICTED: odorant receptor 33b-like [Fopius arisanus]
MLLDVELEERLVKAQSILHHVSRPVALMERSASKSGDALINYGMYIFNMLTIMFANCYMGQCLQDEALKL
CDAFYERDWADLPLPYQRALIICIIHAQTQKCEIKAGKFYKFSLSAYTNILKSVMGFYSMLRATT

>XP_011312059.1 PREDICTED: odorant receptor 94b-like [Fopius arisanus]
MTDLFQPIIEKHIAIIRVSKALDDCSHTFLLYELLDTIVVIGLLTYYLIAHGSASAGAVIVNYSLSMCNI
LVLVFMNCYMGQCLENESNNLLGVFYDSNWNDMPLSHQKGFIICMQYARKPLTMTAGKFYKFSLEGFTSI

LKSSMAFVSMLRTTI

>XP_011311471.1 PREDICTED: uncharacterized protein LOC105271559, partial [Fopius arisanus]
SAKSQEKVHTTLYLLFFRLHLERLHIKAGQDRNDPSNNLQLIKNLKHCSDLQTQILNFVDNLESSYNIAL
LIIVGVNILTVVLTGMTAIIKKSHPNEMSRMAFVSAGALCHLFWISWMGHALQIQSEKVFLSAYQSRWYD
MPWRLQLMIIPIMMRSLRPCQLTAGRVYIMSMESFGNALRLMMSIFTVFNSMR

>XP_011311406.1 PREDICTED: putative odorant receptor 85e [Fopius arisanus]
MTVSISYIVFFDNLFATFVNHACGVFEILKLHVEKLHVKADGNRGHPLNNFQMIMKNLKHCSDLQTQILK
FVKDLESSYNIALLIIVGVNILTVVLTGMTAIIKKTEPGEMLRMAFVSGGALCHLFWISWLGHALEVQSE
KVFISAYQGEWYDMPCQLQLMIIPIMIRSLRPCRLTAGKFYIMSMESFGNALKMMISSFTVLNSMR

>XP_011311291.1 PREDICTED: uncharacterized protein LOC105271436 [Fopius arisanus]
MVLLRENFIQLQYVGLWQPPCWPPNSFKSRAYLIYTIHLLTILNCFMISEALGLFTIIENLEDFSDSCFM
MLTIFSVCVKSMVVLLKRSDIIDILSSLEMNPYKPMNIHEEKIQEFFNRRIRFFTFLYGGVVEISVWIMS
ISAFFQGIPFGVLPYKVWLPFDYSQPILYWSTFCAQLFVITLGANICIGCDTVIPGFYTLYES

>XP_011311290.1 PREDICTED: odorant receptor 42b-like [Fopius arisanus]
MQISVLCAVANEATFECENVSSGIYHMNWYLLPRRAKSYLILMMVRTLRPVVFMSGHVIVLSLNAFSNLL
KRSYSAYTMLQQNSN

>XP_011311289.1 PREDICTED: uncharacterized protein LOC105271434 [Fopius arisanus]
MVEKMVETMSLLKENFNHLQYIGLWCPVKWSSNSWKSRVYRIYTISIIGALYWFGVTESISLFTIVDSVE
DFSDSSFMLLTAIVVCVKVDMILMKKSEIIALITTLEGHPHKPINTDEEVILDEFIGRIRLISWIYGGVV
EASVWAMTISVFFQDIPYGDLPYKAWIPYNYSTPIFYWLTFCLQLLVVIFVANVACGFDTALLGFFMLVC
AQFNILRDRLEKAVGELETAIVKSHEDIFGAMEICERRIVDCVKYHRAIFEYVG

>XP_011311251.1 PREDICTED: uncharacterized protein LOC105271410 [Fopius arisanus]
MVLLRENFIQLQIIGLWSPVVWPTDSFKSRAYWFYTAFLITATYWFGITEFVNLFVVVDSIDDFSDNSFM
LLTTIVVCFKIDMILSKKSEVVALVRTFETYPHEPINTDEESICEKFNARIRLISSVYGGLGEVSVFTMT
ISVFFQGIPYGDLPYKAWIPYDYSTPILYWFTFSLQLLVVIFLASVAFGFDTVLIGLFTLICAQFNMLKN
RLEKAIDEFKATEILKSQKETSDAAKICENRIKHCIKYHRAIFEYVG

>XP_011310442.1 PREDICTED: odorant receptor 94a [Fopius arisanus]
MTPVIFSPDVHYIEFSIKYLTLCGLWNPYKDYRKWLYDIYTIFIVVFMYFTRIDAYFLAAQAFKQNFLQE
TLIMFLESAFVVGIFKIINILWHRADIEFIATTLTWERTLLCSKNVAVYRDQVVGETLRISKKITLIWLG
LVVVDLVLFYRDNLIGNRRSIDDLPLGLTFLGRFLQKLNFFVILAIDYSTLVLSCAIIMSNDCLCLTLML
HIETQLKILNFRLRRCTTQTTSYFNPNQELIMCIQHYQKIFRMVRILQRVYGSLLLPQLLSSIILISVVG
IHIFVTKSVDPTDPVSAGVVIMTLISGLTQLGIYCLGGNNILVESDRTSVMVYDSYWYMADTEFKKNMII
FLSMVENPLSIKAVGLFELSYVTFKSVSYITYN

>XP_011310375.1 PREDICTED: odorant receptor 7a-like isoform X2 [Fopius arisanus]
MIDKVAPGKSYIAFNLKCLTYLGTWNPWNDRRRWVYNVYTFVMLFVMASRINGYIITARALQHNFLQQTL
MLVVISTFSVGLLKHINTLWHHSNVLFLARLLAWERSTICSTQVALYRDEVLRKTMKFTKNVTLIWLCLG
TFDLPLFYRECLDENHRTINHLPISYTVLRNLLEPLNFLVILLIDFLTFLIYCCSIISNDCLFLALMLHI
AAQFKILNFRLKTCTAVDDDYQIGDEFDTNSIGTKGPKHSAPYSSPNHQLIICIQHYQKIFGMMRVLRKI
YGLILLPQLISSTSLITFVGIHVFVTKTVNLGDPSSAAVVVLTLFSALIQLGIYCLAGNSIIVASDLTAL
AAYNCQWYREDSAFKTKMLIFLSMSKRPMSISAIGLFELSCITLKNVLAKSYSAMAVLQKSAE

>XP_011310373.1 PREDICTED: uncharacterized protein LOC105270851 isoform X2 [Fopius arisanus]
MTDKVLPPGKSYIAFNLKCLTYLGNWNPWNHRRGWVYHVYTFVMLFMMSSRIHGYFVAVRELQQDFLQQT

LMLVLISTFSVGVLKHINILWHHSDVLFLTRSLTWERSLICSKKVALYRNELLRKVIKFTKDLTVIWLWL
VGFDLPLFYREFLDGSHRTVDHLSIRMMRVLRKIYGLILLPQLISSTSLITFVGIHVFVTKTVNLGDPSS
AGVVVLTLFSSLIQLGVYCLAGNSIIVASDLTTFAAYNCQWYREDAAFKKKMSIFLSMSKKPMSISAIGL
FELSCVTLKNVLTKSYSAMAVLQKSAE

>XP_011310371.1 PREDICTED: odorant receptor 49a-like isoform X1 [Fopius arisanus]
MTDKVLPPGKSYIAFNLKCLTYLGNWNPWNHRRGWVYHVYTFVMLFMMSSRIHGYFVAVRELQQDFLQQT
LMLVLISTFSVGVLKHINILWHHSDVLFLTRSLTWERSLICSKKVALYRNELLRKVIKFTKDLTVIWLWL
VGFDLPLFYREFLDGSHRTVDHLSISYTIFGFLLAPFNFLVVFVIDYVTFVMFVWSVILNDCVFLALMLH
IAAQFEILNFRLKMCTAVDNDYQIGDEFDTNSIGTKGRDYSAPYSSPNHQLIICIQHYQKIFGMMRVLRK
IYGLILLPQLISSTSLITFVGIHVFVTKTVNLGDPSSAGVVVLTLFSSLIQLGVYCLAGNSIIVASDLTT
FAAYNCQWYREDAAFKKKMSIFLSMSKKPMSISAIGLFELSCVTLKNVLTKSYSAMAVLQKSAE

>XP_011309953.1 PREDICTED: uncharacterized protein LOC105270609 [Fopius arisanus]
MNIGEISKPPDDPNILQSWFQHCFSGDIFMLQLTGLYSARHIFTRDVPISSYWEITPFIFCGGGLSFGLI
CDMRNAFELAQTDIMLTIEIVAACLSSFLSIFKGLRIWLYRRELYDLIRLCYLRWKIQVSRKNITTYMVK
NARTIRQFRIVYSIVVAALLTSYVVRPIRAYLLFTFSQSNGSFHFSETVYPAHYPFVLNSARPFFLCIAL
ETVGIFYLGLYWVTADALFAQLTTHLSIQFQILGNEIRHMRASTEFSSYDTEEIMKGLKRNVDEYLELFG
YVHSLEKIYNPILFATVLINGIDLCTCLYSLQYRIGESHWGDVGKDAVHASGIALQTLMFCVSAQRLNEE
AINNINSL

>XP_011309945.1 PREDICTED: odorant receptor 47a-like isoform X1 [Fopius arisanus]
MEDMTSTRSHFRECLGGDVFILQCMGLYSMGAIFKDDKPQWHYWETIPFIIGLGTITVAVIFDIRNVYDT
AQTDFMMSIEVAAAVFTVALAVFKGYRLWFHRKELYNLLTVCHRRWNILSSRNHLREDSIGIARNTRLFR
ICYTVAVVGTVGSYNLRPYVLYLKFHLAQTNDSFDFSETVYPAHYPFTLNTPIQYFLLTTWENCGLYFLD
LWWIAADCLFAQLTTHFAIQFNVLSGEIRRIEITPTSTPLGIKKIIRQLKRIIREHVELLSYVHAVEEWY
NPIIFATILLNGLNLCTCLYSLQYRLARNNWRDAVKNTTHATTIALQTFLFCSYAQRLNDEINGFRQAIY
ECSWVNFNMATKTMILVLMIHTEREYVYSAYGFFNVNMPQVTVIFSTAMRFFTLLRSVS

>XP_011309931.1 PREDICTED: odorant receptor 67a-like [Fopius arisanus]
MYFLSLFWVAADGLFAQLATHLSIQFQILANEIRYMHPTANNSPYITARISERLKKIVQDHLELFAYVKL
LEKMYNPLLFATILVNGIDLCTCLYSLQFRLDEANWGDVERNLVHAMSILLQTSMFCTFAQRLNDEIAGV
HAAAYDFPWTDFNMPIKVLILLIMIQTQQEYVYSAYGFLHLNMPQLTTILSAAMRYFTLLRSVT

>XP_011309816.1 PREDICTED: odorant receptor 13a-like [Fopius arisanus]
MDWSNCEWTIEIFTNQGEKRYRVVETIKSVQHESFIRSIMIERSLNVNEEFLDSAQDVINILYPILKFGG
LWPLDPPGFRYLFFLVPYIITKLFGFIDLINVFGNLELTIENIAEETLVIVTVSRIFWVRFSPLLPSLIN
TVSDGMRAENFDSDEERQLYLEYNVTTKMVVKFVIAMGMAGTIAYHVRPLIFRMIASFQGETNISYVLPY
RAHLFFDYQHLGFYSLVYLLQSITIFIHFFVTATIGLLFMLCFHCCGELSALQHKIKTIDFDEQLMTHNL
PRPDHVFKDFLQRHMKIIEIAKNIDKASQVVLLEELATMTIILALSSYCAMLNVTNAEVFLSFSMWCTST
LSIIYAYCYIGEALMTESFNVQQAYYASRWYDFSQEYKRSLAICMIGSSRPLILSGGGFYTYSKFSFTAI
LKSAAAYLSMLRNLAEP

>XP_011309813.1 PREDICTED: odorant receptor 47a-like isoform X1 [Fopius arisanus]
MSLEYCEIEGNERIDEDFRRIGTLLIWQKYFLTIGGLWPMERTYIRCSIWTVYLALHLVMEYIELFTSYG
NFNYAVISVLESTMQSMVFIKLIAFRYSKTLYRLLEAMREEFSGDMCDNREEKKLYLKFHQLSRWYYKLS
VPYIMCGATLYVGRPMITSFFSGSFGDNSSTMVFPFQIKLPFAVLDMQTYMITYAYLIPMVYLLACQNAW
ICLLITIQLHICGQLSVVEYRLRNLADENHFSRNSHTIFKSSVDLHSRAIWMSKSFDNNFHLVLLVDLVV
MTLMLGLISYIIVIADALEDSSTGPVFIFCGFATLALIYGYCIVGEALSVESSKIHTAYSECMWHESSGN
FRKAVMIGLLGSQEPLRMTAGKFFIFSLSGFTNVI

>XP_011309751.1 PREDICTED: uncharacterized protein LOC105270483 [Fopius arisanus]
MKAVNIFSCGYYKNNRILMTIIGIWPESSIKIKILLRCTVLVLLISILIPQWAFLLRKCENMDDMIFGII

AQITISIGFTKYYFVMSIMKQLEVVIEKISEDWRIFQDSLAIDTLHVYASRGTSATKFYMITIFSSCIFF
SIAPLKQVILDGLAPLNESRPKIFMLDVDYSIYGMDADDQYYLILIHSCVTTMIVMDMLVSVDTFILVHI
EGNGAGGIWGKAMPDNFPCGGNSSSSYFAIIKMDELSEVTRFGTFAAGQLVHLLFMTLPGQELLDHSTRV
SEQTPGGVVASVVRKPSSSEERTLEYLLQMNGDNIFSDGYYKTNRIAMIIIGIWPESSLSEKIIVRIVMS
IILLSMLIPQYVYLFRKCDTLDDMIFGLIAQVSVAIGFTKYYFIVSNMKQIQMAIDQMRQDWRMFDDSPA
VESIHIYATRGAFITKSYISIFCAVYLVFSVAPLKQVILDVLIPLNESRPKLFLFDVDYTIYGIDALEYY
YPTLVHSWFSCMIVIHMIVTIDTFVLIMVEHCCGLFETIGDILKETELEKSGVRQFKIICRAVVVHHRAI
SLAEFIESSFNMMYAFVVLASMVLISTTGLEAIIKMDEVAEMTRFLAFTVGQLFHLLFISLPGQELLDHS
TRVAEQVYSCDWSRISADGKKLISIMLMRSMKPLRMTAGGFYLMNIENYGNVLRTSFSYFMVMLSTR

>XP_011309687.1 PREDICTED: odorant receptor 30a-like [Fopius arisanus]
MAELVDSTFTNMYALLVVLNTVLISVTGLETILMMDQPSEMARFFLCAISFAAHLMILSVPSQEILDHSS
QLSEAIYASNWMEMSIKDRQLISIMLMRSRKPLCLTAGRFYHIHVENFSQVLKKSFSYFTVLISSR

>XP_011309685.1 PREDICTED: uncharacterized protein LOC105270454 [Fopius arisanus]
MFEMDEPAKMAGFLIFAVGQITHLLFLSIPGQELYDHSSRVFNTIYSTNWIKLSTEHQKMILIMLMRSMN
PSYITAGNFYRMNVENFKNVSLLCHSTVINNDRTIIYSRYCKPQCRTSQYSSQCDNHDDLIK

>XP_011309282.1 PREDICTED: uncharacterized protein LOC105270196 [Fopius arisanus]
MNNSDIPMQNFRRCSNLQTRILKFVKNLESSYNIALLIIVGVNILTIVLTGMTAIIKRSQPSEMIRMAFI
SAGGFCHLFWISWLGHALEVQSEKIFISAYQNRWYCLPHQLQLMLLPIMMRSLIPCHLTAGKFYIMSMES
FGKTLKMIMSSFTVLNSMR

>XP_011308752.1 PREDICTED: odorant receptor 13a-like [Fopius arisanus]
MGDIFDSRHYRILKYSLQSIGHWPFQSSKEKLFFRSLTLFIISTIVIPKVPFLFLFWFVTNLESCYSIAL
LIIVGVNMLLIVFAGVTVVIKISSLDEMIRIIFISLAALSHLFWISWLGHILIVKSEKVFISTYQSEWYR
LPPRLQLMIIPIMMRSLKPCEITAGKIYVMSMQSFGTALKTMMSFFTVLNSMR

>XP_011308637.1 PREDICTED: odorant receptor 82a-like [Fopius arisanus]
MQYPRITRNESKAIELTRWLLTPLGVWAFIKTSPSPKEIISAIFQQFICYSIILFALVPSIFHILFREKN
LSAKIALCGPVGFFMMNLLKYSAIIHHRRNIKMCIEHVKTDWLRIDNDLEIDIMRKNVLIGRKLTIVCAS
FIYTGGMSYHTVMPFLMGSPASSVHFDEPRPMVFPGYDVIFDPYITPVYEIVYCSHCLAALIIYTITTVS
CNLAANFVMHASGQIQIIIGKLNGLVGDEYKSDDKCLGEKIGDIVRDHGRVLRFTIKIERVLREICLVEV
FASAIIICLLEYYCLTTWNESETIGIITYFLLLMSLCFNIFIFCYIGEVLKKQCYEPGHTAYMIDWHRLP
PNKVSALILIIANAQVPRKITAGGMLDLSLSNFLTVMKTSLVYLNLLRTAETS

>XP_011308636.1 PREDICTED: odorant receptor 22c-like [Fopius arisanus]
MTVNRVMEPIQLTRWLLMSLGVWPFVTDTSTTIDIIIGITVQVFSYGLLIFTIVPTTYHIFIREQNMDAR
ITLIGPICFFITNLIKYCAIIYHREDIKQCINSIKGDWQRVTDQRDRDIMMKNVSTGRNLTVLFAIFMYS
GGMFYHTFMPFLIPSPVTGTRNTTSRPLVYAGYDLAFDPYVSPVYEIVFLSHCLTAMVVHTIVTVSCNLA
ASFVAHACGQVQVIVSRINCLIDDESIEDNCCLQRKMASIIQCHARVIRFTVHIEVILREICLIEVVAST
FVICLLEYYILATFGGSEVIGILTYLVLLIALSVNIFIFCFIGEKLTQQCSEAGKAIYMIKWYRLPPNVA
IALVLFLASAQVPRRLTAGGYMSLSLNSFVQVIKTSVVYLNLLRTIES

>XP_011308634.1 PREDICTED: odorant receptor 82a-like, partial [Fopius arisanus]
LLTYFTLLMSLTFNIFVFCFIGELLKEQWNDTGKSAYMVDWHRMSPRIAATMMLIIATAQVPKKITAGGL
LDLTYASFTSVLHLQSILHPSFNFDTFSGDEIISHIP

>XP_011308633.1 PREDICTED: uncharacterized protein LOC105269802 [Fopius arisanus]
MFQDSLRRISSTREMDFGGIKPIKYTRWLLTIVGIWPTIKRHSTAKDQIVAILLRIVCYILAFFAMIPGL
YHMFFRKTTSMKDKVALIGPVGFWVMVTIKYTIMIYRQRGIKKCIEHIESDWERISKHEEYEILVKNFLT
GRKITIICASFIYTGGLSYHTVVPFVIGSAVTTEREDRPLIFPGYDAAFDVYATPVYHFVFLSHCVGAFV
MYTVTIIGCNLAASFVTHACSQLEIIILKLNGLVDHRYDCSDSLKCKLKSIVQDHVRVQRFHRYAGDIEL
VLREICLVEILTSVIIICWLEYSVLKVDTIAMKFLKQAKQHFFSWKILELISDVGRE

>XP_011308632.1 PREDICTED: uncharacterized protein LOC105269801 [Fopius arisanus]
MSLLSQKTHGEESIKFTKWLLSILGVWPFVKRQSTTIEIRLAILLQLFCHILLAFVIIPSVFHIFLREVN
LNVQIALFGPVGFVITNLLKYSAIIYHRDSIRRCINYIDSDWRAIRDQDHRDIMMENVSTAKNLTILCAS
FMYTGGMMYHTVMPFLLTNPIAADRNSSNRPIVYPGYDIVFNPYVSPAYEMIFISHCLAAMIIYTITTVS
CNLAASFVTHACGQIQILIARLHYLMDNDEDKSEKYLQREIGGIIRYHARILRFTVEIEKILREICLVEV
VASTLIICLLEYYCLTTWNESETIGICTYLILLISLSFNIFIFCFIGELLQEQVFTVKYYPFMLLQFSNA
GTSIYMTEWYRLPPNSVLMLILMIAIAQVPRKITAGGLIDLSRNSFITVIKTSLVYLNLLRTVEA

>XP_011308631.1 PREDICTED: uncharacterized protein LOC105269800 [Fopius arisanus]
MFSIIKIKTMEERLAFIGPLGFYFVNNLKYFFIIHHADLMKKCINHMRGDWMEAVGESEQTVMLESADVG
RKLTQVCALFMYSGAIFFQAEKMIEPAPVNSLNVTIRRHIVPRYDYVVDSQISPVYELAYGTHLMYAIFL
YTIEVAVCNLATVFVGHICGQVQVMRLKLEQLEQCAIYNNHKGIDDFIASVIQCHVKIFTGNVRTALSEI
CLVEAIYSVGIICWLEFFCLKGLNDSDILSIITYSTLFLSLSINIFILCYIGEILKNQSESVAELTYRID
WCQIPQRKLSPFILLISMARYPRNITAGGMMEMTLGSFGVENFVRIFPNVADSHRPGYIIFIIILRRKLI
NYDYPEVTNKYN

>XP_011308630.1 PREDICTED: odorant receptor 13a-like [Fopius arisanus]
MKTMSDRFANLGPLGFHLTNPLKYIFMVHHSDVMKECISLMRSDWVEAVLKEEQAVMLRSANLGRSLTKV
CALFMYSGGIFFQAMTIFKPTEIDEFNITIRQHVLPRYDYFVDSQISPVFEIVYGIHVFCGLFLYTIEIA
TCNLAGVFVGHVCGQVQVMRLKLATLENLTNLGNDREIDDSIASVIHCHMTSEWCRFAGNIRKALREICL
VEVIYSTVAMCWLEFFCLNEWSNSEILSLVTYIMTFISLTFNIFILCYIGEILKNQCESVAQLTYAIDWY
KVSQRKLLSFILIIAMAKYPRNITAGGMLDLTLRSFGDVLKTSLAYFQMLRTITM

>XP_011308629.1 PREDICTED: odorant receptor 13a-like [Fopius arisanus]
MCAFFPIGIFILTFIASVIPCVMFASIKMKTMSDRLVFLGPLGFHVTNSLKYILIIYHSDVMKKCVTQVT
ADWMEAQTNEEQNVMWKSANLGRSLTRMCAFFMFSGAIFFQAMTMLKPKEIDKFNVTIRQHVLPRYDYFV
DSQMSPVFEITYGTHTILLVFLYTIEIAICNLAAVFVSHICGQVRVMKLKLKRLEESACERSNDLDDAIW
SVIHCHVKILKLIENTRNGLWEICLIEVTYSAIVMCWLEFYCLREWSNSDSLALVTYASLFISLTFNIFT
LCLIGEVAKNECSSVAELTYNIAWYHIPPKQLTSFILIIAMARYSRNLSAGGMVEMTLQSFATVLKTSFM
YFQMLRTVTG

>XP_011308627.1 PREDICTED: uncharacterized protein LOC105269797 [Fopius arisanus]
MWKSADLGRSLTRFCALFMYSGAIFFQAKTILAPEGVDHLNITMRQHVLPRYDYIVDSQITPVFEIAYGT
HTLLMVFLYTIEIAMCNLAAVFVGHICGQVQVMKLKLNHLENIANAENPNVIDDAISSLVLCHVKTITLF
ENIHNALCEICLIEVIYSIVVMCWLEFYCLREWRNNNILSFIAYITLFVALSFNILILCYIGEVAKNECE
SVARLAYDIAWYRLPAKKLSSFILIMAVARYPRSITAGGMMQMTLRNFTTVSN

>XP_011308626.1 PREDICTED: uncharacterized protein LOC105269796 [Fopius arisanus]
MSSKYSEPVLGNSPLEYQTIELQYTQWLLTALGVWSMVSNNVSRKSRVSSLPSVCVILFAIAFTIVPCLM
FVTIRMKNLRSRLVFFGPLGFRITNLLKYLFIIYRADILKVCVNHIEADWAEAATKEDQLVMLKSAELGR
KLTRMCALFMYSSGIFFHAMTFLRPRQVDAFNVTIRPHILPGYDFFVNPQLTPTYEIIFGINCLCGAAIY
TIVVATCNLAAVFVGHVRGQVQVIRLNLLRLEEYDHRYENNKDISKGIAFVIRCHVNILRFVGNIRDVLR
EICLVEVVHSTAVICWLEFFCLTGWKNSEVIFIITYFLLLVSLTFNIFMFCLIGEILDNEVCHV

>XP_011308561.1 PREDICTED: odorant receptor 13a-like isoform X2 [Fopius arisanus]
MERLETTPTKYKNTDCETNINYVVKISRTLLTPIGIYPLHGSGTALSNFLVTIQIIIIFSLMLFLLVPHL
IYTYWDAEDLTKLMKIIAAQVFNSLALIKFWTMIINKDSLRWCLAEIQDSWRNVSCDEDKQIPLPYPCDY
VFFVPQDSPGYEMLFVTQIIISSLILSTNCGIYSLIATYIVHACCLFEIVGRHLDEVGKTVEGEVLQKLT
QVIDKHTFAMKYALTLEKSLNIVFLSEMVGCTIIICFLEYGILLDFGEKNYLGMVTYVVLMTSILVNVFI
LSFIGDRLKEQSSKISEYTYDLEWHSMPKHMVHNLLFIMIRSNQPVTLTAGKLFDVSLQGFADVVKTSAA
YLNFLRAVV

>XP_011308560.1 PREDICTED: odorant receptor 13a-like isoform X1 [Fopius arisanus]
MERLETTPTKYKNTDCETNINYVVKISRTLLTPIGIYPLHGSGTALSNFLVTIQIIIIFSLMLFLLVPHL
IYTYWDAEDLTKLMKIIAAQVFNSLALIKFWTMIINKDSLRWCLAEIQDSWRNVSCDEDKQVMIKNAKTG
RFLTVAYLSLSYGGALPYHIVMPLVAERIVKPDNTTQIPLPYPCDYVFFVPQDSPGYEMLFVTQIIISSL
ILSTNCGIYSLIATYIVHACCLFEIVGRHLDEVGKTVEGEVLQKLTQVIDKHTFAMKYALTLEKSLNIVF
LSEMVGCTIIICFLEYGILLDFGEKNYLGMVTYVVLMTSILVNVFILSFIGDRLKEQSSKISEYTYDLEW
HSMPKHMVHNLLFIMIRSNQPVTLTAGKLFDVSLQGFADVVKTSAAYLNFLRAVV

>XP_011308558.1 PREDICTED: odorant receptor 13a-like isoform X1 [Fopius arisanus]
MSLEAPSKNTRVNQDVEHAIEIIRWLLKPLGLWPSNRQTPIEKMTSIFLMSVCTFLLGFLIIPGSLFAFV
KIKNPAVRVRLTGPLSFCVMGILKYYFLFIERRNIATSIRHMIADWQKAVAVRDHRIMLTYAQFGRYGST
ICAGFMYSGGLFYALILPYVSVGGKNEKNITIRPLAYPSHYLLFDPQESPAYEIVFTTHCFCAFVMHSIT
SATCSLAVVFVMHACGQLEILIMWLKDLVDEGAGKRFPKVIDQHVRTLDFIIRTEKILREICLVEICGCT
LNICFIGYYLMMEWGEADAIGITTYTILLISFVFNIFLFCYVGELLTEECKKVGETTYMLEWYRLSEKKT
VELTLIISTAQHPITITAGRMIALSLNSFCTVIRTSVAYLNLLRALID

>XP_011308557.1 PREDICTED: odorant receptor 67c-like [Fopius arisanus]
MRDKVEILGTVPKPSKRSKADVKYSTELNRWFLTLIGIWPTRSDAHILKKILSEILFFLSCFLLFFAMVP
AVLHTILKEKDPQRKMKMTGPLIFHAIVIIKHFILVMKKREIRECLEHMDVDWRRVAHSDEREIMLMKAK
FGRFIFLLDAVFMYSGGFFHSVIVPLSMDSFVTPDNITIRPLSYAVYEPLFSTQTKTAYEIVFTIQYVSA
FVANTITIGCSGLAGLFVLHICGQFNIVRSRLESWVRGRTDESKNVEERMAEIIYLHLRALKFGAQVEMI
LTEICLIDFVGSIIHICFLGYYFITVMGKIYLDNLWRWVEMSLSDEFEQNAAICIATYCVLLVSLTFNIF
IFCYIGELLTQQGEKIGTTAYMIPWHELPPKNSRAIIILILAMTKQPATITAGKMVELSIRSFSTVLRTAA
AYLNLLRTISI

>XP_011308551.1 PREDICTED: uncharacterized protein LOC105269754 isoform X3 [Fopius arisanus]
MRNQSSIVGRKSVPAKKSKSDFKEATNISRCLLTLLGLWLSENPTKLLKKVLLDLSIVICYFLIFFLLIP
CALHTFIIEKKPKKQMKMIGPMSFCVMALIKYFFMIIRREKIRGCLHHIEIDWRRVESLEDREIMVKNAK
IGRFITSLCATFMYSGGFFYRTILPFALPRKLLPDNTTMRPLPYPVYRPLFNSQNTPVYEIVFTTQWFGG
FVIYTITVAACSLAAVLTLHACGQLKIVMSRLNDFVENSVGTDKTLTSKLGEIVDLHFRALQFAVKIEGL
LNEICFVEFIGCTMNICFLGYYLITELEQGEKSWDNCIHDKLVRTSREECEWFDYTSGHVQLSSDNYSWK
NGGVVICHFLQCGVSRILFIFSTDNEDPEALTVDLLQVLKTAMAYFNLLKSVIL

>XP_011308549.1 PREDICTED: odorant receptor 85c-like isoform X1 [Fopius arisanus]
MRNQSSIVGRKSVPAKKSKSDFKEATNISRCLLTLLGLWLSENPTKLLKKVLLDLSIVICYFLIFFLLIP
CALHTFIIEKKPKKQMKMIGPMSFCVMALIKYFFMIIRREKIRGCLHHIEIDWRRVESLEDREIMVKNAK
IGRFITSLCATFMYSGGFFYRTILPFALPRKLLPDNTTMRPLPYPVYRPLFNSQNTPVYEIVFTTQWFGG
FVIYTITVAACSLAAVLTLHACGQLKIVMSRLNDFVENSVGTDKTLTSKLGEIVDLHFRALQFAVKIEGL
LNEICFVEFIGCTMNICFLGYYLITELEQGKSSTIAVVTYLFLITSFTFNIFIYCHIGELLNQQGKKVGT
TAYMINWYELPGKNASGLIIILLAMSNCPVTITAGKMVELSYATFCNVECHGFYLFSQQTMKIRKH

>XP_011308548.1 PREDICTED: uncharacterized protein LOC105269753 [Fopius arisanus]
MIFCTTALTKYNFLVGHRQAISKCIEHMNIDFRRAKSDNDRSIVLNYARTGRTIATVSTSLMYAAGFFYR
TILPLTRPVIVTSNNLTIRPLTAPIYDPLFSAYTYRSWTLVFIGQWFSGYVMYTTAVGACNLATVFVLHA
CGQLKLVMSRLESCVEGSKHVPASVEDRVAEIVELHVRSLCFAFRVETILNKVCFAEVGGCTLVICFLGY
YLITDVNSHETAISLLTYYVLIVSFTFNIFIYCYIGELLTQQGMRVGTVAYLIDWHKLPVSSARGIMLII
SMSAYPTSITAGRMMQLSYSSFLSIMKTSVAYLNVLREMTD

>XP_011308547.1 PREDICTED: odorant receptor 85b-like [Fopius arisanus]
MSDNDNSITYELQYTRWLLSVLGIWPLISTNVTRLGKLSSLLLMALSIFAISFVLIPLIIYTLTSVKTLR
GKFTFIGPVCFRISNLLKLLTMAHRADLIKNCIEQMGNDWLEVIIQEDREVMLKNVNVGRSLTVICAGFM
FSSGTFFHVAMPLLRPRKLNAFNITIRPHLYPGYDIFVDSQATPAYEIIFAAHCFSAAGGYTIVTAACNL
AAVFVSHVSGQVEVIGLKLQRLHAPGDGQKIDTLGEQIASIVQGHVKILKFSGSIKTVLREICLVEVVLS

TLVICWLEFYCLTEWHNSETISIITYFLLLMSLTFNIFIYCYIGQILKDKCESVGLMAYLVDWHRIPTKY
ILSLAFIISMTRYPRTISAGGLMELTIQSFGDVMKTSLAYLNMMRTLTM

>XP_011308545.1 PREDICTED: odorant receptor 2a-like isoform X1 [Fopius arisanus]
MYPPFYFSSVRALIALFIRCWSKVKMPSKSSKQKLSHDHDSAVEYELQYTRWVLKTLGIWPMLSDRSTSL
DRIVSFFLIILNLFAIAFILIPCVLHVLFREKNPRKQLLLLGPIGFRVSNILKYFSIMLRVDTIKQCFNH
VKNDWLEMGSEVEESLVLKNITMGRNLTRFCAVFMFSAGVFYHTVMPLLRKKKVNAFNVTIRPHAYPGYD
FFVDPQASPAYEIIFGAHCLFAVANYLITIAACNFAATFVSHICGQVQVMNLRLQGLNQCNEEKFTVAER
IASIIKCHVKLLRLSKMIEKILREICMVEVVASTLIICLLEYYCMTEWKNSDPALLIAYLLLLTSLTFNI
FIFCYIGEILKHQCEGVGEMTYSINWYQIPCENILKLSLIIAISRTPQKITAGGMMDLTIRSFGGVIKTS
VAYLNMLRAMADS

>XP_011308543.1 PREDICTED: odorant receptor 13a-like isoform X1 [Fopius arisanus]
MMEWSKSETLSLATYLMLFISLTFNIFILCYIGEILKNQCESVAQLVYGIDWYRVPQRKLQSFILIIAMA
RYPRNITAGGMMELTLRSFGDVLKTSLAYFQMLRTVTV

>XP_011308322.1 PREDICTED: odorant receptor Or1-like [Fopius arisanus]
MVASAFLLMTNSVHAYKIFLILGNQKRIQRLLDTMTSDNFCKDFDKFERIFTWYAWQGMYHHMRYQSFGT
MAVFCWGFTPIADAISGHARRLPMEAWYPYDTKATPAFQLTCAHQSLAVILGCFHNISMDTLITGLINVA
CCQLEVVKRNLLELDVKTGSRESGEMKLREELHRCIQHSVDVMGFIEEVRNIFGNVILAQLLVNCIIICL
TAFHVTQMTVFVPVETLGMMTYMCCMTYQIFIYCWHGNELTLQSQSLSSTAFASNWWKFNKKLNNDLRIL
ITRSHKPIIFTAGPIMKLSLQTFVGVSAISYLIINLQIMFTWRKNSLCVVIVLLLEHHQGRGECDADIPG
DMEVETYRSGLERLIQGFGGIFRGVSGDDGGTGPDTFGSLLDKFKIQVQAIFPGTLWCGTGDIAPNSSYL
GPFEITDACCRSHDECATIIATGDSYGPLRNNGIFTRSACGCDHAFFQCLKRANTIISSSIGNTYFNILR
PQCFTCDYPIIGCAQRDSKQIFEKKCLVYDLDSSEPKRLQWFDNPNY

>XP_011308210.1 PREDICTED: odorant receptor Or1-like [Fopius arisanus]
MLPYSIALLRAWGVWVPQDWWPTWRKKLYPAYTVFIICFVYANTLSQIIDLFTTYESLKGFINKSFILLS
TIAGGVKGTHCILHRQQFINLAKTLDSYPCRPETPEEQMIQDEFKKKIKFMNRRYITMHLFTITTLTIAS
VLRDVPKGELYYKAWIPFNYTSPSRFWPAYVHQVIAHYFDLCMHAGYDTLAPGMMIHGCAQFAILGHRFR
AIPEHIKGRLQHSDHIQYRELKALEKKKLTECVRHHLQIFEFAKTNNGIFSGMIFMQYTVSSLVLCMSTL
RLSQMRAFSPTLLSIFLYFLSMIVQIFMPCFSGNQITIASEKLCDEIYGMDWTVLSTSTKRSLTLIITRA
QKPLRFTSGYILTLSIESFNSVVKTSYSAFNVLHGSSEF

>XP_011308185.1 PREDICTED: odorant receptor Or1 [Fopius arisanus]
MLPYSFTVLGLWGVWLPPNWSGWKSRIYHLYTGFMIILVYTNTLSQVIDLLLTYKSLKHFINNAFILLST
IGAGVKAAHCLYIRRKIIGIKEKLNIYPCKPQDHEEKAILHRFSRIVKILNIYYIALYVCTITALTTVSF
FRDVPKQQLYYRAWLPFNYSSPGRFWAVYMHQVIAHGFDACMHAAYDTVAPGVMIQACAQFAILDHRFQL
LPKSVNQFRDKSALDFPQDDSNQRRRVLRFEAKKLAECVEHHLQIFQLTKENNEIFGIPIFLQYSLSSVI
ICLSVLRLSQVNTFHPALISVILYLICMISQVFMPCYSGHQITLQSSKVSDAIFSMDWPGLGVASKKSLI
LIMQRSQKPLLFTTGYIITLSINSFNSLIKLSYSVFNVLHN

>XP_011308177.1 PREDICTED: odorant receptor 46a, isoform A-like [Fopius arisanus]
MLWCIDIWLEFCIFSIYWFTISGSIYLFVISDDVDDFSDTSFMLLSMLAICAKVMIAISKRSDIIGVVTA
LENQPHKPVNLETQLLQDRTDERVRLFTVCYGCLTEATVCYGTIAPFFQNIPFGYLPYKAWIPHDYSTPA
VYWCTYCQQLISVFVAANLNIGFDTIIFGSLMQICGQFNMLKCRLEMIIDEFDAKQIGSQSTTNPALLRT
YERYIEDCIKYHIAIFKISERINSIFNSIIFVQYSASSIIICVSVLLISHMPVSSPKFMTIAMVRA

>XP_011308084.1 PREDICTED: odorant receptor Or1-like [Fopius arisanus]
MAILSENFRFLYYFGIWPSVYWHSRSWKTAVYSIYTSYIISSIYWFTISGSIYLLVITDDVEDFSDASFM
LLSLLALCAKIVVTISKRSEIVEVIAALENYPYKPVDPDTQMVQDKVDEYIRLCTIIYGGLGEASVCCGT
IAPFFQNIPFGFFPYKAWVPYDYSDPGIYWFTFCQQLATVFIAANINIGFDTVVFGLLMQVCAQFKMLKH
RLEMIIHEYSEERIMTGDKINFALMKNYEECITECIKSHVAIFRLFDKINSIFSSLIFIQYSVSSIIICS
SVLLLSHIPVSSSKFQLIMMYMLCMLSQIFIWCAAGNEATLEGQSIARAIYNTEWYYLTDNMKKKLEFMM
FRSMKPIIFVSYHIVVLSLQSFCVLLKTSYTAYTMLQQFSY

>XP_011308083.1 PREDICTED: odorant receptor 46a, isoform A-like [Fopius arisanus]
MALLRESFLSLHYFGIWPSVNWKPKTWKTIAYSLYTSYIIVSIYWFTISGSIYLFVISDDVEDFSDASFM
LLSMLAMCVKVIIAISKRSEIIGVITALENHPHKPVNPETQLLQDKTDEWIRFFTICYGGLTEAAVSYST
IEPFFQNLPFGILPYKAWVPYPYSTPAIYWFTYCQQLISVFVAANLNIGFDTVIFGILMQICTQFNMLKC
RLEMIIDEFDAKQIASSVQITPTSSQMYEQYILDCIKYHIAIFSMQHPPSLYA

>XP_011307733.1 PREDICTED: odorant receptor 13a-like [Fopius arisanus]
MGKDLLKSYTSYAKNVKRISIISGIWPSEKPNIFYKSLPYFIISVLLTISCATMNFAYVNIHNMNRVMKG
MSISLSYLNAIMKVICYLVHREKLIELNDTINELLRRQQGDEQLLSRTLSSLRFFKVLSMLLMWSASIVV
GMYFITPLMIMINQYSHGVKPIRYLLPYPAVYPYKILGGSALYALHYAIESYGCLCLFSTTASIDNIFAL
YSSQIIGQLRALTYEMKRFTFKVGYEKHLQGLIEVHRKLIRCCQLLQAIHGPIVLTMMVITAIILCCLIF
QISQMKTVSVKQVMFFVVYISVKLLQTLIYAWAGTLITTECDNFRNEVYATGWENLGLKSAGHHVKIILM
QRPIRLKACSYTFISMNLFTGILNTTLSYFFLLQALDDEQ

>XP_011307732.1 PREDICTED: uncharacterized protein LOC105269295 [Fopius arisanus]
MFSASMKFLMTCLRMLMLNYIIIPLGFITYQLVANKRPVVLILPYPGSYPECFTNDPIIFAGVFFLEAVN
VVVGASISLGADCYFGLIVFQMSAILNIMSNRVLRAEANEELFVTLRECIDQHSQLMDFGHRLEPIYGLM
ILSQRLTDAVVLCAVIYQIHEVCTDELNLNGSTS

>XP_011307602.1 PREDICTED: odorant receptor 9a-like [Fopius arisanus]
MGLWPVKNPSLLYRSLPYICFFASAFSGLAVIRFIYHYITRVSVTLKGMTIGTSLMLSMLKISSLMVHRK
QGIELHYTLDGYFSAALNDERLTQRVLVGITTVRRLCWIMIPTILITVGGYVMRPIVSILSQKQRRIDSV
EYTLIYPGLYPWTVSDGFFYQIHLTLEFIASITVWCVTCGMDALFAYYVFQIVGQLRVMSYRLTHLEDQK
DIDVVIKDCTQQYVVLLKCRDALQEIFGPVIMWVMWTTAIVLCALIYQLSSQLRDLSIGRWIWTLAYMIP
KITQAYIYGWCGSYLNAESEKYRSAIYHTNWSVSNRNTASSIIIMLSQRGINLVVYKFFYLTVNMFLMIL
KTTVSYYFLLKHLEQPS

>XP_011307601.1 PREDICTED: uncharacterized protein LOC105269224 [Fopius arisanus]
MDPHRIPSHPENYADLHLQLVGILSNCTGDKIFIRIITVECDMNHLASSQSDEYREAIYQVDWFSKKSVM
TSVIIMLSQKPLKLTACKFFVVSVEMFVMILQTTISYFFLLKTLDESK

>XP_011307600.1 PREDICTED: uncharacterized protein LOC105269222 [Fopius arisanus]
MKYIHQKRDGQYPIKCPLIYPGMYPWDTSEYGIAYQLHLISEILASEKENIRVVIKECIQQYEILLKCRD
LINGIFGPVIFWMMGTNAIVLCALIFQLSQMWGLSTIQIISIVNYLIMKLTQTYLYTWSGTYLITQSEKY
RQAIYHIDWLRERSIMPWIVIMLSQRSFYIKAFGFIISMDVFVMIFKTAISYFGLLKTMEQRV

>XP_011307599.1 PREDICTED: uncharacterized protein LOC105269221 [Fopius arisanus]
MSDDEGVQQFLEIRETMRKIALYMGLWPFENPGFLYRMIPYCLVSLFVYAILIISNSIAHHISDARLVTV
CFGIITINILCILKVLRISRFGKELLWFNRTVTELCNEFLSDGELKKFVLDDVKIFRSFFRVHASLCAFS
GTVPIITSLVSVVYQMRHDLHPIKYSLLLPGTYPWNVSVNQLVHGLHFGFEAYSLMWNFYVGALVDVLFS
FTILQMTIPLRGMSHALLNLRDDGDYGYVLHKCLIQYRILIKCRCIIQKTYGPIIVFIAVTGPVALCSLA
WQVTQMDSMTTLQKMRFAAHGIAKTLQVWSYSWSATNLRGQSETFLDNVYCSQWLGDRRFMNTVLTVLMQ
RPLIIQAGHMPDVSLNMFVFVMKTTSSYYLLLQTLD

>XP_011307597.1 PREDICTED: odorant receptor 23a isoform X2 [Fopius arisanus]
MSDSKKLETYVAYREHLRWLLDFAGLWPSEDSTSAYRMLPYLQIFVGCGAAMKIGNFIAHHITSIRIVTR
AMSIMTSIILNMFRPVKYPLILLGMYPWTVPDNIFIYTVHYIFEAFALFTVFYVSSGTDAFLPLLVFQVK
GKLRAMAYRLTEIGEKDSIDEEMGQCIRDYTALMECRDILEKTFGPIILLLMSNNAIILCALIFQFTQMK
AITIVQIIQFAAYICGKTTQTFLYSWSGTLLSSKSEEYLGAVYAANWYGNRRGMNSVLITLIQKPLTMTA
WHMSVVSVDMFVMVLNTTMSYFLLLQTIEQG

>XP_011307596.1 PREDICTED: odorant receptor 23a isoform X1 [Fopius arisanus]
MSDSKKLETYVAYREHLRWLLDFAGLWPSEDSTSAYRMLPYLQIFVGCGAAMKIGNFIAHHITSIRIVTR
AMSIMTSIILNMFRVVCLARNREPLIKARKILDSYFDELLVNEKFREVVLHDVKLFRRLSLFYTVLTFFA
LFGYVLTPLIIIIKQHVHHVKPVKYPLILLGMYPWTVPDNIFIYTVHYIFEAFALFTVFYVSSGTDAFLP
LLVFQVKGKLRAMAYRLTEIGEKDSIDEEMGQCIRDYTALMECRDILEKTFGPIILLLMSNNAIILCALI
FQFTQMKAITIVQIIQFAAYICGKTTQTFLYSWSGTLLSSKSEEYLGAVYAANWYGNRRGMNSVLITLIQ
KPLTMTAWHMSVVSVDMFVMVLNTTMSYFLLLQTIEQG

>XP_011307594.1 PREDICTED: odorant receptor 47a-like isoform X1 [Fopius arisanus]
MDSSEKRIEVENEFKRLETIERQLKNRMIFIGTWPVSNPNIFYRAISIFDIICIILLGIMVMRFASANIS
NINLMVRGFSLGGSFFTIAFKIGLFVFHKETALELKVILRKYQTELLAHKKFKYLVLENFGGFRRVITLL
DIFVFLGCLMYSLTPIVMMIIRVQKHGQPVKYLLPTPALYPWKIPPGGLLYIITYICETYIIWCMYPVTA
GMDPMFAYFIYQITSQLRVMSYQMKNLPSAEDLNDFIRKWMMKFLVMKKCCDNLQKIYGPLILWQVITNS
TVICTILFQILQGGASAVQIILVLGHCAGKIMQTYTYAWAGSQLTTESQALTESVYFSDWMGPDRRIFRK
SILLILTQPALQVTAAGWVTVSLDLFTMTLNTAVSYFFLLQTFEEQQS

>XP_011307591.1 PREDICTED: uncharacterized protein LOC105269216 isoform X1 [Fopius arisanus]
MVKGFSLGTSFVSIALKVFLFTFYRESSIELHTTLHNYHKESMSDENLHQFVLKRVTGFSRLTLILTVLV
YNGCFMYLFIPLITIIVEKLQHVPITYLLPVTALYPWEISPGGLLYTLTYIYEGYNILCLGIVTCGVDSL
FGYYIFHIIGQLRVLSYDIMTIKSACNVDVLIRKWIKKFVVLKGCCNILQKIYGPIIVWQIVTNSAVICT
VLFQISQAKRLSLGRYILIIGYSGTKIIQTYIYSWAGSVLMLESEGLSESVYFSNWLEQCQRFKTSILFI
LTQKPLKITAASCMTVSTEMFIMVIFPPKHIVKLNVFIYLFQTLNTAVSYFFLLKTFEEQQS

>XP_011307413.1 PREDICTED: odorant receptor Or2-like isoform X1 [Fopius arisanus]
MEFWSQSYYRCMKFMSYTLGVWPYQSNKEAFLYRCLAAMGFFGTVTPQFLALRHYINDMPLVIESLSVFL
IDLTFVCDGITYFLNWEKMKAILNKVRENWETFPPDKQGREILHQYSNQGQTLAVLYSAGCLTTGVVFTS
KSLQLKLVYKLLKINATIPQFYMPLEYPMIDVDKYYYLIMITIEMSIIILIMMIISYDLFFFLCAQHVCG
LFAASGCYIQHTPEDDNAYNHLKIFIQVHNRAIEFTKLLDNTFLWNNMVLLGVSAINISIIGLQMARHLD
RIGESVQFGLFVFTVLSRFFVICFFAQRVIDHSRSIEMSLGSLQWYNIPLKAQNLVQLAILRSQTASTLG
AGKTLVLSFELFVTATKASASYFTVLLAMQ

>XP_011306868.1 PREDICTED: uncharacterized protein LOC105268753 isoform X2 [Fopius arisanus]
MTGKWNSNAEYDLGFYKLINRVLGIYPLECHEIFSTIRMIVVLTINWIMLYEQAIDLISGCGKTEEIIES
IVGVLSYPVAIAKIILPRYYWRNMKEILESAVEDWSRNIDTAARKTMMEWVAIGRFIFILEITSLCMLLT
YTTISHFPFFILNQNLLENDTIDRNLPWGSGCWLPPNTSNQIFLIVYFATCVQQIVGSLSNMGFDVSLFI
ILSHMCGQIEILKVDRGMGTDATMFDPSTNPQKVYLLNTFVAWYYQNIAMVIKAVFELMIIYVQLFVYCY
AGEKLTNQMENLRDKVYSSPWYTLSKQSMRDVSFILRKLNDPFHLTCGKFYRMNMDNFKNIVKVTASYCS
VIRLMFYD

>XP_011306867.1 PREDICTED: odorant receptor 46a, isoform B-like isoform X1 [Fopius arisanus]
MTGKWNSNAEYDLGFYKLINRVLGIYPLECHEIFSTIRMIVVLTINWIMLYEQAIDLISGCGKTEEIIES
IVGVLSYPVAIAKIILPRYYWRNMKEILESAVEDWSRNIDTAARKTMMEWVAIGRFIFILEITSLCMLLT
YTTISHFPFFILNQNLLENDTIDRNLPWGSGCWLPPNTSNQIFLIVYFATCVQQIVGSLSNMGFDVSLFI
ILSHMCGQIEILKVDRGMGTDATMFDPSTNPQKVYREFINRHNHLLNLCNLFERTFNIIILIHMVLYLGL
VTILLLNTFVAWYYQNIAMVIKAVFELMIIYVQLFVYCYAGEKLTNQMENLRDKVYSSPWYTLSKQSMRD
VSFILRKLNDPFHLTCGKFYRMNMDNFKNIVKVTASYCSVIRLMFYD

>XP_011306605.1 PREDICTED: odorant receptor 2a-like [Fopius arisanus]
MISKLFELMERADLTIRFPQAFIEEKIMKSSDKEISHEGKDFNWEVGLIKTGLKLVGLWPKAAHQTFLFI
LNQLMQNYLLGVFLRSIILEPSVSRKINKIFVLTGIILTCVNHFIFRLQWCSVRPLIESIQRNWRDLEGS
SKDIKDIMVLYGSLERRITRCICIAVGVSLVVFTLQPVILHLVLPSKFNLEHPSNPRHPFNATASPVFEI
VYIIQGVFNCSAALIFASIDCCIIWMIFHCCGQLEVLEYMMKRFRGTRHHSSGSVDDDPVIVGCSCLQCI

VHHHLQIFKIISLFDDAFHVTMLARIIVCTVHFTSIGYTIAKLREDGNFAFLLVHILFLLTMNSSLFVYC
WVGDKLREKSANIADAAFNRYLFVNKEVSKDLIVIIRQTQVRPCHITAGKFFVMSLHLFKEYIFKSISYL
SVVIALRFNDKY

>XP_011306488.1 PREDICTED: odorant receptor 2a-like [Fopius arisanus]
MPSGTIQNQEEFDKAIRVLSWNKWLLSALGLWPKNPNSLIFIVNFGYFVYNMLCEYLDLFLFIDNLEHVI
ENLTENMAFTQILVRMAMLRRYNQQLGEVINEALKDYDARMYRSPEESKVFLDYMNKAKLFVKLLCAFVT
MTATSYYAKPITSPPPPPQSGEISSEDNATGQFLLPYRFHIFHEVNDFRTYTITYASQFPFVFVSGLGQT
AADCLMVTLVFHVSGRLSVLAMRISSLKTGADNCRAELGNIIVEHNRLLKMGQTIEEAFSETLLAHLVGA
TALVCILGYQLLVNYAKGQGADLATFFVFIFLVFLVLYAHCVVGESLITESNKVCEAYYDCWWYEMPKET
EKLIILCMARSQKPLGLTAGKLGSFCLSTLTDIIKASAGYLSLLRTVM

>XP_011305875.1 PREDICTED: odorant receptor 82a-like [Fopius arisanus]
MIVISMTAVQVVTNLNALARLFKSFVYTVALTVHLFIQCFMSQQIVDSSLLAKQSLMNAKWYLARKPTKQ
LLHFMIMRSHVPCQLTAGKIMLMSLNTFSSVIRTSATYFTVFLDMQ

>XP_011305783.1 PREDICTED: odorant receptor Or1-like isoform X2 [Fopius arisanus]
MNKSEYKPVNAFKLNLTIWKYAGIWPAGVKNKPLRYIYFIYNSISPTIWFGSFFVLQFIHIMKVITKLEE
VMHSIYLMISYCAMACKFHSILWYRRRLEKLFNTLNDPIFKPRLPSHFEVMNESMRIVRRDSIIFLSSGL
CATIFWTFWPLIDRTSEENQLAYQMWCPLNISTSPTFELVYTYQTIAITYNTMLNTMTDTVICGLLKMMS
GHLEMLAEDYGSIFEEAFICPGEDAPRTVAGRKMIEKATGENEKRFEKMEKQKILNDKAPEIPPVIIPQI
SQEDMKARVSACVEFALAIERFANELKDIFQAGILVQFIASCLIICASLFALAIFGDLSDAIYTCDWTTC
DESFRKSMDIVMSRSQKPVVLLIGGLFSLSVETFISIIKSGYSFFMFFSEVGGIDK

>XP_011305781.1 PREDICTED: odorant receptor Or1-like isoform X1 [Fopius arisanus]
MNKSEYKPVNAFKLNLTIWKYAGIWPAGVKNKPLRYIYFIYNSISPTIWFGSFFVLQFIHIMKVITKLEE
VMHSIYLMISYCAMACKFHSILWYRRRLEKLFNTLNDPIFKPRLPSHFEVMNESMRIVRRDSIIFLSSGL
CATIFWTFWPLIDRTSEENQLAYQMWCPLNISTSPTFELVYTYQTIAITYNTMLNTMTDTVICGLLKMMS
GHLEMLAEDYGSIFEEAFICPGEDAPRTVAGRKMIEKATGENEKRFEKMEKQKILNDKAPEIPPVIIPQI
SQEDMKARVSACVEFALAIERFANELKDIFQAGILVQFIASCLIICASLFALAILPPASFQFFSLAQYLA
CMLIQIFLYCWRGNEVSLKFGDLSDAIYTCDWTTCDESFRKSMDIVMSRSQKPVVLLIGGLFSLSVETFI
SIIKSGYSFFMFFSEVGGIDK

>XP_011305747.1 PREDICTED: uncharacterized protein LOC105268145 [Fopius arisanus]
MSDRKRLPELALTYLKYGTVLLGTWPPNSRATKIEKFLIESRWWMLCILAWSLQLPVLYTCYVIRGNFMD
LTKNICFGASIAHGIIKMIICRIHRKKYERLIEEIETYLGHATPRERVILDICVKKAAPIYLTFNVIGFV
AAASYVCGPLVLEQDLPMDLLYPFEIHYYPVFQIIYTIQAIATMQCAAVGPIDAQVCMLFWFAIARLKLL
AHDFKNVMSVDDLNACIRAHQRILRFIYFLNYRLITLWLQIQWKSL

>XP_011305746.1 PREDICTED: uncharacterized protein LOC105268144 [Fopius arisanus]
MDRRKLPELAIAYVKIGTTLLCTWPLTSGATNLQRKFNQVKWLSLLLLLCSLEFSILYTAYLSRANFMEL
TRFICFAASSGHAIIKMIVCRWHQQEYRELIEEMEIFLTYATCREREVLDVCVRKAAPVHLTFNIIGLVA
GISYICGPIILDQNLPTETVYPFATDHYPALQIIYLMQSISVLQCCTVGPLDGQVCMPFWFNIARLKLLA
SDMRKISGVDDLNACIKVHQDILRSQNLFYFSR

>XP_011305745.1 PREDICTED: uncharacterized protein LOC105268143 [Fopius arisanus]
MVMTTVAMATISVVFGAVHVIGNEPLEVKAQFVGLNVGYGLELYLSAWAAENFMKAMENVKWAIYDSCWT
QGPRRTTRSLIIVLQKLNRIPKISVGGFIPELSLNYYALYLSKTMSFFTTLHIMLQKMEESVEETRFNDS
Q

>XP_011305744.1 PREDICTED: odorant receptor 82a-like [Fopius arisanus]
MTSDFWDHPYYKPARICTSIVGQWPQQTKQQLILHRSVLFSIVTLQITSRVLAVIINSDDPEVLVDAIGP
LIIDVVFAIKLGNSCYHFKKMKFLSDKIQENWNIFAPNNGLQQLHYHSNFGRQISTLYLVGVYMGTVSFT

TEPLQQRMIYPLLYPNLTIPKRFPTPMYFGSLDLDTYYYPLFFMSTLCTFLVMTVVGSCDVLLFMYAEHA
CGLFKGLGYAIENLPPQEDNETSDLGFYYLRSCVVVHKRAIEFAEGIRDIYLWNFFGVIGLNMILMSVTG
VQVVTNLDAMEKVVKYAVFVFMQMVHLFIECLAAQRLMDASFELKETLTNAKWYMASKKTHKLILLMLMR
SQIPIVLSAGKMIVMNMDTYAIVLKTAASYFTVFLAMQ

>XP_011305718.1 PREDICTED: uncharacterized protein LOC105268123 [Fopius arisanus]
MGRELTPEFAITYTKLTTGILFTWPPSSKASRLTEISFQIGWWIFWLVSVVLIGPLLSTAFQQRANFIQL
TKHLCITVCCIQVVVKMVIGKHHYHRFQYLIEEMETFVKKANNYERKVLTQYVNRIAPFHYRYNMVSFAG
TLAFVFGPLIRDQPFPTEAEYPIPVDQHPVYEIVYLLESIGAVQCGCTGPFDCQGCLLIWYAAIRLQFLI
EKIETVSSADELKECIRMHQHILWYIDETIKAVRPVVAATVVLATLSIACGAIHLVGNAPIEEKIQFVGI
DFGYSLELLCVAWATENLTLACEEVGWALYKSPWIKRSKEFKRIIFFVMQKCHKPPKIAIGGLLPELSIS
YYATYMSKTFSFFTTLHVMLKKFEENV

>XP_011305700.1 PREDICTED: odorant receptor 82a-like isoform X1 [Fopius arisanus]
MATSPDFWDHPYYRLVKLLATVTGTWPRQSLKEIIMYRFAIFVVVVIQVTPQIVAFWKYRDNMEIILETI
SPFIIDLALTVKFMNAWWFFKRSTYLLEQIRENWNIFPRNNGRYMLHYHSEFARKLSLVYLAGVYMAGIV
FSTEPLQQRALSLLLHTNITAPNRFSMPMDFGSINLDVWYYPLFLMSGVCIIIIITVIGSCDLMFLMYAE
HACGLFKGLGYAIIHLPPHDPKDRVDYGFEYMRKCAIIHRRVIDFAEDIKDIFMWSFLGIIGLNMIIISV
TAVQVMINLKSMEKIVKFILFVSMQMVHLFVECFVAQRLMDASFELTESLTNANWYNASRKTQRLIPIML
MRSQNPIILTAGKIIVMNMNTYAVQRRSALETRDDQQKSGSSSLSINGLESIYVPIG

>XP_011305636.1 PREDICTED: odorant receptor 46a, isoform A-like isoform X1 [Fopius arisanus]
MSFFSLNILSFRVLGLWFPEESSTWKLILYRIYNAFAVTAMCTFVLSQFFALLSCFHDTKELTNASFMLV
TMIAVAGKMFNMTIYQYEIRRMVELFDLEPFKSLDDNEVMIKSKFHRSVKRFISVYGTLGTATCTVITLF
SLIRDMPRRQLLFKARYPFDDTESIGYWISYIHQLYSHYMGAVVNMTFDTFVSALMLATSAQLEVLKYRF
VMMPNLMENERSGSGTKGNPEEIERMETKYLGNHTNHHLAIYEFSRATNDTFTGSIFLQYCASSLVLCVS
VFTLTHLKPLSKEFNSLLMYVGCMLVQIFIFCDAANDVTIRSETMGDGIYKMDWTSLSVKSQKSLVLIMA
RTLRPIRYTSGHVVSLSLVSFSSLLKLSYSVYNILNQSSD

>XP_011305557.1 PREDICTED: odorant receptor 82a-like [Fopius arisanus]
MNIRLKNPETRDTSSFGYFFSTDVLLLQFIGASSIVDIFKDSPRQERWWEVIAFIFGITIPSFVLFCEFV
SVYLAAQKDLNYMVEALTATFSGLLSIMKGIRLWTHRVEIYNLLHDLSKLWESARQNDLVTLDMIKTAER
TKLIRFYYCCVVIILALSYCLRPYILLISHCLRNTNETYDFSISIYPATYPFFYGEYTRYLICLGYEVIV
MICVISYWITCDGLFAQITMHLSLQFEILAEELARIGRIETSQVKDDDDARRLAALAERHSDMFSHCFVA
EEFFNPIIFLTVLINGAHCCFCVYSLEKEISNGNLNEIMKNLFHTMALSGQTVVYCGYADRLTYQSAKIS
DAIYNSHWMDKSKKFKLTLLTMLMRAQKEYVYTSYGLITLNLQRVTTIANAAMRYFTLLRSMP

>XP_011305516.1 PREDICTED: odorant receptor 9a-like isoform X1 [Fopius arisanus]
MKKADIFNDGYYKRNRILMTFFGLWPEYPQSQMIFIQFCVLMLLLSIMLPQYAFICKMYKNIDELTAGLV
EDMIVMLGITYYYVFATNMDWVRFTINNMRKDWKTMERSSTIRIMHTYGKKGYSTTTFYLVMLYTATCLY
ILLPMQAIISDVITAANQSESKLLIVKADYMIYGININNHYAAMLIHQLFEAVLLMSLIMTISTFMLIVV
EHCCGLFVAVGELLQHVKGEFSTEQQDRIIFEAVEVHQRALTWADFIESLSTVMYGVLVLISMISISITG
LQLIIRFNEPTEAVRFVACIGGQLVHLLFISIPGQEIYDHSIRLSMEIYNCNWFEVSPKAKKSILMMLMN
SAKPVYLTAGKFYVLSLESYGKVLKASFSFLTMMRSSR

>XP_011305452.1 PREDICTED: uncharacterized protein LOC105267953 [Fopius arisanus]
MWLTAVFNTTYNVLFFEMMMRVVTQVKILKNRFRVMMATLLKRNRTKKRFIDGNLLMEHELFRNCVQYHI
AIRRMARDINSVFSTIILIQYLITSLILCSTVYLMSEITVFSGDFLNLGLYFLAIFLQITMLCCAGHRTC
LEVIRSDN

>XP_011305341.1 PREDICTED: odorant receptor Or1-like [Fopius arisanus]
MPHRDNALFHKETLLLLQYIGLWKPQGLSQWKSIVYDIYAAMMVIVTVTFGFSQFLAVVFLRKQNVKEFM
EHIFMALTTSCVSFKVVNLLYCRSKISNILRTLTGAPFRCENAEEHEIRYQFSRKVRNVSISSFAYFMVA

LLYYSFEALMESFPERRLPVASWLPYDYSSITSWWFSSVYLLFAMIVGGLITVGFNVLFFEVMMQIVAQV
KILKNRLAKMIDTLQEADGRKNNSDERLQECRLFRRCVEYHIDILRIAKETNGIFAAIVLIQYSITSLVL
CSTIYLMSGATTSTGHFLKFGAYFGCMCHQILILCYAGHYTFLEFSSIRDVLYSSDWTELSIINKQSLRL
MLVNAEKPLVFDCGGILQLDVEALKNVLKLAYSIYNIL

>XP_011305105.1 PREDICTED: uncharacterized protein LOC105267739 [Fopius arisanus]
MYRLLHILCPHNVHDINDVIFIILNMTGENSNSLQSFIICRDSSKGLRRESPKDQKMSWFYQLWNRQFEN
YSGDDNYTWEIGFTRFVFTIAGFWPGSKKINLITFPISLTIIFIFLILPQILEVPDINNERELFISLSFI
FYRAYSIGCMLCLRFKIDIFKSILEDFRQHWNTRNHTEDRKIMHHYAKLSKIISIITSVTIGIIAYNKLL
SPLAQDIIKGKKLKMIPGHSSYPFPLSDPLVFAACYLAEFIVITGFSMITIGMDNCFAVLIFHACGQLKI
LGNKIKRYQGKVRHPGRNDAPGDCNCLKCIVDFHVKTCELVKKINNFYNSVAFLKLWVSVSYFCFIGFNI
SKAVESKNYTGLFSNALNMIFMMVALFLYCQLGGMCQEINNGISDIAFNSEWMQNPRNSRDIILIIRQAN
IPLKITAANFFVLSRSFFKEYIVTSMSYLSVLITVRTSK

>XP_011305065.1 PREDICTED: odorant receptor 13a-like [Fopius arisanus]
MTRCSLPSFQYFGRDIFELLGNLRVWSCHSRDRRVFHLIFVAYLFALNFLLIISEILDVGNYHDLSSFAA
HINPLFLHFLGLFKWSYCIIRRADIVGLVECMERCHLLAQQINLQDVDCWEYRLKLNRCQKYSTLFMNVW
LFVAIGGVCQWCTNPIVYDFYEQYYSGKIIESSAMRRLPYPGLFPWEINSISRYVMCFAFQLWGGIASTV
GVAVFDILNLTFMMYACAQLNHLKDTLIAKSEKPDESYNKIQIHQLGEKLMRCIRHHCQVLIFLKKLEEF
SSGPMFVQCLGIIVALCLISFEASTIQFNGSIEPVMKTIMMFEYWCSIVAELFLYCYMASELGQLGMEVS
QAIYACRWEQFYVNYSSDYESKRMISVITHIIRFSIMRSQRPILITGGFYVLCLPTFKALVGLSVSNALI
LRQLTGD

>XP_011305064.1 PREDICTED: odorant receptor 2a-like [Fopius arisanus]
MKRPPRRINDITPEETTLPFKAFISHVFATLSHLKISKNSNSSSLINLIDTCATTCLITLNFLLIISEAM
DVKNSYDLPSFAVRLNPILFHCQGFIKWTYCLTHYVEIQEIIDSMEICHEMCQKIDRNDKGTSRYEIKMA
DCQKNSVAFVNVWMMVCVFGVVQWCANPIIYDLYDDLYGVKNINSTFTRHLPYPGIFPWAVNGFKMYLIT
FTFQFVGGIGAGIGIGAYDILSITFIMYTCSYLEYLNETLTKEIVNRRDDPAKIGSFERKLKICFRQHRQ
ILEAFERIQIITSFPMFIQCFNTIIALCLVSLEASTTEINSSVECVMKLLSTAEYWSGINIELFFYCYFG
TKIQELGSRLSDAFYCCNWELITGDYESNRLGINKMVVLGIMRAQRPMEMAGGPFYILSLETFRALISLS
LSNAIILRQLSN

>XP_011304742.1 PREDICTED: odorant receptor 22c-like [Fopius arisanus]
MHLVGQLESLNHSLDDVDHLESQEIDDKLAKLSMRHSLLLDLAKGFHTCTNVVIFLILGSNTFIICLSEV
MLLWAFNAGDNQTSVDMIIRISLMSVQIFLYSYIGEKFSIQSSKLKLTLYNCPWYKIPPRVARNLQFIMM
RNNIPLRLTAE

>XP_011304532.1 PREDICTED: odorant receptor 13a-like [Fopius arisanus]
MMAITRDINNFVGINTKILRLVGLYPKNYNRYISCVFFMSLIILPEVLEIYYHRNNFDVVLETSSVLMTI
VFAIFKSSIWMTRGDTEYFVDFLVNDYWRIANRLGDPEDIRKFAKLAKIITVSYSFLICNSLLFFYSLPL
LTPFLNSNDTRLRVPFVATYPEFCYHSPAYEIVYLSQLLATSTCGLIIILGTDTLIATALLHTCGHFAIIE
RNISRLNFFESVENWEPRVKMIIRHHQVVIRFSDRLEVLFNPLMFLQVFASTLIICLVGFQAQTGKVDKF
PQYCSYMMVALFQLLLFCWPGDKLITQSLGVSTATIISNWYAAPYHLRRDLQFIILRSQKPNFLSAGKLS
SMNLENFCAILSSSLSYFMLLRSVSEN

>XP_011304237.1 PREDICTED: odorant receptor 43a-like isoform X1 [Fopius arisanus]
MRINYWRGFISENIMSLWRNLQNTKNEEIHPENGFDVEVKYLRVPMKILGVWPPDYRGKQWKIEAIFEYS
KTLIHMFILAKVFLFKFYQREFGEMYESMENNWRQAKYLNSEKKRIMSDDLQQSQKLVKYWALHFSAFLV
LVLVLPLLRILILHEPLKLNIDVWDSFHTSPGFELAMVLQTVVELYNCLALISLECSFLITTSHCSAQLK
ILCHSIEQYDGLITHDNCEEQLNCACIRCVVRRHEEIMKFFGLLRKCFNSAISLNLILAESYLCVFIAMG
SKQAEHQSGLYPAICWSFTMIMIVVGFIYCHIGEKITQESLNVSNAFFNCHWYESEASKAKYYVTPMIIS
RSIPMEITVGIFYKLSHELFKNCIFLAYSVFSLFRTISEKQNAIVK

>XP_011304112.1 PREDICTED: putative odorant receptor 85d [Fopius arisanus]
MVTSASLCTEAPRGILPYQAWYPYNTSTLVGFWSAYLHQIIAHAYGAFTNAACDTLMYGFIMQICPQFGI

LQHRFQCLPKSFAGITENVHQCEKNQLRNCVKHHLQILHYAEECNRVFDFLICLQFFVSSTVLCVSVYRL
AQINLTSPDFAIIVMYLLCMLSQIFILCISGSYVTSESHNMVDGIYSMDWTSLNPQTQKSLVFIIIKCLR
PIKFKSGNILLLSISSFNKLIRLSYSAFNVLQQSSGVYH

>XP_011304111.1 PREDICTED: odorant receptor Or1-like [Fopius arisanus]
MLPYSFTILQILGFCRSVNWQKGWKTTLYDCYTYTMLFLLYTNAVFQSIYVFSSIESVDALIDNTFILLT
TISSGFKATRFVMRREEIIRLLRIYRNHPCAPQDQAEEFIQSKYHRVIHNLSLCYISFTMATVTFQTSAQ
WRLAIPQRELIYKSWLPYDNSHPLAFWLSHVHQMICQFADASVSCTYGVIVAAMMINISGQFVILSHRLE
NLRESVGYQDKEGITKIRDHGTHDEIVDMERKKLAECIQHHLHIFDCFERSNDVFSGTMLGQYMASAFIL
CGTIYKVSKMTGLDPELIGIVMYLTCMLFDIFLPCYFGHEVTTESFRVSHGVYNMDWSELKIPTQKSLIL
IMNRSLKPLTFTSGYIVQLSLDSFGGLIKTSYSIFNLLKQQSN

>XP_011303571.1 PREDICTED: odorant receptor Or2-like [Fopius arisanus]
MASTKKERWPIKITYFFLSLVGFGVADTKNKRQLLNIALHTSLPLSLVSCWFAGSNFYLTITGDGELSDK
IDAAFLVLVVFLANSKLYMIAIKRQPYEHLLEVTDKTLWIQSWTDYGKDVLDECERQAIVVLFVFVILGH
ASGAGWVIAPLVLNIKGNSSDPDDRHLPVKPILGPISPFDSPYFELIFALDVFGSWVVTLLYFCFDYYLV
LVNIFVVGQFGILKQRFEDIYTVGSFSLVTEFRLNDDKYAEKYKSMDYILREFKDCVKQHQFLIAIVEEV
EGVHKLMNLMQVLVFSLIICIVGYQLLMSGSAVTLVKNGVFISSCLIQLFAMTLSCHNIMIASSDISQGI
YSSGWYLENHTHVGRSLSKDFMMVLMKTKYPCYLTGCGFFPITLDTLKSVNIGLSLLYEHRLSQLLFRT

>XP_011303490.1 PREDICTED: odorant receptor Or2-like isoform X2 [Fopius arisanus]
MTVEIPEQWPVTLIYFFLSCAGFGIAENRSQRRILNISLAIFITMCAVAMYVMSMNLLYNIREGSDSQEI
LTILFSTVIAALVTFKLISFLTRRTAYEGLLLITREKLWRNSWTDYGILVLEKCGHQAKVFLFAFVIIGH
ICGATFILEPIYLMRNENITSASHRRLPFEIRIGLPGHETPYYELFFFIDFDILKDRFEDIYRNRREKIS
GDGMGSALMEFKQCVMQHQFLIKLVNDVESLYKIMNLFQLLVYSFVICMVSYQLLTATNAFFVFKFASFG
TGNLLQLFAITLTCHNIKIASEEVAVGIYRSSWYHENFSKKGRELSRNFLLVFMKTQYPCYLSGCGFFPI
TLDTLKSIITTAFSYLTLIRESMK

>XP_011303489.1 PREDICTED: odorant receptor Or2-like isoform X1 [Fopius arisanus]
MTVEIPEQWPVTLIYFFLSCAGFGIAENRSQRRILNISLAIFITMCAVAMYVMSMNLLYNIREGSDSQEI
LTILFSTVIAALVTFKLISFLTRRTAYEGLLLITREKLWRNSWTDYGILVLEKCGHQAKVFLFAFVIIGH
ICGATFILEPIYLMRNENITSASHRRLPFEIRIGLPGHETPYYELFFFIDAFGIYIVTILYFCFDYYLVL
VNIFIVGQFDILKDRFEDIYRNRREKISGDGMGSALMEFKQCVMQHQFLIKLVNDVESLYKIMNLFQLLV
YSFVICMVSYQLLTATNAFFVFKFASFGTGNLLQLFAITLTCHNIKIASEEVAVGIYRSSWYHENFSKKG
RELSRNFLLVFMKTQYPCYLSGCGFFPITLDTLKSIITTAFSYLTLIRESMK

>XP_011303488.1 PREDICTED: uncharacterized protein LOC105266778 isoform X5 [Fopius arisanus]
MTSNLRRQWPIRDTIVASFPLLTSTLTHVKLILFIRQRGPYERLLRITNATLWMESFTDHGKIVLKNCEQ
QAKLFLFIFVILGHASGAGWIFEPLILNMKANSSAPDDRHLPTEVIVGVPLFESPNYEIIFAIDVLGVYI
ITILYFCFDYYLVLVNIFIAGIQRLRDAASIFNLHRRRDREHLQLHEPSGSDHDQFDYLSRFVSTPHAWE
CTDCCEKWSLRLQLSCSTFYLHPILS

>XP_011303487.1 PREDICTED: uncharacterized protein LOC105266778 isoform X4 [Fopius arisanus]
MTSNLRRQWPIRDTIVASFPLLTSTLTHVKLILFIRQRGPYERLLRITNATLWMESFTDHGKIVLKNCEQ
QAKLFLFIFVILGHASGAGWIFEPLILNMKANSSAPDDRHLPTEVIVGVPLFESPNYEIIFAIDVLGVYI
ITILYFCFDYYLVLVNIFIAGIQRLRDAASIFNLHRRRDREHLQLHEPSGSDHDQFDYLSRFVSTPHVVL
FNFLPSSYPVITSWSPVLISPMGYIFRDGTRIIRTSDDRCPRVL

>XP_011303484.1 PREDICTED: odorant receptor 83a-like isoform X1 [Fopius arisanus]
MTSNLRRQWPIRDTIVASFPLLTSTLTHVKLILFIRQRGPYERLLRITNATLWMESFTDHGKIVLKNCEQ
QAKLFLFIFVILGHASGAGWIFEPLILNMKANSSAPDDRHLPTEVIVGVPLFESPNYEIIFAIDVLGVYI
ITILYFCFDYYLVLVNIFIAGQFEILKRRFATIYSVKSVQLMRESQKNMQNGPINDIFKEFKDCVMQHQF
LIFIVDEIESIYNFMNLVEVIMISLIICLVSYPLLMPGSALTVVKNGVFVCSCLVQLFTFILSCHNIMVS

SSDLSNGIYFSRWYENYQDIGRSLSTSFMIVFVRTQCPCYITAWGFFPITMDTLKSELLEGWGWQQSDQM
LPWDHRQLCSTLGSLIINASWCIEF

>XP_011303220.1 PREDICTED: uncharacterized protein LOC105266609 [Fopius arisanus]
MMCTMSSSFAAGYLCTVLKNINLIMRQSDILFVLKKFNWERSVSCTRDIVESGWSVDSLPISNTPLRTIA
KAHNFWIVCALDFITWSIFTYTVLAHDSFFASLMLQTSTQLDILNHKLKNCTNFEMSTQNETPEVDVIHF
DCIEINPKIKNLNPNEELIKCIQHYQRIFQIVKTLQSIFGCILLPQLISSLAMMSLIGFHVFVIKTIDLN
RPSKAIFFSVFFGSIFYQLFAFCWSGGHIIEESDKTSVALYSSCWYQGNRSFRVNVMVFASLVQRPMVMS
AVGLFDLSIVMYKNIMMKSYSAITILKNAYN

>XP_011302983.1 PREDICTED: odorant receptor 13a-like [Fopius arisanus]
MDASSEAEFEKVKCLLDRISWPMTLLGIWPKNLTRSGQLKFTIFITYFTIHLSMQLLDLANVMGNLELVV
LNLTESAFQLMAMIRMLIIRLDKTTCRIIDAIEEDLEIKNFRNIEEIRILAQYSSIAVKFYRLGNRFVTV
TAVIYYVTPLQSYLIAKMTNGTAILVNPYRIYHFIDLSPIERTAVVYAVQFPMIYTGLFYVTSYSLLLGF
VMNLCGQLAVLSRRITTMKNDDADPRIVFRRHTQRHIKIIVTAQWLNDTFHAALLYELLATTVLLGLVVY
QFLLNIDDANALGVCKLATYILSMIILVYVNCFMGECLNTECNALLNAYYQCNWYEMSPFYKKALIICME
TTQEPIRLTAGKFYVFSLEGFTQIMKSSMVYVSLLRTMI

>XP_011302836.1 PREDICTED: odorant receptor 2a-like isoform X2 [Fopius arisanus]
MGLIVYRFMTNLDSADAVGCFGFATYIFSMLVVVYVNCFMGEYLKTECNALLNAYYECNWCDMSMGYKKA
LIICMQTTQEPIRITAGRFYIFSLETFTKTMKSSMVYVQMLRKTM

>XP_011302835.1 PREDICTED: odorant receptor 2a-like isoform X1 [Fopius arisanus]
MGLIVYRFMTNLDSADAVGCFGFATYIFSMLVVVYVNCFMGEYLKTECNALLNAYYECNWCDMSMGYKKA
LIICMQTTQEPIRITAGRFYIFSLETFTKVRPLLQQEIPEIPTNRDINRFSDNEVVDGVRTNVEKNNVIH
SLGIPNRRRKNCSSFGGEMENFCYFLTFPSIKILNEKYCG

>XP_011302460.1 PREDICTED: odorant receptor 67c-like [Fopius arisanus]
MDFWDQDYFRAAKITTSLVGQWPLQTSKELIYRRGAIFILVLLLLIPRIRAVILYGDDPEVVLSATSPLI
IDAVFVIKIIINSCNLERMKNLFKKFYENWKILSKDELKILHLHTAEGNKVSIIYLGCVLGSAGLFLTQP
LQLGLLEIIMRTNDTIRRFPSPVDYGSINVEKYYWWICVSIAFIGLVVALGIVACDLLFFMYSYHVFGLF
ATLGYIIDHLPVDDDNGWSDEMYVKRCIQLHCRVIEFAKELEGLYVWSFFGVIGFNMILISVIGVQIVIS
VGATEKIVQYGSLTISPFGHLFVECLMGQRLIDYSLATQVYISNAQWYKMSDKAQKMIKLFLLRSQLPCQ
LTAGRFLVMNFETFNMIIRTSASYFTVLMATQ

>XP_011302416.1 PREDICTED: uncharacterized protein LOC105266165 isoform X2 [Fopius arisanus]
MHSEWKEDVHYALGFTKLWCQALGIWPWDRNIKFSILRRTFAFATQVASSVLLMERLSVYGHCGLRISLI
DALGITSAFLISATKILILSLQKDRLRDILQSLINDWVNIRGASDLQIMKERALWGRSAFIVQIGVAVVA
VIDLSVTRYPSFGGPVGNDTTRTIIMGPSCWIPDDMSFGAYLVIYYLMFISLWVCCGVYTGCGALMFSVA
LHICGQFEILNTSLDDLTDDDNHYQQRRKIKEYSRRHNELLLVGVAIMMQVKNGNVDKDIINYALRMGVA
YTGLLLYSYVGEQLFIQAEKSRLTIYQCPWYNFSPGMIKDFKFIMMRNDSFCYLTSGGISIINYEQIKDL
TRIMFSYFSVFKVMLE

>XP_011302415.1 PREDICTED: uncharacterized protein LOC105266165 isoform X1 [Fopius arisanus]
MHSEWKEDVHYALGFTKLWCQALGIWPWDRNIKFSILRRTFAFATQVASSVLLMERLSVYGHCGLRISLI
DALGITSAFLISATKILILSLQKDRLRDILQSLINDWVNIRGASDLQIMKERALWGRSAFIVQIGVAVVA
VIDLSVTRYPSFGGPVGNDTTRTIIMGPSCWIPDDMSFGAYLVIYYLMFISLWVCCGVYTGCGALMFSVA
LHICGQFEILNTSLDDLTDDDNHYQQRRKIKEYSRRHNELLLVGNQLNHMLYLIIFTEILGSCFLVCISG
VAIMMQVKNGNVDKDIINYALRMGVAYTGLLLYSYVGEQLFIQAEKSRLTIYQCPWYNFSPGMIKDFKFI
MMRNDSFCYLTSGGISIINYEQIKDLTRIMFSYFSVFKVMLE

>XP_011302414.1 PREDICTED: putative odorant receptor 92a [Fopius arisanus]

MLGPPCWIPVAMPVGLYLTQYYLIFFGLWVVAFLYSSCDAFIISNALHICGQFEILNMTFDDWRPEESDS
NARYRIRKYSKRHNELLLLGNQFNQLVNISILLGIFGNCFIVCSSGIALLISMKNGNALEDVINCVVRIY
VGYMSLFMYSYFGEKMFYQAENSRMTAYGCPWYIMPSDIIKDIQFIIMRNNSFCHLTIGGVLIMNYESFK
QLTRLMFSSFSILKLMIE

>XP_011302250.1 PREDICTED: odorant receptor 22c-like [Fopius arisanus]
MDFWDQNYFRLAKITSCIVGQWPNQSVKELILTRGLLLTLTIIQLIPRIRAVVLHSDDPQIVFDASGPFM
IDTILVIKIINNCYNFKKMKTLLSKVKENWTIFSKDEMQVLHEYTAAGRRVSMIYLVSLFCGAVVFATEP
LQLRLIHTFIKTNASLPLFPMPVDYGSIDVDKYYWGLFSLAEITTCLIVIGILSCDLLFFIYAYHACGLF
ATLGYTIEHLPSDRDPNSSRGIRHVKRCVQIHHRATEFANELAGLYIYNFLAVIGLNMIIISITGVQTVV
HLDATEKIVQYGSLTITQLGHLFMECLLGQRLMDHSLSIQEHIGNAEWYYSSVKSQKMLSLLLMRSQLPC
TLTAGKFLIMNLETFNLIVRTSASYFTVLLATQ

>XP_011302152.1 PREDICTED: odorant receptor 67c-like [Fopius arisanus]
MGFYQPIVESVKSKCLSVASRNHRSLDKPIDLEIDFDDEVKYLRKSMKSLGIWPFPYRRKWIILCIVSFL
LSTVLPLTTKTYFTDWKNERCPQLLTVIFMEFVTTKVLIFKIHRRNVQGIFEFMKSDWERAKYLTPERKN
LMLESVKQSQFILKILTVVFATAFTFTAIRPWLTSYIHQKPITFYADVWHPFTLSPISGVIMILEITTSL
WTVVAFVSLEAYFIILAKHCCAQLKILGSTIEQYKGRTLHNIPAERYNCACTGCVVTRHVEIMQFFKSIK
KSFNLFLSANLLAIQTYGIVTVIISFQQTGSTSTFFLTICGIFAILGFSTEFLFCDIGDKLRQESLNLGD
AVFNSQWYDFKRTDAKSYLMTMMISKNMPMQLTAGAFFTVSLKLFKECVFLVLSVFSLFRSINEKKNREL
MGL

>XP_011302148.1 PREDICTED: odorant receptor 13a-like [Fopius arisanus]
MISAYHCCVCYFCFDGFLCQINITLVGQFLILQEDLRNICGHPEDDSAPENETRIYLRFRECVIKHQKLI
NFINTIKELYKNTILGIVVVLSILICLQLYQLMTTVGELFSQIHSFVYVCNTVVQLFFFLLTCNDLSEAS
TDLSQAAYDVKWFFMKSDALKKRLANDLTIVIRRSQKPCNLAVGEFSSVTLRTFTSICNTSFSYLTLMRQ
TVQHD

>XP_011302145.1 PREDICTED: odorant receptor Or2-like [Fopius arisanus]
MTFDRQEILPVRITYSLLFLAGFGTPTTDQERRTLTILLIYTLWTIFFGGILIFIDAYYVWGSFEEMLYT
AVNIMTVLICGIKFISTVLQRTRYENFLRLARESLWKQAESDDDKKVMEKCTKQALIFVCAFVMLAGGSG
VLYILEAIYYNLVNDITNFTSVRERAFIMKLWCDWPFYEAPKFHLMFLVQSFTIMHYGLLYCCFENFLVL
TNIFLTGQFTILKYRLEVLNSELTTGRIDDGRVGSSKDSLNTMSQEFKNCIKQHQFLIKATQQLEELYTL
KNLASIMAYSSMICLSGYQTMTPGNPLVRKIKYGIYVTGCLSQLFCFTFTCDELSLGSVSVSDGPYNSGW
YGGNWSEKGRSLTKDLSIMIMRAQRPCRLTAGGFFNVTLNILKSVLTTAFSYLTLIRQSAK

>XP_011302065.1 PREDICTED: LOW QUALITY PROTEIN: odorant receptor Or2-like [Fopius arisanus]
MRPFIFYQDLMYCLATLLCDAIINAKFFTFMFNRQQYQGLLRMARDTLWSGFDTEYGRKVLKQCENQAMF
FVIAFATFTQCSGILYSLEPILLNIQNNSTDVKERLFPLKMWFDLPIYESPNFEIWFLIEMFVIYHSCIL
YFCFDNYLILVNILMTGQFSLLRNRLETLYNREVIESITSTGHLDEKTNKDSLILLSREFKSCVKXHQFL
IAFVDQVENVYTLMNLISVLIFSVIICLAGYQLIMPGNTIIRRIKFVSFISGCLAQLLSFSLTCNNVSVS
SADLSDGPYNSAWYTKNCSSRGRSLTKDFMIMIIRAQRPCYFTGAGFFPVTLDTLKSVLTTAFSYLTLIR
QSTL

>XP_011302064.1 PREDICTED: odorant receptor Or2-like [Fopius arisanus]
MTYSGITMLTILIITIKFYAALVNRTWYENLLRLAPQALWGDVETDYDKGVMKKCEKQAMIFVCAFASLA
GSSGCLYISEPIIFNILNNITIPKDRTLIFKIWQDWPVYETPNYEIIFFIQALITTHLCVLYSCFESFLV
LINTFITGHFTILKNRMEFLYSHQIITRMRRNSVEEAEKDSLADVSREFRSCIRKHQFLIEVIDQVEGLY
AIMNLASVMVHSIIICLCGYQTIMPGNPILRRIKFGIYVTGCLSQLFFFAFTCNNLTLGSMSVSDGPYNS
SWYSGQSSQKGRSLTKDYVIMIMRAQRPCRLTGAGFFDITLDTVKSVITTAFSYLTLIRQSAS

>XP_011302061.1 PREDICTED: odorant receptor Or2-like isoform X1 [Fopius arisanus]
MRIRRASGKDRVIMSQNAVETLPIRLTKFFLSAAGFTIATTRREKILVDVIVTYSTIALAFACFVTGVDI
YNCWGSFYEFVYSFIGFGTCCIVQSKFAVFMVKRKKYLNLMRYTSDILWTRHHTEYGKGVIKDCERQAMY
FIVLFTFLAQGVSLCYTVQPILLNIGRNETDRLFPFTFWVDLPIYVSPWFEIAFVVQVLSCYHTSICYFC
FDNYLALTNIFITGQFKILKNRLEVLFDADHVNLQEPEGNRRTSWYLTKSRRMNRLVHEYKLCIKQHQLL

IDLVDQVEDLYSFINLLQVLVFSFLICLVGYQLILPGNSQMRRILFIVYFGGCFTQLFTFAFTCNNLTLA
SLDVGEGPWHSKWTTETRWREGRAIVQDLQMIIMRARRPCRLTAYGFFPVTLNTFKSMLSTAFSYLTLMR
NSEEDIQG

>XP_011301745.1 PREDICTED: putative odorant receptor 85d [Fopius arisanus]
MPKLIKLLESTKDIDIAMECVPMLCVHSVGLVKFFNWAINQDQMTRLLLAIERDWKSLKLQRDIKLLSQF
TDRGRKINSIYATAMYGILVVYLLSPGIPKILDIILPLNQSRPRVFLYQTEYFCDQNEYYVHILIHAYVT
VPLSVTVLVYFDNLFAIFINHTCGMCEVLKNYLEDIGADDADILERIKMCANLQTNIIEFISDLESAYTI
ALLVIVGLNMMVVTTTGMMAVIKVNEPSEVIRYTTFNSGTIFHLFWSSWQGHNLIVQSETVFTSVYQNNW
YRIPCKYQKMLLPLMMRSLTPCQITAGKLYVMSMNSFGGAMRMTLSFLTLLLSVR

>XP_011301743.1 PREDICTED: uncharacterized protein LOC105265752 [Fopius arisanus]
MAQMLLSIENDWKRLKLQRDIDILTDFTDRGRKTNILYAIAMYGVMTVYLASPGIPKILDVFLPLNESRP
HVFLYQTEYFCDQDKYYTHILLHAYFTVPISLTSIIYFDNLFGILVNHTCGMCGVLKSHLEAIGTQNPRE
TLESLKFCANLQTSIIEFVNNLESACTIAFLLLVGLNMMVVTTTGVMLAQMLLSIENDWKRLKLQRDIDI
LTDFTDRGWKINILYASTDYKQLEQ

>XP_011301635.1 PREDICTED: uncharacterized protein LOC105265696 [Fopius arisanus]
MDRVVTPYHRINRIFLRIIGLWPLQPPISRYSFYAFTLFVTLTHGYLQTAGMVAAIVDVDVFLEAIPTVL
ADVVCYFKYLNFTINAGKMKKLLLIMEEDWKRYTYGEELDILKEYAQFGRKVTVYYAGALYGSLVPLMMT
PLVPIVLDIVMPMNVSYPKHLMFQQIEFLFDFEPYLFPLILHGYIGTAGYLTIIIAIDTMLMVYIQHACA
QFSIIRLMLDRLARVDPDADRHTPEFDEIDYKNMAACIERHNHAIAFCDLIEEANYMSFTFIVGINMLMM
TSSALVAVFKMDNPDVAGKFVAFTIGEMFHLFYSNWQGQLLQEHSESIFSDVYRARWNYTSVRTQRLIIP
LLMRSTKPCTMTAGKMYTMSLRSFSEVVKTSFSFLTVFTSMRA

>XP_011301634.1 PREDICTED: odorant receptor 13a-like [Fopius arisanus]
MENMLDSDYYRPTRWALKLMGQWPFQSPRRNKIFKCLTLFLITSISLPMITKFAESLNDIDVVMECVPMA
GSYGTSYLKYFTWAFNHDRMKGLLLSMERDWRDLKTERDIQLLKKFSERARKMNNIYAMTIYGLLFLFLG
APAIPILMDICIPLNQSRPRIFLYQTEYWIDQNEYYYFIVLHAYLTVPVLFGPVIFFDNLFSIFISHTCG
MCAILKSHLESVHIEDSTGDGESACKRLQLCADMQTNILEYVQYLESTCTLLFLLLVGMSILVITLTGMM
IVIKGGEVSEIIRFASFNVGTIYHLFWSSWQGHTLIVESESIFLSVYQSEWYTLSPKAQRMLLPIMMRSA
NPCQITAGKCYVMSMESFGAAMRATMSFFTLLLSTR

>XP_011301588.1 PREDICTED: odorant receptor 85c-like isoform X1 [Fopius arisanus]
MGGGDHLRFHWNLTSNCLRIIALWPLSVVDNFRGKIVDYGHLIVVGSLALLQGVNMMAMCYVSCENFVAA
AESIGPMMSLFIAFGKICIYRYNKSSLRNLSKKLEVCLNNRNSQEYNYLRMYMKYGNIASASVFGSIFIL
ITGYFSIPLILTTHELPFIAYYPWNWRNSTLGYAASYSHQVILTSVLSCACSTEFTYVWYISHCCARLKV
VQDKLKRLSTGNRTKKCGKDVQRILSGITRLHADVLDDVILINTAFTYIVLLLFLAAVIAICCAGLTITS
KTTESTIILLFLVLAVYYAQQLLFYYLPGDLLAREALAVGKAAYSSGWEIFDVECKKTIGLILFRSNSPP
RLLAGDLSPLLLENYMSFLATTASYFTSIRAVTDKN

>XP_011301215.1 PREDICTED: uncharacterized protein LOC105265440 [Fopius arisanus]
MAPERVYQILKFFGRLSCTWPLEDNPGKWWRIVNELQFLFTIVNVMGLLVPLVAGAHHHRHHVGTAMKAL
SELTALGDVLFNLILCRVQRHRLRELLMEVSTYTGRIEGAEKEIFHRSVNHYLPFCALVGLSYLQTAIAF
SFGPLVMSSILPADTWYPFPIEIFSTLYFLVYIQQVFTIIQTGMCITVDFMVAFLLSYLSARLQTLNIEF
RRAHGERELHACIAQHLDFIRFAGELRVAVRFIILKGIVTMALAAIFGAFPIIENEPLPVISQFILMVVA
GCLRLYVSAWPADDVREMSEKIAWSSYASPWIKSPRRMRKTISIVVHRAHRPLVIAVHGILPALTLRFYA
SFLSSTLSYFMTLRAVLRN

>XP_011301212.1 PREDICTED: odorant receptor 13a-like [Fopius arisanus]
MENDWAVPLSEERLNVMLEMAKIGRRISIFSGSMAYAANTIGIVVQKLYNLEAQHIPNPDPRLVTDLFWI
VWLPYNTTNPISYAVSFVLQLYSSVYAALFYFIFDSFIVMIVLHLCGQLGDLQISLQHLHENDIHKSTFQ
VRLKRIVQKHEKLVRLAEDLENYFNFVLLLQLLGCTLMFCFQGYGIIRLLTQGTLNLFQVAFAITVVFAT

AVHFFFCCWAGEILVSQSTSVGLAVYNCQWYRLPPSEARSLVLIGLSKFRPLKLTAGKFSVLTFNLFLHV
LKTSMSYLSMLLAVQNL

>XP_011301211.1 PREDICTED: odorant receptor 13a-like [Fopius arisanus]
METNSGFDYARGWNKINFMILGMWPKTNLRGFRKYHALLNAITVFSLLTLPRLAALYLFWGEVDAMIQCF
STHMPFAIGIVKLVILYRQQQALGEFMEEMECDWRSPLPKKHQEMMMPMARMSRQISIFSGLTVYMANSF
GVVVQKWYNLESVYIQQPDSRLTLNLFWVVFWPVDTSENINFAVALLLQLYTSFTGSLAYFVFDSLIAMM
TLHLCGQLECLQQSFGELCDHNSSHEAKKFERDLKNIVQRHYKLIRLTNNLDDSFNFVLFLSFIGCTLTF
CFQGYAIIRLLDAGKLNFFQVAFSVTVTLGAISHFFICCWVGDFLIIQSAAVGQGFYKNQWYKLSNCQAR
SLMIIGFKNFRPLQLTAGRFAPISLNLFLNVIKSSMSYLSMLLAVQNRRV

>XP_011301210.1 PREDICTED: odorant receptor 85b-like [Fopius arisanus]
MFKLMESTPGFKYACGWNKFNFDLLGIWPQQNVNRIKKYRALINAGSILFILTLPRLAALSLFWGEIDAM
IQCFSTHMPFAIGIMKFTILHFQREVLARFLQAMEVDWSLVKSEKYFESMIKMARIGRIISIISGSMAHT
ANTFGFVVQKWYNLESYYIQDPDPRLTMNLFWLVYIPIDTSNTANYICLLSLQFYASCIGCVIYCCFDSF
VVMLVLHLCGQLQCVEISVAEIGTHGSTNSCFRTDLRRIVQRHESIMRLAGDLEDSFSLVLLLSLIGCTL
TFCFEGYALIRLLDRGQLGFFQLGFSMTVASGAFLHLFFCCWAGDFLSEQSMAVGKAIHCSKWYNLSDTE
ARTLMMMGFKAFVPLELTAGKFAALSLNLFVNVVKTSMSYLSVLLACQGSKV

>XP_011301209.1 PREDICTED: odorant receptor 22c-like [Fopius arisanus]
MSYSRDFKYAIGWNRFSLDVVGAWPRRHAGIFHRERSLLSALGILVLIFLPQSISVIVHWENMDAVIECL
SVNCPVFLALAKLLFFRYRRKEIRMCIDFMSDQWTISRASEEREVMMSAARTSRLISIGSGVITNALFMA
FIFYQLYVGTEIKKLDPTQSVALLYPGWVPFDTRKIEYFIPMWIAQCITTFLSMTIYAVFDCMISTIILH
ICGQLSVLKLALSGIADSEDPRHPEKFNEKLAVIVKHHEQLNGLVAVIENSFNSILLPQMLVCTMTFCFQ
GFAVFTNLLDPNAGKISFFQMAFPITFVFYTVLHLFIYCYVGDELLMKSSSLSYSAYQSQWYNLSALEAR
SLLVVGFQSFRPLQITAGKFCPFSRNLFIKVLKTSMGYLSMLLTVKQRMSN

>XP_011301208.1 PREDICTED: odorant receptor 22c-like [Fopius arisanus]
MSYTQDFQYAIGWNRFSLDILGAWPRRNCGIIHRERSLACAVAIIVLIYLPQSASVIVHWGNMDAVIECL
SVNGPVFLAFAKLLLFRYRRKELRMLIDSMRDDWETQRNLEERQVMLKTAKMSRVISLGSGVITHTLFVA
YIFYKIYFGIEEMKRTDLDPRLAVGLLHPAWLPFDTKKPEYFIPMWIGQCFCTFFSMTMYAVFDCMISAM
VLHICGQLSVIGLSLRNLADSDVSSPPNLFRKKLIPIIKRHEKLIDSIAVIENSFSAILLPQMLICTFTF
CFQGYALIASLLGSSSGRITFLETAFSITYVFYTVLHLFVYCYIGDQLLVESSSISYSAYDSQWFNLPAR
DARFLLFVGYRSYRPLKITAGKYCGFSRNLFIIVLKTSLGYLSMLLTVKQRMID

>XP_011301060.1 PREDICTED: odorant receptor 13a-like [Fopius arisanus]
MTKTGGKSRVNVHDNADFVYSYGWNRTMMHAIGGWPEENDNIIERNRIFFNAFALILFVVLPQSASLSVF
WGDLNAVIECFSVTLAINLSLLKLLWLGVQRPTVQNLLEMMAQDWLEPRTLEENEEMMKMTKIVRLISSL
SFYGTISLFVAYVSVQILIGFEMRNDPNVDPRLSIGFLYTSVFPFDTSSLSIFIPIWIFQFFMTYVSMAS
YSSPDSFIGMFVFHQCGQLKLLRRKLETIVDKETIENPQKFWKKLGEIVKRHEHLNERASDIEENFNKVF
LAVTLVSIFATCTQGFAVITLLRETDGNFPVLKMIFLLVFTTYDLGHFFVYCLAGDILMTESSNFGVSLY
NSRWYDLSPKEAKSVLIFTSRCIIPQQITGGKFVVLSLPLFATVVKTSASFLSVLLAMKM

>XP_011301057.1 PREDICTED: odorant receptor 22c-like [Fopius arisanus]
MEADLSLELSKENYKPVFETAKIARTISIFLGTCTICILITGIFSLKLYQLDSFYIKNPDPRLSINFFFV
AYLPFNSTPIITYMTVLSAQFYVSVISTGIHLVLDSFVVMLVLHTCGQLGVLQESLALLCACGNDKSDFG
TGNFRRAFQKIFIKHQAIGRFTRNLDSLFNFTWFPELFSCTLTLCCQGYIVQKLGRGDQSSMWQIGFPLC
STLGLVTQFFLNCWAGEYLTSQSAEIGYAYYRNEWYTLKSSDARLFLMIGYQATRPSTLSTWKFSILSLR
LFLQVMKASFSYLSMLLVITDS

>XP_011301055.1 PREDICTED: odorant receptor 46a, isoform B-like [Fopius arisanus]
MLILHCCGQLKLVELSLTQLGINYVKNGQSFQVVLRHIVKKHQSVCQFVKSLEKLFNFPWFVELVGCTLM
FCFQGYNVLKLLHADGSGVFQIGFIIFSTFGILIQFLLNCWAGECLISQSSKIGYAFYTSQWYTLRPSDA
RSLMMMGFQKTRPLTLTTWKFSVLSIRLFLRVMKTSLSYLSVLLVMTDF

>XP_011301054.1 PREDICTED: odorant receptor 22c-like [Fopius arisanus]
MRIDFEYSCGWNRLVMDSFGLWPKCHMNFIEKNWSLIIAIMISIVVIIPRFAAIFLFWNETDAVVQSFST

QLLFMTIVFKLLILHFRNEVLADLLTAMEADLSQRLTDIKYLAVLRTARIGRIISIVLTSGTFCILITGV
IATKFYHLDSLYIKNPDPRLSLNFFWVTYLPFSTENTISYVTVLSLQLLASLMAGFYFIFDGFVVMLILH
VCGQLQLIQISLQHLVDNLPLIRDLRVTLRKIMEHHQRMHRFVSSIDQCFNMMWFLELTSCTLTFCFQGY
ILLKLLRADQSSTFQMSFPVCAAIGLLSKFFLNCWAGEYLISQSSEIGSAFYEAQWYKLVPSDARSLMMI
GHKKIRPFTLSTAKFSVLSFRLFVQMLKTSGSYLSMLLVVAES

>XP_011300802.1 PREDICTED: odorant receptor Or2-like isoform X2 [Fopius arisanus]
MVYTLINAFTNAIVTAKFVTFMIRRDDYVKLLQLSCDSLWRPDATGDEAPVLKQCEKQAKFCVIFFAIFA
QITGWVYITEPIISHFTILRNRLTALYNREVNGSKGNHDRNRNDLNLVFSEFKGCVRQHQFLIRVVEQVE
SVYTLMNLASVLIYSIIICLIGYQLIMPGNTVLRRIKFTGFVSTCLSQLLSFAFTCNNVSLASVDVSAGP
YNSDWYATDCSKLGRMLRKDFIIIMLRSQRPCYLTGAGFFPVTLNTLKSVLSTALSYLTLIRQRSMQQ

>XP_011300801.1 PREDICTED: odorant receptor Or2-like isoform X1 [Fopius arisanus]
MVYTLINAFTNAIVTAKFVTFMIRRDDYVKLLQLSCDSLWRPDATGDEAPVLKQCEKQAKFCVIFFAIFA
QITGWVYITEPIIINLLNNSTDPKDRVFPFDVWLEVPVYETPFFEILFFIQSAMTYHVCILYCCFDNYLA
IANIFIAGHFTILRNRLTALYNREVNGSKGNHDRNRNDLNLVFSEFKGCVRQHQFLIRVVEQVESVYTLM
NLASVLIYSIIICLIGYQLIMPGNTVLRRIKFTGFVSTCLSQLLSFAFTCNNVSLASVDVSAGPYNSDWY
ATDCSKLGRMLRKDFIIIMLRSQRPCYLTGAGFFPVTLNTLKSVLSTALSYLTLIRQRSMQQ

>XP_011300122.1 PREDICTED: odorant receptor 24a-like [Fopius arisanus]
MTKGNCGTITELVDAISLMTTSLMAVLKILLPWSHRRRVASLVISVVQDWSQVNSKKCQEVMLRYAFIGR
VVFIVQMSGAYMTIIPLILTRLPRSVELIDADNRSLFLRNIPIGPRCWVSLEISTFTYSLYYIFVCVHLF
ILATSYLGGDVFAFGLAMHLCGQFELLYRSFEKFDGNETSVIQRKIVSQFAKRHNSLLNLADDFETAFSL
LILFELGANAFIISISEIILLRAIKIGDTQIISAMAIRIYLMYIQIFMYSYIGEHLSRQAEKLQVSIYSS
RWYKLPPIIVRDLKFIMMRNNHLFHLTAGKIWNMDYQNFKFIVKSMFSYFSILRLMIKD

>XP_011300121.1 PREDICTED: uncharacterized protein LOC105264737 [Fopius arisanus]
MTEPTGWNKNTVYALSLYKYVAENAGLWPLDNRKTLRVSIVTIMHIVTLIVIIKDWIIKGNCGSITEMVD
AFALFTLSLMNLIKIILSRRHQTKITSIFHSAVQDWVDTENQKDVRIMIHHAYVGRIVCIVQMIGAYLAV
VPMILMHFPSSFETYDSNNDTIIVRSVPVGPKCWLPSDISFTTYLGYYSLICVQLFLLANMFLGVDVFAF
GLAMHLCGQFQVLNRSLGEFDGGEPVFEQKIKVSKFARRHYSLLQLADDFEVAFNM

>XP_011300073.1 PREDICTED: uncharacterized protein LOC105264707 isoform X2 [Fopius arisanus]
MSSFYLNIFTLRMLGFWFPDETSATWRTNLYRIYNAFAVTAMCGFVLSQFFAALSCLHDTKELTNASFLL
VTIIAVAGKMFNLTIYQYKIRRMVGLFDLQPFKSLDDNEVLIKSKFHRSVKRFIFVYATMSIVTCTLITI
FSLIRDMPRRQLLFKARYPFDDSTAIGYWISYMHQHYSHYLGAIFNTFFDTFIGALMLDTSAQLEILKYR
LIMMPSAMENERSQSGCKGSPEEVQKIESKHLENHTNHHLAILEILKNDQRNIHCLNFSPILREYSSSLH
QCLHHDEPEAFQQRVQLSADSENMSEGIYRMDWTCLSIASQKSLVLIMARTLKPIRYTSGHVVNLSLVSF
GSLLKLSYSVYNILSQSSE

>XP_011300072.1 PREDICTED: odorant receptor 46a, isoform A-like isoform X1 [Fopius arisanus]
MSSFYLNIFTLRMLGFWFPDETSATWRTNLYRIYNAFAVTAMCGFVLSQFFAALSCLHDTKELTNASFLL
VTIIAVAGKMFNLTIYQYKIRRMVGLFDLQPFKSLDDNEVLIKSKFHRSVKRFIFVYATMSIVTCTLITI
FSLIRDMPRRQLLFKARYPFDDSTAIGYWISYMHQHYSHYLGAIFNTFFDTFIGALMLDTSAQLEILKYR
LIMMPSAMENERSQSGCKGSPEEVQKIESKHLENHTNHHLAILEFSKMTNETFTVSIFLQYCASTLVLCI
SVFTMTNLKPFSKEFNSLLMYVSCMLVQIFVICDAANDVTVRSENMSEGIYRMDWTCLSIASQKSLVLIM
ARTLKPIRYTSGHVVNLSLVSFGSLLKLSYSVYNILSQSSE

>XP_011300023.1 PREDICTED: odorant receptor 13a-like [Fopius arisanus]
MEALRTDTWSPDTTYALGYYRLLGRSLGIWPLDSARFAPTAKISFVILTQLWMSTTLTKKLLSNGNCGSV
TDTVDALSLVFCGFLTVMKVAIPRVHREDFLAVVNSAVNDWSMATTKSRLVMIKYAQMGRLVFVVQMCGA
YAVGFPLVFLRLPIFRGLWNDLENETFRELPIGPSCWVSQDQSSYQYWGEFILQSVALFIVCTAYIGCDA
YFFGIAMHVCGQFKLLGENLENLETRDDQEKRRKIQGFVRRHNHLLQLVYNFEEMYHVIILAQVGVDTLL

ICISGIALLMSLDSGDAVLISCLIIRIYLVYVQLFMYSYVGENLSSYAETLAINVYNSPWYDMPQDIIKD
IKFIIMRTNYRFNLTAGKLYSMNIENFRTMVTKIASFFSVLRLVFQESP

>XP_011299493.1 PREDICTED: odorant receptor 85f-like isoform X1 [Fopius arisanus]
MKKTMEVLRTEHRDILNMLLTVGRIGGIWSAVDPPLLIQRFLYSIYGFIAKSTLWILAGTMSADIISNLD
DILAISDAGAMLAGLSAVIAKVVVFQRHSMKIRQLIEMVYRPIDTMVMAKEGPVYGLMNFHVTVEKVIEY
GWAGMAVQLVGAMLVSPVIFAGGNSSLPLRSKYPFDTTDDMKHNAALGLQIITAIFNFTAMFALDGLMRG
FCRWTSFQMQILNSNFRHCDPIWPHSGKEFTVEKLTYMSDFESKINCFVLFHPVEITRESDSFLKRFETC
IQHHQRIIIIINTMNQVFGYYIFAEFSSSTFIMCLTGFQILLGKRSTTNVIKFMLYFNAAFVHLATCCFF
GQMLSNEGNKIADSMWASGWEKEANMSHAGYLMIVALMRAKQLLELKALGFYAVSMETFLMVNHSPLAHS
MSELSLIFPSSHLIANELITHNP

>XP_011298933.1 PREDICTED: odorant receptor 43a-like [Fopius arisanus]
MAFADLVLAFGDLEQVISNLTETALQCMIIMKMVLIKYSGTLRETIMMATNELMEDAFSQSAEKKIYLFY
ITVAKKFFKVPCIFAICATTAYHWMPMQAQIYAALTNQTVPMLLPYRTHVPIELTDLTTYIFIWIYQLPV
IYLNTFQIPGVSLGITLVLNASGHLAILKTRIRRIERINGVVNVQVELVDSIRRYLQIARYVKLINKSFR
IILLEEVVIMTVCTALGCYTLLVYSDLSNGIYFMVFIVYMVGMLLPLYGYCFVGEYLITQSEHVHDAYYH
CDWLNLSLSVRKSLILCLIGTKNPLQLTAGGFYRFSLGGFTTIIKTAMAYFSMLRTLAL

>XP_011298924.1 PREDICTED: odorant receptor 13a-like [Fopius arisanus]
MPNFIEALTGITVFMVTAMKILLPWLQQERMRHIIQSAMEDWSNVYDKRSRQIMSQYAFLGRSAYIIQMI
AAFLIVLEMTLSRSPNFTVEEYKNDSSSTRNIILGPACWITTAMPMSLYLVRYYIILIGLWCAALIYAGC
DAFMFNIALHICGQFEILNASVEGVKYEDSFMNQRRTINNYAKRHNELLVLGHQLNDILNVIIFSELLSN
GFLICISGIAILANIKKGHVDNDDINFGVRIYVWYMELFMYTYVGEKLSQQAEKSQMAVYNCPWYNMSPH
ISKDVKFILMRNSSFCYLTAGGIFVMNYEAFKEITRLMFSGFSVLKLVLE

>XP_011298922.1 PREDICTED: odorant receptor 22c-like [Fopius arisanus]
MTITRPPSFVVESSENGSLVMRNIILGPPCWISSSMSTTLYLMHYYIVFCGLCGAALIYSGFDAFMFSAA
LHICGQFEILNSSTETITDEGGYWKQKHRIKEYSKRHAKLLALGTQLNEIVNFIIFSELISNGFLICISG
ISILTSIKNGNVDNDDLNFGVRIFVWYMGLFMYTFVGERLSNQAEKLQIAIYNIPWYNMPTTIARDIQFI
IMRNNSPCHLTACGIMNVNYEAFKDITRLMFSCFSVLKLVLE

>XP_011298921.1 PREDICTED: odorant receptor 30a-like [Fopius arisanus]
MSFGLYLMYYYATFIGIAVAALMYVGCDGFMFNVALHVCGQFEVLNTSVEEWSDVSNYLEQRQIIGEYCK
RHDRLLRLNSQTAHLVKSIILCDLFSNAFLICISGMAIVINIRIGNAEKDIVTLAIRIFICYVGIFMYSY
VGEILYSQAQKLRISIYNCPWYNMPVSIAKDINFIIMRSNSICTLTAGGVYVMNLESFKNITKLIFSYFS
VLKLMFE

>XP_011298918.1 PREDICTED: odorant receptor 82a-like [Fopius arisanus]
MTKEKNLKYNEIVSYAIRPYKLIAQSLGIWCMTCHNAITKIQLTIICILLVTNALSLFHELNPKCGVLKD
KLIPVSTGICCCLSIMKILSIRLNRENMIQIITSIIDNWSSNTCVDDLRIMDNASKVGRKIYYFQLVSTL
TSVTPMYLCGMGNAMVFDPRRNVTEIVKAIPLANPCFYGNLFVDYYSGIYLLQILQVTVTIIGNVGCDCY
FFGITMLLVGQIRCFASDIEKLEPQECEQQNRQILRLVIRRHTHLIRMADHLERTFSAVVALQVMANIYQ
VSMGGLQILLSIRIKDGATAINFTIFISIFLVQLSIYSYAGECLSSSISYLQTCLYCCPWYQMTPKTNKD
FVFIIARCGKPFHLTAGKIIPMNLESYTDILKTLISYFSVMRAMFDK

>XP_011298826.1 PREDICTED: odorant receptor 43a-like [Fopius arisanus]
MNSRKENIPWNDDTTYALGLYRRYMNLLGVWPLECNKVLSTFRLVLFVIIQMSLNITYITKLITIGYGDR
ILDLVEIITILSLSSISVIKLMVPWKYSDKMHFVLNSMVEDWAGDYNRKSHDTMMKYAYKGRIVCVLQLF
ITGFLILRNILHKLPATLETVDASGNFTTLSRIVPYGPACWVSTTMSVHYYNAYYSLICIHWLSASLAYI
GTDIYLFGLGMHMCGQFKLLCDELDGISGNESSEEQHQKIAKFIRRHNHLLNVAESIEDVFNIPILIEVL
NNTVIISISGIILLWAFKLRNRAMAIPKLIRIYLFYVEIFMVSYVGQALSGETEKLQKAVYNIPWYKIPT
NHMKSVIIIMMRVKYPFQLTAGKIANMNYETYKNIIKSTFSYFSFFRLALEQ

>XP_011298812.1 PREDICTED: odorant receptor 85f-like isoform X2 [Fopius arisanus]
MEHQDWTVDTSYELLVYKLIMWPLGIWPLNKGELFSEIRLLLAASTQAATFICLTLEILNNCRGVEIVLD

LFVLSVFSLLACVKGLIVHHHIHNMSENVRSAIADWSLLSQTDNSQNKELMTKYARIGKLVCFSLMIPAS
GGTLSWIILALPLPMFLPANSSNVIKNFPLQTACTFDPITTSKFYYLIFGLQMYQLVTTCLGNCGNDVFF
FGMSMHTCGQLEILQNDLTDIEIEKEESNNWRKLKGMVKRHVHLIELVDKLEGTFNMIILAQLIMSAVLI
CIMGLQVIIALKTGDMFAAVKANIVLSSLMSQLFLYSYGGDCLTTQNASVALAVDKSPWYKASAVTMKNV
AFIMMRANKPIYVTAGKFFDMTLSTFMEILKASVSYMSVLRIAIDV

>XP_011298811.1 PREDICTED: odorant receptor 85f-like isoform X1 [Fopius arisanus]
MEHQDWTVDTSYELLVYKLIMWPLGIWPLNKGELFSEIRLLLAASTQAATFICLTLEILNNCRGVEIVLD
LFVLSVFSLLACVKGLIVHHHIHNMSENVRSAIADWSLLSQTDNSQNKELMTKYARIGKLVCFSLMIPAS
GGTLSWIILALPLPMFLPANSSNVIKNFPLQTACTFDPITTSKFYYLIFGLQMYQLVTTCLGNCGNDVFF
FGMSMHTCGQLEILQNDLTDIEIEKEESNNWRKLKGMVKRHVHLIELVDKLEGTFNMIILAQLIMSAVLI
CIMGESICKRYYRQLIIDISFGIGLQVIIALKTGDMFAAVKANIVLSSLMSQLFLYSYGGDCLTTQNASV
ALAVDKSPWYKASAVTMKNVAFIMMRANKPIYVTAGKFFDMTLSTFMEILKASVSYMSVLRIAIDV

>XP_011298809.1 PREDICTED: odorant receptor 9a-like isoform X1 [Fopius arisanus]
MFLHLLVTIKMISCISNLETEKDLLLQIENQRKFYSLSSEKKVLNKYANRSGTITRLYVYYIYASVALYM
CSPLVPKILDCILHLNESSPIIFLYEAEYFVDRREYTTWILLHSYVVTLLEATVVVAFDSLYFHLAEHAC
SLFSIACKRMDRLAHDIEFQKSSDQSISAKRDDVGDAVVLCIMQHMKALKFADLLGTVYTKALFFILGIN
MLAMSITGFQTVMKLDEPSESVRLGCFTIAQLIHLYFLSWPGQRLMDHSQEIHSAAYQGSWYNMPVHLRK
LLMPIMMRGSKPCVVSAGGFYVMSLQSFSMVLKTSASYFTVLMSCQ

>XP_011298807.1 PREDICTED: uncharacterized protein LOC105263969 isoform X1 [Fopius arisanus]
MRQRSVTPARTMREQSEVSDSDVRYAYSLYEIIYRVMGLWPFTCQNIGSWGLTIFACTTQITMILFMTGE
TIISASQVDPQLMSFLSCNFLSLTKIIVVRANWSKMQLVIKDLISATNFLHDSKALAIVRARAKLGRTIC
IYQLGGTYFVCLPTIIATLPIFATIVNETVNDTSSGLRSFPLQTSRLFGGLSTSLYVIMFGYQIIQLFFT
CTGNVGNDVCFFGIALNLTAQFECLALNCESISEPDTDVSRENIFSGTIKRQVELIELAKHLERTFNLII
LCIVSANCSQICFYGILIIRSARAGDLVVCINTIICVIILTLQMFMYSYAGDSLSRAIENVGTTAYKCLW
YEMSPANKQNVLFILVRTSKIFSLTAGKIYRMDLDSFKNIIKAIGSYFSVMQAMFNE

>XP_011298806.1 PREDICTED: odorant receptor 47a-like [Fopius arisanus]
MTCHNAISKIQLMIICILLVMNALSLFYELNPKCGVLIDTFTPVSVGMSASLSVIKVLSIRMNSESMLRI
ITSILEDWTSRAFNDDLVIIDEASQIGRRICYFQLGFAAVSTIPMFFCWLGNGEAFNPETNMTDVVRAIP
LANPCFYGNLFADFYVEIYLLQIVQITVTILGNVGSDCYFFGITMLLVGQIQSFASDIEKFDSLECEQQN
RQILRLVIRRHTHLIRMADHLERIFSAIIAFQVMANVYQICMAGLQILLSIRIKDGATAITFTIFISIFL
VQLSIYSYAGECLSSSISYLQTCLYCCPWYQMTPNSNKDFVFIIARCGKPFHLTAGKVINMNLESYKSII
KTLGSYFSVIRAMFDE

>XP_011298606.1 PREDICTED: uncharacterized protein LOC105263837 [Fopius arisanus]
MSVGRIDDFVDTIYILAVGCNLSYKAVNILTNRQGIIELLRLLDSKECQTLSPEEDLLRIKYHRRVRKST
IILCSCLEFTVANMIVSSVLVDIPERTFPLKIWLPSNFSEDFGFWTIYCSQLIAFTYAGTFHVFYDTFVI
GIMITLSGQLQILQCRNPKMSAPDVKCTSRRNRDREIGEI

>XP_011298605.1 PREDICTED: odorant receptor 46a, isoform A-like [Fopius arisanus]
MIQGTLGGWNPQRTSLRQSLPVLPDNSIEKNSTEMTTRALSPNFRIFTRFGVWLPPEWSSGWRRILYQTY
GIFVAVMLYSMFSFQFIGVFGSIGRINDLVESLYTCATVFNLGWKIFNLLKKRREIVQLLLLLEADICQT
KNPVEEEIQKTCNQRIWNRTKFLCAYFETTCVTMILTSMVKNIPERTLPLKAWVPFSYDDGLYWPVYLHQ
VLGLIYVGMIHASYDTFIVGVMLTLCSQLKILQYRNSEMSLLTTKSTVDRSTLEKEFVISSIKHHQLLQQ
FAESFNNIIVYVIFIQYSVSSFVICVSAYKLVNLEVGDPEFTFAVLYCSSMIIQTFFYCWYGNEVILESE
NIGNSLYTSKWYSLNVGTVRDVTMVIHKMQYSIKFSCSRLCILSVESFKNLMKVTYSTFSVLAKS

>XP_011298091.1 PREDICTED: odorant receptor 46a, isoform B-like [Fopius arisanus]
MLTQERDGVKGFLPGSWWVFVATGLWRPRSWKSPFLVYLYKVFSIFTIFLVYTFTTTSILGIIANHGGIA

AVMSDFLLLSFIACCGKSVNMIVCRETIIDVIDTLQTDPCMPRDPTEEDMQYKRDHFVWINTLIYGILTE
VTAMMVSVGSLLQHPEEGELPFNTWLPYYHDSGFGYRFAYGQQIISIWFSASMAVAYDTAVPGMMMEICA
KLDILKYRFLNFKSLLETSDNLSAYASERKLVSECVECHLIILRLAETINDVFNAVVFLQYSLSTLLICV
SIYNLANTNIMSSEGSGIILYLGCMLMEIFIICAAGNELTLVSESISDAIYEMDWTELNGSTIQSLILIM
TRTTHPIVFKCGSIVEMSLESFKSLVKLSYSTFNLLQQTSA

>XP_011297871.1 PREDICTED: odorant receptor 22c-like [Fopius arisanus]
MPRKTALAKGAFGKSKELLKKFSVFSDNDYLSKIGFNEMFRVPDTLLRTLGLSGIHSVFDKTPDGARHRI
HNFATGDKGTDFDSEVVLLPVRYPFKIDTDLKYVLCAIFEVSCTIFLAMTYLGVDSLFHQSTTIVCLLLQ
TVGKKFGEVAVYAKGNMVDRRVWRQLHHIGEQHCELLLYCRRIEEIFNPLIFLMTLFTSANLCVSVISLQ
LELSSLQLGNIVILLTQVAAIVLQPFIYCNSAENISHRTKNIATSIYMCQWPEQTEKFKKIVLLIMLRSQ
HNYKFGQYGLFKVNHHLLTQLIHTAWNFLMILKKWEMPL

>XP_011297812.1 PREDICTED: odorant receptor 13a-like isoform X1 [Fopius arisanus]
MDFWEKLYFKPMKIVSCWAGQWPYQSAVERTVIRILLVSAVGSQIISQIAAVAEHSNDLDFFMELVPPFV
VELTCFTKMINCFFFLGKIKILLEQLKENWEFFPSGRENELLREHSKFGFQISMLFLGTLYGSGFIFATQ
PFQTIILHLVLNSSHSIPHPFLIPANWGPIDPDKYYFPLITLSAVSIYCVVTMLVAIDCVFYTCSSHVCG
LFAALGYSIERFEFNDNCRKDDGFAFLRRCIQIHNRALEFTSIMEDVLTFNFLLMLGGVTMAMSLTAVQM
VINFEAIVKLLKNLAFAITQITQLAIKCWMAQRMMDMSLNIKFSLINSKWYLATPRAQKLIGLMIMRSEI
PCEITAGKVIVMCLETFSSIVRVSASYFTMFLALR

>XP_011297624.1 PREDICTED: uncharacterized protein LOC105263243 [Fopius arisanus]
MHGYWKMLNLFQIYVSKKWTDLRGKCPEEDRRSKINFNRMFIVSNILLRKAAVSDLYEVFLDTHEKTYQS
AIIFLTISAISFFCLLGLINNFIHEWDRDMYKALESMPMILSAVLAWVEGVVLCVSSKKIKGLLKKIQIL
WTRELKDDIDDNIFVTAERSIFFTAFYAILIFVIGGLYLVVPLVRLVGTFCTTDDDVYTFDFERRLLPIR
YPFRVDNILMYITCTAFEVISVFFLIIQYTSGDILFFQSTTILSLLLQVTGKKFAEVTEWDDDNKFNRPL
LKKLNDIGKQHSELLKSCRMIEEVFNPVILLMIICSSANLCACVIAFESKLSTFELADAVILVIHFIAMI
LQPLIYCNSAENISHWTQNIYHTIYTCQWPDQKKKFKHIVFFIMMRSRHNYKFKAYGLCDVNRHLFYRLV
RTAYNIFVLLRKTNT

>XP_011297540.1 PREDICTED: putative odorant receptor 98b [Fopius arisanus]
MMLEKLKDPFLRSNFPPEFEKLFNVCRIALRASGILAINSIFYKSKETPNLRIVYYFLGILPVVFVFICE
TLSIIHFWGSNVYLALEIATSVLSGFVVIVQGFCMYYSRESFLDLIASIRDLWKARIHDIDDNAVRKVKA
AHFFTQFYAILVIALASSYTLRPFCLLLGHIVTSKNETYDLSQTAYPAIYPFRIDSLNKYVICISVEFLI
FISVGAWWIAADMVFLQLATHLSLQYQILNDELMTLATQNGSSTASNNALIHQLDSIGKRHTHLLLLSKK
FHRIFSPILLVLMLVTSANICICIINLQEALAHRNYAEVNKCVVHTAVTMIQPAIYCIYADELVERVEET
TTAAYECAWVGQSQNFQKSVRLVAMCAQKGLNFRTFGFFNVDLTQLTQVRSPE

>XP_011297502.1 PREDICTED: odorant receptor 13a-like isoform X1 [Fopius arisanus]
MFDKSEHPVPLPETDSPIDFARLFNVCRVTLRALGVFTINEIFSKSKRKIWDMKALYYIFWLFPTFFANF
SEFRSVLGFWETDVYYALEVTTAVLSGVIVMAQGFFVYHSRNDLLILIAEIRQLWQKQLARNVADVIVKK
VKRAKFFTQVYATLIILLAITYSVRPFLLLLTHVISGKNETYDMSQTVFQAIYPWKLDNFYKYIVHITAE
TVVFVNVSASWIGADMIFLQLATHLAAQYQILHDDLIAVGSGDDPATIQYTPVEQLNSMGKRHAHLLLLS
DKFQKIFSPILLVLMLVTSVNICICIINLQEELMQQNLAGVNKCAVHTIIAMIQPAIYCVYANDLVEWAE
LTAIAAYTCQWVDKTRYFRHSVRLITMRAQEALPFRIYGFFSVDLNLLTQVGPIIRIKIISIFEQRFFIV
VQKLPERDL

>XP_011297500.1 PREDICTED: odorant receptor 94a-like isoform X1 [Fopius arisanus]
MGRRKQTSVNTNNDSVYFEKLFNVCRVALRLAGIIGVDSMFSKSERRVSDLRGIYYVVGILPPILSTSCE
VRSFVHCWRTDIYVGLEICTVILSSLVVITQGFIMYLSRRELLNLISQVCELWDKQLMSIENNIVKKARK
AQSLTKVYAALVVFLGSSFTLRPFFSIFIQFITSTENDTYDLSQTAYPAMYPFATDTVGKYLACITVELL
LFVSVAAWWTGADMVFLQLATHLSLQYEILCKSLTEMITEDFAPTDSSLIQQLSSIGQRHSQLILLSLKL
KQIFSPILFCLMIVTGANICICVLSLEKEILSSNTAGIIKCLIHTSLTLIQPAIYCKYANDLNESAELIG

NAAFQCNWINKSKNFKKQLQLMTLNAQKGIAFRIWGFFTVNLNQLTQIGSTAASFFALVNSVS

>XP_011297385.1 PREDICTED: uncharacterized protein LOC105263093 isoform X1 [Fopius arisanus]
MAKGKIFPIDQSYISLNLKYIAVVGAWNPYEAGIKYQLYQIYILFYNYGIFPVKCIADLLQSLHARTLPE
FTQGFASVIVHTAGGFKTFSIVYNHPEILLLARLFNWERHMACTPQMAYFRDVLMTKAASSLRTITAVIA
GVVLLNSISDLYSTLTVDNCTITDYPLSYLPLKILSSPDNCRSVAVVDYLCHNFASANIIIIDNFLMAVM
TYITVQLKILNFRLKNCHKTFVEELIIPEEERNFKITEYECCRMSKGQLTLKPNEELIKCIENHQKMREI
FKTFELIYSNILLPQLFSSLMILSLMGCQLVLTKYDTDEEVHDMVHPLVFVIWGTMELYIYCAGGNNILV
ESENISSSAYNTEWYNNDKEFRENLSIFLALVKKPLGLHIGGVVSLSKDTFKNILTKAYSFMAILKNSTD
Q

>XP_011297384.1 PREDICTED: odorant receptor 22c-like [Fopius arisanus]
MGRTESQVDKKKVFPIDKSYLCFTLNVLSCLGIWNPSMEKGRKYWLYQAYTMYYVIVIMLLRILTILVRA
INASNSTEFSQEISLLAVLICDAIKSFTIIVHRPELLTILGIFNWERKILATDQIKYYRNRVITTAMLAT
KIFTIAVAIFLTNYFLFYFYVIWKYSDYEIKKLPIGSPPLKYCFILTNYWVAISIDFVIFTALGLSAVCH
DAFIMGVMIHVMGQLKILNFRVERSSMTSLIKTSDDEVEENSKVIQFECVERESNESVRLDPNEELINCI
RHHQQILKILEIFRKVYNVLLLPQLSVSLILITISGLQMILVKDGGNLDGTLVALVFCITAVIQLFAFCW
GGNIILVESDKTSFAIYSAQWYDRDIVYRNNIKIFLSLVRNPLIVSAGGLFDLSAVTFKNILTKAYSGVA
VLQNVQD

>XP_011297383.1 PREDICTED: odorant receptor 45b-like [Fopius arisanus]
MSASGNGIRTKSVFPLEKSYLYFNFKCMTLLGLWNPYENGFKHWLYLIYTIWILGVIVIMRTISTFVRTL
TADTLIAFNRESSVFAAEIAQIIKCTAFLLHRAEISELSNLFNWERRALSGEELRNWRDEVLSNSLIVSK
TFTIVIIISAIQYFVFYFYSAFVQSDYQVEKLPMGSVPTKYLIASREFWFVFVVDYLLGTLQGIIALSQD
AFLMTVMINIAAQLKILNFRVERCHVAHPVIEIKFNHKNLRIHFESIQDDNSPLVDPTQKLINCIKFHQQ
ILKMLILFKVIYNKILLPQVLISLSMIVLQLQLIMGKQMDGSDALVAYLFLFGVIVQLLAYCWGGNLILV
ESEKTSDAIYASHWYNQNKIFRNNLKIFLTATKNPLVVTAGDLIALTAETFKNILSKGYSGAALLKNFE

>XP_011297381.1 PREDICTED: uncharacterized protein LOC105263090 isoform X1 [Fopius arisanus]
MTTTEAGTKRERVFPINKSYLYFNLNCLTVFGIWNPYKEGLKYWLYRIYSTYFVGVVMITRVLSLAVQAM
TADNASQFLQDFSLLAVETADTLKGIAFLIHRVEVLELSKTFNWERHLSSTRQMTHYRNEVLTNSLHTSK
KFTTTIIIALIQYFTLHFYRTLCQSDHKVENLPIGSIPLRYFFILSNFWVAFICDYVTFTSLGLVAVCHD
SFIMSVMINITAQLRLLNFRLEKCHMATITIRSGDANETSSRIHFECVEKEMSGESLVLDPNEELVNCIK
CHQQIVRMVNIFKHMYNKVLLPQLFNSLLLITISGLQMILGQQGNLTDSLVACGFLIPVILQLLAFCWGG
NFILVESDKTSYSLYAAHWYNNDKIFQNNLKIFLGAVRNPLIVSAGGLVDLTAETFKNILTKAYSGVAVL
KNVND

>XP_011297380.1 PREDICTED: odorant receptor 42b-like [Fopius arisanus]
MEKGRKYWLYQAYTMYYVIVIMMLRILTVLVRALNTTNSTEFSQEFSLLVVLICDAAKGLTFVFHRSDVL
KILDIFNWERHILGTEQIRHYRNKVITTAMLASKIFTIAVAIILTQYFVFYFYVIWKYSDYDIHNLPIGS
PPLKYCFILTNYWVAISIDFVIFTSLGLNAVCHDAFIMGVLINLMGQLKILNYRVERSSMTTLSKCSDEE
IEENSRVTQFECAERNSSDESTILDPNEELINCIRHHQQILKILDIFKHIYNKVLLPQLSISLLLIIVCG
LQMILIKEGGSLDGTLVALGFMLSAVLQLFAFCWGGNIILVESDKTSFSIYSSQWYNQDTKYKMNIKIFL
SLVRNPLVVSAGGLFDLSAVTFKNILAKAYSGVAVLQNMED

>XP_011297377.1 PREDICTED: odorant receptor coreceptor-like isoform X1 [Fopius arisanus]
MSSKTELAPKQKNVFPLTKSYLAFNFKWLTILGIWNPYKTGPKYWIYQSYWVYVVVVVMAARLFTLLVRT
IIAENAATFLNEFSLLAVELVDTLKYIALAVHEKEVVELSEAFNWERQLPGADRFKYYRNKVVTRALSSS
RTFTIAIMVALVEYYPFYYYSILTHSDYKVEKLPVGTPPLKYCFITSNFWAAFIFDWFTCTWLGLLAVVE
DALIMAVLINLTAQLEILNYRLERCHLTTYVLHDDKDNYDKSHTFFEHFDGTIENSSDDALVIDPNEELI
NCIKFHQHIKRMLKLFGTIFDKALLPQLFTSLLLITLLLLQMILGQQGNLLDSIIAMAFLFCVLLQLLAF
CFGGNFILVESDRCSLAAGSSQWYNMDITFRKNMKIFLGAVRNPIIVSAGGLVDLSAETFKNLLTKAYSG
SAVLQNVSS

>XP_011297376.1 PREDICTED: odorant receptor 47b-like [Fopius arisanus]

MSTVKSNMRERVFPIDKSYLYFNLNVLSVMGIWNPWEEGYKYWVYKIYEWYVIIVIMAMRLTAILVRGIS
AEDTTQLLQEFSLLAVEAADTIKYIAYIFHREEVLELCSAFNWDRHLLGTEEITQFRNKVLTHSLSSSKT
FTIAIVVAVILYYCFYYYAALGYSEYQLDKLPIRTVPLKYCFILTNFWVAFICDYITFTWLGLIAVTHDA
FIMALMINITSQLEILNFRLEKCHREIFEISNDKIPQVPNLIHFECVEREMNGKSVTLDPNAELINCIKF
HQHIIKMLNMFRAIYDKALLPQLLISLLLVSMSAFQMILGQQGDQSTSDSVGALVFLIACILQLLAFCWG
GNIILVESEKTSDSLYASHWYYRNTTFRNNVKIFLGAIKNPLIVSAGGLVDLSAETFKNILTKGYSASTV
LKNTA

>XP_011297374.1 PREDICTED: odorant receptor 22a-like isoform X1 [Fopius arisanus]
MFFNIRTSSLILTFGMARKIFFPIDKSYLHFNLQCLSVLGLWNPYKRGAKFWLYQIYWAYVVNVIMAMRL
INIIVLLINAPNSTLFLQQFSLLTVESANIIMYIAFFFHRTEVWELADAFNWERHLPGTEEITQYRDTVL
TNLLSSSKTFTVSIGSAVIPYFIFSYYAIFEIAEYKLERLPIGVAPMKYSLVSYNFWVAFICDYWTFTQL
ALTAVIQNAFIMAVMMNVKAQLKILSFRLQRCHIRNYEINNGATDEHPGVIHFECVERQIDEKSRSLDPN
KELINCIKFHQEIVRIFRIFKEIYNQALLPQLFIMLLLITVLLLQMVLGKEGNHADAIVALEFLIPVMLQ
LLTLCWGGNISLVESDKLSNSLYEARWYDVDREFRSNVKIFLGAVKDPLIIRAGGICDLTAVTFKNILSK
AYSGVAVLNNMSQ

>XP_011297373.1 PREDICTED: uncharacterized protein LOC105263084 [Fopius arisanus]
MIPPKVDMQKNCYPLNKSYLHFNLRCLTWFGIWNPYEREDGKKYWLYQVYWVFAVNIIMAMRVGNIFMEQ
VHVENSAEFLDAFSHLGVIIADVITYLAFFFHRVEVWDLAEAFNWERHLPGTHEISRYRNTVVTSSMNSS
KLFAIVIFISPIQYFTLAYYSIFYEADYNIDNLPIGIAPMKYSLISKNFWFAFFWDYLSFTHLGLITVGQ
NAFIMAIMINIKAQLKMLNYRMERCHLTTYEMDNSGDYDDSDVIHFECIERPLTDKSVSLNPNEELINCI
RLHQQILWILETFKTIYNKALLPQLGINLLIITVILLRIALGRRNNSGDAVIAIGYLLPVVLQFFTYCWG
GNIILVESDKSTNSLYASHWYNCDKEFRENVKIFFDVIRNPLIIKVGGLYDLSALTFKNVITKAYSGVAV
LQNMSE

>XP_011297372.1 PREDICTED: uncharacterized protein LOC105263083 [Fopius arisanus]
MVAPKIVMKRKNGFPIEKSYLHFNFRCLTWLGIWNPYEKGIKYWLYQIFWAYVVIVVMALRLMNILVRGV
NADNSAQFLQEFALLAVETADTIKYIAFLFHRDQVLELSDAFNWERHLPGTDEIRQYRNRVLTRSLRSSK
TFTIIIIITLLQYFTFSFYSAFCEADYKVEKLPIGVPPMKYCFILKNFWFAFVCDYISFTQLGLIAVAHD
AFIMAVMINVKAQLKILNFRMERCHLVVHEDPKDDEESDIIDFECDVKRNSKECMSNPNEELIDCIKFHQ
QIVRTLGIFKKIYNKALLPQLTISLLLITVLLLQMILGREGNSADAVVAVGFLIPVLLQLLTFCWGGNMI
LVESDKSSNSLYMSHWYEGNRTFRNNVKIFIGAIRKPLIVSAGGLCDLSAVTFKNILSKAYSGVAVLQNM
NE

>XP_011297157.1 PREDICTED: uncharacterized protein LOC105262946 [Fopius arisanus]
MQYVTDYHHDYQKKITDTGVQTLLKTTINNEKILLYFFIVLAGFLVIALMLFVPKAPGELPIKTSFPFDT
TISPGHEIAMYLQTAAVTFGLYSIVAMDSLAINICRCLSIQLLALSSNYEKCMIQTYDRCHLITSRSIKP
SKRSDLQSREKTIVIRKFALFRKQEEREESDSFVKRFRLCEIHHQRIISMINEFNSIFSSCMLMQIFSSF
SMICFAGFQAVLVSGC

>XP_011296908.1 PREDICTED: odorant receptor coreceptor [Fopius arisanus]
MMKTKHQGLVADLMPNIRLMQISGHFMFNYYGEGKKLMHKVYCSVHLFLIVLQFALCGINLAMESSDVDD
LTANTITVLFFLHSVVKVIYFAVRSKLFYRTLAIWNNPNSHPLFAESNARYHSIALTKMRRLLFCVGAAT
ILSVLCWTGITFFEDPHKKIIDPITNETTMVEIPRLMVRSFYPFDARHGVAHIAMLVFQFYWLLVCMVDA
NSLDVLFCSWLLFACEQLQHLKAIMKPLMELSATLDTVVPNSSELFKAGSADHLRETNGTTQPSATPQQG
DNMLDLDLRGIYSNRQDFTATFRQAAGMQFNGGVGPNGLTKKQEMLVRSAIKYWVERHKHVVRLVTAIGD
AYGVALLFHMLITTITLTLLAYQATKVNGVNVYAATTIGYLLYSLGQVFLFCIFGNRLIEESSSVMEAAY
SCHWYDGSEEAKTFVQIVCQQCQKAMSISGAKFFTVSLDLFASVLGAVVTYFMVLVQLK

>XP_011315403.1 PREDICTED: odorant receptor 67a-like [Fopius arisanus]
MILSLNFFVLQIMGFWRPSEGSPLKIIFYKFYTCLMCLILYSVTLTQLIEMIRSPGTVDDVINNSRVLLT
MMNACAKSLSFVARRDDILKAIGIITSPPCCPRNSAEAVIQERYDRSIRMNSLIYGAFIQTSVNTVILQT
ALECIPIRILPFKCWLPYDINSETTFWLTYIQQSVATYVTSYVNICYDTIVPGCMVQTCAQLDIFKSRLK

DLSGHNHRSHGKDVETHGDKNKILVSDCVEHHLNILKFAELSNNIFAPTIFTQFAISSLVICVSVYDLST
AAPFSPDFVEVMLYLMSMMLEIYLFCFYGNNVTVQSNHVGGNIYDLDWTSLDISVQKSLLIVMTRAMKPL
TFTSGHVVVLSLASFTSLLKMSYSAYNVLQQAS

>XP_011314698.1 PREDICTED: odorant receptor Or2-like isoform X2 [Fopius arisanus]
MEDIFRDSPFYFYIRVMKWFGQWPFQSKMESNIRKYLIISLLLSIFVPSIIKFYEFRNDVYKMMDCIPML
GLHLASISKFINWSYHHEKIEQLLMHMQYDWSNLRNKFDADILEKFRRPSQILYVIYAISVTCLVAFDQM
CAMFIQHACGMLEILKMHLQNLHATFLANDGTALSLREEAVAKEIIYCVRIHNRIFKFLEIVEHWDQVVL
LIVGAGNTMTLTACGVGAILNKPDFLGFVRLTLFNFGGTMHLFYNCWQGHLILEQGESIFILAYQNEWYE
LPCHLQRMLLPIMAKSMKPREITACKLFPMSLATFGMAMRASLSYFTLFKSMK

>XP_011314697.1 PREDICTED: odorant receptor Or2-like isoform X1 [Fopius arisanus]
MEDIFRDSPFYFYIRVMKWFGQWPFQSKMESNIRKYLIISLLLSIFVPSIIKFYEFRNDVYKMMDCIPML
GLHLASISKFINWSYHHEKIEQLLMHMQYDWSNLRNKFDADILEKFRRPSQILYVIYAIIGIYGTTAVYL
VTPLVPIILNIVLPLNESRPHVYVYHTEYFVDQNKYYYPIQLHAYMTIPVSVTCLVAFDQMCAMFIQHAC
GMLEILKMHLQNLHATFLANDGTALSLREEAVAKEIIYCVRIHNRIFKFLEIVEHWDQVVLLIVGAGNTM
TLTACGVGAILNKPDFLGFVRLTLFNFGGTMHLFYNCWQGHLILEQGESIFILAYQNEWYELPCHLQRML
LPIMAKSMKPREITACKLFPMSLATFGMAMRASLSYFTLFKSMK

>XP_011314327.1 PREDICTED: uncharacterized protein LOC105273529 [Fopius arisanus]
MGNSLGNHRAPLGASLFTLRFCGLWSSSDDSHPIKKFIYPIYSMVMTSLIWIFIATILGDLFSNFEDLLV
ITDDGCFLAGISVIVFKHIIFCLRRREIIKLIHEIYRPVDYLAKSSDEGALILVKVSTFYDELHCYSFIG
IGCCLVLALVTIVPTDNGSLPIRAKYPFDSTIEPLHSIAFVIQASAVATGVAGILGMDGVVTSICRYITL
QLEILGSNYRHCQTKWPRGEIHLSIGKPKSRETEINCFIPFSPREAHEANDSFVRRFKICIRHHQRLIKI
VNNVNVVFGSSMFCQLFASSAMICFTGFQAALGARESANLIKFAMYCGTAFSQLLSWCLIGNMLLHESLT
LTNSQWQSGWENEQYFDFAYLIIFAMVRGNKSLELRAINFYSVSMDTFIVILRASYSFFTLLVTVV

>XP_011311721.1 PREDICTED: odorant receptor 46a, isoform A-like isoform X1 [Fopius arisanus]
MSLFSLSIFVFRILGLWFPDENPSTWRTKLYRIFNIFTVTMMCTFTLTQFLALPSCIHDAEELTNASFML
LTMIAVTGKMWNMTLYRKEISRMVKLFDLEPFKPLDDHEVAIKRKVRRSIRLFTIMYGTLTEVTCSMLTL
SSFIRDIPRRQLLFKSWYPFDRSTLIGFWIAYVHQTISHYIGANVNVAFDTFIPGLMMATTAQLEIIKYR
FITMPKTIENKCSESVCEVNEDELMKIETKYLADYTRHHLKILRFSKTTNNTFTGSIFLQYCASSLVLCV
SVFSLSTLKPLSKEFNSLVMYVACMLVQIFIFCDAANEVTIKSGTIADAIYEMDWTSLSTTSKKSLVLIM
VRTLRPIMYSSGHVVSLSLVSFSSLLKLSYSVFNILNHSSQGST

>XP_011311655.1 PREDICTED: odorant receptor 46a, isoform A-like [Fopius arisanus]
MVKIFDLEPFKPLDEPEVAIQKKVHRSIRFYTILYGTLTNTTCSMLTLSSFIRDIPRRQLLFKSWFPFDR
TTPVGFWSTYVYQTIYHYMGANVNIAFDTFLPGLMMATTAQMEILKYRFITLPRMIENKCFESVCEVNAD
ELIKIETKYLADYTRHHLKILRFSKATNDTFTVSIFLQYCASSLVICVSVFSLSTLKPLSKEFNSLVMYV
ACMLIQIYIFCDAANEATIKSGTIADAIYEMDWTALSTTSQKSLVLMMARTLRPIMYTSGHMISLSLVSF
NSLLKLSYSIWNILNRSSQRSD

>XP_011301555.1 PREDICTED: uncharacterized protein LOC105265655 [Fopius arisanus]
MNENMQRLARIVSFAIFMCHLTICIHYVRPLLVNRGRNKSDREFPFRLYSNLPIKLSPWYEILYIAQVFS
TWPCGYTYFCFDNFLCLMNITATAQFVILQKELREICDDVSEYDKSNEHLIRSRFTRCISRHQKLIAFVE
DIKELYRSVMVGVVILLSFLICLEMFQLMTVRSSFTKFYSIIHEM

>XP_011301542.1 PREDICTED: odorant receptor 13a-like [Fopius arisanus]
MIFFTGAFGMYKVSSFTIKRKLFLNLIRYAQKHFWYVEYEDYGNAVMRECMRRLTGIVTFAIFMCHLTVV
LHYITPLIENRGRNESDRIFPFRLYSDFPITLSPWYEILYIGQVIATWPCCYCYFCFDNFLCQMNITAVA
QFVILQKELREICDNENQSTVLNGNHVRLRFIKCIQKHQQLIAFVEAIKDLYRSVMLGVVILLSFLICLE
MFQLMTSTNSSRISTFHYCVYVGGSITQLYFYTMTCDNLTGASLAISDAAYDVRWFLIECETTRKRLVKD

LSMVNMRAQKACTLTVGGFAPVTLQTFTSICHTAFSFLALIRQTLQEK

>XP_011301142.1 PREDICTED: odorant receptor 67c-like [Fopius arisanus]
MPFEFGPIDVDTWYYYLWFGTTCGIFTRLTTIGACDLLLFTLSEHANGFLKALGYATENLPPYDESSSED
ESFNYLRRCVIIHDRAINFAESVRDVYMWSFFGIIGMNMLIMSVTGIQIVNSLDNSKKLIKFSVNMLTQM
LHLFVECFVGQRVMDASFDLKETITNAKWYNASKKTQKMIPLMLLRAQKPVVLTAGKVFFMCMSTYAVVL
KTAMSYFTVFLAVR

>HsOr2
MTNTIVLKNVKSKSDHDYSLQLNRWYLKPIGARPLTDASNVIGRLIVLLQNIICSIVCFITIPCFLHMILEAE
DVQSKLNVFGPLLHRIMGLMNYMTLLKHRSDIRDSIQQMEKDLLVERVDDREIMLQQAKLGRLIALISGIIMQ
GGTLLYSTAKAMKPVTIVVNNDTLTMYPMTCPSYRKFIDTRFSPTNEIMLVMQFMSTLVVNSSTVGVCTLAAV
FATHIYGQLNVLYKRLDELDKEDERGNNTTKENMIDIVKHHLRILSFITRMENIFHDASLVEVTGSTITMCLL
GYYSIMNWANFDGAKIAAYVIVYVSQGFNLFIFCYIGEILTEQCKNVGQTAYMTNWYNLNKNTARGVILIIPR
WNKLIRITAGKLFQLSIATFGNVLRTSIAYLNILRTLT
>HsOr3
KQIDFENVNDYSLQINRWFLQPIGAWPQINSSSMVKRIFMSIQIFTCAMTVAIITVPCILYVSFEKESFKSKL
NVMGSLLHRIMGTVNYYVLLKRSKDIHDCIRHMEIDWQIVQRINDREVMLQHAKIGRFIAGISAAFMQGGAIL
FTLGRAMKTTTFVIDNETYTMHPMTCPAYSKLINTRFSPINEIMLIVQVFSAFVVSSSTVGMCSFAAVFAMHA
CGQLSVLYTWLNELVDDDDKEERSINKNLAVVVEHHLRILSFIARMENIMYKACFTELMGSTLNMCLLGYYFI
VNWSAFDATKVVSYIILYLSMCFNVFIFCYVGEILTEQCKHVGEMAYMTNWYKLPKKSALCLILIIARSNNII
KITAGKLFHLSIATFGDVMKTSMIYLNILRTMTT
>HsOr4
SQQDASDYSLQLNRWFLKSVGAWPRSATTTVGEKLRSTIQIIICYSLIAFVVVPCILNILFEETDIHMKLKAV
GPLSHWCMGGMNYFSLLFRSREIRRCMQHIKTDWRTVTEIEDRRVMLKYAKFGRFVASLCAVFMHSGVFSHSL
LQGITPTIAYIGNKSVIVHVLPCPSYSKFVDIRYSPEGEIALVLQLISSIVVNSITISACSLAAVFAMHACGQ
LNVLMRWLDHLVDDRRQFDAVQRRLAIIVEHHLRVLSFVMRMEALLNQICFVELLGCTFNLCMLGYYTITSWN
VIEKQTSIAYMIIYMSMSFNIFIFCYIGEVLTEQCKQIGETAYMTEWYRLPHKTALGLILIISRSSMVIKLTA
GKLVHLSIATFGDVMKTSMIYLNMLRTMT
>HsOr5
MTKHGRMPTDIEFAYKSNNDYSLQLTRWFLMPIAAWPYASTSTIRRLSSRVHILTCIFLITIIMVPCLLYVSL
EEKDVQMKLIAMGPLSHWIMGTINYWLLLTRSGDIRECVQHMEMDWKLARKMSDQGVMLRYAKIGRFVAGFCA
IFMQSGTFLFTVAKAMTSMPVVIGNETMSMHPMTCPIYSKFIDTRFSPANEIMLAVQLVSCFIVNSVTVGACS
LAAVFAMHAYGQLSMLFSWLNKLVADEDKENDLANQRLAAIVDHHLRVLSFISRMENIMQYICLVELVGCTMN
MCLLAYYSITNWSDFDGAKITSYIMVYISMAFNIFIFCYIGEILTEQCKNVGEQAYMTNWYELSHKTALGLVL
IIARSNNVIKVTAGKLFQLSIATFGDVIKTSVVYLNILRTMTP
>HsOr6
MTASERVLSNTGITCKSNNDYSLQLTRWFLMPIGIWPRTTTTTRMARLSSHAHILTCSSLIAIIAVPCLLYVS
LEEKDVQVKISIFGPLSHWFMGMINYWLLVAHGDDIQECVRHMEADWRLVQKIEDQEVMLRHARIGRFVSGFC
AVFMQSGTFLFSIAKSLTTTIVVVGNETVSMHPMTCPIYNKFIDVRFSPANEIMIVVQLLSCFVVNSVTVGAC
SLDAVFAMHACGQLNMLFSWLNGLVVDRNEGGSADQRLALIVEHHLRVLSFISRMENVMQHVCLVELLGCTMN
MCLLAYYFITNWNTFDAPKTMSYVIIYLSMAFNIFIFCYIGEILTEQCRSVGEKAYMINWYELPHKTAFGLVL
IILRSSNVIKMTAGKLFQLSIATFGDVIKTSMVYLNMLRTMTPS
>HsOr7
MCQSVIRDLHDHSVQLNRWFLKPIGAWPRSSVSTRSEKAVSRALIFTCYFFIAFTVIPCALNVFFEEKDLDLK
LRAIGPLSHWLMGGMNYCSLLLRNADILRCVQHMETDWKVIKRPQDRDIMAKNAKLGRFMAGFCAVFMHGGVF
SYSIVSGMTKIEVSVGNGSVSMLQLPCPFYSKFVDTRFSPANEIVLLMQLISCFIVNSTTVGACSLAAVFAMH
ACGQLDILNLRLNKLVESQDAKKNESAQQRMANIVDHHLRVLRFIAYIEDVMNQICLVELVGCTFNLCMLGYY
SITWWNKIDTKSIAAYIIVYISMSFNIFIFCYIGEILTQQCKKVGETAYMTDWYRLPHKTALGLILIISRSST
VIKITAGKLIQLSIATFSDVIKTSLVYLNMLRTVT
>HsOr8
QHNDYSLQLNRWFLKPIGIWPASTSTEKILSHAIQFICYSLITFTVIPSVLYIFLEDQDVDIRVKAIGPVSHW
MMGILNYLSLLLRGDEIRRCIEHMEVDWQLVNRAEDREVMLKSAKFGRFVAGFCAVFMHCGVFSYSMMKGMSS
LVIVIDNQTVTIPRLPCPFYSNFLDTTVKLTNQIVLMIQFLSGFIVNSITVGACSLAAVFAMHACGQFNVLFL
WLNELVGDDGEEERKLVEYKLANIVQHHLRVLSFVSRIEQVMYQICLVQLVGCTLNMCMLGYYSLTEWNTEDT
KNLVTYGILFVSITFNIFIFCYIGELLSEQCKKVGETAYLTEWYRLPQKTALSLVLIISRSSAVIKMTAGKLI
ELSLITFGDVIKTSLAYLNILRTVSL
>HsOr9

MRSSGKITADVPRRNNNYSLQINRWFLRPIGAWPELPTSSATERTLSKILRLICHILIALTVVPSILYILFEE
KDFRLKLKAVGPTSHWLMGGINYCSLLYQKKQIRRSIEHMETDWRMVKRERDRELMLRNAKVGRAIASLCALI
MQGGIFSYNVARGTSPILVVIGNETVAIGRLPCPSFNKIVDTRFSPVYEVVLTLQCLSTIVVNNTTVGACGLA
AVFAMHACGQLSVVMSRLEELDGEKDECHIVQRKLANLVERHLRALRFLSRMEAIMRQVCLVELLGCTFNLCM
LGYYTITEWHEESTNTLITYLIVLTSMMFNIFIFCYIGDLVTEQCKKVGEAAYMTNWYQLPRKTVLGLILIIL
RSSIVIKITAGKIFHMSIPTFGDVIKTSVAYLNMLRTLTV
>HsOr10
KSQPVTVMYDHEKDMQLSIQLNRWILKPIGAWPRSADSSWIKRCVHILVNLIYTCLIGFIFVPCAFYIVLEIE
DTYNTLKFTGPLSFCIMVIAKYYSLILHSSNIRRCIDYIKDDWINTRHHGDRMIMIRNAEFGRRLVAISAVFT
YGGAVFYHIAMPISIGKVTEEAENLTYRPLVYPVARAIVDTRQSPVSEIFFCIQCVAGFFTQAVTASGSSLAA
VFAMHACGRLEILMQWIVHLVDGREDLCDNVDERLTMIVQQHVRILRFISLTEKVLHEISLVEIVGCTVNMCF
LGYYVIMEWETGELASYVTYTILLISFTFNIFIFCYIGELLAEQCRKVSEISYMIDWHRLPGKRALAIVLLIA
MSNSSIKLTAGNLVELSLSSFGDVIKTSVAYLSMLRTLTS
>HsOr11
MKCVSHEPSLDHYRRDNDYSLQLNRWVLKPIGAWPELPTNSLIRNMLKKLLRFTCHSLIAFTMVPSILYILFE
EKDFRLKLKAVGPTSHFLMGSINYCSLLYQNERIRRSIEHMETDWRIAKREHDREVMLRNARIGRIIAGICAL
IMQGGVLCYNLARGMSPIIMTIGNETVAIGRLPCPSYNKIVDTRFSPVYEVVLVLQCLSTIIVNNTTIGACGL
AAVFAMHACGQLNVVMFRLEELVDEKRDILQLRLANIIERHLRALRFLSRMETIMRQICFVELVGCTFNLCML
GYYTITEWHEESTNTIITYIMILTAMMFNIFIFCFIGELITEQCSKVGEAAYMTNWYHLPHKTALGIILIILR
SSIAVKITAGKIFHMSIATFGVVIKTSVAYLNMLRTLTM
>HsOr12
MSHSFRVFPKHNVLYEQDSEYSIQLNRWFLKPIGAWPDDANQPVSLAKKLLSRCIQFICHSLIAFTVVPCALY
IMFEPDVHLKLKAFGPMIHWLMGGMNYCSLLLRSYEIRKCVDHMRADWRLVKGTHDREVMLRNAKFGRFVSTF
CVIFMQGGVCSYSIITSFTPAIVRIDNITIAMHQLPCPFYTELVDARYSPTNEIVLGLQLLSTFIVNTVTVGA
CSLAAVFAMHACGQLNILMLRLDELVDVSSAKDEYESTRRKLAAIVEHHLRALSFVSQIETIMHQICLVELLG
CTTDICMLGYYTITEWELHDTKNLLTYFTIFIAMSCNIFIFCYIAEILTDQCQKVGDVAYMTDWYQLHHKIAI
DLILIISRSNTVIKITAGKMIQLSIATFGDVMKTAFAYLNILRTVA
>HsOr13
VATVHDYKKDMQQSIQLNRWIMKPIGVWPRSIESSWAEKSSYILVNVICMSLIGFLFIPCVIYLVLEVEDNYD
KLRLSGPVNFCIMVVFKYFSLIGRENDIRRGFKHIENDWMNTQYYSDRNIMIRNAKFGRRLVVICALFMYGGA
MFYYVAMPFSHSKITEPGSNLTYRPLVYPAARLIVDARYSPVSEIFFLLQCVSGFVAHSVLTVACSLAAVFVV
HACGRLKILIKCIEHLVDGREDFCCAVDERLAMIVQHHVRVLRFVSLTDRILREISIVEIVGCTSNMCFLGYY
VITEWETGELTNYVTYTIILISLIFNIFIFCYIGELIAEQCKKIGEMSYMIEWHRLPKKKGLALVLMIVMSNS
SIKLTAGNVFELSLSTFGNVIKTCVAYVNMLRTLTS
>HsOr14
VATVHDYKKDMQLSIQLNRWIMKPIGVWPRSIEISWAERLVYMLVNVICVSLIGFLFIPCAIYIALEVEEIYD
MLRLFGPMSFCLMAVVKYSSLICRENDIRRGIEHIENDWMNTRHYDDRSIMNKNAKFGRRLVAIFAFFMYGGA
VFFYLSMPFSHGKITELGSNLTYRPPVYPVARLIVDSRYSPVSEIFFSLQCVSGFLTHSITTVACSLAAVFVM
HAYGRMEVLMKWIEHLVDGREDFCSNVDDRLAMIVQQHVRVLHFVSLTDRILREISIVEIVGCTLNMCFLGYY
VIMEWETVELASYVTYTILFTSLTFNIFIFCYIGELIAGQCKKIGEMSYMIEWHRLPRKNGLCLVLMIVMSNS
SVKLTAGNYVELSLNTFGDVVKTSVAYLNMLRTLTS
>HsOr15
MVSTVHDYKKDMQLSIQLNRWIMKPIGVWPRSIEISWAERSVYMLVNVICISLISFLLIPCAMYITLEVEDTY
DMLRLSGPVSFCFMAIIKYSSLICHEDDIRRGIEHIEKDWMNTRHYGDRNIMIRNAKFGRCLVAICAFFMYGG
AVFYYLAMPFSHGKITELDSNFTYRPLVYPVAKVLIDARYSPISEIFFWLQCVSGFLTHSVTTIACSLAAVFV
MHACGRMKVLTKWIEHLVDGREDFGSDVDERLAMIVQQHVRVLRFISLTDRILREISVMEVVGCSLQMCCLGY
CIIMEWETGELTNYVTYTIILISLTFNIFIFCYIGELAAKQCKKIGEVSYMIEWHRLPRKEGLAIVLMIIISN
SSAKLTAGNLFELSLSTFGDVIKTSVAYLNMLRTLT
>HsOr16
VAAVHDYKKDMQLSIQLNRWIMKPIGVWPRSIEISWPEKSACMLVNVICISLIGFLFIPCAIYIVLEVKDTYD
MLKLSGPLSFCLMAIIKYSSLICRENDIRRGIEHIENDWMNTRHYDDRSIMIRNAKFGRRLVIICAFFMYGGA
VFYYLAMPFSHGKITELGSNLTYRPLVYPVARLIVDARHSPVSEIFFWLQCVSGFLAHSTTTVACSLAAVFVM
HACGRMKVLMKWIEHLVDGREDFCNDVEERLAMIVQQHVRVLRFVSLTDRILREISIVEIVGCTLNMCFLGYY
VIMEWETGELANYVTYTIILVSLTFNIFIFCYIGELIAEQCKKIGEMSYMIEWHRLPKKKGLALVLIIVMSNA
SIKLSAGNLFELSLSTFGSVVKTSVAYLNMLRTLTS
>HsOr17
VVTVHDYKKDMQLSIQLNRWMMKPIGVWPRSIDVSWAERSVYILLNVICVSLIGFLFIPCAIYIALEVENIYD
MLRLSGPVILCVTAVAKYFLLIYRENDIRRNIEHIENDWINTQHYDDRSIMIKNAKFGRRLVMICAFFMYSGA

VFYYLAMPFSHGKITELGSNLTYRPLVYPVARMLIDVRYSPISEIFFWLQCISGFLAHSITAIACSLAAVFVM
HAYGRMEILMKWIEHLVDGRKDFGTDVDERLAMIVKQHVQVIRFVLLTDKILREISVTEIIGCTLQMCCLGYC
IIMEWQTGELANYVTYIIILIALTFNIFIFCYIGELIVEQFKKIGEVSYMIEWYRLPRRKGLALVLMIIISNS
SIKLTAGNLFELSLSTFGDVIKTSVAYLNMFRTLT
>HsOr18
MESPPRYPDNRSGPYNQHYKDDLHVSVQLNMWTLKPIGTWPKPVNYSWLEMLWCRLLNLSCYLLLAFILIPCS
MYIVLEIKDFYNQLKLGSALSFFMMAVIKYCALILRENDIRRCIEFIEGDWKNVRCAEDRKIMLENANFGRRL
VVICGFFMYGSVLFYYVALPLTRAKIVDEDSNMTYRRLVYPVPKMIADTRRSPINEIVYIIQLLSGFVTHNIA
VAACGLAALLAMHACGQLQVLMSWMNHLVDGREGVNDTLDERLANIIQLHVRILNFISLTEELLHEISLVEVV
GCTLNICFVGYYCMMEWDFKQPVSGLTYLILVISITFNIFIFCYIGELLAEQTTKVRENSYMIDWYRLPAKES
LAIILIICMSNATTRLTAGNIVELSISSFGDVIKSAVAYLNMLRTFAT
>HsOr19
MFDFMSLRNSNYKNDFRYTVQIHKFILGLIGVWPVLEQSWHRKRLLKRLLNCVCYFLLSFSLIPWALCMFLII
DTFKARLKMCGAFCLYTMAPAVYCTLVYQEDRIKECLRHVEEDWRNVRDVNDRKIMLDKAKAGRFILITTTLF
LFTCGFTYRLIQPIARGNIIVNENLTIRQLVQGNYYIFFDPQQSPAYEIVFSVQCMAGIVIYMITASVCGITA
LFTMHACGQLQMLISWLENLADDDEIWKNHVASRRLASIIMHHVRICKFLHRIQDIVGETCFIEIVGYTLILC
LLGYYIITGWERNDAISMLTYTIMLISFTFNIFILCYIGEVLSSQGNKLSTTCCTVNWYCLPNKKARYFIFVI
AMANCPMKIRANKFIDLSFSSFGAVIRTAMAYFNLLRTVTM
>HsOr20
MDQSVKRSAKTIWTDRQENDLNYAVQVSRVILRMIGVWPISKIASNAERIAIRLLNVSCYLLFAFILVPGLLL
IFLKERDLKRRVRLLGPLLNCLIGCVKYSLLVCNEKKIQACLEQVRQDWQHIDNWDDRKVMLSKAKMGRKLAL
FSAAFMFIGGLSYRTIVPLSKGRMLTPMNSTVRALACPSYFVKFDEQASPTYEIVFTLQFFAGLITYSVTSGA
AGLAAFLIMHVCGQLAILIGKFQRLNNMPQPEDRAVATLLADIVEHQIKIRSFLKQVEEALRYIWLVEIMGST
LLLCLVEYYVIMEWEGSDFTATLTMFVMLTSFTFSIFTVCYVGQLLTDQSVKVGLMTSTTNWYRLPHKRARAL
ILIMAISNIPTKISAGRLIEMSLPTFSNIVKTSMAYFNLLRKFI
>HsOr21
MHINRPHHDDIRRIVQVTRAVLNLIDVWPSFGDSSAFRLVMKSLRVLCQGLLYFIFVPGLLKIFLKELKARRR
LKAIGPMCNYLMAMHVVLICWNNRIKDCIRHIEEDWKQVSLSEDRRIMTDNSRFGRSLMIICIAFVYVSGFSY
RTIVPLSNGIVVTLQNVTIRXLGFSEYYVFIDPQKTPAYEIIFTIQFLSGFMQYSVTSGACSLAALLVLHACG
QFKILITRMEDLTLTKHFSEGKANGKLAAIVRQHIRIKRYFLGKVEYILQYMCLVEVIGCTFILSLLGYYIIM
EWEDSDAVSVFTYSILLVTFIFNIFILCFIGELLTDRXLRFSLNKYMYITSCTLDWYRIPYKTARSLVLVIAV
SSTPVKINAGKFMDLSLNSFGAIVRMSITYLNVLRTSSI
>HsOr22
MFANKNYESDIKYTIELNRFICRLLGIWPRMHAEAPFIENIKNILLILICYFLLCSELIPTILYVIIVEKRTR
IRLKLISSVMFTTLAVLKYCSLVFNRNRMKSCLMRVQNDWRNVASANARDLMLNKSRTARNLLILCSAFMYMS
GLYFRTVVPLSKGKFVTDQNITIRYLPCPSYFIFFDGRISPAYEIIFLIQFFSGFVKYTITVSICSLAALFVM
HMCAQLEILMMLMNNLVNEQEVKNLNRKLAMTVEYQIRTRGFLQLVQNTLEHTSLLELLGCTMIVCLLGHDII
TEWEDHNVVAVGSYLILLTSIGFNIFIFCFIGEQLSTKGEKLSMTVCTLDWYRLPDEKARALILIIAMSNIPT
KLRAGKFVDLSIRTFGDVVKTAVTYFNLIRKVME
>HsOr23
DIMYITRPTRNIMSIFGIWPSISRKKTLCGKVYKFLLITISYTLLTCNLIPGFLYWLTQGDTRTRLQMIPFLI
YDTMTVSQYGIFIIRDNQIRECLKHVQEDWENILSADVRNMMLKFARIAKRLVTVCATFMFSGAVLFRTILPL
SQGDIVTEQNVTIRPLACPSYLVFIDVQITPIYEIFFTIQCISGLITASIATGACGLTAIFVIHASGQLRILR
DLMTRLVEERWQKEYEVNKKLADIVEHQVRVRGFLRMVQHTLHQIFLMEIMVNTITLCIVMYFIIIEWQSNNT
AALCVYLLCVGNLILHLFMYCYTGEQLTEQAEKVATASCELEWYRLPDKKARYIVLLMIMSNAPTKISAGNLF
ELSLKTFGDAIKTAGAYFNMLRNITD
>HsOr24
MSHNEYLSRDIMYITQPTCIIMRVLGIWPSISRKKTLCERIYKFLLITIFCAFLTCNLISGLLYWIIQGDART
RIQMIPFLMYAATTISQYGIFIIRDDQIRQCLKHVQEDWKNILSADIRNMVLKFMRIAKHLATICGAFMFSGA
ILFQTILPLSQGDIVTEQNVTIRPLSCPSNLIFIDVQITPVYEIIFTIQCFSGVISAAIATSACGLTAIFVAH
ASGQLRILRNLMTRLVEEQWQMEYEVNNKLADIIEHQVRMRSFLQMVQNTLQQIFLIEIMVDTLSLCIMMYYI
IVEWQNTNITGVFVYLFSAGDITVHLFLVCYTGEQLIVEAEKVAIVSCELEWFRLPNKKARYIVLLMIMSNAP
TKMSAGNLFDLSLRTFGDTIKTAGAYFNMLRSV
>HsOr25
MPRNVHSRHDIVYVKQPTRNLMLAIGVWPFRSEKRSMFQKVYNFFLNFSSNLLFACEIIPGIIYLLLEESIRI
RLQLIPLLLYVFLCAFQYDIILSRNDNIRQCWKHVEEDWRNAFGTDDRNIMFRCAKAARLLILICGVVMYSGV
AMYRIILPLSRGDIVTNQNVTIRPLACPVNFLFIDVQTSPFYEIVFTFQSLTGLLIVSITTSTYGLLAYFVEH
ASGQMKILICLMKNLVQEQWQKEEVDKKLSEVVEHQVRVHSFLQMVQYTMQEICLAEIMVDTLTICLLLYFA

ILDWDTSNPGIVCSYACSIINIIIHIFLFCYTGEQLNDQAEKVAIETRELEWYHLPEKKMRSVILLMIMSNYP
PKISACKIVELSLKTFGDVIKTSGAYFNMLRNVIE
>HsOr27
MLRNVHTQNDIVYVMQPTRNLMLAVGVWAPRSGRMSVFQKIYNLFLNFSTNFLLACEFIPGSIYWLLEESARM
RLQMCPLLFYVFMSVIQYYILLSRNGRIRQCWKHVEEDWENVFSMDARNVMFKGAKSAKFLILICAALMYTGS
ITFRIILPLSQGKIITDQNITIRPLACPVNFLFIDAQASPFYEILFVFQAITGFIIVSVATSAYGLLAYFVEH
ASGQMKIQIWLMKCLVQEQRKKEHEIDKKLAEVVEHQIRVRSFLQMVQYTMQEICLIEIMESTLTICVLLYFI
ILDWDSHNFGVIGSYALNIVNVTIHIFLFCYTGEQLNDQAEKVAIESRELEWYNLPEKKMRSIVLLMIMSNYP
PKISAGKIMVLSLKTFGDIIKTSGAYFNVLRSVTE
>HsOr28
MPRNLYSQEDIVYIKQPTRNLMVIIGVWPSSNERRSLFRKIYNFVLNLVSNFLFACEIIPGIIYWLMEESARV
RLQVTPIILYAIMCAIQYDIILARKSNIGQCWKHVEEDWQNVFVANDRNIMFRCAKTARLLILICGVLMYFGV
TTYRIILPLSRGNLITDQNITIRPLACPVNFLFIDVQVSPFYEIIFTFQSLTGFLVVSTTTSAYGLLAYFVEH
ATGQMKILICLMKDLVQGQWQKEQEIDKKLAKVVEHQIRVHNFLQVVQYTMQEICLVEIVVNTLSICFMLYFT
LLDWDIKNLGVVGSYAFNIINITIHIFLFCYTGEQLNNQAEKVAIESRVLEWYHLPEKKMRSVILLMIMSNYP
PKICAGKIMELSFKTFGNIIKTSGAYFNMLRNVTE
>HsOr30
MERHLPQAHINMSYEQDIKYVMKPVQYLLRILGIWPLLERDLSTFEMMGKILLITLCCLLVCIIVLPGFLYSV
FFSEDEPHAKLMTIEPVIYSFVAFIKYGTLITYENKFKTCLRHVKDDWKFVALPSAREIMIEKAKIGRNVFTV
CCIMLYCAGLSFHTVVPLTSRRVLTDGNVTFRPLAYAGYYIVFDEQRTPAYEIMFLLQLFSGFVMYSVTIVLY
GLIALLVMHACAQMKILMMLMEELVSEQICTEENVNEKLSMVVEHQIRIRNFLYLVEDTMRHSSLFEILGSTL
MLCLVGYCILIEWRVGNAANTCTFFIVLVSDAINIFLLCYVGEHVTEASEEVAWKTHMLGWYRLPSRKTRDMV
LIIIISHIPLKITAGKFIVLSFKTFGDVVKSAVVYFNLLCTVSE
>HsOr32
VPRNEHYQRDIKNILELNRSVLLALGVWPKFDGDKSICEKINKFLRISISYFLLYFSLVPGALYWIFEKRARA
RLRTIPIMLYGFMSLGKYYSLIIYESQIRRCLKHLEEDWRNVNGMNARKVMLESARTGKRLVIISAVFLYSSG
ITLRTILPLSKGKIVTPENITIRPLPYPTHSLLFDAQSSPIYEVVFVMHCLSGLVTMSISVSICGLIIIFVTH
TCGQLKVLIDLIKDLVEKKQRDEYKVNKKLAIIVDHQTRIRSFLQLVESTLQQMCFIEITGVTVIICVLCYCI
LMEWENSNALALCSYFMALSGMIINMLMFCYTGEQLITQAEKVAVTSCELEWYRLPDNKARGIILVMIASNMP
TKITAGKLFDLSFRTFGDILKTAAAYLNVLRNVT
>HsOr33
MFINKNYERDIKYTYKLNQFVFSLLGIWPYARARTNFWLPQELKRIALLLGWYFLLCSDLISMLLYVFMVQKE
TNARLKVIAAMIYSIVTIFKYSNLVYIKNRVRNYLVRVEEDFRSVHSPAARNTMLFHMKTSRRLFILCSIFMY
SAGIAYRVIIPVSKGKIVTAENITIRPLPCAAHFIVFDPQISPAYEIVFFVQCFTAFIKNTITVAACGIAALF
TMHIVAQLDILMAAMNNLTSETKLENVNRELAVIVEHQMKTRDLIRMVQEVIQFSSLVEVVTCSFLVCFLSYY
LLMEWEDSNAIAMCSYFITILSFSFNVFIYCFIGEQLSEKAEQVAVTACTLDWYLLPDTKARALIMIMLISNV
PLTLKAGNFVDLSLRTFGLVVRTAVGYLNLLRSIMD
>HsOr34
MYTNKNYERDINYTYELNRFVFRLLGIWPYATTNVWLLQKMEKIVLVFGSFVLLIFELISMLLYIFIVLKETR
ARIKVTATLLFTIVSILKYSNLLYVKNQLKSCVTRVEEDFQSVISPATRNTMLFHARTGRRLFILCSIFMYSA
GFSFRTLIPLSRGKIVTAQNITIRPLPSPAYYVVFDPQISPAYEIVFFMQCFAGFLKYTITVAACGIAALFTM
HIVARLDILVTLMNNLTHEHEVENINRKLSVIVEHQIRTRDFLHMVQGAIQFSSLVEVMTCTSILCLIMYYVI
MEWEASNVVATCTYFIVLISLTFNIFIYCFIGEQLSEKGEQVALTACTLDWHLLPNTQARALILIIILVSNTPL
KLKAGNFVELSFKTFGAIIKTVLGYLNLLRSVVD
>HsOr35
MPNNERYQDDIIYITHATRNILNVLGVWPSLTGESSRRRICRLLLITISWMLLYCVLIPGVLFCIREKRMRIK
IQTIPLLIFGFMAVGKYGNLILREGQIKRCLKHIEEDWKNVISLDARNTMIQSAKTGRRLVALCGTFMYGSGL
SFRSILPLFKGKIVTAQNVTLKPLPCPGYFFSLDAQVSPTYEMIFAMQFFSGLVTFSITTGVCGLTAVFVMHA
CGQLQILINLMRHLVKEQWDEGRNVDRKLAEMVEYHIRIRSFLRLVEHTMRQICLIEVMGCTMIVCILGYCII
MEWENSNKIAMCSYFMSLTSIMINVFMFCYTGEQLTTQAEKVANTSCDLEWYRLPDKKAREIVLVMIMSNSPT
KITAGKIIDLSFKTFGDVVKTSITYFNMLRNVAD
>HsOr36
MPFNEHYQNDIMYITQITRNVLSLIGVWPLLNRERSTGEKIRQFLLIIISYALLYCVLIPGVLFWLIEHRAHI
RIQTIPLVLYGFMATSKYGNLIYREKQIKRCLKHIEEDWKNVMGVDTRDTMIESAKTGRRLVALCGVFMYGSG
LSFRSFLQLTKQKIVTAQNITIRPLPCPAYFVFFDVQISPAYEMVFLIQFLSGIVTYSITVGVCGLAAVFVMH
ACSQLKILVELMRNLVEEKWKEKDDVDKKLAEMVEHQIRVRNFLQLVERTLQQVCLIELLGCTIILCLLGYCI
ITEWENSNPLAICFYFISLSSMVINMFLFCYTGEQLSVQADKVASTSCDLEWYRLPDKKAREIVLVMIMSNLP
TKITAGKIMDLSFKTFGDVVKTSVTYFNMIRNVT

>HsOr37
MPFNEHYQDDIIYITQLTRNVLSLLGVWPSMNRQRSTSEKICKFLLITISYILLYCVLIPGLLFWLIEQRTRV
RIQTIPLIFYGFMATSKYGNLIYREREIKRCLKHIEEDWKNIMSVNARDTMIEAAKTGRRLVALCGAFMYGSG
LSFRSILPLSKGKIVTPLNLTIKPLPCPAYFVFFDVQVSPAYETVFLIQFLSGIVTYSITVGMCGLAAVFVMH
ACGQLKILVDLMRNLVEEKWNEKNDVDRKLAKMVEHQIRVRNFLQLVEHTLQQACLIELLGCTMIVCLLGYCI
IMEWENSNGIAMCSYFITITSLMINMFLFCYTGEQLTVQAEKVASTSCDLEWYRLPDKKARGIVLVMIISNLP
TKITAGKVMDLSFKTFGDVVKTSLTYFNMLRNVTD
>HsOr38
NENHEQDINYTFELSRIVFRLLGVWPFERVKSWFPETLETASVVFISYILLTCELVPAILYMVLVQKESRARL
KVMGTVIFTVLAMAKYVQLLSRKSQVKNCLLHVREDWQNVVSTDKRSVMMEKARTGRRLLILCGIFMYSTGVS
FRTIIPLSKGKIVTAENITIRHLPCPTHFVLFDVQFSPAYEIVFLLQFFSGFVKCTITTAVCGLAGLCVMHIC
AQLDILITLMNDLVKERELKNVNDKLAVIVKHQIKTRNFLQLVQNTLQYSSLMEITGCTIIVCLIGYYVIMEW
EDNNAAALCSYIIGLISIGFNIFIFCFIGEQLSTKGEKVALTACTLEWYRLPDAQARSLILVIAMSNIPMKFR
GGKFIDLSFRTFGNVVKTAVTYLNLLRSIIG
>HsOr39
MFRNSHYQEDVKYVLQQNYVVLRMLGIWPSTNRLPNAIEKISNISLVVICYFLLHCDIVPGILYYVFADDQPI
EKRKMMLPILYSIIAIAKYSNLLIFGGEIRRCLRYIKEDWQTMLISGARDIIMGNAHISRRLFIICCTFMYCS
GVSYNTIMPLSKGKIVTDQNVTIRSLSYPGYYVFFDPQNSPNYEIVFLLQCLCGIVMYTITVTICGLATLFVL
HACAQMEVLMRLTEDLVGESHSGQRDVAAKLAAIIEHQIRIRNFLHLVETTLRYSNLVEIIGCSTIICLAGYC
AIAEWEDNNLAALSSYVTGLTSVIINIFILCYIGEYVTTQAEEVNLTFCTLEWYRLPTNIVRDMILVTVASNI
PPKFTAGNIIDLSFRTFGDVIKSSLVYLNILRQLAE
>HsOr40
NARMSTLASDTTKKNKLHISDFRYAVQISACLLKPIGAWPLIEKDISAFKIALHRLSMVIATFLQIFTIVPWI
VLIVKEKWSIILILRTMCPLIFTVTIFARYVLLLWHQDRLKFCVDRVADDWQYTILAQDREIMLTNARLGRTF
GIVSVVFMFSCGILFYMLPLVLPNIITEDNVTIRLHPSPCEFLVFDSKASPAYEVVYCLQFLSGFTVYSAFCG
ICSLMAHFVTHICGQCDVLGSFFKETVDGGQRNGGSIDDRIAAAVSRHLHLLKLVSDVSGLFTEICLMEFINA
SCNICLLGYYIITDLNNNESFMQISMYLFALASIIFNVFIFCYIGDMLTERCQMVGTTCYATEWYRMHPKKAV
ELIIPIAISRYPAVITAGKMMTMTLSTFSDILKTSMAYFNLLREFSSRDAAMT
>HsOr41
MGEPRLTQGDPWFSDFHYATQVSLYLLKPVGIWPLKFENTALGQIAHISTIFLATFLQLFMIIPWIIYIFTAQ
CDLYEILRTACPLIFSITVFLRYMLLLFHRDEIKSCIDHIAEDWRNATLAEDREIMLVNAKSGRLFGIVSVSF
MFGSGLLYCIMPMVAPPIVSTGNVTLRPLPNPCELLILDSQASPVYETVYVMEFLSCFTLFTVFCGISSLTAK
FVTHVCGQCEILKYFFDEIVDGSSRNQGTIDQRISTAVMRHLRILKFVTDVDRIMNEICLAEFLNASCNICLL
GYYVIMDWKNEESILQISTYFLAFVSITFNIYIFCHIGEMLVEQCQMIGTRCYMIEWYRLPHNKARSLIFSII
MSNYPIELTAGKMLTMTMSSFSNILKTSMAYFNLLREVSS
>HsOr42
MSSVDRHTLVRNINYRSDAEYVVKVAKFLLTPVGIWPLYGDGSTFSRVRTVFQTSFIFSLMCFLLVPHIIYTF
FDAENLTRYMKVIAAQVFSLLGIIKFWTMIINKNKIKRCLQQMEIQYRDVECEEDRMVMVRNAKVGRQFTVMY
LGLLYGGALPYHIIMPLVAERIVKEDNTTQLPLPYLSDYIFFVVENSPFYEILFVTQILFSTIILSTNCGVYS
LIATCVMHACCLFEITRRQLEAIVMNGTDDLHERFGRIITQHMQALRFAEMIENSLNIIFLSEVVGCTIIICF
LEYGVFKEWEDNQMFGMVIYTILMVSILVNIFTLSSIGDRLKEESEKIGETSYFIDWYTLPAKNVSNLIMVMI
RSSRPSALTAGKMFDISLQGFCDVCKTSAAYFNFIRMVAA
>HsOr43
MSTSNDSTLFRNINYRSDTEYVVKVAKTLLTPIGIWPLYRGDSASDKIKTYLQTGVMFCLMCFLLIPHVIYTF
FDAEDLTRYMKVIGAQVFSLLAIIKFWTMIVNRDGIRYCLQQMEIQYRDVECEEDRLVMAKSAKIGRLFTVTY
LGLSYGGALPYHIIMPLLAERIVKEDNTTQIPLPYLSDYVFFVVEKSPFYEIIFVSQILISSIILSTNCGVYS
LIATCVMHGCCLFEVVRRQIGMVLNNGTDHLHEQLGRIIENHMRAIRFAEMIEKSLNIVFLCEMVGCTIIICF
LEFGVLKEWEDGENLAMGTYFVLMTSMFVNVYIITFIGDRLKEESEKVGESSYFIEWYNLPTKIVTDLILVMI
RSSRPSTLSAAKIFDLSLQGFCEVCKTSAAYFNFIRAMTT
>HsOr44
MGVSRDVHATSSDINHQQDMRYVFAPSSWFLGLIGIWPISFRGVGQHISKIAIVICNFVLSFAIVPCALHIIY
DQKDLNIRLKLSGLLGFCTTAMMKFLVLVIRRPKIRQCIEHVKDDWWQVKFKSDRDLMLKYADTGRKLSIISA
SSMYIAGFIYHLVLPFCTEHKIGNQTIRPLVYPTYSQFIQTQVSPTYEIVYLAHCMCGYTMYTVTAGSCGLAA
IFATHACGQIDIITSRLEDLPQDKSYEQSTDINQRIAIIVKSHVRVLRFAALVEEILQEVCLLEFASSIFTMC
LPEYFCIVDWQDSDTIGLTTYFLLFISFCFNMYVLCYIGELLMEKSSQIGSICFMIDWYKLPAKSIRNLILVI
AMSSHPIKISAGRIVDLSLATFGNVLKTSLAYLSVLRTLIM
>HsOr45

NVYLADKERNIRLIYWLLKSACLWPYSSKASIYERVLSEFSIVTCFSMLSITMVSAGLFLFVEERGNTDLVMM
HIGPFLCFLMTITKYICLVLHVDDIRSCVDCVELDWNIARSDEDHEVMVRNAKIGRLTATSLAVFMHSAIQCY
GISRCLIKDVVEVDDVNVSIRELPFPFYNKILDVRFSPAYEVVLFVHCSSAFFISGITSVNCGLMAIFVMHAC
GQLKILTMWLGNIVHDDDVADVNMMQKKLGFIIEHHLRVVNFVYHIEEVIYMTSLVELVGGSLTLCLLGYCSI
MAWNQDKKERIPTYCIVGISFTLNIFIICYIGEILSEQYRQVGKATYMTEWYRLPPKTTLGLLLINIRSNYNI
NLTAGKMIDLSLYTFGNVLKASMTYLNMLRQM
>HsOr46
MGVSRKTHATSSDINHQQDMRYVFTPSSWFLGLIGIWPISFRDVGQHISKIAIVICNFVSSFTIVPCALHIIY
DQKDLNIRLKVSGFLAFCATAMLKFFVLVIRRPKIRQCIEYVEDDWWQVKFKSDRELMLKYAGTSRKLSIISA
SSMYIAGFIYHLVLPFCTEHKIGNQTIKPLPYPTYPTYSPFIQTQVIYEIVYLAHCMCGYIMYTVTAGSCGLA
AIFATHVCGQIDVITSRLEDLLQGKSYEQTTNVNQRIAFIVKSHVRVLRFSAFVEEILQEICFLEFISSLFTI
CLLEYFCMVDWQDSDTIGLTTYLLLFVSFCFNIYILCYIGELLMEKSSQIGSMCFMIDWYKLPANSIRSLILV
IAISSHPMKISAGRIVDLSLATFGSVLKTSVAYLSFLRTLVM
>HsOr47
YPADKERNIRVIRWLLKSICLWPRSSNASIVDRVFSECLLVTCFFLLIITMVSCGRVLFVKERADVDLLMTHI
GPFLCYVMTIMKYICLVLHVDDIRSCVKYIEVDWNTVRSNEDYEVMLRNAKIGGLMATSIAAFMHCAVQFYSV
TRCLMKNVVEVDNVSVTIRELPYPFYNEILDVRFSPAYELVLVLHVVSAFVMSGVTSVTCGLMVIFVMHACGQ
LKILIIWLNDIVQDNDAINISTVQRKMGFIVEHHLKVINFVSHIEEVIYLTCFVEVVGGSLTLCLLGYCSIMA
WNQNKRESIATYCIAGCSFSFNIFIICYIAEILSEQYRRVGIATYMLEWYRLPPKTAIGLLLINLRSNFKINL
TAGKMVDLSLYTFGNVLKASMTYLNMLRQV
>HsOr48
MKNSREGYVTSPNVNYQQDMQYVFKPSSWILGSMGIWPITIQGIRKHVSTIAIVVYNFALVFAMVPCILHIIY
DQKDLNLRLKLCGLLGFCLTALTKYFILVIRRSKIQSCIEQVKKDWWQVKFKSDRECMLKYASIGRKLSLICV
TAMYSAGFIYHLILPFCTEHKIGNQTIRPLVYPTYSEFRQSQISPTYEIVYVAHCVCGYTIYSITVGTCGLAA
IFSTHACGQIQMIISRLEDLLNGKNFEQVPNVQQRIAAIVKGNVQIIRFAAVVEEVLQEVCLVEFTSSLCTIC
LLEYYCILDWQEDDRIGLLTYFMLFVSFCFNIYILCYIGELLVEKSSQIGLICYLINWYELPPRSARDLILII
AMSSHPIKISAGRMVDLSLTSFGNVMKSSLAYLSFLKTLVM
>HsOr49
IPWSNVNYEKDIVDALMWSRRILRVLGIWPLIFPDTSTIEKILATISFSVCWSALGFLLIPMTIYTLSDQTLT
SDKIKMLGPLGYVVASVLKYMLLVIRHRSIRRCIHVLSIDWRAVQQEDYRMIMIKDSAKGHVLSKFCIAFIYC
GGLCYNTVIPFLSPRPDNELNTTVGPVTYPGFDIIFDLRFVPAYVFVFCAQLLSSVIMFNITTSVCCLAATFV
AHACGQIGIVMAKVKGFVKNVQSNRTNSKHRMAIIVNHHVQALRFSASIEDVLNELCMVEIVESTLIICLLEY
YCLTEWHNNDGFAVMTYIFLLTSFVFNIFMFCYIGELLTDQCNRVGYTSYEIEWYQLPGKVAVDLTLMISMSH
HPIKITAGKLISLSFTSFGNVLKTSVAYLNLLRTVV
>HsOr50
MAKITTIPWSNVNCEKDIVDALVWSKWILRILGIWPLVFPVTSRVEKILATTSFALSWSALGFLLIPIAIFTL
SDHTVTNDKVKMLGPLGHVLISMLKYLFLVVRHKSIRQCIRGLSFDWRAVQRENYRTIMMKDSMKSHMLSKFC
IAFMYCGGLSYVTVMPFLSQKPDGEKNATVGPVPYLGFDIIFDLRFVPGYVFVFCMQCFSAVVMFNITTAVYC
LAAMFVAHACGQIEIVMARVESFMKDVQSNRINSEHCMAVIVKHHVKALRFSASIENILSEICLVEVVGSTLI
ICLLEYYFLMEWQNSDSIAILAYSFLLTSFVFNIFIFCYISELLTDQYSKVGYTFYKIDWYLLPGKIALDFTL
MISMSHHPIKITAGKLISLSFTSFGAVLKTSVAYLNLLRTVV
>HsOr51
PNVNHEKDVVDTLMWNRWLLRIIGIWPLVYPNTTKIEKILATFAFALCWTVLALLLVLTSMYTFSDQSIMSEK
MKMLGPLGYVFFSMLKYLFLVIRHKSIRGCVQVLSADWRMVQQGYHREIMIREAAKGHVLSKFCIMFMYCGGL
SYNTVMPFLSQTPESELNITVRPMAYLGFDILFNLEFMPVYVFAFCLQCFTGVVMFNITTSVCCLAAMFVAHA
CGQIDIVIDRMENLMKGNEQCSRMKFDRCMAIIVQHHVRALRFSANIEDTLREICLVELVGTTLIMCLVEYSL
ITEWNNSDRIAIFTYFFLLISFIFNIFVFCYIGELLTEQCTKIGHTSYMIEWYNLPGKAALDLMLMITMSRHP
VHITAGRMISLSFANFGNNLTLKTSHALVDLL
>HsOr52
MHNRFQHLGETRQIHNVHHENDIHYTMQLCRWVLKPIGIWHLIYGHFSHYDKLTSVILIVACLSALCFVLIPS
GMHTLLREKDINVKVKLFGPVGFCLTSTIKYCYLGARSASLGKCIHHVEDDWRAVRDEDHRRIMLKNALTGRR
LTTLCALFLYTGGLSYHTILPLSSRRKVNGSFISRPLTYPGYDIFFDPEASPAYEIVFCIHCLFALITYNITT
AACSLAAIFVTHACGQVQILMTLLDDLVEGDRNKGTTVDSRLGIIAKHHVRLLRFSTNVEEVLREICLMELVT
STLTICLLEYYCMTEWENSDAVAILTYFILLISFTFNILIFCYIGELLVEQCSKIGSAAYEVNWYDLSGNKAV
DLVMIIAMSHYPPKLTAGKFVDLSINTFGVVLKSSVVYLNLLRTVTE
>HsOr53
MHDRSRLSGRIQVHNVHYEHDIRYAMQICHWILKPIGIWHFVYSRSSPSEKLFSSTLVFTCVSVLCFVLVPSG
PYVLLYEKDIYMRVKLYGPVGFCLSCTIKYFLLGVRGTAIGRCIEQVEDDWQVIRQADHRKVMLKNAMVGRRL

AILCVILLFSGGLSYHTIMPLSAKIKINENLTIRSLVYPGYDRYFNVQASPVYEIVFGMHCLSCLIQYTITTA
TCSLAAIFASHACGQVQILMTLLDDLVDGKKIEGSSTVGKRLRVIAKHHMRVLRFAADVEEVLREICLIELVA
ATLIICLLEYYFMMEWKKSDAIGIVTYFILLVSFAFNLLIFCYISELLVEEFRKIGSAAYNVNWYDLSGYKAL
DLTMIIMISHYPPRLTAGKFCDLSFNTFSTVLRTSLVYLNLLRTVTE
>HsOr54
MLDRSKSRSIESTTDGWNYSFQLNRWFLKPIGTWPLSLCETTAERISCVILIINSCFLIGFLLLPCTLCTILV
DTDLDSKIRMIGPLSFFLMAAVKHYILIARNKDIGECIRHVHLDWSCVELSHEKDREIMFDSARFGRWLSVVS
ALFMYSGGLFYTTLMPLFAKRTDIINNETVRLHAFPVYRRLFDPRDSPFFEVVQFMQALAGYVIYTNTIGVCS
LAAVFVMHACGQFQILMLKMEDLADGKKRKSASSTSEERLGDIVRCHIRILSFITRTEELLNEICLVDVVGCT
LNICFLGFNMMTEWEHRETLGTMTYCSLLISFTFNIFILCYIGELLAEQCIQIGIKAYMIDWYRLPGKGALGL
TLVMSMSNATIKLTAGKFMDLSLASFCSVVKASVAYLNLLRTF
>HsOr55
YLADKDRNIRLIRWLLKSICLWPRSSNASIVDRVFSELSRLTCFSLLFITVLSIGLDVFVEEHEDVNSMMANI
GPFITFLMALLKYTCLVLHVDDIRNCVHCIELDWNTVRSNKDHEVMLRDGKIGRFMAIFIAAFMHSSVQSYNV
SRCLKEYIIEVDNVNVSLRELPYPFYSKILDARFSPAYEIVSIMHFVSSFIVSGVTSVNCGLMAIFVMHACGQ
LKILTLWLDDIVHDSDIVDDKIVQRKLGFIVEHHLRVISFVSYIEEAIYLTCLVELVGGSLTLCILGYSSITA
WNQDETESILTYCVIALSFTLNIFIICYIAEILSEQYKRVGLATYMTEWYRLPPKTALGLLLINLRCNFNVNL
NAGKMIELSLYTFGNVLKASVAYLNMLRQVIS
>HsOr56
ICETNMKSVSRERSWIALSYMEDNEYSIQPIRWLLKPISVWPVADSSIKERILSDILLITCVFLIVGTMVPCA
LAIFLDETTDMETKVREFGPLSNWMLASLKYCSLLAHVGDIRRCIEHVETDWKIVTKTVDREVMLKNARIGRF
IAFFSATFMHSGVFSYSIFRVLTREDSVAKDNTSVHALPFAFYDKIVDTTMSPAYELVFAMQCLSTFVVNSVG
IATCSLTAVFVMHACGQLKILMSFLVNLTDKENEKKNSMKQKFAVIVNYHLRVLSLVSRLEKITNITCLVEIV
GCSLQMCLLGYYCIMEWNQGDKQNIVTYCIILVSVTFNIFIFCYIGEILSEQCGQVGETAYMTDWYVLPGNTA
VGLVLIIILRSSIIVKITGGKMIELSLSTFGDVIKSALAYLNILRTFI
>HsOr57
NDYSLQLSRWFLISIGAWPQTRTSSIIERLSSIILVPMWLFMVAVITIPCLLYVFFEAKGIQTKLRPLGPLFH
RIMGTLNYWILLTRGKAIHECIKHMEEDWRVTENIQNYEIMIKHAKMSRYMAGVCAMFMQGSALFFNIVGTIK
TVNIVIKNETITIHPLSCPAYRKLIDARFNPANKIMLLMQFMSSYITNSSTVCICSLAIVFAMHACGQLNMLH
TWFNQLVEDHAKGKHLTKRRIGNIVRHHLRVLCFIARMESIIYKACFVELTGCTLNMCLIGYYFVTNWSILNT
AKIMSYIMVYISMAFNIFIFCYIGEIVTEQCKNVGQMAYMTNWYELDYKTARSFILIIVRSNNAIKITAGKLF
NLSIATFGDVIKTSLAYMNVLQAMS
>HsOr58
LKNVKSKSDHDYSLKLNRWYLKPIGAWPLASTSSAAEKVVVLIQILVCWVVVCFIVIPCTLYLFFEKESIRSK
VGAVAPLLNRYMGLMSYWMLLQHSGNIRDCIEHIEMDWRSAQKASDREVMLQHAKFGQLIALICGIIMQGGTF
LYGIAKAAKTATIIVGNETITMHPMTCPAYSKIIDTRFSPVNEIVLIIQILSTFIVSSSTAGICSLAAVFAIH
ASGQLNILYMRLDELTKNRKEENHVAEQRIIDIVEHHLRILSFIARVENIMHKACLAQLTGCTVILCLLGYYI
LMNWGIFDAAQISSYIIVYVSITFNIFIFCYIGEILTEKCKHVGQMAYMTNWYNLHHKSARDLVLIIARSSNV
IKITAGKLIQLSIATFGDVFKTSLAYLNILRTMT
>HsOr60
NITVTLSVHRFVLSCVGIWPTKEKSLFTDLRWLVAMILEAAVTPMCVYFTEIYLHCNGAKESFDKVTPGAAAA
LALTRLITPRIHRRELLEIVTSMMNDWATQRDQRVRWIMKKYATLSRRVTTFTFFLVGVIVGVYIFMAISAVT
AQASQLDHQLENVNVSRETDEVLSCVFRGESMSQAFMVVQAMQMFVTGILTFGTTSFFFGLAMHLCGQFDALA
IKLSDFRVEQAHHAIAEAVERHCHLIRMTDRMEESFNANVLIYLFVTTTLMCIDGFMLIVSLKVGDVPMIIHN
ASVLLLMMIQLSFYTFAGDCLEMRSTALSYATYDCDWHELPASTARDIRFMLMRANIPHQLTAGKFVAMNMIT
FKDILKSTASYLSVLRVMLDE
>HsOr61
MVQARYWNDDAHRALGVYEKVLTIIGVWPLSVGELKSVARCFLAVLIQISTIGSLSLEAYRQCLGTEDMMEAF
LMDLSSVVSLSKLLVVRLTWKHSYVLVNSLIDDWSVSRDERQRDVMLRYTNVGRVVSLTMLYLAYASGTSFLF
MAVPFDVLLPWLNISKPEYNGTTVSTYFLASYCVFGSLPTISHGCVLLLQVAQIFVNATSHCGNDGFFFGLAM
HLCGQFEVLEMDFAELDVRKRACHRKMGMLIGRHCHLIRLADSLEYAFNMAIFAQTLMSVLLLCVEGMQLIIS
LKLRDNIAAIKHVVLILTMLVQLYLYCYAGDQLESVTERLSYSAYSTVWYEFDAKLMRNLPIVMLRGRIPHQI
TAGKFLPMNVFSFKEILKATCSYISVLRVMIDV
>HsOr62
MDSNAQWNDAADATHFLTFYKNLLGIVGIWVLDEKSVFSRTRWFVSTMVEMSTAVTLSLEVIRHCKGHEDALN
AFLTGSSSIVSISKLLLHRVNWRYKLLLVEAVVHDWTSVKDPYSRDIMLKYARIGRFGALVFLYLGLASLVYF
ASVLVFSNVHLPWISEWLMHNRTGERKLALAAYCVFGKYLSSAYVLIGALQLLQVFVDCMSHCGNDGFFFDLT
MHVCGQLEILRTSFTEMNGDEILLRDKLDALLKRHRQLICLAYYLEKAFNMVILAQVLMSVILVCVEGFQLIL

MLTINDVFSAAKHVLFMAVLLVQLFLYCFAGQTLELQSEGLAYAIYESRWYSFDVNVIKDLSFVILRASKPHQ
LTAGKFLAINFMSFKEILKASASYLSVLRVMLET
>HsOr63
IGVFPHAGMPDGRWNNDIAYVFSIHRIFLEIFGVWPLQKKTVFTKVRCSVSLLAQIMILSFLMMDFTMTSHDA
DASIESILYFVSVVLGVIKFISIVYNQRKIGMNINAAIDDWLSAKGDEKTWKIMKKHASRARILTLALLYSAI
GCFSSYILAIVMINLKQIFFTEPNSIDANVTDWMLLVPSGSLGNSITGLQYLVILIVQILQTSTLCVIQCTVD
SFFFNLTVHLAGQLEVLKEKFKRFADEPYSIASCRSRFVGLINRHSELMEYYQNLEDTFHFLILCELTMVTIM
LALLGLRLNLCLSENNQVEFAKSASVLNYFLMVSLVFTYAGDFLQRESESIFHALYATSWFTLPLALMKDLHF
TMMRSSIPFRLTGGKFFYVNRETMMYILKTATSYISVLRIALRD
>HsOr64
MESSVKWNADTAQVLTFYKNVLGILGLWVLDEHNFFSRIRWLMSTMVEMSTMISLSLEVIRHCKDYEDVLNAF
LSAFSSVISISKLLLHRLNWRHKLILVESIVHDWTFVKDASSRDIMLKYARKGRLGAMVFLSLGCASCVFLFS
SLLLSDIKMPWDDEEEQIYNKTHEKKLLLAAYCVFSTYTSFAYGLVEILQSLQIFVNCISQCGNDGFFFTLTM
HTCGQFEVLRMDFAAVSCEGLLYRAQLDNLLKRHHRLIRMAHHLKGAFDTVILAVSMSMLLLCVEGFQLLLAL
ATSETFAAMKHVLYIVILLVQLFIYCFAGQILEFQSEGLAYAIYESPWYCFDVGVMKSLPLMILRATNPHQLT
AGKFLVINFMSFKEILKTSASYLSVMRIILET
>HsOr65
MKETWNSDMDYGFSVIRTIMWILGVWPLEQNELVCTVRWFTIFIVESLTMLNLLIEPFKSCGDSKDALEVCLI
IETGLHAWTNMIFGRVYMKKIASNVISAIDDWSSPSMTRESYLIMTGYARVGRIITMSQVMIGFVAASLYFIA
IIIGNNQQVVVIIDHENVTLWTFVFPSTCLYKGISYTTYKALYAMQMVQAFLLFIAECASDSFFFSITMHLCG
QLELLRIRFAEIAGKIDEKNRCGNSFGPWIKRHCQLITLAKNIEDSFNINILLRLGITTIVIAVSGMRIMMSL
KHQDYTDVLKSMMFIQYFIVQSFLFTHAGDTLQSQSESIVTAIYSTTWHELPPTTMKDLALIMMRTRIPLRLS
AGKFFYITRSTITDILTTALTYISFLQAMEE
>HsOr66
MARWHQDVAYALASYKILAWPLGAWPIDDDAFYSRIRWSFAMISEFLLVTSLAIEIYFTCENSQEDPIDTYVV
IASAMLVLVKLSLLRMRRSTLSISLSCAIQDWCEVKDPRSREIMMQHARTARTISLSLFYSGFFAFMLYMLRL
LPFLNTTDERTYYLPTFCLLETASNVKYVIVTFYQVIQLFITYAGNCCTEGIFVGITLHLCGQFELLMINFRR
IGRHVAKCRGGSIVEELVARHRHLLRLTETIEDSYNMIILTQIFTSAILICVTGFALIESSHIKDTIMMVKSV
VVMIVMLLQSFLYSYAGDNLRDQSEALSFAVYDSNWCDFPSNDVRDLAFIMVKTNIPIRLTAGKFFYVTRATF
TDILKTSVSYLSALRVMIEKQTTTS
>HsOr67
MTSSRRQRDIAYATTPFKLLTWLNGVWPLETSTVNSLIRCILATCCMSAVVMLPSIELQMGCNNAEQNVDSFM
CICCALLSIVKTVCFRVYADNLSVAYNAAIDDYLTIKDPEQRKLMRRFAFIGRTLSCFMVCFAYFACVVYTLI
PLLRDEPASQLNATNEDTVLEYTIPSRCTFEYFHAPTSMYKIITLSESLMMVLTCTCNHGNDSMFLNITLHVC
GQVKILKADFVDIDVTNPHVHSRLNALIERHGHLVRLGNKLADTISFILLTQLFITCILLCIMGFQFVLALKQ
KDVVMLAKSCMVLTSFLMQISVYSFVGDYFKAQMEEVGLFLYQSAWYGLPTKLTRNLTFIIMRSQSPVKLQAG
NFIVVNLATYMSILKTSISYLSVLRVMVDT
>HsOr69
YAMTPFKLLMWPIGAWPLQDYNTWYTPTRIKYMTKYIAIVVILPCIEIYLGCTDADKNVDAGAMIISGSISIT
KMIWFRIYTXNFIKTYRFAVNDYLTVEDAEQRAIMLRHSFTAKTFMCSMVLFSYYDATIYMITPYLGSLISHQ
SNVTQEDVELEYALPSRCALEYLNAPQQNMYATYCIIELIMMLFCMFLLRKGNEXLFIHIVLHICGQVKILKX
KFISFDITAPQIYDRLHMLIQRHDHLIAMTRLLADAVSFILLVQLFVSSLLLCTLGFQVVMALKVNNISMTLK
SFMVLNGCLVQITIYCAVEDYLKSQMEEVSAFTYQIAWYQLSAKLTKSLSFIIMRSQSSVHLQAGNFIVINFE
TYMSILKTSISYXSVLRVMVE
>HsOr70
MTSERWNDDIARAMTPFKLLMWPMGGWPLQVYNIFSLIRCVAVTSGMSIIVILPSIEIYLGCNDAESNVDALM
LIVCGLLAMAKMIWFRIHASNLVKTYKFAVNDYLAIESAEQRDIMLKHAFIAKTLMCIMVSVAYSDSCILFLI
SILGNSDNVTQATNVTQHEVILGYTVPSRCALEYLNAPRNMHVTHCIIELIMLLLVCTSNYGNDSLFLHIALH
ICAQVKILKSNLIDFDFMKPQINKRFNALIQRHGDLMEMTKILSNVVSFILLMQLFLSSILLCILGFQLILAL
KLNNFAMLVKSFMILNTFLAQITIYCVIGDYLKSQMEEVGTFIYQSTWYNLPAKLTKSLSFIIMRSQFPVQLQ
AGNFIVVNLATYMSILKTSISYLSVLRVMVET
>HsOr71
MASEQWNDDIAYAMTPLMLVLWPLGVWPLQVYNIYSLIRCVVTTCCMSIIVILPSIELYLGCTDAEKNVDALM
LICCGILAMMKMICFRIYASNFTKNYSSALNDYFMIENEEQRAIMHRHALIGRTLLCSLISVSYFDCGIYGLV
PFLGSNDFNQTNITSEGIILEYTIPSRCALKYLNAPNMHKIYCFVELLAMLVASTSNYGNDILFLHVALHICG
QVKILKSKLINFDVAGPRVYDRFYMLIQKHSHLLKMTKILANAISLILLMQLFVSSILLCIIGFQFILALKVN
DITMTLKSFMVLNVFLTQITIYSFVGDYLKSQIEEVGTFIYQSIWYDLPGTLTKNLTFIIMRAQSPVQFQAGN
FIVVNLMTYTNILKTSASYLSVLRVMVE

>HsOr72
MPDDRWNDDVAYAFDTHKMFMNAMGLWPLQKMTAFTIMRWSLLTILLLGVFAFISMEFTRTDRDTGKSIETML
FSMCTGATLLKNFCFTVNRKKLAKNINVAIDDWLVARNDAQTYKIMKKYAFQSRLFTSVILYLANICTVLYIM
VIFLLNARQDALGTNVTEANTSMTDLTFVLPSGDLGKKVTSMPLYAVLIVIQSIQITLISESESIMDCFYTNV
TLHLTGQLEVLKMRIKTFASKTDTVANHRKEFAKLIDRHCELMELNHNLENTFNLIIILYQLLIVTLLVALLGI
RILFCIKNHDYVILSKSALGLSYVFTESVVYCYCGDFVQNESEELFHALYTTSWFTLPASLMKDLNFAMMKSS
YAFHLSGGKFFSANRQAMFNVLKTAASYISVLRVTLM
>HsOr73
MAKKRWNKDIAYAMTPFKLLTWPIGVWPLQVFNVFSLIRWIMTTCCLSTVVIMPALEIQFGCTNTDMNVDSLM
LTSCGALAMLKTIMFRVYTEQLADNYNSAINDYLTIEDEEKRVIMRRHASLGRMLACFMVCFSYFASAICTLI
SVLTKNEDTQLNVTNVETVEEYPIPSRCTLEYLHVPTSMAKIIRLFEFMALILTSTSNHGTDALFLNITLHVC
GQVKILKADFNNFDTAGPRAHERFVALIDRHNYLLTMASKLAEAISIVILIQFFISSILLCIIELQLIFALKT
NNFGGVAKDIVILTSFMTQLSSYSFVGDYLKSQIEEVGLSIYQSTWYNLPPSVMKNIVFIMMWDQSPVKLQAG
NFIVVNLSTYMNIIKTSMSYLSVLRVMVD
>HsOr74
MSWKEDVAYAMTPIKLLTIPLGAWPLQEYNKFALARHIVSTVGLAVTVVVQFLELNYTCTGAYAQLDALTLFT
CGILAVLKITWFRIYADNLTCNYSSAMSDYRAIDSEEKRAIMRKHAFLGRIICIIALLISYVDSVIFIVGHSA
TSTREFRLNSSIEGLRSGYAIPSTCTLAHFYISKNVFIAIFMLESILLVTMCLGNHGSDSLFLQITLHICGQL
KILKAEFINFEIEGPKVCERFNALIQRHDHLIKMSRKLAESISFVLLVQLFISSVLICVLGFQFIIALKTSDF
GMMSKSVLVLSAFLAQLTLYSMVGDYLKSQMEEVAQSAYQNTWYDLPAKATKNVTFILMWSQLPMKLQAGNFI
VMDLPTYMSILKTSMSYLSVLRVMIE
>HsOr75
MEWNDDVVYALTPLKLLSMPLGYWPLQKNNMFDLLRYSLCVASLAWVLIGLCIEISYNCIDSYTKIDALLIFT
CALLALLKITWFRIYADNLISIFNSAINDYFAIDNEEKRDIMRRHAFMGRAICYSVACVSYVASLFYMSLPLL
MTVSVNGSVAGFERKYPIPSICTLGSLNISMTLHIVLYAMHSIVLIIICSGNLGGDSFFLAITLHVCGQVELL
RSEFANFGVKSSNSNEDFSKLVTRHHYLLDQAVLLTETISFVLVMQLFVSCVLICVMGFQFILALKVGDIIMI
IKAVIVQCDLLAQMFAYSYVGDYLKYQIEEVAQSIYCSNWYFLSVKLMRSITFVIARSQHPIQLVAGKFLVVN
METFMSIIKTSLSYLSVLRMMVK
>HsOr76
MEWNDDVVYALTPLKLLSMPLGYWPLQKNNMFDLLRYSLAVTSMAWVLIGLTVEIYYNCIDPYGKIDALLLFT
CTFLALLKIIWFRRYGDNLTKNFNSAINDYLAIDSEKKRAVMRRHAFMGRTICYGVTCVSYLASVFYISLPLL
MDNVSVNGSATGFQTKYPIPSTCTLGALDISTTLHIALYVMLSIMLFIIGSGNVGGDSFFLAITLHVCGQVEL
LRADFVNFGVKSSNPNEDFFKLVTRHHYLLDQAVLLTETISFVLVMQLFVSCVLISVIGFQFILALKVGDIIM
IVKTGIVQCDLLTQMFAYSYVGDYLKYQIEEVAQSIYCSNWYFLSVKLMRSITFVIARSQHPIQLVAGKFLVV
NMETFMSIIKTSLSYLSVLRVMVE
>HsOr77
MEWNEDMAYAMTPLKLLAMPLGYWPLQENNVLDLLRYIICSMSLIWVLIGLSIEIYYNCIDPYAKIDALLIFT
CTSLALLKITWFRVYGGNLTNNFNSAINDYLAIDNEEKRVVMRRHAFMGRAICYGVACASYVASVFIITLPLV
TGHSGVIVNGSVLVYDTKYPVPSACTLGSLHISTTWHIVLFVMQSIVLVVICSGNLGGDSLFLAIMLHVCGQV
QLLRVEFVNFGVESSNPSEEFSKILTRHQHLLDQASLLADTISFVLMVQLLISCVLICIIGFQLILALKVGDM
IMIFKTLTVQTTLLTQMFAYSYVGDYMKYQIEEIAHAIFSSNWYWLSFKLMRNILFVIVRSQQPIQLMAGKFL
VVNMQTFMSIIKTSLSYLSVLRVMVE
>HsOr78
LDYSYGWNRHTMTFMGLWPEERNFSQASSYRVLAPVISMLCFICVPQSINLFFIWGDFDLMLENLSMANITIT
ISLLKTVAFWSKGRPLKSLVTSMSRDWNTIVSKQDRSTMLDIACTSRSLSRNSVLLVQFVVVMYITLRFFMIR
HNGRQLFFPAYFPYNWTNSPSYELTFLAQFVATMYAANTYTAVDTFIAMLVLHTCGQLSNLRRELTGLQAVRR
AEFRGKLGNIVRKHEYLNRFAGTIEDSFNKMLLMQMLGCSLQVCVQCLQALTSVIDEANELVIFQFIFLVFYV
IYILLQLYLYCYIGERLLIESTKIGYAAYDCNWYNLSPHEARSLMIIMCRTRSPLHISAGGFCSFNRELYSEI
LKRSVAYMSCIYAVK
>HsOr79
MKIELGWNKYNMWMLGVWPEPTKTSRAKQHLSKLIFWSTSTIALLFVCLPMNAHLFIVPTNLNEVIHNLSVNV
PTFLAFVKQLFLRYHGEALTLLVRQILDDWTRPIPKVERQAMLISAKRSRWISILSSTFTYLLLITFTTMSVW
NNMQHSFEVKLYGMPFPTIFPYNCNKSPNFELTWLGETIGAIIVANGYSCFDTFLAMLTLHLCGQLAVLNINM
RNLVDATRHDDFVNFQERLGLIVHRHEELHRYATVVEDYFNIALLAQTLVGTVTFCISVYRLITNRDQGVADL
MYFVIQVLYTTIHFFFYCYIGEQLLAESTNVAQSAFDCEWYDLPPRKAISLTIIIMRANTSFKLTAGKFSPFS
FELFSTILKTSAGYVSVLLAMNDKVTE
>HsOr80

LSFAIGWNRLNLGLVGIWPDPLQGPGQSGGLSRCRFLIASFLMLGFICIPQSVNLFFIWGNAEMMTENLATAN
IPVTNAFMKAFTMWHHRKALKPLVDFFYQDWHASKTLYEKVVMLRSASVARKISIWCTVLTQTMVTLYIVLRI
SVIVRLQRNDPARPLIYTAYFPFDVSRSPIFELVCLGQILSAYSSTASYTGTVSFISMLVLHVCGQFLSLRER
LKNIADGVKTVEEFRDELAQIVKRHEHLNWFAKTIEDSFNMVLLMQIFSCTVQLCFQGFQVFNIIINGRDESL
TFQLIFLAMFVAFILIHLYVYCYIGEMLLVQSTEIGFSAYESNWFNVPGKEARNLLLIMHRSTMPLCLTAGKF
GVFSLQMFSKV
>HsOr81
LHHTFSLCRQFLRLAGVWPDPDASLSDFRRSKLRFIIIVCILIMYVCVPQFSNVIHSWGNMNLVIQHIASANY
SFLACCKLVVARYYNETLRTLITSITVDWATSKETWQRNTMLKIARRGRSLSLRCYMSCMITVMFYVIFNLIK
FHRTLHQPERKLIYQFAYPYDYEKSPNYEITFFIQMSCGAFTAIVNSSVDSFVSILVLHVCSQLINLRTTLNK
TIDKLAEKSISSSRFRRSLTAIVVRHEHLIRSVERINNCYSTVLFIHMIAATFQLCFETFQIFTIITDKNANV
SIIKMAFLVFYVTIVLTHLYFYCYSAERLLTESTNIAYGVYECKWYDIPSRDAKDLMFIVYRSKIPLKLTVGK
FGIFSLEMFGTTVKSSMGYLSAFLSMSD
>HsOr83
FAVCRRFLRLVGAWPDPDVPLSDFRRPKIRIMISVCIVCIYVYVPQLSNAIHSWGNVNLMIQHIASANYSFLA
CCKLIVTRYHGETLRTLMTSLKTDWVTSKRNWERNTMLKVARTGRNISFSCYMSFICVAIFYVYFNLLKFYRN
ICLSERKLVYQFDYPYDMQKVPYYVITYFIQICAGICAAFINSTVDTFVSLLLLHVCAQLINLRTTLNNLVEK
LAEKSIPSAKYKEGITAIVLRHESLIRNAKIIDNCYSLVLLVHMIATTFQLCFQTFQVYTKITDENSDGLTIG
MVYLMFYVMFVLTHIYIYCYSAEKLLSESIAVAYGVYECKWYDIPSRDAKDLMFIVCRSIIPLRLTAGKFSTF
SIETFGMTVKTSMGYLSMLLAINN
>HsOr84
LHHTFPLCRQLMRLVGVWPDPDAPLSDFGRPNLRFIVTVCILIMYVYVPQFMNAIRSWGNVELMIQYIASVNF
SFLACCKLVVTRYHGETLRTLMTSYRTDWITAKRNWERNTMLKLARTGRSISFSCYTSLICAISLYVYINLLK
FYRNISQSERKLIYQFDYPYDMQKTSYYVITYFIQISGGMYVAMINSTVDIFVALLLLHVCAQLINLRTALND
LVEKLAEKSIPSAKFKEGITAIVLRHESLIRNAKTIDNCYSTVLLVQMMAATFQLCFESFQVYTGMIDKNSER
STIKIAFLICYVTLLLLDAYVYCYAAERLLIESTAIAYGAYECKWYDIPSRDARDLMFIVYRSMIPLKLTAGK
FGIFSIEMFGMTVKTSMGYLSMLLAINN
>HsOr85
LHHTFSLCRQFLRLVGVWPDPDTPLSDFRPYLRFFITMCILSMYVYVPQLMNAIRSWGNMDLVIHYTASANYS
FLACCKLVVTRYHGETLRTLMTSFRTDWMTSKRNWERNTMLKVARTGRSISFCCYMSVICATSVYVYMHLLKF
YRNICLSERKLVYPFDYPYDIQKSPYYVITYFTQMCAGLCAAYTNSTVDTFVSLLLLHVCAQLINLRTMLNNL
VEKLAEKSIPSTKFKEGITAIVLRHESLIRNAKMIDNCYSMVLLVHMMVATFQLCFESFEVYTVSESKHGTTG
SKMGFLVFYVMLLLIQLYMYCYSAEKLLTESTAMAYGVYECKWYDIPSRDARDLMFIVYRSMIPLRLTAGKFS
TFSIEMFGTMVKTSMGYLSMLLAIKN
>HsOr86
LSYAFTLSRQCLRFLGVWPDPYVPLSEFRRPNLRYIFVVCIISFYMFIPQITNTFRVWGNMTLMVEQVASANF
SLMAFCKLVVTRYHGETLRTLMTSFKTDWMTSKRNWERNTMLKVARSGRNISFSCYISLICTTIFYISFNLLK
LYRNISQSERKLVYQFDYPYDMQKTTYYVITYLVQISAGTYAAMINSTVDTFVSMLLLHVCAQLINLRTALNN
LVEKLAEKSICSAKYKEDISATVLRHESLIRNAKAIDNCYSTVLLVHMTAATFQLCFQSFQVYMRITDKNPDS
STVKMAFLMFYVILLLIHLYAYCYSAEKLLTESTAVAYGVYECKWYDIPSRDAKDLMFIVYRSMISLRLTAGK
FSTFSIEMFGTTVKTSMGYLSMLMAINN
>HsOr87
LEWAIGLNRLMLKIIGLWPPDKREPHETIKSKFRLFCSIIIMLFILTIPTFASLIKIWGNMMLMIDNLIYSLT
LLIALYKVFIIRYKQKDLAPLIDMIATDWMKPKIKEERNVMLRLAKISRIIAICGCLLPFIPMIVCFTLIYFE
LTLRHVTNLTDSGKPLMVQTYYLHNVSKSPQFELTVVAQGVALFITCVSYYAIDHFLGLLVLHIYGQMENLHI
RLTHMERYTNFDTVLKYNVQDHIRLIRSIEIINDSFYLLLLGMILHFSIIFCLLGFLIVDILNDEGQLSLMQL
VWFAAMTIFVMLHMCLYCAGGEILVTQCEKIYRATYEYTWYTLDPKVARNLIFILLRANKPLYIMAGKTFPMT
MATFCNLLKTSAGYISVLLANQD
>HsOr88
LEWAIGLNRLMLKIIGLWPPDNQDPHVTIKSKIRLMCSIIIMLFILAIPTFASLIKVWGDMILIVDNFIYSLT
LVIALFKVTMIWYKQKDLVPLIDMIATDWMKPKTKEERHTMLRVAKISRIIAIYGWLLPIISVIVCFTLAYFG
LSLRHVTNLTDPGKPLVVQTYYLHDVSKSPQFEMTLLAQGIALCTTCISYYSIDHFLGLLVLHVYGQMENLHI
RLTHMERYTNFDSVLKYNVQDHSRLIRSIQIIDDSFDLLLIAIVLYFDIIFCLLGFFIINVLNDDSQLSFMQL
IWFGGAAISILLHMCLYCAVGETLVTQCEKIHRAAYAYAWYTSDPKAARSLILIMLRASKPLYITAGKTFPMT
MATFCNLLKTSAGYISVLLANQD
>HsOr89
LKWAIGLNRFMLKIIGLWPPDNRDPHVTIKSKIRLLCSIIIMLFILTIPTFASLIKIWGNMMLMIDNLIYSLS
LLIALFKVFIIWYKQEVSFIYLNINIDWMKPKIKEERDIMLKLTNISRMIAIYGWFLPFISTMICITLPCFGR

TIRYITNLTDPGKPMMVQTYYLHGVSKSPQFELTLLAQGIALCTTCISYYSIDHFLGLLVLHVYGQMENLHIR
LTHMERYTNFDSVLKYNVQDHSRLIRSIQIIDDSFDLLLIAIILYFDIIFCLLGFFIINVLNDDGQLPLMQLV
CVVGAIISVLLHMCLYCVVGEILVTQCEKIHRAAYEYAWYTLDPKAARNLILIMLRASKPLYITAGKTFPMTM
ATFCNLLKTSAGYISVLLANQD
>HsOr90
LEWAIGLNRLVLKIIGLWPSDNRGSHMTMKSKIQLLCSVIMLLFVLAIPSFLSLIRVWGDMTLVVDNLIYSLP
VSIAILKVCILWYKQEDLVLLIDMIATDWMKPKMKEERDVMLRLAKISRMVAIYGWLPPFISGIIYFTPTCFG
RTIRHITNLTDPGKPLMVQAYYLHDVSKSPQFELTLLAQGIALFITGISYYAIDHFFGLLVLHVYGQMENLHI
RLTHMERYTNFDSVLKYNVQDHSRLMRFIEIIDDSFDLLLLVIILYFGIIFCLLGFVIINILNEDGQLSFMQI
VWFVGATISFLLHMYLYCVVGETLVTQCEKIHRAAYEYTWYTLDPKVARNLILIMLRASKPMYITAGKTFPMT
MATFCNLLKTSTGYISVLLANQDQ
>HsOr91
LEWAIGLNRLMLKLIGSWPPDNRDPHKTLKSKIRLLCSIIILLFILAIPSFLSLIRVWGDMILMVDNLIYSLP
VSIAILKVCIIWYKQEDLVSLIDMIATDWMKPKMKEERTVFLGLAKISRMIAIGGCFLPFIPMIVCFALTYFG
LTLRHVTNLTDLGKPLMVQTYYLYNVSKSPQFELTVFAQGIALFIMCISFYAVDHFLGLLVLHVYGQMENLHI
RLARMERYANFDSVLKYNVQDHCRLIRSIEIIDDSFDLLLLVIVVYFGIIFCLLGFLIINILNDNGQISLMQL
GWFAVGTITVLLHMGLYCAVGETLVMQCEKIHRAACDYTWYTLDPKPARKLILIMLRASKPLYITAGKTFPMT
MATFCNLLKTSTGYISVLLANQD
>HsOr92
LEWAIGLNRLMLKIIGLWPPDNRDPHETLKSKIRLLCSIITLLFILAIPSFLSLLRVWGDIILVVDNLIYSLP
VSIAIIKVFIIWYKQEDLVSLIKMIAIDWMKPKMKEERDVMLKFAKISRMIAIGGCFLPFIPMIVCFALIYFG
LTLRHVTNLTDSGKPLMVQTYYLHNVSKSPQFELTVVAPGGALFITCVSYYAIDHFLGLLVLHIYGQMENLHT
RLTHMERYINFDSVLKYNVQDHNRLIRSIEIINDSFDLVLLVIIIYFGIIFCLLGFLIVNVLNDDGQMSLMQL
GWYVAATITILLHMGLYCAVGETLVTQCKKIHRATYDYTWYTLDPKASRNLILIMLRASKPLYITAGKTFPMT
MATFCNLLKTSTGYISVLLAKQD
>HsOr93
LKWAIGINRLMLKIIGLWPPDNRDPHEIIKTKFRLLCSTIMVLFILAIPAFISLIKIWSNMILLVDNLVYSLP
VSIALFKVFTIWYKRKDLASLIDMIATDWMKPKTKEERDVMLKLAKISRMIAICGWLLPCIPTLTCFALAYFG
LTLRYVTNLTDPGKPLMIQTHYFHDVSKSPQFELTFLAQGIAVFITSISYYAVDHFLGLLVLHVYGQMENLHI
RLTHMERYTNFDTVLKYNVQDHIRLIRSIEIIDDSFDLLLLVIVAHFGIIFCLLGFLIVNVLNDDGQLSFMQL
VWFIAMTISVLLHMCLYCGVGEILVTQCEKIHRAAYEYTWYTLDPKVARNLILIMLRASKPLYITAGKTFPMT
MATFCNLLKTSTGYISVLLANQN
>HsOr94
LEWAVGPNRLMLKIIGLWPPDNHDPHEAIKSRFRLICNLTMLLFVLAIPSFASLIRVWGDMILMIDNMIYSLP
MLIFLFKVCIIWYKQEDLVPLIDMMTRDWMKPKMKEEQDVMLKHARTSRMIAMSGWFIIIIIVMINLIYPCFG
TTTRHVTNLTDPGKPLPMQTYYLHDVSKSPQFELTLLAQRITIIISSISYYATDHFLGLLVLHVCGQMENLHI
RLTHMEQYTNFNVVLKYNVQDHIRLIRSIEIIDDSFDLLLLVIILYFGIMFCLLGFLIVNVLNDGQLSLMQLV
WFIAVTITILLHMCLYCAVGEALVRQCEKIHRATYEYTWYILHPKAARNLILIMLRANKPLYITAGKTFPMTM
ATFCNLLKTSAGYISVLLANQ
>HsOr95
LEWAIGLNRLMLKIVGLWPPDNQDPRRALRSKIRLLCSFIMMLFILAIPTFVSLIRVWGDMLLIVDNLIYTLP
LSIALFKVCIIWYKQEALMPLIDMIARDWMKPKLKEERDVMLRLAKTGRMIAICGWPMAIFPTITCFVLPYFG
LTIRHVTNLTDPGKPLAMQAYYLHDVSKSPQFELTLLAQGITMLTTGVSYYAIDHFLGLLVLHMCGQMKNLHM
RLTHMERYTNFDTILKYNIQDHIRLIRSIEIIDDSFDLLMLGIMLYIGLIFCLLGFLIVQVLNDDGQLSFMQL
VWFVAVTVCVLLHMCLYCAVGETLVTQCENIHRAAYEYTWYTLDPKTSRSLILIMLRASKPLYITAGKTFPMT
MATFCNLLKTSAGYISVLLANQD
>HsOr96
LDWAIGLNRLMLKIVGLWPPDNRDCHESMKSKIRLLCSIIMILFILAIPTFVSLIRVWGNMILMIDNLIYSLP
VLIALFKVYIIWYKQEDLVQLIDMIARDWMKPKIKEERDVMLRLAKISRMIAICGWLLAFFLTITFFTLPFFG
LTIRHVTNLTDPGKPLAIQTYYLHDVSESPQFELTVLAQGISMFLTSISYYAVDHFLGLLVLHVCGQMNNLHL
RLIHMGQYTNFNAILKYNVQDHIRLIRSIEIIDDSFDLLLLILVLYFGITFCLQGFLIVNVFNDDGQVSFMQL
LWFVAVTICVLLHMGLYCAVGETLVTQGKKIHRATYEYTWYTINPKAARNLILIMLRASKPLYITAGKTFPMT
MATFCNLLKTSAGYISVLLANQD
>HsOr97
AIGLNRIMLKIIGLWPPDNRDHRKITKSRFRLVYNLTTMFFILTIPAFAALIRVWGDMILMVDNLIYSLPMTI
SVFKVCIIWYKQKALVSLIDMIARDWMKPKLKEERDVMMKLAKISRIIVICGWLMVILPTIISLVCSCFGVTT
RYITNLTDPGKPLALQTYYLHDVSKSPQFELTLLAQGITMFTTGLSYSAVDQFLALLVLHVCGQIENLHLRLI
HMGRYTNFDAILKYNVQDHVRLIRSIGIIDDSFDLLLLVVIVYFGITFCLQGFLIVNVLNKEGKLSIIQLVWF

IAVTVCILLHVYLYCAVGEALATQYEKIHRATYEYAWYTLDPKTARNLILIMLRANKPLYITAGKTFPMTMAT
FCNLLKTSAGYISVLLANQD
>HsOr98
KWAIGLNRLMLKIVGLWPPDNRDAHKIVKSRFRFGYNLITLLFILTIPAFAALVRVWGDMILMIDNLIYTLPL
SIIVLKICILWYKQEALAPLIDMIAKDWMKPKLKEEREVMLKLAKISRMIAMCGCLIIIIPVTISLICPLLGV
TNRHMTNLTDPSKPLAIQTYYLHDVSKSPQFELTLLAQGVTMFTTTISYYAVDHFLALLVLHVCGQMENLHVR
LIHMRRYINFDAVLKYNIQDHVRLIRSIEIIDDSFDLLLLVVVVYFGVTFCLQGFLILNVLNDEGQLSIIQLV
WFSTITVCILMHMGLYCAVGETLVTHYEKVHRATYEYTWYTLDPKVARNLILIMLRASKPLHITAGKTFPMTM
ATFFNLLKTSAGYISVLLANQD
>HsOr100
LKWAIGLNRLMLKIPGLWPPDNQDSREPIKSKIRLLCSIILLLILAIPTFISLTRVWGDIILMVDNLIYSLPL
MIAIFKICVIWYKQKALAPLIDMIATDWIKPKTKEERDVMLRLAKISRMIAICGWLLASFLTITFFTLPFFGV
TTRHVTNLTDPGRPLAIQAYYFYDVSKSPQFELTVLAQGISMFTMGITYYAVDHFLGLLVLHVCGQMENLHIR
LTHMERYKDFNAILKYNIQDHMRLIRSIEIIDDSFDLLLLGIMLYFGIVFCLLGFLIVNLLNDDGHLSFMQLV
WAVAVTVCILLHMCLYCAVGETLVTQSEKIHSATYEYAWYTLDPKAAKNIVLIMLRARKTLRITAGKIFPMTM
ATFCSLLKTSTGYISVLLANQD
>HsOr101
EWAIGLNRIMLKTVGLWPPDTRDTRENIRAKIRLTYNLITLLFILTIPSLIALIRVWGDMILMIENMMYSIPL
LTTVFKVCIIWYKQEDLLPLIDMIERDWTKPKMKEERDVMLRRAKIIRAIATCGWIVTSFTIIFTFGLPSVGM
TIRHVTNLTDPGKPLPIQSYYLHDVSKSPQFELTLLAQLLTLITTGFSYTGIDHFLGLLVLHVCGQLENLCLR
LTHMKMYSDFDAALIYNIKDHIRLIRSVEIIDDTFNVMLLFLVLYLGIVFCLQGFLLINVVNQTGQLSYVHVG
WYATAIVYISLHMCLYCVVGEVLVTQSEKIHHATYKYPWYNIDPKTSKNLMLTMLRASKPLYITAGRTFPMTM
STFCNLLKTSAGYISVLHANQD
>HsOr102
ERVININRFMLRTVGLWPPDNRSSRKVMKSKIRLLHTFSMVFFVLTIPALISLIRVWGDIILMIENMQYSLPL
LGAISKICILSQKQADLLPLIEMIENDWVKPKMKEEQDVMLRRARIIRVITICGFFTGILTIIITFGFPCFGL
MLRQVTNLTDSGKPLLIPSYYFHDVSKSPQFELTLLAQGISMIACGCSHIGVDHLLGLLVLHVCGQLENLHLR
LSRLEKYSDFNAALEYNVQDHVRLIRSIEIIDHTFNLLLLILVLYFGILFCVQAFLIVQVVNQKGQLSLTQLI
WFISAIVYVSLHMFLYCAVGEILVIQSEKIHQATYEYSWYNKHPKVVKGLMLIMLRANKPLHITAGKTFPMTM
STFCNLLKTSAGYVSVLLTTQ
>HsOr103
ERVVSIIRLMLRIIGLWPPDNQKDHTVIKSKIRLLYSFSMVLFVLAIPALVSLIRVWGDMVLMIENLQFTLPI
MGATLKIFILSRKQADLLPLIEMIKRDWIKLKMKEERDIMLKRARIIRALTICGLLCGVVLTIIIIFTFPCFG
LMLRQVTNLTDSGKPLPIPSYYLHDVSKSPQFELTFLAQGIAVIVCACSYTGTDHLLSLLILHICGQLENLYL
RLTHLEKYGSFDVALKYNVQDHIRLIKSVETIDHTFDLMLLVLVLYFGILFCLQAFLLVDVINQEGQLSLTQL
IWFTSAIVYVSLHMFLYCAVGEILVIQSENIHQATYEYPWYNKEAKVARSLMPIMLCASKPLYITAGKIFPMT
MSTFCNLLKTSAGYVSVLLTNQ
>HsOr104
ERVVSINRLMLRIIGLWPPDNQDTRQVMKSKLRLLCSFNMVFFVLTIPALISLIRVWGDMILMIENMQYSIPL
LNTIFKFYIIWQKQTDLLPLIDMIKRDWVKPKAKEERDVMLRRARITRAIAMYGLFAAAGTIIITLGFPCFGL
ILRQVTNLTDSGKPLPIPSYYLHDISQSPQFELTFLAQGFALIACGCSYTGTDHFLGLLVLHVCGQLENLYLR
LIHMGKYENFDIALKYNVQDHIRLIRSVEIIDHTFDSMLLVLVLYFGILFCLQAFLIVDVVNQKDQLSLIQLV
WFVSAIIYVSLHMFLYCAVGEILMTQSEKIHQATYEYSWYNKKPKVAKNLMLIMLCANKPLHITAGKTFPMTM
STFCNLLKTSAGYVSVLLTNQ
>HsOr105
EWALSVNRFILRIAGLWPPDNYDTREALKSKIWLLYNFLTILFILTIPMLISLIRVWGDMILMIENMQYSLPC
IGTIFKICIIWYKQADLLPLINMIERDWMKPKMKEERDVMLNRARIVRVIAICGISGALCGIIITFSFPYLGL
RLRQVTNLTDSGKPLPVPSYFLHDVSKSPLYELTLLMQSITAVACGCSYTAVDHFLGLLILHICGQLENLHLR
LSRMKKYPNFNAALEYNVQDHIRIIRSVDIIDRTFNSMLLVLVLYFGVLFCFQAFLIVEVVNRKGQLSLTQMI
WFVADTVYILMHMCLYCVVGEILIMQSEKIYQVAYEYPWYNKEPKVAKSLMLIMLRASKPLYITAGRTFPMTM
TTFCNLLKTSAGYVSVLLAN
>HsOr106
EWAVSLNRFILRIVGLWPPDNCDTREAVKSKIRLLYNFMTMLFILTIPTIISLIRIWGDMTLMIENMQYSLPF
ISTIFKVSIIWYKQADLLPLINMMERDWMKPKIKEERDVMLSRARIIRAITICGMSGAIFAIIIIFGFPCLGL
RLRQVTNLTDSGKPLPIPSYYLHNVSKSPQFELSFLAQVFTMTACGCSYTAIDQFLGLLVLHICGQLENLHLR
LSRMKKYPNFNAALEYNVQDHIRIIRSVEIIDHTFNWMLLILMLYFGVLFCLQAFLIVQVVNEKGQLSLTQLI
WYVTATVYVLMHMCIYCAVGEILVIQSEKIHQAAYEFSWYNKEPKVAKSLMLIMLRASKPLYITAGKIFPMTM
STFCNLLKTSAGYVSVLLTKQ

>HsOr107
KWAISLDRFMLRVVGLWPPDNYDTREAVKSKIRLLYNIITMLFVLIIPTLISLIRVWGDMILMIENMQYSLPF
MSTIFKISIIWYKQADLLPLINMMERDWMKPKTKEERDVMLNRARIIRVIAICGISWALCGITITFSFPYFGL
RLRQVTNLTDFGKPLPIPSYYLHNVSKSPQYELTLLMQSITATACGCSYTTIDHFLGLLVLHICGQLENLHLR
LSRMEKYSNFNAALEYNVQDHIRIIRSVDIIDHTFNSMLLVLVLYFGVLFCFQAFLIVEVVNRKGQLSLTQMI
WFVAAIIYILMHMCLYCIVGEILVMQSEKIHQATYKYPWYNKDPKVAKSLMLIMLRASKPLYITAGKTFPMTM
ATFCNLLKTSAGYVSVLLAN
>HsOr108
EWAVSLNRSMLRVVGLWPPDNCDTREAIKSKIRLLYNFMTMFFILTIPILISLIRVWGDMTLMIENMQFSIPF
VNTTFKISVIWYKQADLLPLINMIERDWMKPKMKEERDVMLRHARIIRAIAMCGMFGAVFAIIVISGFPSLGL
QLRQVTNLTDSGKPLPIPSYYLHDVSKSPQYELSFLAQVFAMIACGCFYTAIDHFLGLLVLHVCGQLENLYLR
LSRMEKYSNINAALEYNVQDHVRIIRSVEIIDVTFNWMLLILVLYFGVLFCLQAFLIVKVINEKGQISLTQLI
WYVAATVYVLMHMCIYCAVGEILVIQSEKIHQAAYEYSWYNKEPKVAKNLMLIMLRANKPLYITAGKTFPMTM
STFCNLLKTSAGYVSVLLTKQ
>HsOr109
EWAVSRNRLMLRIAGLWPPDNYDARKAVKSKIRLLYNFSIVFIVLAMPALISLIRVWGDMILMIENMQYTLPI
MGTLFKICILWQKQKDLQTIVDMVASDWLKSKTNEEREVMLKRARIVRTIAMYGLFSTILTVTTAFSFTCFEL
IFRHLTNRTDAGKSLPIPLDYLYDVTKSPQYELTLVAQIFASVTCGCSYTGVDHFLGLLVLHVCGQLENLHSR
LTYMDKYPNFTAALQHNVQDHVRLIRHANIVSIEIIDNAFDSMLLILVLYFAIIFCLQGFLIVDVVNRKGQLS
MIQLLWFVVATLYVLLHMCFYCIVGEILVIQSEKIYQATYEYPWYNKEPEVAKSLILIMLRASKPLQITAGKT
FPMTMATFCNLLKTSAGYISVLLANQN
>HsOr110
EWAVSINRLMLRIAGLWPPDNHDTREAIKSKIRLLYSFIMVIIILAIPALISLIRVWGDMILMIENLQYTLPI
MGTIFKICILWQKQKDLQTIVDMIATDWLKPKTEVEKAVMLKRARIIRIMAMCGFSGALLTIVIAFGFTCFEL
IFKDMTNLTDSGKSLPLPLHYLYDVSKSPQYELTLLAQGFTTVTCGCSYTGVDQFLGLLVLHVCGQLENLHFR
LTHMDKYANFMAALQYNVQDHVRLIRSIEIIDHTFDSMLLILLLYFAILFCFQGFLIVDVVNQKGQLSLKQLI
WIVAAIVYILMHMCLYCVVGEILVIQSEKIYQATYEYPWYNKEPKVAKSLMLIMLRASKPLQITAGKTFPMTM
ATFCNLLKTSAGYISVLLANQD
>HsOr111
EWAVGINRFMLRVVGLWPPDNHGARATVDSKFRILCSFITLFIVLTIPALISLIKVWGDMTLMIENMQYTLPI
LGAVFKICIISQKQADLLPLVEMIERDWVKPKSEEERGVMLRRARTVRAIAMCGLFSTILTIISVFSFTCFEL
IFKHVTNLTNSGRSLPIPLDYLHDISKSPQYELTLAAQLFAVVTSCCSYTAVDYFLGLLVLHVCGQLENLYLR
LTRMEKYTDFSTALKHNVQDHVRLIRSIKIIDHTFDSMLLVLLLHFAILFCFQGFLIVDVVNRKGQLSMIQLI
WFVAAIVYALIHMCLYCVVGEILVIQYEKIHQATYEYPWYNKESKVAKNLILIMLCASKPLLITAGKIFPMTM
ATFCNLLKTSAGYISILLANQN
>HsOr112
EWAVRINRFILRIVGLWPSDNHGARAAVDSKFRLLCSFITLFIVLTIPALMSLIKVWGNMILMIENMQYTLPI
LGAVFKICIISQKQADLLPLIEMMERDWVKSKSEEERSVMLKRARIVRTIAMCGLSSTILSIISAFSFTCFEL
IFRHVTNLTNSEKSLPLPLYYFHNVSKSPQYELTLVAQIFASVTCGCSYTGVDHFLGLLVLHVCGQLENLHSR
LIYMDKYPNFMAALQYNVQDHVRLIRSIEIIDNAFDSMLLVLVLYFAIIFCLQGFLIVDVVNRKGQLSMIELL
WFIAAIVYVLFNMCLYCVVGEILVTQSEKIYHATYEYPWYNKESKVAKSLMLIMLRASKPLQITAGKTFPMTM
PMFCNLLKTSAGYISVLLANQN
>HsOr113
EWAISLNRFMLKIVGLWPPDNRHARGAIKSKIRLLYSIITMLFILTIPTLISLTRVWGDMMLMIENMQYSIPF
LITILKVCIIWYKQADLLPLIYMIERDWMKPKMKEERDVMLRRAKTVRAIAMCGMFNSILLSIITFGFPCFGL
VLRHVTNLTDPGGKPLLVPSYYLHDVSGSPQFELTYLTQGITLFACGFSYTAVDQFLGLLILHVCGQLEGLHL
RLTRMEQYTNFNAALEYNIQDHVRLIRSIEIIDDVFDSMLLVLVLYFAIIFCLQGFLIVDVINRKGQLPLTQL
IWFVVATAYVLLHMCLYCAVGEILVTQSEKIHEATYEYSWYNKEPKVAKSLMLIMLRASKPLHITAGKTFPMT
MATFCNLLKTSAGYISVLLANQD
>HsOr114
LEWTINLNRFMLRIAGLWPPDHHDASEAVKSKIQLLYNFITILIVLAIPSLLSLIRVWGDTILMIENMQYSIP
YLSTIFKICIIWYRQADLLPLIHMIEKDWAKPKMKEERNIMLKYARTIRLIAMCGLSSTLLALISTCGYSCFE
IIFRHVANLTNPTKLPLPVHYLHDVSKSPQYELTLLAQITTLFICGFSYTAVDQLFGLLVLHVCGQLESLNLR
LTRMEQYTNFDATLKYNVQDHVRLIKSIEIIDDAFDLMLLVLIFYFAIIFCLEGFLIVNIINRKDQLPLKQFI
WFITGIVYILIHTCLYCAIGEIIVTQSEKISQATYEHPWYNLKPRAAKNLMLIMHRASKPLQITAGKIFPMTM
IMFSNLLKTSASYISVLLAIQD
>HsOr115

EWAISLNQLMLKIIGLWPPDNHIIRKKMKSKIRLFYSITTILFILIIPVLISLMRVWGDMILMIENMQYSLPL
LATISKLIIIWYKQADLLSVIHMIEEDWTKPKMKEERDVMLKRAINTRTIAMWGLFVAIFTIVITFGFSYFGL
ILRHVTNLTDSGKPMLIPSYYFHDVSKSPQFELTFLAQGITLIASGCSYTAVDHLLGLLVLHVCGQLENLHFR
LTHMRKYPNFLANLKYNVLDHIRLIRSIEIIDNTFDSLLLVVMLYFGIIFCLKGFLIVEVVNRGGKLSLMQLL
WFAAAIIYILLHMCLYSAVGEILVTQSEKIHQATYKYPWYNVEPKAAKSLMLIMLRASKPLHITAGKIFPMTM
ATFGNLLKTSAGYISVLLAKQN
>HsOr117
EWAVNLNRHMLKIVGLWPPDNRDAREIVKSKLRRLYNIVTLFFILTIPILMALMRVWGDMILMVENMQYALPI
LCAVFKVCILWYKQKDLLSLIDMIEIDWTKSKTKKERDVMLRRARITRAIVMSGWVITILTLIITFGLPCFGL
TARHTTNRTDFGRPLPIQSYYLYNISTSPRFELTLLAQGFTMITTSLSYCGFDHFLGLLILHVCGQLENLHLR
LMHMEMYSNFNVALKYNVQDHIRLIRSVELIDNIFGLMLLVIVIFFSIMFCLQGFLLVNVVHQKSQVSPIQLI
WFVTALVYPFLRMFLCCVVGEIIVTQGTRLSEKIHRATYEYAWYDTDPKAAKNLIMITLRANKTLCITAGKMF
PMTMASFCNLLKTSAGYISVLLAKQN
>HsOr118
LEWAIGLNRLMLKIIGLWPPDSRDPHETIKSKIRLLCSVVILLFVLAIPIFLSLIKVWGDMILMVDNLIYNLP
VSIALFKIYIIWYKQEALMPLIDMIARDWMKPKIKEERNVMLRLARISRMIAISGWLVPFFLAIISFVLTCFG
ITTRSVTNLTDPGKPLMVQAYYLHNISKSPQFELILLAQGIALFITGISYYAIDHFFGLLVLHVHGQMENLHI
RLVHMERYTNFDAVLKYNIQDHIRLIRSIEMIDNSFHLLLLGIILYFGIIFCLLGFFIVNVLNDDGQLSVMQL
IWFVAVTICVLLHMCLYCAVGEILVTQCEKIHSATYEYTWYTLDPKAARNLIFIMLRASKPLYITAGKIFPMT
MATFCNLLKTSAGYISVLLANQD
>HsOr119
LEWAIGLNRFMLKIIGLWPPDNRDPREAIKSRFRLLYNLITLLFVLAIPSLASLIRVWGNMILMIDNLIYSLP
ISIAVFKVCIFWYKQEALAPLIDMIVRDWIKPKLKEERDVMLRLAKISRMIAMCGCLIVIVPMVLSVICSLFG
ITNRHLTNLTDPGKPLAIQSYYLHDVSKSPQFELTLLAQEITLITTGISYYSIDHFLGLLVLHVCGQMENLHL
RLTNVRKCTDFDVTLKYNVQDHVRLIRSVEIIDDSFYIMMLILVLYFGIIFCLQGFLIINVLNDDGQLSFMQL
VWFIALTTCVLVHMCLYCAVGESLVTQCKKIHRATYEYAWYNINPKAARNLILIMLRASKPIHITAGKIFPMT
MATFCNLLKTSASYISVLFANQN
>HsOr120
LEWAIGLNRYVLKIIGLWPPDSNDTRECLSSRFRRLFNIIMLIFVLTIPSLMSLIRVLRIRGDMLLIIDNLQY
TLPLLITILKVSIMWGKKKALMPLIDMITRDWIKTKIDTERNVMLRQARISRSFSMCGGLMILSTLIFGLILP
CFGLTLRHVTNLTDPGKPLPIQSYYLHDVSKSPQFELTLLIQGVGLTLSGLTYTGVDTFLGLLILHICGQMEN
LHMRLTNLGKDTNYKAAVKKNVNDHVRLIRSIQIIDKTFHLMLLGLFFLFGILFCLHGFLIINVVNQDGHLSY
TQLCGFLAASLCVLLHTCLYCAAGEFLVTQSEKVHGATYKSVWYTIEPQMARDLALIMLRANKPLYITAGKTF
PLTMATFCNLLKTSAGYISVLLANRD
>HsOr121
LEWAIGLNRRMLKIVGLWPLENKDTNVALLSKLQLLFNVATLIFVLIPALWSLIRVWGDMMLMIDNLQYTLPL
LITALKVSILWSKREVLMPLIDMIAKDWMKAKLEKERNVMLNRARITRVLAMCGGFMILLTLLITIILPCFGL
TLRHVTNLTDPGKPLPIQSYYLYDVSKSPQFELMLLIQGIGLGLSGLSYTGVDTFLGLLILHICGQLENLHVR
LTNLGESSNYKVALKYNVKDHVRLIRSIQVIDDTFDLMLLGLVFFFGVLFCLHGFLIINVVNRGNGLSFMQLC
WFVSASVCVLLHMCLYCAVGEFLVTQVSRI
>HsOr122
LEWAVGINRTSLKILGLWPDDKLTRRQSLVADARAFSIFLLMMLTAILPQMFAMFHVWGDIMAFTDNLQLSVP
FSVTVMKFFIMWIRKEELKPLVNMIVNDWFKTKTALERATMIKQARIARTIVMLGGIMMTLASIILIIPPCFG
YSMRYVTNITDLGKPLLLQSYFFREIESPFFEVAFIGQALSISLAAISYTGIDNFLGLVVFHICAQLDLLKYR
LLNLHNFRDFNASLSYNVKDHLRLIRLSVDAIDNTFNLMLLALLLFFGILFCMQGFLIISIIDGNGGDVSIMR
ICWLVSILINTFAHMCLYCIVGEILIAKCDGVYYATYGLAWYTLKPNQARDMMLMMIRAEKPLYITAGRIFPM
TMSLFCSLIKTSAGYISVLLTNR
>HsOr123
MFAGTFTRAIEVGLQVIGIWPGAPCAIISRILWTTSMLAAQTFQYRHMLSHLRSEDLSLLMDGLSATLSYSLL
CVKLIVFWTKQRIFHDVIASISTDWEECGETGDALCNMINVASLSHRFSNLIIGLHSMAVLFYCVGVVALRTS
GAGQDDVERELFLKMDLPFESDASPVYELVMTTQFLHQMTSATVIGVLSALLVTLVLHVGGQIDILRERLLEI
LPKDRKPTVSVITMGSLIRKHQSIIVFTGKIENLYSHIALAQFISNTLVICCLGFIIVKSINDDQSFSMLVRS
LLFYVVINLEAFIFCFAGEYLSVKSKMIGDAAYESLWYDLTPSETRILLLLIMRSQKQLTITVGSFTNLSLQQ
FANIIKSSASYVSVLYAL
>HsOr124
KSTINRTVKILLILFGMWPDISSVMFFRVFWGVSLGIIQVCQCRYLLTHFYTSDMFDLMDCFSVIIGFVKFSL
KILVFWWNQRIFNQILQMMAEDWKDCADNDIKIRKVTSKARISHYISNAVVTTHTIAICFYGIGVVLTDVDIT
DHTIEIPHIYKMEVLFEINTQSTYRFTLIMELIHLLLCSLCLAILNVLILILTLHVGDQIDILIFWLTELTFV

ENKHRFRMMRKIIQKHQKIIHFAENIEKLYTIIALMQFVSNVVMICILGFLIIIALDNPNATEKIMRPLAYYS
VTNLEAFIFCYAGEYLINKSRAIGYAAYESAWYDMEPKYSRILLLIISRSQKQLTLTIGKLMDLSLPRFASIM
NSAGSYMSVLLAMQ
>HsOr125
VMARKSTISRMVKCLLVLYGIWPDASCITCCKVFWSISLAVVLISLYRYLCAHFYSSDIFDLMDCLSTFVGYL
KLFIKVVIFWWNQQIFNQILRTMEEDWKDCANNDIEMRKVISKAKISRYVSNAIITLHTVAVLFYGISIILTN
VDITDRTIEIPHIMRMEFPFNIKTQSTYKFVLIMELIQLIMSSWGMGMINALLITLTLHIGGQIDILRCWLTE
LEFVEIADKRRSNMIKRIIQKHQKIIYFTENIETLYTFIALLQFVSNVMMICILGFLIITALDNPNATEKIMR
SLSYYSVTNLEAFIFCYAGEYLINKSKVIGYAAYDSAWYDMEPKDRRILLLIILRSQKQLTLTIGKLMDLSLQ
RFTSIMNSAGSYISVLMAMQ
>HsOr126
VMARKSTISGMVKCLLVLYGIWPDTSCITICKVFWCISLAVVQVSLYRYLCAHIYSNDIFDLMDCLGTFIGYF
KLSIKIIVFWWNQQIFNQILKMMAEDWRDCANNDIEMRKVISKAKISHYVSSAIITLHTVAVLSFGVNIILTD
VDITDRSIEIPHIFRIEFPFDIKSQSMYKFVLIIELIQLVMSSWGMGVINALLIALTLHIGGQIDILRSWLTE
LQSAESADKHRNKMIKKIIRKHQKIIYFTENIETLYTFIALLQFVSNVMMICVLGFLIITALGSPNATEKIIR
SLPYYSVTNLEAFIFCYAGEYLINKSKEIGYAAYDSAWYNMEPKYRHILLFIILRSQKYLTLTIGKLMDLSLR
RFTSIMNSAGSFISVLLAMQ
>HsOr129
KATKVVACQSTISHIVKYLLILYGIWPDTSCITLCKVFWSISLTIIQFWHYQYFLTHFYSNDIFDLMDCLSTF
AGYFKLLIKIIVFWWNQQVFNQILRTMAEDWKDCANNDIEMRKVISKAKISHYVSNAIIYLHTITVLFYGINI
ILTDVDITDRTIEIPHIFRIELPFDIKTQSMYKFVLIIELIQLVMSSWGNGVISALLIALTLHIGGQIDILHC
WLTGLEFARSTDKHRINVIKKIIQKHQKIIYFTENIETLYIFIALLQFVSNVIMICILGFLVITALDNPNATE
KIMRSLSYYSVTNLEAFIFCYAGEYLINKSKVIGYAAYDSAWYDMEPKDRRILLLIILRSQKQLTLTIGKLMD
LSLQRFTSIMNSAGSYISVLMAMQ
>HsOr130
ESTINLTVKILLILFGIWPGTSSVTFFRVFWSISLGMIEICHYRYLLLHFYTSDVFDLMECFNTFIGFVKFSF
KISIFWWNQRIFNRILKMMAEDWKDCANNDIEIRKVTSKARISHFISNAIAITHTVSICFYGIGIILTDVDVT
DHTIEIPHMYKIEVLFEINTQNTYRLTLIMELLHLLLGSLGMGILNALLLVLILHIGGQIDILISWLTELMSV
ESANKHRFNMMKKIIRKHQKIIYFAENIEKLYTIIALLHFVSNVAMICILGFLIITALDNPNASEKIVRSLAS
YSVTNLEAFIFCYAGEYLINKSKAIGYAAYDITWYDMDPKHTRILSLIILRSQKQLTLTIGKLMDLSLQRFAS
IMNSAGSYVSVLLAMQ
>HsOr132
KSAQSRRMISWPLEMLLRLFGIWPGVSYGPLCKVIWMIIMTTNMFLQYVFVIIQARTVNLTIFMYAIAVSLAG
SMKLFKLATFWCNQRRFNEMWAVMSSYWELSDAYTSNIRKMYVMRHMPNFIVAFNSLSVIMTSVSILLSGIHY
DEASNTTRPYIMMMHLPFDINKQSVYLVVIFLQFFYLLLITTGAATLNSIFIILMLYLGDQIDIVCRSLTKLP
QGENERMTTMKQIIQKHQSIINFSENIESLYTYIALGLLLLNTLLTCVLGFILVTSIGSPHFTKMLMKNLLFY
CAVNIETFVFCFAGEYLSTKSREISEAAYNSFWYQSKFHSRVVLFMIMRSQNQLTITMGKFMDLSLEQFSTIV
KTSASYISVLLAMY
>HsOr133
MKVSTVCFSIKFVLRIFGVWPNASCVVLRRLFWSVLLIVTQVFQYAYFVVHLRTDDLPDLMDSLSCALAYTLL
LIKLIIFWVNERKFHEVLTMIASDWKECANYPFGMHVTTSTATMSYRVSNAIIGLHMVAVLTYSFGVLLSDVE
NAGFNASTVPARPLIIKMELPFDSNSSPVYELVMVVQFFQLMSNACAIDVLNALILTLILHVGGQIDILREWL
TNIFPSGGMYGTSGITIKTIIGKHQKIIMFSESVESLYSYIALMQFVSNTLIICSIGFVIASSLGNPDVSTIL
VKTLLFYIVMNLEAFIFCFAGEYLSTKSLNIADAAYGSHWYDMRVNESRPITFLILRSQKRLTITIGKIMDLS
LERFASIIKASASYVSMLLAMS
>HsOr134
MKMSTLSRVMEIGLQACGVWPYLPSTVLYRLLWVVTLGIVQVLQYQYLLIHYHSDNFADFMDGVSSTMAYTLL
FIKLIIILWANQRTFDKILTMMAVDWEKCANTEFSMFAMTSKAKVSHWLANWTITLYSIAVVLYSSDVLLYRLN
ISRISNSSARPFILKMEIPFDSDKRFVYESIIITQFFHLVSCSCTFAAINVLLIILILHVAGQIDILCEWLVE
IFPLARKCDLNLIMIRKVIKTHQKIITFSEHIEDLYSGIAMVIFISDTLIICCLGFVIVASVGTPGASKIIIK
TFLFYIVMNLEAFIFCFAGEYLSAKSQTVGDAAYDSLWYESNPKNSRLMLLLIMRSQNQLTITIGKVMDLSLE
RFSGIVKASASYISVLLAMY
>HsOr135
MKMSTLSRVMEIGLQACGVWPYLPSTVLYRLLWVVTLGIVQVLQYQYLLIHYHSDNFSDFMDGMSSTMAYTLL
FIKLTILWANQRSFSDILQMMSTDWKNCSLTDHSLHITTSRAKLSHRFSHWIIGLQLIAIVLYICGVLAVNAD
EMRRMNVSARQHILKMKLPFTVNTSPVYALVMILQFFLLVMCACGVSIVNSLVVSLILHIGGQIDILRNWLLR
GFSQNVINTVNRITIRALITKHQRIVVFSQNIENLYTYIALMLFVSDTLIICCLGFIIVSSIGTPEGPAILVR

SMLFYIMVNLEAFIYCFAGEYLSAKSQMIGDAAYDSLWYNLTSRKNRITLLMILRSQKRLTITIGKIMDLSLE
RFASVVKASASYVSVLLAI
>HsOr136
MKMSTLSRVVEVGLHACGIWPYLPSTVICRLFWIVLLGTAQVLQYQYLLIHYHSDNFSDFMDGMSSAMAYTLL
FIKLTILWANQRTFSDILQMMSTDWKNCGLTDHSLYITTSRAKLSHRFSHWIIGLQVIAIVLYSCGVLAVNAD
EVRQMNVSAREHILKMKLPFTVSTSPVYALIMILQFFHLVMCGYGISLVNSLVVTLIVHIGGQIDILRDWLLR
AFSKNVVNTVNGITIRTLITKHQRIVVFSQNIENLYTYIALMLFVSDTLIICCLGFIIVTSIGTPDGPAILVR
SVLFYIVMNLETFIYCFAGEYLSAKSQMIGDAAYDSLWYDLTSRKSRVALLVILRSQKRLTMTIGKIMDLSLE
RFTNVVKASASYVSVLLAM
>HsOr137
MRTSTFSRIVKIGLHFYGVWPYLPSTVLFRSYCIIAMTTAQVFQYGYVVMNNDTDNFSEFMDGVSSAMTHSLF
FIKLIIILWINQRKFSDILRIMMMNWQDHTSNHHNLSIMTKATLVARRISRIIFHTNFFSVFFYSLGIFAANAG
DPKKWEPYNRELILKMKLPFEISTYPIYVTVTVVQIVHLMFIGWGIAIVNTLLVTLVLHIGGQIDVLRDWLTR
AFSKTESDEITSRLLIMKHQQIILLSENIENLYTYIAFMILLSDTLIICCLGFVIATSLGEPNAAAILVKSLM
FYVTMNLDVFVYCFAGEYLSAKSKMIGDAAYDSFWYNVAAKDSQIVLFIVLRSQKRLTITCGKVMDLSLERFT
SVVKASASYMSVLLAMY
>HsOr139
STFSQIVKIGLHFFGVWPYLPSTVLFRSYCIMALSTAQVFQYGYVVVNSDTDNFSEFMDGVSSAMASSLLFIK
LIIILWINQRKFSEILQIMTMNWQDHSSNHHSLSIMTKTTFLARRISRIIILMQFFSVFFYSLGVLAANAGDPE
KWEPYNRELILKMKLPFEISTYRIYVTITVVQFVHLMFVGWGITVVNTLLVTLILHIGGQIDILRDWLTRAFS
RNESEPSDKITSRLLITKHQQIILLSENIENLYTYIAFMILFSDTLIICCLGFVIATSVGEPNAAAILVKSFM
FYVTMNLDVFIYCYAGEYLSAKSKMIGDAAYDSLWYNVAAKDSQIVLFIILRSQKRLTITCGKIMDLSLERFT
SVVKASASYMSVLLAM
>HsOr140
STLSRLVKFGLHIYGILPYLPTTVLFRSYWIMVLCTAQIFEYRYVVYVYMVMGNFPLLIEGLSSAMVSSLLFTK
LNILWHNQRTFQEILQVMDADWRNYTSNDFGSRVMTKTAHVAHRVSKLIISLQVVSVLFYMYGTLVSNAHDPY
KVANPGAREQFLKVDFPINMTNSIYWGLIGVQFVHLLSIACGITFINSLLLSLILHDCGQIDILCEWVTKMFS
KDTSQCKTDEITISSIVFKHQQIIKFSTNINNIYAYIALMILLSDTLITCCLGFIIVSSIGTPNGGVIVVKSI
LFYITMNLEVFIYCFAGEYLSFKSIKIIEAAYNCLWYDAPXKYTSIISFIIMRAQKQLTITNGKIMDLSLELF
TSVIKASGSYISVLLAM
>HsOr141
MVTSTLSRAVKIGLHFYGIRPYLPTTVLFRLYWIMALSTAQVFQYRYVIVNIDTDDFSEYMDGVSSAMASSLL
FIKLAILWSNQRTFQKILQMMDADWRNYTSNHFGLNLMTKTAYVAHRASRLIIGLQVVAVFFYTYGVLAANAG
DPNRWEPYARELILKMDFPFNISTNSLYLSITIVQFVHLMLVACGITVINSLLVTLILHDCGQIDILCEWLTK
MFSRDTSQCNTEEFTTRSVVIKHQRIIEFSDNIENVYTYIAMMILLSDTLITCCLGFIIVTSIGTPNGGAILV
KSVLFYITMNLEVFIYCFAGEHLSAKSRKITDAAYDSLWYNTPSKYKYVMLFIILKAQRRLTITSGKIMDLSL
ERFTSVVKASASYISVLLAMY
>HsOr142
STMCRSVELGLRLVGIWPGTSMSRKFSYIALMALFQIFQYRYLIAHFGEHDLSLFMDVLSTTMGYSLLLTKLI
VLASKARLLDEIIACSIEDWKERDMFDEQAMTRMTHAARLFSNLIIFSNATSVVFYAIGTLMWHQNDNQTDTE
RELFLKMDLPFHVESESVYVAVLVTQFIHQMSAVSIIGVLDSLLLTLASENVLHVCGQIDSVQHKLSAITRKD
IERDACGSIVKMLIIRHQRIIAFSKNIEVLFSNIALIQFLSNTLIICCLGFLIVISIDLPNGSTILVKSVLFY
VGVSIQAFTFCFVGEYLSSKSKMIGDAAYEALWYDLKPNQNRDLFFMIVRSQKHLTLTAGKFVVLSLKQFGNI
VKASASYVSVLHAMY
>HsOr143
STFIRAVKNGPHFYRIWPYLPTTVLFRLYWIMAPSTVQVFQYWYVIVNISTDNFSEYIDGVSNKKASXLFIKL
AILWSNQRKFQEILQMMDADWGNYTSLGSLGHFGSNLMTNTAYVAHRSSSLIIGLQLTSVVFYSCGVLVINAD
XRQWEPYVRELMLKTDFPFNVSTNLIYLSITIVQFVQLMLVSWSITIVNYLLVALTLQGDSQVDIMCRGLLEI
SSSDNAFDSPGPVVNALIRRHQKIIAXVETFFCYIALIQFLNTLVICSLGFVIVTSIGDTQNSTMLIKSLFFY
IVITLETFIFCYAGEYLSAKGRMIGDAAYDAKWYNSSPTQSRIMLLLILRSQRKLTISIGKFMDLSLERFTTI
IKASASYVSVLHAM
>HsOr145
LVGLVSRSIEIGLRVIGVWPDSSFSSLRRAFWMITLTIAQTFQYRYFVVHVRTDDLSRLMDGLSTTMSYSLLL
LKLTIFWIHRRIFHHILATMARDRSDCATGWAACSMSRTIYVSHRSSNLIIGLYSMSVLLYGTGVLVGHTDES
DEAEDEQFSEPTREQFLKMELPFESNISPVYEVVMVVQFFHQLAAATIVGVLNALIVSLILHVGGQVDIMCRG
LLEISSSDNAFDSPGPVVNALIRRHQRIIALSSDIETLFCYIALMQFLWNTLVICSLGFVIVTSIGDTQNSTM
LIKSLFFYIVITLETFIFCYAGEYLSAKGRMIGDAAYDAKWYNSSPTQSRIMLLLILRSQRKLTISIGKFMDL
SLERFTTIIKASASYVSVLHAMS

>HsOr146
MRSASISTSVEIGLRLVGIWPGLSYGNFYWSAYMTSLVVALYFQYAYILDHFDVHNISNLIDALSITLAYSLG
FLKLISLWSNRRIFCDILLTMDKDWRDASAYDNNYDNSLLCIMTDNASLSRRCSNMLISINATAATCYAMSGL
MRQSGDLKEDLNVSSRELPIKMEFPFKVDASPLFEFLVVVQCLHEVSIAALVATINSLVVTLVLHVSGQIDIL
RRQLSMIHYDASRPHESIVANIRLLVARHQRIITLSDNIDELYSSIALMQFMSNTVVICCIGFTFISSFAEVG
VSLVLMKSVIFYIAVTLEAFIFCFAGEYLGAKSKSIGDAAYESLWYKMTPAECRFLLLLILRSQKRLTISAGN
VMDLSLEGFTTVMKASASYISVLHAIY
>HsOr147
RMTTVSIPVEMGLGFIGIWPDSPYGNFQWFVYMTLVVIVLYFQYTHIFKNFDMNDPSVLIDALSXTLAYILAF
LKLKMSMDANNRVFHEILTTMDEDWKEYITTDSRVYPMVIYANLSRRYCNILMSINASAAIFYALGGFVRRSA
KDEDNPRGTKFDFPVQMDFPFEVNESAIFEITAMVQFLHELSLSSLVAMINLLIVGELISCFANSYLTSVFRN
VRLTKISSESYENIPFPSEMKVLILKHQRIITLSDNIEDLFSWIALMQFLSNTLVICYLGFMIIITIVNKLGT
VELTKSILFYVAITLEAFVFCFAGEYLSAKSKSIGDAASVWYDITPSQCRTLLFVIVRLQKRLTITAGKMMDL
SLEGFTSIMKAYVSYISVLHAMY
>HsOr148
MIFTSTVSPSLRNGLRFLGIWPDVSHAAVNQLLYISSILIMQYFQYLYVFAHCKPGELQNLVDGLPATLDYSL
TTIKLLTLWANRRVVHEILAAMDADWRECVNVDRYSQVMTAKAGISYFCSNAMLNFNTIAGVLYLLGDYAVGI
VYHAEDDNTTSRPFPIKLLFPLEAEQSPIYELLVVVVFVHVMLNTYTVAILNALIFTLVLHASGQIDIICQEF
RNNSEETSHYGFSRYTIGMLVERHNKVISFSENIDKLFSFMSVMQVFWNTLVICCLGLVVMTSIHDETGVDLV
KTIFAYCAIMMEIFIFCFAGEYLSFKSRSLADAAYESLWYNMSPNHGKNVLFIIMRSQKQLNITAGGMTSLSL
EAFASIMKASASYVSVLHAMY
>HsOr149
MIIRSSVSPLLKVGLQFFGVWPDVPYATFRRLIHVSSILLVQYFQYVHVVAHFNLSELQNLVDSLPAAFYYSL
TGFKVMAMWKYHRVVRELLAAVDTDWRECVKVDQHLYLMTTKARISQFCSYIVLIFNVTAGALYFGAENLTAI
VQLLAGYNVTSRPLPVRIQLPFDAEQSPIYELLVIVLFLHSMTLVFPVNLLSGLVFSLVFHACGQIDIICQEL
KNISEKMSHYGSSTYTTGMLIERHNRVISFSKNIDKVFSFIALMQILGNTLVICFLGLVLMTVSIASHTDIDI
GLLKTMFAYMGISLEVFVVCFVGEYLCIKSKSLGDAAYESLWYNMSPSHVKKLLLIIMRSQKQLTITAGGITN
LSLESFTSIMKASASYMSVLKAMY
>HsOr150
MIVRSSISPLLKVGLQFFGMWPDVSYATVRRMIHLSIMLFIQYFQYLYVITHFNMSELENLVDCLPATFYYSL
TATKVITMWKYRRVVRELLAAVDTDWRECGNVDQHLYSMTTKARLAKVCTYSLLVFNSMAGILYFRGENLITV
MRLMLGFNVTSRPLPIKLLLPFDAEQSPIYEVLFVALFVHSMSIMYTVNFLSGLVLTLVFHVSGQIDIIHRKF
KSFSEKISYYGSSETIGMLVEKHNKVILFSENVDKLFSFIALMQVLGNTLVICFLGLVFTTVSIAATGADADL
LRTIFAYAGITVEIFMFCFLGEYLGLKNKSLGDAAYESLWYNMSPGHGRNILFVIMRSQKHLTITLGGITNLS
LEAFTSIMKASASYMSVLNAMY
>HsOr151
MVTSTISPLLKNGLQLFGLWPDMRYVTVHQLILMSSILILQYFQYLYAFTYCRFNDLPHFVETLTVMFYYSLT
LIKLTTLWTNRRVVREILAAIDSDWDECVNVNQHLHMMRTKASASYFCSRGWYIFNTFAGGLYFGGDCAMAML
TSGNNTARPFPTKLLLPFETEQSPVYEVLAFSLFLHGMLMVYIVATLNALICTLVLHASGQINIICEEIKTTS
ENMIDPESFASTTKMLIEKHNRIITFSENVDRLFSFIALMQVLSNTSSTCLLVLATMSFLDDENNVGLLKTIF
AYTGMTTEIFIYCFVGEYLSHKSRSLADAAYESLWYNMSSNHGKDLLLVIMRSQKQLTITAGGMANLSLEAFT
SIMKASASYISVLHAM
>HsOr152
TSTVSSSLKSGLQFLSVWPDVSYAAVFRLTYMSFILIVQYFQYLYVFAHLRLSELQDLVDSLPPTLDSSLTIM
KLMTLWTNRRVVREILSSMDTDWRECVNVDQQLRIMTTKASVSHFCSNALLSFNTFVAVLYYVGDYTLALMHF
ATAGNDTSRPFPMKILFPFEAERSPIYELLVVALFLHAMLNVYTLTTLNAFIFTLVLHVSGQIDIICQEFQTI
TEKVTYGSTGHAIGMLIEKHNKVIGFSENLDKLFSFMALMQIFWNTLVICSLGLLVITAVHNEAGVGLVRTLL
AYGAIMMEIFIFCFAGEYLSIKSKLLADAAYDSLWYNMSSSEGKNILFIIMRSQKQLTITAGGITNLSLEAFA
SIMKASASYVSVLNATY
>HsOr153
MSTMNPLLKIGLRLLGIWPNVRYASVYRLIYMLSILILQYCQYLYIYSHFKLSELPNLVDSLPAALDYTLAVF
KLLTLWTHRRVIYQILAAMDDDWCMSMHVEQHLHLMTAKANVSHFFSNTVFSFATVAGLLFLLGEYVLNFLHL
TESANASSRHFPIKLQFPFEHADESPIFELLFVMIFLHNMLNGYIIVILDALIFSLVLHASGQIDIICHEFKS
ISGDFAPLGSSTATIGMLIERHNRVIAFSSNVHKISSFIALMLVLCNSLIFVYLGFLIVTSVHGGAGVMLLVK
AAFASSVILVEIFIFCFAGEYLSYKSKLIAEAAYNSLWYDMSSDQGKMMSFVIMRSQKQITLTAGGLANLSLE
TFANIIKTSASYISVMLAMY
>HsOr154

TTSTISSPLKIGLGLIGMWPDSSYATVLWLLYMLSLAAMQYFQYSYVYSHLSMNDFSKLMDGLSVTLDYTLTF
MKLLSLWHNRRIFSDILVAMDDDWHGDCVTDSYTCVMTSKAALAHRCSNAMMTLNTLSTVFYFIGSYLSHRTI
SADEPRGFPVQMQFPFNATKSPIFEFIVLGSFLHVWETAVVIAMLNSLILALVLHVSGQIDIMCQGLREISVT
SKSVTSATRLLIERHQRIISLSNNIDKFFSFVAMIQFLWNTVVICTIGFMIVISLSTDMDGKSGILIQSIIPY
IAVTLEAFVFCFAGEYLSIKSRSIGDAAYETLWYDLSTKECRILLLIIVRSQKRLTITAGNVMDLTLEGFTSV
MKASASYISILHAMY
>HsOr155
STISPSLKIGLGLVGMWPGTSHGTLYWLFYMASLVVMQYFQYSYVYSHLDFNNFTKLMDGLGLTLDYTLTILK
LLCLWFNRRVFADILAAMDDDWNDRSSKLRECVMSDKANLAHRCSNVIMSINAMATVLYFVDSQVRRRMVSKD
GKYREFPVQVQLPFDVQESPTFELVLVGLFFHVLETATVIAMLNALILTLVLHVSGQIDIMCQELKAIPSMCK
ANPSATKSLVRLHQKIISLSNNIENFFSFVALLQFVWNTLVICSIGFMVSFGMNMESKSGVLIQFIIPYLAVT
IEAFVFCFAGEYLSTKSRSIGDAAYETVWYNLSISECRVLLFVILRSQKRLTITAGKVMDLSLEGFTSVMKAS
ASYISVLHAMY
>HsOr156
MVRTTTISRWVEIGLRFIGMWPNSAYPDLYWTSYMMFLMIVQYYQYSYVIVHFDAHNLSLLMDCLGLSLANSL
AMLKLFTLRWNRRIFHYILSAMNKDWNDYIDCDSYRSTMMAAADQSRRCSYFLIGVHAMAGFSLSVGAYLFRP
TRNSDIRELPVKMEFSFDFTKSPLFECILVGQFLCILTIASIVGMINALLASLVLHVGSQIDILRQDLMQISI
KNDPCESLFIIKDLVFKHRKIIALSDKIENVFTAIALMQLLWNTLIICCSGLMIVITLSTNKNVIVLAKSMLL
YVAKTIEVFVFCYAGEFLSAKSKSISDAVYESLWYDMLPTTSRVLLFIIVRSQKRLTITAGKVVDLTLDGFMS
VMKASASYMSVLHAMY
>HsOr157
MKRANTISRTVEICLRVAGVWPDSAYADLHWLGYMTSVAIVQYYQYAYVLAHFELNNLSTLVDCLSLTMGYTL
AFIKLFVLWWNRRTFYYVLRTIDRDWRECDFNQSYLSTMMSMADVARRFANIVFTMNASVAFFLSIGEHFVQS
MNEDNRLANSTRELPIKMEFPFEVSKSPIFECFLIGQFLYDMVIACIVGMINALLVSLVLHLSGQIDIMRQDL
EEFSNNKSDPDTFFIIIKNLIVKHQKIISLSENIENLFSSIALMQILWNTVVICLTGFVIIITIDKDVAITGL
IKSVSYYIAIMLEAFVFCFAGEFLSAKSKSISDALYETLWYNMSPSDSRILLFMILRSQKRLTITAGKVIDLT
LEGFTTIMKASVSYMSVLNAMY
>HsOr159
FSDNRRTMKRTSTISRLVEIGLRFVGMWPDSEYATLYWLIYMTTIVIVQYYQYAYVLAHFDLSDLSLLMDCLG
LTLAYTLAGLKLFVLWWNRRIFYYILTAMDRDWRECVISGSYVSTMMSVADLSRRCSNVMISINALAAFFLSI
GEHMIHTIGDVSRVANASSRELPIKMEFPFEVSKSPVFECFLVGQFLYELLTASVVGMVNALLVSLILHVSGQ
IDIMRQDLAEISGNKYDAGTSLIVIKDLIYRHQKIISLSENIENLFSYIALMQLLWNTLVICCTGFVIIITIG
TDVGITTLFKSVSFYIAITLEAFIFCFAGEFLSAKSKSIGDAVYESLWYDMPSSDSRILLFVILRSQKRLTIT
AGKVVDLTLEGFTSIMKASASYVSVLNAMY
>HsOr160
RTSTISESVEAGLRLIGMWPYCVHADVNWWTYIASVAVVQYFQYSYVFAHFDMGNFSDTIDGLSITFGYSLAF
FKLINLWFNRRKLYVILAAMEQDWSDEIAIDPNIATMTHHADLSRQCSNVMITTNALAVFFYTIGGPILRSTI
NKGDHAATRELPLKMEFPFDVYKSPVFEVVRVAQLLHDLSVACIIAILNSLIVTLVLHVSGQIDIIRRGLLEI
SRNKHASKSSLAAIKLLVGRHQRIIDLSDNIEDLFSSIALLQFVWNTLVICCIGFVIVISIGTEEGATVITKS
LIFYVAITLEAFVFCYAGEHLSAKSKSIGEAAYESLWYNLTPNECRILLFLILRSQKRLTITAGKVTDLSLES
FTTIMKSSASYISVLRAFY
>HsOr161
QREKVKMPASVFALSMELGLRYVGMWPDAPCALFCRCVWILTTTIVQTCQYWYLILHFRTEDLLNLTDSLSVA
LEYTVMFSKLIIILWLNSRIFNDVLASMAIDWREAALNDVQIMTGKASLSRYFSNLIIGLHSAAAFSYGLGVLV
QSSRSDETNANGVPIREFTLKLQLPFECNQSPRYEVVQCLEFLHQLSASAVTGMLNSLVVTFVLHTCGQIDIL
CDALKNLSPGKYTHRLTSSVAGELVVRHQKIIDFSDKIERIFCYIALMQFMSSTLVICCLGYMVVTSISNVQD
SESVDSPALMKAIIFCMAATVEAFIFCFCGEYLSAKSKIIGDAAYKSLWYDLKPEQNRFILLIMLRSQKRLTI
TVGKMSDLSLEGFTTIIKASVSYISVLHAM
>HsOr162
MIPTSTVSKPVKIGLHLTGVWPNTSVLFKLLWTLVMGTGLIFQYQYLLNHFSTNDLPNLIDGLSTTLPYNLLF
FKLVVLWINNRVFVDILRAMSKDWRKYSNMYAMIDKAVLSHRCSKLIIGIYSTAVLLYSTASIDLRNNGGSCR
EMLIKMELPFASCESPIYEIVMFVQFIHLLAVAWSIGVIDALIVTLMLHIGGQIDIIHQQLDEICPNDEHYDL
SSGVARSLVSKHHKIIAFSESIESLFSQIALMQFLSNTMIMCCIGFLVVTTLGTDDGIRMLIKTSFFYIAMTM
ESFIFCFAGEYLSNKSKTVGDAAYESLWYILKPRDGRILLLMIMRSQRRLTITAGKFMDLSLQGFTNSLKASA
SYISVLYAMY
>HsOr163
MSFAITTISPSVEFGLRAIGVWPGFPYTFLCRMLWTITLGVAQTFQFRYIIACAKANDFLNLVDSVSTTLPYS
LLCLKLIILWLNQRLVNKILTLMSQDWRDGGAVAFNVRTMTDKARLSRRCSMLIIGVYSIAVVVYVSVIIEFN

HIHSDEFSNKERQFFLKMKFPFDYDVSPIHEIILFIQFLQLLSNASVIGMLDAFIITLMLHISGQVDIVCYNL
CKLFSEKYEHKSYGEAIGMIIRKHQNLIALSNNIENLFTYIALMQFFTNTFVICCIAVVIVTSLESKQGYILL
LKSLFFYIAITLEAFIFCFSGEYLSNKSKSIANAAYEVLWYNAQPSKSRILLTLMLRSQKRLTLTIGKFNDLS
LEVFANILKASASYVSVLLAMS
>HsOr164
MVPTSTIGGPMELTLRLIGIWPDSSYKNLQLVLWTMMMLTSQIFQYWYLFTHIGSDTLPDLLDCLSLCFSNSL
LFLKLNILWWNRRIIFDIFATMIEDWNVCTSVSSKTQMINKAILSHRFSKCIIGAYTMTLILLGTSNVLAQKS
AGSDQADEERQLFVKMRLPFEYSVSPTYEIVIITQFLLQYTLAIMAGMLNIFIVTLILHIAGQIEIMCQGLLE
MLVAKDKPHIATLRDLVIKHQRIIKFADNIENVFCYTALLQFLSNTLVICFLGLLIVTQPVFELQSLDSNEVL
IKAIPYYVVVNIEAFILCFTGEYLSSKSRSITQSAYASLWYKLKPAESRILMLLIVKSQKLLTMTAGKFVDLS
LESFTSILKASASYVSVLHAMY
>HsOr165
MLPTSTIDGPMGFTLRLIGIWPDCLCKNLLPILWTIVMLASQIFQYWYLFTHIGSDTLNDLAHSLSLCFSNSL
LFLKLNILWWNRRIIFDIFATMAEDWNICTSINLKTKMINKAILSHRFSKCIIGAYTMPLLLLGATNILIQKS
ASFDQADEERQLFIQMNLPFVYSVSPTYEIVMVTQFLLQYTLALMAGMLNVFIVTLILHIAGQIEIMCQRLLE
ILVAEDEYESHIATLRALVIKHQRIIIFADNIENVFCYAALLQFLSNTLVICFLGFWIVTSLDSNGVLMTAIS
YYVIVIMEAFILCYSGEYLSSKSRSLTESAYASLWYELKPAQSRMLMLLILRSQKQLTVTAGKFVDLSLESFT
SIIKASASYVSVLHAMY
>HsOr166
MLPTSTIDGSMEFTLRMIGIWPDSLCKNLPPVLWTIVMLAWQIFQYWYLFTHIGSDTLIDLAYCLSQYLSNSL
LFLKLSILWWNRRIIFDIFATMIEDWNVCTSVSSKTQMINKAILCHRFSKCTIGAYTMNLLLFGITNIFVQKS
VSFDQVDEERQLLIKMKLPFMYNTSPIYEIVMITQFLLQYTLALMAGMLNVFIVTLILHIAGQIEIMCQGLLE
MLVSEDKNESHIATLRDLVIKHQRIITFADNIENVFCYAALLQFLSNTLVICFLGFLIVTQPVFELQSLDSNE
VLMKTIPYYLIVNIEAFILCCTGEYLKSKSRFITRSAYTSLWYELKPAQSRILMLLILKSQRQLTVTAGKFVD
LSLETFTNILKASASYVSVLHTMY
>HsOr167
MEKSRSVQSRRIISWPLKMLLCLFGIWPGLTYGLVCKAFWLIVLVIGTILQYTFLVIHARTIDFTIFMYACAL
TLAMSTKFMKIAIFWYNQRRFNEMWRTMSMNWEFGSACTSAIDNAGKMHVLRHLPNFIVAFNSMSVIMTSANT
LSNAIHYDEASNTTRSYILMMHLSFDINKQSVYLVVIFLQFFYMLIITAGAATINAVLVILMLYLGGQVDVIC
RCLTHMPQGEHVGMTDVMVVKEIIRKHQSVITFSEHIESLYTYIALILLLLNTLITCGLGFILVTSVGSPIFA
KMVMKNLMFYCVINMETFIFCFAGEYISSKSREIGEAAYNSPWYQSKFHSRMILFMIMRSQHQLTITMGKFMD
LSLERFSTITKASASYMSVLLAMY
>HsOr168
GIWPGLTYGLICKASWLIVMVVCTSLQYTFLFIHARTINFTIFMYALAASLAMSMKFIKIAIFWYNQRRFNEM
WKTMSTNWELGCTSAIDSGGKMHVLRHLPNFIVAFNSVSVIMTSANTLSNAIHYDEASDTARSYILMMHLSFE
INKQSVYLVVIFLQFFYLLIVSAGAATINSVLVTLMLYLSGQIDVICRCLTHMPQGEHVRITDVTVVKEIIRK
HESVITFSEHIESLYTYIALVLLLLNTLITCGLGFILVTSVGSPIFAKMIMKNIMFYCVINIESFVFCFAGEY
ISSKSRKIGEAAYNSPWYQSKFHGRTILFMIMRSQHQLTITMGKFMNLSLELFSTITKASASYMSVLLAMY
>HsOr169
MTRKNTINRTTKVILTLCGMWPGACCVMVCRAYWIIALATDEFCHYRYFLLHWRTDDLFDLTDCFSSCIAQVK
VITKFLVFWFNQRKFVNILTMMAEDWRDSADSDVDMRETTCKAKLSSRITNAMVTLHAITIVVYSSGVILADV
DINDRENMPPLLLKVEVPIDIHSQYRYKVLLAAQFLYLFIAGCGAGLVNALLLTLILHVGGQMNILRCWLTKL
VPKENERKRESIVIMTNKIIQKHQKIIIFSEYIEDLYTYIALVQFVLNTLLICTLGFIIVTSIGNPDTKEQLR
KSLLFYTVTTLEAFIFCFAGEYLKNKSKAVGTAAYDSSWYELKPENSRTLILVILRAQKQLTLTVGKIMDLSL
ESFTSIMKASGSYLSVLLAMQ
>HsOr170
MARRSTINRTLQLMLTVCGVWPTTSGIAICRAYWVIALGIDEVCHYRYLLLHLHSNDLFDLMDCFSSFLTQVK
FSTKLIIFWWNQRKFMRILMMMAEDWDDCANSEVDVCEPTCKAKLSSRITNMMFTLHMLTIVAYSAGILLADV
DFTDRQAEWPLLLKVNLPVDISTKRTYRLLLSAQFVHLIMSGCGTGLLNSLLLALTLHVGGQMDVLCCWLTEL
APEERRVERRESIVDRTSKIIRKHQRIIHFSRCIEDLYTYIALVQFTSNTVLICSLGFLIVTAIGSPDATEQI
VRSLLFYTVTNLEAFIFCFAGEYLKNKSKAIGTAAYNSAWYELKPENSRPLIFVILRAQKQLTLTVGKIMDLS
LESFTNIMKASGSYLSVLLAMQ
>HsOr172
KSTISYTLKCLLILYGIWPDVSCITFCKMFWSISLPIVLLHHQYCFAHFYSNDMFDLMDCLGTFLGYFKLLIK
VIVFWWNQQGVWIFNQILKMXTEDWKDCANNDIEMRKVISEAKISHYVSNAIITLHTVAVLLYGINVILTNVD
ITDRTIEIQHIMRTEYPFDIKTQSIYKFVLIIELIQLLMSSLGAAMVNALLITLTLHVGGQIDVLHCWLTELE
SADKCRGNMIKKIIQKHQNIIYFIENMETLYTFIALLQFVSNVIMICVLGFLIITAIGNPNATEKIIRSLPYY

SITNLEAFIFCYAGEYLIDKSTAIGYAAYDSAQYNMEPKYRHILLFIILRSQKYLTLTIGKLMDLSLRRFTNM
MNSAGSFISVLLAMQ
>HsOr173
VVACKSTISRIVKCLLILYGIWPNIPCITFCKIFWSISFIIIQVWHYQYLLAHFYSNDIFDLMDCLSTFIGYF
KLLIKIIVFWWNQQIFNQILRTMAEDWKECANNDIEMRKAISKAKISHYVSNAIITLHTVAVLSYGINIILTD
VDITDRMIEIPHIMRMEFPFDIKTQSMYKFVLIIELIQLLMSSWGMGVINALLIALTLHIGGQIDILRCWLTE
LESVESTDKHRSNMIKKIIRKHQRIIYFIENMETLYTLIALLQFVSNVMMICVLGFSIITALGSPNATEKIIR
SLPYYSVTNLEAFIFCYAGEYLINKSKAIGYAAYDSAWYNMEPKDRHILLFIILRSQKCLTLTIGKMMDLSLR
RFTSIMNSAGSFISVLLAMQ
>HsOr175
MIRRSTINRTIEFMFTLFGIWPGASCVLLRRAFWIVTIVIVQYCHYRYLLTHFYSDDLFNLMDCLSSLLAYAK
VMSKLVMFWLNQRKFVETLVMVAEDWDGNANDELGMRETMCKAKLSDRITNAIITLHTLSAIAYSTRAILADV
DINDRTTEPLYVHKVEIPFNVNTQRMYKTILTAQFVYVVMGSWAAGAVNSLLLTLILHIGGQMDIVTCWLMNL
ASQETGKKRESNVVRTGEIVEKHQRIINLSENVENLYTHIALLQFASNTIMICSLAFLIVSAIGTPDAVEQIM
RSLLFYAITNLEAFVFCFAGEYLRNKSKAVGVAAYNCAWYNLKPEDSRPLLFVILRSQKRLALTVGKMTELSL
EYFASIMNASGSYLSVMLAMQ
>HsOr176
MTRESTISRAVEMLLPLYGVWPGRWCVVIFRVFWVVSVMFIIFCHYRYFVSHASIKNFFDLVDCLSSFLAHVK
VIMKFIMFWINERKLMEMLALMTDDWRDRANKNDGGMSVMVSKAKTSDRITNAIMIIHILGVMMYSIGIIIAD
ADITDETIEAPYINKLDFPFRIDTQSMYRFVLIVECIHMLVINMGAGIANAILLSLTLHVGGQIDILQRQLSE
LEVADLESGLRSIVGTASEIIRKHHRIIYFSENIEILYTYIALVLFASNIIMICSLGFLIVTAIGTPDAKKQI
IRSLMFYTLTNLEAFIFCFAGEYLNNKSRAVGFAAYNCAWYNLKPKESRILLFIILRSQRQLTLTAGKMIDLT
LESFASIMNASGSYLSVLLAMQ
>HsOr177
MKSEKYIGDEYDDFIKPVRMASKLISIWPLEKNHTANALFLNNCYLIWLLFVLYSTCVTVTADAIYHADDLDE
LTECALLCSAFYLSVIRLIIYSLHRKDMLYVVEIMRKDWVTSSYEDRIVLKKKCVYAFRLTKYFTIMVAANVI
MFSCVPILETYLLDAKEKVLPFRGYFFINQTVSPVYQCLYVFNVMAGSYGATTISSVTGFNLIAITHGSAKFA
VLRRKLELMSSDDPDVKKVVVDCVKCHQDAITFADTLERLINVLVLGQFVISTGLLCFAGFQLTSMLKNKGRL
MKYSVFLNAAILEIFMFSFSGNDLMTESDAVGESAYSSGWIGGTFGRSLQIMMLRSMVPSRITAAKFYSMSLQ
SFTQVLSTSFSYMMVLMATSDE
>HsOr178
TEFTRAFKIISWNRRFLIILGIWPTKVNRVAFVFFLVYVAIHCTMGGAHLVKYFSKPEYIVANLTENVLFTMI
IGKMLICERSRRIMVYFLNTIQPDFSAKSYSNAREKALYLHYNEFALIFIKVSLGLATLTTILYYIMGFLENW
TGKLSTRNASYELPYRTYPFFEIQDSTTYFCLCAYQIIALPTIVCGYSAPDSFVLSMALHICGQLAVLSCKIE
QLLKDHENYNHHIGTIVLRHRQLIKLAEILENNFSMIFLQQTLGTVFLLCLTTYHMFANSVNGASTNVVTFLL
YTSCVISTIFAYCYIGECLIKESVRLRDTFYNTDWYNTSPSCGKILSICMIRPEKPLVLTAGKFYPLSLNTFT
SIVKTSMAYLSILRNFL
>HsOr179
LQTDVGHIEDLFVHLERIFSIGGIWPFERTYVRFAIYILYFMLYLVMAYANFCEVFGDLELMVMNLVETMAYT
TTFTMVCMIRCSGLLKRIIVVMRQDMMEQKFEDPEEERIYYSYNYTSKIFLYGSIVGMFVTVMLLYFRPLMSF
SADDQEFINGTGSFVLPYRIRTFFDIASTRTYVLVYLYLCPMFYNSICHMAAICLLVILVFHICGELSILSYR
IKNVEAYSRDALVVRIRSFVQMHLKIIWMAKSVDNAFNMVLMSELFGLSVILIISMYQVLMNLDISELATCCT
FIFFSVISIVMLYGCCLIGEQLTQQCISVLDACYQCNWYEMPLNCKRNLLMCMIRSQVMLYLTAGKFYVFSLN
GFTDIIKTSLAYLSVLRTLL
>HsOr180
LQDQKDLERAAQVLSWNKRLMSTLGLWPFRSNDLIFTVNFGYFSLLMILEYLDLFLFIGDLERVVMNLTENMA
FSQIFVRMSALRLYNGQIGEVITEAMKDFDRTCYRTFEEMKTFISYNARSKIFVKLLMVFVALTATSYYLTPI
IIIFGGGLPRVAIGENATRVIYLLPYRFHMFHVVEDMRTYVITYGLQLPFVFVSGFGQSAADCIMVTLVFHIC
GQMSVLALRINNIDTDPFSCQREIRNVVRMHLRLLRMGRAIEKAFSVTLLAHLLGATSLVCILGYQILTNVAK
GESGVLVTFLIFQFLVLLILYAHCTVGESLLTESAKVCEAFYDCHWYNMSTENARIIVLCMARSQKPLCLRAG
KFNTFCLSTLTDVLRTSMGYLSVLRSFL
>HsOr181
MDFLPMNFCVFRLCGAWKKRKESNLIIRFASFCHRYAIVISIYYFTASMIIRLVHIRNNIEELMESLFITLTF
VHLCLKYTNFLARQFEVRALLDSFHAKLCQPKNSAEESILKRYSRKANQIAWFYMILCQMTGLFFIISPVLAS
DKRPLPYKVYVPYSIAPLFPYVLTYIQQATTLTCSIALNTSCDTLIYGLIIYTCGQIELLCHRMMETFRYLGE
NEIEARKINAIENVVIAECVKHHISMYNIIYKIQSLFVWTVAILFFFSLVTLCTTIYQMSKKNLFSLEFITLT
LYLGAMLFQTFLYCWYSNELDLKAKYIAHAIYASNWTVISAKQRKSLLLVMMISQRGKILSTYGIFALLLSTF
TWILKTSYAAFNLLQQTSS

>HsOr182
MDILPLNFRVLSFCGAWSEQKGSNSFVRFLSLCYRYSIVLLIYEFTISEVIELVRTRDHVEDITEGLFVTLTY
VALCFKYGNFLVRRDEMSALLNCFRDETCQPKNSEEQMILIKYDRKARWCVRTFMSMSQATCLAFVFAPIVGP
QDTDRPLPFKTYIPYSISDWYIYVLTYLQQTAAPFYGVLLSVSFDALVYGFTLHACGQIELLCHRLSEIFEDQ
FNVNQYRIKSNKTVVIGECVRHHLRVHELVSRIQSLFVWTVTILFIFSMVTLCTSIFQMSKKRLLSVGFLSLV
LYFGCMLFQVFFYCWYGNELQLKSKSISDAIYSSNWTTATLQDRRSLLFVMTISQRGLKLSYYGIFNLALDTF
TWILKTSYSVFNVLQQASV
>HsOr184
MDILPVNFKVLWFCGAWKERKDENIVLAFLYTCYRYAVFLLIYESTIFEVIELFRTRNHMSDVTEGLFLTSTF
VTLCLKYANFLLRRNELSALLEYLRMKICQPRNFKERLIIDAHSRKAKWSALSFLIISQATSLALMIAPVLET
NAEERVLPLKLYVPYSITRLFPYAATYLQHVLTLFYAVMLNVSFDSLVYGLTIHACGQIELLGRRLKDDLAAS
ETLEMRPGSNTSIEECVRHHVLMHAFVKRVGALFVWTVAVLFFFSLIIFCTSIFLITKTKLFSVEFFSLILYF
SGIMLQIFFYCWYGNELELKSSGIARAIYFSNWTMVTPQERKSLMLIMINCQKGFVFSYHGTFTLCLDTFTWI
FKTSYSAFNLLQQAS
>HsOr185
MRLRLETFYDGQVLSWSKLLLSMLGLWPESRNDLQFAFFAVYIINVMALLVVGLLQNMHDTDRLMRNVTFTFP
TSLIVLKNLMFRWKMGKLSPLLAAVRSNVREGLYRSLAESRKVIWYNAAATLFTVSSAASLFFVPTLFYIIPI
LSCVRSSSGNCTLPYELPARVNLLYEISGMRSYALFCAILLPSSTFLTIGATAADSLLVSLTFYLCGQLSILG
HRVRNTEPRSLRYHREMKLLVTRHAELLRLASILADAFSSLMFVQTVGLIFSLCIVVYQLLMTAESGKGLNEM
VHFMIYSAAVMLLAFCYCFLGECLIGESVALDWACYSTNWHELPTESVRLLMICIARSRKPLYLTAGKFYVFS
LEMFGAIMKASMAYLSVLKTI
>HsOr186
LQTDVGHEDLFVHLERIFSVGGIWPFERTYVRFAIYILYFTLYLVMAYANFYEVLGDLELMVMNLVETVAYTI
TFTIVWVIRCSDLLKRIIIVVRQDMMERKFEDPEEKRIYYNYNYMSKIFLYGSIIGMFITVILLYFRPLMNFS
ISNQEFNNNTGSFVLPYKIHMFFDITNTRTYVLMYLYLFPMIYNSICHMAAICLLVILVFHICGELSILSYRI
KNVQAYSRDALVARIRSFVQMHLKLIWMAKSVDNTFNIVLMDELFGNSIILAISMYYVLINLEVSELATCCTF
IFFAIIALVMLYGYCLIGEQLTQQCINVLDACYQCNWYEMPLNCKRSLLICMIRSQIMLYLTAGKFYVFSLNG
FTDIIKTSLAYLSVLRTLL
>HsOr187
TAFTRAFRIISWNKRFLITLGIWPMKVNRVAFVFFFVYMVIHCTMGGAHLVKYFSKPEYIVANLTENILFTMI
IGKMFICERSRGIMVYFLNTIQPDFSAKSYSSAREKTLYLHYNKFALIFIKVSLGMATLAGTLYYIRRFLESW
TVSTHNASYELPYRTYPFFEIQDVTTYFCLCAYQIIALPTIVCGYSAPDSFVLSMALHICGQFAVLSYKIEEL
LKDHENYNRHIGAIVLRHRQLITLAEILENNFNMIFLQQTLGTIFLLCLTTYHMLANSEYGDSTNVVTFLLYI
CCVVSTIFAYCYIGECLINESTRLRDTFYNTDWYNTSPSCGKILSICMIRSEKPLVLTAGKFYMLSLDTLTSI
VKTSIAYLSILRNFM
>HsOr188
SRAGMPRRIAAGCLDKFRKLSALHTTYLKYIGLWALDSNSSVTLKYLYFVYNKVILTIMFAFAITLLADICSS
FDDLSIVTDDGCIFAGITVVLFKVMIGQFYREKIARLLCKTIDNCHRLCKFPTGDEEEILDKYLLISRVTFYG
FSTLGFCLVLALLFLVPVENGGLPVRARYPFDTTRYPGHAVGYFVEACSVSVGITAIIGMDSLHTNLCNLFLV
QLEILNAHFSKCGAPAVADSRADGDTHYAIPSTIETGGKDNYDPDAILGRDDFAERLRRSVRGHQRLGALMDD
FNEMFSAGMFVQMLSSTSMICLTGFQATLVIGHNSNICKFAIYLAAAVSQLFYICWVGNEVIYQSARLAQSQW
LSGWNDELSAKTGRLLILSMIFSKRTLNLKAGVFYVLSMETFTAIMRGSYSFFALLSTMQSGGEQ
>HsOr189
MTGNREYRSVSITRLFMKMVGLWHVETPRERLFLRVAFGYAVWAILFAILVEGVDLYHCMGDFYAVTSNLCAT
LLLVMILVKLGSFMLYHDNVMKLIRFAEKHFWAVTYDEVGTRILEEYDKLGMTMTYTFTLIVYVATFNYIFAP
FFEPRATNETQKALPFKLWLEFPYHSPYYEVTYTIQSLSTIHSGICTFCFDNFVSTFNIHAAAQLKILAHKVE
IVAESCIEGATSDKSSQSEAEIVALTFERLQDCVRQHYTLIQYLHNMQHVFAIILLGQLLLSSVVICFGGFQF
LATDVAIRKCIFAFHFVGGLIQLLIYTWTCNDIIVQSAAISDAAYNSKWYLLPGSGPGMALRKGLIMIMIRAR
RPCTLTAGHFAVMSLDTFTGILSTAMSYFTLLRQMSEE
>HsOr190
MAAERKEHDYASMQMARFLMKTVGFWTSDKKGEERLLKGILGYTLFAIVLALWIESTEFYFSIGDFYAVTYTA
CSSMPVVIVLMKILFFLTYREEMLTMLRYTQERFWYAQYDDYGRKLMDDINRKGMIIMCTFTFFVQVTVVTYM
LTPIIENRGRNESERILPFNWYVGIDTHVSPNFEIIFVFEIIALVHAGICFCCFDNLLGLLGMHTAGQFKLLQ
HRLETILERIGQAGDLGSLDEKTKRAVHEEIRGCVLQHQELIWYSEKMEGLFMYTTLCQLLVSSVMICVAGFQ
VFLSRGTLMRRMIFIAHTNGSVGQLFVVTFTAHHLLEESRAVGDSAYSANWEVLSHQDNRSIRNAVLMLMVRS
THACSISAGGFFPVSLETFMAVMSTAASYFALLRNFI
>HsOr191

DVPVHCYRSYARRIHRILYLGGVLQVRSVVQSYVIGILVIFMCFSQVVFVINFCRDYSDNLMIVSKCFGLASS
IFTPVLMSCCFLVKRKKLLKLHKTLNDLFEQELARDQRTKTTMLASLYTFARPSYVLSFMLGFTILSYVLPSV
INVIIRPVIFRSAIAVRYKWPYPVKFPWSVPSSGPLFYLQYLYQVLSSWWMVFTIAAVDNLFGFYTFQISSIL
HAMSARLMSPQPDDVFLVVLKTCTETYQQLLQSVRLLEDVYGIIILRMILANAVLMCALIFEVYPFTDMTVNQ
FCSFGSFMVMKLLQTFIYAWYGSLVTSASEHFREGIYFSEWSNSKLDCHVRVGVVVTMLQKPIVITALKVSSV
NVNMFINILNTTMSYFFLLQSLDE
>HsOr192
MDFRNLNPLNIHANFLSGNLLPLESGVSRFSIAWKLYSVLIWLLLLAQIIVLIPGLVMVPWEKALIDGTVVIV
VNIEIFFMVGRIYARRELVDRLIRKLNEMLRNEDEIMRNVVTTIFQPMNAPLKFYWLAGSLSVFLWCCVPFLL
VSQKTSFRYEDYRIPAVFSKQPFSVGVFLMGNIFLLIGNMYTFLKKGSVDIYMIHLVMMMTAQYRYTAKKLAA
IFRDANLRSKMEQSSLEADGWTEKEMKALCRHYTAVQCLSALLRQLLSLSFSMIYINSVLRFCFIGIMLGTVL
ATTFIEGFVIVTFTCGSVLQFYMLCLCLQQLRDASKDMTEEAFHEEWYRFGSSIKRTFMLMALACNLECKIST
NDKFNLSLPSFMTILNRSYSIALLLL
>HsOr193
MDFRNVNLLNVRTNLLSGNLLPLAPGDSQFSMAWKLYAALMWLVQAIQVIVLIPGIIMVPRQKALVDGTVTAV
LTIEVFFNIGRIHMRRKLVGQVIQRINDILRNEDETMKRIVTATLQPMKAPLRFYWVAGGLSVILWGCIPLPL
IFEKTSFRYEDYRMPAVFSKQPFSVEIFLMGSIFVLIGNTYIFLKKGGMDIYMIHLVLLITAQYRYTTTKLNA
VFRNAKLQGELKQSRPEVDQWVEKEIKALCRHHIAVLHLSSLLKQLLSLSFSMIYVNSVLRFCFIGVMLSTVP
KAAFLEGFPIFMYTCGSIVQFYILCSCVQQLQDASAGLTDEAFHEKWYRFGSPIKRTFLLMMLGTNLGCKIST
NNKFNLSLSSFMSILNQSYSIALVLL
>HsOr194
MDFRDISKLNRWMNLISGNLLPMSSDDSSFPFFWRLNSVVGWSFVLAYLCGLIAGFFFVPGDKSLKDGMISIV
IIMEVSVIIIRIYTQKRLVQELIRKLNDNLHIQDEMMQDVLTMTLKPMKTPLQFYWVVSTVAAIVWCCVPLPL
TLHKNIFYYLDLQSPVVYSKEPFSVTSFVLINVVVLLNNIYLITKKVAVDMYMTHLVLLITVQHLYTSQKLVS
IFREGNQLDDYEDLHKDYSKAGRTMVMALKTLCQHHISAIKLTLMLKKLLSLNFSLIYVNGVLRFCFLAVMIL
SIPSAELAVIVERLAICTFACSEIVQLYVVCASVHKLLEASTKITDEAFHEDWYRFGPCAKRMFMLIIMASNL
GFKLSTFERFNLSLPSFMTILNQSYSIALLIL
>HsOr195
MDFESVNPFNVRMNKIAGNLLPMTADRSSFPILWKIYSSLIWLLELIQMIILIPGCMLVPKEKALKDGLIGVA
VTLEVLFVVARIHMRRGLVQRIIQRVNEILYLDDEMMQSIVTASLKSMEIPLKFYWSAGVMSIVLWSGTPLLM
ILKKNSFSYVDYRMPVAYGKEPISTGSFVIGSLIVMSSSALIFTKKVAVDTYTIHLILLITAQYRYIASKLSI
ILQNDSSDPAGNKSYPKSNLSAEKEIKAVCRHHNAIVHIMSMLKELLSLNFNLLYINSVLRFCSIGIMLLAVP
STSLLEGYLIVMYASGGIVQLYILCSCVQQLLDATVEITNKAFHEEWYLHKPSVKRTFILLIMANNLDCKLAT
FEKFNLSLPSFMAILNQSYSIALLLL
>HsOr196
MDFESANPFNVRVNKISGNLLPMTADGSSFPILWKIYSCLIWLLELTQMIILIPGCMLVPKEKALKDGLIGVA
VTLEVAFVVARIHMRRGLVQRMIQKLNEILCLDDEMMRYIVTTNLRSMEIPLKIYWSAGIMSVVLWSGTSLLM
IFKRNSFSYMDYRMPVAYGKEPISTGSFVIGSLIIMFSNMLIFTKKVAVDSYTIHLTLLITSQYRYIASKLSV
IFRGKASRNDSNDSTGNKNYSTSDLSAKKEIKAVCRHHNAIVHVMSMLKELLSLNFNFLYVNSVFRFCFIGIM
VLAIPSTNLLEGYLILTYASGGVVELYILCSCVQQLLDAAAEITNKAFHQEWYLHESSVKRTFMSLIMANKLD
CKLATFEKFNLSLPSLMSILNQSYSVALLILRTN
>HsOr197
MDFESANPFNVRVNKISGNLLPMRIDGSSFSTLWKIYSSLIWLLELIQMIILIPGCMLVPKAKALKDGSVSAA
ITLEVVFVVVRIHMHRRLVQRIIEKLNEILRLDDEMMRSIVMASLKSMEVPLKFYWSAGVISIILWSSTPLLM
IFEKNSFFYVDFRMPIAYSKEPISTGSFVIGSLIIMFSSIFIFTKKVAADSYTIHLTLLITAQYRYIASKLSI
IFQNNTLRNGSNDPFGNKNYLTTDLSAQNEIKAMCRHHNAVVRVMSMLKELLSLNFNFLYINSVFRFCFIGIM
ILAVPSTNLLEGSLIIMYASGGIVQLYILCSCVQQLLDAAVEITNKAFHEKWYQHEPSVKRTFLLLIMANNLD
CKLATFEKFNLSLPSFMAILNQSYSIALLIF
>HsOr198
MDFESANPFNVLVNKISGNLLPMTIDGSPFPILWKIYSSLIWLLELTEMMILLPGCILVPKGKALKDGVVAIA
VTLEVLFVVTRIHMRRGLVQRIIQRVNEILYLDDEMMQNIVTASLKSMEIPLKFYWSAGVMSIVLWSGTPLLM
IFKKNSFSYVDYRMPVAYGKEPISTDSFVIGSLIVMSSSVLMFTKKVAVDSYMIHLILLITAQYRYIASKLSV
IFRDETTRRDSNDPIGDKNYSTSDLSAQKEIKAVCRHHNTIVRLMSMLKELLSLNFNFLYINSVFRFCFIGIM
ILAIPSTNLLEGSLIIIYVSGGIVQLYILCSCVQQLLDAAVEITNKAFHEKWYLHEPSVKRMFLLLIMANNLD
CKLATFEKFNLSLPSFMAILNQSYSIALLVFRT
>HsOr199
FIESMDFKNVNPLNVRLNAISGNILPMAVGNELFPATWRMYGALVWLLQLVQLAALFLGMMHVPAEKALIDST
TTIAVSAELFFIIARIHMYRHLVVRLIDKLNDILGADDDIMKNIVQGTLRPTSRPLKFYWATAVFAIVFWNGT

AIAVVFKKNVFTYEDYSMPVAFSRQPFSTSTFLMGSLVVLIGTVYNCTKKVSVDVYMIHLVLLMTAQYRYIAI
KLAEIFRGGNLERDDEFCCKMNHWARREMRMLCRRHNAVVQMMFMLKKLLSLIFSLIYVNSVFRFCFIAVMFS
TIPSQTVLIGFLILVYTSGAMVQLYTLCSRVQQLLDASTTLTDEAFHEDWYRFGPSIKRSFMFLIMANNMECK
LATFGKFNLSLPSLMTILNQSYSVALLFLKV
>HsOr200
MNFQSVNPLNVRLNKVSGNLLPVTADGSSFPIYWQVYAIVIWIMEIIQMSILIPGCFQVPMQNALKDGMIGIA
ITLEVFFIVIRLHLCKGLIERLIKRLNDIMLLDDELMRNTVRTTIKSMDKPLKFCWLAGVVSIILWCSPPYAL
IFQKDLFVYLDYKMPVAFGTEPFSTGVFVVGSLIVSISSAYIFTKKIAVDTYMINLVLLVTAQYRYISLKLSL
VIRDRSPENNLNGPVEKERYFEIDRYVKEQVKSLCRHHNTMLYVTLILKQLLSLNFSLIYINNVFRFCCIAFM
ILVVSSMDFVEGSGVILYACGGVVQLYLLCYCVQQLLDASIEITDKTFHEEWYKHDTFIKRIFVMLIMANNLE
CRLSRFEKFNLSLPSFMAILNQSYSIALLLL
>HsOr201
MNFQNVNPLNARLNKLSGNLLPMTGDDLLFPTAWRIYSAVVWVVEVVQVSAIIPAVMYVSKEKILQDMTVGVV
ISVEAFFLFIRIHSRRNLVKRLIRQLNDILSVSDQTMESIVRSTVRPMQAPLKFYWIAGSLSVTVWCCMSFVP
ILKRNYFYYEDYRVPVAFSRQPFSMNVFLVGNLVVSVTSVYIFMRKVALDVYTINLILLMTAQYRYVAMRLAT
IFRKNISRNQREEFRKSYSDANTWLETEMRILCQHHNAIVHMTPLLKKLLSVNLSLIYLNNVFRFCFNGIMLM
IAVSSAPLEALMIFTYMCGGLVQFYILCLCAHQLLDASIEVTDEAFHEKWYQFEPSLKHTFLSIIAANNLECK
LAALEKFNLSLPSFMTVLNNSYSIALLFLRVR
>HsOr202
MNFRNANSLNNRVNLLSGNLLPIEDSDSKFPLSWRVHNVVIWLIELIHTVALVFGLILVPREKALNDGTVCVV
VLMEASFMLSRLYARRKLLSRVVDTLDSILREADETMKDLVRSTLSPITKMFTVYCLVSVTTITIWTVQPVTI
AFDKGAFFYVDYNLPAAFTSEPFSRGVLIASSVVMTIGSVFLFLKKFGVDVYMMHIVLILTVQYRYIGRKLTL
LFRDLQGDNRVDGLCDKRFHLKADWRTKRELRALCQHHNTVLRISIMLKKLLSVNFSLLYVNSVFRFCFISIL
MSTVPSMNLSEGISVLLFAFGSAMQFFLLCFSVQTLSDASTEITDTAFNENWYQYGSSIKRIFLLLIMTNNLE
CKIAAIEKFNLSLPSFMTIMNQSYSIALLFLRA
>HsOr203
MNFQSVNLINVRLNMVSGNLLPITSDNRFSLFWRMYSMLVWLLQLVSAXGFTPGLIAVSRXKIFMDAMISIVF
ITEVIFLNMQILRHNKLMRRFILQLENILDAGDEVMRNMVTMTMKPIMILLNIYWMIGLVSVIIWYIIPFTLL
SKRSSFYEDYRIPVAFSQQPFSATVSVLDSLVVSFGSLFMSLKKVSVDIYMXIYMVLSMTVQYRHMATKLALI
FREGASWNKEDPCHGEXYSSGMNAWMEKELKMLCRHHNTVIHIWNAYITYLRTLRFNLSFVLKKLLSWNLNLI
YLTSVIRFCFGAIQLSTVLEVDFMLGLSITLFLFVGVLQFYILCSSVQKLSDVNTEMTNMAFHENWYQFTPSI
KRVFLLMALSNNLGCEITMCEKLRLSLPSFMSVLNEAYSVALILLKMK
>HsOr204
MNFQSVNLINVRLNMVSGNLLPVTSDKTFSLFWCVYSMLTWLLQLLRMGAFIPGFMEVSTEKIFLDAMLAIVF
TTEVIFLNVQIQRHSKLMRRFILQLNNILDAGDEMMRNIVTMTMKPIIIPLNFYWLTGLATVSIWCLVPLNLL
SKKSSFSYEDYRMPVAFGKQPFSATVFVLGTLVISISSIGMYLKKVSVDIYMVNMVLLMTLQYRYISTKLAQI
FREGASWNEGDNAPAGKYSSGVNXWMEELKVLCRYHNSVVHLSLVLKKLLSWNLNIIYITSIFRFCFGALQLT
SVLKVHFILGLCFILFFFGGVLQFYILCSSVQKLSDASTGMTDMAFHENWYQFTPSFKRVFLLMVLSNNLECE
ITMCEKLRLSLPSFMSVLNQAYSVALLLLLRLK
>HsOr205
MNFDSVNHINVQLNKVSGNLLPMTNDSGFSVFWLTYGVVVWLFELARAIGFIFGFMAVSIEKIILDTMISIVY
TTEVIFLNVQIQRHKVLMRQFILQLNNILDVGDEVMRDVVMTTMKPVMIPLNFYWLTGVVTVSIWCLMPLNLL
SKKSSFYYEDYRMPVAFIKQPFSATVFVLDTLVISISSVGMYLKKVGVDIYMVNVVLLMTVQYRYITTKLALI
FRKGASWDEGNDTAAGNYSSRVNMSVEKELKMLCRYYNTVVSGMHTHILVPAMLSLNLNITYIISIFRFCFGA
IQLSSVLVAHFIVGLSITLFQIGGVMQFYILCSSVQKLSDASTEMTNMAFHENWYQFTPSIKRIFMLMVLSNN
LGCEITTCEKLRLSLSAFMSV
>HsOr206
IIANMNFRNVNPLNVYSNYMSGNLLPLAPRDSRFSLASVLFGGFMWLLQAVQLCVLVPGLMMVSWEKAIVDGT
ITFVLIIEVSFIAGQIQTHKKLTDRLIQKLNDILRIEDEMMENVVKTTLKPIDAPLRFYVVAGSLSVFVWFSL
PLLMILERETFFYEDFRLPAVFSTQPFSSQTFVLGSILLVMGNVQIFLKKCGLDIYMIHLVLMMTAQYRYTGK
KLASIFRDASKRCELGQVGCEVDRWTENALKALCRHHNSVVRLSSVLRDLLSSSLNLIYINSVIRFCFTAILF
NAVLSATFLEGFLVSMYSCGSVMEFYMLCSCMQQLHDASTDITDEAFHEKWYQFGPSVKRTFMLMILANNLGC
KLSTCNKFNLSLPSFMTILNQSYSIALVLL
>HsOr207
IITNMDFRNVNPLNIHSNYLSGNLLPLTPCGSQYSTAWKLYGILTWLIQIAQLCSLIAGILLTSQEEAFQGGT
LINLVLIIEVLFIVRQIQSHRKLVDQLIQKLNDILQIKDDNMKYIVSTTLKPMNTPLKYYVVAGAVSLAVWFA
LGLLLIFEKDTFFYEDFKMVVALSKQPFSARTFILGTFLILISNVHIFLKKCNLYIYMIHFALMITAQYRYTG
KKLAMIFREPYVEQCKNGQVSFEADQWANDAIKALCRHHTTIVRLSRMLRETLTSSLTLIYLNSIFRFCFTAI

MFTTVLSYNPIEFCLFIIYSCGSVVEFYMLCSCMQQLQDASKNITDEAFHEKWYQFGPSVRRIFMLMVLGSNL
GCKLSTCEKFNLSLPSFLSILNQSYSIALVLL
>HsOr208
IITNMDFRNVNPLNIHSNYLSGNLLPLTQRDSQFSTAWKLYGTLMWLIQIAQLCTLTIGIISTSREKAFQEGF
ITFVLIIEVFFIIGQIQSHKKLVDQLIQKLNDILQIGDDLMENMVRTTLKPMNAPFKYYSMLGAICVTVWFSL
RLLLVFEKDTFFYEDFRIPAFFSNQPFSARIFILGSILILIGNIHIFLKKCSLYIYMVHLVLLITVQYRYTGK
RLVLIFREADEQCNNGQINSEADQWANDAIKALCRHHTTAVRLSRMLRETLTSSLTLIYLNSIFRFCFTAIIF
GSVLSTTLIEFCLVSIYSSGSVVEFYMLCSYIQQLQDASKDFMDEAFHEKWYQFGPSVKRMFMLMILGSNLGC
KLSTCDKFNLSLPSFLTILNQSYSIALVLL
>HsOr209
IIMQAKVTLKKVIAVVKLSLLVIWFWPLPQNTSTCKMLCMKLYQFISVILIMVELISILYTLVMNLDDPNLLV
KSILGLFPCGHIVWNILCRIIIYQRLQCVTFEMENFCSLIEFHEEAIIQKNYIDKYANFYGFCIASFYIALFI
LFLGPIVLDQPFPTPTDFPFDTSRQPLRAIIYVHQIIVGLQVAANLSVNVFMALLLWLASARFRLLTEDLRGT
TNIYDFEKCIEKHQYLLKYASEITLTVRPFALGTVLFSTMALLVIGLIIIAGASLQLKIQLILIVFSALSEVF
MYAWPAEHLIHISSDVAQAAFEMEWYDESDNLRKNVQMVILRSQKPIIVALPCGLPSLSLRYYASYLSTIFSY
FTTMRLIFEEQSDQL
>HsOr210
KLTLAKVIYVVKLSLFVICFWPQPKDAIKLKVICVALYQYFSILLHLSLALSLMNTVKNHLNNHIIMAEAIII
ICATMHAICNIVVYRINLHHLQSIIFEMENFYKLIKPHEEAILQKYISKCVIFYGGSLINVYLFTIIFICGPV
TLNQPFPTMAEYPFDVSYQPMKTIVYAHQSICALQAASHICINIFTSLLLWFTSARFELLTENLRAIRNIYDL
MKCIQEHQKLLKYTEQVIRVIRPFAFIVVCCSTSALIILGLVFITEQRLSIKIQCVGLICSGLSEVFMHTWPA
EHLIYICNKIGEAVYSAKWYKHSVELQKSVQIMLMRAQKPLTIMIPCVMPSLSLSYYASYLSTIFSYFTTLRI
FMQSN
>HsOr211
KIKFTKVINFVKLSVFITCFWPQPKDAIKLKIICVALYQYFSILLYLALTLALINTIKNHLNDPAIMVKVAIF
ICPTTHVICNIVVYRIIWHRLQSITFEMENFYKLIKPHEETILQKYINKCVIFYGGSIIGIYLISIIIISGPV
TLNQPFPIMAEYPFDVFHQPMRTIAYIHQSICILQASAHICINTYTTLLLWFSSARLELLTDNLHAIRNIYDL
TKCVQEHQKLLKYAEQVIRAIRPFAFIVMCFSTLGLIIVGLIFVTDQPLSMKIQCAGIAFNGLSEVFMYTWPA
EHLIHVNNKIEKAIYSVEWYEHSVELQRSIQIILIRAQKPLLITIPCVMPPLSLNYYTKYLSTIFSYFTALRI
FMQ
>HsOr212
IIMLKNRALKQIIHMVKLSVFITWCWPLPKDTIKLKVVCVSLYQYLCLISSFGVTIGLMNAVKNHLDDPLIMA
KSLTIMCPTIHIICNIVSCKVYSYRLQLVTFEMENFCESLKSHEEPIFDQYIGKCVYFYGGSIIWIYISCLII
ISGPATLDQPFPTTAEYPFDVYQQPLRSILYIHQAFVFTQAAAQLSMNVFIAMLLWFISVRFELLNEELRTIT
DIYGLMKCVRKHQKLLKYADEVIAVVRPFALSTITLSTTALIIVGLIFITGQPLSMKIQCVGLTFSGLSEVFM
YTWPAENLIYISSEVAQAIYDAQWYEQSIQLRKSLQMIILRAQKSVVISIPCVMPSLSLNYYTSYLSTIFSYF
TTLRVVMQNE
>HsOr213
KVTPKVAMAFLKVIIVLCFSWPLLKTASRFQVIRFKTLRFLFCMNVIVLIVPMTYTIYYNDYDLPKITKLWCL
LGAFVQVPLEISLLGVQYDRLQNLISEMEYNFECAQVHERDVYQRYTDKCVPFYAAFTFAVFLTAIVMSIVTP
LVEADQAFPTEAKYPFDVEREPVKTFIFVHQFIAIWQCFSTVCLSSFIGFLIWFTAARFEILSQQFRAVNGTD
EIIACVHRHIKLLRYAQEVIIAFRSVILALIIICTWTFIASGLTIVSQSTTADKTHFMILWITGLIEVYACAW
PADHLMDASTDVAQAVYDSVWYNQNVNFQKSIWFILLRSQVPTAVSVSSFIPAISLRYYASYVSTAFSYLMTL
RIVFLEDDATKS
>HsOr214
MEKLIAFLKVDLMFACCWPLPPTATKYERIRNKIFRWLSVLHGMIMVVTIIYTIYINSSDVFLIMKLCCELCT
TSEVPLQIMCFSIQYNRLQYVIYELENYCKRAKPEEKAVFHHYIDSCKLIYVGSMCAFTITGLLIIISPLVEP
HPFPIDIEYPFDVDYQPMKTIIYLHHILLLYQSYAQVCSNIFVALLLWFISARCDILSGRFRAVTKFEELRAC
LIEHQELLRYGRKVTLSIRYVVLASLAISTIVIIFTGCTFLSRQPISVKSTFLIFFIASLAKVYLCAWPADHL
LSASTNIAHAAYDSMWYNREVGLQKIFLHAVLRSQYPIAVNVPCMLPTVSLNYYASYVLTAFSYLTTFRVLFE
DDDN
>HsOr215
LVSMSVFVKNDKILRALRICGILTSVWPLEDGAGRFKVISYKVLLWVYLVNDFSMVPTLIWNMSHHRNDFANV
MKAIMELMYVSEEIVNILYCMVREKRFRKLLAELECADDERSPYEEILRREFMFYARVVYTCAVIIYFVCAIT
FALSPLVLSQRRQIPLNTIYPLSMSEMWAWCLVYTLNVFSIIQAFSVLQIDLMIITILWNATFKFSLVGIQMH
SVFTVAKLRAAIDAHQKVFAYTQEMEYVIGFLIMKSVVVVFVNSINCSLLVINNLPFWELIQFLSIIFLCLMR
LCIGCWAAEMMTNMAYEVSWRIYDIPWMYATPSIRRDVFMIMQRCQKPVTVKAYIIPTISVNFCGKVFSLGFS
YFLTLRTIL

>HsOr216
MKDAPTLDKIITFLRVHLKFACCWPLSRTATSFQVTLDRIFRFLSVVNGVLVVVELTYSISNHLDDVFLIMQL
ACALGIFCEVPLQTCLFTQKYDRLQNVICEMEEYYERTTVEERDVIQQYCNKYTSFYGITLALTTASLVGSLV
VPLMRSHTFPLEIEYPFRVDYQPMTAIIYFHQALGMYQVYCQVCSNVFLALLLWFTTARFEILSNKFRTAADY
AECKAYIQKHQDILRYAREVTNSIAHVVLSSLGVSTAALVFGGVTFLSRLPPSIKIQYIIVCSSSLTKVLLCT
WPADHLMRTSANIAESAYNSLWYNQGIDTRKIILYTLLRCQQPIVISVPGLLKALTFQHYASYISTAFSYLTT
FRIILSED
>HsOr217
VIMQAKVTLKRVIDIVKLSLFVIWFWPLPQNVSTCKMRCMKLYQFVSATLTVSVISSILYALVKNLNDPNLAV
QSALGLFPCSHVLWNILCHVIIYQRLQCVTSEMENFCSLIEPHEEAIVQRNYIDKYASFYGLCIASFYMTLFA
LFAGPIVLDQPLPVLADFPFDASRQPLRAITYVHQIVVGLQIAAQLSVNAFMALLLWLTSARFKLLTEDIRAI
TNIYDFIKCIEKHQHLLKYAREIALTVRPFALGTVFFSTVSLIVFGLIMIAGASLPLKIQFIFLAFSALSEVF
MYAWPAEHLIHISSDIAQAAFEMNWYDESDNFRKNVQIIILRSQKPIIVALPCGLPSLSLHYYASYLSTIFSY
FTTMRMMFEEQND
>HsOr218
VTMQLKVTLKKVIAIVKLSLFLIWFWPLPKNASTGKMLCMKLYQLISMIFIVTGMASMLYAVVNNLYDLNLFV
KSVLGVFPCIHIIWNILCHVIIYRRLQCVTYEMENFCSLIEPHEEAIIQRNYIDKYASFYGFCIVSLYVALFA
LIMGPIVQDQSLPTPADFPFDESRQPLRVITYVHQIIIILQLAGHFSVNVFMALLLWLASARFRLLTENLRTI
TNIYDFVKCIEKHQHLLEYTTEIALTVRPFALGTVFFTTLSLITIGLIIIAGTSLLLTIQFVFLAFNGLMEVF
MYTWPAEHLIHTSSGMAQAAFEMDWYEESNHFRKSVQMVILRSQKPIIVALPCGLPTLSLHYYASYLSTIFSY
LTAMRVMFDD
>HsOr219
ITPNFHLRMSLSIVYILGTWPPRAGKYRILYLFYTACIFTFMLGVFLVTEIANLFLNLGDPVKIIAGATLLMT
NCIHASKVIILLCRQARIQTLLDSANSRAFNCHDKAHRNLLMKYTWKGLLHHATYQSFGATAVLCWGFTPIAD
LIAGRARRLPMEGWYPYNVTRTPAFEITAGHQGVACIIACCHNVAMDTLITGLITVACCQLAILEQNIISIDN
KRSIHDPNALLHIPNAKNESNEISENEHLSYQQLKRGVMHNNMIFYFTREIQDIFGTVIFLQFLSNCVIICLI
AFTVSQMKEYIPAVLIGMLMYMCCMTYQIFIFCWHGNELHLHSLRIVTAAYSSKWFSGTEGFKRGLQIMMTRA
HRPFTLSAGNIMLLSLDTFVQILKMSYSIFTVLQSSAA
>HsOr220
MRVMRFTLSILALAGCWRPFSWTSLVKHVLYNIYTLLVISVLYSFTFSQFMEIVLNVSNPNDFTNILYTMMAM
IVACCKVLSMWMNHESFVVLIRKLNEGTFRPLVPAEREIRRKCDETIRNNAMCYAILIEITCAGNVLVSLLTN
FRNRKLTFPEWVPYDYSPYVIYCLTYTYQYVSVITASFVNVACDSFIVGFLLHVCCQISILNYRLKNITSHNT
LNYCVRHHYDIIVYARMVNTRFTGIILFQFVTSTFVICSNLYQLTKTTIGMYHIALITYTFCVMAQIFLYCWF
GNKLKVTSLQVLDTIFEMEWTRLDNKIKKSLLIVMSRATVPIEFNSAYLLNMNLDSFVGLLKTSYSVYNLLVQ
VQE
>HsOr221
MRVLRLTLRIVMFAGCFRPLSWLSRFKRTAYNIYRTLIITFLYTFATLQFMDIVLKVDNPDDFTDNLYMMLNV
SVSGYKLFIMWVNYEDIVTIIKSLTEEPLKPLDPGEMEIRCKFDSLIRANTLRYALLIETSWTCTGLTSLLVD
FRQGRLTYREWVPYDYYASYALFFVTYAHQFLSTFYCATVNVACDTLICGLLMHVCCQMEILEYRLKKISQQD
DLSYCIRHHNSIFQFARAVNEGFARIIEFQFVTSTLIICSNLFQLSKSTMGANNIALVVYTCCMLTEIFIYCW
FGNRVKSKSLQLADSVFQLHWLPLNNSVKKSLLIIMKRATVPIEIVTAYILPLNLDSFVAILKTSYSAYNLLV
QAQE
>HsOr222
MHVLEFTLTVLTVAGVWRPRSWKSSQTKQMLYDAYTVVLSVLILMFTMSQFMELLLNTSNADELTEILYMVLI
GVVGNNLKIVTISINHQTFTAMVDNLTVEPFKPIGKNETIIRRRFDKMITNITFRYLMLIVTTAIYITLLSLF
TDFRRRDLTYKAWFPFDYSVSAIYYFLYVHQMFSMLFTSFLNVACDSLFSGLLLHLSCQIDILDYRLSKIANN
ENMLRKCVFHHNRIFEFATTLNEKLAVVIGSQFIGICLVVCCLLFRLAVSSSSVMYIQTLMCLSCALLAMFYY
CWFANEVKLRSLEFSDNIYKMEWPDLNNNVRKCIVIIMSRAMSSPIKFTSARIVPLNLESFVMVLKTSYTVFN
LMRQ
>HsOr223
MRVLKFTLVICALAGCWQPSSWTSLCKRVVYKIYAIFLLSSLYIFSVSQFMNIILNVENSDEFTDALYMMLTV
LVAAYKQTFMLLDRGNITMIIDQLTVRPFAPCESHEVTIRRKFDKMIQSNVLRYLILVTMSITSILMTSFFTE
LTKRNLTYKAWVPFDYSSSNVVFLLVYTHQLIGMSTSGIVNVACESLICGFLLHICCQLEILEYRLTKISRDH
DVLGDCVYHHNRIFTYAYEVNNMFAKIIAIQFAVSMLVVCSNLYRIAMAEDYMSIVPLMFYTSAILAQIFIYC
WFGNEVKLKSLHVMNSIYEMAWPALSNDNQKALLLVMKRTMVPVEFSSAYIITMNLDSFVALLKMSYSIFNLL
RQTHE
>HsOr224

MHVLEFTLSILTVAGAWRPLSCTSLVKNVIYNAYTILLSTAVFMFTTTQFMYLIFNANNADEVTETLYVALLG
LIGNFKIITINLNHQSFAAMLDNLSEKPFKPMEQNETIIRAKFDKMIKTNAIVYLTLIVLTDIYMTSTSLLMD
FRRRDLTFKAWFPFDYSVSVIYYFLYIHQMISVTLCGFLNVASDCFFSGLLLHICCQIEILEYRLSRIANNQA
KLRECVLHHYRIFEFADMLNDKLVIVIGSQFIGACLVVCCLLLRLTISTSSFMYIETVMCTGCALIAIFYYCW
FGNEVRLKSIQLSENIYKMEWPDFNNDVKKCFLIIMNRATSLPIKFTSAHIVPLNLESFVVVLKTSYSVFNLM
RPTQEE
>HsOr225
MNVLQFTLTICAIAGCWQPLTWISIFKHIMYNVYRALLISVLFTFNITQLMNIILNIDNSEVFANTIYMMLTM
SVAFSKVIIMWLSCKNVTGIITCLMNEPFAPIESSEVTIRKKYDKIAQNTMLWYLLLVMTTVVCMIFTSVFVD
FMRGSLTYPAWLPFNYTAPALFFLVYVHQTIAVVIGATVNVACDSLICGLLLHICCQLEILEYRLMQIPQKHH
VLPECVRHHDRIFEYAYTVNSQFVKIIALQFGVSTLVVCSNLFQLAMATIDVNIVPLILYTACMLSQIFIYCW
FGNEVKIK
>HsOr226
MQILSLNFLIYSVGGVWRPIEWSSSATKFLYNAFTLVTLVPLYFLVLTQFMDIVLVVDNMDDFATNSLMFMTI
VGVCCKATVAVLRRGAIIDLVGMLLTEPCKPRDEAEAAIQARFDEFIRSCSIKYTLLATGSITSFTIRSMLNV
AQGHLPYRVWLPYDLNSSPLFWITSIHQMITLTFATIINVGTETLIFGLFLQTCVQLEIFEYRMHKLLMSRTS
RSCRGFIPRGVSDQVGTILSGHIRHHLNIYKYAKTLNSIFNQVLFFQFFGSILVLCTSVYYMSTHITGSEAAT
LPAYTVCMFVQIFVYCWSGNEVILKSANIGSAIYHMDWPSLSIGEKKDLLMIMKRSTIPIKFTSSFLITFSLD
SYSNILKTSYSAFNVLQQ
>HsOr227
MQILRLNFLIYTVGGVWRPIAWSSNTAKFLYNAFTVCTIVPLYFLMLTQLMDIVLIVDNMDDFTTNSLMFMTI
VAVCCKATIAVLRRNGIIGLVQMLLTEPCRPRDEEEVAIQTKFNKFIRSWSIRYSLLASGSVTGVTIKSVLNI
MHGILPYRVWLPYDPSVSLMFWTTSVMQIVSVIFTTIINVGTETLVFGFFLQTCAQFEILESRLQKLVIGRTR
SQLKKYTSHKTETAISKHVHHHLRIFEYARTVNSIFNQILFVQFFSSILILCTSVYYLSSHITESESATLIIY
TICMFVQIYVYCWSGNEVILKSANIGDAVYQMDWPALSVSEKKNLLMIMKRSTVPIKFTSSFLITLSLQSYSN
ILKTSYSTYNVLQR
>HsOr228
MELLSLNFFIYTMGGIWRPVEWTSGYAKVLYSVFSFCTIVPIYFLMLSQFMDLVLVVDNVDDFITNSLMFMTI
VAVWCKATTALLRRDVIIDLMQTLSREPCKPQDADEAAIQMKFDEFIRSCSIMYSLLATSSVTGVTVRSVLNT
MQGVLPYRAWLPYDSTLFLAFWITSIQQMPSLIFATIINAGTETLVFGLFLQTCAQLEILERRLHKLVIDRTA
GKAAGGQRKWSSVAPPLREKGMISGYIHHHLRIYEYARTVNGVFNQILFVQFFGSILTLCTSVYYLSSHIMES
AAATLVIYTICMFVQIYVYCWSGNEVILKSTNVGDAIYHTDWPLLTISEKKDLLMIMRRSAIPIKFTSSFLIT
LSLQSYSNILKTSYSAFNVLQQ
>HsOr229
MELLSLNFFLYTMGGIWQPVEWSSGSAKLLYNVFSFCTIVPMYFLVLTQFMDLVLVVDNVDDFITSSLMFMTI
VAVCCKATTALLRRDVSIDLVQTLSRGPCKPQYADEAAIQTKFDEFIRQVALLDEGSCSIMYSLLATSSVTGV
TVRSVLNPMQGVLPYRVWLPYDSTLSLAFWITSIQQMISVIFAAIINIGTETLMFGLFLQTCAQLEIFERRLH
KLVIYRTAGKAAGGQRGWSSSAPPLHEKGTISGYIHHHLRIYEYAKTVNGVFNQIVFVQFFGSILRLCTSVYY
LSSHIMEITAATMVIYTVGMFVQIYVYCWAGNEVILKSTKVGDAIYSTDWPLLTISEKKDLLMIMRRSTIPIK
FTSSFLITLSLQSYSNILKTSYSAFNVLQQ
>HsOr230
MELLSLNFFLYTMGGIWRPVEWTSGCAKLLYNVFSFCTIVPIYFFMLTQFMDLVLVVDNVDDFITNSIMFMTI
VAICCKAATVMLRRDVIIGLVQTLSREPCKPQDAVEVAIQTKFDEFIRSCSIKYSLLATSSLTCVTVRSVLNA
MQGILPYRAWLPYDLTLSFAFWITSIQQMMSVTFATIISTGTETLVFGLFLQTCAQLEIFERRLHKLVIGRTA
GKAAGGQPECSSADPPLHEKEMISGYIRHHLGIYEYARAVNSVFNQIFFVQFFGSILVLCTNVYYLSSHITEF
ATATLVIYTICMFVQIYVYCWSGNEVILKSTNVGDAIYHTDWPLLTISEKKDLLMIMIRSTIPIKFTSSFLIT
LSLQSYSNILKTSNSAFNVLQQ
>HsOr231
MELLALNFFIYTMGGIWRPVEWTSGCAKLLYSVFSFCTIVPMYLFMLTQFMDLVLVVDNVDDFITNSLMFMTI
VAVCCRATIAMLRRDVIIDLVQTLSRGPCKPQDADEVAIQTKFDEFIRSCSIKYSILATSSLSCVTIRSVLNA
MQGILPYRAWLPYDSTLSLAFWITSIQQMISLVFATIISVGTETVVCGLFLQTCAQLEIFERRLHKLVIGRTV
EKAANGQRGWYSADPQLHDKRMISGNMRHHLGIYEYAKAVNSAFNQILFVQFFCSILLLCTSVYYLSSHITEF
ATATLVIYTIGMFVQIYVYCWSGNEVILKSTKVGDAISHTDWPLLTISEQKAVLMIMRRSTIPIKFTSSLLIT
LSLQSYSNILKTSYSAFKVMQQ
>HsOr232
MELLSFNFFLYTMGGIWRPVEWTSSCAKLLYNVFSFCTIVPMYFLMVTQFMDLVLVVDKVEDFITNSLIFMTI
VIVCCKATTAMVRPDMIIDLVQTLSRKPCKPQDADEAAIQTKFDEFIRSCSIKYSLLATGSVTGVTIRSVLNA
MQGILPYRAWLPYDSTLSLAFWITSIQQIFSSIFATVINISTDTLVFGLLLQTCAQLKILERRLHKLVISRTA

GEVAGKQPGCSSVAPPLHEKGMISGYIRHHLCIYEYARTVNNVFNQILFVQFFGSILILCTSVYYLSSHIMES
AAATMVIYTICMFVQIYVYCWSGNEVILKSTNVGDAIYHTDWPLLTIREKKDLLMIMIRSTIPIKFTSSSLIT
LSLQSYSNV
>HsOr234
MQLLSLNFLIYSLSGVWRPISWSSSCAKLLYSAFTVFTVVPLYFLVLTQLMDIVLIVDNIDDFTNNSLMLVSI
ISVCCKATVVVLRRGAITGLVEMLLTEPCRPRDEDELAIQARFDEFIRSCSIKYLLLAMSSVTSVTLRSVISI
IEGHLPYRVWLPYDSRKSSLFLITSIHQIVSLIFATFINVGTETLVFGFILQTCAQLDILKCRLNKIVNNETA
GYRANQPLSLPNKKQMVISEHIRHHLRIYTYAKMVNRVFNQVLFVQFFGSILVLCTSVYYMSAHITELGALGI
VVYTICMFAQILVYCWSGNEVILKSASLADAVYHVNWFSLPVSKRKDLLMIMMRSTIPIKFTSSFLITLSLQS
YSNILRTSYSAFNVLQQ
>HsOr235
MQLFSLNFFIYSLSGIWRPVGWSSSCAKLLYSAFTVFTVVPLYFMMLTQLMDVVLVVDNMDDFTNNSLLLVSV
ISVCCKATVAILRRKAIIGSVEMLLTEPCKPRDEDELAIQARFDAFIRSCSIKYLLLAVTSLTSIILRSLLNI
IQGQLPYRVWLPYDSRKPSLFLITSIHQIVTSIFATFINVGTETLVFGFFLQTCAQLDILKCRLNKVVNNEAA
DYRANRPLSLPKMKQTVISQHIRHHLRIYKYAKMMNRVFNQVLFMQFSGSILVLCTSVYYISTHLMEPDAMGY
IVYTICMFTQIFVYCWSGNEVILKSASLAAAVYHADWSSLPVSERKHLLMIMMRSTIPIKFTSSFLITLTLRS
YSNILKTSYSAFNVLQQ
>HsOr236
MQILSFNFFIYSLSGVWRPISWSSNCAKLLYSAFTVFAVVPLYFFLLTQFMDIILVVDNIDDFTNNSLVLVGM
ISVCCKATVIVLRRKEIIGLVEMLLTEPCKPRDEDELAIQARFDKFIRSCSIIYMLMAMSSIASDMLRSVLSI
TQDQIPHRVWVPYDLNKRSLFLITSIHQIVTLFFATFMNVGTETLVFGLILQTCVQLDILTSRLNKIVDREAV
GYRMNRPLALSKNQQTVISEHIHHHLIIYKYAKMVNRVFNQLLFVQFFGSIIVLCTSVYYISTHITEPEAMGI
VVYTICMFAQIFIYCWAGNEVILKSASLTDVVYHVNWSSLPVSKRKDLLMIMMRSTIPFKFTSSFLINLSLQS
YGNILKTSYSVFSVLQHS
>HsOr237
MQMLALNFLIYSASGIWRPVEWSSSSARLLYSLYSSCAIATIVILLITQFVDIVLVVNNVDDFIANALLFMGT
VAVGCKITIAITRRDAIIGLVHTLSSEPCRPRDEEEVAIQTKFDEFIRTWSIRFALLATGSVTGVTIKSVLNI
MQGLLPYRVWLPYDPNVTLMFWITSIQQIMSVIFATVVNVGTETLIFGFFLQTCAQLEILGSRLRKFVASSTT
RRHRKDVPPSTNAAKSMISQQIRHHLSIYDYAKTANNIYNQILFFQFFASILVLCTSVYYMSTHFTEAESTTM
LIYIFCMFVQIFLYCWSGNEVILKSMSLGDMAYNMDWYLLSISEKKDLLMIMKRSTIPIRFTSSFLITLSLES
YCNILKTSYSAFNVLQQ
>HsOr239
MRILSLNFSLYAASGMWRPIEWSSVGSTLLYSVYSVFMVYLMIFLLLTQFLDIVLVVDNVKDFTTNSLLFVSV
VSAICKIVTALTGRDRIISLIEVLQRPPCKLVDQEEMDIQARFDRSIRSHSLSYLFLATSSAIGALMGAVLAI
LQGQLPLRVWVPYDYSSPLSFWLTSIQLLLAVVFGTIVNISTETLVYGLCLQTCAQLEILTHRLQKVMLPAES
PGARERPDDVWSNGASRLSRHICHHLCIIRYAESLNEIYNYVFFTQFFASILVLCSSLYHLSSHLTFEDVATM
VVYTFCMFVQILVYCWAGNEVMLKSIDLSDAIYQMDWILMTISEQKELLMIMRRSTRPIKFTSSFLVTLSLNS
YGNLLKASYSAFNVLQQS
>HsOr240
MHILSLTFALLTYTGYWRPVHWPVNSIKYWMYNFYSAFMFTWLQLFALYGLADIFLSTNMQEFVDRCFLYPSI
LGVFIKIVYIFMHRQQIMDLDKMLLKEDCIPRDADEESIQQKFDRNARQITIACELLNVSSAFVLTFGQFFLL
LKRRTLPVYHWAPFDLSSTVVFVLVLLFQSVGLCLCANSSVAHETLISGLMIQICAQFKILCHRAHILPILLM
QAEKDGGSEQDLRAREKLIMRDLIRYHLYVYKIAYTVNDVFALIMFVQFSIISFVLCLSVYKMSNMTSLFTPE
FAHLFSYLASMLTQIFLYCWYGNEVTLKSAEVCNAIYEMNWTTLRVQVMKDLTIIMMCASKPFTMSSGHVVTL
TLDSFMSILKISYSTYNFLKDS
>HsOr241
MHILSVSFALLTYTGYWRPVNWPVNSIKYWMYNVYSAFMFILLQLFTFYGLVDTLLSVNLQEFVDKCFLYLSI
LGVSIKVVYIFMHRQEIIDLDKMLLKENCIPRDADEESIQQKFDRNARRITIACEVLNECCVMFATFVRFYPL
LKTRTLPVYNWAPFDLSSMAVFLPMLIYQSFALCLCANSSVAHETLISGLMIQICAQFEILCHRAHIMPILLT
QAEKDSKSEQDLRAREKSIIRDLVQHHLHVYKFAHMVNDVFALIMFIQFVMISLVLCMSVYRMSNMTSLFTPE
FAHIFSYLASMLMQIFLYCWYGNEVTLKSAEVCHAIYEMNWTPLRIRIMKDLNIIMMRASKPFTMSSGYVVTL
TVDSFMSILKISYSTYNFLKDS
>HsOr242
MHILPVSFALLTYTGYWRPVHWPVNSIKYWMYNIYSSFMLTLLQSFVFYGLVDTFSSASLQEFVDKLYLFLSV
FGVSIKLLHLFIRRRRIIGLGDMLLKENCIPRDADEKLIQQKFDRNARRITIACGVLNESCAMFATFAQFYPL
LKTSTLPVYNWAPFDLSSMAVFLPMLIYQSFALCLCANSSVAHETLISGLMIQICAQFEILCHRAHILPILLK
QAEKDSESKQDLRTREKLIVRDLIQHHLYVYKFAYTVNAVFTLIIFIQFSIISLVLCMSVYKISHMTSLFTLD

FAYIFSYLGAMLMQIFLYCWYGNEVTLKSVEIRNAVYGMNWTPLRVQVMKDLTIIMMRTSKPFKMSSGYVVTL
SIDSFMSILKVSYSAYNFLKDS
>HsOr243
MNSVDLFFQQSHYNIIRVLLSISGLWPYHTTSKRRAIYFGFLLVFGSGLTFEVLGMIEVWPDSYEVVDCLPLL
VLGAVSMIKLVCAVYTLPEITILLTKMQDYCLSLKSNEETNILHSYALYGRNLGYAYMGILFSHTVVFVFATL
LTKLLDTGSSNNNSSSITHETQVGLFYHVNYIVDVETYYVPIFIHSATCVVFYTLLMITFDVLYLTLVQHCCG
LFQALRYRLEYATKLENENGDLILVSGQDKIHSNILYSIRRHAEAIKFVTIMEAIYRIPLFVHVGANISILSI
LGFQVITNTENINRVLKYASYLSALLVNTFFENWQGQKIIDSSEKVFESAYNAEWYKMSPEQRKFLIMIMMKS
KKPLQIIAGNLVALSYVNFNAVIRTSSSYFMLLRSIQ
>HsOr244
MNFFDGHNYRINKILLSTVGQWPYQSSRTSHVIVIVIATIACSQFLTKLCGMFSYIHDMDVVIECLIPIMIDI
SGITKITNSILYADEIRAMLDRIQADFCSLNNFDDIVILQKYANNGKRFSTIYICVMYSVTILFMLTPFQPLI
LHVVNATSRPMLHRVEYYVDMDTYYFPILIHGYFTVIICMTSIVAVDAIFIILVQHVCGLFMIISTRIERVIQ
EKHLVGDADPPVVRDKTYQNMVRCIYDHKAAIRFADLMEIAYSKHFLFNAGLNMMAMSVTGVGALAKIDDPSE
FFKLIAISWALLFHLCFECSNAQRLMDYSEYLHMKLISLNWYDASPRTKKLIILMMMKVQQPCVLTAGGIFVL
CMETFATIVKTAISYFAFLRS
>HsOr245
MDSVQGQYMRINHFLMYLIGQWPYQENWEKALIQIVFAPLVFAQAVIQGGGMITALLAGDIDVAMESSSPFVI
SLMCICKYINYTYNHKQMKRLTVIMTDDWNMHSKSSQEYDILCRNYEIGRKITIGYAASLYGSMTPFLVVPVI
LNTASYMGLYNVSEGRPVMFRSDYLLDVEKYYYPLLVHSYIGTLGFVSIVVAIDSMLVFHVQHECGMCEILGY
RLARIVDATTLDTDLRPSREEDIAYKHAKDCVIMHNHIIEYAKSLENANTTSYFFQLGFNMIGMTFTIFQAVV
KLTDPKQALRYASFTVTLLSVLFLESWPGQQLSDYTDKIFAFITSGRWYQSSLRVRRIISIMLIRSYKPIRIT
AGKLYTLNLENFSSVVRTSFSYFTVLCSM
>HsOr246
IQVMGMIQIWPDIFEVLDCLPLLFYAIIAIIKAICMIYSIPKIKILLIKMKKYWQSPKSDREMKILQSYAIYG
EKFGYVYTGIMFSHSALYLFMTTLKKFVYTQFNEESEASSKIQNAQEGVPYRVNYMVDINTYYIPILLHTATC
EVLYLFLIISQDVLYLTFVQHCCSLLAALRYLLEDTFEFKDDGDDPVKAIANSNRCYANIVYSIQRHTEAIQF
IDIMESLFTLPLFLHLGCVISIISVVGFQVIRNAKDVAVLTRHGIYFCGAVINAFFENWQGQKIIDYSEKVYE
YAYNVQWYDMPNATKKLLILIMLRSIKPSQITAGKVIVMSYLNFNAVSRI
>HsOr247
MVCFEIEYWNINRILLLIIGMWPYQQSKLVQFQIILCYGILISIIVVQLSAFLTTECTVNFFIKNLSAVLYFC
LYVIFYNSFWVNISFVRHLLNYIQYIYDELKDENEIAIIESYGYEAKRCTAAFTLLGVSCLLIAIVQPWIPCI
LNTFLLINDSQPYRSVYIITEYFVDKEKYFYLILLHVNVVFCLGTFVLVGTGTMLLACSRCICGMFKIASYRI
EQALEINIAVNLKNDIMIYNKIIHAVDIHRKATMIIKSLISNFEGSFVCTIMFCVTCLSLNLFQISQYISFGG
SVEQLMLHGIAICIILVYIFISNYFAQEMLDHNSHIFATVYNTRWYTAPLYIQKMILFLLQRGTKVLTLNVGG
LFSASLESIATLISASISYFAVLCSTQY
>HsOr248
TEYLSMNRILLLMIGMWPYQQSKVVQLQIILFYGIFISMIIFQFTVFLTVESTFTFVIDVLSETMFFFLYVII
YNSFWFNNQSVKYFLEEFQHFIKTLEIFCINNGRNDGDYKINKYEMLIEMFGICCVVILVAQPWCPYLLDIIL
LVNNSRPHPVTYIITEYFIDKERYFYINMLHMNIVISLGTVVLLATGTMLIACAKCICGIFRIASYRIERAME
INIACIVYVKDDIMIHNEIIHAVDIHRKAMTFTKFLLASFEGSFFCIMTCGVITLSLNLYRIFQIMSFGGSVE
ELIVHYIFTGVIFMYILVSNYFAQEILDHNNQVFAAVYNIQWYTTPLYIQRMILFLLQNGTKTLTLNIGGLFA
ASLEGTATLISAAMSYFTVLCSTQ
>HsOr249
YLNISRVLMLAVGMWPYKQSKFISLQVILCYVLLVSIIVVQFTPFLTTKNIPDRVIKILSVILFSCIYIIIYN
SLQINKHIIRSFLDQLQCIFNTIKDDHEIAILERYGYNARCYQFACTVFGMCCLFIVNIQPWWPRILNVLPFI
NVSESYHEMYIITEYFVDEEKYLYLITLHINVTVCVATFIILAAGTLFIAYIKCICGMFTIASYRIEHAMDIY
IPQKIDLKKEIIICSKIIHAVDIHRKAMKLTGDFISTLEYTGFCIIIISVVSLSLNFYRIFQVVSFDGNVEEF
IVHSTVVSIIFIYIFIINYFAQEVLNHCNEIFITVYNTRWYTTSLCVQNMILFLLQRGTKGFNLMIGGLFLAC
LESSATVSNKS
>HsOr250
MICFETEYLNIGRVLLLAVGMWPYKQSKLISLQVILCYTLLLSIMIVQFTPFLTAECSPDLVIKILSMVLFCS
TYTIIYNSFQINKHIIRSLMDHLQRIFNTIKDDYEIAILRKYGYNARCYEFALFSMCCFFIVFMQPWWPRILN
ILPFINISESYHAMYFITEYFVDDERYLYLIMLHINMVFCVGTFVVLAAGTFLIACAKCNCGMFTIASYRVEH
AMDIYIPQRIDLKNEFMITNKIIQAVDIHRKSMKLIRDFISTFERTLFCIIIISVVCLSLNFYRIFQVVSFDG
NVEEFIVHSTVVSIIFIYIFIINYFAQEILDHNNEIFIAVYNTRWYTAPLCIQKMILFLLQRGTRGFNLKIGG
LFLACLESSATLISTSMSYFTVLCS
>HsOr251

ETQYLNISRVLMLAVGMWPYKQSKFISLQVILCYVLLVSIIVVQFTPFLTTKCTPDFAIKVLSLALFCSIYVI
IYNSLQINKYIIRSFFDQIQYIFNAIKDDYEIAILERYGYNARCYQFACAVFGMCCLFIVNIQPWWPRILNVL
PFINVSESYHEMYIITEYFVDEEKYLYLITLHINVTLCAATFIMLAAGTLFIAYVKCTCGIFRVATYRVEHAM
NIHIPQSVDLKNEIMICNKIIHIVDIHRKAMKLIRDFISTLESTVFCVIIISVVCLSLNLYRIFQVVSFDGNV
EEFIVHSTVVSIIFIYIFIINYFAQEILDHNNEIFIAVYNIRWYTAPLCIQKMILFLLQRGTRGFNLKVGGLF
LACLESSATLISTSMSYFTVLCSVR
>HsOr252
QYFKLNRILLFTLGLWPYQQPKNARVKVILYLGILVSSIIYQLQIFLTAEVTDGFVIKVSSLILCHIISGIQY
ISFWINIDTVRSLFEYMRHMCNKLKDKNEIAIIEGCGRIVKRYTIIFTLVSIFYIFILIVQSFWVQNFDVHLV
MNESRSHSTVHFIRNYLDNRIKWLNLHLLYTILIICIELTITIATGGAFIFCFQYSCGMFDIASHRINQALSI
KQKTQNDTNLENEIKIHIAIIYAVDIHRKAIDYATFLITSFEGTFFFMILFGVISLSLNLYRVYQIVLYGYED
KELLIHLGYLHAIVIYMFASNYIGQQITDHYNNIFITAYNIRWYMKSLPIQRLLVLLLQRGNKTFFLNIGGLF
SASLECFATLISASMSYFTVMYS
>HsOr253
MLQNEMRYFKLNRILLLLILGLWPYQDSKSARLRITLIFSILMSSILFQLRVFMTDDVTSNLIIKVLSATLCHI
ITGIQYISFWINMKTVRWSIEYLRHICNNLKDENEIAIIEIYSRNMRRYTLGLTLFGVCCMLVLIVGSILGQN
NDIFLFTNGSRPYRVWMIIDYFVDRKKNLCMILLHTNVTICIGLTIILATGTMLLGHLQYVCGMFSIASYRIS
QAINIDQKAQNDINLKNEIMIYKEIICAVDIHRKAMEYSTFLINNFEGTFFFLIILGVTSLSFNLYRITSIEY
DVKELLLHLTYLNIILLYMFLSNYIGQQVTDHYNYIFLTAYSVQWYVTPLTIQKLLVLLLQRGNKNFALIIGR
LFAASLECFATLSSASMSYFTVMYSVQ
>HsOr254
TQYFKLNRILLLALGLWPYQRQKCARLQIILIFSIHMSSILFQLQTLMTAEYTLDLITKVLSIVSCHIIGGIQ
YISFWVNIKTVRCLLEYIWHMCLKDENEIAIIESCNRITRRYTISLLNITIHTAASLNTFXPAYCVIFFISQS
IWTQNPQRTMQMLTDYLTNREKDLCLILLHMSVSACITIYVTIATGTLIIGYLVYTCGLFDIASYRMKRAFNT
SDKDYISPKNEIMIRREIIYAVDIHRKAMDFSTFMISNFEKTYFLMIVLGVICLTFNLYRIFQIISSFEGNYK
ELFMHLIHLTCILLYMFLSNYFGQEVTDHYSCIFFSAYNVRWYMASLPVQRLVLFLLQRGNKTYSLNIGGLFS
ASLECFATLSGASISYFTVIYSVQK
>HsOr255
QIKTCFKTIENDWLSLNTDSEKTILQRQTVYGQYLTIFYAVFMQLTGFLYMLKSIVLILIEESSNSTKLAVSK
LPFRVEYGHKIDQHFYPILLHSYLTIFSHVTATVATDTLYFALIQHACGMFSVVGHSLEHIGKDSDSNFNLKP
EKTEDDNYKKALDCLRRHLHVIEFAELIESTFTNIFLISISLNMIGGSICGIQVLINLNDAKDIVAPLAIYIA
QLIHMFLQFWQAQYLLDYSAIPYESICRGSWYYTSRRCRKLLLLIMNRTVSPCKITAGKIMSLSIESFGVVLK
TSLSYFTMLRSFH
>HsOr256
KSMEASWSHYYGIIQNLSLLTGMWPYLKPRTRLLRVVLLTTIVLTIFVPQIAHQFTCKQDLQCIFESMTSCLL
TSVGMVKVWSFQLNIRQIKSFTEHLFVDWKELETPQEYEIMRSYAENCRRFSLLYPVYCFSAVYIFMSVSIVP
PILDIVSPLNESRPVLLPYPGYYFVDSRKYFFYIFGHSIFSWEMVMAGIVAHDCMFVSYVEHLCSMFAVVGSR
FEDLFRNSDDAVEDAKGNLDDVYRKKIALFVHTHRKTLKFAQTLESTFTVPFAVQLLIIIGTMSCTLLQITQQ
EAGMLELIRYILYVIGQLFHLFCLSFEGQKLIDHSLRMRDKIYNSLWYKAPLKSRKLLILTMMQSLRPTVLSA
GKVYIFSLESFTTVLQTSMSYFTVLSTFQ
>HsOr257
YYKLCRKLLLLTGLWPYDKLRNRLLHMGVIIVFDISIMFAQIANYFVCDTIQCIFKTLPMNIMTVIVVGKLLT
YHFNRQKIKDLLEHLHSDWNALCYEEEFVIMRKYAETGRSYSFYYATSIYSITLIFGCVVLVPRFMDVVSPLN
ESRPIMMPCPVNYFVDEEEYFYYIFVHLIIGAFVCITGLLAHDCNFFTFTEHVCGLFEIVGFRLENLLYKRNI
TEKNMIELSDDVYVKNIEFSIQAHRRALHFVALLENTFCLSFAIQLLIVTIIMSITLIQVSCFTKNVVRYVPF
VIGQLAHLLIYSYQGQKMINHSLGLCEKIYNGLWYTIPVKSQRLLLFAMRKNIVPSFVTAGKIYIFCLENFTS
VMQCSISYFTVLSSFN
>HsOr258
YKLCKKLLLLSGLWPYEKLRRRLLRMGVIIVCDISIMFVQIANYFVCDTIQCVFQTLPLNMMTVMVLVKIFTY
HFNKQKIKGLLEHLHSDWNVLHCEEEFVIMKKYAETGRSYSFYYATSVYSLTLIFGCTVLVPRFMDIVSPLNE
SRPIILPCPANYFVDEEEYFYYIFVHLIIGAFVCITGLVAHDCNFLTFVEHVCGLFEIVGFRLEHLLYKRNIM
KRNVIELSDDVYVKNIEFSIQAHRRALHFVTLLENTFCLSFAIQLLIITTNMSITLIQVKLQGDMVEMVRLVP
YIIGQLAHLFIYSYQGQKLINHSLELCQKIYNGLWYTIPVKSQRLLLFVMRKNIEPSFMTAGKVYIFCLESFT
SVMQCSLSFFTVLSSFN
>HsOr259
YKLCRKLLLLTGLWPYDKFRNRLLHMGVIIVFDISIMFAQIANYFVCDTIQCVFQTLPLNMMTVMVLVKIFTY
HFNRQKIKGLLEHLHSDWNVLHCEEEFVIMKKYAETGRSYSFYYASKNFLFNKWYFRFEHLLYKRNITEKSLI
EFSDNIYVKNIEFSIEAHQRALHFAALLENTFCLSFAVQLLIATTGMSISLLQVSKLQGDMIEVVRFVPFVIG

QLGHLLIYSYEGQKMINHSLELCQKIYNGLWYNIPVKSQRLLLFAMRKTIEPSFVSAGKIYIFCLESFTSVLQ
CSLSYFTVLSTFN
>HsOr260
MLVHVLREYNTNRIFLSCLGCWPFQSKLGKCFLPIFCFALEISYLPFEILTLYEHRSSGKIVFESCYQMVITF
AFLIKLLNQLWNRDKFRRMYEIMEHHWNTLTSDLETRVLRDYSDISQKFTKYYAIIIYCMMSMFITIPTLTPS
LLDILLPRNESRPRQLAIYAEFGVDKDEYFAPIFSYTSIMIIVGISIMVAADTMHMACTAHACGLFHMIGQQI
ENVSSCVKAIDDRVKVSSLSYVVVDVDAKFESLNERMIYQEYIVCLKKHQLALEYVDILNDTHRIVAISFSLL
IGATFSLVGIRIVYVLDQLEEMIRFTFIIMGALLQLMIVCYSGQKLMDESQNIFHRAYAAEWYRFSPRLKSLL
IITLYRSIVPCKLTAGNLYPLSMTIAATVIRTGMSYFTAFLSLKE
>HsOr261
IMLAHVLREYNINRILLSRLGLWPFQSKLARSLLPIFCLLLEMSYYPFEILMFYDHRDDAQMIFESCYQLVIT
TAFLVRLWNEIWNRDKFQHLYEAMNAHWDVFTDDSEIRILKEYSTVSRKFTIFYATMMYILMSMFVVIPLIPV
FLDIVLPLNESRPRILAIDVEFRVDTSEYFTPIFCYTTAIIVVGVSIMVGVDTMHFTCTTHACGLFFIVGEKI
ENILKAADESKCCGCCRQRNVNATELGLSNERAIYDRYVDCLRKYQHALEFVNILNSTHQTVAVFFLLLIGAT
LSLIGVRIVYELNQMKEMFRFTCIVMGALLQLMVMCYSGQKLMDESQNVFHRTYAAEWYTFSPRLKSLMIVTL
YRSVVPCSLTAGKMLPLSMTTYAGVVRTGVSYFTAFLSLKD
>HsOr262
IMLAHVLRQYNINRMLLSWLGIWPFQCKVARCLLPFCALMIEICYFPIEIMMLHEHWRDAQVLCESCYQCVLL
TVFISKIVNEIWNRDKIRHLYEAINDHWNILTSESEVQVLKDYTIVSRKFTIFYSTLLCFMAMLFLSAPLIPI
ILDIVQPLNESRSRVFAVLIKVRLLDQDKYYVPIFCYHIGVVGLGVISMVSIDSMYVAFIVHACSLLTIINRQ
LEEMTPRLNDERDHLAKFDVPNERALYKRYIECLKKHQLALEFINTFNSAYQMISLVLLLLNGATISVVGVRI
VYILDQTKDTVRYSFIIVTMLIQLLIVCYCGQKLMDESQEVVHRAYAAEWYRFSPRLKSLLIITVHRGIVPCG
LTAGKMIPLSMTTYAAVVRTAASYFMTFLSLK
>HsOr263
LKLFISEYKLDTFLKVLSLDMIMIIITIKYITFYAIVDNVKIFRQYISESWSNLIDEREIQIIYKQGKAGKIS
TIVLATLVYVIILFCIFTQYVPNIFDIIKPLNVSRPCRYFILAEYFIDQQKYFHIIAMNINIGLFTLGTILLA
TETFALANALYAFGLFKIASYRMKYILNGNHLQISATKKYVASRDKIIAAVDMHRRAIQYSELLKNSFGPTYL
ILFMGVIFSTSINMFYLFRIITTKQNMLEIIEHSLLIVLHIISLIAANYAGQQFINCDSHVHRTICNTQWYNA
PLKTQKLILFLIQKTTKCYKVDAGGMFSPSLEGLATGLSMSASYFMVFCS
>HsOr264
IESQHFNLNRILFLAIGLWPYQQSRLAQLQTIFLFSILTSYIVFQLTPFITAEFSLKLVIKVCSSIFCFIVFV
IKYSSFWIKTQTVKWTLEQLQHMCNELKDENEIAIIERYGYYAKCSTFRFTLLTVCGLFIIILQPILPHTFYA
FLFVNTSYSRYKIPITTEYFVDEERYFYLLLLHVDIALCIGMIVLVATGTMLIASFKHACGMFKIASYRIERA
ISITMVMNINLVDKITIYKSLIHAIDIHRKTMQFSTDLMSNFEITFLFLIVFTVLSLSLNLYQIFQIISYGAD
AKELLYHVSYVVITLIYMFMANYVGQEITDHNNHVFFTVYNIRWYIAPLHVQKLILFLLQRGGKTFGLTVGKL
FISSLDCFATLTNASMSYFTLIYSIRQ
>HsOr265
MICIETQHFNLNRILLFVTGLWPYRRSSLVRFQFALFLCIMISFIIFQMTTFITTECTIDLLVNVICSILFFI
PFLIKYCSFYINIQTLKYLLEKLQHICNGLEDKNEVAIIEKYGYDGKRYTTNIIMLTVCGVFIILVQPILPRI
IGMLLCINTSQSHNTFWISEYFVEQDKYFYLILLYVNAAFCIGMFTIAATGSMLLALLKHACGIFSVASYRLG
QIMTTDARRNHLTHKLTIKKRIIHAVDIHCKAMEYSEFLISNFEGSFFLLIIFGVLCLSFNLFRIFQLVSTTN
NMYEFSMRVLCASAVIIYMFMANYIGQQIVDHNEHVHFTAYNVQWYTASLDVQRMILFVLQRGSRIFGLHVGG
LFVASLECFAALASASVSYFTVIYSTQR
>HsOr266
MISIETQRFNINRILLLIIGLWPYQRSRLVRFQTIVFFGILMSFIICQSTVFLTAKCTTDLLINVFSSALLCF
LLLIQFISFWSNTRIVKYLLEQLQEICNELKDENEIAIIKKYGDKAKHVTVFLTLLIVCTQVIIIVQPIWSRI
LGIFLFLNESRSRGEMFIVTEYFVDQQKYSHLVLLHINVAICIGLFAMIATGTMLLVFLKHACGMFSIASYRI
KRAIANNMPQNTNLSDQSIICKKIICAVDIHRKAMQYSDFLISSFEGSFFLLIAFGVTSMSLNLFRLFQLALS
GDNKQELLVRFICAILILLYMFLANEIGQEITDHNDQIYFTAYIVRWYTAPLHVQKLILFLIQRGSKPFGLHV
GRLFVASLECFATLASASISYFTVMYSVQK
>HsOr267
IESQHFSLNRILLLAVGLWPYQRTKFVRLRVVLLFSILIGSIVFQLTPFMTVQLSLDLVVKVFSTMLLLATFA
IKYNSFLVNIRNVKYLMEQLQRICDELRDENEIAILEKYGRDAKRYTINILLLTATSLVILIVQPLGRCIYNI
LPFTNGSRSYCTVPIITEYFIDQEKYFYIIILHMSAAFSIGTIILIATGTWLAVSLKHACGIFKIASYRIERA
ITNNSLENSNLKNKIMIYKRIIFAVEIHRKAMQFAGNVINRFNSTFFFLITCGVACLSFNLFRIFQIMSFRKN
MEEMILHLLLGIVIIIYMFLANYAGQEITDHNNHVYLTAYNVCWYIAPLHVQKLILFLLQRGSKTFSLTVGGL
FTASLECFASLASASVSYFTVMYSMQQ
>HsOr269

IEFQHFSLNRILLLAVGLWPYQRTKFVRLRVVLLFSILIGSIVFQLTPFMTVQLSLDLVVKVFSTMLLLATFA
IKYNSFLVNIQSVKYLMEQLQRICDELRDENEIAILEKYGRDAKRYTINILLLTATSLVILIVQPLGRCIYNI
LPFTNGSRSYCTVPIITEYFIDQEKYFYIIILHMSAAFSIGTIILIATGTWLAVSLKHACGIFKIASYRIERA
ITNNSLENSNLKNKIMIYKRIIFAVEIHRKAMHFCCNFSRVHKFTNSKQLDRYNVWWYIAPLHVQKLILFLLQ
RGSKTFSLTVGGLFTASLECFASLASASVSYFTVMYSTQK
>HsOr270
INIESQHFSINRLLLLAVGLWPYQRTTFVRFRVILFSSILMSSIIFQLTALITGPLSLNSVVNVFCTAKSFST
FVIKFNSFWINVQSVKCLIEQLQRICDELKDKNEIAILEKYGRDAKRYTINILLMTVCSLAVVIVQPFWPYIY
NVLPFMNGSRSCCTLPIVTEYFIDQEKYFYIIILHMDVAFCIGAITMIGTGTWLVVSLKHACSVFKIANYRIE
LAIINIFFKNNMVDEITIYKRITYAVDIHCKAMQFAKNVITSFDSIFLFLIVVGVASLSFNLFRVFQVMSSGN
DMEEMILRLLTGMIIIIYMFLANYAGQEITDHNDYIYLTAYNVYYIAPLHVQKLILFLLQRGSKTFSLTIGGL
FTVSLECFAS
>HsOr271
MINIESQNFSLNRILLLAVGLWPYQRTKFVRFRVILFSSILMSSIIFQLTALIAEQLSAQLVIKVSGAVLFFS
IFVIKFNSFWINLQSVKCLMEQLQRICDELKDKNEIAILEKYGRDAKRYTINILLVTVCSLAVIIVQPFWPYI
YNVLPFMNGSRSCCTLPIVTEYFIDQEKYFYIIILHMDVAFCIGAIAMIGTGTWLVVSLKHACSIFKIASYRI
ELAIINIFFKNNMVDEITIYKRITYAVDIHCKAMQFAKNVITSFDSIFLFLIVVGVASLSFNLFRILSSGNDM
EEMILRLLSGIIIITYMFLANYAGQEITDHNNHVYFTAYNVCWYITPLHVQKLILFLLQRGSKTFSLTVGGLF
TASLECFASLAGASVSYFTVMYSTQQ
>HsOr272
IESQHFSLNRMFLLAVGLWPYQRTKFVRFRVILFFIILMSFIIFQLTALITEQLSVQLIIEVFCASLFFXLAI
KFNSFWINIQSVKCLMEQLQRICDELIKNMGEMRNVIQLIFYVRTYLLTLIILLTVFSVVVLIVQPFWPYIYN
IVPFINVSRSCYTVPLITEYFIDQEKYFCIIMLHMDAAVCIGAIAMAGTETWLLASFKHACQEIVYSYRIKLA
IINSPFKSNLENEITIYKKITYAVHIHRTAMQYAKNVITSFDGTFFFLIVVGVACLSFNLFRVSLLCWFKYXI
KTNCIDKIEIHYTIIMFLSNYAGQEITDHNNHVYLTANWYIAPLHVQKLILFLLQRGSKMFSLTVRGLFTASL
ECFSSLAGASVSYFTVMYSTQQ
>HsOr273
IQTHLKLNRVLLLVLGLWPYQRLKIVRCQFVFVFGILLTYILSQVCVFITLQCTTDLVLNILFVSFFTVTSVI
KYISFYINSEAVKSLIEQIQRNYDKLKDERETAILNKYGNKAKYYTTVFTVLFIFLSSICFLLQLWPCIFNMF
TPANTTRSIHRLRFISEYFIGQEKHFYIIILLHANIAMFISSLTLLSTGVMLLSFGLHICGMFKIVSYHIKHAT
RINGSRPISPHKKNFTYVKMVYAVDIHRKTLQFSESLMSTFALWFFLLIGAGVISMSLNLMQVSIFITLSSGH
NVEELTIQVVFLFVQYTYMFLANYVAQEVTDHNNHVFTTIYNIQWYVTPLHMQKMILFLLQRGTKPYYLQLGI
LFVGSLEGFASLMSATISYFTVIYSTR
>HsOr274
LNLNRVLLLAVGLWPYQQSKLISLQFVLLFSILLTFILSQLGTLVISRCTTDLVLKILFIVFLFGVFVINYAS
FYVNGDAVKSLTQQIQHNYDELKDEGEIAIMNKYGNRTKYFTSMCIVLFICTIYVLLLSQFWPCILDVIQPAN
KTRPINSLRFISEYYIDNQKYLYLIVLYTNAAFFIACLTTLSTGTMLLAYGQYICGMFRIASYRIQYAMRPNV
VQPSNLQKKNPIYERIIYAVDIHRKTLQFSETLMSTFALWFFLLIGAGVISMSLNLMQIFLTLTSGHNVVELT
IQVILLFIQYFYMFMANYVAQEVADHNNHVFTTVYNIQWYVTPLHVQKMILFLLRRGAKPYYLQLGSVFVGSL
EGFASLTSATISYFAVIYSTQQ
>HsOr275
YLNLNRVLLLAIGLWPYQQSKLISLQFILFYSILLTFIMSQLGTLVVSRCTTDLVLKILFIAFFFSTCVINYT
SFYVNIDAVKSLIEQIRHNYDELKDEGEIAIMNKYGNRTKYRTSLSMVLLICSLYIFFLSQFWPCILDVIQPA
NKTRPINILRIINEYCLDKERYLYLILLHTNAAFCIAGLTMLSTGTMLLAYGQYICGMFRIASYRMQHAIRPN
MMQPINLQEKNPTYERIIYAIDIHRKTLQFSETLMSTFALWFFLLIGAGVISMSLNLMQIFLTLTSGHNVVEL
TIQVILLFIQYFYMFMANYVAQEVADHNNHVFTTVYNIQWYVTPLHVQKMILFMLQRGTKPYHLQLGSIFVGS
SEGFTSLVSATISYFTVIYSIQ
>HsOr276
YLNLNRVLLLAIGLWPYQRSKLISLQFVLLFSILLSFILLQVRTLVILRCTTDLVLKILFIAFFFSTCVINYT
SFYVNIDAVKSLTQQIQHNYDKLKDEGEIAIMNKYGNRTKHFTSMCIVLFICTIYVLLLSQFWPCILDVIQSA
NKTRPINSLRFISEYYIDNQKYLYLIVLYTNAAFFIACLTTLSTGTMLLAYGQYICGMLKIASYRIQYAMRPN
VVQPSNLQKKNPIYERIIYAVDIHRKTLQFSESLMSTFALWFFLLIGAGVISMSLNLIQIFLILSSGYNVVEL
TIQVIFLFCQYFYMFMANYVAQEVADHNNHVFTSIYNIQWYVTPLHVQKMILFMLQRGTKPYHLQLGSVFVGS
LEGFASLMSASITYFTVIYSIR
>HsOr277
YFILNQHLLHAIGLWPYQRSKFSQFQFILFFGIPTTFILSQLSVFFTSEYTAEVVVKILFISFFSSIGIISYV
MFYVNSRAIRCLTGQIQYRYNRLTDENEIAIMDEYGNKIKRVTIIFTVFFICGLFIICLLQFWPRILNVILPT
NETRPIYLLQILNGYFLDPERYIYLILFHMNATISICGFTMVSTGAMLLVYVQHICGMLKITSYRIQHAIKLN

VPRSAQKQWNENLAYKRIIYAVDIHREILQFADIFMSTFNLWFSLLIFTVVISTSLNFMRIYMTLTSGCNIEE
LTISVILLIVQYLYQFIGNYSGQQVIDHYNQVFTMVYNIQWYMTSLQMQKMILFLLQRGTKVYSINIRGLFVG
SLEGFTTLLSASISYFTVIYSTR
>HsOr278
MISIEVQYFNISKNLLLAIGLWPYRQTRITQLQYVLFFGILLIAIMCHLTTFIVTEFTVDVVVEVFSSMFLFL
NSMIQYSTFRMNKVTIKIVLEELQRVCDKMKDKNELAIIKKYGNISRRYAFWLTTLAVLGEFGFILMQCWSHM
LNFIQPRNISMQHTLLFPMEYLVDQQRYFYLIMLHMNAAVCIGITASLATGTLFIACQKCICGLFIVASYRIK
HAIRIDASEKINSCSQNLAVIKIARAVEIHRNAMKFSRFLVNKFQLMLSVLILAGVSSLSLNLFRIFSIMSSR
YHNFKELLLPAGYIILIAIYLFLANYIAQDLTDHNKNIFVTAYNVCWYKAPINVQRVILFLLQRGTKEFTVKV
IGWLNGSLECFASLTNISISYFTVLYSTQ
>HsOr279
MINIEDQYFSYNKYFLLALGLWPYWQTRLAYIQYIFLLGFLSTAIIFHFTTFITAKCTVYLVIKVLSSAFLFI
DIMVQYFSFHTNNVTVKVLLEELQRICDEIKDNNELAIIRRYGNVSRCYAMWFMVSIFCLNLILILVSSWPSI
LDFVQPRNESRLRNLLFPMEYFVDQHRYYYLILLYQNTIICIGTVALLSTGSLFFSFQKYICGMFTIASYRIK
QSIKSDTTQKTKLQCRNPIAEIVSAVEIHRRAMKFSKHMVNEFEVMLCILVATGVCSLSLNLFQIFLIISSEY
NINELFLPTGAAMIIAVYMFLANYIGQDLTDHNRQIFIAAYNICWYKTPLNVQRLILFLLQRGVKDFMVSIGG
LINASLECFAMMANVSVSYFSVLYSTQ
>HsOr280
MIDIETLYFSFSKTLLRSVGLWPHQHMRLIQLQYILSFGILLTVIVFHFTTFMTAECTSDLIVKVLASMFVFI
NIIVKYSSFWVNRVTMKILLEQLQKICSELKDENELAIIAKYGNISRSYTIWFTVHFICGESFFILIQSWSSI
LDFVRPQNESRPRIILFPMEYFVDQQRYFCLILLHINIALCIAGITLVGALALLFACQECVCGMFTIANYRIK
YSMRYNSLRNVDLTDKNLISERITRAVDIHRKAMKYSKFLMSRFEVAFGFLTMTVVITLTLNLFRIFQIMSSE
FNIMELILPVAWAISIILYMFLVNYIGQDLTDHHTQIFVTVYNIQWYKAPLHVQKSILFLLQKGSKDFTLTVG
SIINGSLKSFAMLTNTSISYFTVLYS
>HsOr281
MICVKSLQLDLNRILLLAVGLWPYEQSKFVRVQLIVFYVILVTSIIFQFTAFITAECNSQLVIEIFSNAFFFI
TLAIEYNTFSFNMDTVKYLLEVLQYTYNELRDDGEIAIIKKYGTIAKRYTYALTIMSMFGIFSVMLLPFWPCF
LDNVFSMNESRSHVSLYIVTEYFVNQEKYFYLILLHVNLAFCIGWIALLAVGTMTITYWQYARGMYQVASYRV
EQAIMGNALKVNGINDIDLKKIICGIDMHRKALTFVKYLLSKVEVSYFCLMIGVVIVLSLNLYRIFDGLSSGV
NIEKLISPTVFLVTNYILMFLVNYIAQQVKDHNNLLFITIYNIQWYITPLYIQRIILFLLQMGTKPFNMNLAG
LMVGSLESFNMLTSTSLSYFAVIYS
>HsOr282
NRYLSFNRFLLRTIGLWPYEQSKIIRLWLVCLYGVLISSIIFQLTTFFTSECTPQFIIEILSPVTFFTIYIIK
YNSYSLNMDTIKYLLELLQYTFDELKDEGEIAIIEEYGNVGKRYTIALTGKTTHLFGVCGITVLTMLSFWPTI
LDIVLPANESRPHPQLQIMTEYFIDPEKYFFFILLHANAAYCIGGLVMLAIGTMNIAYIQLICGIFKISSYRM
EQIMMICTVNNAGNQNLIHKGIVYAVKMHRRAMRFIKVYISKMEVPFFLIIVAGVISSSSLNLFREISVGFDLR
KMILPTIFLYIFYIYVILANNCAQQIMDHNDHVFATVYNIEWYEAPLTIQKMILFMLQKGNKPFLVNLGGVFT
ASLESAA
>HsOr283
MICVKSLQLDLNRALLLMVGLWPYKQSKFARVQLIVFYIILISSIIFQFTTFITAECNSQMVIEILSNAFFFI
TLAIKYNMFNFNMDTVKYLLEVLQYTYNELRDDGEIAIIKKYGTIAKRYTYALTILSVFGIFSVMLLPFWPCF
LDNVFSMNESRSHVSLYIVTEYFVNQEKYFYLILLHVNSAFCIGWIALLAVGTMTIACWQYACGMFQIASYRM
EQAMMDDALKINDTYNIGLKRIICGIDMHRKALAFVKYFLSKIEVSYFFLMTGVVITLSLNLYRVFDGLSSGV
DIEKLISPAVFLATNYVLMFLSNYTAQQVKDHNDLLFITAYNIQWYVTPLYIQRIILFLLQKGIKSFNMNLVG
LMVGSLEGFSTLASASISYFAVIYSVRQ
>HsOr284
MICVKSLQLDLNRVLLLAVGLWPYEQSIFVRLQLILFYGILITSFVAQLATFLTSKCTLQFVMEILSAVSFFI
CFMIKYITFCFNTDIVKRFLEIMQHTYNELTDENEIAIIEKYGTIAKHCNYIITLLCMSAIFNFIFLPFWPRV
LNVVWLANESHSLPLLQIKTEYFCNQEKYFYLILLHTNAVFCIGEVVLLATGAIIIAYLYHACGMFKVASYRM
EQAVKINTMKINDMMNENSTYKMIVHAIDMHRNAIKLVCIFISDLNVCLFFLIAFGTISTSLNLYRIFHELTT
ECDFEKLITPILFLLTLYVYMIFSNYCGQIVIDHNEEMFATVYSIQWYSTPLRIQKIILFLLQKGTKTFYMNV
GGLFVGSLKGLSTLVSTSVSYFMIMYST
>HsOr285
MICVKSLQLDLNQVLLLAVGLWPFKQSIFVRLQLILFYSILIISIVCQLASFLTTKCTSQFVIEVLSVVNFFI
FFMIKYITFCFNIDTVKYFLKIMQHTYNELKDENEIVIIKKYGTLAKHCNYIITLFGIVGIFSFMFLPFWPRL
LDVIWPTNESRPHSLLQFKAEYFCNQEKYFYLILFHTNAVFYIGETILLATGTIIIAYLHYVCGMFKVASYRI
DQILKTNALKMADMENEKFTCRKIICAIDMHRNAIKSIDFFMSKLNACFFFLIISATISTSLNLLWIFHELTT

KCDFEKLVTPILILITLYVYMFLSNYCGQIVIDHNEEVFTTLYSIQWYCAPLRIQKIILFLLQKGTKTFHMNI
GGLFVASLKGFSTLASTSTSYFTIIYST
>HsOr286
MICVKPLQLSLNRILLLTIGLWPFERSKFTRLPMIVSYSVLITSIVFPFAKFATPKCTSQCIIEVCSVAFFFI
SFPIKYYAFWSNMGTVKLALKIIQSTYDELTDDSEIAIIKKYGTMAKRYTYVLTLLVTNSMFVFILLPFWPRI
LDVVWPANESRPHLPLQITMEYFLDQERYFYLFVLHANVIFCVGETTLLATGTIIIAYVQYTCGMFRVASYRM
EQVMIGTLKRSDTEYKSLIYKRLVYAIDMHRKATKFLKLILSKFEIFFFILLITIIITLSLNLYRVFYELSTG
YNIEKLLISFIFLSSHYVYMFISNLNAQQIIDHNEHVFATVYNIQWYTAPLHIQKMILFLLQRGTKAFYITIA
GLFAGSLEGFTMLTSMSISYFTFVYSTSR
>HsOr287
MVCLRTRYFRVNRLLLLMVGLWPYKQSKITRLQLILFQSILLSLVLFQLSSFLTSKCTAQFAIEVFSGVFPII
GFMIKYNMYSLNFNYVKYLLELIQYTCDELKDKSEVAILEEYGKTGKRYINVLAPIGIIGLLIFLSYPFLMII
LDIISPTNKPGSLTQLQFVTEYFVDREKYFYLILLHTNIAYCIGYSALLAVGTMNLTYFQLICGMFKISSYRV
KKAMMIYTLEISNTRNQQLVQQNISYGVDMHRKAMWIINVYTSKMEIFYFFLIISGIASCSLNLFRIFQLLSS
DINIVKLTFPCITLIVFYIYTIIAMNCAQQITDHNDDMFATAYNVKWYMAPLNIQKTILFMLQKGTKPFHVNL
GGIFIASLENCTKLMSISMSYFTVLCS
>HsOr288
MICVSTRYFSVHRILLLLLGLWPYEQSKFTLLQKILLNGTLMSFILAQFATFATANCTPQFVIEVLSIVFLFI
IYAIKYTSYCVHLKTIKYLLELLQRIYDELKDEVEMSIMEEYGNMARYHTGILTLISTFGVSVFILCPFWLRI
FDIVWPANEVRPHSWIHIPTEYFIDQEKYYYFIVLHTNVAFCVGSTALVAVGTTSMSYFLHICGMFKVASYRV
EQAMMICKLEVGSAGSETLIHKQIVCAIDMHRKATWSIRYFISKLQVFYFLLLLCTVISISLNLFRVITNGFD
FEKLVIPCICLGSLKLYIVIANNNAQQVMDHNEDMFVTVYNVKWYNAPLRIQKMILFMLQRGSKPFYVIIGGM
FIGSLESAAKLLSASMSYFTVLYSTRQN
>HsOr289
MICVESFHIDLNRLLLLAVGLWPYQQSKLVQLQLILFFSILATFILSQLTSIVTSEYTSDLLINVSSTTLFYI
TFVIKYSSFRVNIETMKHLLELLQRIYNELRDENEINIIKKYGQLSRRYTAALIIFSVFILIPIITYSFCPLI
LDILRPLNETRPQPSLEFKYVVNQKKYFYLIMLHAITAFIAGGLAMVSTGAILIAFIQNACGMFRISSYRIEH
AMAIETAQSSNAGNGNMMYEGLIYAVDIHRKAMQFSDLSISKFKVSFFLLIVTGVACGSLNLLRIFQAVTVGY
TNVGMLSIPLVFFVIHMIYMIVSNSVAQDIMDHNSHVFDTIYDVRWYVAPLNVQKMILFLLQRSTRVFSLNIG
GIIVGSLENAATIFSTIVSYFTFFYSTWNK
>HsOr290
MICVESLHINLNRLLLLAVGLWPYQQTKIVQLQLVLSFSILTTFILSQLTSIVTSEYTSDLLINVSSTTLFYI
TFVIKYSSFRVNIETMRHLLELLQRIYNELRDENEINIIKKYGQLSRRYTAAIMTIATFTIIPIITYLFCPLI
LDILRPLNETRPQPSVEIKYFVNKKKYFYLIMLHAITAFIVGGLAMISTGAILIAFLQHACGMFRISSYRIEH
AMAIGTVQSGKAENNNMMYKDLIYAVDIHRKAMQFSDMTISKFKVSFFFLIIAGVICGSLNLFRIFQAMTVGY
SNVGMLLIPLVFFVAHLIYMIVGNSIAQDIMDHNNHVFDTIYDVRWYVAPLNVQKMILFLLQRSTKVFSLNIG
GIIVGSLENAVTIFSTSLSYFTFLYST
>HsOr292
MICVESFHIDLNRLLLLAVGLWPYQQSKLVQLQLILLFSILATFILSQLMSIVTSEYTPDLLINVSSTTLFYI
TFVIKYSSFRINIETMRHLLELLQRIYNELRDENEINIIRKYGQLSKRYTTAIITFSICAVISIVVYSFCPLI
LDILWPLNDTRPQSPLEFKYVVNQKKYFYLILLHAITAFIVGGLAMVATGTILIAFLQHAWGMFRISSYRIEH
AMAIRTVQSGNAGNDNTMYKGLIYAVDIHRKAMQVSDMSISKFKVSFLLLIIAGVICGSLNLLRIFQAVTVGC
TNVGMLLIPLTSFAIHIIYMIVGNYVAQDIMDHNSHVFNTIYGLRWYMAPLNVQKMILFLLQRNTKGFSLNIG
GVIVGSLETAATIFSTIVSYFTFFYSTWNK
>HsOr293
MICVELLHIDLNRLLLLAVGLWPYEQTKIVQLQLILLFSILTTFILSQLTSIVTSEYTSELLINVSSTTLFYI
SFVIKYSSFRVNIETMRHLLELLQRIYNELRDENEINIMRKYGQLSKRYTAAIMIIATCTTTSIIAYLFCPLI
LDIMRPLNETRPQPSLEFEYFVNQKKYFYLILLHAITAFIVGGLAMILTGAILIAFLQHAWGMFRISSYRIEH
AMAIGTVQSGNVGNNNRMYKGLIYAIDIHRKAMQFSDMSISNFRVSFFLLIIAGVICGSLNLFRIFQTMTAVH
SNVGMLLIPLAFIVIHLMYMIIGNYVAQEIMDHNNHVFATIYDVRWYVAPLNIQKMILFLLQRSTKVFTLNIG
GVIVGSLENAATMVSTSVSYFTFLYST
>HsOr294
QIILISSILISFIVLQLATFLTAECTVDLVINILSITFLFMIHLIKYNAFILKIKNVKYLIDQLEQICSELKN
EKEIAIVENYAIVVKRYTIVILLLSIFYLVSINLLPMWPRMLGIFLLINDTRPRRMLLIMNEYFLDQEKYFYF
ILLHTNATLFAGTLIVLATGTMFVGYFKHICGIFRVASYRIEQAMDINVLKNINPKNEIMICKKIICAVDLHR
QAMRSSKVALSMFEGMFFLLLLFGVSCLSLNLFRVFQIVTFEDNIEEFMLRIAFVITILMYIFLMNYIAQEVT
DHNEHVFEATYNIRWYIAPLHIQKMILFLLQKGTKAFTLNLGGLFVASLESASMLASASVSYFTILYSTRQ
>HsOr295

IQTQHFNVNRILLLAIGLWPYQRTKLVRIQIILVSVILTSSVLFNLTTFITRECTTELVINVFSFAFICIIFL
VKYNAFLFKIQNMRFLLEQLQQICNELKSEKEIAIIKNYACGVERYTVILLVLAFCYIFLINAQPMWSRILGV
FLLINETQLQQTMPITTEYFVDREKHFYIFLLHTNVTLCLGTFVTLGIGTMFVGYLRHVCGMLKIASYRIEQA
MGTESSQDTEVKNEIMICKEIIYAVDIHRKALRYSSSKSITSTFEGMFFLLLAFGVICLSLNLFRIFQIVSFE
NDIQELLLRLAIVNAILLYIFLVSYTAQEITDHNNHVFETVYNIPWYMAPLHIQKMILFLLQRGSKTFTLSLG
GLFVASLESASTLASASISYFTILYSTQQ
>HsOr296
FNINRILLLAIGLWPYQRSKLVHQCQLLLIYTILISFIAFQLTTFVTAECTLNITIKILSSTLFFIMHMVHYN
SFLTNGQSVKHLMEQLQRICNDLKDEKEIAIIKKYGNNAKLYTIALTGASISGTFVIFSLPIWSYILDVVLST
NKSTVYRTTQIMTEYFIDAEKYFYFILLHMDASYFIGCTIVIATGTLFVAYVQYACGIFKIASYRIEQALSYD
ILRRIGPKNEIMIHKEIIYAVDIHRKAMKFSEFMITSFKGLFSFLIVTGVISLSLNLFLILQILSFGGDMGEL
LFHLSYLIIVVVYMFIASYISQEIMDHNNHVFITTYNVRWYLASLHTQKMILFLLQRGNKSFTLNLGNIFIGS
LQSFASMVNISISYFTVIYSTQK
>HsOr297
QIIVSFSIIVNFTVFQFTALLTTECTSDFVITVFSSTTVTIATMIIYNSFVVNKHSIKYLLEQLQHVCNELKD
ENEIAIIKDYGSNAQRHAAKITFCAAAFFIIVQIMWFPGFNIFLSKNESGRHQALFIVTEYFFDQEKYFYLIL
LHMDATFLIMLLGTVATGTTILIFLKHICGMLKIASYRIEQTMEINVQYNINLKNVIMIYKNIVYAVDIHRKA
MMLSTSMMKEFEGSFFFLVIILVSGLSLNLYRIFQIISFEYNLMKIVIHSAYVSATLTYIFMSNYIAQQVTDH
NEDLFVTVYNIRWYVTPPHIQKIILLLLQRGSKPFYLRIAKIFVGSLEYFASLTSASMSYFTVIYSTQQ
>HsOr298
QYFTINRILLLLVGLWPHQKSKVVRLQQILIFSILFSFIIFQLTTFMTAKCTANLVIKVLINVFIFIAYVITY
FTFWSKITFMKYLLDELQQIINELRNENEIVIIEKYGYKGRYWTIVLTLISIFYIIFIQLLWIRDLNVSSNLN
GSRSHSVHVMTEYFIDKIKYRYLLWLHMNAAFSIGALTATAAGTMYLGYLYFVCGIFRIASYRIKYAIGINFQ
QNIDLKKEIIIHKDIIYAVDAHRKAMKFIMVIQSNFEVLLFLLIVSFVICLSLCLYRIFQILSFENNIMGFLL
HLAYALCIVLFMFLFNYIGQEIMDHNNHIFHTAYNICWYIAPLHVQKIILLLLQRNTKPFIISFGNIFIATLE
HFSSLMSASMSYFTVLYSTQQ
>HsOr299
FDLQEYFSINRTLLVTIGLWPYQKTKVAQLQKILCFGILTSIIVFQLTMFIITDYTLDFAVEVLSIVLIFITY
FVLYSTFCINAKTVIQLLDQLQYICEELKDENEVAIIKKYAYNAKRFTTIILLFSFLCFLILQLMWPRILHIL
LFLKETHRMEHVVIEYFVDPEKYIYLILLYVFAAVYVGVITILGVGTTLFIYCRHLCGMFQIASYRIKQAINT
DIQRNVSQMKHQFHGLVFAVHIHRKAIEYTETLTSNFESSFFVLIIILVICLSLNMYRVSYSDSQTHPYTLSL
HVTYIMSIIVFILLSNYTMQDITNHNNYVFLTVYKVHWYITPLRIQRLILLLIQRGNKPLIPKLGGIFTASLQ
NFAAMMSMSMSYFTVLYSTQ
>HsOr301
MTCFDLHQHFAINRILLLLVGLWPYQQSSIVHLQKILFFSILSSFILFQLTSFLTAKCTPDFIVKISCSVLLF
AIYLILYNTFHFNTKHMIHSLDQLQHVCNELQDENEIAIIERYACIAERKTIILTVFSSVFVILVQPTWPRIF
DTFLLTNNSLPRHKIQIFTEYFIDQERYFYLIALHTFTAFLIGVIVTVATGTMILTNVKHICGMFLVASYRIQ
QAIKIDDRRIQKNTICKDIICAINIHRKAMKFAEFMIKNIEITLFVLVVLSVSCLSLNLFRIFQIVSFGGDIA
EFWLHLAYTSAIILYMFEANLCGQEVIDHNRNLFHAVYNVYWYITPLSIQKLIILLLQRGNKPFFLNVGGLFI
MSLENFAMLLSASISYFTVIYSTQ
>HsOr302
MICIKSQHLGLNRILLLMIGLWPYQRTRLVRLQRILVSSIIMTYVTFQAIQSLTTFITTECTPDFVMKVLCSV
IFYSTLLVKYNAFRVNVGIVKNSLELIQHTCDELQDVNEIDIVKKYGNIARRYTATFTLVGIVIVSIIICIPI
WPRIVDIFLPMNESRPLFMAHIMIEYFIDREMYSYLVVLHSSIAISVGAIVEVATGTMLIGYYKHVCAMFKIA
SYRIEQAMKINAQQYIDPRREITILMMNRIIHAVDIHRKAMMSAEYLITNFEVSFFILIALGVMCLSLNLFRV
MSFENDTNGVYLHLIYVNIVLLYMFTANYIAQQITDCNHDVFVTVYNIRWYTAPLQIQKTILFLLQRGTKPFS
LTLGGLFVASLKSAATLASTSISYFSVLYSTR
>HsOr304
MSFYDNSYYYLNKRLLSIIGQWPYQSRLESNTMLGITLFFTGSLTFLEGWGLVSGMTDLSIIMENASPLLVNS
LIFVKLINYFFNKYKMKELLDHVEETWQMIQVGPRNEILRSYAEQTKVYTIRYTLALYAMWVLYSTMPLIVSW
TYKLLPINATYTARFLYRLEHVCDVDKYFNLLMLHGFISVFYIVSVPIAVDTMFVLCIQHVCAFYLCMLFPTE
RNRRKSPQEFYMFQLQIFFFLSDAVKFPVNILFSELLSFAYATSYLFLLGSVIICISFSSAELLMVDVQFDEI
ARFSASNIAQLLHIYYLSWMCQRLLDYSGDLHKVIYSCNWYTISMRSR
>HsOr305
MSFYDNSYYYLNKRLLSIIGQWPYQSRLESNTMLGITLFFTGSLTFLEGWGLVSGMTDLSIIMENASPLLVNS
LIFVKLINYFFNKYKMKELLDHVEETWQMIQVGPRNEILRSYAEQTKVYTIRYTLALYAMWILYSTTPLIVSW
TYKLLPINATYTARFLYRLEHVCDVDKYFNLLMLHGFISVFYIVSVPIAVDTMFVLCIQHVCALFEIIKYDME
HIQGSDFVTLELDIADDIEYHKIIECIKLHEQAFKFSELLSFAYATSYLFLLGNVIICLSFSLAELIMVDVQF

DEIVRISAASIAQLLHIYYLSWMCQRLLDHSGGLHKVIYSCNWYTISMRSRQLLKFMLMRAIKPCQIKAGKIY
VMSMENFSSILQVSVSYFTMLTSLQ
>HsOr306
SVEDCYYKFNRILLKSLGLWPYQDSKWACVQATFMTIMYLSAIFVQLAVFITHECTMDVLIKVFSFVFPTIFM
FYTYYLYYLKSDTIKEIMENMYQDWKFFKNKAELDILNDYANLAKQFTEIFLFCLCVGIITTMMCHIIPITLD
IVSPVNVSRAEDLHVAVEYFVDQSKYCYVLILHLTVFVCLGLTTVISVGITLYGYALHACALFKVANYRMEIT
MDKDILRIPNPQRQHVIRDRLICAVITHRRAFLFCDLLMQNLQRSFLFITIIGVVSLSINLFRFLKAITAFEE
VIEIIIPSILIFFHFVYMFLGNHVGQQITNTNAEIFNIICSLPWYTASLNVQKLLPFLLQKSSKHFCISLGGI
FNASYEGFTMLSKLSISYFTVLYAVQ
>HsOr307
ERYYNIYRIVLTTIGLWPDQNVSRIQTGFFFIVYFSIIFFQLTTFLTTTTLNLMFIIKYLSYFLPFLIYTVNY
NSFYFNVKAIGQALEHIRMVWSTLTDNDEIEILTKYTYIAYIVSLIVYIILSVAVFGFILFECSPVIFDVLAP
MNVSRPRTIEIPFEFFLDVDQYFFIYIAYEILVTVIGLTAMVGTGTLLLAIGTHSCAEFKIASDLIQNVVTDR
TLQIPASKRIYFMHRNICRAVHIHRRSMQFLNDMLNGFNVWYFFLIMIGVTSLSCNLFRLLNAVIQFNNVHEL
FTCIMMFLGHIIYMFMANLCGQAIIDHSTEMLNATYNTLWYAAPLPIQKLLLLLQKSATNQKLMIGSIFVASL
EGFSTLVTSSISYFTVMYTM
>HsOr308
KMHAIEKPYYKINRIVLMSLGLWPYQQSYLVRVQNVLFIVILTSYIIVQLLVLVTTQYDSNLLLRVLSLTFPN
IFVTIKYCLYVVQANSVKQLLDKIHDDWNSLRNKVEIKIIKKYARYGRFFTMITMLLCYLGIIVCAVFQFLPI
ILDIVSPLNESRPRKLVTDTEYFVNQDKYFYALVMHEVVTGSIGAIAMCITVTTLMMYIHHACALFKITSYRI
ENAIEKNMLMIPGPTRDYVFHQRIVHAVIIHQRATEFIRYLTSDIMTSYVVLIIIGVGSLSLNLFQLLQLVTL
TDNISEMFIFAILVIGHLGYMFIVNYGGQKVMEHGADLFKSIYSVPWYAAPLRTQKLLLFIMQKGTRNVSLTC
GGIFITSLECFASMISTAVSYFMVIYST
>HsOr309
MVFAGQRYYDINQIMLTKIGLWPYQKPSILQTVFILSALILFILSQFAVFLTTECDFIDVINIFSYTLPCFVY
LILYNCFYFNINEIKQLLEQIKMDWNIVEYSEMQILEKYAVGSRIISLILVYVLIFSGTILITVETLPIILDI
IAPMNVSRPRKIVVDLEIFLDQQQYFYVYLIYEIVPVLIGFLTVTAVGSLLLAFLTHSCAIYKIASRRLKFPP
IKKHARLHLSHSIRFIKMFFSFFLMASCYQRFTKFLMNSVDKWFFVLMVVGVLSLSCNLLRFLITITMLKELS
DAILCGGLVCCHFGMLFISNFIGQFVMDHSTEIFTAVCNITWYLAPLQIQKLLLFVMQYSLKANRIMLGGIFV
ASLEGFSSLTTTSISYFTVLYS
>HsOr310
ERYYKINKILLSSLGLWPHRKTRCSRVKLILIDAIFVYALFVQLCTFSNANCNINLLLEVLSNVLPTLICIVK
YNAYYANTITIELMMKQIQRDWDTLNDKREIEILNKYARIMYTCTIALILVTCVCVTIYDFVEVLPIIFDWLV
PLNESRPYHLIMLSEYFVDSEKYFPLILLHESIAVLVGCLIILSTGTISLVYMEHLCAILRIVSFRLEHMLEK
SGLQYFNVQTERIICDRIIRAVNLHRRALKFFDLFLSNFASSFCIMTVIGVASISINLFRILVYCNIKNLSEI
LLASTLLLAHFIYMFGANYIGQIIINHSENIFHVTYNNILWYTAPLQSQKMLLFLMYRTTKHIKPIIGKIFVA
SLEGFATLSTTSLSYFTVIYSLR
>HsOr311
QLLKFVTTEYSLDLVLKVLSFTAPCVVFVLKYISFCLGAESVRSLMENVMNDWNXMKTKVELEIMKKQSDLGR
FYTMFFTLAVYSALFIYILVQLVPIFLDVVATRTRKLPIVAEYFADQQEYYLPIIFHISVIAFVGFTIIVSTE
SLFITYIQHAIGLFEIARIEHAFDEILGVSVTRKYCLYCTQIISAVNIHRRAIKFTEFLRSSFAVSYVFLISL
GVISLSLNMMRLFLATDFEELIISTLIVAGHIYYIFXGNYTGQKLIDHSTDVFHRSYKSQWYVAPLHARHTIA
LFMMQRSMKSTVMSLGGIFVPSLEGFATITSMSLSYFTVIRAV
>HsOr312
NRYYKLNRIFLSPVGLWPHGNRKLKPIQAVVSVVILVSSVVTQLLKLFTTEYDFDFVLTDLSYSIPSMVYVVK
YKTFYSQANRLIRMMELIQEDWNSLTSEEEIHIIQKNTNTGRRYIIVFTLIMCTAMASFISYSLLPNILDFVA
PLNESRPRKFPFLAEYFVDQQKYCNTILTHMIVALFIDLFVFVGTETISLVYVYHIFGMFEVASYRMKHVLDE
SIALTSTMEWNICVRTRIIAAVEVHQRAIQFFDYLNVTLSLSNFIILCMGIASITINLFRVSMCMYTAMLTTN
KEDTVLLIIFVSGQFYYMFLGNYIGQKATDHSIYILKEMQKLISDLPWYKTSVQSQKLLLFIMQRNTRSCKVV
LGGIYTLCLEGFATLSSMSMSYFTVLCSIR
>HsOr313
MEFLGYQYYRLIRIFLSWISLWPYQQTLLKQIQAIVCIIVLVSSIVTQLLKLFTMEHDLNLILTDLAFAIPSV
IYLLKYKTFYIKSREIRKLMERVRRDWNTLKDEQELEIIKKCAESGRIYLYTFAGIIYPGTVLFILWTLLPDI
LDAVAPLNETRPRQLPFMAEYFLDQQKYFHPILLHMQLTAVVGIVTVVSTETLFFAYVHHVCGLFDVASYRIE
HALDESVTVLSASSKINATHMKIIGAVDIHQRAIEFFQYLMSTFSSSYFILIIILGVASLSLNLFRLFQIILIP
GQRESSVPYVIFVSGHFYYMFICNYMGQKVMDYSTGVSKKTYSTQWYAAPVQTQKLLLFVMQRSMKSCKLIIG
GIYPASVEDFTTLASMSLSYFTVIYSVQ
>HsOr314

MDFDGGRYYTINRVMLSTVGLWPYQSVWFTRIQRVFCLVCLVTGVTLQLLTFVTFEYNLGLLLDILSYVIPFL
IVVLKYVTYCAKTESMRKLIETIKHDWNTLRNEIEYKIIHKYTHIGAFYAQMFAFTYAFPLLFASIHLIPNLL
DLVAPLNQSRPHQLIILVEYFVDIDEYFSVILLHLTVTIFVVQNTLMSTTSMYVAYIQHACGMLEIASYRIEH
ALVEDEKDNPTSERRCTACARIIGAVDIHRRAIEFFEFMKDTFVIMYFFLLLLGVASLTVNLYRVSKAVITEG
VFERLLSVLNVCVHLFYFFLVNYAGQMILDHCNDFFTRIYNSKWYDMSLHAQKLILFIMQRGSKNCILLVGGL
YIASLEGFAVTMSTSVSYFMVIYSTQ
>HsOr315
KYYNINRILLLCIGLWPYQKSVYNKIHVIYMFILFTEVIIVQLLVFITSSFNIQLFLEVLATLCLCIVFLIKY
NAIYLNINEIKLFLKDIKCDWDMLRDSAEIEIMMKQADTTRWYIKFFALIMYIFLFLFVATNYLPLILDVVIP
LDKPRPRKLLTHLEVFIDQHKYFHFIMLFIIIASFFGITTVLAAETMLMIFIQHACGLFKVTSYRMMRAFDIS
SVPAINYSKKCTICNRLASAMKIHRTAILFSERITLYLANNYFVLILCAVVSLSSNLYRLTQALAVMKIMDMF
IIFVFICGHFCYMFWCNYAGQELMNSSTEVYHQVYNSQWYKSPVHNQKMMILILCRSAKPCTFVVGHLFLTSL
EGFGTLISMSLSYFTVIYSTQ
>HsOr316
TKYYNINRLLLLGIGLWPYQKYTFNKFHIIFVFIILTQALVLQMAAFFTTGFNVPLLLEVLSTLFITIVFIFK
YNTIYFNMDKVKLFFEHIKHTWYTLKDPVEIEIMKKQTNIGRWYTKYFTLTMYIITFLFVMTHYVPLFLDVVL
PLSEPRQRRILVIGEMFIDQHKYFHVILLNIMMSSFLGMTTVIAAETMLMALVQHACGLLKVTSYRITRTFDN
DYRSVLSRGKKCIVCARLANAVKIHRSAIMFSDYIRDCFGIYYLLLVLFGVASLSINLYRLSLVLAVVNNIDT
LIISAFICGHFLYMFWCNYAGQELMNHSAEVYYQTYNAQWYMSAVHDQKMLILILHRSAKSPVIIGDSSVFVG
SLEGFGTLVSASLSYFAVIYS
>HsOr317
YYNINRLLLLSIGLWPYQKSTSNKIFIVFMLIICTQTCALQMASFLTMDFSVHLFLEVLSTIFITIVFLFKYN
TIYLNMHNIKLFLERIEYDWSTLKDSTEIEIMKKQTNIGRLYTKYFTLTMYIITFLFVMTHYVPFMLDIVIPL
AEPRPRKILIAIEMFIDRHKYFHLILLYIILISSFGMTTIIAAETLLMALMQHTCGLLKVSSYRITCAFDNDY
HSVLAHGKNCVVCTRLASAVKIHRSAIMFTDHIKDCFAISYLILISFGVASLSINLYRLSMVVAVVSDIDILI
ISAFICGHFFYMFWCNYAGQELINHSAEVYYQTYNAQWYMSAVHSQKMLILILRRSAKPLSFSVSNIFVGSLE
GFGTLVSASLSYFAVIYSTR
>HsOr318
YYNINRLLLLSIGLWPYQKSTKKKLTLYTACDKYYAILIFCCMASFLTMDFSVHLFLEVLSTIFITIVFLFKY
NTIYLKMDNIKLFLERIEYDWSTLKDSTEIEIMKKQTNIGRLYTKYFTLTMYIITFLFVMTHYVPFMLDIVIP
LAEPRPRKILIAIEMFIDRHKYFHLILLYIILISSFGMTTIIAAETLLMALVQHTCGLLKVSSYRITRAFDNN
YCSVLRHGKKCIVCSRLASAVKIHRSAILFADYIRDSFGISYLILMTFGVASLSINLYRVMFTVVNGIDILII
FAFICGHFLYMFWCNYASQELMNHSVEVYHRAYNTQWYMSAVHTQKMLILILHRSAKASIIGVSNLFVGSLEG
FGTLVSASLSYFAVIYS
>HsOr320
YYNINRLLLLGIGLWPYQKYTFNKFHIIFVFIILTQALVLQMATFFTTGFNIPLLLEVLSTLFITIVFIFKYN
TIYFNMDKVKLFFEHINYIWDTLKDPVEIEIMKRQANIARWYTKYFALAMYIITFLFVMTHYVPLFLDVVLPL
SEPRQRRILVIGEMFIDQHKYFHVILLNIMISSFVGMSTVIAAETMLMALVQHTCGLLKVTSYRITRAFDSDY
CSVLGHGKKCIVCVKLASAVKIHRSAIMFTDRIRDCFAISYLILISFGVASLSINLYRLSMVSSLVNDIDTYI
ISAFICGHFFYMFWCNYAGQELINHSAALYYQAYNVQWYMSAVHNQKMLILILRRSAKPFLFSVSNVFVGSLE
GFGTLVSMSLSYFTLIYSVR
>HsOr321
RYYNLNRIFLLCNGLWPYQTNMSKKIHLVCILFVFMQFLIFQSMIFITVDFSILLLLLEVLSTLFISVIFMYKY
GTVYYNIDKVKLLFERIQYDWDNLKDPVEYEIMKRQANIARWYTKYFALVIYILEFLFIAMHYVPVILDAVVP
LAKPRPRRILVIGEMFIDQHKYFHMVLLNITISSLIGMTTVIAAETMLMVLVQHVCGLLKVTSYRITRAFDND
CFPVLRHGKKCIVCTKLASAVNIHRSAIMFADCIRDCLAISYLILILFGVASLSLNLYRLSLVLVAMNNIDIF
IISVLICGHFIYMFWCNYAGQELINHSAEVYYQAYNAQWYMSAVHSQKMLILILRRSAKPFLFSVSNVFVGSL
EGFGTLVSMSLSYFTVIYS
>HsOr322
YYNLNRIFLLCNGLWPYQTNMSKKIHLVCILFVFMQFLIFQSMIFITVDFSILLLLLEVLSTLFISLVFVYKYG
TIYFNMDKVKLFFERIQYDWDTLKDPVEYEIMKRQANIAQWYTKYFTLIMYVITFLFITMHYVPLLLDAVVPL
AKPRPRRILVIGEMFIDQHKYFHMVLLNITISSLIGMTTVIAAETMLMVLVQHVCGLLKVTSYRITRAFDHDC
SPVLRHGTKCIVCTKLESAVNMHRKCFRGIEHISGTRLYIMKNVFISYLNLRHFHLTLVLVMVNNIDIIIIFS
FICGHFLYMFWCNYSGQELINHSAQVYYQAYNAQWYMSAVHNQKMLILILRGSAKPLLFSISNVFVGSLEGFG
TLVSMSLSYFTVIYSVR
>HsOr323
MDFFDNEYYGLTRHLMTLIGLWPYQTPMCRVVRLCCMTVVLVSSILFQCMVFVTNEYTVPLFIEVFSFNILCI
IYALKYNTVYFKANHVKQLFNQIQHHWNLIENVDELKILQKYASKIRFFTIFSGSIVYPGTLAFFLTMFVPIV

LDIVAPMDEPRPRQFPIQLELFIDQQKHFYLLCIIIMISGLLGMTVLMATENMFMIFMQHACGLFEIVSYRIT
CAFDKYSPKTTPSKNEWRVCVKLLNACNIHQQCLEFVENVQSKFAASYLVLCILGVASLSVNMFRLFLAFAMH
NVSEIITSGLFVYAHFCYVFWMNYFGQDLIDHSECLFQQICNTQWCTAPLYVQKLLFIVLQRSMKTSKVIIGS
LFVASLEGFASLTSMSLSYCMFIYS
>HsOr324
MDVYDDRFYGFTRRLMISIGLWPYQNPRYRKLLRILVFLVHVSGILSQYMSFFTYQYSMKLLIEIISFNMLLY
IYILKYNAVYLNSTGVKLLLEQINCDCNALTDTREIEIIQKYASRGRQYTIYSGLFVYPGTVAFILSLCVPDI
LNFIAPLDEPRQRQLPTPIECFVDQQKYFYMIVLFMVAIALLGMTTFMATETLFMVLIQHACGLFTISSYRLA
HAFDDCKNVSVITAKLIRAIRIHERSLQFVKFINSCFAVSYACLTALGMISLVANMLRLLQAIESNIVNEIFA
SALYVIAHFCYVFWVNYFGQYLSDHSDSIFDNTYNTRWYMAPQHAQTLLFVVLQRSLRTCIFNTFGGLLAASM
EGFASLISLSVSYFTMLYSF
>HsOr325
EMHLFTSRYFNVTRLLMLSVGLWPYQKMAYRRFQMLMICFVLVQGLMVQLANFITTEYSVDLLIKVFSFIFIS
IVYMFKYNTIYFKSEQVRYLFEQVRYDWHTLADAEEIEIIQKYANKGRLYSFLAGLIVYSGTLLFLISLFIPD
FLDIIAPLDEPRRHEIPIMIECFVDQEKYFYIIVLSLAIYAMTGMTVLLAAETLYMIYIQHACGLFKLTSYRI
LNAFDDHSQQMSTSTTKCSICVKLCGAIKIHRRSLEFIDYFSTTFSGSYFILCGFAVASLSINLFRFMHAARN
DALNEAISSGLFVYAHFCHVIWNNYFGQDLTDHSADMFAQIYNAQWYTAPLHVQKMILFILHRSAKNISFNVG
SIFVLSLEGVATLINMSMSYCMLIYSTRT
>HsOr326
SVENRYYKCTRILLKSLGLWPYQDSKWTCVQVIFMTTISLSAIFVQLAVFITHECTMDVLIKVFSFIFPTIFQ
LYIYHLYYFKSDTIREIIENIYYDWKFFKNKAELDIMNDYANIAKQFTKMCLLCLCIGIAAIIICYTMPIILD
IVSPVNVSRAEDLHFAVEYFVDQSKYCYILILHLTVFICLGFITVISIGIMCYSYALHACALFKVTNYRMEST
MDKDVLRIPNPQRQHVIRDRLICAVIIHRRAFLFCDILMQNLQRSLLFLAVIGVVSLSINLFRLLKAITAFEE
IFEIIIPSILVFFHFFYMFLGNHVGQQISNINAEIFNIICSLPWYTASLNVQKLLLFLLQKNSKNFYISLGGI
FDASYEGFTMLSKLSISYFTVLHAVQ
>HsOr327
DRYYKFNRILLKLLGLWPYQDSKWACIQAIFMTIMYFSTLFVQLAVLITHECTMDLLIKVFSFIFVTIFELYI
YHLYHLKSDTSVIKPENTNYICNDITNLTRYRIRGQQGKYVYTCINSTMMSFLSLISFCLGYETTICNIMPII
CDIISPVNVSRMENLYFAVEYFVDQSKYCYIIMLHLTVSICLGLVTGTSTSVTLYGYALHACALFKVANYRME
STMDKDVLRIPNPQRQHVIRDRLICAVIIHRRAFLFCEILLLNLRRSLLFLIVIGVVSLSINLFRVTITAPKE
IIEIMTTSFVIFFHFFYMFLGNLIGQQITNINAEIFNIICNLPWYTASLSVQKLLLFLLQKNSKNFYISLGGI
LDASYESFTMLSKLSVSYFSVLHAI
>HsOr328
MVFAGQRYYDIYRIMLTKIGLWPYQKPSILQTVFFFSAYISFILFQLTVFLTTKCDFGDVINIFSYILPSFAY
FIFYNCFYFNTNEIKQLLEQIKMDWNTVEDSEMRILEKYAVGSRTISLILVSVLTFGGVVLIIVEMLPVILDV
IAPMNVSRPRKITVDFEMFLDQQQYFYVYLIHEIVVVMVGFSTIVATGSLLLAFLTHSCATYKIASILIGNIV
TKQTFEIPTDQKTREMYRRITRAVFMQRKAIQFTKFLINSVDKWFFVLMGTGVLSLSCNLLRVLTVLKEISNA
IISSGVVFGHFAIMFIGNFIGQIVTDHSAGMFTAVCNTTWYLAPLPIQKMLLFVMQYSLKANTVMVGGIFIAS
LEGFSTLVTTSISYFTMLYS
>HsOr329
SQLSCTMHATEKPYYKINRVVLKSLGLWPYQKSCLVRVQNVLFIVILTSFIIVQLLVLITTQYNVNLLLRVLS
FMLPNIFITVKYCLYIIQANSVRHLLDNIHYDWNSLRDKMEINIIKEYAHNGRFFTMIIMLFCYFGIIICGVF
QFLPIILDIVSPLNESRPRKLVTDTEYFVNQDKYFYALVMHEIVTGSIGMFATCSTVATLMMYIHHACALFKI
TSYRIENAMENNVLLIPAPAEEYFLHRRIVHAVVIHQRAAKFVEYLKSDIMAPYTLLIIIGVSSLSLNLFRFL
QLIILMDNINELFIFAVLLMTHFGYMFIPNYGGQEMLEHGTELFRTTYNVPWYAAPLRTQKLLLIIMQKGTRN
VTLTCGGIFIASLECLTSLISTAVSYFTVIYST
>HsOr330
VILRILGLWPYQQSYLVRLQNALFIFTLTTXIYYSLLVLVTTQYNFDFLLRILAYTFPNIFVTMKYCLFVIQA
DSLTKPFIHFVANRNLMKDKLEVDILKKYASTSRLITIVIIFLLFFILLGVIFQMLPLILDVILPLNESRPCQ
HIVITEYFISQDKYFYAMLXHELICCYIEATTLSTTGTTFMLYIYHACALFKIARHRIEYAFENSMLATRSPK
XMSYRRIVHAVVIHRKATKFIENLTSEFVTMFFILLLVGVFTLSLNLFQFCQLITLANNTREMFIFALLIINN
LICMFVANYGSQEITEHGIQLLRITYNVPRTWYAAPLQVQKLILFMMRKETKNVSLTCIGIIDASLEGFASMV
STAVPYFIVIY
>HsOr331
YYTINKAFLKALGIWPVDKSYLNLLQQVLIFILTITYIGMQVAAFITTKIDINLFVKIMSTTLPYVIVEVIYC
SFIFKSETLMQMHRRIQEDWEILHSKAENEIMLKHIQKSLTYDIILFLLSGFIIFIGIFIQFYPIILDVISPL
DKPRIHKLIISAEYFTSENKYFYTKVLHEFVIIVTFVSILLSTGTQLLTFTHHCLGLFKIASYRLHHSIEDSM
LRMSNLEWEHAFCKKITHAVIAHRKAMEFADFMVASFNISYCILTIIAVISLSFNIYQQFIFVNILCISNNIA

DILLSFFISLFHLIYMLMANYLGEMISDYHHEIFTSSYDISWYVIPISSRKLLLFLMQKASKDFHFMIGYIFV
AKIQNFVTLANTALSYVTVMYSV
>HsOr332
LIIEKPHYRINRWILLISGVLPCESSMLATAQKLFFYSILASTFYVQLTVFITHELSAIQFIDFLTFMFPTSV
VMIKYYYFYLNTELVTRLLETVQSDWNSLKDEREREIIEKYAVEVKFLTMTMTVFCCSMVVGYLILSVTPYIF
SVYSSHNNTFASCMYVPFDYCIDREKYFYIILTHFILFLSIGSIGVVAMATLMALFLKHACGLLKIASYRLER
AMNTKDIQQTSSLMKERIMRVRVMQAVNIQQQALKFTSRVMSNFALLYFILIVIGAGSISICMFRIIYTLQTT
CDWRELFVIIAMLMAHFIYMFFANYMAQEVTDHCNNLFLAAYSSMWYVAPLRVQRLILFLLQYGTKNYTIVIG
GFYVSSLEGFTTLLSTSLSYFMVIYSTQINDEKI
>HsOr333
IKHIIKCIRDEWTISKTPDEEVDILRKYANISRQFMLVIMVILNFFLAVYILFLFLSNILDIMVLFNESHIFI
TEYFIDQNRYFQAILIHGIITTCVGSVATIIAGGTLVGYVLHIRAMLKIAGHRFKYINDKIPALSISEKDRII
RERIINAIDIHRRALEFAEFIQSIFTIFYFFMIVIGISSLSLNMFQFFQYSISVNDMSDANGMFLCSVMLITH
LIYLFICNLLGQIVTDHNTDIFYKAYGVQWYIISIAQKLLLVILRKNTKNYYFVIGGIFIGSLEGFTIITYTL
LSYFMAIYS
>HsOr334
EFTGYHYYKINRIFLSMFKLWPYHRSRFAIINHQLDTMRYFLHFHRFQLXSFLTYRCTMDFLIVVLSYAVPSL
INIVLCCEFYMNPNSKLIVKQIKNIRDTWSILKNGAEREIMRKYSYTGKLCTTLLLCLLFSFIYRKIKFGKLF
IMIIXPYASLYCNICNYSIHTGYGYRILCHLIGIFVNYFVTIYIITLNLAYIEHIYLAMKLTEYIYSYRIAHS
INQYISHNLRKKEDRMIAQNIGAPVNVHRKALKCNVIISTTFVPYLFLAVIIAVLSLSLNFFRLLNSITGEKD
MNEMFSSAILIFMHIIVIFLDNYYGQKITEYNDEIFYNNSNVFHMNWVKVLLQILFLCIINNTTKPYIMDIDN
VIVGSIEGFAKI
>HsOr335
KKGNTLPNNHYYIINQILLSAFHLWPYQCSRFIIINHIINLLLSLSFCFFQLTSFLTYQISMDLLIMVLSYAV
PSFICTIQYCSFCIKSNAVKQILKDIHNSWSVLKNEMERDIMRKYSFSGQLCTILLFLCLVINLILYAFLQLL
PDILNIINPLNETRPHQTYAVTEYFVDPKQYYYPILCHWLFSIFFNSSIMIATTTLHIVYVEHICGMLSVASY
RIEHSIEKHASSNSTDKEASVIFQNISTAVELHRKAVQCSTFLSTTFALDFFLMVVASVLSLSLNLMRLFKAI
SLLKDVNELISSVLLVGAQLLFMYLDNYYGQKLTDHNNKIFYNAYNTPWYTVSTKIQKLFLFIMKYTTKTYIL
NIGNLIMASIEGFAKLISLSISYFTVIYSL
>HsOr336
HYYRINQILLSTLQLWPYQRSKYAVINHFISLIIMMSFVFFQLTSFFTYQLTVDFLIMLLSYVLPSTACIIQY
YSFYRNPDAVRQILKNIHDSWSALKNEMERDIMQKYSFSGQRCTIISLLLVIGSFIVYGFFQLLPSILDIIFP
LNETRPFQPYALTEYFIDERTYYNLLLCHWLFGVFFNSFILVATMTLHLVYIEHICGICSVANYRIEHSTDHY
ASHNLLKKKDHMTVQNISAAVNLHQKALMYGTFLSTTFALDFFLMVVVGVLSLSLNLFRLLNAISMQRNMNEL
FTSIILVSIHFLIMYLNNYYGQKLTDHNNKMFYNTYKIRWYTVPVRIQKLFLFIMTRTTKTYVLNIGNLIMAS
IEGFSKLASLSISYFAAIYSL
>HsOr337
MLLNREETDSTSENHYYRINQILLSTLHMWPCQRSTYTMINNFIILTILMSFVFFQLTSFLTYQWTMDFFIML
LSYVLPSIICIVQYRSFYAKLDIVKQILKDIRNSWSVLENETERCIMQKYSFSGQRCTILLILSGFICLIVYG
FFQFLPSILDMIFPLNETRPRQPYAITEYFVDKRRYHNLLLCHWLVGVYLSFFVLITTITLHIVYIEHICGML
SVASYRIQHSIDHYVSHNPPKKEDVIFQNINVAVDLHRKALMCSLFLSTAFASDFFFTIVLSVLSFSLNLLRL
IKVILLLKNISELLMSIILVSAHFVIIFLEHYYGQKITNHNDKIFYNAYNTEWYTVPVRIQKLFLFIMKNTTK
AYVLNIGNFIMASLEGFTKLMSLSISYFTVMYSL
>HsOr338
MYVFDDRNHKFNQTLLKLLGLWPYGRTKFQRLRAICFFVLLLSYVVVQLAQFAVADIDSDAVIRILSDALPST
GYFIKFTLFFYNDETQITNNWRMLTDMREIKIAEYYARRGRHVTIFLTFCYNTCCIFYNLASVMLGVFGFIVM
EMMPDILDFALPLNESRVRYLPPMNEYRGYQGAYFYPIFLYVNISIIIGGLTVASVGSMLIVIVLHCCAILKI
SSYRMEHSVSEEIIARPDREHLIIERIIKSVKLHQNAQNLIEMLMSRLSVFFLTLLTLGIISLSINLFRLLNA
IMHMDNSLEFVAALGLVSGLQIYLFAANYSGQLIVDHGNELFNAIYKSLWYVAPVTMQRLLLFMMRCNIKGCK
LCANNLFIASLEGFTTISSMSMSYFTVIYSTQ
>HsOr339
MIVVESWYYNVNRILLLICGLWPYENTKFRYVRAVFIFGILVSFVVDKLVTLFIQEYSFRLILKIFSDILPIG
ICILKFIAFLLNKKQVKHLLDRICEEWSALKDSEEVKIAKKYGKLTRQMTTILLLYGISGWLLLVMIRFIPMI
IGNFATANKSLARQDSVSLGRFDNCIYVFVVKFIGVIVITATITMILAYLRHICMMLKITCYRIKHSMDEYVS
HMTIHQQNRLICQKLINAVKIQRRAIQYTEHVLSSVKYSISPLIGMGVTSLTINLFHTLQAILGLTDMNDLIL
PVFYVIMHFIYIFVMNYGGQLITDHSADILKTLYEVQWYVAPIHVQKLILFLMLKNTKSYGLIIGGIYVASLE
GFVTV
>HsOr340

MFPIVSKYYDLNRMLLLASGLWPYRKSKSKFRHVHAASVLVVILSFIVFQLTVLINTDCTINLVTKVLSIVLL
MFVCVLKYNIFFFNADKMKLLMDLMWYHWTIIQDKREIAILEKHSRFTRRFTMVMTQIFSLGICVVAAGHCLP
AILDIIVPLNSSRPRHFYILMEYFVDQERYFLPILLHVIVSLSVGSIIILSVGTMIMAYMQHACAMLKIASFR
IGNAVPDSAQTSFSRNDVICERIISGVQIHRKAIEFIDLILSNFKVPFFFVMSAGVASIAFSILQVFVALLEK
DMNEIIITIIFVIVQFFYMYIGNYAGQLVADHYIEVFDVAYCSYWYVAPLRAQKFLLFIMQRTTKNFYFVFGG
IFMVSLKGFSTLASMSISYFTVIYS
>HsOr341
MDFAGKQYYKLNRLLLLSLGLWPYQNSILKKIQIVFFQTLFLSFFLPQLNTFFVRQYNIDLFIKVMVFMVVNS
IYIIKYNAFLLFTDNIRYIFERIQRDWNMLKSQAELKIIKKYAKNARLYTIQFFTLVIFFMLSYIVLNCIPII
LNVIVPKNESRSRPILITMEFFVDQDTYFYAILTYSFLINYAGCVTVVAVATILIAYALHTCALFKITSYRIE
QIFDKSVLQEPENIKQHIFYEKLIYAVYIHRRAMDLANILTNSFTIFYFVLLGFGVALMSLSLYHLYNALILM
VDPFELLMSSGIITLELYYIFLGNYVGQDVIDTSTEICEITYSIPWHIAPLWMQKSLLFVMQRSGRRSALTAG
GLFQASLEGFAMLMSASISYVMLLLSVRTQQK
>HsOr342
MNFIYQHYYTANRILLEIMGLWPYNNSVFVHIYRVILFTIFASSSVTQFAKLHSTIHDFDRFLYNLTYAVPSL
IYILKFLTYIVYSNQVKQLIERIQHDWDIFKDEKEREIIRCNATSAMRYTIALYVMSFFALFGYVVMLYLPTF
LDLVYPLNESRPKKLPILSEYFFLDEQDYYYPLLVHQSMVIALGITIVIVTETMNMVCIQHGCGLFEITSYRL
KHVFDNEIFPSDLSDYEKYSIIHSKIIKAVMIHKRTIEFLDYFKSTFNPSYMILLILGVASITINLFRLLQAV
TVMNDWEELMASGSFVLGQYGYMFTINYFGQKLNDRSFYVFKMTYEASWYTAPVQAQKFLILMQQKMIKGYIL
NINSLITASLETFASLTSMTLSYLTVIYSVR
>HsOr343
MDFLGNKYYKLNRRLLLLVGLWPYDHCLFKYCQMMLCNVIVILTMASQVAKLITLRQNIGFMLQIVSPIVLGI
VFIIKYQTFCLIPGKLRRLMDHIERDWNMLKDKRELDIIGRYTYIGSMCTLSFTIFGAVATIVCILLPFIPII
HDILTPLNISRPRQLLFPGEYYIDQRKYFYVISLYLDLALILIIITLLGTETLYVTHVQHACGLFQIASYRMN
QAFDESLSQVYTPEKRTIFVYQRIIEAVHIHKRALEFSEFLWSSLAVSYSILLTIGIMSLIINLFCLFQAVLN
VKEMNEVVRLSIFIVGHVFYIFLGNYVGQILIDHSASIFEKTYLTRWYSAPLRVQKLLPIIMQRSTRSCKMTI
GGMFVSSLEGFAALMSTTMSYFTVLWSVNK
>HsOr344
MNFLEDRYYKLSRRFLLIVGLWPYEQSIFKYCRMILYNVIVSLLVVSQIAYLITLRHNIDLVLNILAPTVPCL
IYAVKYQTFSTIPEKLKHLMEHVKKDWNMLKDKRELEIIEKYIYIGNMCCMGCAILGVMGIIIFLLTPFVPNI
LDILAPINVSRTRQLPIPGQYFVDQQKYFYAIVLHLDINVIIIVTTLLGTESLYIMHVQHACGLFRIASYRVS
RAFDDNLLQAYAPRKRMIIVYQRIIEAVHIHKRALEFSESLWSSLSVSYSILLTIGIISLIINLFCLLQAVLL
VKEINEIIRLAIITFSHILYIFLGNYIGQMLIDHSADVSEKTYLTQWHCAPLQAQRLLPFMMQRSMKSCKMVI
GGIFTSSLEGFATVMSTTMSYFMVLWSARK
>HsOr345
MDFNSEHYYKINRIILSAIGLWPHRHITLRQIQAALLSFLLISVTFPQLTKLIVEEYNVDVVVRVLSSTLPFL
LFIVKYIAFYFVTEDIKGLMQYIQNDWNALKDNNEHEIIHQYAKASVLITKSLAILIYVCIAIVICIQYAPSF
LNVVMPLNKSRRAELIFWVEYFVDQDKYYHMIQFHLDVGLIVAATTILATESFCLMVAIHAYGMFKIASYRME
HIIDAPNIFVGKRYVSYDSVVAAVNSHRRAIEFSEIIRSTFAIPYLALILLGVTSSSINLFLLFQVIMSAGAM
DDIIKSIVYVCCHFLYMFLTNYAGQKFIDHDIEIYKKICSLRWYKAPLRIQRQILFIMQKTAKSYHMNVGGLY
NPSLEGFTTLASATLSYFTVLCSIR
>HsOr346
MEFPENQYYRVNRILLSVVGLWPYDNFKVRRLRFTLTLLILITFSSTQLLTLFNSEYDKIHLLKILSFDLICI
ACIMKYISFYAVMENVKLLRQYVFDNWRILADEREIEIILRHANIGKYFTLIILTIGYITAFFCILIQYIPII
LDIMKPLNVSRPHVFFIEAEYFIDEQKYFHIIAIHINIAICVGSTSVISAETFVLANAFHAFGLFKVTSYRMQ
QISSGYDSQICTTEKYVIFRNRITAATNMHKRAIKFSELLKESFGPMYLILVSGLIISISINLFYVSNILSTE
GNILEIVKCIIFLFLHVIALFAGNYAGQEFINCDTNIYRMICNTQWYNAPLRTQKLILFLIQKTTKCYKVDTV
GLFYPSLEGLASGLSMSISYFMVFCS
>HsOr348
MVFPEERYYRLNHILLSVTGLWPYDDNKVRQVRFILLLLIIMLIISPQLLKLFISEYKLDTFLKVLSLDIVMI
IVTIKYITFYAIVDNIKIFRQYISESWSNLIDEREIQIIYKQGKAGKISTIVLATLVYFMLLFCIFTQYVPNL
FDIIKPLNVSRPCRYFILAEYFIDQQKYFHIIAMNINIGLFTLGTILLATETFALANALYAFGLFKIASYRMK
YILNGNHLQISATKKYVASRDKIIAAVDMHRRAIQYSELLKNSFGPTYLILFMGVIFSTSINMFYVSSMLLIV
TENIYLIIFTKYSNQCNTQWYNAPLKTQKLILFLIQKTTKCYKVDAGGMFSPSLEGLATGLSMSASYFMV
>HsOr349
MGFPGERYYRFNHILLSLIGLWPYSSKKIRWISFILTTLVFISYLLIQLLKLVFWEYNLELRLRMLSFDLLIS
QWALKFITIYAAIKNVKKIHEYIQSNWSILRDNTEIEIIRKEANNGRRYTIAIAVTVCSLASIYMLIHCIPNS
FLIIMPLNESHRCLFLNTTKCFIGQQQYVYISILQMTFGIIVATITGIATETFSLSNALHAFALFKLASYRME

RILIGNDLQMSATKKYVLYRDKIAAAVDIHKRATEFSNLLVATFGTPYLFLFILSECSASICLFLILTMRLRP
LEIIMSVLCIFCNFLFLIIGNFVGQIFINYDTYIYERLCNIRWYNIPLKAQKLILFMMQKATRSYKVDAGGMF
NPSLKGLATTMSLMYSFVMMLTSMQ
>HsOr350
DTCTEMDDTMSNYYTINRILLLIIGLWPYQRCYLRYIMITFVTTILLSGIIVQLMSLITMEYSMDFLLKILAY
NIPWMGFTIKYNILCFNIKDMRRLLELAQNDWKQMSDAQDIEIIKKYWAIGRLITSVSAIFIYVSTVCFVLVQ
LSLNFVGTTTIINGTRLRCLPAVAEYFVDQQKYFYPILLHMVLVVLFGVTTMVATETLYMSYTQHACGMFHIV
STRIEQVLRGGATRDAVSVAERNLMMCHGIIDAVNIHKKAIEFIEMAKTNFAFAYMMALPLGVLSLSINLYRL
SRLIMTDNYHETIISSVFISGHFWYIFFCNYIGQEVIDHSGDIFYKIYNARWYASSLKAQKLLLLVMQRSMRH
CTIIMGGLFVPSLEGFATLTNMSVSYFMVIHSV
>HsOr351
FQMSPLVVNEFSVDILLNVFSVCVPTIFHICKYYNMILNGYKVKILIKRIKDDWKSLKDEEEFRIIQERAKVG
KTITIIAIMFIYVCLFIFLLAYLSPTILDAIMPLNGSRKPIFNIPFAMEKYIHWEKFVYVLQLYTSLMVFFTI
SSILATEVLALACMQHTCAMFMITSYRIKRAVNKNQTKLSLSLAKNPNDYKSITEAIDCHQRAIEFNNFFRSI
SGIIYLILIPLIIISLSINTYYFCRYVLSSNISDGIFCFFILLAYVLYMFFNNFIGQQIIDYSQFVFETICNT
RWYATSIYTQKCLLLMMERSMKGSNLIVGGLFVPSFEGFATLISTSLSYFMVIYSTQQL
>HsOr352
YYKLCRKLLLLTGLWPYETLRRSLLHLGFIILYDISFFIVQTAHYFICETIQCIFKTLPMNMMAVILLVKLFT
YYFNRQKIKGLLEHLHSDWNTLCYEEEFIIMKKYAETGRFYALYYAISIYLATVLFAFSSITPRIMDVVSPLN
ESRLIIMPCPAYYFVDETEYYYYIFVHMIIGAFVCITGIIAHDCNFFAFVEHVCGLFAIVGFRLEHLLYRQNI
TEKSLIDSSDDVYVRNIKFSIQAHRNALHFVDLLENTFYISFAAQMLIVTIGMSISLIQVKLQGDIIELIRYF
FFIVAQLGHLFIYSYEGQKLINHSLETSEKLYNGLWYNIPVKSQRLLLFAMRKNMEPSIVTAGKIYIFCLESF
TSVLQCSLSYFTV
>HsOr353
SMEINWNYYYGIVEKLSCYTGLWPFLKPRRRLICLSLMIVIFITLFIPEFAFLNICEEGIQCNFLSVTAYMLT
IMSIVKMATFQLNIQKFRELTKRLFIDRKSLETPEECEIAKSHAQACRQFSILYPVYVLGTVYIFMSVSIVPP
ILDVISPLNESRPILLPYPGYYFVDSKKYFYYIYGHSIVSWQMVMTVIVTHDCLYVGYVEHVCSMFAVIGYRF
EYVFCNRNEALKDSKDDLDDVYCKKIALFVHNHQKALEFAQLLENAFTISFAIQLSITVITMSCTLLQITRES
GFLDLVRYVMYTGGQLFHLFCLSFEGQKLIDHSLQMRDKIYSSSWYEAPLKSRKLLIITMIQSIRPISLTAGK
IYVFSLESFTTILQTSMSYFTVLASFQ
>HsOr354
IKGLLEHLHSDWNVLHCEEEFLIMKKYAETGRSYSFYYATSIYSITFFIFGCTVLVSRFMDIVSPLNESRPII
LPCPANYFVDEEEYFYYIFVHLIIGAFVCITGLLAHDCNFFTFTEHVCGLFEIVGFRFEHLLYERNIKEKSLI
EFSDNIYVKNIEFSIEAHRRALHFVALLENTFCLSFAVQLLIVTTGMSITLLYVKLQGDMMEVVRFVPFVIGQ
LGHLLIYSYEGQKIINHSLELCQKIYNGLWYTIPVNSQRLLLFALRKTIEPSFVSAGKIYIFCLESFTSVLQC
SLSYFTVLSTFN
>HsOr355
METVKITWSHYYGTLQKISLLAGLWPFLQLRTRLFRIGLQIVNMITISVPQMAFQFRCRDIHCFLLSLTAILL
SIVAVVKVYTFQLNILKFKELTEHLYVDWKNLETPEEYEIMKSYAENSRRFSIIYPVYCIVGTTVFMSVSIVP
HIFDVVLPLNESRPLLLPYPGYYFVDSREYFTYIFWHSFLSWQIVIIGIFAHDCMFVCYVEHVSSMFAVVGYR
FESLIYDRKDALSKNENADLDEKLRRRVAYLVHTHREALELAQLLESIFNIPFAVQMGISTVGMSITLLQLTQ
ESNILKIIKFVFYVFGQLFHLFFLNFEGQKLLDRSIETRDKIYNSSWYKTPRNLRKLLILIMMKSLQPSLLTA
GKIYIFSLENFATVLQTSMSYFTVLASF
>HsOr356
TMDNIWNHYYGVAYKLSLVCGMWPHLERRTRIFRISVLTAIMVTIVIPQLAFQYRCKGDIQCTLTSTLGYLLS
LVILVKVYTFQLNIHELKILTEHLYVDWKMVENSEEQEIMKSYANHTRLYSLIYSVYCVIGLYILMCVCLVPF
ILDVILPLNESRPMLSPHPAYYFVDEKKYFIYIFIHALLGWKIVLVGVISHDCMFVSYVQHICSMFAVTGYRF
KYLFYECNEMIKDTSDDFNEKYHARFTYLVQTHRETLRLAQLLENTFTKPFGLQIVIATIGLSITLQQITVES
NLLTVFRYILYVIGQLIHLFCLNFQGQKLLDNSIQICDKIYNSPWYKLPIKMRKLLMLVMMKTYRPTFFSAAK
LYVFSLENFAMVLQTSMSYFTVLASF
>HsOr357
TMEVTWNHYYGTVRKLSSFTGIWPFMSMRAKIFRLCFLTVTMLSLFVPQIAYQFTCKQNLQCIFESMTSYLLT
SIAALKVYTFVVNIRKIREMTKHLYADWKLLESVQEYEIMKSYAENCRQFSLIYPIYCFVGLCIFFSMSFIPQ
TLDIVLPLNECRPMLLQYPGYYFVDYKEYFIYIFCHSFVAFPIVQVGLFAHNCMFLSYVEHVCSIFAVLGYRF
EYLFCNQDKIIENTKADLDSIYRKKIASFVRMQQEALKFAQVLESXFTIPFAIQLLVGTIGMSITLLQITQSS
NFLKILRYALYVGGQLFHLFVLSFEGQKLMDHSYQTRDKIYSSTWYEAPMKSQKLLIMVMIRSLQPTCLSAGK
VYIFSLESFSMVLQTSMSYFTVLASF
>HsOr358

TMEVTWNHYYGFMKKLSSFSGIWPFLNVRAKNLRLCSLTLIMLSVFVPQIAYQFTCKDLQCTCESMTSYLFTF
VAVLKMYTFVLNIRKVREMTEHLFADWKLLESVEEYEIMKSYAENIRQFSLVYTIYCLVCAGIFMSMSLIPQI
LDIAFPLNESRPVLLPYPGYYFVDYKQYFLYIFCHSFVAIEIILAGIVAHDCMFISYVEHVCNIFAVLGYRFE
YLFCNQDKTIEDTKTDLDIIYRKKIASFMRMHQEALKFAQILESVFTIPFTVQLFINTIGISANLLQVAQSSD
FFKILRYMLYVFSQLFHLFIFSFEGQRLMDHSLQTHDKIYNSPWYEAPMKSQKLIVMVMLRSLQPTCLSAGKI
YIFSLQSFSAVLQTSMSYFTVLASF
>HsOr359
TMEVTWNHYYGIVKKLSSLTGIWPFLSVRAKVLRLCFLTVIILSVFVPQIAYQSTCKHDLQCIFESMTSYLLT
SIAVLKVYTFVVNIRKVREMTEHLFADWKLLESVEEYEIMKSYAEYCRQFSLIYPIYCLACVCVFMSVSLIPQ
ILDIIFPLNGSRPMLLPYPGYYFVDYKEYFVYIFWHSLVAFEVMLAGLVAHDCMFMSYVEHVCNIFALIGYRF
EYLFCNRDKTMEDTKSDLDSIYRKKIASFVCMHREALKFAQVLESILPYLSQFQLLLSTIGISVTLLQITQSS
DFLKILRYTLYVGGQLFHLFILSFEGQKLMDHSLETRDKIYSSSWYEAPKKSQKLLIMVMIRSLQPTCLSAGK
VYIFSLESFSAVLQTSMLYFTVLASF
>HsOr360
TMEATWNHYYGIVKKLSSFTGMWPFLSMRAKILRLCFLTVTMLSLFVPQIAYAFTCKQDLQCTFESMTSFLIT
ISASMKVYTFVVNIRKVREMTEHFFADWKLLESAEEYEIMKSYAXNCRQFSLIYPIYYLVSACIFLSVSLIPQ
VLDIVLPLNESRPIVLPYPGYYFVDYKEYFIYILCHSFVAFAIIIVGITAHDCMFMSYVEHICNIFAVLGYRF
EYLFCKQNKTMEDTKIDLDSIYRRKIDSSVRMHQEAVKFAQVLESILPYLSQFQLLLSTIGISVTLLQITQSS
DFLKILRYTLYVSAQLFHLFIFSFGGQRLINHSLQTHNKIYNSSWYEAPMKSQKLIIIGMIRSLQPTCLSAGK
IYIFSLQSFSAVLQTSMSYFTLLASMQ
>HsOr361
TMEVTWNHYYGIVKKFSSYSGIWPFLSVKAKIFRLCFLTIIMLSLFVPQIAYQFTCKQDLQCKFESMTSYLFT
IIVSVKVYTFVVNIRKVREMTEHLFADWKLLKGVQEYEIMKSYAENFRQFSLIYPIYCFVGLCIFFSMSFIPQ
TLDIVLPLNECRPMLLQYPGYYFVDYKEYFIYIFCHSFVAFPIVQVGLFAHNCMFLSYVEHVCSIFAVLGYRL
ECLFCNQDKTMEDTKINVDSIYRKEVASFVRMHQEALEFAQILESIFFIPLAVQLCIITINISITLSSTASIS
DFVKILKYTLYASGQLFHLFIFSFEGQRLINHSLQTRNKIYSNSWYEASIKSQKLIIMVIIRSLQPTCLSAGK
VYIFSLESFSAVLQTSMSYFTLLASLQ
>HsOr362
MDRRWNRYYGVVEKVSSLAGQWPYQRRRMRIFSLSIVTLSIFSVIVSQIAKMFECDGDRQCLFPTVAAYMLSS
IALVKMYTCHLSSRKIRNLTDHLLVDWDLLKSPEEYEIMKSYAENGRWYSLGYTLYCLISLCLFLSMSLIPHF
LDVVLPLNESRPLVMPHPAYYFVDDREYFYYIFWYSTLTWEITVLGVIAHDCLLVTYIEHVCGIFAIVGFRFE
RLSCERSEPATNVRARPEVSYHKRIMFSVQAHRRAICFAQLLEDTFSLTLALQIAIITVLMSITLLQITLQVN
DVIELIRYILYVVAQLIHLFCLSFEGQKLMDHSLQTREKIYNSPWYEVPVKTQKMLLFMMRRSIQPSFMSAGI
YIFSMENFSKILQTSMSYFTVLASME
>HsOr363
MDCRWNRYYGVVKKVSSLAGQWPYQRSRMRTFSFSMVILSIFSVIVPQIAKMFECDGDRQCLFPTVASFMLTS
IALVKIYTCHFSRNKIRDLIDHLLVDWNLLKSPEEYEIMKSYTKNGRRYSLTYTLYCFITMYLFLSMSLIPHF
LDVVLPLNESRPLVMPHPAYYFVDDREYFYYIYWYSILSWEIVLFGVIAHDCLLVTYVEHVCGVFAIVGFQFD
RLLYEHMNPATIVRARPEVSYHKRIAFSVEVHRRGIRFAQLLEDTFSLTLALQVVIITVLISITLMQITLQVN
DMIGLIRYILYVVAQLIHLFCLSFEGQKLMDHSLQTREKIYNSRWYEVPIRTQKMLLFVMRRSIQPSCISAGI
YVFSMENFSMILQTSMSYFTVLASME
>HsOr364
MEFTWDRYYSVMRRLSSSIGQWPYQNKREKLFKMSMMTTALLVMNIPQTCKFVYCNKDLECILGTTPSYLLVL
LIMMNVYTSSSSKIKTLTDRVFVDWGRLQNPEEYEIMKTYVANARWIALIYFAFCLAGCTLFVLVALIPYVLD
IMSPLNKSRSIVLPYEAYYFVDERKYFFYIFLHGLVAADIVIMGLIVHDTMFIFFVEHACGIFAVAGFRFERL
VHEDTCLMKTVDNELDNKYSKKIAYSVHAHRAALEFAEYLENIFSLSFGIELALMTIGMSITLFQITLQSDDI
WEVTRYVIYASAQLVHLFVFCWEGQRLIDHSLQIRDKMYDCTWYKIPAESQRLILHVQKRSLRPKFLSAGKIF
TFSLQGFTTVVQTSMSYFTVLSSVQ
>HsOr365
DHYYSFMKKLSSFGGQWPYQNKREKLFKISMITTALLIINIPQTCKFVYCNKDLGCILGIMPSYMLIILIMMN
MCTSSSSKIKALTDRVFIDWGKLQNPEEYEIMKSYIANARWSALIYFAVCFGGCTIFVLMALIPYILDIVLPL
NESRPIVLPYEAYYFVDERKYFLYIFLQGLIALHVVIMGLIAHDTMFIFFVEHACGIFAVAGFRFEHLVHKDT
GVMKTVDNELDNMYNKRIACSVHAHRTALEFAEYLENMFSLIFGIEILMVTVGMSITLFQITLQSDDIWEVIR
YVIYVGAQLVHLFVFCWEGQRLIDHSLQIRDKMYGCTWYKIPAESQRLILHVLKRSLQPKFLSAGKIFIFSLQ
GFTTVVQTSMSYFTVLSSVQ
>HsOr366
MEFTWDRYYSFMKKLLSFVGQWPYQNKREKLFKMSMMTTALLVMNIPQTCKFVYCHKDLECVLGTTPSYLLVL
FITMNVYTSSSSEIKTLTDRVFVDWGRLQSPEEYEIMKTYVANAKWIALIYFAFCLAGTTLFVLVAFIPYMLN

VVLPLNESRPIVLPYEAYYFVDERKYFLYIFLQGLIALHVVIMGLIAHDTMFMFFVEHACGTFAVAGFRFERL
VHEDTDLMKTVNNDLDMYNKKIACSVHAHRAALEFAEHLENIFSLTFGIEILMMTGGMSITLFKITLQSDDIW
EVIRYVIYVGAQLVHLFVFCWEGQRLIDHSLQIRDKMYDCTWYKIPAESQRLILHVLKRSLQPKFLSAGKIFI
FSLQGFTTVVQTSMSYFTVLSSVQ
>HsOr367
MESTWDSYYGVTKKFLLLTGQWPHQDRNDKVLRLSVITMAILVMLVPQICMFARCDRDFDCIFRIMPAYILII
VVFLKLYVGQMKSSGIKALTDRVFIDWEGLRKNPEEYKIMKTYVANARWIVFMYSTVCLVSVTVFVMVSLVPY
ILDRVLPLNESRPIVLPYEAYYFVDERKYFFYIFSQGFVAAEIVVIGLVAYDTMFMTFVEHLCGIFSVTGFRF
ERLVREDTDVMEIVRNDLGVVYNKRIAYCVEMHCAAIEFAGHLEETFSLNFGIELLLVTIVMSITLFQVSVTS
QSNIVEAMRYLIYVIAQLVHLFVFCWEGQRLIDHSLQIRDKIYSCNWYKIPAKSQRLILHVMKRSLRPKFLTA
SSVFIFSLEGFTAVVQTSVSYFTVLSS
>HsOr368
MKSTWNRYYDVTKRLSLLTGQWPYQNKRNRMLRTGVIMSNVLVINVPQMCKFIQCDGDVECILRVMPSYMLMI
VIMLKTCLCQYKSSKMRILTKRVIDDWENLQTPEEYEIMKMYATGAKWLVLAYSAVCLIGVAIFMAMALISRV
LDIVLPLNESRPVTLPFEAYYFVDERKYFFYIFSQGFLAGVVSMMGLVAYDTMFMTFVEHVCGTFAVAGFRFE
HLMCESIDIIRVVNNTLDKVYNQRMAHCVKIHWAALEFAELLESIFTISFGIELLILTVGLSMTLFQITLHSG
NNIEVIRYALYVIAQLVHLFVFCWEGQRLIDHSLQIRDKIYNCSWYEIPVKSRKLMLHVITKSLQSNTLSASR
IFIFSLQGFTTVLQTSMSYFTVLSSVQ
>HsOr369
MEHFEKRYCKLYQFSLTVSGLCPYQSIRKARMIRAFITILILSSVFVQLMSIFTSKFTMDFFIIIIPAFIPTL
GTLIHLYMRAMRMDELKGMFDRIRDDWALQKTDDEVKIMYNSAATSRLFTCFFMLQIYGSVLVYSIYLYMPDI
LDIVSPMNESRSRKQYFKLEYLVPEEQHVFFYRMHIIFVMVFIATIFFANIVQFVTLAQHVCGMCELLGYRAE
RLFCVVESTTGRDLGRSKKLIYRNTAIFIQLHHNTIQFVGTIERFYTIPFLLDFLGVVITLSLTLSQVQIFQV
VSNDVEQLVRSMCLTNMQISYITLSNYMGQQIIDKSSNIYDKVYNSEWYNAIVSQQKPLLLIMRQRFLPLVIT
SCKFYVMSLANLGKIFQMTASYCMFIRQV
>HsOr370
MQSAVKQYYNINKIFMSRIGGWPTQSIFMKILLPTVILSFVFSIGFLEFVRLLETWHTIMQDCECLIIVLIAI
GGHVKLFGIVLNNKNIERLLSLIDYHWQIFTHSLEKQIMHDYAIVGKKMTISYAVSLYTLMSLYMLIPIIPKL
LDLVKPLNKSRPNEYLFDVDYSFDRDEYYYVSLLHSYLTTVMTISIMVIIDTIYIVFAQHVCSLFAAIGYKRR
LENLGINSKESDWLIENIEMTRERRGSMDCILLNKDDEVYRELIVILQKHQLSIEYVSILDSTFALYSLILLA
LNIIVTSLLGLQILSLMDHKKQMIRFASMCIGAFMHLFVLSYPGQKIIDHSTDVFHKAYNMQWYKMSRRSIQL
LSILLYRCYVPCTLTAGKVYVLSMANYASMVQTSISYFTAFLSL
>HsOr371
AGNFDYAVAINRWSMRLLGMWPLDTSRLSHLRCGLSFGTMLVINCPAIVYLFMITNWVAIMNQANLTFPLFFA
YVKFVLMKTKAKNLRLILRSMSRDWANYRYLSARDRRAMFHYAKKGRQINVFCFVWMMLAVGGERNHETLYII
TPLVESAWLSGQLANATSSRLPTDVFFSLEQPYAYEMMYVGQSIIGLISGTVIFSVDCFICIVVFHACGQLDV
LAATLERYDGTVEHDGTKDACFRSCACLPYMNVVEKSFNSFILFQLLLTTVILALQGFQIVLFVRDNNVTGFL
SYIIYGVLFTYYMFIYCYVGECIIEKSENIGRTAYNLKWINFPPNYARPIILLITRSRRPCKITAGKFAIMSL
SYYKTVSFLSTTKINL
>HsOr372
IIYTLPLSFALLMYIGYRLVGCSLTSIKYRPYNFYSAIMIFLLHSFAFCSLVDCILIEDLDIFIKRFSLFLSV
LGVCCKVMNLXIRRDRIVDLTEILLNEICVPRNNYEANIQQRFDRSAQSAYRRSLCEILNELAVFLATVSQFR
YFANIHILLLSDVSYNITPMNNFWATMLHTIGLMVAXNASVAHETLIFGFMLQTCSQLDIHVLCHRARMLPNT
LRRDCASKRNMKTHERWIIRKFVRHHRYIYGLAERINSVFAVMIFVQFAVNSTVLCLSIYKMSTKSLLSFEFX
SLSYLGCVLMQIYLYRWFDNEVMLKAGHFKISNSNAKIDTAVYEIDWEIFPVGLMKSLLLISKHSKKXSFRIM
SHIIVLSNDSFMKIIKISYSTYNVLKDS
>HsOr373
MRILHLNFLFYTVSGVWRPIEWXSNCSKLLYSLFTVMTIYLLNFAMLTQLMDIILVIDNMDDFATTNILFFSS
ALCKAITAVIRRDQIVNLTNILQGEPCKACNEEETAIQTRYDRLIRSCSIGYIVLASLSATGATVGEVFAVLQ
GELPHRGWVPYDYNSPFLFLLTSLQEMLSLILGTTVNIATETLVLGFCLQTCAQLEILTHRLHEMTRSRGSKE
ASKSLSNKASQLARHIHHHLCIIRFAKMVNEVFSEVVFVQFFGSILILCSTVYYLSSHVTLEDFANLAVYTIC
MFVQILFYCWAGNEVILKSTGLSEAVYHTDWMLLTISERKDLLMIMKRSTRPIQFNSSFLVTLSLESYGNILK
TSYSAFNVLQQ
>HsOr374
QVLRLTLKILMIVGCWSPDSRTSFHKRTIYNAYTVFVILLLYTFMLSQLMDIILNVNNPDEFADTFYIMLAIV
ISCCKMTGLLINRKNIETFTDILSEKPFIPLETDEMQIRCKYDRTIQNSTLYYTILVEMTCACIAVTSLFTDF
RKGNLTYREWTPYNYSSRIVFCTIYACQLVSSTLGSMVNVACDSLICGLLLHVCCQLEILECRLKKISLGRSS

FRECVRQFALLMNKKFKVIIAIQFIVSTLVVCSNLYQLAKITLNVQSFPLILYTCSMLTQILIYCWYGNEVKL
KSVQLSPKIFEMGWLSLDKNIKEGMLIAMNRSLVPIEFSSAYILTMNLDSFMNLLKTSYSAYNILQQMQ
>HsOr375
MKRFFDTHYYRATKLLSSIVGLWPYQKRLKRKVILSFNLFVLLSYLPPQLTRLYQVWGKDIDIVCMCMPPLLT
ILICTAKTMTIIWFDSEMKTLLCQIETKWIEINMEEENKILTNYASKTKTFCIIYAVIMYNGLMCFIVTPMIS
PMLDIILPRNETRVRSLGFDLDYGVNMQTYWFWLWLHTCVAGTMVICNIVGADVIYVTLTVHASCLFEITRNK
LRHMTDLIRKYSLQFPKDDIGNSKYNIYQCEESRLKLEKQAIVVRECVLSHKKAIEYTRVLQYIYAWCIFFVV
ILNLISISISAVQLVSKMFRWTEMTMSGVYIIGELMHLFFLSLMAQFILDQSLNVHESAYSSAWYNLSTKFQR
DFVLILIRSNVQCQLTIGNMFVLSLETFCMMLQTSLSYFTMLVSFR
>HsOr376
MDSIDKFFQESHYNIIRVLLGTIGLWPFHSVTKRYAIYFGFLLLIASGLIFQVLGMIEVWTDSFEVIDCLPLL
VLSVLCVSKVICLIYTLPKIKYLLIQMERYWYSPKSDNENKILHSYAINGRKFSCAYTIVLLGHSPFFLSATL
LTKFIHTGVNKKFDGSTNIVDAQAGVPYRVNYMVDLDTYFVPIFLHTAMCEISYMIFLVAVDVLYMTFVQHCC
GLLAALRYTLENSFEFEGNVDGLMTTYMKHSKNNRSYSIIAYCIRRHSEAIQFIDILESLFRLPLLMHMGCII
TIISIVGFQVITNAESMSRVLRHCIYFSGSVLNAFFENWQGQKIIDYSEKVYEAAYNAQWYEMPNAARKLLIM
IMLRSIKPSQVTAGKIIIMSYMNFNAVMRLSSSYFMLLQSM
>HsOr377
MYPYNENQINIDDYVLINKKVLKFVGLYPTNIVRYIVCCVCMFTIIIPQTVQIYQDWQDLAIVLETSSVLLTI
LLAILKSLIWMYNRGKMDPFIDYMLTDYWRIMTTQITKYADVYATYARKLTKGYVFLICNSLLFFFSLPLIEI
FITTLAGFNDNSTKHFPFLALYPKSYYNFPIYEIVYLSQMVATSLCGLIILGTDTLIATALFHTCGQFKILQE
KIKNITAKVIFVQHTHSEENSVQKVKLQITHIIKHHYTILRFCDYMEMVFSPMLFLQTLASSLIICLVGLQVT
TATEKSKLIKYFSYLIMALFQLLLFCIPGDILINESLAINKTLYSTNWYKLPVSFKNELCLLMLRSQKPSKIT
AGKFYIMHLENFNSVLSNALSYFMLLRSVSLKENN
>LalbOrco
MMKFKQQGLVADLSPNIMLMKISGHFLFNYYKDGSSKFIHKVFCIVHLSLVLLQFGLCGINLMLESDDVDVLT
ANTITMLFFTHSVVKLIYFAVRTKLFYRTFAIWNNPNSHPLFAESNARYHQIAIKKMRILLMAVMGATIVSTI
SWTTLTFIGDSVKKVVDPVTNETSFVEVPRLMVYSFYPFDARHGVAHIMMLIFQFYFLLFTMLDANLFDTLFC
SWLLFACEQIQHLKNIMKPLMEFSATLDTVVPNSGELFKASSAKPVAEIDAPPPPPITPTGGDSMLDMDLRRI
YNNRQDFTANFRPNAGGQYNGTIGPNGLTKNQEMLVRSAIKYWVERHKHIVRLVTAVGDAYGVALLLHMLATT
ITLTLLAYQATKINGVNVYSASTIGYLLYSLGQVFMLCIFGNRLIEESSSVMEAAYSCHWYGGLVLGAMVTYF
MVLVQLK
>LalbOr2
KWAVTLNRYPLKLLGLWPPENNREESLLDKLRALVVFILMNVILVLPLACMMLLKSNDFMMYLEYTGYFIPCV
ISVMKFVIMYSNKTNLVPLLNMMANDWENLKTDIERDVMIRCISISRGFMKFCYATFGPIIFTMTVLPKLGIS
YLMATNESAMFPLPTYYVVDVSHSPYFEIVYATQLIIILATSFCYLGVDAFFGTVVLHITAQLEILHNRLTSI
QSSDQFDHALMDVVVDHIRLSSAIEVIENTYMLLLLVLLLYFGIFNCVYIFEIVYALTGKSKSDSAGIYYYVG
SYTNTLAQMTIYCLAGQLLTTQSEGIYNAAYECNWTDLKSNEARNLILIMVRAKKPFYLTAGKLFPMNMLTLC
NILKISLSYISFLLTK
>LalbOr3
MSKETLNYRRFTNNVERLLTVCGLFPHQQSIVKYLSILVLVSYCLLFYVIFNFFRIHIANISAIIACFSLLTT
TITVAIKIVQFLRRREKLARIGDTLATLFEETSLKQPSSSPVFQYLQPCYRLVCYEFACMFLTVILYTSKPLI
DIAIRNASPSTPFPSVYPWPIVSTASFAGHYLYETSMSLSFPIITTGVDAFFILCSFRVCSVFRAMAVELKES
PKRHTEDWEQLLRKCIERHTTLIECRDGIQEIYGPVVLSVTLTNGLGMCALIFEVLRVSMVAEDIPLEKATAL
SLYLFGKIIQTLSYAWPGDMITSEDEVFRREVYCNNWYEHSNVRITKLSITILSQRLMVLKAYNLLVISLDLF
AKIMNRTLSYYFLLVTLDD
>LalbOr4
MPKESPLYQEYADFVKRCLLMCGLYPYQQSIASKYLPIFWILGYCLDFCALINFFRLHITEISAITACLSVLS
GIVNMTIRASRFVICKEKLVQVTDTLEALFEETSMQQPSSSPAFQFMLPFYRLGYYHYVCMVSTAFLYSTKPL
IGVTIRHANRSTPFPAIYPLPIDSTAFFVAQYLLEVSICYSFCIITSGVDAFFTLCAFRMSSVFRAMAVELEE
FPKGRNNVESNEQVLRRCIDRHVTLIKCRDIIQEIYGPIIFSVMMTNCLGMCALIFEVSKANAAIAVEKAVAI
TLYLTGKILQTFMYTWPGHVVTSESDIFRQEVYCNNWYEDLNTKTGKLTLTILMQRSVILRACKLMAVTVDLF
AKIMNRTISYYFLLVTLDD
>LalbOr5
KTVITSPIVFSLRMIGAEPRSSRRILQRITCSILMMSSLSMQFWYVASYSKNEELTDLLDGLSVILSNGGTFV
KMVIIWWNHRMFFNALTIIFEDWKNLDSIGRNKRFMVDKALMSLRISNLLIVAYSMSVICYTISSLFISGDSG
EEELLPSKRKLLLKMRFPFEVMFSPVYEIVIVAQFLLEYFIAFVAGMFMALLAALVLHVGSQIDIMCQELIDL
PNHQQEERLSMFKSLVVQHQKIIHLGECIRDLFINIALVQFLSNILVICCLGFLLVNSLGTEGGPSIILKFLP

FYVAANSEAFVLCYTGEYLISKSDDVRRAVCDMDWYKLNNRDMQLLLLILLRSQKELTLSAGKFVVLSVEAFA
TMLKASASYISILLAM
>LalbOr6
MEVFQGNYKIYQTVMCLSGLWPYDNSILTWVHRIAYILLVLGCMVIQAMTIKNVNMSLNEVIAMISFGAQLLL
YFIRYFTTILTFSTTKYVIDNMQNDFTMLKDPIEVELLLKDSILAKRILMLYVGLLCTGSLCILGTLGISTFL
KSDIQIRFLYLVGYFYNEKNFRTTLICWYIIITTIHGLLVLVCSEGTVAVLGTHLSGLLEITSYRMRSAIDKV
ATSTTVRRIDIKPAVDIHLKAADKKYMDDMMLPSMLMILVGVSSFGVNIYRLYISITEVKEIDTVIVSVQVFL
AYLAFIAIDNYSGQIVMDNSIQIIEEIYNSLWYRIPTKMQKMVLFMFMNCHNLVTFNLAGLFILCLQGLAMMS
KSSFSYFTLLCSFQ
>LalbOr7
MDVFRKNYSTYCSVLCMTGLWPGNESLLIKMQRVAFILLNLSCIVVQVSTLKSVELSLYNILIMLSYSFPMIL
YFLRYSWFVINMSEIKAVFQSIGNDCRTFKHPLESELFMKHIAETRRVIMAYAVLAALLGAYIIVAILLPTIL
HSKLQLRYLRMFGFFYNEGSLGTDWAGVHLVVVSVLGQMALVGTEASVAVFSAYLCGLFEITSFRIHTAVNDV
VHSISPEQIDIKPAVRTHQQAIELLGKCMTYPYLMAILTVVLSFAVNLYRLLLAARNGEDLENLVLSFCLTLS
HILITFLDNHSGQKLVSTSVEVFHETYNSLWYCLPVRSQKLLLFVMMKSMVEVQYNFSGLFVPCYEGFSMIMS
SSFSYFTMLISVQ
>LalbOr8
YGGEQYDRLIKPIMTTGRIISIWPLPADSSRSTILFRRFHLFCMFFLVVVMSIALTADVIYNLSNLDEATECA
LICTAFYLCVVRLLVYTAHQKDMLYVVNTMREDWTVSSYEDRMILSEKTMFAYRLAKFFISTVAVTIVIFMCV
PLLEIYVAGNDKILPFRGYFYVNHTVSPIFECFYVFNVTAGGFGGSMIAGATSFNLVVIMHGSAKFAVLRKRL
EAISGDDPDSSSEMTECIIRHQQAIEFADALERIINVLALGQFVISTGLICFAGFQITSMMEDMGRLMKYSTF
LNSAILELFMFSFSGDGLIEESENVGLSAYSCEWIKSHYCPNLMIMMMRSMVPSQITAAKFYSMSLQSFSTVL
STSFSYFTVLTATKED
>LalbOr9
MAPKPGHQVLGSLRRLLKVQLSYLTYCGLWVLNPNTESVLRCGYFAWKVFIIATMYIFTITLLADIYTSIDDI
SVATDSGCICAGMIVVVFKTMNYQLNQKNIERVTDIIKRHYTFNKIIFHGFRAIGCFLVIALLLFSPVKDGLP
IRAKYPFNTTIFPYREIALAVESVAVSSGVLAILSMDGLTLLMCNYTTMQFDILNVNFENCNRRTANNTGSVY
RRPNDTFMTRYKTCLRFHQQLMSTTNDYNNIFSPSVFVQMLSSTSIICLTGFQAVVVGGQNSDIIKFGVYLSA
ATSQLFYICWMGNELTYSCSVLDRSQWLSNWHQERSSSIMQLFVLSTMSVRHPLYMKAGPFYVCSLKTYIAII
RSSYSVFTLLNNMQTTD
>LalbOr10
NNGLLSFLRICCRLFGYWPSNPSSPKWRVIVPEVTYWIHFLNEFFGALAIVNGLYCCFASVGAIVKSFVEIVF
MLETMFNLIYCRRRSSQFERLIIKMEEFFRPSDPRDRIIRQNYLNRTKVAHYSLISTFTVGGLLFIFSPLVAE
SKVTPVNTAYFAIPREEFWGFCVTFALNILHIVNAASVLFLDLLIICLIWHAACKFVILGEAFRNINQKRRVE
MKKWIREHQDAISYANEVNHVIAPLAIKSTIAVALYVIVSCLLLTHDLLLLELSKFFIVTAFSMLRFLACSWA
ADTMTKNAQDIGWMVYATPWTHLAPVFRKERIIIIQMCQKPITINAGRFLTAWSLHFCGQVYYTIFSTFWTIR
AIL
>LalbOr11
MFGTVTPEKAITFVRLSIALICGWPQPSVSTKAQVLCSRISKVLAIISALALFLPVLSATILHSGDANVFSKA
AFMAIATGQVFIYALLTAIQHDRFQRLVENITVSIKDAKSYERNVYQRYVDTYYKFYGLTVMWYYMSAMIVIV
GTIFLPQPFPTISEYPFDVDYEPVRIMIFIHQAIVGFQCSASVSLNMFAALLILNAAARYEMLMIDLKEANSV
DALIVCVKKYHAVTRFAKDVINGAQYIAVGTVLLSSMKIVLCGLNIIGRTSIVIKLQFMALSWTALMEVFMCA
LPADALINVSTNAVRSVYESTWYDQVLSVQKTVLHILVPQEPMAISVKVFIPALSLEYYCSYLSHAVSLFTAL
RMVLGED
>LalbOr12
MFENVMPEKAMVFIRHSVVLICGWPLPSMATKAQVLCYRILKVLSAISALALFLPVLNAAILHFGDPTNFSKA
VIMAIACAHVLTQIVVSSIQHDRWQRLIEHMTASLKNAKSYERNVYQRYVDSYCRFYALTVMWFYMSPIVFVV
GTIFLPQPFPAISEYPFRVNYEPVKTIIILHQALVGFQCSASVSMNMFAALLLLYVAARYEILMIDLRESTSI
DALTMCVKEYYSVTRFAKDVTDATQYVVWFTILYSSIDLVLCGLNIIGRQPVVVKLQFITLSWTAMMEVFMCA
LPADTLINMSTNAVRSVYESTWYNQVLGVQKTVLRILVPQAPIIISLKCVMPRLSLEYYCSYISNSVSLFTTM
RMVLGDDAD
>LalbOr13
MFENVTPEKAMVFVRLSIALICGWPLPSTATKAQVLCYRILKVLCGISAIALLLPVLNAAILYYGDATHFSLA
SIMTIACAHLLTQVLINTFQHDHWQRLIEHMTASLKNAKSYERNVYQRYVNTYYRYYGLTVMWNYMCTMVVIV
ASIFLPQPFPTISEYPFDVYYEPVRTIIFLHQAFVGFQCSASVSLNMFAALLLLFAAARYEILMIDLRESTSI
DALTMCVKKYYAVTRLAMDVIDATQYIVWFTILYSSIDLVLCGLNIIGRQPFAIKLQFIFMAGTILMQVLMCA
LPADVLINMSTNAVRSAYESTWYDQVLGVQKTVLRILVPQAPIIISIKCFMPRLSLEYYCSYISNSVSLFTAM
RMVVGEDED

>LalbOr14
MLERVTPDKAMAFVRVSIALICGWPLPSVATRAQVLCYRILKVLSALSALALFFPVLNATILYVDDFPAFSKA
VVMAISALQVFFFTLLCTIKHDDFQRLIEEMKASLKNAQSYERNIYQRYVDTYYKFYGLTVMWSYVSPVVVVV
GSIFLPQPFPTVSEYPFRVDYEPVRIIIFVHQAIAGFQCSAAVSLNMFAALLLLYTAAKYEILMIDMRRATSV
DALIACVEKYQAVNRFAKDVVTVTRYIVCTTITCSSTNIILCGFNIIGRQPFIVKLQFVVLSWTGLMEVFMCA
LPADRLINMSANAVRSVYESTWYDQVLEVQKTVLHILVPQKPMAISIKCFIPVVCLEYYCSYISNAVSLFTTL
RVVLLDEDD
>LalbOr16
VTPKKAITFTKYSVILCCGWPLPSSAPKSHFYFYVIAKVLIALSNISVILPAFWSLLFYIHDPVRTGKTFGLL
TAMCQTIAYIIVCSFQHDHYQRLIEEMTDYLEKAKLYEMVIFQRYLDTYSTFLGITTISFYIAAGVIVVTTLF
TDQPFPVMAKYPFRVDYEPVRSTIFLNDAFVALQNVASVSLNTLTALLILFAAARYDIVMLELQSAVTVADLK
ESMKKYHEVKRYSVAKFVVPSVKIILVFSSSVILVNAGLNLIGRRDFLIKCQYVFVATTALLEVFSCVLPADR
LIEVVSCTMRSVYDVKWYERDLSVQRTVYHMLVPQKPVIISFLFVVPQLSLNYYCSYISNAFSLFTALRLVLS
ET
>LalbOr17
TDFTINLMIPFMKYFGFWMVTGREKIFSHIIIFITYLQLIYLIIAAMYDAMFLSPDFSTQLYYMINAVTIFNS
LFKLIAIHPYKRKFFNLILHLQHEFIDSEYTEHERRMLLVCQRTAKIFIWSFTILVHVTVLGYSAIPFFDNIG
RNESDRVLIGNMWMSVIAFSPYYEIVYIIQIFCVIKIGVVYHSYDNFQCLMNLHVATQFRILHHRLESLESTY
CSGRRLVQMSSNSLSNDSDYGADCYAMFKTYIQQHKALLAYCKKLNEVFNIYALGQVILFTLILCLDAYQLLM
ADALPSRKMTFAFHMLGCFAQLLMFTYSCDLLIAESLKVAEWAYGIPWTYFPMNKHGKMMRNDLIFLLVRSSE
PCCITAFKFFPVSLETYTTVLSTGVSYFTLLKNSSET
>LalbOr18
MEVFQGNYKIYQSVMCLSGLWPYDNSILTWIHRIAYTLLLLGCMVVQVATIKNVKMSLNETIGMISFGAQLLL
YFIRYSTTILTFSTTKYVLDNMQNDFTMLKDPIEAEMLLKDSILAKRILTLYVGLTCTGSLCIIGTLGISTFL
ESDIQIRFLYLLGYFYDEKSLRTNLTCLHIIISTMHGLLTMVCSEGTITVLGSHLSGLLHVTSYRLRNAIDKV
ATSTTVRRIDIQPAVDLHRKAIDKKYADDMMMTSVVMILVVVSSFGVNIYRLYISITETKEIDAVILSLQFVF
AYTAFIFGNNYSGQIVLDNSIKVIEEIYNSLWYRIPTKMQKVVLFTFMKCQSLVAFNLAGLFILCLEGLAMMF
SSSFSYFTLLCSVQ
>LalbOr19
LKKPVSLNIELFYDENVLSWSKRLLSLSGLWPDNRNDVRFFFYITYVMIFTWLEIVALLQSVHDLEKTLKNIT
LSFPTILIVLKTVMFRLNMHHVLPLLAVVKRDVEHGLYQSQEEQRTVVWYNVAATMFSTSSALSLFFVPTLFY
AKPIVSCFLSNYDNCTLPYELPMKVNPVYEVTEMYTYAFFCVCLVPVSMMLTIGATGADSLLVTLNFYLCSQL
SILSQRVRNIDSDPQKHVSNMRILVERHIELLRLASTLAETFSSLMFVQTVGLIFSLCIVIYQLLMTSESSGG
DVNTIHFIIYSCAVVLLAFCYCFLGECLIAESSDMQLACYFTNWYELPNEYAKALMFCIARSQKPSHLTAGKF
YVFSLETFGVIMKASMAYLSVLRNI
>LalbOr20
MGYRVTPRIAIAFTRASVALTRAWPPPLQASRTELFFFNVSWCASFISSIGLLLPLFNAICEYYDEPIILGKN
VSLFAAVAQVAIKMIVCRLMQQQFQRLFFEMEEFCKHATEKENAVLQRHVDRYKYVHGVYTLWCFLTTIFVIC
GPLYSPQAFPTHAKYPFPVEEQPLRSIIFFHQSLVGFQASAGMAIDAQVALLLRYATARFEILAMKLERVQSE
SQLNACILEHARLLRYTKSIYRAVRFICFATAVTTNLAVIFGSLNLVTKQPLPLKILYALVVVSASGELFMYA
WPADSLIHQSTGTAMSVYNSTWFEQNVKQQKKVYFMISRSQKREAIQITGFLPQISLFYYAKFLYTAFSYFTA
LRVIVE
>LalbOr21
MDAFQGNYKIYYFLMCLLGLWPYDRSIRARIKRIFFSTLMVSCLINQLLSLRNIEPSLYDVVVLLSNACPVMM
YCLRYISSVCMFSTLKYLLDNLQHDFVTLNDPLEVQMLMKDSIVQKRIVTVDIALSTTGTLLVGGLLLVPTLM
QSDAQLRYLRLIGYFYNERSMHTNLTCLHMTMTTLYGLLTFVCTEGTLTIVSTYVSGLLHIARYHVQSKTVKI
LKDFKMSSDRMKPLDIRSAVDMHRQAIKLIKNYTDGVMMASLALVLVAVVSFGVNIYRVSFIRILKKNKTDNT
MFNIQVVLAHLAIIFGSNYSGQIAMDTSVKIVHATYDSLWYRLPPKMQKLILFMMIKAQTKLEISFAGLFTPC
FEGFSTMMSTSFSYFTLLYSV
>LalbOr22
MESSQYECLKVNKYFLCSIGQWPFQSPLEKFLVGVVFVPVILTQTLFQVGGMITAYNVDDIDAFTEGFAPAII
SFMCLMKYINFFNRGAMKDLLCIMQDDWRTYMKTREDYDILRRHCTQARKVTFTYAVSIYGSMVPFMILPTVL
TAISKLHAVNGTYERQMMFRTEYFLNTEKYYYPIMIHNYIGTLAFIGIVVACDSMVVQFIRHECGLCEILGRR
LQHLMESNNLDIELYPARQNDDAYETLKTCAMLHKHIIQFANTLETTSSMPYFFQLGFNMVGVSFTQFQAIVN
LSTPSKSFRFASFTCCLLNILLLMSLPGQHLTDYTARIYDYITASNWYQLSIDGRKLLHIMLTKSIKPVQLTA
GKIYTFNLMNFSAVLKTSFSYCMVLCSF
>LalbOr23

VTLCYFHLGMMQVWDFYDSLHDSKAMLKNATEMPVTLSVFFSFILIRTNKKLIVVVEHIQRDIKRTIIFGNAE
EKRLYRKYNTISVYFGKYVTVLAFVVGYMIYIRPLIDLLIYPRTDGENNTRPFVLPFRSHVPFDYRYNAKMYT
LLYIYQFPIAFTAIYHAAEASLIVTSTLHVCARLSVLARRIRTTLTESPEHFQKRMKMMVIEHLELTELSGFL
NDCFRHLLLIEYVNCSVRLGISMYVVLISLGKDTVITVNFLLYTIIVMAWLYLYSYIGEQLTYESQSVGDAFY
DTDWTEIASRDKRSLVMCLINGQRAQHLMAGEFYKYTLFGFIDIVKTSLAFFSVLR
>LalbOr24
MEEFRKHFSLYSSVLCFTALWPYDESLLGKIQRVMLPVVFLSVLVIQISTLKWVEKSLYNILMMLSVTCPMTL
YLLRYVGFTLNRPTVRVMFDSFANDYSATKDPVELNIFAKHKTDAERVFLAFLGLTAVLLGYMVVVLVIPTIL
RSKMQLQYLHIFGFFYPEMSRQTDLVSILFMFVMAMGLLSIATTESSIAVTSAYLCGMLEIASYRIEVAIDNV
VHSNTSELLQVQPVVELHKRAATTLYDVMNAMLVPYLIAIVAVIISFAVNLFRLLLAVQKMDDFSNVFFSLND
VLLHFVIMYLNNLSGQRLINTSSYIFDKICCSAWYCIPLRSQKILLFMLMNCVKDMHCNLAGLYELGYEGFSM
MMSSSFSYFTVMCSAQ
>LalbOr25
MEGFRKNFSLYSSMLCFTALWPYDESLLGKIQRVTLSLLYLSLLAIQVSTIRWVEISLYNVLMMLSYTCPMAL
CFLRYVGFAVNRPLMRAVFDSFSNDYTATKDPVELDIFKRQIIVARRVILAYLGLSAVLIGYIIVVLLVPTIL
RSNTQLQYLHIFGFFYPEMSRQTDWVSILFIFGNGMGLLSVASTESAVAVSSAYLCGLLEIASYRIEVAIDKV
VHSNTSELIQVRPVVELHKRAAATLDDIMSNLMISYLIAIVTVVISFAVNLYRLLLAVQNLEDIANLFFSVSD
ILLHFAIMYLNNLSGQRLIDTSSHIFNKICSSSWYCIPLRSQKILLFMLMRSIKEMHCDLGGLYDLGYEGFSM
MMSSSFSYFTVMCSTQ
>LalbOr26
DFSIAVISFLTKCTGVWMHKNRGELYRRYLVILYTCYHLCFGIAMQTIASIHNTDNIGEFIYTFFLTLATVLG
LLKLILVYVHRQDFFALVKYLHEKFLHSEYDTREQELLNKCKRLSAIGIGCFTSFTYVTVLSFMVTPLIDNIG
KNESDRLLPFAFAINLPLSMTPYYEITFIIQFLMLYQIAVCYTSFDYFLCLFNMHVATQFRILQYRLSHLNYT
NIEGISDGKVEKPQPDASERCYAAFRTNIQYHQSLISFCGKLEEVFSLIVLAEILSSSLVLTLIGIQLLGVTS
QNGGTLTFISYLVTSLIQLLMFTYTCDGLISESLNVAEAIFNAPWYTLLMNKYGGMLRKDLTLVILKSRTPCC
ITAKGFFPISLETYTKVWTTTFSYFTLLREAM
>LalbOr27
DLMYVYGWNYYTMNFMGMWPRERQWHQLSSYLILSPVLIMFLFVCVPQTANIPNVYGDMEMLVENLSMANISV
TISMLKTIVFWVNGKPLKSLLKCMARDWSNTTKPSERVAMMEVARIARKTISSSTVMATIMVLAYILRYYCAA
KYTDQKLFFQSKFPYDVDTSPNLQLTVCGQLAGGIYAAIGYTAVDTFIAMLVLHICGQLTILRQEIMNLRENG
DEKLGVVLKRIVQRHQYINTFASTIEDCFNMMLLLQMLGCTLQLCFQCFQAIMTLGGNEKIVMYTQILFLVLY
VLYIMAQLYLYCYVGDKLTMESREITNAAYNCAWYNLSSRNAKLLIIIMCRDILPLRITAGRFCSFTLQLYSE
ILKRSMGYISVLYTMQNE
>LalbOr28
AIGWNRFILRIVGIWPEPVMLHERLANLRAFVSVFCIMMFANIWQSVDLFMVGNDMSQITSILSLANIPGYNS
VFMLVFVWYYRKDLQQLIQSFYDDWYAPKTEEERRTMVDSARICKHLAIWCTVLTQCMLFAYIGTRSYAIAKC
DINTEPSDHLTIYPAYYPVDLRRTTYRVVANVSQVVAAYCAVIPYTSVDIFIATLVLHTCGQFTNLRRKLENL
MDGSNGKTVRDSSDIQEELAAIVKRHEHLNWAAARIDECFSMLLFLQMLLSTVEICFQGFLFFNVILQSNEGI
LNFQFLFFVLFILFIIVHMFVYCYVGEMLRVQSTDMAVAAYEARWYNAAPPEAKCLLFVMLRSTQPLCLTAGK
FGTFSMEMFSTV
>LalbOr29
MEFAVGWNQFNLTLLGVWPEPREISTLSRRVSAFVFWFTSVVTFTFICAPQTVNLVLMSTSLDEVIENLSINV
PIAFALAKQIVLRYHRKALTFLVSEILTDWSQPLSGPDHQTMLKNAKLSRMISIVCSTLTYLMLFAFLSLQIW
SNAQSASEVDLGGLLHPATFPYDTKKSPNFEITWLGQLIGTVLTAICYSCFDTFLAVLVLHLCGQLSVLRAAI
EHIAETNVQGVKFNERLGYIVLRHNQLSRFAVIVEDCFNLTLLVQTLICTAMLCLTGYRMMSSVDQEEEDVPI
VGMMFFIIHVIYTMLHLFIYCYVGEALVGESTGLAQSAYNCIWYNLPPKQSVSLIMLICRSRISFRITAGKFS
PFSLELFNAVVRTSAGYLSVLLAMKN
>LalbOr31
DWAVGINRICLRLVCLWPRESHGKGKLLEDLCVMTIVLILCGFVVIPGVLLLLKQNELKSTIDDGVYSLTVLT
LIPKILIIHGKRSVMLKILNVMADDWEKPKTDEEKNIMYRYAKMARIVAVLGFIFAGIAILMVIVLPKFGIYI
RHTTDGIDVFPFPTYYVYDVTRSPYYEIIYFGQIFMLVITLLAYNGINILFGTIFLHICGQAENLRSRIASQR
KFGDFGQALASIVSDHVRLIRTVKMTESTFSVIMLTMLLMFAAITCTVMTSIFSIFAEKDGFSLTRVLYLVLL
ITTNFIQMFFYFITGQTLLNESEGIYDATYECGWLNLKSNEAKSLILVMARSKKPLFVSAGKLFPMSYLTFGN
VIKISFSYMSFLV
>LalbOr32
MEAFEGNYKLYYSVMWFTGLWPYNDSFLLRLYRAAILLMISGCLVNQLCTMKNSKMTISEIVVQISFSFTLFL
YGTRYVSGLVSMKAHRHNVFNIQKDHAAVKDSIELELLLKHSIVGKRVMQLYCGLVCCGTTCLFATLGLSTIL
QSDLQLRFLHLLGFYFDERSMRTNLTCWLIIMSVAFGLLTLAGTEGSITIYSTYISGLLEIASYRMQNTINSV

ANSDALNVIDLRPIVEIHCDAIKHSKQYANDIMIPSLVIVVGVAAGLGVNIYRLYMSITMEGEYDEVLITLFF
VSAYVSFIGANNYCGQIMLDTSHNFYHQTCNTLWYRIPPKMQKMVLLALVRIQVAVEFNLAGLFVPCYEGLTM
MMSSSFSYFTLLCSVQ
>LalbOr34
PNKHLHISLSMIYYMGMWPARGRYRCLYLVYTVCSFSFLLGVFLTTEIAHIVVKRHNMDEVVAGATVLMTNAT
HAYKIIFILRRHNRIQQLIDITECEMFSRDNVKYERVVTHYTWQGIFHHITYQFFGTMAVVSWGAKPVIDLLS
KRSKELPLLGWYPYNTTESPAFEITSLYQAVAIILCCFNNIAIDTLVTGLITIACCQLTILNRNIASLNSETR
QSINVEKDNDIKASNKQYENLKVCVEHSNMIFNFSKEVQNIFGTSIFFQFLVNCNIICLIAFNIAQMKVYVPH
ILFGMLMYMCCMIYQIFIFCWHGNELYLHSLDISLAAYTNKWWQKTESFKQALQIIITRAQRPLILSVGSIME
LSLQNFVRILRMSYSIFTVLQTSTS
>LalbOr36
IKDSFVYCERLFAIGSVLPHQHHPKLFKLVISYYVFLVAMLLWDMYDNLHDFQTSLVRATEWPFMLSMLISLV
LIRTNKKLIMVFKKIQQEIRGSLLFESDAEKRLYRKYNIIADKVGKFTSVFVIANAYLYYARPLIGLLIHPRT
VPLNAENNTRPFVLPFRMHMSLNYRYNAKVYTLLYIYQLPATYASLCHAAEASLIVTSTLHVCARLSMLACRI
RKSLTSSPAHFQQRIRLMVTEHLELTELSGFLNDCFKDILLIEYLSCSFRLAISLYVLLITLGNDTAAFVNFC
LYSALVTGWLYLYSYIGEQLSHESLNVSDAFYDTDWTDIAARDRKSLVICMLNGQQAQHLMAGEFYKYTLFGF
SEIVKSSMAFLSVLRKTI
>LalbOr37
MAILTPAFMLLTIGGCWRPQLWTTRCERILYAFYTVFVLLLLHSFGVSQLLNVLLNVQTTDDLSDSFYMFIAT
VLACVKIITLLINDKNIKMLCEKLQTQPCEPKNSEELAIQRNYDRKIGSVTICYAILVETTVFCMIVSSVLTD
FRYRKLAYNVWLPFNYSSEKLYYVAYVHQLVGLFGTSILNVSCDVIFCGLCVHACSQQEILQLRLKEFPKQDR
PKIGPIVEHHDYLYRYVSTMHEKFHAVIGIQLMASTFVVCFILYQLTNTPLMSLRYLQFVMYMVCMMTQIFFY
CWYGNALKLKSIELVDTIFEIDWTSLDNSTKRSLIIIMRRAMNPIELTSAFVFTMDLNTFVRILRMSYSAYNL
LQR
>LalbOr38
MRVLRTVTRTLIMCGCWHPESWSSPSKTLLYQLYRVIVIFVVYSITFYQIMDLLLVVETQDEFADNLYMLTAM
MIGCQKLTNMLLSHEKILELMDTFQKEPFAPRNKEEVEIRAKFEKTTHTNTLMYASLLTITGSSISAMVCFKS
EKWPLSFRIWLPYNYSAVPLYLPTVIQQSLGLHYGALNHAACDSLFCGLMIHICSQFEILGHRLNQITKGDSA
MIKSCIHLHDKVYRYAQELNNQFKMIIFMQFLASAVIVSFNLYRLTQSTNLGPKLFVIIMYSQCIMTQIFIYC
WYGNEVKLKSLEISDAAHNMNWMELDNIQRKMILMMMRRASVPIEFSSIYIATLNLQSYMAILKTSYSAYNVL
Q
>LalbOr41
LAYVCKILGVGGCWIPDSWTSPSKRILYSTYSAFIWFVMYWWTVSQILDLIIQVKTQDEFTDIFYATMANCCI
CAKFTNVLLTRTYIANMIDRIEKEPFASLNVEEEEIRTRFDKLARRNAVTYTIIISCYILPTCAMGLFVGFRD
HKLLSRAWLPYDYSSNVSFSTTLIHQVGAQVYAGCITTGSDCLFNGFLMHTYCHFEILNHRLKMIEADKKYTL
KRCTYLHDYIYTYATTVNRRFKIIIMIQSLVSIVTVCCTLYQLTQVHLGSRFIEVALYLTNTLIQIFYYCWYG
NEIKLKSLDVSEFVFQCNWTTLDNDGRKLLVMMIKRASVPIEFSAIHIVSMDLEMFTTILKTSYSAYNLL
>LalbOr42
MPTLRWTTTVLTTIGCRYPSTWTSTTKIFLYKVYGIIVVFLVQSVTLTTILDIVFNVRSQDEFGDNLYLTVPM
VISCCKLCSFLAHRESILMLMHAMREKPYLPADDHEMEIEMKYDTMNEISTLYIMSTELCILTRWVTSLMKDP
QDRVLPFRVWLPYNYSSPILYSVTYAHQAVTVTIASLMNNAYDSLFSGLLFCIYSQLEILGHRLQQVKDHSDL
ARECARQHNFIYRFATKVNEDFQVVLCVQFMASMTIICFTLYRITQTDLGSRLAETVMYAFCMLMQIFYYCWY
GNEVRLKSLEIPDLIFASNWANLNENTRKTLLMIMLRSTFPIEFSSAHIVSVNLESFMAVLKTSYSAYSLLQ
>LalbOr43
MPTLRWTSMVLTVAGCRYPPSWTSGTKKTLYKIYGSFVFFLTYSLTITSLLDMVFNVRNQNDFGENLYMSAPM
VITCCKLCTLLANRDGIMILMAATRRKPYLPVDEEELRIETKFDIINERLSTFYTLYTELFMLLTWTSSLFRD
PKNRALAFRAWIPYDYSTLVLYPITYAHQAVSMIISSTANVGFDCLFSGLIVSIYSQLDILGHRLRNIKDDTV
SVNDCARHHKFIYAFAMKINEDFQTALFIQFFGIMAIVCFSLYRITQLGLGEKMFEILVYATCFLMQLFYYCW
FGNEVKLKSLEIADLIFSSNWISLSSNTKKTLLMIMVRSTYPIEFTSGHIVSVNLESFMTVIKTSYSTYNLLQ
>LalbOr44
MPTLRCASMVLTAIGCYPPVTWTSKPKRLFYKLYGYLILMAVQALTLSTVLDIVLNVKTQDEFSENLVLTIPM
LITCCKFCSFLAYRDSIMRLINCQQRKPCKPVDEDERNIESRFDRYNELITAVYTCSTEMCVVVTVASSLITN
PGNKALTFRSWLPYDYSSPSVYAVTFILQTVSVTISSLMNVTYDSLFSGLMFCIYSQLEILGHRLQNIIKTGD
ASAKECARHHNFIYEFAAKVNEDFQAVLCVQFLASITIICFTLYRFTQTDLGSGTIEALMYAYVMLLQIFYYC
WYGNEVRLKGLEIPNMIFASNWTSLDNRTRRILWMIMLRATYPIQITGARLLSVNIESFMAVLKTSYSVYNVL
Q
>LalbOr45

MPTLQCASTILTVIGCHRPPSWTSEPMKFLYKVYRIIILVLVQVLTLITILDIVFNVKNQDEISDNLVLTIPM
LITCCKFCSFLARGDNIMVLINTLQQKPYSPENILEKDVETKFDKHNERVTALYTFSTEACIVMILLTTLITN
GKNKMLAYRLWIPFDYSAPVVYAIIFALQAVSVIISSLMNVTYDALFSGLMFCIYSQLEILGLRLQNVVKNGK
DTARECARHHNFLYEFAAKVNEDFQAVLCIQFMASIAIICFTLYRITQTDLGERLAATLVYAFCMLMQIFYYC
WYGNEVKRKSLEIPDLIFASNWAHLDTNCKKTLLMIMVRATFPIEFTTARILSVNIDSFMAVLKTSYSVYNVL
Q
>LalbOr46
MPTLRMTFTVLTLLGCQRPATWTTSTKKFLYKVYSTVVFIFLQVLLLMSILDMIFNVEDQDEFSDNFYALLPE
ITSFCKLCSFLANHKSIMILVHSMQRKPYSPVDAAEMMIETRFDAINEKLTILFMSMFGMCGVLIWIPSLIRD
PKNRALAFRAWIPYDYSSPTLYAITYVHQAVSVFIGSFMNVAYDTLFSGLMFCIYSQLEILGHRLRNINKDQK
ESAKQCVRHHNFLFELVEKVNKGFRVVLFIQFLSSMLIICFILYRILKRGIASRMLETFVYAFCMLMQIFYYC
WYGNEVRLKSLEIPVFVMNSNWVSLDENTKKILWMIMMRSTFPMQFTSAHIVSMNLDSFMTVLKTSYSAYNVL
QR
>LalbOr47
MPTLRITFTMLTFLGCHRPPTWTSRTKKFLYKMYSAAVFIFLPVLLLMLILDMIFNVENQDEFSDNFYALMPG
ITSFCKFCSFLANHENILILIHSMQRKPYSPVDADEMMIEARFDAINEKLTILFMSMFGMCGVLIWIPSLIRD
PKNRALAFRAWVPYDYSSPTLYAITYVHQAVSVFIGSFMNVAYDTLFSGLMFCIYSQLEILGHRLRNINKDQK
ESAKQCVRHHNFLFEFVEKVNERFRVVLFIQFLSSMVLICFIFYRIVKMGIASKTLETFVYAFCMLLQIFYYC
WYGNEVRLKSLEIPVFIMNSNWVGLDENTKKILWMIMMRSTFPMQFTSGHVVSMNLDSFMTVLKTSYSAYNVL
>LalbOr48
MTAFRYTFLVFTVLGCLPPASWTSTAKRRLYKVYSFLMLIPMQTLMLTSVLDIVFNVKTQAEFCDNFYITVAM
MISIYKMFNFLGKRDDILLLLRKIEREPFKPANEKERSMKMKFDKEIENTSLVFFFVLNVYLLVGLVSSMGRD
LKHRNLFNPRVWLPYNCNHLALYTITYCHQASSLVALTYMHYANDTLISGLMILIRGQLTLLQHRLQNIVGDQ
NLSVKACAKYHEDIYQLAADVNKHFHGIMGFQFIASTPMACLTLFRITTMEIGSQMYECVLYIVCTLVQLSYY
CWYGNEVKLKSLEIPDVIFASNWPDLDNTSKKMLMIIMKRATSPIEFTSAHIVALNLNTFMTVLKTSYSVFNV
LHTT
>LalbOr49
MTGFRYTFIAFTVLGYLPPASWTSTAKRRLYNVYSFLMVMPVHVRTLTSLLDIVFNVQTQAEFCDNFYITVAM
IISIYKMLNFLGKRDDILFLLDKMEREPFKPANEEERSIKMKFDKEIGNTSLVFLWVMNVYLLVTLGSSVMRD
LEHRNLFNPRVWLPYDYNPLGLYTITYCHQASSLIELTYMQYANDTLISGLMILICGQLTLLQHRLQNIVGDQ
HLSLKACAKHHNDIYQLAAEVNKHFHGIMGFQFMASTVMACLTLFRITRMKIGTKMYECVMYIACTLIQLVYY
CWYGNEVKLKSLEIPDVIFSSNWPDLDNSSKKMLMIIMKRATSPIEFTSAHIVALNLDTFMTVLKTSYSAFNV
LQ
>LalbOr50
MLTVCGCLRPATWTSSFKKFVYLVYTVLAYGLMQSLILSNVVEILVTVRNHKDFSDVLNMLMSVTGCICKMTT
ILMNRKNIVSLINVLRREPFLPVDEEEMDIKNRLDKIIDMNSIQFMGAIQITLFIGWVGTPIMEGKNRQLAFR
SWFPYNYSSPTLYAVTYLYQVVAITYHTVVHVACDTLFSGMLICIYGQLEILGHRLQRITNDEGKSVIKCVRH
HHQLYEYAASVNKTFHAVLFSQMNMSVLLVCIGIFQLTQTHFVADMIKKFLYMCCPVLQIFYYCWYGNEVKRK
SLEVSDMIFAGNWIGLDKNTKRTLLMIMLRSTFPMEIKSAHIMYMNVESFMSVIKTSYSAYNLL
>LalbOr51
MASFRVTLSLLTVCGCLRPATWTSSFKKFVYLVYSVIVYGFMQSVMLTHLVEIVFHVKTQKDFSDVFYIMASV
VGGVCKMTMILLNRKNIVTLLKILRREPFLPVDEEELDIKNRLDKIINMNSIQFMAAIQITLFIGWIGTLVME
GKNRKLTFRAWFPYNYSSPTLYAVTYLYQFVAINFETTVHAACDTLFSGMLICIYGQLEILGHRLQKITNDEG
TSVIKCAKHHHQLYVYAAYVNKTFQVVLFCQMIMFVSQVCFAIFQLSQAHLFADVIKQCSYVFCPVLQIFYYC
WYGNEVKRKSLEVSDMIFAGNWITLDKNTKRTLLMIMLRSTFPIEIRTAHIMYMNVESFMSMIKTSYSAFNVL
TR
>LalbOr52
MLTVCGCLRPITWTSSFKKFVYLVYTVIAYSLMQSFMLSCLLELLSNVRSQKDFSDIVIIMTALAGSVCKMTT
ILVNRKNIVTLVTILRREPFLPVDEEEMDIKNRLDKIIDMNSIQFMVAMQIALFIGWIGTLIIEGKNRQLAFR
SWFPYNYSSPTLYAITYLYQFVAINFETTVHAACDTLFSGMLICIYGQLEMLGHRLQRITNDEKSIIKCARHH
HQLYVYAANVNKTFQVVLFLQMLMSVSMVCFAIFQLSQTKLLSDVIKQCFYVFCPVLQIFYYCWYGNEVKRKS
LEVSDMIFACNWVGLDKNTKRTLLMIMLRSTFPMEIKTAHIMSMNVESFMSVIKTSYSAYNLL
>LalbOr53
LLTVLGCMRPVTWTSSFKKCVYLVYTVIAYGLMQSLMLSCLLELLLNVRSQKDFSDIAIVVTAVAGSLCKMTT
ILVNRKNIVILLKILRREPFLPVDEEEMDMKNRLDKIIDMNSILFMGAIQITLFIGWIGTLIMEGKNRKLAIR
SWFPYNYSSPTLYAVTYLYQFVAIHFETTVHAACDTLFSGMLTCIYGQLEMLGHRLQKITDDQGNSVIECAKH
HHQLYVYAANVNKTFQVVLFFQMLMSVSMVCFAIFQLSQTQLLTDVIRQCFYVLCAVLQIFYYCWYGNEVKRK
SLEVSDMIFASNWISFDKNTRRTLLMIMLRSTFPIEIRTAHIMYMNVESFMSVIKTSYSAYNLL

>LalbOr54
LLTVCGCLRPITWTSSFKKFVYLVYTVIAYGLMQSMMLSTIVEVLLNERSEKDFTIIIIMMTSVTVSVCKMTT
ILMNRKNIVTLVTILRREPFLPVDEEEIDIKNRLDKIINMNSILFLGAMQTTLLVGWIRTPIVEGKNRTLAFH
TWFPYNYSSATLYAVTYLYQFVAITVEITVHAACDTLFSGMLICIYGQLEMLGHRLQKITDDQGNSVIKCAKH
HHQLYVYAATVNKTFQVVLFFQMLMSVSMVCFGIFQLSQTRLLGQMIENFMYIFCPMLEIFYYCWYGNEVKRK
SLEVADMIFACNWIGLDKNTKRSLLMIMFRSTFPMEFKSARIMSMDVETFMTVIKTSYSAYNLL
>LalbOr55
MTSLRIALSMLAVLGCLRPVTWTSSFKKFLYLVYTLIAYGLMQSLMLSYLLELLFNVRSQKDFSDIVIMLTSV
AGSVCKMTTILLNRKNIATLLKILRREPFLPVDEDEMDIKNRLDKIIDMNSIQFMVAIQITLFIGWIGTLVME
GKNRQLPFRSWFPYNYTSPTLYAVTYLYQFVAITFETAIHAACDTLFSGMLICIYGQLEMLGHRLQNITDDQG
KSVIKCAQHHHQLYVYAASVNKTFQAVLFSQMIMSVSLVCFAIFQLTQTRLLADVIKQCFFVFCPVLQIFYYC
WYGNEVKRKSLEVSDMIFASNWISLDKNTKRTLLIIMLRSTFPIEIRSAHIMYMNVESFMSVIKTSYSAFNLL
TR
>LalbOr56
MASFRIALSMLTVCGCLRPITWTSSFKKFVYLVYTVIAYGLMQSLMLSHLVELLFNVRSQKDFSDIAHIMTSV
TGGICKMTTILMNRKNIAALLKILRREPFLPVDEEEMDIKNRLDKIIDMNSILFMGAIQITLFIGWISTPIME
GQNRQLVFRAWFPYNYSSPTLYAITYLYQFVATTFETTVHVACDTLFSGMLICIYGQLEMLGHRLKKITDDEG
KSVIKCAQHHHQLYVYAASVNKTFHAVLFSQMIMSVSLVCFSIFQLTQTQLLADMIKNSLYMFCPVLQIFYYC
WYGNEVKRKSLEVSDMIFASNWISLDKNTKRTLLMIMLRSTFPMEITSAHIMSMNLESFMTVIKTSYSAYNLL
T
>LalbOr57
MLTVCGCLRPATWTSSFKKFVYLVYSVIAYGFMQSVMLTHLVELMFNVRTQKDFSDIVYIMASVAGGVCKMTM
ILLNRKNIVTLLNILRREPFLPVDEDEMDIKSRLDKIIDMNSIQFMGAIQITLLVGWISTPIVEGKNRKLAFR
AWFPYNYSSPTLYAVTYLYQFVAITFETTVHVACDTLFSGMLICIYGQLEMLGHRLKKITDDEGKSVIKCAQH
HHQLYVYAAYVNKTFQVVLFFQMFMSVSMVCFSIFQLSQTRLLGKMVENFMLIFCPMLQIFYYCWYGNEVKQK
SLEVSDMIYASNWISLNENTKRTLLMIMLRSTFPMEIKSAHVMSMNVDAFMGVIKTSYSAYNVL
>LalbOr59
MTSLRVPLSMLSALGCLRPITWTSSFKKFVYLVYSVMAFGLIHSIVLTHVLDLILNVRSQDDLSDNLYITMSV
LGCVCKMITFWLNRKKIILLIKMLQKEPFSPRNDEELDIKMRFDKIIDMNSIQFMTVVQASMMVVWIGTPIME
GKNRKLAFRAWIPYNYSSAMLYTLTYLYQAVGLTFDTTMHVACDSLFNGLLICIYGQLEILGHRLQNIKGDQK
KSAKKCAWHHHQLYLYTASVNKNFRAVLCFQLIAAVSMVCFSIFQLTQTRLDGKVVEHVMFTICALLQIFYYC
WYGNEVRRKSLEVPDIIFASNWMHLDKNTKRILLVIMLRSKFPMEFKSARIMYVNLESFMAVIKTSYSAYNVL
KQ
>LalbOr60
MHTLPLSFALLTYVGFWRPTTWPVNSLKYWLYNLYSVMMTFLLYIFTFYALVDAIISKDLDTTTDKFSLSISV
LGVSIKVANLFIQREKIVRVVNSLLLKNHVPRDEEEVVIQRKFDAHARKLALYCEILNESAASFGTVAQIDHF
ISTKTLPISNWAPYDLTSPPIYFASLIQQSLGLMVCANTSVGHETLISGLMIQISAQFEIFCHRARALPALLT
QAERESGSIAELKMKRQRILRDLIEHQMRIYKFAENVNALFTFVIFLQFTVSSTVLCLTIYKMSTASSFGLNF
AWSLSYLCCMLMQVYLYCWYGNEVTLKSKNVCDAIYEMDWTSLPANMMKDLLMIMMRSRKPVKMSSAHIITLS
TESFVTIIKLSYSSFNILKDS
>LalbOr61
NIMNVENYISINLFVLKFVGLYPINIIRYIISVCCIILIVIPLAAHIYKNLDSLNILLETSSVLLTISLAILK
SLIWFSKRKKLELFIDFMLTDYWRLGETTVFEHLQEYAIHAKKVTRSYLFLIVNALLFFFSVPIIEIFINAAY
VSNNNSTVLRDFPFVASYPLAFYSFPFYETVYVSQMVATSVCGLMILATDTLIASALLHTCGHFQILKENLKQ
SYFGKFCDNMEKNFHLMLLMQTVASSLILCFVALQVTTTLMDHSKVMKYASHLMMALFQLLLFCFPGDLLISQ
SSGISQAIYSIQWYRVSAFLRCEASMVMLRSQKPSYITAGKLYIMHLENFSATLSTALSYFMMFRSFNAEA
>LalbOr62
YPNYYHMLRITGLWPDDQTFFKKIQRTIYVTLVVCTIMFQASTLRYADITMKSLIHMLSYASSILVTTLRYIG
IIIYFPIYKEMFDSMERDRNAIEDPVEMELVLNHVTQTRNVITALKALVYAAVVILITFFILVPAIFLPQSQI
RYLFVGTETTMVAIYLVFFSTAILVVIICTELLLAVMSSYFCGLFEIVSYRLETTVNKMAESRYQNGLIDIRS
PVDLHQHVLELTALFMEGVTLPYLMTIPVVIGSFSLSIYRLYLSFMALEDVVNFLIGLIIFSAHVLIIFLNNH
SGQKLMNSSIEIFIKTYNALWYRIPPKSQKMLLFVLMKTVTEVQFNLAGLFNPCFTGFATMMSSSFSYFTALL
SF
>LalbOr63
MEVFEGNLKLYHTLTCYTGLWPYDKSILTSIQRFAFVVMSFTCIANQISTIKYTKLSLEDIIVLISFGCTLLL
YFSRYSTSLMIFPLMKFVFDSMQKDYAAVKNNPVEVQMLMEDSIVAKRIVQAYFSMVCTGGLCLVGTLGVSAL
VGSDVQLHFLNLLGFFYTERSLLSAISCWHIVLTTTYGLLILTVTEGGITVFATYISGLLKIASYRLKNAINA
VAYSDTIKEIDIRAAMDAHCRAMLYTKKYLADLTVFALFLAILVTVSYAINIYRFFISITEFSDKDAVLLSIH

FVVTYTTFICGNNYSGQILLDTSAALFHDTYNSLWYRIPPRMQKMVLFTMMKTQAAVELNCAGLFTPCYEGFA
TMMSSSFSYFTLLCSV
>LalbOr64
MEAFQKNYSAYYSVLCFIGLWPYEQSTLSTIKRTVVSLLTLCCIAVQVFSIKYVEMTLHNLLTMLSYTGPMLL
FFLRYVGFVVNFAVMKSFLENMQHDCDVLVNPNEVKILLKYINKSRLIVYMFLGMSFVGILAVLVLFLIPTLL
HLKYQVRIIQLFGFFYTERDTRTDLICLHLALTSVLGLLSVSCTEASLAVYSFYLSGLFEIVGELTSRYLYSY
VTVTIYSYIISLLYYVYHVSDVMEFNERLVIALGKNVIISYMLAILTVVGSFAVNLYRVFLSIMENYSPEEIV
VPILVTLIHLLIMFLNNYSGQSVIDSSSQIFIESYNSVWYLIPPESQKMLLLVLVNSAKAVKINLAGLFIPSY
EGFSMMMSSSFSYFTVLLSV
>LalbOr65
MEAFQKNYSVYYSVLCLIGLWPYEESTLSTIKRTVISLLTLCCIAVQVFSIKYVEITLHNLLAMLSYTGPMML
FFLRYVGFIVNFSVMKSFLENMQHDCDVLVNPNEAKILFKYIDKSRLIVYMFLGIPVVGILFVLVVFLIPTLL
HLKYQVRIIQLLGFFYTERDTRTDLICLHIALTTVLGILLVSCTEASFAVYSFYLSGLFEIVGYRIRSTVEKA
ARLASTDPIDIGPAVDMHHRAHGLANGFSKNIVVSYTLAILTVVGSFAVNLYRVFLSIVENYSPEEIVVPILV
TVIHVLIMFLNNYSGQSVIDTSLQISTESYNSVWYLIPPKSQKMLLLVLVNSLESVKFNLAGLFIPSYEGFSM
MMSSSFSYFTVLMSV
>LalbOr66
MHKIRSLFIYNRLYLHGQFMWEYFYNFFENIPCFQVFSIKYVEMTLHNLLTMLSYTGPMLLFFLRYVGFIVNF
SVMRCILENMQHDCDVLVNPNEVKIFFKYINKSRLIVYMFLGMSFVGILAVLVMFLIPTLLQFKYQVRIMQLF
GFFYTEYDTRTDLICLHITLTTVLGLLSVSCTEAALAVFSFYLSGLFEIVGYVQNNMHRLFKFIREFLLINVS
DDCITRLASTDPIDIGPAVDMHHRAHGLAIAFAKNILVPYMFAIITVVGSFAVNLYRVFLSVTENDSWEQIAV
PILVTLIHLLIMFLNNYSGQSVIDTSSQIFVESYNSVWYLIPPKSQKMLLLVLVNSLEAVKINLAGLFIPSYE
GFSMMMSSSFSYFTVLLSV
>LalbOr67
MDIFRQKYSIYYNVLYVTGLWPYNQSIPSKILRMVFPLLTLCCIAIQVSTLRLIEMSLYNILMLLSYCFPMML
FFLRYVGFVVNFPVIRCLYENIVKDYTTLSNPIESEMLTKQIVETRRVVLLLLALSLGVVLFVVATLLVPTLL
HSKFQTHYLRIFGFFCNETGLQTDLICYQLVLVSTIGVIALAGTEATLAVFSFYLCGLFEIASYRIQTAVKEM
SLSTTPTSLNISSAVKIHQRAIKLAADLTRNMMVSYLVAIVTVIVSFAVNLYRLFLAVMQLDELDNVLISFQF
VLVHVIIMFSNNYSGQKLMSTSGQVFHGTYNSMWYRIPPKSQKLLLFILMKSAPEVQFNLSGLFVPCYQGFTA
MMSSSFSYFTVLYSAQ
>LalbOr68
MDTFKQNYTTYYNVLNFTGLWPYNHSLVAKVQRVVFAVLTLCCIVIQITTLRMVDMSVYNCVMVLSYGFPMLL
YFLRYVGFVVNFPVIRSVFENIEKDCATLKNPVEAEMLMNQIAETRRVIAALVGLMCLGVLILFSALIVPTIL
ESQLQIYYLNVFGFLYHERNHQTDIVCCQIVYISTIGLLSVACTEASLAVFSSYLCGLFEIASYRIQTAVNTM
ANSEKSNMLIDIRSAVEIHRRAVELSSDLTSNMMLSYLVAIVAVVASFAVNLYRLLLAMSELSKPGNLVITLM
IVLVHVIIMLLNNYSGQKLMTISVEVFHNTYNSLWYCLPPKSQKILLFILMKTATEVQFNLAGLFVPCYQGFT
TMMSSSFSYFTVLLSV
>LalbOr69
MDTFRKNYNTYYNVLCFTGLWPYDQTLLTKVQRVCYILLTVCCIGVQVSTLKWVDLSLYNMLQLLSFAFPMLL
FALRYIGFVVNFPVIRELFDNYDSDCASMNNPIEAKIITNQIIEAKRVIMALLGLSCAGICLAILVLLVPVML
QSSFQIRFLNTLGFFYLERSQETNLVCCHIFIVTSFGLLTVTCTESSLAVFSSYLCGLFQITSYRIETTVNAM
AQSGNQNGLIDIRSAVKLHQRAVQLATNLTDNMMLSYLVAIIAVVVSFAVNLYRFYLAVLELEKVDNFIVSLQ
IFLVHVLIMFLNNYSGQRLISTSVEVFHKTYNSPWYRVSPKSQKMLLFVLMKSITEVQFNMAGLFTPSFEGFT
MVIN
>LalbOr70
MKTNFQSVNHWNNLMNMLSGNFLPVTSEGTKMPVILKLYVAMIWATELTYLSACIAGIFYVPKEKALRDSTVN
LVVTFDIFFFLPILYSNKDSLKRLIGTLNKIFAANGPMLRTIVTDLMAPVMRVQQIYIIGSTAALFAWTMLPL
ITIFQKNQFYCTDYQVPMILAKEPFTVAIFVAGTALQVVGGVITFIRKASLDVYTIHWIRLMTAQYKYIELKF
ISILEMDSTSMAKDTVTQEVRSLTRHHKTVVKMAAVLKQIFSPNIAALYINNVFRFCFLSIMVITSADAVLRG
FILLYTCGALMQLYMMCFCIQHLLEASTTMMDSVFHKKWYDHDISLQRSVMSMTLATKLKCQLSRIRSIDLTL
PSFMSILNQAYSVCLLFLKSRR
>LalbOr71
MRGRSSNQVKSFNDDYENDLNYTLGMCRLLLKPLGVWTFIYDRGSRLERVLSIPLMLICFSSLFFIILPSVYN
MLFMEEDLQNIVKLLGPISFCMFSTIKYCLLGMKGNTLGQCILHLERDWKMVQDPDHRAIMLKQASTSRFLIT
ICVGFLYSGGMSYHTVMQFLSKDRTNTNVTLRPITYPCFNFLDTQSSPTYELVFFTHCVTAMVMQTITTAGYS
LAATFVTHMCGQIQIQISRLDNLIGEHREKNTFQERIAVIVRDHAEVLRFSKDVEEGLREICLTEIVESTLIM
CLLEYYCLMEWANNDTVAILTYIMLLVSFTFIIFIFCYIGELLSEQCSQMGPAAYKIHWYNLSSKNSYDLILL
CAVALYPPKLSAGKIMDLSINTFGTVVKTSVIYLNLLRT

>LalbOr72
TMMIERELDSSSDYSLQVNRWYLKSIGAWPSSSFAPRFERIVSVVLIIICYCSMLFTGIPCILHILLEDEDLR
KKLRATGPLINMFMGSIKYSTLLFRGNEIRDCVEHIQVDWQIVSRVRHRLVMTSYAKFGRYVSVFCAAFTQLG
VASYCFVNALSTRSVQIGNETRIVHVLPLEFYKKLLNIDESPTYEIVFVSQFISTSIVNFSSVGAFSLAVTLA
AHACGQLNILMSRIIETVNGERDRDAKILLNETGVVVENHLRILSFISQIEEVMNKICFTEMFHTSLCTCMLG
YYILTEWDDRDYQNLSCYFMILFSMTFNVFLLCYIGQTLTEQCKKVGEVVYMTNWYFLPRRCILDLRMIIVRS
SVMIKITASKMFHMSVYTFGQIMKSSFAYFNLLR
>LalbOr73
FDHSYPDGHFTNPSYKSDVKYTLEMCRWVTKPIGVWPFVYSRTSKVEKLVAIVLLTVCFSDLLFATIPPGHYL
IFVEKNVYVIVKFLGPLGFCISSAIKYLYLVFKGNLFKRCLVHVERDWKTVVDQRYRRIMVRQSSVSRQLIVL
CAIFLYSGGMSYHTIMPFVSKPKMRGNQTLRPLIYPGYDAFFDSQASPTYEIIFSMHCLSGFIKYSITTGAYS
LAATFVTHICGQVQIQIARLENLVESIQKNSDRNPVAVIVREHVEILRFSKNVEEALREICLTEIVESTFILC
VLEYYCLVEWQNSDVVAIFTYVMLLISFTFNITIFCYIGELLTEECSKIGPAAYEIDWYNLPYKKAHDLILLS
VVSNYPPKLTAGKIFTLSLNTFSSVMQSSVIYLNLLRTVMD
>LalbOr74
KPRNPYYEKDIVHIFKISRWILNSIGIWPSLLENSKQFLPKIVIGLCNVLLLFAIIPFSLYLSIEVKDVVMRL
KLIGLFSFCSVALMKYWALTACKPNLKYCIEFVQHDWKQVEHSEDRELMLKYGNVGRNLTILCLVFMYSGGFM
YHTIMQYAIGTFVDEHNRTIKPLVYPTYSGLFDSQASPFYELVYAVHSVTGYVIYSITVGACGLAAMFATHIC
GQIDIMILRLQNLAQLEENTDLHPRLVRIVQHHIRMLRFSTVVGTLLQEVCFLEFIGSTFMICLLEYYTITDW
EVNNTLSLITYTMLLLSLMFNIFILCYIGDLLVEKTGNVGSLCFMIDWYKFPVETMRSLILIIAMSNNPAKIT
AGHIADLNLSTFGGILKTSLAYLSFLRTAVM
>LalbOr75
QNTDKPRNPYYEKDIVYIFKIIRWILNSLGIWPSLFENSNQFQQNVSIGLCNVVLLFAIVPFTLHLTVEEKSW
KARVKLIGLLTFCWISLFKYWSLVSCKPNLKYCIEFIQHDWREVEQREDRQLMLKYGSVGRNLTILCLAFMYA
GGFMYHTIAQYAIGSYVNEHNETIKPLVYPTYSGLFDPQAWPFYELVYAVHSVNGYIIYSITVGACGLAALFA
THICGQIDIMILRLQDLAQVEVYADLHPRLVRIVQHHIRTIRFSTMVGTLLQEVCFLEFIGSTFIICLLEYYT
ITDWEVNNTISLITYTTLLLSLMFNIFILCYIGDLLVEKTGNVGSLCFMIDWYKFPVDTMRSLIMIMAMSNIP
TKITAGHIADLNLSTFGGILKTSLAYLSFLRTAEM
>LalbOr76
MHDRSRDTVVRTLKKYHEEDDIDYALEMCRWVLKPLGMWSLVYNRTSRWERTVSIILLVTCFLSVILLIVPLY
WEIILGDIGGKARIKRLGATCNCTLSAVKYMYLVLRRATFASCIEYLENDWRTVKNQHHRAIMMKQATFSRSL
VMLCIIFIFCGGISFHVMLPLSRRRVVGNYTLKALAYPGYDRFFDIQSSPTYEILYCIQCFGGFVRHNVTTAT
FALAVFFVTHICGQVQIQILKLEELNIDSEEKNCDMVGEIIQSHTEILRYAKIIAESFSEIIFMEIMASTFLI
CLVEYCLLTEWIDSNAIAITTYAVYMISLTFNAMIFCYIGQILTDQCSQIGFASYQVNWYNLPPTQARNFVLL
GTISLYPPKLSGGKVTELSLNTFTSILKSSMVYLNLLRTVT
>LalbOr77
NSNYKSDVNYTLEMCRWVTKPIGVWPFVYSRTSKLEKLMSIVLLTVCFSDLLFSIIPPGHYVIFVEKNVYVIV
KLLCPIAFGSSSAIKYLYLVFKGNVFKRCLVHVERDWKTVVDQRYRRIMVRQSSVSRQLIVLCVIFLYSAGMS
YQTIMPFVSKPKMRGNQTLRPLIYPGYDAFFDSQASPTYEIIFSMLCFSGFIRYSITTSAYSLAATFVTHICG
QVQIQIARLENLAASSQRNTGRNPVAIIVREHVEILRFSKNVEEALREICLTEIVGSTFILCMLEYYCLVEWQ
NSDVAAIFTYVLFLISFTFNITIFCYIGELLTEQCSKIGPAVYEIDWYNLPYKKAHDLILLSVVSNHPPKLTA
GKMFTLSLNTFSSVMQSSVVYLNLLRRVID
>LalbOr78
KPRNPYYEKDIVYIFKHSRWILNSIGIWPSFLKNNNQFQQNFSVGLCNIVLLFAILPFTLYLAVEEKNLMIRV
KLFGFLSFCWICLLKYWVLIACKPNLKNCIEYVQHDWREVEQREDREVMLKYGNVGRNLTLLCVVFMSSGGFM
YHTIAQYAIGSHVNEQNRTIKPLVYPTYSRLFDPQPRPFYELVYVIHSLNGYILSAITMGACGLAALFATHIC
GQIDIMILRLQNLTQLGANTNLHPKLVKIVQHHVRTLRFSTAVGTLLQDICFLEFIGSTFIICLLEYYTITDW
EVNNTISLITYTTLLLSLMFNIFILCYIGDLLVEKTGNVGSLCFMIDWYKFPVETMRSLILIIAISNNPAKIT
AGHIVDLNLATFGGILKTSLAYLSFLRTSVM
>LalbOr80
NPNYENDINHVFGLSRCILKLIGVWSLFEKQSNTVEHVVSFLVRITLLCIQAFFVIPICLHIFFVEQNPIMKI
KLFGPPSNCVFTVIKFFCMVFNGTTIGRCIEHIKNDWKKVRNPQHRDIMLKQISISKNLTILCFIFIYTAGIS
YYTIIPYMNKHRLKGNYTMRKLAHPGYDRFFDVKSSPTFELIYSAHLLSGFFRYNVTAASFSLTVIFVTHVCG
QIQIQLLRLKELYQENMEKNDGPDPLAIIIHDHGSTLKLASDFRESLNELLLTEIFGCTFSMCLAEYCCLMEW
NDMNPVAITTYLIYCTSFSFNAMIFCYIGELLTEECSQVGFASYDVSWYNLPPSKASGFVLLNLTSLFPPRLV
AGKVIKLSLNTFSVIVKTTVVYLNLLRTVT
>LalbOr81

ENSNYARDVNYTLEMCRWLLKILGVWTLVYHRISKSDRALCTATLATCIGILSFTILPSLYSVIFTKNSTDTI
IKLLGPVGLCSFCTVKYMYLILKGKSLGRCVQHLERDWEMVTHPGHRSIMLKYSSISRKLIVLCVVFLYSGGL
SYHTVKQLITKDESTLNSTERPFVYPCFEFINPQASPNYEIIFFVHCLSGLIQYTITSAMYSLAAIFVTHICG
RIEIQISRLDELIEGSEEKPIFYDRLGIIIQEHAEVLRFSKDVEAALRELNLTEVAESAVIMCVLEFYCMIEW
RRSDAIALLTYFTLLVSFTFNIMIFCYVGELLSEQCSRMGPASYDIQWYNLPPRRAYNLILLSAISLEPPKLT
AGNIIDLSINTFGAVVRTSVIYLNLLRTVT
>LalbOr82
MRDRSCGQVKTYNVHYGSDLNYTLEMCRWLLKPLGVWTLIYHHVSTAERVVSLFLQLTCFSSLLFVILPSAYN
MFFMEEDVQNIVKLFGPVGFCTFSTIKYCILGIKRNMFGRCIKHVEKDWKIVRDPYHRAIMLKQASISRFLIT
VCVLFLYTGGMSYHTVMQFLSKDKTNANVTVRPITYPCFDFLNTQSSPTYEIVFFLHCVSALVQYTITSAGYS
LAAIFVTHICGQIQIQITRLNELIDDDGGEKNNFPDCIGNIVRDHAEVLRFSKDVEEALREVCLTEVIESTLI
MCLLEYYCLMEWANSDTIAILTYVMLLVSFTFNIFIFCYIGEILSEQCSRMGPAVYGIQWYNLPPRKAYNLIL
LNAIALYPPKLTAGKIIDLSLSMFGTVVKTSVIYLNLLRT
>LalbOr83
FYSVQINRWLLKPIGIWPLTLCSGTAEKISIIMLELISIFLIGYLLVPCTLCAILEKNGDLDAKIKMIGPLSF
CVMAAVKYCILLSRGEQISECIRKIWSDWGRCVSPGDNQNREIMIESARFGRFLAVFCAGFMYSGGFFYTTVM
PLCSKRTEIIGNETVRSLAFPIYRGLIDPRTSPSFEIAQLMQCLAGFVIYSVTVGACSLAAVFVMHACGQFKI
LSVRLNQLVNSATVSTHRKSAEDCLGDIVQHHLRILGFISKVEGLLNEICFVEFVGCTLNICFLGYFVLTEWE
QSDTVGAMTYCILLISFTFNIFILCYIGEILSEECIKLGLTAYMINWYRLPGRKALGLVLMLAISNSSTKLTA
GKIVNLSLGSFCRVLKSSLAYLSLLRTLTT
>LalbOr84
LGSPSDYSLQVNRWYLKPIGAWPPSMSTSRLERFASVMLIILCYCCILFTVIPCMLHILLEDIEVNKKLRAFG
PLMHWFMGGVNYTVLLIRGNKISDCVQHLQADWQIVTKVQHRLVMTRYAKFGRYVSVFCAVFMQGGVLSYCFV
TALSTRLVEIGNETRIVHILPCEFYQKLLNTDESPANEIILVSQFLSAFVVNSSAVGAFSLAAALAAHACGQL
NILMTQITEMVNRSQDHDTNILLTETGIIVENHLRILSFISQIEAVMNKICFSEMFHSSVCTCMLGYYILTEW
DDRDYQNLTTYFMILFSMTFNVFLLCYIGQILTEQCKKVGEVVYMTDWYCLPHRCILDLGMIIARSSVMIKIT
ASKMFHMSIYTFGQTMKSSFAYFNLLRQT
>LalbOr85
LDSPSDYSLQWNRWYLKPIGAWPPSASTSRLERFISFVLVIACYGSILFTVIPWTLQICLEEKDLHKKLRALG
PLVHWIMGGVKYTTLLTSGKEILHCVEHLQADWQIMSKVQHHQVMLRYAKVGRYVSVFCGAFMQGGVLSYCLI
TGMATRPVEIGNETRIVHVLPFAFYQKLLNTDLSPANEIVLASQLVSGFIVNSSAVGAFSLAAVLAAHACGQL
NILMIRITEMVHEARDRDTKIFFEQIGVVVENHLRILNFISHIEDVISPICLSELFHNSLSTCMIGYYILTEW
DDHDYQNLTTYFMILFSMTFNIFLLCYIGEVLTDQCEKVGKVVYMSEWYYLPYKCILDLRMITVRSSVIMKIT
ASKMVHMSVYTFSQVMKSSFAYFNLLRETT
>LalbOr86
SDYSLQLSRWNLAPIGFWPPSSSQSRLKRVASIAMVVVSYTLIMLTVIPSMLYIVLSDDNVRQKLRVLGPLTH
WFVGGCDLTVLLMKSKQIRLCIDHVEADWQIITRLQDQQVMQKYTKFSRFISMVLTIWWHSSVVISCAATAMT
IQAIKIGNETRLVHPLPCGFYLKLLDVYTSPANEIALAVQFSSVCIVNAAILGSHSLVAVLVAHACGQLKVLT
SWITEFVNDFGQDRKDEFFMEIGIIVEHHLRALSLMSRIEDVMNRICFMGLYECTLDVCLLGYYILTEWADHN
IPNLSGYFGIIIAIIFDVFVVCYIGEMMSEQSIQIGDVVYMTNWYCLPSKTVLDLTLIIRRSSVLIKMTAGKL
IHMSIYTFADVIKTSFAYLNFLRQTT
>LalbOr87
VHYIKDYNYSVQVNRWFLKPIGIWPIGPDATRSERIFSSLLNIACHSLVVFTFAPCVMFILFEETSLDARIQA
IGPMSHWLMGELNYCCLSFRTNDIFRCIRHMKTDWKTIEKSSDRELMLKDAKVGRSIATIAAVCMNLGVFSYN
FVTGFQKLEFHVGNDTYSMLRLPCPFYSNLMDVRHSPANEIVYFLQLLSGLIVNSVTVGACGMAGVFAMHACG
QLNVITSQLNNLVTPNEEQTFAKKKLTSIVERHLRTLNFVWYIEKIMHMICLVELMGCTLNICMLEYYMLTED
STERMATYAILYVSMIFNIFIFCYIGEKLTTQCLQVGEKAYMTQWYRLPHKTATGLILLISRSRMVTRITAGK
LLPISISTFGDVFKTSFVYFDMLRRLTM
>LalbOr88
DNDYKKDVDLSIEWNRWILKPMGVWPRSRHVSQLEVCFNWLINAVCYSLISFVFAPCYLYVSLEVEEVYEKLK
LSAPLIFCVMAYIKYYSLMAHTNDIRECIKCIEWDWRNVSHSEDREIMVANAIFGRRLVKICTFFMFSGFVFF
YIAVPMSVGKIAIEGANRTFIPLMYPVSKLIVDTQRNPSNQIFFSIQFVGGILMNANAAGACCLAVAFAVHAC
GQMQVLMCWMSHLVDGRVDMSNTTNGRIAKIVSQHVRIQKFLTVMEKALTQVSLVELLGCTVDICLLGYYIIV
ESRAKDMTAVATYAIILVSLIFNIFIFCYIGELVAEQYRKVGQMSYMIDWYRLPENTKLSVVLIMAMSSSPTN
LTAGRLVKLSLVSFSDVMKTSVAFLNMLRTLT
>LalbOr89
DNNYKKDVDLSIKWNRWILKPMGVWPRSRHVSQREVCLNWLINAVCYSLISFLFAPGYLYVSLEVEEVYDKLK
LYGPLMFYVMAYIKYYSLMAHTNDIRECIKCIDCDWRNVIHSEDREIMVANAIFGRRLVKICTFFMFSGFVFF

YIAVPMSAGKIAIEGTNRTFIPLAFPVSKLIVDTQRNPSNQIFFSIQLFGGMLIHSIAAGVCCLAVAFAVHAC
GQMQVLMCWMSHLVDGRVDMSNTTNGRIAKIVNQHVRIQKFLTVMEKALTQASLVELLGCTVGICLLGYYIIV
ESRAMNMTAIATYAMILGSLIFNIFIFCYIGELVDDQYRKVGQMSYMIDWYRLPKNTKLSVVLIMAMSSLPTK
LTAGRLVKLSLASFSNVMKTSVAFLNVLRSLT
>LalbOr91
SNTLTRHVDYKKDVDLSIKWNRWILKPMGVWPRSRHVSQLEMCLNWLINAVCYSLISFLFAPCYLYVSLEVEE
VYDKLKLYGLLMFCVVAYLKYYSLMAHTNDIRECIKCIEWDWRNVSHSEDRGIMVANAIFGRRLVKICTFFLF
CGFVFYYIAVPMSAGKIAIEGTNQTFIPLAFPVSKLIVDTQRNPSNQIFFSIQFFSGMIVHAIAAGACCLAVV
FAVHACGQMQVLMCWMSHLVDGRVDMSNTTNGRIAKIVNQHVRIQKFLTMMEKALNQVSLVELLESTVGICLL
GYYIIVESSSEDMTVVARYAMILASLIFNIFIFCYIGELVAEQCRKVGQMSYMIDWYRLPENTKLSVVLIMAM
SSSSTKLTAGRLIKLSLASFSDVMKTSVAFLNVLRTLT
>LalbOr92
ISLTKEFEHRTNVNLSIQWNRWILRPMGVWPSSGTDSGFEKCSNRLMNLVCYSLISFLFVPCWLFMMLEVENL
YDKLKLFGPLSFCVMAYMKYYSLMTHANDIRECIKYIEWDWRNVKYSKDREIMVANAKFGRRLVKICMFFMYS
GFVFYYIALPIRVGRITVEEQNVTFIPMVFPMTKFMPDTRHRPLNEIFFSIQFFGGIVIHGVAAATCSLVAAL
AVHACGQVKILLRWLEHLIEGRPDMSKSVDKRIAGIVSQHVRILKFLSITEMAMRQISFVEFTGCTLNLCLLG
YYAIMEWDPNDLTATVTYITILVSLGFNIFIFCYIGDLVAEHCNRVGEVAYMIDWYQLWGNRKRCLILIIAMS
NCSTKLTAGKMVELSLSTFGDVVKTAVGFLNMLLALT
>LalbOr93
KNMDLSIGWNRRILETLGVWPLSYSNSGIHRCIRWLKYVVSYFFVSVIFIPFGLFGIFEVEGAYNKLKLFGPT
SYFLMVYMKYFNLINKISHFRTCVEQIESDWKNMSYSEDRDIMVESAISGRRLVKICCFLSYIGFVFYYVAIP
ITTGRVTDPIHNLSFVPTSFPVAQIVADIRYSPVNEIFLAGQVLGGFVVHGIAVGACTYAAVFAVHACGQVKI
LLSWLEHLTGGRTDMSESVDDRIASIVSQHVRILKFLSAAEEALQQVSFVEFIGCTLNLCFLGYYILMEWDIH
DITTVVSYGVILISIGFNLFIFCYIGELIAEQCSRVGEVAYMIDWYNLQGKKKQCLIMIIAMSNSSSKFTAGG
MVELSLSTFGDVVKTAIAYMNMLLALT
>LalbOr94
MSKAEKPKGYQNIHHKSDFVFTIRVPRILLMLPGIWPLHRGDSILGDVKSVIQVGVMFLLMCYLLVPHVIYTF
HDSEDLTRYMKVIAAQVFSLLGIIKFWVMIFKKKRFRACITEMDLQYANVESEEDRLVMKNSAKVARLFTTIY
LGLCFGGAFPYHMIMPFLSEKVFKTDNTTQIPLPYLSNYIFFVIEDSPMYEITFAVQIAISNMILFINCGTNS
LIAFAELIESNLNTVFLTEMVGCTLIICFLEYGVIMELEDKNMLSMVIYSLLMTWMFLNVYILSYIGDCLKQE
SLKVKLMAYQMPWYEYSEEVKKNLRMIMFRPTRPTCFTAAKFFELSLQAFCDVVKTSAAYLNFLRTMT
>LalbOr95
TNYRNIHYESDIAFTVRVAKTLLTPIGIWPLRKSDTFLDRLKLYAHIGVTFTLMCYLLVPHVIYTFHDAEDLT
RYMKVIAAQVFSLLAIMKFWTIIINRKDIRYCLQQMEIQYRDIQCEEDRIVMKNSAHVGRFFTTLYLGLSYGG
ALPYHIILPLMSERVVKRDNTTQIPLPYLSNYVFYVIQDSPFYEITFVSQILISSIILSTNCGIYSMIATIAT
HCCGLFEVVSRQIETMLEYKEAEPYERLKEIIRYHLKAIEFAEMIENALNTVFLSEMLGCTVIICFLEYGVIT
EWSDHKILSTMTYVVLMTSIFVNVFIISFIGDRLKQQSARVGETSYFVSWYDLPENIINGLKMIILRTSRPTN
LTAAKLFDVSLLAFCDVCKTSAAYLNFLRAMT
>LalbOr96
YYPLYYNALRLTGLWPYDETLLMKALRFIYLILVACTMIIQASTLRYVDITVSNLIVLLSFGFCMVLTILRYI
GFTVNFPVFREMFDNMERDSNAIKDPAETELVMKHVIETRRVIVLLLVMLVMFFIVIPALFLPNSQTEYLHML
GFFYPETSMESTVVAIQIVFVTTIALFGVSCTELSLAVFSSYFCGLFEVASYRFETAVNMLANSENQNVLIDI
RSAVKLHQRALELTSMFTNELTLSYLAAILAVVASFAISLYRLYLAFLMLEDVENFLISLQIFMIHVLIMFVN
NYSGQKLMNSSIEIFTKTYNSLWYSIPPQSQKLMLFVLMKSITAVQFNLAGLFNPCFEGFNMMMSSSFSYFTA
LLSF
>LalbOr97
MEEFRKHFSLYSSLLCFTALSPYDQSLLGKIQRVVLPTIFLSALVIQVSTLKSVEKSLYNIMMMLSLSCPILL
MIQRYIGFAVNHKLMRTVFDNFSNDYSATKDSVELSIFERQMIVAKRVIFAFLGLTVVLIGYLFVVLLIPTLL
RSEVQLRYLHIFGFFYPEMSRQTDFVSILFILVMVMGLLSIASTESAVAVTSAYLCGQIEIASYRIEVAIDNV
LRSNTSELIQVRPVVEAHERAIELVSSLLYHMMNDMTVSYLIAIFTVVVSFAVNLYRLLLAVQNTQDFANIFF
SINDVLLHFVIMYLNNLSGQRVINSSSSIFNKICSSSWYCIPLRSQKLLLFMLIRTAKEMRCNLGGLYDLGYE
GFSMMMSSSFSYFTVMSSTQ
>LalbOr98
MQAFRRYYTVYYCALCFTGLWPYNESILSKIHRIAVSLLTFCCIVVQVTSIAHVEKTLHNLLTMLSYTCPMML
FLIRYIGMLANFSVVKSFMRSMQNDCDMLDNPIETQILIKHIEKGRRIIFIFLESKSMADYVNMVGFFYSQKD
AHIKLIYVHISVTLFMGLLTLACTEGLLSVVSHYLCGLFEIVGYRIRSTVDNAAMLISTDLINLGPAVDMHQR
AHELSDALGKGMLMSYLITIIITAVVSFAVNLYRTFLSLVKMEVTHPEEIFVCMLVTSVHVLIIFLNNYSGQAV
VNTSSQIFYDTYNSAWYRIPPSSQKMLLLVLLNSLNGVQISFAGLFIPSYEGLSTMLSSSFSYFTVLYSFQ

>LalbOr99
MDTIRKNCRIYYSMLYYAGLWPYDHSIRARVQRVVFIVLLISCIVLQVLTLRNVEHSMKNVVTMLSYTFLMLV
YFLRYSGFIANFDLLKSFLEDIDRDYKRLRNETEIEILSKQMQNSNRLSFVVCGQVVIVFLVYIVFLLGPTLL
QSKYYQLHYLHFIGYFYNDHSRTNDIVCLQIVVSGLIGILAIASTEATLGVYAFYLSALFKIVSYRIRKAVDD
SAMLISPGCIDLRSAVIMHQRAFRRADSVGTDMMISSLSALLAAVLSFSANLYHAFLSITEDSPIDEVILTVV
MVVGHALIIFVDNYSGQTLMNNSNKVFKETHNSLWYCVPVGSQKMLLFILMRSSTEVQFNLAGLFVPCYEGFA
TMMSSSFSYFTVLYSV
>LalbOr100
MDVFRRHYNTYYLMLRVTGLWPYDESIFPKIQRTVFSISTLCCILIQVSTIRQVEMSLSNILTLLSYTCPMLL
YWLRYLGFATVFPLVKTIVQSIQDSCIAIQNPVEAKILAKYIEKTRRIILMFLGLSIVGMSFIALRVLTPTVR
RSKYQLYSLRFYGFFYSEHSKQADWASVHVTLTSAIGLLTIACTEGFLAVFSLYLCGLFEIVGYRIRQTVDNA
AKLTASKEIDIGPAMEVHQTAFKLKAMMLSYLLAIVVVIVSFAVNLYRASLLMLEMEQLDEAVMSFLVVMTHL
VVMFLNNYSGQQLANISVNVFNETYNSKWYCIPPKSQKLLLLVLMKTANELKFNLAGLFSPCYEGFTMMMSSS
FSYFTVLYSV
>LalbOr101
MDVFQRNYNTYYLVLRVTGLWPFDESIFPKIQRIVFSIFMLCCILIQVSTLHHVKMTLGNILTMLSYTGPMLL
YFLRYLGFAANFAIVKNFVQYLQNDCTTIQSPVERKILAKYMEKTRHIFLCFLGVSIVGITFVSVRIIIPTIL
RSKYQLYSLQSFGFFYSKQTRESDWASIHLTVTSAIGLLTIACTEGSLAVFSLYLCGLFEIVGYRLRGAIDNA
HLITTKEIDIGPAMEMHQRALKLAQSVGSGMLVSYLLAIIVVIVSFAVNLYRASLLILEGQQLDETLFAILVV
LTHLVVMFLNNYSGQQLANISVNVFNETYNSMWYTMPPKSQKLLLLVLMKSATEVQFNLAGLFTPCYQGFTTM
MSSSFSYFTVLYSV
>LalbOr102
MGVIEKEYKTYCFLLRMMALWPFDNSPGSKIRRILLALVPLTSIIIQISLIPSEVTFKNILMVLTFGLPMLLF
FCRYVGIIYTFPTIRFIFENLQSDYNVLQNPIEVEILLKRAYSSRRVVHAFVCKNHSTVYGHVIICGPILLDF
LKPMNETRPHVLHSFGFFVHEGLSVYLVSLSLLATIIAGTLTIICSESTLFVVSQYCCGLFEIANYRFRSLID
ASVYERDEARQRYLMKGSVRHAVEIHTKAIDFVRMFGYDARIPYLIAIVVVVVSMSLNLFRSILAILEMEDSK
ELCISFFFGIFHIVAVFMGNYIGQEVIDSSVAISYATYDSLWYSSPVKIQKMVLLIMQRSSAGTMLDFSGLFI
PSNKGFASMMSSSFSYFTTICSLK
>LalbOr103
HHKLIKFLLSAVGLWPYERTYFVYVKRAAYALLVLSVIVFQVAAFLHEAVYTQNTIVMYVTNVTLVFIIMYKH
TSFLYRIEFVRELCDSVARHRSSITDENERRIIFTHIADVNAKNRSFAIIFYLSVCGYLLYELLTNNYNNFKD
SLDIHIKMYYFTDNPKFSYVLYLHLILLMLPAMAGLVGPELTSIVLGHHACALFHIVRYRVKTAFVESSGMSD
FEKDRLAMDKLISATKLHKVTIKYAGQLSKCLSSSYFLLMILGVISLSLNLLRMAQSLQQQGGLKELMVQLVL
ISAHIMYLCLINYNCQIVMNSSAELFTELCNCRWYTVSVRTQKLLLFFIHHSTKTYTFLVGGLFVPSFEGFGM
VTK
>LalbOr104
HYAVNKFLLTCIGLWPYERTWFTYAKRVAYLLIMVSALMIQVLSVLFFEVLSTNIIFDTLTNLTFTVLVLLKH
LLYLCNLEGVKEVLEMVRADWRRINDPYEMRILLKNADDGKLMGMILAAGIHVMVMSYVVFMTTMHGVSNSTA
SADDYMVYSHLKLIYTKNDEKYYLISLIHTYISVLFGLMALGGTEATTFLCGHHGFAIVQILRYHRIRKAFND
DEMDVIAPKKDRATVAKIIDVAIVHMRILRYKELLNDSLLVGYFYLLVLVMASMSLNLLRFAHAMLRFRKLAQ
IFPTTAVILGHFLYIGLTNHLGQTVINNSAEFFSDTYAGAWYRATVPEQKLLLFIMCHSINDAAIVIGGIFRP
CHEGFAAMTKLAVSYFMVMYS
>LalbOr105
LMTILGLWPYVSRNFRRIQSAVVNCICTSFVTVQITPLTTGEVDLTLLLQCLCFIVIFMGSIVKFNTYMAQIG
TVRTVMDGIKSTWDTEHEDTLEILRRRANLGKQQGVIYALFIYPSALLLVVFRTSSYFMVVTAPPNSNLTDPF
PVMSDYLIDEEKYFFLIMIHQNLTILFGATAYVATETLYIMWLQHSISLLELASYHIEKGVSERPPYISQEVM
EVYRKKCLATAVTYHIEAKAFLTYLKSKFTFSYAVLIVIAVFSLAINFFRLSTALSVTHNVEEMVLASFLSIS
ELTYMFYLNYVMQKVVDYADDLPQIIYSTPWYNRSVAIQKNLLIIILTRCSDPIVFDFYGFYTASIEGFSALVK
TTVSYFMMITSVQ
>LalbOr106
HYKLSIRLMKILGLWPYVNETFRRVQSIVVNLICTILIIAQIAFLRNDEIDLTMLLQSVCFIMITMGAAVKFN
TYMAQIGTTRIIMDDIKRMWNTTHEGTLKILVKRAILGKQQGAIYAIFIYPSAMLLAVVRVLSYAFVVTASSD
ANVTDPFPILTNYLIDEEKYFFFIMIHQNFTIFFVATTYVATETLYIMWLQHSTSLFELASYHIEKGVSERPP
NISQEVMEVYRKKCLATAVKYHIEAKAFLTYLKRKFTFSYAMLIVFGVISLAINFFRLSNALSVTHNVEEMLL
ASFLSISELTYMFYVNYVLQKVVDYAADIPRIIYSTSWYNTSVPIQKTLLIIILLRCSEPLVFDFYGFYNASLE
GFSGLLRATVSYFMMMTSLQ
>LalbOr107

HYKFTIRLMKILGLWPYVNVNYRRVQVILVNAFCTYFVTVQITPFTHDDADVFLVLQNMSLIIMTMGSIVKFN
AFFFAADTVRFVMDEVRSKWNTTHKDTLEILEKRAILGKQQGALYAIFIYPSALLLVVFRTSSYVLLVTASPD
SNVTDPFPVVTDYMIDQEKYFFFIIMHQNVTFFFVATIYVASETLFIMWLQYGIGLFELMSYHIEKGLCDRPS
HVSKKSMDAYRKQSFATAVTYHTEAKTFLRSLKNTFSFSYSVLLVFAVGSLAINLFRLSTAICETHDIEEMIL
SFLFSVSELVYMFYLNYVVQQSLDYADVLPTIIYSTPWYNRSVTVQKNLLIILIRCSTEPIIFEFYGFYNASV
EGFSGV
>LalbOr108
YKLSVRLMTILGLWPYVNVNVRRIQSTLVNCVCTTLVAAQIIPLRNNDMELVQVLQCLSTMIMVLGSVVKFNA
FLSGIGAVKSMMDSVKDRWNTTHQDTLDILERRAILGKEQGAIYAIFIYPSALFTILFRIASYVLVVTAPANS
NQTDPFPVITDYLVDGEKYFIPIIIHQCFAFFFISTIYVATETLFIMWLQHSIALFELGSYHIEKGISVGPFY
KEVSMEVMEVYRKKCLATAVSYHNEGKIFAMNMKDSFSFSYAVLLVFAVISLSINFFRLSFAICVSHNVEEML
LSLLFSISELVYMFYLNYVVQQLLDYAEEFATIVYATPWYQMSVTIQKNLLLILLRCNEPIIFDFYGFYNASV
EGFAGLVRATMSYFMMMTSLQ
>LalbOr109
MEEFKAIHSQLYNLQDFCGLWPISEPQNIFVYRVKQVVINATTILCIVIQYNTLANVELTLDTVLSTMSFSGP
MVLFFLRYGSYLCASKTMCRIFQEMADDFVKWKNTEEMKLLLEEVDEAKLLYYYYIRLVIFSVVGLSLAFIPI
ITQPKHQVAYLRYFGFYFTQQSIKTTLVCFQILFVYGVGIFCVLGTEASLSAICHHVCGLLKITCYKIKKTVN
DCSKSPTPLKVDVGPALELHYKAMSLELGLSYMVTIGTAIVSFALNLCRIFMAITQGKLDETVAPSLIILSHI
IMLFLGNHVGQRVTSTSHGIFNAVYSSEWYNIPLHERKLVNFIMLKSSKEIALCLISLYTANYEGFSMVIN
>LalbOr110
MKEFKEIHDDIYRLLCFTGLWPVHDSQFALQMMVKRIFIPTVTFACLVIQYKTLFTVELTLENVLSTMSFSGP
MVLFFVRYMSFVFALSTIRKLFIAVQNDFVTWRNTIEMDMFNEYIMKAKVLYYYYFLSFTVITMSLAMIPIIT
QPKHQAAYLRYFGFYYTESNINTACVCFHILFVYGIGIFCILGTEASMSVCCHHLCGLLKVTSYKITTAINDV
AKSTTQETVNIAPAIKLHLHAIELDLGVSYLVTIGTAVVSFSLNLCRIFLVLVETNHLDEAVVPFLIILSHVI
MIFLGNHCAQTVTNTSLEVFNATYNTFWYYIPLNARKLVIFIMMRSAKELECSFLSLYTAGYEGCSMMISTSF
SYFTVLYSV
>LalbOr111
MEEPAKSSIDYYILPNRIFCGMAGMWPIEDNSSTFSKLFAYFRLLFALTAVSSVFVPEILAIAVNWGDLKVLA
GVGCVLTSVGQLLFKMMYLLSRRKRMYQLYYELRRLWDSTDDPDERQPYVQLASWARILTIVFYSSCMCNVIT
FATAAAIDYFYMNHNVSEASNDNRHLPFEVWYGTDITESPKFEIAFICQFIASLICCAGISGLDATFMTIILH
VCGQFKLINIWINKICCDDNCLPQFKVDLLKCVQHHQRVINVVSEVNNLLAPIIFVQFLTSGVEICLSGFAVF
DNGTGTDLLKFISYLTSMGIQLVLWCWPGEILVQESQQIGHVIYLNLPWYNLPPIYRRQLCLIIVRAQQQCSI
SALTFQTISIRTLTSVFNTAASYFTLMRQMQQ
>LalbOr112
TNKDFSYAMRPMKTLSWPVGTWPLQKYNFSSGLRSFVSVILVLLMLLILHVEMYLDSSDAEKNLDAVLLIACG
SLAVWKVVSFRIHSKGLVSNFLSAQKDYAELEEQEKRLIVRHHAYMGRLACGSVIFFAYFSSTLYTTVALLAR
EDEDAEQILNVTQEDSFKYPLPSECTLDLLNLPASLYHVVYIGQYVLMLLTSTGNVGSDSLFFGIIFHLCGQA
EVLKLDFSKFVEAREKKARFDVLVARHQQLLKLSDHLKDIISSIMILQLFSSCLLICTSGFQFIRSLSTNNVV
MMVKTLVLVGTMLAQLFAYSYIGEYLKNQFEDVGYYAYCSTWYNLPQKLSFDLVFVVMRSEYPVHLKAGSFFV
INMETYMSIVKTSISYLSVLRVMI
>LalbOr114
NGKDFSYAMRPMKILSWPVGTWPLQKYNFFSGLRFFSRFLQLLMLLIVHIEMYLDSSDAEKNLDAVVLIACGF
LAVWKVVSFRIRSKGLVSNFLSAVKDYAELEEPQKRLIVRQHAYMGRLACGSVIFFSYFGSTLFTTAPMLARE
DEDAVQTLNVTQEDSFSYPMPSECTLDLLSLPASLYPVIYIGEYVLLLVTSTGNLGSDSLFFGIIFHLCGQAE
VLKLDFNKFVEAGEKKARFDVLVARHQQLLKLSDRLNDTISSIMILQLFSSCLLICTTGFQFVLSLSSNNVVM
IVKTLIVVSTLLAQLFAYSYIGEYLKNQFEDVGYCAYCSTWYNLPRKLSFDLVFVVMRSEYPVHLKAGNFFVV
NMETYMSIVKTSMSYLSVLRVMV
>LalbOr115
MELPTDKDFAYGMTPLKIVSWPLGTWPLQEYNFATGLKSVVTLILLLLMMLILHTEIYLDHGNAEKNLDAVVI
MACGMLAISKVASFRFRPAGLIANFVSAVRDYQELNDEETRAIVKRHAHMSRMASANILFFSYFSATLFMLVP
LFAGEEDTVENFNQTRKFRLNYPMPSDRIMELLQVSGNMYAIIYIGEFLLLVLLAAGNLGSDTLFFGIVFHLC
GQVEVLKLDFSRFLDDKENSGTRFNALIKRHHHLLTLVDHLNDTIGYILVLQLFSSCLLICITGFQFILSLHV
HNLVMLMKTFIVVNTLLTQMFAYSYVGEYSKNQFGGIGHLLYCSDWYNAPCNLSKNILFVLMKSQSPVHLRAG
RFFVINLETYMSILRTSMSYLSVLRVMI
>LalbOr116
IFELSVQLLMILFLHTEIYLDFGNAEKNLDAVVIMACGMLAISKVASFRFRPAGLIANFVSAVRDYQELNDQE
TRAIVKRHAHMARMLASANILFLSYFSATQFMMVSLFADEEDTEENFNHTGKFRLNYPLPSDCTMEFLQVSGN
MYAIIYIGEFLLLMLLAAGNLATCSSMIFLLRYHLNYANCGQVEVLKLDFSRFLDDKENSAKRFNALIKRHHH

LLTLVDHLNDTIGYILVLQLFSSCLLICISGFQFILSLHVHNLVMLMKTFIVVNTLLTQLFAYSYVGEYSKNQ
FEGIGHLIYCSDWYNAPCNLSKNILFVLMKTQSPVQLKAGKFFVINIETYMSILRTSMSYLSVLRVMI
>LalbOr117
MNLPKDKDFAYEMAPMKIVSWPVGTWPLQQYNYLTGAKSVTALFLLMIMMLIVHTEVYLDFGNAEKNLDALVI
ITCGVLAISKVACFRFRPAGLIANFFSAVSDYQELRGEDRRAIVKRHAQMGRTASANMLFFSCFSATLFSMVP
MFAGDDDTTEGFNQTRKDQLNYPIPSECTLELLQVPGNLYALIYIGEYILLLVTATGNLGSDSLFLGIVFHLC
GQVEVLKLDFSRFLEDEEDSKKRLTTLVNRHRHLLALAEHLNDTIEYILILQLFSSCLLICTTGFQFILSLHV
HNFVMVIKTFIVMSTLLIQTFAYSYVGEYSKNQFGGVGYFAYSSDWYNAPCCISKNILFVLVKTQCPVYLRAG
RFFVVNLETYMSILRTSMSYLSVLRVMI
>LalbOr119
MKLPEDKDFAYEMAPLKIVSWPVGTWPLQDYNFWTGAKSVIALILLVLTVLILQTELCLGEGDPEKNLDSVVI
GSCGLLAISKVAYFRFRPTGLIANFVSAVKDYQDLQDVERRAIVKRHAYMGRMASVNMLFFIYFGATMCVVTP
MFLRRGGVMESVNRTEEAKRSYPMPSTFTLELLHVPESMYIWIFLGEYILLLTIAAGNLGSDTLFFGIVFHLC
GQVEVLKLDFSRFLDERKNSVRRFNELVRRHRHLLLLADHLNDTIGYVLILQLLTSCILICVTGLQFILSVQV
HNVVMVIKTFIAMGTLLTQTFAYSYVGEYSKNQFGGVGYLAYSSDWYNAPCILTRNIIFVIMETRTPVQLKAG
HFFVVNLETYMSILKTSISYLSVLRLMV
>LalbOr120
DFAISLASIFLRCAGLWTSKNRLEQYSRTVATCFTIFLISLCIFVLSLDVFRGDQDVGGLLYALATMLCTTND
LFKVCVLVAYKKDFLDLLVHLRQHFLESEYDTNEKTILDACKRTCAIFVGSFTLFTNVTLIGYILVPLFLSIG
KHGSDRFFPFALPESPLYISPYYEIAFFVQVALIIYAIDLCHHSVDNILCIMNFHVATQFRILQYRLSNLKSE
NVCDGPLKRVPSAAENSYATFKRCVQQHKMLIAYCNKLEEVFGLVGFSQIIIFSMLICLNGLQVLMDSNVGRR
YTALFFLMTSTMQLLMFTYSCDGVIQESLKVAPAVYSSPWAYLPMTISGKMLRKDLVLVILRSRIPCCLTAKG
FFPISLETYTKVWTTAMSYFTLLRQTM
>LalbOr121
DFAISLASIFLRLTGIWTSKNRLEQYSRSVATCFTILMTTLCILMECLDVIRGDLDAGGLLYALVTILCTAND
LFKVCVLVAYKKDFLDLLVHLRQHFLESQYDTNEKTILDACKRTCAIFVGSFTFFTHVTLTGYILVPLFLSIG
KNESERLFPYALPQSPLYITPYYEIAFFVQALITYSIGLCHFSVDNILCIMNLHVATQFRILQYRLSNLKSEN
VCDGPLKRVPSAAENSYATFKRCVQHHKMLIAYCNKLEEVFSLVGFSQIFIFSVLICLNGLQVLMDTNVGRRY
TALFLLVTFFVQLLMFTYSCDGLIQESLKVAPAVYSSPWAYLPMSISGKMLRKDVVLVILRSRIPCCLTAKGF
FPISLETYTKVWTTAMSYFTLLRQTMDD
>LalbOr122
KGFSIVLSAILMKCVGIWFADNPFEERLRNGTLIYTVTAIVFGVWVQAEDLFHTWDDFGARTYIACNIMCLNI
ALLKISIISLHKKKFLDLVEYMDRNFWHSNYAPNEQIIFMSWKRICTYFICIFTFFTEASIMGYAIRPIVANL
GKNDSDRILPFNMWINLPWTLTPYFEITFVLQVLSLYHVGVCYICCDNFLCIMNLHVAGQFRILQNRLKNIHR
SQSVDGKHEKWNDCAEKRYYTFKRYVQQHQALIAYCAELEHIFSPIVLAQMLTFSLLICFVGYQAILADVPIT
KRIIFVNLLTSSMCQLLMFTYSCDCLIRESAAIATAAYSVPWMKFSMDKFGKQLRRDLHFVVMRTKRPCCLTA
NKFFAVSLETYTSVLSTAMSYFTLLKRN
>LalbOr123
MFGIGTDDFAISLASIFLRFAGLWTSKNRVEQYSRTVGACYTIFMVSLCILVESLDVFRGDQDAGGLLYSLVN
VLCTANDLFKMCVLLACKKDFLDLLTHLRQHFLESQYDTNEKSILDACKRTCAIFVGSFTFFSHMTILGYILV
PLFLSIGKNESERLFPFAVPESPLYVTPYYEIAFFVQALIVYEIGLCHFIVDNILCIMNLHVATQFRILQYRL
SNLNTENDCDGRITLESSATENSYMTFKRCVQHHKMLIAYCNKLEELFSPVMLSQIIIFSVLICLNGLQVLVD
TNVGRRYTALFLLATFFVQLLMFTYSCDGLIQESLKVAPAVYSSPWAYSSMTEYGKMLRKDVVLVIMRSRIPC
CLTAKGFFPVSLETYTKVWTTAVSYFTLLRQTMDD
>LalbOr124
MFGIDTDDFSISLASIFMKITGIWTSKNRIEQCSRTFAAYYTFFMEALCIFVQSLELVHGGLDAAGILYAAIN
ILCAMNDLFKLVVLFVYKKNFLSLLVHLQRQFLESEYDTNEKTILDACKSTCAIFIGSFTFFTHVTLIGYVLG
PLIVSIGKNESDRLFPFALPQVPFYVTPYYEITFFVQALLVYQAGICHFCVDNILCIMNLHVATQFRILQYRL
SNLKSENVGDDPVTRVQSAAENSYMTFKRCVQHHKMLIAYCNKLEEVFNLIAFSQILIFSVLICLNGLQILVD
TSVGRRYTSVFFLATFFVQLLMFTYSCDGLIQESLKVAPAVYSSPWAYLPMTIYGKMLRKDLVLVIMRSRIPC
CLTAKGFFPISLETYTKVWSTAVSYFTLLRQTMDD
>LalbOr125
DLSIAVTAFFMKATGLWTSKNRVEKWSRDVAAFYTIFTMSLFILVQSTDFIRGGLDFSAFLYSLVSILSVGNG
LFKMCVLFVYKKDFLTLVVHLRRQFLESDYDTYERPLVDATKKTCAVFIVLFTFFTNITALCYIVGPLMGNIG
RNETDRLLPFNLWQVPFSYTPYYEVTFFVQALLIYQSGICHFCFDNVLCIMNLHVATQFRILQYRLSNLKSST
VLEDSDTRVPSAAENSHSLFRRYVQQHQTLIAFCNKLEDVFNMIVLGQMLIFSLVICLNGLQVLMATTVAGRR
YTFLFFLMTSFVQLLMFTYSCDGLIQESLNVATAVYSSPWTYLPMSEHGKMLRNDVVMVIMRSRIPCCLTAKG
FFPISLETYTKVWSTAASYFTLLRQTMD

>LalbOr126
DFSIAITSFFMRVVGFWLSVDPVEQRRRDITLTYTVVLLLFAVYLHVADFYYTEDDFTAYLFISVNILTVFGG
LFKIFVLLPHRRDFFSLVHHLQHNFLNSDYDHHEKDIVNRCSSLCTIFIGLLTLSAHLTAFCYAASPLIANIG
RNESDRVLPFNLWVDLPVSMTPYYEIIFIIEVLSLYQICVAYFCFDNFLCIMNLHVASQFRVLQYRLTNLRYK
QDELNRLGDVKICMLHSTKDVYITFKSYVRQHQVLIAYCKKLEAIFNLIVLGDILTFSVVICLNGCQALVVGT
ITRRFIFLFFLLTSFVHLLMLTYSCDGVMRESVKVAAGIYNSPWCYLRFNKYGRMLRKDAILIIMRARVPCYI
TARGFFPITLETYTKVWSTAASYFTLLRQTLDT
>LalbOr128
SLFVTSFYMKLTGIWSATNPAEERQRRNSFVLAMTLLMTAVFVELMDLYHIYSTSFQEIIYIACNLATVGLVL
VKMSIVYLHKKQFVEIISYAENNFWHTNYDPCEKAFLDKCKHMCIVFICSFNFFAQGTAFSYVIEPCVEYFSN
NVTIRRLPFNFYNDFFLKPYIFECTFIFQTSCMIMYGIGYLCVDNFLCITNLQAATQFRILQYRIANIRKDND
TKEHSGISRSLPAYHADHCYAVFKDCIKQHQALISYCRKVEELFTLIVLGQVLIFSILICLDGYLMLLEGASP
YRRVVFTFHLIGTITQLLMFTYSCDCLLHESLEIAESVYTAQWPLLPMNNSGKMLRSDMQLVIMRSREPCSMS
AGGFFTVSLETYTKVLSTAASYFTLLRN
>LalbOr129
LAQTRDISIEWTCLLMKSVGLWLAADEAEQRRRNAALIYTINAISIATCIAIRDIYFSWGNISDCVFIMCNIM
YLMFVLFKIAVLYMHRAEFYELVRYTQKNFWHSDYSYEEKQILDDCKRMSGIFIIVLSFCTQGTCAGYMVTPL
IANIGKNDTDRLLPFNMWVSFPTGKSPYFETLFTIQILCVYHVGICYICFDNFLCLVNLQVASQFRILQRRLN
NLNSIVEAQTRNKESLSVYAKVYQTKLAHCIRYHQSLCEYCKSVENVFTLIILGQVLFLSLIICLVGYQLFLA
DTPSSRNVSLIMNLIGTLCQLFMFTYSCDGIIQQSSDVSKAILFGPWSIMPMDKAGRNLRKTVLLMILRSSRS
SCLTASGFFPVSLETYTGVLSTAMSYFTLLR
>LalbOr130
MLAKESDDFSRVVTAFFLKLAGIWPAYNPAEERQRRKAFMLTMTFMSFGTCVVCRELYYSYGVMFEEFVFSLC
NIVTITLILSKLMTVFLRKPQFFKLIAYMENRFWHTNYDSYEQALLDKCKHLSVVFICSFNFFAQGTAFSYSL
ESSVEYFSATNVTTRSLVFNLYTDVVMKPFIFEIILLLQYFVLVACGIGYICVDNFICISNFHVATQFRILQY
RILNVHKRTQTEIGIGTPSKLSADRADYCYTVFKDCIRQHQALIRYCEDMEEVFTIAALAQILLFSVLICLDG
YLMLLGESSPYRRLSFTFHLIGTMCQLWMFTYSCDCVLQESMRIADTVYETIWKNLPMNTSGKMFRTDLQVVM
IRSRVPCCLTASGFFIVSLETYTKVLSTAASYFTLLRQY
>LalbOr132
QMNFTESQYCKFPRLVCISLGLWPYTYKRHRFRPFCVYVILLSGVICQLLPLARCDRTAETSTALGILIISIA
FVSKFHGYYSNMEKIRHVFDYIEDDWKMFTNEGELETIMKYANRGKLLNLYMLGKRLEFSDRRIFSALVALFI
AYPFVAMAWTHQAQSLQIPIYIDYIVDHDRYVYWILLYIDVQTIVGTSTLLVADFASVIFMFHLCGVFRIVRY
SVLNARNGNKYSSSVRKQLIAALELHQRLLELIQYLNKSFSKHYFYLVFVGVAALSINLYVVAVFISLKKITE
KLLVAITLVCCSVFHMYCDNYLGQKVIDKGNELFMSTYSTRWYESPLPMQKTLLFVRLRCMKGSSYISTTLFV
ASMECLFVLTNTAVSYFMMLYT
>LalbOr133
TNSFSRSVEIGLQITGIWPSPYENLFRCVWVIIMCPTSIFQFWYIRNHLNHDSLVDLVECVSTTLPYILMCFK
LVVLWNKHGIFKNILIAMATDWTDTSITKDNIVTMVEKANLSNRCSQLVLSMYAMAVFLYSIVYINVFRNAGR
NSILTESADLLIKMQLPSAAYQRLNYQYTMIAQFIQLLFVAITIAIVNALMITLVLHVGGQVEVMHQALAVIH
TNGKGRILLKRAIAYLLEKHQKSIEFIKYINSLFSFIALIQILCNTINICCIGFLMVISFDSDQNVRIMIKIL
FFYLCITSEAFIFCVSGEYLSTKSTSINVAAYNSLWYLLKPADSRAMVLLMLRSQKQSTITAGRIVDLSLQRF
AGIMKASASYISILYTMY
>LalbOr134
MKTSSTVSKSVKFGLHFIGIWPGTPLSGLHKILWVIAMVLFQGYQYKSIISRCRTDSLVSLIDSLSIVLPVTL
VSIKLTVSWIHHDVLCKLLTTMEEDCEKYAAIDTHKLIPKAADLSYRLTTVTAVLYPLSAGFYAVGTFAFQQS
NDSTTRPLVVIMDLPFDTNESPVYELVVTTQFLFLTSSAFTFGIFSALLLMMVSENECIQSFKSHRKRQRASL
QIHAVRESCSKNLRIRAYGSANTDLGTRVAICETVGRTYVPAFVGYFRSLPLSGELLFCILKTVLLFCIICFL
VYMHCIFIFHEHVFSSKLIGNTAYESLWYNMQPINSRQLVLLILRAQKGLPLTFGKFSNLNLESFTRV
>LalbOr135
MTIRSTVSKSVRYGLHFIGIWPDTPFPGLHKFYWISTTIIWQFYQCKYMITHFKTDDLTKTIDCLSMTLPYTL
LIAKLSVVWINHNVLSEMLSTMEEDCVKYGMIDANNVISKTAEFSYRITSLVTVLYLSSASGVAAGNLAFPRY
NETVDRQLLSNMDLPFNTNESPTYELVVTAQVLHQMASAYVFGVFSALLVMLILHVGCIIDILCDTIVRISSK
EIEKLRLVALRHQEIIVFADRIEQLFTYIALCQLLLNTLVTCCLGFLAITAIKTEHGLPLLLKSSSFYAVLCL
EIFIYCFAGEYLNVKNQMIIDAAYQMTWYDLQPDISRQIVFIMLKSQKGLPLTFGKFSTLSLESFTGV
>LalbOr136
STISQPVKYGLHFVGIWPGTPFPGLHKLYWIVSMAVWQFSQYKYIVTHFKSNAFMEMIDCLSITLPYTLLFFK
LGIIWTNHGVLCEIISTMEEDCVKYAVIDTNNVISKTANFSYRLTGTIAFLYLASATFYAAGGLAFLRSNDTM
PRELLLNMDLPFDVTESPSYELVVTAQVLHQTASSYTFGMFSALLVMMILHVGCLIDILCDILAQISAQEEDK

LRFVAIRHQEIVDFAERIEKLFTHISLCQLMSNTLVTCCLGYLVITAVKSDNGLPLLLRSFSAYTVICLEIFI
YCCAGEYLNNKSTMIIEAAYQMAWYDLHPNISRQLVLLILKSQKGLPLTFGKFSTLSLRSFSRIMKASASYMS
VLLAM
>LalbOr137
MVSPSTVSKSVKFGLHFAGVWPGTPLPSVHKVFWIFFMVVLQTYQYEYIITRYKTESLMILIDSLSVAMPITL
VFIKLIVSWMNQGVLRDILSTMEEDCEKYAAMDTNNLISKTSVLSFRLTSVIIFSSVVSGVSYTAGTLAYPQG
SNSTTRALIFKMDLPFDTNESPVHELVVTAQFLHLASSGVTFGTFIGLLLMATLHVGCHVDILCNRVLDPSVT
EIEQVRFFISRQQEIIMFVEKIEKLFTYIALSQLLSNTFITCCVGYLLVISLSAETGLPMLIKCIVFYYVICL
EAFIYCFVGEFLDIKSEMIAKTAFDIPWYNLQPDVSRQLVLLILRAQRGLPLTFGKFSIMSLESFTGIMKASA
SYVSVLLAM
>LalbOr138
STVSKLVKFGLHFAGVWPGTPLPSVHKAVWIFVMGVLQTYQYEYIITRYKTESLMTLIDCFSITMPFTLLCIK
LIVSWTNQGVLRDILSTMEEDCEKYAAIDTNNLISKTSVLSFRLTSAIVSSCMVSSVAYTAGTLAYQEGINST
TRELLYKMDLPFDTNESPVHELVVTAQFLHLATAALTFATFIGLLLMATLHVGCHVDILCNRMLDPPVTDNEQ
VRFFISRQQEIIMFVEKIEKLFTYIALSQLLSNTFITCCVGYLLVISLSAENGLPMLMKCIVFYYVICLEAFI
YCFVGEFLDIKSKTIANTAFDIPWYNLHPDVSRQLVLLILRAQKGLPLTFGKFSMMNLESFTGIMKASASYVS
VLLAM
>LalbOr140
STVSQPVKYGLYFVGIWPGTPFPGLHKFFWVLSTTLCQIYQYKYVIAHFNTDGLADMVNCLCFALPYTMTMVK
LIIVWTYHRVLCDILSTMEEDCVKYAVVDANNLISKTADLSYRLTSTLGFFYLAAVSCHSATILAIPRSNNTM
DEKLVMAMDLPFDISESPTYEIVIAAQVIHLTSTAYTFGLFSALLLMMILHVGCVVDILCDVLAHVSAEGTGQ
LRFVAAEHQQVILFTEKIEQLFTYISFCQLLSNTLVTCCLGFLAISALETENALPLLFKFFSAYAAICVEIFI
YCFAGEYLNIKSQMIVDAAYQIAWYELQPNISRQLVLLILKCQRGLPLTFGKFSTLSLDSFTSIMKTSASYMS
VLLAMS
>LalbOr141
MRFRSTVSQSIKYGLNIVGVWPGTSFPGFRKFLWILFTVIPLFYQYMYVIRHFYSEKLPQLFTCLAVLFSYIL
LSVKLVVVSVNHGVLSDILTSVEEDCVKYSVADTESLVSKAAELSQVITRTVVILYAGTTGFYVVSTVAPRQL
NDSIPRRLVMNMDLPFDTVQSPNYELVVILQTISIMATSYVYSIFTALLLMLILHMGCHIDILCHVITKITSS
KDKKYFRFVAVRHQELILFSERIEQLFTFISLAQLMSNTIVSCCLGYLVIIGLQQGSDLPELLKFGVCYVAIC
IEIFICCFAGEYLNNNMIVEAVYQISWYNLHPSTSRQVLLLLLRAQKGIQLTFGKFSALSLNTFTRIMKASAS
YMSVFLAI
>LalbOr142
MTFPSTVSQSIKYGLHIVGALPTAKLPVYNKFFWMTCTAISLFYQYMYVVTHFYSEGLPELFDCFTLIFTYFL
LFVKFTVVSFNRGVLRDIITSMEEDCVKYSVDDSKNLISNAADLSQSVSRTVIIIYVASAGFYAVTTLAAPHS
NDTIPRRLLLNMDLPFNTVESPNYELVVMLQVISILMTSYAYSVFSALLLMMIVHMGCHIDILCLAMSKIATS
KDEKQFRFVAVRYQELIAFSERIEELFAFISFGQLISNTICTCCVGYLVVVTIQEGGKFSQQIKFIVCYLAIC
IEIFTCCFAGEYLNMKNDMIVEAAYEISWYNMRPSMSRQVILLLSRSQKGLCLTFGKFSALSLETFTWIMKAS
ASYMSVFLAM
>LalbOr143
MTSRNTISQSVKYGLHFVGIWPGTPLPGLRKVFWVVSTVFCQIYQYKYMITHFKTKGFIEMIDSLSIALTYTL
LIGKLILAWINHGVLRQILSTMEDDCARYAALDKNNVISKTADLSFRITSTIPALFMISVCCNAVGSLAIPQS
NDSSVRQLLINMDLPFDTNQSPTYELVVAAQIIYQVMSAYTFSIFNALLLMMILHAGCVIDILCYVITHISCQ
DERQFRFVAMRHQEIILFTQRIERLFTYIALCQLLSNTLIICCLGFLFITALQIENGLPLILKSFFFYIVICL
EIFMYCYAGEYLDTKSKMIVDAICETSWYDLQPTTSRCLVLLILKSQEGLPLTFGKFSSLSLESFANIMKASA
SYMSVLLAM
>LalbOr144
MISRNTISQSVRYGLHFVGIWPGTPLSGLRKVLWMVSTVFCQIYQYKYMITHFETDGFIETIYTLSITVAYTL
LIGKLVVAWMNHGVLREIISTMEEDCARYAALDKNNVISKTADLSFRLTSTIPALFMVSVSCNAVGALAISQS
NDSSARQFLMNMDLPFDTNQSPTYELVVAAQIIFQVMSGFTFSMISSCLVMTILHAGCVIDILCYIITHISCL
DERQFRFVAMRHQEIIQFTQRIERMFTYIALGQLLTNTLIICCLGFLMITALQIENGLPLILKSSVYYVAICM
DIFVYCFAGEYLNTKSKLIVDAIYESSWYDLRSTTNRRLVLFLLKSQKGLPLTFGKFSSLSLESFANIMKASA
SYMSVLLAMS
>LalbOr145
MDQSELKYFNICRLHMLLAGQWPYQTKITSVPTRIIMFTELITFGLEKTCRLIKKGNNMDIFIEDIPYFFMWL
CAAVTYVYCHCQSELFKELLERIKHDWHCAVLNKEDHILESFTYQGWKETIFILSLTYILSMFYCIFQFAPDF
LTDKLPWNTTHPRQPFIGAFLLDPDKNYPLRLPYTYIIVLGTVLGLCALSVQFHMLIAHARGMFSIFGHRLEH
AYHDTSEKRIENNRVHTAFVGIIEQHWNIMEYVDILTSYYSALCCIAGLGCVTTVSSLLVNVCIYIRNERNIE

LAILSLLNLGGIFLNLFFFCASSQELLNVSSDVFRQMYSGEWYTASISNQKTLLLIMCNSMQPVAVAFSTILT
MSLETYYLLLETSLSYFTVLYSLQ
>LalbOr146
MDQAELQYFNICRLHLLLIGQWPYQTNITAVPTRIIVVIQIISFGIEKTWRLIKFGTNIQIFIEDIPFFCLWL
CVVVGYGCFWFHLDLLKDMLETIKHDWHCAMLNKEDHILEAVTKQGWKQSIFILSLSYAIASIFSTFLFAPEY
VNNLLPWKTNTSYPRQPFVGAFLLDPNVHFPLRFPYEYAILMGTVLAVSAMILQFHMLIIHACGMFTIFGHRL
EHVFHDNSEKCIKDVRIHTVLVRILKHHKQIMQYVDTLNCYYAACYCFIGIGVIFTISSLLVNVCIYIKDDRK
IDLAILSALNLIGVFFNLHFFCSSAQDLLNVSNHVFRQMYWGEWYTASNSNQKILLLLMYKCTEPARIAFFTI
FSMSLETFYKYLETSVSYFTVLYSLR
>LalbOr147
MDLFEVKYFKIWRLHLLVIGQWPYQKNIIAVSARLIIAAALISFGIEKICRLIKLGNDVRLLIEDIPYCCLWI
SIAAMYISFWAQLELLKDILGKVKDDWHCAMLNKEDHILEAITKQGWKKSIHFLLLTYILASLYMTVQFTPKF
LSNLVQWNTTNIREPFVGEFLLDPDKNYPLRMPYEYGVVIGTVLVPSIMFLQFNMLVMHACGMFTIFGQRLEH
AFQNTSEKRIKGDGVHTAFVRIIEQHKHIMQYIDSLNSYYGVQTCFMGLVSVLTVSSLLVNVCTYMRIERNIE
MAVISSLNFGGVFFNTYYFCDSGQELLNVGSHVFRQTYYGKWYTASISNQKILLLIMCNCIQPAVISFSSIFA
VSLETYYNLLEAAMSYFTVLYSLQ
>LalbOr148
MDLFELKYFKICRLHLLVIGQWPYQGKINAVFWRLIGCVQIISFGLEKICRLIKLRNDVRIIIEDIPYCCLWI
ILIAMYMSFWAQLKLLKEILEKVKDDWHCAMLNKEDHILEAITKQGWKKSIRCLLLTYILASLYMTVQYAPML
MSNSVPWNTTNIREPFVGEFLLNPDKNYPLRIPYEYVVVIGTVLLPSIMFLQFNMLIMHACGMFTIFGHRLEH
AFQNTSEKRMEDDRVHTAFVRIIEQHKQIMQYIDSVNCYYGAYICIIAVLSVLTVSSFLVNVCIYIKKEGNIE
MAVISSVFCCAVLLHGYLLCGTAQELLNSSAHVFRQSYYGKWYTASNSNQKILLLIMCNCIQPVTIAFSSIFT
VSLETYYLLLEAAMSYFTVLYSLQ
>LalbOr149
MDLFGLKYFKTCRLHLLAIGQWPYQRKITAVPARLIIFAQLISFGFEKICRLIKLGNDVRILTEDIPYCCIWI
TTVGIYMSCWVQLELLKEILEKVIDDWHCAMSDKEDHILEAVTKQGWKKSIYFLSLTYILACLYVTVQFAPEF
LSNLFPWNSTNIREPFVGVFLLDPDKHYALRIPYECGIVIVMVLLPSVMFLQFNMLFTHACGMFTILGQRLEH
AFQNTSEVRIKDDGVHTVIVRIIEQHKHIIQYIDSLNSYYGAICCFLGLISVLTISSLLANVCTYIIKEGNIE
QTVIAALNSGGVLFIGYYFCDSAQELLNVGSHVFRQTYYGKWYTASISNQKILLLIMCNCIQPAALGFSTFLT
VSLETFYVYMKTAMSYFAVLYSLQ
>LalbOr150
MDLFELKYFKICRLHLMAIGQWPYQRKITAIPARLIVSAQLISFGFEKICRLVKLGNDVRILIEEIPYCCLWM
SILTIYIFFWVQLDMLKEILEKVKDDWHCAMLNKEDHILQAVSEQGWKKSLYFLSLMYILASLYITLQFAPEF
LCNLLPWNSTNIREPFVGVFLLDPDKNYPLRIPYEYVVVIGTVLLPSIMFLQFNTLVMHACGMLTIFGQRLEH
AFQNTSEKRMEDDRVHTAFVRIIEQHQQIMQYIDSLNSYFAAQICFLGIVSVLTVSSLLVNVCTYMRKDGNIE
MTVIASVHFGGVLFNTYYFCDSAQDLLNVGSHTFRQTYYGKWYTASISNQKMLLLIMCKCIHPVAVTFSSIFT
ASLETFYVLLQAAMSYFTVLYSLQ
>LalbOr152
MDLFERKYFKICRLHLLVIGQWPYQRKITAIPARLIVSAIMISFGYEKICRLIKLGNDVRILIEDIPYCCLWI
ISTSVYIPSWVQLELLNEILEKVKDDWHCAVLNNEGHILEAITKQGWEKSIYFLSLTYIMASLYMILQFAPEF
LSNLFPWNSTNIREPLVGEFLLDPDKTYPLRTQYEYSVVIGAVLLPSITFVQFNMLVTHACGMFTIFGQRLEH
SFQNTSEKRIKDDRVHTAFVRIIEQHKQIMQYIDLLNSYYAAQCCFIGMVFVLLVSSLLVNVCTYMRIERNIE
MAVISSLNFGGVFFNVYYFCGSGQELLNVGSHVFRQTYYGKWYTASISNQKILLLIMCNCIQPAVISFSSIFA
VSLETYYKLLEAAMSYFTVLYSLQ
>LalbOr153
MDFAMGFTKYNLILLGVWPDPRDTTERSRQISSFLFWSTVFMTIMFISLPQMSYLVLRSTNLDEAIENLSVNV
PVLFALSKHASLRYHRKALALLLDQLLADWSAPHSKSDHRVMLKNAKISRSISIVLSSFTYIMFKSPNLQVTW
LGQFIGIASMALCYSSFDTLLVVLMLHLCGQLSILRNALEDLANRNHQTKFRERLVYIVHRHNELSRFAVTVE
ECFNLMLLVQTVMITLMFCMTGYRLINSVDQEDEKVPIYETIFCFMHVTNSMLHFFIYCYAGEMLVQESTGIA
QSAYDCIWYDLPPKQATLMIMIIHRAGISLKITAGKFSPFSFELFNAVIKTSAGYLSFLLAMKD
>LalbOr154
IKDALVYSQRTFLLSGVWPQRPNPVLFAGTLCYQSLVIVLCAWAAYDVRNNFQMLLLDMMEWIILVSSTLALI
LVWLSDSSKVVIDETKREIRDGMFLRNDEERRLYHRYNSISWKFGKYATVFQSALVLLMFLRPAIPLLLHAGN
YDLHIKSNATLSYKLPFQAHIFFDYQNHTMRYFLLYVYQIPMIYSGMFHLAEVSYVVTLVLHVCGKFSILCVR
IRNVPTKPQSLFRHHIKSVVNQHLELKETSEILNDNFYLLLLKEYVSCSSRLSLSMYVALTTLETDPMAATNF
IMYSLDVIGYLYLYSYIGEQLRYEVRSVRDALYDIEWTDASNEDQKILLTCLINGCQANYLTAGKFYQFSLFG
FTKIVKLSMAFLSVLR
>LalbOr155

MFDKATPEKAVNFARLAVCLNMYWPLEPTTNKFWRVLYNVLKALSLICTIAVFLPLLYSVYLNLKRNDYTHFS
KAACMTLASIHVMGQAAVSFFQHDQLQYLVEEMVDYFRNMKPYEREVYQRYMRKYSPFYGVSTGWNALSAFFV
MIGPFVTGQPFPMAAEYPFPVDHEIVRSTIYLQHSYVCVQCMCTLNSNMMAAILILFAAGRFEILMIDLRAAS
SIDDLRNCIEQCYVIRKFAQDVVHGVEYITIITLIVSSGNLVLSGLNIIGRQPIMVKVQFIFLAGSALIEVYM
CAWPVDALMELSETAVRDVYEAKWYDQNVRVQKTVLQMLIPQKPIVISLRFVMAALSLNYYCSYASNAFSLFT
ALRVVM
>LalbOr156
KDAFVYSQRTFLLGGVWPQRPNPVLFAGTLCYQSLIVVLCAWAAYDVRNNIQLLFQGLMEWIIDVALVFTLIL
VWLSDSSKLIIEEIKREIRDGLFLRNDEERRLYHGYNNISCKFGKYGTVFQSAIIALMYLRPFIPLLLHAGNY
NSTFSYKLPSQIHIFFDYQNNTMLYFLLYVCQIPAAYSGMFHVAQVSYVVTMGLHVCGKFSILCVRIRNMPTK
PASLFRDHIKSLVNQHLELKQMSEILNDNFYLMLLIEYGSCSSRLSLAMYVALTVWDPATLAATTNFIMYSLI
VIGYLYLYSYIGEQLRYEVRSVRDALFDIEWTDASNEDQKILLTCLITGCQANYLTAGKFYQFSLFGFTKV
>LalbOr157
IKDALKYSQRTFVIGGVWPQRPNHVFFAGIMCYQTWVLGLSGWAAYDARNNSELLLLDISEWAIILTTAVALV
IVWLSDSWKPIIDEIRRDIREGVFLRNDEERRLYHRYNNISCKFGKYATAFQCALLLLMFLRPALDLLLHAGN
YLNINNSATRSYKLPFQGHIFFDYENHTIRYFLLYVYQIPMLYTGMFHIAEVSYVITMVLHVCGKFSVLRSRI
RNMPTKPSSLFRDHIKSVVNQHIELRQTSEIMNDNLQLLLLKDYVSCVIRLSLSMYVALTTFETDRMAATNFV
MYCVIVISYLYFYSYIGEQLRYEVQSIRDALYDIKWTDASNEDQKLLLTCLINGCQANYLTAGKFYQFSLFGF
TKIIKLCMAFLSVLR
>LalbOr158
FVVPEKVMRLLGIWPLNSDDILRSSLGRWCFAMLTQVSAICSLTTAVYRHCLDIDDTMDAFLMDLSAMVSLSK
VFILRFNWKHTYALVNSIVEDWSNVKDLGHRNIMAKYEEKGQLVSMLLLYMGYASGLSFAVKTLPFHLLPFQV
FQFPSNSTSTVDPTLKNKYFLSTYCAFGPLPWSLRSCVLAVQGVTVFVNVVGHCGNDVFFFSLTMHLCGQFEV
LKTKLSEIEIEKPDYRKKIGSLVQRHCRLVLLADDLERSFNVVILVQLLMSILLLCIEGFLMLVFLKANDRAG
ELKCLLIATTLLIQLYAYTYAGDALESQSAGISFAAYDSTWYPYDSTWYRSRGHRARDLALIIHRGNSPYCIT
AGKFMPMNLLTFKEILKASASYMSVLKVMMD
>XP_012231789.1 PREDICTED: odorant receptor Or2-like [Linepithema humile]
MTIPVATDPMLIVCVQHACGLFAAIGYQLENMNRNDNIDVNVYPLIEDDEHYRIICDSIAKHREILQFTK
SLASSYSTTFLVLTILNVAIVTFSGVQTVTNLDRPTEAFRFAVTGVCLMMHFLFLSLPGQKLIDHSSSMH
QAIYAGEWYESPSKSKKLLNMIMLRSTIPCTLTVGKIMVLSLPSFSANSQYNVIRILLSIAGLWPYHSAV
RRYVTYVVTALILGSGFTFQIMGVIKVWENTLEVVDSLPLLLFAVANIFKLIALVIKLPKIKLLLIKIQE
YCVSSKSDGEYKIQQSHATYGRNFGIVYISIILFHDILLVITTGMGGFTRARYANRTSNSGVFPGLPYRV
DYMVDIDTNYMPIFIHTHTCHIGYMALIIICDTFYLTLVEYCCGLFDALRFRLEHVYDLKDKYNGLSMSS
AKNKCYSNVAYSIRRHAEAIEFVAILESAYSVPLFIHVGCSVLIMSCIGYQILTYSGNLSHLLHHISYLN
GLIINVFFENWQGQKIIDSSEKVFDSPYNAEWYSMPIETRKLLVIIIMKSEKPLTLSAANKLIVMSYVTF
NAMMRMSMSYFTLLRSMQ
>XP_012231784.1 PREDICTED: odorant receptor 13a-like [Linepithema humile]
MMLTGFLYMLKSIVLILIEDSSNSTKLAVTKLPFRVEYGYKIDQYFYPILVHCYLTVFSHVTATVAADTL
YISLVQHACGMFSVVGHSLENIGKDSDASFILKPDKIKDDNYSEALKCLRKHLDIIKFAELIESTFTKIF
LVSISLNMVGGSICGIQVLINLNDAKDIIAPLAIYIAQLGHLFLQFWQAQFLLDYSAVPYESICRGNWYN
TSDRCRKLLLLIMSRTILPCRITAGKVVTLSIETFSVVLKTSMSYFTMLRRNLHGRDVNVQFSQANGKHY
RLQTIVRKVFLKMAERKDIWQSRYYTIPRAYMRLIGIWPYHAIHIKYLLFVPMFTFSVSILVPQLLYLII
AAANLDDVFSSTPSIWITIIFSFKIASLMANNRKLETCLKTIEDDWSSLNTDAERVILQQHTAYGRYITL
TYGVFMQFVGILLIIKSFAVILIEDTSDATISTLVAGSKLPFRVEYGEKLGQYLYPMAIHCYLAVFAHIS
ITIAVDSFYIVLVRHACGMFAIVGHTLEHIGKDSDDSFELKPDKVKDHNYSRALSCLRRHLHVIQFAELI
ESTFSNIFLVSVCLNMIGGSMIGIQVILNLNDAKDIVEPLAIYIAQLIHLFLQFWPAQFLLDYSIVPYES
ICKSDWYYTSGRCRKLLFLIMNRSVLPCKVTAGKVVTLSIESFGTVLKTSMSYLTMLRSFN
>XP_012229380.1 PREDICTED: odorant receptor 46a, isoform B-like
[Linepithema humile]
MSMSQMTAVLLILVPLLSQDERLLPCKMYVPYSITALLPYALTYLLQVIALVYGVLLNVSFDSLVYGLII
QTCGQIELLCRRLTESFQFRWKNDEENGEKRIEAIENFAIAEYVRHHILVHNIMQRIQSLFVWTIAILFF
FSLVTLCTSIFQMSKKELFSAEFFGFILYLSSMMFQVFSYCWYGNELDLKNKSISYAIYTSNWMAISIKQ
RKKLLLVMMMSQKGRIISFYGTCALIVGSFTWIIKTSYSAFNLLQQTYIMDVLPLNFRVLWFCGAWSEVN
DNNPLVRCLSFCYRYAIVILIYGFTILEVIELVRTRDHIEDLTEGLFLALTYVALCVKYGNFLARQNEMS
MLLDCFRGETCQPKDSEEKMILIKYDRKVKWCVRAFMSLSQATCIALVLAPIVGPQNTDRPLPFKTYLPY
SISGLYPYLATYLQHVGAIFYGVLLNVSFDSLVYGFTIHVCGQLELLCYRLSEIFKNYSDVKQCEIDSDK
SAMISECVKHHLHVHEIVRRIQSLFVWTVTILFIFSMVTLCTSIFQMSKKRLLSVGFLSLILYLGSMLFQ
VFCYCWYGNELQLKSRNVADAIYSSNWTSATMRDRRSLLFVMIISQKGLKLSQYGIFSLALDTFTWILKT

SYSAFNVLQQTSI
>XP_012228785.1 PREDICTED: odorant receptor Or1-like [Linepithema humile]
MLSQLMDIVFTVNNTDDFTDNFYVMLAMFVSCCKMFSLLKNRNNIAMLINILMEKPCKPIEHDEIEIRQK
FDKFVQTNTLYYAILVELTCAFALTTSYFRDYRKQRLAFRAWLPFNYSSPMLFQIAFLHQFTSLIIGSIL
HIACDSLICGLLVHICSQIEILECHLRKVVNKPHFLRECVTQHIYISKFAFMVNQKFKLTITIQFIVSTL
VVCFNLYQMTQTSMLSAKYIQIVLYMLCMLIQISFYCWYGNEVKLKSQQLISNAFEIEWFTLDHNMQKNL
LMIMTRSTIPIQLSSALVIPMNLESFVGLLKTSYSAYNILHTFTLSQFLDLILVVDNTDDFNDNFYMLLA
MIVSCFKMLSLLANHSNIALLTDILTKKPCTPLEPDELEIRQKFDKLIEMNTLHYAILVEFSSSYTVIQS
LFTAYWKEKLTFRAWLPFDYSSTMLFNFTYFHQLISLLVGAILHVACDSLICGLLLHICCQIEILNSRFR
KVMYNPEILRDCVIQHNLIMKFAFMVNKKFRLTIAFQFIVSTLVVCVTLYQLTNTNARVIELGLYMSCML
TQIFLYCWYGNEVKLKSLQLIDDLFEIEWIALEQDAKKNLLTITRCGVVPIELTSAYIIPMNLDSFVSLL
KTSYSTYNILQQMRDASIEIE
>XP_012219955.1 PREDICTED: odorant receptor 22c-like [Linepithema humile]
MVSVCDWSADVKVGSHPIFMLIFRSVSSGERAAVTHKALSGTFAQFVRSVDEANFAMIPISTVSRPVEIG
LRLTGIWPNSCIFFRLIWSAVMGTGLIFQYHYLLTHFSTKELPNLIDGLSTTLPYSLLFFKLIVLWVNNR
YAFLSFLHCRIFISILTAMSEDWYEYSSMQAMINKGIIAHRCSKLIIGVYSTAVLLYSTASVSFRRQAGD
DCRELLIKMELPFVFCESPIYEIVICVQFVHLMAVASSIGMLDALIVTLMLHIGGQIDIMHQQLEKICPT
DNERDLSTAIMKSLINKHQKIISFSENIESLFSHIALMQFFSNTLIICCIGFLIVTSLGTDEGIRMLVKT
VFFYIAITLEAFIFCFAGEYLSNKSKMVGDAAYESLWYTFKPRDCRTLLLVIMRSQKRLTISAGKFMDLS
LEGFTTILHTSGQIDILCDALEEISPEKNDHRLAVAITKELIGKHQKIIIFSNKIEKIFCYIALIQFMSS
TLVTCCLGFMIVTSIGTMEDSDSVDSSALMKAIVFYMAVTVEAFIFCFSGEYLSAKSKMIGDAAYKSIWY
NLNPNESKLILLIMLRSQRRLTITAGKIMDLSLEAFTSIIKASVSYVSVLHAMY
>XP_012230725.1 PREDICTED: odorant receptor Or1-like [Linepithema humile]
MYKAWLPFDYTISALFYLAYLHQILSFIFIGLVHPGTDNFICGLLLHACCQLEILEYRITNIANGRESLR
NCVRHHLCIFEYAYTLNERFSRIIPGEFIMIMVVTCYELIHMATTSSSNITYLQDMMAVACTLAAIFYYC
WFGNEIKHKSLQLSDNIWCNMEWTILSDNVKKGLIMIMNRAMIPIEFSSSHLMPMNLDSFVMITVLCIIV
NAVFMDLRKGNLTYRAWFPFDYTPPTIFYLVFTHQMVGMSITAVMNVACDSLIFGLLQQICYQIEILEYR
LTKISLGQHIFRDCVRHHNHIYEYACTVNHRFAPLIALQFAVSMLVVCANLYKIAAMKINAGSVVLILYT
ACMLSQIFIYCWFGNELKLKSIDLANNIYNMDWYTLDTNSKKGLLFIMKRAMVPIEFNSAIIITLNLNSF
VSLLKSSYSAYNVLKTAQ
>XP_012230269.1 PREDICTED: odorant receptor 49b-like [Linepithema humile]
MILLLNTFLLSQFMDLIMTVENADDFSDNFFVLLAMLISCCKLFSMLANRKNIIKFTNILTEKPCKPLKS
NEIKILSKFDKHIETNTWRFVYLVIVTFSFIVLTSLSLNFRKRKLTYRAWLPFNYSSTTMFYLTYSHQLL
SLFAGGFLNVGCDTLICGLLVHISCQIEILAYRLREIMSSKNILPDCVRQHYNIFKLAFIINATFRLIIS
IQFMISMLIVCFSLYQLTKTTVKAKFIELTLYMICMLTQIFLYCWYGNEVKLKSRQLVDDIFEMEWLSLD
KSKKKSLMIIMKRAIVPIQITSAYIIPINLDSFMGVCIISFYYRSEIGTEKYMQTDYFIRNFTENFYATL
ASVVSCSKMFSLLVNRNNINMLTNKLIKDPYKPLEIDEINIRYKFDRLIHTNTLCYTILVESTCICITMT
SLLMEFRKGHLTYRAWIPYNYHSSTIIFCLTYAHQLISLTAGSLVNVACDSLICGLLVHICCQFEILEYR
LNKISKDSEVLRDCVRHHDSIFEYAIKLNDKFKMTIAMQFMVSTMVVCSNLYQMTKSTSLNASILPLLLY
MSCMLTQIFIYCWYGNEVKLKSIQLLDNIFRMDWVSLDKNHKESLLIIMNRAAVPIEFTSAYVLSMNLDS
FVGLLKTSYSAYNILKQV
>XP_012234085.1 PREDICTED: odorant receptor 85b-like [Linepithema humile]
MMHLVLLLTAQYRYTSVKLTMLFQDPQNSSDAFPKRRYPISNQWVQRELRALCRHQNSVLRISVMLRKLL
SVNFSLLYINNIFRFCFIGILMTAIPYLTFMEGISIFLFAIGSMTQFFLLCFSVQTLSDASTKITDTAFD
EGWYQFEPLIKRTFLLLIMANNLECKIAAVDKFNLSLPSFMTIMNQSYSIALLFLRAK
>XP_012234078.1 PREDICTED: odorant receptor 49a-like [Linepithema humile]
MDFQNVNPLNVRLNLISGNLLPLTSDDSDFSITWKLYGICTWLIEVIQIIVLIPGMVLAPWEKVLRDGTV
TSVLTVEVFFMITRIYSRRKLTTQLIQRLNDILRVADKTMKCIVTTTLKPMEFPLKFYWISGWSSVFAWS
CTPFLLILEKASFLYEDYRMPVVFSKQPFSTEIFLLGNIFMLFGNMYIFIKKVGLDVYMIHLVLLITAQY
RYISARLVAIFQEESLRNKFNESSRIYPSEIDRSAEKEMKMICQHYRTVIHLSLTLKKLLSLNFSLIYVN
SVFRFCFIGIMVRTVADTTGSTTFVDGLSIFMYACGSIVQFYVLCFSVQQLLDASTEITKKAFHEKWYHY
GPSIRRTFMMMILANNLECKVAACDKFNLSLPSFMTIMNQSYSICLLLLRSR
>XP_012232769.1 PREDICTED: odorant receptor Or2-like [Linepithema humile]
MIFLLHSFAFCGIVDCFMIKDLETFIEKFSLFISVAGVCCKVMNLVLRRDKIIGLTDMLLKDMCVPKDNH
EADIQRRFDRSAKTITIYCEILNESAVFFATVAQLRHFVNTRTLPLSDWVPYDISSTPVYWATVLHQTIG
LMVCANASVAHETLISGFMIQACAQLDILCHRARTLPESLQEIRKHSTSGQDLKAREQQLVRELVHHHRY
VYRFAKRINTVFTLMIFIQFTISSTVLCLSIYKMSTKSLLSLEFAWSLSYLGCMLMQIYLYCWFGNEVTL
KSAEVSSAIYEMDWPMLSTDLMKTLLIIIVRSKKPIIITSGQIVTLSNDSFMKIIRISYSAYNVLKSS

>XP_012232545.1 PREDICTED: odorant receptor 47a-like, partial [Linepithema humile]
METFYDRQVLSWSKLLLSISGLWPESHNDFRFFFYATYIISCTLLLIVSLVQNMYDIEKMMRNITFTLPS
ILIVLKNMMFRWKKDQLLPLLTVVRGNVKKGLYRNSDEKDIMIWYNVAATLFTTSSMTSLFFVPTLYYIK
PILECILSSSGNCTLPYELPIHINSIYEISGMRSYALFCIYLMPSSTLLTIGATGADSVFVSLTFYLCGQ
LSILGYRLKNIDYMSPKCDYEIKTFTEQHAELLRLANILTNTFSSLMFIQTMGLIFSLCIVAYQLLMTAK
NGKEMNEIIYFLIYSGAVVLLAFCYCFIGECLISESTALDWICYSTKWYELPIESVRLLMICMTRSQKPL
YLTAGKFYVFSLETFGMILKASAAYLSVLKTITMSKLGINPDKELLEDDLLKDMDMEQDIQLQDAAEKIK
KLEEELESLKKK
>XP_012232343.1 PREDICTED: odorant receptor 83a-like isoform X1 [Linepithema humile]
MAFFLVIALLFLVPVENGELPVRARYPFDTTKYPWHGIGFFVEACTISVGLTGIIGMDSLHTNLCNLFLV
QLEILNAHYKNCSNNDQCDAPSDNIDRQENLHATNRSARCKIFCTFGARYEKENYDPDKIRRGGFTRQFR
RSVRNHQRLLAIIDDFNEVFSAGMFVQMLSSTTMICLTGFQAALVSSRDKSKCRRGQIVIAVSLCFEQVR
GQSSNTYKFSIYLAAAVSQLFYICWVGNEVMYQSMSLTQSQWLSKWSDELTVKTGRLLILSMIFSKRTLN
LKAGVFYVLSMETFTAVSICRKKATFNDLSYSQTGRVSRDKSGFIRKIDCSN
>XP_012232067.1 PREDICTED: odorant receptor 13a-like [Linepithema humile]
MHTTACLFVLKPVMLTLLSEDIVNVTKSSITTASKLPFRVEYGKRLDQYLYPITIHCYVAVFAHSFATIA
ADALYYTLIQHACGMFSIIGYVLGNIGQNNNMDFDLNSSKLRDNDYRKILDCLRRHLHIIEFAELIESIY
STIFLLNINVNMVGGSLAGVQVLMNLDKSAKDIAGPVTIYIAQVIHLFLHFWQAQFLLDYSVLPYESICR
ANWYYTSRRCQKLFLLIMNRTLSPCRITAGKIMTLSIESFGTVNKTAISSSIFIAAM
>XP_012231993.1 PREDICTED: odorant receptor 4-like [Linepithema humile]
MVLICAFDTLYLSLVEHCCGLFAALRFRLEHAFELENNDSILTCTTTSKKNKSYSNVVYSIRRHAEAIQF
VTIMESTYSIPLFIHMGCTMLVLSVLGYQVLTNMGNLNLVLHHSSYLNGLLINLFFENWQGQKIIDSSEK
VFDSAYNTEWYNMPIMARKLLIMIMMNSKKPLIVSGGHRFIVMSYVTFNAILRMSMSYFMLLRSV
>XP_012231990.1 PREDICTED: odorant receptor 4-like [Linepithema humile]
MDVDQFFLNSHYKLCRTQLSISGLWPFQSKIRRYATYFALILLLASGFFVQLMGVIEVRYDSVEVIDSLP
MLNMAVVMLFKVIYAMYTLSQIKLLLIKMQEYCLSPKSDEEHQIHNSYTQFGQKIGYLYTYIILGHSVAY
NLAPLLTKLMHKPLEENVTSSNKMIEAQVGVPYRVNYMVDLDTYYVPIFIHMVTYNVYNVFIVVTFDVFY
ISLVEHCRGVFAAFRYRLENACLIENDSNASTKTSTKDKSYVNVAYSIRRHAETIQFVGIMNSVYSPPLF
IHVGITILTISAIGYQVMTNTQNINRTLKNLSYMNAILMNVFFENWQGQKIIDSSEKIFESAYNVEWYNL
PIAARKLLVLVMRRSMQPLVLTAGKFMVLSYITFNAVIRTSSSYFMLLRSVS
>XP_012231987.1 PREDICTED: odorant receptor 13a-like [Linepithema humile]
MTSIFNTRYYRVTKFFTSLVGIWPLLSQSRRRFQQLLSVFIMLTFFPPQIKKLYDVWGVDLDTVCAGIAP
MVTILMSLAKVITSAAFQPKIEMLLRQIEMDWEIDPEGEEHKILVKYSLRTRTLSIAYSTMLCNGILSFI
LTPVIVPIMDIIAPENETREKHLAFEMEYFGIDQQAYWLWLWLHTNTTSVTCILNIVGADLLYAMFILYA
CYLFVCVRHKLESTANTIEQTFENSIMQPFENYNSKYAYEDEPWQLKLKKSVAIIKKCAISHQKAIKYVQ
ELQNIYRWAFFAILGWNLFLLTFLAVQLVSRLGNWQLVMMSFMYICGTLSHLFFLSLMAQFVMDESSNVY
ESTYACCWYALPLECQQNVKLILIRSRTPCRMMAGKMFVMSLENFSSIVQSAVSYFTVLLSFR
>XP_012231794.1 PREDICTED: odorant receptor 9a-like [Linepithema humile]
MDFFDGRNYRVNKILLSAIGQWPYQSSKTSRTIVIVIAAIACTQFFTKICGMLSYIHDMDIVIECLIPIM
VDISGMTKIMNSMLCVNELRALLDRIRCDFHSVRRSKDIEILQKYADNGKKFSTIYVCVMFTLTSVFMMM
PLQELILHVTNETTRPMLHRVEYYIDMDKYYFPILIHGYLTVVICVTAIVAADAIFVIFVQHVCGLLIMT
GSRIERAIQEEYFTGNDNPPIMKDKAYRNMVQCVHDHKAAISITFPSIYPRFVDLMELTYSKHFLFHAGL
NMVAMSITGVGAVSKSDDPSELLRLVAVSWALLFHLCFECMNAQRLIDYSGYLHINLINLNWYDASPRTR
KLILFMMMKTQPPCVLTAGGMFVLCMETFATIVKTAVSYFTFLRSAK
>XP_012231793.1 PREDICTED: odorant receptor 13a-like [Linepithema humile]
MKKLAVVMVEDWNIYSKFSNEYNILCRNYAMGRKVTIAYAVSLYGSMTPFLLVPVLLNTANYMGLYNMSD
DRPLMFRTEYFLDSQKYYYPLLVHSYIGTLGFVSIVVAIDSMLVFHVQHECGMCEILGYRLARIVEATTL
DINLYPSREEDTAYKRAKSCVIMHNHIIEYAKCIENANTTSYFFQLGCNMMGMTFTIFQAVVKLTDPKEA
LRYASFTVTLFSVLFLESWPGQQLSDYTDKIFRYITDGQWYQSPLSVRKVICIMLLKSYVPIRITAGKLY
ALNLQNFSAVMRTSFSYFTVLCSMQ
>XP_012231792.1 PREDICTED: odorant receptor 13a-like [Linepithema humile]
MDTSNDVFQTRLYRLNRVFLSLLGQWPFQKNRERLAIFIAISLAGITQAIAQALALLTLRGDLSATLECI
PPLIVDCACIVKLTNLLCNREKIKILLIHIQKDWQLWTIRSEFEILHKFAENGRSITIGYASGMYAFGSL
FSSLAILPKILGRSVASNYSTRPVGFPYHVEYYIDLEKYYYPILIHNYLAVAIRLTTVVASDTCVAILVQ
HCCGLFSIVRYRMEYIRESIEQDKELSCFEEDDKIYKNFAYCIRKHEDALQFANCLETIYTKAFFVEVGL

IIMAMSLSALQATSETLTLQLAIRHSGFIIAQLLHLYIACWLGQQIIDYSDYIYTSVYQGKWYESPPKSK
KLLNMMMLRSATPCTLTVGKLMVLSLPSFSAVVRVSASYFTVLQSVQ

>XP_012231776.1 PREDICTED: odorant receptor 4-like [Linepithema humile]
MLSTGIMLIAYLQHACGMFKIASYRIEHVVQIHTLQDIIILPNENVVYKGIIRAIDIHRKAMMFSKYLIE
QFEISFMFLIVFGVITLSLNIFRIFQIITVNYDIEEFFMAFVIVLIAILYMFLSNYIGQEIIDHNNHIFH
TAYNVRWYMTPLSVQKLILLILQRGNKPFGLNVGGLFIASLHCFATLANASISYFTVMYSVR
>XP_012230587.1 PREDICTED: odorant receptor 22c-like [Linepithema humile]
MEITWLHYYDIIYKVASLTGMWPFLKSRIKIFRVTLLTLITLTVLVPQIAYQLRCHKSDISCICESMTSY
LLSVVILVKIYTFQLNIRAIRSFTQRLFLDWEELKNPEEYEIMKSYATNSRRFSLIYSVYCLSASPIFMS
MSLVPFVLDITSPLNESRPTLPPYPGYYFVNIREYFFQILWHAIIAWEILIAGIVAHDCMFVMYVEHICS
KFAVVGFAQLLENIFTIPFAIQMLIATIGISITLLQVTQQNSDVVKSIRYVLYIVGQLIHLFFLSFEGQK
LIDHSMQMRDRIYNSCWYEASMRSQKLLVLIMLKSLQPTFLSAGTIYIFSLESFTMVLQTSMSYFTVLAS
FQ
>XP_012230552.1 PREDICTED: odorant receptor 13a-like [Linepithema humile]
MDVNNYVLINRKVLKFVGLYPTSIVRYIVCCVCMFTIIIPQAIQIFEDWQDLAVVLETSSVLLTILLAVL
KSLVWISNRRKMDLFIEYMLTDYWELMTAQVPNVYIVYVKKGHLFTKGYLFLICNALVFFFSLPVIETLV
AVIKDTGDNSTKHFPFVALYPEPCYHFPMYEVVYFSQIVATSLCGLIILGTDTLIATALFYTCGHFKVLQ
TKLENIGAEIDLMQRVHNKGNVVQKIKLQVIDIIKHHYTILWFCDYMETIFSPMLFLQTIASSLIICLVG
LQVATANMDAGIISKSVKYISYLLMALFQLLLFCIPGDALINESSMISRVVYTIAWYELPAWFKTEICLL
MLRSQKPSKITAGKFYVMHLENFNAVLSTAVSYFMLLRSFGSDETNEKL
>XP_012230157.1 PREDICTED: odorant receptor 49a-like [Linepithema humile]
MHANTFYRDDIRRTVHVTRGVLNLIGVWPSRDDSSALKIVRTKMLRVLCQVLLYFIFVPGVLKIFLKESN
ARRRFKMVGPMCNSLIAILKHMVLIYRDDRIKDCIRHIEEDWEKVNPAEDRKIMMNNSRIGRSLAILCIA
FVYGSGFSYRTIVPLLRGVVVTSQNVTIRPLGIAGYYVFIDPQKTPAYEIIFTMQFLSGFVQYSITSGAC
SLAALLVLHACSQLKILIARMEDLTKTEHFSAKIANQKLAAIVKQHIRIKSFLNKVEEIFQYTCLVEVIG
CTFILCLLGYYIILEWKDNNAVSMLTYSILLLTFAFNIFIFCFIGELLTDQNMKMYITSCTLDWYYIPHK
TARGLILMIAVSSIPIKITAGKFMDLSLNSFGAIVRTSVAYLNILRTTSI
>XP_012230153.1 PREDICTED: odorant receptor 13a-like [Linepithema humile]
MTENNDRTLFRNLNYRSDTEYVVKVAKTLLTPVGIWPLYRGNSTSDKIKTFIQTGIIFCLMCFLLIPHVV
FTYFDAEDLTKYMKVIAAQVFSLLAIIKFWTMIINKEGIRYCLQQMEMQYRDVECEEDRLVMTNSAKIGR
LFTVTYLGLSYGGALPYHIIMPLLADREIKADNTTQIPLPYLSDYIFFVVENSPLYEIIFVTQILISSII
LSTNCGVYSLIATCVMHSCCLFEVVRRQMETVLSDGIDNVHQRLGRIIEHHMQAIRFAEMIEKSLNIVFL
CEMVGCTIIICFLEFGVLKEWEDGKVLNMGTYFVLMTSIFVNVYIISAIGDRLKEESEKVGETSYFIEWY
NLPTKIISNLILVMVRSSRPSTLTAAKIFDLSLQGFSEVCKTSAAYFNFIRTMTT
>XP_012230139.1 PREDICTED: odorant receptor 46a, isoform A-like
[Linepithema humile]
MCQSVTKDLHDHSVQLNRWFLKPIGAWPQFSTISSREKALSRIVIFTCYSLIAFTVVPCVLNIFFEERDI
ELKLRAIGPLSHWLMGGMNYSSLLLHSTDIRQCVRHMEMDWRIIKRSQDREIMARNAKLGRFVAGFCAIF
MHSGVFSYSIVSGMTTVSVSIGVNRSVSMLQLPCPSYSKFVDARFSPANEIVLVMQLLSCFIVNSTTVGA
CSLAAVFAMHACGQLDILTLRLDKLVEGEGAKENKSAQKRLADIVNHHLRVLRFIARIEDIMHQICLIEL
VGCTFNLCMLGYYSITWWNKIDTKSIAAYIIVYISMSFNIFIFCYIGETLTQQCKKIGETAYMIDWYRLP
HKTALGLILIISRSSAVIKITAGKLIQLSIATFSDVIKTSLIYLNMLRTVTT
>XP_012230138.1 PREDICTED: odorant receptor 4-like [Linepithema humile]
MISPSNKPMSALHHRNEYHEYSLQLNRWFLRPIGVWPQLHSNTTAERLLSKIIQITCYTLIAFTVFPCML
YFYFEEQDLDIKMDSVGPVSHWIMSGMNYSSLLWRNKDIRRCIEHIKSDWCMVSKVEDRELMLKYAKFGR
FVAGFCAMFMHCGVFSYSVVYSLSPITIVIGNQSVSMRRLPCPFYSKLLDTNHDPANHIVLATQFLSGFI
ANSITVGACSLAAVFATHACGQFAVLYSHLNKLVEKEEKLSAEHKLANIVEHHLRVLNFLSCFEKIMNQI
CMVELIGCTLNLCLLGFCSIKEWKARNTKTLTTYGILFVSLSFNIFIFCYIGELITEQCKKVGEAAYFTD
WYHLPHKTALGMVLIISRSSAAIKITAGKLVQLSLVTFGDVIKTSAAYLNILRTLTM
>XP_012230137.1 PREDICTED: odorant receptor 4-like [Linepithema humile]
MKFANKYYERDIEYAFEMNRFFFRILGIWPFARTNSVLFDLIETVSLVSVCFFLLLCEFMPILLYVFLVL
KDMRLRLKVLGNAIFGLVAIIKYGYVLLYKNQVRNCMLLIDEDWRNVVSSSDRNSMIDRVRIGKRLIVMC
AVFVYMSGVGVRMAMPLAAGKIVTPQNITIRPLPSAAYFVIFDVQCSPAYEIVYCIQFFAGFVKYTITVA
TFGLVTLCVTHFCAQLDILLTLMDNFVNENQLENLNKKLAIVVEHQIKTHNFLQLIQNIIQYPSLVEVLG
STVMVCFAGFYIIMEWEDRNIIRLCTYSIALVMFCFNVFIYCYMGEQVVVQGDKVALKACTLDWYRLPTI
KARALILIIAISDIPIKLSAGKFIDLSLRTFGNVVKMSVTYLNLLRTVD
>XP_012230135.1 PREDICTED: odorant receptor 63a-like [Linepithema humile]

MRLSVQLNRWILKPIGAWPKSANLSSIEKLLYLLINIICTGLIGFLFIPCAAFIVLEVEDTYNTLKLSGP
LSFCLMAVIKYSSLIFRENDIRKGIEHIESDWMNTRHYSDRTIMIKNAKFGRRLVMICAFFMYGGAVFYY
LAMPFGNGTVTDDENLTYRPLVYPVARVIVDARHSPISEIFFWLQCLSGFIAHSITTGACSLAAVFAMHA
YGRLEVLMQWIDHLVEGREDFCASVDERLTMIVQQHVRILHFISLTDKVLREISLVEIVGCTLNMCFLGY
YTIMEWESKEPASYITYIVLLISLTFNIFIFCYIGELVTEQCKKIGEVSYMIDWHRLPGKKGLALVLMIA
MANSSVKLTAGNLFELSLSTFGDVVKTSVGYLNMLRTLTT
>XP_012230133.1 PREDICTED: odorant receptor 85f-like [Linepithema humile]
MESPSHYSDAYNKPELHNRNFKNDLQVSMQLNVWTLKPIGTWPKSLGHSRLETLWCRVSNITCYGLLVLI
LIPGGMYMVLEMKDFYSQLKLGSALSFFMTAVMKYCAFILRENDLRRCVEYIEGDWKNVKYTEDRKIMLK
NANIGRRLIVICGIFMYGAVLFYYVAVPFTRAKIVEEDGNLTYRRLVYPVPKVLVDARRSPINEIFYFIQ
LLSGFVAHNITVAACGLAALLAMHACGQLQVLISWLNHLVDGREGINDTVDERLANIIELHVRILNFIAQ
TEELLHEISLIEVVGSTMNICFLGYHCMMEWNFQQPVSGLTYVILLVSLTFNIFMFCYIGELLTEQTMKV
RESSYMIDWHRLPERKSLAIIILIICMSDATTRLTAGNIIELSVSSFGNVIKSSVAYLNILRTLTT

>XP_012230130.1 PREDICTED: odorant receptor 22c-like [Linepithema humile]
MPFNKYYQDDIMYITQLTRTVLITLGVWPSFNKKRSTSKRISKFLLISASYIFLCFVLIPGILFWLIEKR
TRVRIQTIPLLLYGLMAISKYGNLIFRERQIKRCLKHIEEDWKNVISTDAREIMIESAKTGRHLVALCGV
FMYGSGLSFRSILPLSKGKMVTAQNVTIKPLPCPGYFFSFNAQVSPNYEIIFAMQFLSGLVTYSITTGAC
GLAAVFAMHACGQLRILISLMKHLVEEKWQETQDVDVKLAKIVEHQIRIRSFLQLVEHTLQQICLIELMG
CTIIVCVLGYCMIMEWEKSNAIAMSSYFIALLSLMINMFMFCYTGEQLTTQAEKVATTSGELEWYRLPDK
KAREIVLVMIMSNLPTKITAGKMMDLSFKTYGDVRTYNTLKCTILSVQKFEIIMIK
>XP_012230129.1 PREDICTED: odorant receptor 13a-like [Linepithema humile]
MLRNKHYQDDIMYITQLTRNVLSLLGVWPSFNKRRSISEKAWKFLLILVSYVLLYGVLIPGVLFWLIEQR
TRVRIQTIPLILYGFMASGKYSSLILREREIRRCLKHIEEDWKFVTSMDARDTMIESARTGRRLVALCAA
FMYGSGLSFRSILPLSKGKIVTPQNITIKPLPCPAYFFSFDIQVSPAYELIFAMQFLSGIVTYSITIGIC
GLAAVFVMHACGQLKILVDLMKNVVEEQWEEKQEIDRKLAKMVEHQIRIRNFLQLVEETLQQACLIELLG
CTIIVCLLGYCIIMEWENSNPIAMCSYFITITSLMINMFMFCYTGEQLTAQAERVASTSCELEWYRLPDK
KARGIVLVMIMSNMPTKITAGKIMDLSFKTYGDVVKTAVTYFNMLLNVTD
>XP_012230128.1 PREDICTED: odorant receptor 13a-like [Linepithema humile]
MLYNEHYQEDIMYITQLTRIILMTLGVWPSFNRKSANKKLYKFLLICVSYILLYCVLLPGTFFWLLEKRT
YVKIQLMPLLLFGLMAISKYGNLIFREGQIKRCLKHIEEDWKNVISTDAREIMIKSAKTGRHLVALCGIF
MYGSGLSFRTILPFVKGKIVNAQNITIKPLPCPGYFFFFNAQVSPNYEIIFALQVLSGLITFSITTGVCG
LAAVFVMHACGQLRILMDLLKRLVDQEWQDSQVIDKKLAEIVEHQIRVRNFLRLVEETMQQVCLIDLMGC
TIIVCIIGYCIIMEWEKHNPIAMCSYFITLTSMLINMFLFCYTGEQLTVQAEKVAITSCELEWYRLPDKK
ARGIVLVIIMSNLPTKITAGKIMDLSFKTYGDVVKTAVTYFNMLRNVVD
>XP_012230122.1 PREDICTED: odorant receptor 13a-like [Linepithema humile]
MTLSKGLRDMSQLFLTCQYSEEGYGEADFGEKSRKTNWKGESDGRDNEKKEKAVACEKLEDLVRMLGPLL
NCGMGSIKYSLLVYHSKEIRDCEHWSKVCGFFCRFHVCRRAVLPNYRAALKRTNADTDEHYGFLTYSVTC
GAAGLAAFFIMHVCGQLSILIGKLQHLNDMKPEDRAVAILLADIVEHQIKVKNFLKQVEEAMQYIWLVEI
VGSTVLLCLTGYYVIMEWERSDSTAMLTMFVILTSFTFSIFTICYVGQLLTDQSIKVGLMTSMTDWHRLP
HKRARTLILIIIAISNIPAKISAGKMIEMSLPTFGNIIKTSMAYFNLLRKFIT
>XP_012230121.1 PREDICTED: odorant receptor Or2-like [Linepithema humile]
MKPIAAHWSKRLQINSHKNDFNYAARVVRVILRIIGAWPISKFASKAEKIAVCLQNVLCYFLFAFILVPG
LLLVFLKERDFKRKVRMLAPLLNCGMGTLKYSLLVYHAKEIQDCIEQVRQDWKNAENKNDRKMMLSKASI
GRKFAIFSASIMYIGGMSYRTIVPLSKGRMLTPMNTTVRALACPSYFIRIDEQASPAYEIIFTLQFFAGF
FTYSVTCGAAGLAAFFIMHVCGQLSILVSKLQRLNDMEPEDRAIATLLADIVEHQIKVKNFSKQIEEVMQ
YIWLVEIVGSTVLLCLSGYYIIMEWERSDSTASLTMFVMLTSFTFSIFTFCYVGQLLTDQSIKIGLMIST
TNWYRFPHKRARTLIIIIAISNIPAKISAGKMIEMSLVTFGNIVKTSLAYFNLLRKFT
>XP_012230116.1 PREDICTED: odorant receptor 46a, isoform A-like
[Linepithema humile]
MSPTRNIAYERDSEYSIQLNRWFLRPIGAWPSTSSNIAEKILSRSIQLICHLLIAFTVIPCVLYIIFEPD
VHLKLKAFGPMIHWLMGGANYCSLLLRSYEIRKCINHMCADWRTVERTCDREVMLRNAKFGRFVSTFCAV
FMQGGVCSYSVITAVTPAIVQIGNITITMHQLPCPFYMELVDARYSPMNEIVLGLQLLSTIIVNSITVGA
CSLAAVFAMHACGQLNILVMKLDELVDGTENKRETTHQKLVIIVEHHLRVLSFVSQIETVMHQICLIELL
GCTTDMCMLGYYTITEWELHDTKNLLTYATIFIAMSCNVFIFCYIAEILTEQCRKIGDMVYMTKWYRLHH
KTALDLILIISRSNAVIRITAGKMIQLSISTFGDVSLFDNLLYTIYIYE
>XP_012230114.1 PREDICTED: odorant receptor 4-like [Linepithema humile]
MLEFYYRCDNDHDDHDHCARLIIPILILACSIPSHQKLYSEHRADTILIIQVTFRTDRELMLKYATTGRN

LTIIGVSFMYIAGIVYHMILPFCSEHKINNQTIRPLVYPVYSKFYQSQISPIYELVYVAHCMCGYTTCSV
TAGTCGLAALFTTHACGQIQMIIARLKNLLDGDKFEQTLNVQQRIAAIVKGHVRVIRFAAVIEEVLQEVC
LVEFASSVCTICLLEYYCIVDWQADDRIGLTTYFMLFVSLCFNIYILCYIGELLMEKSSQIGSICFLINW
YELSPKSARSLILILAMSGHPIKITAGRMADLSLTTFGNVLKTSLAYLSFLRTLVM
>XP_012229844.1 PREDICTED: odorant receptor 46a, isoform A-like isoform X1 [Linepithema humile]
MDILPVNFKVLWFCGAWKERKNKNIILAFLHACYKYAILILIYQFTIFEVIELFRTRNRIHELTEALFLA
ATFVTLCVKYANFLLRKKELSVVLNCLRVKMCQPRNSMEELIMEEHGRKAKWSALSFLIISHATTIGFII
TTVLSLKKGEWILPTRSYVPYSISKLFPYLATYLQQIAVLFYAVMLNVSFDSLVYGFTIHACGQIELICR
RLTDNFRASKTLREELGSSEIEECIRHHVLVQIFVKKVGALFVWTVMVLFFFSLIILCTSIFLISKTKLF
TAEFLSLCLYLGSMLLQIFFYCWYGNELNLKSKGIANAIFSSNWTIATTRERKTLMLLMLISQNGLVFSY
HGICTLSLKTFTWIFKTSYSAFNLLQQASN
>XP_012229841.1 PREDICTED: odorant receptor 4-like isoform X2 [Linepithema humile]
MDILPINFSVFRFCGIWKERQSSSPIIRFISFCHRYTIAIVLYHFTISEIIEVIRMRNDMENLTECLFLA
LTYIITCLKYLNFSMRQSELRALLNCFRVKLSQPKNSAEELILKQYDRIVSLLSQDERLLPCKMYVPYSI
TALLPYALTYLLQVVALIYGVLLNVSSDCLVHGLIIQTCGQIELLCHRLTKSFQFLQKNNEGNKEKKIDT
IENFAIAECVRHHILVHNIMQRIQSLFVWTIAVLFFFSLVTLCTSIYQMSKKEVLSAEFFSFILYLSSIM
FQVFLYCWYGNELDLKNKSISYAIYISNWMAISIKQRKKLLLVMIMSQKGRIISFYGACALIVSTFTWIM
KTSYSAFQLLQQASN
>XP_012229840.1 PREDICTED: odorant receptor 82a-like isoform X1 [Linepithema humile]
MDILPINFSVFRFCGIWKERQSSSPIIRFISFCHRYTIAIVLYHFTISEIIEVIRMRNDMENLTECLFLA
LTYIITCLKYLNFSMRQSELRALLNCFRVKLSQPKNSAEELILKQYDRIAKRSTCFYMSMCQSTAVLFIL
VSLLSQDERLLPCKMYVPYSITALLPYALTYLLQVVALIYGVLLNVSSDCLVHGLIIQTCGQIELLCHRL
TKSFQFLQKNNEGNKEKKIDTIENFAIAECVRHHILVHNIMQRIQSLFVWTIAVLFFFSLVTLCTSIYQM
SKKEVLSAEFFSFILYLSSIMFQVFLYCWYGNELDLKNKSISYAIYISNWMAISIKQRKKLLLVMIMSQK
GRIISFYGACALIVSTFTWIMKTSYSAFQLLQQASN
>XP_012228796.1 PREDICTED: odorant receptor Or1-like [Linepithema humile]
MLLAMIVSCCKMLSMLANRKNIATLTSILTEKPCKPLEPAEIEIHDRFDKGVQSNTIHYAILVETTCVCI
TLTSLLTDFRRRTLTFRAWLPYDYSSPILFYITYAHQLISLIVGSVLHVACDGLICGLLVHICCQIQILE
SRLRSIASKPGILDDCILQHNRIFDFAFKVNEKFRFTIAIQFAVSTLVVCFNLYQLTRATTSNAKYIQLA
LYMCSMLTQIFFYCWYGNEVKVKSRQLANNIFEMEWYNLDKNVKETLLLIMRRSAVPIEFTSAYIISMNL
DSFVGLLKTSYSAYNILQKV
>XP_012228795.1 PREDICTED: odorant receptor Or1-like [Linepithema humile]
MHILEFTFKILTICGCGRPDSWTTPYKRFVYHVYTVFILLLIHTFMLSQLLDIILTVDNSDDFTDNFYML
LAMIVSCCKMFALLINRSNIETLIDILTKKPFQPSEPDEVAIRQKYERLIQSNTLHYVILVESTCLCVTM
TSLLTEFKKGKLTFRAWLPFDYSSSSRFLFVYAHQLISFTVGSVHHVACDSLICGLLVHICCQIEILECR
LRKSVYNPEILRECAFQHNRIFKFAQMVNDKFKFTIFIQFVVSTLVMCFNLYQFTKSTALRAKYIQLILY
MCSMLSQIFFYCWYGNEVKLRSRQLVTNIFEMEWFELDNDAKKTLLTIMRRGTVPIEFTSATVISMNLDS
FVGLLKTSYSAYNILKQTQE
>XP_012228790.1 PREDICTED: LOW QUALITY PROTEIN: odorant receptor Or1 [Linepithema humile]
MLSQLLDLILTVRNADDFTDNFYMLLAMIVSCCKMITLLINRSNIVMLIGILIRRPCKPVQPDEIEIQQK
FDKLVQTNTLSYAILVETTCLCIAVTSLLTEFNKGRLTFRAWLPFDYTSPSLFRLVYMHQLIGLTAGSVL
HVACDGLICGLLVHVCCQISIIECRLKKIACNRGILRECVLQHNHIFKFARLVNEKFRLTILIQFIVSTL
VVCFILYQFTKSTALKAKYMQLIMYMGCMLSQIFFYCWYGNEVKLRSRQLVNNIFEMEWFELDIPTKQSL
LMIMRRGTVPIELTSAYVISMNLDSFVGLLKTSYSAYNILKQM
>XP_012228783.1 PREDICTED: odorant receptor Or1-like [Linepithema humile]
MRILGFTFKILTSCGCWMPLSWTSPYRRLMYHIYTIFILMLINTFTLSQFLDIILTVDNPEDFMDNFYML
LAMIVSCFKMFSLLMNNGNIAMLTNILINGPCRPLDVVEVEIRQKFDKLIETNTLCYMILVELTCASTAI
ASLLTDYRKEKLTFRAWLPFDYYSSTTVFHFTYFHQLISLTVGSVLHVACDGLICGLLLHICCQIEILSY
RLKKIVRNPKTLRDCVIQHNVIFKFSSLLNKKFRFTITFQFIVSTLVVCFTLYQLTKTSGKFVELGMYMS
CMLTQIFLYCWYGNEVKLKSLQLVNDLFKIEWFTLEQNVKKELLIITKRCTVPIEFSSAYVIPMNLDSFV
GLLKTSYSTYNILQQMQDTSNEDA
>XP_012228782.1 PREDICTED: odorant receptor 94a-like [Linepithema humile]
MLSLLVNRKSIAKLTNILIEEPCRPLRPNEVKIQQKFDKGIQTNTWRFVYLAIVTVSFIVLTSLSLNFKK
RKLTYRAWLPFNYSSTTMFCLTYSHQLLSLCAGGFLNVACDTLICGLLVHISCQIEILAYRLREIMSSKN

ILPDCVRQHYNIFKLAFIINATFRLIISIQFMISMLIVCFSLYQLTKTTVKAKFIELTLYMMCMLTQIFL
YCWYGNEVKLKSRQLVDDIFEMEWLSLDKSKKKSLMIIMKRAIVPIQITSAYIIPINLDSFMGLLKTSYS
TYNLLQKMRE
>XP_012228640.1 PREDICTED: odorant receptor 85f-like [Linepithema humile]
MLIVYLKFSCGMFTIASYRIKQAMRVNILQKNPKNTIIIYKKMIYAVDIHRKAMNYAEHVINNFEGSFFS
LITISVCCLSLNLFEIFQTMSLKNIDQFLLHVVFIGIIVLYMFLANYAGQEITDHHNHVFSTAYSVQWYT
APLHTQKLILFLLQRTNKTFSLNIGGLFVASLQCFASLTSASMSYFTVIYSTQQ
>XP_012228639.1 PREDICTED: odorant receptor 4-like [Linepithema humile]
MLIVYLKHACGMFRIASYRIKQAMTINILQKCQQNDTLIYKEIICAIDIHRKAMKFAECLISNFEGSFFF
LIAVSVSCLSLNFVRIFQIMTLNIDIDEFLLHSVVIGIIILYMFLANYAGQEITDHNNDVFSAAYNVQWY
IAPLNIQKLILFLLQRGSKTFSLNVGGLFVASLRCFASLTSTSISYFTMMYSTQQ

>XP_012228637.1 PREDICTED: odorant receptor 30a-like [Linepithema humile]
MVGTGTWLIAYLQYTCGMFSIACYRIKQALTADIIQMRSLKSEKEIHKEIICAVDIHRKAMKYTDYFITN
FERSFLFLIAAGMSSLCLNLVCIAMFDGNIEQLVLSIRMSIVLYYYLFLSNYVAQEITEHNNYVFATAYD
NQWYVAHLNIQKMVLFLLQRGTKSFNVILGGIFVASMESAASMLSASISYCTVLYSTRKN
>XP_012228364.1 PREDICTED: odorant receptor 30a-like [Linepithema humile]
MVGTGTWLIAYLQYICGMFSIACYRIKQAMTADVIKMRSLKSEKEIHKEIICAIDIHRKAMKYTDFFVTH
FETSFFFVILVGMSSLCLNLVRIAMFDGNIEQFVLSIVMATILYIYLFLSNYVAQEITEHNNYVFVTAYD
NEWYMAPLNIQKMVLFLLQRGTKSFNVILGGIFVASMESAASLISASISYCTVLYSTRKN
>XP_012226909.1 PREDICTED: odorant receptor 67a-like [Linepithema humile]
MARFATIIMGALLQLMIMCYSGQKLIDESQNIFYRAYAAEWYMFSPRLKTLLIITFYRSITPCSLTAGKL
LPLSMTTYAAVVRAGMSYFTAFLSIKN
>XP_012226904.1 PREDICTED: odorant receptor 67c-like [Linepithema humile]
MLANFQREYNINKILLSSIGLWPYQNKLIRYLILIPCFASEVSYVLLEILTLFDYWEDMDIVFEVCYQCV
ATSAIIGQIFNVFWQREKIKRMYKAIDNHWNIFTSKLEVQTLKDYSNITRKLTFAYTVMIYSMTIIFILA
PLTPVFLDMIHPLNESRSRLFPLTAKFRIDDEKYYTQLYCYIVSMIVFSILGLAASNSTFLICIIHACSL
FSIISQQFEEVIKKLHANKNIRKFGCRMKTKLEFENEQIIYQRYIICLKKYQLALEFVDMLNSAYKIISL
ITLLLNGAVVTFIGIIIINVLDRVREVMRYTFVAIGILIQLLMLCYSGQKLRDESQNVFYRAYAAEWYKC
SPRLKSLLIVTLYRSVAPCSLTAGNMIPLSIETYGFVVRTAGSYFMTFLSLKM
>XP_012226902.1 PREDICTED: odorant receptor 63a-like [Linepithema humile]
MLEHFEREYSVNKKYLYWLGAWPYQSKLSSNLITISCWVYELSYYPLQILMLYDYWNDPELVFEICYQFV
SLTGFIVKMFNPIWNREKLKRICILMNDHWNIFTSKMEVQTLKDSSNISRKLTVGYTTWISFLTVFYVIT
PLTPVFLDIIDPLNETRPRLFPVTANFRIDEEKYYAQLYVYIVSMILIGVSNAITTDCIYLVSIIHACSL
FSTISRQLEEIVTKFSVKKDTRRIECCIQTKLEFENEQMIYQEYIKCLKKYQLALEFVDILNSAYEMISL
FLLLLYGATATFIAIQILNILDRFEDVLRYMFEVVAMLVQLIILCYSGQKLRDESQNVFHRAYAAEWYNC
SPRLKSLLIITLYRSSIPCGLTAGNMMPLSIETYGFVVRTILSYFMTFLSLKEAIS
>XP_012226900.1 PREDICTED: odorant receptor 63a isoform X1 [Linepithema humile]
MLEHFERQYNVIKIYLSYLGVWPYQSKLSRNLISISCLVVQISYYPFLILMLHDYWKNSQVVFEICYQFA
ALNSTVGKTFNLIWNRQMFKRMCTAMNDHWNIFTSKLEVQTLKDYSNMSRKLTIFYSTWIFCLTIIFMII
PLTSVFLDIIRPLNETRPRLLPMTTKFRIDEEKYYTQLYCYIVSLIMVSIISMVANDTTFLVCTIHACSL
FAIISRQLEEIITKLHVNKDTRKIECCIKTKLEFENERMIYQEYIICLKKYQLALEFVDMLNSTYQIISL
FLLMLYGAIITFLGIQIINVLDQLEGVIRYIFVIIAILIQLMISCYSGQKLRDESQNVFYRAYAAEWYNC
SPRLKSLLIVTLYRSVVPCGLTAGNMVPLSMVTYGLVVRTALSYFMTFLSFNEAIS
>XP_012226056.1 PREDICTED: putative odorant receptor 85d [Linepithema humile]
MYIQQFYNEGFSVDNLFSFLMLESKPAGIYKILKVVFQCRSVSLKTMEVTWNHYYDIVCKITSLTGMWPY
MKPKLKRFTIILLVISMMTVIIPQINYQLTCEGDLQCIIRAVTSYVMSAICVVKVCTFQLNTRAIKNLTH
HLFEDWKQLKTAEEYEIMKSYAKTGRQYCLIYFVYSCLAVFAFLSMTLIPYSLDIFLPLNESRPVLMPFF
ANWFVDFREYFFPIYVHSVVAWELIMSGLVAHDCLIMTFIEHICSIFSVVGFRFEYLFHNCNAEVEVVNI
IPNDIYCKRIALLVHTHRQALELAQLLEKLCTVSFFIQMFIVVVALSTTMTEVIRMETDGILESTRYALY
SIGQLLHLFFLSFEGQKLIDHSLQIREKIYSSLWYNASTKSKKLLVLLMMKGLQPSFISAGKIYIFSLES
FAMVLQTSMSYCTILASA
>XP_012224803.1 PREDICTED: odorant receptor coreceptor-like [Linepithema humile]
MLPYKVHTFIDITNTRTYILMYLYLFPMFYASICHMAAICLVVILVFHICGELSILSYRIRNIETYPQDM
VVDRIRSFVRIHLKIVWMAKSVDDTFNLILLDELLGNSIVLAISMYYVTMNLEVSEVATCCTFIFFAIIA

LVMLYGYCLIGDQLTQQCINVQDAYYQCDWYEMPLNCKRCLLICMIRGQVMLYLTAGKFYIFSLSSFTDV
IKTSLAYLSMLRTVI
>XP_012224802.1 PREDICTED: odorant receptor 67c-like [Linepithema humile]
MQDQKDLDRAAEVLTWNKRLMLTLGLWPFRSSDLIFSINFGYFSFWMVMEYLDLFLFIGDLEHVIMNLTE
NMAFSQIFVRMSMLRLYNGQIGEVIADAMKDFDRSSYKTIEEMKMFITYNARSKIFVKLLMVFVALTASS
YYLTPILIILGSGLPKIVINENVTQIIYLLPYRFHLFYSVENMSVYTFTYALQLPFVFVSGFGQSAADCI
MVTLVFHICGQMSVLALRINNIDTDPGICKREVRHVVLMHIRLLRMGRTIEKAFSATLLAHLFGATSLVC
ILGYQILTNVAKGERGILVTFLIFQFLVLLILYAHCTVGESLLTESAKVCEAFYACRWYDMSMGNARMVI
LCMARSQKPLCLTAGSFTTFCLSTLTDVLRTSMGYLSVLRSFL
>XP_012224801.1 PREDICTED: odorant receptor 22c-like [Linepithema humile]
MCSRSVCYTRSTHVEVWQVMQVTELTRASNIIIWNRWFLTCLGIWPTKVNQFLFLVFIGYMITYSIMAVN
HLINHINYPEHVVANLAANILLVMILGKMCVCRRSCGIMADKFLKVIEKDFLTESYDSAQEKTAYLRYNH
IALLFVKLSMTMTITAATLYYCKVFFINWNTMVSGNFTFDELPYPVDPFFEVKDSSTYTYVCIYLALAVP
LIICGYGAPDAFVLSMTFHICGQLAALSCKIDILLKDRENYHRHISNIIIRHHQLIALAEILENNFNLMF
LQQTLGTVFLLCLTIYHVLAMSEYGADINLITFILYTGCVLSTILAYCYVGECLINESAGLRETFYNSEW
YSKPPLYVKSVSICMTRIEKPLILTNGKFIPLSLNTFTSIVKTSMAYLSMLRNFM
>XP_012224261.1 PREDICTED: putative odorant receptor 85d [Linepithema
humile]
MAVIMFMSMSLIPYALDIVLPLNESRPFLPPYRGYYFVDMQDYFFEILSHSIVAWAVTVSGLVAHDCMFV
TFVEHVCSMFAVVGKYVYYTFRYTNIDSDNRDEHFFATSMLLFITKMVLALQEKLQCFFFEIAQGNEDTL
EAIRYIFYVFGQMIHLFFLSFEGQRLIDHSLQMRDKIYNSCWYSASIKLQKLIIILVMMKSLYPSFLSAGK
IYILSLESFTTVLQTSMSYLTVLTSMQ
>XP_012224114.1 PREDICTED: putative odorant receptor 92a [Linepithema
humile]
MENYMEYANLEEQNVFQRYVNKCKLFYGMTMSVLTVTLIAMIFGPLLLSQPFPIEVEYPFYVDVQPIKTI
IYLHHVMAVYQSYVQVCGNIFIALLLWFVAARFEILCHNFRKVTNISEFLMCVELHQHLLRYAKDVTMTV
RYIALTSIVFSTVAVVFSGLTFLSRQPLTIKAQFLTIGASSLVEVFICAWPADYILRMSNNVGHAAYDSS
WYNKEIMLQKDLPYLIMRCQQPVTVTVPCMLPTLSLNYYASYLSTTFSYLTTFRAIFAVEDGLQI
>XP_012224112.1 PREDICTED: odorant receptor 30a-like [Linepithema humile]
MIYTICKRSDDILLVMKVGCQLSVSAQIPLQMLLFAMQNKRLQSITSIMEDYYRQALKYEREIFQKYIDK
CKLLYGGILSWLMMTGISVAIGPLFLPQPFPGEAEYPFDAQYQPVKSIIYAHHLLLIYQSVTQVGANTFA
ALLLWFVAARFDILSVRIRMITNIKQLVKCTHEHNMLLRFAKEVTYAIRYVALLCVTFSTGGVIFGCLTF
MSRQPWSVKWTFLMIAFCSFVELYMFAWPADNVINTSNDVASAAYDSLWYNNNVAMQKILIYVILRSQRS
VTISIPLALPNLSMNYYASYISTVFSYMAFVRIIMGEE
>XP_012224109.1 PREDICTED: odorant receptor 45b-like isoform X6
[Linepithema humile]
MADTLITHGMCCFVTIHKRTNKTRSSCTIYYVQSGCDMHSSKHSCVHTSFSNLRQDIYVWITKDKAYVYF
TAVGCVIATLMPQFFDLIKPLNQSRPVILLYPAEYFVDVNNNFTYIFMHMTIALQFALLVLIAADSMFIC
YTEYANGLFAIIGYRFQYLLYKYSTGNILCQNLTLYKSNNIYQETLVDTIQMHLRAINFVKLLESTYTVP
FAITLGLNTIIMSCTLLQIVLVSEQTEEVVKYLVYVFAQIFHLFCISFQGQKIIDGSIRVTDKIYNGLWY
MMPAKSQKNLLIPLRRCLEPCRLSAGKIYVFSLRSFTTVMQTSMSYFTVLSSFQ
>XP_012224107.1 PREDICTED: odorant receptor 45b-like isoform X4
[Linepithema humile]
MADTLITHGMCCFVTIHKRTNKTRSSCTIYYVQSGCDMHSSKHSCVHTSFSNLRQDIYVWITKDKEWKIL
KLKDEIEIMEKYAKNGRRYIIFYAAYVYFTAVGCVIATLMPQFFDLIKPLNQSRPVILLYPAEYFVDVNN
NFTYIFMHMTIALQFALLVLIAADSMFICYTEYANGLFAIIGYRFQYLLYKYSTGNILCQNLTLYKSNNI
YQETLVDTIQMHLRAINFVKLLESTYTVPFAITLGLNTIIMSCTLLQIVLVSEQTEEVVKYLVYVFAQIF
HLFCISFQGQKIIDGSIRVTDKIYNGLWYMMPAKSQKNLLIPLRRCLEPCRLSAGKIYVFSLRSFTTVMQ
TSMSYFTVLSSFQ
>XP_012224104.1 PREDICTED: odorant receptor 45b-like isoform X1
[Linepithema humile]
MDFHTHSYYKINEYFLSAAGIWPYQTRRRKMLLCFISTFAVLSIQAAQVAQFITCNRDVICILQNTPVFI
LHSVIYVKIYTYGLQKTKIKTLLELMFAEWKILKLKDEIEIMEKYAKNGRRYIIFYAAYVYFTAVGCVIA
TLMPQFFDLIKPLNQSRPVILLYPAEYFVDVNNNFTYIFMHMTIALQFALLVLIAADSMFICYTEYANGL
FAIIGYRFQYLLYKYSTGNILCQNLTLYKSNNIYQETLVDTIQMHLRAINFVKLLESTYTVPFAITLGLN
TIIMSCTLLQIVLVSEQTEEVVKYLVYVFAQIFHLFCISFQGQKIIDGSIRVTDKIYNGLWYMMPAKSQK
NLLIPLRRCLEPCRLSAGKIYVFSLRSFTTVMQTSMSYFTVLSSFQ
>XP_012223782.1 PREDICTED: odorant receptor 13a-like [Linepithema humile]

MLKIARRGRSLSFRCYVSAMFTVAFYVWFNVLKFQRSMHQPQRILAYQFAYPYNTQKSPNYEITFFVQVT
GGAYSALINCTVDSFVSIFVLQVCAQLQNLRSALNNIVDELANKTISTLKFKKGLAAIVIRHEHLIKTAN
TIDECYSGVLFVHMLCATFQLCFETFQVFTIITDNLDVSIIKVAFLSFYVTLVLTHLYIYCYSAERLLTE
STSMAYGVYECKWYNLPPKNAKDLMIIAHRSRIPLKLTAGKFGIFSLEMFGTTVKTSMGYLSALLTMKD
>XP_012223780.1 PREDICTED: odorant receptor 63a-like [Linepithema humile]
MLKIARRGRSLSLRCYISITCACMFHLWICYIKFRRSMHQSQRVLVYQFNYPYNSQKSPNYEITFLIQLC
GGLLVALTNCTIDSFVSMFLLHVCAQLINLRVALNNTVDKLVNRTMSTLKFKEGLIAIVLRHEHLIRNAK
TINDCYSRVLFLQMLAAAFQLCSQTFQIYTMITDHASDTPILKLVFLFFYACLVLTHFYFYCYASEKLIT
ESTYMAYGVYQCKWYDLPSKDARDLMIIVYRSRIPLKLTAESFGIFSMELFGNTLKTSMGYLSALLTIKD
G
>XP_012223779.1 PREDICTED: odorant receptor 13a-like [Linepithema humile]
MDVDRAMKTLMSYMARDWSAAVDKRDRRTMLHITKISRNLSITSTLMAYGVVIAYVTFRCIEIQQTGCRQ
MLLRAYFPYNATESPLYELTFLGQYVAITYVATSYAAVDTFIATLVLHTCGQLSNLRHELTNLHACTRAE
FQKKLGNIVRKHIHLNRFAETIEDSFNMMLLMQMLGCSMQLCFQCLQAFMSVIDEANELFVFQFTFLMIF
VVYILLQLYLYCYIGETLLVESTKIADAAYECSWYNLSAREAKSLVIIMCRARSPLHITAGRFCSFNREL
YSEVLKRSMGYMSCIYALKGKAFEN
>XP_012223777.1 PREDICTED: putative odorant receptor 98b [Linepithema
humile]
MIRVWGNVARMVEYVASANFSLMALCKLITTWYHGKTLRALMTSVMRDWVTSKSDWERNTMLKIARRGRS
LSIRCYAWSTCTVSFYLCFNFLKFYRNIQQSQRSLVYHFVYPYNIQKTPNYIITYFTQMCGGIYTALINC
TVDSFISILLLHVCAQLINLRTTLNNVVNGLADRSTSSMRFKDGLAAIAVRHEHLIRNAKTIDDCYSGVL
FVHMLCATFQLCFETFQVFTIITDHLEVSILKMAFLLFYAILVLMHLYLYSYSAEKLLTESTNIAYGVYE
CKWYDLPSKNAKDLMFIAHRSKIPLRLTAGKFGVFSMEMFGTTVKTSMGYVSALLTMKD
>XP_012223764.1 PREDICTED: odorant receptor 4-like [Linepithema humile]
MHQPHRILVYQFNYPYNSQKSPNYEITFFIQLCGSIFAALTNCTVDSFVSIFLLHVCAQLINLRVALNNT
IDELANRTMSTSRFKEGLATIVLRHEHLIKNAKTINDCYSKVLFQQMLATTFQLCFQSFQIYTIITDHTS
KIPIIKMAFLFFYTGIVLTPFYIYCYTAEKLFTESINMAYGVYQCNWYDLPSKNAKDLMIIVHRSRIPLK
LTAESFGVFSMELFGNTIKTSMGYLSALLAMKD
>XP_012223763.1 PREDICTED: odorant receptor 13a-like [Linepithema humile]
MLKIAGRGRSLSFRCYASSMLTISFYLCFNLLKFYRNMQHSQRNLVYHFVYPYNIQKSPSYVITYFTQIS
GGVYSVLINCTVDSFVSILVLHVCAQLINLRTALKNLVDELTDKSISSLRFTEGLAAIIIRHEHLIRNAK
TIDECYRTALFVHMLTATFQLCFQTFQVYTLVTDHLNVSFIRMAFLSFAVILVLTHLYIYCYSAERLSTK
TTSIAYGIYECKWYDLPSKNARELMFIVCRSMVPLKVTAGKFGIFSIEMFGIMIKTSMGYLSALLTMRN
>XP_012223629.1 PREDICTED: odorant receptor 13a-like [Linepithema humile]
MVKNITSLAGQWPYQKQRTKLLCLSLVTLSTCSIMAPQGLVDCAMSKTKDSSVNMNVTSRYYKTVKNVSS
LCGQWPYQRQRTRLLCLSWVTLSIFSVIIPQMTKFVTCDGNIQCIFTTSAASALIILTLVKLYTCFFNRY
KMKTLIDKLLEEWNRINNPEECKILKKYAENTRLLSFGYAFYYFSTTVMFMSVSFIPPLLDIVLPLNNSR
PLLPPHPAYYFVDEETYFYYIYWHAVLGWEIAVIGIVSHDCLLLMYIEHICCIFTIVGFRFEQLITHKDT
NPEKNLHPYPNVKYRQQVAFSVNTHQEALKFAELIEDTFSLSLGIQVALSTVTISITLLQIAQDTADLLI
VTRYVVYVAAQLIHLFCLSFEGQRLIDHSLQMRNKIYNSFWYQTSPKLQKILLFIMRKSLQPISLSAGKI
FIFSMESFTSIVQTSMSYFTVLATVE
>XP_012223415.1 PREDICTED: odorant receptor 82a-like [Linepithema humile]
MAILENNPQTHQKRATIDVLLLCYRAYTRRIRKLLYLGGVLHDRKRSAVWSYIMSFIVIFMCFGQCIFMI
NFCRDHTDNLVLLSKIFGLTCSFIAPVLMSACFLVKRKKLIELHETLNDLFEQELASDRENKNVLATLHA
FDRPSYLLCFILGSTTLFYVCPSMIFIVRQIVQHSESKIYKLPIPVKLPWLAPINGGPVFYLLCLYQYFT
CWWMIFTIGSVDSLFGFYAFQISAILRAMSIRLANPRPNEVFSKVLGTCVETHHRIERCGRMLEDVWGLI
ILRVIIANAILMCALIFEVSPFTDLTINQIFSFISYMALKLLQTFIYAWYGSLITSASEHFREGIYFSNW
PDSKLDRHMRANIILAMMQKPLTIRALKVSHVNVNMFTNILNTAMSYFFLLQSLDEGG
>XP_012223402.1 PREDICTED: odorant receptor 13a-like [Linepithema humile]
MEILSLNFLLYTMSGVWRPIEWTSIPAKLLYGIFTCSTLYLLNFSMMTQLMDILLVVDNMDDFATTSLMF
LTAIGAFCKAVTAVIRRDEIINLIKILQDKPCKPDNDDEINIQMKYDRLIRLCSISYTLLASFSATGATV
GEIFAVLQGELPYRGWVPYDINNSFVNFLLTSLQEMLALVFGTIVNIATETLVLGFCLQTCAQLEILKCR
LEMVTKFTGNEEILEKSLNRVSNRTNRLSEHIRYHLCIIRFAEMANDIFSQVLFVQFFGSILILCSTVYY
LSSHMTVADFVKLIIYTFCMFVQIFVYCWAGNEVILKSTGLSEAVYEIDWMLLTISEQKDLLMIMKRSTR
PIKFTSSFLVTLSLDSYTSILKTSYSAFNLLQQY
>XP_012223386.1 PREDICTED: odorant receptor 46a, isoform A-like
[Linepithema humile]
MQILSVNFLIFTISGMWRPVEWSSNCAKLLYNVLTYVILISEYFLVTTQFMDVILVVDNIDDFSINILMC

LTIIAVCCKATVIVVRRNAVIDLVQMLLKEPCKFQNEDEIAIQTKFDQFIRTSTIKYLLLVMISVISVTI
GSVLDTMQGHLPYRVWLPFDTNTSLIFWIISIQQIVTVAIAGLINAGMETLVFGLFLQTCAQLEIFENRL
HKLVINTTASYLKHVPASLNKYKTMLSECIYHHLTIYKYAKMVNNIFNQVFFVQFFSSILVLCTSVYYLA
AHVMETKAATLLVYTIGMFVQIYFLCWSGNEVMLMSMKTGHAIYHTNWPLLSVREKKDLLMIMMRSTRPI
KFTSSFLITLSLESYSNILKTSYSAFNVLQQS
>XP_012223385.1 PREDICTED: odorant receptor Or2-like [Linepithema humile]
MQILSVNFLIFTISGVWRPVEWSSNCAKLLYNALTFVILILEYFLVTTQFMDIILVVDNIDDFSTNILMC
LTIIGVCCKATVIVVRQNAVIDLVQMLLLEPCKIQNEDEIAIQTKFDEFIRTWSIKYSLLAMISVISVTI
GSVLNAMQGHLPYRVWLPFDTNTSLIFWITSIQQIVIVIFATFHAIGMETLLFGLFLQTCAQLEIFENRF
HKLVINKTASYLKHVPTSLNKDKAILSECIYHHLTIYKYAKMVNIIFNQVFFVQFFSSILVLCTSIYYLA
EHITETRAATLLLYTIGMIVQIYLYCWCGNEVMLMSMKTGHAIYHTDWPLLSVSEKKDLLIIMIRSTRPI
KFTSSFLITLSLDSYSNILKTSYSAFNVLQQS
>XP_012223384.1 PREDICTED: LOW QUALITY PROTEIN: odorant receptor 46a, isoform A-like [Linepithema humile]
MQILSLNFKMFTIGGVWRPIEWSSNIAKLLYNIFTFIVLALIYFLAITQFTDIVLVIDNLDDFATNALMF
PTIVAVCCKGTIVVARRKAIIKLMQALLKEPYKPRDEDEIAIQRKFDRFIRSCSIKYSLLATSSVTGVTV
RSVLNVMQGHLPYRVWLPYDYNVFPMFWIVSIQQIVAVVFVTIINVGTETLVFGLFLQTCAQLEIFETRL
HKLIINRTSKYLKHVSASSDKKKEIISEYIRHHLSIYKFAKTVNVIFNQALFVQFFGSIIVLCTSVYYLA
SHITETESVTLVVYTICMFVQIYVYCWSGNEVILKSMSIGDAIYRMNWPFLSINEKKELLMIMIRSSSPI
KFTSSFLITLSLQSYTNILKTSYSAFNVLQK
>XP_012223383.1 PREDICTED: LOW QUALITY PROTEIN: putative odorant receptor 71a [Linepithema humile]
MQILSLNFSLYTIGGLWRPIEWSSRCSKLLYGIFTVCTLYLMLFLMLSQLLEIILIIDNVDDIITNSLTC
ISLISVVCKVFVAAIRRDDIINVLQIIQAKPHKACNKEEIDIQMKFDRTIRSLSISYLLLCAFSMMGAVA
GGVLETLEGRLPYRGWVPWDCTSLFLFWFTSLQGILAIIFGTIMNVATDTIVLGLCLQICAQFEILSYRL
QRVVKSSVKKNIAESLLNGAPSKSDRLSEHISHHLCIVRLAEMINDTFSQVIFVQFFVSILVLCSSLYYL
SSHTKLTEMTTFVVYTIAMFLQIFVYCWAGNEIIIKVYIKETIRTKDSDTKRIHAETFANHIAMTQFTSN
XKIFVQSTDLGNTVYEMDWIFMTISERKDLLMIMMRSTRPIKFTSSFLVTLSLNSYSNLLKASYSAFNML
KQD
>XP_012223371.1 PREDICTED: odorant receptor 46a, isoform B-like [Linepithema humile]
MKILTLNFLIYTIGGIWSPIEWSSNGAKLLYNIFTFVILFMLYFLMLTQFTDIIFVVDNVDDFVSNSLMF
VSIVAVCCKATIIVTRRSAVINLLQMLLEKPCKPENENEMGIQTKFDEFIRSWTIKYLLLALGSLTSVTI
GSVLNAMQGHLPCRVWLPFDTNTPLIFWITSIQQIVTVLFATIINIGTETLVFGLFLQTCAQLEIFEHRL
HNLIANKTVGYIEYLTISSSKKEIEISEYVRHHLEIYKYAKMVNVIFNPVLFFQFFGSILVLCTSIYYIS
IHIKEAASFLVYTICMFVQIYLYCWSGNEVMLKSKSIGDAIYCTDWPSLSVSEKKDLLIIMKRSTMPITF
TSSFLITLTLQSYTTILKTSYSAFNVLQQS
>XP_012223370.1 PREDICTED: odorant receptor 46a, isoform A-like [Linepithema humile]
MQLLTLNFLIYTIGGVWRPIEWSSNGAKLSYDTFTFIILFMLYFLVLSQFLYIALVVDNIDDFVTNSFIL
VGAIAVCCKATIVTKRRQAIINLVQLLLKEPCKPRNENEILIQAKFDDFIKSCTIKYLLLATGSVTSVTI
GSVLAVMQGHLPYRVWLPFDTNIPLIFWIISIQQIMTVIFATIINIGTETLVFGLFLQTCVQFEIFECRL
LKLVTDKTRFMEQYPTLSNKKIIISDYIRHHLIIYKYAKTVNAVFNQALFLQFCGSILILCSCVYYISIH
ITESETVTLLTYIICMFVQIYLYCWSGNEVILKSMSVGDAVYNSDWPSLSVSEKKDLWMIMMRSTIPTKF
SSSFLITLSLQAYSNILKTSYSAFNVLQQS
>XP_012223369.1 PREDICTED: odorant receptor Or2-like [Linepithema humile]
MKLLTLNFLIYTISGLWRPIDWSSNSAKFLYNIFTFLILFLIYFFLITLLLDIILVVDSVEDYINNSFIS
VSVFVICCKATVMIVRRSAIINLIQMLLEEPCKPQDEKETAIQAKFDDFIRLWTVRYAILVSSSLTSFTI
KSVLLVLQGQFPYRAWLPYNSTIPIMFWITSMQQMICLVYGSIINVAAETFVFGLFLQTCAQLEIFEYRL
YKFVTKRTKQAKCLEQLLTSPSKKKAVISDHIRHHLTIYKYAKMVNVILNEILFSQFFASILILCTNIYY
MSAHITEYEIATFFFMYTICMFVQIYFFCWPGNEVILKSNSVGDTIYHMDWPSLSVSEKKDLLLIMIRTT
RPIKFTSSFLIILSLESYSNVSLFIIK

>XP_012223367.1 PREDICTED: putative odorant receptor 85d [Linepithema humile]
MQILSLNFLFYTIGGVWRPIEWSSKCSIYLYNAYTFFVMYLMNFCALAFCMDIILVVDNVDDFTSTSLIF
FSLVVTICKGAIAVIRRGEIINLVKILQEKPCKPCDEEEIDIQKKYDRLIRSCSISYVLLASMSVTAGTI
AEIFVTLQGELPFRAWVPYDYNSLFLFWLTSLHEIVAMIFATLVNIATETLVLGFCLQTCAKLEILKHRL

QEVVKFNDKIEICKNFNNMPKKTSRLSEHICYHLCIIRFAELVNDIFNQVVFIQFFTSIIVICSSVYYLS
SHIMFIDFMHLAIFTFCMFVQIFVYCWAGNEVILKSVELSEAVYQIDWMLLTISEQKDLLMIMKRSTKPI
KFTSSFLVTLSLDSYTNILKTSYSAFNLLQQS
>XP_012222836.1 PREDICTED: odorant receptor 22c-like [Linepithema humile]
MKKAKDWNSEVYYAINFYEKVLSIIGVWPLNAGEFKSIVRCFLAVLIQISTIISLSLEAYRQCLGTEDMM
EAFLMDLSSVVSLSKLLVVRLTWRHTYALVTSLIDDWSISRDTQQREIMMKYTNVGRMVSLTILYLGYAS
GVSFLFMALPFDSLIPWLNISKVNDNDTALSTYFLATYCVFGTLPTIAHSCILLLQVAQIFVNATSHCGN
DGFFFGLTMHLCGQFEVLEMDFANIEVEKRACKQRLRMLIGRHCRLIRLADSLEYAFNMAIFAQLLMSVL
LLCLEGMQLIISLKINDNIAAIKHVVLILTMLVQLYLYCYAGDQLESITGRLAYNAYSSPWYNFDVKVMK
DLPMVMLRGELAHQITAGKFLPMNLFSFKEILKATGSYLSVLRVMIDV
>XP_012222766.1 PREDICTED: putative odorant receptor 71a [Linepithema humile]
MVYWNKDAAYALSSCKILAWPVGAWPIEDDTFYSRIRWLFAIISEILLVTTLVMDVYLACKNTTEDPIDT
YVVLASAILVIVKLTLLRLQRSTLSINIYSAVQDWYSVKNVKSRDIMIYHARTARIISLSLFYSGFFAFM
LYMLKLLPFVNGTNERVFYLPTSCLFKSVSNLQYVLITFYQVVQLLITYAGNCCTEGIFVGITMHLCGQL
ELLMIDFRQIDQHKKECKRGSIFKELVVRHRQLLRLTETIEDSYNMIILTQTLTSAILICITGFGLIESL
YIHDTIMMVKSIVIMIVMLLQSFLYSYAGDNLRDQSEALSFALYDSNWCDFSPNDIRDLAFIMLKTNISI
RLTAGKFFYVTRATFTDILKTAVSYLSALRVMIEKQETNSH
>XP_012222757.1 PREDICTED: odorant receptor 67a-like [Linepithema humile]
MISCYSGQRLMDESQNIFDRAYATEWYNFSPRIKSLLIITLYRSSVPCILKAGNMVPLSVTTYASVVRMG
MSYFTTFLSLKE
>XP_012221808.1 PREDICTED: odorant receptor 43a-like [Linepithema humile]
MIGLWPDEKLSRRQKFWASLRAFIIFTTLVSAILVSIIPAIVSLIRVWGDVIAIIDNFQITLPISVIAMK
IIIMWLRRKDLSTVVSMIVADWIRTKTKEERDTMIKQARIARTIIVFGCIMMVLAIVIIVIPPCFGYSMR
YLTNITDPGKPLLLQASVDIIENTFNLMLLALLVYFAILFCLQGFLIISRDDDIDLDWAIGINRISLKII
GLWPTNKLTRQQRILADVRAFIIFVTMMSASILPGILALLRVWGDVMAMSDNLQIGLPFTATGIKYIIMW
FRRSDLVPIINMIVEDWMREKTAQERNAMIKQARFARAIVMFGAIAMSFASIVLIIPPCFGYTSRYLTNL
TDPVKERPFLLQTYYLRDSSQSPYYEIAFTAQAISIVMAAISYTGIDTFLGLVVFHICAQLDILKERMLN
LNNFKDFRTGLRFNIKDHLRLIRSVDVIDNTFNLMLLALLVFFAMLFCLQGFLIVSIIDGEGDLSYMRIC
WLVSILINTFVHMCLYCVVGEILIAKAEGVYYAAYNYEWYLLEPKEAKNLLMIMIRAEKPLYITAGRIFP
MTLSLFCSVRHVFFDKNISWLYICIAR
>XP_012221387.1 PREDICTED: odorant receptor 47a-like [Linepithema humile]
MCLSNHGSDSLFLQITLHICGQLKILKASFMNFDATGPKVDERFNALILRHDHLIQMARKLAEIISFVLI
IQLFISSMLICIVGFAFIIALTTNDFGMMSKSFMVLSAFLAQLTVYSVVGDYLKSQMEEVALSVYQCNWY
NLPTKVARNVVFIMMWSQLPVKLQAGNFIVVDLGTYMSILRTSVSYLSVLRVMLDT
>XP_012221302.1 PREDICTED: odorant receptor 13a-like [Linepithema humile]
MIGFTCVLALIKHLMFRVYAENLTHNYSSAINDYCAIDTEEKRTIMRQHAFLGRMVFYFVTAMAFTCAVC
LIMTPIIEYSNNVQVNASTNKHTSRYPVPSDCTWGKLDVSMRTYLLLFVLQFIQIGISSCAYVGNDSLFL
AITLHVCGQMELLKIEFRKNFAMETKNIMEDFSKLMTRHCCLLRLAQQLTDAISVVLLMQFFVSSILICI
MGFQLILALKVIDVVMIIKATIMLLTFLSQLFAYSVVGEYLKSQTEKVAYSIYCSNWHCLSAKFMRNILF
IIARSQQPVTFLAGNYLVVNLPTYMSVLQTSFSYLSVLRMMVDN
>XP_012221301.1 PREDICTED: odorant receptor 13a-like [Linepithema humile]
MELLRIEFSNLSAEDKNVNKDFLNLVTKHTFLLNQAKLLKDTIGFSLSVQLLMSCILISIIGFQFILALK
VGDIIMTAKTIMVLLTFLTQLFVYSVVSEYLKYQVEEFAHSIYYCNWHCLSVRLMKNVLFIIARSQQPVQ
FAAGNFLVVNMGTFTSILKTSFSYLSVLRMTLDM
>XP_012221300.1 PREDICTED: putative odorant receptor 85d [Linepithema humile]
MELLRIEFSDFDMESKNINQDFMKLTKRHCYLLNHIKLLTDAVNLVLLAQLLISGFLICLIGFQLILALK
GGDLVMIAKTTMALMTFLLQLFAYSVVGHYLKSQTENMAHSIYCCNWHCFSLKMMKNVLFIITRSQQSIP
FTAGKFIIVNLETYMSILKTSFSYLSVLRVMLDT
>XP_012221299.1 PREDICTED: odorant receptor 7a-like [Linepithema humile]
MLWNEDVAYAMTPVKLLTAPVGGWPLQEYNKFALARYIISICSMSVELIMSYLDIYYGCSDIIRWRKVAH
SIFFCNWHCMSVKLMKNILFVIAQSQQPIQLTAGKFLVVNLQTYMSILKTSFSYLSVLRVTLDT
>XP_012220551.1 PREDICTED: odorant receptor 22c-like [Linepithema humile]
MIADWKDYRCLPKRNQQIMVYYARKSRMLNIFSITLMGFSLAGYMLTPIINTWNSDVPWNTTTRSLPIRG
FFPFSFKESPVFEILYTMQSIVGFFACTMIVSIDCFLCTVMLHACGQFDILAATLERYDSSTRHDYTKDM
RSRTCACLSCFVKRHVHILKYMDIVQRSFSDFHLIQLLTYCFNLVLCGHQLITYFQDHNFYDFITFIIYV
LTITCIIFIYCYLSECVMEKSKNIGTIAYNLEWCRFPRDSNLAIIIARSRIPCKITAGKFVTMSFSYFTT

IIISIISYISVLAAFK
>XP_012220527.1 PREDICTED: odorant receptor 9a-like [Linepithema humile]
MLTGFIIPCLHSLIRIHSDIILAIDNLQTTLPGITCILRLVIFWWKKQALIPVVNMMVEDWKKSKNQTYE
RETMIRWALRARIVIICIYSIMGMAYILFVGMPIFGKAIRLTPNITDPGRPLPLQTYYLYDVTKRPQHEL
TFIFQAISTFIAMLCYTGIDTFLGLLTFHICAQLDILKNRLMHLHECESFHDALKDVVMYHIRLLRMIFA
VEDAYNIILLVLLSYFAILFAFYGFLLISLFDEGNGIPITRLLYLVIIVITVLLHMCLYCAVGEMLMTQC
DGIYYAICNYKWYSLDPKKARHMIIFMIKASEPVYLTAGKVFPMTLSLFCNVIKTSAGYMSFLLTTRN
>XP_012220526.1 PREDICTED: putative odorant receptor 71a [Linepithema humile]
MIAEDWIKTKKAEERSMMIKKAQNARIIIITCGFCLMGFACIFIIVLPIFGFSIRLTPNITDPGRPMPIQT
YYIYDVTKRPQYELTFVVQAVYIVLAMMSYTGIDNFLSLLVFHISGQLNILNRRLSYLDKYINTYDALKC
CVAKHMRLLRTIAVIEDTYNITLLALFLYFALFFAFCGFRIINLFDEGNDMSIIHLIYFFALIVNAIVHM
CLYCALGEILVGQCNEVYYAGYNNKWYSMDPKITKDLLIFLIRGNKPVYLTAGKYFL
>XP_012220524.1 PREDICTED: odorant receptor 43a-like [Linepithema humile]
MRISGDILLMLDNLQFTLPAISCTIRIVIFWWKKEAIVPIINMIAEDWINSKSEQDRSMMIKRAQIARII
ITCAYCIMGVGCFFIIILPGFGLSMRVTTNITDPGRPMPLQTYYIYDITKTPQYELTFIVQAVYIVLAMM
SYTGVDNFLGLLVFHICGQLDILKNRLRHLDTYINSQDMLKSCIAKHIRLLRAISIIEDTYNITLLALFI
YFAILFSFYGFRIINLFDDGNDLSITHLVYFLSNVFNIFAHMCLYCALGEILLGQCNEIYSAAYDNKWYS
VDPKIAKDLLLLLIRGTKPVYLTAGKIFPMTMATFCGLIKTSAGYISVLHTTRN
>XP_012220513.1 PREDICTED: odorant receptor 43a-like [Linepithema humile]
MVEDWLRSKSAQERNVMMRRAESARKIVAIEYCLMGLAYVFVVILPICGIPIKYLTNVTDPGRPTPGPIQ
TYYIYDVMKTPQYELTYITYSITLFFAILCYAGIDNFLGLVVFHICGQLDILRHRFTHLDKYMNFHIDLK
NCVMDHTRLLRAITIVDDLYNVLFLILFLCFAVLFAFYGFVIISLITEEDKVSIVRLIYLVSNVINLFAH
MCLFCAVGEFLMAQCDTIYYAVYNQEWYTLGSNKAKNLIPLIIKSRKPVYLTAGKVFPMTLATFSNLLKT
SAGYISVLFGMSV
>XP_012220512.1 PREDICTED: odorant receptor 43a-like [Linepithema humile]
MMADDWIQTKNVQERSMMIRKAKIPRIIITCGFCLMIIACCFIIGLPIFGMSMRLITNFTDPGRPMPLQT
YYIYNVTKTPHYELTFFMQAIYVMLAMTAYAGIDNFLGLLVFHICGQLDILNGRLTRLDKYINSQAMLRD
CVTKHISLLRNIAVIEDAYNITLLALFVYFAILFAFYGFRIINLFDGENDMSIIQLIYLVSNIFNVFVHM
CLYCALGEILVAQCNEIYYAAYSNEWYSMDSKMAKDLMSLLIRGTKPIYLTAGKIFPMTMATFCGLIKTS
AGYISVLHTTRN
>XP_012220511.1 PREDICTED: odorant receptor 43a-like [Linepithema humile]
MMDNLQLTLPAISSSIRIAIFWWKKEAIIPVVNMMADDWIQTKNVQERSMMIRKAKIPRIIITCGFCLMI
IAGCFIIGLPIFGMSMRLTTNFTDPGRPMPLQTYYIYNVTKRPHYELTFFMQAIYSMVAMTAYAGIDNFL
GLLVFHICGQLDILNGRLTRLDKYINSQAMLRDCVTKHISLLRNIAVIEDAYNITLLALFVYFALFFAFF
GFRIINLFDEGNDMSIIQLVYFFACIVNAIVHMCLYCALGEILVGQCNEVYYAGYNVKWYSMDPKITKDL
LIFLIRGNKPVYLTAGKIFPMTMATFCSLIKTSVGYISVLHTTRV
>XP_012220509.1 PREDICTED: odorant receptor 43a-like [Linepithema humile]
MMLGLVFFFGILICLFGFLLFTVFSDDETSNMSPSRICFVMLGTVTMFAHSFLYCSAGEIIAKRCEAVYR
AMCDLKWYKLQPKNSRAVILIMLRTSEPFRITAGNIFPLTMTTFCSLLKTSAGYTSFLLAKR
>XP_012220006.1 PREDICTED: odorant receptor 13a-like [Linepithema humile]
MSGVANLSHRFSNLIIGLHSTAVLVYCIGVVALRSDPADRELFLKMELPFDSGTSPTYELVMTTQFLHQM
TAATMIGVLSALLVTLVLHVGGQIDILRDRLLEILPKDKKPTVSMVTIGSLIRRHQNIIIFTERIESLYS
YIALAQFISNTIVICCLGFIIVNVSNRNSFHYIDQY
>XP_012220003.1 PREDICTED: odorant receptor 13a-like [Linepithema humile]
MNATEKKYNVVGRKNTVNRVVKFMLISLGVWPDASCVTFCRIFWSITIAIVQFWHYRYFLTHYHSIDMFD
FTEWFSSFLGYWKLFTKFVCFWLNQRIFGKILTMMTEDWSDCSNNDIEMRETADKAKTSDYVTNALIAFH
SFALLSYGTSIILADVDVTNRTIELPHLHKMEVPFDINTQRTYKIVLITEVMYMLAAGVGINIPNILLLT
LVLHTGGQINILRGWITKLQPETNENKRESFVIATNKIIRKHQKIIYFSENVKNLYSIIALLQFASNTVM
ICLLGFLVVTALGSPGATGKIVRSLSYYSVTNIEAFIFCYAGEYLINKSRAIGLAAYNCQWYDLAPKQSR
VLLFILLRSQKQLTLTVGKMTDLSLRCFASIMNSAASYLSVLLAMQ
>XP_012219963.1 PREDICTED: odorant receptor 45b-like [Linepithema humile]
MTNKAILSRRCSMLIIGVYCMAVVVYISVIMEFNSIVSDELGKEGQQLFLKMKFPFVYEFSPVYEIVMFV
QFVQLLGHALVIGMLDALIVTLSIDGNQGFIMLLKSLFFYTAITLEAFIFCFAGEYLSNKSKSIANAAYE
AFWYNAEPSKSRNLLILMLRSQKRLTLTIGKFNDLSLQVFASVRY
>XP_012219962.1 PREDICTED: odorant receptor 4-like [Linepithema humile]
MCHELLEIPVADDKRDSRTAALRGVVHRHQRIIAFADSIENVCCYMALMQFLSNTFVICFLGFVIVTSLD
SVDANTVLLKVIPYYVVVNVEAFILCFTGEYLSSKSKTISQAAYDSLWYKLNPSESKILLLLLMVRSQRQL

TMTAGKFMDLSLEGFTSVRDLRFSFSFYPTVIHYKCRT
>XP_012219961.1 PREDICTED: odorant receptor 82a-like [Linepithema humile]
MNRDWKDCVINDSYTSTMMSVADLSHRCTNVMISINALAAFFLSIGEHLLQSMNDADRVDNNSRQLPIKM
EFPFDVSKSPVFECFLAGQFLYELLLASVVGMVNALLVSFVLHVSGQIDIMRQDLNEISNSKYDSSTSLI
VIKGLIHRHQKIITLSENIESLFCYIALMQLLWNTLVICCTGFVIVISKTINDAVYESFWYNMPPTDSRI
LLFMLLRSQKRLTITAGKVVDLTLEGFTSIMRASASYVSVLNATY
>XP_012219960.1 PREDICTED: odorant receptor 82a-like [Linepithema humile]
MDSLGLALANTLAFLKLLTLRWNRRIFHNILTAMERDWSNCGSCESIMMTVKSLSRHYSIVLIGVHALAA
FFLSVAAYVFRALSASDDFRELPVKMEFSFEVTKSPLFECLLIGQFLYELSLASVAGMINALLATLVLHV
GGQIDIMRQAIIEMSSSTSPDISLTVIRNLIHKHRKIITLSEDIENLFSSISLMQLLWNTLIICCAGFMI
IITLSHKSTAGLIKSVLLYVAKTLEVFVFCYAGEYLSAKSRSISDAIYESLWFNMEPSNSRVLLFIMVRS
QKRLTITAGKIFDLTLEGFMSVMKTSASYMSVLHAMY
>XP_012219959.1 PREDICTED: odorant receptor 82a-like [Linepithema humile]
MKRANTISFLVEIGLRFIGLWPDSPYATFYWLSFVTTLAIVQYYQYVYVFTHFELNNIPLLMECLSLTLA
YTLFFLKLVILWRNRRIFHYIVKTMDEDWKECIINDLYASTMMNMADMSRRFSNAVFVLNASGAFFLSIG
DHLFQLMNDANQFGNSSRELPIKMEFPFDVSKTPTFECFLIGQFLYDLVVALLVGLFNSLLVTLIFHVSG
QIDIMRQDLVEIANSKCDRNTFLNVIKHLICKHQKIISLSESIESLYTHLSLLQLLWNTIVMCCTGFVII
LIIGTGQDTLGLIKTVSYYLAIILEAFVFCFAGEFLSAKSKSIGDALYAAVWYNMPPSDSRIILFMIVRC
QKRLTITAGRVIDLTFEGFTSIMKASVSYISVINAMY
>XP_012219956.1 PREDICTED: odorant receptor 82a-like isoform X1
[Linepithema humile]
MSGLGKSTMGRASTISESVEAGLRFIGMWPRCIYANINWWTYIVSVAVVQYFQYSYILAHFDMKDLSVTI
DGLSITLGYSLSFLKLINLWFNRRKLYVILDTMDKDWSDGIQSDVSTMIRHANLSRQCSNIMITTNALAV
FFYTIGGPILRSIINKSNQETTTRELPIKMEFPFNVDNSPIFELVLVIQLFHDLSVACIIAMLNALLVTL
VLHVSGQIDIMRKGLLEISPEKNTPEASLAAIKILINRHQRIIDLSDNIEDLFSNIALLQFIWNTLVICC
IGFLIVISVGTDEGATVITKSLIFYVAITLEAFVFCYAGEYLSAKSKSISDAAYECFWYDLTPSECRVLM
FLMLRSQKRLTITAGKMTDLSLEGFTTIMKSSASYISVLRALY
>XP_012219954.1 PREDICTED: odorant receptor 22c-like [Linepithema humile]
MATSTISPSLKIGLRLLGVWPDVSYSTVYWVIYMSSLLIIQYFQYLYVFTHFKLSELSNLVDSLPMTLDY
SLSIFKLTSLWLQRRIMQQILIAMDKDWRECMDVNQYLYVMTIKANVSHFFSNTILSFYGISGVFYVLGD
YAIHIMHLVSDNNDTLRQLPMKVQLPFETEQSPIFEVLVVTLFLHVMANSFTIALLNGLIFSLVLHVSGQ
IDIICEEFRIISEKILLYESSASTLRTLIERHNKVILFSDNIEKLFSFIALMQVVWNTLVMCCLGFIIII
SIHNEGSLFVLIKTILAYIAMMIEAFIFCFAGEYLSLKSNAIAEAAYDVLWYNLPSNQSKIIIFVIMRSY
RRLTITAGKIMDLSLETFASSSTRNSGSYK
>XP_012219953.1 PREDICTED: odorant receptor 4-like [Linepithema humile]
MTVSSIISPSLRIGLRFLGLWPNESYPTLYIYMTSILIAQYFQYLYISTHFKFSEISNLVDGVMSTLFYS
LMFLKLASLWIHRPVIHKILAAIDNDWRECINVEQHLYMMTNKANISHFYSNCMLGITIFAGILYGLGDY
VIHVIHFIKDHNESGRQLPLKVQLPFKTDQSPIFEFLFIILFLLMITTSLTMAIINATILSLVLHVSGQI
DIMCQEFSIVSKQISIYNSSESPLKMLIKRHNRIIIFSDNIEKFFSFIALMQVVWNTLILCCIGIMIIIS
LYNEAGIIALVKMSVSYFTVLLEIFILCFAGEYLSFQSESITDAAYNMLWYNMSSKQVKFIILIIMKSQS
QLTITAGRFMNLSLETFTNIMKSSLSFMSVFHAMY
>XP_012219952.1 PREDICTED: odorant receptor 4-like [Linepithema humile]
MDLPFDSDKRLVYEIVMFSQFLHLLLCACLDGTLNALLIALILHVSDQIDILCGWLLEIFPKKEKSAQST
TKKVITTHQKIIVFSKNIENLYSFVMMVQLLSITLITCSLGVILVASIGTPNALVIIIRTLLFYSVMNME
AFTFCFAGEYLSNKSKAIADAAYNSLWYESNSKDNQIILFLIMRSQNQLTLKVGRFMDLTLSRFSDIIKA
SASYVSVLLAMY
>XP_012219951.1 PREDICTED: odorant receptor 2a-like [Linepithema humile]
MQVLCNTIVSCCLGFIFIISIYNETNVFVLVKAILAYITIMLELFILCFAGEYLSLKNESIADAAYDSLW
YDMPSNYGKIITFIIMRSQSQLKITAGKFMDLSLEAFANILKASASYISVFNAIY
>XP_012219949.1 PREDICTED: odorant receptor 4-like [Linepithema humile]
MMAADWEQKNSTDFSVTINTATLSHYFSNIIVGFHMLTATLYSIMIVFGANNHKMTKVSERPFILKMDLP
FDSDTQFSYGLVLIVQFSYLLITSCALSTLNALLIVLVLHLVGQINILDKWLTEMYSKENGFKPSLTLIR
KLIKKHQRIIDFSENIETIYSNIALMLFVSDTLIICCVGFVIVTSIGTPNAVATIIKSLAFYAVINLEAF
MFCFAGEYLSVKSKTIGDAAYNSQWYETKFQDSRIILLLIMRSQNQLTITVGKFADLSLERFASFVRGAA
SYVSVMLAMY
>XP_012219948.1 PREDICTED: odorant receptor 22c-like [Linepithema humile]
MLIEKHNKVISLSDNIKKLLSIFCLMQIILNTLLICCIGFIIVISLSNEAGIFVLVKVVVGYFAIMTETF
ILCFAGEYLSHKSISIADAAYDSLWYDLPSYQSKIITFIIMRSQSRLTISAGKFTNLSLEAFTTIVKASA

SYISVFLTIY
>XP_012219947.1 PREDICTED: odorant receptor 2a-like [Linepithema humile]
MEILELITDDWTDFANSDTSMRTITRKAKTSAHITNGMVILHTTAIVAYCLGVITAGADVTNQTTELPYF
NKLDLPFSVTTQRMYRLVLMSEFLHLILSNCLAGVVNAILLSLVLHVGGQIEILQCWLEQLTVKNIENRE
EQSIVIAIRKIILKHQKIINFTEMIENIYTYIGLILFVSNSMLICSIGFLIVAAIGTDDAAEQIIKCVLF
FTLTNLEAFIFCYAGEYLKTKSKAIEFATYSCAWYNLKPKDSRVLLFIISRSQKQLTLTAGKMMDLNLES
FASIMNASGSYLSVLLAMQ
>XP_012219946.1 PREDICTED: LOW QUALITY PROTEIN: odorant receptor 4-like [Linepithema humile]
MSEDWIKCGNRGVALSETIRKTKASSRICNGLIILHTFGAIAYVAGILLADVDVTDQTSELPLVMKMEYP
FVIDTRSKYDLVLATQFVYAMVCSWEAGLFNALFLTLTLHLGSQVNILLCWLAEMEPNKIEEENKSFVVS
ITNIIQKHQRVINLSKNIESLYSYIVLLQFTSNTLQICSLGFLIITAIGNPNATEMIGRSLLFYTLTNLE
TFIFCFAGEYLSNKSKAIGNAAYNSAWYDMKTKESNILLFIILRSQKQLKFTTGKMMDLSFESFTSIMKA
SASYLSVLLAMK
>XP_012219944.1 PREDICTED: odorant receptor coreceptor-like [Linepithema humile]
MSTLSPVIKIGLHIFGVWPYLPSTILFRSYWIVMLSTAQVFQYRYVLVNIGMDDFSQFMDGVSSAMASSL
LYIKLVILWVNQRIFFDLLQIMDADWRDYTSNGSRIMKSSADLAHRASRWIVGLQIGSVTFYSLGVLAAN
VNDPGKIEPYTRELILKMALPFNISTEAIYIAVQSVQFYHLFLVGLGITIVNSLLVTLSIGEPNGAAIMV
KSLLFYITMNLEAFIYCFCGEYLSAKSEMIGHAAYDSLWYDFPAKEGRIMLLVIVRSQKRLKITSGKVVD
LSLERFTSVVKASASYISVLLAMQ
>XP_012219943.1 PREDICTED: odorant receptor 4-like [Linepithema humile]
MHLHSNDLFDLMDCFSSFLTQVKFMIKLIIFWMNERKFMKLLTMMAEDWNDCADNDVSMHETTCKAKLAD
RIINAMFTLHTLTIVAYSVGILFADVDVTNHEGELPLLLKVKLPIDITTKQAYRLFLSMQFVHLIMSGCG
TGLLNSLLLALTLHVGGQMDILSNWLNELVPKENEERRESIVITTNKIIQKHQKIISFSEHIEDLYTYIA
LVQFTSNTVLICSLGFLIVTAIGNPDATEQIVRSLLFYTVTNLEAFIFCFAGEYLKNKSKAIGNAAYNSA
WYKLKPENSRKLIFVILRAQKQLTLTIGKIMDLSLESFTSIMKASGSYLSVLLAMQ
>XP_012219942.1 PREDICTED: odorant receptor 4-like [Linepithema humile]
MLTFYGILPGISCVLFCRIFWIVTIILCQFYHYRYLSTHFYSGDLFNLMDCLSSLLAYAKVMSKFIVFWL
NQGKLVKILTMMTEDWNDCANSEINMRETMCKAKLSDRITNAMIILHTMSVIAYSTRIILTDVDITDCMS
EPLYIHKMELPFDVNTQRVYRMVLITQFIYCITGGLAAGTVNALLLTLTLHVGGQMDILHYWLTNLTPKK
NSQKKTESVTANEIIQKHQKIIKFSETIENLYTYIALLQFASNTIMICSLAFLIVSAIGTPDAVEQIIRS
LLFYTITNLEAFIYCYAGEYLNNKSKAIGLAAYNSTWYNLKPKDTRILTFIILRSQKQLSLTVGKMMNLS
LEYFATIMNASGSYLSVMLAMQ
>XP_012219941.1 PREDICTED: odorant receptor 13a-like [Linepithema humile]
MARKSTLNGTGKFILTLCGIWPDASCVLFCRMLWVIASIIILLCHFRYFLTHVYTADFFDLIDCLSSFLA
FLKVFVKCLVFWFNQRKFIEILAIMTNDWKDCAHSDISMQKAMSKAKISDRIINGIFILHAMTIVTYCVG
LIFADVDVTDQTTELPHFSKIDIPLDIKTLRVYRLLLITQFFHLLLCAWMAGITNALLLSLTLHAGGQFE
ILCYWLTQLVPYENENKHKSIVPTTDKIIQKHQKIIHFAEKIESLYTYIALLQFTSNTIMICTLGFLIVT
AIGSPNAIEVIIRSILFYVITSLEAFIFCYAGEYLKNKSNAVSLAVYDTPWYNLKPKDSRVLLFIMKRSQ
KQLMLTAGKMMDLSLESFTSIINASGSYLSMLLAMQ
>XP_012219940.1 PREDICTED: odorant receptor 4-like [Linepithema humile]
MARKSTFNRTLKFMLTLCGIWPGASYVLFCRMFWIVATAIILFCHCRYFLTHVHSAEILDLMDCLSSFLA
YSKVIIKCFVFWLNERKFIKIMATMTEDWNDCANSDISMRETVSKAKISDRITNAIITLHTMTIIAYCIG
IVLIDVDITDSSTELPFFNKLEMPFNINTLRMYRCVLLAQFVNMLMCGWAAGITNSLLLTLTLHAAGQID
ILRYWLTQLVPRENENKHESIAITTNKIIQKHQKIINFSENIESIYTYIALLQFVSNTIMICSLAFLIVT
AIGSPNAAEQIMKCLLFYTITNLEAFIFCFAGEYLSNKSKEIGIAAYNAAWYDLNPKESRVLLFIILRSQ
KQLTLTAGKMMELSLKSFTSIMNASGSYLSMLLAMQ
>XP_012219939.1 PREDICTED: odorant receptor 22c-like [Linepithema humile]
MIVTSTVSPSLKIGLRLLGVWPGVSYSIISWLIYMSSIVILQYFQYLYVFAHFSLNELSNLVDSLPTTLN
YTLTFLKLSSLWINRRIIHQIVDTMNNDWQECVGIEHHLYLMTIKASISHFCSNAMLSFYIIAGILYLLG
EYAIHAVHLAGDYNDTSRQLPVKIQLPFETEQSPIFEVLALMLSLHVISNVCTVSVINALIFTLVLHLGG
QIDIMCQDFKNINEKISFRKSSASTIEMLVERHNKIIFLSDNMEKLFSFIALMQVFWNTLVMCCLGIIVI
ISVNNGAGIIMIVKTIFAYFGIMIEIFNICFAGEYLSLKSKSIADAAYDSLWYNLPLNKSKLISFIIMRS
QKRLAITAGKMTNLSLEAFTSIMKASVSYVSVLYAMY
>XP_012219880.1 PREDICTED: odorant receptor 4-like [Linepithema humile]
MALMQVFGNTLVICCLGFIVVISVGNENSVFMLVKSAIVYVAVMVEAFIFCFAGEYLSHKSKLIADAAYE
SLWYDMPLSQDDESVGIVYICLKCNVLMLTKKSTAIC

>XP_012219879.1 PREDICTED: odorant receptor 22c-like [Linepithema humile]
MLIERHNKVILFAENIENFVSSIALMQVVSNTSVISCLGFIVVISLSNEDGAFMLLKVISPYIALLMEIF
LFCFAGEYLSHKSKTIADAAYDSLWYNMPLNRSKIIAFIIMRSQTRLTITAGKIISLSLESFASISELMN
LVDSISVTLEYSLTVLKLISLRIQHR
>XP_012219877.1 PREDICTED: odorant receptor 85b-like [Linepithema humile]
MRATTISTSVEIGLRFVGIWPGLPYGSFTWFTYMSSVAVVLYFQYAYIFGHFNANDVSNLIDALSNTLAY
SLTFLKLFSLWSNRRTFYNILLTMNEDWSNVNRDRSVLCAMTDNANLSRRCSNILITINIIAATCYAAIS
LIHLSTVSEENLNVSLKVLPIKMELPFEVNATPFFELLVAGVFLHEVSVATLNAVTISLILSLVLHVSGQ
IDILRRDLLTICDSGSPQRDSNVKLLTIQHQRIITFSDNIEELYSNIALMQFLSNTVVICCIGLTLINAL
DKDGVSVLLLKSVSFYIAITLEAFIFCFAGEYLSAKSKSIGDAAYESLWYNMTPTVSRILMLIMLRSQKR
LTITAGNVMDLSLEAFTVVMKASASYMSVLHAMY
>XP_012219875.1 PREDICTED: odorant receptor 4-like [Linepithema humile]
MTTSTVCFSIKSILYIFGVWPDTSCLALRRLFWTVLLTVTLILQYTYVALHFHTDSLTDLMDTVSTCLAY
NLLFIKLIIFWINQRKFHNILTRIAADWKECANDPFSMQVTASMASLSHRVSNTIIGIHMAAVVTYSLGA
FLSNSGDETFNASTIPVRVFIINMEYPFDSSSSPVYELVVIMQFIQLVLNACAIDVINALIMTLVLHVSG
QIDILQEWLTNIFSKDSIHGMTGTTIKNLIGKHQKIILFAEDVENLYCYIALMQFIAITLIICSIGFVIV
SSLDSPDASTMLVKTLMFYIVMNLEAFTFCFAGEYLSSKSQNIANAAYGSLWYNVHVNKSQIIIFLILRS
QKRLTITIGKVADLSLERFASIMKASASYISVLLAMS
>XP_012218439.1 PREDICTED: odorant receptor Or1-like isoform X3
[Linepithema humile]
MIISWGDMSKIVAGATLLMTNCTHASKIVVFLREQARIQALLDIANSPVFHRHDKTHQDLLKSYTKKSIF
HHAVYQSFGAIAVFCWGFTPLADLIAGRSRRLPMEGWYPYNTTTTPAFEITAGHQGVAVIIACFHNVAMD
TLMTGLITVACCQLAILERNIMSINNEESVSKMQNKNTLFITDEKALCYQRLKKCTVHSNIIFHFTKEIQ
DIFGTVIFFQFLSNCVIICLIAFNVSQMKVYIPAVLIGMLTYLCCMTYQIFIFCWHEHTYSHSCVLEWLV
LRHGEIQTQFTDCDDQSPSSIYFKRWQHHVTVFGHFRTDTTDVLLDFYGTSRFSCLTRD
>XP_012218435.1 PREDICTED: odorant receptor Or1-like isoform X1
[Linepithema humile]
MIISWGDMSKIVAGATLLMTNCTHASKIVVFLREQARIQALLDIANSPVFHRHDKTHQDLLKSYTKKSIF
HHAVYQSFGAIAVFCWGFTPLADLIAGRSRRLPMEGWYPYNTTTTPAFEITAGHQGVAVIIACFHNVAMD
TLMTGLITVACCQLAILERNIMSINNEESVSKMQNKNTLFITDEKALCYQRLKKCTVHSNIIFHFTKEIQ
DIFGTVIFFQFLSNCVIICLIAFNVSQMKVYIPAVLIGMLTYLCCMTYQIFIFCWHGNELHLHSIRIVTA
AYSSGWFSGTERFKRSLQIVMIRAHRPFILSAGNIMLLSLDTFVQFLYILDTTDVLLDFYGTSRFSCLTR
D
>XP_012215916.1 PREDICTED: odorant receptor Or2-like [Linepithema humile]
MDRSNGYNDLEWAIGLNRRMLKLVGLWPQNSTISYEVIFSKFCLLFNIITLIFVLTIPALASLIRVWGDM
ILMIDNLQYTLPLLITILKVSIMWCKREALSPLIDMVIKDWTKVKMEEERNVMLKQARITRILAMCGGFM
IFLTLLITFSSLFFGLTLRHVTNLTDPGKPLPLQTYYLHDVSKSPQFELTFFTQGIAVTTSGLSYTAVDN
FLGLLLLHICGQIENLHLRLLNLGKNSNFIATLKFNVKDHIRLISSIQIIDNTFNLMLLGLIFFFGVLFC
LQGFLIINVLKQGENLTLLHFIWYISANVCVLLHMCLYCAIGEFLVTQSEKIHSATYEYLWYTLEPKAAK
HLILVMLRAKKPLYITAGKTFPMTMATFCNLLKTSAGYVSVLLANRD
>XP_012214674.1 PREDICTED: odorant receptor Or2-like [Linepithema humile]
MCNRSRLPERTLEVHNVHHEQDIHYTMQLCRWVLKPIGIWHLIYGHPSQNEKLTSIALIVACFSALCFVL
IPAGLYTLFREKDINVKVKLFGPVGFCLTSTIKYCYLGARGAAFGNCIQHVENDWRVVRDQDHRTIMLRN
ALMGRRLTTLCAIFLYTGGLSYHTIMPLSSTRRINENVTSRMHTYPGYDLFFDPVASPAYEIVFCVHCLF
ALVTYNITTAACSLAAIFVTHACGQAEILMALLGDLVEGKKSKGTVEKRLTVIAKHHVRILRFSANVEEV
LREICLMELVASTLIICLLEYYCMTEWKNSDAVAILTYFILLISFTFNILIFCYIGELLVEQYSKIGSAV
YEISWYNLSGNKARNLVLIIAMSHYPPKLTAGKFFDLSINTFGVVLRTSVAYLNLLRTVTQ
>XP_012214673.1 PREDICTED: odorant receptor 4-like [Linepithema humile]
MGNSGVEYTTLPNANYQQDMQYVFRPCSWILGSIGIWPLAIRGIGRNISKIAIIICNFALSFAIVPCILH
IVYDEKDLNIRLKLSGLLGFCLMSMMKYCVLALRRPKIMRCIEHVKSDWWQVKFSSDRELMLKYAGIGRK
LSIISASSMYIAGFIYHTILPFCTVHEVNNQTIRPLVYPTYSKFYQTQMSPVYEIVYLAHCVCGYTMYSV
TAGLCGLAAIFVTHACGQIDIIASRLEDLSQGKNFEKSSNVNQRIANIVKSHVQVLRFSVAVEEILQEVC
LLEFVSSIFTMCLPEYYCIVDWQDSDTVGLTTYFLLFISFCFNMYILCYTGELLMEKSSQIGSMCFMIDW
YQLPPKSIRSLILVIAMSSHPIKISAGNMFDLSLATFGNVLKTSLAYLSFLRTLVM
>XP_012214672.1 PREDICTED: odorant receptor 4-like [Linepithema humile]
MSSVHDEHATSQNSSYQRDIRYIFKLNSWILGSLGIWPIAIRGIGQHASKIVIVIFNLALSFAIVPCALH
IIYDEKDITMRLKLCGLLVFCLTAMTKYCILAIRRPKILRCIEYVKSDWWQVTFRTDRELMLKYATTGRN
LTIIGVSFMYIAGIVYHMILPFCSEHKINNQTIRPLVYPVYSKFYQSQISPIYELVYVAHCMCGYTICSV

TAGACGLAALFTTHACGQIQMIIAQLENLLDGDKFEQTLNVQQRIAAIVKGHVRVIRFAAVIEEVLQEVC
LVEFASSVCTICLLEYYCIVDWQADDRIGLTTYFMLFVSFCFNIYILCYIGELLMEKSSQIGSICYMINW
YELSPKSARSFILILAMSGHPIKITAGRMADLSLTTFGNVLKTSLAYLSFLRTLVM
>XP_012214671.1 PREDICTED: odorant receptor 22c-like [Linepithema humile]
MLDLSESKSLKPIANGWNYSIQLNRWFLKPIGAWPLTLCETTMEKFSCVILTIISCFLICFLLIPCTLCT
ILVDTDLDTKIRMIGPVSFILMAAIKQYILIARSENIAACIRHVHVDWNRIALGREKDREIMLDNAKFGR
WLSSVSAVFMYSAGIFFTTVMPICARRTEIIDNETVRSLSFPIYRGLFDPRTTPSFEIAQFAQALAGYVI
YTITISVCSLAAVFVMHACGQFQILMMKLEDLADGKERKSARNTPEKRLGDIVEYHIRILGFITDTEQLL
NEIFFVDVVGCTLNICFLGFNMITEWEHRETLGTITFSSLLISFTFNIFIFCYIGEILSEQCIQIGTKSY
MIDWYRIPHKGALGLTLVMTMSNTTIKLTAGKFMDLSLTSFVSIVKAAVAYLNLLRKFYV
>XP_012214668.1 PREDICTED: odorant receptor 4-like [Linepithema humile]
MTNPKLVPTSVEFKGHNDYSLQLNRWFLKPIGAWPQMDASHTIWKILVSLQIFICSTMVAVITVPCLLYI
LLEESDIKLKLGALGPLFHRIMGSINYWVLLKRSDDIRDCIRHMETDWRLIRRTQDREVMLQHAKFGRFV
AGICGVIMQGGTFMFSIARAMKTTTITIDNQTFTTYPMTCPIYSNLIDIRFSPVNEIALVIQFLSTFVVS
SSTVGACSLAAVFAMHACAQLNVLYIWLDELVTNEKKENRKLVEQKLTIIVEHHLRALSFISRIESIMHK
IALVELTGCTINMCLIGYYSIMAWQVFDAAKITSYIIVYVSLCFNIFIFCYIGEIITEQCKHVGEMAYMT
DWYKLHHKTALGLVLIIARSSNVIKITAGKLFQLSISTFGDVIKTSMVYLNLLRALTM
>XP_012214663.1 PREDICTED: odorant receptor 4-like isoform X1
[Linepithema humile]
MNAHPQRAAKSADSKNISDNEIKYQYKWLLLSIGAWPQTTIVKKILVSIQIVISASSVAVVMIPCLLYVL
FDDDSIQIRLGALVSLLFRIMSTATYWVIITRQKDIYDCILHMSADWKRIQRTTDRKLMIKYDKIGNFLV
KLSTSFVFFGSHVFIIARAAKTITFTIGNETFKTHPMACPIYKKIIDVRFSPANEIFLFLQFLSSLVSNC
IIVAFFSIAAVCAMHACGQLQVLYTWLNEVVQNHEENNLAQEKIGDIVEHHLRIFSFTTNLESIMSRTCL
ITIMACTLQLCFLGYYAIMNWAAFDAAKMASYICVYTSISYCIFVYCYIGEVVTEQCKQVAEVAYMTEWY
KLPYTIARNYVLIILRSNQAVKMTAGKMLQLSIATFADVSTYIIVIMPVLEKPLTKLIFY
>XP_012214662.1 PREDICTED: odorant receptor 22c-like [Linepithema humile]
MRFHEVMMNAHPKRAVKSANSENDNKNDNKIKYKYKWLLLSIGAWPQTTIVKKILVSIQIFISASSVAII
MIPCMLYILFDDDHIKIRLGALVSLLFRIMSSTNYCLIILHKKSIFHCIRHMDTDWKLIQKTNERKLMDQ
YDKTGSFMLKFYMTFLFCGSNLFLIARAIKTTTFTIGNETFTTHPMACPIYKKIIDVRFNPTNEIFLVLQ
LISSLVSSFIALTSFSIIAVSAMHACGQLQILYTWLNEVVQNHKQKNSAEKKIAIIMEHHIRIFSFIANL
ESIMSPISLSTIMTCTLELCLLGYYAIMNWAAFDAAKMISYLVVYITVFYNIYVVYCYIGEILTEQCKNVG
EIAYMTDWYKLPSTTARSYILIIILRSNHVVKMTAGKILQLSIATFADIVKTSMVYLNFLRTMTA
>XP_012214661.1 PREDICTED: odorant receptor 82a-like [Linepithema humile]
MNTNSKRDLKSFDFENISDYSLQLNRWFLISLGAWPQGTSSTVKRIVVSMQILIFSSSIAIITIPCMLYV
SFEREDIQTKLSAVVPLLHRIMGSVNYWTLLTRHKDIYNCIRHMETDWKLVQKIDDREVMLQYAKIGRFM
AGFCAVFMHGSAFFFTVVRAIKTTTITVGNETFTMHPMTCPVYSKIIDARFSPANEIFLGVQVFSAFVVG
SSTVAVCSLAAVFAMHACGQLNVLYMWLNELVKYDEKKNLAEQRLAAIVEHHLRVLSFIANMESIMHKAC
LAELMGCTLNMCLLGYYSIMNWAAFDAAKILSYVTVYMSMSFNIFIFCYIGEILTEQCKHVGEIAYMTNW
YKLPHKTALGLVLIIMRSSLVIKITAGKLFQLSIATFGDIIKTSMAYLNILRTMTT
>XP_012233382.1 PREDICTED: odorant receptor 4-like [Linepithema humile]
MSVFALFVGPIVLDEPLPAYAEFPFDVSHQPLRAITYIHQIIVGLYIAAHLSVNAFMAFLLWLASARLKL
LIEELRIITNIYDFIKCIKKHQQLLEYAGEVAFTVRSFAFGTVFSSTVSLIVFGLIFITGASLPLKIQCT
FLAISALLEVFMYAWPAEHLIHICNHVAQTTFEIEWYNELGNLRKNIQMIILRSQKPILVVLPCGLPSLS
LRYYASYLSTIFSYFTTMRIMFEGQSDVL
>XP_012229928.1 PREDICTED: odorant receptor Or2-like [Linepithema humile]
MKETTREKLMANVRVFILIIMVTFVCVIPCIHSLIRVWGDLMSMTDNLQFTLPLVSMVMKLIIMWNKKAV
LAPILCMITEDWLKLKSDKERKTMIRCARIPRMIIICGFVIMFASFILLFILPSFGITMRYITNVTDPGK
PLPLQTYYFYDTDTSPYFELTFIAQGVTLMVSAMGYTAIDSLFGLLIFHVCGQLQNLKGRLMDRREKDSN
FVRVLADAIRDHVRLIRCIKDIESTFTLMLLGLFLYFGTLFSLYGFLLVTIVTDGRHLSLVRLLFLVTVV
MNIFAHMCLYCAVGEFLIAQCEGVYQAACEYHWYELEPKEARSLILLMARANKPLYVTVGKIFPLTMNAF
CSLLKTSGGYISVLLARRD
>XP_012227125.1 PREDICTED: odorant receptor Or2-like [Linepithema humile]
MHQTNFLRFTALTLCANSSVAHETLISGMMIQTCAQFEILSYRTHALPTLLTEAEKNSKSDKDLMTRERM
LIRDLIHHHLYVYKFAHTVNAVFMLMMFIQFSISSLVLCMIVYKLSTMTSLFTLDFAYLSSYLCAMLMQI
YLYCWYGNEVTLKSIDIGNAVYEMDWTTLKVRVMKDLLIIMMRASKPVKMSSGYVVTLTLESFMSILKLS
YSAYNFLKDS
>XP_012221815.1 PREDICTED: odorant receptor Or2-like [Linepithema humile]
MDSWRTITEDCECFIIFVLVTGGYIKISSVVLNTKNINQLLSVIDYHWRVFVHSVEIQIMHDYAVAGRKI

TIFFIVTFYSLLCLFMAIPPLPKLLNIIKPLNESRPNIYMFDVDYSFDREEYYFTVLLHSYITAVIGISM
LLSVDSLYIVLTQHGCSLFAAVGQRLENLNSAISSWKDNELEKTHKSGLANCVQYDRDEKIYRELIVILW
KHQLCLEYVNLLNFSFGHFSFMYIFLNMMAISLLLMQILALMDRKGQMIRFVAMLIGAFGHLFILNFPGQ
RIMDHSSDIFDKAYNVLWYKLSRKSTQLLSILLYRTLSPCTLTAGNMYVLSLINYASMAQTSFSFFTTLS
SLQ
>XP_012231460.1 PREDICTED: odorant receptor Or2-like [Linepithema humile]
MNYKDVKTIINKLNEEPFQPLDAGELEIREKFDKIIRFNTLRYSTLIEMSWSCSGLTSLLVDFRHTRLTY
REWIPYNSSSYIVFCITFAHQFLSTFYCATINVACDTLICGLLMHVCCQIEILEYRLKKLVKDQDTLGYC
IYHHNNIFEFAQLINTKFSQIIGFQFITSTLIICSNLFQLSKSSISADYIALFIYTCCMLTEIFIYCWFG
NKVKSKSIELANSIYQMEWPVLNNNIKKDLLVIMKRALVPIEFTTVHIISLNLDSFVSLLKTSYSAYNLL
VRMQEE
>XP_012230984.1 PREDICTED: odorant receptor 46a, isoform B-like
[Linepithema humile]
MRVLKFTLLVCAFAGCWQPSSWTSLFKHIIYKTYAMFLVSALYIFSISQFMNMIFNVENSDEFTNSLYMM
LTVFVAGYKQVYMWVDRKNIMVIFNVLNEKPFAACETREVMIQQKFEKIIQNSILRYLFIVLMAIVSTIL
TSLVTEFKKGNLTYKAWVPFDYSSFVIYILVYIHQLIGMATSGIVNVACEGVICALLLHICCQLEILEYR
LTKMTQNQDILRDCISHHIRIFEYAYAVNNMFAKIIALQFAVSMLVVCSNLYRLAMATDYVTLISLSLYT
GAILTQIFIYCWFGNEVKMKSLQLANNIYNMEWSMLNKSNKKGLLLIMKRATIPIEFNSMYIITMNLESF
VALLKMSYSAFNLLHQTSE
>XP_012230862.1 PREDICTED: odorant receptor 46a, isoform A-like
[Linepithema humile]
MHIVLNVDNPDGFTDALYMMLTVFVAAYKQLYMWIDRKHLMSLLNVFTEKPFAPYETQEVMIQEKFEKLV
QNNTLRYLIIVMMSITSIVLTSVFMDFTNRRLTYKAWVPFNYSSTGSYYLVYTHQMIAMSTSGIVNVACE
SIIFGLLLHICCQFEILEYRLTKITHDEDILRDCINHHNRIFEYAYIVNNTFAKIIALQFAVSMLVVCSN
LYRIAMATDYMSFIPLIMYTSAILVQIFIYCWFGNEVKLKSLHVMNSIYEMDWPALSNSNKKALLLIMKR
TTTPIEFSSAYIITMNLDSFVALLKISYSISNVLRQTQV
>XP_012230606.1 PREDICTED: odorant receptor 46a, isoform A-like
[Linepithema humile]
MAQLMDIILNVDNADDFTNNLNMMLTSSAACYKMFIMWLNYDKVAALISCLTEEPFKPLDPGEMRIRQQF
NQTTRNNTLRYTILIETTCSFIALMSLLTDFRHRRLTYREWVPYNYSSHMAFCFTYAQQMISTFHCATVN
VACDTLICGFLTHVCCQLEILEYRLKRLSNNNVTLGYCIRHHNRIFEFAKLVNIRFSLIIGFQFMASMMV
ICSNLYQLTKSALSPDHVPLIMYTSCMLTQIFIYCWFGNKVKTKSVKVVDSIFQMEWSILNNSIKKSLFV
IMQRALIPIEISTAYILTLNLDSFVSLLKTSYSAYNLLVQV
>XP_012230503.1 PREDICTED: odorant receptor Or1-like [Linepithema humile]
MCRRIVYNTYTVFISLLLFTFMLPQLMDIVLNVDNPNDFTDTFYVMLAMVIACCKMLSLLLNRKHIKILT
GALIEKPFRPLEPDEIKIRQKFDNIIQINTIWYIILVETTCASITLTSLLTDFRKRNLTYREWMPYGYSS
DIVYYIIYFRQLISLTTASVVNVACDSVICGLLLHICCQIEILECRLKKSLRDRVDLGECIRLHDRIYKF
AYMINEKFRFIIVVQFAVSTLVVCSNLYRLAETTLSAKYIPLILYTCCMFTQILIYCWYGNEVKLKSVQF
ADEVFGMNWVTENKKVKDNLIMIMNRSTMPIEFSSAHLNLNLDSFVKLLKISYSAYSILQQM
>XP_012230397.1 PREDICTED: odorant receptor Or2-like [Linepithema humile]
MLSQLMDLVLNVDNTDDFTDTFYIMLAMVISCCKMTGLLINRKNIGTFTSILTQKPFIPLEPDEKKIRYK
FDKTIQTNTLRYTILVEATCACTALISLLTDFRKGKLTYREWTPYNYSSKVVFSIIYARQLISSTFGSMV
NVACDSLICGLLLHVCCQLEILEYRLNKISLGQNKLRECVRQHAHIFKFAFMVNEKFKIIIAIQFIISTL
VVCSNLYQLAKITISAQSFPLILYAFAMLTQILIYCWYGNEVKLKSVQLAANIFKMKWPTLNQSVKASLL
IIMNRSLVPIEFSSAYILTMNLDSFVSLLRISYSAYTLLQQMQVT
>AGI62937.2 olfactory coreceptor [Macrocentrus cingulum]
MKFKQQGLVADLMPNIRLMQISGHFMFNYYSDGKKFMHKIYCSVHLFLILLQFALCGLNLAMEADDVDQL
TANTVTVLFFLHAIVKIGYFGVRSKLFYRTLAIWNNPNSHPLFAESNARYHSIALTKMRRLLFCVGAATV
LTIIAWTGITFAENPVRILTDKVTNETTTIELPRLMVRSWYPFNAKSGMAHIGMLIFQFYWLTITMVDCN
SLDVLFCSWLLFACEQLQHLKAIMKPLMELSATLDTVVPNSSELFKAGSADHLRDTAGSVPSATQPNGES
MLDLDLRGIYSNRQDFTATFRPTAGTQFTGGVGPNGLTKKQEMLVRSAIKYWVERHKHVVRLVTAIGDAY
GVALLFHMLITTISLTLLAYQATKVNGLNVYAATTIGYFSYALAQVFLFCIFGNRLIEESSSVMEAAYSC
HWYDGSEEAKTFVQIVCQQCQKAMSISGAKFFTVSLDLFASVLGAVVTYFMVLEQLK
>AID59301.1 odorant receptor 1 [Macrocentrus cingulum]
MEQSWRASNYLTLQEFFTTMFRHLISGLAFFTLHACAHARNCRIQFIKLGRTYDKEAIVQCILRHQRLLE
YSRDIEDVFSCVYLIQTVLSISVICGFSFTLLLLGKDEYIKYIVLIIGAMAQLWIYCWPPHMLIVETTLV
AEAAYNLKWETWSKEQRKIVEIIIFRSQIPAKLTAGKFNTISIDTFSSICSTAMSFLRY
>AID59302.1 odorant receptor 2 [Macrocentrus cingulum]

MELLKVSFTVLQLSGFWCPVTWSGWKMWLYKIYTILVIFTLYSVTISQLIELLRSIDDAQEFIKNSLILL
TTTNACAKVANILQKRSDILKLVDMLQSEPCCPCNDTEHSIQNRFNHIISRNSLLYTTLTEVSVFFVALG
TILSDTPQRRLAFKA
>AID59303.1 odorant receptor 3 [Macrocentrus cingulum]
MPREEKLSEYFSYRTTVVRFLTIVGLWPKQQTELWYKLLPVLQLIANISSVLATAMFVRKHVNNLDRVIK
GMSSMMSYLSNVLKTTCVIINREDAAKLHQMLDAQFFMLLNRPEVSKIILSDVTTFRRLSVMMSFLTYLS
CTIYASRPVVIIIYQHVKQIYPITYGLIFPGVYPWKISPNGALYKFHYGLETVSTILAFSVCAGIDLLFT
LYIFQMIAQLREMSHCMSNIDTTKNGHKAVENCVIKYEQLMKMS
>AID59304.1 odorant receptor 6, partial [Macrocentrus cingulum]
MMQTCAQFEILKSRFRTLSRVPRCKEDDKTWLNDFRDEEKKYEFERKVIANCVEHHLQIFRFAEFSNETF
LSTIFFQYSVSLVIICASVYGLSNVAPFTAKSLRFLSIFFVCQCRYSFSVSTGDKVTNESINLHETSISW
DGVTLGSNTAKKPAEQNGPGPLKAHPIFQWKTRGGGLPPQTSFQRGLFKKLPHIFHLLKNRFLKKKKPPP
PKKGGPFL
>AID59305.1 odorant receptor 7 [Macrocentrus cingulum]
MKIVLPIIYQESMYFIVHSAVNDWASVGHGKHREIMIRYAYIGRIVFMVQMIGAYMTIIPLIFGNLPSIN
PVLNGANDNTTIVYRNIPIGPNCWVSAHLSTNVYLAYYFLITLHLIILCTAYIGGDVYIFGIAMHVCGQF
QLLYDNMEKLNANDIYFVQRINLSRLCQRHNHLVKLTNEFERTFNFVLLIQLLRVPSLLAFQVFFFYWAS
KMENNDIGIPSLISIYLLYFHLFFIII
>AID59306.1 odorant receptor 8 [Macrocentrus cingulum]
MLKYAMMGKVMTVLGMLGASFSLAFYHLPLIFGIVPRTITNLTDQPGALLPLQSVYLYNISTPLRFYLTE
VSELIGGICAITAYTGIDVLFGVIVLHACGQLENLAKRVEVIVGETNFSDVLRLHVQNHCRLIQFVMKIE
QSCSLMLLGLFASVALTFCVLGFQLIEACTDKNLDISMPQVIFYIQFLSYCMFLMFVYWLGRPKSSQLR
>MdemOrco
MMKTKHQGLVADLMPNIRLMQISGHFMFNYYGEGKKLMHKIYCSVHLFLILLQFGFVAINLVKEKEDVDDLTA
NTITILFFLHTLIKIIYFAARSKLFYRTLAIWNNPNSHPLFAESNARYHSIALTKMRRLLFCVGAATVATTIS
WTTLTFFEDPHVERINKETNETYIEEIPRLLVRSWYPFDARHGMAHIGMLIYQIYWLFICTVDANSIDVLFCS
WLLFACEQLQHLKAIMKPLMELSATLDTVVPNSGELFKAGSADHLRDNDGVPAEPAMNGDNMLDMDLRGIYSN
RQDFTATFRPTAGTQYNGGVGPNQLTKKQEMLVRSAIKYWVERHKHIVRLVTAIGDAYGVALLFHMLITTITL
TLLAYQATKVNGVNVYAASTIGYLLYSLGQVFLFCIFGNRLIEESSSVMEAAYSCHWYDGSEEAKTFVQIVCQ
QCQKAMSISGAKFFTVSLDLFASVLGAVVTYFMVLVQLK
>MdemOr2
GCWPLNRSIKQTILFVVSITYYLIHLSMSYTDLINVFGNLELMTINLMETAVQMIAIIRLVYLKLSPRVKKTI
ISYKESLNIERTNDSTEVKIYDYYRSVAKSYFSIIMPFSAMTSFIWYLMPLQNCLIAWLKNEPVILVPPYKVV
YVFFNLTSMEQVIVAYIYQAPMSFLPLGFIGIICLKIVIIANVCSQLAVLSYRIKNLNFKDSNKKFSVFGYIV
RKHWELLRFIMDIDDTWTLIFCFELVLSTILCALVTYNAVMAIGNKDKIEIISLFTYVISSLLILFANCLMGE
MLKFESENLQEALYSCDWFEMSINHKRSLLICMTRSQIPLRLTAGKCYVVYSFNAYVQIVKSAMGCVSLLRTL
>MdemOr3
IINLVGAWPIQRTLKRNIIFGVYLLYHNLYLVTSYSYLVIVFGNLELMTGNLMESAIQSMTLIRLLTINLSPQ
LKKNIFVVRQNLTKKHYEDSTEKNLYNRYYLIAKKYCKFTMIFTIVTSVSWYFIPIQTCAISWFLNKPVVLVA
PYKVHLFFNVSSLKRIVILYIFQSPLQYPPICCAATINVNVIIITNICGQMAIFSHRIKNLKTNDSSKKVFNN
IVNKHLELVRLTKNFEESWTIIFGFELIVTTILCALVLYNALMAVSADDKIGLISLVAYVYASLLFLFANCLM
GELLKFESENLMKSLYICDWYNMSISNRRALLICMTRAQLPLEITAGKFYVVYSYNAYIQIIKSAMAYVSMLRT
L
>MdemOr4
GVWPIEPTLKRQILFYIFIVYYIVYLSMGYNDLFEIIGNLDLITENLMTTTMQTIMLVRMLYVKFSKNIPEII
MAVKVGILEKNGYSQLEKEIFFGYYSLAGKYHRVTIVFTFMAGFSWCLLPLQNYVISLLLNRPAILIAPYRVK
IFFLNATSFKSIVVIYILETPLPLPPVCYVAIVNLQVVLVIYICAHMAILSQRICNFRLENESSYREFSEIIN
IHLKIIKLAKKIENTWAPVYLFEMVVLTPIVALVMYYGLLAFDAGEKVTFISLCMYVCAAMSCLFANCLMGEM
LKTECDRLLDAYYHCNWYDMPISDRRVVLTCMTHTKNPLTITAGKVYTFSFNAFASILKSSLAYVSILRTLL
>MdemOr6
MIQILNAWPIDRTTRGLLLFIFYLIYENIYLSMAYNDFFSIFGDLQLMTANLLGTLVQTVMMTRLTFVRFSKS
LKNIIIEVLDNFCDKNYEDPNEKRIYVQYSSMAVKFYKVTMMFGAGSAFGFGLLPLQKCFISWLLHKPVVLEL
PFKIKVFYHSNLSADQTLLLYMYELPLPINACCFIASINLQFMIIMNICARTAILRNLIKHTNVKERTSNQKL
FFKQITLKHLVLLKLIDAFEQTWTPIFCFETIILVPLTSLVMFSVLAIDGIDTIAILTLITYVASMLSCLFAN
CWMGQLLLDERDQLLEALYGCSWYEMSNEGRKFWLICTVNCGNIPMRITAGKIYIFSFNSFTGILKSSLGYVS
LLRTLTL
>MdemOr7

LFGKIAWMIQILNAWPIDRTTRGLLLFIFYLIYENIYLSMAYNDFFSIFGDLQLMTANLLGTLVQTVMMTRLT
FVRFSKSLKNIIIEVLDNFCDKNYEDPNEKRIYVQYSSMAVKFYKVTMMFGAGSAFGFGLLPLQKCFISCKKI
QNLGFSLMLLPFKIKVFYHSNLSADQTLLLYMYELPLPINACCFIASINLQFMIIMNICARTAILRNLITNMN
IKGKTIREKLFFKQIALKHLVLLKLTEAFEQTWAPIFCFETIIFVPLMSLVMFSVLALDATETVAILTLVTYV
GSVMSCLFANCWMGQLLLDERDQLLDAFYLCKWYEMSNECKRFWLICMINCANIPMRITAGKIYIFSVNGFTG
ILKSSLGYVSLLRTL
>MdemOr8
WPLNPTNTKLILFLVYLIYENIYLLIAYNDFFFIFGDLQLMTTNLIGSLVQTIMMIRLIFVKFSKKVKNIIIE
IQDDVCEKNYENLDDKKIYVQYSSLAITFYKVTMGFGTGAGLSFFILPLQNCILSYEAYPVASSKIILPYRVK
LFNSNETSFKESILLYIFEAPLPVTALCFFASVNMQFIIITNICARIAILTSKIKKVRGEVKSVSAITHIVIK
HLELIRLTRKVDEIWTPVFCVEIIVLIPLLALVMFSAILALEANETIAFMTLFTYVGAVLSCLFANCLMGELL
LTERAQLLEAFYLCNWYKMSIESKRSWLICMLNCARIPMHMTAGKIYIYSFNGFTGILKSSMGYVSLLRTL
>MdemOr9
MFNRNKKSDDLTTTSCEELWNLDITISRAVGVSFRNAFGGEYRKPNLLETTLYIVGLIVHSLFICFALCTLYV
VKNIDLDSITNLMPVVFGISMTILKGSTLWFHRRELFTILKEFHTRWSYTRTKVHSQNKIHMLINTSKKVRYC
YMTAILVTSLSFGLRPYVILLTYYFETVVLQSNTTIDFSVVIYPLVYPFACQTMNRYVLLLMYEQSVMFFAIC
YVTCETIFIQLMTHTSINFLVLADDFRNMNQHIDINNNDSDNNNVGDQHMIELVNKHRSMLSICEKIESSYSL
VVFFTMVMNGLDLCLCILSLDKQLSEGNWELVIKNVVHAFAIFVQIIIYCNFSNTATEMVSIISNILYDNS
>MdemOr10
INLDNLYFSRFSGIWISDSSTPLRKLISLFCRLLSFFVLTSYILTSIADLVVNCNDLIVIVDDSCFIAGAGSA
FFKICTVIFKYQKFEKLITGIHDPVDVLRQSNDKGVMKIIKRCVFFETLDYYLWSNATFVLGFATIFLVTSQK
GQLPSRAIFPFDITKPSMYAIALSIQIYTVIYGLISLMAIEITMWGLLRWTTVQIQVLSYNYKNCDRNLSRRK
SRVLSRSQFDGIEKLNLSETDNEEMEIKNFLLFENDKSNCQVDINCFQWRLRYCIKHHQRIVEIVNRLNDTMS
TCLMVQFAVSTMIFCLNGFLAVTFPHDKKRLMRSIFFLLVGFIQIFYWCRFGNELKFQADYLTTSQWMSGWES
NFNSNLKNYLTTAMIRTMKPVEIRAGGLFILSMETFISILKNSYSVFVLLTTV
>MdemOr11
WRLTSILLSFLGVWPFQKRRHQIIFGSLTFFFINSIFIPQIVDLIRVWPDLEMIAERVTIVGFIIVSLKFYTY
VINMKKIKILLQKIYDDFHSQDLSEEEIQMLWKNAAKNSKSIRIYIWYIYVTLVTFLCLPMIPKIMDTVKPLN
HTRDVFNLYEVEYGIDREKYQLLIYAHAYLITPYPGTLVVACDSLYGNGLFHAWSMFDIIGYKLRNIATHRFI
THDERANIKDEADEIYQALTACIKMHKKNLMYTQYFEKTFSLSLGLILAANIIALCITLLQSLLRINELGEVA
RFGGYALGQILHIFFLSYYGQKLIDAGEELSDSLYQSEWYSLPLKSRKLFVALLIKFNEPVCLTVGKLYKMSN
ENFSVIIKTATSYFMVLNSI
>MdemOr12
MEHYYKKLTDPKDKETLNVQYAIGYTEWFINLLAIPNSNIKLSIKQKIFSNIKFFIIIVLFWWNISFRFIDML
INARGFDEQILLVAPLSFSIVILLKYIIILYRRKEIMEFIGHIRLDWKRIEFQQEKSLMVKNAQISNKITIIF
MILMYMSGLFYNFLMPIILPILFHTKTNTSDRLPIFQGYDVIFNAQVSPLYEIAFFFEVYAVIIGFSILIFMC
HLTAVSVTHACSQIQTIKYLLKCLIHDLTKKKTFHIKISAIVSRHVRILVFSNNIRKSLFELCLLEIGASTIL
FCIDEYCFLKMLEKNDFANMIPYLMLFAALNFNILVLCYFSELLDSQFAEIGIHSCMTEWYRFPLKIRKYLIL
IICMSQRSQKLTAGGIIDISYTTYVQIIKAGFTYLQLLR
>MdemOr13
YTQLLMKILAIPQSEFKSLSKKKIFSIIHSIIVISLSVWNIFFRCLYTYLNVNDFDEQIIVITPVIFIFVILI
KYFIILYRHKEINQSIEYIKKDWKRIKSREEKSFMIDNAQISNQLSFLIIVLMYTSGMFYNVVMPLILPIVFK
TNTHSNTSERLPIFPGYDVAFNAKISPVYEITYVFFICAVIVVFSTITLMYILTIVLVSHVRGQIQIIINLLK
LSINNVTDEKILLMKMSTLIRHHMRTLTFSNHIRKTLYELCFVDLWASAIFFCLDEYCFLKMLDQNDFMNMIP
YAMLFISLNFNILIMCYSSELLDSQFVEIGEETYKTEWYKFSVKIRTYFILIISISQRSQKITAGGIVELSYA
TYLSIIKTGFAYMQILR
>MdemOr15
MTVLENSFFLFTCVGFWRPVKWNGLKAILYNFYTVFVVITNSLFFISAFMDIDYIHFDFFGSMDLITLMLQFV
ENTPKVLCVMNDRNVILDVLRHFQSDPLNPKNKYEKLIQQKFDNFNRRVNLWFPVLGFTSIMWYTINHISLME
SPTVLPYDGKIPYNYSSNKKIYLLTAVNQIYSVFSLASINAAFNTVFPTMMFQLCAKLNILEYRFKLMLKKLE
CDKNNNSTKFGKKNFNEIRNNLIGNWVENHIRLLNLFDSVNSLFAKAVFIHYIINSFLLCTIAYIFSHTPFGG
MASVSYLFYFTVKCSQQFLQCASAHQVTVEFENLRDTIFDTNWYTTKRAVQKSMIIIMSKTMIPVVFVCGYFV
DLSLDSFKSIMKLSYTIYNVLE
>MdemOr16
LRSLNVLTYAGVWKPVEWKGLKGRVYDSYTIIIIIISHLTFLISGLMDMGFDDFEFAAMVDHLSLIVGYVQNFP
KITSIVGYRRDLIDIIDKLNSYPLKLRNDEEKLIHAKYDRLDRIITFWFPTLGMTSIAWYTMRHFVAIDTAYG
LPYRGYFPYNYSSNPIVYWCTAVQQMYSVICLAVVNAGFNVFLPTMMFQVCAKLSVLQHRFKLLIQKLGTIST
HEKNNHQLRLSKIEQQLIRDWVENHIAILNLFKFSNLLFSKAVFIHYITNTVVLCTVAYMLSQTPPSDLNFIT

NVFFFMVMCSQQFLQCITAQQVTIEFEYLNNEIFNTNWYTVRTTTIKSMLIIMFKTSRPVVFVTGHFVNLSLD
SFKSIMKLSYTIFNV
>MdemOr17
RFLNSIFRLFNLTILVLLTLIMSADAITNINEISLITDNLCFLIGCFETLAKGFKCCIEYENIKKLVNDIYEP
IDIIKKKNNVQVMKWITEIADFENRQFFIFLGVIILLASARIFSADIKNREFPVRIFVPFDASEPPYYHLMYV
AISYGVLIVDYSLFGVDIMVIVIIRYLTVQLEILKANCRHCRSDSIKRAINLSSNKDSKIFRRSAMTNALIDG
DNNEDIEIKEFVMFEVQEKNVYDEDTFDWRFKHCVTQHQKIINMLNVVNNCFSFCAVVQITTSTILVCLNGFQ
ILLSYDNLHLLIRRILAIIVVLFQLFVWCWYGNKMSAAAESLTSNLWMCGWENEHKHGIRNIVSIPMILSLQS
LELRAVGVVPLSLQTFVSV
>MdemOr18
KILNLLFRLFNLTIVVLLTLIMSADAIVNINEISLITDNLCFLIGCFETLAKGFKCCIEYKTIIQLINDIYEP
IDIIKKRKNVEVMKWITKLSHFEHRQFLTLFGIVFTLVLARIFSADFKNREFPVRILVPFNASETPYYHLVYA
GISYAILLVDYSLFGVDVMVIVLMRYITVQLDILIANCRHCHNSSIRSVINLPLNKNPKTFERIKNAPDDDNN
NESNQIKNFVMFEMQENSVFDKNNFDWRFKHCVIHHQKVIDILNVTNDCFSFCVVVQILTSTILICLNGFQII
LVSGTLSKKPRFYVAITVVLIQLLFWCWYGNKMSSAAESLTINLWMCGWENEYKYGIRNTVSIPMILSLQSFE
LRALGLVPLSLQTFVSV
>MdemOr19
LPYSFGILQCLGLWQTTECISGWKIYLYIVHRNLMIIFIYLCVLFELVALIASFDDFDEFINNLIVLLTMVGV
CGKIVNVILKRKEILRLINILETYPCLCQNLEEKYIQDEFDKSIKLRTLAYFGLTESAVAMVVSVSILCDTPN
RSLPFKTWLPFDSKTFFGYWTAYNHQILAHVFGALVNVAYDTLIPGLMLKICAQLSILEHRLKLIPKKIYSSD
LFIDVKRREINEISNCVKHHLKIFQFAEITNTVFSSVIFLQFSISTIVICVTVYKLSQIQINNPEFAPMAFYF
TCMLSQVFVICFASSECNLKSYDIAMAVYQTDWYNLSVSTQKSLLFIMMRSLRPLKFTAGYFIDVSLNSYNQV
>MdemOr20
MHLPYSFGILQYLGLWHTETISRWRLCLQMIYSYFMIIFIYLDALFESIALVSNYNDFQQFVDNSIILVTMIG
ICGKMFNVIIKSKEIIQVINILNSYLCCCQDSEEKKIQDKFDKMINYRTLAFLVVTESAVILTVSVSVMRDTP
NRIMFFNTWLPFDASTFFGYWIAYTHQVLAHFFSALANVAYDTLIPGLMLKICAQLSILEYRLKLLAKNNYSS
ESLNNIKQREINAVSKCVKHHLKIFQLAEKTNQVFSTIMFVQFSISTFVICVTIYRLSQVEISDPEFASMASY
FMCLLSQIFGTCLASTECSIKSFDIAMAVYQTDWYNLTTATQKSLLLIMTRSSRPLRFNAGYFIDISLNSFNQ
LIKLSYSAYNVLK
>MdemOr21
LPYSFGVLWCLGLWRTEIISRWKLCLRIIYSYFMIIFIYSDALFESIALVKNYNDFHKFIDGSIVLLTMIGVC
GKIANAVIKRKEILQLINILKADPCLCQNDEEKTIQDKFDKTINYRTLAFLAVTESVIIISVGASVLQDTPNR
SLFFNTWLPFDTSTFFGYWIAYIHQVLAHFCCALAGAAYDTLIPGLIIKICSQLSILEYRLKLLAKNNYSSEP
LNDIERREINAVSKCVKHHLKIFQLAEKTNKVFNTIMFVQFSISTFVICVTIYRLSQVEINDPEFAEMAVYFI
CMLAQIFGTCLASTECSIKSFDIAMAVYQTDWYNLTTATQKSLLLIMTRSSRPLRFNAGYFIDISLNSFNQLI
KLSYSAYNVLQ
>MdemOr22
LPYTFGILWGLGLWHMENNSGWRIFFQVIHCYVVIFIVYAEVLFESIALFGTFNDLEQFVGSSIIYLTMIGVC
GKMANIVIKRKEILNLINILKADPCWYRSAEEKNIQDKFDKTINYRTLAFLVVTESVIVSTVSISIVRDTPNR
IMFYNSWLPFDTSTFFGYWIAYIHQVVAHFCGALVNVAYDTLIPGLMLKICSQLSLLEYRLKLLCDNNYSSKS
SGNVKQKEMDAISECVRHHLQIFQLTERANKIFNIVMFLQFSISTFVICVTIYRLSQVEISDPEFISMIIYGL
CMLAQIFGSCLASTECNLKSFDIATAVYQTSWYNLNVDTQKSLFIIMTRSSRPLRFNAGNFIDVSLNSYNQLI
KLSYSAYNVLK
>MdemOr23
LNESVNWTEDSIKALGLYKIITQIICSWPLEPRNIYWKIRITLLLIFLVTSGFILSEELFDHCGPTGDIVFIF
GLFISTVSAVSKIILLNLYHNNMTIIIDNIINDWWDAKDNAYLKAIMHKYSSLNRILFYMILTPLLLYIFKIS
SERIPYTTMIDNITVLIRTTPVSSECWNYADAPIIIYITRFVSRIIECCIYNIASGGVDLYFFVIAMHICGQV
EIISSRFQKFHLNNQIFDRNKLLIIINRQKCLLELIDCLLGAFEFLMLVVLLSSTIQLNVIAYTVLFYMKENN
ITAAIEITSGTFFYVFTELYIYCYIGDKLSSQIDKLCFAVYDCCWYNFPINALKDIIFVMMKNNRGFSLTAGK
IYIMNLENFKNIVKTMGSFFSVMRLMV
>MdemOr24
IDKKLNWTKDSINALGVYKIITQIMCLWPLESRTIYWKIRISLLIIYLILSCIFVSKEMLEHCGSMKDRILLS
AHLLASVTSIGKIIILNLNTKNLSTIITSAVDDWEKTNDVNLRKIMNRYALINRTLLYMMLSPALLYVIKVTS
DRIPYTLIIDNVTVLIRTTPLSSECSNYADAHIIIYIVRFSFRVAECFIYNLTNNGLDSFFFLLAMHLCSQLE
ILNIYFRQFTIDEAGVFMKDKFCKLIARQQHLYYLLNILDDSFNLMILIVLISDFFYLSIIAFTSLVYIKEQK
IMEAVVITAAISLNVSFELFLYCFVGEKLTNEVDKSSFAMYSTSWYNLKPNVWKNIMFVTLKCSKEFRITGGK
LFKMNLENFKTIVKTLGSFLSVIRLVV
>MdemOr25

MKKKVIDKELNWTEDSINALGVYKIITQIMCLWPLESRTICWTIRVTLLIIFLILSCIFVSKEMLEHCGSMKD
RILLSAHLITSITSVAKIIILNLNRKNLSTIITNAVDDWEKTNDVDFKKIMNRYALINRTLLYMMLSPALLYV
IKVTSDRMPYTLIIDNDTVLIRTTPLSSECSNYADAHIIIYIVRFSFRVAECFIYNLTCTGLDSFFFLLAMHL
CSQLEILNIYFRQFIIDEAGVFMKDKFCKLIARQQHLYYLLNILDDSFNLMILIVLISDFFYLIDKSSFAMYS
TSWYNLKPNVWKNIMFVTLKCSKEFRITGGKLFQMNLENFKTIVKTLGSFFSVIRLVV
>MdemOr27
EKNIEYAIQINRWLLIPLGIWPLSSKSNLLEKVINYLIIIICSLLLIFIIVPGSLFTYVKIKNPAIRIKLTGA
LSFCVMAIIKYYSLVSGRKNIADCIEHLVSDWLRINPLNNREIMLKYAQLGRYGSIICALFMYGGGLFYAGIL
PHVSASVKNENNVTIRPIAYPSYYIIFDPQESPAYEVVYSLHCCCAFVMHSVTSAACSLAVVFVMHACGQLDI
LIAWLNELVDENEDRNEVSKRFSDIIEQHTRTLRFIASTENVLCEICLVEVIGCTLNICFLGYYLMLEWQQSD
AIGIMTYTILLISFIFNIFLFCYIGELLKAKCSEVGERTFMIDWYRLPEKKALGLTLTIATAQNPITITAGKV
FNLSLNGFCTVIRTSVTYLNLLRTFMS
>MdemOr28
YALQINRWTLKILGIWQFVIESSNFSKIVAVCLMIVCINFLTFVIIPGALFAFVLIKDPAIRLRITGALSYIV
MGIIKYYYLTTQRYKISNCINYLIYDWKKLNEFDDKNIMIDYAKFGRHSSILSAIFMYSSSMFYVYILPFEST
GINNKGNLTTLSFVYPCHFIIFNQYESPAYEIVYIMHCSCAFVLASITNATCNLATVFIMHACGQLEIMTLWL
NDIVTPENTKKKTYSKYFAIIEQHTKTLRFVEEIKELFQHICFVEVIGCTLNICLLGYYILLEWKNNETGGMT
TYMLLLVSFVYNIFLYCYVAELLTEKCQDVSNVVYMSEWYQLPENLARGFVLTIVIAQNYEPIKAGKLVYLSI
NTFGTIIRTALAYLNILRT
>MdemOr29
YSTEMNRWFLQPIGIWPASSNIVEKIISEILVIVCYFLICFLLIPCGLHTFLNEKDPRLKMKMIGPLSFCLMA
ISKYCFLVMRRKQIRECLKHIYVDWRRVRLPEDRTVMLINAKIGRFIASLSAVFMYSGGFFYHTIMPLSAGNF
ITPDNITIRPLTYPVYAPLFAALTTPSYEIVFTIQWFSGFVLYSITIGACSLAAIFVLHTCGQLKIVMSRLEN
LIDDKKKFTDSLENRIAEIVQIHLRALNFIVRTEKILNEVCLIEFVGCTMNICFLGYYFMTEFERAETIATVT
YCVLLVSFTFNIFILCYIGEMLTQQGVKVGLTAYTINWYELSGKKARDLILLLAMSNYPNSITAGKMAELSYN
SFCGVLRSAAAYLNLLRT
>MdemOr33
KDMKYTIGITESIKHTRWLFSLLGLWTLMSDYTTTAEKHLNKITRIICLGLMTFVIVPALYHLFYYEKDYRVK
IALFGPIGVCIACALKYLAVIYRRKEIIKCFNELEKDWTKINNDTERDLLFKNIQKGNKVTVYFAMFMFGGGI
SHQAAAPFLPGTPLSLVRNSTDKPLVFPTSMFNNIFDTQIMSIYVLIYISHILMGAVVCIMTVGTCNLGAIFV
THLCGQIQIMIFRIKALLNTNSDMKNDYSQKQIIQIVILHNRVITLSQNIQSILNEVCFVEILASTIIICVDE
YYCMMVWRHNEMVGFAIYFTLLIAFISSVLIFCYIGELLKEQGEKIGQSVYMANWYLLPSKTAREFILIIGMT
QHPGKITAGGLIELSCYGFTSV
>MdemOr34
YVFKYTYWFLNIIAIWPLMFQSLTLKKIISIIHAFIVTVLFLWQIFIRCIHMYFYAKDFNEQVTLIAPIAFIF
VVLLKYLAIIYRCNTIMKSIEHIKIDWSMIKCQEEEKIMIKNAKMSNKITFVFSVLMYTSGALYNFFMPHVIP
LLFRNDYGNTSERLVIFPGYNVVDCVNSSPLYEITFFFHVIAIFFCFTVLVSTCNLTVVLVTHANSQIQIVTV
QLKSMINDFLDGIKFSYPKMSIMIRNHVRVLEFSNKILRKALLEICLVEIGASSILLCIDEFCFLRVNTQDIA
NTIPYIMLFFALSLNILILCYFSELLNSQFIEIGNQCYMTDWYKIPLKARKYFILIMNMSQRPQKISAGGIID
LSYITYVQIIKTGFAYLQILK
>MdemOr35
RWLFYILGLWPFMSNHLTTSKIFYALIIQIICYSVLMFTIVPSFYHMFWYDKSIKIKIALFGPTGVSVACAFK
YLAVIYHLKNIKNCINQMRKDWEISKSEEDWKIMINYAKKGNNVTYFCIAVMYGGGVSHQSAAPFLPSSPTSL
LRNSSDRPLLYPTSLFDPYFNTQNSPIYEIFYLSHIIMGVVVCTMMIGTCNLGAVLITHICGQIEIIIIKLQN
LIEHPSNNGNFDKNMSAIILRHNKIIKLSVYTEQILREMCLIEVVAATLFICMDEYYCLMAWKNDNRIGLTIY
STLLVAFIFNIYIFCHIGELLKQQFGKIGDSIYMINWYNFSPKNASNLIMMIAISQNPQKITAGGLMELSFKG
FSNVIKTSVAYFNILRMV
>MdemOr36
NDNSNKYYAVKYTYWFLNLLGIWPIIFPALVIRKIIALIHVLIFISICIWEFTFRYIHMYYYVDDFDEQITLI
APILLIFVIFLKFMAVIYRRNTIMKCINHITADWNMINCQEEKEIMTKNLNTCNNITFVFTVSMYISAAFYNC
IMPHVIPFLLGSSNNQNTSERMIIFPGYNVVFNVEFFPLYQITYIFHILTAFLCYTVLIATCNLTVILVTHVS
GQTQIIISQLNSLINDFSKGKKFSYPKLSLTIRRHVRALEFSNNILKKTLFEICLVEIGASSALLCIDEFCIL
RMLDKNDFANMVPYFMIFLSLSLNILILCYFSELLDSQFMEIGVQSYATDWYKIPLQARRYLHLMINMSQRPQ
RISAGRIIDLSFLTYVQIIKTGFAYLQILR
>MdemOr38
MIRKAAINEPKTIHWTEDSIKAIGLYKYIIQIICSWPLEQRNIYWKIRISSLFVYLIISGIFIIQELLDHCGT
SYEIASLFGMIIATISAITKISLLNIYHKNISFIVNNVINDWWIINDDNCRTVMRRYSSLNRILFYGILTPLL
LYTLKFTIDRIPHTVLTNENTTVYVRTTPISSECWNLADIPTVFYIIRFACRTFEFVIYNIVSCGIDLYFFVL

AMHICGQMEISNMNIQNFLVANNGRFERDKFYKLIDRQKYMLGLMDELRESFNYITLTVLLISGIHLNIMIIM
IFVALKDNNLNAVFEDATGTILYFSAQIFIYCYAGDKLSSKVKNSCLAVYSCSWYNFSINTRKDIVYIMLRVN
KEFHLTAGKFYYMNLPNFTNIVKTMVSFFSVMRLVI
>MdemOr39
MKNKIVINDTSTVHWTKDSTKAIGLYKYIIQIICSWPLEQRNIYWKIRSILFVAYLITSDIFLAQELLNHCGT
SYDITSLFGIIFAAVSGHIKILFLHIYNDNIKFIVTNFINDWSVIEDEKSRKIMHRYSSLNRILFFCILTSMF
CYTLKLTIEGLPQKIIKDNITVIERLLPLSAKCWDFSNTPIVIYAFQFACRIFEFIIYNITSCGIDLYFFILT
MHICGQMEITNSNLKNFKTISNGVFDKKKFYGIIVRQKYLFDLINLLRESFNYTILTVLLISGIHLNIIIIMI
FIALKNNDINSVIRDAAGTIFYFFSQIFIYCYAGEKLSSVIQNSCFSVYSCYWYNFSIKTRKDIKYIMLRNSQ
EFHLTAGKFYHMNLENFTSIVKTLVSFFSVMRLVI
>MdemOr40
MEQLASNFLLLSIAGIWKPRGWHGIKAFLYNIYKFSIMIANHLFLISGILDLEFRNVELNSLIDNVSLLLAMA
VVRQKIGCFILNRTAIKAMVDTFDKVPFKPRDGKEKVISMRFNRLARNILVLQPMIFSGALLFYSSSRKNIID
PPYILPYRGWIPYSYTSPGVYWGTTIFQFYAIYTATAINIAVDPLLSAIICQMCAQIHILRHRFEEMVNQLKV
IDSYDSDTVFAERKLIAQWVEYHINVLSLVKYMNKIFSSVIFVQFTVSSLVLCTLSFLLSHTETMTTNFVGYF
GFLTAMYVQIFLPCYCTHMLTTECLNLSSGVFETDWFKLSTNVRKSIIIIVSKCYKPVLITSSFFIVLSLESF
TKIIKLSYSIYNILD
>MdemOr41
MKVLTFNFFLLSITGVWKPRRWGGIRAILYYIYQTFVVIINLKFLLSNIMDLKLENVNLEAFADNLSLIFALS
ITQKKINCVIENRTSIKHIIDLLHRNPFKLRDQQEELIFSQFDKFARSIFTCYVLAHTGFLSIYSLGRMTLMD
PPHILPYNGWFPYNYTRTTRTYWTTAVFQFYAVFSLGFIDLLLDLLLPCIICYMCGHIHILRYRFQVMIETLL
IMSENNEPYGKIVAAERKLMAEWVKYHIDIINLVKFTNDIFEGVIFVQYTVISLLLCTIAYFLSHTKSGTMMS
FAGSFAFLAGMIIQILLPCFFADQLTIEFLDISKGVYNTNWYKLSNNIRRSVVIILRKAYQPVTITSIFFIIL
SLESFMKVIKVAYTIYNVLE
>MdemOr42
MEILTTNFFVLTLVGLWKPRGWSGIKAILYHCYSAIVIFANISFLISGIMDLEFTNIDIAAFIDNVSLLLSLV
TIRQKTACAIGNRGDIKEIIDSLGRSPFKPQDKEEENIVKRFDDLTGYILKYYPVLFTVAITWYSIGHMFIMD
PPYVLPYRGWFPYNYTTTGVYWLTAVYQLYAICSAASINLAFDPLLPCIICHMCAQVHILRYRFGVMLKKLEV
ISDNEPRCVITSAERKLMGEWVDYHISILNLIKYVNSTYSKVIFVQYTASSLILCTVAYVLSHMDALSMNFAG
NFFYFIAMNFQIFFQCFCANQVTLEFLDITTALYDTNWFNLSNNVRRSMTIILCESFRPVLFTSSFFIVLSLE
SFTKVIKCAYTVYNVLQ
>MdemOr43
MENNIRNIEEEELPTCDKLFSLELLAFKLIGLGSLQSAFEKNNKIAVTFLEIFLFLCGFGVLIVLVFSASYTV
SIFLSIDLTLACEILTFIFSTTITLGKVLRLWIYRMDLIKILREFNDLWEENARKRQDLKEEIYKIINDSKPI
RYSYFIVAGLLSMSYGMRPYFLMLCYFLKQSENKTMDLKETVYPIIYPIPSGTWPGWLSCVTYEQGIIFFGMI
YWIACDTLFILLTSHICVHFMIISNDLYKLHDNHHENNNDNYKLIGELSRRHQKMFVLCQKIETLYSPIVLLT
VLFNGVDLCFCIFALDKEISEGHWIKVARSITHALTLFIQIIIYCNFAHMATEQTKTVSDAIYNSSWLNCDTK
MKKIMGIIMMRANKEYKFAAYGLLILDREQMTRIVKTTMSYFTLLRSFS
>MdemOr44
MKENIPSCIEICAIESFVFNLIGLSSLNTAFSLNKDQLLPKLKSWEKFFFYAAFFPFMFLFFSLVTTIPQYVG
KDFSLTCDNVSVLFTTIIITTKLLRLWSCRLDFFLVLKKLNKLWEEKIVNRLDLESEINEIVNNSKPIRYCYM
ISGLSLGACYALRPHLLMLKHFLLPSENSSIDYSDTVYSAIIYPIKPQTLSGYLLLLTLEQEIAGFGIIYFII
TDILFIFLSSQISVNFTVLAKDFENIYDVYKNNNSNHHTKTLGKTIQHHCVLLSLCQKIEYLFSPIVLLTMLL
NGIDLCCCIFSFEQDLASGDSTGSIIRNLPHAVTLFIQIVIYCNYAHIATETTQVLREAIYNSSWINYNRKMV
KIMIIMMIGAKNEYHFTAYGLITLNREQMARIFNTTISYFTLLRS
>MdemOr45
ELPPCEKFFNPELSLFKLIGFGILERAFIKNNDKIYVKIFEILLIISGFMIMIVLVFSEFRTLNKYFFDDLTR
TGEVIAMIFGSTIIITKLFRFWMSRTDLILILKEFDDLWEINVRKRLDLKDKVNKIINASKPIRYCYFIAGGS
LITSYGVRPYFLMLRYFLKQSENKTIDLTETVYPIIYPIPSGTWPGWLSCVTYEQGIIFFGIIYWIACDTLFI
LLTSHICIHFMIISNDLNNLHVNYLKKNNESFELIRDLSRRHQEMFVLCQKIETLYSPIVLLTVLFNGVDLCF
CIFTLDKELSGGHWVTVARSVTHALTLFIQILIYCEFSHVATQEVILLINSIYNSSWTYFDNKIKKMLLIIMM
RASKEYKFSTYGILILNREQFTNIVKTTMSYFTMLRSFS
>MdemOr46
MQNENEELPTCEKLFSLELTVLKLVGLGSLKNGFRQKNANLLVKFYEIFLFIYSASVLTTFFISSVFTVHVLL
HSDFILACEIATFIFAGAITVSKVLRIWSYRIELIEILKEFNDLWEDITKHRLNLKENICDILNASKPIRYGY
CLIASSLSLSYALRPYFQMLFYFIKQSENKTYDLTVTVYPLIYPIQRGTWLGYLLCLAYEQSILFFGAVYWIM
WDTLFILLTSHICVHFMIMSNDFNNLHVNYDKNDNESFKSIEDLSRRHQKMFILCQKIETLYSPIILLTVVFN

GIDLCLCIFALDKDLSNGNWAKVASSVTHALTLFVQIVIYCEFSHVATEETSRISEAIYNSSWIYFDKKMKKM
LLIIMMRASREYKFSVYGILILDREQLTKIVKTTMSYFTMLRSFS
>MdemOr47
LWNIELIVMQLIGLKSLREAFGEEKYRSASFGENLVSSLGMTVFIIYIFSGFWTLHLVGYEDISYTAEVITVI
LSASMIMIKGLTLSFHRRQLFVILRDLNILWEDARTRNNLSSEINELIDSSKPARYCYMLAAISVGLSYGLRP
YYLLMGHYLHKNNSTIDYSTTIYPLLYPFPYHSFLSYNLCLIYEQCVNYFAVCYWISCDTIFIQLTTHICIHL
LVLADDFNFRPQNDTEVTNYSHSERVIALSKKHRHILGMCKQIEKLFNPIVFFTILFNGLDLCCCIFTLDKEL
SEGHWPKFARSVAHAMTLLIQIIIYCNFAHRATELTKKIADSIYNSQWINYDKKVQKSLMIIMMRASKEYKFM
AYGLLILDREQMTR
>MdemOr48
MQVLTFIFFLLSIMGVWKPQEWRGIKATLYYIYRAIIIIVNHLSILSFLLDIQFKNIKIGYLIDNLSLLFILL
IIRQKIVCIVGNRPGIIHIIVSLKNSPFKFEDRKEEVIFSRFEKIARNIITYYPMIYLSNLSMQCTGFISVMD
PPYALPYKGWFPYNYTRTTKAYWATAVYQMSAAFVLGTINAISDMLLPCIMCYMCGHIHILRYRFRVMTEKLQ
KMSENNEPKDEIISTEQKLMADWVKFHIDILNLFKITNDIFLTVIFVQYTISSLILCAIAYLLSHMEPMTMEF
VGNLAFLTAMFIQILLPCYFADKLTFEFLDISTGIYDTNWYQLSNNIRKSIVIILRKSHRPVTMTSSFFIVLS
LESFTKVIKLAYTIYNVL
>MdemOr49
MEVLKFNFYLLSIMGVWKPRGWRGIKAIFYYIYRSFVVIITINLLISGILDLKLKTVQLDAFVDNLSLTLAWV
VVRQKLVCVIENRCGITYIIDSLEKYPFKLRDYREELIFQRFKKLARTIFIYYPLLFMSSLLAYSSGHMSVMD
PPYNLPYQGWFPYNYTRRTQIYWATAIYQLYAVFTTATINLILDLLLPCIMCYIYGHIHILRHRFEVMTRKLH
IMSGNDEPQDKIITAERKMMAEWVENHSDILNLVKFINNIFERVIFVQYTVSSVLLCTIAFLLSHTEPWTMKF
AGNLSFLTAMFFQILLPCYCADKLTYEFLDISTTIYDTNWYHLNKNARKSVILILRQSYRPVMITSSFLIVLS
LESFTKVIKLAYTIYNVLQ
>MdemOr50
MQVLTIIFFLLSITGVWKPRGWRGMKAIFFYVYRSIVIIVNYIFLITGSLDLEFKNIDLEAFIDNFSLILSTF
IAQKKINCVIENRTKLIHITDSFNNSSFKLRDRQEEVIFSRFEKLARNIFMYYFLIFFGALLIYSSDHLSVMN
PPYTLPYNGWFPYNYTRSNKIYWATCVQQVYAVYNSATIDLILDLLLPCIMCYMCGYIHILRYRFGVMTEKLQ
IMSENNKSQEEIVYTERKMMAEWVEYHIEILRLVRFANGIFSSVIFMQYTSSSLLLCTIAYLLSHTDPATMSF
AGNFGFFFAMVIQILLPCYCADKLTFEFLDISTGIYATNWHYLSNNIRRSVVVIIQKTHRPVMITSGYFVILS
LESFIKVIKLAYTIYNVLE
>MdemOr51
MKILPYSFKILEYFGGWKPLEWNTNRKGTLYNIYTLTIALTLATFCISCLVDLFHTTNFEDIVQNMSMSLTFI
IGCSKLILLIVKRIEIINVLQLFDLNICRVRCAEEANILLKYDTKEKFYGGSVCIAVVVINSASVLNNIPTKT
LPISGWFPYRHTNSTGFWVAYFHQMIALSITSMIAFSFDTVVYGVFLRNCGQLKILKNRLENFVEIINGEKLN
NKTSGSVCTIRKSERNFIRQCIHHHWIILKFSQQSNDLFAPIIFIQYSFSSLILCLIVQSSTKLVFMSPEFIF
MVIYLGCMLLQIFLFCWYGNEVTLESSGIHLAIYNMNWQVLITRSQKDLLIMKTRAILPIRFTTGYLIELSLD
SFTKLIKFSYSAYNLL
>MdemOr52
MKTLPYSFMILEYLGGWKPLEWSTSIKGKLYDIYTLTITLAISTFCMSCVTDLFYTENFEDIVHNMSMSFTLI
VVCSKLALLKVKRNEIINIIKLLEIKVCRVRCATEANIKFMYDQKAKNIVRKYGGMVLIAVFTITSASIFNNI
PTKTLPISGWFPYRHTNSSGFWIAYFHQIIAHFFTAMIGFSFDSVIYGIMIQNCCQLKILKNRLENFVEIVDE
EKLNNKINRLSRTIRECEHKFIKECIHHHWIILQFSQQSNDLFAPIIFMQYSSSSLVLCLSVQSSTKLVFMSP
EFIFMILYLGCMLSQIYLFCWYGNEVTRESSGIRFAIYNMDWQVLTTRSQKDLLIMKTRAILPIRFTSGHLIE
LSLDSFTKLIKFSYSTYNLL
>MdemOr53
MKTLPYSFIVLEYVGGWKPLEWSTNIKGKLYDIYALIISLTMVTFCMSCVIQLFHTEKFEDMVKNMSMSLSFI
VSCSKLILFNVKHDEIKSVLQLFDLSICRIRCAEEAHIQLTYAKIEKNLVRKFGGMVCVAVFAINSGSILNDI
PSRTLLINGWFPYRHTNSTGFWIAYFHQLIVFSVAPMILFSFDCAVFGVFLRNCCQLKILKNRLENFVEIINE
EKLNMKTKGLGCTTREIERNIIEQCIRHHWIILQFYRQSNDLFALVIFMQYSSSSLVLCLSVQSSTKLVFMSP
EFISMILYLGCMLSEIFLFCWYGNEVTQESLGIHLAIYKMDWQILTTRSQKDLLIMKTRAILPIRFTSGHLIE
LSLDSFTKLIKFSYSAYNLL
>MdemOr57
YAVGWNQFMLNFIGAWPRSSKGISKYKTWFCFFCVLIFIWIPLIASIYPLWGNMDAVIECLSISIPLIIAMMK
LVIFYYYKKEMKIFIDLIAKDWTINRRDNEKIPMMKMANLSRAISIISGLLISSTVIAFILFKVVWMATQIQK
KQIDHDSNVAIDLLYPAYLPYSTKDLKYYLLTWVIQCFATLFSPTAYASFDSFMSMILLHICGQLAIVGISIK
NLVNETNFKNIYKFRWSLGQIVKRHEELNQFIELIENSFCIVLLPQMLVCTLAFCFQGYMLTMVNNNTKNYAN
NNMSILEIMFFSSYMIYTILHLFIYCYIGDLLSFQSTLITQTCYESNWFNLPFREARLLMIINHRSYKPLTIT
AGKFCSFSRHLFIMILKTSFGYLSMLLAVK

>MdemOr62
YACQWSEISLRILGIWPNSKPSFFNKIKAHFYVIFVLATYYLPQSATIVIIFGDIDSMVNLLSYNVSGLVAIA
KYIVIYKHRKVLMKIFEIMAKDWAAHKSAYDLTIMIKNAKIGRLLAFISILYAPLIVILHILTFLKPDRLLLY
GSIPTIANNLMWPSYFPFNSSRKYIFELTWLCQITATMGASLIYGTFDTFIAVIILHLCSQLAIVRSRLRNIN
EDINQGLLFHDSVKKKLRAIIKRHEQLISMAETIEKTFNVILLPQLICYPLIFCFQGYAMLTSMAKTQVSSLQ
ILYYLSYDTYTLYHLFIFCWIGEYLQNESTSVVYSYYESPWYNFSYTDARSLMFIGYRATRPFYISAGKFSRF
SLSLFVSILKTSFGYLSMLRAVQSR
>MdemOr63
GFNYACQWSELSLRILGIWPLYKFTLLKKLRVYFNVIFVLAVFYLPHTGTIIIIWGDIDSMVNLLSYNVSAIV
AIGKYIVLYNQRHVLASIIEIMADDWSSVKSQYNIKIMMNEAKIGRILAFISILYAPLIVFLHILVTKLFLYG
STPSIASNLMWPSYFPFNTSRNYIFEITWICQITATMFTSLVYGTFDTFMTVLVLHLCSQLAIVRNQLKNLYN
VNKHPAVDIFVAEKLRDIVQRHEYLINLAETIEKTFTLILLPQLICYPLIFCFQGYAMLTVSSMAKTQVSSLQ
ILYYLSYDTYTLYHLFIFCWIGEHLYNESTKIGYAYYESMWYNHSFTHAYSLMIIGYRALRPLHITAGKFARF
SLGLFVSIIKTSMGYLNLLRAVQSR
>MdemOr64
HYECGVNKFILGLIGVWPESNQKFIRKHRTLVDAIVLFITLWIPRAAAFILLWGEIDALAQAGATNVPIALSV
VKLLILYRRKNDFSRILDEMKADWKKPMTTEEYKAVMKMATLGRRISILSTFLTLNAIWLGAIVQTTIVENPD
PRLTLNLFWVVYLPYDTSKTTNYVLTWLVHFYTSIMSAVVYGSFDGFVVVLVIHLCGQLDLLKIWARKLADQP
DLENFKRKVKFIVNRHEQLNEIADTLESSCNFVFLPQLMSCALTFCFQGFAIATKMFTGQFNPLQIAFALFYT
GVMVTHLFFCCLAGELLLSKSQDLGYATYECRWYNLPPKAARSLVMIFQKSAKPFILTAGKFMPLTYNLFLTV
LKTSMSYLSVMFAMS
>MdemOr65
FEYAIKWSKISLTLLGIWPNINYSISQKINVCFDVIFFLTTTYLPNLGAIILMWGDIDSVVHLLSYNMATTVS
IIKYIVFYKKRNVLASLIEFMKEDWINAESSGNVNKMMSNAKIGRTLAFISIVYTPITIFLYIIVETMFLYGQ
HPKILTNIVWPSYLPFNLSIYTLELMRVCQVTGLLGASLVYGTFDTFLAVLILNLCSQLSIVQNQLRHVCSDS
NDTALTKYTFHKKLRTIFQRHEHLMKIGETIEETFNLVLLPQLICYTLNFCFQGYSMLMVIIMAKMKVSFLQM
AFYISYDTNTLYHLFIFCWMGELLYNESTSVSYAYYQSLWYNYLNVNIKLFMFIGYRCSRPLRISAGKFGYFS
FNLFVAVIKTSVGYLSMLRAVQS
>MdemOr66
WAVGLNRTVLKILGLWYYNNQTRWQTLTFNVQTFIFVIGIFTFVTFPQTLALIKIWGDLTLIVDNLIVNLPIT
TCELKIFILWWHKKVLVALFDEIEKDWHGIKNENERNIMIKYATISRIMTICGLCGAFFSLILYQGPLTFGIV
LRTITNLTDDPDNLFSLQAVYLYDTSTVYSYYLTRISQIVGCGLSAVAYTNADVLFGMIVLHVCGQLEILASR
IKIIADKPQYFSRLLREHVKNHVRLIRFVQDIEKMFSLMLLALFGSVAITFCIQGFQFINVFTDTTIDLPITQ
IVFYMQFLSYCLFLTFVYSWVGETLLTQSSSIYMAACDCNWISCDHNESKDIILMIFRSQYPLQITAGNMIPL
SMNTFLQLLKTSGGYISFLLA
>MdemOr67
KINTFDSELGLLVQCLKLNGLWPYTDKYKNIKFLLCFIISLCGVLNGIFTIFNQKNNHDRILSTFQATLYIHT
FTQRCVFKWNQLNFKIVFNYMNSDWQESNHLKTNDLNIMFNYVKKSRHVVKMIFITFFIAGFGWATNLLIEVL
VNNKNDLLIFEKWNSETWDLLSSPKLLRLTFQLLAGFNGLFGAATIDSCIVLLILHCCGQLEILCEKIKKSRG
LIRHNLINKSIYCRNTIRIINNCSCLQCIVDKHNYLIHFVKLLDESIHSLMLLQLIPGTISFCTNGYGLIKSY
NNANVSLMIYHIIYILAIMPIFLTFCWVSNDLLEKNENIKHMLYNLEWYNYNNKTRKFFIIMINHANQAIKIT
GGGFFVISLKCYKNWFINGVEYLSVLAAIMTRS
>MdemOr68
LLPESFFILKCIGLWQPVHWEGFKTWIYKLFTVFSLVILFTDTGSQIVDCFVTCKTVSDYADHSFILLTMIGI
CIKMASLVKNRYRIAELMERLSTGVFRTNNLEEENILAKFDNIMKWRTVLFCIFVQMGTVNLIISSVIYLAPH
HITITKLYLPWDIKTDAGYWTAWVLQAISRLFGGPVNVACDSLVSGVMYRASAQFKILASRLRGFLLEPANFE
NKKYDYHRVEKHEAKKMAELVKEHLEIIQIITKLNDIFGLVIFMQYCVSSTVLCVTVFVFAQVRQFDIHCAMM
ITYTGCLYFQIYLLCSAGTEMTSESLNFVSYIYTFDWNELKLSTKKSLLIMMLRCHRPLKFRSSQIIELSIES
FSQIVKFSYSAYSV
>MdemOr70
LLKMRVKPVILLRVIKVLGEVSGIWPIFDSQVSIRVIKELVWFVSIVNITLIIIPTILRIHKDINENQPLNAL
HSLSALPVYSETFVNLIIWKYNKSQIQGLLVEMFDHIKSVNGRDKNILSKSINHHLPLYIFVVVFCTIACVSH
LSVPIFTPKKLLPSIAYYPFSIEQYTTTYYLVYTQQAIANLQCGLHHTVPPIIIAILLSYVTVKLKVLSLALK
NVDNVDQFNNWIRQHRDCIRYFEQLDKTVRVIIFKSFVIITLSVILGGIKIVHTSSIIETTKFLFIVIAGCLA
VYVYIPPADDLHELSEKVSWSLYESSWIDKPIVIRKSIIIMMCRSQRPLTISFRGIIPALNKNFYSTYLSAAM
SYYMTLR
>MdemOr72

LKMRVRPEFLLHVIKILGEVSGIWPIFESKLWIRIIKELLWFVSIVNISLIIIPMVLRIRGNIYEGQQLNALH
PLIALTVYSEPFVNLIIWKYNKNQIQGLLVEMFDYIKLVKGKDKKILSKSINDHLPLYMFVAVFCTITCVSHV
FVPIFTPQKFLPSAAHYPFSIELYTTIYYLVYTQQAIANFQAGLHHTVAPIIIAILLSYVVAKLKVLSLALKS
VGDVDQFNDWIRQHRDCIRYFEQLDKTVRFIIFKSFVIITVSVILGGIKIVHASSLIETTKFLCMVITGCLAV
FVYIPPADDLYELSEKVSWSLYESSWVEKPIVIRKSIIIMMCRSQKPLTISFRGIIPALNKNFYSTYLSFAVS
YYMTLRVL
>MdemOr73
VEKEFNNVSSLLLWNKRFLTIGGIWPLDKTYKRATIWTVYLTLHLIMEYAKLLAVFGDLEMMVLSVLESVMQS
MVYAKLFVFRYSIMLRHLICAIIEDFEDDYYDNWEEKKIYLEYNALAKLFYKISMPYIIVAASLYFLRPILTY
YFRDFEGNLTLNASVYNFTFLVLPFQLTTFIPIETTQSYFIMYGYLSPMVYLLVCHNAWICLLITMVLHICGQ
LAVLNHRIKKISVDIDEDKVQIIFRRLVRRHMRSVWMAKTLDDTFHFVLLIDLVGTTLLLGLMSYIVIIGSSM
SESSFGYVYIICIIATAFLIYGYCIVGECLLNESRKVNEAYYECLWYKASHKFRKSLLICLIETQEPLKITAG
KFFIFSLSGFTDVLKTAMSYLSMLR
>MdemOr75
IFKATPEFAIAFTKLITLLGTSWPNPKDAPKWKHVLFQIRWWLTFCLSISAFLPMCYAAYNHWKNILSFTKSV
FDAANTVQTFAKMIFCKIHFKKMQYLLEEMEDYVTRAQAHERALFIMYIKRCGRLHATLMISGLMTIFIIIIA
PIGVPQPFPNIVEYPFSTEGHPIFELIYLHQSAATIHCLSILTFDCQIALLLWYAGARLELLSQEFHDVTHYK
QLNECVEKHQHLLLYIQDIVISSRHILATTVITCVLAAITSGIHIVGNEPIAAKVPFIVASMIMGVVLYVTAW
PSEHLAQMCEKVGIALYESSWIQNSKTLNTSILIIIQRSQKAASIDVAGILPTLSLPYYATFLSKIFSYFTTL
RVLL
>MdemOr76
KATPEFAIAFTKLTSILGSSWPHYKNATKFQFIVFNIKWWFFWFISITTFLIMCYTAYNNTKNILNFTKILCD
AANCSQAFIKMLLCKIHYRKLQFLFYEMEKYVEQARAHERELFISYIKRCGRLHVSIMISAVITAVIIIMAPI
GMPQQFPNVAEYPFPVDGRPTFEIIYLHQSIATIHCISIPVFDCQIALLLWYAGARLELLGDEFQKVTDNQQF
IACVKKHQYLLWFIQEIIMSSRYILATTVVMCTIAVITSGVHIVGNEPVADKVPFVIISMGLSVVLYLCAWPS
EHLAQMCENIGKALYQSSWIKTTKELNKSIFIVIQRSQKPATIEVAGILPMLSLPYYAAFLSKIFSYFTTLRV
VL
>MdemOr79
DKKISDYLIYRTVIKKLVISVGLWPLDKPNTFYNLIPYIQIIINVFMCFGMIGYVRANFTNIALVTRGLSIMT
SFLSTIVKVGSFVMNRKDAIDLHNTLDLYFNKMLNSKQLPWVILNNITTVRPLTWTFLILVSISCGVRIIDPF
SSVISQLSHKISPIKYPLLYPSVFPWSLVPGDTLYNCEFVIELLATMTLWLITMSVDCLFTFYVFQMIGQLRE
IADCFDNLDGDDDYDCRNIVRKCVNQYQTIIKCRDLLQKIYGPIILWIMVTNAIILCTVAFSATKMDSIPIGK
GLILLSYILLKLVQTFMYAWSGSLLMAESEECREAIYAAKWFGNKRLMTAIIIILSQKPLSLTACNFSVVSVD
IFQAVVNTTVSYFFLLQTIEPDS
>MdemOr80
MTKSRISDYTSLYQLTKKLGTVIGVWPYEKSNIFYRLVPYIPMTLHLIISLAIFGYVGEHFPNIALVSRGLSI
STSFVTTILKIVCLKINHKDLTELHEHLHPYFNKLLKDSKLSELILKDVEMFKLVSWSLTICVATCLGSYILT
PLSFIISCYIHHIEITKYPLIYPCSYPWTITSNGIVYQIHFVFETCCGLAMFFVSTSVDILFPFYIFQMIAQF
REISYNITHIDDDDDDNSILDKCVTQYERLMRCRDILEKIWGPIILWSVTTNAIVLCTVLFQISKMRSITIVK
GLLLVAYVGLKLGQTFIYAWSGSCLIDESEVYRDAIYAANWFGNKRLMTSIVMMLTQRPLTLTACNFSIVSVK
IFITVR
>MdemOr81
MSQKKISDYLIYREIVKKLLIVVGLWPYDNPSIFYNFLPYIQILVNVFLCFGMLGFVQKNFTNIGLVTKGMSI
MTSLMSTIIKVVCFIRNRSDAMELHKNLDPHFNEILQDLKLSKFVLKRFPVVRHLSCSFTIIVTISCALRLII
PISIMIKQIKHNIHPVRYPLLFPSVFPWKVTPESYIYEFEFAIESFAVVTLCFITLSVDCLFTFYVYQMIGQL
REISYCFKNLTEKSDSQSILRKCISQYQVLLKSRDILQRVYGPIVLWIMVTNAVIMCTIAFQVTQMDSITLGR
GLLIFTWIALKILQTFMYAWSGSCLTLESEECRDSVYACEWYGNKRLMTSIVIILSQRPLILTACNFSVVSVE
IFQMVINTTVSYFFLLQTLEPEA
>MdemOr82
KSRISEYRAFHDFTKRLLTIGGLWPYDNTNIFYRLLPYIQIFLNLGMALVVYGFVQKHFSNVAVVTRGFGIMT
SFVTAILKVMCLVINHNDLSKLHKNLHPYFEELLKNPKLLEHILKKVNIFRFLSWALGCCVFTVIAFYIITPL
SFIINLIYLGSYPWKIPSSGFVYKAHFIFDTLGSFALFFITTSVDSLFTLYVFRIFGQLREISYCITHINHES
NDGDFVIYKCIAQYEQLIKCREILEKIYGPVIIWIMGTNAVILCALLFQVSQMKSITVIQALLFTTCSILKMV
QTFMYAWSGSCLTEKSENYKDATYSANWFGNKRFMTSIVILLTQRPLTLTACNFSIVSLRIFISVVNTTVSYF
FLLQTLD
>MdemOr83
ISDYRAFHKLTKRLTTVVGLWPYENSCIFYRLLPYFQISLNLILVLAMLGFVCKHFSNISLVTSSLSIMTTFM
TVIIKLFCLVVNRKDLTELHKNLDPYFNELLNHSKLSKLIYKKMNIFKSISWTLFACVFFSSALCIITPIVSI

CYYTNEMKTKKYRSICPSDLCKITSNRLFYQIYLAFETLASFESFFVTTAADSLFMLYVFQIIAKLREISHCI
THIKDKNENSVVGKYVSQYVKLIRCRDLLEKIYGPIILWIMGTNTIVFCSLLFQISQIESISIVQGLLLIMCI
ALKMIQIFMYSWSGSWLTEESEDCRDAVYAANWYGNKRLMTSVVIILMQRPLTLTLYNFSSVSVRNFITVFNA
AISYFFSLQSL
>MdemOr85
LKIKMSSYFIFREVIKGPLLALGLWPNKNPSLFYRFLSYIQLPLNFGMFLAIFNFVRLHVNNITLLTKSFGVM
TTYLSTTLKITCLLINRREALELHRTLDPHFSKLAQDVEMKKIIFKKFTMLKLITWLFFSCVFITLSAMIITP
LIDIAMQYHKYGKNNKYPLIYPSKYPWQTSMNGWPYKVTYLFESLATMSLFCITSSVDSLFLFYIFQIIGQLR
EMSYSITNTNGFNDDNEKDVVLRKTIYQYQTLIKCREIIEKIYGPIILWIMCTNAVIMCVFIFQFMQMKNLPI
IRLMIIFTYVLSKVLQTFIYAWSGTCLTVESEDYRKAVYNMNWYGNKRIMTSVIIMLAQKPMVVTACDFSIVT
VNIFVMVLKTTLSYFFLLQTLEE
>MdemOr86
KVYAYLIYRKIMKRLLLSLGLWPKENPTLFYRLLSYLQLPLNFGMSLAISNFVRLHANSIKFLTKSFGVMTTY
FSSTLKIACFLINHKEALYLHKTLDSHFNKLTQDDQMKKIIFKKFTLLISIIWIFFFTVFINLSVIIMTPLID
IVIQQNGNDENMKYSLVYPSEFPWQISSNGWSYKITFFFESLATISLFCITMRVDSLFFFYIFQIIAQLREMS
YRITNTDGFSDNEEIYFVLRKTIFQYQTLTKCREIIEKIYGPIILCIMCTNAVIMCVFIYQLMEMKNLSIRRL
MIIFTYILTKVLQTFIYAWSGTCLTAESEEYRKAVYNINWYGNKRIMNSVIIMLSQKPMVVTACSFSPVTVNI
FIMVLKTTASYYFLLETFEE
>MdemOr88
ENKVSDYIMYREVTKKLMTIIGLWPYENSSFFYRLVPYIQMGLNLGMALAIFGFVLEHFPNIALVTRGLSIMT
SFITVILKVACLVINQKDLTELHTNLDPYFNRLLNNSKLTKLILKKVKIYKFLSWALTFCVFTCLAAYVVAPL
VFVINCYFNKTKITKYPLVYPSTYPWKIISNGFVYKLHFIFETLATSALFFVTTSVDSLFTLYVFQMAGQLRE
MSYCITHIENESDGHAVISKCVNQYEKLMRCREILETIYGPVILWIMGTNAIVLCTLLFQISQMKSISIIRGL
LIGAYVGLKMVQTFMYAWSGSCLTEESEDYRDAVYAANWYGDKHFMDSVIITLSQKPLTLTACNFAFVTVDIF
VKVLNTTISYFFLLQTLDE
>MdemOr89
KVSDYTTYREITKNLMTISGLWPYENSNIFYRLIPYIQISLNLGMALVILGFVRQHFTNIALVTRGLSIMTSF
LTVIIKVICMLINQKDLVELHNNLDPYFNELLKNSKLSKLILKKVKIFRLLSLALIFCVLFSIIFYVVTPLIF
IIKRHYHQMKITKYPLIYPSGYPWKITSSGFIYQIHFAFETLASLALFFVTTSIDSLFTLYIFQMIGQLREIS
YCITHIDEDENENKDSVVRKCVTQYEKLMRCREILETIYGPVILWIMGTNAIVLCTLLFQISQMKSISVIRGL
LFITYIALKMIQTFMYAWSGSSLTEESENYKHAVYAADWYGNKRLMDSVIITLSQKPLTLTACNFSVVSVDIF
VMVLNTTVSYFFLLQTLDE
>MdemOr90
YIAFHKLTKGLLTIGGLWRYEDSGFFYRLLPYKQLAISIGLSLAVLNYVFENFSNLSLVARGLSITTSFLSAA
FKVICFMIRREDLTNLHKTLDPYFNEMLKDSKLSKFILRKVNMFRYISLVYAVLLSLCSVFYIVAPLIAIFYC
RHHKINLTKYPLIYPTTYPWKITSTGVVYQIHYIFEILASLTLFFVTTSVDSLFTFYIFQMIGQLREISYCIT
NINDGDDEKKAVCKCLTQFEKILKCREILEKIYGPIILWIMTTNAIVLCTLLFQISQMKSITVIQGILFTTYF
VLKMIQTFMYAWSGSCLIEESEDCRDSVYAANWYGNKRFMSSIVIMLSQRPLILTACNFSVVSVKIFVMILNT
TISYFFLLQTLDD
>MdemOr91
RISDYKAYRGLTKTLLTVGGLWPYSNSNIFYRFLPYIQIILNLGMALAILGFVHDHFSNIALVTRGMSVMTSF
LTTILKVTCLVINKKDLMELHENLDPYLNDLLKNSPSTEVILKDINSFKFLSWGLTFFAFIAMCFLIFTPLLF
ILHCHQHEIELINYPLIYPSVYPWKIISNGWIYKLNYIFETLASLILFFVTASVDSLFTLYVFQMVGLLREIS
YCITRLDEKNVDKDSVICKCITQYEKLMRCREILEKIYGPVILWIMTTNAVVLCTLLFQISQMKSISVTRGLL
FTTYITLKMIQTFMYAWSGSCLTDESENYKDAVYAAHWYGNKRFMTSVIIMLAQRPLTLTACNFSTVSLKIFV
MVLNTTVSYFFLLQTLD
>MdemOr92
EYMKFQTLIRRLLLVIGFWSSKNSNILFQSLLFLHLSFFIIPIIGVFNFFKTHISNIFLATKGLSVLVGYSTV
IMKILCFIINRKDINDLHTILDPYIDELIKKSGLSNIILNGITSFRRLCKIATGFVTISCTSYAIAPIISIIS
QWRHHIRPIKYNLIYPTDYPWEHPPSGLLYNCHFLNEYLTTFSIISVTASIDSLFVYYVFQIIGMIRDISHHM
SCFNEENSEATIRHCVLQYEILIECRRKIEKVYGPIILWSMGTNAIILCAAKSIPFTVMVLCAAYAGLKLTQI
FIFAWAGSQLTTESENLIDTIYAANWFGNKRTMTSIIIMLSQKPLVLTACSLSTVSIDMFVSVINTTISYYLL
LKTFDPDS
>MdemOr93
FRSLLLVHLSFFILPLIGVVNFFKTHISNIFLATKGLSVLVGYSTVIMKMICFMVNREDLNELHAILEPYIDG
LIEKSGVSKDLFKGVSIFRGLCAGLTGCVTTSSTLYAIIPIMTIISQYRHQVRPFKFLHLYPVIYPWERSPSG
VIFYCQILNEYFTTFSIITVTASVDSLFVYYVFQMVGMIRDISYNISTLTDENCEATVRQCVRQYEVLLECRK

KVDKVFGPIILWSMGTNAIVLCGLIFQLSHAKAIPFLTMVLCAAYVSFKVTQIFMFAWAASKFTTESNKLRDT
IYAANWIGNKRVSTSIIIMLTQRPLVVTACSYSTISIEMFSAVMNTTISYFLLLKNFD
>MdemOr94
EYVKFQSIIKRLLFIVGLWTKEDSSLLFRSLFHIYLSFFIVPIIGVVNFLITNITNINLATKSLSTLLGFSTV
IIKGICFIINRKDVDALHTILDPYFDELLKTPEVSTLVLNKISTFRRLPTFITTFITIVCISYAIVPIISIIN
QCRQRIWPISYNLIYLTIYPWEISPNSLMYNFHFFDEYLLTISIVLITSSVDSLYTYYIFQIIGMLREISYRI
SIFDEKDSESIIRQCVDKYDILTQCCGKIEKVYGPIILWTMNVNAIVLCAVIFQLSHAKSIPFISMVLCIAHA
CLKLTQVFIFAWAGSLLTTESEKFRDTIYASRWLGNNRLKSSIIIMLSQKPLILTACNLLYVTIDMFVKVINT
TISYYLLLKTFEQGA
>MdemOr95
VADKQLEFVKFKNSIKRLLFIVGLWTKDDSNLIHRSLLHIYLSFFLIPIVGVFNFFITNIANISLATRSLSTL
LGFSTVMLKVSCFIINRKDVDDLHTVLDPCFDKLLQSPEMSNLVLNKVTTFRRWPIFITTFVTIVCISYAIDP
IIFIINQCRHRIWPIEYNLIFLTIYPLKAQRNSLLYNFYFIDEYLLTASMIFITSSLDSLYTYYIFQLIGMLR
EISYHILTLDKNNSELTIRQCVDKYEILLKCCKKVQKIYGPIILWTMNVNAIVLCAVIFQLSHAKTIPIISMI
LCSAHACLKLIQVYIFAWAGSQLTIESEKFRECIYAANWVGNNRMKSSIIIMLAQKPLILTACNLLHVTIDMF
VKVINTTVSCYLLLKTFE
>MdemOr96
EYKKFKNILKYLLAIVGLWSSKKSNFLFRSLLCLHISFFLIPIIGAFNFLKTHISNVNLATKSLSILVGFSTI
IMKVLCIQVKRKDVNELHETLDSYYDDLIRKPKLSNIVSEGITTFRRLPILITGFVTLTCISYAIVPILSIIN
QWRHDIHPIKYNLIFPTAYPWYPSPHTFIYNFHFVNEYLTTFSIIFITGSFDSLFIYHIFQIIGILREIAHDI
SLIDQDTNSEIIVRQCVIKYEILLECRHKMEKIYGPIILWTMKTNAIILCAVIFQLSHAKTIPIMVAMICAGH
AGLKLTQVYLYAWSGTRLTTESDKLRDAIYNANWIGNKQLINSLIIMLIQKPLVMRAYHFTTVSIDMFSAVIN
TTISYYLLLKTFEPN
>MdemOr97
KLSEYIAYENVIKVLLNLIGLWPPQKSTLLSRSIIYLQMSTCLLPFIGVFNFFKTHFLNVSLATKSFSLLASF
LTVIVKGMCVVLNRNDVKDLHEILDSRYNKIIRNPKMTNIMLKQITTFRWLSYVAIFFVVVACMSYIIIPIIF
IISQLIRGSEHIKYPLPFPSIYNWNIPPNGFRYRLHFLTESMTILSIICITIGVDNLYSLHVFQMIGFLREIS
YRLTHADDNRDKEDSSDLIVRECVYQYETLIKCCRKLQRIYGPIILWTMGTNAVILCAVTFQLSQMSSISVGR
IILFTAYAGVKLVQVFIYAWAGSRLTAESEDCRAAIYAANWVGKKRYMRSIIIMLSQKPLILTACNYSIVSVE
MFGSVLNTTASYFLLLNTFEQES
>MdemOr98
QDELSSYIKYENFIKIILSILGLKTSANDSNSIINRSILHLQMSMSLLPMIGIFNFLKTYINNVFFVTRGLSI
LVSFLTIILKGFCIILNRQDVNELNEILSSHYHKLISNPKMKKAILKQVTTFRRLSYTMTIFVAVSCLSYVVI
PLIAMINQSINGIKPIKHILPFPAIYSWNIPPDSYRFKLHFLNESLTVLSVICITVGVDNLYTHYVFQMIGFL
RVIAYRMINFNEQNKESSEIVVRECMSQYETLINCRNKLQKIYGPIILWTMGTNAIILCAVIFQLSQVKNIMN
SISISRIILFTTYAGAKFSQVFIYAWASSLLTAESDKCRAAIYAANWVGNKRFMHSIIIMLSQKPLILTACNF
TVVSMDVFMSVLNTTLSYFLLLNTFAE
>MdemOr99
KKTINHHASIKITRFCMSGIGMWHVEKPRDKIISNIVLCYTIATTIIALIVEGFDIYHCLGDLHAISYTAPCT
ITVIIELFKLTKFVTNRTEVMAFNDYTFRKFWSIPYIDSERKILDDCNKKSIKIIIIYIIILQLILWQYITIP
IIESYGKTTSERTLPFNLWFTFIPFKETPYYEIFYTLQTAATMSVCACATTFATFLFTINLYVTGQFKILQQR
LETACCTTYNEKVDPIEKSYTNLRKSVELHHVLLNYIARIEKLYCQIMLVQTLGSVLQICFSGFQILLVIDNS
ILRTMLSAVFFSGSISQLLLYSWSCHEIIIESLEVAEAAYRAYWYSLSWSRYGKSYRQALLIIIARSRRPCIL
TVGKFVPMSLETFTSVFNTSLSYFTILLQMNE
>MdemOr100
DHASIKITCFCMNGIGMWSIEKRRDRIISNIVVCYTVATLIFGFIVEAIDIYYCIGDLREMSYVAPCLLNEIV
ELLMMGKFIINRSEVMDFNNYTFRKFWSVPYVDSERKILDECNKKSFKIIIAYIIVIQLVVWQYITIPIIESY
GKNSSERTLPYNLWFTFIPFRETPYYEIIFFLQSAATLTTGVCATAFATFLFTINIYATGQFKILQLRLETSC
QGYKIEKINSVEQINLIAAESYSNLRKCVELHKVLLQYIIRLENLYCQIMMVQTLTSVFLICLTGFQIGVDTS
ILRTSRSAVYFACLITQLLLYSWSCHEIIIESLEVAEAAYRAYWYSLSWSKYGKLCRQALSIIMARSRRPCIL
TVGKFVPMSLETFTSVFNSSLSYFTILRQMNEE
>MdemOr101
QHTSIQLMRLCMNGIGIWSIEKRRDEILSNFVICYTVVTLIFGLIVETTDIYYCMGDLREMSYTAPCFLNVIV
ELFLMGKFIINRSEVIAFNDYTIREFWNIPYIDPERKILDECNKKSFKIIIAFIIVIQLVVWQYITMPIIESY
GKNSSERTLPYNLWFTFIPFRETPYYEIIFFLQSAATLTTGVCATAFATFLFTINIYATGQFKILQRRLETSC
QGYNIEKINSVEQLNLIAAESYSNLRKCIELHKVLLQYIIRLENLYCQIMLVETLACVFLICLTGFQIVLVID
TSILRTSRSALYFCCLVTQLLLYSWSCHEIIIESLEVAEAAYRAYWYSLSWSKYGKSFRQALLIIITRSRRPC
VLTVGKFVPMSLETFTAVFNSSLSYFTILRQMNEE

>MdemOr102
WNGDTRYALGLYKYITKSVGIWPCDSQTIPSKIQTLLVIVLEITMAISILSEINLNCGEVNDVIQMYSLAACC
AIVIIKITSLRYNNIKILAIISSSIHDWQTVTHPKALEIMKRNAKLARSVCLFQMISAYLTSVVIIIGPLPYL
SLQVNATLNSSDMILRPLPLRTVCFYGEMSTKMYSAIYILQAIQTLSTCTSNIGCDCYFFGITLHVAAQFEWL
GVEFQTLNTDASEKECKKSLATLVSKHNRLMQLSDYLEDSFHICIFLVLLVNTVQICLNVMQLIISVRFGDTA
TAINAVIIIYVMNLQIFLYSYAGDRLTSGIENLHVAIYGSSWYDLPQKTIKDLSFIMLRVNKTFNITAGKIYP
MNIDSFKNILKAMLSYFSVMQAMFE
>MdemOr103
MTFFDSLNWRITKVLLSCFGAWPSQSPRIRKILRFLVFFLIASIFLPEVNGIISWGDINKVVECAPIFALHAL
TIVKMCNCLFNIDKIKILLIKTQTDWKSPMSDVELQMLRKYGNDNRKITHAYIYFIYAVTVMFLTTPMVPKIL
DYYMPLNQTRPQISLYQTEYFVDPVQYEIPILIHAYIVSPFPTTVIVAFDSLYANFVNHACSMFVVIGYRLKN
VANNKYVWNKRKKVIREDRMHQLMIDYIKKHNNILEYCRILKSTYNSCLLFIVMINMLALSITGFQTVMKMNE
TGEMIRFGTFSIGQVVHLFFLSWPGQRLLDHSLSIHQLVYSGEWYNISTKTKKLGIFIMMRSRKPCILSAGKM
YTMSCESFSKVIKASMSYFTLLTSLK
>MdemOr104
WNSGTSYELTIYKIIMWPLGIWPLNRGELFSDIRLFLAAITQASTCICLHIEMWLNCQGLEDILDIFVLSVFA
LLACMKGIIVRYHQDKLHCNVTSAVNDWFTLSLKANLRNRKIMMEHARIGRIVCISLMAPASGGTLSWIVFAL
PLPMFIPENSTDVIRNYPLQTACTFQSVTLSGYYHIIFIIQIYQLIATCLGNCGNDVFFFGLGMHICGQLEIL
KNEFRELKTTNNKLEDRKIFQNVVRRHSHLMSLIYKLESSFNLVILAQLIMSGILICIMGLQVIIALKTENLF
AGVKASVVLSSLMSQLFLYSYGGDYLTSQCEAIAFAAYESPWHKSSVKRIKDIQFIILRAEKLIYVTAGKFFY
MTLGTFMDIIKLSVSYMSVLRVAIDV
>MdemOr105
SMDEQVPANYGNLDQEVYLTRSFLLFCGLWPGTTYLRYFSVIIFLFLVLWMPFAAYLYFFFSDTIENVVETTG
YLMSVKYVILEIFIFRSNKNKITSIVEHMSDNWLSMTFIPEKEKKIIYKNLLESRFAVRLAFSFVTFCLIFYS
IMPYIIALILGKEFPRQAARVAWYPFPLENLYVIFLTSSVTLAAIFLIMLIEVAIDAFLILLVYHSVGQLRLL
RNTIGKYRGLIRHPTESSPKFSRGFKKSCPCLKCIVDHHVDIEMFVEKINNSFSTIIFIKISVTIIKFCTMGF
FVVQSAHTKNISGIIEHALYVFWGGFSLIIYCWLGETLSKRSEDIARAVCEIDWSIKKSKKSLALMLIIIRCR
KPFAITAGKIFTMNLIFYKECILASMSYLSVLIAI
>MdemOr107
MDNSVDSFLRINRLFLSSLGQWPEQEQVSKIFALINAVFFLLTQAYFQTGGMIAAKCDQPIFMESIAPVLISF
MCLVKFVNFNYNADKMKKLLECIQADWNSISDEEEMKILHSWAKDSRKNTIMYAGALYGTMAPFMLGPLVPIF
CRLMPAGFLPENSSIVLEKPVLFHVEYFYDLDKYYYPLVIHSYFGTTAYMTVAVAIDSMFMVYVQHACAIFAI
IGNRLEHLADDSSINFCYHPHISNDEPYKRMIKCIVQHSKALKYAQMIQSANSLSFFFQLGFNMLTITFSGFQ
AATKLDRPDEAFRYAGFTITQTFHLFFQSWPSQRLADESARISETTTRCAWYLTSMRSRKLLQLFIMRSSVPC
QLTAGSFYTLNMQNFSAVVRTSMSYFTVLTSVQ
>MdemOr108
MDNTLDSLLRVNRLLLSSLGQWPQQDRVSKTCTIISAIFFLITQGYLQTGGMITALCDPPIFMESIPPVLISV
MCVIKYINFNYNADKMRTLLESIQADWNSISDEEEMKILSSWTKDSRKNTIMYAGAVYGSMVPFMLGPLVPIF
IKLMPEGLLPANNSMASAKPVMFHVEYLYDLDKYYYPLVIHSYFGTMTYITVVVAIDSMFMVYVQHACAIFAI
VGNRLERLVDHDSININFNPHISNDEPYKRMIECIVKHSQGLEYAQMIESANSLSFLFQLGINMVTISFTGFQ
AATKLDRPDEAFRYATFTIAQTFHLFFESWPSQRLADESARINEYTTKCAWYSTSMRSRKLLQLFIMRSSVPC
QLTAGSFYDLNMQNFSAVVRTSMSYFTVLTSMQ
>MdemOr109
MDNTLDSLLRVNRLLLSSLGQWPQQDRVSKTCTIISAIFFLITQGYLQTGGMIAAWCDPPIFMESIPPVLISI
VCVFKFINFNYNADKMRTLLESIQADWNSISDEEEMKILSSWTKDSRKNTIMYAGAVYGSMVPFMLGPLVPIF
IKLMPEGLLPANNSMASAKPVMFHVEYLYDLDKYYYPLVIHSYFGTMTYITVVIAVDTMFMVYVQHACAIFVV
VGNRLEHLVDNDNIDINSKPNILNDESYNRMVKCIIEHSKALDYAQLIESANSFSFFFQLGFNMITISFTGFQ
AITKLDRPDEAFRYATFTIAQTFVLFFLSWPSQRLADESTRIIESTTRSAWYLTSIRSRKLLQLFILRSSVPC
QLTAGSFYILNMENFSAVVRTSMSYFMVLCSMQ
>MdemOr110
MDIFKNRHFINYRFIISCYGVWPHQSGCNKYLLRFIITTGIFSIFTPKTIKFIEYFGDIDGMIQCIPMLCVHL
LGFVKFINYIFNAKSIKRLFSLIERDGKTLKSEEDKEIMERWLIRTRKITSAYTAAMFPILGLFLASPAIPKV
LDIIKPLNETRNLIYLYETEYYVDQDEYYVPILLHAYFTVPLSVGGIVFFDNMLGTFIHHACAMLEILSTYLS
KIHIDNDAKKIYDKEKLDRIREKIIHCINMHQNSLEFALELDASINIAYFIVLGLSLLVLTVTGMTTVIKLHQ
PDEAIKFGAFTMGAFFHLFYSSFQGQILITQSEKVFNSIYESEWYNLPRYHQNLLNIILLKSIKPFTVSAGKL
YILSMDTFSIVLKNAMSFFTVLSSMR
>MdemOr111

MDILKNRHFKNYKFIFTCYGQWPYQSHFQKNSIRFLITMGVLSILTPKTIKFVEYFGDVDGMIQCIPMIGVHL
LGLVKYYNYTFNYNSIKRLFSLIERDGKNLKSQEDKNIMEKWLLRVRKITSAYTAAMFPILGLFLASPAIPKI
LDIIKPLNETRNLIYLYETEYFVDQDEYYVPILIHTYLTVPLSVGSIVFFDNMLGTFIHHSCAMLEILSTYLE
KIHVDKIEKIDDQIRLERIRRKIIHCVHMHQNCLEFTSELDTSINIAWLIVLGLNLIILTVTGMTTVIKMNEP
DEAIKFSAFTMGSLFHLFYNSFQGQILITQSEKVFHAIYESKWYDLPCYHQKLLSIIIMKSVRPNAVTAGKLY
ILSMDTFSIVLKNAMSFFTVLSSMR
>MdemOr112
KIALLVLGQWPSQSPCQRYCARFVVLLAIFSIITPKMIKLIESIKDVDEVIECLPMVILHLVSLTKYFNWIFN
EKKVNHLFVLIDRDGKKLTADQDVKIMKKWLEHIRKISLSYTTAMFSILVLYLSSPVFPKLMDMIAPLNEPRG
RIYLYQTEYFVDQDKYYVHILIHAYMTVPFSLAVIVYFDNMLATNVAHACAIFEILSTYLENIKYDVSKITII
NAEKHSRKIRKSIIRCVDMHKNALRFANDLESSVSFAWLIVLFYNLTIISITGMVTAMKLDQPNEAFKFFAFT
IGAVFHLFYTSFQGQELIQQSERVFHAVYLSQWYNIPPQYQRLLIPIMMKSLKPCTVTAGKKFVLSMDTFSMV
IQKAMSFFTVLSSTR
>MdemOr113
KVVLTLLGQWPSQSPLQRYCARFLVLLAIFSILTPKMIKFFEVIRDIDGVIECLPMIFVHLASLTKYLNWIFN
ADKFKKLLIIIERDGKTLESYQDVEIMTVWLKRIKSITTAYATAMFSILALYLVSPAIPKIMDAISPLNESRP
LIYLYQTEYYVDQEVYYYVHILIHAYMTVPISVTVLVYFDILLGTHVHHACAMFKVLRYQLLIGYLENFTSITY
LETIHVDIYNTQLNDEEISNKIRKKIMHCIDMHRNTLEFANALEASYNIAWLILLTYCLIALTFTGMATVMKL
NQTSEAIKFIAFSIGHVFHLFFTSFQGQLLIEESEAVFYSTYLSEWYNIPLFYKKLLIPIMMRSVTPCRISAG
KVYILSLDTFSSVIQKAMSFFTVLSSMR
>MdemOr114
KVVLTLLGQWPSQSPLQRYCARFLVLLAIFSILTPKIIKCIVVINDIDGVIECLPMIFVHLTSLTKYFNWILN
ADKFKNLIDLIERDGNSLQVRQDIEIMDDWLRRIKSITTAYTTAMFSILGLFLVSPAIPKIMDFVNPLNESRP
LINLYETEYFVDQEKYYVLILMHAYMTVPVSMGILIYFDILLGTHVFHACAMFEILRYDQIIIIVSFLVISTY
LQSVHVSICNTEISDKAKSDKIIHCVHMHKNTLEFADEMDASYSTAWFVLLTICLVGLTFTGTATVLKLKETS
EAIKFIAFSSGHIFHLFFTSFQGQLLIEKSEAIFYSTYSSEWYNMPLFYKKLLIPIMVRSMTPCRISAGKVYI
LSMDTFSSVIQKAMSFFTVLSSMR
>MdemOr115
MNILQSRHYRGFKVVLTLLGQWPTQPPLQRFCARFVILLAIFSILTPKVIKFFEVIDDVNGIIECLPMIILHL
VSLTKYFNWILNEKKVNNLFVLIDRDGKALKLDRDVEIMNKWLQRISTVSTNYSTAMFSILILYLASPGIPII
LDMVSPLNRTRERIYLYQTEYFIDQQEYYLHILIHAYMTVPISVAVLVYFDNVLGTKVHHACAMFEILSTYLE
NINARKITTRGDKKMSHAVRHYIVHCIKMHKDILQFSNKLESSVSLAWLIILFYSLTIISVTGTVTVMKLNQP
SEAFKFFAFTIGAVFHLFYTSFQGQELLQQSERVFHAIYESKWYDLTCYDQKLLSIIIMRSAKPSTLTAGKLY
ILSLGTFSIVLKNAMSFFTVLSSMR
>MdemOr116
METNTTPYHRINKFFLSVIGQWPHQSTKSKWICYLILFFFCSTQGYLQTAGMIAARIDIDVFLEAIPTVLADV
ICGIKMFNFSVNAAKMKKLLLTMEEDWKMYASGPENKILNEYAHFGRKVTIYYTGALYGTLAPLVIVPITPLI
LDVIAPMNESYPKHLMFQQIEFLVDAEKYFYPLFIHNYMGTVAFLTIIIAIDTMLMVYIQHGCAKFAILGLCL
ERIAKNDQNIDRSTAEFDDIDYREIVKCIAIHNRAIEFANLIEDANHLSFLIVIGINIIMMTTSALVAVFKLA
MNDTEIAGRFAFFTLGEICHIFYSSWQGELILKHSESIFYFGYQANWNNTSTRSQKLMVPLLLRSAIPCRITA
GKMFEMSLKSFSMIVKTSFSYLTVFASMRV
>MdemOr118
YYKLPRNFARSIGRWPYQSERQRFTIAIAIISAFILQVGPKILADIVHSDDQELIFETLAPTITDVASFAKYM
NTFINAKMIKKILHRIKDDWTYMSNDSEKLILKKYAGLGKLMATGYVGFVYMTTVMFITEPLLPMIINLILKT
NISAPHEFPVPMEWIVIDKEKYYWLLFSNSSVCIMVILTVLISYDVIFITFIHHAYGLFAITGYQFFPNLFEF
DSRVNASSNSDDVHYKHLVSCIRIHKRALEYADLIESTFAGCFGVVIGLNLPLMSITGIQIIAETSTFQQKIK
YVMFTGAQMLHLFFECFLSQQLTDISLRVQQHMYICTNGKWYNISSKSQKLLILMTMRCQVPCTLTAAKIMGL
SIESFGMV
>MdemOr119
KMDTYEESYYKIMINFLCFLGLWPYQPRLKRVLYPTALWITHIVQFIPQIITVFVDSYDIDLMFELLTTVVQE
IDCMIKFLNLINRAKTIKIFLDRIPDDWKILQNDEEKDVFKYHLNIGKLMSAGYVAGMLIATVLYLMDPVITI
LVNTISKSNESAPHKFLVPMKFIIFDEEEYYWPVLSFSSICISLYSIVYVCYDVIFISLVQHVCGIFAVVGFR
LENSPNVESYDSTIEEKKIFTNLDDTMYNHFVSCVRDHKRALESSKLIEEIYTLSLGALVVLNLPIISATGVK
ILSPSATIEDIVKNISDVSAQLVYLFFTCYMAQKLTDMSSYVHQCITRVNWYNNSAKSKKLLFLMILRSQVPC
KLTAGKIIELSIENFGKMVKTAGSYFTVLLSM
>MdemOr120
MDIYERPYYKISKNFASFIGQWPYQSRLHSFMFGSVLWTLFIIQVIPQIIAAVVNSDDEELLLESLSPFITDV
IYIAKYVNTIKKAKMIRTLFERVRDDWKIPKNNYEKLVLESHLKMGRYLSIGYAGFVNVTLLIFITDPVLPII

INIISKSNDSVAFKFCVPMQFIIFDEEKYYWLLSSLSNICIIFIINVIVCCDVIFITFVQHVCGIFAVVGFRL
EHSPSDTISHDLLEGTRFSINSQDIYYKYVVSCIRDHRRALEFAELIESTFAISFGIVVGLNLPLMSITGVQI
LTQSNSMQGTMKYIMFTGAQILHLFFDCYMSQKLTDMSSQIQHSIAKANWYENSAKSRKLLILMTLRSQVPCK
LTAGKIMELSIENFGMMMKTAGSYFTVFMSMR
>MdemOr121
YYKLLRSFARSIGRWPEQSRLQTFVIGVIIITAFILNLTPIITADVVHFDDQKLIFETIGSLLTNAATLTVYF
NTFLNGKKLRILFKRINDDWKLVTNRREIIILKKYAKLGSVISAGYAGFVYIATFIFVIEPALPMIINFIFKT
NISAPYNFSCPMEWVIIDKRKYYWILFTNSTVCVMLILSVLISCDVIFITFVHHACSLFAIAGYRLQNLPTDV
NLNSKLTQSYLLRNSHDVHYIHLVSSIKIHRRAIEYFNLIENTFTVGIGVVVFLNVPLMSITGVQLIVQTNTL
QQKIKLIVFAVAQIIHLFFECFLSQQLTDMSLQIQRHIANGKWYNISKKSQKLLILMTLRSQVPCILTAGKIM
ELSIESFGMMMKTSGSYFTVLLSMQ
>MdemOr122
YYKLPRNFGRSIGRWPDQTRLQTLLIGIFIVSAFILQFVPIIIADVVHSDDQKLIFETLAPAITDFASFAKYM
NTFIKRKTLKILLQRINDDWNVVTSCDEKCIMEKYAKLGNLMAAGYAGFVYVSTVMYVTEPALPMIINFFFKT
NLSAPHKFSVPMEWVVIDKEKYYWILLNNSGVCITVILSVLVSCDVIFITVVFHACGLFAVGGYRIKNLTNAK
YYKNNLTELKLSTNADDAHYVHIVSCIRIHRRALEYFNLIESTFAGCFGVVIGLNLPIISITGVQIITQTNTM
QELIKLVLFTAGQIMHLFFECFLSQQLTNMGLQIQKDIVNGEWYNISKKSQKLLILMTLKSQVPCILTAGKIM
GLSIESFGMMMKTSGSYFTVLLSMQ
>MdemOr123
DIYDKPYYKITKISASLIGRWPYQSSRQSLVIVAVIWIAFILQAIPQIIAIVTHFDDREVLLEALAPFIIDIV
CVAKYMNSFYNAELIKTLFERVKKDWRLLPKDREKKILEYHVDLGRLMSVGYAGKLLFQGFAFITTVIFVTEP
ILPRIINKLTNSNESVPLKFALPLEYIIFEKENHYWLMLIITNIFAINMIVVIISCDIMFITFVQHVCGLFAV
VGFRIQSSPTGKITGKNKKEASSRKSSQDVAYKHLVSCIRSHRRALEFAEIIETTFSTSFGVVVALNLPIMSI
TGLQLITQSNTVEQMLKYLMFALAQMLHLFFDCFLSQNLTDMSSQIPHCIANVKWYDNSKESQKLILLMIMRS
QVPSKLTAGKIMELSIENFGVMVKTSGSYFTML
>MdemOr124
TMDIYDKPYYKITKISASLIGRWPYQSSRQSLVIVAVIWIAFILQAIPQIIAIVTHFDDREVLLEALAPFIID
IMFVAKYMNSIYNAELMINLFERMKQDWKLLSRAKEKCILEYHTNIGRLISTGYAGFAYTTTAIFLSEPILPR
VINYFSKSNESVPLKFALPLEYIIFEKENHYWLMLAITNMFAINMIIVTISCDIMFITFVQHVCGLFAVVGFR
IENSPTGKISDSRQEGVSLRKNSHDVSYKHLVSCIRSHRRALEFVKLLEETFTGTFGVVVALNLPMISITGLQ
SLNKIFWSKRFMAIKIFAIIFTSYYYLPLNFQKFHNITYQFLLRANMKWYNISKKSQKLILLMTMRSQTPCKL
TAGKIMELSIENFGMMMKTSGSYFTMLLSMQ
>MdemOr125
YYRVVKIWSCLTGQWPSQSPTKKFITITIIWIAFFMQFIPQIIAFVVHIKERDVVFEAFSGLVIDIGFIIKYM
NAICNADLMKQLFERIRRDWKLLLNEEEKVPLQYQSTLGYLFSASYVGICFGTATIYTTEPVFPRFLNIVLGR
NESVPLKMALPLEYIIIDKEKHYWLMLTISNICVYNIIAVIVSCDVVFITYVQHTCGLFAVIGCRLKNTPVDK
DYLEGHKGRDFLSKSKNISYKHLVSCIKGHRRALEFADLLENAYCLNFGLAAVMSLPLISITGVQVVTQANTA
EQLLKTTTFALSQTAHLFFICLMSQQLTDKSLQMQESIVGVSWYDISVKSQKLLILMTMRSQVPCKLTAAKIM
DLSIENFATMLKTSASYLTMLLSFQ
>MdemOr126
MDIFDAPYYRVVKNSSHLIGQWPNQSPQKKMMITIIIWTGFFMQFIPQVIAIVMHLDDPDILFEAFSSMVIDL
AFVFKYSNAIYKANLMKKLYSRMVSDWKLLLNDVEKSTLQHHTNLGCIFSSGYAGFAYMSAMIFIMEPIFPRI
INMFIETNETVPHKFALPLEYIIIDKEKHYWLMLTITNLLIVNIAVVIISCDITFITFVQHVCGLFAVVGCRL
VNTPIDENDGEIHKRGDYLSNVKDVPYKHLVSCIRSHRRALEFAELLENAYCVSFGLLVGLNLPVISVTGFQI
ITQSNTIQQLLKYTSFTFTEILHLFFECFMSQQLTDMSLQIQENIAKVRWFNNSIKSRKLLILMTMRSQVPCK
LTAAKIMDLSIENFGMMVKTSGSYFTMLLSTQ
>MdemOr127
YYRLMKYSAKIIGLWPYQSRRTRIFIIFMVWVFVFAQLIPQGISFFEYLTLVLHYLFKCTVVVIYSLNIKFIQ
MKNLHDRIKADWGMVRNDEEKNTQAKHANLGSIISSVWAGVAYISATVFVFDPIIPKILKIFISINETDPYKF
ALPLEYIVIDKEKHYPIMLFFTSLFVYNIIIILISNDILYINFVQHACGLFAVTGCRLENTPMDENDGEIHKR
GDYLSNVKDVPYKHLVSCIKSHRRALEYAELLEDTYCSSFGMLVGINAPLISITGFQIITKSNTIEQLLKYLT
FTLSQIFHLFFLCYMSQRLTDMSLEIQKNIANVKWFHNSIKSRNLLILMTMRSHVPCKLTAAKIMDLTIENFA
MMVKTSGSYFTMLLSMQ
>MdemOr131
DLFDDLNWRHTKLLMSSFGAWPFQSRAKRLILGTTGHFMLESMLIPEIIKLVTIRNNLKMVADCIPLLLILHIM
VLIKILSCHQNTQRLKNLLMRVQRDWQLNLDESEIKILRNDGHNHKIFMDFYYISIYSAAVIYMLVPIIPKIL
DYIVPLNESRPSLPFYQAEYFVDPNKYSTIIYICSCLVTPITPTVFVAFDSIYSCLIQHSCSMFTIIGRRLQN
LTNDLNISRNEKNDDDKFKLLVNCIRIHKDVLEFVALVEMNFTNYLFVLLGIIVIGISFTGFQVLIMANKLVD

KIRIGWFGMCQVVHLYVLSYFGQKLIDHSEYIHKSVCLTHWYSFPHKTKPLIVLILLRCKILSKITAGKLYVM
SVENFTSVMKKSISYFTVLTSVQ
>MdemOr132
MDVFDSMHWRPTKKLMSALGAWPFQPVIQQKILGSLFYFIIQSIYVVEISKLMVVWGNLTETLDCLPVFVYHS
MVQIKMSNCLLNLKKAKILLSKVKRDWESNLEESEIKILCNDGRIHKSVMDIYSSGLSSVAVIYAVVPLVPIF
LDIVVPLNETRPKILPYPTEYFVDIENNFYALYIHGFIVTPIALFYFLAFDSLYAGLAQHACSMFTILGRRLE
KLTADQNIINIKRNPVNEENGIKSLVICIKMHKEILRFAQLLEENYSNYFFLLLGTIVLGLTVTGFQFVVLAT
EIGEKIRCLWYGTGQIIHLFFLSYLGQQLINHSEYINESICAAKWYNYPKKMKLLIIVMLMRGKIVSTITAGK
IYIMSVENFSSVMKTSMSYFTVLTSVQ
>MdemOr134
LHIDNYVMINRKILKLVGLYPVDLRRYFFSICCLLLIIIPEVLRIFFNQKDLDVILETSSVLSTILLAVIKST
IWIIFRRQDTTILINFLFNDYWDIVVTYCSANEIKYIDKNAKIAKRITVSYIVLIVNALLIFYSLPLPILKSD
VQDPKFNVSLNFPFIAAYPQFCYKSPFYEVVYFSQMLATSMCGLIILAMDTLIATAIFHSCGHFNVLCSKLQH
INFQNNYHLTDELITIIKHHQLAIRFSDHLEYAFNPLMLFQVIASSIIICLVGFQVNTTLKHDNKDKLVEYIC
YLMMALFQLLLFCWPGDKLINDSLRTNEAAYSTNWYSHHFSHTIKTELILMILRSQRPSYLSAGKFHLMSLEN
FSTILSTSVSYFMLLQNL
>MdemOr135
LEPYYNIKVSLTLTKYIGTWPPILEPYRSIYLLYTCVSFIFILGIYLTVQTVNLFVIWGNIELMIATAFLLMT
NSIHAYKVFVILGNQKRIQVLLDKLSTTNYYHNDDKYERVFTYYAWQGLYHHIAYQSFGTVAVLCWGLTPLAD
AVAGNTRRLPMEAWYPYDTKKSPAFEITSGHQAVAVIIACFHNIGMDTLVTGLINAACCQLEIIKQNLKNVGS
DFDYNSNNECDYEDFMNNEINKMVEHSNEIYNFVKEIEDIFGLIILFQLTVNCLIICITAFHITKMTVFIPTE
FFGMMIYLCCMTYQIFIYCYHGNELAIQSASIGSAAFASNWWKFHKNYNRSLGLLITRFNKSIVFTAGPLMRL
SLQLFMSV
>MdemOr136
MEVLKLNFRLLSIMGIWKPRGWRGIKAIFYYIYRSFVVINILNLLVSCILDIRLKNVQLDTFVDNVSLTFAWF
IARQKVFCVIENRCGIIRIMDSLAKNSFKLRDYREELIFQRFEKFARTIFTYYPLLFISSLLTDSLGHISAMD
PPYSLPYPGWFPYNYTRSTQIYWGTAVYQVYAILTSGTINLILDLLLPCIMCYLCGHIHILRHRFQVITEKLQ
IMSENDKPQEEITTVERKMIAEWVENHRDILNLVKFINNLFERVIFVQYTVSSLLLCTIAFLLSHAQPGTVKF
AGNLAFFATMFFQILLPCYCADKLSYEFLDISTTIYDTNWYHLSNNARKSVVIIIRQSYRPVMITSSFFIVLS
LESFTKVIKLAYTIFNVLQ
>MdemOr137
RYSGIWPLLPSVNIFWKILHFIFRIFNYSAIIIQIAGMSAIAINNITDLTVAGEIGGFIIGLIMCLLKFLKFT
LSYDEIMNHVDVVFNPINILQQSSVMCIKSCAFKEEAQVTLFFVPPSFLPILMLFFGHKQKVGLPLKTQYPIN
TAMSPNRELAIFFQSYLVAYCITILLASDMLVIGLIRWSTMQFAALTSNYQNCNSKLVKRATIVSPKETFDIL
NKCDAMKITDEDMEIHTFLLFDENEIEKNIDDSFSLRFITCIKNHQRLMKVIRDLNATFSAFMLLQFASSLTV
ICLNGFQMILNLDDVKNLMQFAIFIAAMLEELFIFCWYGNEFTWMANSLSYNQWLSGWEYVNDKNNNSDRSSN
NNYDNNKLSNLITISMIQTMRPLEFKAVGIFILSMPTFLSVVKSSYSALALLITI
>MdemOr138
MKSPNHRSSDIAYALGLYKILGRTLGIWPLDCHNISSFIRITFVTVTQLSMSISFTKHLLVKGNCGKITDVVD
VLSLIACGVVTVIKVIIPRIYYNKMYVIVSTAINDWKTVNNEKARQTMLRYAYIGRVVFIVQIIGAYATGFQL
IVSRLPFMMEWNNDRNYSTPLYTVPIGPSCWISSDISSYHYTVYYTFQCIQLFVVCTAYIGADTYFFGIAMHV
CGQFELLSNSLTNIYSDGKISNQKQNFSKFVERHKHLLLLANNFEETYNLIILSQVAIDALLICISGIVLLMT
LHTEDLFIIIGLIIRIYLVYVQLFLYSYVGEQLHAQANKMQLTIYNCPWYNMSPKITKDLVFTIMRTNYSFNL
TAGKMYFMNMENFKNIIKTMGSFFSVLRLMF
>MdemOr139
MAILERNIFILTLVGVWKPEHWKGFKAALYYFYICLVTIVNHSFLLSGVLDFELRNIDVVVIIDNLSLLSCLF
TVRYKIVTILYYRKLIEEFVNRFQRYPFKAQDDEEEKIYVKFDKLTKMISMLYPGLFTVAVSWYSVGHIFRMS
PPNVMPYQGWFPYNYTIYKYYWPTAIYQLYAICSAACVNLAYDSIFCSILYYICAQTHILKYRFSVLVENLQK
INEGNDASVNVREIERKMIGDWVDYHNDILDLVKFVKSLFSTAIFVQYAASSLLICSIAYTLSHTETRSMNFA
GDFFYLTAMTIQIFFQCIAADRVTVEFADITDALYSTNWYNSSNNVRKAMTIILAEPLKPTLINSGYFVILSL
ESFTKVIKLSYTIYNVL
>MdemOr140
MAILERNIFILTLVGVWKPEHWKGFKAALYYFYICLVTIVNHSFLLSGVLDFELRNIDVVVIIDNLSLLICVF
TIRYKIITVLYYRKFIEEFVNCFQRDPFKAKDKEEEKIYIKFDKFTKTISILYAGLFTVAVSWYSVGHILRMS
PPHVMPYQGWFPYNYTIYKYYWPTVLYQLYAVCSGAWVNLAYDSIFCSILYYICAQTHILKHRFSVLAENLQK
INENNDGSVNIRGFERKMIGDWVDYHINILDLVKFVKSLFSTAIFVQYAASSLLICSIAYTLSHTETRSMNFA
GNFFYLIAMTIQIFFQCIAANQVTVEFADMTNALYNTNWYNLSNNGQKAMTIILTEPLKPTLINSGYFVILSL
ESFTKVIKLSYTIYNVLE

>MdemOr141
MAILERNIFILTLVGVWKPEHWKGFKAALYYFYICLVTIVNHSFLLSGVLDFELRNIDVVVIIDNLSLLICVF
TIRYKIITVLYYRKSIEEFITRFERDPFKAKDKEEEKIYIKFGKFTKTTSILYAGLFTSVVSWYSVGRIFRMS
PPDTMPYQGWFPYNYTIYKYYWPTAIYQLYAICSGAWVNLAYDSIFCSILYYLCAQTHILKHRFSVLVENLQK
INNGNNGSVNIREIERKMIGDWVGYHNNILNLVEFVKFSFSTAIFVQYAGSSLLLCSIAYTLSHTETRSIDFV
SSFFYLTAMTIQIFFQCIAANQVTVEFADITNALHNTNWYNLSNNVRKAMTIILAKLLKPILINSGYFVILSL
ESFTKVIKLSYTIYNVL
>MdemOr143
MDEMEMLNSVPFESFFRTEIRILRYMGLSYFNRVFSNKEENEKWWEKITPLFGILIMSLLISMEIMKIIRVIS
ISITLAAGIFTAMLSGMLCTFKAVRCWTHRKELFDFIRQLKVFWDTASSNKLITKNELKSALYARSLRNYVIV
LVFALAFSYAFPVYVGICKHFLYHRDEYFFTFSRIMYPVKYPFTINSFSRYFMCLFFEQVSEILAIVYWLCGD
ILFIQLTTHVSIQCEILVNRLHDLNKDNSSDDEGSRRYFVDIVLQHNQLFSHCQWLQRFFSPIAFFVTLINGL
NLCFSLYRVDQQVSQGNWSHLIENTIHLINVFGQTILYCMHADLLTEKLGEVNNAIYYSNWVQSNKKLKMMVL
MIMRRAQKEYRFTVYGIITLNLRQFTKIVNTAMSYFTLLRSFG
>MdemOr144
MEGGKLINSITFEEFLNGEILILRCMGLSYFNRVFSHSEEIEKWWEKIIPFCGILIVCLSTYLELIRVIRVID
VDFTHAIDILSAMSSGMLCTFKAIRCWTHRKELFDFLRQLKTLWDSADSNKYISEDVLNIVIYARSFRNYLTV
IVIALAVSYAIPAYTVLGSHLIFHRHEYFFNFSMIMFPVTYPFTIDSYSIYFSCLLFEQLVELLAIPYWLCGE
VLFVQLTTHVGAQCTILVNRLRGINKDGNGSDNQENHRQLADIISIHHKLYLHYCWLQRFFNPILFFVTLVNG
ANLCFSLYRVDGEINERNWDILLSKVLYLVAVLGQTFLFCKHADVLTEKLDEISYAVYYCDWTNCDKKFKTMM
LMIIKRAQNGYRFTTYGIITLDIMEFTKIVNAAMSYFAILRSFG
>MdemOr145
MDEGEIKNSMLFEDFLNGDVLVLRCMGLSYFNRVFSHNKETEKWWEKIIPLCGILTMCLLISLEIIRVLRVIV
VDITLATEILTAMLSGMLCTFKAIRCWTHRKELFDFLRQLKTLWESADSKKYITEDVLNIVLYARSFRNYLTA
VVISLAVSYGFPAYMVLGNHLIFHRDEYFFNLSMIMYPVAYPFTINSYSVYFSCLLFEQIAELLAIVFWLCGD
VLFIQLTTHVGAQCTILVNRLRGINKDGNGSDNQENHRQLADIISIHHKLYLHYCWLQRFFNPIVLFVTLING
ANLCFSLYRVDGEINERNWSNLLINVLHLIAVLGQTFLYCKHADVLTQKVDEISYAVYYCDWTHCDKKFKTMM
LMIIRRAQKGYRFTIYGIITLDLMEFTKIVNAAMSYFALLRSFG
>MdemOr146
MSKVEIINSVPFEEFFNAEIRMLRCMGLSWFNCVFSNDENRREKWWEKITPLFGILTLSLMLYFLTVALIKII
TIDITLAPEMFTALLSSSLCCFKAIRCWTYRKELFDFIRQLKFLWDTASSNKFITQNIFDSVVYAKSLRNQLA
VGLFLLVVTFGFSPYVILCSHFLSHRDEYFFNFSVIVYPISYPFEVKTFPRYFICLFFEQTVILLCYIYWLCG
DVMFIQLTTHVGIHSTILVNRLHNLDRVAISDPKNKNIYHELSDIVESHYQQFIYCHWLQKFFSPIVLFVTLI
NGANLCFSLFRVDEEINNRNWSGLIVNVVHLLAVFGQTILYCMHAEILTVKLEEINYAAYFCDWTNADEKFKA
MILMIIKRAQKEFKFTVYGIITFNLNQFTKIVNAAMSYFTLLRSFG
>MdemOr149
FIDYEWAIGMTKLGLKVCGVWPDLQRAKWLRVLISLRVTIATLIVVIFTLIPGLIALTRVWGNMTLIIDNLII
TLPFFTAVFKLNVLWYKERDLQTLFNKINEDWSIPRNTLEHSLMIKNATVARNVTLFAYILVIWVILIHHLPL
WIGGIVPRTPTNITDGNRALVMQTIYFYDVTESPIFELTGMFQFMSSLVAGSAYTTIDCVFGTLILHVSGQLQ
VLELRVNNLCNSYNKKTNYVDKTSFQKDLNTTAKVHQRLIDFVEKMEDIFSMMLLEQFTAFAIIFATEGFNII
SIFAGDVEGSFSTMSFSISYFIYALFLIFLYSAAGEFLSENSTKIYHAAYNCEWVNLEKRDMYQIVMIINRAQ
KPLVVTAGKFAPISLSTFASLIKTSAGYMSVLLAVR
>MdemOr151
LIILNTADFIVNFADLGEVSYNLGYIFPMVTATMKAYTLIKHQKSIRKLLDDIHEPITKLKYSSDLGALTTVR
IGLTYQNMDFIIFIIACTFVVLTLVLPLPIILKTRQLPIRVVVPFDKEPDPIFQLVYLFELYGLCTECMWTVI
YDPMVMGLIRWIVVQTIILEANYGHCNSLEIPRATFSMDEQEYRTIKNYKYLDVTSEQTKIHAFVPFSSKEAH
VKNDSFVKRFTLCVKHHRRLLSLIDRFNSTYSVVLFAQISSDCLLTCIGLFQMALALKKKRNIFRDVIMLGSG
LIHLVYWCILGNRLIIEDDKLWKSIYTSGWENNIGSKSKQLVLNGLLQGLTPLEMKAGYFFVFSMKTYLSVIQ
KSYSYFAILNTVMD
>MdemOr152
MDFWRNSDWRFLRFQLCTLGVWPFQKSILQKAIGFFVILSVQSITLPEVIKFTYIWRDMEEFADCFPLIGIHC
VCTVKWMCCVVNMDKIIALLNVIKSDNLSAELTEDEHQILRDTGRLNRLFVLAYSIWIYVIAILFLVFVPLIP
STMDFFMPLNESRPKIVIYHTEYLFDSVEYSWVILLHQCIISPFPTIIIIATGSLYCNCCQHACGMFEVIGYR
LKNLDMTIKETMNEKNLGYRTNNEIFFKSLRTCEQMHQKMLQYVEKFQDIYSVTLCFSMTISIITLCITGLQA
IIKKDEFFEVIRYVTCGLAEIADVFILCWYGQKLIDSSDYLYFCACQTNWYKYSAKSQKMLISMIVKLSTPCS
ITLGKLYRVSLECFSTIMKTTMSYFTVINSLR
>MdemOr153

VSVIITTGIDTVRNINNIPIVSNNICYIFPMTTILTKATFSFFNEQKMKDLIKRVYKPIDTLRFSSDIGVLKM
IRTALSYQFFDSAIYIFVCISVSASTFLLANYHNRELPIRGVFPINSTDTPNYQIIYGLQVYAILVNCVWVYS
FDMILLGMIRWMTIDLKILQLNYRMCPYYQEPRTNFVVPKEGFSVIEKFSYFAEIKLDDEISRFVAFSEKEAN
VKDDSFLKRFLSCVMHQRKLIANINDTNDLFSVVLFVQTFSNCSLTCMGLFGLVGSIKKHRNPMNDIVMLQIG
FLNLLYWCVFGHFLIVQHDCLIDSIYECGWEDHLTNKKVPQLIINTILHGMQPIVITAGHFFDLSMKTYLEVL
NKSYSFFAILNTV
>MdemOr154
MLNIYWKFFMAVGLWRPIEWHGLKAHLYNCYTMFVMLMNYLFFMTGVMDINFTNIDFFGSIDIITLMLQSIEN
FAKIFCLVKKRNDLLNLNIYLHSEPFNLRNDDEILIQKKYDDINRIVNFVCLTLGLLSTLWYTGFNAFQMIMP
TVLPYRSKMPFNYSEPKIFIITAASQCYTVVTIGIINTTFGILFPSMMLQICAKICILQYRFRMIITKLETND
YKGNDNLSVEKTNENSPKYLIAKWVESHIALLHLFKCANSLFAETVFIHYVINSFVMCTLTLLLSRSSFDISL
IVNAFYFVLKCVQQFGQCASAHQVTLEFEDLRHVIFSTNWYAIDVGVQKLLTIIMSKTVEEVIFVSGYFVDLS
LDSFKSIMKLSYTIFNVIDQ
>MdemOr155
DQKELDEGVKAFYWAKRMSRCIGLWPATPNYYWFNICIFYLTIFVSLQLLHLYNSVYDFDKVIDDFTENLAST
HMYTRILMLRVHNYKIGEMITQAMKEYNTNAFKNSDEIKVFMKFINKGKFLIKGLIIFIMSTEISWFLKPLTT
PSSSDDLMINNNKTLPQFILPFHVSLFYEVNSLKRYILTYLSLMPMVYVTGIGHSAVDCILVLLVFYISGKLS
VLTTRINALKNNHYDCRKELREIIAEHSRLLKMGDEVKDVYSTGLLVYLVNGNLLICIIGYQILINYMTGPNS
DLLQYFVYIGATYFMISNFCIISEHLTAESNKVCEAYWNCEWYNMPQDCVKDIIYCILRSQRPLALQAGKFST
FSSVTLTDVTKTALSYLSVLRNFL
>MdemOr157
LNEGVKVFDWARWISVGIGVWPLALNNYIFNIGFFYFTIVMIFKYIDLFVNIYDLKAVMENLSENIAFTIVYM
YTFLIRVNIKKFIQVFEQVLMDYNSASAFKNIKEVKVFMIYANNTKVFTKYIIISIAMSEFLWFIQPLTTSTT
SNNSANALAIDNEMTTYILPYHIYVFYDINSFKNYVITYVSLSLFTVINALGIMSLNIFLIILVFHVSGRLAV
LAIRIDELQKNIIKPRDQFVEIITEHIKVLKLGNDIADVFSAPLLVYFLLTNLLLCILGYEILLNFMSGLNSD
IMQFIVLISTVYLILFVICMNSENLASESDNVCQAFYNCNWYNLPPNTVKDIIYCLARAQKPIFLKAGKFSTF
SYSTLTDVTTTAMGYFSVLRNIL
>MdemOr159
DQKDLNEGVKIFNWGKWVSKGLGMWPLAPNNYIFIITFFYFTIVMTLEWLDFYNSLNDLDKVISNLSDSLAFT
HTYVRGLILRFHVNEMRMLIQNSMRDFNVNTFKNSWEVEIFMDCINKGKRVTKYIIILFIAITEIIWFLQPLAI
PEVEIQFYFFCVVVNDNETIRYILPYQFHIVYEIKNFKSYVLTYILYTPHMFISGFSHSSTECFLITLVYYLS
GRFVILADRINALSKKPNLRKSDFNDIIAEYIRLLKLGETVINSYTTSLLVYMMNSTILLCIIGYQILIHIMI
GPKIKLIHCFVYIFTIYLVITVFCIVSERLKAENNKVCEAFWNCEWYNLSPKQVRVIMFCIMRSQKPLALKIG
KFSYFGISTLTNVTKTAMGYLSVLRHFI
>MdemOr160
LKDQEDLHEGLKIFDWIKRGSKGIGLWPLAPNNFFFIIAFIYFSIVIALEWMDFYYSLDDFDKVLDNLIESLS
LSQIYIRLIILKLQINKLGQVIKDTMDNFSVEKFKSSSEIEIFLDYINEGKFFVKSIILFLAMTLITWYFGPL
TAPALIADDNETIIYILPYRVPSVLKINDFKSYVLMYIFYGPFILIVCSTHASLECFLITLVYYLRGRFVILA
DRIDSLYDKSEVGNNGVKDIIVEHSKLLRLGETFINVYSTSLMVYMTTATILLCVIGYKILISFIKGSNADVV
TYLICILTIYLIISLFCIVSERLTSECDKVNEAFWNCKWYNMPPNMIKDIMYCILRSQNPLALKIGKFSYFGN
NTLTIVTKTAMGYLSVLRHFL
>MdemOr161
DQKDLDEGLKIFDWSRRVSKFIGLWPLAPNNFIFFTTFSYFSVVMILEWMDFYYSLNDFDKILDNLTENLSFS
QIYIRGIILKLQVNKLRQIIKVSMDGFNVNKFKNSSEIEIFLSFINKGKFFVKSIISFMAMTIITWYISPLTA
PAPIADDNETIIYILPYRFHVVFEINDFKSYMLTYISYAPHSLIDCCSHASIECFLITLIYYLRGRLVILAGR
INALNNKPKVEKYEINDIIAEHSRLLKFGETIISIYNTSLMVYMMSVTVLLCVIGYKILINFMTGPNADLVQY
LIYIFITYLIIGLFCIVSERLISECNKVSEAFWNCEWYNMPPNTIKDIMFCILRSQKPLALQIGKFSYFNNNT
LTIVTKTAMGYLSVLRHFV
>MdemOr162
DQNDLDEGLKIFNWIKYVSRTLGLWPLARNNFIFFTTFSYFSVVMILEWMDVYYSLNDFDRVLDNLLENLSYS
HIYVRGIILRLKITKIRQLIEDTINNFNVDKFKNSSEVKIFLSYINEGKFFVKSFISFMAMTVITWYISPLTA
PAPVADDNETIIYILPYRFHVVFEINDFKSYMLTYISYAPYTFIHGCSHASVECILITLIYYLRGRLTILAGR
INALTNKPKVKRSEINEIIMEHIELLKFGETVISTYSTSLMFYMMSATMALCVICYKILINFMIGPNKDLVQY
LIYILATYLIIGVLSTVSEGLISECNKVNEAFWNCQWYNMPPDLINDIMFCIFRSQKPLALKIGKFATFGNNT
MTSVQKSAMGYLSVLRNFM
>MdemOr168
MKINPEKIFRMALVLRKMLLLLPPDNYENKYIKIIYHMIWWIFLINQTIIIIILATEAYGKSDDINLLVDAIE
KVSVRVYVLFDYIFWKLNEPQLNQLVSNVTKYFNELSLNNKKIFTECINRHISIILINGSFYFLAIMTCVFLP

LFSSRLLPLTEAYPFEIKKYTMVYFIIYLTHIISFFQVNVCFVTIFCMTIMYSHILTELKLLSLKLKNINNDD
CEFKFCREKHLNCIKYAQSLNHVVKFLTLKIYVFPTVIIIFSAVPILIYRNIFEMIKGISIVGSCLLSVFAFT
LPADDLHAMSMDVGQAIYDSNWIGSSLSIQKSMVIMMCRAQKPLVMNVKVVLPEITCKFYATFLSFTMTYFMT
LRALIYRYND
>MdemOr169
MKVLELNFYLLSILGVWKPRHWHGIKAIFYHIYRSIVVIINHFLLLSGILDLEFKNVDLDAFVDNLSLIFAMV
VVRQKVICMIENRTSITYVLDSLGKGSFELRDHQEEAIFKRFTDFIRNIIIYYPLVIMSTLMTYSAGHMSVMD
SPQVLPYNGWFPYNYSRTNKLYWVTAVYQLYSVFSMSTIYLILDLLLPCIMCYMCGHIHILRYRFRVMTEKLK
VMLENNNTQDEIIVAERKLMAEWVRYHIDILSLVKFINEIFSSVIFVQYTVSSLLLCTIAYLLSHTKPTTMSF
AGNFAFITAMFFQILLPCYCADKLTIEFSDISTAIYDSNWYYLSNNIRRSVVIILRQSYRPVAMTSSFFIILS
LESFTKVIKVAYTIYNVLQ
>MdemOr170
MIKRPIETHRKYLDKCIHCIRFCGIWEFDSSASIYHKLMNLFGKIFNPTILIFHFLTLFADIVVNYNDLIIVA
DDGCFLAGSFTVCFKAYEFHLLNNIYMKIINDVHNSVDVLQKSCDLGVLTIIKQYIFFETLDFFLLIYVVTVL
GVGLIVLLPLTRGGLPVRAIFPFDVTKPLMHKIAFFIQAYNISFGLVTIVALEYLSWGLMRWTIVQLKVLSSN
YQNCNSDKVPIASFNVTKNTYNKIKNFNILKVDDEDTEIHNFIVFEEKELNNINDCFNWRFRTCIRHHQRLIK
IIYDLNDIFTISLLIQLGVSTFLMCLNGYLAFMFPHDNQRLIRSVLYLVAGFVQLLYWCGFGNELKFQANDLT
ISQWMSGWEDKFDSGIRNLVTTAMIRTMQPLEVRAGGLFILSMETFLSILKTSYSVFVLLTTVSDDE
>MdemOr171
FRYVGIWSLHSTVPTVLRIFHSVFRLCTFITYVLLLIGSGVDGIDNYKDLTKVTENLCLFIGSGTTIVKVLIF
CLRQNIILKHVEDVTHSIDILQHSSDSQVLNVIKITVRQDTKEFYSLTIIITIVSLIRILAGRKRENRLPLPV
YHPFDTSESPIHQIMYILHSYCIVYNLTAILMGDFLIVSCIKWLTVQLKIVASNYQNCDSSLFERSGFTSTSL
ESKILNNQENIHTDINDIEIKTFVPLEQVHQDHNNNDDINDCFYERFKICVKQHQKIIKTVDDLNATFSVYML
MQFAISTGLLCLNGFQVIICKDSSAIIKFFLGLVIALIQLLFWCYYGNNFVSMADSLTNSQWFSGWENSYNGS
LKNLLSISMIQSLRPLEFHAIGLFKFTIPTFVDIIRKSYSVFILLKQTVTTD
>MdemOr172
IYIYRYSGVWPLLPTAKTGWKVFNFIYRIFNLAVFIFYLITLGADAVTNYNDLTIFGNDGCYFFGTCMCLFKG
CKFWASYHKIIKLIVDVYDPIDVLIQSSDPGILMNIKQSYYQENIAFWGFWILCSFFNFSVIFLIPREKGILP
IRAIYPFDTTISPNYELAILYQAYCLAYALCIAIALDITTIGFIRWSTLQIAALTSNYKNSSPNVAKRASLVT
STTDSRKIIEKLNKIKITDDDVEIGTFVPFNLHEAKCFIDDTFLSRFTTCIKNHQRLMKIIHDLNAILSSLML
VQFATSTCIICLNGYQMILSTKNSANFTKFTAYLVISFAELYFWCWYGNEFSYIADTLTHNQWISGWEDAYND
NNYYYFNNNNNKISNFITISMIPTMHRLQFKSVGIFVLSMPTFLSIVKSSYSLLIIILNSFTSAK
>MdemOr174
RYSGIWPFLPTEKIGWKIFNFIYKIFNFVVFIFYLITVGADAVDNITDLTIVGYDGCFFFGTCAFVYKSCKYS
ALHDKIMKLVDEVYQPIDRLLQSSVVTNIKYELLIETLLLSCAFSNFVFMSFAIIFLIPRDKGVLPVRAIFPF
DTTTSPNYELAILYQAYCVFYCMMMMLVLDATAIGLMRWLTIQVMILTTNYKSCNNELTKRANIVVPSESIKT
YKNIIASKVTDEELEISNFIPFQESESKEINDDFNWRFITCIKNHQRISNVVNNFNSIYSSIFLAQFAVSATI
ICLNGFVLILNTSNAANLVRFGIYFALCFGQLFIWCWYGNQLTSTAETMTYNQWMSGWEYAYGKLKSNELRNL
VTIAMMPTMKPFEFTAVGLFALSMPTLLAVVKSSYSMLILLTTV
>MdemOr175
RLSGIWPLLPSAKTGWKIFNFIYRFFNLILFIFYLMTLVADALDNVTDLAIVGCDGCFFFATTMIVFKAYTFS
LSYDEILRIIDHVHDPIHILSRTSDFGITISIRNCIFREKLEISFFCVSCSLFAILFLVPPEKGAIPIRSVYP
FDTDKSPNHQLVLLHQSYIIIYSLTVLIAMDAMSVGFIRWSTIQIQVLTSNYQNCNVHSIRRAVLISPLLTTE
SVKIENKFNNIKKYDEDTEICEFLPFYYNEIENIVEDSFFARFKTCIINHQRIIGMINGLNEIFSSSMLVQFA
TSTTIICLSGFQLVVNTNDTSMFIKFSSYWISCFSQLLFWCYYGNQFTSLANTLTYNQWMSGWEYVFEKNKNI
ELRNLVTISMIPSLRPLEFKAVGLFVLSMPTFLVIVKSSYSFLVLLTSVTAD
>MdemOr176
RYGAVWPCLPSTGIIFKILNSFYILLNFSLLVFYLITLCTDLYYNYTDMNVCGNDGCYFLGTVMIMFKGYKFR
TMYDKILKLIDDVYGPIDTLMRTSDRGIIMNIKHSIFFENLHFYAFLVGCLSLSFTIIVLTPREKGELPVRAV
YPFNSTISPYNEMALFFQAYCVLYCLLVIIALDIMTIAFIRWSTIQMKALTANYKNCNAKSIKRATLISPSET
IKIINEIKPWKITDEDLEIRTFLPFAQSEADDTEDSFVWRFKTCIQNHQRLLKLMIGVNTTLSPFLLVQFSTS
TLIICMSGFQWISNTGSHGNLIKYTVFLGICFAELLFWCFYGDAFNYSADAITYSQWMSGWENAFTKKNIYKM
SNLMTIPMNQSIRRLEIKAFGMFSLSMPTFLSVVKSSYSVLVLMTTANSDE
>MdemOr177
MKNSFETHRLFLNKSISILRHCGIFDFNNSTPMYHKILTYFIILFSQSMTLIYGLTIVADAVANCTDLMIVAS
DGCIIAGWILVYFKIQKSYSVRHKILKLIDDIRDPIDVLLKSNDLGVLILVKEYTIFETMDCYLVISCTVFLG
TVLIFLSLLTGDLPCRAIFPFDTTISPYFEIAYFIQSYATAFNLVSLMTLEFISLEFLRWTTVQLKILSSNYQ
NCTSVPLKPILSFGFTRDTIDNITVFNVLNIEDEQKKINSFVAFEEREIDIINDCFKWRFKNCIKHHWRLITI

INNLNNTFSSCLMAQLGGSLFIMCLNGYLAVTFSNNKQVLVRAVIYLLGGFLHLLYWCALGNELKFQADFLTT
SQWMSGWENKYDSSIKNLITTSMIKTMQPLEMRAGGLFVLTMETFISILKSSYSVFVLLTSVSN
>MdemOr178
MKKSPIDTHRRYLNKALTILRYFGVLTYDSSALMHRKFLNKFMRIINIIISIIFVSPMIADLIVNFNDLIIFA
DDGCCIVGTFVAFTKEQLFYRKRHQICKLIDDIHNPVDVLSQSLDLEVLVNVKECMLFEEFDCYLLLIFVLLM
IMSLSGLSALAGQLPGRAIFPFDTTTSPYYQIAFFIQAYNIVYHLINLISAEFISWQLIRYTTLQLRVLSFNY
KNCKGDSKKISSFNSTRETIDAIKIYNLFDIEDEQKEITSFIAFDDKEINNIENSFTWRFKTCINHHQKLIRI
VKDLNDIFQNSLMVQLGGSLLLMCLNGYLAVMFPHDKKILIRATMYLLSGITQLLYWCAFGNQLKFQADNLTT
GQWMSGWENNYDSGIKNLVTTAMIKTMQPLEIRAGGLFVLTLETFISV
>MdemOr179
RIIGILDYTPSTLFLRIKGNAVKVFNWAVALCVTTSLAVDVSQYYSDLEAITNDVGYLFPMLGILLKTLAVNL
GQKNIVGLIEAIHAPILKLRYSSELGVLTEIRTTIFYQSLDFIIFAAILGATTLTIVVMAVISDTKLPLRGVF
PLNETVLPGYAVVFYIQSWTVMMCSLWILLIETSVIELIRWKNVQLIILQRNYENCCNWMEPRANFEMSDETY
EKIKNYSYFKLKDEDFKINLFVPFKEDEVNVKNDSFILRYKTCLKHHRRIINNVYEYNDFFSVLQFFTVFITC
LFVCLCLFQIVVNKAKKAAVLNTGILMCSEMCHLGYWCIFGNLLMNEADIIHQSQYNSGWEKELNSEVRHLVI
NSLIESKEPLKITAGKFFVLSLATYLTVIQKSYSYFAILNTV
>MdemOr180
MTSTPIETHRRFLDKSIKILRYCGVLTFDSTASMYQKFFNNTIRIFNYIIAVFYFITLITDIIMNYKDLMTIA
DDGCFIAGWIVTYFKIHKFYTQRHRICKLIDDVHNPVDVLRQSCDLGVLTTMKTYMFFDALDFFLFSNCAVTL
GIALVILVPREKGKLPVRAVFPFDIKKSPNYELALFIQLYTLIFGLISVAMEEFISLGFLRWTTLQLKVLSAN
YKNCNSYSIECADFNLTQDTYDCIKKFKISNLLDDQMEINKFVEFDRSKDKKKIEKVVDCFKWRFKTCVKHHQ
RITHIIDDLNDVFDSCLIVQFAVSLFLICLNGFLIVMCADDRKKLISAFTYLSVGFLQLLYWCGFGNELSFQA
NSLTTSQMMSGWENWFEIGLKNLVTTAMIKTMQPLEMRAGGIFVLSLDTFINILKNSYSIFVLMTTVTSD
>MdemOr181
RLVGIFDYDSPKLYLRIINFFTKIANWIIVILTLGTMAADIVVNIKDLEAVTNDVGYLLPMLGILIKTFAVTR
GQKNIRRLIEDIHKPIKKLCYCSDVGVLTKIRTTLFYQNFDYAAIAMILAGTVIALTAMSADSETKLSLRGIF
PCDETVSPVYEIVFFMQFWTVYMSCLWILIIETSVIELIRWINVQLVVLQANYEQCHSFTESRATFQMDNKTH
DTIINYRYFGVTDEQMVIRSYIPFDETEINVKDDSFILRYKTCLKHHRRIINNVNEYNDFLSVLQFFTVFNTC
SFVCLCLFQIVLVSILKSINFIRTIQERAAELSHLFYWCMFGNSLMDEAEAVHQSQYNSGWEKEVNSEVRHLL
INSLTESKEPLKVTAGKFFVLSLATYLAVIQNSYSYFAILNTV
>MdemOr182
FSTIIVTIYFSTILADLIVNYKNLNILSDDGCFLCGVYVILFKAYKYNRFQSRIHNLYDEIHKPIDILRQSCD
LGTLITIKKTIFLENLDFYMFSSFATTLGLSTTFLVPRKKGELPVRALFPFDTRISPMHEIAFFIQIYSIAFG
LTNVVTLEFISLGFIRWTTVQLIVLTSNYKNCRTDIIRRATYYPSSETIKLVKNFGITKLINKKMEIRKFMPF
MKYEADDIVDDCYLWRFKTCIKHHQRLTLIVKELNSLFSSSMFTQLATSATMICLAGFQAVLGSNDKSSFMKF
TVYLGATFTQLLYWCWFSNQLLTQAISLTSGVWMSGWEHQLPLNVKNLLTISILRTLKPLQLKAGNFFTLSLE
LFVSILKSSYSFFALLSS
>MdemOr183
RLCGIWSLDSSSPIFLKLLHKISNIFGIIALIIFVGTLAIDLILNSNDLLIATDDGCYLAGISVIVFQVYNFR
RHHKRIKNLIYATYRPIYVLQKYTDVGLNRILDTTAYYQDLEFWFFVLLGSSLTIALMFFVPTKDGSLPIRAA
YPFDTTKSPMHEIAFVLQFYAVAYGIMAIVFIDTVGLGLIKWINIQCVILASNYQNCRINESKLISSESSDNL
SMIASIAEDINNTNDDYDEKEPNITTFCPFDDQDTEGISNCFVARFKRCTKNHQQLLDTVDQLNDCFSSSMLM
QLFASFSMICLTGFQAVLGATTKTSLIKFVLYLGAACTQLFNWSWVGNELLYQKVTLLTSQWTSGWENELDSN
IKPLLIMSMIKTMRPMALQAGGFFTMSMATFLSIIKSSYSFFALLITLID
>MdemOr184
RLCGCWNYDSDKLSLKFINVLVRFINWIIVTFILISLFIDMTQNITNIEAVSDVIGFLAPLIVTLLKSIILQV
LQKDIDKLINSIHKPIERLRYSSDLGVLTKIRTAIYYQNFDFTLFLSVLSVVSIVIFFISSEVSDTGLAMRGY
FPFNETVSPMFETIFFMQLWTIFINCIWVALFDLSLLGLIRWINVQIHILQYNYKNCDPSFSKRDDLKTSKDT
YLKIENYDFFKVPSDQFKINLFVPFETSEMNVVDDSFILRFKTCLKHHQRIINNIDNYNTIFSFVLCVQIITT
NAVACFYLYEAAWAIKHNKNYLKCFVMFGSVMVELFYYCIFGSQLIIQAEMLRYSQYDSLWHYFLNRQVRDLV
VNALLISMKPLEMRAGYFFIFSVETFVSVLQKSYSYFAILNSLME
>MdemOr185
GIWSLDSSSPIFLKLLHKISNIFGIIALIIFVGTLTTDLILNSNDLLIATDDGCYLAGISVIVFKVYKFHRLH
KKIKNLTDATYRPIYVFWKSTDIGVKTVLKTNKFYEDLGFTFFVTLGGLLVIALIFFVPKQEGALPIRGVYPF
NTTISPMHELAFTLQIYAVAYGLMAILMMDTVGLGIMRWLNVQCILLASNYRNCRINKNELVYSESHDNSLMI
VSIDENNNNNAGYDETESNITTFCPFSEQDTAGISDCFVGRFKRCIKNHQQLLNTIDELNECFSSSMLMQLFA
SFSMICLTGFQAVLGATTKTSLIKFVLYLGAAFSQLLYWCWFGNELLYEKVTLLTSQWTSGWENELDSNIQPL
LIVSMIKTMRPLELHAGAFFIMSMETFISV

>MdemOr186
IRFFGFWDYNSSKFSLKLVNFLSRTVNWACCLCILLSLAADIIKNIKNLEIISNDVGYLAPIYSIFFKSIKVQ
TLQKKIGELINSVHKPIDKLRYSSDVGVLTKIRTAICYQNFDYSVYASILGFVFCAVIIIFSSISDTGLPMRG
YFPFNETISPTFQIVFFIQFWTILMNCIWVLLVDLLLIGLIRWINVQLYVLQNNYENCRPDISDRDNFSISHD
NYTLIKNYNFFKVPEEQFKIRSFVAFQMDEINVKNDSFALRFKTCIKHHQRIIDSVNNYNDLFNSMLFVQIFN
NQSVACLFLFQGVLAMKQNEDFINYFICSASSVTELFYLCFFGSQLIIEAEKLHESQWKSGWDENICPEVRDL
MLNAQLQSIKPLKLMAGYFFVLSVETYVLVLQKAYSYFAILNTVIDEKV
>MdemOr187
RLLGLWKFNNPTSYLKFIYNCYNKISFILVLIFVSTLTADLIINYDDLSIITDDGCYIAGITVIIFKLYKFNS
EHEKIKILVDEIHRPLDLLSQSPDPGVRKIIKRTAFFENLMDKFFIILGCFLAVALTFFVPKDNGALPIRAVF
PFDTTETPLHELAFIIQAFSIFYGLLTIVFMDELIITLIMWINCQLVILNNNYKNCSGVAEDQTSIEKKNGLS
EEIYELKNEKFSIRTFVLFDDETDGRIDENFLGKFKYCIKHHQRLIVTVDHLNEIFSSSMLLQLFASFSMICL
TGFQAVLGVNETENLLKFIIYLGAAFSQLLNWCWFGNELLQKSASLTNGQWSSGWEKHISNNVKSFMIISLLR
TQKILQLQAGNFYNMSLHTFTMVLKNSYSFFALL
>MdemOr189
MHHKLINILSKILRYNGIWPVESATTISFKLLNLILRFINFFIFVFLTSIIMADAIANHSDLSLITDNLCFLI
GCFETMSKAFKFYTEYNNIIKLINDIYEPIDKLKKVNNIEIMTRVNELSRFECRQFYILSGIVVLLISARVFG
ADRANREFPVRAIFPFDGKKSPNYLLIYILISYGVAFIDVSLFTLDLMIVVIMRYLTFQLEILISNYKHCHVR
STRNIARNILPNGLETVERFNEIATVNDYDDDDNGDDEIKNFVIFEIHQKDINNINTFDWRLKQCIKHHQKIV
QMLVVLNDCFSFCVIVQILTSTILICLNGFQILLGNDDRHLLIRRIIAINAVLLQLFFWCWYGNKMSTVADSL
TYNQWMCGWESEFKRGVSNSVTTSMILSLRSLELRAIGLVPLSLQTFVSAIKKSYSVLILLLTVVED
>MdemOr191
MEILPECFFIFTCIGLWKPAGWTGYKSIAYNLYTVFMISIPCLFVVSGFLDLILLTTDVSDISDNVFLVLTIM
AGCGKMFNIILNRNMIFYIIDCLQNKPLRPQNDKEINIKNRYHRISRFITFSYSTMTGVGVTLFVLGKIIENG
PQRILPYRGWLPYNYSQPVVYWLSAGQQALSMIIAGGVNAAFDTFFPGMMFLVCAQINIFKHRFKIMLNSLEI
NDNNNNNDIIDNDYRKVNYIFGESVKHHNYIFQLFDNINNVFSTVIFVQYSVGSVIFCTSIYHMSDMKITTVE
FVSNIFYIGSMLSQIFLLCVSANQVTLEFEDLNTALYDSKWFAVNNSARKCLIIMMINSQKHITFTTGYIVTL
SLDSFTSLVKLSYTIYNVLQQ
>MdemOr192
ILPQSFFILSCVGLWRPIEWQGCKCILYKAYCCFIVLNNILFSLTEFLDLILVSSTINEFTNDLAKMLGQVGA
FGKIIGVLCNHRMIAYVVQLLDQKPFTLLDLYEINIKKKYIYIYSFVIRFYFMFFTVGVTGVLFTQISMAEFL
NVLPYRGWFPYNYSRPNIFIITAAHQLFTFFIGAYVHVAFDTLFLGMMLYVSIQINVLQYRFKHIVRTMVKFN
VVNRNGDEENSPDKKLIAAWVDHHNDVLSLSDYVHRIFSGSIFFQYCLSSTQICVTVYALSSISVFSVEFMSG
MVFLLGTTLQIFVLCMAAHQVTLEFADLSDSTYNSDWCDLSIRNKKSVLIIVLQTLKPVVFTTGYFVTLSLES
FKNVIKLSYSIYNFLQ
>MdemOr193
ILPQSFFILTCIGLWRPVDWQGWKCILYDMYSYFVVVTNFAFSLSQLLDIILVRTSINELTNNMTMLLVMVTA
CGKILGILRNRNEILQIIKSLEEKPFKPRDQYEIDIRNKYIYVSSVLTRSYCVYLTIGISGILLTRIPEAKLP
DVLPFSSWLPYNYSNPKIYLLTAAQQIINSLISTYAQVGFDTLFPGMMMYVSAQTNILQYRFKKVIKALEKIN
LKNCADQVKNHKDAEKNIITEWVECHIAVLSFSDYVYEIFSKPVFLQYCSSSIMLCGTVYYFSSIPMDYVECI
STMVFIIGTILQIFMCCMSAHQLTLQFADLNDEMYNTDWFNLSINAKKSMIIIVLKTFQPVVFMSGYIVTLSI
ESF
>MdemOr194
ILPQCFFILSWVGLWRPVDWQGWKCILYNAYSWFVVLNNILFIVTKFLDMILVSTTANEFINDLASMTGQIGA
FGKIIGVLYKHRMITHVIQLLDEKPFLLFDLFENDIKKKYDDIVVIRLYCLFFTAAVSGVLITQTLMAEFPTI
LPFKGWFPYNYSEPSIFIITAAHQLFTFFHGAYVHVAFDTLFLGMMLYVSVQVNVLQYRFKHTIRTVVKFDVI
NQNSDEENNPNKKLIAEWVDHHNHVLSLSNYVHGIYSGPVFLQYCLSSTQICVTVYGMTSVPIFSVEFMSRIF
FLCGTTLQIFMLCMAAHQVTLEFADLSNSTYNTDWCDLDIRHRKSIIIIVSQALKPVIFTSGYFVTLSLELFK
NVIKLSYSIYNFLQ
>MdemOr195
MAILKESFFVLTCIGLWRPVEWQGIKGAFYNCYTLLVILNSVTFVISESMELIFFNDGIFDFFNNSSMLITII
GMCGKTITVISNRGTIIKMIEKFNGNPFSPRDNQEEIIHKNFNRMIRFNTFVYAVFFEVSVSVFTVGKVFEDR
PPGVLPCRAWLPYDYSNNIIIYWVTASQQLLTIFMSANVDIAYDTLFPGMMMQVCAQINVLKHRFRLTLDALE
NVSVDKMDPVMVKTVEKKIFAECVEYHVEILRMVDIMSHVFGPMIFIQYSFSSIILCSSVYALSQMVPFSPEF
IACSVYILCMFFQIFVICMSGNRVTLEFAELSTAMYNTNWFALSNNAQKHIVMMMMSSIKPIIFASGHVVTLS
LESFKRLLKLSYTIYNVFQQ
>MdemOr196

VVMTIITTMSADAIINFRDLEAVTNDAGFLFPIITILLKTIRLHSRQKQINQLMNAVHNPIEKLRYSSDVGVL
TIIRTAISYQNFDFCFFSIMLGSVFCIIFAITSDSEQLALRGYYFCNETVSPGYEIAFFLQLYTIFMCCLWVV
IIDTTMLGLIRWINLQLYIIRFNYKNCKHDKIDRADFTMTREVYEVIKNYNYFKVTNDQTTIRLFIPFDMNEV
NIKIDSFTIRFKLCIKHYQKLTENINDFNEIFSSILFIQIFLVIFFTCLCLFQAVLTMNQKAKFIKYIFLLGA
ELVHLFYYCFFGNEFLYQGEAIMDSIWNSGWVDNMSTEIKDLMINALLQTTKPTVINAGYFFTLSVKTFLSII
QKSYSYFAILVTMMNGSN
>MdemOr198
LQFTKMNVYFVSSWPLSKSDSRSSKIWFNVRWWTAFISILLLFLPLTNGVYVYRNDSLVMVKAACLSSASAQA
LLKLIVCRFQSNRLQLLHEEMEIIMKNADALEKKYLLKYIKKSGFFHVFGSFSVWAVCLSFIIEPILLSHPYP
TDTAYPFVVESQVLRIILYIQQIIALFFVGAAVIIDFQVATLLWFTCVRFEVLSHYFREVSNEAELVECIKKH
QKVLWYANEVKYAIRYITLSTIATTTIGVICGCLALLSHQPISVKLRVANIVGNAATELFIYAWPADNLIQVS
QAIGQSAYNSKWQKGSVALVKTTMSVIHRSQLPSTISIRGVMPSLCLRYYTSFLSTTFSYFTTLRV
>MdemOr199
FNVTPEKAVQFTKITVFLTCAWPPSNESRLFKLFIYFSIFLSIALFLPLVVSIIKFCDNFFIVMKSVIFICGI
TNYVAKVITVRIYQKEFQQLGSAVDDFINKASVSEKQVLQKYVDKCWKFQLFMTCSYYLTTTGIIIGPLVLPQ
KFPTDAVYPFPVDNQIISLMVYLHQCIVGYQCSAGMALDCQAALFIWYLSAKFELLISETKNVVTYNELRHYI
KKHQDLLTYAEELIRPTRVMAFSTVSVTKIGMIFGGVFLISDEPLAVKIQFGIMVISTTVNIYVCTWAADYLI
TISSSTLSDEIFSTTWMHGPKLRKLWLIVLHRAQKPVVINIPGLLKTLSNEYYSAFLSAAFSCFATIRVVV
>MdemOr200
KVTPEAAIEFTRTSVLLTYIWSPPSANKVRFMLFKIFMYTSVFFSIIFILSLLLSVIKYFDNLLIVMKSMAVL
CGVTNFVAKVIIVRIYCKEFEQIELTLNDFVKNANESERVILQKYVNKCWKFQLLVTCGSYATTTLVMLGPLV
LPQKFPTDAVYPFPVENKIVSCIVYLHQSIIGYHCSAAILLDCQMALFLWYLCARFESLGSEIKNVTNYQELR
YIIRKHQSLLVYAEKVTEPIHAVVFSTVTITKFIMICGAFFLLSDEPIAGKIQFGIMVMSTTLNIYTSIWPAD
CLLDISSKLITNEIYNMCWTWTQQMRKSCLLFIRRTQKPVMIKIPGLLETLSNVYYSSFLSAAVSGFTALRVI
VN
>MdemOr203
MYIEPATVIRILMIFGKLTSTWPPDPNSNKFSKLAKEFLWTIFFLNVIGLLVPLFLGAYYNRREIILMMKSLS
ELTALGDVFFNLLLCRIQRRQLFVLLKEIADYIKTIKENEKVVFRKYVSRYFPFCTFVGLSYFQTAIAFSCGP
LFMKQILPADTWYPFGIKPFTTRHYIIYIQQILAILQTGMGITVDFMVALLLSYSSTKLELLSNKLQDVTNNE
ELKICIYEHQNCIRFVRQLCHSVKFIILKSNATMAMAVIFGAIPLIYAILSQFILMVFGGCMRLYISAWPADD
LREMSEKVAWSVYESSWIGNSKKLQQSTAIIIRRAQKPLVISIVGILPALTLQFYAT
>AGG17934.1 olfactory receptor 1 [Microplitis mediator]
MVTVYEYEEMMKPIKMVNRLISIWPLEENDNSILSRLRIFHRISMFILILIQTVAVTADIVHHWGSMKEV
TECALIATAFYLCVLRLTVYTIHDKDLQTSVQIMKNDWIKFSGEDELTLKEKCLPIIKLAKFFIMTVFST
IGLFMVAPILEVKILGMEEKKLPFRGYFFENQTITPAYGGLYLLGVTAGGFGGSMIAGATTLNLILVMHG
AAKFMVVRKNIESLKSNSENSITFIDCVRGHQDAILFAERVENTINVLVLGQFVISTGLVCFAGFQITEM
AEDRGQLMKYTSFLNSAIFELFLFSYSGNELLTESDAISQSCYASNWVGTSFAKSMQIVMTRSLSPCKIT
AVKFYDMSLANFSSIFSFSFSYLTVLRTMEAE
>AGG17936.1 olfactory receptor 3 [Microplitis mediator]
MDIYERPYYKISKNFASFIGQWPYQSRLHSFMCGSVLWTLFIIQVIPQIIAAVVNSDDQELLLESVSPFI
TDGIYIAKYVNTIRKAKMIRRLFEKVREDWKVPKNNDEKLVLESYLKMGRFLSIGYAAFVNMGVIIYIMD
PVLSAIVNIISKSNDSMPLKFSVPMRFIMFDEEKYYWLLLILSNTCVIFIINVIICCDVIFITVVQHVCG
IFAVVGFRLEHSPSDTVSPDLIEGTRFSMNSQDISYKHFVSCIRDHRRALEFSELIESTFAISFGISVGL
NLPLMSITGVQLLTQSESMRATLKYIMFTGGQILHLFFDCYMSQKLTDMSSRIQHSVARANWYENSVKSR
KLLILMTLRSQVPCKLTAGKIMELSIENFGMMMKTAGSYFTVFLSMR
>AGG17937.1 olfactory receptor 4 [Microplitis mediator]
MDFFDSHYYKGMKILLCLIGRWPYQTLKERVLITIILSILWSYTMFHRMLVYSNYDNYSSKHEVILETIS
PLIVDTITFAKYITTICKMDTIINLLESIKQDWKIYTNKEEKKILEYYANLGKILSLGYVGAVYMVTVLF
MTEPIVEQTFFKLFQNETIPKRFSIPIYWKTPDIEKYYYYLISFQTLNTNFIISITCASDAMFINLLQHV
TGLFSVTGYLLENVPIEENSEENGQKKIKDVAYEHYVRCMRSHKRALEFAENLESIYVWCFGIVITLNMP
VMSVTAMQLTTGTSNVIQSMKYGTFAGVQLLHLFFYCFMSQKLLTSSSVIPECAMNGKWYLCSVKAQRLV
TLVIMRSQISCQLTAGKILVLSMETFTSIVKTSGSYFTMLVQMRNV
>AGG17938.1 olfactory receptor 5 [Microplitis mediator]
MDFWGNSDWRFLRFQLCTLGVWPFQKSNFKRVVGFFVILSVQSITLPEVIKFTYIWHDMEEFADCFPLIG
IHFVCTIKWMCCVVNMDKIIALLNMIKSDNLSKELTEEEHQILRDTGKINRLFVLVYSIWIYVIAILFLV
FLPLIPVTLDIILPLNESRPKIELYHTDYLFGPVKYSWVISLHQCIISPFPTIIIIATGSLYCNCCQHAC
GMFEVIGYRLKNLDITIEAAMNKKNLGYYADTEIFYKSLVTCEQMHQRMLGYVEKFQDIYSLTLCFSMTT
SIITLCITGLEAIIKKDQFFEVIRYVTCGLAEIADVFILCWYGQKLIDSSDYLYLCACQGNWYKYSAKSQ

KMFISMIVRFSTPCSITLGKLYRVSLECFSTIMKTTMSYFTVINSLREEE
>AGG17939.1 olfactory receptor 6 [Microplitis mediator]
MTLIESCWMVFTWLGLFRPIKWKGLKARIYDLYTAIVLFFNYSFFICGVMDIDFTHLNFFADIDLITLML
QYIENTPKILCMILNRNALIEIDFKLQHDHFKIKDEDEKKIQNKFDKFSRYVLLAYSALQATSLVYYTTG
RILAMESPVILPYRSRIPFNYSSSGKIYMLTALDQLYSVSSLICINGAFNLVFTSTMYQICTKIRILKHR
FKVIIQQLEHDGELGNNDNKNLRDVMRKNDATTDKFESQLIANWVESHIALINLYDYAKSVFAKAVFIHY
VINSIVMCTLAYILSHCEIDNIFFGNVCYFSVKCTQQFLQCSSAHQITLEFEDLRDMIFSTNWFATKITI
QKSIIIIMFKSIVPIEFVSGYFVTLSLDSFKRILKLSYTIYNVLEG
>AGG17940.1 olfactory receptor 7 [Microplitis mediator]
MDIYDKPYYKITKISASLIGRWPYQSSRQSLVIVTVIWSAFILQAIPQIIAIVTHFDDREVLLEALAPFI
IDIMFVAKYMNSIYNAELMVTLFERMKKDWKLLSSAKEKRILEYHANIGRLISTGYAGFAYTTTAIFLSE
PILPRIINYFSKSNESVPLKFALPLEYIIFEKENHYWLMLAITNMFAINMIIVTISCDIMFITFVQHVCG
LFAVVGFRIENSPTGKITDSNHRAVSLRKNSQDFSYKHLVSCIRSHRRALEFVKLLEETFTGTFGVVVAL
NLPMISITGLQLITQSNTVEQTLKYLMFALAQVLHLFFDCFLSQNLTNMSSRIPQCIANMKWYNISKNSQ
KLTLLMTMRSQTPCKLTAGKIMELSIENFGMMMKTSGSYFTMLLSMQ
>AGG17941.1 olfactory receptor 8 [Microplitis mediator]
MDVLQENFSVLFYLGVWKPLDCTGIKSFLYNLYTLFITSISYTFLLSQILDLIISTKTVSDFTNNIFIVS
AILTGCLKIFRFIRSRNTFINIINNFKRGLFKPANNDEIIIWNKYARITRLVTIGATTSLIIGLIVMSYA
LCSFNIPQRQLLYRAWLPYNYSSLPIIYWLSSMEQLATVHILAGINFSFDLIFFGTMLNICAQINILKLR
YKVALSHIYSINDSINNNDLGELRDVSKLIREYTDSHDSIIKLFNSAHHLFSTIVTIQYCTSSVAICTSA
FNVTKMKFFSFQFFSTALYINNVMIELFILCVSCNEVTLEFADLGNTFYDCQWYAINNANKKSVAIMMTN
TIKPIYFTCGYVIHLSLDSFTSVLKLSYSIYNVLQSAD
>AGG17943.1 olfactory receptor 9 [Microplitis mediator]
MYSSNSTQVIADFFKQPCYRVNVLTLRICGLWPYQSKFEGKLLRFLWAFFVSSQMIPQVCVCITDFGMDT
LTSTIPPFTVAIIAGAKMIISTLKVNQIIELLVTIQKDWSKLKSETECDIMKKHLDQGKKLTLFISAWYY
NSMVVFLLLPMRPKVMTWLGLSKGPADFDFPYPVNYGVDHDKYFYAIETHISFCSTLVITTIIAADTLFI
VFVQHICGVFKIISYRLENLVSSSSLDIDLHPNKINDRAFWVISDCIKKHNKAIQFAELLENSYCWIFFI
SVGFETLLMTFSGVQLVSQMGNIDGLFRYGPFAFGQLVHVFIENAISQQLINYSGEINDAISMIKWYTLS
MRSRKLLTFMIMRSQVICSVSAGKMFIMSMETFGMILKTTMSYFTILSSMQDD
>AGG17942.1 olfactory receptor 10 [Microplitis mediator]
MSTENTQLDADIEYNLKLTKWILKPLGVWSIVTKDYRFISKLSSVFIIILNSFIMFITFMPCCVHMIYRE
TDPAVKIILMGPFGFCLTNCCKYYFIIFRSNIITHCLENLKSDWARPKTKQDRLEMIKNVNIGHNITKLC
AIFMFSGGVSYHTIMPIWSGSTVNEANETIRPLVYPGNEIFVNCQETPIYEFIFILHLTCGMVMQTITTA
ACHLAAVFASHVCGQVDILKSQLQNLVDKESLKIDGTIENRIASIIQSHVRILDFSTNIEKMLREICLVE
VGASTLIICLLEYYCMTEWSNSETINILTYLMLLVALSFNIFIFCYIGELLKKQCNSIGESTYMINWYKI
PRNKGKQLMLIIASSNNQRKLTAGGMMELSLRSFGNIIKTSVAYLNMLRTVTE
>AGG17944.1 olfactory receptor 11 [Microplitis mediator]
MQNEKEELPSCEKLFSLELAALKFVGLSSLRNGFRKENMNIPVKFYEIFLFICAASILSGFFISSALTVH
MLLQSDFILACEIATFIFAGAVTVSKVLRIWSYRIELIEILRELNELWEKIVKHRLNLKENILNMLNESR
PIRYGYCFIASSLNLSYALRPYFHMLVYFVKQSENKTIDLTVTTYPLLYPIERGTWLGYLLCVTYEQSIL
YFGGIYWIMCDTLFILLTSHICVHFMIISNDFNNLHINYDKNNNESFQLIEDLSRRHQKMFVLCQRIETL
FSPIILLTVVFNGIDLCLCIFALDKDLSDGNWAKVASSVTHALTLFFQIVIYCEFSHVATEETSKVGEAI
YNSSWIYFDKKMKKMLLIIMMRASKEYKFSVFGILILDREQLTQIVKTTMSYFTMLRSFS
>AGG17946.1 olfactory receptor 13 [Microplitis mediator]
MEEERSRDERDLESSVRAFYWGKWMSKWIGVWPLAPNFYLFNVTFAYFTAVMLLEYVDLFFCLPNFEKVL
DNLTENLSFTIIYVRTLMLRVHNYKLGTAIRECLKDSSVSAFRNSKEIDIFVQYTKEGKFFAKFVIAFAA
MTETSWYLRPITSHTAIRVIADNETLNNVTLKFSLPFHFYVFYEINSIKTYALTYLSHGPFVPINGFGTA
SANCFLIALSFHISGRLAVLAERIKTLKDNPDSYKRELKLIIDEHIRLLRMGEDVKISYGVNLLVYLLNG
TILLCIIGYQILLTLTVGPRTNIMPFIVYIMTMYMVISIFCILSENLIAESNKVCEAFWACGWYDMPPDC
ISDVYYCIARSQKPLALTAGKFLTFGYGTITDVTRTAVGYLSVLRNFLLLEE
>AKO89978.1 odorant receptor 14 [Microplitis mediator]
MEKKTINDHTSIKLMRLCMNGIGMWSIEKRRDEIISNIVICYTIATLTVGLIVETTDIYYCLGDLREMSY
VAPCLLNVIVELFLMGTFVINRSEVIAFSDYTTREFWSIPYIESERKLLDDCNRKSVKIIIAFIVVIQLV
VWQYITIPIIESYGKNASERTLPYNLWFTFIPFKETPYYEICFFLQSAATLTTGVCATAFATFLFTINLY
ATGQFKILQQRLESSCQVYNIEKIKSVEQINLIAEESYANLRKCVELHNVLLKYITRLENLYCQIMLVET
LACVFLICTTGFQIVLGVDSSILRTSRSALYFCCLVTQLLLYSWSCHEIIIESLEVAEAAYRAYWYSLSW
SKYGKSFRQALLIIIITRSRRPCVLTVGKFVPMSLETFTAVFNSALSYFTILRQMTEEMENS
>AKO89979.1 odorant receptor 15 [Microplitis mediator]

MEAFRLHLFFLSILGVWKPQGWHGIKAFLYSIYGSTVVIFNHIFILSGILNLTKFKHVSLDVFIDNFSQM
LALIVVRQRIICVIENRNSISQIIESTDKYPFKLRDRQEKLIFSKFSKLAKNILIYYPIVHMCIILVHTV
GHISVMDPPYALPFQGWFPYNYTRKTKIYWATATYQLYAIFSEGSIDLILDLLLPCILCYMCGHIHILRH
RFGVMTEKLQIMSENNEPREKIDSAERKMTAEWVEYHIDILRLVELVKKIFERMIFVQYTVSSLLLCTLA
YLLSHTKCTTMTFAANFSFFMAMFIQILLPCYCADKLTFEFLDISTGIYNSNWYQLSNNIRRSVVVILRN
TYQPVTITSGFFIILSLESFTKIIKLAYTIYNLLE
>AKO89980.1 odorant receptor 16 [Microplitis mediator]
MKLPAHTKGYKVTPEGAVNFIRVTVYLTCISFPLTEKTKVRINYEIILWLSIFLSISLFAPLLASIIKYS
DDTFIVMKSFILMSAISNYVIKVIIVRIYHKELQQLGSALDEFIKKANESEKVILQRYVDNTWKFHGFMT
CSYYLTATAVLLGPLILPQKFPTDAVYPFPVDNQIISYIVYLHQCIVGYQCSAGMALDCQAALFLWYLSA
RFEILISEAKNVETFDELRNYIKKHQIILLYAKELIRPTRLIAFVTVVMTKIGMIFGGIVLISDEPVVIK
IQFAILVISTTINIYVCAWAADNLITVSSTAMSNAIFEISWMHAPKLRNFLQTVIHRTQKPVVIKIPGLL
ETLSNEYYAQFLSAAFSCFAAARVVVSS
>AKO89981.1 odorant receptor 17 [Microplitis mediator]
MENIPIDIHRKFLNINITILRYSGVWPLLPTAKIGWKVFNFIYRIFNLTVFIFYLITLGADAVTNYKDLT
IFGSDGCYFFGTCMCVFKACKFWASYHKIIKLIVDVYDPIDVLVRSADPGILMNIKKSYYQESIAFWGFS
TLCSFFHFSVIFLIPREKGILPIRAIYPFDTKISPNYELAIIYQAYCLAYALCVTIALDITTIGFIRWST
LQIAALTSNYKNSNPNVTKRASLVTSSSDARKIIEKLNKIKITDDDVEIETFLPLDYHETKYFINDLFLS
RFTTCIKNHQRLIKIIRDLNAVLSPLMLVQFATSTCIICLNGYQMILAETMTYNQWMSGWECAYGKVKSN
ELRNLVTIAMMPAIKPFAFNAVGLFALSMPTLLAVVKSSYFMLILLTTVTED
>AKO89982.1 odorant receptor 18 [Microplitis mediator]
MYSILPGSFVVLQAIGLWKPPEYNNSPILNYYYRLRTFITFFLIYSFTITGITGLILTTKDIADVTSDCF
ILLSIFAICGKIANIIWSRNEIIWIIDTLNSEPCKPLNNDEIIIQQKVDRLIWHSTLFYGILTEITVFMV
TFGTLLLQLPIGTLPYNTYLPWDYSHGYLYWVAYGYQIISVCLSANSDIGFDTLVPGLMLQITAKLEILK
YRFINLVDTLKLTQWNGVNDKSYHNFRIENKLIADYVKCHLIILKLADTINKTFDKVILLQFFISSIVLC
ISVYNLAFLDVFTTEFTSIILYLCCMLMEIFILCAAGNQVTIVSSTLSDAIYHTDWINLDTSAVKSLMII
MNRGLKPIIFSSGHIIKISYDSFKTPIKLSYSSYNVLQRT
>AKO89983.1 odorant receptor 19 [Microplitis mediator]
MDVFEEPFYKMIKNFSHLIGQWPYQSSRKKFTIVTLIWIAFFMQFIPQIIAIVIHFDDRDVLFEAFSSMV
IDFAFIIKYLNAIYRAGLMKELWESIRRDWTLLLNDVEKRTLQHHANLGNFFSMGYAGLAYMSTTIFVTE
PIFPRIVNIFVETNETIPLKLALPLEYIIIDIDKHYWLILTITNIFVFNIIIVIISCDIVLITFVQHVCG
LFAVVGCRLESTPFDENYLEGQKGEDFLSNSNDIPYKHLVSCIKGHKRALEYAERLERAYTLSNGIVSGL
NAPVMSITGFLMITESSTIEQLLKYATFAISQMSHLFFLCFMSQRLADMSLRIQENIGNATWYNNSLKSQ
KLLVLMLMRSQVPCKLTAAKLMDLFIENFAVVVKTFASYITMLLSM
>AKO89984.1 odorant receptor 20 [Microplitis mediator]
MENTSIESRQSVLSMAIAGLRLCGIWSLDSSSPIFLKLLHNISNIFGIITLIIFVGTLTIDLLLNSNDLL
IATDDGCYLAGISVIVFKVYQFHRHHKRIKNLTDATYQPIYVFWKSTDIGVKTVLRTNKFYEDLGFTFFV
SLGGFLVIALIFFVPTEEGALPIRGAYPFNTTISPMHEVAFCLQIYAVTYGLMVIVLMDGMGLGIMRWLN
VQCIILASNYRNCRTNQNNSFYLESRDDLSKIASIEDDNNNVTDIYDEPDSNITTFCPFDEQDHAGMSDC
FIGRFKKCIKNHQRLLNTIDELNACFSSCMLMQLFASFSMICLTGFQAVLGATTKTSLIKFVLYLGAAFS
QLLYWCWFGNELLYEVFILPHD
>AKO89985.1 odorant receptor 21 [Microplitis mediator]
MDYLSSPYCRLNKILLSCLGEWPYQTSTQRRFIRSTIYFFSASIIIPKIIKLIKVWGNLDMIIECIPMLL
LDAVNFVKVVNGFINFRKMRELFDRIQDDWGLNYSKREFEIMQNYAEDGKKLSQFYASYMYATMLIYFCM
PIIPKVLDIVLPLNTTRPELYLFEAEYFVDQHKFYYPILIHAYITCAVAVSMLVAFDTEYAIQALHGSGI
FSALRYKLENLVIKDDEADYKNDEKIKQSTYNMVVQCAVLHKRALDYADLLESSRVTCFFFVLLVNIAAI
SITGVQTVMKLDQPTEAIRFGVYTLAQITHIFYNSYPAQMLFDNSWKTSDAIFAGNWYRAGSKSKNLLHM
MIMRSRIPCKLTAGKIYLMSLENFTGVVKTSMSYFTVLLSFR
>AKO89986.1 odorant receptor 22 [Microplitis mediator]
MDNSLDSFLRINRLFLSSLGQWPQQEQISKIFTLINAVFFLITQAYFQTGGMIAAKCDQPIFMESIAPVL
ISFMCLVKFVNFNYNADKMRKLLEIIQADWNSINDLEELKILNSWAKDSRKNTIMYAGALYGTMAPFMLG
PLVPIFCKLMPAGVLPANSSIVLEKPVLFHVEYFYDLEKYYYPLLIHSYFGTMAYMTVAVAIDSMFMVYV
QHACAIFAVIGNRLEHLADDSSINFYYNPHILNDEPYKRMIECIVQHSKALQYAQMIQSANSLSFFFNWD
SICSLLLSVDFNWPSQRLADESARISETTTRCAWYLTSMRSRKLLQLFIMRSSVPCQLTAGSFYTLNMQN
FSAVVRTSMSYFTVLTSVQ
>AKO89987.1 odorant receptor 23 [Microplitis mediator]
MFKINPEFAIAYTKLTVTLVCSWPPGRNSSRLDFLLFRIKWWISWLMGIFLVIPLIYAAYIDRRNVLEFT
KSLCLAVSCGQCAVKMFFCKLQHHRIKFLLDEMEEYVKVAEPFEREIFLGYIKNCGLVHVTLNVCSLVAS

VGVILGPLVLPQSLPTEAKYPFSVENHPNYEIIYIHQAFAGILCSSIGSIDCQIAMLLWFSIARLELLSL
EMKNITNVYQFHNCVRKHQFLLWFVDEVIKAGRNLVATTVIMTTFAVILGGVHIVGNEPMLVKLQFVIIV
GGFSMLLYVTAWPSEILTRMCQNIGWTIYNSEWIRNSKELNKGIEFVIQRSNKPAVIYISGIFPAISLNY
YATFLSKTFSYFTTFRIILAKLE
>AKO89988.1 odorant receptor 24 [Microplitis mediator]
MELDNIKLRHLEPYYNIKVSLTLTKYIGTWPPVLEPYRSIYLLYTCVSFIFILGIYLTVQTVNLFVIWGN
IELMIATGFLLMTNSIHAYKVFVILGNQKRIQVLLDKLSTTNYYHNDDKYERVFTYYAWQGLYHHIAYQS
FGTVAVLCWGLTPLADAVAGNTRRLPMEAWYPYNTKKNPAFEITSGHQAVAVLIACVHNIGMDTLVTGLI
NAACCQLEIIKQNLKNVDLDFEYQIDKCDYEDFMNKQINKIIKHSNEIYK
>AKO89989.1 odorant receptor 25 [Microplitis mediator]
MIFKATPEFAIAFTKFSTLVGTCWPNYKNAPKWKFVLFQIRWWLTFCLSVSAFLPMCYAAYNHWRNILSF
TKSLFDAANTSQTFIKMILSKIHYKRLQYLLYEMENYVTNAREDERELFIVYIKRCGKLHLFVMIFGFMA
ILIIIVAPIGLPQPFPNIADYPFPVDESPAFELVYAHQSAATLHCLSIPVFDMQIALLLWYSGARLELLA
REFKTVTDNKHFVECVKKHQYLLWYIQEIIISSRYILATTSVTCVIAVITSGVHIAGNEPVGFKIPFAGA
SSIIAIILYISAWPSEHLIHMCEGVGTALYESEWVQNSKALNNSMLIVMHRAQKPSTIEVIGVMPILSLP
YYATFLSKTFSYFTTLRVLLSKVEMD
>AKO89990.1 odorant receptor 26 [Microplitis mediator]
MDIFDAPYYRIMKNSAKLIGQWPYQSREKRIIIIIIVWAFFFMQFVPQIIAIVVHIDDPDILFEACSNMA
VDFVTAIKYINTICKTNLIKKLHDRVIIDWSLMLNDEEKSTLEKHTNLGYLFSSGWAGFAYMSATIFVLE
PVFPRILNVFISVNATDPFKLALPLEYIIIDREKHYWIMLFVSTVFVYNIIIVLVSCDIMYITFVQHVCG
LFAVVGCRLVNTPINENYSERHKAGDYLSNSKDIPYKHLVSCIRSHRRALEFAELLEDAYCISFGLTVGL
NLPVISVTGFQIITQFNTIQQLLKYASFTITEILHLFFECFMSQRLTDMSLEMQKSIAEVQWFDNSIKSR
KLLIIMTMRCQVPCKLTAAKIMDLTIENFGMMVKTSGSYFTMLLSMQ
>AKO89991.1 odorant receptor 27 [Microplitis mediator]
MKFFEQSYFTLPRNFARSIGRWPYQSSLQSFLIGIVIISAFILQVGPKILADIVHSDDQELILETLAPTI
TNVMAFAKYINTFVNARMLKILFERIKDDWESVTDKKEKIILESYAGFGKLMATGYAGFVYAATVQFITE
PVLPIILNNILRTNLSAPHKFADPMEWIIIDKEKYYWILLSNSSVCIMVILTVLISYDVIFITFVYHACG
LFAITGHRIENLPHDENFKIINRNTNSLKNSRDVHYKHLVSCIRIHRMALKYVDLIESTFAGCFGVVVGL
NLPLMSITGVQGMKFFRLLHNRMTLQQKIKYVMFTGAQMLHLFFECFLSQQLTDMSLRVQQHIANGNWYD
ISTKSQKLLILMTMRSQVPCILTAGKIMELSVESFGMMMKTSGSYFTVLLSMQ
>AKO89992.1 odorant receptor 28 [Microplitis mediator]
MTRSLKLQDQETFDQVAKVLKWNKWLLSTLGLWPQSPNTFIFTVNFSYFVYHMAMEYLDLFLFIDNLEHV
IENLTENMAFTQILVRIAMLKKYNRQLGEVVNEAFKDYDARIYRTDEERQVFIDYMKKAKLFIKLLCAFV
TMTATSYYAKPITSPPPPPEGELDVEMENATMSFILPYRFHLFYQVNDSRTWALTYLSHFPFVFVSGFGQ
TAADCLMVTLVFHVSGKLASLAIRISEINTEPGVCKQELRSIIIEHDRLLKMGQSIEEAFSETLLAHLIG
ATSLVCILGYQLLVNYARGQGADLVTFFVFIFLVFLVLYAHCVVGESLITESFKVCEAYYDCLWYKMPKE
SSKTIVLCMARSQKPLGLTAGKFGAFCLSTLTDVVKTAMAYLSVLRTFLVIE
>AKO89993.1 odorant receptor 29 [Microplitis mediator]
MYHKLLNKLVRILRYNGIWPVESTVRSYKLLNLIFRLFNLSIIVIMMLLTIADAIANFNDISLITDNLCF
FVGCSEALTKGIKYCIEYKNIVKLMNDIYGPIDIINKKNNTEVMKGINEIARFENRQFKIIFGIVSLLIV
ARVLGADFKNKGFPIRALFPFDATATPYYHLIYLLISYGVLLVDYTLLGVDLMVVVIMRYLTIQVDILRA
NCRHCDIESTRRNIVINGYDDKNNENTDIRNFVGFEIEHEDSDGKDSFDDRLKRCIIHHQKVIYMLNGLN
DCFSFCVVVQILGTTVLLCLNGFQIIMGRDIHLFMRRVLASTAALLQLLLWCWYGNKLSAAADSLTINLW
MCGWEDNYKHGLRNFISIPMTLSLQTLELRAIGVVPLSLQTFVSAIKTSYSVLVLLLTVAKDE
>AKO89994.1 odorant receptor 30 [Microplitis mediator]
MLFKATPEFALKFTKLIALLGTSWPNYEGTPKWKLVVFQIRWWSTFFLAITACLTMCYAACNQYQNILNL
TKSLFDISNTSQTFVKMFFCKVHYKRMQYLLCDMEKYVTKAKPHERDLFIKYIKRCGKLHLTVMGSGLLI
IHIIILAPIALPQPFPNIAEYPFPIDGHPTYELLYLHQSCATIHCLSIPAFDCQIAMLLWYAGARLELLS
EECKTITDNKQFVECIKQHQYLLWYIQEITTSSRHILATTGCTCILTAISSGVHIVSNEPVAFKVPFMIS
WVIVSSTLYITSWPAENVLQMCEQVGMALYESPWVQNSKELNSSILFVVQRSQKPSTIEVPGILPVLSLR
YFAMFLSRTFSYFTTLRVLLDKINLDMEIPAED
>AKO89995.1 odorant receptor 31 [Microplitis mediator]
MLFKATPEFAIAFTKLTSILGSSWPHYKNATKCQLIVFNIKWWFFWFMSITAFLPMCYAAYNNTKNILSF
TKSLCDAANCSQAFIKMLLCKIHYRKLQFLFYEMEKYVEQARANERELFISYIKRCGRLHVSIMISAVMA
AVIIIIAPIGMPQPFPNVAEYPFPVDGHPTFEIIYLQQSIATIHCMSIPVFDCQIALLLWYAGARLELLG
DEFRKVTDNQQFVACVKKHQYLLWFIQEIIMSSRHILATTVVMCTIAVITSGVHIVGKEPLADKVTSVIL
STGLSAVLYLCAWPAEHLAQMCENVGAALYCSTWIKNSKESNKNIFIVIQRSQKPETIQVPGILPILSLT
YYATFLSKTFSYFTTLRVVLDKMED

>AKO89996.1 odorant receptor 32 [Microplitis mediator]
METTATKNDNTNQNKNELQLTKTNKYENVDYKSDADYAVVVARTLLTPLGIYPLHGSDTSLSKFLIAIQI
IIVFGLMLFLLVPHFIWTFFDAEDLKKLMKIIAAQIFNSLALIKFWTMIIHKKELRNCLIQLENNWKNVL
CEEDRVIMIKNAKIGRFFTIAYLSLSYGGALPYHILLPLTAERIVKEDNSTQIPLPYPTDYVFFVPEDSP
GYEMLFVTHIIISTMILSTNCGIYSLIATYIMHACCLFEVVCRHLDEFSKNNTNNFKTELTWIVENHNRA
IQFAETLESSLNIVFLCEMVGCTVIICFLEYGVIVDWEDGKLLGLVTYVILMTSIFVNCFIISFAGERLK
EQSIKIGESAYFAEWYLLPKGLVYDFMLIMIRSSKPASLSTGKVSDLSLAGFAGLVKTSAAYLNFIRAVV
>AKO89997.1 odorant receptor 33 [Microplitis mediator]
MQVLTFNFFLLSIMGVWKPRGWHGIKAALYYIYQTITIILNHLCLLSLLLDLQFKNIELGDLIDNLALVF
TIIIIRQKIVCIIGNRPGITHILDSLKNSPFKLEDSKEEFIFSRFEKLARNIITYYPLIYLSTLSVHSTG
FISVMDPPYTLPYKGWFPYNYTRTTKTYWVTAVYQIYIVLTMGSINAISDILLPCIICYMCGHIHILRYR
FQVMAEKLRIMSKNNEPKDKIISTERKLMGDWVKYHIDILNLVKFTNEIFSSVIFIQYTVSSLLLCTIAY
LLSHMEPTTMRFAGNSAFLTAMFFEILLPCYCADKLTFEFLDISTGIYDTNWYHLSNNIRKSIVIILRKS
YRPVTMTSGFFIVLSLESFTKVIKLAYTIYNVLE
>AKO89998.1 odorant receptor 34 [Microplitis mediator]
MEDLTQEQKDLNEGAKMFDWGKWISKGIGVWPLAPNDYLFTTTFLYFTAVMTLEWVDLYTCLGDFEKVVD
NLTENLAFVHIYVRTLMLRVHIDKLRDVMTESLKDYRTSAFKNSTEIKLFMTHINKGKVFAKVVITFIAM
TEVTWYLQPLTTPSPPVDNRDNETISILLPYHFYVFYEINDFKTYVLTYLSHGPHVVISGFGHATSDCFL
IILVFHLSGRLAVLAERINALKNKPEMNGIQIKSIIAEHIRLLKMGENIRSAFATALLAYLFNGTILLCM
IGYQILVNFMTGPNSDLMQYFIFILATYFIITVFCIVSERLIFESTKVCEAYWNCGWYNMPREHINDIMY
CIVRSQKPLALQAGKFAYFGNSTLTDVTRTAMGYLSVLRNFLIVN
>AKO89999.1 odorant receptor 35 [Microplitis mediator]
MAILKESFCVLTGIGLWRPVEWQGIKGAFYNCYTLLVLLNSITFIISESMELIFFNDGIFDFFNNSSMLI
TVIGMCGKLITVVTNRETIIKMIERFHRSPFSPRDYEEEIIHKNFNQKIRFNTLAYIVFFEASVTVYTVG
KIFEDRPPGVLPCRAWLPYDYSNNIIIYWMTASQQLLTVVMTANVDIAYDTLFPGMMMQVCIQINVLKHR
FRLTLDALENISDDKMDPVMVKTVEKKFFSEMVDTISHVFGPMIFIQYSFSSVVLCSSVYALSQMVPFSP
EFCACSVYILCMFFQILYICLSGNRVTLEFAKLGTAMYDTYWFALSNNAQKHIIMMMMSSVKPIIFASGH
VVTLSLESFKRLLKLSYTIYNVFQQSS
>AKO90001.1 odorant receptor 37 [Microplitis mediator]
MEVLTFNFFLLSIMGVWKPRGWRGIKAILYNINRSIVVIVNHIFLLSGILDLEFKNVDLDAFVDNLALIL
AMVVVRQKIVCVIQNRTGVKHILDSLAKGPFKLRTHQEKLIFSRFDDFARNIFTYYPLVFMSSLLTYSSG
HMAVMDPPYVLPYKGWFPYNYTRTTKIYWTTAIYQLYAVFTTATINLILDLLLPCIMCYMCGHIHILKHR
FKEMIEKLLVMSENNVPQEKIISTERKLLGEWIEYHIDILRLVKFTNELFSSVVFVQYTVSALLLCTIAY
LMSHTDTMTMSFAGNLAFFTAMFIQILLPCYCADKLSYEFLDISTGIYDTNWHYLSNNIRKSVVIILRKS
YRPVIITSSFFIVLSLESFTKVVKLAYTIYNVLE
>AKO90002.1 odorant receptor 38 [Microplitis mediator]
MKTLPYSFMILEYFGGWKPLKWGTSIKGKLYNMYTLTVAVTLVTFCLSCIIDLFYTANIEDIVHNMSMSF
TLIVVCSKLTLVTVKRNEIIRIIKLLDINICRVRCAKEANIQLKYDEKAKNIVKKYGGLVCGAVFAVTGA
SILENIPTKTLPFNGWFPYRHDNSTGFWVAYFHQNIAHFYVAMIGFSFDTVVYGVLLQNCSQLQILKNRL
ENFVEIINEEKLKDKINGLSRTIRECEHKFIKQCTHHHWIILQFSQESNDLFAPIIFLQYLSSSLILCLC
VQLLTKLAFMSPEFIFIVVYLGCMLTQIFLFCWYGNEVIMESSEISSAIYKMNWQVLTNKTKKDLLFMKT
QSILPIKFTSGHLIELSLDSFTKLIKFSYSAYNLLHQK
>AKO90004.1 odorant receptor 40 [Microplitis mediator]
MSMQYSQDFKYALAWNRTSLKIVGLWPEDDDGIITKSLGWFCACLIIIIIYLPQSASVYVYWGNMDAVIE
CLSVNGPVFITIVKIIIFRYYRRVLKRVIDTMAEDWSSSRSNEEYAVMLKTAKISRAISVTSTIITNSLF
VAYIFFKIWEGLEMSKRTDLDPRLSVGLLHPAYFPYDTKKIKFFVPTWIAQLVATLFSMTAYAVFDTFVS
CMVLHICGQLAVVGISLQNLINEDTNSDPNYFWSKFSKTVERHEKLNELANVIEDSFSSILLPQMIICTV
TFCFQGFAMITSFIDPLAGKVSILEMLFSIVYVFYTVLHLFVYCYVGDYLSFESSIIGQSYYKSEWYKLS
QDKSRSLMFIGHRARRPLKITAGKFCAFSRNLFIRILKTSFGYLSMLLAVKRDKSA
>AKO90005.1 odorant receptor 41 [Microplitis mediator]
MKQTATKEGLGRKGFSVTPETAISFTRITVYLTCIWSPSTSSTKLTFILFEIFLWFSIFLSLGLLFPLIV
SIIKFIDDTFVVMKSFILISGIVNFVIKVIVCRIYRQELQELGASLNDFVRNSNENEKFYLQKYVDKCWK
FHGYMTCSYYLTTSAVLMGPLVLPEKFPSDAVYPFPVDHPVVATIVYLHQCIVGYQCAAGMALDCQAALF
LWYLSARFEILISETVNIASHKEIQDFIKTHQDILKFGKQVIRPIRLIVLTTVTMTKVGMIFGAIVLISD
EPITVKVQFAILVVSATVNIYVCTWAADNLLTVSSSTISNEIFHVSAIHPPAMKKLWLTVIHRALKPITI
EVPGFLETLSNEFYSNFLSTAFSYFAAMHAVVNS
>AKO90008.1 odorant receptor 44 [Microplitis mediator]
MENDQKELDEGVKAFYWAERMSRCIGLWPVTPNYYLFNICLLYFSVLMVLELIDLYNSVYDIDKLIDNFT

ENLASTHMYARILMLRVHNYRIGEMITQAMKDYRISAFKNSYEIKVFMEFVNKGKFLIKGLFIFIMSTEI
SWFLKPLTTPSSSDNSIVNANKTFPQFILPYNVYIFYEVNSIKRYVLTYLSFMPMVYVSGIGHSAVDCIL
VLLVFYISGKLSVLTMRIDALKNNQYDCRKELKEIIAEHSRLLKMGDEVKDVYSTGLLVYLVNGNLLICI
IGYQILINYMTGPNSDLLQYFVYIGATYFMIANFCIISEHLTAESNKVCEAYWNCEWYNMPQDCVKDIIY
CIVRSQRPLALQAGKFSTFSIVTLTDVTKTALSYLSVLRNFLIAE
>AKO90009.1 odorant receptor 45 [Microplitis mediator]
MDVFDSMHWRTTKKLLSAIGLWPFQPVIQRIVVGAIVYFIIQSIFICVFLKLIVAWGNLAETLYSVPILV
YFSMIQVKITNCHLNLHKAKFLLLRVKRDWESKLEDSEFEILRNDGRIHKIIMDVYFSGLICVATVYIVL
PLMSPLLDIIIPLNETRERILPYPAEYFIDIQKNFFMLYPHGAIVTPIALTVLVGFDSLYAGFVQHACSM
FTIIGRRLENLTVDRNNIDEEKNSLNERHGLQSFITCVKMHKDILQFVKLVEKYYSNYFFVLLGVIVVGL
SAAGFQFVVLLSGVGEKVRCLWYAMGQVTHLFFLSYVGQKLIDHSQVINASLSAAKWYDYPQKMKPLIIL
MLMRGKRVSTVSAGKIYVMSIENFSSVIKASMSYFAVLTSMES
>AKO90010.1 odorant receptor 46 [Microplitis mediator]
MKVNPSDQNSTVIQNQSSEPENIVNNEQDFEWAIHYHRKVLKFCGIWIYSNSNRWYLKLITDLHSLFIIG
GVLICLTTPEAMALVKIWGNLTLIVDNLLSSAALISTQIKLFVLWTRRKAIARIVEAVKSDWLEPKTEAE
RKIMRRYARIARIMMVCGLSNIAYNLITFHGSVLFGFVYRTVNNITDIEGYLIPTQSVFPFDITIGYRCW
IIRIVQALQCFGAGITYTAIDVFCGMSVLHNCGQLEILADKIKDLVNPDEPRVFQELLKTIVLRHYRIIG
LIEEIRNIFATVLLLLVLCFGILFSVIGFLIASSFESDGTKVPVSQMNFYIGYILFFVGLLFVYSWVGEN
LLSHSEEIHVAVYSCNWTDLEPHQIAQLIIILVRAQRPLEITIGKFAPATLNTFAQILKTSAGYISVLLA
RNG
>AKO90011.1 odorant receptor 47 [Microplitis mediator]
MSEKATNSIITSKIVNSKYALDANRWILKILGVWHFAIDSSYFHKVIALCHIIICTFLLSFVVIPGILFI
FVIVKDVTTRLRISGVFSFCVMGVIKYYYLIKSNKQIGNCVKQFNSDWAQINDIKDKTIMVKYARFGRNS
SIICAAFMYGSCMFYACILPHVSGVFKNKNDPTERTFAYPCHFIVFNQYESPAYEIVFSIHCCCAFVLAS
ISNAACNLITVLITHACGQLEILMVWLNDLDSCQQNEVYAEKYSKIIKQHVKTIRFIVKIENLFQQICFV
EVVGCTLIICLVGYYVLLDWNQKDTGGMTTYVMLLISFVYNIFLYCYVCELLTAKCKLISESTYLTRWYQ
IPENFARGLVLTIAISQNSNPIKAGKLIPLSINTFGTVMRTSVVYLNFLRKLME
>AKO90012.1 odorant receptor 48 [Microplitis mediator]
MEVHSRDQRDLDKGARLFTWARFISKGIGVWPLEPNYYLFNICIFYFTYIMITEYIDLYYCLPNLKKVIN
NLTESLAFTQMYVRAVMIRVHIKKLHHLMSEALKDYHVSAYKNSDEVYEFMSYVKRGRFFVKSVTIFILS
TTTSWFLRPITSSTPSTSMSAPDNETAAKFTYILPYKFHVFYEINNYRTYVLTYISHGPFPYVSVLGAIT
SAVFLIILSFHVSGRLAILARRINALNCKNEGFRTDLTDIIAEHTRLLEMGEEIKISYAVALLVYLVVGT
TQLCIIGYQILVLITMGKQHSLMPFFVFILTTYLLISIYCILSEHLLAESKKVSEAFYSCEWYDMPQDCI
KDISFCILRSQKTLGLTAGAFLTFSNSTLTDVTKTSMGYLSILRNFLLNEQ
>AKO90013.1 odorant receptor 49 [Microplitis mediator]
MEVHSRDQRDLDKGVRVFTWARLMSKCIGVWPLEPNYHLFNICFFYFTYIMITEYINLYYCLPNFKKVLG
NLVENLAFTHIYVRTLMLRIHIDKLRDIISESLKDYHASAYKNSDEVNEFLIYVRKGKFFVKAAGIFVIS
TATSWYIRPITSPSLPNNETAAFTYILPYKFHIFYKISNYRTYVLTYLSHAPFAIISGVGAVTSAWLLIM
LSFHVTGRLAILAKRINSLKDKNGGYRSHLDEIISEHSRLLEMGEGIKSSYAIALLIYFVNGTILLCIIG
YQILVTITLGVKHNLMPYFVFILTVYLVISIFCILSENLLAQSNKVSEAFWSCEWYKMPQDCVKDITFCI
LRSQKTLGLTAGAFLTFSNSTLTDVTKTAMGYLSILRNFLIVE
>AKO90014.1 odorant receptor 50 [Microplitis mediator]
MKFFNHPDWRIGKLMLCSFGGWPYQSQHSRRILNFISIFTVQSIFIPEIFRLTRIWNSMEMIVECVPMIS
LHIVANIKMFNCIINLNKVKVLLEDIERYYQSDLCKSELRILYKDRHSHKKVISVYVFYIYSIAVVFASI
PVLSKVLDLIAPLNESRPKVYLYPAEYFVDQDKYSTYIYIHGYMALPITMTLCTAYDFLYSACGHHVCSM
FKIAGSRLKNFIDNKIAWSKNESLDRNYRQDEVYKSLVECVKMHKFILNYVDCYQETFSDSLFLVIGVNM
LALCITSLQSLITMNQFHDAVSYIVFAFGQLTHLFLLNYQGQNIINHSENFYNDAYQTNWHEFSRKSRKL
YILIIMRSSEFSVIRAGKIYVMSSDSFSNVLKTAMSYLMVLNSLR
>AKO90015.1 odorant receptor 51 [Microplitis mediator]
MKRKEKKNSQTKKKINLTVFDDIEYTQQLLSILAIWPLVTKSSTIKKILSWIHLLVIIGSLIWNILFRCI
FIYIYVKKFDDQIMLIGPTFFRVIILLKYLAIIYHRKTIKKIFNHIQTDRSGVECQEQQNIMMRNVQINR
HVTLVFAIFMYSSGTFYNFVMPHILPLLTHRGSRNTSQRLTIFPGYNVLSDVQKSPIYEITFFFHIFSVF
AGFSVLVLACNIAVVLVTHACGQIQIIIGQLNSLIDDFTNNEKTLYIKFSSIISRHIRVIQFSNNIIKDA
LYETCLVEIGASSVLICIVEFLLLKMIENQNYANMIPYAMLLASLTLSILIICYFSELLESQFIEAGIQA
YSINWYQLPPKARKYLILIIIISQRSYKITAGGIIDLSYIAFVQILKTGFAYLQLLRATK
>AKO90016.1 odorant receptor 52 [Microplitis mediator]
MEYNQDFKYAVAWNRTSLRFVGLWPEPNDGFFTKLKGWLGAWSIFMTIYLPQSTLAYVNWGDMNAVIESL
SINGPILIAIIKIIIFRHYRDVLKLAIVTMTKDWNELRSKEEYKVMLKTAKISRIISVTSTIITNTLFIA

FVFFKIWIGMQLMKRTDLDPRLSVGLLYPGYLPFDSRIMTYFIPTWIAQCFATCFSMTAYAAFDTFVSCM
VLHICGQLAVIGVSLKNLINDDVKVDSKVFWIKFSEIIKHHEEINKLGLMIENSFNSILLPQMFVCTVTF
CLQGFAMITSFIDPSAGEISIFEMLFSIVYVFYTVMHLFVYCYVGDYLSFESTLIGQSYYKSKWYELPVI
KSRSLMFIGHRARRPLLLTAGKFCAFSRNLFLAVLKTSFGYLSMLLAVKQEKISDT
>AKO90017.1 odorant receptor 53 [Microplitis mediator]
MVVLKDIFTILFYVGLWKPATWHGRKSILYTLYTSCIVIMASTFLITEVMDLIFVTSNIVEFTNNVFMMS
AVISSFIKTIIIIRHRKIIADIIDVLKIYLSKISGNEEIIIIDRYTRLIKFMNRSFLCTALFGVSLMVYV
ASSQNISQHILFYRAWLPYNYSQPMAYWMTTTGQVLTIYVLTIIYTVFILLFSGIMFNICAHINIFKYHL
QITFSDEYYHSRNDKRRCISKKENDKKIIHDCVETYLSIRRLFNTVKNLFSSIMLYQYSVGSIIFCTSAY
NMLQVEIFSAHFFSITLYMLNMMTELFIICITCNQITLEFQGVPNALYHSLWYVTDNNNRKSIVIMMSYT
LKPVYFTCGYVIDLSLDSFTNVLKLSYSIYNVLQSTF

>MphaOr2
IIIMQKNITLKKAIAVVKLSLFVIWFWPLPLNTSKCKMLCMKMYQYICILLTIAVLLSMIYALVKNLDDLDLL
IKSSLGLFPCSHVIGNILCHLTVYKRLQCVTLGMEKFCALIKPHEEAIVQREYVDRYSKFYGFCLSLFYMSLF
GLFMGPIMLDEPLPAPAEFPFDASQQPLRAITYMHQIVVGLFIAAHLCVNAFMALLLWLTSARFKLLTEELRT
VTNIYDFAKCIEKHQQLLVYAGEVALTVRPFALVTVFFSTVSLIVFGLIFIAKVSFSLKMQCVLLATSALLEV
FMYSWPAEHLIHISTNIAQAVFEMDWYDESEYLRKNIQIVILRSQKPILVVLPCGLPALSLRYYASYLSTIFS
YFTTMRIMFEE
>MphaOr3
MKLVHLNFHQIFQFLKICNSITGTWPYGSDVGKLMFIFQKIIYGFTVLNSIFIQITLMRAIYKYWYDITIIMK
SLTEFVCVTDFLIKLFICRLKSECIKHILEEIKDFLLISNDQEKIILQKYINRYAFFTMFVIVNFFLAPITFL
CTPIFASRKFPTEGLYPFSTESLFMTLIIYATQIYGTMQVAFSIGVEFMFAVFFLYLTARLEMLCLEIQTAKN
ERQLNSCITKHQEIITFFDKTRNIVQCSLFWMNIMMAICAICGTIPIFRQSLGISSSFIFLVTAGCEKVYIIA
WPADDLIENSEQVATALYNMSWIGRSQRIVKNIFFMIQRSQKPLVINMSLLSTLSLKYFAKYVITLLSYFTTM
RAIIRD
>MphaOr4
MKVQNTVSQTIEIWLRIFGIWPNTSCVLFRRLFWSVALAIEQIFQYRYVVRNFHLFDFSEMVDVLSATMAYTI
FFIKLVIFWFKQRTFNRILMMMAIDWEKCSTTKFNMFATTYNAKLSQRFANMTVIFYSTAVILFSSNILIRLA
DDGKSSNVSTRLLILQMDLPFEINQRFVYELIIIAQFLHLIVCSDAIGLLNALLINLILHIGGQIDILRVNLT
EIFSKKKKNSLNNFIVEEVIKKHQKIIIFSQCIEDLYSYIALVLFISDTLIICCLGFTIVTSVGQPDATKMII
KNILFYLAMNMEAFIFCFAGEYLSSKSKSIGDAAYDSLWYESDSRDSQVIQFLIMRSQNQLTITIGKIMNLSL
ERFGSIVKASASYISVLLAM
>MphaOr5
MEMSTVSRVVKIGLCACGIWPYLPSTVLFRLFWIVMLGTAQYFQYQYIAVHFSTDNFSDLMDGVSSSMAYSLL
FIKLSILWANQRTFSNILQMMAMDWKNCVSTDCSLRITTNKAKLSHRFSNWIIGLQLTAIVLYSCGVLAVNTG
DVQRMNVSAREHILKMKLPFQIDTSPIYMLVTILEFLHLVMCGCGISLVNSLIVTLILHISGQIDILCDWLLN
VFSKDMTHIVDEITMKTLIIKHQQIIMFSENIENLYTYITLILFVSDTLIICCLGFIIVTSIGTPDGPAILVR
SVLFYVVINLEAFIYCFAGEYLTAKSQMIGNAAYDSLWYDICTIKKSRIILFIILRSQRRLTITIGKIMDLSL
ERFTSVVKASASYISVLLA
>MphaOr6
MTTLSRLVRFGLHIYGIRPYVASTVVFRLYWIIMLSMAQVFQYRYVVMNIHMDDFSEYMDGVSSAMASSLLYI
KLIILWTHQRIFSDLLQIMSSDWQDYTLNHYSSHVMTKAANLARRTSRWIVGMQMASGTFYSVGVLASNANSP
EKLEPYERELILKMGLPFNISTDFIYKAVQTAQFYHLSLICYGITIVNSLLVTLILHVCGQIDILRECLTKIF
SKNSANSINEIMMQSLIRKHQRIIVFAENIETLYTYIALMMLLSNTIIICCLGFIIVTSLDTPNAAAILVKSM
LFYISMNVEAFIYCFSGEYLSAKSKMIGNAAYDSLWYDFPAKKSRTVLFLIVRSQKRLTITSGRIVDLSLEQF
TSVIKASLSYISVLLAMY
>MphaOr7
MVGQNPLNRMVKFILTLCGVWPGGSSVLFCRILFTVSLTFAQYCNYRYIMIHMHSIAIEDVVTWLTVVLAHCK
VFFKCLIFWLNQRKFIEVLTMMQEDWSDCAKNDVCMRETVRKAKMSERITNMLIIILHTTSIVSHSMGVFLANV
DVTSNTTEILFFTKIDYPFDINTQSTYRFVLVTDFFFLLTCSCSAAITNALLVILILHTAGQINILRYWLMEL
TSFKEKNKNESIAVIMTKIIQKHQKIILFTENIESLYTYIALVLFASNMILICLVAFLVVKRISALNAELIIK
NLLFFTITNIEAYIYCYLGEYLRDKSRKIDFAVYSSTWYDMKNKDSSTLLFVLLRSQKQLTLTIGKMMDLSLQ
TFTSIMNASGSYLSVLLAMQ
>MphaOr8
MIVLQFTLILCAAGGCWQPLTWTSISKRIMYNSYRIMLIYLISVCMISQIMNIALNINNLNEMSDNIYMTLTV
FIGTYKIITMWTMKEHVRMIINFLTKEPFKPLESAEVTIRQKYDKMIRQYAFLYYSLVQITIICVFINSIFTD

FMKGNLTYKAWVPFDYTPTIIFYFVYTHQMVGVSMTGVVNVACDSLICGLLQEICCQLEILEYRLTKISHDQH
VLHDCIRHHDHIYEYARIVNRKFAKIIALQFAVSMLVVCANLYKLASISLTINGDLITLLMYTVCMLSQIFLY
CWFGNELKLKSVGVTNSIYNMQWQMLDNKSKKDLLLLMKRSMVPIEFNSAVIITLNLDSFVSLLKASYSAYNL
LKRSQ
>MphaOr9
MEHQVYADKEYDDLIKPIMIVSKIASFWPLERDHSISMKLFKTCHVFFFFVLFTIMCTSIMAEILHNFNNLDE
VTACGLMLIKFSLDVIRLIIFSTHQKDMSYAVETMRKDWTCSFQENRAILKEKCLFAFWLSKCFIIMVLVTVS
TITCTPILEAYFLGKKKIFPYRGYYFANQTVSPYYECFYIFNIIGGTFGAMMILGATTFYLIAVTHGSAKFAV
LRKNLEAINTNNPDVDEAMVNCIKEHQDAITFADTLERILNVLALAQFLLSTGLVCFAGFQITSMMKNKERLI
EYSMFLNSAILELYMFSISGNALIDEAIFSDAVGESAYCSRWFGSTFGRNLQIMMIRSKVPSRITAAKFYSMS
LGSFSRVLSTSFSYIMVLLSTSEK
>MphaOr10
LFKHTFTTMTVVGCWQPLSWLSPIKYKLYSIYTLFLTLMLLIFAISQFMIIVLNAANPEDFTSVLYMMTAMCM
GIFKISNMWLNRKNYADIINTLIDEPFRPVIADEIKIRQNFDRMIWNNTLFCLVLVGSTCGIIVLTSLFTNFR
TGELTYKAWLPFNYSSPALFFFVYTHQLISIITGALVNLACDCFICGLLLHICCQIEILDYRLNKSHTWENLR
NCVRHHDLIFNFASVVNKSFTQIIAVQFISSMLVVCSNLYQLAQTTLNAEYFSLILYTFCMLIEIFIYCWFGN
EVKLKSLQVIDRVFEMDWTELNNSFKKAFLMMTNRATIPIEFSSAYIFSMNLESFVGVLRLSYSAYTLLQRL
>MphaOr11
QHVLKLPRTIFMVSGCFRPQSWTSLFKRTIYNIYKLYIITMLYTFTILQIMDIVLYVDNTDDFTSNLNVMINA
LVACYKMFVMSVNYENIIALIKCLTEEPFKPLDSEEIKIRRQFDRIIRNNTLLYTVSVVISVTFVILSSLFTD
FRYKKLKFREWIPYDYSSTTMYCFTYFQQMLGACHSVVVTIATDNLMCGFLMHICCQIEILEYRLKKILNNRL
TLGYCIRHHNQIFEFAQIINLKFTKIIGFQFLASTMVMCCNLYQVTKSSLNANNLWLIMFTNCILTQIFIYCW
FGNKVKLKSLQLIDSIFQMNWPILDNSVKKSLVIIMKRAMIPIEISTIFILTLNLDSFVALLKTSYSIYNLL
>MphaOr12
MIVFKSWYYNANRIFLLICGLWPYQKKFRYVRAIFILGILVFYIISQFRVLFAYKYNFKFALKIFSNIFPAIL
CLLQFIAFLINPKEVRKLLDQISEEWNALKDSKEIEIVRRYGTFTRRLTEALLLCALLSWVFLILLYLIPISV
NDSTIANESLLKHDISMQELTKEEYIFFFIVVFIGVFIMTATTSMILAYMRHICAMLKITCYRIEHSLDGNIL
HMSISQQNRLMCQKLVSAVNIHRRAMEFTSRALDSFKESYFFLIGIGVVSLAINLLHALQAALIVDNLNELAI
STFYVIIHFVYIFIGNYGGQTITDHSADVLKALYHVQWYIAPVQVQKLILFLMLRNTKHYGLVIGGIYIASLE
GFITLTSMSLSYFMVIYSTQ
>MphaOr13
MKRTSTISLPVEIGLRFIGMWPDSAYATLYWLIYMSTMVIVQYYQYAYVLTHFDLNDISLLMDCLGLTLAYTL
AFLKLLALWWNRRIFYYIVKAMDEDWKECTINDSYESTMMSVADLSRRCSNVMISINALAAFFLSIGEHLLQS
VDDASRVDNNPRELPIKMEFPFDVSKSPIFECFLIGQFLYELVLASIVGMMNALLISLILHVSGQIDIMRQDI
NEISHGKYNPSASLVVIKSLICKHQKIISLSEHIESLYTYIALMQLLWNTLVICCTGFVIIITIRTSDSGTTS
IKSVSFYVAITLEVFILCFAGEFLSAKSRSISDAVYESLWYDMPPTNSRILLFVILRSQKRLTITAGKVVDLT
LEGFTSVRHYIINYI
>MphaOr14
MLELGKGAMGRTSTISESVEAGLRFIGMWPRCVYANINWWTYIVSVAIVQYFQYSYVLAHFDINDLSVFVDGL
SITLGYSLSFLKLINLWFNRRKLYAILDKMDKDWNDGLAIHSDVSTMIRHANLSRQCSNVMITTNALAVFFYV
IGGPIFRSMIQKNDLESSTRELPIKMEFPFKIDNSPIFELVLIAQFFHDLSVACIIGMLNALLVTLVLHVSGQ
IDIMRQGFVEISSKERTRSSLVIIKDLINRHQKIIDLSDDIEDLFSNIALLQFIWNTMVICCIGFVIVISLGT
EEGTTMITKSLIFYVAITLEAFVFCYAGEYLSAKSKSIGDAAYECLWYDLTPSECRVLLFLILRSQKRLTITA
GKITDLSLEGFTTIMKSSASYISVLSALY
>MphaOr15
TNTITLLVQIGLRIIGFWPNTPCVLLLRGFWILTIAMVLTGQYWWLIVHFKTDDMFHLIEGFSVTIEYTKLSI
IFIILWLNSRIFYDVLAMMAADWRKATNREMDTMMSKANLSRRFSKVLIGVQSIGVCCFGIEVLLAHTDDYDA
NEMEYPMETPVRAFTLKLQLPLRCNESPLYELVAFLEFFHQLLSSSVMGVINSLLVTLILHTSGQIEILCDAL
RDIFLEKNNQQISFSVKELISKHQKIIIFSDKIERIFSYIALVEFLASILLTCCVGFTVITSFSTIQDSDTTD
SAALLKAIVFYIAALIEAFIFCFCGEYLSSKSKMISDAAYKSIWYDFKPNDSKLILLIILRSQKRLTITAGRI
MDLSLEGFTNIMKASVSYGSVLHAMY
>MphaOr16
MLFYNNHYYYFNKTSLCIIGQWPFQSRKKNNVMFALTMISTCTLAGFEFLGFITGMSDLDIIMDNCSPMSGVI
FIIIKLLSFFSNKYKVPQIKQLLIQIEETWKLLSEGPENEILRHHGEQSKTFTTRYAIIIYLTGFFYCTPPVI
ISGINMFLPTNETQPPKFLYRLEHVLDVDKYYNLLMLHAFISVFSIIAIATAIDGMFILCIEHICALFTCLKY
NIERIQSSELVLLNLNIADDETYHTIIRCIKLHTRILKFFDLFASTYASVFMTVVGIIMIPFCLSLAAAEFVM
VDTQFDESVRIVSTNFAQTVHIYVLCFMSQRLIDYSSKFQDVIYNCNWYKISLRSRQLLRFTLMCAIKPCRIR
AGNVFVISMETFSGLLKITMSYFMMLTSLK

>MphaOr17
MSFYNNHYYHFNKISLCIIGQWPFQSRLKNNVMFALTLIFTCSLIGLEFWGFVTGATDLEIIMDNLSPMFLDL
VTVVKLIMFSFYKYELQVKQLLIQIEEVWKLLGEGPENEILQRHGEQSKIYTSRYAIGLYLSGIFYLTPPVIT
SGINMFLSTNETQPPRFLYRLEHVLDVDKYYNLLMVHTFISIFGIVAIATAVDGMFIFCIEHICALFNCAKYN
MARIQSSELILLNPSIADDEAYHNIIRCIQLHTRILKFFDLLSSVYSTIFMTVVGSVIICLSLGAAEFVMVDT
NFIESVRILAANSAESIHIYVMSYICQRLINYSSEFQDVIYNCNWYKISLRSRQLLRFTLMRAFKPCQIRAGN
VFVVSMETFGSLLKMTVSYFTMLTSL
>MphaOr18
FMWAVEIHRLSLEIIGLWPKNDTIATKNLWSKLHTGIILILLLILVSNVPMILAIMQAWNDMVLVIDILHITIP
WITVLLKYIIMQWKQEVFLSIVNMMAEDWMAFKSNVEKSVMIKQAQTARLIVTIGYLFCAIAVISVIIFPYFG
IQVIHITNLTEQEKPLPLETYHFYNTNKSPQFELTYFIHAVTVFIATIIYISVDIILLLIIILHICGQLENFRC
RLVSLVSCKNFNKALNDIVTSHLRLIRFTDKIENMYCAMMLIMVLYFGIVFCLTGFLFTILLIDREINKATIA
QVYYSIIFFIMILANIFLYCFAGELIKEQCNGVYRAICDLEWYKLKSRKARNITLLMLRVKHPLQITAGKIIP
LTFGTFCSILKTSSGYISFLLTK
>MphaOr19
FVWAVEIHRLGLEITGLWPKNDTAAMKKFWSKFHPCIILTLIIFISNVPMIYAVMQAWGDMILVIDILRIAIP
LLVVPLKYIIVRWKRTVLLSIVNMMAEDWMTFKLDSERAVMIKRARTGRLIMIVGYVIAITAIVTVVTPTYFG
IQVMRVTNFTDGNKALLLDTYPFYDTDKSPQFELTFFFQTITLLLAITIYMTVDIFLLLIIILHICGQLENFRY
RLIRLVSCKNFNNTLNNIIASHLRLIRFIDNIENAYSLMMLILLFYFALVFCLSGFLFTIILNERKNEAIVTQ
VYFSIIHFTILLMNTLVYCSAGELILKQCDAVHRATCDLEWYKLESRKARSLILLMIRTHHPLRITAGKIIPL
TMATFCSI
>MphaOr20
MSIIRDMSVTLPNSSYQRDIRYVFKLNNWILGSLGIWPVAIRGIRQHMFKIAITIWNLALSFAIVPCALHIVY
DEKDIILRMKLCGLLAFCLTAMTKYCILVIRRPKIHRCIEHVKNDWWQVTFRSDRELMLRYATTGRNLTIIGV
SFMYAAGIIYHFILPFCSEHKINNQTIRPLVYPTYSKFSQSQISPVYEIVYVAHCMCGYTIYSVTAGACGLAA
LFATHACGQIQILVSRLEDVLLGERFEQIPNVHRRIAAIVQNHVQIIRFAAVVEEVLQEVCLVEFTSSVCTIC
LLEYYCIVDWKADDRIGLATYLLLLVSFCFNVYILCYIGEVLMEKSSQIGSICYMINWYQLSPKSTRSLILIL
AMSSHPIKISAGRMVDLSLTTFGNVLKTSVAYLSFLRTLVM
>MphaOr21
YQQDIRYVFKLNKWILGSIGIWPVSEQGFGRHASKIAIALGNLTLSFAMVPFALHIVYDEKDIIMRLKLSGLL
AFCIVSMTKYCILAIRRPKIQQCIEYVKNDWWKVTFKSNRELMLKYATIGRNLTIISASFMYTAGIIYFMILI
PFFSETKINNQTVRPLVYPIYNAFYQSQISPIYEIVYVAHCLCGYTTYSITVGACGLAAVFVTHACGQIHMIL
LQLEDLLNGENSDIHQRIAIIVKDHVRVMRFSAVIEEILQEVCLVEFSSSVCTICLLEYYCIVDWQANNKLSL
ANYFLLFISFCFNIYILCYIGELLIEKSSQIGSVCFMINWYQLSPKSVRSLIMIIAISSNPIKLSAGRMVDLS
LTTFGNVLKTSVAYLSFLRTLVM
>MphaOr22
MSNSNYATENGPKKYNDYSLQLNRWFLKPIGAWPQINASNSIWKFFILIQIFTCSSLVAVITVPCLLHVLFEK
VNINAKLNALGPLIHRIMGSINYWVLLKYSGDIRELIQHMETDWRLIHRTDNRDVMLQHAKFGRFVTMICGVI
MLGGSLLFSLARAMKTATIVVGNETFTTHPMTCPIYSKIIDTRFNPVNEIALVLQFLSTIVVSSSTAGACSLA
AAFAIHACGQLNVLYTWLHELVENQEKGKHVPEQKLTAIVEHHLRILSFISRIESIMNTVSLVELMGCTINMC
LLGYYTIVAWETLDKAKLTSYAIMYLSMSFNIFIFCYIGEIITEQCKYVGEMAYMTDWYNLHHKTALGLVLVI
LRSNNVIKITAGKLINLSIATFGDVIKTSMVYLNLLRTVT
>MphaOr23
MSNSNCTLKSDQKKYHDYSLQLNRWLLKPIGAWPQINSSLILKIFTTIQIFTCSSLVAAVTIPCLLHVLLEKE
SFSAKLDAIGPLIHRTVGSINYYILVNRSADIYKLIRHMEVDWSLIRRIDNRKIMVQYAKFGRSLTMIYGVFM
QSSTLFFGVAKAMKTATIIIGNETFTTRPLTCPIYNKIIDARFHPANEVALVIQFLAVFIFSSCTVGACSLAA
VFAIHACGQLKVLYAWLNELVENQENGKHVAEKKLAAIVEHHLRVLSFISRVESIMNTVSFVELMECTICLCL
LGYYTLVAWETFDTAKITSYVILYLSMGFSIFIFCYIGEIITEQCKYVGEMAYMTDWYNLHHKTALGLVLIIL
RSNNVMKITAGKLIHLSIATFGDVIKTSIVYLNLLRVM
>MphaOr24
MEDDLADSTNVSTYLIDKYNEYSIQIIRWILKAISAWPRSANVSIVDKIWSDFAIFMCYFLIIAIMIPNGLSM
LMDSQMSYETKLQNFGPLTFWFIAMVNYSCLLMHVDDIHDCVEHVKTDWRIIRSFEDRRVMLKTARLGRFIAG
FCAVFMHCGVFSYNIVQGLSKSVLQVDNSSIMVRNLPYPFYNKILNAHFSPAYECVFFVQFLSSFVVNCVTVA
TCSVAAVFVMHACGQMKIMISWLENLIDNKNEERTSVKQRFAIIVNHHLRILSFVSRTEKIMNIICLVELIGC
TMHICCLGYYCLMDYIQDNKQSILSHSMVLISITFNIFIFCYIGEILSEQGEQIGKCAYMTKWYLLPGKSAQG
LILIILRSNAVLKITAGKIVQLSFSTFGDVIKSALAYLNILRTVLV
>MphaOr26

MYDRSRLPDKIYNIHYKQDIHYAMQLCRWILKPIGIWHLTYDRSSRRDKLLSFVLIFVCFSGLCFVLVPAGPY
ILFREKDVNIKVKLFGPVGFCLTAAIKYCFLGARVSAIGRCVKHLESDWRAVQHQDHRRMMLRNSLVGRRLTT
LCVIFLYTGGMSYHAIMPLSSRTRTNGSFTSRPLVYPGYDLYVDSQASPAYETIFCMHCLSAIIQYSCTTAAC
SLAASFATHACGQVQILMTLLDDLVDGERTKGTVEKRLSLIVRHHVRVLRFTADVEEVLREICLMELVAATLI
ICLLEYYCMMEWANSDAVAILTYFILLISLTFNILIFCYIGELLVEEYSKIGSAAYEINWYDLPGHRAVDLVM
IITMSHYPPKLTAGKFVELSLNTFSAVLKTSVVYLNLLRTVT
>MphaOr27
MRDRSQLPDRIHNAYYDQDIRYVLQLCQWILKPIGLWHLIYDRSQSSRSEKFLSFVLILVCFFCLCFVIVPAG
PYILFREKDTYTKIKLFGPFGFCLTSAIKYCFIGSRVSAIGRCVEYVECDWQGVRHRDHRRIMLSNAMVGRRL
SILFMIFLSTGGLSYYTVMPLIAKTNSNLTIRPLVYPGYDIYYDSQASPAYEITFSMHFLACLVQAFATTAAC
SLAAIFAAHACGQVQILMTLLSDLVDGKRTKSTVEERLSFITKHHVRVLRFTNDIETVLCEICLMELVATTLL
ICLVQYYCLVEWANSDTMAIFTYFIILISLTFNILIFCYIGELLMEKFGKIGSAAYKVNWYDLPGHKAVDLVM
MITMSHYPPKLTAGKFCDLSLNTFKTVMQISIVYLNLLRTV
>MphaOr28
MESSRDHYYDISKKYLILVGQWPYQKPKESLFFFILILIFDANVLIPQVANFFTCNNMQCIFETLPPHLLAAI
VPVKIFAYRFNSRKIKQLTDSLFVDWDMLETEKERDIMRKYAENGRWYALIYSSYVYISTVSFTTTSLVPRIL
DVIFPLNISRPVMLPYPAYYFVDENRYFYYIFLHMIIGAVACVTGLIAHDCMFFTYIEHICGLFAIVRYRFEN
VSCEHSNMKKSTLDCPNYHKKIVISIHAHRKALQYVKLLEDTFSISFAVQLLLVTGCLSITLISTHLHDLAEA
MRYFVFFMAQLFHLFCFSFQGQKLINHSLETCDNIYHSLWYKIPVKEQRLLLFVMRKGIEASALTAGKIYVFS
LENFTMVVQSSMSYFTLLSSFDV
>MphaOr29
MESEQNYYYRFTKGMLTIIGQWPYQEERTRLFLLSLLTVTAISVIIPQVNKIIQCDGDMRCIMLVTPALMLIF
IILVKLYTCQINNNKIKRLTEHLFNDWKNLEDAEEYEIMKKHGANARLISLTYTTCVSAVSVLYVSISLIPKV
LDVVLPLNKSRPIIMPYESYYFVDSEKYFYHIFLHIGVSLAILVPAVLAHDCLLLTYIEHACSIFAVTGFRLE
AVSRNSHNNSMNNGSHSVYNPKIAVSVYAHWRALRFAELLEDIFCISFVVQLMLVIITLTVTLLYVALQMDEV
VQALRMSGFVGTQTTHLFCYCLQGQRLIDHSVRIREKIYNSSWYNIPAKSQRLLLYVMKRSMQPNFLSAGKIF
VFSLKSFTTVLQSSVSYFTVLSSFQ
>MphaOr30
MQILSLNFSMYTVGGIWRPVEWSSNGAKLLYSIFTFIVVLSQYFLVLTQFMDIIFVVNNIDDFATNTLMFFTM
IAVCCKATVVIVRRNELFNLVQGLLEAPYKPQDEDEIAIQMKFDKFIRSCSIKYALLATCSVTGLSLGAVLNA
LHGHLPYRIWLPWNYNNNVFWILSIYQIFIIVFAAIINVGTETLVFGLFIETCAQLEIFENRVHKFITNKIVG
RALTSLSENKIELPECVRYHLSIYKFAKMVNVLFNQVLFVQFFASIIVLCTSVYYLSNHSSDISGLVFILAYT
ICMFAQIFVYCWSGNEVILKSLSTGDTIYRMDWPLLPNKEKKGLLMIMMRSTIPIKFTSSFLIILSLQSYNNI
LKASYSTFNVLK
>MphaOr31
MQILSLNFLMYTFCGMWRPIKWSSNGAKLLYSIFTVFVMFLEYFWVLTQFLDIVLVIDNIDDFVANSLMLVSV
IAVSCKATIVVIRRNAIISLVQILINEPCKPQDKDEVTIQTKFDQFIRSCSIKYSFLATSSLTGVVMGGILNV
MHGHLPYRIWLPWDYNNVSLTFWIISIQQIVTIIFATIINVGTETLVFGLFLQTCAQFEIFESRLCKVIDVKN
MKCLHTFPSSSKDKTKISESIHHHLTIYKYAKTVNVIFNQVLFIQFFGSILVLCTSVYYLSTHISELSGLATL
LAYTICMFVQIYLYCWSGNEVILKSLNIGDAIYHMKWPLLPVSEKKELLMIMIRSTIPIKFTSSFLITLSLQS
YSNILKFSYSAFNVLQK
>MphaOr33
MRATTISTSVEIGLRFVGIWPGLPYGTITWFAYMISLVVALYFQYAYIFGHFDVYNIANLMDALSITLSYSLG
FMKLISLWSNRRIFYDILLAMEEDWNSVDICDKRVSCIMASNADLSRRCSNVLISINASAAISYSLSTLLRRS
ADFKDDRNVSSRMLPIKMEFPFEVDASPLFELLAVGQVLHVVSIAALVAMMNCLIITLVLHTSGQIDILRREL
LKICGNGTSQCDSIVASVRLLIVRHQRIITLSDNIEGLYSAIALMQFLSNTIVICCIGFTIIGSIGSGTVVML
KSVIFYVAVTLEAFIFCFAGEYLSAKSKSIGDAVYEALWYNVTPAECRILLFVILRSQKRLTITAGNIMDLSL
EGFTSVMKASASYMSVLHAMY
>MphaOr34
IVKSTVSPALKIGLRLLGVWPGVAHSTVYWLIFMSSILVIQYFQYLYILEHFKISELSNLVDGLSLTLDYSLT
FLKLASLWIHRRIFHKILAAMNDDWRECVNIDQHLSTMTVKASVSHFYSNAMLSFNGVAAVLYVLGDYVIRFI
YSSKNYNNTLRQLPIKMLLPFETEKSPVFELLVITLFLHIILISFIVAVLNGLIFTLVLHVSGQIDIICQEFR
NISKNAFPYRSSVFTLGTLIERHNKVYAFSENIEKLFSFIALMQVVWKTLVICCLGFIIIISIHNESDVFVFM
KAILAYFAVMIETFIVCFAGEYLSLKSKSIADAAYESLWYDMPPNQGKIISFMIMRSQRRLSITAGKMMDMSF
EALTNIMKASASYVSVLHAMY
>MphaOr35
MTITSTISPVLKIGLRFLGIWPGVPYSIIYWLSLTLSTIIMQYFQYWYIIKHLKISELSNLIDGLTVTLDYSL
TIFKLIGLWLHRRVFHQLLAAMDNDWRECIKFDQHLYIMTINANISHFFSNAILSVVTIFAIFYLLGEYVIRF

VFLIEDQNDTLQFPIKIYFPFETQQWLVFEFLFVTIFFHVILQVCVLCLINGLIFTLVLHVSGQIDIMCHEFK
NISKSIFLHKSSATLFGMLIERHNRIISFSENIEKFFSFIALMQVVWNTLVICCLGFIIIISFHNGTDVFITI
KTIFAYVAVTFEVFIICFIGDHLRLKGELIANATYDSLWYNMSPRQNKIILFIIIRSQKRLIITAGKMMDMSL
ETFTNIMKASVSYASVLNAMY
>MphaOr36
MISSSISPALKVGLHLLGMWPGKSYSAINFMSFMLCMLIMQYFQYVYLFDHLKISEISNLVDCLTATLDYGLT
IFKLTSLWIHRRAFHTMLAAMDNDWRECINIDKHLHVMTIKAKMSHSISNTLLSFIAFVAVPYLLSDYVIHYV
FLIENHNDTLRQFPIKIQFPSQTQQSPIFEVLAVTIFFHAMLHSFTIALLNGLILTLVFHLSGQIDIMCYEFR
NISKNTFLHESSVSFGKLIKRHNKVILFSKNIETLFSFMVLMQIIWNTLVICFMGFFIIISIHAENGIFVLVK
AVGGYLAIIIEVFVICFAGEYLSSKSKSVADAIYETLWYDMPSHQSKIIVFIIMRSQKRLTITAEKVMDMSFE
TFTSIMKASVSYVSVLYAMY
>MphaOr37
IILSSISPSLKVGLQFLGMWPGNSYSVINWLSFMLSLLIIQYFQYMYIFDHLKINEVSNLLDGLIAALDYSLT
IFKLTSLWIHRRTFHKILAAMDNDWRECINIDKHLHEMITKANMSHFICNILLSFTAIAGILYLLSGYVIHFV
FSTENYNDTLRQFPLKVQFPFETQQSPIFEVLVATIFVHAMLHSCTVAVLNGLILTLVLHLSGQIDIMCYKFR
NISKNTLHKSSVFLFGMLIERHNKIVLFSKNIETLFSFIALMQVIWNTLVICCLGFVIIISIHNETGIIVLIK
TVGAYLAIMIEAFVICFAGEYLSLKSKSIAGAIYETLWYDMPSHQSKIIVLVIMRSQKRLTITAGKMIDMSFE
TFTSIMKASVSYVSVLYAMY
>MphaOr38
MTIRSTISPVLKIGLQFLGIWPDVSYSTIYWLSLTLSILIMQYFQYWYILKHLKFSELSNLIDGLTVTLDYSL
TIFKLIGLWLHRRVFHQLLAAMDNDWRECINFDQHLYIMTINANISHFFSNAVLSVVTIIGILYLLGEYVIRN
FVLLIEDHNDTLRQFPIKIYFPFETQQWPVFEFLFVTIFLHVILHVCVVFLINGLIFTLVLHISGQIDIMCHE
FKNISESILLYKSSATLFGLLIKRHNRIISFSENIEKFFSFVALMQVVWNTLVICCLGFVIIISFHNGTGIFV
TVKTIFACFAIIFEVFIVCFVGDYLRLKGELIASATYDSLWYNMSPRQSKIMLFIIIRSQKRLTFTAGKMMDM
SLETFTNIMKASISYASVLNAMY
>MphaOr39
MIIPSSISPALKVGLQFLGIWPGNSYSAITLFSFMLTMLIIQYFQYVYIFNHLKISELSNLIDCLIVALDYSL
TTFKLTSLWINRRSFHKILVAMDNDWRECININEHLYVMTNKANTSHFFSNTLLSLNAIVGVLYLLGDYVTHF
VFLNENYNGTLRQFPIRIQFPIETQQSPVFEILVVALFIHVMIHSGTIALLNGLILTLVLHLSGQIDIMCYEF
RNISKHTHKSSLSLFGMLIEKHKKIVLFSNNIETFFSFIALMQVVWNTLVICCLGFIIIISIHNQTGIFILVK
TIGGYLVVMTEAFVICSAGEYLSLKSKSIADAIYETLWYNMPSHQSKVIFIIMRSQKRLTITAGKMMDMSFET
FTSIMKASASYVSVLYAMY
>MphaOr40
MTITSTISPALKFGLQFFGFWPGVSYSTIYWSSFMSSMLIVQYFQYLYVFDHLKMSELSNLVDSLVVTLDYSL
SFLKLASLWLHRRVLHKILADMDNDWRECINIEQHLYLMTIKANISHFFSNALLSFNVIVATLYLLGDYVIRF
VFVTEKYNDTVRQLPIKIQFPFDIQQSPIFEFTAVTVFIHTMLQLWTIAIVNGLIFTLVLHVSGQIDIICEEF
KNISKNILFYGSSVPFGMLIERHNKVISFSDNIEQLFSFIALMQVVWNTLVICCLGFVFIISIYNGAGVFMLM
KTVLAYLAITTEAFLICFVGEYLSHKGKLITSATYETLWYNMSSNQCKIIMLIMMRSQKRLAITAGNMLYMSF
ETFTSIMKASASYVSVLIAMY
>MphaOr41
MIISSTVSPVLKIGLQFLGIWPGVSYSYIIYLLSFMLSMIIIQYFQYLYIINHVNVSELLNLVDSLITTLVYS
LTFIKSISLWIHRRIFYQILIAMNNDWQDCINSDEHLCVMQVKANISHFCCNAVLSFNTITTVLYFLGDYVIR
LIFLTKDYNDTLRRFPINIEFPHETQQSPLFELLFMILSLHVVLNSSIIAIVNGLIFTLILHVSGQIDIICQE
FKNICENTLLYKSSIPSFRMLIEKHNKIISFSENIEKLFSFIALMQIIWNVLIICCLGFIILIFIHKETGIFA
IVKTGLAYLGIMGEAFLMCFAGEYLSYKTKSISNATYNTLWYNMLPNQSKIIIFIIMRCQKRVGITAGKMMDM
SFETFGNIVKATASYISVLNAMY
>MphaOr42
NLYYGHIEKFLSLIGQWPYQTKKTRLFRLFILTIVAISLLIPQIAGAVQCDGDTACLSFIILLFPLMPLIIMS
VYTCQYANDKIKQLLKYLNSDWEIPKGDKELSIMKTYAANGKRIFYVYSGMIYLAVMFCFLIVPLIPKALDIV
LPLNESRPASLPYEANYFVDSDKYYYFIILHIFVCLEILATLVIAKDNIVLGCIQHVCSIFTVAGHRFDVVSR
YSDNNSTNSLHAINMYNKKIALSVYAHQQALRFVDLLEDTFCYPSLIHMFIIVVALTFTLMMLAVKFDDQIID
VPRKTIVLLCLLSQIFYYCMQGQRLIDHSEEAREKIYNSSWYNIPPKSQKMLLFVMLTNMEPNTITAGKIFTF
SLETFARVVQSSVSYYTVLASM
>MphaOr43
HYYDIVYKISSLTGTWPYMKPRARIFRVALLTITMLTIFVPQIAYQFMCKTNIQCMCEAMTSYLLTFVALLKV
YTFQLNIRTIKGLTQHLFVDWEGLEKTTKEYEIMKLYAENSRRFCLIYVVYCITAVFTFMSMSLLPFAFDVVW
PLNESRPVLAPYPGYYFVDIQEYFFKIFWHSLIAWEIIMAGIVAHDCMFVTYVEHVCSMFAMVGHFKNLFCN
HDKAIKVTDDSNNTYCKKIGFFVHAHQEALRFAEVLENTFTVPFAIQMLIVTLGISITLLQITQQDGDILESI

RYGLYIFGQLIHLFFLSFEGQKLIDHSLQMSDKIYGSSWYEVSRRSQKLIMLVMMKSVRPACLSAGKIYIFSL
ESFTTVLQTSMSYFTVLASVQ
>MphaOr44
MTRSEAYRSVNITRLFMKVVGLWYVETPRERLLLRAAFGYAVWAIVFAILVEGIDLYHCIGDFYAVTSNLCAT
LLLIMILVKLGSFMFYRDFIMDMIHFAERNFWSVTYDETGAQILGGYDKLGMTMIYTFTFIVLIATFNYIFAP
FFEPQEKNETEKILPFKLWVDFPYHSPYYEITYVIQSLSTMHSGICTFCFDNFVSTFNIHAAAQLKILAHKVE
VVAENCIEDTIDQKLPLETDATIRTFKKLRDCLQQHLTLICYVRNMQRVFATILLGQLLLSSVVICFGGFQFL
ATDVTIRKCIFAFHFVGGLIQLLIYTWTCNDIIVQSTAICDAAYNSKWYLLPDTGLGRAVKKGLIMMMIRARR
PCILTAGHFAVMSLDTFMGILSTAMSYFTLLRQMSENNI
>MphaOr45
KKRDFLSIRMVRVLMKVVGFWPAKSKIEKRLLNGLVGYTILVCVIGVWIEGTELYLGKGDFYAITYTACSVMP
LIIILLKTFLFYHHRKELLNMLKYTEDNFWYIEYDAYGSKLLEKINERALLLMCMFTFFAQGTVCTYMLTPII
ENIGKNESDRILPFNVWAGVPTTVSPYFEIIFTIEIIALIHCGIIFCCFDNLLCLLNLHSAGQFKILRHQMGA
ILDKIERVGTVNLFDEKRRQMVYEKLRKCVRFHQELIWYNEKMEQIFTYTTLCQLLVSGIMLCVAGFQMFLAR
GTLIRRMIFIAHTNGCFNQLFVITLTSNDLMLGSRAVGDAAYNTNWQILSHEENRAVRKTILLIIMRSARACS
ISAGGFFPVSLETFMTVLSSSASYFTLLRKFVE
>MphaOr46
ITRKSTFSRIVFLMLPLFGVSSDRLVILITRLFWIAITAFIELCHYLYFTTHLSSENFFNLVDCVCSFLAHAK
VFIKLVAFWVNQRKFEETLALITDDWSDYAKNDIGMRVMIGKAKISDRITHIIIILHTINIFAYCLGVIIADA
DVTETIELPLVNKLELPFSINTQHMYRFVLIVEFIHMILCNWVAGLYNAVLLTLVFHVGGQIDILQCWLAKFI
PKDIENKQESIVMTNKFILKHQKIIQFSENIESLYTYIALLLFASNTMLICLLAFIIVTAIGTADAMEQIIKC
ILFFTTTNVEAFIFCYAGEYLNNKSKEIGFATYNCAWYNLKPKDSRILSFIILRSQKQLTLTAGKMMDLTLQT
FASIMNASGSYLSVLLAMQ
>MphaOr47
MISVVDKHFSLNRILLLLIGLWPYQKSKLARFYLFCCYSIIATIIIFQFTTFLTSKCTTNHIIKICSISFNFF
NYAIQYNSFYVNNDTIKLLMEQLQLIYNDLKDNNEIAIFERYGNISKRYTIAFITCLLCSLFIVINAQLWPDF
INIILFINESRPRRLLIETEYFIDQEKYYYLILFHFNASTCIGLLTIVATGSMLFAYLQFACGVFKIASYRIK
NAIEIYTQSINLRNKIVVYENLIYGVHIHRKAIMFSKYLISNFEMSFMFLIAFGVLTLSLNTFRILSSTFINI
EELSVTCVVTYCCIIYMFLANFIGQEITDHYNLMFITTYEVQWYIAPLYIQRFILFLLLR
>MphaOr48
FEWAVKLNRITLNMLGLWPKTARSSREKLICNFRVFVAFFGIMFCILIPAIHSLIRIYGDLMLMIDNLNFTLP
ATSSALRIAIFWWKKETIIPIINMIEKDWTKSKSMQERNIMIKKAQTLRIIVSCAYFIIGIACFFIVIPPVFG
VSMRFASNITDIGRPMLLQSYYIYDVSKSPLYELTFISQAAFISLGIMTYTGIDNFLGLLIFHISGQLDILKD
RLIQFDKCSNSDMLKICVIEHVRLLRAIAIIEDTYNVTLLALFTYFAILFAFYGFWIIYIFNNGNNVSLIFLI
CFICIIILNLLGHMCLYCALGEFLMIQCNEMYYAAYNNKWYSVNPKVTQDLLLLITRGVKPTYLTAGKIFPLTM
STFCSLVKTSAGYISILLTTK
>MphaOr49
FEWAVKLNRFTLKVIGLWPKSKYNIQEKLTYNSRPLITLIILICNLIPLIHSLIRVHSDITLVIDNLQFTLPT
ITCAVRIIIFWWKKKDILSILNMIMEDWLRLKNKYERTTMIRRAQNARIIITFAYGVMTIGFVFIVVLPICGI
SIRHLTNVTDPGKLLPLQTYYPYDVSKRPQYELIFFTQCIAIFLSIVSYTGVDNFLGLLVFHICGQLEILRYR
IMHMNNCINFHDVLKSSVINHIRLLRAIAIIDDTYNIILLVLFLYFAIFSACYGFLIINLIEKKNDVSITDLT
YKICVFINVFGHMCVYCAVGEILVAQCDRIYSAINNYEWYNLDPQSARCLIFLLIRSNKSSYLTAGKVFPMTM
ATFCNLIKTSASYMSVLLTTKT
>MphaOr50
MIYLEKQHFKLNRLLLLVIGLWPQQQSKFAQVQFIVLFSILVTFIVFQFTTFITSKCTTVFIIKVLSSTFFFI
CLAIKYNSFRINADTMRFSLEQLQYICNKLENKNEIAIIEKYGRIGKYQTIVIMIIGACSILIFLLLPMWSYI
INVILSVNNSQSRPAFQIATEYFVNQENCFYLILLHANLALCIGLTGMVATGTMLIVYLKHICGMFSIASFRI
EKAMAIMQQNVNREKMTIIYKGIICAVDIHRKATEYSKVLIKSFEGSFFFLIAAGMVCLSSTLFQIALYNDSI
DQLVIPFIYLSILYTYLFLSNYTAQEVTDHNEYVFTTVYNVHWYVAPLHIQKMMLFLLQKGTKAFHLILGGIF
IASMESAATV
>MphaOr51
MIRTDSWHISLNRFLLLIVGLWPYQQSKYVQLQLILLIGILVSFILFQFTVFVTLKYTPVLLINVLSSALYYI
LYVIKYIFFSINTNNAKCLLDEIQHIYNELTDENEINILKEYGNYVKRVTIVFSSLLMFIILFLIVNLFWPYI
FNILFPTNRTQSCSSLPLMTEYFVDQEKYSCLISFHANAATGIGTTAIIAIGTIFLVYLKHACGMFKIASYRI
KRAMAFETLQKNSLEYENLIYNKLIYAVNMHRKAMTFCNSLMSRFQLMFSCMIVVGVLSASLNIFQIFQMISH
EYDIVELMLHSLFVNIHFLYLILGNYFAQEIIDHNKDVFATAYNIQWYVAPLQVQKMVLFLLQRGNKAYNLSI
AGLFVGSLEGASTLFSAIISYFTVLYSTQ
>MphaOr52

LAEMILESFRLNRFLLLVIGLWPYQKSKFAKVQFSVFFSILITFIGFQFTTFVTSDCTTDLIIKVLSSAFFFI
CLAIKYNSFWINANAVRLSLDQIQYTYKKLKNKNEIAILEKYGNIAKLYTATLIILCSCSISVFIILSIWPHI
VSIILFMNNTRLCPRIQFTTEYFVNKENCCYFIFLHTNVALCIGATAMVATGTTLLVYFKHICGMFSIASFRI
EKAMMTNMLQDINQEKEILIYKGIIYGIDIHRKATEFSQLFMKNFEGSFFFLIVAGMISVSNTLVQVVSINNI
EELLLPLLIMLFLYIYLFISNYTAQEVMDHNNRVFATVYDVQWYIVPLRIQKMLLFLLQRGTKAFNLNLGGLF
IGSLESAATV
>MphaOr53
MIHIDTLQISLNRFLLLVVGLWPYQQSILVRLQLILLLGILVSFIAFQFTVFMTLKYSLDLLINVLSYALFYT
YFAIQYISFSINNKSIKDLLEQVQRTYNKLIDKNEISIMEKYSTYAKRYTIAFSLIVIFVGITLIFYIFWPQI
FDILFPINGTWSHSLLPLMTEYFIDQEKYFYLILFHATIAFIIGALAALAIGTILIVCYEHACGMFRIASYRI
ERAMEIQILKRKNSLEKKNLIYKKLICAVVMHRNAIEFSEMILSKVKVMMYCMLLDGMITTSINLFRTFQMIS
YGFKTMELFLHVIFCIIDFTYIIIINYFGQKIIDNNNKVFVTVYDVQWYVAPLQVQKMILFLLQRSNKTFTMN
IDGLMTGSLENAASLLSAAVSYFAILHSTQ
>MphaOr54
MICINSLNIGLNRFLLLAVGLWPYQQSKLVRLQLISFFCILTTFILCQLTTLLTSQCTPDFIMKILSSVLFFM
MFVIKYRYFNVNIKFVQNFLEQLQHICNELTDKNEINIIKQYASSAKRYTTTLTLLAVLIISAFILYQTGLYI
FNILLPIHNKSRPCSLLFVTEYFVDQERYFYLITLHTYAAFGIGSAVMLATGTILIVCMQYACGMFQIARIAY
SYRIARTTNTLEKSNLKNENLTYKGLIYAVNMHCKAMKFCDLILSRFQVMFFLLILIGVICGSLNFFLIFQVI
SFKYDTSELLLPLIGVIVHFSYSSIGNYIAQEVIDHNNNIFVTVYNIQWYLAPLQVQRMLLFLLQRGTKEFTM
NIAGLFVGSLESAATLMCTAVSYFTFLLSTRN
>MphaOr55
MICIKTRHFHVNRILLLVLGLWPYYQSNFVRFQIILCLGILTSFIIFQLTTFLTAECTPYLIIKVFSTVMFCA
TYIINYNSFWINTHNVKRLMERLQQICNELKDENEINIMKEYGDSVKRYTAVITMFAMLCLFIAISISMSAII
CDNVLHMNGSRTRQMLFFTSEYFIDQEKYGYIILLHSNAVLYIGITTVTAIGTMLRGYFIHACGLFKIASYRI
EQALAIKIVKNISIENEMMIYKEIICAIDIHCKAIKYSKFLLSSFKGSFFLLMAIHVICLSFNLFAIFRIAPL
GNKEEFLYHFVLVSVMLSYMFLANYTGQEVMDHNNHIYITAYNVRWYVASVHTQKLILFILRRGCKTFTLAIG
NIFVASLECFATLLKASMSYFTFMYSTQQ
>MphaOr56
MICIDCLHINLNRLLLLAVGLWPYQRSKLVQFQLVLFFGILATFVLFQFTVLVTLKCTPDLVINVFSSALFYI
IFVIKYISFSINTEVIKCLLEQLQHIYDGLTDKNEISIIRKYGSFAKRCTAMLLLLMVLNISILILYLYWPYI
FHTLFSTNKTRLYSSLPFMIEYFVDQKKYLYLILLHASAAFIIGGITLLATGTMLIVYLQYVCGMFQIASYRM
MRAMMKLEIQQKKNLQNENLIYKEIICAIDMHRKAMKFSDSSIYRFEVMFFFLIVVGVICGSLNFFRVYQVIS
YEYDIMKLSLPIVFIIIQFMYMIMGCYFAQEVMDHNNDVFVTVYGIQWYIAPLQIQKVILFLLQRNTKAFHIK
IAGMFVASLEGAATLLNIAMSYFTVLYSTR
>MphaOr57
MFFHRDITVTLSVHRFALSCVGIWPVRERNIFMDLRWIIAVFLEAIPLCVYFTEIYLHCNGAKKSFDWVTPGA
AAALALTRLIIPRMHREELLEIITSMMDDRTMPKDKKVRWVIKKYATMSTRVTVLTFILVGIIMGVYIAMAIS
AITAKHPDDDEISGVNVSHQEGSQSCVFRSASSHQAFMIVQAMQMFTTCVVTFGTTSFFFGLAMHLCAQFDAL
GIELSEFQVSNARRMITAAVQRHCQLIRLAECMEESFNASILVYLFVTSTLMCIDGYMLIASLPLGDLPMIIH
SVCVLLLMLIQLSFYTFAGDYLEMKSAALSHATYDCDWYELPASIAKNFHIIIMRASIPHQLTAGKFIVMNMI
TFKDILKSTASYLSVLRVMLD
>MphaOr58
MKKIRNWNDESHYALNVYEKVLSIIGVWPLDAGEFKSIARCALAIVIQISTIVSLSLEAYWQCLGTEDMMEAF
LMDLSSIVSLSKLLVIRLSWRNTYVLVTSVINDWSMSWDTRQREIMMRYTHIGRVVSLTMLYLGYASGVSFLF
MAIPFDSLLPWLNVSTTNDNDTVVTTYFLATYCVFGSLSTTAYSCVLLLQVAQIFVNATSHCGNDGFFFGLAM
HLCGQFEILQMDFAGIKADEQTCKNKIRMLIGRHCHLIRLADSFEYSFNMAIFAQVSMSVLLLCVEGMQLIIS
LKLNDNVAAIKHIVLILTMLVQLYLYCYAGDQLEYVTGRIGYSVYDSPWYNFDVKIMKKLPMVMYRGKTPHLT
TAGKFLRMNLFSFKEILKATGSYLSVLRVMINV
>MphaOr59
IIRVLPLAKMTDKRWNDDIAYVFSTHRQFLRIYGLWPLQAKTVFTKIRWGFCMIAQFAIIPFVMMDIFWSNQN
TNSSIDHVVFIISTITGITRSLCITANQKKLSTNINAAIDDWLSAEYNKEIKEIMKKYAIKAKMLTFTLLYSL
IVVYGLFVSVIIFINLKQVFFTDTSVNVNTTNWLFFIPSGSLSSSITGSQYAIILTIQIIQIAILAFILCLTD
SFFFNITIHLCGQLEILKNKFGTFTNKPDTKINYRKKFISLINRHKELTELYQNLEDCFHFLILFQLVESTLL
LAIIGLRLTVYLNEKIYIEATKSIFMLNYCIMQPMVFTYGGEFLQKKSEDIFYALYTTSWYTLPLALLKDLHF
AMMRLSIPFRLTGGKFFYINRETMMSIFKTAVSYVSVLRIAIK
>MphaOr60
LKMSVEWNADPTYILTLYKYFLGVIGLWVLDDNVFSRIRWFISTMIELSTTISLSLEVTRHCRGHEDALEAFL
SVSSSITSIAKLLLHRVNWRHKLILVESIINDWNLVKNSHSRDIMLKYARISRMGSFVFLYFACASCIFIFSS

FIFANFNLLGTSEKEFYNKTSERRLLLPAYCVFGSYTSLTYDFMRILQILQILVNCFSQCGNDGFFFNLTMHV
CGQFEVLRVDFIKIDSRKFFNRKRLAILLRRHHCLIYLAHHLQKAFSLVIFVQLLLSVTLLCIEGFQLILTLS
MHNTYAVMKHILFIIVLLIQLFLYCFAGQTLEFQSRELASAIYDTPWYTFDVSVMKSLPLMILRAANPHCL
>MphaOr61
MKRAGISRPVEIGLRFIGMWPESAYPNLYLFMYMTMIAVMQYYQYLYVVARFDMTNLTVLTDCLGLALANTLV
FFKLSCLAWNRRIFHNILAAMDRDWNECIINDSSIMMAVANQSCRNSIVIIGIPIIAGFFVSIGIYIYRMMSS
EISRDLPVKMQFSFDALESPVFECILALQMFYVMSLASVAGIINALLASLVIHMGGQVDILRRVITEIHSSNN
ELGTSINLLSDLIKKHQKIISLSDDIENLFSFIALLQLLWNTLIICCSGFMIIFALSTHKGVMVLAKSIFFYI
AKTLEVFVFCYAGEFLSFKSKSISDAVYESHWYNLMPSDTRVLLFVMLRSQKRLTITAGKIFDLTLEGFMSVM
KASASYMSVLHAMY
>MphaOr62
MICIETQYFNLNRILLLIVGLWPYQRSKFTHLQSIFCLGILTTIILFQLTSFLTSKCTSDVIIKIVCPVPFLI
IFIIKYIAFAVNINNIKDLLMQLQHTFNQLKDESENAIIKKYGQNGKRYTVVLIILGVCSICIVIIVQFLSII
LDIVLPRNVSQLHHMLIVTEYFIDQEKYFYLIMLHICAAFYIGMLTMISTGTMLIVYFQYICGLFKVSSYRIE
RAMKINTLQNITLRNENLILKGIIYAIDIHRQAMRLCKLLLSKFETMLFCLIIVGVICLSLNLFQIFQIASSG
ENIKEIFIPVILSVCSIVYMFTANYIAQDLINHNNHIYITAYGIQWYMAPLYIQKLILFLLQRGVKDLSLNVG
GLFTGSLECFATLVKASVSYFTFMYSTR
>MphaOr63
LQNKVGNVENLFVHLERIFSIGGIWPLQRTYARFVIYMSYYSLHLVMAYTDLYDVFGNLELMVMNLVETVVYS
ITFPVMFLIKCSNLLKLLIGVIKRDMVEHKFENSEEERLYYNYNYISKIFIYGSTVSMFSTVLLMYLRPLIYL
VTTKALHRNSSESFTLPFRVHTFFEISNTRTYVLTYLYLMPPIYTSICHMAAVGLLIVLVFHICGEMSILSYR
IRHVGEYSQTMIIDRIRSFVQMHVKIIWMAKSVDDIFNFILLSELVGNSIVLAISLYYVIMNLEVAELATCCT
FIFFALTALLMLFGYCLIGDQLTQQCINIQNAYYECSWYEMPLICKKCLLICMLRGQIMLYLTAGRFYIFSLN
SFTDV
>MphaOr64
QDQKDLDRAAELLSWNKRLMSMLGVWPFHSSDLIFSINFGYFSFLMILEYLDLLLCIDNLEEVIMNLTENMAF
SQIFVRIITLRLYNNQFGEIITEAMKDFDRTSYKTTEEMKAFITYNSRSKIFVKLLMLFVALTASSYYLTPII
IILGTGSLPQIPISENATQIIYLLPYRFHLFYVVENMRTYVITYALQMPFVFVSGFGQSASDCIMVTLVFHIC
GQMSVLASRINNIGTNNELCDCRVEVRHVVLMHLRLLRMGTTIRQIFSMTLLVHLLGATSLVCILGYQILTNF
AKGERGVLITFLIFQFLVLLILYAHCTVGESLLTESAKVCEAFYDCQWYNMSKSNARMIILCMARSQKPLCLT
AGKFTNICLSTLTDVLKTAMGYLSVLRSFL
>MphaOr65
DLVRASNIVTWNKWFLNFLGLCPTKVNQPVFVVVIVYMVLHCIIAANHLIRNINHPDLVVANLTDNVFISMIL
GKMSLCRWNSKIMAKFLKSIEADFTTEMYGNVREKMAYLYYNEIALIFVKVSMFMTGFTASIYFFWKIFVNWS
AGNLESNFTNSLPYPVHPFFEIKDITTYICVSFYLGIMLPVILCGYGGPDAYVLSMSFHICGQFAALSCKIDN
LLRDHKNYHRHISNIILRHRHLIRLAQIVENNFNMMYLQQTVCSVFLLCLTLYHMMAKSDYEENSKIALYALY
TFCVSGTILTYCYTGECLLTESVKLRETFYNTDWYNISPSYAKLIGICLIRSERPMILTAGKFCVLSLNTFTS
IVKTSMAYLSVLRNFM
>MphaOr66
MNCIEAKYFSINKILLLAIGLWPYRQTKLIRFRFIVYLSILITAVLFQLTAFLTSKCTSDLIIKILSVALFFV
VFITKYISFAFNMEAVKYLLVQLQHVYNKLQDEYENAIMEKYGYIGKRYTVALATIALICGIFSVFVSIAVEF
LLSTSDVLSINVSREHQMQFTTEYFVNQEKYFFLILLHIYVAYCLGVVIIVAIGTILIAYLQLTCGLFKISSY
RIKRAMRINILKNISTKNDKFVFEGIICAVDIHRQAMKLSKLVVSKFEIMLFCLITVGVISLSLNLFRIFQIN
ASDGNSKEYLFPTIFTTICVIYMFMSNYIGQDIIDHNNHVFVTAYSVQWYVAPLNTQRIILFLLQRGAKNFTL
DVGGLFVGSLECFATLVKTSVTYFTVMNS
>MphaOr67
ITFIPKWQNCSMNRYYTMNRILLLCVGLWPYQKSSSKYIIIPFMTIMLTSGVIAQLMTFVTTEYSTDILLHIL
TYTIPWLGYTLKYNALCLNMKQMRDLMERVCCDWNELNNIQEIEIIKEYSAFGRFITLVLTLFMYTCISCFIL
VQFLSNILIDIMSSKNESHLRQFPILIECFIDQQKYFFPILLQLCVVVICGLTTVLAAETINMLYTQHVCGLF
EIASYRIEQALHPDIIQSIAPTKRNSMIYQEIINGFNMYRRATEFMDLLKINIKRTYSLLLPLGVLSLSINLY
RFSRLIDSKDYYEMIVSFIFIIGHFGYMLFGNYLGQKIIDHSSDIFYKTYNVQWYTAPLRAQKLLLLVMQRGM
RHCTIVIGGLFIPSFEGFATLTSMSISYFAMILSLF
>MphaOr68
HGTCTEMTDSNTADTYYTINRNLLLCIGLWPYQSFGFRCVLITMMTIILISGVVFQFTTFVTREYSMDLLLKI
LAYSMPWLTYLLRYNILCLNTKKMQSLIERVHIDWNEINNARELEIIKKYSAIGRLITLVTTLSIYLSTFSFI
LIQLLSNFVLDITKAVNESRLRQFPAEIECFVDQQKYFTPLLLYLFFVVLCSLTVLIAVETLFMLYTQHACGL
FEVANCRIEQTLHRGMVQDVISVAEKNSIIYQGIISAVDIHRKAIEFIEMSKENFKWVFFTALPLTVLSLSIN

LYRLSRQITTKEHQETVTTLLFVMGQFGYLFFCNYLGQKVIDHSGDIFHKTYNVQWYMAPLKAQKLLLLVMQR
SMRYCTITIGGLFIPSLEGFATLTSMSLSYFMVIYSIQ
>MphaOr69
LYINKYCLQFMKLFLPECTFDLLLEVLAVIFLVLMWFIKYITFSAVMGNIKQLRNHVQSNWSIIVNDQETAIM
HKYATIGRQFTIAIGICVCFGCFGFTIIQYIPNILDIVKPLNESRPRLLLYQAHYFVKQQKYFYFVIIHDAVG
VLISGITGIAAETFSLVNALHAFGMFKITSYRMKHMLSVDISQLSTAKSYIIFHDKIIAAVDTHRRALEFSDL
LKASFGLSYLFMITAGLCTATISFFRLFRILTMQQEKMETIKLVCYIIFVFLFLIIGNFVGQEFINCDEHVYH
MICNTKWYNAPLKIQKLIFFLIRKGTKSYKVDAAGLFSPCLEGLATALSLLLSFFTLLCSM
>MphaOr70
FVWAIEIHRLGLEIIGLWPKYDKFDANNLWSKFRISIVLILLIFVNNVPSVHAVTQVWGNMILVIGHVRFALP
LLMASIKYIIMLWKRTVLTSIVNMMAEDWMEFKSNIERSMMIKRARTARLIMIIGYVFILIAVLSTIIPPTFG
IQLISGANLTKRKLPLFTSHFYNTDRSPQFELTFFIQSISVLFATIIYMSIDIFLILTVLHMCGQLENFRYRL
FNLISSKNFNKTLNNVITSHLRITRFADKIENTYSLMMLIMILHFVIVFCLSGFLFTIFLTDREMDEIIITQI
FHSIAVFSALLTNTFLYCGTGELITEQCNAVYQAMCNLEWYKLESRNAKNIILLMIRARHPFCITAGKIIPLT
MVTFCSVLKTSTGYISFLLAK
>MphaOr71
FEWAVEIHRLGLETMGLWPKYDKYNENNLWLKLRVGIILILLIFITNVPMIHAIVQVWGNMVLVIENLRIAIP
LLITSVKYIIMLWKQTVFLSIINMMVEDWMVFKLNTERAIMIKRAQTARLIMIIGYIITVMVLLLWTISSSFG
IQTVLAMNFTDRRKFLPLFAYHFYDVNKSPQFELTFFIQTISMLFAITIYMSVDALLVLMVLHICGQLENFRC
QLVNLISCNNFNKTLNDIVMSHLRIIRFIDKIENTYSLMMLIMVLHFVIVFCLSGFLFTIFLIEREMDKIIIT
KIYFSIIVLFTLLMNTFLYCAAGELITEQCNAVYSTVCDLKWYKLKSRKARSLILLMIRMRHPLFITAGKIFP
LTMATFCSILKTSTGYISFLLTKR
>MphaOr72
FEWAVKIHRLSLETMGLWPKYDKYNKNNLWLKLRVGIILVLLIFVTNVPMIHAIIQVWGNMVLVIENLRSILP
LLIASVKYVIMLWKQTVFLSIINMMMEDWMAFKFNTERAVMIKQAQTARIIMIIGYIIIMTVLLLTISSSFG
IQTVLAINFTDRRKFLPLFTYHFYDVNKSPQFELTFFIQTISILFATTIYMSVDILLALLVLHICGQMENFRC
QLVNLISCKKFNRTLNDIVMSHLRIIRFVDKIENTYSLMMLIMIFHFVIVFCLSGFLFTIFLIDREMDKVITT
KIYFLIIMLFILLMNTFLYCGAGELITEQCNAVYRTMCDLEWYKLESRKVKGFILLMIRMRHPLCISAGKIIP
LTMATFCSILKTSTGYISFLLTKR
>MphaOr73
FMWTIELNRFGLKLVGLWPKTDGAIRDDTSDLRMVIIFIIITFICGIPLVSSLIRVRHDMILVIDNLQTTLPI
IVVSLKLVIMRWKRTALLHIIKMMEEDWLALKLNAERNVMLKRARTARMIVICGYVMMILAFIMVIAFPLFGI
PFRRLTNLTDRNKPLPLQTYYFYDTDKSPQFEITFIIQALTIFLSAIIYTSVDAFLGLAILHICGQLENFRRR
LVNLVLCKDFNSTLRNNVVTHLRLIRFADKIENTFSLMMLGLVFYFGTVFCLYGFLLLTIIADNETSNIPFLR
VFYVMLGVATLLVHTFLYCGAGELLREQCEAMYRALHDLEWYRLESKKAKSLILLMTRASEPFRITAGKIIPL
TMTTFCSLLKSSAGYISILLAKQ
>MphaOr74
LNYAFALTGQCLRILGVWPDPLVPLSKFQRPNIRFIVVTCILSLYVFVPQVTNMILAWGNVSRMVEYLSSANY
CLIALCKLIATWYHGKALQTVMASVITDWTTSKNQGRTIMLNMARRGRILSFRCYASMSCTVAIFIFFNILKF
IRNISQTQRSLVYRFVLLYHSQKSPNYEITFFIQLSGGAYAGVINSTVDSYISILLLHISAQMINLRTSLNNL
VEQLAEGSISSSIFKKGLAAITTRHEHLIRNARTINDCYSAVLFVHMLSATFQLCFESFQVFTIITSNMDVPI
IKVTFLSFYFVAVVMHLYTYCYSAERLSTESTNMAYGVYECKWYDISSKDAKNLMFMVHRSTIPLRLMAGKFG
TFSLEMFGTTVKTSMGYLSALLTLTD
>MphaOr75
LNYAFALSRQCLRMLGVWPDPLVPLSEFQRPNIRFIVVTCILSLYVFVPQVTNMILAWGNVSRMVEYLSSANF
SLMALCKLIGTWYHGEALRTLMASVITDWTNSKNQERNIMLNIARRGRILSFRCYVSMTCTVLFYISLNVLKF
IRNVSQTQRSLVYRFNYPYNTQKSPNYEITFFTQLSGGAYSAIINCTVDSYISILLLHICAQLINLRISLYNL
VEELAAGSISSLIFKKELAAITIRHEHLIKNAKTINDCYSAVLFVHMLAATFQLCFESFQVFTIITSNMSVSI
IKLAFLSFYFVLVLTHLYIYCYSAEQLLTESTNMAYGVYECKWYNISSKDAKNLMFMVHRSTIPLRLTAGKFG
IFSLEMFGTTVKTSMGYLSALLTLMD
>MphaOr76
YMFTVTRSLLRVFGIWPDPYAPSKNLCRQNIRLVFIVCHITMYVSMPQFVNVFRAWGNLSKMVETFIATNYTF
MAIVKMSVTWYHIAKLQLLLMSMVNDWNILNKWERDVMLKMTRRSRNLSHRYFVAIINLIIFTFSAYTPKFFK
NIHQPVRYLVYRIDQIQKTPIYEIVYTIQVISATWAVLGSFMVDSFVTILVLHISSQLIILRMTLNNLVDDLA
NKSISSSGFKKGLAAIIERHQHLIRSVKTIDNCYSTILFVNVLFVTLQMCFIAFQIFTMITSSTNTPVIRIVW
LTVYISNILMHLYTYCYSSEKLIAESTKMAYSVFKCKWYDIPPEIAKDLMLIVYQSTIPLKLTARKFGTFSLE
MFGNAVKTAMGYLSALVTI
>MphaOr77

LNYVFTLSRQCLWIFGIWPDPQIPLREFRRPNIRFIIITCTVSLYVSTPQMMNVIRAWGDVTRMVENFASANF
SLLAVCKLLVTWYHGETLRPLMASIMHDWITSTRDWERNTMLKIARRGRSLSFRCCVAAMGTIVFYISFHSLR
LFKNIHESHRHLVYRLENIQKSPTYEITYFIQLSGGTYSVLVNYTVDSFVSILVLHVCAQLINLRTTLNNLVN
ELTTKSISSSSFRKGIATIVERHEHLIRNAKTIDGCYSSVLFVHMLSATFQMCVLTFQVFTIITDNLKVPFVR
MIFLTFYFSLVLTNMYAYCYSAEKLIEESTKLAYGVYECKWYNLPPKDVKSLMFIIYRSTIPLRLTAGKFGIF
SLQMF
>MphaOr78
MEFPEEQYFKLNYTLLSIVGLWPYDDFKVRYVRFILCLLIILSFISTQLIKLFILECSFDLFVNVFSYNIIFT
ICFVKCITFYAVLKNIKKIREHVRNNWSILTDKQEIAIICKQGHIGKLFSIFLIVSIYAVLLFGSVFAQCISI
LFDIVIPLNESRPRKLLFPAEYFIDQQQYFYVITIHIAVSIFFMTTSVMAAELFAFSNALHAFGLFKIASYRM
KNILNGINQTEMCLNKKYIIFYNRIIAAVDFHRRAIELSDLLKASFGPSYLILLLMGVCSATINLINVSSTEK
EILEIIRAIVFIILHLVYFSVGNYAGQEFINCDTHVYRMICNMKWYNAPLKIQKLILFLIQKTTRCYRVSAGG
MFNPCLEGFATVRNLT
>MphaOr79
MNFVGNVYYKLNRRLLLLVGLWPYEHSIYKYCQMILYNIIIIFTLICQITKLITLKNVKSRNTDMMLKLLSLI
VFDIIFITKYQTFCIVARKIRYLMDHVKEDWNMLKDKRELEIITKYTYIGSICTLILTILGFAGTLICIFLPL
IPDILNIIVPLNISRPQQLLFPGEYFVDQQKFYYAIVLQLDVTVGLIIFTLISTESLYVTYIQHACGMFEIAS
YRMDQAFNNKLLQSYTSEKQAVIIYKRIIEAIHIHKRAIEFSEFLWSTLAISYSILLIIGITSLIINLFCLFQ
TILFVKAIDEVITSFLFTIGHIIYLLVGNYVGQILIDHSVSIFQNIYITRWQGAPLQVQKLLLMIMQRSMKSC
KMVVGGMFVPSLEGFATLMSTTLSYFTVLWSVQK
>MphaOr80
MNFDGQHYYKVNRFFLEIMGLWPYNNSMYIYIYRAFTSFILVSSCITQFGKMQSTIHDLDHFLYNLTYSVPCL
IYILKFFSYIININNVKEIMSKIQHDWNTLKDEKEREIIRYYTIIGMRYTIVFFALTLPFLFCFILSFYLPGC
LDFIHPLNESRLRKLPILAEYYIIDEQKYFYPVLVHQSLLIILGIMIVLATETLNMIYLHHACGLFEVASYRL
KHIFDDEIFSVAKNVQYTIIHARIVKVINIHRRTVEFLDYLSSTFTWTYFIMLVLGIIAITINTFRLIQAILI
IHDFKELIGAVSFICGQYGFLFFANYFGQKLTNRSFLMFHKVYDVPWYTAPVHIQKLFAFILQRTVKGYVLNI
SGVIMASLEGFASMTSMTLSYLTMIYSVR
>MphaOr81
CNTFLVKKCSIQYIIKSLMFIVINCIYIIKYNGCLLLADNIKYIFDRVRYDWNILKTQAELEVIRRYASIARL
YTVYFIFLATFIVPGLIIITCLPRILNMILPMNESRSEQLIITVEYFIDRETYFFAIIHIIVAQYAGCITITS
VATILIAYVMHTCAMFQIASHRIKHIFDENLELISKDIKQYTLYNKLIHAVYVHRRAIDLTNILTNSFATLYF
VLLGFGVASMSLSFFNFLNAVIFLEVDTIVICSFVILIHFYYIFLGNYVGQSIIDNSTNICQMTYNTQWYAAS
LYMQKLILFIIQRSNKRSALIAGGLFDASLEGFATLMSMSISYLMVFQSVAMHKEKENNHI
>MphaOr82
MQSAVRRYYNINKILMSKLGSWPTQSTFMKILLPTIVTSFIFCIGFLEFVRLSEIWKNVTEDCESFIMVIITI
GGYVKLFIIVLKSENIEQLLSLIDYHWRVFTHSLEVQIMHEYAIVGRKLTISYAVMIYSLMTLYMLIPVTPQL
LDLIVPLNKSRPYKYLFDIDYSFDREVYYYPVLLHAYLTTVIAMSLMIITDTCYISLTQHACSLFAAIGHRLE
NLTSEISLKKDRFDEDIEVTCDKRKSRDCDEKIYRELVLLLWKHQLSIQYAQMLDSLFEMYSFLLLFVTIIVM
SLLGIQILSLMDHKEEMIRYVSMVIGAFLHLFVLSYPGQMIMDHSGDLFHKAYNMLWYRMSRRTTQLLSILLY
RCLVPCTLTAGKIYVLSMANYASMMQAALSYFTALSSFQ
>MphaOr83
MTDKQYQAYKTYQRFTQICLTIGGCWYMPTKSGKPAYYWPVCVVCLTIMNAVIRFNLIYISRHNLVHMMKFVG
NLMSAIGCLIKVISFLINRGSLINHHRTLNESFEEEFMQNKKIRTMILSSQRKIYIVTYTYTTILVTLLLMIY
AASYIHIIRDLFHLRLTINYTLPMSKGYGYFWSVPDNFLYHLHLLIETILSSLGVVTASGVDSIFGFYVYQFV
STLRFMTFRLSNNLSIESFSDQLRTYIKKHQKLLQCRNTLEHVYGPIILWHVITNAMFMCTMMYEMIHLSNFN
LRYVLIFLMYTSVKLFQTFIYAWCGTILTNASEDFRNGIYFGKWHNSKLDHYVRVNVILMLMQKPMTIKAIFL
SVDIVMFANLIHATVSYFFLLKSL
>MphaOr84
ITPNFHLKLSLTIIYFLGTWPPATGKSRLIYLLYTALSFTFVLGILLATEIANVLVHWGDMTKLAAVATLLMT
NSTHASKVITLLRCQTRIQTLLDAANSPMFSRYDKKHEDILVRYTWKAIFHHAIYQSFGGIAVFCWGFTPISD
LIAGRSRRLPMEGWYPYNTTATPAFEITAAHQGIAIIIACCHNVAMDTLMTGLITVACSQLAILERNITSIDN
EGNVRRMRNNDKGKSLAIEGRVLSYQLLKRCAVHNKLIFDFTNEIQDIFGTVIFFQFLSNCVIICLIAFNVSQ
MKVYIPAVLIGMLTYMCCMTYQIFIFCWHGNELHLHSMRLVTAAYSSNWFSNTERFKRGLQIVMTRAHRPLTL
SAGRIMTLSLNTFVQIMRMSYSIFTVLQGSAA
>MphaOr85
MLETTMKRTNPVATVYNHEKDLQLSIQLNRWILKPIGAWPKLSEISRMERYAYGLVNVICTSLIGFLFVPSAI
FMVLEMDDTYHILKLSGPLSFCLMAVIKYSSLIFRENDIRIGIQHIENDWISTRHYGDRIIMIRNAKFGRRLV
SICAFFMYGGAVFYYLALPFSKGKITEYDGNLTYRPLVYPVAKVIVDARHSPISEILFWLQCLSGFIAHSITA

GACSLAAVFAMHAYGRLEVLIQWIEHLVDGREDFYDNVDERLAMIVQQHVRILRFISLTDKVLREISVVEIVG
CTLNMCFLGYYTLTEWESKETASYITYIVLLISLTFNIFIFCYIGELVAEKCKKIGKVSYMIDWHRLSEKKGL
ALVLMIAMSNASIKLTAGNLFELSLSTFGDVVKTSVAYLNMLRTLT
>MphaOr87
MIGHTDLRNSNYEDDIKYTVQVHRLILGLIGVWPNFEKPQKWARFLRGLLRIVCCFLLSFNLIPWILYMFLIV
DTFKGRLKMLGALFFYSMVPAMYCTLMLRQDRIKECMRHLQEDWRNVQDVNDRRIMLDKARAGRFILICTTVF
LFTSGFTYRLIKPIVRGKITIGNVTIRPLVQGNYYIFFDPQKSPAYEIVFSMHLVTGIVIYIVTTSVCGITAL
FTMHVCGQLKMLTIWLENLPNEDHWSKECAVAQRLATIVMHHVRIRKFLHQIQGVVGEMCFIEIIGSTLILCL
LGYYVITGWEQNDALSSMTYAIMLVSFTFNIFILCYIGEVLSSQGSEVNTTCCTIDWYCLPSKEARYLILVIA
MARYPVKLTAGKVIDLSFNSFGAIVRTAMAYLNLLRTVTM
>MphaOr88
KTSRYENDFNYAVQVTRVILRAIGAWPVSNYASTVERIAIRLQNFICYFLFAFIIIPGLLRVFLKEHEFKRRV
RLLAPLLNCGMGWMKYSLLINHAREIQSCLKQARHDWRDTVDEDDRKTMLSTAKVGRKFGIFSAVFMYVGGLS
YRTLVPLSKGRMLTPMNTTVRALACPSYFIKFDGEVSPAYEVVFTLQFFAGLITYSVRVGAAGLAAFFIMHVC
GQLRIIMGKLQHLNDMPNPNDRAVAILLADIVEHQIKVKSFLKQVEETMRYVWLVELMGSAVLLCLSGYYIIM
EWENNDSTAMLTMSVMLTSFTFSIFTNCYVGQQLTNQSISLGLKTSTINWYSLQYKRARSLILIMAISNIPEK
ISAGRMIEMSLPTFSNIVKTSVAYFNMLVKF
>MphaOr89
MYRNKNYRNDIKYTTQLNRFICQLLGIWPDAEPSFLKNLRSISLILACFFLLGCELIPITLYVVFIEKRTRVR
LKVISSITFTTVAILKYFCLVLSKNQVRNCLARVKDDWQNVVSANSRNSMIDKARISRHLFILCSIFMYTAGV
YYRIIVPLSRGKTVTKQNITIRHLPSPGYYILFNEQNSPAYELLFIIQVCAGFIKYTITVAICSLTALFVMHI
CGQLEILMILLDNLVNKTEEKNLNKMLALVVEHQIKIRNFLQLVQNTLENASLLEIVGRTIDVCFLGYNIIVE
WENQNVVSMCSYVILLTSMTFNIFIFCFIGEELSVEGEKLTLTVCTLTWYRLPNAKARALLSIIAMSIVPTKL
RAGKFFDLSIKTFGDVVKTSVAYLNFMRKM
>MphaOr90
MYPNKNYTNDIKYTTQLNRIICHLLGVWPDAEFSFLKNLKSNSLILSCFFLLGCELIPTILYVVFIEKRTRVR
LKLISSILFTTVAVLKYFFLVLSKNQVRNCLAQVKDDWQNVVSARSRNSMINKAKIAKRLLVLCGIFMYTSGL
YFRTIVPLSKGKSVTDQNITIRHLPCPSYYVLFNGQISPAYEIMFFIQFFSGFIKYTITVAICSLAALFVMHL
CGQLEILMALIDNLINETEETNLNRRLALVVEHQIKMRNFLRLVQNTLEHTSLLEVMGCTIVVCLLGHDIITE
WEDQNVISMCSYIILLTSIGFNIYIFCFIGEQLSIEGEKLALTVCTLAWYRLPNAKARSLILIIAMSIVPKKL
RAGKFFDLSIRTFGDVVKTAVTYLNFIRKMME
>MphaOr91
MPRNEHHQNDVLYIMQPTSKILRAIGVWPSIRKERSVYTNAQNLLLICISYTLLSSDLIPGTLYWLMGGTTQV
RLQLIPPLFYDIMSMIQYSIFIFRCDQLRQCLKHVEEDWKNIFSVDVRNIMLKSGRTGKRLVTICGIFMYSGA
ITFRTILPLAQEKIVTDQNVTLRNLACPGYFFSLDVQVSPFYETVFVIQFLSSIVTVSIVTSACGLTAIFVMH
ACGQLKILINLMRDLVQKQWQKEHEVDKKLAEIIEHQIRVRNFLRLVQHTLQEIYLMEILVNSITICILVYFM
TMDWQNRNITSVCTYLISIVNVTIHIFLFCYTGEQLTSQAEKVAIASCELEWYRLPDRKARSVILLMIMSNAP
TKISGGKFIDLSLKTFGGVVKTSGAYFNMLRNVI
>MphaOr92
NLLLDKHPNKNSEQDIEYVTKQSNFLLRILGVWPLLDRDLSVAELACKILLMTICCSLVCFESIPILLYCMFV
TVEEPRVKLMMVAPTIYSFTALAKYAALIVNENEIRNCLKHIKDDWKFIAVSNARDIMMEKAKIGRTMFTICC
VFLYSAGFSYHTIVPLSRGKFVTDDNVTIRPLAFAGYFVLFDEQRSPAYEIVFALQFFGGFVMYSVTIVTYGL
AALLVMHACAQMKILMVLMEELVDERAYDEKNTNEKLVAVIERQIRIRNFLYLVEDTLQKSNLFEILANTIMM
CFMGYCILTEWQDGNAANAFTFVVALTSDIVNIFLLCYIGEYITATADEVAWRTSMLEWYRLPAKRTRDMVLI
ISVSHMPLRITAGKFIVLSLKTFGDVMKSAVVYFNILRTVAD
>MphaOr93
MFANKYYERDIKNAFALNRFFFRVIGIWPFAYANSLLPELFETIALVLACFVFLICELIPMLLYVFMVLTDVR
IRLKFTGSLIFTAVQIIKYVYMLLYRSHVRNCLMLVDADWRNVVNPSDYISMIDRVKIGKRLIVMCAVFVYLN
GVALRMVIPLSMGKIVTPQNITIRPLPSAAYLIILDVQRSPVYEILYFIQFLAGFIKYTIIVACFGFVTLCAM
HFCAQADILVTLMNDFVNENRPENLNKRLATVVEHQIRIRNFLQLVQSIAQYPSLVEVVGSSVMLCFAGYYII
MGWEDHNIIILCSYILSLIMLMFNIFIYCYMGEQIVEQGEKIALTTCTLDWHRLPNTKARALILLITISENPL
KLKAGNFIDLSLKTFGNITKMAVTYLNLLRSV
>MphaOr94
MFANKNYERDIENVFVLNRFFSRMIGIWPFVHANSLLPELLETITVVLTCFVFIICELIPMLLYMFMVLTDIS
KRLKFMGSVVFTVVQIIKYVYMLLYRSHVRNCLMLVDADWRNVVNPSDRISMIDRIRIGKRLIVMCAVFVYLT
GVALRMAIPLSMGKIVTPQNITIRPLSGPAYLIILDVQRSPVYEIVYFIQFLSGFIKYTITVATFSFVTLCAM
HFCAQADILMTLMNDFINENRPEYLNRRLATVVERQIKIRNFLQLVQNITQYPSLAEVLGSSAMLCFAGYHII

MGWENHNIVVLCSYILALIMLMFNIFIYCYMGEQVIEQGEKIALTTCTLDWHRLPNTKARALILLITISEIPL
KLKAGNFIELSLRTFGNITKMAVTYLNLIRS
>MphaOr95
MLYNEHYKDDMIYITQLTRNVLFSLGVWPYYNRRRSIREKVWKYFLISISYTLLFCVLIPGALFWLIEKRTRV
RLQTIPLLLFGFTASGKYGNLIFREKSIRRCLKHIEEDYKFATVKTRDIMIESAKIGRRLVTLCAIFMYGSGL
SFRSILPFAKGKIVNSQNITIKPLPCPGYFFSFDVQVSPAYELIFVLQVLSGLVTYSITTGLCGLAAVFVMHA
CGQLKILVNLMRNFAEGQWQEKQEVNRKLAVMVEHQIRIRSFLQSVENTLQQACLIELTGCTGVACLLGYFII
MEWENSNSIAMCSYFSALVSMMINMFMICYTGEQLTVQAEKVARTSCVLEWYRLPRKETRGIVLVIIMSNMPT
KLTAGRIMDLSFKTYGDV
>MphaOr96
MIRNEHYKDDMVYVTKLTRNVLSLLGIWPYYNRRSSHGEKLWKYFLILLCWILLYCVLIPGMLFWVLEKRTRV
RVQTIPLLINVFMATGKYGNLIYREKNISLCLKHIEEDYKIATNAKTRDTMIKSAKIGRRLVTLCAIFMYGSG
LSFRLILPFAKGKIVTPQNITIKPLPCPAYLFSFNMQVTPVYELIFALQVLSGVITFSITTGLCGLAAVFVMH
ACGQLKILTNLMRNLVEEQCQEKQEVNRKLAIMVEHQIRIRSFLQLVENTLQQACLIELMGCTAIVCLLGYFI
IMEWENSNVIAMCSYCSSLLSMMINMFMFCYTGEQLTVQAEEVAKTSCVLEWYHLPNKEARGFVLVVIMSNLP
TKITAGKIMDLSLKTYGDVVKTAVTYFNMLL
>MphaOr98
NTLAVNVTKQNKYVSDFRYAVQISIYLLKPLGAWPLANDETSRFKIAQHRTSMIITMFLMSFLVVPWIACIVK
EKWSAYLIFRTTCPLLFSTTLTVRYLLLLWNQDRLKFCVDHVADDWRCTTIAEDRDIMLTNARIGRTFGIISV
VFIFSCNGLYTTLPILMPNPINEDNVTVRLHPSPSELLVFNAKASPVYEIVYIIQAASCYVLCSTFCGICSLM
ANFVMHVCGQCDVLMSILEEIVDGGKHISGSIEDRIATAITRHLRLLRLVSNVSDLFTEICVMEFMNASCNIG
LICYYIVTDMNNNEPFALIFMNGFGLVSIIFNMYIFCYIGDLLIERCQQVGTACYAIEWYRMPPKKAVELLMP
IAMSQYPVMLTAGKMITMTLATFSYILKTSMAYFNLLREFISRDITTT
>MphaOr99
LDWAIGINRRCLKIVGLWPDDKLSRRQKFITNLRATAFFATMLCVSVIPGIMAFVRVWGYMMAMTDNMHINLP
FSVNMLKFIIMWFHREELEPLSRMVIEDWLREKTEEERNIMIKHARITRIIIMFGCTMMIIACFLLIIILPMFG
YTMRYLTNLTDPGRPFLVQTYYLCDITETPYYEIVLVAQATSIIVGAISYTGIDTFLSLVVFHICAQLEILEG
RFLNLDKYKDFKTGLTINIKDHLRLIRLSVDVVDNTFNLMLLTLLISFGMLFCIQGFLIVSIIDGTTDVSFWR
ICWLVSILINTGSHMCLYCVVGEILIAKGEGIYKAVYNYTWYLRPPTEVRNLLLIIVRAEKPLYITAGRMFPM
TLSMFCNLIKTSAGYVSVLLANQ
>MphaOr100
MEGKITLDRVIAFLRVYLTFACCWPLPPNATKLQRLVRSAFQCFCLTNSIVFAIAAIWTLCKHSDDVLLVIKL
GCQLSAIVQIPLQMILFAMQNKRLQFIVLEMESYYQQAQEYEKKIFQLYIDKCKPFYGIILCWLAMTGISVII
TPLFSSQSFPSETEYPINMHSQPLKIIIYAHHVLIAYQSVIQVSTNTFPALLLWFVAARFDILSIRFRTMKNM
KELTKYTHEHRLLLGYAKEVTRAIRYVALLCVTFSTGAVIFGYLTFMSRDIPWTVKSTFLMIAFCGFVELYMY
AWPADNVMSTSSGIASAVYESLWYNDDLTRTRKILIHIILRSQRPVTVSIPCALPNLSMNYYASYISTVFSYM
AFVRAHIVMGQE
>MphaOr101
MKKTPISTINRAIEIPLRIFGIWPGSSYIPFCRLFWTIAIAATQSFQYRYVIKHLYTVDLSDLMECLGATLSF
TQFFFKIINFWVNQRTFDKILAMMAMDWKKSNNTDFSTRAIIRKAILSHNISNLCFGSVIIVVTLYSASIFTY
NSFNLDETDISMRPLILKMDFPFSGDTRFVYELILLSQILCTVLAGCGNVMLNTLLIVLVLHLAGQIEILSKW
ITDIISEEDEYTLNLTTIRKIIKKHQNIITFSKNIENIFSDIALVLFVSDTLSICALSFVLVTSIGRPDTATI
VMKTLGFYFVINFEVFMFCLAGEYLSSKSKVIGDAAYCSRWYQCRFQDSRIILFLIMRSQNQMTITVGKFMDL
SFERFVSIMKASASYVSVMLALY
>MphaOr102
MSILSFNFLLYTICGLWRPIEWSSKWSKLLYSVFTFTTMYLIACLSLTNLLFVIFVAENIDEFSLSSPIFFSS
ITVIFKTIAMIIYRDQIINLIKTLEEEPCKPCNEDETAIQIKFDYTIRSYSIYYAILCTFSVTGGLTAGVFKI
LKRQFPFNIWVPWDCTSYLLFLFTSIQVIVAITVATVINVATETTVLGFCLQVCAQFEILKHRLQRIVKDRKE
EMSLKRSLYNASHKMNTLSKHICHHLCIIRLAERINNVYRYVIFIQFFVSILVLCLIVYYLSSHVTVVDIATW
IGFVFSMFVQIFVYCWAGNEIILQSTGLGEAVYHVDWVLMTLSELKDLMIIMKRSTRPIKITSSFLVTLSLES
YTNLLKATFSTFNLLKQL
>MphaOr103
MQMFSLNFLMYTVGGVWRPVKWSSNIAKLLYNMFTFIAIFLLYFLMITQFMDIILVVDNIDDFATNTLMFLTI
VAVSCKATIVVARRNAIIALVQVLMTAPCKPQNEDEVAIQTKFDKFIRSCSIKYSLLATCSCSGVTIRSFLNA
VNGYLPYRVWLPYDFNTTPYVFWITSLQQIIAVILCTIINVGTETLVFGMFLQTCAQFEIFENRLHKLIINKT
NKSMTNKITYLENSSPSSNKGKAIISKYVHHHLEIYKYARTVNAIFNQVLFVQFFGSVLVLCTSVYYLSTHST
LSETATLIIYTICMFVQIYVYCWSGNEVILKSVRVGDTIYNMNWPLLSVNDRKELLMIMLRSTLPMKFTSSFL
ITLSLQSYSNILKISYSAFNVLQK

>MphaOr104
MQILSLNFLMHTFCGLWRPIKWSSNSAKLLYNVFTFIVIFSHYLLMLTQFLDIVLIVDNIDDFATNTLMFLTI
VAVCCKATVVVVRRNAITNLVQVLTKTPCKPCDEDEAAIQTKFDMFIRSCSIKYLILTTSSVTGTTIGSVLNI
MQGHLPYRIWLPYSDNVPMMFWIISIYQIITVIFSAMINTGTDTLVVGLLLQTCAQFEIFESRLHKLIISKTI
KYLGHAFSSSNQDKIGISECIRHHLNIYKYAKTVNVIFNQVLFVQFFASMLVLCTSVYYLSIHITELSEAATF
LVYTIGMFVQIYVYCWSGNEVILKSVSVGDAIYSMDWPLLSVNEKKELMMIMIRSTIPVKFTSSFFVTLSLQS
YSNILKTSYSAFNILQK
>MphaOr105
MHLMKFTLIICALAGCWQPLMWTSLFKHIIYKAYAMFLISSLYIFLFSQFVHVILNVGSSDEFTDALYMMLTV
FVAGYKQIYLWVDHKNIMATINVLLEKPFAPYEAHELMIQEKFEKRIRNNTRRYLIIVMISIISIISMSISID
FMRRNLTYKAWIPFDYSSPAIYFVVYIHQLIAMSTSGIVNVACESLLCGFMLHICCQFEILGYRLTKLTHDQN
NIRNYVCHHNRIFEYAYTVNNMFAKIIAIQFAVSMLVVCSNLYKLAMTTDYMNFIPLMMYTSAILMQIFILCW
FGNEVKLKSLQLMNNIFDIEWSALSSSNKKHLLLIMKRTMIPVEFTSAHIVSMNLESFVALLKMSYSIFNLLH
QTNE
>MphaOr106
MGVLKFTLQVCTFAGCWQPNSWTSLFKHLIYKTYAMFLLSALFIFSISQFMNIIINVENSDEFTDSLYMMLTV
SVAGYKQFYVWIKRKNVMMIINILNKKPFAVRESHELMIRQKFDRIAQNNTLRYLSLITVTVIIVVVTSVFTV
FTNRNLTYKAWVPFNYSYPALYVLVYTHQLIGMGTSGFVNVACDSIICGLLLHICCQLEILEYRLTKVVHGED
ILRDCICHHNRIFEYAYMVNNMFTKIIGIQFSASMLVICSNLYRIAMAKDSVIFISLVMYTGCILAQIFIYCW
FGNEVRVKSLQFMNNIYYDIEWSVLSNSNKKGLLIIMKRAMIPIEFSSAYIITMNLDSFVALLKMSYSAFNLL
HQTQE
>MphaOr108
MSFFDNHYYYFNKILLRIIGQWPFQSRMESNMMLAVTSLFIFSLTAFEFWGLAAGITDLSIIMENFSPLLVNT
MAITKLINCVFTNDKMKVLLEDIEETWKMKSGAEKEILQLYAEQSRTFTIRYTIALYVAWLSYSTTPIVVSGM
YKILPTNETYTARFLYRMEHVLDMDKYYNLLMLHGFISVFYIVSVPIAIDSIFTICTQHICALFECLKYNIER
IRCSDIMLLELNVKNDESYHDIIGCIESYKHALKLSEVLASNYAVSFLFLLGNGIICLSFSAAELIMIEIQLD
EMIRILSANLGEVLHMYLLCLLCQRLIDYSSGVREAIYNCDWYKMSIKSRYLVRFTLLRTTKPCQIKAGKMFL
MSMENFSSILQVSLSYFTMLTSLQ
>MphaOr109
MSFFDNHYYYLNKIFLRIIGQWPFQSRLESNLTFVAASLSILTLGALESWGLVAGISNLSIIMDNAAPLLVIS
IVNIKLYNCVFTRNKMKMLLEDIEETWKMKRSEPEKKILQHYAEESKDFTIRYAIALYAAWLFYCGTPLVITW
IDKLLPKHTNETYTARFLYRIEHVLDMDKYYNLLMLHAFIGVFYLLSVLIAIDSIFTVCIQHICALFECIRYN
IEQTRTLDTVLLQPNIENDEAYRNLIGCIKSYKHVLKLSHVLSSNYAVSFLFLLGNVVISLSFGWAQFIIVDT
QLDEKIRILFGNMTQLLHIYGLCSISQRLINHSCEFQGVIYSCNWYRMSLRSRHFLRFTLLRSMKPCELKAGK
VFVMSMEIFSSILKVCLSYITMLTSLQ
>MphaOr110
MDVFDTRYLQINKLLLSYIGLWPMQSNRNTIFICFTILGLLSMTLPQIAFLFTRLRDLSDFYDILPTLMGSFI
CTFKSLGLHWQNKKFKILLQHVRQDWCLLMKHKDIWILMEYSEKSRMFTLLYLIVTTVGIASFVAIPLMIPLF
DLVFASNVTRSKRLPHPAEFFVDTEKYYYILLTIMFASYVVCVLIIVACDTIYVVLLQHTCGILTMLSNRLKK
WEIDDKLKYVRCNPVSKGDKDIENIVQCIQLQVRTERLIELIESTFAICLFIDFGFGILLQCSSCVMIVFHTD
VIKNCVLLFIQTSRLFFNYWMGQRLLDHNFQISIAAYNGMWYEMSLKAKKVLLFLMMKCQKPCYITVAKIYVI
CMQSYSTLMRTSASYVTLMISLNSDDV
>MphaOr111
IDVFDTRYLRINKLLLSFIGLWPMQSNRNIIFVCCTILGVLIITLPQIAFLFIRLRDLNDFYEIFPTLMGSCI
VTFKSLGLHWQIEKFKMLIQHVRHDWCSLMKHSDIWILMEYSEKSRMFTLVYLIFTNISVVSFATKPLIIQLF
DFIFESNVTRPKRMPHPSEFLVDMEKYYYILLTITLASYAVCVTIVNASDTIYVALLQHTCGILAILSYRLKK
WEINDKSKCVDRNPVLKGNRDIENIVQCIQLQVRTERLIQLIESTFAICLFSDFAFGMLFQCSSVVMMVSYTD
VLKNCVFVLLQSTRFFFNSWIGQRLIDHNSQISTAIYNGMWYERSLKAKKMLLFLIMKCQKPCYITVAKIYVI
CMENYSTLMRTSASYVTLMISLNSD
>MphaOr112
TKAMNANWIHYYSVVKKISSLSGQWPYQKPITRLLCAILVTLSTISIIIPQIAKFVRCDGNLQCIFETMTSYM
LTTVTLVKLYTCYFNRCKMKVLIDQLFVDWDELETPEEYEIMKRYAKNSRRYSLGYSLYCYFAVYVFMSISLI
PQILDVILPLNESRPILPTYPGYYFVDERKYFFYIFSHAIMAWEIAMSGIVSHDCMLLTYIEHICSIFALVGF
RFERLMCKHNDAMNVLHSRTSDMYRKQIAFSVYTHRKALKFAQLIEDTFSLTLAIQIALNTVMISITLLQQNV
SILEVIRYAMYVAGQLIHLFCLSFEGQKLIDHSLQTRDKIYNSLWYETPPKLQRMLVFVMQKSLQPIFLSAGK
VYIFSMENFTMIMQTSMSYFTVLSSVE
>MphaOr113

FDRAIAITRYGMQLLGFWPQDTRLLCDLRCGVIFALIGFFTFPTALRTYTVVYTVTDWDKVMHQALETVPAIP
LLARFIFMKVMARNFRLVLCAMTADWTDYRYLTKRDRRIMVHYARRGRRFSILSTILLALVLIGFMLTPIVNM
WYSGGFWNVTTRILPQEGLYPFHNKKSPIYEILYVAQLLMTFLCATALATVECFLYIIVFHVCGQFDILAALL
KRYDSSIRHHYAKNTRNCACLVCIVKRHVHILNYVDLIEKSFRDFLLLQLLGYWASLILYGRELILHIQHSNI
QGIITCILYMVTIMYYIFIYCYVSECIIERSENIGKIAHDLEWQHFPRDSNLSIIVTRTRISCKLTAGRFLTL
SFPCYTTILTTTISYISVLAAFK
>MphaOr114
MEHQVYADKEYDNLIKPIMIVTKIASFWPLERDHSTSMKLFKICHVLFLVALFTIMCTVIIADIIHHLNDLDE
LTACALTACAFFLDIIRLIVFSTHQKDMSYVVETMRKDWTCSVHENRAILKEKCLFAFWVSKCFITMVTVTII
IFACSPILETYFHGKERIFPYRGYFFANQTVSPYYEYIYIFNIMGGLFGCSLICGATTFNFIVVTHGSAKFAV
LRKNLEAINSNNSDANIAMVNCIKEHQDVIAFADALERIINVLALAQFILSTGLFCFAGFQITSLLQDKHRLM
QYTMFLNSAILELFMFSISGNALIDEANSSDAVGESAYCSRWFGGTFGRNLQIVMMRSKVPSKITAAKFYSMS
LRSFSQYTILSIKVSYIKIIK
>MphaOr115
ISMDFQNVNSLNVKLNLLSGNLLPISNDSPFPFFWKIYSAFTWLLQIILGIVLILGCISNVSLEKIISDTIIT
FVVLIEVLFMIMRIYIHKDLIYLLIQKLNQILYTADETMKNIVTVTLEPIKVPLNIYWVTGAMSTFTWSCMPL
LLIFERNQFYYEDYRAPVVFSKQSFSLSIFLLGSLFLTISMTNMFLKKASVDVYMMHLVLMITAQYRYFALKI
AIIFQEENKGDDFQEKYRLNRKKEEEIKVLCRHHNDMIYISSLLKQLLSLNFSLFYLTSIFRFCFIGIMASNV
TVTTFWEAISIVMYTTGAIMQLYITCSCVQQLLDASTEVTDKAFHEKWYLLQPSTKCIFMLIIMANSLECKIA
AFENFNLSLSSFMKILNQSYSIALLFLRM
>MphaOr116
MDFQNRNSLNVRLNRVSGNLLPMTSNDSPFPVAWRIYSAVIWLIETIYISAIIPGILYVPIDKALRDGTVGMV
VIIEVFFLLRQMHVHRDLVTQLIQKLNEILRVGDKTMKFIVDSTLRPVDVPLKFYCIAGTISLAMWCCISFAS
ILKKKYFFYEDYRVPIVLSKQPFSIEIFLLGNCIVTIASVYIFIKKVALNVYMINLVLLVTAQYRYIAVKLAT
IFRENILQSQHDEFEKEYSNVNSLAILKLKALCRHHNAIVRITLMLKKLLSLNMSLIYLTNVFILCFLDVMLI
SAILSMDFLEGIMIVMYISGALVELYILCLCVNQLLDASVEITDKAFHEEWYQYESSLKHMFRMMIITNNLGC
KLSVSDKFNLSLPSLMTILNQSYSFALLFLRIK
>MphaOr117
LQLLDTIAIRHDFFAVIHSLPLILGTIIIINKTICSIYNLPQIKILLTMMYEYSISLKSDEEIKLHNLRVQNG
RKLCNAYMGFIVSHSILYYIISSLMIFYTKYIETEELSNNTLHDQISGIPNSINSTLVDEDTESLPIFIYSIV
SDCVIMFTITVSDVLYVTMTEYCCGLFAALRYRLEFALEFDNDELTMLDNKSYLNVVYSIRRHTETIQFVTNL
EAIFSSNLMVHVGLLVLILACLVYEVTVIGKVMGTNYIIQTIAYLNFILLNIFFENWQGQKIIDSSEKVFESA
YNAEWYSMPIASRKLLIMLMMKTEKPLMLRMGKLVVLSYVTFNAVLRTSSSYFMLLQSL
>MphaOr118
MHDIDQFFQDSDYNILRVLLGAVGLWPFHTRNRRYTIYIVIMLLLGSALLFQLLDIIAIRNDFFAVINTIPFI
LHFIIVINKAICSIYKLPQIKILLIMMYEYRISPKSDEETKLQNLHSQYGRKLSYTYMGCLIGFYALYYLIDT
LSVRTIRTKYNETEELSNKTLHYQISGISSSINSTLVDEDTESLPIFIHFFMCDCVAIILIIVSDVLYVTVTE
YCCGLFAALRYRLEFALIFDNNELTITEDKSYSNIVYSIRRHIESIQFVTNMESVLSLNLFVQTGSFVLIIAL
LVYEVTNIGIFMGRSYIVKAVAYLNFVLINVFFENWLGQKIIDSSEKVFESVYNAEWYSMPIITRKLLIMLMM
KTEKPLMLKMGKLFELSFATFNTILRTSSSYFMLLQSL
>MphaOr119
FEWATKLNRLTLNIVGIWPNIDEDASDRFLSNLRAMFTLLLFIFVGMIPTVHSLIRTWGDDMMTTIDNLQFTL
PLITTTVKLVIIWWKKPDLILVLNTIADDWLKAKTNKELRVMTKRAQNARVITIFGYIFMCGAFGFLVFLPCF
GTSLRYITNVTDPIKILPLQSHFLYDKDQSPYFELTFAAQTLLLIMASACYSGVDNLLGLLVFHLCGQMENLK
ERLINIKQLKNFNGNLAFIIKDHVRLIKYFNIVENTFTLLLLGLLLYFATLFCVYGFLIIAILTEGREMSTIR
LIYLLSAVLTVCGHMCLYCVVGEILIAQCEGIYHAAYNYEWYMLKPEEARTLIIIMVRVNKPLNITAGKMFPM
TLSMFCNLIKTSGGYVSVLLAR
>MphaOr120
MHTLPLSFALLTYTGYWRPVHLTSVKYWAYVVYSIAMIFLLYSFTVCGLVDCFIIKDLDTFIEKFSLFLSVFG
VCCKVTNLALRRDEIIDLTDMLLKDICIPRNEYETNIQRRFDRNAKIITICCEILNESAVFFATVAQFRYFVS
TRTLPLSDWVPYDISSTTVFWATMMHQTIGLIVCANASVAHETLISGFMIQTCAQLDILCHRARTLSDSLQEA
QRRNASKEDLKALERRLIHELIHHHRYVYRFAERINAVFTLMIFVQFTVSSTVLCLSIYKMLTKNLLSLEFAW
SSSYLGCMLMQIYLYCWFGNEVTLKSTKIGSAIYEMDWPMLPTDLMKTLLVIITRSKRPIQITSGYIITLSND
SFMKIIKISYSAYNVLQGS
>MphaOr121
MSILIFTFQILTICGCWPPNSWTSRYKRIIYDVYTIIIVLLIHTFTLSQLMDLILTVDNADDFAENFYVTLAN
FVACCKMFSLLRNRNNIAMLIDILMKKPCKPTEHDEIEVRKKFDKLRNSKIILYRTNTLCYAILIEMTCAFAL
ATSVFKDYRKHRLAFRAWLPFNYTSPALFRIAYVHQSISLTMGSILHIACDSLICGLLMHVCSQLEILECHLK

KIIDKPYFLRECVVQHASIFQFAFMVNEKFRLTITIQFLVSMLVVCFNLYQLVQTSASSAKFIQIILYMFCML
TQISFYCWYGNEVKLKSRQLISDVFQTKWFGLDYHLQKSLLIIMTRCTIPIEFTSAYVISMNLESFVSLLKTS
YSAYNILQKM
>MphaOr122
MCLLGFTFKILSSCGCWIPDSCTSPQRRLMYHVYTIFILLLINTFMLSQLLDIILIVDNSDDFTDNFYMLLAM
IVSCFKMFSLLINRSNIAMLTDILMNKPCRACNPIEVEIQQKYDKLIETNTLYYMILVELSCAFTAVVSLLTD
YRNEKLTFRAWLPFDYSSSSTLFQFTYFHQLISLTVGSVLHIACDSLICGLLLHICCQLEILSCRLKNIVHNP
KILRDCVIQHNLLFKFAFLLNKKFRFTIVFQFIVSTLVVCFTLYQITKTNSGKFIELVMYMSCMLIQIFLYCW
YGNEVKLKSLQIISDLFEMEWFTLDQNTRKDLLIIALRGRMPIEFSSAYVIPMNLNSFVGLLKTSYSTYNILQ
QTQN
>MphaOr123
MQILDLTFRILMICGCWMPDTWTTPYKRLVYYVYTIFIMLLVHSFMLSQLMDLILTVDNTDDFTDNFYMLLAM
FVSCCKMLTLLINRSNIAMLIDILVRKPCKPVQSDEIEIQQKFDKHVQTNTLWYAFFVETTCLCIAVTSLLTE
FRKKRLTFRAWLPFDYSSSSLFRIVYVHQLISLTAGSILHIACDGLICGFLVHICCQIEIIECRLRKVAHDRN
ILRQSVLQHDNIFKFANLVNEKFRFTIIIQFIVSTLVVCFNLFQFTKSTGLKAQYMQLIMYTGCMLTQIFFYC
WYGNEVMLKSRYLVNCIFEMEWFGLNKHTKQSLLMIMRRSSKPIELTSAYVISMNLDSFVGV
>MphaOr124
LEWAIGLNRCLLKIVGLWPEKNKEQRKEFLSKIRLLFNIFTLIFVLTIPALVSLIRVWGDMILMIDNLQYTLP
LLITTLKVFIMWYKKGALSPLVNMIVKDWMKVKMKEERNVMLRQAKIIRLLAMCGGLMILCTLLVTVCSFLLG
RTLRLVTNLTDPDGKPLPIQTYYLHDVSTSPKYEITYLIQIISLTLSGVSYTAVDNFLGLLILHICGQMENLY
LRLLNLGKDPNFQAVLKYNVKDHVRLIRSIKVIDNTFNLMLLGLLLLFSILFCLHGFLIINVVNRGGKLSFMQ
FILYLSASVCVLMHMCLYCAVGELLVTQSEKLHRATYEYVWYTLEPKAAKNLTLIMLRAKKPFNITAGRIFPM
TMSTFCNLLKTSAGYVSVLLANRD
>MphaOr125
MIPISTVSRPVEIGLRLTGIWPNSPIFFRLLWTLVMGTGLIFQYHYLLTHFSTKDLPNLIDGLSTTLPYSLLF
FKLIVLWVKNRIFNNILTAMSNDWHEYSGMYTMIDKAVLAHRCSKLTIGVYSTAVMLYSTASINFRKQSNNNC
RELLIKMELPFEFCESPIYEIVAGVQFVHLMAVASAIGMLDALMVTLMLHIGGQIDIMRQEADEICPKDNKYD
LPVAIVKSLISKHHKIIAFSESIESLFSHIAFMQFFSNTIIICCIGFLIVTSLGTDEGVRMLVKTMFFYIAIT
LEAFIFCFAGEYLSNKSKTIGDAVYESVWYNLKPQDCRILLFVIMRSQRRLTITAGKFIDLSLEGFTNSLKAS
ASYVSVLYAMY
>MphaOr126
ESIYSHYYDIVYKISSLTGMWPYLKTKIRIFRIALLTIILLTILVPQIAYQFMCKKNLQCTFQAMTAYLLSIV
AVLKMYTFQFNVHTIKDLTRHLFCDWKELHTSEEYEIMKSYAANSRRFSVIYSVYCFAALLVFMSMSLVPHIL
DIISPLNESRPILPPYRGYYFVDIRDHFFQIFWHGVVAWEIVIAGIISHDCLFVTYVEHVCSKFAITGFHYEH
LFQDCSNEMEIITSMKLDAMYSKRVAFLVRKHLEALEYAQLLEDTFTIPFAMQILIATVGMSITLLQITQDTS
DILEATRYVFYIIGQLIHLFFLSFEGQRLIDHSLQVRDKMYSSSWYKASIKLQKIVTLMMMKSLHPSFLSAGK
VYIFSLQSFTMVLQTSMSYFTVLASFQ
>MphaOr127
MTDRKDIWKTRYYLILRTYMTISGIWPYSRLRKRYIRFIPTFIFCFSILIPQLLYVVIAPNINEIIESVTAAF
ISIIFSVKVASIMFDKNVKSCFKIIEKDWQSLKTDVERSILQHHTEYGQYLTTSYAVFMHMTQVFYLLKPVIL
TLLETDVTNSTSVSKLPFRVEYGVDVDRYFYPITIHCYLAVFAHVFSTVAVDSLYCTPIQHACGMFSIIGHVL
EEIGKNNDANFSINLNKIEDDNYKKAFDCLRRHLQVIEFADLIESTFTKMLFLSVNLNVIGGSMTGIAMVMNL
GKGFHEIAAPMAIYIAQLVHIFLQFWQAQFLLDYSTVPCEAIYRANWYYTSKRCQKLFLLIMTRTISPCKITA
GKIVVLSIEGFGTVLKTMMSYFTVLRSFQ
>MphaOr128
MEFFVDQQYRLIRLILSIYGLWPYDSQTINTKIKWVFTTFCLIFIAVGQIIKYCKTDLTIDFVFECLPVFIIS
ISAIQQYVMCIVINDKLKGLLEQIKIDWKCARSQDEINIMQINTTNVKKATKMFLVYILVSLNIYMLINFMPQ
ILDVFMPLNESRPWRHPIQIELFLDDERDFYTVRLLQYIGITYTAVFLFANGTMFIINTQHASGMLAILGHRA
EQLFNDKQKLFKSQFNWEKDYKNISIFVEDHRSTLQFVAIIQSCYSLKMFLEFFNLMFLLGTTLVQIIKFKQS
ERLRAIFYVCAQIIYMFMCSYMGQQLIDKGTQLFMKIYFSRWHSTVVWKQRMIIFIMLKCMRMITINMYNTFI
ISLQTVSGILQFAMSFCMVL
>MphaOr129
PVLKFTLTVLAVAGCWRPISWTSLFKYIIYDAYTALIIILILYTFAITQVMGLILNPDSETFGDALFNSVISLL
ACYKAIILRRNHDSITILIDNLVEKPFKPIDLNESIIREKFDKRITNNTLCYLVLVFVTALYMIIISLFTDFK
NGILMYKAWLPFDYSISVLFYFAYIHQILTLICIGLVHPTCDNLICGLLLHICCQIEILEYRLSNIANGQQNL
RDCVRHHIRIYEYAYILNDTFSKIVPSEFAMIVIVMSYNLINMAFKSSSTASYIQDVMVVASTLFPIFYYCWF
GNEIKLKSLQLSDSIYNIEWTIFNNNIKKGLLMIMNRATIPIEFTSADIISVNLDSFVLKTSYSLFNVLIRSQ
K

>MphaOr130
MICTKTQYFNLNRILLLIAGLWPYQRSKFIHLQCIFFFIILTTMILFQFTTFISSKCTFNFIIKVGCNLSIFI
LCIITYIAFAVNTNAIKNLLMQIEHIFDELKDEPENVIIKKYGQSAKRYAVMLIIILAFCGICIFTVVQFLSII
LDTVLPRNGSRLHHGLIVTEYFINKETFFYLIELHMCAALYIGIISLVSTGTLIIAYLQYVCGLFKISSYRIE
RAMDFNLLHNITLKNENLIFERIIYAVNIHRQAMRLSKLMMTKIEIMCCCLIITGVIGLSFSLFRIFQIVSSE
ENLKEKLMPFLVTICIITYMFVANYICQNVTDHNNHIYITAYDVQWYMAPLHIQKLILFLLQNGTKDLPLRVG
VLFTGSLEGFATVRK
>MphaOr131
MKRETSSETTVDVYRRDNDYSLQFSRWILKSIGAWPEFSTNSITKNVLMSILRLMCHSLIVFTIVSSILYILF
EEKDFRLRLKAIGPTSHILMGGINYCSLLHHNNRIRVSIEHMETDWQTVKREHDREVMLRNARVGRIIAAVCA
LIMQGGVICYNIARGMSRITVVIGNRTIETGRLPCPSFNKIVDTRLSPVYEVVLALQCLSTIVVNNITIGACG
LAAVFAMHASGQLNVVMLRLEELVAEKQDLQLRLADIVEHHLRALRFLSHLETIMRQICFVELVGCTFNLCML
GYYTITEWHEESINTIITYIMVLTGMMFNIFIFCFIGELVTDQCKKVGEAAYMTNWYQLPHKTVISLILIIILR
SRIVIKITAGKIFHMSIQTFGLVIKTSVAYLNMLRTLTM
>MphaOr132
MLHSFRVSSTRNIAYQKDSEYSIQLNRWFLKPIGAWPGTNVGIADKVLSRGIQFICHSLIAFTVIPCVLYIMF
EPNVYLKLKAFGPMIHWLMGGANYCSLLVRSYEIRKCVDHMRADWQYVGRTRDREIMLQNAKFGRFVSAFCAV
FMQGGVCSYSIITTLTPATIQIENVTMTTHQLPCPFYTELVDTRYSPANEIVIILQLLSTVIVNSVTVGACSL
AAVFAMHACGQLSILMMELNELVDETKSKYNVVQRKLAIIVEHHLRILSFVSQIETIMHQICLVELLGCTIDI
CMLGYYTITEWKLHDTKNLLTYFTIFVAMSCNIFIFCYIAEILTNQCQKIGEMAYMTEWYRLHHKIALDLILI
ISRSNVVIRITAGKLVQLSIATFGDVMKSSFAYLNILRTVTT
>MphaOr133
MTDKQYQAYQTYQRFIRIWLTIGGCWYMPTKSGKPTYYWPVFVLWLTIANTVIKMNLIYISRHNLAHMMKFIG
NSLSTIGSLIKVISFLINRGSLIKYHQTLNESFEEELMQNKKIRTMILSSQRKIYIVTYTYITIITAFVLMAY
MFPYIYIIRDLCHLRLTTNYTLPMSKGYGYFWPVPDNFLYHFYLLIKTILVMLAVTTSTGVDSIFGLYVVQFV
STLRFMTFRLSNLSIESFSDQLRTCIKKHQKLLQCRNTLEHIYGPIILWHIITNAVLICTLIYEMMQSSNFNL
RNLFVFLIYTSIKLFQMFIYAWCGTILTNASDNFRNGIYFGKWHNSNLDHHVRVNVILMLMQKPMTIYAIFSP
VDVVLFGNLINTTVTYFFLLKSVSDK
>MphaOr134
MYIFEDKKFNRCQKLNKILLRLIGLWPCGRTKYDRFRAICCFTFLISCLIIEFAQFVVFDFNVNVAVRILSYT
LPTFLCFLNFNMIFFGAKELKQMFEEVSNTRRMLTNVQEIEIIEYYIHQGEKITILLTSGRLFFLHVPLQTIT
IFSFLALELLPDVFDFFKPINKSRSHYLIDVTEYHINEGIQYYFFFFCAVTSILIASSATILVISIILSVCLH
CCALFKICSYRIKQFVDEKVITSSNKRIVIVERQLQRIVELHQKTKHFTISYLSILVIGICSFAVSLYSLLQA
ITNDTINITEVLTAIVSFIGHKLILFLVSFGGQLLINHADDLFIAIYMSLWYETPVTVQKLLLFIMQISSKSL
VISCGGIFVIAMETFASITRTAISFMMVISSI
>MphaOr135
MYIFKDEKFNRRQKLNEILLRLLGLWPYEKTKCERFRAMCCFVILISHIITELAQLIIVDFNANVITRILSDT
LPTTLCFINFNMIFFGTKELKQLLEEVINTRQMLTDAQEIKILEYYAHQAEKFPIIIIRKIVSFTSTVNIIST
LQAFTILSLLIMELLPDILDFLRPLNESRAHYLIVMNEYDINEGIQFYSFLLYTISGIIIGTSTIIFVISTIL
SICLHCCALYKICSYRIKQFVDKEIIASFNKRNIIVERIIRIVELHIKAKNFTISYLSMLVIGMCGFAMRLYR
LLYIITYTHDSVELLIEAAYVIGCEFTLLTISFIGQLMINHSDEIFNTLYMSLWYEAPVTIQKLLLFIMQISS
KSLVVNFGGVFILAMETFTSITSTTISFMMVISSIQ
>MphaOr136
MTDKKDIWKSRYYRILRTYMSLLGIWPYDRLCKRYIRFIPTFIFSFSILIPQLAYTVAIASDFNDVIDSVTSA
IISIIFSFKIASIMFDKNVKTCLKIIEKDWLSLKTNVEKAILQRYTEHGQYLSTSYAVFMHTAQVFYMVKPVV
LSLLENDMTNSTSALKLPYLVEYGVDIDRYFYPITIHCYLAIFVHISSTIAVDSLYYALIQHACGMFSIIGHV
LEEIGKNNSANFSLNLNKIKDDNYKKVLDCLRKHLQVIEFADLIETTFTKILFISVNLNVIGGSITGLAMVIN
LDKGFHEFAAHLAVYLAQFIHIFLQFWQAQFLLDYSTVPCKSICRTNWYYTSKRCQKLFLLIMTRTTSPCGLT
AGKIVTLSIENLGTVLKTMMSYFTVLRSFQ
>MphaOr137
MIRSVIQHHKFNRILLLAVGLWPYHQSKFTRIQFIFLSVILSASVIFQLTPLIILKCTSDLIIKVLSSVSFFM
LFLIKYNSCHLNIEAVKYLFMELQDVFNNLKDENEIAIVEKYIYVTKRFTFVLTAFAVCGVFACIMAESWPII
VSAVSLNVSQSRHIPIMTEYFIDREKYFYLIILHILVTICIGTVIMLGTGTMFIIYLQHTCGIFEIASYRIEH
AININITQKNKILMTEGVIYGVDMHRQAMKLSKHFISTFEIMIFCLTGCTIICLSLNLFQLSRIASSKNNITE
FFIPYMYASTSIAYMFVANYIGQNVTDLNNNLFITAYNVEWYRTPLHVQKIILFLLQRGTKKFILKVGGIVDG
SIENFTMVAKASISYFTVM
>MphaOr138

FKYNYSLNRILLLATGLWPYQRSKLTQFQFILFCSILLSLVVFQLSPLIKLKCTTDLVIRVLSSILITIMFVI
KYSGYFMNLDTVKYLMMQLEHVHNKLKDKNEITIVYEYNCIAKYYTVTLTVFGACVLFACPIVEFWSNINIST
NVSQSHLIILLPYFVEEKYYHLFLLYIFTIVCIGTTILVGTGTMFITHIQHICGMFKIASYRIKHAININILK
NITLKNKVLMTESLICAVDMHRQAIRLSNYFVSKFDLMITCFTACLVISFSLNLFQLSHVQLSVNNISQIFPS
FLYANISIMYLFISNYLGQIVFDHNNHVFVTAYNVQWYRTPLHIQRIILFMLQKGTKGYFLKIGNIFDSSMEG
LATLLKASISYFTVINS
>MphaOr139
KEEYFSINKILLLMIGLWPYQQSNFTRFQHILVIITLIAIIIFQLTVFLTLSCTLDLLMKILSSIFFFAIFII
KYNAFCFNIKGVKNLMMQLQNVHNRLKDQNEITIMRKYNYNAKRYTIIITTLGICGIFGDIILQFWMNMVDNS
AKNSSQSFHFLITTEYFIDQKKYFYLIFLHINAAICVGVISIIAIGTVNIVYIQHICGMFRIASYRIECVINI
NNVSLKNENWMAKGLIYAVDIHRQAMILAKYFTSTFQKMFACLIVCGVALCSLSLFQLSSLKNDIQKLFISFL
SVLMIIIYMFLTNLMGQNITDHNNYVLTTVYNVKWYKTPLHIQKMILFLLQIGAKEFTLNIAGLIHGSMEGFA
MLTKASISYFTVIHSL
>MphaOr140
MIRLEVQYFNLNRILLLAVGLWPYQQSKLTRLQFIFLCSILTTSIIFQLTSLITLRCTSVLVAKVVSSASFFA
VCLVTYSSFCANINNAKNLMVQCQHVYDELKDEKEIAIIKEYGYYTKCCTIAFMVMGIHFIIVIIIIQYWTDT
FDLALSINVSQPHRIQFITEYFIDQEKYFFLILLHINVAFCVGIVAIIAIGTILIGYINFIFGMFKISSYRIE
RIIEINIPQNFILKKKNFKSESLICAVNIHRKAIRLSKYLVITFEIMFLSLAAVLIISLCFNFLRIFQIMSSG
ESIEKLFFPVTFVSVDILYMFLCNFFGQNIIDHNNHVFITVYNIKWYVAPLQIQRMILFLLLNNAKDFTIIVG
GIFVGSMENFAMLIKAAISYFTVILSV
>MphaOr141
MISSNERYYNVYRVILTAIGLWPYQTPIVMQIQALFFLGVYCFILVFQFTTFLTATCNLEFIFTELSYIFITM
LYIVTYNSYYFNSKGIKYLLEQIKLDWNTIKDNNEIRVLKKYAFETHLLSLILIFFVASAVFCIIIIELIPVI
LDAVTPMNESRPRKIKINFEFFIDEQQYFYIYLIHEIILVIIGMFTIIGTGTLSMAFLKHCCATCKIASNLIE
NTVTKHTLSIPTCQKTHVMYQKINRAVHIHRKSLQFSTFLMNIMNKWFFFMVMISVMSLSCNLFRLLNALTAL
NKFSEIIMSIGLVVANFIYIFVPNFVGQSFTDHSAKIFNAAYNTMWYLAPLSIQKLLLFVMQNSLKAHNLTIG
GVYISSLEGFSTVINI
>MphaOr142
SSNRMVFTWERCYKFHQITLTILGLWPYQKSSVLRIQTIFFFSMFFAFLVFQFTIFVTTECDMEFIITELTYI
FPTIIHVLSYNCFYFNSKIIKQLLEHIKLDWKTMKDNELKIIEKYTFNCYMFALYINILAGLGMLIFTIIEFI
PVILDAIAPLNESRPRKLKVSYELFIDEEEYYFLYLLFEVLTVTVGIWSTITTGTFFIIIGGHCCATLKIASN
LIQKAVTKNLLQLPAPHRIYFMYQNICRAVHIHRRTIKIMSTLLDSIHMWYFPLLLFGVISSSCIIFRLLNAI
MRLNNLSELFVPFVLFIGHFVYMFMANYCGQIIIDHSAEMLKAAYNTLWYVAPLSIQKLLLFLLKSVKDFKLE
IGGMFVPSLEGFSTLVTSVMSYFTVIYSM
>MphaOr143
YKMHAIERYYKINRILLKTVGLWPYDQSYVLQLQKVLFISIPVTFIFVQLLAFITKQYNTNLFLQILSFIFPV
LINIIKYCLFIIQADNLKQLLEQIQADWNSLRDKLEINIIEKYACNARLFTIVLIVYCHFGILFCGIFQLLPM
ILDIIVPLNESRPYQLFVVTEYFLNEEKYFYITLLHEALAYTIGTTTLCATGAMIMICILHACALFEIASYRI
ENAIQKSTLMMPSSRREYFLYRKIVYAVVMHQRATKFIELFTSSFAPLFCILIIILGVSSLSFNLFQFLQLITL
TKNICQTSVVAIFILLHLNYMFVANYGGQELLNHGLKMFKATYNGLWYAAPLRTQKLLLFIMQKGTTNIGLTF
GGIYVASLEGFATLVNATVSYFTLIYS
>MphaOr144
SESVRPHQDASDYSIQLNRWFLKPVGAWPSSASTTIREKVISTILIIICYSLISFTVVPCILNILFEETDVYK
KLKAIGPLSHWCMGGMNYFSLLFRNRDIRRCVQHMKTDWRTVTESKDRQVMLKYAKAGRLVAGLCAIFMHGGV
FSHSVLQGTTPTFECIGNVSVSVLVLPCPSFSKFVDATQSPAGEIALTLQLISSIVVNSVTVGACSLAAVFAM
HACGQLNVLMRWLDQLDDRKQQDSVQKRLAIIVEHHLRVLSFVAQMEALLNQICFVELLGCTFNLCMLGYYVI
TGWNVIEKKTSIAYMIIYISMSFNIFIFCYIGEIVTEQCKQVGETVYMTNWYHLPHKTARGLILIISRSSNVI
KLTAGKLVHLSISTFGDVMKTSMVYLNMLRTMTAS
>MphaOr145
YSERVLTNIKFTYKSNNDYSLQLTRWFLMPIAAWPRPTATTTVERISLQAQVLACSSLIAVVMIPCILYVSLE
EKDVQIKLSVMGPLSHWIMGTINYWLLLTRSDDIRECVQHMEMDWRLVRRTDDQDVMLRYAKIGRFVAGFCAV
FMQSGTFLFVVAKAMTSITIIVGNETTSMHPMTCPIYSKFIDTRFSPTNEIMLFVELLSCFIVNSITVGACSL
AAVFAMHAYGQLSMLFSWLNNLVEDEKKANKLVERKLATIVEHHLRALSFISRIENIMQNICLVELVGCTLNM
CLLAYYSITNWSDFDAAKITSYIIVYVSMAFNIFIFCYIGEILTEQCKKVGEKAYMINWYELPHKTALGLVLI
IARSSNVIKITAGKLFQLSIATFGDVIKTSVVYLNILRTMT
>MphaOr147
MTYSEHVMTRSIELPYESDYSLQLARWFLIPIGAWPRMCTATRVERFSSHVHVLACTSLIAIIMVPCLLYVSL
EEKDIEIKLSVMGPLSHWIMNMINYCLLVTRSDDIRECVLHMETDWRHVRKIEDRQVMMRQARIGRFVAGFCA

VFMQSGTFLFVIAKSLSTVTVIVGNETLSVHPMTCPFYNKLIDTRFSPANEIMMIVEWLSCFIVNSVTVGACS
LDAIFAMHAYGQLNMLFLWLNKLVVDEDKTDECVEQKLAIIVEHHLRVLSFISRIETVMRYICLVELVGCTMN
MCLLAYYFITNMDSFDAAKSTSYVIIYLSMAFNIFIFCYIGEILTEQCKNVGERAYMINWYKLPHKTALGLIL
IIARSNNVIKMTAGKLFHLSIATFGDVIKTSVVYLNMLRTM
>MphaOr148
RSIELPYASDYSLQLVRWFLIPIGAWPRMCTATKVERLSSYVHVFACMFLIAIIMVPGLLYVSLEEKDSETKL
RVIGPLSHWIMNIINYCLLVAGSDDIRKCVLHMERDWRHVRKIEDRQVMMRQAKIGRFVTGFCAVFMQSGTFL
VVIPKSLSPITVIVGNETLSMHPMTCPFYTELIDTRFSPANEIIMIIEWLSCFIVNCVEVGACSLDAIFATHA
YGQLNMLFSWLNELIVDEDKRSECAQQRLAIIVEHHLRVLSFISRIETAMRYICLVELLGCTMNMCLLAYYFI
TNIDSFDAAKSTSYVVVYLSMAFNIFIFCYIGETLTEQCKNVGERAYMINWYELPKKTALGLILVIARSSNVI
KITAGKLFQLSIATFGDVIKTSLVYLNMLRTM
>MphaOr149
MCRSVTKDLHDHSVQLNRWFLKPIGAWPRASATSIGEKTLSRILIFTCYSLMMFTVIPSALNVFFEEKDTEMK
LRAIGPLSHWLMGILNYCSLLLRNVEIRQCVQHMEMDWRIIRQPRDREIMIRNAKLGRFVAGFCAIFMHSGVF
AYSLVSGTTKVTVSIGDNQSVSMLQLPCSFYSKIIDTRFSPANEIVLVLQLVSGFIVNSTIVGACSLAAVFAM
HACGQLDILILRLDMLVEGKGAKKRESVHQRLANIVDHHLRVLKFIARIEDVMHQICLVELLGCTFNLCMLGY
YSITWWNKIDKKGIIAYIIVYISMSFNIFIMCYIGEILTQQCKKVGETAYMTDWYRLPHKTALGLILIITRSS
AVIKITAGKLVQLSIVTFSDVIKTSLVYLNMLRTITT
>MphaOr150
MDFQSTNPLNTWLNLLSGNLLPITENSSFPLFWKIHSAFVWLLELIYIVTFVPAYFLVPKDMSDGLNGSVIID
ASDMLLEYFQIFAQITLVKELIRKLNKALSIEDENMRHIVIMNLKPLKIPFTIYLVTGIFIAFSWLCMPFLLI
FEKSTFYYTDYKIPAIFSKEPFSVGVFLLGNVIILLNGVYVFLRKASLDVYTAHLISLVSAQYQYVSLRLVLI
FRSNEQQDNACCREHNSKMDFFVAKEIKKLCRQHIDVMNLTSMLKKLLSVNIFFIYVNSVFRICFLGLIAIKL
ISSSLLEGFMAIIYASGIITQFYVLCACFQKLSDASSEIANKAFNEDWNRFNLSIKRAFLLMITASNNIEIKL
SLYDKFNLSLPSFMSALNSAYSIALLILKI
>MphaOr151
LDLLPINFYIFRICGMWREREDGLIVRFVSFCTRYTIAALIYFCTLSEIIELVRKRNNVEDFTEALFLTMSFV
TMCFKYINFFMQQCELRVLLDCFRTDLCQPRDSMERSILKQYSRKAKQTTCIFMSLCQSTGLLILAVPLFTKD
KRLPMKVYVPYSIATFLSYVLTYLQQCAALIFGILVNASFDSLVYGLIIHTCGQIELLCYRLTEAFRCLQKND
EKQRHSAIENLAITECVEHHILVYDIISRIQSLFVWTTTSLFMLSLLTVCTSIYQISKKELFSPEFITFIMYL
VCVNSQVFIYCWYGNELDLKTKSITHAIYNSNWVIISAKQRKNLCLVMMMSQRGRIISSFGFCALVLNTFTWV
>MphaOr152
EWAVKLNRIILEIIGLWPKAAQNSRQKLICNFRVLIVFLAVTCGVLIPSVHSLIRIYGDIMLMTDNLLFTLPA
ISCSIRIAIFWWKKEAVIPIINMMAEDWIKSKNAQDKNIMIKRAQTARIIITCAYYIMGIACFFIIVLPGVFG
ISMRMTPNITDPGRPMPIQTYYIYDITKRPQYELTLISQSIYILLSIMAYTGIDNFLGLVIFHICGQLDILKN
RLIHLDKYTNSRDTLKKCVTKHIRLLSAINIIEDTYNITLLMLFVYFAILFAFYGFRIINLFDGKNDLSVTQL
IYFITNVFNIFMHMCLYCALGEILMARCSEIYYAAYCNEWYCMDPKITKDFLFLFLRGTKPVYLTAGKVFPMT
MATFCSLIKTSAGYISVLHTTK
>MphaOr153
EWAVKLNRITLDFIGLWPETIQNPWQKLMCNFRAFVVFVAITFGVLVPSLHSFIRIYGNIMLMIDNLQFTLPA
ISCSIRIIIFWWKKEAIIPIIEMIAEDWVKPKSAHERNMMIRRARTERIILTCTYCIMIVGCLFTIVLPALGM
PIRLTSNITDPGRIVPLQTHYIYDISKRPQYELTYISQAVYIIILAVLSYTGIDHFLGLVVFHISGQLDILKNH
LIHLNKYIYSHKVLKKCIARHIRLLRAITIIEDTYNITLLLLFVYFAILFAFYGFRIINIFDEGNNLSLIHLV
FFISTIFNLFIHMCMYCALGEFLIAQCNEIYTAAYNNEWYSLDPKIVQDLLFLLIRGTKPLYLTAGKVFPMTM
TTFCSV
>MphaOr154
LLRYRMYTRRVRRMLYLGCVLQDRTRSATWSYITGFLVILMCFSQCVFLINFCRDHTDNLVLILKCFGMTCSF
IAPVLMSACFLVKRKKLMELHETLNELFERELARDQETKTTVLAAVCAFDRPSYVLCFTLGSTALLVLYPSLI
SIVHQIIRREEPKRYRLPLPNKFPWPVPVDGGVSFYLHLLYQIFTLWWVVITVGSVDSLFGYYAFQISSILRA
MSARLANPRIREVFTESLATCIQTHHRLLQCGHILSDIWGLIIIRMVVMNALLICALIFEASPFTHLTISKIF
LFISYMALKLLQTFIYAWYGSLITSASEHFRDGIYFSEWPESSLDRNIRTNVILTMMQKPIIIKALKLSSVNV
NMFTTIVNTAMSYFFLLQSLDE
>MphaOr155
MARKSTFNRTLKFMLTLCGIWPGTPYVLLCRIFWVVSMAVTLFCHYRYFFTHVHSAEILDLMDCLSTFLAYSK
IIFKFFVFWLNQRKFVEILAVMTEDWSDCANSDISMREAARKAKISDRITNAVVTLHTMTIVAYCIGIIILADA
DVTDTTRELPLINKLEIPFDIKTQSMYRIVLITEFLLMILCGWAAGITNSLLLTLILHTAGQIEIMSHWLAQL
VPRIKNKDKHKSFATTTGKIIQKHQKIISFSKNIESLYSSIALLQFVSNTIMICSIGFLIVTAIGSPNALEQI

MKSFLFFTITNLEAFIFCYAGEYLNNKSNEIGIAAYNCEWYDLKSTESSFLLFIILRSQKQLTLTVGKMMDLS
LQTFTSV
>MphaOr156
MTRKSTINRTLKLMLTLCGIWPGTSCVIICRAYWIIALATDEICHYRYLLMHLHSNDLFDLMDCFSSFLTQVK
FSIKLIIFWLNERKFIEILTMMTEDWNDCNGSDVNMRETLCKAKLAARITNAMFTLHTLTIVAYSIGIFLADV
DVTNQSELPLLLKVELPVNINTERTYRTLLTMQFVHLIMSGCGTGLLNALLLTLTLHVGGQMDILRCWLHELV
PRENKGTAESVAFTTNKIIRKHQRVINFSEYIEDLYTYIALVQFTSNTVLICSLGFLIVTANSAIGSPDATEH
IIRSLLFYTVTNLEAFIFCYAGEYLKNKSKAVGIAAYNSAWYEMKPENSRNLIFVILRAQKQLTLTVGKIMDL
SLESFTSIMKASGSYLSVLLAMQ
>MphaOr157
MGSKRTISRTVEFFLTAFGVWPGAPIFYRMFSVFLLGTTVLLHCWYIMMHFNLDNLFNLMDCLSSFLAFLKVT
CNIIIFTLKYQKFNEILTTIMEDWDDCGNSDVALREVTNKAKLSSRISNGLILLHTFGGLAYVISILLIVIEV
DVTDQSMDEIPFMMKMEYPFVINTQRKYRLVLVTQFIGVMLCTWGAGLFNGLFLTLTLHVGSQINILLYSLTA
IGSKDIRKKDSFLTVMTKIIEKHKKIIILSENIENVFAPISLMKFLSNTTMICSLGFLIVTALDGPGAAEKIL
RSLTFFCVTNVEAFILCFAGEYMSNKSKEIGNAAYNSSWYDMNVKNSRLLLFIILRSQKQLQFTAGKMVVLSL
ECFTNIMKASGSYLSVLLAMR
>MphaOr158
MGSKRTINRTVELMLTMFGVWPGIPCVQLYRVFWIITLAINQYFHYLYFATHFHFDNLFDLMDCLSSFLAHVK
LTFKLVIFSLKQRKFVEILTMTKEDWDDCGNSDVALRETASKTKLSSRICNALIIILHTIAAVAYVIGILLADA
DVTDRTAELPLMMKMEYPFVIDTLRKYRLVLATQFVFVMVCSWGAGLFNGLFLTLTLHVGSQINILLRWLTEV
GSIDIEKKRDSFVTVMTEIIRKHQKIINLSENIENLYSYIALLQFTSNTVMICSLGFLIVTAIGSPDATEQIV
RSLLFYAVTNIEAFIFCFAGEYLSNKSKAIGNAAYNSTWYNMRAKDNRILLFIILRSQRQLKFTAGKMADLSL
EYFTNIMKASGSYLSVLLAMK
>MphaOr159
FMWAIELNQFGLKLIGLWPNMTVKNSFISDLRVGIIFFIVTFLSIIPLLCSFVRVWGDMILVVDNLQVTLPLL
VVSLKLIIIRWKRKAIALFIQMMAEDWMSLKIDAEREVMIRRAQTARLVVICGYSLMIFVFIVLIVLPSFGLH
FRYITNNTIQERLLPFQAYYFYETDKSPQFEVALIIQAMTMFLGAITYTSVDAFLGLVILHICGQLENFKHRL
VNLTSCTNFDNVLRNNIQTHQRIIRFADNIEDTFTLMMLGLVFYFGIVFCLFGFLFVSVITGDEIGHMSLTRV
CFLVIGIFTLLAHTFLYCGAGEIIAEQCEAVYDAICKLEWYKLEPRNEKNLILLMMRSNEPFRITAGKIFPLT
MTTFCSLLKTSAGYISFLLAKR
>MphaOr160
FTWAVELNRLGLELIGLWPKNNDVPRNKFASDLRIVIIFVIVTFVSGIPLACSLIRVWGDMILMVDNIQITLP
LLVVSLKLVIMRWKQTAMLSIIKMMAEDWMELRTVKERDVMIGRAQIARKIVIGGYVLMVLAFIMVIILPYFG
LSLRHVTNLTDLGRPLPLQTYYFYDTHNSPQFELTYLIQAITIFLAAVTYTSVDAFLGLVILHLSGQLENFKD
RVANLVSYQNFIYTLSNNVMSHMRLIRFTNIIENIFTLMMLGLILYFSILFCLHGFLLLTVITEESDFSFARL
CYPLISISILLSHAFLHCGAGEIVTMQCGKVYRAMCDLEWYKLEPKKARLLILLTMRTSQPLRITAGKILPLT
MITFCSMLKTSAGYISFLLANR
>MphaOr161
KRMDFESMNLLNSRVNVLSGNLLPIAGNNSQFSVICRVHSIFVWLTQIILTIALISGFILTPKEKALKDGMVC
IVVLIEASFMLSRFYSRQTLIREMIEKMNNILQNADEIMADIAKAAIKPIIMPFTIYGVTSGLSITIWTIQPV
LLVFEKSTFFYVDYNLPTAFTTEPFSSRVLIFSTIIMTLGSVYLFLKKFGVDVYMMHLVLMLTAQYRYTAAKL
MILFQDLQNSSHDKSQDKRHLVEDQWIEKELKKICQHQNTILHMSFILKKLLSVNFSLLYINNVFRFCFIGIL
LISIPSLSLAEGISVMSFATGSLMQFFLLCYSVQTLSDASTKITDKAFDEGWYQFGSPTKRTFLLLIMANNLE
CKIAAIGKFNLSLPSFMTIMNQSYSIALLVLRAK
>MphaOr162
KRMDFENANLLNSRANIFSGNLLPISTDNSRFSVIWRTHSVVVWLIELTHVIALILGIILSPKEKGLKDGTVA
VVVHLETFFMLASLYSRKKLMKEMVQKMNSILQSADEIMMDIVKSAIRPIIMPFIIYGVTSMISVAIWTIQPI
LLVFEKSTFFYVDYNLPAAFSTEPFSSRILIPTTIIMTIGSLYQFLRKFSVDIYMMHLVLMLTAQYRYMAAKL
SILFRDLQNCHDEFRNGFHSEKDRWTEIELRKLCQHQNIVLNMSLILKNLLSVNFSLLYVNNVLRFCFIGIMM
TTVPSQSFAEGMSVTFFAMGSLMQFFLLCFSVQTLSDASTEITDKAFNEGWHQFGPSMKRTFILLIMTNNLEC
RIAAIEKFNLSLPSFMAIINQSYSVALLFLRTK
>MphaOr163
EDLMLFQNHNYKADTEYVVKVAKTLLTPVGIWPLYRGNSKSDKIKNFLQTSVIFGLMCFLLVPHVIYTFFDAE
DLIRYMKVIAAQVFSLLAIIKFWTMIINRDGIRYCLQQMEIQYKNVECEEDRLVMTKSAKIGRFFTVTYLGLS
YGGALPYHIIMPLLADRIVKEDNTTQIPLPYLSDYVFFVVEDSPLYEILFVSQILISSIILSTNCGVYSLISS
CVMHSCCLFEVVRRQMETVLKDGTSDLHRRLGKVIKHHMQAIKFAEMIEKSLNIVFLCEMVGCTIIICFLEIG
VLKEWEDGKILEMGTYFVLMTSIFVNVYIISTIGDRLKEESEKVGESSYSIKWYNLSPEIVRNLILVMLRSNR
PSTLTAAKIFDLSLQGFCQVCKTSAAYFNFIRTVTT

>MphaOr164
MIAGMSPADDRALFRNFNYRSDAEYVVRVAKIMLTPIGIWPLYGGDSTFDKIKYYLQTSIVFCLMCFLLVPHI
IYTFFDAEDLTRYMKVIAAQVFSLLGILKFWTMIINKKDIKCCLEEMEIQYRDVETEEDRLVMMKNAKVGRQF
TMIYMGLLYGGALPYHIIMPLVAEKIVREDNTTHLPLPYLSNYVFFVVEDSPFYEILFVVQIVFSTMILSTNC
GVYSLISSCVMHACCLFEVARKRIETLLVGGTDNLHDQFRWIITHHLKALRYVEMIENSLNIVFLSEMVGCTI
IICFLEYGVLKEWEDNQMFGVIIYFILVISIFVNVFTLSTIGDRLKEESIKIGEASYSIDWYMIPTKNVNSLM
MVMIRSNRPATLTAAKMFDISLQSFCEVCKTSMAYLNFIRMVT
>MphaOr165
MDILPFNFRVLWFCGAWSEENNNNNNFFIRFLSFCYRYVIVILIYEFTISEVIELVRTHDDIEDMTEGLFLAL
TYVALCFKYGNFLARQNEISMLLDCFRRKTCQPRNFEEKMILLKYDRKAKWCVRTFMSISQATCIALILAPVV
GPQKTDRPLPFKTYLPYSISNFYPYLATYLQHVGAIFYGVLLNVSFDSLVYGFTLHTCGQIELLCYRLSKLFK
YHPDQYRINLGKGAMISECVRHHLYVHEIVRRIKSLFVWTVTILFIFSLVTLCTSIFQMSKKKILSVEFLSLT
LYLGCMLFQVFFYCWYGNELQLKSKGIGDAIYLSDWTTATTRDRRSLLFVMAISQRGLKLSYYGIFNLALDTF
TWILKTSYSAFNVLQQTSM
>MphaOr166
MDLLPLNFYVLRFCGMWKERKDRNLIVRFITFCYRYIIVALIYYFTISEIIELIRTRNDVDDLTEGLFLALTY
ISLCLKYLNILIRQCKLRELVNCFRVKHCQPKNSVEELILRRYHIKAKKITCVFMFMSQTTGLLMIITPLMVE
NKRQLPVKMYVPYSVAELFPYLLTYLKQVAGIFYGVLLNVTFDCLVYGFIIQTCGQIELLCYRLSETFRLLQK
NSEEKKDDTIEKFAIAECVRHHISVCNITHRIQSLFMWATTILFFCSLLILCTSIYQMSKTKLFGVEFFTFVL
YFGSMLFQVFSYCWYGNELDLKNKNIAYAIYSTNWFSISVKQRKCLSFVMMISQRGQIISFYGVCSLVLSTFT
WILKTSYSAFNLLQQASN
>MphaOr168
MEFLGYQYYKLYRIFLLSLGLWPYDSSFFKRIYMIFCIFTLLFSIITQFLKLFTTECNLVFVLTDLSFVIPCI
AYLLKYSTFCIQSQKIKKLMEHIRIDWTVLQDEEEIEIIQKYAKTARRYMYAFVGVAYPGTIVYISIPILPDF
LDIVSPLNESRKRYLPFLAEYFLDEQKYFYPILLHMAITIVIGVVTVVTTETLTFAYVCHVCGMFEIVSYRIT
CALDKSISILLNEENMIRVKLIGAVEIHQKAFKFFEYLISTFSLSYFILIIILGVVSLSLNLFRLFQTATLPDQ
RKDFAPYVIFVLAHFYYMFICNYMGQKVIDSSTDIFRKAYDTQWYMTPIQTQKLLLFVMQRTMKSCKIIMGNL
YCASVEQFTTLASMSLSYFTVIYSVQ
>MphaOr169
MMKNLTLKKVIAVTKISLFATWCWPLPKNTVKLKVICVRLYQYLCLILTLSLTAGLMNTVKNHVDEPLIMAKS
IIVMSPTVHVICNIICCKIYSRRLQLVTFEMENFSELLKPREEEIVQRYIDKCVYFYGGSILCIYLSAIVIIT
GPVTLDQPFPSNAEYPFVMYKQPLKSIIFVHQSFVFIQAAGQICMNVFIALLLWTTSARFELLTIELRAVTDI
HGLIKCIREHKKLLQYAKEVVTIARPFALSTISLSTFALIIIGLILVTNQPLSIKIQCVGLTFSGLSEVFMYT
WPAEHLMHISGEIRQAVFDTQWYKQSIALRKYLQIIMLKAQNPIVIAIPCVMPALSLNYYAS
>MphaOr170
MQRKVTLKEIIFIIKLSVLPIWCWPLSKNATKLKTFCMKLYQFWCIIIAIALELPLLYSVSNHINDAAFIIQQ
LVVSSSSIHIICNFISYQMKYHNIQNVTHEMEKFCDLMKPREKVVIQRYIDKCLVFYCAIMCIFYSVVIFMIT
LLPILQHHPFPTLAEYPFDVSYQPLKTIIYLYQSIVGIMAAAHLCLNVYIALLLWFTSARFEILIENIRETTN
IYQLFKCIKVHQELLEYAKEVVLIVRPFVSIAIFCGTFCTILVFLVFVTHQPMALMCQFTGLALTTLGEVYIY
AWPGEQLISMASINIAQAAFDIFRLKGQSIKMHKCLQIIIMKSQKPIIVSIPCFLPELSNMYFSMYCSTILSY
FTTLRVFILDE
>MphaOr171
MQRKVTLKEIFFILKLSLGPIWCWPLPKNATKLKMFCMKLYQFWCIIMAICLDLSLLYGVSNHINNIAYVVQQ
LLLSSSSIHIICNFISYQMKYHNIQNVTHEMEKFCDLIKPREKVVIQRYIDKCLVFYCTIMCIFYSVAIFTIV
LLPTLQHQPFPTLAEYPFDVSHQPLKTIIYLCQSVVGIMTSAHLCLNVYMALLLWFTSAKFEMLIEKMRETTN
VYQLFKCIKVHQELLKYAEEVIFVVRPFAFIAVSCATFSAILVFLVFVTHQSITLMCQFAGLFIITLAEVYMY
AWPGEQLISMSSTKIAQAAFDIFRAKGQSIKMHKCLQIIIMKSRKPIIVSIPCFLPELSHKYFSTFCSTIMSY
FTTLRIIILED
>MphaOr172
MIYTETQYFNLNRILLLIIGLWPYQRSKFSHLRFICFFSILATLIFFQLTSFVSSRKCTFDFIIKVVCSLSVF
IACITKYIGFAVNINNMKDLLMQLQHIFNELKDDSENAIIKKYGQNAKRITIILIIILGFCSMCILTVAQFLSI
VIDILSKNVSQVHHVPIVTEYYIDQEKYFYLIVLHICAALYIGIITLVSTGSVLVVYLQYICGLFRISSYRIE
RAMEFNMLRNITLKNENFIFKGIIYSVDIHRQAIRLCKLVVSKFETTLFYLIIIGVICLSLNLFRVFHIVSSE
ENVKEIFMPFIFSICIIVYMFTANYIGQDLINHNNHIYITAYNVQWYMAPLHIQKLILFLLQRGAKDLTLSIG
GLFNASLEGFATVRK
>MphaOr173
KYAYGWNRYTMIFIGIWPENRSLSQASSYTVLFPVFTMFCFICAPQSANLPYVWSDFDLLVDNLSTGNVTITI
SLLKTIIFWSNGGPLKTLMSYMSKDWSVTVNKQDRKTMLGVANITRKLSIRSTVLVIIVVLSYVGFRFREIQR

TGRRLIFQANFPYNTTSSPSYELTLFGQLVGTMYAAVTYTAVDTFIATLVLHICGQLSNLHRELISLSAYTKV
EFQTKLKNIVRKHEYLNRFAEIIEDSFNKMLLLQMLGCTMQLCFQALQAFMTIHFADKMDEMLMFKISFLFLY
VSYILLQLYLYCYIGEKLLVKSTQIAYAAYDCSWYNLSARDARSLVTIMCRARTPLQITAGRFCSFNQELFSE
VLKKSMAYMSCIYAMNS
>MphaOr174
MISVADKHFNLNRNLLLLIGLWPYKNSKFARFHLFCCYSAIASIIIFQFTAFLTSKCTTDHIIKICSLLFGYI
NVAIQYNSFYINSNTIKFLMEYLQLIYSDLKDNNEIAIFEKYGNISKRYTIAFITCLLCSLFILINAQLWPDF
IDIIFSVNESQPRRLSIAMEYFIDQEKYYYLMLIHFNASTCIGFFTLVATGSMLFAYLQHACGMFKIASYRIK
NAIEIYIQSINLKNKNLVYKSLIYGIHLHRKAIMFSEYLISNFEMSFMFLIAFGVLTLSLNTFRLFQLLSFKF
NIGEFSVACVATYSCFVYMFLANFIGQEIINHYNLIFITAYDVQWYITPLYIQKFILFLLLRGNKSFGLNVGG
LFVSSLQCFATLVNASLSYFVLMYSMG
>MphaOr175
MINIVDKYFSFNRTLLLIIGLWPYEKSKLAQLQVICIFSILITGIVFQLTTILTSQCTTELIIEILSSAIATS
GFIIKYNAFYVNNETVKYLMEEVQHIYDDLTNNNEIAIIERYGNKAKSYTIEVFIFFFFGAITFVGIQLWSHF
IHIVLTANVSRSRRLQIATEYFIDQERYFYLLFFHINTVMIAGCVIVGATGTILVACLYHACGMFKIASYRIE
RAIQANVKNNINLQNEFLAHKRMVRAVDIHRKALTFSNDLISTFEISFMLLIAVGVLSLSLNIFRVFQIVLFG
CNKEEFLVNFAIAVLITVYMFMANFIGQEIIDHNNHIFVTAYKVRWYTTPLRIQKLILFLLQRGNKSFGLCVG
GLFVASLECFATLANASISYFTIMYSMR
>MphaOr176
MINIVDKYFSFNRTLLLIIGLWPYEKSKLTQLQVICVFSILITGIVFQITPILTSQCTADLIIEILSSTVAST
AFFIKYNAFYVNNKTVKYLMEEVQHIYNNVTNNNEIAIIERYGNKAKNYTVGLSVSLFLGAITFVGIQLWSHF
IHTVLFANVSRPRRLHIVTEYFIDQERYFYLLFFHINTVMITGCFTLGATGTMLVAYLHHACGMFKIASYRIE
RAIHIYIKKSINLQNEFLAHKSMVCAVDIHRKALTFSNDLISRFEISFMFLIAVGVLSLSLNIFRVFQIILFG
YNKEEFLVNIAIAFLITLYMFTANFIGQQIIDHNNHIFVTAYEVRWYITPLRIQKLILFLLQRGNKSFGLCVG
GLFVASLECFATLTNASISYFTIMYS
>MphaOr177
QRIQFHNFKMLTIIYIIQYCFQLMSFVTIEYSRNLLTKVFSFSFVILICTVKYNMLYFKSEQVKYLFEQVLYD
WHALTDAEEIKIIQKFANKGRSYTIYTGLILYIGISIFVVLFFIPDILDIVAPLKEPRRHQLPVVIEPFFDQE
KYFFFIIINFLIIAFISLTIILTTETLYMIYVQHACGLLKLTSYRILNAFDNRLQQISMPNNKSKCITCIKLS
KAIKIHKRSLEFIECLCSTFSASFFIMCVFGVASLSINLFEFLKAIESKYTSQAIHFGIFVYAHLCYIFWINY
FGQALIDNSADVFTQTYNVQWYMVSTHIQKKVLFILHRSSKNVIFDVGKMFAFSLDGIATLINMSLSYSMLLY
STR
>MphaOr178
MDFFNNEYYFSRYLMELVGLWPYERPERRIVRGLCVSFVLTQGLLLQLTTFITHEFNMSLFIDVFSFSFIFII
LILRYNIVYFNSNHAKNLLDQIQDDWNLINNVDELKIIKKYVYKTKIISIISTSIVYPGTFVFILIIFIPDFL
NILAPLNEPRPRSLPIQIECFLNQEKYFYFILLVFAVMVFVGMTVLVATESMYMIFVQHSCTLLELISYRLTC
AFDTRLSKTTLSKTKRKLYINTKLLSAFNIHQHCLEFLENVQHKFALYYFFLFTVGVASMSVNMLRFFEAVTT
HMLSEAILIGLIMYAHICYGFYVSYFGQDVIDHSEHFFQQIYNTQWYTAPLYAQKLLLMTLRQSIKNNKIIVG
GLIVVSLESLATFLSMSLSYCMVMFSI
>MphaOr179
MLWNDDITYAMTPFKLLTLPLGVWPLQKYNTFSLFRSIVSGISMTVMTIILFLEINFGSGDAYVKLDALMLMS
CTVLCALKVLSFRLYADNMIRNFSSAVKDYLAIDNERTRTIMRRHAFMGRMICYSIIFSTYAASVIFFLLPII
ADDGEDIQINVTIKNQGAELPVPITFLGDLHIPTILYFTINTMQLFMLIFIGTGNCGSDSLFLGITVHLCGQM
ELLKVEFFKYGMKSGNLNKDFSVLASRHSYLIEHAKFLTEVISSVLLVQVFSSCLIISLIGFQLIIALKAHDA
VMIIKSITVLSSLLLQLFLYSFVGDYLKCQMEEIADSIYGCKWHSLQTKLMRNVVFVTMRSQRPVQLFAGKFL
VVNLKTYMTILKSSLSYLSVLRVMMD
>MphaOr180
MSWNDDIAYAMTPYKVFTLPLGVWPLQKYNMFSLFRSIVCGISLIAMIIMLFLEINFGSSDAYVKLDDFMLMN
YAIICILKILSFRLYADNMIRNFSSAVKDYLAIDNEGTRIIMRRHAFMGRIICFNIVFCTYLTSAFFFLLPII
AGEGEDIHINVTIKNQGAELPVPITFLGDIHIPTILYFVISTMQLFMLMFIGTSNCGNDSLFLGIVLHICGQI
EVLKVELFKYVTKSKNLSKDFSVLVARHCYLIEHAELLTELISFILIVQLLSNCLIISLIGFQFILALKANDA
VMIIKTIAMLFTMLLQLFFYSFVGDYLKCQMEDVADSIYSCKWYSLQTNLIKNVVFVIMRSQQPVQLFAGNFF
VVNLKTFMTILKSSLSYLSVLRVMMD
>MphaOr181
MPPNDDIAYAMNILKFITLPLGIWPLQKYNTFSLILCILSGSSLAVLMITMFLEVNFSNDNAYIKLDKLMLTC
CATLSVLKIFSFRFYADKLILNFTSAVTDYLAIDTEEKRTIMRRHAYMGRIISYGISILAYVAATLFTLKPIL
ESNTQVNVSIKNQLADLPLPLTWTLGNLGHISTTLYFTLALVQYYLLLLNVNGNVGNDSLFYAITLHICGQME
LLKIEFTNYGVKSENLSKELFVLISRHRHLMEHAELLVDVISFVLLVQLLFSCIIICFIGLNLIVALKLHDAM

MISKCLSVIITLLLQLFFYSFVGDYLKCQMEDIAYSIYSCNWSSFPLKLMRNVLFVIMRSQQPVQLLAGRYFV
VTIETYMSILKSSLSYLSVLRIMID
>MphaOr183
MPPNDDIAYAMNFLKFVTLPLGTWPLQKYDTFSLIRSIICSSSSAVLMITMFLELNFNNGNAYMKLDELIIVC
CALLSVLKILFFRLYADKLIHNFTSAVIDYLAIDTEEKRTIMRRHAFLGRIIAYSTVVSGYLATTLFILMPMI
IGDKNAEVNVSIKNQAADLPVPLTWTLGNYYISTTSYFTIILVQYYLLLLNCNGNVGSDSLFLAITLHICGQM
ELLKTDFANYGTKSKNINKDFSLLISRHCYLMEHAKLLTEVISAVLLVQMLCSCFVICFVGLNLVLALKAHDT
VMFTRCISAMMSFLLQLFSYSFVGDYLMCQMKDIADSMYSCNWYCFPFKFMRNFLFVIMRSQQPIQLLAGRYF
VVNIETYMTILKSSLSYLSVLRVMMD
>MphaOr184
MPWNDDIVYAMNPLKFLTLPLGTWPLQKYNTFSLIRSIICGSSSAVLMITMFLEVNFNNDNAYVKLDELMLMC
CALLSVLKILFFRLYADKLIRNFTSAVTDYLAIDTEEKRTIMRRHAFMGRIISYSTIILGYLASTLFILMPMI
TGDKNAEVNVSIKNQAADLPLPLTWTLGNYYISTALYFMIILVQYYLLLLNSNGNVGSDSLFLAITLHICGQM
ELLKIDFANYGRKSKNMNKDFSLLISRHRYLMEHAELLTEVISIVLLVQMFCSCFVICFVGLNLVLALKAHDT
IMFTKCISAMMTFLVQLFFYSFVGDYLKCQMKDIADSMYSCNWYCFPFKFVRNFLFVIMRSQQPVQLLAGRYF
VVNIETYMTILKSSLSYLSVLRVMIDA
>MphaOr185
MLWNEDTSYSMTLVKHLTVPIGGWPLQEYNKFTLLRHILCSFGLSVVVIVQYLELYYNCTSANANLDALTLFV
CGILALTKIIWFRIYADNLICNYSSAMNDYLAMDTEEKRIIMRKHAFWGRIICIIALLIAYVDSVIFIMGHAQ
ISSEEAKVNISILGHQAGYAIPSTCTLAHFHISTSSYLVIFALEYIYLVIMCLSNHGSDSVFLHIVLHVCGQL
KILKANFINFDVKGPQVHERFNALIMRHDHLIQMTRKLAEIISFVLTVQLFISSMLIIIVGFQFIIALTTSDY
GMMSKSFLVLSAFLVQLTLYSVVGDYLKTQMEEVAQSVYQSVWYDLPAKVTKNIVFIMMWNQLPIKLQAGNFI
VIDLGTYMSILKTSLSYLSVLRVIVE
>MphaOr186
MKTNWNRGIDYGFSTIKSMMWILGLWPLQRNNVVYTIQWFIIFTAGSLTLINVLVEPFKSCDAIRDGLEILRI
IESAMHGWLNIVFPRIYIRKLAININYAIDDWSSPSMKKESHVVMTTYARVGRLIALIQLIAGIISGIVWYGS
VFLSNKQEAANIDNGTVTTWNFVLPSTCLYKGVSYSTYKILFLMQVVQGFLILISECACDSFFFSITMHLCGQ
LELLRIQFLEISKRYKEKCHGNILRPLVKRHCQLIALSKNIEDAFNINILIRLLIISIVIAASGVGVILALKQ
QNYKEMMKMLIPIQFYIIQTLLYTYAGDILQTRSESIVYAIYSSTWHKMSPIMTKDLKFIMMKMQTPLRISAG
KFFYLTRNTMTDILKTTLTYMSFLQVSLN
>MphaOr187
MSDDVWNDGIAYEFSTLRTFMQILGIWPLQKKTVFTVILWNIITILQLSPLILLYMELTETKNDAGRNMDAIT
YCVCTVSINLKYLFIIANREKLAKNINDAIADWLSAKNNEKSYKIMKKYASTSRLCTFIMVYLCYIVGSLYIL
TVVVINVKEIFFEDRMNITDGINTRNRMFIIPCGELGNKMTELQYAIFTIVNTVQLLVICTTQSITDCFFINV
TLHISGQLKVLKTKFKSFTSKPDTHINYRKQFINLVNRHCKLMEHNQNVEDTFHLCILFQFVSVTLLLALLGL
RILLFFKNGDYIESIKTILFLNYMFIESLLFCYGGDFVQKGSEGIFRAMYMTSWVTFPVTLMKDLKFAIMRSS
YPFRLTGGKFFYVNCQTMVYILKTAASYISVLRVALKD
>MphaOr188
MASKRWKNDVAYAMTPFKILSWPVGVWPLQVYNGFSLIRCILATCCMSTLVILPSMEFQMGCTNTEQNIDGLM
LACCGVLGVLKTICFRIYAKNLTDNYSSARNDFLTIENTDHRTIMRRHAFIGRILSCFMVCFSYVSVLIYSLI
PLLGEEQINDQAKQINITDEDIILDYPMPSRCALEYLHVPRSMYEITCLLEFIVLILTCTCNHGNDSLFLNIT
LHMCGQVKILKANFIEFSVSSPQIYDRFNALIQRHKYLIELAKELAESISLVLITQLFISSVLLCIMGFQFIL
ALKTHNIVVMGKSFMVLCTFLTQLSVYSFVGDYLKSQMEEVGLYIYQSTWYNFPVKLAKNLTFIIMRTQLPVK
LQAGNVIVVNLATYMSILKTSMSYLSVLRVMIE
>MphaOr189
MASCEKWNKEMSREFSLYKRLMWPLGSWPLDRSRNFANLRALFLTLTQAIMVIYISVDIINSKDFTLGVIIDH
FVMGSCASLAIVKSTLIRLHRDNLSKNFGNAASDWTYIKKQDLRQVMFRYTNLARFVLFSQMGFSYFVTVLLL
IRPLLFPMPLPPSQNVTLTMTSEKEIRNIELPYIMICPFDTQIACYSLYIFQMVQVISTATGNVGSDVFLFSV
CMHLCGQLEVLGLELLRFHEGKENGYWKRTKMITLIERHCLLLNLAKDIVDMLNMIMVAQLILHALLVCIVGL
QIILSLADHDFFHCMSGLTTFNIFMIQLFLYCYMGETLSSNTQAITRSIYFSKWYDLPTNVARDIRFITARSN
VPVRIRAGKFYTIDFNSFTNVLKVSVSYFSVLKIIV
>MphaOr190
MDFFDGHNYRVNKILLSAIGQWPYQSSKTSHAIVSIIVAIVATQLVAKLCGIVPYIHDMDILIECLVPIMVDV
SGMTKIMNSMLCVNEIRALLDQIRDDFYLLKNSDDSVILQKYANTGKRFSTIYLCVMCMLTVVFILLPFQPLI
LRVANATTRPLLHRVEYYVDMDKYYFPILIHGYFTAIICVTSIVATDAIFITFMQHACGLFIITGSRIEQAIQ
EVYQIGNTNPPVTKDTAYQNLVQCVHNHRGALRFAGLMEVAYSKHFLFHAGLNMIAISVTSFGVVTKSDDLFE
FFRLAAVSCALLFHLCFECINAQKLIDFSGYLHTNLINLNWYDASPRTRKLVLFMMMKTQPPCVLTAGGMFVL
CMETFATIVKTAMSYFTFLRSAH

>MphaOr191
MESVQSQYMRINQMLMCLIGQWPYQENWEKFLIQLVFVPVVFAQAVVQGGGMITAWFADDIDAFMESSSPFVI
SLMCIFKHINYTYNHDQMKRLVFIMVDDWKSYSKLSHEYDILCRNYAMGKKVAVAYAVSLYGSMTPFLVVPVV
LNTAGYMGFYNLSEGRPLMFRTEYFIDSEKYYYPLLVHSYIGTLGFVSIVVAIDSMLVFHVQHECGMCEILGY
RLARLIDGDNLDINLYPSKEEVVSYNCVKNCVIMHNHIIEYARRIENANTTSYFFQLGCNMLGITFTIFQAVV
KLSDPNEALRYASFTICLLSVLFLESWPGQQLSDYTDKIFMYTSNGKWYQSSLRVRKVISIMLMRSYAPIKIT
AGKLYTLNLANFSAVLRTSFSYFTVLCSM
>MphaOr192
RNGVAGMTRGTTTGRSLEQFRKLSALHLTYLKYIGLWAEFDSNSSRFLRYLYHVYNKFILAVILVFMVTLFAD
ICLSFDDLSIVTDDGCIFAGIVVVFFKVMIFQTRREQIVRLLHETIDGCNRLCKFPIGDEGEIVDKYLLISRV
TFYGFSTLAFFLVISLLFVVPVEDGELPIRARYPFNTTVYPWHGIGFFVEACTVSVGLTAIIGIDSLLTNLCN
LFLVQLEILNTHFKNCGNDRQHDATPNGIDKRDNSGTTSRNVCYEVSLTFEANCGKDNYVPDAIRRGDFSVRF
GRSIRNHQRLLAVICDFNKIFSAGMFVQIVSSTSMICLTGFQAVLVIGQNSNILKFMVYLMAALSQLFYFCWI
GNEVLYQSALLTQNQWLSKWDEELSAKTGRLLILSLIFSKKAVKLKAGVFFALSMETYTSILKGSYSFFALLS
TMHLEDDQ
>MphaOr193
MIDKRDVVQSRHYSVLRVYLTISGLWPYHRLRDRCICFVPMFALCFLILIPQVLYLLIGPITLDDIFECVPST
MISIIFSFKILVIMLNSEKVKTSLNMIQTDWMSLNTDVEKIILEQHTKYGQYLATFYAVFMHTTACLFVLKPV
MLTLLEDNIFNVTKSSVPYASRLPFRVEYGKRFNQYMYPIAVHCYMAVFAHSFATIAVDGLYYTLIQHVCGMF
SIIGNVLENIGKNNEDNFNAKPGKIKDDDYNKTLHCLRRHLLVIELTEHIESLYTKIFLVNLNLNMVGGSLAG
IQVLMNLNKGASDIAGPVTIYIAQLIHLFLHFWQAQFLLDYSILPYESICKANWYYTSQRCRKLLLLMLYRMI
TPCKITAGKIVILSIDTFATVVKTSLSYLTVFRSFQ
>MphaOr194
MNERKNIWQSRFYTVPRAYMSLVGIWPYHTFRDRCLLFVPMFTFSLTIIIPQFLYLLIAAADLDDVFSCTPSM
YITIIFSFKLGWLMINNKKLKTCLKMMDDDWLSLSTDAERTILQRHTTYGRYITLTYGVFMQCVGILLILKSL
VVILLEDTSDATVSTLVAEAKLPLRVEYGETLDRYLFPMAVHIYLAVFSHISITIAVDCCYIALIRHACGMFA
IVGHTLEHIGKDSDGNFDLKPDKIRDDNYNRALNCLRRHLHVIQFAELIESTFTNIFLVSVCLNMVGGSMIGI
QVILNLNDAKDIVEPFAIYIAQLIHLFLQFWPAQFLIDYSTLPYESICRSNWYYTSVRCRKLLFLIMNRSVLP
CKITAGKIVPLTIENFGRVLKTMMSYFTMLRSFN
>MphaOr195
MAERKDIWQSRYYAIPRLYMTLAGLWPYYSIRDRYLHFVPTFIICSIILIPMLLYVQIAMTDLNDLFECMPTI
LITIIFSFKLASLMANSEKIKTCLKTIEDDWWSLNTDSEKAILQRHTTYGQYLTIFYAAFMMMTGVLYMFKSL
VLIMIEEDTSNSTRLAVTKLPFRVEYGYKIDQYFYLILVHCYLTVYSHVTATVAADTFYFILIQHACGMFSVV
GHMLERIGEDININFNSKPNKKNDDNYIKVFNCLRKHLHVIEFAELIESTFTKIFLVSISLNMIGGSICGIQV
LMNLNDAKDIIAPLAIYVAQLTHLFFQFWQAQFLLDYSVTPYESICRANWYYTSERCKKLLLLIMNRTVSPCR
ITAGKVATLSIESFGVVLKTSMSYFTVLRSF
>MphaOr196
MQSYLQRYYHINKILMSWVGTWPYQNRTIRILILSTLVVTAVIFNIAGVIRMVNTWGNIDIAVEWVINLIVFF
GCFIKLFNLNFNIKKLQHLFTLIEYHWHVLTDSIDVEIMQNYVTLGRKVVIFYATYIFTTALLFMLLPLTPLI
MDIVMPLNESRPRKLLFEVEYYVDQQKYYYLLLLHSYMVGLIHASIMVSVDTTYITYVQHGCSLFAVSGYRLK
HIVSKEHISRTSYVVKSKGRIRQDIEAENICFKEKAIFSEFVACLRKHQLAIEYAGVLESSFSKATALLLLIN
MIGISFMGIQVISNLNNTKNAVRYSCLCLAAFIHLLIMSLPGQRLMDHSLDVFNNICRSHWYEFSLKTKKMLP
TLLYRSNIPCTLTASKVYVMSMANYGMVVKTAMSYFTAFSSL
>MphaOr197
MNSEMEIYYATNKLFLSRIGGWPYQRKPLKVLIPCFFTMVHFSVIVTQALLLYDTWGDIDIVVECIITLTLLF
AASIKLINIVVNNDKLRRLLQHMNKHWELFNSELERQILRYYGSIGQKVTNYYAAYFSATLLSYVLIPLVPRI
LDVIIPLNESRPLGYVYQAEYRVDKEKYYYPILFHTYLASTITTTILFTVDTTYIVCVLHTCSLFTAVSQQLE
NITRKTDITSSDKKVHKEMHYHIFMEKHNSIGNDYIALINCLKKHQLALESILDSMFTHATFIMLSINVLILS
IIGIQLINNLENIEETVRYVFISCASFIHLITMCIPGQLLIDKSTEVFIKTYGSAWYTFSVRTKKLLGILLYR
SFTPCTLTAGRTFIMSMTMCSSVMQTAMSYFTAFSSMK
>MphaOr198
MNREIEMYYGTNILFLSRIGGWPYQQKALKILKLCLFAIIQFSAVVTQVLLLYDTWGDLNIAVDCLVNFVIII
GGTIKLTNIVVNNNKFRRLLQHMSEHWELFNSEFEQHILRYYANIGQKIRSYYGVYVIVALTLYLSMPLVPRI
LDVVIPLNESRPLMYVYEAEYRVDKERYYYPILFHSCVATSITAIILFTVDTTYIMCVLHACSLFTAVSQRLE
NITGKTDIKSDDNENIQIEMYYHVFMEKRGSIGNNYRELIMCLKKHQLALESILNSMFTDVTFVLLFTNMLIL
SIIGIQLINNIKNTGESIRYICILFGVFLHLACMCIPGQLLINHSIEIFDKAYNLRWYTFSIETRRLLTMLLY
RSLIPCKLTAGKSFVMSITMLSTMIRTAMSYFTTFLSMR
>MphaOr199

LNYAFALSRQCLRILGVWPDPLVSLSEFQRPNLRFMVITCILSLYIFMPQMTNVILAWGNVSLMVEYLSSANY
SLLALCKLFGTWYHGKALRTVMASVMTDWTTSKNQERIIMLNIARRGRILSFRCYASMLCLVLVYIFFNVLKF
IRSISQTQRSLVYRFILPYNIQKSPNYEITFFIQLSGGVYTAMINSTVDSYISILLLHICAQLMNLRTSLNNL
VNELAEGSISSSIFKKGLAAITTRHEHLIRNAKTINDCYSSVLLVHMLAATFQLCFESFQVFTIITRNMDVSI
LKLVFLSLYLVLVLTHLYIYCYSAERLSTESTNMAYGMYECKWYNISSKDAKNLMFMVHRSTIPLRLTAGKFG
TFSLEMFGTTIKTSMGYLSALLTLMD
>MphaOr200
FILDLFGLWPKDIQNSWEKLMCNFRVFIILLGLILCLIIPSIHSLIIICGDLMLMIDNLQFTLPILCSLIKIM
IFWWKKEAIVSIMNMIAKDWTKSVNDQEKSLMVRRAQISRIIIACSYCIMGVQCFFVVVLPVFGMSMRLTPNI
TDPGRPMPIQTHYFYDITKRPQYELTFISQSIYVAIAMMAYIGIDNFLSLVIFHICGQLNIIENYLTHLNKYI
NYHKILKYCVAKHIQLIRSINIIEDTYNIMLLSLFIYFAVLFAFYAFHIISLFDGENDLPFIRLMFLILTIFN
LFIHMCLYCVLGEILMAQCNKIYYAAYCNEWYTMDPKIGKDLLFLITRSSKSIYLTVGKISPVTMATFCSLVK
TSVGYISVLHATR
>MphaOr201
FEWAVKLNRFILNLIGLWPITAQNSRQKLMCNFRVLVVFMGVTFGLLIPSIHSLTRIFGDIILMLDNLQFTLP
TISCSIRIVIFWWKKEAIIPIMNMIVEDWVKLKNAQERNLMIRRARCARIIITFASCIMASGCFFLIIILPSFG
ISMRTTTNITDPGKPMPLQTHYIYDVTNSPQYELTFISQSIYIVVAMMSYSGIDNFLGLLVLHICGQLDILKN
HLTSLDKCVNSHEVLKSCITRHIRLLRAIDIIEDTYNVMLLFLFVYFAILFAFYGFRIITLFDEESDISFPHL
MYFASTVINIFAHMCLYCALGEILVAQCNKIYYAAYSNKWYTMNPKVTHNLLLLMIRGSKPIYLTAGKIFPVT
MATFCSLVKTSVGYMSVLHTTRS
>MphaOr202
FEWAVKLNRFTLNFIGLWPNIAQNPRQKLMCNFRVLVTLMGLISLLIPSIHSLIKIFGNSLLMLDNLQFTLPV
ISCLIRIVILWWKKEAIIPIMNMITKDWIKLKSNSERRLMIRRAHSARIIIICSYCIMGLGCFFVAILPIFGV
TMRLTPNITDPGKPMPLQTYYIYDITKRPQYELTLISQIIYVAIAMMAYTGIDNFLSLVVFHICGQLDILKNR
LQQLDKYINYHEMLKCCVAKHIRLLRAIDIIEDTYNIILFSLFIYFAILFAFYGFRMITLFDEGNDMSFAHLL
YFVSTVFNIFGHMCIYCILGEMLMAQCNKIYYAAYSNKWYTLNTKTVDDLFFMMTRGSKPIYLTVGKVSPVTM
ATFCSLVKTSVGYISVLHTTRN
>MphaOr203
MLENFLREYNINRILLSITGLWPYQHKLVRNLLWMFCFLLEISYYPFEILLLYDHPNDAQLIFEGCYQILILT
IFLIRHINVVLQKDKMQWMYEAIDEHWNIFTDDIEIRIMKEYSMLSRKFVKYYSILFFSSLSMVIIAPLTPIF
LDIFMPLNKSRPRFFAVEVEFRVNKEEYFLPIFCYTTVIIVVGSFIALGFDAMHITFAAHACSLFVVVSKQIE
NVMLKVNNNDNIRQNKYVSNKFDLLNEEKIYREYIICIKKYQFAIEFVDILNSTYQGFALALLVVIIGILSLI
GLRIIHVIDQLGTLIKFVLIFGTTLFCLMIPCYSGQKLIDESQTIFYGAYATEWYKFSRRLKSLLVITLYRSN
KPCILRAGNAIPLSITTFAKVIRMSMSYYTALLSMQ
>MphaOr204
DLARARNIVIWNKWFLNFLGVWPAKVNQPVFLLVSTYMIIYYAMAVNHLIQNFGNLALMVANLTDNVLIFMIL
GKIAICRRSSNTMTTFLESIETDFTTEMYNNVREKMAYLHYNKVALIFIRFSLSLGAFTAEAYYLKKLPEHWD
SMMSGNFTYDELPYPVHPFFEIKDTTTYICICLYLAVMVPIILCGYGATDVFLISMAFHMCGQFAALSCKINN
LLKDHENYHRHISSIILRHYHLIRLVEILENSFNMICLQQTLGTVLLLCLTLYHTISVSSVATKSNIVVFILY
YLSVFSTIFAYCYTGECLIIESAGLSETFYNTDWYNNSPSHTKLIVICMIRADRPMMLTAGKFCALSLNMFVS
IVKTSMAYLSMLRQFV
>MphaOr205
MICTETQYFNLNRILLLIIGLWPYQRSKFSHLRFICFFSILATLIFFQFTSFVSSIYTFDFIIKIVCALSIFF
FFIIKYIAFAVNINNMKDLLMQLQHIFNELKDESENAIVKRYAENAKRYTVMLIIILGFCSTGILTVVQFLSTI
LDIVLPRNGSRLHHVLIVTEYFIDQEKYFYLIVLHVSVAFYIGVIILISTGTLLIAYFQHICGLFRISSYRIE
RAMEFNLLHNITLKNENFRFKGIIYAVDIHRQAMRLCKLLLSKIETMLFCLIIIGVIGLSFSLFRIFQIISSE
ENVEEIFMPFLLSICIIVYMFIANYIGQDLIDHNSHIYITAYDIQWYMAPLHIQKLILFLLQNGTKDLSLSVG
GLFTGSLECFATLIKASVSYFTFMYSTR
>MphaOr206
MFILTFTFKILTFCGCGRMDSWPVSSKRLVYHVYTIIIMLLIHSFMLSQLLDLIMVVDNSDDFTDNFYVLLAM
IVSCCKMIALLINRSNIEKLIKTLTSKPFRPVEPDEVKIQQKFEKLIQSNTLHYTILVETSCLSVTVTSLLTE
FRKGNLTFRGWLPFNYSSPLLFPIVYAHQLISFTMGSVHHVACDSLICGFLVHICCQIEILEHRLRKSAHNPK
SLRECVLQHNHIFKFASRVNKKFRITIFIQFVVSTLVMCFNLYQFTKPTALKTRYMHLILYTCSMLSQIFLYC
WYGNEVELKSRHLINNVFEMEWFNFSENDQKTLLMIVRRAAIPIEFTSACVISMNLDSFVGLLKMSYSAYNIL
KQT
>MphaOr207
MVYWNKDAIYALASYKILAWPVGVWPIEDNIFCSKMRYLFAIVTEIFLTITLLMDVYLACENSSVDPIDTYVV
TSSAVLVFVKLTMLQIQRSAFSTCLHSAIQDWCSIKDPKSREIMIQHARAAKIISLSLFYCGFVSLMFYLLRL

LPFVNATANERTFVLPMSCLFKSISNLQYVFISFYQVIQLLIAYAGNCCTDGMFVGVTMHLCGQFELLMIDFQ
QIARRKYKRKGGSIVEEFVVRHRKLLKLTANIENTYSIIILMQIITSAILICMTGFGFIISWHIHDTFMTMKS
IVIMIVMLLQCFLYSYAGDNLRDQSEALSFALYDCNWCDFSPNDIRDLAFIMIKTNIPIRLTAGKFFYVTRAT
FTDILKTAVSYLSALRVMIEKQETN
>MphaOr208
MKQTPTSTINRAIEIPLRIFGIWPGSSYILFCRLFWTITIAAIQSFQYRYVIKRLYTVDLSDLMECLSVTLTY
SQFFFKIINFWLNQRTLDKILAMMAMDWKKSSNTDFSKRATIGKAILSHNISNLCFGSVVIAVIFYTVSVFTY
NSFNLDETDISMRPLILKMDFPFSGDTRFAYEIILLSQVFCTVLTACGNVMLNTLLIVLVLHLGGQIEILSKW
ITEVISDENEYKLNLTTVKTIIEKHQNIITFSKSIENIFSDITLVLFVADTLTICSIGFVLVTSIGRPDAVSI
VMKILGFYFIINFEVFMFCFAGEYLSSKSKVIGDAAYSSRWYQCKFQDSRVILFLIMRSQNQMSITVGKFMDL
SFERFVSILKASASYVSFMLALY
>MphaOr209
MLKNFQREYNINRIILSTSGLWPFQNKYVKNSLLIFCILVEISTYPFEVVLLLYDHWDDPKMIFDGCFQMVAV
TSFIAKIVHHIWHEDKLQQLCIAIDKHWDIFTNDADIKIMKYYSTLAQRFTILTATLFYSIISLMIVVPLVPV
LLDILMPLNESRPRFFAVEIEFRVNEEDYFLPIHCYITIVVVMGVNIALGFDTIHIVFSAHACSLFATVSKHM
KNIIPKANNSCKIRKNKYYINIELDPLNEKKIYREYIICLKHHQLAIDFVNMLDSSFREYSLFILLLLLANIS
LTGIRVLNVLDHMQELIKYTLVFLAMVSVLMIICYSGQKILDESQNIFYQAYAAEWYKFSPRLKSLLM
>MphaOr211
MDFQSVNPLNVRLNLLSGNLLPLKSGNSQFSITWRIYSLLIWLIEFIQTIILFPGLFFVSRGKALEDGTVVSV
VTIEVFFMIARIHSHKQLVSRLIQKLNDILHCADETMKNVVTTTLQPMENPLKFYWLSGSLSILVWACLPFLL
VFKKVSFFYDDYRTPAVFSKQPFSINVFLLGSIFLLISNIYIFVKKVGLDVYMIHLVLLITAQYRYIAIKLEA
IFRDGNSLSEFDESHQKYYSGINRWVEKEVTALCRHHNTVIHLSSMLKKLLSLNFSLIYVNSVLRFCFVGIML
STIPSTNLLEAFLIIMYSCGSIVQFYMLCSCLQQLLDASKEMTNNAFHEKWYQFGPSVKRTFMSMILSNNLEC
KLSMCDKFNLSLSSFMTILNQSYSITLLLLRAQ
>MphaOr212
MDFQNVNLLNVRINLLSGNLFPMTTDNSRFPIGWKIYSAVIWLMILAIVIAFFIGFNMVSKMKAISDGMIGTV
FVAEVFFMVVRIHAHRDLIVQLIRNMNDILRVQDETMRCIVMASLKLMHSPFKFYWLSGVTTVVIWIGVPLTA
VFRKNSFFYEDYRLPFAISKQPFSTEIFLLGGFMLVLCSVYVIFKKAAVDIYMLNFVMLMTAQYRYIAVKLHK
LFQEKYSQDKRANSRMKNIDLGAETEMIAICRHFNAVVHMSSMLKKLLSSNFCLIYMSSILRFSFVGVMLSNT
RLLTTVVERIAMIFFASGEILQFYILCSNVQKLLDASTEMTYMAFHENWYQSRLSIKRKFMLLILGNSLKCRI
AAFEKFTISLPSFMTVRSHFFKTLLKIFQILNQAYSISVLCLKIVKK
>MphaOr213
FKLIMEGSSKMNKHENEFTYAVQVTRVILRMIGAWPLSNYVSNVERIATRLQNICCYFLFAFTIIPGFLLMFL
KERDYKRKVKLLAPILNSWASCVKYSLLVYHAREIHSCLKQARQDWMNTVDLNSRKAMLSNAKIGRQFAIVSA
IFMYISGLFFRVIVPLSKGRMLTPMNTTVRALPCPSYFVKFNEQASPAYEIVFILQFFAGLITYSVTVGVAGL
AAFFVMHICGQLSVLIGKLKCLNNILEPEDRTVTILLADIVEHHIRVKGFLKQVEETMRFVWLLEIVSSTLFL
CLTGYYVIMEWENNNSTATFTYSVMLASLTFSIFTNCYVGQLLTDQSSKFGLMAATMKWYRLPHKRSRSLILI
MAVSNIPAKISAGKLIDMSLLTFSNIVRTSVVYFNLLRKF
>MphaOr215
IKLIMESSSKTNENEDDFNYAVQITRVIMRALGLWPIPKYASMVERIAIRLQNVICYFLFAFIVVPGLLLIFL
KEHDIKRRVRLLGPILNCWMGLMKYSLFIYHAKEIQFCLEQARQDWRDTVDWRNRKAMLSKAKIGRKFAIFSA
IFMYVGGLSYRTIVPLSKGRMLTPMNTTVRALACPSYFVKFDEQATPAYEIVFTLQFFAGLLTYSITVGAAGL
AAFFVLHGCGQLKVLIDKIQRINDILDADDRTVVMLLADIVEHQIKVKNFLKEVERAMRYVLLVEIVGCTLLL
CLTGYYIIMEWENSDSTAMLTMTVILTSFVISIFTNCYVGQLLTDQSIKVGSITSTTNWYRLPYKKARGLILI
MAISNIPAKITAGKMMEMSLPTFSNIVKTAVAYFNLLRKFM
>MphaOr216
ITETAMPVAIIYDHKKNMQLSIQLNRWILKPLGAWPKSAKISRTERYVYALVNVICVSLIGFLFVPSAIFMVL
EMDDMYNILKLFGPLNFCIMNIIKYLSLIFRGNDIRSGIEHIESDWMNTRHNGDREIMIRNAKFGRHLVSICA
FFMYGGVVFYYFALPFSTGKITENDGNLTYRPLVYPVAKMIVDTRHSPISEIFFWVQFLSGFIVHSINAGASS
LAAAFAMHAYGRLEVLIQWIEHFVDGREDLYDNVNERLAMIVQQHVRILNFISLMDKILREISILEVVGCTIS
MCLLGYYFVMEWKLKNATSYVTYIVLYTSLTFNIFIYCYIGELVAEKCKEIGEMSYMTEWHRLSGKKSLGLIL
MIAMSNTSVKLTAGNIFELSLSTFSDVVKTSFGYLNMLRTLTS
>MphaOr217
LREYNINKILLSHIGLWPLQSKFVRRLLPISLLILLISFFLLQITTMFDCWKDKDIIFDICLIVAAMVIFIAK
LWNELFNYDTAQRLYEAMENHWNIFTSESEVRILKNNSSKSRKCIKMYAILLYTAITIFLLTPFKPILLDIIS
PLNESRERYFATAFEVKIDMNKYYVPLLCYNTSVILAGVAVLIGTDSIFITRIFHAYSLFYIINIKDSEYYGH
TNVYVNNEYYMNAMLKSTREQEIYQKFVICLKKHQIALEFVDLLNSIYRLGTLITILTPCMVISLSGIQLIYV

LDQIELVIKNVMIIVSVLTNLFVFCYFGQQLLNESQNVFHQAYAIEWYIFSLRIKSLLMITLQKSIIPCTLTA
GKVLPLTMATYATMVRGAISYFMTFLSLKD
>MphaOr218
IMLVHVLREYNVNKIFLSRLGLWPYQSKLVRNSLPILYLIFEISFFSSEILMLYDHQDDSQMIFEGFYQFVVS
NAFIVRLWNEIWNRDKFRHLYETMNNHWDIFTNELEVRILKNYSNISRKFTIFYSTMMYLLSSMFIVIPLTPV
FLDIILPLNESRPRILAIEVELKVDTDEYFVPIFLYTTAIIVIGISIMVGVDTMHFTCTTHACSLFSIVGEEI
ENITSTQYLQKCKCGLNKHHELSREQMIYQQYVTCLRKYQLALKFVDILNSTHQTAAIFFLLLIGASLSLTGV
RVVYVLDQLEEMIRFLFIIMGALMQLLIMCYAGQKLMDESQNVFHRAYAAEWYLFSPRLKSLLIITLYRSLVP
CSLTVGKFFPLSMSTYATVVRGAMSYFTAFLSLKN
>MphaOr219
FEWAVKLNRFTLDFLGLWPKNIQTSWQKLICNLRALAAVMGILFCLLIPSIHSLIKIFGDSLLMLDNLQFTLP
AASCSIRIIILWSKKEAITSIINMIAKDWMKPKSDQERDIMIQKAHIARIIIICSYCIMGVQCFFVVVLPVFG
MSMRLTPNITDPGRPMPFQTHYFYDITKRPQYELTFISQAISVTIGMMAYTGIDNFLSLLIFHICGQLNILQN
RLQHLDKYKNYHKVLKCCIAKHIRLLGAIEIIENTYNTILLSLFIYFVILFSFYGFRLISLFNEGNNMSFTHL
IYFISSVFNIFIHMCLYCVLGEILVAQCNNIYYAAYSNEWYTMDSKIIKDLLLLMTRGSKPIYLTVGKVFPVT
MATFCDV
>MphaOr220
EWAVKLNRITLNFIGLWPKIAENPRQKLMCNFRALVAFMAMIFGILVPSIHSFIRIYGDVLLMIDNLQFTLPT
ISCAIRIIVFWWKKEAVIPVIDMIAEDWIKSKSAEERNVMIRKAQIARIILTYAYCITTVGCLFIVVLPVFGK
TIRLTSNITDPGKLMPLQTYYIYDVTKRPQYELTYISQIIYIIFAVLSYTGVDHFLALLVLHISGQMNILKDR
LINLDKYVNSYEMLKKCLAQHIRLLRAVIIIEDTYNATLLSLFVYFAILFAFYGFRIINLFDEGNNLSLIRLV
FFISTNFNLFLHIYLYCALGEFLVAQCDEVYSAAYNNEWYSMDPKIVQDLLFLLIRGSKPIYLTIGKMFPMTM
STFCSLIKTSVGYISVLHTTRS
>MphaOr221
VSQFLCEMYTFEERYYKVNRIVLKTLGLWPYQQSYITQMHKVIFASILLTFILVQLLVFITRQYNTNLLFKVL
SFVFPCLFATIKYFLFIIEANRFKQLLKQIQDDWNSLNDKLEIEIIKKYASKTKLLTISWMTFCIIGIFSFGL
IQHLPIILDVVLPLNESRPYQLFVITEYFVSRNKYIHVMMLHEFLVIYVGCTTVFGTGSTLVRFVAHICALLK
IASYRIENAIEKNILEISSSTREYFLHQRIVHAIIMHQRAIEFNEFLTSICLLPFAILIVVGVSSLTFNLFLF
FQLMALNKNLCMACIAATSIFFHLMYLFTANYCGQVVINNGINLFEATYNGLWYAAPLSTQKLILFILQRGRI
YVTISCYSVFVASLEGFATLTSMAVSYFTVIYST
>MphaOr222
MYLAEEYYSNSFNKTVLKILGIWPHDKSRLVFLQRVLFIILAVTYIIFQLSVFITSQYNMALFIKIMSLAFPA
IITTLKYCVFIFKLETIKILNYHIQEDWTIMQNKIEYDIIKKHAHNCRNYITFIFRFGILFLIVIHIIQFFPI
IFDFVLPLDKPRPRKLLIEVEYFVVQDNYFYIIVFHELIIIVLYTFVMLAIGTQLLLFAHHSFGMFKIASHRI
KHSIDDVIYMPNCEKERTFCKKIFHVVIAHHRAMEFSNIMISSFNLPYCVIAIIGVFSLSINLFRFVQAITVL
KNMEEILVSFFAILGHLLYMFIATYIGQKATDYNNEIFRLTYDTSWYLMPVTSQKLILFLITRTGKDFYYTIG
FIFVAKIENFAMFINTALSYVAVMYSVE
>MphaOr223
IEEQYYKINRFLLLLLGIWPYQSSILAKAQRLFVFSFLVSGICVELAIFITHEFNTAVIIDVLSFIFPTLVTV
IKYYYFYIKAETIKQLLECIQLDWNLLKDKHERKIVESYAENGRLVTVVVTLFIIGIMIIYMTTPIVLYTINF
NLFNNNTYSQYIYIPIDYFINQEKYFYVIFLHFITFTFFGMITTVAIATFMGLYLHHASSLLKIASYRIEHAI
DTECVQQIAPLKREYVMYMRVMRAVNIQQKGLKFTNCLLSSFSGMYFILIIILGAGAVSVYMFQFLRNLQTNRD
SREMFLILSMLVGHFLYMFLANNRAQDFSDHCNNVFFAAYNSMWYIAPLRIQKSILFLLQYCTKNYTVIFGGC
FVSSLEGFATV
>MphaOr224
MCILKFNFKLLTIFGCWRPDSWSSLYKCIAYHIYTSIIIFLVNTFMLSQLIDVILSMNNVDDFFDNIFMLIAV
FNACCKLFMLLINRKSILNLLNILMEKPCRPLRLTETKILYKFDKNIQILTKCYAYLGMITISCIILSSLSIN
FRNRRLTYRAWLPFNYSSTALFCVMYTHQLIGFAIAGFVHVGCDAIICGLLVHICCQIEILTYRLKNIACYSN
LRDCVHQHYHIFRFAFIVNTRFRITIAIQFMISIMIISFCLYQLNRTTTKSNYFEMALYISCMLTQIFLYCWY
GNEVKVKSSEMVDNIFKMEWMTLDENRKKSLMIIMRRSLVPIQISCAYIIPMNLESFMSILKISYSTYN
>MphaOr225
MYILKITFNLLTIFGCWRPYSWSSLYKRLVYDIYTGIIFLILNTFMLSQLIDIIITLSNAEDISDNFFVLIAM
CITCCKLFILLINRKNIIMLINILMEKPCRPSRSIEMKILHKFDNSIQINTWRYVQLGIGTILCVSLASFHIN
FRNKKLIYKTWVPFDYSSTVLFRITYIHQVISFIAAMFVNIGCDTLICGLLVHICCQLEILTYRLRKMILYSN
VIRDCVYQHYYIFRFAVIVNAKFKWTIAIQFIMSTMMICFSLYHLSKTTSKFKYIETTLYMSCMLTQIFLYCW
YGNEVRVKSYEIIDKIFEMEWLTLNKNNKKSLIMIMRRALVPIQINCAYIIPINLNSFMNILKMSYSTFNLLQ
QMSK
>MphaOr227

MHVLQLTFKILTIVGCWRPKSCSSFYLRLIYDAYTVFMIILLYTFLVSQFLDIIWNVDNAKDFTENFYATLAS
VVSCSKMLSLLINRKNINKLTNVFLERPYRPSEMDEMKIRYKFDRHIYINTLYYTILVQTTGACYIITSLLTV
FRKGNLTYRAWLPYDYYSSTIVYCLTYAHQLISLAIGSLVNVACDSLICGLLVHVCCQIEILECRLSKISNNY
DTLCDCIRHHISILEYALKLNNNFKMTIAMQFVVSTLVVCSNLYQMTKSKNINASYLPLLLYMSCMLTQIFIY
CWYGNEVKLKSTQLLSNVFAMDWVTMNKSLKESLLMIMNRAVVPIEFTSAYVLSMNLDSFVGLLKTSYSAYNI
LKR
>MphaOr228
MQVLELTLNILTVVGCWPPNSWTSLCKRTIYNIYTVSLILLLFPYSLSQFMDVILNVDNTDDFTDTFYVMLAT
IISCCKMISILINRKNIGIFTNILTQKPFIPLEADEIEIRHKFDKTIQTNTLWYTILVESTCACIVLTSLFTN
FRKGNLTYREWTPYNYTSEVLFCFIYSRQLISATVGSIVNIGCDSLICGLLLHICCQIEILECRLKKISNDQN
NLRECVRQHNCIFKFAIMVNEKFKIIIVIQFVVSTLVVCSNLYQLTRITLNAQCFPLILYTCSMLAQILIYCW
FGNEVKLKSVQLTTNIFGMEWLMMKQSRKKNLLIIMNRSLTPIEFSSAYILTLNLDSFVSLLKTSYSAYNILQ
QM
>MphaOr229
MQVMQFPLKILTVAGCRPPISWSSLCKRTVYNAYTILMCLLMLTLLLPQLIDIIVNVDNAEDFAETFYVMIAL
INACNKMISLLLNRKNIEILTKALVEKPFRPLEPDEIDIREKYNNMVRTFSIFYTILIVTTIGYMSLTSLLTD
FRRGDLAYREWVPYAWSDTMYYITYFRQLISSVVGSILNLAGDVLICGLLLHIYCQIEILECRLKKSLRNQND
LGECVRLHDHIYKYARTMNENFKITIGVQFIASMLVVCFNLYRLAKTTLSVKYIPLISYTICMCMEILIYCWH
GNEVKLKSIQFCDEIFAMDWVIADNKARKNLILIMNRSLIPIEFSSAHIVTVNLDSFVKLLQMSYSIFNLL
>MphaOr230
MQVMQFPLKILTVAGCRPPISWSSLCKRTVYNAYTILMCLLMFTFLLPQLIDIIINVDNADDFADTFYLMIAM
VNSCYKMISLLLNRKNIEMLTEALVEKPFRPLEPDEIKIREKYNNIVRTYSIFYTILVEMTCGCMNLTSLLTD
FRRGDLAYREWVPYAWSDTMYYFTYFRQLISSTFGSIINVACDILICGLLLHIYCQIEILECRLKKSLRNQND
LGECVRLHDHIYKYARTMNENFKITIGVQFTASMLVVCSNLYRLAKTTLSAKYIPLMSYTICMCMQILIYCWH
GNEVKLKSIKFCDEIFAMDWVTADKKARKNLILIMNRSLIPIEFSSAHIVTVNLDSFVKLLKMSYSIFNLL
>MphaOr232
MKRITLEKVIAFLKVDLMFACCWPVSRNATKFQIICNKIFRLISGLHGLLLLIELIYTIIYRNDNIRMFLQSC
CAIGILLEIPLQILLFTLQHDRLQTVIFQMEDYYQRAKSEERDVFQKLIDKYIYFYVITLGMITLALFSSLLE
PLFHGSNSFPLVIKYPFSVDQQPLYAIVYFHHTLGLYQAYCQVSSFVFLALLLWFTSARFEILINKFRMVTKY
SEWQSCIREHQELLRFAQEVSLSVSYLVLASLGISTYSLVFAGITILSRLPLAIKAEFFGVCISTLAKILLCA
WPADYLMTVSSDIGDAAYDSLWYKHDIDSQKIMLYTLLRCQRPVIITVPGLLSALTFQHYSSYVSTAFSFLTT
FRIILSEDN
>MphaOr233
MRMLSRHFFAYTIGGLWRPIEWSSKYSKSLYNVFTFFTMYLFTYLLLTHGLHIIFVVDNLESLISSSSVFFSF
VGLMCKATTVVVRRDRIINLLEIIQEEPCKVCDEDEIDIQMKFDRLLGLYSMPYIILCAISAVGTLIGCIIYM
MEGVIPIGTTWVPWDCTSPFIFYLTSLQVVAALMLAIFVNIGSETTILGFCLQTCAQFEILKHRLQRMMKNNK
NNPMSSLNNALNRTSRISEHVFYHLCIIRLAKTINDVFSPIIFVQFFVSILLLCSVIYRLSSQMVIADLASLS
TYTIILFIQIFYYCWGGNEVMLKSVELGQEIYHMDWISMAKSEQKDLLMIMKRSTKPIRFTSSFLVTMSLESY
GNLLKAAFSAFNLMQQ
>MphaOr234
MSTNIIARSIEIGLRVAGIWPGAAYVIVKRFLWTITMIAAQIFQYRHMALHLNSEDISQLMDGLSATLSYSLL
FVKLIVFWTKQRIFNDVLASITMDWEECRDTLCTMSGVANLSQRFSNLIIGLHSMAVLFYCIGVVALRSDDVA
DRELFLKMELPFESSTSPIYEVVLTVQFLHQMTAATVIGVLSALLVTLVLHAGGQIDILRERLLEILPRKKKP
TISVITIGSLIRRHQNIIVFTEKIESLYSYIALVQFISNTLVICCLGFIIVNSIGDDQGSSMLVRSLLFYVVI
NLEAFIFCFAGEYLSVKSKMIGDAAYECMWYDLTPSENKILLFLIMRSQKQLTITVGKFMNLSLNQFANIIKS
SASYVSVLHAL
>MphaOr235
MIRKNTINRTIEVMFTLFGIWPDTSCIPFCRIFWIVSIIIVQFYHYQYLQTHLYSDDLFNLMDCLSSFLAYMK
VMTKFIVFWLNQRKFIITLKMITEDWDSCANNEINMHETIYKAKLSSRITNAMIILHTISAVAYSVRIILADV
DVTDRTSDLPYIHKMELPFDVNTQRTYKMILIIQTVYVVMCSWAAGAVNALLLTLVLHTGGQMDILRCWLLNF
ASQENEKNESITTGKIIQKHQKIINFSKNIENLYTYIALLQFTSNTIMICSLAFLIVSAIGTPDATGQIIRSL
LFYAITNLEAFVFCYAGEYLNNKSKAVGHAAYNSAWYNLKPEDTRILSFIILRSQKQLTLTVGKMANLSLEYF
ASIMNASGSYLSVMLAMQ
>MphaOr236
MDFAGNRYYRINQILLTCLGLWPYYTQRTRYIYCIFLFLLLLSNVFLQLSLFFTVEYSVDLLLNICSIFGLTI
YYTIVYFVLFINGNKVRKLMEKVRFDWNSLKEKEELKIIHNCANVGRFYTIITLICLYTSYFVFAFILFSPNI
LDIVMPLNESRQSVWYLTSTMNFFIEREKYVGLIILYFFLTIFIGITTTVTAETFLLMNAQHICAIFQITSYR
IEHFINIYSQLKSLISSKKFNNDRNIVEVVNSHRNAIKSVDFLKSTFSIIRFFTIPIGVVSLSINLYRLCERV

IEKDISGIIALFLFVAVHFGYMFFCNCIGQTVIDHSEGVFKKICNTQWYAAPLNIQKCIMMIMRRSMKTSTLI
YCLGLFLSSLEGFATVVSSSLSYFMIIYSMRQ
>MphaOr237
MDFAGSRYYRINRILLICLGLWPYYTQRMKYVYCIFLFLFLLCNVFFQLSAFITKEYSVDLLLNILSVIGPII
YYTFLYFATLVNGNKIRELIEKVQYDWNSLKEEQELKIIHNCANVGRFYSIIVLVTIYTTYFLFVLIFFSPNI
LDIIMPLNESRQSLWHLSYAIDIFIGRDKYVELLTWYLILTSFIGMTIMITAETFMLMNVQHICAMFQITSYR
IERTINKSQIKSLASSRKYNCHKNIVEAIDSHRNAIKFVEFLKSTFSVTNFLAIPIGVGSLSINLYRLSEHVM
ARDINGTSLMFLQVFLHFCYMFLCNYIGQEIIDHSDNILKKISNTRWYATPLNIQKCLVMIIRRSIKTSTLAV
HFSLFVPSLQGYATLVSSSLSYFTVIYSMRR
>MphaOr238
MVVLHFTLILCAVSGCWQPLTWSSMSKQIMYNSYRILLICLISTFMMSQVMNIALNINNFSEISDNLYMTLTV
FIATYKIITMWITKKHVKMIINFLTKEPFKPLESAEVTIRQKYNKIIRQYALWYYGLVQITVICTILNSITMD
FMKEKLTYKAWMPFDYKLPVIFCFVFTHQMIGISITASVNVACDSLVSGLLQEICCQLEILEYRLTKIFYDQN
VLHDCVRHHDRIYEYAYMVNRRFANIIALQFAVSMLVVCANLYKLVSISLLVINGNLIALMLYTACMLSQIFL
YCWFGNELKLKSTGIANSIYNMKWQVLDNKSKKDLLLIMRRSMIPIEFNSAVIITLNLDSFMSLLKASYTAYN
LLK
>MphaOr239
LEWAIGLQRSMLKIVGLWPQQSKNQRKEFLSKFQFLFTTIVTIFVLTIPALAALRRVWGDLMQMVDNLQLSLP
LLVTVLKIFIMWYKKGALSLLINMIMKDWMRIKMEEERSVMLKKARITRLFATIGAFVTFIAASSRTSAVFFK
EYFEHDSTTSNLTNFERSFPVPAYYLYDVSSSPTYELMYLLQTIALFTCALTYIAIDNFLGLLIFHVCGQMEN
LHLRLLNLGKNSNFKAVLKYNVTDHIRLIRSVEVIDDTFYLLLLSMLLIFSILFSLHGFLIINVVNQDTHQLS
IMQMTSYIFTSISISMHTCLYCAVGELLVIQSEKIHSATYKCLWYTLEPKTIKCLIFIMLCAKKPLNITAGKI
VPMTMSTFCNLLKTAASYLSVLLASRN
>MphaOr240
MSQSLCQTRALQERYYKIYRIILKTLGLWPYQQTYLTRLHKVLFIGILLSFLLVQLLVFITKQCNVNLLLKVF
SFIFPTIFVTINYVIFIIQADDLKTLFKQIQDDWNLLKDKLEIDIIVKYAYNMKLFTISVIVYCYLSLLCVTI
LQSFPLILNIILPLNKSRPFELIVLTEYFVNREKYIHYLLLHELLTAYIALIIILCGLGVIILMSLMHSCILFK
IASYRIENAVDKSVLIVPKRKYLLYQRIVHAITMHQRAIEYTDFIISVYATILSILIVVGVSSLSINLFQLFQ
LITIQKDICNICVTVILIMCHLTYMFILNYVGQEIINHGLQFFKASYNGLWYKAPLHIQKLLLFIMQKGTVNT
VFICGKIYVGSLEGFATLTSTAVSYFTMIYS
>MphaOr241
MICTETQHFNLNRILLLIIGLWPYQRSKFSHLRFICFLSILATIIFLQFTLFVSPKCTFNFIIKVVCTLSIFI
FFIIKYIAFAININDMKDLLMQLQYIFNELKDESENAIIKKYGQYAERYTITLVALGFCSMCILTVVQFLSTV
LDIVLSKNVSQVHHVLIVTEYHIDQEKYFYLIVLYIYAAFYIGVIALVSTGSVLVAYLQYICGLFRISSYRIE
RVMEFNMLHNITLNENFIFKSIICSVDIHRQAIRLCKLLVYKFETMLFCLIIIGVICLSLNLFRIVSSEENVK
EIFMPFISSICIIVYMFTANYIGQDLINHNNHIYITAYDMQWYMAPLYIQKLILFLLQKGIKDLSLSVGGLFI
ASLECFATLVKASVSYFTFIYSTR
>MsexOR-1
MIFMDDPLSKSIKDPRDYRYMKLFRSTLRLIGSWPGRDLKEEGATKYEIAPLYWVLVIKITCFLLTIIYLIEN
TNKLGFFEIGHVYITVFMTMITLSRSITLSLNPKYRRVMTKYITKMHLFYYKDMSDIALKTHIRVHKLSHFFT
MYLSTQVVLGTVTFNIVPMYNNYKVGRFENNILVNDSYELSIYFKTPTKFLSTLNGYIAITTFNWYSSYICSN
FFCMFDLALSLLIFTVSGHFKILIHNLNNFPLPAVVSDSSKVLKTDEIQAPLYNKTEKKDITLRLKQCIDYHR
EVLEFTQDISEAFGPMLFVYYLFHQVSGCLLLLECSQMDAAALMRYGLLTAVLFQQLIQLSVVVESVGTVTGY
LKDAVYNVPWEYMDTQDRKTVCIFLMNVQEPVHINALGLAKVGVQAMAGILKTSFSYFAFLRTVSN
>MsexOR-3
MTSAGSSVAPHLRCLRMVGFCRLSAGTAGATGRGRWRRRAHGAYHAFALSATSTYVLQQLIYAYQERGDMEKL
SRVMFIMLCFVTCVVKQIVFHVDATRIDMLVAGLAEPLFNPAAEWARALLRETARAARRQALAYAGCAVATCV
LWIVFPVLDRARGRHVHFSFWAPIDCTHTAKFLTLLLYSFYATSLVGVGNTTMDSFVATVLHQCKTQLTILRI
NLETLVERARAACGDARDQYDRVVYKLLVESLVHYQKITQTCALLQDIFGAALLVQFGVGGWILCMAAYQIVS
LDVMSIEFASTTLFIVCILTELFLYCYYGNEVAVESDLVLQSMYALEWLAVPRRVRRALLIAMERAQRPMRPA
AGAVLPLTLDTYVKILKSSYSFYAVLRQTK
>MsexOR-4
MKFFVDGSEIAHITKPQDIQYMQMLKFFTNSLAGWPIEAVEGIDGKKNFYWRNGLVVIAYAYFFGQVFYIYRY
INDYTFLVMGHSYITVLMTIVTIARHTLPYFKCYDDTTADFVHNIHLFNYRNKPGYYKEFHLKIHKISHAFSV
YLCTLLVTGPSMFNGIPLYNNYASGAFSFNRSPNVTYEQAVSLLLPFDDTNNFKGYFVVFLANCCVSYISSCC
LCIYDLLLSLMVFHLWGHLKILTKTLDNFPKPGFLNPQAIEADPNKSLKFSDEELKVIHKKLGECVAHHQLIS
NFSTRMSNTFGLSLFIYYGFHQLSGCLLLLECAQLEAAAIICYGPLTLVVFQQLIQLSFIFELIGTVNEGLTD
SVYCLPWEAMDQGNKKIVFTFLRQSQKSMNLKALNMLSIGVQTMAKILKTTMTYFLMLQTIAKDES

>MsexOR-5
MKVPLKKFRPTETTKLLDELNKLFYIFAFRGFWVEEVKLPKTFVKIYDAMYPALNVSLVIFCALQIGAHFTQK
HLNIQQKININIMGIAQPLTKLCCLNCMYYKEECKQVLYHLFVAVKEIYNDEETEKMLVKKLKFSLWVYMFSS
VSTTLLYGMYALIEMVRSGSTFVGTVTAWPDTTDTSALAACARVYLYFFWIVYTSTGSMVILMVLTFFMGLTY
QYKNLQKYFENLNSIFEDKQVTHEELEMNFEKALQKGIKAHSDTLWCVNQCQTICKRINMAIVLINTGILIVL
MQGFLDSKDDILKVGTCLIVLGMCLMILAFFMWNAGDVTVEAQKLSDAIYSSGWYNCYGKRSARIRSLVVIAM
IQAQEPVVFTAFGVIELSYETYVAIIKSAYSVFSVLY
>MsexOR-6
MEETKKYAPTDTTKLLDKFNKILYVFGFRGFWMGDVKLPKTVMKIIDILYPVLIITQGTFGTLQLGAYFTQKH
LNSVQKIDALVMGFGQPMMLLYCLNCRYYKKACREVFYHLFVVLKTVYNDKETEENMVKRLKLYFGAYLIFSL
LVVLLYGSYASVETVRKGATFVTVVTAWPDVTDTSKLASYARVGIFMYWSVYSFTTSVVIIIVLIIFLGLTYQ
YINLQRYFENLNSIFENDRLSHEEMEKNFEKALQNGIKAHSETLWCVKQCRMICSAINAGVILLTTGTLVILM
PAILGSKDDLLEAMMYLMVSNIVLMIMAFFMCNAGDTTVEAQKLPNAIYSCGWYNCYGKRSARIRSLVVVAMI
QAQEPVVFTGFGVIELSYENFVTIIKSAYSLISVFH
>MsexOR-7
MDEPEFKPFHKTYQIITFALSVGMIYPNPATDKMRLASIPISILTILPLACMIFLEMYQCWTQGDIVNIIRHT
TVLGPFLGGLFKMFLMYHKRKQAKQILDEFERDYHLYNSFTGDYLNIARDGIRNSLIYSERGWAITVTTCVMT
FPVMAILLNMYNYTFKFQATKYMIHDLNKPGASPEARFDSPYYEIMFFYMMYCSLLYVINFIGYDGFFGLSIN
HACMKTNLYCKMLEDAWKAEPNERYRRVVAVIDEQCRMFEFVNLIQDTFNIWLGIIFLATMIQICTCLYHITE
GYGFDLRYMIFVSGATIHIYLPCRYAAKLKAMAAETATHFYSSGWERVNDRRLRNMLLFMVARAQTPLQITAF
NMITFDMELFVSIMQSSYSMFTLLRS
>MsexOR-8
MAQTLFDKSLSKLSMVFRWSGTNIAIGEAAPTNTKRNRCIYSFNFILQNTDVLGAIYWFISGLKTGKSYTELT
FIAPCIIISILSVMKSMSIIIYEKKVYQLMENLRMMEAHERNRENTAERRKIIEKGVNFLNLVINVLCGLYLI
MFVCFALSPLVLMILKYMKTNEIEFKLPFFIAYPFDPYNIKVWPMVYLRQLGTEIVTVSNMCVADFIFCIFCS
YITIQFRLLQYDIEHVITGTRESIYNDEMHGGKIRNKLVEIIKWHQELIMCVNLLENIYSVSMLFNFISSSVI
ICLTGFNVTENHDIVLVITFITFLFMGLVEIFLLCFFGDMLIDASSDVSDAVYNCKWYLAPPKMRKTLLLIQT
RAHIPCKLTAYGFADVSLKAFMKVLSTSWSYFALLKTIYSTE
>MsexOR-9
MTSPDSKIKEFFRKFTLITYLCGLADFWIEDLDLPTQFIKCYDTFCKIFNNFLYLYVIAQILSLFTQHNLTEK
QRNYQLMFCVAHPFVVTFPTLIAKHRKKLQSVLFRLVVSLKLVYNDLDIEKEMIKRIKFYLFAVSAPFWISMI
SYCVDSYVQVVTSGTTFNIMILAWPLVDDHSVIASLARFIYHIIWILFETRVTAAYVMVISLTTCISYQYRNL
RSYFESIHRVFEEDLTQKEKEKKYEKLLKIGIQAHSDTLRVTKDGTEACSAVLSAQVLFNTVFLVILMYQAVV
VNENRSLVKMFSTLCTVITLLFSTWFFTCNAGDVTYEASLIGTAMYTSGWQNCRGLSSLSIRKLLVIALARAQ
EPIILKGFWILTLSHQSFLSIVKFSYSIFSLLY
>MsexOR-10
MALQFDDTIKKIDFVFRYAGINIKCGERKKKETIKARCMYVFNFFWLNVDVSGSIFWFIDGVRMGKDFIGLTY
IAPCISLSTLANIKSVFLISSEKHIYKLIDNLRELEMRENARPRSVQKEEIINTEIKFLNYVLKTINILYVVL
VMTFALSPLIIIAIKYMQTGEVELILPFLILYPFDPYNIKLWPFVYLHQFWSECVVTLNICCSYIRIQFRLLQ
HDFERIISAPSGNRRVRENDFKAKFIELVKWHQDAIESVSLLETIYSKSILFNFMSSSLIICLTGFNITVVND
FAFVVTFLSFLFMGLVQVFFLCFFADLLSESSVEVSNAVYNSQWYMADSNIGKQLLLVQTRAQKPCKLTAWGF
ADVNLNSFMRVLSTAWSYFALLQTVYGQ
>MsexOR-11
MPRRYRNDNKKEIMNNVVLVLTNFMRKMGRQNGYQSGNVHWLAKFAILCYLSTYILQLIAVFLAKGDTERLFE
CVSVACFCGVGLLKLYLLLSKHSRWSFLINQITLLENKQLNSEEDTFCLDYKSEDEDSENQFTPYIISYNKRF
VAISTILVRIYVSTGVVYAVSPFAEYSFRKVFLNETLGYPHILPCWAVLDELSFLGYLLTIVAEAVAAIYCVV
VHITFDITAVGIMIFICGQFESLRRCSESIGGKGKVCNVTAERDARARFRIKKCHRIHVILIHSIKELKELIK
NILGVYFFVATFKLCSLAVRLKTENMSKMQLVTLVQYLGASITQLFLLCYYGDAVFNESAITMGQGPFGAAIW
CVSPKIRRDIVILGMGMMKPHSLQAGPFNVLNLPSFIQIVRTAYSCYAVIGPK
>MsexOR-12
MEQAKREIDESLKLSAFCMRRIGLSFEKHKNASAHLRQQLMFALSVCSICYHVFSEIMYIGLTLANSPRVEDV
VPLFHTFGYGALSIAKVFALWYKKDVFSQLLRELVGIWPTPPLEDEAQAIKDKSLDALRITHKWYFAVNVLGV
WFYNVTPIAVYFYRLWQDGDAQVGYVWVSWYPFDKHQTIAHVAVYIFEIFAGQTCVWIMVSTDLLLSGMASHI
SMLLRMLKRRLESLASTEKTDDEYYHEILENIKLHQRLITYCYDLEDAFSLSNLVNIVLSSLNICCVVFVIVL
LEPFVAVSNKLFLGSALIQIGMLCWYADDIFHANADVAAAAYNSGWYSTNARCRRALLFLMQRAQKPIAFTAM
KFTNISLVTYSAILTRSYSYFALLYTMYNEN
>MsexOR-13

MSTFDLKFETVFKVTMTCLHLNRAHPNIPRNIYWMAQFTPILALSILSFLLLGNSFLFHDVQSGNYTEASKNG
VMAIVAFTIIVKYVILLYRQKDIKNLIATVNDDYEIAKDFCREEQDIVRKYAQKGVTACKFWLVSTFMTSSIF
PIKAFVLMAYYYIYKGEVHLVPMFDMTYPGTMERDKNIIPIFCLLFFLCFLFDCFAGTMYIGFDPLVPIFMLH
TCGQLELLSRRIIKLFADNKNPRIIEKELKLIIIKLQGLYRFVDLIKTYFAVLFEYNMKTTTFMMPLTAFQVL
EALRRGQLNLEFTSFLTACMLQFYLPCYYSDLLMENNRNFRQAIYSCGWEKQPDKGVRQLILFMMTRASLTLG
ITTVFYEICLDTFAEMCRQSYAIFNLMNAAWS
>MsexOR-15
MMDELYLQQPPKKQLFYKVLSFTLSIAGLYWYELWDYKFPKLVKFFLCIVHYTVIIFTPICVTLQMLYIYTRW
NNIAFSTHGTIFTIVPITFLILTKVLSAQKESYRRLMKTFLQEIHLCNFGSNSYMKQRAVEVEKYSRYLATFF
FVFLVVDWALWTIVPIVFNLKNSKAIENKEIPLKTCFYMWLPFDYEHEYKYWVITHIANSTLIALGCIVVTSY
DTINYSIVFHLIGHIKSLKHLIKTNISQNLSDDETKRGLVEVIRYHCFVLKIFGEIERAFGINVTGNYLYNLI
ADSLLLYRLMLGDKENKLMYGVMLLVFMGGLIVMTLILEEVRRQTFDIPQTVYDMPWEKMSVSNQKIVVIMLA
RTQPTLEYKSAGGLKAGVNPTIQIIKSTFSYYVMLKSSL
>MsexOR-16
MGIFVQNVNRSLRFCLTMLKLTGFLVPNGVIEFTVFHNIYWFFWMMFVVGINVITQTGDLIQVWGNLSLMTSA
AFLLLSDVAMMMKIINVVMRGRVIQTVIDGMDLELRSEARAKGRKIIKECDDQTTRHLYLFLCLSGVTVLGWA
GSAEHNKLPLRAWYPYDTSTSPAYELTYIQQNQLITTGQNTIDLRLRSCVLKHQAALRAASQIQECFSEPILT
QFTASTVIICVTAYQLRIEIHQSNLVRVISMMAYLLCMMLQVFLYCLQGNQLAEESSNIAEAVYECPWYRLPL
PLRRSLLLIMVRSRRVAQLTAGGIATLSLACFTSIIKVSYTFFTVLQSVED
>MsexOR-17
MLKTVDSLKQELLFEMDYVNNIGQKIFAYPFIGRSKTAFFFYRTTYFLVVLTAIQLFATLCLTKFKDWFEIIN
IAPNFGVCLMIVIKYSKIYSHRSVYDKILRHFRYDLWDVVSDSKDHRAILQQYTKTTRLIVRFQYYYTLILIV
IVDLFPRIIMLYEAEFLGNEDQYLYPFDAWYPFDKVKWYYPAYIWESFMTAVVIFVYVFANMIHISYTRHICM
ELKILGNSMENLITAEDIVTITKNKDVDKLHENIKSKLKVIIKRHQYLAEITSELDDLLGDAMLLTYIFGSVF
ICLTAFTATVVGDIYMTVRYVSFFLSLLVEVFVQCIIGQILIDHSEKFERAIYSADWPHSELKTKKMLLILLT
RAQKPFVYSANGYLVMNLDTFCGICSLSYQFFNLLRTAYN
>MsexOR-18
MLSFLKNLEDSERPLLGPNYWILKKMGLLLPKNKISKIFYILVHEIVTLFVVSQYMELYVIRSDLDLVLTNLK
ISMLSVVCVVKVNTFVFWQSDWKQVLEYVTKADMFERQNQDVPKSNIINSYTKYCRRLTHFYWGLVFTTFLTT
TNTPFMRYLSSSNFRNNFANGTEMFPHIFSSWMPFNKNHSPGCWITILWHVLLCAYGAAIMAAYDTCVVVIMV
FFGGKLYLLRERCSNMFKNSDENASEATVKQLHGIHIQLIKYSRLFNSLLSPVMFFYMVMCSLMLCASAYQLT
SATNAAQKLLMAEYLVFGVAQLFLFCWHSNDVLIKNENVTLGPYESNWWSASLRQKKNVLILSGQLRISNKFS
AGPFADLTLATFITIIKGAYSYYTLLRK
>MsexOR-19
MTSHVTAYIQYPQTLLTFFGLWPTNRTKFSNVKAKLAIFEQYLFLVLNGVYIVMMRGDVQAMCDASYLFFTQL
ALCCRATLFFLNREMVKNIVDMMNDDIFKPECEDHEMKLMKYATKCKNLYKVLIMGFTSTLTWWIMASLISDE
RRYPYNEIWFPVDMQKSPQYELAYVYEVLAIASSGSIFLSVDTFTVASMLFSCAQLEILREKMNRIMPVPLTT
KKNKSEMQKKLRDNNDLLIACIKHHQAVKRLVLKIEDTFHFSVFCQLCTAVLIICIIGIRLSQDSPNNASFYG
IVSYLTLILSQLYLNCWCGTEITDRSLDFRDWLYEVPWYDQDKRFTSTLSVLLQCTKKPLEFRAGHYVLLARA
TFVSVLRCSYSYFAVLQQANEG
>MsexOR-20
MAVSVLKRLLTYGDNIFEFNIKYLMLVGLWHNENWTRNQKLLYKIYDNTLDVLGLIYLTLTAIGIHGNMDDIT
AALAGVDKSLVAYNFMLKIIVFHFRKHQLRKLVKEIIASGDVVPEEHKILVAKLSLATTTITTIIVTIFTGIS
VLAGELPAKVWLPFDTSKNFMNLLAGVQICLVTFGVPICYRGLALKCFVSTMIFYLRDQLIDLQVKFKELDNF
KDAEEEVRTNFKKIVKKHIRLIRYSKTIDNLLREYFLIQNLAITIEVCMNSVTLTLSEGISSAAYNTSWTSWP
VDMQKDLLTVIIVAQSHFKLTAGGMAVMSLETYAQTLYNGYSIFAVLIDAVN
>MsexOR-21
MLVGFWHNENWTRNQKLLYKIYDLTMHVFGITYISLSAVGIYRSRHDLTAALAGVDKALVAYNFMFKIIVFHF
RKDQLKKLVKEMIASGDVVPENHKILMAKLSVATTITSTIIVVIFTGSSMMVGDLPARVWLPFDISKSFMHLL
AGVQFCLVIFAIPVCYRGLALQCFVSTLIFYLRDQLIDLQDKFKELENFQNVETELRRNFKKIVKKHIRLIRY
SKEMDYLLREYFLIQNVAFTTEVCMNAVILTLIGFSQKTLAINFIAFLITALIHAFVYCYLGDEIIEQSEGIS
FAAYNTSWTSWPIDMQKNLLTVIIAAQSHLKLTAGGMAVMSLETYAQTLYNGYSIFAVLADAVH
>MsexOR-22
MGWIERIKGFILKKSFDFDRPDICLYNFHPQLRILFALKGIFFNKQNSKLKIILPTYFNLLTILGMVFEGMFA
HRGLTIKDYSFAIESFLYFIILTSTPLVYLCLFYHKDKIIQLLDDMNEEFKFVCSLGPRHRTPFLKGQLLIWK
LCYAWYTLSISTGTAFMMFPVMALVYQTLFVTHTEKTIRPLAFTMWLPNDDPYRTPNYEIFLFFEMNYCVIIV
QTFGVYIYTLFHLLLHHYFILDMMILDFEAIFDGLDESVAALPSKHPRRREVQLILNARIKRIVTWHNSVIKT

INTLSIVYKPALVFQILLSSIMVCLIGYQIAESLDNGVIDFLFIMLGVCTCMQVWIPCYLGTLLRNKVFAVRE
ACWNCGWHRNSLGTLIRLDILIIIQRTQVPLLIKLSDMSTVELETFSSIMSAAYSYFNMLRNSN
>MsexOR-24
MGIPAYEDFLRQIKINMWVSGIPFGDTKIYIRYYILLSLLISMIIAEGSFFVSRISSENFLELTQLAPCACIG
LLSFLKILPITVKRRKIFDLTERLGRLYENILDDTTKKAIVKRELHLLSLLTKYFFVLNAVLISVYNFSSPII
MLYQYIAKNKVVFVLPYAVLLPFPTDGWLSWFLAFVYSATCGCICVLFFTTIDVLYCVLTSHVCNNFSIISDQ
LQHLQVNNVNIIGNIVKEHQYILKLADDLEDIFTAPNLFNVLVGSVEICALGFNLTTGNLAQLPGTILFLTSV
LLQILVMSVFGENIITESRKIGESAFLCKWYEMDEKSKKMILTIMIRSRKPQILTAYKFSIISYGSFSKIIST
SWSYFTILQTVYKPPEINREE
>MsexOR-25
MELCKTIWRIITPTKALQQSSGHLETLFFESVYRVSYLMGLSTSDHDMFYLLYSTTVKFMITLLVCGELWYIF
TETSSLDGIASSINVTLIQFITIYRYKNMMDHKDIYKKLATSMESPYFDTSNEKRKQLVVFWAKRNEKYLKLL
LFLGNCTLAAWYLYPLVDDIEYNLIIGFHTPFSFKTPLRYPVVYLVVVIAFTYISHFVMVTDLIMQAHLIHLL
CQFTVLADCFENLLDDCQHGFEDVPRNMLVNNKQFAAKYIRRLGHLVEQHKKILKHTVNLRNTLSRPMLGQLA
ASGTLICCIGYQATTTMTESIVKCLMSLFYLGYNSFELYIICLWCEEITTQSMNIGDAIYCSGWECGVTKLPG
VRSTIMLVLARANKPLVLTAGGMYNLSLTAYTTLVKTSYSALTVLLRFRHE
>MsexOR-26
MASYEGNKTKDFFYKVALLTYLFGLPNFWIEDFKLPKWFMRSFDIFTKIINNVLYFFILMEMIAFFTQENLSE
KQKSDLLVYGISHPILYSYRVFISYKEDNLRAVLLDLVVTLKKVYNDVKVERQMIKKSLLYSSALVFSCILAM
FFYTFDSILHVIRTGATFNVVITTWPKVEDRSTLANAGRIVFYILWWFFMSRVSGAYTTVICLTTCLSHQYTN
LRSYFENLNNIFETNFDQAVKEQKYEDGFKVGIALHLDTLRCTRECHSICQGVFSGQIILNILLLVVLMSQMI
NSERTLVTAFATASSASAVLISTGYFMWNAGDVTVEASRLSSAMYLSGWHNCHGRSSITIRKLVVITMFNAQK
PVILKGLGIVDLSYQSYLSIVKSSYSVLSLLY
>MsexOR-27
MTICTETVKKKLSFLSIILPYGVVESWDNLDPGLYHAVHIYWLRFYGMWYNNFSWKTIYFWAQMMYTLSVLWL
VCFFPGIGEVVYLLKRRENIGNVAEGLYLFLSEMYTYVKVVVFWFNKKKVVNLLEFLHCKEFKAKEMEHREII
HKSIKRARFVMTFYSTMCVGAVSVGIVMPLTENFNILPTNVEYPHFNVYNSSTYVAIYLHHIYYKPATCIIDA
VMDTMLAAFVASAIGQIEILTFNLRNFDVVAERRRKKAIKDNKYIGLQNNNNYFMKCILKECIVHHNHIMRYV
SMIENAFSLASALQFMLSVMVLCLIGIQFLSIENPSEYPMQMVWMAIYLTCMLVEVFILCWFGNELIWKSTGL
RQAAFDAPWLATDPKNAKYITLFMERCKRPMKVTAGKIFTLSLDTYTALINWSYKAFAVMSNMKK
>MsexOR-28_partial
YCDLPTMLWNVAFLLRGITLNIDSRHKKRIPFILYVITVVITLSYFYVFLVSMAWFVFIRSPQTGDLLAAMVV
LSLGISSEIGTLKFFYTFVYINKVQKIVKDYLECDSLIVPGSRFSGNLLRALRNVKKRAIVYWLVIIINGITY
VTKPMFMRGRHHMEDRYVIYGLEPMFESPNYEFAYFLMTAGLCFICYPPANVTVFLIVVVGYTEAQMLALSKE
LLHLWTDANEHYQKNINQHETTLINAQASKNKIINDYVRYRLKEIIKMHAFNIHLVRQVEFVFRGAIAIGYVF
LTLGLIAELLGGLENTYLQIPFALIQVAVDCYTGQKVTDASLIFERAVYDCKWENFDKMNMKTVLLLLQNSQK
TMTISAGGITMLNFSCLMSVIKSIYSAYTTLRTTMK
>MsexOR-29
MKNYYILKTYCSKIFLLGSGDFWYENKVIGDDKRILYRMYSCALFFIYGFMTVLEIMAALMGEFPTDEKRDSV
TFAVSHAIVMFKIISVVFQKELVKTLNRKMVTICEHYEEQALMSEKYRIMKINVIVYFLIVYGSAACFVFEGL
RKLFDGSHFVTVVTYYPDYEDNSMFANSVRILATVILFLMLMNMIVCVDSFTMVYLIMYKYKFITLRHYFENL
SVTIDKLNTPGNEEVVAKMLTDGLVEGVEMHSKLLRLSKDIDKAFGTVMALQLCQSSGSAVSLLLQIALSDQL
TFVASLKIVFFVMALFFLLGLFLCNAGEITYQAAQLSDAIFYSGWHVCRPRHSSRHVRKIVLLAIMQAQQPLV
MKAFKMLELTYGTFILVVRATYSVFALFYAQDK
>MsexOR-30
MRLLKKLENAERPLLGPNVKALQFWGLLLPKYRLKRYFYLTMHFLVTLFTFTECVDVYFVKSDMNLLLNNLKI
TMLATVSVCKVNTFLYWQKRWTDIIAYVTRADLNQRNTKDEKKLALIKKFTLYCRKITYLYWFLMYTTVVIVV
AQPIFKYAFSKNYRDNVRMGTETYLQVVSSWVPFDKNTIIGYIAASIYQTYAAIYGGGWITSFDTNAMVTMVF
FKAELELLRIDSGNIFGNIGRPVDKSIVLRRLKNCHRRHLDLVKYAQLFDACLSPIMLLYMFVCSVMLCVTAY
QITIETNPMQRFFTTEFLVFGVAQLFIYCWHSNDVLIASEELMKGPYESTWWSHTEHRKDLYILISQFKKSIV
FSAGPFAKLTVATFISILKGAYSYYTLLSQSQMK
>MsexOR-31
MAQNTELFLGRPKKILTFFGIWLPSKQYQDLVKIYMILVMITQYSFVLFEIVYIINVWGDIEVVSEASYLLFT
QASVCYKTTAFLIKKRKLVLLLEHMEQEIFASQSKDHEKILLELSLRIKRLCTFFLTSAITTCTLWAMIPLFD
DAGHKTFPFRIWMPVTADESPGYELGYLYQMVSIYISAFLFIAVDSVALSMIMFGCAQLDIIKDKMQKVREVP
ICLNVDEKKEKLKSNNDLFTECVRHYQNVISFVELVEETYHANIFFQLSGTVAIICNIGLRISIVDHHSVQFF
SMLNYMVTMLSQLFLYCWCGNELTIRSEVLRDWMYQCPWYDQDTKFKRTLWITMERMKKPIIFKAGHYISLSR
PTFVAILRCSYSYFAVLNRANT

>MsexOR-32
MDVCIMYLAVSEQLTMTLNSEDLYLNRAKFVMKFLGVWVPPNDENIIRKLYRYFMISLQYLFLIFQIIYIIQV
WGDLEAVSQASYLLFTQACLCFKVTVFHVNVDLLKELLLQMNGETFRAQSFDHEKILESQAARIKRLLLAFMI
SSQLTCGMWALKPLFDDVGSRKFPFDMWMPVTPESSPHYHIGYTFQLVTICMSAYMYFGVDSVALSSVIFGCA
QIEIIKDKLMSIQPVDKNANDAINAFSDNYQQLVNCIKHHQAIVRFTDTVENAYHTFLLFQLVGSVGIICMSA
LRILVVDPRSMQFFSIVSYLSVMISQLFVSCWCGHELTATSEDLHTTLYMCAWKDQNTKFQKELCFAMLRMSR
PLVLRAGHYIALSRQTFIMILRMSYSYFAVLNQTVN
>MsexOR-33
MAETLLDKLLSKINILFRCSGTNIAIGKAAPTNTKRNRCVYVINLILLNTDVLGSTCWFISGLKSGKSFTELT
YIAPCIILSILSDMKSMSIIIYEKKVYQLMENLRMMEAHERIRENTAERGKIIEKGVNFLNLVINVLYVLNII
LLVCFAFHPLVVMMLNYKKTNEIELLLPFPIVYPFDAYNIKVWPVIYLRQIWSEIVAVFNICAADFAFYNFCS
YITIQFRLLQYDIEHVITGTQKSIYNDEMRSGRIRNKLVEIIKRHQELITCVNLLENIYSVSMLYNFISSSVI
ICLTGFNVTENKDMILVITFFTFLFMGLLQILFLSFFGDMLMDASIEVSNAVYNSKWYLVPPKTRKTLLLILT
RAQKPCKLTAYGFADVSLKAFMKVLSTSWSYFALLKTVYSTE
>MsexOR-34
MKIFINNANGTLWLSLNLLRWVGFLVPDNFEGRKKKLLPFYSFFWFMFIVGIYVIVQTGDLIQVWGDITLMTG
TSFLLFTNMAFITKIINVMVRRDAVLAIIDEGDEVLRSERRIEGKAIVKSSNQETSRLLYLYGLLTVVTVFGW
AASAEKGSLPLRAWYPYDTSKSPAYELTYLHQSVAVILLAFLNVSLDVLVTSLAAVCRCRFQLLALSLRTLCH
DIPIDEKHLVSPEHKQIVHERLRLCILQHQSILESAAKIKTCFSGHILAQFTVSIVIICVTAYTLALETHDNP
IRLIAMFSYLLGMMMQVFLYCYQGDYLSEESSDIADAAYECPWYACPIPLRRSLLVIMARSRRVAILTAGGFS
TLSLACFVSIIKASYSFFTVLQQVEE
>MsexOR-35
MVVIEKILSFLEDPRYPSVGPHIRLLSLTGLWHPDLKSPKSRFKIIIFLVTVAFFLSQYVKCIIKFNTDDLKL
ILQYAPSHMGIIKTCLFQKDYKIWEELIDFISSVELKQISRKDENLDKVMKAYIRRNRRVSYFFWALAFFSNF
SIFTEPYQKNNFNVNGTSTYLYIFDGYTPFSREPNGYYFSMCIQTMLGHIVSAYVIAWDTLVVSIMIFFAGQL
KITRLYCTRMITANNEESHRNIAECHRFHTSLVKYQKTFNCLISSVMFVYLVVISVNLGVCIKQIAEIEDDLP
TLVSSCVFLMACLIQLLLFYWHSNEVTIESDLVSYSTFQSNWAQSNKNIQKEVALLALTTRKKLVFRAGPFNV
MSLSTFVSILRASYSFYTLLKGTN
>MsexOR-36
MAETLLDKSLSKINILFRCSGTNIAIGKAAPTHTKRNRCIYSFNFIWQNTDVLGAIYWFISGLKSGKSFTELT
FIAPCIILSFLSVMKSISIIIYEKKVYQLIKNLRMMEAHERNRENTTERGKITEKGVNFLNLVINVLYVFNML
VLVCFALNPLILMILNYMKTNEIDFILPFPIAYPFDPYNIKVWPMVYLRQIWTEIVVVLNTIAPDFVFCIFCS
HITIQFRLLQYDIEHVITGTRKSIYNDEMRGGRIRNKLIEIIKWHQELITCVNLLENIYSVSTLYNFISSSVI
ICLSGFNVTENKDIVLVISFIAFLFMGLLQIFFFCFFGDMLIHASIEVSDAVYNSKWYLAPPKMRKTLLLIQT
RAQLPCKLTAYGFADVSLKAFMKVLSTSWSYFALLKTIYSTK
>MsexOR-38_partial
MDLNFDKMYILTVFAMRINRSHPLIAKDTKWFLQLLPMYGIFSIMFALLINCIIHYDLKAKDFSSTCRNGCLC
VLYFISTLSYYVMLVHQTTLKTIINTMNEDYAQALKFKADEQKVVLDYAKKGLYVCRQWLAMSIMGVGLFFVK
NVLLYTYNYYVDDMKLVPLHDMTYPRIIEEKRDDIIVYASLYALTVYYGVFAAIMYMSFVPLGPVFMLHCCGQ
LELIVMRLENLFIKYTHEEANEKLKDIIRHLQNIYGFVKNIEKCFTGFYELTLKATTITLPIAVYEIIESCHR
RELRMEFLVFIFGAMIISSSPCYYSHLLMEKGEQVRLAVYCSGWEVVQSRAARSTLMVILVRALQPIAIRTMF
RTICLDALTDLLNQSYALFNLMNAMWA
>MsexOR-39
MEGTIRTFHRILSLLGISIFAKEEWNSKKWLALQIFTFINGFITSILTSIFVIANVSDLFLFIQGACIWTTSV
IMSISLGVCLLFRKAFRNFLDEMVFEDAVLEIPLIKLVVMAEGGEKMAELKTLVLESQEKLLKYTKILLKTYM
AIVWLCAILYICTPVYLMITTDDKSLRLLGFDMWFPWSLDNFKVYIASFVFDAYGASLCCISYPGFQSTIILL
VGQEKLSPDTTKYYVHEFCFIQVVLMFCLLGQHVENELEVAVLEKWHIFNKPHQTNVRIFHTAVSQRMPIYIF
GTIPLSLPTFTWEKLSPDTTKYYVHEFCFIQVVLMFCLLGQHVENEKWHIFNKPHQTNVRIFHTAVSQRMPIY
IFGTIPLSLPTFTWVS
>MsexOR-40
MTYKNRQHQELEMELSHLPGDYLKPLIACFDLLARCNIGFFHGNSSYFKKYWRYSYIISCVVAYYSSLTVYAL
KIFLGQMELFELAYVVPVFVVCTQAILKAIIVIIHKGEIRALVLQLGETWRTDNLTTRQLNKKNLLLKKLNFC
YGVFRIVYSYLGTEFLLISLCSHLSTEFCLLREDLLNVKPVGNRRFRILQDSSNIELHDIVVKHQKLIKFSEQ
LNEIFNKMIFVNLSSVTITVCFFAFATKVARGPVDMANNFMAVMALILPIFNLCYYAEMLINASAGNKQESAY
HSLWYVANKQYQMSIWFIIRRSQKPCCLTSLKFSPVALHTFTAVLSTTWSYFSLASSLFENEN
>MsexOR-41
MEFHQIDCFAINMKFFKLLGICIRNYPFRFYNVYSIMLIFTFIIFYDLLCTINLFYLPVQLDLIIEELVFYFM
ELAAISKVLTFVFFRDKLAKILDALEDPMFQASNGKEQKIIDGAKRFNKRYWKIVATVSLTSHATHILSPIVE

RLFLSVPLQLPTCRYSFLSENTIQQFIYPLYLYQTLGMHCHLWFNANIDSFFLGLMILIIAQLQILDLRLRTA
TDVKKNDDIGQANTSEARANYSLTQLNKCIVHFDEVGKFCSLVEDTFSMTLFMQFSMSSCILCVVLFRFTLPA
PFEYYIFLGTYMSVMISFIFVPCWFGTRVMELSVLLCSSVYECDWTAMPKKFKSNLQLFVERAKRPLTITGGK
MFMLSLTTFTSIMNSSYSFFTLLRNVQTHD
>MsexOR-42
MASLWRKYFTKEIQVLKRVYEKSDYEDTYETPRKYLKWSGIRMKHNISKSLSICWLVYYWFCFVNIVFASVAE
IIAMCMTASAGTFDDAIAIFQMMPCNGFCGLSLVKSFKMVKHRPVFENLITEIGNMWPQRLVDEEEHKIISSA
LREIKIFVKGYHWCNNYLMLSFLYPPFWELIKRLSGEKWEPKLHFIYWLPFDPLQPVYYECMLALQTWQAMTV
IWTNMSGDFMFCLFLSHITTQFNLLSVRIRKLIYVPVDQQLIESYPLGQYSEEYLRKNKEPVDSYTPQQWEEK
HFKEITEIVLQHQALIRLSRDIENMYSLTLLVNVVNSPLVICFCGFCSVVVEKWNETAYKSFLVTALSQTWFV
CWYGQKLLDSSEGVAEAVYNSGWYRASKKIRRSLMIMLHGSQKGVGVTTYGFSIISLASYSTIIKTSWSYFTL
LLNFSNK
>MsexOR-43
MLERLSKYLENPNHPLLGPTLYGLKCWGMWQPLGVNRIIYNAIHFFAILFVISQYVELWFIRSNMELAIRNLS
VTMLSTVCVIKAGTFVFWQKSWNDVIDYVSGLENIQLSKRDRITNSVISEYTKYSRSITYSYWVLVTATVFTV
ILAPLVGFLSSSDKDLMLNGTLAYPEIMSSWLPFNRSRGFGYWVAAIEHSLICFYGGGVVANYDSNAIVLMSF
FAGQLKLLSINCARLFNDNEVLKYSDTMKRIKDCHHHHVEIVKFSMVLNSLLSPVMFLYVIICSLMICASAIQ
LTAEGTSNMQRIWISEYLMALIAQLFIYCWHSNEVLHMSSKVDEGVYASHWSAQNVRVRRSVVLLGGQLRRPI
VFTAGPFTKLTISTFVAILKGSYSYYTLLSKKED
>MsexOR-46
KKLTPTKSLEQSSGTLETSFYESVYRIAYLAGLSSSDYGILYIIYSTSVKIMVALFVIGELWYASTEPLSLDE
IAACINAIVIHLITLYKLKNLMDHKDVYKRLATSMESPFFDVSTKRRQEIVNFWVQRHERYLKLLLALGNSAL
TAWYMYPLLDELEYNLMVGVSLPFKYNTPFRYVITYILVGIAFNYTSHFCMVTDLIMQSHLIPLICQYSVLAD
CFTNLVSDCEVGFEGIAREHLVNNKKFVKLYLRKLGNLVEQHKFILNHSIELRTILSVPMLGQLAASGILICF
VGYQATTTISVNITKCLMSLFYLGYNMFTLYIICRWCEEITIQSQNIGEAIYCSGWEQGMSKIRGVRTTILLV
MLRSSKPLIFSAGGMYKLSLTSYTTLVKTSYSALTFLLRIRRE
>MsexOR-47
MQIIKIIKMCRDRLFENSCVNLLRIIDFLPSLAGFSLRRSKFSVTFWIIHTVLLFYVYVSGTIIYQKYYAKEF
VDLINSFFNISIFILIFVNSWWLLSRRSTLKELLDLVIINDDHITESGRLSSQHLQLLNKIKKILFACYVFHF
VNDVCIFIPSRTVIIDDFVMASCVGLKPQDSSLDRIICMGILTVQEITSIMVVASYDMTLLFLFSHTTAMFQI
LYEDVTNFRELAENYYDSSEMKSAVFERLKNLLIRHSSILRTVQKMQDVYSVVVGIGFGLNAISMCLFFVLPI
DVCLNFAPLIFHSLFVFFLYCYQGQRLTTASEKFEIAVYSCGWEHLGVRDQKTILLMLIQAQKPVIMMAAGVI
PIRIRTFAYTLQNIYKFVTLFKI
>MsexOR-49_partial
VLPGTRYYNNIMKTLRIVKRRAVGYWVTIVMNGIIYNITPIFRSGRHLSEDLMVIYGLEPTIETPNYEISSLL
MTVAVSFVCFTVASTSGIIIIILVGYVEANMLSLSVEIKEIWNDAVADKKEKKEYQHLKNQPMKIVNMYVKRRL
VNIIERHKANINLLSDVEHLFRIPIGAGFLCQTISIVAELLGGLKNTYLEVPFGLVQITMDCYIGQKVMDASL
IFEEAVYDCKWEKFDNSNMKLVLTILQNSQKT
>MsexOR-50
MLWNVAFLLRPLTLNIDSRHKKPIPLLFYVLTVVITSSYFYVYLISMSWFVFVRSLETKELIPAMVVLSLGIS
SEIGTLKFFYTFVYIDKVRKIVDEYLECDALVVPFSRFSKNLLTTLRFVKKRAIIYWLVIIGNGFAYWSKPLF
MKGRHHLEDNLVIYGLEPMLESPKYEIAYFLMTAGVCFICYPPANVTILLIVVVGYTEAQMLALSEEILNIWD
DANEYYNNLPSTNQNTNELKSKIINKYIKDRLKDITKSHARNINLLRQVEFVFRGAIAIGYVFLILGLIAELL
GGLENTFLQIPFAFIQVAIDCFTGQRVMDANIVFERAVYDCKWENFDKTNMKTVLLLLQNSQKTLTLSAGGVT
MLNFRCLMSVIKSIYSAYTTLRTTMN
>MsexOR-51
MGQDYYLQPPRTQLFYTILGHISTIFGTCQMEWWGPQYKISKWLAWLYFLQRHSVRTFGKVVCLSQSVFMVLN
FNTLSSSVLIVILTITPVGYLVAVKAETAKLKSYEKVMKAFMDKIHIYNCYVRNKDSEYVKTKVKQVEFLSRF
TTWFLTFFLYICWSAWMLQPTLYNIRNIDAILNKTQDFQYYIYLWTPLEYHHNLRNYLIIHTVCVYLGVTAIT
VIITFDCLNMIIVYHIVGHIYILKHNFRTKLADDFTDEKAKEFLVDTIKYHSFIITTFKNVQNAFGINVAANY
LQNLFEDGLCLYQMMKGDMENFVKYGLMLVIYLGSLIMLSVVLEEIKRQNSDLSEVVYSIPWQRMSVSNQKTV
MLLLFRTQPDLEFRAACGMKAGVQPALSIIKSMFSYYVMIKSRM
>MsexOR-52
MLSKLKARLNNINERFRDVSFDSLMWIVNIAPNLVGFSVRGDKAGAAFWFIHLFLLTYVYGVGSVVYQWKFAH
TIGDYIKSYINVSLVIFIANNSWWFISKRVQVKDVLEKIALSDQLACQTDASQKKYKKMLKIIKHIIIGFYAV
NYIDELFIYLPHRVDVSNDYSLTPCVGLEPLTQSPNKEICSFILCLQECTIATAVLNYQALLLLLIAHTAAMY
RILSAEMISFNDYDNLEEHQARAKKILPSLIERHVLILCVIDKLKSLYSVSLGVNFGSHAVCLLLLLYLPIRE

SFMFIPIFAYCFSVFFLYCFLCQKLVNASEDFERSVYCCGWENFCLKERRMVYFMLCEAQRPVEILAADIVPV
NIYTFATTLQAMYKFVTVVKF
>MsexOR-57_partial
IRTFHRILSLLGISFFAEEGWDSKKWLALQIFTFINGLIIFIFTSGFVIANVSDLVMFIDGACIWCTGVIMAI
SLGVCLVFRKGFRNFLDEMVFEDAVLEIPLIKFVVMAEGGVKMAELKTLVLESQEKLLKYTKILLKTHLFAAS
LCVTLYICTPVYLMIMTDDKSLRLLAFDIWFPWSLENFKVYIASFVFHAYGASLCCITNPGFQSTIILLVGQL
IRQLRILTFILLNLNELVIELVGNKNPRWQAHCTSVLVQCVKHYIKLKRFSNGLNYICRPFYLTLILVSTMLV
CMCSVKIATSEKLTPDTIKYYVHEFCFILIVLMFCLLGQQVENECRELEVAALEKWYIFNKQHQSNVRIFHIA
VSQRMPIYIFGTIPLSLPTFTWFMKT
>MsexOR-62
MEIKTDKPREKKFKTFNETFSLCAFALAFAFLYPNRTNAVKRAVIITLIILFNGGQLFWFITYTLKCLYTLDI
LNFARNMTLAVVLILFFIKTYYVIYATKDFAPILIKMSNDLLAANELEEDYQAIYEEHIRQGKVGQISWLLIP
IVLSAQFPIYAGICMSIESIKTDNFTRLMVHDMELLFVEDIQSETPFFQCMFAYNCCQCIVLVPNYCGFDGSF
CIATTHLRMKLKLMTHKVHRAFAHAQNTNELRKMVNEAIQDHQDALKFYKEMQHVYGPWLFAVFMLTSFMISF
NLYMIYLLKRVDPKYTLFGLVGVLHIYLPCHYASTLTKVGEEIGPDLYDTPWEKWADPEVTKLLIFMIARAQK
TLIVTGNGLVVFNMELFKSIIQSSYSFFTLITA
>MsexOR-64_partial
ATNYIHYIILPLKIVACWDWFPKPKRNREILMNNIYLGMVLFVLTYMPVALAVHLYTEWQDIMSSLDKIADCL
PLLVSVAIVSYYALYRKDLYTLVDYMEKNFKHHSARGLTNMTMWGSYCKARNFGRIYTACTMFSVTMYVTLPL
ILHLWTKEPIQSWVYSDVTQSPFLEFVFLRSFLTQFFVGLAMGQFGFFAANSILICGQLDLLCCSLRNARYSA
LLRRGVRYSALFAARAEFTADERHNYIYNVAETINSDFHYDRKVVTTCALESYCDMYSSEYDEATVDALCDCA
RLCQIIKQYKEMFETFVSPLLVLRVVQVTLYLCTLLYAATLKFDMVTVEYLA
>MsexOR-65
MALKYPWNKSKEWFERIVFWAYLSGLPNFWIEDLNFSKHFMKFYDKYARALDIISCIFVLLELLSVFTQHELS
KKQQTIQILFCVGHPFLCLYSALMGHYKEKTRGVLLELVVTLKQVHNDPNVEKMMIKQCNMYSIAFTFSCFCS
MLFYCVDSLIQVLKTGVTFNVVIPVWPIIADNSVIANVARVLYHIVWIIFMAKVACVYIIVISLSTCISHQYK
NLQKYFESLNEIFLDENLKQDEKKAKYEEKFKIGVKAHAQTLRVTKESHEICNGVISGQIVFNIIMLTIIMYQ
TFIMTRERSLVKMMSTATTAMTVLCSTGFFMWNAGDITYEAAHTGTSMYSSGWQNCCRQSSIRVRKLLVVAIA
HAQNPVIMRGLGIMELSHQSFVSIVKCSYSLFSLLY
>MsexOR-67
MRPTRYFAVHYLLLRVLGLGWWHHPDEDNKSNFPGWYLYYSIVTQMVWVVGFVGLETIDPFVGEKDLDRFMFS
LSFVITHDLTLIKLCIFYFRNAELQDIVHTLEIDLYQYYQNDNKNRKTIKLSRIFTGAFLFFGWMTIGNANVY
GIIQDLRWKSIIKNLNDTAIKPVRTMPQPIFIPWPHQSDKSYIPTFALETVGLLWTGHIVMTIDTFIASVILH
MSSQFAILREALVTYDRTMAQLATNDSDGNLSINADTNYEEIDTEANEKIVKRFYTGPEIEKALENSLLNCLR
QHQMLIGCVEKFSRTYSYGFMTQLLSSMAAICVVMVQVSQDASSFKSVRLVTSLAFFFAMIIQLAIQCFTGNE
LTLQAERISDAVMQSKWERMPPRLRRLLIIMMMRAQRPLRLTAAGFANMDNACFLSILKAAYSYYAVLSQKEV
QV
>MsexOR-68
MSLKTKRLLKYFCKYVYFAGAGNYWYEDIYQETTLYKMYNVISFSVYTTMIFLENLAALFGNFPEVEKNSAVM
FSAIHNIVLAKMFLLLYHKKSIRKLNYEMATVGENLEVEFVMRRQYRKVKLGIILYIISVYLSLIAYGVESTR
RAIIEGTPFYTVVTYLPFYDSTTVFASFLRIFFYITWLYMMLPMMSADCMPITHLIAMTYKFITLCRHYKRIR
TEFDRNMLTLGVRESAARLKAGCLEGIRMHQKLMALADEIHRVFGIIMSLQVCESSAVAVLLLLLRLALSPHLD
LTNAFMTYTFVGSLFLLLALNLWNAGEITYQASLLSNAMFYCGWHLCVTEKESHREIKTFVLIGCAQAQKPLI
LKAFGIQDLSYETFVSK
>MsexOR-69_partial
ITLTERLEDPKNPLLGPTLKGLYLFGLWQTGGKIQTICYNVFHCSTVVFVVSQFTDLYLVRNDITKVLNNISL
TALSVVCVVKCFSYVLRQAKWKELVKEISQEELLQIKKQDEVTMKLMAKYTEYTRVITYMYWMMVFFTNALLL
TTPLIKYLSSKKYREEIKGGSEPPHIMNSWLPLNTSEMPGYLIEIVVHVIMGCQGAAVVAVYDMNAISIMSYL
KGQLLILKAKCKGIFDDGSRVDVLNRIKECHRHHTVLLKHYRLFNSLLSPTMFVYVLICSITICCSVIQFSSK
EATPSQRIWVIQYTTSQVAQLFLYCWHSNEITTESRKLDRGIYDSDWWK
>MsexOR-70
MDQKRLFENCLNRVYFFLKLIGVGIDKDDRQRTFFQRLCGRRIYILHFIQLNLNLSGEVAWLVNSLIFGAGTL
EQYTHLLPGLTLGILGDLKTFFMIRYAHHVLDLIESFKSQYTNMIVSNETQSEILNKKLSFLAVTTRVIAFPY
VFGVSMFAVGPVIVMISIYYTTGKVELILPFIVLYPFDSFKLPIYPIIYVAQIWSGMIFYYVNHVCYLRNIKN
VFLIFLINFTSKYPLLYLIENLLSVNCKNISNLPIFVSDSICFFFQLRLILFTGPDCFYYTACMFIHIQFDHQ
HDVEQFIEKYRNGRTSHHQSHYSEFVELLNRHRELIRCVDLLEIIYKTLFNFVSSLIICVTGFNIMVIKDLPL
AVAFTFFFVVGIFQICIFCYYGEILIQSSSKVADAIYNSKWYECSVADMKDYLFILTRAQKPRKLTAYGFSVI
SLNSFAKILSKSWSYFALLLTLYKEPQTSRH

>MsexOR-71_partial
MASYFKRIFSRKQLDANKPTVYTREDYDNTYSVPMAVLKLVGLRLTKEDLSCARLCWNVFYWFEFANLFIVTW
LELINMVQTAKGGSFQDAVEIFRMMPCVGYLLLAMAKSYKIVYYRPVYENLVSELRDMWPRGVVSEEEHLIIS
TALKQLYYVVQGYYWCNNALLVIFLPPVEIIKRAAGDEVPLILPFFYWFPFDPFQRIFYEIILIAQTWHGLIT
IWFMLCGDLLFCIFLSHITTQFDLLSVRIKRLVYVPIDKQLVESYPLGEYSKDFLKKDKHINLDSFTEKEWEN
RYQKEVTEIVLRHHALIRLSGDVENLFSFALLVNFFNSSIIICFCGFCCVIVEKWNEMVYKSFLTTALSQTWL
LCWYGQRLLESSEGVSSAIYNSGWYMATRKIKSSLLIMLHRAQKEVYVTTYGFSVISLSSYTTIIKTAW
>MsexOR-72
PIFAANGRENEKYLLKNIKEMHRLSKIMNIFVTIAAVCWLASNVVQRLRDESTAQVQLKMIKHNLKHLFDETS
LNKSSGLYDDVTEKKLYKKFVYYVKRYERLVWYITEVNGVFNPSMTFQFLTSSITMCIVIYEMSETSVLSMEF
IFLMVLIVILLIQVFLYCYYGNLVRHESESMNTCLYESEWTSSSPRFRRAMLIAMARWSKSMTTRVARIIPLA
LDTFISIIKFSYTLYTVMSSNKDKNIGTISILNSYLVNTILSHKCFNKYINYES
>MsexOR-73
MGRSAGYVTLKVKQSKDLLGDKRAAACLLLRDPAPGSNPVKPRKPAKCGNEVIPGQSKNFGEGILKYSEVFAD
RGEYAYVAGQMINSASIEDFVGSYLHIAGYDTMSFGKLITIWYKRSTFRQLVSELADIWPVSIKDKEAAQIKR
DSLSALKHRQIWVISVCCMLGSDLLFMTMSSHISMLLRLLQVQIRRLGTTGSSNENLENCYQDIAAVVQIHQR
LIKYINHLEDAFSVVNLINVLLSSVNICCVVFTIVFLEPWIEMSNKFFLGAALTQMGIVCWYADDIYRASVGV
SDAVYESGWYNSDARSRRLLLVVLQRSQKPLYFTALKFRSITMSTYSSILFDGPIGVPEDSGRAEEARPSLAT
NVSGPNLTSTRKSALVPLHTTE
>MsexOR-74
MNLKQSHPQKYYMKLVFRCLYYFGLGDGWYEQTNRSANAKKLYLIWAVIANLYVVGIICNECLANFRSDLSDL
EKNDLVQFTFAHLSILAKIMSFYFQKSKIRIIFEKLLEETRSVFYSEELDKQCVKQYIRYSLALIGVSYLTLF
TTTINGIRVHFMEGIPIRTEVTFYPRASDSGYLINILRFFIEFHWWYIVGIMVVIDCLGVASLVFVSYKFKLL
QLYFKQMREKVLENHGKVERKVLEDEFKKDFVVGVKLHAHALWCARNVQQGLGSIYSVLVFESVSLMVMCLVK
LVASLLSTAIFHCGWEFIRADTKLRVLTVFAIQQSQVPVYMMAFGIMTLSYNNFISKSQRCVSLGFEPADIRP
GMAVRSTSNQAIAAK
>MsexOR-75
MFTQLKARSVAVRRERTSAGVASLMRYALSSITAKCMSSFCQTDIFKPNLFFWKCFGIWGGRTENKNYKYYSF
SYLFVTLFVYNILLTINLIYTPLKIESLIREVIFYFTEIAVTAKVLMILVMRSKIIEAFNLLDCKEFKGDDEE
SKRIIETNNSFYRTCWKLYAVLSNVSYSSQVFAPLFINLIWTAKIELPVCKYYFLSDEMRDKYFIFWFIYQSF
GIYGHMMYNVNVDSFIAGLILMAITQLKVLNAKFTNFNLEKRHKKRRILIQDKIQIMRLNRYLKHYDCVLRYC
ATIQDILSVTMFVQFGMASAIMCVVMCGLLLPSTTETFVFMVTYLFAMMMQIFVPAWLGTQLSHESCGLVFAA
YNCEWIPRSMSFKRSIMIFVERANNPIQLTGLKMFPLSLATFTSIMKTAYSFFTLFRNLQDHDDGAN
>MsexOR-76
MQLLRWCGYCRLDGGKCGGGADNESLHRVYRAFTLALTIAYLLQECVYAYQERHDMDKVARVMFLLLCHITSI
AKQLVFYLDADRVEHLVTGLDDPLYNQQAGGARRLLEATATWASRFVRMYSGCAVVTCTLWIIFPVIYHLRGQ
QVEFPFWIGVDYSNSHWFAAVLLYSYYVTTLVGIANTTMDAFMATVLNQCKTQLTILRLNFKSLPERAAAAAG
AGAVCRARHDAALMTALLQCLAHYKHIVDTAGLLQRIFGTAILIQFAIGGWILCMAAYKIVSLSMLSIEFASM
VLFTSCILTELFLYCYYGNEVTVESNRLVESIYSMEWVETGVRFKRALLIVMERAKRPLRPAAGLIIPLSLDT
FVKIIKSSYTFYAVLRQTK
>MsexOR-78_partial
CYSLISFMFLLGTYLIIQVVDLFLIWGDLPLMTGTAFLLFTNVAQFAKIINILSRQERIQRIIDDADAVLTSV
RDAEAKEIVRTCDRETKLQLGMYSCITLVTAVGWAASAEKNKLPLRAWYPYDTSKSPAYELTYVHQIGALFIA
AGLNVCKDSLVSALVAQCRCRLKLLGLVLSADLPGVDQNHLTSEQEAVVSARLRECVVEHQAALDAARELQAC
FSAPTFAQFTVSLIIICVTAFQLVSM
>MsexOR-79_partial
IVKRRAVGYWVTIVLIGIIYNSTPLFRSGRHLSEDLFVIYGLEPAMASPNYEISNLLMTVASIFDCFTVASTS
GFVIILVGYVEANMLSLSVEIKDIWNDAVANKREKKEYQHLKNQPMKINIIERHKANINLLSDVEHLFRIPIG
AGFLCQSISLVAELLGGIKKTYLEIPFALIQITMDCYIGQKVMDASLIFEKAVYNCKWEKFDNSNMKLMLTIL
QNSQKTMRLSAGGMATMSFSCLATVLNTIYSTYTALYPKIRKNTRRN
>MsexOR-80
MSDLMFDQSMSKIEILFRITGINITSKGTPNTRKNRIIYILNFILLNTDVLGAVCWFISGLLHGKNLTQLTYV
APCVTLSFLGDFKTIFLVLYEKNINQLIQNLRKMELREKTRERTDAKDAMIKKKMNFLNAVVNVSYVLNILLL
VAFAINPLVLMGLKYMKTGELEFLLPFLIVYPFNAYDIKVWPMVYIRQIWSEVVVVLDVCVADFMFYIFCTHI
TIQFQLLQHKIEKIIDTPKNINTKILYNEHFRTKLIEIIKWHQELIWSTNLLETIYTKSTLYNFVSSSIIICL
TGFNVTAINDIALVISFLTFLFMGLLQIFFLCFFGDMLMKASMDVSDAVHNSYWYLAHPRIGKHLLLVQTRAQ
KPCKLTASGFADVNLRAFMKILSTSWSYFALLQTLYSPPRE
>MsexOR-82

MDTMFLLLTNSDSIYKQVVLWRKADKIGELLAVMKGFIFNSPSACPQKLLLKTAFQARLLLRIFNIVALVTCL
LWVLYPVILYSQGKPVELAIWLPFDTNISPHFYAAIIYIWIQTSWLAYCNTTMDAFVSFFFAQTKTQLRILRH
NLENVIKVCKLESKKNKIPLELIFDSHIRKILLHYEEIIIFSNKILNIFGGALLYQFLVSGWIICTTAYRIVN
IHPASMEFVSMIMYMCCILTEIFLFCFFGNEVTHESERLKESVYEMNWLEIPLKYRRTLIIFMERIKRPIDPM
AGAIIPLSNSTFLSVVKSSYTFYTFLKNTEK
>MsexOR-83
MYLNFDEFYRWPILAMKLNRSHPHIKRDKKWFMQFFILHGMFTIIFLLIIYCVIFHDLKNNDFSATCRNGCLS
VMYFVVSFNYAVMLANQDLLTSMFEIMKADYKETQSFITEEQKVVLKYAEQSKWVCTQWLAISIAGVVLFPLR
NFILYAYYYYKDDFKLVPVYDMTFPRIIEENKESNFFVYLLTYFLIMYFGLYGSFMYAAFVPIGPIFMLHSCA
RLEVI
>MsexOR-84_partial
MTHSEEFESTIRLTQFTLLISGIKLYRKKWNFLIDILVQKFLFYLNMITLYPVLIAEIYLLIEALQKGGSFVE
HSLMTPCITVRILGTVKVCFLYKNSDVLLEIIDKLSEIHPKFNNEVEIANEDRKTTNEEKVTKDSMRLLNLNM
LVLIGSGVCVVTMFCLMPFILMMIGYYQDGVLKIQYPYLVKYFFDAYSIRLWYLEYIHQVYATGIVFANVVGT
DTLLLAFCTFIEMHFKLLSFRIENLRATSSEETKRNFVTSVIRHQELIQLVSKIEMVYTKSMLFNIVTSSLLI
CLSGFNMT
>MsexOR-85
MFRCSGTNISIGEAAPTNTKRNRCVYGFNFIWQNTDLIGAISWFLEGIISGKSFTELTYIAPCIILSILSVIK
SISIIIYEKKVYQLMQNLRIMEAHERNRENTTEKEKIIKKGVNFLNLVINGLYVFNILLLACFALNPLALMIL
NYMKTNEIEFLLPFLITYPFDPYNIKVWPIVYVRQIWTG
>MsexOR-86
MDSDSRFIFRISGEVSEIFGVLLLMHIMSSSVIICFFGFLAIVYGSPADKVSYLLTVFSAVLMIFVLALAGQI
LYDASSRVAEAAYESLWFESDINVRRAMLFIIIRGQQPASLSALGFSDMTLRTFSKIMSSGWTYFNLLIHIFE
DI
>MsexOR-87
MAVSVLKRLLTYGDNIFEFNMKYLMLVGLWHSENWTRNQKLLYKIYDNTLDVLGLIYLTLTTIGIHESMDDIT
AALAGVDKSLVAYNFMLKIIVFHFRKHQLRKLVKEIIASGDVVPEEHKILVAKLSLATTIITTIIVTIFTGIS
VMAGELPAKVWLPFDTSKNFMNLLAGVQICLVTFGVPICYRGLALKCFVSTMIFYLRDQLIDLQVKFKELDNF
KDAEEEVRTYFKKIVKKHIRLIRYSKTIDNLLREYFLIQNLAITIEVCMNSVTLTLIGFSQKSLAINFIAFLI
MALIHAFVYCYLGDEIIEQSKGISLAAYNTSWTSWPVDMQKDLLTVIIAAQSHFKLTAGGMAVMSLETYAQTL
YNGYSIFAVLIDAVN
>MsexOR-88
MKIIYFFKRFFDKLCLENDDPVEAAKIMITFIQQFVGIHNVFGDEGRSYSLLSVIAVVIFGGTFVYVGTFSQI
VYLVHVFPDKSETFKVFNCLSATSVPLSKYIFMWTSRSRLKTVFQMCKDGLSSIPADSTSHVKMMKTLQKARF
VSWLVVGNQAIVHVAYLVVPFLFTVFGNNRYLPSTPGDSYGLSPKYETPYYETTFALTCIGTVFSGINQTGYI
VLFITLCAHELAHFYAITEMLKDVHQILTKENNLARTKIVNEKLRFCVKHHQFLMQYHIKIRELYKIIFGAHF
LMMTIVLVTTLQTMNSWDMRNTILTAVTGIMPLFLYCFGGELLITAGLDMSTAVYTCGWELMDGKQARLVLLM
LCLSQRPLSLTAADIFIMNRETFGKVVQVVYKIYAVFN
>MsexORCO
MMAKVKTQGLVTDVMPNIKLLQLSGHFLFNYYADNSGMTMLLRKMYSTVHAILIFVQFVCMGVNMAMYADEVN
ELTANTITVLFFAHSIIKLGFLAFTSKSFYRTMAVWNQSNSHPLFTESDARYHQIALTKMRRLTYFICFMTVM
SVVSWVTITFFGESVRMIANKETNETLTEPAPRLPLKAWYPFDTMSGSMYVFVFVFQIYWLLFSMSMANLLDV
LFCAWLIFACEQLQHLKAIMKPLMELSASLDTYRPNTAELFRVSSTDKSEKVPDPVDMDIRGIYSTQQDFGMT
LRGTGGKLQNFVQNTVNPNGLTQKQEMLARSAIKYWVERHKHVVRLVASIGDTYGTALLFHMLVSTITLTLLA
YQATKINSINVYAFSTIGYLCYTLGQVFHFCIFGNRLIEESSSVMEAAYSCQWYDGSEEAKTFVQIVCQQCQK
ALTISGAKFFTVSLDLFASVLGAVVTYFMVLVQLK
>NvOr3
MAEMKRMEDVFAYYDERMKKPGPSCSNEKFEEDVKYATALNRRIANAIGIWPIFTSTGARLGFDICVKTLKNA
AVYILLSFLLVPGILHIVVEEGKLKAKILKTGPMILNTMALLKYSVMLFRKSQIQECLKQLESDWRKAGNDEL
RALMRRNTAVGHRLSRVCVATFYVGGIFYRLIKTLLTPIRYTKDGLMIKPLPSPLYKGLFRFNTSASPVYETI
FATQMMSGFVVHSTTVTTCSYAVLLATHACGQLDIVVYLLKRLIEDDGDNGRLTRVGNEAVDRKLRVIVQLHL
KVLRFISSVEDLMNQICLVEILGGSTILCLTSFYFIVDLQSNDALGLFTYMVMITSLIALLFTYCYVGEIVSD
KAKKVGAKTYMINWYDLPPKKGLCIGLIISVAHSPVQLTAGKMLELSMYNFGCIMKSTAGYLNLLRTITD
>NvOr8
MYSAMDNHDIKDLINAEDFEYAIQILRWLFQPMGIWPLKSAAYPSFLRPISIVISFWSAAFLIIPGILSVIRV
QNDFALRLRLIGPVSFCLVTSFKYFSFLVKNRQFYAYLINVALDWREMKKNNHNRIIMLRKTQISRFFMTSCS
ICMYLSGMSYNILLPLTKAPTQVGNVTFKNLPYIGYYIFFDQYADPYYYVVFVMQCMSSFFCYSTCCGVCCIS
IQSVLHISGRCDITSIMIKNLNGNCNEKALKAVVEFQLQSLKFAREIEKLLNQMFLVEFVGSTFNICLLVYYF

MGDFKENDTVGTMTYVLLFISFTFNIFIFCYLGEHLTEQCASAGAAVYTMDWFRFSAKKSRDLFLIVLFCQRP
VVITAGKMVNFSLLSFASLMKASAAYLNMLYKMG
>NvOr11FIX
MTSSVSWQHLVVVGRFEGIDMVTDSKTESGYDIAVGPCRFFLRIFGIWPDPFVESSALEHLRALIVSSTMVFF
ATITQTAKNVQVWGDLNVVTEISINSEVPTSTAAVKVAGIWYYRRALRKLMIQITNDWRSPHGPIELEIMEEN
ARFSRLVSATCIFLADATFAAQFIIAVLRIDAQNRLQANGTDIDRPLYMRASFPYDSQISPNYELTLAGQLFS
NFFAATTYTSIDSLFIVLMLHLCGQLSVLRLKFPGLKGCSGNEFAEKIAYIHRRHDQLTVCANTIEEVFNKTF
LLQIITSSFVLCMLGYQIMTITNGNNIPFMELVFMIYYVICFLFITFTYCYMAEKLREKSVDISDAAYQCDWY
DLTPQQSKQLIIIMLRARRPLQVTAGKFVAFSLSLYCSILKSSGGYMSMLLAIQQRLGTS
>NvOr12
MADKKGYEEAIEATRAVLRAFGVWPNRHKISENWLSRSHFLAPAFLIICFINIPQTLKIIKVWRNLNEVLDIL
VTANIPSFVALIKLLCVRYNKKVIGLLLVSMENDWKSLKTLVETRIMWKNGKLGSLITLVIYTLTCGSYVAYV
IMITYINVGGSKQEDVITLNESKKLRPLYMRSYFIYDVQKTPVYEIIWIFQFVSMGVATFTFMAVDSLFAVLM
MHLCGQLINLQERLKNFTNMLGQTKTRNFSYQLSTIVSRHEQLNRFAKAIENAFNTMFLVQMLLSGMVLCLQG
YQIVIILTGRDTVQIIELLFMVYYTLCFAFSLFVYCYIAEILRIESMEIGNAAYHCDWYDLSAFERRLFILTI
IRSKTPFEITAGKFAAFSLEFYCSILKTSGGYLSVLLAVQDRLAA
>NvOr13
MTVEIAEDSMERIVALSDDQNVDGYNHAIGPCRFFLRLLGTWPDPYGNVDSWTTSARCLVITATMFLFATISQ
TVKMALSYKDLNLVTEILTNCNIPTTIATIKIASIWYYRWVLRDLVRQIIEDWEMSHDRHESAIMWRSAKISR
IFSIGCMFMTEGTLLTQCVVGLFRPISYAFKTDLNQSIEWPLYMKGSFPYDVQSSPNYELSILGQLLSNVFAS
TSFSSADSFFIVLMFHLIGQLSILKLTILDLPSKIENSDDRSKFIDRFAFVHMRHNRLWRFSMAIEESFNTMF
LIQMIPCIFALCTQGYQLIMIMDADNVSLMELIFMIYFLVLFLFTIFTYCYVTEILRCKSLELSYAVYDCDWT
ILPAKEARILLLILVRTQHPFEITAGKFASFSLPFYCRILKTSAGYLSMLLAVKKRSEQVASKVVL
>NvOr16
MDDKEGFEVAVKASRTILRVLGIWPNHHERTESWLSRSYFIMPTFILVYFTSFPQTMEIIKVWGDLNSVLELL
TTFDIPNLISLIKILSVWYNKKVLGLLIMAMENDWKSLKTVFELRVMWKNVKLGRLITLAIYLLTYSTVATYV
VMAVYITANAYKQEFILTPDNSTKLRPQYMRAHFAYDVQKSPVYEIVWIFQCIAMHLAGLSFMAIDSLFSILV
LHLCGQLINLQERLKNVTENLTKRHNLSYQLSRIVMRHEQLDRFAKAIENAFNTMFLAQILLSGVVLCLQGYQ
IVIILTSRDTVQVTELLFMIYFILCIAFSLFIYCYIAEILRTESTEIGNAAYECNWYDLPACETRLFILTMIR
SKTPFEITAGKFTAFSLQLYCSILKTSGGYLSMLLAVKERLAL
>NvOr18NTE
GFDVAIGATRAVLCFLGVWPDIKRKKLISRTRFLLPTLIMLYFAIIPQTTMAIKVWGDLNAVLEVLTISDISI
AIALFKMLGLWHNKDVLGQLVVSMSEDWRSSKTEKEFQVMWQNARMSRLMSITIIILAEGTIMAHFTMALYFT
MLESKQYSLTKLNATTRFRPLYMSAEFFYDIQSSPNYEIIWLFQFLSTMFAASAFSSVDAFFAVLVLHLCGQL
NNLKEKLKNFPKRHEEGEIQSFLHMFSDIVVRHEALNKFANTIEESFNIVFLVQMVASSLMLILQGYQIVILA
TSEGSIPFFELIFMAYFTCCFTFSLFVYCYVAEVLRTESMEIGNAAYQCNWYTLPPSEAKCLTLIMIRAKQPF
EITAGKFAAFSLELYCRILKSSGGYLSMLLAVKDRLAV
>NvOr19
MTTKEEGFDVAIGITRFVMRTHGIWPGFSVSKAGIMRYAYLPAALMLLLFVIIPQTVQVIFVSRDLNAVLNVL
TLGNVPVGIALAKLLGVSYKQNVLHQLILSVCEDWKHTTKESELVVMRLNARKSRMFSIICIVLSEGTAMAYS
ARMFYAAFSTHTKAQATGIDDCEKPLFFIGKFPFDPQSYPNYQITWTLQIIATFLAAGAFSSVDALFVTLVLH
LCGQLTNLQAAFSEIGEENAEKGTMFVSKLSKLIERHRKINVFADIIEYSFNMMFLVQVLSSTLLLCLQGYLF
MIILSGQDGLLVEMIFISYFTICFTFSIFVYCYVAELLQEKSLQLGYAIFYSKWYNLPAKKARLLIISIVRCK
RPLEISAGKFCIFSLNLFCNIVRTSAGYMSVLLAVKDKIT
>NvOr20
MARKEEGFDVAVGFSRFFMRLHGIWPGDTSSKFTWARFAFVPPAVIILMFINIPQTVQIFFVGGDLNAILDIL
TLANVPLGIALAKILGVSYNHNILRQLIVSVSGDWKHTTKKSELQVMWRNARISRTFSILFIGLAEVTVLANT
ARMFYILYSTRSEAESSGIKNYKKPLYYTGKFPYDAQSSPNFEITWVMQILATILAAGSFMAVDALFVTLVLH
LCAQLTNLQTAFRKIGEDKHEKEVDFMSKLSKLMKRHRKINEFADIIEYSFNMMFLFQVMSSTFLLCLQGYLF
VILISSQKVILVELIFMVYFIICSSCSIFVYCYVAEILREESLQLGNAIFYSKWYNLPANKARLLIIAILRVQ
KPLELSAGKFCIFSLNLFCNIVKTSAGYISVLLAVRDKIVQP
>NvOr22
MMANNNKLGFDESVGVTRWTMNVIGLWTLDERRDLQTRFRSLLPAFLILFFIVIPQTRKATLAHDDLNLMLEI
LTTADIIEGICLLKIFGLWYNKADLKKLVIQISEDWTHTNNDEQGIMWSNARLSKFVCLFCISSSSGSVLTHA
IVFLVTNVGANETRSLFLISQFPFNTQHSPVYEIVCFCQFAGALLSTFIFSSFDGFFVFSILHFSSQLSNLNI
RIRSLTEKTSGDKCQFVESLKSVVKHHQHLISYTDIIEYNFNKIFLVQIFATSIVLCLQGYQFVMIISESGTK
LLTSLIFILVFTTGNVLSLFMYCYIAEIIRNESQRLLRAVYEMKWYTLPAKDSCLLLIVMCRLKMPVEITVGK
FAPFSLEYFASVVKTSVGYLSVLLAVRNKIND

>NvOr23
MEVKTLVKSDTQISISNNLNGLSGFDHSVKVTRVISRMCGVWPGFEEKKSFTERFFFIVPGMVTFFSITLPQL
RRVMIHRKDLSTVLELMTTGIVMELISILKLLAIRYNQSGLRWLLRRMVDDWKIYDKGQYYKIMWVYARHTNT
IVTICIALTTGNIAAQIIRQYAIYIIERHYSSANETVIKPTILKSDFYFNEQIEGIYELVVAAQILGGFSVAF
SFTAFDGFFVCSIMHVSGQIHKLQMQIEDLVQCYERREGAFSEVLGPIVHRHRDLRGYAAVIEENFNKIFLVQ
MLVTSVFLCLQGFEFAMVVAEGGTEMVPHLIFIVCFVASNLVSIFTYCFVAEQLRTQSNQLFRSIFQIRWYDL
TPKDSRLLIIIMVQTKKPIEITVGKFVPFSLDYFCSVLKTSAGYLSVLLSMKDRL
>NvOr24
MEESPGFLHAFGICRTCLTMSGLWSDTHFKKSKKFVISVLYAANVFVILTFMNVAQTVKLFLIWGDFDEMSQI
ISTSDFSVGMLVVKMFVFRSYRKALALLIEFVEKDWLDLKTISEEETMEQNAHTANKIYLTCFFLGNSAVNSY
TLLRLGQEMSFLPGPPDKRQPLFDAYFPYDDKRSPAYEITWLMQYAGIALANLAFTGMYCLFVGLMLHLCGQF
ANLRIKLIEAVSRKEGESEKKSDGAKTFRERLAFIVERHNSLNKYAQVIEKIYHWIFFVEILSSTIQMCSQWF
MLVTVISNTQGGLPYLQIGFLLIFTAHSGFHLFACCYAAERLQNESLSIFEAAYSCEWYNLSPQDAKMLLFIM
QRTKTPLRVTAGKLCVFGLELFAKILKTAGGYLSILLAMRDRLVIDEEPI
>NvOr25
MDGKRGFDHAFSLCRINLGTVGLWPNSKNGKGHQEVASLIFFIISLFTIIVFVNLAQTVKLIMIWGDLNHMID
NISTANLPIAVVVFKMLTFRRYKKTLTRLLGIAMDDWCTKKTSREAENMSKNARTARKMSLVCVVLGFGSVNG
QLAVRISQELDILPGQTEKRLPMLSSYIPYEYQTSPAYEITWFMQYLGAVLATLVYSGVYCVFVGLVLHLRGQ
VANLRFMFESVDDPEEDKGKNFRRRLRSLVERHESLNRFAEDIENIFTLMFLAEILSCTIQICLQVFLLVTLM
SNDNGGVPILQILFMMVYAMHVGTHVFICCYVADKLRDESLSICDLAYNYEWYRLPARDARLLLFIMLRAERP
LEVTAGKFCAFSLRLYAQILKTSGGYLSMLLAVKDRSTNF
>NvOr26
MDKKRGFDHTFGMCSINLGIVGLWPNSKNTKFQEFRSNVSFVFAIFSVSVFISMSQTAKLIMIWGDLYQMIEN
ISTANLPITVTVFKMLIFRSHKKVLGELLALAIGDWCTKKTEEETANMCANARLAHRISMICVFLAGGTVSIH
AVLRTCQELDIMPGPPEKRLPLFSSSYVPYDYKSSPIYQVTWLMQLTGTSCATLVFSGVYCAFVGMVLHLRGQ
VANLRLKLENICEIREKGEGLVEARRDFRKKLGFIVERHLVLNRFAADLETVFTLMFLAELLSCTIQICLQVF
LLVTLLSNIKHGFPILELFFLMVYIMHVGTHVFICCFVADKLREESLLICNSVYNYQWYKLSAQDAKMLIFVM
HRGDRPLAMTAGKFCAFSLQLYAQILKTSGGYLSMLLALKDQS
>NvOr27
METKAVAMTDSRAQVSNYFPDSSGFHKSINITRTISRVCGIWPELEEKKSIAARYYFIVPTIVIFFTMTVPQV
RRAVLHRKDLSAVLELMTTGIVMELIALLKLLGIRLNESGLRWLLRRMIDDWKTSNSKERNIMQEYSNLTRFI
MTLCITLTIGNVVAQTTKQFAIYFMERYQSMANETVIKPTFLKSDFYFNEQPEGIYEAVVAAQILGGFYVAFA
FTACDGFFVFSILHVSGQICNLQLQIEGLVQNHEQRRCSFIKVLAPIVVRHRDLRGYAAVIEENFNKIFLVQM
IATSIFLCLQGFEFAMVITKSGSEMVPYLMFILCFVASNLVSIFTYCYVAERLREQSENLFRAIFEIRWYDLA
PNDSKLLIIIMTQTKTPIEITVGKFVAFSLGYFCSVLKTSAGYLSMLLAVQDRL
>NvOr28
MDGEKGFLYAFGMCKKSLTLIGLWPKSKSSNYAEAVVVLRFTLTTLLIVSFVNIVQTIKLLAVWGDLDAMTDI
ISTANLPIAVAVFKMMVFYKHRKAFEPLLSFVEADWKSYKTDSDMTNMWSNAQTTRRISMICVILGAGTVNGH
LFIRLGQEAKILPGKDGATRLSFVDSYFPYDYSPTPIYEITWAIQYIGAALATCAYSGIYCLFVALMLHLCGQ
FSNLRKKLRRVVTNEDDKRKFVEKLAEIVKRHENLNNFARVIEKIFNLMFLAEILGCTIQFCMQGFFLLTLSS
KEGMGLPILHILFMVIYVLHIGTHLFICCYVSEKLQDESVSIVRAAYNCEWYNLSAKDAMLLVMIMNRAKKPL
RITAGKFCAFSLSLYAQIFKTSGGYLSMLLAVRDRIT
>NvOr29
MDDKKGTSFIHAFGLCRINLTVLGIWPTLRSSKRDETAALFRLVLSLTIIILFINTVQTIKLFIMWGDLDAMT
DIISTANLPIGLMVFKTFVFLYHKEALVPLLSFVQTDWSNFKTVSEAANMWSNALAARKISLLCVVIGWVTVN
CHLAIRIGQELRFMSGKNGLTRLPFFDSYFPYDYTPSPVYEITFVIQYIATMLATFGYSGLYSLFVALMLHLC
GQFANLRDRLYTVTQKKAGVTFQQRLGYIVMRHQCLYNFAQVVEKMFNLMFLAEILGCTIQFCMQGFFLLTLS
SKEGMGLPILHIMFMVVYYVAHIGTHLFICCYVAEKLQDESVSIAKAAYECQWYHLSPKDVMLLIMIINRAKDP
IEMTAGKFCTFSLSLYAQIFKNSGGYLSMLLAMRDKIT
>NvOr30PSE
MSKSIEKATSMDNNKGFDYGFGICRMALTIYGLWPRLKNATYYDKSSSRSFRLIVWFILNILFINLVQTIQLI
MWGDLFAMTDIISTANLPIGLVVFKTFVFIYHKKALLPLLLCAQSDWDSPKSASEDMNMWSNTRIARRLSLIC
LTIGISTVNCHLVVRVCQEIKFIPGKTTSKLELFFNSYFSFDYTHSPIYELTVTIQYVATILITIGYSGLYGL
FVALMLHFCGQFANLRAKLDRIAQEVDGTKFCKNLEEIVIRHQFLYDCAQEIENIFNIIFLGEIIGCTVQFCL
QGFYMFTLNTDDMAALILYIIFMIFFMGHIGSHLFICCYVSEKLKVESTSIAKAAYECQWYDLSPKDIMLLVM
VINRAKDPIEITAGKFCTYSLSLYAQIFKTSGGYLSMLLAVRDKIT
>NvOr31

MDDKDGFEYAFGVCRKELIIFGMWPKPNDTMDHKVFAIFRLVLCIALNFIFINLVQTIQLFIMWGDLFAMTDI
ISKASLPIGLVLFKTLVFIYYREALLPLLAYASSDWKKPKSSLEAANMWSNARTARQLSITCLFIGLSAVNYH
MAVRICQELRIIPGKTKVERELYFNAYFPYNYTESPAYELTFAMQYFATVLATFSYSGLYGLFVGLMLHLCGQ
FANLRVKMDKVAKQADSAKFRQNLTAIIIRHQFLFRFSQIIEKIFNVIFLGEILGCTIQFCLQGFFLCTLSTE
DVGLLVMYIFFMVFFIGHIGSHLFICCYVSERLQDESVSIANAAYKCQWYHLPAKDVMLLVMVINRAKDPIQI
TAGKFCVFSLSLLAQIFKTSGGYLSMLLAVRDKIT
>NvOr32NTE
EANFAVNLSRLCLCFVGVWPNLQCSRFFEIVSNAVFSVSSILIIYFIVIPQTTKLFYSDRDLDIIVDILCTAD
IPIIVALIKMFVLRYDSNVMRYLLDQLFKDWKSPQTESRRATMRDSAKSGRMIAAACMGIAYGTSNVFFFLRL
IMLRQKQARTGHRAFLFESHFPFDVFNSPWFEVTWIIQAFCTFSATTAYSGIDSFFAVLVLHLCGQLIILGDE
LNSLAFQKDTEGIKEDIAKIVKRHYELNKYAETIETTFNKMFLAQIIACIVQFCLQGYQIISILIDKSLELPY
FQLIFMVMYISYMSLDLYIYCYVAEKLRGKSIEIADAAYNCKWYNFQKKEVSDLILIMKRAQYPLDITAGKFC
SLSLRLFAIIGKTSLGYLSMLLAVKERIN
>NvOr33PSE
MEIQDFDFVISWNRRILNIVGIWPEPETKNSLFSKCNFVWAAFLIFYFVSAPQTADLFIICSNMDLVVENLST
ANIAITTTLLKLFVFWFNREDLVPLIAYMRKDWIKTRKREHLAEMRAVGRISRSMTIHLVILTQIMVFVYMLL
QIYLNVSGFFPTRRLFYRSYYPYDTSYTPVYEITWISQYYGASISVIAYVGVDSFIGTSILHVCRQXAILKYE
LEHLLDPADDEKYNLLNSVKKKIKFITRRHEHLNEFAGTVEDIFNMMFLVQMTSLTILLRFQGFQVINSMSSG
KGTGLFQIIFMFLFIISVILLSYLYWYVGEQLNVKTTELGIAAFDCDWFTLPTSEVKSLGFILLRTKNPLALT
AGKFCSFTMVTFCE
>NvOr34PSE
ALTQLYHRIPLVVDNLTTSCAALTSTIKLFLLWNSRQAFQPIIHTAGLDWSRLKTTREQETMIRQARRARLLA
IIGYAIMFVSAXSSRLPPYASSTTSPTSPRTGSYRCRRADPFETSRSSYFELTYATQLVAGSFVGFSISVPDN
FFGALVFRASAQCEILGEKMKGLLDGQVDGRMLFRQRLGRLVDTHVHLLRXIDGEKEIVTLILTQVLCLSLRI
VCCLGFGVIRGLVPTQYTLKKIGVYYVMHRLQSIASDDNESPPIVHEILAEHACYSSPASYARDLIPIMVRSN
VPVQLTAGKFFALSLNAYAXILKTSAGYISMLLAASVVNKHVDRLAESAYCDVLFYRNR
>NvOr35
MSDKIEDAQKKLETREQRLRDFKWALGLNRLSLRLMGVWPGDDEAEGLGRLAILLRVPFMIAAMFFCLFLPQM
GALALVIHELPLVIDNLMTSCAAFTCCIKLYFVWRSKQVLRPVIQSVSADWLRPKLDWEREAMIREASRARIF
TVSGYAVLAGCYTGFAFAPLFGFDIRMISNITDYGEKHLLVQSYFPYDYSKSPNYEITQVSQLIAGFFIGMSV
SVPDNYFGALLFHASAQFEILGANLENLVRQDDKALRSRQFNRRFGIFVDRHVHLMTMVTAVEYSFSFVIMAQ
IFCMSIMVCSLGFQILGMIEGTTADKPSLLQVLTLLGTLFTLMMHTLVDCFACETLELRSAGIFENVYNSRWY
TVPKQSVAKDVIPMMVVSKNPRKLTAGKIFTLSLATYCSILKSTAGYISMLIAVNRR
>NvOr36
MERSQKNQLQDFDWALGLNRFSLRLMGIWPADQDESSKSLLTVSRIPLMILVLLCGLFLPQMWALALVIEQLP
LAIDNLMTSCPAFTSCIKLFFIWRSKTILQPVIDSALQDYLRPKSKSEETAMQREALRGRLVTIADYSIMASC
YVGFIFMPMLGFNVRIINNLTDCDTQRVLLVQSYFPYDYARSPAFELTHLLQLAASFFVGMAISIPDDYFCAL
LFHASAQFEILGLQIESLPIDGSKSGRLLSGFIERHVHLNRMVSAVERSFEFVIAAQIFCMSIMVCCLGFQVL
RMLDSAAEKPTPVQILTLGGTLFTMLLHTFVDCFASENLAARSSELFFKIYSSRWYSLSWSKMRCLVPMMLVA
KTPRQIRAGKILSMSLATYCSIIKSTAGYISMLIAVSGR
>NvOr37
MESLEKYRSQEFDWALGINRVSLRLLGIWPADQDESSKSLLTVSRIPLMVLVIFGGLFLPQMWALALIIEQLP
LAIDNLMTSCPAFTSCIKLFFIWRSKTILQPVIESVLQDYLRPKSEWEELTMRREASKGRLITIADYSLMTIC
CVGFIILPTLGFHVRIVNNVTDYASYGNRALLVQSYYPYDYYESPAFELTNLVQLTAAFFVGMTVAIPDDYFC
ALMFHVSGQFEILGLQIENLMGKDDAKEGVDWSLLGSFVERHVHLNRMVATLEKSFEFLIAAQILLVTVMVCC
MGVQVLRTLNGAGEKPSPFQILTLSGTVFYLLLHTFVDCFVSESLTSRSSEIFFKIYSCRWCALPWNKVRCLL
PMMLAAKTPRQIRAGRIMPLSLATYCSIVKSTTGYISMLVAVSGR
>NvOr38
MKSNNAHESFFAYLNWAIGLNRLSLRLMGIWPDDSVAETKLFTTILRIPLIISVMMLCIVVPQMYALILVRNN
LLLIIDNFMTSFPTLIGCAKFYFLWRSKEVLRPVVCSVTEDWLRPKSDLECQKMRDAAVVARLFTVGGYSLIT
GSLMGFIIAPLCGLNIRVEQNITDYGRQPLLVQSYYPYDYSQSPNFEITHSSQIVAACFVAMSLAVPDNYFGA
LVFHISGQFQLLGLNFEHFIKQNEKIVGIMAVRDFNKSLGVYVDRHVHLIRMVAIVEKSFNFIILIQIFCLCV
MACCLGVRILSAIGNPNDKTAVIQIINLGATLISLMIFAFSNCYASETLASRSAEIFQQVYSSDWYKIPKRST
CCYLIMIMIMSKNPQMLSAGKILYLSLSTFCIILKSIAGYLSVLIAQSN
>NvOr40PSE
MDRRRSTLRTDSTKGNQDNNQGIVLREYFEWAFSLNRICLKAVGVWPSELDELDTRGVSNNRNHLRIPIMLFT
LWFGLITPQLYALAKVYRQLQLVLDAHXYCTLTSFIKLFFLWRSRRVYGLILRSALIDWSGSRSSDWKYETMY
KQAYRAQIFTLGGYLVMYLLLRLLHVLSIFGXRIRIINNITDPGGEYRFLPIAYYPFRYDRSPYFEITYATQM

LVGCCAASTFTIPDYFFGVLVFHASAQCEILAAGIRELCLEELDTSSTEKPRELGIFRDIXFVNNIKKSFNMV
ILAQTFKLVLVICCLGFNIVKATGNNSESLPILPIVTLGGSTITMMCHMLVYCFASEVLAMHXRTIGTACYGS
DWHLLTVDCARTIIPIIARAQHPLQLSAGKFFYLSLNAFLSVVKTSFGZVSVLLAVGA
>NvOr41
MRTRVKRLKVSRSSKTKGGFSYEFRIYKIITWPAGLWPLERDNIFNVLRFLLAASSQMFIVVAALVEIYRKCG
NVADVLDYYALSIAFWLSFVRLVLVRIHLAKIHKICYNARKTWARIKDPDLVKIMISHAKTGKRFYYLQMSIA
FVIVTLYVFNPLILRRYDAANLPMQTVCTFNNADVLKHTAVYFIETLSFVYLAVGFISIDLLFLGIAMHLCGQ
LKILQKEFSEIVGKSTSQADCIRYVISLSRRFQRVVELTDDIRKTFSEILLVNFVVNLFLITSQSVTLLLALK
INNYFLAVKCSQTFPILLIEMFLYCYVGELLRHAFDDIPRAIYSSRWYLLPPKIRRGYLLHVMAQASKTFDLT
AGKMIRMNMCTFIQLVRSIVSFFSLLLLMFDK
>NvOr42PSE
IFIVTLVSEEIYGTCGTADDLLDYYAFFGCFIMSLIKLVCIRLHFIEMYRNCLYAQKNWTKVADTNVLKIMQR
YANVAKKFYHVQMFAAYCVVSFYVANPFILPRTDASNLPIQTVCSFRNLTDREYVIVYVVQSMSLLYLAFGLI
GVDVFFFGIVMHLCAQLKILQIEFNTIDKLTWRLDNIRLMALYSKKHYQLIMLANDLKTKFRVSYSCDNLYXS
ATFFIALKINNKFLAFKSIQSFPILFLEVFLYCYAGQLLQYEFSNITNSIYNSRWYILPPKLRQYYLLFGMTA
GNKVFKLTAGQLLNVDMMLFIYFVKTMFSFFSLLLLMLDKRAEKLILCL
>NvOr43
MKCFDSYDKSNFKTDPQHAINFFKKLGRFWTIWPVSANASRFTKVYHECSLWFIIINLFFASLTLWMSVCVYH
KYPILMAKNLSQLMIISDSFTHLVLYRINRSELQVLVQEVFDFMKNSKQNEKYIMRKHYNRFLGHYTILIVLY
VIASLAFFCGAFILGKKFPMDASYPFSTDSILVSSIIFTHQTFSIVQNSVLIMIDLLVITLFWYAGARIKILG
YKFKIVDSNEKLKNCIKEHQKIIQYVASIVKAVRFILYKTISIVAIIIISAGLQLLYYDAKVVISQFSLIIIV
ACFRIITYSSTIEEMNQLNEDLRWTVYKSSWFCITSEMKQCLQIFIHRCQIPLVTIDGQLLNIMSLAFFAKLI
YSTVSHLTTLRAIIERS
>NvOr44
MAFKISPEKAFTFTKLSVFFTAIWPPNCNDSSFKIKLANIFWIYSIISAMCLLIPMLASVLVYKDNPMIVSKS
ICLSCAVIQVIAKAIVCRHHQKKLQFLVKELTHFLKKAKKEERQLIEKYINRRAIFHMTFTLCCFGSSFFVIC
GPIFLPFSLPADAVYPFSVNSSPIWEIIYVHQASVGIQASSGMCVDNLVAYILWYTGVRFESLYYKFKHIKDS
KEMLQCIKEHQYLLRYGTTVADTFRYVIFTTVLSVTAGLAFAGIYLFSPQPIFVKGQFVVVSISVVVNLYVTA
LPSNNLISMCHKVGDVVYESLWVGDSPSIMKHWIFIIQRCQKPVVIAIPGLIKELSLQFYSSVLCSTFSYFSA
LHVIMTKE
>NvOr45
MKFLIRPKFFFKTLRLIGDMIAIWPKHIGAKKTMIMFHEVKWWLSFTNATGLLIPLVLGVYYFRNDSITMTKT
LSELTALCEVFINLIQCRLQEKKFQVILYEIENFIENSNEQEESLLQDYLNRYKTLQLFVGSSFISTAILFSC
IPIFTSQLLPADAWYPFSVEYFPIRVFLYITQVLAIFQTGFGICVDLTVATMLWYSAVQIELLEKNVHKAVSK
AELRECARRHQEIIEFTDNIKKGIKFIILKTNATMIIVVICGAFQLIHHEPLEVLLRFTLMVLAGCLRLYVSA
KPADDLKENSEQLARTAFQTALMQKSTSNSKIGLMLAFRCQKPIVLSVTAVIRAYTLQYYASFLSRTVTYFVN
LRAVLDD
>NvOr48
MMINKKVTDKLVTIVSTLLNIFLHIDYLFHSFKILKFFSRLFALYPLNSDCTKLEILYDNFVWLFIHFHFWVA
AAATLVAIYKARSDLSIWLIAFSELIIIIEIIVAMILYRLQRSRLKILMHIFEDFVKDPDDSKIQLIRRNAKE
HIKVFSILALLFIIVVIMYVYRALSTRPYQLLLSGYYPCTSDSLIIWLVVFFHQCILVIYSPSTFASDSIVTV
LIFAAIIKLQKIRPRFRNIENYAQLVGCINEHQHIIWFVQEINYVIRLFVFKSIFCLAALQLGVGVILFMPNI
SVFTRIQFLLLFTVTIFRIYIYSYCAEILTKSGLDLGFAVYSSRWYDQRRKMVLAKSIIICRCQKPLLIAING
IIPALGMRYLARFLYLTFSYIITLQAMTRT
>NvOr49PSE
MIGNKFTNKLVTIVPVTLIDIISYLDYLFYNFKVLKLFARLCALYPLDSGCSKLEIXYDNFVWYYIQFNLWTA
LVATFTAMYKARSDLSSWLNSFSESIIFIEIILLMILYRLQRPHLQILLQIFDDLIKDPDDFKLLLIRQSARK
HLKGFSTVAVLFTMLAVIYVYKSFSTKPIQFILSGYYPYTSNSMMLWIIVLYHQLTILLYVPSIFASDFIVTL
LIFGAIIKLRNIRPEFENIRNHNQLICCIDEHQHIIWFVHKINYVIRFFILKTIFCLGALQLGVGVILLRPDI
CAITRIQFLVIFAITISRIYIYAYCAEILTKSGSDLGFAVYSSRWYVQRHNMVLAKSMIICRCQKPLVISVAG
IIPVLGMGYLXRFLYLIFSYIIALQAITKNHYL
>NvOr50
MFKKIKPNFTVQKNFNILCNCMKILGTWPVHRRHNKIFTCLNHSLWWFYVVNHMMLLLPTMQTFYNTTKDIIS
ASYSLIEITGIVESMVILITFKLQGSRIQLLLQIIKNQIVVKKPKPALNNRNVHASVFAIIAVLYVIVVYMYI
HKPATLINKGFIMTTCYAFPTEDIRTKIAIYCNQLIALMHTSVVLVTDGVAVLFIYTCAIKLKTLEIRLKKAP
DWTKLKYDIAEHQTILLVIEETNSLAGVLVVKTVICFMCYSISAGVQIINQHVVTAQMLHQFIIIAIVYLRIY
ICAETAEMLLTVNGDMLFTVYSIAFSTPNIVKVKSLIIMRCQKVPKIYVNGLMAALNRAYLRSISYATFSYFM
TIRAIVSK

>NvOr51
MRIHLNAKIAYQYLRLSATLMATWPLSDATKKYRNVFYNMLWWLYLTNHLIILYLTLNTIITHNKNHLTVFYT
WLEISFMTENIIVLVSYKLQETKWKQLLYTSKMTINRTEDNIRLENSDLYPKVFATLFIFFIVIIISYVNKAE
TYERGLIMTTRYPFEIKSIGLKLFLNLSQFITLLHASSILITDAIVVLLLYTCTIRLKIVEQKFRACKYYRHL
KLHIYEHQKTLLLIEDTNLLVSKTVLKSIASFMSYSIGGGLVLYNKNTSPLQLVQICLVICVIYLRVYVCAEI
AEKMISANESIGFTIYFTKWYEESAKDINAKNIIIQRCQKLPRIYINGFMQSLNRNYVRMITYATFSYFMTIR
KIINKTANCVDC
>NvOr52PSE
MQNKYEKSGSKNRSMEMVGYTVISLCLKCLGVYTVSSTNDKLWIRVYSSLWWFYLLNHVVFFLATAYAFFTSK
LDVISLSYSFMEGLFVLECIILLLHFKFQGPRLRNLLVNANVQVSRKKTLTILNNNNTYILIASLIALIYVIS
LSLYANKPKTVYYRKYFTQARYPFEINSTFATILIWCHQSIAVLHMLLIPTSDALVVLLLYACTVQFKILESN
IRKGRSRKKLQAYVREYNDIFLTVEETNRLVRVIVIKTVVYFMTFSISTGIQILNXSFKHSFRKMQLMVTAIV
SLRFYVCAESAEKMVSSVESIGLAMYFTSWYEEKLKFLIAKLMIIRRCQSLPKIYLSGFMPKLDRQYLGTVAY
TTYSYFTMIRSLVYKYAIENDQPTLSATFNKGRIQ
>NvOr54PSE
MNIYIPHNKSFCSLRFIGTCLGIWLTDNKKWTCKNDYLFWFCILNYIVSILPLFYSLYLNRRNVSAALYTWIE
LSGVIKMMAVITIYSKFQRHRLNARLIALFVPTEQLLAIDKVKIVKKYANTYTLMFLAVLLLYMITMSIYLAI
EQTSNWIRIRXMTAVYPFRFYCYAVKVLTCANZAFVQMHSVILPTFDGISILLIFMCTYRIKVLDHDFKNAZN
FIELRKCIQEHENILXMVKEINLIIRFMIFKTVITFMSNVIACDLHILNNVPVTQSTFQVSTIFLVCTEIILC
AECADNMTTAGEDMEFTIYSTPWYEEEPKIMIIKSIILSKCQKAPVISINGVMSTLDRKYLATIMYATVSYIT
TLRAVIENDKIL
>NvOr55PSE
MRIYISPKKSFRVLRFVGIILRVWPVNDTKWNYRTDIFFWFCIINCALLWVPLCNALYLNHKNAHGNSCYFMD
XVSWFAEIIFVLTYSKYKKRQLYVCKVIKVKYVERCVYLKKTNIIKQYATIYAMIFFSVMLFYLLAFFVYMSI
ERPNTGYNTLITTAVYPFDINSYXVAKAILYCNQIFLLTYAAIAPTFDGISVLLLFVCTHRIKILDHNFRLAK
TLSDLAQCIREHDDILYTIKETNSIVRIVVFKTVCSFMSNVIPAGLQILNSVGLSQSILHICTILLVYTRIVL
CSESAGNMTNAAKDLVFTIYSSLWYNEEPKIVLTKTFILQKCQNVPTIHINGLMSGLGRKYLLTIVYSTFSYL
TTLRAVTSNERQ
>NvOr57PSE
MSVYISPHRSFRVLRFLGTHLRIWPDDNKKWNFKTDVFFCLINYVLLLLPLCNALYLNRKNVIAASNTWIELS
GYAEVLAAFIYIKYKRNQLYARKTNIVAKYFLLKKSMIIKEYANTYAKIFLLVILFYLFAVLVYSSVEKPITV
SPIYYFLKTFVXRTFSNYNLFDEPKIVSMKIFIIQKCQNIPAIHITGVMSGLGRKYLLLIMYSTFSYLTTLRT
ITRNEAQ
>NvOr58
MTIQSVLRRKVDVLLKAIALNKMCVSPKMILLVIKFAAMYLAIWPLDSSGKHWNTAFDCLWWFYVVNNVLVII
PTLLAFYSSRRDIIAAMFSWLEILALLEALIIILANFRYYRSRMQPILKEAVDYIGSANSRRQLCLEKRASIIT
TTFGVIVALYIAGIIIYIYRPAVTEWDGMLTTAYYPASMRSPFADVFIYITQLTALLHNGVLIVSDAFTVLLL
YVCTVRLEVLQKNILRVADYDELKLWIREHERVLRLVTDTNMVVRINISKTVISFVGYSVGAGLQIISPTVTI
VSFQRFALVIAMNAMRLFFSATFADDLVNSSNSLINTIYSTIWYKDNRDMKIGKIIIMLRCQKLLRISVGGIM
PVLGKPYLTKILYTSVSYFMTFRAITGN
>NvOr59
MSRNLYHDAKIKCKMNVITYLLKCLNFLRFMGKIYAVWPLKTDDNIRWRFVYECLWWFYFLNYLVAASFTLNT
CGHASDDITIASFSWLEFVSMVESIIIILINYKCYHVTLQLLLTEVEDYLTLADEKKQWVLKEKASIFAVMMCI
ITFLYFVLVVLYFTNPAITAWETFLTTSYYPPAIRSPVMDVFLFSNQLIVMCHTSVIVNLDAMVVLLIYICSV
RLKVLAADLESVNDDEELKQRIREHQHILCLAKKTNIAVRLVVSKTVICFISYTVGAGLQLVNPTATVASLQR
FGIVLLINYVRLIMNATSADELLTVSRNVGLSIYSTDWYGESKIVTSSKFIVMLRCQKLVRIHVDGVMPALTL
TFITGIISTSISYYTTLRAVTRQN
>NvOr60
MSRVLQSDSSSREGKHVWSKDAKFALMLNKFIVWPLGLWPLECDDAFSRFRNFYAVVSQVWMIGTQATAAYLG
CGDVADTVDFVMMTACALMALSKIVTIRLHMSKVHTVFVSALDDWLAVDVDKSRDVLIPFAKTGRFVFYLQMV
SAYMSNTLIIIGALPFLIPPAANGTWANVSETLQSRQLPMRTGCMFAGYRDEIYGSLYVYESVMIMITAHGNV
GCDVLFFILAMHLCGQIELLKTDVLKIGEDEKVPGEWKNKIVECVHRHIRLLGMAKALNKVVSGVLVIQLLLN
AGLNLMLGIRMLIEIKRGSIFNAVRPMIGFNVLMLQLYLLSYASDRLSSQAESILDAVYDSYWYKLPAKLRRD
LYFVTMRANKPIYFMAGHFYAMNIENFMNILKASFSYFSILRIMFQA
>NvOr61
MGGKSEVDEAFAVYRAFLWAIGVWPLEEKSFSQILRYIVAAVVQVTFLLHTFTEILLNNGKVSDMVDVFFFSS
AAFLTFAKHTYLHLHKDAIRENLRCYLDDWSNTKDEHFLRIMREHVKIYKYQFHIYNLCGYVGTTLFMCRSIL
INILAKRQLGPGESYNYQFICQTSYLSQDTLAKYYPIIMAIQYIQCMYCCTSGACTDCFFFGLVFHLCAQFEI

LKIKWERLGTKDFGVTAVHDRVKVNALIARHKELVKLGENLESGFNNTILVQLMISIVLICMSGCSILVAIMR
NDHVTMLISTNSISFMVTETLIYGYASDYLVTQSESIVQAVYSSSWYDMDSSVKKDIVFVMMRAKIPLHITAG
KFFCVTRNTIVQLLKTSVSYLSVLRLTLEMSHQEGQL
>NvOr62
MCANIFIGHHQFGLRVVGSWPGKSQLPGFYFAIGIMLFFLIFEILNITEVYHDLEELMDNLVSTIGVVLGLFK
FITVRVKRRKLKTVINKIFDDWKTDSQFVSEMMVKNCTRSQLVSKFVIFLYNSMNFTYFLRTVISHIFDEVQD
RKFLAQVTFPIVDGRQTPLYEIIIFFQFITASVCFNSQALVEGLLATLVLHACSKVDVVRREILNFSTICKTD
KNDKKDILKTLRKLSEEHFKFIEFSEDIQDIFSYVSFFHIFFLTLIQVVSGYMFIDGLERGTKPVNLIHYAIL
TTSFLVSAGYYCIAGEYLTSQSEIIFNELYNCYWYEFPSSYKKAICFMLLKARKPVKLTVGKFSTLSLIYLTS
IMKTSFSYLSLVRAVR
>NvOr63PSE
MMKTNIIKKPHRIGLKMIGVWPGVTGWSGFMFIMGWLFFTLIFMIWDVFIIYHDLELLMNNLLLNCFGXSSKQ
SPVFEVVCFFQFAIAVISANAHALIEGVLTVLVLHAGTKVHLLEKEIQKFSAICQSKTDKEITSKAIRGLIDK
HLNFIKFVKEVKDIYYFVSFVHVFLSLFXHVIVGYMFIDTLESGDRSIKLFLYGLFTTRALASTTIYCIVGEY
FMNQSMRIYGELYNTAWYDFDVKNIKAVTFMIMKARNATSLKSASFGQLSLFYLTGVIRTSFSILSLTRATR
>NvOr64
MSDKIVMRHVRVALQVIGLWPGYTSSVGFVIAITWLLTCLTFQLWHAAVVFSKLDALMGNLGATMAVATATLK
LIAFHVKGRNVKIVIKEILNDWAYENRSSNCEVMVQNTKRAKYLTKWITGAYNATVITYLVNAIIAYCSGITE
QRLYVLPSKFPSFCKQSPVFEIVCFFQFSAALISTNVQVLVEGMLTVLVLHAGTKVFLLQKEIQKLSVICQSK
TNNKEVISKSTIALINKHLNFIKFVKEVKDIYYFISFVHVFTFTFLHVIVGYMFIDTLERGDRSIKLFLYGLF
TTRALASTTIYCIAGEYLMNQSMRIFDELYNSAWYEFDVPNIKAITFMIMKARNATSLTPASFGQLSLFYLTS
VIRTSFSILSLTRATR
>NvOr65
MSILSRHVKIGLYAIDAWPGVSSSGLFFLVMAYMTFSLIFQILNTTEMITQLDLLMNNLQTTMPVILVVLKLS
VFRVKCRSARLIIADMLSDWKCINETKERKVMMKNAKIAFYLSSTIAICYNGLILSYLLKAILAYETENIYDR
KYVMQATFPINAKSSPVFEMLCLFQFTVSVFAANGHAILEGLLTTSVLHANTKAFGVCQEITKFAKSCEANKS
RKNIVEAKRRLIKRHLYFINFAEKIQETYAYISFFHLFLMTLINCIVGYMFINLTINKDNISALLLCIAYMFT
ALSAVGSYCIAGEYLMSQGSLIFEKLYDCPWYKFKPVDTKTFIIMLMKSRHSVTITAGNFGDLSLVYFTNIIK
TSVSYLSLVRAATN
>NvOr68
MKIPIIGIPLEYTLKLAGLWPDQSNILGSIVMGSALVTMIPFQVWDTINVSDNLVMVMDNLSNILSEVLLYTN
FIVLLLNKSYLDDLLREIADDYKNNIVTEKWLKLDQNSRRFCNYDYGMYLGACCLFYLQFALMYTQMPSEDRI
MLLKAYYPFDYKSSPVFEIMCFIQVIQGLLMCSIQALSESLLIALVSHVSGHIDLMNKQINVVSKSYDGQNSL
TLKLVIKSHLKVLNLVNKIESVYTYVSLTQVCLSTFIICVTGFVVLTMNSANEIVVMIKYIMLYFTLLWQSFS
FCFAGQHLLNKSDMIPYQVYDALWYKAEATEMKAILFIIKRAQTPLSLSAGKFIALSAQTFTLIIKTSFSYLS
VLKASYA
>NvOr69
MKIPMVYWPLEYTLRINGLWPGENNILGSIVTASGMVLILPFQVWDAIKTIDNPILLMDSLSDIMTEIALYAK
LIIMWFNRRYVVDVLKEISNDCNQNDVSQNWTLLNYNARRFCKYDYSWYISATLLYYIQLVTMYIEVPVDGRE
MLLKSYYPFDYKSSPTYEIMLFLQIILAMSMAIANAMTESLFIVLILHACSYVDLLLDEIKIFSDNCNKKVLN
ITDSNNMRFYVHVILKRHIQLLESVKKIENIYSNVSLVQMFFSVITICVTGFVMITALESKDIVLLIKFATFI
WFLLWQIFSFCFAGQYLLNKGETITGAMYDSDWYNIESNDVKAISFIIKKTQRPLSVTAGKYIPLSVTSFAAI
VKTSFSYLSVLRASYVE
>NvOr70PSE
ALMALINKILDDRQAKKTIDESRVMINNFERSKSLGTFIIWLYNDILLTITLKPVISYIYDSVVNRQLIAPVA
FPKFFGDPKQSPTYEIVLIIIGQIGTTLFAVNIVILTYAILEGLLALSVFHACSIMDSVRQEIVKFSNVCRLQ
SGNKVLIMTAMRRLIQVHIYSDEFSDDVDDLFTVIAFFHIFFVTLAQFFCAYMFICNIEDGGDAVKTIHYGVM
TIIMLISCGYYCITGEYLTSQNELFTVEIYNCFZNDFPFSQQKAIKFVLAESQRPVCLTFAKFDQLNFLFLIK
XNFSSFSYLSLVRKVH
>NvOr71
MYDEIFIRPHKISLKLIGAWPGYAKLTGFFLVIGSSSVLLFFALWNTIEVFGNLELLVDNLVNVIGIIVGFFK
LTTLRVKRRNLIFMVDTMFEDWQTSKKTIEELNAMKDHFERSKWLCKSIIMLYNSLILTFLLKPVRSYMNDSI
EGRQYLAPVSFPKFIDAKQSPIYEIVIIGEIGTAFFCINSHALVEGLLASTVLHASAKIAAVRQEIIRFSKVC
RSQNSNKRLIISATRRLVQVHLSCNEFSETIVDIFAVISFFSILLMTLAQVFSGYMFIFNIENGGETVQTLHY
GFLTIVFLVSSGYFCIAGEHLANQSELLTMEIYNCFWSEFRIPEQKAIRFILAQSQRPVRLTLGKFDELNLVY
LTKIIKTSFSYLSLVRRVR
>NvOr72

MDDEIFIRPYQISLKLVGAWPGCAKLSGFFFVTGWSSILLFFALWNTTEVYENLDFLVDNLVNVIAVVVGLLK
LTTLRVKRRTLMTILNKMLEDWQTMKMIEEFKAMTDNFERSKWICKSIVMLYNSLILTFLLKPAISYMNDSVE
HREYLAPVSFPKFMDAKQSPMYEIITAGEIVTTFLCLNSHALIEGLLASSVLHACSKVDAVRQEIVKFSDVCR
TQSGDKMLKLTAIRRLVNVHVNCDEFSENVENIFTVISFFHISLLTLMQVLSGYMFILNLEEGGEILQTLHHG
LIIIVILVSCGYYCIAGEYLTNQNELLNVEIYNCFWTEFPVPQQKAIKFILAKSQRPVRLTFGKFDQLNLLCL
TKIIKTSFSYLSLVRQVH
>NvOr73PSE
MGDTYNKNMQSIFITIILMRLLGMWYVSNKREKIIADVLLGIVLASMIFSCMVQTNEFYHARKSLMLICSAAP
TFFVLFSEQMKLLVFCKNRERVMKLNKYTYEHFWNGKYNRKETKIFNDCNSWCIKALACFLVTMQCISVHLML
APYLEPSDNSTSGEKIFPFPVYVNYSIFETPTYEILYVLEVLGLYGVVLCVMSFPIFLLVTNMFTAVQFKMLN
LRMRSLCQFPRNKNDDVNLQQQTNAYEKLKECIRKHQSLIHYVNEMENLYCYAMLGQILASIFQLSSTSITIL
LSNQGEESLNKAVLRILILVASLMQFYFYAYSSHEILTESEKISEAIYSSDWYQITHPRYRKNFSLLVQIVMR
MSMSFFTVMRQTIG
>NvOr74PSE
MGDTYNKNMQSIFITIILMRLLGMWYVSNKREKIIADLLLGIVLVSMIFSCIVQTNEFYHARKSVMLLCSAAP
TFSSLSSELMKLFVCFKNRERVMQLNEYMYENFWNAKYNREEXKIFNECNSWCIEALACFLVTMQCISVHLML
APHLEPSDNSTSGEKIFPFPLYVNYPIFNSPVYEILFVLEVLGLYGVVLRVLSFPIFLLVTNMFTAVQFNMLN
LQMQLLNELPKNKNGNVNLQVQEHAYEKIKDCILEHQLLIHYVDEIESLYSYAMLVQIFASILQLSSASITIL
LSNQGEESLNKAVLRISMLVGSMMQVYFYAYSSHEILTESEKISDGIYSTGSYQITHPRYQNYFSLLVQIIMR
MSMSSDSIYSIIERVSISFVCKLILTSYTEYKFVKRY
>NvOr75PSE
MEKSNVSKKMQSIVITVVLMKVLGMWYVSNRKEKMIADALLSAVTASVLFSFVIQTNEFYNARNSLMMMCSVA
PTFCSLSSELIKLLVFCKNRQRVMKLNKYMYENFWNVEYNKEETVLFNKCNNWCIKALICYLILLQIIVVHLT
IAPYLEPRHNTTSREIELPFPIYIDYPIHETPVYEILFILEVFGLQGVILCLLAFPIFLLVTNMFMAVQFKML
SLRMRSLCKFPNNDNSDNVDIKLQMHTYEKLKDCIRKHQLLIHHVDEMENLYCYAMLGQIFASICQVSSTSIT
ILLSNQGEESMNYAVARIEILAASTLQFYIYAYSSHAILTESEKISEGIYSSDWFRITHPSYQKHFSLLVQIV
MRMSMSFFTLLRQTIA
>NvOr78
MARSFASFDEYTFLNRWGLTFLGIWKSDAEARGGPLRRFLHRLHVTILFTLLMLLLLPQWMDMYVLWGNIDAN
AETFVLNVFTITALLKLWCFLSARQIFEQVIDTMKENWRRTMSGDEPGRKTHREILLDMAGKARDYTKRYGLL
MYSTATMYFVSPFVGMQRDNVRIRKYPFFGWYYFDRFSNLYYGICYASQVIIGIVVGTSNYAMDSIFLVAIYH
TCARLQMLQHDLKKIGEDRENRSPEEIVQLIRLHQREIRDAKRLTKIFNGSSLQQLLVSCVIICIIGFKLIIA
LNDGGFEFLVYVAFMFVALLQIFLYCRPGDELIVQSTAVGYAAYQSHWTSLEAESIRKIMFMILRSQTSLKMT
AGNFYVLSLPNFTMILRMSMSFLSLLRAMYRKSDGFG
>NvOr79
MRIGARRASRMESTTEASGIMREYDDCIFLNRLGLTMVGIWPLEHNASRLRIVLRRIHLGAIYVLMLSVVIPQ
WFDIYCLWGNIDANTETFMSNVFMIAVMIKISNFLNSMRLFEDVLRTMRLNWLDVMRLSSGELEKKEIMQGLS
MKARSRGRVYGLVVVMTGAMYGLMPLIGSNKVASLRDRSYPFFGRYLFDRNSDTVYRLCYLSQLMSGSVTAVA
NFATDAIFLFCVYHFCAQLRILQTDLLKLGGPRFDSREALVQLIRRHQKEIRNVRALQSLFSISSLQQLFLSC
LMICLNGFKLIVSLCNREVDILMYIVCLPVTLFQILFYCQPGNELIVQSQSLDEAIQQSHWVNLDRLSKRQLF
FMIQRSQKPLAITAGKIYVLSLENFMRIVKTAMSALSVLQAMYRKTGS
>NvOr80
MHCSYSFFLVTAFAMWRPRSWDDSKILTALYTLYSILSFTVYYTFLISQILDIVLLAENIQQITENMIQLINV
VNVSQKSLCFFLKRKKIIRFMDYFFEDMTLPQSPREKEIQKSFDDESKGNSQKLFVLYSVSVVMYVYMPFFIS
KREDRVLPYRAWRPYSLDNVNYYYLAYLHQSWSVTIAATGNAATETLVSGFMIQICAQFEILEHRFMQLPKIL
KEMRENGESESTVLATERSIIIKLIHHHWRIFEMTELFNDIFVFVILSQFVTSITVLCVSTYNLALCKSVNND
FVTIFMYLLCMLLQIFMYTWYGNEITLRSCDLGNRIFLSEWRSLNPPTVKNLLIIAQRTMKPIILSSGYVITL
SNVAFTSIVKTSYSVFNVLNV
>NvOr82
MRVLPITFGILTVCGFWRPISLESSIPKQMYNCYSIFMCFLIYTFTLSHLIDIVISAADFESLTGSCFMLLSM
MNVCCKMTNILYFRKNIVELLQILASDHCTAKDVVERDIEKKFHKRARSVTLCYWILTETTCMLITLRTFFGS
SKQILPFKAWIPYEITGLAVYWTTFFHQTIAHVAAANLQIANETLICGLMIQACSQLEILKYRLKKIPDESKI
DKFPLQSTVNNAQNTNKKDTKTLLVNCIDHHRRIIEFSEKLNSTFNVILFVQFAISSLVLCSSVYLLSKMKLV
SVHFMSLSLYLSCMLYQIFLFCWYGNEVILQSLDLGNAVYHMDWTILSTEDKKKLLIVILLVRKPIQFTSSFL
VSLSIESYCKILKTSYSVFNLLQRTSI
>NvOr83PSE
LSACGTWQPLHWQERRRLRYLYELYSAIAVTCVNVIVLLQGALLFLNEFDVNFVADILFTLLCAVSIATKSLN
FLIKRDRVIDLANMLTRSCCLPRSAVEIQMEKESDEFLSXFAIHFNGLAQVAVALLAAMPLVQEPGERDLPYR

MWLLYDIHDALNYWITYAVIAGVIIIGVLINVSVDVVISGFVLKACMQLDMLKHRLNRLSGIVRRAEEKGAAS
RQALISFEQATLRQAVLHHDYIIKSRDIRRGDSRTVFAGALAFSIICXYILTIGKVAVLEKILAIVYLICTMG
ELFAYCWFGNEITVKSLEFSTDIYNIDWTALSQKTKKMLIIIMMRTNKPIVISYGYLIVLNIDSFKSILKVTY
TAFNVIKESS
>NvOr85
MRSLTFTFKVLSLCGIWLPLHWQSHRRLRLFYKIFSISTVVLTNIFILLQGLLLALSEFDWQFLAEILFTLLT
AFSVSFKATNFLMRRDKIICLADMLLKSWCIPRNAVEIEMESRINEFLRVFTIYFNALAQLSLACLLIMPLVQ
DPDKRELPFRMWLPYDIRNQWNYWSTYVIEVGPMIVGILLNVTTDVVVSGFVLQACIQLDMLKHRLNKLPNIV
KVAKRKRLASEEVVRSFERKTLHQAARHHDYIIKYAKVVTETFDVVIVEQFFAGALIFSVIIYVLTIGKVPIL
QKLMSVGYLICMLGELFAYCWFGNEITLKSLEFSDDIYKIDWMALSDSSNKKLIFIMMRATQPIIMSYGHLVI
LNIESFKSILKITYTAFNILKESTSTT
>NvOr86
MLELPYKLLILTGIWMPEDWTHKHQKLGWLIFSIISIGLVFMQFSSLVIFLMISKSCAQFFERVFLIPAGVSS
LQKIYIFITHRKELIDLGKMLLKDYCIPRNFEELSIQHRYEELIRVLTLVCFVLVNITMMNLLVLPLVTNGEN
RTLPMNVWLPYPVDSDASYWLTYTHQTLGTLLLGTGAVGSTLMINGFMHQVCCQFEILSSRFQKLPQIIKRLQ
LLKKPNHLIYEYEKKSMKQYVQHHLYIFRVADTINDIFKSVIFQQFCISSIVVSASIFQLSTRPDKDMEFIMV
FCYLICVLVEFLIYSWFGNELMLESLHFQTSVYQIDWTALSIGSGKDLVFIMMRASKPVIMYCGHFIILSLES
YLGILKASYSVFNILRRSSN
>NvOr88
MHTILQLPFKLMTLTGIWMPKEFTSQYEKQGWTLYSIASITLMAIQSLTLSITLILSENSEQFFETLFIVPTG
LQNLQKIYVVVAHRKKLMDLEKMFSNDYCIPRNVEELLIQRKYDENIRILTLSCIILMNLTVANLIASPLFDA
YFTTMNTRTLPMRIWLPYKMDLNIIFWLTFIQQSVGVIFVGYCIISTTLMINGFMYHVCCQFRILSCRFKKLP
QVIDYFRSLKKPYNVIYQYERRAIKQNVQHHLCIFRIAENINDTFKSVIFQQFCISSIVVSASIFQLSTRQEI
DMEFFMVLFYLICVLVDFYIYSWFGNQLMLESLNFQRSIYEIDWTTLSTNAGKDLVFIMMRASKPILMYCGHF
VVLSLESYVGILKVSYSVLNLFRRSK
>NvOr89
MEIIEQLEKMRILQVPFKVLTWSGVWMPEDWTQNQRKLKYNLFSFVCIGLMTIQSCSLTVYLMMSKTWSQFVE
TLFLIPPGLSNLQKIFVIMLHRKKVIDLVNMFENGHCIPRTADEWSIQQRYDATIRVVTLVCFVLVNVTMVNM
VTTPLFLKADERILPMKVWLPYSIETDFFYWLSYMHQTLGVTLVGSGIIGSTLLINGFVYQVCCQFEILSSRL
EKLPQIIRNLRSLKKSDHLVHQYELKLIKQIVQHHLYLFSIAETVNEIFKSVIFQQFCVSSIVVSASIFQLST
KPDTKTEFIMVLFYSICLLVELFIYCWFGNKLMFESLNFHQAVYDADWTVLSNESGKDLMFIMMRASKPIIMY
CGHFIVLSLETFLSILKVSYSVFNVLRRSHG
>NvOr90PSE
QALPAEVWTPYNHTSTSSLYWLSYAINSICAVSMGNLSIGTDVAIYGLMLATNAQLELLAHRLVNLSNZANTE
KGIMLTGPSRVRRENFLLGQSVRYHVFIYDIYQLTKDTSASSPAFYVMLFYLSGLMTQLFMFCWFGNEVTVRS
QELGMTILEASWTGLSTQSLKAILLISLQRLGLFLYLEGTLLRSLWRRLKRILKVSYTAFNVLRDSTYEE
>NvOr91PSE
MDGFNPAFEILTCFGFWKPTSWPTPWSERFYDGIRVMLVSLLYYLALGQILRLLLDTISIDEMADTLFSMMST
VNACCKLTNMHLRNKQIDELMDMLRIEWTKPGNKEENVIYNGFNDIIGYVTLVEPATLLDLSIPIIVGKAEQS
LPLEVWTPYNXNVRRIDGQLVHWHRRYYIYGLMLATSAQLELLAHRLVNLSKYANKEEGMMLSRSSRLRKENY
LLAQSVRHHVFIYDVHQLTKDASASSPEFYMMLFYLSSVMTQLFLFCWFGNELMIRSQELETTILESDWTTLS
LQSTRSILLISLRTSKPILISRGYFVPFSLETFKRILKVSYTAFNELRDSSYYDE
>NvOr93
MHVLPESFMMFTCAGVWQPVHWSACDSRFLLYKLYTLFSIVLVYTLTISELMGAILLTQSLEDFTDISFLLIS
TISVCCKIASIIARRDRVIHLTEMLLEVQCIPKNVRELEITRKFDKIARFTALSCIVLAEATVVVMSTGPLFQ
KAENRTLPFKSWLPYDSTTTPCTFWLSYVHQTAAIVLCATVNVANDSLICGFMTHSCSQLELLNRRLLELPRA
VKLKMKKLPRRLMCNVEAMIVSRHVKHHVHIFKFAENINVIFTPVILVQFCMSSIVLSLSVYQLAVRSANGIQ
FITMVMYLTCMLVQFFMYCWFGNEVTLKSVEFGQAIYNIEWTSLQVQTSKDLMIMMIRAKRPIIMSSGALVTL
SIKSFTSILKASYSTFNVLQRSSHN
>NvOr94
MHVLPEAFNLATYIGLWEPTHLESSIARCFYKFYTCLSFALIIILTMITQILAMLFFTKTLDEFAETAYMLLSA
INASVKGVVILLRRKHVIDLAEMLLKKECVPINATEKRVCSYFNKISRYTVLSCIVLAEGTISALALLPVVFE
QGELVLPLRAWYPYNAGSGLGYWLSYLHQAMALTLIAAYDVANDTIITGFMVQACAQLELMTCRFHRFSWRGS
NAVMRNGARHQLRLFEKRMVAQSVRHHLLIFRFTEIINSIFAPVILVQFCLSSGVLCITVYQMSASKSNGLKV
IVLSLYLVSMLVEFFLYCWFGNEVTLKSLGFNIAVCEMDWTAMHVQTLKELLIIMVRSTSPIFLSCGPLIKLS
LESFTNILKISYSAFNVLKQFD
>NvOr95PSE

MLSVQFNVMTIAGIWCPPNWPSTWMRILYKLYSFVVNMLMYTLGLFQLARLIFVKQSFKEFNDTFFLLLSTNF
TCIKTACHLLIQKRVINLTNMFKEDCCIVRRDGEIKVQKKFYDISRXPYDLSSPVVYZLSFLHQSIAAILLAA
IGIINDALIAGFMLQVSDQLEISAHRIQEMPNYVTEALKVRKSSDEVRLLEKRLLEENVKHHNHIFRIADSLC
MTFYEIVVCQFLVSVLAICVSVYQISVGSSNKVELITFVLYLVCMLQQLFVYCYFGCEITKQSKNIGSEILGM
DWSSLNNSVVLSIXLTCHTFYNIKQKSLHIDSYIV
>NvOr99
MKFQDSIEYQLLPIPFMVLTLCGTWCPENWSKKRKRIYKCVTTVLVSLGIILLVEMLVFIIVKSGKDNIDLEN
IFATICIAVGLYKKINILYHRPKLMNFISNYTKNEWNKPKNFEEATIHLNILSETRYISYAYAAFILVSIIFR
SITPILESGTFIILPLDACYPYNADNFIAFSLTYLHQIISGVTLTCMHIGTDTLFVGLLLQMNYQLHILKNRL
RQLGNSKTYKNNTQTIKDRELFIKSKISQRVREHESIFRFGYDLQKTFKPILMAQMVVVVPSVIINVYFLSIY
TDRLNLKYFMTFFFALVSLMQIYMFCWYGNEILLSSSDVGDALYESNWFALDQSTKKIMLTMITRSSKIFLIS
AVAIIPLDIDTFIKIMKTSYSAFNLLQRTTAQ
>NvOr100
MHEKLIAIQKANVEYELLPFQFLLLTIWGIWHPKDWPVRLKNISNIIFIVVFCLDIIICFEMSIYLVLSIGTN
DFKLVNIFFTSATITGIYKAIKTMQIRESFRTILLNYFNYEHLCSLNTKERMIRESNQAQIRKVTVIYSASMA
GIFALNAIAPALSQPDSTMQLPVDAWYPYSIQKSLNYWLTYFHQIILGSSLICVHIGTDTLFVGLLLKLVCQI
NILRYRLQSLTSLCSKNFEHFNAMGRKFIYRYIHHQNEIYEFSKVLNNKFQAVLLIQVITSIPNLCINVYTLS
KYSGIINMDYISIFFNTTSSLIQLFITCWYGNEVLLSSLQIKKSIYEMDWTKLDVPTKKLLIVIMARSLRPIA
FSVAHVIPMNIESFIKIIKISNSAFNVLQQT
>NvOr103
MDLSQCLEYRALPMQFYIFTLSGVWCPSNWTSLLKLSYNMYTTTIAISGILFWASMFVNLIITKNESEYFYEN
VFAISTLTYAMYKEFFVLKKRKEIQQMLKLSFDDEWYRPFDNREIQIIDHYAHETRWVTQVYAIGIIAGLATK
AIMPMLNSNSAWVLPIEAWYPYNTSNLKNYLFAYTQQLMGGIPLICLHISVDSLFVGLILQMCIQLKLLQYRL
QKTFSTDIDLQEEKNIERNIKISDVIIANYAFKHQCIFRLGNYLNQEFRGILAGQVMITIPNICINVYLLSQH
RGGITLHLVDSFLCFTTCLMQIFLYCWYGNKIILLSIDVANTAYTTNWLSLNISSKKKLLTIMVRATRSIQFA
AGTFIMNIDSFIEIIKTSYSAYRVLQKTS
>NvOr104PSE
MEKYTNPGVTRILKSNIEYKLLPFQFLLLTFWGIWRPQTWSLQIKNVHEMYFALIFFLDSRISIEMLIYFILS
FGTDNFKLINVFFVSANITGVYKAIKIMKNRENIRNLIKSYFDYEWMNPRDAHEDKIHKKSZHENXZVTMTYS
TSMIVIVFLKALSPLAEVNIIIPLPVEAWYPYPIGNPYWFWITYLNQVILGSSAVSAHVGIDTLFVGLLLKSS
CQIDVLKYRLQNLTPINIKIKLIKNEHLKIXIFECIHYHERICRFGDDLNSQFQDMLIILKIPNICINVYALS
AYRDYINLQYIATFFCTTSAFLEFFIVCWYGNDVSLNSIQVKNAIYEMDWTICCMFQLKKFIIIMARSLKPIQ
FSVGSVLPMNLDSFIM
>NvOr105
MIIRKTLEHQVLPIPFHILTLWGIWCPEHVQPRLRRFYFAFTCIVIISEILLTTEVFINLIIIIRNKRFELDV
FFILTSLMNGLYKALNILLTRKRIAKLITIGFEDRWRFPRDDSEKKILQNYKFESWRIHLIYAGACLAGVTIK
LVGPMMKQNADIEFPAPAWYPYDTNKPVYFWLAYVQQMFVGGATISMHIGADTMLSGLMLQSCIQLKLLKHRF
KHFFQHYEQVKGRLHLSSTKRKVEIALMKQYICDHQFVYSYANKINRNFSGWLIAVLIVVVPNICINVYLLSF
SKIGLNVDFITSLGLFSISLFQIYLPCWYGNEVMLHSSEIANSIYDMDWVRLSPTARKTLIIVMIRSSKPIQI
RAGYFVSMNLRSFLSIMKTSYSALSVLQQTT
>NvOr106
MTTTTIIERTGSADVYGIENRLFSISFNVIKLSGFWRPTTFRKPFDYLYEMYTLFCLVGILMLIATIIVDNVV
TEKSIRSLIENLYLILTVSNGISKLCNIYHRRDRVISMLQRSSEDRWSVHRDEEEARIVEESIESESYIIRFC
IYLVTINNVSNALNPILNPDPEHDLMVDAYSPCDRSKSALCFWTAYLYQVFGYVSTSLVHVGCDCLVFNFVDR
LCAHLKILEHRILQLPDLVEANACDEIRYLKSCIEDHHSICEGIKELNDTFYETIFIQFVTSISVLCTNIYLL
SMQDLFSAEFIAVFVYLCCAFVQNFFYCWYGYKVSVNTLHISDAIFNMNWCILKRESKKILSYVMMKTSQKVF
LFNSAVVTLTPESFVNILKVSYSAFNILQQTK
>NvOr107
MEVMDTIKSTDIMPLPFFYLKLSGAWKPSSWPSYLRLIYDSYTILMTFFIMKVIIVTEILYVIFAEENQSKVL
KDNVYIICTFINGWFKMFNLICRRKNIANLVKGCIAKQWNPPRDNYESSVLAATKQTSRKITLAHASVVGSCV
VSTLLNSVLSSPPFLPVDAWYPCNITLPICFWTSFVHQSIGYTVTAIVHVANDNIVVGFMMQICAQLNVLNRR
LLLVHVEVEKAARQQKDQSQITSLETTLVNDCIVNYRDILKFAEQLSETFIETIFIQFCAGLSVICTSVYVLT
TLNIFSFEFFGMFLYLWCMLGQMFLYCWFGNEVVLNSSKLFHSIYNMDWIKLQSQTQTKLLFMMLVASSPIQL
FRGAIIRVNLDAFINILKFSYSAFNILH
>NvOr108PSE
YITLAYATSVGTCVLIIILLNPTVSADAWATPVYSWIPCNINFSSCFWACYLHQSVGTATIAIVHVACETLVTG
FMLQICAQLNVLNHRVLSIDLKIRNLTLREKDENRILMAETFLTNACVADHNGILKFSKLLSETFIQVLVIQF

CASLTVFCTSIYMLTKIKVNTLDFLLMTTYLICMLNQILLYCWYGNEVMLNSQKLVQSIYNADWIALHGKTQK
TLLLMMLVASSPIQLFEGAIIKVNLDAFLNILKFSYSAFNILHSEDHN
>NvOr109PSE
YISLSYATLVCGNCLNFIIIPIVSATWTPPLYAWYPCNMSISICYWSCYLHQSTGFLTIGVVHVACETLVTGF
ILQICAQLEVLNHRVWSINFKIQNLALREKDKNTIFLYESFLTNACITDHNSILKFAELLSETFLQVLFIQFC
ASLSVIGTSIYLLTTIKVYSADFVITTLYLICLLNQMLLYCWYSNQVLLNSRELFRSIYNMDWISLHSKTQKT
LLLMMLMASSPIQLFKGAIIKVNLDAFLNVLKFSYSAFNILKNPSKDLN
>NvOr112PSE
SNVGDVEKKTFPRTFALLIIGGLWAPTTIKZRALFICYQIYAVFYFISALMMIITIVIDNILSDDKSMEYLME
NWZKLIVFFNGLQRITNLAVRRDKILHLLRSNIMSGRWQIIEKSRISEIFILKIWGSLIFVNGISSWLNPIVH
ENPENKLMYECYSPRDRRTPTCFWIAYAYQLFGYLVLSAANVGTDCLIYNFIDRINAHMMIFLNRLLKLPTRV
RDTAAQDDVVAFHYENKYIRECIEDHHDIYESIEELNRIFNELISIQFLSCISLLCMNIYFLSKQELFSLPFI
AVSAFL
>NvOr114
MLRTEELLANSRANERRNERVGIEDQVFPATFLLLKAAGVWTPTTLKLRSQYMCYRIYSAFCFISVLALVVTV
SIENVVSSNASILESWYMLVIFSHGLLKIKNLQWRRVKVIHLLKECIMNERWSIARNQDERAIINESKRAEKF
ITHLWLSLLLVNGLGNALNPLIHENPNNSLIFECYSPCDRSLPSCFWTAYAYQLFGYAISSTVHVGCDCLIFN
FIERINAHTMIFIDRLQKLPSRVVEGKNEGCLDASRHEARLLKECIQDHRRIYESVEELNNTFYEVVTIQFLT
TISIVCTNIYFLSKQELFSADFIGVLVFLVCVLTQNFIFCWYGYKLSESSSYIVNAIFNMDWLVLNKRSKGLL
LFAMMSASNEIKIFHNALVNLSPETFLQFVKMSYSAFNLLQQSN
>NvOr115
MHQAIKIANGRIDADGIPNVEGLEKRVFPRTFLLLIVGGMWAPTTIKSRALFACYQLYTVFCFVSVCMLIITI
LIDNVLSDDKTMESLVEYAYMLIVFSNGLVRIINLVSRRDKILRLLQGNIMLDRWQSLRDDEELAIIAESKVS
EKLVLKIWGSLILMNGISNAVNPIIHENPDNTLMFECYSPCDRSVSYCFWMTYSYQLFGYVIMSVAHLGVDCL
IYNIIDQINSHYKIFLNRLLKLPARVREKARDDVAAALRYENNYIKECVADHHSIYKAAGELNDIFNELVFIQ
FISCISLLCTNIYFLSKQELFSPPFIAVFAFLCCALTQNFFFCLFGNKLSQTGSEIAGAIFGMDWQELQKETR
RKLLFIMLLTSKGIALFNNAVVNLSPETFLKLVKVSYSAFNLLNQSTHK
>NvOr116PSE
MRRTSEIEVADIDGVEKKIFRRTFILLIAGGLWTPTTTKSQALSTCNQIYTVFIFVSVLMLIVTILIDDTMEA
LIEYGHVLIVNLARSNIVLNKWLNCRDDEELSDNCQIRNLGXLVLKIWGSLILVNDISNTLNPIIRENPENTL
MFECYSPCDRSISSCFWMTYSYQLFGLYXNAAHVGVDCLIFNFIDRIDAHYKIFLXQVAQIAGSSQGKARDDV
AAALLYENNYIKECVADHHSIYXSSKIADAIFGIDWQDFQKETRRKLLFIMLLTSKEIDLFNNAIVNLSPETF
LRLLKVSYSAFNLTRMTIPPRRTLFYIQL
>NvOr121PSE
MDILPLNFQTCRMCGMWFESNSYYLPKMIYRLLVFLIIFQFTLSQIIELLTMHGSVDDFTEVLFLTLTFVALC
LKVLNFTTZRNEMVDMLDDFRTPICLAKSPEEKDIIQKCSKTTKKIFLSIMSLSQSTGLVLLVIPFLTSETRE
IPLPFKSYQPYEISSATNFWITYALQIFAAIYGVLLNVSMDTLVYGFIIMATGQFEINCYRLENSTCSMRDCI
EHHVLIQDIVYKIQHFFIYVVVPLFLFSLVTLCTSIFQMSQKQVASFEFLSLTMYLACMLCQVFLYCWFGNEL
ELKSNAVANAIYCSAWTDFTRREKRNISFIMLSAKSGSKISYHGQCSLSISTFAWIVKTSYASLNLLQQASSD
K
>NvOr123PSE
MDIVPLSFRTLYYCGIWHEKDEVMTFPKLLYQYFVTLTIFYFTFIQGAVIFEAQLDVNEFTDALFLTLTFVTL
CFKIINMIYGRSEINSFLRDFRNERCKPLKSDEADILDVYSKKAKKIYKVMMSMAIIDTLFFVVVNIIKVLNN
ISNKYVFFITNFIQIGTMCXAEYKHVLRYNDTAGVLMFTAGQFKLNAYRLAQLDQNNIHLLNQYIAHNRLING
IVTKIQSFFIRAVIPFFLFSLISICSTTFQVSQHSIFSFKFLGLITFATCVLLQVFLYCWFGNKMKIKSTAIL
SAAYNCDWITLDPKTRRSLYIIMLCNQNGRTVSYSGQCSLTLDTFVWIVKTSYAMZASCNK
>NvOr127INT
MDVLPLNFRILQYCGIWYEYPEHLWMVKTVYKTFVVVVLFSLTLSELIELGLISNNVHESTECLFLSLTFLTI
CFKIINFMCRQDSLKEILDAYRVDIFRPKTAEEKQIIVNYQNVISTFFVIYLTMALMAGTCMILVPIISNEDL
MLYSITYFHQILSFLFGILINVCMDMLVCGFVILACCQLDLCGHRIGQNQMDIPAKDHITHHILIGDVVKKVQ
SFFIVVVVLLFSCSLIILCTSLFQMPQQNIMTLEFFTLFMYLMSVLYQIFVYCWFGNQLQLKSKSISDAIYDS
NWADLTPHKRKDYLFSMFMSQNGFTISFHGQCSISIQTYVWIVKTSYGAYNLLQKTSA
>NvOr130
MEIYDSRYFVFNKRFQMALGIWPYQSRVKNSITYAGLVLVMIIMLIPQFIRLNTYLGKDIEKTMENIFIFFYV
FGIFVKLFTAHFAEDKLKILYESTAKNFETYTDAVEAEIMKRYSERGRLLTFVFLLYMISAVAVSVVLPMCPI
VLDSTDPLDQPRPRMFILNGEYIVDKYEYYFQIYTLDIISVFLMICILCATDPMYAAIVEHCLGLFSICKYRL
RNFNKSCGLQMVEHAEAERYGGDYAYAALVRAILLHKDIIKFTEIIQTSYSLYFLLEMGATIGILTSSSVVVV

MKLKQPLELLRWSLFLFGVILHIFFLTWPGQKLIDFSSDIFQEAYLNDWYKSSLKCQNLLKFMSLRCSRPCEL
SGGGLYIMNFINFATILKTSASYITVFSSV
>NvOr131PSE
MEIYDSKYFFFNKRFQQALGIWPYESRAKKNAHVRNPGADRHRCTLVPQIARLSTSIGKDLEKTTENVVILLY
TYXIYIKLFVAVYSEDQMKTMYESTARNFQVYTDETEKKILKDYSLFLCKLIGHQLYMIQALMIFISLPLTPV
SLDIIMPRPRMFILNGDYFVDNDIYYYQIYLFDSIACAATVFIMVSTDPCMYAATVEHCLALFSICKYRLEIF
NRNQNDSKTSITEKPRHDYAYYALKEAIILHKEILKYHEILQASYSLYFLLIMGATIGSVTMNSAMILVKTHN
PFVLIRYTLVLIGIMGQLFYLSXPGQKLIDYSSEIYRHIYLNDWYNSSLKCQNLLIFMTLRCTRPCTLAGGGI
YVMNFINZAAILKTSASYIMTVFASL
>NvOr132
MEIYDSRYFVYNKRFQTALGVWPYQSRVKNAIICGFLLLVMIALVVPQIIRLKMYIGKDKDKSMENVFGLFYI
FAIYVKLFTAVYAEDRLKILYESTARNFQIYTDKMEKKILHENSERGRLITLVFIMYMMTALIVFILLPMYPI
MTDVIVPLDHPRARMFILNGDYLVDRDEYYFQIYVFESTSAALTVFILCSTDPMYAAIVEHCLGLFCICKYRL
NNFNKPRRTEIIEKANAESQVDEYAYTALVEAIQLHKNILKYTKIIQTSYSLYFLLEMGATMGLLTSTSIIIV
MKLYRPLDCIRYFLVLIGLLLHIFFLSWPGQKLINVSGDIFQDTYHNDWYESSLRCQRLLRFMSLNCSKPCQL
SGGGLYVMNFVNFARILKTSASYITVFSSF
>NvOr133
MEIYDSRYFIHNKRFQKALGVWPYQSRAKNIVVCGLLLLLMLGMLLPQIVRLKKYAGKDSDKMMENIFILFYI
FGIYIKLFTAVYAENRLKVLYESTAKNFQIYTGEAERRILYEYSERGRLLTLAFIVYMLPAVTVYVMLPMCPI
IMDAAKPLDHPRYRMFILNGDYLVDEYDYYFYIYAFDSMAAIVTVAIMCATDPMYAAIVEHCLGLFSICKLRL
KNFNKPNGTKAIEKTYYYSETCGDEYAYAALVKVVQLHKDIFKYTEIMQASYSLYFLLEMGVTMGVVVCNSVI
IVMKLSQPLELVRWSLVLIGGLLHIFFLTWPGQKLINFSGDIFQDTYLNDWYESSLRCQKLLKFMSLRCLKPC
ELSGGGLYVMNFINFATILKTSASYITVFSSF
>NvOr135
MELFDSRYFIINNTCMKLLGIWPYSSHVKNYLRRCGLGLFLLSCYLPQFIPLYMYFGEDMDQMIQNIGVILYV
FGTSVKLITGVTAKDRMKIVYEKTARDFQTIVDKEERNILFEYSERGRTLSITFIIYMWIALAIYVGLPMGPL
VLDYFIPLQNGSRERGFVWKGEYLVDPDKYYLTIYAVELFSSVLSVTILSSVGPMYQAIVEHCLGLFVIVKFR
LQICTRGGKKAEEESYRLIVKIIRLHNDIIEFTRIIEASYTSYFFIEMDITISLVTLISVNLISRLDYLFDSI
RHIFILLGVMIHMFYLTWPSQKMINHSTDLFHDTYSNEWYNCSIRCQNLLKFVALRCVEPSQLTARGLYVMNF
ENYASLVKTSASYITVLLSFR
>NvOr136PSE
MEIFDNRYVVINKTCMKLLGLWPYSSRVKNYCRRCGLGIFLFSCYLPQFIRLYKYFGEDMDEMIQNIGVILYI
FGISVKLITGVTAEERMKTVYENTARDFQTIVDKDERNIFLEYAERGRTLSITFIXIKTHTLIAFVCLPLGPL
VLDYFIRLQSGSRERVFIWKGEYXVDPDKHYYKIYAIEMFSSLLTVAILSSVGPMYQAIVEHCLTSIZCFXYR
LQIYTRGGKKADEETYGLIVRTIRLHTEIVEFTRIIEASYTSYFLIEMGITILLVTLVLAILISRLDRQFDSL
RHIMILLGVMIHMFYLIWPSQKMINHSTDLFHDTYSKEWYKSSIRCQNLLKLMALRCAEPSQQTARGLYVMNF
ENYASLVKTSASYITVLLSFR
>NvOr137
MDIFDRRYFVLNKALLRSTGLWPYEDRRKKLYIRTFVNLILGICVIFPQIVRIYNYFGVNMNMVLEHAAVLMY
ITSIYLKFLTSVYYEEKLRVVYDNIAKNWQVIKDENEINILIQYSENGRLLTIGYTMYIIAAFCSYVFLPIVP
VLLDVFNPLNQTRSRFYILGGEYFIINNVEDYGKVYAFDCLAVIVTVWLISAVDSMYAASIEHCLGLFAIVKL
RLQTCTRSICDGQKDECYKMIVRLIRMHKDIIKFTDILESSYSSSFLILVGINVIFLSFECIIVLTRFGQAME
MMRYSMIMVGIVVHLFYISWPGQKLIDFSLGLFQDAYLNEWYTCPTRAQKLLGLMTLRCSKPCQLTAGGMYVM
NFSNFAKIVKTSMSYMTVLASFR
>NvOr138INT
MEIFERRYFTLNKTLLSSAGLWPYQNRRKKFCIRSFVNLIIGIFVIFPQIVRIYSYLGVNMDLMVEHSAVLLY
IMTIYLKFLTSVFYEEKLRVVMNLFAFQYIIAACLAYILLPMAPILLDVFVPLNQSRPRFYILGGEYFIIDKV
EDYGKVYMFDVLAVIVSVWLICAVDSMYAASIEHCLGLFAIVKLRLRMCTQPSCEGPRGRDASYKLIVRLIRM
HKDIINFTDILESSYSSSFLILVGINVIFLSFECIIVLTRFGQAMEMMRYSMIMFGIVVHLFYISWPGQKLID
LSLGLFQDTYLNEWYACPTRAQKLLGLMTLRCSKPCHLTAGGMYVMNLSNFAKIVKTSLSYMTVLASFR
>NvOr139
MEIFDQHYFSVNKALLKSTGLWPYESRRRKFCIRTFINLILGVFVIFPQLVRIYNYFGVNMDMVLEHAAILLF
ILTTYLKFLTSVYYEEKLKVVYDNIAKNWQAIKDENEVNILSQYSESGWFLTISYIMYIVIAASAYSLLPMAP
VLLDMIDPLNETRPRLYILGGEYFIVDNVEDYGKVYAFELVPAAVTVWLICAVDSMYAASIEHCLGLLAIVKL
RLQMCTQPSCDSRKDVSYRLIVQLIRLHKDIINFTDILESSYSSSFLILVGVNVLFLSFECIIVLTRFGQTME
LIRYSMIMVGIVVHLFYLSWPGQKLTDLSIGLFQDAYLNEWYTCSTRAQKLLNLMILRCSKPCQLTAGGIYVM
NFSNFAKIVKTSMSYMTVFASFR
>NvOr140

MEIYDSRYFIINKTLMTKMGLWPYQHPLKKFLVRTFLVVFIFVSSMPQLYGLKKNFGVHMDKIIEHLALLMYI
YGIKLKLVTSILSEKKLKKVYENIMENWQQIKDVHERAILVEYSERGRTLTIGYIMYMTSALLFFIILPITPM
VLNVIKPLNESRPWDFIMHGEFPVNDMHAHYGEIYLFDSLACIATVLVFCTVDSMYATCIEHCIGLFAIVKSR
LDLSTKFVNRQGALGIKRDDKVYDLIVKTIKLHKKIINFTHILESSYSTSFLILMGMNMLYCSLVSVLLIIKS
DALMERIRYGTILLGLLIHLFYISWPGQKIIDLSTGLFEDAYSNEWYETSIRSQNLLKFMRLRCLTPCQLTAG
GIYVMNFANFASIIKTSTSYITVFASFT
>NvOr141
MDIYDTYVLNINKKLLSFVGIWPYEEKKKNKFTRVFYLITMFCIIVPQMIGFYQHFGVDIDELLENTGTIFFT
LSIYTKLFTSIIFENKLKILYDSVAKNWKNITEKHEREILVKYSERGRMLTLGYITYNFAAVIVYTTMPLMPF
LLDIILPLNESRPSMFILNGQFYVDKHEHYKKLYAFDCLCIFVIVPAALAVDTMYVACTEHCLGLFAIIKYRL
AMSDKFISTRDIYLTEEKDSSYRWMIHTIRMHIDILKFANILDKSYSSSFVILMLINTVYVSVLCVLVLISLD
KPLNLIRYYMLLVAICIHLFYLSWPGQKLIDHSEGLFRDAYNNQWYEGSAKSKTLLKILTLRCVEPCLITAGG
LVTMNFATYLTIMKKSVSFITVFSSFR
>NvOr142
MTMDFYNSRYFSINRRMMTIMGLWPYQDFKTKLFIRTFLAIVLGIALIPQIISIVKYTNEDSDKVIQGIATLL
YVTGITLKILTTITSEKKIEIVYRNIVDNWKLLDDENEIRTMTEYSEFGRLLTIGYVTYMFFALGLFVTMPML
PMMIDVISPINGSRPRIFILDGEYIADKNENYGKVYIFESLTCIMSVFVFSTVDSTYAVCVEQCVGLMAVVRL
RLKLATAKAARMKYKSDSDEHDIPYQLVSSSAKLHIKAISFARILDSSYSVNFLLSMGSNVMILSVGSVVILI
NLGRPMEFIRYSMIFIGLMIHMFYLSWPGQKVIDSSQGILYDAYNNEWYECSKKTKTLLKFMMLRCIEPCQLT
AGGLYVMNIANFGSLAKTSMSYITVFASFR
>NvOr143
MDFLDSRYFILNKKMLHILGIWPYQKRLERYAIRSVYFFFMGVSFVPQILCVKKYFKVDSDKFIRGVTTLLYL
SGVSLKLTIAILMNGKIQIVYSKVADNWKMFTDKDEIKTLLEYSEVGRMLTLGYVVYMVLAVIVFITMPYLPV
VIDIVFPINGTRPRLFVLDGEYIVDKYENYNKIYIFESVCSVVSVPIFCTIDSTYAVCVQQCVALLAIVKLRL
KVATKYTKNYLRDHKYNDASQQLIIKSADLHNKVIEFAQILETSYSMVFLLLMGMNCLILSVGTLVILVNLNN
PLELSRYIMIFIGLMMHMFYVSYPGQQLIDRSSAIFNDAYNNEWYECSIKSQRLLAFMMLRCTKPCELTAGGI
YTMNLENFGSLVKTSISYIAVFASFT
>NvOr144PSE
FKVMYDDIRRNWDTITEKNERNILIEYSERGRLLTIGYIXQLKAQTLPFRFDVLTCSTSAFVVMAVDTMYATC
TERCIGLFALVKYRLQLPNKLTVNEYSDGLKKEDESYRWMIDTIRLHSKILKFTNTIETAYSASFLLIMGINI
LYYGILSVLVLVNLDNPLNSIRYIGLMIGPMVHLFYLSWPGQKLIDHSTGLYRDAYCNDWYEGLVQSKKLLSF
FTLRCSEPCLLTAGGLVICMNMENFMMIMKKSVSYITVFSSFR
>NvOr145
MDKLKQSTVDIDTINNIFGNTYFKINKELQELVGLWPYQKGFSVRVVQTIMLFVLSFIMIPHLNGIRVWCGKD
LGICSENIAATIYLSGCFLKYLVVLLCKRDISKVYEKIAINWLTINDPNERVILDKFSSLGKLKSIGYTVYVS
AAGIGFSQFALLPFAFDYFSPLQNGSRPKIRIVRAEFFVDPIEYYWHIYATYCIVTFVSAFTIISIDTSYTAV
VHQNLGIFNIVKYRLSLAKKAVGTSKDLAYEQIISAVRLHQDSLGFNNLIEVTYRVCFLLLILVCISFLTFGA
ITILENSDNWIDIVRLGSIEVGAVIHLFYLSWPGQLVVSESEELYYYTYNNEWYNLSAESKTLLHFMMLRCIN
PCCLTAAGLYVMNFENYGAIIKSTVSYITVLSSFRE
>NvOr146
MNGFETSSLNSKIINEVFDNTYFKINKILQELIGIWPYQKRFDALIKQFIVILILSMVAMPHINGIRVWCGKD
LGLCAENLAGVTYASGVCTKYFVVTRSKDQMTKVYEKITSNWLTITDPDERVILNKFALLGKYKSIGYVGYIT
VAAICFSQLGLLPILIDVVLPLQNGTREKLRVVKAEFGVDPYDYYWHIYGAYCAISVVSSGVLMAIDTSYTAV
VHQNLAIFNIVKYRLTQAKRAVNTVKDVAYEQIISAIRLHQDSLEFNNLIEHTYDVSFLILILICVTFLTFGA
ITIMEESDSYLDMFRLSLLECGVCIHLFYLSWPGQLVVTESEDLYYYTYNNEWYNLSEKSKTLLKFMMLRCMK
PCCLTAAGLYVMNFENYGAIIKSTVSYITVVASFRED
>NvOr147
MTNEISQEKLNRVFDSHYFYLNKKLQIVSGLWPYQSRKRKFIHKLTMLCFLGTALVFLLNGLRHWCGVDIDVC
GENLVGLIYVISVLSKLFITSLYEEKFKIIYTRLAINWLELTDPQEHNILISFARQAKIKTVVYFVYMAAAGV
GFCQIPMIPVFLDFINPLNETRPKILFVKAEFIFDPYKYFYQLYAFFIGCAASAVFIVCSIDTTFTAVVHQII
GVVSIIKYRLNCATVSFNPNKDVSYKLIVHAIQLHKEVLQFSDLIEKSYNIFFLVLTGLTVVFLSTGAIVMLV
RVGAMLDLIRLVLVLIGAILHFLFLTWPGQNLIDHTSDLFAAIYATEWYNVSERSKKLLSIIMLRSLKPCVFT
AGGLYVMNLENFGSIMKTAVSYMAVVSSFR
>NvOr148PSE
MKKEEVAFDDSWLKINKELNVFNRPERGKIFRSIIVLAVFITGFLSHVFGTALQCRRILVLHRHCSGIIAKFI
VLMVSKEKALESILLNELLSKISLDWNGITDPYERSILEECSKSGRPKMWLYFVYCVLVGFALCQMFALASLM
RIILPLNESRPKILAIKAECPFNYYYELYSIYCDVAVTSVSVTDQTYVMILQQSLGLFQIVKHKMQKTTMYNN

QDEVMVLAITLHKSVLQFLELTESTYQSAFLLFMVATVAFLSFGLVIMIKHSKEIIDLIRMTMIVXSHIFPQQ
QARTISYZSZYPTKWYNVL
>NvOr149
MNKEEVDEAFNDSLLKINKELNIFNGLWPHRPDGDKLFRRIIVLTVLISVTLPHVLGMFIQCGRNMALCGENI
CGFCYCSGVIAKFIVPIVSKEKFITLYEKIALNWKEITDPYEQSILEEFSKLGRLKSWLYFVYCAVAGFAFCQ
MTALPALMDIILPLNESRPKILVTKAEYPFDPFEYYYELYFLYCTAAVVSVSVLASTDSTYSVIIHQSLGIFG
IVNHRLQKAAKHKNQEESYRVMVSAIELHKSALEFLELIESTYQSAFLIFIFVTVAFLSFGSLIIVEHSEEII
DLIRMTLIEFGAMIHIFFISWPGQLVIDHSENLFLSTYTTKWYNMSKKGKMLLLFMMMRCLKPSFLTAGGFYI
MNFENYGSIVKTTLSYVTVALSFH
>NvOr150CTE
MDVQKVEEVYDQHLRINKTLQIHSGLWPYRPIKEKLIRRTIVFIMMAIFIFPHINGMRTWCGKNMDLVLCGEN
FEGVLYLGVVTLKYYVTILSEPRIMQIHKQIAKNWSTLTDSEEQSILIKHSKQGKIVTILYLVYLYAAGFAFS
QVSMVPVILDYIWPLNESRPKILIVHAEYIIDPLQYYFELYGAYCILTFVNLTVLISIDTAFSSIVYQNLGVF
SIVKNRLNAVTKRPNEKKDNAYELIVGAIRLHNSALQFNDLIEMSYRKTFLIILGYTILFLSVGGVILIEQTN
IMQFIRLAMIEVGVMIHLLYLSYPGQKVLDYSSSICQESYTNEWYLISKKSKILLKFMMLRCIKPSVLTAGGL
YIMNYENYGI
>NvOr151
MDEREIDLLYDNYYFKLNKKLQIITGLWPYKSRKYKLGIRAVVYATLSLVMIPLCNGFRTWCGVNLDICGENL
VGICYTMLIFLKYWVTTHSEERLKNVYRLVAKNWMEITDPHEHEILVDYAKQGRLKTIGYTAYVVVAGIGFCQ
IPMISVILDIIIPLNVSRTKILFKGGEFILDPYQHFYKLYVYFVITSFVIMTIIIAIDTNYTIIIHQILGLLT
IVKHRLQRLAIPMNLKKDNSYHAIIKAIHLHNDALQFVDLIESSYSCLFLVFIGFTIIIISISTSIMMAQIGK
LLNMIRVAMFVLGASLHFLYINWTGQQMIDHSKELYLNVYSNEWYNLTKEAKTLLKIVMLRCLKPSKFTAGGL
YTLNLESFGTIMESALTYVAIMSSFR
>NvOr155INT
MDIFNSHFYKTNSFYLKLLGLWPLGDVSNNRIKRVTIISLTISLMIPQVIRLVEEWGNDMDIVIEVIGSLIYF
TGSQIKYITCVSVESQMKFLYEEIARHWKTLTSEEEKKILSQYAQDGYDLALGYLISINIILVGYITLPLAPM
LLDIIDPLNETRPKAFPYFAEYFIDDQKYYFELTVHGWIVCILSVQIYGTFDATYTQLVQHSCALFAIVEYRL
NKATKIEPSISSDFVGLIEKCYTFVFFGVITLNTAVVSLAAVDTMLNLDKGNHKQMIRIGLLYIGFSFHLLYN
MYPGQKVIDSSTRIQQAAFHSDWFNTSPKTKQLIRIIMLRSMMPCTLTAKTLVVLDLESFAFVFKKSISYVTM
IGSMR
>NvOr156
MSESKTLKIFESDYYRTYKNSVKLIGLWPHENIHKKRITRFFITALLTTFMILQGIRLYEELGNDIDIVLELI
GSIAYFSGCICKYLTTIKAQAALQFLYEQIQGHWDTITNKRERQILEQSASESQFLSKFYMGASYVALVVYTA
SPVLVPIILDIALPLNESRRKTFPYFIEYFIDTEFYYYQLMVHGTICFTISVLVYISIDTMYAACCQHLCGLF
DIVEHRLKEAVKTNSNRINLEPDRTDILMHKLLNEAITLHQDSIEFAVLIENTYALCYLLVLGLNLAVIVLAA
VDIVINLDDTNQIIRLSILYIAFSFHLFFNSVPGQKIHDKSVNVMNSAYFSEWYNLPLNARKLIQLIIHRSLN
PCQFTAGGLFVLNIENFGSIMKSSMSYITVLASIR
>NvOr157
MDIFNSRFYRTNCFFLKLLGLWPLGDVSNNRIKRVTVVSLVVSLIIPMVIKLVQEWGNDIDIVIEVIGSLIYL
SGSQLKYISCASVQSQIKFLYTEIERHWNTLTNEEEKKILKQYARDGYNLSFGYLMLLNVILVGYLLVPFTPM
LLDLIDPLNETRPKAFPYFAEYFIDNQKYYFELTVHGWIICILSVQIYGTFDATYTQLVQHSCALFAIVEYRL
GQATKMVASDEDSSHKDTDKVAYNMMVGAINYHKQAIQFVGLIEKCYSLLSFLIIILNTAVVSLAAVVTMLHI
EKGNQKQAIRIGMLYVAFSFHLLYNSYPGQKVIDSSTRIQEAAFHCEWFNTSSKTKQLIKIIMLRSMVPCTLT
AKTLVVLDLESFAFVFKKSISYITVIGSMR
>NvOr158
MSESKRLEIFESDYYRMYTNALRLIGLWPFECTYRQRIIRFFIIILLITFTILQGIRLYEEFGQNLDIVLELI
GSITFFIGCILKYIVTIQTQSMFQFLYEQIQSHWEIVTNRERRILEQSANDSQFFTKLYMGAAYGALIVYVST
PIIVPNVLDVVIPLNESRAKTFPYFIEYFIDTEVYYYQLMAHGTLCFTISALVYVSIDTMYATCSQHLCSLFD
VVEYRLEKASKTDSKMNVNLDLNGNDKNIYKLLNEAIVLHQDSLEFALFIENTYASCFLPVLGLYLTSIVIVA
VDIVINLGDMNQIIRLSILYFAFSFHVFFNCVPGQKIHDKSVSIMNSAYFSEWYNLPLEAKKLIQIIIHRSSI
PCKFTAGGIVVLNVENFIVIMKSTLSYITLLSSIR
>NvOr159
MSVRKDMHVFESQYYRIYKNSVKIIGLWPYENIQIKRVIRISIILLLISLVILQAIRLYEELGRDLDIVLELI
ASLSYFAGCLSKYITTIRAQSAFRFLYDLIAGHWQIITDIKEREILEESTRQSQTLCLSYMVAAYSALVVYST
MPAIAPAVLDIVIPLNESRKKTFPYYAEYFIDDEAYYYQLMGHGTIVFTVSVMVYVSIDTMYACCAQHLCGLF
SIVEYRLQEALRTDDKLHLEPPERDKLTHKKLHEAIILHKDSIEFAFLIENTYALCFLLVMGLNLTVIVFTAV
VIIINLGDMKQMIRLTLLFGAFSFHLFFNCVPGQKVHDKSISIMNSAYFSEWYNVSLKSRKLIKFVMHRSLNP
CQFTAGGLFVMNMENFGSIMKSSMSYVTVIASIR

>NvOr160
MSVYSSDYWKMPVLAQKFMGVWPFNNRQYDKCMRVFVYVALYSLIVPIGIRLVEELGVNTAIAIENLVGQMYL
NAAVIKFSMTILFKEKHKQIYELIARDWKMTSDKEELEIMEKHAAIGRTISLAYGICCCSTAGAFLMIPTLLP
LLDYVAPLDNSSRPVVLPYYAEYYIDQRKYYLPLMLKALVAGMISMTVFITYDMAFAMCVQHVCSLFDIINLR
LQRASQLGSQLGSQLGRSARTSASYDSGVFRLIQKAIELHQIVIENVSSLENAYNLNWFFILLLNTTAVGGAL
LVVLLKLGHPEDLVRYGMFFAAIFIHKYFIFLPGQKIINYSLEVFEYSYSCEWYNLSAECKVLIKIMMLRSIR
PLNLTGGKMFLLCMETYSAMLKAGMSYFTVFASTQSF
>NvOr162
MFDSKINTQDDFNLDIFETTDYKLYKDGMKLIGLWPFESSTKKTLKRAFVLISMISVLISIQIRFVEELNQNI
DIVLQSAGSEILSIGCIAKFVTTLRAEDSFRVLFIQIAKQWASITDETECKILADNVKLCHPLCTFYRVMAVF
ALSSYACLPSFGPVIMNILLPLNETRQKRIPAPAEYFVDEEKYFYILFSHGMILYMLVCVLYVTIDSMYSCIV
HHTVGLVGIVTYRLQNIIDLDITSSPKNHTNNLEIRRRLRRAITLHKESIEFAENIEATYSLCFIIVMFVNLF
SMVFTAACGIRTLHYDKVESFRWLMLYGSIIFHLFFNSNPGQNLFDKTSEIINTLYFTAWYDSGISTSNKRII
QIMMIRCLRPCQLTAGGLLVLNMFNFGAIVKTSFSYITMLLSVG
>NvOr164PSE
VMSVFALAAYASLPSFGPVIMNILFPLNETRRKQLPAPAEYFVDEEKYLYILFLHGMICCIFVPILYVTIDSM
HVLCXYTVGLVGIVTYRLQNIIDLEVTSYQEYHANNLEIRKRLKRSITLHKEFIEFAEKIETTYSLCFIIVMF
VNLFAMVFTAACAIRSMYIDKADSFRWFILYGGIIFHLFFNSNPGQNLYDKSSQIVDMLYLTEWYXLGVSTGN
KRIMQIMMIRCSQPSQLTAGGLLVLNMENFGAILKTSFSYITVLISKG
>NvOr166
MLSFKIQAKVNNGDVLGVGYWKLNKLMLKSVGLWPYQKSSTKMCIRTFIFIAIYSMMIPQIIRTFEEWGKNSE
IVIENITGFLYFQVVITKYVTSCIAESNLQYLYVRITEDWNHFRDEGEQKVLSHFASHGRFLTIGYSVYLYTA
GIAFTTLPCLIPAVLDLIIPLNDSRQKVLCFYGEYFIDQRVYYYELLLHTFVCVMCTIMLFTTIDAAYACCIE
HVIGLFNIVDYRLNQAFNLVKDKYDTKSEVMRSEIHKCVLRSIEVHNHSIEIVELIQTTYTTCFFFTTGISLI
CLSLGTVDMMLSVNNYINFARVFFAWCGIVIYFFYISMPGQRIIDASSDIFNSVYFSGWYDFPLKTQRLLKFM
MMRCSVPCQFTAGPLLVLNLENCGVILKTAMSYCTFVFAIS
>NvOr168
MLDSNEVFNNKYFILNKRLLMVNFIWPYQTKHMKKFVNTFIIVAIHTMMIPQIIRSVFEWQLEERNVEILVEN
FAGFLYFLGTLSKYYTTIYLEKKLLQLYQQITYDWKGLQDDNEQAVLLRSWDIGRTLTIFYMGYMFTACISFV
TLPSAVPLMLDLILPLNESRGVNLCYYAEYFIDQQKYFIYLLLHTFICVSFTILIVTAVDSTFVSIVYHAVGL
FDILEYRMRNVLRFVEDDYQNNKFLSKEELHKYVVDSVKMHKKTIEFSEIVQRIYNDCFFFVTVLVLVCLSVG
TIDLVMNLNNPINLIRVGFVWLGIIMYTFFISWPGQKLINCSHELFNTVYACGWYGCPTKTKYLIKFMMMRCT
VPCRLTAGPLLTMDLVTSGNILRTALSYFTVVASFS
>NvOr169INT
MELMVEKKTKSEIKEEYIFSNKYFIFNKMMLKSTGLWPYQDVWLKRGMMILYITGFFSLMIPQMRFIYEEITW
NWGEINDSGERAVLGRFCNIGRILSIFYTAYIHICMFFFVWSPALGPMILNTFSNATYKKSICIDAEYFVDQD
EYFSYIFSHTVFVSLLSASILTAFDSTFVLITQHGIGLLHVLKYRLNKISKIFEMSEYNFKDNTIIHTKIVSC
IILHKKSLQFLKVIGSTYDLYHFFIYSLLLASLSIFTIDVVVNLQDFATVLRLLITWSALVMYMFYFNWPGQK
IINSSEEIFEVIYLSNWYSYPLKTQKLIMFMLLRTGKRIFLKAGIFEMSFVTVGH
>NvOr170
MELKVERKAKSGLREEDIFNNKYFILNKKLLALVGLWPYQDARLKRVVRILLVLCIYSMMIPQMMKGIEECRE
KNPNPEIILENISGFFYFQGVTAKFLTAILTEDKLKYVYEEVMKDWKRFTDKNEIAILCKFAHVGRVLTVVWS
IYAAMSCLLFVTLPAVIPMILNIILTRNETFKKSLCIYCEYYIDQDKYFFYIFLHHIIAGIATIFLTIGIDTS
YVNCVQHVLALFNVSRYRLKVAFDTIHHSKKNDYNLKTLENNVHSYVVSSIRLHQRSIKFVDTIQSAYNIVFF
IVCALLLFGISIITVDLVWNVHNPINLIRIACLWMGTIMYMFYSNWPGQKLIDSSNELFDAIYTCGWFEFPMK
TKILIRFMLLRSIDPCRLTAGPLLQMNFESCSLILRSAMSYFTVLVTTG
>NvOr171PSE
MIKGIKECQEKNPSPEICLENLSGFFXSQIISTKFVTSILTEHKLKYVYEXKDWKELSNENEVAILRTFAIIG
RLLTLIWSVYTIISDLAFVTMPAVIPMLLNAILARNKTFDKSLCIYCEYFVDQVKYFFYIFTYHIITGIVTIF
MTLGIDTSYMNCIQNILALLNITRYRLKVAFDTISHYTAKKELDLKVIENQTHDYVVSSIRLHQRSTXFIDTI
QSAYNVVFFIVCVILLFGISTLTADLIWNSHIPINLMRITCLWMGAILYIFYS
>NvOr172PSE
MLDSSEIFSNKYFILNKKMLIVIFIWPYZKKYLRIFVNTFIILAIHTMMIPQINGMRTWCGKNMDLVLCGENF
EGVLYLGVVTLKYYVTILSEPRVYLYAAGFAFSQVSMVPVILDYIWPLNESRPKILIVHAEYIIDPLQYYFEL
YGAYCILTFVNLTVLISIDTAFSSIVYQNLGVFSIVKNRLNAVTKRPNEKKDNAYELIVGAIRLHNSALQFND
LIEMSYRKTFLIILGYTILFLSVGGVILIEQTNIMQFIRLAMIEVGVMIHLLYLSYPGQKVLDYSSSICQESY
TNEWYLISKKSKILLKFMMLRCIKPSVLTAGGLYIMNYENYGI
>NvOr173

MDIFDGRYYKTSKWFLEFLGLWPFQSNRRRYVTCFIFVFMTATVVFPQVLLLIELKTSNFNILIENSLSIIFG
FACLLKYGVTFASRSRLQTLLTQIASDWQRLTDKAEIDILSQYGEEGRYLVLFYTVYVFLAWVTCNFVPFIPP
LLDILLPLENGTHDLVYPFYADYVFFKQTDYHYESCLHVFFVYFGTTSLFAGMDTIYVATVKHSCGLFAITWL
ETMARTGKSNRSNYSIKPNSVVHHDMVEAIVMHNETIRFVELLEDSFSLCFLMVQCMIVAGLATLCFYMMRIY
DKTFNMCQFSTFTVGLVIHLLYLHWVGQKIIDSSDKVFYSTYYSDWYLISRNERQLTKIILARSLYPCQLTAG
KISVLSMETFGALMKTSMSYCTVLLSVS
>NvOr174PSE
MEIFDSRYYKSCKVLMEMLGLWPYQALKRKRLTYIIFYSLHGSLLLPQVIRLLQTKDFNIIVENTISILFLFV
CIAKYAVSYTSGPNFKLLFTQIAADWQNITDKSEREILSKYGNDGRFASLFYTFYVFWAWATYNMMPFLPPIL
DIVLPLPNGSHPLLLPFYANYIFFEQLDYHYETALHAFFVYFFATSLFAGIDTIYVSTVKHTCGLFAIIZLET
LAKTDENKASIEFRSDKVVHHDLKNTIIMHNDTIRSCRLLEESFSLCFLLIQSLCVVTIALGGFYMLCIYNDA
YKLLRVVAFYTGTVLHLLYLHYVGQQIIDSSEKIFNSAYSSEWYLISTNARKLTKVIMIRSLYPCHLTAGKIT
SLSMETFGSLMKTSMSYFTVLLSLE
>NvOr176PSE
ALMLIQKWGKDFTITFENSGSLLFACASLSKYLVTYSSAKRLELLFLQISKDWQRITDIKERQILMKFYEEAK
SLVVFYTIYAFAAWLVYTSIPFIPFILDLVHPLENGTRPLFMPFYADYVFFDQNDYHYSACGHVAVIYFTSFL
LYSGVDGIYVLTVKHTCGLFAITCARFICRGSIYSCIIRKYYVSIFRYHFLKEDILYFYENTFLLLRVGAFLF
GVILHLLYLNWIGQKIIESSSQVFDSTYKGNWYTVSIRSRKFINMIMYRSLKLCELTAGTMTTLSMETFGILM
KTSMSYFTVFLSVT
>NvOr178PSE
MDLFDSQYFKINKRVLTICGLWPYQSKLGKRINFASLASSTILFIFTLIAGIISQSPVEFVNTVETSMGISFC
STGLLKCAIFYKQQNKVKELYERIATDWMKLTDDSERVILQSFFXVGRSLNFITMVYSFAAFVVFSCFEFLPR
IFYKESZYHKPHSFPYYFRPMVIHKKFYDLQVAVHTTVIVIYSGLTYLSAIATYIFSVKHVCALYEIARYRLQ
NAIVYDISNKPLQELIDDTSTIPKLIKVINMHKQALRATQRIEKVFSTDFFVLDASSLIALAIGIFXLNYYKG
NFREFVRALLVVSIIIIYLFFVNQSGDRVIQASNNIHITAYNIDWYKSSSRVHLFVLMIMRRTLKPEKLTAGT
IMMLSIENFTA
>NvOr180
MDLFYNQYFNINKHVSMICGLWPFQSQFGRRISYMIFAMSTFSMIFSLTAGIISQLNPDLLNILETCVALFFC
VCGFLVCTILYNQKNQIKRLYERIAADWENLTDDLERDVLRTFLLEGRKLIFITLVYSFPAFSLFACITFLPR
MFSEESTKLCLHSFPYYLESMVIDKNLCNLQVSLHYSVALGYVGLSFLSVGATYICSVKHVCALYEIARLRLE
NATVRYGNYDPLGELTDETSIIHNLIEAIDMHKNALRGIQIIEQVFSTGFFIIQIFGLSLLAILICELKYHEG
EITEMIRFMLVLSVFVIYLFFMNWSGEQVIQSCDDIQKTAYDIDWHRISSRTRIFVLMIMQRTLKPVHLTAGN
MMILSIQNFGTILKSAWSFGTILLTTQKSV
>NvOr182
MDLFDSQYFKINKRVLMICGLWPYQSILGRRIAFAMLANSIFLFVFTLIAGVISQSQLDIINTEDTIIITFFC
LLGLLKCTMFYNQQNKIKNLYECIATDWNKLTDSSEHNILRSFLLDGRKINFITMVFCSSAFMIYSCIDFLLR
IFNKESEYQRQHSFPYYFKPMVIYEKLYDWQVALHVTVIVIYSGLAYLSAIVTYISSVKHVCALYEIARHRLQ
NAIIACDKINHPLQKCIEDISLIPKLIKVIEMHEQAVRGIRIIKKVFGADFFVLTVFCISALTIGTFELNFCR
ADIHSFIRVLLLMPIIMIYLFYVNYSGEQVIQACDDMYTTAYNIDWYKTSSKTRIFVLMIMRRTLKSEYLTAG
TMIMILSIKNFATIIKTAWSFGTLLLTTQKHRKNEDANFVAENTLI
>NvOr183
MTLINSHYFKLNKLLLTFCGLWPYQTKLKRRINYTTFAIITLSMIFSLAGGIQSELNTGFMNISESIIALLFF
STGFLKCTIFYNQRNQLKILYEQTAHDLKKMTHHLERDILQAFLLEARNFNVVSLVYSIPIYIVFAIATYLPQ
VFGFTNESAKYELHFFLYYKPMIIHESVQDLQVLIHATISTIYVGSAYLCVSATYISSVKHVCALFEIARYRL
KNVIVDHSNNNRRGLMKNASVISNLIKVIDIHEKALRGVQRIDNVFNASLFILEVTALSAVTILIFHLNYHQG
NFRQMTRYSTILSAFVSYLFFCNWFGEQVIQSCNDIRETAYNVNWYNMSLRARMFVLMIMQRTLKPVHLTAGT
VVILSMENFSAFLKTAWSFGTLLLTTQKPSPKENSIFFEY
>NvOr185PSE
GCGIPAFLLFVCITFLLRIFSKQSLDQCLQGFPYYLRSMVINEKTCNLQICVHCSVAIILASFAFLSISATCI
TSVKHVCALYSIAXHRLRNAEVYFKDNGPLKDSIEDTPIIPSLIKVIDIHKKALRGIQRIEKYFSLGFFSXIG
AVLAFLAVLMFEVKYHGGNVNELLRFSPILLIAVIYVFFINWCGEQVRESCNDLRISTYNIDWYRISSRVHIF
VLMIMQRTAKPKHLTAGTVMMLSIENFATIIKTAWSFGMLLLTTQKQGRNKGANFLEY
>NvOr187
MKLNGYDKFYYTLHKYVLTCIGLWPYQSRMSKRFFFITYGISSCSLIIALIAGLSEKWSTDPVIILENMLGII
FLTSSTAESSILYMHESKIIEFYDKIKTDWKKLTNKKEIEILQMHTRKGQFVSTAYIVYGIPAFAIFGFVTFL
PPILDPPSRVEYSHIFPYYFYCMIINEDFRYYQIVLHCMVSFSYASVSYLAVNCTFAKCVNHVCGIYAIICYR
LQNAIEPTVVRGPFNKLKNSKLIRFNLIDVIAKHREVIHGVDMIEQIFSTGFLVIEIAGFSGIALVIADILYN

QKNAYQLFRIMVVTAIFLVYIFYINWMGEQIIQVSDDVRLTAYFIDWFTLSIEAQEIIHMIVWRSCKTNKLTA
GSFVALSLENFLSMLKTSWSVATVLLSAHRSQKNAHFTGYGITNSFTNSSST
>NvOr188
MNIFDFPQRHLLTCLGLWPYQSKFTQRIFFTCAILSFFSLFVAMAAGLGEEWSTELVIIYETIVALFVIFGGL
AKCIVLFCRKHQMKSLYDQIRKDWQELTNEKEAAILQSFMLKGKAQIILYVVCAIPGYFIFVALTYVPIISSE
DASKDYSHTFPYYTDLLILSKRFRVYQVFIHAGLGIFCGGITYVAFMAMYITCVRHVCALYAIVRYRLENMVK
SQDKLMDKLNDDEEVIPGLLEIICTHKRAIKRVRLINRIFSRTFFMVEICLLICLALLIFDVKYNQHNVRLVI
RMLMIALMFIVHVFCMNYCGEQVIQFSTDVQYAAYFMEWYMISSRAQKILIMILCRSSNPDYLTAGNMALSLK
NFASIVRTSWSMATVLLTTQKVNRPSYSIS
>NvOr190
MPMEEPDSSIPYVKIQKFLMNCCGIWPYNSRLVNCLIYSFFVVFSFTTMTPLSLGLNEEANNDIVTYFETLVA
VVAIFGGFAQITMLGIRNHLLKCLYRKISTDWRTLKDARETEILSAFSIEGRSLTFLFMLITISSYVFYLLLT
YIPLINDEVASSDYSEIFPYYSNDWIISDRMRHLQVILHGCFGIFYGGAAYIIVMALYICSFKHICGMYAIVG
YRLKRLVTSCKLTSSGELRDDDAVSKLYAIIDQHEEAIKGVRLLVRLFSRFFFIIELFLLICLAFLIFVLQYD
IRSSKVIVRIFLASIILVTHVFFMNYGGEQIIHYSSKIHTTTHFMQWYLLSVKCRRILLMVIQRSCQSEQLSA
GIMTLSLENFTSIIRASWSMGTVLMSAHRKE
>NvOr191
MDIFQSSYYIRCNRYLSFCGHWPYQSLRNRIRNFVLLMFLMSTILIPQIIKFWQLRHNIHVFVAALPSMLYYC
AFLFKNSFSMLQSKEIKKVLEKIKSDFQRYKDEDLKILHKYSGQANKINTFYTVYMFMAVGGYSMLPLTLHVM
DIALPKNESRLPTKPRLINYNIEAFDENIFFIIIHGVIVDTAVIVFIIGFETLCFSFSYHVCALFVIVTNKIR
DSIDERITSKHSEVDQDIFYRNFVKIVIMHKDALDFVDTVETALSVLNLFAIGFAMMPLTITGFEFILSKGNV
GEMARWSLFAFGEIVHLFYYNWPGQKIRDHSLCVYQSCYAIEWYKEEIPDKCKKLLNLMMLRGQKPCSLTAGK
VYILGLENFAAVMKVSMSYFTVLSSVM
>NvOr193
MEDNVLDGPYYVYCKNYLSSFGTWPLQSYKKKVLLRTLMYLGCSSALIPHVTKAYELRNHLEYFFLCIPSIIF
YVQVLTKISCMILNEDKCKELIKQIKSDFQSYTGDNLRILNEYAEQARKVNHVYIYYFMGTVVVYNTSAFVPL
LLDLLVPLNETRPRPVLRLMKYNIQRIENNFFVTTLHGFVLNILGMMLIMGFDTLLLNCSQHACALFQIVMTE
LKDTIDKHKIEATSDTAKDTNSRDVFYQEVVKVIIKHKHAIEFVDLVESIYAMANLLVIGITLGSITLAEFET
VQHKDNHEIAFRYAIFTSGELLHILFHNYPGQRIKDHSLMVYQSCYNCEWYREGITDECKKLLSFMMLRSQKP
SCLTGGGLYVLGLENYATILKASLSYFTFLSSV
>NvOr194
MDIINGPDFVYSRICLRPFGLWPFQDPKSKLISRVITLMAVSTVLIPHIMKTYEFRNDFHILLMCIPSLLYYA
HYITKFLYIAFREEKVFRNVLERIKDDFVTFRGESLNHLTNYSEEARKFNTFYMMYLCSTVVIYNVTAFIPHM
LDFVFPLENATRPRHAARLVKYNIHQIDNNFYFVLIHGMIFDVVAIAIIIGFDALFINCAQHACALFKIVVVE
LRKSTKLDEKMSNSASDLVTLQCRQDIFYAKLVRTIIAHKHAIEFTDNLESTYALVNFLMIGIAVATITLTEF
ETIVHVNEVDIMCRFAFFSGGELISMLYQNWPGQRIKDHSLRVHASCFECEWYREDVSYKSKRLLMFMMLKSE
VPSALTAGKLFILDLQNYVKIFKASLSYFAFLSSVAKVSSN
>NvOr195
MKMKDNVLDGPYYVYSKNNLSPFGMWPLQSYKKKVLLRTLIYLGCNSVFIPHVCKAYEVRNNFQYFFICIPSV
IFYIHVMLKMACMILNEDKCKELFKQIKNDFEIYTGESLRILNKYAEQARKVNRVYVYYFMGTIVAYNTLAFM
PLFLDFLVPLNETRPRIITKHMKYNIKRIENNYFVTTLHGYVFNILGMMVVVGFDSLLLSYTQHACALFQVVR
NELKDTIDKHEIEVTSHAAKDANSRDVFYQEVVKVIIKHKHAIEFADLVESTYAVTNLLVIGITLGFITLVEF
ETVQHKDNRALGIRFAIITIVELLHILFHNYPGQRIKDHSLRVHQSCYDCEWYREGITDECKKLLSFMMLRSQ
KPSCLTGGGLYVLGMENYATILKASLSYFTFLSSV
>NvOr196
MAYAEEPRENILESSTFLYSKSSLRVFGLWPYQEPKQRLICRTSTAVLIGSLLIPTICIVLEQWRNFDYVVLG
LPSFLYYVEFVTKYMYLAANQKKLEQIFGHIKNDFDTRKDRKLEILKDYATETRLFNHIYAAYLIIVVVMFNL
SFYQPHFLDLIMPLNESRPRPIPRLARYYVTSLDESFNFVVLHGLVIDWYSMIFFLGHDTLLVNCAQHACALF
KIVINDIQDCLVVQKNDKADDEDQFYRRISNTIDLHKWALEYTAMVDKMYMYVNLCIIGVSLLAITLSQYQTA
IHLDNTDLVIRYSFFSIGELVHILYFNWPGQRIRDHSLSIYQACYNCEWYRDDISYRCKKLLKLMMARSQLPS
NLSAGKLYVLGYENFAQVLKASLSFFTVLLSVN
>NvOr197PSE
LDNSKIMLENTVALIFTTVCMSKYCITYSSTKQLDLLLLQIVKDWKTFEDKTERKILEKNTGQGTTLVLIYTY
YLLFAWMIYTFMPFTPYFLDQIFPLRNGTRPIQLPFYADYVIFNQLDYHYWCCGHIAVVYFTSFFLYSGVDGA
YVLTVKHVCGLFAITCNRIETMGNSQKTDEARYEQKNYSTVKGEMAGAVILHNETIRSVDLLENSFSKCFLIV
EGLCVIGLALESVYMLFIENEIFKFLRSGAFIIGVIIHLLYLNWVGQQVIDSSSKIFYSS
>NvOr198

MDKERNMDRMNFYYIYSRISMLSLGLWPYQSWSSMMTLRSLWIIQHISIMLPELIKIYENRGHFNLLIESLPP
FTYNIVMAIKFTNGVLNQRKLKSILEKIKYDWNKFTDKKEIEMLCYYSHRGKSLNTVYIGLVAVVLLSYMLLP
MLPAVLDLINPLNESRPKSPLYMVEFYIDQDKYFYSVLTHAYITSLAGVLPLFATDLLFSNCAHHACGIIKIL
GRRIENILSEEPALKRSYKVDDEKKAIACVIEHQNIIKYCESINSLYTTSFFLILSISIGLMSVTGFVTLIKM
NEEFKDCIRFAMFTFAQIFHQFCYYFLGQSVLNHEEKLKDYVSNFNWYKASPKTKFIIKFMIMRTLKPTKIRA
MIFPLTLENFTSLMKTTMSYFTVIKSTR
>NvOr199PSE
GIWPLQSKSSKVIFEFLWITQEFIITGAESIKLVEIYKEIDLXPFIYNRLNDIVFANGIINQHKMKILRRKIQ
NHWDSLTDESEIQILAKTVKFGMLINVGFIXIIPLLPLVLDIVZSLNESRPLKSPFTCEFFVDPDKYYFTILA
QNYISALASPLPLVGMDLFFINCCNHICRIIGILGFRIDNITKTEALNKESKSTYKQLCDCMDMHQIIFDFCD
SINDTFSTNFLVMLFINMSLMSFTGVAAVIKMDDFSQINDVMRFGVFTTAQIFHLFCFNYMGQSILTAIEILE
EKIYNVNWYELPLESRVLVQVMILKNYRPTVIQGGIFPLSLPSFTAVMKTSISYFTVIKSTR
>NvOr200PSE
MSILGFWPSRIVLRCFWVFQHMSIMIPEAIKLYENRNNIDLVIEGTAPFTYNTTMLIKFLNGIFNLEKTKSVL
EKVKNDWNTLLDDRZTEILSYNCSSGKFLNTVYIYMAWLTLVSYMFFPLTPMILDFISPLNETRPKSPLYLVE
LYIDQDKXYCDNINEIYNISFFIILIVELLEMSVTGTAXVSRCQSHTAIFSTSHHTQIFHVFCYFFLGQNVLN
YSENLRDTAYNLNWYAASTKTKYLIQFIIMKSLKPCLISVSIFPLTLENFTTLMKTVFSYFTVMNSTR
>NvOr201
MDKERNMKFYFVYTRLSMLCLGIWPYQSWSSMLTLRSLWVIQHISILLPEGIKIYKNRKNLNSIIDGLPPFIY
NVVIAIKFINGIINQHKIKSILEKIKNDWNQLSEKKEIEMLRDYSDTGKAFNTVYLSLVTVILLSYMLIPMLP
AALDLVNPLNESRPTSPLYLVELYIDQDKYFYSVLTHAYITSLAGILPLFAIDSLFSSCAHHACGMIEILGGR
LENIINEEASIKEIDNNEEEKNAIACVIEHRGVIKYCESINSLYSTSFFFVLSFAIVMMSVTGFVAVIKMGEE
FKDSIRFAMFTFAQIFHMFCYYFLGEIVLHHEEKLKDYASNLNWYKASPKTKYIIKFMIMRALKPTTMRAIIF
PLTLENFTTLMKTTMSYFTVIKSTR
>NvOr202
MEQHYSIRTYFKLNRVFMVSSGVWPYQPLHVARIIRLLWITQHISIMTPEIIKLIEVRGMADLLLECIPSVFY
NIVIAVLYGTTIHHQRKIKELIEKIQKNWITISKKSEVEILTRYSNMGIRIGWLYIGALYFTLFIFCLFPLSP
IVMDYVNPLNVSRQRLPLYRVQFFVDDKKYYWTILMHAYTTTMIGIIPLLTVDLFLANCTQHICGMMLILGKR
LEKTMETTKLVVNKLDDNIYKDIRKCTILHTEILDFIEDINYIFSTAFGILLAVLTFLTSFTGIVVLIKWGDW
NEVIRFGMFTMAELFHAFCYSYHSQDVIDHNNQIHKSIMNSGWYKSSMRTRVLVQMMFLRSNKPCLINCIIFP
LSMENFTTILKTMFSYFTVVKSCRF
>NvOr203
MDGKQCLQTYFIVNKVFLFSCGTWPYQHTIFAKTFRYFWITQQIVIMAAKSIKLYEIKNDTDLVIEAVASFFY
NISITIKFVNQVINEHKVKIILEKIQDDWKSLEDDSEIKILSYYARLGKLFNFMYIGAVYSALISYMVLPLTP
IILDFIVPLNESRPKQPLIMAEFFIDQDKYFYPLMIHAYLSVLYGIIPLLGTDTLYMNCVHHSCGMLKILGNR
IRNILNSSSRELSNKIKYEKMVKCIIQHQNIIEFCNNINETYSTSFLIVLCFSITLMSFSGVATVIKLGDNFN
DVIRFGFFSVAQIFHLLCYNYMGQNVLNYGEELRAQIYNTNWYEASLKTQRLVKFMMAKNMHPIILRANIVPL
CLPNFTRVIKTSMSYFTVLQSTR
>NvOr204
MEKENDCIRAYYKLNKFSMTLLGHWPYQSENSVKIVTFLWIFQHLSILLPELIKFVEIRNNVDYVILAFSPII
YNIVVGIKFVNGSLNRHKIKITLDTIQSDWKSLRTEEEARILANYSSFGKLCTVGWAWICTTTIICYLLFPFT
PFVLDLIRPLNETRPRQLIYMVEFFIDEDKYFYEIQIHSYATTLIGFIPLISIDTFYAASVQHACGMFAILGH
RLRRINGAMSKSKKRSDEDAYREIVSCAIQHDKILQYCDNLNDTYTDSFFYIMGCNMISLSFCGVLLILMWGR
IYDMLRNGIFTFAQIFHLFYYSFQGQVLSDRSLMISDCVYDSEWYTASLRTRKIMTMVSMRSLKPFLLTAKVY
VMSLPNFTLVIKTSMSYFTVLKSSR
>NvOr205PSE
MILEQTDIRDGIGPKTYFKYNSMFARGIGVWPFQSKLSKAVYFTLWVAQELILAIAELIKFTQIYTDVDLLLE
AVPPFLYNRGNDVLLANGVINQKKIKALLIKIQKHYNSLTDESELQILDETTKFGHFLNLGYIGLIYAAVLQW
MLLPLAPLLLDYIKPLNESRSLQPLFMVEFYIDPDKYFYTILAQNYVCALVSPISIIAMDLIFMNCANHICGM
IWILGFRIDNISKNVEMKGQSKEREYKQLCDYIIMHQNIFDFCDDINTAFSTSFLLALFINMVVMSFTGMAMV
IKMDDFSQFNDVMRFGVFTVAQIFHLFCFNYMGQSILTAIEVLDLKXHDVKWYNVSLKSQVLVLLMILKNYRP
TVMQAFLFPLCQETLKSFMKTSMFYFTVLKSTR
>NvOr206PSE
LYIFQEKYLIYFAWQMVSTLKNGADDSPPFLRIVKKMTTRLIYFKNLKINIFMIXYFEHVYCNNIGNIFYQNI
IVSWNHTSKEKEIILTRLYTECCLKTMIVHTISINILFQRRPVLYDIIKYLKESNITCYNKELPFCFEVFADQ
EKYFDYICMLLFLMSCTLLIALSHELIFLQSVQDIIGSFEITEXGLSKIVKDMALIZYZKKLKCKMLACIFRV
VELYNAILXNIQLINTAFCTNYYEHLLFNCLIFRAFXXCSHEWYKLPKRLKGLLRLMILRRAIKCHFTVGSTF
VLSSETLCR

>NvOr207
MIFSFKDKFFPYYAWQTVTVLEKAAGWWPFQNRTKNMTLRLMHFGNLLLIIIMCSVRLFQEYKQKKLYIVVEN
TVILVMIICTKMKVIMFFINEKQRKIFYENVLIHWKDTNDEEEMMILKQYAKLGLKTIINYAISVNILFQGVP
VLSDVINYLNDPNITYLKKELPIYIEVYIDQEKYFYQLYVVLFFMTCAALLLAFSHELTFFQSVQHINAMFKI
IEIRIIRLSKIVKRTECGLDTFKEADRKIFVCISRAVDLHNAVLNNIKFINSSFGATYLVVLLFNCLIFGASL
FLIFNNSDQKIHLIRYGLVFLGLSVHFFIIFWPGQKIMDGSESLFNVCCSCDWYKLSKRSKNLLRIMMLKSVM
QCQITASGMFVLSFETYVKLFKTGLSFVAVFS
>NvOr208PSE
MHXILFKGENQYFLYHPWQVVTSLEEANGTWPFQNKRKNIILRLLHMGNITAMFIMFSIRLWQEYKNKNFHII
MENSLALIICFCVQVKIIMLCINEKQRYSFYKQVLALWKDTSDEEEIMILKQHADFGFKTLKIYIIISSTIYQ
VIPILSGVLRYLTDPNITYYQKELPFYIEIYIDQEKYYYHIFIAMFIMILAAAIITFSHELTFLQSVQHIIAS
FKIIEFSLIRLSKIAGEVESGLNTLKDTDKIVLTSISKIVDLHNATLNNIKYIDQAFGTTYFIILLFNSLLFG
SSLVLINNNLGDTLYVIRYALIFTGLTTHFFIIFWPSQRITDANESLFQVCCSCEWYKLSKRPINSLRIIMLK
SSMQCEITAIGMFVLSLETFSKLFKTGLSFFAVFARN
>NvOr209PSE
FKDKYFLYYAZQAVTVLZKVSDCWPFQNQTKNVILRSMHFGNIMMIILVAXLMSRTIIMKNNKFKYYLRIRTS
HTMPFENQIKKMIIRLIHFRKRFYEQIFVQWKDTSDEEEIMILRQYATSGLKTIIRYTTSISIYFQTVTMLLT
LMKYLMNPNITNYQKQLPFYAEYFVDQEKYFYQLYLLLYFMIGAAFIVTFSHELTYFQLVQHTMASFKIIEXL
RLSRLLKIVKSVDCCLGRFNDADQKIFTSIRGAVDLHNVTLKNIKIINRSFGTTYLFVLLLNSVMVGSGLFVI
SKNIDDIFDLIRYGLSFLGLTTHFFIIFWPGQKIIDSSESLFNFYYSCKWYKLSKQSKRTISIMMLRSFGQCQ
ITASGMFVISLETYGKLFKVGLSIFAVFSQN
>NvOr210PSE
MRKIFSLNRDXDDFFPSQYWHLLKRLEKLAGFWPYQNKIKNTLIQSLNMIFLTIMILAFVARIPIEIKLQNHA
IAIEASVGAMTAFCVVMKSCIMSVNDKSKKLLYAHVVSHWRNVSLKNEKSILSRYAHNGHRIGVTYVVCLAIV
CVFNVPIGITYIEYWLAENRTDLKIQTLFYAEYFVDQDKYFYEILTFNVIVTINGYFANCAHDFAFIVTIQYA
SSLFKIIIXXTVAYIENIFNLSWYFVISTNIMILAPGLVLIMRVZSIEEGIPYILALVVYFTHLFTIFWPGQI
VSNSSSLLYYACWNCQWYRLSRRSKFLIQMMMRRSSKCCILTGGSIFVLNLETFKKVFQTALSFFSVFANI
>NvOr211
MGKWRESKSEFYEDRLFVINKKFLQILGRWPYQAKRSRIFLLSLYFVGILTVVVAELIHFVRVIRIKDIPKII
DCLPALIFTYGAMVMIFNSMIKFAEMRQILGKIEENWLSARDAEEYALLKEFAEEGRFLIIGYTLCIIGSLAT
YSLEPLVPQILDKLSPLNESRPIKTFLEVEYLVDHRRYYTAIYLHNMQAACWVILTVLAIDAYFVMIVQHACS
MFAVLGLRIGKIGSGSPERIASGRIIECLKLHKSCIEFAGLIESSFTASLLLQLFLNMVVISVTGVQTINRRE
QPGEMLKFSLSSLSVMTRLFYISWPGQKMIDHSLRVRELVCSVAWYELPAGSRRLLLLMLLRSSRACHITAGG
MFPMNFETYCRLMQTSMSYFTMILSTQ
>NvOr212PSE
FAGIIYYAHQDIWVKALENFVAILFFGAILSEFNLNYLLKERVRLLYKKIAYDWVMLSDKNELDILHTYSRFS
RTLTMIYXNYALLAFILILFVPLAPIIMDNIMSLKSGTRIRVETIHPYYFDMIDVSKYYYHAYVLHFQLVGSY
DZIAYLSINSMHATCIQHVRGLFAIVCYRLEKLERCDNISETSEKTSIGLIDDRKVIQNIKAINTLYSSSMXY
IHMIANSFGLNFIMFISFTVVGISIIMFDLLFYHDHPFEKIILLGLIIGILMFFFVLSWIIQKLTDRSEKVFI
QV
>NvOr213PSE
MRLPITGYSVFDGPYYWINKILLDGFGLWPETSRERKFMSFVFFNISTFSLLIPTFAGMIFYGKRKIWVKALE
NFVAILFFGAILSEYNLNYLLGKRVKLLYKKIADDWKMLSDKAELDILHKYSRFSKTITMIYICYALLAFILI
LFVPVVPVIMDQIIPLKNGIRIRVETIHPYYFDIFDINKYYYHMYVFHFLLVGSYGSIAYLAINSMHAACIQH
VRGLFAIVCYRLEKLGKSDSMAEAPCGLINDSKVVHNIKAVNILYNSSIXCIHMIGNSFGANFIVFISFTVIG
IAILMFDLLFYQDHPIQKILVVGIIVGVLMFFFVLVWITQQLTDSNENVLLQVYSCHWYNLSVKGQKLVYLLM
MRTFKTTKIRAGGLIEINIHLFESVLKTSLSYAMVMQKLNIS
>NvOr214PSE
MALLSTDFSVFDGPYYRINKILLKAYGLWPETCNKTTKRTIFGFLNLATFGLIIFIFAGMMHYVNENIWVKAL
ENFVAILYLIAIVLEYNINYLLENKVKLLYEKIEYDWKMLTDQNELDILHKYAQFSRKLITIYIYYALVACIL
FLILPLAPIAMDYIIPLKSGIRIRIQTIHPYYFNIFDVNKYYYYVHVFHVSAVGFYVSAAYLTLNSMHVACIQ
HVRALFAVACYRIEKLGHHDNTSETSAGVIDNKSVNQNIRSVNNIYNNCKSX
>NvOr215PSE
MRKQSVNLAIFNGSYYGIIKTLHVSYGLWPELGKTRKFISLILYFTTTLSLYIPIIAGIIYYLHRSEWVILLE
NVVGILYSLVTLLKYISCHMFEERIKRLYNRIVCDWENLTNKNEVDILHNYSKYSRTFAIFYIIYSAIGWIII
LCLPFIPIIMDQLAPLQNETRIRIELYHPYYFGIIDMNKYYYHXYYAYIFHGQVIYGISTIAIVTVDTMFAAC
MQHVCGLFAILCYRLKQIGSNTKGNEMRVSDDKQVYRDIKFANEMYNNCRXFIDGIWNSFGITILNFVSSIIF

ATAVLLFDLLFYRIHLYERIILSILIMGILLLFLLLSWLFQHVSNSSEXVFKAVCCSCWYNLSVKEQKLMYLL
MLRTFNSIKLRAGGLIQINLRQYAWVVKTAISYAMVMQSANT
>NvOr216
MTISETDLAVFDGPHYSLNKKLLIMFGLWPTLSRTRKVICLIFFTMIDLSLYASFADGINYYRKQKKWMYVIE
DTISIIYLSVTWIKYVTSYIFESRIKLIYEQIAADWKSLIDEEEIQILNNYSAFARLLTVLYVFYAIIATTLF
YVLPFLTIVIDRIKPLENGTRFRAQPYHQHYFDLIDNEKYYYHMYIGHGYVVSIIVTVAVMAIDTMYAANIQH
ACGLFAIVRHRLSKIGILNGEREYEFRVVDDKKVYESIRAVCEMHKNSIKIVELIWDSFSISFLIFMGCSLVG
MGMLMFNYIFNMIHPIEKVVGTGLLLGIAILLFYMNWIAQQLTNSSDEIFIAVYSNRWYNLSIKGQKLIYSLL
QSNANSVTLRAGGIAEMNLQQFAAILKTAMSYATVMMSMNG
>NvOr218
MPVETKDQRKSYDFLSISSSHLTFMRLSSFLPLKGKSFFHPLSLLLQLWDHFIVIGYNVMWQGYGIRMIQRGD
VEVDFICEDIITVGFTIRYLLLCAKREKLCCLVESCEKLWDLLKDGEVIFVRQFARKGYYFRNFILINAMLMA
ALYSVTAPFVRLPPIEANGTERKILPFRFFMDIQKEPAYSIVFAFQSILLQFIDLMIVSTETVSLYLIMMACG
YLRSVRNRLLSFKGNDDNTSEKGEAALKFVIDCAHFHQQIMIFCEDIERMTRTLFFFACFCPIYNVSITGIVL
FNNDEDKYKFLPLLCYNFFQFFLCQWAPEHLAVESEDIALAAYSASLRPQAPSHREKINRILYFMMMRAQKPV
QLTAGGFVDLSIETFGAMTKSAFSFFMVLRKFRS
>NvOr219
MSTKAEDSHTFQSISASHLNYLRLCSLLPWQGKGFSHPFSLLFQLWNHLAIFGFNAMWHGYGIRMLQRGDVEI
DLICEEITVLDITARYFLLLIKREKLGRHIETCRKLWSYLKAGEDMFVSQFERKGYYLRNFVMINSVMVTTAF
IITATFVRLPPLEANGTERRMLPARFFMDVQEDPAYSIVFASQSILLLSVDVMIGSTQTVSLYPIMMACGYLR
SVRNRLLSLEGSDNGTDAKGEATFKFVVDCAHFHQQIIIFCEDIERMTRMLFFFACFCPIYNVSIAGIVILNS
NEDKIKFVLLLVYNFFLFFLCQWAPEHLTVESRAIAEAAYFASLQPLASSYREKINWILYFMVVRAQKPIQLT
AGGFAPLSIQTFGAMTKSAFSFFMVLRNFRT
>NvOr220PSE
MSIKTEDSDSFQSISSSHLNFLRLCSLLPLQGKSFSHPLSLLLQFWNHFAVFGFNAMWHGYGIRMLQRGDMEI
DLISEEITVLDITARYFLLLFKREQLRHHIESCKKLWGYLZEGEDMFVSQFERKGYYLRNIVVINSALVSTAF
IITATFVRLPPLEANGTERRILPARFFMDVQEEPAYSIVFASQTIILMFINFIIGSTQTVSLYPIMMACGYLR
SVRNRLLSLEGCDDDSNEKGEATYKFVVDCAHFHQQIIIFCQDIERMTRLLFFFACFGPIYNVSIAGIVILIS
DDDKFKFAPLLVYNFFQFFLCQWAPEHLAEESQVIAEAAYFASLQPLASRYRQKINRILYFMVVRAQKPVQLT
AGGFATLSIQTFGAMTKSAFSFFMVLRNFRT
>NvOr221
MRGAMSAEPKDLRESFTFLSISSSHLIFMRMFSYLPLKGKSFSHPLSRLLQLWNHFAVFGFNAMWQGYGIRMI
QRGDVEVDFLCEDIITIGFTIRYIVMRIKREQLCRLVESCEKLWDLLEDGEAVFVRKFERKGYYFRTFILCNA
LLMAGSYSIAAPFVRLPPLEANGTERKILPFRFFMDVQEEPAYSIVFVLQSIALQFLDFMMVMTETISLYLIM
MACGYLRSVRNRLLNLKGSDSDPSEKGEAALKAVVGCAHFHQQIMIYCEDLSKMTETLFLISCFCPIYNVSVT
CLVILNTEEDNLKFVPLMLYNFFQFFLCQWAPEHLTVESDNIAEAAYFASLQPQAPSHREKINRILYYMMMRA
QKPVQLTAGGFAPLSIKTFGAMTKNAFSFFMVLRNFKN
>NvOr222
MTTKTKDGYTFLSISSSHLIFLRLSSFLPLKDKSFSHPLSILLQLWDHFVVMVYNSMWTGYGIRMILRGEMEI
DFICENVVVMGFTVWYIVIQMKRQQFCSLVKFCEKLWSYLEVGEEVVVRQFERKGHYFRNFMLFNLLLMCTLF
ITTAHFIKLPPLEANGTERKILPFRFFMDVQEEPAYSAMYTLQFFVCYFVVFMIASAETVSLYLIMMACGYLR
SVRNRLLSLEGNDDDTGEKGEAAFKLVVGCAYFHQQILIFCKDIERMTRTLFLFACFCPIYNASITGIVLLNN
DEDKFKFILNLFYNFFQFFLCQWAPEYLSEESEVIAEAAYFASLQPLASSHRQKINRILFFMMMRAQKPVQLT
AGGFVKLSIETFGAMSKNAFSFFMVLQNFRS
>NvOr223PSE
VRGATSIKTKDGHTFLSIFSSHMIFLRLSSFLPLEGKSFSHPLSLLLQLWGHFLVVMFNVMWHGYGVRMMQRG
DMEIDFIWEEIVTIGFTRRYLVLFINRRQLCHLVESCEKLZDLLEDGETVFXGYYFRNLVMFNMMFMTTVFSV
TAVFVRLPPLEANGTERRMLPFRFFMEVHEEPPYSAVFVSQVVFVYFLSVVFVSVDTVSLNLIMMACGYLRSV
RNRLLNLRVNNDDKSEKGEATFKIVAGCARFHQQVLIYCENIGRMTQTLFLISSFCPIYNASVAGIKLLDNDK
DKYKFMANLVYNFFIFFLCQWTPHYLTEESEDIAAAAYSASLQPZAPSHRQKINGILYYMMMRAQKPVQLTAG
GFVQLSIETFGAMTKNAFSFFMVLQNFRT
>NvOr224
MPGAMSAETKNTFLSISSSHLIFLRLASFLPLRSKSFSHPLSLLLQLWDHWSVLAGNMMWSGYGIRMTLRGEM
EIDFICEDIIMVGFTMRYILLATKRKKLCHLVESCEKLWDYLEIGEDALVRQFERRGYYYRNFMMLNLLLMCT
LYIVTAHFATLPPLEANGTERRMLPFKFFMDVQEEPAYSIAFVSQSVVTYFICFMFVSTETVPLYLILMACGY
LRSVRDRLLSIEGSDDDTSERGEVAFKFVAGCAHFHQQIMIFCEDIKHTMRTIFLFACFCPIYNLSITGIKLL
DNDEDKFKFIVILVYNFFQFFLCQWAPEYLIEESEDIAAAAYSASLQPQALSHREKINGILYFMMMRAQKPMQ
LTAGGFVRLSVETFGAMTKNAFSFFMVLRNFSS

>NvOr225
MSTEMEDPHESYTFLSISSSHLIFMQLSSFLPLKNKRFTHPLSLLLQLWGHFVVFASNVFWTGYGIHMVMHGE
VEVDFICEEIVVLDFTARYILLIVNREQLCCLVKSCGRLCSYLEAGEDIFVRQFERKVYYFRNFVIINSLLVS
TVFDVTAYFTRLPALEANGTERRMLPARFFMDVQEQPAYVVTFVMQVILDYYLDFLIASTGAAPFYLIMMACG
YLRSLRNRLLNFKGGDYDTSEQGEAALDTVIGCAHFHQQMMIFCKNIERMTQTLFLFACFCPIYNVSITGIAI
LNSDEDILKFTPLLVYNFIQFFICQWASEYLAEESEAIAEAAYFASLQPQVPSHRERINRILYFMMMRAQKPV
QLTAGGLVNLSIQTFGAMTKSAFSFFMVLRD
>NvOr226
MSAKKKVQKEGDTFLSLSWSHILFLRVASFLPLKGKSFSHPLSLLLQLWDHINVIAFTSLWQGYGYRMIKRGE
MEIDFICENIITIGFTLRYIILCLNRELLCHLVESCEKLWDLLEDGETVFVRQFERKGYNFRNFFFGNLMFMA
TLYTITAAFVKLPPMEPNGTETRMLPFRFFMDVQENPGYAAAFVFQDVVVFYTDVIFASAETVPLYLVLMACG
YLRAVRNRLLKIEGNDNDSSEKGEAALKVVVGCAHFHQQIMIYCEEIGQMTKTLFLVSCFAPIYNVSIAGIKL
LENDEDKFKFIVILVYNYFQFFICQWAPEYLTEESESIAVAAYSASLRPQAPSHRQKINGILYFMIMRAQKPV
QLTAGGFVNLSIQTFGAMTKSAFSFFTVLRNFSG
>NvOr228PSE
MTTEMDNAKPEATETYTFLSISLSHLWFLRLASFLQLKDKSFSHPLSLLLQLWDHVSVVIFNSMWTGFGIRMI
IRHEVDVEYICEEVAVNAFCLRYLVVCFKRVQLCRLIEFCERLWEYLEVGEDMVVRQFERKGHFFRQLILINV
LAVATLYVITAHFIRLPPLEANGTERRMLPYRFFLEIQEEPAFSIVYVMQIVVVYFVNTMFASVETVSLYLIM
MACGYLRSIQNRLLNLAGSYDDSDAKGEAAFKRVTDCARFHQQIMXFCEEVDRMTRMLFLFSCFCPIYNLSIT
GIKLLENDEGKFKFAAILSLNLFQFFACQWAPEFLINESEAVGTAAYFASLQPFASSHRERINRILYFMMMRA
QKPIQLTAGGFIPLSIQTFGAMVRSAFSFFMVLRSFRT
>NvOr230
MEREPIKYEDISRLYYRLFRTMGILPSSSSRRTTLLRVYFHVTIVLYYSMSMFDGLRMLGHNDIEIEYVFEEV
VIHGICARFLILSCRREELAELLLSCEKLWRMLKPGEDRVVKSYEKIARYLAHYITWTTLVAIFFYIVAARIV
KLPPAEVNGTERRMLPFRFYVDVQRQPWYDIVTVLEIVVVLNIAMIVSTIETTGPFLITMACGYLRSIRNRLL
AIADEAEGRGEISRLSTIRVVSCVKFHQKIMRFCQDIEKLTSSVLLVQVVCTAYNISLVGFRILKNDPNAVKF
VPLLLLNLLQLFTAQWIPEHLLSESKAIANAAYSASLLHPEYEPRANRALLFVMLRANRPVQITAGGYMKLSL
ETFKRMLTSALSFFTVLRSINDGAGDEGE
>NvOr231PSE
ISAEKSVRPSFDELTSLYVRLFRLVGLLPFEAGSFDFLGTRLLAVNFLLFAGLDCSMFICYTINHLKQGERDV
EIFCEIVPVVGTFVKYAILLSNRNELREIFVDGREIWECCTDSERQVVEGFEKKIRMLFKCLFSTTMLNIIFY
FMKMLTTRLPPLEPGGPERKVLLFKYYVDEVSTSPAYELVVTLQTAVCYNFAIVLLVQDIFVPLLIMISCGYL
RVVQNRLLNICELDSKDDALDEGIDDCCKFHQSTLDMCLKIKKLSPTLYLVHILCTGYVLSLFGLKFSXSDPD
RFKSLSSMMILVVQLYICQWAPDQLIRESEAVSTAAYFANLSRIGSPGNRKILSIVMQRAHKPVILTAAGFVR
LSIESFGSMVTSAVSFFAALRSISIRDEEQ
>NvOr232
MRLHEINSFERVPASGTIRKFVEFRETDGSLRIFSPPHRGFTFGEPRLQLSLLQDASHLLRVYWPTQQCSGVV
EVDSIFVMVMAVSTIMRYYILVYHRFDFRDTMDACRVIWNDCTPNEHQIVRWFERKTWMLFKLLAGSGMFINV
FCSIGSIVVRLPPDEPNGTERRLLPYRWFIEDREYHWLGYELIFGLQVLITHHLTVIAATVDTAGPLLMMISC
GFFKALQERFFAAAARNEMILCKDKLEFKQTIVSCSKFHQSVLVLCKKIEVMTRMIFMVQLICLGYNISLIGL
KLTGTDPERFQYIPNLVLCLCQLFITQWASDYLLEQSEEVATAAYFATLMSLDARIGGLLLTMVIRAQKPVQM
TAGGVIKLSIERFGSLITNAISFFMVLRNFTTQV
>NvOr233
MRVHEINSFERALASDEIRKVAEFQESDGGLRIFPSTHRGVTFGKPRLQLSQLPDASHLLHVHRTPHQRHGAV
ELDSVFVTVMATSTIVRYYILVYHRFEFRDAIDACRDIWEDCTPSEHQIVRWFERKSWILFKLLAGSGLLINI
FCSIGSIVVRLPPDEPNGTERRMLPYKWFIEDREYYWMGYELIFGLQVLILHHLTVLTATVDTAGPLLMLLSC
GFLKALQERFFAAAVSNEKFFIEDKLSYQPLLTSCSKFHQNVLNLCRKIEVIMRMIFMVQLMCLGYNISLIGL
KLAGNDPERFQYIPNLVLCLCQLFITQWAADYLLEQSEGVATAAYFTTLMSLDPRIGGLLLTVITRAQKPVQI
TAGGVINLSVERFGNLITNAISFFMVLRSFTA
>NvOr234PSE
MSEKSRQDECGKLHVFLLKLIGLLSFDGRSLNFPGTLLLSLYVHCSHLLVTSMYVCNVAKTGLGDGEYDIETV
AETVGLCGVHCRFLIMFFNRERIAKLLNESKKLWTELDEFEVAIIRFISYZVXSLPYPFYADVQSTLWYELSV
TGQMLGMIGICLSSSGVDTAAPFFIMVACGHHRSLCDRLQNLASAVDDDDQVVKVDAVKKIIECIVYHQRMIK
FCKEVEKLTNSLFMVQLISTTYNMSLIGLKLVGDDPEKFKYLTVLSLLMCQLFMCQWAPDLLVSESESLARSA
YFVPGSGDESKKLAKLIHILMMRSQRPLQLTAGGWINLSMECFGSMITSAMSFFTVLRSIH
>NvOr237PSE
MSSKELAKYRKYAQNARNLLLPVGLWPYESSGFIYRFLSVFAGMSLVLLSYTALNYCFINVTDITKVTRSFSW
CISIWTMLMKIVLFVVHRDRLLDINETLSKTFERELKSFGLESVMLQRLSVFTGVYYTMVVALAMAFSLFAVI

PLIFMITDRXFGVIYYLIYAYEALLAFLFFCVGCGTDTAFGFWVFQICGQLRILAVKFSKLEPGKDYAAGLKE
CLEKHEMLLRCRDHLQRVFGFLVIWLYVTISIVLCEYIYKMSKMTLQLSFWQSVMIFTFATKFLQAFTYAYCG
SIIDIESEKLLYAIYDCHWPGSGDRRIMSDVLSLLTHKSMALRSYNFFIVSMEMFMKIVNAAVSYFFLLRTFD
T
>NvOr238PSE
MEVELAKYKRYKRDLRFMLICSGLWPNYEKHPRIFRAFLSFCSGFLNGLVSFGILSFCIINATNINLLTRGLG
LFFSFSSAFLKVCTLSLHQNDLQKLNDGVSASFARDLEIPENRPHLLAHFRTFSKFFYTFDYSVAMNVLLYAL
IPLSLLRHGKYIRMYPQVFFYIYDYEAGGAIHWTLYAFELLAGFFLWSVTCGVDSFFGLYSLHVVGEMRLLSH
RFQNLKSSKEYAKDLKDCVERHVELIKXSVCWERVFGLSGPFGWAITCAVVLCALIFQATETKNLSTLKVCYL
ICYSFLKLVQAYSYAWFGNIVAVESKSCLEAMYNAQWPGSGDTRFMNDVLIVLTQKPFAFKARNVMLLEMDMF
QKIVNTSVSYFFLLRTLEETLDHDS
>NvOr239PSE
MESGLLKYRTYSRNVRAFLAFSGIWPETNRLLSRILAFAALFSNSVVALTSZXVIIFLLHHKELAHLNGQLSK
RYTEDLSNPDNRSRLLIRVAPFSSILYTLIITGCLTILMYRLAQTRQIPPDVPAYEPGGVLHWSXYGLELVAG
MFLCSVTTGVDCSFGMYSLQVCGELRLLZLKRFEELGASKEYKKDIKQCVGRHHVIVSAKNKLESTFGLLTIW
IAVSAGTVLRVRLXFQFTEAIKVRSTGLHMGVMSMYIVLKFLQGVFTYAWYGDIIAEESDLCLHSIYDSYWPD
AFDPHFRKGVLFVLLRKPLTLXAKGCMHIQLDMFSKIVNTTVSYFFLLQTLNDESEQ
>NvOr242
MIMEKEVEKYKKYKSNLKFMIVSNGVWPDYEKHPYCVRKFLNFCSISSISMTNYCMMLFVIATTTDVRSFTSF
FGLLLGGFGNLFKVCALTMNQKELHALNEGISASFERNLRVPENRPHLLANFPMFSKFFNFLSYSTLGTIGFL
TVIPLLHLRHGTYSRMWPILLPFSYEPGGTIHWIIFVFELVVSFFAWITTCGVDCLFGLYSLHIVGEMRLLSS
RFQKLEWSENYRKDIRSCVKSHLLLLKTLSQMQEAFGDLAVWFAFNSAASLCTLVFQFSQLTVMNPARVLYLL
CHTCIKLVQAYSYSWYGNIITVESEVCLNAAYNSHWPNHGDKHFMRDVLIILLQRPMVFKAKSFIALRLDLFA
RIANTTLSYFFLLQTLDEKV
>NvOr244PSE
MDLKLLKCKAYERNVIWLFEAAGLWPEAHPVSKKILSLVTLFSSFVVMVTATNYSFQNAINIKMLTKGMILAV
SASSVFWKIALFILHQDDLLYLNRHLTGGFMHDMKNSENRSDLLNNVKTFNKFLFTHAVSVAIAMSMYSITPL
LALRKHGKYIRVFPGIYPFAYEPGGLLHWIYAVEVAATFVTVSAGVYNLFGVYSLZVCGELRVLAHRFRDLGA
GNNYKEKLKDCTERHQVLINAKNKLEDIFGLIAIWLALSGALVLCSLIFQVSELIKAAVSYLRXHASAYLLPK
FLQIFLYAWCGNLIAEESKTCLYAMYNSHWPDPYDANIKSYVRIVLSQEPLFVVAKGCMIIQLDMFANIVKTS
VSYFFLLRTLSMENE
>NvOr249
MESKVARYAKYKRDIKCLIVASGIWPHYEKHPHVLRKLLSFCSAFCSGSTFYCIVAFCFKYATNINIFTSCLG
LMIGFFTTFIKIVILSMRQEDLQSLNEGVSKSFENNLKLPENQPHLLYHFPSFSRFFYLYAYVVGISFVFLAS
TPLSIMLRYGKYVRMYPQLMPFAYEPGGSVHWAVFGFEMFTGFYLWSVTIGVDSIFGLYALHMVGQLRLLGSR
FQNLKSSSNYDKELGECVRSHIQLMKSRHKLQRVFGFLAIWLAVTCAIALCSQVFQALHMRNTTPVRALYLFG
HWFIKIVQAYSYSWYGNIIAVESDLCLNSMYYSHWPGSGDKRFMADVLIILSQKPLVFKAKQLMELRLDMFLK
IVHTSLSYFFLLRTLDENPKAGT
>NvOr250
MELELAKYKSYARHVIFRLIFAGLWPESNPKIKRMLSFVTFTSTLTVMVTAINFGIHNASNVILLTKGIGLAS
GFSSVFSKALMLPLHQEDIVFLKKRLTSKFMSDMETIEYRADLLSSVHVFSAFVNMHEAMMAFAMSMYCFVPL
YVLFKHGTYMRTYPCLYPFSYTPGGLAHWLIYALEVAGAISVWTITIGADCGFVMYALELCGEFKILARKFTE
LKAGDDYKKKLKECIERHHLIIEAKNRLEDAYGIIAIWLALSGAFLLCSLIFQITELYDNNGSYVRIAHLCSH
LLAKYLQIFMYAWYGNLIADESKAFLDAMYGSHWTEACDKRFKNDILIVLTQEPLIVVAKGCMYVQLDMFTKI
VKTAMSYFFLIQTLAS
>NvOr256
METELRKYERYSRDLKCLLVLSGIWPDFHPIIQPLLGCFAAFVCFVTVIAFLNFSIHHITNVVVLTKSFGLVI
SFFSSFLKICVFLWHHDDLVYLKAALTDRFNTDNLNKSFRRFTLAKVNVFANLFYILTIAVGLTTGMAVVLLI
ISLRHGKYVMLYPSIFPFSYEPGSRVYWILLMVELFANLFVWAVTSGVDSVFGWYTLQICGEFRVLAHKFQNL
KSSENYRDDLKECVERHYVLMKTRDVLQDVFGFLTILLALTSAIVQCMLVFQAIQVFKNLSLGMMVFLIAYIT
LKVVQAFIYAWYGQLIAEESEVCLGAIYNARWAGSGDTRFMSDVVIVLSQKPLIFRANGCMSLKMDIFIKILN
TSVSYFFLLQTLDEGSEHHQH
>NvOr257
METELRKYERYSRDLKWLLVLSGVWPDFHPVIQPMLGCFAVLVCSLTAIAVLNFSIHHITNFVVMTKSCSIAI
GLCLSTLKLCACLWHHDDLVYLNTSLAASFNADNQNKSFRRFTLAKVNVFANLFYILTIAVGLVIVMGLVFMI
LSLLHGKYVLVWPSIFPFSYEPGGWVYWILLTVQLLANFFAWTVPSGVDSVFGWYTLQICGEFRVLAHKFQNL
KISENYQDDLKECLERHYALMKSGEVLQDVFGFLAILVGLSSAIIQCMLIFQAIQVFQQLSFGMMILIFAFIT

LKHVQVFIYAWYGQLIADESEDCIEAMYNAQWAGSGNIRFMRDVLVVLSQKPMIFRAKGCMLLKMDMFIKVLN
TSVSYFFLLQTLDEGLQN
>NvOr259PSE
MENELRKYKTYRRDIKCLLCVLSGIWPDFHSIIQPMFGVFAAVVSLITVTAFLNFSIHHITNVNVLCKSFSLV
ISFFSTFLKICVFLZHHSDLVYLNTILNARFEEDTRNESYRRFTLARVNIFTKTFYAVSISVGLATGTAFVLL
IISLGHGKYIMLYPSIFPFGYESGGRLYWILLSIELFTNFFVCAVTSDXDSIFGMYTMQICREFRVFAHKFEI
LKTSENYRNDLKECVERHHELIRTKEVLHDVFGLLSIWLAIRSAVILCILIFQAIQVFKTMGLGVIYIVGYIM
LKLVQSFIYAWYGKLIADESENCIEAIYNARWAGSGDARFMTDVIIVLSQNPLVFRAKGCMSLKMDIFIKIFN
TSVSYFFLLQTLEEGSQH
>NvOr261
MEAKLAKYARYRNVVRRLLLLSGIWPHLEDTCRLYRVLTFSATFVIAALGAKVFAYCIDNIAHVSLFAKGMSN
AFSFYTSVLVYLVYRKDLVMLNDCLGRRFEDELKREDRRPLLLQSISVYTRFMCIVAGLTATALVFYTLVPLV
FIFKYKKLTQIYQGRYPFAVEPGGRVYWCVCFVESISVVFVWNVVCSVDNAFGLHSFRMCGLLRSLADRFAKL
QPDDPGYIVELRDCVRTHQLVLRAKEALQRVYGLVVLWTYVTSAIIMCSILYQADQAKKHMTVTRVIFFTSYI
TLKLLQSFTYAYYGSLVSQESEKCQNAIYTSNWPGSGDLRLMKDVLIIQSQRPIVLRANGFFIVSMEMFEKIV
NTTISYFFLLQAVEEK
>NvOr262
MMMELELLRYKAYTQNVIWFLKLAGLWPESHPVPKKILSTITLSSILVIVLTVSNFSFHNLGNIMVFTSGMCM
AASSTSAFSKVALFLLHREDVVYLNKHLSGGFMRDMDEPDNRPDLLSNVKTFERFMVTHVISVAIAMFTYSVR
PLLVLRKHGKYIRSFPAVYPFAYEPGGLVHWILYAVEVSGTASLWTVTIGVDCVFGVYALQVCGELRILSRKF
RELRADDNYKEKLKDCIRRHHVLINAKNKLENIYGLISILLITSTTLVLCSLVFQVSELMKTNSYLRTAHLCV
YLIPKFLQIFTYAWYGNLIAEESGACLDAMYGSHWTDSCDKNFKNDILIVLAQEPLALVAMGCMVLQLDLFAK
TVKTAVSYFFLLRTMNEGSE
>NvOr268
MLPPTEDDFEYYFAFNLKLLSLVGFKCSLEKNEQSLSFINKLPSYMMCMHGIILSMCEVYFIRDIYSNENKTL
VMQILSQGINNTLCISKGFFLAYSIERMQNVLQEIQFLWKTYRPSQDNRKIILADAHRTFLFCKIYFCVLASC
CSSYFLCYLPALFNLAQQYRNRDANNHTYDFSQRLVLLKYPFEIPNIPTYFLIELEEGFYLFYSAALFFVSGD
TLFAQTVTHICLQFKILKYDIDETFNSESTGERDHSILVNFVKRHRDLLRICALIEEAFSPIILSMMLLSSLS
LCVGLVGVRGTMAKHSYEETAVVVTLMMLTFLQILFYCTFAEKITEETRSLADAMYSCDWTVKNYKLGLYIQL
IILRAQKPFQCTACGFFPIGHSQLMTIINTAFSYYMMLQTTS
>NvOr269
MKTSVNQPTEDDLEYYIGFNLKLLSSIGLKCSLEKNLKSLGLINKLPTFIMCIHGLIFFIFENYFIRDIWSSD
KTLAMQILSQEVSNIQCISKGFFLAFAIERMQNVFQEMQYLWKTYRPSQDNRKKILLGAHQTFSFCKIYFFVL
LSCSISYFLCLIPSLFNLAQQYRNREANNYTYDFSQRLGLVKYPFEIPNIPTYLLIVFQEAFYLFYTAALFWV
SGDTLFAQSVTHICLQFKILKYDIDATFNREDMRDHLSLVTIVKRHRDLLRICKLIEEVFSPIILSIMLLSSL
NLCVNVVGIRGTIAKENYQETAINVTIFMLTFLQILFYCTFAEKISEETRSLADTIYNCDWTVKNYKLRFYIQ
LIIMRCQKPFYCTAYGFFPIGHLQLTTVLNTAFSYYMMLQTMN
>NvOr270PSE
ALAAQTLGQLVCNTQCITKGLLFAFSIEKLQPVFHEIRILWEKYQPEEVIQKSIVKDADRTLTFCKCYVTANF
SCLVSFSLPMGVQLFLQCQSRVATNHTYDVSQTMLLVKYPFEISDTSTFAIVIFLEEFLLVMNVVFWVSSDAT
FAQTTTHLCLQFKVLKRDIEKMFNYEGPDGKEILLKLVHRHRELLRICMLIEDIFSPIIFFHRVLXSINMCVN
VIGTRETISNRVYSDTGVYATMLLMTIFQIFFFCVFAEKISETTTSLANMVYNLNWTSKDNKLGFYXFIIIRA
QRPFYCTAYGFFPIGHQRLTSIIRASFSYYMMLQTTDNK
>NvOr271
MKMTAINPEDYFGLNIKLMSLCGLRCSMTKTIGSFINKVPTFLANLVGIIYLVFQATFVMEAVRLRDVALTSQ
ILSQLVSNIQCITKGFLFAVSIEKMQSILYEIRSLWERYQPDIEIQESILDDADRTLNFCKYYVIANFSCVLA
YALPLVLNLFMQYQARESTNHTYDLSQMILLVKYPFEVTKVSRFIILVLLEEYLLVVSVIIWVSSDTLFAQTT
THICLQFKVLKQDIEKTFNYGGPNSKEILLKLVHRHRELLRMCMLLEDVFSPIIFFTVFLSSVNMCVNVIGTR
ETISDKTYLNTGIYATILTMTIFQILFFCIFAEKISEETTSLADMVYNLNWTAKDNQLGFYIYFIIVRAQRPF
YCTAYRFFPIGHQRLTSIIRASFSYYMMLQTTDNK
>NvOr273
MQTNTEEKAITAVDAEYYFDLNIKLMSLIGLKCSMTETVTKFIYKIPTFLTNVLGIIYLIFQISYVREAVRSH
DTSLAAQILSQTVCNIQCNSKGFLFVISIAKVQAILHEIRILWETYPPDDEIQKSILLVADKTVTFCKYYVTA
NLSCVLAYALQMGLNFFMQYQAREATNHTYDFSHIILLVKYPFVVTEIPTFITLFLSEEFLLIMGATLWAIID
TLFAQVTTHICLQFKILKRDIQEKFNTEGSNDKEILLKLLRRHRNLLRICMMIEDIFSPIIFFTVILSSVNMC
VNVIGARETIASKAYFETCIYASIFLMTIFQIFFFCIFAEKLSDETTSIADTVYDLNWTTKDYKLRLYLRFII
VRAQKPFYCTAYGFFPIGHQRLTAIIRASYSYYMMLQTTDGK
>NvOr274PSE

MQTKERHKSKVLNIEYYFYLNIQVMSLIGLRCNGPTITGFVHKIPTYVANVLSVIYLMSEIYFVSDPVYSSDM
GFIIQTLSQSVSNIQCFTKXFLFATAIAKLQVIYNEIKFLXQKHPPKDNIRVLIFNLADQTMNFSKNYMMAIC
SCILZYYTPIAINLVKHALQDSSSNHTYDFTFPIYSIILFIEAVFLIVQTVFWVDSDTLFTQITTHLCLQFXV
LKHDIQSTFNIEGSKSKEILIGLIKRHRKLIKICMLTEDIFSPMIFXVMLLSSVNMCVNVIGTSAAVKDESYL
DAGIYATMLLITVFQFFFFCILQKELXEETRSLADIVYNLNWAMKDYDLRLYILLIIMRAQKPFYCTAYVFXP
IVHQKLTSXLKLLYDLYMMLRTTADK
>NvOr275
MQSKERDKPKVLDIEYYFDLNIRVMSLIGLRCDGPKITGFVHRIPTYTSNTIAILILIFEICLMSDPVCSSNM
ELTIQTASQTVSNIQCVSKGFLFVNAIEKLQVVYNELQVLSQKYPLEDEIQVLVFDIAEKTMNFCKYYAIAIC
SCILFYYTPIVVNVIVYILQDPSTNHTFDFTQTLFYLKYPFTIKTFPIYSTIVSIEAVNLIAQGIFWFLGDTL
FAQVTTHICIQFKILKHDIQKTFNDEGSKSKEILIGLIKRHRQLISMCMLTEDIFSPVIFSVMILSSTNLCVN
IIGASTAINDGDYMNAGVYATILLITVFQIFFYCIFAEKTTEETRSLADTVYHLNWAMKDHHIRLHILLIIMR
AQKPFYCTAYGFFPIGHQKLTSILSTAYSYYMMLRTTANV
>NvOr276
MQTKEETIEVNAQYYFSLNLNLMSMIGLKCNMTENVGRFYHRIPTFITNVCALMYQSMTVYYLVEAISAKNTS
LSIQIISQLVSNIQCFTKGFFLAFGINKIQFILQEKQILWKKYPPNNNNHHTILGIAQQTLTFCKFYVVAIFS
CVMSYDVPLAINIFMQYLKRESTNYTYDLSRRVILVKYPFEVTEISTYVILCLQEALFVFIQCIFWVNSDTLF
AQVTTHIGLQFKILKCDIEAAFNRDDAKNKEILIELVNRHRELLRICMLIEDVFSPIIFCTVFLSSINICVNV
IGVRETISEKAYLDTGIYFTMLLITLFQIFFFCIFAEKLTEETRSLADAVYNLNWTIKDYKLRVYINLIIMRA
QKPFYCTAYGFFPIGHQKLTGIISTSYSYYMMLQTTDK
>NvOr277
MKRLAKVQSTKDDIEYYFGFNLKLLSQIGLKFSMNEKTDKFTFLQKLPSYIFLVEGMILFILEVYLIRDTIQS
DTLLSIQIMSQIISNLQSVSKGFLVLNKAKSIKNVLETLGIIWRKYPLNNSDRALLNAPSKIISLSKIYWGIA
VALLVIYDLPPFVIFFMQYQNRDAMNHTYDLSQTILLLKYPFNITRKSTFFFLISQEAFVLYASGVYWIGSDA
LFAQFTTHICLQFKILKCNTKEVFNRGSKEAHSSLIDLIKRHRELLKICEMTEEIYSPIIFSTMLFSAINMCV
NVVGVRETITRGFYQETGVYLFLFLVTFAQILLFCIFAERITEETKSLADLAYNLEWTKEDHKLRVYILFIIL
RAQKPFSCTAYGFFPIGHKKLSSIINASFSYYMMLQTMS
>NvOr278
MKSQENIFTKDDIEYYFDFIFKSLNTLDLKFSISKKTDEFKFRHKLPTIIGCLIGLIIFFLEIYFIRDALHNH
TILPIQIFSQVISNFQSISKVILIVYKVNKIQQILEKIGVLWKTYTPDEGNRAVLYNTLQRTLSICKIYYAVL
IATVLIYYVQPIVNFVGQYGARNSINHTYDYSQTLVIIKLPFKVTQKRYFFVISQEAYLLYMSGVYWGCSDTF
FACFTTQICYHFKILKYHTKAFFDEKNNNSRLNLVTLIKRHQELLRLCVLIEDVFSPIIFSTILFSAMNLCVN
VIGVQETILNGSYRQAGIYLFLFIITFSQILFYCAFAEATMEEAWSLADLAYNLEWTSKDYKLRYYIHVIILR
AQKPFHFTAYGFFPIGIQKLTSIINASFSYYMMLQTVS
>NvOr279
MKSRENIFTKDDIEYYLNFILKSLRTVGLKLSLSKKIDEFKFRHKLPTIIGCSIGIIIFFLQIYFIGDALHNH
TILPIQIISQVISNLQAVSKGFLVVYKINKIQRILEQIGVLWKMYTPDESNRATLYNILQRTRSICKTYYAVL
IATVSIYYLQPIANFMGQYGARNGINHTYDYTKTLLIIKVPFQVTLKRYFFIISQEAVLLYMSALYWACSDAF
FACFTTQICYHFKILKYHTKVCFDVKNENSRLNLVTLIKRHQKLLRLCELTEDVFSPIIFSTMLSSAMNLCVN
VIGVKETISNGSYRQTGMHLFLFIITFSQILFYCAFAEAMTEEACTLADLAYNLEWTSKDYKLRYYIQVIILR
AHKPIYCTAYGFFPIGIQKLTSIINASFSYYMMLKTVS
>NvOr280
MKTQENDLSIEDDIECNYSGYIFKAFHFMGLKLSLKKKTDGFKFVHKLPTTIGILQSIVVFFLQMNFIRDVVQ
CDSNPPIQIISQVISNIQAGLKTLLVFKKIEDIQRMLETLGEFWKKYSPDKNYRVVLFRELGKTSSLCKYYFG
TLVGIMIAYDVQPLVYFLTYYFEQNATNHTYDLSRRILLVKYPFEITRKSTYCFLLSQEAYLLYITAIYWANG
DTLFAQFTTHICLQLKILKYETGKFFNQSNQEGRSDLLILIRRHQELLSMCDMIEDIFSPIIFSTMLLSAINM
CVNVIGVTETIAAGSYEETGIYTFIFIATFLQIIFYCVFAETLTEETRSLSDFVYNLEWTSKDYRLRFLIQVI
ILRAQTPVYCTAYGFFPIGHQKLTSIINASFSYYMMLQTVK
>NvOr281
MKSQEDTKDDIEYYLGFILKSLHTAGLKLSISRKTDEFKFYHKLPTIIGCSIGIIIFFLQIYFIRDALHNHTI
LPIQIISQVITNLQSISKGFLVVLKINKIQRILEQIGVLWKSYTPDESNRATLYSILQRTLSICKTYCAVLFV
TLLIYYLQPIANFLVQYRERNGLNHTYDYTKTLLIIKVPFQVTLKRYFFIISHEAVLLYTSALYWGVSDTFFA
CFTTQICYHFKILKYYTKVFFDVKNNNSRLNLVTLIKRHQDLLRLCELTEDVFSPIIFSTMLSSAMNLCVNVI
GVRETISNGSYRQTGMHLFLFVITFIQILFYCTFAEAMTEEACTLADLAYNLEWTSKDYKLRCYIQVIILRAQ
KPIYCTAYGFFPIGIQKLTSVINASFSYYMMLQTVS
>NvOr282PSE
MRINEKRPKQAVPDIEYFFRANLKLLSSVGFKCSVDQKCEKFKFRHRIPTYITNIEGIVLFALEIYFIVDVFK
TDTLLTMQIVSQIISNIQCICKGYLMANTIDEIQQIFENLGTCWRKYNTKEADKVLILGRAYTAMKFCKLXRM

FIQYLNREATNHTYDFSTRILLLKYPFEISNVSIYILTNFLEGNLVFICTIFWASSDSLFATVTSHICVQFKV
LKNDITSAFNDSTLKERFILVNMVNRHRELLRMCYLIEDIYSPIIFSTTLLSAVNMCVNVYCFRETIAKGYYL
ETGVSLSLFIGTFLQIFFYCIFAESLTEETRSIADTVYNLKWTTRDHKLRFYIQLIIMRAQNPFYCTAYGFFP
IGHKQLTSILNTAFSYYMMLNTMSN
>NvOr283
MQIRVVENLTLTKHDIKYFFKENLKLLSKIGFKCSLTKKSEKFKFHHKIPTYIANFCGLIVFALQIYFVIDKI
QTNTVLAMQSLSYAVINVQSILKGFMTANSIENIQQIFENLGIFWQKYMSRKPGRELILDRAYKTISLCKFFF
VMAIVCYFLFVMQFLIKFSIQYLNREATNHTYDFSNTVDLIKYPFEIPNLPVYFLLISVEINYLFVCIVFWCN
TDSLFVTLTSHVYVQFKALKLDTTLAFNNSMLKERSILIDMVNRHRELLRMCYLIEDTYSPIIFSTTLLSALN
MCVTVYAVREYIDKGYYLEMGIPLFLFIGASLQILFYCIFAESLTDETRSVADSVYNLKWTTKDNKIKFYIQM
IIMRCQKPFYCTAYGFFPIGHQQLTSIISAAFSYYMMLQTMSN
>NvOr284PSE
MQSTEVKKMASLNPEYYFDLNIKLMSLCGIKCSMTETIGSFINKIPTILSNLVGLVYLMFQVNLLREIREHLR
DTVLAVQILSQIVCNVQCNIKGLFFLLSINKVQYMLHEIRILWESYPPDDELQKSIFLIADQTLTFCKYYITV
NLSCILAYALQMGXNFFRQYRTHQHGSNHTYDLSHIIILVKZPFVVTGVPTFITLFFTEEFLLIMYATFWAII
IDTLFAQVTTHMGLQFKILKRDIEEKFNNKSSNRKEFLLDQVRRDRNLLXVCRMIEDXFSPIVFFTVFLSSVN
MCFNVIGTRETILNKDYFDMGIYATILTMTIFQIFFFCIFAEKLSDEXTILADTVYNLNWTTKDYKLRFYLQV
IILRAQKPFYCTAYGFFPIGDXRSILRASCSYYMMLQTTDSK
>NvOr287PSE
MTRSYETLESPSKDQGIRRYLGLNLKMLLFIGMEFSLNNDRTIKKSRFVQILPILMTNVISLSVRIILKYSLY
SQIYIIQRQRDSYYYSNNCNCRCYLGVVISGITAFGLPPIYNFVYHYFIREVKKQTMAYSKRVFLLRYPFRVK
NEPIFFSVLAKEEYLGXSCDTLFVQLTTHISLQFKILHYDVRTLINREVPYRLKMNIIIFIKRHQGLLRTCRL
IEDIFSPVIYSXVLLSALNICVNIFEARAMNAEGNYAGTVLYVILVLILFFQILFYCSFAETLTDKTSAIAES
VYNCKWTEKNHKLRFYLQMIIIKSQRPFYCTTZGFFPIGHARLASIISTSFSYLMMLQSVTQ
>NvOr292
MNLYESDVNQKSLTKCKDDLKNASKILTWNKRLLLLLGLWPESPMDFLFCASAVYYIFYLGLDFVSFVLFLRK
KILNVSIFIQLLAYGHISARLLLLRRHNKTFGILFTEIKQDYELRNFESDQELRMFLKYNRPAKTMIKLLFIC
STFFGVVFYVRPFLTTYFVHRAIRKAHRNVTAPFFWNTYFYKFHKITTINVYAMHYVSEFPFSILTGIISCAT
DCLVLTLGCHLSGRLAALSHRIRNVNFRNGSQEFKAVIRLHQQVLRIAEMIEDSLSSLMLCHILVASILMCIV
LYKTLICLRPGKRIHLINTVILLFLNIVRLYSHCCVGEFLIQESRAVQAAFYECKWYTMPLQDRKLIIILNLLR
SQRPIRFTAGSLGTFSIQLFSEVLKSSLGYLSVLRNIV
>NvOr293
MEFVRKAYGDKQRKRQASSKCISRADRVFKRCVFFHKFVGIWLEKDRSQRLLDRLKGYVSAAFTLGICIFQIV
MLSVESSSVVTVLQNNLLILIRKTKEIAAPLHASKIKERKIVDRWLNNQDKILKILLTSYTFTFSSYSLFPLL
KENGLPFTGRLPAICYVNPWYPTIFAAQLVFIIFRFFCVLSNDILCITFLCQLCSELELVKHLIVELGNGKDR
NVKQIIIRHAMVLDYGEIICETYSATLIMQHLNCSIFLCLSGLVTMKTSDMFALLKIGSLSLIGIITMLIICF
VGEMVMSSSLEIASTIESSVYKDYRNDVANLKLLNFMLMRAQKPLCMMVCTQGKLSLRFFSENINKVASFFIY
LKTLVE
>NvOr295
MGLIEVLESRKIFLWICGLWPKEYQHKPKLSQMKLYFIWFNMLMMCLLVFAGLVAIATPDNIPQASIRLPRKR
MMKVIMETLKLEETSKFENDNDLEIIRSWRRTRDGVLKYHMRIYGFISVAYCFLPIISQVNTYPAQTIIQASL
FVSPWYEFFYGFHCAQLFLYLFIIIATDGLSMILIFKLCEELQRFECLLFERHRADDATLSEKYKSRGEFLRC
IIRKHCTILDYGESICNLLTGALFTQYFLLSGTLCFSVFTILSSNSSAMANQMSIMAGTCIVQLFMISLAGEL
VSTRSLALADALLQSDFCCSIFGELKSSELRQTVLMQLRMQKPLKLSIGTLGVINIEFFSRIMKGVYSFTMLL
RTSYV
>NvOr296
MNVGEEYDKLALPMTLSSRVVGSWPSRAELEGQGGRSVLVHRLHRYLAIVSIYLMSMGVAAEVIVFFGEDMNE
TIECALISSAFFMALTRIITFASHQPEMLYVVETMREDWIRSTDEERAILRDKCLFAFKLAKFFAISVTITCS
AFILMPMLELKFVENAKRMLPYRGYFFFNHTVPGVYEYVYLVNSMLGVLGCSTIACATSFSLITSIHGAAKFA
IVQKDFERIDQVTWNNSEIVGRCVRRHQECIRFAETVENIINVLALAQFVISTGLICFAGFQMTTMLTDRARF
TKYASFLNAAVTELFIFSYGGQSLKSESEEVAEGVYSSNWIGSALSSNLRLIVLRSRKPCTITAGKFYDMSFE
SFLKVLSSSFSYFTVLLAMEEE
>NvOr297PSE
MTTAVEEYDNLALPMIISGQACGSWPMRAELEGERSLRVLLHRLHRFLAILFLYLISAGVTEVEVIVFFGDDMN
ETIESALVSSAFYMTFARVLTFARYQPQMLYVVZTMREDWLRSTSEERAILRKKCLLAFKLTKFFALSVVTTG
ISFVIIPLLELRFKEDAKKVLPYRGYFFYNYTQPGVYGYTYLANSMVGALGCMSIAYGTSFSLISTIHGAAKF
AIVKRDFEKIDRTTWTDHKIVGKCVRRHQECIRFAETVEDIINILALAHFVISTGLMCFGGFQLTTMLEDRAR

LTKYTSFLNTAVTELFIFSFSGQSLKSESEDVAEFAYTSNWIGSVLSTNLRMIILRSRKPCTITAGKFYDMSL
ESFLKVMSSSFSYFTVLLAMREED
>NvOr298
MLGKSSLNSKIPIRERDFNYSMKLSRITLSIIGLWPFRENIRCSNFKFVVILVSILMTLLSSLTFVYQTDDDD
KMFHSLINSLYMLMTLVKLLMMRCKNDKLEVILSEMRIDWRKYERFSDGNKRLVDLYTGKARTSSFVCIFFME
FSITTYFISRVAYALQQPAKIREWDLPYTAVYPFEVTSSLFVPMYLWQVFSAMCLGSVTISIDCLLVTTACHA
TGQLAALCENIKSYGHEQRHRDETLSSEIECSCIRCIIERHVDIVRYCRLVEDAYNLILLTEFIGTTFQFCLQ
MYIIVEHSHDKNIVGLLSFCIYLLVFNFRLFMYCNVFDAMVEMGEKVGASAYDISWYDFHPEAVRQLMFCILR
ANKPLNVTAGKFFSLNRNSYKNVIMTSSSYASVLLSIK
>NvOr299
MRTYNFRRVYSAVKLRLLKVVSLLKMKTESSNARLKGKNVTFCTEVFECDEILQLDNELGLIQWSLKLMGIWP
FWTRFSNVKFFLCGAILAFNVVGCFSGILNVNSDIEQFIECLLYFNVNLATLLKFLIVKYKRRSIEFILRCIL
DDCSRYSHLSVSCRSRVAGNIKKRKLMTLTLALFILAPVAGKRSFLSMLWSTAYIFSAFAITKYIEYRDDLAM
IRELPIFSALPTFVRHSQIFYLALLSGLFGILMSTLVIVTIDSVFAILMIHATNQFIVLSEELKAYREDHLDA
CYKISKNMRNCKCMRCIIDGHVNILRYTLNFYWYSKFILYKIDSVFSIVHIAVILLSMNLHLFMYCVTSKSMT
DASEQIGIRAFKMKWYSFQKTTVRSIVLMTLRSQIPCYVTVAKFINLSLETYTSVLKTSISYASVIIIAREHL
VNER
>NvOr301FIX
MFVRIIIILNEINFLGNMDSEIYDSEYYHEVKLLLTYFGLWPNLSRFRKVVSFIAMVAMPISLVIPMSFGLKRA
IRLKEPIQIIEDTIGILYFLAITTKYICIFIFEGRMIVVYEQIASDWKKIKDKNELEYLHGRAKEGKIITILY
LGYGAVGCTIFASTPYLPLFLDLVIPLNVSRDKIYPYYADYVIVDSEKYFYTLYTLHGIFIIILVTMSAISID
CLFIMMVKHSVGLFQIVCYRLKKIGEEHNEKPHECKRLMDDKIIHTRMKEIFDSHKSSIECVDAIQASFDVSF
LFIMTMSGVGVSLILFDLLLNLDDLTQILRINSMMFGVYIAVFVICYAAQMTLNSSEIVFNDTYCGYWYNISP
NARKYTQMVMVRSMKPCIITAGGLINMNLQSFFAILKTSVSYATVMLSMQEESNMQN
>PbOr2FIX
MSRNDSILFRNQNYKSDTEYVVKVAKTLLTPIGIWPLYRGNSRSDKIKNYIQTSLIFCLMCFLLIPHVIYTFF
DAEDLTRYMKVIAAQVFSLLAIIKFWTMIINREGIRYCLQQMEIQYRDVECEEDRLVMTKNAKIGRLFTITYL
GLSYGGALPYHIIMPLLADRIVKEDNTTQIPLPYLSDYIFFVVEDSPLYEIIFVSQILISSVILSTNCGVYSL
IATCVMHSCCLFEVVRRQMETILNDGTDNLHERLGRIIENHIQAIKFAEMIEKSLNIVFLCEMVGCTIIICFL
EFGVLKEWEDGKILPMGTYFVLMTSIFVNVYIISAIGDRLKEESEKVGESSYSIYWYD
LPTKIVSDLILIILRSSRPSTLTAAKIFDLSLQGFCEVCKTSAAYFNFIRAMTT
>PbOr3
MISTILPADDRALFRNFNYRSDVEYAIKVAKILLTPIGIWPLYGDDSTLDRIKYVLQTSVTFTLMCFLLVPHV
IYTFFDAEDLTRYMKVIAAQVFSLLGIIKFWTMIINKNDIKCCLQEMEIQYRNVESEEDRLVMIKNAKAGRQF
TMMYLVLLYGGALPYHIIMPLVAEKIVREDNTTHLPLPYLSDYVFFVVENWNSPFYEILFVIQIIFSTMILST
NCGVYSLIASCVMHACCLFEIARRHMETLLVGGTDNLHERFGWIITHHMRALRYVEMIENSLSFVFLSEMVGC
TIIICFLEYGVLKEWEDNKLFGVIIYFILVISIFVNVFTLASIGDRLKEESVKIGEAS
YSIDWYTIPTKNINSLIMVMIRSNRPSTLTAAKMFDVSLYSFCDVCKTSMAYFNFIRMVTM
>PbOr4
MDDSNLAQKNERLLDFHYAAYISFMLLKPIGVWPLRPRATTLEIIVHGFSIAVATILQLFMIIPWIICIIVAK
WSFYEIIRTACPLIFAITVFIRYLLLLFHRDEIKLCIDRVAEDWSNTMISEDRKIMLENAKSGRFFGILSMTF
MYGSGLPFASMPFVLSLFAAESNITIRELPVPCELIFLDVQVSPVYEVTYILEFFTFFVLYAVFSGICSLTAK
FVMHACGQCDILMYILEELIDGGDRNQGTLDQRISTVILRHLQILRFISAIDKILNEICLAEFVNASCNICLL
GYYVIMDWNNGESMLQIYVYSLAFVSITFNIYVFCHIGEQLVERWQKIGNKCYMIDWY
RLPQNKARNLIFPIIMSNYSVKLTAGKILQITIDTFSNILKTSMAYLNILREVSSREIM
>PbOr5
MVDRKRENTFVNTLAVGVRTKQNESHVLDFRYAVQISVCLLKPIGAWPLASDETSRLKIVLHKASMVIVTFLL
VFMIVPWIILIVKEKWGVFLILRTMCPLIFITTVFARYVLLLWHQDRLKVCIDRMADDWRFAIIAEDRDVMLA
NARLGRTFGMVSVVFMFSSGALYYALPLTMPKMINEDNVTVRSHPSPCEFLVFDAKVSPVYEIVYFLQLLSGY
TAYSTFSGICSLIANFVTHVCGQYDMLISIFEETVDGGKHNSGSIKDRIAIAINRHLRLLRLVSNVSSLFTEI
CFVEFISASCNICLFGYYIITDMNNNESFVQIGMFTFALLSTIFNIFMFCYIGDLLKE
RCQRVGIACYAIEWYRMPSRKALELLMPMAMSQYPMTLTAGKMITMTLSTFSDILKTSMAYFNLLREFSSRDI
TRT
>PbOr6
MILNEHHEADVKYVVEQSNVILRMLGIWPSTDRRPDIIEKITNIVLVIICYLLLHCDMVPGVLYYAFVDDEPR
ERVKMMPPILYSIMAIAKYSNLIIHECNIRSCLRHIKEDWKTIAIGNEREMMLNKASKGRRLFTLCCTFMYCG
GLSYNTVVPLSRGSIVINENITIRPFSCPGYYVFFDPQNSPAYEIVFLLQVLCGFVMYTITVAICGLAAVFVM

HACAQMEILMRLMENLVDESESFGQRNVKAKLALIIEHQIRIRNFLQLVENTLRYGNLVEIVGCTTIICLCGY
CIIGEWEDRNVPAMISYVTALTSVIINIFILCFIGEYITSQAEEVSLTSCTLNWYRLP
TNIARDMILITAASNVAPRITAGKFIDLSFRTFGDVIKSSVVYFNMLRQLTE
>PbOr7PSE
MSNKNYKSDINYVFELNRFVFHLLSIWPSARSLXFPETLERITMILAFYIVLICELVPAILYMIIVQKETRHR
LKVIVTVIFTIXAMAKYGQLLFGRNQVKNCLRQVEDDWRNVTDLRDNIMIDKAKTGXHLLIICAALMYSTGVS
FZTIIPLSHGKIITEQNITIRHLPCPXLFCSVFEQLSPAYEIVFLMQFFSGFVKCTVTTAMYXLAGLFVMHIC
IQLEILTILMNNLVNESELKNVNKRSADIVRHQIKTRNFLQLVQDNXQYTSLMKVTGCTLIAYLLGYFIIMEW
EDNNTTALCSYTIGXSITCNIFIFCFIGEQLLAKEEKVALTACTLEWYRLPNAKARSL
ILIMIMSYSPTKLGGGKFIDLSFRTFGNVVKTAVTYLNLSVIG
>PbOr8
MLTNIHYKDDMIYITYLTRIILNCLGVWPSVGRRRTTSEKIWKYFLITISYTLLYFVLLPGGLFWFVEKRTRV
RIRTIPLLLYSLMASGKYISLIFREKHIRRCLRHIEEDWKIVPSAEARDMMLESAKIGRRLVTLCAIFMYGNG
VTFRSILPFAKGKIVNAQNVTIKPLPSPAYFFSLDVQASPTYEMIFVMQILSGLVTYSITTGLCGLAAVFAMH
ACGQLKILMNLMTNLVEKQWQEKREVDRRMARMVEHQIRIRSFLQLVENTLQQACLIELLGCTVIVCLLGYFI
IMEWENSNPIAMCSYFVALISLMINMFMFCYTGEQLTVQAERVAITSCELEWYRLPEK
KARGIVLVIIMSNLPIRITAGKIMDLSLKTYGDVVKTAVTYFNMLLNMID
>PbOr9
MFANKYYERDIEYVFATNRFFFRLVGIWPFASVNSFIPELVKTVPLILTCFVSLLGEIIPIMLYIFMVLTDVR
LRLKVVGSAIFAIVELIKYSYVLLYKDQVRNCLMLVDEDWRNIVSPSVRDSMIDRVRTGKRLIVICAVLVYIT
GLSTRLAMPLAVGKIVTPENITIRPLPCVAHLVILDVQQTPIYELMYFIQSLSGFIKYTITVATFGFVTVCGM
HFCAQSEMLMQLMNNFVNESRSENLNKRLATVVEHQIRIRNFLELVQNVTQFTSLIEIVGSTIMICFAGYYII
MEWEDNNIVRLCTYLSALIMFCFNVFIYCYMGEQIIEQGEKIALTACTLEWYDLPDDK
ARSIILIIIMSNSPKKLQGGKFIDLSFRTFGNIVKMAVTYLNLLRSVVE
>PbOr10
MLRNKRCKDNMIYITRLTRQVLSLLGVWPSYDRKRSTGESIWKYFLISMSYILLYCVLIPGGFFWIIEKRTRV
RIQTIPLLLYGFMACGKYGNLIFREKNIRRCLKHIEEDWRIVTNVDARDTMIESANSGRRLVTLCAVFMYGSG
LSFRSILPFAKGKIVTAQNITIKPLPCPAYFFSFDIQVSPFYELVFAIQFLSGIVTFSITTALCGLAAVFVMH
ACGQLKILVDLMRNLVEDQWQEKEEVDKKLARMVEHQIRIRNFLKLIENTLQQACLIELLGCTIIVCLLGYFI
IMEWENSNSVAMCSYFISFTSMMINMFIFCYTGEQLTVQAESVARASCELEWYRLPEK
KARGIVLVIIMSNLPIRITAGKMVDLSLKTYGNVVKTAVTYFNMLLNITD
>PbOr11PSE
MYANKYYECDIEYVFAVNRFFFRLLGIWPFACVNSFIPKLVKTVSLVLTCFVFLSCEIIPMMLYIFMVLTNVR
LRLQVVGSAMSAIIHFIKYNYMLLRKDQIRNCLMFIDEDWRNVVSPSDRFLIIVRVKNSKRLIVKFVLFVYLT
GFICRVVLPLSIGKIVTSENITIRPLPSAASLVXDMQQTPFYEFMYFVQFFSGLIKYAIIVATFDFVXVCEMH
FCAQSDILMKLMNNFVNVEQKXLNKWLNAIVEHQIRIRNFLKLIXKCEFSNLIEXLGIKYVXCLAGYDILLGZ
ENYNVIYMCIFIMTLIMLCFNXFIYCYVGKQIIEQEEKVACTLAXELPDDKARSIILI
TIMSNSSKKLXQGKFIDLSFKTFGNXVKTSVTYLNLLHSFGN
>PbOr12
MLHNQHYKDDMIYITRLTRQVLSLLGVWPSYDRKRSTGESIWKYFLISMSYILLYCVLIPGSFFWIIEKRTRV
RVQTIPLLFYGFMASGKYGNLIFREKNIRKCLKHIEEDWRIVNNMDARSTMIESANIGRRLVTLCAVFMYGSG
LSFRSILPFAKGKIVTAQNITIKPLPCPAYFFSFDIQVSPFYELVFAIQFLSGIVTYSITIALCGLAAVFVMH
ACGQLKILVDLMRNLVEDQWEEKEDVDRKLARMVEHQIRIRSFLQLVENTLQQACLIELLGCTMIVCLLGYFI
IMEWENSNAVAMCSYFITVTSLMINMFMFCYTGEQLSVQAERVASASCELEWYRLPDK
KARGIVLVIIMSNLPTKVTAGKIMELSFKTYGDVVKTAVTYFNMLLNVAD
>PbOr13
MENLILEKHPNENYKQDIKYVIKQNHILLRILGIWPLLERDLSVYETICKILLIIICCTLICFEGIPSMLYCL
LVPEGEPGSKLKMIAPTVYSFTALAKYGALIVYENEIRSCLRHIKDDWRFVAIVSNARDIMLEKTKVARSMFT
ICCVFLYCAGLSYQTIVPLSRGGIVTHDNVTIRPLAYAGYFVLFDEQRSPAYEIVFTLQFFGGFVMYSVTIVT
YGLAALFVMHACAQMKILMMLMEDLVDERVCKEKTVDEKLIAVVERQIRIRNFLCLAEDTLQKSSLFEILGNT
IMMCFVGYCILMEWRDGNTANVCTFLVALASDTVNIFLLCYIGEHITATADEVALKTN
MLEWYRLPARRTRYMVLIIIMSHIPSKITAGKFIVLSLKTFGDVMKSAVVYFNILRTVAD
>PbOr14
MYPNKNYENDIKYTIQLNRFIFRLLGIWPESEPSFFENLKRILLILGCYFLLASELIPTILHIILVEKRARVR
LKLISSVMFTVLAVLKYSSLVLSKNQVGDCLTRVKDDWRNVVSANARNSMIEKARTARRLLILCGIFMYISGL
YFRTIVPLSKGKSVTDQNITIRHLPCPSYFVFFNGQISPGYEIMFFIQFFSGFIKYTITVAICSLAALFAMHI
CAQLEILMMLIDNLIDETEEKNLNGRLAVTVEHQIKMRNFIRLVQNTLEHTSLLEVMGCTIIVCLLGHDIITE
WEDQNVISMCSYLILLTSIGFNIFIFCYIGEQLSLEGEKLALIVCTFAWYRLPNEKAR

ALILVIAMSIVPMKLRAGKFFDLSIRTFGDVVKTAVTYLNIIRKMME
>PbOr15PSE
MRVNILHHDDITRTVHVTRGVLNLIGVWPSPSDSFALKIVRTKMLRIFCQALLYFIFVPGILKMFLKESNARR
RLKMIGPMCNCLMAVLKHTVLICRSDQIKDCIEHIEEDWRTVSLAEDCKIMRGNSRIGRSLAILCVAFVYGSG
FSYRTIVPLTRGVIVTPQNVTIRPLGFDGYYIFIDPQKTPAYEIIFTIQFLSRFVQYSVTSGACNLAALLVLH
ACGQLKILITRMEDLTHAKYFSDKNANRKLAAVVRQHIRIKSFLSKVEDILQYTCLVEVVGCTFILCLLGYYI
IMEWENNDAISLLTYTILLLTXFILCFIGELLMDQSMKVYITFCTLDWYQIPHKTARG
LVLIIAMSSIPIKLTAGKFMDLSLNSFGAIMRTSAAYLNILRTTSI
>PbOr16
MKLYAKDSLKMNSRENDFNYAVQVTRVIMRMIGVWPISRYASNVERIVIRLQNVFCYFLFAFILVPGLLLIFL
KERDFKRRVKIFAPLLNCWISCMKYSLFILHAREIQFCLKQAQQDWRDIIDWHNRKAMLIKAKIGRKFAICSA
AFMYVGGLSYRIIVPLSKGRMLTPMNTTVRALACPSYFIKFDEQLSPAYEIVFTLQFFAGLVTYSVTCSAAGL
VAFFVMHVCGQMSILISKLQHLNDIAKSEDRTIAILLADIVEHQIKVKTFLKQVEESMRDVWLVEIVGSTILL
CFAGYHVILEWENSDSTAMLTIFVIFTSLTFSLFTNCYVGQLLTDESIKVGSMTSAIN
WHHLPHKRARNLILIMAVSNIPAKISAGKMIDMSLPTFSNIIRTSMAYFNLLRKFIT
>PbOr17
MKLFAEDLKTSSRENDFNHAVQVTRVIMRMIGAWPIPRYASNVERIVIRLQNVFCYFLFAFILVPGLLLIFLK
EHDFKRRVRILGPLLNCWMGCMKYSLLVFHAKEIEFCLKQARQDWRDIIDWHNRKAMLIKAKIGRKFAICSAA
FMYVGGLSYRTIVPLSKGRMLTPMNTTVRALACPSYFVMFDEQASPAYEIVFVLQFFAGLVTYSVTCGAAGLA
AFFVMHVCGQMSILISKLQHLNDIPEDIPEPEDRAVAILLADIVEHQIKVKTFLKRVEESMRYVWLVEIVGST
ILLCFTGYYVILEWENSDSTAVLTMFVIFTSFIFSLFTNCYVGQLLTDESIKVGSMTS
AINWHCLPHKKARNLIPIIAISNIPAKISAGKMIEMSLPTFSNIIKTSMAYFNLLRKFIT
>PbOr18
MIEQAYLRNSNYEDDIRYTVQVHRLILGLIGVWPNLEPGKSQKRTTLLKGFLRIVCCFFLLFNLIPWVLYMFL
IMDTFKSRLRMTGAFCFYSMVPAMYFILMLRGDHIKECMRHLEEDWRNVGDANDRRIMLDKARNGRFILICTT
LFLFTSGFTYRLIQPIMRGKIVIGNITIRPLVQGHYFIFFDPQQSPAYEIVSSMHMLTGIIIYIVTASVCGIT
ALLTMHACGQLKMLAVWLENLANEDQWSKNRIVAQRLAAIVIHHVRIRKFLHQIQDVVGDMCFIEIIGSTLIL
CLLGYYVITGWERNDAVSSMTYAIMLVSFTFNIFILCYIGEVLTSQGSKVNTTCCTID
WYCLPSKEARYLILVIAMARYPTKLTAGKVIDLSFSSFGAVIRTAMAYLNLLRTVTL
>PbOr19
MMKENSFHYLHDVHSESKSYNRIYKEDLRSSMQLNIWVMKIIGTWPLRQSWTETLWNRVLNVICYVLLAFLLI
PSGMFIVLEIKDFYNQLKLGSALTFFFMAVMKYCALLLREGDIRRCVDYIEGDWKNVRYTEDRKIMLENANFS
RRLVIICGIFMYGGVLFYFVAVPLTRAKIVEEDGNITYRRLVFPVPNVIVDARRSPVNEIFYFIQLFAGFVAH
NITVAACGLAALVAMHACGQLQVLMSWISHLVDGREGVNDTTDERLAKIVQLHVRILNFISLAEELLHEISLV
EVVGCTLNICFLGYYCMMEWNFKQPVSGLTYLILLISLTFNIFIFCYIGELLTEQTIK
IRESSYMIDWYRLPEKKSLAIILIICMSNATTRLTAGNIIELSISSFGDVIKSSVAYLNMLRTFTT
>PbOr20
MHGIVVKKANSVATVHDYEKDIELSVQLNRWILKPIGVWPKSTEISWVERYTYTLVNVICTSLIGFLFIPTAV
FMALELDNTYLILKLSGPLSFCLMAIVKYSSLIFRENDIRSGIEHIESDWMNTPHHGDRVIMIKNAKFGRRLV
AICAFFMYGGAVFYYLALPFSNGRITEDDGNLTYRPLVYPVARVIVDARHSPISEILFWTQCLSGFIAHSITA
GACSLAAVFAMHAYGRLEVLAQWIEHLVDGREDSYDDVDERLTMIVQQHVRILCFISLTDKVLREISVVEIIG
CTLNMCFLGYYTITEWEIKNSASYITYIVLLISLTFNIFIFCYIGELVAEKCKKVGEI
SYMIDWHRLSGKKGLALVLMIAMSNASIKLTAGNLFELSLSTFGDVVKTSVAYLNMLRTLTA
>PbOr21
MSHSFRVSSTHNLAYQLDSEYSIQLNRWFLKPISAWPGTSTGLAGKILSRGIQFVCHSLIAFTVVPCALYIIF
EPNVYLKLKAFGPMIHWIMGGANYCSLLSRSYEIRKCVDHMRADWRYVGNTHDREVMLQNAKFGRFVCTFCAV
FMQGGVCSYSIITTLTSSIVRIGNVTITTHQLPCPFYTGLVDTRYSPANEIVIVFQLLSTVIVNTVTVGACSL
AAVFATHACGQLNILMMRLDELVDGKESEHKATRQKLAAIVEHHLRVLSFVSQIETFMNQICLVELMGCTINI
CMLGYYTITEWKLHDTKNLLTYFTIFVAKSCNVFIYCYIAEILMDQCQKIGEMAYMTE
WYRLHHKIALDLILIISRSNAVIKITAGKMIQLSIATFGDVMKTAFAYLNILRTVAT
>PbOr22
MKRVSSDISVEIYRRDNDYSLQLNRWFLRPIGAWPELQTNSITKNVLMNILRLTCHFLIAFTWLPSILYIIFE
EKDFRLRLKAVGPTSHLIMGGINYCSLLHNNEQIRKSIEHMETDWRMAKREHDREVMLRNARVGRVIAGVCAL
IMQGGVICYNIARGMSRISVVIGNETIETGRLPCPSFNKIVDTRLSPVYEVVLALQCLSTIIVNNITIGACGL
AAVFAMHASGQLNVVMLRIQELVTEKHEVLQLRLANIVEHHLRALRFLSRMEAIMRQICFVELVGCTFNLCML
GYYTITEWQEESLNTIITYVMILTSMMFNIFIFCFIGELVTDQCKKVGEAAYMTDWYQ
LPHKTVLGLILIIILRSRTVIKITAGKIFHMSIPTFGVVIKTSVAYLNMLRTLTV
>PbOr23

MYSIPMQKSSSVAAATIHDEKDMQLSMQLNRWMLKPIGAWPKSDDLSRIEKLIYLLLNTICSGLIVFLFVPAA
VFMVFEVDDVYHRVKLFGHLLFCVMAIVKYSAMMCRENDIRRGIQEIEDDWISTRHYDDQIIMIESAKFGRRL
IVISGIFMYGGAAFYYFALPLMDGKIIEDDGNLTYWPLVYPVARVIVDVRYSPANDIFFSVQCLSGVVAHCIT
AGACSLAATFAMHAHGRLRVLAQWIKHLIDGREDLCSSMNKRLAMIVQQHVRILRFISLTDKILREMAFIDIF
GCTLNMCFLGYYTITEWEDKNSVVIYIILLLSLAFNIFIFCYIGELVTEQCKKIGDVS
YMIDWHRLSGNKGLAIILISAMSNSSIKLTVANIIELSLSTFGNVINTSVAYLNMLRTLT
>PbOr24
MMRGSPTVQKSNPVAIVCDHEKYVRLSVQKTRWILKSIGVWPKALESSSLMKRYVRVLINVICIGLVTFLFVP
CVFYVVLEVEDTYNTLKFTGPLSFCIMVFMKYILLIARGNDIRVCIEHIRNDWMNTQHHGDRAIMIRNAEFGS
RLVMICSFMSYGGAVFYHIAMPIGMGKITDMDNLSYHLLGYPVARVIADTRYSPINEIFLLIQCISGLTTQAV
TAGACSLTAVLAMHAYGRLEILVQWIMHLVDGREDLCNNVDKRLAIIVQEHVRILRFISLTEKILHEIFLVEI
VGCTMNMCFLGYYIIMEWEIGESASYITYSVLLISFTFNIFIFCYIGELVAMQSRRVS
EVAYMIEWHRLPKREALAIILMIATSNSPIKFTAGNIIDLSLSSFGDVIKTSVAYLNILRTLTS
>PbOr25
MMRGSPTVQKSNPVAIVCDHEKYVRLSVQKTRWILKSIGVWPKALESSSLIKKYVRLLINVIYIGLVTFIFIP
CIFYVVLEVEGTYNALRFTGPLSFCTMVLMKYISLIIRGRDIRICIEHIKSDWMNTWHHGDRAIMIRNAEFGS
RLVMICSFMSYGGAVFYHIAMPIGMGKITDMDNLSYHLLGYPVARVIADTRYSPINEIFLLIQCISGLTTQAV
TAGACSLTAVLAMHAYGRLEILMEWIVHLVDGREDLCKNVDERLAVIVQEHVRIMRFISLMQKVLHEISLVEI
IGCTLNICFLGYYIIMEWEIAEFASHVTYIILLTSVTFNIFIFCYIGELVAQQSKRVS
EISYMIDWHRLPGRKALAVILMIAMSNSSVKLTAGNIIDLSISSFGDVIKTSIAYLNMLRTLTS
>PbOr26
MVSSDKPKTDVYRRNNNYSLQLNRWILKPIGVWPELPSSSTTEKILSKLLRLTCHSLVALTVVPSILYILFEE
KDVRLKLKAVGPTSHWLMGGINYCSLLYQKRQIRKSIEHMEADWQMAKRECDQELMLRNARMGRVIAGVCALI
MQGGIFSYNVARGTSPILVTIGNETIAIGRLPCPSFNKIVDTRFSPVYEVVFTLQCLSSLVVNNTTVGACGLA
AVFAMHACGQLGVIMSRLEELVEEKERNVIQRKLANLVERHLRALRFLSRMEIIMRQVCLVELAGCTFNLCML
GYYTITEWHEESTNTLITYLIVLTSMMFNIFIFCYIGDLVTEQSKKVGEAAYMTNWYQ
LPHKHVLGLILIIILRSSSVIKITAGKIFHMSISTFGDVIKTSVAYLNMLRTLTM
>PbOr27
MTITSDFDKPSFSHCYNEYHSYSLQLNRWFLKPIGAWPESYATTIAKRLLPRIIQITCYILIAFVVVPSLLYF
YLEEQDLDTKLDLVGPVSHWIMSAINYTSLLWRGKDIRCCIKHMENDWYAVSRSEDCEAMIKYAKFGRSVAGF
CAVFMHCGVFSYSAVNSLTPIVTVIGNETISARKLPCPFYSKLLDTSRTPANEIVLALQFLSGFIVNSVTVGA
CSLAAVFAMHACGQFAVLYMWLNELVDEEKKEQYAEYKLANIVEHHLRVLNFLSRVENIMNQICLVEVVGCTL
NLCLLGFCSIKEWNAGNTKTIATYSIVFISVSFNIFIFCYIGEIITEQCKKVGEMAYF
TNWYRLPHKTALGMVLIISRSSAVIKITAGKLIHLSLMTFGDVIKTSAAYLNILRTITM
>PbOr28
MVTMCRSVTEDLHDHSVQLNRWFLKPIGAWPRFSSSTGHEKAMSRALIVTCFSLIAFTVIPCALNVLFEEKNI
ELKLRAIGPLSHWLMGGINYCSLLLRSADIRRCVRHMRMDWQIIRRSEDREIMARNAKLGRFVAGFCAICMHG
GVFAYSIVSGMTTVMMPIDENRSVEMLQLPCPSYSKFVDARFSPANEIVLVMQLISGFIVNSTTVGACSLAAV
FAMHACGQLDILILRLYRLVEGDGKKRGESAQKRLAEIVDHHLRVLRFIARIEDVMHQVCLVELVGCTFNLCM
LGYYSITWWNKIDRKSIAAYIVVYISMSFNIFIFCYIGEILTQQCKKVGEIAYMTDWY
RLPHKTALGLILVISRSSVVIKITAGKLIQLSIATFSDVIKTSLVYLNMLRTVTT
>PbOr29PSE
MYPSITEDTISTIIACSSZQLNRWFLKLLSAZPRSSATSSSEKIMSYALIFVCYFLIAFTVRAEXIFFEEXTI
DLQLRAIGPMSHWLMGGTNYTSLLXEIQRCMZHMEMDWQIIRRPQDREIMARNAKLGRFMADFCAMFMHGDVL
SYSVXGTIVIMSIDYNQSVSMXSSCPSYSKFIDARFSPANEIVLVMQFISCFIXNSTTVGACSLAAVFAMHAC
DQLDILISRLDSLVEGERTKENESAQQRLADIVDHHLCVLXNFIACIENIMHQICLVELVGCTFNLCMLGYFC
LXGIERVQKAAYSIVYISMSFNIFIFCYIGEILTQQCKRVGEIAYMTDXIVPHKTLGL
ILIISRSSAIIKITAEKLIQFSIGTFSDVIKTSFVYLNMLRTVTT
>PbOr30
MTYSKRVPRSTELSYKSDNDYSLQLTRWFLMPIGVWQRVHVTTKIDKFFSHIHILVCTFVIAIIVIPCFLHLS
LEEKDMENKLNIIGPLSHWTMGMINYYLLLARSDDIRECVLHMETDWRLVRKIEDRQIMLRQAKIGRFIAGFC
AVFMQGGTLLFNVAKPTTTIIVDNETLSIHPMTCPLYTKFIDTRYSPANEIMVLIELFSGFIVNSVTVCACSL
DAVFAMHAYGQLDILFSWLNELVVDGDKGNEYAEQKLAVIVKHHLRVLSFISRLETVMQQICLVELLGCTMNM
CLLAYYFITNIDSFDRAKTLSYVIIYLSMAFNIFIFCFIGEILTEQCKNVGERAYMIK
WYELPNKTALGLVLMIARSSNVIKITAGKLFQLSIATFGDVIKTSMVYLNMLRTMT
>PbOr31FIX
MTYSERVPRSIEMYKNDSNYSLQLTRWFLMPIGIWPRMSTATRAERLSSYIHILICTSLIAIIMVPCLLHVSL
EEKDMGIKLTVMGPLSHWVMGMINYCLLLARGNDIRECVLHIEADWRLVQKIEDRQIMLRQAKIGRFVAGFCA

VFMQSGTFLFAVAKSLSTTIVIVGNETVSMHPMTCPMYTKFIDVRFSPANEIMVLIEWISCFIVNSVTVGACS
LDAVFAMHAYGQLNMLFSWLNELVVDEDKRNERAEQKLAVIVEHHLRVLSFISRMETVMQQICLVELLGCTMN
MCLLAYYFITNIDSFDGAKTMSYVIIYLSMAFNIFIFCYIGEILTEQCKNVGERAYMI
NWYELPHKTALGLILVIARSSNVIKMTAGKLFQLSIATFGDVIKTSMVYLNMLRTMT
>PbOr32
MTYSERIPTSMKFTYQSNNDYSLQLTRWFLMPIAAWPRASTSTTVEKVSLQAHVLACLFLIAIVMVPCLLYVS
LEEKDVQMKLSVMGPLSHWIMGTINYWLLLARSDDIRKCVQHMETDWRLIYRTDDQNVMLRYAKIGRFVAGFC
AVFMQSGTFLFTIAKAMTSVPIVVGNETMLMHPMTCPIYSKFIDTRFSPVNEIMLAVELLSCFIVNSITVGAC
SLAAVFAMHACGQLSMLFSWLNNLVAEDDKGNDFAEQKLATIVEHHLRVLSFISRMENIMQNICLVELVGCTM
NMCLLAYYSITNWSDFDAAKITSYIIVYISMAFNIFIFCYIGEILTEQCKNVGEKAYM
INWYDLPHKTALGLILIIARSSNVIKITAGKLFQLSVATFGDVIKTSMVYLNILRTMTPAT

>PbOr33
MIMEPVKSDKDASDYSIQLNRWFLKPVGAWPSSSTTTIKEKVISTILIIICYSLIAYTVVPCILNILFEETDV
HKKLRAVGPLSHWCMGGMNYFSLLFRSRDIRRCVQHMRADWRTVTKIEDRQVMLRYAKVGRFVAGLCAIFMHG
GVFSHSVLQGTTPTFEIIDNVSVSVHVLPCPSYSKFMDTTQSPAGEIALTLQLISSIVVNSVTVGACSLAAVF
AMHACGQLNVLMRWLDQLDDRRQEETVQRRLAIIVEHHLRVLSFVARMEALLNQICFVELLGCTFNLCMLGYY
VITGWNVIEKKTSIAYMIIYISMSFNIFIFCYIGEVLTEQCKQVGEIAYMTNWYRLPY
KTARGLILIISRSSTVIKLTAGKLVHLSIATFGDVMKTSMIYLNMLRTMTAS
>PbOr34
MSDSKRILINTEFENCNDYSIQLNRWFLLSLGAWPQAGASSRIKKFVVSMQIFIFSTAVAVITIPCVLYVWFE
KEDIEIKLITLVPLIHRTMGSINYWILLTRKKDIHHCIRHMETDWEFIRRIDNREVMLQYAKIGRFMAGLCAI
FMHGCTFAFTAVRAMKVTVTVGNETFKMYPMTCPMYSKIIDARFSPANEILLSIQLLSAFVVASSTVAICSLA
IVFAMHACGQLNVLYMWLSELAEEEKIDEAEQKLAVIVEHHWRILSFVAQIESIMHKACLAELMGCTLNMCLL
GYYSIMNWAAFDAVKILSYIITYVSMSFNIFIFCYIGEVLAEQCKNVGEIAYMTNWYK
LPRKTALSLILIIMQSSHIVKITAGKLFQLSISTFGDIIKTSLAYLNMLRTIIA
>PbOr35PSE
MLNFKSAPESGVSKKHNDYSLQLMVXGFSSGAGLQINMSSSLWKIFVLQIIICSFLIACIMXVRSLFVFFEKV
NIKLKLNAIGPLLHRVTGSINYWVLLTRSDDICQLMQHMKADWGIIRNINDREVMLKYAKFGRFITIICRVIM
QGGAFLFSIVQAMKTATIIVGNEXFLHSMTCPVYSKIIDARFSPVNEIALVLQFLSTIVASCSIVGACSLAAV
FAMHACGQLNMCYTHDCEISLRIKRRETIZLNXKLAIIIEHHLMSFISRLESIMNKISLGKLMGCTINLCLLG
YYTIMVWETLDKAKIIYYCIXLSMSFNIFIYCYIGEIVTEQXHKTALGLVLIIAZSSN
VIKITVGKLIHLSIATFDNVIKTSVVYLNLLRTSI
>PbOr36FIX
MPNFKSAPESDESKKHNDYSLQLNRWFLMSIGAWPQINASNSLWKIFVFLQIIICWSMVASITVPCLFYVLFE
DDNIKLKLNAVGPLLHRVMGSINYWVLLKRSDDIWQLMQHMEADWRNIQKIDDREVMLNYAKFGRFITVICGV
IMQGGTFLFSLARAMKTATIIVNNQTFTTHPMTCPIYSKIIDTRFSPVNEIALVVQFLSTFVVSSSTVGACSL
AAVFAMHACGQLNVLYAWLRDLVENQEKENHAEKEMATVVEHHLRILSFISRLESIMHKVSLGELMGCTINMC
LLGYYTIMAWEMLDKAKITSYVIVYLSMAFNIFIFCYIGEIITEQCKLVGEMAYMTDW
YKLHHKTALGFVLIIARSGNVIKITAGKLVHLSIATFGDVMKTSIAYLNLLRTLT
>PbOr37
MKRLSQSSPIIFNYIKNKEYSIQLLRWLLKPISVWPVTNSSIADRILSVVLLILCTFLIIGTLVPCALAIFFD
ETKDLEMKIRDFGPLSNWMLASLKYTSLLTHVGDIRRCIDYIEADWRAVTKFEEQEMMLNSARIGRFIVIFSA
SFVHSGVFSYSIFRGMTMDKSGVEDDNVSARSLPFAFYDKILDTTISPMYEIVFAIQFLSTFVVNSVVVAACS
ITAVFVLHACGQLKILMSLLDNFTDERNEKKDSIQKFSVIVEHHLRILSFVSHIEKITNIVCLIEIVGCTLHM
CLLGYYCILDWNQDEKQGVVAYGIILISVTFNIFIFCYIGEMLSEQCGQVGETAYMTN
WYLLPGNTALDFVLIIILKSSITVKITAGKMIDLSLSTFGNVIKSALAYLNILRTLII
>PbOr38
MGDDFTNSRSLSASIYLADKYNEYSIQIIRWILKAISLWPLPVDASFVEKIRFDFAVFICYFLIIAVMIPSGL
SIFVESQESFETRFRSFGPLTFWFFGLLNYSCLLIHIDDIRICVDHVKTDWRNMTKVEDQKLMLRSAKKGRFF
GAFCAIFMHSGVFSYNVSRSFGKDILHVGNSSIEVRALPYPFYSKILNAHYSPAYEFVFLIQCLSSFVVNSVT
VATAGLAAALIFHACGQLKILMSRLDNLVDETDEKNTSLKQRYGIIVRHHLRALSLVTRVEKIMNFICLVELF
GCTFHICIVGYYCMMDYIHGNNSNVVSYSMIITSVTLNIFVFCLIGEMLSEQGGQIAK
SAYMTKWYLLTGNNANGLVLIIILRSNVTVKITAGKVAQLSIATFGDVIKSALAYLNILRTVMVM
>PbOr39
MLDHPKSKSVELVTDNWKYSIQMNRWFLKPIGAWPLTLCETTSEKLGCMILTVVSCFLICFLLVPCTLCTILV
DTDLDTKIRMIGPTSFILMAAVKQYILIARSENISECIRHVRADWNHVASGREKDREIMLINAKFGRWLSSFS
AIFMYSAGIFFTTVMPICARRTEIIDNETVRSLSFPIYRGLFDPRSTPSFEIAQFTQALAGYVIYTITIGVCS

LAAVFVMHACGQLRILMLKMDDLADGKERKSASNTHKGRLGDIVKHHMRILSFITRTEDLLNEICLVDVVGCT
LNICFLGFNMMTEWEHRETLGTMTFCSLLISFTFNIFILCYIGELLAEQCIQIGIKSY
MIDWYRIPNKGALGLTLVMAMSNATIKLTAGKFMDLSLASFCSIVKASVAYLNLLRTFYV
>PbOr40
MENDFTDSHNVSTSMYMADKYNEYSIQIMRWILRAISLWPLSVDASIIEKICSDFTICLCYFLMIVIMVPNGL
SIFIESQESYETIIRNIGPITFWFIAMVNYSCLLMHIDDIRACVEHVKADWRVMSKTQNRQVMLKTARIGRFI
TGFCAVFMHSGVFSYNVAQGLSKDILQVGNSSIEVRVLPYPFYSKILDTHYSPAYECMFFVQCLSSFVVNSVT
VATCSIGAVFVMHACGQLRIMMSLLENLVDERNEEKTSVKQRFVIIVEHHLRVLSFVSRIEKIMNIVCLVELI
GCTMHICLVGYYFILDLIQGKDTESVVSYSMLLSSITFNIFIFCYIGETLSEQGGQIG
KSVYMTNWYLLPGKTARGLILIIILRSNADLKLTAGKIVQLSFSTFGDVIKSALAYLNILRTILIIK
>PbOr41
MLDRSKSKSIEPTMDGWNYSIQLNRWFLKPIGAWPLTLCETTAEKLGCVILTIISCFLICFLAIPCTLCTILV
DTDLDTKVRMIGPVSFILRAAVKQYILISRSKNISECIREIRTDWDRVALNYVKDREIMLDKAKFGRWLSSIS
ALFMYSAGIFFTTVMPICAKRTEIIDNETVRSLSFPIYRGLFDPRTTPSFEIAQFMQAVAGYVIYTITVSVCS
LAALFAMHACGQFRILMLKMEDFADGKERKSASTMHEGRLGDIVKYHIKILSFITRTEKLLNEVCLVDVVGGT
LDICFLGFNMMTEWEHREALGTITFSSLLISFIFNIFILCYIGELLAEQCTQIGIKSY
MIDWYRIPNKGALGLILVMSMSNATIKLTAGKFMDLSLSSFCNIMKASLAYLNLLRTFYV
>PbOr42
MRDRFQLSDRIYNRHYEHDIRYAMQLCQWILKPIGIWHMIYGRSQSEKFLSIILIIISFFLLCFVLVPSGPYI
LFREKNIDVKVKLIAPIGFCLTSAIKYCFLGLRGLAIGRCVEHIERDWRVVRYWDHRKMMLRNALVGRRLTIL
SVLFLYSGGMSYYAIMPFSSRTKINGSYTSRPLIYPGYDRYVDPQVSPAWEIIFCMHCLCAIVQFTVTTAACS
VVAIFATHACGQVQILMTLLDDLIVGERNKDTTVEKRLSLIAKHHVRVLRFTANVEEVLREICLLELMSSTLI
ICLLEYYFLKEWENSDAVAIFTYFILVISLTFNILIFCYIGELIVEEFSKVDLAAYEL
NWYDLPGYKAVDLVLIIMMSHYPPKLTAGKICDLSLDTFSSVLKTSLAYLNLLRTVT
>PbOr43
MHDRFQLPDRIYNVHYEHDIRYAMQLCRRILKPIGIWHMIYSRSSQRQKFLSIILILISFSLLCFVLVPSGPY
TLFREKDIDMKVKLFGPIGFCLTSAVKYCFLGMRGSAIGRCVEHVERDWRVVQHRDHRKMMLRNALVGRRLTI
LSVLFLYTGGMSYHTIMPLSSRTEINGSYSSRPLVYPGYDLYVDPQISPAWEIIFCMHCLSAMIQYTATTAAC
SLAAIFATHACGQVQILMTLLDDLVIGERNKDTTVEKRLSSIAKHHVRVLRFTANVEEVLREICLMELVAATL
IICLLEYYCLKEWENSDAVAIFTYFILLISLTFNILIFCYIGELLVEEFSKVGSAAYE
LNWYDLPGHKAVDLILIITMSQYPPKLTAGKFCDLSLNTFSTVLKTSVVYLNLLRTVT
>PbOr44
MHDRSRLSGKKFEEYNVHQENDIRYTMQLCRWVLKPIGMWHLIYGHPSQSERLISIALIVTCFSALCFVLIPA
GLYTLFREKNINIKVKLFGPVGFCLTSTIKYCYLGARAAAFGKCIRHVEDDWRVIRHQDHREMMLKNALMGRR
LTTLCVLFLYTGGLSYHTIMPLSSRRKINENYTIRLHTYPGYDIFFDPGASPAYEIVFCIHCLFALITYNVTT
AACSLAAIFVTHACGQIQILMTLLDDLVDGKWSNGMTVKNRLKVIARHHVRLLRFSNNVEEVLREICLMEIVA
STLIICLLEYYCLTEWKNSDAVAILTYFILLISFTFNILIFCYIGELLVEQYSKIGLA
VYGINWYNLSGNKALGLTLIIAMSHYPPKLTAGKIVDLSINTFGAVLKTSVVYLNLLRTVTE
>PbOr45PSE
MSKITMZSNVSYKKDIIDMLIWNRLLRIFGIWPLIYANTTTIEKILATFLFASCWTVLSLFLILISIYTFTDQ
SIMNEKMKXGPLNYVFFSILKYLFLLVRSTRISEMLQVVTIGWRMVQEKYHREIMIRDAEKGHLLSXFCIMFM
YYGDLSYNTVMPFLSNSPDANEZNFTIRPMVYLGFDIIFNLQLMPIYVFAFTLQCFTGVVMFNITTSVCCLVT
MFVAHACGQINIVIVRVEDLXEMHSDYHVQHHVRPLRFSASIEKNFARXCLVKGTMLIICLVEYSLIXEZEQZ
RQYSDLHILFSIRFIRLQNIFIFCYIGKLLTKQXEGHTYNTSYKIEYNLPEKIALNLM
LMINISRHSIQITAGRLINLSFANFGNAXKTSVAYLNLLRTAV
>PbOr46
MAKLNISWSNVNCERDILNTLIWNRWILRVLGIWPLVYPDTSTIEKILASISFAFCWSVLSFLLIPLTIFTFS
KHSATYDRVKMLGPLVYVCISMLNYLFLVIRHKNIRQCIRVLSTDWRVVQQEDHRKIMIRNAAKGHVLSKFCI
VFMYCGGLSYHTLMPFLSHAPTDEQNGTVRPLPWKGFDSIFDLHFMPVYIFVFCAQCCSGIVMFNITTGVCCL
AAMFVAHACGQIEIVMDHIENFIKSVQKSPKQRMAVIIKHHVQILRFSISIDSILREICLVEIVGSTICICTL
EYYCMTEWNNNDSIAILTYLFLLVSFVFNIFMFCYIGEQLTEQCSKIGYSSYEIEWYR
LPGKIALDLTLIISISHRPIKITAGKLINLSFSSFGNAIKTSIAYFNLLRTFVD
>PbOr47
MPGARDEKDPNHQQDIRYVFKLNNWILGSIGIWPISIRGIGRHVSKVAIVFGNFTLSFAMVPCALHIIYDEKN
IIMKLKLCGLMAFCLTAMTKYCILVIRRPKILRCIECIKNDWWQVMFTSDREMMLKYATIGRNLTIISASFMY
TAGIIYYMILIPFFSETKINNQTVRPFVYPTYSEFRQSQISPIYEIVYGAHCICGYIMYSITVGACGLAALFA
THACGQIQVIVSRLEELLNDESPNIHQRIAVIVKDHVRIIRFSTVVEEVLQEVCLVEFASSVCTICLLEYYCI
DWQDDNKLSLAIYFMLFISFCFNIYILCYIGELLMEKSSQIGSICYMIKWYQLSPRSV

RSLILIIVMSSHPIKLSAGRMADLSLSTFGNVLKTSVAYLSFLRTLVM
>PbOr48
MSSIHHEPATLQNSSYQRDIQYVFKLNTWILSSLGIWPVAIRGIGRHVSKIAIAICNLALSFAMIPCALHIVY
DQKDIIIRLKLFGLLAFCLTAMTKYSILVIRRPKILRCIEYVKNDWWQVTFESDREQMLRYAATGRRLTIISA
SFMYTAGIIYHLILPFCSEHKINNQTIRPLVYPTYSGFRQSQISPIYEIVYAVHCVCGYTIYSVTAGTCGMAA
LFATHACGQIQVLVSRLEDLLTGKKFKQNPYVHYRIATIVKNHIKVIRFAAVVEELLQEVCLVEFTSSLCTIC
LLEYYCIDWQANDKIGLVTYLMLFVSFCFNIYILCYIGELLMEKSSYIGSICYLINWY
ELSPRSARDLLLILAMSSHPIKISAGRMVDLSLTTFGNVMKTSVAYLSFLRTLVI
>PbOr49
MENSQNEHTISPNINYQRDIQYIFKPGNWVLGSIGIWPLAIRGIGRHASKIAILLCNFALIFAIVPSVLHMIY
DQKDLNIRLKLFGLLGFCSTALMKYCVLTMRRPKIMRCIEHVKSDWWQVKFNSDREMMLKYATTGRRLSMISV
TSMYLAGFIFHTILPFCTEHKIGNQTIRPLVYPPYSKFFNTQTSPVYEMVYLAHCLCGYTMYSVTAGSCGLAA
IFATHACGQIQVIESRLEDLPRGKNFEQFVDVNQRIAHIVKSHVRILRFSAAVQEVLQEVCLLEFASSIFTMC
LPEYYCIVDWQDSDAVGLTTYFLLFISFCFNMYILCYIGEQLVQKSSQIGTKCFMTTW
YQLPAKSIRGLVLLIAMSNHPIKFSAGRMVDLSLATFGNVLKTSLAYLSFLRTLVV
>PbOr50
MENSHEEHTISPNINYQRDIQYIFKPGNWILGSIGIWPLTIRGIGRHASKIAILLCNFALIFAIVPCFLNMIY
DQKDLNIKLKLFGVLGFCSITLMKYCVLTIYRPKISRCIEHVKSDWWQVKFSSDREMMLKYATIGRTLSMISA
TSMCLPEVIYHTILPFCTEHKIGNQTIRPLVYPTYSKFFNTQVSPVYEIVYLAHCMCGYTIYSVTAGTCGLAA
ILATHACGQIQVIISRLEDLSRGKNFEQLLDVNQRIAHIVKSHVRILRFSTAVEELLQEVCLLEFASSIFTMC
LPEYYCIVDWQNSDAVGLTTYFLLFISFCFNMYILCYIGEQLVEKSSQIGTKCFTTTW
YKLPTKSIRNLVLVIAMSSHPIKITAGRMIDLSLATFGNVLKTSLAYLSLLRTLVV
>PbOr51
MENSQDKHTRSSNVNYQQDIQYIFKPGNWILGSIGIWPHAIGGIGLHASKIAILLCNFALIFAIVPCILYMIY
DEKDLNLRLKLFGLLGFCCTALMKYCVLMIHRPKILRCIEHVKSDWWQVKFDSDREMMLKYATTGRRLSMISA
TSLYLPEFIYHTILPFCTEHKVGNQTIRPLVYPTYSKFLNTQMSPVYEMTYLAHWMCGYTICSVTAGSCGLAA
IFATHACGQIQMIVSRLEDLSRDKNFEQLLDVNQRIAHIVKSHVRILRFSSAVEEILQEVCLLEFISSIFTMC
LPEYYCIVDWQDSDALGLLIYFMLFISFCFNMYILCYIGEQLVEKSSQIGTRCFMTTW
YQLPTKSIRNLVLVIAMSSHPIKLTAGRMIDLSLATFGNVLKTSFAYLSLLRTLVV
>PbOr52
MIFHRDVTVTLSVHRFFLSCVGIWPVREKNIFMDLRWIIAVVLEVIPVSIYLTEIYLHCNGAKKSFDWITPSA
AGMLALTRLITPRIHREELVEIVTSMMDDWVMQKDKKIRWVMKKYATMSTRVTVLTFILVGIIVAVYIAMAIS
AITAKIEQFDNNNVSTSHKDGIQSCVFHSASSHQAFMIVQAMQMFVTGVLTFGTTSFFFGLAMYLCSQFDALS
IKLSEFQINTAHRAIAEAVQRHCHLIRLAECMEESFNASVLVYLFVTSTLMCIDGYMLIASLPIGDLPMIIHS
ASVLLLMLIQLSFYTFAGDYLEMRSSALSYATYDCNWYELPASTAKNFQIILMRASIP
HQLTAGKFVIMNMITFKDILKSTASYLSVLRIMLDE
>PbOr53
MNETKEWNEKSHYALSVYKRVLSIIGVWPLTAGEFKSIARCSLAILLQISTIGSLSLEAYRQCLGTEDMMEAF
LMDLSSVVSLSKLLVIRFTWRHTYVLVTSLIDDWSISRDSRQREIMMRYTHVGRIVSLTILYLGYASGVSFLF
MAVPFDRLIPWLNMTKTMNDNDTMVSTYFLATYCVFGSLPMTVHICVLLLQVAQIFVNATSHCGNDGFFFGLA
MHLCGQFEVLQMDFARIEVEERACKRKIRMLINRHSHLISLADSFEYAFNMAIFAQLLMSVLLLCVEGMQLII
SLKLNDNIAAIKHVVLILTMLVQLYLYCYAGDQLEYITGKIGYSVYNSPWYNFDVKIM
KDMPMIMFRGRIPHQITAGKFLRMNLFSFKEILKATGSYLSVLRVMIDV
>PbOr54FIX
MRMESDMNWNTDTEKVLKFHKHFLGIIGLWVLDEKSVFSKIRWFISTMVEMSTSVTLSLEMIRHCNGHEDAMD
AFLVGSSSIISMIKLLLHRVYWRQKSILVKSIIRDWTYVENSHSRDIMWKYAYIGRMGSSTFFYFGCASVTSF
VTTLVLANIDLSWISEKQMYNETHERKLMLAAYCTFGKYTSSFAYCATEILQFVQILVNGISQCGNDGFFFDL
TMHICGQFAILRTNFSKLGSEDFLYRSKFDILLKRHYRLICLSHCLERAFTLVILAQVLMSVLVLCVEGFLLL
FSLKTNDSITATKHGVFIVSLLVQLFLYCFAGQTLEYQSQGLAHAIYNSSWYTFDVKM
MKNLPLIILRAANPQQLTAGKFVAMNFMTFKEILKASASYLSVLRVMMDT
>PbOr55
MIIRVLPLARMTDKRWNDDIAYSFFTHRIFLRIYGLWPLQTQTVFTNIRWNLFLIAQFMILPFLLVDSFWSDK
NAGSNIENILYFTSTVTGIIKNVCVRINKKKLATNINAAIDDWLSAKDNKETQMIMKKYALKARMLTFTLLYS
AVGCFSIYVLAIVFINLQQIYFMDKNLVDANTTNWILFIPCGPLSKSINGLQFVIILVIQILQTISLCIILSI
VDTFFFTMTIHLAGQLEVLRKKFTTFANESDIEANYRKKFVSLINRHSKLTEFYQNLEDTFNFLILSQLVSTT
IMIALIGMRINLCINEKNHIELTKSVLVLNYLFMQSLIFTYAGDFLQTKSEDIFHALY
ATSWFTLPLALMKDLHFAMMRSSVLLRLTGGKFFYVNRETMMYMLKTSASYVSVLRIALRN
>PbOr56FIX

MTSTVKWNANIAYVLMLYKHLLGLIGLWVLDEKKIFSRIRWFLSTLVELSATISLSLEVIRHCRGHEDALDAF
LSASTSVISMSKLLLHRVNWKHKLILVESIIYDWTFVNNSYSRNIMLKYVRRSRLGTLVFFYFGCASCVFLFS
PFIYANFDLLWTSEEQIFNKTSERRLLLAAYCVFGSYTSLTYGFVEILQTLQILVNCLSQCGNDGFFFVLTMH
VCGQFEVLRMNFSEIDDKVFFSRNKFDVLLKKHRRLIYLAHHLQKAFSLVILLQLLMSVLLLCVEGFQLILTL
SMHNTYAAMKHVLFIAVLLIQLFLYCFAGQTLEFQSQGLAFAIYDSPWYTFDISIMKN
LPLMILRTIHPHQLTAGKFLAINFVSFKEILKASASYLSVLRVMLET
>PbOr57PSE
MIYWNQDAVYALSSYKVLAWPEVGVRPIDDDIFYSKIRCIFAIILLRITLLMDVYLACENSSEDPIDTYVVAS
SAILVFIKLTLLRMQRSTLSTNLYAAIRVVLKMRXSREIMIQYALTARIISLSLFYSGFFAFTFYLLRLLPFV
NAIANGRTYFLPMXCLFESISNLQYVFITFYQVIQLFIAYAGNYCTEGIFVGITMHLCGZLELLMIDFQRLDH
SKYKRQRGNIIERFVIRHRQLIKLTETXEDSYSIIIFAQIFTSAILICITGLIESFHINDTIMIVKSIVIMIV
MLLQCFLYSYAGDNLRDQSEALSFALYDSNWYDFSPNDVRDLAFIMIKTNIPIRLTVG
KFFYVTRATFTDILKSAVSYLSALHVMIEKQETNN
>PbOr58
MRCIDAPSMPVIDSSYSHYLDPMASEQWKNDVAYAMTPFKIISWPIGVWPLQVYNVYSFIRCVLGTLSAALMV
VMPSIEIYLGCTNTETNVDCLMLICCGILGMVKTIWFRIYARNLTNNYGSAVNDYLTIENTRERAIMRKHAFM
GRFLLCLILGFAYISCVIYALIPLLDDDEIEQVNITNEDITLEYPMPSKCTIKYLNTSMNMYRIFCLIETIAL
ILASTTNHGNDALFLNITLHVCGQIKILKNNFLNFDTKSPEIYDRFTVLIKRHNYLIKLTRELAEIISFVLLV
ELFIISILLCIMGLQLILALKVNNIIMIGKSLTVLITFLIQLTLYGIIGNYLKSQMEE
IGLAVYQSAWYNFPAKLTRHLVFIIMRSDSPVTLQAGNFIVVNLATYMSILKASLSYLSVLRVLVET
>PbOr59
MIRDRSIMSIYRVFPVNGISYSHYLDSMANEQWKDDVAYAMTPFKIISWPIGVWPLQVYNVYSFIRCILATCC
AALMIVMPSFEFYLGCTDADTNIDCLELVCCGTLGMVKTICFRIYARNLTNNYGSAINDYLTIENTKERAIMR
KHAFMGRFLCCLILGFAYIGGLMYVLIPLLDVKGIEQVNITNEDIMLEYPIPSKCAMKYFNAPVSMYRIFCLV
DAIAVMIATTTNIGNDALFLNIILHICGQIQILKVNFFDFDVESPQMYDRFNDLIKRHNYLIKLTRELAEMIS
FVLLIELFIIGLLLCVIGLQFILALKVNNIIIVAKTIIVQVTFLIQLTLYGLTGNYLK
IQMEEIGIAVYQSAWYKLPAKLARHLNFIIMRSYSPVTLQAGNFIVINLSTYVSILKASLSYLSVLRVLVEA
>PbOr60FP
MNRNRDCHVYAPSALSAIGISYSHYLDSMGSGQWNDDVAYAMTPFKLISWPIGVWPLQIYNIYSLMRCILATC
CSAFTFLMSSIEFFLGCTDTETNIDCLMIVGCAILGMVKTIWFRIYAKNLTHNYSSAVNDYLTIENAKERTIM
RKYTFMGRALCSLILSFAYISTMMYALIPFLDFDEIEQVNITNEDKLEYPIPSKCALKYFNVPVSMYRTFCLI
ETVAIMVACTNNIGNDVLFLNIVLHICGQIEILKNNFHHFDVKSPQIYDRFNVLIKRHNYLIKLTRELTEMIS
FVLLVKLFIISILLCIIGLKLILALKMNXHVVTITKAIIVQTTFLTQLTLYCFTGNHL
KSQMEEIGIAVYQSTWYNLPTKLTKHLNFIIMRSYASISLQAGNFIVLNLTTYVGILKTSFSYLSVLRILVEA
>PbOr61FP
XALSVIGISYSHYLDSMASGQWSDDVAYAMTPFKLISWPIGVWPLQVYNVYSLIRCTLATCCAALVIVIPSFE
VYLGCTDPGTKIDCLMLACAGILGMVKTLSFRIYARKMANNYDSAVNDYLTIENTKERAIMRRHTFMGRFLCC
VLICFSYFSCMMYVLNPFLSNNKQVNITNEDIMLEYPMPSKCAMKYFNAPVSMYSIFCLMESIALFLTGTANQ
GNDALFLNITLHVCGQVKILKNNFLDFXMSKSPQIYDRFNVLINRHNYLIKLSRELAEIINFVLLIEFFLITI
LLCVLGFQFILALKVNNIIVMTKSLMVQITFLLQLTLYCSIGNYLISQMEEIRLAVYQ
SAWYYFPAKLTRHLIFIIMRSDSPVSLQAGNFIVVNLATCISIIKASLSYLSVLRVLVET
>PbOr62PSE
MGSEZWKNDVAYAMTPFKLITXGVWPLQVYNIYSLIXCAFTTYCAAFVIAMPSFEFYLGCTDAKRNVDCLMLV
CCGILGMVKTVWFRIYARNLVNKYGSAMTDYLTIENTEERAIMRKYTFVERFLCCSLISFPYVSCIIYVLIPF
LDNKTGNXYVNVTNENVMLEYPMPSKCAMKYFNAPVSMYRMYLLFHGVHNYLIKLTRGIAEMMSFVLLIELFI
XGFQLILALTVNNMMMITNILIIEITFFIELILFGSIGNYLISQMEEIGLAVYQNAWXDFPAKLTKHLVFIII
RSESPVTLQAGNFIVVNLTTCMNIIRVSLZYLSVI
>PbOr63
MASERWKNDVSYAMTPFKLLTWPIGVWPLQVYNIFSLSRCIFATCCVSLMVILPSIELYMGCTDAEQNVDALM
LICCGILGMLKTVWFRIYARNLTHNYDSALNDYLMIENAKQRAIMRKHASTGRILCCSMLCFSYFSCIIYALI
PLFGGDISKQINVTNEIVLEYTVPSRCALEYFNTPTSMHKIFCLIEAISLILTSTTNHGNDSMFLNITLHVCG
QMEVLKTKFIDFDVTRPQVYERFNLLIKRHSYLIRMARELADAISFVLVIQLFIISVQLCITGFQFILAIKVN
DTVMATKSIMVQGTFLIQLSLYSFIGDYLKSQMEEIGPSIYQNVWYNFPTKLMKDLIF
IIMRTECPVTLRAGNFIVVNLSTYMSILKASVSYLSVLRVMVET
>PbOr64
MASEQWKNDVAYAMTPFKLLSWPLGVWPLQVYNIYSLIRCILATSCMSIIVILPSMEFHLGCTNAEQNIDSLM
LICCGVLGVFKTICFRIYAKNLTDNYGSALNDYLTIENKQHRAIMRRHAFMGRILSCFMVCFSYVSVILYSLI
PLLDETLDDNQDKQINVTDEDTILDYPMPSRCALEYLNAPISMYRIICLMEFIILVLTCTCNHGNDSLFLNIT

LHMCGQVKILKANFSNFDVSNPQVQDRFNTLIQRHNYLIELSKKLAETISFILLTQLFISSIQLCLMGFQLIL
ALKMNNVVVMGKSCMVLCTFLTQLSVYSFVGDYLKSQMEEVAFSIYQNIWYDLPTKLT
RNIIFIIMRAQSPAKLLAGNFIVVNLATYMSILKTSISYLSVLRVMVEI
>PbOr65
MSDDIWNDDIAYVFSTHRTLMRLFKLWPLEKRTIFTIIQWSLMTILQLSPFIFVFMEFGNQSDAGANIEAMLF
FSVTVALTLKNFCITINQRKLAKNINAAITDWLSAKNDEKSYKIMKKHASKSRQLKLMILSTCYVAGGIYILF
VILLNLKQIFLQTQMINISKGDNITGLQYAMLITFQSFQLLLICLLQTAADSFYISIIFHLTGQLKVLKTKFE
IFGSKPDTPKNYRKQLANLVNRHCELMELNQNLEDTFHLLILFQLVIITLLLALLGLRILLCLKNNDYVEMAK
SIIVLNFLFVQSIMYCYGGDLVQKGSESIFYAIFTTSWFTLPVRLTKDLNFAMMKSSY
PFRLSGGKFCYVDRETIMFILKTAASYISVLRIALKDKS

>PbOr66FIX
MKTNWNNGIDYGFSMIKSMMWMLGLWPLQRNSVVYTFQWLLIFTAGSLTMINVLIESFKYCDAVRDGLEILRL
IESSMHAWLNIIFPRIYMRKIAVNVNSAIDDWSSSSMKEESRAIMMVYARAGRLIALTHLIIGTAAGTLWFVS
VFLSNKQQAAIMDNDTIAAWNFVIPSTCLYKGVSYSTYRMLFGMQIIQGSLILIAECACDSFFFSITMHLCGQ
LELLRVQFIEISKKHHEQKHGNFLRPLVKRHCQLIELAKNIEDTFNINILLRLLIISVVIATSGVGIILSIKT
QDYKEIIKMLMSIQFYMVQTFLYTYAGDTLQNRSESIVYAIYDSAWHKMSPIVVKDLI
FIMMRMKTPLRISAGKFFYLTRNTITDILKTTMTYVSFLQVTVDK
>PbOr67
MSWNDDISYAMTLVRHLTIPIGGWPLQKYNKFTLVRHVLSSFGLSVVVIVQYLELYYNCTSAYANLDALTLLA
CGILALTKITWFRIYADNLTCNYSSAINDYRAIDTEKKRIIMREHAFWGRVICIIALLISYVDSVIFIVGHAQ
LSSEEAKVNMTIFGHQAGYAIPSTCTLAHFHISTSLYLVIFALEYIYLVIMCISNHGSDSVFLHIALHVCGQL
KILKANFINFDVTSPEVHERFNTLILRHDHLIRMTRKLAEIISFVLMVQLFVSSMLICIVGFQFIIALTTSDY
GMMSKSFLVLSAFLIQLTLYSVVGDYLKSQMEEVAMSVYQSAWYDFPAKVTRNIAFIM
MWAQLPIKLQAGNFIVMDLGTYVSILKTSASYLSVLRVMVET
>PbOr68
MSWNDDIAYAMNPHKFLTLPLGNWPLQKYNLFALIRSIVCGSSLTVMMVVLFLEIVFGKADMYLKLDALMVMF
CNVLSVLKLLSFRIYAKNLIRNFSSAVNDYLAIDTEEKRIIMRRHAYIGRIVCYSILFFAYFASCIFVVVPLI
LGDNNVQVNKSNINPASELPMPLTWTLQNYKISATLYLTISLVQHVLLMLNSTCNCGNDSLFLAIVLHLCGQL
ELLKIDFTKYDVESKDLSKDFSKLATRHRYLIELAELLIDVISFVLLVQMLFSCLIICLIGFQLIIALKSHDA
TLIIKTTTVLSALLLQLFFYSYTGDYLKCQMEDIAQSIYSCNWYCLPANMIKNVRFII
MRAQQPTTLLAGKFFIVNIETYMTILKSSLSYLSVLRVMVDA
>PbOr69
MSRNKDIDYAMDPHKFIILPLGVWPLQKYNTFALIRCIVCGVSLTVMMSVLFFEIVYGDASTYDKLDALMVMF
CNLLSILKLLSFRIYAKNLIRNFSSAVDDYHAIDTEEKRIIMRRHAYMGRMVCFSIMSFSYFVPLIFVWIPLL
LDKNNDNNIKVQVNVSKNPASELPIPLTWTLGNYEISPALYLVISFTIYVLLMLTCNSNCGSDAFYVAIVLHV
CGQMELLKIDFTNYGMESNDLSKDFLKLATRHRYLMELAELLVDVISFVLLIQLLCSCLIICLMGFSLIIAMK
TNDVAMISKTISVLSALLLQLFFYSFAGDYLKSQMEDIANSIYTCNWYYLPANMMKNL
LFVIMRAQQPVMLLAGKFFLVNIETFMVILKSSLSYLSVLRVMVDA
>PbOr71FN
LSYVFALSYQCLRIFGVWPDPFVPLSDIRWPNIRFIIVMCTLFFYVIIPQTTNIILAWGNVIRMVESLSSANF
SLMALCKLGCTWYHGKTLRTLMTSIMTDWMTSKNHERNTMLNLARCGRSLSIKCYMASTCTVLFYLFLNLMKF
YRSMHEPQRNLVYHFSYPYNIQKSPNYEITYFIQLFGGIYTAMIMSTIDSFISILLLHICAQLINLRMALKNL
VDELAKESISSSEFKKGLAAIVVRHEHLIRNASTINDCYSAVLLMHMLAITLQLCFETFQVFTIITNNLNVSV
IKMCFLSFYAILVLMHLYMYCYCAERLLVESTNMAWAAYECNWYDISFQDAKDLIFII
HRSTIPLRLTAGKFGIFSIEMFGITVKTSMGYVSALLTLMD
>PbOr72NTE
LNYMFSLTGQWLWVFGIWPDPHISLSDFRRPCIRFVIVTCTISLYVFGPQMLNVIRAWGDVTRMAENFVSANL
SLLAACKLFITWYHGETLRSLMASILADWITSKGNWERATMLKIARRGRSLSFRCCVAAMGTITFYLSFHLTR
FFKNIHQPHRHLVYRFENIQNSPNYEITYIIQLSGGMYSVLANYVVDSFVSILVLHVCAQLINLRTALNNLIN
ELANKSISSSRFRKGLAGIVIRHEHLIRNAKTIDGCYSIVLFIHILVATLQMCFITFQVFTIITDNMHVPIIR
MIFLSFYISLVLTNLYAYCYAAERLILESTKMMYGVFECKWYELPAKDAKDLMFIIYR
STIPLRLTAGKFGIFSLELFGKALKTSMGYLSALLTIRD
>PbOr73NTE
LNFAIGWNRYNLNIVGIWPNPLENSKQKRLSLCRFMIASFFMLGFMCIPQSANLVFIWGNTNLMTENLATANI
PVATAFVKAFTMWRYRKVLKSLVDFFYQDWHSPKTSEERTIMLKNAKLVRRISIWCTILTQVMVTIYIILKVS
MVAKFSRNDPARPMLYLAYFPFDITPRPIFELISTCQCLSAYTAAVSYTGSDSFISMLVLHTCGQFQNLHGRL

KNLANEPGGIKTSKEFKSELKQIVKRHEHLNWFAKTIEESFNMVLLIQMLSCTIQLCFQGFQVFRILMNEEEN
GSVTFELIFLLSYIAFILVHLYIYCYIGEKLIVQSTNMGFAAYESNWFNVAGKEARDL
LFIMRRSATRPLCLTAGKFSIFSLQMFSTIVRTSLGYLSVLLTVTDRKQ
>PbOr74NTE
LNFAIGWNRFNLNLVGIWPDPLQNSKQKGLSSFRFMVASFFMLGFMCIPQSANLIFIWGNVDMMTENLATANI
PVANAFMKAFTIWRHRKEVLKPLVDFFYQDWHSSKTSEERTIMLKNAKLVRKISIWCTILTQVMVTIYIVLRI
SMIAKFPRNDTARPMLYTAYFPFDITRSPIFELICVCQILSAYSATVSYTGSDSFISMLVLHTCGQFQNLHDR
LKNLANDTGGMKTSKEFKSELKQIVKRHEHLNWFAKTIEESFNMVLLIQMLSCTIQLCFQGFQVFRILIMEEE
NGSMTFQLIFLIIFVIFILVHLYIYCYVGENLIVQSTEMGFSAYESNWFNVAGKEARN
LLFIMRRSAARPLCVTAGKFSIFSLQMFSTIVRTSLGYLSVLLTVTERK
>PbOr75NTE
AEYAYGWNRYIMTFIGIWPENRNLNQASSYTAIIAILAMLCFCCAPQSANLPFIWGDFDSVVDNLAMGNITIS
ISLLKTIMFWSNGAHLKTLVSCMAKDWSVIEDKRDRKTMSDIANFTRKLAISSVVLVQFVLIAYVVFRYLQAR
ETGHMILFRAYHPYNVTSSPFYELTLFGQFLGMIYAANSYTVVDTFVAILVLYTCGQLSNLRCKLTNLRADTT
AEFQMKLRTVVRRHEYLNRFAETIEDCFNMMLLLQMMGCSLQLCFQCLQVFMSLMDKMDEVFFSQFSFLTIYL
VYIMLQLYLYCYIGEKLFVESTKIAYAAYDCSWYNLSVHEARSLMIIMCRARTPLQIT
AGRFCSFNRQLYSEVLKRSVAYMSCICAINSVG
>PbOr76NTE
LEWAIGINRFTLEMIGLWPDEKLSSRQKFFASLRVFIIFNTIVIVSIIPAIFSLIRIWNDMIAIIDNLQITLP
MTATSIKIIIMWLRKEDLMSVVNMVVADWMKTKTLEERDTMIKQARVARTIVVFGCVMMVLAIIIIIIPPCFG
YSMRYLTNITDAPGKPLLLQTYYLRDTTESPYFEIAFVAQTAAVIMAAFSYTGIDNFLGLIVFHICAQMEILK
KRLLNLQESKNFYVGLSMNVQDHIRLIRSIDIIDNTFNLMLLALLIYFAILFCLQGFLIISIIDDGGNVSFPR
ICWLVSVLVNTFVHMCLYCVVGEILIAKCEGIYHAVYNYEWYSLKSSEARNLMLIMIR
ADKSLYITAGKIFPMTLSMFCSLIKTSAGYISVLLANR
>PbOr77NTE
LEWAIGINRISLKIIGLWPDDKLSRRQRFLADLRAIIIFITMLFVSVIPGIFALLRVWGDIMAMTDNLQIGLP
FTATVVKFIIMWFHKKDLIPIINMVTEDWIRRKTVQERNTMIKQARIARAMVMFGCTMMSLACILLIIPPCFG
YSMRYLTNITDAPGKPMLVQTYYFREIKESPYYEITIGAQATSIVMAAISYTGIDTFLSLLVFHICAQLDILK
ERLLNLDGFKDFRTELSLNIQDHLRLIRSVDIIDNTFNLMLLALLIFFATLFCLQGFLIVNIVDGVDKVSFMR
ICWLVSILINTFVHMCLYCVVGEILISKAEGIFYAVYNYTWYLLTPNEARNLLLIMIR
TEKPLYITAGKIFPMTLSMFCSLIKTSAGYVSVLLANR
>PbOr78NIC
FEWAVNLNRFTLNLLGLWPKTVRNSRQKLICNSRVFITSLGMIFFIIIPCIHSLIKIFGDMLLMLDNLQFTLA
GISSVIRIINFWWKKEAIIPIMNMFAKDWIKPKSAQGRNFMIRRAQSARTLLICSYSIMGITCIYITIPPIFG
MTIRLTSNITDPGRPMPLQTHYIYDITKSPQYEFTFISQAIYIPIAMMAYVGIDNFLSLLIFHICGQLDILQN
RLTHLDKYENYHKVCNAIYYAAYNNKWYTMESKAVRDLLFLMTRGSKPIYLTAGRVSPVTMVTFCG
>PbOr79FN
FEWEVNLNRFTLNLLGLWPEIVRNSRQKLICNSRVFIALLGMIFCIIIPSIHSLIKIFGDLLLMLDNLQFTLP
GISCFIRIVIFWWKKEAIIPIMNMFAKDWIKPKRVQERIFMIQRAQSARILLICSYSIMGITCFYITILPVFG
ITMRLTPNITDPGRPMPLQTHYIYDITKSPQYELTFISQAIYIPVAMMAYVGIDNFLSLLIFHICGQLDILHY
RLTHLDKYENYHDELKDSLIKHIRLLRAIDVIEDTYNIILLFLFIYFAISFAFYGFRLISLFSEGNDMSFSHL
VFFLSTVFNIFIHMCLYCVLGEILMDQCNAIYYAAYSTKWYTMDSEVIRDLLLLMTRG
SKPIYLTAGKMSPITMATFCGLVKTSVGYMSVLHTMRS

>PbOr80FN
FEWAVNLNRFTLNLLGLWPKTVRNSRQKLICNSRVFITSLGMIFFIIIPSIHSLIKIFGDLLLMLDNLQFTLP
GISCFIRIVIFWWKKEALIPIMNMFAKDWMMSKSTQERNLMIQRAQKARILIICSYGIMGITFFFSTILPIFG
ITMRLTPNITDPGRPMPLQTHYIYDITKSPQYELTFISQAIYIPIAMMAYAGVDNFLSLLIFHICGQLDILQN
RLIHLDKYINYHDVLKTSLAKHIDLLRAIDIIEDTYNITLLFLFIYFAILFAFYGFRLISLFDEENDMTFSHL
VFFFSTIFNIFIHMCLYCVLGEILMNQCNKIYYAAYNNKWYTMESKVVRDLLLLMIRG
SKPIYLTAGKVSPIIMTTFCSLVKTSVGYISVLHTTRS
>PbOr81NC
FEWAVQLNRFSLKLIGLWPEENVSSYEKMSSNLRLLCVIITISCVCTTPSVHLLLKVWGDITAMIDNILFTLP
LLTVSLKLLIMWWKKETLAPLMTMIVHDWIKLKSTDERDVMIYRAQNARLIIAFSYFIMILSLIILIVASAFG
YTLRHITNITDTDRPLPLQAYYMYDTSVSPQFEFTFFIQCISLLMVALSYTAIDNFLGLLIFHICGQLENLTK
RLCRMHESKDFIMALRININDHTRLIR
>PbOr82NP

YKWVITSNRXLLGIWPKSDKNPRRKLMINVRVIITLNIIIWSCAIPTFHSLXRIWDNIISMIDNLQYSLPLTI
AIMKIILMWQNKXXFVYFGILFALFGFLFIIXITQDRNLSIFRLIYILTAFINAFTHMCLHCAIGEILVIQXG
ISEATYRFKWYNLKPKRAKNLLIIMMLVNRPLYLTAGKFFSMTMSTFCNLLKTSDGYISVLLAYRN
>PbOr83NF
YEWAIRLNRISMKFLGIWPENNETKQKIIMSNIRTGIILNMSLWCLTVPTIHSLFRIWGDIMSMIDNLQYSLP
LLMSTTKLSIIWQKKKDILPLLNMIKNDWLMLKTAKERDVMIKYARIARTLTIFGYIIMLLSFILIMVPPLFG
ISIRYLTNKTDPDKLLPMQTYYIYDQNKTPFYETTYILQTFAITTMGIAYSASDCFLGSLVFHICGQLENLKV
RIIELDKFDNFEKALICNVQNHIRLIRSVAIIENTFTLMLLVLFLYFGTMVSTYGFLVVIVIFEEGNVSIKRA
TFLIMVICNAFTHMCIYCAVGELLVIKYEEIYNAVYTCKWYAVKTMEAKNLILLLVRT
SKPLFITAGKILPMTMSTFCSILKTSSGYISVLLANRN
>PbOr84NTE
FEWAVKLNRITLDFIGLWPNTVQSSQEKLMCNFRVFIVFLTITLGILIPCIHSFIRIYGDIMLMIDNLQFTLP
AVSCIIKIIIFWWRKEVIIPIINMIAEDWIISKNMEERNVMIRRAQSARLIIICAYCLDAVGCFFLIIILPRFG
ISIRLTSNITDPGRLMPLQTHYIYDITKRLQYEVTFISQSIYIFLAVISYTGIDNFLGLLVFHISGQLDILKN
RLTRLDKYINSHDILKNCIVRHIRLFRTIAVIEDTYNIILLSLFIYFAILFAFYGFRIINLFDEENNLSLTRL
MFCISTIFNLFGQMCLYCALGEFLMAQYNNVYYAAYNNKWYAMNKKTVQNLLFLQIRG
NKPVYLTAGKMFPMTLTTFCGLIKTSLGYISVLHTMKRY
>PbOr85NTE
FEWAVKLNRFTLDFLGLWPKTAQNSRQKLMCNFRVLTVLLGLIFGLLIPSLHSLTRIFGDVLLMLDNLQFTLP
TISCTIRIMILWWKKTAIIPVMNMIAKDWMKSKSAHERSLMIRRAQSARIIIICSYCIMGTACFFVVVLPAFG
MSMRLTPNITDPGKPMPLQTYYIYDITKRPQYELTFISQAIYVSIAMMSYTSIDNFLSLLIFHMCGQLDILKN
RLIRLDKYMNYHNMLKYLVTKHIRLLRAIDIIEDTYNMILLSLFIYFAILFAFYGFRMISLFDEESDTSLVHI
IYFASTVFNIFVHMCLYCVLGEMLVAQCNKIYHAAYSNQWYIMDLKFTQDLLFLMTRG
SKPIYLTVGKVFPVTMTTFCGLIKTSVGYISVLHTMRS
>PbOr86NC
FEWAVKLNRIILDVIGLWPKAAQSTREKLMCNFRVLIVFLAVTCGVLIPSLHSLIRIYGDLMLMIDNLQFTLP
AISCSIRIVIFWWKKEAIIPVMNMIVEDWVKSKSAQDRNVMIRKAQNARIIITCAYCIMGIACFFIIILPGFG
ISMRLTPNITDPGRPMPLQTYYIYDITERPQYELTFISQTIYILLAIMSYTGIDNFLGLLIFHICGQLDILKN
RLICLDKYINFHDMLKSCITKHIRLLRAIVIIEDTYNITLLALFVYFAILFAFYGFRIINLFDEGNDLSITHL
IYFVSNVFNIFAHMCLYCALGEILVAQ
>PbOr87NC
FVWAVELNRFGLELIGLWPNTNKAVKNTFISDLRMGMIFFVVTFLSVVPLLCSLVQVWGDMILIVDNLQVTLP
LTVVSLKLVVMRWKRTAVSHIVKMMAEDWMGLKIDAERNVMLRQARAARLIVTCGYFLMIFVFIVLIILPSFG
LHFRYITNHTNHERLLPFQAYYFCDVDKSPQFELALVVQAMTMFFGAITYTSVDAFLGLAILHICGQLENFKY
RLINLESSENFTPALRNYVQTHLRLIR
>PbOr88NC
FTWAVKLNRLGLELVGLWPKNDQVIKNKFASDLRMAIIFIIVTFVSGIPLTCSLIRVWGDMMLVVDNLQITLP
LLVVSLKLVIMRWKQTALVSIIKMMAEDWMELKTTKERDVMMRQAQIARTIVISGYILMVLAFIVVIVLPYFG
LSLRHLTNLTDPGKPLPLQTYYFYNIDKSPQFELTYIVQAITIFLAAVTYTSVDAFLGLAILHFSGQLENFKG
RISGLISCQNFICAL
>PbOr89FNC
YDWAIGLSRTSLKFFGVWPENNETKREKYISNIYIFLIINIAFCGCLIPSVHSLIKIWGDLMSMIDNVQYTLA
FVIVIIKLLVLWLKKRDILPLLNMIKNDWLKPKTANERDVMIKQARAARMLTLLGFFIMLLTFILGVILPVFG
ISTRYMTNKTDPGKLVPMQTYYIYDRDKSPLYEITFIVQSIGLSTVALVYTSTDSFLGLLIFHICGQLENLKA
CIIHLGKFSNFESGLSHIVQDHVRLVRSMNTINNTFMLMLLGTVFFFIVLFAFNGFLLSV
>PbOr90NC
FEWATKLNRLTLNIVGIWPNIYKNTSDKFLSDLRATFSLLVIIFVGIIPAMHSLVRTWGDMIAIIDNLQFSLP
LMITIMKLVIVRWKKPDLILALNMIADDWLKIKTDKELHIMIKHAQNARAIIIFGYVLMILAVSLLVFLPCFG
TSLRYMTNITDPIKILPLQTYYFYDKNQSPYYELTFAAQVVLLIMASISYTGIDNLLGLLVFHLCGQMENLKE
QLINIKRFETFNNGLAFIVKDHIRLIKYFKIVENTFTLLLLGLLLYFGILFCLYGFLTIAILTEGREMSIMRL
IYLISAALNVCGHMCLYCIVGEILVTQCEGIYRAVYDYEWYTLEPEEARTLIIIMIRA
NKPLYITAGKMFPMTLSMFCN
>PbOr91NCP
FEWATHLNRLILNYIGIWPSMHKNTYDKFLSNLRAMFTFIVLLFIGIIPAIHSLMRTWGDMMLIIDNLQYTLP
MIMAMLKLVIVWWRKRDLAVIINMIAEDWIKSKTDKEZHIMIKHAQNARILTMIGYGFMILSIALIIIILPLFG
KSVRYLTNITDPIKILPLQTYYIYDKDKSPYFEFTFVAQIFLILMCFASYSGVDTLFGLLVFHLCGQMENLKE
KLINIEQFKNYNKGLAFIVKDHIRLIKCFDIIENTFTLLLLGLLIYFGILFCLYGFLILAILTEGKKMSLMRF
LYLVSVAMNICCHMCLFCAVGEILIAQCENIYHATYEHKWYKLEPKEMKNLLLIMIRT

NKPFHITAGKIFPMTMSMFCN
>PbOr92NTE
FEWAVKLNRYSLEFIGLWPKMEETAREKLTANIRVFLLIIMVGFVCVIPCIHSLIRVWGDLMSMTDNLQFTLP
LVSMIMKLIIMWSKKAALASILYMITKDWLKLKSDEERKIMIRCARIPRMIIICGFVIMFASFILLFILPCFG
ITMRYITNVTDPGKPLPLQTYYFYDTDISPYFELTFIAQGITLMVSAMGYTAIDSLFGLLIFHVCGQLENLKS
RLTYPEKNSNFNYVLADVIMDHIRLIRCVKIIEGTFTFMLLGLFLYFGTLFSLYGFLLVTIITDGRHLSFVRL
SFLVTVIVNIFAHMCLYCVVGECLIIQCEGIYQAACEYCWYELKPKQAKNLILLMIHS
NNPLYITVGKIFPLTMNTFCNLLKTSGGYISVLLARHD
>PbOr93NTE
FVWAVELNRFGLELVGLWPKMNEAVENDFTSDLRIGIIFIIVTFVSGIPLICSLIRVRHDMILVIDNLQITLP
LMVVSMKLIMMRWKRTAILPIIKMMAEDWMALKPDIERNVMLKRGRTARLIIICGYMLMISAFTMIIIFPCFG
VPFRRLTNLTDRNKPLPLQTYYFYDTDKSPQFEFTLIIQAMTIFLAAITYTSVDAFLGLSILHICGQLENFRR
RLVNLVSCKNFDNALCENVIAHLRLIRFANRIEDTFSLMMLGLVFYFGIVFCLYGFLLLTVITDNETNDVTFS
RIFYVISGVITLLVHTFLYCGAGELITGQCEAIYRTLNDLEWYKLDSKNARCLILLII
RTSEPFRITAGKIIPLTMTTFCSLLKTSAGYISFLLAKRN
>PbOr94FIN
FVWAVELHRKGLEIIGLWPKIDKSVKEGLWPKIRVCIILSLLIFVCNIPMLYAVVQTWGNMILVIDNLRTTLP
MLIASVKYVIIRYKQAVLSSMISMMAEDWIKFKLDTERNVMIERARTSRLIMIIGYIFTIIGLITMTIFPFFG
VQVGYVMNITGRSKMLPIKTYHFYDADKSPQFELTFFFQAVTCFFAAGSYMFIDILLLLMIFHICGQLENFRH
QLIRLILCKNFNRTLNNIITTHSRLIRFADNIENTYSLMSLILVLHFGIEFCLSGFLLITCEAVYHAVCDLEW
YNLNSKKARNIILLMIRTSHPFHITAGKIVPLTMASFCSILKTSSGYITYLLAKYG
>PbOr95NJ
FIWAVELNRFGLRLIGLWPNINKTIKNTFISDLRVSIIFFIITFLSIIPLLCSLIQVWGDMILIVDNLQVTLP
LAIISLKLVIMRWKRTAVSVIVKMMAEDWMELKIDAERNVMLRQARTARLIVTCGYILMIFMFSFLIIILPSFG
LHFRYIANFTNQERLLPFQAYYFCDINRSPQFELALMAQTVTIFVGTIIYTSVDAFFGLAVLHVCGQLENFKY
RLINLASSRDFAHALRNNIRTHLRLIRFADNIKDIFTFMMLGLVFYFGIVFCLYGFVLVTVITGDDINHMSVP
RVCYMIIGVFTLLSYTFLYCGAGEILAGHCEAVYRALCNLKWYKLEPQKGKNIILLLI
RANEPFRITAGKIFSLRMTTFCSLLKTSAGYISFLLAMRD
>PbOr97NC
YEWAIGLNRISLKFFGIWPENNETKREKFMSNLRIIFVLKIAICGCLIPTIHSLFKIWGDLMSMIDNLQYTLP
FSMAIIKLFIMWLNKKDILPLLNMIKNDWLKSKTANERDVMIKQARIARIFTILGFFIMLLSLILAVILPAFG
ISMRYLTNKTDPGKLLPIQTYYIYDRDKSPLYEITYIVQSIGLSTLAMVYTSTDSFLGLLVFHVCGQLENLKK
RIICLNKFHFESALSCSVQDHIRLIRSINMIDNIFTLMLLSALLYFGILFAFYGFLLGT
>PbOr98NFI
LEWAIGLNRFMLRIVGLWPQESKNQREELLSKLRLLLHVIMLIFVLTIPALASLIRVWGDMLLMIDNLQFTLP
LLITTLKVFIMWYNKEALSPLIKMIVKDWIKKKMKEERSVMLKQAKITRLLATCGLFMIFLTVLVSLGSMLSG
RTLRYVTNFTDPGKPLSIQAYYLHDVSSSPKYELTYLAQIISQIISGLSYTAVDNFLSLLILHICGQMENLHL
RLLNLKNDANFKTVLKFIVNDHIRLIRSIKIIDNIFDLMLLGLLFLFGILFCLHGFLIINVVSRGGHLSFMQL
IWYISATVCVLMHMCLYCVVGEFLVSQLLKTSAGYVSVLLARQD
>PbOr99NCF
IEWAIGLNRFMLKIVGLWPQESKNQHEELLSKLRLLFSIIMLLFILIPALVALIRVWGDMILMIDNLQYTLPL
LIVTLKIFIMWYKKKALSPLIKMIVKDWTKVKIKEERNVMIKQARITRLLAMCGLSIVFLTFLIAVGSIPFGF
SLREVTNLTDPGKPLALQSYYFYDISSSPKYELTYLGQVISLIISGLFYTGVDNFLGLLILHICGQMENLHLR
LLNLKNDENFKTVLKFTVNDHIRLIRSIKVIDNTFNLILLGLLFIFGILFCLHGFLIISVIRRGQLSFVQLFW
YLSATLCVLLHTCLYCVVGEFLVSQ
>PbOr100NFI
VEWAIGLNRFMLKIVGLWPQGSKNQREELSSKFRLFFNVITLIFILTIPALVSLIRVWGDMILVIDNLQYTLP
LLITTLKVFIMWYNKEALSPLIKMIVKDWTKIKMKEEKSVMLKQAKITRFLAMCGLFMILLTLLITLSSMFSG
RTLRYVTNLTDPGRPLAIQVYYLHDTSNSPKYELTYLVQMVALTTSGLSYTAVDNFLGLLILHICGQMENLHL
KLLNLKDPNFKTVLRFIVNDHIRLIRSDKIHRATYEYVWYTLEPKAARNLILIMLRAKKPLNITAGKTFPMTM
ATFCNLLKTSADYISVLLARRAIARDQ
>PbOr101NTE
LEWAIGLNRSMLKIIGLWPQENKNQREELLSKFRLSINLITLIFVLTIPALVSLIRIWGDMILMIDNLQYTLP
CLITTLKIFIMWYNKEALAPLIKMIVKDWTKMKMKEERGVMIKQARITRLLTICGLFLILLTILVTLGSMLSG
LTFRYVTNFTDPGKPLAIQAYYLHDVSSSPKYELTYLVQLISLTMCGLTFTAVDNFLGLLVLHICGQMENLHL
RLLNLGKSANFKAALKSNVKDHIRLIRAIKIIDNVFDLMLLGLLLFFGIFFCLHGFLIINVINGTGQLSYIQL
IWYFSAHICLLLHMCLYCVIGEFLVTQSEGIHRATYEYVWYTLEPGAARNLMLIMLHA
KKPLNITAGKTFPMTMATFCNLLKTSAGYVSVLLTRRDQ

>PbOr103NPJ
YEWAIGLNRISLKFFGVWPKNSKTKWEKFMSNIRTILILNIAIWICAIPTIHSLFKIWGDIMSMIDNLQYTLS
LLMATIKLFIIZLKKEDISPLLNMIKNDWLKPKTARKRERCYDKTSPHGTYAHNIRIFIMLLSIILASFLPIF
GISTRYLTNKTDPGKLLPLQTYYIYDREKSPLYNLYHVXSIGMAGFAAAYTSTDNFLGLLVFHMCGQLEDLKT
RIIHLDKFQNFESALSHNVQDHIRQVRSINMIDDIFTLMLLGALFYYGVLFAFYGFLLATIMTQDYDVSLLRI
MFIMIIFLNTLAHTCLYCAIGEILIAQCEGVYEAACEYKWYTLEPKKARNLILIIIRA
NKALYLTAGKLFPLTMSTFCTIIKTSGGYVSVLLARQLEKNYFRSIYTFHIHIL
>PbOr104
MIPTSTIDRTMEFTLRLIGVWPDSSCRFLPRVVWTMTMIISQFLQYWYLF
THIGSDTLPDLLDSLSLCLSNSLLFLKLSLLWFNGRIIYNLFATMAEDWN
ECASTRLKMQSMINKAILSRRFSRFSIGAYSCSIFLFGVGNMVIQKSTDS
EQLVEERQLIVKMELPPECSISPLYEIVSVTQFLVQSTLALVAGMLNAFI
VTLILHVAGQIDIICHELLEIPIAEDKCDMRIVALRSIVNRHQRIIAFAD
SIENVFCYMSLMQFFSNMFVICFLGFIIVTSLNSPDVDLLLMRIMPYYIV
MNLEAFVLCYSGEYLNSKSKSINEAAYNSFWYELKSTESKIIFFLIMKSQ
KELTMTIGKFMNLSLEGFTSILQASASYVSVLHAMY

>PbOr105FIX
MGLTTTVSPTVEFGLRAIGIWSGSYSILYRTLWTISLGLAQILQFKYIAT
CVETANFPDLVDSVSTTLPYSLLCLKLIMLWLNQRLFNDILSSMSRDWCN
CGTVAFNVCTMTSKANLSHRCSMLIIGVYSIAVVVYVSVILEFNSINDEF
SKGDQQLFLKMEFPFVYEFSPVYEIVMFVQFVQLLSNASVIGMLDAFIVT
LILHVSGQIDIVCRGLFELFSKDCECKSYNDAKIAIIRRHQDIITFSNNI
QTLFSYIALMQFLTNTLVICCIAFTIVTSIGNNKGYAMLLKSLFFYIAIT
LEAFIFCFAGEYLSNKSKSVANAAYEALWYNAKPSESRFLLILILRSQKR
LTLTIGKFNDLSLEVFANILKASASYVSVLLAMS

>PbOr106
MEENASKLHRRMIPTSTVSRPVKIGLRLTGIWPNSPIFFRLLWTSVMGTG
LIFQYHYLLMHFSTKELPNLIDGLSTTLPYSLLFFKLIVLWVNNRIFNDI
LTAMSNDWREYSSMYAMIDKAVLAHRCSKLIIGVYSTAVMLYSTASVNFR
KQTDDDCPELLIKMELPFVFCESPIYGIVVSVQFVHLMAVASTIGMLDAL
MVTLMLHIGGQIDLMQQQVGEISPKKYDEYLSTNIVRSLINKHQKIIDFS
ENIESLFSHIALMQLFSNTMIICCIGFLIVTSLGTDEGIRMLVKTVFFYI
AITLEAFIFCFAGEYLSNKSKMIGDAVYESVWYTLKPRDCRILLFVIVRS
QRRLTITAGKFMDLSLEGFTNILKASASYVSVLYAMY

>PbOr107
MTQQPTSTIAFSVQIGLRIVGIWPNAPYALLFRGIWILTISIVQIFQYWW
IVLHFGDDMSHLLDGLSVTTEYTVMSLKLIIILWLNSRIFYDALAAMAADW
KETTIGEMHTMTTMMSKANLSRRFSNVIIGLHSIAACSYGIGVLVSHTDN
ADGIETPVREFTLKLQLPFECNESPFYELVMCLEFLHQLASSATTGVLNS
LIITLILHASGQIEILCDALRDVSPEKNNRRLAVSIMKELIGKHQKIISF
SDKIERIFCYIALIQFLSSTLVTCCLGFMIVTSIGTAQGSDMIDSSALMK
AIVFYMAATVEAFIFCFCGEYLSAKSKMIGDAAYRSIWYDLEPHESKLVL
LIILRSQRRLTITAGKIMDLSLEGFTSIIKASVSYVSILHAMY

>PbOr108
MLELGKNTTRIGRASTISESVEAGLRFIGIWPHCVYANINWWTYIVSVAI
VQYFQYSYIMVHFDINDLSVLVDGLSITLGYSLSFLKLINLWFNRRKLYV
ILDTMDKDWSDGIAVHSDISTMIRHANLSRQCSNVMITTNALAVFFYTIG
GPILRSMIQKNEDTTRELPIKMEFPFNVDNSPIFELILILQFFHDLSVAC
IIAMLNALLITLVLHVSGQIDIMRQNFLKISSKKPTSESSLATIKLLINR
HQRIIDLSDNIEDLFSNIALLQFIWNTLVICCIGFVIVISIGTEEGATMI
TKSLIFYIAITLEAFVFCYAGEYLSAKSKSISDAAYECFWYDLTPSECRV
LMFLMLRSQKRLTITAGKMTDLSLESFTTIMKSSASYISVLRALY

>PbOr109

MKRTSTISRPVEIGLRFIGMWPDSAYATLYWLIYMTTMVIVQYYQYAYVL
THFDLSDISLLMDCLGLTLAYTLAFLKLFALWWNRRTFYYIVKAMDQDWR
ECVLNDSYESTMTGVADLSRRCSNVMISINALAAFFLSIGEHLLQSMGDA
SGVDIINNSRELPIKMEFPFDVSESPIFECFLVGQFLYELLLASIVGMIN
ALLVSLILHVSGQIDIMRQDINEISNRKYNSSASLIIIKGLICKHQKIIS
LSENIENLYTYIALMQLLWNTLVICCTGFVIVITIGTDESATTSIKSVSF
YIAITLEVFILCFAGEFLSAKSKSISDAIYESLWYDMPPTNSRILLFVIL
RSQKRLTITAGKVIDLTLEGFTSIMKASASYVSVLNAMY

>PbOr110
MTRASTISRSIEIGLRFIGMWPDSAYPNLYWFSYMTSVAIVQYYQYAYVF
IHFELDDLGLLMDSLSLTLAYSLAFLKLLVLWRNRRIFYYIVKIIDQDWS
ECVINDSYKSTMTGMADLSRRFANTVFSIYAFSSFFLSIGEHLLQSTNDI
NQFGNSSRELPIKMEFPFDVSKSPIFECFLIGQFFYDMVIAFVVCLINAL
LVALILHVSGQIDIMRQDLDEISNGKYDRSTFLVIIKGLICRHQKIITLS
ENIENLYTHIALMQVLWNTLVMCCTGFVIIITIDAGDRASLIKSVSYYIA
IVLEAFIYCFAGEFLSAKSKSIGDAIYASLWYNMSSSDSRIILFMILRCQ
KRLTITAGRVMDLTLEGFTSIMKASVSYISVLNAMS

>PbOr112
MKHASTINRGVEIGLRFIGMWPDSAYPTLYWSTYMTVIAVLQYFQYSYVI
AHFDISNLQILTDCLGLALATTLAFFKLFVLMWNRKIFYNILAAMDRDWN
ECVISGYGSTMVIAASQSRRSSVVLIGIHALAGFFFSVGAYVIRTVNNIH
TDEPGTIPREFPVKMDVPPEVLETPLFECILVGQFLYELSLASVVGMIDA
LFAALIIHVGGQIDIMRQVVMEISNSNDLDRSLSVIKDLIRRHRKIITLA
NDIEDLFSSIALMQLLWNTLIICCSGFMIILALNMKKGTAILIKSTLLYA
CKTLEVFVFCYAGEFLSSKSKSISDAIYESLWYKLVPSNSRALLFIMLRS
QKRLTITAGKIVDLTLEGFMSVMKASVSYMSVLHAMY

>PbOr113PSE
XYIGIWPDASHGTLRWVFYMATLFVMQYFQYSYVYAHLDFNNLTKLMDGL
GLVLDYTLTMLKLLSFWINRRXLAAMEDDWKDCVTDFRKYVMMDKANLTS
NVVLTFWYPSMLFLLFYILLTATYVDVCLPYGQFQEFPVQIQFSPEVYXL
IYELVGMGVFFHVLETAIVIALLNALILTXVLHVSGQIEIMCQELKDIPF
MSKSSLLFTKSLVARHQKIISLSKNIENFFSFVALLQFIWNTFIICALEF
MAVISLDMNMESKSGILIQFIMLYLAVTMEAFIFCFAGEYLSTKSKSIGD
AVYEMVWYNLSTSECRILLFVILRSQKQLTTAGKVMDLTLESFTTIMKAS
ASYISVLHAIY

>PbOr114
MSTSTISPSLKIGLALIGMWPGSSYGTFFWLFYMGSLIAMQYFQYSYFFA
HLGTNDFSKLMDGLSITLDYTLTFFKLLSLWHNRRIFSDILGTMNDDWNN
CPTDLDTCVMMGKASLAHRCSNVMIILNSLSVIFYFVGNYVSHRSANGDL
REFPVQIQFPFNATNSPIFELIVLGLFLHVWETATVIALLNSLILTLVLH
VSGQIDIMCQGLKEISTKQKSHLSRSLIEKHQRIISLSNNIDNFFSFVAL
IQFIWNTVVICSIGIMIVISLGTDMEGKSGILIQSIIPYIAVTLEAFVFC
FAGEYLSTKSKSIGDAAYETLWYDLSTNECRILLLIIVRSQKRLTITAGK
VMDLTLEGFTTVMKASASYVSVLHAMY

>PbOr115
MLITSSISPAMKMCLQFFGMWPDVSYSIIYWLSFMLSMLVMQCFQYMYVF
EHIKISELLNLVDGLTVAMDYSLTIFKLTSLWINRRVLHQMLAAMDNDWR
ECINTEQHLYIMTIKANISHFICNFILSLNAIVTIFYFLGSYAFRFMFLT
EEYNDTLRQFPVKIQLPFDTQSPIFELVAITTILHVMIHSCAISVLNGLI
FTVVLHVSGQIDIICQEMKNISKNILYGSSKFSFGMLIERHNRVISFSQN
IEKLFSFIALMQVIWNTLVICSLGLVIIISIHNEIDVFVLVKTIFGYCAI
MMEAFVICFAGEYLSMKSKSIATAIYEIVWYNMSSNQSKIIIFIIMRSQK
SLTITAGKMMDMSFDAFASIVKASASYISILNAMY

>PbOr116
MILTSTVSPSLKIGLQFLGMWPNVAHSTVYWLSVMLSILIMQCFQYLYIF
KHLKVSELLNLVDSLTVAMDYSLTMFKLICLWINRRVFHQILTAMDDDWH
ECINNDQHLYIMTVKANISHFCFNALLSFNAIATILYFLGDYIVRFMFLN
EEYNDTLRQLPVKIQLPFDIQQSPIFEFLALTTISHVMLHASIVAILNGL
IFTLVLHVSGQIDIICQELKNISENILYKSSMFSLGMLIERHNKVILFSE
NMEKFFSFFALMQVVWNTLVISSIGFVFIIFMHSETGIFILIKTIFAYIG
IMIEAFIICFAGEYLSLKGNLIASATYEILWYDMPPNQSKIIMFVIMRSQ
KQLRITAGKMMDMSIETFASIIKASASYLSVLNAIY

>PbOr117FI
MIIASTISPVLKMGLQCLGMWPGVPYSTAYILHQILAIIDNDWRECINTD
LHLNIMMNKANVSHFICNIMLSLNVIAAVFYIFGDYIISSLFLTENYNNT
LRQFPIKVQFPFDVQQSPTFEYIFVIIFVHIMINASIMALINGLMFTLVL
HASGQIDILCQELKNISKNILVHKSSMSVLHVLIKRHKRIISFSYNIEKL
FSSISLMQIVVDTLVICCLGFLIIISIHNETGTSMIMKIILSYIALTTEV
FVICFAGEYLSLKGDSIISSTYDTLWYDMPSTQARIILFVIMRSQKRLTI
TAGKMIDMSFETFTNIMKASVSYISVLNAMY

>PbOr118
MIIESTVSPVLKIGLQCLGIWPGVPYSTVYWLSFLLTLLIAQYFQYSYAF
RHLKMSEFSNLFENLIASLEYTMTIFKLSTLWIHRQVLHQILTIIDNDWR
ECINTDLYLNIMTDKANVSHFICHTALGINVITAAFYLLGDYVISSLFQM
ESYNNTLRQLPIKLHFPFDVQQSPTFECIFVIICLHIMLHAITLALINGL
IFTLVFHASGQIDIICQEFKNISQNVLVHKSSVPLFSILIERHEKVILFS
DNIEKLFSFIALIQVVWNTLVICCVGFFIIISIHNETGTIIVAKTILVYI
AVTVEIFAMCFIGEYLSFKSESITSSTYDALWYDMPTTQARIILFILMRS
QRQITITAGKITDMSFETFTNIMKATVSYISVLNAMH

>PbOr119PSE
MIVESTVSPVLKIGLQCLGIWPGIPYSTVLZAZFYIDLADCSVFPVFHHL
KISKLSNLVDNLTAILEYTMTIFKLSNLWIHRQVLHXLTIIDNDWRECIN
TDLYLNIMTDKANVSXFICHTALGINVITAAFYLLSDYVISSLFLMENYN
NTLRQLPVKLQFLFDVQQSTFECIFXNIHIMLNAISIAIINGCXTLVLHV
SGQIDIICQEFKNISKNILVHKSAVXXSKDIIFIVMRSQKZLTITTRKMM
DMSFESFTNIMKASASYILNAMY

>PbOr120
MTVTRMTTVSSSVEMGLRFIGMWPGSSYGNFQWFVYMTSVAVVMYFQYMY
IIKNFDMSNFSVLIDALSITIAYSLAFFKLLSLWLNRRVFHEILVTMNED
WREYITASSRMYPMIIYANLSRRYSNILMSINASAAVFYALGGLVRRSTK
NEDDPRGTRFDLPVKMELPFEVNESPIFEITAIVQFLHELSLSSLVAMIN
SLVVTLILHVSGQIDIIRQGLTQVSSKSYQSSSFLPEIKVLILKHQRIIS
LSDNIEDLFSWIALMQFLSNTLVICCLGCMIIITIGSNQGAIILTKSILF
YVAITLEAFVFCFAGEYLSAKSKSIADAVYESVWYNMTPSQCRTLLLVIV
RSQKRLTITAGKVIDLSLEGFTSVMKASASYISVLHAMY

>PbOr121
MRATSISTSVEIALRCVGLWPDLPYGTVTWYTYMTSLVFALYFQYAYIFD
HFEISNMSNLIDALSITLSYSLGFLKLLSLWSNRRIFYDILLAIKKDWSD
VNICDKSVSSYIMTSNAEMSRLCSNMLISINATAAICYSMANLLRSTDFK
EDLNISLRVLPVKMEFPFEVDASPLFEMLAVGQILHVVSIASIVGMINCL
VISLVLHVSGQIDILCQELLTICGDGTLQDNLIVASVRFLIIKHQKIITL
SENIEELYSDIALMQILSNTVVICCIGFTIIGSLTKEGATVMMLKSAIFY
VAITLEAFIFCFAGEYLSAKSKSIGDAAYEVLWYDMTPAECRILLFVILR
SQKRLTITAGNVMDLSLEGFTTVMKASASYVSVLHAMY

>PbOr122FIX
MDPKIPKMNSMIPDGTVSRSIEIGLRVIGVWPDSSYSVLRRAFWMITLTM
AQTFQYRYFVIHVRTDDLSHLMDGLSTTMSYSLLLLKLTIFWINRRIFHD
ILATMARDRSECVTEWAVYSMSKTTDVSHRSSNLIIGLYSMSVFLYGTGV
LVAHAEEPDEAEEQITVPARELFLKMELPFESNASPAYELVMITQFFHQL
AAATIVGVLNALIVSLILHVGGQIDIMCRGLVEISSGDDTFDLRTSTIKA
LICRHQRIIALSADIETLFSYIALMQFLWNTLVICCLGFLIVSSLTKEGA
TVMMLKSAIFYVAITLEAFIFCFAGEYLSAKSKSIGDAVYGTLWYDMTPA
ECRILLFVILRSQKRLTITAGNVMDLSLEGFTSVMKASASYMSVLHAMS

>PbOr123FIX
MENEMKHTVCRSVEYGLRLIGVWPGTSFAILRKFLSILSVIVFQIFQYQH
VIVHFGEEDLTVLMDALSVTVAYTILFIKMIIFSFNARLLNEIIASMSKD
WKECDISNEYTMTRIAYVSRWVSNVIIFSHMMSVFLYATGTLLKIKSKNQ
TNTRELIIKMEIPFEIETTPQHVIILVTQFIHQTTAAGMVGVLDSLLIIL
VLHICGQIDLMRQKLSEVTRKNIERKNVDQDVNESIVKALIIRHQQIITF
SKNIENLYSNIALIQFFSNTLVICCLGFLIVISIGVPGGSMMLIKSVFFY
IVMSLEAFIYCFVGEHLSTKSEMIGDSAYESLWYESSPSQNRDIHIMIIR
SQKHLTLTIGKVAELSLRQFANIIKASASYVSVLHAMY

>PbOr125
MNKETKSINTVCRPVEFGLRVIGVWPNTSYAILRRVFCISSVAVFQIFQY
RHIILHFGEEDLLLLMDVLSATLAYSLLLIKLIIFAFNTRVLSKIIANII
KDWKECDVCDECTMTRMAYISRRFSNFIIALYAISVFLYATGTLMKYKSN
NQTDTRELILKMELPFEIKSTSVHIVVLITQFVHQTSAASMVGVINSLLL
TSVLHLCGQIDIVRQKLSEIRRKDIERDMNRNIVKMLIVRHQNIISFSKN
IEALFSNIALLQFVSNTLVICCLGFVIVISIGRPGGSSMLIKSVFFYIVM
SMEAFIFCFLGEYLSTKSQKIGDAVYESLWYELNPNQNQDILIMIVRSQK
HLKLTIGKVADLSLKQFASIVKASASYVSVLHAMY

>PbOr126FIX
MTTISRLVKFGLHIYGIWPYVPSTVIFRLYWIIMLSMAQTFQYRYVVVNI
HMDDFSQYMDGVSSAMASSLFYIKLVILWTHQRIFFDVLQMISVDWRDYN
TLNHYSSRIMTDSANLARRASRWIVGLQIVSVFLYSAGVLTANANSPERS
EPYARELILKMELPFNISANFIYTAVQTVQFYHLFLVAYGISIINSLLVI
LIIHICGQIDILREWLANIFSKKMAGSMDEITMRSLIIKHQRIIIFAENI
ENLYTYIALMMLLSDTLIICCLGFIIVTSLDTPNAAAILVKSVLFYITIN
LEAFIYCFSGEYLSAKSKMIGNAAYDSLWYNFPAKQSRIVLFLIVRSQKR
LTITSGKIVDLSLEQFTSVIKASLSYISVLLAMY

>PbOr127
MTTLNRLVRIGLHVYGIWPYVPSTVIFRLYWIMMLSMAQVFQYRYVVVNI
HMDDFSQYMDGVSSAMASSLLYIKLVILWSHQRIFFDLLQMMSADWQDYI
LNRYNSRVMADTANLARRASRWIVGMQVASVSLYSVGVLASNTNNPERSE
PYARELILKMELPFNISTNSIYTAVQSVQFYHLFLVGGGISIINSLLVIL
ILHVCGQIDILREWLTKAFSKDTIGATDEITIRSMIIKHQKIIAFAENIE
NMYTYIALMMLLSDTIIICCLGFIIVTSLDAPAVIVKSLLFYITMNLEAF
IYCFFGEYLSAKSKMIGNAAYDSLWYDFPAKESRIVLFLIIRSQKRLTIT
SGKMMDLSLYRFTSVIKASLSYISVLLAMT

>PbOr128
MEMSTVSRVVKIGLCVCGIWPYLPSTILYRFFWIVMLSTVQFFQYHYVAV
HYYTDSFSNFMDGVSSAMTYSLLFIKLGILWVNQRTFSDILQMMVADWKN
CILTDCSLHITTNKARLSHRFTNWIIGFQLTAIVLYSCGVLAVNVGDIQR
MNVSAREHILKLKLPFQIDTSRTYVLVTIFEFLHLVMCGCGISMINSLIV
TLILHIGGQIDILRDWLLNIFSKNMIKNDMDGITMKTLITKHQRIITFSE
NIESLYTYIALILFVSDTLIICCLGFIIVTSIGTPEGPAILTRSVLFYLV
MNLEAFIYCFAGEYLTAKSKMIGDAAYDSLWYDVTSEKSRLMLLVILRSQ

KRLTITVGKIMDLSLERFTSVVKASASYISVLLASTEY

>PbOr129JF
MKVKNTISYTIEIWLRIFGIWPNVSCVLLRRLFWFVALLIEQVFQYRYIV
KQFHLIDFSEIMNILSATIAYLILLIKLVIFWCKQRTFNRILTMMAIDWE
KCSTTEFSMFVTTYNAKLSHRFANTIVIFYSTAVVLFSSKIFLRLDDGTA
SNVSTQMLILDMELPFDTNQRFIYELVLIVQFLHLILCSDVIGLLNALLI
NLVLHIGGQIDILCKGLITIFAEKRSLNHFTVKKIIKKHQKIIIFSKQIE
DLYSYIAIVLFVLNTLIICCLGFVIVASIGRPEASKSIIRTLLFYLAMNM
EPFAFCFAGEYLSAKSNTIGDAAYDSLWYESNPKDSRIILFMIMRSQNQL
TITIGKVMNLSLERFSSVVKASASYISVLLAMY

>PbOr130
MSATSTICFSIKFVLRAFGVWPDVSFTALRRLFWSATLIIVQICQYAYIV
LHYRTDELMDLMDNLSSSLAYSLLLFKLIIFWTHQRKFRSILTMIAADWK
ECAGDSFSMHTTTNMANLSYRVSNAIIGLHMAAVVTYSLGVILSNTAGDN
FNISTVPVRALILRMEFPFDSNSSPVHELVMIAQFFHLVTQACAIDVLNA
LIMTLVFHIGGQIDILRERLMNVFSKKSMHTLTKITMSNLIGKHQKIILY
IENVENLYCYIALMQFVSNTLIICSIGFVIVISINSPDLSTILVKTLLFY
IVMNLEAFSFCFAGEYLSTKSQHIADAAYESLWYDVHVNESRTISFLILR
SQKRLTITIGKVMDLSLECFTSIVKASASYISVLLAVS

>PbOr131
MEQTPTSTINRFVEIPLRIFGIWPGSPYIAFYRLFWTIVLAVAQFFQYRY
FVTHLRTIDLADLMDSLSATLGFSQFVSKLVIFWLNQRTFEKILAMMAMD
WKKNSNADFNMRVIISKAVLSQNFSNFVFGAFSIAITLYSITVLTFDTNN
IEKVDISMRPLILKMDFPFNNDTRFVYGLILILQFLCVVICGSAVVTVNT
LLIILVLHLSGQIEILRKWLTEIFSRENEYRPSLIMIRKIIKKHQNIIIF
SKSIENLYSHIALVLFLSDTLIICGLGFILVTSIGKSNATTIIIKSLGFY
LIMNFEVFMFCFAGEYLSVKNKEIGDAAYDSHWYQCKIQDSRIILFLIMR
SQNQLTITVGKFMDLSFERFISIIKASASYMSVMLALY

>PbOr132FI
MTNKRTINRTVELMLTMFGVWPDISCVVFYRVFWMITLAINQFFHYRYFV
THFHIDNLFDLMDCLSSFLAHVKLTVKLIIFSLMQRVEILTMTTEDWSDC
DNNDIALRETTQKTKLSSRICNGLIILHTVAAFAYVIGILLADADITDRT
AELPLMMKMEYPFVIDTLRKYRLVLATQFVFVMVCSWGAGLFNALFLTLT
LHVGSQINILLRWLMKIGSKDIEEKHNSIVIVMSKIIRKHQKIIHLSENI
ENLYSYIALLQFTSNTVMICSLGFLIVTAIGSPDATEQIVRSLLFYAVTN
LEAFIFCFAGEYLSNKSKAIGNAAYNSDWYDMKAKDSRILLFIILRSQRQ
LKLTAGKMTDLSLECFTNIMKASGSYLSVLLAMR

>PbOr133
MKRKSTINRTLKLMLTLCGVWPGTTCVIICRAYWIIALATDEICHYRYLL
MHLHTHDLFDLMDCFSSFLTQVKFTVKLIVFWLNERKFLEVLTMMAEDWN
DCTDNNVNMRETACKAKLANRITNAMFTLHTITIVAYSIGILLADVDVTA
QSEVPLLLKVELPININTKRKYKMLLAMQFVHLIMAGCGNGLLNTLLLTL
TFHVGGQMDILRCWLNVPIGNKERSESISNMTSKIIRKHQKIINFSEYIE
DLYTYIALVQFTSNTVLICSLGFLIITAIGSPDATEHIVRSLLFYIVTNL
EAFIFCYAGEYLKNKSKAIGMAAYNSAWYEMKPENSRNLIFVILRAQKQL
TLTVGKIMDLSLESFTNIMKASGSYLSVLLAMQ

>PbOr134
MTRRSTFNRTLKFMLILCGIWPGQSCVLFCRIFWIVSLTVTLFCHYRYFL
THIYSAEILDLMDCLSSFLAYSKIIVKFFVFWLNQRKFIEILTAMTEDWN
ECTNNDVNMRETTRKAKMSDRITNAIISLHTMTIVAYCIGIILADVDVTD
STKELPFVNKLEIPFKIKTQFVYRTVLITEFLFMILCGWAAGITNSLLLT
LILHTAGQIEIMRYWLMQLVPRDNKNNHTFDKSISTNKIIQKHQKIINFS

KNIENLYSYIALVQFVSNTIMICTLGFVIVTAIESPNAVEQIMKSFLFYT
ITNLEAFIFCYAGEYLNNKSKEIGIAAYNCEWYDLKSTESRVLMFIILRS
QKQLTLTVGKMMDLSLESFTSIMNASGSYLSVLLAMQ

>PbOr135
MTRKTTPNYTLKFMLTLCGIWPGTSCILFYRIFWIVSMPITLFCHLRYFV
THVHSAEILDLMDCLSSFLAYSKIIIKFFIFWLNQQKFVEILTEMTEDWN
DCANSDIGMRETIHKAKISDRITNAVISLHTVTIIAYCIGIILADVDVTE
TTELPFSTKIDIPFDIKTQSMYRTLLITEFLHMILCAWAAGITNSLLLTL
VLHTAGQIEIMRYLIHLIPHNNENKHKSITASKIIQKHQRIINFSKNIES
LYSYIALLQFVSNSIMICSIGYLIVTAIGSPNAIKQIMKSFLFYTITNLE
AFIFCYAGEYLNNKSEEIGIAVYNCEWYNLKSEESRVLLFIMLRSQKQLT
LTVGKMTDLSLKTFTSIMNASGSYLSVLLAMQ

>PbOr136FIX
MIRKNTPNNTSKFMLTLCGIWPGASCVFLFRTLWVVSMGVSSFFHCRYFA
SHIYSAEIMDLMECLSTLIAHTKIMFKFFVFWFNQRKFIEILTDMAEDWS
DSANSDISMRETLHKAKLSDRITNTIIILHTMTIVTFCIGIFLANVDVND
EIELPFATKIDIPFEIKTQFTYRIILLIEFLHLICCGWAAGVTNSLLLTL
VLHTVGQIEITRHWLVNLVSHENNHTSFIETTSKIIEKHKKIINFSRNIE
SLYSYIALLQFVSNTLMICMLGFLIVTAIGSPDAVKQILKSFLFYTITNL
EAFIFCYAGEYLHNKSKEIGVAAYDSKWYNLKSKKSRILLFIMLRSQKQL
SLTVGKMTDLSLETFTSIMNASGSYLSMLLAMQ

>PbOr138
MARQNVLNRAVKFILTLSGVWPGVSCVLIRRTFWVVSVILVQFFHYRYLI
IRMHSAEFLDIMDCLAELLANTKMFVKCLMFWLNQQKFIEILTMMKEDWA
DCVDNNINMLQTASKAKTSNRITNAILIFHTISVVGYSTGVLLADVDVSN
HTGELPLITKIELSFDINAQRTYKSIVMSEFILLILSTWTAGAMNALILT
MTLHVAGQINILRYWLKRLAPSDDEGKNESIAIETTRIIRKHKKIIKFSE
NIESLFTYIALVLFASNMIMIGCLAFVVVTAVGGPNAVEQIIKSFLFYVL
TNVEAYIFCYAGEHLKTKSKEIGLAAYNSTWYNMKSKNSRVLLFIILRSQ
KQLTLTAGKMMELSLQSFTSIINASGSYLSILLEMQ

>PbOr139
MSRKSTISHLIFPILHWFGICPNKSLLLISRLFWIVTIGFIEFCHYLYFS
RHLNSENFFNLVDCLCSFVSHAKVMTKLVAFWINERKFVETLALITEDWN
DCAKSDISERMATCKARISDRITHVILVSHTMTCVAYCMGVIIADADITD
ETIDEVPLVNKLELPFSVNTRNTYRLVLITELIHMTFCNLAAGAVNAILL
AMVLHIGGQIDILQYWLAQLTPEEMENKQLSVVIATQKIILKHQKIIQFS
ENIESLYTQIALMLFASNTMMICSLGFLIVTAIGTPDAMEQIIKSILFFA
TTNMEAFLFCYAGEYLNNKSKEVGFAIYNCPWYNLKPKDSRILLFIILRS
QRQLALTAGKIMDLTLQSFASIMNASGSYLTVLLAMQ

>PbOr140
MSRKSTINRVIFMMLPWFGICSNKLVLLISRVFWIVTIAFIEFCHYLYFS
KHLSSDNFFNLVDCLCSFLAHAKVIAKLIAFWINQRKFAEILALITDDWN
DCAKSDISVRMATYKAKISDRITNVILVLYTVTIVAYCMGVIIADADITD
KTMVELPFVNKLELPFSINTRNMYRSVLIMEFLHMILCNLAAGTVNAILL
AMVLHIGGQIDILQYWLAQLTPEEIKNKELSIVIATKKIILKHQKIIQFS
ENIESLYTQIALVLFASNTMLICSLGFLIVTAIGTPDVMEQIIKCLLFFT
TTNLEAFIFCYAGEYLNNKSKQVGFATYSCAWYNLKPKDSRILLFIILRS
QRQLTLTAGKMMDLTLQSFASIMNASGSYLSVLLAMQ

>PbOr141
MIRKSTINRTIEIMFTLFGIWPHTSCVPFCRIFWIISIIAVQFYHYRYLL
THFHSDDLFNLMDCLSSFLAYMKVMIKFITFWLNQGKFIKTLEMVSQDWD
SCADNENNMYETIDKAKLSSRITNSMIILHTVSAVAYSTRIILANVDVTD

QSFEPPYIHKMQLPFDVNTQRTYQAVLITQCVYVVMCSWAAGAVNALLLT
LVLHIGGQMDILCYWLTNFASKSGKKHESITKDKIIQKHQKIINLSENVE
HLYTYIALLQFASNTIMICSLGFLIVSAIGTPNATEQIMRSLLFYTITNL
EAFVFCFAGEYLKNKSKAIGIAAYNSAWYNLKPEDTHILSFIILRSQKQL
TLTVGKMANLSLEYFASIMNASGSYLSVMLAMQ

>PbOr142FIX
MKTRNSTINLTLKIILILFGVWPDATCVTFCRMFWGSTAIIFIILQYQYL
MIHYRIDSIFELIDCISSFVWCIKLVCKFFLLWFNQRILKELLTIMAKDW
KDCAKSDIEMRQAVNKAKISDRVANTVVIYQTVAVTLYGIGIVFGDVDVT
QSNLPHIVKMEFPFQITTQRMYRSIVTIELIYIIMTGWCSALVNVLILTL
SLHVGGQIDIVCCWLAKLMPKPNMKENEFVAATISKIIQKHQKIIYFSQQ
IENMYSFIALILFLGNTIMICFLAFLFVTALDQPDTTDKIFRSLPFYGVT
NIEAFIFCYAGEYIINKSKALELAAYNSAWYDLEPKFGRMLLFVILRSQK
RMTLTVGKMIDLSLQCYARIMNSSGSYLTMLLAMQ

>PbOr143
MSANTITRSIEIGLRVVGIWPGAAYAIAKRLFWTATMIAAQIFQYRHVAL
HLNSKDISQLMDGLSATLSYSLLFVKLIVFWTKQRIFNDVLTSITTDWEE
CRDSLYNMNSVANMSHRFSNLIIGLHSTAVLFYGIGVVALRNDDVTDTAD
RELFLKMELPFESGTSPIYEVVMTTQFLHQMTAATVIGVLSALLVTLVLH
AGGQIDILREKLLEILPKEKKPTISAITMGSLIRKHQNIIVFTEKIESLY
SYIALAQFISNTLVICCLGFIIVNSIGGDQGSSMLVRSLLFYVVINLEAF
IFCFAGEYLSVKSKLIGDAAYESLWYDLTPSENRILLFLIMRSQKQLTIT
VGRFTNLSLQQFANIIKSSASYVSVLHAL

>PbOr144
MTRNSTINRTLKFILCLFGIWPDMSCAILCRTFWAITAIIFIICQYRYLS
LYFRTDDIFELVDCISSFVWYFKLMGKLIFFSINQRTLNEVLTMMTKDWK
DCAKSDIETRQALSKAKISDRVANIVIVFQTIAVLSYGTGIVLADVDVTD
QTIRLPHIHKMDLPFHISTQRMYRFIVASELIYVTMAGWCSGLLNVLLLT
LSLHVGGQIDIVCYWLTKLVPESNMKETESVDVTIYKIIQKHQKIIYFSK
KIEKIYTFIALILFVTNMIMICFLGFLFVTALDNPDAMEKIARSFSFFGV
TNIEAFIFCYAGEYIINKSDALELAAYNCAWYDLEPKLSRMVLFVILRAQ
KQLTLTVGKMTNLSLQCFATIMNSSGSYMTMLLAMQ

>PbOr145
MESQVSADKEYNDLIKPIMLSGKFICVWPLEKDRPIHKKLLHICHLFWLL
FVLLSMCVTVTTDTILHFDDLVEFTECALWSSVFYLTIVRLIIFMTHQKD
MAYVVETMRKDWICSSYEDRVVLKEKCHFAFRLSKIFISMVYGTVICFSC
LPILEIFIQGGQRILPFHGYFFINQTVSPVYECIYVFNVMGGFFAGGTIC
GATSFSLMATIHGSAKFVVLQKKLEAITSNDPHVDRALANCVKRHQDAIA
FADALERTINVLALAQFVISTGLVCFAGFQITSMIQNKALLMEYGMFLQA
AVLELFMFSFSGNALIEESDAVGESAYRSGWIGGTFGRSLQIVIMRSKVP
SRITAAKFYSMSLETFSRVLSTSFSYITVLMAAKEE

>PbOr146
MQVVRKLTTSTDLVQASKLIVWNRRFIGFLGLWPSKVNQPVFMFVVIYMI
IYCIMGARHLIKNFNYPERVVANLTDNVLFAMILGKMFILRGSCGVMTKF
LKSIEIDFSTEMYNNVQEKMAYLYYNEIAIIFLKFSTFMAGLAASLYYLK
TFIKNWSAFVHGNFSYELPYPVHPFFEITDTTTYVCFCLYLSICIPIIVC
GYSAPDAYVLSLTLHVCGQLATLSCKINNLLKDQKNYQHHVGNIVVRHRH
LIRLADTLENKFNLIFLQQTLGTTFLLCVTLYHMLATSENGENYNVLAFV
SYIICVISTILAYCYIGECLITESSGLRDAFYNTDWYNNPPRYTKLISIC
MIRAERPLLMTAGKFCALSLNTFTSIVKTSMAYLSVLRNFM

>PbOr147
MDINNYVIINRKVLKFVGLYPISIVRYVLCCACMIAIIIPQIMQIYQNWQ

DLSTVLETSSVLFTILLAILKSLVWVNNRRKMDSFIEYMLTDYWKIMTAH
ISKKRRDVCAIYVEKGLSYTKGYLFLICNSLIFFFSLPIIEIFVTVIKDT
NNNSTLTSKHLPFLALYPESYYSFPMYEIIYLSQMVATSLCGLVILGTDT
LIATALFHTCGHFKVLQKKIKNIENEIDLVQNAYIEKNITQRIKLHVIDI
IEHHYIILWFCDYMETIFSPMLFLQTLASSLIICLVGLQIASTNITASTI
SKSIKYISYLIMALFQLLLFCIPGDALIYESSMIYKSVYTITWYKLPVLF
KTEICLLMLRSQKPSKITAGKFYIMHLENFNAVLSTAASYFMLLRSFSSD
GTTINL

>PbOr148
MKNKDDHIEDLFVHLERIFSFGGIWPFKKTYIRFAIYITHYFFYLVMAYA
DFYKVFGNLELMVMNIVETVAYSITFPLMWLIRCSNMLKLIIKEIKKDLA
ERKFENLEEERIYYNYNSISKLFTFTSIVGMFITVMLLYFRPLVYLLLSN
QALRNSTESFMLPYRVHAFFDTNNIHTYILVYLYLCPLIYISICHMAAIC
LLVILVFHICGELSILSYRIRNIGECSQSEVIIDRIRSFIRMHLKIIWMA
KSVDNTFNLILLDELLGNSIVLAISMYYVIVNLDISEIATCCTFIFFAII
ALVMLFGYCLIGDQLTQQCINVQDAYYQCNWYEMPFNCKKCLLICMIRGR
VMLYLTAGKFYIFSLSSFTDVLKTSLAYLSMLRTLL

>PbOr149
MQDQKDLDRAAEVLAWNKRLMSTLGLWPFQSNDFIFSINFGYFGFFMILE
YLDFFLFIGDLEHVIMNLTENMAFSQIFVGMSMLRLYNNEIGEIIMEAMK
DFDRTNYKTVEEIKTFLIYNARSKIFVKLLMAFVALTASSYYLTPIIIIL
GSGLPKIMINENVTQIIYLLPYRFHLFYAIENVHAYIIAYVVEMPFVFVS
GFGQSATDCIMVTLVFHICGQMSVLALRINNINTEFRNCKREVRHVVLMH
IRLLRMGRTIEKAFNATLLAQLLGATSLICILGYQILTNYAKGERGVLVT
FLIFQFLVLLILYAHCTVGENLLTESAKVCEAFYDCHWYDMPKTNARMMI
LCMARAQKPLSLTAGKFTNICLSTLTNVLKTSMGYLSVLRSFL

>PbOr150PSE
LWPKSYNFRFFFYITXFAFPSILIIILKNVMFRXRDQVLSLLIVMRKNMKX
LYRNSIEKCXNIMYNIISTLFITSFLTVLFHLKSIVSYILSXNTTSDEAC
YFTKZYEMPIESIRFLMICIVEZQKPLCLTAGQFYVFSIETFDIIIKAMV
YLSILRPIT

>PbOr151
MVMKRSSQTGMRQGTAASRLEQFRKINVVHITCLKYIGLWALDSNASTLL
KCLYYVYSRCVLAIILVFMITLFADICLSFDDLSIVTDDGCIFAGIFVVF
FKVMIFQIRREQIARLLCKTIDGCKELCKFPVGGEDKILGKYLLLSRVSM
YGFSVLGFFLVIALLFLVPVENGELPVRARYPFDTTVYPWHGIGFFVEAC
AVSVGMAAIIGMDSLNTNLCNLFLVKLEILNAHFESCGNDDRHDDATTPD
VDKRDGDSSNAGGRNGVETCYGIFPTAEASCGKDNYDPDAIRRGGFAERL
GRSIRNHQRLLAIIDDFNELFSVGMFVQMLSSTTMICLTGFQATLVIGQS
SNTCKFSIYLMAALSQLFYICWQGNEVMYQSASLTQSQWLSKWDDEFSTK
IGRLLILSIIFSRRTINLKAGVFYVLSMRTFITILKGSYSFFALLNTMHS
EVDQ

>PbOr152
MVPNHDSFASVIRVNVALLRLSGIASYKNGSIQLEDRSSSRNVLSAIAYG
CLFAYAGLYTYEFALRTVHLDTWMESFAMILSLVGGQARFTILLLFRSRF
QRLLMICEKLWSALNVAERKYVRDYVKMTRHLTYYYLFGCAFTIFFYAVA
SLFMGHHDDSSNATVRTLPYACPVAVDRTPYYEIMYIAQLFSMTNVGLTC
AAADTVGPVLILTVCGHFKVLNTRLLHLRDRDRVYSQVVSEESQSTPTES
LEMTMLEEKRNVAKNLNLIKSDLEACVYYHQTMLELCKEVERLTTSIFLT
QLLGSTYNVSLVGFKLAGDDPDKFKYTTQLSIAMIQLFLCNWPANVLLVE
SADVARAIYFMPWYRFSHQIKRSLNIITMRSQKPTQLTAGYIIPLSLQTA
SMVSSAASFFTMIRGMN

>PbOr153
MIVKKKRNKFLSIRMVRFLMKFIGFWPAESKTEERLLNAILGYTICMIAM
GLWIEATEMYLGKGDFYAITYTACSTMPIIIIMLKICIFLSNRKELLNML
RYTEDNFWCAQYDDYGNKVLERINKKGIILICMFTFFVQGTVFTYMLTPI
IENIGKNESDRILPFNLWIGIPTSVSPNFEILFVCESSTLIYCGVCFCCF
DNLLGLLNIHTAGQFKILQHRLGTILKRVEGTGIVRSFDEKGNRKVYQEI
RECIMLHHDLIWYSEKMGGIFMYTTLCQLLVSSVMLCVAGFQMFLARGTF
IRRLIFIAHTNGCLFQLFVITLTANDLMDESRAVGDAAYNANWQVLPHEE
NRDVRKAILLIMMRSAHACSISAGGFFPVSLETFMAVLSTAASYFTLLRK
FLE

>PbOr154
MTQSKEYRSVSITRLFMKLVGLWYVRTPREQLILRAAFVYAVSAIAFAIL
VMGVDLYHCLGDFYAVTSNLCATLLLVMILVKLGSFMYYRDMIMDIIHFA
EENFWNVNYDKVGTQILEKYDKLGMTMVYTFTFIVYFATFNYIFAPFFES
QETNVTEKILPFKLWIEFPYHSPYYEITYTIQSLSTLHSGICTCCFDNFV
STFNIHAAAQLKILAHKVEVIVEDCIVDIMGQKCPLETDAAALTFKKLQE
CVQHHLTLIRYVRKMQRAFAIILLGQLLLSSVVICFGGFQFLAADVAIRK
CIFAFHLVGGLIQLLIYTWTCNDIIVQSAAISDAAYNSKWYLLSNDGPGR
AVRKGLIMIMIRARRPCMLTAGHFAVMSLETFTGILSTAMSYFTLLRQMS
EDNV

>PbOr155
MRNLANFVRYFSMFHDYCNEQSSEATIDMTVLRYRVYTRRVRRMLYLGGI
LQDRKRSAICSYITGLLIIFTCFGQCIFTINFCRDHTDNLVLLSRSFGLT
CSLIAPVLMSICFLVKREKLMNLHETLNGIFERELTRNRETEETILATLH
TFDRPSYILCFTLGSTVLLILCPPLVSTVRQIIYHTESKRYRLPLPAKFP
WSISIDGNFSFYLSFLYQIFICWWMVFTVGSVDSLFGYYAFQISSILQAM
SFRLANPRPREVFAEVLRTCVQTHHRLLRCGHMLNDIWDLIIIRMILTNA
ILMCVLIFEASPFTHLTIGQILLFVSYMALKLLQTFIYAWYGNLVTNASE
HFRQGIYFSEWSNSNLNHHIRTSIIITMMQKPMIIKALKLSSVNVNMFTN
IVNTAMSYFFLLQSLDQNR

>PbOr156FIX
MTDKQLLAYNRYQRFLQVLLTGCGCWHAPTKSGKSTYYWSVCILPMLFAY
AMLNIRISYMYRHHLAIMMKNIGVSITTMGTILKVSSFLINRRSLIDYHR
TLTDLFEQELKQNEKIRMVMFSPLHGISTFAYIYFGIIITLLLTYLMPTL
TVITHGILRFQLITNYTLPYSRGYGYLWTVPTGHLRHFHLFFEIAMITIN
CITCIGVDSVFSFYTYLLSSMMRAMTFRLTNPLPNDKFYDVLSTCVVKHQ
KLMRYCNALTHVYGPIIFWHTITNAILLCALIFETVQLSEISIGKVFNFV
TYSTLKLLQMFIYAWYGTVITSADEDLRNGIYFSEWPNSSLNCHVRTNVL
LIMMQKPMAIKAFFSTVDIVMFTNFMNTTISYSFIGIYRR

>PbOr157
MTDKQIKAYHTYQRFLRVLLLICGCWHAPSKSGKSTYYWSVCILPMLFAF
GMLTLRVSYMFRHHLAIMMKNIGIFITLAGTILKITIFLINRRYLIDYHG
TMSDLFEEELKRDEKVRRVMLAPLRRISTLAYTHTSLSITLIMTYLMASF
VVIVRGIFHLHLPTDYTLPYSRGYGYFWTVPNNFLRHFHLLFEWWALITQ
AITNASVDSAFGFYVYLLASTMRAMTFRLTNPLPNDKYSDVLRMCVTKHL
KLIQCRDMLKRIYSVIILWHIVTNAILLCAVIYEALQLSNITIRMVFNII
SYSVVKLLQTFIYAWYGTVITNADEDFRNGIYFSEWHNSNLDRHVRTSIL
LMMMQKPMAIKVFSISIDVVIFTNLVNTTMSYLFLLESISDKNE

>PbOr158FIX
MTDKQIKAYHRYQRFLRVLLLICGCWHAPSKSGKSTYYWSVCMLPLLFTY
GVLNLRISYIFRHHLAIMMKNIGVFISTSGTLLKIAIFLINRRYLINYHG
TMSDLFEEELKRDEKVRRVMLAPLRGISTLAYTHSSIMLILIMTYVMPPF
VVIIRGIFHLYLPTDYTLPYSRGYGYFWTVPNNFLRHFHLLYEWWVIITQ

AVTNASVDSAFGFYVYLLASTMRAMTFRLTNPLPNDKYSDVLRVCVTKHL
KLMQCRDMLKRIYSVIILWHIVTNAVLLCAVIYEAMQLPEISIGIAFHFM
SYSVVKLLQTFIYAWYGTIITNADEDFRNGIYFSEWHNSSLDRHIRTNIL
LMMMQKPMTIKVFSVSIDVVIFTNLVNTTMSYLFLLESISDKNG

>PbOr159FIX
MIDKQLKAYYRYQRFLRVLLLICGCWHVPTKSGKSTYYWSICILSMLFAY
MILNIRISYMYKHHLAIMMKSIGVLLTTTGTLLKIACFLINRRSLINYHR
TMSDLFEEELKRNEKVRTVMLAPLRGISILAYTYSGIILIVVVTYLMPSF
VIIIRGMFHLYLSTNYTLPYSRGYGYFWTVPNNFLRHFHLLFEWWAIITH
GVTNASIDCAFGFYIYLLASTMRAMTFRLTNPLATEKYSDVLRICVAKHQ
KLIQYRDTLKRIYSVIILWHIVTNAVLLCALIYEALQLSDITIRIIFNII
SYAVVKFLQTFTYAWYGTVITNADENFRNGIYFSEWPNFGLNRHMRTNIL
LIMMQKPMIIKVFSVSVDVLIFTNFVNTTMSYFFLLKSIDDKSA

>PbOr160FIX
MTDKQLQAYHRYQRFLQVLLTGCGCWHAPTKSGKSTYYWSVCILPMLFAY
AMLNIRVSYMYRHHLAIMMKNIGVSITTMGTLLKIISFLINRRSLINYHR
TLNGLFEEELEKDEKIRTVMLTPLRGISTLAYIYAGCVFTLFVMYLMPTF
VVITRGILHSHLPTNYSLPYTKQGHGYFWTVPTGFLRHFHLLFEMSALIL
QTFTTIGVDNAFGFYIYLLASTMRAMTFRLTNPLPDDKFSDVLRVCVAKH
QKLMRCRDTLTRVYSVIIFWHILTSALLLCALIYEAMQISEFTIHIIFNI
ATYSTIKLLQMFTYAWYGTVITNSDEDFRNGIYFSEWLNSNLDHHVRTNV
LLIMMQKPMVIKAFTISVDFVMFTNFVNTTMSYFFLLESVGDKGG

>PbOr161FI
MTDKRLQAYHKYQRFLRGLLITCGCWYAPTKSGKSTYYWSVCILLSLFAF
SSLSLRISYMYRHHLSYMMKNIGIMLTTLGIFLKVSCFLINRRSLINYHR
TLSSLFEEELTQNEKVRTVMLSPLRTISTLAYTYSGCTITSFFKVSCFLI
NRRSLINYHRTLSSLFEEELTQNEKVRTVMLSPLRTISTLAYTYSGCTIT
CITTTGVDSVFGFYIYLLSSTMRAMTFRLTNPLPNDKFSDVLRVCVAKHL
KLIQCRDTLKRVYSLIILWHIVTNAVLLCALIFEAMQDEDFRKGVYFSEW
PNSSLNHHVRTNILLIMMQKPMTIKVFSASVDVVMFTNFVNTTMSYFFLL
QSLGDKGG

>PbOr162
MTDKQLQRYRRYQSFLRRMLVICGCWHTSTKSGKTTQYYSFIVLILLVMF
ITIKLHILYQFRHNLVHLMKNLSLLMISLGSILKVSCFLINRRFLIAYHR
TLNNLFEKELSQSEKVRTVMLSPLRTIATLAYAYSAIVITLSMTYFLPTY
IIIIRGMIHLHIPSNYTLPYTRGHGYGYFWTVPPGILRHLHMFFESYLTV
INSTTCTGVDSVFGFYIYLLTSTMRAMTFKLTNPSPNDKFYDVLKINVAK
HKKLMQCRDILTRVYSPIILWHIITNAVFLCSLIFDAVKLSNRSFDKIVN
FVTFSMLKLLQMFIYTWYGTIITTTDEDFRNGIYFSEWPNSSLNRHVRTN
ILLMMMQKPMIIKVFTTTVDVVLFTNFVNTTMSYFFLLQSFE

>PbOr163
MKQINFENVNLLNSRVNLLSGNLLPIAIDNSRFSIIWRIYSVIVWLIELI
HTIALIFGIILVPKEKALKDGTVGSVVILETSFMLLWLYSHKKLMKQIIQ
KMNDIFQNTDEIMKGIVKSALKPITMPFVIYGVTGAISVMIWTVRPVTLV
FEKTNFFYVDYNLPAAFSTEPFSSQVLISSTILMTIGSVYLFLKKFGVDI
YMIHLVLMLTVQYRYTAAKLTILCQDQNFQNKSPNKYSMKNRWIKKELRE
LCRHQNTVLNISFMLKKLLSINFSLLYINNVFRFCFIGILLSTVPSSTFA
EGISIILFTLGSLTQFFLLCSSVQTLSDASTEMTDKAFNEGWYQFGSSNK
RTFISLIMANNLECKIAAIGKFNLSLPSFMTIMNQSYSIALLFLRAK

>PbOr164FIX
MDFQIVNPLNVRLNKISGNLLPITGDNSSFPIAWKIYSIIVWLIELFQTS
TTICGFVLVAKRENLQDGGTLSIVVSIEVFVLLTRMHAYRGLANQLIKKL

NDILHSEDETMKSIVRSTLKPIETPLEVYSIAGTGSVILWCCIPLVLIFK
KKYFFYEDFGLTAAYSKQPFSINIFVLGNVIQIIASALMFLKKVAMDVYM
INLVLLLTAQYRYIAVKLATIFRNHTSQDKPNEYQKEYSMNSLIEKEMKT
LCRHHTVTLHMTTVLKKFLSLNMSLIYLNNIFSFCFTDVILINAIVSRAF
LQGFLLIAYICGALIQLYILCFCIHQLLDASTEITDAAFHEKWYQFGPSI
KHMFRMIIIANNLKNKFSISEKFSLSLPSFLSILNQSYSIALLLLRVK

>PbOr165CTE
MNFQNVNQLNSYLNLLSGNLLPITPNNFPILWQIYGAIVLIVQVVYKTGL
FLGCAVLPSKTAVSDNIVGTVYFIELLFTLIYFYAHKKMIIQVIQMMNEI
LRVREETMTNMIKSILKPIMMPLKLHGVICVTITFLWFVQPLILIFEKNT
FYYADYNSPAVYSTEPFSTSVFISSSIVIIGGVTEFLKKFSLDVYMTHI
VLLLTALYRYLAVKLTMIFQNSENYHDRNGSKIDEWAEKELRTLCYYQKT
IGRISFLVKRLLSINFSLLYVSSVVKFCFMGIMMSTITVLPLFHGILITF
YMISTLIQFFFICYAVQKLGDAIMQQSYSVYLLFFKIKLGRLMVIYGLYL
VLTDLKGTIRDKKYLINQYLI

>PbOr166
MSRDLKSMNFQNVNPLNNLLNLLSGNLLPVTPNNFPIFWQIYCAIVSILQ
VVHLTGMFLGLILIASKRALEDGLISIVIVIEVFIMLIYLYAHKKMIIQV
IQMMNDIMQDGDETMQNIANTVLKPVMVPFKLHGMISVIITFFWGIQPVT
LIFEKNTFYYSDYNSPAAYSTEPFSTSIFVSSNIVIATGAVIGYLKRLSL
DVYMTHLVLLLTVLYRYLAVKLAMIFQNSENYCDKNSSRMDEWAEKKLRT
LCRYQKTIGRISVLVKKLLSLNFSLLYVTSVFKFCFIGIMISTVTVESFF
FVILIIILFTMSSLTQFFLICYAIQKLSDASTEITDEAFHEGWYQLKPSLQ
RIFLFLISSNIECKISAIEKFNLSLSSFIAIIQQSYSVCLLFLRFSWID

>PbOr167
MDFQSVNLLNVRLNKFSGNLLPMTSNDSLFPVSWRIYSAIIWLIEAIQTS
AVIPGILSVPKDKALQDGTVSLVITIEVFFLLRQMHVHKNLVNQLIEKLN
EILHAEDKSMKIIVKSTLKPVEVPLKFYCIAGTGSLIVWCGVSFALIFKK
KYFYYDDYRVPIVLSKEPFSAEIFFLGNCIVSIASIFMFLKKVALDVYMI
NFVLLVTSQYRYMAIKLATIFRKNSPQNESQQEYSSVNSLALTEMKALCR
HYNAVVQITYMLKKLLSLNMSLLYLTNIFTFCFLDIMLISAILSTDLLEG
VMIIMYICGGLVQLYIICLCVHQLLDASTEISDKAFHEKWYQSEPLLKHM
FRMMIITNNLECKLSVSEKFNLSLPSFMTILNQSYSFALLFLRIK

>PbOr168
MSMDFQNVNLLNIRLNLLSGNLLPMTNLNSSFPFFWKIHSIFTWMLTTIM
AILIIPGCIYVSIEKALKDGMICLAIFTEMSFMILRIHSRKDVVYELIRK
LNEILHTADEAMKNVVTATLEPVRVPLNFYWSGGVASIIVWICMPLLLVF
ERNLFFYEDYRIPVVFSVQPFSFKIFLLGSLFLVIGSIFLFLKKVGVDVY
MVHLILMMTAQYRYIATKIAIIFQEEYNSVESQKKHLSGLDRRKEKKIKA
LCRHHNEIIYTTSLLKKLLSLNFSLIYVISVFRFCFLGIMLSSITSTTFW
EAILIVIYSSGAVVQLYILCSCVQQLLDASTEMTDKAFHEKWHLSQSSMK
RIFILIIMANNLECKVATFEKFNLSLPSFMMILNQSYSIALLFLRVKS

>PbOr169PSE
MIVICYESINIAYMDFQNXNPLNVRLDIMSGNLLPMTADNSPFPIFCKIY
SILVARNSSNVRLTPWMHSCIEREGSKRRLDRSCGYYRSSLYVIRIHSHK
KLIQQLIQKLNDLLHIKDEMMKSVVMVTVKPIEMPLKFYWTSGVMSIILW
SCSFILIFKRNFFFYVDYRMPVVYAKDPFSTSIFLLGSVIVMTSSVYIFT
KKVAVDSYIINLMLLITAQYRYTAIKLSTIFQNKSLQNNYNKSNLQKNVI
LIQIIIIMIMLRELLSLNISLIYLNTVFRFCCIAIMMISKMPSINLWEKF
SLITYAIGGVVQLYILCYCMQQLLDASIEITNYAFHXKWYQYGASIKQMF
MFMIMANNLEIKLSTFERYNLSLSSFIX

>PbOr170

MNVQNVNPLNVRLNMISGNLLPMTADNSSFSIFWKIYSILIWLLEVIQTC
VLIPGCIFVPKEKALKDGIIGIVITIEVVSVFIRIHTRRELVQQVIQKLN
DILRMEDDMMRSVVMATIKPIEIPLKFYWMAGVMSIVLWSGTPLILILQR
RSFFYVDYRMPVVYSKEPFSTGIFVLGSIIVLTSSAYIFTKKVSMDSYII
NLMLLITAQYKYIALKLSTIFQDGLHQSNNNKLNKDGHFNKDYYAERNIK
AICRHHNTVIHIMIMMRELLSLNISLMYIISVFRFCCIAILMISIPSVTL
WEIFLQLMYASGGVVQLYILCYCMQQLLDASIEITDYAFHEEWYQYGESI
KRTFMFMIMANNLEIKLSTFEKYNLSLASFMAILHIVSMLLIIQTKYKS

>PbOr171PSE
MDFQNINSLNVRLNLLSSNLLSMTSDSSFPIFWRIYNIFVWLFELFYTVS
LISSFFFIPKEKAINDGMISVVIIEIVIMITRIHSQTTLVQQLIRKLNKA
LSIEDENMRNIVITNLKRMDTPFKFYLIISTVFYISFCCMSLPLVFEKNT
FYYSDFKVPAIYSKEPFSSNIFVMGSILILISNLYIFIKKVSVDIYTTHL
ISLITAQYQYIASRFILIFRNDQWDNNNLQKNNSRVNSSIKKEIKILCRQ
HNAVICITLMLKKLLSLNILLIYINNVFRFCFIGLILTKISTSFLEGCMI
IMYGSGSIKQFYILCSCVQQLSEASTELTDKAFHEDWYRFDLSVKHIFLL
VIIASSTXLELKLSTFDKFNLSLSAFMSVLNQSYSIALLILRMK

>PbOr172
MDFQSVNSLNVRINALSGNLLPLKPDDSRFPITLKMYSVLVWLIEIIQTI
VLIPGLILVSREKALKDGTVVCVVTVEVFFMAARIHSRRQLVNRLIQKIN
DILRFADETMKNVVTATLQPMDNPLKFYWLSGWMGVFVWAILPFLLIFKK
VSFFYEDYRMPAAFSKQPFSFNVFLLGSILILIGNMYIFIKKVGLDVYMI
HLVLLITAQYRYIATRLVTIFRDGNPRSEFNESLQKYSSNINQRVEKEII
LLCRHHNAVVHLSSMLKKLLSLNFSMIYVNSVLRFCFIGIMLSTVPSTTL
PEAFSIVMFACGSVVQFYMLCSCVQQLLDASKTMTNEAFHEKWYQFGPSV
KRIFMLMILGNNLECKLSSCDKFNLSLPSFMTILNQSYSIALLLLRVQN

>PbOr173
MNFQSVNLLNVRLNTFSGNLLPMTSDDTQFSIVWKIYSGFVWLIELIRAV
SLIPGIMHVSRGKTLEDATVAIVFTIEVIFMVMRIQFRGTLVREFIQKLN
DILRTEDEIMKNVVTMTMEPMKIPLNFYWFTGLVAVIVWGSIPLMLLFKK
TSFYYEDYRMPVAFSKQPFSTEVFLLGSLFISIASMYMFSKKVGLDVYMI
HLVLMITTQYRYIAVKLEEIFRNGNSEDECNSSKERHSKRKDLWTERELR
AICRHHNTVIHLSFTLKKLLSLNFSLIYVNSILRFSFIGIMLSTVLSTIF
IEGFSILIFACAEIVQFYVLCSSVQQLLDASTKMTDLAFHKNWYQFGTPI
KRTFMLMILGNNLECKLAAFEKFNLSLPSFMTVMNQAYSIALLFLKMK

>PbOr174PSE
MDFQRMNLLNVHLNLLTSNLLPMTDNSSFPIFZRIHSVFAWLIELVYMVT
LIPAYFFVSIEKALNDSVISXIIVESAIMIIRIHSQRILVQQLIQKLNKV
LSIGGENMRNIVTTNLRNINNPFKFYLIIGAFSVLFWGCMSLXVFEKSTF
CYADLKTPAIYSKEPFSLDIFVIFLIFATVISLISIYIYFFKESZCGRLY
NTLISASSLITAQYQYIALKFIAIFRNSNSQATRASNZQFNLKNYSKVDF
SIKKEMKILXITLMLKKLVSLNIFXLLYINNIFRFCIIDIMLTQISSSFL
EGCMIIMYGSASITQFYILCSCVQKLSEXXTNLTDKAFHEDGIDLIITHT
FXLVIVANNNLEIKLSMFEKFKLSLSAFIXILNQSYSIALLILKMK

>PbOr175
MDFQNVNLLNVRTNMISGNLFPMTADNSSFRIGWKIYSAVIWLILIIIVI
GFFFGFMLVPKAKAISDGMIGTVFIVEVFFMVVRIHTKRNLVKKFIQNMN
DILHVQDETMRHIVISSLKLMHSPFKFYWLSGIITSTIWIGVPFTSVFKK
SSFFYEDFRLPFAISKQPFSTEIFLLGEIMLTICSIFIVGKKVAVDIYML
NFVLLMTAQYRYIAMKLENIFQEEYSEDNYNNSQQKHRPKTKSWAETEIK
AVCRHHNTVVYLSSMLKKLLSLNFSLIYVNSVLRFSFVGVMLSNTALLTT
FVERIAMVFFASGEIIQFYILCSNVQKLLDASIKMTEVAFHENWYQFESS
LKRKFMLMILANNLECKLAAFEKFNISLPSFMTIMNQAYSIAVLCLKIVK

KQ

>PbOr176
MISKITSIEAIHFIKISVALLCAWPPSPNATKFEIIVFKIRWFTCYLSSI
LLLLPLLNSIYEYRDDPEILGKSVCLSCGCIQVTIKMMICATRYTQFQIL
YHEMETFCKQADEKTNVMLQRYVDKYKSIYGIYSLWCYLTAIGIICGPLF
LPQEFPTYAKYPFSVEHPIKSIIYLHQSLVGLQASSGICIECSIAVLLFY
SAARLDLLAQKMRNIKKENELNACIKLHDEILKYTCKVTNTVAPLVFTTI
FTTAMAIIFGSLNMVTEQPITIKILYTIVAFSASLELFMCALPADNLMHT
STNICMGAYDSQWFQGNISMQKKIIQIIFRSQEPIVIRINAILPALSLRY
YAGFLYTSCSYFTAVRMMVNENIKN

>PbOr177
MREKIILKEVMFVVRLSLFPVWGWPVSKDAKFKIFCVKMYQTLCIIISMC
HEIPLIYGALNNLNKPIILVQQLLLASGCIHVIFDFIFYRLNYHHLQDVT
FKMTDYFDLKLKSTEEVIIKKYIDKCLIFYGFCMFMFYLITIVSLVAPSV
LEQDFPTLAEYPFNVSNQPLKMIIYVHQCISGLITAGQLCTNNYMALLLW
FTSARFEMLTEELRSSTDIHQLFKCIKTHQELLKYAAKVALIVRPFAFTT
ICCSTFCIIIVLLLLITRHPVVQLIQFFGLVLICLSEVFMFTWPAEYLMY
KSNATAQAAFDAFQCNQSIKMWNCLQIIVMRSQKPIRIRIACLMPTLCFN
YFTTYCSTIISYFTTLRVVMNEDN

>PbOr178
MKRVTLEKVIAFLKIELMFACCWPVSRDATKCQIVCDKIFRLISALHAIL
LVIEIMYTLIYRIDDVRMFMQSSCLMVVILEVPVQIFLYTLQHDRLQEVI
FQMEDYYRHSKLEERDIFQKFIDKHIKFYAIILGVITAALATATLGPMFK
DSFPIVIKYPFHVEQQPLHAIVYLHHSFGIYQSYCQVCSTVFVALLLWFT
SARFEILSNKFRMASKYCEWQSCIREHQELLRFSENMSLSISYIVLSSLG
FSTCTLVFGGVTVLSRLPLSVKVEFVIGCMSSLGKVFLSAWPADYLMAAS
SDIGDAAYDSLWYEHGTDSQKIMLYTLLRCQRPVIITVPGLLKALSFQHY
SAYISTAFSYLTTFRVILSEDDAS

>PbOr179
MEKFIAFLKVDLLFACCWPLPPTATKCEKIRNKIFRCLSILHGILMMVEI
IYTIYSNRSDLFLIMKLCCNLCTTTEVPLQIMCYSLQYDRLQYVIYALED
YCKQAKPEEHIIFHQYINSCKPVYIGAVCGFTITGILLIISPIIGPHPFP
MDIEYPFSIHFTPVKIIIYLHHIFIIYQSYVQVCSMIFIALLLWFVSARC
DILSNKFRAVTKFSELRECLKEHQELLWYGKMVVLSIRYVMLAALGVSTI
VIIFTGCTFLSKQPMSVKSTFLIFFISSLAKVFLCAWPADHLLSASANIA
YAAYDSIWYNEKADFQKNFVNTLLRAQQSIVVNVPPMLPTVSLNYYASYV
STAFSYLATFRVIFAEEDD

>PbOr180
MEGKLTLDKVIAFLRIYLTFACCWPLPPSATKLQRLLRSALQYFCLTNAI
AIAIAAIWTLYRHSNDVLLVMKLGCQLSAIVQIPLQMILFAMQNKRLQFV
ILEMENYYRQAQEYEKEIFQQYIDKCKPFYGSILCWLAMTGISVIVTPLF
SSQSFPCEAEYPFDVQYQPIKTIIYMHHILAAYQSVLQVSTNTFPALLLW
FVAARFDILSVQFRTVTNMKELIKYTREHSLLLRYAKEVTRAIRYVALLC
VTFSTGAVIFGYLTFMSHQPWSVKWTFLMIAFCGFVELYMYAWPADNVMS
MSSNIASAMYESLWYDGDLTRRKILMYVILRSQHPVTVSIPCALPNLSMT
YYASYISTVFSYMAFVRIIMDQE

>PbOr181
MKLLHLKINRVFRFLQICNIITCTWPHDPDVNKLVYIFQKINWIFSLSNM
IIIEATLIRTIYDQNDIMTVMKALSEVACGMELLFNIVICTLKRKHIQHI
LKEIKDFLRTYNDHERIIIQKYINRYTFFSIFVIICYGVSITAFSCIPIF
TSKKFPIEGSYPFSTESLVVTSIIYTIQVYAVMQSGLCVSVDLMFAVFFL
YSSARLEMLCLEIQTAKNKRQINSCIKKHQKIIRFVDKTKDVVQFSLFKT

NILMAVMSICGTFPIIHKQSLEVSSQFLCLVLAGYQRLYITAWPANDLME
NSERVAIEIYNMSWLGKSQYIIKDMCFIMQRSQKPLLISMGMFLPTLTLK
YYAKYFMTTLSYFATMRAIIGD

>PbOr182NEW
MIIMQEKITLKKAIAVVKLSLFVIWFWPLPQNTNKRKILCMKLYQHVCII
LTIAVLASMMYALVQNLDDLDLLIKSALGLFPCSHVIGNILCHLIIYQRL
QYVTFEMENFYAVIKPHENAIIQREYVDKYAKFYGFCISLFYMSLFGLFV
GPIVLDEPLPAPADFPFDASHQPLRAITYIHQIVVGLYIAAHLCVNAFMA
LLLWLASARFKLLIEELRTITNMYDFSKCIEKHQQLLEYAAEVALTVRPF
ALVTVFFSTVSLIVFGLIFITKVSLSLKIQCVFLATSALLEVFMYSWPAE
HLIHISTNVAQVAFEMDWYDDSEYFQKNIQMIILRSQKPILVVLPCGLPS
LSLRYYASYLSTIFSYFTTMRIMFEETNSGI

>PbOr183PSE
LMPIZSZPLPRDATRFKIILSRLHHYMCTGFIIILMSPLTYTITMILTPH
ZZIIELSCCLHAMHNIIFFRINHHHIQVFIDIFDHXNVTFEISNFCALMK
PNEEVIIQXNKCIVFYSISLTIFYLTGRAGVAIVPTIKHQTFPSSAEYPF
DAYKEPLKTIVYMDQCIIEMIVTVQIYMNIYVAFLLWFLSARFEILTEEL
RAITNIYDLMKCIKKHQELFKXSEIINVGHYFVLTSICCGTITTIIVGIS
FLCXLFDNKNPIYYHGFNVFSGSIYVCIWPAEHLIFIXMMAQAAFDVYMY
NQSVELWKSLHIIMMRSQKPIMLGILCVTPSISFIYFTSYCSTIMSYFTT
L

>PbOr184NEW
MWWSKLTLKKVIAVVKLSLLITWCWPLPKNTIKLKVICAGIYQYFCLTVT
FSVAVGLMNAVWNHLDDPVIMAKSISVLCPALQVVCNIMYCKINFYRLKL
VTFEMENFCELLKSHEETIVQRYIHKCAYFYGGSMIWIYLSAVFIMSGPL
TLDQSFPTNAEYPFNVDYQPLKSIIFIQQTIACMQGAAQLCMNIFIALLL
WFTSARFEILIEKFREITNIYELKIYIQEHQNLLKYTEEVIILARPFALS
TIYFSTIALIIVGLILITDQPLSMKIQCIGIIFSGLSVVFMFTWPAEHLI
HISNEIGQAVFDTQWYEQSIGLRKDLQIIMLRAQKPVIISVPCLMPALSL
RYYASYLSTIFSYFTTLRVAMQDD

>PbOr185
MQRKVTLKEVISVIKLSLIPIWGWPVSKDATKYKMFCVKLYHSLCIIMAI
MLAIPIIFGAINHLKEPIYLVQELVISSSTLHVIANFIAYKVNYNDLQDI
TTKVEHFCDFMKPHEEIVIQRYIDKCVIFYGFSLFIFYFFTLITITLLPP
VLHQPFPTLAEYPFDVIQQPLRTIIYMQQSLTGILVSGQLCTSVYIAFIL
WFTSARFEILCEELRNVGTDIYQLFKWIKLHQELLKYAVKISITVRAFVF
ITVACSTFCIIIILCLLLTHQSFIQLFQFFGLSLVCLAEVFMYTWPGEYL
INMSSNVSLAAFDALQTDQPVIMWKCLQIIIMRSQKPVTVKIPCFLPALC
YTYFTTYCSTILSYFTTLRVMINE

>PbOr186PSE
MQRKVMLKKVISVIKLSLIPIZGZPVPKDATRYKMLCIKLYHYSYILTVI
LLVTSFIYGTINHLKDPVFLVNQLVMTNSTLHVIANFTFLRLNYDLQNVT
IVMERFCNFMKPHEEIVIQQYINKCIKFYTLFLYFSLFIFYFIAFITVLL
LLPVMHQKFPSFAEYPFDVTQQQRTIIFIHQGLCDVLVSEQLCANVYIAL
LLWFTSAKFEVLLEEIRTSTRIYQFFKCIKIHRELLKYAEVAMTVRPFAF
ITVCCSMFCLSFAYFSXHPPIIAFIQFSALTVVGLAEVFMYTWSGQYLIY
MSSNITQAAIEYAFQYDQQIIIWKCLQIIIMRDQKPTVRILCLMPALSFN
YIXTTMLILWHFS

>PbOr187
MIKITPEATVSFTKIIVALSFSWPLSKSASKLQVLRFKILRFLVCMNATL
LIVPVAYTLYCNDVDLPKITKLWCLLGAIIQVPLEITQCALQYDRLQYLI
SEMDYTIKCAGHHEKVVYQRYVDKCAMFYASSTLAVFSTAFVSMIAPLIT

ADQSFPTDSRYPFNVEYEPVKSIIFLHEIIVIWQCFSIVCHCCFIGLLIL
FAAARFDILSQQFRKVTNIHDITLCIRQHIKLLRYAQEVITAVRSLILTV
IIICTWVIIASGLTIVGKSTFADKVQFMILCITGLMEVYACAWPADHLID
ASTNVAQAVYELLWYDQNKIIQKNVCFILLRSQTPMIVSISVILPTLSLQ
YYASYVSTALSYLMTLRIVFLENDEIKN

>PbOr188FIX
MTGILTLEKIIAFLKVDLVFACCWPLQVKATKFQKICNKIFRLFCCLNGI
LMSVSLFYTLSNKCYNMLLVMKVGCELSAFLQIPIQITLFTFQSDRLQII
ICEMEDYINRAKSEERSIFNQYIKKCKLFYITTICWLTITASAMIFGPIL
LSQPFPIEVDYPFDVNKQPLRTIIYLHHAMAVYQSYVQVSSNIFVALLLW
FVAARFDILSQKFQKISSISEFMMCVQLHQQLLRYAKNVTMTIRYIALSS
IGFSTVAVVFSGLTFLSRQPFAIKIQFFTVGASALIEVFVCAWPAEYLLR
TSNDIGHAGYESSWYTKEVSLQKNMLYVVSRCQHPVYLSVPCMLPSLSLN
YYASYLSTTFSYLTTFRAIFAVEDGLQI

>PbOr189
MLSKITPEVAVKFTKIIVALSFSWPLPKNASKFQVICFKIFHALTHINII
LLIVPMIYTCYYSNDYDLLKISKLWCLLGVFLQIQLQITMCALHHDRLQY
LISEMEHNFKCGKLHEKMVYQQYVDKYATFFASSTFAVFLTASGITLMPL
VTDEIFPLDTKYPFDIERGAFKPIIYLQQASVFWQTFSIVCLSIFFGLLI
LFTAARFDILSQQFRTVTDICGIIECIRQHTKLLKYAQDVIISVRLIILC
IIICCTWAVVIGGLTIISRSTITEKVQFIILCTAGLMEVFTYALPADHLI
DASFNVAQAAYESLWYNQDVIFQKKLCFILLKSQVPVALSVYSILPTISL
QYYASYVSTAFSYLMTLRVVFVEELN

>PbOr190FIX
MRTAPTLDKVIAFLKIDLLFACCWPLPRTATKFQIIRDRIFRYLSIVNGA
LIVIELIYSITNHLDNVFLIMQLACALGIFCEVPLQIYLFTQQHDRLQNV
IYKMEDYYEQANAQEKDVFQQYINKYITLYGTTLTLTTASLAGSLIVPLI
RSRMFPLEIEYPFRVDYQPMTAIIYFHQALGMYQVYCQVSANVFLALLLW
FTTARFEILTNKFHMITKYCDWKTCIQEHQEMLRFAKEVSNSIAHVVLSS
LGVSTVALVFGGVTFLSQFPPSVKIQYMIVCSTSLTKVLLCAWPADHLMR
TNSNIAEAAYNSLWYNQNIESRRIMLYTLLRCQRAVVISVPGLLRALTFQ
HYASYVSTAFSYLTTFRIILSDET

>PbOr191FIX
MRSICYVHICLCNIFALTIIYKYTTINKIAIFLLCNLIFEMSIFMNYNKL
IYGIRVWGRFTTTWPLNSKVGKVKIFLYKFFWCIYTVNMICQCYLLANRI
FPFNRKNFIDTMRTLVEMTYSLETTFNLLYCRIREKQLQELLFDIDQCIK
VWMPYEKTMRRKFMYNTCAICLSLIIIYIICAWLFILSPVMIDRILPLNV
IYPFSLSKFWVYCVAYAINVFSILSCSIIIIVDIMVIATLWQAVFKFSLL
GMQIRSAVTVDKLRACIIIHQNTFDYVRGIDCTFSHIIFKLTIVVSIYII
SSGLLILNNAPILEISLFLLITSFSIWRLFVCCWSAQAVTDMSYSICWQI
YDSPWLNASVNVRRTIFIIIQRCQKPIGISGTNLISIISIRFCGKVLSTT
FSYFMTLRTILYLE

>PbOr192
MIMQKNLTLKKVIGAIKLSMFVTWCWPLPKDTVKLKIICVTLYQYLCLIL
TLGLTVGLINTVKNHIAEPLIMAKSIIVMCPTIHVICNIICCKVNSHRLQ
YVTIEMENFCELLKPHEETIVRQYIDKCIHFYGGSILWIYLSAVIIITGP
VTLNQPFPTNAEYPFRVIYEQPLKSIIFIHQSLVFIQAASQLCMNVFITL
LLWITSVKFELLNEELRSITNIHDLIHCIRKHQRLLKYAEEVIVVVRPFA
LTTISLSTFALIIVGLVFITDPPLSMKMQCVGLTFSGLAEVFMYTWPAEY
LIQTSGEIGQAVFDAQWYQQSIALQKYLQMIMLKAKYPLVVSIPCVMPSL
SLNYYASYLSTIFSYFTTLRVVMQNE

>PbOr193JOI

MATIHLKFQKMFYILRLFGIFTNTWPPQPNIGKIELFLRNFYYYVAIFIL
MAVWIPMIISIYNSRNDDIGIMMKNVSHAAAVIEAILNSILCRIKKRQLQ
ILLLNIEKFIKASSDREKVILQKYVDRYATFILFVAISFIMAGVTVICAP
LFMPLELPIAVWYPFSTEPLLRKFILYIMQIFIVAHTVLCLGVDVMIAVL
LFYSTARLQMLSFEIEQATNEIHIISCVKKHQEIICFVDDVQCTVRYLIC
KSNITMASAVIFGAFPLIYKQPSTVTAQFVCMVIGGCERLYITAWPADDL
AEMSQQISWSAYSIMPWSEKSHGSIKNILIFIQRSKKPLLISMGSIFPAL
SIQYYANFLKTISSYFMTMRAVITE

>PbOr194JF
MVLVHIKIHQILYILQICGTFTSTWPPEFTAGKREIFLRNLGWTLAILNI
LGSMPPLILGAWYSGNDVIRMMKALSELTALMEVLFNLILCRIERSRLQQ
IKDFLKHSESHERIIIQKYVTRYLSFSIFVGMSYILAAIAFSCGPLFLSI
TLPMEAWYPFSIETTYAKGILYILQVFAILQTGLCITVDFMIAMFFWYPA
ARLEMLRQEMQQITHENQIRTYIQKHQEIIRFTSKTQEAIQYVLFKTTLT
MGFTVISGGFPMLYLQSHALIPQFISMIMAALQRMYITAWAADDLKEVST
QFIWSVYNIWIGKSQKMKGNILIMLQRSQQPLLISMSALLPALTLEYYAS
FVTSVLSYFMTMRIVIAT

>PbOr195JF
MAIIHLKFQQMLNILRILGTFTNTWPPYPKFGRKELILRNLYYYIAIFIL
MAVWIPMMISTCKNRNNVGVLMKNMSHIAALTEAILNSILCTIKRKQLQS
LLMNIEMFAKVSKSHEKVILQKYVNRYIIFISTVAISFIIAGITVIFSPL
FLSLEFPVDVWYPFSTEPLFRKFILYLMQIFTVAHTVFCLGVDVMIAVIL
FYSSARLEILAFEMQHVTNAIHMILCIRKHQEIIEFISKTQQAIQHILFK
TTLTMGFTVISGCFPMLYLQSHVLIPQFLSMIFAALQRMYITAWAANDLK
EVSIQLTWSIYSASWIGKSLKMKNDIFMMLQRSHKPLLISMNGLLPALTL
EYYGSFLTSVLSYFMTMRAAITT

>PbOr196JF
MVYFRLKPQQLLRILEFLGVLSSTSPKSISGRFIILKNVVWYLTFSNLIF
QLIGETLYIYHCQDTIMILKTIFVAACVTDAVLNLIICHTQRERLQHLLK
EIENYLQNADENEINILQKHVDRYTLILVANTLLLSCAGLIICLRPLITK
EYFPVDVWYPSFMYSTSSRRCLIYISQIFSGIECVLCFNTDISIAMFFCY
STAKLEVLQQKLQCTKTKDYIRKCIKQHQDIIRFVDLTQVAVQYLILKLN
VTMGTAAVCSLFPLIIDQPLVVKGQFIFTFLSACERFYISSWSATDLSEM
SKLIAYSSSSDDYISPRTVNDILLLIQRSQKPLTISMASFLPVLSVEYFG
NFLTSVLSYFMTMRAAITT

>PbOr197
MRKERGMSVPNFHLRVSLTIVYFLGTWPPHTGTYRFLYLLYTVCSFTFIL
GILLAAEIANVFMNWGDMSKLVAVATLLMTNCTHASKVIVLLRRQMRIQA
LLDTANSSAFSRYDKEQKDLLIRYTWKGIFHHVVYQSFGAIAVFCWGITP
ITDLVAGRSRRLPMEGWYPYNTTMTPAFEITAGHQGIAIIIACFHNVAMD
TLITGLITVACCQLAILERHIISIDNDKKSTCKVQSDEHKLSVVKGKIRS
YQLLKRCAVHSNMISHFTSEIQNIFGTIIFFQFLSNCVIICLIAFNLSQM
KVYIPAVLIGMLTYMCCMTYQIFIFCWHGNELYLHSMRLVTAAYSSNWIS
NTEAFKRSLQIMMIKAHRPLTLSAGKVMLLSLDTFVQIMRMSYSIFTVLQ
GSAA

>PbOr198FIX
MHILPISFGMLTYFGYWQPIGLTSIKYWTYIIYSVVMNFVLYSFAFCGLV
DCFIIEDLETFVDKFSLSVSVVSVSCKVLNLIVHRDKIIGLTDMLLNDIC
IPRNDYEMNIQRRFDHNTKIVTIWYEILNEISVLFAVIAQLWNSIDARIL
TLAKWVPYDLSSDFAFWATTLHQTIGLIACAHASTAHETLISGFMIQTCA
QLDILCHRARTLPDKLREVRKYCTSKEEIKSKERQLVRELIHHHRYVYRL
AERINSVFTIMIFIQFIVSSSVLCITIYKMLIKEVGAEFIWVASYLAVML
GQIFLYCWFSNELTLKSTEVGSAVYEMDWPMLPNDIMKSLLVIIMRAKKP

IVMTSGYIVTLSNESFTKIIKISYSAFNVMHGS

>PbOr199FIX
MHTLPLSFALLTYTGYWRPIGLTSIKYWAYIVYSIIMNFLLYSYAFCGLV
DCFIFKDLETFIEKFSLCVSVVSVSFKVLNLVVNRDKIVSLTDMLLDKVC
IPRNDHEMNIRRRFDHNAKIITLWFEILNEFCVMFATIVQLRYSISTRTL
TLAQWVPYDISSDFAFWATTLHQAIALIVCANASVAHETLISGFMIQTCA
QLDILCHRARTLPDMLREIRKHCTSKEEIKAKERQLIREFIYHHRYVYRL
AERVNTVFTVMIFMQFTVSSIVLCLTIYKMLMKNLLSAEFVWISTYLGCM
LGQIFLYCWFSNEVTLKSTEVGNAVYEMDWTMLPNELMKSLLVIIIRSKK
PIVITSGHIVTLSNESFMKVIKVSYSAFNVLQGS

>PbOr200
MHTLPLSFALLTYTGYWRPVGLTSIKYWAYIVYSIIMNFLLYSFTLCGLV
DCFIIEDLETFIEKFSLFLSVLGVSCKVMNLLLHRDKIIGLTDMLLKDIC
VPRNDHEMDIQRRFDRSAKTITICCEILNESAVFFATVAQFRYFVSTRTL
PLSDWVPYDISSTPAFWATMLHQTIGLMVCANASVAHETLISGFMIQACA
QLDILCHRARTLPDSLREVRKHDTSKEEIKAREQQLVRELIHHHRYVYRF
AERINAVFTLMIFVQFTISSTVLCLSIYKMLTKNLLSLEFAWSSSYLGCM
LMQIYLYCWFGNEVTLKSTEIGSAIYEMDWPMFPIGLMKTLLIIITRAKR
PIKITSGHIVTLSNNSFMKIIKISYSAYNVLQGS

>PbOr202FIX
MQILSLNFLLYSMSGLWRPIQWSSKYSKLLYSLFTLMSIYLLIYLMLTIL
LYIIFVSDNVEDFASSSLFFMSMISVFFKVIVAIFRRGRIINLIKILQEK
PCRACNEEEINIQIKFDRSIRSYSMRYVVFSTFSAMGAVTGGMLDILDGQ
LPFNTWVPWHCTSFLLYWFTSLQEIVAVIIATVVNVATETTILGFCLQTC
AQFAILKHRLQKMAKSREKELSRKNSLNNASHQTGKLSEHISHHLCIIRL
AEMINDVFSQVIFVQFFVSILVLCSCLYHLSSHLTFTDVTTLIVFIFSMF
VQIFIYCWAGNEVMLKSAEMSEAIYHMDWILMTIDERKDLLMIMRRSTKP
VKFTSSFLVTLTLESYANLLKASFSAFNLLQQF

>PbOr203
MQMFSLNFLMYTIGGVWRPVEWSSNVAKLLYNVFTSIVLILLYFLMITQF
MDIIFVVDNIDDFATNTLMFLTIVAVTCKATIVVVRRNAIINLVQVLLTA
PCKPRGEDEMAIQTKFDKFIRSCSIKYSLLATSSVTGVTIRSVLNVIQGH
LPYRVWLPYNFNNTSPMFLITSIQQIITVIFVTIINVGTETLVFGLFLQT
CAQFEIFENRLHKLIANKTSEKLIKDKIRCIEKSLSSPNNNKAIISKYVY
HHLKIYKYAKTVNVIFNQVLFVQFFGSILVLCTSVYYLSTHSTLSETATL
IVYTICMFVQIYVYCWSGNEVILKSMSVGDTIYCMNWPLLSVNDRKELLM
IMLRSTLPIKFTSSFLITLSLQSYSNILKISYSAFNVLQK

>PbOr204FP
MQILSLNFSIYTFCGIWRPVEWSSNGAKLLYSIFTFIIIFSEYFLVLTQF
MDIILIVDNIDDFATNTLMFLTIVAVCCKATVVVIRRDAIINLVQVLLTK
PCKPRDEDEMTIQTKYDKFIRSCSIKYSLLATSSVTGVTVGSIFNVMHGH
LPYRIWLPWDYNVPLTFWIISIHQMITLIFATMINVGTETLVFGLLLQTC
AQLELFESRLHKSIISKTVIYLEHAFSSPNGGKTEISECIHHHLNIYKYA
RTVNIIFNQVLXFNFLVVYWYYVRVYIYLSTHITDLYGTASLLVYTICMF
VQIYVYCWSGNEVILKSTSIGDAIFRMDWPLLSINEKKKLLMIMIRSTIP
IKFTSSFLITLSLQSYSNILKTSYSAFNLLQK

>PbOr205PSE
MQILSLNFLIYSLCEXWRPIEWSSNVAKLLYNTFSFIIIFSAYFLMLFQF
MDIIFVVNNIDDFATNTLMFXVCCKSVVVIRRNVIINLVQVLLTEPCKPR
DEDEITIQTKFNNFIXSYSIKYSLLATSSVTGVTVGSVFSVMHDRLPFRI
WLPWDYNVSLMFWIISLHLXIITVIFAIMINVGTDTLVFGLCLQTCAQLE
LFESRLHKLIVKXVRYWKDALPSLNKDRAEISECIHHHLSIYKYAKIVNA

IFNQILFVQFFGSILVLCVYYLSMHIMDMLEAGSLLVYTIIMFIQIYVYC
WCGNEVILKSLNVGKAIYCMDWPLLSVSEKKELLMIMIRSTIPIRTSSFL
IILSLQSYIXILKTSYSAFNIL

>PbOr206FIX
MAITFSNFLMYTVGGIWRPIEWSSSGAKLLYSIFTFIIVLSEYFLVLTQF
MDIIFVVNNIDDFATNTLMFFTIVAVCCKATVVVLRRNAIINLVKILLEA
PYKPRDEGEIAIQTKFDKFIKSCSIKYSLLATCSITGLTLGSVLNVMHGH
LPYRIWLPWDYHVPMRLWIISIHQILTVIFAAIINVGTETLVFGLFIQTC
AQLEIFENRLHKLIINNTIRYLERALSSSSENRRNISECIYHHLCIYKYA
KTVNVIFNEVLFVQFFGSILVLCTSVYFLSMHITELSAIANLLVYTICMF
VQIFVYCWSGNEVILKSTNVGETVYRMNWPLLPVNEKKALLMIMMRSTIP
IKFTSSFLITLSLQSYSNILKTSYSAFNVLQK

>PbOr207
MQLLTLNFLIYTVAGIWRPIEWSSNGAKLLYNIFTSVILFLEYFLVISQF
MDILLVVDNVDDFVANSLMFASIVAVTSKATLVVIRRNAIVKLVQMLLTI
PCKPQDESEEVIQTKFDKFIRSWSIRYLLLAMSSLTSVTLRSVLNVIEGQ
LPYRVWIPYDFNIPLVFWMVSIQQIIALIFATFINVGTETLVFGLFLQTC
AQFEIFENRLYKFVSNKTVKLKHSFISPIKEKSTISEYIHHHLSIYKYAK
TLNIIFNQVLFIQFFSSILILCTSVFYMSTHISESGTMTLLIYTICMFVQ
IYVYCWSGNEVMLKSTSIGDAIYDLDWPSLSISERKELLMIMMRSTIPIK
FTSSFLITISLQSYSNILKASYSAFNLLQR

>PbOr208PSE
MNVLKFTFKILTICGCWPPNXWTSPYKRIMYNVYTVLILLLINTFTLTQL
MDIILTMDNVDDFTENFYVTLAMLVSCYKMFSLLRNRNNIEMLIDILMXK
PCRPTEHDEIEIRQTFDKLVLTNTLYYATLVELTCAFALAASVFKDYRKH
KLAFRAWLPFNYSSPMLLRIAYAHQSISLTAGSVLQIACXSLICGLLMHV
CSQLEIVECHLKKIVDKPYFLQECVVQHICIFXFALMINEKFRSIITIQF
LVSMLVVCFNLYQLTQSTLSAKYIQIILYMFYMLTQISFYCWYGNEVKLK
SQQLVNDMFEAERYMVGLXICKKSLSLIITRSSIPIEFTSAYVIPMNLES
FVNLLKTSYSAYNILQQMRE

>PbOr209
MRVLGLTLKILTSCGCWIPDSWTSPYKRVLYYAYTGFIFVLINTFTLSQF
LDLVLIVDNADEFTDNFYILIAMMISCSKMFSLLINRNNIALLIEILRKK
SCKPLELDELEIRQKFDKIIEGNTLHYAILIEFSCTYSVIQSFFTAYLKE
KLAFRAWLPFDYSTTVLFHFMYFHQLIALLVAALLQVACDSMICGLLMHI
CCQIEILNSRLKRIIHNPKILRDCVIQHNLLIKFALLVNEKFRLIITLQF
IVSTLVVCVILYQLTKTDANIIQLGLYMSCMLIQIFLYCWYGNEVRLKSI
KVINDVFEIEWLQLEPDVRKDLLIITRCGTIPIEFTSAYVIPMNLDSFVD
LLKTSYSVYNILQQMRDTSI

>PbOr210
MRVLGLTLKILTSCGCWIPNSWTSPYKRAMYYAYTTFIFLLINTFTLSQF
LDLILIVDNADDFIDNFYILLAMIISCSKMFSLLINRNNIVLLTEILTKN
PCKPVEDDELEIRQKFDKIIEINTLHYAILVEFSCSYLAVQSFFTAYCEE
KLTFRAWLPFDYSTTVLFHFTYFHQLIALFVGAFLHVACDSIICGLLLHI
CCQIEILNSRLKRIIQTPKILRDCVIQHNLLIRFALFVNKKFRLIITLQF
IVSTLVVCVVLYELTKANAKIIQLGLYMSCMLTQIFLYCWYGHEVRLKSL
KVINDIFEIEWLQLEPDVRKDLLMLTKCGSVPIEFTSAYIIPMNLDSFVA
LLKTSYSVFNILQQMRDTSIEE

>PbOr211JF
MQILDFTFKILMICGCWIPNSWTTPYKRLVYHVYTIFIMLLIHTFMLSQL
MDLILIVDNADDFTDNFYMLLAMIVSCCKMFTLLMNRNNIAMLIDILVKK
PCRPVQSDEIEIQQKFDKLIQTNTLYYAILVETTCVCIAVTSLLTEFSKR

RLTFRAWLPFNYSSSSLFYIVYVHQLIGLTAGSVLHVACDGLICGLLMHV
CCQIEIIECRLRKVANNQNSLNESILQHNYIFNFAWLVNEKFRMTIVIQF
IVSTLVVCFNLYQFTKSITKYMQLIMYMGCMLSQIFFYCWYGNEVKLKSR
QLVDSIFEMEWFELNKYTKQSLLMIMKRSSRPIELTSAYVISMNLDSFVS
ILKTSYSAYNILKQM

>PbOr212
MFILAFTFKILMICGCGLLHSWKTPYKRLVYRVYTIFIMLLIHSFMLSQL
VDLIMIVDNSNDFTDNFYVLLAMIVSCCKMFALLINRNNIVTLIETLMSK
PFRPVEPDEMKIQQKFEKLIQSNTLHYFILVETTCLSVTVTSLLTEFRKG
NLTFRAWLPFDYSSPLRFFLVYAHQLISFTIGSVHHVACDSLICGFLVHI
CCQIEILEHRLRKSAREPNILRECVLQHNHIFKFANVVNEKFKLTIFLQF
VVSTLVMCFNLYQFTKSTALKTKYMQLILYTCSMLSQIFFYCWYGNEVKL
RSRQLMNNVFEMEWFNLDGNAQKTLLMIVKRATIPIEFTSACVISMNLDS
FVGLLKTSYSAYNILKQTQE

>PbOr213
MQVLNSTFKFLTICGCWRPDSWTSSFKRIIYHIHTFFVFVLINTFTLSQL
LDIILTVDNPDDFTDNFYMLLAMIVSCCKMLSMLLNRKNIATLTNILMEK
PCKPLEPEEMQIYHKFDESVQTNTIHYAILVETTGICITLTSLLTDFRSR
TLTFRAWLPYDYSSPVLFYITYAHQLISLIVGSVLHIACDGLIIGFLVHI
CCQIKILESRLRRIAHEPDILYECVLQHNRIFDFAIMVNAKFRFTIAVQF
MVSTLVMCFNLYQFTRSTTSNAKYVQLILYMCSMLTQIFFYCWYGNEVKT
KSRQLIDNIFKMKWYMMDQNIKKALLLIMKRSIMPIEFTSAYIVSMNLDS
FVGLLKTSYSAYNILQQI

>PbOr214
MHILKLSFKLLTLFGCWRPDSWSSLYKRIAYHVYTSIIIFSINTFTLSQL
MDIILTVDNADDFSDNFFVLIGMLISCCKLFILLINRKNIIKLIDILMEK
PCRPVRSTEMKILYKFDKGIQINTRRFIYLTLITISSIVLTSLSNFRNRK
LTYRAWLPFDYSSTVLFCLTYTHQLISLFVAGFLNIGCDTLICGLLVHIC
CQIEILIYRLRKIISYRDNLRDCVHQHYYIFRLVFIINTKFRLTIAIQFI
MSTLVVCFCLYQLNKVTGKAKFVEMTLYMLCMLTQIFFYCWYGNEVKIKS
TEMIDNIFKMEWLTLDENKKKSLIIIMRRAVVPIQINCVYIIPMNLNSFM
SILKTSYSTYNLLQQMRE

>PbOr215
MRVLQLTFKILTITGCWRPQSCSSCMRIVYDTYTIFMIILLYTFLVSQFL
DIIWNVNNAEDFAENFYATLASVVSCSKMLSLLVNRDNINTLTNVLIEKP
YRPLEMDEMKIRYKFDRLIYINTLCYTILVETTCLCITMTSLFTMFRKGT
LTYRAWLPYDYYSSTIVFCLTYAHQLISLTAGSLINVACDSLICGLLVHI
CCQIEILEYRLSKISNNHDILRDCVRHHDSIFEYAIKLNKKFRMTIAMQF
VVSTLVVCSNLYQMTKSETLNANYLPLLLYMSCMLTQIFIYCWYGNEVKL
KSTQLLQNIFSMDWITLDRHVKESLLIIMNRAAVPIEFTSAYILSMNLDS
FVGILKTSYSAYNILKQV

>PbOr216PI
MQVLEFTLKILTVVGCWPPNSWTSLCKPXYNAYTTFIILLLFTFMLPQLM
DVLLNVDNTDDFTDAFYIMLAMSCKMIGLLVNRKNVEILTNILTKKPFIP
MDSDEVEIRHKFDRTIHTNTLLYTILVETTCTCIALISLFTDXRKSNLTY
KEWTLYNYTSEIIFCVTYVRQLISSTMGSMVNVACDSLICGLLLHVCCQI
EILECRLKKISHSQNHLRECIQQHDCIFKFACMVNEKFKIIIAIQFIINT
LVVCSNLYQLAKITLNAQCFPLILYIYSMLTQILIYCWYGNEVKLKIFKT
SYSAYNTNITDITNASNINQIKI

>PbOr217FIX
MCMQVMQFPFKVLTVAGCWPPISWSSLCKRTVYNAYTVFVCLLLLTFMLP
QFMDIVLIVDNPDDFTETFYVMLAMVIACCKMFSLLLNRKNIEIFTDTLV

EKPFKPLEPDEIEIRQKYNNIIQTYSMCYTIMIETTCACMNLTSLFTDFR
KGDLAYREWLPYECSGVIYYLIYFRQIISLTAASIVNVACDVTICGLLLH
IYCQIEMLECRLKKSVRNRSDLGECARQHDRIYKFAYMINEKFKFIIIIQ
FTASMLVMCFNLYQLAKKTLSAEYIPLILYTLCMCLQVFIYCWYGNEIKL
KSIQFADNIFGMDWVTARQENKKINLIIIMNRCLTPIEFTSAHIITVNLD
TFVKLLKTSYSVYNILTHM

>PbOr218
MNLLKYTLTLMMFAGCWRPTSWVSSFKYKLYNVYTTFLILMLYAFAISQL
MAIVLNAANPEEFTSVLYMMMTVCIAIFKISSMWMNRKNVADIINILTKK
PFKPKIASEIKIRQNFDKMIRNNTLACFVLVETTCMCIALTSLFTNFRTG
TLTYKAWLPFNYSPPVLFSLTYTHQLISMATCALINLACDCFICGLLLHI
CCQIEILEYRLNKSYTWKNLRDCVCHHELIFDFASIINERFAKIIAIQFI
TSMLVVCSNLYQLAQITLSAEYLPLVLYTICMLIEIFIYCWFGNEVKLKS
LQLIDRIFEMEWPELNNSFKKAFLMIMNRTMIPIEFTSAHLFSMNLESFV
GVLRMSYSAYTLLQRI

>PbOr219
MHVLKLTRTIVTIAGCFRPLSWTSSFKRTIYNIYRLYIISMLYTFSLSQF
MDIVLNVKNPDDFTDNLNMMMTSSAASCKIFIIWLNYKNITALINYLIEE
PFKPLDLGEIKIRRQYDKIIRNNTLRYTSLIGTTCLFISLVSLFTDFRHR
KLTYREWVPYNYSSYMTYSLTYAQQMISTMHCACVNVGCDTLLCGFLMHI
CCQIEILEYRLKKVLNNQFTLGYCVRHHNRIFEYAQMVNMRFTKIVGFQF
IASTMVICSNLYQLTKSTLSANHVTLIMYTSCMLTQIFIYCWFGNKVRSK
SLQLMENIFQMEWPIVNNTIKKGFIIIMKRAMVPIEISTIYILTINLDSF
VALLKTSYSVYNLLVRVQ

>PbOr221
MHILKLTRTIVIITGCFRPLSWTSLFKRTIYNIYRLYVISMLYTSSMSQF
MDIILNVDNPDDFTDNLNMMLTMSASCYKMFIVWLNYENIVALINCLIEE
PFKPLDLSEMKIRRQYDKIIRNNTLRYMTLVGISCSLNAVVSFLTDFKNR
RLTYREWVPYNYSSYMTYCLTYAQQMITTFHSASVNVACDTLLCGLIMHI
CCQIEILEYRLKKILNNQLTLDYCVRHHNRIFEYTRMVNIRFTKIIGFQF
MASTMIICSNLYQLTKSALSVNHIPLIIYTSCMLTQIFIYCWFGNKVRSK
SLQLMDNIFQMEWTALNNAVKKSLIIIMKRAMIPIEISTIYILTINLDSF
VALLKMSYSVYNLLRVQ

>PbOr222FIX
MPVLKFTLTVLAVAGCWRPTSWTSLFRHIIYNAYTASIILILYIFAITQI
MEMVLNADDSDTFGDALFNALISLLACYKAIIIRINHDNITILIDNLIEK
PFKPMDIDENIIREKFDKRVTNNTLCYIILVLITASYMILISLFTDLKNG
TLMYRAWLPFDYSISSLFYLAYTHQIMALICIGLVHPTCDNLICGLLLHI
CCQIEILEHRLSNIANNRGNLRDCVRHHIRIFEYAYMLNDTFGKIIPGEF
LMIIVVMCYNLIHMAFKSSSTISYIEDIMVVASTMAPIFYYCWFGNEIKL
KSLQLSESIFKMEWTMFSNNIKKGLLMIMNRATIPIEFTSADIIPVNLDS
FVLKTSYSLFNVLIQSRK

>PbOr223JOI
MLVLQFTLKLCAVSGCWQPLSWTSLSKYIIYSCYRILLICLITAFTISQM
LHTGLNLDKHNEISDNLYMTLTVFIATYKIIIMWKTKKHVVRIINALTEE
PFRPLESNEEMIRQKYDKMIKKYAFLYYGLVQVTVICIILNAIFMDFMKG
NLTYKAWIPFDYTSPIIFGFVFTHQMIGMSITAAVNVACDSLVSGLLQEI
CCQLEILEYRLTKISHDQHILRNCIHHHNRIYEYAYIINRRFAKIIALQF
AVSMLVVCANLYKLASIPSLMINGGIITLILYTACMLSQIFLYCWFGNEL
KLKSTGVINSIYHMNWQNLDKASRKDLLLIMRRSMIPIEFTSAVIITLNL
ESFVSLLKASYSAYNLLKRFQ

>PbOr224JOI

MSMLKFTLIICALAGCWQPLSWTSLFKHIIYKTYAMFLISSLYAFLISQF
INTILNVGNSDEFTDALYMMLTVLVAGYKQIYMWIDRKNVLVIINVLTEK
PFAPYETQEVKIQQKFEKMVQNNTRRYLTIVMMSITSIISMSVFTDLMKQ
NLTYKAWIPFNYSSPTIYSAVYTHQLIAMSTSGIVNVACESLICGFLLHI
CCQFEILEHRLTKLIHDKNILSDCICHHNRIFEYAYIVNNMFAKIIAIQF
AVSMLVVCSNLYRIAMATDYMSFIPLIMYTSAILVQIFIYCWFGNEVKLK
SLRLVNSIYDIEWPALSNNNKKALLLIMKRAMIPIEFTSAYIITMNLESF
VALLKMSYSIFNLLHQTHE

>PbOr225
MRVLKFTLLICAFAGCWQPLSWTSLFKHIIYKTYAMFLVSALYIFSISQF
MNIVLNVENSDEFTDSLYMMLTVFVAGYKQVYMWIDRKNVMVKINILMEK
PFAACETREIMIQQKFEKMVQNNTLRYLILVLMTITSIVLTSVCTELKNR
NLTYKAWVPFNYSYPAVYLFVYIHQLIGMAISGIVNVACESVICGLLLHI
CCQIEILEYRLTKITHDESILHDCIYHHNRIFKYAYTVNNMFAKIIALQF
AVSMLVVCSNLYRIAMAKDYAIIVSLMLYTGAILAQIFIYCWFGNEVKIK
SLHLANSIYNNVEWSTLSNRSKKDLLIIMKRAMAPIEFSSMYILTMNLDS
FVALLKMSYSAFNLLHQTQE

>PbOr226JP
MHTLKFTLIICTIAGFWQXLSWTSLFKHIIYKTYAMFLLVIHIFNISTYG
LVLNVDNSDELTDSLYMMLTVFVVCXYKQVCMWLNHZNVTIIIKVLSEKP
FKPCESHEIVIKKKFDKMIENNTLRYLTLVGTAVVSVIVSSIFMDFRKGN
LTYKAWIPFDYSTSATFIFVYINQMIDMSTSDLINVACENLVCGLLLHIC
CQIELLEYRLKKIAQDKNILRDCINHHILIFEYAYXVNNLFAKIIAMQFA
VSLMVVGSNLYCIAMASDYLKFIPLMLYTGCMLAQIFIYCWFGNEIKLXN
LHLTNNIYNVDXLEFSDNCKRMNLDFFIALLKLSYSAFNLLHQSQE

>PbOr227
MDLLPVNFYVLRFCGVWKESKDGNLIIRFISFCYRYTIAVLIYHFTISEI
IELIRMRNNMEDLTECLFLALSFITLCLKYLNFLMRQCELRALLNCFRAK
LCQARDSTEESILKQYDRKAKQTACLFMFMCQTTGLLILTVPLLTDDKRS
LPCKMYVPYSITAFLPYVLTYLQQTTALIYGILLNVSFDSLVYGLIIHTC
GQIELLCYRLTEKIRFLQEHNEEKKLGVIEDYAIAECVKHHISVYNITNR
IQSLFIWTTTTLFFFSLLTLCTSIYQMSKKELFSPEFFSFILYLGSMMFQ
VFIYCWYGNELDLKNKNIAHAIYASDWTVISAKHRKNLLLMMLMSQRGRI
LSSYGFCALILSTFTWIIKTSYSAFNLLQQASN

>PbOr228PSE
MDILPVNFYVFRFCGVWKENKDSNLIIRFVSFYYXXEARRIAFMIISQMM
GLLVLTVLLMAQEKQLPLKMYVPYFMTTLLPYILIYLEQAVAVFYGVLLN
VALDSLVYDLIIHTCGQLCYZLTEAFRYLZENNEEKKISSVEKFAIAEHH
ISVCNITYRIQFLFMWIIATLFFFSLVTLCSSIYQIXNKDLFTVEFFAFM
LYLGSMLFQVFXYCWFGNELDLKYKSITHAIYASDWTVISINQRKSLSFV
MMMSQRGRIIXFYGVCSLLLSTFTXIIKTPYSAFNFNCSKLQFNIFIINL
MSIFIFVSIYSVLSL

>PbOr229
MDILPLNFRVLWFCGAWSEKKDNNLFVRFSSFCYRYAIVILIYEFTLSEV
IELIRTHDDIEDITEGLFLALTYVALCFKYGNFLARRDEIIMLLDCFRKE
TCQPKNTEEKMILVKYDRKAKWCVRFFMSISQATCIALILAPVVGPQDTD
RPLPFKTYLPYSISDLYPYLATYLQHVGAIFYGVLLNVSFDSLVYGFTLH
VCGQIELLCYRLSKIFKDYSKQYQIDSGAMISECVRHHLNVHELVRRIQS
LFVWTVTILFIFSMVTLCTSIFQMSKKKILSVKFLSLTLYLGSMLFQVFF
YCWYGNELQLKSKNIGNAIYSSNWTIATTRDRRSLLFVMIISQRGLRLSY
YGIFNLALDTFTWILKTSYSAFNVLQQTSI

>PbOr230

MDILPINFKALRFCGAWKEREDDNICIGFLRIFYKYAVFLLIYEFTIFDV
VEVIRTRDRIHELTEGLFLGLTFVTLCVKYANFLLRENEMTDLLDSLRVK
MCQPRNSMEKLIMEEHSRKAKWSTILFMAISFATGLGMMVTPALELLMGG
ERTLPLKSYIPYSLSSLLPYLATYLQHFLTLFYAITLNVSFDCLVYGFTI
QVCAQIELMCYRLTDNLRSTGDISFGKKPQTNASIEECIRHHLLVSTVVK
KLRAFFIWTVMIFFFFSLLIVCTSIFLVSKKKLLSFEFLSMFLYLNGILL
QLFYYCWYGNELELKSKDIANAIYSSDWTMVTPRERKSLMLIMISSQKGI
MFSYHGVFALSLNTFTWICRTSYSAYNLLQRASN

>PbOr231
MEQVDPEEHYYKLNRFLLSVSGLWPYQNEWSARLMRAIITFIMLTSTIFQ
LASMFTSDLTLDFIVDLIPLVILTLGTLTNLFSRILHIDKLRELFDNIWK
DWALQKTHDEIKIMHERAKVTRFLTLCYFILIYISIGIYNVWMFVPEILN
IISPLNESRPRRRPFQAEFFIDEERYFFLVRSHICLSIIIVPIVYLAGAT
LFLALAQHICGMCELLGYRAERLFCIRDEGFNLIKGSKVTRGNIAVLIRL
HYNIIQFVDILQIFHTIPFVLDLIGTVILLSMTLSQILNISGIERAIRCI
GIAVVSLCYVFIYNYMGQKVTDLTSDISNRMYNSRWYNAVVSDQKTLLII
MMRRCQPLFLTACKFYVMSLQNFGMILQTTISYCMFIRQV

>PbOr232FIX
MEHPEEHYYKLNRFFLSISGLWPYQSKWSACLMRAILTVVMLSSTIFQIS
SFFTFDVNAEFIVDVIPALIPTLGSLSHMYARVRNIDKLRILLERMWNDW
SLQKTNYEIKIMHEHAETTRLVTVCYSFLMYAAVIIYIIWIFVPEILDVI
APMNESRPRIQPFKVEFFIDEEQYFYLIRSHICIVLFLLPIIFISSVTLY
IALMQHVCGMCELLGYRAERLFCVVKGTAGCGLIRRSKINRGGIAVFVRL
HYNIIQFIDIIEIYHTIPFLMDFLGFVLAMGLALAQILTIGGNIERAFRS
IGFTFATMVYLFISNYMGQKITDKTSNICERVYNSIWYDAVVSEQKLLLL
IMKRRFYPLVLTACKLYVISLQNFGMILQTAMSYCMFMRQT

>PbOr233
MTDEKDVWKSRYYRIPRTYLILSGLWPYRRLCDRYIHFIPTFILCFSILI
PQLLYVLIASINVDDVMESLPSALISLVFSYKVATIMFNNKKVKYCLKRI
EKDWQSLNTDVEKTILQHHSEYGQYLATSYAIFMHIVQVCFLLKPIALTL
LENNITNSTKSISKLPFLVEYGVDIDQYFYLITIHCYLAVFAHVFSTIAV
DTLYYTLIQHACGMFSITGHILEEIGKTNDTNFHLKLNKITDNDYKKALN
CLRRHLRVIKFAELIESTFSNVFLISININMICGSICGIKIILNLGNAED
ITAPLAIYVAQLVHIFLQFWQAQFLLDYSTVPCESICRGNWYFTSERCRK
LFLLIMSRTMSPCKITAGKIIILSIENFATILKTSMSWFTMLRSFQQ

>PbOr234
MTDEKDIWKSRYYLITRTYMILTGLWPYYRLRNRYICFILTFISGISILI
PQLMYVLAATNMNDIMESVPSVILSIIFSWKVVIIMFDIKKVKYCLQSIE
KDWQLLNTNTERTILQRHAEYGQFLATAYAIFVHVMQICYILKPIIPRLL
DNDITNSTKPIPKLVYPVEYGVDIDQYFYPISIHCYLALFAHSFGTIAVD
ILYCILIQHACGMFTIIEHILEEVGKDNNANFQFKLNKTTDIDYKKALNC
LRKHLQVIKFVESIESVFTKVFLISVNLNVICGSISGIQVILNLDNPGTI
LTPLALYTGQLGHLFIQFWQAQFILDYSTIPWESICRGYWYYTSKRCRKL
FFLIMSRTMSPCKITAGKIIILSIENFATILKTSMSWFTMLRSFQQ

>PbOr235PSE
MTDEKGIWKSQYYLIPKTYMILTGLZPYYRLRNRYICFILTFISDTSILI
PQLMYGLAATNMNDIMENVPSVILSIIFSZKVLIIMFDTKKVKYCLQRIE
KDWQLLXDIKRIILQCHAEYGQYLMTSYAIFMHVVQICYLLKPIIPMLLQ
NDITNSTKTISKLLYLVEYGMDIDQYFYPITIHCCLALFTHIFTSIAVDI
XYILEEVGKNNDANFQLKLNKTDTDYKKALNCLRKHLQVIKFAESINSIF
TKIFLISVNLNIICGSISDIQIILNLDNPGIILTPLALNTAQLXHLFIZF
WQAQITVLLDYSTVPWESXCRGNWYYISEKCRKLFLLIMSRTMSPCKITA
GKIIILSIENFAAILKASMSZFAMLCSFQ

>PbOr236PSE
MTDQKDIWKTRYYLIPRTYMILSGLWPYHRFCDRCIHFISIFTFCFSILI
SQLLYLIIDSMNINDVIDSATSMIISIIFSFKMIVLLFNNKRVMSCLKVI
EEDWFTNIETNAEKIILQRHTEYGQYLXSYAVFMHLVQVFYLLKPVVLTL
LEADIMNSTKSLSKLPFFVEYSVDVERYLYPIMIHCYLAVFAHVFSTIAV
DILYYTLIQHACGMFSIIVYVLEEIGKNINANFHLKLNKTTDNDYKKALN
CLREHLQVIKFAEXIESTFTNVFLISINLNVICGNMCGIQIILKLGNAED
ITASLAIYVAQLIHIFLQFWQAQFLLDYSIAPWEAXCRGNWYYTSNRCRK
LFLLIMSRTMSPCRITAGKIINLSIESFAA

>PbOr237
MKGKNAILHNKYYNVLRLYLKISGIWPYHKLCDRCVRFVLVFTFCFGILI
PQTLYLIGPINLDDIFECAPSIIIGIVFSTKILILMLNNKKIKICLNTIE
KDWQSLNTDIEKVILQRHAKYGQYLTTFYAIFMLTTECLILLKPTISILT
RDNILNVTESDTPIMPDLPYHLEYGKRFNQYVILITIHCFGAVFAHMFVT
VAVDNLFYTLIQHACGMFSIIGYMLESIGKNADINFDSKPSKVKDENYNK
ALYSLRKHLQVIEFAQLIESMYTKIFLVNFNLNMLLGSLIGIQILMNLDK
GISNVIGPAAIYIAQLTHLFLLFWQAQFLLDYSVLPYESICRANWYFASE
RCQKILLLIMKRTNSPCKITAGKLVILSIESFGAVVKASVSYLTMFYSLR
K

>PbOr238PSE
MSNTDNFYRCYKICKLFMIICEVWPYDRLFTRYVTXTIYLINFMSLYNIF
ECMPSIFVGIIISIKLMILMLNSEKIKSCLNKIENDWLSLNTDTEKAILQ
QHAKFGQHVSMFYTAFMFTMRFLYCFKSLFVILLEEDTSNATESIPFAVK
LPCHVEFGERINQYEFLTAIHNYAVFAHMLAITTGNSLYYILIHHACGIF
AVIGHVLQNIDKDDDINFDLNLHKTKIXNYRKALHCLRKHLQLVEXFAEH
IEFIYTKIFLVIVNINMFIGNLCGVQILMNLKKEDKSIDDIMGPIAMYSG
QLFHLFLIAWQAZFLLDCSVLTNESACKSNWYYTAKRCRKLLLLIIARGN
SPCKITAGKLMIISLESFAAVVKASVSYLTMFYSFQ

>PbOr239PSE
XSRYYIVLRLXLIISGTWPYHKLRDRCVRFVAIIYILSLVXNTQLLYLTH
PITLDDIFESLPSIFISIVLSFKVITMILNNEKIKICLNTIKEDWLSLNT
DIEKAILQRHAEYGQYLTTFYIVYAHMTACLFVLKPVMLTVMVDDTINVT
KSSISFASKLPYRVEYGQKFNQYIYPITVHCYGAVIAHMFTVIMVDGFYC
TLIQHACGMFSIIGYVLENIGKNAGVTFDSKPDKRKDDNYNRVLQCLRKH
LHVIEFAEQIESLYTKIFLVNLNMNMIGGSIAGIQVLMNLDKTANDVIGP
ITIYVAQLIHMFLHFWQAQFLLDYSVLPYDSICRANWYYTSSRCRKLLLL
IMNRTNSPCRITAGKLMIMSIESFATVVKTSLSYLTMFRSFQR

>PbOr240
MIKKDIWQRQYYILKLYLTISGTWPYHGLYGRYIRFVLIFTLSFTILIPQ
LLYFLVFLNLDNVFECLPTIMIGTVFSFKILTIMLNSEKVKICLNTIKED
WLLLNTSIEKDILQRHTKFGQYIATFYAVFMHMTTCLFVLKPVMLTLLAE
DVLNVTESTIPFASRLPFHVEYGKRLNQYVYPIAVHCYAAILAHAFATIA
IDSLYYTLIQHACGMFSAIGYVLENIGKNSDNNFDLMPDKMKDDNYIKVL
NCLRRHLHVIEFAEHIESMYTKIFLLNLNFNMIGGSLAGIQVLMNLDKNM
NDIAGPITVYIAQFIHLFLHFWQAQFLLDYSVLPYESICRANWYYTSTRC
QKLFLLIMNRTVSPCKITAGKIMVLSIESFAVVVKASLSYLTVFRSLQ

>PbOr241
MDERKNIWQSRFYTVPRAYMSLVGIWPYHAFRDRCLLFVPMFMFSITILV
PQLLYLMIAAVDLDDVFSCTPSMWITIIFSFKLGWLMMHNKKLKTCLETM
ENDWLSLNTNVERDILRKHTAHGRYITLTYGVFMQFVGILLISKSLVVML
LEDTSEATVSSLVAESKLPLRIEYGKILDQYLYPMAIHCYLAVFSHISIT
IAVDSCYIALIRHACGMFAIVGHTLEHIGKDSDGNFDLKPDKVKDDNYNR

ALGCLRRHLHVIQFAELIESTFTNIFLVSVCLNMIGGSMIGIQVVLNLND
AKDIVEPFAIYIAQLIHLFLQFWPAQFLLDYSILPYESICKSNWYYTSGR
CRKLLFLIMNRSVLPCKITAGKVVPLTIENFGTVLKTMMSYFTMLRSFN

>PbOr242
MTERKDIWRSRYYTIPRLYMTLAGLWPYHQISNRYLHFVPTFIICSIILI
PMLLYVLIAMTGLEDLFECMPTILITIIFSFKLASLMANSEKIKACLKTI
ENDWLSLNTDSEKAILQRHAAYGQYLTMFYAVFMVMTGLLYMLKSIVLIM
MEDTSNSTKLAVTKLPFRVEYGQKIDQYFYPILIHCYLTVYSHVTATVAA
DTFYISLIQHACGMFSVVGHTLEHIGKDMNANFNLKPDKINDYNYNKALD
CLRRHLHVMEFAELIESTFTNIFLVSVSLNMIGGSICGIQVLMNLNDAKD
IIAPLAIYIAQLIHLFLQFWQAQFLLDYSNVPYESICRANWYYTSDRCRK
LLLLIMNRTVSPCRITAGKVATLSIESFGVVLKTSMSYFTMLRSFQ

>PbOr243PSE
MQSVILRYYSINKIFMSQIGAWPYQXRNIRILILSSIVLIDIMLPHSXII
TNVRCLXWGNIDIAVECIVSTIILIGCFTKLFNLIXKMKEMRHLLSLIEY
HWNTVFINSTDVEIMQNYVVFCRKVVIFYAIYIYIISMMLYLTIPVLPAD
HGHYSASQZESSAKVRFPLEYHVDREKYYYLISFLYNDYNDYINYGVHGM
VDTTYIAYVQLIAVYLQQXYPIVIKCISHESYRTSSKYLMEYICTERTVL
LIRSESNFXXIRYFLICIATFFHFLCMTLPGQRLMDHSEDVFNSACRSRW
YAFSSQTKKLLQILLYRSNVXLTAGKIYMMSMANYSAMAQTVISYFMAFS
SMK

>PbOr244
MSAVFDTRYYSVTKLFASVVGMWPYQSQLKRRIIQSISMFIMFTFFPPQV
KRLYDVWGKDIDAMCMCVPPIMTILVSMAKVITSARYDTEIKMLLRQIEV
DWKINPNAEEYKILSNYTSKARMLCIVYSTTLCNGLMCFIMTPMISPVLD
IIVPHNETRTRSLAFDLDYGIDLQTHWLWLWMHSSMTSMATIINIIGADL
MYVTLTIHGCCLFAIVRYKLEHVADSIKKHTMQSFENHNHNIKYYEYDER
LKWKDSVQLLHCIYTWGFLTILSLNLINTSISAVQLLSKLFQWDAMAMCI
MFICGQLIHLFFLSLMAQYLLDQSSNVHESTYSGLWYNMPTKIQKDIVLI
LIRSRIPCKLMAGKLFVMSLENFCTIVQTAMSYFTVLASFR

>PbOr245FIX
MNSYMEEYYAINKFFLSQLGGWPYQRKVLRVLIPSLLTMVQYSVIATEVL
LLYDTWGDIDIFVEVIITTIPILASNLKLLNIVVNNGKLRCLLQLMNDHW
KLFNSEFEHHTLRYYANIGRKLTNYFGVYFIIVLTFYLLIPLIPRVLDII
IPLNESRPLAFVYQAEYRVDKEKYYYPIMCHAYISTLIIIIIILFTVDTTY
IVCVLHACSLFTAIGQRLENITGRTNDKKPNTEKYYYLLKESHGSVGSDY
HELITHVQILDSMFTHVTVCLLTMNVLILSVMGIHLINKLDHTGEVIRYV
FVTIAVFTHLICMCVPGQLLIDRSTGVFDKAYNSEWYTFSLKTRNLLRIL
LYRSFIPCTLSAGKLFVMSMTMCSSVMQTAMSYFTAILAMK

>PbOr247
MDLFDTRYFKINKFLLSFIGLWPSQTPFIKLLTLSFAIYGLTTMCLPQIV
YLFKNANELDKMLELMPTLTGTVICIMKVISLTYNVEKFKMLLRQTCEDW
DSLLTIEETQILTRYAENSRKFTLLYSICVIGFVCCYAFLPLIEPILDII
SPLNETRPRKMQHSMDYVVLDLEKHYYLILLNTYLGYIVCLMIAVATDTM
YVVMVVHICGMYNILCNRLEKITTYDNYTYQHDEIGRRVRHCIQLHKRIK
LFIETMESTFALFLLFDIGGGFLLHTSSCIMIVIRIGSSEIVRYVALVIM
QSCRLFFNSWAGQQVIDHSLEVSIAAYKAVWYNISVKTQKLLILLIARSQ
KPTQITMAKLYIINLQGFSTVMRTSVSYCTVMISLRESS

>PbOr248FIX
MDFFDTRYFKFNKFFLASTGVWPYQTPFTKLLGLSLTMYSITMMSVPQSI
YLFKRATNVDEIFEVLPPISGSIICIAKIISLTRNTEKFKILLDQIYDDW
KNLLTSEETQILINYAEKSRKFTLVYAISLSGCVLCYTSEPLIRPILDII

LPLNETRPKEFPTMVDYVINQEKYYYPLLLNTYFSYIISMLFVIGTDTTY
ILLIEHICGMYAILCYRLKNLTTYNELQWMDSDYIHSYTSIYQHIRCCIQ
LHERIRLFIEVMESTIMLFLLFDIGSGLIMHISSCVMFVIQMNQSSKILQ
YVALVMLQSGRLFFNSWTGQEVTDHSLEVSTAAYDGLWYNVPTKAQKLLK
LLIARSQKASRITIAKLYVINLEGFNTVMRTSVSYCTVMISLRDSSKNT

>PbOr249IP
MDFFDTRYFKFNKFFLTSTGVWPYQTPFTKLLGLSLTLYSITMMCVPQDI
ILPLNKTRPKEFPNLVDNVIINQEKYYYPLLLNVYLSYIICILLIIAVDT
TYVLLVEHICGMYAILFVIRTNQSSXEILRYVVLVILQSGZLFFNSWSRQ
EVTDYSMKVSMAAYNGLWYNVPVKAQKLLVLLIARSQKASQITIAKLYXY
INLEGFNMAMRTSVSYCTVMISLREPSEDA

>PbOr250
MSMDFFETRYFRINKLLLSSVGLWPYQTPFMKLLSLSFAIYGIVMMTVPE
TIYLIKHPNLDVVFELMPNISGTLICIAKIICLIRNTKMFEVLLQQTHED
WDNLLTSEETQILTSYAENGRKFTLVYSISISGFVFCYVTLPLADPILDI
ILPLNETRQKKMPHLVDFIVVDQEKHYYALLLNMYIGYTVCVLLGIATDT
MYVLMVVHICGMYGILCHRLENLMVHDELRWIDNNYTYRHDKIGRRVRRC
IQLHERIRLFIETMESTFALFFLSDVGIGFLLQTSSCIMIVVRTGRSSEI
MRYLSLAILQSCRLFFNSWAGQEVTDHSVGVSMAAYNGIWYNVPIKIQKL
LMLLIVRSQKASRITIAKLYVINLEGFNMVMRTSISYCTVMISLREQSDG
A

>PbOr251
MDVFDTRYFRVNKLLLLFTGQWPIQSLYNKNIIFCCVLICTQIVILPQII
YLIKYSKNLNDLYNVLPTLAGAFICFVKGIAFHWNSKKFKMLVQHVCHDW
NLLSQHDDIWILIEYIEKSRIFTLVYIIFISTGAFFYLTAPLTVPILDIL
MGSDVTRPKRLPHVGVFFLDLEKHYYSILGITYIGYIACCMLVIAVDTIY
FALLQHICGVLAILSRRLEKLHDTEDIDHSNAISKRDRDIKNMVQCIQLQ
IRIERLILIQLTESMFAICLFTDIGLGILFQCSASVMIVTHTDTLYLVKN
FPLLLIQTARFFFNCWIGQRIIDHSSQVSFAAYNGKWYQMSLEAKKILLF
LMMKCRKPYRITMTKLYVICMESYSTLMKTSASYVTLMVSLNSNDM

>PbOr252
MDVFSTPYFRINKVLLMVTGLWPIPSSNKIALYYCVIFSILIVLLPQITY
MLKHARRLDEIYDLLSTIAGTSICLVKAISVHRESKKLGMCVQHVRYDWS
LLSDHNDICILIEYIKKSRIFTLAYIIFISMGATCYLTAPFTIRIIDILL
ASNVTRPKRLPNPSEYFLDLERYYYFLLAITYVGYYVCSVIVIATDTIYI
ALLQHACGILAILSHRLENLPMSNKSSNVDYNFIPKWDTNIKDIIQCVQL
QIRVERLIQLIESTFAICLFTDIGFGILFQCSSCVMIVTNVNTMYLIKNC
PLLAMQSIRLFFNSWIGQRIIDHSSQISFAAYNGIWYQMSLEARKMLLFL
IMKCRKPYHITMAKLYIISMEGYSMLMRTSASYITLMISLNTNDA

>PbOr254
MDVFNTRYFRINKVFLTVTGLWPIPSSNKIALYYCVIFGILIIILLPQFAY
IFKYVRRLDDIFDLVPTIAGVCICLVKTINLHLKYKKFRMCIEHVRHDWS
LLADHDDIWILTEYGEKSRLFTLAYIIFVSVGAISFLTAPFTIRIIDILL
ASNVTRPKRLPSASEYFLDLEKYYYFLLAVTYVGYITTVVIIITVDTIYV
ALLQHVCGILALLSHRLKRLATSNQSDNVDRNPKWDKNIENIIQCVQLQI
RVERLIKLIESTFAICLLVDIGFGILFQCSSCVMIVTHTDTTYLMKNCPL
LIVQSVRLFFNSWVGERIIDHSSQMSFAAYNGLWYQMSLEAKKMLLFLIM
KCQKPYHITMAKLYIISLEGYSMLMRTSVSYITLMLQVTSNDV

>PbOr255
MDVFDTRYFRINRMLLSTVRLWPIQSSSKKNTLLYCAILSNILFIILPQI
AYLFKHAKNLDDFYDLLPTLLGATICFIKIIGFHKKLKKFRILVEHVRYD
WNLLADHDDTWILIEYIKRSRIFTLIYLIFLSITAICSVSAPITLPIIDN

IMGSNVTRPKRLPHPSEFFFNLDKYYYTILAISYVGYALCAMIIMATDTI
YFALLQHVCGVLTILSYRLKKLTISNESDNIDHNRMSKWDKNIQNIIQCV
HLQIRIERLIHLIQSTFAVSLFTDIGLGILFQCSSCVMIVMHTDTVYLIK
NCPLLLIQSSRMLFNSWLGQRIIDHSSQISFAIYSGLWYQMSLEAKKMLL
FLIMNCQKPYRITIAKLYAISLEGYSMLMRMSASYVTLMISLNSNDI

>PbOr256PSE
MEVFDTQYFRVNKLLLSFVGLZPFQSSFNKNKNYCTILGLLITLLXQIGY
LFKHTKTLNDIFDVLPTFAGMWLCLLKASSLRWKYKXFTMLIQHVSYDWN
LLSNHDDIWILIKYVERSRIITLSYLXFISTGINFMTTPVTIPIFNIILS
LNVTRPKQMPHPAEFFVDMERYYYILLVITFAGYVACCTVLIAADTIYVA
LLQHVYGVLTVLSHRLEKLAMQNKLEYIDCDLISKVDKNIEKMHTATSQD
RKVHILIKLIESMFAICLFTDIGLGILLQCSAWZXPILLMQTTRIFFNSW
MGQKIIDHSSQIFDAAYNGRWYQMSXQVKKMVLFVMMKSTKPYCLTMAKL
YYIXLESYDSLMRTSASYIMLMLSLTSDNV

>PbOr257FC
MEVFNTRYFRVNKLLLSFIGLWPFQLSFNKNRNYYCTILGLLIILLPQIG
YLFKHTKTLNDIYDILSTFAGVWICLLKASSLRWEYKKFTKLIQQVNYDW
NLLSNHDDIWILIKYVERGRIFTLIYLFFISMSAVSFVTAPIIIPIFDVI
LALNITRPKQMPHPTEFFVDMERYYYILLVITFAGYVACCTVVIATDTIY
VALLQHVCGILAILSYRLEKLDVRNKSEYIDCDLISKGDKNVDKMVECIQ
LQVRIERLIKLIESTFAICLFTDIGLGILLQCSACVMIVTDMDTTKIIRH
CPILLMQTTRIFFNSWVGQKIIDHSSQISNAAYNGKWYQMSLQVKRMMLF
IIMKSKKPYCVTMAKLYVICLESYGS

>PbOr258FIX
MEVFDTRYFRVNKLLLSFIGLWPFRSSFNKNRNYYCTIIGILFALLPQIG
YLFKHTKNLDDINDVLPTLAGVWICLLKASSLHWKYKKFTMLIQHVSYDW
NLLSNHDDIWILIKYVERSRTFTLIYLFFIGTGVVSFVTAPITIPIFDTI
LSLNVTRPKQMPHPAEFFVDMKRYYYILLVITFAGYAACSAVAIATDTIY
VALLQHVCGVLAILSYRLEKLDVHNKSEYTDLISKVDKNVEKMIECIQLQ
IRTERLIKLIESTFAICFLTDIGLGILLQCSACVMIVTDMDTTKIIKHCP
ILLMQSTRIFFNSWVGQKIIDHSSQISDATYNGRWYQMSSQVKRMVLFVM
MKGTKPYCVKMAKLYVICLESYGSLMKASASYITLMLSLTSDDV

>PbOr260FIX
MSFYDNRYYYLNKRFLSIIGQWPFQSRLEGNIMFVTSSLFIFCLTALELW
GLITGITNLSIIMENTSPLLANSSVIMKFINCVYTNNKMKEFLEEIEETW
KIKHGPEKEILQNYAEESKTFSIRYAFVLYMTWIFYCTTPVVITGIYTLL
PTNETYSARFLFRLEHVLDVDKYFNLLMLVAFISVFYIISVPIAIDSMFI
LCTYHVCALFECIRYNMKRIQSGNFILLKPNIKEDKAYHDIIDCIKSYKH
ALKFSDLLSSTYATLFLFLLGNVVIGLSFGAAELIMVDTQIDEIIRIAAA
NSAQLLHIYLLSMTSQRLMDQSSELQEVIYSCDWYKLSLRSRHLLRFTLL
RTTKPCQIKAGKIFVMSIENFSSILQASLSYFMMLTSLQ

>PbOr261
MSFYDNRYYYLNKRFLSIIGQWPFQSRLKNNLMFCIMVFFLFTLTALEFW
GLVAGITDLSIIMENASPLLVNNFIIVKLVNCVCVNYKMQMKKLLEDVEE
TWKVKHEGPEKEILCCYAKECRIFSIRYATAIYAMWLFYTTTPVIISGIY
IFLPTNETYSARFLYRIEHVIDIDKYFNLLMLHGFISVFYVVSVPIAVDT
FFILCIQHVCALFKCIKYNIERIGNSEFVLLKPNIADDKAYHVVISCIKS
YKHVLKFSDLLSSAYATSFLLMLGNVVICLTFSTAELVMVDNQIDEIIRI
LAATLAQLVHIYYLSLTSQRLIDHSSEFYDVIYGCNWYNISLRSRHLLRL
TLLRATKPCQIKAGKLFIMSMETFSTLLKSTMSYFTMLTSIQ

>PbOr263
MNAEHYKINQLFMSWIGQWPEQSALKRIFLSCHVFLIVCSQYCLLILGLV

AAWQDWSLVIECSSPIIIHSLCIIKFANYFFNARKFNSLFIQMRDSWRLA
TFGAQIQILTAYTNEGKKMAKVYAVIIYGSTCLYMILPVTPSIVAILTNP
NQTHIHGLLYHVEAVLDTKKYYYIILLHSYYATFFLMTIPVAADSMMIAY
IQHACGLFQAIGHQLENVKQNDDLKINIYPLYKDDEHYRIISDSVSKHKQ
VLQFAELLASSYSISFVALIVLNVAIMTFSGVQVVNNRNQPSEAFRFLLT
GLCLSAHLLFLCLPGQKLIDHSFNMHKSIYAANWYAISLRARKSLNLMLI
RCKTPCQLTAGKMSVMCLRSFSGVVQASMSYFTVLTSMQ

>PbOr264
MDTSNNIFQSRLYSLNRTLLSLLGQWPFQKDKERRVFFITISLIGITQAI
AQVLAVVTLRDDFDAIIECIPPLIIDCACIIKITNLMCNTEMMKILLIHI
QKDWQSWSIRSEFEILNRFAESGKSITVGYATGMYIFGSGFSLMEIIPVI
IGKNVTSNYATRPKGYPYHVEYYVDLNKYYYPVLIHSYLSTAVRLTIVVA
FDTFVAILVQHCCALFSVVRYRLEHIRISIEQDKELALLEENEKMYKNFV
YCIQKHKHALWFAKCLEIVYTKAFFMEVGLIVLAMSLSALQATNNTLTPH
LAIRHASYIIAQLLHLYIACWLGQQIIDHSDRVYTSIYRGEWYESSPKSR
KLLNMIMLRSTLPCTLTVGKIMILSLPSFSAVVRVSASYFTVLQSVR

>PbOr265FC
MGDIDQFFQESHYNIIRVLLNISGLWPFHTINRRYAIYFAMLLILGSGFI
FEMLGIIEVRHDSFEVIDALPLFFFAIVTLSKMFCVVYTLPKIKMLLIKM
REICLSAKSDEETKIQNSHALHGRKLGYAYTGHSILYMLSTLVTRFFHMR
SNKIDKPPAKESNSQVGLPHRVNYMIDLDTYYVPIFIHSAICDFSYTVLL
VVFDVLYLTLVEYCCGLYASLSYRLETALVFQNENNGLMMTIKDKSYSNI
VYSIRRHVETIQFVAVLESVYSLPLFIHVGLTVFILSVLEYQVINNSENI
TRITLILKPAAYLNGLLINIFFENWQGQKIIDSSEKVYNSEWYNMPIATR
KLLIMMMMRSEKPSILRIGKIVALSYVTFNT

>PbOr266
MHNVDEFFRESCYSNLRILLIVCGIWPFQASNIRYAMYAVFAALFGFTIV
FESMCTIEVWPDFFETFDCLTMLFYALLSTIKLIYTVYKLPKIKMLLVKI
HEHWCSSKADKEAKILHSNELVARKFGFIYAGGLGVYAVIYVFTPLVIMF
LYNAEDASNKTQDSETRDSTRHINYMIDLQMQYIPLYIQGSMCEIYFTII
IVAINILYLASVQHCCGLFAALCYHLENAFEFEDDDDIMTSTQNKCYLHI
TYGIRRHAEAIQFSRAIESLYRLPFFLHMTINVTLLSIVGFQVMAYPENI
NRMLPYGSYLSGLVLNTFFENWQGQKMIDCNEKVYNSAYKLEWYKMPIAS
QKLLIMIMLRSNKPLTITAGKVLVLSYVTFNAAMRTGFSYLMLLRSMQ

>PbOr267
MDSVQGQYMRINQMLMCLIGQWPYQEVWERFLIQFVFVPAVFSQAVLQGG
GMITAWFADDIDAFMESSSPFVISLMCICKHINYTYNHEQMRRLTVIMAD
DWNIYSKLSYEYDILCRNYAMGRKITIAYAVSLYGSMTPFLVVPVVLNTA
SFMGLYNISEGRPLMFRTEYFVDSEKYYYPLLVHSYIGTLGFVSIVVAID
SMLVFHIQHECGMCEILGYQLGQIVDEDTLDINLYPSKKEAASYMYIKNC
VIMHNHIIEYAKRLETANTTSYFFQLGFNMMGMTFTIFQAVVKLSDPNEA
LRYASFTVCLLSVLFLESWPGQQLSDYTDKIFAFTTNGRWYQSSLRVRKV
ISIMLSRSYVPIRITAGKLYTLNLANFSAVVRTSFSYFTVLLSMQ

>PbOr268
MDFFDGYNYKVNKILLLAIGQWPYQSSKTSHIILIVIATIVCSQFLTKIC
GMVAYIHDMDIVIECLIPIMVDVSGMTKMLNSILCVNEIRALLDRIQNDF
NLLRNSSDVKLLQKYADNGKKSSTTYICVMYMLTVVFMLMPFQPLILRMA
NATTRPMLHRVEYYVDMDKYYFPILIHGYFTAVICVTTIVATDAMFVIFV
QHVCGLFIVTGSRIEQAIQEIHSIGDANPPVTKDKAYQNMIQCVHNHRAA
IRFANLMELVYSKHFLFHAGLNMIAMSVTGVAAVTKSDDLSELLRLVAVS
CALLFHLCFECINAQRLMDYSGYLHVNLINLNWYDASPRTKKLVLFMMMK
TQPPCVLTAGGMFVLCMETFATIVKTAVSYFTFLRSAH

>PbOr269
MQFFDGSNYRINKVLLSCIGQWPYQTNRSSNAIIIIIVSLAGSQFVAKVC
GLLSIDNIDVFIDSLSPLVVDIGCGVKLITCILKATKIRALFDQIQYDWQ
LLTTSPYIKILNNYAQNGRTFTIIYASAFYSALVFFMLVPLQPLLLGSSS
NDTTRPLLHQVEYYIDMEKYYFPILIHGYLTAIICVSIAIAADTMYVIVV
QHVCGLFMIIGQQLGNIIKDDNLEINFNPLIEDDKPYESIISNIYAHKRA
LRFASLIEGVFNQMFLVIAGFNMLIISMTGVTAVTNMDKPEEFFRQITFS
CALLVHLFFESFQAQRLIDHSIYIHTNLTNIAWYQTSSRTRKILIFMIMK
TREPCVLTAGKMFVISMDTFSTIARTSVSYFTMLRSMQ

>PbOr270
MEIISNHYYDLIYKLSSLTGMWPYLKLKTKVFRIALLTIILLTILVPQIA
YQFNNQCNKNLHCTFQAMTAYLLSIVAMVKIYTFLFNTRTIKDLTQHLFC
DWKELNTSEEYKIMKSYATNSRRFTLIYSVYCFSAMLIFLLMSLIPYTLD
IILPLNESRPILLPYRGYYFVDIREYFFQILWHAVVAWEVVIAGVVTHDC
LFVTYVEHVCSIFAVIGLRYEHLFHDDDGGKMEIVNILDNIHCKKVALLV
HKHREALQYAQLLESIFTGPFAVQILIVTIGMSISLLQDNSDILESTRYV
FYVIGQLIHLFFLSFEGQRLIDHSLQICDKIYNSFWYKTSMKLQKIAMMV
MMKSVYPSFLSAGKVYIFSLESFSMVLQTSMSYFTVLASFQ

>PbOr271
MTKTVNVEWNQYYSVMKKLSSLSGQWPYQKPITRLFCVILLTLSTISMII
PQIAKFVTCNGNLQCIFETMTSYMLTTVTLVKLYTCYFNQYKIKVLVDQL
FVDWDELQTPEEYEIMKKYAKNGRRYSLGYSVYCYMAVYTFMSLSLIPQV
LDVVLPLNESRPILPTYPGYYFIDERKYFFYIFSHAIMAWEIAMTGIVTH
DCMLLTYIEHVCSIFALVGFRFERLTNKYNDYQTIKVLHPRASDTYHERI
AFSVYTHRKALKFAQLIEDTFSLTLAIQIMLNTIMISITLLQNEAGILEV
IRYAMYVIGQLIHLFCLSFEGQKLIDHSLQTRDKIYNSLWYEAPAKSQKM
LLFVMQKSLRPISLSAGKIYIFSMENFTTIVQTSMSYFTVLASLE

>PbOr272
MDTEYEYRGWNHYYNVVKRVSSLAGQWPYQRLKIRLLCVILVTLSIFSMN
VPQMARFVICNGDLQCIFETMTSFMLATMCLVKLYTCYLNRYKMKDLTNH
LFVDWNALETSEEYEIMSRYAKNGRLYSLGYSLYCFVALCVFLSMSLIPQ
LLDVIMPLNKTRPILLTYPGYYFVDEKEYFFYIFPHAVVCWEIAISGIVA
HDCIFVTYVEHVCSMFAVVGFRLNHLFNDCNNKVKTTNMDDIYRKRVAFI
VNTHREALRYAQLLEDTFNVPFAMQILIVTIGMSITLLQRNDNSNILETM
RYALYVIGELIHLFFLSFEGQKLIDHSLQMRDKVYNSCWYEVSMKSQKLI
VLVMMKSLRLSFLSAGKVYIFSLESFTTVLQTSMSYCTVLASFQ

>PbOr273
MKLQTKEYISFTAMDSTSNYYGVVKKITSLAGQWPYQKRKTRIICLILVT
FSTFSMIIPQIAKFVICEGDAQCIFQTMTAHMLTIITLIKLYTCYFNQCK
IKVLIDWLLDGWDELETPEECEIMKRYAETSRRYSLGYSLYCFISAFLFL
CMSLIPKLLDVVLPLNESRPILPVHPGYYFVDETEYFFYIFVHAIVAWEI
AITGIVAHDCMLLTYIEHVCSIFALVGSRFERLIRKRNDAIKVLHSRNSS
GTYRKQIAFSVCMHRKALEFAQLIEETFSLTLAMQIALNTVMISITLLQQ
NANVFVIIRYVVYVLSQLIHLFCLSFEGQKLIDHSLQTRDKIYNSHWYET
PTKSQRMLLFVMQKTLQPIFLSACKIYIFSLENFTTVVQTSMSYFTVLAS
ME

>PbOr274
MEPNRTYYYEITKRYLTIVGQWPYQKPKESLLFMIVILVLDATAVITQAA
RFFVCDDMQCIFETLPPYMLAVIIPVKILTYRFNSQKIKDLTDRLFVDWD
MLETKIERDIMRKYAENGRWYSLIYGPYVYVSTISFTTTSLVPRILDVLR
PLNTSRPVRLAYPAYYFVDENQYFYYMFLHMLSVATICITGLIAHDCMFF
TYVEHICGLFAIVRFRFEHVSYQRDNTEKSLIGCPNDMYFKNVVFSIHAH
RKALQFTKLLEDTFSTSFVIQLLIVTFCLSITLLQLHDFTEAMRYFVFFC

AQLFHLFCFSFQGQKLINHSLETCDRIFHSSWYKIPVKEQRLLLFVMRKS
IEASALTAGKIYVFSLESFTTVVQSSMSYFTLLSSFNV

>PbOr275
MKNAPNYYYNVTKRMLLLVGQWPYQERKSRLFRVSLITAMTFSMTVPQVG
KIIQCDGNAQCITLVIPSCILMVLITVKVYTCQFNSNKIKNFIDNLHSDW
TKLESAEEYEIMETYAKKANFISLAYCLYYHISAGVFVSLSLIPHIANLV
LPVNESWTTFMPYEAYYFVDSEKYFFYIFVHALVGAEVCLAGVMAHDCML
LTFIEHVCSVFALAGFRFESITHNDHVEKSTLDDKYVRKIAISVHAHWQA
LHLAEFLENIFRISLAIQVLIITIGMSITLLQVALRLNETAEMLRYISFV
TSELINVFCISVQGQKLTDHSVKIADQIYNSFWYELPTKSQKMLLSVMRR
SGQPNFISAGKIYVFSLKSFTTFVQSSVSYFTVLASFQ

>PbOr276PSE
MTFSIIITKRMLLLAGQWPYZEKKSRFFNLSLTTIIIFSMTVPQIGKIIQ
CDGNAQCITLIILSCTZQFNSNXIKNFIDNLYSDWAKLESAEEYEIMKTY
AKKANLLSLAYCXLLYVSAAVFVSFSLIPHVANLVLXEFWAVFLPYEAYY
FVDSEKYFFYIFFPYXVGIDICLLZIMTHEYVLVTFIKHICSVFAVAXFC
FEXVIDSDHVRKSRLNNRYXDSIHVHWQALYLAEFLENTFRVSLAIQMLI
LTIAMSITLLQIALRLNKITETMRYTAYIFCQTISLFCVSIKDIKVDHSI
NIRDKIYNFFWYELPVKLQKMLLSAMRRSLQPNFLNAGKIYVFSLKSFIT
VVQSSVSCFIVLASFQ

>PbOr277FIX
MKDSPNYYYNITKRMLLFAGQWPYQGRKSRIFRISFITVITFSITVPQIG
KIIQCDGNVQCIMLVIPSCCMIAMILVKLYTCQFNSSKIKSFFDYLHSDW
MKLECAEEHEIMKTYAKRANLISLIYCLYYYISGAVFISISLVPHIANLV
LPVNESWPIFMPYEAYYFVDGEKYFFIFFFHILVSLEICIAGVMAHDCML
LTFIEHICSVFAVAGFRFGDLVRNDSDHVEKNRLNDKYNRKIAVSVHAHW
QALHLAEFLERTFRVGLAIQVLIVTLALSITLLQIVLRLNEIAEVVRYTA
YVICQVINLLCISVQSQKLMDHSVQIRDKVYNSSWYKLPTKSQKMLLSVM
RRSLQPNFLSAGKIYVFSLKSFTTVVQSSMSYFTVLASFQ

>PbOr278PSE
MESSRDYYYDICKKYLILIGQWPYQKPREKFIFLSSMILLLATVIITQIA
RFVVCDSPQCIYETLPPHMLAIMILVKIFTXQFNSRKIKDLTDRLFVDWD
MLESKEEHEIMRRYAENGRWYALMYGLYVYLSALSFATTSLIPRILDIIF
PLNTSRPLMMAYPAYYFVDENQYFYYIFLHMALSAGVCMTGLIAHDSMFF
TYVEHTCGLFAVVGCRFEHVAYKCRNIKXLINYPDDIYYKNIEHSIYVHR
QALQFAKLFEDIFSIAFAVQLFIVTVGLSITLVQXHDLTEAMRYFVFIIA
QLFHLFCLSFQGQKVIDYSLEICDKIYHGLWYTIPMKEQRLLLFVMKRSL
EASVVIAGKMYVFSLENFTTIVQSSMSYFTLLSSFEVS

>PbOr279
MKIWNRYYNIVYKISSLTGTWPYLKPRTRIFRVFLLTLTMLTIVIPQIAY
QYMCKRNDVQCIFESMTSYLLTAVAALKVYTFQLNTRTIKSFTQHLYVDW
EGLKTPEEYEIMKTYAENIYCLVAVFTFMSMSFIPFILDVIWPLNESRPI
LPPYPGYYFVDTREYFFKIFCHSVIGWEIIMAGIVAHDCMFVTYVEHVCS
MFAMAGFHFENLFCYHDEEMEITEKSNNTFFRKKISFFVRAHREALKFAE
LLEDTFTMPFAVEMLIATIGMSITLLQITQQNGDILESVRYVLYIIGQLI
HLFFLSFEGQKLIDHSVQTCDKIYNSTWYEVSMTSQKLIVLVMMKSVRPS
FLSAGKIYIFSLESFTTVLQTSMSYFTVLASFR

>PbOr280
MTKSARSHYYSVTRKMLLLAGQWPFQERRTKLFGVSLLTIINISFLMPQM
IKLIHCNEDVSCILLVVPGCLFIIILLIKLYTCQFNNNKIKDLTEYLYSD
WEKLKDTEEYEIMETHAMKARLLSLIYCIYIYIASIMFVSVSLIPHIVNN
MLPVNESWPIFMPYESDYCVDGEEYFFYIFSHAFVGILIIIITGLIGHDCV

IMTYIEHVCSVFAVAEFRFKTMVRDTHDVTMVNNLDKEYNRKIAISVHAH
WRALRFAELLEETFCIGLGMQIMITTITISVTLLQVAKANLFAETARNAV
FVLGQIIHLFIISLQGQKLMDYSTQIGDNIYSSSWYKMPVKSQRMLLFAM
RRSLQPNFLSAGKIYIFSLKSFTTVVQTSVSYFTVLASFQ

>PbOr281NTE
YRMGIMIDKNILRISSPEREYIICKRIICAVGIHLRSFQFCKLLLSNIEN
LFAALIVIGVSSLSINLFRLLQEITLYDNMVDIFLSVGIIIIHFLYFFFG
NHVGQLVMNNNGDIYNTIYNLPWYIAPSNIQKLILFLLFNSHKTFYLSIQ
GVFNASYESFTTLITMTMSYFTVIHSTQQN

>PbOr282JI
MKLQIANDAIEQHYKINRIILKLLGLWPYQQSYITQIQRVLFISILLTFI
LVQYRMGIVIDKNILRSSSPEREYIVCERIICAVGIHLRSFQFCKLLISN
LGNPLITAILIGVISLNINZFRLLQEITLYDNMVDIFVSVSLIVLHICYI
FFGSHLGQLVTNNNADIYNTIYNLPWYLAPSNIQKLTLFLLLKGNKTFYL
SIQGIFDASYDSFTTXMATSISYFTVIHSTQ

>PbOr283FIX
MSHSSSGTIFLGKLSYYNFHRITLSALGLWPYQKLSILRIQAIFFFGIFF
SLLFFQFTIFLTTECDIEFIIKELTYIFPSLIHTLNYNCFYFNSKIIKCS
MEQIKLDWKTFKNNNELKILEKYLFDSYLFAVIVNIIAGSAMFIFTIIEF
LPIILDAVAPINETRARNMKVPFEFFVDKEQYYVWILIFEIITVTTGIWS
TVTTCTFLVMLGTHNCATFKIASDLIQNSVSDYTLQIPASQRIYFMYQNI
RRAVHIHKRSIQFLTNLLHSVHIWFLPLLVFGVVSASCIIFRLLNAIIRL
NDFCELFISIMLFWGHFVYMFMANYCGQTIINHSAEILQAVYDTLWYLAP
LSIQKLLLILLKSTKSYSLNIGGIFTPSLEGFSTLITSAISYFTVMYTM

>PbOr284JP
MSSVINRYYKINEIILKAIGLWPYQNSTXGHCTSKIITGLFLLLLFVQXS
NSFYHECTLNVLIDVLSLTFPNIFILLMYDFYYFNVNFLKEAMDFILYDW
KSCKDNAELIIMNNYSALAKIFTQLLFFGGITTIIVLIMIHVVPLVLDIV
LPLNESRPQKIYIXVEYFLDPNKYYYLFAFHLVVTCYIGIVTVIGVSSIL
LCLGFHACALFKITXYRMGIVIDKNILRSFSPZRKCIIYERIICAVGIHL
RSFQFCKLLISNLGNPLIIVILIGVISLSINSFRLLQEITLYDNMVDIFV
SVSLIVLHICYIFFGSHLSQLVTNNNADIYNTIYNLPWYIAPSNIQKLTL
FLLLKGKKTFYLSIQGIFDASYESFTTFMATSISYFTVIHSTQ

>PbOr285FC
MSSVIDRYYKITEIILEALGLWPYQSSTWMRCIKAMIIRSLFLSGLLVQL
AVFITHEFTLNLLIDVLSLIIPNIYIIIVYDFYYFNVDFFKEAMDFILRD
WKSCKDHAELTIMNNYSTITKILTQLFFFGGVVPGIIVLIMVHVVPLVLD
IVLPLNESRPQKIYILVEYFLDPNKYYYLFAFHLVLTGCIAIVVMIGSSS
MFLCLGFHACALFKITK

>PbOr286NTE
LLAFITNQYNTSLLLQILSFVFPILIDVIKYYLFIIQADNLKQLLEQIQD
DWSSLKDKLEINIIEKYACNARFFTIIVMVYCYFGLLLCGIFQCLPIFLD
IILPLNESRPCQLFVITEYFINQEKYFYATLLHEGLAYTIGTTALCGTSV
TIMICILHACALFEIASCRIENAIEENILMIPSPQREYILYRKIVHAVVM
HQRASKFAELLTSSFTTLFSILIVIGISSLTFNLFQFLQLITIMKNTTQT
FIIAVLIILHLNYMFIVNYGGQELLNHGLKLFKATYNGLWYAAPLHTQKL
LLFIMQKGTMNIGLTCGGIFVVSLEGFATLTNAAISYFTVIYSTQ

>PbOr287FIX
MHALHERYYKIYRVIFKLLGLWPYQRSYLIWLYNMLVVAILLTFIVVQFL
AFVTKQYDTHFLLEILSFFFPSVYVIIKYVGFIILADDLKRLLDLIQDDW
NSLKDKLEIAIIEEYACNMRQLTIFIIICLYIGFLYLIILVFSPLILDVM

LPLNKSRTLYSLAITKYFFYQETYTYYIILYELLIVMIGTTLLIGILLLI
VMYFVHACALFKIASYRIENAIEKDILMIPNPRRDYLLHQRVVHAIVMHQ
RAFEFTDFINSTFVTLLSILFAIGIISLSLSLFEFSQFLILMDTLKILSF
AGILILQFIYLFAINYGAQMLTDHALQIFNACYNGLWYTAPLQTQKLLLL
ILQKGRTNVNIMCFGIFVMSMDGFATSASTAISYFMMMYSVGK

>PbOr288FC
MSQFSCKTHALHEHYYKIYRIIFKILGLWPYQQSYLTRLHNLLFASILLT
SIIAQLLAFVTRQYDANLFFEIITFVFPLMYIIIKYIIFIIQADDLKQLL
DQIKDDWNSLKDKLEINIIEEYAYDMRLFIVAITMFTCFVLFFCIIFESL
PLILDVVLPLNESRQFHSVTITEYFVNEEKYIYYIVLHELLTGIIGTILL
IGILLLIVMYMMHACALFKIASYRIENTIEKSVLMIPSPRKEDLLYRKIV
YAIVMHQRALQ

>PbOr289FP
MYLIEEHYYTINKTFLKILGIWPHDRSRFILLZRLLIIFLTTIHIGMQLS
VFITMKYNMALFIKITSCAFPCILITMKYVLFILKSKSVQNLHYLIREDW
RIIRTELESKIILKHAHNSQIYNIYIFLSINFVIIGIVMVQFLPNILDIV
LPLDEPRPRKPIIMAEYFVLHDKYFYTKILHEIVLIVIYTSILFATSSQL
LVFSCHSFGMFKITSHRIEYSIEDSVLHVPNPGKKSAIYRRIMHVVIAHR
RAMDFCNIMISSFNIPYCIITIVGLISLSINLYGFVEAVIISKNMENFTM
YFFTTTGHLVYMFVANYIGQKVIDYNNELFKLSYDTSWYLMPVSSQKLIL
FLMQKTGKEFYFTIGSMFIAKLESFATLLNAALSYVAVMYNYMC

>PbOr291PSE
MFVIEERYYKINRVVLKTLGLWPYQQSYFTQIHKILFASILLTFILLXLL
VFITTQYNTELLFNTLSFVLSYLFVTVKYFLFVIQTDNVKQLLEQIRADW
NLFKTKLEIGIIEKYTCNLRLFTVIIMVCCYFAIFLFGILQYLPLILNII
LPLNESRPYRPVITITGYFMNQDKYIHIILLHELLASYVGLTTLCGTGAI
IILYVTHICILLKITSCRIENAIEKNVLAIPNPTKKYLLHRRIVYAIIIH
QKALEFNDLMVSNFTIPYAILITIGICSLSLNLFQFLQLITLNNFEKAFI
VALLILIHLMYLFAGNYCGQIIINHGVKLFQATYNGLWYTAPLHTQKLLL
FIMQRGRINITLTCCSIFVASLDGFATLTSMAISYFMVIYSTYNK

>PbOr292FIX
MHAIGEYYYKINRILLLMIGIWPYQTSIFSKLQKLFVVIILASIICIELA
AFITHEVNSIFFIEIISYTFPTLVAILKYYIFYIRTKIIKRLFERIQADW
NLLQDEHEREIIASYAEYGRLITIFSTIFCCCILIVYVTSPMTLYILNVY
LSNHTCNYTYSQLMYIPMKYSVDREKYFYVILPHFIVYACIALLIIIAIA
TFIYLHIKHVCGLLKIASYRMERVMDMENMQQISPLKREHIMCVRIMRAV
NIQQKAMKFMDHFNSCFGIMFFLLIILGAISVSVSMFRLFLILQTSSNDL
CESFIILSVLIGHFLYLFLLNNAGQEFLDHCSNVCLATYNSIWYIAPLRI
QKLILFLLQYNTKNFTIIIGGFFVTSLEGFTTILSTSLSYFMVLYSTQLN
DEKMKN

>PbOr294
MVSFEYHYYEINRRLLTIMGLWPYQKWRFRIIQITLFPIVLISFLIAQLA
TFVTSGFNVNIMLRVLSQAFPCLIYFLTYISFLLNSRKIKIFYERLHYEW
TVLKVVGELDILRKYAAISRQFGIAITILLILSLNVFGLFQFLPNFLDII
APLNESRERYLSFSAEYFIDQKQYFYLILTHGLLAVYIGSAGVLATGSSL
IGFISHICAMLKIASYRLEHLSDNLSSVSELEKDCIIRKRIINVVDIHRR
AIEFAEFILSSFATLYFILFGVGISSLTVNMFQFLQLMMLTNNTNGILAC
GLLVIAHFIYIFVANFAGQIVTDHNTNILTTTYAIQWYAVSLRAQKLLFF
IMQRNTKNYYFMIGGLFTASLEGFATMMSMSVSYFTVIYSTR

>PbOr295
MFLYSNGTDFAEYHYYKLNQIFLSVINLWPYQRSKFTNIIHTFHFIIMVS
FVFFQFTSFITFPHTLDVVVMILSHAVPSFVYIVQYYSFYARSNAVKRIW

RCMRDNWSLIKNETERNIMGQHSSSGELCTIILSSSIIISLILYAFMEML
PCILDLIFPLNETRSREFYAVTEYFVDKRTYYYPILCHWLIGVSFGGFVV
IATCTFQIVFLEHICGLLNIASYRIERSIDEYTLCNSAHKKNCVAHRNII
AAVNIHRKAFKCSELMTNTFNPFIFLIILIGVLSASLNLFQLLKAIILRN
VKELISSTIFIGMHFILMFLGNYYGQKITDYNNKIFKNAYNVQWYKAPIK
IQKLLLIIMQNTTMPYILSIGNLIMASVEGFAKLISLSMSYFTIIYTVM

>PbOr296
MYMFDDRSHKLNQILLKIFGLWPYKKTKFDRFRATCFYMLLISNVIIQLI
QFTVADFNINVIIRILSDAIPAFLFIVNFNIFFLNTKKLLTDAQEIKIVE
HYIHQGKYITVIFTCNKIMFTFKKLLLISLVFALTIMTFLPDILDFLWPL
NEPRTHYLIFISEYHIDQRTQFYFSSYIFMTGFIGTLSVVAINSMLVLIM
LHCCAAFKICSYRIERFIDKKAFSNEKNVMIKKIIRTVELHQNAHKLSKL
LITSFTINFLIILIVGMCSLIINLLRLLHSFTCMDNFIEFLVAISYVLGH
EIYLLGSSYGGQQITDHANELFNAIYMSLWYKAPTTVQKLLLFMMQMCSK
DVKVSLGGIYVAVMESFSSVTSTAISFMMILYSLQ

>PbOr297
MFVIESWYYNLNRILLLICGLWPYEKTKFRYVRAVFILGILIAYMIYQFN
ILFAYEYNFKLALKICSDIFPAILCTLQFIAFLIKPGEVRGLLDDICKEW
NALKDSTEIEIAKKYGKFTRRLTEVLLLCAILSWFLLVLMNLIPISINGI
VVANESLLRYDNISIHQFDRERYILFFIVIFIGVIVITTTTTMVLAYMRH
ICAMLKITCYRIEHSLDENLLYMSISQKNRLMCQKLISAVNIQRRAMKFM
QRALASFKESYFFLIGIGVISLAINLLHALQAALFLDNINELAISTFYVI
LHFLIIFIGNYGGQTITDHSADVLKMLYHVQWYMAPVQVQKLILFLMLRN
TRYFSLVIGGIYVASLEGFITLTSMSVSYFMVIYSTQ

>PbOr298FI
MINPMTKYYDVNRLLLLICGLWPYEKSKFRYIQAIFYITIILSFIVFQFM
TLIDADCSINLITKLLSIVLPMCVCLLKYGAFLYNNQKMKHLMDLIWYHW
SIIQDKQEVAILKKYTKFSRRFTLFMLHIFSLSIFVIIMGHMLPMILDII
IPLNSSRPRHFYMIMEYFIDQERYFLWLLLHTSMTLLVAALMVLSIGTML
MSCVFHACAMFKIAFTDLITCDFKISYFILLATGVASLALNIFQVFISLS
TSNVNELFTTFTFVVAHFCYLYVGNHAGQIVTDHHVDVFNATCRSYWYTA
PLRAQKLLLFIMQRTSKNFSFVFGGIFVVSLKGFSTLASMSISYFTVIYS
MIYSR

>PbOr299
MNFIGRRYYRLNKFLLLCLGLWPYQTSVWKKIQIIFFQTLFISFLVCQCN
TFLINQYSIDHIVRISLFIVINCIYIIKYNACLLLTDNIKYIFDRVQYDW
NMLKAQAELKMIQRYANDAKLYTVCFTILSHLCLLGLLILYCIPYVFDVT
IPMNDSQPQIVLIRVEYFIDKEKYFFAILTHIILTQYVGCITITAIATIL
IAYVLHTCAMFRIASYRIEHMFDENIRQIPKNIREHILYEKLIHAVYIHR
RAVDLVDIMTNSFATLYFVLLGFGIASTSLSFVNLFNTIVSLHELELILS
IGIVVLHLYYIFLGNYVGQNIIDNSISICEATYNMQWYAAPLWMQKLMLF
IMQRSNKKSALTAGGLFDASLEGFATLMSMSISYVMLLQSMETHKKNEDD
NSHI

>PbOr300PSE
MNFDGQRYYKVNRFFLKMMGLWPYNNSTZTYIYRVFTILVLASSCITXVK
EMMDRIQHDWNTLKDEKEYEIVRYYTIIGMRYTIVFFAASFPLLLCFILG
FYLPGFLDFVHPLNEFRSRRLSILAEYYILDEQKHFYPILMHQSLLALLG
VTTILATETLNLIYVHHACGLFEVISYHLNMLLMTKCFRXKIYHNSRQNC
TSYKYIHTRTIGSLDYLVSTFILTYLCXTSLGVIAITINMFRLIEAILAM
NDFXRIIGSFGVFIGGQYGFLFFANYFGQKVTDHSILVFDKAYDVAWYAA
PVRIQKLLIFILQRAIKSYVLGIGDVIMASLEGFAZVVSMMLSYLTVIYS
VR

>PbOr301
MNFVGNEYYKLNRRLLMLVGLWPYEHSIYKYCQMIFCNVIVMYMTISQIA
ILITLKRNINIMIKFLSPIILCIIYIIKYQTFCIVARKIKHLMDHVEEDW
NMLKDKKELEIIKSYTYVGSMCSLSFTILGSMAMITFFILSLIPTILDII
APLNISRPRQFLFPGEYFIDQQKFFYAILLQTGISLGLIVTTLIGTESLY
VTYVQHACGMFRIASYRMDQAFNDKSLHSYTSGKQTIIICKRIIEAIYIH
KRALEFSEFLWSTLAISYTILLIIGITSLIINLFCLFQTILFKHEINDII
ILVSFIFGHIIYLFLGNYVGQILIDHSGSIFENIYIARWQGAPLPAQRLL
PLIMQRSMRSCKMVVGGMFVPSFEGFATLMSTTLSYFTVLWSVQN

>PbOr303
MDFIGSKYYELNRMLLLSIGLWPYDESIFKRIKMLVSSSLIVSCTTAMIA
KIITSKCDMVITLRVLSLTFPCFIIIVKYVTFLLLPTSIKELMEWVQNDW
NTLKDVNELKIIHKYANFGRLCTITFSAFAYICAITFLINMYLPNILNFV
MALNFSREQKLPVDVEYFIDEQKYFYIIFYHIYITVIFGMTIALGTETLY
LAYVNHACGMFKIASYRLQHAYDQNILQLSPFVKNTMIHKKIVAAIKMHK
RALEFHEFLWSKLAVSYFILILIGVVSLTVNLFRVFQAIVVIKDTDELLT
SSICVLIHFVYIFCGNYAGQVFIDHSMSVHENVYDTRWYRIPIREQKLII
FILHRSMHSCKLITGGNFVLSLEGFTTLLATSISYFTVLSSVQY

>PbOr304FIX
MEFPEERYYKLNRILLSIIGLWPYDDFRVRHFRFILSLLIMISFTGTQWM
KLCVSEYSSDLLLKVLAYNIIFIVFFVKYITFYAVFKNIKEFRDHVRNNW
SLLADNYEIEIMSKQGSIGKLFTILIIIILVYIIIVFFILTQYISILLDIV
IPLNESRPRKFLFPIEYFIDQQKYFYIITIHIAIGLSCIATSGVATESFS
LANALHAFGLFKIASYRMKYILSDINPQMHTAKKYAMSRNRIIAAVDFHR
KAIEFSELLKTSFGKMYLVLFVACVCSASINLFNLSRIITMEKEIVEVII
SVLFIFLHIVYLTLANYAGQEFINYDTDFYRTICNTKWYNAPLKTQKLLL
FLIQKTTKCYKVDAGGMFSPCLEGLATGLSMSVSYFMVLRSA

>PbOr305
MEFPEERYYGLNRILLSTVGLWPYDNFKVRYFRFILTLLINISFLSTQWT
KLCVSEHNSDLFLKILSYNIVFILFFIKYITFYAILKNIKEFRERIRNNW
DTLIDKHEIEIICKHGNIGKLLTLVLTISVYIIIILFYILSQYVSILLDI
VSPLNESRPRKFVFPVEYFIDHDKYFYIITLHIAIGLLIATTNALATESF
SLTNALHAFSLFKIASYRMKYILSEINSQMCITEKYAISHKRIVAAVDFH
RRAIEFSELLKTSFGPAYLILFVIGVCSVSINLFNLFQIIMIERDILEII
KSSLFIAIHILALTLANYAGQEFINCDMHFYRTVCNTEWYNTPLKTQKLM
LFLIQKTTKCYKVDAGGMFSPCLEGLATSLSMALSYFMVLCSI

>PbOr306PSE
MDFDSYHYYKFNRIFLSTIGLWPHRYITLRQIQDIISSFILISVTSLKLI
KLFTMKYDIDLVLRDLSSALPFMLFTVKYVTFYFVTENIKELMQQIQNDZ
NALKDNNELEIIRRYKSSKIIXIILYICIIAILCIQYVPIFLDIVAPLNK
SRQTDLLFRVKYFLDEKKYYHTIQFHLDVGLMVAAMTILSTESFCLTLAI
HAFGMFKISSYRMERIIDKSVPNTFTKNHYAFYENIIAAINGHRRAIEFS
ETFKTIFAIPYLALILLGVTSSSVNLFLLFQIVISTNAMDDLRXSVVIVF
CHFVYMFSTNYAGQKFIDHDSDISEX

>PbOr307
MDFSEEKYYRLNYILLSSIGLWPYDNLSIRRIQIILILLIYTLTIITQLA
KLFVLEYNLDLFLEMSALFFINLIWIIKYITFASVITNIKQFRKNIQNNW
SILTDNREIDIMHKYTNFGKLSTIAIAIIIYFGFFVCILIQYIPILFDII
MPLNESRPRELLMKASYLIDEQKYFHILVINEIIGLLALTTTGVATESFI
LANALHVFGMFKIASYRMEHMLSINVLQTPIAKSYNIFYDKIVAAVDIHR
RALEFSELLQVSFGLSYLFIIVIAICLATVSLFRMFRIITMEQEKLEILK
FFLIVLFIFIFLVIGNFVGQEFINCDSNVHQIICNTQWYNAPLKIQKFIL
FLIRKTIKSYRVNAAGLFSPSLEGLATTMSLLLSFLTILCSI

>PbOr308PSE
MESNEFSFFRVNQMLLIILDLWPYNDTIYRCIQRILFGLLISFSIFLXXY
KIVTNVSNMEALLQVLSFVILCLTVVLKYVTFCIKAEDXFLYLSFSAFLT
LQISPKLFHIVVPLNKSRZLKLLGLATYLFDQEKYFVPIFIHMIVTLFVE
STMIVATETMZYIYCILVNYSXFRIKHANIANIDCKIQISVLEKHKLYYT
NIINAIIVHNRALEYFEFFNSCFEVSYFFLLVLDVFSLSVNMFXXEFLSV
GLLIIIHFIYVFLNNLGTQLVTNYSAGIXYDLPWHLSLSDYKNYXVYNAK
KYRKYIYNVQYLPDNFNVLLQZLANVSLSYFTFFWSLQH

>PbOr309
MRHNTCTEMTCAADSYYSINRILLLIVGLWPYQKPAFRHFLITFITILLI
SSVVFQLTTFITTEYSMDLLLKVLAYTIPWMSYLLKYNILCVNKKKMRSL
MERVRCDWNELNNALELEIIKKYSTLGRLITLGTTLFVYISTFGFIVIQL
LSNFILNIAVATNESHPRQLPAPFECFVDEQKYFTLLLLFIFILALCGLT
TMVATETLFMSYTQHACGLFEVANCRIEQALHRGMARNVSSVAEQNSIMC
QGIISMIDMHKKAIEFIELSNANFKLAYLFTLPMATLTLSINLYRLSRLL
MTKEYHEIITTGTFVLGQFWYLFFCNYVGQEVIDHSGNIFHKTYNVQWYM
VPLKVQKLLLFVMQRSMRHCTIMIGGLFIPSLEGFATLTSMSLSYFMVIY
SVQ

>PbOr310PSE
MRYNICIKXDCLLNSYYIINRILSLCIDLWPYQTSNFKHIXVIFTTLILI
LSIIFXXMTFITTEYNIDVLLHVFAYIIPZLACTIKYNVLCLNLRQMQDL
MQRVHYDWNELNDTQEVEIIKKYZKIEKFTTLITAXFLYVFISCFILVQY
LTNFVVNFTTAINESRPWQLLVLIECFIDQQRYFLPILLNVCFAILCALT
MVVITETLHMSYAQYACGLFEIASXVEZALHKNAIKAVVSSTKKYSIICQ
GIINGFNIYKRAIEXKLKTSYKWAYSLLLPLAILSLSINLAZFSRLTFKD
YYEMSFSFLFIVDHFGYMFFYNXGQEIIDHNSNIFYKTYNVQZYIAPLKA
QKLLLLLMQRSMKHCTIVIDGLFIPSLEGSVXXASMSTSYFAMIFSLF

>PbOr311
MTFAPKQDCSVDSYYTINRILLLCVGLWPYQKSNWRYIIITFLTIIFISF
IVFQLMTFITAQYSIDILLHILASCVPWVAYTLKYNFLCLNSRSIRRLME
RVYYDWTELNNVQEFELIKKYSAFGKLLTLIVTLIIYTTIFCFIIIMFLP
NIFLNVSTTMNESRQVQLPVLTEYFIDQETYIVPIVCHMGFVHLCAFTSM
IASETFLMSCVQHACGLFEIASYRIERALHKDMVHGITSFTKRNSIICRG
IINGFSMYKKAIEFIEILKVYCKWAYSSLLPLGVLSLSINLYRFSHLVTS
EEHYDMIINFMFIVGHFGYMFFCNYVGQELIDHSSDVFYKTYNLRWYVIP
LKAQKLLLLVMQRSMRHCTIVLGGLFIMSLQGFATIASMSISYFTVLFSL
F

>PbOr312FIX
MTFVPKQDCSVDSYYTINRILLLCIGLWPYQKANWRYIIITFFNITFISF
IVFQLMAFITTQYSVNILLHILSSCISWIAYTLKYNLLCLNSRVIRRLME
RLYHDWTEFNNVQELEIIKKYSAFGKLLTLIVTLIVYTTLFCFILMMFLT
NFILNIPTAMNESRHIQLPLLIEYFIDQDTYICLIICHMSFVHLCAFTSM
IATETLFMSCVQHTCGLFEIASYRIERALHKDMVQGITFFTKRNSIIYQG
IINGFSMYIKAIEFIEILKIYCKWAYSSLLPLGVLSLSINLYRFSQLVTS
EEHYEMILRFMFIVAHFGYMFFCNYVGQELIDHSSDIFYKTYNLRWYVIP
LKAQKLLLLVMQRSMRHCTVVIGGLFIISLEGFATIASMSISYFTVLFSL
F

>PbOr315
MAFAPKQDCSVDNYYAINRILLLCVGLWPYQKSNWRYIIITFFTITFILS
IVFQLTTFITTQYNTDILLHVLAYSVPWLAYTLKYNALCLNLRHIRDLMD
RIYYDWNELNNVQELEIIKKYSALGRFITLITTLFIYISISCFVLILFLS
NFLLNIPTTMNESHPRQLPVLMEYFVDQQKYFVPIFVHICVIIVCGLTTV

AATETLFMSYIQHTCGLFEIACCRIEQALHKDTIQGITSFTKRNSVICQG
IINGFYMYKKAIEFIEILKTNCKLAYSLLMPLGVLSLSVNLYRLSRLITS
REYYEMIISFTFIVGHFWYMFFCNYVGQELIDHSSDIFYRIYNARWYVAP
LRAQKLLLLVMQRSMRHCTIVIDGLFIPSLEGFATLTSMSISYFAMILSL
F

>PbOr316
MDFTGYHYYRINRILLISLGLWPYHTQRVRYVHCALLFLLLLSNTVFQFS
SFITMEYNTDLLLHVLSLLCFTMYYAIKYISGFINSNKMKELIEQIQYDW
NSLKEEEELKIIRNRANIARSLTILLLTITSIVIFLFMLMFFSPNILDIV
IPLNESRQPWQLPPAMKFFFDQHKYVYLLTIYFLLTLIIGTFTVIGIETF
MIIFVQHICAMFQITSYRIERIVNKSQMKSLISSEKLNSDKSIVEAIDSH
LKATKFVDSLKSTLAGLHLFALPLGVISLSVNLYRLCVYIVTENIGAMSI
SLLYVVCHFGYLFFFNYLGQQVINYSDNVFKKICSTRWYAAPLNTQKYFM
MIMHRSMKTSTLMVYVGLFLPSLEGFASLVSTSLSYCMVIYSIHQP

>PbOr317FI
MKVYSSFLLSLGLWSNDSTCFKQIRVIFCILMFIFLIIAQLLKLFTMEYD
LDFILADLSFAIPSLAYFLKYSTFYIQSHKIRQLMEYIRNDWNALQNEQE
INIIRKYAVSAKRYMYAFVGVSYPGTIGFLLISLLPDILDIIVPLNESRT
RHLPFMVEYFLNEQKYFYLLFLHIAVTVILGITTVIATEALMLAYVYHIC
GMFKVIRYRIERALNASTSISPMLRENMIHIKIIDAIEIHQRTIELIERQ
AVKSLEKSSNNLFNGARLIGHVYISQWYEAPLHAQKLLLFMMQQSIKGTA
MSVGGIFVPSLEGFATLTSMSVSYFTVICSIK

>PbOr318FC
MNFTGNRYYNIHRILLSLVGLWPYQQSNMRKVQWIISSIVLASSIIIQFM
KFLTTEYSLDLLLKVLSFAVPCMVFVLKYVSFYIGSETVRYLMESIISDW
NSLKIDIEIEIIKKHSDFGRLYALFFTLAVYSGLFFYMLIQFLPNFLDII
ASRNESRLHNIPLTAEYFADQQKYYLPILLHVDIIALVGFTIVISTESLI
TAYIRHAIGMFEVASYRIEHAFDEVLGIMTSRKCCLYCTKIINAIHIHRR
AIKFIEFLRSSFAISYFFLVCLGITSLTANLLRLYLATQYLNNLEECITA
VLLVLGHIYYIFFGNYTSQKLIDQSIDVFHKM

>PbOr319
MDFDGSRYYALNRMMLSSIGLWPYQNPWYIRIQQFSCLSCFMFGIVMQLL
TLVTFEYSLDFLFELLSSVIPCFIVVLKYVTYSIRIESIRKLMEIIKYDW
KTLKNKVEYKIIKKYTSIGTFYARLFASYTFPIFVGCIHFIPNLLDFVAP
LNHSRPHQLLILTDYFIDIDEYFYFVVLHVIITVFLIQMTLMSTTSIYVA
FIQHACGMFEIARYRIERALNASTSISPMLRENMIHIKIIDAIEIHQRTI
EFFEYLSSTFSLSYFILMILGVISLSLNLFRLFQVAILPDQKKNLASFVI
FVSGHFCYMFICNYLGQKIIDNSTEIYNEIYNTQWYAAPIKTQKLLLFIM
QRSIKSCKLVLGGIYYVSVENFTTLASMSLSYFTVLYSMQK

>PbOr320
MYKINLFESHYFNLNRLLMSLIGLWPYQKLIFRRIKMFLILSFLTIAIIA
QLMSFTTSEYSRDLLIKVFSFSFIVILCTMKYNTLYFKSEEVKYLLEHVL
YDWHALNDAEEIEIIQKYANKGRLYTILAGFLVYLGIMVFVVLFFVPDIL
DIVAPLNESRQHQLPIAIETFFDQEKYFRFIVLNFSVISFLSVTVFLTAE
TLYMICVQHACGLLKVTSYRILNAFDNRIQQINTSKIKCIICTKLSEAIK
LHRRSLEFIEYLCSTFSLSYFILCVFGVASLSINLFEFLKAVELKYTDQA
ICFGLVVCAHICYMLWVNNVGQDLIDNSIDVFEQTYNVQWYMVSTHIKKD
ILFILHRSSKTICFNVGSIFVFSLEGFATLINMSLSYSMLLYSTRT

>PbOr321PFC
MDFLNEYYGITRLDLMESIGLWPYEKPERRVIRMLCVSFILTQGILLQXA
TFITHEYSTSLFIDVFSFNILCIIYVLKYNIVLIPIMZVKXLFDQIQYDW
NLIKTADELEIIRKYASKHKTRFYTIFSGSIVYPETLFIQGLFLLIIFVP

DVLNVTNTNVXPIQIECFFDQEKHFYLISFVLMISAFLGMTILVATEIMT
MIFIQHACGLFNLISYRLTCAFDISKTIPSKTKPRFYTKLLSAFSTHQHC
LEXFLDNMQSKFSISYLILCAFGVVSLSVNMFRLLQAVVTHNIFEVIATG
LFVYAHLCFVFYMNYFEQDIIDHSEYLFQQIYSTQWYTAPLYVQKLLLIA
LZQSVKNSKMICRLIYLLHRLK

>PbOr322
MEFPEERYYGVNHILLSIIGLWPYDNFKIRYLRFILSLLLLLSFLSAQWM
KLCVSEYSSDLLLKVLAYNIIFIVFFVKYITFYAVFKNIKEFRERVRNNW
NALIDNYEIEIMCKHGSIGKLFTILLIIFINVISTSFYILSQYVSILLDI
ISPLNESRPRKFVVPVEYFIDQDKYFYIITLHIAIGILIATISGVATESF
SLTNALHAFGLFKIASYRMKHVLNGINPQMCITKKYTISHKRIIAAVDFH
RRAIEFSELLKTSFGRVYLFLFVIGVCSASINLLNLSRIIMTEREIVELI
KSISFIALHFFYLTVANYAGQEFINSDTHFYRTICNTEWYNAPLKTQKLI
LFLIQKTTKCYKVDAGGMFNPCLEGLATSLSMTVSYFMVLYSI

>PbOr323FIX
MEFSEEKYYRLNYILLSSIGLWPYNKFSIKQIRVILILLIYTYIIIIQLA
KLFVSEYNLDLLTEILAFFFFTLIWFIKYITVASIMGNIKQFRKYIQNNW
SILTDNREIDIMHKYTNFGKLSTIFITIYVYFGIFVYILIQYIPDLFDIV
MPLNESRPRHLLIKASYWIDEEKYFHILVIHEMIELFVVATTGIATESFT
LANAIHIFGMFKIASYRMEHMLKTNALRIPVTKSYKIFYDKIIAAVDIHR
RALEFSELLQASFGLSYLFMFIVALFSATVSLFRLFRILTMEEEKLELIK
FLLYVLFIFLFLIAGNFVGQEFTDCDNHIHRIICNTQWYDAPLKIQKFIL
FLIRKTTKSYRVKAAGMFSPSLEGFTTAMSLIFSFLTLLCSLH

>PbOr324PSE
MEFPEERYYGLNHILLSIIGLWPYDNFKIRYLPYYHXLIIISFLSTQWIK
LCVSEYNSDFLLKVLAYNTLFTVCFIKYVTFYAVFKNIKEFREHVRNNWN
ALIDNYEIEIICKHGSLGKLFTIFLIISINIISTSFYVLSQYVSILLDIV
SPLNESRPRKFLIPAEYFIDHEKYFYIITLHIAIGTLIVTFSGVATESFS
ITNALHAFGLFKIAXYHMKHILNGINPQICVTKKYAIFHKRIIAAVDFHR
RAIEFSELXKRVFGRAYLILFVIIVCSASVNLFNLFRIIMTETDILELIK
SISFIALHIFYLTAANFAGQEFINGDTHFYRTISNTEWYNAPLKTQKLIL
FLMQKTTKYYKIDADGMFSLCLEGFXSLSMTVSYFTILYSV

>PbOr325FIX
MIGLETQYFGLNRILLLALGLWPYQQSRFTQFQFFFLFSILTTYIIFQLT
IFMTLKCTTELVIRILSSVSFYMIFMIKYYAFRVNIKAVKKLLIKLQHIC
NTIKDSNEIAIIEKYGYIAKCYTAALLVVGICVIPFGTIGLYLINVSDVT
SINISFSQPYHMQSLIEYFVDRRKYFFFIILHGITALYIGAFSMMATGTM
LIAYFKLICGMFKISSYRIDHAVKINILQNITIKNKILRSEGLIYAVDIH
RQAMKLSKCLLSKFEIMFFCIAELFVISLCLNFVRIFQILSSLENLKEAL
LPLICAFVNILYLFISNSIGQDITDHNNYIFATVYKVEWYITPISIQKML
LFLLQKGAKDFTMNVGGLFVPSLECFATLVKASVSYFTVILSTQ

>PbOr326
MICLKTQYFSLNRILLLAIGLWPYQQSKFTQCQFIFLFGILSTCIIFMMT
PFITFKCTGKLVITIVSTASFFIILLIKYCAFRVNIKAVKKLLIKLQDIC
NELKDKNEIAIIEEYGYVAKRYTLALITISVCGVFIYIISQSWLTMPDIF
LMMNVSRLHYVELIIEYFFDQEQYFSFIILQINIAVLIGITALIAIGTTF
HAYFALICGIFKVSSYRIECAIKINILHNFTSKSKISRSENLISAVQIHR
QAIKLTKRMLIKFNIMFFCLAEVFVISLCCNFVQNLSSEKTSKEVYLPII
IVGANIIYLFLANSIGQDITDHNAYVFATVYKVQWYVTPIGIQKMLLFLL
QKGTKDFIMNVGGFFFPSLECFAMLMKASVSYFTVILSTQ

>PbOr327
MIRLEVQYFNLNKILLLAIGLWPYQQSKFTRFQFIFLSSILTASIIFQLT

SFITLKCTFDLIAKVVSSASLFTIYMIHYNSFCINIKIVKNLLTQLQYVY
NDLTDKNEIAIIRKYNYVAKRYTVLLTASGICGMCIISISPYLSNIFDVT
LSINISQSRMIFITEYFVDQEKYFVFILLHINTAFCIGTIATIATGTMLI
GNLQFIFGMFKISSYRIERAINNNNLQNITLKNKISRSKDLMYAVEIHRQ
AMRLSKYLVTQFELMFLSLTAVFVISLCFNFLRIFQIMSSEEAIIEAVFP
VIFIIINILYLFICNLFGQNILDHNNQVFTTAYNVQWYIAPLHIQKMILF
LLLKGAKDFTITVGGIFVSSIECFATLVKASISYFTVMLSMQ

>PbOr328NTE
LTTLTTLKCTTNLVAKVLSSVTIFIMLMSKYISFRLNIEIVKDLLMQLQH
MYNEIKDENEIAIIEKYDCIAKRFTNSFTTIAVSGIFFGLIIDMWLIINV
DLSINASQLYQLPIMMEYFINQEKTFYLTLLHVNVAACIGIIITLAISSM
FITYTLHTCGIFRIASYRIENAMNIKVLQNITMEGSTFTYNGIICAVNIH
RQALKLFKRFQSAIKIMVICLMVCGVIALSLNLYQIASFTNNVKELLLSS
CYVFLIILYMFLSNYMGQNILEHNKHVFVTVYNVQWYEAPLYIQKIILFL
LQKGVKEYSITIAGLFHMSLEYFATLLKTSASYFTVIYSIQ

>PbOr329
MISPEIRFCTFIKFQFLVIGLWPYQQSEFTRLQFIFFSVILSTFVIFQVT
PLITLKHTSYLIAKVLSSASFFTMFITNYFWFRFNIEVVKDLLIQLQHKH
NKIKDENEIAIVEKYDCIARRYTNSIIILGVCGLLVVFIIEFWLNIIASD
LPMNTTRLLIMMEYFIDQEKSFYLALLHINIAIYVGSATMVTIGSLLITY
LQHTFGMFRIASYRIQNAMDIGSLQNTTIKNSIFMYDGIICAVNIHREAL
KLSKNLLSILEVMIICLIICGVTSLSLSLFHITSVKNNIQEILLSSIFVS
VIVFYMFMANQMGQMALEHNTHIFTTVYNVQWYKAPLRIQKMILFLLQRG
AKEYTLNIGGLIDGSMECFATLLKTSVSYFTVIYSTQ

>PbOr330FIX
MSSPETQYCTFNKFQFLAIGLWPYQQSEFARFQFIFFCVILLTSVIFQLT
ALITLKCTSNLVTKILSSTLIFTSFLIKYVSFHFNIEIVKDLLTQLQHTH
NEIKDENEIVIIEKYDCIARRYTNSFTTFAVCGIIFGLTLNVWLLINIDV
PINTTQLYQFPIIMEYFFDHEMYFYFSLLHVNAAVCIGATALLAIGSLMI
TYIIHVCGMFKIASYRIENAMDINILQNITMENDIFMYDGIICAVKIHRQ
ALKLSKNFLSAFEVMAFGLLVCGVISLSLNLFQIASFKNNVKELLLSLLY
VFIIVLYMFLANYMGQNVLEHNNHVFVTAYNVQWYKASLRVQKMILFLLR
KGVKEFTLNIGGFLHGSMECFATLLRTSVSYFTVIYSMQ

>PbOr331
MSSPETQFDTFIKFQFLAIGLWPYQQSEFTRFQFILFSVILLTFIIFQLT
PLITLKRTSNLITKVLSSASFFSLFTTKYISFRFNIEVVKDLLMQLQHTH
NEIKDENEIAIVERYKCIAKRYTNSLTVVAICGVLVSLIMEIWLNIADAN
LSTNMTRFPIMEYFIDQEKSFYLILFHVTIATYVGTAAMLTIGSTLITYF
LHTCGMFKIASYRIENAMNIKILQNITMEGSIFMYDGIICAVNIHRQALK
LSKNLQSAVQVMIFCLVVCGVTSLSLNLYQIASFKNDVQELLLSSLFVFV
IVLYMFIANYIGQKALEHNNHIFATAYNVQWYKAPLHVQKMILFLLQKGV
KEHTLNLGGLLHGSMECFATLLKTSVSYFTVIHSMQ

>PbOr332FI
MIYLQAHYYSLNKILLLAVGLWPYQQSKVTQFQCIFIFSTLSACIAFQVV
VICGTIIIIIIQFLSIMVNVDISTNVSVSSRLLIMTEYFIDQEKYLYLIL
LHVCMALCIGTVAMLAIGTLLITYLQHTCGMFRIASYRIKQAIKIDILQN
VNQKSKILITRDIICAINIHRQAINLSKHLLSIFEIMFFCLIVVGVTCLS
LNLFQIASSVNNIGELFPPLLYSSVSILYMFLANYMGQDIINHNDDVYVT
AYNAQWYKAPLNIQKMILFLLQRRAKEHTLDVGGLFHASIECFATLVKTS
VSYFTVIYSTR

>PbOr333
MICLQVHYNLNRILLLAVGLWPYQQSKFTQFQCIFIFSTLSACIVFQLTP

LISSKFTSDLLVKVLSSVSFFTMFMIKFIAFHLNIDAVKDLLKQLQYIHN
EIQDENEIAIVTKYNCIANRYTVTLTICAILTICVSIIFQFLDIADTGLS
VNISRSYHLPILMEYFIDQEKYFYLILLHINITLYIGIVALLAIGSIFIT
YLQHTCGMFKIASYRIKHAMKIDFLQNINLKNKILIIKGIICAVIIHREA
MKLSKQLLSVFEIMFFCLIVDGVTCLSLNLFQIVSENNIKELCYPFFYSM
AILLYMFLANYVGQNVTDHNSLIYIAAYNIPWYRAPMQIQRMILFLLQRD
TKEFIFTFGGIFDASMECFAMLVKASVSYFTVIYSTH

>PbOr334FIX
MICIETEDFNLNRILLFIVGLWPYQQSKLTRFQLICFLSILITAIFFQFT
ALLTSKRTSDLVLKIVCSVFYFTNFIIKYISFAVNTDNIKDLLAQLQYTC
SELKDDCESAIIRKYSQNARRYTVALTILGVSSVFILIIVQFWANILNVI
LPRNISRPRHIPIMTEYFIDQEKYFYLIVLHIHVSICIGAIVIVSTGTMM
IAYLEYICGLFRISSYRIERAMGTNVLQNITVKNKTLIFEGIIYAVDIHR
QAMRLSELILSKFEVMLFGLITFGVISLSLNLFRIASTERDIKEILMPLV
FAISIIVYMFIANYVGQNVTNHNNCIYVTAYNVQWYIAPLNIQRLILFLL
QRSAKDFSLNVGGIFYASIECFATLLKTSVSYFTVIYSTQQ

>PbOr335INT
MICIETQYFSLNRTLLLAFGLWPYQQSKLTRLQFILFFGILTSSILFQFT
AFLTFKHGSDYVVTILSTAIFFLNFIIKYVAFAFNIKNVFQMDSSIDNIR
ELIFPVLFIFISIFYMFIANYIGQDVMDHTNQVFVTVYNVQWYVVPLHIQ
RIILFLLQRGTKDFTLRVGGLFIGSLECFATLVKASVSYFTVMRSMR

>PbOr336NP
XYLLLLALNFCIHLISYRNVSKPRRLFAIEYFVNQEKYFSLILFHINAAF
CIGMFIIVSTGTILIVHIQLLCGMFRISXYRIKHTMEINMKNVSAKNENL
IFEGLICAIDIHRQAMKLSENMINKFENTMLSLIVFGIISLNLNLLRIGL
SQNNIKEFVFPFFFVTVCILYMFLGNYSRQNIIDHNNDIFVVAYSVQWYA
APLYIQKMIFFLLQRSAKNFFLNLGKLFVVSLECFATLIXTLVSYSTVIY
STR

>PbOr337
MIRAKNRYFSLNRIFLLMLGLWPYEQSKLVWLQLILHSGILGSFIIFQFT
TFATSKCDLQLVIEILSITSFFFNFVIVYLSFCFNMDVVKHLLELLQHTY
DELKDEDEIAIIENYWNIAKRYTEVLTLLIVLCVSFFIFSPFLPYIFDVV
FSTNESWPHPSLQIVTEYFIDEDRYFYLIILHADTAFFIGALAMLGTGTF
LIMYIQHVCGMLKIASYRIERVMTISIQRMSGVRNTNLIHKSIICAIDMH
RKALKFIKIFISSFNLSFFVLMLDGLLYASLNLYRMYQEILSGCISEKLI
QSVLIMNMYYTYTFLANHMAQQVMDHNNNVFATVYNIQWYTAPLQVQKVI
LFLLQRGSKTLKIMIGGMFMGSLEGFATLASASMSYFTFIYYTSQQRLDQ

>PbOr338
MICLAQNFKLNRLLLLAIGLWPLQRSKFAQIQFIILFSILITFIAFQFTT
FITSKCTIDLIINVLSSAFFFICLAIKYNSFWVNADIVKFSLERLQLTCN
ELKNKNEIAIIEKYNCIGKYETIAILLFGISSMFTFFTMSIWPCILDIII
SMNNSWQRPTYQIIATEYFVDYDNYFYLILLHTNIALCIGTIAIVSTGTM
LIMYLKHVCGMFSIASFRIQKAMMTNMLQNVNQQKKIQICKGIICAIDIH
RKAIEFSQFLIKNFAGSFFCLIAAGMVCLSSSLVQLFKTSSDNINIVQLI
QSFMFIIILYIYMFLSNYTAQEITDHNEYVFTTVYNAQWYMAPLHIQKMM
LFLLQKGTKAFYLILGGIFVASMESAATLMGTSISYFTVLYSTS

>PbOr339
MICLETQHFKLNRLLLLVIGLWPYQESKLAQIHFIVFFSILTTFIAFQFT
TFITSKCTADLIIKVLSSAFFFICLTIKYSSFRINAKIVKLSLERLQHTC
NELQNENEIAIIEKYGNIGKLYTSSVLILGIFMFFILPIWPLIFDVILPM
NFSRSRPAIQITTEYFVNQQNYFYLILLHTNAAIFIGGTAIIATGTMLLV
YFKHICGMFNIASFRLETAMMINMLKNVNQEKETLIYKRLIYAIDIHRKA

TEYSHFLIKNFEGSFFFLIAAGTICLSSSLVQLTSSINNFEELLPPIIII
FALYIYLLISNYTAQQVMDHNNYVFATVYNVQWYVAPLRIQKMLLFMLQR
GTKAFNLNLGGLFVGSLESAAMLTSASISYFTVLHSSQQH

>PbOr340FIX
MICIDSLHVSLNWFLLLIFGLWPYQQSVLVRLQLTILFSILTTFIVFQFT
IFVTLKCTPDLLINVLSSAFYTIFFIIKYISFSINTEIVKYLLDQLQHIR
NELTDEGEISIIRKYGRYAKRYTIAFLSFAVLVVLAFFMFSFWPHIFDVL
FFINRTQSNHLFLFKTEYFIDQERYFYLITLHANMASIIGIGVVMATGTL
FIFYQQHACGMFQIASYRIKQAMIFKTLRNNNLQNKYLIYKKLIYAVDMH
CKAMRFSELIISRFKVMFCVLIIFAVLCGSINIFWIFQTMSSEYEIAELL
FHFMIIIIEIVYILTANYLAQEIIDHNNNIYVTVYNIQWYITPLQIQKMI
LFLLQRRTKEFNLNIAGLFVGSLEGAATMFSAILSYFTVLYSTQR

>PbOr341FIX
MICVDSLHISLNRTLLIIIGLWPYQQSKLARIQLIVFYGILTTIILFQFT
TFITSKCTFDLLINVLASVLFYIIFIITYGSFSINIEVVKDLLEQLQHMY
NELSDENEINIIKKYGSNAKRYTATLLILSMSLTSVFTLYSFWPFMFDIL
FPINETRPHLGLLLVTEYFIDQEKYYYLILIHINAATFIGIITMLATGTI
FIVYQQHACGMFRIASYRIEQAMAIDTLYKNNLIKETLIYKGLICAVDMH
RKAMKFSDSSVSGFKVMFFLMIVAGVISTAINFFRLFQIVTFGYNIEKLL
FPVIFLITNALYMFLGSYFGQKIMDHNNDVLVTVYNVRWYTAPLHIQKMI
LFLLQRGSKVFNLNIAGLFVGSFEGAATLVSATLSYFTVLHSTQSR

>PbOr342
MISTDSLQINLNRILLLTIGLWPYQQSKLVRVQLILFLGILSTSILFQFT
IFLTSKYTMDLLISVLSSTLYYTFLIINYGAFSMNVEIVKYLLEQQQHIY
NELSDHNEIDIIKKYGSYAKRYTIALISLALFVTSILFFYQFWPLIFDIL
FPINETRSHLSLFFITEYFVDQEKYYYLILIHINAASFIGIIAMVATGTM
INVYQQLACGMFRIASYRIKRAMAIDMVYENNLKKKILVHKRLICGVDMH
RKAMKFSDLSVSKFKGMFFLMIVAGMMCTAINYFRIFQIVSFGFDTEKFL
SSIVFAQTYVLYLCIGCHIGQQIIDHNKLVFETVYNVQWYIAPLQIQKMI
LFLLQRGSKTFTLNIAGLFIDSLEGAATLMKAILSYFTFLHSTQR

>PbOr343PSE
ISSDSLHINLNRTLLFTIDLZSYQQSKLICVQLILFLSILSTSVLFQFII
FLTSKYTLDLLIKVLSSALYYTVFIIIYGSFSMNIEVVKYLLEHIXHICD
DLSDENEIDIIKKYGSNAKRYTIALILFCVFGISILLLNQFWSILYDILF
LINETRFHLSLLFITEYFVDQEKYYYLILIHINAVTFIGIITMZLATGIL
LIVYEQHAWXIFRIVSYRIERAMAINMLHKXXSDLSISKFKVMFFLMIVX
SNTCTTINYFXXVSFXILRSFYSSMLFAHFVFINFFIESYIEQQMINHNK
LVFAILYNVRZYRTPLQIQKMILFLLQRDSKAFILNVAGLFIGSLEGAAX
XMNAIPSYFILFHSMQNK

>PbOr344
MISTDPLHIKLNRTLLLTIGLWPYQQSKLVRVQLILFFGILSTSILFQFT
IFLTSKCTMDLLINVLSSALYYTVFIIVYGSLSMNIEVVKYFLEQLQHIC
DDLSDENEIDIIKKYGSNAKHYTITLIVFVVFGIFVLFLNQFWPIVCDIL
FPINETRFHLSLLFITEYFVDQEKYYYLILIHINAATFIGMITMVGTGTL
LIIYQQHACGMFRIASYRIEQAMAMDMSQKNNLKTLIYKGLIDGVDMHRE
AMKFSDLSISKFKVMFSLMMLSGIWCTAINYFQIFQLVSFGFNFKKFLIP
MMSVLTLVLCILISGYIGQQIIDHNNDVYDTVYNVRWYTAPLQIQKMILF
LLQRSSKVFNLNIGGLVVASVEGAATLMNATLSYFVLLHSTQNK

>PbOr345FP
MVSIDFLHINFKVNZILLLIIGLWPYQQSKLVRVQFALILLFSILTTFIL
FQLMTFLTSKCIMDLLINVFSSVLFYIVFIIKYGSFSRNIEIVKSLMEQL
QHTXNELSDENEIDIIEKYVSSAKRYTITLLIVVVFAISFLLLNQFWPIV

CDILFPINESRPHLSLLFIIEYFVDQEKYYLILIHINVVTFIWITSMLAT
GTLLIVYLQYACGMFPNXSYRFEQAMAIEVLQAVCKNSLDKKALIYKKLI
YTVDMHRKAMXIQDLKZSRFKVMXFLMMVTGTICTAINYFRIFQTVSFEF
XVAKFLLPLFFAVTHTTYLFIGGHIGQQIIRSQYLVFDIXYNVQWYIAPL
QIQKMILFLLQRDRKVFYLNIAELFVESFERAATILNATLSYFAFLHSTQ
SR

>PbOr346
MISIDSLHINLNRTLLLIIGLWPYRQSKLIRVQLIVSLSIMMTFILFQFT
IFLTSKYTSDLFINVLSSTLFYIILTINYGSVSMNIQIIKTLMEQLQYIC
NELSDKNEIDIIKRYGSNAKRYTVTLLILVVLAIPVFILYQLWPLICDIL
FPINETRPLSLLFITEYFVDQEKYYYLILIHANAATFIGVIALIATGTMF
IAYLLLACGMFRIASYRIERAMAIDMLHKNSLEKKSPIYKGLICGVDMHR
KAMKFSDLSISKFKVMFFLMLVVGVICTAINYFRIFQLISIEFNIEKLVL
HVIFAFTQTVYFFIDGHLGQQVIDHNNYVLDTIYNVRWYIAPLQIQKMIL
FLLQRGSRTFNINIAGLIIASLEGVATWMSSIFSSFTVLYSTQSK

>PbOr347
MNSIDSLDISLNRILLLTIGLWPYQQSKLVRVQRIIFFAILTTFILFQFT
IFLTSKCTSNLLINVLSSTLFYIVLIIKYGSFSMNIKVIKDLLEQIQYIC
NELSNDEIDIMKRYGCNVKHYTITLLLLVIFVTCVFILYPFWPLLSDILF
PINETRSHLSLLFITEYFVDQEKYYYLIVIHANAATFIGTVAMLATGTIL
IAYQLLACGMFRIASYRFEQAMTIDTLHKNNLEKKILIYKGLICGVDMHR
KAMKFSDLSVSKFKVMFFLMIIAGVMCTAINYFLIFQMVSFGFNIEKFFL
PVIFALVHTLYLFIGGQIGQQIIDHNNDVLDTVYNIRWYIAPLQIQKMIL
FLLQKGCKVFNLNIAGLIVGSFEGVATLMSTTLSYFTVLYSTQNK

>PbOr349INT
MICIKTRHFNIHRLLLLAVGLWPYCKSNFVQLQIIFNFAVLISFIIFQLT
VFLTAECTSFLIIKVLCTTTFSMLYVIKYNSFWVNAHNVRCLMEQLQQIC
NELKDENEINIMKEYGYNTKRYTTLISIFLTASLGNKEECIFHFLVVDAF
LVYLFLTCYFVQQVLDHNNNVYDTVYNARWYVTSIQTQKLILFILQKGSK
VFTFKIAKLFDASLECFGMLMKISMSYFTFICSMQQ

>PbOr351FIX
MICIKTRYFGIHRILLLAVGLWPYSKSNFVQFQIILNFAILISFIIFQLT
IFLTAECTSLLIIKVLCITTFSMLYVIKYNSFWVNAHNVRRLMKQLQQIC
NELKDEDEINIMKEYGYNTKRCTTLLSLFSICCICIIFVTPILKPVLNIV
LHTNKSQSRELLHFKIIQEYFIDQEKYWFVIMLHMNTFICIGAITVTATG
TMLLGYIIHTCGIFKIASYRIEQAIMTKACEKICIKSEKMIYKEIIHAVD
IHRKAIKYINFIVSSFEGSFFLLIVICVICLSLNLFAIFLAVSVGNKEDC
IIHFLIIDSFLVYIFLGCYFIQQVLDHNNNVYDTAYNVQWYVASIHTQKL
IFFILQKGSKVFTLKIGKLFDVSLECFGTLIKLSMSYFTFIYSMQQ

>PbOr352INT
MICIKTKYFSIHRILLLAIGLWPYSKSNFVQFQITFNFAILISFVIFQVR
CLMEQLQQICNELRDENEINIMKEYGYNTKRYTTLLSLFCIFCVFISVIM
PILNSISREFLNIVLHTNKSRELLHFMIMQEYFYQYFKEKYWFILTLYMN
TFLCIGATTITATGTMLLGYVLHACGIFRIASYRIERAMAIKSCRKICTK
NENMIYKEIIYAVDIHRKAVKYVESVIYSFEGSFIAILIICTISLSINLF
AIFLAISAGNKEEYISHFLIIEIFLLYIFLACYFAQLVLDHNNNVYDTAY
NVQWYITSIHTQKLILFILQKGSKVFTLKIAKLFDASLEGFGTLIKLATS
YFTFICSIVTTM

>PbOr353
MICIKTRYFNIHRILLLAVGLWPYCKSNLVQFQIMLCFTILISFIIFQLT
IFLTTECTSLLVIKVLCTTTFCIIYVIKYNSFWINARNVKHLMERLQQIC
NELKDENEINIMKKYGNNTKHYTTAITLFCICTVLIPTIIPILKPIFNIV

LHVNKSQSREIIHFTIIQEYFIDQEKYSFLIMLHMNTFICIGAITLTGTG
TMLLGYMEHGCGMFKIASYRMEQAMRIKVFKKISTKDEIMIHKEIIYAVD
IHRKAIKYVELLLSNFEGSFVLLILVGVISLSLNLFAIFWAISVGNKEDC
IFHFIFVSCVLVYMFLANYFAQEVLNHNNNVHASAYNVHWYIAPIQTQKL
ILFILQKESKIFVLKLGKLFSASLESFAMLIKLSISYFTFMYSMQQ

>PbOr354INT
MICIKTRYFNIHRILLLAIGLWPYYKSNVAQFQIILCLAVLISFIISQYR
MEQAIIIKXKKISTKNKIMFQKGITYAIDIHRKAVKYVEFLLSTFEGSFI
LLVLFGVFSLSVNLFAIFWAISNGNKEECSFYFVFVSGILAYMFLASYLA
QEILDHNSNVYATAYNIQWYVTSVQTQKLILFILQKGNKVFTLKIGKLFH
GSLESFGTLIKVSMSYVTFMYSIQQ

>PbOr356INT
MICIKTRYFNIHRILLLAVGLWPYCKSNLVQFQIMLCFTILISFIIFQLT
IFLTAECTSLLVIKVLCTTTFSIIYVIKYNSFWINARNFQKGITYAIDIH
RKAVKYVEFLLSTFEGSFILLILFSVFSLSVNLFAIFWAISNGNKEECIF
YFVFVSSILAYMFLASYLAQEILDHNSNVYATAYNIQWYVTSVQTQKLIL
FILQKGTKVFTLKIGKLFDASLECFGTLIKISMSYVTFMYSIQQ

>PbOr357FIX
MICIKTRYFNIHRILLLAIGLWPYYKSNVAQFQIILCLAVLISFIISQLT
IFFTTECTSLLIIKVLCTTTFSILYVIKYNSFLINARNVKCLMERLQQIC
NELKNEDEINIMKEHGNNTKRYTTVITLFCICCVFILIIMPILKPLLNII
LHINKSESHKTLRFIINQEYFIDQEKYSFLILLHINTFICIGSITVTATG
TMLLGYMVHGCGMFKIASYRMEQAMTIKVFKKISTKDEIMIHKEIIYAVD
IHRKAIKYVELLLSNFEGSFVLLILVGVISLSLNLFAIFWAISVGNKEDC
IFHFIFVSCVLVYMFLANYFAQEVLNHNNNVHASAYNVHWYIAPIQTQKL
ILFILQKGNKIFVLKLGKLFGASLESFAMLIKLSMSYFTFMYSMQQ

>PbOr358
MICITARYFSLNKILLLALGLWPYYQSNFVRFQIVLILGILISFIIFQFT
IFLTAECTSLLIIKLLSFSTFFIMYIIKYNSFWINSDNVRLLLERLQEIY
NELKDENEINIMEEHGNNTKHYTIILIIFTICAVSIHTTTPFLMPVLNII
LHTNKSQTYQIHVITEYFIDQEKYSYLILLHVNIVVCIGAATVTATGTML
RGCLIHACGMFKIASYRIEQAMTMKFKKFSTNNEIMIYKEIGCAIDIHRK
AMKYSEFLLSSFEGSFASLILVGVISLSLNLFSVFQTASLGNNEECVMHI
VILFVILLYMFLANYVGQEVTDHNNYVYSTAYNVRWYIAPIHTQKLILFI
LQKGSKVFVLNVGKLFGASLESFATLIKASMSYFTFMCSMQ

>PbOr360FC
MISITTRYFSLNKILLLALGLWPYYQSNFVRFQIVLILGILISFIIFQFT
TFLTTECTPLLIIKVLSFSVFFIIQIIKYNSFWINSDNVRLLLERLQEIC
NELKDENEINIMEEYGNNTKRYTIIIIFFTICSVSIITTTPLLMPVLNII
LYTNKSESYQIHVMTEYFIDQEKYSYLILLHLNIIVCIGAATITATGTML
RGCLIHACGMFKIASYRIEQAMTMKFKKISTNNEIMIYKKIGCAIDIHRK
AMK

>PbOr364
MICIITKYFSLNKILLLALGLWPYNQSNFVRFQIILILSILTSSIIFQLT
TFLTAECTPLVIIKVLSVTIFSSIYIIKYNSFWINSANVKLLLERLQEIC
NELKDENEINIMEEYGNNTKRYTAVIIFFTICSLFIAYITPFLMPMLSII
LHINKSEPYQMLYFMTEYFIDQEKYSYLILLHINIVLSIGATTVTATGTM
LRGCLIHACGMFKIASYRFEQAMTMKAFENVNTKNEFMIYKEISYAIDIQ
RKAIKYSEFLLSSFQGSFFLIILVSVISLSLNLFSVFQSASLGNNQECVL
HVVILSVLLLYMFLANYVGQEVADHNNYVYTTAYNVGWYVAPIHTQKLIL
FILQKGSKVFVLNVGKLFGVSLECFAVLIKASISYFTFMYSLQ

>PbOr365FIX
MIETHYFSLNRILLIAIGLWPYQQTRLVRIQLTLLFSILISCIVFQFTTF
LTAECTLEFTIKIFSYISFFISFLIKYNSFLVNTQTVKSLLEQLQHIYSE
LKDENEIAIIEKYGYNAKRYTIRIIKITLCSGFTVIFLPIWPCILDIIAP
MNNSRPSFVMPIMTEYFIDQKKYFYLIMLHINAATCIGATAYIATGTMLI
VYLKHACGLFKIASYRIERAMMTNILENNVKNEIVIYKAIIYAVDIHRKA
IKFAEFFISNFEGSFFILIAVSVCCLSLNLFRIFQTVSTFNNNKEEFLLH
FLIVTVILLYMFLANYAGQEITDHNNRIFSTAYNVRWYTAPLSIQKLILF
LLQRGNKTFSLNVGGLFAASLKCFASLTSASISYFTVIYSTQQ

>PbOr366FP
IISLETQHFNLSRILLLMIGLWPYKQSKLILFQQIILFSVLSSFVVFQFT
VFLTAECTFGFTIKILSYISIFASFIIKYNSFWINNHIVKNLWDQLQYIC
NELEDQNEINVMKKYGNDAEHFTTKLIMIMIVSVTSFIFLTIWPCILDIV
KPKNESRPHPTMHIMTEYFIDQERYFYLIVFHMNAALCIGAIAVIATGSM
LIGYLKHSCGMFRIASYRIEKAMAINNLQNIRKNYIVTYKGIIYAQEIHR
KAMEFSKFLISSFEGSFFLLIAVGVLNLSLNLCRMFQTISNKVNKEEFIP
HFISMSVTLLYMFLANYAGQEITDHNSHVFSTAYNVRWHNAPLRIQKLIL
FLLQMGNKTFGLNVGGLFMASLSCFTSLMSASISYFTVMYSTLQ

>PbOr367FIX
MISLETQHFNLSRILLLTIGLWPYERSKFVLFQQIILFGILTSFVAFQFT
VFLTTKCTINFIIRILSFILFFIAFIIKYNSFWINSQSVKNFLEELKHVC
NDLKDENEINVMKKYGKNTESYTAKIILITIISTVSIIFASIWPLILDII
LPKNESRPHLAMHIMTEYFIDQKNNYYLILFHMNAAFCIGAITMIATGTM
LIGYLKHACGMFRIASYRFEKAMIINNLQNRFDNEIIIYKGIIYAVNIHR
KALQFSKSLISCFEGSFFFLIAVGVLNLSFNLCRIFQIVSFGGNKEEFIL
HLITITVTLLYMFLANYAGQEITDHNNHVFFTVYNVRWYIAPLHIQKLIL
FLLQIGNKAFGLNVGGLFVASLNCFASLMSASISYFTVMYSTQQ

>PbOr368PC
MLMHVLZEYNFNKILLSHLGFZPFZSKLVRSLLPICYLVLQISCYPLLIL
MLYGHWKNARLVFSKAATKLFZLLTIFVTKLFNNFWNHDKIRCLYEIIEN
HWNTFTNEFEMRILKDYSIISRKFTIFYSNXLMYSMLLIFFMTPLTSILF
DIVRPLNESRVRFFAVSIEWRIDKDKYFVXNMSIIVTGVIIMIVDIMHVT
CTVHACLFSIIDQFEKIISKLDVNKEINKYGYCINTKLDHQELDYXKEIL
YHEYVICLKKYQIALEFVDILNLMYRTVALIMLLLTGATVSLVGIRIVYV
LDQLEKVMRFVLVIIGMLIQLLILYYSGQKLIDKSQNVFYRV

>PbOr369FNC
MSIFMILGNVQVIFETSHHAIGMTMFIVKLFNEFWNRDKIQHLYEIMENH
WNIFTNEFEINILKDYSIISRKFSIFYSTIISSMAAIFILVPFKPILLDI
VRPLNESRPRVFALSIKWRIDKDKYFVPLFCYNISAIVAGIIILTGTDSV
YVTRIVHASSLFSIIRQQFEKITSKLVINEETNKHGYYFKLTYEQMIYQE
YIICIKKYQLALQFVDLLNSIYQTAVLILLFQITVIISLIGIRIVHCLDQ
LEEAIRYSFMMIGVLLQLLLLCYFGQMLIDESQNVFYRM

>PbOr370PN
ISILYDYWDNVQVIFETCHHIVNXAMFSVKLFNEFWNHDKIQRLZIMENH
WNIFTNEFETNMLKDYSIISQKFSIFYSTIFIIVPFKSILLEIZIDFTFY
RTXLNESRPRVFALSIEWRIDKEKYXLFCYNISAIVTNIIIVTSIDSAHV
TRIVHASSLFSIIRQQFEKITSKLVINEENXXYAAEWYAYSPRLKSLLIT
TFHRSFIPZGLTAGNIISLSIATYTAMLRAGMSYLMTFLSLRN

>PbOr371PSE
MLVHVLREYNVNKILLSHIGLWPFQNKFIRNFLPILYFICETSYFLLEII
TLYDHLENTEVIFETCYHIAALTMFITKLSYEFWNRDKIKCLYETMENHW
NIFTNDFERNVLTDYSIISQKFSMFYSXLIISITHPFKSIFLDIIRPLNE

SRPRNFPVPVEWRVDRDKYFVPIFCHTTIVMMMGVIILIGTDTAYVTRTV
HACSLFSIISQQLEKIIPNLVMNEKTSNCENYYKSTREQIIYQEYIICLK
KYQFALQFVNLLNSIYQPVTLILLFLICMFISLLGIRILYVNQLEQLALC
YFGFIGMLLQLLILCHFGQMLIDENQNVFYRIYASEWYKFSSKLKSLLII
TLHRSFIPCILTAGNIIPLSMTTFTGVLRASMSYFTTFSSLKN

>PbOr372
MLVHILREHNVNKILLSHLGLWPFQNNFIRNFLPILYLVCETSYFLLEVS
VLYNYWEDAQVTFETFYHIVSLAMFIIKLLNEFLNRDKIQRLYETMEKHW
NIFTSEFERSILKDYSITSRKFSIFYLTIIYSMSAIFIIVPLKPVLFDIV
RPFNESRPRVFAISIDWIIDKDKYFVPLLCYSTSVIMAGVMIIVGTDSVH
VTRTVHACSLFSIISQQFENIISKLIINEKINESCSKSTCEQTIYQEYVI
CLKKYQLALQFVDLLNSIYRTTALILLFLIAAFISLVGVRVVSVLDQLEE
AIRYSFGIIGMLVQLLVLCYFGQMLIDESQNVFHRIYAAEWYKFSPRLKS
LLIITLHRSFIPCSLTAGNIIPLSMTTYAMVLRASMSYFTTFLSLRD

>PbOr373FIX
MMLVHFLREYNVNKIFLSRLGLWPFQSKLVRNSLPIFCLVLEISFYPFEI
LLLYDHRDDAQMIFEGCYQIVITTAFIVRLWNEIWNRDKFRCLYEAMNDH
WEIFTDELEVRILKNYSTISRKFSIFYSTMMYLLSSMFIIIPLTPVFLDI
VLPLNESRPRVLALEVEFRVDTDEYFVPIFCYISVIIVVGMSIMVCADTL
HFTCTIHACSLFSIIGEQIENITMTLRTEKCERYESKEVVLSKEQAIYQE
YVSCLKKYQLAIKYVDILNSTHQTVAVFFLFLIGATLSLIGVRIVYVLDQ
LDEMIRFTFIIMGALLQLMIMCYSGQKLMDESQNIFHRAYATEWYLFSPR
LKSLLIITLYRSFVPCSLSVGKLFPLSMSTYAAVVRTGMSYFTAFLSLKD

>PbOr374
MLVNILREYNINKIFLSCLGLWPFQYKLARRMLPLFCFILEISYLPFEIL
TLYKHRHNGQIIFDSLYQMVVTIAFLVKLLNQLWYRDKFRRLYEIMENHW
NIFTNDLEVRILKTYSHISQKFVVLYSILIYVMMSMFITIPSIGPMLLDI
VLPLNKSRPRHIAIYSEYGVDQDKYFTSIFLYTSIMIIVGMTIMVAVDTM
HITCTAHACSLFELIGQQVENIISNVHIGDRRCNVGRCKNVGYKMFSEEM
IYREYIVCLKKHQLALEYVNILNDTHKIVGISFSLLIGAIFSLLGVRIVY
VLDQLEEMIRFTFIIMGALLQLIIVCYSGQKLMDESQNIFHRAYAAEWYK
FSPRLRSLLIIIILYRSVVPSKLTAGNLFPLSMTVFATVVRTGVSYFTAFL
SFKN

>PbOr375INT
MLENFLREYNTNRILLSLTGLWPFQNKPVRKLLWTFCLLLEISYFPFEIL
LLYDHSDDAQLICESCYQFLILTAFLVRYLNELLNYDKMQWIYEAIDEHW
NIFTNDMEVEIMKEYSMLSRKFMKYYSILICLSLAVLVIMPLTPIFLDII
MPLNESRPKFFAIEVEFRVNKEDYFLLIYCYITTVTVVGANITMGFDTMH
IACTAHACSLFAAVSKQIENIILKVENNNKINENAYGLIRKLDLSSEEII
YQEYIACLKKHQLAIKFVEVLNSSYQGYALAILILILLLLSLLGLRYAAE
WYKFSPRLKSLLIITLYRSDKSCGLKAGNVIPLSIATYAKVIRMSMSYYT
ALLSVQN

>PbOr376FI
MLANFLREYNINRIFLSIVGLWPFQSKMGNCFGIFFFLIEITYWPFEIIM
MYDHWDDTRIIFDACYQSAMLSSFIGRQLHQFWNHSKLRWLYKAIDEHWD
IFTDDTEVQVMKNYSTLSRKFTKYYSMLMCSIMLILLSSPLIPVLLDIVM
PLNNSRARFFAVEVEFRVDENEYFAPIFCYTSIVIITGVATTMSVDAMHI
ACSAHACSLFAAVSYNAEWYKFSPRLKSLLMITLYRSNIPCGLKAGNMFP
LSIATYASVIRVAMSYFTAFTSFKD

>PbOr377
MLSNFLREYNLNRILLCISGLWPYQNNHIRYPLRTFCLLLELSYLPFEIL
LLHDHWNDPQIIFDASYQILLFIALIARALHDIWNHDKLQWLYEAIDEHW

NIFTNDMEVCILKDYSALSQKLTKYYSTIVYLMCLICMTIPLAPPLLDIV
MPLNESRSRFFIVEVEFRMDKNENFVLIYCYTTVTIMVGINIMVGVDAMH
VMCTIHACSLFAAISKQLENMFVKANNSKNISKSKHCINTKFDSLSEEIV
YHEYIVCLKKHQLAIEFVNVLESSYQGLSLLILLLIIGALSLVGIRIVYV
LDQLQEAIKFIFIVIALLVTLMIISYSGQRIMDESQDIFYRAYAAEWYNF
SPRLKSLMIITLYRSNMPCGLKAGNMVPLSIATYASVIRMAMSYFTAFLS
IQE

>PbOr378FI
MLSNFLREYNMSRIFLSNIGCWPFQSKLVRNSLWTFCFLLEISYCPFEIL
LLYDHWNDARMIFEAGYQIAIITSFILRQANQFWNYDKFRQLYKTIDEHW
DIFTNDIEIQILKDYSSLSRKFTKYYSTSIYLMCTVYIIIPLVPPLLDIV
IPLNESRSRFFMMEVEFRIDKNENFLLIYCYTTAVIMVGMSIMVGTDGMH
AICTAHVCSLFAAISEQIENIFLKANNNKNIGKHHVNAELNSLNEEIIYR
EYIVYLKKHQLAIEYAAEWYNFSPRIKSLMIITLYRTNVPCGLKAGNMVP
LSIATYASVVKMAMSYFTAFLSLQE

>PbOr379INT
MLSNFLREYNINRVFLSITGCWPFQNKLVRNSLRTLCFLLEISYCPFEKQ
LENMFLKADNSKNISKPKHYINTEFDSLSEEIVYREYIVCLKKHQLAIEF
VNVLESSYQGFSLLILLLIIGALSLIGIRIIYVLDQLQEAIKLIFILIGI
LLTLTIISYSGQRIMDESQDIFYRAYAAEWYNFSPRLKSLMIITLYRSNV
PCGLKAGNMIPLSIATYASVIKMAMSYFTAFLSLQE

>PbOr380
MLSNFLREYNINRVFLSIIGCWPFQNKLVRNSLRTLCFLLEISYCPFEIL
LLYDHWNDGQMIFEGCYQIVVSASFIVRQVNEFWNYDKFRQLYKTIDEHW
DIFTNDIEIRILKDYSMLSRKFTKYYSMLMYIMMSIFITIPLTPMILDII
VPLNESRPRFFALVVEFRVDKDEYFLPIFFYTTTIIVVGTNVTMAIDAMH
IACTAHACSLFAAISKQVENIIPKTNNSEISEHKCCIDLDSSNEKKIYQE
YIICLKKHQLAIEFVDVLDSSYQGISLVMLILVIGTISLIGIRIVNVLDQ
LGELTRSLFILTGSFVTLMIICYSGQKLMDESQNIFYQAYAAEWYNFSPR
LKSLLIIMLYKTNTPCGLKAGSMIPLSIATYATVLRTAMSYFTAFLSLQD

>PbOr381FIX
MLENFLREYNVNRIFLSNIGLWPFQNRLTRNLLWTFCFLLEISYCPFEIL
LLYDHWDNTQTIFEGLYQIVLSASFLVRIMNEFWNRDKIRRLYQTIDEHW
NMFTNDVEVRILKDYSTLSRKFTKYYAMLMYVMMSGFIVIPLTPAFLDVI
MPVNESRPRFLAIEVEFRVNKDEYFLLIFCYTTAICVVGISIMVSVDAMH
ITCTAHACSLFAVVSKQVENIISKIDNNNENEYRYLTTELNLSSEEMIYR
EYITCLKKHQLAIEFVNILESSYQELSLVLLILVIATLSLIGIRIVYVLN
QLEETTRFMFIIMGALMTLMIVCYSGQRLMDESQNVFYRAYAAKWYNFSP
RLKSLLLITLYRSNIPCGLKAGNIVPLSIATYATVVRMGMSYFTAFLSLK
DK

>PbOr382
MQSAVKRYYNINKIFMSKLGGWPTQSRFMKILLPTIITSFIFSIGFLEFV
KLAETWSDLTEDCECVITMLITIGGYVKLFVLVLNNKDIQRLLSLIDYHW
RIFTHSLEIQIMHEYAIIGRKITISYAVLIYSLTSLYMLIPITPQLLDLL
KPLNKSRPHKYLFDIDYSFDREVYYYPVLLHSYLTTVITMSVMIITDTIY
MAFAQHACSLFAAIGHRLENLTSGISSEKNGSSAGMICNKHEPTVYVLHN
KDDEIYCELVLLLRKHQLSLKFVRMLDSFFALYSFMLLFITIIIMSLLGI
QIISLLDHKEEMIRYVSISIGAFMHLFVLSYPGQRIMDHSTDIFYKAYNM
LWYRMSRRSTQLLSILLYRCFVPCTLTAGKIYVLSMQNYATMVQAAVSYF
TTLSSFR

>PbOr383
MELVLDQYYKLNRQVLSIYGLWPYQNTTNAWIMRTFAILFMSWATFGQIF

RICHSKLTTDFILDCLPIIIPNIGAIVQVVNRIIINDKLKSLFNQMKIHW
ACARSQNEIEILQTTAETTRLTTKLVLLYLFLGATTYMFSTLIPQILDVF
LPLNESRSREHPFHAEWFLNEEKYFYVIRSLMYIALLFILGVVLVNGSVF
VAYMQHANGMFTILGHRAEQSFNGDKHLLKNRSIREEDYGRIVVFIEDHR
NILEFVDIIQSCYGLSLFLEFLSLMILIGATLVQIIKFTGLSDRSFRSVL
YIAGQLMYMFMYSYMGQQLIDTSTQLFMKIYYIKWYNISVWKQKLMLFVM
LKCMRTLSINAYNIYIFSLESFSTIVQSAVSVGMLLRHV

>PbOr385FJC
MEFSLDCYYRLNRQMLSIYGLWPYQNATNAWIKRIISILLMIFAAISQAI
KICMTDLTIDFILECIPILIPNVVAILQFINRIIINNKLRSLCDQIKIDW
KCARSQDEIKIMQTTAMNTQIIIKLFMEFSLDCYYRLNRQMLSIYGLWPY
QNATNAWIKRIISILLMIFAAISQAIKICMTDLTIDFILECIPILIPNVV
AILQFINRIIINNKLRSLCDQIKIDWKCARSQDEIKIMQTTAMNTQIIIK
LF

>PbOr387FN
AIKICMTDLTIDFILECIPILIPNIVAILQFINRIIINNKLRSLCDQIKI
DWKCARSQDEIKIMQTTAMNTQIIIKLFLFYSFIGICMHMLSTFLPQILD
VFLPLNESRSREPPFHVEMFVDEQKHFYTVRLIMYFYAVLILGEILANGT
MFVVYMQHVSGMFIILGHRAKQSFSNGQLPKNRPNQGKDFGSIAIFIEDH
HNVLQFVDLIQSCYGLSLFVELLDTVILIGLTMVQIIKFTGTLYRSIRSL
FYVVGQLAYIFMYSYMGQQLIDMNTHLFKQIYYTKWHNISIWKQKMMLFV
MLKCMRMVTINAYNIFILSLKSFSTIVHSAVSVCMLLRRI

>PbOr389NC
IAFLGISMYMLSTLLPQILDVFLPLNESRSREPPFHVEMFMDQQKHFYTV
RLIMYFYILFILGEIIANGTMFVVYMHHISGMFIILGHRAKQSFSNGQLP
KNRPNQGKDFGSIAIFIEDHHNVLQFADLIQSCYGLSLFVEFLDAIILIG
LTMVQIIKFTGTSDRSIRSIFYVMGQLTYIFMYSYMGQQLIDMNTHLFKQ
MYYTKWHNISIWKQKMMLFVMLKCMRTVTINASNIFILSLESFST

>PbOr390INT
MTNVVDKYFSVNRTLLLAVLTTIMTSKCNANLIIKVFSTSFAVITFIIKY
NSFNVNRQTIKYLMEQIQHIYNNLRDNNEIAIVERYGSNAKHYTRYLAIL
FASSASVIMSALSWPVFYDIILFDTNESQPRRLQITTEYFIDQERYFYLL
LFHLNATISIGLAVMLATGAMLVAYYEHACGMFKIASYRIDKAIQIYISK
DISLQKDILLYKGIIHAIDIHRKAMKFCIYLISRFEISFMLLIIFGVISL
SINIFRIFQIVLSGYNTGEFLMCLVIVVICIFYMFAANLVGQQIIDHNNH
VFIMAYKVRWYIAPLNIQKMILLLLQRGNKSFGLSVGGLFIASLECFATL
INASVSYFTVMYSMQ

>PbOr391
MINVVDRYFNLNRTLLLVVGLWPYQKSKLSQLQLICTFIILITHVIFQLT
ATITSQKCNANFIIKILSNVFILLGVIIKYNSFIVNRNTIRHLMEQIKHI
YKGLRDINEIAIVERYGSNVKYYTSSLTVLFASSVSVFLSALSWPVFYDI
ILRANESQPRHLQIPVEYFIDQERYFYLLLLHLNITVGLGFIITLATLTM
LIAYYEHACGMFKIASYRIDRAIYILKDIRLPNEILVYKNIVCAVDIHRK
AMRFSIYLISKFEISFGVLIVVGVTSLSLNIFRIFQIILSEYDIKEFFIC
LIFVFMCTFYMFLVNLVGQQIIDHNNHVFITAYKIRWYIAPLNIQKMILF
LLQRGNKSFGLTVGGLFIASLECFATLISASVSYFTVMFSMQQ

>PbOr392IP
MNVVDRYFSLNRXLLLIIGLWPYQKSKFSQLQLICIFSILITYVVFQFSS
YLISKFEISFMLLIVVGVISLSLNFFRIFQIILSGYNTGEFFICFVFAFM
CIFYMFAANLVGQQIIDHNNHVFITAYKIRWYIAPLNVQKMILLLLQRGN
KSFGLSVGGLFIASLECFATLASASVSYFTVMFSMQQ

>PbOr393
MIADTYFKLNRFLLLTVGLWPFYKSKFTRLQFICIFSMLTTCIIFQLTTF
LTSKYTADLVIKVFSSTCYIIILEIKYLSFCINSNIVKYLMEYTQHIYNT
LKDNNEIVIIKKYGSKAKRCTIMLTIFWIGFVIIHIGVQLWPNFYNIVSP
VNESRHHHLQIVTEYFIDQEKYFYLILLHTNMSLYIGSFILLATGAMLIA
YAQYACGMFRIASYRIENAMKIYLSQDVLEKRFFIYKSIICAVDIQRKAI
MFSKYLISKFEITFILLIVFGVINLSFNIFQTFQITTSEYNIDEFLLPFV
ITVCSLTYMFLSNLMGQEVTDHYNHVFHVTYNIQWYLAPLNIQKLILILI
QRGNKNFGLNVGGLFLASLECFATLANTSVSYFIVMHSMR

>PbOr394
MISHTFFNLNRILLLIVGLWPYEKSKFSQFQFICISSILTTCIIFQLTTF
ITSKCTADLIIKLFSFAIGIISLEIKYMSFCINSKTVKRLMEYLDHIHNS
LKDNNEIVIIERYASKAKRYTIILIMLWASLGFFHISIQIWSNFYNAVSV
NGSHRHHLQILTEYFIDQQRYFYLLLLHINSAFYIGIFALVATGTMLISY
LQYACGMFRIASYRIENAMKIQISQNIILQNISLPHKRIICAVDIQRKAM
MFSEYLISQFEISFMLLIVVGVLTLSLNIFRVFQIITSKFVNFEELSIPF
VSSISCLVYMFLSNYVGQEITDHHNHVFNTTYKIQWYLAPLQIQKLILFL
MQRSNKSFGLNVGGLFVASLECFATLANTSISYFVVIYSTR

>PbOr395FIX
MSCIVERYFNLNRILLLLVGLWPYQKSKLAQFQLICFFSILVTFIVFQLT
TFMTLKCTINNIIKILSDTFSILILVIKYNSFYINSNTVKYSMEQLQRIH
DELKDSNEIAIIEKYGNDAKRYTTILTMFFICSLSIAVSAQLWPDLMYVI
SFANKSRYHLYFSTEYFIDQERYFYLLLFHTNAAVCIGLIVLLATGTLLI
AYFQHACGMFKIASYRIENALEIYVSKINLKNEYLVYRSLIYAVHIHQKA
MMFSEYVISRFEISFMLLIFFGVITMSLKIYQIFQIISTTYNIEELLLTF
IGTLFCLLYMFLANLVGQNITDHCNLVSTTAYEVRWYITPPQIQKMILFL
LQRGTKTFGLNVGGLFIASLECFATLANASLSYFTVMYSMQ

>PbOr396FIX
MICVTTQYFNLNRIILLLVGLWPYQQSKLVYMQTIFNFGFLISCIVYQFS
VFISADYTPDLLVKVLSIASFFFIYVIEYSSFRINNQIFKWSLEQLQHIH
NELKDEREIDIMKKYGNNAKRATAIFILVNISMILIIVLLPFVIYALNDI
LFINEHDMKSAMQTMIPKHFVGREHYFHLIYIYMSIALSIGGTVIVAIGM
LFIACIVHACGMFKIVRYRIEQAITVNGLNNNLKDEIPMYKKIIHAVDIH
RKAIKFTELFFSGFDQSRLTLIMFAVICLSLNLYGISEIILLGGDFQQFI
IHFLLVIIIFLYFFLENYVGQELINHNDRIFSTAYNVRWYIAPLHVQKLI
LFLLQRGTKAVSLNLGGIFVFSLEFFGTLMKASISYFTVICALEY

>PbOr397FI
MICVTTQYFNLNRIILLLVGLWPYQQSKLVYMQIIFILGLLISCIVYQMK
WSLEQLQHIYNELKDEKEIDIIKRYGNNAKHFTAIVILFHINNLILGFFM
PFALYAFNGILNKQDIKRVIQTIIPKHFVGREHYFYLIYLHIGMAVTVGG
TATVATGMMLIAYIIHACGIFRIARYRIEKAIAINTLNNINLQNEITMYK
QIIHAVDIHRKAIKFTALFFSGFDQSRLALIMFAVICLSLNLYGISETIL
FGNNFQQFIIHFLLVLSIFVYCFATNYAGQELINHNDRIFSTAYNVRWYI
APLHIQKLILFLLQRGTKTVSLNIGGMFVLSVEFFGMLMKASISYFTVIC
SMEH

>PbOr398FIX
MICVTTQYFNLNRIILLLVGLWPYQRSKLVYMQIIFILGLLISYIVYQFS
AFVFADYTPDLVVKVFSITSFVLMFVIEYSSFRMNNQIMKWSLEQLQHIY
NELKDEKEIDIIKRYGNNAKRFTAIVILFHINNLVLVFFMPFALYAFNGI
LNKQDIKRVIQAIIPKHFVGREHYFYLIYLHMGIALTVGGTAIVATGMMI
IAYIIHACGIFRIASYRIEKAIAINTLNNVNLQNEITMYKQIIHAVNIHR
KAIKFTALFFSGCDQSRLALIMFAVICLSVNLYGISEAILFGDFQQFIIH
FLLVLSIFVYGFAANYAGQELINHNNRIFSTAYNVRWYIAPLHIQKLILF

LLQRGTKTVSLNIGGMFVLSVELFGMLVKASISYFTVICSMEH
>PbOr399
MICITQHFSFNRILLLSIGLWPYKRSVLVQLHLCLFFGILISAVIFQLGV
FLTVECTSDLVIKVLSIALFFVMYVINYNSFVINFETVKRSLEYLQYIYS
TLKDENEIAIIKEYGNNAKRYTAIITLFHISNQLSIMSMPMLPRILGIFL
FINGTDMRAVIENVMPKYFFNQDNYFYLIMLHVEAAVCIGGTTMVATGMM
LIAYLKHACGIFKIASYRIEKAITINMQENISFEDEIIIYREMIYAIDIH
RKAIKFSTFLLSSFQGSHFCLIIVGVVCLSLNLYGISVTASLGYDIDQCL
VHLVIVLTIFLYLLLANYAGQEVTDHNENVYSSAYNVRWYVTPLHVQRLI
LFLLQRGSKAISLNLGGVFVMSLELFASLIKASVSYFTVVCSTQQ
>SinvOr2
MIPTSTIGGPMEFTLRFIGVWPDSSCRFLMRVIWMTAMVISQLLQYWYLFTHLGSDSLLDVMQAMSLCLSNSL
LFFKLSLFWLNGRIICDILAMMAEDWNECESAHTKLQLMISKAILSHRVSKGSIAAYTATVLLFGVCDVIIQK
TAGPEQLVEEKGFIVKMQLPSECSTSPFYEITMATQFLMQFTLALVAGMLNALIVTLILHIAGQIDIMCHELL
EIPVAEDKYVSRMVALRSIVNRHQRIIAFADNVEDLFCYMALMQFLSNMFVICFLGFVIVISLDSSEANIVIM
KIVPYYIVVNLEAFILCFSGEYLSSKSKRIYCAAYNSLWYELKPMESKILFLLIIRSQKELTMTAGKFIDLSL
ESFTSIIKASASYVSVLHAMY
>SinvOr3
MGLTTTVSPLVEFGLRTIGIWPGSYNILYRAFWTITLGLAQTFQLRYIAACIQTANLLDLVDSVSTTLPYSLL
CLKLIILWQNKRLFNNILILMSQDWRNCGTTAINVRIMTSKANLSHRCSLLIIGVYAMAVVVYVSVIMEYNTI
GKGSDKEENQLFLKMDFPFVYEFSPVYEIVMFVQFVQLLSNASVIGMLDAFIITLVLHISGQIDIVCRGLLEL
FSKDCESKSYKDAKSAIIRRHQNVIAFSDNVESLFSYIALMQFLTNTLVICCIAFVIVITINANKGYIMLLKS
LFFYIAITLEAFIFCFAGEYLSNKSKSVANAAYEAIWYDVKPCESRILLILILRSQKRLTLTIGKFNDLSLKV
FASILKASASSVSVLLAMS
>SinvOr4
RTMIPISTISRPVEIGLRLTGIWPNSLVLFRMLWTLVMGIVLIFQYHYLLIHFSTEELPNLIDGLSTTLPYNL
LFIKMIVLWVNNRIFNDVLKAMSNDWREYSGMYAMIDKAVLAHRCSKLTIGVYSTAVLLYSTASINFRKQSND
SCRELLIKMELPFNFCESPVYEIVMWVQFVHLMAVASSIGMLDGLMVTLMLHIGGQIDLMRQEVEEICPNDDK
YDLPITIVRSLINKHQKIIAFTENIESLFSHIALMQFFSNTIIICCIGFLIVTSMGTDEGIRMLIKTMFFYIA
ITLEAFIFCFAGEYLSNKSKTIGDAVYESVWYNLKPRDCRVLLFVIMRSQKRLTITAGKFMELSLQGFTNSMK
ASASYVSVLYAMY
>SinvOr5
MIPTSTVSQPVEIGLRIIGIWPNSSILFRLLWTLGIGTSLTFQYYYFLTHFSTEELPNLIDSLSTILPHSLLF
FKLFVLWINNRIFNDILMAMSNDWREYSSMCVMIDKAVLAHRCSKLTISVYSIAVLLYSIASVNFRRQADDVC
RELLIKMKFPFEFCKSPIYEIVVCMQFVDLMAISSAIGVLDALMVTLMLHIGGQIDLIRQEVEGISFQNNKYD
LPTAIVRSLITRHHKIITFSENIESLFSHIALMQFISNTMIICCIGFLIVTSLGTDEGIRMLVKTVFFYIAIT
MEAFIFCFAGEYLSNKSKTIGDAAYESIWYNLKPQHCRVLLFMIMRSQKRLTITAGKFMDLSLEGFTNSLKAS
ASYISVLYAMY
>SinvOr6
TNAVALTVQIGLRSIGFWPNTPCALLFRAFWILTIGIVQTCQYWWIIIHFRTDNMFYLMDGLSVAIEYTVMSL
KLIILWLNSRIFYDVLAAMAADWREAAISEMDTMMSKANLSRRFSKVIVGLHSIAVVCFGIEVLVSAHTDDYD
VDGMETPVRAFTLKLQRPLQFNESPLYEIVVCLEFLHQLASSTVSGVLNCLLITLILHTSGQIEILCDALRDI
SFENKNEQLGFFMKELIVKHQKIIIFSDKIERIFSYIALIQFLSSTLLICCVGFTVITSISTIQDSDKIDSTA
LIKAIMFYMTAMVEAFIFCFCGEYLSAKSKMIGDAAYKSLWYDFKPNESKFILLIIILRSQRRLTITAGKIMDL
SLEGFTTIMKASVSYISVLHAMY
>SinvOr8
MKRVSTISLPVEIGLHFISMWPDSTYASLLMYMTLIVTVQYYQYAYVVAHFDLNNIPLLMDCLGLTLAHSLCL
LKLSTLWWNRRTFYYIVKTMDEDWKERNVNDFYASTMVGMANLSRRCSIAMISFNAFGAFFFTIGEHMLHSLV
ANKGDHKPRVLPLKMQVPFDVSKSPIFEYFLLGQFLYEVVLASVVAMINSLLVSLILHVSGQIDIMRQEINEI
SHGKYDSSTFLMDIKGLICKHQKIITLSKKIESLFTYIALMQLLWNTLIICCSGFMLILGLSNNKEAMVLIKT
AFLYFAKALEVFVFCYAGEFLSYKNKSICDAVYESLWYNLMPSNCRVLLVIMIRSQKQLTITAGKILDLTLDG
FMSVMKASASYMSVIHAM
>SinvOr10
MKRAYTISRSIEIGLRFIGMWPDSAYPNLYWFSYVTTVAIVQYYQYTYIFVHFDSNNLWLLMDCLSLSLAYSL
AFLKLLVLWWNRRIFHYIVKMIDQDWNEYIINDLHRSIMTSMAGLSRRFANITFSFYAFSAFFLTIGEHLIQS
MDDGNQFSNNSRELPIKMEFPFDVSKSPIFECLLIGQFLYDMVIAFVVGLINALLVASILHVTGQIEIMQQDL
IEISNGKYDRATFILVIKSLICKHQKIITLSENIETLFTHIALMQVLWNTLVMCCTGFVIVVIVSNGEDTTNL

IKSVSYYIAIIMEVFVYCFAGEFLSAKSKSIGDAVYESLWYNLPPSDSRIILFMMLRCQKRLTITAGRVFDLT
LEGFTSIMKASVSYVSVLNAMY
>SinvOr11
MKRASTISLPVEIGLRFIGMWPESAYSNLYWSMYMTLIVVAQYYQYSYVVARFDVNNLIILTDCLGLALANTL
VLLKLVCLFWNRRIFYNILAAMDRDWRECIINDSSIMMTVADQSRRCSLVLIGIHVLAGFFLSIGLYIIRMMN
SNVSRDLPVKMQFPFGALESPIFEFILAGQMFYIMSTASMAGMVNALLASLVIHMGGQVDILRRVVMELHCSN
DDGLGTSVTLFSDLIHRHRKIISLSNDIENLFSSIALLQLLWNTLIICCSGFMIVLALSSKKGAMVLTKSTFL
YIAKTLEVFVFCYAGEFLSFKSKSISDAVYESLWYNLMPSDCRALLLIMMRSQKRLTITAGKIFDLTLDGFMS
VMRASASYMSVLHAMY
>SinvOr12
MLTSTISPSLKLGLRMIGMWPDSSCKTFFWLFYMITIVVMQYFQYLYFFAQLGTNNFSKLMDGLSVTLDYTLT
FLKLLSLWHNRRIFSDILSAMNDDWNDCSTDSHVYVMTSKANLAHRCSNVMLVLNTLSTFFYFIGSFLSHRTI
SKNGDLREFPMQVQFPFDAATDSPIFELIVLGLFLHVWETATVIALLNSLILTLVLHVSGQIDIMCQGLREIS
TTQKPLYAIRSLIVRHQRIISLSNNIDNFFSFVALIQFVWNTVVICSIGIMIMISLGTGVEGKSGLLIQSIIP
YIAVTLEAFVFCFAGEYLSTKSKSISDAAYDTIWYDLSISECRILLLIIIRSQKRLTITAGKVMDLTLEGFTT
VMKASASYMSVLHAMY
>SinvOr13
MTITNTVSPALKIGLQLLGLWPGISYSIIYWSSFMLSMIVMQYFQYLYIFNHIKFSELLNLVDSMPAALDYSL
TIFKLISLWLHRRVLHEILTAMDNDWRECINVDWQLYVMQVKANISHICCNAILSFNAIATLLYFLGNYIIHI
MFLTEDYNDTLRQLPFKTQLPYETQQSPLFEFVFAILLLHVILHSSTVGIVNGLIFTLVLHVGGQIDIICQKF
KNTSENTLLSKTSVPSFGMLIERHNRIISASDNIEKLFSFIALMQVLWNTLVICSLGFAFTFFIHNGANGFAL
VKTIFAYFGVIMEAFVICFAGEYLSHKGKLIANASYEMMWYDMPSKQSKIIIFIIMRFQKRLTITAGKMTDMT
FGAFTSIIKTSASYVSVLNAMY
>SinvOr14
MTIPSSISPALKVSLRFLGMWPGDTFSAIYWLSFMLSMLIIQYFQYVYIFDNLKISELSNLVDGLIVTLDYSL
TVFKLTSLWIQRRIFHQILAAMDNDWRRSFSFEKHLYTMTVKASISHFISNALLSINAIVAVPYLLGDYAIRY
VLTENHTDTLRQLPIKIQFPFDAQQSPIFEILVVALFLHVLIHACTIAVLNGLILTLVLHVSGQIDIICCEFR
NLSKDTVLHKSSVPLFGMLLERHNRIISFSKNLETLFSFISLMQVVWNTLVICCLGFVIIISIDNGTGIFVLV
KTVSGYFVVMVEAFVICFAGEYLSLKSKSVGDAIYETLWYDMPTHQSKIIIFIIMRSQKRLAITAGKMVDMSF
ETFTSIMKASASYVSVLYAMY
>SinvOr15
MTIPSAVSPALKVCLQLLGLWPGDSTSAIYLLSYLLSMLIIQYFQYVYILDHLKISELTNLVDCLSLALDYTL
TMFKLIVLWIQRRVFHKIVTIMDNDWCECTKIDNHLHMMKIKADISHFVSNSLLSFNAIIAVLYLLGDYIMHS
IFSSVNRNITLRQLPVKIQFPFDTQQSPTYDILVMALFLHVFIHAGILATVNGLIFTLVFHVSGQIDVICYEF
TNISKNTLLHKSSVSLIGMLIKRHNRIISFSKNIETLFSLIALMQVIWNTLVICCLGLVIIVSVHNNTGVFVL
VKTGVAYIAMTIETFVICFAGEYLSLKSKSIADATYKSLWYDMPLRQSKIILFIIMRSQKRLGIHAGKMLDMS
FQTFTNIMKASASYVSVLFAMY
>SinvOr16
MTIPSSISPALKVGLQLLGMWPGDSTSAIYLLSFLLNMLIVLYFQYVYIFDHLKISELTNLVDSLSVALNYTL
TIFKVIILWIQRRVFHKILATMDHDWRKCINIDKHLHMMTIKAKVAHFISNTLLSMTASIGVTYLLGEYIIHS
VFLSEDYNDTLRQLPIKVQFPFETQQSPTFEILLAVIFVNVMMNVCVVALINGLIFSLVFHASGQIDIMCYEF
RNISNNKSTLLRESSVTSLGMLIERHNRIISFSNDIETLFSFIALMQIVWNTLVICCLGFVLIISIHTKAGIF
VLIKTATAYLAIMMEAFVICFTGEYLSMKSKSITDATYKMPWYNMSLHQNKILLFMLMRSQKRLCITAANMMD
MSFETFTIMMKASGSYVSVLYAMY
>SinvOr17
MVITSTVSPVLKIGLQCLGVWPNVPYSAIYWLSFMLSTLIMQYFQYWYILEHLKFSELSNLIDALTVTLDYSL
TFFKLIGLWIHRRVFHQILTDMDNDWRECVNSDQYLYIMTINANISHFFSNTILSVVTTATVLYLLGDYVIQY
MFITEDYNITLRQLPIKIQFPFETQQSPIFEFLVVTIFLHVMLHVCMLCLINGLIFTLVLHVSGQMDIMCHEF
NRISKDILLHKSSASLFGMVIEKHNRIISFCENIKTLFSFIALMQVVWNTLVICCLGFCIIISFHNKTGIFVL
VKPVLAYIAVMTEAFIVCFAGEHLSLKSELITNAIYELLWYDMSPRYGKIILFIIMRSQKRLTITAGKMMIMS
FETFTNIMKASVSYMSVLNALY
>SinvOr19
MIPDGTVSRSIEIGLRVIGVWPDSSYTVLRRAFWMITLAMAQTFQYRYFVIHVRTDDLSHLMDGLSTTMSYSL
LLLKLTIFWIKRRIFYDILAMMARDRSECVSEWAVGLMSKTIDISHRSSNLIIGLYSMSVFLYGAGVLVAHPD
EPEDQLTTMPPRELFLKMELPFESNASPAYELVMITQFFHQLAAATIVGVLNALIVSLILHVGGQIDIMCRGL
VEISSGDDTFDLQTSTIKALIRQHQRIIALSSDIERVFSYIALMQFLWNTLVICCLGFLIVTSIGSTQGSTTL
IKSLFFYVVITLEAFIFCYAGEYLSAKSRMIGDAAYEAKWYSSHPTQSRILLLLILRSQKRLTITIGKFMDLS
LERFTTIIKASGSYVSVLHAMS

>SinvOr20
MENKIDSINTVCRSVEYGLRIIGVWPGMPCAILFKVFSISTMIVSQIFQYRYAIVRFGEEDLMVLMDGLSVTF
AYSLLLVKMLIFAFNSRLLNEIVTCIAKDWRGSDISGEYMMTKLADSTRWLCNVIIFSHMMSVFLYAIGTIMK
IRNENQTEARELIIKMEIPFEIQSTSVHVAVLVTQFIHQTTAAAMVGVLNSFLIILVLHVCGQIDIIRQKLSE
ITQKSIVQKNIEQGVNESVIKTLIVRHQQIIAFSKNIETLYSNIALIQFVSNTLVICCLGFLIVISIGAPNGS
MMLIKSVFFYIVMSLEAFIYCFVGEHLSTKSEMIGYSAYESLWYDLSPRQNRDIQIMIVRSQKNLTLTIGKVV
ELSLRQFANIVKASASYVSVLHAMY
>SinvOr21
MNKGIKSMNTVCGSVEFGLRVIGVWPGTSYAILRRVCCVFSMAVFQTFQYRYLIVHFSESNIFLLMDVMSATL
TYSLLFIKLIIIMFNVRLLDDIIAHVVEDWKNCDVSDEYTMNRMANISRWFSNLIIGSHAVSVFLYAIGTLLK
IETQELILKMELPFKMESTLVYLSVLVTQFVHQVSAASMMAVLNCLLLTLVLHACGQIDIIRQKLSEITGKNV
ERDANESVAKMLIVRHQKIISFSKNIETLFSNIALLQFVSNTLVLCSLGFLIVISIGVPGGSGMLVKSVFFYI
LVNLEAFIYCFLGEYLSTKSKMIGDAAYEALWYELNPIQNRDILFIIVRSQKYLTLTIGKVADLSLRQFTNIV
KASASYMSVLHAMY
>SinvOr22
MSSSIESVNTVCRPVKFGLRMIGVWPSTSYAILRRVFCISSMAVFQTFQYRHLIMHFGEEDLLLLMDVLSTTM
AYTLLMIKLIIFTFNTRLLNEMIARVIEDWKQRDVCDKYTMTRMAYISRRFSNFIIGLYALSVFLYATGTLLR
YRSSNQTDTRELILKLELPFEIKSTTVYVAVLVTQFIHLTSAASMVGVMNSLLITLVLHVCGQIDIVRQKLSE
ITRKDVKRDVNESIVKMLIIRHQRIISFSKNIEAFFSNIALIQFVSNTLVICCLGFLIVISIGVPGGSSMLIK
SVFFYIVISMEAFIFCFLGEYLSTKSQKIGDAVYESLWYELDPNQNRDILIMIIRSQKHLTLTVGKVMDLSLK
QFASIVKASASYVSVLHAMY
>SinvOr23
MTTLSRLVRFGLHVYGIRPYVTSTVVFRLYWIIMLSTAQVFQYRYVVMNIHMDDFSEYMDGVSSAMASSLLYI
KLIIILWTHERIFSDLLQMMSTDWQDYISTRHSSRIMTNAANLARRTSRWIVGMQVASGTFYSVGVLASNANNP
EKLEPYTRELILKMELPFNISTEFIYTAVQSVQFYHLSLVCYGITIVNSLLVTLILHICGQIDILRECLLKVF
SKNSAESTDEITMRSLIAKHQRIIIFAEHIETLYTYIALMMLLSDTIIICCLGFIIVISLDSPNAAAILVKSM
LFYISMNVEAFIYCFSGEYLSAKSKMIGNAAYDSLWYDFPAKESRTVLFLIVRSQKRLTITSGKIVDLSLERF
TSVVKASLSYISVLLAMY
>SinvOr24
MKIQNTVSQVMKIWLRMFGIWPNMSCVLFCRLFWIVALIVEQILQYRYVVNHFHLIEFSEIMSILGAIVAYTV
FLIKLIIFWVKQRTFNKILMMMAIDWEKCSSTKFSMFTMTCNAKRSQRFVNMTAIFYAIAVTLFSSNVLVKHV
DDGKPSNVSTRMFILQMDLPFDVNRRFVYESVIIVQFLHLILCSEAIGLLNALLINLILHVGGQIDILCQNLM
EMFPKKGKSGPNNFMIKEIIEKHQKIITFSRYIEDLYSYIALVLFISDTLIICFLGFTIVTSIGRSDGMESIM
KNLAFYLNMNMEAFIFCFSGEYLSAKSKSIGDAAYDSLWYKSDSRDSRTVQFLIMRSQNQLTITIGKIMNLSL
DRFSSIVKASASYISFLLAM
>SinvOr25
MEQTPTTTINRTVEIPLRIFGIWPGSPYITFYRLFWAIVFAAAQTFQYRYIIMNLYTDDFPDLMDGLSASLGF
SQFLSKLIVLWFNKRTFNEILTMIAMDWKKNKDDNFSIRVTISKAILSHNLAKFVFGFFSIAITLYSASVFTF
NTSNLEEPDTSARPLIVKMDFPFDSDTRFVYGLILVIQFFVVAFGGSAVVMINTLLVVLVLHLAGQIEILNKW
LTEIVSKEKDGYRLSLIMIKKIIEKHQNIIAFSKGIENLYSNIALIIFVSDTLIICGLGFILVTALGRPDAAT
ILIKTVGFYFIMNFEVFMLCFAGECLSTKSKAIGDAAYNSHWYQCKFQESRIILFLIMRSQNQLTITVGKFMD
LSFEQFLGIMKASASYVSFMLAVY
>SinvOr26
MGSKRTINRTVELMLTMFGVWPGVSCVVLNRAFWLVTLVINQYLHYWYFVTHFRVENLFDLMDCLSSLLAHVK
LTFKLIVFSLKQRIFVEILTTTAEDWKDCDNSEIALRETASKTKLSSRICNGLIIILHTIAALAYVIGILLADA
DVTDQTVELPLMMKMEYPFVIDTLHKYKLVLATQFVFVMVNSWGAGLFNALFLTLTLHVGSQINILLHWLTEV
GPTDETKRGSFVTVMTKIIQKHQKIINLSENIENLYSYIALLQFTSNTVMICSLGFLIVTAIGSPDATEQIIR
SLLFYAVTNLEAFIFCFSGEYLSNKSKAVGNAAYNSSWYNMRAKDSRLLLFIILRSQRQLKLTAGKMTVLSLE
CFTNIIKASGSYLSVLLAMK
>SinvOr28
MARKNTFNRTLKFMLTLCGIWPGTPYVLLCRIFWVVSMTVILYFHYRYFYWHVRSAEILDLMDCLSSFIAYSK
IIIKFFVFWLNERKFVEILAKMAEDWSDCANNDVGLRETARKAKLSDRITNSIVILHTMTIVLYCIGIILTDV
DVTDTSKELPFMNKLDIPMDINTLFKYRIVLTVQFLYMMLSSWAAGITNSLLLTLILHTAGQIEIMRHWLAQL
VPRKSEDKHKSITATASKIIQKHQKIISFTKNIENLYSYIALLQFVSNTIMICSLGFLIVTAIGSSNAVEQIM
KSFLFYTITNLEAFIFCYAGEYLNNKSKEIGVAAYNCEWYDLKCTESRVLLFIILRSQKQLSLTVGKMMDLSL
EAFTSIMNASGSYLSVLLAMQ
>SinvOr29

MIRENPLNRMAKFMLTLNGVWPGASTALFWRMVFIVSIPFAQYCHYRYLKAHMHSITIGDFIDCLSSFIAHCK
VLFKCLVFWLNQRKFIEVLEVMREDWSDCANNDICMRETVRKAKVSERVTKVMMILHTTTIVTYSIGTILADI
DVTNNTTELLFYTKVEFPFDVNTQRTYRFILAIDFFFLLMCSWCAGIMNTLLLILTIHVAGQINIVRHWLMQL
ASPENKSKNVSIATTKIIQKHQKIIRFTENIESLYTYIALMLFVSNMIMICLLAFVVVTAIGSPNATEVIMRS
LLFYIITNFEAYIFCYAGEYLKNKSKEIGFAAYSCSWYNMKCEKTRTLSFVILRSQKQLTFTIGKMMDLSMQS
FTSIMNTSGSYLSVLLAMQ
>SinvOr30
MTRKSTINRIAFLMLPWFGISSGRLIILITRLFWIVTTAFVEYCHYLYFSTHLNSENFFNLVDCFCSFLAHAK
VITKLVAFWVNQRKFEETLALITDDWSDYAKNDIGMRVMTGKAKVSDRITYIILILHTMTIVLYSMGIIIADA
DVTETIELPFINKLVLPFSINTQHMYRFVLIAEFIHMMLSNFVAGVYNAILLAMVFHTGGQIDILQCWLAQLQ
PKNIENKQKSIVVMANKIVLKHQKIIEFSENIESLYTYLAMLLFALNTLLICTIAFIIVTALGTADAMEQIIK
CILFFTITNLEAFVFCYAGEYLSNKSREVGFATYNCEWYNLKSKDSQILLFIILRSQKQLTLTAGKIMDLTLQ
SFASIMNASGSYLSMLLAMQ
>SinvOr31
MSANTITRSIEIGLRVAGIWPGAAHAIVNRFFWTATMITAQIFQYRHMVVHLNSNDISQLMDGLSATLSYSLL
FVKLIVFWTKQRIFNDVLASIATDWEKCGDTLCSMSAVANLTHRFSNLIIGLHSTAVLFYCIGVVALGGDDVA
DRELFLKMELPFESGTSPIYEVVLATQFLHQMTAATVIGVLTALLVTLVLHAGGQIDILREKLLEILPRKKAP
PISVITMGSLIRRHQNIIVFTEKIESLYSYIALVQFISNTIVICCLGFIIVNSIGDDQGSSMLVKSILFYVVI
NLEAFIFCYAGEYLSVKSKSIGDAAYESLWYDLSPSENRILLFLIMRSQKQLTITVGKFMNLSLQQFANIIKS
SASYISVLHAL
>SinvOr32
MQIFSLNFLIHTLCGIWRPIEWSSNGVKLLYNVFTFIVIFSHYFLALTQFMDIILIVDNIDDFATNTLMFLTV
VAVTCKATVVVMRRNAITNLIQVLTKTPCKPRDEDEAAIQTKFDMFIRSCSLKYLLLTTTSVTGTTIGSVLNV
MHGHLPYRIWLPWNNNAPMVFWIISIHQIITVIFSAMINVGTDTLIIGLILQTCTQFEIFESRLHKLIISKTV
GYLGRAFSSSNQNKTEISDCICHHLSIYKYAKTVNIIFNQVLFVQFFASILVLCTSVYYLSIHIAELFGAATF
LVYTIGMFVQIYIYCWSGNEVILKSMSVEDAIYCMNWPLLSINEKKELMMIMIRSTIPIKFTSSFFITLSLQS
YNNILKSSYSAFNILQK
>SinvOr33
MLSKEHYKDDMVYITQLTRHILSLLGIWPIYNRSKSTGDKFWKNFLISLCNILLYCVLIPGLLFWLIEKRTRV
RVQTMPLLIFVIMACSKYSTLIYRETNIRRCLMHIEEDYKTAINEKTRATMLESAKIGRRLVTLCAIFMYTSG
LSFRLVLPFARGKIVTAQNITIKPLPCPAYFIVFDVQVSPVYELIFALQVLSGLITYSITTGLCGLAAVFVMH
ACGQLKILVTLMKNLVEEQWQEKQEVNRKLATMVQHQIRIRNFLQLVENTLQQACLLELFGCTTIVCLLGYFI
IMEWENSNLIAMCSYFSSLISMMINMFMFCYTGEQLTVQAEKVARTSCMLEWYRLPNKEARGIVLVVIMSNLP
LKITAGKIMDLSLKTYGDVVKTAVTYFNMLL
>SinvOr34
ERQMDADKEYDDLIKHIKRMGKFISIWPLERDPPTNVKLFRICHVLWFFFVLIAISVAVTVDLVRNINDLDNL
TACALMASTTYLSIIRLTVFSTHQKDMLYVVETMKKDWVCSSYEDKAFLKEKCLFAFRVAKCFLIMVTITTFF
FGCSPVLEMHFLGKERMFPCRGYFFANQMVSPFYELLYVFNIMAAVFSSSTIMGATTFNFVAVTHGSVKFALL
RRKLELINNNDPDVDGAMVDCIKDHQDAITFADTLERIVNILTLAQFVISSGMLCFAGFQITAMVKNEERLMQ
YIMYLSAAILELFMFSFSGNLLIDEAISNAVGESAYCSCWIGSTFGRSLQIVMMRSKVPSKITAAKFYSMSLE
SFARVLSASFSYIMVLLSRKE
>SinvOr35
MFFHRDVTVTLSVHRFALSCVGIWPVRERNIFMDLRWIIAIFLEASTAIPMCVYFAEIYIHCNGAKRSFDWVT
PGAAATLALTRLITPRIHRGELLEIVTSMVDDWTMQKEKKIRWIMKKYATMSTRVTVLTFIMVAIILGVYIAM
AISAVTTKRQHPDNEINVSVSDEAESQSCVFRSESSHQAFMVIQAMQMFTTCIITFGTTSFFFGLAMHLCAQF
DALSIKLTEFRVSNAHRVISEAVQRHCQLIRLAECMEESFNANVLMYLFVTSFLMCIDGYMLIASLPVGDVPT
IVHSASILLLMLIQLSFYTFAGDYLETRSTALAYTTYNCDWYELPASRAKDFQIIIMRASIPHQLTAGKFVVM
NMITFKDILKSTASYLSVLRVMLD
>SinvOr36
MKEARNWNDESRYALNVYKKVLNIIGVWPLNAEEFTSVARCSLAILIQISTIVSLSLEAYWQCLGPEDMMEAF
LMDLSSVVSLSKLLVIRLSWRHTYVLVTSIIDDWSISRDTQRREIMMRYTHAGRAVSLTMLYLGYASGFSFIF
MAIPFDSFIPWLNVSEVNNNDTMVTPYFLATYCVFGSLRTIAYTCVLLLQVAQIFVNATSHCGNDGFFFGLAM
HLCGQFEVLQMDFAGIKAEEQTCKKEIKMLISRHCDLIRLADSFEYAFNMAIFAQVSMSVLLLCVEGMQLIIS
LKLNDNIAAIKHIVLILTMLVQLYLYCYAGDQLEYVTGRIGYSLYDSPWYNFDLKAMKNLPMVMHRGRMPHQT
TAGRFLRMNLFSFKEILKATGSYLSVLRVMIDV
>SinvOr37
RMKSNVKWNTDTTNILKFHKNFLGIIGLWVLNDKNIFSRIRWFLSTMVEMSTSITMSIEMIRHCNGHEDAMDA
FLLSSSSLISMVKLLLHRVYWRQKLILVESVIHDWTYVKNSHSRDIMLKYARIGRLGSSIFFYFGCASVVSFV

SSVVLANVDLPWTSGKQTFNETYERKLMLAAYCIFGKDTSLFAYCAIEALQFVQIVVNGISQCGNDGFFFDLT
MHMCGQFAILRMNFTKLGCEDFSYRSKLNILLKRHYQLICLSHYLERAFTMIILAQVLMSMIVICVEGFLLLL
SLEMNDALTAAKHSVFILSLCMQLFLYCFAGQTLEFQSKELACAIYESPWYTFDVSMMKTLPLIILRTAHPQQ
LTAGKFVAINFMTFKEILKASASYLSVLRVMIKT
>SinvOr38
IPLIRMTDKRWNDDIADVFYVHRVFLKVYGLWPLQTQTVFTKIRWGLCMTAQFTILPFVMMDLWFNQNTNSNI
ECIIFFTGSVLGIAKCLCIAANQKKLSMNINAVIDDWISVKNNKKKTIMKKSAAKAKMLTNTLLFSLIITFSF
YISGIIFMNRKQIFFMDDSVNVNTSNWIFIIPSGPLSSSITGSQFAIILPIQIIQIGIMAFMIYMADTFIFNI
TIHLTGQLEMLKNKFKIFANKMDTELDYQKKFVNLINRFNELTELYQNLEDSFTFLILFQLVITTVLLILIGL
LLEIRLNEQNYIESVTFALGLAFLLTQSLIPSYSSEYLQEESESIFYALYETSWFTLPLALQKDLHFAMMRSR
IPFRFTGGKCFFINCETMKHILKTAVSYISILRIVLK
>SinvOr39
MKSSIKWNAETKYVLTLYKYLLGTIGLWVLDENKFSRIRWFMSTIIELSATISLSLEIIRHCRGHEDALDAFL
SVSTSVTSIAKLLLHRVSWRYKLILVESIIHDWSFVKKPYSRNIMLKYARISRMGSLVFFYFGCASCVFLFSP
LIFANFNLLSISEEQLHNRTSEKRLLLAAYCIFESYTSSAYSFILVLQILQILVNCTSQCGNDGFFFNLTMHV
CGQFEVLRMDFAEIDSKEFFNGKKLGILLKRHNRLIYLAHYLQKAFSLVILSQLLMSVILLCVEGFQLILTLS
MHNTYAAMKHFLFIVVLLVQLFLYCFAGQTLEFQSRELASAIYDTPWYNFDVSVMKSLPLMILRASNPHHLTA
GKFLAINFMCFKEILKASASYLSVLRVILET
>SinvOr40
MANIRWKDDVSYAMTPFKLLTWPIGVWPLQVYNIYSLLRCIFATCCVSLMVILPSMEIYMGCTDAEQSVDSLM
LICCGILGIMKTIWFRKHARNLTKNYSSALNDYVMVEDTKHRAIMRKHAFTGRMLCCCMLGFSYFSCIIYALI
PFLGAATNNQINVTHEDILEYTVPSRCAVEYFFNAPARMHTIMCLIEAVSIILTSTTNHGNDSMFINITLHVC
GQVEVLKTKLINFDVTSPQVYERFITLIKRHSYLIRMARELADTINFVLIIQLFIISIQLCITGFQFILALKV
NDPVMAGKSVMVQSTFLSQLTLYSFMGDYLKTQMEEVGLCIYQNYWYNFPPKIMKNLIFVIMRTETPVTLQAG
NIVVNLSTYMSILKASISYLSVLRVMVET
>SinvOr41
MARERWKNDVAYAMTPFKLLAWPVGVWPLQVYNVSSLIRCILATCCMSLIVIFPSMEFHMGCTNAEQNIDGLM
LACCGVLGVLKLICFRIYAKNLTDNYGSARNDYLTIENTEHRAIMRRHAFLGRIVSCFMVCFSYVSVMIYSLI
PLLGEEPTNEQDNQINITDEIVLDYPMPSRCALEYLHVPTSMYKIICLLEFIVLVLTCTCNHGNDSLFLNITL
HMCGQVKILKANFIDFGASSPQIYNRFNDLIQRHNYLIELAKELAESISFVLLTQLFISSVLLCIMGFQFILA
LKAHNIVLMGKSCMVLCTFLTQLSVYSFVGDYLKSQMEEVGLYIYQSTWYDLPVKLAKNLIFIIMRTRIPVKL
QAGNFIVVNLATYMSILKTSISYLSVLRVMIE
>SinvOr42
MSNDVWNDDIAYEFSTHRTLMQILGIWPLQKKTVFTIIHWSVIIILQLLSIILIYMDLTEKYGDTGRTMDAII
YISSTVCLILKYSCLAANQRKLAKNINAASDDWLCAKNNEEAYKIMKKHSFESRVYTFVMLYSTYTCGSLYIM
SVVVVNIKEIFFQAQMVNASNAYIFPCGSFGDNMTTLQYTMFTIFQIVLLLVVCTAQSITDSFYISLTLHIAG
QLKILETKFKNLSSKPDNQLYYRKQFIKLVNRHCELRDHNQNVEDTFHLVILYQLVTVTLLLALSGIRILIYL
RSKIYFESLKTALIINFLFMESLLYCIGGDFVQRGSTGIFHAMYTTSWYTLPLTLMKDMNFALMRSARSFQLT
GGKFFYVNRMAMVYVLRTAASYISFLRVALKE
>SinvOr43
MSNDVWNDDIAYAFSTHKTFMQILGIWPLQKKTIFTIIHWSVITILQFLSLLFLYMELTAKHGDTGRDMDAII
YAVCVVSMKLKYFCVVANQQKLAQNINAAFVDWLSAKNNEKAYKIMKEQSSRSRLYTFIMIYSCYICGFLYII
SVAVVNVKEIIFKAQMMNTSNERIYIIPSGDIGDNMTTLQYTMFTILHTVQLLSICTAISTLDSFFINVTLHI
SGQLKVLETKFKTLTSKPDALLIYRKQFIKLINRHCELMEHNQNIEDTFHLIILYQLVTVTLLFALSGIRILI
YLRNGIYFESAKTGLIINYLFMQSLLYCWGGDFIQRGSTGIFHAMYTTSWYTLPLTLMKDMNFALMRSARSFQ
LTGGKFFYVNRMAMVYVLRTAASYISFLRVALRE
>SinvOr44
MLWNDDTAYSMTLVKHLTVPIGAWPLQEYNKFALLRHILSSFGLTVAVIVQYLELYFNCTSATANLDALTLFA
CGIVALTKIISFRIYADNLICNYSSAMNDYLAIDTEEKRIIMRKHAFWGRIICIFAIFIAYVDSGIFIVGHAQ
LSSEEAKTNISILGHQAGYAIPSTCTLAHFHISTSSYLVIFVLEYVCLIIMCLSNHGNDSVFLHIVLHVCGQL
KILKASFVNFDVESPKVHERFNALIRRHDHLIRMTRKLAEIISFVLVVQFFVSSMLIIIVGFQFIIALTSNDF
GMVSKSFMVLSAFLVQLTLYSVVGDYLKVQMEEVAQSVYQSGWYDLPVKVTKNIVFIMMWNQLPIKLQAGNFI
VVDLGTYMSILKTSISYLSVLRVMVE
>SinvOr45
MPRNDDVAYAMDPLKFLTVPLGIWPLQKYGIFPLIRSIVSVFSLVVWLITLFLEVNYSNSDAYVKLDQLMLLS
CAILSTLKIVFFRLYADNLICNFFSAVSDYLAIDTEEKRTIMRRHAFIGRMISYSTISLAYVAATMFILLPML
SDENAQVNVSKKQAADLPLPMTWILGEYHFSTSLYYTIFIVQYYLLLLNANANVGNDSLFLAIVLHICGQMEF
LKTEFTNYGVKSKNLNEDFLVLISRHRYLMEHAERLVDVISFVLLVQVLISCIIICVIGLSFIVALKTHDMMM

ITKCGSVLSALLLQLFFYSFVSDYLKCQMEDVAHSIYSCDWYSFPLKLMKNVLFVIMRSQQPIQLLAGKFFVV
NIETYMTILKTSMSYLSVLRVMID
>SinvOr46
MSQNDDLAYAMTIYKFLTLPLGVWPLQKYNTFSLVRSIICGFSITVMLITLLIEINFGSDDVYLKLDALMLII
CNSLAVTKLLAFRLYADNLTRNYSSAVNDYLTIDTKEKRTIMRRHAFLGRMICYSILFFAYLGSITFIFTPMF
AGDSENIQVNVTAKNKISELPVPVTFLGELCLPTSVYVVISTVQVFIMILTSTSNCGNDSLYFAITLHVCGQI
KILKIEFLKYGMKSKNLNEDFSILVLRHRYLIKHAEYLADAISSVLLIQLLLSCIIISLVGFQLIWALKMNDA
VIIIKTTSVLSALLLQLFFYSCVGDYLKCQTEEIANSIYSCNWYNLPTKLIRNVLFVIMRSQQPVQLLAGRCF
VVNIKTYMTILKSSLSYLSVLRVMMN
>SinvOr47
MAWNKDVAYAMTPAKLFTLPLGVWPLQKYNTFALVRVLVCGFLMSSNMITLFLEINFGTSDAYAKVDALMLMS
CNILCVLKLIMYRFYADNLIHNFSSAVKDYLAIDNEWKRIIMRRHAFMGRIFCYVCVLTTYVCSLIWSLMPII
ADDGEDIQVNVTIKNQVSELPLPVTFLGDPDIPTGIYFAIVTMQLFILMLTGTSNCGNDSLVFAITFHICGQL
ELLKTEFNKYGTTDKDVNKNFSVLASRYDYLMENAKLLTDVISFVLLVNLLFNCLIISLLGFQFILAMKINDA
VMMIKSLAVMLSLMFQLFFYSFVGDYLKCQIEDIADSIYTSTWYRLPTKLMKNVLFVMVRSQQPVQLLAGNFF
VVNIKTFMNILKTSLSYLSVLRVMMDS
>SinvOr48
MSWNDDIAYAMTPSKLFTLPLGVWPLQKYNTFALVRVLVCGFLMSSMMITLFLEINFGSSDAYVKVDALMLMS
CNILCVLKLILYRFYADNLIRNFSSAVKDYLAIDNEWKRIIMRRHAFMGRIVCYICVLSTYVCALIWTIMPML
ADDGEDIQVNVTIKNQVSELPVPVTFLGDPDIPTGIYFVIVTMQLFILMLTGTSNCGNDSLFFAIVLHICGQM
ELLKTEFNKYGIIDKDISDNFSILASRHHYLMENAKLLTDVISSVLLVQLVFSCLIISFIGFQFILALKVNDA
VMIIKSVAVLTSLTLQLFFYSFVGDYLKCQMEDIADSIYTSNWYRLPTKLMRNVLFVMVRSQQPVQLLAGNFF
LVNMKTFMNILKSSLSYLSVLRVMVE
>SinvOr50
MHTLPLSFALLTYVGYWRPVNLTSVKYWAYIVYSIAMIFLLYSFTFCGLVDCFITKDLDKFIEKFSLFLSVLG
VCCKVANLALRRNDIIDLTEMLLTNICVPRNEHETNIQRRFDRSAKTITICCEILNESCVFFGTIAQLQYFIS
TRTLPQCDWVPYDISSTIAYWITVVHQIIALIVCANSSVAHETLIAGFMIQTCAQLDILCHRARTLPDSLREA
RKSSTSKENFKAQELRLVRELVHHHRFVYRFAERITSIFTLMIFVQFTISSTVIGLIIYKMLTRSLFSIEFAW
HSSYLGSMLVQIYLYCWFGNEVTLKSTEIGSAIYEMDWPMLPTDLMKILLIIITRSKKPIQITSGYIITLSND
SFMKIIKLSYSAYNVLKG
>SinvOr51
NYAFGVSRHSLRIFGVWPDPLVSLSDFHRPSIRFIIAMSIVLFYAIMPTTINTIRAWGNVIRMVEGIATTNFC
MLALCKIFGTWYHRKTLRTLMTSIMIDWTSKNNEERNTMLHITRRGRILCITCYAMMIILISFFINSNLIKIF
RNISQPQRTLVYPFNYFYNSQKSPNYEITCLFQLIAGACSCFINSTIDVFISLLLLHICAQLINLRTALNNLV
DKLAEGSISSSSFKKGLAEITTRHEHLIRNTKIVNNCYSIILFLHMFATTFQLCFKSFQFFTIIIRHLDVPII
TKIYALLYFIYELMHLYIYCYSAERLLTESVNMMHGAYECKWYDLPSKDAKNLIFMIRRSAIPFRLKAGKFGT
FSIEMFGNTVKTSMGYLSLLLTLME
>SinvOr52
LNYVFTLSRQWLWIFGIWPDPRIPLREFRRPNIRFIIIACTVCLYVTTPQMMNVIRAWGDVSRMVENFASANF
SLLAFCKLIVTWYHGEKLRPLMASIMDDWMTSTSDWERNTMLKIARRGRSLSFRCCIAATGTIIFYISFHSLR
LFKNIHEPHRHLVYRLENIQKSPTYEITYFIQLSGGTYSVLANYTIDCFVSILVLHVCAQLINLRTTLNNLVN
ELASKSISSTRFREGIAAIVERHEHLIRNAKTIDGCYSSVLFVHVLAATFQMCVITFQVFTIITDNLKVPFVR
MIFLTFYVSAVLTNLYAYCYSAERLVAESTKMAYGVYECKWYDLQAKDVKNLMFIAYRSTIPLRLTAGKFGMF
SLEMF
>SinvOr53
LNFAVGWNRFNLSLIGIWPGPLRNSKQKVGLSRCRFIIAGFFMLGFMCIPQSVNLIFIWGNADMMTENLATAN
IPVANSFMKAFTTWRQRKALKLLVNFFYQDWYTPKTSAERTNMLKTAKLGRRISIWCTVLTQTMVTIYIVLRL
SIIIRIPRSDPARPMLYTAYFPFDINRSPIFELICVCQILSAYSATVSYTGNDCFISMLVLHICGQFRNLRER
LKNLVNESNAKTPDKFKKELRRIIKRHEHLNWFAKTIEDSFNMMMLIQMLSCTVQLCFQGFQVFRILINKEEH
GSVTFQLIFLVSFVAFVLTHLYIYCHIGEMLLVQSTEIGFSTYESNWFNLRGKQAKNLLFIMQRSTTPLTLTA
GKFSAFSLQMFSTV
>SinvOr54
MICTDCLCISLNRLLLLTIGLWPYQQSILVRLQFFLFFGILTTAILFQFTVLFTYKYTLNLLIEVLSSALFFA
VLSIKYFSFNFKTEIIKCLLEQLQHMYDELIDKNEIDIIRKQASFAKRYAIALLLAFILIIFTLSLCYFWPLI
SNILFSTNVTQLRPMPIMFEYFIDQEKYSYLILFHLSAAIIIGSTAIIATGAMLIIYEKYVCGMFQIASYRIT
RVMTFENQENENLQNENLICKKIIRAVDMQRKAMQFCDTLISTFHVTFCVMVVIGITCASLNLFQICQIISFE
YDTVKLILNVAFLLALHIYMIICNYCAQEITDYSNDIYVAVYRIQWYMAPIKIQKIIIFLLQRSIKIFHIQIG
GIFVASLEGAATILSTVVSYFTVLYSVKQ

>SinvOr55
MSLNDDVAYAMTPFKLLTLPLGVWPLQKYNAFSLVRFIVCIFSLTVLMIMLFLEINFGSSDAYVKLDALMVMF
CAVLSILKLLSFRLYADNLICNFSSAVTDYHAIDNEEKRTIMRQHAFMGRMIFYSILFFAYVASSIFVLTPMI
AGNKDVQVNLSIKSQAAQLPLPMTWTLKESNISTNLFLLISAVQYFLMVLNSTSDCGGDAVFLAIVLHVCGQI
ELLKIDFINYGVNNQTGDISKLTSRHCYLIKHAKLLVEVISSVLLVQVLFSCLIISLIGFQLILALKSHDVIM
ITKTITVLSALLLQLFFFCFVGDYLKCQMEDIADSIYNSNWYFLSTKLMRNVLFVIMRSQKPVQLLAGNFFLV
NIKTFMNILKSSLSYLSVLRVMVDA
>SinvOr56
MSWNDDIAYAMTPFKFLTLPLGVWPLQKYNTFSLARCFVCGIGMAALAIVTFFEFNFGSSDAYVKIEDLLCIY
ANIIGILKLLTLRLHAENLTYYFSSAVNDYLAIDNEEKRIIMRRHAFMGRIICYSLVPLTYLAILFITLTPMV
THYNEDIQVNVTIKFHLSELPFPITFLGHMQIPTGIYFAISTMEFLITAVIVASNCGNDALFAAIILHICGQM
ELLKIEFTKYGMTNKNENENFLVLVLRHRFLMEHAELLSDVISFVVLTQILFNCVLISLYGYQLFLAMKVVLI
VRTTILLIVLLLEMFVYTFVGDYLKCQMEEIADSIYSSNWYCLPAKLMRNVLFVIMRSQQPVQLLAGKFFIVN
IKTFMTILRTSLSYFSVLRVMMDA
>SinvOr57
MSWNDDIAYAMNPIKFLTPLGVWPLQKYNTSSLVRYIVCIINMIVMTIVMFLELNFGNDDAYIKIHALYAIYS
TVLCILKLLTFRLYADNLTRNFSSAVNDYLAIDNKEKRIIMRRHAFMGRMICYSIVLLIYLAVLIIILTPTIT
SYSEAIQVNVTIKIHVSELPVPLTFLGDFQIPTNVYLALSMMELLICIINATCNCVFNDSLFLAITLHVCGQM
ELLKIEFNKYNMTNKNDNKKFSILILRHRYLMENAELLADVISFVLLMEVLFNCLFISLYGFQLLLKLKVVLT
ISTTTVVIVLLLQLFSYCCVGDYLKCQMEEIADSIYSSNWYCLPAKLMRNILLVIMRSQQPVQLLAGKFFTVN
IKTYMTILKTSLSYFSVLRVMMD
>SinvOr58
MSWNDDITYAMTPLKLLTLPLGVWPLQKYNTFSIVRSFVCGFCMITMIFVLFLEINFGTSNAYVKLDALMIMF
CNILCVLKLISFRIYADNLTRNFSSAVKDYLAIDNEGKRTIMRRHAFMGRMICYSIVLFAYIASLIFTLAPII
AGDGEDIQVNVTIKNQASELPIPVTFLGDLQLPTSVFFTISMMQLFILMLTATGNCGNDSLFLAIVLHVCGQM
ELLKMEFVKHDTTKKDVNKNFIVLASRHCYLMEHAELLANVISFVLLVQVLISCLIISFIGFQLILALKAHDA
VMIIKTITVLSTLLLQLFYYSFASDYLKCQMEDIADSIYSSNWYCLPTTLMWNVLFVIMRSQQPVQLLAGKFL
IVNIKTFMNILKSSLSYLSVLRVMVDA
>SinvOr59
MSWNDDIAYAMTPFKLLTLPLGVWPLQKYNTFSLVRSIVCGVSMTAMMIMLFLEINFGSSDAYVKLDDLMLMS
CNILCVLKLLSYRLYADNLIRNYSSAVKDYLAIDDEWKRIIMRRHAYMGRIICYICILSTYGCSLIWTVMPML
AADGEDIQINVTIENQASELPVPVTFLGDVHIPAGVYFVISSMESFILLLTGTSNCGNDALFFAIVLHVCGQM
ELLKIEFTKYGKTNKNENENFSLLGSRHRYLMEHAKLLTDVISFVLLVQVLFSCLIISLIGFQFILALKVNDA
VMIIKSVSVLASFLFQLFFYSFVGDYLKCQMEDIADSIYSSNWYCLSTKLMRNVLFVTMRSQQPVQLLAGKFF
IINIRTYMTILKSSLSYLSVLRVMMD
>SinvOr60
MEFSVDQQYRLNRQVLSFYGLWPYHSQTTKIRIKQIFSILFLIYATVVQAVRIFMTELTTDFILDCLPIVIPS
LGAVVQFLNRIIINDRLKDLFDQIKIDWKCARSQDEIEIMQTNIINTRLTSKVFLLSAFTGITLYMFSTFIPQ
ILDIFLPLNESRPWVHPFHAEFFLDEEKDFYIIRFIMYFGIVFVLGVVIANGTIFVIYTQHATGMFTILGHRA
EQLFCNEQLSKNRTIRGKNHGNIALFVKDHRSILQFVDIVQSCYGLSLFIEFLSLVVLLGLTMVQIIKLSGLD
RPFRSIMYITGQIAYMFMYSYMGQQLIDKSTQLSLKIYSIKWHSIAVWKQKMMIFIILKCMHTITINAYNIFF
LSLESFSTIMQSALSFGMMLR
>SinvOr61
MEFSVDQQYRLNRLVLSIYGLWPYHNRTTDARIKRVIAILLMFYITFIQVTKICMTELTANFILDVLPILIPS
AGVIIQIINRIIINDELRNLFDQIKIDWESVRSQHEIEIMQRSVKNALLTTKVVLLDMFIGMTLYMLTTFSPQ
ILDVLLPLNESRPWEHPFHVEFFFLDNERDLYIIRFILYFGAVFGLGVILANGSIFVIYTQHATGMFTILGHR
AKQTFSDEQLPNSQFIHEKDYKSIALFVEDHRSILQFVDMVRSCYGSSLFLEFFSLMILLGTTMVQIIKFSGM
SDRPMRSAAYVTGQIMYMFMYSYMGQQLIDKSSQLSMKIYSVRWQSIAVCKQKMMLFVMLKCMHTITINAYNI
FYLSLESFSSIVQSAMSVCMLLRQV
>SinvOr62
MDERKNIWQSHFYIVPRAYMSLIGIWPYHAFRVRCLLFVPMFTFSLTIIVPQLLYLLIAAANLDDVFSCTPSM
WITIIFSFKLGWLMVNNRKLKTCLKTMEDDWLSLNTDVEKSILQRHTTYGRYITLTYGVFMQGVGILLILKSV
VVMLLEDTSDATVSTLVAEAKLPLRVEYGEALDRYLYPMAIHTYLAVFSHISITIAVDSCYIALIRHACGMFA
IVGHTLEHIGKDNDGNFDLKPDKVKDDNYNRALDCLRKHLHVIQFAELIESTFTNIFLVSVCLNMIGGSMIGI
QVVLNLNDAKDIVEPLAIYIAQLIHLFLQFWPAQFLIDYSVLPYESVCRSNWYYTSGRCRKLLFLIMNRSVLP
CRITAGKVVPLTIENFGTVLKTMMSYFTMMRSFN
>SinvOr63

MAEQKDIWQSRYYAIPRLYMTLAGLWPYHPIRDRYLRFVPTFIICSIILIPMLLYVSLAMEDLDDLFECMPTI
LITIIFSFKLASLMANSEKIKVCLKTIEDDWLSLNDKEKAILQRHTTYGQNLTIFYAVFMVMTGLLYMFKSIV
LIMIEDTSNSTKLAVTKLPFRVEYGSTIDQYFYPILVHCYLTVYSHVTATVAADSFYFILIQHACGMFSVVGY
MLEHIGEDNDTNFDTKPNKINDNNYKRALYCLRKHLHVIEFAELIESTFTKIFLVSVSLNMIGGSICGIQVLM
NLNDAKDIVAPLAIYVAQLTHLFFQFWQAQFLLDYSLAPYESICRSNWYYTSERCKKLLLLIMTRTVSPCRIT
VGKVATLSIESFGVVLKTSMSYFTMLRSF
>SinvOr64
MNSEMEMYYATNKLFLYRIGVWPYQRRVLKVMIPCFIVIVHISVVISEALLLYDTWGDINVAVDCIVNLILLF
TADVKLINMVVNNRKFRRLLELMNKHWELLNNKIESHILKYYASISQKLTSYYAVYLIVIIIFYLLIPLTPKI
LDFVVPLNESRPLAYIYQGEYRVDKEKYYYPILFHSYLATACTMTILFTCDTTYIICVLHACSLFTAIGEQLE
NITSKAGTTSNNDGEIHTEMQYHTFIKKHSFIGNDYKILITCLKKHQLAVEFTLNSMFLHVTFILLSMNMLVL
SIIGIQLINNLENTKETIRYICLTGATFIHLVCMCIPGQLLIDKSTEILDKTYGSEWYTFSNKTKKLLSVLLY
KSLVPCTLTAGKMFVMSMTLCSSVMQTSLSYFTAFLSMR
>SinvOr65
MNPEMETYFATNKLFLSRIGSWPYQKKMLKILIPCFVVIVHSSAIVTQALLLFDTWGDIDIAVEIMINFVIVF
GGTVKLMNIVLNNDKFRQLLQHMSEHWELFNSEFEHHILKYYASIGQKITSYYGVYVIVTLTLYLSIPLAPRV
LDVVIPLNESRPLIYVYPAEYRVDKEKYYYPILFHSCLACSITGIILFTVDTTYIVCVLHACSLFTAVSKRLE
NIIGESNIKSDDDENVHTEMHYHIFMEKHISIGNEYRQLITCLKRHQLALESILNSVFTCATTILLGTNIMVL
SAIGVQLINYIHTGEVIRYIFILFGTFLHLVCMCIPGQLLINRSAEIFDKAYSSEWYTFSKETRKLLTILLYR
SLVPCKLTAGKIFVMSMTMISTMMRTAMSYFTAFLSMR
>SinvOr66
MSVFDNRYYSLNKRLLSLIGQWPFQSRWEGNMRLAFASLFVFSLISFEFWGLLAGLSDLSIIMENGSPLLINC
YIITRLINCVFTKNKVCTLRSANCKDYQIMKILLEDIEETWKIKCAGAEKEILQRYAKISRTFTRRLTFAIYL
TWVIYVLTPLVVTWIYKLLPIKATYTARFLYRIEHVLDVDKYYNLLMLHGAVSIFYILSASVAISTAFIACTL
HICALFECLRYNVERVQSTDPVLLEPNIKDDETYHDLVDCIKSYKHALKLSDVLSSNYTVSYFISLGNVMLSL
SFGTAELVMVDLQMDEIIRIVTSNLAQLLHLYCLCTIAQRLTDRSSEFQDVIYSCDWYNMSLRSKRLVMITLM
RANKPCYVKAGKLFVMSLETFSSILKLSLSYFTVLTSLQ
>SinvOr67
MSFFDNRYYYLNKRLLSVIGQWPFQSRLEGNIMFAVTSLFMFSVTAFEFWGLAAGITDLNIIMENVSALLVNC
LGIIKLINCVITNDKVRILCSSTCKANPIMKVLYEDIEQTWKKKCTSAEKEILQRYAEKSRSFTIRYAIALYV
AWLCYCTTPLVITWIYKLLPTNETYAARFLYRVEHVLDMDKYFNLLMLHGIIGIFFILTIGVALESTFVFCTY
HICALFECIRYNVDCIQSTDLVLLEPNIKDDKTYHDLVDCIKSYKHVLKLSDVLESNYAVSFLFLLGNVIICL
SFGAAEIIMIDIQFDELIRLLASDIAQLTHIYLLSMISQSLIDHSSAFQDVIYNCDWYKMSIRSRHLLRLTLM
RTIKPCQIKAGKLFVMSMENFSSILKVSLSYFTMLTSLQ
>SinvOr68
MSFFDNRYYYVNKRLLSVIGQWPFQSRLESNMMFAVTSLFIFSLTAFEFWGLAAGITDLNIIMENASPLLVNC
MMIIKFINCVITNDKLQMKVLYEDIEQTWKKKCTGAEKEILQRYAEKSRSFTIRYAIALYATWLFYSTTPLVI
TWIYKLLPTNETYTARFLYRMEHVLDMDKYFNLLMLHGFISVFYIVQVPIALESTFVFCTHHICALLECIRYN
IECLQSTDFVLLEPNIKDDKTYHDLVDCIKSYKHVLKLSDVLASNYAVSFLFLLGNIIICLSFGAAEIIIIDI
QLDELIRILAADLAQVAHIYVLSMISQRLIDHSSAFQDVIYSCDWYNMSIRSRRLVMFTIMRTTKPCEIKAGK
IFVMSMENFSSILKVSLSYFTMLTSLQ
>SinvOr69
MSFYDNHHYYHFNKTSLCIIGQWPFQSRLRNNVMFALAVFFISSLTVLEFWGLIAGITDLSIIMENTSPLLVN
NFIIIKLTSCWLNQYNLQMKDLLEQVKETWKVIHKGPENEILQYYAEATKNYSMRYATGLYAMWLFYCLPPVI
ITKIYTLLPTNETYSAKFLYRLEHVLDVDKYYNLLMLHAFISVFYIVSVPIAVDSMFILCTEHVGALFGCIKY
NMQRIEDSELFLLNPNIAHDEAYDIIVSCIKLHKRILKFFDRLASTYAMSFLLLLANVVICSSFNMAELVMVD
NQPDEIVRILASNAAQLAHIYYLSATSQRLTDYSTEFQEVIYSCKWYQISLRSRHLLRLTLLRSTKPCQIKAG
NMFIMSLETFSELLKMTMSYFTMLTSM
>SinvOr70
MDAQHYKINQLFMSWIGQWPEQSTFKRFLLSVHLLFTVSSQFCLLVLGLVTAWQDWNLVIECSSAIIIHLVCI
IKYLNFLFNNRKFKSLFNQMKSSWKLATFDAQMQILTTYTNEGKTMIKVYTVILYSSMILYMTLPVMPSIIAS
FMSANQTQIYGLLFHVEAVLDTKKYYYYILLHSYYTTFFMITIPVAVDSMMIAYVQHACGLFQAIGYQLKNVK
RSGDLDINIYPLREDDEHYRTIIDSIVKHKEVLQFVELLTSSYSISFLLLTILNMAVMAFSAVQAINNRDQPT
EALRFLTTCISLSMHFLFLSLPGQKLIDHSSNMHESICATNWYAISLRARRLLNLMLVRCKVPCKMTAGKMRV
MSLQSFSGVMQASMSYFTVLTSVQ
>SinvOr71
MSDIDQSLKNSHYNIIRILLGISGLWPFHTRNRRYAIYLTMILVLGTGYIFQILGIVEVWNDSFELINAIPFL
FFTTIALGKMICTVYTLPQIKVLLTRMREYRLSPKSNEETKIQNSHAQYGRKFGYAYTGFLLGHSVLYLLSSL

SLKIFHTPSNETDELSDKESNYRIGLPHRANYIVDVDTYYVPIFIHSAICDFTCTYLLTVFDVLYLTVVEYCC
GLFASLRYRLEIALVFNDDKLRTLKDKSYANIVYCIRRHAESMQFVSIVESVYSLSLLLHVGSFVLVISFLLY
QVLNDLEVNANTVVRTTAYLNAVLFNVFFENWQGQKIIDSSEKVFESAYNTEWYNMPIATRKLLIIMMIKSEK
PLMLKMGKVVLLSYITFNTVLRTSSSYFMLLRSL
>SinvOr72
MDDIDQFFQNSHYNIIRVLLGITGLWPFYSRKRRYAMYLLLILLLGSGFIFQILGIIEVWDDSYELINAIPML
FYTLVALSKIISTMYTLPQIKILLIKMQEYCLSPKSDEETKIYNSHAEYGRKFGCAYTGFLLGHSVLYVLTSL
SAKIFYTEFNKTDELSDKVPNSQIGLPHRANYIVDINTYYVPIFIHAGMCDFISTFIITVFDVLYLTVIEYCC
GLFAALRYRLEIALEFNNNKLTMSKDKSYSNVVYSIRRHTESIQFVAIVESIYSLSLSIQMGLVILILSLLLY
QVLNDIGNIDRILKPIAYLNGALFNVFFENWQGQKVIDSSEKVFDSAYNTEWYNMPIAARKLLIMLMMKSKTP
LMLRLSKMVVLSYITFNTVLRTSSSYFMLLRSL
>SinvOr73
MRNIDKFFRQSCYSNLRVLLTWCGIWPFHVPSKRYAIYVAFTMFCGFCIPFQTLCMIKVWPDFFEVFDCSTML
VYSLSTTIKLIYAVYKLPEIKMLLLNIREHWCSPKSDEEAKILRSHVLFARKFGYVYSGVLFVYSVIYAFTPL
VARFYYNKMNSLNNTQDAETNSERQISYVLDLQIRYIPLFIQGASAEIYFTFIISSFNVLYLMCVQHCCGLFE
ALKYHLENAFEFGNNDDDIVTISTQNNCHSNIAYGVRRHIEAVQFAVALEALYRLPFFMHMTINVSLLSIVGS
QVMTDTENIGRLMPYGSYLSGLVLNTFFENWQGQKIIDCNEKVFNAAYNLKWYKMPIVSQKLLIMIMMRSKKP
LTITAGKILVLSHMTFNTAMRTSYSYLMLLQSM
>SinvOr74
MDSDQGQHMRINQMLMCLIGQWPYQENWEKFLIQLVFVPAVFAQAVLQGGGMITAYFAGDIDAFMESSSPFVI
SLMCVCKHINYTYNHDQMKRLAFTMVDDWKIYSKLSHEYDILCRNYAMGRKVTIAYAVSLYGSMTPFLVVPVV
LNTASYMGLYNISDGRPLMFRTEYFIDSEKYYYPLLVHSYIGTLGFVSIVVAIDSMLVFHVQHECSMCEILGY
RLARIVAEDTLDINLYPNKEEAISYDHIKNCVIIHNHIIEYAKRIENANTTSYFFQLGFNMMGMTFTIFQAVV
KLSDPNEALRYASFTVCLISVLFLESWPGQQLSDYTDKIFAFITNGRWYQSSLRVRKVISIMLMRSYKPIKIT
AGKLYTLNLANFSAVVRTSFSYFTVLCSMQ
>SinvOr75
MDFFEHNYRVNKFLLSAIGQWPYQSSKTSHGIVIILVTIVCTQLLAKLCGIVPYIHDMDIMIECLIPIMVDVS
GMTKIMNSMLCVNEIRALLDRIRDDFYSLKNSSDSVILQKYANTGKKFSAVYVSVIYMLTVVFMLMPFQPLIL
QVANATTRPMLHRVEYYVDMDKYYFPILLHGYVTAIICVTSIVATDAIFLIFMQHACGLFIITGLRIEQAIQD
VYLIGNANPSITKDMAYQNLVKCVHDHRSAIRFADLMEIAYSKHILFHAGLNMMAISVTSFGALTKSDELFEL
FRLAAVTCAVSFHLCFECINAQKLIDYSGYLHTNLINLNWYDASPRTKKLVLFMMMKTQPPCVLTAGGMFVLC
METFATIVKTAVSYFTFLRSAH
>SinvOr76
IYMFDDHYYKFNKTLLRLVGLWPYETTKCERFRSIFFYMLLLSRVIAEFAQFLIADFNINVIIKILLNASTVF
LQFVLFNRVFFNTKVKINHNRRILTGSEEIKILHYYAHQEEKFMSILLCNNNAKSSFIFKKFCLLIFGCLTLF
ISGLLPDIFDFLLPLNESRPHNLLIMTEYQINVGIQYYIFYLYSMISTITGLFTTTIILSAIIFIYLHCCAIL
KICSYQIKQILDKNVLAYSNNTNIIIKRIIRVVELHRKIKKLLKLITTYFAMTYLLVLIVGICAFAMSLYTLL
NVIKYMNNFAEALIPTSYIIGYEVGIFILSFTGQLVINHSDELFNALYMSLWYEALITIQKSLLFIMIISKKS
LIVNLGGIYVITMETFTSITSKSISVLMVFLSI
>SinvOr77
IYMFDDHYNKFNETLLRFLGLWPYGRTGCERFRAICFHMILISHMIAEFAQLIIAEFSVNVIIKILLDALTAS
LYVLTFNMFFFNSKKLKQILEIIDNNRRMLTNSQEIKILEYYAHQGEKFITIVICNNVSFFIIERLQICILLT
FVIMGVLPDILDFLRPLNESRAHYLLFMNEYRINVGIQYYIFFYYSVISIIIGNSTVVFIVSSILSICLHCCA
IFKICSYRIKEFVDNKIITCKKNGIVEKIIKIVELHRKAKNFTMSYLSIVAVGTCGFAMSLYRFLNAIKCMHD
MVEFLLAMAYIIGYEFCLSSVSFIGQLMINHADELFNALYMSLWYKAPITIQKSLLFIMQISSTKLVMNIGGI
FVITMETFTSITSTSMSFMAVIYSVQ
>SinvOr78
FEWAIELNRYSLEFIGLWPKMKETTREKLTANIRVFLLIIMVAFVCVIPCIHSLIRVWGDLMSVADNLQFTLP
MVSMIMKLIIMWSKKAALAPLLYMIAKDWLRQKSIKERKIMIRCARIPRMIIICGFVIMFASFILLFILPCFG
ITMRYITNVTDPGKPLPLQTYYFYDTDASPYFELTFVAQGVTLMVSAMGYTAIDSFFGLLIFHVCGQLETLKG
RLTMNSEKNPNFNHVLADVIMDHVRLIRCVKIIESTFTLMLLGLFLYFGTLFCLYGFLLVTCEGVYQAACEYR
WYKLEPKQARNLILLISRANKPLYVTVGKIFPLTMNAFCSLLKTSGGYISVLLARRE
>SinvOr79
FMWAVEIHRLGFEMIGLWPKNNTVAMKSLWSKLHASIILTLLIFVSNVPMISAAMQSWGNMVVVIDILRIALP
LLIVPLKYVIMRWKQTVLLSMVNTMAEDWMAFKSDIERGVMIKRAQTARLIMIVGYVTSIIAVVTVVTLTYFG
NEVMFLTNFTSGNKALLLDTYHFYDVDKSPQFELTFFIQTITILLSITIYMTVDIFLLLLILHICGQLENFRY
RLVNLVACKNFNEALNNIIASHLRLIRFADNIENTYCLMMLISIFYFTIVFCLSGFLFTVILNEKKMNEAIIT

QIYFSSIHLFILLMNTLVYCSAGELVLKQCNELHRAVCDLKWYKLESKKARNLVLLMIRAHQPFRITAGKIIP
LTMASFCSILKTSSGYISFLLTK
>SinvOr80
FVWAVEMHRLGLEIIGLWPKNDRMKSLWSRLHAGIILILLIFVSNVPMIFAIMHAWSDMVLVIDILHITLPLL
TVPVKYIIMRTKRKVFLSIVDMMAEDWMAFKSNKERNVMIKRAQTARLVVIIGYIFSIIAMSTVIIFPSFGIQ
VIHISNLTENQKPLPLEAYHFYDTNKSPQFELTYFLHSITIFFATIIYMSVDIFLLLVTLHICGQLENFKCRL
VNLVSCTNFSKALNDVVSSHLRLIRFADNIENMYCAMMLIMVLHFGIVFCLSGFLFTLLLTNRKMNKTILAQV
YYSIILLIMLLANTFIYCFAGELIMEECDGVYRATCDLEWYKLKARKARNIILLMIRLNHPLQITAGKIIPLT
MATFCSIIKTSSGYISFLLTK
>SinvOr81
FVWAVEIHRLGLKLMGLWPKNNKFDTNNLWPKLHVGIILILLIFVVNIPMIHAILQVWGDMVLVVNNLRIALP
LLIALAKYVIMLWKRKVLLSIINMMAEDWMALKLNTERSVMIKQAQTGRLIMIIGYISAILAVSIMIIPSIFG
IQMISERNLTDRHKSLPLAAYHFYDTDRSPQYELTFCIHTISLIFTNTIYMFADIFLIVLVLHICGQLENFRC
RLVNLKSCKSFNIILNNIVTSHLRMSRFTDNIESTYSIMMLIMMLHFVIVFCLSGFLFTTFLIDRKMDEAVMS
KIYLSIMAITILLMTTFLYCGAGELISEKCNAVYRAICDLEWYKWESKNARNLILLMIRVRHPFRITAGKIIP
LTMATFCSVLKTSTGYISFLLTK
>SinvOr82
FVWAVEIHRLGLEIMGLWPKNDKSDTNNLWPKLRVGIILILLISVTNVPMIHAVMQVWGNMVMVVNNLRIALP
MLISSMKYVIMLWKQRVLLSIINMMAEDWTAFKSNTERDVMIKQAQTARFIMIIGYVVAITASLTVIIPCFFG
IQVISQTNLTDQHKLLPLAAYRFYDTNKSPQYELTFCIHAIAILVSTTIYMSVDIFLVLMALHICGQLENFRC
RLFNLISRKNFNKILNNIVASHLRMIRFTDNIENTYSLMMLIMVLHFVIVFCLTGFLFTVFLIEKEMNKVIMS
NIYLSIMMLIALLMNTFMYCGAGELITEQCNAVYREIYNLEWYKLESKNGRNLILLMIRTRHPFCITAGKIIP
LTMATFCSVLKTSSGYISFLMAKR
>SinvOr83
FMWAVEIHRLGLEIIGLWPKKDKFNNLLPNLRVSIILILIIFVINVPTIQGILQVWGNMVLVVDNLRIALPPL
IASLKYVIMLWKRTVLLSIINMMAEDWMAFKSNIERTVMIKQAQTARLIMVVGYVIVIIGLLTVTIPSLFGIQ
IMSGLNLTDRQKSLPFLTYHFYDTDKSPQFELMFCIQTISFSFLAIAYMSTDIFLVLIVLHICGQLENFRCRL
INLMLYKSFNKTLNNIVASHLRIIRFADNIENTYSLMMLIMVLHFIIVFCLTGFCLLIEMDEVVMSKIYISIM
AIITLLMNTFLYCGAGEIISEQCNTVYRAMCNLEWYKLESRKARNLILLMMRARHPCCITAGKIIPLTMATFC
NVLKTSTGYISFLLAKR
>SinvOr84
FVWAVEIHRLSFEIMGLWPEGDKFETSNLWLKLRVIIILSLIIFVTIIPMIHSVIQVWGNMVLVVDNLRLTLL
FIITLMKYVIMLSKRTVILSIINMMVEDWIVFKSNIERAVMIKQAQIARVIIVIGYFIMIIGAFGTIISAFFG
IQIASVTNRTDQHKTLLFLTYDFYDTDKSPQFELTFCIHIISLVTATTVFMSVDNFLVLLVFHICGQLKNFRC
RLVNMTSCKNYNKTLNNIVTSHLRMIRFADNIENTYSLMMLFMLLHFIIVFCLCGFCFILEMDEVTTKIYISI
ITLIIILLMNTFLYCGAGELLTEQSNAVYRAICNLEWYKLESKNARNIILLIVRTHHPICITAGRIIPLTMSTF
CSVLKTSSGYITFLLAKHG
>SinvOr85
FVWAIEMHRLNFQTMGLWPEGDKFETSNVWLRLRISIILSLIIFVIVIPMIRAVIQVWGNMLLVVDNLRVALP
LTISIIKYVIMLWKRTVLLSIINTIAEDWMAFKSNTERAVMIKQAQTARVIMVIGFVSTIIGCLGAIVPAFFG
IQIISVTNRTDQHKTLLFVTYDLYDTDKSPQYELTFCIHTISLWFATNVYMSVDSFLGLLVLHICGQLKNFRR
RLVNMTSCKNYNKTLNDIVTAHLRMIRFADNIENTYSLMMLIMLLHFIIVFCLTGFCLILEMDEVITTKIYFS
ITMLTILLINTFLYCGAGELLTEQCNAVYRAMCNLEWYKLESRNARNLILLMLRARHPVCITAGKIIPLTMAT
YSSVLKTSSGYITFLLAKR
>SinvOr86
FVWAVEIHRVSFEIMGLWPTGDKFETSNLWSKLRVGIILSLITFVTIIPMIHVIIQVWGNMVLVVDNLRIVFP
LIMSLMKYIIILWKRTVLLSIINVMVEDWMAFKSNTERAVMIKQAQTARVIMVIGYFIMLIAILGAVVPAFFG
IQMMSVTNRTDQHKTLLFETYDLYDTDKSPQYELTFCIHTISFVIAATVFMSVDSFLLLLVLHICGQLKNFKC
RLVNMTSCNNYNKVLNNIVTSHLRMIRFADNIENTYNLIMLIVLLHFVIVFCLSGFCLILEMDEVITAKIYFS
IVMLIIILLMNTFLYCGAGELLTEQCNAVYRAMCNLEWYKLESRKAKNLILLMIRARHPVCITAGKIIPLTMAT
FSSVLKTSCGYITFLLAKRG
>SinvOr87
FVWAVEIHRLGFEIMGLWPKNDKFDTSNLWLKLRVGIFLSLIVFVIIIPMILTVIQVWGNLVLVVDNLRIALS
LTMSLIKYIILLWKRTVLLSIINVMAEDWMAFKSNTERTVMIKQAQTARVIMVIGYFIMIIGIFGAVVPAFFG
MQMMSTTNRTDQHKTLLFVTYDLYDTDKSPQYELTFCIHTISFVIGALAFMSVDCFLLLLVLHICGQLKNFRR
RLVNMTSCKNYNEILNNIVTSHLRMIRFADNIENTYTLIMLIVVLHFVIVFCLSGFCLILEMDEGTTAKICFS
IIMLIVLLMNTFLYCGAGELLTEQCNAVYRAMCNLEWYKLESRKARNLILLMIRARHPVCITAGKIIPLTMAT
FSSVLKTSCGYITFLLAKRD

>SinvOr89
FMWAVEIHRVSFEIMGLWPKEDKFNRSNLWSKLRVGIILCLLIFVINVPVIHGVIQVWGNMILVIQNLRSALP
LIISSMKYVIMLWKRIVFLSIINMMAEDWMAFKSNKEKTVMIKQAQTARLIMVIGYLFMIIGVLGAIIPAFFG
IQTLSITNRTNQHKTLLFLTYDFYDSDKSPQYELTFCIQAISSVFVGSVYMSIDNFLVLLVLHICGQLKNFRD
RLLHIVSCNNFDKTLNNIVASHLRMIRFADNIENAYSLMMLIMLLHFVIAFCLSGFCFVLIITTKIWFSIMNL
IVLLMNTFLFCGAGEFLTEHCNAVYRAICNLEWYKLESKNARNLILLLIRARHPCCITAGKIIPLTMATFCSV
LKTSCGYITFLLAKR
>SinvOr90
FVWAIKMHRLNFEILGLWPKDEKYETNNLWSKFRISIILCLIIFVTFVPTVHAVIQVWSNMVLVVDNLRIALT
FLTILLKYVTMLRKRTVLLSIINMMAEDWITFKSNTERDMMKKQAQTARLVTVIGYFFMIIGVLGAIIPAIFG
IPIISITNRTDQHKALLFLTYDFYDSDKSPQFELTFCIHTVSLVLVANVYMSVDSFLVLIILHVCGQLENFRC
RLINMVSCKNFDRKLNNIVASHLRVIRFADKIENTYALMMLIMLLHFIIVFCLSGFCLLLEMEAITTKIYFSI
IMLTILLINTFLYCGAGELLTEQCNAVYRAICNLEWYKLESRNAKNLILLIIRARHPCCITAGKIIPLTVATF
STVLKTSCGYITFLLAKR
>SinvOr92
FVWAIELNRFGLKLVGLWPKIHEVIEDNYTSDLRIVIIFVIITFISGIPLICSLLRVRHDMILVIDNLQITLP
LMIVSLKLVVMRWKRTALLSIINMMEEDWIALKTDAERNVMIKRARTARMIVICGCVLMIMAFTSIIVFPIFG
VPFRRLTNITDRDKPLPLQTYYFYDTDKSPQFEITLVIQAITIFLAAVTYTSVDAFLGLTVLHICGQLENFKY
RLDNLISCKDFNSALRSNVIIHLRLIRFAYKMEDTFALMLLGLVFYFGIVFCLYGFLLLTIIAENETSKISIV
RVLYVAVGVITLLVHTFLYCGAGELITHQSRKAKSLILLMTRTSEPFRLTAGYIIPLTMATFCSLLKTSAGYI
SFLLAKRD
>SinvOr93
FVWAIELNRFGLKLIGLWPKIDNVIKGNYTSDLRVVLIFVMITFITGIPLVWSLLRVRHDMILVIDNLQITLP
LMIVSLKLFVMRWKRTVFLSLINMMEEDWIASKSNIERNVMMKRARTARTIVICGYVLTTLGFVMVIIFPLVG
VPYRRLTNLTDRDKPLPIQTYYFYDTDKSPQFEITVAIQAITCFFAAVAYTSIDAFLGLAVFHICGQLENFKY
RLDNLISCKNFYSALRSNVITHQRLIRFANKMEDTFALMLLGLVFYFGIVFCLYGFLLLTTVADDGTSKIPSR
VYFVMFGVTILLVHTFLYCGAGELISEQCEAIYRTLHNLKWYKLESRNAKSLILLMARTREPFRITAGYIIPL
TMATFCSLLKTSAGYISFLLAKRD
>SinvOr94
LQWAMKLNRIILDFIGLWPKAAQNSRQKLLCNFRVLIVFLAVTCGVLIPSIHSLIRIYGDVMLMLDNLQFTLP
AISCSIRIVIFWWKKEAIIPIMNMISEDWIKSKNAQDRNVMIKRAQTARIIITCAYCIMGVACFFIIILPSFG
ISLRLTPNITDPGRPMPIQTHYIYDVTKRPQYELTFISQSIYILLAIMSYTGIDNFLGLLTFHICGQLDILHN
RLTHLDKYTNSYGMLKSCVIKHMRLLSAIDIIEDTYNITLLALFVYFAILFAFYGFRIINLFDEENDLSVTHL
VYFISNVFNIFMHMCLYCVLGEILMARCSDIYYAAYNSEWYSMDPKIAKDLLPLLIRGAKPVYLTAGKVFPMT
MATFCSLVKTSAGYISVLHTTRN
>SinvOr96
FEWAVKLNRFTLDILGLWPKNIQNPWQKLICNLRALISAIGILICLFIPSIYSLIKIFGDSLLMLDNLQITLP
VVSCSIRIILFWWKKEAIIPIMNMVAKDWMKPKSDQERELMIRYAHIARIIIIFSYCIMGLQYFFLVVLPIFG
FSLRLTTNITDPGRPMPVQSHYFYDITKRPQYELTFISQAIYMSIGMMAYTGIDNFLSLLVFHICGQLDILKN
RLQHLEKFINYHKVLKYCIAKHIRLLRAIEIIEDTYNIILLSLFIYFMILFAFYGFRLISLFDEGNDMSVIHL
IYFISSVINIFVHMCLYCVLGEILVAQCNKMYYAAYSNKWYTMDPKVAKDLLFLITRGSKPIYLTVGKVTPVT
MATFCGV
>SinvOr97
FEWEVKLNRFTLNVIGLWPWIAQNFRQRLMCNLRVLVALLAITCGVLIPSIHSLIRIYGDIMLMIDNLQFTLP
AISCSIKIVIFWWKREALVPIMNMILEDWLKLKSVQEKNLMIRRARISRIIIIFSYCIMGTACVFVIILPIFG
ISMRTTTNITDPGKPLPLQTHYIYDVTKSPQYELTFISQSIYIIVAMMSYSGIDNFLGLLVFHICGQLDILKN
RLTSFDKYINSHEILKSCITRHIHLLRAIDIIEDAYNVILLFLFVYFAMHFAFYGFMIISLFDEGNDISLSHL
AYFVSTVINIFAHMCLYCAVGEILVAECNKIYYAAYSNKWYAMSPQVLRSILFLMIRGSKPVNLTAGKIFPLT
MTTFCSLLKTSVGYVSVLHTVRS
>SinvOr98
FEWAVKLNRFTLNLIGLWPNSAQTSRQKLMCNFRVLAVFLSITFGLLIPLLHSLTRIFGNLVLMLDNLQFTLP
LMSCSIRIAIFWWKKEAIVPIMNMILEDWLKLKSVQERNLMIRRARISRIIIIFSYCIMGTTCFFVIILPIFG
ISMRTMTNITDPGKPLPLQTHFIYDVTKSPQYELTYISQSIYIIFAMMSYSGIDNFLGLMVFHICGQLDILKN
RLTSFDKYINSHEILKSCITRHIYLLRAIDIIEDAYNIILLFLFVYFAILFAFYGFMIMSLFDEGNDISLSHL
AYFVSTVINIFAHMCLYCAVGEILVAKCNKIYYAAYSNKWYAMSLQVLRSLLFLMIRGSKPVYLTAGKIFPLT
MTTFCSLLKTSVGYISVLHTVRS
>SinvOr99

FILDLLGLWPKNIQNPWKKLVCNFQVLVICLGILFCLLIPSINSLIIIFGDLALMIDNLQITLPILCSFIKVI
IFWWKKEAIVSIMNMIAEDWIKSSDQERRLMIRRAQIARIIITCSYSIMALQCFFNVVLPIFGTSVRMISNIT
DPGRPMPVQSHYFYDITKKPQFELTFISQAVFIIIAMLSYIGIDNFLSLLIFHVCGQLDIIENYLTHLDKYSN
YHEVLKRCIAKHIRLLRSIVIIEDTYNVMLLSLFIYFAVLFAFYAFQITSLFDGETDLPFTRLLFFVLTIFNL
FVHMCLYCVLGEILMAKCNKIYYAAYSNKWYTMNPKIEKDLLFLMTRGSKSIYLTVGKASPVTMATFCSLVKT
SVGYLSVLHTTK
>SinvOr101
FEWAVKLNCITLNFLGLWPKIARNSREKLMCNFRVLVACLGLMLCVLIPSIHSLIRIFGDIILMIDNLNFTLP
AISCMLRIIIFWWKKEAIIPIINMMAEDWTKSKSAQERNIMIKRAQTARIIVTCAYCIIGTACFFIVVPSIFG
VSMRFASNITDPDKPMILQTYYIYDVSKSPHYELTFICQAIFICVGIMSYTSIDNFLGLLIFHICGQLNILKN
RLICFDKCIKSHMLKTCVIDHVRLLRAIAVIEDTYNTILLALFIYFAILFAFYGFWIISLFGNGNDISLIYLI
CFIFIIFNLLGHMCLYCALGEFLIAQCNEMYYAAYNNKWYSADPKVAQDLLLLITQGAKPIYLTAGKVFPLTM
ATFCSLVKTSGGYISILLTTR
>SinvOr102
YEWAVKLHQCSLEIIGLWSKSGQTTWEKHMCNLRALLTFLMLMFVVVIPAIHSMIRVHSDILLLSDNLQFTLP
ATTCVIRLAIFWWKKEAVTSIIDMIENDWLKTRTSQEEARMVARAQIARIIIISAYSLMAIAYIVLVILPICG
YSVRYQPNNTDLGRPFPLMAHYVYDTNKSPQYELTYTVQSIAVFYNVLCYTGIDNFLSLSVFHISGQLDILKN
RLMHLHTVKNYNDVLKSCVVEHLRLLRAIDRIEDIFHVILMILFLYFGILFAFYGFWIMNMLETGQHITIVHL
IYVVSIVINAVCHMCLYCAVGEMLTAQCDGIHYAVYYNKWYSMDLKNARNLILLMIKTNKPFHLNIGKIFPLT
MGTFCNLLKTSAGYVSVLLTTRN
>SinvOr103
YNWVITLNRLCLSLVGVWPESNETPRRKLIMNVRVITILNVIIWSSIIPTVHSLIRIWGNIMSMIDNLQYSLP
LLISAMKLVLLWRKKEVLISVLKMVKEDWIKLKTEEERAVMIRQARIARLIMIWGYMIMILSFVFVVIFPSFN
ISMRYLTNITDPGKILPLQTYYLYNVSDSPFYETTFILQGFSLMAAAAIYSGADTFMSNLIFHVCSQLENFKT
RILNLDKHSHFDEALSSSVQNHIRLIRFIKIVDNTFNLMLLGLLVYFGILFALFGFLFVITQGRNLSIARLVY
LLTAFINTFTHMCLYCVVGEFLVIQCDGVYEAVYHYKWYNLKPKQARNLLIIMTLANRPLHLTAGKLFPMTMA
TFCNLLKTSGGYISVLLAHR
>SinvOr104
YKWAVGINQVILTLCGIWPKNNETKQEKLLSNIRVILMLNILICGCFIPTIHSLFKIWGDIMCMIDNMQYTLP
LFIILIKLSTMWIRKKDILLLLNMIKDDWLKPKKIKERDVMKKRARLARIFTIFGYFMMLVSYILVVVLPIFG
ISMRYLTNKTDPDKLTPLQSYYIYDKNKSPFFEVSYIMQSLGLMVAGVTYSSVDSFFGLLVFHVCGQLENLKM
RIIHLDKFKNFEIALSHSVQDHIRLIRFINMIDDIFTLMLLSVLLYFGIVFACYGFLLGTMVNQGRNLPVPRL
ILIIILASVNSFAHTCLYCAVGEILVTQCEGVYEAACKYKWYNLDPKKTKSLMMIMIRANKPSYLSAGKLFPMT
MSTFCNMIKTSGGYISVLLAHRE
>SinvOr105
FEWATRLNRLLLNIIGIWPNAHRNAYDKFFSNVRAAFTVIVILFVGTIPGIHSLVRTWGDLMAMIDNLQFTLP
ITMTIIKLIDIWWKKTDLLMAINMIAEDWIKDKTSKERCIMIKQAQNARIITIVCCSFMFLGSTLAIILPCFG
MTVRYITNVTDPVKILPLQAHYIYDKNQSPYFEIIFAAQSLVILMCAASYTGVDNLLGLLIFHLCGQLEILKE
KLINVKLFKNYNESIALIVEEHIRLIKCFRIIESTYTLLLLGLLMYFGIIFCLYGFLTLAILTEGKGMSMFRF
IYLVSIVLNVSTHMCLFCAVGEILVTKCEALYQAAYEHKWYTLEPAKAKNLLLIMIRANKSLYITAGKMFPMT
MSMFCNVIKTSAGYVSILFA
>SinvOr106
IVIMQEKITLKKAFATVKLSLFVIWFWPLPLNTSKRKMLCMKLYQYVCILLTTTVLMSMIYAVVKNFNDLDLF
IKSSLGLFPCSHVISNILCHLTTYKRLQYVTVEMEKFYTLIKPREEMIVQREYLDKCSKFYGFCIGLFYMSLV
GLFVGPIVLDEPLPAPAEFPFDASQQPLRAITYMHQIVVGMYIASHLCVNAFMALLLWLVSARFKLLTEELRT
ITNIYDFAKCIEKHQQLLKYAGEVSLTVRPFALVTIFFSTVSLIVFGLIFIADVSSSLKIQCVLLSTSALIEV
FMYAWPAEHLIHISTNIGQTAFEINWYDESEYFRKNIQIIILRSQKPILVVLPCGLPSLSLRYYASYLSTIFS
YFTTMRIMFEE
>SinvOr107
SKLTLKKVISIIKLSLFITWCWPLPKDTIKLKVICAETYQYFCLIVTFGVAAGLINAVRNHFDDPVIMAKSIG
VLCPAIQVIFNIVFCKVYSYRLQLVTFEMENFCELLEPREETVVQRYINKCVYFYGGAMFWIYLSAVFILSGP
LTLDQPFPTNAEYPFNVYRQPLKSIIFIQQAIACMQGAAQLCMSIFIALLIWFTTARFEILIEKLQEITNIYE
LKKCIQEHQNLLKYAEQVVFLVRPFALSTVWLSTIALIIVGLILLTDQPLSIKIQCVGIIFSGMSVVFMYTWP
AEHLIQISDEIGQTAFDTEWYQQPVALRKDLHIIMLRAQKPIVISVPCVMPTLSLKYYATYLSTIFSYFTTLR
VAMQ
>SinvOr108
MVDKRLQEYRKYQRITKILLIISGCWYMPTKSGKSTRYWPVCVILLMIMFITLTLHTAYVFRHKLVNMMKMIG
LTISAVSATIKVSSFLINRSSLINNHKTLNDFYEEELVQNEKIRTIIFSPLRATYTLAYTYVIIMIGLIVGIF

LSPYIFIIRNLSHFHSATNYTLPLSRGYGYFWTVPDNILCHLHLILETAMVMLSCITTCGVECVFSLFAYQLA
STARGMIYRLTNPLPNEKFSDLLKTCIKKHQKLLRCRNILEKVYGPILLWHIISNAMLLCSLIYDEFKFTFFN
ILMSLTYSLIKLLQMFMYAWNGTVITNAGDNFRNGVYFGEWPKSDLDRHVRTNVILTMMQKPMTIYAIFSPVE
IVMFTNFVNATISYFFLLQSIDDKS
>SinvOr109
MLDKRLQEYRKYQQITKILLIVSGCWYMSKKSGKSTRYWPVCAFLLMIMYLTMSFRAVYIFRHKFTIVMKMVG
VVMSSVSATIKVSIFMINRNSLINNHRTLNDFYEEELVQNEKIRSIIFSSLRTTYTIGYTYVILLIGLVMAYI
VPPYIFIIRNISHFNSITNYTLPISRGYGYFWTVPDNFLYHLHLIVETIIATVSCITACAVENVFGFFAYQLA
STMRGMTYRLTNPLPNEQFSDLLKTCIEKHQKLLRCRDILEKVYGPIIFWHIITNAMLFCSLIYDITTQFEFT
VVSTLTFLIWSLIKLLQMFIYAWNGTVIQMRDDFRNGIYFGEWPKSDLDRHVRTNVMLTMMQKPMTIYAIFSR
VEIVLFTNFVNTTMSYFFLLQSIGDKS
>SinvOr110
MPDKRLQAYRKYQRITKTLLIISGCWYMPTKSDKSTRYWPVCVFLLMIMYTTMTFRTTYLFRHKLANMMKMVG
MATSAVSAIVKVSSFMINRSSLINNHRTLNDFYEEELMQNEEIRTIIFSLLRATYTLTYTYVIIMIGLITAYL
VPPYIFIIRNLSHFHSTTNYTLPLSRGYGYFWTVPDNFWYHLHLIVETIFGSLSCITACGVESVFSLFAYQLA
STARGMIYRLTNPLPNKKFSDLLKTCIEKHQKLLQCRDILEKVYAPISFWHIISNAIVLCSLIYDITTQQFKF
TFSNILVFLMYSLIKLLQMFIYAWNGTVITNASDDFRNGIYFGEWPKSELDRHVRTNVILMMMQKPMTMYAIF
SPVEIVMFTNFVNATMSYFFLLQSVGDKS
>SinvOr111
MIASMSPADNRALFQNFNYRSDAEYVVRIAKIMLTPIGIWPLYGDSMLDKIKYHLQTSIVFCLMCFLLVPHII
YTFFDAEDLTRYMKVIAAQVFSLLGIIKFWTMIINKNDIKCCLQEMEIQYRDVESEEDRLVMVKSAKLGRQFT
MIYIGLLYCGALPYHIIMPLVAEKVVRKDNTTHLPLPYLSNYVFFEVEEMDSPFYEILFVVQILFSTMILSTN
CGVYSLISSFVMHACCMFEVARKRIEILLINRTDNLHERFRWIIMQHLRALRYVEMIENSLNLVFLSEMVGCT
IIICFLEYGVLKEWEDNQLFGVIIYFILATSIFVNVFTLSTIGEHLKEESIKIGEASYSIDWYMIPTKNVNSL
MMVMIKSNRPSTLTAAKMFDISLETFCNVCKTSMAYLNFIRMITM
>SinvOr112
STLAVDVTKQNEAYISDFRYAVQISVWLLKPIGVWPLFNETSRLKIALHKALMVIGTFIVLFMVVPWTVYIVK
KKLDVFLIIRTICPLLFSTTTSVRYILLLWNQDRLKFCMEHMADDWRCATIAKDRDVMLANARDGRTFGIISM
VFMFSCGGLYYTLPIVMPNPINENNVTVRLHPSPSELLVFDTQASPVYEIVYFLQILSGYTIYSAFSGTCSLI
ANFVTHVSGQCDLLTTIFEETVDGGEHNSGSIENRIATAITRHMRLLRLVSDVSNLFTEICLVEFINASCNIC
LIIYYIVTDIRNNEPFMPTFMFGFGLMSIIFNLYMVCYIGDLLKERCQQVGNACYAIEWYRMPYKNAMRLLMP
IAMSQYPTTLTAGKMVTMTVTTFSDILKTSMAYFNLLREFTSQDITKT
>SinvOr113
MFANKNYKRDINYVFELSRFVFRLLGIWPYARTKTWFPETIERLVVILVSYILLTCELVPAILYMAIVQKETR
ARLKVVATVIFTMVAMAKYGQLVFSRDRVRSCLAQVEDDWRNVADSRNRDVMTEKAKTGRRLLVICAIFMYST
GVSFRTIIPLSRGKIVTEQNITIRHLPCPNYFVLFDVQLSPAYEIVFLMQFFSGVVKCTVTTAVCGLAGLCVM
HVCAQLEILMVLMNNLVNERELKNVNERLAIIVKHQIKARNFLQLVQNTIQYTSLLEVIGCTIIVCLLGYFVI
MEWEDNNSIALCSYLIGLTSISFNIFIFCFIGEQLSTKGEKVALTACTLEWYRLPDAKARSLILIMIMSNLPT
KIRGGKFMDLSLRTFGNVVRTAVAYLNLLRS
>SinvOr115
MLPNEQYKEDMIYVTKLTRHVLSLLGVWPSYNRRSVGEKAWKYFLISISYMLIYFVLIPGISFSLIEKTKVTV
RTIPLLFYGFMASGKYSNLVFREKNIRRCLKHIEEDYRIITSTEARDTMIESAKIGRRLVTLCAIFMYGTGLF
ARSILPFAKGKIVTAQNITIKPLPCPAYFIVFDAQVSPVYELTFTLQVVSGIITYSITTGLCGLAAVFVMHAC
GQLKILVDMMKNLVEEQWEEKQEVDRKLARMVEHQIRIRSFLHLVENTLQQACLIELMGCTAILCLIGYFIIM
EWESNTNSIAVCTYFITMTSLMINMFLFCYTGEQLTVQAERVARTSCELEWYRLPDKKARAIVLVIIISNMPI
KITAGKIMDLSFKTYGDVMKTAVTYFNMLVNIAD
>SinvOr116
MLCNEHYKDDMIYVTQLTRNISSLLGIWPVYKRRATSEKVWKYFLICISYALLLWVLIPGALFWLIEKRARVR
VRTIPLLFYGIMACSKYGTLIYHETNIRRCLMHIEEDYKTAINEKTRATMLESAKIGRRLVTLCAIFMYGSGL
FFRSILPFAKGKIVTAQNITIKPLPCPANFIVFDAQVNSVYKLIFALQILSGLITFSITTGLCGLAAVFVMHS
CGQLKILVTLMKSLVEEQWQKEQEVNRKLATLVQHQIRIRNFLQLVENTLQQACLLELFGCTMILCLLGYFII
MEWEDSNLIAMCSYFSTLTSMLINMFIFCYTGEQLTVQAEKVARTSCMLEWYHLPNKEARGIVLVVIMSNLPL
KITAGKFMDLSLKTYGDVVKTAVMYFNMLVKVTD
>SinvOr117
MLRNEHYKDDIVYITQLTRTVLYLLGVWPAYKRRSTSEKVWKYFLIVISYALLFCVLIPGALFWLIEKRTRVR
LQTIPLLLFGFTASGKYGNLIIHENSIRRCLKHIEEDYKSIRSEKARNTMIESAKIGRRLVILCAIFMYGSGL
SFRSILPFAKGKIVTAQNVTIRPLPCPGYFLSFDVQISPAYELVFAIQVLSGLVTYSITTGLCGLAAVFVMHA
CGQLKILMNLMRNLVEEQWQEKREVNKKLAVMIEHQIRIRSFLQSVETTLQQACLMELTGCTAVACLLGYFII

MEWENSNSIAMCSYFTALVSIMINMFMFCYTGEQLTVQAEKVARTSCVLEWYRLPKKEARGIVLIIIMSNMPM
KITAGRIMDLSFKTYGDV
>SinvOr118
MFANKYYERDIGNAFAMHRFFFRMIGLWPFAHANSLLPEQLETIVVVFVCFACLIVEAVPTLLYVFMVLTDIR
VKLKVMGSAMFTTVEIMKYVYMLFYKSQMRNCLILVDEDWQNVVSPSDRTSMIEKVKICKRLVVLCAVILYSL
NIIVRIVIPLSVGKIVTPQNITIRPMPHVAYLVILDVQQSPVYEITYIMQLLGGFFKYTIVVTTFSFVTLCAM
HFCSQSNILITLINDFVNESRPENLNKKLSIVVEHQIRIKNFLQLVQSVTQYPSLVEVLGSTVMLCFVGYCII
TEWEDQNILRLCVYSLILVMFVFNVFIYCYMGEQIIEHAGQVALTACTLEWYRLPDTQARALILLIIVSETPF
KLKAGSFIDLSLRTFGNITKMALTYFNILRSLL
>SinvOr119
NLLLEKHPNKNYKQDIEYVTKQTNFLLRILGIWPLLDRDLSVVEIACKILLIIICCILVCFESIPSMLYCIFV
TVEEPRMKLMMIAPTIYSFTSLTKYGALIVYENEIRKCFRHIKDDWKFIAMSGARDIMMEKVKTARSMFTICC
TCLYTAALSYHTIVPLTRGKVITSNNVTIRPLAYAGYFVLFDEQRSPAYEIVFTLQFFGGFVMYSVTIVTYGL
AALLVMHACAQMKILMMLMEELVDERACKEKNTDEKLVAVVERQIRIRNFLCLVEDTLQISNLFEILANTTMM
CFMGYCILTEWQDGNTANAYTFVVALASDIVNIFLLCYIGEYIIDTADKVAWKTNMLEWYRLPTERTRDMVLV
ILVSHMPSKLTVGKFIVLSLKTFGDVMKSAVVYFNILRTVTE
>SinvOr120
MPQNGQHQNDILYITQPTRKILRALGAWPSISKGRSIYPKAHNLLLICIAYALLSSDIIPGSLFWVMEKTTRI
KLQIIPALIYDFMSAIQYGIFIVRYDQVRRCLKHVEEDWKNILSADVRNIMLRSGRTGKRLVTICGAFMYSGA
LTFRTILPLSMGKTVTDQNVTLRHLACPGYFFSLDVQVSPVYETVFIIQLLTSIVTVSIVTAACGLTAIFVMH
ACGQLKILINLMKSLVQKQWLEEREVDKKLSEIVEHQIRVRKFLRLVQHTLQEIYLMEILVNSITICILVYFM
SMDWQNRNVTSVCSYMISIANVTIHMFLFCYTGEQLTSQAEKVAIASCELEWYRLPDRRARTVILLMIMSNAP
TKISAGKFVDLSLKTFGDVVKTAGAYFNMLRNVIE
>SinvOr121
MYPNKNYENDIKYTTQLNRVICNLLGIWPDIETSFLKNFKNVSLILSCIFLLITELIPTILYVVYIEKRFRIR
MILIPSIMFTTSAVIKYFSLMLNKNQVGNCLTQVKDDWRNVVSASARRSMINKARTARHLIVLCCIFMYSAGF
YFRTIVPLYKGKSVTDQNITIRHLPCASYFILFNGQISPAYEIIFFMQFASGFIKYTITVAICSLAALFVMHV
CGQLEILMALIENLVNETDEKNLDRMLGMAVEHQIKMRNFLRLVQSTLEHTSLLEITGCTIFICLLGHSIITE
WENQNVVSMCSYVILLTSMSFNIYIFCFIGEQLSIEGEKLAVTVCTLAWYRFPNAKARELILVIAMSIVPTKL
KAGKFFELSIRTFGDVVKMAVTYLNFIRKSME
>SinvOr122
MYPNKNYENDIKYTTQLNRVICRLLGIWPDTEPSFFKNLKSVSLILSCSFLLASELIPTILYVVFIEKRFRVR
LKLISSILFTTTAVLKYFTLVLSKNQVRNCLTRVKDDWRNVVSASARHSMINKARTAKRLLILCGIFMYTSGL
YFRTIVPLSKGKSVTDQNITIKHLPCPSYFVLFNGQISPAYEIIFFIQFFSGFIKYTITVAICSLAALFVMHV
CGQLEILMALIDNLVNKTEERDLDRMLAMTVEHQINMRNFLRLVQSTLEHTSMLEVTGCTIIICLLGHDIITE
WEDQNVVSMCSYIILLTSIGFNIYIFCFIGEQLSVEGEKLAITVCTLAWYRLPNEKARALILVIAMSIVPTKL
KAGKFFDLSIRTFGDVVKMAVTYLNFIRKMME
>SinvOr123
KTNKNENDFNYAVQVTRVILRAIGAWPIPNFASNMERITTRLQNLICYFLFAFIIIPGLLRVFLKEHEFKRRI
RLLAPLLNCGMGWLKYNLLVNHAREIKSCLKQARQDWSNTIDEDNRKMMLSTAKIGRRFAIFSAAFMYIGGLS
YRTLVPLSKGRMLTPMNITVRALACPSYFIKFDGEVSPAYEIVFTLQFFAGLITYSVRVGAAGLAAFFIMHVC
GQLRIIIGKLQYLNNMPNLNDRAVGILLADIVEHQIKVKSFLKQVEETMRYVWLVELMGSAVLLCLSGYYIIM
EWENNNSTAMLTMSVMLTSFTFSIFTNCYVGQQLTNQSLKLGLMTTTINWYHLQYKRARSLILIMAVSNIPEK
ISAGRMIEMSLPTFSNIVKTSMAYFNMLLKF
>SinvOr124
MIENAGPQNSNFEDDIRYTVQVHRLILGLIGVWPYLEKPRKWTRFLRGLLRTMCCFLLSFNLIPWLLYMILIM
DTFKGRLKMLGALFFYIMVPAMYCALMLREDRIKECIRHVQEDWRNVRNANDRRIMLDKARAGRFILISTTLF
LLTSGFTYRLIKPIMSSKITIGNVTIRPLVQGNYYIFFDPQKSPAYEIVFSMHLVTGIVVYIVTTSVCGITAL
FTMHACGQLKLLAAWLENLPKEAQWSKECAVARRLATIVMHHVRIRKFLHQIQGVVGEMCFIEFIGSTLILCL
LGYYVVTGWERNDALSSMTYAIMLVSFTFNIFILCYIGEVLNTQGSEVNTTCCTIDWYCLPSKEARYLILVIA
MARYPTKLTAGKVINLSYSSFSAVVRTAMAYLNLLRTVTV
>SinvOr125
DTCNGSEPYNQNYKNDLQNSMQLNIWVMKVIGTWPLRHSWTETLGKKVLNVICYMLLAFLLIPSSMYIVLEIK
DFYNQLKLGSALTFFFMAVMKYCALLLRENDIRRCVDYIEGDWRNVRYTEERRIMLENASFGRRLVFICSVFM
YGGVLFYFVAVPLTRAKIIEEDGNLTYRRLAFPVPSVIVDARRSPINEILYFIQLFGGFVAHNITVAACSLAA
LLAMHACGQLQVLISWINHLVDGREGVNETTDERLAKIIQLHVRILNFISLTEELLHEISLVEIVGCTLNICF
LGYYCMMEWDFKQPVSGLTYLILLISLTFNIFIFCYIGELLTEQTIKVRESSYMIDWYRLPEKKSLAIILIIC
MSNATTRLTAGNIIELSISSFGDVIKSSLAYLNILRTFTT

>SinvOr126
MVGTMMERTNPVATVYNHDKDIQLSIQLNRWLLKPIGVWPNLTDISRMERYAYGLINVICTSLIGFLFIPSAI
YMALEMDNMYYILKLSGPLSFCLMAVVKYSSLIIRENDIRHGIKHIESDWISTQHYGDRIIMIRNAKFGRRLV
SICAFFMYGGAVFYYLALPFSKGKITDYDGNLTYRPLVYPVARVIVDARYSPISEIFFWLQCLSGFIAHSITT
GACSLAAVFAMHAYGRLEVLIQWIEHLVDGREDFCDNVDERLTMIVQQHVRILRFISLTDKVLREISMVEIVG
CTLNMCFLGYYTLTEWENREPASYITYIVLLISLTFNIFIFCYIGELVAEKCKKIGQISYMIDWHRLSGRKGL
ALVLMIAMSNSSVKLTAGNFFELSLSTFGDVVRMSVAYLNMLRTLT
>SinvOr127
VAIIYDHKKDVQLSIQLNRWILKPLGAWPKSIKISRAERCAYALVNIICICLIAFLFIPCAIFVALEMDDIYN
ILKLTGPLNFCLMATVKYSAMIFRENDIRSGIEHITNDWMNTRHYGDHKIMTRSAKFGQRLVKICAFFMYGGA
TFYYLALPFSLGKITENDGNLTYRPLMYPVASVIVDARRNPINEIFFWMQFFSGFIVHSITTGACSIAAVFAM
HAYGRLEVLMQWIEHFVDGREDLYDNVDERLAMIVQQHVRILNFISLTDKILREISLVEVVGCTMSMCLLGYY
IVTEWESKDMTSYVTYIVLYTSLTFNIFIFCYIGDLVDKKCKEIGEISYMTDWHRLSGRKSLSLILMIAVSNT
SVKLTAGNMFVLSLNTFGDVVKASFGYLNMLRTVTS
>SinvOr128
KTKKNEDDFNYAVQVIRVIMRMIGAWPISSYASNVERFAIRLQNIICQFLFAFVIVPTLLLIFLKERDFKRRV
RLLGPLLNCLMGWIKYNLLIYHMREIQSCLKQARQDWRDTVDWGDRKAMLSKAKIGRRFAIFSAAFMYIGGLS
YRTLVPLSKGRMLTPMNTTVRALACPSYFVKFDEQATPAYEIVFTLQFFSGLLTYSVTVGAAGLAAFFVLHVC
GQLQLLIGKFQRLNDMSEPNDRSVAILFADIVEHQIKVKNFLKEVEKTMRYVLLVEIMGSTILLCLVGYYVIL
EWETSDSTATLTMFVIFTSFVISIFINCYVGQLLTDQSIKFGSVTSTTNWHRLPYKRARTLILIMAVSNIPAK
ISAGKIMEMSLPTFGTIVKTSMAYFNLLRKFI
>SinvOr129
KKNRNEDDFNYAVQVTRIVMRMMGVWPIPSYASNVEKILTRLQNVICYFLFAVVVVPGLLQLFLKEHDFKRRV
RLLGPILNCFIGCTKYSLLLHHAREIQYCLKQARQDWRDTVDSKDRNAMLSKAKVGRRFAIFTAAFMYVGGLS
YRTIFPLVKGRVLTPMNTTVRVLACPSYFIKFDEQATPAYEIIYTLQFFSGLLAYSVTVGGVGLAAFFILHVC
GQLRVLIGKIQRINDMSEPDDRVVEILFANIVEHQIKVKNFLKEVEETMRYVLLVEVVGCTIILCLAGYYVLW
EWETSGATAMLVMFVVFISFIISIFINCYVGQLLMDESMKFGSMTSTTNWHRLFHKRARSLILIMAVSNIPEK
ISAGKLIEMSLPTFNNIMRTSMAYFNLLRKFI
>SinvOr130
IKLIMEVSSKMSRRKNDFTYAVQVTRVILRAIGAWPISSYVSNVEKIATRLQNICCYFLFAFTIVPGLLLMFL
KERDVKRRVKLLAPILNSWTSCVKYSLLVYHAREIHSCLKQTRQDWMNVVNSRDRKTMLSKAKIGRKFAIVSA
LFMYISGLSFRVIVPLSRGRMLTPMNTTVRALACPSYFVKFDEQASPAYEIVFTLQVFAGLLTYSVTVGIAGL
VAFFVMHICGQLSILIAKLQRLDNILEPEDRVIKMLLADIVQHHIKVKGFLKQVEETMQYVWLVEIVSSTIFL
CLTGYNVIMEWENSNSTAMLTYTVMLTSLTFSIFTNCYVAQLLTNQSIKFGLMTSTMNWYRLPHKRARTLILI
MAVSNIPVKISAGKMIEMSLPTFSNIVRTSMAYFNLLRKFAT
>SinvOr131
MERANPIASVYDPKKGTQLSIELNRWILKPIGVWPKSVHLSWMGKFVYLLINVICITFVCFLLLPAAINVELD
KEEFTYRMLRLSSIRGFSIMTIIKYLSLIFRENDIRNGIQHIEIDWMNTQRYEDRIIMIKNAKFGRRIVTIVA
FVTYGSAFFYYIALPLNSIMMTKNDENLTFQLQMNPAATETIDKKYNFNDIFFFWMQLVSGFIAHSVYAGTLS
LAAVFAVHAYSRLEVLMQWIQHLVDGREDLCDNVDERLMTIVQQHVRIIRFISLTDKVLREISFLDITCSTLI
MILLGYFCIKEWETREITNYLTFIVLLMCMTFNIFVLCYIGDIVTDRCKKIGEVSYMTDWYRLPERKNLALVL
MIAMSNASIKLTAGIFELSLSTFGEVIKVSLAYLNVLRTVMT
>SinvOr133
MRSYHESKIDVYKQNNNYSLQWTRWILKPIGVWPELSTSSAIEKILSKILRLTCHTLIALTLIPSILYIIFEE
KDSQLKLKALGPTSHWLMGGINYCSLLYQKDQIRKSIKHIETDWRMAKRQCDQEMMLKNARAGRIIAGVCVLF
MQGGVFTFNVARGTSSILVAMGNETVAMGRLPCPSFNKIVDTRFTPVFEIVFTLQLLSALVVNNTTVGACGLA
AVFAMHACGQLSVVISRFEELVEEKQHDVIEKKLANLVEHHLRTLRQGLFLSRMEIIMRQVCFVELTGCTFNL
CMLGYYAITQWHEETTNTIIAYFVLFISMTFNIFIFCYIGDLVTEQCRKVGEAVYMTNWYQLPQQTMLSLILI
ILRSSIVIKMTAGKIVHMSISTFGDVMKTSVTYLNMLRTLTM
>SinvOr134
MTHSDHVTRSIELPHGSDYSLQLTRWFLIPIGAWPRMSTTTRVKRISSHMHIFVCTSLIAIIMVPCLLYVSLE
EKDTEIKLSVIGPLSHWIMGMINYCLLLTRSNDIRECVLHMEMDWRLVRKIEDRQIMMRQAKIGRFVAGFCAM
FMQSGTFLFAVRKSLSTTIVIVGNETVSMHPMTCPFYNKLIDTRFSPANEIMVVVEWLSCFIVNSVTVGACSL
DAVFAMHAYGQLNMLFSWLNKLVIDEDKENKCVEQRLAIIVEHHLRVLSFISRMETVMRHICLVELLGCTMNM
CFLAYYFITNMDSFDGAKTTSYVIIYLSMAFNIFIFCYIGEILTEQCKNVGERAYMINWYELPHKTALGLILV
IARSNNIIKMTAGKLFHLSIATFGDVIKTSVVYLNMLRTM
>SinvOr135

MTHSERLLTNIKFTYKSNNDYSLQLTRWFLLPIAAWPRPTTSTTVERISLQAHVFACVFLILIILIPCILYVW
LEEKDIQIKLSVMGPLSHWIMGTINYWLLLAHSDDIRECVQHMETDWRMVRGTDDRDVMLRYAKIGRLVAGFC
AVFMQSGTLLFMVAKAVTSMTVIIGNDTVTMHPMTCPIYSKFIDARFSPANEIMILTQILSCFIVNSITVGAC
SLAAVFAMHAYGQLSMLFSWLNNLVEDEDKENEFAEQKLATIVEHHLRVLSFISRIENIMQTICLVELVGCTL
NMCLLAYYSITNWSDFDAAKITSYVIVYLSMAFNIFIFCFIGEILTEQCKNVGEKAYMTNWYELPHKTALGLI
LIIARSSNVIKITAGKLFHLSIATFGDVIKTSMVYLNILRTMTPAT
>SinvOr136
VKPYQDPSDYSIQLNRWFLTPVGAWPRSASTTFREKVISTILIIICYSLISFTVLPCILNILFEETDMHKKLK
AIGPLSHWSMGGMNFFSLLFRNRQIGRCIQHMKIDWQTVKNSEDRQVMLKYAKIGRFVAGICAVFMHGGVFMH
SLLQGITPTFEYIGNVSVSVIVLPCPTYSKFIDIGQSPENKIALTIQLMSSIVVNSVTVGACSLAAVFAMHAC
GQLNVLMRWLYQLDDQKQQNTVQRRLANIVEHHLRVLSFVAQMEELLNQICFVELLGCTFNLCMLGYYVITGW
NVIEKTTCLSLMLIYISMCFNIFIFCYIGEVVTEQCKQVGETAYMTNWYNLHHKTARGLILIISRSSTVIKLT
AGKLVHLSIATFGAVIKTSMVYLNMLRTMTVS
>SinvOr137
QDVTDYSTQFNRWLLKPMGAWPSEDSTPIKDKVISIILAITCYSLIVYALVPCILNILFEETDMHKKITTLGP
LSHWSIGGVNYFFLLIRSRDINRCVQHMRNDWRTVTDSEDRGAMMRYAKVGRFVAGLCAIVMPSGVIAHTLLT
GTTPVIVHIGNKSVSTYLLPCPSYTKFVDTTKSPAFEITLILQLMSSIIVTFIRVGTCSLAAVFATHACGQLT
MLMRWLDQLENRKKQNTVQHRLGNIVEHHLKVLSFVAQMEALMNQLCFVEVLGCTFDLCLLGYYIITGWNVMG
RKLSISYVIIYVGMAFNIFILCYIGEIVTEQCKQVGETAYMTNWYDLAHTTARDLILIISRSSTVTKLTAGKL
VHLSISTFGDVMKTSMAYLNMLRTMTAS
>SinvOr138
MSNYNRAPESNQKKYHGYSLQLNRWFLILIGAWPQINASSKIFILIQVLFCWSVVASMTVPCLIYVLFEKISI
KTKLKALGPLIHRAMGIVNYCVLLKRSGDISKLIRHMEADWSLIQKTDNCEVMLQHAKLGRFITIICGVIMQG
GVFLFSLARAMKTVPITVDNQTFMTHPMTCPIYSKIIDTRFSPVNEVALALQFVSMFIMNCSSVGVCSLAAVF
AIHACGQLNVLYAWLHKLVEEQEKSKQTTKRKLAAIVEHHLRVLSFVSLIEDIMNTVSLAQLMGCTTVMCLIG
YYMIMAWETLDAANIISCVFTYLSYGFNIFIFCYIGEIVTEKCKYVGEMAYMTNWHDLHHKTARGLVLIILQS
SNVVKITAGKLINLSFITFGDVIKSSMVYLNILRAMT
>SinvOr139
QNSPVAFNYIENKEYSIQPIRWLLKPISVWPVTDSSIVERILSVVLLIVCIFLIASTLVPCALAIFLDETKDL
EMKMRDFGPLSNWILASLKYVSLLTHVGDIHRCIKHIETDWRAVTKLEEQEMMLRNARIGRFIAIFSATIMHS
GVFSYGIFRGMTLSAQSTESNNVTVRPLPFPFYEKIVDTTTSPMYEIVFVTQCLSTFVVNSVGVGTCSLTAVF
VMHACGQLNILMSLLDNLIDKENEKRNSSQKFAVIIEHHLKVLSFVSHIDKITNVVCLVEVVGCTLHMCLLGY
YCILDWNQGEMEGMVSYAIILISVIFNIFMFCYIGEILSEQCGQIGETAYMTNWYLLPGNTALSLVLIILKSS
IAVKITAGKMIDLSLSTFGTVVKSALAYLNILRTLI
>SinvOr140
MENELTNNPSVSVPKHLIEYKYNEYSIQIIRWILKAINAWPRPADISIVEKIHSDFARFMCYFLIIAVMVPNG
LSMFLDKQLPYADKLQNFGPFTFWAIAIVNYSCLLIHIDDIRDCVEHVKVDWRIIKGLEDRKVMLKTARLGRF
FAGFCAVFMHCGVFSYNIVQGTLKTALQMENGSVTVRTLPYPFYHKILNAYYSPAYECVFFMQFLSSFIVNSV
TVATCSLAAVFVMHACGQIKIMISWLENFIDNRNEEGTSMRQKYAIIVNHHLRIISFVSRIEKIMNIICLVEL
LGCTMHICCLGYYCMMDYFSGDKRSILSHGMVLCSITFNIFIFCYIGEILSEQGEQIGKSAYMTNWHLLPGKS
AQGIILIIILRSNTVLKITAGKIVQLSFATFGDVIKSALAYLNMLRTILVL
>SinvOr141
MLDHSKLKSSSLEPVTGDWNYSIQLNRWFLKPIGAWPLTLCETTMEKIICIVLTIGSAFLICFLLIPCTLCSI
LVDTDLDTKIKTIGPNSFILMAAVKQYILITRSENISKCIRDIRADWDHVALGHREIMMTTAKFGRWLSCVSA
IFMYSAGIFFTTIMPICARRTQVINNETVRSLSFPIYRGLFDPRATPFFEIAQFTQALAGYVIYTITIGVCSL
AAVLVMHACGQFQIVMSKMEDLASEKEKKSASRHEGRLGDIVEHHIRILSFIARTEKLLNEICLVDVIGCTLN
ICFVGFNMMTEWENRETLGTMTYLSFVISFTFNIFILCYIGELLAEQCIEIGIKSYMINWYRIPNKGALGLIL
IMTMSNATIKLTAGKFMSLSLASFCSVMKASLAYLNLLRTFYV
>SinvOr143
PKSSSLEPVADFNYSIQVNRLFMKSIGAWPFSLCETTMEKIGQIVLAMTSCFLLLFMFIPCTLCIILAKIDLK
AKIAMVGPDSFILMGVCKQFALLTKSKSISECIRDIRADWDYIAMDSKKNREIMSTTAKFGRWLSTVSAIFMF
SAGLFFTTITPIFAGKTEIIDNETVRSLAVPVYREILDPRSTPYFEIVLLTEAVGCNAIYSFTVSVYSMAAIF
VMHTCGQFQILMLKMDDLADGKESKLANNTYQGRLGEIVKLHVRILSFIARIEELLNGICLVDVIGCTLNVCF
LGFYLITEWENSEAIGTIMYGLLMTSFTFNIFILCYIGDLLADQCVEIGTKAYMIEWYRIHPNKGALGLMLMM
TKSNSTLKLTAGKFFSLSLDTFGAVMRTSMAYLNLLRTFF
>SinvOr144
MHDRFQPDKIHNVRYEQDVHYAMQLCRWILKPIGIWHLIHERSSQSEKFLSFILILACLSGLCFVLVPAGPYI
LFREKDINIKVKLFGPVGFCLTSAIKYCFLGARVSAIGRCIKHVESDWRVVRYQDHRKMMLKNSLVGRRLTTL

CVIFLYTGGMSYHTIMPLSSGTKTNGSFTSRPLVYPGYDLYVDPQASPAYETIFFMHCLSAMIQYSCTTAACS
LAASFATHACGQVQILMTLLDDLVDGKRIKGTVAKRLSLIAKHHVRVLRFTADVEEILREICLMELVAATLII
CLLEYYCMMEWANSDAVAILTYFILLISLTFNILIFCYIGELLMEEYGKIGSAAYEINWYDLPGHKAADLILI
ITMSHYPPKLTAGKFCDLSLNTFSTVLKSSVVYLNLLRTVT
>SinvOr145
WSNANHEKDVIDILIWNRWLLRVLGIWPLIYPNTTTIEKILATFSFASCWTVLGLFLVLTSIYTFTDRSIMGE
KLKMLGPLGYVFFSMLKYFFLVIRHKSIRRCIRNLSTDWHKVQEEYHREIMMRDAEKGHLLSKFCIVFMYCGG
LSYNTVMPFLSQTHDANEQNITIRPMAYLGFDIVFNLQLMPIYVFAFSLQCFTGIVMFNITTSVCCLAAMFVA
HACGQIDIVIARVESLFKEERNHIKFEKCMAIIVQHHVRALKFSASIEDTLRELCLIELVGTTLIMCLVEYSL
ITEWNNSDSIAIFTYFFLLVSFVFNIFIFCYIGELLTEQCSKVGYTSYKVEWYNLPGKIALDLLFMINTSRYP
VQITAGRLISLSLASFGNVLKTSVAYLNLLRTA
>SinvOr146
TYQRDIRYVFKLNKWILGSIGIWPVSIRGIGRYASKIAIALGNLALSFAIVPCALHIVYDEKDIIMRLKLSGL
LAFCLVSMIKYCILAIRRPKILRCIEYVKNDWWQATFGSDRELMLKYATLGRNLTIISASFMYTGGIIYYMIL
IPFFSENKINNRTVRPLVYPTFSKFRQSQISPIYEIVYVVHCMCGYTTYSITAGACGLAAVFVTHACGQIQVI
VSRLEALLNGENPNIHQRIAVIVKDHVRIMRFSVVVEEILQEVCLVEFSSSVCTICLLEYYCIVDWQDDNKLS
LANYFLLFVSFCFNIYMLCYIGELLIEKSSQIGSMCYMINWYQLSPRSVRSLILIIAMSSHPIKLSAGRMVDL
SLTTFGTVLKTSVAYLSFLRTLVM
>SinvOr147
MSNIRDKSTTLHNSSYEQDIRYAFKVNNWILGSIGLWPVVIRGIGQHVPKIAIAFWNFALSFAIMPCVLHIVY
DQKDTITRLKIGGLLTFCLTVKAKYCILVIRCPRIHRCIEHVKNDWWQVTFRSDRELMLKYANTGRNLTIIGV
SLMYGAGIIYNIILPFCSEHKIGNQTIRPLIYPIYSKFSQLQISPVYEIVYMAHCMCGYTLCTVTAGACGLAA
LFVTHACGQIQILISRLEDLLTGKRFKQNPNVHRRIAVIVQNHVQIIRFAATVEEVLQEACLIEFTSSICTIC
ILEFLCIVDWKANDTVSLATYLTLFVSFCFNVYILCYIGELLMDKSSQIGSICYMINWYQLSPKSARSLVLIL
AMSSHPIKISAGRMVDLSLMTFGNVLKTSVAYMSFLRTLVM
>SinvOr148
MENSQNERVMSSNASYQQNIRYIFKPGNWILGSIGIWPIYGIGRHASKIAIVLCNFALAFAIIPCVLHMIYDQ
KDLNIKLKLFGLLGFCTTAMMKYCILVIRRPKIMHCIEHVKNDWWQAKFSSDRELMLKYATTGCRLSMISATS
MYIAGFIFHLVLPFCIEHKVDNQTIRPLVYPTYSKFFKAQASPIYEIVYLAHCVCGYTMYSITTGSCGLAAVF
VTHACGQIQVIVSRLEALLNGENPNIHQRIAVIVKDHVRIMRFAGVVEEILQEVCLVEFSSSVCTICLLEYYC
IVDWQDDNRLSLANYFLLFVSFCFNIYMLCYIGELLIEKSSQIGSMCYMINWYQLSPRSVRSLILIIAMSSHP
IKLSAGRMVDLSLTTFGTVLKTSVAYLSFLRTLVM
>SinvOr151
MLVNILREHNINKRFLWWLGLWPFQYKLMRCWLPMLLFAIQITYLPFEIVTLYMHRHNGQIIFESLYQIVVSL
AFITKLLNQLWNRDKFRRLYEIMEDHWNIFTNEMDVRILKNYSHISHKFTILYSTLTYTMMSLFIMIPSLGPM
FLDIVLPLNKSRPRNIALYSEYGIDRDKYFFPIFLHTSIVIIFGVSIMVAVDSMHIVCTAHACSLFQIIGRQI
ENIISNMNTDNKMNKLERYPNEEYKILNEKIIYREYITCIKKHQLTLEYVIVLNDTHKIVGFSFLFLIGVCFS
LLGIRIVYVLDQIEEMIRFMCIITGALLQLMIVCYSGQKLMDESQNIFHRAYAAEWYNFSPRLKSLLIIIILHK
SIVPCKLTAGNLFPLSMAVFAAVIRTGISYFTAFLSIKN
>SinvOr152
IMLVHVQREYNVNKIFLSRLGLWPFQSKLVRNLLPIVCLILEISYYSFEILMLHDHRDDSQMVFESCYQFVIT
TAFVVRLWNEVWNRDKFRRLYEAMNDHWDIFTNEIEVRILKHYSNISRKFTIVYSTMMYLLCSMFIIIPLTPT
FLDIILPLNESRPRILAIEVEFRVDTDEYFVLIFCYTTAIIVVGVSIMVAVDAMHFTCTTHACSLFSIVGEQI
ENITSTQHMRKCECDLNEEHELSRKEQVLYQQYVMCVKKYQIALKFVDILNSTHQTVAVFFLLLICATLSLIG
VRIVYVLGQLEEMIRFTFIIVGALLQLLIMCYSGQKLIDESENIFHRAYAAKWYLFSPRLKSLLFITLYRSIV
PCNLTVGKFCPLSMSTYATVIRAAMSYFTAFLSFKN
>SinvOr153
MLVHVLREYHVNKIFLSHVGLWPLQNKFIKNLIPISLFVLHLSNFPFEILMLYDHWEDKQLIFETFHFTTSLV
MFIAKLFNEFWNYDKVQRLYQAMENHWNTFTNEFEVRILKDYSSQSRKIIIIYSIVMYLAITTYIMPSFTPIL
LDIISPLNESRPRIFSMSFEWRIDMDKYYVPIVCYNTITLVTGIIICIGIDSMYITRIFHACSLFSIVSQQLE
MIQYCGCYINENVNRYCKNAIFKSANEHMIYQEYVICLKKYQIALEFVDVLNSQYRTMTLISLAITCMILSLG
GIQFVYVLGQLDNVIKNGVVISGMLLQFMLLCYPGQKLLDESQKVFYKIYAVEWYAFPLKLKSLLIITLQRSS
ISCGLTAGNLFSLSMVTYGTMVQAGISYFMTFLSLKD
>SinvOr154
MLVHIIREYNINKTLLSRFGLWPFQNKFVRYLLPISLFVLEISGIQLEIIFLCDDWKDTELIFESFFITIAMI
MFIMKLCNEFWNYNKVRHLYEIMENHWNIFTKESEMRILKNYSGLSRKLIIIYTLLVFLAIFSYVTIPLKPLL
FDIIWPLNESRPRKFPMPIKKITDLDKYYVPIYCYLTTTLLLGVGVFCAVDSVYITRILHACSLFSIVNQQIE
MISKPDINMKTSNNCKCCINTNDSKYCMKIDTYKSTSEEVTYKEYIICLKKYQIAREFVSLLNSMYYVGTFFL

LLLNILIFSLSIIRLVYELEKIVNAISHIVIITAILMHLLVICYSGQIIMDHSQKVFYRIYAVEWYALSWRFK
SLMLITLRKSFTPCGLKTAIFSLSLETYAAMVKAGVSYFMTFLSFKD
>SinvOr155
MLVHIEREYSINKIFLSCLGVWPFQSKLARSLIPICYIVYETSYYSIEILMLYDHWKDARIVFDCCYQIVLLT
TFVSKLLNEFLNHDKVQRLIETMDNHWNIFTSESELRTLKDYTNISRKFTIFYSIMMYSMVTTFLLIPLTPVF
LDIIRPLNESRPRFFAVAVELRIDQEKYYTPLFCYNISIIMLGTIIMVGVDTMYVAYTAHACSLFSIISQQFE
EILSKLDVTKQVSKYEPYLDEDLELLSEKEIYQQYIICLKKYQLTLEFVNIIDSAYQVLTLFLLLLNGITISL
VGIRIAYVLDELQEMIRFGFIIIGMLTQLLIICYSGQKLMDESQNVFYQIYAVKWYKFSPRLKTLLIITLYRS
IIPSGLTAANIVPLSMATYATVVRAALSYFITFLSFK
>SinvOr156
MLVHVQREYKINKIFLSFLGIWPFQNKLARNLIPIFCLAYIISYYSLEIMMLYDHWKDTRLVFDCCYESVLIT
TCNAKLLNQFLLHDKIQHLYETINNHWNIFTNKSELQILKNYSNISRKFTIIYSIMICSMVTLFFLIPLTPVL
LDIIRPLNDSRPRLFAMSLKLRIDQEKYYVPLYCYQYSITVLGAIVIACFDTTYVVKTAHACSLFSIINQQFE
EILSKLEITKQVSKYNLDEDLELLSEKEIYQQYIICLKKYQFALEFVNLLNSTYQVVALFLLILNGMVISLVG
IRIVYVLDQLQEVIRFSFVIVTMLIQLMIGCYSGQKLMDESQNIFYQIYAMKWYKFSPRLKSLLMITLYRSIT
PCSLTAGNMIPLSMTTYATVVRTALSYFTTFLSLK
>SinvOr157
MDFAGSRYYKIIQIILICLGLWPYTQRTRYVYSIFVLLLLSSKIFFELSTFIIVEYSVDLLLNVCSIIGPTIY
YTLAYFATFISGNKIKELMEHVQYNWNSLKEEEELKIIHNRANIGRFYTIITLICLYITFISFLLIFFSPNIL
DIIVPLNETRQPIWRLTSIMNAFIDTQRQIRTELLTWYFFLTIFIGMNTLIATETLFLMNVQHACAMFQITSY
RIERTVNNNQMKSLTSLEKFSIDKNIVEAIDSHRRATKFIDSLKSTFSIVHLFAIPIVVGSLSINLYRLCDSI
TNKDVNGTIVLSLLVACHFAFMFLINYIGQIVIDHSEDTFKKICNTRWYATPLNMQKCLVMIMHRSMKTSTLM
FCLGLFLPSLQGFAKLISKSLSYFMVIYSMHR
>SinvOr158
MNFAGSRYYRINQILLTFLGLWPYYTPRAKYIYNIFLFLLLFSNIFFQLASFILMDYNVDLFLNVCSVIGPII
YYTFTYFGMLTNQNKTKEFIEQVRYSWNSLKEKEELKIIHTHANIGRFYAIIGSITIYTTYFLIMLIFFSPNI
LDIIMPLNTSRESLWHLSTTMNFFIAQDKYVGLLTTYLMLTFFIGMTTLLTSKIFLLMNVQHICAMFQITSYR
IERIVNKSQVKSLVFPEKFNNYKNIIEAVDLHRNALKFFESLKSAFSVENFVGIPIGVISLSINLYRLCDRII
AEDIGGAILMCLTVIIHFGVMFLSNYIGQMVIDHSEDIFKKICCSRWYATPLNMQKCLMMIMHRSLKPSTLMI
YFGLFLPSLQGFAKLISKSLSYFMVIYSMHR
>SinvOr159
LTTFITTKYNIDILLRVLTYTIPWLAYMLKYNAQCLNIKRIRSIMERVLYDWKELSNAQEIEIIKEYADIGRF
ITLLTTLTIYIGVFSFIVIQFLPNILQAIISSKNNTDSRQLPILIECFIDQQKYFFLLLLAIFFCVACGLTTV
AATETLVMSYVHHACGLFEIASYRIERALFKDTAKDITSSTQRSSIICQGIINGFTMYKRAIEFIDMIQITYK
WAYSILLPLGVLSLSVNLFTFSRLVISKEYYNMCISFMFIFGHFGYMLFTNYLGQKIIDHSSNVFHRLYNVQW
YVAPLKAQKLLLLLMQRSIRNCTIVVDGLFISSFEGFATLASTSISYFAVIFSLF
>SinvOr160
KITPKAAIAFTKAITALCFTWPLPKSASKFQVIRFKILRFLLCINATILAVPVAYTLYLNDYDLPRLTKLWCL
LAAFIQIPLETTQCALQYDRLQYLISEMEHNIECAEPYEKILYQRYVDKCAIFYASATATVFFGAFITIIAPF
IEADHIFPSDAKYPFEVEHEPVKLIIYLHQFVVVWQCFSTVCLCSFIGLLIWFATARFEILSQQFRMITDLYG
MIVCIRQHMKLIRYTQELINALRSVILSIVIICTWAILACGLTIVSVNSYFQKKIISLLFLLKNVLIVLRSPT
MLSIYVTKDTSVAQGVYESLWYNQDKIFQKNLLFVSKRSQIPIVVSVSSILPVLSLEFYALYVSTAFSYLMTM
RIVFVEDN
>SinvOr161
MTETLTLEKIISFLKVDLTFACCWPVPIKATKSQKIRDKLFRFLCCLNGILMSIFLIYTLSNKCDNMILIMKV
GCELSAFLQIPVQITLFTFQSDRLQIIICEMEDYIQQAKSEERNVFQQYINKCKLFYGTTLCWITTTATIMIF
GPLVLSQPFPIEVEYPFAVDKQPLKTIIYLHHIMVVYQSCAQVCSNVFVALLLWFVAARFEILSHKFQKITSI
SEFINCVQLHQRLLRYAKEVTMSIRYIALSTIGFSTIAVVFSGLTFLSRQPLTIKTQFFTVAASALIEVFVCA
WPADYLLRTSNDIGHAGYKSSWYNQELSLQKNMLYIVSRCQHPVTLTVPCMLPILSLNYYASYLSTTFSFLTT
FRAIFAVEDGLNM
>SinvOr162
MKTVPTLDQVITFLKIDLLFACCWPLPRTATKGQIIRDKIFRYLSIVNGAFIVIELIYSINNHLDNVSLIMQL
ACALGIFCEIPLQIFLFTQQHDRLQNVICEMEDYYKQANAEEKDIFQQYINKYIFLYGTTLSLTAASLAGSLI
VPLIQSRMFPLEIEYPFHVDYQPMKTIIYFHQALGMYQVYCQVSANVFLALLLWFTTARFEILANKFRIITKY
SDWITCIQEHQETLRYAKEMSNSIAHVILASLGISTAALVFGGVTFLSRFPPSVKIQYIIICSTSLTKVLLCA
WPADHLMRTSSNIAEAAYNSLWYNQSIKTRKIMLYTLLRCQQAVIISVPGLLKALSFQHYASYVTTAFSYLTT
FRIILSEET
>SinvOr163

MRKKVTFKEVISIAKFSMFPLWFWPSKNETKLKTFCRKLYNYWCILMGLCLELPSLYGASQHTDDPALLAQTL
VMSSTSWHIMFNCIYQLVYNHRIQSITYEMENFCDLMKPHEEVVIQRYIDKCGVFYGAAIIEYYALIVVQIVV
LPPLMHQSFPTLVEYPFDVSYQPLKTIIYLQQSIVGTYMGAQLCLNIFMNLLFWFVTARFAILTEELKDATNI
NSLFKCIKTHQKLLKYATEVTHVLRPFAFSSICCSTYSIIILFVLLITHQPIALVIQFVGLAITCLAEVYMYV
WPGENLINACDDIVHAAYSVYNKHSIKAQKCLQFIMLRSQKPVTLSIPCFLDALSNNYFTSYCSTILSYFTTV
RVMMLEDN
>SinvOr164
KVTAKKIFSVVRLSLIPVWGWPIRNATKFDMFCVKLQHCFSIIMTIGLVLPLVYGITDNFNNISDLVTQIVVL
GGLIHSISNFIFHMINYQILQKLTFEMVDFCDLMESHEEVIIQRYIDKCVLFHSLSMSIFYFMPMFTALLVPS
LTDQPFPILTKCPFDTLQQPLRTIIYLQQTIVGLFVTGALCMNIYMALLIWYATARFEILAEELRKAINIYEL
YECIKKHQKLIEYANEITVVARPIAFSTVCCCMIGVIISFCLVVTHQPIGIIFQFTTASMTGISEVFMYTWPA
EYLIHMCKEVGEAAFYLLENNDLLQIWKCLQIIIIRSQKPITISIPCFMPTLSLNYFASFISTILSYFTTLRI
MMLE
>SinvOr165
MICTNSLHIGLNKSLLLIVGLWPYQQSKLIQLQLTLFISVLMTFILVQFTAFFTLRCTSELIVNILASALFYI
FFAIKYSSFSVNLEAVKYMLEQLQHLYNELTDENEINIIKQYAIYAKRYTIGLTLLLILMILIFILNSTWLHI
FYILFSINKTRSHFSSLLVTEYFVDHEKYFYLILFHTNAAFFIGLVTMLATGSMFLVYLHYARGMFQITSYRI
AEAITQNTLQNKNLIYEKLICAVDMHRRALRFSDLIISKFKLMFSLMMIIGVICLALNAFRAFHLTFFEFDIG
EFSLRLMFMNSLLFYGLISNYLSQGIIDQNNNVFVSVYNIHWYIAPMQIQKIILFFLQRGSKEYAINIAGLYV
ASLQGVASLINTALSYFTVLYSVQ
>SinvOr166
MICTNTLHIGLNRRLLLIVGLWPYQQSKFVQLQLTLFISVLMTFILVQFMAFLTSQCTSEFVINVLGSASFCV
IFAIKYSSFSVNLEVVKCILEQLQHMYNELTDKNEINIIKQYAIYAKRYTIVLTLLFILMILIFILHSTWLQI
FDILLPINETRSHLSSPLVTEYLVDHEKYFYLIFFHTNAAFFTGSIAMLATGTMFIVYAHYAYGMFRITCYRI
AEAITHKTLQKNNLQNKNLIYKELICAVDMHRRAMKFSDMMISKFKLMFSLLIIVGVICGGFNMFRAFQLTFF
EYNIGELSLRLIFLFILFGYVLLGNYLAQGIIDHNNDVFATAYNVQWYIAPLQIQKIILFFLLRQNKEFTLNI
AGLYVASLEGAASIINTGLSCFTVLYATR
>SinvOr167
CEMRFFDGRNYRINKVLLSCVGQWPYQTNRSSNAIIIIMVSLAGTQFIAKICGLFSIDNADVFIDSFSPLIVD
LACGVKLITCILKATEIKALFNQIQSDWQLLTTPSYIKVLNNYAQNGRTFTIIYASVFYSALILFMLIPLQPL
LLGPSSNDTSRPALLHQVEYYIDMEKYYFPILIHGYITAVICVSIAIAADTMYVIVVQHVCGLFMIIGQQLEN
AIKEDNIEINFKPSITDDKPYGNIISSIRAHKRALRFASLIEATFSPMFLVVAGFNMVIISMTGVTAVSNMDK
PEEFFRQITFSCALLVHLFFESFQAHRLIDHSTYIHTSLINLAWYETSFRTRKLLIFMLMKTREPCVLTAGKM
FVISMDTFSTV
>SinvOr168
KRMDFKNVNLLNSRVNMLSGNLLPIAGDNSRFSVFWRAHSAIVWLIELIHTIALIFGLILSPTEKSLKDGTVC
VVVAVEAFFMLSRLYSRRKMIEEMIQKMNDILQDTDEIMADIVKSAIRPIIKPFIIYGVTSAMSIAIWTVQPV
ALALEKSTFFYVDYNLPTAFSAEPFSSHVLISSSIFMTIGSVYLFLKKFGVDVYMMHLVLMLTAQYRYIAAKL
TILFQDIRNCHDESQNKYHSAKNRWTKRELRKLCQHQNTVLHMSFILRKLLSVNFSLLYINNVFRFCFIGIMV
ISIPSLSFTEGMSVMLFAMGSLMQFFLLCSSVQTLSDASTEITDKAFDEGWYHCGSSMKRTFILLIMTNNLEC
KIAAIGKFNLSLPSFMTIMNQSYSIALLFLRAK
>SinvOr169
KRMDFENVNLLNSRVNMLSGNLLPIATDSSRFSVIWRTHSVAVWLIELTHIIALIVGIILTPKEKGLKDGTVA
VVVHLEALFMLMSLYSRKKLMEEMIQKMNKILQSADEIMKDIVKSAIKPIIMPFMIYGVTSMISVAIWTIQPV
LLVFEKSTFFYVDYNLPAAFSPEPFSSRILIPTTIIMTIGSLYQFLRKFSVDIYMMHLVLMLTAQYRYMAAKL
VILFKTLQNGHNEFRNERHSAEDRWMERELKKLCQHQNTVLNMSFILKKLLSVNFSLLYVNNVFRFCFIGIMM
TTVPSLSFAEGISVTFFAMGSLLQFFLLCSSVQTLSDASTEITDKAFDEGWHQFGPSMKRTFILLIMANNLEC
RIAAIEKFNLSLPSFMTIMNQSYSIALLFLRAK
>SinvOr170
MDVQNVNPLNVRLNMISGNLLPITDEDLSFAVPWRIYNIVIWLIQVIQICATIYGFVSVANREALQDAGTMGI
LVSVEVLILLTRMYANRNLGRQLIQKLNSLLRTEDESMKNIVHSTLKPLEIPLEFYYIGGTGSVIIYCCLPFM
LIFKKKYFFYEDFVMTAAFSKQPFSTKVFVLGNLFETISCAFIFLKKVAMDVYMINLVLLLTTQYRYVAVKLA
TILRNDSSQNEDHECQKNDRPADLLVEKKMKALCQHYSSVIFTTLMLKKFLFLNMSFIYLNNIFRFCFIDLLF
INAIRSGAIYEVALLIIYVCGALVQLYIVCFCVHQLLDASKEVTDIAFHEQWYEFKPSVKHMFRMIIMANNLK
IKLSVSENFSLSLPSFLAILNQSYSVALLLLKVK
>SinvOr171
MDFQNTNLFNVRLNLLSGNLLPMTDKSDKFSFSLFWKVYCAFVWLLELVYMISLILAYFLVPNKIALSDGFDG
LFVIEEVGFVIDYSRILIQKTLVQQLIRKLNEALSIEDENMQRIVTTNLKQIKNPFIYYLVTGTGSVIFWCCT

TLPLILERNTFYHADYNAPAVFSKEPFSVGVFLLGNMMIMVCNTYLFLKVISVDVYTAHLISLITAQYQYISS
RLVLIFRNNKHPNNRSSREGNSEVDSFTTKEIKNLCRQHVNVMHITLMLKKLISVNIFLIYLINVFRFCFIGL
MVNKISESYLVGCFVILYASGSVTQFYVLCACFQRLSEASSEITNEAFHEDWNQFSSSIKRTFLLITLASNNI
KVKLSLFERFNLSLPSFMSILNKAYSIAVLILELS
>SinvOr172
MDFQNVNPLNIRLNLLSGNLLPLKPGDAQFPMIWRVYSSLVWLIDIIQTIVLIPGLFLASREKVIKDGTVLCV
VTLEVFFIATRFHSRKQLVSRLIQKLNDILQFADETMKNVVITTLQPMDNPLKFYWLSGTLAVFLWACLPFVL
IFKEESFFYDDYRTPAVFSYQPFSFNVFLLGSILLLMGNMYIFVKKVGLDVYMIHIVLLITAQYRYIATKLTA
IFRDGNPLSEFDGSRQKYCSEVDRWVEKEIRALCRHHNTVVHLSSMLKQLLSVNFTLIYVNSVFRFCFIGIML
STVPSTTLPEALAIVMFSCGSIVQFYMLCSCVQQLLDASKQMTNSAFHEKWYQFGPSVKRTFMLMMLGNNLEC
KLSMCDKFNLSLPSFMAILNQSYSIALLFLRVK
>SinvOr173
MDFQNVNLLNVRINMLSGNLFPMTADNSRFSIGWKIYSAITWLMILTVVIAFFVGFNLVSKMKAISDGMIGTV
FTIEVFFMAVRIHICRDLIVQFIRNMNDILRVQDETMRCAVMTSLELMHSRFLKFYWLSGITTVIIWIGMPLK
AVFIKNSFFYEDYRLPFIISKQPFSMEIFLTGGLLLILCSVYVLLKKAAIDIYTLNFVMLMTAQYRYIAVKLE
ELFREKNSLNEYNFQMGRTDSWAEAEMKAICKHHHTVVYMSSRLKKILGPNFCLIYLTSILRFSFVGVMISNT
TLLTTVVERVAMVFFAMGEIIQFYILCLNVQKLLDASTEMTDMAFHENWYQLRPSIKRKFMLLIFGNNLTCRI
TSVEKFDISLPSFMKARSRFYYITYLFLIMDQAYSIAVLCLKIVKK
>SinvOr174
EWATRLNRLVLNIIGIWPSAHKNAYDKFLSNLHVTFVIVIILFVGEIPTIHSLVRTWGDLISMIDNLQFSLPL
TIAIIKLIDIWWKKTDLLMAINMIAEDWIKEKTNKERCIMIKQAQNARIITIVGCCFMFLGIFLVLVLPCFGT
TVRYITNVTDPVKILPLQTHYIYDKNQSPYFELTYAAQVVLLIVSAASYSGIDNLLGLLIFHLCGQMENLKER
ITNIKQFKNFNGDLAVIVEDHIRLIKFFDIVESTFTILLLGLLLYFGTLFCLYGFLIVAILTEGKEMSLFRLI
YLISVVLNVCGHMCLYCVVGEILVAQCEGIYRAAYNYEWYKLNPEEARSLIIIMIRANKTLHITAGKMFPMTL
SMFCNVSK
>SinvOr175
VSQFSCEMYAIVERYYKINRISLTMLGLWPYQQSYLTQIQQILFASILLAFMLAQLLVFITRQYNAELLFMNL
SLIFPTVLVTMLYLFFTVKINSVKLLLEQIQDDCNLLKDKLEIDILEKYASNFGFFIQITMALCYFGAFSFGI
LQYLPLILDVILPSNKSQPLQLFVYTEYLINPNKYIHFMILHEWLTICIGLTTVGSSGLTCLVYVAHLCMLLK
IASYRIENVTENMLAISNPRREHLLHQRVVHAVIIHQRAIELVSLFRVSDIFSILFNLILNCFLSNNLLQCSL
QFSTSNDKSMILINVGLISVYLIYLFTLNYCGQILIDNGLNFFEATYNGLWYAAPLSIQKLLLFVMQRGRIPL
TLTYYDLITSSIDGFATVLKAVVSYFMVLNST
>SinvOr176
VSQFLFEMYSIMERYYKIIPTVLRSLGLWLYQQSYFTQIHQILFASILLTFILVQLLVFITRQYNMEFFFMVL
TFVCPNLFATIKYFTFITKANSLKQLLEQIQNDCNLLKDKLEIDIVEKYNRNAGLFILSLLGFSYILLLTFST
LQYLPFILHKMLPFNKSRSIQLVTSTEYLIIPDKYIYVRILHEFLTFYVGLTTISATGLTIIIYCVHMCVLLE
IASYRIKNIVQMNTLVIPSPRREQIVHQKIVNAIIIHQRAIEYHELFMSVFLIPFGILCIIGVGCVVSALFLV
SIASNNMGKAIVTFGFLQICLIYMLVGSYLGQRIIDNGMNLFKDTYNGLWYAAPLSTQKLLLFVMQRARMYFS
LTFYGVIAASFDGLATLLKLAVSYFMVL
>SinvOr177
IMERYYKIIPIVLKSLGLWPYQQSYFTQVHQILFASILLTFILVQLLVFIKRQCNMELVFMVLSFVCPNLFAT
IKYFTFITKANSLKQLLEQIQNDCNLLKDKLEIHILEKYNRNARLAVLSLLGFSYAILFIFSTLQYLPFIFHN
MSPFNKSRSLQLITSTEYLIIPDKYIYVRMLHEFLAFYVGLITISATGLTALIYCVHMCVLLEIASYRIKNVI
QMNTLVIPSPKREQVVHQRIVNAIIIHQRAIEYHKFFMIAFLVPFGIVCIVGMSSLILALFLVIASNNMGMAS
VAFGLLQVLLIYMLVSCYYGQKIIDNGIYFFEETYNGLWYTAPLSTQKLLLFVMRRAKTYFSLTFYGVIAASF
DGLTTLLKLAMSYFMVLNSVQ
>SinvOr178
MYSIMERYYKIISILLKGLGLWPYQQSYFTQVHQILFSSILLTFILVQLLVFFKRQCNMELLFMVLSFVCPNL
FAIIKYFTFITKANSLKQLLEQIQNDFNLLKDKLEIDILEKYNRNAGLFILTVLGICYAILFIFSTLQYLPFI
LHKMLPFNKSRSLQLVTSTEYLIIPDKYIYVRMLHEFLTFYVGLTTICATGLTLLIYCVHMCVLLEIASYRIK
NVIQMNMLVIPSPRRDQIVYQRIVNAIIIHQRAIESVFLYFLLLNFLFSTIIFMYIFMYVISKITVQMFTENE
LQIYIIFASNNMGMASVAFGLLQILLMYLMITCYCGQKVIDNGINFFEEAYNGLWYAAPLSTQKLLLFVMQKA
RTYFSLTFHGVIAASFDGLVTLLKLAISYFMVLNSVQ
>SinvOr179
SQSLFEMYSIMERYYKIIPIVLKSLGLWPYQQSYFTQVHQILYASILLSFILVQLLVFIKRQCNTELFFMVLS
FVCPNIFATIKYFTFITKANSLKQLLEQIQNDCNLLKDKLEINILEKYNRNARLVILSLLGFCYTILFIFSTL
QYLPFILHKMLPFNKSRSLQLVTSTEYLIIPDKYIYVRMLHEFLAFYVGLITICATGLTALIYCVHMCVLLEI
ASYRIKNVIQMNTLVIPSPRREQIVHQRIVNAIIMHQRAIEYHKLFMFVFLIPFGILCTVWMGSIILALFLFS

QQVISNNMGMAFVAFGLLQVLLIYLLITCYCGQKVIDNGINFFEDTYNGLWYAAPLSTQKLLLFVMQRGRAYF
SLTFYGVIAASFDGLTTLLKLAVSYFMVL
>SinvOr180
QFSCEMYAIVERYYKINRIVLKMLGLWPYQQSYFKQIHQVLFASILLTFILVQLLVFTKQYNTELFFMIMSFV
FPNIFATIKYFIFITKANSLKQLLEQIQDDCNLLKDKLEIDILENYASNVRLFTQSVLGFCCIILFIFSTLQY
LPFILHILPFNKSQSLQLLSFTEYLIIPDKYIYVMMLHEFLTIYVGLATVLGTGLTIMIYVVHICVLLKIASY
RIENIIERNILVISSPRREHLVYRRIIHAIVIHQRAIEYHKFFMSVFLKPFGFLVIIGIGSIIFALFLFSQQM
AANNKGMALASFALIQIYLIYLFVGNYWGQTVIDNGIIFSEAIYNGLWYAAPLSTQKLLLFVMQRGRAYLSLT
FYNVIVASFDGFATLMKMSVSYFMVLNST
>SinvOr181
KMNLFESRYFNLNRLLMSLIGLWPYQKLESRRIKMIFISFFMAIGVIAQLLSFVTIEYSRDLLTKVFSFSFII
LICTIKYNMLYFKSEQVKYLFEQILYDWHAFTDAEEMKIIQKYANKGRSLTIYTGLVIYIGILVFIVLFFIPD
ILDIVTPLNEPRRHQLPVTIETLFDQEKHFLFIVLNFFIITFVMLTVLWTIETLYMMCIQHACGLLKLTSYRI
LNAFDNRLQQISIPNKKLKCTICIKLSKAIKIHRRSIEFIECLCSTFSVSYFILCVFIVASLSINLFEFLKAV
ESKHTSQAIHIGMFVYAHLVYIFWINYFGQALIDNSADVFTQTYNVKWYMVSTHIQKKILFILHRSAKDIMFD
VGNIFTFSLDGVALLINKSLSYSMLLYSTR
>SinvOr182
MDFDGSRYFTLNRIMLSSVGLWPYQNKWHVRIQRMFCLLSFLSCIIVQLFTFLTLEYNLDLLVEVLSFIFPCL
IVILKYTTYWAKIKSIKKLMEMIKYDWNTIRNEVEYEIIKKHTYTGAFYSQIFAVMTYTMPTFIAFMHLIPNF
LDFVAPLNQSRPHHWLILTEYFVDSDEYFYPIFLHLIANLFLIQNTLMSTTSIYVAFIQHTCGMFEIASYRIE
HALNNYKQRNLMFGRRCSTCTKIISAVDIHRRVIEFFEFLKETFIVMYFFLLGLGIASLTVSLYRAVITKGII
KNIMPITCVFIHIFYFIFVTYSGQKVLNHSNDFLLRTYNSNWYMMPLHSQKLMLFVIKRSLKNCILLVGGIFV
ASLEGFATSMSLSLSYFMVLYS
>SinvOr183
MEFLGYDYYKLYRIFLLSLGLWPYGDSFFKRIYAIFCIFTLLVLITAQLLKLFTMEFNLEFVLNDLSFAIPSI
VYLLKYSTFYIQSQKIKKLMEHIRIDWNALQDEEEIEIIRKNAKIARRYMYAFVGLAYPTTVVYVSIPFWPDI
LDIVAPLNESRKRHLPFLVEYFLDEQKYFYPILLHMNLTLVVGIVTVVATETLFFAYIFHICGMFEIVSYRIT
CALDKSMSILSTLNKEDTIRVKLIDAIQIHQRTFEFFEYLTSTLSLSYFILVILGVVSLSINLFRLFQVATLP
DQRKDLGPFVVFVSAHFYYMFVCNYMGQKIIDNSTDIFRKAYDTQWYTAPVQMQKILFFITQRSMKSCKIVMG
GLYSASLEQFTTLASMSLSYFTIIYSVQ
>SinvOr184
MDVIGNIHRYYNIHRILLSSVGLWPYQDPKMKKVQWIISSIVLSSSIIIQFMTFLTTEYSLDLLLKILSFAIP
CMVFVLKYVSFCIGSNSVKDLMESVISDWNLLKTDAEFEIIKKHSNFGRLYGLFFALAVYCGLFFYSLIQFSP
NFLDIISPRNKSRLHNIPLAAEYFIDQQEYYLPILLHIDIIAIVGFTTVISTESLITAYIRHAVGMFEVASYR
IEHAFDEVLGIMTSQKCCSYCTKIINAVHIHRRATQFIEFLRTTFVISYFFLVFLGVTSLTANLIRFFLATQY
LNNLEECITALLLVLGHIYYIFFCNYTSQKLIDQSIDVFYKTYMSQWYDAPLHTQKLLLFMMQQAIKGTAMSV
GGIFVASLEGFATITSTSVSYFTVIYSV
>SinvOr185
MNFIGARYYKINKLLLCILGLWPYQITKRIQIQPILFYSIYISFLFAELCSFFILKCDLIVVLKILSHLFPTI
LELMEYNSFYTNAKNIKLLLDKIQNDWNALDDKKEIEIIEKYTYRMHIFTSIVTLFSCFGLIVFYFMELWPII
LDNILPLNESRPRNTFAVTEYFIDREKYFIPIMLHEMVIVSAGALTIISTGTTLMAYTQHSCGMLKIVSFRIA
HLLDKNISQRSSHQIDRIICNRIAHAVDLHRKIANFLDTLVSTFVVQFTILTFIGVVSMSINLYRVSTKFNDS
NDETELFASVVLIFGHYFYMFMANYVGQLIIDHSEDVFRTTCNVLWYVVPKSSQKMLLFLMHRTMKNLKICGG
FNAITASYEAFATVICIAI
>SinvOr186
RYYKINKLLLCILGLWPYQITKRIQVRPILFYSIYISFVFAQLCPFFILKCNLIVVLKILSHLFPTLLEFVKY
NSFYTNAKNIKLLLDEIQNDWNALDDKKETEIIEKYAYRMHIFTSIVTLFSSIGLIVFYFMELWPIILDNILP
LNESRPRNTFVVTEYFIDREKYFIPIMLHEMVVVSVGALTIISTGTILMACTQHSCGMLKIVSFRIVHLLDKN
ILQRSSLQIDRIICNRIAHAVDLHRKIANFLDLLVSTFTVQYSILIFIGVASISLNLYCVSTKFNDSNDNTEL
FASVVLIVAHFFYMFMANYVGQIIIDHSEDIFRVTCNVLWYVVPISSQKILLFLMHGTMKNLKLSGLNVFTSS
YEGFATVI
>SinvOr187
LCYSSDRMVFIGERCLNLHRITLSLLGLWPYQKPSVLRTVFFFGIFFASLVFQLTVFITTKYDTEFIITELTR
FCPFVIYFLNYNCFYFNSKSIKQLLEHIKLDWKIMEDSEMKIIKKYSYIGYFFSFSVNILLTLVMLIFTIIEF
IPIILDAIAPLNESRPHRFKVYYELFIDEEEYYFLYLIVEVVTVTIGIYSTVMTGTFLLTIGGHFCATFKIAS
NLIKETVTENMIQMPAPYKIYFMYRNICRAVHIHRRTVQIISNMSNSLTIWYLPLLLFGVISSSCIIFRFLNA
IRRLNDFSELFVSFSLFMAHFIYMFIANLGGQVITDHSSEMLKSVYNTLWYVAPLSIQKLILFLLTSVKDYKL
LVGGIFVPSFEGFSTLITSAISYFTVIYTM

>SinvOr188
QFSCEMHTIERYYKLNRILLETMGLWPYHYTYVTQLQRTLFITVLITFIIVQLLAFFTNQYTTHLLLQILSFV
FPIFINTVQYCLFIVQTDNLKQLLEQIQDDWNSLKDKLEINILDRYACNARFCTKLLIVYCCFGLLFCGLFQL
LPIILDVIAPLNESRPCELIVVTEYFVNKKKYFYITLLHETLGYLVGAAIFCSTSAIVMICILHACALFEIAS
CRIENAIPKSTLMIPTSKREYFLYRKIVYAVIMHQRAIKFVKLMTTSFATLFLILIILGISSLSFNLFQFLQL
ITLRKNSSQTSTVGILILLHLNYMFITNYGGQELLNHGLKLFKVTYNGLWYIAPLRTQKLLLFIMQKGTINIG
LTCGSIFVASLEGFATLANVTVSYFTLIY
>SinvOr189
MSESMYKTRAQERYNQVYRIILKSLGIWPYQQTCFVRLQQMFNTGVLVLFILAQSLVFITNQYDINLLLKIFS
FLFPNLFATVNYVGFIVMADNLKILLDQIQNDWNLLKDKLEIDIIKKYACNMRLFAISVIVFCYLVMLSCGIF
ESIPLILDIILPLNESRPIKLMAMTEYFIDQEKYIYYLMLHESLTGYIGMLSLCGIGVILVMVCMHSCALFKI
ASYRIENVIDKSVLPRREYLLYQRIVHAVVMHQRAIEYLDFVNSIFATYFVILIIIGVSSFSINLFQLFQYIR
YSNDIRITIISLVITMFHLTYMFTLNYPGQEMINQGLYFFKACYNNLWYVAPLHIQKLLLFIMQRGSIDTTFV
IGKIYVGSFEGFATLMSTAISYFTVIYST
>SinvOr190
FLCEMYVIVERYYKVNRVVLKALGLWPSQQSYFTQILQILYASILLTFILVQLLVFITRQYNTDLLFKILSFV
CPCLFATIKYFLFIIETKNFKKLLEQIQDDWNSLKDKFEINIIEGYACKMKFYTTSSVASCYIGILLLGILQH
LPLILDIMLPLNESRPYQLIVITEYFVNQDKYIHVMMLHEFLVIFVGSFIIIGTGSILVIYIIHICALLKIAS
YRIENAIEKNILEIPSPTREYLLHRRIVHAIVIHRRAIEFNEFLTSICLLPFATLIVVGVSSLTFNLFLFIHL
ITANKNIMACVTATSIIFHLIYLFVANYCGQAVINHAINWFEAIYNGLWYAAPSSTQKLILFIMQRGRIYLTI
SCYSVFVASLEGFATLTSTAVSYFTVIYST
>SinvOr191
MYLVEEHYYTINKTVLKGLGIWPHDKSRLAFLRRMLFLTLAVTYIALQLAVFITSKYNMNLFVKTMSLAFPSI
LFTLKYITFMFRTKTMKNLHHRMREDWKLMQNKIEHDIIKKCAHDCQNYIIFISQSIAVLIIILVIIQFLPVT
LDFIWPLDEPRSHKPIITVEYFIFQDDHFYIATFHQTIIVALFTAIMIATGTTLLLFALHSFGMFKIASHRIK
HSIDNYVIRMPNCEMERTIYKRIIHVIIAHRRAMEFSNILVSALNVPYCTIVIIGVFSLSINLFRFVQAVTIS
MDIEDILVSFSAVFGHLVYMFIAIYVGQKATDHNDELFRLTYDTSWYLIPVTSQKLILFLITRTGKEFYYTLG
YIFVAKIENFAMLLNTALSYVAVMYSIQ
>SinvOr192
VEEHYYTINEAFLKILGIWPHNKSRLVFYQRMLFMTLAVTGIFLQLAVFITTTYDITLFVKIMSIVCPCILIT
LKYFVFIFKLKTIKFLHHFIQEDWKIMRNKLENDIIQKHAHECRNYIIFIFRCSSVLMVILVLIQAFPSILDF
VLPLDESRPFKFIVEVEYFISQDTYFYIILLHLVIVLALYASIILATATQLLLFSYHSLGMFKIASHRIEYFI
DDHIICMSKYEKERSICKEITHIVKAHRRAIEFSNILTSSFNLPYCIIAIVGVLSLSINLFRFFQAITISRNI
GEILISFFIVFGHLIYMFIANYMGQQVTDYNNKVFRSTYNTSWYLMPVTSQKLILFLMTRTSKDFYYSIGYIF
VAKIENFAMLVNTALSYVAVMFSI
>SinvOr193
IEDHYYKINRILLLIIGIWPYENSILVKAQRLIVFSLLVSGICVELAIFTTREFNIAVTLEVLSLVFPTLVPI
IKYYFFCVNADALKQMLERVQIDWNLLKNKHERKIIESYAEYGRFMTVVTSLFIVGIMIIYMISPIVLYTINI
YLFQNNNTYTYYIFIPIDYFVNQEKYFYITFLHFITFAFVGMVITVAIATFLGLYLYHVCGLLKIACYRIEHA
INIEEILQISPLKREYVMCMKIMRAIKIQQEGMKFKTRVLSSVSVMYCAIILLGGGAVSIYLFRFLVNMKTDR
DIREFFLILSMLFGHFLYIFLANNTAQDVSDHCNNVFFAAYSSMWYVAPVRVQKLILFLLQYCTQNFTIIFCG
CFIGSLEGVATV
>SinvOr194
MILFVDQYYDINRKLLSIAGLWPYQKQRFKLLQITLFPIMLISFLTAQLATFTTSEFNVNIMLTVLSQAFPCS
IYFLFYISFLLNNKDIKIFFDLIHHEWMTLKAADEIDILRRYANFSKRFNVVIFVLLMFIVNIFCLYQFFPDF
LDVIIPLNESRDHHLTFVAEYFVDQQRYIYPILIHSLLAIYMGVIAVLSTGTTLIAFLLHICAMLKIASYRLE
CVNDNIPLVSKSEKNNIIRERIINAVNIHRRALEFAESILSSFSTFYFIMIGVGISSLTINMFQFMQLMMFTS
DTNGMLASGFLILSHFTYMFIGNLAGQIVTDHNNNIFNAMYILKWYTMSIEAQKLLLFIIQTNTKTYYLIIGN
IFAASLEGFATLMSMTVSYFTMIYSTR
>SinvOr195
MFLNREKSDFAENHYYKLNQIFLAAISLWPYQQSKFIIISHIFHFIIMGSFVFFQLTSFLTFQFTLDLFVMVL
SNAVPSCLYIVQYYSFCIQSNTIKRFWKDIRDSWNLLKNETERNIMRQQSFFGELCTILLSLSISISIILYAF
IELLPTILDLICPLNETRPRELHALAEYFIDKQTYFYPILCHWLICLAFGGFVFMATGTFEMVYIDNVCGLFK
IASYRIECSIKKYASYSSVQKKDYEAQRNIIAAVAIHTRALECSEFFSKSFTSYFFVLIVIGVSSTSLNLFRL
LRAIISHNMFEFISSILFIAFHFIFLFLGNYYGQKITDCNNEVFNSVYNVQWYKTSVKIQKCLLFIMQNTTIP
YVINIGNLVWASVEGFAKLVSTSMSYFTIIYTLL
>SinvOr196

MYMFDDHYHKLNQIFLRILGLWPYGKTKYDHFRATCFFIILVSHTFVQLAQLLIADFSIIVIIRILSEFLPIL
MCLVKFNMFFLETKKVKMTNNRRILTEAQEIEIIEHYANQGRIFTIIYTCNNVNFTYKKLLMTFILYILATIE
LFPSILDCFWPLNESRTHNMLFLTQYHISEGIQYYSCFLYFSISVSIGCFSVICITTMFTVAMLHCCAIFKIC
SYRIKQSVDEKIIMCSNKRNVIVKRIIRTIELHQNAKRLFKLCIPNFGIYFFIMVVVGTCSCVANLYRLLYSI
ICMDNFPEIMLALGYIITHEIYLVATTYMGQNLVNHADELFNAIYMSLWYKAPVTIQKLLLFMMQVASKSVVP
KVGGIYCSVMESFTTVMSTVISYMMVLYSV
>SinvOr197
MDVVGERYYKLNKNFLLCLGLWPCQTSTLKKTQIIFCQTLFISFLVCQWNAMFVRKYSINYIIKICMFITCNC
LYIVKYNAFLLLDDKIKYISSRIQYDWNMLKSQMEWEVIQKYADTAKLYTIYFIFLASISMPGMLILFSLPRI
LDVIAPMNESRPGQSIILVEYFVDEETYYFTILIHIVVTQYVGAMTIASVATILMAFVLHACAMFQIASHRIE
HIFDDLQQIPKDIKKYMLYNKLIHAVYVHRRAVDLTNIMTNTFATLYFILLGFGLASTSFSFLNFIYAVLSME
IIDIVLFSYIIFIHMYYIFLANYVGQNIIDNSIDFHRATYNAQWYVAPVYMQKLILFIMQRSNKRSALIAGGL
FDASLEGFATLMSMTVSYVMVLQSV
>SinvOr198
MDFVGERYYKLNKILLLCLGLWPCQTSTLKKTQIIFFQTLFISFLVCQWNSMLVQKYSIEFIIKITMFITINC
IFIVKYNAFLLLDNKIKYIFDRIQYDWNMLKAQVELEMIRKYANTAKLFTVCFIILVTTIIPGLLVILSVPRI
LDVIVPMNESRQRQSLIQLEYFIDEETYFFASLIYIILTISVGTITIAAIATIHMAYVLHACAMFQIASHRIE
HIFDNLQQIPKDIKKYMLCNKLIHAVYVHRRAVDFTKILTNTFATLYFVLLGLGVVSMSLSFLNFIYAVLSLK
IWDVLICSYSIFVHMYYIFVGNFIGQNVIDTSIDICRASYNAQWYVAPLYMQKLILFIMQRSNKRSALIAGGL
FDASLEGFATLMSMSVSYVMVLQSIGHKENYEKKNDHI
>SinvOr199
MNFDGQRYYRANKFFLEIMGLWPYNNSTYVYIYRAFTAFVLVSSGITQFSKLRSTYHDLDLLLYNLTYSVPCI
IYVSKFFSFIIKMDNVKEAMNIIQHDWNTLKDEEREIIRYYTIIGMRYTVTFFALSMPCLFCYTISFFVPGTL
DLIYPLNESRSRKLPILAEYFILDEQKYFYPILVHQLLVIAIGITIVLSTETLSMMYLHHTFGLFDVARYHLK
HVFDNETLSVTKNEKYAILHARIVKVINIHRKTIEFADYLLSTFYVTYFIMLVTGIIAITINTFRLIQAVLAM
NDFKELIGSVSFIGGQYGFLFFVNFFGQKISDRSFLMSEQVYDVPWYTAPVEIQKLFVFILQKTIKGYELGIN
GVIVASLENFASLASMTLSYLTMIYSMR
>SinvOr201
MEFPEDRYYGINRILLSAIGLWPYDNFKSRRFRLIFSLLIMVSLICAQWIKLFTSEYSPDLVLKILSVNLILI
FCPIKCITFYAVFENIKEFRERVRNNWDALKDNREDEIICKQGRYGKLFTIFIISKLSIYSFSIFYIFTQYLA
VLLNIIIPLNVSRPKKLMFPAEYFIDHEKYFYIISIYLTFALIIGMSCIIATESFSLTNALHAFGLFKIASYR
IKNVLSGINPHMCLTKRYAISHNRIIAAVDFHRRAIEFSELLKASFGQAYLGLFVMCVCSASINLFNLSRIIT
MEKEMLEIIKSIYLIIVHIIYITLANFAGQEFINNDTQFYRTVCNTEWYNAPVKTQKLIFFLIQKTTKCYKVD
AGGMFRPCLEGLATSLSMTVSYFMVLHSV
>SinvOr202
MEFPEDRYYRINRILLSAIGLWPYDNFKSRRFRFIFSLLIMVSLLTTQWIKLYTSKFSSDIVLKILSYNIILI
ICFVKYITFYAVFDNIKEFQERVRYNWDILKDKQEIEIISKHGSYGKLFTIFLTMSAYTFCIFHMFTQYLAVF
LNIIIPLNVSRPRKLMILTEYFIDPEKYFYIISIYLTIALTLSIVVWAATESFSLTNALHAFGLFKIASYRIK
NILSGINPHMCLTKRYIISHDRIIAAVDFHRRAIEFSDLLEASFGRVYLAILVIGVCSASINLCYLFRIITTE
KEILEIIKSILLIMIHIIYVTLANFAGQEFINYDTEFYCMVCSTKWYNAPIKTQKLILFLMQKTTKCYKVDAG
GMFSPCLEGLARSLSMIVSYFMVLYS
>SinvOr203
MNFNGDDYYKLNRIFLSTIGLWPHRYITLRQIQCVVSSFILISVTFPQLMKLITMKYDVDLILRVLSSALPFT
LFTVKYVTFYFVTKKVKELMQHIQNDWNTLKDNNELEIIHRYAKVAKLFTTSLATLIYVCIFAVICNQHVPSF
LDIIAPLNNSRQTELLFQVEYFLDQDRYYQTIQFHMDIGLIVAAMTILSTETFCLILAIHAFGLFKITSYRME
CIINKSAPNTFMEKHCAFQNNVVTAVNCHRRAIEFSENVKSTFAIPYLALILLGVSSSSINLYLLFQVIMSTS
TTDDLIRSIIYVFCHFVYMFSTNYAGQKFIDHDAEVYNKICNIQWYNTPLRTQKQILFIMQKTIKSYHVDVGG
LYNPSLQGFTALASASLSYFTVLCSVQ
>SinvOr204
RIQQVLLTILGLWPYSDVLYRRIQKVFVTILLLFSIFLQVAKIATSASDLESLLQLLSYAIPCLIVTVKYASF
SIKSEDFKKIIEQIHHDWNSLKCKEELKILRQRLNIARLMTMSLGGLFYIGVCLTLITQILPKFLDIVMPLNE
SRPLKLLGLATLFFDQDKYFFPILIHMTVALFVEASTIIATEGITAICLQHACSLFKITSFRIKHAFDCKTQI
SDLKKHKIYYTNIVNAIIIHNKAVEFFDYFNSCIEVSYFLLLLLGVVSLSLNLLRLFQAGLKKELEEFFVIGI
FVIVHFVYIFLCTFMAQILANDSADIFYDICQLPWYAASIPLQKLLQFIMQRSLKHCKFSIFIFIGSLELFTT
VVSTSLSYFTIFLSL
>SinvOr205
HGTCTEMTTADSYYTVNRYLLLIIGLWPYQNFGFRVIVITFLTIVLTSGVIYRFQFTTFVTKEYSTDLLLRIL
AYSMPWITHLLRYNILCLNAKKTQDLIERVRINWNELNNARELEIIKKYSAFGKLITLVSTWVINLGVFGVII

MTVLVNYVLNVPTSANESRQREFPAEIECFVDHQKYFAPLLVYMFFVVWSSLTVLAASETLFMSYTQHACGLF
EIVNCRIEQSLNRGTPQNLTSVAEKNSIICQGIISAIDIHKKAIEFVEMLKDNFKWVFFTALPLTVLSLSINL
YRLSRQVTSDEHHETITTLLFATGQFGYLFFCNYLGQEVIDHSGDIFHKTYNIQWYMAPLKAQKLLLLVMQRS
MQYCTITIGGLFIPSLEGFASLSSMSLSYFMVIYSIQ
>SinvOr206
VTFTPRLQNCPINRYYRINRILLLCVGLWPYQKSSFRYIAIAFMTIMLVSGVVFQLTTFITREYNIDILLRIL
AYSIPWLGYTLKYNVLCFNVRQIRNLMERVRCDWNELNNTQEIEIIKEYSAVGRFITLVITLFMYTCISCFIL
VQFFTNFLLNIISSKNESHQLPVLIECFIDEQKYFFPILLQLCVVAICGLTTVIAAETINTLYVQHVCGLFEI
ASYRIEQALHKDSVKSIASSTKRNSIIYQEIINGFNMYIKANEFANLLKISFKWPYSLLLPLGVLSLSINLYR
FSRLIDAKEYYEMILSFMFINGHFGYMLFGNYLGQKIIDHSSDIFYKTYNVQWYIAPLKAQKLLLLMMLRGMR
HCSFVVAGLFIPSFEGFATLVSMSISYFAIILSLF
>SinvOr207
LEWAIELHRYMLTIVGLWPQSSQQIKDEYKKFSAKFRMLLNVFILSFVLIIPTVAHLIKVWGDLLQTIENLQF
SLPFLVTELKIFIMWHKKEVLSMLINMVIKDWTRVKMDEERNVMLEKARIARLLLKLGVFVTALAVFIRLGSV
FFIEYIWHANNDTNHKRALPILTYYWYDLSSSPKYELAYLAQTIGCCLATLTYMGIDDFLALLILHICGQLEN
LHLRLLNLGKDPDFKAALKYNVKDHIRLIRSVEVIDNTFNLMLLSVIGFFCIFFSLQGFLALNANRDNHLPMS
LLLFYIFGSLCVLIHTCLYCVAGELLVIQNEKIHRVTCECIWYTLEPKEARNLTLIMVCAKKPLNITAGKIFP
MTMSTFCNILKTSAGYISMLLAVRN
>SinvOr208
XISLHRNMLQIIGLWPQQTEDQQDKFFLTFRMLLNVFIITSIVTVPALVQLMKVWGDMLQIIDNLQYTLPFLV
IELKIFVIWYKRRVLSLLINMIIKDWLKVKIDQERNVMLKNARITRLLIKSGIFISVFAGLSRISSTFFRQYF
EHMRNLTNPEGSFPIPTYYWFDVTSSPKYELIYLAQIIGMIAGGLTYVAVDNFLGLLILHVCGQMENLHLRLM
NLRKDPKFRTVLKYNIKEHIRLISRSVEAIDSTFNLLLLAMIGFFSILFSLHGFLIINAVNRDSNLSITQLAF
YISAGVTVLMNTCLYCAAGELLVIQNEKIHSATWECIWYILEPKATRNLILIMLCAKKPLNITAGKIFPMTMS
TYCNLLKTSAGYMSMLLALRN
>SinvOr209
LRWAIGLHRYMLKIIGLWPQQIKDQHKEYFSIFRMLLNIIIITFVMTVPALVQLIKVWGDMLQIIDNLQYTLP
FLVTELKIFVMWYKKGVLSLLINMIVKDWLGVKIDQERNVMLKHARITRLLIKSGIFIAVFAGLSSMSSAFFK
QFFGHIRNLTNPERSLPMPTYYWYDVYSSPKYELTYLAQIIGMIASGITYIAVDNFLGLLILHVCGQMEILHL
RLMNLGKNPDFRAILKYNIKEHIRLISRSVEAIDSTFNLLLLAMIGFFSILFSLHGFLIINAVSRDNHLSITQ
LVFYISSSVSFLMHTCLYCAVGELLVIQNEKIHSATWECIWYTLKPKAAKNLILIMLCAKKPLNITAGKIFPM
TMSTFCSLLKTSMGYVSMLLAVRN
>SinvOr210
LEWAIGLHRCILEIVGLWPQQNKDQYKEFFSKFRKLFNATAIIYILIIPGLAQLINVWGDMIQIIDNLQFTLT
FLVTILKVYIMWYKKGALLLLINKIIKDWTRVKMDEERNVMVKKAKITRLILQIGLFITISATFSRPGSIIFK
EYIWHVNNLTNHERSLPVPAHYWYDVTSSPKYELTYLAQTIGLVTCAITYVGVDNFLGLLILHVCGQMENLHL
RLLHLGNDSDFKAVLKYNVKDHICLIRSVKIIDDTFNLLLLAMIGFFCVLFSLFGFLVLTAVNQDTQLSFAQM
SWYFLASGTLLMHTCLYCAVGEHLVNKCEEVHSATYECVWYTLEPKATRNLAVIMVCAKKPLNITAGKMFPMT
MSTFCSLIKTSAGYLSMLLAIRN
>SinvOr211
LEWAIGLHRCMLKIIGLWPEQNRKQYKEFFSKFRMLFNATAIVFILIIPALAQLINVWGDMIQIIDNLQFTLP
FLVTILKVFIMWYKKADLLLLTNKIIKDWTRVKMDKERNVMLKKARITRLILKVGVFVTVLAVFSKLSSVFFK
EYIWHVNNLTNHERSLPFPTYNWYDVTSSPKYELTYLAQTIGAIAVAVTYVAVDNFLGLLILHICGQMENLNL
RLLNLGKHPDFKAVLKYNIKDHIRLIRSVEIIDDSFNLLLLAVICYFSILFSLFGFLVLTAFNQDTQLSIAQM
SWYFFAGGSLLMHTCLYCAVGEHLVNKSEEVHSATYECEWYTLEPKAVRNLAVIMVCAKKPLNITAGKMFPMT
MSTFCSLLKTSAGYLSMLLAVRN
>SinvOr212
LEWAIGLHRCMLKIVGLWPQQNKDQYKDYFSKFRMLFNATIIICVLTIPALAHLINVWGDMIQIIDNLQLTLP
FLITTFKIFIMWYKKGVLLLLINKIMKDWTRVKMDEERNVMLKNSKITRLILQIGLFFTVLAAFSRPGSIFFK
EYIWHVNNLTNHERPLPVPTHYWYDVNSSPNYELTYLAQAIGLYACAITYIAVDNFLGLLILHICGQMENLHL
RLLHLGKDPDFKAVLKYNVKDHIRLIRSVEIIDDTFNLLLLAMIGFFCILFSLFGFLALTAVNQDTQLSFAQL
SWYFFAGGSLLMHTCLYCAVGEHLVNQCEKVHSATYECIWYTLKPKVARNLAVIMVCGKKPLNITAGKMFPMT
MSTFCSLLKTSAGYLSMLLAVRN
>SinvOr214
LEWAIGLHRCMLKIIGLWPQQSRDQYKEFFSKFRMLFNATAIVFILTIPALAQLINVWGDMIQIIDNLQFTLA
FLVTILKVFIMWYKKEDLLLLTNKIMKDWTRVKMDEERKVMLKKARITRLILKVGVFLSVLALLSKLSSAFLK
EYIWRVNNFTNHERSLPLPTYNWYDVTSSPKYELTYLAQAIGLFTGSFTYIAVDNFLGLLILHVCGQMENLHQ
RLLHLGKNPDFKAVLKYNVKDHISLIRSVEIIDDTFNLLLLAVIGFFCILFSLFGFLALSAVNQDTQLSIAQM

SWYFFAGGSLLMHTCLYCAVGEHLVNQSEKVHSATYECIWYTLKPKVARNLAVIMVCGKKPLNITAGKMFPMT
MSTFCSLLKTSAGYLSMLLAIRN
>SinvOr215
LEWAIGLHRCMLEIVGLWPQQNKDQYKEFFSKLRMLFNAMIITCMLTIPALAQLINVWGDMIQIIDNLQLTIS
FLITTLKIFIMWYKKTALLLLINKIMKDWTRVKMDEERNVMLKKARITRLILKVGAFVTALALLSKLSSVFFK
EYIWRVNNLTNHERSLSVPTHYWYDVTSSPKYELTYLAQAIGLFACGITYVGVDNFFGLLILHVCGQMENLHQ
RLLHLRKDPDFKAVLKYNVKDHIRLIRSVEIIDDTFNLLLLAAIGFFVILFSLFGFLALTAVNLDTQLSFAQM
SWYFFAGGALLIHTCLYCAVGEHLVTQCEKIHSATYECVWYTLEPKAARNLTVIMVCAKKRLNITAGKMFPMT
MSTFCSLLKTSAGYISMLLAVRN
>SinvOr216
LEWAIGLHRCMLKIVGLWPQQSRDQYEKFFSKFRMLFNATAVVFILIIPALAQLINVWGDMIQIIDNLQITLP
FLVTILKVFIMWYKKKDLLVLTNKIMKDWTRVKMDEERNVMVKKAKITRLILKVGIFVTALALLSKLSSEFFK
EYIWRVNNLTNYERSLPLPTYYWYDVTSSPKYELTYLAQAIGLFTGGITYIAVDNFLGLLILHVCGQMENLHL
RLLHFGKDPDFKAVLKYNVKDHIRLIRSVEIIDDSFNLLLLAMIGFFCILFSLFGFLALTSFNQDTQLSIAQM
SWYFFAGGSLLMHTCLYCAVGEHLVNKCEEIHSATYECVWYTLEPKTTRNLAVIMVCTKKPLNITAGKIFPMT
MSTFCSLLKTSAGYLSMLLAVRN
>SinvOr217
LEWAIGLHRCMLKIVGLWPQQSRDQYKEFFSNFRMLFNATAIVFILIIPGLAQLINLWGDIILIIDNLQFTLP
FLVTILKIFIMWYKKGDLLLLTNKIMKDWTRVKMDKERNVMLKKARITRLILKVGVFISVLTLLSILSSAFLK
EYIWRVNNLTNHEKSLPFPTYNWYDVTSSPKYELTFLAQAIVLFTCAITYIGVDNFFGLLILHVCGQMENLHV
RLLHLRKDPDFKAVLKYNVKDHIRLIRSVEIIDDTFNLLLLAIIGYFCILFSLFGFLVLTAFNQDTQLSIAQM
SWYFLGSVTLLMHTCLYCAVGEHLVNKCEEVHSATYECVWYTLEPKTARNLAIIMVCAKKPLNITAGKIFPMT
MLTFCSLLKTSAGYLSMLLAVRN
>SinvOr218
LEWAIGLHRCMLKIVGLWPQQSRDQYKEFFSNFRMLFNATAMVFILIIPALAQLINVWGDMIQIIDNLQITLP
FLVTVLKIFIMWFKKRDLLLLTNKIMKDWTRVKMEEERNVMLKKAKITRLILKVGVFVTALALLSKLSSVFFK
EYIWHVNNLTNHERSLPFPTYNWYDVASSPKYELTYLAQTIGATAVAVTYVAVDNFLGLLILHICGQMENLNL
RLLHLGKDPDFKAVLKYNIKDHIRLIRSVEIIDDTFNLLLLAIIGYFCILFSLFGFLILTAFNQDTQLSIAQM
SWYFLGSVTLLMHTCLYCAVGEHLVNKCEEVHSTTYECVWYTLEPKAARNLVVIMVCAKKPLNITAGKIFPMT
MSTFCSLLKSSAGYLSMLLA
>SinvOr219
LEWAIGLHRCMLKIIGLWPQQSKNQHKEFFSKFRMLFNATAIVLILTIPALAQLIKVWGDMIQIIDNLQFTLP
ILVATLKVFIMWYKKRALLLLTNMIIKDWSRVKMDEERNVMLKNARITRLFAKSGILITVLAALSRISSVFFK
QYFGHVRNLTNLERSLPIPAYYWYDIFSSPKYELTYFTQIIGMAGCGLTYIGVDNFFGLLILHVCGQMENLHL
RLLHLGKDPDFKAVLKYNVKDHIRLIRSVEIIDDAFNLLLLGMIGYFCILFSLFGFLILTTVNQDTQLSIAQM
SWYFSAGVSLLMHTCLYCAVGEHLVNQCEKIHSATYECIWYTLEPKAARNLAVIMVCAKKPLNITAGKIFPMT
MSTFCSLLKTSAGYLSMLLAVRN
>SinvOr220
LEWAIGLHRSVLNMIGLWPQQNKDQHDEFFSKFRMLFNTTLIIFVLIIPALAQLIKIWGDMIQIIDNLQFTLP
FLLSILKIFIMWYKKKVLSLLINIIIKDWMRVKMNKERNVMLRNARITRILIKGGIFVAIFAAFIRMSPTFFK
QYFGHVKNLTNLEKSLPIPTHYWYDISNSPIYELTYLAQTISVFTCALTYVVIDNFLGLLILHVCGQMESLHL
RLLNLGKDPDFRAVLKYNVKDHIRLIRFVEAIDDTFNLLLLGIIGFSCILISLLGFLMVNAINQDSHLSIPKI
TWYFSAIASMLLQSGLYCTVGELLVNQSEKIHRTTYECIWYTLNLKTAKDLTLIMLCAKRQLNITAGKIFPMT
MTTFCSILKTSMGYISMLLAVRN
>SinvOr221
LEWAIGLNRRMLKIVGLWPEESKNHREELLSKLRFLVNVMTLIFILIIPALVSLIRVWGDMILMIDNLQFTLP
LLITALKVFIMWYKKGALSPLINMIVKDWMRIKIEEERNIMLKQARIIRLLAMCGVLMIISTILISICCFLFG
WTLRHVTNLTDPMGKPLSIQSYYLHDVSSSPNYELTYAIQVIGQSVSGLTYTAVDNFLGLLVLHICGQMENLH
LRLLNLGKDSKFKAVLKYNIIDHIRLIRSIEIIDDTFNLMLLGLLFLFGILFCLHGFLIINVVNRGGHLPIGQ
LSFYVAATLCVLMHMCLYCAVGEFLVTQSERIHWATYEYMWYTLNPKVARNLILIMLRSKKSLNITAGKIFPM
TMAMFCNLLKTSAGYVSVLLAHRN
>SinvOr222
LKWAIGLNRCLLKIVGLWPEEIKDKREELLSNLGFLFNVITLIFVLTIPALVSLMRVWGDMILMIDNLQYTLP
LVITTLKVFIMWYKKGALSPLINMIVKDWMRAKIKEERNVMLKQARIVRLLAICGGIMILFTLLVTVCSFLFG
RTLRYVTNFTDPFGKPLPIQTYYLHDVSTSPKYEITYLIQIISLTISGVSYTAVDNFLGLLILHVCGQMENLH
LRLLNLGKNQNFQALLKYNVNDHIRLIRSIHVIDNTFNLMLLGLLFFFGILFCLHGFLIINVVNQGGHLSLMQ
FILYISASVAVLMHTCLYCAVGELLTTQSEKIHRATYEYVWYTLKPKAAKNLTLIMLRAKKPLTITAGKTFPM
TMSTFCNLLKTSAGYVSVLLANRD

>SinvOr223
MNTSEIQHFSLNRILLLSIGLWPNKQSKLVLFQQILFFGLLISFTVYQIILYFNTETTDDFITILSFILFYIV
FIIEYNSFLINGQVVKNLLEQFIYVYNELKDENEIDVMKRYISKTKRYIATIPYISTFVSTVGIQRAVYKNVF
KIYIPNNIKYPTMIVHTQGFVKYYFINYKNITLRININRYCMSIWTTYIKNYNTVLILSVRTRENVFTIYYYR
IERAMTINNLYKNFEHETEIYKGIINAVNIHRKTMKICNYFMSSFNGTWSVLIVVGVLSLSLNIYQVFHCVLF
GRNKQEFILHFVIVAACLLYMFLTNYFGQEIIDHNNHIFITVYNGDWYIAPLHVQRLILFLLQVGNKTFGLRV
GGLFTGSLNTFASLMNASMSYFTFMYSTQK
>SinvOr224
MNTSDIQHFNLNRILLLSIGLWPYNQSKLVLFQQILLFGLLISFVIYQLIIFFITEFTIGFTITVFSFILFYI
AFIIEYNSFWSNGQAVKNLLEQFMHVCNELKDENEINVMKKYASNTKRYTTGMILIVLAALPITIAASIWPYI
LNIVKPKNESRSDFMIQLLTTHFADPDKYRYLMLLYMHITICIGGITKIATGTMLIGYLKYACGMFRIASYRI
ERAMAINNLHGNLENKNVTYKGIIYAVVVHRKAMELCKSLMFSFEGTFFALIAVDVLNLSLNLYRVFQSVSVG
DSKEETIIHVIIVLITLLYMFLANYAGQEITDHNNHLFSTAYNVHWYIAPLHVQKLILFLLQMGHKTFGLNVN
GLFTASLNCFASLANASISYFTVLYSTQK
>SinvOr225
MICTNSLHIGLNKCLLLIVGLWPYQQSKLIQLQLTLFISVLMTFIWVQFTVFFTSECTSQLLINVLGSALFYV
TLAIKYSSFSVKLEVVKYMLDQLQHMYNELTDENEINIIKQYAIYAKRYTIIFTLLVALMILIFILHSTWLSI
CDIVLPINETRTHLSSLATEYFVDHERYFYLIFFHTNAAFIIGLLTMLATGTMFIVYLHYACGMFQITCYRIA
EAITHETLQKNNLQNENLIYKELICAVDMHRRALKFSDLIISKFKLMFSLLILVGVICGTFNVFRVFQLTSSE
YDIEELSLRLIFLIVHFSYMFVGNYLAQEIIDRNNDVFVTAYNVQWYIAPLQIQKIILFLLQRGNKEYTINIA
GLFVASLEGAASLISTGLSILTVLYSTRS
>SinvOr226
MICVDCLQISLNRLLLLAIGLWPYQQSVLVRLQFFLLFGILMTFILFQFTALLTSKCTPDLLIEVLSSALFCV
FLAIKYFSFNINTEDIKCLLEQLQHMYDELIDKNEIDIIKRHASFAKRYTIGLLLTAIFFTFSLILYLFWPYI
FNLLFATNVTQSRSMMFITEYFIDQEKYYYLILFHAIAAVAVGGTALLATGAMFITYLKYVCGMFQIVSYRIT
RVITFKNEKNNLQNENLICKGIIRAVDMHRKAMQFCDTLISRFNVSYFFFIAFGVISESLNLFQIFQIISFKC
DIMKLMLHVVFFLIELIYLIIGSNFAQEIIDYNDDVAVYGVPWYVAPIKIQKMIIFLLQRRTKAFHIQIGGIF
VASLEGATTLLSATVSYFTVLYSTR
>SinvOr227
MICIKSRYFHLNRIFLLALGLWPYSHQSNFVRFQIILCFSFLASSIIVQLTVFFTTECTLYLIIKVSSTAMYC
STFVINYNSFWINAHNVKTLMERLQEICNELKDENEINIMKDYGNSVKFYTFVLTMLTAISTCTIVSVSLFMP
IVLHMNESQTRQLIYFTSEYFIDQEKYAYLILIHSNIVLCIGITTVSTIGTMLRGCCIHACGLFKVASYRIEH
ALAMKMFKNINIENEIIYKEIICAVDIHRKAIKYSEFLLSSFKGTLFSVMVIHVICLSLNLFAVFQTATLGKK
EEFSYHCLIVIVILLYMFLANYTGQEIIDHNNDIYIAAYNARWYVAPVHIQKLILFLLQRGGKPFNLSVASIF
VASLECFATLLKASMSYFTFICSAQQ
>SinvOr228
MICSLHIGLNRFLLLTVGLWPYQQSKLVQFQLILFFSILTTFISFQFTTFMTSQCTPDLVINVLSSILFFFTF
MIKYSCFSINTEVIKYLLEQLQHICNELTDENEINIIKQYASNAKRYTATFMLLAIVIASLLLLYPIWPCIFD
ILLPINETRPRPSPLFATEYFVDQERYFYLIILHANAAFVIGGVAMLATATIIIFYLQHACGMFRIASYRFKR
AITTETLQKNSLKHKNLIYKELIYAVDMHRKAMKFSDSTISKFKVMFFLLIVAGVICASMNFFRMFHVMYFEY
NTSEFFLRLIYIVVHMSYLFAGNYLAQEVIDHNNDIFVTVYNVQWYVAPLQVQKMILFLLQRGTKVFNLNIAG
LFVGSLEGAATLFSSIISYFTILYSTRN
>SinvOr230
MICADSLHVGLNRFLLLTIGLWPYQQSKLVRLQVILCIGILASFILVQLTVFVTLKYTLDLLINVLSSALLYT
MFAIKYISFSINTDIVKYLLEQLQDICNELTDENEINIIKKYSSYAKRYTLLLLLFAISCISILTLYSLFPQI
FNILLFTNATRLYSLPLMTEYFINQEKHFYLILCHANAATGIGTTIMLGTGTMFIFYQQHACGMFQIASYRIK
QAITFDTLEKNSLEKENLIYKRLICAVDMHRKAMNFSDLIISRFKVMFSLLIIAGVISMTLNMFRIFQMVSFG
YNGDIIQLLTPILYAMIILMYIMTANYFAQEIIDHNNNLFVTVYKVQWYIAPLHIQKLILFLLQRGTKAFNMN
IAGLFVGSLQGAATLISTILSYFTVLHS
>SinvOr232
MICTDSLNISLNRFLLLIVGLWPYQQNIFVWLQLILLIGILTSFILVQFTVFLTSKCTPVLFINVLSSALYYI
LYVIKYIFFSINTNNARCLLEQMQLIYNELTDENEINILKKYSNYTKYVTVVFSLCLMCIAPVSIVYLFWPYI
FNILFSTNGTQSCSSLPFITEYFVDQQKYSCLISFHANAATCIGAIAVLAIGSIFLVYIYHACGMFCIASYRI
KSAMPYETLRKNSLEYENLIYNRLIYAVDMHRKAMKFCNMIISRFQVMFCCMIVIGVISASLNMFQIFQMISH
EYDIVELMLHSFFVNVHFLYLILGNYFAQEIIDHNNCVFVTAYNVQWYVAPLQIQKMILFLLQRGNKTFNLTI
AGLFVGSLEGAATLFSSIISYFTVLYSTR
>SinvOr233

MVSLETERFRFNRLLLLAIGLWPYQESKLAKVQFLILFSILITFIVFQFTTFITSECTTNFIIKVLSSAFFFI
CLAIEYNSFWINADIVKLSLDQIQHTYNKLKSKNEIAILTSYGNIAQLYTAVLTIIVAWSVFVFLILQMWPFI
IGVIMTMNNSGLYPRIYITTEYFVNQENCYYFTLLHLNAAMCIGVTAIIATGTMMLMYFKHICGMFNIASFRI
EKAMMTSVLQNITQEKEILIYKGIIYAIDIHRKATEFSQFFIKNFEGSFFFLIAAGMVSLSSSFVEVASSNNI
EELILPTLIIFTLYVYLVISNYTAQEVTDHNNRVFATVYNVQWYIAPLRIQKMLIFLLQRGNKAFNLNLGGLF
VGSLESAAMLTSASISYFSVLYSTR
>SinvOr234
MVRLEIERFRLNRLLLLAIGLWPFQKSKFAQVQFPVFLIILITFIVFQFTTFVTSDCTTDFIIKIISFTTFFI
FLTVKYNSFWINADTVKLSLEQILYTYNELKNKNEIDILENYGKVAQLYTAILTIISLCLISVSFILQMWPLI
IGVILSMNNSRLHPRNYITTEYFVNQEDYHFLIFLHLNAAICIGITAILAVGTMFLTYFWYICGMFNIASLRI
EKAMMTSILQNITQENEILIYKGIIYAIDIHRKATEFSQFFIKSFEGSFFFLIAFGIVSLSSSFVEIASVNNV
EEFLQFSIIIVTVYVYLVLANYTAQEIIDHNERVFATVYNVQWYTAPLHIQKILIFLLLRGNKAFNLNLGGLF
VGSLESAAMV
>SinvOr235
MVRLEIERFRLNRLLLLAIGLWPFQKSKFAQVQFPVFLIILITFIVFQFTTFVTSDCTTDFIIKILSFAFFFI
FLTIKYNSFWINADAVKLSLDQILHTYNELKNKNEITVLENYGKVAQLYTAILTMFGVFYWCVFLTLQMWPFI
IAVILPMNNSQLHPRIYITTEYFVNQEDYYFLIFLHLNVAICIGLTAIIGVGTMFLTYFWYICGMFNIASLRI
EKAMMTSILQNITQENEILIYKGMIYAIDIHRKATQFSQHFLKIFEGPFFFIIASGMISFSCTLVEIVSINNF
EEFIPFSIIILTVFLYMIMANYTAQEVMDHNNRVFATAYNVQWYIAPLRIQKMLIFLLLRGNKAFNLNLGGLF
VGSLESAAMLTSASITYFTVLYS
>SinvOr236
MVRLEIERFRFNRLLLLAIGLWPFQKSKFAQVQFPVFLIILITFIVFQFTTFVTSDCTTEFIIKILSFACFFI
LLTIKYNSFWINADVVKFSLEQILHTYKELKNKNEIDILENYGKVAQLYTAVLTIFGVCYMSVFYILQMWPFI
IGVILSMNNSRLHPRIYIRTEYFVNQEDYYFLIFLHLNAAMWIGVTAILAVGTMLLTYFWYICGMFNIASFRI
EKAMMTSILQNITQENEILIYKGIIYAIDIHRKATEFSQYFIKSFEGSFFCIIATVMVSLSCTLVEIASVNNI
EEFLPFSIIIVTVYVYMVLGNYTAQEVIDHNKRVFATVYNVQWYIAPLRIQKMLIFLLLRGNKAFNLNLGGLF
VGSLESAAMLSSASVTYFTVLYSIQ
>SinvOr237
MVRLEIERFRFNRLLLLAIGLWPFQKSKFAQVQFPVFLTILITFIVFQFTTFVTSDCTTNFIIKILSFAFFFI
FVTVKYNSFWINADVVKLSLEQILHTYKELKNKNEIDILENYGKVAQIYTAILTIIGVCFMSVLFISQMWPLI
IGVMLSMNNSRLHPRIYIATEYFVNQEDYYFLIFLHLNASMWIGVTAILAVGTMFLTYFWYICGMFNIASLRI
EKAMMTSILQNVTQENKILIYKGIIYAIDIHRKATQFSQYFIKSFEGSFFFIIASGMASASCSLVEIASIKNI
EEFVLFSVIIFILYLYMAMANYTAQEVMDHNNRVFATAYNVKWYIAPLRIQKMLIFLLLRGNKAFNLNLGGLF
VGSLESAAMV
>SinvOr238
MVPLELKRFRFNRFLLLAIGLWPYQKPKFAQFQFLVLFSILITFIIFQFTTFLTSDCTIDFIIKILSYAFFFI
VLAIHYNSFWINTETVKLSLEQLQHTYTELKNKNEIAILQNYSNKGQLYTVILMIISVTGTVVFIILQMWPLV
VGVILSMNNSRLYPRIYIKTEYFVNQENYCYLIFLHSNVAICIGVTALIATGTMMLMYIKHICGMLSIASFRF
EKAMMTSILQNITQEKEILIYKGIIYAIDIHRKATQFSQYFVKSFEGSFFLIIATGMVSFTCSLVEIVSVNNI
EGIMPFSIIVFSLYTYMVIGNYTAQEVTDHNNRVFATVYNVQWYIAPLRIQKMLLFLLKRGNKTFNLNLGGLF
VGSLESIAMLTSASITYFTVLYS
>SinvOr239
MICLETRHFNINRILLLIVGLWPYQRTPFVQFQLLLFFGTLTTFIIFQFTTFLTSKCTPELILKVFSTTFFFI
CLAIKYNSFWINGDIVRSLLEQLQEICNELKDENEIAIIEKYGSKAKYYTTVLILFGSFGEFTAIFIQIWPYI
QYFVLPNNGSRVNHSLSIVTEYFIDQEKYFYLSVLHGIAACHVGAIAMVATGSMLIAYLQHACGMFSIASYRI
KHAVNILQKDVKSESKVSKEIIYAVHIHRKAMEVTDFMISNFEGSFFFLIAVGISCLSLNLLRVASYSGNIEQ
IVLPLLIVTILYVYLFLSNYTAQEIIDHNEYVFATVYNVEWYVAPIHIQKMILFLLQRGTKAFYMLLGGIFVA
SLESAAALMSTSISYFTLLYSSKKD
>SinvOr240
MICAKDKYFSLNRILLLTVGLWPHTKSKFLQLQLVLQYGILGSFIVFQLTAFATSKCDVLLIIEVLSVTAFYF
NFVLTYLSFNCNMDVVKHLLEHFQHTYDELRDEYEVAIIEKYWGFSKRFTVGLTLLVIFNMICFLLNPFVPYI
FDIALSANKSRPHPSVHIITEYFIDQEKYFYLIIILHADVAFCVGALAMLATGTTSLAFFQHACGMFKIAGYRM
EKAITIGIRQMNGTRNATFIYKGIIVAVDMHRKAIKIVEIFAASFNVTFSLLIFDGILYASLNLYRTFREMSS
GDISEKLLQSITFIGVYYVYTFLANYGAQQVTDHSNSMFITVYNVQWYLAPLHTQKLILFMLQRGTKILNIKI
GGLFVGSLEGFSTLASATMSYFTFIYYTN
>SinvOr241
MVYMKNKYFSLNRILLLTVGLWPYTKSKFVRLQLGLQYGILGSFIVFQLTTFATSKCDVPLVIEIFSVTAFYF
NFVLIYLSFSCNMNVVKHLLEHFQHTYDELRDEYEVAIIEKYWGFSKRFTKGIMLFIIFNVSCFLLIPFVPYM

IDIVLSANKSRPHPSVHIITKYFIDQEKYFYLIILHADVAYCVGALAMLATGTTSLAFLQHACGMFKIAGYRM
EKAITIGIRQMNGTRNATFIYKGIIVAIDMHRKAIKFVEIYAASFNVTFALLIFGGILYASLNLYRTFREMSS
GDISEKLLQPITFVGVYYTYIFIANCAAQQITDHNNSLFVTVYNAEWYLTPLYIQKLILFMLQRGTKALNIKI
GGLFVGSLEGFSTLTSATMSYFTFIYYTN
>SinvOr242
MIYIETEHFTLNRTLLLVIGLWPYQQSKFIFRLKFIFLFSILTTIILFQFTALASSKCTSDLIKIVCTILFFT
TFMIKYIAFVINTNNMKDLLIQLQHICNELKDESENAIIKKYGQNAKRYTVALIILGTGSLFILIIVQFWTNI
VDIVLPKNESRSRHVLIMTEYFIDQEKYFYLIVLHVHTAIWIGAFIIVSTGSMLIAYIQYMCGLFRISSYRFE
RAMGFNMLRNITCKNENLIFKGVICAVDIHRQAMRLSELIVCKFETMLFCLITVGVTCLSLNLFRMFQVASSG
ENIKEILLPFIFSVCSVLYMFIANYIGQDIINYNNRIYIAAYNVQWYMAPLHVQKLILFVLQRSAKEFSLGVG
GLFIGSLECFATLVKASVSYFTFIYSAQ
>SinvOr243
QYYSINRFQLLLVGLWPYQQSTFTRFQFICFSLILTTGIIFQLTPLIILKCTSNLVAKVLSSVSFYAVFVIKY
NAFHFKIEVMKNLLMELQCVFNELKDEHEVAIVEEYTCIGKRYTIGLAAFGFCGLSVSILAQFWSKIFAFVIL
SKNVSQPYHLHVVTEYFIDQEKYFYLIVFHMYLVFSIGGTVLIAIGAMLITYLQHTCGMFRIASYRIKHAMSF
DILQNITLKNKNLMTEGIIYAVEIHRQAMKLCKLLVSSFETMMFSLISCGVLVLSLNLFQIAVIENSFVELFF
PSFFVLATVLYMFIANYMGQIVTDHNNHIFSTAYNVQWYRAPIHIQRMILFLLQRETREFTINIGGLFNASME
CFATLVKTSVSYFTVIHSTR
>SinvOr244
YYSINRFQLLLVGLWPYQQSTFTRFQFIFIFFMLMTGIIFQLTPLIILKCTSKLVAKILSSVSFYTVFVIKHN
AFHLKIEVMKTLLMELQYVFNKLKDEHEIAIVEKYSCIGKRFTIGLTAFAFCGLAIGTLTQFWSEILTLVVVS
INVSQPNNLLIMTEYFIDQEKYFYLILFHIHSVFCIGCYIVVAVGSMVITYFHHTCGMFTIASYRIEHAVNLD
ILQNITLKDKILMTEGIINAIDIHRQAMKLCKHMISALETMMFSLIVCGVLVLSLNLFQIAMIENSLVELFFP
SFFVLSIVLYMFIANYIGQIVTDHNNHIFSTAYNVQWYRVPIHVQRMILFLLQRETREFALNVGGLFNASMEC
FAMLVKASVSYFTVIYST
>SinvOr246
THYYNLNRILLLLIGLWPYQQSIFTRFQFIFFSVILTAGIVFQLTPLIVLKCTSDHLVKVLSSVSFYTIFAIK
YISFRFNIKVVKNLLMDLQHAFNKLKDENEVAIVEKYSFIGKRYTIVLIAFGICGISICILAQFWSKIIASFV
VSMNVSSQSYQLLIKTEYFIDQEKYFYLIVLHIYAMICIGAIAMVSIGTMLITYLQHICGMFRIASYRIEHAI
SINILQNITLKNKILMTKGIAYGVDIHRQAMKLSKQMLSTFEIMFFSLIVCGVSCLSVNLFQMASSKNTFEEL
CLPMVYVFVTVLYMFVSNYVGQDVVDHNNSIFSTAYNVQWYKAPIHVQKMILFLLQRETKEFTLNVGRLFNAS
MECFATLVKTSISYFTVIYS
>SinvOr247
MIRPVIQYCRLNKILLLIIGLWPYQQSIFTRFQFIFLSFILTASIIFQLTLLVILKCTSNLVLKVLSSVSFYT
MFVITYNSFHFNMEDMENLLTELLHVFNNLKDENEIAIFKKYNFIAKCYTFGLTAFGIFSIFIYILIEYWWNI
VDTPMNVSVPRHLLIITGDFVEEKYFSVITLYMYTVLCISLIIMLATGTMMLTYLYYICGMFKIASYRIEHAI
NINTLQNITLKSKTLMTQGVIYAVDIHRQAIKLSKRFISILNTMILSFTGCVVICFSLNLFQVKLSGNNINEF
CLPFLYATISMIYIFLANYIGQNVFDHNIHVFVTVYNVQWYKAPMHIQKMILFLLRRGTKKFTLNVGGLLDGT
IENFATLTKASISYFTVIYS
>SinvOr248
YHKLNRILLLIIGLWPYQQSTFTRLQFIFLFLILTAGIIFQVKKKYHNYLVVKVLSSVSFYTLSIIKYTSFGL
NIEGVKNLQTDLQYVLNDLKDKNEIAIFKRYNFIAKRWTFMLTDKTILIGFNIIEHIFARNYVFVDTSMNVSK
PHHLLMTEYFIEEKYFYVIMLYMYTSFCISIIIMVATGTIMLTILYHVCGMFKIASYRIENININILQNVTLK
SKTLMTEGVIYAVDIHRQAIKMSKRFTSIFNELILGFTGCVIICFSLNLFQVKLSGNNINKFCLPFLYATISI
IYIFLANYIGQNIFDDNIHVFVTAYNIQWYKAPLHIQKMILFLLRRGTKKFTLNVGGLLDGTIENFAALTKAS
ISYFTVIYST
>SinvOr249
SLNKVLLLAVGIWPYHQSKFTQFQFVFFSATLSASIILIPLIKFECTSDLVVKVMSSVSFCIVLIIKYILFHF
NIEAVKNLFTNLQHVLNDLKNENEIAILEKYNFIAKCYTFVLTALGVGVLFSCIIAQFWSDISNIILSLNTSQ
SRHILIPSEYFIDQEKYFYWIVLHISSTFCIGTIIMVGIGTILVTYIQHTCGIFRIASYRIEHAVNINILQNT
LKNKILMMESIIQAINIHRQAIKLSKRLVSTFDIMFFCFTGCLIACLALNLFQLLQVASSKNIIDLFLPFTYA
TTSFLYIFISNYIGQNVTDHNNHLFVTAYNIQWYRTPLHIQKMILFLLQSGTKKFTLKVGGMIHGSIENFAMV
IKTSISYFTVIYSTR
>SinvOr250
SQYCNLNKILLLALGFWPYKQSKLTRFHCNIFRVILSTGIVFQITPLITLKCTSELVIKVLSSVSLCTMFLIQ
YHSYYLNIEVVKNLLIQLEHIHNKLKDKNEIAIVYKYSCIGKRYTIALTAIGFCGILVLSTSQIWLNLNDTNL
SIMNVSGPHYFFVMEYFIDQKKYFYLILLHACAVLYIGGTVLLAIGTILITYIEHTCGMFKIASYRIEHAVNM
DLQNTTLKSKILMTEGIICAVHIHRQAIKLSKRILSTFEAMISCFTGCVVICFSLNLFQLFQITSFKNNVHEF

ILHFTYATISILHMFIANYIGQSVIDHNNHVYIAAYNVQWYRTPLHIQRMVLFLLQRGAIQHTLNIGGLFDAS
IEGFATLIRASISYFTLINSVQ
>SinvOr251
SQYCNLNKILLLAIGLWPYKQSKLTRFHCIIFRVILLTGIIFQVTPLITLKCTSELVIKVLSSVSFCAMFVIQ
YHSYYLNIEVVKNLITQLEHVHNKLKDKNEIAIVHEYSCIGKRYTIALTAFGICGQFILTITQIWLNAVNTNL
SIMNISEPHYFFVMEYFIDQKKYFYLILLHTCAVLYIGGAIMLAIGTLLITYIEHTCGMFRIASYRIEHSVNL
DILQHTVGKCKSLMTEGIICAVHIHRQALKLSKRIVSTFETIISCLIGCVIICLSLNLFQLFQITSFKHNLNE
FVLHSTHLAAFYASVCILYMFIANYVGQNVIDHNNHVYCTAYNVQWYKTPLHIQRMILFLLQRGTLHYSLNIG
GLFDASIEGFATLIKASMSYFTLMNSIR
>SinvOr252
MTRISQYCININRILLIAVGLWPYKQSKLTRFQCIFFCVILSSGIIFQVTPLITLKCTMELVIKVLSSVSFCL
IFLIQYNTCCLKIELVKNLLMQLEHIHNKLTDKNEIAIVYKYSGIGQRYTVMFTALAIGGLFIITISQIWLIA
AVDSSINASESHYVFIMEYFIDQEKYFYLVLLHTYVVICVGVIVIVTTSTMVITYVEHTCGMFKIASYRIEHA
VHMSIQNTLQNKILMTKGIICAVDIHRQAMKMSEHFVALFEKCIFVLTGCSLMCFTLNLFQLSQTTSFTNNYN
KLFLPSMYAIISVLYMLIANYIGQSVIDHNNDLFVTVYNVQWYKSPLYVQRMILFLLQRGTKKFTLNICGLYD
GSIEWFATLMKAAVSYFTLMHSVQ
>SinvOr253
EQQYFNINRILLLMVGLWPYQQSIVTGLQHIFITATLIAIIIFQQLTTFLTLNYTLDLLMKVLSSASFFAFYT
IKYYAFYFNIKSVKELMMQLQCVHNKLKDKNEITIIKKYDCLAQRFTTIITTLGICGICSSITMQLWMNIDVN
TSTNVSRSFNFLITTEYFIDQNKYFYLIFLHINAAICVGTISIVAVGTINIAYVQHICGMFRIASYRIEYAIN
IRILHHVSLRDKIWMAKGVIYAVNIHRQAMILAKHFISTFQKMFACLIVCGVASCSLNLFQLSSFQNDVQKFL
FSFTISFMIIIYMFLTNLMGQKIIDHNNHVLITAYNVKWYKTPVHIQRMILFLLQIGAKEFTLNIGGLIHGSM
EGFAMLMKATISYFTVIHSIR
>SinvOr254
LLIVGLWPYQQSSFTTFQQIFVTTTFITFILFQQLTSFLTLSCTSEHLMKVFSSASFFGAVMIKYNSICFNIE
GMKDLLIQLQRVHDKIKDKNEIAINKKYHCIAERYVTGLIDYKICKVLCVMSSLFYAFSMVWIFLNLFTQTTA
VGLREYSTRQLLMAYWQNLNMLNTRICTLSSATDRYLNFLDIQCHYVDINICSYRIEYVINININILQNVTQKHK
IWMADNIGYAVDIHRQAMKLMKQLMSILETMMFCLIICGVAALSLNLFQIVLFRNNVENIIMPFVFATICMLY
MFLANLMGQNIIDHNHHVLNTAYNVQWYKSSLQMQKMILFLLQRGTKEFTLNVAGLFDASMECFATLVKASVS
YFTVIYS
>SinvOr255
MRVLKVTLQVCTFAGCWQPDSRTSLFKHIIYKAYAMFLLSALYLFAFSQFMNLILNVENSDEFTDSLYMMLTV
FIAGCKQVYMWIKRKNFMMIINVFNKKPFAVCESHELMIQQKFEKMAQNITLRYLTLIMVTVTSIVFVSIFTI
FTNRELTYKAWVPFNYSIPVLYVLVYAHQLIGMGTSGFVNVACDSVLCGLLLHICSQLEILEYRFTKMMHCED
ILRDCVCHHNYIFEYAHTVNNLFTKVIGMQFSVSLLVICSNLYRISMAPDFATFITVMLYTGCILAQIFIYCW
FGNEVKIKSLQLMNNIEWSALSNNSKKGLLIIMKRSMIPIEFTSAHLITINLESFVALLKMSYSVFNLLRQTQ
E
>SinvOr256
MRLMKFTLMICALAGCWQPLMWTSLFKHIIYKAYAIFMISSLHIFLFSQFVHMMLNVANSDEFTDALYMMLTI
LVAGYKQIYLWVDRKNVMAIINVLIEKPFAPYETHEIAIQEKFEKMIQNNTRRYLTIVVMSISSIILMSVSTD
FMKRNLTYKAWIPFDYSSPAIYFIVYIHQLIAMSTSGIVNVACESLLCGFLLHICCQFEILGYRLTKLTHDQN
SLRDCVCHHNRIFEYVYTVNNMFAKIIAIQFAVSMLVVCSNLYRIAMATDYMSFIPLMMYTSAILVQIFILCW
FGNEVKLKSLQLVNSIFDIEWPALSNSNKRNLLLIMKRAMTPVEFTSAYIITMNLDSFVALLKMSYSVFNLLH
QTNE
>SinvOr257
MIVLQFTLKLCTVSGCWQPLRWTTMSARIIYDSYRVLLICLISAFTMSQFINIALNIDNFNEISDNIYMMLTV
FIATYKLISMWISKKHVTTIINIFTEKPFKPLESSEVMIRQKYEKTIRQYAFWYYGLVQITVICIIINAFAMD
FMTGNLTYKAWVPFDYRPSVIFFFVFIHQLIGMIIAAAVNVACDSLVSGLLQEICCQLEILEYRLTKIFHDQN
VLHDCVRHHNRIYECAYMVNGKFAKIIAIQFAVSMLVVCANLYKLASISLAMIDGGLLTLILYTCCMLSQIFL
YCWFGNELKLKSIGLANSIYNMKWADLENKNKKDLLLIMRRSMVPIEFSSAVIITLNLDSFGSLLKASYSAYN
VLKRSQ
>SinvOr258
MHILKLPCKIFTISGCFRPQSWTSLFKRTVYNIYKLYTFTMIYTVSILQIMDIVLYVDNINDLTDNLNVMINS
LVSCYKIFIVSLSYENIVALINCLTEEPFKPLDSDEMKILQHYDEIIRKNTLRYTSFVTISAGFVFLSSLFTD
FRYKQLKFREWIPYDYSSNTMYCITYFQQMLSLSHCVIVNISTDNIMCGFIMHICCQIEILKYRLKKMLSKQL
TLDYCIRHHNQIFKFAQIVNLKFTKIIGFQFIASTMVICCNLYQVSQSTLNVNNLWIIMFTNCILMQIFVYCW
FGNELKLKSLQLIDSIFQMEWPILNNSIKKSLLLIMKRAMTPIEITTIFIFTMNLETFVALLKTSYSVYNLL
>SinvOr259

MQVMQFPFKILTVAGCRPPTSWSSSLGKRIVYHAYTIIMCLLLFTVMVPQFMDIILNVNNADDFTDCFYIMLA
TFIACCKILSLLLNRKNIELLTEALVEKPFRPLEPDEIEIREKCNKTVRTNSIIYTVLIELTCGSMNLTSLLT
DFRRGKLAYREWIPYKWSDTVYYFTYFRQIISLTVASIVNVACDILICGLLLHIYCQIEILECRLKKSLRNRG
DLGECVRLHDRIYKYARTMNEKFRLIITVQFIASMLVVCSSLYRLAKTTLSPKYIPLMLYTICMCIQILLYCW
FGNEVKLKSIQFSDEIFGMDWVTADKKARQSLILIMNRSLLPIEFSSAHIITVNLDSFVKLLKMSYSVYNIL
>SinvOr260
MQVMQFPLKILTVVGCRPPISWSSLYKRMVYNVYTILIFLPLFSFMLPQFMDIILNVDNADDFMDTFYVLVAL
LIACCKMFSLLLNRKNIEMLIEALLEKPFRPLEPDEIEIREKYNNIVWTNSVYYTIMVQMTCGCMNLTSLLTD
FRRGNLAYREWIPYEWSDTMYYLTYFRQLIGLTVASIVNVACDILICGFLKHIYCQIEILECRLKKSLRDQGD
LGECVRLHDHIYKYARSMNEKFRFTIVVQFTASMLVVCSNLYRLAQSSLSAKYIPLMSYTICMCIQILIYCWH
GNEVKLKSIQLSDEIFGMDWVTANKKARKDLILIMNRSLIPIEFSSAHIVTMNLDSFVKLLKMSYSIYNIL
>SinvOr261
MRVLQLTFKILTIVGCWRPQSCSSFYLSIIYDIYTVFMIILLYTFLVSQFLDIIWNVDNAEDFTENFYATLAS
VVSCSKMFSLLVNRKNINMLTNVLVERPYKPSEMDEMRIRYKFDRHIYTNTLCYTILVETTCACITVTSLFTV
FKKGNLTYRAWLPYDYYSSTIVFCLTYIHQLISLTAGSLVNVACDSLICGLLAHICCQIEILECRLSKVSNNH
ETLRDCVRHHNSILEFAFKLNNKFRMTIAMQFVVSTLVVCSNLYQMTKSTDINASYLPLLLYMSCMLTQIFIY
CWYGNEVKLKSTQLLTNIFAMDWVTMDRSLKRNLLLIMNRAVVPIEFTSAYVLSMNLDSFVGLLKTSYSAYNI
LKQ
>SinvOr262
MHILKLTFNLLTIFGCWRPNSWSSLHKRLVYYVYTSIIFLLLNTFMISQLIDVILTLSNAEDISDNFFALIAM
CITCCKVLILLINRKNITMLIDILMEKPCRPSKSTEMKILYKFDKSIQINTWRFVHLGIITVSSVILSSLLIN
FRNRKLTYKAWLPFDYSSAILFYITFTHQMTSLVAAMFINIGCDTLICGLLVHICCQIEILTYRLRKIMSYSN
IIRDCVYQHYYIFRFAVIVNAKFRLTLTVQFVMSMMMICFSLYHLSKTTSKAKHLEIILYICCMLTELFFYCW
YGNEVKLKSYEMIDKIFEIKWLTLDENNKKSLMMIMRRTLVPIQIKYAYITSMNLNSFMNILKMSYSTYNLLQ
HMNK
>SinvOr263
MHILKSTLNLLTIFGCWRPDSWSSLHKRLVYYVYTSIIFLLLNTFMVSQLIDVTLTLSNAEDISDNFFVLIAM
CMTCCKWLILFINRKDITMLIDLLMEKSCRRSSSTEIKILYKFDKSIQINTWRYLHMGIVTVLCVALSSLIIN
FRNRKLTFKAWLPFDYSSEILFYITFTHQLISIVAAVFVNVGCDTLICGLLVHICCQIEILTYRLRKIMSYSN
ILRDCIYQHYHIFRFAVIVNAKFKLTITIQFVMSTVILCFSLYHLSRTTSKAKYLETTLYICCFLTQLFIYCW
YGNEVKVKSYEIIDKIFEIEWLTLDEDSKKALIMIIRRALVPIQIMCTYTIPMNLNSFMNILKMSYSTYNLLQ
HMNK
>SinvOr264
MHILKSNFNLLTIFGCWRPDSWSSLHKRLVYYTYTSIIFLLVHTFMLSQLVDVILTLSNAEDISDNFFTLIAL
CSACCKLFVLLINRKSIIMLVDILMDKPCRPSESTEMKILYKFDKSIQINTWRFVHLAMVTVSIVILSSLFIN
FRNSKLMYKAWLPFDYSSAILFYVTYIHQMMGMIAAIFLSIGCDTLICGLLVHICCQIEILTYRLRKIMSYSN
MLRDCVYQHYHIFRFAVIVNAKFSLTITLQFVMSTVILCFSLYHLSRTTSKAKYLETTLYICCFLTQLFIYCW
YGNEVKVKSYEMIDKIFEMKWLTLDENSKKSLIMIMRQALVPIQIKCAYTVPMNLNSFMNILKMSYSTYNLLQ
HI
>SinvOr265
MCILKFNFKLLTLFGCWRPDSWASLYKRIIYHIHTSIITFLLNSFMLTQLIDVLLTTDNASEFFDNIYILISV
FNVCCKYFVLLINRKNILILINILMEKPCKPSTSTEMKILYKFDKNIQIYTKYYVYLAMISIFNIVLSSLFIN
FGKRKLMFRAWLPFDYTSTVPFCLTYIYQLIGIIIAACVNVGCDTLICGLLFHICCQIEILTYRLRKIISYSN
IIRDCVLQHYNIFRFAFILNTKFNLIIGVQFLMTAIIMCFGLYQISETTAKLKYAESLLCTSCVLTQIFLYCW
YGNEVKVKSYQMVDNIFETKWMRLDKNNQKSLMIIMRRTTVPIQITCAYVIPVNLDSFMGILKMSYSTYNLFQ
QMRQ
>SinvOr266
MRVLKYTFKVLTISGCWRPDSWSSLYKRIAYYIYTSIIILIVITFMLSQLIDVILSIGNVDDVFDNIFVVISL
INVSCKLIMLLINRKNISILIDILMEKACRPSEPTEIKIQYKFEKYIESNTKCCVYMGIVTVLCIALSSLPMN
FKNRKLMYRTWLPFDCTSIVPFYLTYTYQLISVAFSTLITLGCDTLICGLFVHICCQIEILTYRLRKIIFCSD
GFHDCVLQHYNIFRLAFIVKTKLSLIIAISFIMTTMIICFSFYQLDKTTTKAQYFEMILGILFALTEIFLYCW
FGNEVKIKSCQMIDNIFEMEWMTLDKDRKKSLMIIMTRALVPIQITCSYVIPVNLSSFMGILKMSYSIYNLIQ
QMRQ
>SinvOr267
MSVLKYTFKVLTISGCWRPDSWSSLYKRIAYYIYTSVIILLVITFMLSQLIDVILTIGNIDDVFDNIFIVISL
INVSCKLFILLINRKNILILIDILMEKACRPSESTEIKIQYKFEKYMESNSKCCVYMGMVTILCIALSSLPIN
FRNRKLMYRAWLPFDYTSTVPFCLTYTYQLISLAVSTLITLGCNSLICGLFVHICCQIEILTYRLRKIISCSD
RFHNCVLQHYNIFRLAFIVKTKLSLIIAIQFIMTTMIMCFSLYQLDKTTTKAEYFEMILCILFVLTEIFLYCW

FGNEVKIKSCQMIDNIFEMEWMTLDKDRKKSLMIIMTRALVPIQITCSYIIPINLNSFMGILKMSYSIYNLIQ
QIRQ
>SinvOr268
MLILALTFKIMAICGCARLDSWPTSYKRLVYHVYTIFVMLLIHTFMLSQLVDLIMIVDNSDDFTDNFYVLLAM
IVSCCKMFALLVNRSNIKMLIEILTSKPFKPVEPDELKIRQKFEKLIQSNTLHYTILVETTCLSVAVTSLLTE
YRKGNLTFRGWLPFDYTSPQLFPLVYAHQLISFTMGSVHHVACDSLICGFLVHICCQIEILEYRLRKSARNPN
ILRECVLHHNHIFKFASIVNEKFRLTIFIQFVVSTLVMCFNLYQFTKSTALKTKYMQLILYTCSMLSQIFFYC
WYGNEVKLRSRQLINNVFEMEWFKFNENGQKALLMIVRRAAVPIEFTSASVISMNLDSFVGLLKTSYSAYNIL
QQTQ
>SinvOr269
MRILDVTFRILMICGCWVPDSWTTPYKRLVYHVYTIFIMLLIHTFMLSQLMDLILTVDNANDFTDNFYMLLAM
IVSCCKMFTLLINRSNIAMLIDILVRKPCKPVQSDEIEIQQKFDKHVQTNTLVYAFWVETTCLCIAVTSLLTE
FRKGRLTFRAWLPFNYSSSLLFRIVYAHQLISLTAGSVLHVACDGLICGLLVHICCQIEIIECRLRKVAHDQN
ILRESVLQHNHVFKFAHLVNEKFRVTILIQFIVSTLVVCFNLYQFTKSTALRAQYIQLIMYMGCMLTQIFFYC
WYGNEVMLKSRHLVHSIFEMEWCELNKHTKHSLLMIMRRSSKPIELTSAYVISMNLDSFVGV
>SinvOr270
MCVLGFTFKILSSCGCWIPDSWTSPHRRLMYHVYTVFILLLINTFTLSQLLDIILTVNNPDDFTDNFYMLLAM
IVSCFKMFSLLINRSNIATLTDILINGPCRACDPVEVEIQQRYDKLIETNTLYYMILVELTCASTAVASLLTD
YRKEKLTFRAWLPFDYYSSTTLFHFTYFHQLISLTVGSVLHVACDGLICGLLLHICCQLEILSCRLKNIVHNP
KILRNCVIQHNLLFKFAFLLNKKFRFTITFQFIVSTLVVCFTLYQLTKTSGKFVELGMYMSCMLTQIFLYCWY
GNEVKLKSLQLVNDLFEIEWFTLEQNTRKDLLTIALRGKIPIEFSSAYVIPMNLDSFVGLLKTSYSTYNILQQ
TQN
>SinvOr271
MSVLMFTFKVLTICGCWPPNSWTSRCKRILYDIYTILIVLLINTFTLSQLLDLILIVDNADDFAENFYITLAM
FVSCCKMFSLLRNRNNIAMLIDILMKKPCRPTEHDEIEIRQKFDKLVQTNTFYYATMVESTCAFALVTSVFKD
YRKHRLAFRAWLPFNYSSPMLFRIAYAHQAISLTVGSVLHVACDSIICGLLMHICSQLEILECHLKKVINKPH
LLRECVIQHTYIFQFALMVNEKFRFTITVQFLVSMLVVCFNLHQLTQTNVLSAKYIQIVLYMFCMLTQISFYC
WYGNEVKLKSQQLVSNVFGMEWFTLDYHVQKNLLIIMTRCIIPIEFTSAYVISMNLESFVSLLKTSYTVYNVL
QQMR
>SinvOr272
SADLTHASNIVVWNKWFLTFLGLWPERVNQLVFIFFTTYMVIYCTMGMNHLIRHSDQPELVIANFTDNVFLTM
TLGKMLICRRSSRIMATFLKSIEPDFTTRMYDNIQEKQAYLQYNKLALIFVRLSMPMLAFTSTLYYLRLFYEK
WSIMISGNFSYETLPYPVHPFFEIKDTATYVCVCIYLAIMLPIILCGYGGLDAFVLSMALHICGQFAALSYKI
NNLLKDHKNYHRHITNIVLRHRHLIKLAEILENSFNMICLQQTLGTLVLLCLTMFHMLATSAYGDNANVVAFT
LYAVCVSSTILAYCYTGECLFTESAGLSDAFYNTDWYNNSPSSTKLVGICMIRSDRPLILTAGKFCILSLNTF
TSIVKTSMA
>SinvOr273
DLARASNIVIWNKWFLTCLGLWPERVNQPVFILFSIYMVIYCTMGMNHLIRNFDRPELVAANFTDNVLLTMIL
GKMIICRRSSKIMATFLKSIEADFTTAMYDNVREKMAYLYYNNIALIFIRISMFMTGISGGSYFFRKFIENWN
ELISGNFSYELPYPVHPFFEIKDMTTYVWVCLYLFIMVFMIFCGYGGPDSFVLSMVFHVCGQFAALSCKIDNL
LRDHENYHRHISSIVSRHQDLIRLAEILENNFNMICLQQTLGTVFLLCLTLYHMIAKSDFGEDTSVVLFAMYT
SCVISTILVYCYAGECLITESAKLRETFYNTNWYNNSPSQTKLIGLCMVRSEKPLILTAGKFLVLSLNTFTGI
VKTSMAYLSMLRNFL
>SinvOr274
QNQKELDRAAEVLSWNNRLMSLLGVWPSHPNDLIFSINFSYFGFLMILEYLDLFLYIDDLEQVVMNLTENMAF
SQIFVRIFIMRLYNDQIGEILTEVEKDFDKTNYKTAEEIKSFITYNARSKMFMKLLMVFVALTASSYYLTPII
IILGSSLPKIVISENVTQIIYLLPYRFYTFYAVENMRTYVITYALEMPFVFVSGVGQAASDCVMVTLVFHICG
QMSVLALRINSVDADNELYDCRREVRHVVLMHIRLLRMGKRVEQIFSTTLLVHLIGATSLLCVLGYQILTNFA
KGEKGVLVTFLIFQFLVLLILYVLCTVGESLLTESTKVCEAFYDCHWYSMSKNNAQMIILCMARSQKPLCLTA
GKFTTICLSTLTDVLKTSMGYLSVLRSFL
>SinvOr275
LQNKGDHIEDLFVHLERIFSIGGIWPSKRTYVRFAIYISHYALYLVMAWINLYDVFGNLELMVMNIVETVAYS
ITFPLMCLIRCSNLLKLVINVIRKDMVRKFENSEEERIYYNYNYISKVFTYGSVVGMFITVVSLYFRPLVYLL
TTNQALRHNDTEPLVLPYRVHPFLDTSNTHAYILMYLYLFPLIYISVCHMAAICLMVILVFHICGELSILSYR
IKHVGEYSEDLIVGRIGSFVRMHLKIIWLMKSINDTFHLILLDELLGNSIVLAISLYYIIMNLDVTNMATCFT
FTFFAIIALVMLFGYCLMGDQLTQQCVNVQDAYYECNWYEMPPVCKKCLLICMIRSQVMLYLTAGRFYIFSFT
SFTDIIRTSLAYLSMLRTLI
>SinvOr276

MEPRDRYYDISKRYLRWIGQWPSQKPKESLFFFIFILFFDANVIVAQVARFFVCDNMQCIFETLPPHLLAVIV
PVKIFVYRFNRRKIKHLTDRLFLDWNMLKTEKERDIMRKYAATGRWYVSIYGLYIYISTVSFTSTSLAPRILD
IVFPLNTSRPIMLPYPAYYYVDENEYFYYIFLHMIISSTIIVTAIVAHDCMFFTYIEHNCALFAIVRYRFDQV
SRERSNMEKSAINYPDYMYHKNVAISVYAHQKALQYAKLLEDTFSIAFAIQLLLITLCLSITLVQLHDSAEAL
RYIVFIMAQLFHLFCFSFQGQKLINHSLETCDDIYHSLWYKIPVKEQRMLLFVMRKSIEATALTAGKIYVFSL
ENFTTVVQSSMSLF
>SinvOr277
MKSARNHYSSIAKKMLSLAGLWPYQEKRTRLFRVSLMTVITLSIMIPQIAEINHCKGNVQCIFLTIPAIMLMI
ITLVKLYTFQFNSSKIKSLTDHLRNDWKKLKGAEEYKIMKKYAANARLFSLTICLWYYIGCILFLLSGLAPDI
LNAVLRLNESRPSAFPYPAYYFGLDNEKYLFYIYFHMFVCCEIIITVSIAHDCMLLVYVEHACSIFAVAGFRF
EILSRTNCGNKMNNSLNAYNQKIAKSVYAHWRALNFANLLENTFYVAFTVQITIIIVAMSITLLQVAVQVNKI
TELMRYSLYFLSQLIHLFCYSLEGQKLVDHSVEIRDRIYNSFWYNIPVKSQRLLLYVMRRSMKPNSLSAGKIF
VFCLKSFTTVLQSSVSYFTVLESFQ
>SinvOr278
MDSARNQYFSITKTMLSFTGLWPYQEKGTRLFRVSLMTVITLSLMIPQIAEINLCKGNVQRIFLTIPALMLMI
VILVKLYTFQFNSSKIKSLTDHLRNDWKKLKGAEEYKIMKKYAANARLFSFAICVWYHIACFVFIFIGLAPNV
LNAVLRLNESRSIAFPYPAYYFGLDNEKYLFYIYSHMYVCCEIAITVVIAHDCMLLVYIEHACSIFAVAGFRF
KTLLRTNCGDKMNNSLYAYNQKIAKSVYAHCRALNFANFLENTFCVAFTVQIIIVIVAMSITLLQVAEQVNKI
TEIMKYLLYFFSQLVHLFCYSLEGQKLVDHSAEIRDRIYNSSWYNIPVKSQRLLLYVMRRSTKPNSLSAGKIF
VFCLMSFTTVVQSSVSYFTVLQSFQ
>SinvOr279
NYYDIVCKISSLTGLWPYLKPRARIFRVGLLTVTMLTIFIPQIAYQFTCKTNLQCIFEAMTSYLLTVVAFVKV
YTFQFNTHKIKGLTQHLFVDWKRLETPKEYEIMKLYAKNSRRFCMVYAVYYTVATFTFMSMTFIPFVFDVVWP
LNESRPVLPPYPGYYFVDNREYFFKIYCHSLISWEIIMVGIVAHDCMFVTYVEHVCSKFALVGFHFENLFCNC
DEEIKITDNSDDTYRKRIKLFVHEHWEALKFAETLEDTFVVPFAVQILIVTVGISVTLLQITQQEGEVLESIR
YVVYVIGQLIHLFFLSFEGQKLIDHSLQTGDKICNSAWYEVSVRSQRLIMLVMMKSTRPSFLSAGKIYVFSLE
SFTTVLQTSMSYFTVFASVQ
>SinvOr280
RNHYYNITKRMLSLDGLWPYQSRGTRSFRVSIFTVITISMIIPQVIQIIRYDGNARIFLIIPAVLLIVTIQAK
LYTCQFNTSKIKNLIDHLRDNWEKLEDAEEYEIIKTYVARAKLLSLIYFLYVYVSTSLFMLITISSQILNIML
HLNETRPLMMPFDAYYFVDTEQYYFYIIFHIWVSIIIILTGAAAHDCMYLIYIEHVCSLFTLAGFCLKTVSRN
DRNNVSNNSKNGDKIYNQKTAISIHIHWRAIWFTNYFEDFCISFIVISTIITIAISIILLQVCFEEVLQKVKS
IAFIIGQLMHSFYYSMQSQKLMNHSIQLREEIYNSSWYKILVKSQKLFLYMMQRSLEPNFLSAGRIFVFSLKS
FTTVLKSSMSYFTVLASFQ
>SinvOr281
NHYYSVTNRMLSLAGQWPYQSKRTRLFRVCFLTAIAVSLIIPQLAQIFRCNGNGRCIFLIIPAVLLMITILVK
LYTCQFNNSKIKNLTDHLGEDWEQLEGVEEYEIMKTYAARARLLSLIYSLYLYVSLSLFMLITISPQILNVVL
PVINESRPLMMPFDAYYFVNTEQYYFYIIFHIWVGVIIILVGALAHDCLFFTYVEHVCSLFALAGFRLKTVSR
NICNDAANGKNGDKIYNRKIAISIHIHWRAIWFAEHLEETFSISFVVQMMIVVVVMSITLLQIALQFEDVLET
IKSISFIVGQLMHLFCYSMQGQKLMDHSIQLREEIYNSFWYEIPVKSQRLLLYVMQRSLEPNFLSAGKIFVFS
LKSFTTVIQSSMSYFTVLASFQ
>SinvOr282
HYKLLNRMLSLAGLWPYQSKRTRLFRVCFLTAIAVSLIIPQMAQINHCDKNLRCIFLAIPATLLMFMILLKVH
TCQFNISKIKKLTNHLHEDWEKLESVKEYEIMKTYAAHARLLSLTYCLCGYVGAYLYMLITLLPRILNIMLPL
NESRPLIMLYDAYYFVDSEEYYFYIFIHNLVGMSILMVGILAHDCMFITYIEHVCSLFAIAGFHLETVSCNDR
NDAANNSRDAYKTYNRKIAISVHVHWRAIWVAEHLDDTFSSSFVIQMMITTTMISITLLQMALQIEEFIVTLK
CMVYVMAQLIHTFIYSVQGQKLIDHSTQLREKIYNSFWYEIPVKSQKLLLFVMRRGLEPNFLSAGRIYVFSLT
SFTTVLQSAISYFTVLSSF
>SinvOr283
MQILTFNFRLYTISGLWRPIEWSSKSSKLSYSVFTITTIYLLTYFSVTHLLYIIFVIDNVEEFASTSPFFFST
ISLLFKASTAVIYRNQIINLIEILQNKPCKAENKDELDIQMKYDRAIRTYSMRFVLLCSFSMTGAFVAGLLDV
LKGQLPYNMWVPWECTSLFTFLSTSIQEIAGVTIATVVNVATETTVLGFSLQVCAQFEILKHRLQRMMENSNE
KMSPKNSLNNGSHKINKLATHIFHHVCIIRLAEKFNNVYSQVIFIQFFVSVLVLCSIVYHLSSHLTVVDVSTW
VVFTVSMFIQIFIYYWSGNQVIIKSSELGEAVYHMNWMSMTLNERKDLMMIMKRSSKPIKFTSSFLVTLSLES
YTNLLRATFSAFNVLQQF
>SinvOr284
MQMFSLNFSMYTVGGVWRPIEWSSNIAKLLYNVFTFIVVGLLYFLMITQFLDIILVVDNIDDFATNTLMFLTI
VAVTCKATIVVVRRNAIITLVQVLLTPPCKPQNEDEMAIQKKFDKFIKSCSIKYSLLATCSVSGVTVRSVLNA

MQGYLPYRVWLPYDFNTSPMFWITSIQQIVTVVFVTIINVGTETLVFGLFLQTCAQFEIFESRLRKLVIDKTS
KIMTNKVECLKHSSLSSNKKKAIISKYVHHHLEIYKYAKTVNVIFNQVLFVQFFGSILVLCTSVYYLSAHSSL
SETATLIIYTICMFVQIYVYCWSGNEVILKSTKVGDSVYHMNWPLLSVNDRRELLMIMLRSSLPLKFTSSFLI
TLSLQSYSNILKISYSAFNVLNK
>SinvOr285
MQILSLNFLIYTLCGIWRPIKWSSNSAKLLYSVFTFIVIFSQYFLVFTQFMDIILVVDNIDDFATNTLMFLTI
IAVCCKATVVVMRRNEIINLVQVLMREPCKPRDEDEAMIQTKFDKFIRSYSIKYSLLATGSVIGTTIGSIFNI
MRGYLPYRVWLPSKYNVPLVFRIVSIHQIITIILATMINVGTETLVFGLFIQTCAQLEIFESRLHKLINSKSV
GHLGHGFSSLNQDKIEIPDCVRHHLSIFKYAKTINVIFNQVLFVQFFASILVLCTSVYYLSMNITDLSGTAPL
LVYTICMFVQIYVYCWSGNEVILKSMNTTDSIYHTDWPVLSVDEKKKLLMIMIRSTIPIKFTSSFLITLSLQS
YNNILKASYSAFNVLKK
>SinvOr286
MQILTLNFLMYTFGGIWRPIEWSSNAANSLYNIFTCIVLIMEYFLVITQFLDILLVVDNVDDFVINSLMFMSI
INVVSKATVVVVRRNAIINLVQRLLKGLCKPQDEDEITIQTKFDQFIRSWSIKYIILASSSLTGVTIGSILNV
MHGQLPYRAWLPWDYNNVPLVFWVISIHQIIALIFATFINVGTETLVFGLFIQTCAQFEIFENRLHKLIVNKT
IGHLGHALSSSNKDKTEISKCVCHHLSIYKYAKSVNVIFNQILFIQFSCSILILCTSVYYLSIHIAELSGVAS
LIVYTICMFVQIYIYCWSGNEVILKSMNTGNAIYHMDWPLLSVSEKKELLLIMKRSTIPIKFTSSFLITLSLQ
SYSNMLKTSYSAFNLLKQ
>SinvOr287
MQILSLNFLLYTVCGIWRPVEWSSNGAKLLYSVFTFIVVFSQYFLMLGQFMDIILVVDNIDDFATNVLMFLTM
IAVCCKATVVIVRRNAIFNLVQRLLKAPYKPQDEAEVAVQIKFDKFIRSCSIRYLLLASCSVTGLTLGSVLNA
VHGRLPYRIWLPWDYNIPLIFWILAIYQILTVVFAATINVGTETLVFGLFIQTCAQLEIFENRVKKFIINKIV
GRALSSSNTNEIGISECVHHHLSIYKYAKMVNILFNQVLFVQFFASILVLCSSVYYLSLHITEFSGIAFLLMY
TICMFVQIFVYCWSGNEVILKSMSTGDAIYRMDWPLLSSNDRKGLLMIMIRGTIPIKFSSSFLVILSLQSYST
ILKASYSTFNVLR
>SinvOr288
MQILALNFLFYTIGGVWRPIKWSSKCSICLYSVLNFFSLYLLTFFVLTQLIDTIFIVDNIDDFTTNLSLLLSA
IAVYCKAVTATARRSEFISLIKMLQEKPCKACNEEEINIQMKYDRLIRQVSYTMSYSILASFSLTGITIGEVL
IALQGELPIRAWIPYDYTSTFLFWLTSLKLIVAMALSTFVNVATETVILGFCLQICAQFDILICRLRKVIESD
EKQENELNSATNKTSRLSENIHYHLYIIRFAKMVNKVFSQIVFIQFFVSILVLCTSVYYLSSHKTVTDFIKLG
IYTSCMFVQIFMYCWAGNEIILKSIGLSEAVYKMEWILLTISERKDLLMIMMRSTKPIKFTSSFLVTLSLESY
ATILKASYSAFNILQ
>SinvOr289
MVNNVDRYFSLNRILLLTVGLWPYQKSIFARLHLFCFFSTVTSFIILQFTTFVTSRCTLDHIIKICSFSFVIT
CTAIKYSSFYINSDIIKNLIDQLQHIHNDLKDNNEIAIAEKYGSIANRYTILFTVTLFCSIFVIISSSLWPDL
TNIILSTNESRPRRLQFSTEYFIDQEKYYYILLFHINATFCIALTVLTATGSMLNACLHHTCGMFSIASYRIK
NAIEIYVQNINLKNKNFVYRNLICGIHIHRKAMMYSEYLISNFEVSFMLLIAFGVLSLSLNTFRIVSSKFDIG
ELLLTFIVMLVCFIYMFLSNFIGQEIIDHYDFVSFTAYEVQWYITPIYIQKLILFLLLRGNKSFGLNVGGLFV
SSLKCFATLANASLSYFIVMYSMR
>SinvOr290
IYMFDDHCKFNETLLRFFGLWPYERTVCERLRAICFNMLFISHVIAEFAQFAIVDFFNVNITIRILRDAITTF
LIFVIFNMFFLNTKKVNSQEIEILDYYAYKGKKIIVTILCNNVNFTIYHSVISLVMCITFMTFVSGLLPDIFD
VLWPLNESRVHDLLVMKEYKINEGIQYYIFCYYSIICINIGGIVTTFIISTLLSINLHCCAIYKVCSYRIKQF
VDKEVIACSSKGNVNERIIKTVELHRKAKKLFKLITTSFTMSYLIMAVAGMCGFAMSLYGLLNVIIYKYDLTE
LLTTACFIIGYEFCLFTITIMGQLLINHSDELFKSLYMSLWYEAPITVQKSILFIMQISVKSLVMNLGGVLEV
AMQTFTSITNMSISFMMIFFSIQ
>SinvOr291
MYTFNNQYYKLNEILLRFLGLWPFEKTDGERFRAICFYILLISYMIAQFAQFIIADFSINVTIEILLDAVTAF
VFLDKRDLTTRIVRFLLSRKIKTRVLEYAINFLAWATLIVLLQYKDLRLQIFVLFTFLITGLQPDILDFLWPL
NESRAHNLLFMKQYQINVGMQYYLFFLYSVISIAIGTSIVIYVISTLLLICLHCCAMFKICSYQIKQIVDKEI
LTHSNKKKIITEKIIRTVELHQKVKKLFKCFTTSFTMSFLTIFIVGTCSFAMCLYRLKNAITRMHDLVELLIS
IAYIIAYEFCLFTMSLMGQLVINHSDELFNAIYKSLWYEACVTIQKSLLFIMQISAKRLIINFGGVCVINMET
FTTVTNTAISFMTVIFSIQ
>SinvOr292
IYMFDDHYKFNETLLRFLGLWPFRRNECERCRAICFYILLISRVVAEFAQFIIADFSINVTIKILLDALTAFL
YAVTFNMFFFNTEKVINNNWRMLSDSQEIKILYYYSQQGEKFIIITICNNTSFFLIIRLLQVLVLFIFFITGL
LPDIFNFLRPLNESRTHYLLFMNEYRINVGIQYYLFFLYTIISVIIGTSTAIFIISTVLYINLHCCAMFKICS
YRMKHCVDTKIIACSNKRNIIVGRIIKTVHIHQKANKLFKLVTTSFTMSFLTMLMIGTCSFAMCLYRLMNAIT

RMHDMVEILISAAYIIGYQFCFFTVSFMGQLVTNHSDELFNAIYMSLWYETFITIQKSLLFIMHIWSKRIIIN
FGGVCAISMETFTSITNMSISFMMMFLSIQ
>SinvOr293
DSFASVIRVNVALLRLSGVVSYKNGLIRRSSSQNILSAIAYGCLFAYSGLYTYEFALHTIHLDIWMESFAMIL
SLVGGQVRFTILLLYRSRFQRLLTVCEELWTALNATEKKHVRDYVKRTRHLTYYYLLGCVFTIFFYAVASLFM
GQHDDSLNATMRTLPYACPIQVHRSPYYEIMYIAQLCSMINVGLTCVATDTVGPVLVLTVCGHFKVLNTRISH
LTERYQEHSEVHAERSRSTPTESFEIIREGNDGDVSKNRVKLNLEACVHYHQTMLELCKEVESLNATIFLTQL
LGSTYNVSLVGFKLAGDDPDKFKYTTQLLIAMIQLFLSNWAANVLLTESQDVARAIYFTPWYRFPYQLKRSLN
IITMRSQKPAQLTAGYIIPLSLQTFASMVSSAASFFTMIRSMN
>SinvOr294
MEGKITLDKVIVFLKVYLTFACCWPLPSNATKSQRLVRSAFQCICLTNSIVFVIAAIWTLCKYSDNALMVMKL
GCQLSAIVQIPLQMILFAMQNKRLQFIVLEMENYYQQAQEYEKKIFQLYVDKCKPFYGNILCWLAMTGISVIF
TPLFSSQSFPSEAEYPFDMESQPLKTIIYAHHILIAYQSVIQVSTNTFPALLLWFVAARFDILSVQFRTMTNI
KELMKYTHEHRLLLRYAREVTRAIRYVALLCVTFSTGAVIFGYLTFMSHAPLSVKSTFLMIAFCGFVELYMYA
WPADNVMSTSSDIASAVYESLWYNNDLIKTRKILIYIILRSQRPVTVSIPCALPNLSMNYYASYISTVFSYMA
FIRAHVIMNQE
>SinvOr295
RNEVAGMARKTTTGSSLEQFRKLSVLHLTYLKYIGLWYKFDSNVSIFLRGLYYVYNKFVLISIIAFSATIFAD
VCSSLDDLSSVTDNGCMFTGLIVVVFKLMIFQTRSDRIIQLLHKSIDSYNQLCKFPIGNEKKILDKYLLFCRM
IFYAFSVMGVCLGIGILFLVPLEKGQLPIKARYPFNTTVYPGHGISYSFEVYCVFIAMTSIVGMESFVINLCS
MFLVQFAILNVHFKSCGNGQHHAMSNGIGKRDSLGTIVKNACFEVYSTIEASCGKDNYDSDAICRGGFDERFG
RSIRNHQRLLAIVDDFNEIFSSCLFVQMFSSTSMICLTCFQFTLVIGQSSNPFKTTVYMVTAFSQLFFFCLVG
NEVIHQSALLMTSQWLSHWNDELSAKSGRLLILTLIFSKRTISLKAGAFFSLSMETFITIVKCAYSFFTLLST
MN
>SinvOr296
KIDVYKRNNNYSLQWTRWILKPIGVWPELPTSSAIEKILSKILRLTCHTLIALTVIPSILYIIFEEKDIELKL
KAVGPASHWLMGGVNYCSLLYQKGQIRKSIKHMETDWRMAKRQCDQEMMLKNARAGRIIAGVCALIMQGGLFT
YNVARGTSPILVAIGNETVTMNRLPCPSFNKIVDTRFTPVFEIVFTLQLLSTLVVNNTIVGACGLAAVFAMHA
CGQLSVVMSRFEELVEEKRHNVIQRKLANLVEHHLRTLRQDFTVKYINFRFLSRMEIIMRQVCFVELTGCTFN
LCMLGYYAITQWHEESTNTIIAYFVIFTSMTFNIFIFCYIGDLVTEQCKKVGEAVYMTNWYKLPQKTVLSLIL
IILRSSIVIKMTAGNLVHMSISTFGDVMKTSIAYLNMLRTLTM
>SinvOr297
DIEQFFQDSHYNIIRVLLNISGLWPFHTRNRRYATYLAMILILGSGFIFEMLGTIEVRHDSFEVIDALPLLFF
AIVTVSRTFCAVYTLPKMKILLIKMQEHCLSPKSDEETKIQNSHAQYGRKLGYAYTGFLLGHSVLYILSTALT
RLFYTKSKEDDGTSDNVQIGLPHRVNYMVDLDKYYVPIFIHCAICDFSFTFLLVVFDVLYLTVVEYCCGLFAA
LRYRLETALVFKNDRLTMTTTNDQCYANIVYSVRRHTETMQFVAIMESIYSLPLFIHIGLTVLILSVLGYQVI
SNTEDISAILKPIAYLNGLLINVFFENWQGQKIIDSSEKVFESAYNSEWYSMPIAARKLLIMIMMRSERPSVL
RMGKIVILSFVTFNAVLRTSSSYFMLLRSL
>SinvOr298
LEWAIGLHRCMLKIVGLWPQQSKDQYKKFFSKFRMLFNATAIVFILTIPALAQLIKVWGDMIQIIDNLQFTLP
FLVTTLKVFIMWYKKGALLLLTNMIMKDWTRVKMDEERNVMLKKAKTTRLLLKIGVFFTVLAVFSRLGSVFFK
EYIWRVNNLTNHERSLPVPTYYWYDVTSSPKYELTYLAQTIGAFAIALTYVAVDNFLGLLILHVCGQMENLHL
RLLHLGKDPDFRTVLKFNVKDHIRLIRSVEIIDDAFNLLLLGMIGFFSILFSLYGFLVLTAVNQNTQLSFAQM
SWYFSAGVSLLMHTCLYCAVGEHLVNQCEKIHSATYECVWYTLEPKAARNLTVIMVCAKKPLNITAGKMFPMT
MSTFCSLLKTSAGYLS
>SinvOr300
MDLLPLNFYVLRFCGMWEEHMENNLIVRFANFCYRNIIIALICHFTICEIIELIRMRNNVDDLTEGLFIATTY
ITFCLKYLNFAMGQSELRALLNCFRAKPCQPKNSTEESILRQYHVKATKIACVYMLMSHTAGLLLLITPIMAQ
DERQLPMKMYVPYSMVKLLPYLLTYLEQAVAIIYGISIDIVLHSLVYGFTILACGQIDLLCYRLSETFRFLQL
QDNSEEKGDDTIEKFAIAECVRHHNSVCNIIHRIQSLFMWSTAILFFTSFITLCSSIYQISNKELFSIEFLFF
VVYVVSISFQLFLYCWYGNELDLKYKDIASTIYASNWTAISVKQRRSLSFIMMISQRGQIISFYGFCSLVIST
FTGILQTSYSAFNLLQRVSN
>SinvOr301
MDLLPVNFYVLRFCGVWKERKDDNLIIRFISFCYRYTIVALIYHFTICEVIELVRMRNNVDDLTEGLFMVLTY
ICLCLKYLNILMRQSELHALLNCFRIKLCQPKDSTEKLILKQYHLQAKKVICAFMLISQITGLLVLTAPLMSQ
NKRQLPLKMYVPYSMAELLPYLLTYLQQVGAVIYGVLLNVTFDSLVYGLIIHTCGQIELLCHRLSETFRFLQE
NSEEKRHGAIEKFAITECVRHHISVCSITQRIQSLFMWIVATLFFFSLVTLCTSIYQMSNKKLFGVEFFAFVM

YLGSMLFQVFSYCWFGNELDLKNKSIAYAIYASNWTIVSVKQRKSLLFIMMISQRGRIISFYGICSLVLSTFT
WILKTSYSAFNLLQQASN
>SinvOr302
FERATRLNRLLLNIIGIWPNAHKNAYDKFFSNLRAAFTFIVIFFVGTIPAIHSLVRTWGDLMSMIDNLQVTLP
LTMAAIKLIDIWWKKTDLLMAINMIAEDWIKDKTSKERCIMIKQAQNARIITIVCCCFMFLGSTLVVILPCFG
MTVRYITNVTDPVKILPFQTHYIYDKNQSPYFEIIFAAQFLVALMCVASYTGVDNLLGLLIFHLCGQMDILKE
KLINIKQFKNYNDGVALIVKEHIRLIKCFCIIESTYTLLLLGQLIYFGIIFCLYGFLILVILTEGKHMSLMRF
MYLVSVAINICGHMCLFCAVGEMLISKCDDLHHAAYEHKWYKLNPKKAKILVLIMIRANKPLYITAGKMFPMT
MSTFCNLIKTSAGYVSILLA
>SinvOr305
FEWATKLNRFTLNVAGLWPNIHENASNRFLSNLRAMFLLFLLVFVGIIPGLHSLVRTWGDMMATIDNLQFTLP
LLTTIIKLVVIWWKKPDLVLVLNTIANDWSNVKTDKELHVMTKRAQSARVVTIFGYILMVVAMFLLICLPFFG
TSLRYRTNITDPDKVLPLQTHYFYDKDQSPYYELTYVAQALLLLMSSASYTGVDNLLGLLIFHLCGQMENLKE
RIINTKQFNNFNGDLAVIVNEHIRLIKFFDIVESTFTVLLLGLLLYFGTLFCLYGFLIIAVLTEGREMSMIRL
IYLISGALNVCGHMCLYCVVGEILVAQISGKCEGIYRAAYNYEWYKLNPEEARSLIIIMIRANKTLHITAGKM
FPMTLSMFCNLIKTSAGYVSILLAKQ
>SinvOr308
MIRVELQYFNLNRILLLILGLWPYQQSTFTRLQFFFFSIILTAGIVFQLTSLMTSTYTLNFVNKVLSSVSFCT
FSSVKCNLFWINIGNMKDLLMQLQHVHNQLKDKNEVAIIKGYNCTARRYTIVLTIFGACIPFIFIISQYWYII
GDLPKNVSRFRRLPIMTQYFIDQEKYFNLILLHIFAIICTGTIAILATGTMFIAYFMHICGLFKIACYRIEHA
INIHIQQNNITQKNKIWMTNGIIYAVDIHRTAMKLSTHLTSTLETMMFLLIASAVTCTTLNMLQIFQIISSKN
FEELFLPTVIITIILIYMFVGNYIGQLVIDHNSHVFFTTYNVQWYRAPLHIQRMILFLLQKESKKFTLTVGGL
ITASMECFATLIKASVSYFTVIYSMQ
>SinvOr310
MIRLEVQYFNLNRILLLAVGLWPYQQSKFTRFQFIFLSSILTASIIFQLTSLITLRCTTDLVAKVVSSASFFT
VYLITYNSFCVNMKSAKNLMIQCQRVFDELKDEKELAIIQKYGYNTKLYPIVLTVMGTCSSIVIIVFQYWTNM
FDIALSVNVSQPRRIQFVMEYFLDQEKYFFLSVLHINVAFCIGIVAVIAVGTLLLGYLNFIFGMFRISSYRIR
RVVEINIPQNFVFKKKFFKCESLKCAIDIHRKAMRLSRYLVITFEIMFLSLTAIFMISLSSSFLRIFQIVSSR
EPIEEIIIPIFFVTTDIIYLFICNFFGQNIIDHNNHVFTTAYNIKWYVAPLHIQRMILFFMLNNAKDFTIIVG
GIFVASIENFAMLVKAAISYFTVVLSM
>SinvOr311
EGQYFGINRILMLTIGLWPYQQSNFTRFQYFINTAILIAMITFQLTTFLTLKLTVDVIMRVLSSVLFFILFTL
EYNSFYFNIKNLKDLLIQLQHVHNELKDKNEITIIKKYDYAGKCYIVMLIIGAFFAVSLGFIVQFWMNTDVVD
LSINISQTHRFILITEYFIDQEKYFYLILLHINLAICIGILGALATGTLFIMYFHHTCGMFKIASYRIEYAMN
INVLQNFTLKNEILMTESIICAVKIHRKAMKLSQHMLSKFKIMVSCITLCGVACLSFNLFQMASFQNNVNEIL
LPFVFSTICMLYMFLANFMAQKISDHNNYVLTTAYNVQWYKTPLYIQKMILFLLQRGTREFTLNIGGLIHGSM
ECFASLVKTSVSYFTIIYS
>SinvOr312
MTSKEHQYFSINRILLLIVGSWPYQQSIFAKFQYIVICVILLSNIIFQQLTSFLTLHCTVEVIVRILAIVTFF
SVAIIQYIIFHFNIEGVKDLLMQLQHIHNELKNKNEIAIIKKYDCIAKRYVIGLIAVGICGIFLTVITELILS
IIDIQSSTNASRSHFFLLKMEYFIDQEKYFYFIWVHINATISIITITLVANGTMLIAYIQHVCGVFRIASYRM
KYGININVLQNVMLKNKVWMIKHISYAVDIHRQAMKLMKQLMIILNKMMFCLIVFGVATLSLNLYLLVLAKNN
IHIFLLTCVIVFVCILYMFLANNMGQIIIDHNNHVFISAYNAQWYKTPIQVQKMILFILQRRSKAFTLNVGRL
FDASMEGFATLVKAAVSYFTVMYQIQ
>SinvOr313
MDFQNTNLFNVRLNLLSGNLLPMTDKSDKSSFSLFWKVLSIFLWLLILFYIISLIFAYFSVSNEIELSDGLDG
LYVMEEIGFIICYSRILVQKTLLQQMIQKLNEALSIEDENMRCIVTTNLKPLKNPFRYCLVIGVGSTIFWCCS
TLPLILERNTFYHADFVTPAAFSKEPFSVDVFVLGNVIILVCNIHFFLKIIGVDVYTTHLISLITAQYQYISS
RLVLAFRNNKHPNNSNSRQGNSKVDSLTAKEIKNLCRQHVNVMHVMLMLKKLISVNTFYIYIISVFRFCFMGL
VMINVSEIFSDACFMFFYASGSVTQFYVLYACFQRLSEASSEITNEAFHEDWNQFNLPIKRMFLLMTMASNNI
EIKLSLLDRFILSLPSFMSVLNQAYSIALLILKIS
>SinvOr314
MDFQNINLFNVRLNLLSGNLLPMIDKSSFSLFWKVHMTFVWLLQLVYFTSLILAYFSASNENELSDGSDGLYV
IEASGFIICYLRIFTQKTLLQQMIRKLNDALNIEDENMRRIVITNLKLIKNPFRYYFVISGCSSIFWCCSTLP
LILERNTFYHADFVTPAVFSKEPFSVGVFLLGNMIILGSNIYFFLKIIGMDVYTAHLISLITAQYQYISSRLV
LAFRNNKHPDNSNSREGNSEVDSLTAKEIKNLCRQHVNVMYITLMLKKLISVNFFLIYLFNVFRFCFLILMAI
KISENFFEGFSVILYTSGIAAQFYVLCACVQRLSEASSEITNKAFHEDWNQFNLSIKRTFLLMTMASNNIEIK
LSLLDRFNLSLPSFMSVLNQAYSIALLILKMS

>SinvOr315
KSSFSLFWKVHCIFVWLLKLVYMISIILAYSSVPNDTGLSDGFDGLFFIEEVGFAIDYLRILSQKKLVQQLIR
KLNEALRIEDENMRRIVITSLKQIKNPFKYYLVTGACSIIFWCCMTLPLIFERNNFYHADYVTPAVFTKEPFS
VGVFLLGNMMILVCNTYFFLKVISVDIYTAHLISLITAQYQYISSRLVLIFRNNKHPNNSSFRKGSSKVDFFT
AKEINNLCRQHVNVMYITLMLKKLISVNIFFIYLINVFRFCFMGLIATKVAETYIEMCFAILYVSGSLTQFYV
LCACFQRLSDASSEITNKAFHEDWNQFNLSIKRTFLLMTMASYNIEIKFSLFHKFNLSLPSFMSVLNQAYTVA
LLILRI
>SinvOr316
MTQSKEYQSVNITRIFMKMVGLWYVETPKDRLLLRAAFGYAIWAIVFAILVMGVDLYHCLGDFYAVTTNLCAT
LLLVMVLVKLGSFMFYRDKIMNLIHFAERNFWNATHGETDTQILKQYDKLGMFLIYTFTFIVTVATFNYIFAP
FFEPQEKNETKKILPFKLWFDFPYHSPYYEITYVIQSLSTIHSGICTFCFDNFVSTFNIHAAAQLNILAHRVK
VVIENCIEDTMDQKLSLETDATVLSFKKLHNCIQQHLTLIRYVRNMQRVFATMLLGQLLFSSVIICFGGFQML
AADVIIRKCIFASYFVGGVLQLLIYTWTCNDIIVQSTSISDAAYNSKWYLLPDNSLGRSVKKGLIMMMIRARR
PCELTAGPFAVMSLDLFTSILSTAMSYFTLLRQMSED
>SinvOr317
PEENYYKLNRFLLSASGLEPYQSKWNARLIRAFITIVMMSSSIFQISSWFTFEITYEFVVNGVQSLLLILCIL
TNLHLRTGNVDKVKILFDRISKDWALQKTHGEIEIMREHAEFSKLFTFCCTILSYVSMVGYCIWLCIPEILNI
IMPMNESRPPRQPFAAEFFIDEERYFFLIRSHLYLVLLTLPIVLLSGFTIFVTLAQHVCGMCQVLGSRAECLF
SVVEDRTGCDLIKKSQIRNENMTIFIQQHYNIIQFVDMIETCHTAPFLSDLTAIMITLSIALIQILTISDIEG
AIRSIGISVTSLCYLFVCCYMGQKITDESSSAYEKIYNSTWYNAVVSEQKTLLMILIRRCHPLVITASKFYTM
SLQNFGKGKKKILQTSLSYCMFVRQI
>SinvOr318
MLENFLREYNINRILLSITGLWPYQNKVVRSLLWIFCFLLEISYYPFEILLFHDHSDDAQLIFEGGYQTLILT
IFVIRHLSDVLHNDKMRCLYEAIDEHWNIFTDDIEIRIMKKYSTLSQKIVIYYSMLYFSSLMVIIILPITPIF
LDIIMPLNESRPRFFAMEVEFRVNKDEYFLPIFCYTNLVIVVGMFIALGLDTMHITCTAHACGLFAAISKQIE
NVLLKVDDNNNIKKSKHVNKKLELLNEEITYRKYIICLKKHQLAIEFVEILNSSYQECALAILIVLLGILSLV
GIRIIYVLHELGTLIKFVFIFITTLCVLLVPCYSGQRIMDESQNIFYRAYATEWYKFSHRLKSLLLITLYRSN
KPCGLKAGNMIPLSIATYATVVRMSMSYYTALLSMQD
>SinvOr320
MDFQNTNLFNVRLNLLSGNLLPMTDKSDKSSFSLFWKVYCAFVWLLELVYMISLILAYFLAPNEATLSDSFDG
LFVIEEVGFVIDYSRILTQKTLVQKLIRNLNKALSIKDENMRRIVTTNLKSIKNPFIYYLVTGTGSLIFWCCT
TLPLILERNTFYHADYNTPAVFYKEPFSLGVFLLGNMMIMVCNIYLFLKVISVDVYTAHLISLITAQYQYISS
RLVLIFQNNKHPNNRSSREGNSEVDSFTATEIKNLCRQHVNVMHITLMLKKLISVNIFLIYLINVFRFCFIGL
IATTMSESYLKICFVFLYASGSATQFYVLCACFQRLSESSSEITNEAFHENWNQLNLSVKRTFLLMMMASNNI
EIKLSLFDRFNLSLPSFMSVLNQAYSVALLILRM
>SinvOr321
TFFQISSFFTVDNMNTDYIIDLLPVLIMTFGIVGHMYLRIMHVDELRELFERISNDWRFQKTHYEIKIMHEHA
EITRLFTFYSFLIHFVALVVYFIWISIPEILDVISPMNDSRLRIQPIKAEFFIDEKRYFYFIRFYVFILILTM
QITGITYLMISLAFVQHVCGMCVLLGYRAERLFSVAKKTAKSDLIRSKTYCGNIAVLVRLHYNIMKFIDLIEI
SHTMPFFMETVGLIFITSIAFMQILVINDVERAYRCFYISTAAIVCLFLLNFLGQRITDANLNVYEKLYNSMW
YNANISEQKLLILILKRRFYPTVITACKFYVMSLPNFGKICHTILSYFMFMRQ
>SinvOr322
MEYPEERYYKLHRLLLTSVGLWPYQSKWSTYLIRTVITIISLLGIIVQMSSFFTVDNIDTDYISDLLPILLCT
FGIVGHMYLRIMHIDKIRELFERISNDWKFQKTRYEIKVMHKHAEITRLFTFYYLLANLIIIVAYYIWMSVPE
ILDVISPMNESRLRIQPFKAKFFIDEERYFYFIRFLICALIFTILITSITYLVTSVAFVQHVCGMCELLGYRA
ERLFSVAKETAKSDLIRSKTYCGNIVVFIRLHYNIIKFIDLIEIIHTIPFFMDTLGLIFVTSIALMQILFIDD
IERVYRFLYTSTMAMVILFSFNFLGQKITDANLNACEKLYNSMWYNANISEQKLLILILKRRFYPTVITACKF
YVMSLQSFGKISQTILSYFMFMRQ
>SinvOr323
MEYPEENYYKLNRFLLSACGLEPYQSKWNVRLIRAFITVVLMSSSIFQISSWFTFEITYEFVVNGVPSLLVTL
CNLNSLHLRIGNVDKVKILFDRISKDWALQKTRGEIKIMREHAEFSKLFTFCWTILSYVSMVGYCIWLCTPEI
LNVLMPMNESRPRRQPFNVEFFLDEERYFILIRSHMCFVLLTIPIVFVSGFTLFMTLTQHVCGMCKLLGSRAE
RLFSIVQDKTGCDLIKKSQIRNKNMTVFIQQHYNIIQFVDIIETCYTTLFLSDLTGIMIMLSLTLIQILTISD
IEGAIRSIGISCATLCQLFVCCYMGQKITDESLSVYEKMCNSTWYNAVVSEQKTLLIIILVRRCHPFVITASKF
YTMSLQNFGKIFQTSLSYCMFVRQ
>SinvOr324
RGIIAPNFHLKLSLTIIYFLGTWPPATGKFRLLYLLYTACCLIFMLGILLAAEIANVLVHWGDMTKFVAVATL
LMTNSSHASKALILLRRQTRIQALLDTANSPAFSRYDEEYQDILVNYTWKAIFHHVVYQSFGGIAVFCWGFTP

ISDLIAGRSRRLPMEGWYPYNVTATPAFEITAAHQGIAIIIACFHNVAMDTLMTGLIAVACSQLAILERNITS
INNEGNIRGMRNNDTGKSLAMEKTVLSYQLLKRCAVHSNIIFDFTSEIQNIFGTAIFFQFLSNCMIICLIAFN
VSQMKVYIPAVLIGMLTYMCCMTYQIFIYCWHGNELHLHSMRLVTAAYSNNWFSNTEEFKRGLQIIMTRAHRP
LTLSAGRVMTLSLDTFVQIMRTSYSIFTVLQGSAA
>SinvOr325
MIRYRVYTRHIRRMLYLGGVLQDRTRSATWSYITGFLVILVCFSQCVFLINFCRDHIDNLVLISRLFGMACSY
IAPVLMSACFLAKREKLMELHETLNDLFERELTRDRETEATLLATLYAFDKPSYILCFTMGSTGILILCPRLI
PIVRNIVHHAEPERYRLPVPNKFPWPMPIGGGFSFYLHVLYQMSTFWWVIFTVGSVDSLFGYYAFQISSILRA
TTARLTNLRTPEVFTEVLGTCVQTHHRLLRCGRLLSDIWGLIIIRMLFTNALLMCALIFEASPFTHLTVGQIF
LFISYMTLKLLQTFIYAWYGSLITSASEHFREGIYFSEWPKSRLDRNVRTNIIVTMMQKPMIIKALNLSSVNV
NMFTKIVNTAMSYFFLLQSLDEG
>SinvOr326
MIDKQLQAYRKYQRFIKTILTVTGCWYMPTKSGKSTYYWPICVLLLMNVYLILILHTTYVFRHNLVNMMKMTG
VAISSISAIIKVTTFMVHRKSLKNCHRILSDFFEEELTQNKQIRRIMFSSLRATYTMAYAYFIILISLISGYA
APPIISMIRDLCHLRLTTNYNLPVGRGHLYFWTVPDNLLYHLHFFYELTQTSLSSMLACAIESVFGFYTYLFA
STVRVMIFRLTNPLPTDKFSDVLKMCIKKHQKLLKCRDTLERVFGPIVLWHVVSNSILLCSLIYETMEFSFAN
ITVIVPYSSIKLLQMFIYAQYGTVLTNASDDFRKGIYFSEWPNSDLDCHVKTNVILMLMQKPMTIYAIFSPVE
MIMFTNVI
>SinvOr327
MDINNYISINKKVLKFVGLYPICIVRYIMCCVCMITIVIPQGMQIYQNRQDLSTVLETSSVLFTILLALFKSL
VWVSNRRKMDPFIKYMLTDYWDIMTTCISEKHTDVHIICVKKAHLFTKGYLSMICLSLTFFFSTPIIEIFIMV
KSTNDNSTKHFPFLALYPESYYNFPMYEIIYLSQMVATISCGLVILGTDTLIATALFHTCGHFKVLQKKIKDI
NNEIDFVQDAYIKKNVMQKIKLHMIDIIKHHYTIIWFCDYMETVFSRMLFLQTLASSLIICLVGFQIATANIT
VSMISKLFKYISYLIMALFQLLLFCIPGDALIYESSMISKIVYTIAWYELPVLFKTEICLLMLRSQKPCKITA
GKFYVMHLENFNAVLSTAASYFMLLRSFSSDETTVKL
>SinvOr328
MTDKKDIWKSRYYLILRTYMTISGLWPYRSLRERCIHFIPTFALCFSILIPQSMYVLIASDINEVIEGLTSAL
ISIAFSWKVMCIMFDKNVKSCLKTIKKDWLSLKTDVERSILQRHVEYGRYLTTSYAVFMHTIQVSYILKPFLL
TLLEINTTNSTKTSASKLPFHVEYGVDADQYFYPIAIHCYLAIFAHIFSTIAIDSLYCTLIQHACGMFSIIGH
VLEDIGKNNNANFSLNLNKISDDDYKKTLDCLRKHLQVLQFTDLIESTFTKILFVSVNLNVICGSVTGIAMIM
NLNKGIHEIAAPLSVYVAQLVHIFLQFWQAQFLLDYSIVPCESICRANWYFTSKRCQKLFLLIMTRTVTPCRI
TAGKIVTLSIESFGSVLKTMMSYFTMLRSFQ
>SinvOr329
VTKTMNTNWNHYYSLVKKLSLLCGQWPYQKPVTRLFCLILMTLSTLSMIIPQIAKFVTCDGNLQCILQTMSAY
LLTTITLVKLYTCYFNRCKMKVLIDQLFVDWDELETPEEYEILKKYASNGRRYSLGYSLYYYFGVYVFVSMSL
VPQVLDVVSPLNQSRPILPVYPGYYFIDERKYFFYIYTHAMMAWEIAVTVIVSHDCMLLIYIEHVCSIFTLVG
FRFEQIIYNDAMKILHSHTSDAYLRKHIAFSVYTHRKALKFAQLIGDTFSLTLTIQLALNTVMISITLLQQQA
DILQLIRYVMYVVGQLVHLFCLSFEGQKLIDHSLQTRDKIYNSLWYETPPKSQKMLLFVMQKSLQPIFLSASK
IYIFSMENFTMIMQTSMSYFTVLSSLE
>SinvOr330
LKWAIGINQTCLKLMGLWPDSELSRRQKFVTDVRAIIFFTIMMCVAVIPGLMAFVRVWGNMMAMVDNMQISLP
FSATTLKFIIMWLHKKELEPLTKMIIEDWLRPKPKKERNIMIKHARIARIIIMFGCIMMTLACFILIIPPLFG
ASLRYLTNLTDPGRPFLVQTYYLNDFFSTDSPYYEIVFAAQATSIIMAAISYTGIDTFLSLLVFHICAQLEIL
KGRFLNLNSFKDFNTGLAINIENHLRLIRLSVDVIENTFNSMLLTLLVFFGMLFCVQGFLIVSIIDGTTTNVS
FWRICWLVSILINTGNHMCLYCVVGEILIAKAEAVYSAVYNYTWYLRPPKEARDLMMIMIRAEKPLYITAGRM
FPMTMSMFCSLIKTSAGYISVMLANR
>SinvOr331
LKWAIGINRTALKIVGLWPDCTLSHQQKRMTNAMALIFFFIMICVSVIPGMLAFVRVWGRLMELLDNVQIGLP
FSVTTFKFIVMWFHKDELEPLTKMIIEDWLRAKTTAERNIMIKHARIARILIIFGCFMMVIACFNLMVPPFFG
ASLRYLTNLTDPGRPFLVQTYYLNDFFSTDSPYYEIVYASQATAIFIAAISYTGIDNFLSLVVFHVCAQLEIL
KRRLLDLDSFRDFNTGLAINIEDHLRLIRSVDILDNTFNSMLLALLVFFASLFALQGFLIVSIVDGTATDVSF
WRMCWLITILINIASHMCLYCVVGDILIDKAEEVYNAVYNYTWYMRPPKEARDLMMMMIRAEKPLYITAGRMF
PMTLSTFCSLIKTSAGYISVMLANR
>SinvOr332
LEWAIGINRFTLQCVGLWPDEKLSSRQKFLANVRAFVIFTTLVTVSIIPSIFSLTRVWDDMIAIIDNLQILLP
ISATAMKIIIMWLRKEDLTLVVSMVIADWIKKKTEEERDTMMKQARIARRLVQLGCIIMVSAIVIIIIPPCFG
YSMRYLTNVTDPGKPLLLQTYYFQDTTQSPYFEIAFVAQAAAVVMAAFSYTGIDNFLGLIVFHICAQMEILKE
RFLSLKEYKDFNIGLSTNVQNHLRLIRSIDIIESTFNLMLLALVVYFGIIFCLQGFLIISIIDDGGNVSFPRI

CWLVSVVINTFVHMLLYCVVGEILISKCESIYHAVYDFAWYTLKPNEAKNLMLIMIRADKPLYITAGKMFPMT
LSTFCSLIKTSAGYISVLLANR
>SinvOr333
FDRAITITRYGMRLLGFWPQDTRLLYDLWCGAIFMLISSFTFPIALRTYTVVYAVTDWNSVMHQALEIIPTIP
LLARFIFMKMMAKNFRLILCMMTADWADYRYLTKRNRQIMIYYAKRGRRFSILSIILMALALIGFILTPVINM
WRNDSLWNVTTRILPHEGLYPFHNKKSPVYEMLYVAQLLVMFLCAMALATVDCFLYVIVFHVCGQFDILATIL
KRYDGSIRHHYMKDARNCACLSCIVKRHVHILSFVDIIEKSFRDFLLLQLLGYWFSLVLQGRELILHVRHSNV
DGIITCILYVLTIMYYIFIYCYVSECIIEKSEDIGRIAHDLEWQHFPRDSNLPIIAARTRIPCKLTVGRFLTL
SFPCYNTIITTTISYISVLAAFK
>ALG36144.1 odorant receptor 1, partial [Sclerodermus sp. MQW-2015]
MMKFKQQGLVADLMPNIRLMQISGHFLFNYYNDGGTKFMHRIYCCVHLFLIVLQYGLMGVNLILESGDVD
DLTANTITMLFFTHSIVKVIYFAVRSKLFYRTLGIWNNPNSHPLFAESNARYHSIALTKMRRLLFCVGAA
TVFSVLAWTGITFLEDPWKKVIDPVTNETTLVEMPRLMLRAWYPFDVKHGMSHVLILIFQFYWLLFAMTD
ANSLDVLFCSWLLFACEQLQHLKQIMKPLMELSATLDTVVPNSSELFKAGSADHLRDTQGTQPALPQPAQ
GENMLDLDLRGIYSNRQDFTATFRPTAGMTFNGGVGPNGLTKKQEMLVRSAIKYWVERHKHVVRLVTSIG
DSYGIALLLHMLATTITLTLLAYQATKVHAVDVYAATVIGYLLYSLGQVFLFCIFGNRLIEESSSVMEAA
YSCHWYDGSEEAKTFVQIVCQQCQKAMSISGAKFFTVSLDLFASVLGAVVTYFMVLVQLK
>ALG36145.1 odorant receptor 2, partial [Sclerodermus sp. MQW-2015]
MVLSLCGFWRPENSSKFFYGAYSFFMLIIVFTNAISMTMDLVINVKNFDDFSKNALMLFSMINVFSKGSS
LVLRHNDIIKLTETLFEYPCRSTNEDEVRVESKFNRIARYNSNRFMQFGLIVVSLIILESLYTCFSGKLI
LPFRAWYPIDYDGSLFIFFVCWTHQMIAITVGGMSSSSLDTIIAGLMLQVCAQVEIMKVRLHKWPVIRKN
LNVDGQDLRFQETRYLTACIHHHRHIFIFARQLNKLLNIVLFMQFFIGAILICSLVYHISVISVTSTEFY
VVMLYFSGIAAEVFIYCWFGNECILSSLSVMNAVYTMDWTLLTSEGQKSLLMIMLRAAIPIKFTSSFL
>ALG36146.1 odorant receptor 3, partial [Sclerodermus sp. MQW-2015]
MSHLYLQFNILRVLGFWEPSDWSSSMSLKLLGYRFFTCFMMFCMCSFNLTQILDLAFNVKNVDEFIGNSF
MLLTIFIVCCKMANALQNRRNILRLLRILQQRPCELLDQEELEIQKRFDRG
>ALG36147.1 odorant receptor 4 [Sclerodermus sp. MQW-2015]
MFLELQFSILTYCCLWLPRDWESPWLTIPYKVMAILVTFLIYTLTLSEFLDVVLAVENYEDLAGTLFMLL
SFLAVCTKVANALHSRRDILDLTEFFLTEPCKPFSEREIVIQRGYDKMIR
>ALG36148.1 odorant receptor 5 [Sclerodermus sp. MQW-2015]
MVFVLVTSFMSQILDTFLPLNESRPHQFPFEFEVFVDKQKHFILIIILMFHTMAVVYVMFAVANETMFMVH
MDHVLGMFAVLGYGINVNRGKRSTNFLKFSSFLIRHRLEHALPNDVESLKNKESVINLKYYHSIILCIKR
HQKASEYAAILESYYAPCLIVSITMSILILSPAFVQLSEIQLMKPQDVFEAVFVVCFWFVHNYMGQRLID
LSSETATKVYFSQWYLCSQRLQKMLPMIMSQCEKPCQISVYDLYISSLYCFRQVMQMGISYCTVIKQLR
>ALG36149.1 odorant receptor 6, partial [Sclerodermus sp. MQW-2015]
MVGPIFTYFADIKNYISVNSTQVEQWIPLPVPMVCTFRKYSPTKFVYSMIYFSQGLTAFCMVCGNIGMDA
FFIGIALHICGQIEILKRKLKLLGTGKNNQWLAFMELKRRHEELLNISDLLIQPTNFALNFQFFLSSLSM
ITIGGSVIDYAP
>ALG36150.1 odorant receptor 7 [Sclerodermus sp. MQW-2015]
MLQLGLSMILAVMDNFNEVVQYAIITSGVLLNLLFNSYPGQILMDHSIDISNKAYSGNWYDAPPKFQRLF
LMIIRRGSEPCCISAGKMVILTLSSFSTVNFHSIDRFLRSSQISE
>ALG36151.1 odorant receptor 8, partial [Sclerodermus sp. MQW-2015]
MLKNAKIGRFIAGFCAVFVNSGVFSYNVIMPLNSHVVLVNNETVTVKPLPFSFYNKIIDARFTPAYDIIF
VIQCLSGFVVNCITCSACGLAAVFVMHTCGQLKVLESWLQTLVEEWQTRGKQAVQDKLVTIVVQHLRILQ
SESIQNSFCTLLLFYHFITFSSNFSFISQIESIMNEICLV
>OXU26421.1 hypothetical protein TSAR_014901 [Trichomalopsis sarcophagae]
MELEVRKYENYSRDIKRLLIVSGIWPNFYPVLQRFVAVLAIFSTAMTFMGAFNFCLEHVSNVVVLTRGMG
LLFTLLSTGMKICVFLHHQKDLIHLNQHLSARFLDDLRNKAYQSHVLVRLPAFSELFYSLTYTIGSTAFL
TTIVIPLLALRHGKYIQVCPSIFPFEYAPGGLVYWLLQLTEAFAAFFVWSVTSGVDSAFGLYTLQMCGEL
RILGSKFESLRVSDKYREELRECIERHHLLMKARDSMEKTFGLLAIWLAVSSAVIQCTLVFQAMEVAKSM
NPLRIGFFFLYIVLKLLQAFMYAWYGNLIAEESAMCLNAIYNARWAGCGNSRFMTDVLIILSQKPLVFTA
KGCMSLKMEIFSKIVNTSNELRKYKTYRRDIKCLLVLSGIWPDFHPIIQPMLGVFAAVVSLITVTAFLNF
SIHHITNVNVLCKSFGLVISFFSTFLKICVFLWHHDDLVYLNTILNARFEEDTRNESYRRFTLARVNVFT
KTFYAVSICVGLATGMAFVLLIMSLSHGKYIMLYPSIFPFGYEPGGRLYWILLSIELFTNFFVWAVTSGV
DSIFGMYTMQICGEFRVLAHKFEILKTSENYRNDLKECVERHHELIRTKEVLQDVFGLLSIWLAITSAVI
LCMIIFQAIQVFKTMGLGVIYIVGYIMLKLVQSFIYAWYGQLIADESEDCIEAMYNARWAGSGDARFMTD
VIIVLSQNPLVFRAKGCMSLKMDIFIKLRKYARYSRALKCLLVLSGVWPDFHPVVQPMLGCFAVFVCSLT
AMATLNFSIHHITNFVVMTKSLTIAIGLCLSTIKIVVCLWHHDGLVYLNSSLTACFDADNQNKSFRRFTL

AKVNVFANLFYTLTIAVGLVTGMGVVFLILALLHGKYVMVWPSIFPFSYEPGGRVYWILLLVELSANLFA
WTVPSGVDSVFGWYTLQICGEFRVLAHKFQNLKTSENYQDDLKECLERHYALMKSGEVLQDVFGFLAIMV
AVTSAVIQCMLIFQAIQVFKRLSLGMMILIMAFIIIKHIQAFIYAWYGQLIADESEDCIEAMYCAQWAGS
GDIRFMSDVLIVLSQKPMVFRAKGCMSLKMDMFIKILNTSVSYFFLLQTLDEGLQKLRKYERYSRDLKWL
LVLSGIWPDFHPVIQPMLGWFAVLVCTLTAMATLNFSIHHITNFVVMTKSCSLAIGLCLSTLKLCACLWH
HGDLVYLNTSLAASFNADNQNKSFRRFTLAKVNVFANLFYILTIAVGLVIGMGIVFLILSLLRGKYVLVW
PSIFPFSYEPGGWVYWILLTVQLFANFFAWTVPSGVDSFFGWYTLQICGEFRVLAHKFQNLKISENYPDD
LKECLERHYALMKSGEVLQEVFGFLAILVVLSSAVIQCMLIFQAIQVFQQLSFGMMILIFAFITLKHVQV
FIYAWYGQLIADESEDCIEAMYNAQWAGSGNIRFMRDVLVVLSQKPMMFRAKGCMSLKMDIFIKTELRKY
ERYSRDLKCLLVLSGIWPDFHPIIQPLLGCFAAFVCFVSVIAFLNFSIHHITNVVVLTKSFGLVISFFSS
FLKICVFLWHHDDLVYLKAVLTDRFNTDNLNKSFRRFTLAKVNVFANLFYILTIAVGLTTGMAVVLLIIS
LRHGKYVMLYPSIFPFSYEPGSRVYWILLIVELFANLFVWAVTSGVDSIFGWYTLQICGEFRVLAHKFQN
LKSSENYRDDLKECVERHYVLMKTREVLQDLFGFLTILLAVTSAIVQCMLVFQTIQVFKNLSLGMMIFLI
AYITLKVVQAFIYAWYGQLIAEELRKYERYSRDLKCLLVLSGIWPDFHPIIQPVIGCFAATVCFITVIAV
FNFTIHHITNVVVVTKSFGLVISFFSTFLKVCACDRFLFQEYEDMMVNTKVYSSKICVFLWHHDDLVYLN
TALTDRFNADNQNKSFRCFTLGRVNVFANLFYILSVAVGLTTSMSVVIYFLTLRHGKYVMLHPSIFPFSY
EPGGQVYWFLFMVEFFASLFIWTVTSGVDSAFGWYTMQICGEFRLLAHKFQSLKSSDNYRDELKECVERH
YFLMKTKDVLQDVFGFLAIWLAVTSAVIQCTIIFQALQAFKNLSFGMIFIVGYIILKLVQAFIYTWYGQL
IAEESELCLGAMYNARWAGCGDTGFMSDVIIVLSQKPLIFRAKGCMSLKMDIFIKILNTSVSYFFLLQTL
DEEMQKLLNNLENSCSSNNPLDRSSTKFRPLPTFTCPYLDITM
>OXU22547.1 hypothetical protein TSAR_005521 [Trichomalopsis sarcophagae]
MEKEVEKYKKYKSNLKFMIVSNGVWPDYEKHPYCVRKFLNFCSISSISMTNYCMMLFVIATTTDVRSFTS
FFGLLLGGFGNLFKVCALTVNQKELQALNEGISASFERNLRVPENRPHLLANFPMFSKFFNFLSYSTLGT
IGFLTVIPLLHLRHGTYSRMWPILLPFSYEPGTVHWIIFAFELVVSFFAWITTCGVDCLFGLYSLHIVGE
MRLLSSRFQKLEWSENYRKDIRSCVKSHLLLLKTLSQMQEAFGGLAVWFAFNSAASLCTLVFQFSQVCHQ
VSSFDFHIICKLISELLNVVNGNEPCKSAVLVMPYLHKVGAGIFVFLVRKYHHCREKYMCMHQSEVCLNA
AYNSHWPNHGDKHFMRDVLIILLQRPMIFKAKSFIALRLDLFARIANTTLSYFFLLQTLDEKLELLRYKA
YTHNVIWYLKSAGLWPEGHPVSRKIRSMVTLFSTFVVMVTVSNFSFQNVGNVMVLTRGMSLAVSFSSAFS
KVALFLLHHDDLVYLNEHLTGIFERDMKEPEHRPDLLKNVKTFHRFMYTHVASLTFTLIMYVIGPLLALR
KHGKYVRVFPAIYPFKYEPGGLVHWILYVLEVLGAMCLWSVTSGVDCVFGVYALQVCGELRILAKKFKEL
RATENYREKLSDCIQRHHVLIKAKNKLDNIFGLISIWLAISGALVLCSLIFQITELIKSKSSYLRVVHLS
VYLLPKFLQIFSYAWYGNLIAEESTGCLEAMYDSHWTDSLDKNFKSDILIVLVQEPLTLIAMGCMVIQLD
MFTKIVKTSAIMDPKLLKYKDYERNVIWLLEAAGLWPEAHPVSKMILSLVTLFSSFVVMVTATNYSFQNA
INIKMLTKGMSLAVSASSVFSKIALFILHQDDLLYLNKHLTGGFMRDMKNSENRSDLLNNVKTFNKFLFT
HAVSVAIAMSMYSITPLLALRKHGKYIRVFPGIYPFAYEPGGLLHWIIYAVEVACAATFVTVSAGVDNLF
GVYSLQVCGELRMLAHRFRDLRAGDNYKDKLKDCIERHQVLINAKNKLEDIFGLIAIWLAISGALVLCSL
IFQVSELIKANASYLRVAHVCAYLLPKFLQIFLYAWCGNLIAEESKTCLYAMYNSNWPDPYDANIKSDVL
IVLSQEPLSVVAKGCMIIQLDMFANRFKYKAYERNVIWLLESAGLWPEAHPVPRKILSLVTLFTSFVVMV
TATNYSFQNVGNVRMLTKGMSLAVSFSSVFSKIALFILHQEDLLYLNKHLTGGFMRDMKRPENGPALLSN
VKTFNRFLYTHAVSVAIAMIMYSITPLLVLRKHGKYIRTFPSIYPFAYELGGLVHWIIYAVEVSAAATFV
TVSAGVDNLFGFYALQMCGELRMLAHRFRDLRAGDNYKDKLKDCIERHHVLINAKSKLEDIFGLITIWLA
ISGALVLCSLIFQVSELIKNHVSYLRIAHVCAYLLPKFLQIFLYAWCGNLIAEESKICLYAMYDSHWPDS
HNTNSKRDILIVMSQEPLSVVAMGCMVIQLDMFAKRIMEHDVRKYFKYKRGIVFMLSASGVWPNSASHPA
AVRLFLNICSALASGSMFYCIVNFCLKYATNINAFTSCLGLMIGFFSTFIKVIILPMQKEDLQSLNEGVS
ASYERNLRIVEFRHHLLAHFPMFSRFFYLYSYSVGISVLLLTVMPLLALRHGKYVRMYPQLVPFSYEPGG
ALHWSIYAFEVFCGFYLWSVSSGVDSVFGLYALHMVGELRLLNVRFQMLKSSKNYAKDLKSCVHSHIMLM
ESRHKLQRVFGFLAIWLAITCAIALCALVFQALQAKHATIVRVIYLCGHCFLKLLQAYFYAWYGNIIAIE
SDACQSAIYESQWPGSGDKRFMNDVLVVLSQTPMIFKAKQWMPLRLDMFSKLELAKYKSYARHVITRLIF
AGIWPESNKTIKTILYFISFASTLTVSVTSINFGIQNANNVILLTKGIGLASAFSSVFSKALLLPLHQED
IIFLKNRLTTKFMSDMETIEYRADLLSSVHVFSAFFNMHEAMVAFAMFMYCFVPLYVLFKHGTYLRTYPC
LYPFSYTPGGLVHWLIYALEVAGAISVWTITVGADCGFLMYALELCGEFKILARKFTELKAGDDYKKKLK
ECIERHHLIIEAKNRLEDSYGLIVIWLALSGAFLLCSLIFQITELYDNHGSYVRIAHLCSHLVAKNLQIF
MYAWYGNLIADESKAFLNAMYDSHWPEACDKSFKNDILIVLTQEPLVVVAKGCMYVQLDMFTKIVKTSMS
YFFLIQTLAN
>OXU31131.1 hypothetical protein TSAR_010114 [Trichomalopsis sarcophagae]
MCANIFIGHHQFGLRVVGSWPGKSQLPGFYFAIGIMLFFLIFEILNITEVYHDLEELMDNLVSTIGVVLG
LFKFITVRVKRRKLKTVINKIFNDWKTDRQFVSEMMVENCTRSQLVSKFVIFLYNSMNFTYFLRTVISRI
FDEVQDRKFLAQVTFPIIDGRQTPLYEIIIFFQFVTASVCFNSQALVEGLLATLVLHACSKVDVVRREIL

NFSTFCKTDKNDKKDIIKTLSKLSEEHLKFVEFSEDIQDIFSYVSFFHIFFLTLIQVVSGNPSLYEDLPK
QFLRINSVVSADVPRKRPILRVTTRQNYQWYTVVLTEQSLRWQNERGTKPVNLIHYAILTISFLVSAGYY
CIAGEYLTSQSEIIFNELYNCYWYEYSSSYKKAICFMLLKARKPVKLTVGKFSTLSLIYLTSIMKTSFSY
LSLVRAPHRIGLKMIGVWPGVTGWSGFAFIMGWLFFTLIFMIWDVFIIYHDLELLMNNLLNTVGIVMGLL
KILTFRVKRRNIEFMINTILDDWLVENNETANGIMPKNLSRSELLCKMLLISYNCMNVTYITKTLFSYLF
DEVKDRQFLAQSSYPIDARVSPMYEIVFAEQIIAAILCVNSHALIESFLGVLALHACIKMDSVRREIINY
SNICRVQQHDKRLALMTLRKLWHKHLQFIEFAENIPNVFSYISFFNIAFIVLIQVICIFIFMNMIENEEL
SIHSIHCIACNVSCLLSSGYYCVIGEYLTYQSELILNYLYECPWYEFEASDVKILTFMLMRARKLIHLKF
GKFNDLSLEFLTTVSMQYFTYTLKSHYKQIILLFRYTKLPIMSAKIVMRHVQLGLQVIGLWPGYTSSVGF
FITILLLLTCLAFQLWHAAIVFSKLDALMGNLGATLAVATAILKLIAFHSKGRNVIIVINEILNDWTIEN
RFSNCEVMIKNTKRAKHLTKWITGACNGTVITYLVSAIIAYSSGVVEKRLYVLPSKFPLYCKQSPVFEVV
CFFQFAIAVISANAHALIEGVLTVLVLHAGTKVHLLEREIQKFSVICQSKTNKEITSKATRGLIDKHLNF
IKFVKEVKDIYYFVSFVHVFTFTFLHVIVGYMFIDALESGDRSIKLFLYGLFTIRALASTTIYCIAGEYL
MNQSMRIYGELYNTAWYDFDVKNIKAVTFMIMKARNATSLTSASFGQLSLFYLTGTNSNLANSKKIMSDK
IVMRHVRVGLQVIGLWPGYTSSVGFVIAITWLLTCLTFQLWHAAVVFSKLDALMDNLGGTVAVATATLKL
IAFHAKGRNVKIVIKEILNDWAYENRSSNCEVMVQNTKRAKYLTKWITGAYNATVITYLVNAIIAYCSGI
TEQRLYVMPSKFPFFCKQSPVFEIVCFFQLSAALISTNVQALVEGMLTALTLERGDRSIKLFLYGLFTTR
ALASTTIYCIAGEYLMNQSMRIFDELYNSAWYEFDVPNIKAITFMIMKARKATSLTPASFGQLSLFYLTS
DRTQRSFIAIHHHKTKYTMSILSRHVRIGLYAIDAWPGVSSSGLYFLVMAYMTFSLIFQILNTTEVITQL
DLLMNNLQTTMPVILVVLKLFVFRVKCRSARLLIADMLSDWKCINETKERKVMMKNAKIAFHLSSTIAIC
YNGLILSYLLKAILAYKTENIYDRKYVMQATFPINAKRSPVFEMLCLFQFTVSVFAANGHAILEGLLTTS
VLHANTKAFGVCQEITKFAESCEANKSRKNIVEAKRRLIKRHLYFINFAEKIQETYAYISFFNLFLMTLI
NCIVGYMFINLTIKKDNISSLLLCIAYMFTALSAGSLIFEKLYDCPWYKFKPVDTKTFIIMLMKSRHSVT
ITAGNFGDLSLVYFTKSIKHLL
>OXU29017.1 hypothetical protein TSAR_005145 [Trichomalopsis sarcophagae]
MHLFVHDEFDCRPMEDQLSSQSMNLFYFRSTRIYMSILGIWPYQFWSSRIVLRCFWIFQHMSIMIPEAIK
LYENRSNIDLVIEGTAPFTYNTTMLIKFFNGIFNLEKTKSVLEKVKNDWNTLLDDGETEILSYNCSYAMF
ITAQIFHVFCYFFLGQNVLNYSENLRDKAYNFNWYAASTKTKYLIQFIIMKSLKPCLISVSIFPLTLENF
TTERNMKFYLVYTRLSMLCLGIWPYQSWSSMLTLRSLWGIKIYKNRKNLNSIIDGLPPFIYNVVIAIKFI
NGIINQHKVRTCNDDMQTWLLIKSILEKIKNDWNQLSDKKEIEMLRDYSDTGKAFNTVYLSLVTVILLSY
MLIPMLPAALDLVNPLNESRHKSPLYLVELYIDQDKYFYSVLTHAYITSLAGILPLFAIDSLFSSCAHHA
CGMIEILGGRLDNIINEEASIKEIDNNEEEKNAIACVIEHRGVIKLSVAERKILREHQFVVQYVLFFCAE
LCDRDDERYWICGILFSEKFIDNAIQSAVIKMGEEFKDSIRFAMFTFAQIFHMFCYYFLGEIVLHHEEKL
KDYASNLNWYKASPKTKYIIKFMIMRALKPTTMRAIIFPLTLENFTTIIKLYEVRRLTDLVMECIPPIFY
NFGISAIFINSLYHKRKIKNVIQKVHKNWTRLSDKSEIEILTQYSDTGLIMDRFYVGILYSVLLFFALLS
LTPIMLDYVHPLNVSRQRTPIYRVEFFVDEEKYYWTILLHAGVVSILGMIPLTTMDIFVANSVQHTCGMM
KILIKRLENTMKAISTTKKDIYDDIRECIILHAEILDFFNDINNIIGTVFGLLLIILISLTSLTGIVVLI
KWGEWHQVIRFGIFTMAELFHALCFSYHSQDLINHNNRLYISIMNSGWYESSMETKVLVQMMLLRCNKPF
LINCIIFPMSMENFTLLIKTMFSYFTVIKSCRVFMVSSGVWPYQPQNVARILRLLWITQHISIMTPERCN
RRLINCHNLLLYVMKARVIDIWLLKFKCYVSIFLQIIKLIEVRGIADLLLECIPFIWNYNSSSTKGKSLI
KELIEKIQKNWITISKKSEVEILTRYSNMGIRIGWLYIGALYFTLFIFCLFPLSPIVMDYVNPLNVSRQR
LPLYRVQFFVDDKKYYWTILMHAYTTTMIGIIPLVTVDLFLANCTQHVCGMMLILGKRLEKTMETTKLVV
NKLDDNIYKDIRKCTILHTEILDFIEDINYIFGTAFGILLAILMFLTSFTGIVVLIKWGDWNEVIRFGMF
TMAELFHAFCYSYHSQDVIDHNNQLHKSIMNSGWYKSSMRTRVLVQMMFLRSNKPCIINCIIFPLSMENF
TTSIKLYEIKNDTDLVIEAVASFFYNISITIKFINQVINEHKVKIILEKIQDDWKSLEDDSEIKILSHYA
RLGKLFNFMYIGAVYSALISYMVLPLTPIILDLIIPLNESRPKQPLIMAEFFIDQDKYFYPLMIHAYLSV
LYGIIPLLGTDTLYMNCVHHSCGMLKILGNRIRNILNSSSRELSTKIKYEKMIKCIIQHQNIIELCNNIN
ETYSTSFLIVLFFSITLMSFSGVATVIKLGDNFNDVIRFGFFSVAQIFHLLCYNYMGQNVLNYGEELRVQ
IYNTNWYEASLKTQRLVKFMMAKNMHPIILRANIIPLCLPNFTRVIKTSMSYFTVLQSTR
>OXU26754.1 hypothetical protein TSAR_007005 [Trichomalopsis sarcophagae]
MDADNVSLMKLIFMIYFLVLFLFTIFTYCYVTEILRCKSLELSYAVYDCDWTILPAKEARILLLILVRTQ
HPFEITAGKFASFSLPLYCRILKTSAGYLSMLLAVKKRSEQAASFDVAVGPSRAFLRFVGVWPNPEGSET
PFETIQCIIVTLTMIIFANIAQTVKVFMVWGNLNSVIEILTTADMPIFVALMKFLVAWYNRKVLKGLVIL
MMDDWSRSYSSSNLDSMWRTARFSRKLSAVCIGLAQGTITAQFIMVVVFDVNNKGEAERTLYMISYFPYD
TQVSPNYEITWLGQCFSNIFAAGAFSAVDAFFAVLVLHLCCQLSILRKELVMLAGHHKKQGDNGEEFSRK
LARIVEKHEYFNSFAKTIEDSFNTMFLSQMIASSLALCLQGYQLVIIITNTEGKLPVFQLIHMIYFTCCF
SFSLFVYCYVAEELRFESTELDYAAYDSDWYNLPPKDTKLLLLLMHRSRKPLEITAGKFCAFSLRLYCSI
LKTSGGYLSMLLAVKDRLSLGFHTAFSVTRFVMRFQGIWPGVDKPRTGISRFQFIPAALMMGFFINAVQT

MEMTRVGGDLNVIIDILTFADIPIFIALVKHVGIAYNNKVLYKLLYLISEDWKEVTKESEKKVMWQKARL
SRIFTMIEVSLGLGRLFIHTIRMTYAMLHPTSFDPTGKPIRPSYMRGYFIYDSQSTPIYEITWGCQFVAT
AFGGCAFASADALFVALVFHLCGQLTNLQIEFREVGKNTSRNKLEFVRSLARIIKKHRRICHMVDKVEYC
FNKIYLLQVSSSSVIFCLHGYSLVTILFYQDDVVVELIFMTFFTFGFIYSMFVYCYVAECLSTEASASLA
LSSAIFDNTWYDLPPKHAKLLLLPLQRTGKPLIVTAGKFVVFSLNLFSNIIKTSAGYLSMLLALREKFFP
QTMEIIKVWGDLNSVLELLTTSDIPNLISLIKILSVWYNKKVLGLLIMSMENDWKSLKTVFELRVMWKNA
KLGRLITLAIYSLTYSTVVTYVVMAVYITAIAYKQEFILTPDNSTKLRPQYMRAHFAYDVQKSPVYEIVW
IFQCIAMHVAGLSFMAIDSLFSVLVLHLCGQLINLQERLKNVTQNLTKRHNLSYQLSRIVTRHEQLDRFA
KAIENAFNTMFLAQILLSGVVLCLQGYQIVIILTSRDTVQVTELLFMIYFTLCFAFSLFIYCYIAEILRT
EVCILKVHLQIIIMINPFVSIINLKIELTQSTEVGNSAYECNWYDLPACETRLFILTMIRSKTPFEITAG
KFTAFSLQLYCSVYFENLGWLLINAACRFDVAIQTSRTILRFLGVWPDLKRKESWMYSGHFLIPAIVMFY
FVNIPQTMMVTKVWGDLNAVLEVLTTSDIPIGIALFKMLGIWYNRDVLGQLVVSMSEDWKSVKSPEERDV
MWKNARLSRLLSVTIIGLAEGTIVAQFAMVIYFNVLEARQYSLTKDNVTARFRPLYMSAQFFYDVQKSPN
YEIHWLFQCSSTIFAASAFSSVDAFFAVLVLHLCGQLNNLRKKLKKLPKQISDKGGGAFVEKLSEIVTRH
DHLDRFGNAIEDAFNVMFLVQMVASSMVLCLQGYQLVMITTAGDGIPLFELIFMIYFTCCFTFSLFVYCF
VAEVLRTESMEVGNAAYESNWYDLPSCETKLLMLVIIRAKKPFKITAGKFAAFSLGLYCSILRSSGGYLS
MLLAMKDRLAKALITFIIIGARFFLGTLNYK
>OXU24529.1 hypothetical protein TSAR_001686 [Trichomalopsis sarcophagae]
MSSKELAKYRKYAQNTRNLLLPVGLWPYDSSSFIYRFSSVFAGMSLVLLSYTALNYCFINITDITKVTRS
FSWCISIWTMLMKIVLFVVHRDRLLDINETLSKTFERELKSLGLEQALLVYPGKYPFNFEPFGVIYYLIY
AYEALLAFLFFCVGCGTDTAFGFWVFQICGQLRILAVKFSKLEPGKDYAAGLKECLEKHEMLLRCRDHLQ
RVFGFLVIWLYVTISIVLCEYIYKMSKMTLQLSFWQSVMIFTFATKFLQAFTYAYCGSIIDIESEKLLYA
IYDCHWPGSGDRRIMSDVLSLLTQKSMALRSYNFFIVSMEMFMKIVNAAVSYFFLLRTYKRDLRFMLICS
GLWPNYQKHPRMFRAFLSFCSAFLNGVVSFGILSFCISNATNINLLTRGLGLFFSFSSAFLKVCTLSLHQ
KDLQKLNDGVSASFARDLEVAENRPHLLAHFRTFSKFFYTFDYSVAMNVLLYALIPLSLLRHGKYIRMYP
QHFFYAYDYEPGGAVHWTLYAFELLAGFFLWSVSCGVDSFFGLYSLHVVGEMRLLSHRFQNLKSSKEYAK
DLKGCVERHVELIKSQHLLERVFGFLAIWLAVTCAVVLCALIFQATETKNLSTLKVCYLICYSFLKLVQA
YSYAWFGNIVAAESKLCLEAMYNAQWPGSGDTRFMNDVLIVLTQKPFAFKARNVMLLEMDMFQKSQLLKY
RTYSRNVRAFLAFSGIWPETNRLLSRILAFAALFSNSVVALTVLNFCIHHASDIMILTKGMGMVISMSTS
CLKVIIFLLHHKDLAHLNCQLSKRYTEDLSNPDNRSRLLIRVASFSSILYTLIITGCLTILMYGLIPIFA
WLKHGKFLRMYPAIYPFAYEPGGVVHWSIYGLELVAGMFLCSVTTGVDCSFGMYSLQVCGELRLLAKRFE
ELGTGKEYKKDIEQCVERHHMIVSAKNKLESTFGLLTIWIAVSAGTVLCTLIFQFTEAIKVRSTGLHMGV
MSMYIVLKFLQVFTYAWYGDIIAEESDLCLHSIYESYWPDASDPHFRKDVLFVLLQKPLTLKAKGCMYIQ
LDMFSKKYKKYKSDIKFMLIGSGLWPDFHQHPKHLRTFLSVCSAFASGATSYGIVAFCVDNVTNINVLTR
GLGLMISFSSTWLKVVMLAVHKNDMLSLNKGVSAQFEKDLEKPEYRPHLLAYFSSFSRFFYVFDYSVALN
IGMMVFKPLLSLRQGKYVRTYPVLVPFKYEPGGVMHWSIYAFEALSGYYCWSITVGVDSVFGLYALHMVG
ELRLLSHKMENLKASSNYKRDLKECVERQMMLMRSQQLMQRVFGFLAIWLAVTCAIVMCAIIFQATEIRS
MSAYRAIYLVCYCFLKLVQAYSYAWFGNVIAVESELCLSAIYNSHWTDSGNLSFMNDVLIIQSQSVMRFN
AIGCMIVRLDMFSKIVQTSVSYFFLLRTLDESLLELLRYEAYTHNVIWFLKSAGLWPEAHPVPRKILSMV
TLCSTFVVMVTVSNFSFQNVGNVMVLTRGMSLAVSFSSAFSKVALFLLHHDDLVYLNKHLTGGFMRDMKE
PENRPDLLNNVKTFNRFMITHAISVAIAMSMYSIGPLLALRKHGKYIRAFPAIYPFAYEPGGLVHWILYA
LEVSGAASLWTVTVGVDCVFGLYALQVCGELRILAKKFRELRATENYREKLHDCIQRHHVLINAKNKLDN
IFGLISIWLAISGALVLCSLIFQVTELLKTNNSYLRAAHLCAYLLPKFLQIFTYAWYGNLIAEESGACLD
AMYGSHWTDSCDKNFKSDILIVLAQEPLALVAMGCMVIQLDMFTKIVKTSVSYFFLLRTLNEENE
>OXU31622.1 hypothetical protein TSAR_016112 [Trichomalopsis sarcophagae]
MIESVYKLKMNEDNKQNLERNQGFDYAVQLTRLLLMPCGIWPAKFSSRVQRFLRPFLIVACCFVMLFLLV
PVCLFMFLIVRDVRIRIKLLGPLGFSLMSLFKYAVVIIRSREIEKCIQNMLDDWQQVASDEDRDTMFENA
RTGRVLTMVCMFLMYGGGMPYVTIVPLAKGATMIGNVSYRALAYPSYFIFFNPYIRPVYDVVFLTQCLCG
FTRYTITCGVYSIVIICVMHICSRIAVTSSMLQRLADNYDNKLMGIVVKHHLKFLNFAAKLDNIFREIFL
VEVMGSTGVICLLGYYFITEYEQRESIATITYFFLLMSFVFNIFILCYIGQVLTEKCESIAKAAYTTKWY
QLTGKEARSIVFIVSCNHRPVALTAGKLLKLSLNSFSSIIKAAAGYLNILRTAIPMGIWPLKSAAYPSFL
RPISIVISFWSAAFLIIPGILSVIRVQNDIVLRIRLLGPVSFCLVTSFKYFSFLVKNRQFYAYLINVALD
WREMKKNNHNRIIMLRKTQISRFCMTSCSICMYLSGMTYNILLPLMKAPTQVGNVTFRNLPYTGYYIFFD
QYADPYYYVVFVMQCMSSFFCYSTCCGVCCISIQSVLHISGRCDITSVMIKNLNGNCNEKAVKAVVEFQL
QSLKFAREFEKLLNQMFLVEFVGSTLNICLLVYYFMANFKENDTIGAVAYALLFISFTFNIFIFCYLGEH
LTEQCASVGAAVYTMDWFRFSAKKSRDLFLIVLFCQRPVVITAGKMVNFSLLSFASLMKASAAYLNMLYK
MDVSAIKNAQGYAYAVQLTRWLLLPLGLWPTKSIIYQKILRPVAILLCLFIMLFVIIPLCLFIFLVVKDL
GIRLKLIGPLGFGLMSLFKYVVVIVKQRDVASCFLGMAVDWQELSSLSDRKVMLRNAKTGRLLTIICVIF

MIFGGMPYITVLPLTKGPIMRGNVSLRPLAYPSYFVFFNPQIRPIWDYVFVTHCMCGLVRYSVTCGVYSI
AILCIMHICSQITITSSMLDRLVENFDNMLLGKIVTQHLRFLKFASKLEDLFNQICLVEVLGSTCIICFL
GYYLITEYEQREPIATVTYFLLLCSFVFNIFILCYIGEILTEQCESIGTTAYMIHWYHLSGKEARNVVLI
IASTQRPVVMTAGKMVNLSLQSFTNLYHIITNNKIFGQTSLGDKSFSNMSKQNLISNSIELKSIRNAQDF
EYAVQMTRWLLQPLGIWPMKTSTFFSSILRSLSIATCTFLLGFLLVPCCLHMFLIEKDLGVRLKMIGPLS
FCLMNIFKYYAVLIKDGQISSCIVEMAGDWHRLEGSEQRGYMLENAKTARVFTTICALFMYGGGLPYSTI
LPLTRDAIIVGNDSYRHLAYPSYFIFFNPHIRPIYDLVFFAHCLCGFVMYSVTCGVCSIAILCIMHICSQ
CSITSVSLRSLTGDVDEKTFGKIVTQHLRSLKYTGLQVFASKLEKILNDMCLVELIGCTFNICMLGYYFI
TEFEQSETVGTITYSLLLISLTFNIFIFCYIGDLLTEQCENIGEVAYMINWYQFSGKDARNIILIVASTQ
RPVVLTAGKMVIKASVTYLNMLRTLTASES
>OXU29131.1 hypothetical protein TSAR_009914 [Trichomalopsis sarcophagae]
MLGTKSPASYLNNNTSNIDSADVGIENKIFPTTFSLLIGAGIWRPTTMKSRLSHVSYNVYTAFCFIGVVM
LILTILLNNMSDESSLIESWYMVVIFSHGLLKFTNLLCRRAKIIDLLEKCIMDEGWTVARNKDESAIMVT
SENSEKFITHLWLSLLLVNGLGNALNPLIHENPNNSLMFECYSPCDRSIPSCFWTAYAYQLFGHAISSTA
HVGSDCLIFNLIERINTHVKIFVDRLHKLPARVESGKLKEGFDVSQSERLLLRECIQDHRKIYESVRDLN
DTFYDLITIQFVTTISIICTNIYFLSKQELFSPDFIAVCIFLGCVLTQNFIFCWYGYKLPETSARVVDAI
FNMDWFILGEKTKKTLLFMMMSASNEVKLFNNVLVNLSPETFLKLVKLSYSAFNLLQQSKTEELLANSRA
NERRSERIAIEDQVFPTTFLLLKAAGVWTPTTLKSRRSQYMCYRIYSAFCFVSVLALVVTISIENVVSSN
ASILESWYMLVIFSHGLLKIKNLQWRRVKIIHLLKECIMDERWSIARNQDERAIINESKRAEKFITHLWL
SLLLVNGLGNALNPLIHENPNNSLIFECYSPCDRSIPSCFWTAYAYQLFGYTISSMVHVSCDCLIFNFIE
RINAHMMIFIDRLQKLPSRVVEGKNEGCLDPSRHEARLLKECIQDHRGIYESVEELNNAFYEVITIQFVT
SISIVCTNIYFLSKQELFSADFIGVLIFLGCVLTQNFIFCWYGYKLSESSSYIVNGIFNMDWLVLNKRSK
GLLLFAMMSASNEIKIFHNALVNLSPETFLQVPIKIANDRIDEDGIPDVEGLEKRVFPRTFLLLIVGGLW
APTTIKSRSLFACYQVYTVFCFISLCMLIITILIDNVLSDDKTMESLVEYAYMLIVFINGLVRIINLVSR
RDKILRLLQGNIMLDRWQSHRDDEELAIIAESKISEKYVYIRDQSQYPTGIIAKPCFRLVLKIWGSLILM
NGISNSLNPIIHENPDNTLMFECYSPCDRSVSSCFWMTYSYQLFGYFILSVAHVGVDCLIYNFIDRINSH
YKIFLNRLLKLPARVREEARNDVAAALLYENNYIKECVADHHSVYKATEELNGIFNELVFVQFMSCISLL
CTNIYFLSKQELFSPPFIAVFVFLCCALTQNFFFCLYGNKLSQTGSEISCAIFGMDWQELQQETKRKLLF
IMLLASKEIALFNNAVVNLSPETWSKYRTRLLICSTNQPTKIEVADIDGVEKKIFRRTFILLIAGGLWTP
TTTKSQALYTCYQIYTVFSFVGVLMLIVTILIDDTMRALIEYGHVLIVITNGLVRIINLASRRDKILHLL
RSNIMLNKWQNCRDDEELAIIAKSEISEKYVYIRDQSQYPTGIIAKPCFRLVLKIWGSLILMNDISNTLN
PIIRENPENTLMFKCYSPCDRSISSCFWMTYSYQLFGFYILNAAHVGVDCLIFNFIDRIDAHYKIFLNRL
LKLPARVREKARDDVAAALLYENKYIKECVADHHSIYKKSSEIADAIFGMDWQDLQKETRRKLLFIMLLA
SKEIALFNNAIVNLSPETFLKLLKVSYSAFNLLNQSTKK
>OXU28886.1 hypothetical protein TSAR_009575 [Trichomalopsis sarcophagae]
MDLLPVHFRTFQFFGLWYNDPRSYRLIKLVHRSLIVLLIVHLSLFQMIALFSAKRSVDEYTNTLFLALTY
FVHIYKTLVFMAKNRSVNEMLDEFRSDICRTRGPEEEHILAKHVQRANWAYSGRMILTLFAGSIRVVLPI
LIGFSTGKLELLPFDTYFFNVKHLAQYALVYVLQTLAIITVIVTDVCLDSTPCACMILACAQLEICRHRI
KHDNMVLYGNSKDGPGRGCNEEMALKEYVKHYVLIQEAVHRIQSVFISIVLPIFSSALLTLCTSIFQLAQ
KNHTTGEYCFIILYLCCLLVQTFSLCWFGNELQSKGEIVTSAVYETDWTVLKPCLKKNYRYLMFMGQNKF
IISFHGQCTLSLQTFIWMIKTSYGAFNLLKQVADTNMDLLPMHFRTFRFFGLWYDDPRSYELAKLVHRSL
LVVLIVHMSLFQMIALFSVKHSVDEYTNTLFIALTYFVNIYKILAFMAKNRSVNEMLDKFRLDVCRTRGP
EEKRILAQYLHTANWTYSARMILTLCTGVIQIVVPFLIGHFTGKVGLLPFDTFFFNVEDLAQYALVYALQ
AVAIVTVVITDVTLDSTPCACMILACAQLEICRYRIKHDINVIANEVTGDGKMNSECKPGKELREKMALK
KYVKHYVLIREIVDRIQSVFISIVLPIFCSALLTLCFSTFQLAQKNQTTGEYCFIITYLCCLLVQIFCLC
WFGNELQFKGEIVSNAVYETDWTVMKPRIKKSYWYLMFMGQNKFIISFHGQCTLTLQTFIWMIKASYEMD
LLPMHFRTFQFFGLWYSDWRSYRFIKLVHRSLELLLIVHVCLLQIIALFSVKHSVEEYTNSLFIGLTHFA
NIYKTVVFMVKNQSINEMLDKFRLDICQARGPEEEKILAKYLHKANWTYSARMILTLFCGSISIVVPILV
GIFTGKLELLPLDTYFFNVNDLKQWTLAYVLQSLTVITVVVTDVCLDSTPCAFMILACAQLEICRHRIKH
DNMASHEIGQDVPRKEYKEEMALKEYVKHYVLIREVVYQIQSVFISIILPIFCSALLTLCTSIFELAQNY
HTTGEYCFIMSYLCCLLVQIFCLCWFGNELQLKGEIVSNAIYETDWTVMKPCTKRDYWYLMFMGQNKFII
SFHGQCTLTLQTFIWMIKTSYEMDLLPVHFRTFQLFGLWYDDPRSYRLAKLVHRSLVVLLIIHFSLFQMI
ALFSVKRSVDEYTNTLFFAMTFFVHIYKMLVFLAKNRSVKEMLDEFRSDICRTRGPEEENILAKYVRRAN
WIYGARMILTLLCASIRCVLPILIGLSIGQIDELPFDTYFFNVSDSLQFALAYVLQTLAVVAAIVTDVCL
DSTTCASMILTCAQLEICCHRIRCDNSNKAVDADSDIAEVFIRKDKDENCFKEYARHHITIQKIAQRIQS
TFIPIVLPIFCSALLTLCTSIFQLAQKNHSVVEYCFITIYLCCLLFQIYCFCWYGNEVQLKGELVSDAIY
QTDWITIKPSTRKIFWYIMFMGRNKLVISFHGQCALTLQTFIWFACSHTKLDKTFNQSPTEVITLNKIQY
LFYNYEVTWEEARLICTGYESQLATIDTVEQALSITKQTSESSLADEEFWIGSKMQSKGFWIWISNNKKL

MPNEFFRQIIMSPSYTNTSSESDFRCLTIFRRNHSIPLFSPLRCREQRPFICQRGIVDELPFESQIEDLF
TVKIENKDFTLFSAKLTWLESVVQCRQRGLQIAEVSSTAEAQLLAIKMIKARPSNWHDTISNMNDEKYKY
IIIHKRNFTYSTGSIENAWIGGYVKDRLWKWLPTGSDIARNQMWYDENMISGECLLLDRHVCKFPMYVST
KCNRKRNFLCQTPVEKLVRFEPVAVFVDTCQYWISFKPQKWIDASQSCTKLNATLVVIDSLTTLNHITNI
MIDNRGNLQHIWIDGRRRYEVTEDRVSDGIWYWNSSGVRIPNLKESFIPWYEDPRLRENNTDCLNMDREN
FDVPIVYGLDCNIEQTYICQKSTVSCNQIKTKNVFNSSYGSTENPNEKLLLNLKDDSNIRPENVKSKTDN
LLNNNPTNKTVYDFLTPTNFSTVTERNFKHGLDIENSTLSDNYTENNLSISARFLLDDFITRKQSNHANI
NRETTTISILGFQDIFELMAKNVVENKNLSNILLSNTEGNESIQSSLENVDMESYDKIDKQLQILLNDSI
LKIVQSSINKNLNKSNFVPSSTFTTPIPKNLSKKKFLNNTLQTDKNLRILQPSKLPVSSSKNVASLKSEG
NQYKKTINRSEILKDDAKNMYNLGEIMKSMFSPRRYPSSKIDNMHTTRKEKDMTLETSTTETNFRLYNVK
TSANKKKNFARRKMYSSKNTIQSIRKPSNDNKDKVNVTDIVDALKNDFDKYNSKPLSLGIMNEMPFIGVK
KNVKPWNALLQHSMSKWSYSLSISISYCENRRFSGQAKNNTMGRKQNYYLPKMIYRLLVFLIIFQFTLSQ
IIELLTMHGSVDDFTEVLFLTLTFVALCLKVLNFTT
>OXU27948.1 hypothetical protein TSAR_005503 [Trichomalopsis sarcophagae]
MSEDWRSSKTEKEFQVMWQNARISRLMSITIITLAEGTIMAHFTMALYFTVLESKQYSLTKLNATTRFRP
LYMSAEFFYNIQSSPNYEIIWLFQFLSTMFAASAFSSVDAFFAVLVLHLCGQLNNLKEKLKNLPKRHEEG
EIQSFLHMLSDIVVRHEALNKFANTIEESFNIVFLVQLVASSLMLILQGYQIVILATSEGSIPFFELIFM
AYFTCCFTFSLFVYCYVAEVLRTEINKLTQSMEIGNAAYQCNWYTLPPSEAKCLTLIMIRAKQPFEITAG
KFAAFSLELYCRILKSSGFDVAIGITRFVMRTHGIWPGFSASKAGTMRYAYLPAALMLLLFVIIPQTVQL
IFVSRDLNAILNVLTLGNVPVGIALAKILGVSYKQNGSTLTRLHFLVTVLHQLILLVCEDWKHTTKESEL
VVMRLNARKCRMFSIICIVLSEGAAMTYSARMFYAAFSTHTKAQATGIDDCEKPLFFIAFLAAGTFSSVD
ALFVTLVLHLCGQLTNLHAAFSEFGEENAEKGTMFVSKLSKLIERHRKINAFADIIEYSFNMMFLIQVLS
STLLLCLQGYLFVIILSRQDGLLIEMIFISYFTMCFTFSIFVYCYVAELLQEKSLQLGYAIFYSKWYNLP
AKKARLLIISMVRCKRPLEISAGKFCIFSLNLFCNIVRTSAGYMSVLLANFDDCFKKSGFDVAVGFTRFF
MRLHGIWPGDTSSKFTWARFTFVPPAVIILMFINIPQTVQIFFVGGDLNAILDILTLANVPLGIALAKIL
GVGYNHNILHQLIVSLSGDWKHTTKESELQVMWRNARISRTFSFLFIGLAEVTVLANTARMFYVLYSTRS
EAESSGIKNYKKPLYYTGKFPYDEQSSPNFEITWAMQILATILAAGSFMSVDALFVTLVLHLCAQLTNLQ
TAFRKIGEDKHEKEVDFMSRLSKLMKRHRKINEFADIIEYSFNMMFLFQVMSSTFLLCLQGYLFVIVSKL
SYSIIPNLFIPNMNYIFILANIKSKSYTSRINFYGLFYHLLLVQHIHVLLCCGNFARRSKRKMYYIQFSV
SIQGIHWTKWYNLPAKKARLLIIAILRVQKPLELSAGKFCIFSLNLFCNIVKTSAGYISVLLAVRDKIVQ
PMKIRKSGYDECVGFTRLIMTIIGTWPGAEYSQHWYARYIFSIPLFFSMFFMIIPQTRMLLHVKDDLNYI
IEILTTADVMIIVACLKLIGVWYNKKDLRYLLNEIEKDWKITEKEEQHPMWENVKLGKFIMNGYAVLTYG
TVVLYAAGMLLLMNSQKMEDFDNENITQSRLMFVRSKFPFETQGSPTFEIIWFLQFLAAVMSIAAFTTFD
GFFIFSILHVSAQLVNLQSNFRNLISRCRLTKRTFVQHMRNLVERHIHLQRFTKIIENNFNKVFLMQMIG
YSVTLCLQGYQLVISLTENSEQNFITIAYILVYTTANILSLFVYCYVAEKLRKESTEIFYAVCDMPWHEM
KPEESKMIVNIMYAAKHPFEITAGKFAVLSFSYFVKVLTNQSV
>OXU26417.1 hypothetical protein TSAR_014897 [Trichomalopsis sarcophagae]
MEEDIQTYKVCLQNVVICLIVSGVWPATHPVLKKIAFFVTFFSTFSIMAHTLNFSLHNAQNVRILVRGLA
AASSFLSISSKVFLFFQHQDDLVYLNDYLSKKFTSDMQNPENLPDLLANVRTFAVFVTMYKTTAAFIASM
YSVVPIVAFLKYGKYLRVYPCLYPFSYAPGGVVHWLLYGWESAGALSAWAITVGTDCIFGMYAIQICGEQ
RILARKLKDLRVGSNYKKQLQDCMERHHFIITVKNKFEDLYGLISIWLAISGAIVLCSLIFQVTEYLEND
GGHVRAIIFFAHFSSKMMQVFMYSWYGNLINEESLAFPRAIYSSHWTDCCDTRFKNDILIVLAQRPLIVT
ALGCMNVQLDMFAKIVQSSISYFFLLQTLKAKGEENYSNTIIWSLICSGLWPKGHYVLKKILSCISFLSI
TTIMTTAINFSFQNARNVQLMTKGMGTAVSFSSVFSKIVMVLYHQNDFIYLRKHLTTRFKRDLEQTENRQ
DLLFNVHIFTKFVNTHEASMAFAMFMYCIGPILALYRHGKYVRTFPCLYPFHYEPGDVVHWMIYGLEVTG
ATVIWFITIGVDCGFCMYALELCGEFKVLGRKFRELRVANDYKEKLRDCIERHHLIINAKNRLEDAFGIM
AIWLALSGAFLLCSLIFQITEILENHGSYLKIAHLCSHLLAKYLQIFMYAWYGNLIADESQSFLYSMYSS
HWIDACDKRFKSDILIVLVLQEPLMLVAKGCMNIQLDMFVKEVVKYKAYSSNIIWCLSFAGLWPHAHPVPK
RIFSFVSFFSTLAILMSTTNFVFQNGRNVILVAKGLCPAVLLFLLHQDDLAYLNKHLTSKFMIDMEVSEY
RKEQLSIMRFFAKFVRAHEASLTAAMSMYILVPIIIFLKHGLYVRTYPCLYPFSYEPGGLIHWLLYTLEA
VGAVCIWSTSIGVDCGFAMYTLQLCGELKILAKKFKELKLGKDYKEKLRDCIERHHVIISAKKRLEEAFG
LVSIWLAISGALVLCSLIFQISELSENKNSFLHMGHLCAQFLAKFLQIFMYAWYGNLVAEESLAFLDAMY
SSHWPDSCDNQFKNDILIVLVQKPLVLVAKGCMNIKLDMFAKIVKSSMSYFFLLQTLNADTDLELKKYKR
YYRDIKLLLVVSGIWPNFYPILDRVVSIVAAISTLLMTMALLNFCAHHVANVMILTKSMGIAISFFSSFL
KICIFLSHHDDLVYLNDYLTSSHTSDLSNPDERSHLLEKFSSFSKFFYTLTIAVALTFVLITITPFFALK
RGKYLHIYPVIFPFDYEPGGSVYWSLICLELTAGFFVWSVTSGVDSVFGLYALQMCGELRVLAKRFEELR
ATGDYRMRMRECMDRHHLLMRSRDILEKVFGFLAIWLAVTSALVQCSLVFQAKVEFKTLSPFKIGFFFFY
ILMKLVQAFTYAWYGNLIAEESALCLNAMYNAHWPGSGDIRFMNDVLIVLSQKPLIFKAKSCMSLHMDVF

TKIMNTAVSYFFLLQTLDEGSVRHL
>OXU23915.1 hypothetical protein TSAR_015812, partial [Trichomalopsis sarcophagae]
TKGVVEVDSIFVMVMAVSTIMRYYILVYHRFDFRDTMDACRVIWNDCTPNEHQIVRWFERKTWMLFKLLA
GSGMFINVFCSIGSIVVRLPPDEPNGTERRLLPYRWFIEDREYHWLGYELIFGLQVLITHHLTVIAATVD
TAGPLLMMISCGFFKALQERFFAAAAKNEMILCIDKLEFKRTIVSCSKFHQSVLALCKKIEVMTRMIFMV
QLMCLGYNISLIGLKLTGVRHILHSPSASTILISLLKHRNSTAQTDPERFQYIPNLVLCLCQLFITQWAS
DYLLEQSEEVATATYFATLMSLDARIGGLLLIMVTRAQKPVQMTAGGVIKLSIERFGNLRHLDVSVTIRS
SFLKDYIATTIMTKSEKSLSFKSLTAVFVFFHRLIGVLPLENRDFNFPSCQMLLLYFMFIGLLISVMFLR
YCITRLIQGSVELDSVFVTVMATSTIVRYYILVYHRFEFRDAIDACRDIWDDCTPNEHQIVRWFERKSWI
LFKLLAGSGLLINVFCSIGSIVVRLPPDEPNGTERRMLPYKWFIEDREYYWMGYELIFGLQVLILHHLTV
ITATVDTAGPLLMLLSCGFFKALQERFFAAAVSKEKFLIEDELRYQPLLTSCSKFHQNVLNLCRKIEVIM
RMIFMVQLMCLGYNISLIGLKLAGNDPERFQYIPNLVLCLCQLFITQWAADYLLEQSEGVATAAYFTTLM
SLDPRIGGLLLTVITRAQKPVQITAGGVINLSVERFGNLITNAISFFMVLRINSNIMSEKSRQDECGKLH
VFLLKLIGLLSFDGRSLNFPGTVLLSIYVHCSHLLVTSMYVCNVAKTGLGDGEYDIETVAETVGLCGVHF
RFLIMFFNRKRIAKLLNESKKLWTELDEFEVAIIRSFVEKSLRLTHFYLGANGVMCAFYLTVSQFSVHNS
LNSTFTRSLPYPFYADVQSTPWYELSVTGQMLGMIGICLSSSGVDTAAPFFIMVACGHHRSLCDRLQNLA
SAVDDDDQVAKVDAVKKIIECIVYHQRMIKFCKEVEKLTNSLFMVQLISTTYNMSLIGLKLVGDDPEKLK
YLTVLSLLMCQLFMCQWAPDLLVSESESLARSAYFVPGSGDESKKLAKLIHILMMRSQRPLQLTAGGWIN
LSMECFGSMITSAMSFFTIVHRTSLYLRTRRTIYPLSAFAATTEHNSRRGIKFTSRRRKKKLVKYSSSTP
TLSGNATQRTQYELGTYRSLIYTAMSSSRASKSFNASSRIQLSLFRLIGVLSFEKSRPLTSRLLSGWFYL
CYCFFGSMFLNTCVHNCLRGYYNLEQISETVILLGSVGRFLLLIGSRREMERLLTSAEDLWRVLEQGERT
LVARFADISRKVVYAYLAASCLMCSFYIGITPILQGNATWRVLPFEFYVEVQSTPWYELVLILEGIAMFS
LALVSSLVDITAGPYLILMGCGHLRTLGHRLRNIDSRLDGRSAASSRQYLAELTSCIRYHQMIFSYCDQV
QRVLGGVFVTQLISTTYNISLLGLKVIGSDPDKSKYVMLIGVLMLQLLFLQWAPDLLVEESKRLASDSFL
VPPIGRENRQIGQLICIFAMRSQRPIEMKAAGYLELSMESFGAMLTNILSFFTVLHSIN
>OXU16806.1 hypothetical protein TSAR_016509 [Trichomalopsis sarcophagae]
MEVMETLKITDIMPLSFFYLKLSGAWKPSSWPSYLRLMTFFIMKVMIVAEILYIFAEENQLKVLKGNVYI
ICTLINGWLKMFNLICRRKNIANLVNSCIAKPWNPPRDNYESSVLAATKHTSSRKITVVGSCVVSTLLNS
VLSSTPVLTLDAWYPCNITLPICFWTSFVHQSIGYTVTAIVHVAKDKIVVGFMMQICAQLNVLNRRLLLV
HVEVEKAARQQKDQSQITCLETTLVNECIVNYRDILKQVYICYTRVSTLMYVNXRLIYDSYTILMTFFIM
KVIIVTEILYVIFAEENQSKVLKDNVYIICTMINGWFKMFNLICRRKNIANLVNGCIAKQWNPPRDSYES
SVLAATKQTSSRKITLAHASVVGSCVVSTLLNSVLSSPPFLPVDAWYPCNITLPICFWTSFVHQSIGYTV
TAIVHVANDNIVVGFMMQICAQLNVLNRRLLLVHVEVEKAARQQKDQSQITCLETTLVNECIVNYRDILK
QVYICYTRVSTLIFAEQLSETFIEMIFIQFCAGLSVICTSVYLLTTLNICSFEFFGMFLYLWCMLGQMFL
YCWFGNEVVLNNQTQXLLNSVLSSPPFLPVDAWYPCNITLPICFWTSFVHQSIGYTVTAIVHVANDNIVV
GFMMQICAQLNVLNRRLLLVHVEVEKAARQQKDQSQITSLETTLVNECIVNYRDILKQVFAEQLSETFIE
TIFIQFCAGLSVICTSVYVLTTLNIFSFEFFGMFLYLWCMLGQMFLYCWFGNEVVLNSSKLFHSIYNMDW
IKLQSRTQTKLLFMMLVASSPIQLFRGAIIRVNLDAFINILKFSYSAFAEQLSETFIETIFIQFCAGLSV
ICTSVYVLTTLNIFSFEFFGMFLYLWCMLGQMFLYCWFGNEVVLNFEALPLDLYNMDWIKLQNQTQTKLL
FMMLVASSPIQLFRGANHPSEFGCFHQRKSFFLNSIRCCTFAEQLSETFIEMIFIQFCAGLSVICTSVYL
LTTLNICSFEFFGMFLYLWCMLGQMFLYCWFGNEVVLNFEALPLDLYNMDWIKLQNQTQTKLLFMMLVAS
SPIQLFRGANHPSEFGCFHQHPQVFLLGVQYSTLKS
>OXU28884.1 hypothetical protein TSAR_009573 [Trichomalopsis sarcophagae]
MDILPLNFRILRYCGIWYELPEHLWLVKIVYKTFVVVVIFSFTLSELIELALTYDDLQNLTECLFLTLTF
LALCFKMINFMCRQESLEALLNTFRDEICQPKTLKEKDIIEKNRSMLRLFCISYFSLGILSGSTLVFVPF
ASFKSSKIELPIKTYQPYDVEDFALFSLTYFHQILSMYFGVLINVSLDMLVCGFIFLTCGQLDLCYYRIV
SSNMYTMNDNIRHHAVTKDIVKKMQSFSIVVVVPLFIFSLITLCTSLFLMPEKEIMSFEFITLFIYLTCM
LTQIFLYCWFGNELQLKSKTISDAVYHSNWTRLTPKLRRNLLFTMFISQNGLMISFHGQCSLSINTYVSI
LKTSYAAFNLLRKTSNTLVKSTMDMLPSFRVLAYLGIWIEEGSRFVFLRRLCGLFLSSTIFYFTLTEVIE
LYLLRNNIEELVDVMFLTVTFAMLCLKILNFNFRHKGLLNLLTDFRMDVCKARSPEEENILNKYTTKILN
IFQNILVTGIFFCVLPFITLEPADYEIPYKTYQFYDDTTTMGFTITCVIQFIALIFGIFINVSMDTMIYG
FIILSTGQFELISYRINKSSKENDRALLKQCIMHHNCMNNLVKKTTNLFMTVIAPLFFFSLLTLCASIFQ
MSQNDIISLEFLGFALYLSCMLCQVFLYCWYGNELKLKSADLVNEVFGSDWTVLEYTEKKTLYLLMLSAQ
RPCDISWRGQCTLSLETFVWIIMDVLPLNFRSLQYCGIWYEFPEHLWLIKTVYRTFIVVVIFSFTLSELI
ELALTYDDLQNLTECLFLALTFLALCFKMINFMCRQESLEELLNTFRDEICQPKTLEEKGILAKYQNILK
RVFIFYMSLGLMSGSSLLIVPLVSMENSRISTLPMKAYQPYDVQDSTLLNITYFYQVFSTWIGIIINVSL
DLMVCGFIILTCGQLDLCCYRILCTKTKMFHDNNVRHHAVIAEVVRRVKSFFIVVIVPLFIFSLITLCTS

LFQMPEKEVLSLEFFSLFMYLSCMLFQIFIYCWFGNELQLKSKTIVDAVYQSDWTDLTPKLRRHLLFTMF
ISQNGLTISFHGQCSLSINTYVSILKTSYAVFNLLQKTSNI
>OXU27738.1 hypothetical protein TSAR_003157 [Trichomalopsis sarcophagae]
MEIYDSRYFIFNKRFQMALGIWPYQSRVKNSITYAGLVLVMIIMLIPQFIRLNTYLGKDIEKTMENIFIF
FYVFGIFVKLFTAHFAEDKLKILYESTAKNFETYTDAVEAEIMKRYSERGRLLTFVFLLYMISAVAVSVV
LPMCPIILDSTDPLDQPRPRMFILNGEYIVDKYEYYFQIYTVDIISVFLMICILCATDPMYAAIVEHCLG
LFSICKHVSLTFSCTRDLCELTREYRLRNFNKSCGLRMVEHAEAKRYGGDYAYAALVRAILLHKDIIKFT
EIIQTSYSLYFLLEMGATIGILTSSSVVVVKLLVLQYIAKVVMKLKQPLELLRWSLFLFGVILHIFFLTW
PGQKLIDFSSDIFQEAYLNDWYKSSLKCQNLLKFMSLRCSRPCELSGGGLYIMNFINFATILKTSASYIT
VFSSMFSSLHGDGYIHQESKAVMEIYDSKYFFFNKRFQQALGIWPYESRAKKKLMCGILVLIAIAVLVPQ
IARLSTSIGKDPEKTMENVVILLYIFGIYIKLFVAVYSEDQMKTMYESTARNFQVYTDETEKKILKDYSE
RGRFITIGYICMCLFFCVNSLDIIYMIQALMIFISLPLTPIFLDIIMPQDQPRPRMFILNGDYFVDNDVY
YYQIYLFDSIACAATVFIMVSTDPMYAATVEHCLALFSICKYVLGDFFTVSLLCPSTSKTRSFSIVKFDI
LEIRISTKAYSMLLHFRYRLEIFNRNQNDNKTSITEKPRHDYAYYALKEAIILHKEILKYHEILQASYSL
YFLLIMGATMGSVTMNSVMILMKTHNPFELIRYTLVLIGIMGQLFYLSWPGQKLIDYSSEIYRHIYLNDW
YNSSLKCQNLLKFMTLRCTRPCTLTGGGIYVMNFINYAAVMYFVYNKRFQTALGVWPYQSRLKNAIICGF
LLLVMIAIVVSQIIRLKKYIGKDKDKSMENVFILFYIFGIYIKLFTAVYAKERLKILYESTARNFQIYTD
KMEKKILHEYSERGRLITLAFIIYMMTALIVFVLIPMYPIITDVIAPLDHPRVRMFILNGDYLVDRDEYY
FQIYVFDSICGALTVLILCSTDPMYAAIVEHCLGLFCICKYRLKNFNKPRRTDIIEKANAESQVDEYAYT
ALVEAIQLHKNILKYTKIIQTSYSLYFLLEMGATMGLLTSISIIVDELTTLCHILLGMNFSVDRNETGSA
TGMHQIFSCLNWIVAAHILSELAGSEVDKRQRYHNDWYESSLRCQRLLRFMSLSCSKPCQLSGGGLYVMN
FINFARVIFQPSRSLVSMSNALGVWPYQSREKNMIVCGLLLLLMLGMLLPQIVRLKKYAGKDSDKMLENI
FILFYIFGIYIKLFTAVYAEKRLKVLYESTAKNFQIYCNDEAERRILYEYSERGRLLTLAFIVYMLPAVT
VYVMLPMCPIIMDAAKPLDHPRYRMFILNGDYLVDEYDYYFYIYAFDSMAAIVTVAIMCATDPMYAAIVE
HCLGLFSICKLRLKYFNKPNGTKAIEKTYYSETCGDDKPHSCNCSTDDYTDALFRYTEIMQASYSLYFLL
EMGVTMGVVVCNSVIVGTHLSRHIIQNNVDMTLHLA
>OXU27556.1 hypothetical protein TSAR_016892 [Trichomalopsis sarcophagae]
MPTGMDSLNATFQSISSSHLMFLQLSLFLPLENKSFSQRLISNLLQLWNHLMVIVYNVSYAGYGIGMALR
RDIEIDYICEQIVVETFSARYILLCFKRAQLRRLIESCKRLWGYLEVGEDIVVRRFERKGHFFRHFLILS
SLMAVTSYVVTAHFIRLPPLEANGTERKMLPFRFFMDVQEGPPFNAVYALQIINSYYLVFMFASVETVSL
YLIMMACGYLRSLQDRLLSLIMEMNEDDLSKNGEAMFNVVMGCAHFHQKIMIFCKDVDRMTRTLFLFACF
CPIYNMSITGIKLLESDEDKFKYASLLFVNLFQFFSCQWAPEFLIIESEAIATAAYFASLQPFAPTHREK
INRILYFMMMRAQKPIRLTASGFIELSIETFGAPEATETYTFLAISSSHLWFLRLASFLQLKDKSFSHPL
SLLLQLWDHVSVVIFNSMWTGFGIRMIIRHEVDVEYICEEVAVNAFCLRYLVVCFKRVQLCRLVEFCERL
WDYLEVGEDMVVRQFERKGHFFRQLILINVLAVATLYVITAHFIRLPPLEANGTERRMLPYRFFLEIQEE
PAFSIVYVMQIVVVYFVNTIFCEEVDRMTRMLFLFSCFCPIYNLSITGIKLLENDEGKFKFAAILSLNLF
QFFACQWAPEFLINESEAVGTAAYFASLQPFASSHRERINRILYFMMMRAQKPIQLTAGGFIPLSIQTFG
ATESYSFLSISSSHLTFLRMAAFLPLDNRSFHHPYSRLLQLYGHICIFVYNTMWTGYGYRMISRREVEID
YICEQMVVEGVCLRYIVLCAKREQLCALVESCKRLWSYLRSGEDVTVRQFERKAYFFRNFMLINSILVVM
LFIGTASFVRLPPLEANGTERKVLPFRFYVDVQEDPMFSAVYALQAVVCTTIPFVIASIETVSLYLIMMA
CGYLRSLRNRLLSLTENEDDAILAGETSFRLVVGCAHFHQQIMIFCEEVDRMTRTLFLFACFCTIYNMSI
TGIKLLENDENKFKFGAILVLNLFQFFTCQWAPEFLIIESEAIGKAAYFASLQPMASSHRERINRILYFM
MMRAQKPIQLTAGGFIKLSIQTFGAMVKSAFSFFAVLRSFSST
>OXU26755.1 hypothetical protein TSAR_007006 [Trichomalopsis sarcophagae]
MTSSVSWQHLVVVGRFEGIDMVTDSKAESDLCDLGCKPMTIGFIGYDIAVGPCRFFLRIFGIWPDPFIES
SALEHLRAFIASSMMVFFATITQTAKNVQAWGDLNVVTEISINSEVPTSTAAVRVAGIWYYRRVVSNNMR
CITFLSAALQKLMIQITNDWRSPHGPIELEIMEENARFSRLVSATCIFLAHATFGSQFTIGVLRIDAQNR
LQANGTDIDRPLYTRASFPYDSQISPNYELTLAGQMFCNFVSALTYTSIDSLFIVLMLHLCGQLSVLRLE
FPGLKGCSGNEFAEKIAYIHRRHDQLTVFANAIEEVFNKTFLLQIITSSFVLCILGYQIMTITNGNNIPF
MELIFMIYYVICFLFITFTYCYMAEKLHEKVSFVIVLSNFKRRIIDLFMQSVDISDAAYQCDWYDLTPQQ
SKQLIIIMLRARRPFQVTAGKFVAFSLSLYCSVRYEEAIKATRAVLRAFGVWPNRHKMSENWFSRSHFLA
PAFLIICFINIPQTLKIIKVWRNLNEVLDILATANIPSFVALIKLLCVRYNKKGLLLVSMEKDWKSLKTL
AETRIMWKNGKLGSLITFVIYTLTCGSYVAYVIMITYTNASGSKQEDVITLNQSKKLRPLYMRSYFIYDV
QKTPVYEIIWIFQFVSMGVATFTFMAVDSLFAVLMMHLCGQLINLQELLKNFTNMLGQTKTRNFVYQLST
IVSRHEQLNRFAKAIENAFNTMFLVQMLLSGMVLCLQGYQIVIILTGRDTVQIIELLFMVYYTLCFAFSL
FVYCYIAEILRTESMEIGNAAYHCDWYYLSAFERRLFILTIIRSKTPFEITAGKFAAFSLEFYCSMTVEI
AEDSMERIVALSDDQNVDGYNHAIGPCRFFLRLLGTWPDPYGNVDSWTTSARCLVITATMFLFATVSQTV
KMALSYKDLNLVTEILTNCNIPTTIATIKIASIWYYRWVLRDLVRQIIEDWEMYHDRHESAIMWRSAKIS

RIFSIGCIFMTEGTLLTQCIVGLFRPVSYAFKTGLNQSIEWPLYMKGSFPYDVQASPNYELSILGQLLSN
VFASTSFSSADSFFIVLMFHLIGQLSILKLTILNLPSKIENSDDRSKFMDRFAFVHTRHNRLWRFSMAIE
ESFNTMFLIQMIPCIFALCTQGYQLIMASAFE
>OXU25120.1 hypothetical protein TSAR_002718 [Trichomalopsis sarcophagae]
MSDEIEDVQNELDTHEQRLRDFNWALGLNRLSLRLMGVWPGDDEAEGLERLAILLRVPFMIAAMFFCLFL
PQMAALTLVIHELPLVIDNLMTSCAAFTSCIKLYFIWRSKQGDQVLRPVIESVSADWLRPKLDWERETMI
REASRARIFTVSGYAVMTGCYTGFAFAPLFGFDIRIISNITDYGEKHMLVQSYFPYDYSKSPNYEITHVS
QLIAGLFIGMTVSVPDNYFGALVFHASAQFEILGANLENLIRQDDKGLRSGQFDRRIGIFVDRHVHLMTM
VTAVEYSFSFVIMAQIFCMSIMVCSLGFQILGMIEGTTGDKPSLLQVLTLVGTLFTLMMHTLVDCFACET
LELRVDFDWALGLNRFSLRLMGIWPADQDDSSTSLGIIILRIPLMILVLLCGLFLPQMWALALVIEQLPLA
IDNVMTSCPVFTSCIKLFFVWRSKTILQPVIDSALQDYLRPKSKSEETTMQRAAFRGRLVTIADYSIMAS
CYVGFIFMPMLGFNVRIINNLTDYDTQRVLLVQSYFPYDYARSPAFELTHLLQLVASFFVGMAISIPDDY
FCALLFHASAQFEILGLQIESLPIDESKNGRLLSGFIERHVHLNSGGEIVRVRYRRADILHVHHGLLFGL
SGSEEFRAVFQNLQQQVVLAIVEQDALPRADDARSQDSETDKGRKDFVHVSGYLLLQFDWALGINRVSLQ
LLGIWPADQDESSKSLLTVSRIPLMVLVIFVGLFLPQMWALALVIEQLPLAIDNLMTSCPGLTSCVKLFF
IRRSKTILQPVIESVLQDYLRPKSEWEELTMRREASKARLITIADYSLITICCVGFIILPTLGFHVRIVN
NVTDYASYGNRALLVQSYYPYDYYESPAFELTNLVQLTAAFFVGMTVAIPDDYFCALMFHVSAQFEILGL
QIENLMGKDDDNEGVDWSLLGSFVERHVHLNRMVATLEKSFEFLIAAQILLVTVMVCCMGVQVLRTLSGA
GEKPSPFQILTLSGTVFYLLLHTFVDCFVSERLTSRSSEIFFKIYSCRWCALPWNKVRCLLPMMLAAKTP
RQIRAGRIMPLSLSTYCSVR
>OXU20489.1 hypothetical protein TSAR_000489 [Trichomalopsis sarcophagae]
MICSAAPTFFVLFSEQIKLLVFCKNRERVMKLNKYMYENFWNAKYNREETKIFNECNSCCIKALACFLVT
MQCISVHLMLAPYLEPSDNSTSGEKIFPFPLYVNYPIFETPTYEILYVLEVLGLYGVVLCVMSFPIFLLV
TNMFTAVQFKMLNLRMRSLCQFPINKNGDVNLPQQMNAYEKLKECIRKHQSLIHYVNEMENLYCYAMLGQ
ILASIFQLSSTSITILLSNQGAESVNKAVLRMLILVASLLQFYFYAYSSHEILTESERISEAIYSSDWYQ
ITHPRYRKNFSLLVQIVSARTQRPCILTVGKYCPLTLNTFTTLLCSAAPTFSSLSSELMKLFVFFKNRER
VMQLNEYMYENFWNAKYNREETNIFNECNGWCIKALACFLVTMQCISVHLMLAPYLEFINEFNLKIKHPH
ILQEPSDNSTSGEKIFPFPLYVNYPIFESPVYEILFVLEVLGLYGVVLCVLSFPIFLLVTNMFTAVQFKM
LNLQMQSLNELPKNKNGNVNLQVQEHAYEKIKDCILKHQLLIHYVDEIESLYSYAMMVQIFASILQLSSA
SITILLSNQGEESLNKAVLRISMLVASMMQVYFYAYSSHEILTESEKISEGIYSTGWYQITHPRYQKNFS
LLMMCSVAPTFCSLSSELIKLLVFCKNRQRVMKLNKYMYENFWNAEYNKEETLLFNKCNNWCIKALICYL
ILLQIIVVHLTIAPYLEPRDNTTWREIELPFPIYIDYPIHETPVYEILFILEVFGLQGVILCLLAFPIFL
LVTNMFMAVQFKMLSLRMRSLCKFPNNDNSDNVDIKLQMHTYEKLKDCIRKHQLLIHYVDEMENLYCYAM
LGQIFASICQVSSTSITILLSNQGEESMNHAVARIEILAASTLQFYIYAYSSHAILTESEKISEGIYSSD
WFRITHPSYQKHFSLLVQIVSVRAQRPCILTVGKYCPLTLITFQSRTKQSLTVYSFVPGNEDVDVVLHSF
TPDDCIIHG
>OXU31133.1 hypothetical protein TSAR_010116 [Trichomalopsis sarcophagae]
MGSALVTMIPFQVWDTINVSDNLVMVMDNLSNILSEVLLYTKFIVLLLNKSYLDDLLREIADDYKNNIVT
EKWLKLDQISRRFCNYDYGMYLGASCLFYLQFALMYTQMPSEDRIMLLKAYYPFDYKSSPVFEIMCFIQV
IQGLLMCSIQALSESLLIALVSHVSGHIDLMNKQINLVSKSYDGQNSLTLKLVIKSHLKVLNLVNKIESV
YTYVSLSQVFFNTFIICVTGFVVLTMNSANEIVVMIKYIMLYFTLLWQSFSFCFAGQHLLNKSDMIPYQV
YDALWYKAEATEMKAILFIIKRAQTPLSLSAGKFIALSAQTFTLMKIPMVYWPLEYTLRINGLWPGENNI
LGSIVTASGMVLILPFQVWGAIKTIDNPILLMDSLSDIMTEIALYAKLIIMWFNRRYVVDVLKEISNDCN
QNNVSQNWTLLNYNARRFCKYDYSWYISATLLYYIQLVTMYIEVPVDGREMLLKSYYPFDYKSSPTYEIM
LFLQIILAMSMAIANAMTESLFIVLILHTCSYVDLLLDEIKIFSDNCNKKVLNITDSNKMRFYVHVILKR
HIQLLESVKKIENIYSNVSLVQMFFSVITICVTGFVMITVFLI
>OXU31132.1 hypothetical protein TSAR_010115 [Trichomalopsis sarcophagae]
MIPIFNKPLKCCLKVAGFWPYDFNMLGPAAITSMLVTTLPFQCWNAFALTKNLVVLMDSLSDIFTEVLIY
IKIFILWNHRREIRDLLEEIGKDWSIKSIPTEWANIADYCRIICNIDVILYASASILYYPDLLMSYFGKP
VNERHMLFQSYYPFDYRRSPIYEVINIVYFFQGILMIIADSVSKTLFVSMIFHVSSQIYELRNNLEQYSR
HSNDGYENNFKRLKLVVQQHLKILSLVRRIDHIYSYVALFQIVFSSIIICVTGFVIITAMESANIMLLVK
FTTFIIAMLAQVSYFCFAGQYLLNKGESIVEMINSSYWYNSQCKDVKVLIFVLTNAQKPLTIVKTSASYL
SVLRAILKLSGFWPFEFNIIGSLALISTLVTTLPFQCWQAFNFTNDFVLLMDSLSDILAEVLIFLKLFAM
WKSKSCITIILREIFNDWGTEKIPDEWKTLAYYSRMFCNIDTLVYVSAAVSYYPDLLISYFGKPIENRKM
IFQSCYPFNYLGSPTYELINLMQIIQAVAMMAADSLSKTLLVALILHVIANIDLLKNEIRIYSTNIANTC
NHTNNKKSTVDLKQVISQHRKILYLVQSIDNAYSYVSLFQIVFSTIIICVTGFVIVTAMESANIILLFKF
ILYIIVMLSQAFTFCIAGQYLRNEGESIIHEIYDCLWYHTEPKEIKSLIFVLKSAQIPLTLGGGKLFELS
TNSFTMIVKTSVSYLSVLRARAYGQINRTFLDP

>OXU31081.1 hypothetical protein TSAR_014238 [Trichomalopsis sarcophagae]
MNKEEVDEAFNQSLLKINKELNVFNGLWPNRSSKDKIFRQMIVLVVLIIITLPHILGIVIQCERNMTLCG
ENICGICYCSGLLTKFLVPVVSKRKFVTLYEKIALNWKDITDPYEQSILEEFSKLGRLKTVLYFGYCIVA
GFAFCQMTALPALLDIILPLNESRPKILVTKAEYPFDPFKYYYELFFMYCIASVVSVSILASTDSTYSAI
IHQSLGIFAIVKHRVQEAAKHTNQDKSYRVMVSAINLHKSALELSRYPSSFTTQIPHKKNYSIHLYIRFI
GLIESTYQSAFLVIIFDTVAFLSFGSLVIVERSKEIIDLIRLTMMEFGVLMHIFFLSWPGQLVIDHSENL
FLSTYTTQWYNMSRKGKRLLQFMMMRCSKPSFLTAGGFYILNFENYGSIVKTTLSYVDKAFNDSWLKINK
ELNVFNGLWPNRPNGDKIFRRFIVLTVLITVTLPHVLGIVMQCGSNMALCGENVCGFCYCSGVIAKFIVP
IVSKNKFVTLYEKIALNWKNITDPYEQSILEEFSKLGRLKTWFYFVAGFAFCQMTALPALMDIILPLNES
RPKILVTKAEYPFNPFDYYYELYFIYCTAAVVSVSVLVSTDSTYSVIIHQSLGIFSIVNHRLQKAAQHKN
PDESYRVMVSAIKLHKSALQFLELIESTYQSVFLMFIFVTVAFLSFGSLIIVEHSEEIIDLIRMTMIELG
AMIHIFFISWPGQLVIDHSENLFLSTYTTKWYNMSMKGKILLQFMMMRCFKPSFLTAGGFYIMNFENYGS
IVKTTLSYVTVALSFH
>OXU28980.1 hypothetical protein TSAR_002357 [Trichomalopsis sarcophagae]
MDILKSSYYIRCNKYLRFYGHWPYQNVIIKIRNQIVIMLLIMSIFLPQLIKMFEIRHYFHMFILSISALL
YYVQFLAKNVFAFIGRKEIKNILDKIQQDFQVYKGEDLAVLHEYSKKAQLFNKFYTVYMFTVVGAYSMLP
FTLYMLDTFVPLNYSRLPYKPRLMTYCITSLDDSTLFMIIHGAIVDMMSIVFVIGFDTLFLNFAYHICAL
FVIVTHKIRDAVDIDLDSNNNTKSSESSENVYYRNFVKTITLHKYVLELCIIRVIDQLSEFHRFIETIEM
AFSPLNMISIALAMIPLTIMGFQAVMNKGNPGEMLRYAMYAIAEMVHLFYYNWPGQKIRDHSMLIYDACY
INCYVMNLMMIRSQKPSYLTAGKIYVLGLENFAASSYYIRCNKYLSFYGHWPYQSVKVKIRNQILIMLLI
MSIYLPQLLLQFMKMIEIRHYFHYFILSLPSLLYYTQFIAKNVFAFVGRNQIKNVLDKIQQDFQVYKGED
LAVLHEYSKKAQKFNKFYTVYMFMVVGAYSMLPFTLYMLDTFVPLNYSRLPYKPRLVKYCITTFDDNMLF
IIIHGGIADMMAIVFVIGFDTLFLSFAYHICALFVIVTHKIRDAVNDEIDSQNSTISECSNLREDISYRN
FVKTITLHKYVLTFIETIETAFSPLNVISIALAMVPLTITGFEVVMNKGNPGEMLRYAMYAIAEMIHLFY
YNWPGQKIRDHSMLIYEACYATNWYKKDFSIKSKKVMNLMMIRSQKPSYLTAGKIYVLGLENFAAVMRVS
MSYFTVLSSVT
>OXU28885.1 hypothetical protein TSAR_009574 [Trichomalopsis sarcophagae]
MLDNFRTPICLAKSPEEKDIIKKCSKTTKKIFLSIMSLSQSTGLVLLVIPFLTSETREIPLPFKSYQPYE
MSSATNFWITYALQIFAAIYGVLLNVSMDTMVYGFIIMATGQFEINCYRLENSTCSMRDCIEHHVLIQDI
VYKIQHFFIYVVVPLFLFSLVTLCTSIFQMSQKQVASFEFLSLTMYLACMLCQVFLYCWFGNELELKSNA
VANAIYCSAWTDFTRREKRNISFIMLSAKSAYVSICKTDVFQSETGEMDVLPLNFRTLWLCGIWYEENEK
LTVSRIAYRFLVVCLVFYFTFTLSAVVFVENSDVSELTEAIFLAVTYITLCLKIVNFAFRRGEMIKILDD
FRHPYCKAEDSEESEILAGYSNQARKMYIYLMAFVMSDVAYFWSTFAFKVSKNIMDLPYHTYQFYNVSSK
AILFSTAALQATSVLYSVFINISFDTMTAGLLILTTGQLELNALRLSKLAEHNVDSMNGYIAHNVLINGT
VDKIETFIMTVVIPFLFFSLLSICASVFQLSEYSVFSLEFLGLFSFAICILLQVLVYCWFGNELMLKSEA
VTDAIYRSDWTMLSPQNRKSLQVMMICNKDGRTVSFGGQCSLTLETFVWVRIC
>OXU28883.1 hypothetical protein TSAR_009572 [Trichomalopsis sarcophagae]
MDVLPLNFRILQYCGIWYEYPEHLWLVKTAYKTFVVVVLFSLTLSELIELGLISNNVHESTECLFLSLTF
LTICFKIINFMCRQDNLGRLPGGYFSTENSGGEADNRELSERTCMILVPIISSTSNDTELPIKTYQPYNT
QDLILYSITYFHQILSFLFGILINVCMDMLVCGFVILACCQLDLCGHRISLNQMDIPAKDHITHHILIGD
VVKKVQSFFIVVVVLLFSCSLIILCTSLFQMPQKNIMTLEFFTLFMYLMSVLYQIFVYCWFGNQLQLKSK
SISDAIYESNWADLTPHKRKDYLFSMFMSQNGFAISFHGQCSISIQTYVWIVKTSYGAYNLLQKTSASTD
VSEEVRIPLYQRETMDVLHLNFRVLQYCGIWYEYPENLWLVKIVYKTLIVFMLFCFTLSELTELVLNRNN
VHDLTECLFLSLTFLTCCFKMINFLCRQEGLNRILNTYRADVFQPKTTEEKQMTMQYQNLISKFFMVYLI
MALLSGICLSLIPIISSASNETQLPAKSYQPYNTQDSTLYLITYFHQILSIFFGIFINVSMDMLVCGFII
LACCQLDLCCHRISLNKKNTSTNDHVVHHVLIGDAVSRVQSFFILIIVFLFIFNLIVLCTSLFQIPQKNI
MTLEFFTLFMYLVGILFQVFVYCWFGNQLQLKSKTISDAIYESNWPDLTPCKRKDYIFSMFMSQNGFTIS
FHGQCSLSIKTYVWVQCLIVLNCDAIVKTSYAVYNLLQNTST
>OXU28537.1 hypothetical protein TSAR_008982 [Trichomalopsis sarcophagae]
MDLYESPYCTMVKRVLKVCGLWPYQSNFKRRSNYVIFIISTFSLVFAMATGIYTQANAKLLIIIETYCAL
CYCGGSFFAYTLLYIRRVEIKNWYGGVVDGWIRLADASERKIMDNFLLEARHAILCEVIVYNTLGNMPIK
SMSLKFSGYGFPAFGIFVCFTFLPRIFRKESLEQSLQNFPCYFKSIIINENTCDLQISVHFTVTILFATS
AYFSVSAIYLTSVKHVCGLYGIARYRLQHSVVYNKNLDSLRKLKDYSSVIPSLINVIDIHKDALRALQMI
EKNFSLGFFFLEICALALLAVLMFEANYNRGNVNVLIRCLPLLFNFIMYVFFMNWCGEQVIQSCDDLRMS
AYNIDWYGISSSRVRIFILMIMQKTAKPAHLTAGTVMLLSIQNFATDCVVIWDVAINYAKASQYFKSNKR
LLEVCGLWPYQSTLRSISNYAIFTVTLVSMVIAMVAGIYSETKLLHILEGSLPIIFCSGALVAYMLLYSE
KKEIKERTELMATDWMELADDSERKILGTSFLEARSAINITIGYGIPVYGLFFGVTFMPRIFNKKSLEQC
LRSFPYYFKLIVIDESTCDLQVGVHFALAVLFSSFTFLTIGSTYIISVKHICGLYGIAWYHRLRNAELVL

DNESLDALKKSSEDYDTRVIHSLIKVIDVHKEALRGIQIIEKHYSFGFFLLEIGALVVLAILMFEINYHK
EHISELLRFLPLLLLFTTYVFFMNWCGEQVIQSCDDLRISVYNMDWYKKSSRIRIFVLMIMQRTTKPVHL
TAGTVMLLSIKNFATILKTASSFGMLLLTTQKPGKNKDANYLGY
>OXU28144.1 hypothetical protein TSAR_006959 [Trichomalopsis sarcophagae]
MDLFDSQYFKINKRVLTICGLWPYQSKLGKRINFVSLASSIILFIFTLIAGIISQSPIEFVNTVETSMGI
SFCSTGLLKCAIFYKQQNKVKELYERIATDWKKLTDDSERIILRSFFLEGRRLNFITMVYSSAAFVVFSC
FEFLPRIFYKESEYHKPHSFPYYFRPMVAIHTTVIVIYSGLTYLSAIATYISSVKHVCALYEIARYRLQK
AIVYDTSNKPLQELIDDTSTIPKLIKVVNMHKQALRATQRIEKVFSTDFFILEASNLIALAIGIFDLEFN
YYKGNFREFVRALLVVSIITIYLFFVNQLGERVIQASNNIHITAYNIDWYKSSSRERKILKSGVNLNTRD
HADEGNDTHYAVNGITAFKIALKLSKYEFAKTLLCYGARIKDYYAPVDSESRKLAMLFYGFEDIDNQDGD
ELQYFEINKRILTICGLWPYQSKLGKRITFAMLASNTFLFNFTLIAGILTHSPGEFVNTAETLIGIFFCS
TGLLKCAILYNQQNKIKKLYERIATDLKKLTDDSERGILRSFLLEGRRQNFLTMVYGVSAFIVCACVEFL
PRIFNEESEYHRPHSFPYYYRSMVIHEKFYDLQVAVHGTVTMLYSGLTYMSAIATYISSVKHVCALYEIA
RYRLQNAIICDKHNYQLQELTDDISTIPKLINVINMHKRALRVTKKIEKVFSADFFVLEASCLIALASGI
FELNYFRGNVRAIMRPLLVMPIVTIYLFFVNQSGEQVIQACNDIHTTAYNNDWYKASARVRIFVFMIMQR
TLKPENLTAGSILILSIENFATILRAAWSFGTIMLTTQKHSPSRNEDA
>OXU25024.1 hypothetical protein TSAR_005275 [Trichomalopsis sarcophagae]
MISVILDIIMPLNVSRTKILFEGGEFILDPYQHFYKLYVYFVITSFVIMTSIIAIDTNYTIIIHQILGLL
TIVKHRLRRLAIPMNLKKDNSYHAIIKTIHLHNDALQFVDLIESSYSYLFLVFIGFTIIIISISTSIMMA
QIGKLLNMIRVAMFVLGASLHFLYINWTGQQMIDHSKELYLNVYSNEWYNLTKEAKTLLKIVMLRCLKPS
KFTAGGLYTMNLESFGTIMESALTYVAIMSSFLYMLIAGLGFCQIPMIPIVLDLIMPLNESRPKILFVKG
EYIIDPLKHFYKLYLYFAIASLITVTVVSAIDTTYTACVHQILAIFSIIKHRIHTATISFDSKNDISYDI
IIKAIHLHTAALQFIDLIDLSYSLSFLILIGFTIVFISIGTVVVIDHMGAMLDLIRLTLFLIGSLMHFLF
ISWPGQLVIDHSNDLFVSVYANEWYVISARAKTLLKIVMLRCIKPCILTAGGLYVMNLENFGVILKSALS
YMAVVASFR
>OXU23953.1 hypothetical protein TSAR_005004 [Trichomalopsis sarcophagae]
MMRKLTFAFKVLSMCGVWLPVDWTSPRRIFYYKIFSCVVLITCYTIVTLQCLLLSVTEFDFSFIADILFT
FLTSITVCTKTMNFIAKRPNVIRLADMLLLGCCLPRDKEEAAIQKRCDEFIRVFTIVLNIMAQSTLACWL
LIPLFQSPELRSLPFQIWLPYETRDCRNFWMTYAYEIWPMIIGVLVNATVDVVVSGFILQACAQLDILKY
RLTRLPELVKQAEESNAPLDSIRKFERTTLEMASRHHAHIIENVNETFNMIIVEQFFASSLILSVIIYAL
TKRNVSTLQYVMDVGYLVCMLAEFFSYCWFGNEITLKSTELGVGIYRIDWTLLSAEANRNLLFIMLRTSK
PIVMSCGHFVVLNVESFKSGLLLALSEFDWRFLAEILFTLLTAFSVSFKATNFLMRRDKVICLADMLLKS
WCIPRNAVEIEMESRMNEFLRVFTIYFNALAQLSLACLLIMPLVQDPDKRELPFRMWLPYDIGNQWNYWS
TYAIEVGPMIVGILLNVTTDVVVSGFVLQACIQLDMLKQRLNKLPNIVRVAKRKRLASDEVVRSFERKTL
HQAARHHDYIIK
>OXU22694.1 hypothetical protein TSAR_008364 [Trichomalopsis sarcophagae]
MKTKDESLQPNIFLQHYLNINSKMLRYMGLVVRTKGNKTDSKSKILERLPTYSTNIISIIDAFFQMRWIM
DLWQRDNGLVMQITTSGISNIVCICKGFRLAYCREDIQTLFEKLATIWDQTCVPEDIRDTIVKKAQSTLV
FCRCYIAMMLGLGICFALPPMKNFLIQYFARKATNHTYDYSERVFLVRYPFEINSSSIYFSVLFEEQWVL
FCSALYWVCCDTLFAQLTTHTSLHFEILQYDIEAVVNRENDEDRLKQSTIDFVKRHRELLRICHMIEKLF
SPVIFTTMLLTSINICVNVFELREMISEAKLGDALLHGFHLVNIFFQLLVYCMFAERLTQQAGTIANATY
NCKWTEKNHKLRIYLQILIMKSQKPFHCTAYGFFPIDHKTITIGTIILHDVGNNELNKSAPLTPDFEDYT
KINSLFLRCMGMGIGTDGNKKDRRSQIIERVPTALINIVCLLDSVFQVQWVSEIWKTDKKLVLQILTNAL
SNIVCLCKGFQLAYSREDLQRLFEDLAMIWRKRIPHHEIRDEILRGAQKTLVFCRCYISMILVLGLCFGL
PPLKYFILQFTDRNANRTYDYTERIFLVRYPFDVNNLTAYNFIFMEELWVLYSAAIHWMCCDTLFVQLTS
HTSLQLKLLNYDIEASGNTEDERQFKENVMDIIKRHQELLRICDSIEDVFSPVLFVIMLLTAMTMCVNLF
ELREMLLEAQYVGAILHSFHLINLLIYCVYAETLTEQAGSIAEAIYNSKWTENSHEVRTNLRMCIMKSQK
PFYCTAYGFFPIDHRRITYILKTAMSYYMMLHQTTS
>OXU22656.1 hypothetical protein TSAR_005619 [Trichomalopsis sarcophagae]
MSTKKIASSIDSFLWPNRYTLEFLGFWPPEPGTPSISKYFAAFRIVFSILAIGFLFVPEIMMVVVFWGDI
TVLTGVGCVSTTLAQLNFKMLYVLARRRFCRAYRKTRELWSMTDHESELRKGLEKLAGQAKKYSIAFFF
TCFCNNISFTTLSVVVWLNYNAQENKSLLERRLPFDVWFGFDLQRTPNFELVFIGQSISAIFCCFGIVGL
DTAMMALILHVCGHFRVIGARLHAIGQGMHNDAQSKNSAEYLHTSRKLAICQCIQYHQQMIKFAEEVRSL
LSPIIFVQLLTSGLEICLSGYAVIVNSDAGNYGDLVKCTGYFLSVFIQLIIWCWPGQILIQDSSEIGRIV
LHDLPWWDMATEQQRQFVFVIFRTQKECQITALGFQVMSMSKLTDVGVHFVIVRKVMAGRGSSVRIDDYL
WPNRYLLELFGTWPTDYDGRTLASQLFVNFRVCFVFVAITGVLVPEILMIIVYWGDLDVLTGVGCIATPV
SLILFKVAYMIIRRNRFHGVYSNLRRLWLAIDDAEEFEPLEELARLAKRVTIGFFLSCFSNNVSFTTAAV
IDWVNYDETRNDSTPRHLPFDVWFSFDVERSPNFEIAFGCQVISSFYCCTGIVGIDATMMTFILHICGHF

RTIAAKWRAIGSRILDDEKYSKSGQVMSVKKDINQILRQHSEMLRIAEEVRRLLAPIIFMQLLTSGLGIC
LSVYAVTMNGSKGADLFKFIVFFVSIFVGLIIWCWPGQLLMQDSAALGDVVCYELPWHLLGIAEQRNLAF
FIMRAQKECQITALGFQVLSMNKFTEIFNSAGSYFALLRTIHEKQLEAQ
>OXU22549.1 hypothetical protein TSAR_005523, partial [Trichomalopsis sarcophagae]
MESQVARYAKYERDIKCLIVASGIWPHYEKHPHVLRKLLSFCSAFCSGSTFYCIVAFCFKYATNINIFTS
CLGLMIGFFTTFIKISFLEFGILRQIVILSMHQEDLQSLNEGVSKSFEDNLKLPANQPHLLYHFPTFSRF
FYLYAYSVGISFVFLASTPLSIMLRYGKYVRMYPQLMPFAYEPEYLFMFSTICFIHQGGPVHWAIFGFEM
FTGFYLWSVTIGVDSIFGLYALHMVGQLRLLGSRFQNLKSSSNYDKELGECVRSHIQLMKSRHKLQRVFG
FLAIWLAVTCAIALCSQVFQALHMRNTTPVRALYLFGHWFIKIVQAYSYSWYGNIIAVESDLCLNSMYYS
QWPGSGDKRFMTDVLIILSQKPLVFRAKQLMELRLDMFLKLVRMMPKSRKFNKYVNGKAILLLVQIGPGM
KFLAGRNAKETIDIATMFHAVIAQTLGHQDRFYHIFMVMELELAKYKSYARHVISRLIFAGLWPESNPTI
KRMLSFVTFTSTLTVMVTAINFGIHNASNVILLTKGIGLASGFSSVFSKALMLPLHQEDIVFLKKRLTTK
FMSDMETIEYRAELLSSVHVFSAFVNMHEAMMAFAMSMYCFVPLYVLFKHGTYMRTYPCLYPFAYTPGGL
VHWLIYALEVAGAISVWTITVGADCGFLMYALELCGEFKILARKFTELKAGDDYKKKLKECIERHHLIIE
AKNRLEDAYGIIAIWLALSGAFLLCSLIFQITE
>OXU22548.1 hypothetical protein TSAR_005522 [Trichomalopsis sarcophagae]
MTDNEVENYEKYRSLWPDYEKQMPAMSLFLSVQAAFTSFTAFCFIAYSCYLDSADIGAFTSYIGGLVGYL
TTIIKIFVLEMQQKNLKQLNNGISTIFEANLEVPENRQYLLAHLPMFLKFYYAYSISTGSSMVLLALMPL
LLIRHGVYVRMLPLTLPFSYKPGGMVHWIIYLYEILCGWNLWTVAVGTDNLFSLYCLHIVGELKLLSSRF
QNLKSSKSYRKDMRNCIQSHMLLMKTFLKLQNVFGFVAMWFAITCAICLCTLVFQAVEMDKVQAVKVFYL
LIHSFVKLFQAYLYTWCGNIITVESEICLNAAYEADWSDSGDKRFMKDILTVVLQRPMVFKANTFMELRM
ELFLKRLTAFIGLWPDYQKQMPSISLLLSIQAAFSSFTTFCFIGYSCYLDSADIGAFTSYIGGLVGYLTT
FMKIFVLGIQQKNLKKLNNGISASFEANLKVPENRKYLLAHLPMSLRFFYTYAITTGSSLALLVLIPLLL
LRHGVYVRMLPLTLPFSYKPGGMVHWMFYLYEILCGWNLWTVAVGTDNLFSLYCLHIVGELKLLSSRFRN
LKSSKNYRKDMKDCIQSHMLLMKTFLKLQKVFGFVVMWFAITCALCLCSLVFQAVEMDKVSVMRVFYLLN
HSFVKLLQAYLYTWCGNIITVESEICLNAAYEAHWSDSGDKRFMKDILTVVLQRPMVFKANKFMELRMEL
FLKVPLHFSIKFKSSFVK
>OXU21900.1 hypothetical protein TSAR_002411 [Trichomalopsis sarcophagae]
MVLNLIKPLNESRPWDFIMHGEFPVNDMYAHYGEIYLFDSLACIATVLVFCTVDSMYATCIEHCIGLFAI
VKCVFICFYENSCMSDKIRITTTDRRSRLELSTKFVNREGVLGIKRDDMVYDLIVKTIKLHKKIINFTHI
LESSYSTSFLILMGMNMLYCSLVSVLLIIKSDALMERIRYGTILLGLLIHLFYISWPGQKIIDLSTGLFE
DAYSNEWYETSIRSQNLLKFMRLRCLTPCQLTAGGIYVMNFANFASWSSVQRRAPDRASTSYVHLFRLVG
LMPFEARSFDFLGTRLLTVNFVLFVGLDCSMFICYTINHLKQQETSCRGIEKKIRMLFKCLFSTTMLNII
FYFMKMLTTRLPPLEPGGPERKVLLFKYYVDEVSTSPAYELVVTLQTAVCYNFAIVLLVQDIFVPLLIMI
SCGYLRVVQNRLLNIYELDSKDDALDDSIDDCYKFHQSTLDMCLKIKKLSPTLYLVHILCTGYVLSLFGL
KFSTSKSSNSDRLKSLSSMMMLVVQLYIYQWAPDQLIRESEAVSTAAYFANLPRIGSPGNRKILSIVMQR
AHKPVILTAAGFVRLSIESFGSMITSAVSFFAALRSISIRDEEQ
>OXU20855.1 hypothetical protein TSAR_007277 [Trichomalopsis sarcophagae]
MHILSLAFTFFQIYGFWRPLSWKSPTLGFLYDVYTFVMFMIVFTFALSQLMSIILTVQTVDEFTSSSFIL
LSIVSACFKASNLLLKRKSLVRLLNVLISTTCKYQDDDEKMIQDMFDKKARSNTVWYMAMIQSSVFMITL
QSIFVNIPQKTLPFPAWLPYNYSNTGLYAISYTHQVIGNAASATLHAANDALISGIMLQICAQLEILKHR
ILKLPTIVLKMNSGKEAPVNAVASKESELLGNIIKHHNCIFQFSIATSYSFSKDINDTFSMALFAQFFIA
ALVICSSVYELSKMVLLSSDFVALLSYLACILVQIFLYCWYGTAVTMKSWSVGDTIFATDWSPLSMGLKK
SLLTVMIRAKKPIELKTGKIFTLSILTFAKMRVLPITFGILTVCGFWRPISLESSIPKQMYNCYSIFMCF
LIYTFTLSHLIDIVISAADFESLTGSCFMLLSMMNVCCKMTNILYFRKNIVELLQILASDHCTAKDVVER
HIEKKFHKRARSVTLCYWILTETTCMLITLRTFFGSSKQILPFKAWIPYEITSLTVYWTTFFHQTIAHVA
AANLQIANETLICGLMIQACSQLEILKYRLKKIPDEARIDKFPLQSTVNNDQYNNKKDTKTLLVNCIDHH
RRIIEFSEKLNSTFNVILFFQFAISSLVLCSSVYLLSKMKLIFLFCWYGNEVILQSLDLGNAVYHMDWTI
LSTEDKKKLLIVILLVRKPIKFTSSFLVSLSIESYCKVSLNKKDKLLDRLLY
>OXU20625.1 hypothetical protein TSAR_001148, partial [Trichomalopsis sarcophagae]
FEWAFGLNRICLNFIGVWPNEVDANAARVKDSSIKYLRIPAMLILILVGLIVPQMYAITKVYKRLQLVLD
NLTLSYCTITSFIKLFFLWKSRGVYGPMLRSALLDWQSSQGSLWEYEIMRKQARRAQIFTLSGYIVMLFS
FIVFIIFPIFGLSIRIINNITDTAGEDRFLPIQTYYPFDVNRSPYFEMIFVSHVIICSGAASCFSIPDYF
FGALVFHASAQCEILAVKVQQLFPPQVEAGISNTFLENKFFRTKIKQLVQRHVHLISFVETIESSFNLII
LAQTICLTLIICCLGFNIIMEKQINYFQKHNEQKYYSQSLGTESNDSPILPVVSLGGSTLTILMHMLVYC
FASEILAMHSEGISEAAYDCDWYTLPVNCAREIIPIMVRASYPLKLSAGKFFYLSLNAFLSIVKTSFGYV

SVLLALAPERSESAERRVKLNHLSQECRIEFYRTFLIKVVLIYTVRLAHECDKHDFEWAFSLNRICLKAV
GVWPSELDELDTRGVSNNRNHLRIPIMLFTMWFGLITPQLYALAKVYRQLQLVLDNLTLTYCTFTSFIKL
CFLWRSRRESCPTVYGPILRSALIDWSGSRSSDWKYETMYKQAYRAQIFTLGGYLVMTFSFACFMFFPFF
GLSIRIINNITDPGGEYRFLPIQAHYPFRYDRSPYFEITYATQMLIGCCAASTFTIPDYFFGALVFHASA
QCEILAAGIRELCLEELDTSSTEKPRELGIFRTRLRHIVRRHVHLIRFVNNIEKSFNMVILAQTLNLATE
NNSESLPILPIVTLGGSTITMMCHMLVYCFASEVLAMHSESIGTACYGSDWHLLTADCARNVIPIIARAQ
HPLQLSAGKFFYLSLNAFLSVVKTSFGYVSVLLAVGA
>OXU19652.1 hypothetical protein TSAR_000470 [Trichomalopsis sarcophagae]
MFSGNIGECSSADEGCVRVQSRILHDTILSEQRNNATVSVLLVWQLIDDKISFTILTYCGIWQPIYWTSG
WHRISFNFCRVVFRPLPYLLASAQLARIALVDMSFEELTEVIFILLSIVNICCKSVSILMRRADLIKLTK
MLGIVSASPQDSDEFNIQHQYHQFIRYVTLSSLVLVEITAITFLIPPFFQPENNRTLPFKIWLPYDYSMD
KLFWITYFPESITIILASLISVSSNTLIFGFLIEVCGQFELLNHRFMTMPLYIEDFAKGEKMTTYEVCKL
EKQLLSRNIRHHSFIFEFVDVFKKTFSSAIIGQYIVSSLVISTSVYQLSTNTTMDVVFFTNVLYLMCMLL
EFFLYCWFGNELTVKSEDFGRKVFRTNWIALSTKSNKDIFVAMLRSSKPIIVSTGFFAVLSLESFMKPEH
LEDIMGKKKMSDLQVATYEKNLVKQISSYNTSLISSLIISISVYQSSTSKSSNMDFFMKLCYLICVLVEF
FVLCWFLNELTIKSQEFGDSVYNVNWLALRPSSTKCLMFLTLLATKPIVVSYRFFVVLSLEIFTKVEWKR
LICHLKFSRSSEFGSQQMIQDLKAFCTTCAVLHISLCPRLFLMKLNFEEFTETLLISVATLGICFKATSF
VLRRKQIIQLSNMVSSVHALPRNPEEIHFCDSDWHGPNNRSGVAVWTVFPIQEATYLTIQANDIVFFGLI
VVVCGHLKLFTYRLTSLPKRVEKDLIDKKLKHKEIGAYEMAKQNIHHIFIHDLVWVIENLKFLRGRSDSG
CLANFMLIFNRSSIFFDENPIIFLPGFANTIKSAFTLVILTQFISSSLIISINVNYQLSTAANPLIWNFL
LNYVFLYACSWSSSCFVGLEMNQQFKVKNSATSFIRLIEFLRGSNRQSVSYSFTKMHVLPESFMMFTCAG
VWQPVHWSARDSRFLLYKLYMLFSIVLVYTLTISELMGAILLTQNLEDFTDISFLLISTISVCCKIASII
ARRNRIIHLTEMLLESWLPYDSTTTPCTFWLSYIHQTAAIVLCATVNVANDSLICGFMTHSCSQLELLNR
RLLELPRAVKLKMKKLPRRLMCNVEAMIVSRHVKHHVHIFKFAENINVIFTPVILVQFCMSSIVLSLSVY
QLAVRSANGIQFITMVMYLTCMLVQFFMYCWFGNEVTLKSVEFGQAIYNIEWTSLQVQTSKDLMIMMIRA
KRPIIMSSGALVTFQCAKLYKAFILDFKSFLFNF
>OXU32296.1 hypothetical protein TSAR_007072 [Trichomalopsis sarcophagae]
MSVFALAPYACLPNFGPVSMNILFPLNAARRKQLPAPAEYFVDEEKYFYTLFLHGMICYIFVPILYVNID
SMYACIMHHTVGLVGIVTYRLQYIIDLEVTSYQEYYANNLEIRKRLKRSITLHKEFIEFAEKKETTYSLC
FMIVMFVNLFAMIFTAACGYSFRVSRGNKRIMQIMMIRCSQPCQLTAGSLLVLNIENFGAL
>OXU32114.1 hypothetical protein TSAR_002955 [Trichomalopsis sarcophagae]
MTTRLETLAKTDENKASIEFRSDKVVHHDLKNTIIMHNDTIRCLAFRSCRLLEESFSLCFLLIQSLCVVT
IALGGFYILCIYNDAYKLLRVVAFYTGTVLHLLYLHYVGQQIIDSSEKIFNSAYSSEWYLISTNARKLTK
VIMVRSLYPCHLTAGKITSLSMETFGSVRLNITIILYLFYLLDVHEVVFNIIFMALQLMKTSMSYFTVLL
SLE
>OXU32113.1 hypothetical protein TSAR_002954 [Trichomalopsis sarcophagae]
MDIFDGRYYKTSKWFLEFLGLWPFQSNRRRYVTCFIFVFMIATVVFPQVLLLIELKTSNFNILIENSLSI
IFGFACLLKYGVTFASRSRVCDIYCMLLKKTTFILFIKVYISFKQLQTLLTKIASDWQRLTDKTEIDILS
QYGEEGRYLVLFYTVYVFLAWVTYNFLPFIPPLLDILLPLENGTHDLVYPFYADYVFFKQTDYHYESCLH
VFFVYFGTTSLFAGMDTIYVATVKHSCGLFAVTLETMARTGKSNRSNYSIKPNSVVHHDMVEAIVMHNET
IRSVELLEDSFSLCFLMVQCMVVAGLATLCFYMMRIYNQAFNMCRFSTFTIGLVIHLLYLNWVGQQIIDS
SDKVFYSAYYSDWYLISRNERQLTKIILAKSLYPCQLTAGKISVLSMETFGALMKTSIRYYKSCKVLMEM
LGLWPYQTLKRKRLTCIIFYSLHGSLLLPQVIRLLQTKDFNIIVENTISILFLFVCIAKYAVSYASGPNF
KLLFTQIAADWHKITDKSEREILSKYGNDVYVFWAWATYNMMPFLPPILDIVLPLPNGSHPLLLPFYANY
IFFEQLDYHYETALHAFFVYFFATSLFAGIDTIYVSTVKHTCGLFAIIW
>OXU32020.1 hypothetical protein TSAR_001182 [Trichomalopsis sarcophagae]
MKGTEVKRTTIDPRYYFDLNIKLMALCGLRCSMTKTIGSFINKVPTLMANLVGIAVFVSEFNLLVDAVHL
RDSALAAQTLGQLVSNTQCITKGLLFAFSIEKLQPVFHEIRILWEKYQPEEEIQKSIVKDADRTLTFCKC
YVTANFSCLVSFSLPMAVKLFLQYQSRVATNHTYDVSQTMLLVKYPFEITDTSTFAIVIFLEEFLLVMNV
VFWVSSDTTFAQTTTHLCLQFKVLKRDIEKMFNYEGPDGKEILLQLVERHRELLRYRDNPKRVFLKTIIT
MFF
>OXU32019.1 hypothetical protein TSAR_001181 [Trichomalopsis sarcophagae]
MQIKVVENLPLTKHDIEYFFKDNLKLLSKIGFECSLTKKSEKFKFYHRIPTYIANFCGLIFFALQIYFVI
DKIQTNTVLAIQSFSYALINIQSILKGFITANSIEIIQQIFENLGIFWQKYMSRKPGRELILDRAYKIIS
LCKFFFAMAITCCFVFVMQFLIKFSIQYLNREATNHTYDFSNTVALLKYPFEIPNLPVYFLLISGEIIYL
FVCVVFWCNPDSLFVTLTSHVYVQFKALKIDMTSAFNNSTLKERSILIDMVNRHRELLRMCYLIEDTYSP
IIFSTTLLSAVNMCVTIYATREYIDKGYYLEMGIPLFLFIGASFQILFYCVFAETLTDEMIIMRCQKPFY
CTAYGFFPIGHPQLTSIISTAFSYYMMLKTMTVKKLRSIKHDIAYFKENLILLSFVDFKCSITKKGEKFK

LHHKYQLDIEKIVEYISSLILIPSGSNGEELLDKAYKTLLMCRIYLFTSFVTYLALTARLFINFCFPYLN
REATNDTYDFSKRMILMKYPFEIQSVSIYILVTFIEGHFLLITATFWTSGDCLFATVTTQICIQFDKYGG
HYLKHVIPQIIYEVDEKLIHVRAAVHITVHNVHLL
>OXU32017.1 hypothetical protein TSAR_001179 [Trichomalopsis sarcophagae]
MHGIFGDPRLRRKPPGSGELLFDVLKVDFLTGEKSSPNEDIEKYLGLNLKMLSCIGLDVSLENDDVIQER
RILEKMPIFMTNGLGIFAAILQISLITDSMTHNRMYLATQVSSHLFSNMLCISKGYQLATAIAKLEEILR
EIALIWKQNPLKDEFHRNILSGAAKTLLFCKVFVVVTLCAVVGFGLPPIQNLFFQYLHARNSANHTYDYS
QRVFIIEYPFLIQDVLTYSSVLLEEEYLLLASGLYWVCCDTLFAQLTTHISLQLEILQYDIERLINRESA
EDRLNENFIIFVKRHRKLLSICELIESVFSPVILTTVVLSGMNICMNIFELSKTISEGNYAEAALHAFLF
MNTFLQIVFYCTFAEKLTEQTSFVANSIYNCKWTEKNCKFRVYLQMLIIRSQNPFYFTAYGFFPIGHKRL
TMVINTAFSYYMMLQTTS
>OXU32016.1 hypothetical protein TSAR_001178 [Trichomalopsis sarcophagae]
MSQPTEDDLEYYFAFNLKLLSLVGFKCSLDKKEKGLGFVNKLPSYIMCIQGTILSLFEVYLLRDIYKDED
KTVVMQVLSQGVENTLNVCKGFFLAYSIERMENVLQEIKFLWNTYRPSPDNRKIILAEAQQTYSYCKIYF
CVLASCCTSYFLCYLPALLYTFTMYFSKLAQQYRDRQANNYTYDFSQRLLLLKYPFDIPSIPIYFLVELQ
EGFYLFYAAALFFVSGDTLFAQTVTHICLQFKILKFDIDAMFNPENTGEKDHLNLVTFVKRHRDLLRVCA
LIEEVFSPIILSMMLLSSIALCVDLVGIRGTMEKNNYEETAVVITLMMLTLLQILFYCTFAEKITEETRS
LADTMYGCNWTMKNNKLGLYIHLMILRAQKPFQCTAYGFFPIGHSQLTTIINTAFSYYMMLQTTS
>OXU32012.1 hypothetical protein TSAR_001174 [Trichomalopsis sarcophagae]
MPSLLVCVLEESFTRLENTGHLTKHGGKFKTWRKRYFIPKKCALCYWNSQNDIYRKPSTPDPARQSLQVY
VIRGYIFFKDFTVPETDGDLNCLLPMPQRHQLLLHSKKNITNPLTKVKKMASLNPEYYFDLNIKLMSLCG
IKCSMTETIGSFINKIPTILSNLVGLVYLIFQVNLLRDVVHLRDTVLAVQILSEIVCNVQCNIKGLFFVF
SINKVQYMLHEIRILWESYPPDNELQKSIFLTADQTLTFCKYYVTANLSCVLAYALQMGLNFFTQYRTHQ
HGSNHTYDLSHIIILVKYPFVVTGVPTFITLFFTEEFLLIMCATLWAIIDTLFAQVTTHMCLQFKILKRD
IEEKFNSKSLNRKEFLLDQVRRHRNLLRVIEDVFSPIVFFTVFLSSVNMCFNVIGTRETISNKDYFDTGI
YATILTMTIFQIFFFCIFAEKLSDETTSLADTVYNLNWTTKDYKLRFYLQVIIVRAQKPFYCTAYGFFPI
GHQRLTSILRASWSYYMMLQTTSSK
>OXU31759.1 hypothetical protein TSAR_003115 [Trichomalopsis sarcophagae]
MHRSSSSVTVHRVTQRREAVFPISSLASGHFIRRSNWYLRNSFYKQVLALWKDTSDEEEMMILKQHADFG
FKTLKIYIITSSTIYQVIPILSGKYATIAYVEKSTKFIHITICSWFRLIRLSKIAGEVKYGLNTLKDTDN
IVLTSISRIVDLHNATLNNIKYIDHAFGTTYFIILLFNSLLFGSSLVLINNNLGDTFCEWYKLSKRSINS
LRIIMLKSSMQCEITAIGMFVLSLETFSKDKNFPHHAWKVVTLLEKGSGCWPFENQIKKMIIRLIHFVNM
IMMLVMLGIRLWEEFERKNVKIVMENSVAMIMILCTMVKLIMSYFNERQRKRFYEQMFVHWKDTSDEEEI
MILRQYATSGLKTIIRYTIPMLLTLMNYLMNPNITHYQKQLPFYAEYFVDQEKYFYQLYLLLYFMIGAAL
IVTFSHELTYFQSVQHIMASFKIIELRLSRLSKLVKSVDCSLGRFNDADQKIFTSICGAVDLHNATLNCK
WYKLSKQSKRSISIMMLRSFGQCQITASGMFVISLETYGKARRNNVTEMLRDEIQSVSSEISGREKPARD
ETKIGNGMKLAWLDTGEAKSREKLNPLLEQLKRYCGDSSLAGCKYIAEGQRTWFERVMWILLHLVMVGTL
GAIIYNVLDDFKHTPVVTVVDSDNYPSNLLDLPVDKVLDLLRELGYLYESSFDTEHKSTNELDRLLGKYF
DGLYDVTDVMKELTPKCSDMLIKCRFRAEIANCSELFRFRKSQDGFCCVFNYAREMDDLERQGQKNPVHK
AHKIESLGKVNGLSFLMEPFLDDYLFPILPVSGWKITIFSPSDYPDNTSGGVSDALISPQSENFLELNAD
SFFSEKAVHNVDIEQRKCIFHSDAAALYDSYTYSDCIVNCRRPITSPLESHGQSRKMLILQLNLHTNELL
YIQASNIYYATAEKRTTKSCIANTRRVCNTSDIECLQRFKSKWWSVLPHDIARNGQESKVFDVISNASWS
LRCKNCYPSCNDVRYYLRTSSVNLVRDELNTFLKDVNVANQSIVHIFFARYGTVRLKQDIAYYWYELLSN
IGGICGVFIGFSLISAFEFVYFLVLSILALLKSRHPNENQDKTYPMRPIFWDELAPRGKTRNAHADRYSK
RRVLAH
>OXU31621.1 hypothetical protein TSAR_016111 [Trichomalopsis sarcophagae]
METQNTKRIENDRGFNYAVKLTRLLMMSCGIWPAKFSTSFQKCLRPILIIACFFIMFFQLIPFCLFMFLI
IKDMRIRLKLLGPLGFSLTSLFKYVVVVIKNREIAKCVQIMVDDWHQLNSTEDRKAMLINAKTGRVLTMV
CMFLMYGGGMPYVTIVPLAKGVTMVGNVSYRHLAYPSYYIFFNPHVRPIYDVIFTTHCICGFTRYTITCA
VYSIVIICVMHICSRIAITSSMLQGLADDCDGRLLGTAVKHHLDILKFATKLENIFKEIFLAEVLGSTYQ
ICLLGYYFITEYEQRAGIATATYLFLFMSFVFNIFILCYIGQILTEQCESIATTAYTSKWYQLSGREARS
IILIVHWNRRRVVLTAGKMLTLSLESFSSVSIFESSSLISRYQLLASSLCFFSQIVKAAGGYLNILRTAV
ANSN
>OXU31360.1 hypothetical protein TSAR_014933 [Trichomalopsis sarcophagae]
MNKEEVDEAFNDSLLKINKELNVFNGLWPHRPDGDKLFRRIVVLTVLISVTLPHVLGMSIQCGRNMALCG
ENICGFCYCSGVIVKFIVPIVSKEKFITLYEKIALNWKEITDPYEQSILEEFSKLGRLKSWLYFVAGFAF
CQMTALPALMDIILPLNESRPKILVTKAEYPFDPFEYYYELYFLYCTAAVVSVSVLASTDSTYSVTIHQS
LGIFGIVNHRLQKAAKHKNQEESYRVMVSAIELHKSALEFLELIESTYQSAFLIFIFVTVAFLSFGSLIL

VRLYIKQKKMKTNGLINFKIVEHSEEIIDLIRMTLIEFGAMIHIFFISWPGQLVIDHSENLFLTTYTTKW
YNMSQKGKKLLLFMMMRCLKPSFLTAGGFYIMNFENYGSIVKTTLSYVTVALSFH
>OXU31134.1 hypothetical protein TSAR_010117 [Trichomalopsis sarcophagae]
MLQALESKDIVLLIKFATFIWFLLWQIFSFCFAGQYLLNKGETITGAMYDSDWYNIESNDVKAISFIIKK
TQRPLSVTAGKYIPLSVTSFAAILKTSFSYLSVLRATMVAENPATYRYIEIGLKIIGSYPGTSESKGFII
IIILIIINLPFQILNALEAYRDINIFMNSFGPTLVMFNCMDNVPQGEEKQANSSEAADLWDAGIYCAAGE
YLPKQSENIYEDLSKCS
>OXU29589.1 hypothetical protein TSAR_009073 [Trichomalopsis sarcophagae]
MEIFDSRYFVINKTCMKLLGLWPYSSRIKNYFRRCGLGIFLFSCYLPQFIRLYKYFGQDMDEMMQNIGVI
LYVFGISVKLITGVTAEERMKTVYENTARDFQTIVDKAERNILLEYAERGRTLSITFIIYMCSALIVFVC
LPLGPLVLDYFIPLQNGSRERVFIWKGEYLVDPDKHYYKIYAIEMFSSLLTVAILSSVGPMYQAIVEHCL
GLFAIVKHRLQICTRGGKKADEETYGLIVRTIRLHTEIVELSIGDVLRCLSFFNRFTRIIEASYTSYFLI
EMGITILLVTLVSAILISRLDRLFDSLRHIMILVGVMIHMFYLTWPSQKMINHSTDLFHDTYSNEWYKCS
IRCQNLLKFMALRCAEPSQLTARGLYVMNFENYASVLVNKLSNVFRRCSVDFIRFEQIIFVFFYEFQLVK
TSASYITVLLSFR
>OXU29180.1 hypothetical protein TSAR_000295 [Trichomalopsis sarcophagae]
MEEHEILNNEYFKVNRFLLKLTGLWPYQKRHVKLIIRILYICAIHSMMIPQVIRTVEEWGKDFEIVLENI
VGFIYLQCVLAKYVITFMAEPQLVFLYKKMALDWTRYIEAEEQLSLQRAASNGQLMTIIYSVYVNFAGVG
FATLPGTLPTILNIIAPLNESRPTKVLCFYAEYFIDQEEYYYQLLFQTFIGVMSTVFINATVDTLYVICA
HHSDGLFNIVSYRFQKAFNKSQERYQLKSRNLVAAKNLDEEIHEYVLTAINIHNESIEYSILHVVLEFTC
FHLFINLIQSTYTLYFFIQMSLTIISLSLATVVVNYHAMMNLDDIINLIRIFFIWCGIILNLAYISIAGQ
QIIDTSLQIFDSAYFCGWYNHPLKTQRLLKFIMLRCSRQCQITAGPMLVINLESCSNILKSSLSYCTFMI
AVS
>OXU29179.1 hypothetical protein TSAR_000294 [Trichomalopsis sarcophagae]
MLDSNEVFNNKYFILNKRLLMVNFIWPYQKKHIKKFVNTFIVVAIHTMMIPQLIRSVFEWQLEEKNIEIL
VENFTGFFYFQLTLSKYYMSIYLEKKLLKLYQQITHDWRGLQDDNEQAVLLRSWNIGRALTIFYMGYMFT
ACIIFVTSPSAVPLMLDLILPLNESRGVNLCYYAEYFIDQQKYFIYLLLHTFICVSFTILILVAVDSTFV
SIVFHAVGLYNILEYRMRNVSRFVEDYQNNKFLSKEELHKYVVDSVKLHKKIIEFSRVYLLLISYYEIIL
LVTWSFGTFSEIVRRIYNDCFFFVTVAVLICLSVGTIDLVVNLNNPINFIRVGFVWLGIILYTFFISWPG
QKLINCNYDLFNTAYACGWYGCPTKTKYLIKFMMMRCTVPCILTAGPLLTMDLVTTGNILRTAFSYCTVV
ASFS
>OXU29130.1 hypothetical protein TSAR_009913 [Trichomalopsis sarcophagae]
MQQDLYTVLESLGWLFRISGIYPFTLNNSGESRKYALSTISVTFSIVFFIVSAKYFYEYSIIIKNVYDIL
YSSLLRNVLFSSAEDESLAVLMISVRQITSLICIFDASIMKIIYSRRFMLAINNLALQDQILKIMGYSLN
YKLMFRISVLSIPCYLLVSNTLLNSEFLVVSSIDIPAAFRVFGNFSILVQMTNTILFVFLIINVGQRFKG
LNFCILKLVNREYTKLGMPISNKTPAEYMQMAAQVHARLCEIGKAINSVFYITLVGQYTILFIICITKVV
SIVTTIGAFIFSTTMIFYIFTLAKYGFQNVGFSLVFLFFALLFIQIFTVLKIVLSCNWTRRQAAITSKIL
HNLSLSDMCIDDDSLSEMASSHIFDADTSSQFFVYRCGIFSNRFDFNTISKHVLFTFLLNLVLTLFLVCS
WKTVELKSFGTAMMSEIFPTTYFFLKISGFWRPYSLKLPLYLCYQIYTAFSFATVLSLIILLVLYCAFAH
DKSLELLLENMYLVISFSNCISKTSNIILRKKNIEKLLQWTREKQWLAERDSEESCIVAHSKLMEKAILQ
FCTLLVCVNGIGNLMNPIIRAKPDKKLVIEAYPICDRSRPVCFWLTYLHQCFGFVIINVIHLACDCLIYN
FIDRTCAHLKILGHRLQKLPVLVKGIRHQGIDTVEFEKSYVIDCIKNHQGIFIFIRELNDTFCETVFFQF
LSSILVLCTNIFLLSKQELFSPEFVAVFLYFCCVLAQNFFYCWYGYKLSVNSLAFVDAIAKINWVELDLK
TKKMLVYMMLITSNKVELFNNAVVNLSPASFINIVKISYSAFTVLQRTSHKEMKI
>OXU29015.1 hypothetical protein TSAR_005143 [Trichomalopsis sarcophagae]
MMDVREALGPKSNFKYNIFFLSACGIWPLQSKSSKVIFGFLWITQEFIITGAESIKLVEIYKEIDLLLEA
LPPFIYNRLNDIVFANGMINQHKMKILLRKIQNHWDSLTDESEIQILAKTVKFGMLINVGFIGMVYSALW
SCMLLPLLPLVLDIVQPLNESRPLKPLFMCEFFVDPDKYYFTILAQNYMSALASPLPLVGMDLFFINCCN
HICGIIGILGFRIDNITKNEALNKECKSTYKQLCKCMDMHQIIFDFCDSINDTFSTNFLVMLFINMSLMS
FTGVAVSRLFQEL
>OXU28981.1 hypothetical protein TSAR_002358 [Trichomalopsis sarcophagae]
MDIFQSSYYIRCNRYLSFCGHWPYQSLRNRIRNFILLMFLMSTILIPQVNHFFNSTELHHTISSNFGSCD
TIYMFLLLLRYHRCFTTVLFYLKIRSVCYNRKKYDLNLLSCIKKVLEKIKSDFQRYKDEDLKILHKYSGQA
NKINTFYTVYMFMAVGGYSMLPLTLHVMDIALPKNESRLPTKPRLINYNIEAFDENIFFIIIHGVIVDTA
VIVFIIGFETLCFSFSYHVCALFVIVTNKIRDSIDERITSKHSEVDQDIFYRNFVKIVIMHKDALELSTE
KQLLNIVLTNAIFVLTCSFVDTVETALSVLNLFAIGFAMMPLTITGFEFILSKGNVGEMARWSLFAFGEI
VHLFYYNWPGQKIRDHSFYAIEWYKEEIPDKCKKLLNLMMLRGQKPCSLTAGKVYILGLENFAAVMKVSM
SYFTVLSSVM

>OXU28964.1 hypothetical protein TSAR_015794 [Trichomalopsis sarcophagae]
MDEIIDPIKGTDVLPISFLYLKLVGAWKPLDSPKWLRLIYDLFTIFMVILICKLLIISDILCVVFAEENR
FAVFKGIVHVTITHLSGWIKMLHVLSRRRSIVLLVNGCVAKQWNPPRDRHEASILTSFNNSSRRTTIAYT
IQVSAAVSMLVLSPVFSSTWFLPINNWYPCNISSPICFWPSYLHQSMGIVAIAVAHVATDSLIVGFMIQI
CTQLNILNHRLLSIHVKLEDTTRRQKNPEQISAVETLLYHRETTFNASILICIIIICDTDIFFRFADLLS
KTFVEVVFIQFCVGFSVICSIVYLLAKLSILSFDFFGTLFYLGGMLSQMFIYCWYGNEVVLNSTKLFHTI
YNMNWVAFQIKTQKKLLLMMLVALSPIQLFEGAIIKVNLDAFINVLKFSYSAFNILQKSS
>OXU28658.1 hypothetical protein TSAR_004827 [Trichomalopsis sarcophagae]
MGIPDPENPLAIFYTDYYKYNRKLLEICGLWPELGRARKIIMMILFSLLMTSLIIPMVHCYKIVLTHRST
TIFIKGAGAIHYFHKGRIMYVVEDLIGLLYLTVASSKYFTYRAFEGRILRLYHQVGEDWRNTTDEEERKI
LQEYSEFARLLSIIYFVYAAIGAFYFNMSPYLPLGLDRWMPLENNETRVRIRTYHPYYFDLIDVEKYYYE
CYFFHGNSVVYISTMVGLSVESMTAFNVQHICALFHIVGHRLRKIGSTLEINAKGEKIARVDDITVVRQI
KHVCGMHRTSIDSVELLQSSFGMNWLVFLIGTVTGIALLMFDLIFSMKHPLEKMTGLVIFIGIQILIFYV
NWIAQKLTDSTEQIFLAACETSWYNLSVKGQKLVYFMMQKNIIPLTLTAGGIAELNFQQFASVSKTSMSY
AMVILQMNDE
>OXU28628.1 hypothetical protein TSAR_015445, partial [Trichomalopsis sarcophagae]
MDILNGPDFLYSRICLRPFGLWPFQDPKSKLISRVITLMAVSTVLIPHIMKTYEFRNDFHILLMCIPSLL
YYAHYITKFLYIAFCEEKFRNVLERIKDDFVTFRGESLNHLTNYSEEARKFNTFYTSKLHRMRFEFIVIF
REVYLCSTVVIYNVTAFIPHMLDFVFPLENATRPRHAARLVKYNIHQIDNNFYFVLIHGMIFDVVAIAII
IGFDALFINCAQHACALFKIVVVELRKCTKLDKKMSNSASDLVTLQRRQDIFYAKLVRTIVAHKHAIEFT
DNLESTYALVNFLMIGIAVATITLTEFETIVHVNEVDIMCRFAFFSGGELISMLYQNWPGQRIKDHSLRV
HSSWYVSTYVHAKKQSLNNHLAHFDFSFECEWYREDVSYKGKRLLMFMMLKSEVPSALTAGKLFILDLQN
YVKIFKASLSYFAFLSSVAKMKDNVLNGPYYVYSNNYLSPFGMWPLQSYKKKVLLRKLMYVGCNSAFIPH
VCKAYEVRNNFQYFFVCIPSIIFYIQVMLKMACMILNEDKCKELFKQIKNDFEIYTGESLRILNKYAEQA
HYFMGTMVVYNTLAFMPLFLDFLVPLNETRPRIITKHMKYNIKRIENNYFVTTLHGYVLNILGMMVVVGF
DSLLLSYTQHACALFQVVRNELKDTIDKHEIKVTSHAAQYANSRDVFYQDVVKVIIKHKHAIELSIYCPI
TVCFLILIPRHRFADLVESTYAATNLLVIGITLGFITLVEFETVQHKDNRALGIRFAIITIVELLHILFH
NYPGQRIKDHSLMLRLRVVQGGHNRRVQEAFELHDAAESEAELLDWWWTLRSGNGKLCNGAILEKKTVIV
RSTNEHGSLSFLDSKSIAVVLHFPVIRVNGTSHKSSAETVFH
>OXU28627.1 hypothetical protein TSAR_015444 [Trichomalopsis sarcophagae]
MEDNVLDGPYYVYCKNYLSAFGTWPLQSYKIKLLLRTLMYLGCSSAFIPHVTKAYELRNHLEYFFVCIPS
IIFYVQVLTKISCMILNEDKYKELIQQIKSDFQSYTGNNLRILNEYAEQARKYRLRYVFAGYFMGTIVAY
NTSAFVPLLLDLLVPLNETRPRPVLRLMKYNIKRLENNFFVVSLHGFVLNILGMMLIMGFDTLLLNCSQH
ACALFQIVIIAKWSDSVIKKIVEINPYMIFFNIDHVSSFLFEDMKTLLQIIPAIMLNSNQLSFKERRAYL
SGIVPFTISDKKLHIVLSKIQVENGNEEQEDLPNRFLKYYSKSSYKRTRARCLLIVSADKPLPESYTNSI
LYKSWNNQFLDFTMLNVIKNSKTSDVELVKLFYNPFQKLFINEPWNHSSQLFPDKLKDMNGYPLKVPVFQ
LPPYLNLNKNSDGSIRNVNGMHYGFFSTLSNFRNFSIKIIAECKASNNTTLRAVLHNLLHKNEVNLLPVP
QYTVIFGNQSITETSFPVDFISFIALVPVQPYNLFILPHSLFFNLLAVLTMYAIIIITIRLLKFDIKYWS
PIFIIEMLLSVPVLKTPRNLAERIVFISIVFVSFTYTNDGFLKLMDIKIVSDSRNFDTYKELSDSGLDIY
IEAPGYDTLLPSYDKQAKRIISKMKKLSQMNECVNKLILNKNCICLVSEPYGELLLQSYGAYDAKPILKK
SNLKIFFSYVSFHYEKASPYVRRFDEILIRIVESGIPKMWAPIYQRDTNQTVVEETNNISTIQFYSVLAV
VYGMSFVVFCLEFVYDTIDKHKIEATSDTAKDSNSRDVFYQEVVKVIIKHKHAIEFVDLVESTYAMANLL
VIGITLGSITLAEFETVQHKDDHEIAFRYAIFTSGELLHILFHNYPGQRIKDHSLMVYQSYNCEWYREGI
TDECKKLLSFMMLRSQKPSCLTGGGLYVLGLENYATILKASLSYFTFLSSV
>OXU28626.1 hypothetical protein TSAR_015443 [Trichomalopsis sarcophagae]
MSTQQSEYAVQMSRYFLRPIGLWSSSSSSRSQAYLHNLLNLLLIVITYLLIMFILVPCALHTFIEEPNMA
IKMKLIGPMSFALMAVTKYASLTQKTAVIAQCFRHVEEDWESGCAMEKKVMYRHAKIGRSLSIFSGIFMY
GGGIFYHGIMPMAAVQTFSASIDGSLSLNTSSVFEVAKKPRILTFPTYNAWLNIEISPIYEIVYLLQCFS
GYVLDTITVGTCSLAAVFVTHTCAQLELVMMLIRRYIDGDVGPDDVPYPKDATTREARLAVIVTRHIRAL
KFAARVEKYLNGICFVEFIGCTMNICFIGYYCLTEWERKEPISTTTYFILLASFTTNIFIFCYIGELLTE
QCTRVGSIYHAIDWYNLPGKSASDLFFIIAISRYPAKLTAGKFVDLTLVTFSNVMKSAFTYFNLIRTVII
>OXU28468.1 hypothetical protein TSAR_011465 [Trichomalopsis sarcophagae]
MTTAVEEYDNLALPMIISGRACGSWPMRAELEGERSLRVLLHRLHRFLAILFLYLISAGVTVEVIVFFGN
DMNETIESALVSSAFYMTFARVLTFARYQPQMLYVVETMREDWLRSTSEERAILRKKCLLAFKLTKFFAL
SVVTTGISFVIIPLLEVLVYTDNLLKHTEKAQLRFKEDAKKVLPYRGYFFYNYTQPEVYGYTYLANSMVG
ALGCTSIAYGTSFSLISTIHGAAKFAIVKRDFEKIDRTTWTDNKIVGNCVRRHQECIRFAETVEDIINIL
ALAQFVISTGLMCFGGFQLTTMLEDRARLTKYTSFLNTAVTELFIFSFSGQSLKSEVICAKKAAYTLFSY

LRNFHTNIPIVMKSEDVAEFAYTSNWIGSVLSTNLRMIILRSRKPCTITAGKFYDMSLESFLKVMSSSFS
YFTVLLAMRDED
>OXU28192.1 hypothetical protein TSAR_006578 [Trichomalopsis sarcophagae]
MSSEPKIFDNPLLARNKTLLIHYGLWPYQSKLARRCKYSLFSITSISLIIPMLQGLTDDTSTDIVIFFET
LVATLMVFGGFVQCLIMYARENKMRNLFNEIDANWCKLTDYKEREIFLHFARRGRALVTLFTVSTISCYF
VFMIMNNIPTIVGEATNDASKTFPYYSKRWIIDERLRNLQIFLHVIIVTFYSGMVYIVAITTYIYSIKHF
CGIYAIVALKNLTSTRRVMPRNTKYDVLSELFDIVNKHKEAIKGVRSIGSIFRRSFFVIEICFLCAFAMI
LFDIHSNLFNLRLLLRICFVASIFFMHTFYINYGGEMVIHFSDKIRMATFFIQWYSIPSDARKVLVTIMQ
RCQRPDTLGWIHMTYSNANLIQILRSSWSMAAVLIAVHDVK
>OXU28052.1 hypothetical protein TSAR_008866 [Trichomalopsis sarcophagae]
MHILHLPFKLLTLTGIWMPEDWTQKQQKLIWVLYSMVSIGLVFMQLSSQVGYLMQSKTWAQVNERLFFIP
TGISSVHKIFIFIVHRKDLISLGNMLLKEYCIPRNAEELSIQERYNEIIRVLTLACAFLVNVTMMNLVTL
PLVTSGDNRTLPMRVWLPYKVDSDMSYWLSYAHQTVGIVFVGTGAVGSTLMINGFMYQVCCQFEILSSRF
QNLPLIIEKFQSLKKPNQLIYRYEKRVMRQNIHHHLYIFRFAEALNNIFKSVIFQQFCLSSIVVSVSIYQ
LSTRPEKDLEFIMVFFYLVCVLVEFLVYSWFGNELMLESLNFQQTIYEINWTSLSTRSSRDLVLIMMRAS
KPIIMYCGHFIVLSLESYIGVSMYVIEEIFVNSRFNVSIYFCTQILKVSYSVFNILRMSEE
>OXU28001.1 hypothetical protein TSAR_016066 [Trichomalopsis sarcophagae]
MLGKSSLNSKIPSGDRDFNYSMRLSRITLSIIGLWPFRENIRSSNFKFVLTLVSTLTTLLSSLTFVYQTD
DDEKMFDSLINSLYMLMTLVKLLMVRCENDKLKVILSEIKIDWKKYQRFSERNKRLIDLYTSKARTSSFV
CIFFMEFSITTYFISRVAYALQQPAKIREWDLPYTAVYPFEVTSSLFVPMYLWQLFSVMCLGSVNISIDC
LLVTTACHATGQLAALCENIKGYGREQRHRDETLSSEIECSCIRCIIERHVDIVRYCRLVEDAYNLILLT
EFIGTTFQFCLQMYIIVEHSNDKNIVGLLSFCIYLLVFNFRLFMYCNVFGAMVEMGEKVGASAYEISWYD
FHPEAVRQLMFCILRANKPLNVTAGKFFSLNRNSYKNEHNLVEHSYRHPKVH
>OXU27888.1 hypothetical protein TSAR_016340 [Trichomalopsis sarcophagae]
MRNSDLKKWTIKSIVMSSWRMGSIKDIMMNVFMMKVIGVALAIWPLKSEGKRWYYAFLQEMVYRFFHVNF
WLLIIPSLWSIYKIRHNLASVLTSVTQLTIVFEILAIMVLSRRQAARLKTLLTMAYDYVSVADDKVYPVV
YKYVRKAQIIFGIITLAYALILLTYLMQAFIENKPPIYAYYPFDIKSPVVWICVYGNQLLCTSYAAVVII
MDAMMMFMIFVTSIRLELLQKDFKKVKDYPDLVKCIRTHQDIIWYIKEVYCIKKYMVLKMLISIAIYIIC
EGLQLFALNLSWGMRFQVSLLFGIGLFRVYIYAASSQDLISSGLDLGYFVYSSLWYNQSHNVIVAKAFVI
CRCQKSLGIRVCGITDNLNMKFLANFLYRVFSYTMTLRAIIKTLR
>OXU27769.1 hypothetical protein TSAR_009023 [Trichomalopsis sarcophagae]
MDIFDSRYYKTNIFYLKLLGLWPFDDFLNKRVRRILIIIAVVSLIIPQVIRLFEEWGRDIDIVIEVIGSL
IYFSGCQIKYLSFLRVEAKMKYLYNKIAEHWKSLSSKDEIKTLEEYGEIGRGLTLGYIIPINIILVIYIS
LPLLPLLLDVIDPQNETRPKQFPYFAEYFIDDQKYYFELTIHGWIVCILSVQIYGTFDTTYTQCVQHACG
LFGIVEQRLKKATKLASSNEFSTLEEKDEKVYDKFFTKLTSSFFTTCRFVNLIEDCYSFSYFFVVTLNTA
VVSLAAVDTMLNLENGNTKQMVRIGALYIGFSFHLLYNMSPGQRVIDSSTNIQNAAFHCDWFNASSKTKT
LIRIIMLRSLTPCQFTAGKLIVLHLESFAFVFKNSISYVTVVGSMR
>OXU27767.1 hypothetical protein TSAR_009021 [Trichomalopsis sarcophagae]
MEIFYECYYKNCKWFLRGIGLWPYQRRNRRIISYIIFFTINPSLAIPQGIMLIRNLGNSNIMLENTVSLM
FSTVCMSKYSVIYASAKQLDLLFLQIVKDWKIFEDKTERKILEKNTEEGRALVLIYTFVSNYLLFAWIIY
TFMPFMPYFLDQIFPLRNGTRPIQIPFYADYVIFNQLDYHYWCCGHIAVVYFTSFFLYSGVDGAYVLTVK
HTMGNSQKIDEARYEQKNYSIVKREMAGAVILHNETIRCVALLENSFSKCFLIVQGICVLGLALGSVYVK
MLFIANDIFRFSRSVAFIIGIILHLLYLNWVGQQVIDSSSKIFYSSYFSKWYLTSAKARKITQIIMCRSF
NQCELTAGQITTLSMESFGIVNYRLFAYMIYAFMPSTTYFLDQIFPLGNGTRPIQTLFYTDYVIFNQLDY
HYWCCGHISIVYFTTNLNGNCGLFAIYKSFYISQIIHKHEASYQQKNDSIVKREMVGAVILHNETISYFS
KWYLTSAKARKITQIIMCRSYNQCELTAGQITTLSMESFGILMKTSASYFTVFLSVT
>OXU27765.1 hypothetical protein TSAR_009019 [Trichomalopsis sarcophagae]
MEIFDGRYYKHSKWFLRAIGLWPYETHIRRINSFIIFYIIILSLAIPQTIMLVKHWRDFNIMCENSVSLM
FAIVCMSKYAVTYTSAEQLKLLFLQIIKDWQKIKDETERKILQKTTEEGRTLVLMYTIYITYATIMYGLI
PFTPYFLDQILPLQNGTRPKQVAFYADYVIFDQFDYHYWCCGHMILVYFYTSCSLFSGVDGIYLLAVKHA
CGLFLITWIETMGNSQKTDENCYQPKNDAIVRREMTEAVILHNETIRCVDLLEDSFSKCFLIVQGVSVLG
LSLGSLYTLFILDDFYKFVRIVAFIFGIQIHLLYLNWVGQQIIDSSLKVFYSS
>OXU27763.1 hypothetical protein TSAR_009017 [Trichomalopsis sarcophagae]
MKKCIHMAAAYLTTWFFFSGVDTTFVLSVKHTHRLDGILKSEKSVKFEIKLIHIDKTTNSKFLVIIYNLL
TRYLAFLENLFNWCFLVLNCMTVIVLAMIGVYLLYIYNHIYKMIRIGLITLELFIHLLYINWVEQMLIDK
SEKVFETVYFSNWYFISTKFRIFAEVMINRSIHPCPLTAGKITVSSMIRSFGIQPIPGHKTIKML
>OXU27762.1 hypothetical protein TSAR_009016, partial [Trichomalopsis
sarcophagae]

VFDGQYYRTCKWFLHIIGLWPYDKKSRRLFCNFIFCAVNISMTVPQLELLFLQISKDWQRITDIKERQIL
MKFYEEAKSLVVFYTIYAFAAWIVYTSMPFIPFILDLVHPLENGTRPLFMPFYADYVFFDQNDYHYWACG
HVAVIYFTSFLLYSGVDGIYVLTVKHTCGLLERITGFNEDKTSKNDFVVHRSLSEAVTLHTETIGCFDLL
ENSFNKCFLVVQGLSVVALSIAAVYILYIYENTFLLLRVGAFLFGVILHLLYLNWIGQKIIESSSQVFDS
TYKGNWYTVSIRSRKFINMIMYRSFKPCELTAGTITTLSMETFGILMKTSMSYFTVFLSVT
>OXU27755.1 hypothetical protein TSAR_002475 [Trichomalopsis sarcophagae]
MAPDAIVKHFADVETIGARFCILLGVHLGFLCVNFYMKCLGIYPLPSTVSKFWTRVYNLLWCFYLSNHLL
IIFPTFYAFGSTTQDIAVATYSLMEGLCMIECIVLLIHFKYQKSDFKILLSLVHHELNKKKRIITLDNGN
VYIIAFVLIAIMYVLIVFNYIQRPETVHYHKLLTTARYPFSTRAAAIKIILSCHQMVVLLHMTIILTSDG
LAVLLTIICTVRLKNLETKIRNGKRGKLSKRIREHQQIILQVEETNLIVRIIVIKTVFCFMVFSISTGIQ
IFHKFEIIQIFIVMIVFLRFYVSAESADNMATCANNLGIAVYSTAWHEEKTKIRIAKTIIIQRCQNSPRI
FITGFMSELNRKYFLVVAYATYSYFTMIRTLIGKNK
>OXU27739.1 hypothetical protein TSAR_003158 [Trichomalopsis sarcophagae]
MEIYDSKYFIHNKRFQMALGVWPYQNRVKNLSICGVLLLVMFGMLIPQLLRLRTYLGKDIDKTMENVFIL
LYTFGIYIKLFTAHIAENKMKILYESTAKNFETYTDEAEKKIMKQYSERGRLITLAFLLYMVSALILFVL
LPLYPIIMDATIPLDVPRPRMSVLNGDYLVDENDYYFQIYVFDSIACTLTVFIMCSTDPMYAAIVEHCLG
LIKNNTKSYITQSSSNEKARVFRYRLKNFNKSCGMRMVERADAQRYGGDYAYAALVKAILLHKEILKYTQ
IIQTSYSLYFLLEMGATVGILTATSVIIVMKLHRPLDCLRYFLVLIGIMGHMFYLTWPGQKLIDFSGDIF
QDTYLNDWYKSSLKCQNLLRFMTLRCSRPCELSGGGLYVMNFINFAAILKSSASYITVFSSV
>OXU27259.1 hypothetical protein TSAR_002812 [Trichomalopsis sarcophagae]
MDLFYSQYFKINKLALTVYGLWPYQSEIGRIINHVIFAVTSFSMIFAMNVYFTIQKAAGIQSQINAELKN
ILETVVALVFCGAGLVKCMILYNQRNQIKKLYERIAADWEKLTDTSERDILRAFLLEGRHSIVITIVYAV
PAFCLFICVEFLPRIFSKESAKHRLHSFPYYYKSMVISENTYDLQVCVHLMVVIIYVGFSYLCASATYIS
SVKHVCAFQRLRNAIVYRKDSRPLKELIEDTSVIPNLIKVIEMHKEALSTFNDFYLSYRGIQIIEQVFSA
GFFVFEISALTTIAILIFDLNYHQGNPFQMMRVLLILSVFVLYLFFMNWCGEQTIQSCNNVNAQAYNIEW
YGISLKARVFVLMILRRTLKPIHLTAGTIMILSMENFATILKTAWSFGMILLTTQTSVRNKDPIFFGY
>OXU26977.1 hypothetical protein TSAR_008798 [Trichomalopsis sarcophagae]
MEIFERRYFTLNKTLLSSAGLWPYQNRGKKFCIRTFVNLVIGIFVIFPQIVRIYSYLGVNMDLMVEHSAV
LLYIMTIYLKFLTSVFYEEKLRVVYDNIAKNWQVIKDVNEVNILVQYSENGRFLTIGYIMYIIAACLSYI
LLPMVPILLDVLVPLNQSRPRFYILGGEYFIIDKVEDYGKVYMFDVLAVIVSVWLICAVDSMYAASIEHC
LGLFAIVKLRLRMCTQPSCEGPRSRDASYKLIVRLVRLHKDIINFTDILESSYSSSFLILVGINVIFLSF
ECIIVLTRFGQAMEMMRYSMIMFGIVVHLFYISWPGQKLIDLSLGLFQDTYLNEWYACPTRAQKLLGLMT
LRCSKPCQLTAGGMYVMNLSNFAKIVKTSLSQATSMLGLRLIGLVLFASSAAQLSQRLDQQPYVGVRSFR
KELTDPRILAVHNDTLDGHTRLSYVKSAWNMYSEEPADGSTSSFVYNVVIETYVDGLTFRKSCWTNLTAH
ESKENVRLVSLQDSDRAVLMWTETLEPPSLAKLKVYLVDAKNCSWKNVTELTVNLPLNRPFDRNVFVVPY
TRVFDLMFSNLDSCKRRLCRASYDVEGRILGNVSILQDDSYDPSLSSPGSSGHFPHFAPVLPGSAAKGYF
MFVEEADQLKLKTLDSTGELSNSRIAHVVPRLPEEGRPFVTSNSHETFGYCFILLANRSIQCRQYDSGLS
IKSNLTWTLGYNSRRASMLNLASGGFLLLTEDSQYGSQYLTKVESDGRRSDPMELTMGGGKDSQLCGPAM
LYEKSSGDFYCAYGLCWDQPKSSLKVDVRCVPKRYFVARNE
>OXU26944.1 hypothetical protein TSAR_013834 [Trichomalopsis sarcophagae]
MMQNHQLQGQAELDDSSRVFRWNYILMTTLGLWPASLSDVRFFLNFGYFCYEMLLEYLDLFLFIDNFENV
LMNLTENMAFSQIFIRMLMLRVYNSELGEIIGDAKKDFDAKNYTEEERKTFLAYHVKSRTFMKLLITNTA
LTASSYYVKPLLGQMGELMEYANSNGENSTFIFMLPYRFYTFYELNDAQTYFWTYGSQLPFVFISGFGQS
AADCLMVTLVYHVSGQMAVLALRIASIDTHPSKCTQEVQKIVKAHIRLLRCSYKIKLTNALQWLRMGKVI
QRAFSATLLGHLVGATSLVCILGYQILTSLANGERAILISFFAFIFLVLLVLYAHCTVGESLITESERVS
QAYYDCEWYNMSKENARIIILCMARAQKPLQLTAGKFSMFCLQTLTDSIKASMGYLSVLRTVM
>OXU26943.1 hypothetical protein TSAR_013833 [Trichomalopsis sarcophagae]
MSEKSEINHDILKQELCSAELVFVWNKKLFAAFGVNPLQFNSLLYLFWLFYSIMHFILSCAALPQNLHNL
NNMIDAITEVGSMITILAKLALYRINRKSLADLLVEIEKDYSIDKYENELEVRMFIDYVFKAKGFFQATI
PLSIASAVMYFFRPFAMNGFDSANLTTFVLPYRMIFFYPITKLSGYLGALFYLIPVIPITAFGLVTDANL
MLAIVAHICGQLSVLSRSLLHFAKDGTSFQQKLSKTVRQHQRLIRMAKTLDSVFNLLMLQQVLSVTFVIC
FVSYSMLINWSQRESALILTFLVCMVSLSFLLFAYCYAGQCLIDESTFLGNALYTSTWYKLEPSNVKNYI
IFMKRTQKPLVITGGRFYIYSLPSFLAVMKSAMAYLSVLRTLI
>OXU26938.1 hypothetical protein TSAR_013828 [Trichomalopsis sarcophagae]
MNISGSEQTILAKYKNDLQKASKLLTWNRRLLSLLGLWPESPMDLLFCASAIYYVFYLGLTFVSFVLYLK
KKILNVSIFIALLSYSHISARLLLLLRRHNRTFGVLFAEMKQDFELRNYKSDQELRVFLKYNKLAKSMIKF
LLFCSTFFAIVFYVKPLLMTYNIHRAIRKLHRNATAPFVLAQNSFYQFYKITTVKKYAINYVSVLPFSVL
TGFINCATDCLVLTIGCHLSGRLAALSHRIRNVEFCNGSQEFKAVIRLHQQVLRIGDMVENSLNTLMTCH

ILTAGVIMCFILYKTLIYLRPGKRIHLIHIVILLSLNIVRLYSHCCVGEFLMQESRVVHEAFYECKWYTM
LLQDRKLIVLNLLRSQRPIRFAARGLGTFSIELFSEVQYSIYNHMCSEKYLSIFPTMIFLGTKIITRLLI
SSEERYLKCMANQTLCKALNYGSTFYETK
>OXU26416.1 hypothetical protein TSAR_014896, partial [Trichomalopsis sarcophagae]
KFSVDLIYYTFTCIFNDNLNFKLYDNNGSYVRIAHLCSHLLAKYLQIFMYAWYGNLIADESKAFLDAIME
RDIQTYKVCSENVTLCLIFSGVWSATHPVLKKIAFFVTFFSTFSVMAHTLNFSLHNAQNVRILVRGLAAA
SSFLSISSKAFLFLQHQNDLNYLKDYLTEKFMSDMQNPENLPDLLSNVRMFAVFVTMYKTTVAFIMSMYC
IVPLFSFLKYGKYLRVYPCLYPFSYAPGGVVHWLLYGWESTGALSAWAISVGTDCAFGMYAIQICGEQKI
LARKLKALRVGSNYTRELKDCMERHHLIITAKNKFESLYGLISIWLAISGAIVLCSLIFQVTEYLETRGG
YVRAIIFFAHFSGKIIQVFLYAWYGNLINEESLAFPRAIYSSHWTDCCDTRFKNDILIVLAQRPLIVTAL
GCMNVQLDMFAKIVKTSISYFFLLQTLKAKTEEE
>OXU26110.1 hypothetical protein TSAR_016784 [Trichomalopsis sarcophagae]
MEETSAFYRRIRRIQMRVLRLAGLVPFENRTLRFAGTILMSIYVNFAFTAVSSVYIWAFVEDCLNKRFNP
DITSELFSFVGFHFRFMYIFSRRRKLGKMLGYAESLWERVRSEEKVHVRLFVRKVSKLSVCYSGIILTTI
TLYVLSSQLPQLTAAATNETVHRVLPYPFYVDVQSSPRYEILLGAQIVCLLTVTQTSVCVDTAIAFLIMI
ACGHFRLIQVRLGVIARHIEENEDKRRSQRSVAKDGEVIELEVEAEMDEEDFERTDERVRERVKELVMHH
QEILSFCDDIKNLSSEIFMIELISTTYNLSLIGILLAGNMPLAEKFKFAPVLFILTTQLFVCQYPPDLLL
QESEAVANAAYFVPPFRRDRRRIDRILLSLLARSQTPYQLLAGGQIPLSIESFGNMIRGAVSFFTVLRSF
N
>OXU25856.1 hypothetical protein TSAR_010087 [Trichomalopsis sarcophagae]
MTSTDPRDDANTRERTDIGSFGVAVPKNSREPPDKEYCDVRKRKSTLANILETLLKLESKVLIFNTSSPL
KIVLDHNNQTCLILIMKSSRRFWNEFKIYKFVLWPCGVWPSEKRSLIDIFFYLVATISQIFIATLVSEEI
YGTCGTPADLLDYYAFFVCFIMSFIKLLCMRLHFIKMYRNCLYAQKNWTKVVDTNVLKIMQRYANVAKKF
YHVQMFAAICVVSLYVVNPFILPRTDASNLPIQTVCSFRNLTDWEYVIVYVVQSMSLLYLAFGFIGVDVF
FFGIVMHLCAQLKILQIEFNTVGKLTWRLDNIRLMALYSKKHYQLIMLANDLKTTFSATFFIALKINNKF
LALKSTQAFPILFLEVFLYCYAGQLLQYEFSNITNSIYNSRWYALPPKLRQYYLLFGMTAGNKAFRLTAG
QLVNVDMRLFIYFVKTIFSFFSLLLLMFDKRAKK
>OXU25585.1 hypothetical protein TSAR_001228, partial [Trichomalopsis sarcophagae]
MQFYIFTLSGVWCPSNWTSLLKLSYNMYTTIIAISGILFWASMFVNLIITKNESEYFYENVFAISTLTYA
MYKEFFVLKKRKEIQQMLKLSFDDEWYRPFDNHEIQIIDHYAHETRWVTQVYAIGIIAGLATKAITPMLN
SNSAWVLPIEAWYPYNTSNLKNYLFAYTQQLMGGIPLICLHISYRLGNYLNQEFKGILTGQVMITIPNIC
INVYLLSQHRGGITLHLVDSFFCFTTCLMQIFLYCWYGNKIILLSFDVANTVYTTNWLSLNISSKKKLLT
IMVRATRSIQFAAGTFIMNIDSFIE
>OXU25584.1 hypothetical protein TSAR_001227 [Trichomalopsis sarcophagae]
MHSTLAAIVKNNIEYKILPFQFFLLTFLGIWCPSNWSLKSKTAHNVYFTFIFFLDFLICIEMFIHFVSSF
GTDNFKLINFFLVSANITAVYKSIRLMQNREVLRYFIISYFDYEWTKSHDSVEHAINSKIDLRIRRVTVI
YSASMIGIVLLKAMSPIAESNGIPLPVDAWYPYSIEKSKWFWITYLHQVILGSSAVGAHIGIDTLFVGLL
LKSSGQIHLLNYRLRNLMLLKKCNFGKLKEYSEKNVVLRCIYHHKRIYRFGGDLNDKFQEILFILVVSSI
PNICINVYSLSSYKGNINVQYIATIFSTTSALLQFFIACWYGNETTLDVNKSLQVINAVYEMDWTNFHVS
TKRLLIFIMLRASKPLKFSVAYIIPMNLDSFIKTIKASYTAFNLLQQTTN
>OXU25583.1 hypothetical protein TSAR_001226 [Trichomalopsis sarcophagae]
MHLKFVGILRSNIEYELLPFQFVVLTVWNIWCPKDWPPRLKNTSIILFIVIFILNFIMCTEMLIYFILSI
GTEQFKLTNLFFVSASITGVYKSLKIMKNRKIIRCFVRNYFNHQWIKLLDDEENEIHEKINTRIRRITVT
YFISMVSIVLMKDLGPIAESGLVIQLPVDGWYPYDIENSVLFGITYVQQVILGSSVICAHVGIDTLFVGL
LLKLLGQINILKHRLQILGNLLDHKMISLNKFESFQTVQKHLILECIHHHKRIYRFGEDLNKIFQEMLLI
LVVSSLPNICINIYALSSNLKNINMDYIATFFSTTSAFIQFFIACWFGNEVSLNSVEVRNAVYAMDWNKL
DTPTQKLLIIVMARSLKPIEFSVGYIIPMNVDSFLKIIKASYTAFNLLQQTSSSS
>OXU25580.1 hypothetical protein TSAR_001223 [Trichomalopsis sarcophagae]
MKNEKMNEDLAPIPFRILKFCGWWRPLNLSTWSRAVYSCFTVVMLTLLVTITLTVLIGVTQMSATDDLFA
DNVFLMFALINSVFKATNVLLSRRRFIKMLEIVQDTRWRDLRNDEEIEIQDRYRKTIRKISVYFTTAVFI
AIVLRVVAPLLDLSDEIKLPVDAYCPCDIRHSSCYWTLYWHQAFGTGVATLTHAAKDCLISALLLQTCAQ
LEILKNRLLSIADTCVVAGNKTGAADRVEKLEQKLIGECVRDHESIFEFAKILNDSLNVMLFGQIAVTIP
NLCLSIYLLSTQKIASMDFMMTTQFFSAVVIELFFFCWYGNEVTLNSLDVENAISEMNWTLLSTRSKKDL
LMMMVRTSRPILFRVGPIMNMNIDSFLSVSLNMRTFSTNLGEFVSYMIVLSDHENILFCLQRFAVYWRLA
NVYKMIIKDKVLVLKK
>OXU25121.1 hypothetical protein TSAR_002719 [Trichomalopsis sarcophagae]

MFLCVIGFLLTPIFGLSIRVINNITDVASDRFLPLQTCYPIESSRSPYFELIYATQLVAGSFVRFSFSVP
DNFFGALVFHASAQCEILGERMKELLDGQVDGKRLFRRRLGRLVDTHVHLLRYSIPVCFVCVDRFVDAVE
ESFNLVILAQVLCLSLIICCLGFGVIQSIASDDNENPPIVQAATLAGTLLNLMIHMLVYCIASEILAEHA
SLRLYCA
>OXU24974.1 hypothetical protein TSAR_010896 [Trichomalopsis sarcophagae]
MSKSKTLKIFESDYYRTYKNLVKLIGLWPHENIHKKRITRFFITVLLTTFMILQGIRLYEELGNDIDVVL
ELIGSMAYFSGCILKYLTTIKAQAALQFLYEQIQGHWDTITNKRERQILQESASDSQLLSKLYMGAAYVA
LMVYTTSPVLVPIVLDIALPLNESRRKTFPYFIEYFIDTELYYYQLMVHGTICFTVSVLVYISIDTMYAA
CCQHLCGLFDIVEHRLKEAVKTNSNRIYLELDRTDILMHKLLNEAITLHQDSIEFAVLIENTYALCYLLV
LGLNLAAIVLVAVDIVINLDDMNQIIRLSILYIAFSFHLFFNSVPGQKIHDKSINVMNSAYVYAIHNYCE
SRYFTEWYNLPLNARKLIQLVMHRSLNPCQFTAGGLFVLNIESFGSVMGLDDGLKTFRNSRTGFIGDFDL
AATPTDLLLEIADCPLGEFLRRVDFRLVMK
>OXU24808.1 hypothetical protein TSAR_007682 [Trichomalopsis sarcophagae]
MRNLPSTIFRNNTFQNKAQKLRGVRDLLPSTVRIYSTMTSKVSPKLVKAPLAYVNEQYLADTEYVVRVAK
TLLMPIGIWPRYGDNSSLSNAIIYIRVCLIFCLMLFLLTPHFIWTWFKAEDLRKLMKIIAAQVFSSLAVL
KFWTLILNKQDIRYCLEVMENDYRVAESEEDRQIMLKNAKIGRFFTTAYLGLSYGGALPYHIIMPLLQPR
VFKSDNTTMIPLPYPSEYVFFIVEDSPLYEIVFVTQILISSIILSTNTGVYSLIACVVMHCCCLFEVTSN
RAEKLLRGMKYDKSKISPELGKKLSELIDFHVKAIQYAETMENALNIVMLSEMGGCTIIICFLEYGILQD
LEDREYLGMVTYIMLMTSIFVNVFILSYVGDKVKEQSEAIGFSAYSMQWVDLPNEFIMKDLKFVMARANQ
PTRLTAGKLFDLSLQGFCDVAKTSMAYLNFLRTLEIT
>OXU24807.1 hypothetical protein TSAR_007681 [Trichomalopsis sarcophagae]
MTETKTKEDVFAYYDERMKKPSCCNEKFEEDVKYAIALNRRIANAIGIWPIFSSTGARLGFDICVKTVKN
AAVYVLLSFLLVPGILHIVVEEGKLKAKILKVRLEILSYIFRIIFYATRCFSDTPLQTGPMILNTMALLK
YSVMLFRKSQIQECLKQLESDWRKAGNDELRALMRRNTAVGHRLSRVCVATFYVGGIFYRLIKTLLTPIR
YTKDGLMIKPLPSPLYKGLFRFNTSASPVYETIFATQMMSGFVVHSTTVTTCSYAALLATHACGQLDIVM
YLLKRLIEDDGDNGRLTRVGNEAVDRKLRVIVQLHLKVLRFISSVEDLMNQICLVEIFGGSTILCLTSFY
FIMDLQNNDALGLFTYMVMITSLIALLFTYCYVGEIVSDKAKKVGAKTYMINWYDLPPKKGLCIGLIISV
AHSPVQLTAGKMLELSMYNFGCVRIVLAFRFPSRCTLQRFLDITEILFTDNEVDRRLFELTAYDYGLTR
>OXU24777.1 hypothetical protein TSAR_009598 [Trichomalopsis sarcophagae]
MGTRDKRLKVSMSTKTKGGFSYEFRIYKIITWPAGLWPLERDNIFNVLRFLLATSSQMFIVVTSLVEIYR
KCGNVADVLDYYALSIAFWLSFMRLVLVRIHLAKVHKICYNARKTWARIKNPDLVKIMISHAKMGKRFYY
LQMSIAFVIVMLYIFNPLILRRYDAANLPVQTVCTFNNVDALKHTSVYFIEIVSFLYLAVGFISIDLLFL
GIAMHLCGQLRILQKEFSEIVGKSTSQADCIRYVISLSRRFQRVVELTDDIRKTFSEILLVNFVVNLFLI
TSQSTYIRMYSISLIYILSDFRFWPAGVTLLLALKINNYFLAVKCSQSFPILLIEMFLYCYVGELLRHAF
DDIPRAVYSSHWYLLPPKIRRGYLLHVMAQASKTFDLTAGKMIRMNMSTFIQLVRSIVSFFSLLLLMFDK
>OXU24641.1 hypothetical protein TSAR_008047 [Trichomalopsis sarcophagae]
MFLVIALWLIFGLIIFWALLDPVSRVATAAWEVLLPILNSKPVELKSQFGEWAVVTGSTDGIGKAYAKEL
AARGMNLIIISRTLERLEKTKNEILEINPEIEVKIISADFSKGREIFAKIESQLKDIPIGILVNNVGKQY
TYPMYLGEVPEDELWDIINVNVGAVTLMTRLVIDDMKKRGKGAIVNISSGSELQPLPLMTVYAATKVYVR
SFSEALRAEYSKFGVTVQHLTPLFVNTKMNAFSQRLQVSSLFVPDATTYAKNAIATLGKVDTSTGYWAHG
IQNFFTLIPPVWIRMRIGQFMNQMIGFYQHFGVDIDELLENTGTIFFTFAIYTKLFTSIIFENKLKILYD
SVAKNWKIITEKHEREILIKYSERGRMLTLGYITYNFAAVIVYTTMPLMPFLLDIILPLNESRPTMFILN
GQFYVDKHEHYKKLYAFDCLCIFVIVPAALAVDTMYVACTEHCLGLFAIIKYRLAMPDKFISTRNIYLTE
EKDSSYCWMIHTIRMHIDILKFLNKVIRVRVDTYIANIKFDSRFANILDKSYSSSFVILMLINTVYISVL
CVLVLISLDKPLNLIRYYMLLVAVCIHLFYLSWPGQKLIDHSEGLFRDAYNNQWYEGSAKSKTLLKILTL
RCVEPCLITAGDHEEISIVYNLILLLLNPAIEFAKGFWGFLVKLCNFKTIDLRKTYGDWAVITGCTDGIG
KEYAKELAKRKMNLVLISRSLDKLNKTKEEIQVINSTIDLKIIQADFSKGKEALANIKSQLQNIPVGILV
NNVGKINEYPMYLEEYKEEDIWDIINLNISTLTLMTHMLIEKMKISGKGAVVNLSSASSFVPLPLQSIYS
ASKSYVNFFSDALREEYSRYGLTIQCLTPFYIDTHMIGYSKRFKNNPFVPDPATFARNAIETLGKINSTT
GYWVHDILLISVLILPTRYRLKLAFFINETTRRDYFIEQKEKVVE
>OXU24620.1 hypothetical protein TSAR_000941 [Trichomalopsis sarcophagae]
MLPTFLLCESEKGKEVMDIFDRRYFVLNKALLRSTGLWPYEDRRKKLYIRTFVNLILGICVIFPQIVRIY
NYFGVNMNMVLEHAAVLMYITSIYLKFLTSVYYEEKVLTLRLRKFLILMRFIVTHMSHIFTTQLRVVYDN
IAKNWQVIKDENEVSILIQYSENGRLLTIGYTMYIIAAFCSYVFLPIVPVLLDVFNPLNQTRSRFYILGG
EYFIIDNVKDYGKVYAFDCLAVIVTVWLISAVDSMYAASIEHCLGLFAIVKLRLQTCTRSICDGQKDESY
KMIVRLIRMHKDIIKFSMEGILTFDDILESSYSSSFLILVGINVIFLSFECIIVLTRFGQAMEMMRYSMI
MVGIVVHLFYISWPGQKLIDFSLGLFQDAYLNEWYTCPTRAQKLLGLMTLRCLKPCQLTAGGMYVMNFSN
FAKIVKTSMSYMTYTDQLARMNRISSILYLIGIIYCQFNTVLGNPDSNRQPKIVNAYPAQTNQFPYLVSI

YHTGKDHDCGGGIMADQYILTAAHLVDTVDSVHNTGNRIGVEKAYVLKSYQHPRHDKDIGILKLNESLQL
FGNPQRRKLDLPKIGTDYTGEAAVIAGFGRTDPDDRSTMGEHLFYSAAKIVSNKDCEDDHKKSSFFVTEN
HICAKIMQSFAGVHTGVCNGDSGSPLVVDGNTVVGITSPSAANCDERRSPSRYTRVSSYLSFIKAVVTGQ
LNNKIAEYNVPPPPKDDPCASFLTIRNTYHLSHTVAALLDDEGIIGGEEASPNQFPYLVSVYRTNSRHNC
MGGIINDRYILTAAHCIVNPSTGRFFNIPMEIVVNTLDLVNDTGIGINIKKDFAEAGDPNDIGILKLSES
LDLSNNPQRQRLDLPKAGIDYVGRDGSGDSGSPLVIDNTAVGVTIVAEDNCDERLKPALYAQDSRYLPFI
ETVLSGQLNTLLLCLFSIMSSSLSNVLGMPDSGRRTKMIGGKEAKPNQLKYLVSIYHTNPQHNCGGGTTY
AASTVS
>OXU24616.1 hypothetical protein TSAR_008383 [Trichomalopsis sarcophagae]
MDVFKIDQSLTAGLGSAPAPDTLTPEVSFDAGSEFNLSFFDGPEENSGQGFSDPGSIIRSVFEWQLEERN
LEILVENFAGFLYFQGTLSKYYTSIYLEKKLLILYQRITYDWRGLQDDNEQAVLLRSWNIGRALTIFYIG
YMFTACISFVTLPSVVPLMLDLILPLNESRGVNLCYYAEYFIDQQKYFIYLLLHTFICVSFTILIVTAVD
STFVSIVYHAIGLFNILEYRMRNILPFVEDYQNNKILSKEELHKYVVDSVKMHKKTIEFSRVYLLNKFSE
IVQRIYNDCFFFVTVLVLICLSVGTIDLVINLNNPINLIRVGFVWLGIIMYTFFISWPGQKLINCSYDLF
NTVYACGWYGCPTKTKYLIKFMMMRCTVPCRLTAGPLLTMDLVTSGNILRTALSYFTVVASFS
>OXU24418.1 hypothetical protein TSAR_002761 [Trichomalopsis sarcophagae]
MMTFMGLWPYQDFKTKLFIRTFLAIVLGIALIPQIISIVKYTNEDSDKVIQGTATLLYVTGVTLKILTTI
TSEKKIEIVYRNIVENWKLLDDENEIRTMTEYSEFGRLLTIGYVAYMFFALGLFVTMPMLPMMIDVISPI
NGSRPRIFILDGEYIADKYENYGKVYIFESLTCIMSVFVFSTVDSTYAVCVEQCVGLMAVVSFARILDSS
YSVNFLLSMGSNVMILSVGSVVILINLGRPMEFIRYSMIFIGLMIHMFYLSWPGQKVIDSSQGILYDAYN
NEWYECSKKTKTLLKFMMLRCIEPCQLTAGGLYVMNIANFGSQQSKQMKPTQEPVDNLNTANVNVNVITA
HESDSEDEDAGPAGYMPLSQTPAEGEPLEDEDDDDDEDFEWTHAPTEPQQSLPESNEPGEPSVDPETLEV
WSAPGNSSNIDLDADKINQVKSAMASFTLPTTAIPDWANSISEDQWKEQLIHRIKEIQKDKK
>OXU23954.1 hypothetical protein TSAR_005005 [Trichomalopsis sarcophagae]
MSVGYLICMLGELFAYCWFGNEITLKSMGFSDDIYKIDWMALSDSSNKKLIFIMMRATQPIIMSYGHLVI
LNIESFKSVSTYKHILSFNVTPND
>OXU23950.1 hypothetical protein TSAR_009946 [Trichomalopsis sarcophagae]
MVLRMIKFQHLPQNNDIREYLGLNLKMLSFIGLEFNLNNDQPIKKSKFMQILPIFMTNIVSLTIAALEIT
FIAFVLRNHEEHLAVQICSELFSNILCIGKSLRMATAVESIQAAFDEVSILWAKHRPNQHCKMEIMKKAR
STLNFSRWYLGFLITGIAGFALPPIHNFVYQYFIRDANNYTLAFSKRIFLLRYPFEINNVPLFFFVLTEE
GYILLISAMHWVTCDTLFAQITTHTSIQLKILHYDIGALIDHESVEDRLKAKILIIIRRHQCLLRVCRLI
EDIFSPVILTTVLLSALNICVNIFETKAMNAEGNYARAALHANLVLILFLQILFYCSFAETLTDQTSAIA
ESVYNCKWTEKNHKLGFYLQMIMMKSQSPFYCTAYGFFPIGHARMASIISTSFSYLMMLQSMS
>OXU23853.1 hypothetical protein TSAR_010331 [Trichomalopsis sarcophagae]
MNIKSAESSLLQSTTFKYDAIFHKLVGVWPDDDYFLARYSRIRGCVLAAFAVVVCVFQFTALLEANSDDV
PENDFINLMRMTKEINEYSRLTDEEETVHFDWQSVQDKLVKIISRYYFLTVIGLYFVAPMFRNALPLRGI
VPEVLRVTPWFQMIYVLQCLLLLSNVVTSISSDAFSVTFMCQLCKQLELVQCSIKHLGSHTKVNLAETIN
RHAVALDYGQRVCNTLKTMFLLQHIFISMFLCFAGVIVLNTQNSLILIKMIVISVIFVSTLLIICFVGET
ITSSSLNIANATESSNYEIFLGDVSTLRAVSFMLCRAQKPLRMAVSMSGSMNLSFFTETMNKLVSAFMIL
RTMME
>OXU23852.1 hypothetical protein TSAR_010330 [Trichomalopsis sarcophagae]
MEFVRKAYGDKKRKRQASSSKCISRADRVFKHCVFFHKFVGIWLEKERSQRLLDRLKGYVSAAFTLGICI
FQIVMLSVESSSVVTVLHNAMEAMVSIAATVKVAPIVLKRNNLLILIRKTKEIAAPLDASKINERKIVDR
WLNNQDKILKILLTSYTFTFSSYTLFPLLKENGLPFTGRLPAICYVNPWYPTIFAVQLVFIIFRFVCVLS
NDILCITFLCQLCSELELVKHLIVDLGNGKDRNVKQIIIRHAMVLDYGEIVCETYSATLIMQHLNCSMFL
CLSGLVTMKTSDMFALLKIGSLSLIGIITMLIISFVGEMVMSSSLEIASTIESSVYKGYRNDVANLKLLN
FMLMRAQKPLCMMVCTQGKLSLRFFSENINKVASFFIYLKTLVE
>OXU23648.1 hypothetical protein TSAR_014102 [Trichomalopsis sarcophagae]
MDIFDGAYYKSCKWFLSTLGLWPYQTDNRKRISAIIFFVVNISLAIPTGALLYKLWLKSFTVTFENTVSV
MFAIGCCAKYVVTYTSSQRVRKKVDQLVRLFEQIASDWQRITDTTELSILTKYSEKGKFLITFYQVYIWF
GWTVYTLMPFIPYFLDKVSPLNESRPLLMPFYADYIVFEQADYHYTSCFHIAFVYISSALLFCGVDATFV
MSVQHTCGLFAIICHRLEGEKIKKESEYAQNVTKTLSEEELREVVIFHNNCITCSGLLEDSFNLSFLILN
SMSVLGLALSGVYFIYIYEDYYKFVRIMAFFVGLIIHLLYLNWVGQKIIDSSEDVFLSAVHQATINFDSY
CSKWYVISTSARAFIKIIMVRALEPCRLTAGGLSTLCMESFGILIKTSVSYFTVFLSVA
>OXU23403.1 hypothetical protein TSAR_003485 [Trichomalopsis sarcophagae]
MKKTIFQECDKENQKAFDEAKTLISWNKYLMSALGLWPSHRYDFIFVSLFCYYIFHFLLDYAAFYFALRS
FNLIKIIGVTMENVTMAQIFLRLYTMRRYNRQYGEILEEFTKDFSVKNYKSEEERNTFLSYNSRSKFFIK
IVVIFLGVTAILYFTKPLIRQLSLSKNVNTTIAFTYDLPYRIHLLYKITDIQTYIATYISRIPILYIIGF

TQTAMDCLTLTLIAHLCGQLGVLSIRISNLDVVNKSNELNEIIQRHQKLIKIGLRLRRMYRLCLLGHFLG
ATIAICILVYQVLISIAAGQKTNLVTFFVFGFLNIYRLYTHCWVGEYLIHEVMGFYMCKLCKLSFSINVS
HAYYCCNWYKLPLRDQKSFIICIKRSQQPLSLMAGNFSHYSLVMFTNVMKSAMAYLSFLRNFI
>OXU23402.1 hypothetical protein TSAR_003484 [Trichomalopsis sarcophagae]
MVSEMLCLHIHEKDYAFGKIVEKKSKNEVVIHNLELMTMNRGQYYPVAVQIKRRYERIAVDWIKLADDSE
REIIGTFLLEARSAIFVSIGNYEIILYTFLLFACITFLPRIFRKQSMDQCLQGFPYYLRSMVINETTCIF
INQCHVHNKRQTCLRPLHHRLRNAEVYVKDIGPLKDSIEDTPIIPSLIKVIDIHKKALRLNIPRLIGVLA
FLAVLMFEVQYHGGNVNELLRFSPILLIALIYVYFINWCGEQVIESCNDLRITTYNIDWYRISSRVHIFV
LMVMQRTAKPKHLTAGTVIMLSIENFAIIIKTSYGHLACYC
>OXU22945.1 hypothetical protein TSAR_002417 [Trichomalopsis sarcophagae]
MLSEKSHYAVQLNRLFLMPIGVWPIGRDAPLVQRLLKRLAIIGCYFLMSYLLVPTALHTFLEEPDPAIKL
KLIGPMSFHLMAIGKYVSLVGRNEEISACFEHVEEDWKMYSDKNAKPELEMMKRNAKVGRFLIYLCAAFM
YGGGFFYHMIMPLSVGRLVTKQVRAERYLQLLAENASTDNIDVEPVRVLSYPIYGLLAKLDYVTLLLVQF
VAGFVLYTITIASCSLAAVFANHVCGQLEIVMSLLRDFVHDNEDNPRIYALADDAATVERSRSDKFEEIV
QRHLRALNFASRVEKNLNAICFVEFIGCTLNICFLEYYFITVWLKRKIYPQEWENQNTVSTMTYCILLIS
FIFNIFIFCYIGELLTEQSKKIGEVTYAINWYTLSGKRAVDLIMIIMIASCYPARITAGKMVNLSLGSFC
NIYANLIQLV
>OXU22943.1 hypothetical protein TSAR_002415 [Trichomalopsis sarcophagae]
MAYVEEPRENILESSTFLYSKSSLRVFGLWPYQEPKQRLFCRTSTAVLIGSLLIPTICIVLEQWRNFDYV
VLGLPSFLYYVEFVTKYMYLAVNHKKLEQIFGHIKNDFDTRKGRKLEILNDYATETRLFNHIYAVVVMFN
LSFYQSHVLDLIMPLNESRPRPIPRLARYYVTSLDESFNFVVLHGLVIDWYSMIFFLGHDTLLVNCAQHA
CALFKIVINDIQDCLEVQKNDKADDEDQVYRRISNTIDLHKRALEFTMVDKMYMYVNLCIIGVSLLAITL
SQYQTVIHLDNTDLVIRYSFFSIGELVHILYFNWPGQRIRDHSLSIYQAWYIQSRLLQLKNLTAASLEPM
LNIAVTTANGIEMTSPIGARNY
>OXU22911.1 hypothetical protein TSAR_016171 [Trichomalopsis sarcophagae]
MYDYDKFYYTLHKYVLTCIGLWPYQSRMSKRFFFITYGISSCSLILALIAGLSEKWSTDPVIILENMLGI
IFLTSSTAESSILYMHESKVKDKNRFSSIRYYIIIEFYDKIKTDWKKLTNKKEIEILQMHTRKGQFISIA
YICKLNLIFYSKIIIRIWNTSFCNFWFRNVPAADSRPSFQGGIFSYLSLLLLLYDNQRRFSLLSDSSALY
GILFLCRCILFGRQLYVCKMCKPRLWNICYYLNAIEPTVVRRPFNKLKNSELIRFNLIDVIAKHREVIHG
VDMIEQIFSTGFLVIEIAGFSGIALVIADILYNQRNAYQLFRITVVTAIFLVYIFYINWMGEKIIQVSDD
VRLTAYFIDWFTLSIEAQEIIHMIVWRSCKTNKLTAGSFVALSLENFLSILKTSWSVATVLLSAHRSQKH
AHFTGYGITNSFTNSSST
>OXU22406.1 hypothetical protein TSAR_001521 [Trichomalopsis sarcophagae]
MKCTFTFSIISVYETLKYFGLLTSIWPIFSKNRYLWVFMKAVYYFILVNYLCVFIPLILMTLFNINTSAT
VTITAVEQMIIVEAVYNLLYSRFYSDQFKSVVKEIEEFFKNSSPKERYILDRYATTRTFFNIYIAINYFV
AIMSFNFGQLFLKDRPYPLNLWYPFTIKSQVIVVIIYIHQVIVITHTLILIVFDLIVQIFLWTLAARFEL
LQADFKKTASEMDLKCNIQKHQYLIRTTEAVIDFTKYMILKVFLAVTILVISSTLQILHRGPSTIIVQFF
FIMKIASMRAFAYCWAGHSLAEKTGGLARSIYNSYWINQTQRMKTNVLIVMQRCQKPTVVKISGIISSLS
FRFCVNYFYMIYSAFMTLRAVLEVADPLETLEYLRALSRIWTIWPVDSKASWKTKVYHECSIWIIITNLF
ITWLTLWISVCDSYKYPVIMANNLATLMIATDSCSHLIFYRIHRSDLSITAASAFFCGALILNKRFPINE
RYPFSIKSSLAFSIIYAHQIFSIIQNSTIIMIDFLVITLCWYAGARFNILGYKMKLVENKYKTKTIY
>OXU22355.1 hypothetical protein TSAR_004129 [Trichomalopsis sarcophagae]
MEIYDSRYFVHAKRFQELLGFWPYQSRLKNNCSWVILSFLFIAMIIPQIVGLSVHAGIDSKKTLECTFGT
CYMLAIYMKLLVACADKDKAKFIFECTARNFKKINDNDERKILIEYSERGRLIGLVYTIFVLAALGVFVA
VPLAPGILDVILPLENGKRSKFFILNGEFLVDKSEYFIEIYAFDSICCIVTVLIICATDPLYAAILEHCL
AIFAIVKLRLRKYRVRKGLKCLSADEAEYEAIIRAVQLHREIIALKTIQNNCSFYFAFEMGVTLISFTAN
FVLAVLKTPDLFDRLRLAMILFAQAVHLFYIMWPGQKLIDHGEDLFKETNGKKIFSYQRYFNDWYKSSIK
CQKALRFMSLRCSKPCKLSGAGVYVLNFAYTLMILKTSASYITVVAQFEYKIVA
>OXU22316.1 hypothetical protein TSAR_003397 [Trichomalopsis sarcophagae]
MCEGHPDSMSSPLIPQHSSPQSIALRQSKSSTSKCPLKGVMEAKLAKYARYQNVVRRLLLFSGIWPHLED
TCRLYRVLTFSATFVIAALGTKIFAYCIDNIDHVSLFAKGMSVAFSFYTSVLKVLCYLVYRKDLVMLNDC
LGRRFEDELKREDRRPLLLRSISVYTRFMYIVAGLTAAALVFYTLVPLMFIFKYKKLIQIYQARKSLVAQ
LRGLLRRKGKPTIPTFLKNYNFTARTRVFISKHTEPDNIPPQGRYPFAVEPGGRVYWCVCFVESISIVFV
WNVVCSVDNAFGLHSFRMCGLLRSLADRFAKLQPDDPGYIVEMRDCVMTHQLLLRSKEALQRVYGLVVLW
TYVTSAIIMCSILYQADQQSEECQNAIYTSNWPGSGDLRLMKDVLIIQSQRTIVLRANGFFIVSMEMFEK
VARVARIIVNTTISYFFLLQAVEEK
>OXU21660.1 hypothetical protein TSAR_016061 [Trichomalopsis sarcophagae]
MIVNKFTNKLVTIVPVTLIDIISYIDYLFYNFKVLKLFARLCALYPLDSGCSKLEIFYDNFVWYYIQFNL

WTALVATFTAMYKARSDLSSWLNSFSESIIFIEIILLMILYRLQRPHLQILLQIFDDLIKDPDDFKLLLI
RQSARKHLKGFSTVAVLFTMLAVIYVYKSFSTKPIQFILSGYYPYTSNSMMLWIIVLYHQLTILLYVPSI
FASDFIVTLFVHKVNYVIRFFILKTIFCLGALQLGVGVILLRVCMHIKAIKKYDKNPDIPAITRIQFLVI
FAITISRIYIYAHCAEILTKSGSDLGFAVYSSRWYIQRHNMVLAKSMIICRCQKPLVISVAGIIPALGMG
YLGRFLYLIFSYIIALQAITKNHHL
>OXU21334.1 hypothetical protein TSAR_003961 [Trichomalopsis sarcophagae]
MIIKSIILSKCQKYPAISINGVMSTLYRKIYVSPKKSFRVLRFVGIILRVWPVNDKKWNYRTDIFFWFCI
INCALLWVPLCNALYLNHKNVVTAAISWIEVSWFAEITFVLIYSKYKKLQLYVLLSQVERCVYLRKTNII
KQYATIYAMIFFSVMLFYLLAFFVYMSIERPNTGYNTLITTAVYPFDINSYVAKAILYCNQIFLLTYAAI
APTFDGISVLLLFVCTHRINILDHNFRLAKTLSDLAQCIREHDDILYTIKETNSIVRILAFKTVCSFMSN
VIPAGLEILNSVGLSQSILQICTILLVYTRIVLCSESAGNMTNAAEDLVFTIYSSLWYDEEPKIVLTKTF
ILQKCQNIPAIHINGLMSGLDRKYLLANYYTNHDNSLIEKVVLKNKEIKAWNVKTALYKSASVMACVWVY
RVIVRSLHYWEFKVAMRYYRQLRELKNMASIKSLLTLVLLFIARRTYQRARVPIEVDVQEGETDDPSVDD
SENEDAGPSPKRARIEDLRAVHSPGRISLRGVGSDKAIATASRKVNLQIRNDFESFKMKNVLVIDSLALP
PQRVSKEMVEHYQIKTGIVTRAYDASPMLLIGQENCSLILTREHREINSDVYYVVFRCILGWSIHGFPNKP
SKIRQINVVGNSIRALCISENFWLPSLLTFKHANQV
>OXU21333.1 hypothetical protein TSAR_003960 [Trichomalopsis sarcophagae]
MGGYIVPHRSFRVLRFLGTHLRIWPDDNKKCNFKTDVFFWFCVINYVLLLLPLFNALYLNRKNVVAASNT
WIEVSGYAEVLAAFIYSKYKRVQLYVLLCEAEKYLLSKKVTIIKKYANTYAKIFLLVILFYLFTVFVYWS
IEKPITGYEYLITTAVYPFNIRSHPIKGLIYCNQTFNLMYSSILPVFDGISVLLIFNCTHRLKILEHKFK
LAKTSSDLSECVREHDDVSRTIKETNSIVRFLVFKTVFSFTSNVIPGGLQILNNVALSQSICQVCIILLV
YSRIVLCAECAGNMTDAGEDLLFTIYSTLWYNEEPKIVSMKIFIIQKCQNIPAIHINGIMSGLGRKYLLT
IMYSTFSYLTTLRTVTSDEKSYFATSRTST
>OXU21158.1 hypothetical protein TSAR_010022 [Trichomalopsis sarcophagae]
MNIFDFPQRYLLTSLGLWPYQSKFTQRIFFTCAIISFFSLFVAMVSLLARPPRITHSTEFLRVAFKAAGL
GEEWSTELVIIYETIVALFVILGALAKCIVLFCKKHQVKSLYDQIRKDWQELTNEKEAAILQSFMLKGKA
QIIVYVVCTIPGYFIFVALTYVPIISSEDASKDYTHTFPYYTDLLILSKRFRVYQVFIHAGFGIFCGGIT
YVAFMAMYITCVKHVCALYAIVRYRLENMVKSQDELMDKLNNDEEVIPGLLEIICTHKRAIKRVRLINRI
FSRTFFMVEICLLICLALLIFDVKYNQYNVRLVIRMLMIAVMFIVHVFCMNYCGEQVIQFSTDVQYAAYF
MEWYMISSKAQKILIMIICRSSNPDYLTAGNMALSLKNFASIVRTSWSMATVLLTTQKVNRPNYSIS
>OXU21157.1 hypothetical protein TSAR_010021 [Trichomalopsis sarcophagae]
MEEPDLDIPYMKLQKLLMNCCGLWPYNSRLINRLIYSFFLLILISAIVPLGLGLIEEANNDIVTYFESLV
GVVMMFGGVAQITMLRTIRNHLVKHLYKKITADWQTLKDAKEIKILSAFSFKGRSLTFLYMLITMSSYVI
YLMLIYIPLANDKATSWDYSKIFPYYSKHWIINERVRHLQVTLHGCFGIFYGGVAYVVGMALYICSFKHV
CGMYAVVGLKRLIISCEVTSSGKLRDDVLVCNLYAIMDQHKEAIKGVHLLARLFSHSFFIIQLCLLVCLS
FLIFGVQYNIYSHKGMVRMFPASIIFVAHVFFMNYGGEQLIYYSSKIHTTTHFMQWYLMSVKSRRILLML
IRRSCKPEQLNAGMMTLSLIIKASWSMGTVLYIVPMQHELYILFLYECEQRRVRVISQTTARIYVVSFIN
ATKVHFHEPQLDIPYIKLYKFLMNCCGIWPYNSRFVNRLIYSFFLLFLISTMIPLNFTSVMD
>OXU20689.1 hypothetical protein TSAR_013728 [Trichomalopsis sarcophagae]
MDFSNLLSHMMKGIEECREKNPNPEIILENISVFFYFQGVTAKFFTAILTEDKLKYVYEEVMKDWKRFTD
KNEIAILCQFAQVGRVLTVVWSMYTVISCVLFVTMPAVIPMILNIILTRNETFKKSLCIYCEYYIDQDKY
FFYIFLHHIIAGVVTIFLTIGIDTSYVNCIQHVLTLFNVSRLKVAFDTICHFKKKDCNLKTLENHAHSYV
VNSIRLHQRSIKLSSRFVDTIQSAYNVVFFIVCALLLFGISTFTVDLIWNLHNPINLMRIACLWMGTIMY
MFYSNWPGQKLIDSSNELFDAIYNISYTCGWFEFPMKTKILIRFMLLRSIVPCRLTAGPLLQMNFESCSL
VSYTSEDFFNLINSSNILRSAMSYFTVLATTG
>OXU20118.1 hypothetical protein TSAR_003533 [Trichomalopsis sarcophagae]
MIANSFGLNFIVFISFTAVGISILMFDLLFYHDHPFEKIILLGLIIGILMFFFVLSWIIQKLTDSSEKRA
FFFFLYASPLPHNTGKTQAPSPSGAARSPLSEQRRQRSNLPEPAGAQGGRDAVGRECRSGRGPDAGDEAD
WVGQPTSNYECDLQYSCHWYSLSVKAQKLVYLLLMRTSKTTKIRPGGLIEINIHLFESVSYSVFDGPYYW
INKNLLDGFGLWSETSRERKFMSVVFFNISAFSLLIPTVKLLYKKIAYDWKMLSDKTELDVLLKYSRFSK
MITMIDIYYALLAFILILFVPVAAVIMDQIILLKNGIRIRVETIHPYYFDIFDVNKYYYYVHIFHVSAVG
FYVSAAYLTLNSMHVTCIQHVRALFAVACYRIEKLCHHDNTLETSVGVIDNNSVNQNIISLKDKN
>OXU19963.1 hypothetical protein TSAR_011076 [Trichomalopsis sarcophagae]
MKTEDSPSVTPGFEDYTKLNSLLFRCMGMGIGTDGNKRDKRSQIIERVPTVLINIICLLDFVYQMQWIND
IWKTDKKFVLQILTNALSNFVCLCKGFRLVYNREDLQTLFEDMAEIWRRKMPRHEIRDEISREAHKTLLF
CRFYVIMILFLSLSFCLPPLKYFILQFTDRNANRTYDYTERIFFVRYPFEVNNLKVYNFLFIQELWVLYA
AALHWMCCDTLFVQLTSHTSLQFKILHYDTETSDNTKDERQSRKNIVDIIRRHQELLRICDAIEDVFSPI
IFIIMLLSAITMCVNLFELQEMFLEAQYAGIALQSFHFMSVFFQLLVYCDYAETLTEQAGSIAGAVYNSK

WTDNGHVLRMNLQMCIMKSQKPFYCTAYGFFPIDHQRITAILKTAMSYYMMLYQTTIYSVTFGVSPDDQG
VIRYLLRGPENP
>OXU19654.1 hypothetical protein TSAR_014272 [Trichomalopsis sarcophagae]
MGKGKVRSFSDYFWLSQGMLKFCGVLPMPERGHFVNYFLIMLSISSLVFLFFPGFYIIAFHGSEINAAAK
VDIIAGEALEIWVTTIKALALLPCRQTMLSVSRRATRLLVDIEDENELQLAEPYARRGYYLLYGFGGTVF
FALVSIVIKPFGQQVQYGANGTILASKDLPYSIGIVHENQQLFNAWWIGQCFAGIIAIIAIIGIDTTLAI
FVLHACGHFRILRSRFQAVAESKAKCSSGRRMSVSRSWDDRRRLIDLIDKHQEIIQFVSTIVIIRYVIIL
RKWFFFFSIKLLRRNRIRLQSGGTDSNDTQHLSDLRIRLQFAGGKSLYIHGSCDYNRYIYIYRLFKSSQV
GFEAYNLRWYDWIEDDKSLVTFLITRSQKPMLITAGRFTSISLETFSSVLSSAFSFFSILRKTL
>OXU19403.1 hypothetical protein TSAR_008624 [Trichomalopsis sarcophagae]
MDKETSMYMAAALLSFTTLPIAPMVLNFLKPLNESRPKIFILGGEFFTDRLEHYGKIYLFDCVCCSITAI
IICAVDSMYAVCIEHCLGLFAIIKFRLQMSTKFINNKSIESKQKHEDISYELIVQTIQLHKKIIMLHIIA
SSYSSSFLILMGLNMIILSFESVLILIKLNNIIEVVRFGFILLGIVIHLFYISWPGQKLIDFSVGLFQDA
YVNEWYESSIKAQKLLGFMTLRCIRPCQITAGGIYVINFSNFASIVKTSLSYMTLFASLR
>OXU18993.1 hypothetical protein TSAR_009976 [Trichomalopsis sarcophagae]
MYENRGHFNLFIESLPPFTYNIVMAIKFTNGVLNQRKYDWNKFTDKKEIEMLCYYSHRGKSLNTVYIGLV
AVVLLSYMLLPMLPAVLDLINPLNESRPKSPLYMVEFYIDQDKRRIENILSEEPVLKRSYKIDDDKKAIA
YCESINSLYTTSFFLILSISIGLMSVTGFVTLIKMNEEFKDCIRFAMFTFAQIFHQFCYYFLGQSVLNHE
EKLKDYVSNFNWYKASPKTKFIIKFMIMRTLKPTKIRAMIFPLTLENFTSVMREKL
>OXU18896.1 hypothetical protein TSAR_016577 [Trichomalopsis sarcophagae]
MHTIHLPFTILKYIGIWKPNSWKSTWKSYQYDCWSLAVMMIMYSFVTTELIAVVKLISDHYDEISDTLFL
LLTTAGVFIKSMNFLANRNNMAHLGNMLLKSCCIPKNLNELKIQKKFDDINSRFVTLMCITMVYGNIMTM
MTIPLLQPKEHRNLPFNTWLPYDTRSNTLNYWLSYLHQGLGLACCGTMGVMIVNIITGFMQQACAQFEIL
DSRWRNLPKIVEIARSRWSDSAADEHENIMLRQHIRHHIHVYEYMNEFVKTFNIMILVQFCVSSAVITIS
VYQMSTKSLGLEWFMVFGYAVSMLTEFFLYCWFGNEVTLKSMDFALKIYDTEWNLLNIKSWKLILFVTYR
TRKPIVVRCYNYIILSLDTYVNVSNSDINIFYFFNI
>OXU18895.1 hypothetical protein TSAR_016576 [Trichomalopsis sarcophagae]
MLSIHFQVLTISGVWCPNHSSSVQRIFYKCYSFIVVVLMYSLALSQLARIIFVKQSFNEFNDTFFISLST
NFACFKAASNLVNQKQIVSLVNMFKHNCCLAHNDSERSIQKQYNDSCSKIIISLLILVETSAFFVVVAPL
CGTMDNQDLPYQVLLPYDLSNKLCYWLTFVHHSLGAVLFTAISITNDAVITGFMMHVCGQLSILQHRLVL
LSRSLANDVSKKGRTTDFDIMLERDWLRQIVYHQNHISNIAKKICWTFNEIVICQFCISGLEICVSVYQL
SVRNNNTVELCTYAIYLMVMLGQFFVYCYFGNEVTLQSKITHRAIFDMDWTSFSLSLKKDLTLIMLYSSK
PIAMSCGPFVHLTLESFTNVKFHFKQLPFKIILFLAVVDYSEHFFLFQILKTSYSIFSVLKTTT
>OXU18753.1 hypothetical protein TSAR_011572 [Trichomalopsis sarcophagae]
MEIFDSRYFIINKTCMKLLGLWPYSSHVKNYLRRCGLGLFLLSCYLPQVRRNMRFFQINSIVIFLIFTKA
LRIIAFKFIRLYMYFGEDMDEMIQNIGVILYVFGTSVKLITGVTAEDRMKIVYEKTARDFQTIVDKEERN
ILFEYSERGRTLSITFIIYMWIALAIYVGLPMGPLVLDYFIPLQNGSRERGFVWKGEYLVDPHKYYFTIY
AVELFSSVLSVAILSSVGPMYQAIVEHCLGLFAIVKFRLQICTRGGTKAEEETYRLIVKTIRLHNDIIEF
TRIIEASYTSYFFIEMGITISLVTLISVNVSIFDTAPDRLLETMLLIFLISRLDYLFDSIRHILILLGVM
IHMFYLTWPSQKMINHSTDLFHDTYSNEWYNCSIRCQNLLKFMALRCVEPSQLTARGLYVMNFENYASVL
KSSTDFILLNESSLVKTSASYITVLLSFR
>OXU18687.1 hypothetical protein TSAR_001571 [Trichomalopsis sarcophagae]
MDLFVGQYFKLNKIVLTICGLWPYQSKLRRRITFAMLASSTLLFIFTLVAGILSQSKFDFVNTEETFIFI
FYCSAGLLKCTILYNQQNKIKKLYERIETDWKKLTDTSEREILRSFLLEGRKLNFIIMSNYTILLVSCSS
AFIIYSCVDFLPRILKEKSEYHRPHSFPYYFRPMVINEKFYDLQVAVHITVIVFYAGFVYMSAIATYISS
VKHVCALYEIARYRLQNAVSYDKSNNSLLVLVEDTSIVPKLVKVIDMHAQALRGIQIIEKVFSADFFVLE
ASSLTALATDVYELKYCRANVRTFIRALLLTPIVIIYLFFVNYSGEQVIQACNDMRITAYNIDWYRTSSR
ARVFVFMIMRRTLNPKYLTAGTIVMILSIEKFAAIITTAWSIGTILLTTQRHTRNEDTNVIADNNWLC
>OXU18399.1 hypothetical protein TSAR_016109 [Trichomalopsis sarcophagae]
MMKMKQQGLVADLLPNIRVMQGVGHFMFNYYSEGKKFPHRIYCIVTLLMLLMQYGMMAVNLMMESDDVDD
LTANTITMLFFLHPIVKMIYFPVRSKIFYKTLAIWNNPNSHPLFAESNARFHALAITKMRRLLFCVAGAT
IFSVISWTGITFVDESVKRIVDPETNETTIIPIPRLMIRTFYPFNAMSGAGHVFALIYQFYYLIISMAVS
NSLDVLFCSWLLFACEQLQHLKAIMKPLMELSATLDTVVPNSGELFKAGSADHLRDSQGVQPSGNGDNVL
DVDLRGIYSNRQDFTATFRPTAGTTFNGGVGPNGLTKKQEMLVRSAIKYWVERHKHVVRLVTSVGDAYGV
ALLLHMLTTTITLTLLAYQATKVNGVNVYAATVIGYLLYTLGQVFLFCIFGNRLIEEVSSSVMEAAYSCH
WYDGSEEAKTFVQIVCQQCQKAMSISGAKFFTVSLDLFASVLGAVVTYFMVLVQLK
>OXU18384.1 hypothetical protein TSAR_009405 [Trichomalopsis sarcophagae]
MDDDILCLRSLQVNSVSSKIRKMKEMNPAKGAEVLSTSFLYFKLIGAWRPLNLPKWLRVIYDLFTISMVI

LMYEMLIVTEILAIIFAEENRLKVFQDIVHITITHVSGCFKMLFVINRRQSIMLLVNDCVAKQWYPPRNE
LEATIFTEHNNLSRRITLTYATLVGVSLLAAVLNPILYSTKVLTFATWYPCNISLPICYWSSYAHQTMGI
LAMAVAHVATDSLIVGFTIKVCTQLNVLNQRLLSINFQLDNTSARCQKSQDQSLALEAILVNECIVTYND
ILRQVYQTKNYRRSLHAFADLLSRTFIEIVFIQFCVGLTVICSTVYLLAKLSIFSFDFFGLFLYLGCMLI
QMFLFCWFGNEVVLDSMKLFHTIYDINWIELQIQTKSKLLLMMLVASSPIQLFRGAIIKVNLDAFINVNT
VGINIYKNPIFSIKLILYIFKRLIQVLKFSYSAFNLLQKSS
>OXU18350.1 hypothetical protein TSAR_008474, partial [Trichomalopsis sarcophagae]
MPNSGSNYNYISSTGICMCKPVLCILAVALDLRMHLVRLFENHKKKSFKMPASKINTQDQCNLDIFETSD
YKTYKDGMKLIGLWPFENSTKRALKRAFVLISMITTLIFVQVRFVEELNHNINVNDNIDVVLQSAGSIIL
SIGCIAKFITTFRAEDNIKLLFSRIAKQWASITDETECKILADNIKVCHPLCTFYRAFFALVTYACLPSF
GPVIMNIVSPLNETRRKRLPAPAEYFVDEEKYFYVLFSHGMICYMLVCALYVIIDCMYSCIVHHTVGLVG
IVTYRLENIIDLKVTSNQEYQANNSEIRRRLRRAIALHKESLDFAQKIETTYNLCFIIVMSVNLLTMIFT
AACAIRTLFYDKVESFRWFILYGSIIFHLFFNSNPGQNLYDKSNEVINTLYFTEWYTLGISTSNKRTMLI
MMIRCLRPCQLTAGGLLVLNMINFGA
>OXU17923.1 hypothetical protein TSAR_015062 [Trichomalopsis sarcophagae]
MELFESNYWKLTVFLQKLIGLYYFQSLWKNIVAWIYVYMFTLSFIIAIGVRLYQEIGIDINIVTENLIAE
MYLIIVFAKLTTSVVYMKDLKHLYKSIANDWKVMSDEKELKVMHEYTDIGRKSVQLYSGILKIIPTYAGY
MIIGITVFLSLPISAPLWDYIVPLENATRPNALPYYAEYGIDQEKYYFPLMGQAVFGGIGTGMLLVTFDL
GFILTVQHVVALFALVCYRLDQAANLSLSVERGQIDFIKGDGSAYEYTVKAINLHQTVLGYVDLVENCYN
AGWLVVLFMNMLLCGGGLAVLLMKTDRPEELLRYFTVLLAGFIHFYYIFLPGQKIINSSLEVFDKCYASR
WYNLSEKSKNLIKIMMIRSLRRCELTGGKMFILCMDTYCNMMKTGLSVFTVLR
>OXU17576.1 hypothetical protein TSAR_002007 [Trichomalopsis sarcophagae]
MLDSSEVFSNKYFILNKKLLILIFIWPYQKKHLGIFVNTFIILAIHTMMIPQIIRAVLEWQMEVKNVEIL
IENFGGLLYFQGTLSKYYSSIYLQKKLQQLFEQITYDWKGMHDDNEKAVLLRSWNIGRNLTIFYMTYMFF
ACIAFVTLPSVVPLMLDLLLPLNESRQLKLCYFAEYFIDQQKYFVYLFMHTFFGVSFTILIVTAVDSTFV
SIVHHALGLYKVLEYRMKNVLHLIENNYQFNSLLAKDELHKYIVDSVKIHKKTIEFSEIVQRIYSDCFFF
VTILVLLFLSIVTIQLVMNLNNPPDFMRIGFMMMRCAVPCKLTAGPLLTMDLATSGNILRTAMSYFTVVA
SFS
>OXU17525.1 hypothetical protein TSAR_009827, partial [Trichomalopsis sarcophagae]
SLLQNKLLFFKIIGVMASRGCFEQSNQESNSCIFSSIVNYTNGSQLKYISCASVQSQIKFLYTEIEKHWN
TLTNEEEKKILKQYARDGYNLSFGYLIGYLLVPFTPMLLDLIDPLNETRPKAFPYFAEYFIDNQKYYFEL
TIHGWIVCIISVQIYVTFDATYTQLVQHSCALFAIVEYRLGQATKMVTSDEDSSHKDTDKVAYNMMVGAI
NYHKQAIQFVGLIEECYSLLSFLIIIILNTAVVSLAAVVTMLYIEKGNQKQAIRIGMLYVAFSFHLLYNTY
PGQKVIDSSTRIQEAAFHCEWFNTSSKTKQLIKIIMLRSIVPCTLTAKTLVVLDLESFAFVFKKSISYIT
VISSMR
>OXU17519.1 hypothetical protein TSAR_001317 [Trichomalopsis sarcophagae]
MMELELLRYKAYTENVIWFLKLVGLWPESQPLPKKILSTITLSSILVVVVTVSNFSFHNLSNIVVFTSGM
CMAASSTSAFSKIAMFLLHREDVVYLNKYLSGGFMRDMREPNNRPDLLNNVKTFDRLMVTHVICVAIAMF
TYSIRPLLVLRKHGKYIRSFPAIYPFAYEPGGLVHWILYAVEVSGTASLWTVTIGVDCIFGVYALQVCGE
LRVLSRKFRELRASDNYKENLRDCIQRHHVLINAKNKLDNIYGLISILLITSATLVLCSLVFQVSEVCMR
TPVKSLLSSYFPYNRLCHINMYYIRFATTVLFKLIKTNSYLRVAHLCVYLIPKFLQIFTYAWYGNLIAEE
VSV
>OXU17446.1 hypothetical protein TSAR_016928 [Trichomalopsis sarcophagae]
MGEQRITRNMSFYFRSSKTYMSILGIWPYQSWLSRIVLRCFWIFQHTSIMLPEAIRLYENRNNIDLIIES
IPPFSYNTVMLIKFVNGIFNSGTTKWVLENMKNDWNTLVDKREIEILNYYYRSGRFLNTIYIYMVWLTLV
SYMFFPLTPIVLDFINPLNESRPKSSLYLVEFFIDQDKYFYFILIHAYITSLAGVIPVFASDLLFSNCAH
HACAMLEILGRRIENMMMRNQENEDTSKGESKRINEYQRAIDCVVQHQSIIEFCESINKIYNISFFIILI
IELVEMSVTGFAAVMKIEDNFSDSIRFAMFTLAQIFHAFCYFFLGQNVLNYSEKLRNNAYNFNWHTASTK
TKYLIKFIIMRTSKPSVISVSVYPLTLENFTTLMKTIFSYFTVMNSTR
>MpulOR1
MMKTKYQGLVADLMPNIRLMQISGHFMFNYYGEGKKFMHKIYCSIHLFLILLQFALCGLNLAMESDDVDVLTA
NTVTLLFFSHTVIKIIYFAFRSKLFYRTLAIWNNPNSHPLFAESNARYHSIALTKMRRLLFCVGAATVASIIS
WVVLTLVEDDPVREIVDKVTNETTIIPLPRLPVRSFYPFDARHGVAHIAMFAFQLYWLIITMFNANSIDVLFC
SWLLFACEQLQHLKAIMKPLMELSATLDTVVPNSSELFKAGSADHLRDTTGTVPSATQPNGDNMLDLDLRGIY
SNRQDFTATFRPTDRTQYNGGVGPNGLTKKQEMLVRSAIKYWVERHKHVVRVVTAIGDAYGVALLFHMLITTI

TLTLMAYQATKVNGINVYAASTIGYLLYSLGQVFLFCIFGNRLIEESSSVMEAAYSCHWYDGSEEAKTFVQIV
CQQCQKAMSISGAKFFTVSLDLFASVLGAVVTYFMVLVQLK
>MpulOR2
MEAIIEFDEMIRPISLVNRLISIWPLELNRNSSFESRMRPAHRVLMFILVGILSVSATADIIRHWGDMNEVTE
CALIATAFYLSMLRLVVYTSHKRDLEYAVNTVRVDWSESCEADIIVLREKCLFGFQLAKYFIVSVVMALGAFM
ISPALQIIFLGTDERILPFRGFFFQNQTNTPYYECLYVFDVIAGALGGSTISGATSLNLMLVTHGAAKFSLVR
KKLQALKSNDANVDRDLEKCVRLHQEAILFADRAEDIINVLALGQFVISTGLVCFAGFQITSMIEDKGRMMKY
VSFINSAILELFLFSYSGNQLITESEAVGESFYQSDWVGGNYMQNMRILMLRSINPSRITAGKFYDMSLKSFS
SVLSTSFTYFTVLQAVNED
>MpulOR4
MSNKSWNSGTSYELSIYKIIMWPLGIWPLNRGEVFSEVRLLLAAMTQAATFICLDIEMWNNCRGLEDILDIFV
LSIFSLLACVKGLLARYHQDEIYSNVSSAIDDWYALSIIDNSNNRKIMMKHARIGRIVCISLMAPASTGTLSW
IILALPLPIFAPANSTDAIRNFPLQTACTFESFTTSTIYYVIFIVQIYQLIATCLGNCGNDVFFFGLGMHICG
QLEILKNEFRTFVDVDKMTTDRKRFQLLVRRHTHLMDLVENLEESFNLIILSQLVMSGLLICIMGIQVIIALK
IGDLFSGINAGVVLSSLMSQLFLYSYGGDYLTSQNEDLAFAAYESMWYTWPVNTMKDIGFVIFRAGKPINITA
GKFVQMTLATFMDILKLAVSYIKSEVLQFRKESRRDFARDWECVLPPINSTFLYYKLWF
>MpulOR5
MYVFMNEAISHNVPVTITIIIEIVELAKFVINRNEVMSVNAYTQQTFWNALYDASESEILDECNRKSIRMVAT
DFVLAQGFTWLHLCIPLIEYSGYDDSTRELPFRMHFNWPLAETPYYEIAYVVELFSAMGVTTCAVIFAGYLGT
MNFYTAGQFKILQRQLENSIKDMDKMRVEDLMNQVKFNAICGNIKNCIKQHQLLILYIDRIENLFNVIMLIQT
LGSVALLCFSGFQILLDDQSSILRTTMSVVLLIVSIVQLFLFAWPCHEIITESQEVSEAAYRALWFCLPYNSE
GRACRQLLLIVITRARRPCVLTVGKFAPMSLQTFASVFNTAISYFTVLRQMNESTEQ
>MpulOR6
MVNNPELTRSILTNLRNFRLLTWFFTFFQFIFTTLVLVKPLIFLFQQHRHAVDPIVYPLFFATVYFPSLDPPE
LRYKIHFIIELVAIVPYSCVSLSVDTLFMLYGFQMMAQLREMSHRIMNFNDMSDDKIRVQQCVVQHQSMMR
>MpulOR7
MSQTDKFESRPEATTQTRWIFLLLGVQPMTKNSTITEKRISAVMYLICRSLLAFIIIPANIQLFWFERLEIKL
AYFGPLGTAMACALKYFAIISRRDDITNCIQQIEEDWCQSKNKDSLEIMKQHTRQGRRITTFCGSVMYTGGIL
HQIMMPFLPGSVVSIFSNSTKRLLVYPMSEIILDDQVTPTYEILYIIHIVAGIVVCTIALASCHLAILFVTHT
SSLTEIMTLKLENLMTAQGEVKQNLCMSEVIDINHKIIKFSHTVEICLREICLISILEALYVICLAEYYALLT
WNHNETFGAIIYLILLQGFVGSFFIFCHVGEIMKEQVCSLFIHCQ
>MpulOR8
MIAMNTFKSKVEVTKQTRYIFSILGIWPMMTNNTTIIKKRVSAVMRLVCQSLLAFIIIPGLIQFYLLENLEIK
LAYVAPFGIVIACAFKFTAIVNRRKEIKKCVTQIEQDWREISNENENAVMLDNFKKGERITTVCASVMYYGGI
SHQIMMPFLPGSIASIFDNTTKRLLVYPTNDMMFNSQITPVYEILYITHIVIGILICTILVGSCHLAILFICH
ILGQNQTMILKLANLTDEQVDDERKVNQRFTEIIHLHHKILEYVVDWFAIYKYIKYF
>MpulOR9
MSKLVLKGDTLVYCLSWILTFVVGLSTLSYVTIPMISIIYQHSHNIQPIQYTLVYPTGYPWSIDPPGFRYKIH
FIIESVATLALFCITVGIDSLFTLYGWQMVAQFREMSHRITHISDKDDTRNVLRECVLQHQKMIKCRDILQKI
YGPVILWIITTNAVILCSLLFQFSQVKSIGRGISFLTYIMLKLMQTFMYSWTGTQLTTASDDYRDAVYAANWY
QDKSLMSSFLIILTQKPLILTACNFSNVTLDMFIMVLNTTISYFFLLRTLEENV
>MpulOR10
MSHDSAQDYRRYHSIIKRLLLTVGLWPNSDPNIFYQLLPFPYLFVNISVVLGTAVYIQMHLTNVASITKGLSI
MTSFGTVILKMTCIMINRDNVNELHDILDLHFNKMLNDQQISSTILKDFRTFRRLSWSFSFFVTIVILLTAAT
PITSLIYQYFHSVNPIVYPDVYPTAYPWSTDPPGVRYMIHFLLELSTIVPLFCVSPSIDSLFMMYGFQMSGQF
REMSFRLKNIKKMADERKAVRECVLQHQSMLKCRDMIEKIYGPIILWMLVSSAIILCAIMFQLSKIRSFSAIR
ICLLALNLTTRLLMLFIYTWSSSMLTSESEKFRDAIYSSNWPATGQRKFMTSILIMLTQKPLMISACGFVAIS
IDMFPKTINTAISYFFLLKTMEGKGT
>MpulOR11
MTCVMIYHKKYCELHGILDRHFNDLLNDEELSTIILVNFRTFRRLSWALMFSVFVVVSLVAVAPVISLIHQHF
STVYPMTYPLVYPTAYPWSLNRPGLRYKIHLIIELFATLPQFSVTSCIDSLFMLYGFQMTSRFRLMAHRLKTI
QKMTDERKVIRECVMQHQSILRSRDLLSTIFGPIVLWLMTANAISLCTVIFQLSQMKTMTPSTIVHFMAYVIV
KIIQTFMYAYTGTFLTAESENYQQAIYDCNWHANKRLMTSVVIMLSQKPILMTACNFSVVSLDIFVKVLNTAI
SYFFLLQTLS
>MpulOR12
MRNDKLSEYYVLRKFIRIMLMALGLLPLEGACVLYQFVPYIHLFLTVGLAYGLFGFVFTHITNVVIVAKNSAL
MISLFSLCVKLLCMIIYQEDTKLLFHGLDEYFNKLINDQKFTDIVSKGITPTKWVVWFLSFLCFVMCTTNLVI
PIIFILHQRKHHIKPLTYILPNGTKYPWAVVGPGLLYKLHYFYEILAIYSSVTITCAIEPLFSLFTIQMIGQF

REMSFRMIHLDQSDQCEIVVRQCISQYIVLMKHRDRMQKVFGPVVLQMIITNAIVLSLGIFQLSQMKTVSMVH
LTLFITYVCFKILQTFISAWSGTRLTTESENYRDSVYASNWVGNRRVITSVLMMLSQKPLSLTACHFSTISVD
MFVSVLNTAMSCFFLLRTLQHD
>MpulOR13
MVIIFIKDLVTKGNCGAITDLVDAWSLIATSILSVMKLILSRINHKSMHLIVNSAIEDWNNVDTAKTRGTMMK
YAYIGRLVFIVQMSGAYTTVIPLIFKSPPNFDTGNYHENVNITLPFRNIPIGPNCWLPLSISENIYLLYYLLV
TLHLIILCTAYIGGDVYIFGIAMHVCGQFQLLYDSFEKLDGSLNDFVLRNKIHQLIQRHCHLLMLANEFENAF
NLVILVQVAANTFIIGISGEMKYFL
>MpulOR14
MYHLPIAWNTVSRNLNNITDIPGALFAAQTVYPYDVTQKTAYRLTVFSQYISEVSASLTYTGIDVLFSLVILH
LCGQLKILAHKIETMTDDRQNFQKNLKMNAQRHCSLIKYVDRIENIFSLMVLSLIILFSIIFCIESFTLISVS
SSDTSEIPVSQFTFYILFTCYFLMLMFLYS
>MpulOR15
MITRRLRDIAKQHDQLFEYCHLIETFYNPIIFLSVLSSAVNTCCCLYAMEAQVAAGNKNEISKSAFYLIVLAL
QIMIYCGYAETLTQSSMRVFQACTWAKWTDWNKRNKIILFIIMMRAQKEYEYTVYGMIVVNLNRLTLIANGSI
SYFMLLRNFG
>MpulOR16
MSNNRGVEDYKWAVKEIRLGMKCCGVYFKNDLPKWQRSLISVRAALNVIIVMNFILIPSIPALIKVWGDLTLI
IDNLIVSLPYTLSICHFLTMWFNKKEINELFQMIKEDWITSEYDFERNIMMRNTKISRTIVRFAYLIVAWLVA
MHNMPFWILGISPRTYTNLTDGAQPLIAQTVYFYDLTIGINFKLTAIGQTMSSITVGLAYSTAGCIFYTFILH
VGGQLKILKILMEHIFENHEKESNINYKSVPKFIRSVVKRHQRIIRFVDSVENIFNIMFLQQFIGMIITISTE
GFLVILMLVGDVQVPVVVMGFTILYVSYCMIMTLMYCTGGEYLIESSTAIHSAAYESSWYNSAGKYQVELSMI
ILRAQKPLWITAGKFVPLSLSTYADMLKTSASYMSVLLAMKA
>MpulOR17
MIKNARIAKIIVRFTYIFVLWVIAIHNLPFWLFGIVPRTHTNLTDGNQPLIAQTVYIHDMNVDLNVQLTAIGQ
TVCSFTVGISYSTAGCIFFTFILHACGQLQILKMHLENIFDEDVTKIDDRKIPEAIANNVRRHQRLIKFIATV
EKVYHLMFLQQFIGITIALSTEEFLVISILVGDVEISTLGMIFCVFYTIYCAFMTLMYCSGGEFLIENSLELY
SAAYNSRWYNSQRTYRKELTMIIKRAQKPMFITAGKFVPISLNTYARLIKTSASYMSVLLAAKS
>MpulOR18
MKICGVHFANNRPVLLQSLMTLRAALLASIVINFIIFPAILAMIEVWGDLTLVIDNIIIFIPYSMSICHLFTM
WYNRKEISELFSEMKKDWTAQKNILQHEIMVQNAKISMNIIRFAYIIAALLVAMHNIPFWIGGIIPRTLTNLT
DGERPLLMQTYYFYDVSVYMNFRLTAIGQSMSSSTAAMCYTTAGCIFYTFVLHVCGQLKILKHFIENIFNNND
NKNEISEKTLARLIKISVKRHQKLIKFSGIVENIFNMMFLQQFLGMMITISAEGFLIISMLVGDVEVPVVGLG
FVVIYVIYCMIMTLMYCTGGEYLIASSNALHSAAYNSLWYNSGQTFRMEITMIINRAQRPLYITAGKFSPISL
TTFTKFMKTTASYMSVLLAVKS
>MpulOR19
MSQNQGIEDYNWAVNEIHLAMKICGIHFDNDRSKWLQSLMTLRAIFLASIVINFILFPAIPAMIKVWGHLTLV
IDNLIIFIPFVMSICNLFSMWCNRKEVGGLFQEIEKDWMIQRPIAERNIMIKNAKTSMNIIRFAYITATCLIS
THNMQFWVLGIIPRTLTNLTDGERPLLIQTYYMYDVTTDVNFMLTAIGQTLSTMVAALCYTTAGCIFYTFVFH
ICGQLEILKILMENMFGDNEQESDKLDESAAQFFKVVVRRHQELIKFAEMVEDIFNMLFLQQFLGSMISISSE
GFLIISMIVGDVDVPAVAVIFVIIYAIYCIVMTLVYCTGGEYLIASSEAIYTAIYNSHWYNSERLHRMELTVI
INRAQRPLYITIGKFTPISLTTFTNFMKTTGSYMSVLLAVKS
>MpulOR20
MFSILILYLASPSVPTILDIIIPLNESRDRIYLYQTEYFVDQDEYYIPILIHAYMTVPISVSVLVFLDNMFAM
YIHHACGMFAILGSHLEVLHMSNNNENLLDKAIRSRRIHSEIVRCANIHCNAIDFANELESSYAVAYLLMFSV
NLSMITVTGIVTVMKLNQPSEMVKFLAFTVGEIFHVFFSCFQGQELIQQSGNIFEAAYNSEWYKIPVRDRNLL
IPIMVRSMKPCAVTAGKLYPISMGTFSVVLKNAMSFFTVLSSMR
>MpulOR21
MMMNIFETSYFRTSKRLSVLFGQWPFLPPIRRNCIRCVVFMFISSILIPKLIKLVEVIHDIDSIIECVPMIGL
HICSLTKFFNWIVNSKKMKHLLLRMQTDWNNLQYSQDIEIMHEFYKRGRLFTKTYAIAMFSILGLYLASPSIP
KILDIIHPLNESRSRIYLYQTEYFVDQDEYYLMILIHAYMTVPISLGVQVFVDNMFAMYIHHACGLFAALK
>MpulOR22
MHAYMTVPISLGIFVFAENIFAMYTHHACALFTTLRLHLESIHSDIHDYGNIESGMEPTQKLHRKIVMCVNMH
ENVIKFSNELESSGNMSYLIIILMINVSMITITGIVTVMKFNTPSESARFFAFTMGNVVYTFATCLMGQKLIDE
SEIVFHAAYSFEWYTLPMNLQSMVIPIMVKSLNPCQLTAGKIMSLSMDTFSLVLKNAMSLFTVLSSLR
>MpulOR23
MSSEVEQKKSGREQLWNSDTIYSLGLYRTLGKVLGIWPSDSNRLSSYIRVASVTIIQLWMSIRLTEQLLTKGN
CGIITDIVDVLSLITCGLLTALKVLLPRIHKHKMEIIINSAVEDWAERVNRKSRNIMFRFAKIGRVVFITQMI

GAYVAGFPLIFAHLPFISILWTDQLNSSVMVRNVPIGPNCWISPDWPASYYVAHYVLQSIQLLVVCTGYIGGD
TFFFGMAMHVCGQFEVLYTRMKDLQGIRNSKIQRRIIAEFCRRHNHLLMLADNFEKIYNIIILAQVGADTLLT
CITGIVLLISIQSLDFVIVIGLVVRIYLVCIQLFMYSYVGEQLSSQAEKLRMAIYDSPWYDMPPDLMRDMSLI
IMRCDYSYYLTAGKIYTMNICNFKNIIKTMTSYFSILRLMFIDEPE
>MpulOR24
MIWTSDTVNALGLYRMVGRIIGIWPIDHTCTLPKIKMAFVIITLMWMCIDLIKQLVAKGSCGTVTEIVDLISF
FTCGWLSVMKLIALRTHEKYIFEIMNSAIKDWVKLNNRTSRSIMLQYASIGRTVFISQAIGYCCSIVFLISSH
LPSITTLRDSPMNSTVTINGLPLWSECWIPVGISMPQYAIQFILQSFILAVLDICYSSCDSYFFGMAMHLCGQ
LEVLSMYFEDLYEIDNFEVQTCHLIQFVKRHQHLLLLTNMLEKAYNFIILIHVGVATMIICVSGIVLLMSLET
SNFNVIGDMLLRIVLTYLQLFMYCYAGEQLSSQAMKLENTIYNCPWYDMPPDSVRSMSFVIMRCRYPSNLTAG
KIYCMNFQNFVNIIKTTTSYFSVIRLLLKGNR
>MpulOR25
MVWNADTNYALGFYKVIGRFIGIWPLEYNRIPSIIRVIIFVATQVWFCIDLTMQVIFKGNCGTLPEVIDVLSW
ILVGFSTSVKMIFLTIYRCNMKTIMNTAITDWSTVDVPESRQIMLKFATVGRIGSVIQVCSAYSVCVLQITRC
LPAITTTLNHQSNSTVPICIPLWPACWVPQNVFDYTINFINQSFLAIILVICYSGCESVFFGTAMHLSGQFEE
LYRNPKHLDGSGDYSQQLQQLVKFIERHKHLLILAKDFEETYNIIILVHVAVDVLIPCVSGQ
>MpulOR26
MDIFETPYFYYSKRFGVMFGHWPFLSPIRRNCVKCLVLVLISSILLPKLIKLIEVIHDIDGIIECVPMIGLHI
GSLTKFFNWIVNSNKMKHLLMRIQRDWNNLKVTKDIEVMHEFYERGRLFTMAYATAMFSILGLYLASPSIPRL
MDILYPHNESRGRIYLYQTEYFVDQDEYYLLILIHAYMTVPISLGVHVFVDNMFTMYIHHACGLFAALKLRME
SLHINDIDYRENKSTKSAEVFHRIVMCVDMHENVIEYVKRSL
>MpulOR27
MQLDINDEFEKSKSLLDTILRPLHWFGIWSPQRSIMDDVKFSILVTYFTIHLVMMCYDLIMVFGDLESIVLNL
MESGVQSIFMLRVLVLRFRKHFNLIVISLRDEISDGNYKDSFERNTHVEFSSMARFYDRIVMTLGIISSFSWY
LMPLQNYILAWLFNGEMTLNYPYRMFGGNNSSSTKWNVIIYIYQTPVTFFPICYLSSYGLMMMAIIHICEHMA
ILSYRINNLSEYEKTYDVLSNLMGEFVEKRTKALRLTKIIADGFDTVLLYELIITTVLVSLISYNILLNIDNF
AVLEIATSTLFVISIMAVIYGYCFMGQFLITESENLAAACYGCAWHEMPIHFQRSLLIFMKSSQIPLQLTAGR
FYVYSYRNFTNIMKSSAAYVSMLRAVI
>MpulOR28
MNPNIDRKFEEAKGLLDRVSRPLQLVGVWPVKRTSTSNLRFVMFITYFSTHLCMEYCDLLVVFGDIQSMVLNL
MESGLQSMIILRLFCLRYRKIMHQIVIAIQDDVIDDNYDDPNEKKIYIKYSSIAAAYHKFSVWLGWITAINWY
FMPLQNYGFALMQNATIPLITPYRIRTGFDISSMKWTISIYIYQMPMIFTSVCYVATFNFIIVAVTNICAKIA
VLSYNVNNLAVHRVTFTTMKLSDVVEKHLKIIRIVDKLDKAFNLIFFYELINTTVLIGLLLYCAMVNLDNAEP
MALVLSLLYAATMMMLVFVNCLMGEYLATEAAQLLASYYECSWYEMPLSFQRDLIICMMRAQVPMHLTAAKFY
VYSLSSFTSILKSSMAYVSMLRTMI
>MpulOR29
MRIIKSGSVILLAFIHSIHRSSMHKHNEFLCVSDLSTTNQTILLKTPTRINFFYKPDTNFKNIIFYLLEMPVM
YTGLYRVTMISLLMTLVFTACSQLAILSRTVEQIIPGRNYHIFHKIVSKHLHIISMTQSIGRGFDYLLLFELI
VTTVLLGLIAYNGILNFDKADKLSLLSIVIYGSTMLIVIFGNCLIGEYLIQESTNLHKSFAKCVWYEMPIFHR
KCLLMCLNNSNTPLYITAGKFYIFSLSGYTSQILKTSMAYFSMLRTLTKN
>MpulOR30
MALTCWYHFISNFNRAQHTTIEEVQRAMSEKSDISNHFSLKTLRFFMQLMGMWPVGDSRERLISNLALFYTVA
IVTMGVTAQAFDFYYSMGDLQTLSYNIPNTFTAGNELIKLLKFVVNRDEVMNFNAYTEKTFWKAKYDEKEYKI
LNECNRKSMEMVITHFFFAQSVVVLFVSVPIAETSGMNVTERMLPINLYFNFPYTESPYYEITFILEVKYIHE
W
>MpulOR31
MVLVYIFFAQSVVCLFCAIPIFEAVEKNVSERMLPFDLHLDLPLTQTPYYEMAFFVEAAGMMSVGLCTTGFAG
FLCTTNLYTAGQFKILQRKLELACSNGEAVLSHAQTKTNLIEYPSKWWLREQIASNSITKDVSQTLRIDANFS
IDDNRADTEGDVQYNMLKNCIKEHQLLLEYMEKVENLSSAVMLAQALGSILKICFSGFQILLVGGTSILRTIL
SIQFVVGTLFLFFMFSWSSHEIIIESQALAEAVYRTSWYSLPYTTGGKACRQSLLIIIARAHKPCILTVGKFA
PMSLRTFVSVFNTSISYFTILRQMNARLNE
>MpulOR33
MSDVKGIRNHFSLQILRFFMHLVGMWPVKNMREQLISDILLFYIAMVVILALMIETLDFYYSWGDLHAISYNA
PTTITVAIEVLKLTKFMVNRSEVMSFNAYTEKTFWKQTYSPEEYQILNKCNRQSLKIVATHIFLMMCVVWMNL
SIAIVESVGKNMSDRTLPFNLYFNLPFAETPYYEAAFVFESAAAMGVGICTTSFAGFLCTTNLYAAAQFKILQ
RKIEMTCYNNATGIVRTEYSARNNSPSADWWTLNPVIGLDKTDQTNFAKDIGKNENFYLSRLEVANKKNITAD
NARFENKNKETFALLRDCIIQHQRLIHYMERVENLFSTIMLAQALGSILEICFSGFQILLGVGNSIMRTTLSV

QFLIAAMVQLLLFSWSSHEIIIESQEIAEAAYRACWYGLEYTDEGKAFRQSLLIIIARARKPCILTVGKFAPM
SLDTFTSVFNTSISYFTILRQVNEEGMEE
>MpulOR34
MFFPKTMHQALTFNLPSTVSTLVEFWKLCKFIMNRSEVMDFHAFIEKTFWKVGYNTDEYQILDRCNRKCMRLV
IAHFIITQVLICLLISIPIAETSGTNVSSRMLPVNLHFDIPSTESPYYEMIFILEAVVGVGVGLSITISAGYI
STTNFYAAGQFRILQRKLEFAFRDAAGDEVDELLVTMKINPNFPILRDCIKQHQLLILYMERMENFFSMIFFV
ETLASILFLCISGFQLVLGDAKTSLFRYLFGVEFLIIVFVNFFLFAWPCQEIIEESLKVAEGAYR
>MpulOR35
MKIVVIHFLIVQSVEGLYFCIPIVESYGKNSSERSVPFPLHTDIPYTESPYYELIFIFESASALSVGICTTGF
GGFLCAMNLYTAGQFKILQCKLEITCAMGRTKFSQVQSRPDRESSSHDWLRWSLFSDVISKDYTDLIKDVDEN
QKSDYTQFSLYNVKSVGADESNEKKNAPYILLRECIQQHQLLIHYMEKVENLFSGIMLAQTLGAVLELCFAGF
QILLGVGTSKLRTALSIQFLLGSTVLLLFFSWSSHEIIIQSQEIAEAAYRASWYGLGFTDEGRAFRQSLLIII
ARARKPCILTVGKFAPMSLNTFTSVFNTSLSYFTILRQVNEGLDS
>MpulOR36
MVVSLCTTTFATYVGTTNFYTAGQFKILQRKLECAFYVMASDEDEGPIITMSIDPKFSNVRDCVKQHQRLILH
MERIENLFSMILFVQTFGSVINLCFSGFQIVLGDSETTLLRLFFSVDFIIASSVNLFLYAWPCQEIIEESQEI
ATGAYGALWYCLPYTEEGRLCRQSMMIVIMRARKPCVLTVGKFTPMSLQTFGSVFNSALSYFTVLRQMNEEA
>MpulOR37
MPELTPETIFTFTKFSVYLIMSWPSENETTKNGNIIFNIKWWILWMNSILFIAFCVYGAYGDRQDVLSCTEIL
FIITAVIQYPIKMLICRIIREQMQFVVNEMECHIHSAEPSKRIILSNYLKKPGVVYVLYHFVACLGFLGMVLA
AFILQEPLPVKIRYPFSIDTRLVFIFVYFQQFIFGIQAITTTSIDSQIALMLWYVVARLRILQEQMRKISNMY
EFRKCIQQHQYILRLADEVISVGKYILATTVVMSTLSLILGAILIVKAHQMTVRLKFSINVVTVSATLFMSAW
PSEMLISSVSSATNYYIRKILWNITRQIIFSNIFSQESNKLALCLHQILIIVVLQINIYLLDE
>MpulOR38
MMSILPASFGLLQISGLWMPSHWQSPILQLLYKFFTVFVLLLIYWFVVTGLTELIRSPPNAEEFTDNLFILLT
MITVCGKYLNVVICRESIVEMLDILQQNPCAPRNDAEVAIQNKWDRVIWLNTLAYGSLGEVTVFLVTFGTFLQ
DIPIGTLPFSAWLPYNYSHGITYWLAYFQQLMTLIISAHTAAAYDTLVAGMIMQVSAKLHILKYRFSNLSQVL
QFNLEQQDDKSDINKVGMERKLIADCVECHLTIFKLAEMVNTTFDRVVFLQYGLSGLIICVNVYVVAHINLLS
AESAEIVLFLCCMLYQIYIYCDAGDEVTMVSSSVSDAIYNMDWTIMDISTTKSLVVIMSRTLRPIIFTSGSLI
TLSLNSFMSVIKISYSTYNVLQETSS
>MpulOR39
MSSLSYNIFVLRCLGLWYSDDWITGWKAKIYIGYTALIVLILYSFTLSHFIMLYECIDDANEFANTLFMLLTL
IAICGKMFNIVSKRQAIEKIMTALQTDPFTPEDPTELSINSECIRKINFCRLYTILYGILTESTCTMLTLASL
NRDVPRGDLPYKAWLPYTISSPNAYWFAYSHQTFGLYIAATTDVAFDTFVPGLMMMTCGQIQIFNHRFEKIFE
TLKSTTDPNGDALHLEDQAIAEKKLVADCIEHHTAIFESSILFF
>MpulOR40
MSVLPATFRLLQLTGVWLPTHWHSSILRSLYQLFSIVVLFLIYCYVICGLVELAIDPAEMLNTTNDLLILISM
ITNCGKSVNVLICREKIIEILDILQRDPCQPRDEKEIAIQNEWNGIIWSSTFTYGMLLETTGVLGIIRIMALN
LPMGMLPFKEWLPYDYSNGFAYWQAYSQELIALFISTNLCIAYDTLVRGLIMQVTSKLYIFQHRLVTLPETLQ
SMWNNNEEKLEMKKLQMEKKLIADCVHFHLIILKLANTVHTTFNLVVCLQYCLSGIIICFNVYNIAHVNLMSS
GSAEIILFLCSMLYQIYMICQAGDEITLVSGSVSEAIYNMDWTTLNHSTVESLIIMMNRTLRPIVFTSGSLIT
LSRNSFMKVIKLSYSMYNVLQDTSS
>MpulOR41
MYSFTLSQFAMLYEAANDLNDLADAVFMLVMLSVISSKMVNLIVKRKVIAGMMKSLQHDPFYPQDAEEFRIDS
KAADNINFFTLWYGIMTESTCTMLTLASFKRDIPRMELPYKAWMPFSITSPMTFWIAYLHQTLGLYIAANVDI
GFDSFVINMMMMTCVQLKILKHRIGKINHTMNSAQSSYKNLFSIVHQMHLETKLIANCTKHHIAISEFADLQN
DTFGVSVFLQYCAGSLILCASTFSVSQLEPFSADFMSLVIYVMCILIQIFIYCQYANEMTVASESIRDAIYAM
DWTSLTVNAQKSLIIIMARTLQPIQFTIGHISLCLDSFSSVRTIIFSL
>MpulOR42
MSALDSSISIFKYLGMWRPESCFSGWKSHLYNIYLAFIVSIVFSLTFFELVTLAMSFDNVSNAASKCTTFFAI
FTACGKILTIFINRARILELIKILTSDPLNGQDPGEKEALERIHRKIRFRTKLFIWVKTLGLFAMLTMTLVIS
IPQKVVWMNIWVPFHFATDTQYVIIMTLEIFGHYFTAGPAAMAYDTFVPGMMMQISAQLQLLQYRIYMRLKTL
ESHSPSVRQDSNENYTQVKMIAGWLQHHLLIYQYAAELNAIFVPLICLQYSSSVILLCVNVYAMVEVPVLSAK
FIRSLMYNTMLFSQIFLLCLAGTEVMDDSIYFSRSIYNTHWYLLNQRSKKSMNIMMTRSLEPIKFTSIGIIET
SLESFLRLVKISYSAYNCLQRFTPNE
>MpulOR43
MKGTQVNAYHTVLSMSIDGLRLCGIWGLNTSTSIYIKLIHLLSGIIGIIALMLLITTIAADLLYNLDDLLIIT
DNGCYLAGLSVILFKIYKFHNQRNQIQNLTKAIYTPIRNLQRSTDAEISNILRANTFYENIGFTFFSSMGAIL

MIALIFFVPRKDGELPMRAAYPIDITTPPTFQLAFVAQLYAVAYGVAAILLMDTIGLGFMRWINVQLIILSHN
YRNCEPKSFQENDGHSNTFINSAQLLENRRKSKISMDINGDPKITSTIVLFPTSELDNVHKDFVSRFKQCVMN
HQRLIGIVEDINNIFSTSMLMQLFASFTMICLTGFQAVLGVEEKSSLMKFVIYLGAALTQLLYWCWYGNELFY
QTQSLLIAQWMSGWENQLNSASKILIVMSMSRTMQPLELKAGVFFTMSMETFIAVISIEFLPHAFLHIIIFDM
EH
>MpulOR44
MIIFDFKWWISWMNSLLLALPLMYAVYHQTRDVISFVTALGTCASCFQIMIKMVICKFMRQRMQFILDDMERF
VRDAEPLEKEIFLHYIKRCGILHISYLIFTLSSIIAMIITPMVSRIPFPTGAKYPFSVHTHPLFDIIYIQQSA
AALQIVSMIAIDCQIATMLWYIISRLKMLEEATRAIFNAKEFHLCIRQHQHLLWMASEVTSIARYILLTTVTM
ATIALITCGIYIVGNQPIAMKIRIGIVITSYCVLVFVNAWPSEMMMRSCEDIGTAIYESAWIESDFNKNIVLV
VQRCRNPPVISVAGLLPILSMNYYTKFLSKTFSFFTTLRIILAKIEAHNAPILDTK
>MpulOR45
MFEVKPEWAFIFTRFSVALVCAWPPNKNANKREMIIFDIKWWISWINSWLLSIPLAYAAYNDRQDILLFTKSL
CLTACCIKCTIQMFICKIFQKRMQFVLDEMESYVRDAAPAEREIFLHYTKSRGIIHVIYLSYALSTSMGVIMG
PLILPQPLPSDAKYPFRVDKHPLFDIIYIQQSIVGIQITSMGGIDCQMALMLWYIIVRLKVLQTEIRNIKNAN
EFSICIQKHQYLLWLAKEMIFIARYILLTTVIMATLSIILGGVHIVGNQPLIIRIQFTLIVFGFSLLLLLNAW
PSEILIRSCQGIGMAMYESAWAKSNFDKKVVLVIQRSYKPVTIEVTGLLPKLSLNYYATFLSKAFSFFTTLRI
VLSKMETNFPTAQV
>MpulOR46
MKLFETIPESPFAFTKFSVALLCSWPPSRNASKKEKIIFELRWWISWINSMLLVLPTIYTAYVDHREIVQCTK
SLVFFTTCIQINIKMLICKIIRKRMQYILDEMEDYVRNASLDERKTFAYYTKKRGIIYVGYLLIAFVSVVIMM
MGPLVLPQHLPVEVKYPFLVNTSPLFEIIYIQQSTAGFQILCMTAIDCQMAMMLWY
>MpulOR47
MLICKMMRDKMQFVLDEMEFYIRDAVPHEREMFLEHTKKYGIVNVISLFLATCTIIGFIMGPLILPQSFPTEA
KYPFSVDTHPLYDIIYIQQSTVGIQVIAMTAIDCQMSMMLWYIVFRIKILQKQTLKVKNAYEFSIFVQKHEYL
LWLANEITNIVRYILLTTVSMTTLSIIMGGVHIVGNQPMIEKMQFTFVVSAYASLVFLNAWPSEILMRSCEGI
GTAIYESEWIGGNFNKDLVFVIQRCERPPVITIAGFLPILSLNYYTKFLSKTLSFFTTLRIVLAKLEPISI
>MpulOR48
MKLFEIVPESTFTFTKFSVALLCTWPPSGNASRKEKIIFELKWWISWINSILLVLPVIYTAYVDYRDIVLFTK
SLVFFTVCFQITIKMLICKMMQRQMQYVLDEMEDYVRNASMDEKRILLYFTKKCGKVHASLLLGALVTVLGMI
MEPLVLPQPFPAEAKYPFPVNTFPMFGIIYFQHAVTGIQVVCMAAIDCQMAMMLWYIVVRLKILKERTRKITN
AYEFSMCVRQHQYLLRFADEVTHIVRYISLTTVSMTTMAIIMSGIHIVGNQPMVVKVQFIFTVSVCSTLVFMN
AWPSEILIKSCEDIGNAIYNSKWGGNSADRGIVLVILRSSKPVVVTITGFLPILSLNYYAKFLSKTFSFFTTV
RILLAKMESSVAS
>MpulOR49
MNYQIDNHKFRRLKPNYHIIVSLTIKRFIGTWPPIAKPKRYFYSIYTIISFIFTLGIYLIVQAINLSVIWGDI
DRMVASAVLFMTNAVHTYKIIVILRNEIRLRMLLNILKSPLFSRDNKKYERIFSYYSWQGIIHHVIFQSISCV
TVACWGLIPIADAVLGISRRLPMDAWYPFDVKQSPAFELTCAHQFIAVVICCIHNISMDTLITGLLNVACCQI
EVIKHNLLNIDLIEATEECENRIEEEY
>MpulOR50
MDLASNTDNNSSKNQRVDENISIKKYMNTNHELDSNYTVKIARTLLTPIGIYPLHGTDTQMSRFLVNLQIIVI
FGLMCFLLVPHLIWTWFDAEDLKRLMKIIAAQVFNSLALIKFWTLIINKKDLRMCSQQMEDNWRKVGCEEDRM
VMIKNAKIGRLFTIAYLSLSYGGALPYHIILPLVAERIVKEDNTTQIPLPYPTDYVFFVPGDSPGYEMLFISH
IVISTIILSTNCGIYSLIATYVTHSCCLFEVVCRHLDHLMDDGMNKGLSRQLAAIIKRHMEAIQFAETLEKSL
NIVFLCEMVGCTIIICFLEYGVIVDWQDGEVLGLMTYVTLMTSIFVNCFIISYVGERLKEQSLQVGESAYSIN
WYLLPKELVYDLMLIIIRSSQPTCLTAGKISDLSLEGFAGVVKTSAAYLNFIRAVV
>MpulOR51
MAILSESFLLLSICGLWKPNTWSGWKSIIFYLYAIFVFFLHNGFVLAEILDLFLSISTADERIDTIVTLISML
AVTAKMVGIYATRNDILGVCERFDSEWHAPNPDEIQIQQTCDSNFRFYFFVYTSMFEMAVSGNTLGRLRADTP
AFTLPFKIYVPYDYSSPGVFRLTVLFEFLAVLVASNIEAAFDTLFRGVMVQICARIQMLKRRFEVSVDSLQKM
RSEKSSDSREYKKMEQELFSDWIKKHNAILRLSDDIESVFSKVIFVQYYLSSFVVCTTVYILSQTPFSPPFIG
IGGYLFVMTSEIFMICYSANQVTLEFADLSVAMYNTNWFVLSIPAKRYIVIMMTRTLRPIIFTSGHLVTLSLE
SFKSLMKVTYSIYNVLKN
>MpulOR52
MAILTESFLLLSCCGLWRPVTWSTWKSKLYNLYAVFVFILHNGFVLAEITDVLFFDNSVDERIDIIVSLTSMF
AVSVKMIGLHATRDSIIEICEKFENEWRKNDIEEILIQKTYDHNFRLYFLAYTAMLEVTVSGNTLGRLCADSP
PFTLPFKIFLPYDYSTPGIFRLTVLIEFIAVLIATNIEAAFDTLFRGIMVQICVRIKMLKRRFQVAITTLEKK
RREEPFNREEYKAMEEKFLINWIESHNSILSLSDYIESVFSKVIFVQYYLSSFVVCTTVYILSQMPFGGEFLG

IGIYLIAMTVEIFMICFSANQVTLEFADLCVAMYNTNWFVLSISAKRYIVIMMARTLRPIVFTSGHLVTLSMD
SFKSLMKVTYSIYNVLKK
>MpulOR53
MDFLSPSFTVLQYCGVWPPTYYSWWKKIFYSLYTTVIAIAILLFCLSGVIQLCLITRSIDEFIRNSYMLLTTF
AVSMKVVMIIIKRNQVIRTFEILHLNSCKASNEEEKLIERESDPQSSYFLRGYAVLLIAAIVTATVGSIKENI
PSKTLPFSGWFPYNETHSVGYWVAFMHQSVAHCWGGLIAVSVDS
>MpulOR54
MKKYLPQCSTILEFCGAWPSVEYYTGWRKIIYDFYTLVVILLIFTCCLSGAIQLCIFTRTINEFAINGYMLLT
LCTTFSKAFTIILKRDYLAKAFESMENDTYRAQNEEEKQIQDEWNPHSSRSPQGYRFLVMVSIFVVTITSFED
NIPSRTLLFGGWFPYNHTDGIGFGAAFVHQLIVHNWGALIGSSIDAVIYGTLIQICGQLKILGCRLRKIPEVI
RKHTRLSANMNKNDDKTFKIEDWERLVVLDCIRHHLHILQFSKTCNGIYSIVIFFQFLASGVIICICVILLTK
MKFMGSEYIFMLSYLGCMVAQIFFYCWFGNEVILESLNIDTVIYEMDWSELSLSTQRDLLIIMTRSMYPIEFT
SGKMVTLSINSFNAVMKLSYSVYNVLGQKG
>MpulOR55
MKFLPLSSTILEYCGAWPSPKYYSGWKKILYDLYTILIVLSVFTFCLSGIIQVCIFTHSIRQFVSNSYMLLTF
IAVFFKVLVIVAKRDHLSELFESVQAGLCEAKNEEERRIKNEWDPKSSFRVQGFRTLVMSTVIIGVVSSIKGN
APSRTLQYGGWFPFNHTEGVGYWTAFIYQRVAHDWGTLIGSTTDAVIYGIMMQTCAQLKILKCRLRKIPETIR
QYAALSKNENDNYKDAKDTRKWESKIIIDCIKHHLLILQFADICNGIFAIVIFLQFSASGLVVCISVIMLSKL
KFMSPAFTYISFYLSCMLSQIFLYCWFGNEVITESSDIYLAIYEMNWPDLSLNTKKDLLIIMARSVYPIEFTS
GHVVTLSINSFNAVMKLSYSVYNVIGQRG
>MpulOR56
MRFKLSLLSEGEFRHNISFVTAKLKGWVYSNDRMTSCIGNIERKKERNSVSTHCLFIILATIHHCGNTMSHLQ
CLFCFHNFSPLYSSPTNVHFLPLRGGIPSRTTVESTMTFFPPSSTVLEYCGIYPSPAYYSGWKKILYDFYTLS
IVILISTFCLSGSIQLFLFTNSVTEFAANCYMLVTFIAALIKIFIVISKRECLPETFGFTLPISFRPKNQEEI
QIQSKWDPQSRFVFILIMLRNTSTMNIFLWNDSTLLVFPETALVKVV
>MpulOR57
MEPCYPKNNEEKEIQKRCDKVIKRDSLIYGGVTELAVIIGLVCSICRDVPQRRLTYKAWLPYDHDTGITYCTT
YIYQYGALVIGSLINISYDTLVSGCMMQTCVQLEIFKHRMKNLCRIQRESYWKCQPLGMDIKAAIHIMTRRIV
TENIKHHIQIFRFAESANRIFAYTIFFQYSVSSIVICNGAHRLSTVASFSPEFFTIIGYLMCMLMQILIFCYY
GNVVIYKSTNICNEIYAMDWQLLNQPTKKSLLIIMARTFKPIKFTSGHIVVLSLRSFNKLMKLSYSVYNVLHQ
RIS
>MpulOR58
MDTVATPYHRINRIFLSIIGQWPYQTYIYRRLSHIFMVFFTTTHGYFQTAGMIAAWVDLDVFLEAVPTVLADV
VCGVKFINFSYNAEKMKKLLVMMIEDWKLYKSGPENDILNDYARFGRRVTMYYAGALYGTMFPMMLVPLVPIV
LDIVAPMNESYPKHLMFQQIEFLFDYEKHLFLLLIHGYLGTFAFLTIIIAVDTMFMVYIQHACAKFSILGLYL
DRVAKNTDRDIDRHGPDFEDVDYKDMVTCVAKHNDAIEFANLIEEANYFSFVFVIGINMLMMTSSGMVAVFKM
NEPNVAMKFVGFTIGEVFHLFYSSWQGELLLDHSAAIFYKAYDSRWNDTSVRSQRLMVPLLMKSATPCKLTAG
KSFTMSLQSFSMVIKTSFSYLTVFTSMRA
>MpulOR59
MVAHKSSEYHTYRNIIKWLLIMLGLYPLEKAGLFYRSLPYIHVFLNIGTSFGMVGFLRANITNVVVVIKRLGV
LVSFLSVALKVIVMIINRNDTKELFITLDVYFNNLINNPRYTKIVTNGMTAFRRLSWTVSILSCLSGIVIIIT
PVILVIYQHKHHVQPIKFILPNHSIYPWYIEPNGLSYKLHYIFESMATVSLVAVTSSVEPLFTLYVVQMIGRL
REMSYCITHFDETNADSVIRECIFQHKILLRCRDLVQTIFGPLILWMVVTNAIILCLGIFQLSQMKTISAGQA
IFFIAYVGTKMTQTFICGWTGTRLTAESEDYRAAIYAANWQGNKQHMKSVVIMLSQKPLTLTACHFSTISVDL
FVSVLNTTMSYFFLLQTLQHGE
>MpulOR60
MSKLVLKGGTMLQFLSWTLTFAVGAWALNCVAIPVMFIINQHSHHVKPVQYTLVYPTGYPWDITAPGFLYKVH
FIYESAATVALFCITVGVDSLFTMYGWQMVAQFREMSHRITHISEKDDEKDVLRECVLQHQKIIKCRDILQKI
YGPIVLWIITTNAVILCTLIFQVTRVWKFRISCSVL
>MpulOR61
MVAAKLSEYNTYRKITKWLLLLLGLFPLENASLFYRFLPYIHLFLNLGTAFGMLGFVRAHITNILVVSKRLGL
MVSFLTGSLKIVCMIIYHEDAMKLRSLDKHFNILINNPQFTNMAFDGVTAFRRLSWAVSLLVLLSGTVNVITP
IILIIYQQSHHFQPVKYILPYLSIYPWNVEPNGYLYKFHFIFETVATYSLVAITSSIEPLFTLYVIQMIGQLR
EMSYVMEHLNESNDPESSVRDCINQYTNLVECRNMVQKIFGPIILWQIITNAVILCLGIFQLSQASLTIHIVL
VPS
>MpulOR62

MLTPIIFIIFQLQHGVHPITFKLVMAAKYPWSISSPSFLYFCHYTMEVIALSNAIIVTCGLDTYFMLCILQIS
WKFRAMSHRFYNMKDGDDYNATVQDCLKDYKKLLKCRDNIEKIFGPLTLWLLISSAVVLCSNMFQISKVMFMT
KCFGYLILMMFLHFLCY
>MpulOR63
MAKTSRIEQFQSLMRIVRRVLLPLGLWPLKQPNFFYRLLPFFMSTVTASAACAVINFTIAHRKNIPVMTQALG
LSLTLTTITLKLLSCNPNRKCVRILFETLSELTNELSKDTVSKHLISGKFVIGRRLSFALTYITFTSVVAYML
TPIIFIIFQLQHGVHPITFKLVMAAKYPWSISSPSFLYFCHYTMEVIALSNAIIVTCGLDTYFMLCILQISWK
FRAMSYRFYNMKDGDDYNATVQDCLKDYKKLLKCRDNIEKIFGPLTLWLLISSAVVLCSNMFQISKIKSLSVK
QIYNFALYLGARSLQLFIYTWSGSVITIESEKFRDAVYASNWPATGQRKFMTSILIMLTQKPLVISACS
>MpulOR64
MATIDKLREYRSFIRILKKMSLCVGLWPVTETNKLQRLLPTIGFISCAMASYVIMNFVLIYHADIPAMTSGLG
LAGSYMTTALKIFCVDYNREILKVLHATLDRFIDKVLNESTIRSEVLNKFSTFRKLSVAFTCFVFSSVTAHIL
APMFHMISQLIRQVEPIEYASVIPAVYPWTSSGGLLFCCEFVFESLSAYVIFFVATSVNVRFLVYIYQISGTL
TAMSYQLTNLQDNDDDYDAIVRECVKNYVSLLECCGIIETIYGPIVLWMAGTSAIVLCTDIFRISTTDDPSFG
FLCVIIAYMGAEAMQTFMCAWAGSVLTTESEKFAEAIYASNWAGSGRIKFMSSILIMVTQRPLVITVCHFSTV
SIDMFAKILNTAVSYFFLLRTMQDKTS
>MpulOR65
MMCVMINYEKYRELHNTLDHHFEKLLIDQQISSTVLNNFRNFRRLSWALIFLVTIVTLITAGRPIISIISQYL
NSGNPIIFPHFYPTLYPGFRFHFIIEFAATVPFFCVSPSMDSLFMMYIFQMSGQFREMSYRIMHIDQMGDDKI
AIQQCVHQHQTMMRCRDMLQEIFGPILLWVVATNAISLCSLIFQLTQA
>MpulOR66
MATIDELHEYQIFLQRLKNIMVFSGLSPIVEQNLFYRILPYVLFAVNLMSALATLNSCVEYQSNMEIVMKSMG
IFITTTATDLKILCVVLNWRDMRELHETLDHIFLEVTQDRRTQRHFLKRFAVARRLSTTLIIMAFLAIGTFVI
APIASFIFQWTHHIHPIRYILPLPTVYPWTISGGSYSYYFHLSTEMLSCFAIFVVACSVDALFPVYIFQISGQ
LDAMSYQLDALKADDNYDMIIRNCARKHQMLLRCRDIIEAVYGPIIIWMLMTTAIVLCALTFQLSKVGHFKNH
IDWMHIADLIRLSSVGELCFRTSFSRRGLHGLEDVADVHLRLFRHRLNFSE
>MpulOR67
MSHDKWPDYLLYRNIIRKSLLIVGLWPEEKPKILYRFLPFPFLFANTFVTLGIIFYIKMHSLNFNKAMKGLSM
MPSFLTVNLKIICVMIHRDEAITLHNLLDLHYTELLSDQRVSKAILTGSRIFRNLSWVFTLLGVIIISTIAMT
PIISLIDQQSHAVNPIHYPHVYPTKYPWSSDPLNFSHKIHFFIEYVAVVTHFCASHLDALFMMYGYQMICQFR
EMSHRITHIDVMTFDITAIKQCVQQHQLMMKCRDIMQRIFGPILLVVWSANAISMCSFIFQITQIKSISIPMM
MTSTGYVVLKLLQTFIYAFTGTCLTSESVHYQQAIYDADWQRNRRFMTSILIMLTQEPIILTACSFSVISLDL
FIKVSTLLHHVDKIKFPTFTYFQFLNATVSYFFLLQAVS
>MpulOR68
MLCIMVNRNDAHELHNILDRHFNEMVNDDTNSSAKLKFSIFRRIWGISIVCVGISLILIAAAPSISIIQQYRR
SVNPIFYPLVFPTTYPWSLNRPGPRYKIHLIIELTTVVSQFCVTSIDSLFMMYGFQMGAQFREMSHRIMHVDK
TDDVRKIIPECVAQHQAMMRCRDIIQTIFGPILLWVMTTNAISLCSMMFQLSQMKSISIPTILTFGTYITAKT
LQAFTYAYTGMILTSEVSLE
>MpulOR69
MATANTFSEYKMFIKPIKSFMFFAGLWPVKNPCLFYRLVPTLMFALTLLSSCMIINFIVAHRSSILIMTQGLG
SAVTFITLTLKTFYCDPNRRCVKALYETLNPVTNELMNDPVMKNLISGKLTTVRKLSTVLTCLILSSVLAYIL
TPIIYIIYQYIHDIHPFKYRLVMPARYPWSISGGSFLYFCHYTFEVISFFNCFVVTAGVDNYFMACMFQMSWK
LRAMSYQFRNLGHDANYDTAVHECIASYRMLLKCRDNIEKIYGPLILWIFVSGAVILCAIVFQLSKIQSFSIV
RICFFAFYMGARLLQLFIYTWSGTMLTSESEKFRDAIYSSNWPATGQRRFMTSILIML
>MpulOR70
MMTVYREDLRGLRETLDEYFETLIANQDLMKKVLKGGAIIWRLSSVALTTFFVGSVVNVFTPIMAITKQHKLH
IHPIKYFLPNGSVYPWNVTPGGLLWKFHYVCETFSCYTLYAIANSVVSLFCLYVFQMISQLRAMSDRMLHLDE
SSDPDSIVRDCIHRYETLLKCRNDIEKIFGPVVYWLTITNAISMCLAIFQLSQV
>MpulOR71
MTTPRKLHEYRAFTQSLRKMMYSIGLWPVVKPTLFYRLLLIMAFIASFVACFVIFNFVMAYHSDIDTMTKGMG
LGGSFINTMIKILCFAFNRGDVKELYETLDNIFNELLKDTTVQSLILKRFTAVRRLTNIFSYLVFLSIATYAV
TPILHIISQHIHHVHPVIYPLALPAIYPWPISNGGLLYYFHYLFELFSAYTLFLVTCSVDGFFPIYVFQMSGL
LKAMSYRLNNIKKDDDFNGAIRECAKKYAILLRCRDILETLYGPIVLWMLLSSAVVLCALTFQMSKLDGFAFG
RICLIVAYMGSKSLQTYIYAWAGSLLTSEVRRVRIRIEYQRWLL
>MpulOR72
MHLDPYFDKMLSNPNLKDSVLDGVATFRCLSWAVAAFVFISGNVYILAPIIFIIYQYAHHVESIKYLLPYPGV
YPWPISPNGFLYKCHYIYEIFATFSLFSLTSSIEPLFSLYSFQMIGQMREMTYRLAQIDNTNERQLRKCVLQY
TTLMKCRNILQKIYGPIILWMMGTNAIILCALIFQLTQVRSIRYIKRIP

>MpulOR73
MAATNTLREYEAFMRTRKRFLYCVGLWPIKQPSLFYQLLPVFIISMNISGACLTISFIVANSPKIPIMMKGFG
TTITFLTTTFRTFFSMPRRKYVRVLHEDLDHFTNSIVKDSATINLISGRLPLVRKLVINLTCFVMLTAVTYLV
PPVTNMINQYIHDTRPRTYRLVLPMKYPWSIVDGTASYYCAFIFESVNFFVSCMVAAAVDSHFMVYILQLSWE
LRAMSHQFSDLKDHDDYDLVVQDCIRSYKRLIRCRDNVEKIYGPIFLWMMVSSAVTMCACVFQLANIRALSFI
QIFHIGMYLGARLSLLFGYTWSGTVLTTESEALRDTIYSSNWPGSGRQRYMTSILIMLTQKPLVISACSFAAI
SIKLFPKLINTAISYFFLLRTMEDKKK
>MpulOR74
MMLSVTSQTALNFTEMSVYFVSSWPPTKFVNKQQKLWFNFRWWLAFTLLLLFLLPLLNGVYVHHKHPVKMINA
ACLSAATVQALLKMLICRAQWPRLQMLHSEMDKFIKNADDLENKYIRKYIERGYIFHGFVTSSMYLVSLVFII
EPALQSHQFPTDSVYPFRIQHEAIHIALYTQQIVALFLVAAALSVDFQVAILLWFIGIKFEILGDLCERVSTN
LELDKCIIEHQRILWYADQVQRAVCFITFSAVATTTLGVVCGCLALLGNLPLAVKFRVANIVGNAATEIFIYA
WPGDNIITVSQEIGWKIYNCNWLAGSPKMVKNVMSIIQRAQHPVTISISGFIPSLSLQYYTSFLSTTFSYFTT
LRIILSAAG
>MpulOR75
MNTPVEKLQTKRKSIEQSSLEDLEWAISLNRVILQGLGLWTYPNESQLRTALSHIHLGMIGVCMCFLIVPQTM
AMFKVLSVPMLIVDNVGHNLPFLSAEFKLALLWYNKKDVAHIFELIREDWLMEKSSYERDVMIKYAELYKMLT
IGGLLSSLATTTMYHLPIAWNTVSRTVNNITDVPGALFAAQTVYAYDVTQTKTYRLTLISQYVGTVFASLSYT
GVDVLFSMFVMHGCGQLEILAGRIETMASEQPNLKHRLKNNVERHCSLIE
>MpulOR76
MSQSTFVTPTYQKYSYCPYLSHLLIMTTLLPVSFFILKYIGLWQPVQWTGWRAHLYTLFSIFSVIILYTDTGS
QVVDALETSVTVSDYADHSFILLTMIGICIKMGSILKNRKQIIDLVTALQSGSFQTRNSAEDEVLREFEYIMK
WRTILFGIFVQMGTTNLIVRSLIYMAPYRITITKLYLPWDTKTAIGYWTAWLFQAISRWFGGPVNVACDSLVS
GVMYRASAQFKILQIRLRDLTKPVASHETKDDDVKQWETKKMMELVEEHLEIIQIISKLNDIFGFVIFMQYCV
SSTVLCVTVFVFAQVTKFDGYAAMMITYTGCLYFQVYLLCSAGSEMAYQSSKFASHVYASDWDRLQISTQKSL
LLMMMRSHKPLKFTSFNIIELSIDSFSQIVKFSYSAYNVLAHGQ
>MpulOR77
MSSLSYNIFVLRCLGLWYSDNWITGFKAKVYVGYTIFIFTVVYTFTLSHLVMLYQCIHSADDFADASFMLLTF
LVICRKMFNIVHNRKLIAELVASLKNDPFRPQDEVESDIDKEYTWKTKLYTLVYGILSDSTCTMLTIASILRD
VPNRTLPFKAWLPFSIATPTAYYFAYVHQTLGHYMGCNVNVGFDTLVPSMMMMTCAQLKIFKHRVQKIHQTLK
RMKNSSLVEYSVQDQERMEKELVADCAKHHIAIYQFAKFTNQSFDVSIFLQYCASSLILCVSTWTLSQLKPMS
QEFFSFVLYLVCMLTQIFVFCRYATEVRLESEKISDAVYETDWTLLSVRAQKSLVTIMARTLHPIEYKTGHIT
LSLDSFSRLLKLSYSVLNVLQHSS
>MpulOR78
MSGSRQTSNMKKGTNEAKNVASDIDAQYAFQINQWLFEPLGLWPHARTSIIDKMFRAMRIFACLFLLAFVTIP
GFFLFLQVRGVAALTGPFILYVMSSFKYVLLILGQRDFLNCLETIFADWHKLNSTNERNIMIVSAKYGRMATV
ICATCMYSAWILYAGIFPRISGITVTADNETIRTFAFPGYYKFFDPQKSPAYEIIYYLHCCCAFVMLSITCAT
CSLAVAFVMHACGQLEITITWLKGLVAKTGDGEDSNIRLSMIVQHHVKTLRCKNDRISND
>MpulOR79
MDAELIPSLKYASQTTPERSGIDSDVEAHHALQITRWQLKLLGIWPLSLNSSGFEKMFTNVKIILCSFLLVFI
IVPGGLFTYVIIKDFNVRIKLTGALSFCVMAIMKYASLLMRRQEIDNCIRHLLFDWRQVTSANSRIIMKNCAE
FGRWGSIICAIFMYSGGIFYAVILPCVTPSTENEQNVTIKPLAYPSYYVWIESQENIAYIVIFSTHCCCAIVM
HSITSTTCSVAVVFSMHACGQLKIVTSPLSKLINASKKTVRADRILPEVIEHHTNTLRFIDNIEKILNKSCLV
EVGGCTLNICLLGYYFLLEWEDSDAIGIMTYTILLMSFTFNIFLFCYIGDLLTEECRKVGDAVYMIDWLEMSG
NNIRQLILILITAQNPITITAGKFIDLSLVNFCTVIRTSLAYLSFLRKFIE
>MpulOR81
MTINDEKNAILQKWVDRYIPIWIFGIILFSLTAISFSFGPFITSELLPATTWYPFEIKKFTMRHYFFFIQHVI
AIFQTGLGITTDITISFLFCYVSVKLEILKNLISISKKSEAIIECFNEHKKCIQFFKQLTITARLMMLKNNLL
MMITIICGAIPLLSNAPLTESSQFISIVATSYLRIYMNVWPADDVREMSLNTAAAFYECPWIDAPKSVQRSIN
FAISRAQKPLIVSIPGLLPPITLQFYANVNTNFYATINNHRIEFL
>MpulOR82
MLFFLYYMCFVTLLYLIGPLLPILFDQIKPLNQSRPRVILFQVEYFLDEEKYKMPMIIHAFIFTPLPTTVAVA
YDVMFATSIHHASSIFRIVGKRLQFSINDMERHGPKMDILLVQTELRRALVESIRLHRDILQFVDLLQAIFST
SLFIMNILTMINLSMTAYQAVNNSGNSARLVGFASYAIGQLIRLYFRSWHGQMLTNSSESVFDSVYQADWYDL
PFRLRRFIILILARGKKQSKITAGKLFDMSMENFTFVVRTAISYFTVLRSMQ
>MpulOR83
MKHAMSCENIDFFNHLNWRFTKHLLSCFGAWPFQTLRLRRVIRSVVFILVSSLFIPEILRIITVRKNANLLVE
CVPIFVFHFLTVTKMLQCLLNLKKIEQLLIQIRRDWESSLSSIEAEILNSNGQQNRILIHTYVYFIYSVTVVY

MSTPMVPKLFDFIIPLNESRPKVNLYETEYYVDSETYELPILLHSYIITPFPTTIILAFDTLYVSCVHHGSIM
FAIVGFLCFRHRLQNLSGQKNEQNTWENGKTDGTIRNYHSLANCIAKHKEVIEYAELLESTYTTSMIAIVGMN
TLAISITGLQTILKINEPSEMIRFGTYTVGQVVHLFFLSWPSQKLIDESERIFQSTYQGEWYALPPKLRSCYV
LIMMRGGKPCALTAGRYYTMSLQSFSQVVKTAVSYCTVLLSLQ
>MpulOR84
MPQTSIHYTQGFKYALGWNRFNLSCVGAWPDSSDGFFGKHNSLLCGFWILIMVYLPRSASIIIFWGDMDAVIT
CLSINGPLLVTIGKLWIMYYHREVMRMVIAAMAKDWAIPLGKAESEVMWRIARISRGISIGSASLTNVLFVAF
VVFEICLGMQLKNRMELEPRSSIGALYPAYFPYDTRRLEYFLPTWMGQCLCTGLAMIAYAAFDSVMALMVLHV
CGQLELMGISMTDLINASTRMNRQEFSWRFAELIKRHEELNRIAKLIENSFNYIFLPQMVVCTISFCFQGYTM
LSVSDVYENYHGHNIIRLI
>MpulOR85
MGCLQKEKNESKFSDINKRRLLIVATKQTRWIFSLLGVWPMLTKNTKNIEKRLSLVIQLICQSFLFFIIIPAA
IQLYWCEKNMKIKLAYFGALGFTTACAVKYITIVYRRREIRECIEQIEKDWHEVVSEETNQIMLKNIKISQRM
TIICGSFMYCGGVLHQTMIPFLPGSIVSIVDNSSRRLLVYPIYEVMFDDQITPTYEIMYLIHIVTGMIVCTIA
IGSCHLSILFITHVSGQNQIMISKLKNLMNTRDETIENMNQCMREIIYFHHEIIKFSSNVEKCLREICLIAIF
EALLIICLAEYYSMVAWKHNETFSAIIYFILVDAFIFNCFIFCHVGEILQEQFRKVGEAVYMTEWYRLSHRNA
RALMLIIAVIQHPPKLTAGGLIELSYNGFVSIIKSTAAYFNILRMTEF
>MpulOR86
MIKNVPCIRWELENILRLMSLWPDSKNKLACLFIGFSWIITIPFQLMYFYELSNNVTELMSSLFDVANEFALL
FRFIVAWIHRKILISLVVEMSEDWKQSKLKETRETRTAFLFTRGDLYFYCGAMLLLLPQVVINYFTNGVDKRE
FILNTIYPFEDHQSPVYELIIVTQFIHIILVGCAEFFLQALMISVVCHLGVQYNIMNEQIIKFIADAQKEKIL
EREKNLMAGLLVQKHQKNISFAERIEEAFVFISLFQCANSVASICCCSLVFVMTDDPIEILRFSVFLIMRLAQ
TFVLCYAGEYLTDKNSLVADIVYHSDWYELHPKYIKILQLMMVRAQKPLVLTAGKFVVLTSETFTVILKTSAS
YISVLRVIYK
>MpulOR87
MRGKSKNSVTSTKPISRWENDIKNSTKISRWILTMLGIWQINRHVSIIRRILADIRVLICYSLIFFLLIPCAL
HTFFEEKDPHRKVKLFGPVIFYSMGMMKFSFLVARRKNISDCLDHMLVDWKRNSSTEDREIMIANAKISSFIA
TVCTIFSYSSAIFFRLIIPLTMGKRVTANNITIRPLGSPVYRPLFTAIESPSYEIVFTTQTISALLLYAVTVG
ACSLAAVFVLHACGQLRIVMCRLDSYVVGAERADDILERRMAEIVELHLRVLNFIRRIERLLNEVCLIEFVAS
IINICLMLYYMVTEFTKAETIAVIGYCVIVISFTFNIFILCYIGELLNEQVKNVGSTAYMIDWYKLPEKNAKG
LILLLAMTNYPCRITAGKMAELSYRSFSGVLKTSMGYFNLLWKIAS
>MpulOR88
MMADKSKTLWSIPQPTNESNHDIKYCPAVSRGILTTIGIWSIDQQVSIFGRIITTTRVVICYFLMIFLFIPCA
LHTFLEEKDQHKKIKSLGPLIYYLTALAKYSFLVARRNDINQCLNQVSGESQHGSSFTDHKIMLNSAKLSRYI
AVVCIVFTYSSSVFFRVIMPLCAGKRVGPNNITIRPLGTPVYKPLFTADESPTYEIVYTLQTMSSLVIGSVTI
AAGTLTAVLVLHACGQLQLVMNKIDSFVERSERVDDLFENRIAEVVKHHYRTLSFINSVEVTMNELCLVEFLG
CMIEICLIGYYLLTELQRAETIGIITYCVLLISFTFNIFIFCYIGEMLTQQGNKLGLTANMIEWYKLPGKSAR
DLVLLLAMTNSPYNITAGKMAGLSYKSFSDVLKTSMGYFNLLRKVV
>MpulOR89
MKNCTKMNRWFLIPIGIWPIDPDANVVKLILSETRVVICYLLILFLLVPCALHTSLNEKDPRLKMKMIGPLSF
CLMAISKYCFLVTRRHKIRKCLNHVFIDWRRVNNPADREIMLTNAKIGRFIASLCAVFMYGGGFFYHTIMPIS
AGSFVASNNITIRPLTYPVYDPLFAAHSSPSYEIVFVTQWFSGLSCIALQLERAAWLLCLFFMHVGN
>MpulOR90
MITKIPCISWEFEYILRIVSLWPDSYNIPVAVILLLAWVTILPFQFIHMSEILDDVVELMTAICDFSSEVALF
IKLLIAWINRKVLMGLICEMAEDWEKSDVKVTNETRVSCFITRLDLYIYCGAITMFFPKLLIAYFTDSPKNRE
FVLKTSYPFEDYKSPAYEIIIVLHIVQGLLMAAADLVPQAFIVALVCHFGAQCNILQQQLKEFLAIAAKDGAM
NKKNFQQVGELVMMHQKNINFSDRIEEAFSTISLIHCLCNGVAIGCSGLVFVMSRNPFDIGRFSIYLVMKLVQ
TFVLCYAGEYLSNKSISIANVIYESNWYDLDRQYIKILQLITVRSQKPLQLTAGGFAILSSGMFAVILKSSGS
YMSMLRATY
>MpulOR91
MSALDYNFAILKYVGIWRPREWSTGWKYRLYNIYTSFVILIVAVMATAELIDLLLTLNDIDHLAESMFMMLSL
ISVCGKSVTILISRDRIIKLLDILKTEICQAKNPDEDVAQEKFYRRIRFRVKAYSLLVIVTVIVMTIGNMLLI
VIDRILPIGFWTPFNMSRDSVYISVISEEFCGLSITAFVNMSYDTLVPGIMLYSCAQFCILKCRIRNVATIYM
KLEQTNAEQEKVTRDEISNCVRHHLLINQFAEEVNSIFGSAISLQCICSLIIICMSIYKLGHIKGINTEFAST
MVYSSAMLLQIFMLCFAGTELTHESQHFGNAIYDADWYMLSQSNKASMLLMMVRSLRPIIFTSASVIQLSLGF
FVQLIKLSYTTYNFLQQMSV
>MpulOR92

MSSVSHSIFLLRSFGMWYCDDWLTGWKAKLYIVYTVFIFFVVYSFTLSHLLMLYAFINDAREFADASFVLLTC
IATCGKMFNMISKRKTIGALLRTVQEGLFRPRDSEEFAINDESIHRTRLYTCLAGVLAELSCTLLTVLSLARD
FPRRELTHEAWLPFSTATPVAYTIAFLHQIYARYIVASINIGFDCLIPGIMIITSTQLAIFKHRVRKIHDVVE
HARNSYNDTFHLKDKVYMEQKLIGSCVEHHCAIFQ
>MpulOR93
MPRIRLPDEKSTSKKEYSDLTEATKQTRLIFSLLGLWPMMGANIPVIEKYLSFIIHTLCQSLLAFVIFPSFFH
LFCREKNPRMKIALFGPVGVAVGCALKYIAIIYRRGEIKRCVEQMAVDFDEIENEKDHEIIMKNVKMARIMTT
LFAIFMYTGGVSH
>MpulOR94
MGSAEKVDLSTAIQQTRWIFSALGIWPMMTSNTTTLERRLSVPVRIMCHSLLGFIVFPAFFHLFWVKMDVKMK
IALLGPVGFAVGNLLKYLAIINRRKEIRKCIEQMESDWNEIQEDENRKIMMENVEKGKKVTTVCAVFTYTGGV
SHQIMVPFLPGSIISIVGNSTKRMLVYPVYDTMFETQYTPVYEIMYLYHVFTGVIICTICIGACHLSILFITH
VSGQNEILILKLKTLLDNHTEKEMNINDRIGEIVEHHRALIKFSYEVETSLREMCLICIMEALYIICTAQYYS
MTAWRNNESFGALIYFILVNAFVLNCFIFCHVGELSKRQFYELGEAAYMTDWHRLSLRNAKAMFFIIRITQKS
PKLTAGGLMELSYNGFVTVLKTTAAYFNIMRMVEF
>MpulOR95
MWESTRSDNNITAKMARAAKYSKTLTKYYTIGVCFMCSLYIIYPYSVTSTAYFHKEFNSSIEYSANVFPFDIP
YKINSFFIFVISVTFQQIGAIAVCVIWASCDTLFAQLTCHVNIQFTVIANELKVIINNYRDLNDHRTAETKLT
NIVNRYEELFMCCYSIQMFFNPIIFFTIISNGISLCACAYRLNDNVINARWGELPKQTLALLSTSVQTMIYCT
FAEALTESSGAVKDGVYNSHWIHCNRKLKKALIIIMIRAQKEFKYTAYSMIDINYNQLTQIFNGAMSYFMLLR
NLS
>MpulOR96
MLCRQRLHQAVNNTFDYSVSVFPTFYPFTITSFPGFLIFSSYQQLTVILACMYWVSCDALFLQITTHICLQFT
MQAHYLDDVIANANTYGNGADLKHQLRDIVKKHNELFAFCYEVQMFFNPVIFLTILSNGINLCCCLYQMDQQL
VTHEWAEITKNMFHLCSLIIQTLVFCGYAENMTQSSIQMGYSAYHSQWIELNKETKLMLLLIIMRTTKQYNFT
VYGMLSLNLNRVTMVCILNFSDYFEQRPV
>MpulOR97
MKVLTESVQVLCFIGLWKTSTWSGWKSNFYNMYTMSIIFLVGGFTLTQLLDLLFISKTIDEMIDNISTSVTIA
VVCVKFIGIIMKSRELEKIRLDFVTGWLKPTTADEISIQEKYNRFTKITTLITVGVYEASAIVVTAGRIIEVN
LNHTLPYRAYLPFDYSKPAVYYSSVAVQSIIICIAIIIDSGFDTLFFGVMIQISAKIDILKYQFRVAVEALEK
MCEEGANNSKQYRELERRYLGNWICCHNAILSLCSYVESVFSKVIFVQYSVSFLILCSVIYRMARTPMLSVEF
LSGVLYLVSTGSEMFMLCFFSNQVTLNLADLCRTMYDTNWFHLCVPTQRKFVPMMTRTLKPVVFTSGHVVTLS
LESFTAVSKI
>MpulOR98
MEIENSEKNGAVSFAELLEWDVYILRLMGMPSMIGIFSQRELNGKWWEGGPVLIICGILITICMLQVHTISRV
FHDEFSYVVVIGASIVPDLTSIVKIIRLWTKRVELYNILQGIGVMWEFTRSTHSITTEMAETARRTKLLTKYY
AIGVGVMCIVYMIQPYSLTLADYSNREINDSFTYSANVFPFNFPYKIDSFTVYIISVTYQQLGAVAVCIIWAS
CDTLYAQITFHVSVQFMVLANTLQEIVNNCGNSCVDRKIEIELKNIIRKYHQLFTCCNSIQIFFNPIIFFSIM
SNAISLCCCAYRLNTNVTNGHWEEMPKEIFALISISAQTMIFCICADTLTDSVIMLTFLAFY
>AfOr1
MGEEIGNTTNYRNIHYKSDAEYTVHVAKTLLTLIGIWPRRNTFVDNVKFYVQIGIVFFLMCFLLLPHVIYTYF
DCENLTKYMKVIAAQIFSLLAIIKFWTIIINREEIRFCLMEMEIQYRDVECEEDRSVMMNTAKIGRFFTIVYL
SLSYTGALPYHIILPLISERIVKEDNTTRIPLPYLSDYVFFVIEDSPIYEMTFVLQIFISSIILSTNCGTYSL
IASITMHCCGLFEVTNRKIETLCKWDNRNLHDRVIDIVQSHLKAIEYSARVGESLSIVFLSEMLGCTVIICFL
EFGVIMELEDHKTLSTVTYFVLMTSIFVNVFIISFIGDRLKQESERIRETSYFIPWYDFPTEVAKNIKIIILR
ASRPSSLSGAKILDLSLQAFCDVCKTSAAYFNFLRAMTV
>AfOr4
METKHAEKDLKQAFYAQSFLKIIGAWPIAIESPLSSKIQKWFIISFCICLQICIVTPCIFEALLKEKNGKRRI
NLFMLLINTLSQVFKYVITLNRANELRIAIHEIKKDWLTTTSEDRFIFVTNSRIGQRMMLIMAVIMYSSGLGY
RMVLPLLKGKIILPNNVTIRLLPCPTYFTFFNELVSPYYEMIFILQLLARFFIYTILNGTVGISLMLSLHMCS
LLKILTRKMIDLADGSIISEKIMQQKVVDIIEYQIKIKRFLSNTELITQYFCFYDIGSSTCLICFIGYSVIVE
WENNNIAAIMIYFSGLVTCIFMIYIICYIGQLLLDESNNLAQTCITLNWYRFPKKKARYLILMIIMSNYPVKL
TAAKVVDVSLTTFTDVMKAAVSYLNILREVT
>AfOr6/7
METKYVEKDLKQAFYAQPFLKIIGAWPNAIKSPLSSKIQKWFIISFFLCLQICIVTPCIFEALLKEKNAKRKI
HLFMLATNTVNQVFKYVITLNRSSELKIAIREMKNDWLNATPENRYIFITNSKIGQRIMLIIVVTMYASGLGY
RMVLPLLKGKIILPNNVTIRLLPCPTYFTFFNELVSPYYEMIFILQLLAGCFIYTVLSGTIGISLMLSLHMCS
LLKILSKKLVDLADGSITNENIMQEKFVDIVEYQTKIKRFLNNTELITQYFCFYDISFNTCLICFVGYCIILE

LENHNIVAIVVHFMLLGTCIFVTYIVCYIGQLLLDESNNLAKTCITLNWYHFPARKARFLILMIIMSNYPVKL
TAAKVVDVSLTTFTDIMKAAMGYLNMLREVT
>AfOr8/9P
MVQIRNAREGIKHTFWFAYPLSRMLGYWPLSAPSSAFSKILNSFTIFFSYLLPLVVLIPGLLYVFLKERNGRR
KVKMLMPHINSIAQMTKYTIILRRTKELGKLLDEIKKDWSTATQENRQXFSARASIEHKLTTVIAITIYGGGF
FYRLILPLSKGRIVLPNNVTIRLLPCPGYFGSLNVQITPNYEIIFTLQILGGFIIYTALCGVKSSCLMLCMHM
CGLLRILTNKVMELTNDKDERVVQEKIVFIVQYQTRIKEFYNYVDQFVPYVYFIEMIVGVSITCVLGYCIIVE
WEDSDAMAIIAYIVLQITCVFGTFSICYAGQLLVDESENVRQACNTLKWYRLPTKKARSLILLIIMSNYPLKV
TAGRIIDMSLITFTSIIKSAMGYMNILQQVT
>AfOr10
MVQIRNAKEGLKHTFWLAYPFSRMLGHWPLSVSSSAFSKILNSFIIFISYLLQLIVLIPSLLYVFLKEKNPRK
KIKLLMPHVNIIAQMIKYTILLRRMKQIGKLLDEIRKDWSIATEENRQIFSATASIEHKMTSIIAVTVYGGGF
LYRTILPFSKGKLVLSNNVTIRLLPCPGYFGSLNEQVTPNYEIIFTLQVLAGFIIYTAVCSTKSICLMLCLHM
CGLLRILTNKVMELTNDNDERVVQEKIVHIVEYQMRIKEFLNQLDQFIPAICLIEVFIQVLIMCIIGYCIIME
WQENNGMGIITYVLVQMTCLIGAFSVCYVGQLLFDESENVRQAYNTLKWYELPIRKSRSLILLIIMSNYPIKI
TAGKIIDLSLVTFTNIIKTAASYMNMLQQIT
>AfOr15P
MSRTRNAEDDMRHTIWFAYMLLGKLGAWPNRATSSTFSKTWKNCILIFMCYSVQLVILIPGLLHFFLKEKDSR
RKVKILIPLINGYLQLCRYSLVLRSTNKLCYLLNEMKKDWMNISEEDRLIFRKKASIGHKLMSVVAITMYSAG
LGYRTFIPISKGRILLPDNTTIRLLPCPGYYIFFNEQITPNYEIVFTLQVIGGLLSYTIMCGTTSMCAMLCLH
ATSLLRILVKKXNELTKQSDINESAVHIKITDIVRFQTKIKQFLNDVEHITTYLFLLEIIDETGIGCVIGYCA
ITEWEDSDATAAIIYLLLEASVFGVTFTMCYVGQILIDEGNNVRRMSITIDWYRFPAKEARNLILVIIMSSYP
VKLTAGKVVDISLSTYTDIIKATMGYLNMLRKVT
>AfOr17
MKNISGIAKAEEDLKYATRFVKPIMGMIGAWPISPSTSFFSKVLQRSKHIFTYFLFFLIMIPTLMYVFLKEKN
NKMRLKLMPPIINCSIQFFKYTIILWRRKEIQEGLYAIKHDWIKATEEERLIFRSKTKIGRRVVLIVAFTMYG
GGLCYRMILPLLKGTIVTANNTTIRALPCPSYFLILNEQQSPIYEILFVLQIIAGIAIYAVICGFCGIFALLV
LHAWSMLRILVNKIKKLVDKSDMSEVVLQRKIMDIVEYQMKIRGEFLKNIETITEYICLIEMIGSTCMICLVG
YCILMEWENTNTMAIVIYITIQISIIFCVFILCYIGQMLVDENYIVSQASSTVNWYRLSIKNMRCLILIIAMS
NHPMKLKAAKMMEMSLITFTDIMKVSMGYLNILREII
>AfOr36/37/38
MTDDIAAIQKKFGSLNEYSIQLNKWLSKTIGVWPLPSSTSKFEKIMTRILILLCWIIALFDTMSGLLHFALVK
EDIIVKLKSLAPISYIMGGGLNYAVLLLRKNDILYCIEHMETDWKTITRMTDRQIMVKNAKIGRIIACCIAGF
MQIGTLCFCIVLGVFKRTIKIGNESMEIYVLPSPTYKIPVDTNPGHDIILGVQFLAAYITSATVVSAFSFAAV
FACHASGQLTIMITWVKEFINRPQEKDKNICIDEISVIIEHHMRILSFLERAEHLLCPICFMEMFKNILSICL
FSYCILAEWSEHDIGVLCTYTVAVINITLNTFLICYIGEVLTERCKEIGNMVYMTNWYRLPKKDILNLIMIIT
RSSVEFKMTAGKIIDMSVITFGNIIKTVFGYLNILRQTTML
>AfOr41/42P
MADDIAAIQKKFGSLNEYSIQLNRWILKTIGVWPLXTSTSKFEKIMTRILILLCWAITLFITISSLLHFTLVK
EDIISKLKMLGPISYCIGGGLCYAILLLYKDDIRYCIEHMETDWKLITRTDDRQVMFKNAKIGRIISGCIASF
LQGSTISYCTVFGVFKQKIKIGNESMEIYILPFPTYKLPVDTNPGHSIILGFQFLTACIMSATVVITFSLATI
FACHASGQLTIMITWIEEFVNRSQGENKNVRVNEISLIIEHHLRILSFLERTEQLLSPICFTEMFKNILTTCM
LGYCILVEWSGRDIRAITANSFTITNLYLSMFLLCYIGEVLSDKCKEIGNIVYMTNWYRLPDKDILNLIMIIT
RSGVEYKMTAGKIINISMITFGNIIKTTFAYLNILRQVTIL
>AfOr47/48
MTDDISAVQKKFGSLNEYSIQLNRWFSKTIGVWPLSSSTSKFEKIMTKILIFLCWNIALFVIISGLLHFTLVK
EDIVSKLKTLGPISYCFGGGLNYAVLLLRKNDIRYCIDHIETDWNAITRTKDRQVMLKNAKIGRIISGCIAGF
MQVDSICFCTVLGVFKQTIKVGNESIKVYVLPSPTYKIPVDTNPGHGIVLGLQFLSAYIVNATIVISFSLATV
FACHAIGQLTIMVTWIEEFVNRPQEENNNVCIDKISMIIEHHLRILSFLERTEHLLSPICFMEMFKNILSICM
LSYCILAEWSEHDIRILSTYTFAVMNITLSTFLICYIGEVLTERCKEVGNIVYMTNWYRLPDKDILNLIMIIT
RSEVEYKMTAGKIIDMSVITFGNIIKTVFAYLNILRQMTIL
>AfOr54
MHDRLHHNIESQLKNSYYKSDIHYTLQMCQWLLKPIGVWPLICNQTNKFEQFVSIILMIMCFSSLLFIILPSG
HHYFFIEKNLNMKVKALGPVSFCVSSTIKYCYLVLKRSSFEKCIEHMKRDWMIVQDPNHRIIMLKYATISRRL
ITICAVFLYSGGMSYHTIIQFLSKRKNNDNHTIRPLPYIGYDPFFDTQSSPTYEIVFCIHCFTAMMMYSISTV
AYSLAAIFVTHICGQIQIQIARLQDLVENKERKKYESCDPFALIVRDHVEILRFSNNIEEALREICFTEIIEC
TLNMCMLEYYCLIEWSAGDTITLLTFFTLLTSFTFNIFIFCYIGEILTEQCSQIGTVSYEIDWYKLSPKEAYN
LILLISISQYPPKLTAGKIIELSLNTFSSVAKTSVVYLNLLRTVTNW

>AfOr56
MHFSIRNPINEPRNPNYEKDIAYVTKYNKWVLSCIGIWPIVLKNTNKILPKIVIGINNLLCSFILIQSALHII
YEEKDVLLRLKILGLIFFSLISLMKYWALTIHKPEIKYCIEQVQLDWKQVEMENDRELMLKYGMLGRNLTIYS
ILFMYIGSITYMSITQYAMGLQINEHQSNQTIRVLIYPTYGYNIQKSPIYEIIYGIQFMCGYVVDTITSGACG
LAALFVTHACGQIDIITSRLDDIVAGQFYNKNLSPNIRLMAIIKHHIRILKFSAVVETVLQEVFFLEFAGSTF
VICLLEYYCIADWEQKNIISLTSYILLLISLTFNMFLLCYIGDLLIQKSGNIGVVVFMIDWFHLPTKTIQNLI
LIMAMSNNPAKLTVGRIVDLSLSTFGNVLKTTFVYLNFLQTAVMQ
>AfOr64_2
MKTTSNKDFAYAMTPLKFLSWPLGTWPLQVFDTFSIIRAMFTTFLVLLMLAILQVELYLDRSNAENNLDALVL
INGGILAVAKVMCFHVRPVGLISNFTSAVKDYNELNSEENRVIMRRHAYMGRVACASLIFCSYVGSTLFMTVP
MLAGDEEEVINVTEESAIKYPMPSENTLTLINIPENMYFVIFIVEYLMLLLTSTGNLGSDSLFFGIAFHLCGQ
VEILRLEYNKLSNENERTTKHITLLTKRHIYLLKLSDMLNETISSILVVQLFSSCVLICTTGFEFILALSIGN
IVMTIKTFIVMCVLLIQLYLYAYAGDALESRTEEIAQAAFHSFWYQSRGRTARDLILIICRGNSSYHVTAGKF
VFMNIFTFKEILKSSTSYLSVLRVMMDT
>AfOr65
MKTTSNKDFAYAMTPLKFLSWPVGTWPFQVHDTFSLTRTIFSVSLLLLMIAIVQVELYLDRSNAENNLDALLL
INCGILAVAKVMSFRVRSIGLVSNFISAVKDYNESNDEENRVIMRRHAYMGRVACASLICCSYICSTLFITVP
MFAGDEEEVINVTEESAIKYPMPSENTLRLINMPENMYFVIFIVEYSMLLLTSIGNLGSDSVFFGIVFHLCGQ
VEILKLEYSKLFNKNEKITEHFVLLIKRHIYLLNLSKMLNETIGTILLIQLFSSCVLICTTGEYSFQFILALA
IGNIVLTTKILLIMIVLLIQLFAYSYVGEYLKTQTESVGNSVYFCTWYYMPKNISKDIIFIIMKAQRPALLTA
GRIFVVNMETYLSILKTSMSYLSVLRVMVNS
>AfOr66
MKTTLNKDFAYAMTPLKFLSWPVGTWPFQVYDTFSLTRTIFSILLLLLMIVILQVELYLDRSNAENNLEAVLL
INCAILAVAKVMGFRVRSIGLVSNFISAVKDYNELNSEENRVIVRRHAYMGRVACAILIFCSYAGSTLIMTMP
MLAGDEEEVINVTKESAIKYPMPSENTLTLMNMPENMYFVIFIVEYLMLLLTSTGNLGSDSLFLGIAFHLCGQ
VEILRLEYNKLSNENERTRKHITLLTKRHIYLLKLSNMLNETISSILVVQLFLSCVLICTTGFEFILALSIGN
IVRTIRICIAMCVLLIQLFAYSYVGEYLKTQTEDLGNSVYFCTWYDMPKNVSQNIIFIIMRAQHPVLLTAGKF
FVVNMETYMSILKTSMSYLSVLRVMVNS
>AfOr69
MQLLRTIYHLLTSCACWRPPFLSPLKNLAYTVYYCYVILLIYGATFCTFVDLLLIVETEDEFCDNFYLTLAIF
ISCHKMYSMLVNRENIILITSMLESKPFQPETEEEMNMRNKCDKQARLNAICYAILVELSVMSLSFGGLLKAE
SHKLPYRMWLPYNYTSLSAYTFIYTQQVVSLIVSAMIHVACDSFIWALLMHICNQIEIFNCRLKKIKDHEKNE
VTKLCIHYHNLIYRLATTINEQFKMVIFVQFTVSTLTICVNLYILMGTQITFERIMQLAIYSSCMLTQIYIFC
WYGNEVKLKSLDISDMIFKLDWPDLDNTTKHDLLMIMMRASYPIEMTSVHVITMNLDSFVILLKTSYSAYNLL
QSNRE
>AfOr74/86like_1
MLYTVASHIYALYINITDIYHSWGDFNYCMFIISNLLIVILSLSKFIILSFRRREFVKIILYAQKNFWHYKYE
NYEKLLFMKCQTFCKFWTIIAYFFVQSCIFFYVITPFYVNYGKNKSERTLPVRMWIDIPLSTTPYYELLFASQ
LIALQQIGLTYLSNDNYLCVLSMHLNYQFRILQHRMLTIWSNIKEQKDIVSCTEKCYKALKKCIKQHQSLLKY
CKKLDYVYTLTILNHVIVFSLIMCIAFYEILIADVPLTTRFIFVFSGIGILVHILFFTYICGELIEESGNVGL
ATYSGWWTTLPMNKTGKMLRNDMKIIMMKCMRPCYLSAGGFFPVSLETSTSLISSTVSYFTLLRDNVEESNEM
RPLISNLYIQRINAHKRHALTYQPLSGR
>AfOr75P
MRRPQDKDISIIWTSFLMKIVGLWLAADRNEQRCRDFALIYTVGTLFISVCIAFRDIYHSWGNFSDSVFICCN
ILYVAIVLLKISVLYTHKVEFFNLITFTQRNFWRFYNDPQEVLIIAGCKRICNFSIVLIVFCAQGTCAGYMVT
PLIENIGKNESDRALPFNLWIDFPVGLSPYFELLFIIQILCVYHVATCYICFDNLLCIVNLHVAGQFRILQHR
LKNLVADEGRGDTPRYENCCYEKLRDCVEQHQTLIEYCKRLENIFTVMVLGQVMFXAVVICLVGFQLFLAETP
ASKRASLVVNLGGTFFQLLIFTYSCDNLIRQSVNVGNAVFSGPWVNLPMNKAGALVRKNLIIVIMRSQKVCCL
TAGKFFPVSLETSTAVLSTAMSYFTLLKQSSLDADNI
>AfOr76
MKSKEVRDLSITVTAFYMKIAGFWTSNNYVEERRRNAIMSYTLFTIFFAVATEARDLYFSWGNFSDSIYVTCN
IITVALVLIKLLTSFVYNEELLAVIRYARTNFWHSNYDAREKVIMNNCQRTCNYLVFVFTFFAQGTILSFILR
PILVNRGKNESDRILPFNMWLELPLSVTPYFELMFIVQVVLAYHVCVCYHCFDSLLCILNLHTASQFRILQYR
FANTCNEKCKDEVSSLRSSHEYAKLKTYIQQHQALIEYCKKLEEVFNSMIFGQVLLFSLLMCLDGYLILMEDT
PFGRRVTFAFHITGCICQLLMFTYSCDCLIRDSTNVADAAYNCLWSLLPMDKYGKMIRKDLMFVIARSRTPCC
LTACGFFAVSLETYTRVLSTAISIFTLLKRYEKK
>AfOr77

MGFLNTDDISMIMTATFMKLVGLWTAKDRHEQRARKFALIYTLATILFALWIEFTDFYYSFGDFSTCLFNVCN
IMYITMPLLKIFIIVSNKKDFFYLIFYTQNNFYKDNYNNKREQQIFTNCRRQCTIFVCFLTFSTKGTLVCYII
SPLVENIGKNESERVLPFNMWVNLPLSTSPYYEIVFTIQALSLYHVGIGYFCFDNLLCVFNLQLAGQFQILQY
KMANIADLIEEKNEKGIVSSPRFAKKCYAAFKNYVREHQALIAFCEKLEKVFSLIILWQVLMFSLIICLDGYQ
ILLPESPVRRRLIFAFHLAACMCQLLMFTYSCDCIIQESSSIASAVYDGPWPFLFETTSGRTMRKDLTLVILR
SAVPCCLTARGFFVVSLETYTRVLSTAVSYFTLLRQSTLE
>AfOr80
MEAETQLDVSMSLSIFFLKNVGVWISNDPAEKRRMKILVLITLWNTMLGAVVIFRDIYFTFMYNGDILYVITN
FLTVMISFVKIIIIMIYKKEFIDMILYMEKNFWNVKYDFQEEEILNNCRKTCIFFVTSVSTIGICAMISYAIT
PIIEYYTENLSERILPFHVWLNLPIISVTPYYEILFAAEVLSIYCVALCYFCFDNVFCILAVHLAGQFNILKH
KFAKLCDTDSKISKKDEESRLAIEHVQVLYERFKEYVRRHQSLINYCERLENVYTVIILGQVVISSVLICLFG
YQILVNDVSIGRRSIFVFLLLGSMCLLFMFTYSCNSVIVHSENIATGAYSALWTTMPMNKFGRMLRNDLILVI
ERSRRVCCLTANGFFPVSLETYTTILSTAVSYFTLLRNNLEE
>AfOr82P
MQTEGQLDISMNLSTFFLKNVGVWMSDNPSEQRQIKMLLVNTTWILLSGIVINGRDLYFTLLYDGDVLYSITN
NITMIMALIKICVILMYKGXFLNLIVYMQRNFWNVNYDYHEKEILDDCRKICIFFVSSLTIMVICAMISYLII
PFIGRFNNNESERMLPFNMWINLPLSKTPYYEIMFLVQVQCAYXYIGVSSFCFDNIFCIMAVHLAGQFRILRY
RLTKLYDVECEMYKKDSILANRMPKFYEKFKKCVRHHQALIYFYQNLESVYTRIAFGEMLVYSILICLFGYQV
LVATASFARRSIFVFLLSGSTFLLFMVTYSCNGVIEHSDNVAVGAYSALWTIIPMDKFGRMLRKDLIMVIRRS
RRVCCLTANGFFPVSLESYTKILSTALSYFTLLSNRVETTN
>AfOr83
MHTKSELDISISLSTFFLKNVGVWMSDNSGEQRRMKMLFVYTIWMLFCGTIISTRDLYFTLLYNGDILYAMTN
TITTIMALIKICIILMYKGKFLNLIVYMQQNFWNVTYDYREKEILDDCRKICIFFITSVTTIGMCTVISYLTT
PVITQSGSNESERMFPFNIWINLPLVTRTPYYEIIFFIQGVSLYYIGISYFCFDNIFCIMAVHLAGQFRILRY
RLTKLCDTENEIRKKDSQSTLAKQMYKFYEQFKKCVRYHQALIDYYQNLENVYTMITLGQVLVFGVLICLFGY
QVFVATSSTARRFIFVFLLSGSMFLLFMFTYSCNGVIEQSDNVAVGAYSALWTILPMDKFGRMLRNDLIMVMK
RSRRVCCLTANGFFPVSLETYTKILSTAVSYFTLLNNRVENASGS
>AfOr84
MRPTGSRDISIIWTSFLMKIVGLWLAADRCEQRRRDFALIYTVGALFIIVCIGFRDIYFTWGNFSDSVYISCN
NLYLMIVVLKVSVLYAHKMEFFNLVTFTRKNFWRPYRDPEEKLILAECKRICTIFVVVISFCAQGTCTGYMVT
PIIANIGKNESNRELPFNLWVDFPVGLSPYFEILFTIQILCVYHVGVCYICFDNLLCIVNLHVAGQFRILQHR
LRSLSSAEPGDRRDARKLYAKLKSCVLHHQALTKYCKQLENIFTIIVLGQVLFLAVVICLVGFQLFLMDTPAS
RKVSLVLNFAGTLCQLLMFTYSCDDLIQESVKVGNAIFSGPWASLPMDEVGRVLRKNLVIVIMRSHRVCCLTA
GKFFPVSLETSTAILSTAMSYFTLLKHSSLEKMENT
>AfOr87
MGEVSKEThtRVVGVARDSSSRGKDFALLMTAFLMKIVGLWLAESKEEQRRRHLTLMYTVVAILFGVWVQFRD
FYYSWPNFGNCAYTACNILCLIMVLLKLFVLFVHRKEFIDLLVYTHEHFWHTNYTYNELLLVEKCKRICMLCI
TLINVCAQGTIVSYVLTPIVENIGRNHSDRVLPFNMWVDLPKLSVSPYYEILFVLQVLSLYHVGVCYICFDNL
LCLMNLHAATQFRILQHRLSSLGSSGWDTGGRAFDNRDTSRWSSCIENCYATFKLCVKQHQDRITYCQRLNDI
FTIIVLGHILVFSLLMCLVGFQVLMANSPPTRRLIFVFHITGSLCQLLLFTYSCDSLIQESTNVGSAVYSGPW
ICLPMNRTGRTLRRDLKMVIIRSRKPCCLTASRFFPVSLETCTTVLSTAMSYFTLMRQSFAN
>AfOr88
MSARKVRNVSIVVTAFYMKIVGFWVANNYVEKRWRNVAMCITIFFVFIAITIEARDLYFVWGDFEDSIFAGCN
VITLLLVLVKIFILYINNEELLNVVNYAKTNFWHETNYDTHEKKIIDDYKRLCSFLVCSFTFFAQGTVVCFLI
TPVFVNNGKNESDRIHPFNMWFDRSLSLSPYYEIIYTIQVLSAYNVGICYHCFDNLLFVINLYTAGQFRILRY
RFENICGKNGGNNYHKVSKSSYCMYEYKSFKTCVQQHQALIEYCKKLEDVFSVIVLAQVLLFSLLICLDGYLV
LMEDTSRAKRVIFTFHLMGCMCQLLMFTYSCDCLIRDSTNVANAVYNSLWSYLPMDKYGKLLRKDLMFVIMRS
RSPCCLTACGFFPVSLETYTGILSVAVSWFMSLKKYEKKLLQYANQSLKGEESRTIFYHHQS
>AfOr89
MQVEPDVSVNLAAFFLKNIGLWISDDPAYERRRKVILVYTIWCIMLSSVVIGRDVYFTWFYDGDILYVVTNAL
SMMMITVKICMIVIRKEEFINLIVYMQENFWNDNYYDFREKEIFENCKRTCAFFVSLVTTIGICAILSYLATP
LIVQTVSNNSERMLPFNMWLKLPLSESPYYEIMFVYQIMTFYFIGISYFCFDNIFCIMTVHLAGQFQILRYRF
AKLCNVEDQISEKDTGSTLFYERFKAYVRYHQTLIDYCEKIENVYTMIILGQVLVFSVLICLFGYQILLANAP
SARRAIFIFLLIGAMSLLFMFTFSCNGVIEHSDNVAVGAYAMMWTIMPMNKFGRMLRKDLIMVMERSRRVCCL
TANGFFPVSLETYTTILSTAVSYFTLLRNNVEKANEA
>AfOr90
MEKELDISVNLSSFFLKSIGLWIADDSTDERRRKGMLAYTVWCTFFSTVITSRDLYFTWIYNGDILYALTNYT
SVMMILLKICVIVVHKGEFINLIVHMQQYFWNVNYDSREKEILNSCKKTCAFFVSSVTFIGICAILSYLTTPF

TARIGNNESERILPFNMWLNLPLSQTPYYELSFIIQIVTLYCIGICYFCFDNVFCIMAIHLTGQFRILGYRFT
KLCDTEICEKGAESGLAKRVHTCYEKFKEHVRYHQALINFYTKLENVYTMIILGQVILFSVLICLFCYQVLLA
NAPSARRSIFIFLLIGAMSLLFMFTYSCDGVIEQSDNVAVEAYSALWTILPMDKFGRMIRKDLIMVMERSRRV
CCLTANGFFPVSLETYTTILSTAVSYFTLLRNNVENDKNDQ
>AfOr91_1
MGFLESDISVRLTSIFMKLVGLWMAANQYEQRMRNAMITYNVVAILFALWIQTMDIYYSWGNLSACIFSTSNT
LSLILPLLKIFILLSHKEEFFRLIVYMQRNFLRGNYDDYEREVVFGCKRKCTFFICFFTFFTMATIVSYIASP
IVGlaNIGKNESDRVLPFNMWVNLPLSMTPYFEITFTLQTLSLYQIGVSYFCFDNFLCIMNLHVAGQFKVLQY
RISNIADLIIKTEEKREKLIVDSSYFSSKCYTTFKKCIRQHQTLITYCRKLEAVFNLIVLEQVLMFSLLICLD
GYQILMADGDIKTRLIFSLHVLACLCQLLMFSYSCDCIIRESVNVATAAYGGPWTLLPMTTNGKMMRKDLIVV
IMRSSIPCCLTGKFFIVSLETYTSVLSTAASYFTLLRNNIGSADET
>AfOr91_2
MSFLESDISVNLTSIFMKIIGLWMATNRHEKRIRNITIIYSLIAILFALWIQSMNIYYSWGDFSACLFSTSNI
LSLTMPLLKIFILLTHKKDFFHLISYMQRNFLHGNYDDYEREVVFGCKRKCTFFICFFTFLTMATVASYIFSP
LIENIGKNESSRMLPFNMWVNLPLNVTPYFEITYTLQVLSLALSLYHIGVSYFCFDNFLCIMNLHVAGQFQVL
QHRISNIIDSMNKEKEEKREKLTVDSSYLASKCYAIFKKCIRQHQALIAYCRKLEQVFNLIVLEQVLMFSLLI
CLDGYQILLANGDVKTRLIFSFHILGCLCQLLMFSYSCDCIIRKSVNVATAAYGGPWPLLSMTTSGRMIRKDL
ILVIMRSSVPCCLTGKGFFMVSLETYTSVLSTAASYFTLLKQRAEVIS
>AfOr96
MNLKYRKDLAFTVASFYLRVVGFWLTTSRLEECFRIGVVGYTILAITFSAWVQIRGLYFNWGDFSACTYIACD
GLGLVMDFFKIFSLFIHQKKFLGLMAYMQKNFWHYDYDEEENLIVKDTKKTTAYFVCILTFSSLSSIFMYMFR
PLLTNIGRNETDRILIFNMYLDLPLSISPYYEIAYTIQIAALNQAGSCYFCFDNIFCILCLNVACQFRILQYR
IANAPILKKKENLDSSKYPSDECYNAFKNYIQQHQALLHFCETLEQIFTIIVVGQILMFSILFCFLGYQVILA
DLTTSYRVSFISYLFAGMCQLWMFTYSCDCITQESAEIASAAYTSPWIDLPMDKFGKMLRKDLQIVVMRSRRA
CCLTACGFFPISLETYTKIMSTTMSYFTLLKQRTVDAVDT
>AfOr97P
MTYDGKQKYGKNGKYKSTVFDISYYKTFKKYLKFLGQYPNQSRWNKEFNTNVMICSLISFLIPGLLQVYTSIV
EKNLNALMEIIPIVFATISCAIKLLNHRINKKNFDKLFDFMSEEWEMENDRNQTCILDEFTKQGNKFAEIYKN
VLLSALLLFLLLPLFPSFLDVVFPLNETRQQLQIFKMKYFIKEDQYFYPIYFHSVWSSFVIIMITVTIDSLYM
LIIHHASGLFAICGYQIAKATECNDININENELFRQCVITHNKAYKFFEIMNKSSRNNYXLSILLSIIGISIT
AVQIVMYLHKPEEAFRISLFLMAIQFHLFIITLTGQVIADQSSKLASNIYCTTWYRMPPNIQKIFHIIQIKSS
KPCKLTAGGILELNIENFGIALKTCMSYFTIFLSLQD
>AfOr100
MCIHLVFQIYGGQCLDIFEIPYYKSLKKWLTLSGLYPLRNTIIILVVISIISVTLPLIFAIYTSLCAKNIDAM
LECLPPLGVCVVAMFKLQNIYNNSENFKKLFTFVAKQWHQLKLNNEIRVLEEIVMQGNKMAQIYRNTLLVAMV
IFFSVPLIFPILDIVYPLNETRPRQQLYKVNYLIFNHEDYYFYVYFQLVWSSFVCVIIIIIFDWLYILIIHHN
SGMFTVCGYQIQKIFTEEAFSNIRIYEQFKNCLIVHNEAIQFFDILDESSRNTYFFLVGSNIIGTSMSAVQIV
LNLDKLEVAIKSTVFLIAAQFHLFILSIPGQILLNHYSNLTNNIFMSAWYNMPIEVQKMFYVMQIRCNKPCSL
TACGLYEMNMENFGTALKTCMSYITMILSLK
>AfOr101F
KKQHNEIYDIPYYKMMEKYIRFLGQDPRQKKESRNIIVFILVISIASIIIPTTLELYTSLRNKDMDGVIECIP
HFIASSISAVKLLNLHFNRQNYDILLHFVTKKWQQLKSTYELSALDETIMQGKRMAQLYRNTLFSFLILFLLV
PLVSPILDIVHPLNQTR
>AfOr103PC
MNLNSSYSVFQKTERQYPEIFDIPYYKMVEKYFQLLGQDPRLKSNFRNCIVTVVVISISSNIVPTSIELYTSL
CDKNMDAVIEGLPHFIAATISAVKILNIYFYRENFDKLFQFVASEWNKLKLNNELHILDNTVIQXNKMAQLYR
SALLTALVLFLLIPLLSPILDIVLPLNETRPRQQLLKVNYLFFNDDNYFFYVYLQLAWGSIMVVVTIVAVDSL
LILIIHHCSGLFTVCGYQVQKVTRNAKSLNEIVSNNYTYEQIKNCVITHDEAIQF
>AfOr105
MEQISIIPIFYVLQKNNEQEYNAFDIAYYKTLKLYLTICGINPYQNNNISIIIIIMIISVCMSFLCPTSIQLW
EAISNKDFDNIIQNIPQVITVIISMIKILNIYSNKMQFKKLFYSLAQDWKSLESKEELIMLDKFTQYGSKLAL
LYRKSLLTFLVIFLSLPLCNPILDIILPLNETRPRQNIFNVNYIILDNYEYFYIVYVHLSCSAAIIVIIIISV
DSLYISIIYHACGLFAACGYQIQKLTKIHTIEKNGPNISSIEYEEFKQCVIMHYKCLQLYDVLEKCCRNLYLI
QMGLNIMIISVTCVEIVVFLDRPEEAIRAIIYVMAQQFHLYAISLPGEMLLNQSSKLADKIYDSEWYKIPMKV
QKVLHIMQIRSNKPCILTAAGLYEMKIESFGITIKTCMSYFMMFLSLRE
>AfOr106
MDVQRLEERYLKINKIYSIIIGMWPNQNRKMIRCIFVEFIGIAAHLTQGSNIILFFSLSVAMDQVPFLVVATL
LMIKYNFFIINEQKFKELFTSILNDWQKKKTHEEEMIMEKYADKSVFFILIYVVNAYFCSILFLLLPFTPILL

DIFIPLNESRPRVQMYPAYYYIENEADYYYPILIFSIVSLLTAMCVFIATDTMLIYIVQHACGLLALAGHRFR
NSLNDLYSSRKDSKVDGKIYRRLCYAIKTHKRALAYLAEIEDFYSVNIFMQVGASILCLTVTMMKIATITWSM
ETNQYYGYVIAQVVHIFFLTAQGQFVIDSHENVYWDMYEPYWYNLQSKMQAMFVLILRRNLNPPLLTAGGLVQ
LNLNTFAKVVKTSVSYFTVLKSV
>AfOr112_1
MDARTVEKNFLKVNKIFGLITGVWPYQNYRMKMAERFLSVTVMMSGFVTQFAYLVLNPTIDKAATNLPYSIAS
FGTFVKMGNYFLDETKLITILNHIFEDWAAIKTKEEYEIMYKYSRRGLFITISYFLHIGITETFMLMWPMVPP
ILDIIMPLNVSRKRILLYPAYFWLDDEKYYVLLLGHMIITILMICFIFCACDMNYVYAVQHACGLLAIAKYRF
KNVCKDLNDKEDHAMPLEKIKYKSICESIKAHQHALKYLRLIENSYHTYLFVSMGLLIMAISVSLLEVANGKN
GSRKLVQATFLFAQLFHTFILTVQGQFVINELQDVYESIYESPWYTFSPRIRSLYVLSLRSCLNFPILTAGGL
IVLNLQSFAEIIKAAVSYYTVMQTT
>AfOr113
MDLDMLEKRFLKITKRFAKLGGIWPDQNKYLKCISWIFIYVINIPSVVVQVARIVHMSTINVIVEQSSIATAI
FLLLLKETNYILNATKVKSLFNDMYIDWTMDRPKEEIEIMSTYAQRGSFLATFYFLNSFFCALLFLQVPWTAR
LSYMIKSQNTSPPMLNVVPGYYFVEDDHDYYYFIQLHMSLSIITVLIVYVGYDTCYMVFVQHVCGLFAVAGYR
FKHAIDDSASKNSEEKTEMCKRIRSSIQGHEQAIKYLKKIEDAHVNLLFISLGLIIICFSITLLKVVTMDYCL
DFYKYFSFLMIQLIHLCYVMIQGQFVIDSCNEIYCSIYEASWYDISPKIQALYILALRRSLTPPRLTAGGLIE
LNMQTFLEVIKLSVSYYTVLRST
>AfOr116P
MFRKMINYLKKETKLKKINPNKHLQNSLSIIYYVGLWPDRVKYKYLYNLYAICSLIFLVGIIIVSEIIYIIIN
WGKIELMMIGLTILMTNSTYAAKVIYIICQYKRIKNLVDITNSEIFNRDNDKYKHIISYYNWQGIFHYIAYQG
FASISIFFYSCIPLQSVFSEKSKQLPLAGWYPYNVTSTPIFEITWLHQVLVILINCINNIAIDTLITGFIIIT
CCQLTILKKCIARNNNVNIEKSLSKIYNKFYESLKYCVEHSIIIFDFTKQIQDIFGIIIFFQLFVNCIIVCLA
AFNVSQIKNYITPEFFGSLLYMCCMIYQIFIYCWHGNELYLHSMKICLSAYKNNWWNNNKNFNYALLIIMIRT
QIPLIIIVGKVXMELSLQNFLLILRTSYSIFTLLKTFT
>AfOr117
MKKPFNKSIDYYILPNKIFCSIAGMWPIDEKSSMFSKIFAYVRLIFGLIVVSSFFIPEIIIIAMNWKNIKIIA
GIGCVLTTITQVLFKMLYLIVRREKTYSLYYKIRSLWNSSNDSKERPYEEFAYWARIFSIIFYSSCMCNVFTF
SIAAAIDYFKFEYNANNTENNRHLPFIVCIITRYGTDISASPNFEIAFFYQIISASICAAVISGLDTSLMTII
LHISGQFKLINIWINNIGIEINCNPNYIRKLKIDLIKCIRHHQQIIHVVNNVNNLFTPIIFIQLLTSGIEICL
SGYAVLDSDSANADLLKFISYFISMGIQLLLWCWPGEILIQESQEIGHVIYLNIPWYNLPPIYQKYFCLMIVR
TQQYCRITALTFQTLSICTLTSVFNTSISYFTLLRQIQQ
>AfOr119PC
ITYSRDTCTTTLRKLFYLXLVVVGGQVADXASGHEWMKSIRRLTSIKINYLKYSGLGEIDSSCSHILKCAYFV
YKVWMLVTMCILAITVFADIYANMDNLSITTDDGCIFAGIFVVIFKAMNLQIQLESVKEIIDKYYTRNKVMFY
GFCVIGGCLGFALLCFTPMEDGLPIRAKYPLNTTISPWREISFFVETCAVSGGLLGIIVMDSMTTFKCSLITM
LLDALNVNFENCHSDETTTIDVYTDGDKKKRDKNRFLDRYERCLRFHQRLVIISKDYNNIYNLSMFVQMLSST
TIICLTGFQAVVVGGQSSNIMKYGIYLSAAMSQLFYICWLGNELDYASSTLDKSQWFSGWHNERLAGVARIFT
LSTVFTRKSIILRASAFYVLSLETFIVIIKRSYSFFTLLNNMDLTGH
>AfOr122like_1
MDVFDKQYRIYRMVLKIIGLWPYDNSIYVWIQRICLLLYFLSGVIFQIIVLLKSEITLRNYILTLSETFPLLL
FFLRYIYYITIIPYVEFLFNNIRTEEHSLQDMTEIQIQTKYLDISNHIIYFFCCVTCAFIAASIVFLLNPIIL
DFLMPLNESRAHFNVSFLFDDQSMYIKIFLILNCTLNVSFGLLCIMATELCTNIFSYYICRQFHIASYRIRKI
IEDLSMPLSKQIDLKLIDIHRVVDIHNDVIKYIKLATNNAATQYLLAIIVCILSFSINIYRLYNAITTMDDRI
EIFGSAFIVMYHLMIAFYNNHYGQLIIDSSLDIFNELYASTWYRIPLKAQKLLLFMILRSSMGCELGLSGLFT
PSYAGFTSMMSSSFSYCAVIYSIQ
>AfOr122like_2N
MDVFDKQYRIYRMVLKIIGLWPYDNSIYVWIQRICLLLYFLSSVIFQITMLLKSEITLRNYILTLSETFPLLL
FFLRYIYYITNFPHVEFLFNHIRTEEHSLQDTTEIQIQTKYLDISSHIIYFFGCVTFAFIAASIIFLVNPIIL
DSIMPLNESRVHFNVSFLFDDQSLYIKIFLILNCMLNVSLGLLCIMGTELLTNIFSYYICRQFHIASYRIRKI
IEDLSMPLSKQIELKLIDIHRVVDIHNDAIKHINLATSNAATQYLLAIVVCILSFSVNLYRV
>AfOr122like_3
MNVFDNHYHTYRTVLKIVGLWPYDNSIYVWIQRICLLIFFLIIILFQIIVLVKSEISLKNFIVTLSMTCPVLL
YFLRYLHFITIFSFVELLFDKLLTEEHLLQDSIEIQIQTKYLDISSHIIDIFCWMSFFGIAAVIIFLLNPVTL
DIIMPLNESRTHFNVLFLFDDQSPCIKIFLILNFMLIMLFGLLSLIGTESLTNIFSYYICRKFYIASYRIRKI
IEDLGIITMPKQIDLKLTDIHRVVDIHNQAIELINMGTNATATQYLTAVIICILSFSVNLYRLYNAITTMDDR
IEIFGSAFIVVYHLMVAFYNNHYGQLIIDSSLGIFNELHSSTWYRIPLKAQKLLLFMIVRSSMGCELCLSGLF
TPSYAGFMSMISSSFSYCAVIYSLQ

>AfOr122/130like_1
MDVFHKQYRTYRILSKIVGLWPYDDSIYRWIQRLLLLMLFLCGIIFQIMAFVKSKILTLRSCILIFSTICPCV
IFFLRYICVLTMFPFLKNFFGHLRMEQNIIQQDLREAQIQIKCINDSSYMINIFILLNLMISLNESHINYVIY
ITMFCDDRIVCIYMVYLTLILIIFFGLLSLTCTESLFNILSHHLCGLFNIMSYRIRKTIQELNIYTSSSKQIE
LKLKDFHQIVDVHNQAIEFAYASASKMTMQYLLVIVVTTISFAINLQRLFKAIKSMDDPLETFTCIILVLYHL
VFIFINNYNGQLVIDSSLNMFNEIYISTWYSIPLKAQKLLLFIMLRSSIECAFRLSNLFTPCYVGFSTMINTS
FQYFTMMYSIQ
>AfOr130_1P
MDVFDKQYRIFRIVLKIVGLWPYDNSFYVWIQRLWLINLLSSKYNISVMSLLRSEITLENSILILSTICPFTL
FLLRYVGFIVFFPTIKIVFDHIRTEESIVRDSIESQIRMKLIDDSCHIINIFLWMTYTTITFYSACLFYPAIL
DLIIPLNESXYSSYFTTFSHDQTIYLDILCFDVTFITTFGLLSITCSESITGICTYYICMLLKILSYRIQKIV
TYLATFKLSSEQIDSKLVELYRVVDIHNQAIDFINFTVSTAGMHYIIANLLVVISLAINLHRLVNTILIKKNQ
LEMLFCFTLVAIHLVIIFVNNYTGQILMNSSQELFDELYNSMWYSMPLKAQKILLMIILQSSTTYGFNILDLF
IPCHSGFSTMLSSSFSYFTLIYSIQ
>AfOr130_2
MDVFDKQYRTYRILLKIVGLWPYDNSICIWIQRLCLLTYFLGSIIFQVLSLLKSKITLQNFILTSSMTCPFVL
FLLRYVSFMILFPTIKIVFNYIRVEECIVQDLIEAQIRIKFINDSCHLINIFFWMSYATIAFYSLYLLYPVTL
DLIIPLNESRIRLIYYFTTFSHDRIMYLDILCFNITFLIILGVLSIACTETLIGVCSYYICILLKIISYRIQK
IVTYLAMFKLSSKQIDSKLVELYRVVDIHNQAIELTNVAINASAIHYIIASLLAVISLAINLHRLVSVSIIKK
DQLEMSFCFMLVTIHLMVMFLNNYNGQILIDNSQQLFDELYVSLWYSIPLKAQKILLLIMLRSSTVCAFNLFG
LFTPCYTGFSTMLSSSFSYFTLIYSIQ
>AfOr131like_1PC
MIFLSIALFNIFLLYLITMDFIMPLNQYRIFILRYITLFSVNRNIYFYILCLDFIFVVTFGLLSIICTESIIG
FYSYHTGMLFKIISHRIRKIITYLTIFNLSSKQIDSKLTELYRVVDIHNQAIELVNIIINNSGKQFLMSTLLL
VISMAVCLHRLVNAITIKXDHLEILICLIFFANHLVIMFLCNHSGQILIDNSEEFFHELYISVWYFVPLKVQK
ILLLVMIRSSTACMFHIFGVFIPCYAGFTAMLNTSFSYFTLIYSMQ
>AfOr131like_2
MDVFDKHYRTYHIVLKIIGLWPYNNSVYAWIQRLLLLTLYLGNVIFQIVSLLRSEITLRNCILILSTTCPLII
ILLRYINFIMFFPMVKHLFHHIGMEENIVQDSIETEIRTKYINESCQKIDFYFRVIFSTITLFNISLLYFITM
NFIMPVNQYGILILRYITLFSVNRNIYFYILCLDVMFVVTFGLLSIICTESIIIVYSYHTGMLFKIISHRIRE
IITYLTIFNLSSKQIDTKLTELYRVVDIHNQAIELINIMINNSGKQYMMSALLLVISMAVSLHRLVNAITIKK
DQLEILISLIIFANHLMIMFLCNHSGQILIDNSEEFFHELYISVWYFVPLKVQKILLLVMIRSSTACMFHIFG
VFIPCYAGFTAMLNTSFSYFTLIYSIQ
>AfOr131like_3
MDVFDKHYHTYRTVLKIVGLWPYNNSVYVWIQRLLLLTLYLGNVIFQIVSLLRSEITLRNCILILSTTCPFII
VLLRYVSFIIFFPMLKYLFHHMSMEESIIRDSIERRIQTKYIADSCHMIEILLRVTYGTVISYGMFLLYLITS
DFIMPLNEFHTRVLHYFTLFSVNRTVYFYILCLDFLFVVTFGLLSIICTESIIGLCSYHVGILFKIISHRIQK
IIKYLTMFNLSSKQIDSKLIELYRVVDIHNQAIEFISIVINNLGKQFMIPSLLIVISMAVNLHRLVSAIIANK
DQLEILISLIFFTNQLVVVFLNNHNSQIFINNNEEFFHELYISVWYFVPLKVQKILLLIMTRSSTTCMFHIFG
VFFPCYAGFTSMLSTSFSYFTLMYSTQ
>AfOr132like_1
MNVFDSKHYRTYRTIMKIVGLWPYNNSIYVWIQRLLLLAFYLGNVVFQIVSLLTSEITLQNCILILSTTCPLV
IVSLRYVSLILFFPTIKLLFRHMRMEEAMVQDSIEAQILGKYIDDCSCMIDIFFWMIYEIIAFCAILLFYPIT
LNFIIPFESLRYVILFSINQTIFIDILRLNYTFVMIFAILSITCTESTIGLYSYHISMLFKIISHRIQKIVTY
LATFDLSSKQIDSKLTELYRVVDIHNQAIQFIDIMTNNSGKQFTMSALLVVISIAINLHRLINAITIRNNQLE
SLISLIFFVNQLTIIFFCNHICQIVINNSEEFFKELYISVWYFVPLRVQKILLLIMTRSSTTCMFHILGIFFP
CYAGFSKMLSTSFSYFTVMYSMQ
>AfOr134like_1
MDVFDTHYHSYRTVLKIIGLWPYNNSIYVWIQRLLLFTFFLGNVIFQIVSLLRSKITLRNCTLILSTTCPLII
ISLRYVSFIIFFPMIKHLFNHIRMEESIVQDSIETEIRAKYVHDFCHMIEIFLRVTCATFIFYSVLLLYVMIL
DFVMPFNESRIYILDYFTLFSINRTVYFYILCLNFFFVLIFGLLSVISTESILGLFSYHAGVLFKIISYRIQR
NITYLTTFNLSSKQIDTKLAELYRVVDIHNQAIQLINILINNSGKQFLMSTLLTVISIAVNLHRLVNAIIAKK
DQLEIIVSFIFFVNQLVFMFFCNRSAQILINNSEEFFHELYISVWYFVPLNVQKILLLIMTRSSTTCMFHMFG
VFFPCHAGFTTMLSTSFSYFTLMYSIQ
>AfOr135like_3PF
FKNTIEILYNHYKIQYFKPVLTNFXIVSLLRSEITLRNCILILSTTCPLIVILLRYISFIIFFPMIKHLFHHI
SMVESIMQDSIETQIRTKYINDSCQRIHFLFRKKYVNFFYNI
>AfOr136like_1

MDVFDKHYHTYRIVLKIIGLWPYNNSIYVWIQRLWLLIFILGNIIFQIVSLLRSEITLQNCTLILFTTCPLTV
VLLRYVGFVIFFPMIKLLFRHIRMEESMVQDSIETQIRTKYIDDSCHMIDIFFWIIYTTTAFCSISLLYPITL
DFINPLNESRTRIIHYFTMFSHHRIIYIDILCLNYVFVAIISSLSVTCTESIFGLYSYHISVLFKIIGYRIQN
IVTCLTIFNLSSKQIDSKLTELYRVVDIHNQAIELIDAINNSGIHMMPSFLIVISMAMNLHQLVSTIFRKDQL
GILIFLISFIVQLIIIFICNYNSQILINNSEELFHELYISTWYFVPLKVQKILLLIMIRSSTTCMIHILGVFT
PCHAGFSTMLSTSFSYFTLMYSIQ
>AfOr137/138P
MDVFDKNYYTYRTVMKIVGLWPYNNSIYVWIQRLWLFTFFLGNIIFQIVSLLNSEITLQKCILILSTIFPLTI
VLLRYVSFIIFFPMIKLLFHHMRMENNIMQDSTEIEIRTKFXINGSCHMMDIFFWIIYATVALSSLFILYPIT
LDFIMPLNESRIRIIHYITIFSYNRTIYVDILSLNFMFVGIFGSLSITCTESIFGLYSFHTSMLFKIIGYRIQ
KIVTYLAMFDLSSKQIDTKLKELHRVVDIHNEAIGLIDVMINNSGKQFISSNLLCVISVAINLYQLVNAIITK
KDKLEIFIFSVFLMMQLMIIFLYNYNNQILIDKSQELFDELYISMWYFVPLKVQKILLLIMIRSSTACMFHIF
GVFTPCYTGFSKILSTSFSYFTLIYSIQ
>AfOr138P
MDVFDKHYRTYRTVLKILGLWPYNNSVYVWIQRLLLLTLYLGNVIFQIVSLLRSEITLRNCILILSTTCPLII
TLLRYISFIIFFPMIKHLFQHIDMEESIIQDSVERRIRTKYIDDSCHMIEIFLWITYTTIAFYGMLLLYPIIL
DLIMPFNESRMYNYHYFTLFSVNRTIXFYILCLDYIFVITFGLLSILCTESIIGLCSYHAGVLFKIISHRIQK
IIAYLTTFNLSSKQIDTKLGELYRVVDIHNQAIQLINILINNSGKQFLISVLLAVISMAISLHRLVNAIIIKK
DQFEIFLSLIIFTNQLVFIFLCNHSAQILANSSEEFFHELYISVWYFVPLKVQKILLLIMTRSSTTCAFHILG
VFVPCYAGFITMLSTSFSYFTVIYSIQ
>AfOr139like_1F
FYDEIKNDWNSLRNIEEIIHEHCKAIKRVTINFI
>AfOr141P
MIDEKTKREFNKTIDLNLFXLKLCGIVSCGDGFANRNILAWLAFSCLTIYSISYVHEFITNTTNLTTALESIA
MIISIVGGHARYMILLWFRDICQTMLNVCETFWSNLKPHEKKIVQSYTRKTTRLTRWYLASCVLTIAFYAFLV
LFGSLFDHSKDFEHITRNDSGLVPSEAGNVLESTNKRHLPYAFFLDVQKTPWYEIVYALQLIGMFNVGFTCVG
VDTVGALFILITCGYFDTIRSRIENLHSFDTSLPSSSLSLLNILSRKITTAKMSDTKTKTSNSAQMKNLRMCV
IHHQLLLNRFCEDIEHLTSGMFFIQVIASTYNISLVGFKLLEDTPDKFKYITQLIILIIQLFLCNWPADLLLS
KSVDISRATYSMPWYRYSYNLQKITNILMVRSQKAVRLTAGKFIGLSLETFASMISTAASFFTMVRSMN
>AfOr147
MLKQVTPEKGIYFIWLSVALSLCWPLPINSTRKKIVYMKILQIGAIISAFIILLSLIYTIYLNLDNLNIFFKC
VCLSMAVFQHIVQTTICFIKYNTLQRIVEEMMTCIKEIQLYEIMCTYVAKCNILYGAIIVLIYTAATIFVLGP
TFLPITFPWETEYPFQVNYTSRNFIIYMHQFFFTYQCAAHICVSMFVALLLWFTSARFECLVKELQKTTNVEM
LILCLKKQLLLRRYAEDVVNCIRFIIFYTMTISTIVLTLSGIILITTSSLFVKIQFLIICVSMLLEIYIYTWP
ADHMYDMSITVLRSAYDSMWYDQTLNMQKFVLITLIYQKPIIISINVVLPQLTLHYYCSYVSNAFSIFTAIRA
TIYDR
>AfOr149
MLKQVTFEKVIHIIWFSVALTFCWPLPANSTKNQVFVFRILQIISIINAFMLILPLLYSIYLHFNDIAIVFQS
LSILVGLSQMIIQTVILCFVKYNSLQRVIEEMITCVKEAQQYERKIFCKYIDKCNIFYGSSLIFTYMVVIVYI
MGPIVLPAPFPIDTEYPFHVNYTITRIIIYLQQSLLIVQCAGHVCLSVFGALLLWFTAARFECLIVELQKITN
IGMLIICIEKQLRLRRYARNVVNSFRFMIVYAIGVSTFALTLYGIIMIVKAPLIMKIEAITLSFVLLLQIYIY
AWPADYMKDMSINVSKSVYNIIWYKQTLRMQKNLLNVLVYQQPIIFSISCILPELSLRYFCSYVSNIFSIFTA
VRVIIEDDQLDPLSNC
>AfOr150
MLKQLTPEKAIHITWLSVAITFCWPLPANSSKIQVFMFKALQIISIINAFVLLLPLLYSIYLHFDDIVVVFKS
IALCVGLSQMIIQTAICFVKYDSLQRVIEEMITYVKEAQQYERKIFHKYIEKCHIFYGCSIICMYLTGVAFII
GPAFSPASFPADAEYPFQINYKPVKVIIYLQQTLVGFQCTAHICLSVFGALLLWFTAARFECLAVELKKITNI
GMLIVCVKKQLHVRRYAKRVVISFRFIILCAIGVSTFALTLGGVIMIKKAPFIVKVQFITLILTLLTEIYMYT
WPADHMKDMSMNVSQSVYNITWYKQTLRMQKDLLNVLMYQQPIILSINCILPELTLHYYCSYLSNAFSIFTAI
RVIIEDDPS
>AfOr151_1
MTKQVTPEKAIQIIWLSVASTFCWPLSINSSKTQVFVFRVLQITSIINACMLLLPLLYSAYLHFDDIITVSKC
AGLAIALTQIIVQTSICFVKYDVLQHVIEEMITYVKEAQQYERKIICKYIKKCHIFYGCSIVCVYLTAIAFII
GPVFLSTSFPADAEYPFQINYTPVKVIIYLQQSLVAFQCTGHVCISIFGALLLWITAARFECLAVELQKITNI
SMLIACVKKQLHIRRYAKRVVNSLRFMIVYTIGISIVILVLDGIIMIMKASLIVKIQFIGISLTALVEIYIYT
WPADHMKDMSIDVSQSAYNVTWYKQTLKMQKNLLNVLVYQQPIILSVNCILSELSLRYYCSYLSNVFSIFTTI
RVMVEDDP
>AfOr151_2PN

MLKQLTPEKXLSTLLELASTLCWPLSANSSKTQVFIFKALQIISIINAFILLLPLLYSVYLHFDDVIIVSKCV
ALSIGLTQLITQTAICIAKYDSLQHVIEEMIICIKAAQQYERKIFHKYIEKCYTFYACSITCTYLTTTVFIIG
PAFSPSSFPIDAEYPFQINYTPVKTIIYLQQTLVSFQCAGHICISIFGALLLWFTAARFECLAVELQRITNIG
MLXACVKKQLRIRKYTQKKVIISFRFIIVYAIGVSTFVLILDDIIIIMKASLIVKIQFITLSLTVLTEXIYIY
AWPADYMKDMVTNVSKSVYNITWYKQALSMQKNLLNVLMYQQPIILSVNCILSELSLHYYCSYPSNAFSIFTA
IRVMIEDNP
>AfOr152P
MLKRKIIEKAIHITWLSVAITFCWPVPLNSSKTQVFIFKVLQIISIINVSMLLLPLLYSIYLHYDDIIIVSKS
IXLSVGLIQVIAQTAICFVKYDSLQRVVEEMIIYVKQAQQYEKKIFHKYTEKCHTFYGCSIACMYLTATAFVI
GPVFSPASFPADAEYPFQVNRTPVKIIIYLQQSLVAFQCAGHACLSVFGALLLWFASARFECLAVELQKIRDI
GMLIVCVKRQLHVRRYARRVVISFRFIILCAMGVSIFSLTLGGVIMITKAPFIVKIQFITLILTLLTEIYMYA
WPADHMKDMSINVSKSVYNITWYKQTLRMQKNLLNVLMYQQPIILSINCILPELSLRSYCSYLSNAFSIFTAI
RVIIENNPS
>AfOr153
MVKGITPEKAIHITWLSVALTLCWPLSVNSGKTQVFVFRVLQIISIVSACMLLLPLSYSIYLHTDDVIVFLRT
IIVFVGVAQNIIQTVICFVKYYPLQHVIEEMKICVKEAQKYEIEIFQKYIAKLKTVWGCSITCMYLTALAFTI
GPVFMSTPLPCDAEYPFQLNYTPVFAIVYFHQSFLSYQCLAHMCISMFGSLLLWFTAARFECLCVELQNATDI
DMLIVCIKKQLHLRRYAKKVINGFRFIIFNAIGLSTLVLTLAGIIILITNVPKILKYQFVIVCMTLLAEIYMYT
WPADYMTDMSVNVSRKVYDSMWYKQTLKMQKNLLKVLIFQRPVSLSISCILPELTLRYYCSYLSNVFSIFAAL
RVVIEDNI
>AfOr154/155P
MSKRMTPETVIRIAWLSVVITVCWPLPMGSGRGRILGFKILQIVSIISDFMLLFPMIYSIYLHFDDIIVSSQC
VCMSIALTQLIIQTFICFVNHDSLQRVIEEMMTCVKEANQQETEIFYKYIEKCGRFCGGSIAWMYATAICFSL
GPVVLPITFPSKAEYPFRVNYVPVNVIVYMHQAFLSFQGAAHICLSMMGALLLWFAAARFECXAVELQNCANM
PTLIVCVKKQLRLRRYAEQVIDNFRFIVLYAIAVSTFALTLCGIILIINVPILVKMQFITLCFTVLTEIYLYA
WPADYINDMSMNISQSAYNSVWYEQTIELQRNLLSVITYQKPLILSIKCILSELSLRYYCSYLSNTFSIFTAL
RVLIVKNSS
>AfOr157
MSRQVTPEKSVYLVWLSVAITFCWPLPANTARKKIVGMKVLLIASIINGCVVILAMFYWIRLHLDDIISLFKC
VCVVLCLLQYVVQTIVCFVKYDTLQRVVDEMMGCINEAKTDEILRAYASRCNTLYGMSLLSIYICGTSFVFTP
LFLPNPFPFETEYPFDVNTTSRSFIIYASHVLAIFQSTGHMCLCTFGAVLLWFTAARFECLIGELRSVTSIDA
LVVCLKKHSRLKRYAEEVVSCIRFLVFHAILLGTFVLTLCGIVLIINSPLIVKAQFIIICVCILLEIYLYALP
ADYMYDMSMNISRSVYDSMWYEQKLDLQKALLTVLAFQKPIPVSINVLLPELTIRYYCSYVSNALSIFAALRT
VVDDS
>AfOr159C
LSMLVILYPMVFNVILPLNESRKFIVLCDLYYFVDPQEYAIIFLLYTFVLIFILCTIIGTQFTTIIFVQHCCG
FFKVASYRLEHAMDTYKAQNEIEIYYIICAKLIKAIEIYKLAIKFFKYAVDIYKIPYTMTIFLYVLDVSVQLY
YVVYMLQQLQSIHKLCINVILLIGKFCFLFLVTYLGQNIENHSSEVFEKCYDSLWYTAPVATRRLLLIIMINI
MKPCQYEMFGGLFKGNIEGFSKIIRVCISYFMSIYSIQ
>AfOr159like_1PF1
KYQEDHKIILKNASFNYRLIVICFXMHGGMIFYYIILPFTRTKIVEKGSNLIXEGNKRVCPFPKILLHACHST
INEIRYMIQLLCNIIVPACSLAMLXFVIHTCRQLQILMSWFEKILNXRKNNNENL
>AfOr159like_1F2
QTVKMNEKFLHLSNGMCWKKSITISLIISMSYSTIKMIVGNIIE
>AfOr165
MLVLNTLSPSVKFGLHFAGIWPGTAFPYLHKLGWLAAMVALQSYQYRYIVMHYKSDNLMSIIDNLSIAMPFSL
VFIKLIVTWVNYGVFCDILSTMKKDCQKYAVIDINNLISKTGQISFYMTTIVMSSYLVSAVFYITGTLAFQRT
NDSISRELLFKMDLPFETSESPNYEFVVTSQFLIHVSAALTFGTFSALLLMVVLHIGCQIDILCQNLLHISPH
ICASHLKFFIIRYQEIIIFAERVEKLFTYIALSQLVSNTLITCCVGFLIVIAVHEDNSLPLLLKSVLFYMVIC
LEAFIYCFAGEYLRIKSKLIGDTAYEFLWYDLRPNESQLLIPVILRSQKGFTFTFGKFSSLSLESFTGIMKIS
ASYISVLLAVY
>AfOr166_2PC
MTTANTVTQSVKYGLYFAASWPGAPFSILHKLFWTIVFSSLHISQYSYLIMHYKYDALTEIIDNISICLPFSL
VCIKLXTAWTQNTLIRSILSTMEEECQKYAIMDTDNLISKTAYLSYRLTSIIMFSCVASTVCYLIDIFSYQEV
NVTKSRELLLKMDFPFDTNKSPIYEFVIIVQYFYQVSSAFMFGVFAAFLLMIV
>AfOr168
MNFQNLNRLNAFANMISGNFLPMTNINEKSSIVSKIYFVIVWIIQLIYLAACTLGLFNVPWERSLKDGTVNMV
LLFEVIILNIYLHSRRNLLRELIGKLNHVLINEDEMFRDVTISTIKTLEKPLRIYILVNVTSIMVWISSPLIK

LFQKNEFYYEDFTMPVVLSEQPFSTGVFICGVFLQLFGGEYLLFRKISLDLYTMHLNLLITAQYKYLRIKFAT
VLKEKGETFGKDTWQNIPYDDDTTIRQEMKLLTRHFETVIEMTGMLKKLLSPNIGVLYLNYVFRFCFLSFMFA
TTSLLEKLTYTIIVSYTTGALIQFYILCYCIQDLFEASSSIADDVVYEKWYSYDVRFQRVILMISLANELKCK
ISNFQNIDLTLPSFMSILNQAYSICLLFLKTKQD
>AfOr170P
MNFQNLNRLNVLVNMISGNFLPMNNISEKLSIVLKIYFVIAWIIEVIYVTACFLGLFNVSGEKALKDGTVNIA
ISFEVIVFNIYLHSRKKLLHKLIGKLNHLLITEDEIFRSVIIDTVKPLEMPLKIYIIASVASLMIWILSPLIK
LFXKNEFYYEDFIMPAVFSKQPFSNDIFICGVLLQLLGGEDMIMRKISLDIYTIYLCLLITAQYKYLKIKFAI
ILKEKREITKDLYKDIIWQNENVETIRQEMKLLTRHFETVIETTIILKKLISPNIGFLYLSYVFRFCFLSFMF
AMTTAKYFEKCLLVSYTLGALIQFYILCYCIQRLFEASSSIADDVVYEKWYFHDVRFQRVILMISLANELKCK
ISNFQNIDLTLPSFMSILNQAYSVCLLFLKAGQD
>AfOr171
MSKELRLYKKYASFVKLFLLIGGMCPITRELNIIYRYIPIWTIFSCFIELCAVGNSSLQNVENIPLLTASLIL
FGTILNVITKASCFFIHRKKLQQVNDILSSILEEILGEIYIKSVILFYLQKFYRLIYVQAILMFITSIIYSMK
PMIIKMFHDANNITNVQYPLPLLGTFPWRINSILIWQLHYFFDVNILWFIFFVSVSVDAFFGFCMFRISVILR
FLSFEFKRSSIDDKNKRNKEYEKSHQQIFRECVEKHVLLLKCRNIIQEIYGPIILLVTITNAMSMCSIIFQLF
QVNGINIYKIGTFMAYLILKLIQTFLYSWPGDVIFTESEFLRRNVYCSCWYDKNTSFAKYFLLVLAQKPIVLK
ACSLVQVTMDLLAKIMNTTISYYFLLETMDNK
>AfOr172P
MDFEGSQYYNINRILMICVGLWPYERTIYSKLLNIFIFXFFLSTGIFLQAMSFITLQMDINLTLNLLSYISCT
CICIMKYFNYLFYFKDMKDFLDEIKNDWNSLRNVEELRIIHEYSKTIKRITICFVIIIVPLQLVFFLSIFGNN
ILDILIPLNHTRPRTVPITIYYFVDQQKFFYFFGMHLNIISSFAGLGYIAIETISMAMIQHLCGLLKITSFRI
SDTFVANIPRISSAERNIIIRKKVMSIVDLHVRIKNYINWIQEKIIISYDLLILLGLIAFSIFMFELAKSIIS
RTTNEIFSSLVFVICITVYGFIPNHFAQEIINHSSNIFTDAYNTEWYKLPIMEQKLLLFIMQNNLKSLNFVLL
GSMIASYNVYATILRTAFSYFMVIYSMD
>AfOr173P
MDFEGSQYYNINRILMICVGLWPYERTIYSKLLNIFIFLLLSTGIFIQAMSFITLKMNMNLILTSLSYISCTC
ICIMKYFSFLFYIKDIKVLFDEIKKDWNSLKNIEELRIIHEYSKTIKGITISFIIIIVPLQLVFFLSIFGNNI
LDILIPLNHTRPRTVPIIIDYFVDQQRFFYIFGMHLNLITSFGGLGIIATETISMATMQHLCGLKITSYRISH
TFVANIPRVSSVERNIIIRKKVMSIVYLHVRIKKYINSFQEKIIMSYNFLMLXGLIAFSISMFELVKSITFKT
DTNELISSLFFVICFTIYGFIPNHFAQEIINHSSNIFIDAYNTEWYELPVIEQKLILFIMQNNLKHLNFVLLG
SMVASYHVYTTILRTAFSYFMVIYSMI
>AfOr174
MDFEGSQYYNINRILMICVGLWPYERTIYSKLLNIFIFLLLTTAIFIQIMSFITLKMDMSLILTLLSFTSCTC
IFITKYFIFLFYIKDVKNIEELRIIHEYSKTTKGITICFIIIIVPLQLIFFLSIFGNNILDILIPLNHTRPRT
VPIVIDYFVDQQKFFNFFGMHLNLITSFGGLGIIATETISMAMMQHLCGLLKISSFRISHTFVANIPRVSFTE
RNIVIRRKVISIVNLHVKIKNYINWIQEKIIISYDFLILFGLVVFSISMFEFARSVTSKTDANELISSLFYII
CITIYGFVPNHFAQEIINHSSNIFMDTYTNTEWYELPVIEQKLILFIMQNNLKNFNFVLLGSMIASYHVYVTI
LHTAFSYFMVIYSILLKTDE
>AfOr175
MVELLTYYDFTKFLLTTLGLWPYYNAKYRILYNSIISFISFSFIVVQYLIFKTKKNKLKYLSMLIMSLSSFIK
YNINWYQLAIVKEFMDKIKYDWEIIEMNNETLEIMKRHACLGKYYVLRFASIFYSSLAIVVFCHMSTCILDKI
LPSNETHLKKFPILMEYSILDEKYFLFTVIYLYVSLLLIITIFVATESLMILWLQHAASLCEIISFFIQNATL
KSSKDHFDNNIKRYIIKAVIIHRRVIQFLQDIRDYNAITYCVLLILAITSLGINFFRLSQSILVLTKIEETII
SLMCIICELSYMYYMNYICQLVLDCFNNLNITIFHTNWYKMSVPIQKLILNIMLNCNKPLLLTFLSVYCPTME
GFASLLKMSLSYFMMISSMQ
>AfOr176P
MLDNYYYRYVRFYLKLVGSWPFDNSKQQTIRKWFIQAMFVLIIVYQIRILXIXYNIELLECIIILTINVILSI
KYYTCIFNYDVMKILFINIERDWIXELNCINDMNILKKYSRNSYLYEKYLIWTTWIIYGSIIVINIIMNYAVY
FINVLNDLNKIDYYILMIFLNIFIIFSLIILISTDSICTTITYHVCAVLDITRFKHLFDDLRKMNVAHRKYLL
RNRIVGIVRHHWKCIEYINLLDSYYSIPIVLQYLIATIIFTLKLYKIYECVKINHFRKIIIILIDIIQNYVFM
FLYFCPATKLTKHSEILLNSAYNACWYETFVHIQKMFKFVIQNTLKSYHLGILEIFCITFESMGKLVQFSFSC
FILLISLKE
>AfOr177
MDFYETNVVKSYRLFLLLVGVWPYEYSKANEMQRILYILCVISFLLVQIFKLFTDEINYALLYQIITNTLIVS
LILIKYINIWVKIGEFKEFFDYIKVDLSKLTEREKIIIKRTYKKCSYYCQIIFLLGYFLVFVLIIIQVFQIVD
YKIASSNRTNYFNQIIVTEYFIDREKYSYPICIHLSITILIISWMEIGTDVLHAIILTHIIGIFDKIGYLTIH
LFDEPEITCQKIDKYLIYYKRLVYIVNLYKRNLKFCKKYNSSILICGSIQIIWGTLIISALLLKLRNELLPHN

LRIVFIYCFLISSYLITMYMLLLPYQKVYEISETLFFKAYCGHWYSTSAKTQKLLLLLMHSFMTPYKFRFRNL
VIVSNQIYVSMIRFGFSYFMFIHSIQE
>AfOr178C
FYYEIRYDTKWYRQPLKIQKFVLQGLLRTSKQYVQGIGSFYIMSMRGFFSMINISISFFTVMYSIQ
>AfOr179P
MDFEGNQYYNINRILMICIGLWPYERTIYSKLLNIFIFLLLSTGIFIQAMSFIMLKMDMNLILTSLSYISCTC
ICIMKYFSFLFYIKDIKVLFDEIKKDWNSLKIIEELRIIHEYSKTIKGINRIFHXXLLPLQLLFFLSIFGNNI
DILILLNHIRPQTVPIIIDYFVNQQRFFYFFGIHLNLITSFGGLGIIATETISMATMQHLCGLLKITNYRISH
TFVANILXVSSVERNIIIRKKVFIYTNCFFFCXSYINSFQENIIMSYNFLMLFGLIAFNISMFEIAKSITFKT
DTNELISSLFFVICFTIYGFIPNHFAQEIINHSFNIFIDAYNTEWYELPMIEQKLILFITQNNLKNLNFVLLG
SMVASYPVPQQILRTAFSYFMVIYSMI
>DnOr1
MTDARNPPSYRNVHYKVDTEYTVQVAKNLLTPLGVWPLHRGDLFLDNVKSFVHIGTIFSLMCFLLI
PHIIYTYHDVEDLTRYMKVIAAQVFSFLAIAKFWTMVINKKDIRFCLTEMEAHYRDVECEEDRLVM
KNSAKVGRFFTTLYLGLAYGGALPYHIILPLMSERIVKADNTTQIPLPYLSNYIFFVIEDSPIYEI
TFVSQIVISSIILSTNCGIYSLIATIAMHSCGLFVVVGRQLDTLLEYKQDKLYDRLRNIILHHLKA
IEFADTIEMALSTVFLLEMVGCTIIICFLEYGVIMEWEDHKILSMMTYVVLMTSIFVNVFIICFIG
DCLKQESEKVGETSYLLSWYDLPEDIIKSLKMIIVRTSRPANLSASKLFELSLQAFCDVCKTSAAY
LNFLRTMTS
>DnOr2
MMKFKQQGLVADLMPNIQLMKATGHFMFNYYTDNSSKFIHKVFCFVHLFLILLQFGLCGINLMLTS
DDVDVLTANTITMLFFTHSVVKVLYFAIRSKLFYRTLAIWNNPNSHPLFAESNARFHQLAVKKMRI
LLMAVMCTTMLSVIAWTTITFIGDSTMKVVDPVTNETTYVEVPRLMLYSYYPFDPSHGMAHVLTLI
FQFYFLLFAMADANLLDVLFCSWLLFACEQIQHLKDIMKPLMEFSATLDTVVPNSGELFKASSPSH
PAESHEPPPVSDLQGDNMLDMDLRGIYNQRQDFTATFRPTAGTAFNGTVGPNGLTKKQEMLVRSAI
KYWVERHKHTVRLVTAVGDAYGIALLLHMLATTVTLTLLAYQATKINGINVYSASVIGYLLYTLGQ
VFMLCIFGNRLIEESTSVMEAAYSCHWYDGSEEAKTFVQIVCQQCQKAMSISGAKFFTVSLDLFAS
VLGAMVTYFMVLVQLK
>DnOr3
MTDTKNPNTYQNFRHKSDFVFTIRAARILLTPVGIWPLDRSSSITADVKSMIQVGVIFGLMSFLLI
PHVIYTFHDCEDLTRYMKVIAAQVFSLLGIIKYWTMIFKKQRIRSCLVKMQIQYKTVECEENRLVM
KNSAKIARFFTTIYLSLCFGGALPYHIILPLLSEKIVRADNTTQIPLPYLSNYVFFVIENSPFYEI
TFASQIVISTIILFTNCGTNSLIASMTMHSCGMFEVVNRQLETLMLDQRRDEMKASLRNVVQYHLR
AIEFADMIERNLNGIFLSEMVGCTLIICFLEYGVIMEWEDKNILSMVTYFLLMTWMFLNVYILSYI
GNYLKQESERVGVMSYFLPWYDHSEEVNKNLRMIILRATRPTCFTAAKFFDLSLRGFCDVFKTSAA
YLNFLQTMTG
>DnOr20like_1N
MKEHFWTRWHKDYLHELTVRRKWHTSTPTEVKEGALVIVHEDNVPPLCWALGRIVALHPGDDHITR
VVTIKTVAGEYKRSLKKLSLLPL
>DnOr22
MRGPETQVKNSNLSLQWSRWMLKLFGAWPYSSDMSRQKKHMQWLINIVCYFLISFLFVPCGLFVTL
EVEDTQNKLRLFGPLSFCVMAYMKYYSLLAHANDIRECIKRIEWDWRNIEHRQDRDIMMENAIHGG
RLVKICIFFMYSGFVLYYIALPMSRGKVTAENQNLTFIPMVFPISSLMADSRNSPGNEIFFSIQFF
GGIVIHGISAAACSLAAVLAVHVCGQMKVLMCWMGHLINAREDMSRSVDVRIANIVSQHVRILKFL
IVTEEALQNISFVEFVGCTLNLCLLGYYFITEWHSNDLTSAITYLTLLISLGFNIFIFCYIGELIA
EQCNKVGEMAYMVDWYRLQGKKQRCIVLIIAMSNSSTKLTAGNMVQLSLSTFGDVVKTAVGFLNML
LALM
>DnOr24
MQTPTKDPKKSSIRWNEYEENVDLSIQWNRWILKPMGVWPSSHNVSCLEKSFNRLMNVVCYGLISF
LFVPCGLYVMLEVKDTYNKLKLFGPLIFCMTAYIKYYSLMVHANDIRECIRHIEWDWKNVQHQEDR
SIMIANANFGRRLVRVCTFFMYSGFLFYYIAVPVNVGKVVAEEGNRTFVPMVFPFSRLIADTRDSP
TNEILFSIQLLGGMLIHGIAAGACSLAAAFAVHACGQMEVLKCWMEHLVDGREDMSKSVDVRIANI
VSQHVRILKFLALIEKALTQISLAEFLGCTLDICLVGYYIIVEWSSNDMTAAVTYTIILTSLIFNI
FIFCYIGELVAEQCKKVGEMSYMIDWYRLPENKKLGLVLIMAMSNSSIKLTAGNLVKLSLTSFSDV
VKTSVAFLNMLRTLT
>DnOr24like_1
MREEETKASYLSLQWTRWILIPFGVWPHSPNTSKQKKYICRLINVICYFLISFLFVPCGLFVILEV
KNVYYKLKLFGPLNFCAMAYVKYYSLMVHKKSIRECIKHIEWDWKMVNYSEDRNIMVENAIIGSRL

VKIYIFFMYSGFVFYYIALPISNGKVTVEDRNLTFIPMVFPISRLIVDSRLSPGNEIFFLIQFFGG
IVIHGITAAACSLAAALAVHACGQMKVLICWMEHLIDGREDMSRSVDVRIVNIVRQHVRILRFLFT
TEKTLQQISFVEFVGSSINLCLLGYYFFVICMYGRNTKGLNVLNKIHNANAHCSYIGELVAEQCNQ
IGEMAYMIDWYKLRGKKRRFLTLLIIMSNSSTKLTAGNMVALSLRTFGDVVKTAVGFLNILLALT
>DnOr26like_1
MEKGSHVKMVNNVHHAEDYDYSIQVNRWILQPIGVWPSINKLSKAKWFLSFLLNLACHAVIMFTVA
PCLMFILFEDESIHMRMKAIGPMSAMLMGELNYWSLSLKTKDILRCFDYLDTDWKMVGRPQDRRVM
LKNAKVGRMVVTVAAFALNIGVFSHSLMAGFKKMEFNIGNDTYAMLRLPCPCYSKLIDARYSPINE
IVFFVQVLSGLIVNLVTVGAYGFAAVFAMHVCGQLSIAMSYLDELVEPNMDQPNAQEKLGDIVDIH
LRALNFVSHIEDVMHLTCLIELTGCTLNMCMLEYYMITESSKENAVAYGVIYLSMTFNVFIFCYIG
EKISEQGEMVGEKAYMTEWYRLPYKTASGLILVILRSSTVTKITAGKVLPMSIATFASVVKTSFVY
FNMIRTVTM
>DnOr26like_2
MKGDRVDTVSNVHHVEDYNYSVQVNQWILKPSVLYILFEQADTHTRMQAIGPMSHWMLGGVNYCCL
SLKSKDILSCIEHLETDWKMVKRPNDRQLMLKNAKVGRLIVSIAAVCMNVGVLSHTLIAGFKRAVF
QVGNESYSMLRLPCPVYTQLMDVRFSPLNEIVFFLQCWAGLIVNLVTTGACGFAAVFAMHACGQLD
IVMLHLEEMVEQNEDQPSAQKKLGTMVDIHLRALDFVSHIESVMHITCLIELTECTLNMCMLEYYM
ITENSKETAAAYGIIYISMTFNIFIFCYIAEKLTEQCKMVGETAYMTEWYRLPYKTAAGLILVILR
AGSVTRITAGKILPMSLSTFADVVKTSFVYLNMLRTVAE
>DnOr26P
MVNGARVITVNNVHYVKDYNYSIQVNRWSLKPIGIWPSLSEFSRAESILSRLXHALIVFTVVPCVL
YILFEDESMHIRMKAIGPMSHWLMGELNYCSLSLKTKDILRCIDHMETDWKTVKRPHDRQLMLKSA
KIGRIIMTVAALCMNIGVFSYNFVTGLKKTVLHVGNESYSTLRLPCPFYTKLMDVRYSPANEIVFF
LQCFSGLIVNSVTVGACGLAAVFAMHACGQLNIVMSQLEDLVERNKDQPSAQRKMASIVDIHLRAL
NFVSHIENVMHLVCFVELTGCTLNMCMLEYYMLTENSKETIATYGIIYASMTFNIFIFCYIAEKLT
EQCKRVGEKAYMTEWYRLPHKTAAALILVISRSGLITKITAGKILPMSLATFGDVVKTSFMYLNML
RTVAV
>DnOr31
MASESVTFEADNLNDYSLQLSRWYLLPIGAWPSSSSASRLERIVSVTLIVLSYSLIMFTVVPSFLS
IILEDETVRLKLKLLGPLGHWFVGGINYTTLLMRSKEIRHCIEHMGTDWRIITRTKDQQIMMKNAK
FGRYVSAFCAACMQGGVLSYCVVSALTMRVVQVGNETRTVHVLPCSVYKKLLNVDNSPMNEIVLVS
QFLSAFIVNSSAVGAFSLAAVFAAHACGQLDVLMIWITEFVNDSRRQRKNACLNKIGVLVEHHLRV
LSFISRIEDVMTRICFMEMFKCTLDICMLGYYILTEWSDKDVRNLTTYFVILMSMCFNIFTVCYIG
EILSEQCIKVGEVVYMSNWYHLPDKLVLDLVLIITRSSVVVKITAGKLIRMSIYTFGDVMKTSFAY
LNLLRQTT
>DnOr32like_1
MLGEATMIERDLGDPSDYSLQLNRWYLKPIGAWPTLPSTSRLERIVAIVLILLCYCSISFTVVPCM
LHILLEDEDIRMKLRAAGPLSHWFAGGINYTTLLMRSNEIRHCVEHLQTDWRLVTRVKDRQVMLKN
AKNGRYVAVCCAAFMQAGVLSYCAVTAWSTRIVENGNRTGIVHVLPCVLQTSVVGIFSLAAVFTAH
ACGQLNVLMAWITELVNESRGRNARIYLNEIGVVVEHHLRVLSFISRIEDVMTRICFLELVRCSFG
ICMLGYYILTEWSDHNFQNLTTYFMIMGSMTFNVFIVCYIGELLTEQCKKVGEVIYMTEWYCLPHK
CVLDLTMIIARSSLLIKITAAKMIHMSVSTFGDVMKTTFAYLNLLHQMV
>DnOr35
MATTDGDNRGDDWMHSVRINRWLLKPIGVWPVSLCVDTMEKMITVLLVLISASLIGFLLVPCALCT
ILDKTDDLDMKIKMIGPLSFCVMAAIKYCILVSRGGQISKCIRIIRSDWTRTNMDELVGNKEIMIE
YANIGRSLAIFCAGFMYSGGFFYTAVMPLCTKRTEIIDNVTVRSQAFPIYRGLLDPRTSPSFEIVQ
LTQCLAGFVIYSVTVGACSLAAVFVMHACGQFRILGMKLDRLVNCSMGRADRKVTEQCLGDIVQHH
LRILGFITQVEELLNEICFVEFVGCTLNICFLGYYLLTEWEQSDPIGALTYCTLLISFTFNIFILC
YIGEILSEECKKIGLTAYMINWYRLPGKEALDLILIFAVSNSSRKLTAGKLVELSLGSFCSVLKSS
LAYLSLLRTLTT
>DnOr52
MQPKGQNRLNNYDHETDLDYTLEMCRWLLTPIGVWPLVKKCAGKHERAMSIVLLLVCFTCLLFVIL
PSGYNIFFVEKSMQNKVKLLGPVGFCLSSAIKYCYLVAKGTVFGRCIEHVQKDWQTVEQPCHRDIM
LKHASLSRQLIAICAIFLYTGGMSYHTVMQFLSKDRSKRNFTVRPLTYPCFDTFLDTQSSPTYEIV
FSVHCVAAMIMYSVTTAAYSLAATFVTHICGQIQIQMARLENLLGDSREKSSFHDRMTVIVRSHVQ
VLRFSKNVEKALRGICLTEIVESTLIMCLLEYYCMMEWRNSDAIAILTYLTLLISFTFNVLIFCYI
GEVLSEQCSKMGPASYNVEWYNLPAKEAHNLVLVSAISLYPPKLTAGKIIELSLNTFGTVVRTSVI
YLNLLRTVTTW

>DnOr52like_1PN
MRDQADKHSSYPTYKDDANYVLGYCRKLSRIIGLALLYDPVNKKEKIASIMYIVLCFAVLLFATIP
PFYYVIFDEKNTNKRLWALGXVGYNVTSTIKYLFMVYNANLFKNCLIRVDQDWQIVREPSHREIML
KXVTIYRKVSFYYLITFYAATGFNNTIIPFLAPRWHSNNTKKMLRVPGYDKFFDTQSSPTFEILYS
LQLFSSVIKITSTVVATILITTCVCHICSLMQIHIMQLNHFVENRQNLDDRLNPLTMIVHEHAETL
RRIGKVVQLSSFTTYPNIIQIGGTFLQFHHIFQMFHNECYISILLY
>DnOr53/54
MYARSCSRIDKNRPNNNDHETDLNYTLEMCRWLLKPIGMWPLVKKRACKLEKATSIVILFACFTCL
LFVILPSGYSAFIVEKSVQNKVKLLGPVGFCVSSAIKYCYLITKSTVFGRCIEHVQRDWQMVEQPC
HRDIMLKHASFSRQLIAICAIFLYTGGMSYHTVMQFLSKERNKQNFTVRLLAYPCFDTLLDTQSSP
TYEIVFSVHCVAAIILYTVTTAAYSLAATFVTHICGQIQIQMARLENLVADSRGKSSFQDRLTVIV
RSHGEVLRFSNNVEEALREICLTEIVESTLNMCMLEYYCLKEWENSDAIAILTYFTLLISFTFKVL
IFCYIGEVLSEQCSQMAPASYNVEWYNLPPKEAYNLVLVSAISLYPPKLTAGNIIELSLNTFAAVV
KTSVVYLNLLRTVTTW
>DnOr53like_1
MPDRSQRTMIVGQTGNVNYENDVSYVLEVCRFMSKPLGIWSLIYNDTSRLERTISVVLLTAGFCGL
LFIAVPVTLHIIYEEMPLHVRIKKLGPDSYCILALIKYFYLALKGTAIGRCIEHVTNDWRTVKEPR
HRDIMLKQAGVSRNLSKLCIVFIYTAGLSYHTVLPVLAKRRIQSNQTTRVLAYGGYDRFFDVQSSP
NFEILYGLQCVAGFIKYNVTAAIFSLTVIFVTHICGQMQIQISRLGELTNKRREKDVVGSGSIGVI
VQDHVEILRLSKNVGEAFSEIILIEIMGSTFLLCLAEYCCLMEFQAASALAITTYVVYMVSFTFNV
FIFCYIGEILTEQCEQIGVASYEMDWYNLSPRIACNFMLLNVMSLYPPKLTGMNIIDLSLNTFSVV
MKSSVVYLNLLQTVV
>DnOr60
MESFPVQEPNCGEPRNPYYEKDILQILRYNKWILSSIGIWPIRSGDTNRYLPKLVIGLCNGILFFA
IVPCTLHITFELKDTVMRLKLLGLLTFCVTSFFKYWVLAACRSGLRECVECVQNDWKQVERGGDRE
SMLKYGNVGRNLTLLCLVFMYTGGMMYHTLVQYAIGSFVDEQNRTIRPVIYPAYSGLYDPQRTPIY
ELVYGLHFMCGYVIYSVAVGACGLAALFVTHVCGQINIIMSRLENLIDRKKNTDPGQRLIEIVEHH
IRTLRFSTKIERLLQEVCFVEFIGSTFLICLLEYYTITDWEFNNTLSLVTYFILLMSLTFNIYLLC
YIGELLVEKTSNVGLFCFTIDWYCLPTETVRGLVLIIAMSRNPAKITAGRVADLNLYTFVTVLKTS
LAYLSFLRTTVME
>DnOr60like_1
MESFPVQKQNNGIPRNPSYEKDILRILRHCRWILSSVGIWPILLKDTSLYLSKLAMGFWHVVVVVT
MVPCSLHMKYELNDSLRQLQLAGLMVFCMLSMSKYWALVARRLIFIECIERVQDDWKQVDCAGDRK
LMLKYGNMGRNLTLLCLVFMYTGGFMYHTVVQYMIGSYVDEQNRTIKPLVYPPYSGLYDSQRFPIY
QLSCVLQCMCGYVIYSVAISGCGLVAIFVTHVCGQIDIMLSRLENLVDHKENTDPGMRLKEIVKHH
IRTLRFSATIQTLLQEICFCEFAGSTFLICVLEYCTISGWDDSDPIALFTYTMLLISLTFNIFILC
YIGELLVEKTSDVGISCFMIDWYHLPTKTIRCLILIIAMSGNPAKITAGQIADLNLSAFGNVLKTS
LAYLSFLRTSVME
>DnOr62P
MEERLVFSERVLRLLGVWPLCGNDIMXTSLGRWCFAMLTQISTICSLSMEVYRHCLDMDDTMDAFV
MDLSSVICLSKVLILRYNWKHTYSLVNTIAEDWSTVEDSRHRRIMAEYREKGRFVSSTMLYLGYAS
GLSFVVKALPIHDLLPFQMLENSENSTGRVGQSPKKNYFMATYCVFGPQPFTRRAFVLVVQAVCIF
VNAIGHCGNDGFFFSLTMHLCGQFEVLKMNLGEIEIGKAGHRRRMGMLVKRHCRLILLADDLEKSF
NMVMLVQLLMSLLLLCVEDFMVLVYLNSMDNVAVLKCLVIIVTLLTQLYVYTYAGHVLESRTEEIS
YAAYDSPWYRSRGHAARDLALIINRGNSPYRITAGRFVSMNLVTFKEILKASVSYMSVLKVMMDT
>DnOr64
MKTPTNKDFDYAMTPIKVLSWPVGTWPLQTYNFVSGLRCAISIVLLLLMLLIVNTEMYLDHSDPEK
NLDALLFIACGILAIWKTLCFRVCCSGLVSNFTSALKDYNELLEQEKRTIVRQHAKMGRIACASVI
FFSYLDSTIFTTIPMLAGEDEVAFSKSNVTQEDSLNYPIPSEVTLKFLQVPESLYVVIYITEYLFL
LITSTGNLGGDSLFFGIMFHLCGQAEVLKVDFDRFVEGTENLSQRFNALVLRHQELLRLSEQLNET
ISLVMVIQLFLSCILICTTGFQCILSLSNNNIVMTVKTFSVVTTLLIQLFAYSYIGEYMKNQFDTV
GYRAYCSDWYNIPCDLSRDIIFVLMRSQNPVQLKAGSFFVVNMETYMGILKTSMSYLSVLRVMVTS
>DnOr68
MTVLQPAFFLLTICGCWRPSMWTMRYKRVLYTLYTILVISFLHTFCISQFLNVILNVETADELSDS
VYMFIANVLSCFKIITLLMNHNNIEMLCKKLNRKPCKPRNHEEAKIQRSFDNRIGSVTIYYTALVE
TTVLCMILSSIFTDFRHKKLAYNAWLPFNYSSRRLYYVVYGHQIIALIGTSLLNVACDVVICGLCV
HSCSQVEILKHRLEELPMQVRPQIGDIVHFHNYLYGYVDTIQQKFRVIIGVQLVSSTLVVCFILYE
LSNTPLMTAKYLQFLLYMACMMTQIFFYCWYGNGLKLKSVQVVDTISEMDWTRLDNSTKKSLIMIM

RRAMNPIELSSTYVFTMDLNTFVSILKMSYSTYNLLQRKKETNV
>DnOr69
MRILKFTFSVLTVCGCWRPESWTSQCKRLLYTVYTLIVLFLVYSITFYQFMDILVNVETQNEFADN
LYMFLAMMVSCQKIYGLLASRGNIAGLMNSFDEKPFLPENFEELGVRTKYDKKAHVNAFLYMILLL
GTVSCMSMTVFLTSEKWQLVFRLWLPYNYTTIHLYTLTYVQQSLALYLGALLHVACDSLICGMLIH
INSQCEILGCRLKALKNGEYEAVKSCVRYHNRMYRFARALNDEFKMIIFVQFAVSLLVVCFNLYRL
TQTTNPDAKLLEILLYSSCILTQIFIYCWYGNEVKMKSLEISDTVYDVNWAMLDNNEKRILLMIMR
RATVPIEFTSVHIASLKLESFVSILKASYSAFNVLQRGQR
>DnOr70like_1
MRTLRYVCTLLSVCGCLRPSSWTSPSKRILYRVYTAVVWLLIHTLAISQVLDLIVNVENQDDFSDN
FYVTLAALGICLKMCNVLMNQRNIGDMIDRLEKEPFSSVNKEEDEIRTRFDKVSNRNTLAYTMIIK
FYVLSMCLMSVFNDFRYNKLLYRAWLPYDYSTSFRFTVTYVHQIVAIIYCAFITVACDSLFSGVLI
HTYCHFEILGHRLKSIAKNDSESAKQCVYLHDHIYKYATMLNKQFKVIMFSQSLVSINTVCFNLYQ
LTQRDLGSKLFEVILYSFNTLMQIFYYCWYGNEVKLKSLEVPKMIFECDWACFDNDTRMTLLLIMR
RATIPVEFSSIHVVSMDLDTFTKVLKMSYSAYNLLQ
>DnOr71
MYILRRLNTFWMLCGCSRPTSWTSPTKKFLYTVYTIFLLLTIHSFLLTQILDLVLVVDNQDDFGDN
FYVTMSMLVTCCKLNSMLITRENILGLLNTLEGEPFLPVNDEELEIRTKCDRITEWNAMAYSISLL
SCVTWMFLMPVIMEYENRKLTFRVWLPFDYTSAGAYCLAFSHQLLAAIICCVSAIACDGLFSGLLV
HIYSQFETLGNRLRNIHRDENDSVKQCVRHHDEIYKFAGMVNQQFEGIMLIQFLTSTAAMCFDLYR
LTQKDFDLRVVDVLLFMFCTLIQIFYYCWYGNEVKLKSLEVPGMVFESDWLSLSNRSKKSLLMIMG
RASVPIEFTSIYFLSMDLESFKALLKISYSAFNLLQQTR
>DnOr71like_1
MYILRRLNTLRSLCGCSRPTSWTSPTKKFLYTVYTIVVLLTIHSLLLIQILDLVLVVDNQDDFGDN
FYVTLTVLTTCIKWNSMLMTRENILGLMNTLQGEPFSTVNDEELEIRTKFDRIAEWNAMAYTITLV
SCVTWMFVMSLIMEYENRKLTFRVWLPFDYTSAGAYCLAFSHQLLASMICCVSIVACNGLFSGLLV
HIYSQFETLRNRLRNIHRDENDSVKQCARHHNEIYKFAALVNQQFEGIMLVQFVTSTATLCFDLYR
LNRMNLDLNIVDVLMYTACTLLQIFYYSWYGNEVKLKSLEVPGIVFESDWMFLSNSSKKSLLIIMQ
RASVPIEFTCVHLLSMNLDSFKALLKTSYSAFNLLQQTR
>DnOr71like_2
MQMLQWTSGILQFLGCLRPPSWTSSSKKFLYNMYTLFVFTLMHSLLITVLLDIVFNIENQSDFSDN
VYATMSIMISCMKMCCFLASRQHIVVLLDTLEKEPFLPVNAEEMKIKRTFNRTNETISRSYTILLE
SCVTLVFIASLFRDAKNRKLTYRAWMPYDYSSLILYSVTFCHQVVSVVMCSLVTIGYDTLFSGLLF
CINCQLDILGHRLQNLSKDEKDSAKKCARYHNRIYEFAANVNKGFQTVVCVQFLTSSVIICFNLYR
ITQKDLQSTFFETVLYSFCILMQIFYYCWYGNEVKRKSLEIPKVIIESNWSALDDNGKKILLMVMR
RAMFPIEFTSIHVLSVNLESFMTLLKGSYSVYNLLQQRESSKFS
>DnOr71like_3
MHTLRLVSKILSIAGCWPPTSCSRRKKLLFDVYTVVVFLFIHTFTITLILDIFLIVDNLADFNDNF
CITVPLLSTCFKLYSLLANRRNMETLLDTLEQEPFAPVNIDECEIRRRFHRLTEWNTTVYMILVKF
CIMWMFVTSSTNYRLRKLKFRAWLPYDYSSTVLFNLTYFHQIVSALYAGLLGVSSETLYSGLLIHT
HGQLELLEYRLKNIEKNENYSVKRIKSVFIFLPRFAARVNRGFKTIVSTQFLFSTLVVCFQLYQFT
QMKRTMNSELLEMMIFLFTILIQVFYYCWYGNETKLKSLAIPDMIFGSNWTSFNNNTRKILLMIMR
RATVPIEITSVFVVSMNLQTFVTILKNAYSACNLLQKHQR
>DnOr71like_4
MGLSYLFTISTFCGIWRPLSWSSRFKKVLYNMYSVFVVCMNVTFTISQIISVLTVDNLDDFAENTY
MLVPAIVCCCKLINLLVYRGGIIELVKIFLEEPCATLNDKEELIQMRYDKQIRSRTNIIKYTRMVK
SSTVMILFTSFLNDFRNGRLTYRAWIPYNYSSTTAFGLTYIHQMMALAIAGYVHLACDVLLCGLLS
QICCQFDILQCRLNEVKKNETIALRECVRHHNRIYKLAAMVNDTLNLTIFAQFFGSFLALCLSLIK
LIKENTLSTQVISLLFFVVMIMTQSFLYCWYGNEVTLKSIAVADMIFQMNWIETNEQAKKILIITM
SRSRSPIQLETAHVMTCDLDFFVALLKSSYSMYNVLQSTRK
>DnOr73
MHKLPLSFAFLTYSGYWRPTRWSVHSLKYWLYNVYSVFMVLLLYTFTFCAFVDSVISKDLRTTTDK
FSVFISILGVCIKIANLFLQREKIIRVVDILLTENCVPRDEDEMMIQRKSDAYARRLTIYCEILNE
SAASFATVAQFDKLIRTRTLPISDWVPYDLSSRKVHMISLLHQTFGLMICANASVANETLIAGLMI
QVRAQFEIFCHRARSLPVSLLEVQRNSVSFAETVNAIFQYTIFLQFMISSTVLCLSIYTMSTADSF
DVNFVWTFSYLCCMLMQVYLYCWFGNEVTLKSMQVSDAIYEMDWTTLPVNIMKDLLMIIKRSKRPF
RMSSAHIITLSTDSFMAIMKITYSSYNILKDSSKK
>DnOr77N

MSWIKNKDISIAISALLMNCVGIWCADNPSEQRFRNATLMYTVCALIFAVWVQAQDFYHSWGDFGT
CAFIASNILCLNIALLKIFILSIRKKKFLDLVVYMEKHFWHSNYTPHEQTIYNSWKRLCTYFICFF
TFFTEASIVGYVIEPITANIGKNESNRTLPFNIWLDLPLSMTPYFEITFLFQVIINLMNIILTY
>DnOr80like_1
MQTKRHSDISIGSCAFFMKIIGLWTAENEREQLYRNIALVYTHVANVFGACVLFRDLYFCRSDSNL
YAVCNILNKLIVSIKLIVLTAHRGEFLDLLTYMQKHFWNGNYDHHEKTIIATSLKNCILFTTIVTA
ISHITLVCFLITPIIENKGKNESDRMLPFNMWLVNLPLSITPYYEMMFTFQGLLLYYTAICYFCFD
NVLCVMSQHVCGQFRVLQYRFTKLYDSEETIKNQNENYAAKSYVQFRKCVRQHQELLNYCWRLENV
FPVIVLGQVVIFSVLICLYGYQAFLAQSTIARRIIYIWFLVGSASLLFMFTYSCHELAVESENIGD
AVYSAPWTLVPMNKDGKMLRDNLKLTIFRSSKACCFTAYGFFPVYVETFTTVINTAVSYFTLLKQS
LKDSLEA
>DnOr80like_2PC
YKVCVFGFLYCVLLGGGKYPLTFELRLVCSLCILLIFRVXWLDVGLSFRGRFLVAYSLEGFLLYYT
AIYYFCFNNVLCVMSXHVCGQFRVLXYRFTKLYDSEETIKNQSENYAAKSYVQFRKCVXQHQKLLN
YCWRMENMFPVTVLGRVVIFSILICLYGYQAFLAQSTIVRRIMYMWFFLVGSASLLFMFMYSCQEL
VVEIENIGDAVYSAPWTLMPMDKGGKMLRNDLNFIIXRSGRVYCLSACGFFPVYFQIFTTVITENL
LNPVFVIHTIT
>DnOr84
MRLARSDDISIDWTSLLMKIVGLWMAADRAEQRRRNFALIYTITIIVIATSIAFRDIYFSWGNLND
CVFVSCNILYLMFVFFKIVVLYVHRIEFFALVRYTQKNFWHSNYDPREKVILADCKRVCAIFIVVI
SFCTQGTCAGYVMTPLFANVGKNHSDRILPFNMWVKFPVGISPYYEISFLIQTLCVYHVAVGHICF
DYFLSIVNLHVTCQFRILQYRLLSLGNVIETQTDEESLSRYTNVCLAKLRGCIQQHQALTEYCRRL
ENIFTLIILAQVLFLSIVLCLISYQLFLVNVPHFRNISLVMNMIGILCMLFMFTYSCDGLIRQSTN
VGRVTFLAPWSSMSMNKAGKTLRQNILITIMRSNQSCRLTASGYFPISLETYTGVLSTAMSYFTLL
RHKSLNVEVH
>DnOr84like_1C
DDVFVSCNILYLMFVFFKIVVLYVHRIEFFALVRYTQKNFWHSNYDPREKVILADCKRVCAIFIVV
ISFCTQGTCAGYVMTPLLLANVGKNHLDRVLPFNMWVKFPVGISPYYEISFLIQRLCMYHVAVGHI
CFDNFLGIVNLHVTCQFRILQYRLLSLGNVIETQTDETNVYLAKLKGCIQQHQALTEYCRRLENIF
TLIILAQVLMSIVLCLISYQLFLADVPQFRNISFVMNMIGTLCMLFMFTYSCDGLLGQSTNVGRVI
FLALWSSMSMNKAGKILRQNILITIMRSNQSCRLTASGYFPISLETYTGKLRDGSFLPLLQSTCYP
RTVGFFLPKSCHGGSELTM
>DnOr86like_1N
LCIPLSCVLMKPTGCWLANNNGERRRRRFALTYTFALLFFSIYINGTDIYHTWGDFSNDTFTACNF
MRTIMAIAKTVLLCRRKTEFCELVLHMQRKFWHSNYDSREQMFLKDARNFLTLCVVFVCFFTKSTL
VNYVTTSIVGKLNTEVSDTV
>DnOr87
MRTEQSKDYSIIMISFLTKLVGFWPATNPVEKRWRQFAFIYTICLLFFGAYLEMVDMYYAYGDFND
FIYLTCNLVTIILVLYKILVLYVNKKQFAELLLYSEKNFWHAKYDSYEKLALAKCRRTCTRLICIF
SFFAQGTAVSYTIGPIITYIEKNESDRTLPLRLHSNFPLYETPYFETIFTIQVLILYQYGVCYLFV
DNCMCVINLHVATQFRILQYRLKNMYTVSTKKKQGNTSETTSSIYADKCYATFKRYIKQHQALIAY
CNKMENVFTRIVLGQVLLFSLLICLDGFLVLLDEAPRTRRLIFAFHISGCLVQLLMFTYSCDCLMQ
ESTKIASAMYSAPWTHLPMNKSGRMLRNDMRLMILRARNPCCLTASGFLVVSLETYTKVLSTAVSY
FTLLRQY
>DnOr87like_1
MGKLLNLRKINASNHSESPVVSEQSRDISIKMSLFFMKLTGFWTSSSIEEDRLRTLAFVYTHCAIL
YGMFVQLRGLYYTFGDVSEFLYITVITLSITLVLLKTVLLSIHKKGFLDLIVYMDSNFWRANYDSH
EKQIIRDCNKVCSFFILFFTFFANGTVVCFTLQPIVENMGRNKSDRKLPFEMYFTEARWLSVSPYF
EMVFVAEIATLFHIALGYFCFDNFLCIMNIHVTGQFRILQYRLSKLYEEKMLQTDDHTPDKILLQY
TTKCYAALQSCIRQHQALITYCNVLEVVFSKIVLGQVLTFSALICLQGYQLLLSDAHLSRRLLFMC
FLTTTMCQLLMFTYSCDGLVQESSGIGTAAYISPWPLLPMSRQGHKLRDDLRMVVIRSRTPCCLTA
SGFFPISLETYTTVLSTAMSYFTLLRQSTIS
>DnOr90
MMTPVRDQEDKLGRPFTTQAKRHLDISIRSCAFFMKIVGLWTAENEREQLYRNLAIVHTLVSNFCG
YVILFRDLYFSWSDSNLAMYVFCNILTMLLVSVKLFALMAHRGEFLDLLTYMQKHFWNGNYDHHEE
KIIATSRKNCILFTSIVTIVGHATMVSYLLTPIIENIGKNESERLLPFNLWVQNLPLSMFPYYEII
FAIQGILLYYTAIAYFCFDNFLCIMSQHVCGQFRILQYRFTKLYASEETMTNLNENYAAKSYVQFK
KCVRQHQVLLNYCWKMENVFPLVVLGQVVIFSVLICLYGYQVLLAQSTFARRFIFISFLIGSGSLL

FMFTHSCHKLTEESANIGDAVYSASWTIMPMDKDGKMLRNDLNFTILRSRKACCLTACGFFPVQLQ
TFTTIVSTAVSYFTLLKQGLEDAVETQ
>DnOr90like_1
MRTERYSDISTRLCAFFMKIVGLWTAENKREKLVRDLTLIYTFVTHVLATTILYRDLYFSWSDYNV
SILQMALYVVSNCLSLNIVTVKLFTLTARRREFLELLAYMQKHFWHEKYDNHEKQILAATKKKCIL
FTSIVAMFAHATILCYVITPIITERILPFNLWLDNLPLSISPYYEITFFIEIMLLYYNGICYFCFD
NFLCILCQHVCGQFCILQYRFTKLYEADKETKNRNKIINSANFTMKSYTELKKCIQQHQLLLYYCN
KMKNLFPQVVLGQVIIFSALICLYGYQAFLARSTATRRFNFIFIMSEGSYLLFMFTYSCHRLIVES
ENIADAAYLAPWTIMPMDKYGRRFRSDLHLTILRSKRACCLTACGFFPVNMKTFTTVMSTAVSYFT
LLKQRLENTLEA
>DnOr95
MFATQSDDTSIAITVFFMKLAGYWTAVGRAEKCCRFCALVYTIASLIWGIYVHTFDFYYTRNDFDA
SLYAICNLLSVLMCVLKLFVMLSHKKDFFRLFLCLERKFLNSNYDSYETMVMGTCKRSCTAFIGFL
TFVTLATICSYTVSPLITNIGRNESDRVLPFNLWVGVPISMTPYYEIAYVIQILTAYPTGVCYFCF
DNFLCIMNMHVATQFRILQYRLANMKTSNVEEYPRGEFRSCISCSAEMYYDKFKGYVQQHQNLIAY
CDKLEEVFNLIALGQVLLFSMLICLDGYQILMANVGTERRLIFVFHLLTSMAQLLMFTYSCDGLIR
ESLNVATAAYVSPWIYLPMNKYGKTMRNDLILVIMRSRSPCCLTGRGFFVVSLETYTSVLSTAVSY
FTLLQETHDV
>DnOr95like_1
MSWIKNKDISIAISALLMKCVGIWCANNPSVQRFRNATFIYTVCTLVVAVWIQSQDFYHSWGDFGT
CTYIVCNILCLNIAFFKIFFLSIQRKKFLDLVVYMEKHFWHSNYTTYEQTIYDSWRRLCTYFICIF
TFFTEASNISYVIKPIAANIGKNESDRILPFNIWLDLPLSMTPYFEITFLLQVLSLYHVGLCYICC
DNFLCIMNLHVAGQFRILQYRFRSLHTLQIKDKEHQIQNTGSSYIAEEYYNSFKTCIQQHQALTTF
CAGLESVFSQIVLAQVLTFSLLICLVGYQLVLTNSSSTSHVIFVNLLSSTMCQLFMFTYSCDCLIR
ESTDVCTEAYVAPWSELPIDRYGKMLRKDLQFVMMNEALSSRGSSLEARGFYVLHVHC
>DnOr95like_2
MLEKSDDFSLSFTIPIIKMIGFWIAPNRRLQILTNCNLIFSCTQVSFLFYGTMYDIFYHCPDFNYL
IYLLCNLVTVGLCLFKTVIMLTYKRKFFSLVLYLQRNFLKSKYDAYEWSLMVTCKHTCTFFICTFT
ILTYATTSSYVISPLIENIGKNESDRKLVINTWIETLTYSPYYEIMYIVEVLCIMKCGFLHYIYDN
FLCILNLHVANQFRILHHRLENMKSPYDEGLVQEAMTTEDMRKNESRIGAEYYATFKKYIRQHQIL
IAYCENLEEVYNIYALGQILVFSMILCLDAYQLAEAPLTRKFIFLFHLLGSMAQLVMFTYSCDGII
QESLNMATAAYASPYTNLPMNVFGKMIRTDLIIVIMRSGVPCYITACKFFPVSLETYTAVIVVSTG
VSYFTLLRQAGETSP
>DnOr95like_3C
ICFLLLNTFVQNLSITAFIQVLSLYHVGVCYICCDNFLCIMNLHVAGQFRILQYRFRNLHTLQLKD
EEHERPTTGSFYSAQKYYNSFKICIQQHQALTTFCAGLESVFSEIVLAQVLTFSMLICLVGYHLVL
AESSSTSHVIFVNLLSSTMCQLFMFTYSCDCLIRESTNVCSEAFASPWSELPIDRYGKMLRKDLQF
VIMRSKRSCCLTANRFFPVSLQTYTSILSTSMSYFTLLKQNSDDTTNT
>DnOr105like_1C
VATFFTHEYDRRLLSRTAVFILAMTAFLDKYALFYVHMEKVRTLFDRIEYDWSRLANMNEYNILKS
YTENGKNVQRGCIVCVIPILIVFQLYEYSPMILDFVLPLNESRSNELIFKAELFVVDVEEHHMSVH
LFATIVLTMGLGTLVLADGTVTMFILHSCAMFKITGYICLFCRYRIQCAINTENKRLACKRLKDAV
LFHRRSIEFADSVKEFCSTSYFIFLICGSCSITMNLYRVSRNLWNDKKELAEGVYFSIYQFFYLFV
SNYASQMLINHSSHIFPLTYDTEWYSLPLQAQRSIITILGRSMKPSILTLGKMYDPSCEGFAKIYN
MSMSYLMFLCSI
>DnOr105like_2C
YDTEWYSLPLQAQRSIITILGRSMKPSILTLGKMYDPSCEGFAKLLPLTVQFLAILYSL
>DnOr107like_1N
MDEQAIKAEYLGTNIFCGKLVGIWPEQRKSLKYLARTIVLILAIIVAVTQVCTYVTRDPVVAVLHS
AQIENP
>DnOr108like_1N
MINVVTTFLGLVMPLNTSCPHHFVVLIEYTMVQEKSLFLLYAHLLTIYVFGTSMVAATNSLIFLFI
HYRCAMFRI
>DnOr111like_2N
MRLLEEPQYYIMKFFMMYIGLWPYESRKLAMVKQMFAVGLIISIAIPQV
>DnOr113F_C
KFLGQWPNQDPMDRGRRIIFIVFIIIIEVITTVTIQLEYLQSQINSDYKERTSEKELEIMERYW
SQGKQYAKGYMYFLYTATAVYCGFVFETRLLDIISPRNETRPYKFIYPAYFALDEHKYYYLISVFL

SGYILLDIITFIGFDTMLIMYAQYICGLLAFIGNQLEYELYKCTKDGEPIEHQRRAYKSIIEDSVR
THRKTLQLFVNRLDSAFMLPQLLQLGLNMIFLSISLVKVSDNQRGTFETFRHSIYIMGQLIHLLYI
CYQSERLIDHSLAISQNAYNGQWYNIPTETQKMLMLISRRSSIPSYVTAGKIFIFSMQSFSMVIQN
SMSYFMALTSLQ
>DnOr114
MDVKKELTYVYGWNHYTMRFIGIWPDERKWNQASSYLVLTPILMMLCFVCIPQTLNIPIIWKDLYL
VVENLSVANITITIALLKTIAFWFNGKPLKSLLRCMSRDWASDVKPSEREIMMGIARTTRTTIIRS
TLMVNAVVGFYCFLRYYSVNYSENKLLFRGYFPYDQTTSRNILMTAFGQLIATLYAAASYTAVDTF
VAMLVLHVCGQLSILKQDILSLRENGEEKMQIALGRIIRKHVYLNAFVETIEKCFNMMLLLQMLGC
TVQLCFQVFQAIMSLGGEEKWIAIFQILFLALYVVYVTLQLYLYCHVGEKLTVESADIANAAYNSE
WYNLSAKNAKLLVIVICRGQLPLQITARRFFSLTMVIYAEILKRSMGYISVLYAMKNK
>DnOr114like_1
MLITGVWPDPETRNGRSGGYRALISCSCIMSFIVMPQTLNLFRLDGDLELITQTLTLAQIPALNAV
IKIAFAWYRQTGKTMIKSFYDDWYTVRTEEERETMLNHAKVTKNISICCTILTQTMVTAYISLRLF
EILTSDWNEESQHRLVIYPGFFPFNVRPTLICILINMAQVFAAYCAREVDGRNERAKKSYNESMVV
LSSQDILSLSSRNKYNDEHFLFHRRMAASIEKSFNKLLLLQMLLCTVEICFQWFLFFSVIMKDENG
LLNFQMVFFVLFVVFITVHIYLYCYVGELLLVGGSSMAISAYNTSWYNVTPAEARCLLFIMLRSTR
PLCLTAGKFGTFSMELFSSVIRTAMGYLSVLLTVTGSDN
>DnOr115
MGWSQFNLTLLGVWPEPRKVSNISRAISSLVFWSTTFVTFMFICVPQTANLILKSTSQDEVIENLS
INIPIFFALAKQIVLWYYKNDLALLLRQILDDWSEPISGPDRKMMLKTAKLSRMISIFCSTLAYIM
LFAFISLQIWSNMENSSEADLGGLLHPATFPYDTKKSPNFEITWFGQFIGTTLMAICYSCVDTFFA
VLVLHLCGQLTVLRAALENLANATKENDFAKFQERLAFIVCRHNQLSRFAVIVEDCFNLTLLVQIL
IGTAMLCLTGYRMLSSVGEEDADLPVVGLIFYILHVICTMLHLFIYCYVGEALVTESTGLGQSTYD
CNWYDLPPKQAVSLVLIICRAKVSFQITAGKFSPFSLKLFSAVIKTSAGYLSVLRAMKT
>DnOr116
MDVFALERTKRRNITPNKHLNISLLMIYYMGMWPAQGKYRYLYFMYTICCFTFLLGIILTTEIAYL
IVNWGDIAKLAAGATLLMTNTCHACKVIIILCRHERIRCLIDVTRSKLFSRDNIKYERIVTYYTWQ
GIFHHIAYQSFGTMAVISWGVSPIIDLLSERSKQLPIEGWYPYNVTVSPVFEITSFYQAVAIFICC
FNNVAIDTLITGFITIACCQLTLLNRNISSLNPEAEKQSIVSKDDVDVKRSTIETYNRTYDNLKLC
IEHSNMIFDFSKEIQSIFGTAIFFQFLVNCIIICLIAFNMAQMKVYILHVLFGMLMYMCCMTYQIF
IFCWHGNELYLHSLDVVLASYSNDWWQRTKNFKRAIQIIMTRSQRPLILTAGNIMELSLQNFVRIL
RMSYSIFTVLQTSTD
>DnOr117
MKELSKSSIDHYILPNKIFCGMAGMWPIDENSPTYSKVFAYFRLLFALTAISSVFVPEVMAIAANW
GDLKVLAGVGCVLTTVGQLLFKMIYLLARRKKAYRLYYELRDLWITSNDPEERQSCEELAHWARLL
TLIFYISCMCNVVTFSVAAGFDYFKIKYNVSNAAEEIRHLPFDVWYGTDITGSPEFEIAFACQFVA
SMICAAGISGLDATFMTIILHVCGQFKLIKIWITKIGTEISCDSINGNSSRKLEVNLLRCIRHHQR
MLNVVNEVNNLLTPVIFVQLLTSGVEICLSGFAVLDNTTGTDLLKFISYLVSMGVQLLLWCWPGEI
LVEESQQIGHVIYLNVPWYVLPPIYRRELCLIIVRAQQQCSISALTFQTISIRTLTSVFNTAASYL
TLLRQMQQK
>DnOr118
MTVTSMINRPLELSIRLFGVWPNSSRWILKGVTWTTLITTFLVFQYRYCVTHFKSGNLIELLDGIS
ILLSNTLVLFKFVVIWIYRRTFHSILTTMLEDWQDHTSAAKTKQIMVNQATLSSRISNFLFVYYVL
CYMSYVIIFFRPFGTNHGSDFNSRKLLLKMEFPFEVTYSPLYEVILVLQFVMEMFIALAASTMTAL
ITALIIHVGSQVDVFCNKLQEISDSTGKHELPIVEIRDIVLRHQRILSLSRNIEVVFTYMSLMQFL
ANTMVICFMTFVLTLALHSAEGSDIIAKYVPYYIAINFEAFILCYTGEYLTSKGDSITQAVYNFSW
YELRPSDTRVTVFFIILRSQSQLKLTAGKFVTLSLTAFAGVRCFIVCFA
>DnOr119
MFSAMSIFAVTLFADIYSNIDNLSVATDDGCISAGIIVVIFKAIIYQVNREKIVRVIRDVLKCADD
LTEFSNVDRIRDIIKSHYTFNKVISHGFNILGCVLVIALLFFSPTENNLPIRAKYPFNTTISPWHE
IALGIEICAVSGGVLAILSMDSITVLLCNLIIMQFDILNVNFENCRRRVVNDVSKKVNFSSQTNRG
CDEQCYKDFERHVEKTESSVFLSRYKTCIRFYQRLVSVTKDYNKTYSSSLFIQMLSSTSMICLTGF
QVVVVGGQSSDVIKFGIYLSAAVSQIFYSCWIGNELIYSSSVLDRSQWLSDWHQEPLSDIVQVFTL
SMMSARQSTQIKAGGFYVMSLQTFITIIRRSYSIFTLLNNMQVTNS
>DnOr120
MNIENYTFINQLVLKFVGLYPINITRYIICINCMLLIVIPLVAHIYKNWENLNIILETSSVLLTIS
LALTKSLIWIFNRKELESFIDIMLTDYWKLVEPDVFTDLQKYAIYAKNVTKGYLFLIVNALLFFYS

LPVIDSFMSIIRDTNDNSTVPKNFPFVASYPLTFYKFPFYEIVYISQMLGTCICGLMIVATDTLIA
SALLHTCGHFKILKRNLRQLDSDIYYVSLMKFRFCSNMQKNFHSLLFVQTMASSIIICFIGLQVSM
TLMDQSNLMKYASHLMMALFQLLLFCFPGDMLISQSSSISRAVYSIQWYKLPLFIKYETSMIMLRS
QKPSYITAGKIYVMCLESFSSTLSTALSYFMMLRSFNLEA
>DnOr121
DYEWSVILIRYILDAVGLWPRQLSTEQKVLDKIQATVVLISVITIILIPLICALVLLLRDDLLAFL
ESFGFVMAIVTSLVKYVTFYANKEDLLLILKMMEDDWKKPKMNAEREVMIQHARIARAFVSLSYAL
VSWAAIALIFLPKLGMPLRHSTKETEMFLLPTYYIIDISRSPYVEIIYVLQLITALMVACCYVGVD
SFFGLLVLHISAQLENLQTRLANIKTSTHFDRVLKDTVMDHTRLIRAVDVIENTYMLLLLILLTNF
GIFSCLSIFEIVTVINGKDNYSKSILYLQLPSYINTFLQTCLYCIAGQLLVTQSEGVYEAAYDCEW
LNLKPKDAKNLILIMARSKKPLYVTAGKLFPMTMLTFCNLLKISLSYMSFLLTK
>DnOr121like_1
MNILGLWPQELNPEEKVLDKIRAITILLVTIIIVVIPSICTLLILSDDLPSRLEYFEFIIATAMAL
IKYGIVYKNKDFLPVLKMMENDWNEPKTDAERNVMIQYAGIARKFAKFCHGAIVFLTFTLLFLQKL
GMPVRHTTKETEMFIFSTYYVIDVSRRPYFQIILILQLISLFAQIYAYVGVDIFFGMLVLHISAQL
ENLRTRLLNIKTSNRFDRVLMDTVMRHTRLIRAVDVIENTYTLLLLMLLLYFGLFNCLCIFEIITI
INGKDNYSASVLYFQLGSYINTCVQTSLYCITGQFLATQSERVYEAVYDCEWLNLKPKDAKNLILI
MMRSRKPLYVTAGKLFPITMLTFCNVLKVSFSYMSFLLTKV
>DnOr121like_2
MNIAGLWPRELTGREKILDKIRAIAIVLVTIIIVAIPLTCTLLILSDDLPSRLENFEFIIAIVVAL
IKYVVLYKNKTDFLPVLQMIKDDWKEPKTDAERDVMIQRARIARSFIKFCYAAVFLITLTFIFLQT
LGMPLRHTTKETETFLFSTYYVIDVSRRPYFEIIYILQVISVLAIVYSYIGVDIFFAMLVLHISAQ
LENLRMRLANIKTSNCFDRVLKDTVMRHTRLSAVDVIENAYTLLLLILLFYFGVYNCLSIFEILTI
INGKADFPASVLYFQIGCYISVFIQTSFYSIVGQLLATQSELVYEAVYDCEWLNLKPKDAKNLILI
MMRSRKPLYVTAGKLFPITMLTFCNVLKISFSYMSFLLTKNLDTSGHA
>DnOr121like_3
MGTIGLWPQELSPTQKVLDKIRAAAIVLVTISIMIIPLICSLLILDDWHHDLPSCLENFEFLLAIL
TALIKYIFMYKNKQDFLPILKMMEDDWKKPKAAAERDVMIQRARIAKTLAKFCYVVVVLLTFTVIF
LQKLGMPLRHATNETEMFLFSTYYIVDVSRSPYFQIIYVLQAISLSAIVYAYLGADIFFGLLVLHI
SAQLENLQTRLSKIKTSNRFERVVKDAVMHHTRLIRVIDVIENTYTLLLFMLLLYFGVFSCLSISQ
IITIITGQSNYPASVLYFQIGCYATTFVQTSLYCIAGQLLVTQSEGIYAAAYECEWLNLKPNDAKN
LILIMTQSKKPLYVTAGKLFPMTMLTFCNVLKISFSYMSLLLRKL
>DnOr121like_4
MSIVGLWPRELGRRQKVFDKIGAVAFVLVMITVMVIPIICSLLLLSDWQHDLPSCLENFVVLMAIL
AGLIKYIAVYKNKDFLPILKMMEDDWRVAKTDAERDVMIQRARIGQTFAKFCFTAAALLTFTIIFL
QKLGIPLRHTTKETKMFVFSTYYIIDVSGSPYYEIIYVLQIISVFAIVYAYLGVDIFFGMLVLHIS
AQLENLQTRLADIKASNRFERVLKDIVMHHTRLIRAVNVIENTYTLLLFMLLLYFGAFNCFSITQI
IGIITAKGDYPISVLYFQAGCYINTVAQTSLYCIAGQLLATQSEGVYEAAYNCEWLKLKPKDARNL
ILIMIRSKKPLYVTAGKLFPMTMLTFCNVLKISFSYMSLLLRKL
>DnOr122like_1N_C
MSHGKDFYGLSRKFLMIAGLWPFSGRTTKIVIRLSSPLVLDIVSPLNTSRSLHIFLIIEIHSKD
KYFFPIYAYVISSHIAGMVLITAVDCTSSIVVHYHLAMFETSYRMKNAINDWLDPENRTIEKDHQL
YRGICEAMEIYREISRYQIFSHRQLVEFFHSPGQADFSVLYGITTVMLFLYSYRENSISQTYIDSS
LNVFHETCSMRWYELPLKAQKLLILVLQRTSKGFVPVLAGIYVPSRSGYASMLNTSFSYLAVIYSL
RYGS
>DnOr130
MDVIAKECSTYNFMLRVGGLVPYANSFFTKIHRTVFLFILFSCLAIQVIRMLRITKQLLRNMFLAS
TFACPMILFICRYIGFVSTVPIVQVRLCFDSLQKDYYAIKNPVEMGILMKQIDTSKRIILAVLCTS
IAGLFYVYVVLSVPVMLDLLMPLNQSRPRMLHSFGFLVDGSTYIDLVALHIAATTTIGVVTLVCTE
STLVIVVCYICGLFKIANYRIRCAVDKAANLVADSKENTLIHLRICEAVEAHRKAMEFSQETCTSY
LITIVTAMISLALNLYRLFLAVISMDDFKEFIMSVAFVNVHLAIAFMCNIVGQLVINSSAELFYET
YNSDWYSIPVKMQKMLLLIMVRSSVDSMLDLSGLFVPCCEGFGIMMSTSFSYFTMLYSLSS
>DnOr130like_1
MDVFQRNYNMYYNVLCITGLWPFDDSLPSKIQRVVISVLTLWCIGVQLKGLTTVDFTIYNLLTMLS
YFCPMLLYFVRYVGFIYSFPSIRFMFKSIMADCVTLKNPIEVELLMKRMKATRTMFLIYLCKTFCI
LGFSCLAIALISILLLVPIFLQPKLQSRALRVMGFFFDEKSKQLNWICVHVTLTSLIGLLGIACTE
ASLAVFSFYLCGLFEIASYRIRTAINSGVKLATSNPIDVRPAVIMHRKAVESLISTITDLRELDNV
AMSILMVLVHLIIMYLNNYNGQQLINSSIELFDDVRNSLWYKAPLKTQKMLLFILMRSSTEVQFNL

AGLFIPSYTGFSTMISSSFSYFTVLYSVQ
>DnOr130like_2C
LQIRLIFESLQNDYYSLKNPVELEILMKQIDTSRRIILAVVCISSAGVFYVNTILSVPVMLDLLMP
LNESRPRMLHSFGFQVDGNIYVDLISLHVVSTCAVGMVIIVCTESTSVIFVCYICGLFKIANYRIR
SAVDGAANLISGSKESTLMHLRLCDAVEAHRKALDLVKRYGKDTCTSYLLAIIAVIISFALNLFRF
FLAVLAKEELKELLLCFVFVNVHVAIVFLCNIIGQLVINGSRELFSETYNSKWYYVPVRTQKVLLL
IMLRSSVDCMIDLAGLFVPCCEGFGMMMSSSFSYFTVLYSLT
>DnOr130like_3
MDVIAKECSTYYFMLRLAGLVPYANSFFTKIHRTVFLFILFSCLAIQIFIVIPHASLANLFVASTF
SCPIILFFCRYIGFIYTVPMVIAGVFYIYVVFSVPVMLDLLMPLNVSRPRMLHSFGFLLDGSTYVD
LVALHVAATCTIGVVTLVCTESTLVILVHYICGLYKIVNYRLRCAIDEAANLVAGSKESTLIHLRI
CEAVEAHRKAMEFVLLFCTSYLITIVTAMVSLALNLYRLFSAIISMDDYKELIMSVVFVSTHLAIA
FLCNMVGQLVINSSTELFYETYNSNWYCTPVTMQKMLLLIMLRSSTDCKIDLSGLFVPCCEGFGIM
MSTSFSYFTMLYSLSS
>DnOr130like_4
MDAFRKNYNFYYKVLRLTGLWPFSNSLPTKIQRVALCVIILWCMGIQLKGLTTVNLSIYNLLTMLS
YFFPMMLYIVRYIGFICNFSSIRFIFENIEADCCTLKNPIEVELLMKHIRTTRKVFLTYLGFSCLG
IFFMSILLLTPTLLVPKYQIRALRMVGFFFDKNSTQLNWICVHVTVVTAIGLLGIACTESYRIRTA
INNGVKLATSNPIDIRPTVIMHEKALRSANEISTVMVLPYLLAIIVVVVSFAISLCRLSSTITEMA
ELDNIVISIVIVLIHLIIMFLNNYSGQQMMNSSLELFDAVWNSLWYEAPMNTQKMVLYILMRTSTE
VQFSLAGLFIPSIGGFSTMISSSFSYFTVLYSVQ
>DnOr130like_5
MDAFRKNYNFYYKVLMDAFRKNYNFYYKVLRLTGLWPFSNSLPTKIQRVALCVIILWCMGIQLKGL
TTVNLSIYNLLTMLSYFFPMMLYIVRYIGFICNFSSIRSIFENIGADCCTLKNPIEVELLMKHIRT
TRKVFLTYLGFSCLGIFFMSILLLTPTLLVPKYQIRALRMVGFFFDKNSTQLNWICVHVTVVTAIG
LLGIACTEVALSVFSFYLCGLFDIASYRIRTAINNGVKLATSNPIDIRPTVFMHEKALRSLSSTIT
EMTEPDNIVMSIVIVLIHLIIMFLDNYGGQQMMNCSNELFDAVWNSLWYEAPMNTQKMVLYILMRC
STEVQINFAGLFIPSIGGFSTMISSSFSYFTVLYSVQ
>DnOr130like_6PN_PC
MESVNDHYCRISKVLLSTTGLWPYDVTWLTYVKRVLYSLVLLSSFVCQNGVYSQDVVVCCVTQLTF
NIIFLFKHHAFLYRIKNVSRNNLNVLIQTYYFTGDDEVPSHVMYLHVFTFLYLGITGLVGPESA
TIVLASHGCTLFNVIRYRIKNVFNQSPGASVFEKEKSVVDKLINAVKLHNETIRYCKQLNDSFSTS
YFILIMLGVMSLSLNLLYMSQSLQQHWQRKELMLQVMMIFTHLLYIFLANYIXQTVTDSSSKLFTE
LYNCRWYTASVPTQKLLLFIMHHSMKTYTFLLGGIFTPCYEGFSTVIRMSFSYFTVIYSAQL
>DnOr130like_7N_C
LKYRQSTLGKVLLSFIGLWPYEETWFAYAKRMFYSLVIFTSFVFQFAALFHTGVYTQNVIITFMTS
ATLYTIYTFRYYAFLYKIKNVRCFFLMECLIFFVRHYKDKIKTYQTYNIIFLSVNLLRVSTTIVTG
EYRLIISNIYSVCRPNLIVSACFCVLFKMQMSQSLKQRCEFNVLIVQMAIISIHLFYLFLCNYT
CQIITDSSNMLFMETYNCQWYIGPVRSQKLLLFIMHCSMKSYCFQLGGLFVPSYEGFSTVIRMSFS
YFMVMYSTQL
>DnOr132like_1C
RYIDLVQTVINIAYLVLINCGITGLAVALIRIRQSLIVTHDMSGVAFGISQFLLITLYVYGANICG
QELIDLSEEAFRKLANDRTLSIDTPIQIELLLQMKAKDNCQYSIGGGIIISSFDCLANV
>DnOr133like_1N
MRLLEKPQYCLIKFCLKYVGLWPYQNRKLAMVKQTFGVGLIIISIAIPEMILVNTNTCSIRIVDSKL
TITSVLRVMVLFH
>DnOr135like_1PF
NTLIHATLVEECVCQNDFMKFVSCNNFSGIDSPKIKXFRILGFSCLGIFFMSILLLTPTLLVPKYQ
IRALRMVGFFFDKNSTQLNWICVHVTVVTAIGLLGIACTEVALSVFSFYLCGLFDIARYVVSKLWL
AEITRLIPLH
>DnOr138C
MMLTLNKKITFFQFMSLAKTDMSWTNIVKTMTLAGPVTCTLLRYVSFIHNLPISLQIHVSINTLST
ISHTELMPNVFNRSFLTRSVKFFLILDVAGSMMITAAGIISSPIIFDIVTPLNVSRTRALTLIGFF
YNNSSHIDLVSAAVLLITGISFTMVLCTEAVVTVLYYRITGAVVEASKFTSTSKQYRTYYTNIQRA
VDIHLTAIQFVHFFEFHCSLLYRLDPVNSPIWTVSNYNLTLYNTLYNVDGVLIDLSIPTSAYTLVY
QRKYQTVNMIILVSSKKIYIYSSPWYFIPPEMQKSLLIIMQRSRIDCTYDLAGFFTPCYNGFATIM
STSFSYFTVIYSV
>DnOr140TRA

MRLLEEPKYYLMKLYMTYVGLWPYQNRKFAIAKQTLRIFFILSITIPEVAIIEAWGKDYDLTIQCI
PPVFTMCVMTAKYVNVIGKMEQFKTMFALIKSDWDRMTNQPEHQIFRKCAMEGRVCIGALTVLYVL
MVTVMMVHDNLRDRNETEPRQMLYEVEYFIDNEKYYYLLILHALILSLWTVMLLVAGDVIYIILIQ
HACVLFSITGYRLKTVHVLDVNTVCTSEDITEASKRNFSKREHEQIFQKFILCIREHNRALAFADH
LQDTFSVTLFIQMAMNIICVSATGVMILIRSNSTETLLRLALWLLGQLIHLFGICLPGQRLTNFSE
NLYYDALGCMWYICDLKTRILYQFFIMKTMTPQYLVAFKMMTLSMETFQSVVRVAASYFTFLSSSL
>DnOr140TRAlike_1PN
MDFFEQPDYYIVRFYMKYIGLWPYQSTRAATVKHTLVICILWSMVLPGIIAMVKEALTKNYVVFIM
CIPPLTCTTVAVIKYTSASIKTKKQIRTIFESIKSDWNKLMNLPEGQIMCKQAMEGRKFTIYLTFV
IITATVIYMLLSIVLITQDSKNMKNNSEPRVMLFNVEFFVDQQKHYYSFNIYFCLMSMNVVFVILT
IDTLYIVLAEHACVLLAVIGYRLKTVHVLNVNTVCTDEDIQTACDRVFSKEEHEQIYQKFKLCITE
HIRALDSTAVLVRVNSRGFFLNVSHYPVFTNPLXILLRSSSTEDMIRLVMWLTGQLTFLYSICVPG
QRFMNYSEKVHYCAIECMWYICNSKTQTIYRYFLMNTLTPRCLVACKLITLSMDTFLSV
>DnOr140TRAlike_2
MRLLEKPQYCLIKFCLKYVGLWPYQNRKLAMVKQTFGVGLIISIAIPETAMIEAWGKNYDISIQCI
PPVFTMCVAVAKYINVMVKMEKIKTMFESIKNDWDRITEQAEQQLLREYSMEGRKNTITFVSVCVG
AMASLYMLLTTATMVHDNLRDRNETEPRQMLYEVEYFIDSEKYYYLLILHAYILSLWTVILLVAGD
VIYIILIQHACVLYSITGYRLKTVHVLGVNTVCTGEDIMNASNRNFSKREHEQIFQKFKLCIREHN
RALEFADHLQDTFTATLFIQMATNIVCISATGVMILIRSNSTETVLRLAMWLFGQVIHLFGICLPG
QRLTNFSENLYYDALGCMWYICDLKTRILYQFFIMNTITPRYLVAFKLTTMSMDTFQSVIRVAASY
FTLLSSSL
>DnOr140TRAlike_3N_3C
MRLLEKPQYCLIKFCLKYVGLWPYQNRKLAMVKQTFGVGLIISIAIPEQIKTMFASIKNDWDRM
KEQPEQQLLREYAMEGRFVSVCIAVSGSMYMLLMTVTVVHDNLRDRNGTEPRQLLYELQYFIDQEK
YYYLLNLHAYILSLWTVILLVAGDVIYIILIQHACVLYSITGYRLKTVHVLGVNTVCTGEDIMNAS
NRNFSKREHEQIFQKFKLCIREHNRALEFVDHLQDTFSVTLFCQIAANVICISATGVMILIRSNST
ETALRLALWLSGQVIHLFGICLPGQRLTNFSENLYYDALGCMWYICDLKTRILYQFFIMNTITPRY
LVAFKLTTMSMDTFQSVIRVAASYFTLLSSSL
>DnOr142
MGLHVTPEKAIRFTKITVALAFTWPPSINSSNNRLLLFNVLWWAAFVSCLGLLLPLLSAIYVYSNE
PVLLGKTVSLFSAVAQVMIKMIVCRLMQKRFRRLFFDMENFCKYATEEDRIVVQRHMDSYKYSHVV
YAIWCFLTCLFVVSGPLYSPQMFPTLAIYPFPVEYQPMKSIIFFHQILVGCQATAGMAIDAQIALL
LRYATVRFEILAIQLRKAKSGQDLNACIREHVKLLWYTKSVYKSVRFIALATVATTHIAVIFGSLN
LVTKQPLLFKLLFALVVFSASVELIMYAWPADSLIHASNKTAAAAYETNWFEENVDIQRKIRFIIL
RSHRLEAIEISGIMPKITLSYYASFLYTAMTCFTALRIIVEKSNHD
>DnOr146
MFDQVSPEKVINFTRVCVALSFCWPLPTTATRSELRRFKFVRSILALNAIALMSALLYSLYGYYDD
PANFAQAACLMTAITHALMQICFGITQYEHLQWLIEEITAYCEEAKTNERQILQRYVDRYSMFYGI
SAIWFYLTAVMVVVGTLFISQPFPTKAEYPFPVDYEPVRSIIFFSQALAGFQCAAHISFNIFVAML
LFFAVARYEILMMELQNVTRFVDLTNCLRKYSHAKVYAQEVANCAWYVVATTVSVSTVALVVGGVN
FFGHQPLTVKLQYVCILGTGLLEVFMCALPADHLLDMSQKALHSVYEGRWYEQDVKLQKTVLYTLT
YRNPVAVRIKCIVPVLSLKFFGSFVSNAFSFCTALRVIMSDEDGEL
>DnOr146like_1
MFDQLSPEKVMKFTRICVALSFCWPLPTTATRSEHRRFKFVRSILALNATVHMLALLYSLYLNYGD
RANSAQTACLASTVGHVLMQICFGITQYEQLQWLIEEMIAYCEKAKTNERQVLQSYIDRYSMFYFL
VAIWFYMTAVMVFVGAYLLNQPFPTKAEYPFPVDHQPVKSIIFLMQTVIAFQCSAHVCVNVFVGML
LFFSVARYEILMLELQKVTRFVDLTNCLRKYSHAKRLLTRIVITYDSNGILELFKSEISPELPNNQ
PLSVKVQFVGILGTVLLEVFISALPADHLRDMSLKSLHSVYEGRWFEQDVKLQKTVLYTLTYQNPV
EIRINCIVPILSLNYFCSFVSNAFSFFTALRVIMSDEDGEL
>DnOr151N
IRQHFWTRWHKEYLNELTVRQKWHRHRDADIREGTLVILKEDNLPPLCWPLGRITSIHPGEDGIVR
VVTVKTV
>DnOr160
MTVGTKKPISLNVELFYDENVLSWSKRLLSLSGLWPDNGNDVRFFFYITYVMIFTWLEIVTLLQNM
HDLEKSLQNITLSFPTILIIILKAVMFRLNMHLLLPLLAVVKRDVEQGLYQSLEERRTVMWYNIAAT
LFSTSSALSLFFVPTLFYAKPIVSCLLSKFDNCSLPYELPMRVGSVYEVTEMQTYALFCVYLIPTS
LMLTIGATGADSLLVTLTFYLCSQLSILSQRIRNIDMEPKKYLPKMRALVERHTELLRLAATLAET
FSSLMFVQTLGLIFSLCIVVYQLLMTSESDGSDVNTIHFIIYSCAVVLLAFCYCFLGECLITESSE

IQFACYYTKWYDLPEEYVRSLTFCIARSQKPLYLTAGKFYVFSLETFGTIMKASMAYLSVMKSII
>DnOr161
MDFQLYGRREYDALIKPIMITGRIISIWPIAEKSGTATVLFRRFHQFCMFFLVTVMSIAVTADVIH
NLDDLDEATECALICTAFYLCVVRMVVYTIHQKDMLYVVNTMRDDWTTSTYEDRAILAGKTMFAFR
LAKYFISTVAVTIVLFMCVPLLEIYVAGKDKILPFRGYFYINQTVSPTFECLYLFNVSAGGFGGSM
IAGATSFNLIVIMHGSAKFAVLRKRLEAINSEDRDSATVITLYADALERIINVLALGQFVISTGLL
CFAGFQITSMMEDKGRLMKYSTFLNSAILELFMFSFSGDGLIEESEAVGESVYNGGWIGSRYGSSL
MIIMMRSTVPSKITAAKFYSMSLQSFSKVLSTSFSYFTVLTATKDE
>DnOr163
MASKTVITYPIEFCLLLVGVWPRSSYRIFQRIVWSVAMVFTLSSQFWYFAKKDLPDLLDGLSLVFS
NLVAFIKLIILWLNYGIFFNALTIVFEDWDNIALSGRNSRFMLEKALTSFHVSRSLIYIYSAAWIL
YSASAMMITDGTDEGSVSNQRKLLLKMKFPFEATVSPVYEVIVIAQFLLEYTAALIAAMLMALFAA
LVLHIGSQIDVMCQELMEIPNQRKEDRLRVFRSVVVKHQRIIYLSTNVKDLFTYISLVQFLANILV
ICFLGFNLVKSLGTENGSMLIAKMLPYYLATNVEAFILCYTGEYLASMSENISGTACNMDWYKLNP
PEIRLVKLLLLRAQKQLTLTAGKFVNHRNVCQCKMLNASASYISLLLAM
>DnOr165
MKSPSTVSLSVKYGLHFIGIWPGTPCPGLHKVLWVTCMMICQSYQYGYMIVHFKTDSLVTIIDCLS
IALPFTLVFIKLIVAWMNHSVLCDILSTMEEDCEKYARFDTNNFISKTADLSFRITTVIPSLYLIS
AGFYAAGTFAVQNANDSRELLHKMDLPFDSNESPAYELVVTAQFLHQAASAFTFGVFSSLLLMTIL
HVGCHVDVLCQILTTVSPDDNGQLRFVLSRHQEITIFTKKIEKLFTYIALSQLVSNTLITCCVGFL
IAISIQMENGLPLLIKSITFYFVICLEVFIYCFAGEYLDIKSNLLGSAAYNSLWYDSECSKSRQIV
LLLLRSRKGFPLTFGKFSTLNLESFTSIMKASASYMSVLLAM
>DnOr165like_1
MKLPSTVSLSVKYGLHFIGIWPGTPCPVLHKVLWITCMLICQSYQYGYIITHFRTDSLVTIIDCLG
IALPFTLVSIKLIVAWMNHNVLCDILSTMEEDCEKYARLDTNNFISKTADLSVRLTSVILCLYLMS
VGSYVAGTLAFQNTNVSRELLLKMDLPFDISESPAYELTVTAQFLHLTASAFTFGVFTALLLMAIL
HVGCHVDLLCHILTAVSSEDIGQLRFVVSRHQEIIVFTKKVEKLFTYIALSQLVSNTLITCCVGFI
IAISIHMENGIPLLIKSILFYFAICLEVFIYCFAGEYLDIKSDLLGKAAYNSLWYDSESSKSRLIV
PLLLRSQKGFPLTFGKFSTLNLESFTGIMKVSVSYMSVLLAM
>DnOr165like_2
MKSPSTISQSIVFGLHFAGIWPGTPYPGLHKVLWVTCMGICQTYQYKYVITRFRTESLIMLIDSFS
IALPLTMVCIKLIVAWTHRGVLRDILSTMEEDCQKYAVIDTNNVISRTAAISYRVTSTILILYVSS
VGFYAVGTFAFQNGNHSRELLLKMDLPFDINESPAYELVVTAQFLHQVASSITFGAFLGLLLIAVL
HVGCQIDIICQRLTDISIKTKGQLQFFITRHQEIIVFIDRVEKFFTYIALSQLVVNTIITCTVGFL
IVVSLKSENGVALLIKSVMFYIVICLEVFIYCFAGEYLNMKSKLIGDTVYESLWYDLQPSNCRQVV
FLILRSQKGLTLTFGKFSVLSLESFTGIMKASASYMSVLLAMS
>DnOr165like_3C
KTESLVTIIDCLGIALSFTLVTIKLIVAWMNHSVLCDILSTIEEDCEKYAGLDTNNFISKTADLSV
RLTSVILCLYPMSVGCYAASTLAVQNKNHSRELLHKMDLPFHVSETPVYELAVTAQFLHHVAAAIP
FCIFTAFLVMAILHVGCHVDILCHILTTASSEDNGQLRFVLSRHQEIIVFTKKVEKLFTYIALSQL
VTNTLVTCCVGFLVAISIHVENGLPLMIKSLVFYLVLTLEVFIYCFAGEYLDTKSDLLGKAAYNSL
WYDSESSKSRLIVPLLLRSQKGFPLTFGKFSTLNLESFTGIMKVSASYMSVLLAIS
>DnOr166like_2PF
LILVNRHGIIVICIGVLYCFVEEYLITKNKIISDAAHEPQXYDFQPSSSRQLVPXLISKSQKGLSV
TSVKFSALSLESFTDVRHLHFTHDNIYYSL
>DnOr166like_3C
CNIVIFLEVFIYCFAGEFLNTKSKTISDAAYKSQLYDVKPSSSRQPVLLMSTGQVKLPLIFGMFAV
LLV
>DnOr166like_4C
LLLISKSQKGLSVTSVKFSALSLESFTDVTKASASYMSVLLAMSPELFPSSVV
>DnOr167
MISVSRPVEIGLRVTGIWPSSYENVFRFVWILAMCTAQVLQYWYMMNHFNLDDLTDLVECVSTSLP
YSLVCFKLITFWTKRGILKHILLTMTADWENTSVTKHSLDAMIRKTQLSNWCSQLILSVYAIAVFL
YSSVYINVFRKAGRDDVIVDSSQLLMRMELPLASYERPAYQYVMIAQFVQLMFVATAIGTIDAFMI
TLILHVGGQVEMMHQALAAVCSKNKEQCLPKSTVKRLVNRHQKILNFTKYIENLFSYIALMQILCN
TISICCIGFLMSFDTDQNLRNVIKILFFYIAIVLEAFIFCFAGEYLSSKSTSINSAAYNSLWYLWK
PNESRTMLLFMIRSQKRSTITAGNVMELSLEGFATILKASASYVSVLYAMY
>DnOr169

MNTHFQKFNYLNVCVNVFSANMLPTTSEKIRTPGIMKFYVLFVWTIVLACLSATLYGMVHVPNDKM
LKDSTVYTMVSFESILLVVYLHNQKKSLRRLIETLNCLFDIDDKTIRIATTRLVTPFEKPMKIYVI
ASVGAVIVWTFLPLMEIFHRNQFYYEDYQIPMGVSREPFSTGIFIGGIAFQIVGCMYTTVRKASVD
IYTMHIILLMTAQYKYLNMKFTSALARYPVPGDEDIIRQELGGLVQHHKIVLRISNILKNVFTPNV
ALLYINNVFRFCFLTLMLVMNNEGPYVTMCVVSYTIGALIQLYMFCFCIQQLLESSTTMMDDVFHG
KWYLRDVSLQRTIMMMTMTDKLGCKLSRIRNINLTLPSFMSILNQAYSVCLLFLKSRQS
>DnOr169like_1
MNFQKLNNLNILGNIVSANMLPMTGEKTKMPTVLKLYVLVVWTFSMIYFITILYGLLKVPKDKVLK
GGAINMVVMFENLFLMLHFYNNKKVLRQLIRTLNSVLTIPDEILRTVTTKTVMILQKAMKTYIIFS
ISALIFWMPIPVMEAFHRNQFYYEDYQLPMAVSKEPFSTQIFVAGSVFQFFGAVYSLIRKLSLDLY
SMHIILLMTAQYKYLNKKFTSALASDPVPGNENQFRRELGGLIRHHRIVLRMTALLKQVFSLNVTL
LYVTNVFRLCFLSFMIATASEESLVACLVIIYTIGALMQLYMFCFCVQQLVETSTTMMDDVFHQKW
YLYDVPVQRSIALMILATKLDCKLSRYRTLDLTIPSFMSIINQAYSVCLLFLKTRQV
>DnOr171
MATLPLYQTYVSFVNRSLRICGLYPDTIHRSLSSRIIPPFWLLCYGIILYTVTTCLVQNVTDMSKV
TAGLSGIIAIVNAIVKAFRFVTYRGELEHVNGVLETVFEESSDRKPANSPAFRLMGSFYRLGYYQY
VCLLFTSFVFVVRPLLAVRFRNANYSLPVPGVYPWTINSTPVYVIHYVIESHICWSLCVVTIGVDA
FFTLCTFRISSVFKLLALEFEELPKRPIDKENRELTLRTCIRRHSTLIHCYDVIQEIYGPIVLVVA
LGNALSMCSMMFEVFQSKEFTVGKIIATVIYLFGKLIQTFIYAWPGEVVATEEVYCNDWYEHIDDP
SAKFFLMILKQKPIVIRACSILEVSLDLFAKIINRTISYYFLLVTLDDLDDGS
>DnOr172
MFGSAEEEDFPGYQYFRINRLLLDSVGLWSTQNQTITKIKKVLILSILLSVFVLQLTAFITTDISM
VTFLQNFVNITVTLTITLKYLTFLLNMKETQVSDMYNEIRYDWNSLTNEDELRIIHDYYRMMKKLL
PLFVVIILPSGLVITFTGVKSPILDFLYPLNYSRPREMPVQAEYFLDQEKYYYLLCMHEYTVALLG
MTVVFSTETLAMLNLRHICSLLKIISYRLEHVFDPHIPGGSFVQGSCVVYKRIVSIVELQRRVIIY
INIVRTKFMPQYFILFIFGAVCITLAAYHLLKALLLNSGLNEVLASLILVFCSTCYTFTLNHFCQD
LIDHMSSISQQSYNIQWYNASGTEQKLLLFIMFNNSNSKVLTIGGIYIPSYEGFGKVITENYTYF
>DnOr172like_1P
MNFLESRYYRITRVVSMPLGLCPYKRCDATKLLHVLSASLLETXDLILFKFRLQILALTTQERNLN
TILRILSSITPPVVCLVRLFTYYPNIDKVNEIRLLLKYTTQGKFLTLFYTSVFMFIAYPFVSVVFV
REVSSNPNRSSLPHLPILVEYFVDRNRYAYWILLHIDAQAFLGVSVLIATESATVTFIYHVCGVFQ
IVGDRIERTIEKSILNVLRGNKYSFVREEFRTAVELHQRLLELIEYLNKSFAKHYVLLVYIGVTTL
SINLSVLAVSLSLKKITDEFLLALIVICCNLFCLACANYFGQLIIDSSNDLFIKTYLTQWYESPLP
LQKSLLFLRQRCMKCSSYVADTLFVASMECFSSLTNTSMSYFMVLYSTM
>DnOr172like_2
MDFIGYRYYKTIVYFLNLTGLWPYNTSRFKAAHTIVTYIILLSSFVAQKFTYFNRLTVNVPAIRVY
CYAKLKEAMEQLKHHWNILRDSKEVELLERYARFGARCMIVYILHSYVIFSCMFVGIVFSTALTSD
KSITNVSRAFSINMDFFVDEEKYYYWIISYQYILCLMAGTIIACTEGLFLMMILHTQGLYNIASYR
LRKAIDSSSWKIPISEKQMLCHKGIANSMEIYRFIGMLVFLQCIIKNYLISIFITYTGFNDVFINC
ILLFEAFLLIFVTNSLMQRLIDEHIMFFATPYDTPWYDAPLPIQKLLLFLMQGQMKIISIKIYGHL
QPSMETTTMQMKQSFSYFMTLHSTRQL
>DnOr172like_3F
RTETFIGVVIPVIILPSGLVITFTGVKSPILDFLYPLNYSRPREMPVQAEYFLDQEKYYYLLCMHE
YTVALLGMTVVFSTETLAMLNLRHICSLLKIISYRLEHVFDPHIPGGSFVQGSCVVYKRIVSIVEL
QRRVIIFLQFLGPSFALVYSNNLSMHQRCDTRYLQHINHCEIHL
>DnOr172like_4PN
MDKLDFYYKPCRVHLLLVGLWPYGTSKLCKIQNFICFSVFCTAIAIQVERLNXFVFTNPLYLVTYL
QNSRIIFRRTYIGYLMCLVINWSFLLKYYSFWRYSTIVTFFQYIFVTTIVFESTINYRNLRFCNID
YTLPVAQSYLFRITFGGDI
>DnOr174
MFGSAEEENSPGSQYFRINRLLLYSVGLWPPQKQIITKPKNVVILLLLLLSGSVFQLNAFVTIKFSI
KTCLQNIVTITLSLAFTLKYLAFLLNMKEVTTKTVIGDMIPVVVFPSALAVAFNGMKNQILDLVYP
LNYSRPREMPLPAVYFVDQEKYYYLLCTHEYVVMCLGVIVLLGTESTAMMVLRHICSLLKIASYRL
EHMFDPITPGASFAQRSLIIYRRIVSIVELHRRVIKYIHILRAKFSAPYFILLGCGTVCITLTFVQ
LLKALQLKSSLYELLASLLFVFSITCYMFIANHFAQDVLEHISYIYHQSYNSQWYNESTAEQKSLL
FIMLNSFNTNVLKFVTYIPSYEGFGIIMKTCFSYAMVIYSIE
>DnOr174like_1N_C
MILVKFYDNKIHKRLLLTLGLWPGSCPKIQRLRQLIVYFVILAGTLSQVFVVTEFLVIETGECGFK

GTVQIMSAVFPNLIAIVKYMNYHLSLGTVSEPFVAKKLLDVYANIDVIDHPVCVSFYKTMQTVHFN
DTLLSVLGIFWTFVLLILLVLSLHDLPANFVRIHVFSYLGEHIDRQTYTDTVAFSTFVIWIVDIYI
IIATEFSIFILLEHVNVCFKIVGRFVDIIWDTFGTTYWIIIPLGIAGMSANMMNVVQGIKSKEH
ILVITSAVHIITLMFYAFAGTFCGQRLIDYSVSIFYDAYNSRWYEIPVDIQKLYLFILQRSLKCCS
LSNKSIFVSSFEGLVVVKKENTSYYLIVRF
>DnOr180
MRRLRRNGLMNLLKVCCQLFGIWLPDSNASWWRVMFSRAMYWINFWNEFYGALAVLNGLCCCFTSV
SEVAKSFVEMVFMLEMLFNLIYYRWRSSEFQRLIIEMDKFFGTTNPRDRIVRQNYLNRTKTMYNLL
FGVFVVCALSFAFSPFITRNKVTPLNTAYLFLPREKYWGLRVTFALNIFHLMFTTPVILLDLLIIT
IIWHASCKFVEVGEALRTIKAKKLRGWIREHQAAISYADDVNHLMAPLAIKSTIAVTLSVIISGLV
IAHVSVSDFIIRNTGLPLFEMSKFFMLTAFSMLRFLMCSYAADVMIVEAQDIAWRVYDSPWLNATP
GVRKEAVIIIQRCQKPIAIHSAGFLTAWSLRFCGQVFYTIFTYFSTLRAILRV
>DnOr181PC
PLEYPQRTLSVIGVWPEQYRTTKGDIAYSITFCYFAIAITLIAYGAYKNSHVLDVLIIIVLELILT
GTNFIYMYILRASKDLERVIVMMKKEIKDGTIFENEEEKCVYLPYNRLSYYYSKYAITSAFCISYL
MYFVPLIELAVHFHAGNTTRTYEMPFPLYIMFDYEHNAWLYACTYIAEFFFTYVGIIHTSIVSFEV
DVVLHICGKLCIMCHRIRTIPAQPPELFEKSAKRMASSHVKLTKTHPVQFPEVVLQXSFEYKKKCM
CLNLYIFLILQALETNPVAATKFFMYTLQGLSLIFLYSYVGEIVLSENENVENALSDIDWPEVAIK
DRRTFLIFLVNAQQALYLTGGKFYKFSLYGFTDVIKSAMAFLSMLRATM

>HlOr1
MVEEIAKKSNYRNVNYKLDTEYAVRVAKALLTPIGIWPREDSLRDRVRLFAQTGVVFALMCFLLVP
HVIYTYFDCEDLTRYMKVIAAQVFSLLAIIKFWTMIVNRKEIRFCLLEMETQYRDVECEEDRQVMT
NCAKIGRFFTTLYLGLSYGGALPYHIILPLMSERIVKEDNSTQIPLPYLSNYVFFAIEDSPIYEIT
FVSQILISSIILSTNCGIYSLVATIIMHCCGLFEVTSRRMETISVPTGKDIRGRLRDVVQFHLKAI
KFAEMIEKALNIVFLSEMLGCTIIICFLEYGVIVDWADHKTLSMMTYFVLMTSIFVNVFIISFIGD
RLKQVSERVGETSYFMPWYDLPDDLAKNIRTIILRASHPSSLSGAKLFDLSLQAFCDVCKTSAAYF
NFLQAMTG
>HlOr2
MMKFKQQGLVADLMPNIKLMKASGHFLFNYYTDNATKGMHKIYCIVHLVLILIQFGFCGINLMLES
DDVDELTANTITMLFFTHSVVKLGYFAVRSKLFYRTFGIWNNPNSHPLFAESNARYHQIAVKKMRI
LLMAVMGTSVLTVISWTTITFIGDSVKKVVDPVTNETSFVEVPRLMVRSWYPYDPSHGMAHILTLV
FQLYWLIFTIADANLLDVLFCSWLLFACEQIQHLKNIMKPLMEFSATLDTVVPNSGELFKAGANMD
HGKDQDLPPPATPHPQTDNMLDMDLRGIYSNRTDYTTTFRATAGATFNGGVGPNGLTQKQEMLVRS
AIKYWVERHKHIVRLVTSVGDAYGVALLLHMLATTITLTLLAYQATKIHGVDTYAASVIGYLLYSL
GQVFMLCIFGNRLIEESSSVMEAAYSCHWYDGSEEAKTFVQIVCQQCQKAMSISGAKFFTVSLDLF
ASVLGAMVTYFMVLVQLK
>HlOr16_1
MKMSGIARAEEDLRYATRFVKPILGIVGAWPIPVNSSPFTRLVRRIEHVVTYFLFFLMIVPGLLYV
FLKEKNGKIRLKILGPIVNCSIQFFKYSVILYRIDKIQKSLNTIRRDWIEATDENRLIFRSKAKIG
RRVLLIAAATMYGGGLCYRTILPLLRGTIVTPDNVTIRPLPCPVYFVFFNEQRSPNYEIVFILQIM
AGFVTYAVISGTCGMCALFVLHACSMLRILVNKMNALVDKADMTETLVHRKIADIVEYQTKIKRFL
KNIETITEYICLTEMVGGTGLEWENNNTTAVIVYITLQISCTFCVFILCYIGQLLIDQNHVVGLTS
CTVNWYRLPNRHARSMILIIAMSNYPIKLTAGKMFEMSLASFTDVMKVAMGYFNILREVV
>HlOr16_2PF
FCIFILCCIGQLLIDENYVVGLTSCTVNWCRPPNRHVRYLTXLIIAMSNYLIRLASSKVVEINLAT
FTDV
>HlOr18
MRQIFLNRNYKNDLSFNVRLNVWTLKIIGSWPRSIDRSWLENSQHILLNVLSHCLLGFIVIPTGMF
AILVVEDFYDQLKICSALSFFMMAFTKYVIMTVREGDIRKCVEYVEADWRNVEHEEDRKIMLDNAS
FARKLIVICGAFMYGGVIFYYIALPLTRDKVVEEGGNLTYRRLVYPVPKFILDVRYSPVNEIFYTV
QLLSGFVAHNITVGACGLAALLAMHACGQLQILMSWMEKLVDGREDEEKNLDQRLANIVEQHVRVI
NFIALTENLLHEISLIEMVGCTLNMCFLGYYTMIEWDSKDPISSLTYIVLLTSVTFNIFIFCYIGE
VLAEQTVKVGEKSYMIDWYRMPGKKGLVVPLMISMSHSTTKITAGNLIVLSISSFGDVIKTSVTYL
NMLRTFTT
>HlOr20
MQKLATNKEQSPRTDYEYEKNINLSIQWNRWLLKPMGLWPNSRTTTRSRKILYKLINALCYGLISF
LFVPCSMYVFLEVEDLYDKLKLFGPLIFCVMAFVKYYSLIVHKADIRECVRRIERDWRNVRCEKDR

EAMIVNANFGRKLVLLCTFFMYSGFVFYYIAIPISVGRILSENGNLTFIPLVFPFTKYIIDTRYTP
TNEIVFFLQLIAGALMHGITSAACSLAAAFAVHACGQIEVLMNWLQHLIDGRADMSDTVDGRIASI
VSQHVRILKFLTLTERTLQQISFAEFLGCTMDICLVGYYIIVEWKSNDVTSAVTYIILLISLTFNI
FIFCYIGEIVAEQCRKIGEMSYMIEWYRLTGKKKLWSVLTIAMSNSSIKLTAGNMVELSISTFTDV
VKTAVTFLNVLRTLT
>HlOr22_1
MQKPVTDSLSSTRVEYDYKTNVKLSVQLSRWILKPIGVWPNSANISTVERYLYWLINVVCYSLITF
LLVPCSLYVILEVEDVYNRIKLLGPLSFCVMAFLKYHLLILYENDIRECIERIEWDWKNITYSKDK
DIMIENAIFGRRLIMICTFFMYSGFVFYYIAIPFSVGKIPAQDVNVTFIPMVFPFSRFIVDTRYSP
VNEILFSLQIMAGALMHSITSAACSLVAMFAVHACGQMQVLMSWLKHLINGRADMCNNVDRRIAKI
IPKMHPILQFLKVSEKALQQVSFVEFLGCMLNICLLGYYVIVVRKKITTSAVTYFILLMSLTFNIF
IFCYIGELVAEQVKKIGQVSYMIDWYRLTGNKKLCCILIIAMSNSSIKLTAGNMVELCLTTFGDVS
ITITVTIVKELFKT
>HlOr22_2PF
LPLKYNGQHSKVVCIQWNRWILKSMALWFDKSNISPVEKXLHXRLISAVCXGLMGYFLILCSLHVF
LDVENILKKIKLFVPLVLLCLATILKCHLSVLHDDDTYTCGEHIKWEWRYCVTHDEDKKIRDKRDL
LCLLHVQGAKKLMCTDFTFYFFVVSLGLEKMVAEDLSVTSFSGFIIDVRYSPVNEIFFSIRIVAVI
LIHGVTVGACCLTALFAVHDCGQMDVLMAWLEHLMDGRSDMSKNVNGRMVTIVSQHIRILK
>HlOr24like_1
MQKPGGKDEKLQVEDCTYEKRVNLSIPWNRWLLKPTGLWPDKSYVSPIEKNIARLVNTVCYGLIGY
LVIPCSLYVFLDVENIFKEIELFGIFAFCVAALWKYHSLILHGDDIRTCVEHMKYDWKNITHEEDK
EIMALNASFGKRIVTICAFFMYTGFTFYFLVVPISADKMVAEDLNVTFRQMVVPVSRFIVDVRYSP
VNEIFFLIQIIAGVLIHAIAVVACSLAAVFAVHACGQMGILMSWLEHLIDGREDMSKNVDDRIAVI
VNQHVRILKFLELVEKALRQVSFAELLVCTVNLCLVGYYIITEWNSNNLTTTVTYVVLYISLTFNI
FIFCYIGELVSEQCKKVGEVSYMIEWYRLPGNKKLCCILIMAMSNSSVKFTAGNMVELSISTFSSV
VRTSVAFLNALRTFS
>HlOr25
MKKQRSANLASLATENDYQEYLNLSIQLNRWLLKPLGIWPSSRKSSRIGRCFSWLMGFLCYALMSF
ELVSSGLYVVLEVKGAYNTLKMIGPMSFFAMSLLKYSMLTFHEDDILECVERIKWDWKNIRYLEDR
NVMVTYAYYGRKLITICTFFVFSSFVFFYIVVPINAGSVATEDGNHSYIQMPFPFPKIIADVRYSP
FNEIFFSVQFVTGIVLHGVTSAACSMAALLAVHACGQMEVLVSWLGHLIDGRADMSKTLDDRIASI
VTHHVRILNFLALIEKTMQQIAFVEFLGCTIILCLIEYYIITEWNPKELTFAVTYSALLLSFTFNI
FIFCYIGEIVAEHCRKVGEKSYMIEWYRLTGNKKLCCILIIAMSNCSTKFTAGNMVELSVNTFSDV
VKTSMGFLNMLRTLT
>HlOr26
MLDSSRDTLNATRDYEYSIQVNRWFLTPMGAWPRVTETSRAQQLLAKLLRLTCHSLMISSIVPCIL
FILFEDTSLEERMKSIGPMSHFLMGELNYCCLLAKANDILYCVEQVKQDWRNVKKPVHRDLMLKNA
KLGRSIACIAAFCMHTGVGSDEIVTGLRMSTFYVGNESYSMYPLPCTFYTKLMDTRFSPTNEIMFV
LQCLSGFIVNSVTVGACGLGAVLAMHACGQLNVVMSKLDDLVDAKGQEERVAQRKLGFIVEHHLRT
LSFIRNIENVMNLICLVELVGCTLNMCMLEYYLLTEKSKEKLASYVIVYVSMTFNIFIFCYIGETL
TEQCTKVGEKVYMTEWYRLPHKTALGLVMIISRSSMVIKITAGKFLQMSVATFGDVFKASFAYFNM
IRTVAM
>HlOr26like_1C
NIFILCYIGETTKVGGKVYMTEWYRLSQKTALGLVMIISRSSMVIKITAGKFVY
>HlOr27
MTNEFAVVKTNFKSYSDYSLQLNRWFLKPIGAWPPSSSTTTLERNVSIVLNIVCYGSILFTLIPCL
LHVFLDDDSFYMKVKNFGPMSHWIISCANYTLLLLQGKDIRYCVEHIETDWRMVRREKDQQVMMKN
ARFGRYVATFCAAIMQSGVFCFFVSSALNTEIIHVGNETTIVRVLPVTVYKKLLNVDQSPTNEIVV
FMQTWSSIIATTSTVSIFSLAAVFATHAYGQLTVLMSWITEFVNESRNREKTFPFKQIGVIVEHHL
RVLSFISYIEEVMNRICFLELFRCTLAICMLGYYILAEWSTQDIQNLSSYFMILISLSFNIFVICY
IGEILTEQCKKVGDVVYMTDWYYLPDKRILDLILVIARSSVVVQITAGKFFHMSIYTFSDVIKTSF
AYLNLLRQVS
>HlOr27like_1
MSNDKTVVTEINITASSDYSLQWNRWAMKSIGTWPQFSSNLERNISRILNVICYTAILFSIIPCSL
HLMLEKGTFYTKVKILGSLSHWVFCIISYTMLLLRRKTIRHFFNHMETDWRTTTRKEKKEVMLKYA
KFSRYAAISCTIFIHGGILGYCVMTASTTMVVVVGNKTTILRVLPLPMYKGLLPVDTNTMNDIVVL
SQFLSGFIANNSAISVISLTSALTSHASGQLSVVMLSIEDFVSEARRRGKDDHFDEIPTIVEEHLR
VLNFISRIEAMMNKACFLELMRCMIAVCALGYYIFMEWAESDAKILITYFVALVAIVSNTFILCYI

SERLTEQCLKVGEVVYMTDWYYLPQRRILDLILIIKRSNVVVKVTAGKVLHMSLYTFGDVVKTGFA
YMNMLCQMS
>HlOr27like_2N
MSNDKTVVTEINITASSDYSLQWNRWAMKSIGAWPQSSSNLERIISRILNVICYTAVLFTIIPCSL
HVMLEKETFYTKVKILGPLSHWVFCIISYTMLLLRRKTIRHFFNHMETDWRTTMRKEKKEIMLKYA
KFSRYAAISCTIFIHGGILGYCVMTAFTTMVVVVGNETMILRVLPLPMYKGLLPVDTNTMNEIVLL
SQFLSGFIANTSAISVISLTSALTSHASGQLGVVMLSIEDFVSDARRRGKDDHFDEIPTIVEKHLR
VLNFISRIEAMINKACFLELTRCTIAICVLGYYIFMVDVILIVRA
>HlOr27like_3C
EEHLRVLNFISRLEAMMNKACFLELMRCTMAICAIGYYIFMEWTESDAQMLFTYIVTLVAVVSNTF
LICYISDRLTEQCLKVGEVVYMTDWYYLPQRRILDLILIITRSNVVVEITAGKIFHMSLYTFGDVV
KTGFAYLNMLCQMS
>HlOr28like_1P
MTNKSVFVETSFTSHSDYSLQFNRWFLKPIGAWPPSSSTTRLEKIISVILIFICYSTILVTLIPCV
LHMLLEKESLYMKTKVLGPLTHWFVGCINYTTLLLRGRDILSCVEHVENDWRIITREKDQNVMLRY
AKFGRYVAASCAAFVQGGVLCFCLVTVLSTEVIQVGNETRIVHLLPCAVYKELINVNDSPMNEIVL
VVQFVSGFIVNSSTVGIFSLAAVLTAHACGQLNVLMEWITEFVDESRNRKKKAPFKEIGVIVEHHL
RALTFISRIEDVMNRICFLELFRCTLDICLLGYYILMEWAEQDIQNLSTYFMMLIAICFNVFIICY
IGEILTEQCSKVGDVVYMTNWYYLPDKSILDLILIIARXNRREISSNVGLHVRRSSPLIFQVVKTA
FAYLNLLRQMT
>HlOr28like_2C
LDFCYAYLDDILVGYYIFMEWAESDAKILITYFIALVAIVSNTFILCYISDRLTEQCLKVGEVVYM
TDWYYLPQRRILDLILIIKRSNMVVEITAGKVLHMSLYTFGDVVKTGFAYLNMLCQMS
>HlOr31
MANESLVIEANLNSLSDYSLQLNRWFLKPIGAWPPLSLTSKLERIVSYALIVICYCSILFTVIPCL
FHLVLEDESIRTKVKVFGPLSHWFVGGINYTTLLLRSKEIRGCVEHMQTDWRIVTRPEDKQVMLKN
ARIGRYIAIFCAAFMQCGVLGYCVITAFTMQTVEVGNETRIVHLLPCAVYKKMIAVDTSPTNEIVL
VSQFVSGFIVNSSVVGAFSLAAVFAAHACGQLSVLMMWIKEFVNRSRDSNKNVCFDKIGVVVEHHL
RVLSFIARIESVMSEICFMELFKCTMDICVLSYYILTEWTDHDFQSLTTYFMILISMTFNIFIVCY
IGEILTERCKKIGEVVYMTNWYYLPGKDILDLILIISRCSVVIKITAGRIVPMSVYTFGDVMKSAF
AYLNMLRQTT
>HlOr31like_1
MTNNCAINVTANFDSLSDYSLQLNRWFLKPIGAWPLASPTTKFERILSHLLIFLCYCFILSTVIPS
IFHIILVDESLHLKLKLLGPLGHWFIGGINYTTLLLRSKEIRGCVEHMQTDWRIVTRPEDQQVMLK
NAKIGRYVAIFCAAFMQCGVLCYCVITAFTMQTVQVGNETRTVHMLPCKAYKKIVAVDTSPTNEIV
LASQFVSGFIVNSSAVGAFSLAAVFAAHAYGQLSVLMIWITEFVNHSRDQNKNVYFSEIGVVVEHH
LRVLSFIARIEDVMNRICFMELFKCTLDMCMLGYYILTEWSDSDVQTMTTHFMILISMCFNIFTVC
YIGEILTEQCKKVGEVVYMTNWYYLPDKDILNLLLIISRSSLVIKITAGKLIQMSVYTFGDVIKTT
FAYLNILRQTT
>HlOr31like_2
MTDDLESFGSLSDYSLQINRWLLKPIGAWPLSASISRMERIIWFLLIVVCYCLILSTVIPSILHIV
LEAENFHMKLQVLGPLGQWFVGMINYTWLLLHSKDIQGCVQHVQEDWCIVTRLEDQRIMLKNAKYG
RYVAASCAIFMQTSIMCKCLVTAFTTQVIEDGNETRILRMLPCPVYKEIIPVDTNPTNEIFLATQF
LSGFIVTATTVGAFGMTAVFAGHACGQLNVLMSWITEFVNQSRDENKNLYFTEIGVIVEHHLRVLS
FIDRIKNVMSTICFVELFKATLDICMLGYYILTEWESHDIQNLTTYFMILISMCGNIFLMCYIGEI
LTEQCKKVGEVVYMSNWYYLPYKDILDLILIIILRSSVVFKFTAGKILHMSMYTFSDVIKSSFTYLN
LLRQTM
>HlOr31like_3PC
FLSICLSIILTLERHLRPRYISSIEYLKYVIYFVEVKTMKITRVTVRFIEWSDGDVQTMTTHFMIL
ISMCFNIFTVYYIGKILTEQCKKVGEVVYMTNXFYLPDKDILNLLLIISRSSLVIKITASKLIQMS
AYTFDDAIETTFAYLNILR
>HlOr35
MALVTSSTDSTWDTDWMYSVQMNRWFLKPIGVWPLSLCVTTLEKISSVVLALISCFLIGFLLVPCA
LCTVLDKTGDLDTKIKMIGPLSFCMMAAIKYYILISRGGKIGQCVKDIRADWSLTLSHSQEEEREI
MRDSARIGRSLAIFCAGFMYSGGFFYTTVMPLCTVRTEIIDNETVRSQAFPIYRGLLDPRTSPSFE
IVQFMQCLAAFVIYSVTVGACSLAAVFVMHVCGQFRILVTKLDKLVDGVKGRKGLSTHEQRLGDII
EHHLKILGFISQIEGLLNEICFVEVIGCTLNICFLGYYLLTEWEQSETIGTITYCTLLVSFTFNVF
ILCYIGEILSEQCLKVGLSTYMIDWYRLPGKTAQGLILIFAVSNSSIKLTAGKIIDLSLSSFCSVL

KSAFAYLSLLRTLTT
>HlOr51
MRSFSSDRPSNDRYETDIRYTFELCHWILKPIGMYPFVFSRASRRERTASVLLILVCCSILQFIIV
PFSHHVLFSDNDMNTFVKNLGPLTFCLTTFFKYCYFSMKGSAIGRCVKHIERDWKMLRDEDHRVIM
LRYVTMSRNLIKLCAIFLYIGGLSYNTVIPLFSKKSVNGNVTIRPLTYPGYEEFFDVQKSPAYEIV
FCMHCVYVMITANITMAAYSLTTIFATHTCGQIKIQTTRLEDLTKRGMLLEKGVSDRLAVVVSGHV
EILKFTRMIESALHEIFLIEVIVSTVLLCLIEYLLMMEWESSDSIGILTYVTLLTSFTFNILIFCY
VGELLLGQGSEIAHASYDTEWYNLPRRKARDIVLVLAITKHPIKLTAGKIFVLSLNTFGAVRESKL
VLIH
>HlOr52
MYDRSCSSVDGQLRNYHYQNDIHYTLQMCQWLLKPIGVWRLLDQRSSKLEHLVSIVLMTICFSSLF
FIVLPSGHYIFFAEKSIHVKVKVFGPVGFCLSSIVKYCYLCLNGGAFERCIQHVERDZZZZZZZZH
RTIMLRYVTMSRNLTTVCAVFLYAGGMSYHTVMQFLSKDRSRENYTYKPLAYPGYDWFIDTQTSPT
YEIVFFFHCFAAMIMYNVTTAAYSLAAIFVTHICGQIQIQMARLEDLVERTGEKDDGPDPLVVIVR
DHVKVLRFAKNVEETLRDFCLVEIFESTLILCLLEYYCLVEWQNSDTIAMLTFFTLLTSFTFNIFI
FCYIGEILSEQCSQIGSASYEIDWYNLPAKRAYDLILLSVISQYPPKLTAGKIIDLSLNTFSSVVK
TSVVYLNLLRTVTNW
>HlOr53like_1P
MDDTNRNDVANSLKYCRRLLKPIGLWPLIYSHTSKLNKVLSIIILIAWCTLTVLFVLVPCGYFCVFH
VKNVKLKMKLLGPVLYSMLSMLRYSCLVLKTPAFKHCIEDIENDWKLIDNPEHRAIMIKNAMVTQR
MTVAFTIIIYSSAVSYHAIVPFFSNALENKKNNTIRALPIPGYELFVDAYSSPTYEIIRTHLFVYX
ELISLFTRSSSTKLVSYFVAHAYGMLQVQMARLKYLVADNRKESDRSRMLVVIVNGHSNALRYTRR
ILNALETLCFVEVLLSSILLCLCENLCVEGWKTHDMIFGVTIGGFMLSITFNIFVLCHAGELLVEE
AEKFGNAAYNIEWYNLPPNRALDLVLMIGIAKFPPRLTGGKIFDISINTFSAVLKSSVVYLNLYQT
MTEW
>HlOr60
MHLSMREQIDRKPRNLNYKKDIVYVTKHSKWILKSLGIWPAVLDETAGFLPKITIGLSNLVLLFTV
IPCILHIIFEQKDTIIRLKLFGLLSFCLISLMKYWALATRKPRIKECIEEVQSDWKEVESLRDREM
MLKYGQMGRNLTIICAIFMYTGGTIYHTVMQFATGTYVDEYNRTIKPLVYPTYSALFDVQTSPIYE
LVYFVHCMCGYVIYSITAGACGLAALFATHACGQIDIVMSRLGDLVNSKESSDLDKRLIEIVEHHL
RILRFSAIVQTVLQEVCFLEFIGSTLLICLLEYYCITDWESSNTVSLTTYTVLLISVTFNIFILCY
IGEILMEKSSSVGLSCFMISWYHLPTKTIHGLILIIAMSSNPAKISAGKVVDLSLSTFGNVLKTSL
AYLSFLRTTVI
>HlOr62
MKSTEENSGEKIAREFLVQETVLRIIGIWPSSVNNFASIGRWTFAILTHISTIYSLSLEVYRHCLD
LDDTMDAFVMDLSSVISLSKLLVLRMKSKHTYALVDSIVKDWTNVDHSRHERIMTEYFNKGRIVSL
TILYLGYASGLSFVLKALPLHLLPFQKLHSWANSTVNTNDTARLNYFLATHCVFGPLPLQHHVCVL
LLQGMHIFFNAVAHCGNDGLFFSLTMHLCGQFEILKMNLAEIEFEAIACRKRIGVLVKRHCKLAVL
TNDLEQSFSMIILVQLMMSALLICVEGFVFLVALSTKDNVAALRSFVLMVTLLIQLYLYAYAGNTL
ESRTEEIAQAAYDSLWYRCHGHAARDLLLIIHRGNSPYRITAGKFVPMNLFTFKEILKASGSYLSV
MKVMMDA
>HlOr68
MAILRPAFNILTICGCWRPCSCRTTRAKAAYIVYTMFVVLLLHSFCISQFFNVILNLRTADELSES
FYMFIASVLSCCKIVTLLTNHGSIEILRRRLEEEPCKPVSAEEAVIQSKFDRNIGSVTIYYTILVE
LTVLCMILSSLLGDFNERELAYRAWLPFDYSLPTYYYIAYVHQIVALIGTSLSNVACDVIICGLLV
HACGQQEILKHRMKEMTQEQRPNIGKIVRFHDYLYGYVFTMQEKFRWIIGVQLLSSTLVVCFILYE
LANTPPISSKYLQFVLYLACMMTQIFFYCWYGEQLKLKSVEIVRTIFEMDWIPLQNGIKKDLIVVA
RRAMIPIELTCAYMFTMDLNTFVSLLKMSYSTYNLLERRSDK
>HlOr69
MHLLQKQLQFCAMCGLWPPPSSAPLFKRFAYSVFSWYTLILVYITVFSQIMDLIVNVTTQDEFSDN
FYILLACAISLQKAQSVLNSRKNVHRMIDLLNSEPFHPECKEEIEIRSRISEHARSSTYYAILVES
TVMTLSFGALLKPESRPLPYRIWLPCNYTPLITYCSIYTLQCVALALSAMMHVACDSLICGLLLHT
YGQIEILGCRLKTIKENESKTSKLCVRYNNLIYRFTAMINEQFEMAIFTQFAVSTLAICFNLYLLT
GSDVTPIRYVEIIMYSSCMLTQIFIYCWYGNEVKLKSLEISNMIFHLDWTPFDKATKRNILTMMMR
ASSPIEIISVRVLSVNLDSFVALLKTSYSAYNVLQRGEG
>HlOr70like_1
MHTLQLSLKTLTICGYIRPTSWKSPGLKLLYNVHTVTVVVVLSTFFVAQILDIIFNVENQDEFSDN
FCITCCVMNSLGKLYSLLIGREMIQNLIDSLQSDPLRPLDEVENEIRLRHEKTIEKLSMAYTVCTV

LSATGVWMLSAMTAFRIRELPLRVWLPFDYRATLGIFSLTYAYQFVTLIFITTAIMGTDNLFSGLL
IHIYCQFEILQHRLKSIKPDREYSAKQCAFHHYRIYKFAEIINAKFSITMATQFLVTTMTMCFNLF
RLTQTKVYSQVLALIPFMVSVFAQIFYCCWYGNETKVKSLEICDTVFESNWTALSQSTKKTLLTIM
LRSLKPIEFTGAFVFPVNLESFKSLVKTSYSVFNVIQQT
>HlOr70like_2
MKVSVLFTFLSIGGCWRPISWSSTYKTAIYNVYTVLVLIMGVNFMVSQFGSMFTIETVQDFVDLTY
ICVSVFVGCCKLSNVVFYRKDIIDLMNIFMEDPCACSNKEEDQVQARYDDQLRKNAVRYTMVVEMS
VASTILNSLVTNFQHGRLTYRGWFPYDYTTAVLFPLTYALQLLCVLVYSWIHVSVDILFFGLLMQL
CCQFDILFSRFSSITSENKGVLRRCIRHHDRIYRLAEIMNDSLQLTMFAQFFGSFMVICLSLIQLL
NADILSTEFLATIFYLSSILLQSFLYCWYGNEVRTKSVDLADVMFHVDWTGLSEKGRKIILFAMTR
TRSPILFESVHVITVNIDFFVVLVKSSYSIYNVLKNT
>HlOr70like_3P
MDTLSWTFKLLTVNGILAPSAFMSQWQTILYGMYMFFMVSMLYAMECCLILGMIFNVDSQEDFSEN
LYITLILMCSCCKIYMLVTSAKGIKVMVDVLREEPFVPMNREENEIRVRFEEKIDWNAKAYTYFLD
ILVVVLWITSYFTDYRRRKFKFRVWLPYDTTTQPVFTLIYCQQIVCTWYSVNINVICDCLFSGLMI
QICSILRKMKPTRRNCVLVTIIRYTSQSRQEIKRYVXLAQIVNEKLGNVSSIQFAISTGAICFNLY
RMSVIELGPKFVEALTITVCLLAQIFYYCWYGNDRNYDSLEIVDRVTNSNITVSDESSKKILVLMI
RRALEPVEFTGLRIMSVNLESFTSLLKSSYAAFNMLQERREE
>HlOr71
MSVLKSTSLLLKISGCYLPHSWTTPFERSLYNIYGVFYLLLLSSLALSQILDVAINVESQDDFSDN
FSITAVVFLTWFKLSILLIRRGNFIFLIDTLQNKPFSPVDTEENEIRAKFEKITEWNTIGYWSLLL
ICAFWIYVRSLLTDFSSRKLMFRAWLPYDYSPAPIFILTFVHQAIAATICCTSSVTVDSLYTGLLI
NIYCQFEILEHRLRNVKTEQDDSVKQCAQHHDRIYQFSKKVNEEFKLILISQFCISMSVICFNLYR
MTQIKMDTKFVEIILYSFCTITQIFYYCWYGNEVKHKSLQLPYMIFHSDWTSLNNNVTRALLIMMR
RAVRPIEFTSIHVVSVNLESFMAVIKTSYSAFNMIQQSKQS
>HlOr73
MHRLPLPFALLTCCGYWKPTKWPASSLKYWLYDVYSVFMVFLLYFFTFCACIDSLISKDLKTMTDK
FSLFISVFGVSLKVANLFLQRGRIINVMNILLTENCIPRDEQEKIIQRKNDNYARKLTIYCEILNE
SAAFLATVAQYNEFIRSKTLPISDWVPYDLSSQKLYTISLLHQTIGLMVCANTSVANETLIAGLMI
QAGAQFEIFCHRARNLTALLLDTRRNSESFTQTVNTVFQYMIFLQFSISSTVLCLSIYKMSTINPF
SMNFVWCASYLCCMLTQVYLYCWFGNEVTLKSEKVGEAIYEMDWTALPTDIRKDLLLIMARSKRPV
KMTSGHVVVLSAESFMSIMKITYSSYNLLTNSTNK
>HlOr76
MTSNNRDIAVTVTAFYLKIIGFWLADTYEEKRRRKFAQNFTIFMLVCAVVIETRDIYYTWGDFSIV
VYIMCNILTLGISLFKIFLSMWSKKKLLELIKFARTNFWHSDYDSQEQIIVNECKRTCTFLICVFN
FFANGTVSGYIIRPIVASIGKNESDRLLIVNFWIDLPITITPYYEILFTIQIFIIMYVCISYLCID
NFLCMINLHTATQFRILQYRLSNVCGANERSDKISKKTPSNSDECYAKFKNCIQQHQALIEYCNKL
QEVFGIFVLAQVLLFSLLMCLDGYLVMEDTPIMQRLTFLFHLTGCLCQLLMFTYSCDCLIRESLNV
ANAIYDCSWIHLPMDRSGRMLRKDLTFVIGRSRVPCCLTACGFFPVSLETYTSVISTAVSYFTLLR
Q
>HlOr76like_1
MTSKKNRDVSVSVTAFYLKIIGFWLADTYEEKRRRKFAQNCTILMLMCAVLLEVRDIYHIWGDFGA
VVYVMCNILTVGISLFKILVSMRSKEKFLELIKYARTNFWHSDYDSQEQMIVNECKRSCTFLICVF
NFFANGTVSGYIIRPIVESIGKNESDRILIVNFWIDFPTTMTPYYEILFTSQVFIVMYVCISYLCI
DNFLCMINLHTATQFRILQYRLSNVCGANERSDKISKKTPSNSDECYAKFKNCIQQHQALIEYCNE
LQEVFGLFVLAQVLLFSLLMCLDGYLVMEDTPIMQRLTFLFHLTGCLCQLLMFTYSCDCLIRESLN
VANAIYDCSWIHLPMDRSGRMLRKDLTFVIGRSRVPCCLTACGFFPVSLETYTSVIHSTFHTISML
EYFKYFFLYLINVFGKQ
>HlOr84
MHLVQSKDISIVWSFFLMKIVGLWLAADEAEQRRRNFALIYTLNAIFIASCIAMRDIYYSWGNVND
CIYVGCNILYLAIVFFKILVLYKHRIEFYGLIRFTQEKFWHFDYNSREKLILSECKKICTVFIVAF
SFCTQGTCAGYVVTPILANVGKNQSERMLPFNMWVDFPTGLSPYYEVLFIIQTLCVYHVGICYMCF
DNILSLLNLHVATQFRILQYRFMNLSNVIEKQTDGREFYLQLKSCVQYHQALTGYCKKLENIFSLL
VLGQVLFLAMVLCLVGYQLFLTDSPPSRNVGLVLNLAGTLCQLFMFTYSCDGLTRESMDVSRAVFA
RPWANLPMDRNGKSVRQSMLMVIMRSNRCCCLTASGFFPVSLETYTGVLSTAMSYFTLLRQSTMSI
AD
>HlOr86
MQATKYRDISLKCGIFLLKLVGLWMSMNSVEERRRRFTLFGSVFALSYGVYVNVNDILHSFDDLSH

CIFLTCNTLCILLAIFKIFILSNHRTEFKEIVLYAQKHFWHDNYSKEERVLYGDCQRFCKLWVILI
CFLTQASLFYFVTPLTANMSRNKSDRVLPFKMWVDWPLSDTPYYELMFTFQALCVYQIGAAYICSD
TFFCMLNMHVICQFRILNYRLLNMWPVIDERTNMTEYTNKCYTKLIDCIKNHQSLIEFCVKLENVY
TLTILGHIVVFSLLMCFDTYEVVLAKVPTTTRLIFLFHMIGSFIHIIFFTYSCGGLIEESMDIATA
TYAGMWTVLPMNKVGKMIRGNMKLMTVRSLQPCCITAGGFFPVSLKTATALMSSTMSYFTLMRERS
AE
>HlOr87like_1
MVTDTTKYFSIRLTRILMKIIGFWNVQTRREQLILRVNYLYMLLVLSVSITIELLDLYYCRHDLYA
ATYTACNTIPLFVILMKTVVFTIRRKEILWLIEFAQKYFWHDKHSSFGKKAMDNLDKRALIYMSSF
TILIYATVVIYVLSPILENIGKSDEERILPFPFRVFNLPTSLTPYYEILFVIETLSVVHSGICFCC
FDNFLCILSMHVGGQFKILQNKLQTVLSEKSSKEKSAGLYKEFKECVQYHHLLLSYVEKLEYVFCV
PLMIQLLVSSIVFLYYLLFCSILFIRYLVNGVLMKRLLFLNYFLGGVIQIFLITLNCNDIMEQSGA
IGTAIYSCNWERNVYNHFYQFRKDMMIVMVRAKRPCYISAAKFFPISLESFTKVLSATASYYTLLR
TMEMDVIE
>HlOr87like_2P
MSNKEAHSPLAARVTAKLMWITGIWVASTEKEEKIFAILREILALNIFAMSWTIIYEIYVCDSSFY
SITQVASNAMPVFVVTIKTWSLLRNRQRILALIRFTETHFWNARLDKIDADEVKGIDKVNVCWIFV
LVYLNTFNISSNNVGRNASDKVLLYDTYLGLPKTESPFFQINFISEIFLCMYLLLCYIGFDASACV
LIIHAAAXFRILGRLLSYIFTEELYDKEISIQKQMSQKFEKCLRRHYLLIDYVEELNNLFKYTLLL
QMMTSSLLICMSGYECLVYQASLLKRMVYIMYMVASVSELWLIVRTVDELTTHSKAIGDAMYSSDW
GVIFGKLEKKLKFALTTMVIRSIETCSMKAGFFAVSSVTSTAFSYLTLIRTVEVNNVDG
>HlOr87like_3N_C
MTSKKNRDVSVSVTAFYLKIIGFWLADTYEEKRRRKFAQNCTILMLMCAVLLEVRDIYHIWGDFGV
SQVLSLYHVGVCYICFDNLLCLMNLHAATQFRILQYRLSNLGDTNRKQVDDNESSAALSRFAEN
CYKAFKNCVRQHQNHITYCHRLNEIFTVIVLGHILVFSLLICLVGFQVLVTPVTPARRLTFVFHIV
GSLCQLLLFTYSCDGLIEESTNIGTAIYLGPWIHLPMDKAGRRLRRDLTMVIVRSRKSSCLTASGF
FPVSLETCTTVLSTAMSYFTLLRHSFV
>HlOr87N
MRKISEEVKVGVMSARSKAVKKGSHTKDFALLMTSFLMKIVGLWLAKDSKEERKRRYTLIYTVVAI
LFGVWVQFRDFYYSWPNFGDCAYTACNILCLIMVLLKLFVVFIHRKEFIELLVYMYKNFWHLNYDC
NELLLLQDCRRISTICITLINFCAQGTIVSYVLTPIIANIGRNSSDRVLPFNMWVDLPLSVSPTYE
IVFVLQVLSLYHVGVCYICFDNLLCLMNLHAATQFRILQYRLSNLGDTNRKQVDDNESSAALSRFA
ENCYKAFKNCVRQHQNHITYCHRLNEIFTVIVLGHILVFSLLICLVGFQVLVEN
>HlOr90
MQTEKDSDISINLSKFFLRNVGLWRAENHAENRKRKALVAYTIWNMFLAIVVETRDMYFTWFYKGD
ILYVSTNLLSVILTTIKLTVVLIHKVEFMSLIDYMYEHFWNVDYDLHEKRIMDNCKKTCIIFTSSV
TTIGICAIISYIATPFIMHSQSNKSERAFLFDMWLNLPLTVSPYYEGMLAVQISGLYFTGICYFCF
DNIFCIMSVHLSGQFRILQYRLTKLSDVQYQISEKNMESVLEDCTRRSYEKFKSYVRQHQTLIDYY
EKLEHVYTKIILGQVLLFSVLICLFGYQILLANTSLARRSIFMFLICGAMVLLFMFTYSCNMVIEQ
SDNIAFAAYSALWTGMPMNKTGKKLRHDLIMVIGRSRRVCCLTANGFFPVSLETYTTILSTAASYF
TLLRNNVEDVVKA
>HlOr94like_1P
MSLKYRQDVSFRLANFFLYVVGFWFAANRIEEWFRNAAILYTIVTVFFVMWLQLRGLYFSWGNFEV
SLXYMACNSIALVLDIIKIFVLVVRKKKFLGLIEYMQKNFFHLNYDQDENSIIAHAKRTCIYFVCV
FSFCSQSTVFSYIIRPFVSNLGKNESDREHIYDMWLDLPLSVTPYYEITYIIQALSVYQVGVCYLC
FDNIFFIMCLHVAGQFRILQYRIANVSGLSEIVKSNDNTSTAELYSEKCYIKFKNCIQQHQALIEF
CEILEEVFTVIVLGQVLMFSIIICFAGYEVLLITLPFESRVAFTCFLITGVSQLWMFTYSCNCITT
ESLNIAESVYAGPWINLPMDKFGKLLRKDLQVLILRARRPSALTACGFFSITLETFTKIMSTSMSY
FTLLKQSAEDTVNS
>HlOr94like_2
MSLKYRQDVSIKLANFFLYVVGFWFAANRIEEWFRNAAILYTIVTVFFVMWLQLRGLYYAWGNLEV
CMYMAGNGLAMVLDIIKIFSLVVHKKKFLGLIEYMQKNFLHLNYDQYENSVIAQAKRMCIYFVCVL
SFFSQSSVLSYIVRPLVSNLGKNESDREHIYDMWLDLPLSITPYYEITYIIQALSVYQVSACYLCF
DIIFFILCLHVAGQFRILQYRIANVSGLSEIVKSSENPSTDELYSEKCYIKFKNCIQQHQALIEFC
ATLEEVFTVIALGQVLMFSILICFVVYQALLVSIVRYNKYSMMFATMTGVSQLWMFTYSCDCITTE
SLNIAESVYAGPWINLPMDKFGKLLRKDLQVLILRARRPSSLTACGFFSISLETFTKIMSTSMSYF
TLLKQSIDDTVYS
>HlOr94like_3N_C

MSLKYRQDVSVRLANFFLYVVGFWFAANRIEEWFRNAAILYTIVTVFFVMWLQLGGLYFAWGNFEV
CMYMAGNGLAMVLDIIKIFTLVVHKKKFLGLIEYMQKNFWHLNYDQYENSVLAQAKRMCIYFVCVL
SFFSQSSVLSYIIRPLVSNLGKNESDREHIFDMWFDLPLSVTPYYEITYIIQYNGTQNKLSFEW
RVALACFSMTGVSQLWMFTYSCDCITTESLNIAESVYAGPWINLPMDKFGKLLRKDLQVLILRARR
PSSLTACGFFSISLETFTKIMSTSMSYFTLLKQ
>HlOr95
MSSFTTDDISISLTSVFMKIVGLWMASNRFEQRVRNITLCYTLIAILFALWIQMTDLYYSWGDFGA
CLYTACNILSLTIPFLKILVLLAHKNDFFHLILHLQREFLHADYDDYEKKILLGCQRQCTFFVCFF
TLFTKGTVISYIVNPLIANIGKNESERILPFNMWVNLPLSMTPYYEITFSLQVLSLYHIGVSYFCF
DNFLCIMNLHAATQFRVLQYRMANMTDLKDKERKERNMKTSVSSACFATECYNVLKDYIRQHQNLI
AYCNKLEKVFNIIVLGQLLMFSMLICLDGFQILMANTPTRTRLIFIFHIGACMCQLLMFTYSCDCI
IRESTSTAEAVYSGPWTLLPMSADGKMIRKDLMLVIMRSSVPCCLTARGFFVVSLETYTGVLSTAA
SYFTLLKQRSENADSS
>HlOr95like_1
MKLFKTDDLGFSVTSMFVKMIGLWMAKNQFEQHVRNVTLFYTFFFVSFGFYVEATNLYHSWGDLSD
TLFDICTLLSVIMPAIKIIILFINKEEFFRLIVYTERQFMNGDYNEDERKIVFDCKRLCTFFTCFL
SSVTLSCVLSYVVTPIYMNIGRNESDRILPFKMWIDIPTSMTPYFEIFFTLQVLSAYQIGVSYGCF
DNVLCIMNLHLATQFRILQHRLVNINGLKSKQTPEKGETSIIDSTEFADNSYDVFKRCVRQHQSLM
AFCEKLEEMFSMIILGQLLVFSIFMCLDVCQVLLPGASVGKQMIFTFHLAGCMCQLLMFTYSCDCI
ITESLNVTEAVYDGPWPRLPMSTRGRMIRKDLILVIIRSRVPCCLTARGFFVISLETYTKVISTAV
SYFTLLRQRIEESNS
>HlOr104C
NEIFQIQHYQPLQKFLQFLGQDPSQRDGTRGSIVFVMVLSVLGILIPTFIEIYVQLCEKNMDAVIE
CLPNGIAAATSVVKLLNVYLNRENFQKMYDLVTREWEQLRMNDELHVLEDITMQGGKLAQFYRNTL
LSFMVVFLLVPLVFPFLDVVLPLNETRPRQQGFRVNYLYFDEADYFFSVYFQLAWGAVVVVMIIIT
VDSLYMIIIHHASGMFAVCGYQVKTATEYKDSIYGNDISENHTHEQFKRCVTTHDKAIEFYDILNE
SSRNSYLIQVGLNMMGISVTAVQTVLTLDRPAEALRCAVFLVAEQFHLFIISLPGQTLVDHCTKLS
KDIYSSTWYKVPAKFQRVIYTMQIRALKPCVLTAGGLYEMNMENFANTFKTCMSYFTMLMSLRE
>HlOr104like_1C
SAFQTNKRQNYEIFDIPDYQPLEKLLLFLGQHPYQTETTKSVIVLLMTFTLLSMAIPSLIEMYVQL
RKKNMDDMMESFANTIAAATSLVKLLNVYLNRENFRKMYNLVTIEWEQLRMNDEVHVLQEVTLKGS
KLAQLYRNALLFAMCVFLLVPLFGPALDIVLPSNETRPRQQLFRVNYIIFDQADYFFYVYIQLFWC
TIVLVMAIITVDSLYMIIIYHASGMFAICGYQVKKATEFVDSIDNSNISQNHTHEQFKRCVTMHDK
AIKFYDILNESSRNSYLLQVGLSMMSISVTAVQIMLALDRPSEAIRYSVVLGGAQFHLFIISLPGQ
TLVDHCTMLTNDIYSSTWYKVSAKFQRVIYMMQIRALKPCILTAGGLYEMNMENFANTFKTCMSYF
TMLMSFRE
>HlOr104like_2PC
SVFQKRERHEGSIFDFPYYGSFKNNMLFLGQYPYQSKLTEIFNETLMVCTYTSLIILQVIQMFEAI
IVKDIDATMETVPVIVTELVCVIKLLNHIINKGKFDKLYDIMKKEWDMLSLDEQQILEEITKRGSK
LTNIYRSTLFYFSIIFVLFPLIPPILDVVSPLNETRSRIEVIKLQYLFDMEKNFYSLYVYTGWCSF
MTMMVIITIDALYVVIIHHTCGLFAVCGSQVSDKYSYNIXSLKKKKKXRVQTFLRFVIFIHDFSSF
DTINEITSTCYLFQVGLNMIGISVTSVLAFINLDRPEEFARIACFLIAQQLHLLVISVPGQIITDQ
SSKLTIDIYNSMWYQTPVWIQKILHIVQIRSGKPCKLVAGGLYEMNIENFGSTFKTCISYFTMLLS
FK
>HlOr104like_3PC
KRSVLQKSGRAPREVCNIFYYRDIRKYTMFCGVDGNKINNTILIVTICSLMSMVVPAVREIYISVR
EKNRNATIEVLVSITATITAIIKLVNVYAHRQYFKNLCISVVEKWEELRISNELSVLEEVTAQGSK
FVQLYRIVLFSVLFIFLVVPIFPPLLDILKPLNETRARQKIFKVNYLIIDRDEQALITYFHLSWTT
ITLGISVLSSELIYVLLIHHTSGLFAVAGIDTIYKXIQSNDLIVGKNLEDEIYEKFKRCVDTHNEA
IECYNVLQKSSVLSYLFQVGLNIVGLSVSAVQTVGNIDDPTVALRNATVWGAKQIHLFIISIPGQV
LIDHCVELSNDIYSSKWYNNGMEAKKILQVMQQRASKPCILYRVCKINMKLCFNFKTFKTCISYCT
MLLSLRE
>HlOr105like_1
MYSVFQKKSKQAYNVFSMIYYKSMKSYMNILGINPYQDKRAGIALQSTAMFLCVSVMSPMFLKIYV
AQREKDFDGVMQVLPNIIAQTGALVKVMNIRINKANVCIYIVAKKEKGIFVCEKIVMIERISSFEN
YSISWYEDSLWIVLLVYMSFPLINPVLDVVAPLNETRPRRLLLPVSYIFIDEYEHFYSVNFHLYTS
VISCGMMIIAVDMLYITIIFHLCGLFAVCGHRIQVTTEMNIVNKDETKKSEAGYKQFKQCVTMHHE
ALQYCQILERSSQHMYLIQVGLSVFIISVTAAACIMNIDRPDELIRLAMFVVAQKFHLLILSLPGQ

MLLDHSLGLADIIYASKWYELPVKGQKLIYTMQMRSIRPCILTAGGVHEMNIESFGIAIKACMSYF
MMFLSLKE
>HlOr106like_2
MDEEEVINRYLKNVRYYGRILGIWPLDNSWKPRVVPKLLYIRCITSLRLQSARIVVAYSFTVLTEQ
LPYLCIALMMIVKHGNCFLQLSKLRQIVTNMISDWQKKMIDEELAIMNKYAKRGALFSFLYITIYI
GVSVFSTAPLIPFILDLISPLNESRPRLYAYKAYCYFNPDDYYYYVVGYTSACIGMAGLVLVGADI
IMIHLVLYRYRFEYAVATVENSTKEEIYKMRRASYMKLRAAFQSHNAAMEEATYTLNLVPQVFITI
VVFSVTLFQVIAQTVHMLFLTIQGQFVIDSHDTVYNVCMAIWYKMPPKLQLLDVVALRKSLTPPIL
TAGGLMRLDLNSFAQLLKACVSYYTALRPS
>HlOr108like_1F
ESLISGARNDWSIGRPQGEIDIMTKYAERGAFLISTYLVNAIVCVILFVSTPVTPHILDILLPKNE
SRDVAYIYPAYYFTDEHKYDVYIITHMASVILVVFYVYFACDTSYIYVVQHGCGLLAVSGYRYKCA
IVELSSRKRNSVTQDEIYRRVCHSIQGHQHAIKYIDQIAKIHAVYFFICVGVIMICFSVILVKVSN
SEINAEFTKDCTFIMIQLVHILYLTLQGQFVIDANEEIFESM
>HlOr111
MEASDVERNYLYVNKFCGTMAGIWPYGDEKMKWVSRFVILVITVTAMTTQVAYVKVNLSTDTVIEQ
CPFFTMGLGIFLKELNYIIHEQQLRKLLGCVFADWIVKRPKPEMDIMDKYAKRGLFFSRLYVANGI
FCYVLFTAVAITPRVLDLISPKNESRGVGFIYPANYGVDEEEHYYYLMGHMLSVITVVFFVYISCD
TIYMNIVQHACGLLNLSGTRSFMINYTEVDDTYRKVRHSIKAHQHAMEYIMQIEDVHDSYLFIIVA
LIMLSFSITLVRTSMMDPCMEFYKYCAFLVVQLVHLLFLSIQGHFVILSHDMTYDNIYSAKWYNSS
PRTQTLYVLALRRSLTPPLLTAGGLISLNLETFAEILKASVSFFTVMKST
>HlOr111like_1
MEADDVERNYLHVNKRCGTVSGIWPYGSSRSKWIGRITMIFVTVTGMMSQIAYVAVNLSIDNVIEQ
CPFFTIGVGLLIKEVNYILHEEQVNTLLSCIFDDWLIKRPKTEMDIMDKYAKRGRFFSRLYTVNGL
FCYVIFTSVAITPRVLDLISPKNESRGVDFIYPAYYGLDEEEHYYILMGHMLCVITVVFFVYISCD
TIYMSIVQHACGLLNVSGYARRHCFKWAVETVITKNEKMHTEVDDTYRKVRHSIKAHQHAMENRYI
MQIEDVHDSYLFIIVALIMLSFSITLVRTSMMDPCMEFYKYCAFLVVQLVHLLFLSIQGHFVILSH
DMTYDNIYSAKWYNSSPRTQTLYVLALRRSLTPPLLTAGGLISLNLETFAEV
>HlOr111like_2
MDAITLEEQYIKLTKICAQFSGFWPWQSKLSKYVSRIITYTIVFTVLFTQVAQVVCFFSLDVLIDQ
LSFFGAVSGTLLKQGNYIVFKEEYESLISGARNDWSIERPQGEIDIMTKYAERGAFLISTYLVNAI
VCVILFVSTPVTPHILDILLPKNESRDVAYIYPAYYFTDEHKYDVYIITHMASVILVVFYVYFACD
TSYIYVVQHGCGLLAVSGYRYKCAIVELSSRKRNSVTQDEIYRRVCHSIQGHQHAIKSVLKKIRQI
VLNVLCFFSSMLLLRPTIAWKQVSNSEINAEFAKDCTFIVIQLVHILYLTLQGQFVIDANEEIFES
IYSALWYNSDKRTQSLFVLALRNCLSAPQITAGGLITMNLQSFSEIVKTSVSYYMVLKSV
>HlOr111like_4N_C
MDEEEVINRYLKNVRYYGRILGIWPLDNSCVVLIFQVLNWSRSNTAIVVQLLQSARIVVAYSFTVL
TEQLPYLCIALMMIVKHGNCFLQLSKLRQIVTNMISDWQKKMIDEELAIMNKYAKRGALFSFLYSI
TIYIGVSVFSTAPLIPFILDLISPLNESRPRLYAYKAYCYFNPDDYYYYVVGYTSACIGMAGLVLV
GADIIMIHLVRVFITIVVFSVTLFQVLRLCTFLIAQTVHMLFLTIQGQFVIDSHDTVYNVYEAI
WYKMPPKLQLLDVVALRKSLTPPILTAGGLMRLDLNSFAQLLKACVSYYTALRPSY
>HlOr111like_5
MADIFEDRFFKMSRRCISLVGLWPYDDASFDKSFRRVSIALVYLIFAEVQLAKFFTTRFTFDIFLD
TITAILMELLILAKCSIFWFNGEMLKQLLERVKHDWDAVRGREELIIMRKYTKYYRMFGILYTYFY
SCGIGLFVTATLLPIVMCRIQPRNESHVHTIPFQGEYFFDQEKNMVFIILHMILTVTITHCIAVMT
DMILLVLTMHACALFAVVGHRFERLFDNAPGYAATKDALANWNCRFDERQDYCIEYHLRTMKGVQY
AVHRVLKVCCARCSQLFDKLMPLKSVNEVVIPLFHVWAFLVVVFLNIYPAQMVMDTSEVIFYQAYY
GSWYAAPTRAQKLLLLLVLQKCMVPCSLSAAKIVTASLQIYATIVQTSVSYFSVLYALKE
>HlOr111like_6C
LKCIILCKVFIVYLLCTFIMIQLVHVLYLTLPGQFVIDANEEISESIYSALWYNSDKRTQSLFVLA
LRNCLSAPQITAGGLITMNLQSFSEIVKTSVSYYMVLKSV
>HlOr112
MYAETVEERFMKLNKVLGLICGVWPRQNSRRKLITQILSLLLMASSIVTQIANTVLFFSMDNLVDG
IPFNVAAVGTFVKLGNYIINETKLKSMVEQIFDDWTSITSETECKIMVTYARKGQLIALCYAAHLF
LPAAIFLCVPFAPIILDIYAPLNKTRKRVFVYPAYYWMDPEQYYMIMIIHIMFVVIMVCVIFCACD
MNYVYAVQHACGLLAVTRYRFSQAYKTLSINGDENGARFFDKRTYRDVCHSVRGHQRVVQYLKHIE
DSHRIYLFISMGMLMIAISVSLLTMASSHNLPQKVVHGTFFVGQAFHVFFLNVQGQFVINAFDELY
DKIYESQWYNFTPRTQALYVLALRSCLNPPLLTAGGMTTLNLRSFAEIIKASVSYYTVMQTK

>HlOr112like_2
MDAITLEKRYLRINKKLGMLTGVWPYQKSSPKWISRTVVLFVIIPCYVTQYARIVTFPRIHIILND
YPYLITSLGVIIKFGNYFINESEVSAILSKIYEDWRAMTSEDEYKIMTKYAEKGIFSVILYILHIS
LCAALFMCLPFVSPILDILMPLNETRARLFVYPAYYFVDEDKYRYLIVGHMYLVVVMLISVFCACD
ANYVYAVQHACGLLAIAGGYRFKYACYEVIPEDEKEAVKMMKKMYRNVCHSIRIHQRALQYVKEIT
AAHDTCMFISVGLLMMSISTSLLQISEHEHDAEWTLYCTFFTAQLLHMLFLVVQGQFVLDAYDDVY
NTIYESTWYNSSCKTQALYILALRSSLNPPLLTAGGFITLNLKTFSEIIKSSVSYYTVMQS
>HlOr112like_5F1_F2
MYAETVEERFMKLNKVLGLICGVWPRQNSRRKLITQILSLLLMASSIVTQIANTVLFFSMDNLVDG
IPFNVAAVGTFVKLGNYIINETKLKSMVEQIFDNWTSIKSETECKIMVVHGTFFVGQAFHVFFL
NVQGQFVINAFDELYDKYESQWYNFTPRTQALYVLALRSCLNPPLLTAGGMTTLNLRSFAE
>HlOr112like_8N_C
MYAETVEERFMKLNKVLGLICGVWPRQNSRRKLITQILSLLLMASSIVTQIANTVLFFSMDNLVDG
IPFNVAAVGTFVKLGNYIINETKLKSMVEQIFDDWTSITSETECKIMVTYARKGQLIALCYAAHLF
LPAAIFLCVPFAPIILDICAPLNKTRKRVFVYPAYYWMDPEQYYMIMIIHIKLKVVHGTFFVGQ
AFHVFFLNVQGQFVINAFDELYDKIYESQWYNFTPRTQALYVLALRSCLNPPLLTAGGMTTLNLRS
FAEIIKASVSYYTVMQT
>HlOr113
MDAATVQARYVKYTKRFALMAGIWPGQKRLTKYFLRTIVFMVIISSLLAQLARMLQTLTVDVIVEQ
VPFFFAIVLLLCKQGNYIINTDKFEYLLDSMCEDWNQERSYKEIGIMKKYGQRGNLLSRFYVVNAY
ICSFLFIQVPWSTRLVNSYKTQNITPSLPVVVPGYYFVDEVEYYYYIILHGTCTILIVVVVYCACD
TSYMVLVQHTCGLFAVAGYRFKNSIREDNSVKGKYFDHWTKEAYKNACFSIQGHQRAINFVKEIER
GHDTYLFTCLGLVILCFSITLIKLSTSDFDIDFYRYCSFTLCQLMHLFVIMLQGQFVLDSCERIHE
AIYESLWYTSSSKTQALYVVALRRNLTPTCLTAGGLVQLNMQSFAEVVKMSVSYYTVLKTT
>HlOr113like_10PF
KEAYKNACFSIQGHQRAINFVKEIERGHDTYLFTCLGLVILCFSITLIKLSTSDFDIDFYRYCSFT
LCQLMHLFVIMLQGQFVLDSCDRIHEAMXVEGINNRNKRI
>HlOr113like_11C
KFIVYRYESTWYNSSCKTQALYILALRSSLNPPLLTAGGFITLNLKTFSEIIKSSVSYYTVMQSK
>HlOr113like_3
MERNELEETYLYVSKKCGVVAGIWPYGSDRIKWIGRITMMLITITSLITQIANVLVNLSTDTAIEQ
CGYFMMAVGIILKEMNYIIHARELKTLFECTFNDWITSRPKSEMDIMNKYARRGLFFNRLYVVTGI
CCFTMFISAPYIPHVYDVMLSKNESRKLGFIYPAYYGVDQDKYYYYIIAHMTSGGLVVFFTFISCD
TIYMNIVQHACGLLLISGHRFKWAIEEMGGNNEKLNPTMLEGIHRRVCQSVKAHQHAINYIERIEE
MHHTYLFIVVLVVVLAFSITLLRASMMDPCVEFYKDCVFLVVQLVHLLFLAIQGHFVTVSHDQVYD
SMYDAVWYNSTPRTQTLYMLALRRTLTPPLLTAGGLISLNLETFSKILKTSVSWFTVIKST
>HlOr113like_4
MEASDVERNYLYLSKRCGIVSGIWPYGNDRIKWIGRTTVLFIGATSLITIAHVVMNLSTDTMIEQC
AYVVLAVGIILKEMNYIIHAREVRLPYFRIFNDWITRRPKSEMEIMDKYARRGLFFNRLYVANGVF
CYIMFSSAPFIPHFIDVALSKNESRKLSLMYPAYYGIDQDKYYYYLMAHMMSAGAAVFFVFMSCDT
IYMNIVQHACGLLFISGHRFKCAIEDDGADDEKVNPTMLEDMHGRVRHSVKAHQHAINYLGEIEAM
HHTYLFIVVGMVMFAFSITLLRASMMDPCVEFYKDCSFLVVQLVHLLFLSIQGHFVIVSHDQVYDS
IYSATWYKSTPRTQTLYGLALRRGLTPPVLTAGGLISLNLETFAEILKASVSWFTVMKST
>HlOr113like_5
MEAREVEKTYLYLSRRWGSMAGIWPYGNDRTKWIGRVTMILVAASSLLTQIAHILVNRNTDVVIEQ
CPFLMVAIGIILKEANYIIHEKQLKEMIECLFNDWVTTRPKSEIEIMDKYARRGLFFNRLYVANGI
FCYVIFTSVAITPRVLDLISPKNESRGVGFIYPAYYGLDEEEHYYYLMGHMLSVITVVFFVYISCD
TIYMNVVQHACGLLNVSGHRFKWAIAAEKSNAKHAFMMEEAYARVCHSVKAHQHAINYITEIEQIH
GTYLFIAVGMIMMSFSITLVRVSMMNPCVEFYKYCGFLVVQLVHLLFLSIQGHFVIVSHDMTYDNI
CAATWYNSTSRTQALYMLALRRNLTPPLITAGGLISLNLETFAEILKASVSWFTVMKSA
>HlOr113like_6F
SSSLFIFQHSKFSLLARYLLIFRTGLFALFQLARMLQTLTVDMIVEQTPFFCPLVLLLCKQVNYIV
NTDKFEYLLDSMCEDWNQERSYKEIGIMKKYGQRGNLLSRFYVVNAYICSFLFIQVPWTTRMVNSY
KTQNITPSLPVVVPGYYFVDEVEYYYYIILHGTCTVLIVVVVYCACDTSYMVLVQHTCGLFAVAGY
RFKNSICEDNSVKGKYFDHWTKEAYKNACFSIQGHQRAINFVKEIERGHDTYLFTCLALVILCFSI
TLIKLSTSDFDIDFYRYCSFTLCQLMHLFVIMLQGQFVLDSCERIHEAM
>HlOr113like_7C
MNDTKLQLKEMIECLFNDWVTTRPKSEMDIMDKYAKRGRFFSRLYVVNGLFCYVIFTSVAITPRVL

DLISPKNESRGVDFIYPAYYGLDEEEHYYILMGHMLCVITVVFFVYISCDTIYMNIVQHACGLLNV
SGHCFKWAVETVITKNEKMHTEVDDTYRKVRHSIKAHQHAMEYIMQIEDVHVSYLFIMVAVVVLSF
SISLVRTSMMDPCMEFYKYCGFLVVQLVHLLFLSIQGHFIILSHDMTYDNIYSAKWYNSSPRTQTL
YVLALRRSLTPPLLTAGGLISLNLETFAEILKASVSFFTVMKST
>HlOr113like_9C
YITEIEQVHGTYLFIAVGMVMMSFSITLVRVSTMNPCVEFYKYCGFLVVQLVHLLFLSIQGHFVIV
SHDTTYDNIYSATWYNGTPKVQALYVLALRRNLTPPLITAGGLISLNLETFAEV
>HlOr114
MIDLKYVYGWNHYTMRFMGIWPEERKWNQPSSYLALIPVLMMFLFVCVPQTINLSFIWGDFNLVVE
NLSMGNITITISLLKTIAFWINGKPLKSLLKCMADDWSTVSKKDDLDTMSNIARITRKTIISSTVL
CHIVVITYIFLRFLTRKYSGTRLLFRAYFPYDSDVSPNYEFTVIAQIIAAFYAATTYTAVDTFIVM
LILHVCGQLSNLRNEFGKLQACDKVTLQAKLGKIVQKHEYLNRFAETIERCFNMMLLIQMLGCTVQ
LCFQCFQAVMSIDEEIDENMIFQILFLLFYVVYVMLQLFLYCYVGERLSVESVEIANAAYDSEWYN
LSPKNAKLLLIIMRRARLPLQITAGRFATFTLMLYSQILKTSVGKGYVSVLYAMKYKETTL
>HlOr114like_1
MELVGIWPEPSRSDQRWPNFKALLFIFVIIFFGTGPQSVNLLFIWGDLGLVTENLSTANIPGINAM
MKLIIAWHHKAFKPVIKSFYDDWKTSRTKEEKETMLKRAKFAKQISIWCSVLTLTMVTVYLSLRAW
IIYQLDRANQKQDRLLLYPGYFPFDIRPIGVLLSTNFGQVLAAYSAVISYTTVDTFIAMLIMHICG
QFEILRQKLRRLMDDEKKTRSVDEIQKELVLIIERHEHLNWFATMIEDCFNTLLLIQMLLCTVEIC
FQGFLFFDVILENEKGIFNVQLLFFVLFVCFILVHIYIYCYIGEMLLTHSREMSNSAYESNWYNVS
PPEAKCLLFIMHRTARPLCLTAGKFSTFSMEMFSTILKTAMGYLSVLLTVTGSD
>HlOr115
MDFAMGWNRFSLSVLGVWPEPRKMSNLSRFASNFIFWFTTLITVVFICAPQMTHLVLKSTNLDEVI
ENLSVNIPMLFSLGKQIILRYRRKALVFLMSQIFDDWAEPIPEVDRHTMLKTAKISRMISIVCSIL
TYVMVLAFLSLQIWSSMQNTSEFNVGGLLIPATFPYDTNKSPNYEITWLGQFVGTVLSAICYSCFD
TFVAVLVLHICGQLTVLRIALENLANTTIKDNYSKFQERLGYIVHRQNQLFRYAVIVEDCFNIMLL
VQTLMCTAMLCMTGYRMMTSVNQEQEDVPIIGVIFFISHVTYTMLHLFIYCYVGETLLGQSTGIGQ
SAYDCNWYDLPPKHAVSLVIVICRAQVSFQITAGKFSPFSLELFNAVLKTSAGYLSVLLAMKDRLV
EEN
>HlOr116
MDEFAGKQTKLKKIKPNKHLKNCMLLFYYAGIWPGQMKYKRLYYLYGISSFVILLAFTLVTEFAYL
CVHWGDYAEIMSGATIFMTNYSYCLKVIVIVLRRKRIKDLINITEGKLFIRNNDKYERIVTYYTWQ
GIFHHITYQSFGIIAVIFWSCTAVVNLMKRTYQLLLIKAWYPYNVTISPAYEFTILHQVVGVTVNC
INNVAIDTFITGFIITACCQLTLLSYNISSIHYEAEKEFVARTDDISADKSTSKTYNQLYEDLKIC
IEHSRLISDFARKIQDTFGTVIFFQLLVNCITVCLLAYNISQLKYYVLSDLFGMFMHMCCMTYQIF
IYCWHGNELYLHSMNICFAAYMNNWWHNTKDFKRALLVVMSKAQRPIILTVGNIMELSLENFVLIL
RTSYSIFTVLKTSTNA
>HlOr117
MKELSKASIDYYILPNKIFCSIAGMWPIDEKSSTRSKLFAYFRLIFGLTAISSVFVPEIMAIVWNW
GDIRILAGVGCVLTTVGQLLFKMIYLIARRDKTYKLCNEIRDLWDSSDDPKERQSYEELAHWARNL
TIIFYSSCMCNVFTFTIAAAFDYFTIEYSVSDADKSRHLPFEVWYGIDITPSPRFEIAFLCQIVAS
MICAAAISGLDATFMTTILHVSGQFKLINRWISKIGIEINIEPDYPRKLKANLIKCIRHHQRMINV
VNDVNNLLTPIIFMQLLTSGIEICLSGFAVIDNGTGTDLLKFVSYLTSMGVQLLLWCWPGEILVQE
SLEIGHVVYLNIPWYNLPPIYQKQLCLMIIRAQQYCSITALTFQTLSIHTLTAVFNTAASYFTLLR
QMQQT
>HlOr117like_1
MKEPSKTSIDYYILPNKILCGMAGVWPIDEKSSTRTKLFAYFRLIFGLVALNSMLVPEIMMVISNW
GNLRILAGVGCVLTTLGQSLFKTIYLIMRRDKTYRLYYEIRDLWHSTDDPKERQNFENLAYWARTI
TIVFYSACMWNVFTFTFAATHDYFMIEYNISNAEKSKHLPFEVWYGTDITASPLFEIAFVCQLIAV
AFVAASTSGLDGSCMTTILHVSGQFKLISTWVSKMGVETNVKPVYSRKLEVDLIKCIRHHQRMINV
VNDVNNLFTPIVFVQLLTSGIVICLSGFAVFDNDARTDILKYVSYLSSIWLQLLLWCLPGEILVQE
SLEIGHVVYLNVPWYNLPPIYQRKLCMMILRAQQYCSITALTFRTLSIHTLTSVFNTAASYFTLLR
QMATK
>HlOr118
MTSTTIINRPLEYSLRLFGAWPDSSYSFLKSIIWSTIMATFLMFQYWYCITHIRSGLIDLLDGLSI
TLSNSLVFLKLIIIWLHSRTFYGILTTVFEDCNNYASTVENKRIMTDKAMLSSRISNFLIGYFSIT
FLVYSGLALVLFDEDQNGPVSKQRKFLIRMEFPFEATVSPRYEIILVVQFIFEAFIVYGAATSIAL
IAVLILHVGGQIDLLCQNMRDISRNHEKKVPQKLTIKDVVVRHQRIIRLSKNIETVFTYISLCQFL

SNMLVICFISFVLVTSLNTEQTTGLILKCFPYYVAVNCEAFILCYTGEYLTSKSERITQSVYNFLW
YDLKPREARIVLLIILRSQKELILTAGKFVNLSLAAFANMLKASASYVSVLHAMC
>HlOr119
MAYVPGHEWMRSFRQLIHVQISYIKYSGLWKIESKHSSFMTIAYFAYKLWILIMMNVFAVTIFADI
YENMDNLSIISDDGCFLAGILVVIFKAMNYQIHQKSIIKLLDDILNCADDLCRFSNTETNKYIINR
YYKFNKVVFYGFSMLGCVLGIALLFFSPLQNGLPIRAKYPFNTTISPFREIGLAVETFAVSGGLLS
IVAMDNITAMMCSQITAQFDMLNVLFEKCTSQVENKNVNVTDEIFKTETNRINKTHDNTFLRRYKT
CLQFHQRLISITKDYNRIYSSSMFVQMISSTSIICLTGFQAVVVGGQSSDIMKFGIYLSAAISQLL
YICWLGNELSYSSSVIDRSQWLSGWNHEHLTSIVKIFTLSTMFTRQSITLKAGVFYVLSLETFIAI
VRRSYSVFTLLNNMHSTSP
>HlOr120
MNIKNYVFINQLALKFVGLYPISIIRYLICISCIMLIIIPQITMIYTNWDDLNIVLETSSSLLTIS
LATLKSIIWMFNRKKLAVFIEFMLTDYWEIVKTNVSEYLQEYAIYAKNITKGYFISMCNALLFFCS
LPIIEIFVTEHQNSDNLTLKNFPFAATYPTVFYNFPFYEIAYVSQALATSICCLMILATDGLIATA
VLHTCGHFAVLKENLRRLDLCIYRVCVNSFENNSKHINAKLYQIKVQITHLIKHHVVLWFCDNMEK
NFHLMLLLQTMTSSLMICFVGFQVSTTLTEQSKVIKFASHLIVTLFQLLLFCFPGDMLIRQSFSIS
TAAYSIQWCQLPTFLKDEVCMIILRSQRSSYITAGKIYVMHLENFTAILSTACSYFMMLQSFNSEA
>HlOr121P
MNRSSLRIIGLWFEEDHAGTMKFLRKLRNMGIVLIILFVCVLPCLYSLIKQCDSLMAVIDNLSYTI
PLTITLMKYIVILSKKEVLLPLVNMIAEDWAKLKTDFERDVMIQRTRITRATSILSYALIFTAVMF
MIVLPRTGITTRYVTNETNIKKLFPVPTYYIYDISETPYFEIMFLLQMATLVLMALCYIGVDNFFG
ILILHICGQLENLRTRLANIKHLETFDRILVTTVEDHVRLIRAVDVIESIFTMLVLALLFYFGTFA
CIYGLLLMTILTQEEQFSVLRITYLACIFLNTFLQTSLYCITGQILVNQSEEVYVAAYECEWLNLK
SNKAKSLILIMARAQRPLCLTAGKLFPMTMLTFCXRFATSLSYISFLLTKL
>HlOr130like_1
MDVFNKQYRTYRLMLSLLGLWPYSQSIYSTIHRISFATIILSCIIFEIATLVSFERASENILQTCF
VCPLLVLFFERYVSFICNFPFVKSIFECIQTDVYTLQDSNEMKILTKYIDDSSRIITIFLYAAYFG
ITIIVTTLLYPVSMDLLVPLNNSRPRYLGYFVILPSNQIQYIDFMTFYGTLVPFVGLLTVACTESL
LSISAMYLCSLYKITGYRLGKAISNATKTSFMSVQNYTDFYCAVDIHTRIIKLVNILQSKMKVPYM
MASVLAVVSFVLCLSQIVKAIVTMNDQLEILLSIMIVSNHIVFLFFNNYAGQLLINHSEEVFVESY
NSTWYCVPLSAQKLLLFIMMKSTIPCKINLSGIFAPCYKGFSSMISTSFSYVTAIYSTYN
>HlOr140TRA
MDFFEQPAYLLNKRLLTIVGIWPFQSTSSRLLRQTVMLFFCFTLLIAELRGLYRIWGVDYDVVIEC
MPPIISIFLSLTMYLNGIFNMNKIKDVLLFIKYNCNYYMNQPENMILKYYERQGKKITLYYTSYVY
MTLLIYLLLPPMSLIIDDVISSNYSQERNFLFELDYGVDSQQYFYYISIHSYMGTAVVANLIATCD
TTYMLYSQHAYALFAIVCSQLKTVHILDTGNLINVKDSYILEKYKNIELLPGEQKKVCRKLLNCIK
EHQNAIEYSSVLESLFTKSILSQLFCNIICLSITGVETVMKLGNIGDMIRFGSFTFAQAVHIFLIC
LPGQRLVNHSEQVYATACEVMWYTLPKKCHSLYKFLLARSSKPSKITAYKLAPMTMETFLAIIQTA
MSYFTVLLSTT
>HlOr140TRAlike_1
MDFFDQPTFSLNKFLAVLFGTWPFQSITQTVVRNILVSIGIISIIIPEVNHLCKTWGVDKDVSFGC
MSSLLTVIIPMIFQYNSLLRKKGVNIFLIFIKHDYERLINLPEYNVLYSVAIKGRRNSLVYIIVYV
CISATMYFTLSIAVVIFEELNKNRTEPRQTLYPVEYHIDSEKYFYLITAHCYASTVLLLLQTVVHD
TMYIVLVQHGCALFAVTGQVYCLKGAHCLDSKGLLKDDDFEQYKNRCYTLSEQKKIYQRLVLGIKE
HKRAIEYTKLLHAVFSECLFIVLMLNIISLSINGVQALINLHEIQTSIRLILWEIGLFIHLFWLCL
PGQRLTDFSERVYYDVLNCLWYTFYSISHVLYRFLIMNTLKPCELIALKIIPLNMETFLSVTQTAI
SYFTVLSST
>HlOr141
MKGKDEGGEFDRLIGLNLDLLRLCGVIPCGSGFLGRNGLACVAFGCLTVYSISYTYQFVTCMENLD
TIFESFTMIISIVGGQARFSILSWFRGTCRTMLEACEFLWSTLKPREKEIVRSYANKTRLLTRCYL
ASCVFTIFIYGVFALFDQSQPSPENLSENGTVDSTVRRLPYVFFLEVQQTPWYEITCALQLFAMLN
VGITCVGVDTIGPLFVLLTCAHFDVIRSRIESLHAIDRSSSSVQTRNLRVLVIRHRMLLEFCQDIE
RVTNVMFFTQLFGSTYNISLVGFKLIGDDPDKYKYISQLSIAIIQLFLCNWPADFLYSKSEAIGRA
AYTVPWHWYPRCLRKPTNMLMIRGQKPVRLTAGKIVGLSLETFASMISSAVSFFTVVRTMN
>HlOr142
MRKSITPEQAISFTKLSVAVTCSWPPSPLATRTQVLLFNTLWSTAFVSTIALLLPLLSGIYEYRKD
PIILGKTVSLASAVAQVAIKMIMCRFQQKQFQVLYFDMENFCRYATSKEKVVLQRYVDRYKYFHGI
YILWCFLTTIFVISGPLYSPQTFPTHAIYPFQVEHTLFKIVIYFHQSLVGFQASAGMAIDTQIALL

LRYAAARFELLGNEMRNANTHCEFNACIEKHSELLRYTKTIRQSIKFLILTTIATTTVAVIFGSLN
LIANQPLLLKALYAVVVFSASVELFMYAWPADNLMRMSTKLAADIYNTDWFEKDIRLQKKVYYIIQ
RSQRLEAIRINGIVPKLSLPYYAMYLYKSLSYFTALRIMVESETAVESI
>HlOr143
MSLFNANIHRVLHILELAGRFTCTWPLDSNSSKREVVFRNVRWGFTILNVILLLISLILAIFHFRN
DITILMKTISEMTALLEVLMDMILCKMKSAQLQVLVGKVKAFVKVANKNEIKIIQGYVDRYKRFFG
TIAMGYVMTGITFSLAPLFSVQELPADGWIPFSIKPLGVYCLVYFVQVYCILQTAFCISVDFMIAL
LISFSAAKLDILGMKLRKANGHDVLVSCVKEHQEIIGFVEDIKTSVETLLFKTNATMGSAVICGTF
PLIYNQSLTVISQFLPLMLSGCGRLYVISWPADDLKESSITFAKSLIDSPWIGKPRKMTNIVIIMM
QRSQRILLITMGGILPAISLEYYANFLTTVGSYFMAMRTMIES
>HlOr146
MLKYPSPERAISLVQVITAFTCCWPLPSTATKSLVLQFKILRSFLLLNALVLFLPLLYALHVHRND
AENVAKAACLTLALVQIFFQTFVCIGQYDHFQKLIEEMKICCETAKPYERDVFQRYVDKYSLFYVT
CSSWFYLTAVIMVLGSRIISDPFPTNAVYPFPVNFEPLRSIIFMHQSFVGIQCAAHTSMNVLTALL
LLFAAARFEILMMELRNVSDIETLIKCMKKYSIVRRYATDVVTTIRLLVLITVITCTVACVFAGIN
LIGKQPFAVKAQFLTVSGTALLEVLMCSLPADHLIDMSENIMQGIYESKWYERSLKIQKSTVLMLT
PQSPVTVKIKCLIPVLSLNFYCSYVSNVFSLFTALRIVMIDDENGN
>HlOr146like_2
MLKYPSPERAISLVHVITAFTCCWPLPSTATKSLVLQFKILRSFLLLNALVLFLPLLYALHVHRND
AENVAKAACLTLALVQIFFQTFVCIGQYDHFQKLIEEMKICCETAKPYERDVFQRYVNKHSLFYIT
CSSWFYLTAIIMVLGSLLISDPFPTNAVYPFPVNFEPLRSIIFMHQSFVGIQCAAHASMNVLSALL
LLFTTARFEILMTELRNVNDIETLIKSMKKYSTVRSDREVAEEVGAVRRGNRFKRVLRTRTNLLIR
LPKQPFAVKAQFLTVSGTALLEVLMCSLPADHLIDMSENIMQGIYESKWYERSLKIQKSTVLMLTP
QSPVTVKIKCLIPVLSLNFYCSYVSNVFSLFTALRIAMINDETEN
>HlOr152
MLKQLTPEKVIDIIWFSVALAVCWPLSSSASKAQVVAFKILQICSAISALILLLPLLYSTYLHFDD
VIVLSQSICLSMGVFQMIMQTVICFTNYDLLQRIITEMITYVKEAQQFEREILHNYIQKCKLFYGG
SLIWTYLCATCFVLGPVVLPNPFPSSAEYPFRVNRTPMNAIVYLHQSFISFQYSAHICVSIFGALL
LWITAARFHCLTLEVQQSSSIHMLIICVKKQLLLRRYAKEVVDGFRLIVLNAIAMSILALTLSSIT
LIMEIPLIVKVQFVTVSFTVLAEIYMYAWSADYMKDMSAKVSQSVYDLTWYGQTLEMQKNVLYILV
HQEPVILSVSCIVPELSLRYYCSYLSNAFSVFTALRVVLDAKSA
>HlOr159
MKFVDYHCYKSNPIILFIVTLWACDNVWLKRLQNLISFFVLTSALFVQLMPFATQSLKFGDILNDI
LFISLTLTFIIKYVTFYYSTKQVKKLFNSIHRHWDELDNKEERKIMEKYSDNGGFFVMLSIMYLIP
IIIAVILLPFYDIIFNIVTSLNVSREIELPADLEYFTDESKYYPIKLLHVVSHAITGVTVVIFIDS
TLLIFLQYSCALFKTTYSYRIGYAIESYSWLNEPQRYYIILTKLIKAVESHCLAIKFVGQLTNCFK
YCYMFSLLLFVPSVSINLYCLSQSMLTMDDFEDLCRNIFLLMGQLGYLFIINYMGQDLVNHNNEVF
QTAYNSLWYIAPTPVRKLLLTMMLQNVRQRVFKLMGGLYITNIEGFSMILRTCISYFMFMYSTQY
>HlOr159like_1PN_PF
MDFVGNDYNGFIRKLLKFLGLGPYQKVTWFIRIWRLIIAIILINGIIFQVNVIRIPRETFLIYYGN
LVVLRLQIHLFLHHLSQSKRILAXVKSILERDQQDWKTLKNEDEVKIMRNCRKQANLLIVIFSNKE
RILIRYIDITVSIFSATYFVLIGLGVVIVTATLFRVSLRQALISMELTSCLXLPDLCLQLFYIF
ICNYIGQGVKNCSSEVFYDICNSVNWYTAPIAIQKLLLIIMQNTIRNSSITLFGMYISSFDGFFMV
NNTII
>HlOr160
MRRSVSLNVELFYDENVLSWSKRLLGLSGLWPDNRNDVRFFFYISYVVIFTWLEMVTLLQNIHDLE
KSLKNITLSFPTILIVLKAVMFRLNMHLVLPLLEAVRKDVKQGLYRSQEERRTVVWYSVAATLFST
SSAMSLFFVPTLFYTKPIVGCLLSKFSNCTLPYELPMKVNHLYEVTEVRTYALFCVYFVPTSMLLT
IGATGADSLLVTLTFHLCSQFSILSSRVKNIDVEPQKYFPQMKALVERHTELLRLAGILAETFSTL
MFIQTLGLIFSLCIVVFQLLTMSESGEDMNTIHFIIYSCAVILLAFCYCFLGECLINESSEMQQAC
YFSNWYDLPDKYTRSLIFCIARSQKPSYLTAGKFYVFSLETFGIIVKASMAYLSVLKSII
>HlOr161
MNARPYNNKEYDELIRPIMLTAKIISIWPLAENSATGAILFRRFHLLCMFFLVVVMSIAVTADVIH
NIDDLDEATECALICTAFYLCVVRMVVYAIHQKDMLYVVNTMRIDWTESSKEDRVILAEKTMFAFR
LAKYFISTVAATIVMFMCIPILEIYVLGKDKVLPFRGYFFLNQTVSPIFECLYLFNVTAGGFGGTM
IASCTSFNLVVIMHGSAKFAVLRRRLEALSGKDPNSTTVMGDLVIRHQEAIEYADALERIINVLAL
GQFVISTGLICFAGFQITSMMEDKGRLMKYSTFLNSAILELFMFSFSGNGLIDESDAVGESAYGSG
WIGSRFSQSLQIMMMRSRIPCKITAAKFYSMSLESFSKVLSTSFSYFTVLTATKDD

>HlOr162
MDNTQYEYLRFNKYFLFMIGVWPYQTVLQRILVGIIFIPIISAQTILQTGGMVTAMTDGDVESFLE
SFAPLIISLMCLSKYINFFYNYKKMRYLLNIMQEDWRIYKKVKNEFDVLCEQYAVGKKLTTSFAAF
LFGLITPFAAMPLLLNAADTLGFSNVSSDRPLVFRVEHFVDVDKYYYTLLVHSYFGTIAFTAIVVS
INSIIAVYVLHECGLCEILRVKLENFVEDDTINVELHLNKRIDRWYQNFRECVLLHKHIIEFANLL
EEANTTSYLFQLGFNMICVSFTQFQTVINLQDTPKALRYVSVTICLLCDLLFVSWPGQRLSDYTER
IFEYTTNGKWYESSYNCRKLLIIMLSKSLTSLKLTACKIYTLNLESFNAVVQTSFSYTMVLCSLQ
>HlOr162like_1
MDLFQNRYYKISKFLLILIGQWPYDHSWTKMCKQIFIYSYLLSYLGVQMCLLWTSEMNFELLVLVL
SPSLVNILCMAKYLFYSLKGDVKLLENLRHDWTVLNNANETKVLKMHAAVGGKLMLFYIGFVYIGI
FSYAIVQFLPYIIDVIAPLNESRHHVLPYAGEYFVDQQKYFLPIALHMLGTVTLGLTVATAVDSIF
IFFMFHVCAKFNILGDRLEHFMDDNRDKKQPPLHGNAGSHFLQMKIARCVRFHQQTIQLVKLIMDC
YNASYVVQGSLNPIIITVIMLRVLIQMFMCSKLGMFINGRYCHLIQMFMCSKLTFPMQKLLDNSSD
ILYRMFYSRWYDAPLKIQKLVLIIMNRSFEPVGLRIGDYQDVTIENFSSIIQVSFSSFMVLYSLQ
>HlOr165like_1C
GVFSDILSTMEEDCQKYAAIDINKLMSKTGQLSFYLTSIVMSLYMASAAFYITNTIGFQGNNASVS
RELLMKMDLPFETSESPNYELVVTTQVLIHLSVAVAYGAFNALLLMVVLHLGCQIDIMCRNLLDVS
FKDGKQIKFFINRHQEIIVFAERIEKLFTYMALSQLVSNTIIICCVGFLVVIAVHIDNGVPLLLKS
LLFYVVICLEAFIYCFVGEYLSIKSQLIGNTAYEFLWYDLRSTESQLLIPVILRSQKGFTFTFGKF
SSLSLESFTAIMKVSASYISVLLAVY
>HlOr165like_2F
IFHSVLSDILSTMEEDCQKYAAVDINKLMSKTGQLSFYLTSIIMSSYMVSAAFYITGTIGFQENNA
SVSRELLLKMDLPFGTSESPNYELVVTTQVLIHVSAALTFGVFSALLLMVVSMNNLYLFK
>HlOr165like_3N
MILRNTMSQSVKFGLHFVGIWPGTPFPSLHKIGWVIAMAVLQTYQYRYIITHYKSDSLMVTIDNLS
ITMPFTLVLIKLIITWANYG
>HlOr166
MTSINTISQTVKHGLRYAASWPGTPSLILHKFFWTIVMCGLQIYQYGYVIRHYKYNTLVDTVDNLS
ICMPFTLVCIKLFVAWTHRGLLYDIISTMEEDCKKYAVLDTKNLISKTAHLSFRLTNIVICICVSS
TACYAIGVLAPQRTNITAPRELLIKMDLPFNTNNSPIYELVIVLQYLCKVSTAFTFGVFSSLLLMM
VLHLGCQIDIMCQTLKEVPYKNKTQLRFFISRHQQIIIFAERIEKLFTYLALSQLLSNTLITCCVG
YIIAFALQNNNGFKMLIKFVVYYMAVCSEAFIYCFAGEYLNIKSKLIGDTAYEFLWYNMHSKESRL
MLLVILRSQKGFTFTFGKFASLSMESFAGIMKASASYISVLLAMS
>HlOr167
MSHNKSISRPVEIGLRLTGIWPGSSYEIIVRVTWIILMTVVQIFQYRYIIKHLDGSNLANLIDSVG
TTLPYSLLYVKLIIFWTKRRIFENMLVGMSNDWSNSSSTKFDINAMISKAELAYRYSKVIMSTYAI
AVFAYTSVFFEFMRHDNDGEYDLKSRELLMKMDLPFAYYKTPTYQWVFVVQFLQLLINASAIGMLD
ALIITLIFHIGGQIEMLQKALTNISIKDEKHRLSRNTTKSLIHRHHRIIINSDSIESLFSYIALMQ
WLCNTLVICCIGFLIVIVSNDGNIKTFIKTLLFYIAITMEAFIFSFAGEYLSNKSLSISNAAYSSP
WYLLDPQDRHIIILLMIRSQRRLTITAGKFMDLSLEGFANVSLLFSYVMHML
>HlOr169
MDFQSLSQLNMLINKYSGNQLPMTDKKTKLPYILKSYSIMSWLIQLTYLTVCVFGLFNVPRDKALK
DGTVNIVVALEEIVLVIYLHNRKTLVRRLIGELNRVLAVDNEMLRNVTINTVKSLEKPLKIYTIAS
VNAVMLWTALPLVQVFRRSEFYYTDYSVPAALSKQPFSYDVFVGGVVLQIIGGVYTVMKKIGVDIY
TMHLILLMTAQYKYIRGKVAMTFRQYNEAFDDFGNSITGLKVSCEKQKVIKQEMRSASHHYEVIVK
MTIMLKKLLSPNIGILYVNNVFRFCFLSLMIITNSGDNFEKLIIVMYTIGALTQFYVLCFCIQELV
EASTSVADDVVHERWYFCDVLVQRVIAMIILADKMECRLSSFRNIDLTLPSFMSILNQAYSTSLLF
LKAK
>HlOr172
MDFAGSQYYRFNRILLSTIGLSPYNSTKYTKFWNTFIILVLFSGIFVQLMTFITIKMNAKQFLKTL
SYIAISFSFVSKYLTFLIHFDVEKEFLERIEHDWNSLTNKEELRIIHEYSSVTRVLTICFSLIIGP
SLLVLILGIFNNSILDILIPLNYTRPRTIPINSEHFVDQQKYFYPIAAHVVLFVIIGAIVLLSTEI
LIMAIIQHTCGLYTIVGYRIDHTFVANIPGLSPAERRVLIHRKIVSLVNLHKRVKELVSCYQVIVL
LTLQQNNIPNFVAFSYPHKLVKAVTKSKQLDEMLEFLFFACGIICYEFVANYFAQKIINHSSHIFV
HAYNSEWYRIPVAEQKVLLLLMQNSLKDDMILLGGIIPASFQGFSLMIRTSFSYFMVLYSFE
>HlOr172like_1C
IREIYDNIADDWKSLKNMEEIEIIRKYANGGRRFTLAIAISTYCGALVITTLQLFPILLDIVVPLN
ESRPCELYLTAEYFVDMKKYMHYIIAHIITCLLIAATTLTANESLLITNVLHVCGLFKLTCSYRLE

HSLDHVTSQKLGSTKRTYVRLIEAVNIHRKAYETISIHLVSQLLEEVIAMENMKELLAAMLLMISH
FLYTFLANYAGQIVMDHSIDVFACIRRYLIPWYRAPLRIQKYLLFISERSKRVSSLCMYGMIIASF
EFFATFMKAAMSYCTVLYSINN
>HlOr172like_2
MDFSGYHYYKFSNDLLRYLGLWNLNAGRLERLQRFVFCSVFVMGIVVQRAAFFTHEYDLQLLSRSV
VFIVGVYMFLNKYMMLYINMDKVRLLFLRVRYDWNTLQNTDEYNILKKYTESATLVNKIHLMCFIP
VILFICLLEVLPDILDVVAPLNESRSHQYLITAEFFITDSSPNYIVTCVGTDITLIMGVTTIIASE
TFTFLLLQHICAMFKITCYRIQYAINQKEIRLIYTKLINAVVTHQRTIQLVLRFAEITEKTIATHF
AYLLVAGLLSITMNMYNVSILWLLSQVILVTKDAKNFSEAAFFVIYQYFYMLMCNNLGQMITDHSF
DVFTQTYDTQWYRLPLPAQKLVIFIMQRSMKSNVFTVCKLYVPSRQGVAKKNESCIRYFHVYISYS
YMFTVHLLELQ
>HlOr172like_3
MDVEKIPFYSFGRSMNVLTGMWPKTGLLTQIHRCFITILLASNILNQLQLGQFFQLGALVTYKNNY
QICCKIIAFVMPTLVYLLKYTFHIVKVKNNSKLLEYVRTDWNNYTKKGEVEIMTKNTKSERNSIIC
FLSIVFLCLIMIALIPYLPRIFAIVTHQNRTRPLKIPIIVEYHVDHNEYFDLIILHINFCHFIGFC
VVISAEVTNLVLAKHICSLFDLVSYRVRIAVDDTILHSNEYFIRKRAKAVVEIHCRAIFLVDIMNE
MFAFHYATLMVIGVLSLSLNFYVCAQAIFYTGNLEESLLEILLIFSQVLYTFYCCFVGQLIQNKSY
GIFFTVIVLYIYIFRYCTQWYLAPMPTQKLILLVMTRCMRGSKFKTIFILEASLELFSMIMSATMS
YCMVLMSLS
>HlOr172like_4PN_PC
MDFAGEQYYVYVKFFSSIIGLWPYTTGRLKTFCNMFIYILFSMAFLIQVTNLLITSVILSMSPL
ILDIIIPLNTSRPRYFPFTFDFIPNQEEYFXPITIFSGILCICGLAAMISSEGLLMMNVLHVRGLY
NIATSYRLKKAMSKNSLQMPVSEKRVLIQKGITESMEQFCQEIFRMTNYNAVFIYSLVLSESALFV
FYIYSRIQILLDESMRFFDSLYNSEWYCSPLPIQKLLHFIMQGRINLNIFFMYGTFVPSMEGFGAH
AKITWSYFVMLYSTQQR
>HlOr174like_1
MDFLGYEYYSLNQQLLTLVGLWPYKRYKLTFLYNTYMLFFFITAFFLQVLVLFFTTELDVDTILSD
GACIIMLLIYIVKYCTFCKQAKNVSLMVSTVEICSFFILCMIFRFIALNFNNFIIFLIFTISVVFA
LSLISFTLVEFVVHFLDILMPMNVSTRHELPFLIQCFCDQDKFFHYILVFLIVLTILGGSVVLATT
TTYLIFSQHCCGLFKIAGYKIQHLLDNDEFEVSDIDQNLTVYLRIINAVKMHKRALVFLDGIKTTF
QMSYTILVILAVISLILNLFQSMMIMHNTKKSLLILLVIIIHFAIIFFCNYTVQNIIDSSTSIFYD
TYYTKWYEAPVFVQKMYLFLMLRTTKDVKIVLGFYTPSIEGFSSLMNITFSYFMVLYSVRMTRM
>HlOr174like_2
MDSLEHHHYKLSKLFLSINGLWPYENSWFMHVKRLLSFVTVVSCIVLLSQNSSLQTSIQHVLIHYF
LFFLIERFFVSNSSSGFQITTYFMHRKKCSKIILLNTLTYVMLTLVLWISKSGSFVTFIVTVVIYL
ATCIYIYIMYKKPLNLSICESSSNLYFTIDYFVDDEKYFYVKFLHVYLYFSLGSTVILGSEVTTIM
FANFASGLYQVVCYRARSVFSKKVLNASSTSRDSVVHEKLVEVVKMHRRAVELVNLLGESFGSSYF
LLLLLGVACTSINLVHVTQIKMPRSNLQLLILEVSMILACFAYLFLTNHMGQTIMDSSSKLLGEAY
SSGWYTASVPAQKTLLYIMQHSIQDYNFSIGGLFVPSYQGFSTLTRMSFSYFMVMRSK
>HlOr174like_3PF_PC
FHKRLLLLIGLWPGIDFKYRTFRVAFFYTIQLSNMLYQVIIRLINYVVSTYTWFYTSECNLQCTLY
VLSIFLQNVLGIIKYTSGHIWMKTVYTFYSEIEFDWKLLKYDKENVEILRKYAGTTHVFAVAATSK
KLGRNYWFLLFNLNGTVFFLIPRYKRVIKVHMIKLVXQLVTTKGDERIILVMFMYTSNMCNYGV
LTTFFGQRIIDHSSNVFYDACNSHWYDVPLDARKLYLLILQRSMKSSSFSISDLYVSSLQGLSALV
STGMSYFTFLYSVQK
>HlOr175
MGEFITYYKLSIFILSWIGLWPYKKLKFQWLYNVAISLISLSTIIPQVCRSTLNFTWVTMLMKVSS
VLMTLCAFIKYNIFWYQIDAVKENMEQVKYEWQKNRDECLEILRKYASTGKYYSNIFASLIYPSVL
ILILCHMSSFVLDVIVPLNETRTHQFPVSMEYFIDQQKYSLFITFHQWFSFLVVATIIVGIETLIV
MWLEHAASLFTLKAVLEDCAQSPHVTRRYLSANCKQNLVNAVVAHRKAIWFVDHLKDRYVFSYAIV
LLFGVVSLSINLFALLQSIFLSKKIQDTLICVLFIVSELAYMFYMNYMVQRTVDTADNIITFIYSN
NWYETSIPLQKLLLIIMMRSIDSCSYDFFGFYTASIQGFSTLLKNSVSCFMMITSMTNRSPMKL
>HlOr175like_1
MDRVHESLHENYNFTVSTMSAIGLWPYRNINIRRLQNFGINVIFFALLFLEVSLIAVANLRWTLET
FVADMISMTILLGSINKYNAFLCQEKIVKQLMEKIKYDWMTINDEGELRILKNNAYRGKGFTIIFA
LLVYPTIFTMILLLFSSKILDSLLPLNESRPSWSLIRIEYFINEQKYFYLSVIYQTVTILLFGTTY
VATEAIFTMWLQHCISLFEIVSYRIEKLIIQCNAEPYVSQKRLHRNSRKILINAVVMHRRVLQFCE
KLRIHFTLAYSCLLILGVISVSVNLFQLSQTIFLEGNIQQFLTSLSSVFSLMGYMLYINYMMQEWQ

DSSNNILTTIYNSEWYTAPLSIQKLLFLIILRTTKPPISKAVFVYTASMESFTLVKSSVSYFMVIY
SIQ
>HlOr175like_2PN_PC
MQYYNFHKNLLTAIGLWPYHNTKFRTLQNSIVSFIRFSFVAVQCLPLCTTEITVQVLLKNLLLIII
FSGSFFKYNIQWYQITRVTTEYFIDEKKYFIFIISHVYLSLIMHATVYYVATESXVIMWMVHTIG
LYNIIRYRIQKIVRKDLNSRLNTNIKKGVIHAIATHRTAIQFFHDVIRDSIPAYSVLLLFAITSLS
INLYRVTQAVTHLDEIEETLITLMFASGELSYMYYLHYILQKIIDHSDNLVTLIFNSNWYETSLTI
QKMLYTMVLRSSKSMLVTFFGFYYASVQGFSSLLQMSVSYFMMMSSMQ
>HlOr176N_C
METFFESRYFNYVRFCARQIGLWPFDDEKEQRIKRYVVFMFSVLYTLLQVWTLMTLKDDLKTTIAS
LPYVVSSLIITIKYFTCYLNYNMIKKLFVTIGENWMELKCKNEMNLMEKYTHDGYLYTVFFDTV
DSKVSVLLFCVIFIMIIAVMLTTDLIYITLTYHACAMISVTGLQHLFDDVKRMRNTEQKNTLLHVR
VAKAIRYHWKCMKYVNGLESCYTVSLFLQHIMITIVITSKLYRVYSFSMTGRANLIIVSVDIAYTL
GLLFANVYPAERLTDCGQNLYHNVRYNAHWYDTPASTQKVIQIMLQKISEPFYFGISGLFSASFE
>HlOr177
MDFLEQSCIKKYYPLLFHIGVWPFDYSKRKTVQRICFCLYQISFISVQVSVFFTTEISIDLLIDTM
PATLMCMLVVLKYATFWVKNDGVYNQAFEHMRIDCCGLKDVEEINALRRGYSKASRYSTFLLVSSC
LLLFSIVFLHTLSQTTNTITSRNETRANGLIVLTEYFVDYEQYIYPITTHVVVGIFMGFAIQIGSD
LLNMIFMMHVSGMFDAIGIVAEHLFDKYESSDGKIDENLIFHARLLRLVNMHKRNLKLFEIYKDCM
YNSGLIQLVIATIVVTNVFYLHLQMLHIISTSEGNRTKIAALLLISSYLLILYINLLPTERIAESS
GDLFFKAFSGKWYTAPVKSQKTVLFLMQKFIKPCKIEVISTVAAGSETYASIVQIGFSYFMVLYSL
QE
>HlOr177like_1
MDFLEQQSCIKKYYPLLFHMGVWPFDYSKKKTVKRICLCVYHLSFISVQVWCFILTMHINTFNYIQ
AYEHMKIDWCMLKDAEEINALNRVYSSASHCNWLLFVFSYLLTSSILLSQTLVPLTDVNANDLLVR
AEYFVDYEDYFYLITTHTVVENLCAFGMQISSDILNTIIMMHISGMFEVTGLSKEDITRLYGFFFS
RLFKIFEDCMHSSGVFQICLVTVSITTIMLKALEMMSPTGNLQNKFFTVVFIVSYYILLYIDMIPG
EKIIQSSEVLFFKAYCAKWYTTSVKSQKMLLFLMHRFMEPCRLQLKNVLAMGNEMYVVLVQTSFSY
FMVLYSLQE
>HlOr177like_2N_C
MKFFEQSCIKKCYPFLLRAGLWPFDYSKRKVVHRICLCVYHLSFISVQVFVYFTTEINLRLLIDTI
PALMIYMIVFLKYVTIWVKNEAYSNASKYNMLLFVFSFLLLLGFVFLQILPQILGIISPLTEINTN
KLLVRTEYFVDYKDYIYLITIHATIAIMCSVTMQISSDILNNIFMMHISGMFEVIGIVVEHLFDKC
KSADENIDENLIFYTRLLRIMNMHRRNLKLNENFEYKFFVVLIAFVYLSIMYVNLSPAEKIIQS
SEVLFFKTYCAKWYKAPVKSQKMLLFLMHRFMEPCRLKLKSLVAAGNELYATMVQTGFSYFTVLYS
LQE
>HlOr177like_3F
TLSQYNQGYEHMRIDWCKLKDVEEINTLNREYSNASKYNMLLFRKEIQYYSSRETFNPKMIDFSII
PVFSFLLLLGFVFLQILPQILGIISPLTEINTNKLLVRTEYFVDYKDYIYLITIHATIAIMCSVTM
QISSDILNNIFMMHISGMFEVIGIVVEHLFDKCKSADENIDENLIFYTRLLRIMNMHRRNLKSVSV
N
>HlOr180C
FFTKWPLDPNAPKWRHALSKIIYCLHFVYELYGILAIINGLHCCFTTLGEFVKSFVEGIFMLEMLF
NLIYYRLREMEFQRLIIQMEHFFEMSNPSYSSQLRSVYYILPTTFVIGGLLFAFVPLFSENHLTPM
NTVYHLIPREKYWGICLSYALNVGHILNAASVMFLDLLVITIIWHATCKFSILGSKLKTTNEKKLK
MWIREHQNAIDYVREVNCVVAPLAIKSTIAVALYMIVSGLVLIHVRTSSFRNIYLKVLFIEISKFS
VVAMFSMLRFLACTWAADRMTENAHSIAWDIYDSSWINASTKVRSRIVLIIQRCQRPITIHAIGFF
TAWSLKFCGQVLYTIFTFFSALKAVLRE
>HlOr181C
ICDAWPLRKRYVKFILYAIYLIIIIILMYMDLFEVLGNLVLMVENIIHNTIIVTTFFMLFLLRCNK
LIAHVIITVKQEIAEIKFRNKDEMDLYLSYHSIADKFGRYAVSTTAVVAILLYLTPMLHMLITYSG
QSNETRVYELPFPSYHVLPYKHDFRSYIIMYAFQGPLMLVGLNHITTLSLIVSLVLHVCGKFSILS
YRIQNIQIQSKDNLNRKIEDFVTDHIKLILMANSINSALEVILLIDLTQTSIRMAVLIYSALLSPE
ANLVGTFTYVLYILLVTSLLYLYSFIGERLAYESTKVTEAYYDTEWQELSVRNQKLLLLAMRSGRR
TLYLTAGKVYNFSLYGFIHIMKTSFGYVSLLRTLT
