# Supplementary material for: InsectOR—Webserver for sensitive identification of insect olfactory receptor genes from non-model genomes
Source: PLoS One. 2021 Jan 19;16(1):e0245324. doi: 10.1371/journal.pone.0245324 (PMC7815150; doi:10.1371/journal.pone.0245324)
Supplement: S6 File — (PDF) [file pone.0245324.s006.pdf]

Application of InsectOR webserver on other insect species

Table S1. InsectOR prediction of ORs in *Dufourea novaeangliae.* (Known ORs: 112 [1])

OR prediction method	InsectOR
No of predicted genes/gene-fragments	107
Proteins with 7tm_6 domain predictions	95
Complete hits with 7tm_6	80
Partial hits with 7tm_6	15
Normal hits with 7tm_6	86
Pseudogene hits with 7tm_6	9

Table S2. InsectOR prediction of ORs in *Apis florea.* (Known ORs: 180 [2])

OR prediction method	InsectOR
No of predicted genes/gene-fragments	197
Proteins with 7tm_6 domain predictions	170
Complete hits with 7tm_6	167
Partial hits with 7tm_6	3
Normal hits with 7tm_6	143
Pseudogene hits with 7tm_6	27

Table S3. InsectOR Prediction of ORs in *Anopheles gambiae.* (Known ORs: 79 [3])

OR prediction method	InsectOR
No of predicted genes/gene-fragments	100
Proteins with 7tm_6 domain predictions	86

Complete hits with 7tm_6	69
Partial hits with 7tm_6	17
Normal hits with 7tm_6	78
Pseudogene hits with 7tm_6	8

Table S4. InsectOR prediction of ORs in *Leptinotarsa decemlineata.* (Known ORs: 37 [4])

OR prediction method	InsectOR
No of predicted genes/gene-fragments	84
Proteins with 7tm_6 domain predictions	54
Complete hits with 7tm_6	12
Partial hits with 7tm_6	42
Normal hits with 7tm_6	48
Pseudogene hits with 7tm_6	6

References:

1. Karpe SD, Dhingra S, Brockmann A, Sowdhamini R. Computational genome-wide survey of odorant receptors from two solitary bees Dufourea novaeangliae (Hymenoptera: Halictidae) and Habropoda laboriosa (Hymenoptera: Apidae). Sci Rep. 2017;7: 10823. doi:10.1038/s41598-017-11098-z

2. Karpe SD, Jain R, Brockmann A, Sowdhamini R. Identification of Complete Repertoire of Apis florea Odorant Receptors Reveals Complex Orthologous Relationships with Apis mellifera. Genome Biol Evol. 2016;8: 2879–2895. doi:10.1093/gbe/evw202

3. Hill CA, Fox AN, Pitts RJ, Kent LB, Tan PL, Chrystal MA, et al. G protein-coupled receptors in Anopheles gambiae. Science (80-). 2002;298: 176–178. doi:10.1126/science.1076196

4. Liu Y, Sun L, Cao D, Walker WB, Zhang Y, Wang G. Identification of candidate olfactory genes in Leptinotarsa decemlineata by antennal transcriptome analysis. Front Ecol Evol. 2015;3: 60. doi:10.3389/fevo.2015.00060
